# Accuracy of four digital scanners according to scanning strategy in complete-arch impressions

**DOI:** 10.1371/journal.pone.0202916

**Published:** 2018-09-13

**Authors:** Priscilla Medina-Sotomayor, Agustín Pascual M., Isabel Camps A.

**Affiliations:** 1 School of Dentistry, Azogues Campus, Universidad Católica de Cuenca, Azogues, Ecuador; 2 Department of Dentistry, Universitat de Valencia, Valencia, Spain; Klinikum der Johann Wolfgang Goethe-Universitat Frankfurt Klinik fur Nuklearmedizin, GERMANY

## Abstract

**Statement of problem:**

Although there are specific and general digital scanning guidelines depending on the system used, it is important to have the necessary flexibility in the acquisition of three-dimensional (3D) images to adapt to any clinical situation without affecting accuracy.

**Purpose:**

The purpose of this in vitro study was to identify and compare the scanning strategy with the greatest accuracy, in terms of trueness and precision, of four intraoral scanners in the impression of a complete dental arch.

**Material and methods:**

Four digital scanners were evaluated with a 3D measuring software, using a highly accurate reference model obtained from an industrial scanner as a comparator. Four scanning strategies were applied 10 times on a complete maxillary arch cast inside a black methacrylate box. The data were statistically analyzed using one-way analysis of variance (ANOVA) and post hoc comparisons with Tamhane T2 test.

**Results:**

The trueness of the Trios and iTero system showed better results with strategy “D,” Omnicam with strategy “B,” and True Definition with strategy “C”. In terms of precision, both iTero and True Definition showed better results with strategy “D”, while Trios showed best results with strategy “A” and Omnicam with strategy “B”. There were significant differences between the scanning strategies (*p*<0.05) with the iTero scanner, but not with the other scanners (*p*>0.05).

**Conclusions:**

The digital impression systems used in the experiment provided sufficient flexibility for the acquisition of 3D images without this affecting the accuracy of the scanner.

## Introduction

Since the introduction of the first scanner for digital impression in the 1980s, numerous companies have developed in-office scanners that have increased the production of dental restorations with a precise fit. These systems can capture three-dimensional (3D) images of dental preparations from which restorations are directly manufactured. This is called computer-aided design/computer-aided manufacturing (CAD/CAM). [1] Most intraoral scanning systems facilitate the production of real models of the teeth, which are based on the digital capture of information by a stereolithography technique or milling.

In CAD/CAM technology for short-span digital impression, involving a single dental quadrant, the risk of errors is low, but as the scan area increases, precision may be affected. Many studies show significant deviations in precision using different intraoral scanners on the complete arch, [2, 3, 4] with a marginal adjustment value exceeding 165 μm; this may cause the created dental crown to exceed the clinically acceptable marginal adjustment limit of 120 μm. [5, 6]

The first step of all digital work is recording the intraoral optical impression. It allows the quality of the impression to be checked immediately, including the geometry of the abutment and the finish line of the prepared tooth. If the dentist is not satisfied, the impression can be repeated at the same appointment. Hence, this technique contributes to more efficient work at the dental practice. Use of impressions material in trays is avoided, contributing to patient comfort.

The digital impression involves capturing a precise 3D image of teeth (prepared or not), dental implants, and/or any intraoral defect. The dentist must achieve an exact replica of the site, so that the laboratory technician can create a restoration to exactly fit the destination site.

However, it is still unclear is whether the 3D image acquisition method (scanning strategy) of the intraoral digital scanners can affects the definitive accuracy of the digital impression, and if so, which strategy is the best. Recent studies have investigated this variable, even though there are specific and general digital scanning guidelines depending on the system used. [7, 8] Its accuracy in complete dental arch impressions ranges from 5 to 35 μm for the experimental scanners, with no significant differences between the strategies; however, these studies use a single scanning strategy with each experimental scanner, while the present study aims to use four different strategies for each scanner.

The image acquisition method is an important factor to consider and the methods are essentially similar in all systems: [9, 10, 11, 12] placement of the retraction cord to expose the margin of the preparation, drying the area to be scanned, and application of powder (if required) with a special sprayer. The process is usually started with the occlusal surfaces as reference, due to the anatomy. Next, images are taken from various angles to generate precise 3D data of the prepared tooth. Missing or incorrect areas are corrected, and the process is completed in the same manner with the antagonist. Finally, oral lateral exploration is performed to obtain the bite (bite record). Table 1 specifies the strategy recommended for each impression system.

**Table 1:** Digital scanning systems tested.

INTRAORAL SCANNER	COMPANY	IMPRESSION SYSTEM	SCAN PROCEDURE	LIGHT SOURCE	IMAGING TYPE	SURFACE CONDITIONING	IN-OFFICE MILLING	OUTPUT FORMAT
**Trios**	3Shape A/S	Ultrafast Optical Sectioning	Light source provides an illumination pattern to cause a light oscillation on the object. Continuous images are recorded to form the 3D model.	Blue LED light	Video	No	No	Proprietary or STL
**iTero**	Cadent Ltd.	Parallel confocal microscopy.	Illuminates the surface of the object with three beams of different colored light (red, green, or blue) which combine to provide white light, 5 scans of the prepared area are recorded as a single image.	Red Laser	Multiple images	No	Yes	Proprietary or STL
**Omnicam**	Sirona Dental	Active triangulation (Multicolor stripe protection)	Video and continuous images are recorded to create a 3D model.	White light	Video	No	Yes	Proprietary
**True Definition**	3M ESPE	Active wave front sampling	Measuring out-of-plane coordinates of object points by sampling, records continuous images in various positions.	Pulsating blue light	3D in motion video	Yes	Yes	Proprietary or STL

3D, three-dimensional; STL, standard triangle language

Recording complete arch impressions for extensive restorations may however be complicated, as little information is available on the impact of different scan strategies on the accuracy of full-arch scans.

This study aimed to determine the scanning strategy that obtains the best accuracy results, in terms of trueness and precision, for each of the intraoral digital impressions systems included in the study; and the null hypothesis was there are no differences in the accuracy of the intraoral scanner related to scanning strategies”.

## Material and methods

A maxillary master cast was manufactured with Exakto-Form epoxy resin (Bredent, Senden, Germany), a wear-resistant and totally opaque material, at the Silicom Dental laboratory (Silicom Dental, Valencia, Spain).

**Fig 1:**
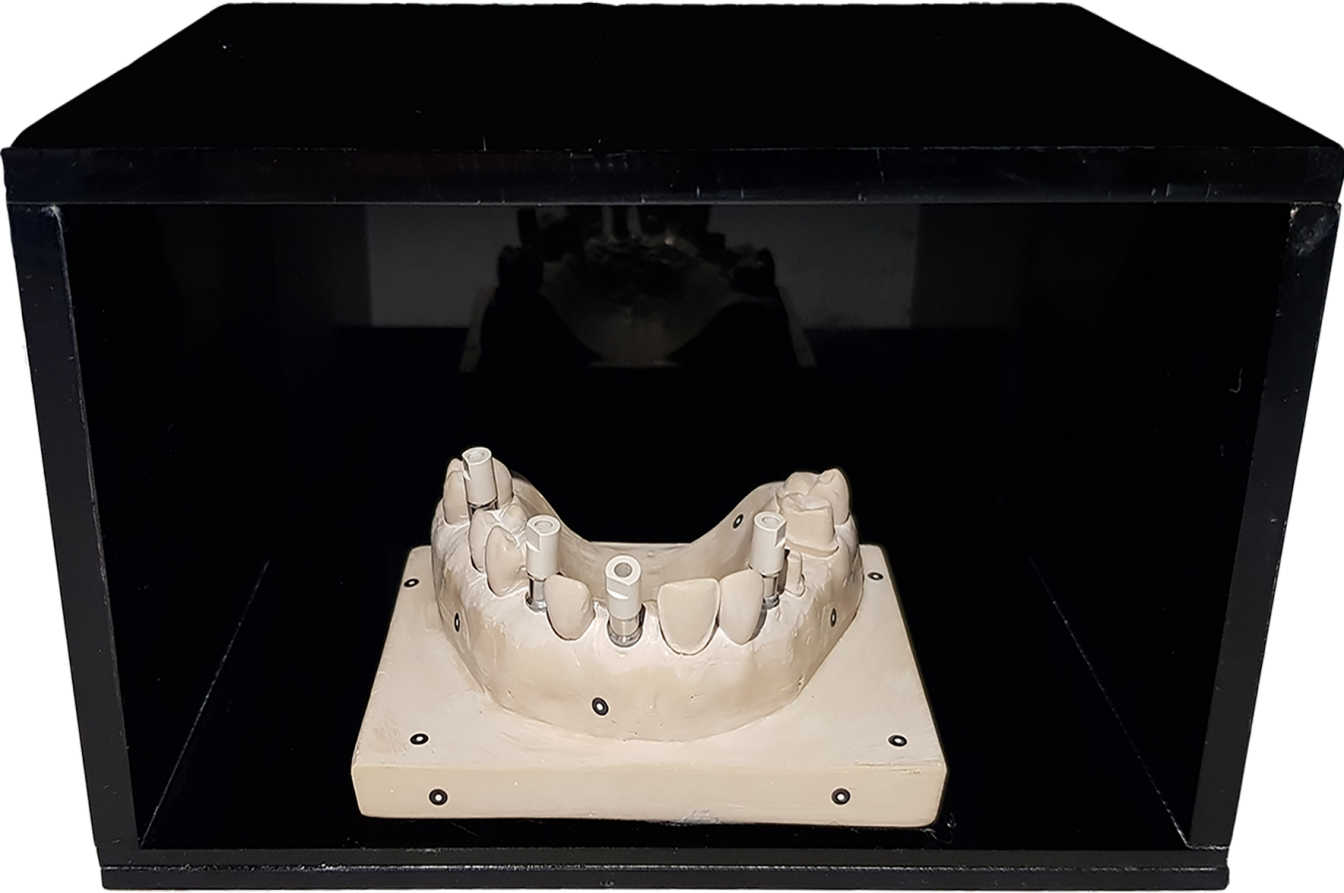
Master cast inside the methacrylate box to record digital scans.

This cast had several dental preparations for onlay, abutment tooth, fixed dental prosthesis (FDP), veneer and Straumann RN anti-rotational Core3D scanbody (Avinent Implant System, Barcelona, Spain), to try to simulate complex clinical situations (Fig 1).

A methacrylate box was created for taking the digital impression. This box was opaque black to avoid light reflection and simulate the oral cavity.

A base measuring 8 cm (width) x 6 cm (depth) was manufactured on the master cast, which was fit into a cut-out of the same dimensions made in the base of the methacrylate box, allowing a controlled environment (stability and temperature control) during the scanning process. (Fig 1)

The impression systems included in the study were Trios (software version 1.4.5.3, 3Shape Dental Systems, Copenhagen, Denmark), iTero (software version OrthoCAD 5.7.0.301 Cadent LTD, Align Technology Inc., San Jose, CA, USA), Cerec AC Omnicam (software version CEREC SW 4.4.4; Sirona, Bensheim, Germany); and True Definition (software version 4.2; 3M ESPE Dental Products, Seefeld, Germany). Table 2 exhibits their features.

**Table 2:** Scanning procedure and indications of the intraoral digital impression systems included in the study.

INTRAORAL SCANNER	COMPANY	INDICATIONS	SCANNING STRATEGY
**Trios**	3SHAPE	Crowns and partial fixed dental prostheses Veneers, onlay, and partial crowns Temporary crowns and virtual diagnostic wax-ups Post and abutment tooth Design of partial removable prosthesis Abutments and implant partial fixed dental prostheses Planning of implants and surgical guides Orthodontics and splints	Stat with the molar, for better identification. The angle of scanning is 45–90 degrees to complete the sweep. The exploration pathway is occlusal, lingual, and buccal. COMPLETE ARCH: Start with the occlusal sweep, then turn toward palatal, buccal, and then 90 degrees to record the contact points.
**iTero**	CADENT	Crowns and partial fixed dental prostheses Orthodontics and splints (Invisalign) Workflows on implants	Each scanning procedure must have crosshairs following the natural shape of the arch (tangential). Buccal and lingual explorations must include occlusal information (scan with a 45-degree angle)
**Omnicam**	SIRONA	Partially or fully edentulous maxillary and mandibular jaws in support of cemented restorations of one or multiple units. Tooth- or tissue-supported implants	The camera moves at a distance of 0–15 mm above the dental surface. Trajectory of exploration: occlusal, vestibular, and palatal in the first quadrant; the second quadrant is recorded in the same manner
**True Definition**	3M ESPE	Crowns and partial fixed dental prostheses, Onlays Workflows on implants Veneers Partial prostheses, Orthodontics and splints,	Trajectory of exploration: occlusal, vestibular, and palatal in the first quadrant; the second quadrant is recorded in the same manner.

Four scanning strategies were carried out 10 times with each digital impression system, to obtain a total of 40 digital files for each intraoral scanner. Each scanner was calibrated using the manufacturer´s guidelines.

**Fig 2:**
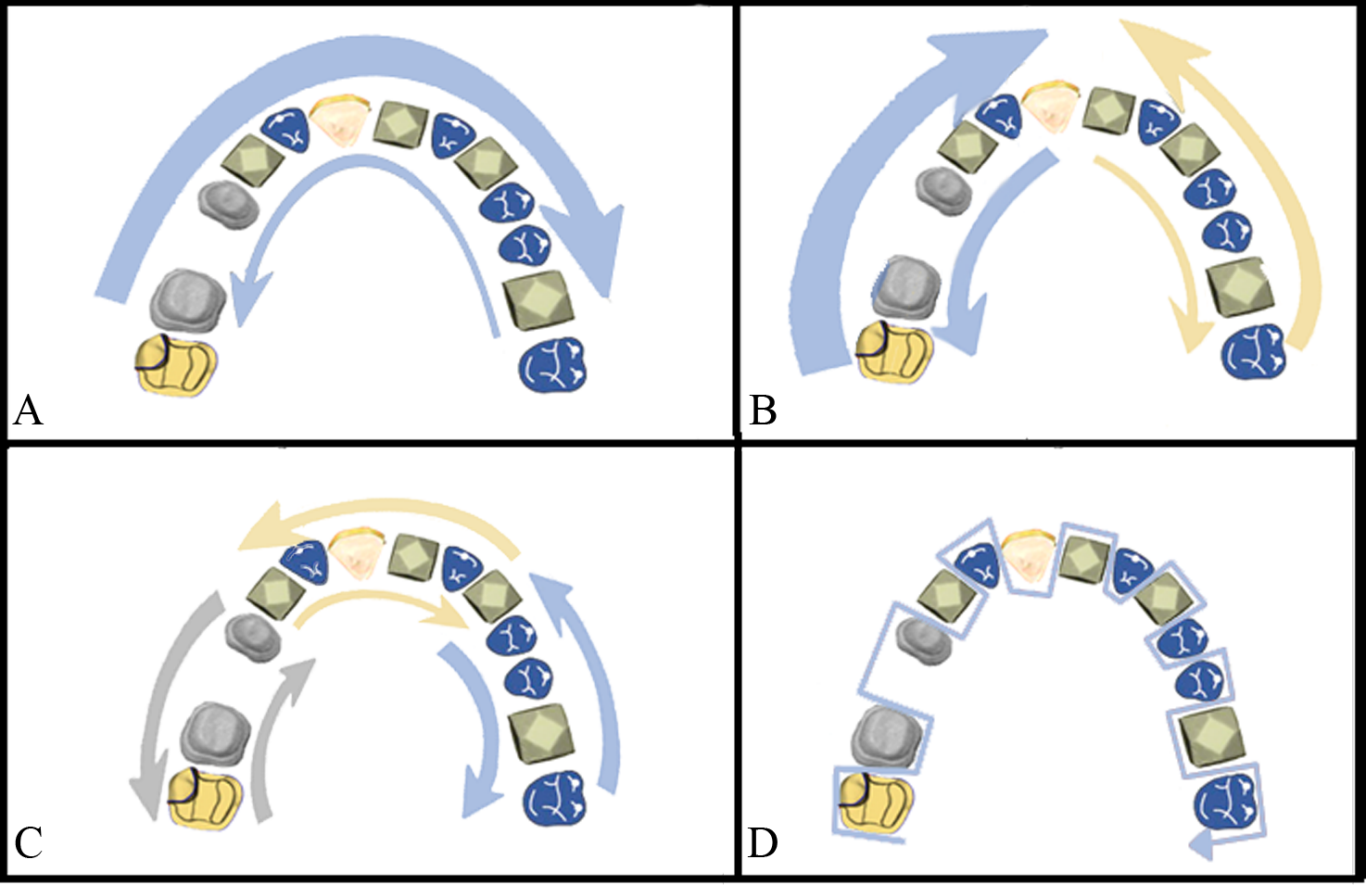
Scanning strategies: (A) Exterior-Interior, (B) Quadrants, (C) Sextants, (D) Sequential.

The strategies, carried out by the same trained investigator, were as follows (Fig 2):

Exterior-interior (A): First, occlusal surfaces, starting with the left second molar and going to the right second molar, returning via the vestibular surfaces and finally a sweep over the palatal surfaces. [7]

Quadrants (B): First, occlusal surfaces, starting with the right central incisor and going toward the right second molar, returning via the vestibular surfaces, and then the palatal surfaces. Second, occlusal surfaces, starting with the left central incisor till the left second molar, returning via the vestibular surfaces, and then the palatal surfaces.

Sextants (C): First, occlusal surfaces, starting with the right second molar until the right first premolar, and returning via the vestibular surfaces, and then the palatal surfaces. Second, occlusal surfaces, starting with the right canine until the left canine, returning via the vestibular surfaces, and then the palatal surfaces. Third, occlusal surfaces, starting with the left first premolar until the left second molar, returning via the vestibular surfaces, and then the palatal surfaces.

Sequential (D): Sequential scanning of the three surfaces of each tooth (occlusal, vestibular, and palatal), performing an “S”-shaped movement from the right second molar in all directions and without returning to the starting point. [7]

The process always started with occlusal surfaces as references while taking the impression, and a final sweep was performed to fill the spaces that did not have digital information, generally the interproximal spaces.

**Fig 3:**
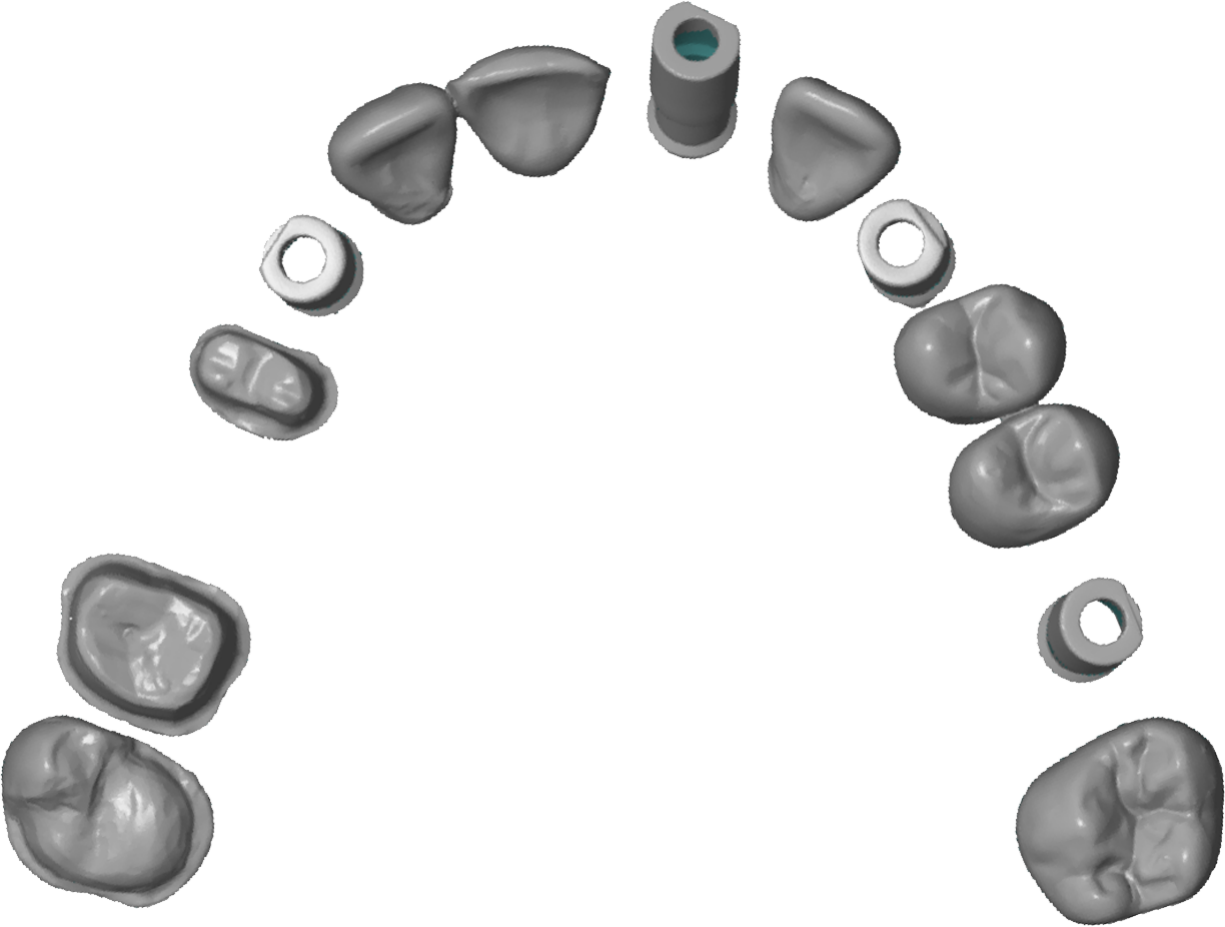
CRM scanned with ATOS II Triple Scan scanner.

A “CAD reference model” (CRM) was created with ATOS II Triple Scan (GOM Technologies, Metronic, Barcelona, Spain), [13, 14] and an industrial structured blue light scanner complying with ISO 12836 and was shown to be accurate up to 3 μm with precision of 2 μm for jaw-sized scans [15,16] (Fig 3).

The files obtained with the scanners in the study were then converted to standard triangle language (STL) format. iTero, Trios, and True Definition systems export in this format, but for the Cerec system, Omnicam, Delcam Exchange 2016 R3 software was used.

Discrepancies were analyzed using Geomagic Control (Geomagic, Morrisville, North Carolina, USA, 2013). This software makes it possible to fully select the parameters to be studied when performing the comparison. [12, 17, 18, 19]

With the “cut with planes” tool, all the soft tissue surrounding the teeth was removed, to reduce data points in the file that may affect the mean distance, and all the study models were aligned with the CRM. Each of the files obtained from the scanners was compared with the CRM obtained with the industrial scanner using the “best fit alignment,” a mathematical algorithm to overlay a digital impression on a digital master objectively measuring variances across the entire experimental model in relation to the master.

Next, all the files were compared, superimposing them on the reference model to calculate the total 3D deviations (X, Y, and Z) between the data sets obtained from the reference scanner and the different intraoral scanners included in the study (trueness and precision), differentiating each of the scanning strategies during the comparison.

**Fig 4:**
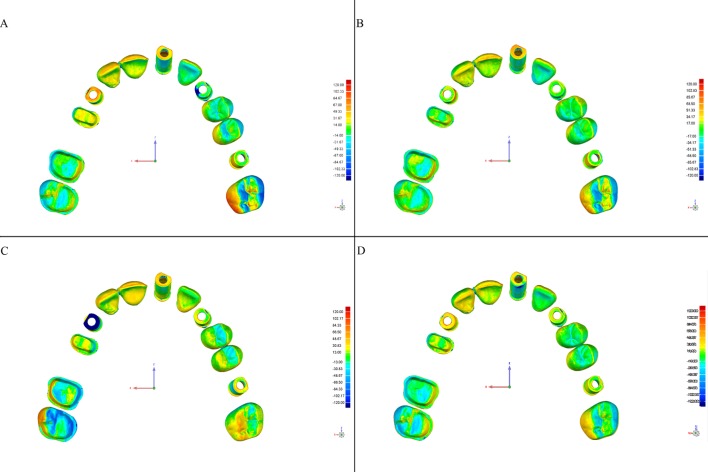
Color-coded map of deviations between the CRM and test models from the Trios scanner according to the scanning strategy (Geomagic Control software). Color degraded from -120 μm (blue) to + 120 μm (red), representing the contraction (blue) and expansion (red). (A) Exterior-Interior, (B) Quadrants, (C) Sextants, and (D) Sequential.

**Fig 5:**
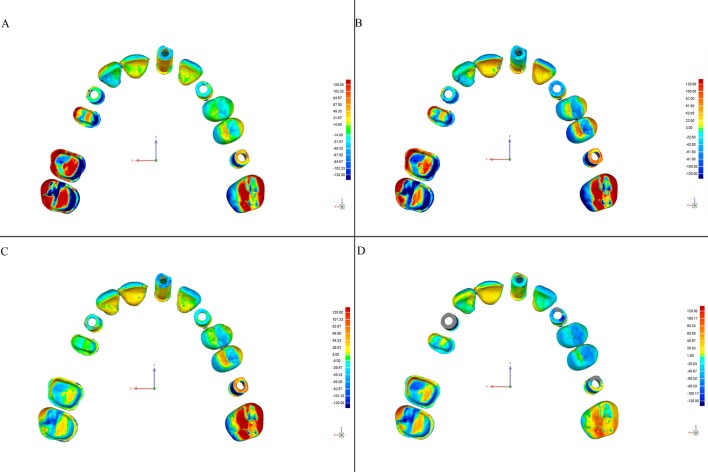
Color-coded map of deviations between the CRM and test models from the iTero scanner according to the scanning strategy (Geomagic Control software). Color degraded from -120 μm (blue) to + 120 μm (red), representing contraction (blue) and expansion (red). (A) Exterior-Interior, (B) Quadrants, (C) Sextants, and (D) Sequential.

**Fig 6:**
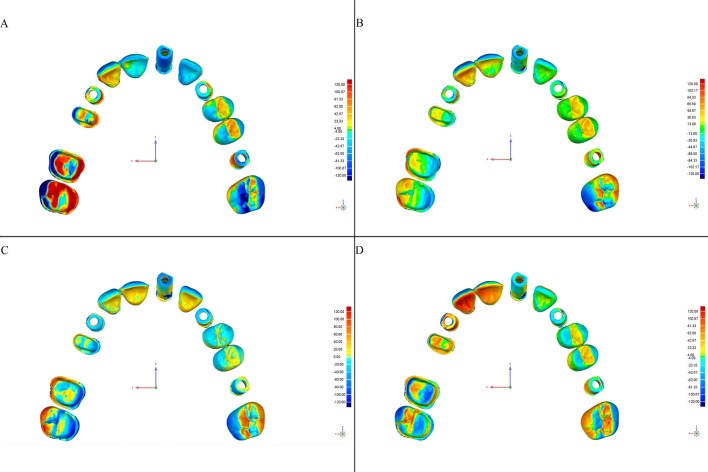
Color-coded map of deviations between the CRM and test models from the Omnicam scanner according to the scanning strategy (Geomagic Control software). Color degraded from -120 μm (blue) to + 120 μm (red), representing contraction (blue) and expansion (red). (A) Exterior-Interior, (B) Quadrants, (C) Sextants, and (D) Sequential.

**Fig 7:**
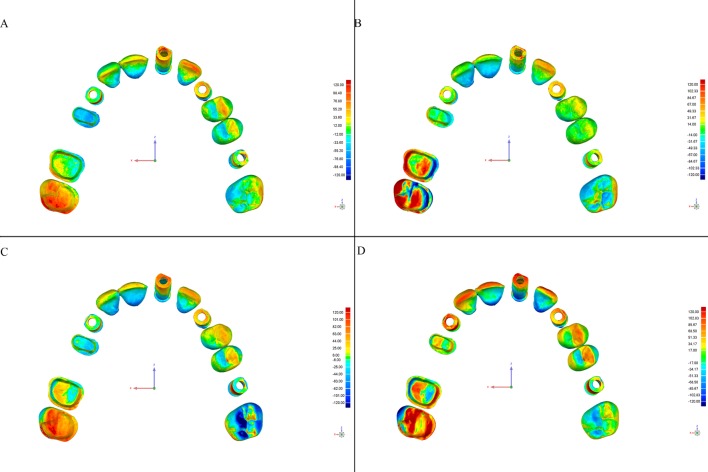
Color-coded map of deviations between the CRM and test models from the True Definition scanner according to the scanning strategy (Geomagic Control software). Color degraded from -120 μm (blue) to + 120 μm (red), representing contraction (blue) and expansion (red). (A) Exterior-Interior, (B) Quadrants, (C) Sextants, and (D) Sequential.

This software allows detection of discrepancies in micrometers, both positive (expansion) and negative (contraction). Deviations are viewed on a color-coded superimposed image (Figs 4–7).

The total mean discrepancy (trueness) indicates the deviation of each point of the STL compared with the CRM on average. They are calculated from the average of the mean internal and external discrepancies without considering the negative or positive signs (contraction or expansion). This value corresponds to the trueness which describes the discrepancy among the measurement values of the CRM and test models.

Meanwhile, the standard deviation indicates the dispersion of the points of the STL around the mean of the CRM; this measure is evaluated by the same software when evaluating the 3D deviation. This value corresponds to the precision which describes the repeatability of the scans (discrepancy between the test models).

The data were statistically analyzed using one-way analysis of variance (ANOVA) and multiple post hoc comparisons with Tamhane T2 test.

## Results

Tables 3 and 4 list the data analyzed. (S1–S17 Tables)

**Table 3:** Raw data of trueness (μm) used for statistical analysis of the four scanners according to the scanning strategy.

	Scanning strategy	N	Mean negative	Mean positive	Mean	Median	SD	Minimum	Maximum
**Trios**	**A**	10	-50,.30	63,88	57.09	55.92	6.75	50.02	70.12
	**B**	10	-49,61	61,66	55.63	55.07	3.72	51.57	65.59
	**C**	10	-51,38	59,76	55.57	55.77	3.01	51.38	60.24
	**D**	10	-40,96	64,95	52.95*	56.32	15.68	10.04	67.42
**iTero**	**A**	10	-106,07	109,29	107.68**	109.70	24.53	66.50	157.10
	**B**	10	-104,82	107,75	106.28**	108.19	16.15	74.98	129.78
	**C**	10	-82,89	95,42	89.15**	89.51	10.70	72.38	106.43
	**D**	10	-71,19	78,77	74.98*	74.47	8.62	64.56	89.82
**Omnicam**	**A**	10	-100,41	104,76	102.58	101.92	12.55	83.22	127.73
	**B**	10	-84,27	94,08	89.17*	87.64	12.47	74.71	112.02
	**C**	10	-87,84	98,70	93.27	88.55	10.83	82.64	112.65
	**D**	10	-108,04	108,52	108.28**	115.56	12.98	91.57	120.56
**True Definition**	**A**	10	-55,09	66,51	35.67	27.24	19.70	19.15	69.06
	**B**	10	-47,23	68,08	34.25	28.36	14.02	20.09	58.18
	**C**	10	-41,00	52,46	28.78*	26.33	10.68	19.67	57.70
	D	10	-43,96	52,72	29.61	28.32	8.72	19,08	44.96

Scanning strategies: Exterior-Interior (A), Quadrants (B), Sextants (C), Sequential (D)

*Strategy with best accuracy within each scanner

**Strategy with statistically significant differences within each scanner.

**Table 4:** Raw data of precision (μm) used for statistical analysis of the four scanners according to the scanning strategy.

	Scanning strategy	N	Mean (SD)	Median	SD	Minimum	Maximum
**Trios**	**A**	10	184.51*	184.09	10.75	167.15	198.55
	**B**	10	194.53	193.81	7.22	181.53	205.47
	**C**	10	193.28	194.00	8.30	175.21	202.45
	**D**	10	205.79**	207.85	10.36	187.54	218.62
**iTero**	**A**	10	269.84**	251.06	53.96	210.03	391.69
	**B**	10	272.21**	267.84	29.95	231.29	311.30
	**C**	10	248.04**	240.86	15.92	233.64	283.84
	**D**	10	197.16*	198.49	25.57	157.17	246.49
**Omnicam**	**A**	10	260.12	275.21	36.23	209.95	299.87
	**B**	10	243.68*	236.24	35.63	191.23	307.49
	**C**	10	259.52	252.81	23.91	232.79	294.70
	**D**	10	283.73	278.19	23.32	253.29	327.42
**True Definition**	**A**	10	109.83	88.25	48.95	64.89	209.94
	**B**	10	111.78	90.35	44.15	73.91	203.24
	**C**	10	90.79	81.30	37.61	59.47	193.31
	D	10	82.83*	79.38	24.88	56.64	132.36

Scanning strategies: Exterior-Interior (A), Quadrants (B), Sextants (C), Sequential (D)

*Strategy with best accuracy within each scanner

**Strategy with statistically significant differences within each scanner.

The Levene`s test applied to the one-way ANOVA for trueness and precision was found to be significant (P = .000). Therefore, and after the check for normal distribution, the nonparametric Kruskal-Wallis test was used to analyze whether there were differences between the scanners, and significant values (P = .000) were obtained for iTero and Omnicam.

Post hoc comparisons for these intraoral scanners were carried out with the Tamhane T2 test.

The Trios scanner did not show any significant differences in relation to the scanning strategy used (P<0.05). The best result was obtained with strategy “D” (sequential), with 52.95 μm trueness and 184.51 μm precision.

The results for the iTero scanner showed significant differences (P<0.05), with best results obtained with strategy “D” (sequential) as the best for both trueness (74.98 μm) and precision (197.16 μm).

The Cerec Omnicam scanner showed best results with strategy “B” (quadrants), for both trueness (89.17 μm) and precision (243.68 μm), with significant differences in trueness with strategy “D” (sequential), which had the worst result, with a value of 283.73 μm (P<0.05).

Finally, for the True Definition scanner, the best scanning strategy for trueness was “C” (sextants) and for precision was “D” (sequential), with values of 28.78 μm (trueness) and 82.83 μm (precision). There were no statistically significant differences between the results with the different scanning strategies (P>0.05).

## Discussion

Accuracy refers to how accurate and precise an object is.

The selected model material was epoxy resin, an opaque and dimensionally stable material with good mechanical and chemical resistance according to the manufacturers. No measurements were conducted to ensure the stability of this material, but in order to maintain a controlled environment, the digital scanning was performed after placing the master model inside a black methacrylate box.

A sample size of 10 for each scanning strategy was determined by using a sample size calculation with 95% confidence level and a margin of error of 5%. A total of 40 impressions were made with each scanner. This is widely confirmed by several authors as sufficient to obtain consistent statistical results. [19, 20, 21, 22] Some authors believe that clinically valid results can be obtained with 5–10 repetitions. [23]

Only the iTero scanner had statistically significant differences; for this scanner, strategy “D” (sequential) was the best strategy.

According to Muller et al. (2016) and Ender et al. (2013), using a different scanning strategy according to the digital system does not influence the accuracy of the digital impressions. This study [7] used the Alicona Infinite Focus Standard scanner, with a resolution of 0.5 μm, to obtain the CRM. Its trueness and precision in complete dental arch impressions ranges from 5 to 35 μm for 3Shape and 32 μm for Omnicam, with no significant differences between the strategies. [7, 8]

The earlier That study used only one strategy for each scanner. By using four different strategies with each of the scanners and comparing the accuracy data among them, it is possible to understand whether or not it is really important to follow a scanning sequence according to the digital impression system used. Therefore, except for the iTero scanner, the other three scanners are able to record an accurate 3D images of the scanned object with any of the strategies used. Hence, the clinician is able to obtain equally satisfactory results, regardless of the clinical difficulty encountered, and is able to scan the dental structures following any scanning strategy, adapting it to the specific situation.

## Conclusions

Within the limitations of this in vitro study, the following can be concluded:

1. The accuracy, in terms of trueness and precision of the Trios, Omnicam, and True Definition scanners are not affected by the different scanning strategies in recording long-span impressions.
2. The accuracy, in terms of trueness and precision, of the iTero scanner depends on the strategy used when recording intraoral impression in long-span impressions; the sequential strategy is best for such impressions.

## Supporting information

S1 Table Raw data of trueness (μm) used for statistical analysis of the four scanners according to scanning strategy and Table Raw data of precision (μm) used for statistical analysis of the four scanners according to scanning strategy. Trios (scanning strategy A).
(ZIP)

S2 Table Raw data of trueness (μm) used for statistical analysis of the four scanners according to scanning strategy and Table Raw data of precision (μm) used for statistical analysis of the four scanners according to scanning strategy. Trios (scanning strategy B).
(ZIP)

S3 Table Raw data of trueness (μm) used for statistical analysis of the four scanners according to scanning strategy and Table Raw data of precision (μm) used for statistical analysis of the four scanners according to scanning strategy. Trios (scanning strategy C).
(ZIP)

S4 Table Raw data of trueness (μm) used for statistical analysis of the four scanners according to scanning strategy and Table Raw data of precision (μm) used for statistical analysis of the four scanners according to scanning strategy. Trios (scanning strategy D).
(ZIP)

S5 Table Raw data of trueness (μm) used for statistical analysis of the four scanners according to scanning strategy and Table Raw data of precision (μm) used for statistical analysis of the four scanners according to scanning strategy. iTero (scanning strategy A).
(ZIP)

S6 Table Raw data of trueness (μm) used for statistical analysis of the four scanners according to scanning strategy and Table Raw data of precision (μm) used for statistical analysis of the four scanners according to scanning strategy. iTero (scanning strategy B).
(ZIP)

S7 Table Raw data of trueness (μm) used for statistical analysis of the four scanners according to scanning strategy and Table Raw data of precision (μm) used for statistical analysis of the four scanners according to scanning strategy. iTero (scanning strategy C).
(ZIP)

S8 Table Raw data of trueness (μm) used for statistical analysis of the four scanners according to scanning strategy and Table Raw data of precision (μm) used for statistical analysis of the four scanners according to scanning strategy. iTero (scanning strategy D).
(ZIP)

S9 Table Raw data of trueness (μm) used for statistical analysis of the four scanners according to scanning strategy and Table Raw data of precision (μm) used for statistical analysis of the four scanners according to scanning strategy. Omnicam (scanning strategy A).
(ZIP)

S10 Table Raw data of trueness (μm) used for statistical analysis of the four scanners according to scanning strategy and Table Raw data of precision (μm) used for statistical analysis of the four scanners according to scanning strategy. Omnicam (scanning strategy B).
(ZIP)

S11 Table Raw data of trueness (μm) used for statistical analysis of the four scanners according to scanning strategy and Table Raw data of precision (μm) used for statistical analysis of the four scanners according to scanning strategy. Omnicam (scanning strategy C).
(ZIP)

S12 Table Raw data of trueness (μm) used for statistical analysis of the four scanners according to scanning strategy and Table Raw data of precision (μm) used for statistical analysis of the four scanners according to scanning strategy. Omnicam (scanning strategy D).
(ZIP)

S13 Table Raw data of trueness (μm) used for statistical analysis of the four scanners according to scanning strategy and Table Raw data of precision (μm) used for statistical analysis of the four scanners according to scanning strategy. True definition (scanning strategy A).
(ZIP)

S14 Table Raw data of trueness (μm) used for statistical analysis of the four scanners according to scanning strategy and Table Raw data of precision (μm) used for statistical analysis of the four scanners according to scanning strategy. True definition (scanning strategy B).
(ZIP)

S15 Table Raw data of trueness (μm) used for statistical analysis of the four scanners according to scanning strategy and Table Raw data of precision (μm) used for statistical analysis of the four scanners according to scanning strategy. True definition (scanning strategy C).
(ZIP)

S16 Table Raw data of trueness (μm) used for statistical analysis of the four scanners according to scanning strategy and Table Raw data of precision (μm) used for statistical analysis of the four scanners according to scanning strategy. True definition (scanning strategy D).
(ZIP)

S17 Table Raw data of trueness (μm) used for statistical analysis of the four scanners according to scanning strategy and Table Raw data of precision (μm) used for statistical analysis of the four scanners according to scanning strategy. N, Mean, Median, SD, Minimum and Maximum.
(PDF)
