# Correction: Accuracy of four digital scanners according to scanning strategy in complete-arch impressions

**DOI:** 10.1371/journal.pone.0209883

**Published:** 2018-12-20

**Authors:** Priscilla Medina-Sotomayor, Agustín Pascual-Moscardó, Isabel Camps

There is an error in the second author’s name. The correct name is: Agustín Pascual-Moscardó.

There is an error in the third author’s name. The correct name is: Isabel Camps.

The correct citation is: Medina-Sotomayor P, Pascual-Moscardó A, Camps I (2018) Accuracy of four digital scanners according to scanning strategy in complete-arch impressions. PLoS ONE 13(9): e0202916. https://doi.org/10.1371/journal.pone.0202916
