# Supplementary material for: Accuracy of four digital scanners according to scanning strategy in complete-arch impressions
Source: PLoS One. 2018 Sep 13;13(9):e0202916. doi: 10.1371/journal.pone.0202916 (PMC6136706; doi:10.1371/journal.pone.0202916)
Supplement: S1 Table — Trios (scanning strategy A). (ZIP) [file pone.0202916.s001.zip › S1/3S8A.pdf]

3D Comparación Resultados

Modelo referencia	MRC
Modelo test	3S8A
Nº de puntos de datos	101088
# Aislados	304

Tipo tolerancia	3D desviación
Unidades	u
Máx. crítico	120.00
Máx. nominal	14.00
Mín. nominal	-14.00
Mín. crítico	-120.00

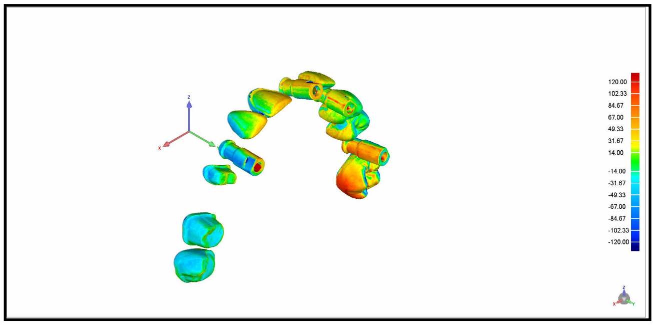

Desviación	
Desviación superior máx.	2660.05
Desviación inferior máx.	-2984.26
Desviación media	73.43 /-59.63
Desviación estándar	198.55

Distribución desviación

>=Min	<Max	# Puntos	%
-120.00	-102.33	992	0.98
-102.33	-84.67	1462	1.45
-84.67	-67.00	2045	2.02
-67.00	-49.33	3764	3.72
-49.33	-31.67	7606	7.52
-31.67	-14.00	13078	12.94
-14.00	14.00	27802	27.50
14.00	31.67	16935	16.75
31.67	49.33	10615	10.50
49.33	67.00	5120	5.06
67.00	84.67	2432	2.41
84.67	102.33	1410	1.39
102.33	120.00	1000	0.99

Fuera del crítico superior	4323	4.28
Fuera del crítico inferior	2504	2.48

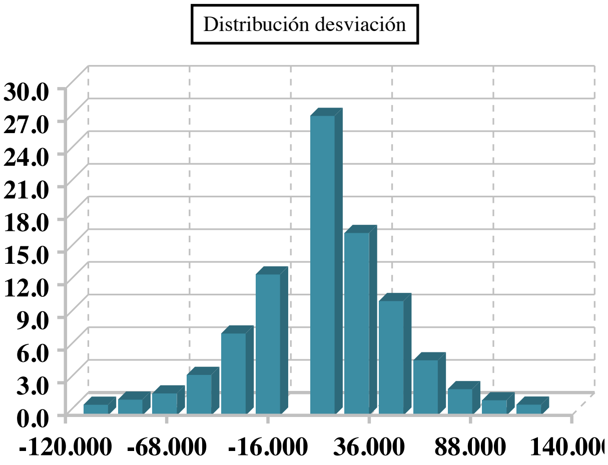

Desviaciones estándar

Distribución (+/-)	# Puntos	%
-6 * Desv. estánd.	545	0.54
-5 * Desv. estánd.	104	0.10
-4 * Desv. estánd.	174	0.17
-3 * Desv. estánd.	225	0.22
-2 * Desv. estánd.	625	0.62
-1 * Desv. estánd.	57772	57.15
1 * Desv. estánd.	38579	38.16
2 * Desv. estánd.	970	0.96
3 * Desv. estánd.	444	0.44
4 * Desv. estánd.	398	0.39
5 * Desv. estánd.	365	0.36
6 * Desv. estánd.	887	0.88

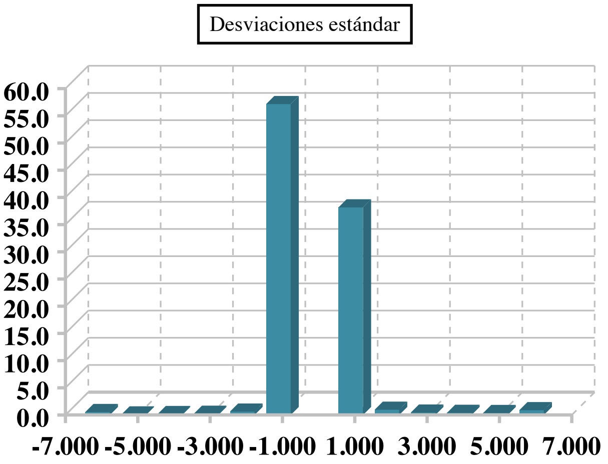

Predefinido: Isométrico

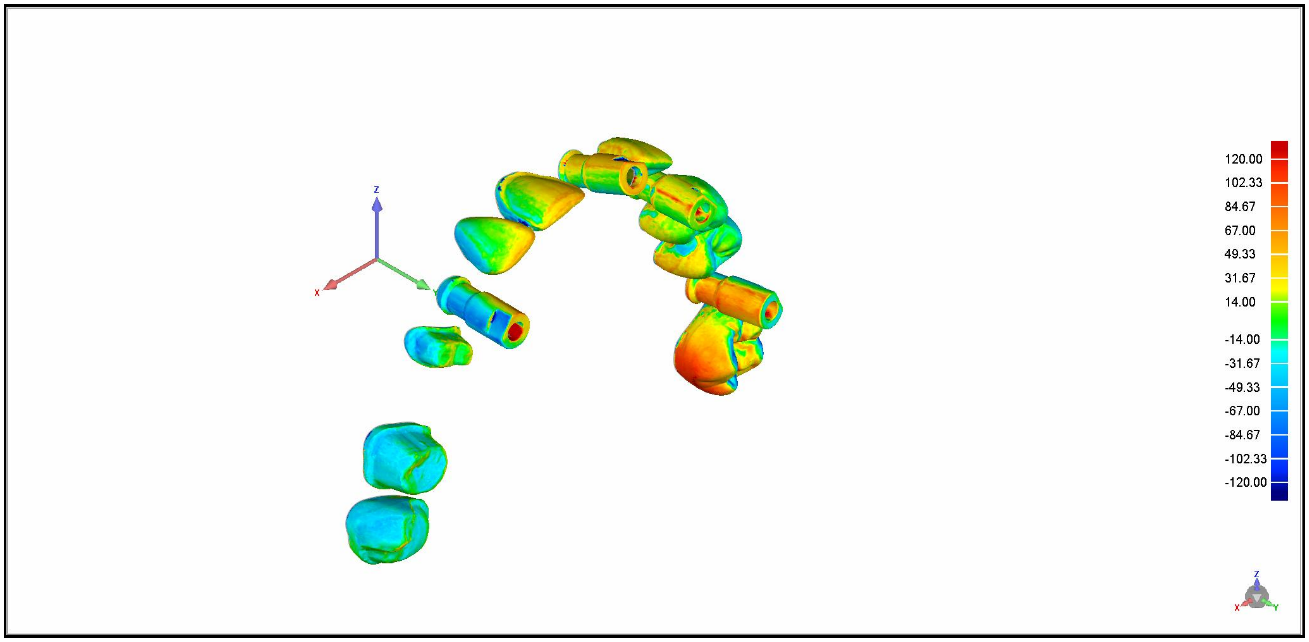

Predefinido: Frente

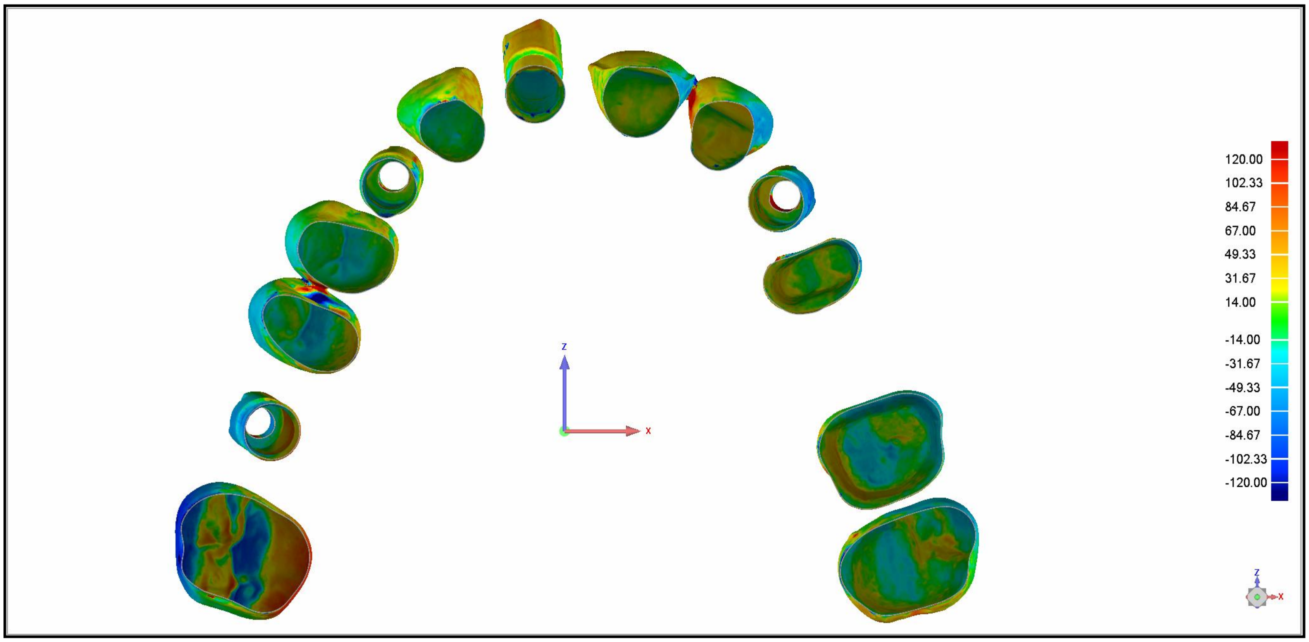

Predefinido: Atrás

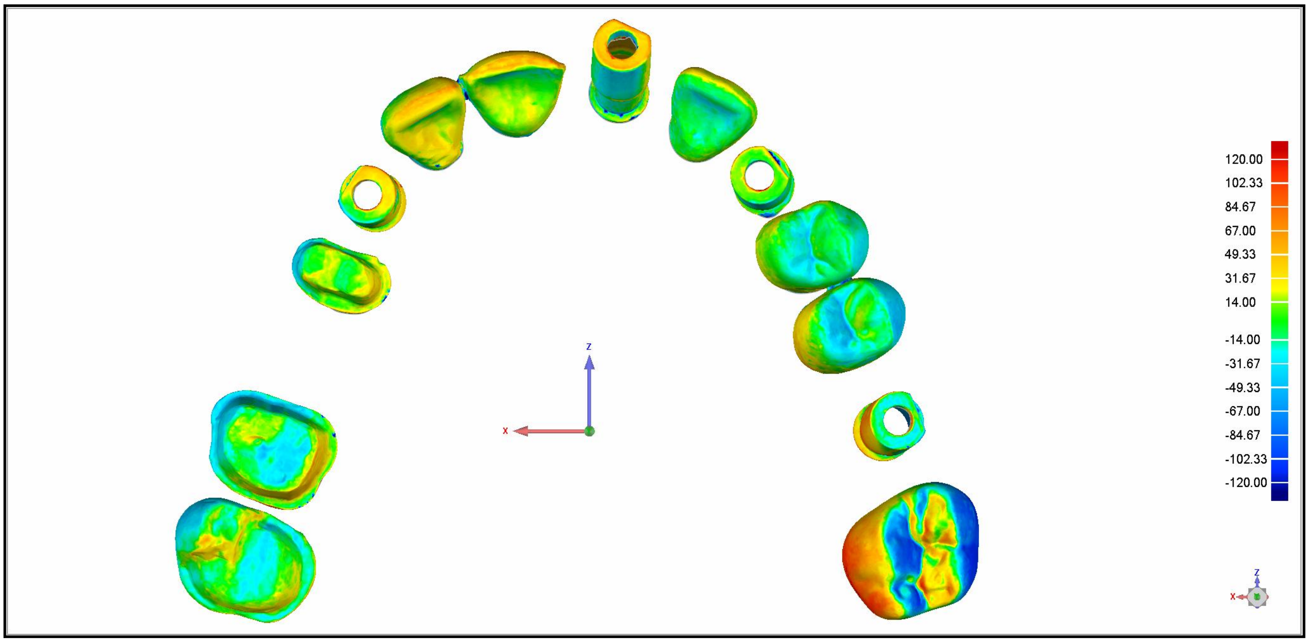

Predefinido: Izquierda

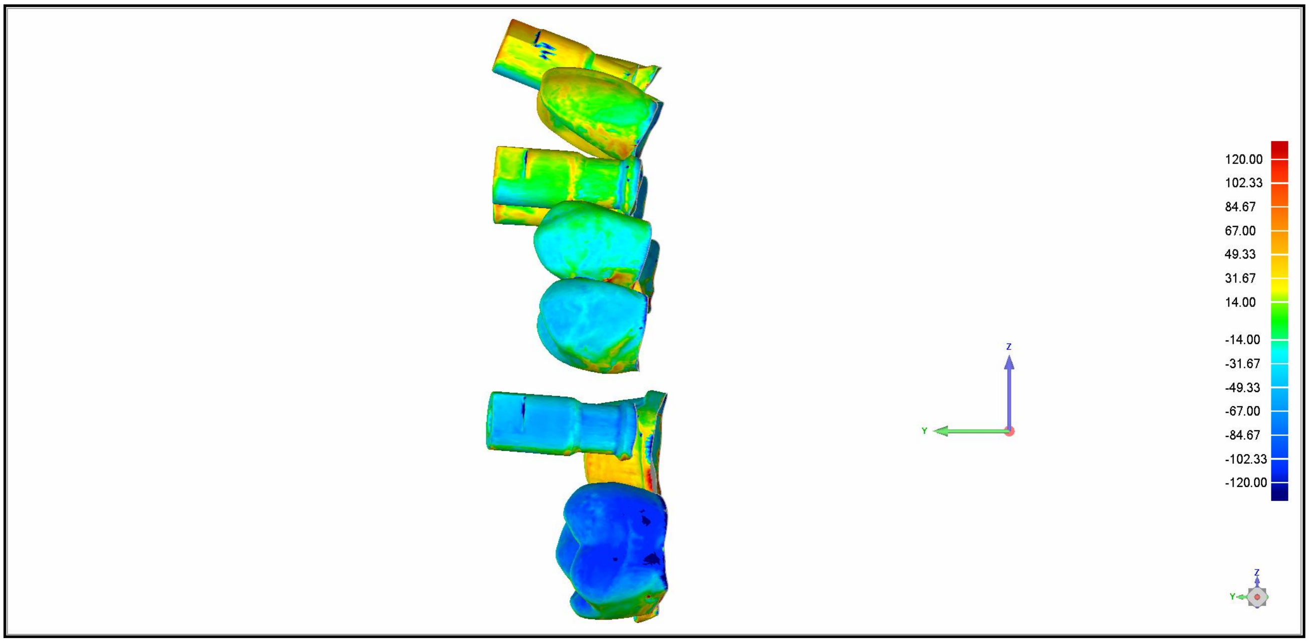

Predefinido: Derecha

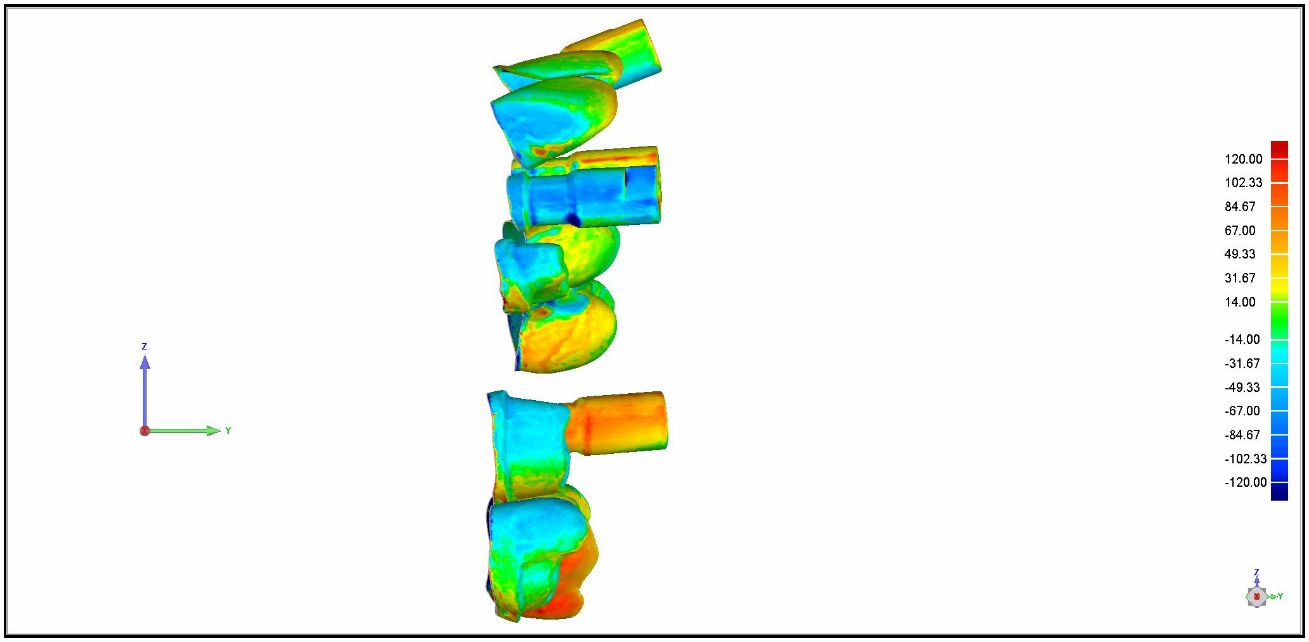

Predefinido: Superior

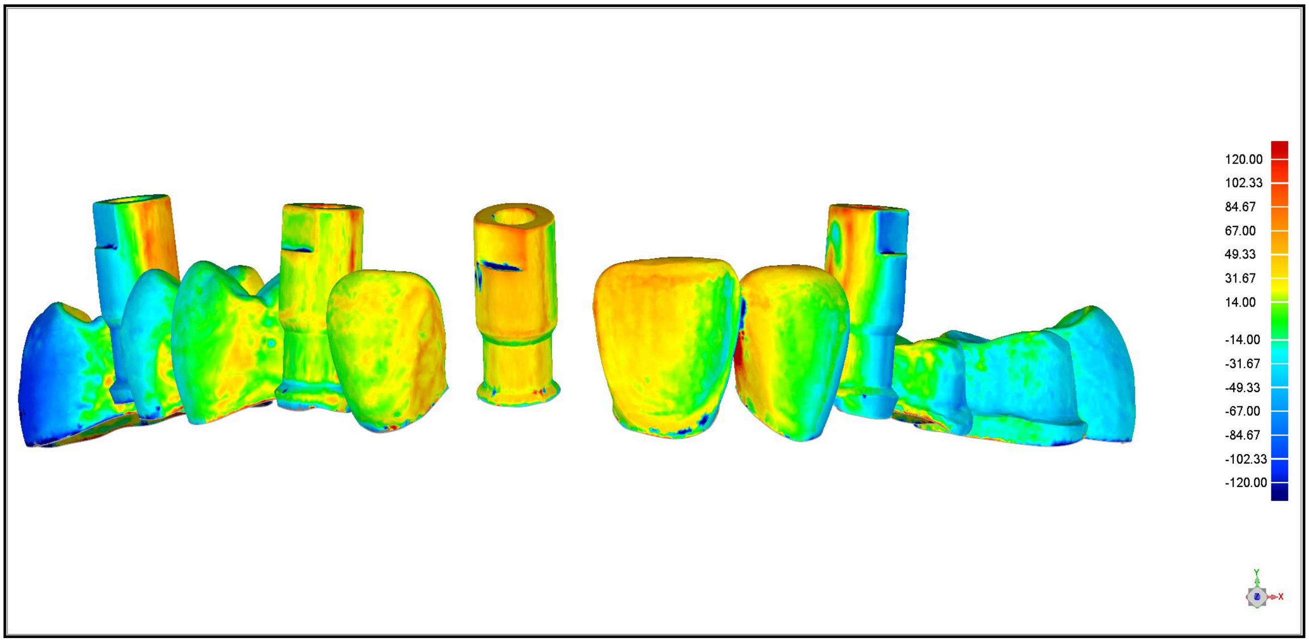

Predefinido: Inferior

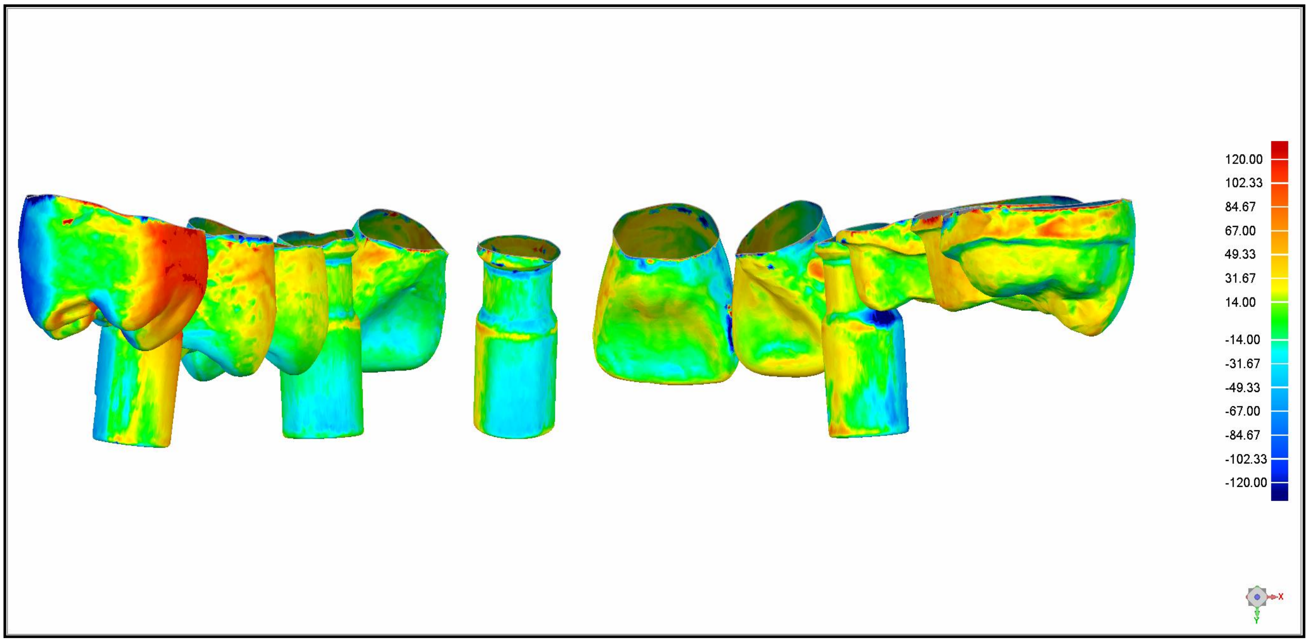

Ajuste de ubicación: Desviaciones superior e inferior

Unidades: u

Nombre	Desv	Estado	Superior Tol	Inferior Tol	Ref X	Ref Y	Ref Z	Radio	Desv X	Desv Y	Desv Z	Medido X	Medido Y	Medido Z	Dir. proy. X	Dir. proy. Y	Dir. proy. Z
Desv. inferior	-2984.26				-20516.36	29366.37	10344.29	n/a	-2834.72	-901.56	-239.44	-23351.08	28464.82	10104.85	0.95	0.30	0.08
Desv. superior	2660.05				14721.30	29902.09	19236.98	n/a	-2317.94	19.62	1304.84	12403.36	29921.71	20541.82	-0.87	0.01	0.49
