# Supplementary material for: Accuracy of four digital scanners according to scanning strategy in complete-arch impressions
Source: PLoS One. 2018 Sep 13;13(9):e0202916. doi: 10.1371/journal.pone.0202916 (PMC6136706; doi:10.1371/journal.pone.0202916)
Supplement: S1 Table — Trios (scanning strategy A). (ZIP) [file pone.0202916.s001.zip › S1/3S4A.pdf]

Geomagic Control

Fecha: 3/6/2017, 12:19 pm

3D Comparación Resultados

Modelo referencia	MRC
Modelo test	3S4A
Nº de puntos de datos	103381
# Aislados	203

Tipo tolerancia	3D desviación
Unidades	u
Máx. crítico	120.00
Máx. nominal	16.00
Mín. nominal	-16.00
Mín. crítico	-120.00

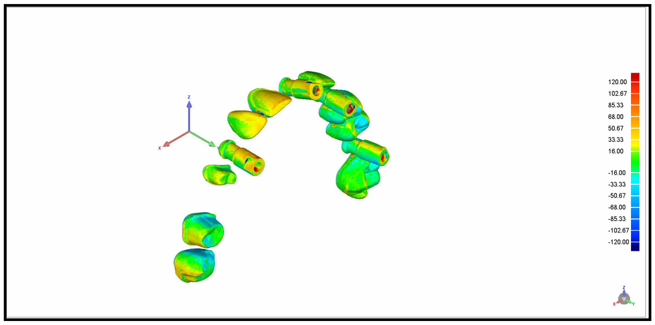

Desviación	
Desviación superior máx.	3121.77
Desviación inferior máx.	-2710.45
Desviación media	60.16 /-45.71
Desviación estándar	184.59

Distribución desviación

>=Min	<Max	# Puntos	%
-120.00	-102.67	329	0.32
-102.67	-85.33	417	0.40
-85.33	-68.00	599	0.58
-68.00	-50.67	1345	1.30
-50.67	-33.33	4679	4.53
-33.33	-16.00	12426	12.02
-16.00	16.00	45475	43.99
16.00	33.33	17233	16.67
33.33	50.67	9229	8.93
50.67	68.00	3401	3.29
68.00	85.33	1418	1.37
85.33	102.67	525	0.51
102.67	120.00	428	0.41

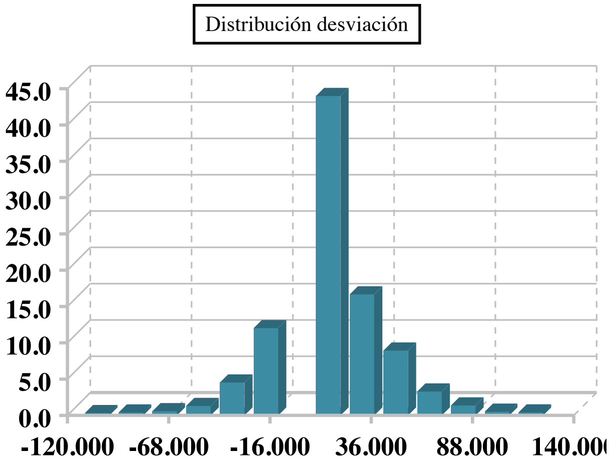

Fuera del crítico superior		3787	3.66
Fuera del crítico inferior		2090	2.02

Desviaciones estándar

Distribución (+/-)	# Puntos	%
-6 * Desv. estánd.	480	0.46
-5 * Desv. estánd.	73	0.07
-4 * Desv. estánd.	96	0.09
-3 * Desv. estánd.	152	0.15
-2 * Desv. estánd.	700	0.68
-1 * Desv. estánd.	65865	63.71
1 * Desv. estánd.	33269	32.18
2 * Desv. estánd.	769	0.74
3 * Desv. estánd.	374	0.36
4 * Desv. estánd.	381	0.37
5 * Desv. estánd.	282	0.27
6 * Desv. estánd.	940	0.91

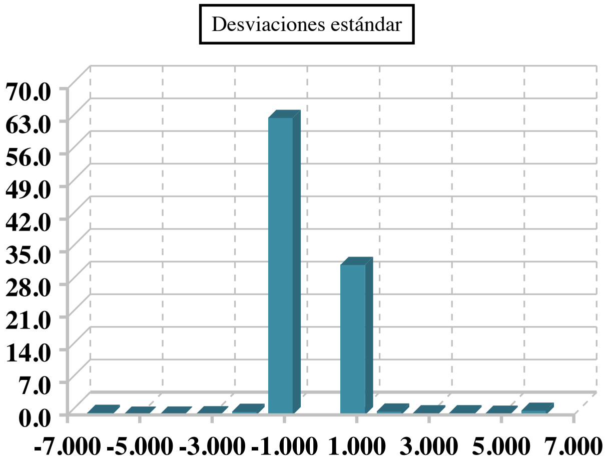

Predefinido: Isométrico

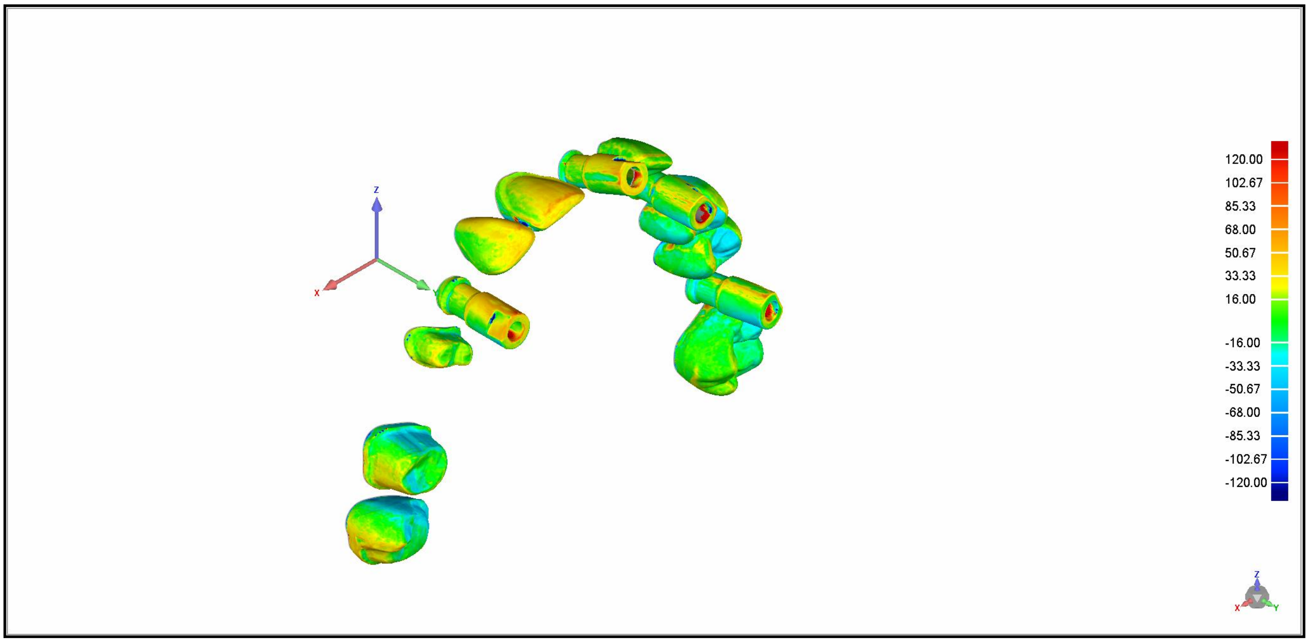

Predefinido: Frente

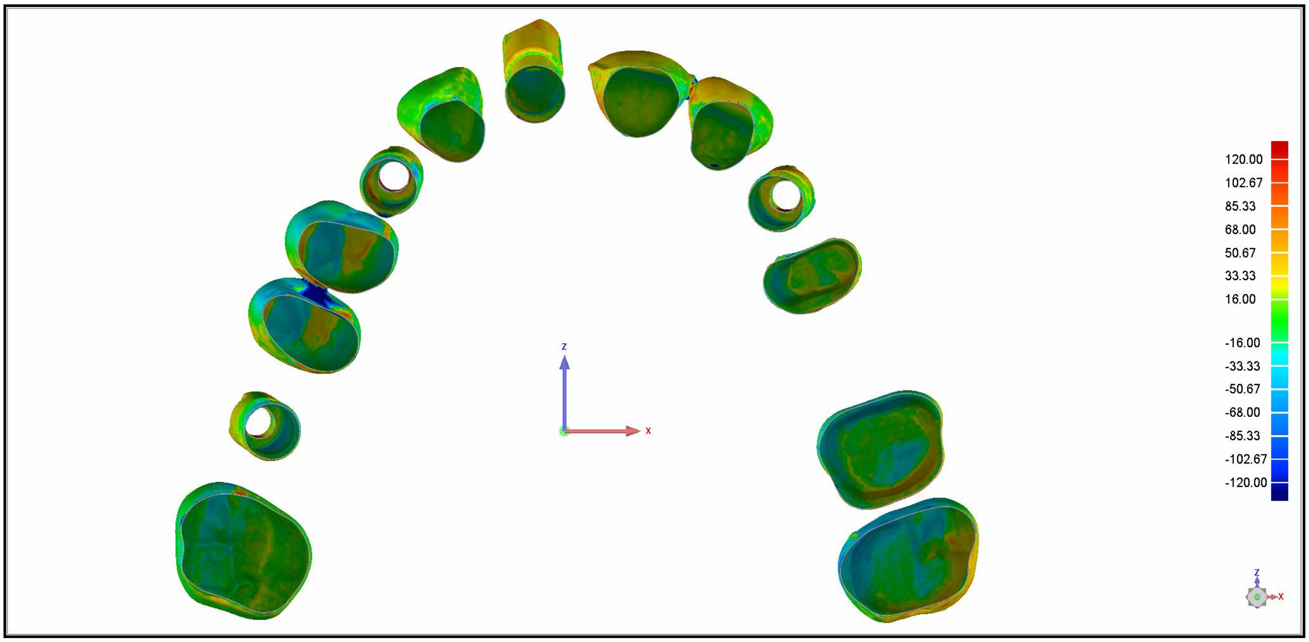

Predefinido: Atrás

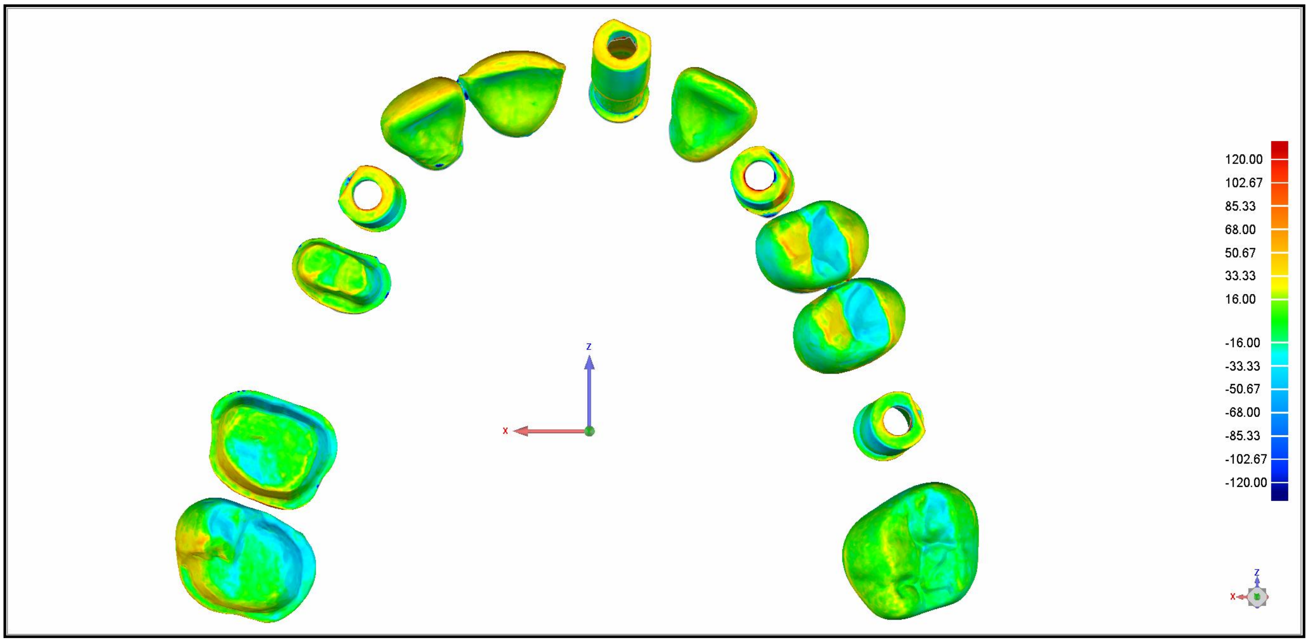

Predefinido: Izquierda

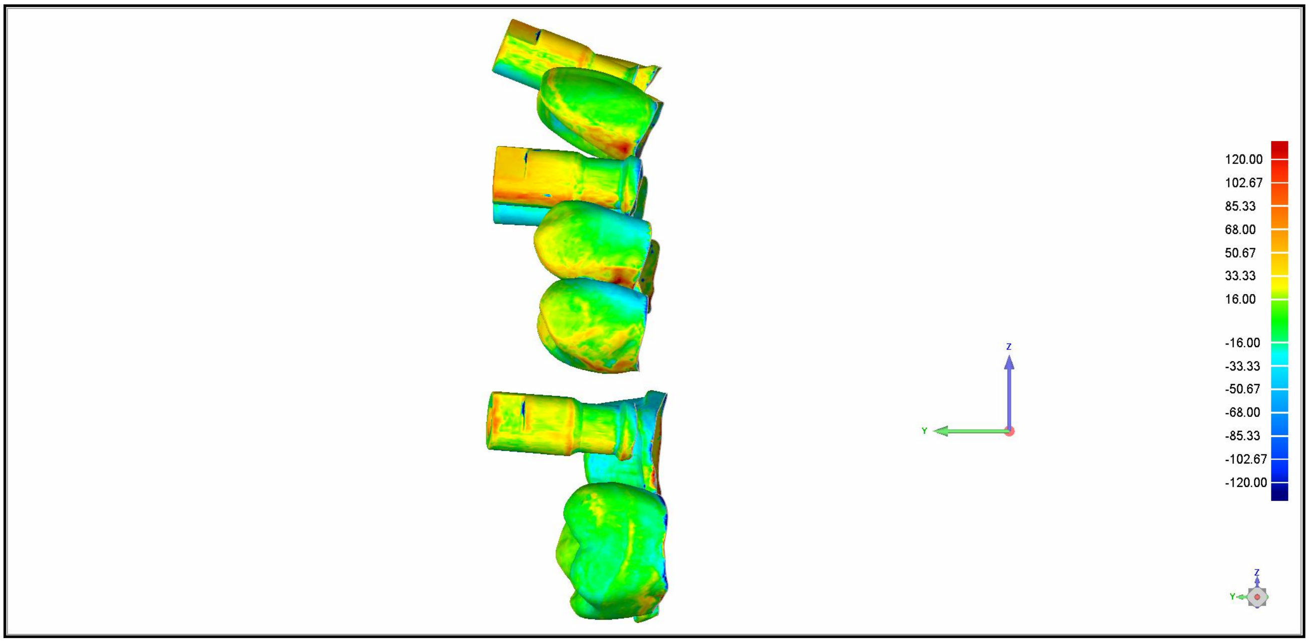

Predefinido: Derecha

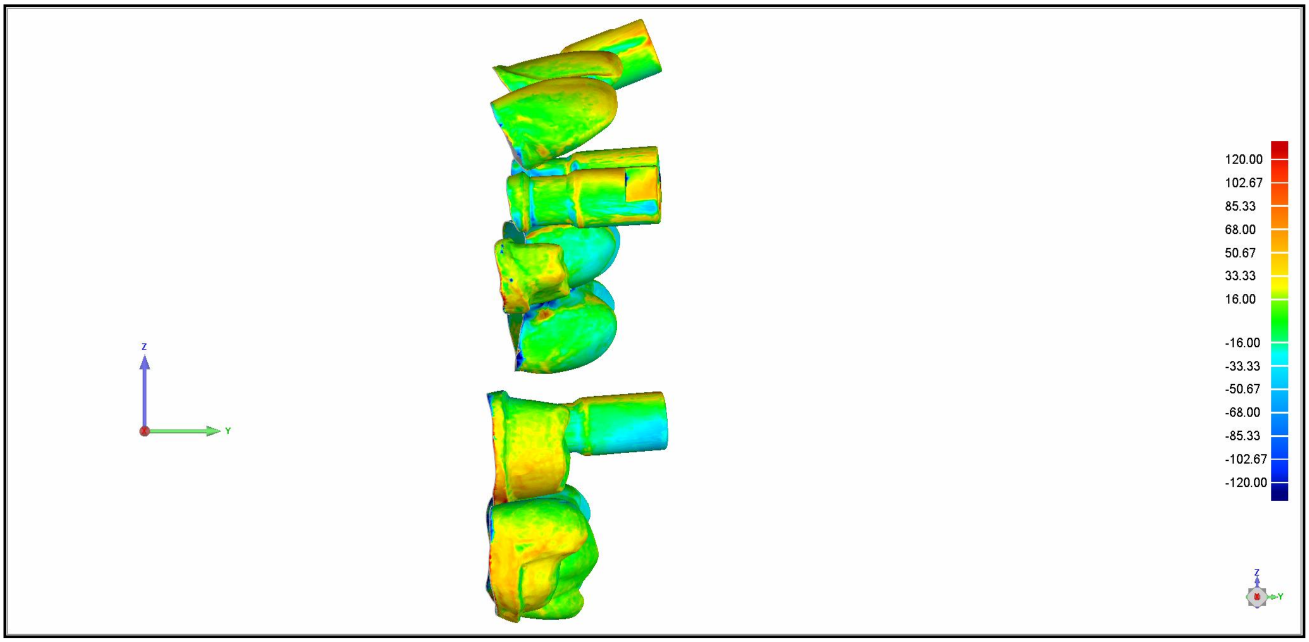

Predefinido: Superior

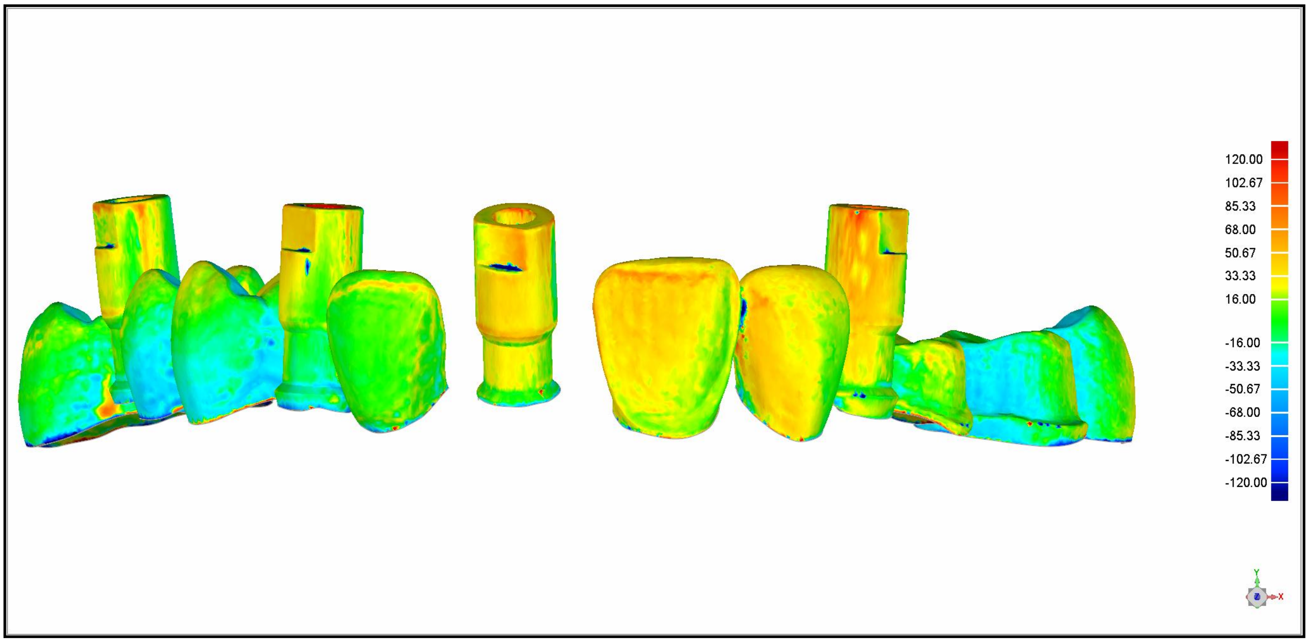

Predefinido: Inferior

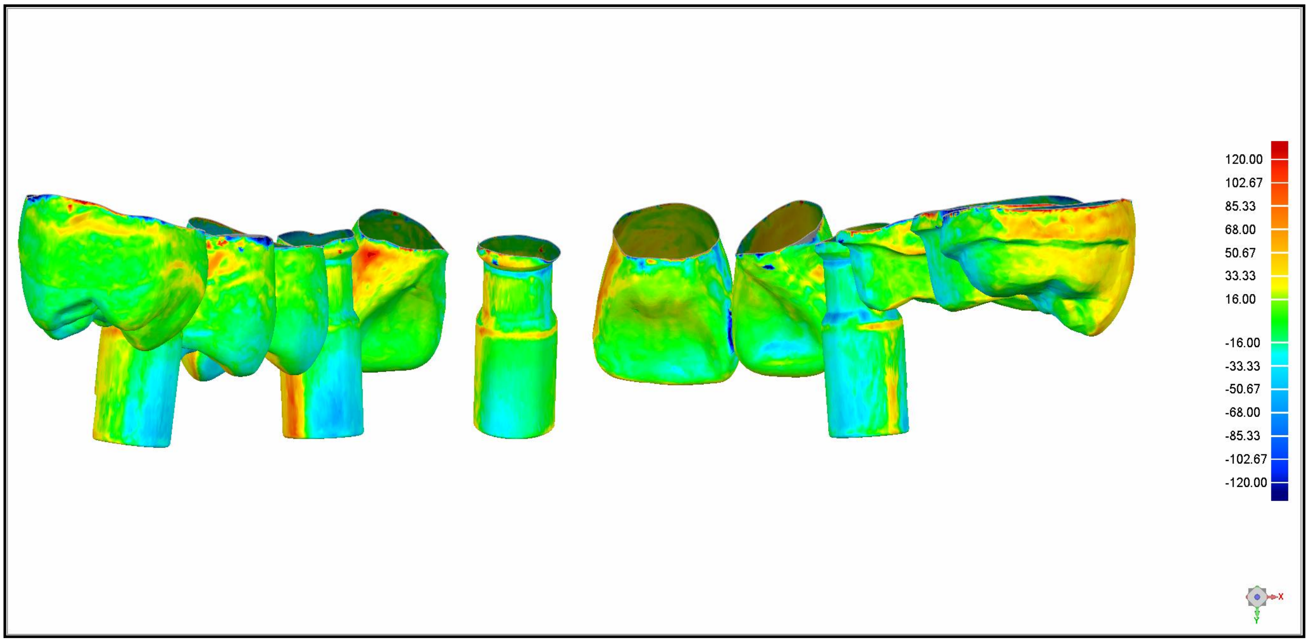

Ajuste de ubicación: Desviaciones superior e inferior

Unidades: u

Nombre	Desv	Estado	Superior Tol	Inferior Tol	Ref X	Ref Y	Ref Z	Radio	Desv X	Desv Y	Desv Z	Medido X	Medido Y	Medido Z	Dir. proy. X	Dir. proy. Y	Dir. proy. Z
Desv. inferior	-2710.45				-3443.63	37025.30	30878.26	n/a	1070.36	68.90	-2489.20	-2373.27	37094.19	28389.06	-0.39	-0.03	0.92
Desv. superior	3121.77				-17902.57	29727.70	11012.38	n/a	3100.16	2.45	366.69	-14802.41	29730.15	11379.07	0.99	0.00	0.12
