# Supplementary material for: Accuracy of four digital scanners according to scanning strategy in complete-arch impressions
Source: PLoS One. 2018 Sep 13;13(9):e0202916. doi: 10.1371/journal.pone.0202916 (PMC6136706; doi:10.1371/journal.pone.0202916)
Supplement: S1 Table — Trios (scanning strategy A). (ZIP) [file pone.0202916.s001.zip › S1/3S5A.pdf]

Geomagic Control

Fecha: 3/6/2017, 12:21 pm

3D Comparación Resultados

Modelo referencia	MRC
Modelo test	3S5A
Nº de puntos de datos	100972
# Aislados	220

Tipo tolerancia	3D desviación
Unidades	u
Máx. crítico	120.00
Máx. nominal	10.00
Mín. nominal	-10.00
Mín. crítico	-120.00

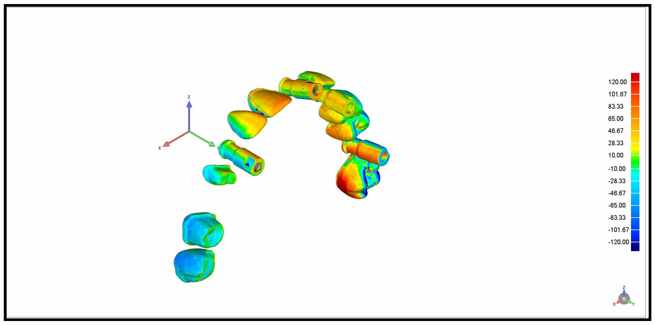

Desviación	
Desviación superior máx.	2741.78
Desviación inferior máx.	-2649.12
Desviación media	75.94 /-64.30
Desviación estándar	198.42

Distribución desviación

>=Min	<Max	# Puntos	%
-120.00	-101.67	1224	1.21
-101.67	-83.33	1601	1.59
-83.33	-65.00	2858	2.83
-65.00	-46.67	5164	5.11
-46.67	-28.33	8818	8.73
-28.33	-10.00	13681	13.55
-10.00	10.00	18438	18.26
10.00	28.33	14854	14.71
28.33	46.67	10816	10.71
46.67	65.00	7391	7.32
65.00	83.33	3921	3.88
83.33	101.67	1906	1.89
101.67	120.00	1029	1.02

Fuera del crítico superior	4654	4.61
Fuera del crítico inferior	4617	4.57

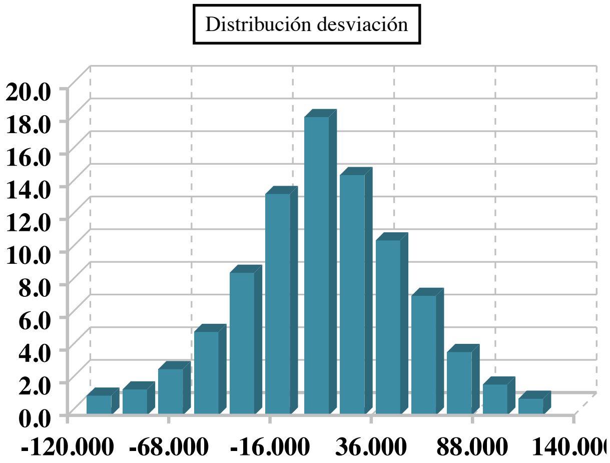

Desviaciones estándar

Distribución (+/-)	# Puntos	%
-6 * Desv. estánd.	509	0.50
-5 * Desv. estánd.	87	0.09
-4 * Desv. estánd.	128	0.13
-3 * Desv. estánd.	142	0.14
-2 * Desv. estánd.	635	0.63
-1 * Desv. estánd.	55323	54.79
1 * Desv. estánd.	41472	41.07
2 * Desv. estánd.	829	0.82
3 * Desv. estánd.	341	0.34
4 * Desv. estánd.	310	0.31
5 * Desv. estánd.	327	0.32
6 * Desv. estánd.	869	0.86

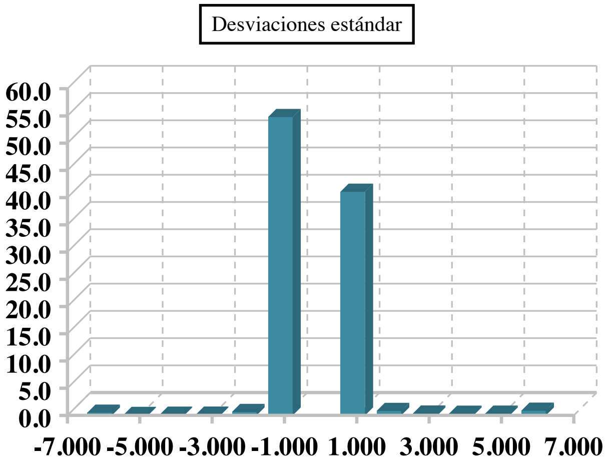

Predefinido: Isométrico

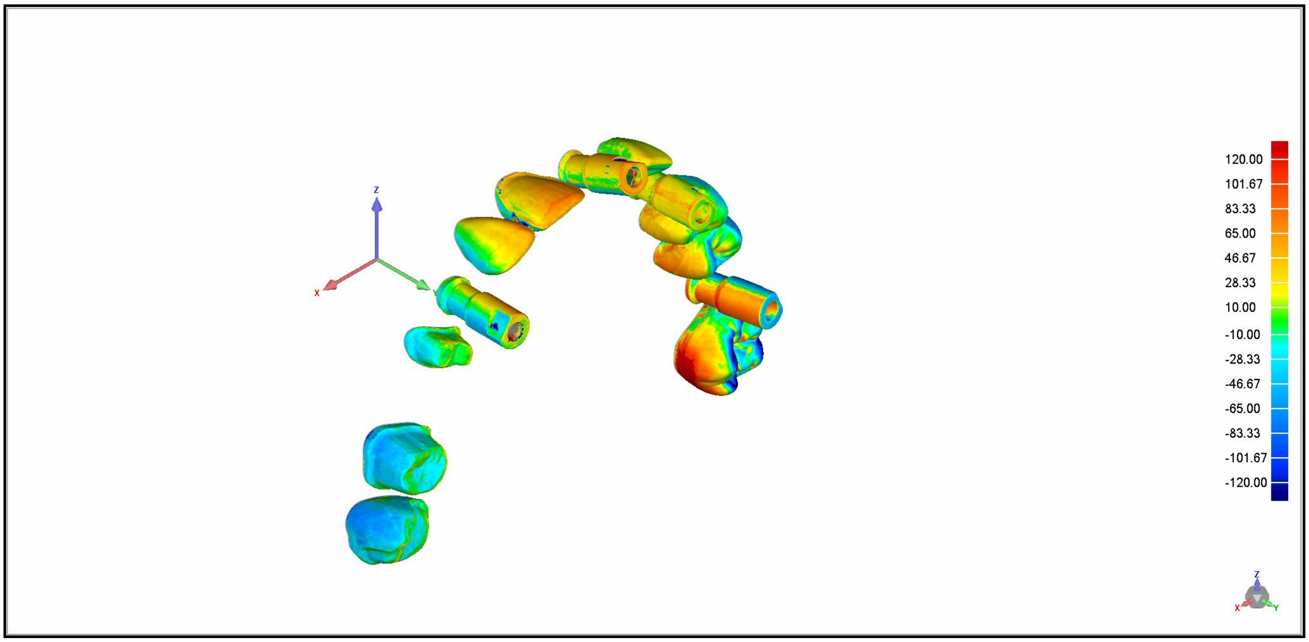

Predefinido: Frente

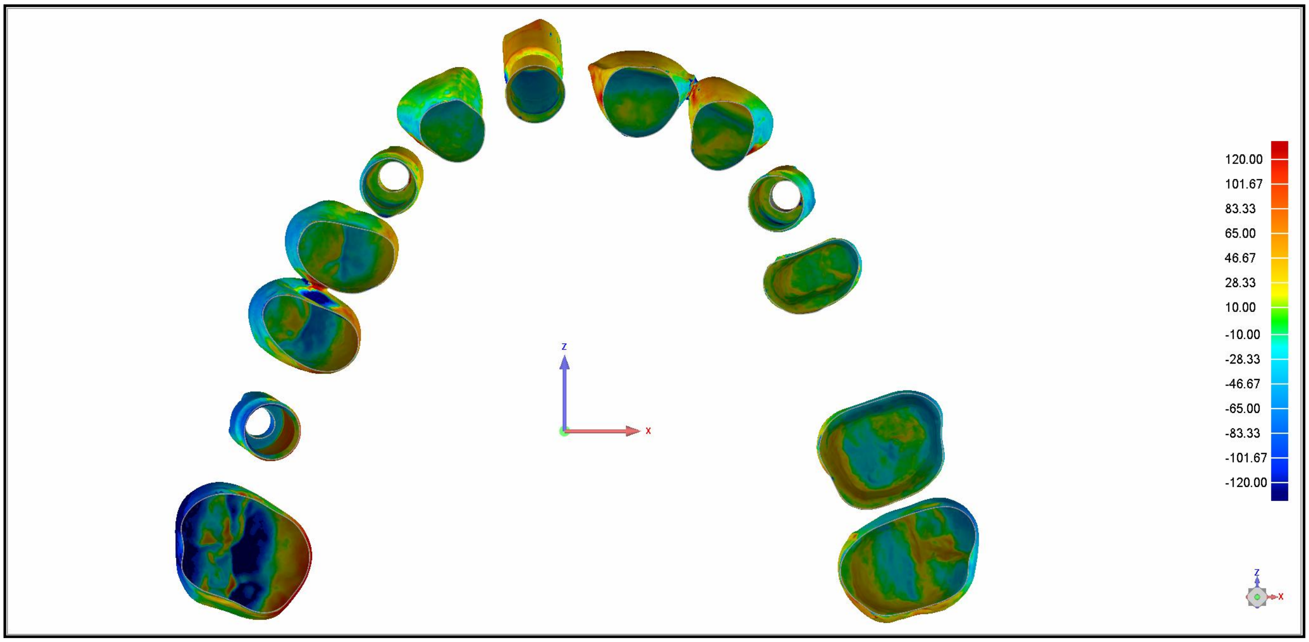

Predefinido: Atrás

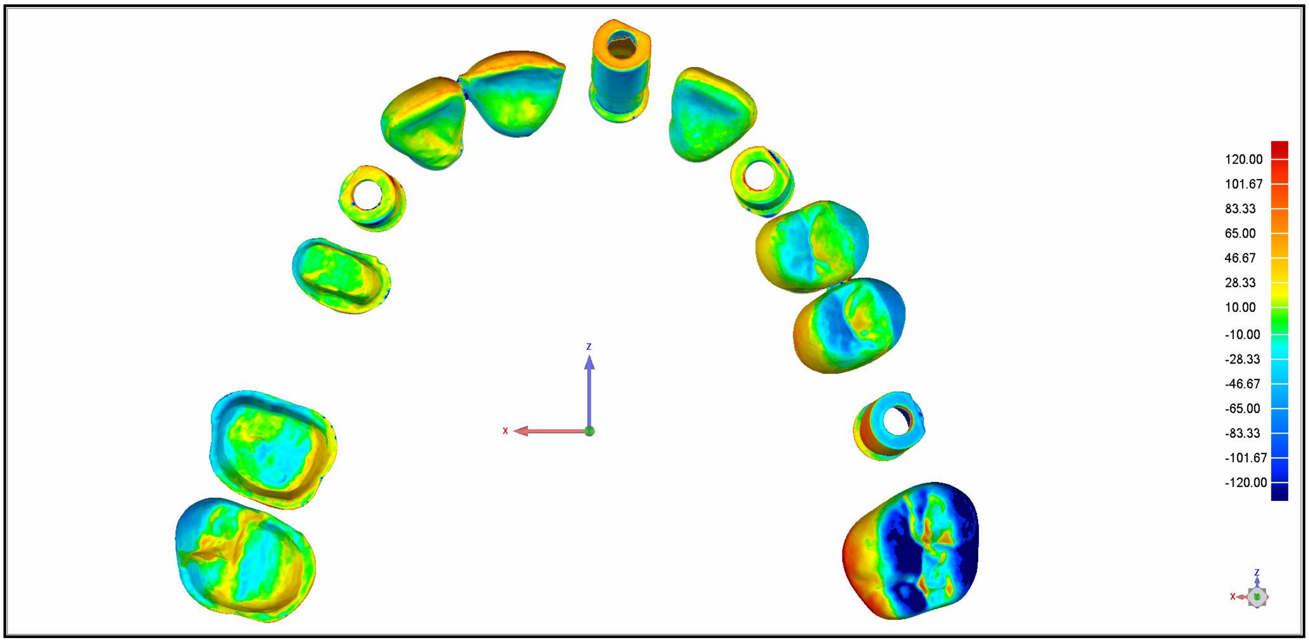

Predefinido: Izquierda

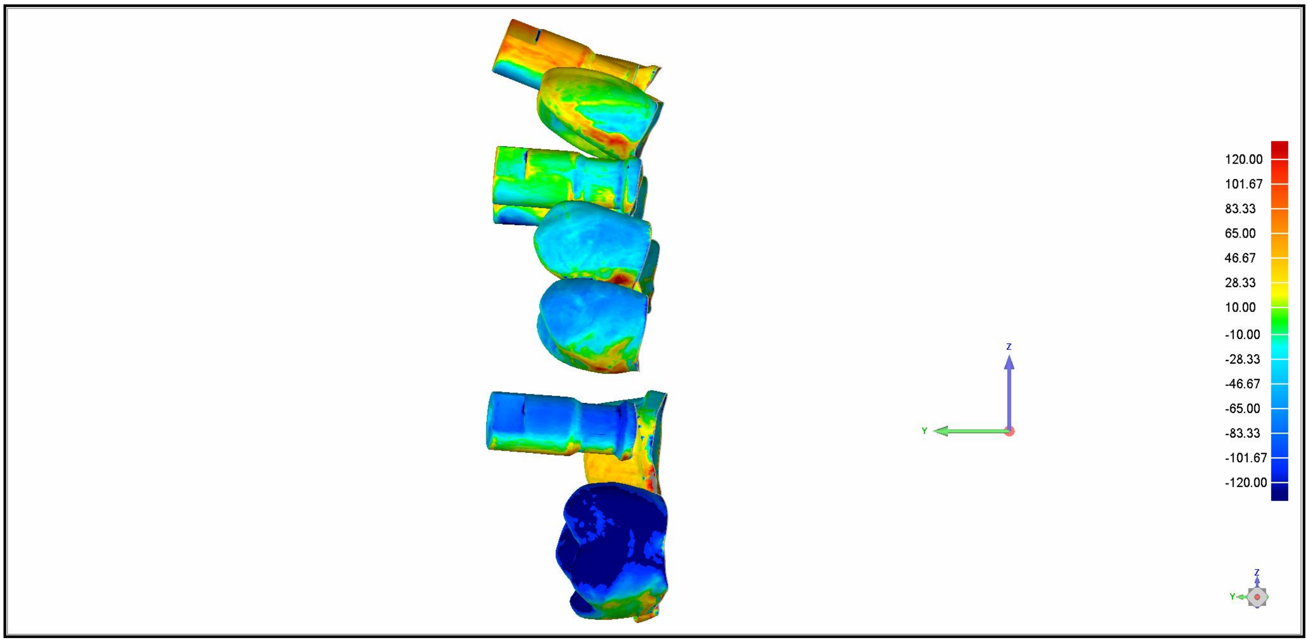

Predefinido: Derecha

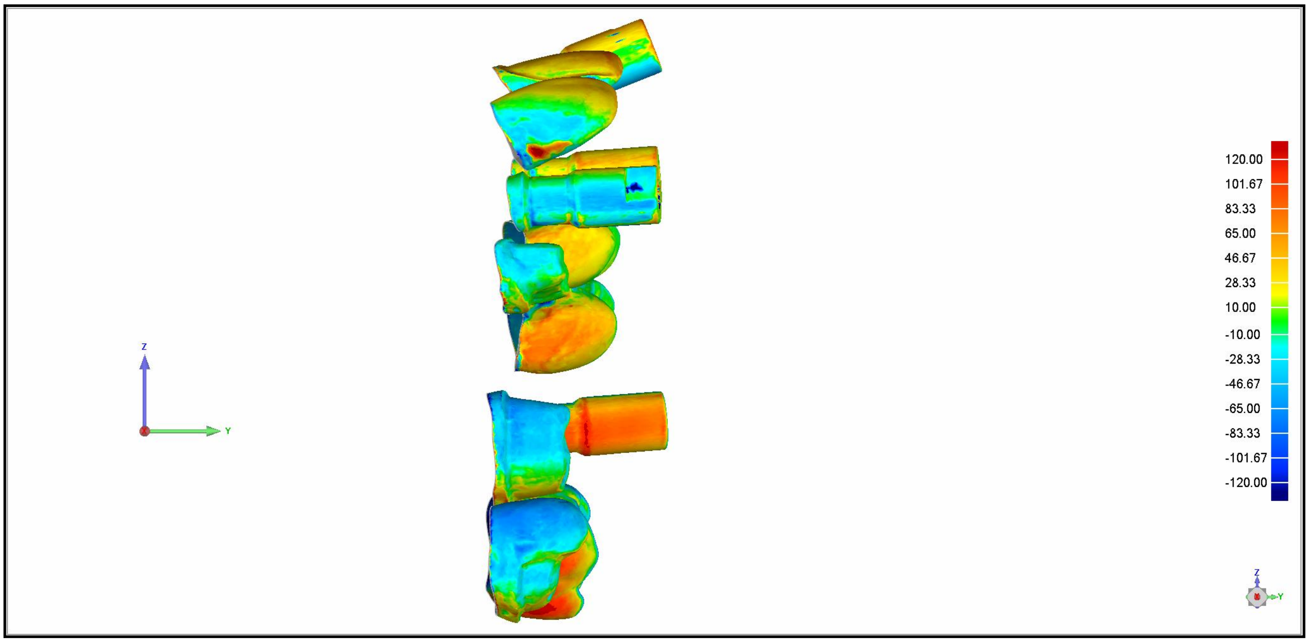

Predefinido: Superior

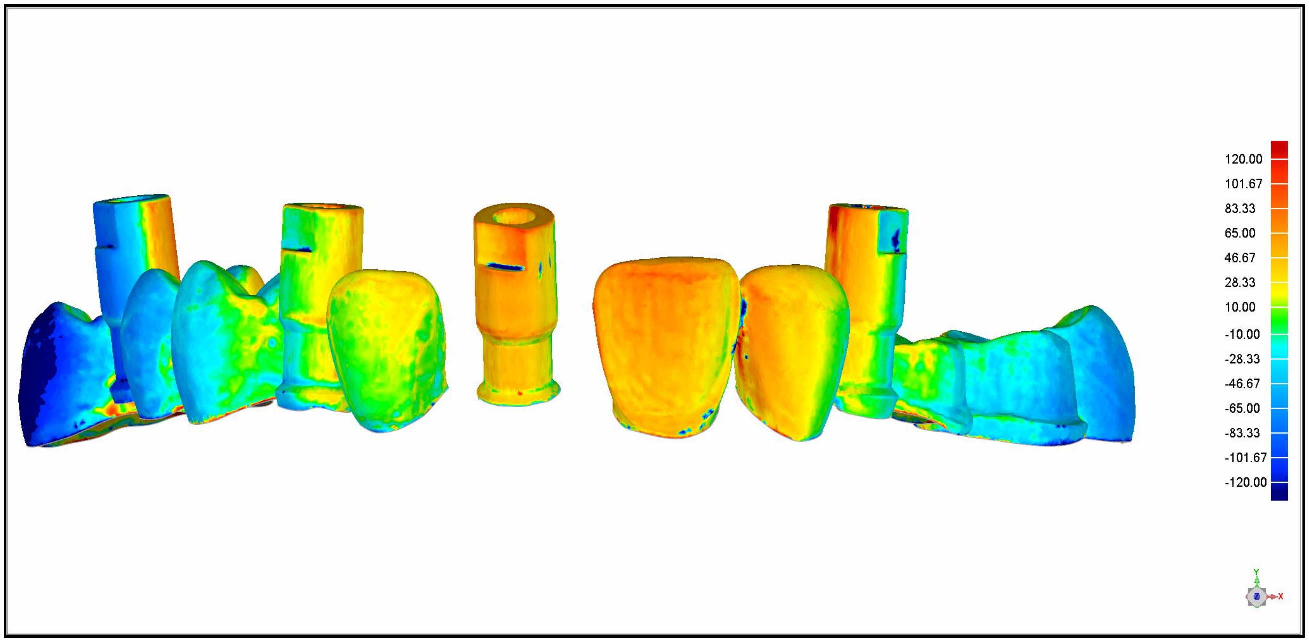

Predefinido: Inferior

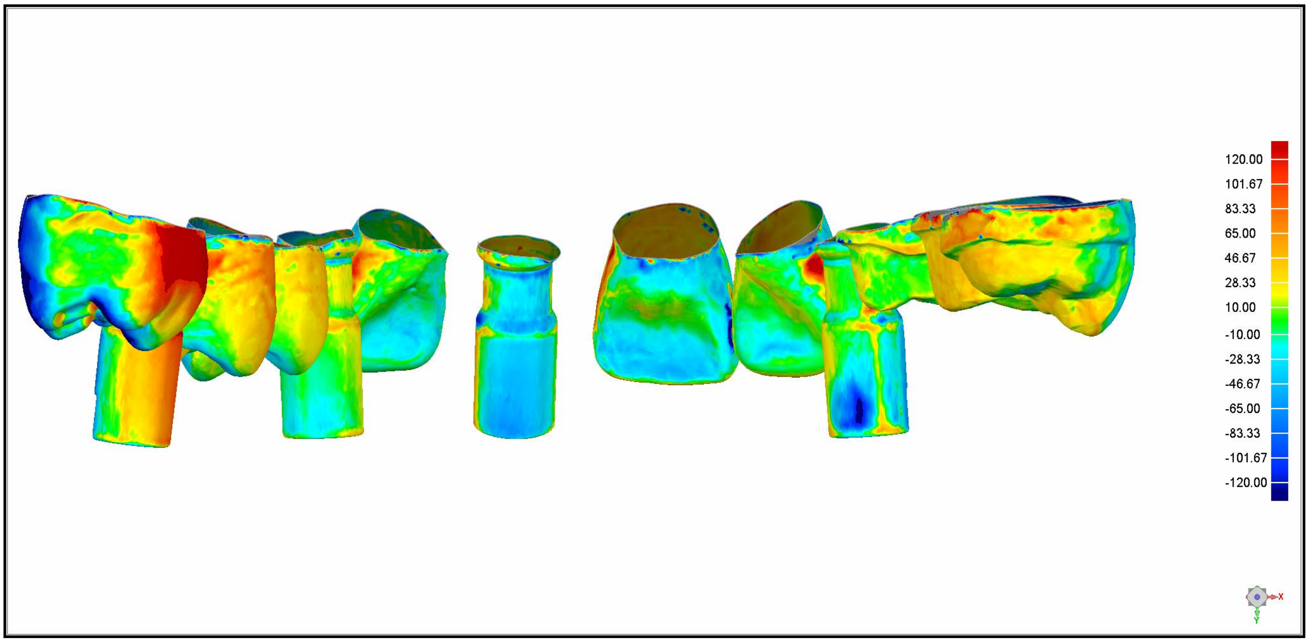

Ajuste de ubicación: Desviaciones superior e inferior

Unidades: u

Nombre	Desv	Estado	Superior Tol	Inferior Tol	Ref X	Ref Y	Ref Z	Radio	Desv X	Desv Y	Desv Z	Medido X	Medido Y	Medido Z	Dir. proy. X	Dir. proy. Y	Dir. proy. Z
Desv. inferior	-2649.12				-3207.86	37006.23	30978.39	n/a	1056.65	-26.32	-2429.12	-2151.21	36979.91	28549.27	-0.40	0.01	0.92
Desv. superior	2741.78				14705.95	29908.45	19207.57	n/a	-2421.77	84.47	1282.67	12284.17	29992.92	20490.24	-0.88	0.03	0.47
