# Supplementary material for: Accuracy of four digital scanners according to scanning strategy in complete-arch impressions
Source: PLoS One. 2018 Sep 13;13(9):e0202916. doi: 10.1371/journal.pone.0202916 (PMC6136706; doi:10.1371/journal.pone.0202916)

Geomagic Control

Fecha: 3/6/2017, 12:42 pm

3D Comparación Resultados

Modelo referencia	MRC
Modelo test	3S9A
Nº de puntos de datos	103054
# Aislados	86

Tipo tolerancia	3D desviación
Unidades	u
Máx. crítico	120.00
Máx. nominal	16.00
Mín. nominal	-16.00
Mín. crítico	-120.00

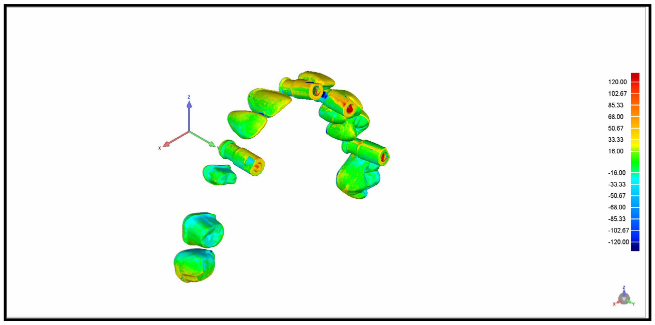

Desviación	
Desviación superior máx.	3133.91
Desviación inferior máx.	-3104.33
Desviación media	61.42 /-47.28
Desviación estándar	196.25

Distribución desviación

>=Min	<Max	# Puntos	%
-120.00	-102.67	323	0.31
-102.67	-85.33	385	0.37
-85.33	-68.00	617	0.60
-68.00	-50.67	1006	0.98
-50.67	-33.33	2723	2.64
-33.33	-16.00	11099	10.77
-16.00	16.00	48705	47.26
16.00	33.33	17702	17.18
33.33	50.67	8399	8.15
50.67	68.00	3063	2.97
68.00	85.33	1288	1.25
85.33	102.67	793	0.77
102.67	120.00	536	0.52

Fuera del crítico superior	4316	4.19
Fuera del crítico inferior	2099	2.04

Distribución desviación

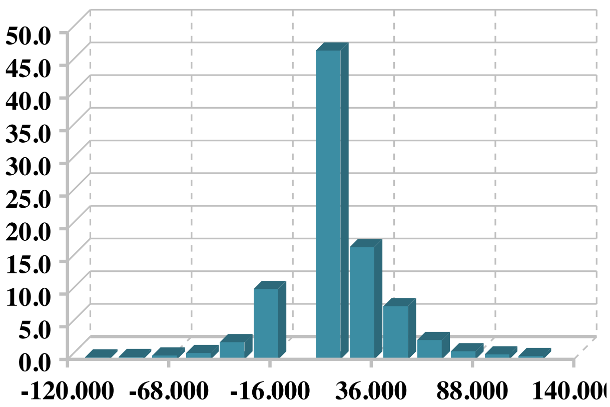

Desviaciones estándar

Distribución (+/-)	# Puntos	%
-6 * Desv. estánd.	570	0.55
-5 * Desv. estánd.	87	0.08
-4 * Desv. estánd.	89	0.09
-3 * Desv. estánd.	145	0.14
-2 * Desv. estánd.	444	0.43
-1 * Desv. estánd.	65898	63.95
1 * Desv. estánd.	32945	31.97
2 * Desv. estánd.	962	0.93
3 * Desv. estánd.	459	0.45
4 * Desv. estánd.	371	0.36
5 * Desv. estánd.	364	0.35
6 * Desv. estánd.	720	0.70

Desviaciones estándar

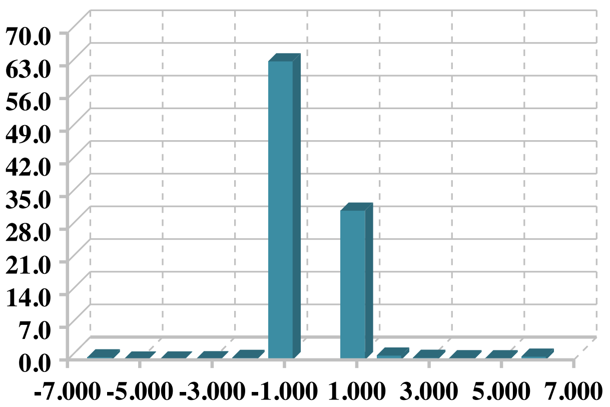

Predefinido: Isométrico

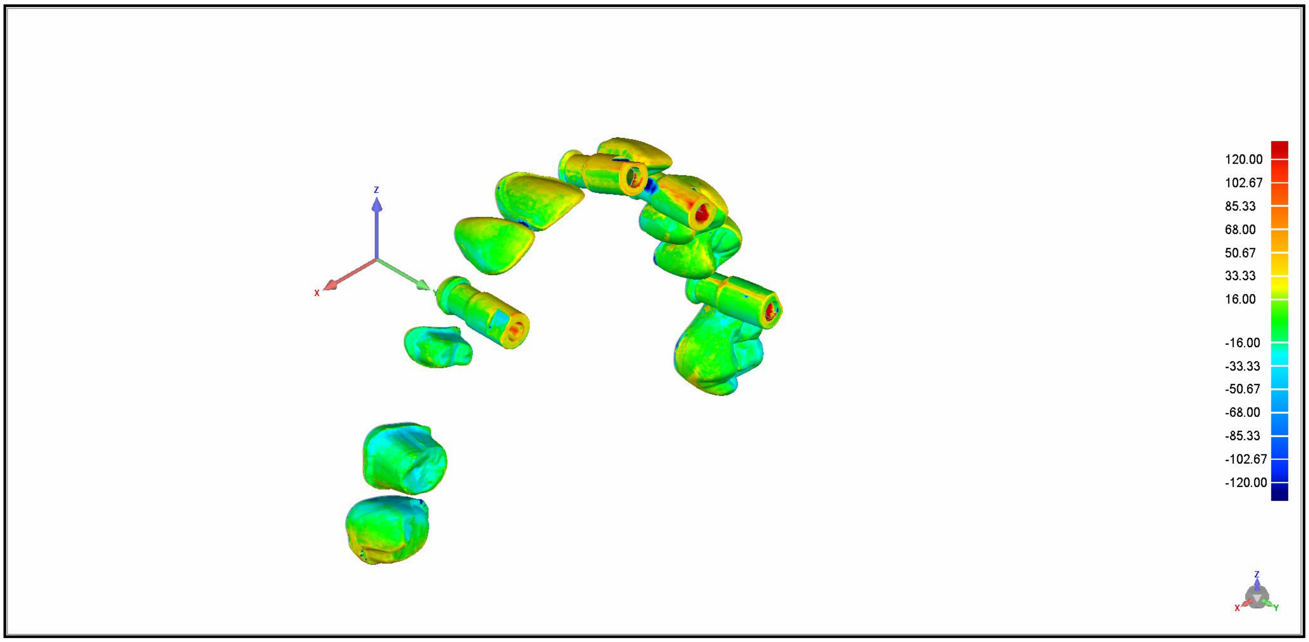

Predefinido: Frente

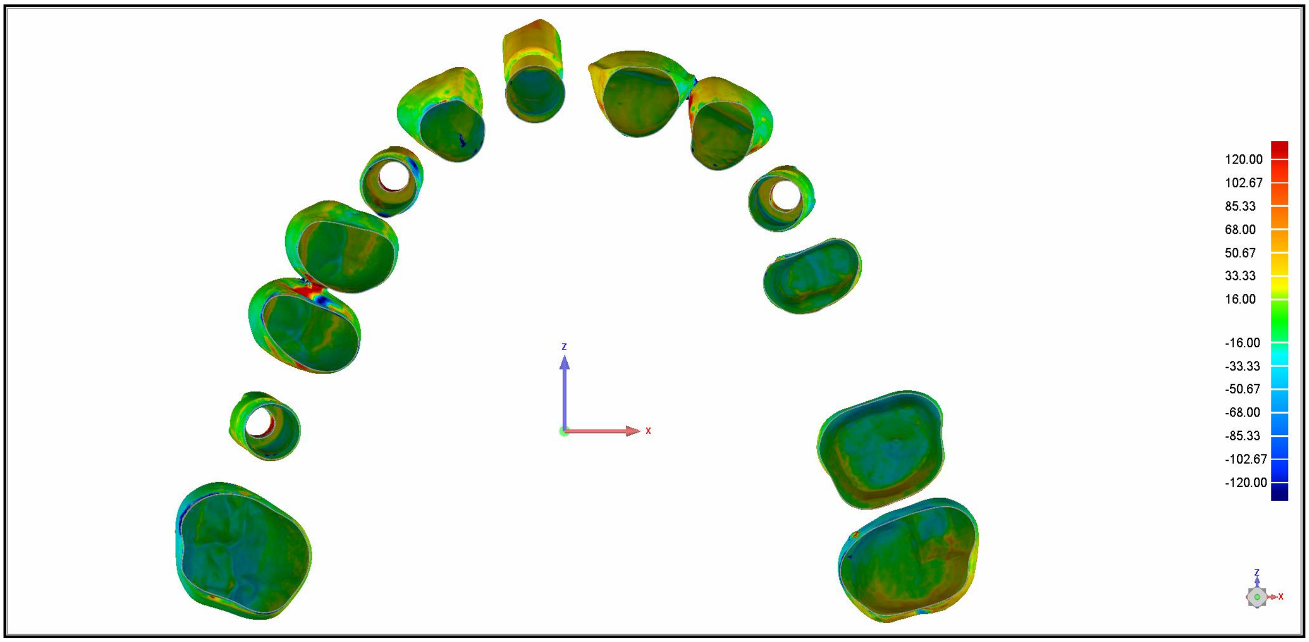

Predefinido: Atrás

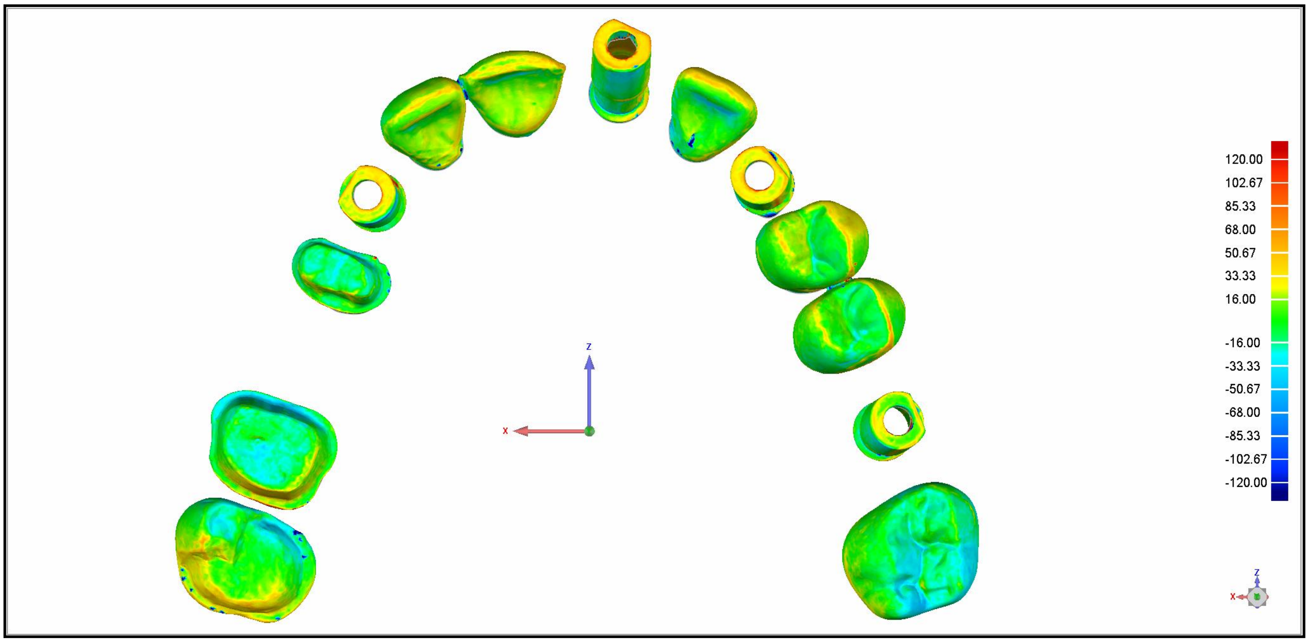

Predefinido: Izquierda

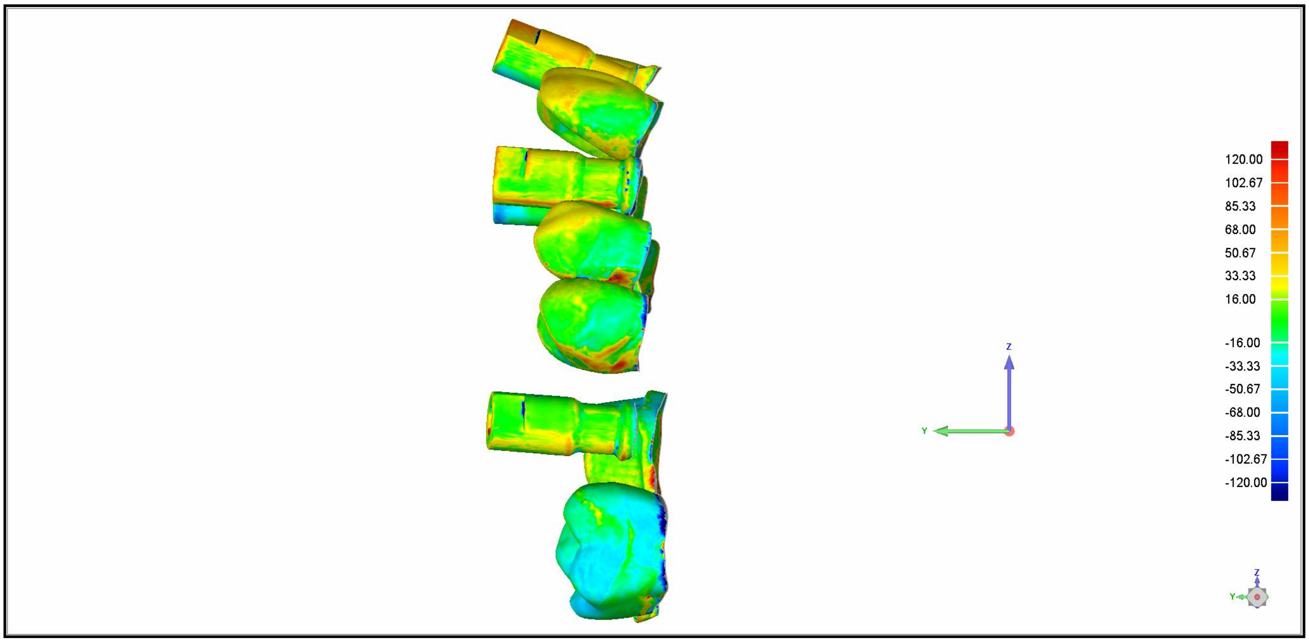

Predefinido: Derecha

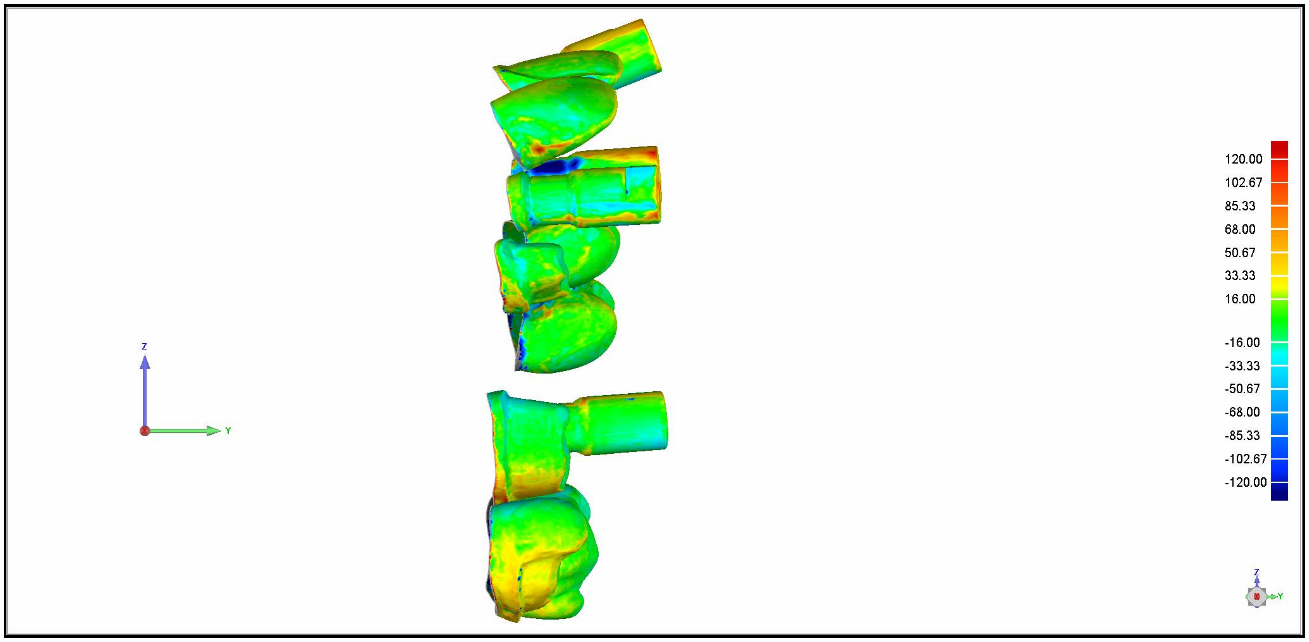

Predefinido: Superior

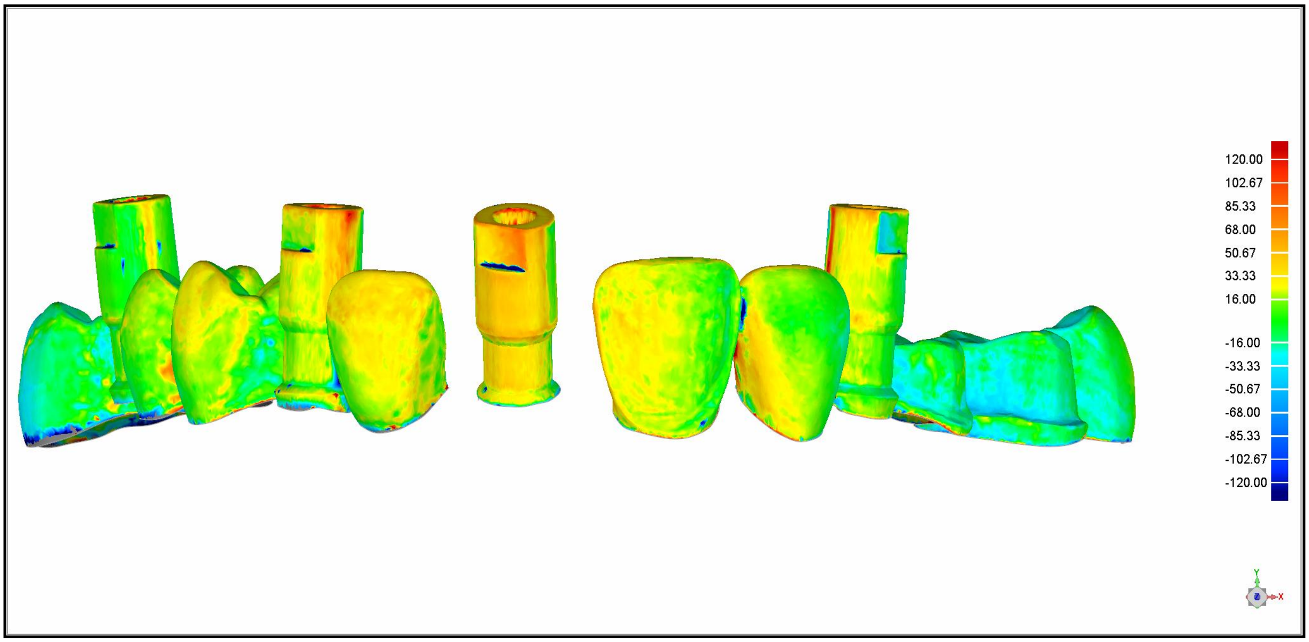

Predefinido: Inferior

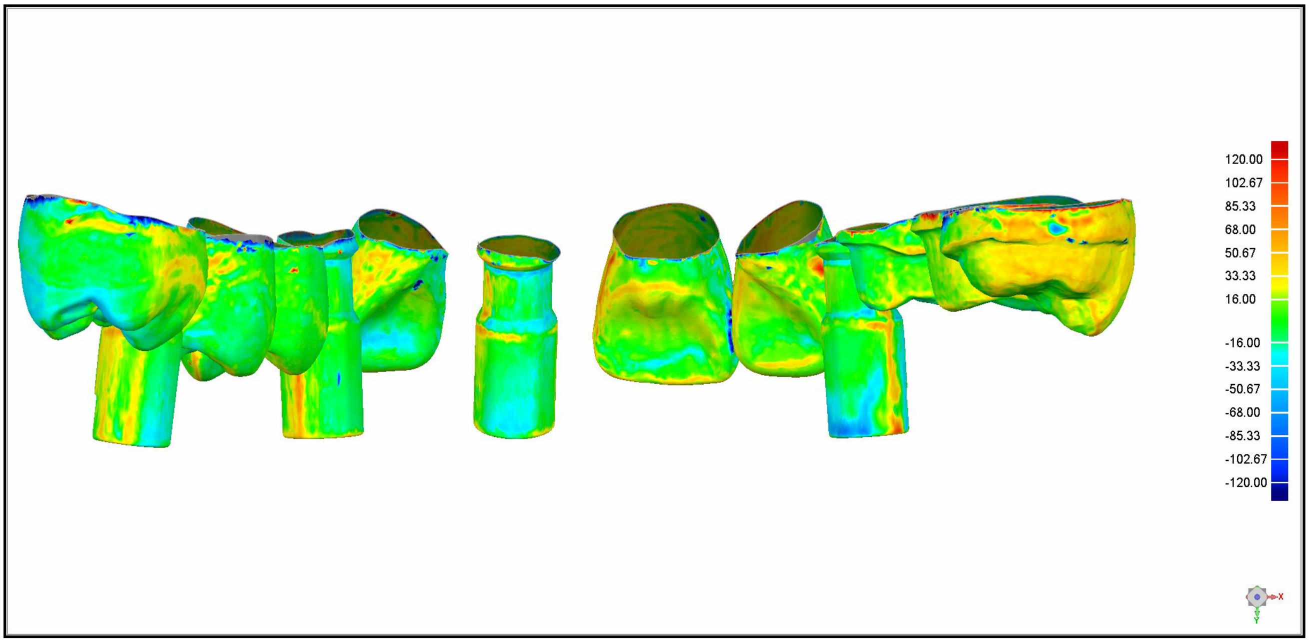

Supplement: S1 Table — Trios (scanning strategy A). (ZIP) [file pone.0202916.s001.zip › S1/3S9A.pdf]
