# Supplementary material for: Accuracy of four digital scanners according to scanning strategy in complete-arch impressions
Source: PLoS One. 2018 Sep 13;13(9):e0202916. doi: 10.1371/journal.pone.0202916 (PMC6136706; doi:10.1371/journal.pone.0202916)
Supplement: S1 Table — Trios (scanning strategy A). (ZIP) [file pone.0202916.s001.zip › S1/3S2A.pdf]

Geomagic Control™

Fecha: 3/6/2017, 12:14 pm

# 3D Comparación  Resultados

| | |
|---|---|
| Modelo referencia | MRC |
| Modelo test | 3S2A |
| Nº de puntos de datos | 108540 |
| # Aislados | 280 |

| | |
|---|---|
| Tipo tolerancia | 3D desviación |
| Unidades | u |
| Máx. crítico | 120.00 |
| Máx. nominal | 17.00 |
| Mín. nominal | -17.00 |
| Mín. crítico | -120.00 |

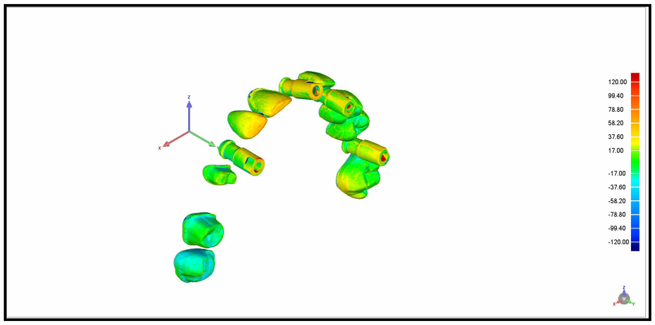

| | |
|---|---|
| Desviación | |
| Desviación superior máx. | 2605.45 |
| Desviación inferior máx. | -2895.40 |
| Desviación media | 56.69 /-43.99 |
| Desviación estándar | 185.78 |

## Distribución desviación

| >=Min | <Max | # Puntos | % |
|---|---|---|---|
| -120.00 | -99.40 | 349 | 0.32 |
| -99.40 | -78.80 | 473 | 0.44 |
| -78.80 | -58.20 | 875 | 0.81 |
| -58.20 | -37.60 | 2084 | 1.92 |
| -37.60 | -17.00 | 10643 | 9.81 |
| -17.00 | 17.00 | 58204 | 53.62 |
| 17.00 | 37.60 | 20356 | 18.75 |
| 37.60 | 58.20 | 5967 | 5.50 |
| 58.20 | 78.80 | 2092 | 1.93 |
| 78.80 | 99.40 | 759 | 0.70 |
| 99.40 | 120.00 | 557 | 0.51 |

| | | |
|---|---|---|
| Fuera del crítico superior | 4290 | 3.95 |
| Fuera del crítico inferior | 1891 | 1.74 |

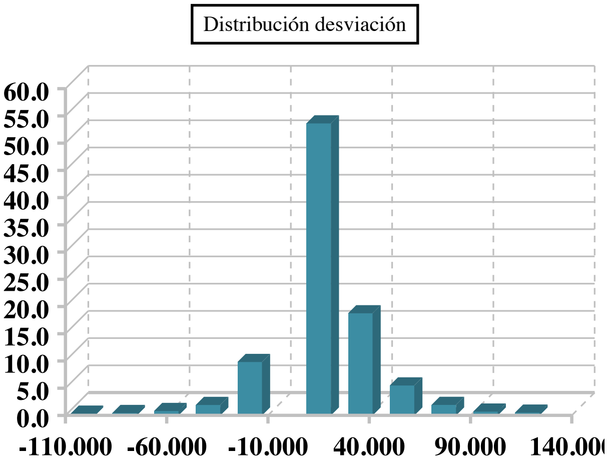


## Desviaciones estándar

| Distribución (+/-) | # Puntos | % |
|---|---|---|
| -6 * Desv. estánd. | 520 | 0.48 |
| -5 * Desv. estánd. | 86 | 0.08 |
| -4 * Desv. estánd. | 123 | 0.11 |
| -3 * Desv. estánd. | 155 | 0.14 |
| -2 * Desv. estánd. | 543 | 0.50 |
| -1 * Desv. estánd. | 74023 | 68.20 |
| 1 * Desv. estánd. | 30129 | 27.76 |
| 2 * Desv. estánd. | 889 | 0.82 |
| 3 * Desv. estánd. | 393 | 0.36 |
| 4 * Desv. estánd. | 355 | 0.33 |
| 5 * Desv. estánd. | 348 | 0.32 |
| 6 * Desv. estánd. | 976 | 0.90 |

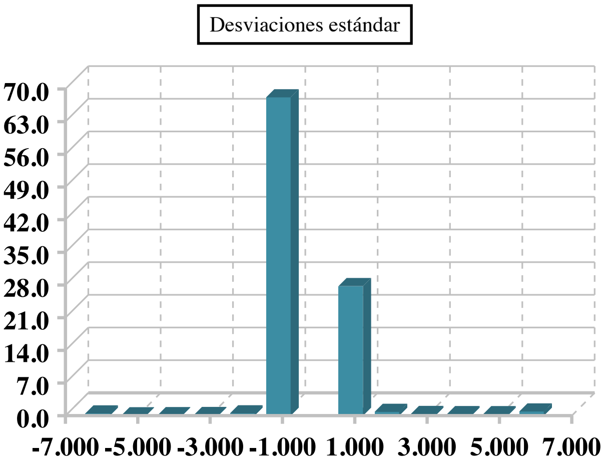

Predefinido: Isométrico

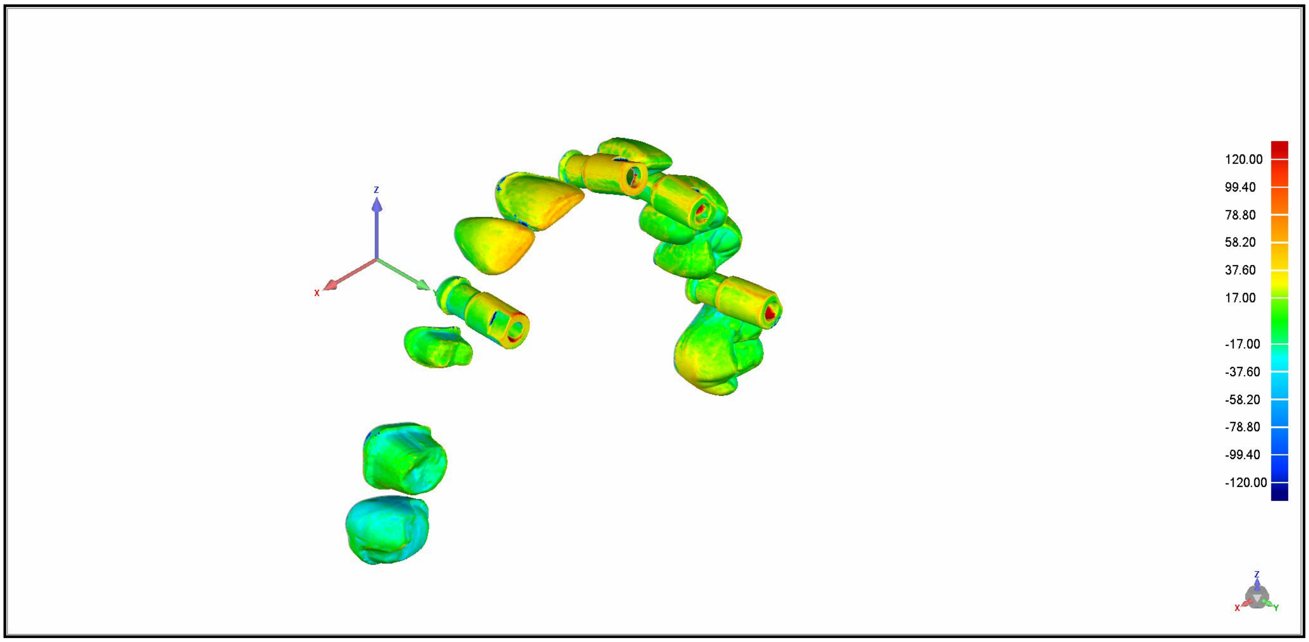

Predefinido: Frente

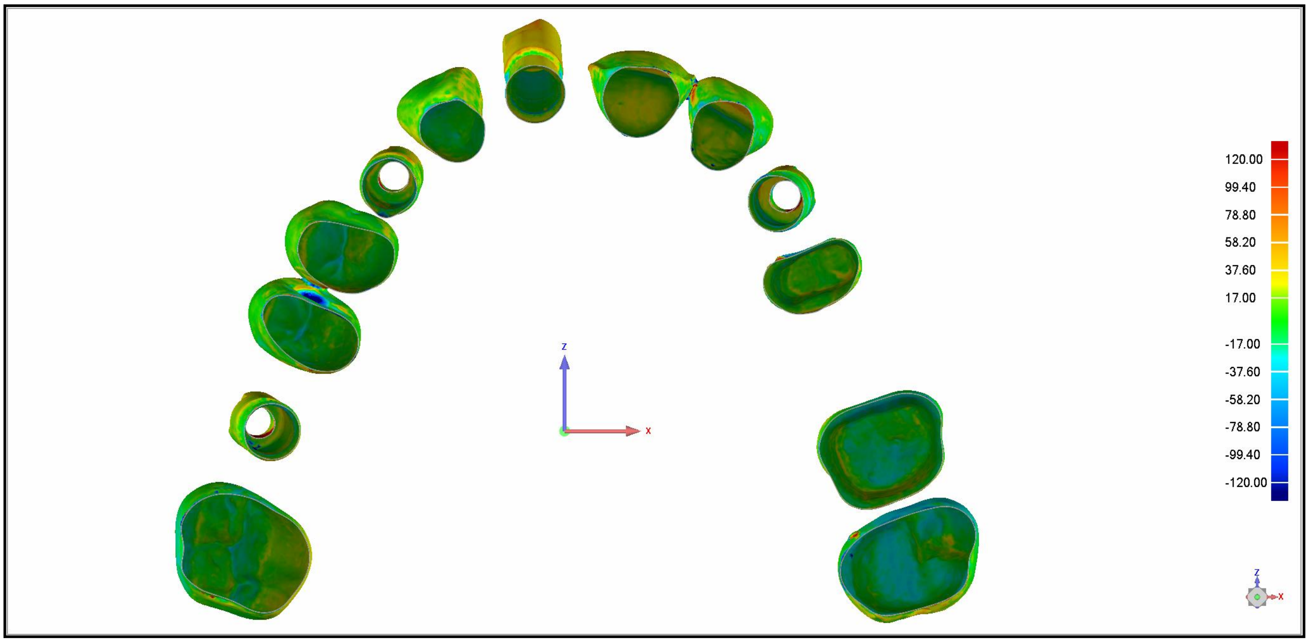

Predefinido: Atrás

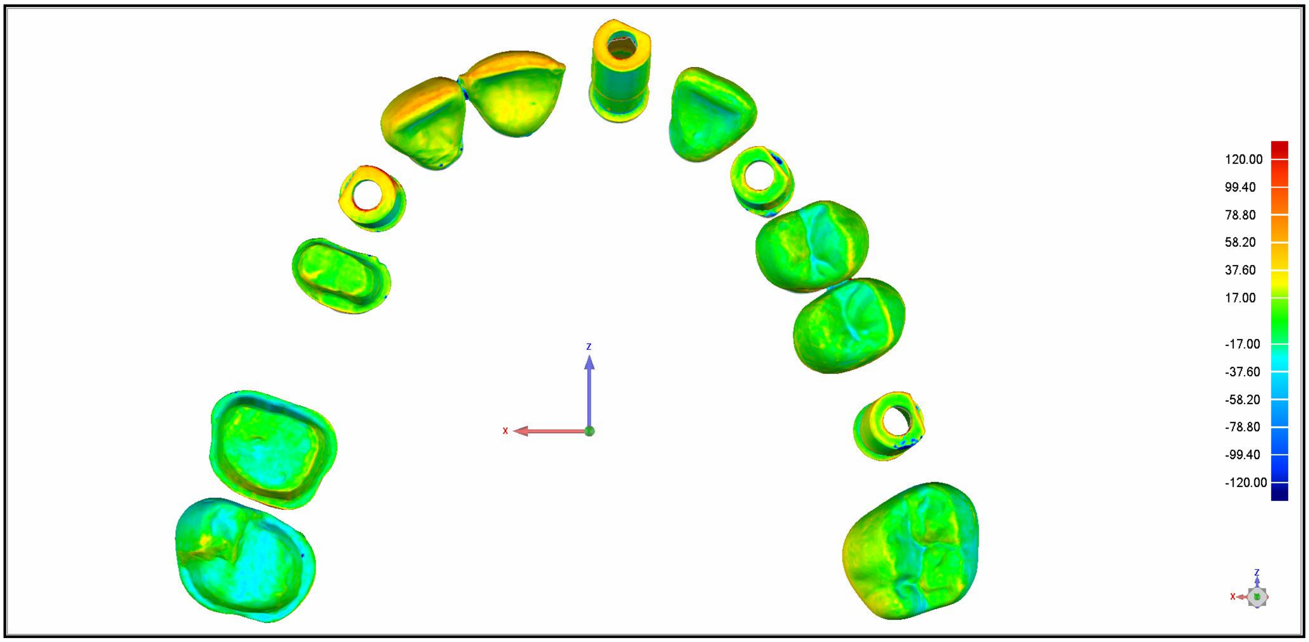

Predefinido: Izquierda

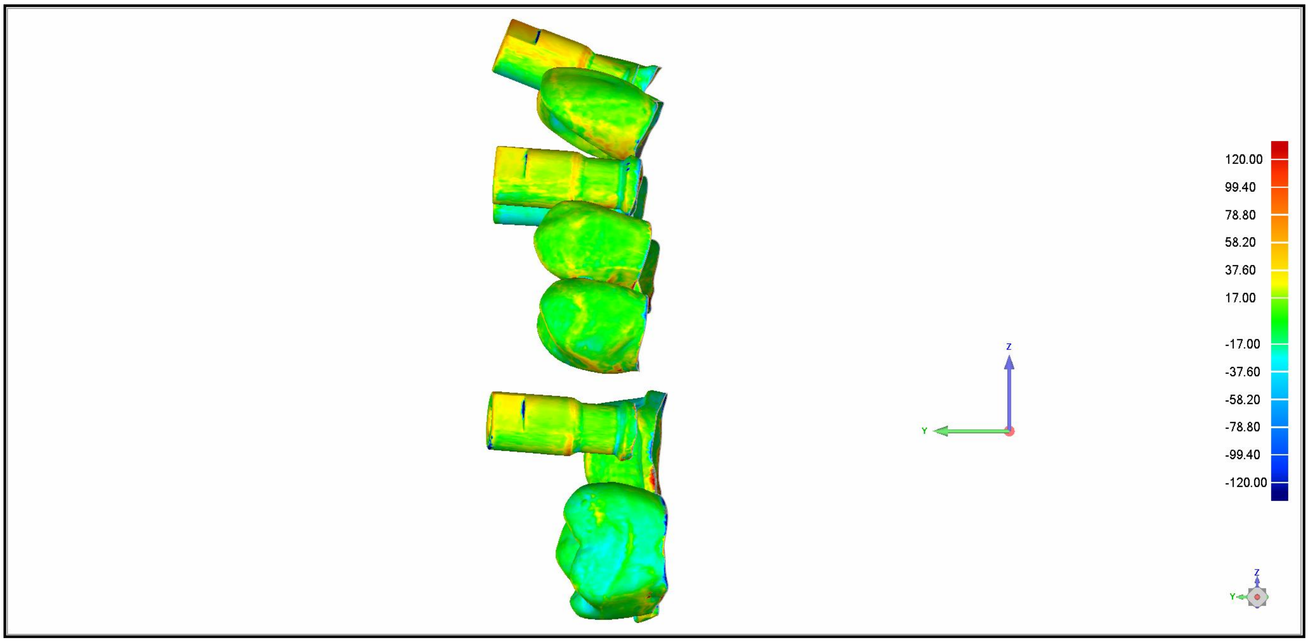

Predefinido: Derecha

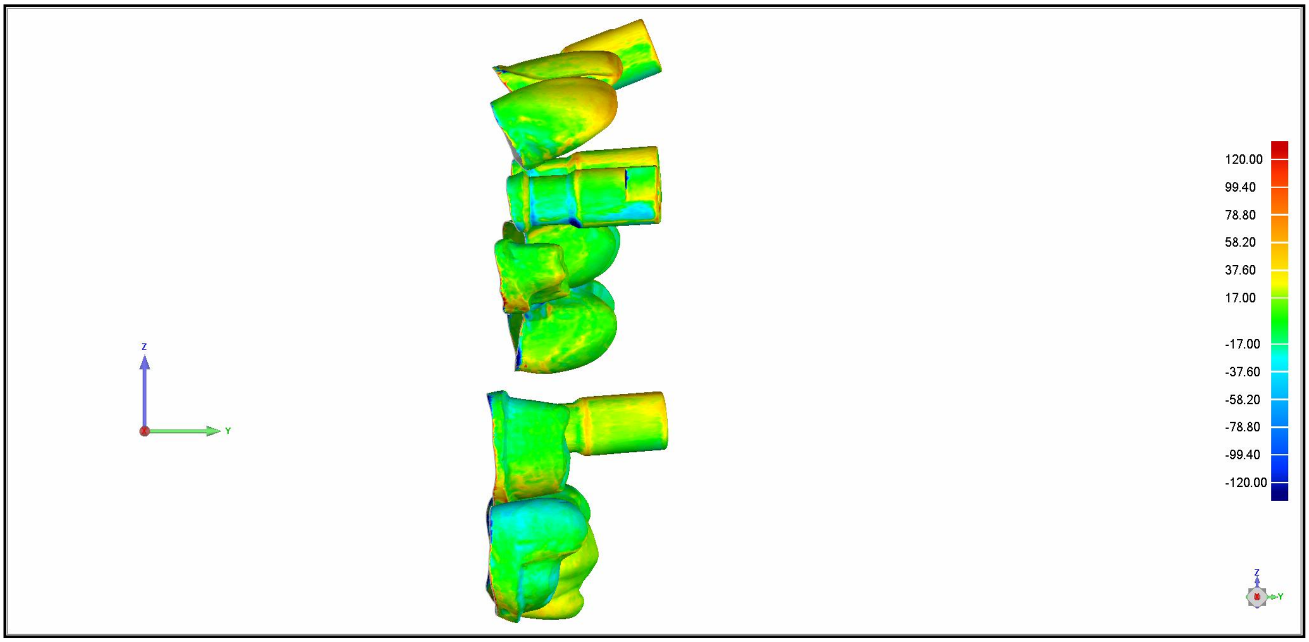

Predefinido: Superior

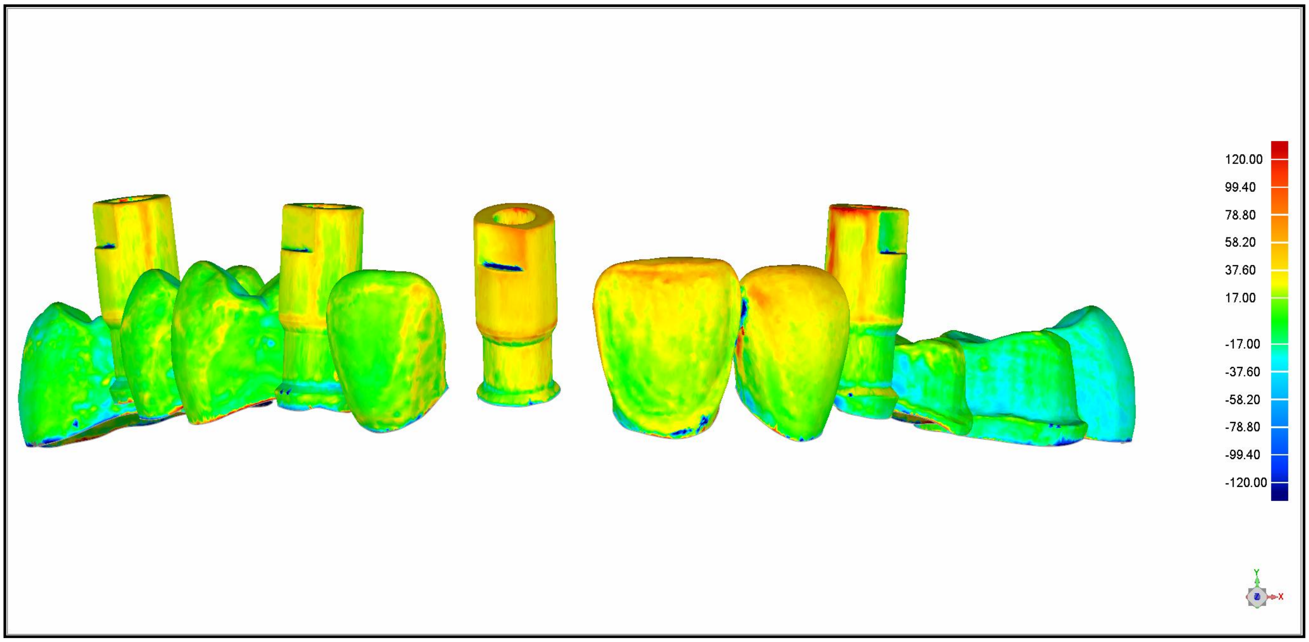

Predefinido: Inferior

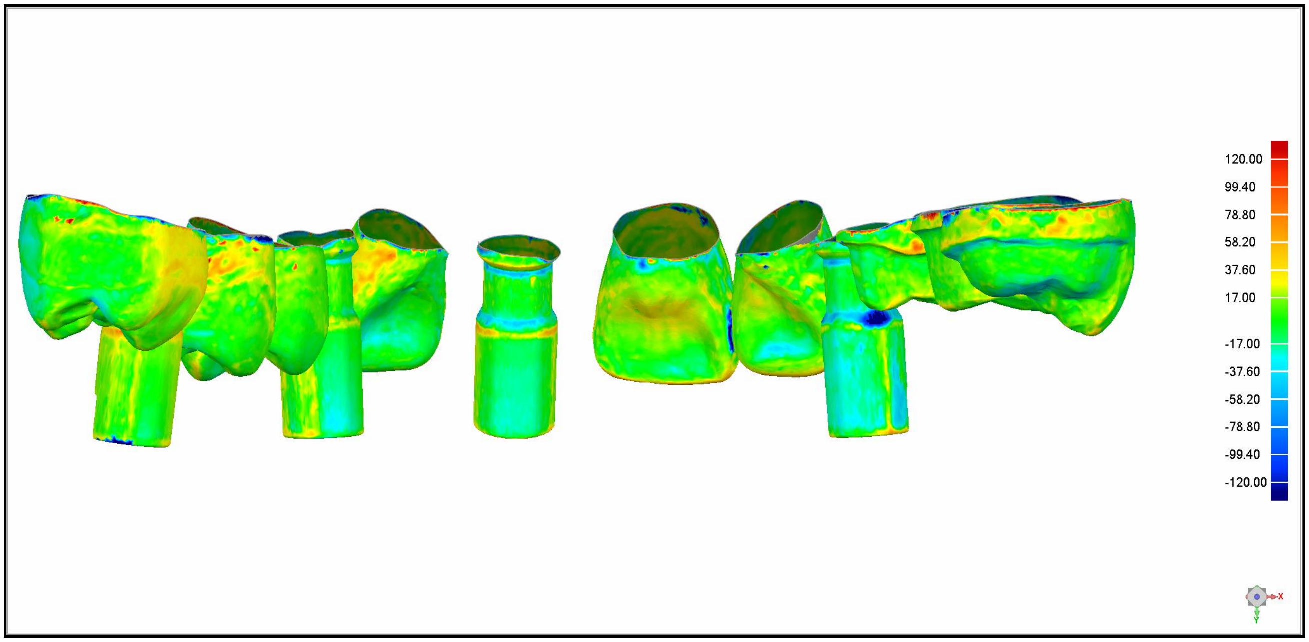

# Ajuste de ubicación: Desviaciones superior e inferior

## Unidades: u

| Nombre | Desv | Estado | Superior Tol | Inferior Tol | Ref X | Ref Y | Ref Z | Radio | Desv X | Desv Y | Desv Z | Medido X | Medido Y | Medido Z | Dir. proy. X | Dir. proy. Y | Dir. proy. Z |
|---|---|---|---|---|---|---|---|---|---|---|---|---|---|---|---|---|---|
| Desv. inferior | -2895.40 | | | | -16597.07 | 29132.89 | 5701.00 | n/a | -2768.13 | 47.40 | -847.69 | -19365.20 | 29180.29 | 4853.31 | 0.96 | -0.02 | 0.29 |
| Desv. superior | 2605.45 | | | | 14721.30 | 29902.09 | 19236.98 | n/a | -2228.22 | 4.86 | 1350.32 | 12493.08 | 29906.95 | 20587.30 | -0.86 | 0.00 | 0.52 |
