# Supplementary material for: Accuracy of four digital scanners according to scanning strategy in complete-arch impressions
Source: PLoS One. 2018 Sep 13;13(9):e0202916. doi: 10.1371/journal.pone.0202916 (PMC6136706; doi:10.1371/journal.pone.0202916)

Geomagic Control

Fecha: 3/6/2017, 12:45 pm

3D Comparación Resultados

Modelo referencia	MRC
Modelo test	3S10A
Nº de puntos de datos	108250
# Aislados	82

Tipo tolerancia	3D desviación
Unidades	u
Máx. crítico	120.00
Máx. nominal	12.00
Mín. nominal	-12.00
Mín. crítico	-120.00

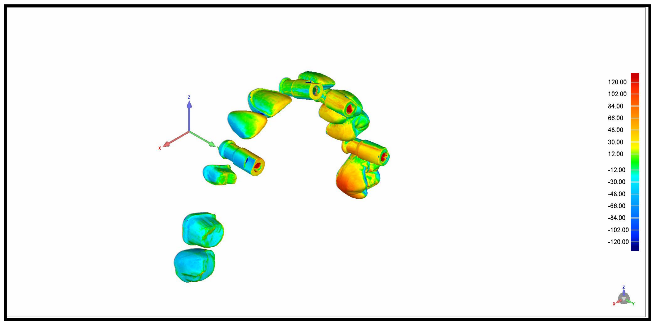

Desviación	
Desviación superior máx.	3129.77
Desviación inferior máx.	-2992.71
Desviación media	66.59 /-48.39
Desviación estándar	183.59

Distribución desviación

>=Min	<Max	# Puntos	%
-120.00	-102.00	333	0.31
-102.00	-84.00	914	0.84
-84.00	-66.00	2226	2.06
-66.00	-48.00	4004	3.70
-48.00	-30.00	8636	7.98
-30.00	-12.00	17649	16.30
-12.00	12.00	30063	27.77
12.00	30.00	18514	17.10
30.00	48.00	10674	9.86
48.00	66.00	4728	4.37
66.00	84.00	2081	1.92
84.00	102.00	1281	1.18
102.00	120.00	670	0.62

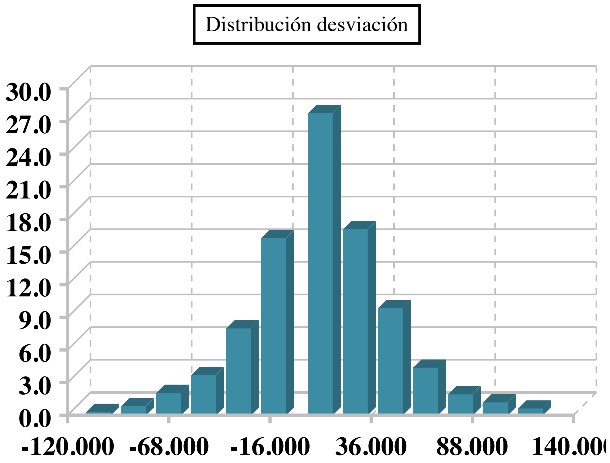

Fuera del crítico superior	4418	4.08
Fuera del crítico inferior	2059	1.90

Desviaciones estándar

Distribución (+/-)	# Puntos	%
-6 * Desv. estánd.	500	0.46
-5 * Desv. estánd.	70	0.06
-4 * Desv. estánd.	137	0.13
-3 * Desv. estánd.	151	0.14
-2 * Desv. estánd.	627	0.58
-1 * Desv. estánd.	65497	60.51
1 * Desv. estánd.	38332	35.41
2 * Desv. estánd.	931	0.86
3 * Desv. estánd.	418	0.39
4 * Desv. estánd.	381	0.35
5 * Desv. estánd.	310	0.29
6 * Desv. estánd.	896	0.83

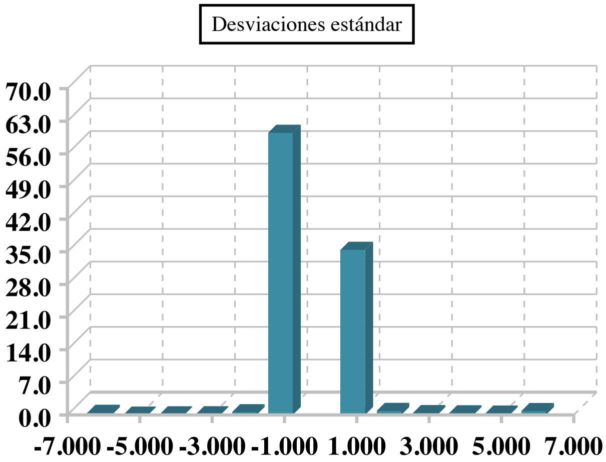

Predefinido: Isométrico

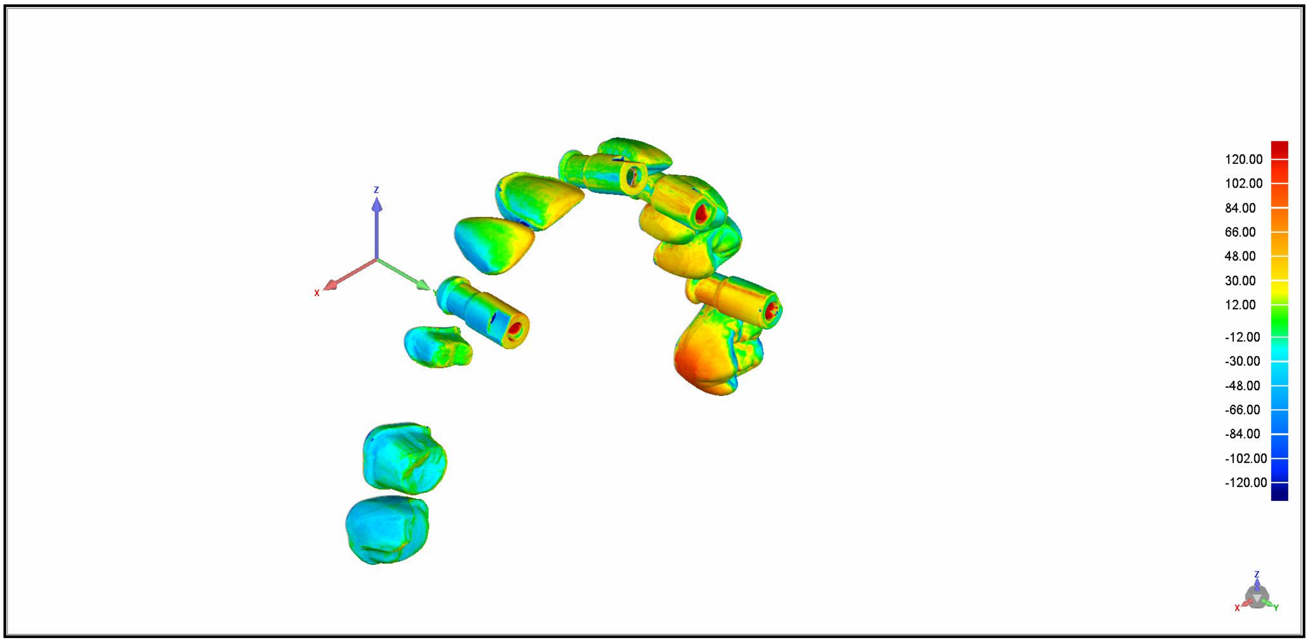

Predefinido: Frente

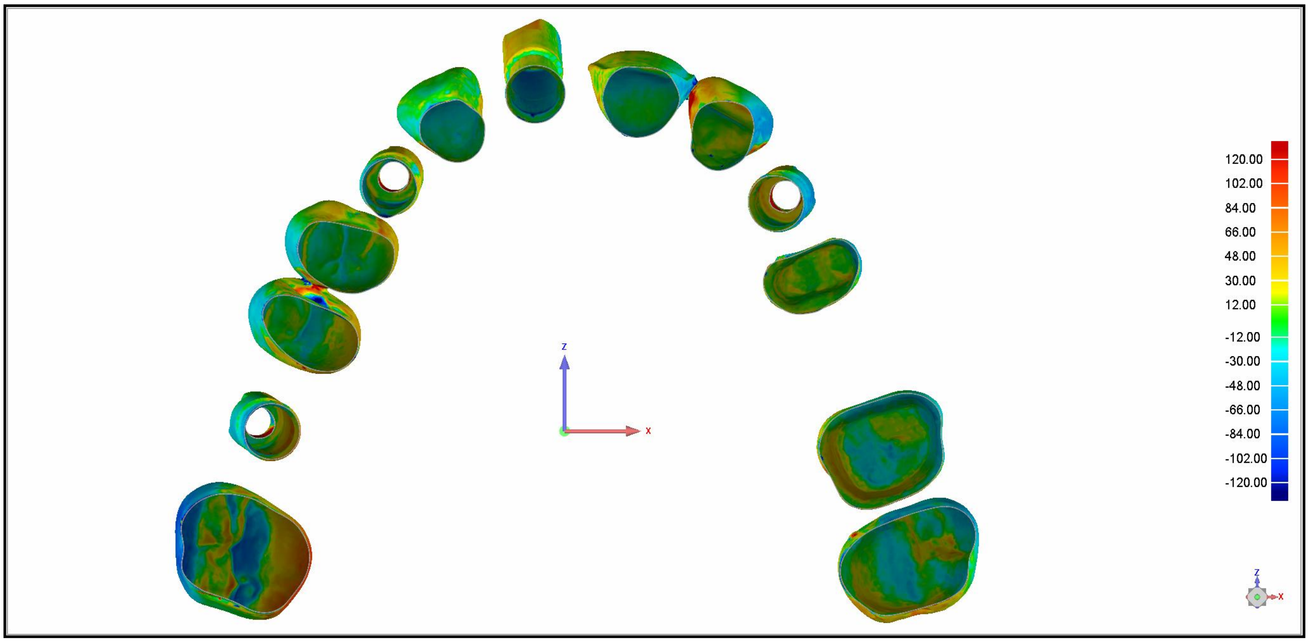

Predefinido: Atrás

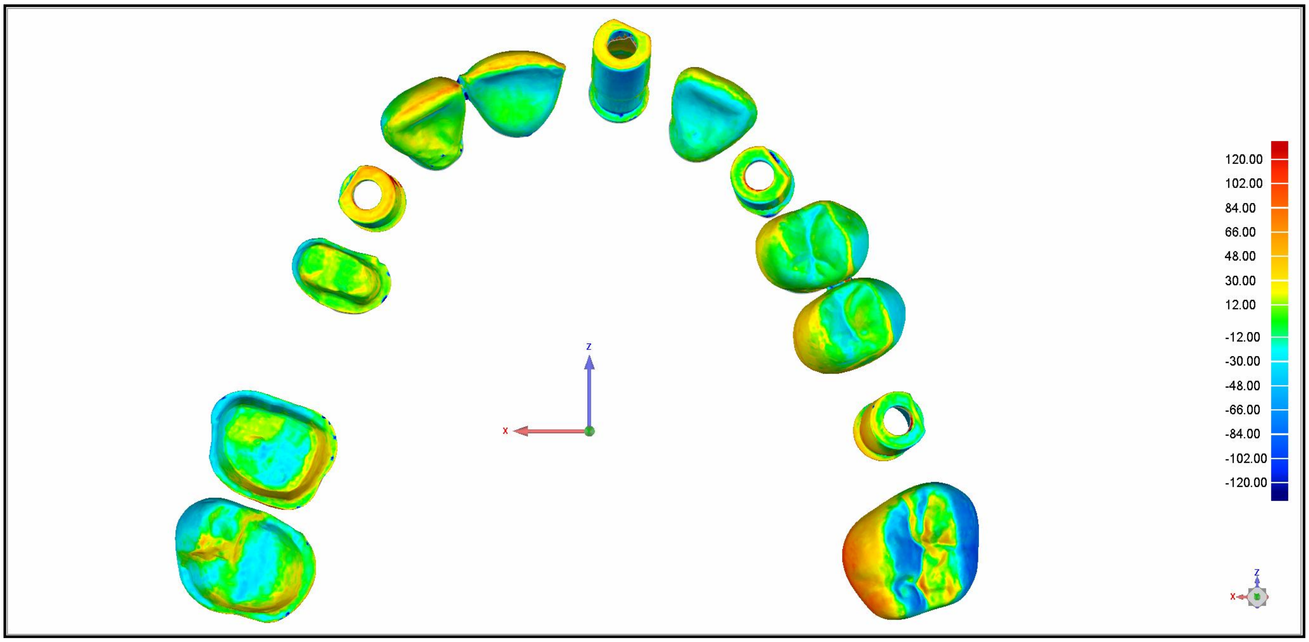

Predefinido: Izquierda

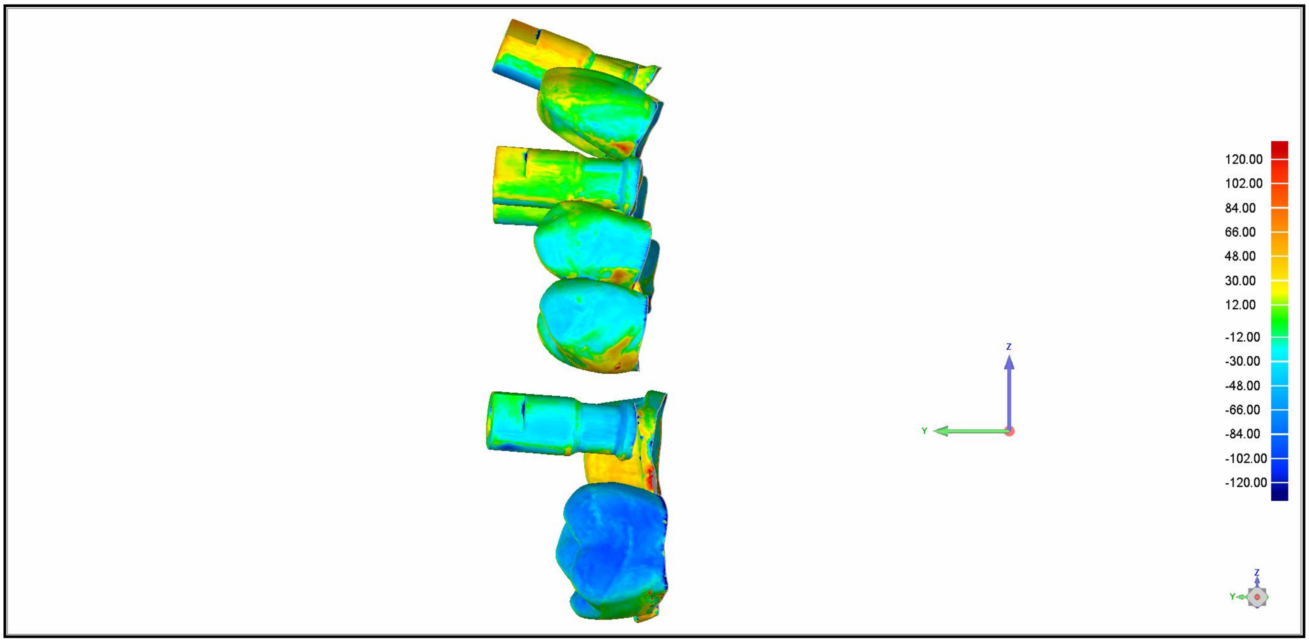

Predefinido: Derecha

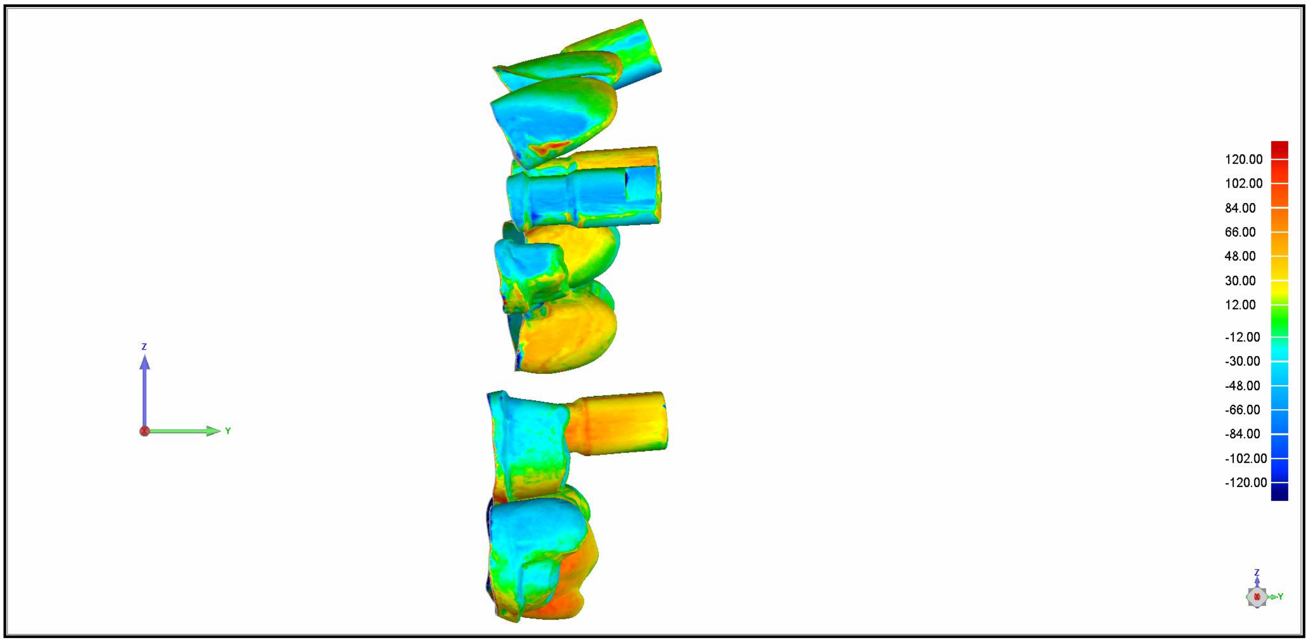

Predefinido: Superior

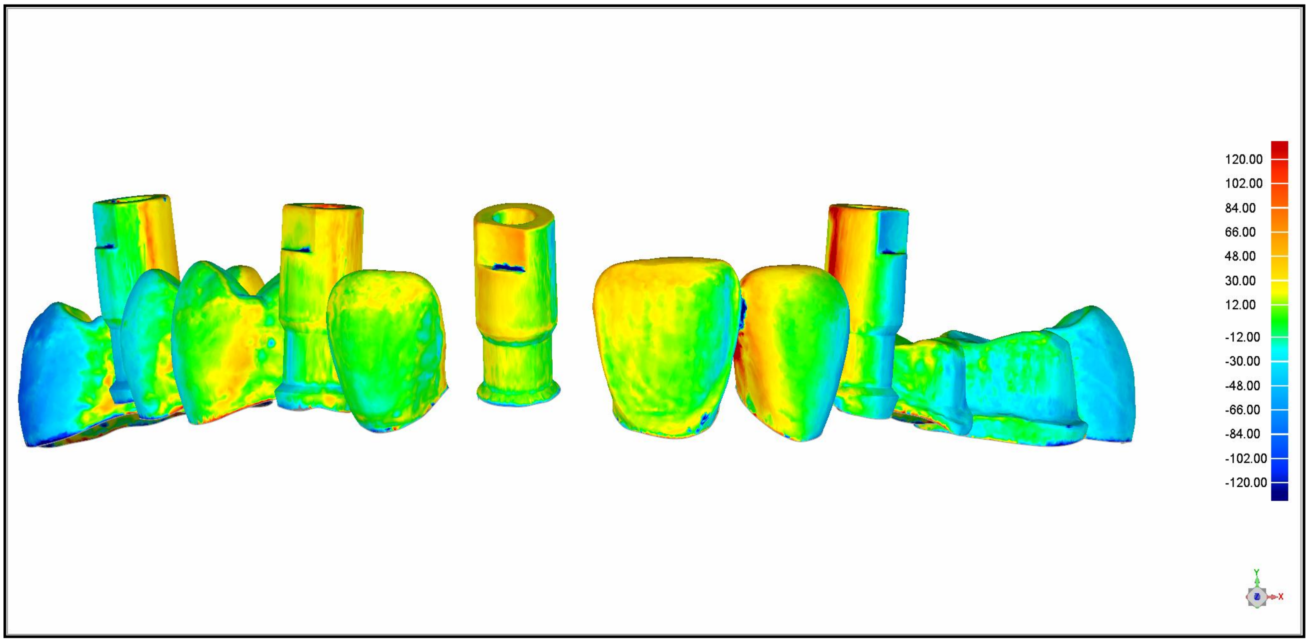

Predefinido: Inferior

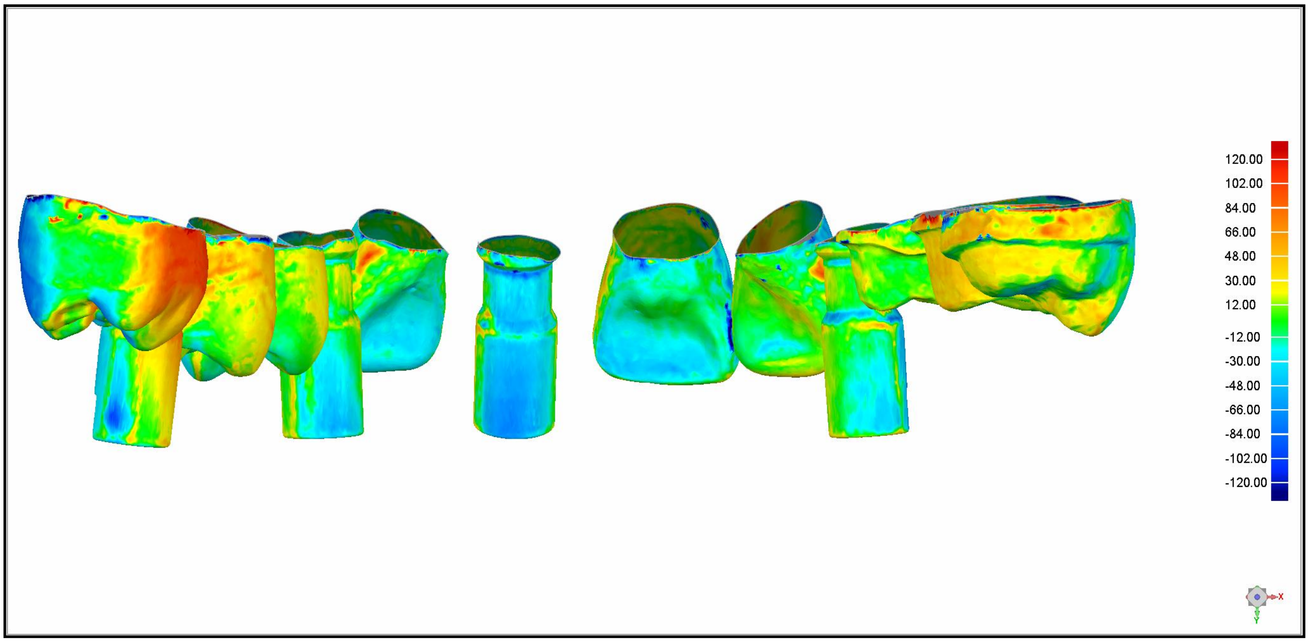

Supplement: S1 Table — Trios (scanning strategy A). (ZIP) [file pone.0202916.s001.zip › S1/3S10A.pdf]
