# Supplementary material for: Accuracy of four digital scanners according to scanning strategy in complete-arch impressions
Source: PLoS One. 2018 Sep 13;13(9):e0202916. doi: 10.1371/journal.pone.0202916 (PMC6136706; doi:10.1371/journal.pone.0202916)
Supplement: S1 Table — Trios (scanning strategy A). (ZIP) [file pone.0202916.s001.zip › S1/3S3A.pdf]

# 3D Comparación  Resultados

| | |
|---|---|
| Modelo referencia | MRC |
| Modelo test | 3S3A |
| Nº de puntos de datos | 104763 |
| # Aislados | 246 |

| | |
|---|---|
| Tipo tolerancia | 3D desviación |
| Unidades | u |
| Máx. crítico | 120.00 |
| Máx. nominal | 13.00 |
| Mín. nominal | -13.00 |
| Mín. crítico | -120.00 |

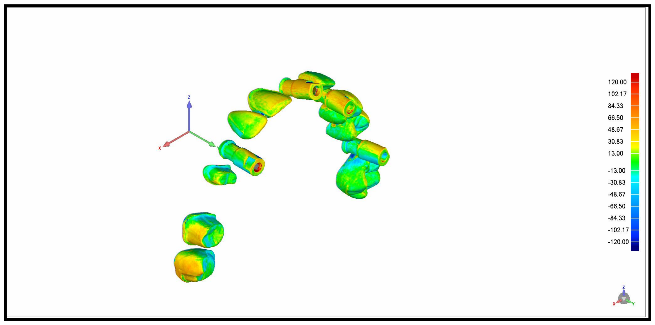

| | |
|---|---|
| Desviación | |
| Desviación superior máx. | 2964.60 |
| Desviación inferior máx. | -2527.09 |
| Desviación media | 54.58 /-49.42 |
| Desviación estándar | 181.86 |

## Distribución desviación

| >=Min | <Max | # Puntos | % |
|---|---|---|---|
| -120.00 | -102.17 | 367 | 0.35 |
| -102.17 | -84.33 | 461 | 0.44 |
| -84.33 | -66.50 | 790 | 0.75 |
| -66.50 | -48.67 | 1867 | 1.78 |
| -48.67 | -30.83 | 5362 | 5.12 |
| -30.83 | -13.00 | 12809 | 12.23 |
| -13.00 | 13.00 | 38483 | 36.73 |
| 13.00 | 30.83 | 22112 | 21.11 |
| 30.83 | 48.67 | 10046 | 9.59 |
| 48.67 | 66.50 | 4061 | 3.88 |
| 66.50 | 84.33 | 1543 | 1.47 |
| 84.33 | 102.17 | 706 | 0.67 |
| 102.17 | 120.00 | 454 | 0.43 |

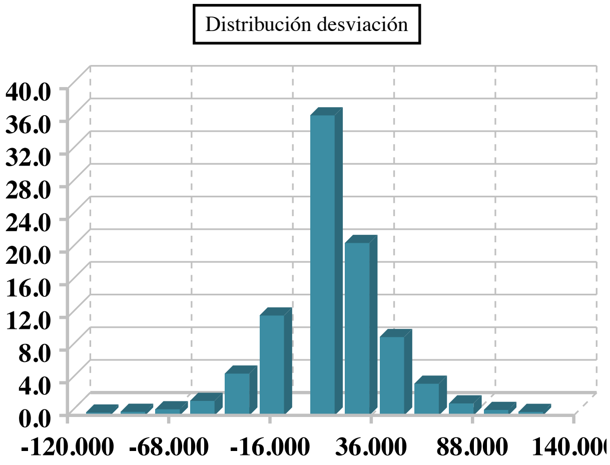


| | | | |
|---|---|---|---|
| Fuera del crítico superior | | 3648 | 3.48 |
| Fuera del crítico inferior | | 2054 | 1.96 |

## Desviaciones estándar

| Distribución (+/-) | # Puntos | % |
|---|---|---|
| -6 * Desv. estánd. | 540 | 0.52 |
| -5 * Desv. estánd. | 67 | 0.06 |
| -4 * Desv. estánd. | 108 | 0.10 |
| -3 * Desv. estánd. | 142 | 0.14 |
| -2 * Desv. estánd. | 557 | 0.53 |
| -1 * Desv. estánd. | 61786 | 58.98 |
| 1 * Desv. estánd. | 39044 | 37.27 |
| 2 * Desv. estánd. | 776 | 0.74 |
| 3 * Desv. estánd. | 327 | 0.31 |
| 4 * Desv. estánd. | 312 | 0.30 |
| 5 * Desv. estánd. | 293 | 0.28 |
| 6 * Desv. estánd. | 811 | 0.77 |

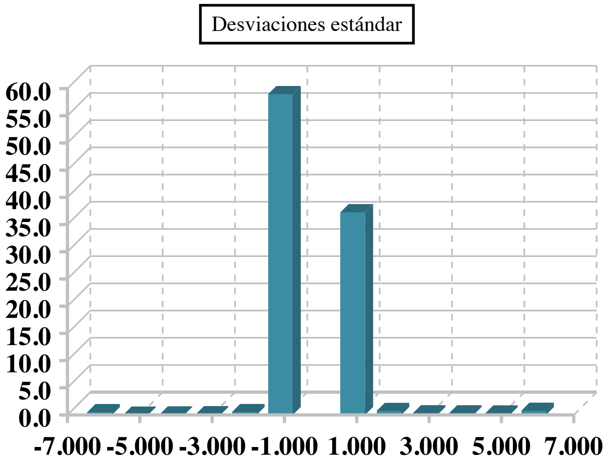

Predefinido: Isométrico

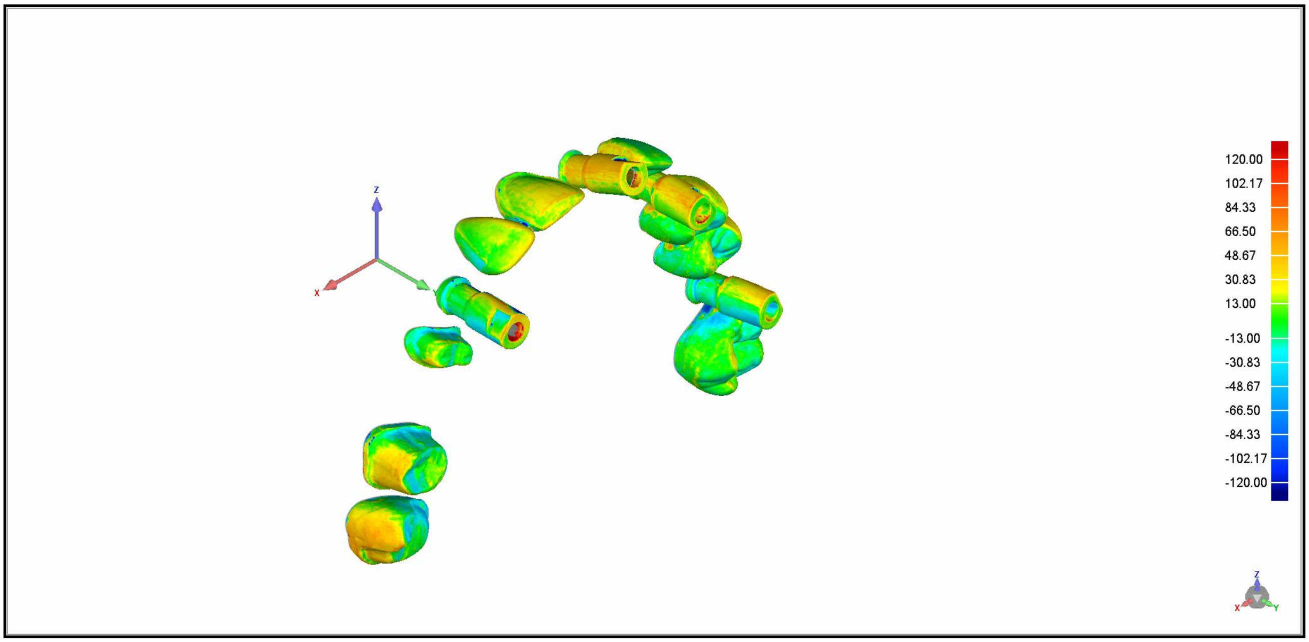

Predefinido: Frente

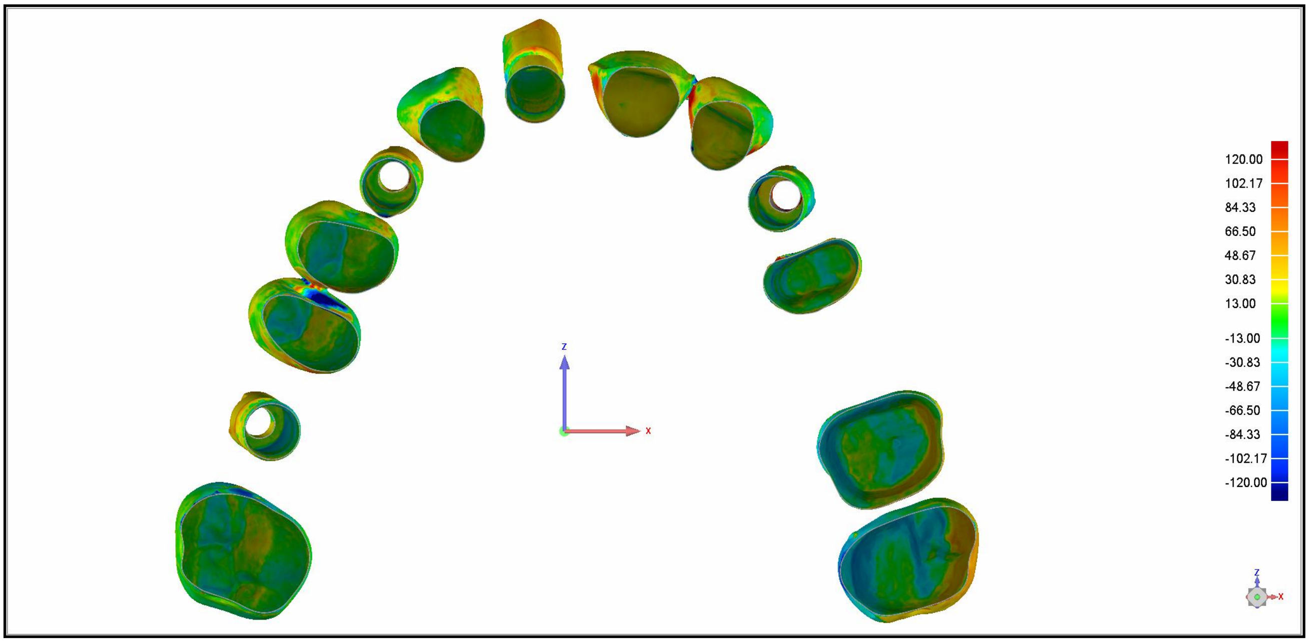

Predefinido: Atrás

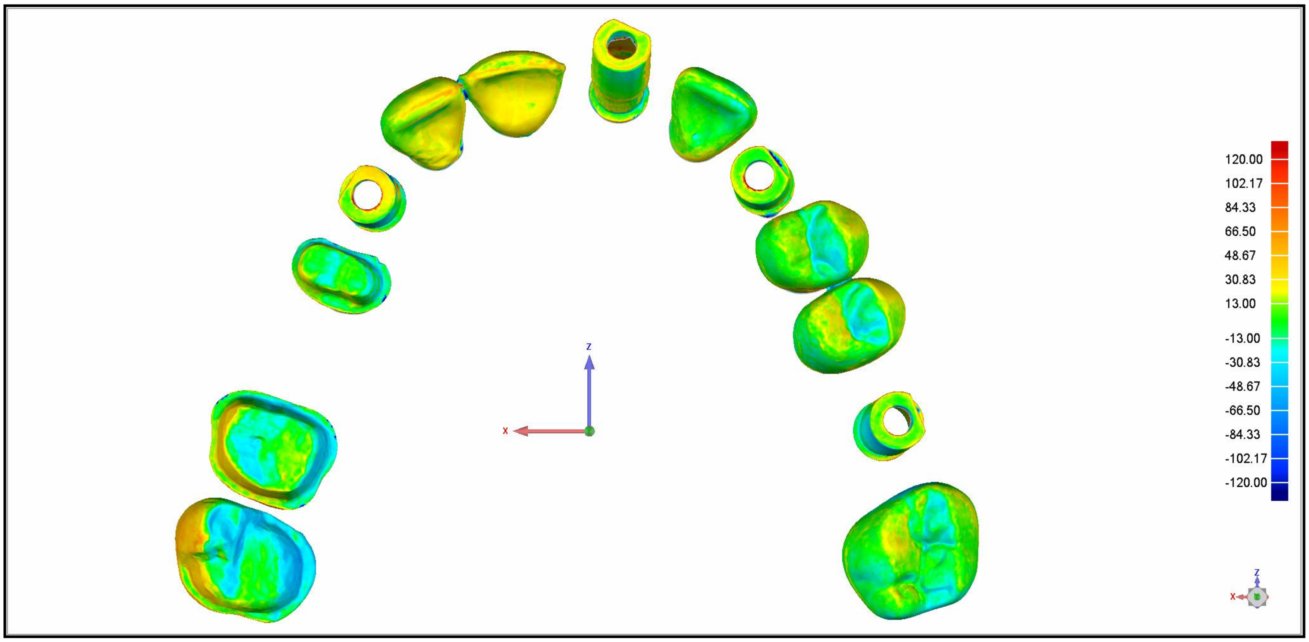

Predefinido: Izquierda

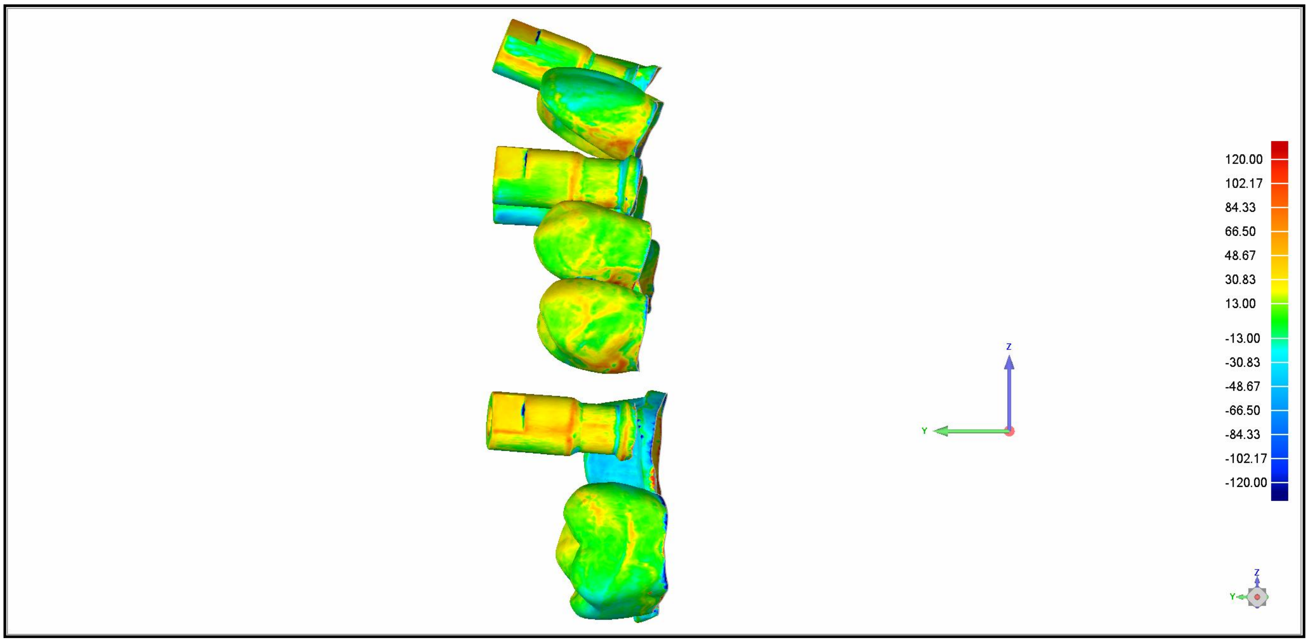

Predefinido: Derecha

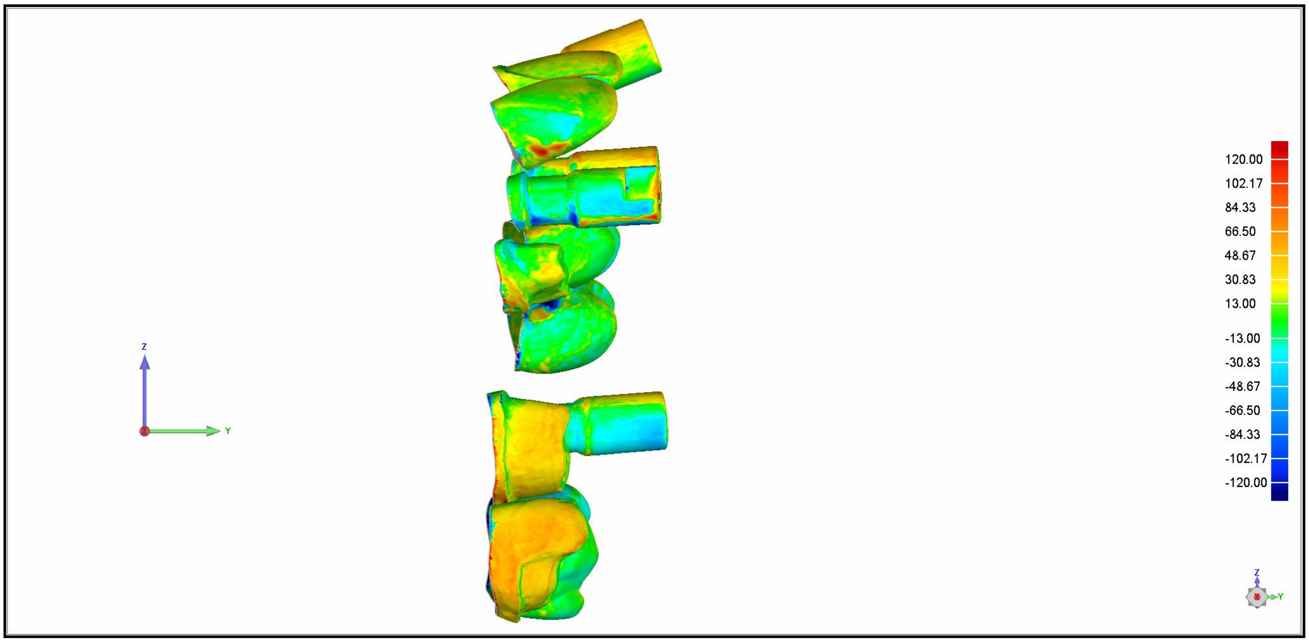

Predefinido: Superior

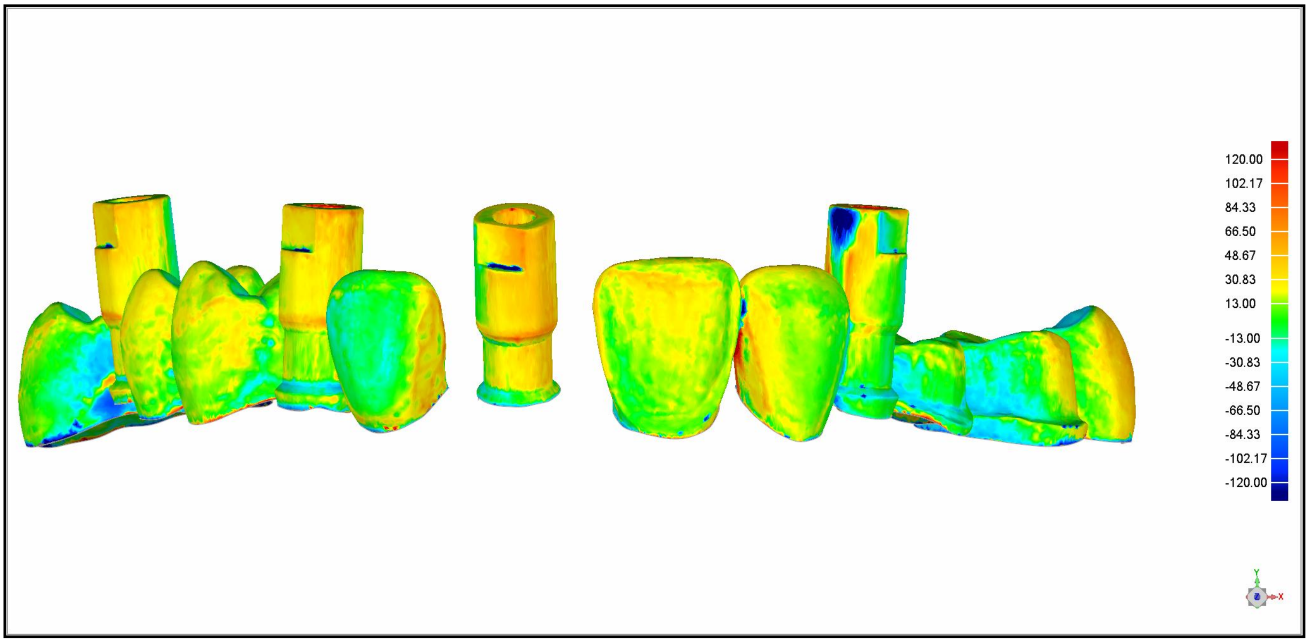

Geomagic Control™



Predefinido: Inferior

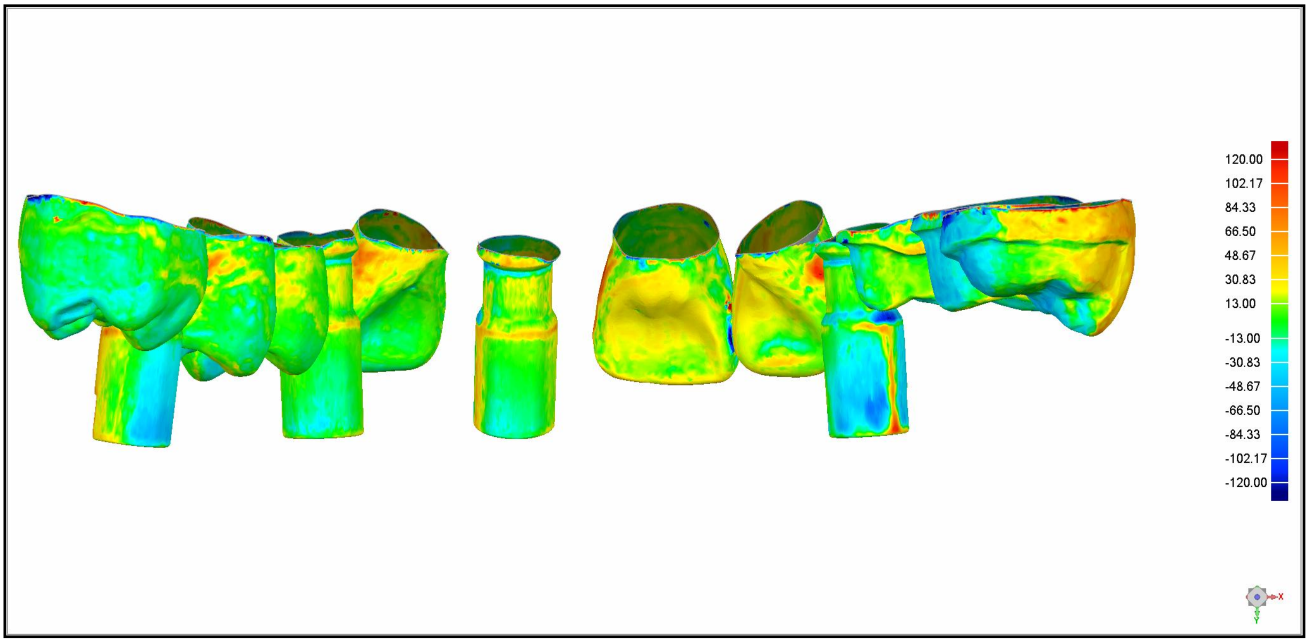

# Ajuste de ubicación: Desviaciones superior e inferior

## Unidades: u

| Nombre | Desv | Estado | Superior Tol | Inferior Tol | Ref X | Ref Y | Ref Z | Radio | Desv X | Desv Y | Desv Z | Medido X | Medido Y | Medido Z | Dir. proy. X | Dir. proy. Y | Dir. proy. Z |
|---|---|---|---|---|---|---|---|---|---|---|---|---|---|---|---|---|---|
| Desv. inferior | -2527.09 | | | | -3526.55 | 37052.94 | 30834.95 | n/a | 1067.28 | -102.27 | -2288.36 | -2459.26 | 36950.66 | 28546.59 | -0.42 | 0.04 | 0.91 |
| Desv. superior | 2964.60 | | | | -6666.84 | 29713.26 | 24874.31 | n/a | -69.27 | 192.17 | -2957.55 | -6736.11 | 29905.43 | 21916.76 | -0.02 | 0.06 | -1.00 |
