# Supplementary material for: Accuracy of four digital scanners according to scanning strategy in complete-arch impressions
Source: PLoS One. 2018 Sep 13;13(9):e0202916. doi: 10.1371/journal.pone.0202916 (PMC6136706; doi:10.1371/journal.pone.0202916)
Supplement: S1 Table — Trios (scanning strategy A). (ZIP) [file pone.0202916.s001.zip › S1/3S1A.pdf]

3D Comparación Resultados

Modelo referencia	MRC
Modelo test	3S1A
Nº de puntos de datos	102521
# Aislados	344

Tipo tolerancia	3D desviación
Unidades	u
Máx. crítico	120.00
Máx. nominal	14.00
Mín. nominal	-14.00
Mín. crítico	-120.00

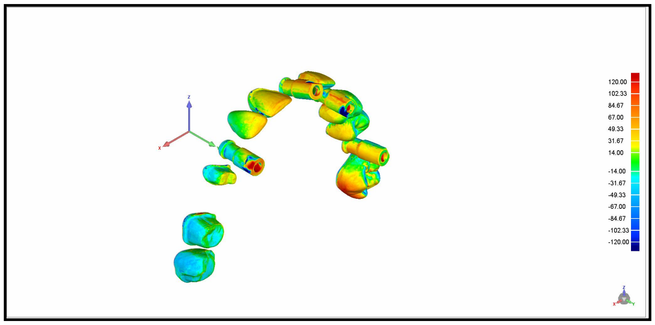

Desviación	
Desviación superior máx.	2753.80
Desviación inferior máx.	-3006.85
Desviación media	62.78 /-52.65
Desviación estándar	174.43

Distribución desviación

>=Min	<Max	# Puntos	%
-120.00	-102.33	383	0.37
-102.33	-84.67	1186	1.16
-84.67	-67.00	1820	1.78
-67.00	-49.33	2527	2.46
-49.33	-31.67	7140	6.96
-31.67	-14.00	13290	12.96
-14.00	14.00	30047	29.31
14.00	31.67	18848	18.38
31.67	49.33	10915	10.65
49.33	67.00	5295	5.16
67.00	84.67	2648	2.58
84.67	102.33	1452	1.42
102.33	120.00	914	0.89

Fuera del crítico superior	3937	3.84
Fuera del crítico inferior	2119	2.07

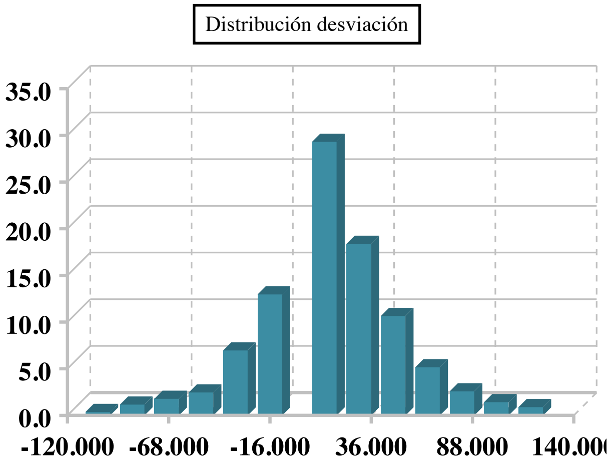

Desviaciones estándar

Distribución (+/-)	# Puntos	%
-6 * Desv. estánd.	492	0.48
-5 * Desv. estánd.	83	0.08
-4 * Desv. estánd.	137	0.13
-3 * Desv. estánd.	240	0.23
-2 * Desv. estánd.	733	0.71
-1 * Desv. estánd.	57548	56.13
1 * Desv. estánd.	40534	39.54
2 * Desv. estánd.	907	0.88
3 * Desv. estánd.	391	0.38
4 * Desv. estánd.	339	0.33
5 * Desv. estánd.	284	0.28
6 * Desv. estánd.	833	0.81

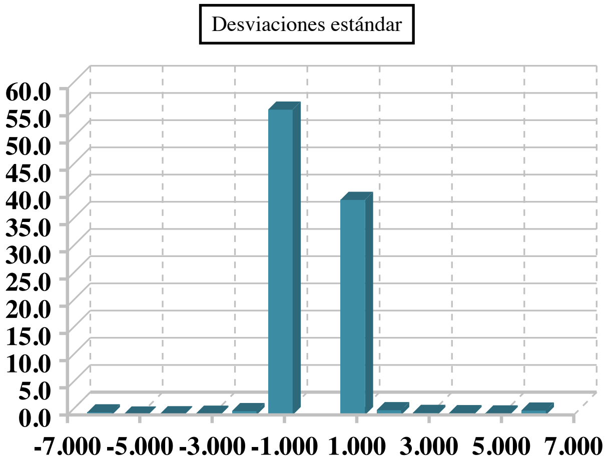

Predefinido: Isométrico

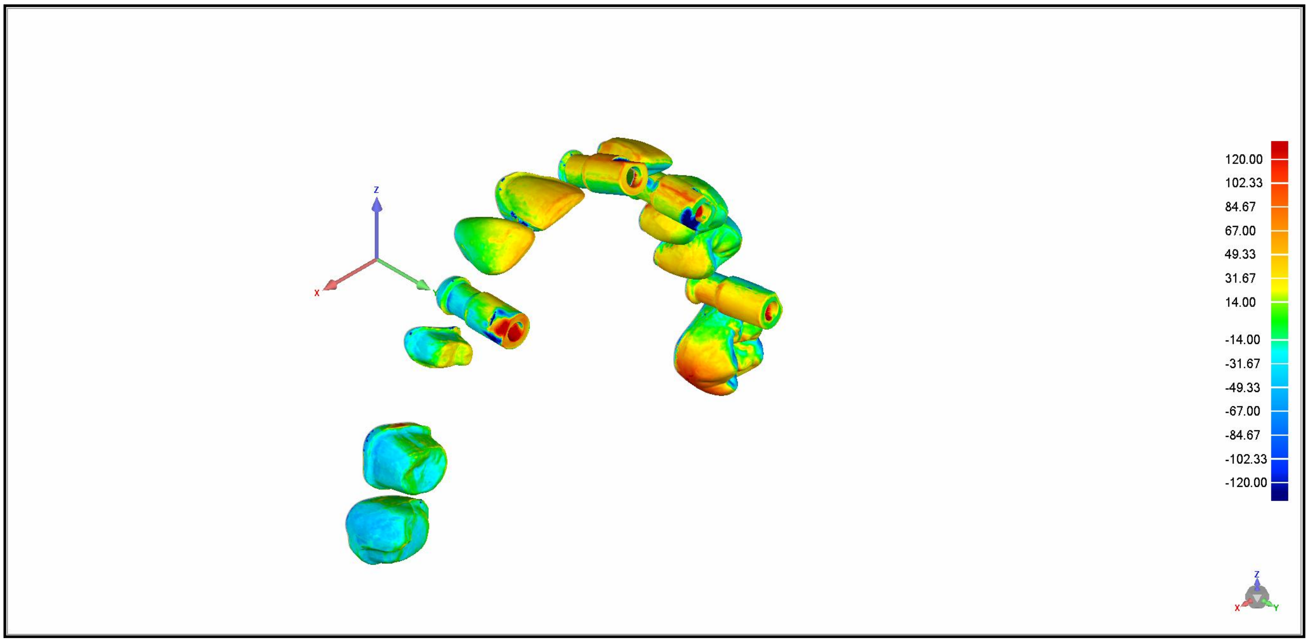

Predefinido: Frente

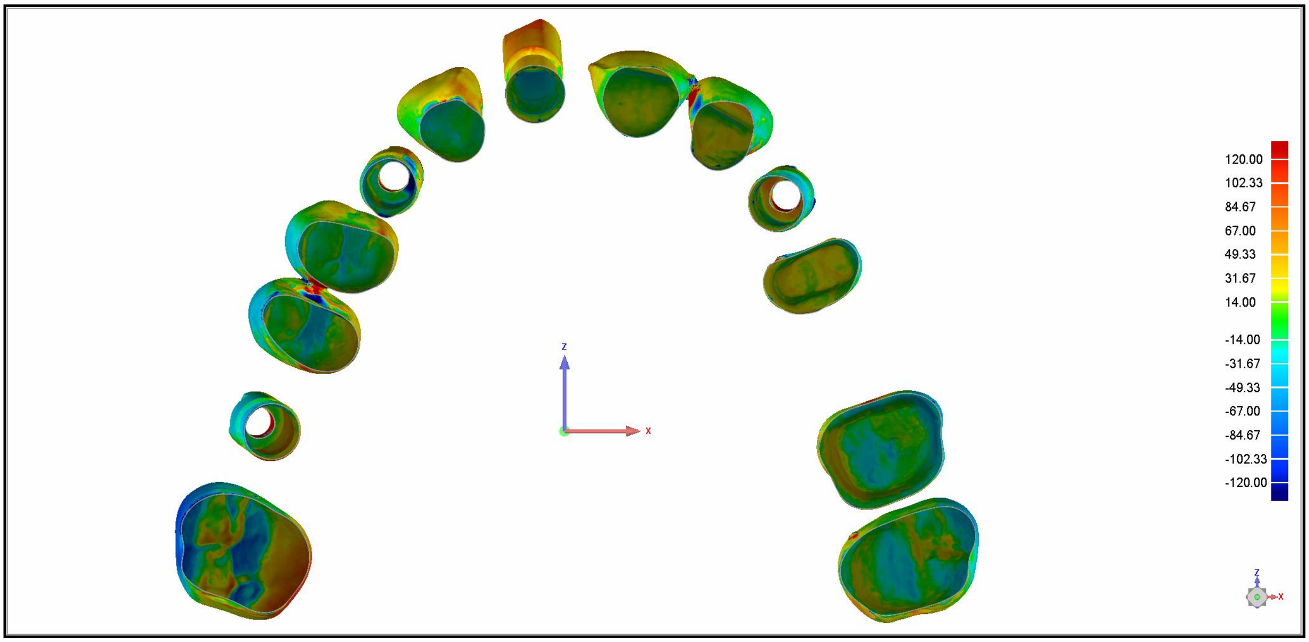

Predefinido: Atrás

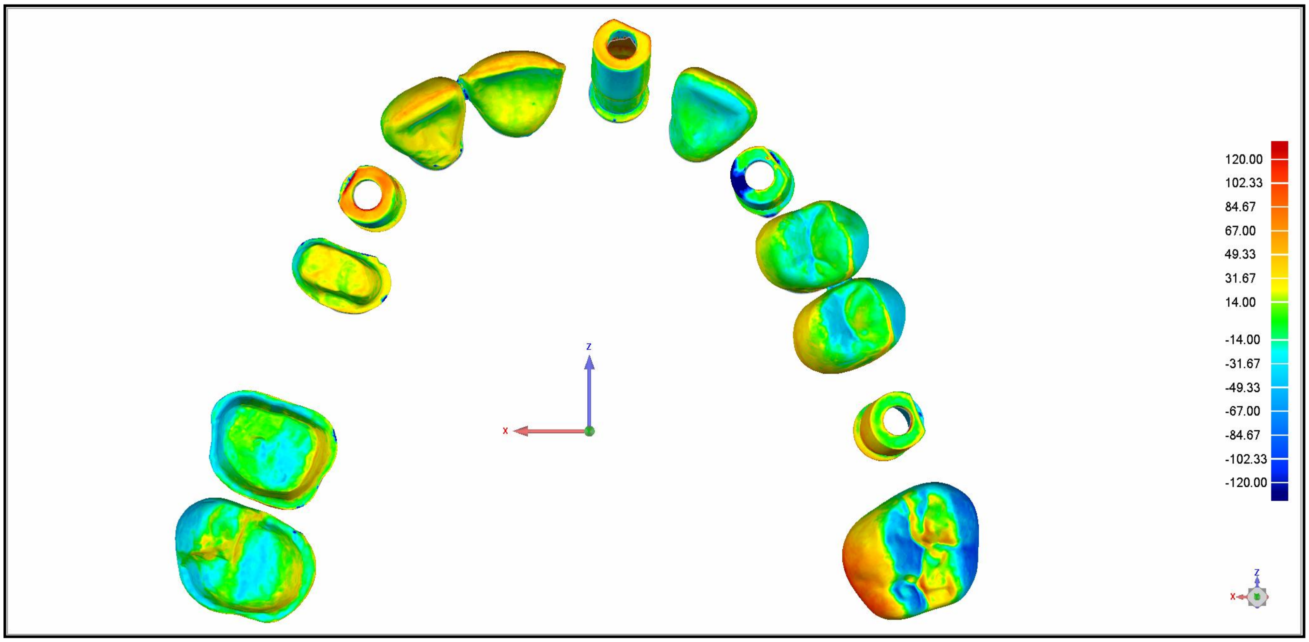

Predefinido: Izquierda

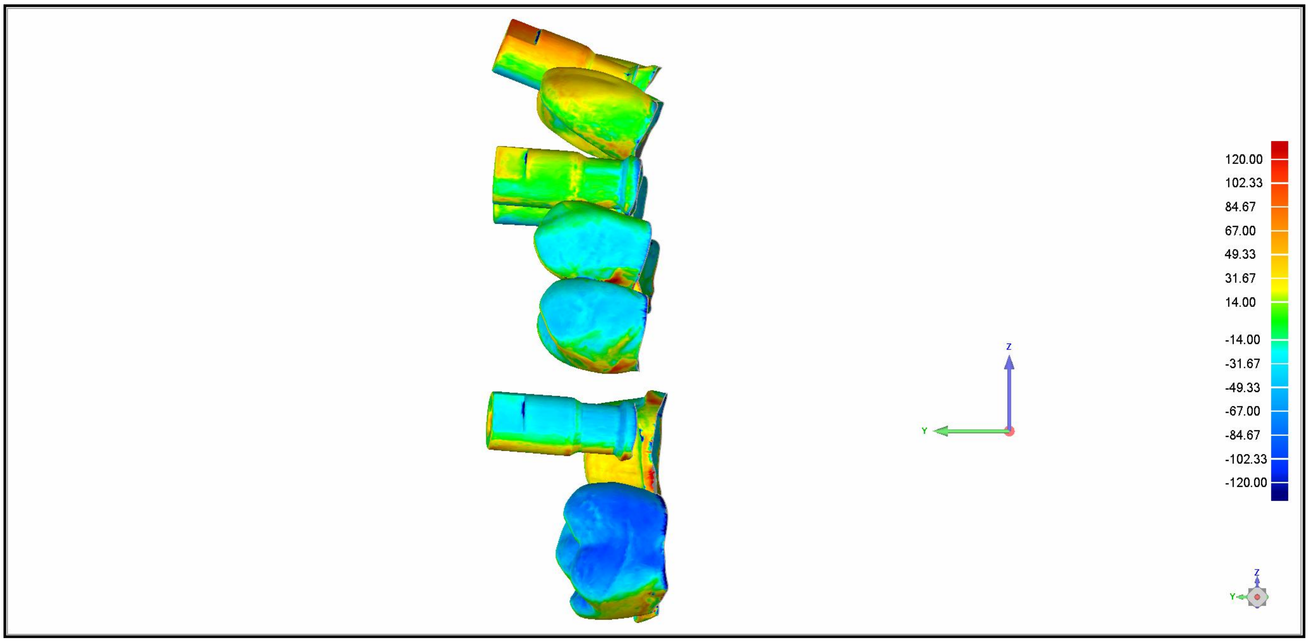

Predefinido: Derecha

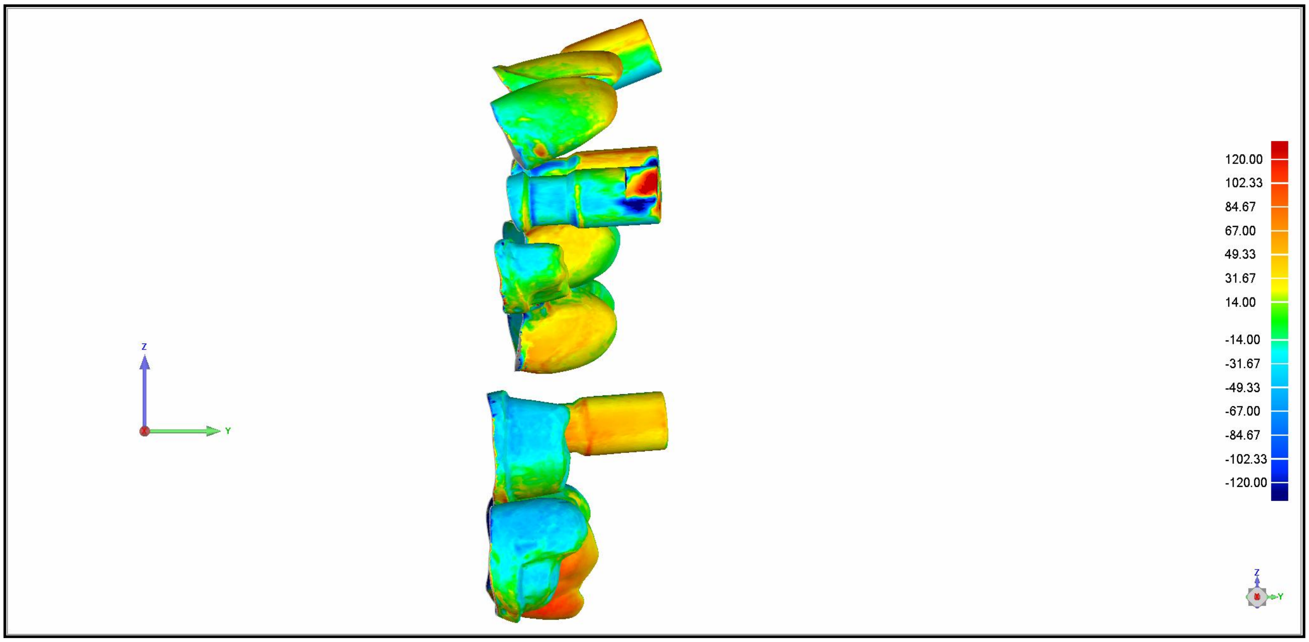

Predefinido: Superior

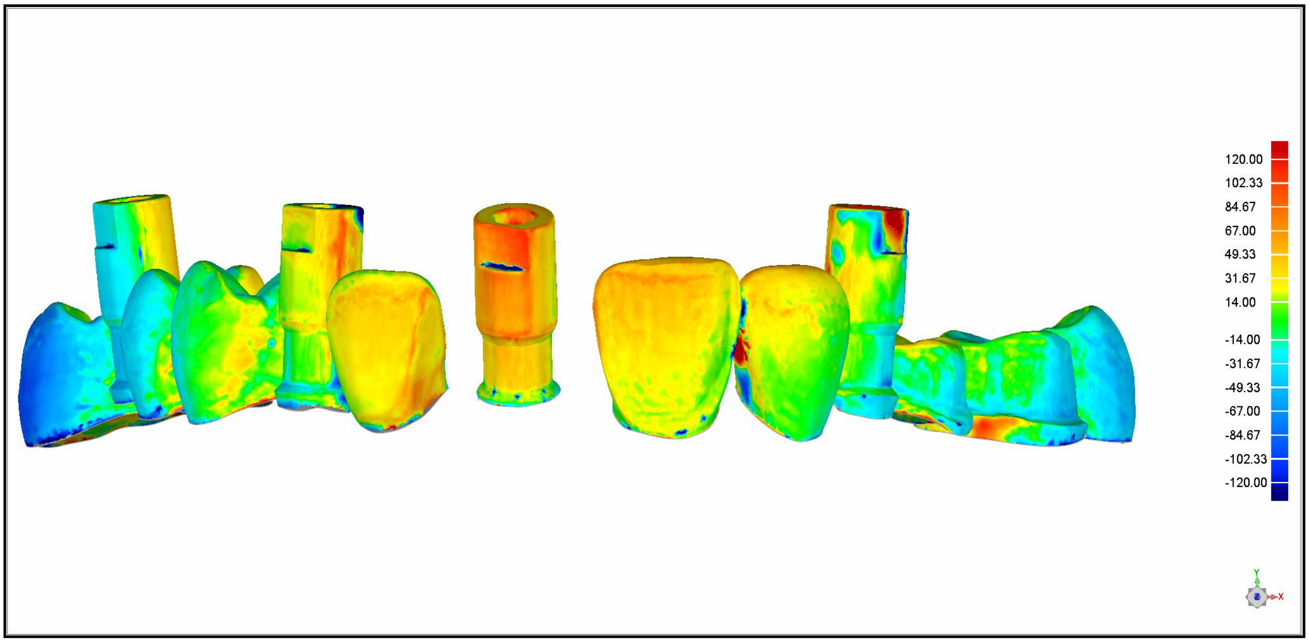

Predefinido: Inferior

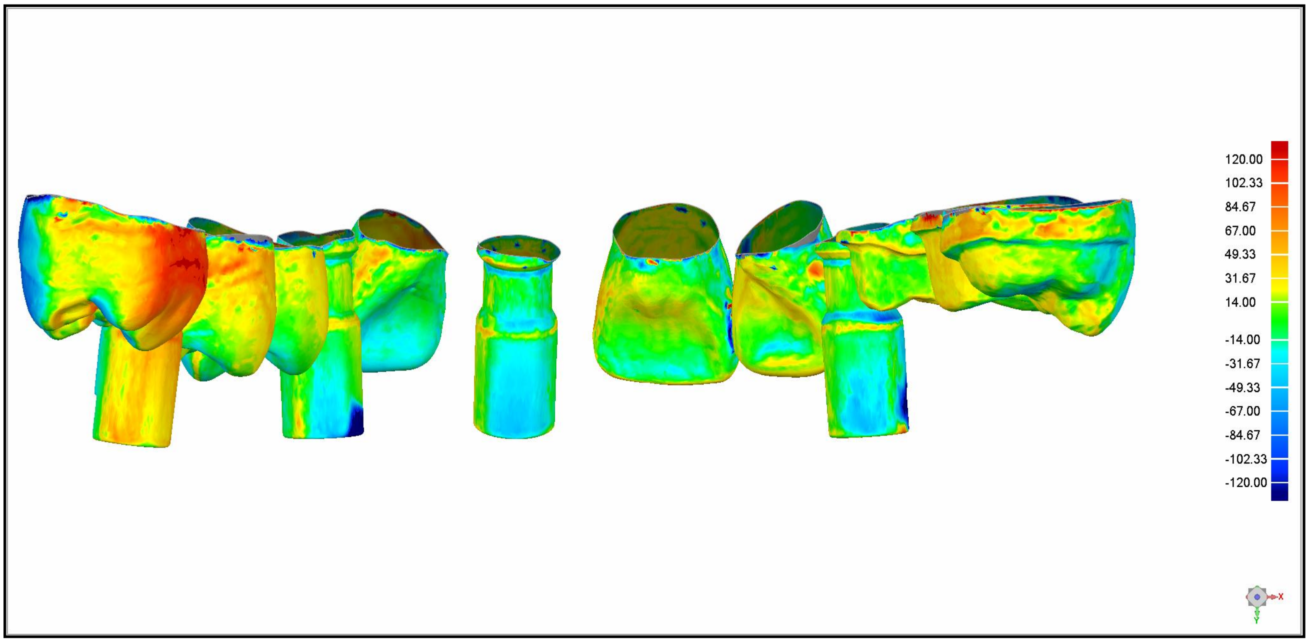

Ajuste de ubicación: Desviaciones superior e inferior

Unidades: u

Nombre	Desv	Estado	Superior Tol	Inferior Tol	Ref X	Ref Y	Ref Z	Radio	Desv X	Desv Y	Desv Z	Medido X	Medido Y	Medido Z	Dir. proy. X	Dir. proy. Y	Dir. proy. Z
Desv. inferior	-3006.85				-29292.33	26884.28	-11910.36	n/a	2611.91	344.12	-1449.38	-26680.43	27228.40	-13359.74	-0.87	-0.11	0.48
Desv. superior	2753.80				-19959.81	29021.59	12242.50	n/a	-2298.05	-307.14	-1485.95	-22257.86	28714.44	10756.55	-0.83	-0.11	-0.54
