# Supplementary material for: Accuracy of four digital scanners according to scanning strategy in complete-arch impressions
Source: PLoS One. 2018 Sep 13;13(9):e0202916. doi: 10.1371/journal.pone.0202916 (PMC6136706; doi:10.1371/journal.pone.0202916)
Supplement: S1 Table — Trios (scanning strategy A). (ZIP) [file pone.0202916.s001.zip › S1/3S6A.pdf]

Geomagic Control™

Fecha: 3/6/2017, 12:36 pm

3D Comparación Resultados

Modelo referencia	MRC
Modelo test	3S6A
Nº de puntos de datos	107289
# Aislados	409

Tipo tolerancia	3D desviación
Unidades	u
Máx. crítico	120.00
Máx. nominal	16.00
Mín. nominal	-16.00
Mín. crítico	-120.00

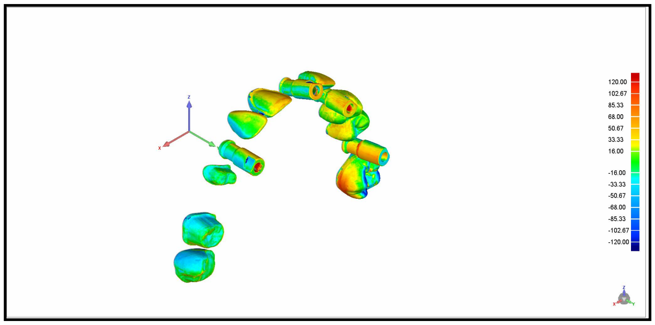

Desviación	
Desviación superior máx.	2091.17
Desviación inferior máx.	-2144.61
Desviación media	69.66 /-49.22
Desviación estándar	174.54

Distribución desviación

>=Min	<Max	# Puntos	%
-120.00	-102.67	1252	1.17
-102.67	-85.33	1508	1.41
-85.33	-68.00	1757	1.64
-68.00	-50.67	2711	2.53
-50.67	-33.33	6854	6.39
-33.33	-16.00	13170	12.28
-16.00	16.00	36982	34.47
16.00	33.33	16644	15.51
33.33	50.67	9918	9.24
50.67	68.00	5388	5.02
68.00	85.33	2362	2.20
85.33	102.67	1292	1.20
102.67	120.00	633	0.59

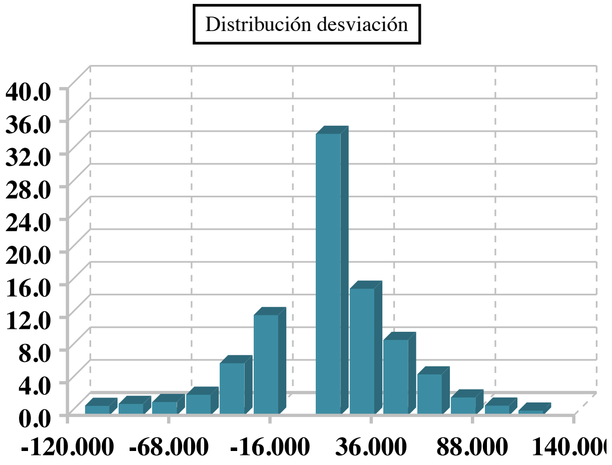

Fuera del crítico superior		4412	4.11
Fuera del crítico inferior		2406	2.24

Desviaciones estándar

Distribución (+/-)	# Puntos	%
-6 * Desv. esténd.	477	0.44
-5 * Desv. esténd.	109	0.10
-4 * Desv. esténd.	90	0.08
-3 * Desv. esténd.	107	0.10
-2 * Desv. esténd.	653	0.61
-1 * Desv. esténd.	65514	61.06
1 * Desv. esténd.	37069	34.55
2 * Desv. esténd.	876	0.82
3 * Desv. esténd.	460	0.43
4 * Desv. esténd.	433	0.40
5 * Desv. esténd.	433	0.40
6 * Desv. esténd.	1068	1.00

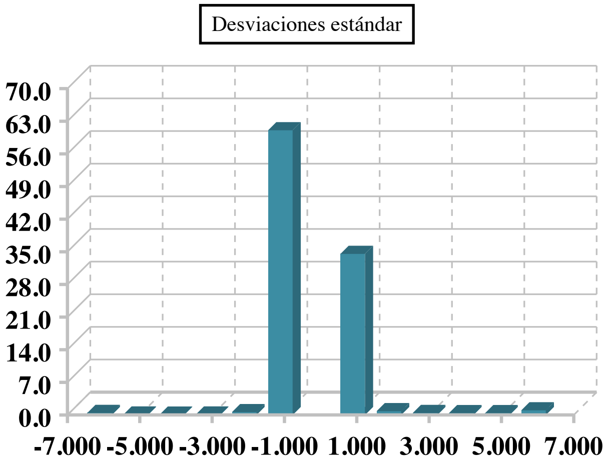

Predefinido: Isométrico

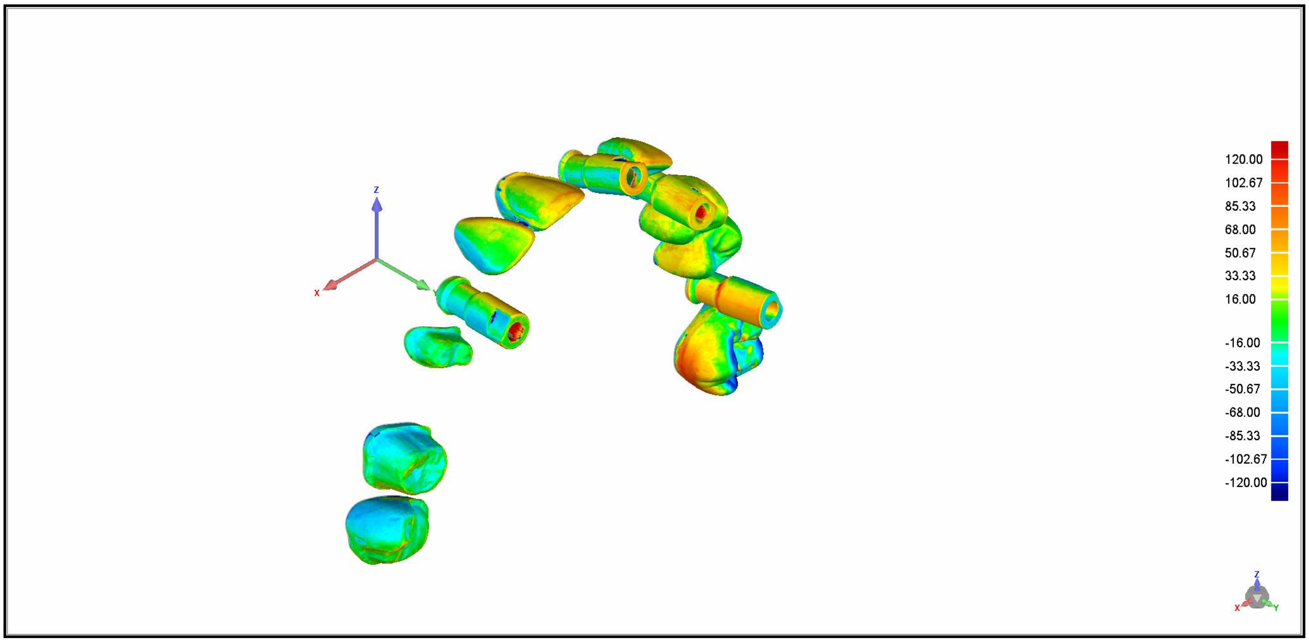

Predefinido: Frente

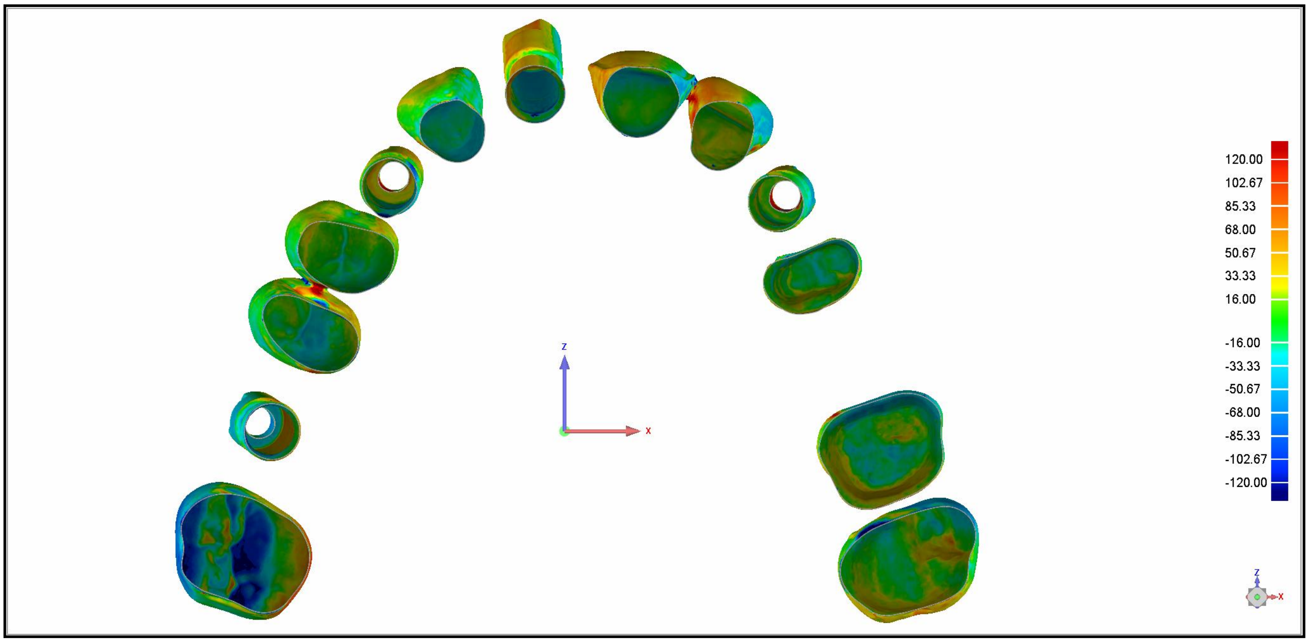

Predefinido: Atrás

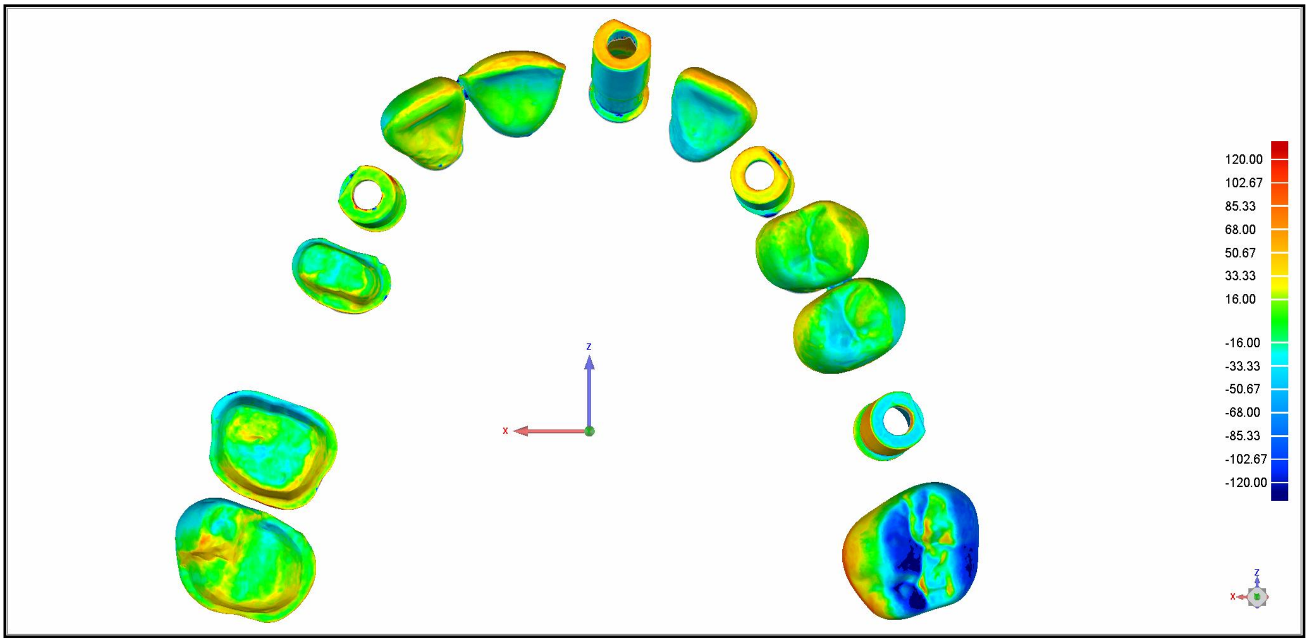

Predefinido: Izquierda

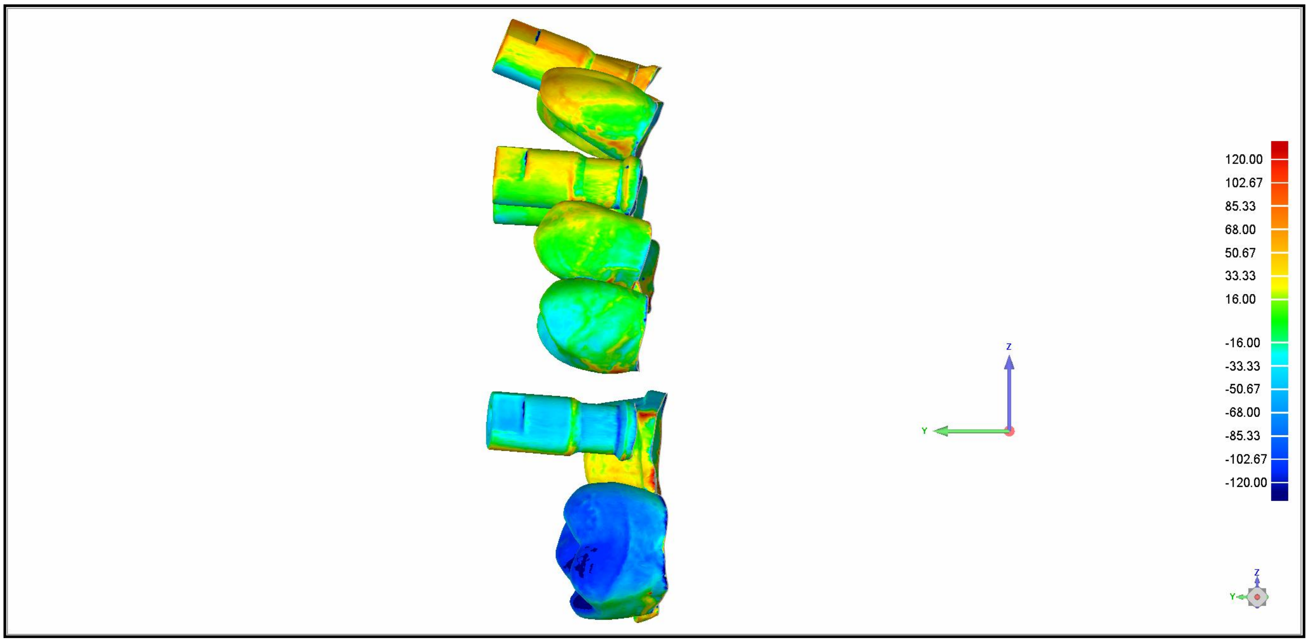

Predefinido: Derecha

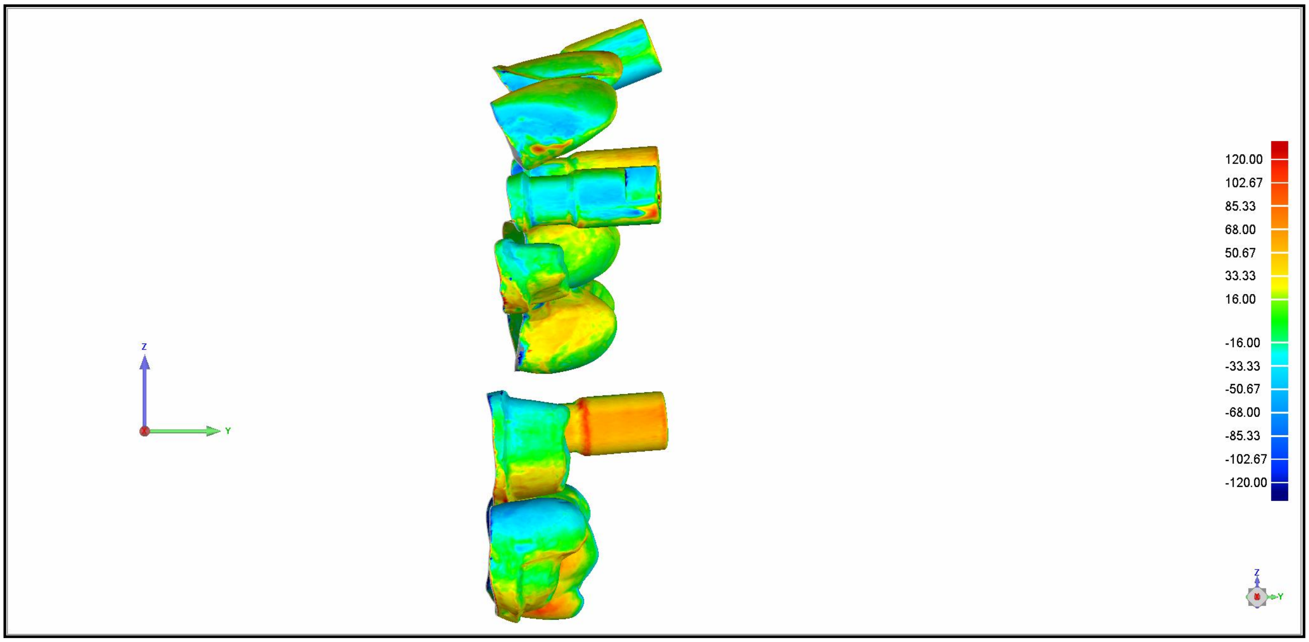

Predefinido: Superior

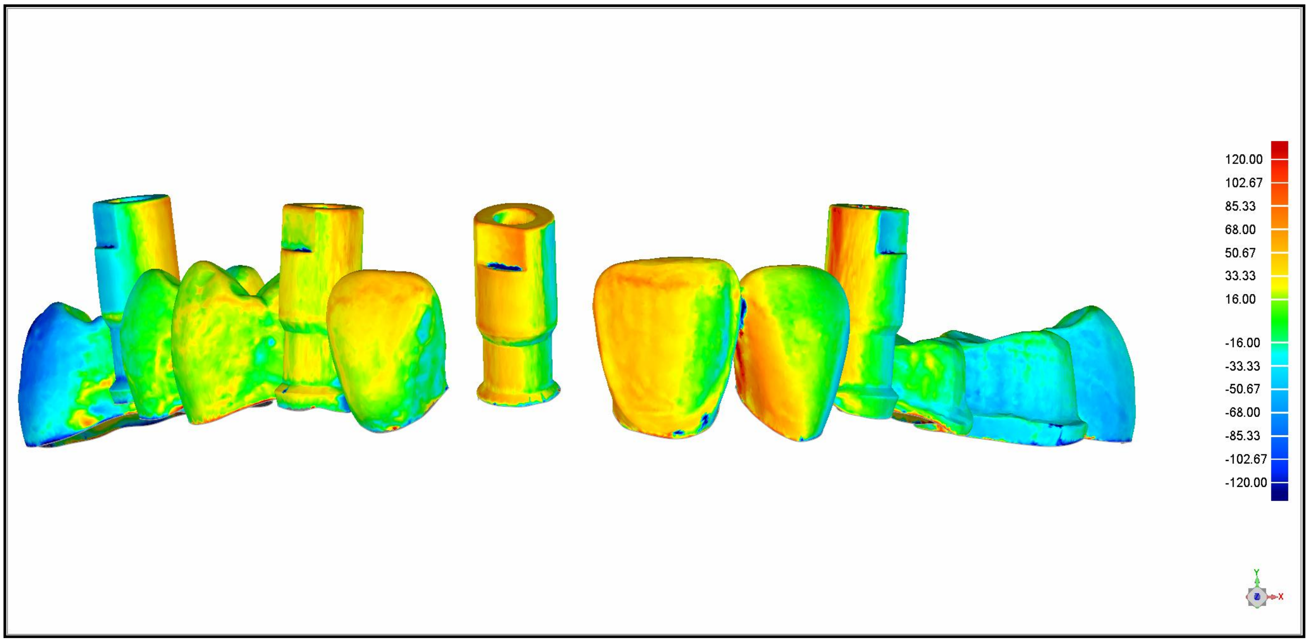

Predefinido: Inferior

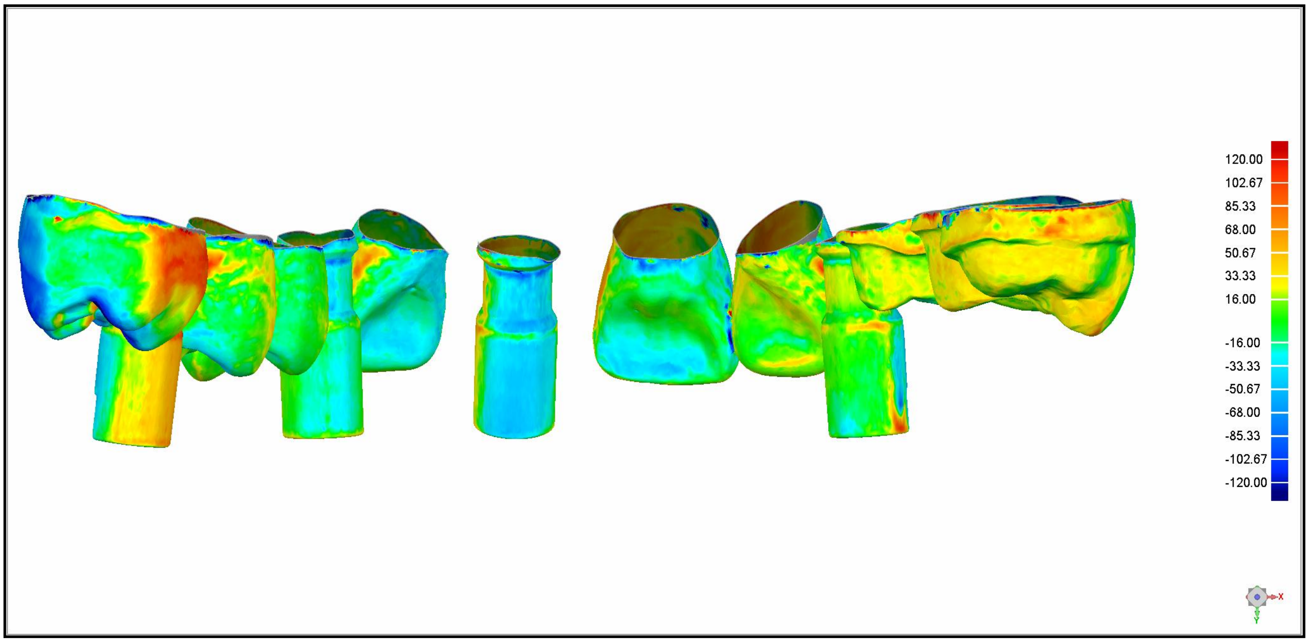

Ajuste de ubicación: Desviaciones superior e inferior

Unidades: u

Nombre	Desv	Estado	Superior Tol	Inferior Tol	Ref X	Ref Y	Ref Z	Radio	Desv X	Desv Y	Desv Z	Medido X	Medido Y	Medido Z	Dir. proy. X	Dir. proy. Y	Dir. proy. Z
Desv. inferior	-2144.61				-12418.65	38556.38	20660.02	n/a	-965.55	-1508.89	-1179.11	-13384.20	37047.49	19480.91	0.45	0.70	0.55
Desv. superior	2091.17				-4595.58	29577.70	26257.71	n/a	1095.65	542.76	-1696.45	-3499.94	30120.46	24561.25	0.52	0.26	-0.81
