# Supplementary material for: Accuracy of four digital scanners according to scanning strategy in complete-arch impressions
Source: PLoS One. 2018 Sep 13;13(9):e0202916. doi: 10.1371/journal.pone.0202916 (PMC6136706; doi:10.1371/journal.pone.0202916)
Supplement: S1 Table — Trios (scanning strategy A). (ZIP) [file pone.0202916.s001.zip › S1/3S7A.pdf]

Geomagic Control™

Fecha: 3/6/2017, 12:38 pm

3D Comparación Resultados

Modelo referencia	MRC
Modelo test	3S7A
Nº de puntos de datos	99465
# Aislados	310

Tipo tolerancia	3D desviación
Unidades	u
Máx. crítico	120.00
Máx. nominal	12.00
Mín. nominal	-12.00
Mín. crítico	-120.00

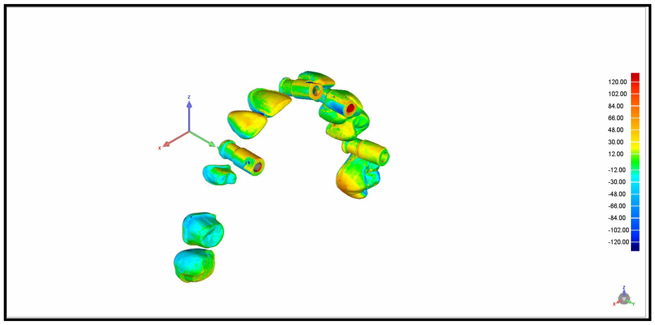

Desviación	
Desviación superior máx.	3068.01
Desviación inferior máx.	-2140.78
Desviación media	57.64 /-42.41
Desviación estándar	167.15

Distribución desviación

>=Min	<Max	# Puntos	%
-120.00	-102.00	365	0.37
-102.00	-84.00	569	0.57
-84.00	-66.00	1255	1.26
-66.00	-48.00	3269	3.29
-48.00	-30.00	6661	6.70
-30.00	-12.00	15102	15.18
-12.00	12.00	32401	32.58
12.00	30.00	18659	18.76
30.00	48.00	9084	9.13
48.00	66.00	4118	4.14
66.00	84.00	1649	1.66
84.00	102.00	751	0.76
102.00	120.00	502	0.50

Fuera del crítico superior	3468	3.49
Fuera del crítico inferior	1612	1.62

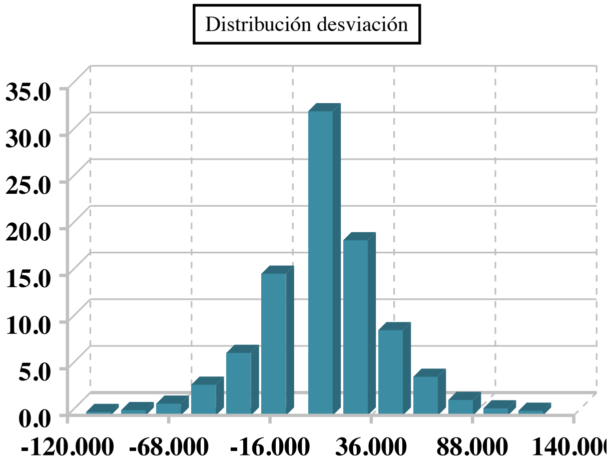

Desviaciones estándar

Distribución (+/-)	# Puntos	%
-6 * Desv. esténd.	422	0.42
-5 * Desv. esténd.	72	0.07
-4 * Desv. esténd.	100	0.10
-3 * Desv. esténd.	101	0.10
-2 * Desv. esténd.	531	0.53
-1 * Desv. esténd.	61076	61.40
1 * Desv. esténd.	34646	34.83
2 * Desv. esténd.	876	0.88
3 * Desv. esténd.	325	0.33
4 * Desv. esténd.	241	0.24
5 * Desv. esténd.	217	0.22
6 * Desv. esténd.	858	0.86

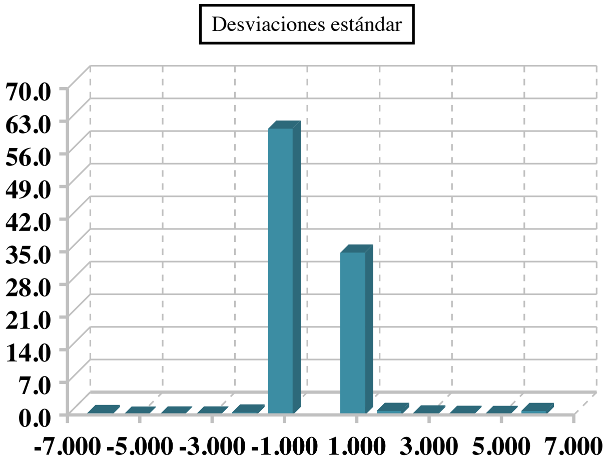

Predefinido: Isométrico

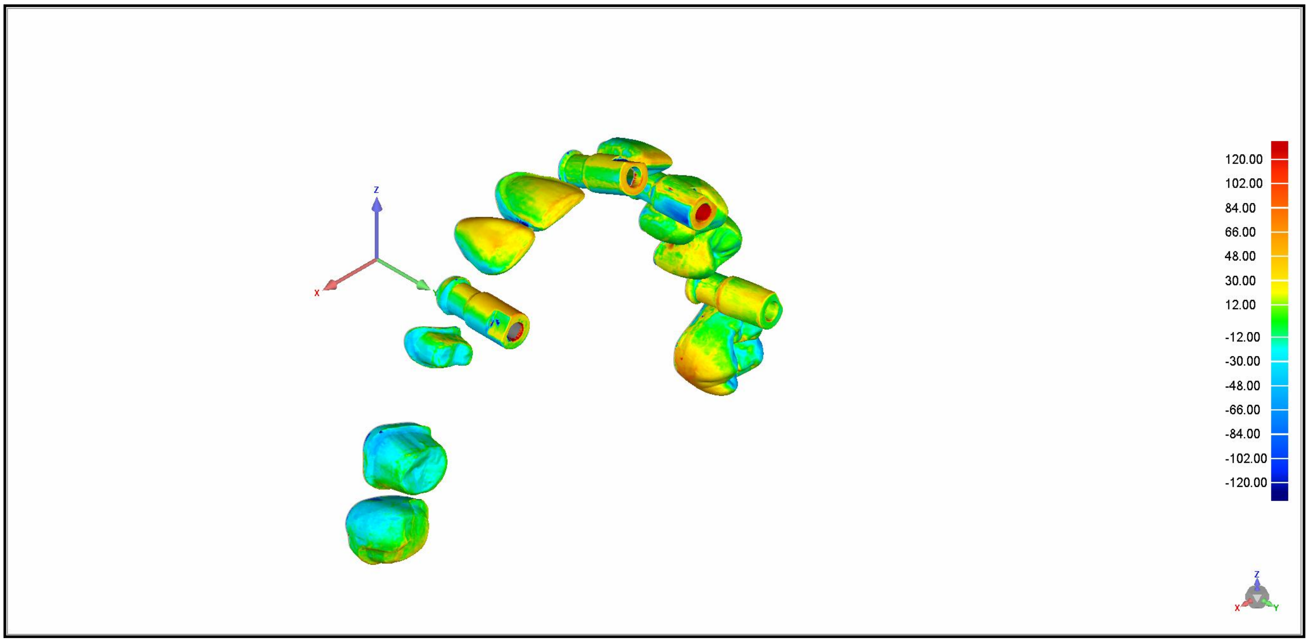

Geomagic Control™

Predefinido: Frente

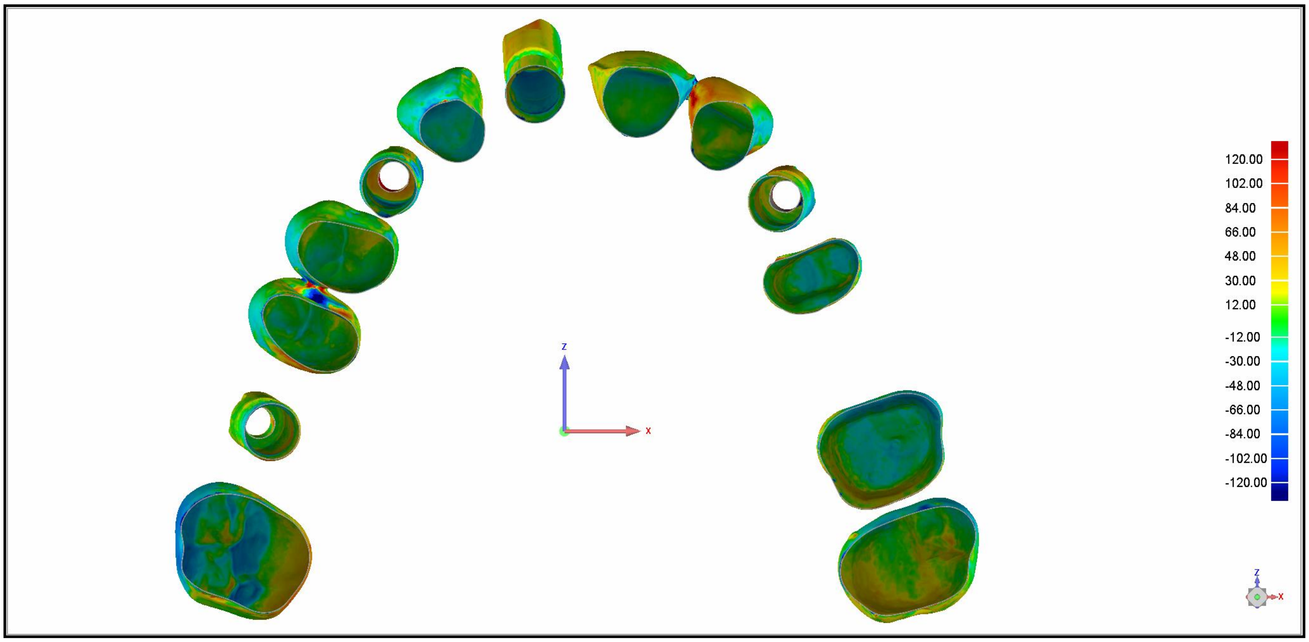

Predefinido: Atrás

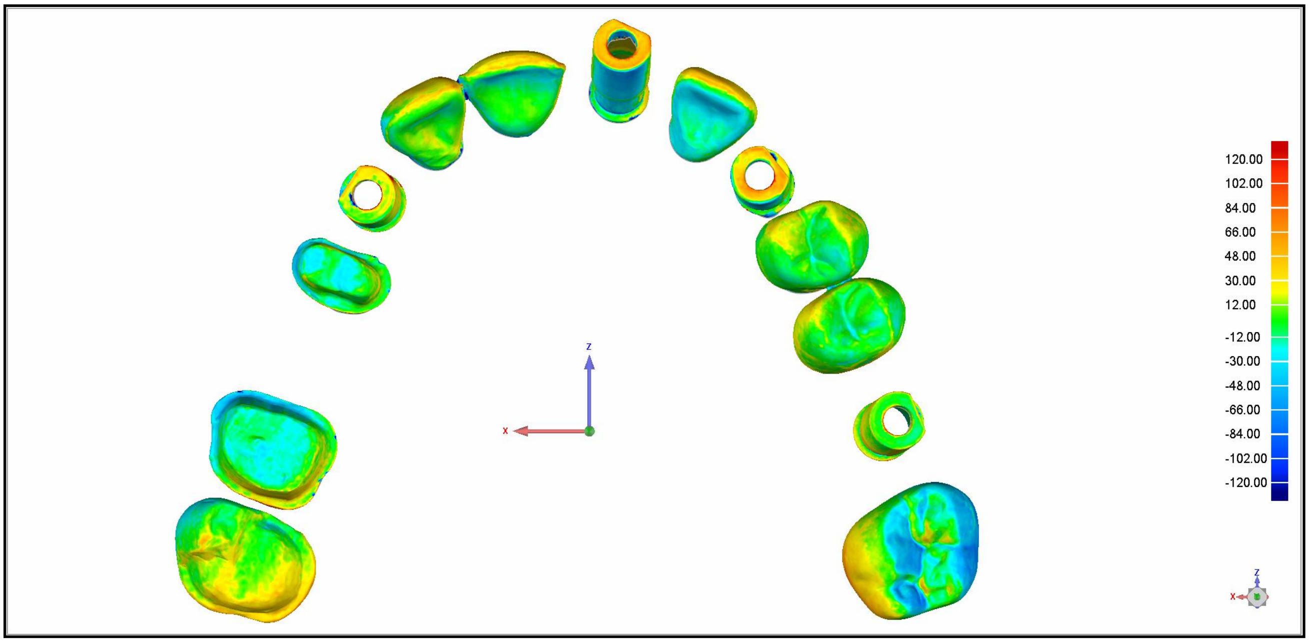

Geomagic Control™

Predefinido: Izquierda

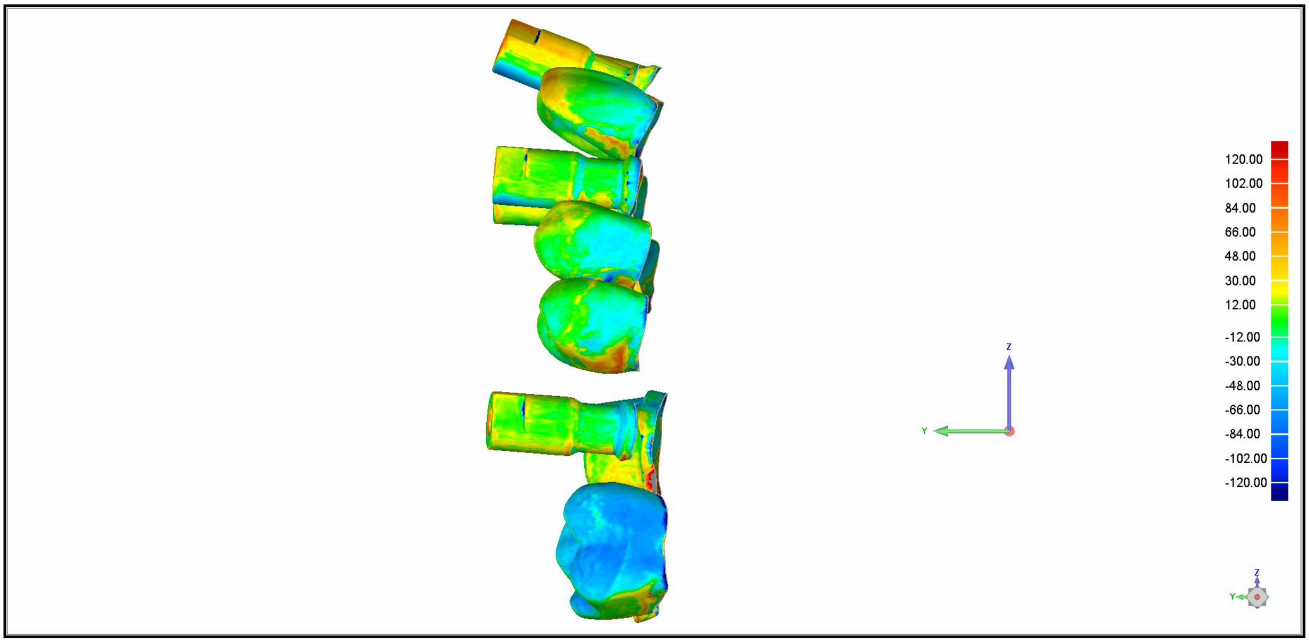

Predefinido: Derecha

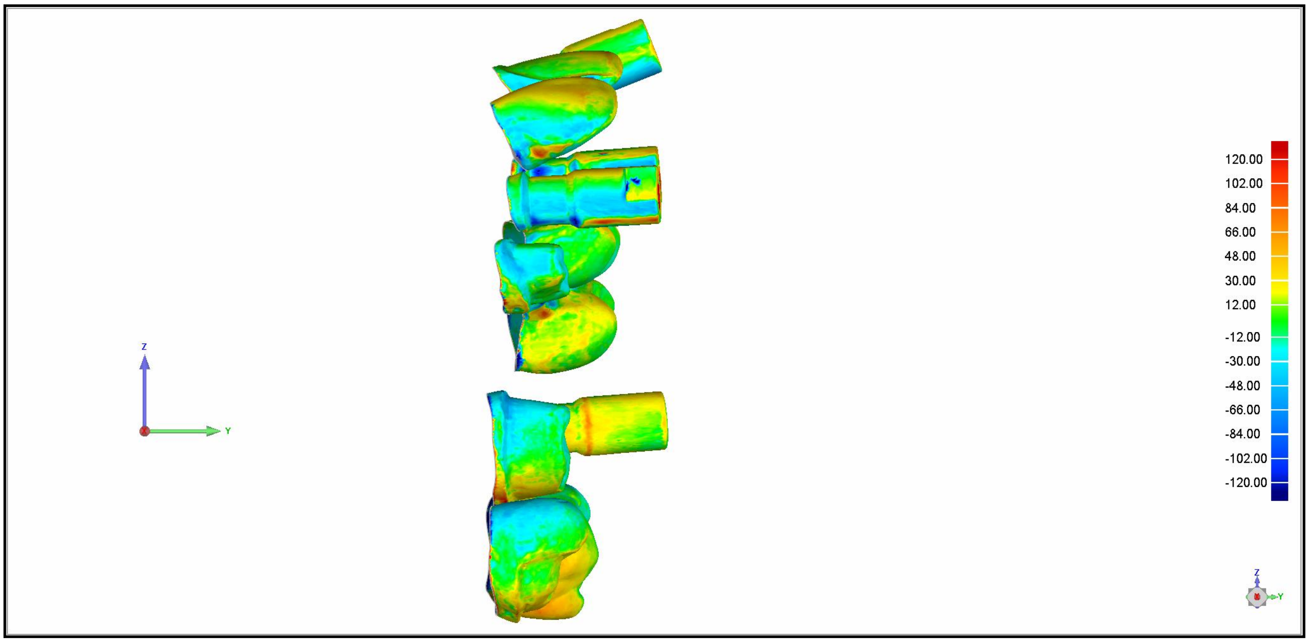

Predefinido: Superior

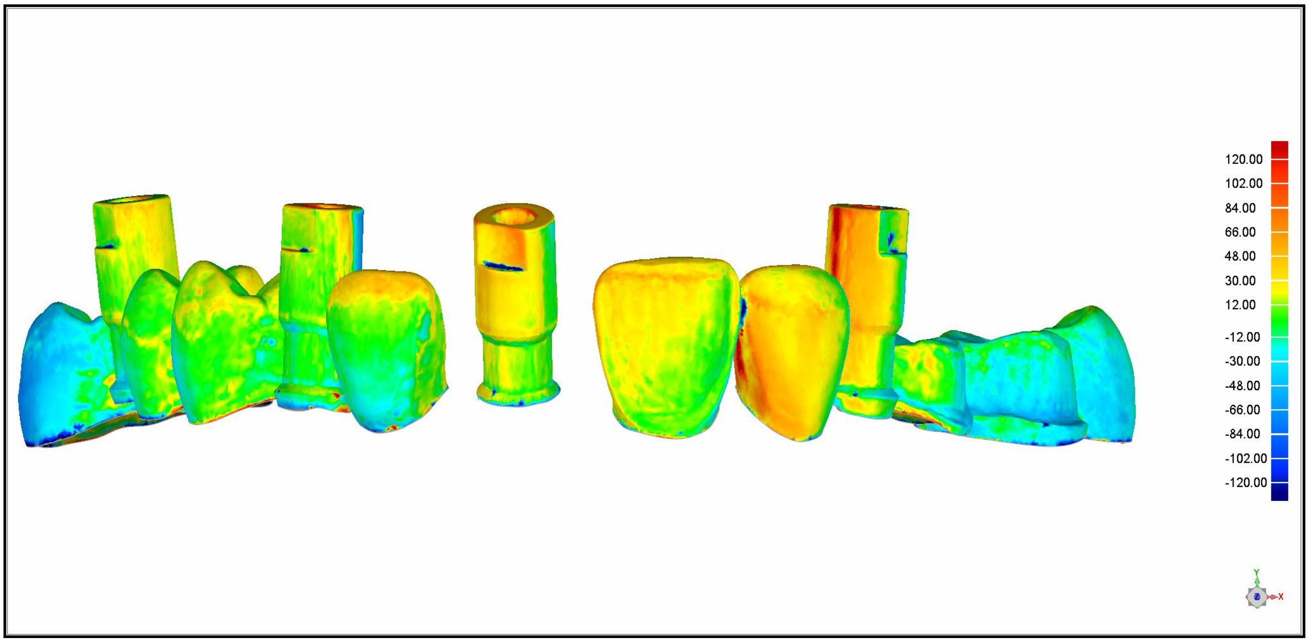

Predefinido: Inferior

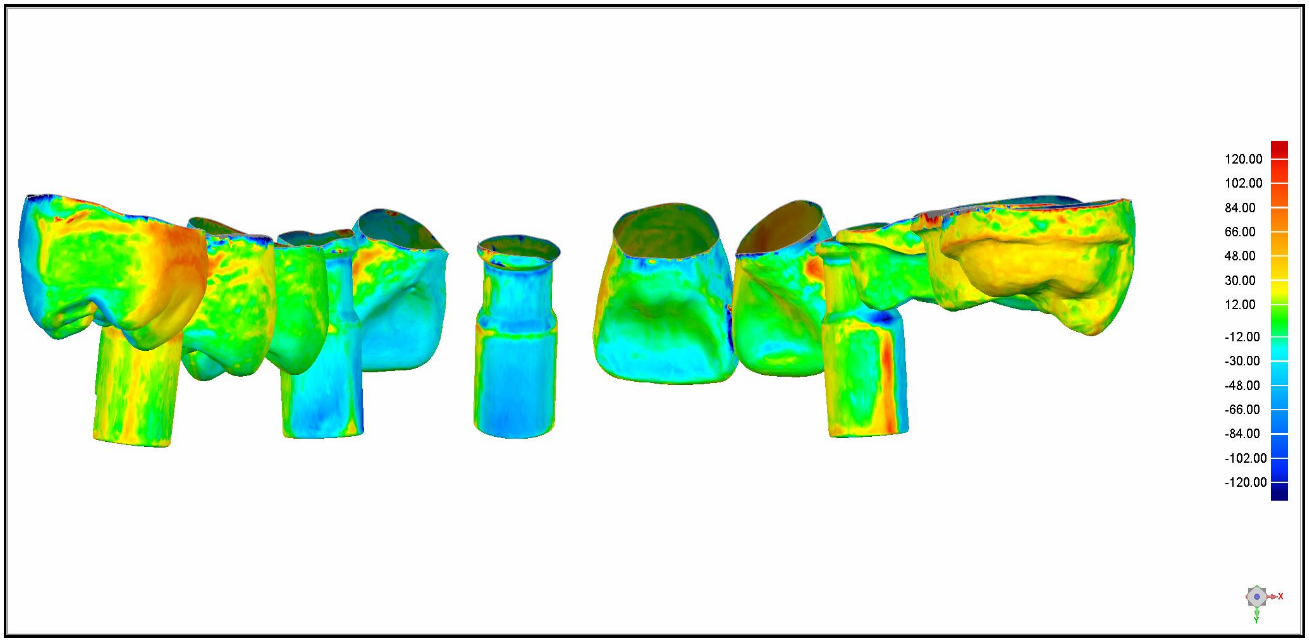

Ajuste de ubicación: Desviaciones superior e inferior

Unidades: u

Nombre	Desv	Estado	Superior Tol	Inferior Tol	Ref X	Ref Y	Ref Z	Radio	Desv X	Desv Y	Desv Z	Medido X	Medido Y	Medido Z	Dir. proy. X	Dir. proy. Y	Dir. proy. Z
Desv. inferior	-2140.78				-14298.94	37919.58	21641.87	n/a	1461.05	-1095.26	-1117.45	-12837.89	36824.32	20524.43	-0.68	0.51	0.52
Desv. superior	3068.01				-12553.67	29785.74	21343.12	n/a	1170.39	-1382.30	2476.31	-11383.27	28403.44	23819.43	0.38	-0.45	0.81
