# Supplementary material for: Accuracy of four digital scanners according to scanning strategy in complete-arch impressions
Source: PLoS One. 2018 Sep 13;13(9):e0202916. doi: 10.1371/journal.pone.0202916 (PMC6136706; doi:10.1371/journal.pone.0202916)

3D Comparación Resultados

Modelo referencia	MRC
Modelo test	3S4B
Nº de puntos de datos	104026
# Aislados	62

Tipo tolerancia	3D desviación
Unidades	u
Máx. crítico	120.00
Máx. nominal	12.00
Mín. nominal	-12.00
Mín. crítico	-120.00

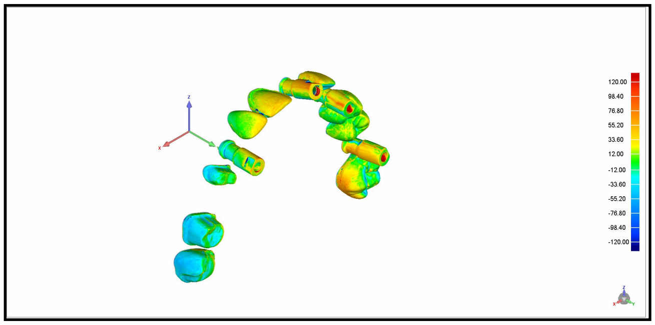

Desviación	
Desviación superior máx.	3122.01
Desviación inferior máx.	-3091.48
Desviación media	61.04 /-46.75
Desviación estándar	181.53

Distribución desviación

>=Min	<Max	# Puntos	%
-120.00	-98.40	348	0.33
-98.40	-76.80	677	0.65
-76.80	-55.20	1585	1.52
-55.20	-33.60	7840	7.54
-33.60	-12.00	15776	15.17
-12.00	12.00	33413	32.12
12.00	33.60	22971	22.08
33.60	55.20	10538	10.13
55.20	76.80	3646	3.50
76.80	98.40	989	0.95
98.40	120.00	475	0.46

Fuera del crítico superior	3937	3.78
Fuera del crítico inferior	1831	1.76

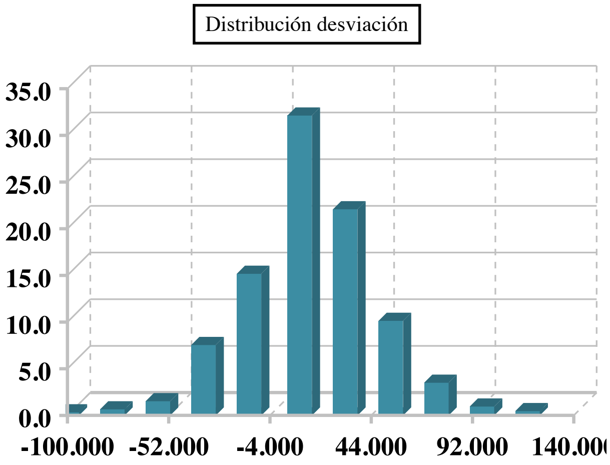

Desviaciones estándar

Distribución (+/-)	# Puntos	%
-6 * Desv. estánd.	384	0.37
-5 * Desv. estánd.	127	0.12
-4 * Desv. estánd.	162	0.16
-3 * Desv. estánd.	158	0.15
-2 * Desv. estánd.	537	0.52
-1 * Desv. estánd.	66650	64.07
1 * Desv. estánd.	33106	31.82
2 * Desv. estánd.	761	0.73
3 * Desv. estánd.	416	0.40
4 * Desv. estánd.	397	0.38
5 * Desv. estánd.	384	0.37
6 * Desv. estánd.	944	0.91

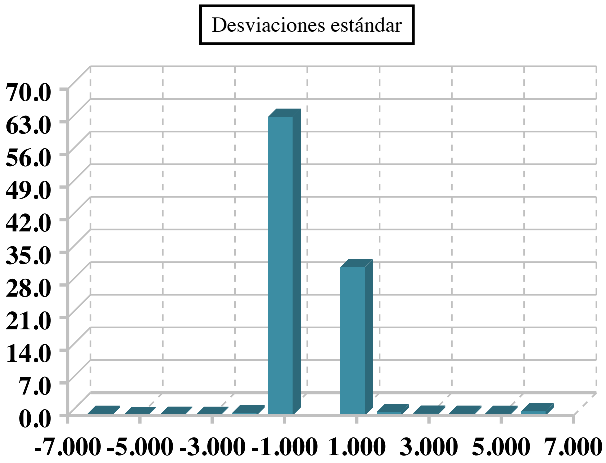

Predefinido: Isométrico

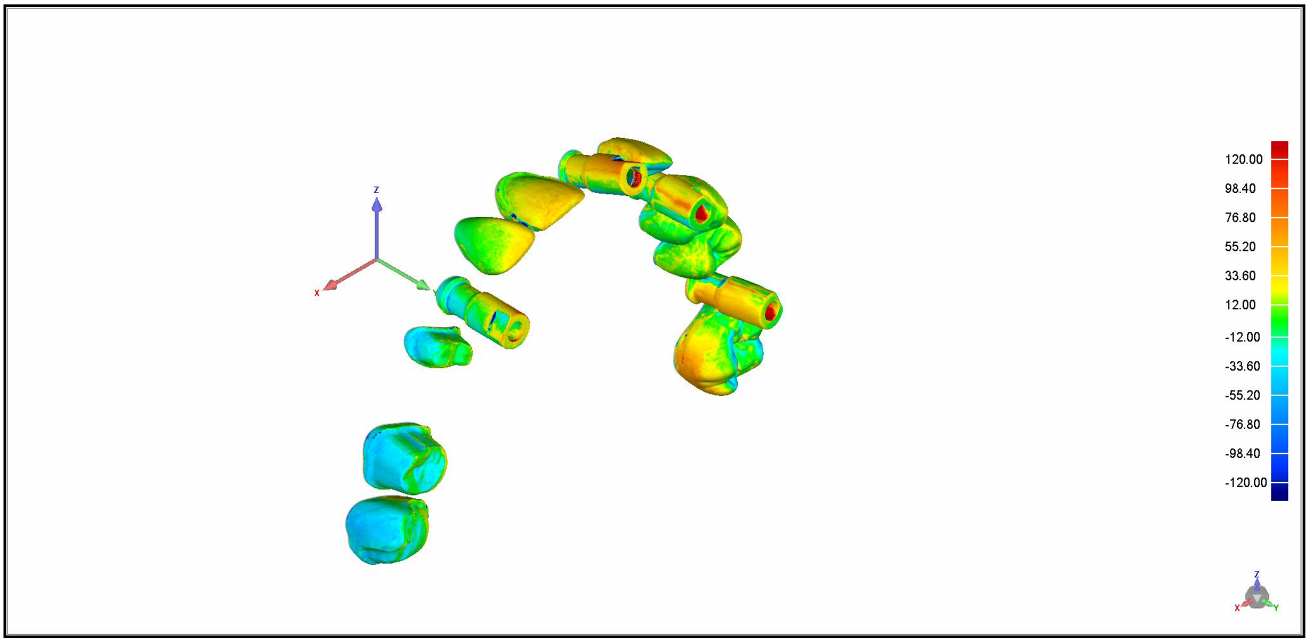

Predefinido: Frente

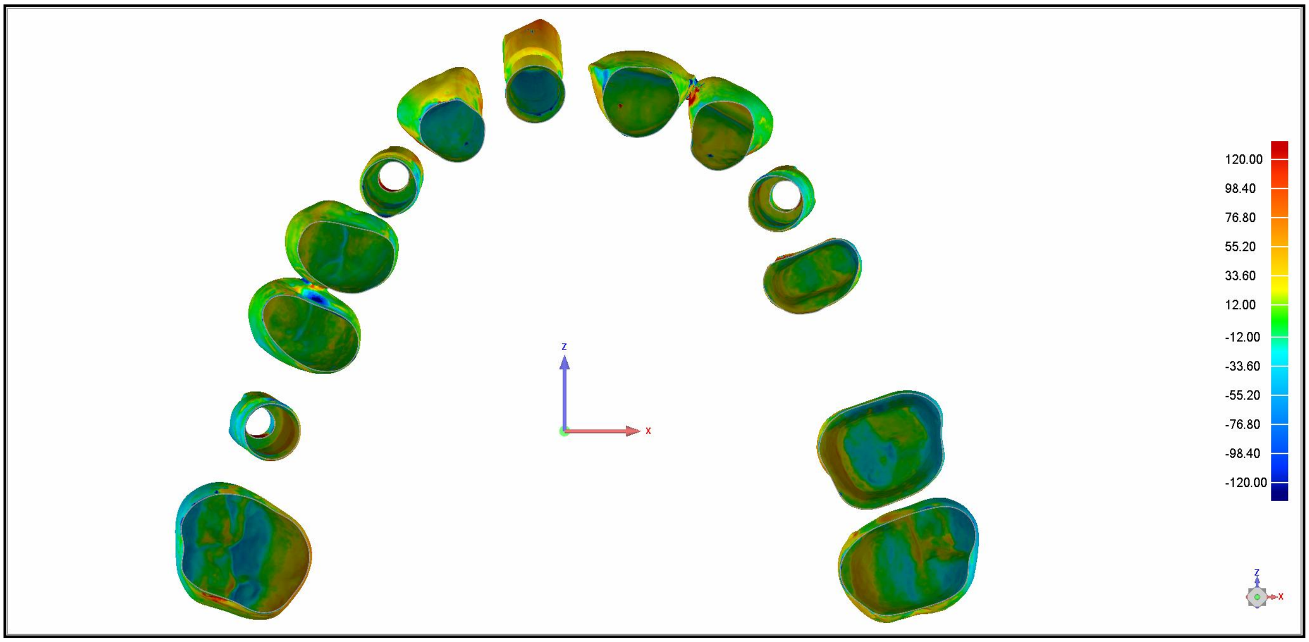

Predefinido: Atrás

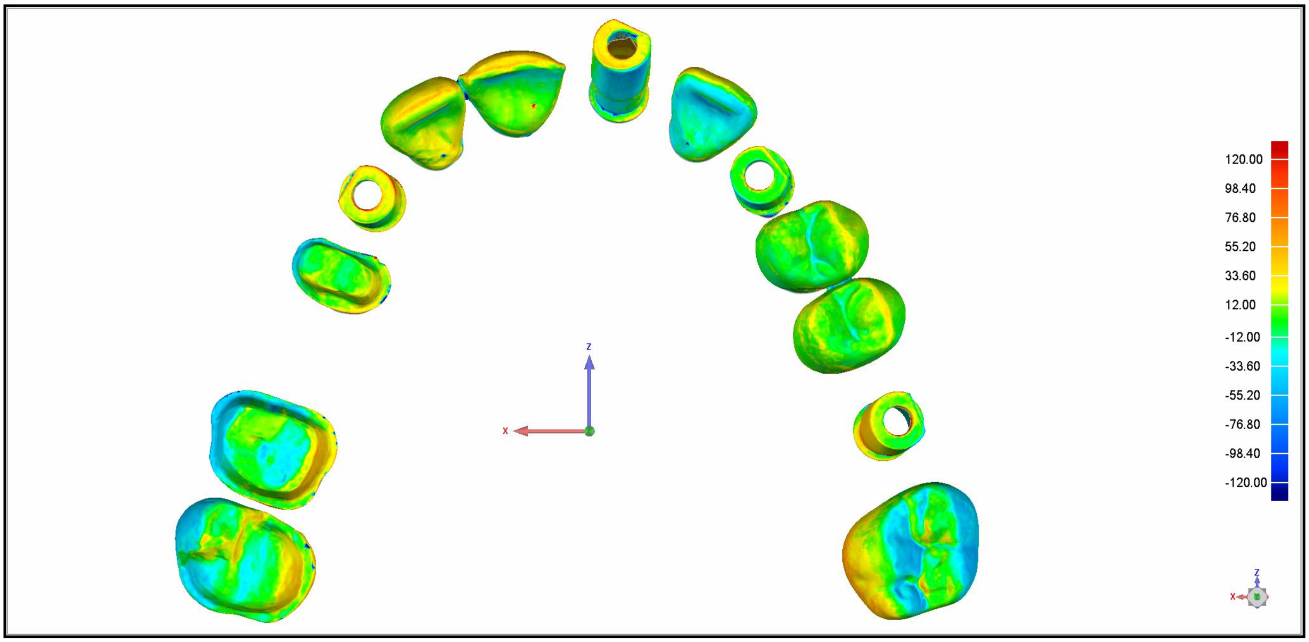

Predefinido: Izquierda

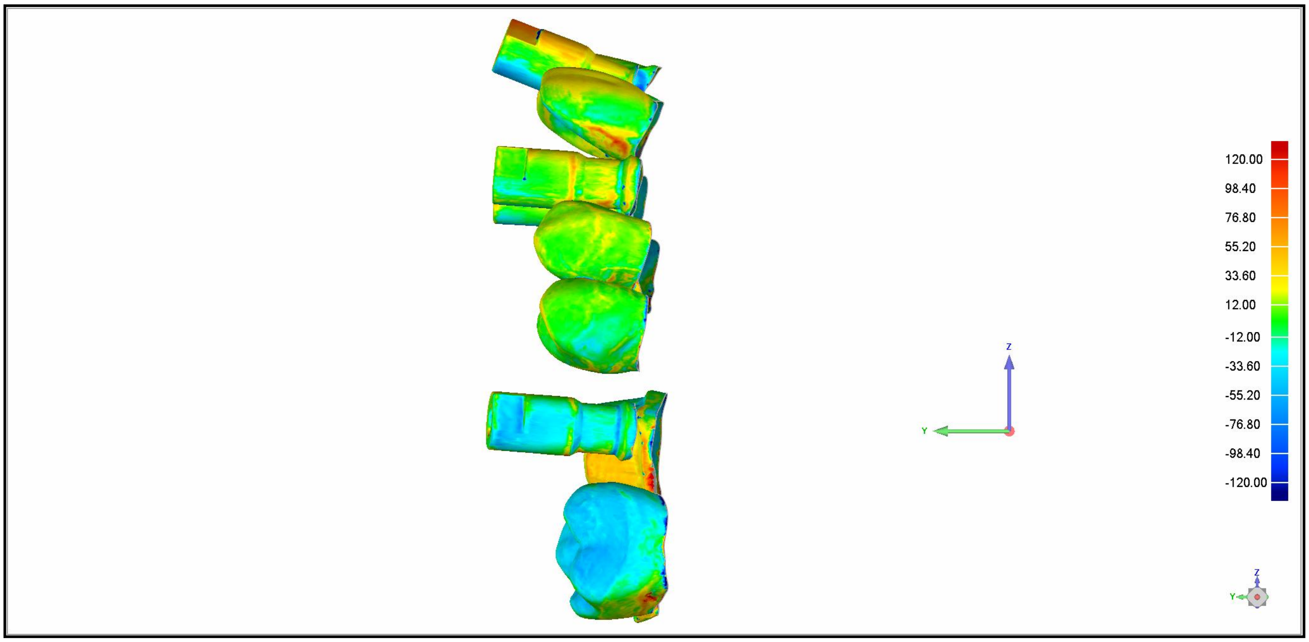

Predefinido: Derecha

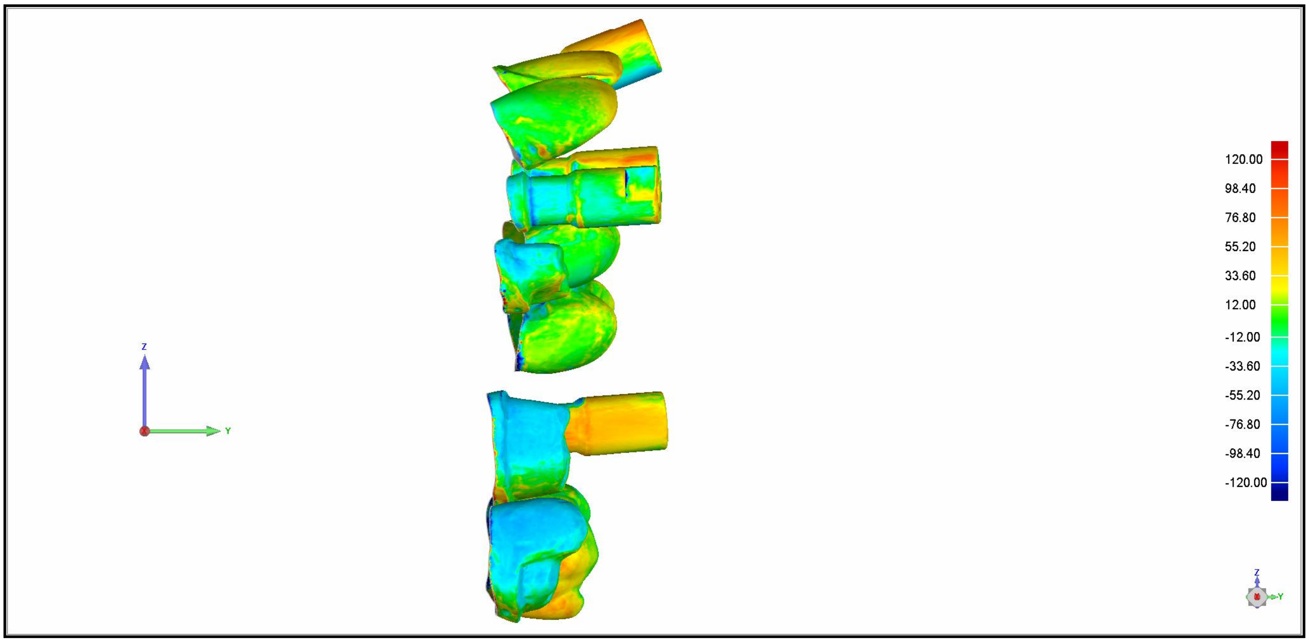

Predefinido: Superior

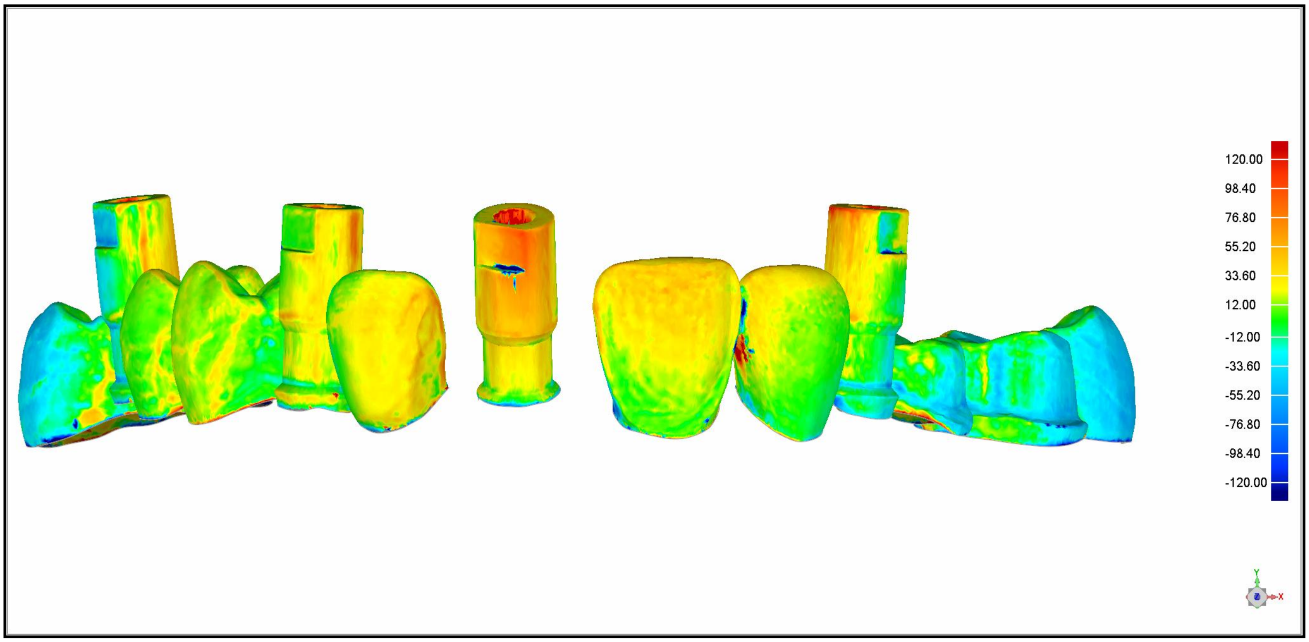

Predefinido: Inferior

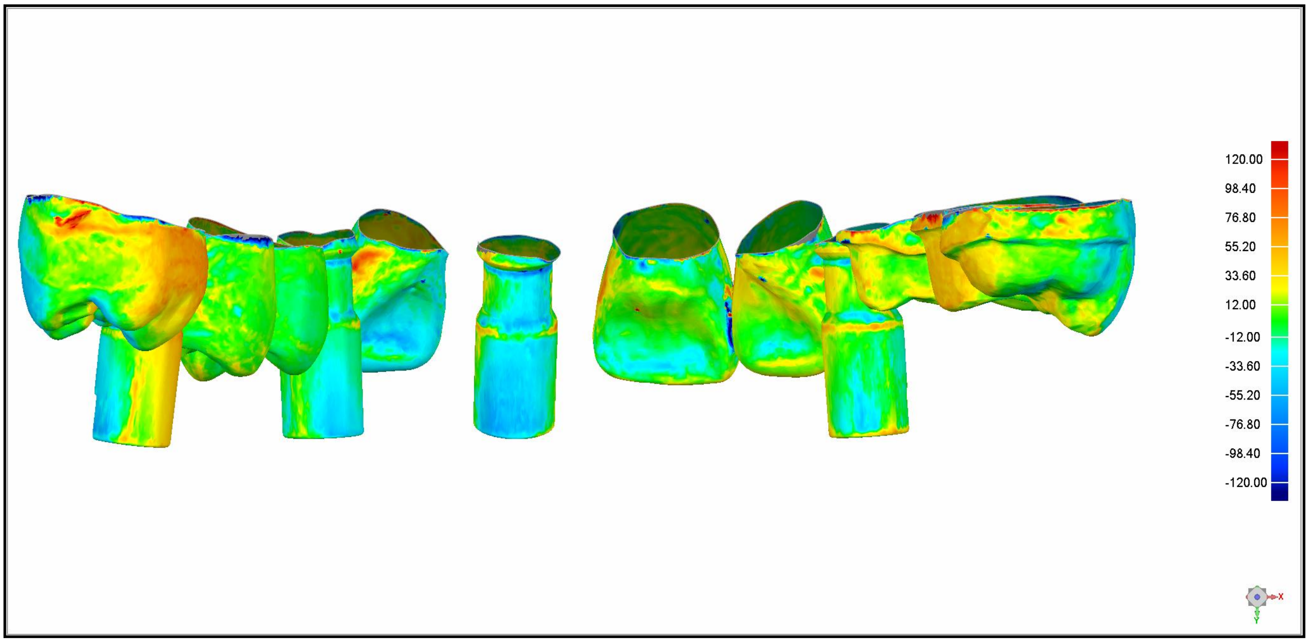

Supplement: S2 Table — Trios (scanning strategy B). (ZIP) [file pone.0202916.s002.zip › S2/3S4B.pdf]
