# Supplementary material for: Accuracy of four digital scanners according to scanning strategy in complete-arch impressions
Source: PLoS One. 2018 Sep 13;13(9):e0202916. doi: 10.1371/journal.pone.0202916 (PMC6136706; doi:10.1371/journal.pone.0202916)

3D Comparación Resultados

Modelo referencia	MRC
Modelo test	3S8B
Nº de puntos de datos	102029
# Aislados	38

Tipo tolerancia	3D desviación
Unidades	u
Máx. crítico	120.00
Máx. nominal	14.00
Mín. nominal	-14.00
Mín. crítico	-120.00

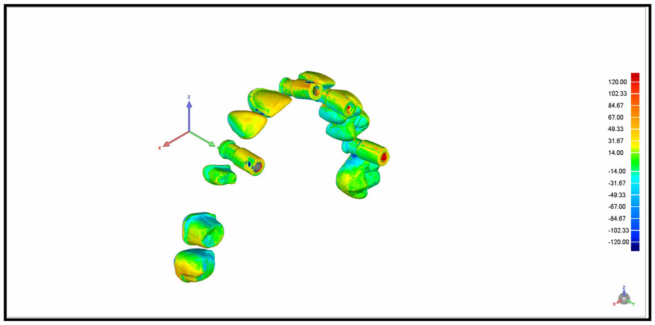

Desviación	
Desviación superior máx.	3043.07
Desviación inferior máx.	-3086.29
Desviación media	58.61 /-44.54
Desviación estándar	188.58

Distribución desviación

>=Min	<Max	# Puntos	%
-120.00	-102.33	333	0.33
-102.33	-84.67	429	0.42
-84.67	-67.00	655	0.64
-67.00	-49.33	1242	1.22
-49.33	-31.67	4282	4.20
-31.67	-14.00	14615	14.32
-14.00	14.00	42563	41.72
14.00	31.67	18602	18.23
31.67	49.33	8055	7.89
49.33	67.00	2930	2.87
67.00	84.67	1284	1.26
84.67	102.33	730	0.72
102.33	120.00	520	0.51

Fuera del crítico superior	3895	3.82
Fuera del crítico inferior	1894	1.86

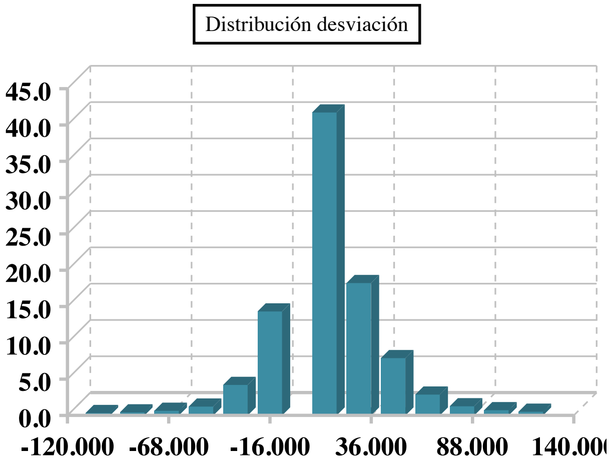

Desviaciones estándar

Distribución (+/-)	# Puntos	%
-6 * Desv. estánd.	489	0.48
-5 * Desv. estánd.	68	0.07
-4 * Desv. estánd.	87	0.09
-3 * Desv. estánd.	136	0.13
-2 * Desv. estánd.	520	0.51
-1 * Desv. estánd.	64770	63.48
1 * Desv. estánd.	33357	32.69
2 * Desv. estánd.	864	0.85
3 * Desv. estánd.	390	0.38
4 * Desv. estánd.	300	0.29
5 * Desv. estánd.	231	0.23
6 * Desv. estánd.	817	0.80

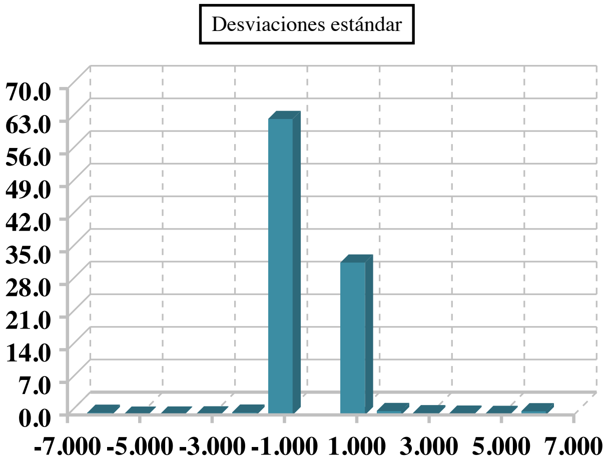

Predefinido: Isométrico

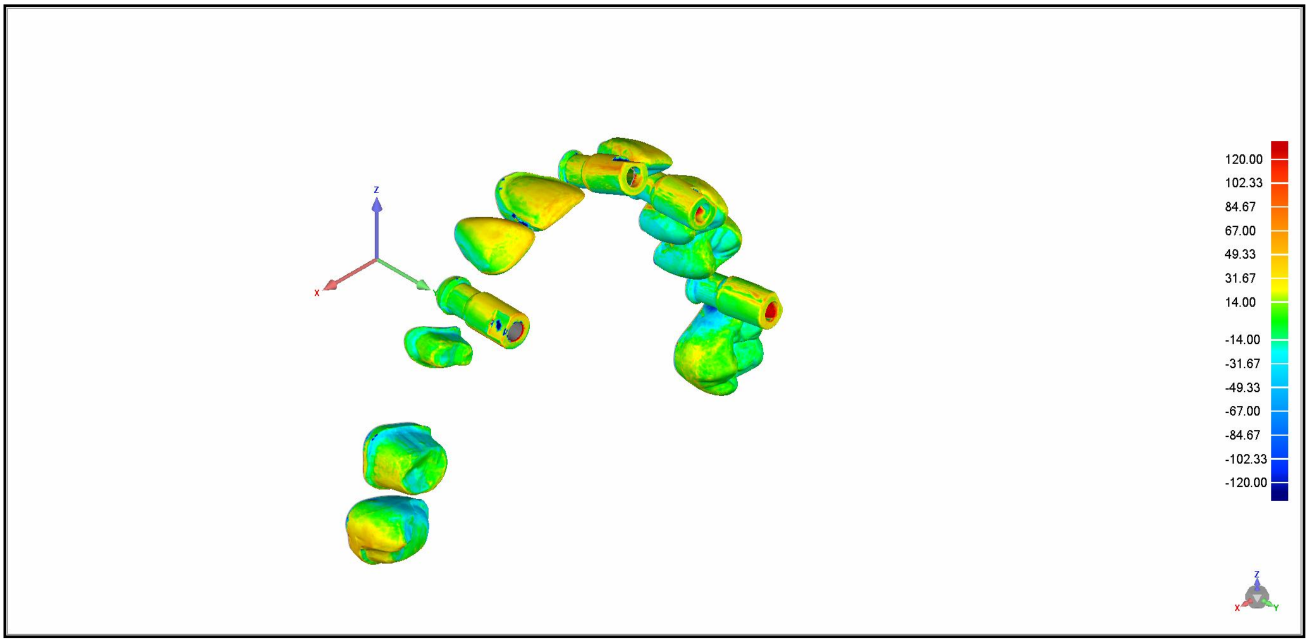

Predefinido: Frente

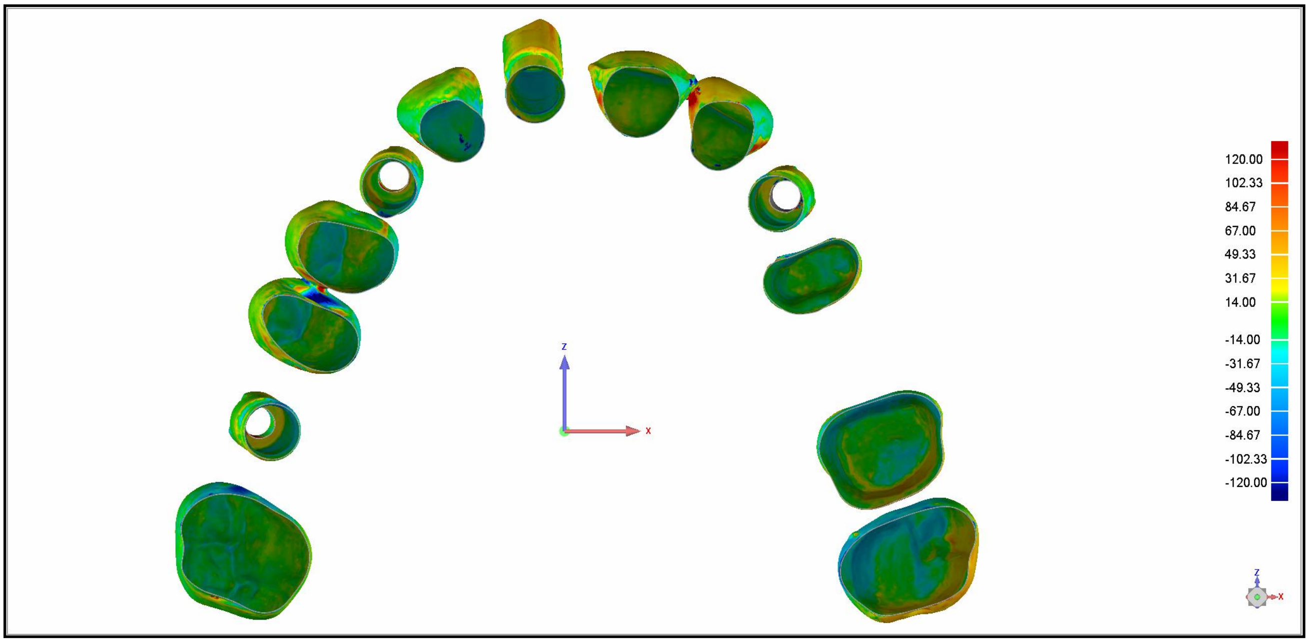

Predefinido: Atrás

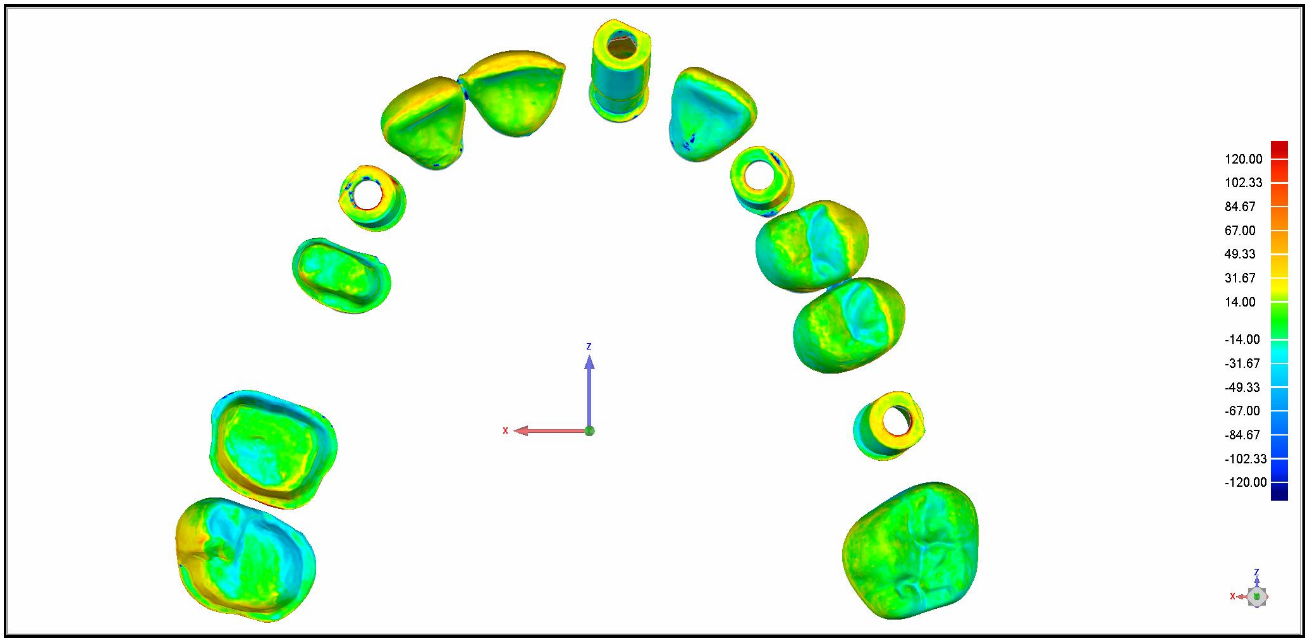

Predefinido: Izquierda

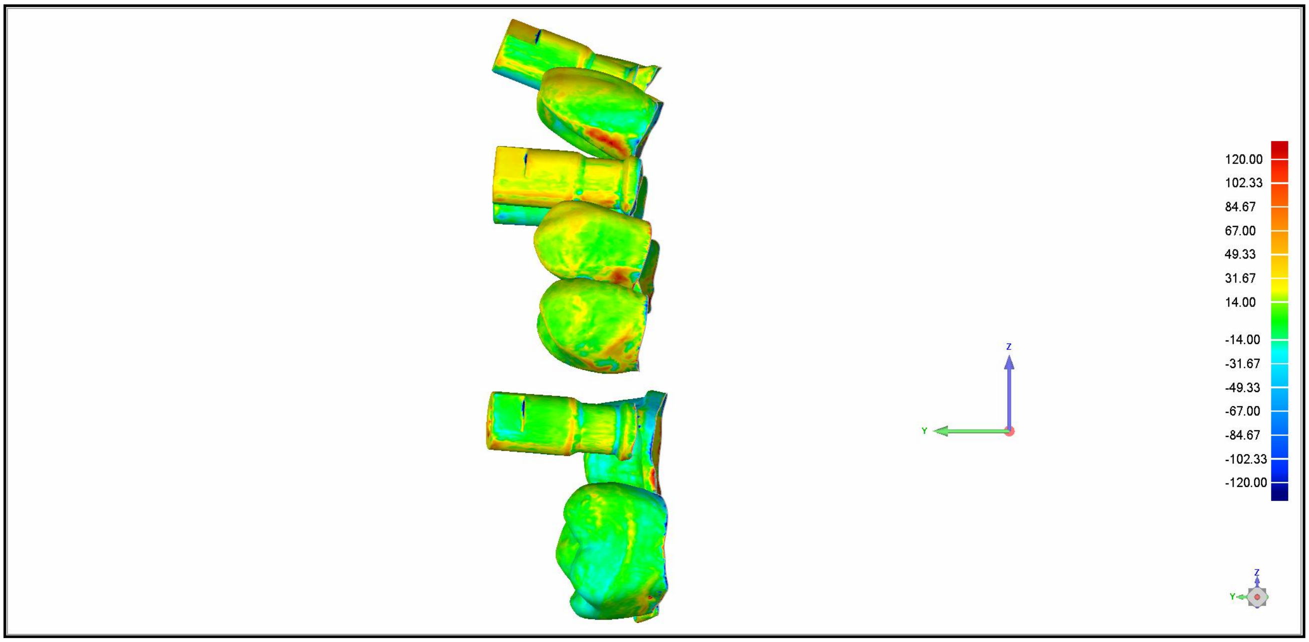

Predefinido: Derecha

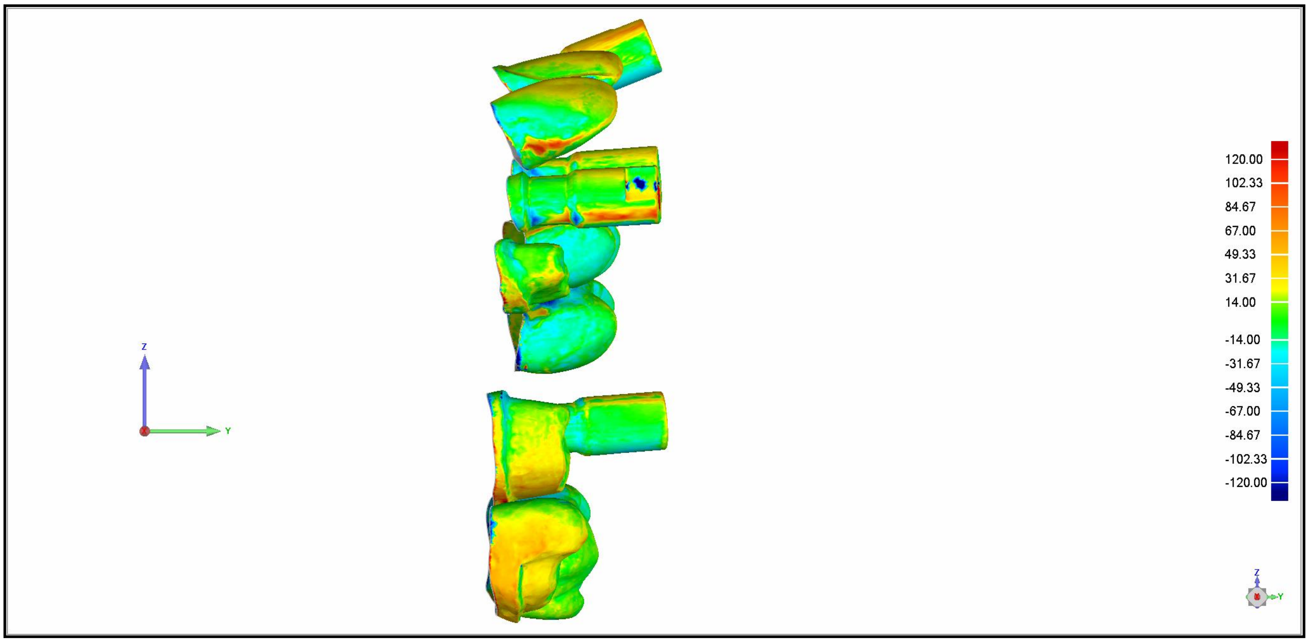

Predefinido: Superior

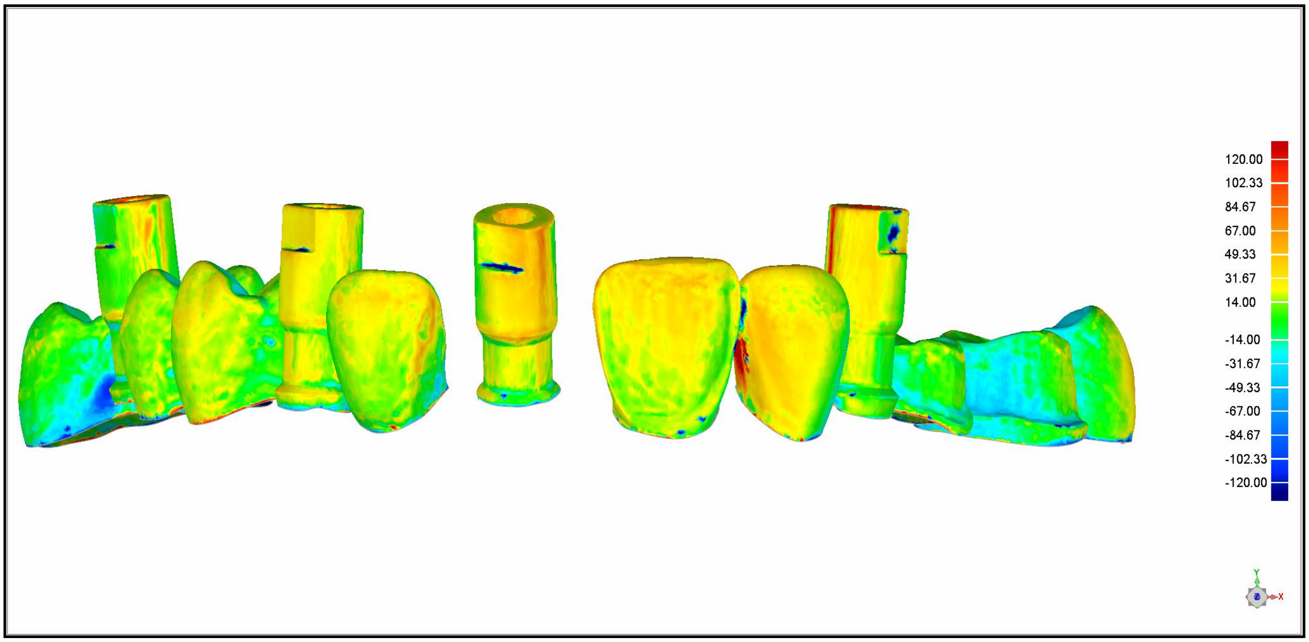

Predefinido: Inferior

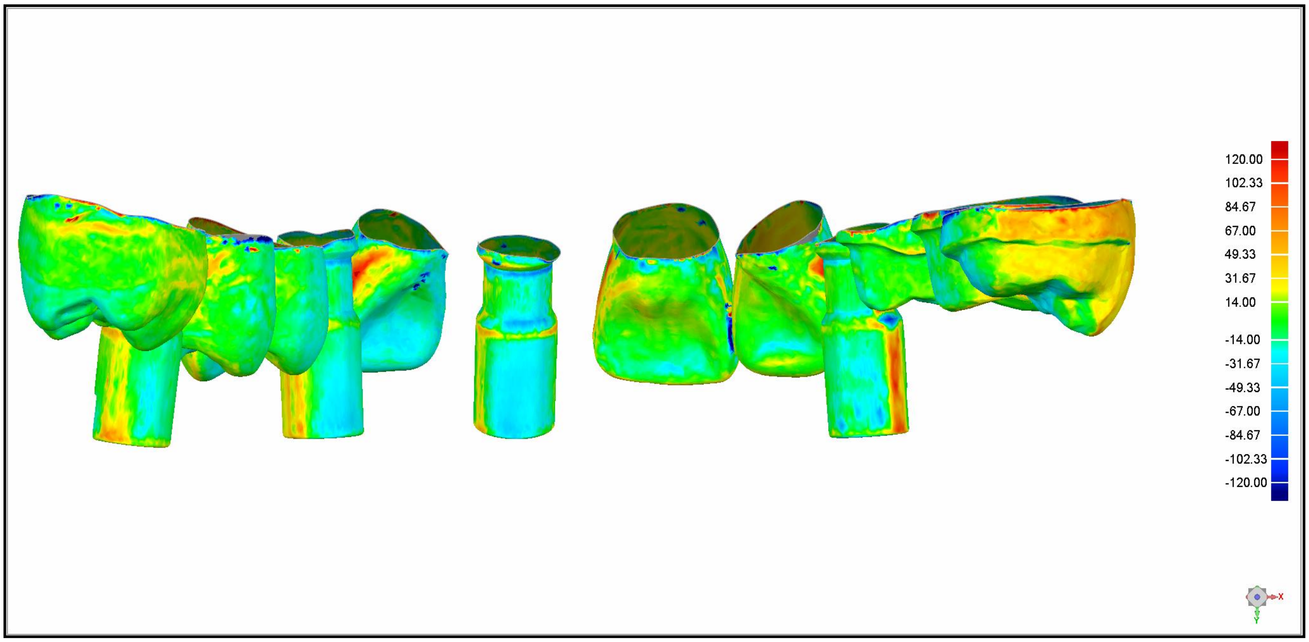

Supplement: S2 Table — Trios (scanning strategy B). (ZIP) [file pone.0202916.s002.zip › S2/3S8B.pdf]
