# Supplementary material for: Accuracy of four digital scanners according to scanning strategy in complete-arch impressions
Source: PLoS One. 2018 Sep 13;13(9):e0202916. doi: 10.1371/journal.pone.0202916 (PMC6136706; doi:10.1371/journal.pone.0202916)

Fecha: 3/6/2017, 12:43 pm

3D Comparación Resultados

Modelo referencia	MRC
Modelo test	3S9B
Nº de puntos de datos	102492
# Aislados	74

Tipo tolerancia	3D desviación
Unidades	u
Máx. crítico	120.00
Máx. nominal	17.00
Mín. nominal	-17.00
Mín. crítico	-120.00

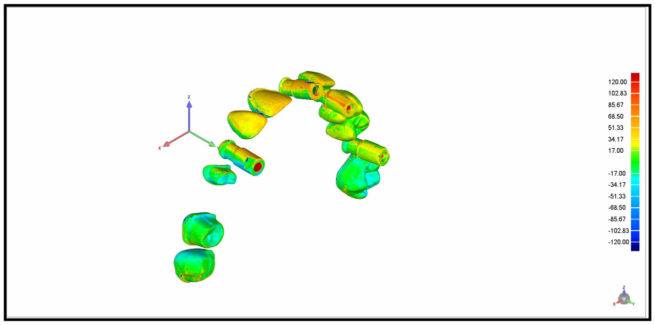

Desviación	
Desviación superior máx.	2968.28
Desviación inferior máx.	-3143.26
Desviación media	60.22 /-50.54
Desviación estándar	200.28

Distribución desviación

>=Min	<Max	# Puntos	%
-120.00	-102.83	267	0.26
-102.83	-85.67	342	0.33
-85.67	-68.50	537	0.52
-68.50	-51.33	950	0.93
-51.33	-34.17	2299	2.24
-34.17	-17.00	9994	9.75
-17.00	17.00	50622	49.39
17.00	34.17	17016	16.60
34.17	51.33	9013	8.79
51.33	68.50	3336	3.25
68.50	85.67	1138	1.11
85.67	102.83	531	0.52
102.83	120.00	392	0.38

Fuera del crítico superior	4069	3.97
Fuera del crítico inferior	1986	1.94

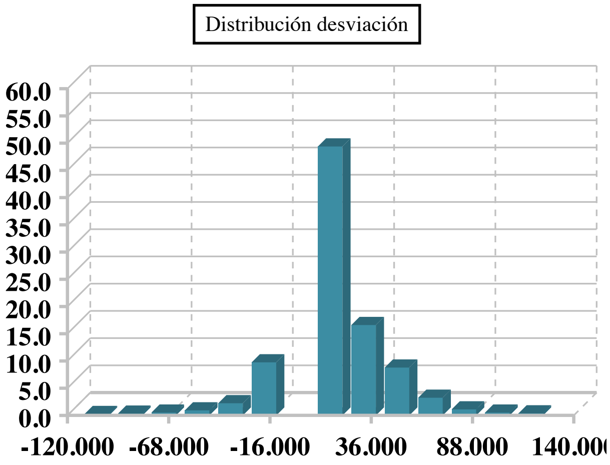

Desviaciones estándar

Distribución (+/-)	# Puntos	%
-6 * Desv. estánd.	528	0.52
-5 * Desv. estánd.	110	0.11
-4 * Desv. estánd.	132	0.13
-3 * Desv. estánd.	181	0.18
-2 * Desv. estánd.	528	0.52
-1 * Desv. estánd.	65912	64.31
1 * Desv. estánd.	32306	31.52
2 * Desv. estánd.	793	0.77
3 * Desv. estánd.	432	0.42
4 * Desv. estánd.	429	0.42
5 * Desv. estánd.	328	0.32
6 * Desv. estánd.	813	0.79

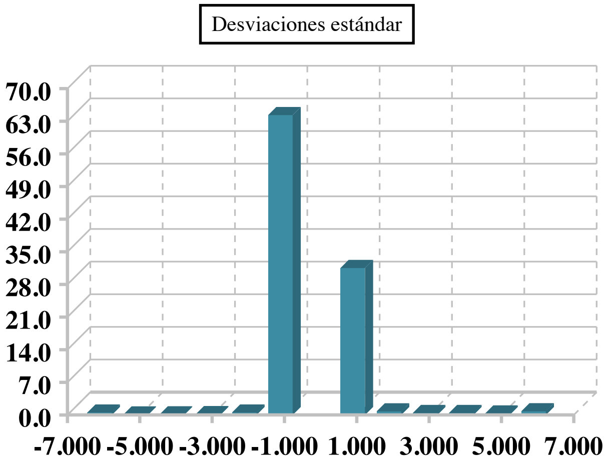

Fecha: 3/6/2017, 12:43 pm

Predefinido: Isométrico

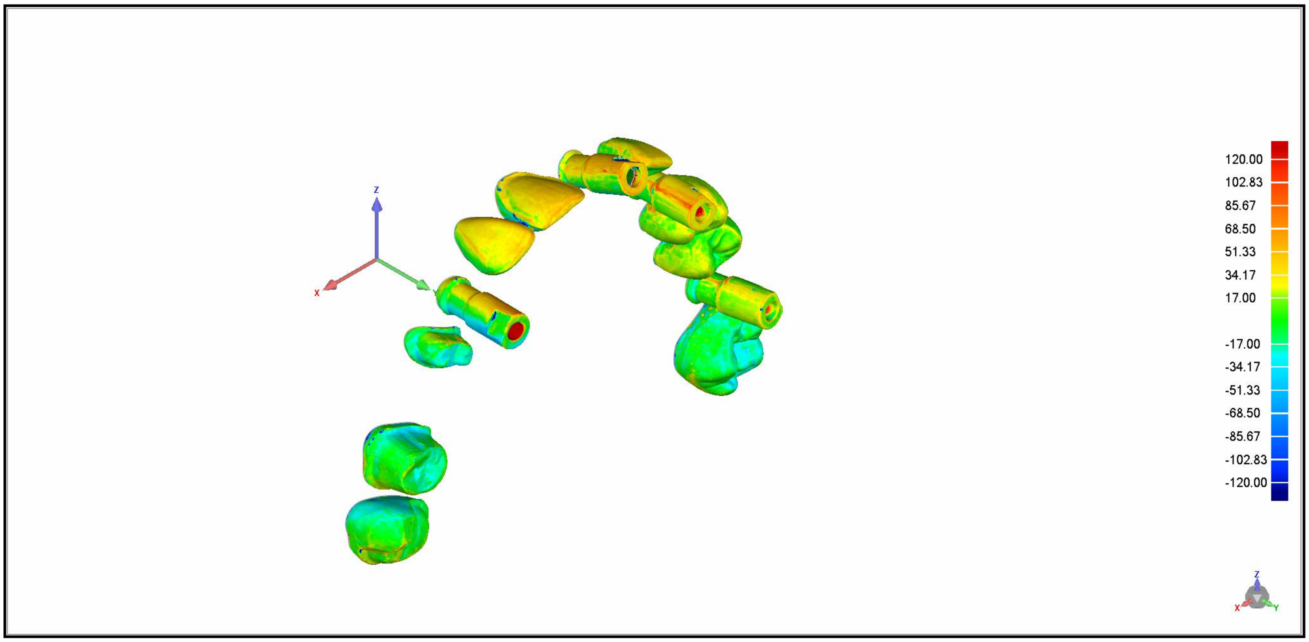

Predefinido: Frente

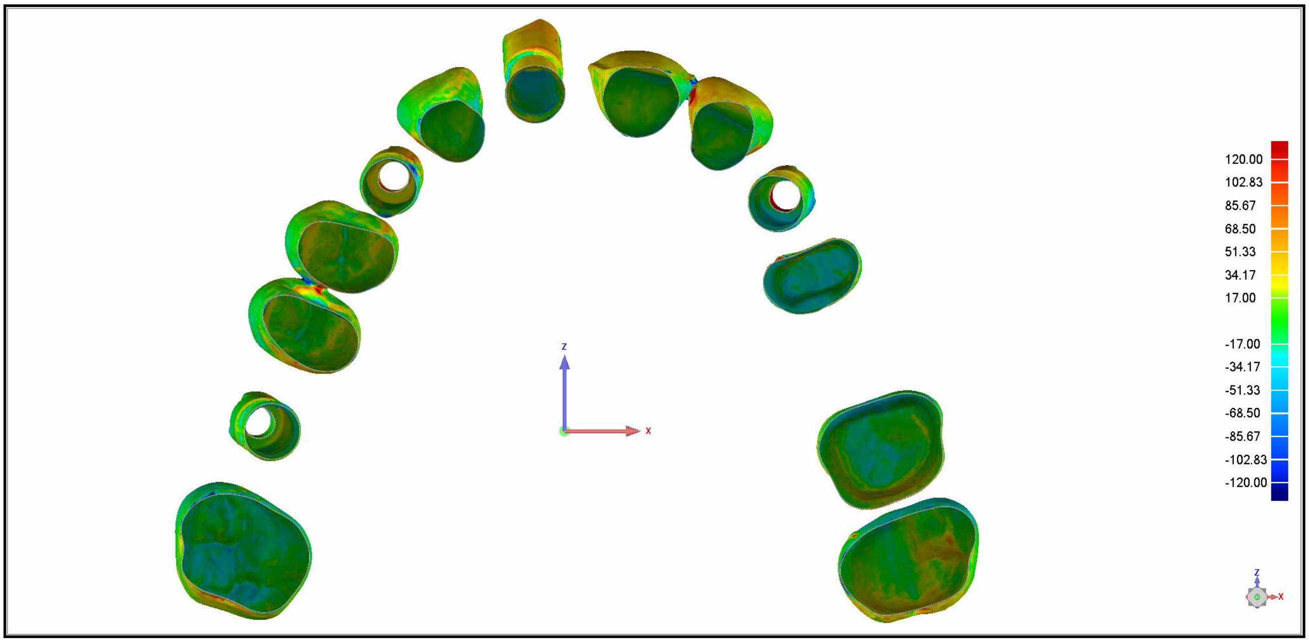

Predefinido: Atrás

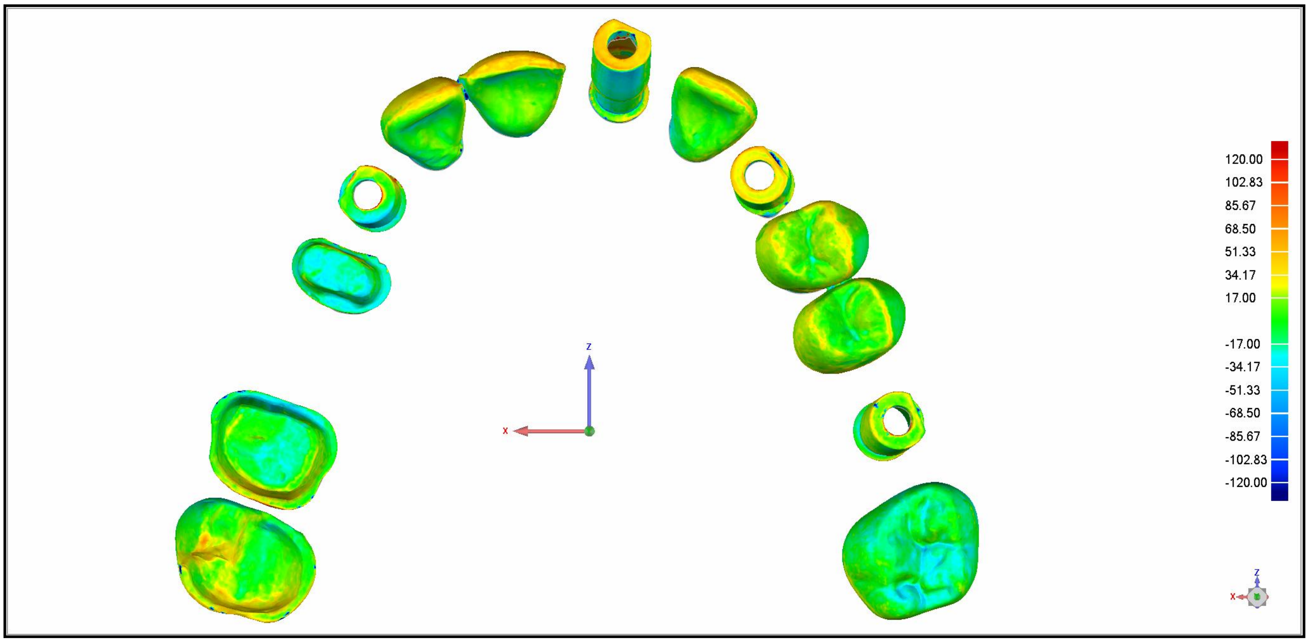

Predefinido: Izquierda

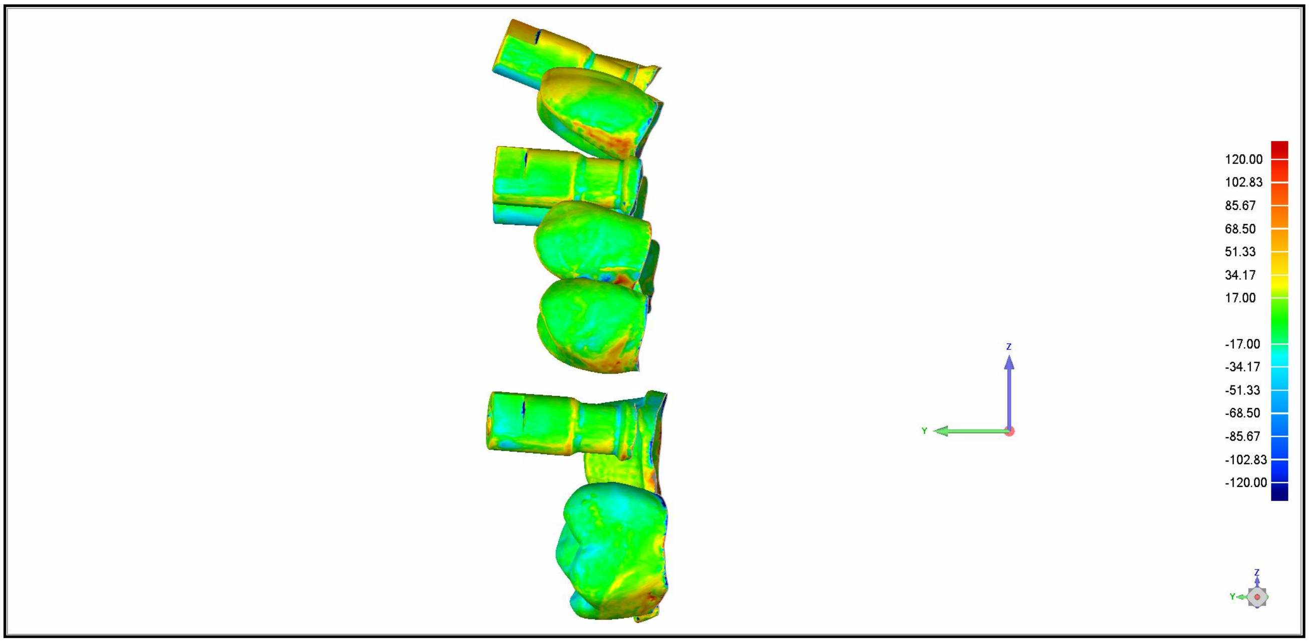

Predefinido: Derecha

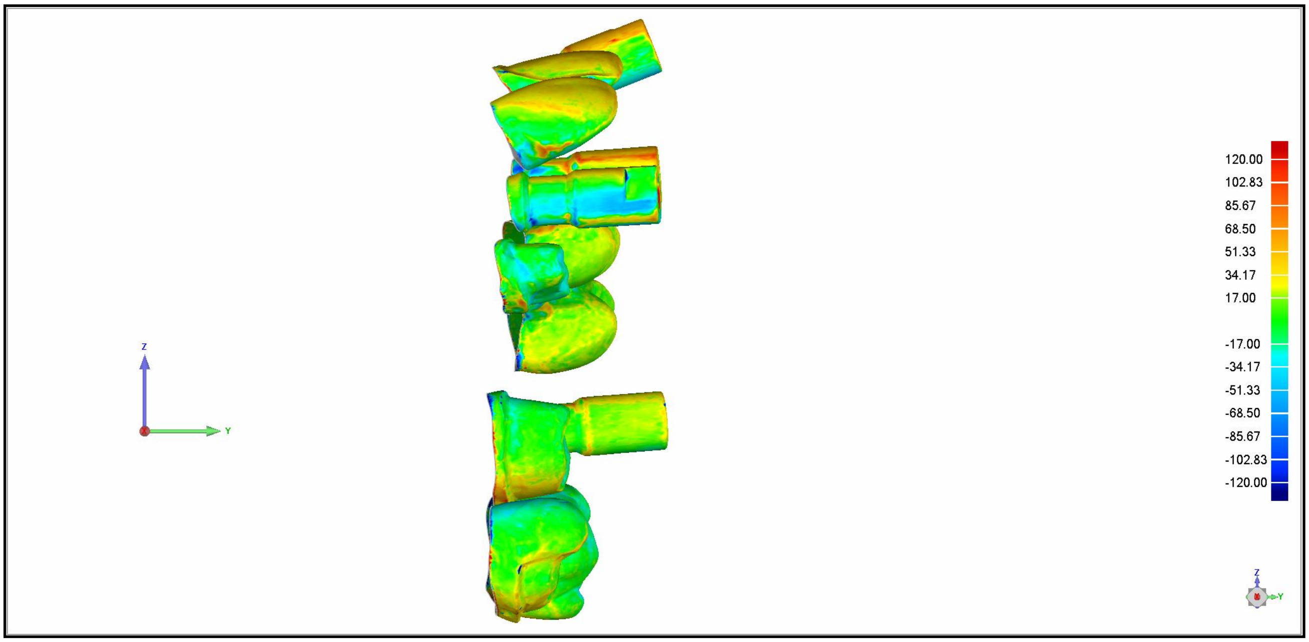

Predefinido: Superior

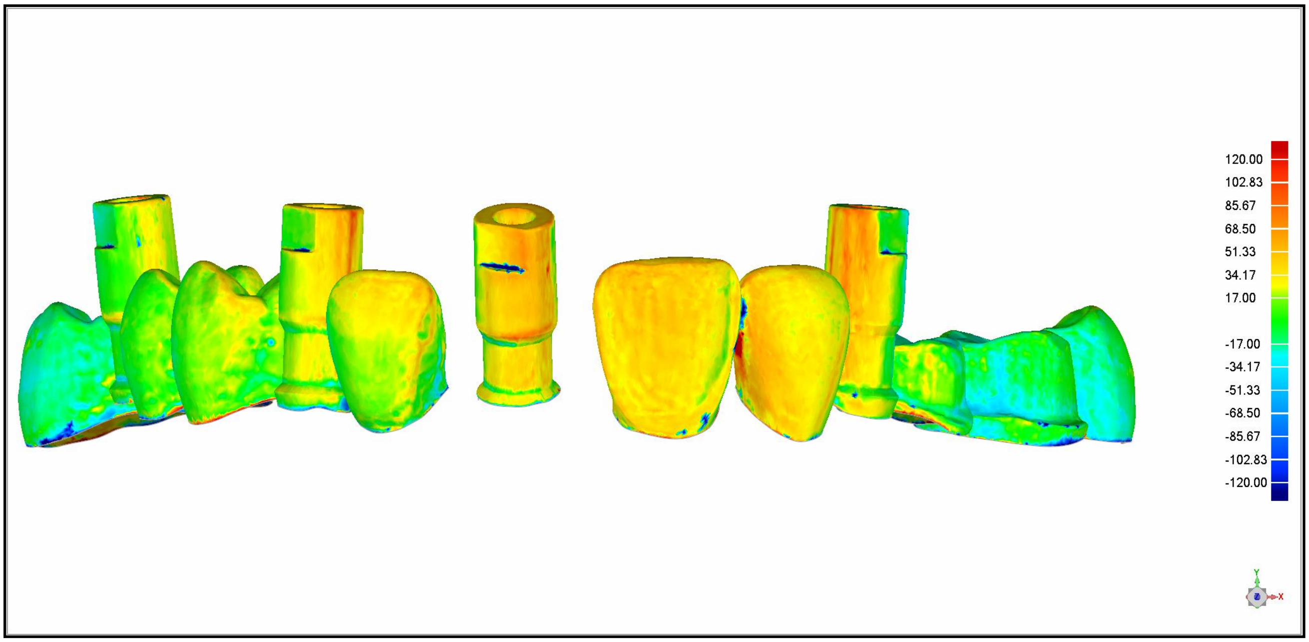

Predefinido: Inferior

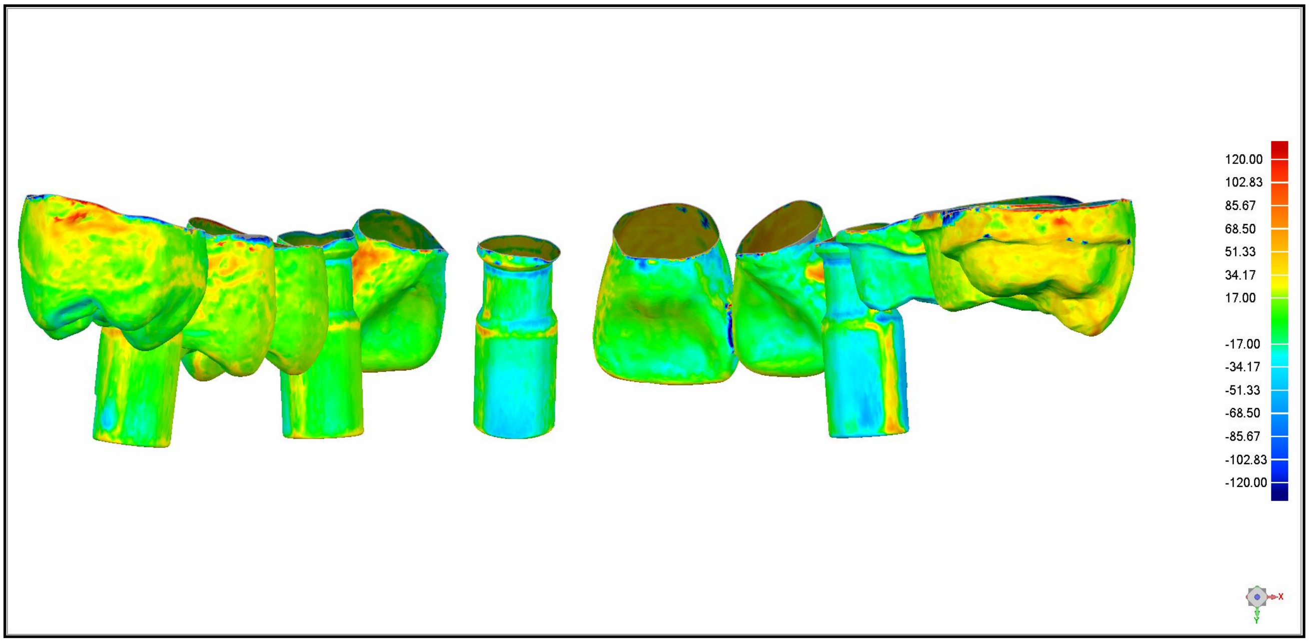

Supplement: S2 Table — Trios (scanning strategy B). (ZIP) [file pone.0202916.s002.zip › S2/3S9B.pdf]
