# Supplementary material for: Accuracy of four digital scanners according to scanning strategy in complete-arch impressions
Source: PLoS One. 2018 Sep 13;13(9):e0202916. doi: 10.1371/journal.pone.0202916 (PMC6136706; doi:10.1371/journal.pone.0202916)

Fecha: 3/6/2017, 12:21 pm

3D Comparación Resultados

Modelo referencia	MRC
Modelo test	3S5B
Nº de puntos de datos	106032
# Aislados	78

Tipo tolerancia	3D desviación
Unidades	u
Máx. crítico	120.00
Máx. nominal	13.00
Mín. nominal	-13.00
Mín. crítico	-120.00

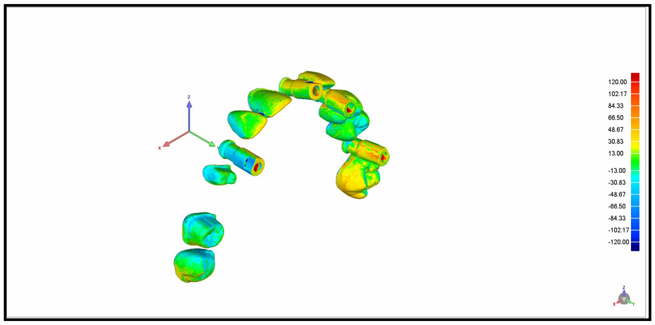

Desviación	
Desviación superior máx.	2980.90
Desviación inferior máx.	-3075.60
Desviación media	61.16 /-48.11
Desviación estándar	194.01

Distribución desviación

>=Min	<Max	# Puntos	%
-120.00	-102.17	340	0.32
-102.17	-84.33	475	0.45
-84.33	-66.50	760	0.72
-66.50	-48.67	1556	1.47
-48.67	-30.83	5924	5.59
-30.83	-13.00	16937	15.97
-13.00	13.00	36843	34.75
13.00	30.83	21274	20.06
30.83	48.67	9998	9.43
48.67	66.50	3238	3.05
66.50	84.33	1190	1.12
84.33	102.17	726	0.68
102.17	120.00	458	0.43

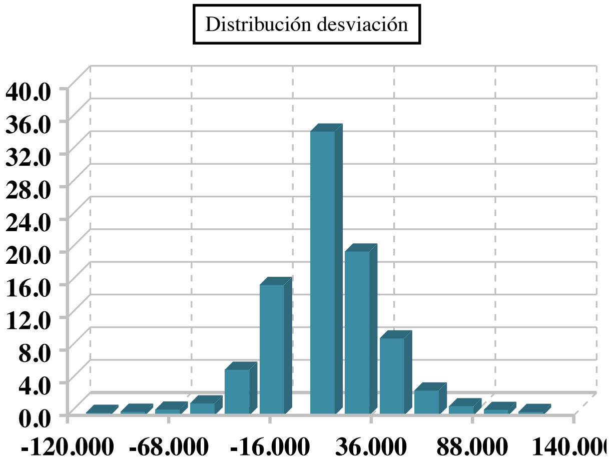

Fuera del crítico superior		4239	4.00
Fuera del crítico inferior		2074	1.96

Desviaciones estándar

Distribución (+/-)	# Puntos	%
-6 * Desv. estánd.	556	0.52
-5 * Desv. estánd.	74	0.07
-4 * Desv. estánd.	97	0.09
-3 * Desv. estánd.	153	0.14
-2 * Desv. estánd.	494	0.47
-1 * Desv. estánd.	64321	60.66
1 * Desv. estánd.	37548	35.41
2 * Desv. estánd.	971	0.92
3 * Desv. estánd.	326	0.31
4 * Desv. estánd.	308	0.29
5 * Desv. estánd.	311	0.29
6 * Desv. estánd.	873	0.82

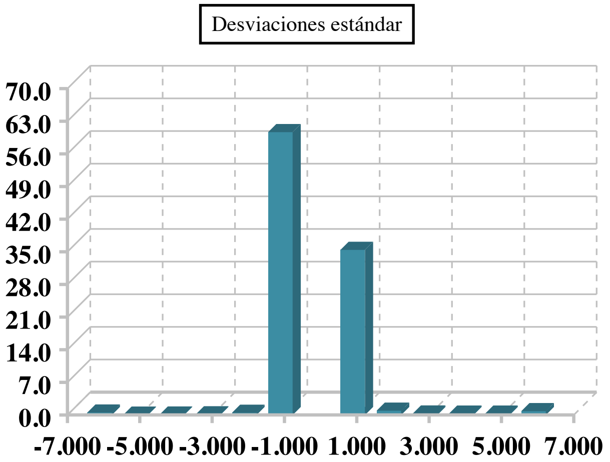

Predefinido: Isométrico

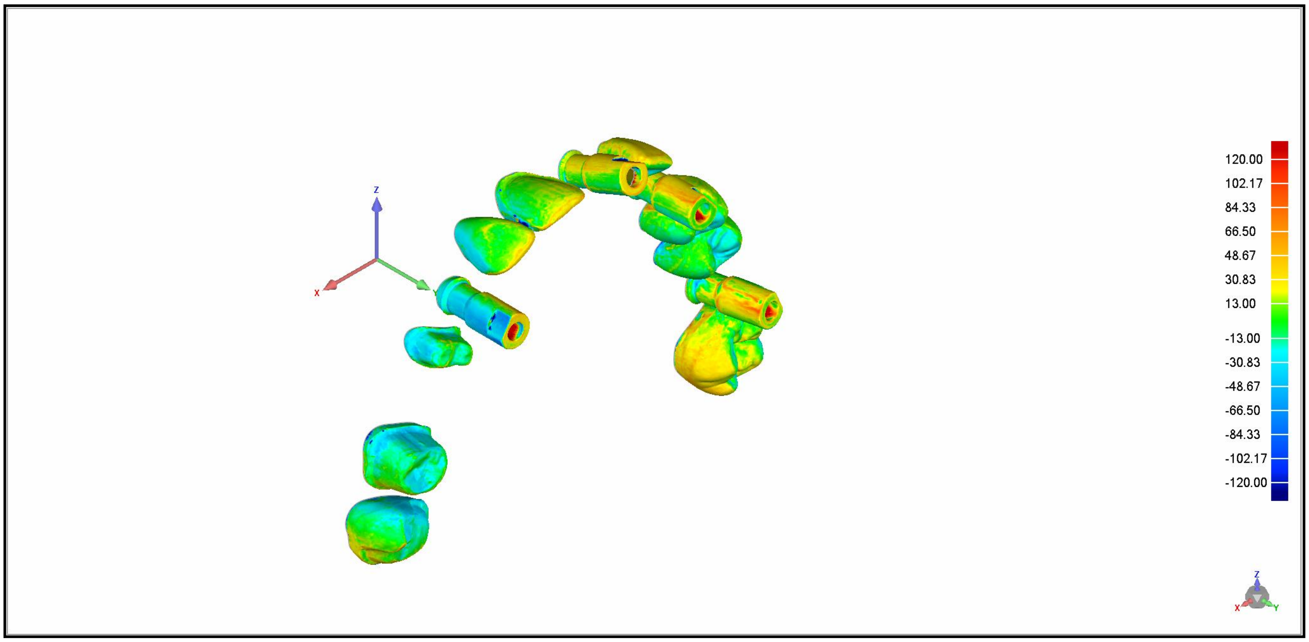

Predefinido: Frente

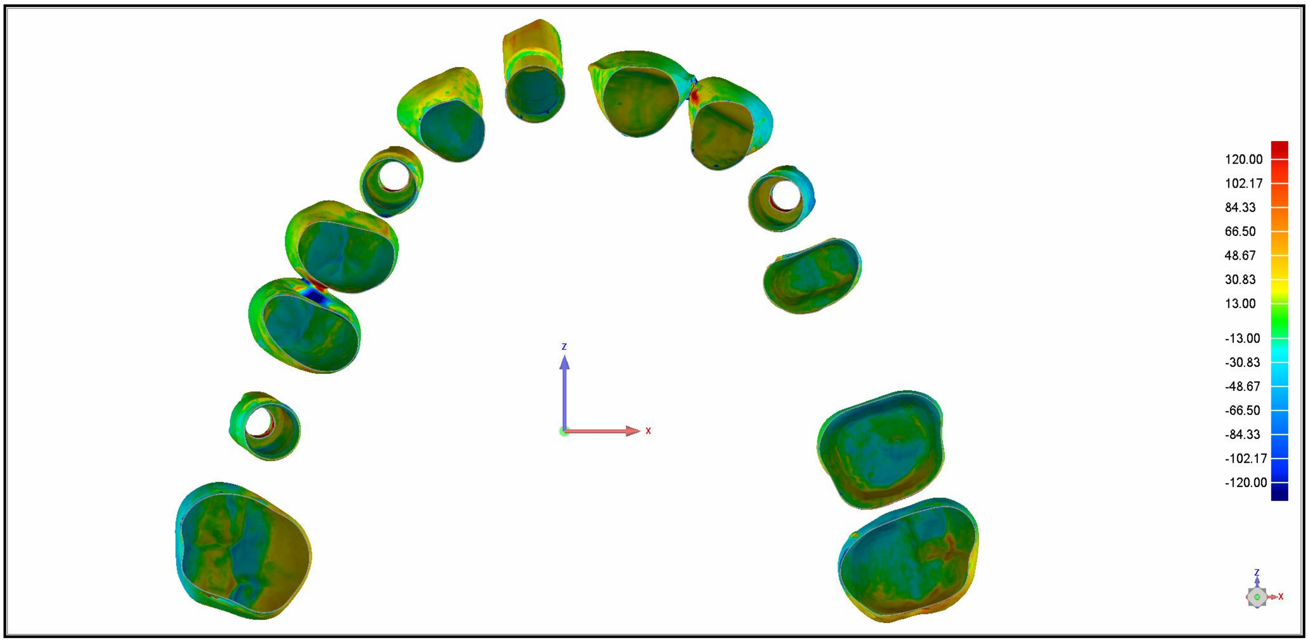

Predefinido: Atrás

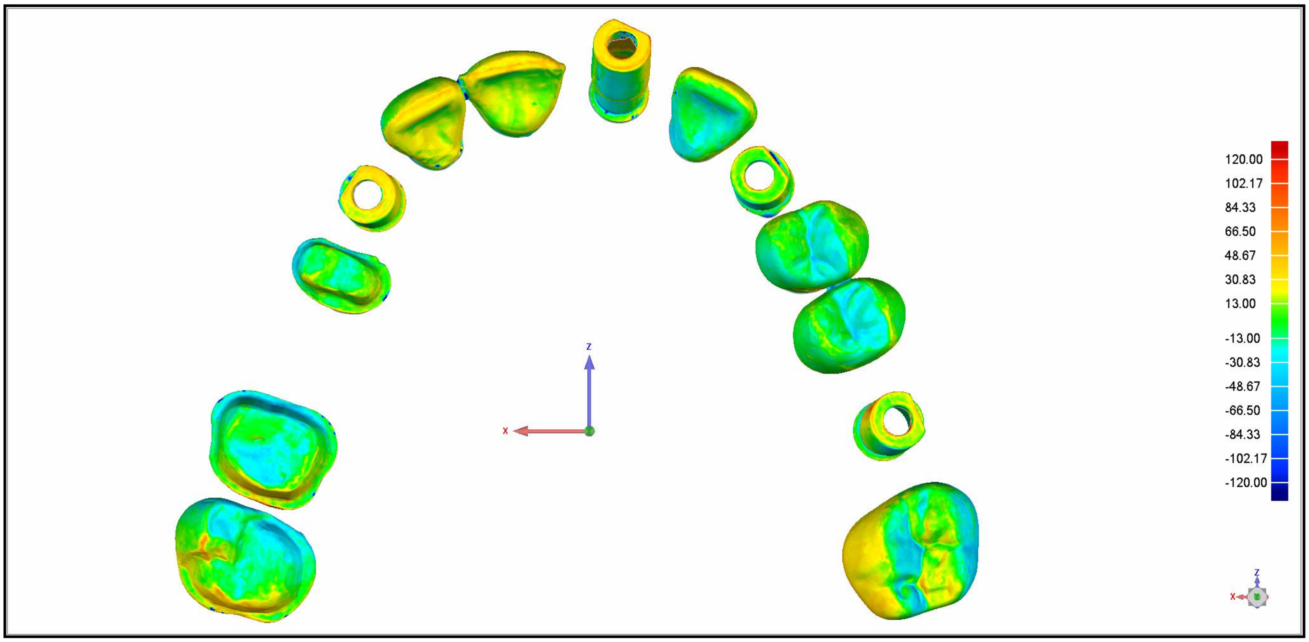

Predefinido: Izquierda

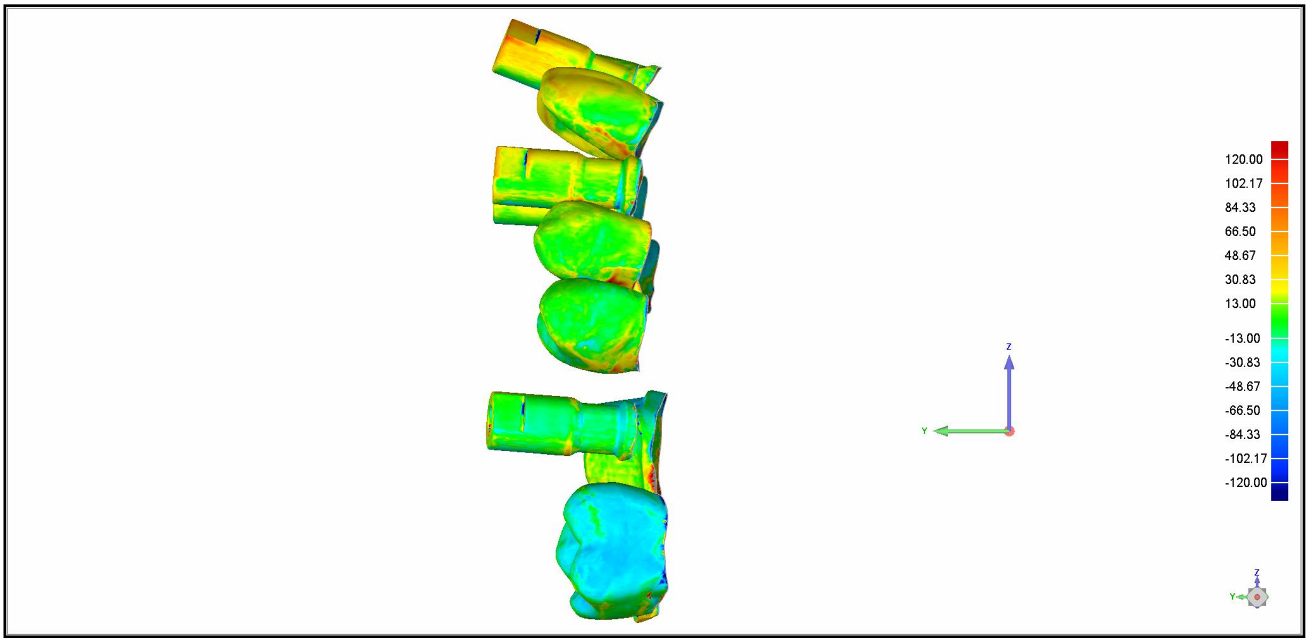

Predefinido: Derecha

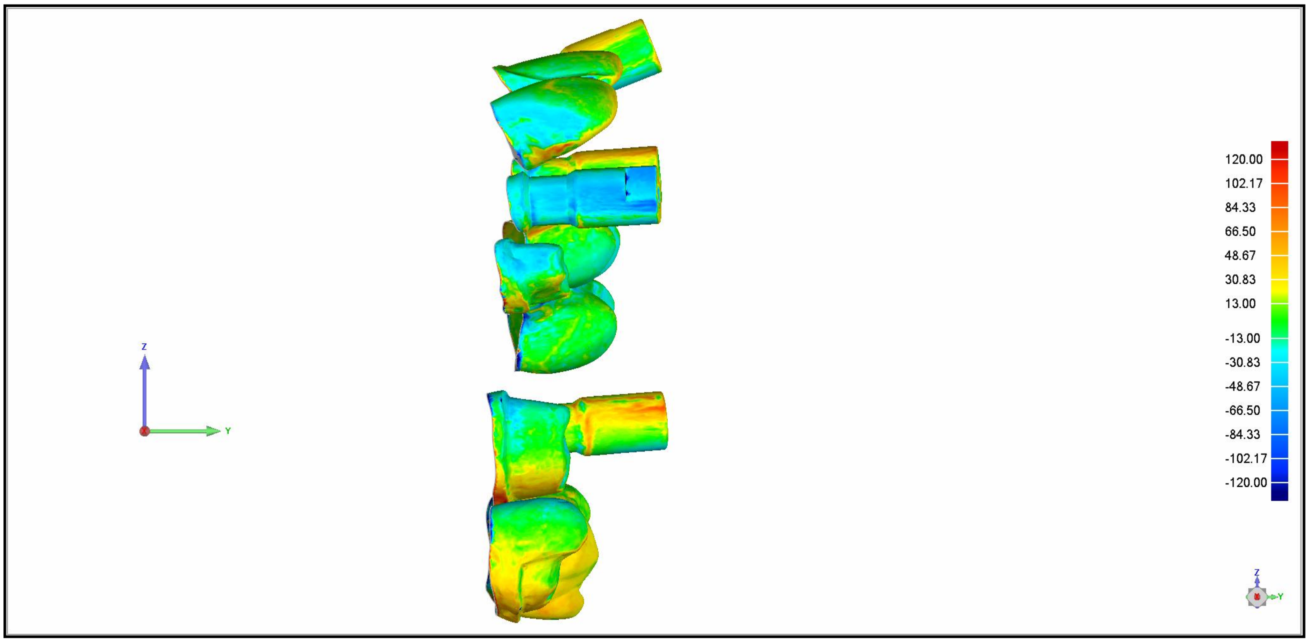

Predefinido: Superior

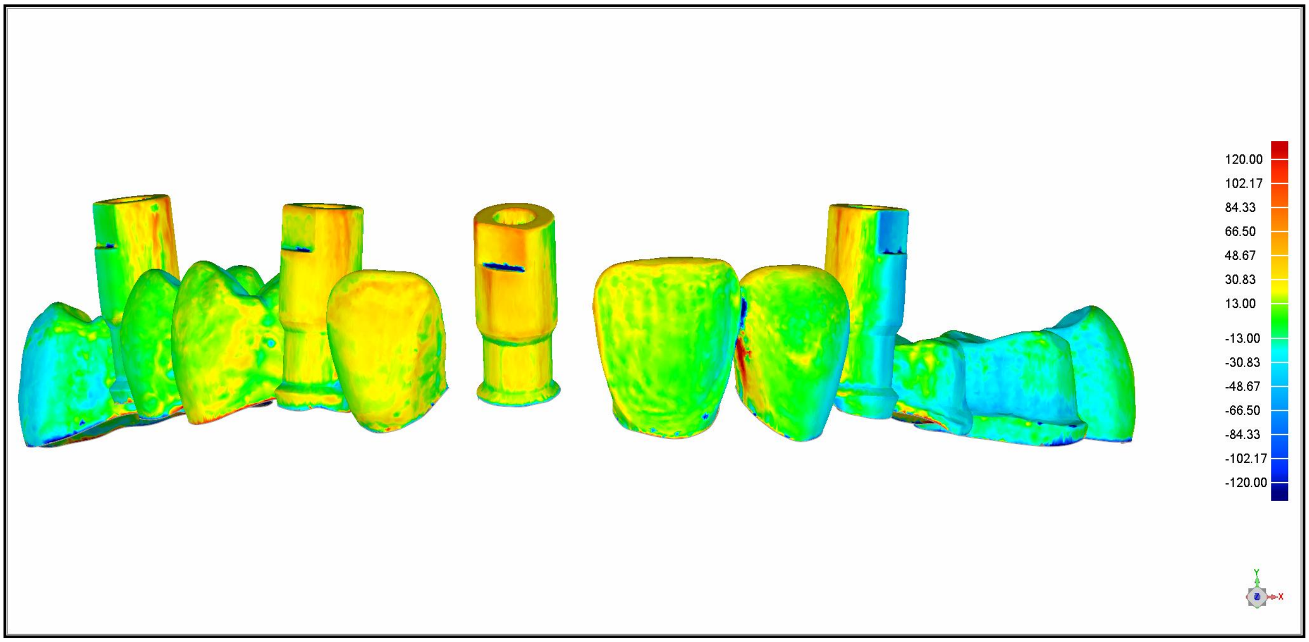

Predefinido: Inferior

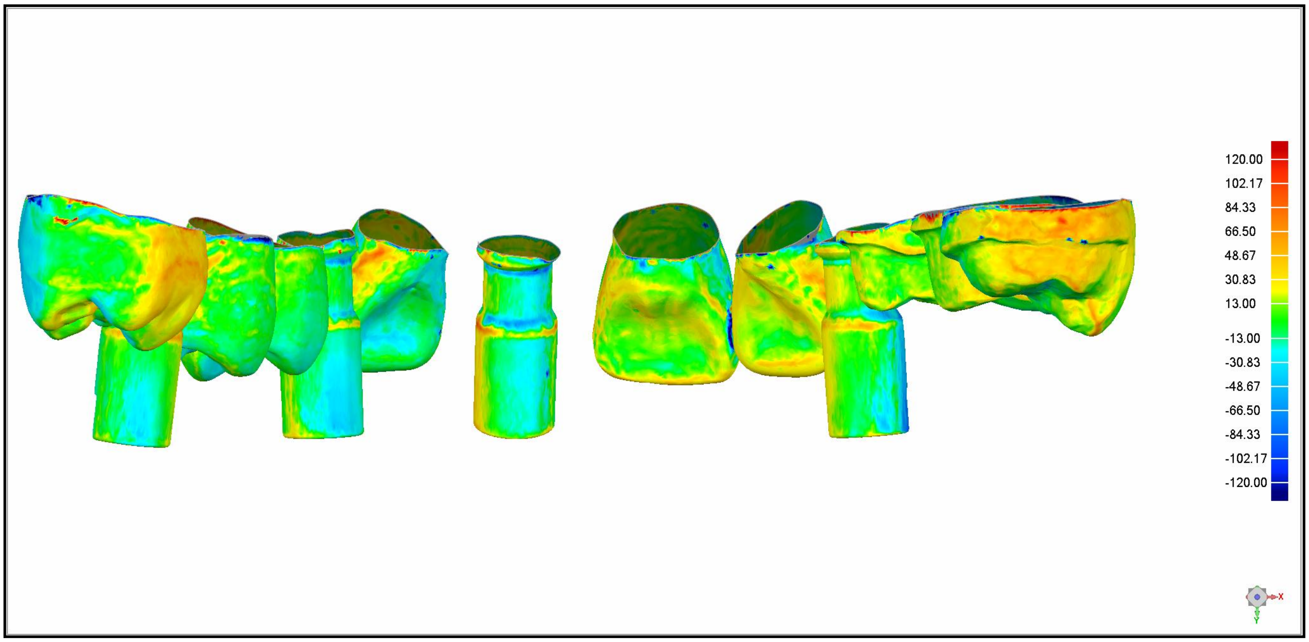

Supplement: S2 Table — Trios (scanning strategy B). (ZIP) [file pone.0202916.s002.zip › S2/3S5B.pdf]
