# Supplementary material for: Accuracy of four digital scanners according to scanning strategy in complete-arch impressions
Source: PLoS One. 2018 Sep 13;13(9):e0202916. doi: 10.1371/journal.pone.0202916 (PMC6136706; doi:10.1371/journal.pone.0202916)

3D Comparación Resultados

Modelo referencia	MRC
Modelo test	3S2B
Nº de puntos de datos	107676
# Aislados	85

Tipo tolerancia	3D desviación
Unidades	u
Máx. crítico	120.00
Máx. nominal	14.00
Mín. nominal	-14.00
Mín. crítico	-120.00

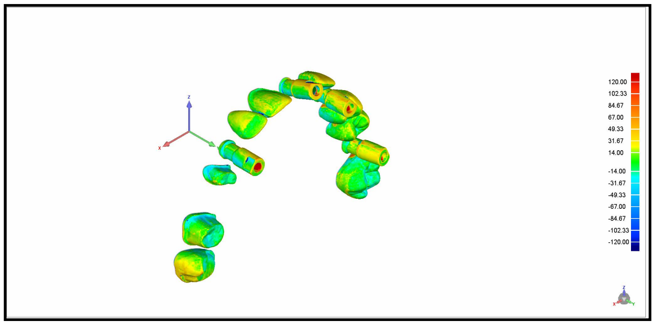

Desviación	
Desviación superior máx.	3154.45
Desviación inferior máx.	-2991.27
Desviación media	58.78 /-51.50
Desviación estándar	203.51

Distribución desviación

>=Min	<Max	# Puntos	%
-120.00	-102.33	296	0.27
-102.33	-84.67	455	0.42
-84.67	-67.00	671	0.62
-67.00	-49.33	1094	1.02
-49.33	-31.67	3335	3.10
-31.67	-14.00	13050	12.12
-14.00	14.00	46344	43.04
14.00	31.67	22460	20.86
31.67	49.33	8698	8.08
49.33	67.00	2767	2.57
67.00	84.67	1113	1.03
84.67	102.33	580	0.54
102.33	120.00	410	0.38

Fuera del crítico superior	4196	3.90
Fuera del crítico inferior	2207	2.05

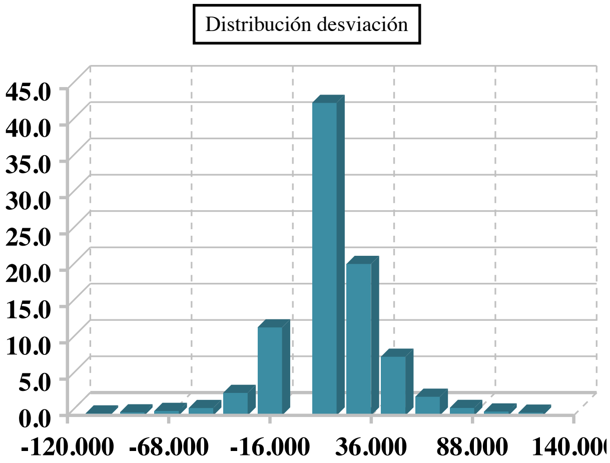

Desviaciones estándar

Distribución (+/-)	# Puntos	%
-6 * Desv. estánd.	599	0.56
-5 * Desv. estánd.	127	0.12
-4 * Desv. estánd.	131	0.12
-3 * Desv. estánd.	174	0.16
-2 * Desv. estánd.	452	0.42
-1 * Desv. estánd.	67447	62.64
1 * Desv. estánd.	35927	33.37
2 * Desv. estánd.	846	0.79
3 * Desv. estánd.	399	0.37
4 * Desv. estánd.	378	0.35
5 * Desv. estánd.	351	0.33
6 * Desv. estánd.	845	0.78

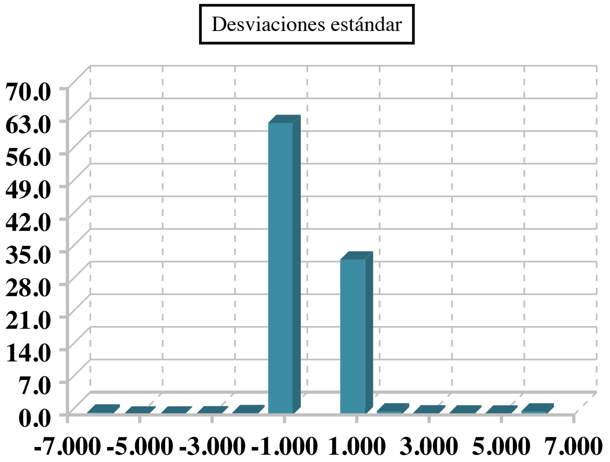

Predefinido: Isométrico

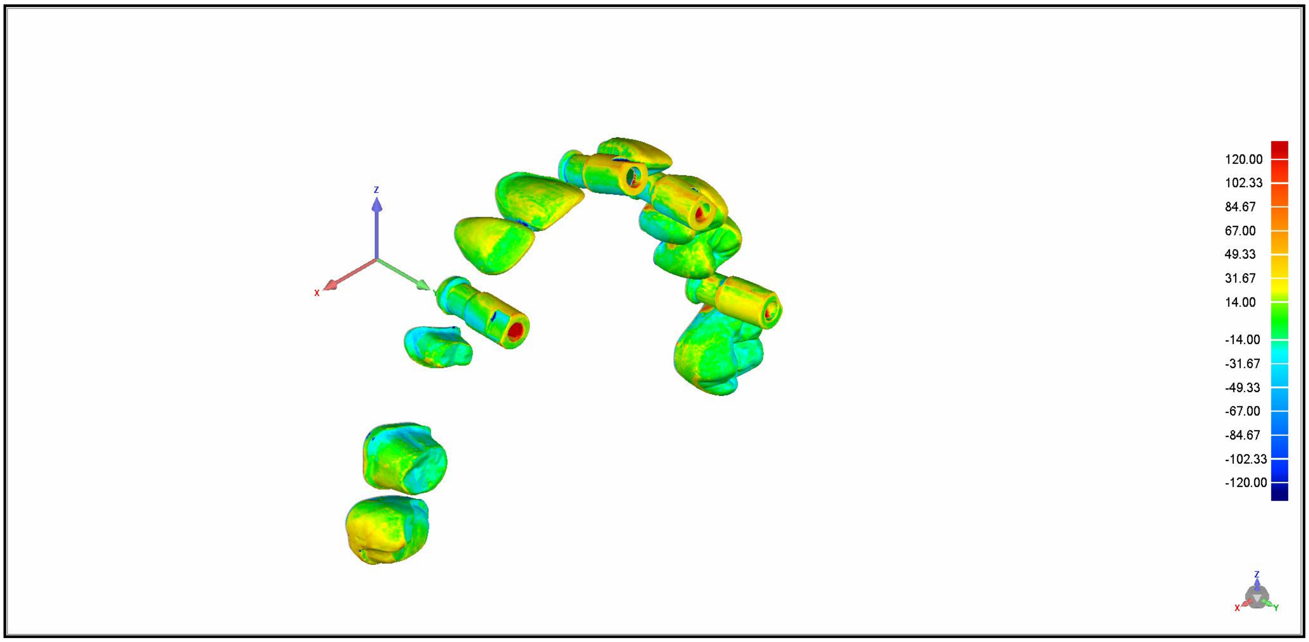

Predefinido: Frente

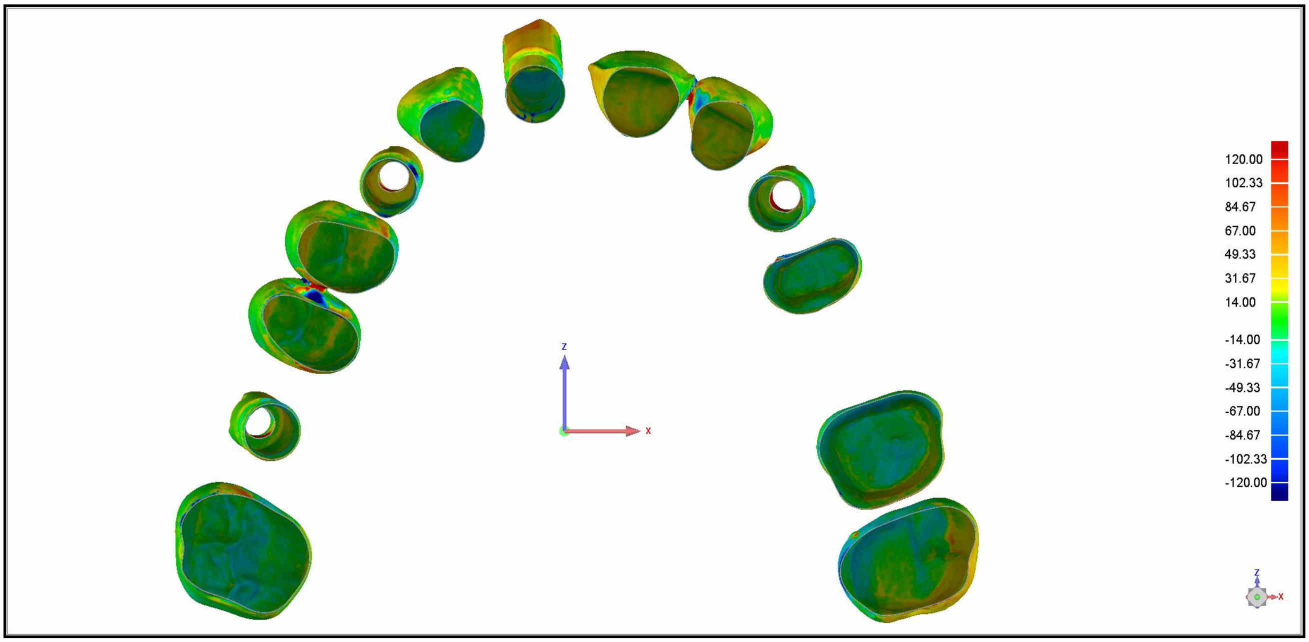

Predefinido: Atrás

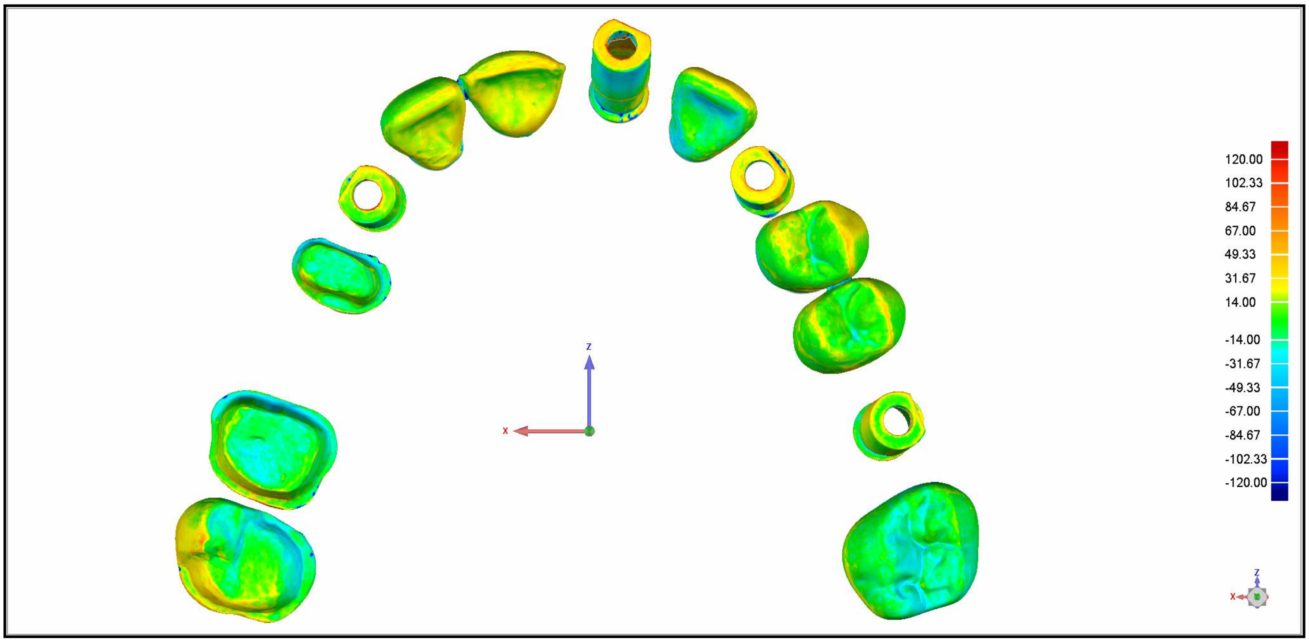

Geomagic Control™

Predefinido: Izquierda

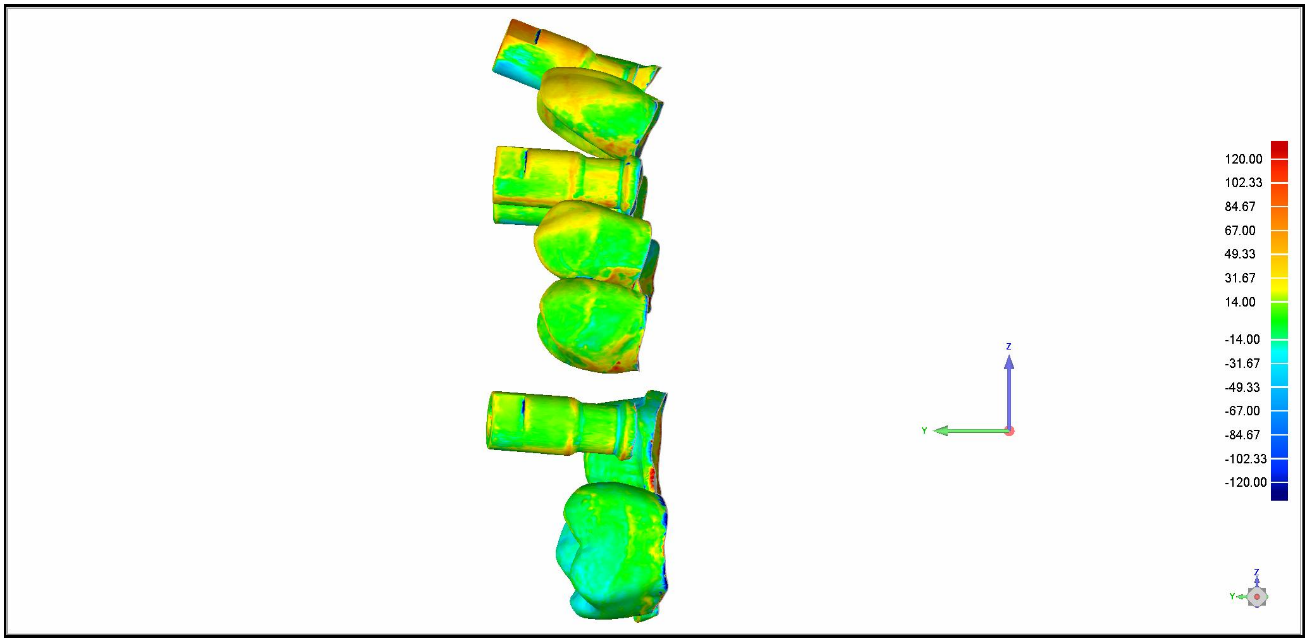

Predefinido: Derecha

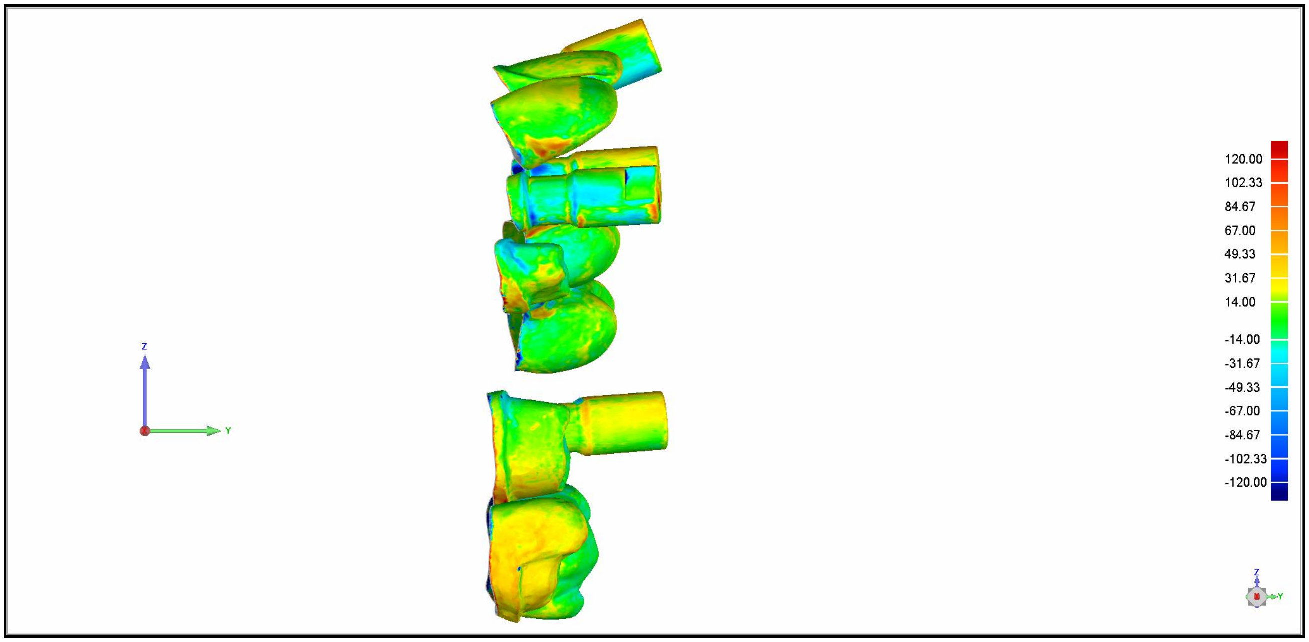

Predefinido: Superior

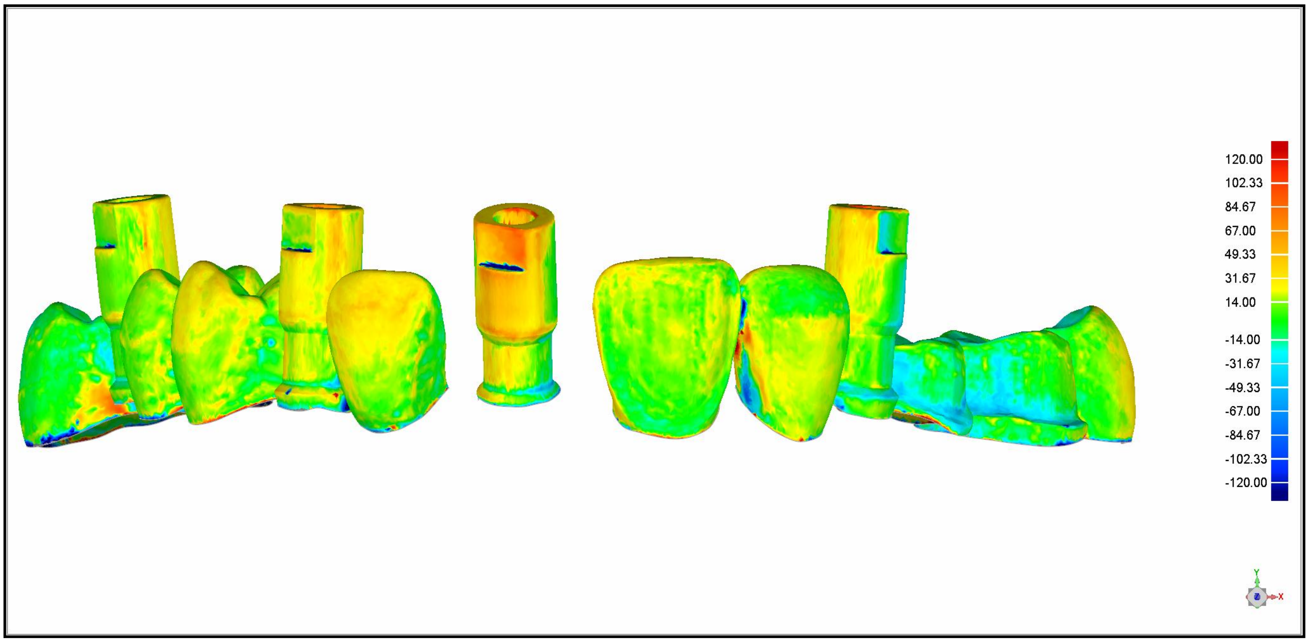

Predefinido: Inferior

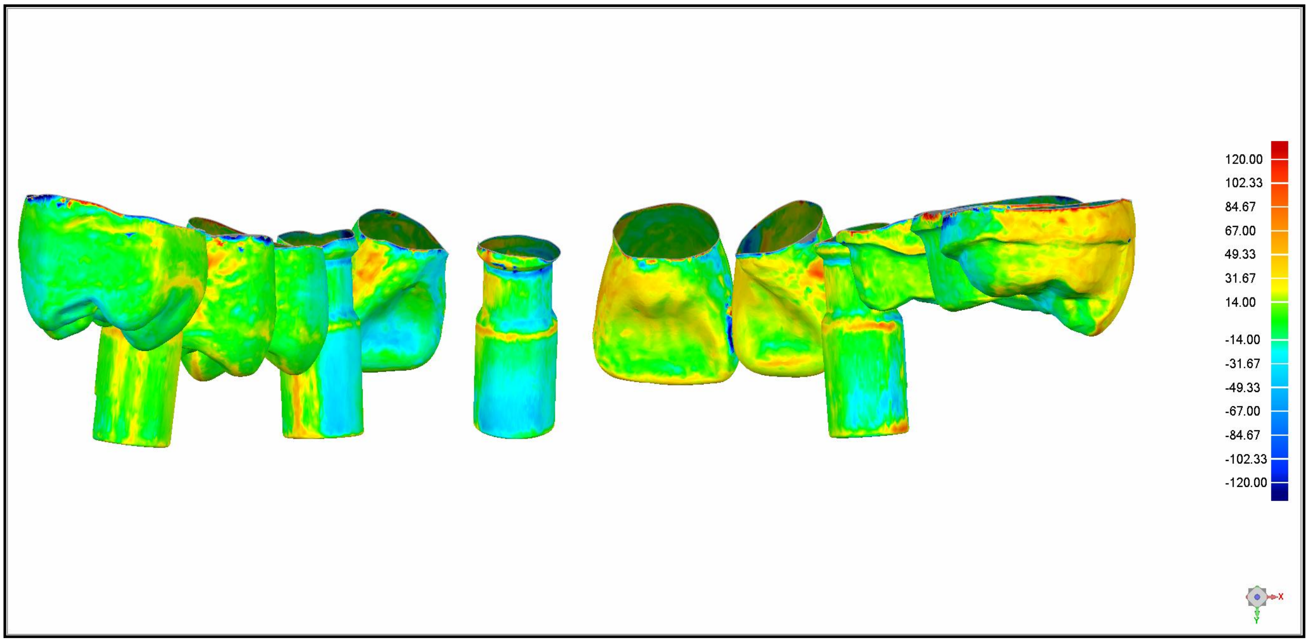

Supplement: S2 Table — Trios (scanning strategy B). (ZIP) [file pone.0202916.s002.zip › S2/3S2B.pdf]
