# Supplementary material for: Accuracy of four digital scanners according to scanning strategy in complete-arch impressions
Source: PLoS One. 2018 Sep 13;13(9):e0202916. doi: 10.1371/journal.pone.0202916 (PMC6136706; doi:10.1371/journal.pone.0202916)

Geomagic Control™

Fecha: 3/6/2017, 12:17 pm

3D Comparación Resultados

Modelo referencia	MRC
Modelo test	3S3B
Nº de puntos de datos	103042
# Aislados	78

Tipo tolerancia	3D desviación
Unidades	u
Máx. crítico	120.00
Máx. nominal	12.00
Mín. nominal	-12.00
Mín. crítico	-120.00

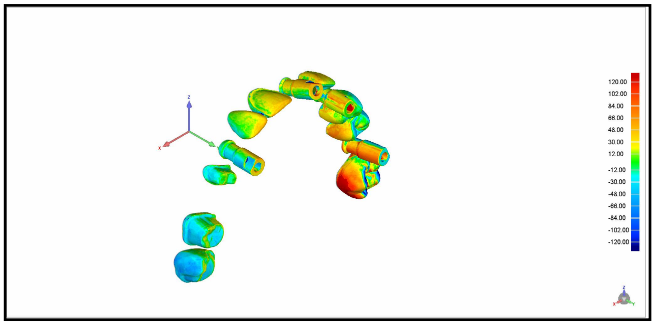

Desviación	
Desviación superior máx.	3140.06
Desviación inferior máx.	-3124.96
Desviación media	70.57 /-60.61
Desviación estándar	196.52

Distribución desviación

>=Min	<Max	# Puntos	%
-120.00	-102.00	986	0.96
-102.00	-84.00	1356	1.32
-84.00	-66.00	2039	1.98
-66.00	-48.00	4112	3.99
-48.00	-30.00	7473	7.25
-30.00	-12.00	13670	13.27
-12.00	12.00	24554	23.83
12.00	30.00	18115	17.58
30.00	48.00	10674	10.36
48.00	66.00	6044	5.87
66.00	84.00	2874	2.79
84.00	102.00	1594	1.55
102.00	120.00	908	0.88

Fuera del crítico superior	4585	4.45
Fuera del crítico inferior	4058	3.94

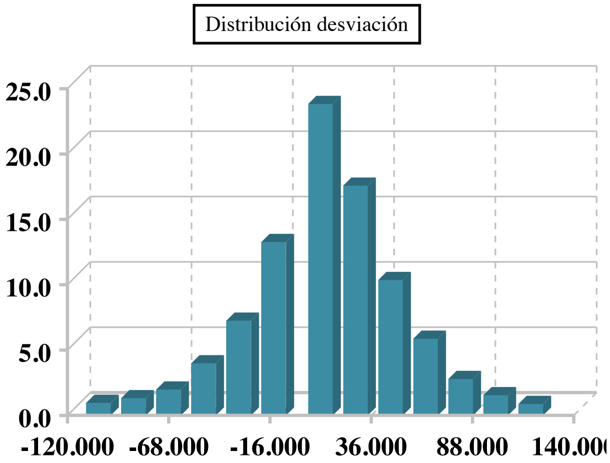

Desviaciones estándar

Distribución (+/-)	# Puntos	%
-6 * Desv. estánd.	492	0.48
-5 * Desv. estánd.	100	0.10
-4 * Desv. estánd.	114	0.11
-3 * Desv. estánd.	139	0.13
-2 * Desv. estánd.	565	0.55
-1 * Desv. estánd.	58010	56.30
1 * Desv. estánd.	40997	39.79
2 * Desv. estánd.	710	0.69
3 * Desv. estánd.	345	0.33
4 * Desv. estánd.	343	0.33
5 * Desv. estánd.	332	0.32
6 * Desv. estánd.	895	0.87

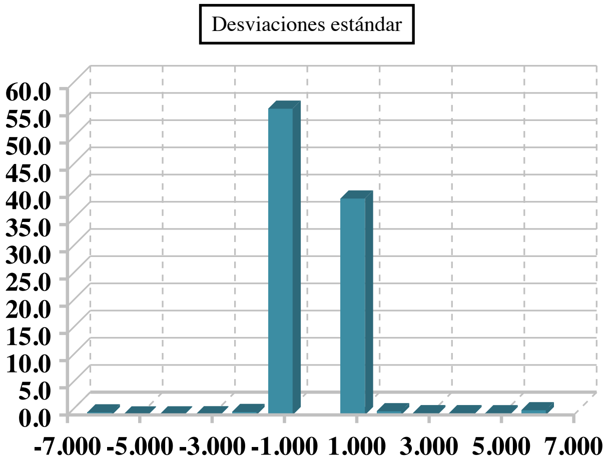

Predefinido: Isométrico

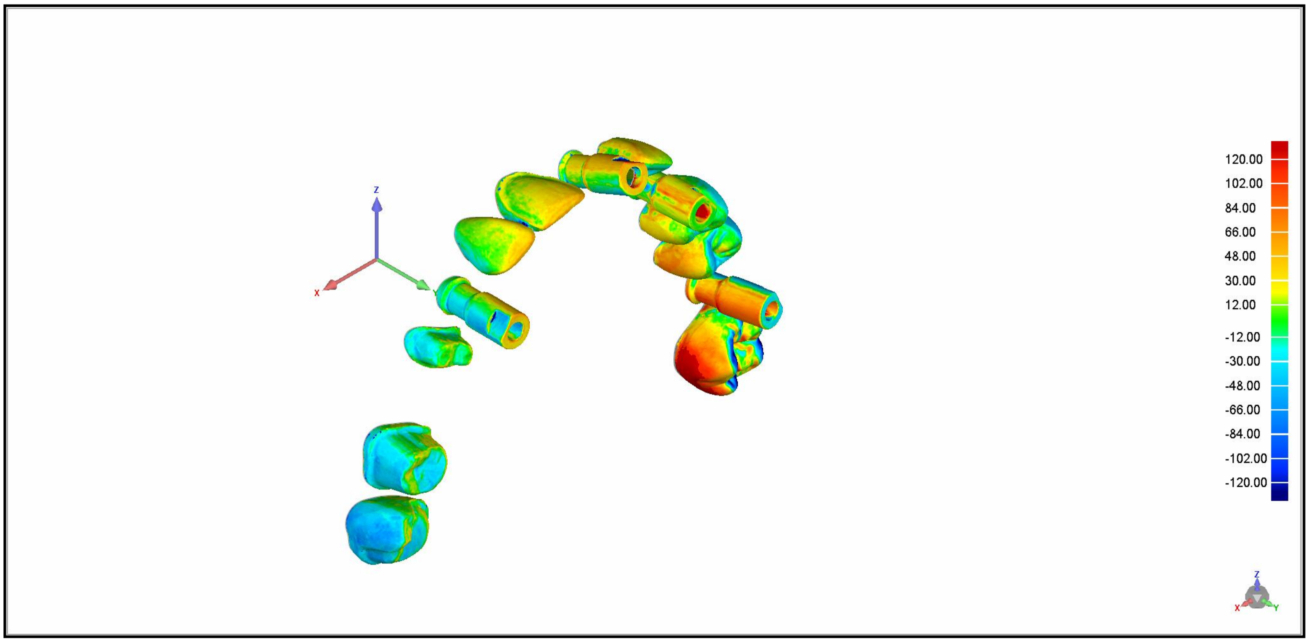

Predefinido: Frente

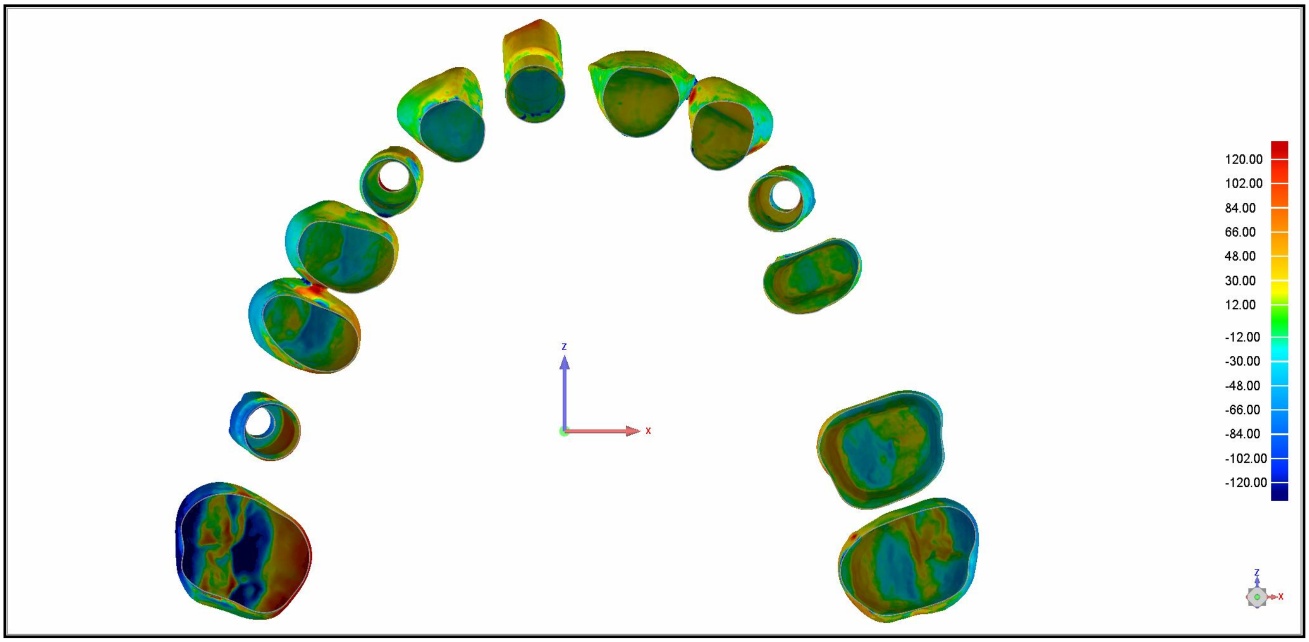

Predefinido: Atrás

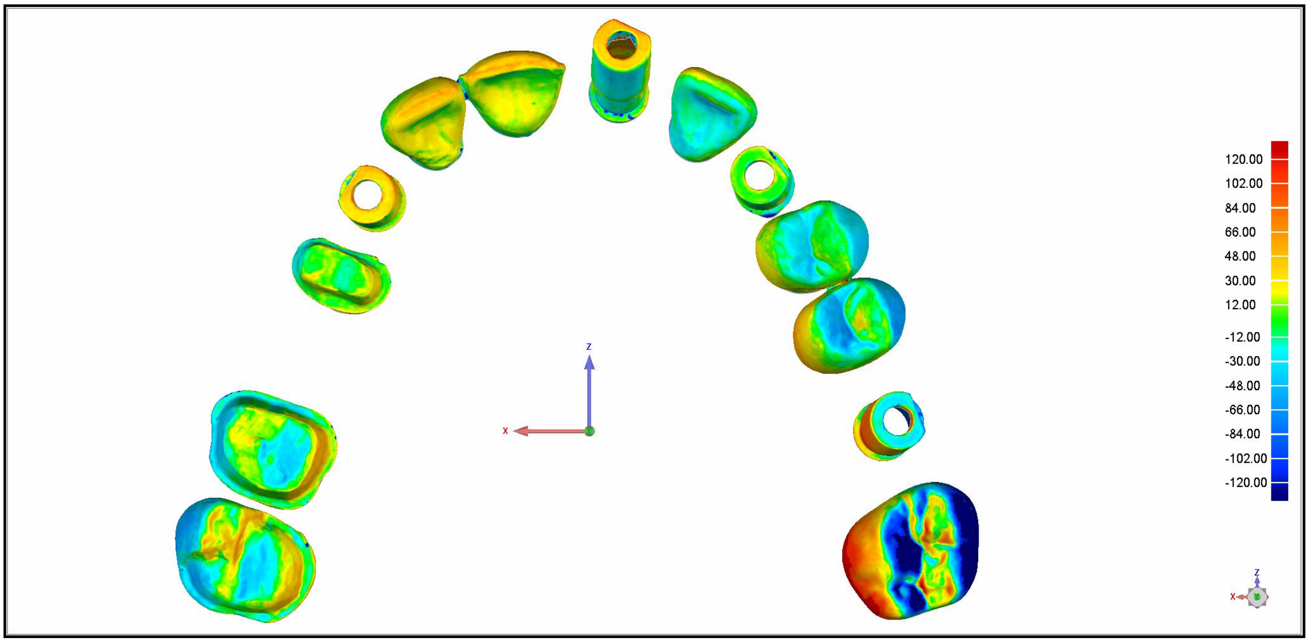

Predefinido: Izquierda

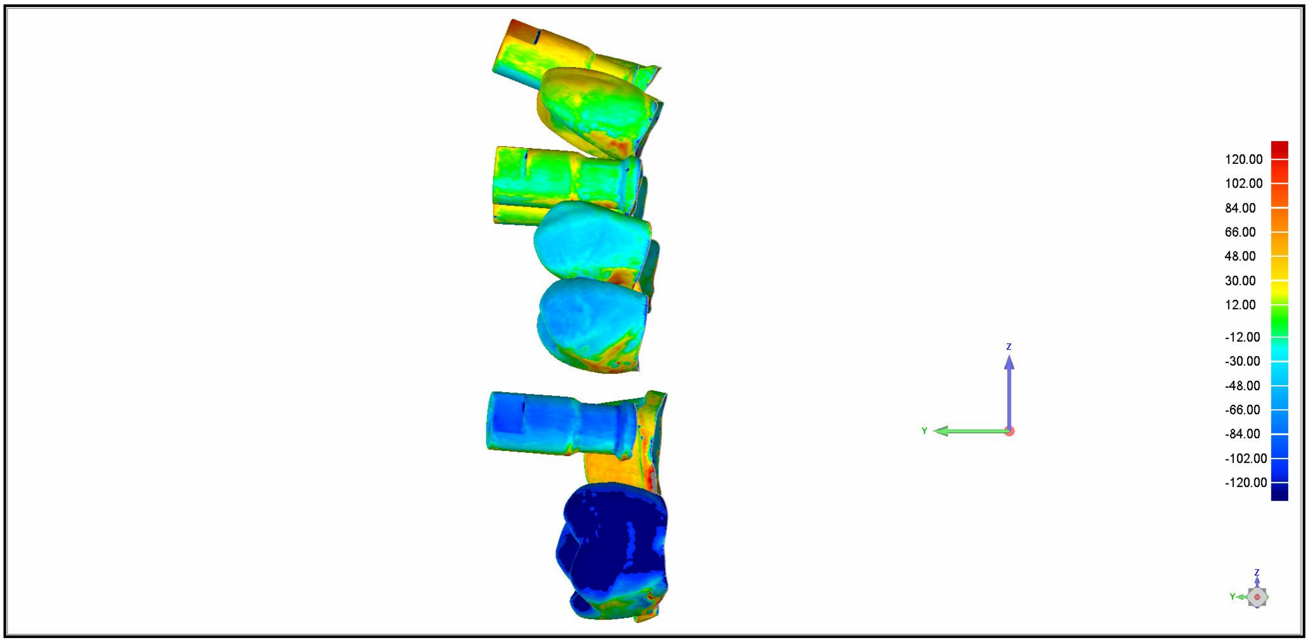

Predefinido: Derecha

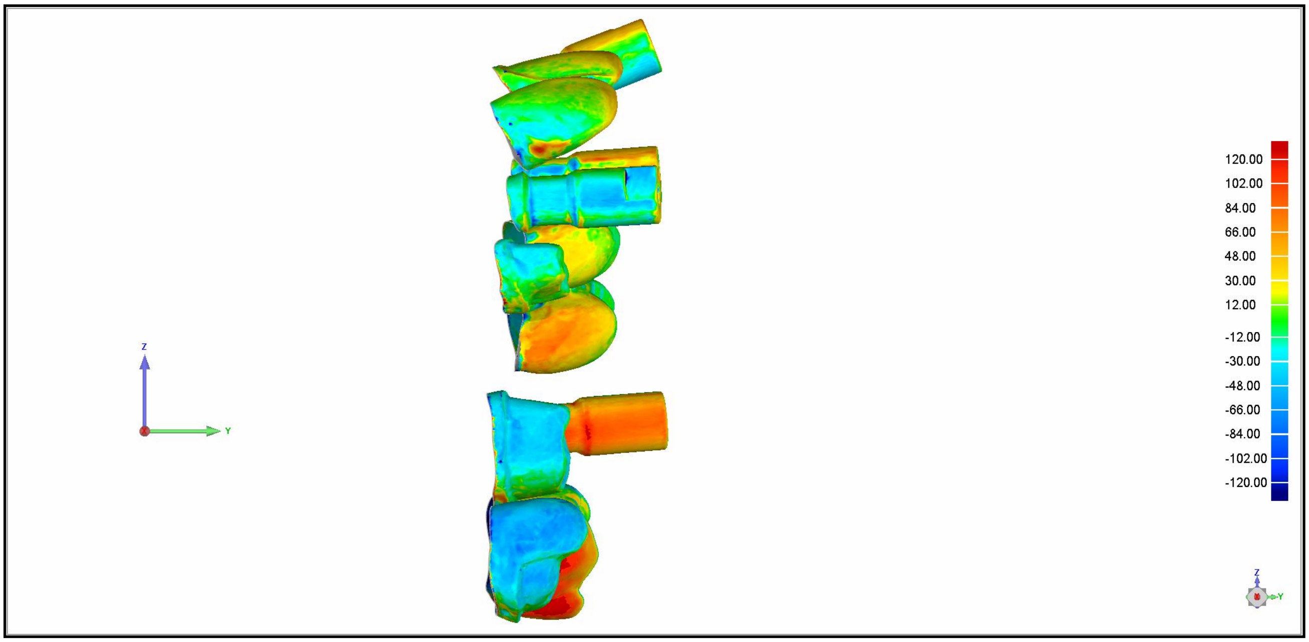

Predefinido: Superior

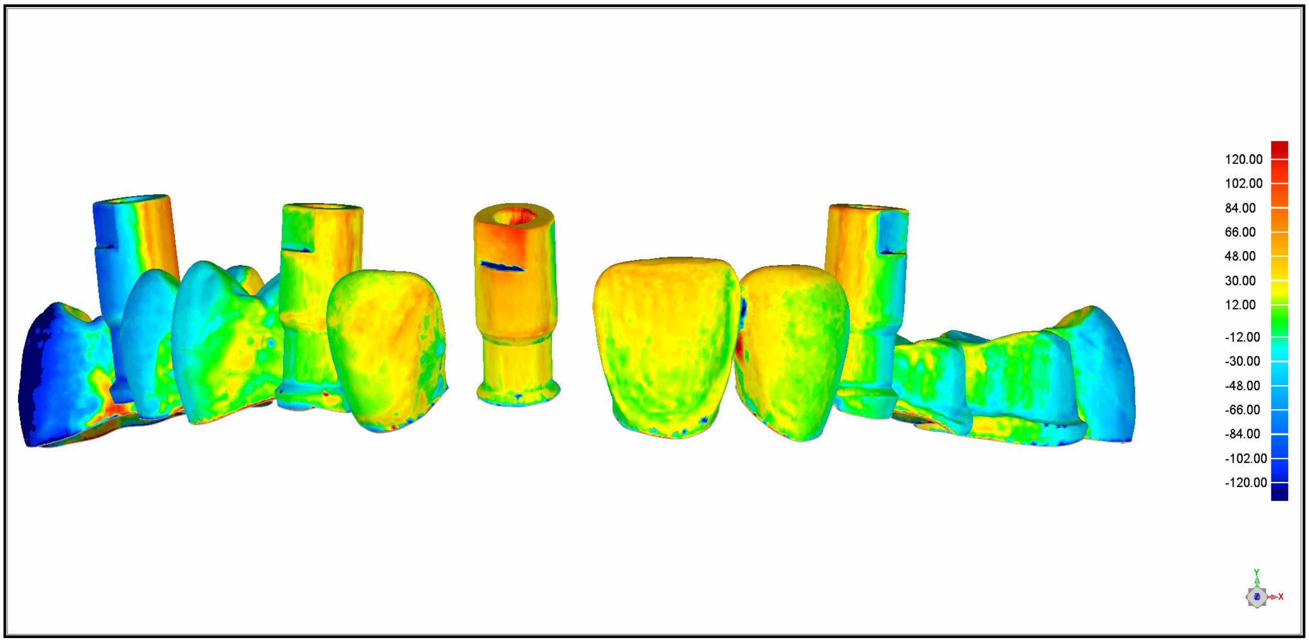

Predefinido: Inferior

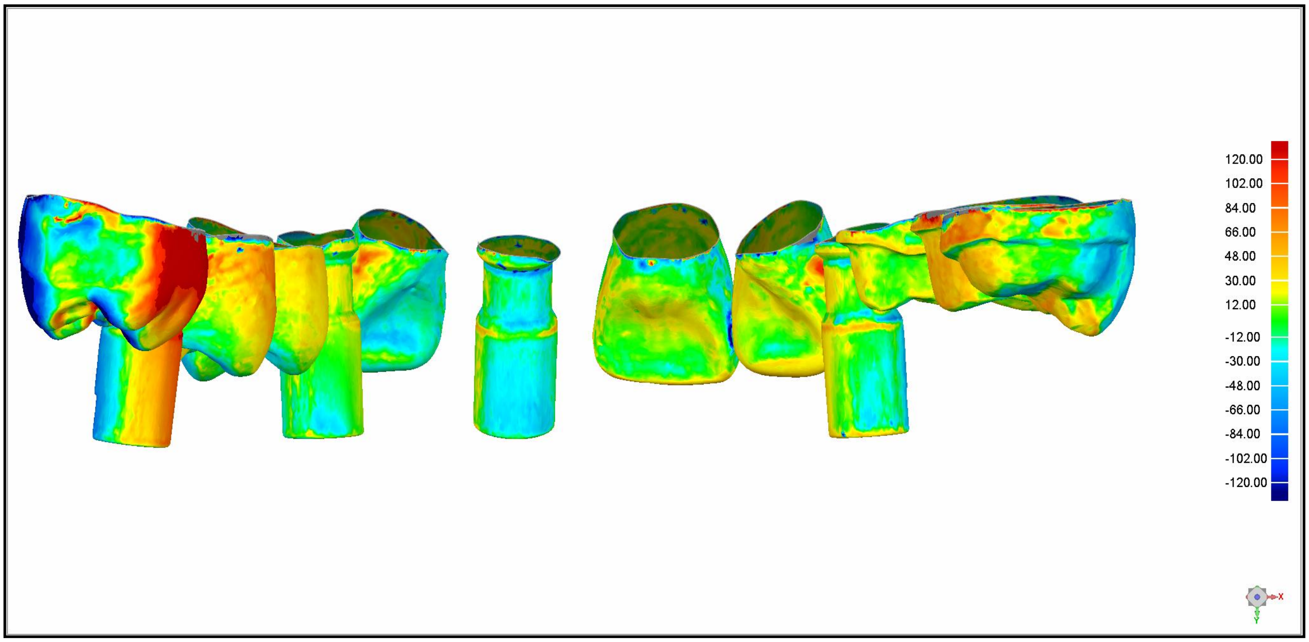

Supplement: S2 Table — Trios (scanning strategy B). (ZIP) [file pone.0202916.s002.zip › S2/3S3B.pdf]
