# Supplementary material for: Accuracy of four digital scanners according to scanning strategy in complete-arch impressions
Source: PLoS One. 2018 Sep 13;13(9):e0202916. doi: 10.1371/journal.pone.0202916 (PMC6136706; doi:10.1371/journal.pone.0202916)

3D Comparación Resultados

Modelo referencia	MRC
Modelo test	3S10B
Nº de puntos de datos	102970
# Aislados	58

Tipo tolerancia	3D desviación
Unidades	u
Máx. crítico	120.00
Máx. nominal	16.00
Mín. nominal	-16.00
Mín. crítico	-120.00

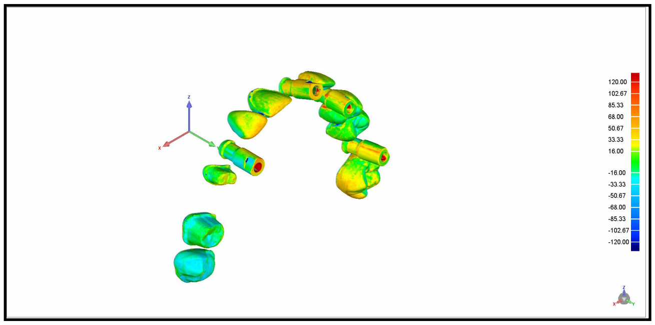

Desviación	
Desviación superior máx.	3151.20
Desviación inferior máx.	-3132.83
Desviación media	62.39 /-49.87
Desviación estándar	205.47

Distribución desviación

>=Min	<Max	# Puntos	%
-120.00	-102.67	299	0.29
-102.67	-85.33	375	0.36
-85.33	-68.00	514	0.50
-68.00	-50.67	841	0.82
-50.67	-33.33	3233	3.14
-33.33	-16.00	12333	11.98
-16.00	16.00	47522	46.15
16.00	33.33	19762	19.19
33.33	50.67	7346	7.13
50.67	68.00	2553	2.48
68.00	85.33	940	0.91
85.33	102.67	499	0.48
102.67	120.00	394	0.38

Fuera del crítico superior	4091	3.97
Fuera del crítico inferior	2268	2.20

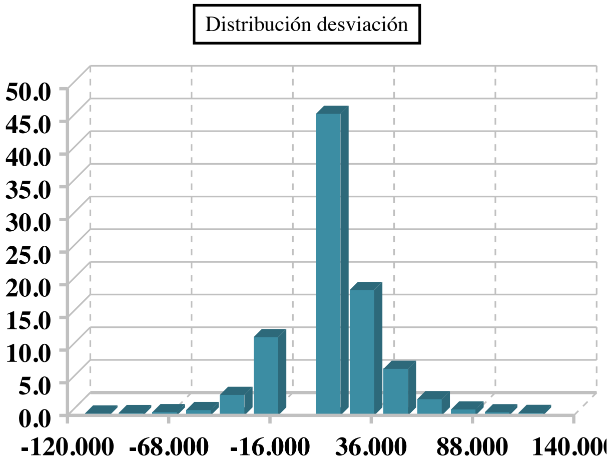

Desviaciones estándar

Distribución (+/-)	# Puntos	%
-6 * Desv. estánd.	508	0.49
-5 * Desv. estánd.	101	0.10
-4 * Desv. estánd.	126	0.12
-3 * Desv. estánd.	168	0.16
-2 * Desv. estánd.	614	0.60
-1 * Desv. estánd.	66514	64.60
1 * Desv. estánd.	32037	31.11
2 * Desv. estánd.	774	0.75
3 * Desv. estánd.	477	0.46
4 * Desv. estánd.	422	0.41
5 * Desv. estánd.	412	0.40
6 * Desv. estánd.	817	0.79

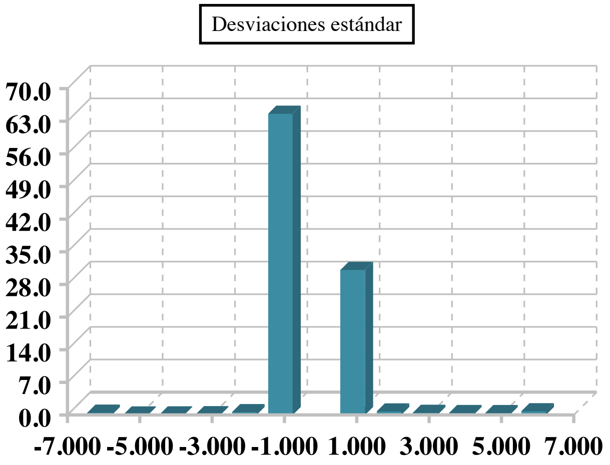

Predefinido: Isométrico

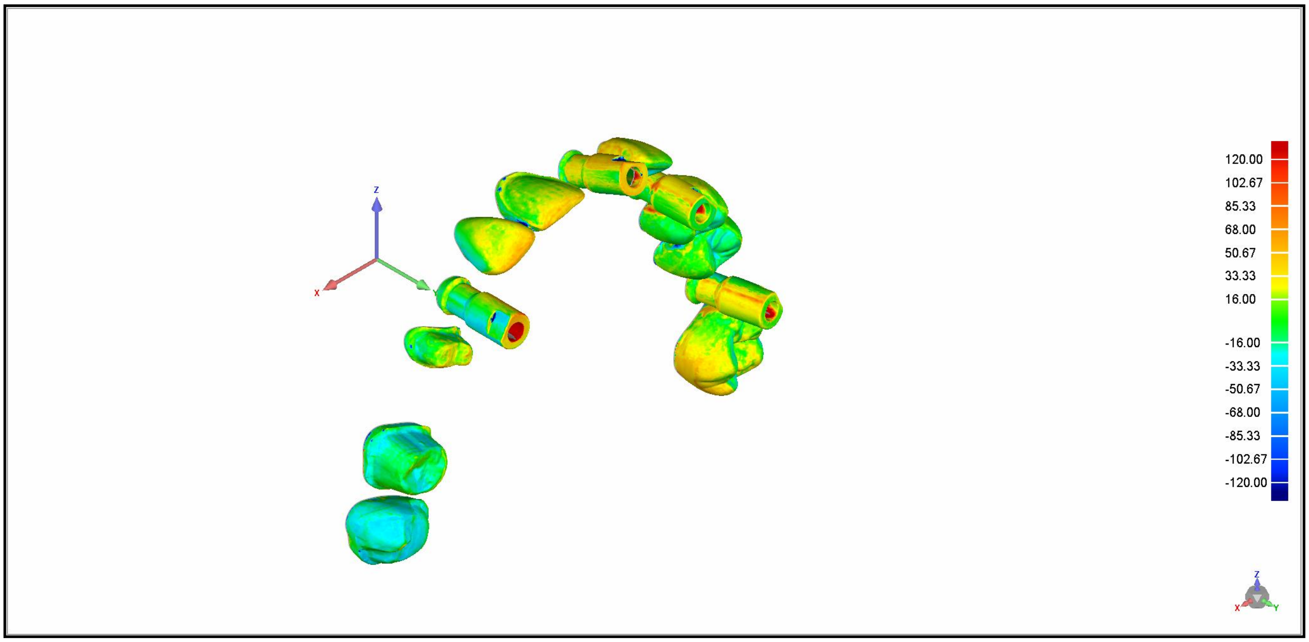

Predefinido: Frente

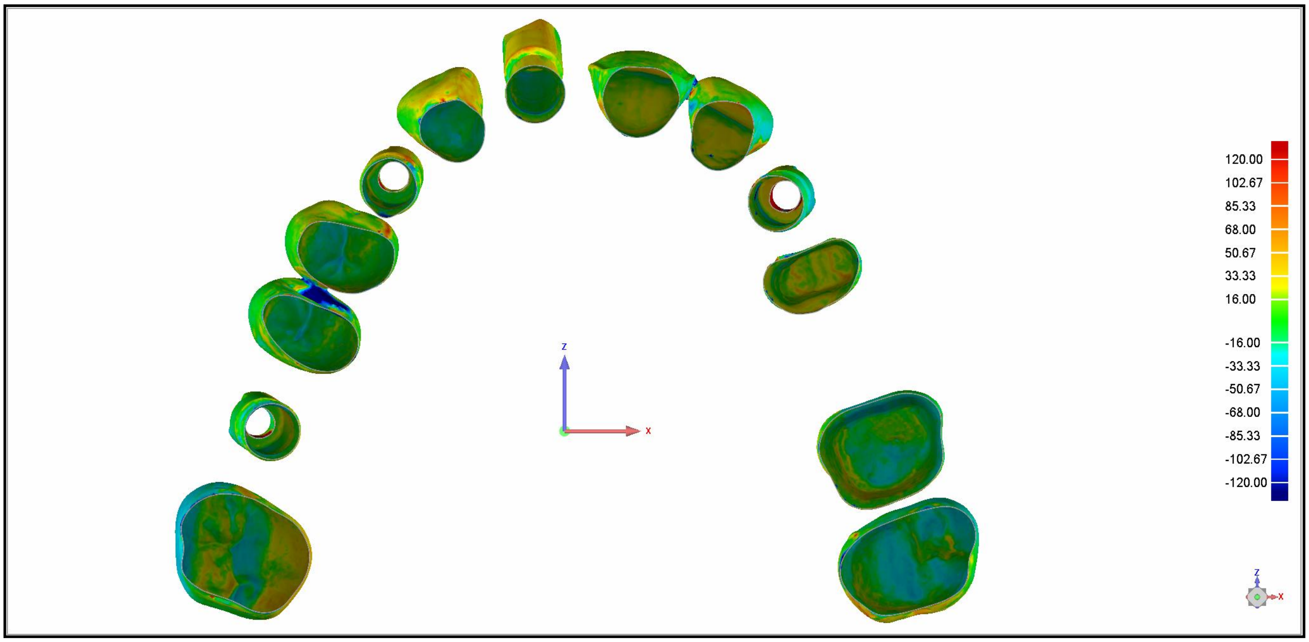

Predefinido: Atrás

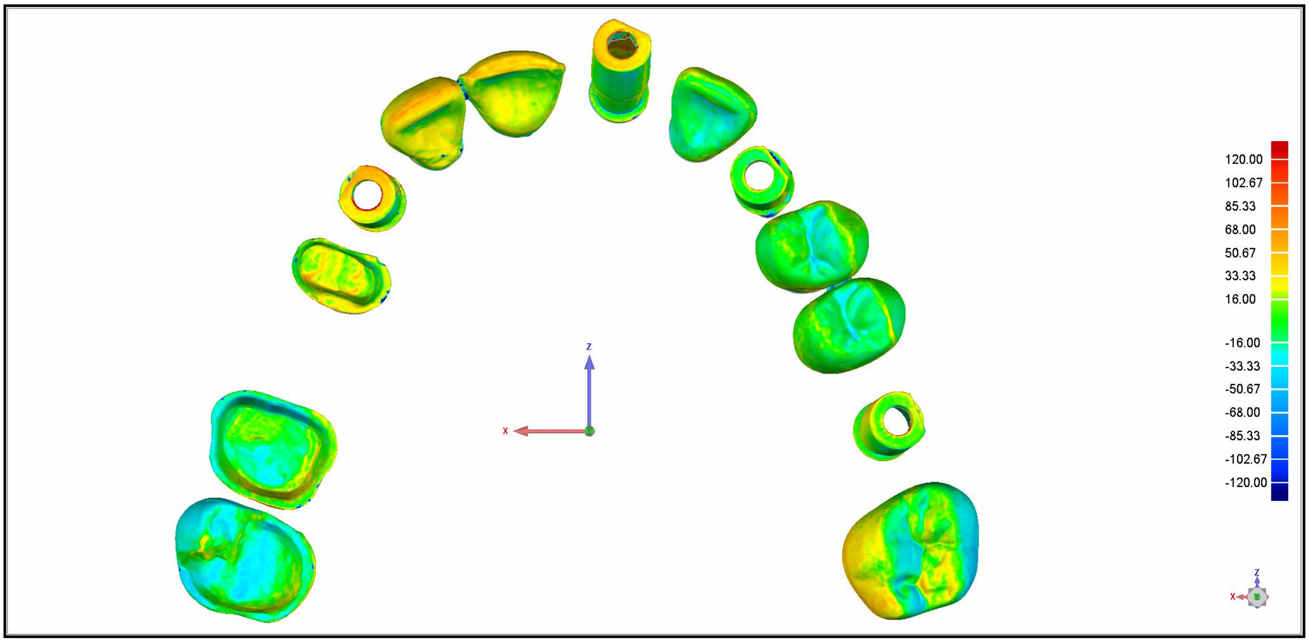

Predefinido: Izquierda

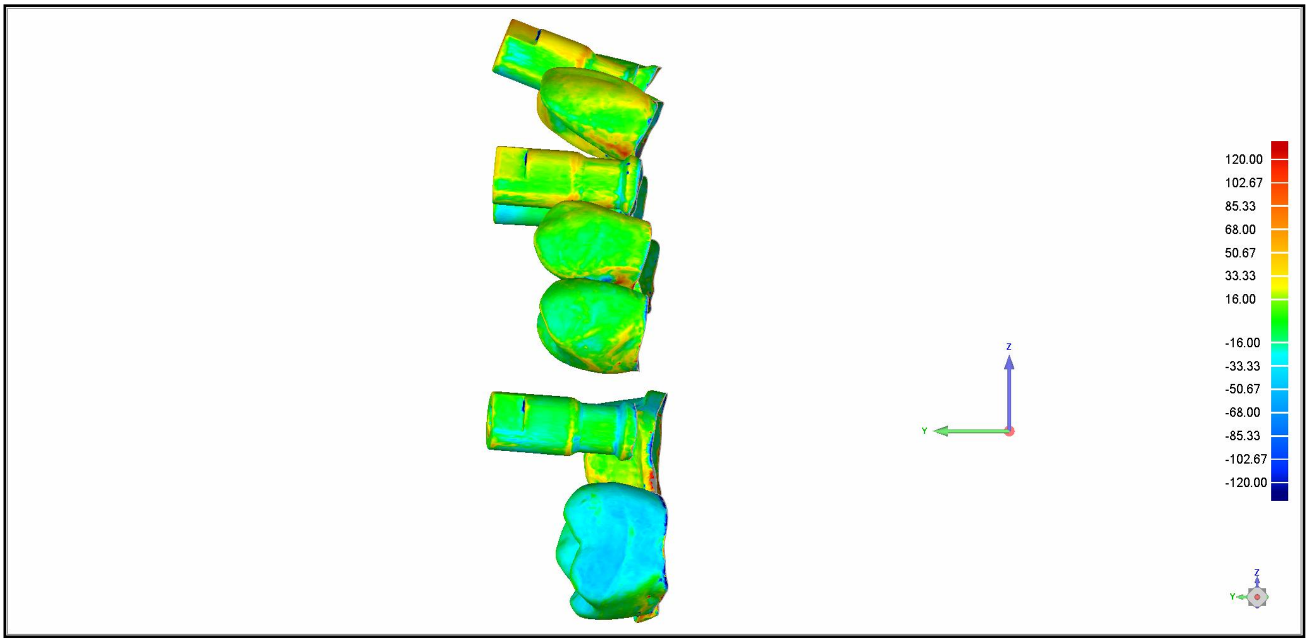

Predefinido: Derecha

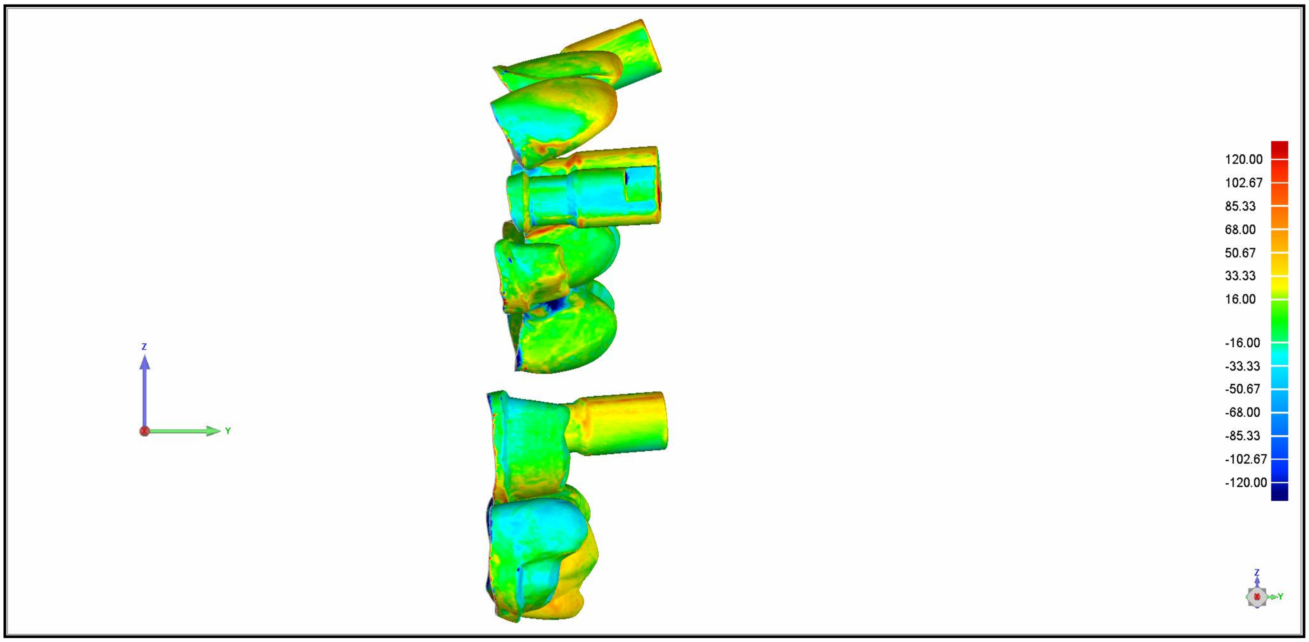

Predefinido: Superior

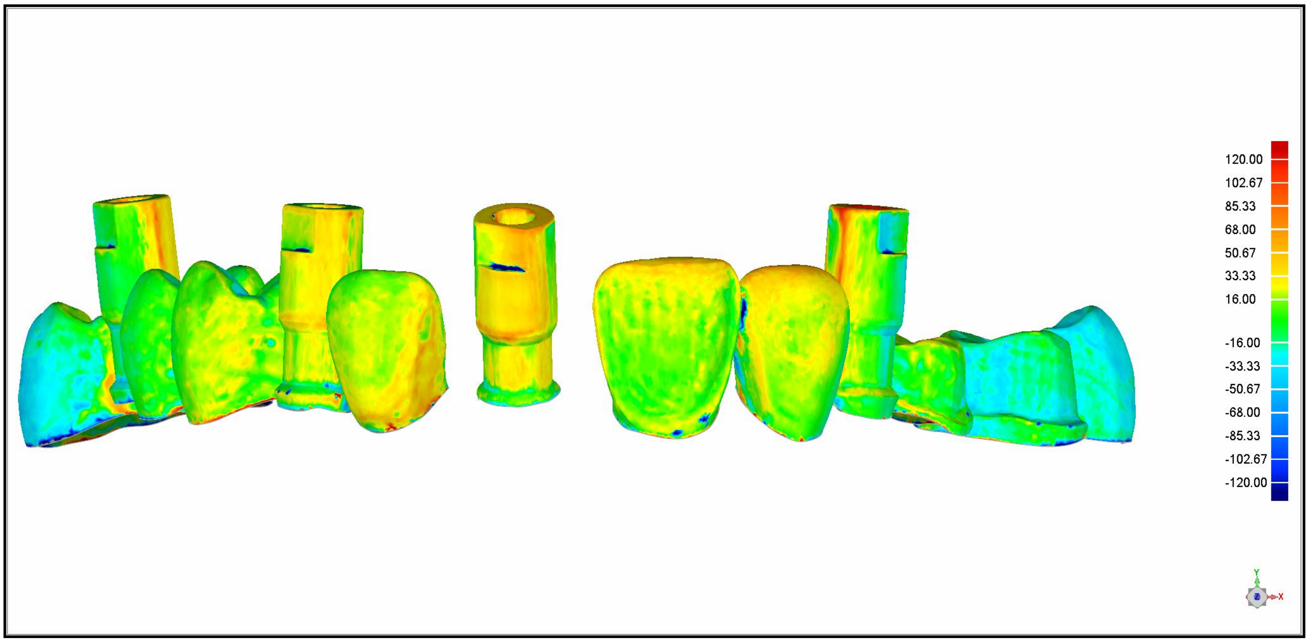

Predefinido: Inferior

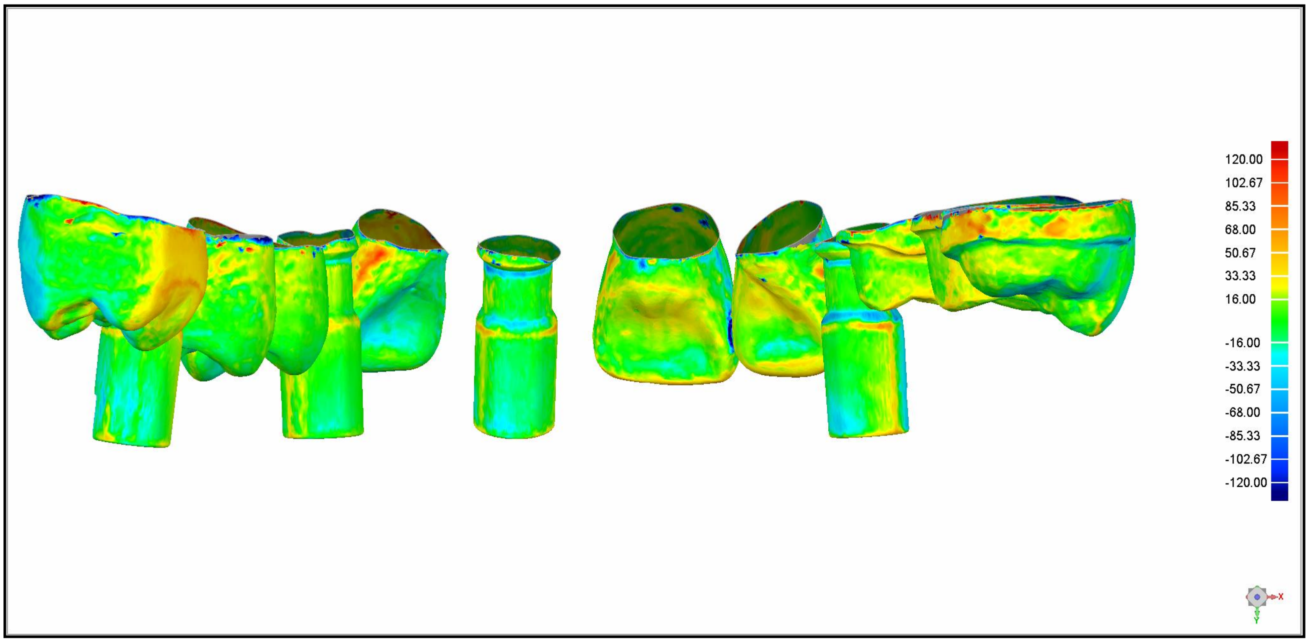

Supplement: S2 Table — Trios (scanning strategy B). (ZIP) [file pone.0202916.s002.zip › S2/3S10B.pdf]
