# Supplementary material for: Accuracy of four digital scanners according to scanning strategy in complete-arch impressions
Source: PLoS One. 2018 Sep 13;13(9):e0202916. doi: 10.1371/journal.pone.0202916 (PMC6136706; doi:10.1371/journal.pone.0202916)

3D Comparación Resultados

Modelo referencia	MRC
Modelo test	3S1B
Nº de puntos de datos	104608
# Aislados	78

Tipo tolerancia	3D desviación
Unidades	u
Máx. crítico	120.00
Máx. nominal	17.00
Mín. nominal	-17.00
Mín. crítico	-120.00

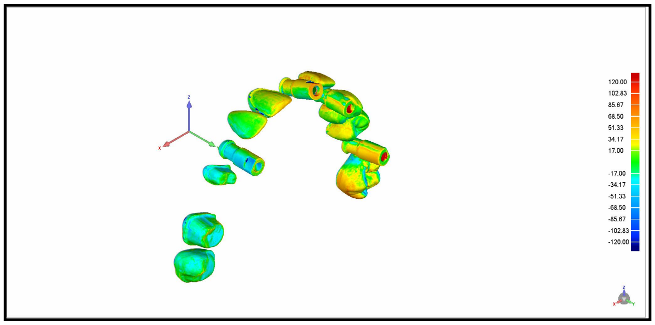

Desviación	
Desviación superior máx.	3067.01
Desviación inferior máx.	-3130.49
Desviación media	63.31 /-46.72
Desviación estándar	191.06

Distribución desviación

>=Min	<Max	# Puntos	%
-120.00	-102.83	286	0.27
-102.83	-85.67	362	0.35
-85.67	-68.50	754	0.72
-68.50	-51.33	2643	2.53
-51.33	-34.17	4805	4.59
-34.17	-17.00	12562	12.01
-17.00	17.00	42086	40.23
17.00	34.17	19253	18.40
34.17	51.33	9468	9.05
51.33	68.50	3667	3.51
68.50	85.67	1444	1.38
85.67	102.83	746	0.71
102.83	120.00	517	0.49

Fuera del crítico superior	4200	4.01
Fuera del crítico inferior	1815	1.74

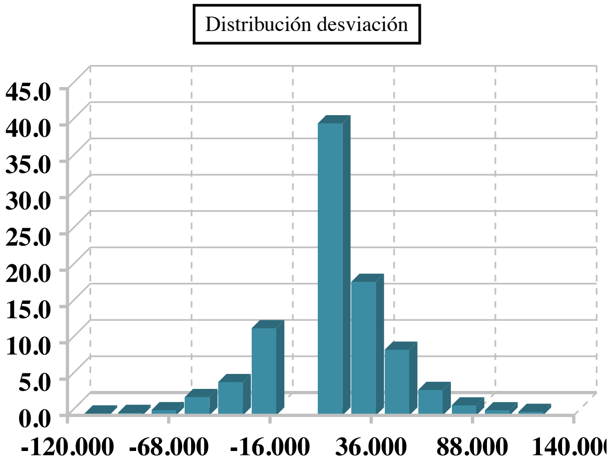

Desviaciones estándar

Distribución (+/-)	# Puntos	%
-6 * Desv. estánd.	416	0.40
-5 * Desv. estánd.	77	0.07
-4 * Desv. estánd.	119	0.11
-3 * Desv. estánd.	142	0.14
-2 * Desv. estánd.	513	0.49
-1 * Desv. estánd.	65042	62.18
1 * Desv. estánd.	35523	33.96
2 * Desv. estánd.	849	0.81
3 * Desv. estánd.	400	0.38
4 * Desv. estánd.	377	0.36
5 * Desv. estánd.	359	0.34
6 * Desv. estánd.	791	0.76

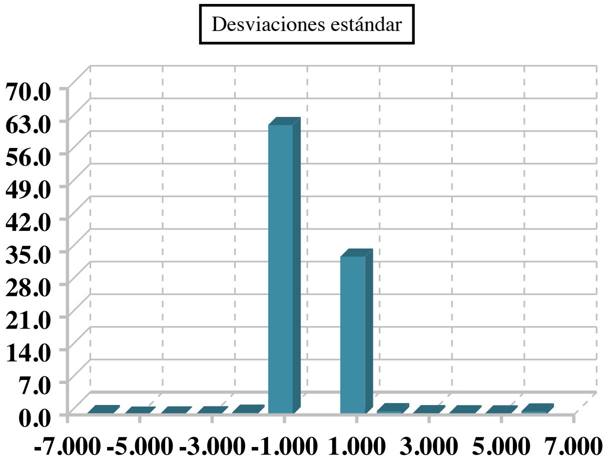

Predefinido: Isométrico

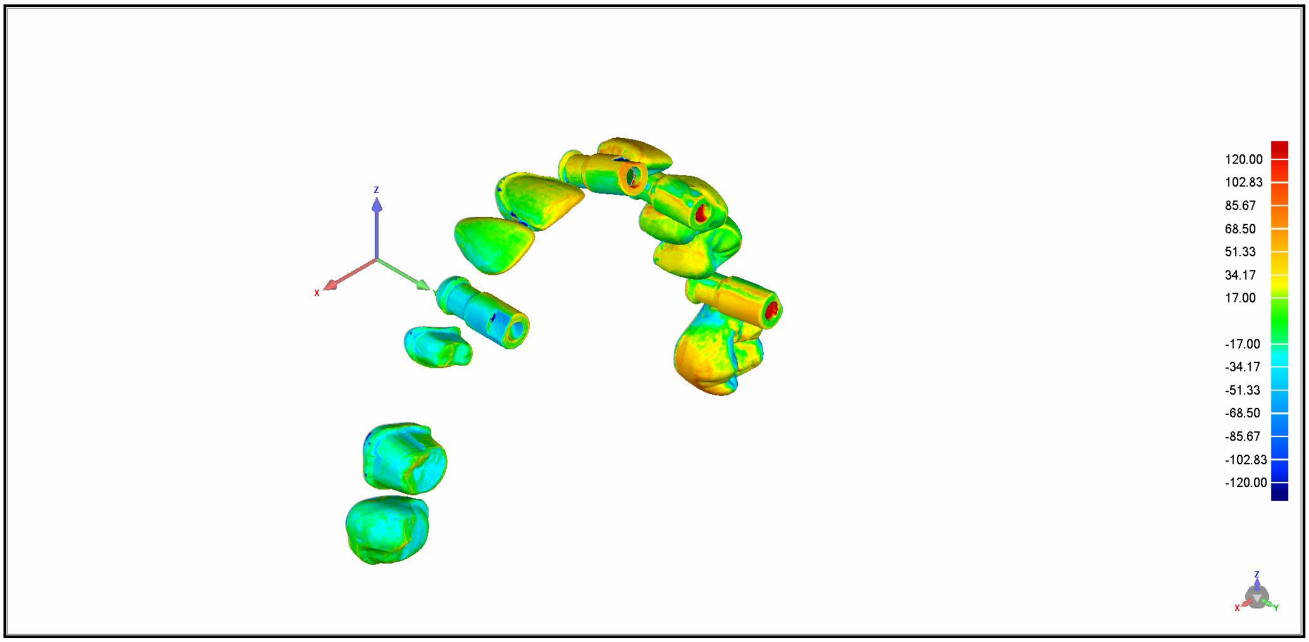

Predefinido: Frente

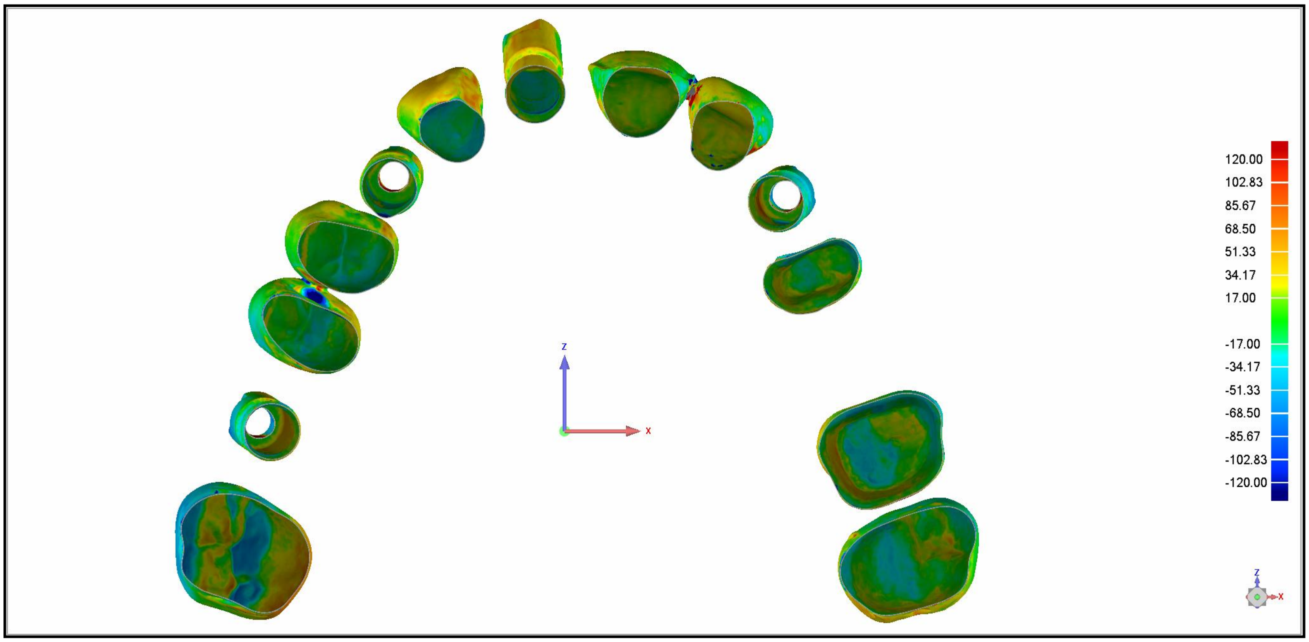

Predefinido: Atrás

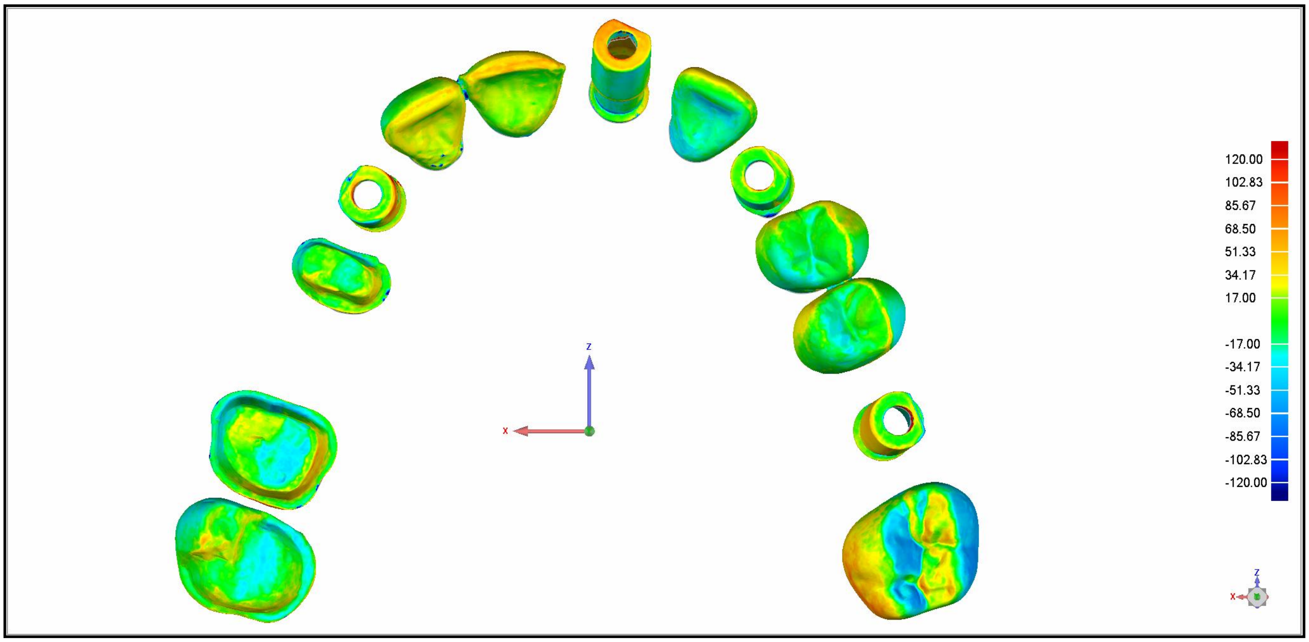

Predefinido: Izquierda

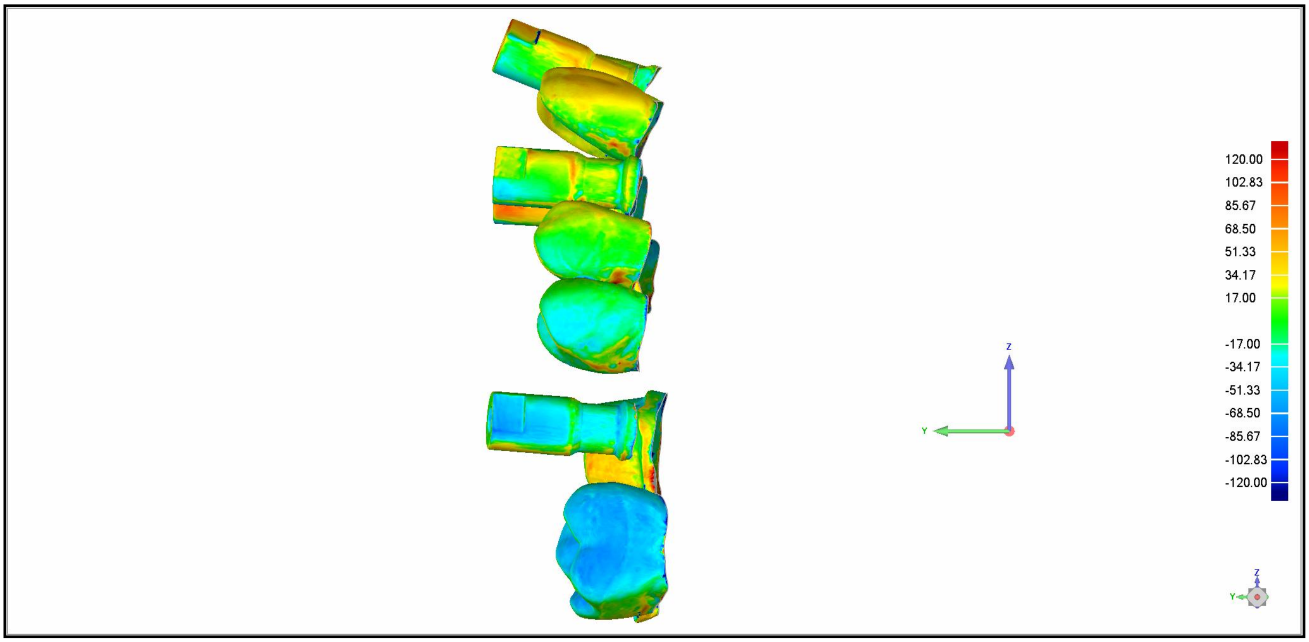

Predefinido: Derecha

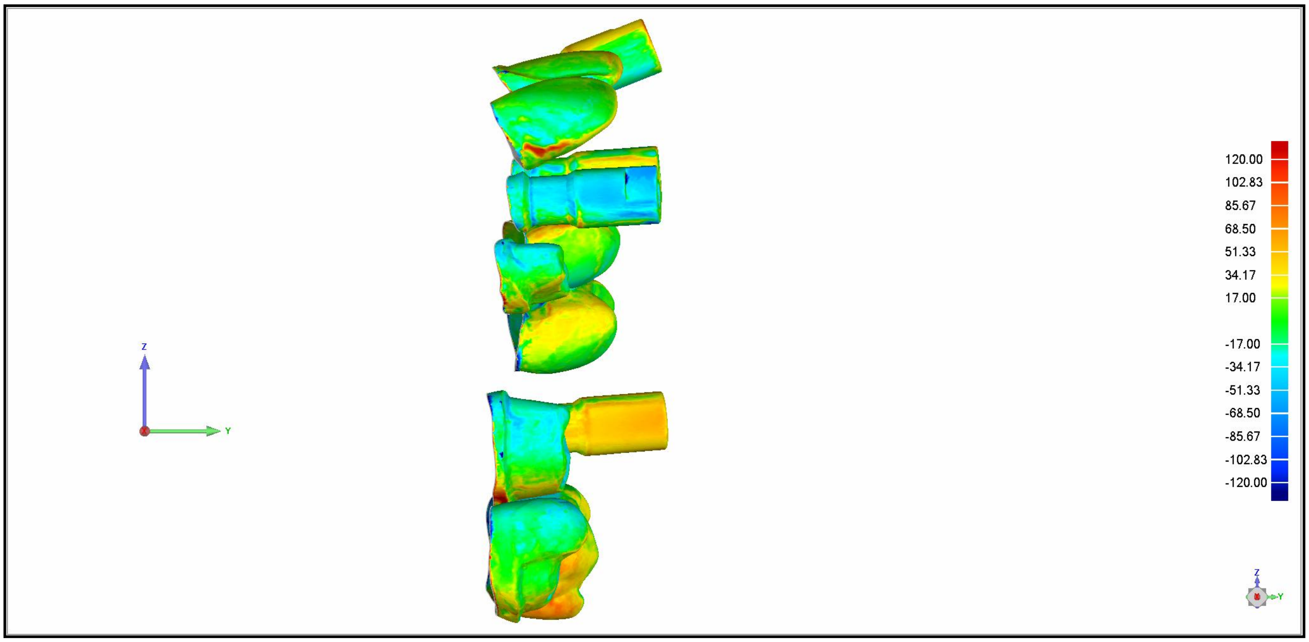

Predefinido: Superior

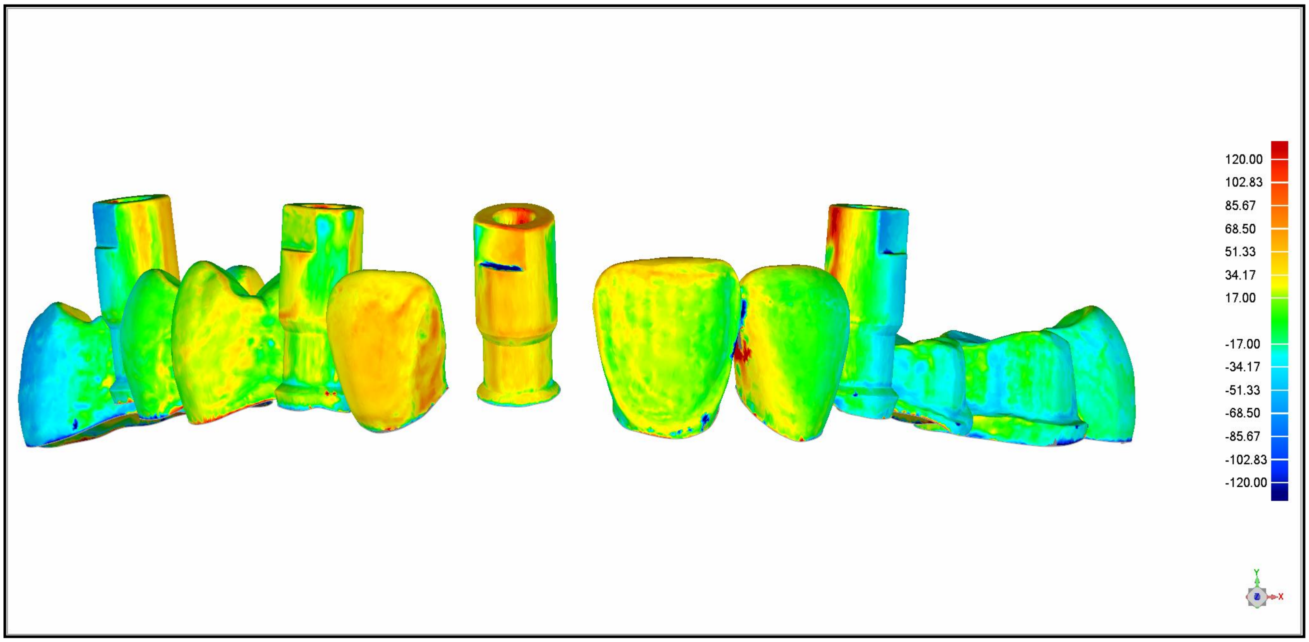

Predefinido: Inferior

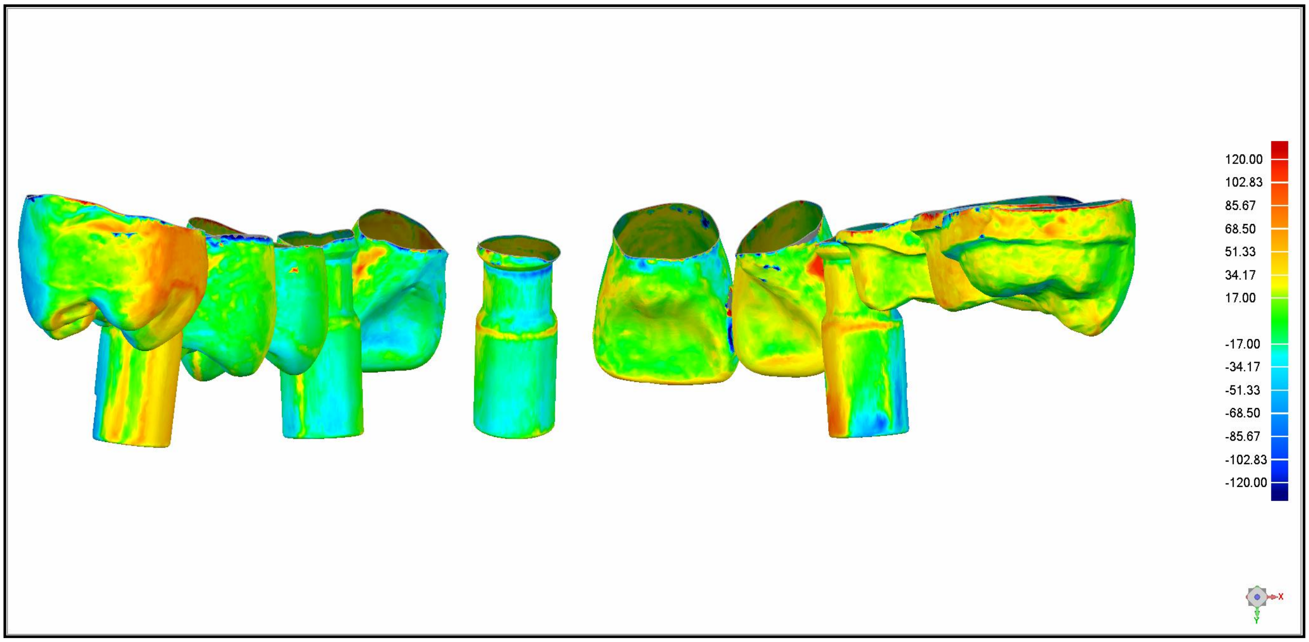

Supplement: S2 Table — Trios (scanning strategy B). (ZIP) [file pone.0202916.s002.zip › S2/3S1B.pdf]
