# Supplementary material for: Accuracy of four digital scanners according to scanning strategy in complete-arch impressions
Source: PLoS One. 2018 Sep 13;13(9):e0202916. doi: 10.1371/journal.pone.0202916 (PMC6136706; doi:10.1371/journal.pone.0202916)

Geomagic Control™

Fecha: 3/6/2017, 12:36 pm

3D Comparación Resultados

Modelo referencia	MRC
Modelo test	3S6B
Nº de puntos de datos	99746
# Aislados	76

Tipo tolerancia	3D desviación
Unidades	u
Máx. crítico	120.00
Máx. nominal	18.00
Mín. nominal	-18.00
Mín. crítico	-120.00

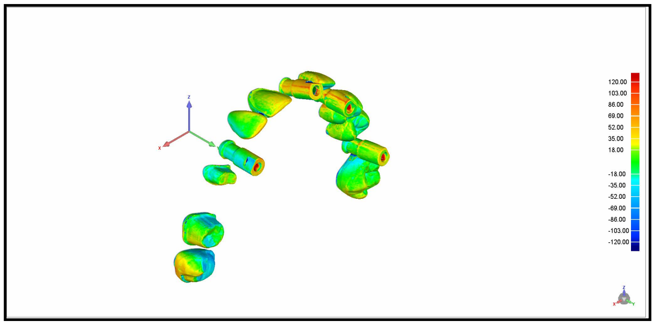

Desviación	
Desviación superior máx.	3104.44
Desviación inferior máx.	-3147.02
Desviación media	60.70 /-50.28
Desviación estándar	190.74

Distribución desviación

>=Min	<Max	# Puntos	%
-120.00	-103.00	308	0.31
-103.00	-86.00	387	0.39
-86.00	-69.00	574	0.58
-69.00	-52.00	1791	1.80
-52.00	-35.00	4046	4.06
-35.00	-18.00	9632	9.66
-18.00	18.00	44480	44.59
18.00	35.00	18620	18.67
35.00	52.00	7883	7.90
52.00	69.00	3423	3.43
69.00	86.00	1394	1.40
86.00	103.00	707	0.71
103.00	120.00	490	0.49

Fuera del crítico superior	4217	4.23
Fuera del crítico inferior	1794	1.80

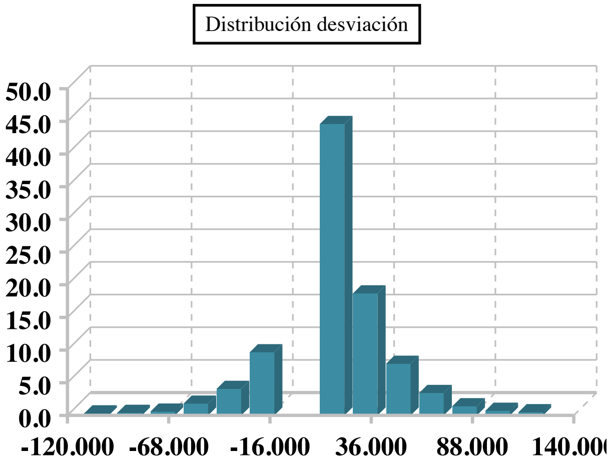

Desviaciones estándar

Distribución (+/-)	# Puntos	%
-6 * Desv. estánd.	471	0.47
-5 * Desv. estánd.	58	0.06
-4 * Desv. estánd.	59	0.06
-3 * Desv. estánd.	121	0.12
-2 * Desv. estánd.	539	0.54
-1 * Desv. estánd.	62898	63.06
1 * Desv. estánd.	32688	32.77
2 * Desv. estánd.	989	0.99
3 * Desv. estánd.	430	0.43
4 * Desv. estánd.	343	0.34
5 * Desv. estánd.	306	0.31
6 * Desv. estánd.	844	0.85

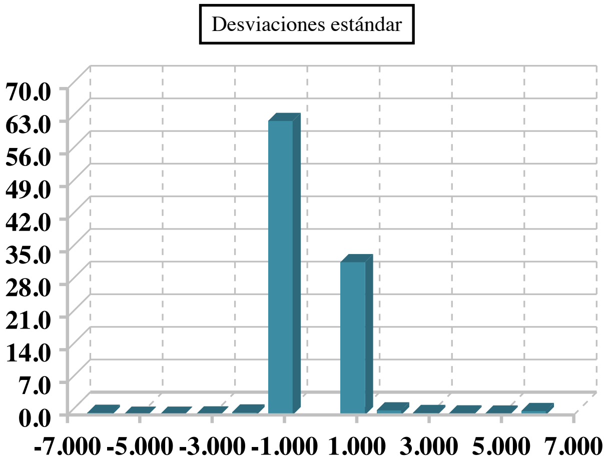

Geomagic Control™

Predefinido: Isométrico

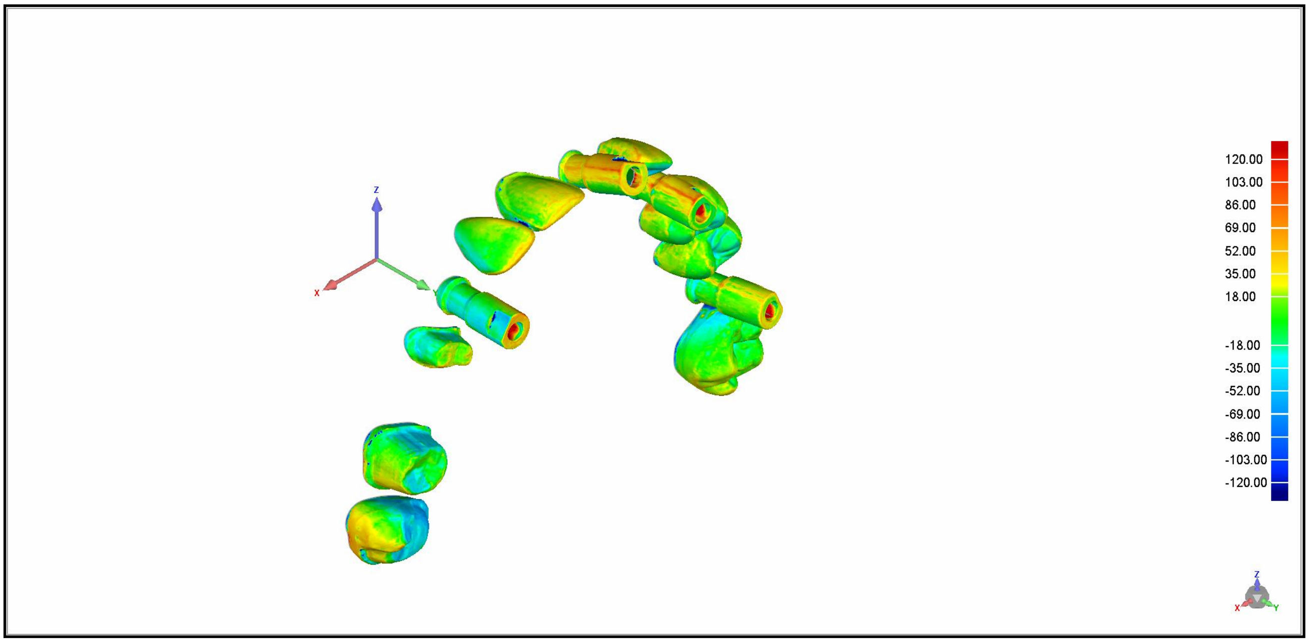

Predefinido: Frente

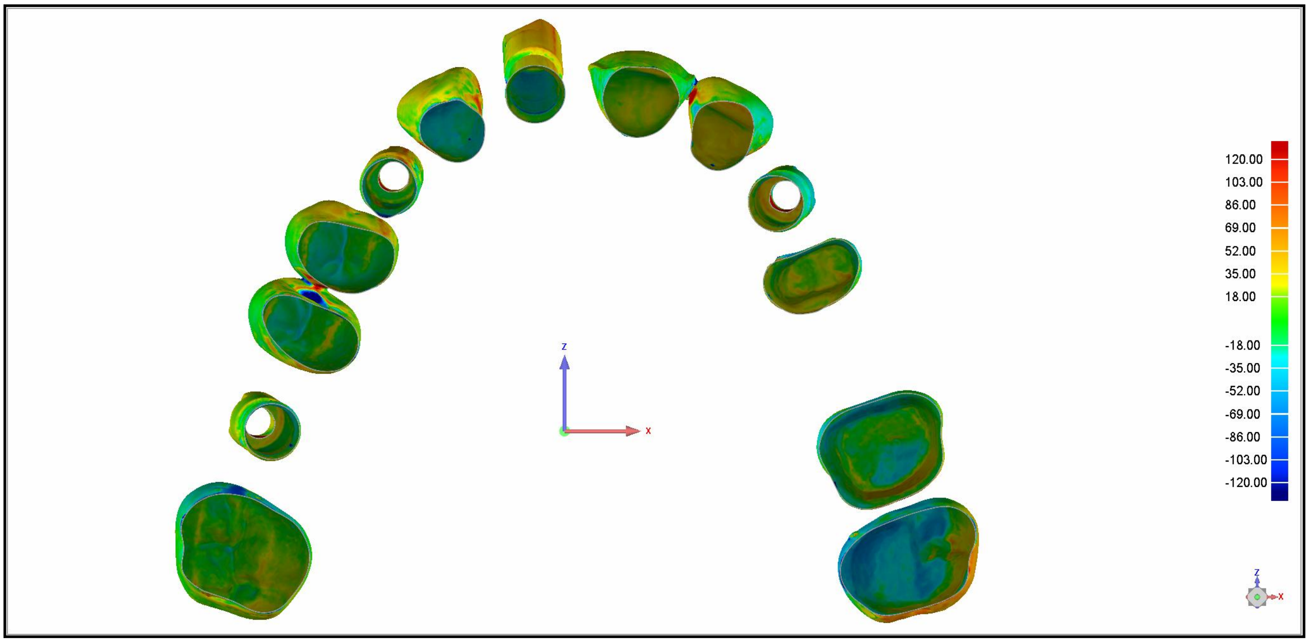

Predefinido: Atrás

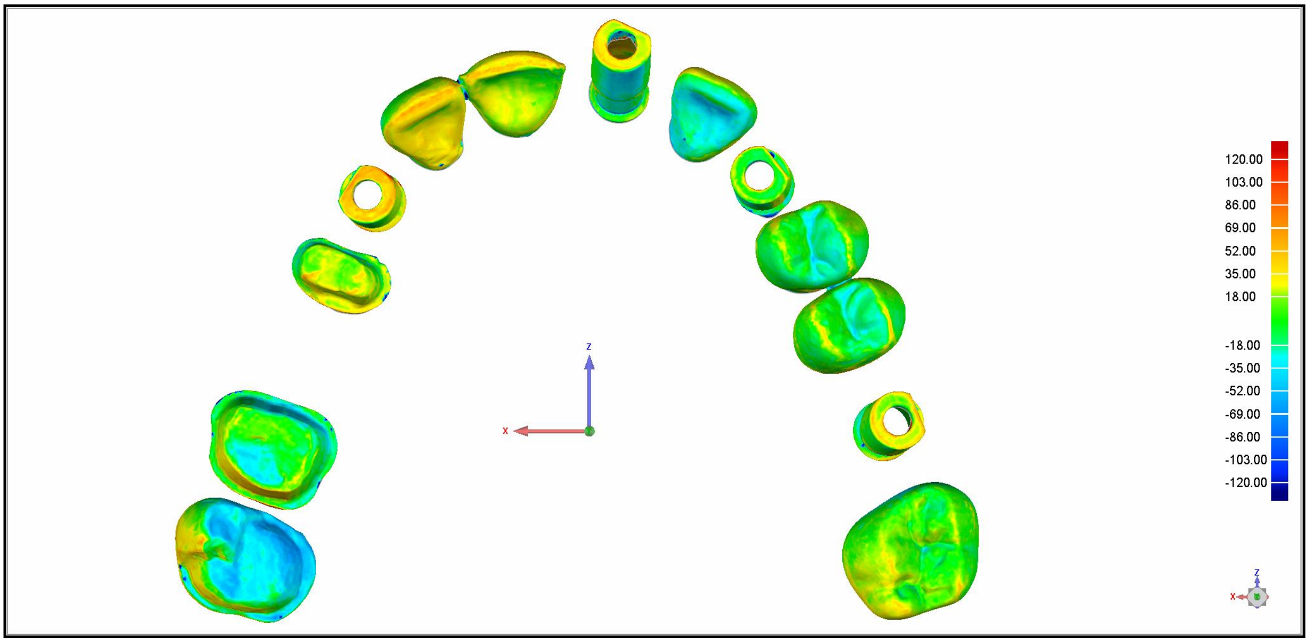

Predefinido: Izquierda

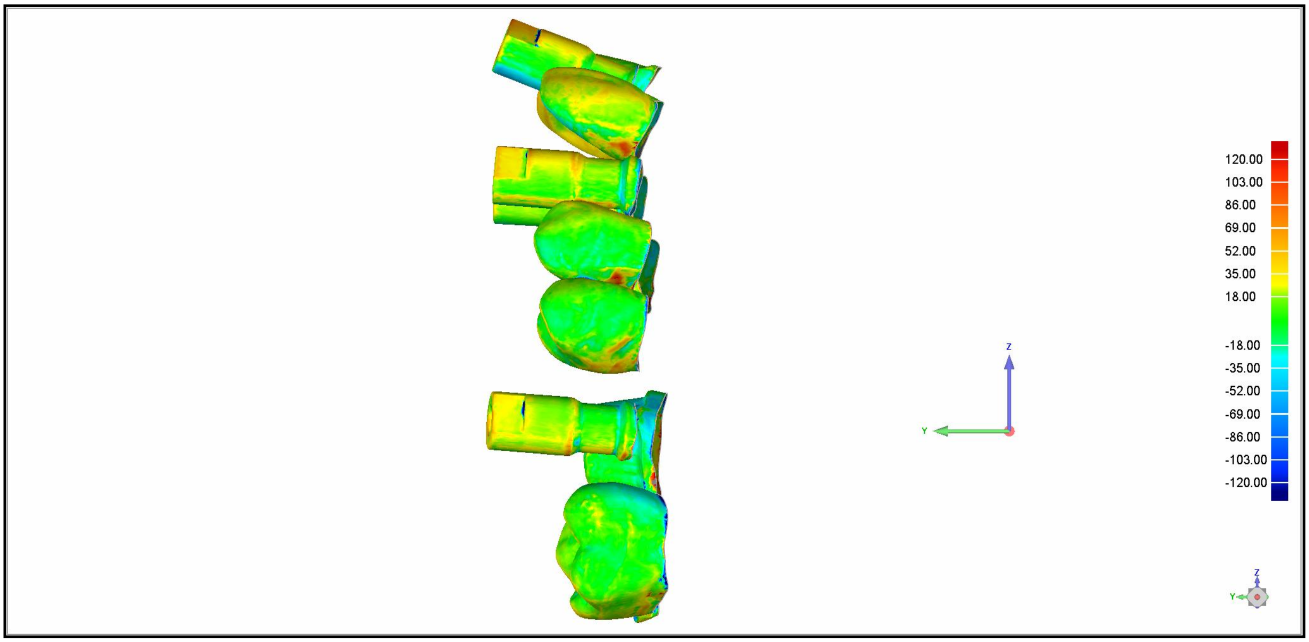

Predefinido: Derecha

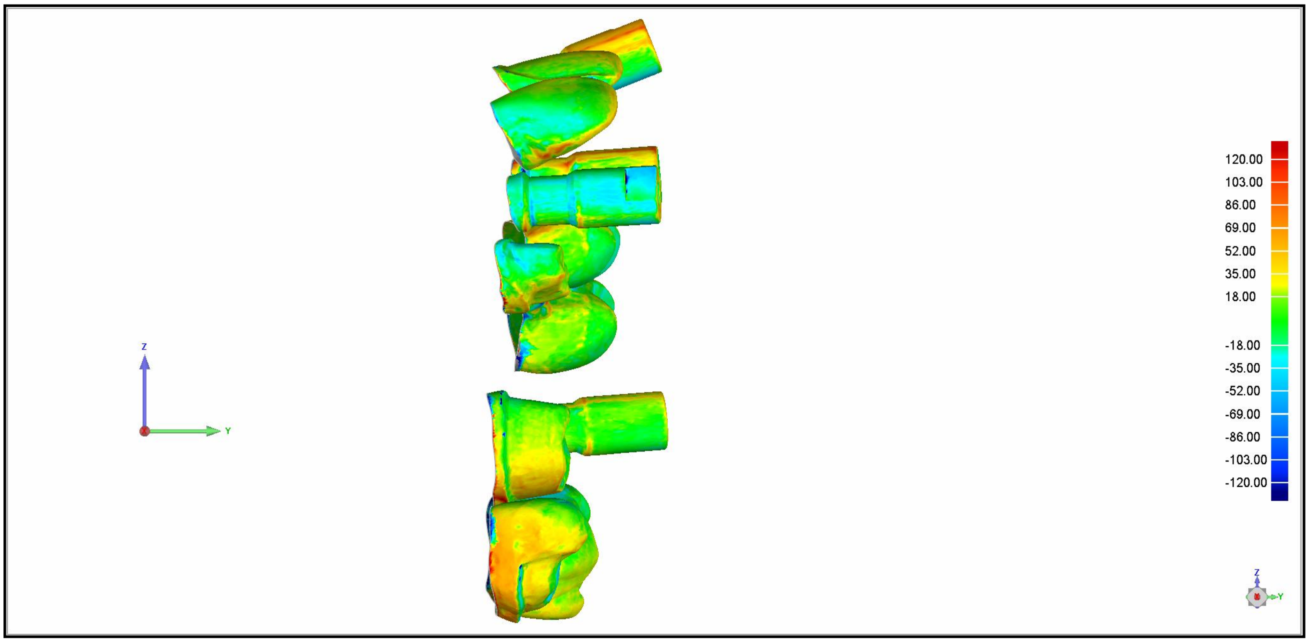

Predefinido: Superior

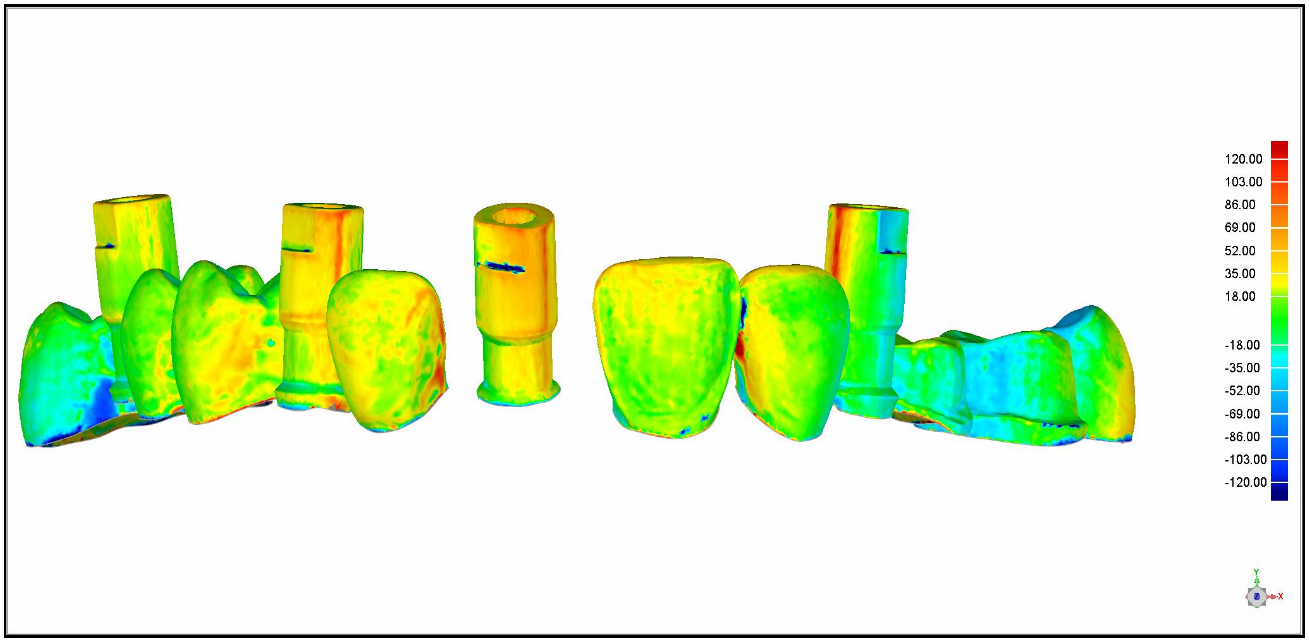

Predefinido: Inferior

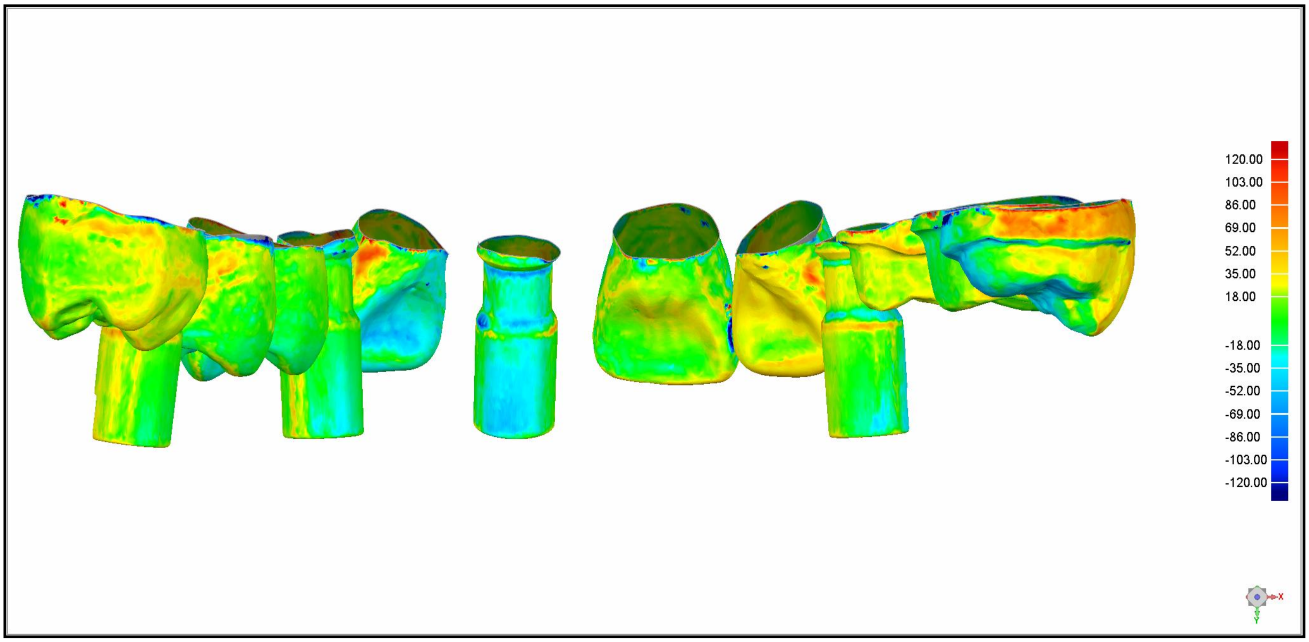

Supplement: S2 Table — Trios (scanning strategy B). (ZIP) [file pone.0202916.s002.zip › S2/3S6B.pdf]
