# Supplementary material for: Accuracy of four digital scanners according to scanning strategy in complete-arch impressions
Source: PLoS One. 2018 Sep 13;13(9):e0202916. doi: 10.1371/journal.pone.0202916 (PMC6136706; doi:10.1371/journal.pone.0202916)

3D Comparación Resultados

Modelo referencia	MRC
Modelo test	3S7B
Nº de puntos de datos	99769
# Aislados	104

Tipo tolerancia	3D desviación
Unidades	u
Máx. crítico	120.00
Máx. nominal	17.00
Mín. nominal	-17.00
Mín. crítico	-120.00

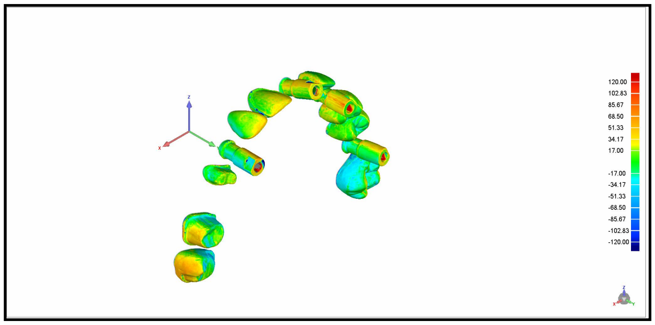

Desviación	
Desviación superior máx.	3154.33
Desviación inferior máx.	-3111.81
Desviación media	59.83 /-47.21
Desviación estándar	193.62

Distribución desviación

>=Min	<Max	# Puntos	%
-120.00	-102.83	261	0.26
-102.83	-85.67	348	0.35
-85.67	-68.50	612	0.61
-68.50	-51.33	1544	1.55
-51.33	-34.17	4031	4.04
-34.17	-17.00	10465	10.49
-17.00	17.00	45034	45.14
17.00	34.17	17473	17.51
34.17	51.33	9262	9.28
51.33	68.50	3443	3.45
68.50	85.67	1046	1.05
85.67	102.83	540	0.54
102.83	120.00	355	0.36

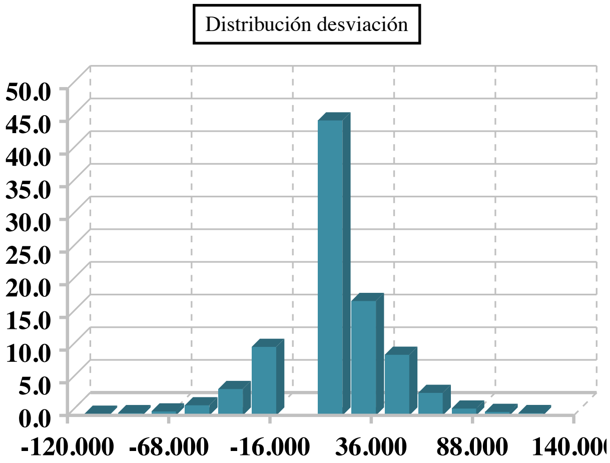

Fuera del crítico superior		3431	3.44
Fuera del crítico inferior		1924	1.93

Desviaciones estándar

Distribución (+/-)	# Puntos	%
-6 * Desv. estánd.	373	0.37
-5 * Desv. estánd.	87	0.09
-4 * Desv. estánd.	115	0.12
-3 * Desv. estánd.	195	0.20
-2 * Desv. estánd.	589	0.59
-1 * Desv. estánd.	63372	63.52
1 * Desv. estánd.	32561	32.64
2 * Desv. estánd.	652	0.65
3 * Desv. estánd.	312	0.31
4 * Desv. estánd.	305	0.31
5 * Desv. estánd.	296	0.30
6 * Desv. estánd.	912	0.91

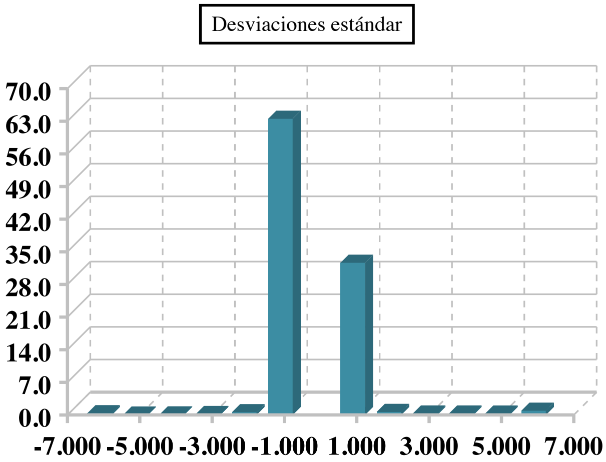

Predefinido: Isométrico

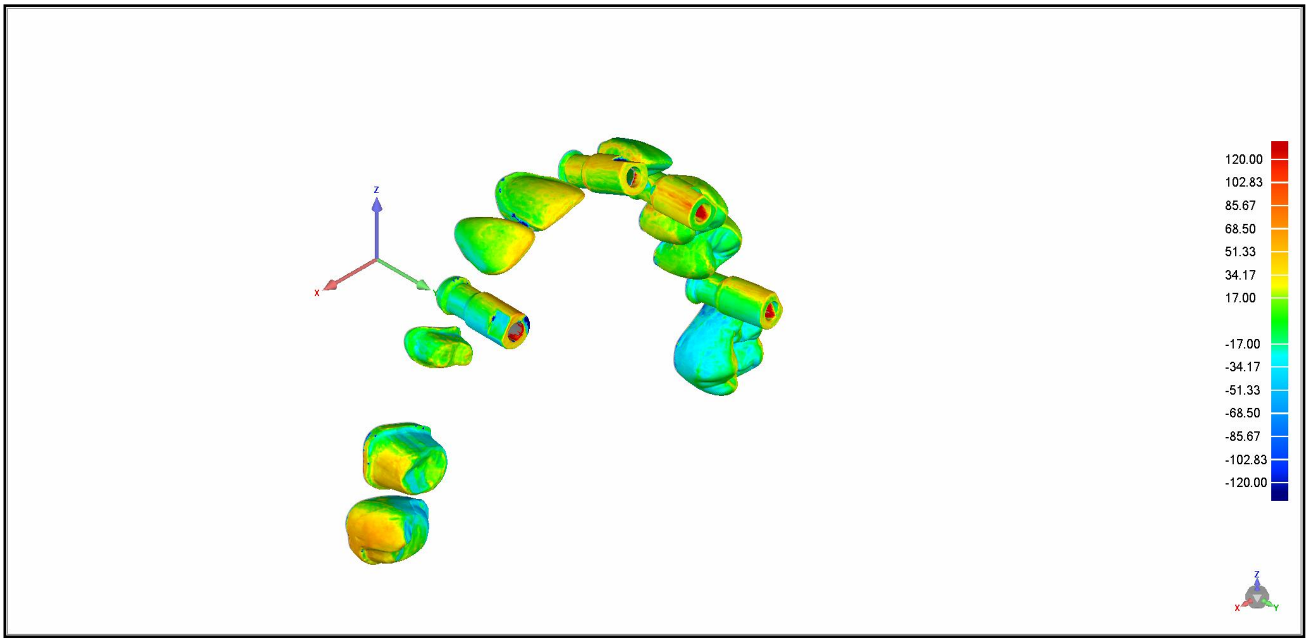

Predefinido: Frente

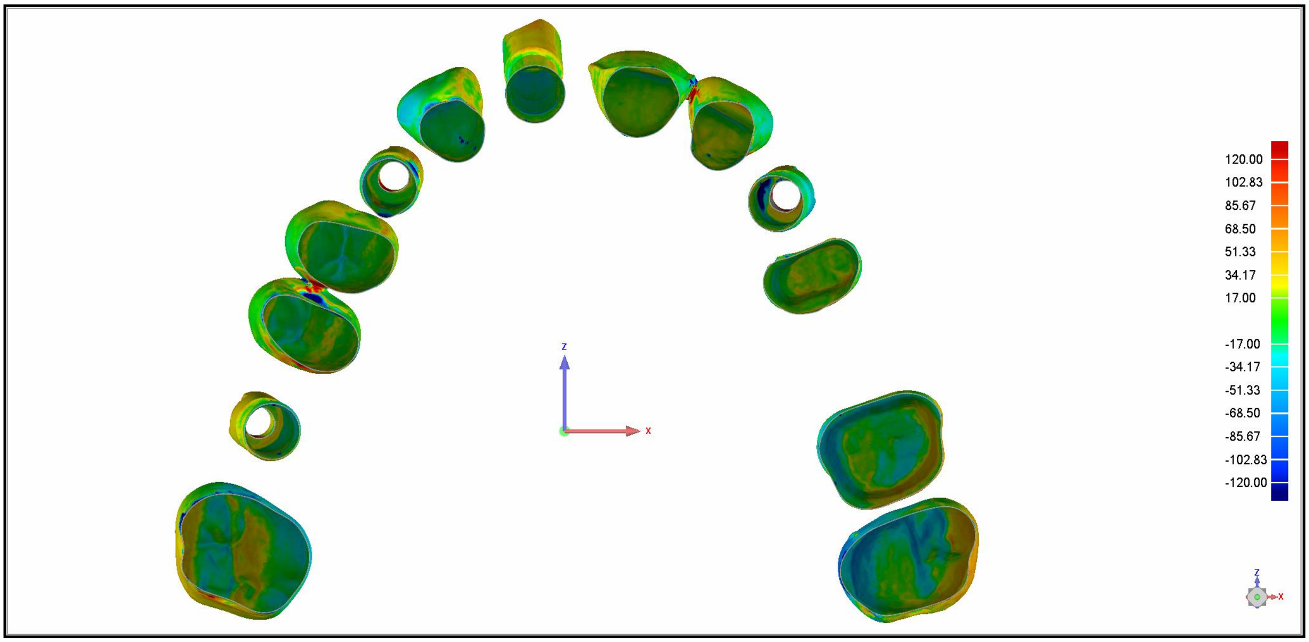

Fecha: 3/6/2017, 12:38 pm

Predefinido: Atrás

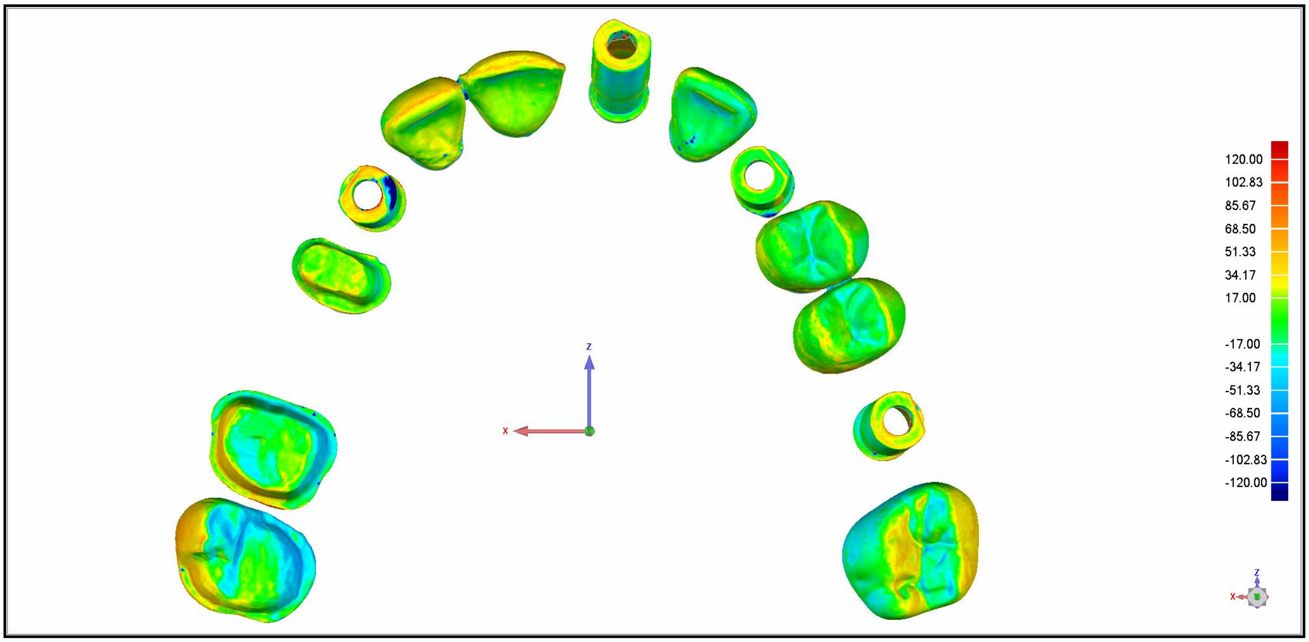

Predefinido: Izquierda

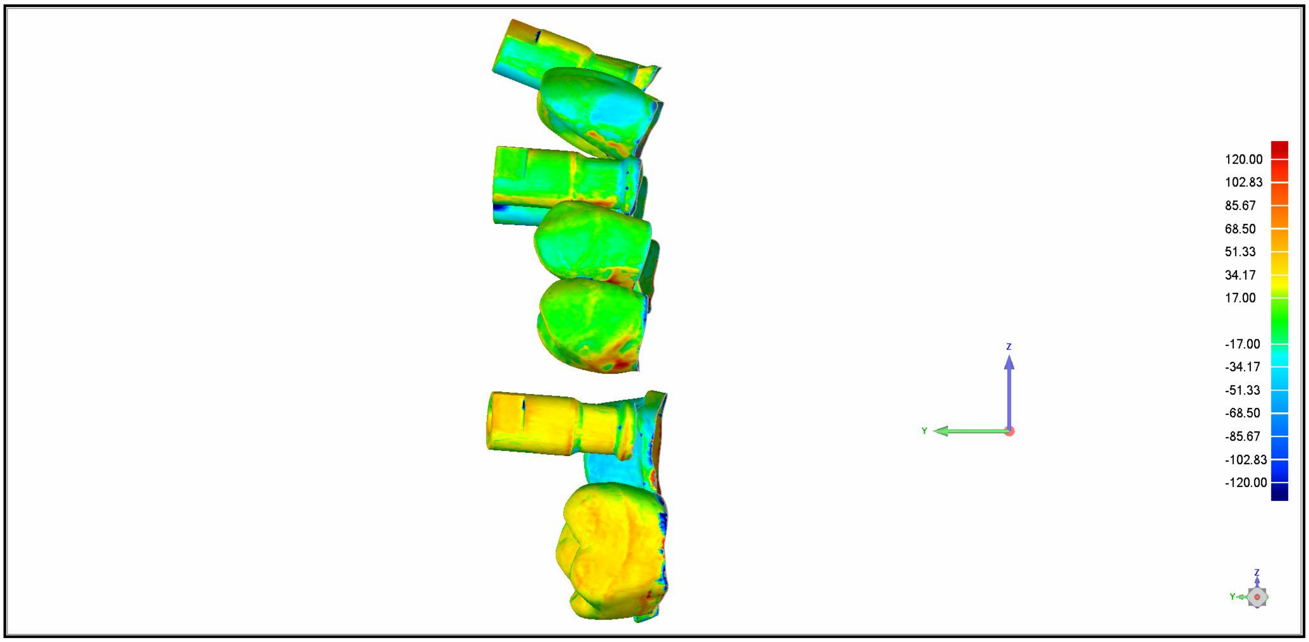

Predefinido: Derecha

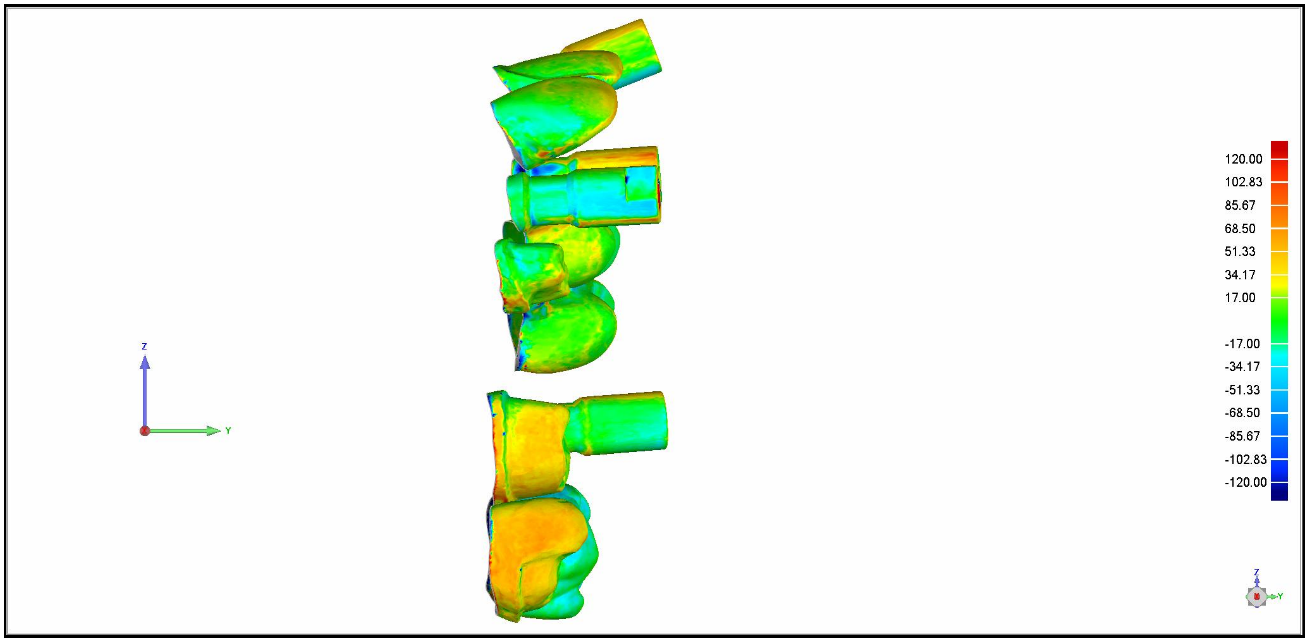

Predefinido: Superior

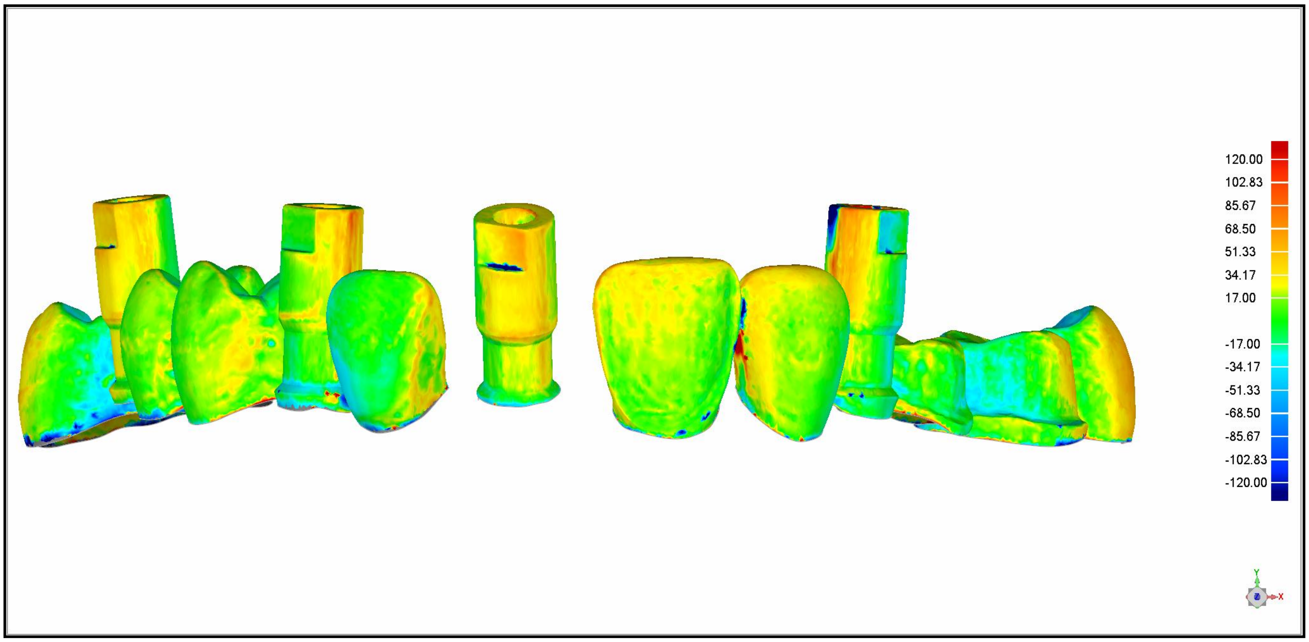

Geomagic Control™

Fecha: 3/6/2017, 12:38 pm

Predefinido: Inferior

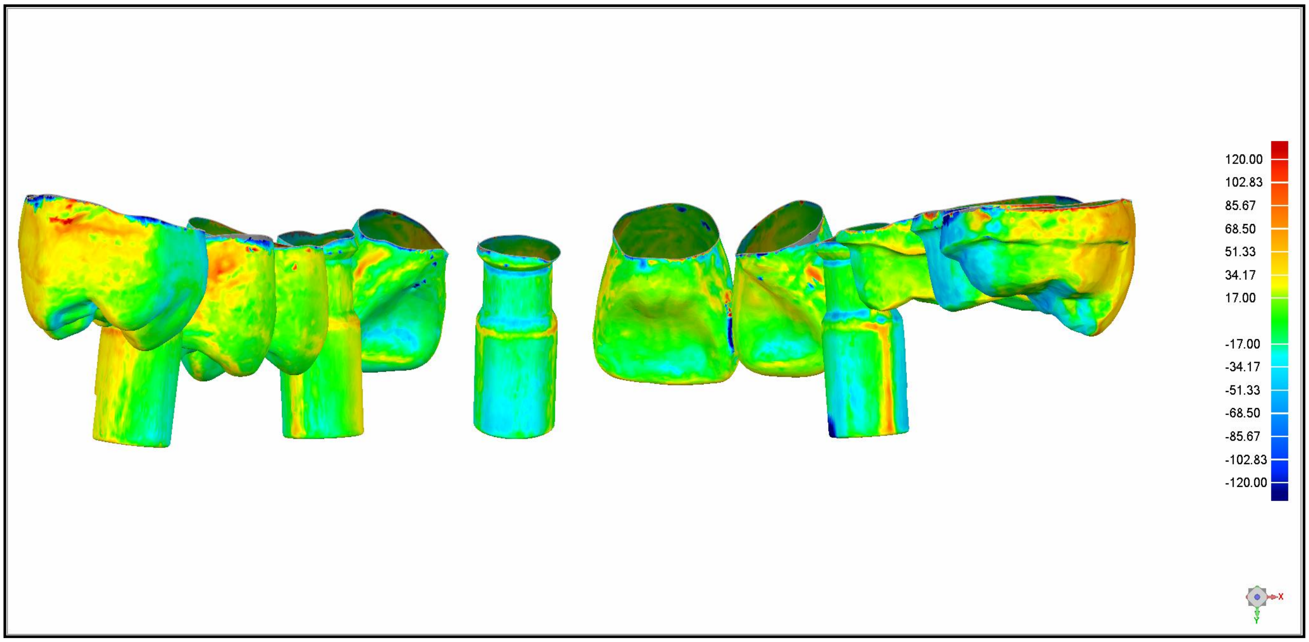

Supplement: S2 Table — Trios (scanning strategy B). (ZIP) [file pone.0202916.s002.zip › S2/3S7B.pdf]
