# Supplementary material for: Accuracy of four digital scanners according to scanning strategy in complete-arch impressions
Source: PLoS One. 2018 Sep 13;13(9):e0202916. doi: 10.1371/journal.pone.0202916 (PMC6136706; doi:10.1371/journal.pone.0202916)

Fecha: 3/6/2017, 12:20 pm

3D Comparación Resultados

Modelo referencia	MRC
Modelo test	3S4C
Nº de puntos de datos	98135
# Aislados	82

Tipo tolerancia	3D desviación
Unidades	u
Máx. crítico	120.00
Máx. nominal	18.00
Mín. nominal	-18.00
Mín. crítico	-120.00

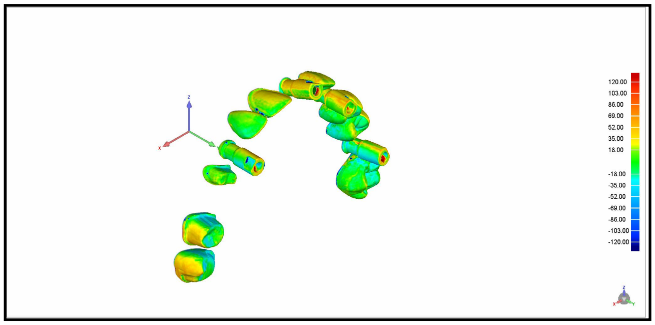

Desviación	
Desviación superior máx.	3153.79
Desviación inferior máx.	-3123.78
Desviación media	55.38 /-50.21
Desviación estándar	175.21

Distribución desviación

>=Min	<Max	# Puntos	%
-120.00	-103.00	293	0.30
-103.00	-86.00	377	0.38
-86.00	-69.00	563	0.57
-69.00	-52.00	1153	1.17
-52.00	-35.00	3220	3.28
-35.00	-18.00	8276	8.43
-18.00	18.00	43450	44.28
18.00	35.00	19977	20.36
35.00	52.00	9248	9.42
52.00	69.00	3379	3.44
69.00	86.00	1306	1.33
86.00	103.00	709	0.72
103.00	120.00	493	0.50

Fuera del crítico superior	3695	3.77
Fuera del crítico inferior	1996	2.03

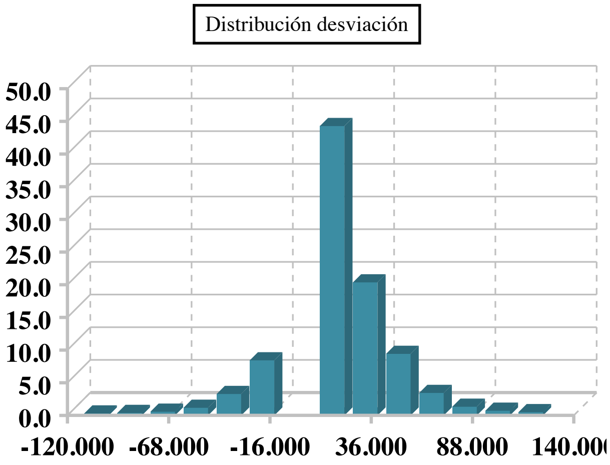

Desviaciones estándar

Distribución (+/-)	# Puntos	%
-6 * Desv. estánd.	462	0.47
-5 * Desv. estánd.	89	0.09
-4 * Desv. estánd.	108	0.11
-3 * Desv. estánd.	173	0.18
-2 * Desv. estánd.	701	0.71
-1 * Desv. estánd.	58682	59.80
1 * Desv. estánd.	35269	35.94
2 * Desv. estánd.	916	0.93
3 * Desv. estánd.	394	0.40
4 * Desv. estánd.	280	0.29
5 * Desv. estánd.	250	0.25
6 * Desv. estánd.	811	0.83

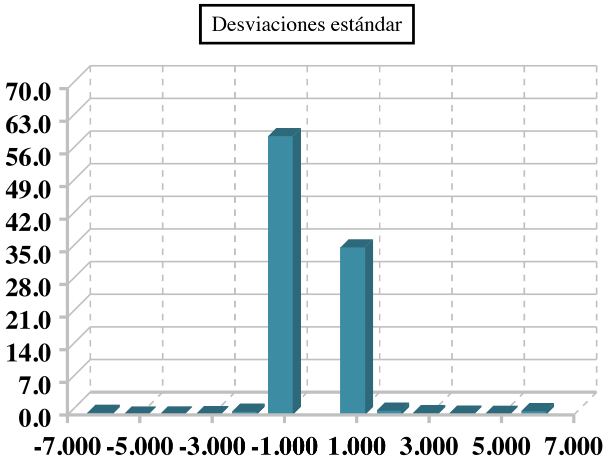

Predefinido: Isométrico

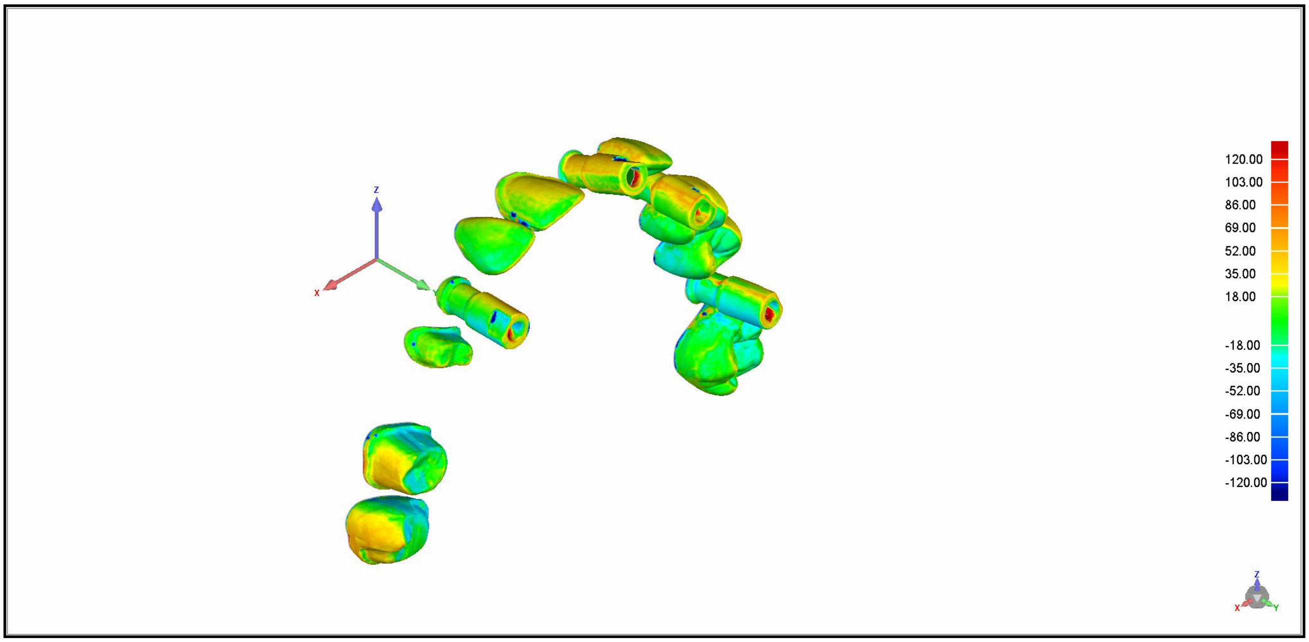

Predefinido: Frente

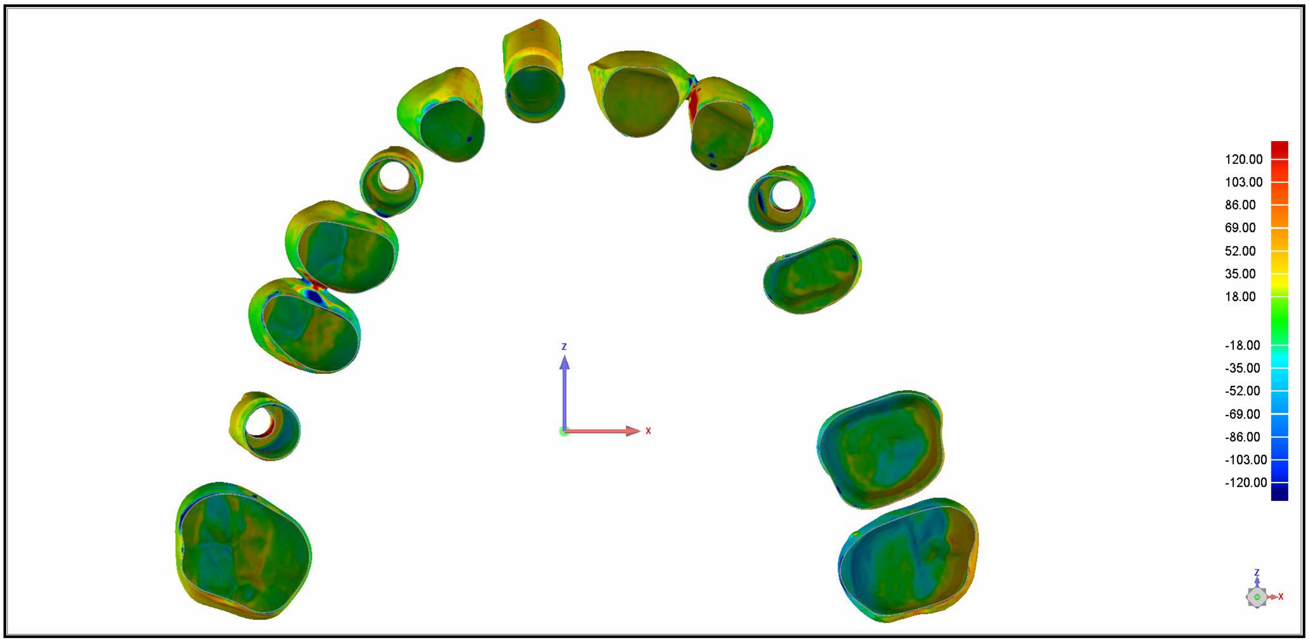

Predefinido: Atrás

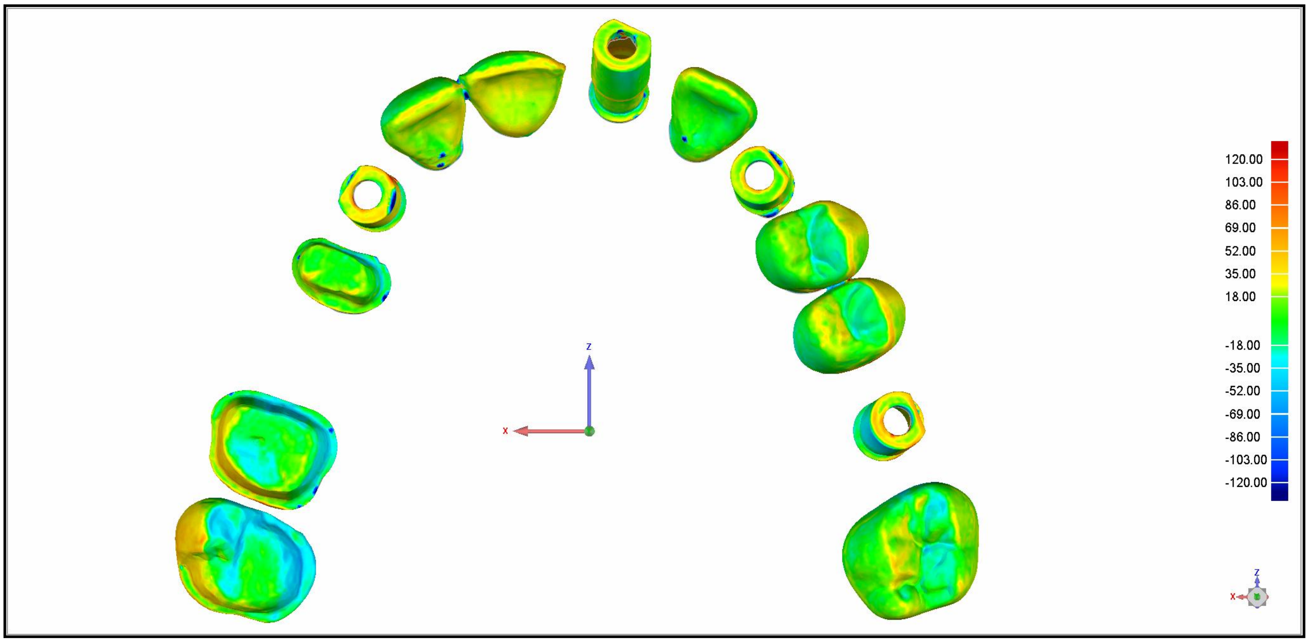

Predefinido: Izquierda

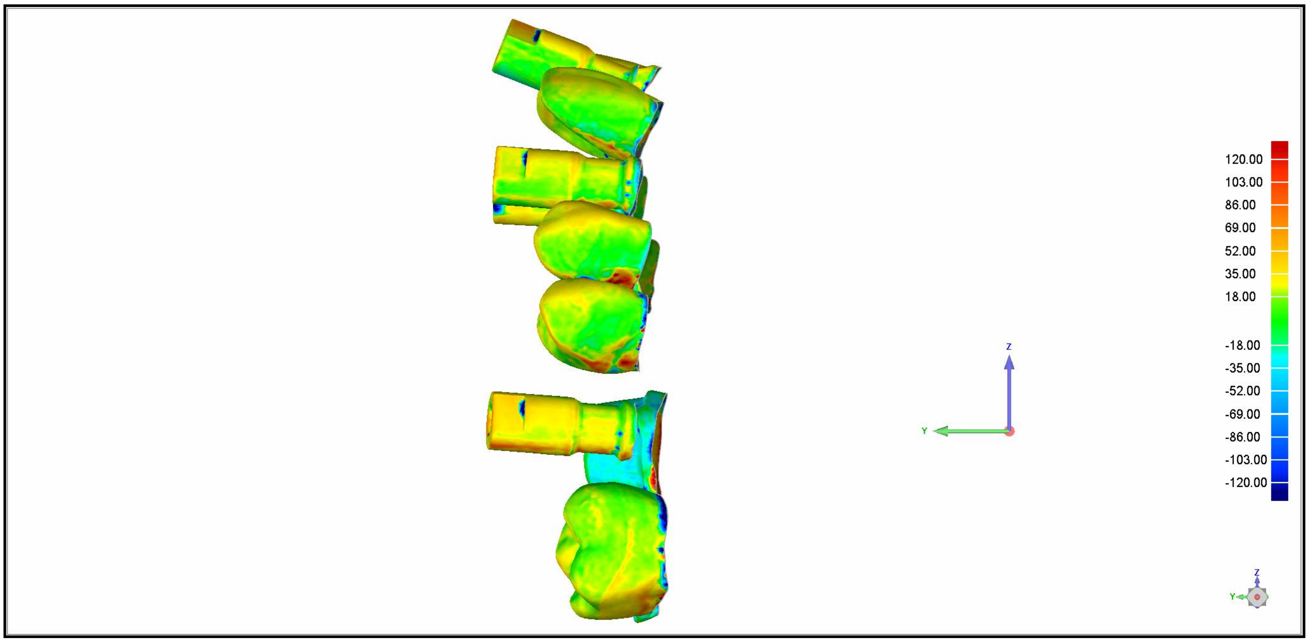

Predefinido: Derecha

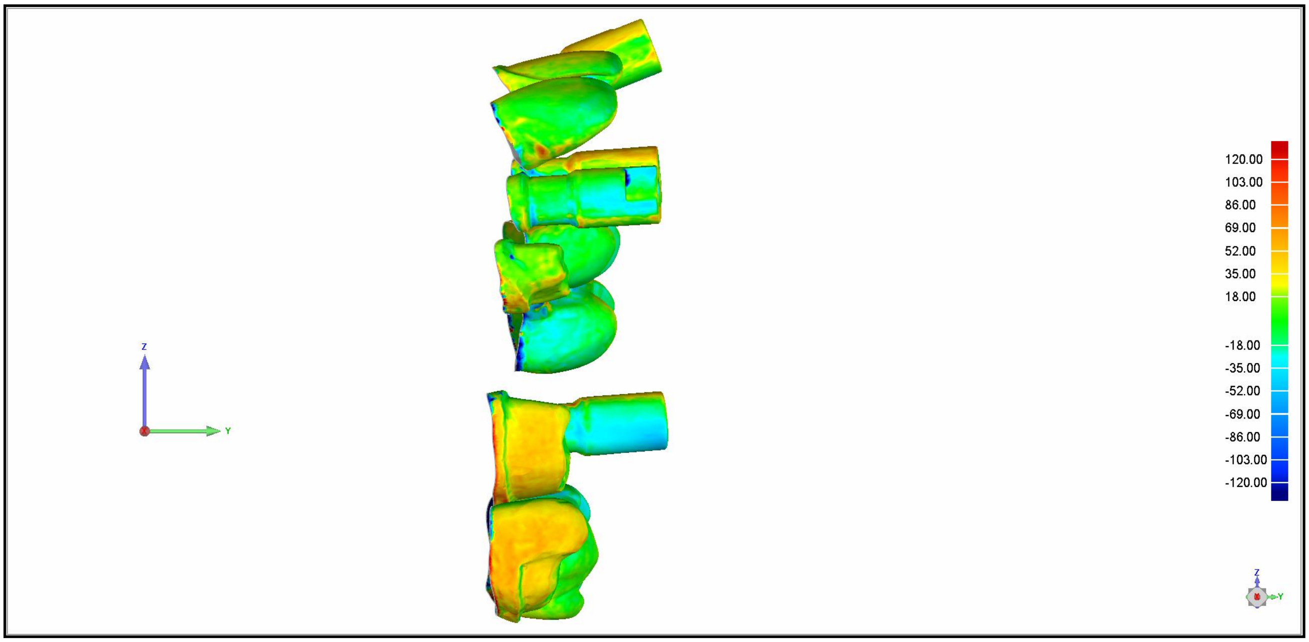

Predefinido: Superior

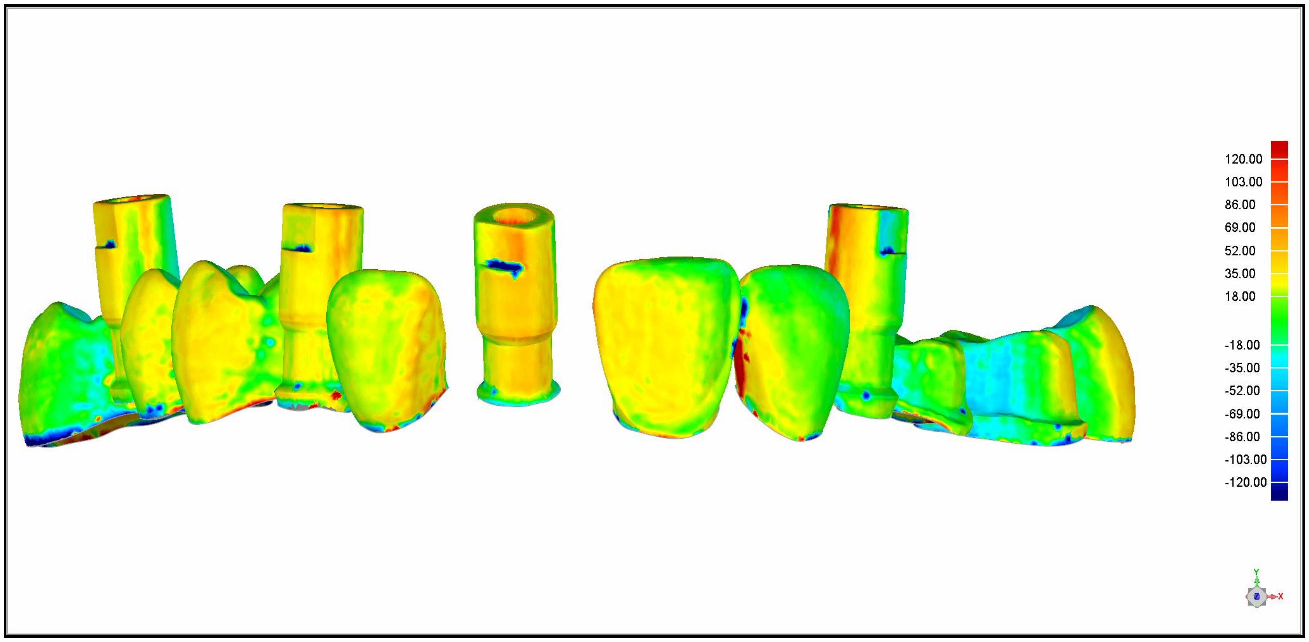

Predefinido: Inferior

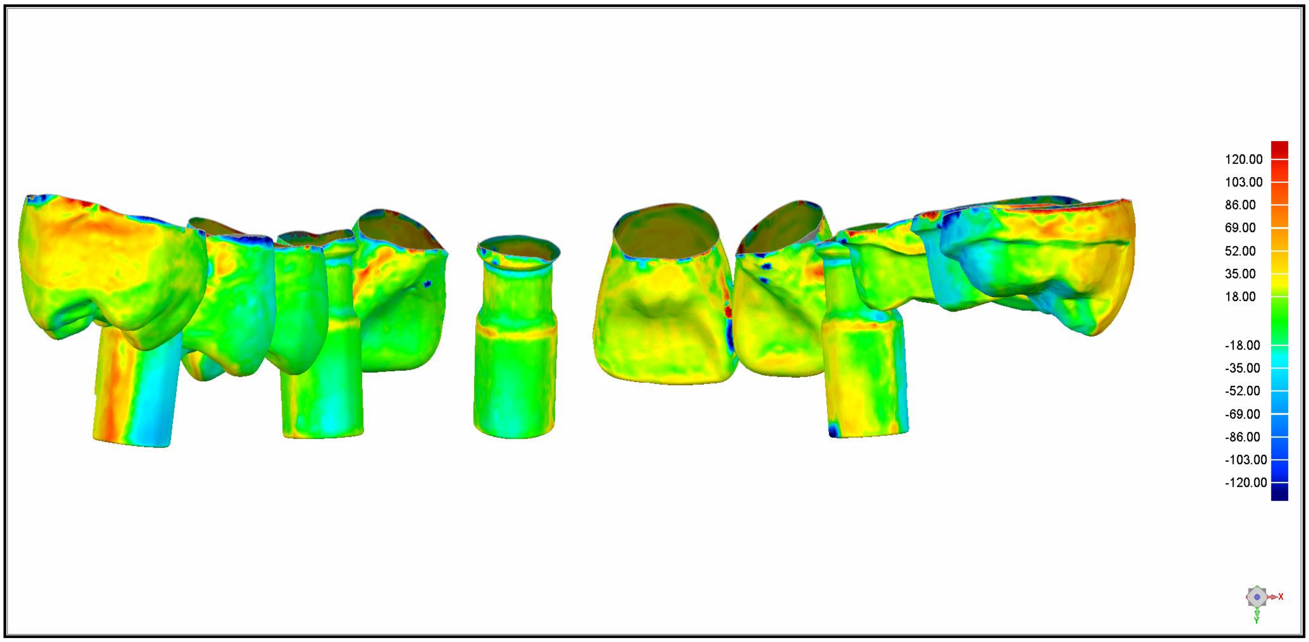

Supplement: S3 Table — Trios (scanning strategy C). (ZIP) [file pone.0202916.s003.zip › S3/3S4C.pdf]
