# Supplementary material for: Accuracy of four digital scanners according to scanning strategy in complete-arch impressions
Source: PLoS One. 2018 Sep 13;13(9):e0202916. doi: 10.1371/journal.pone.0202916 (PMC6136706; doi:10.1371/journal.pone.0202916)

Fecha: 3/6/2017, 12:41 pm

3D Comparación Resultados

Modelo referencia	MRC
Modelo test	3S8C
Nº de puntos de datos	107434
# Aislados	132

Tipo tolerancia	3D desviación
Unidades	u
Máx. crítico	120.00
Máx. nominal	13.00
Mín. nominal	-13.00
Mín. crítico	-120.00

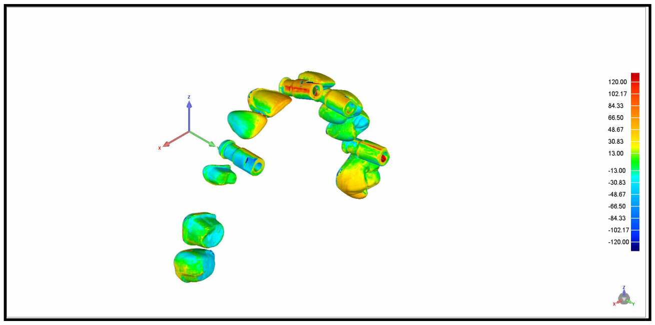

Desviación	
Desviación superior máx.	3110.88
Desviación inferior máx.	-3106.42
Desviación media	61.74 /-51.23
Desviación estándar	196.19

Distribución desviación

>=Min	<Max	# Puntos	%
-120.00	-102.17	333	0.31
-102.17	-84.33	433	0.40
-84.33	-66.50	746	0.69
-66.50	-48.67	1881	1.75
-48.67	-30.83	6675	6.21
-30.83	-13.00	16067	14.96
-13.00	13.00	36359	33.84
13.00	30.83	20667	19.24
30.83	48.67	10424	9.70
48.67	66.50	4607	4.29
66.50	84.33	1789	1.67
84.33	102.17	861	0.80
102.17	120.00	541	0.50

Fuera del crítico superior	3772	3.51
Fuera del crítico inferior	2279	2.12

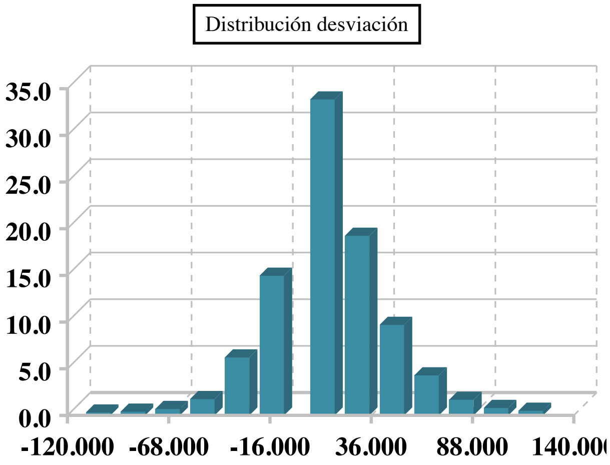

Desviaciones estándar

Distribución (+/-)	# Puntos	%
-6 * Desv. estánd.	619	0.58
-5 * Desv. estánd.	106	0.10
-4 * Desv. estánd.	141	0.13
-3 * Desv. estánd.	185	0.17
-2 * Desv. estánd.	498	0.46
-1 * Desv. estánd.	64090	59.66
1 * Desv. estánd.	39175	36.46
2 * Desv. estánd.	666	0.62
3 * Desv. estánd.	379	0.35
4 * Desv. estánd.	387	0.36
5 * Desv. estánd.	344	0.32
6 * Desv. estánd.	844	0.79

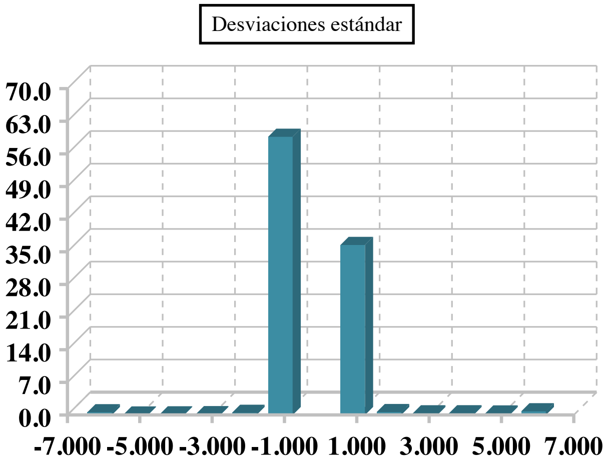

Predefinido: Isométrico

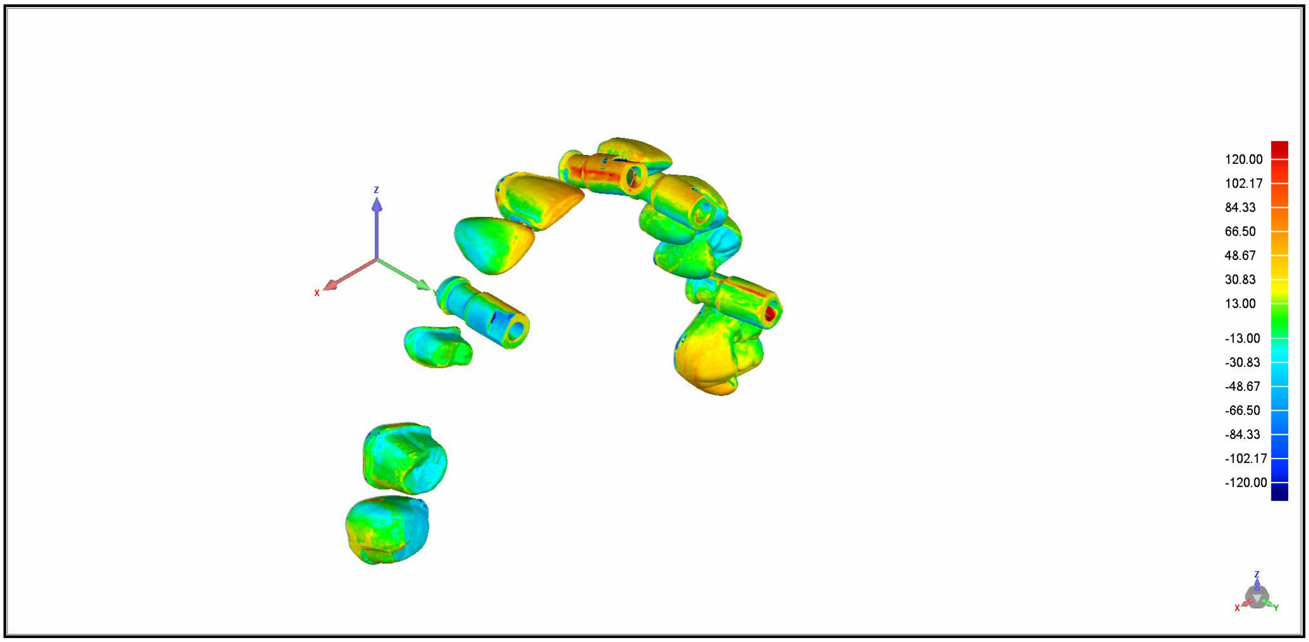

Predefinido: Frente

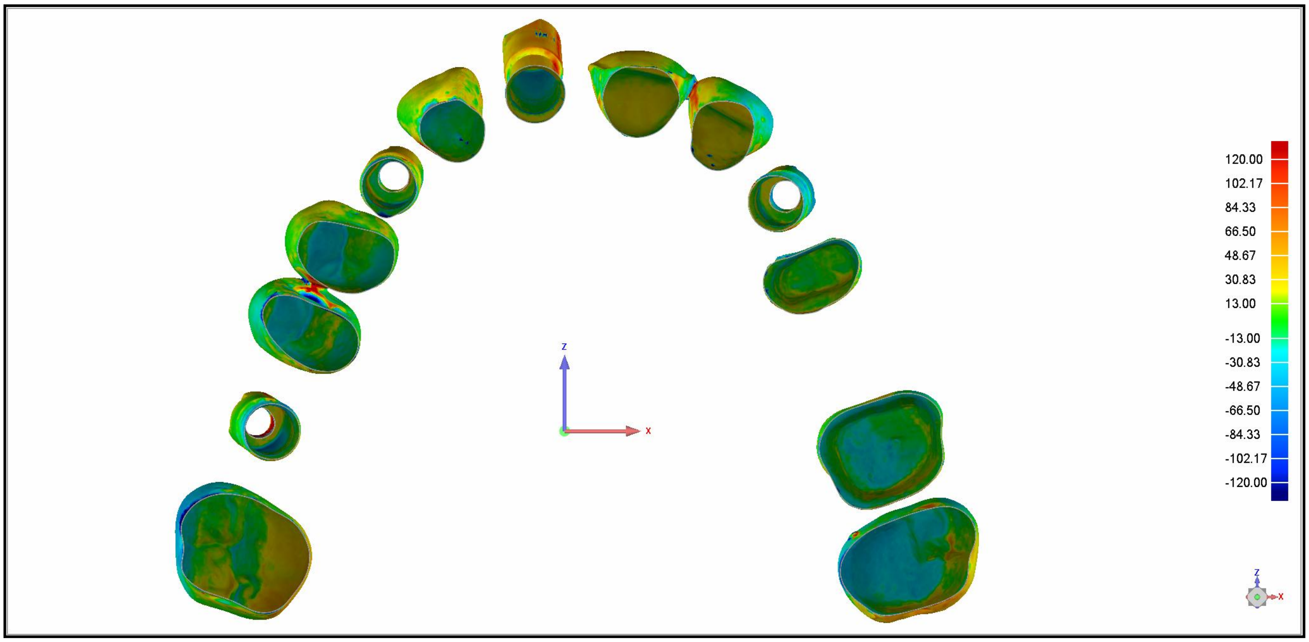

Predefinido: Atrás

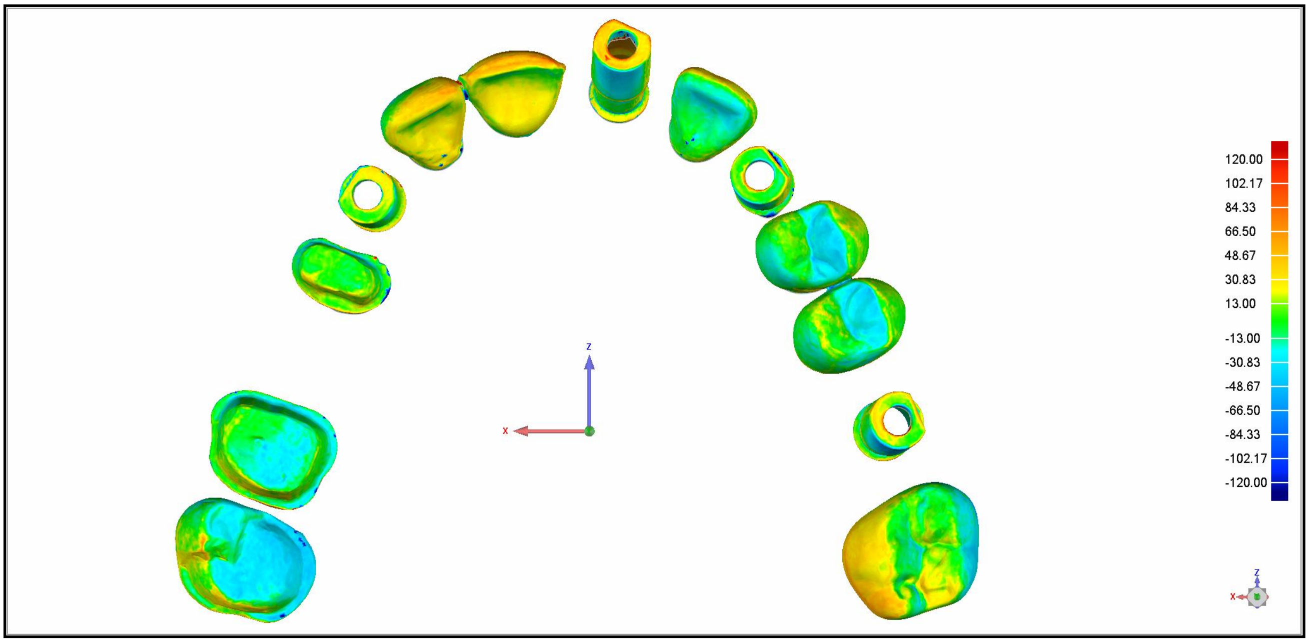

Predefinido: Izquierda

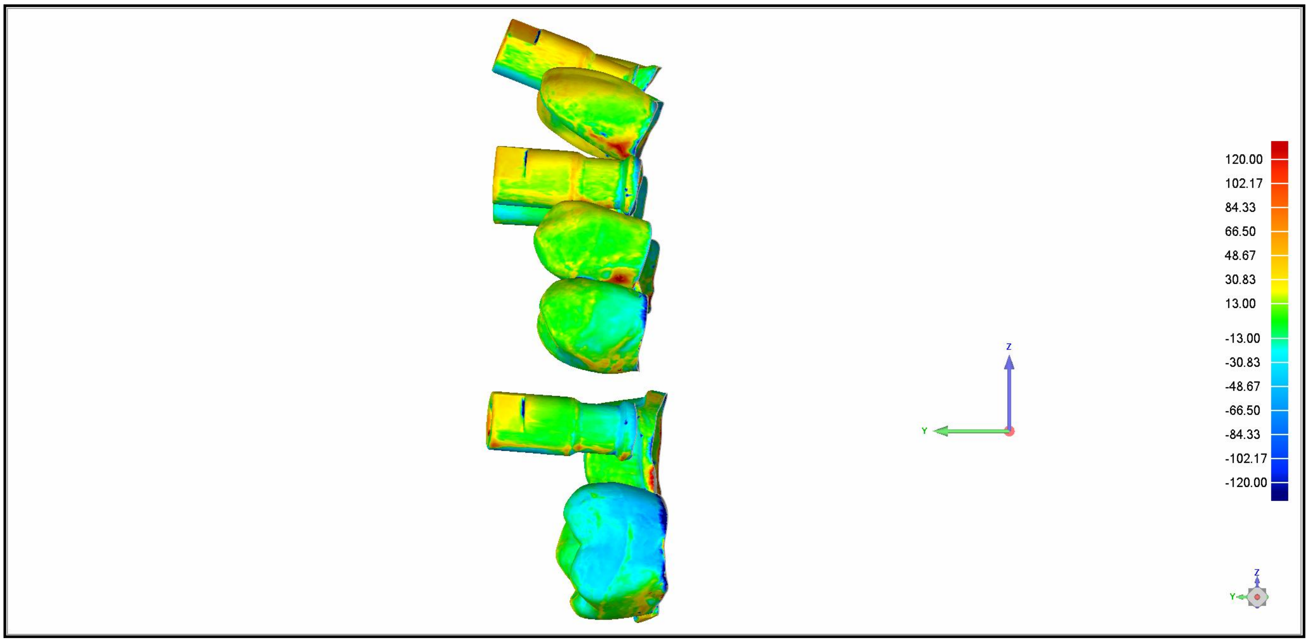

Predefinido: Derecha

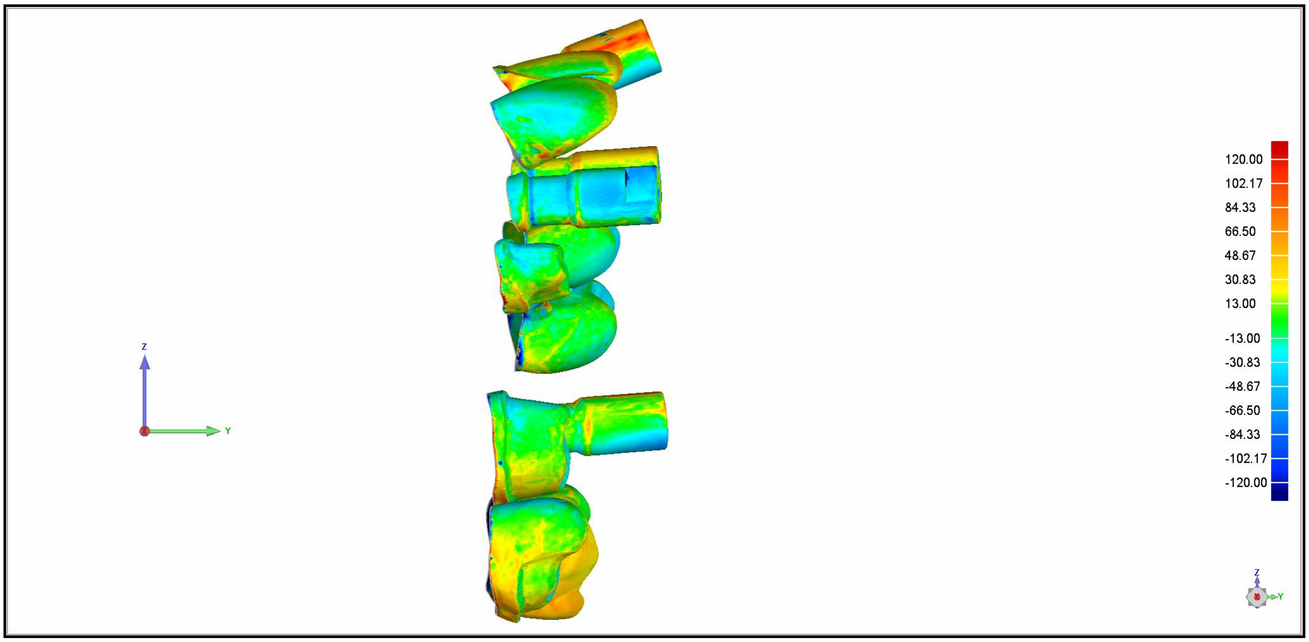

Predefinido: Superior

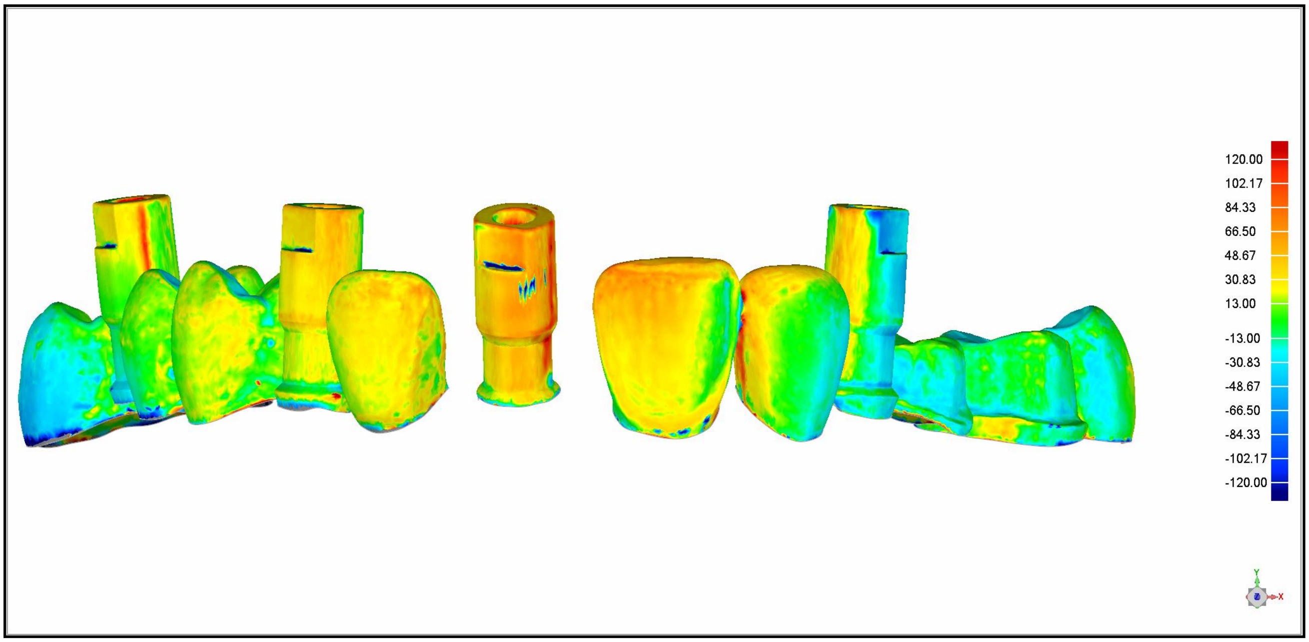

Predefinido: Inferior

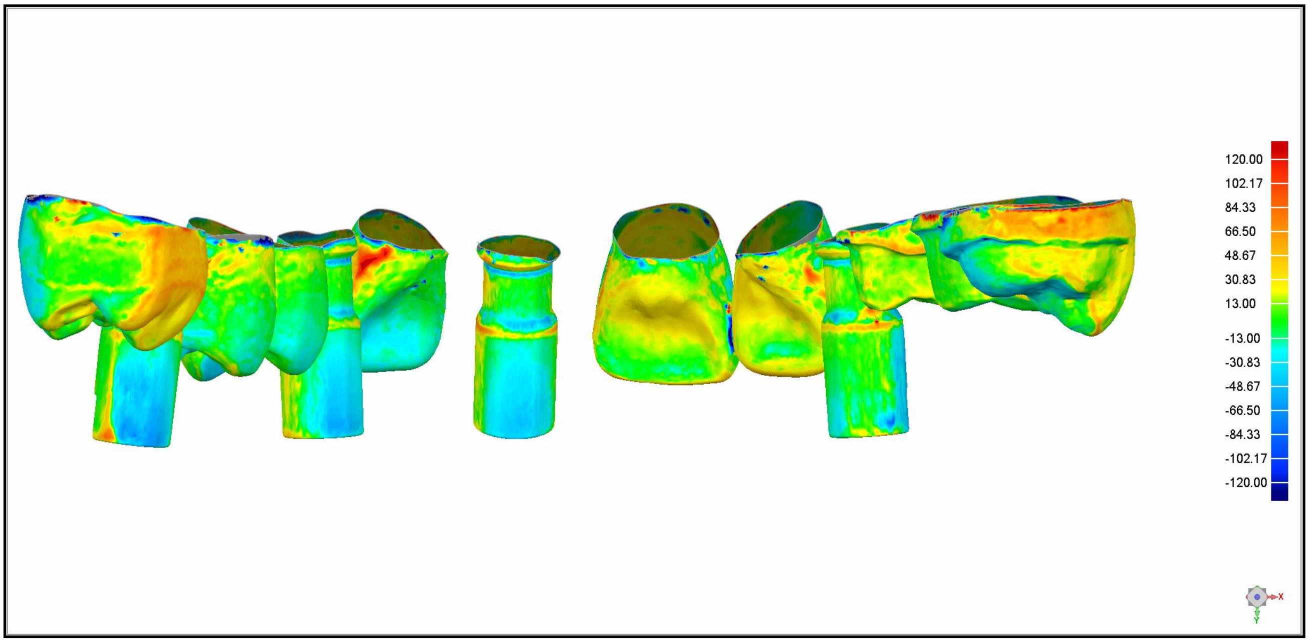

Supplement: S3 Table — Trios (scanning strategy C). (ZIP) [file pone.0202916.s003.zip › S3/3S8C.pdf]
