# Supplementary material for: Accuracy of four digital scanners according to scanning strategy in complete-arch impressions
Source: PLoS One. 2018 Sep 13;13(9):e0202916. doi: 10.1371/journal.pone.0202916 (PMC6136706; doi:10.1371/journal.pone.0202916)

# 3D Comparación  Resultados

| | |
|---|---|
| Modelo referencia | MRC |
| Modelo test | 3S9C |
| Nº de puntos de datos | 106246 |
| # Aislados | 63 |

| | |
|---|---|
| Tipo tolerancia | 3D desviación |
| Unidades | u |
| Máx. crítico | 120.00 |
| Máx. nominal | 17.00 |
| Mín. nominal | -17.00 |
| Mín. crítico | -120.00 |

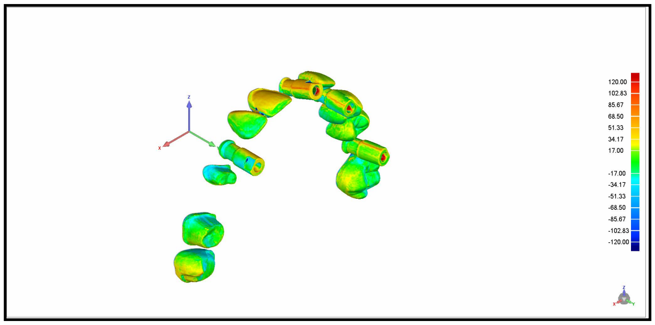

| | |
|---|---|
| Desviación | |
| Desviación superior máx. | 3153.61 |
| Desviación inferior máx. | -3155.89 |
| Desviación media | 59.81 /-42.95 |
| Desviación estándar | 190.14 |

## Distribución desviación

| >=Min | <Max | # Puntos | % |
|---|---|---|---|
| -120.00 | -102.83 | 279 | 0.26 |
| -102.83 | -85.67 | 384 | 0.36 |
| -85.67 | -68.50 | 498 | 0.47 |
| -68.50 | -51.33 | 843 | 0.79 |
| -51.33 | -34.17 | 2970 | 2.80 |
| -34.17 | -17.00 | 11651 | 10.97 |
| -17.00 | 17.00 | 52000 | 48.94 |
| 17.00 | 34.17 | 18288 | 17.21 |
| 34.17 | 51.33 | 7972 | 7.50 |
| 51.33 | 68.50 | 3324 | 3.13 |
| 68.50 | 85.67 | 1285 | 1.21 |
| 85.67 | 102.83 | 634 | 0.60 |
| 102.83 | 120.00 | 402 | 0.38 |

| | | |
|---|---|---|
| Fuera del crítico superior | 3836 | 3.61 |
| Fuera del crítico inferior | 1880 | 1.77 |

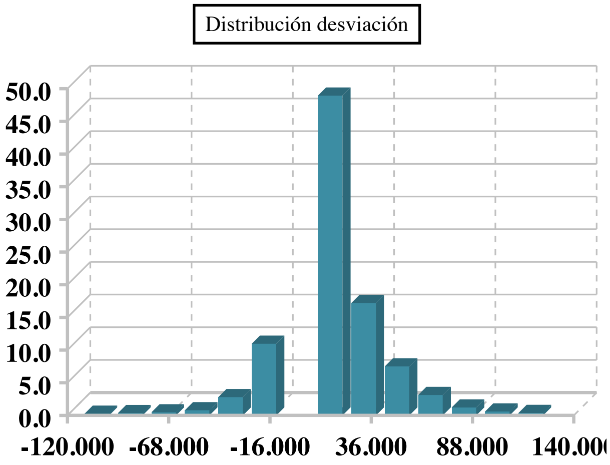


## Desviaciones estándar

| Distribución (+/-) | # Puntos | % |
|---|---|---|
| -6 * Desv. estánd. | 456 | 0.43 |
| -5 * Desv. estánd. | 92 | 0.09 |
| -4 * Desv. estánd. | 123 | 0.12 |
| -3 * Desv. estánd. | 146 | 0.14 |
| -2 * Desv. estánd. | 515 | 0.48 |
| -1 * Desv. estánd. | 70485 | 66.34 |
| 1 * Desv. estánd. | 31751 | 29.88 |
| 2 * Desv. estánd. | 761 | 0.72 |
| 3 * Desv. estánd. | 347 | 0.33 |
| 4 * Desv. estánd. | 314 | 0.30 |
| 5 * Desv. estánd. | 292 | 0.27 |
| 6 * Desv. estánd. | 964 | 0.91 |

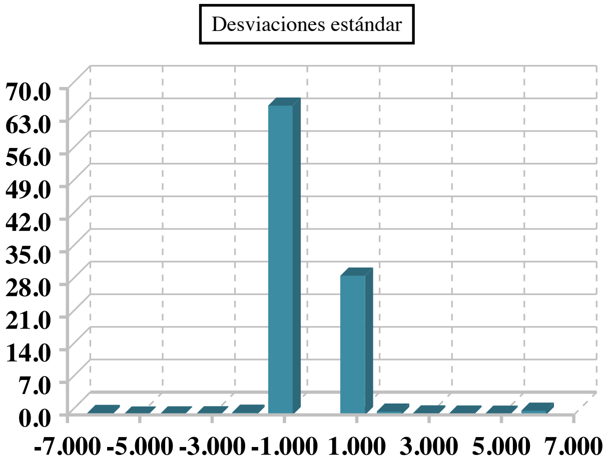

Predefinido: Isométrico

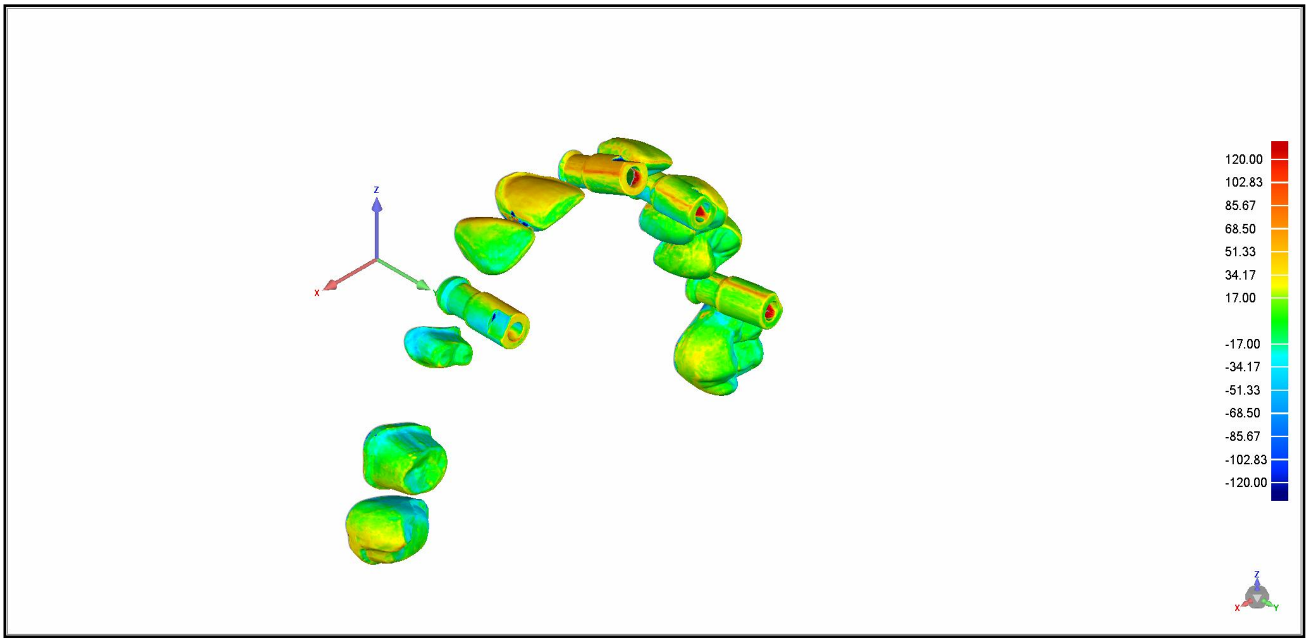

Predefinido: Frente

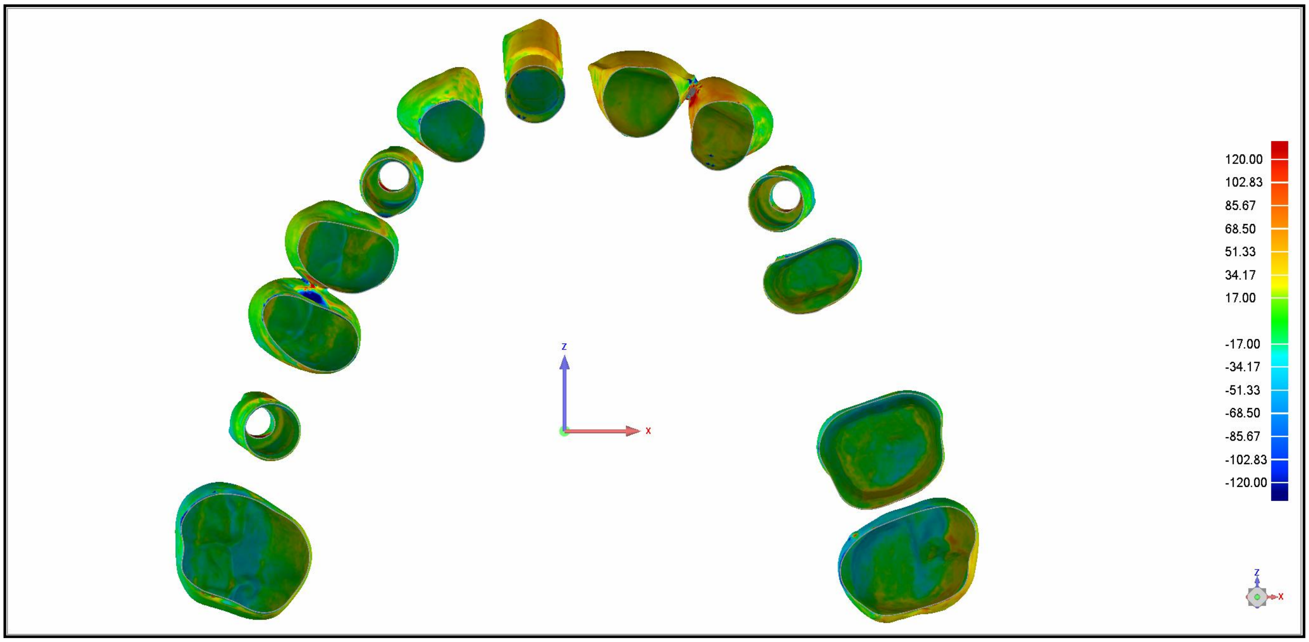

Predefinido: Atrás

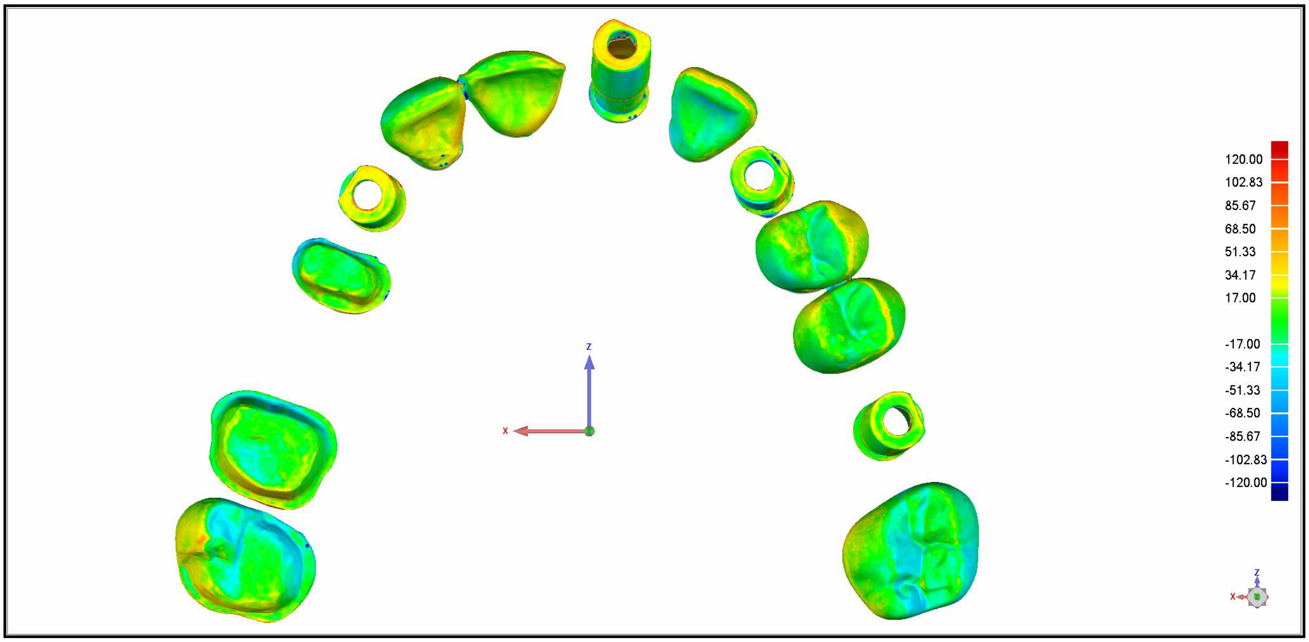

Predefinido: Izquierda

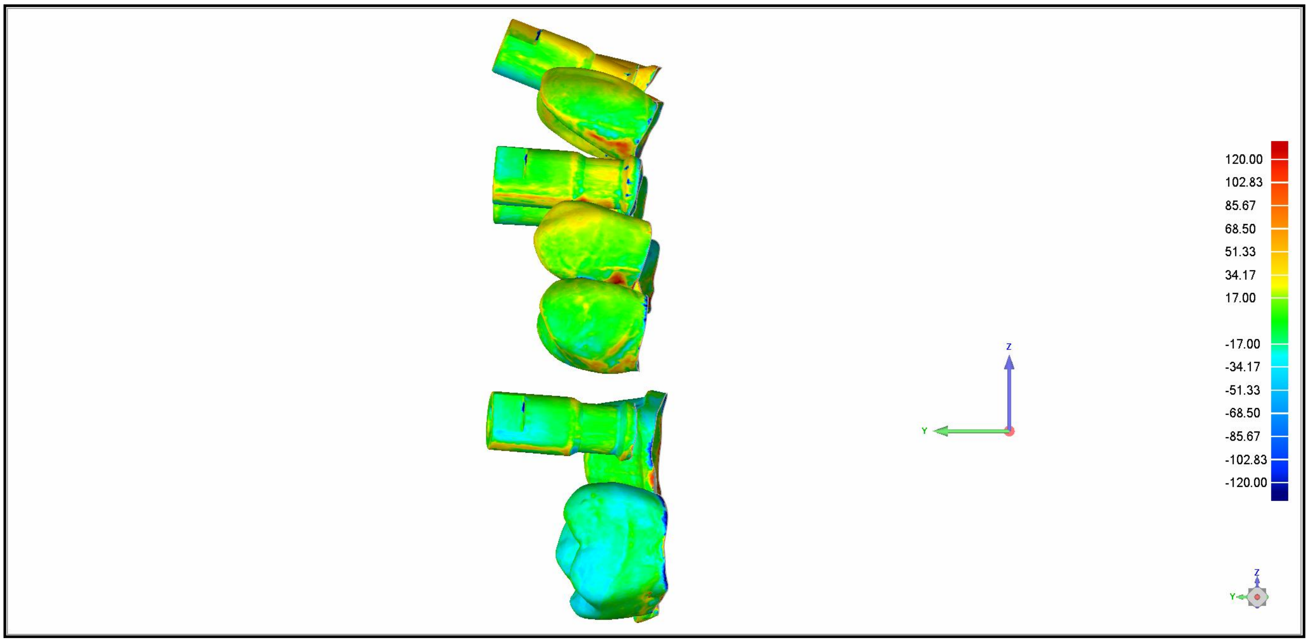

Predefinido: Derecha

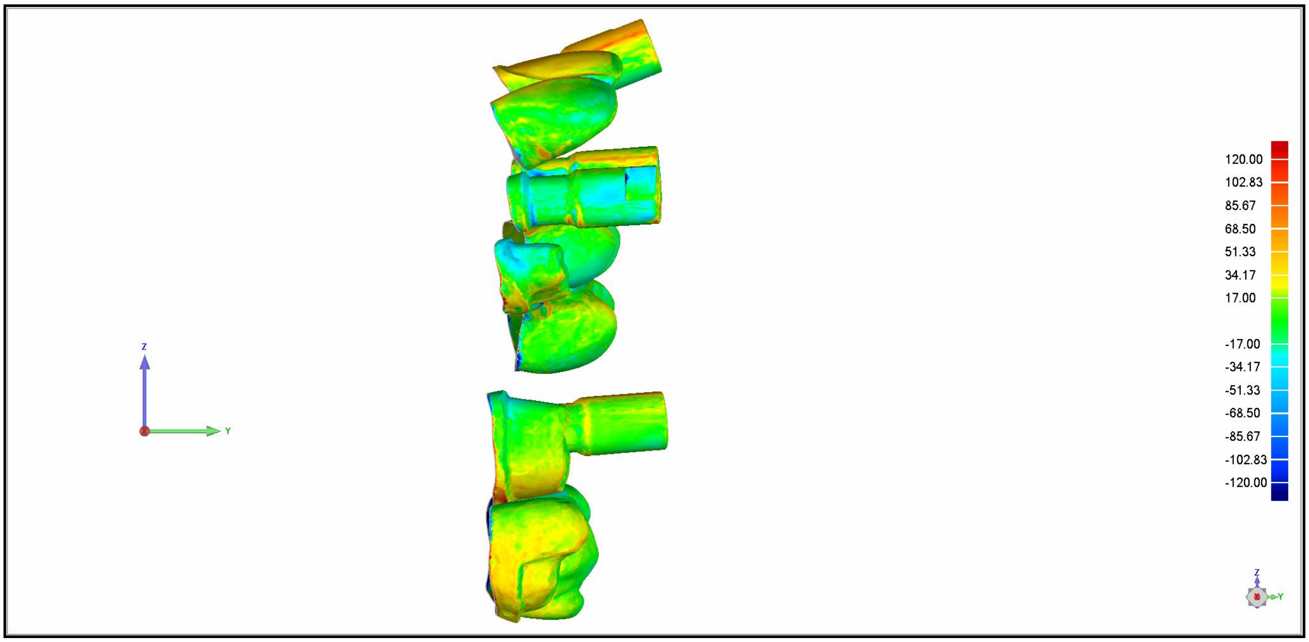

Predefinido: Superior

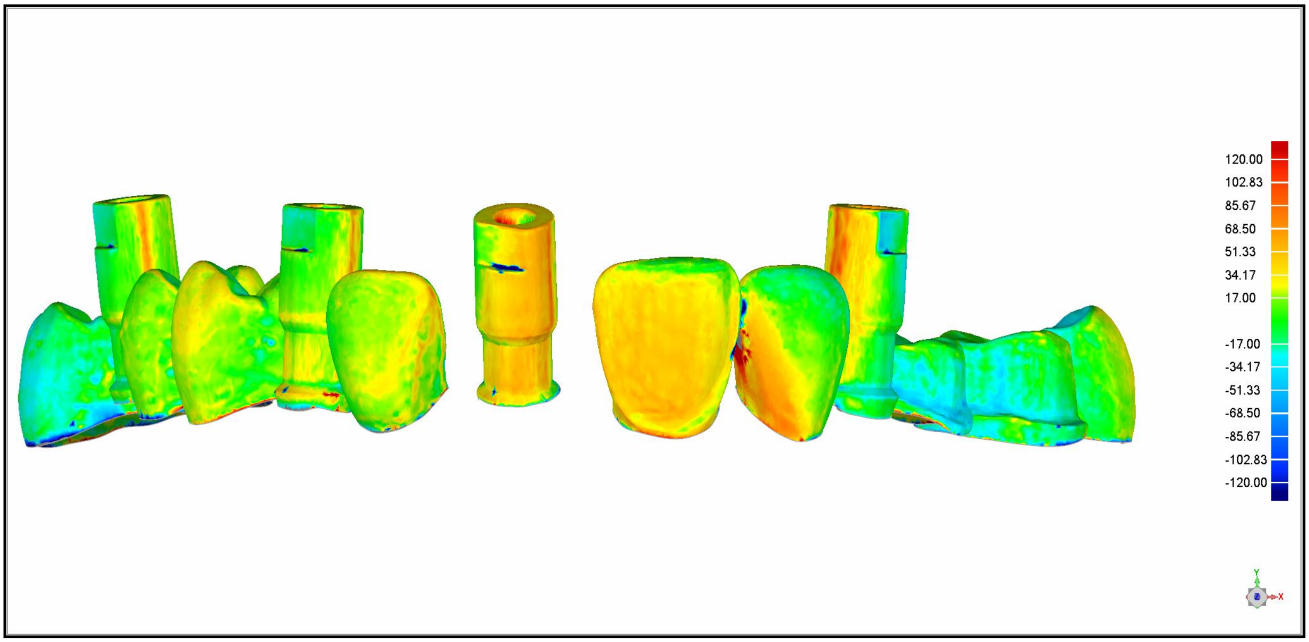

Predefinido: Inferior

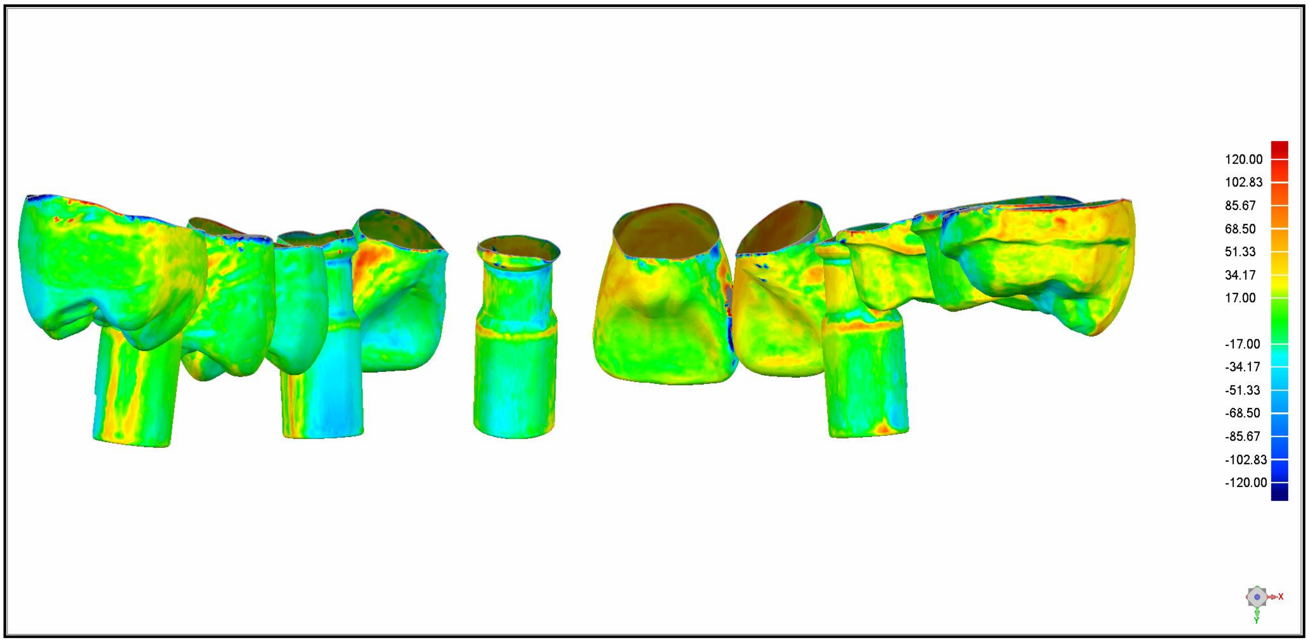

Supplement: S3 Table — Trios (scanning strategy C). (ZIP) [file pone.0202916.s003.zip › S3/3S9C.pdf]
