# Supplementary material for: Accuracy of four digital scanners according to scanning strategy in complete-arch impressions
Source: PLoS One. 2018 Sep 13;13(9):e0202916. doi: 10.1371/journal.pone.0202916 (PMC6136706; doi:10.1371/journal.pone.0202916)

3D Comparación Resultados

Modelo referencia	MRC
Modelo test	3S5C
Nº de puntos de datos	103444
# Aislados	87

Tipo tolerancia	3D desviación
Unidades	u
Máx. crítico	120.00
Máx. nominal	17.00
Mín. nominal	-17.00
Mín. crítico	-120.00

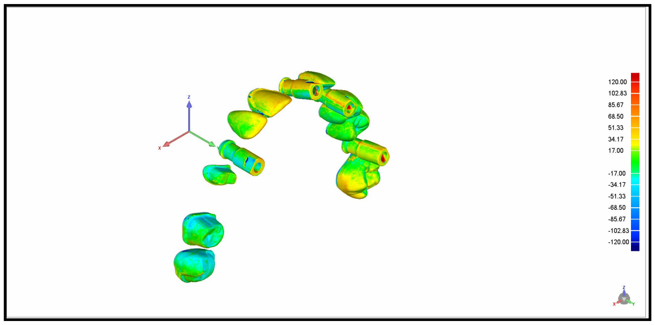

Desviación	
Desviación superior máx.	3133.67
Desviación inferior máx.	-3126.26
Desviación media	61.05 /-53.08
Desviación estándar	202.45

Distribución desviación

>=Min	<Max	# Puntos	%
-120.00	-102.83	286	0.28
-102.83	-85.67	342	0.33
-85.67	-68.50	589	0.57
-68.50	-51.33	1122	1.08
-51.33	-34.17	3962	3.83
-34.17	-17.00	12096	11.69
-17.00	17.00	45672	44.15
17.00	34.17	20479	19.80
34.17	51.33	8262	7.99
51.33	68.50	2704	2.61
68.50	85.67	1089	1.05
85.67	102.83	554	0.54
102.83	120.00	371	0.36

Fuera del crítico superior	3908	3.78
Fuera del crítico inferior	2008	1.94

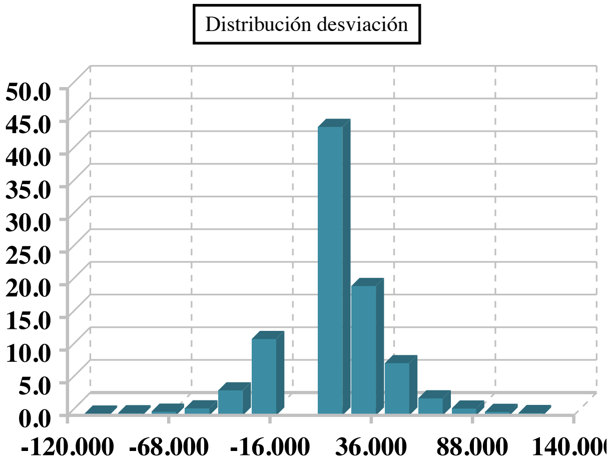

Desviaciones estándar

Distribución (+/-)	# Puntos	%
-6 * Desv. estánd.	541	0.52
-5 * Desv. estánd.	131	0.13
-4 * Desv. estánd.	134	0.13
-3 * Desv. estánd.	173	0.17
-2 * Desv. estánd.	454	0.44
-1 * Desv. estánd.	64909	62.75
1 * Desv. estánd.	34378	33.23
2 * Desv. estánd.	697	0.67
3 * Desv. estánd.	339	0.33
4 * Desv. estánd.	381	0.37
5 * Desv. estánd.	373	0.36
6 * Desv. estánd.	934	0.90

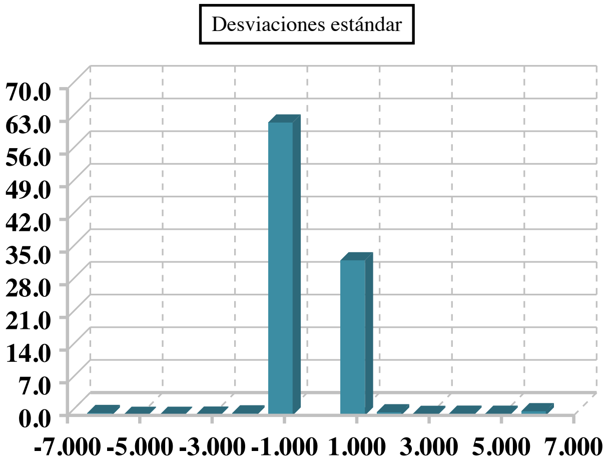

Predefinido: Isométrico

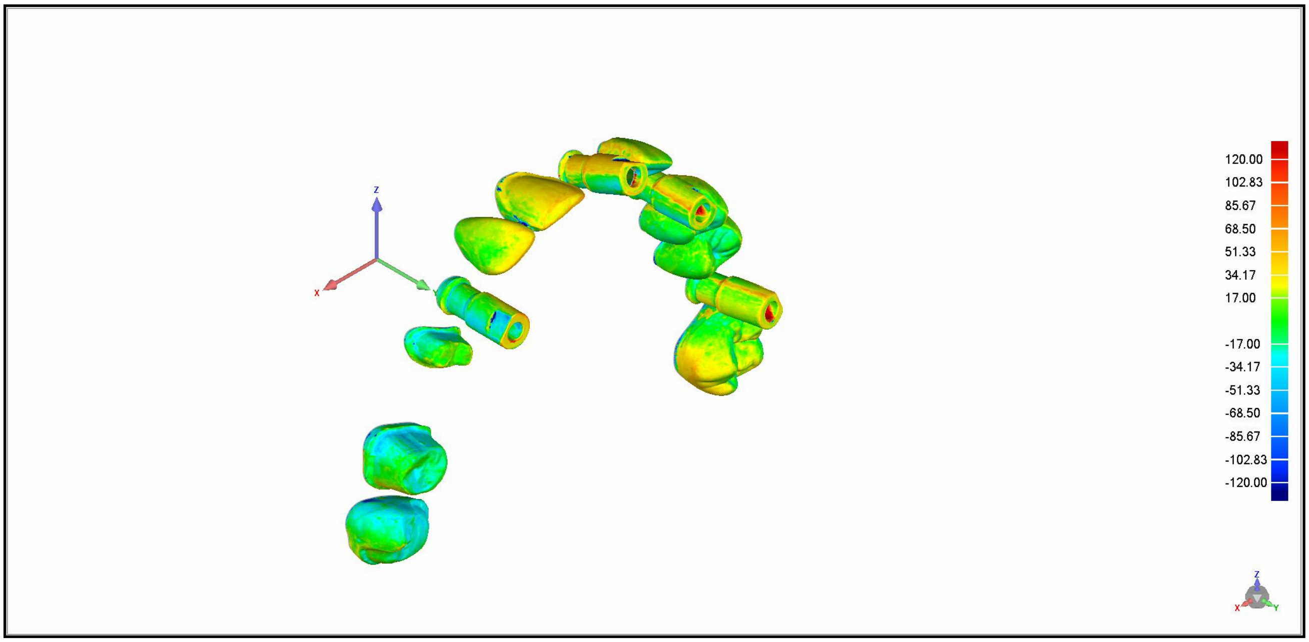

Predefinido: Frente

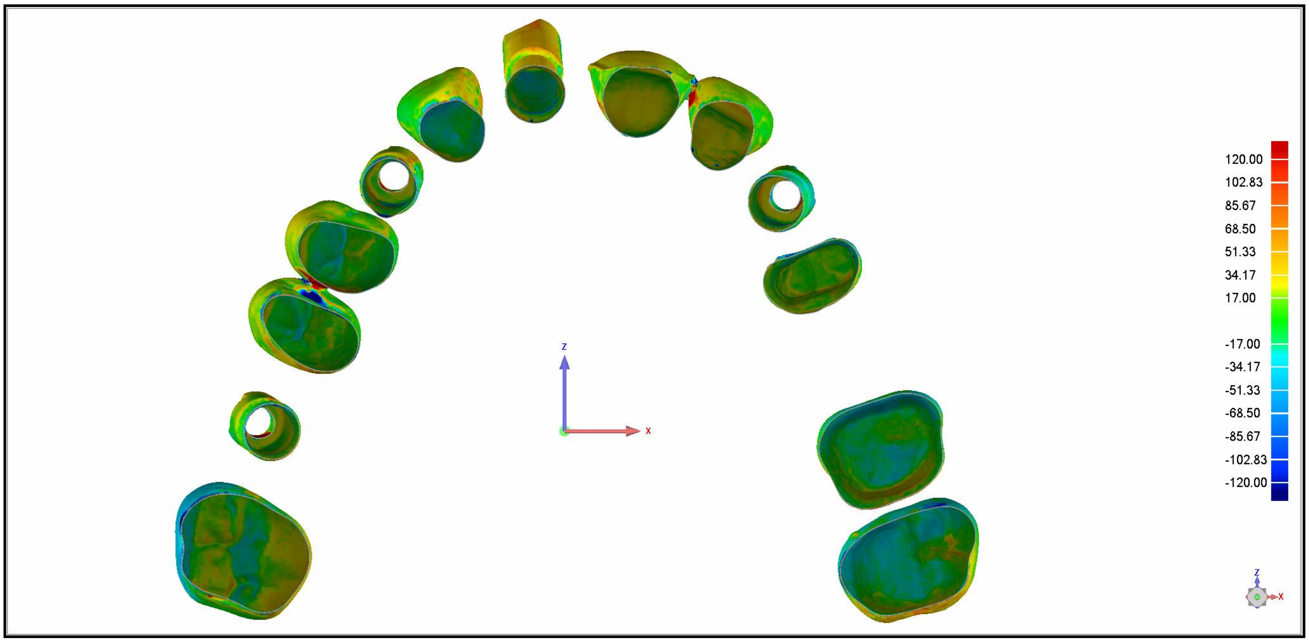

Predefinido: Atrás

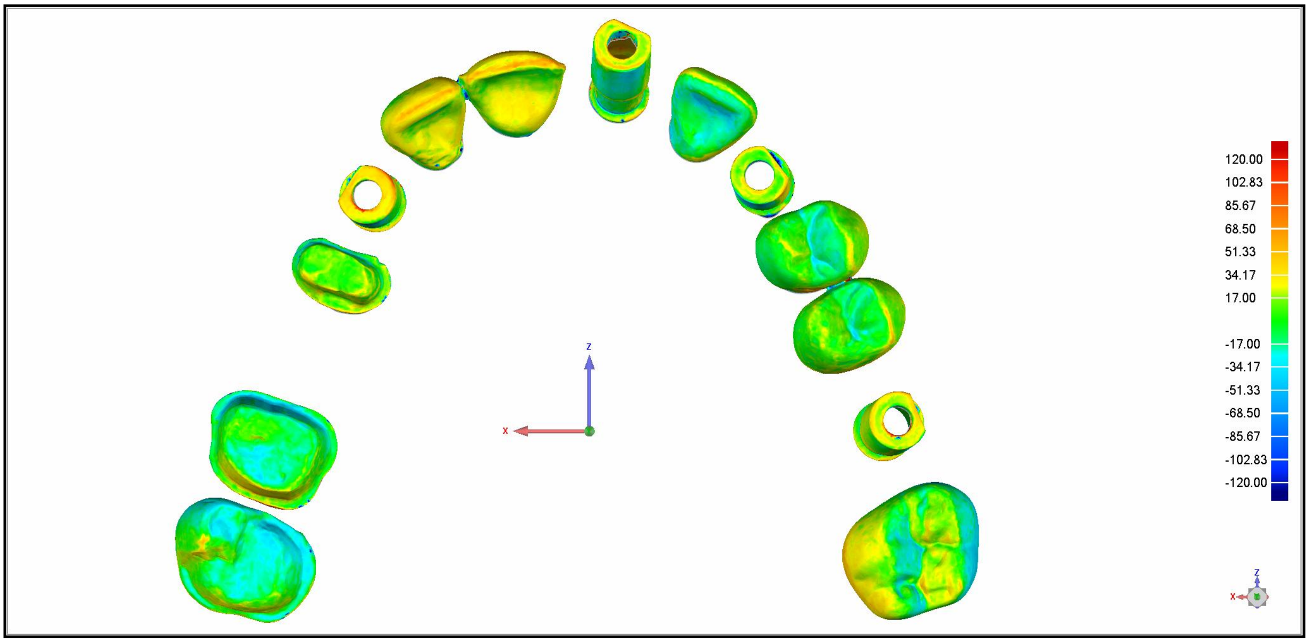

Predefinido: Izquierda

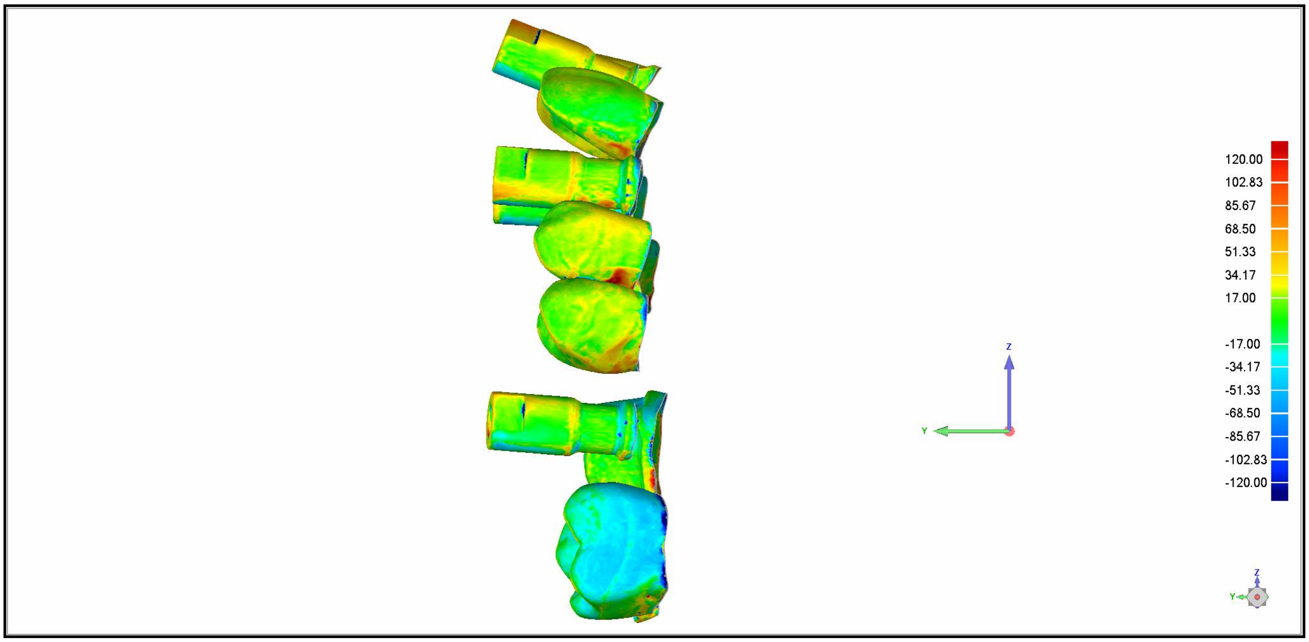

Predefinido: Derecha

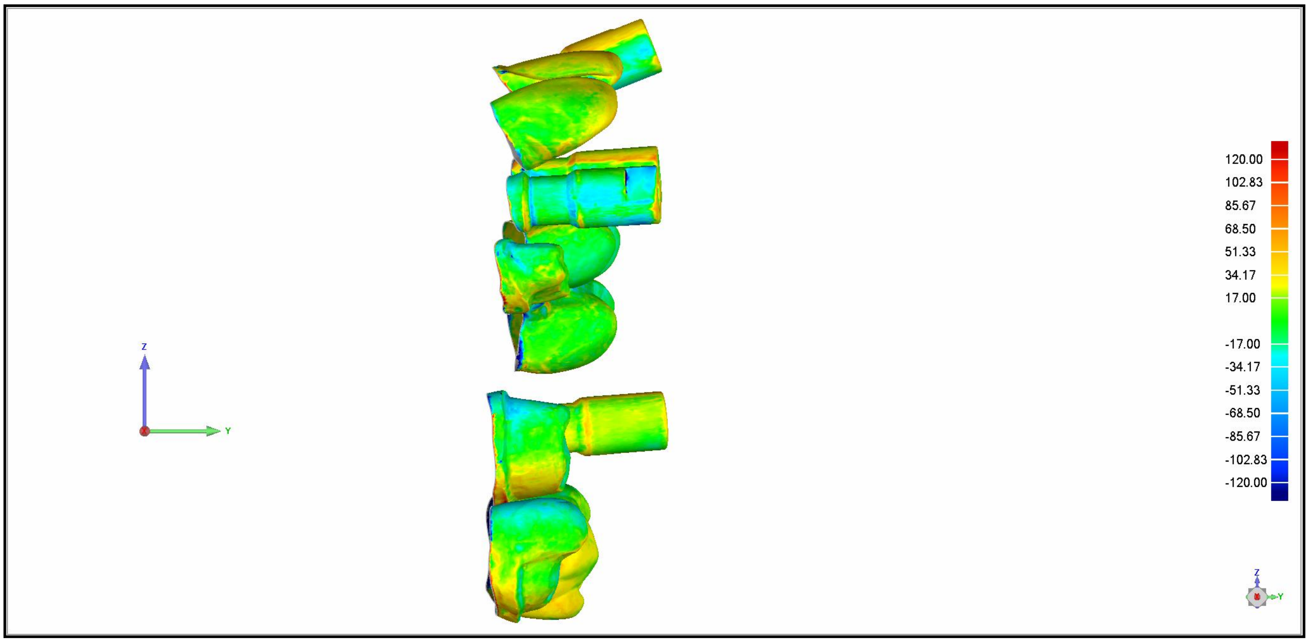

Predefinido: Superior

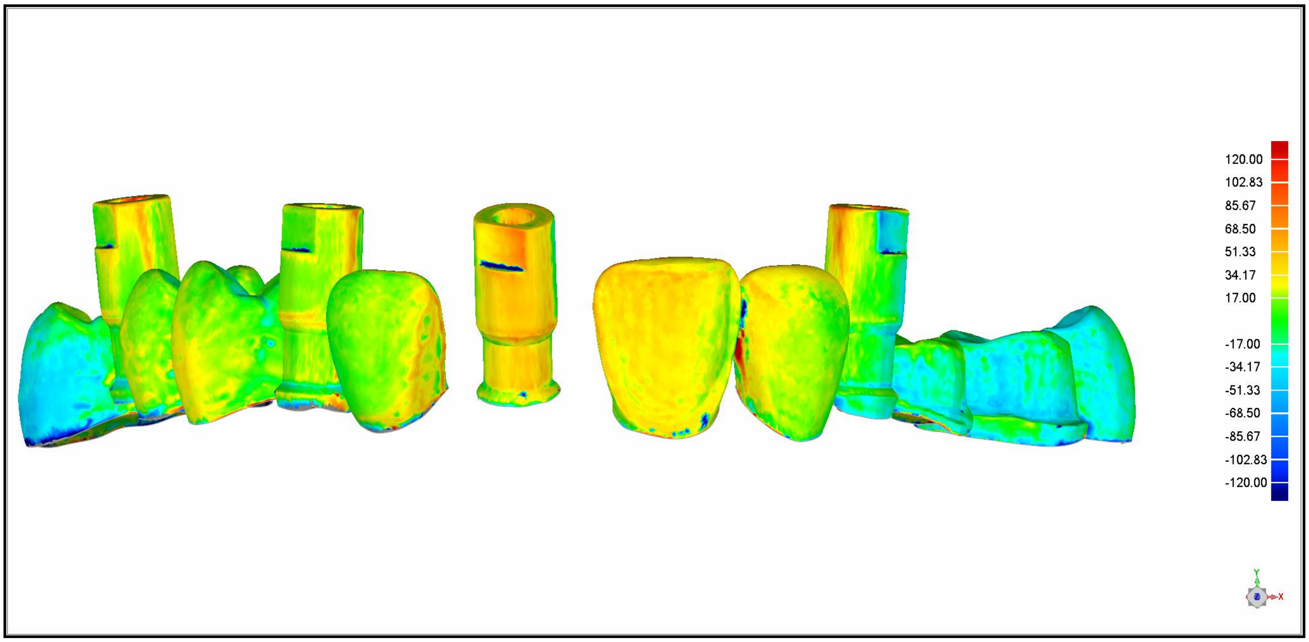

Predefinido: Inferior

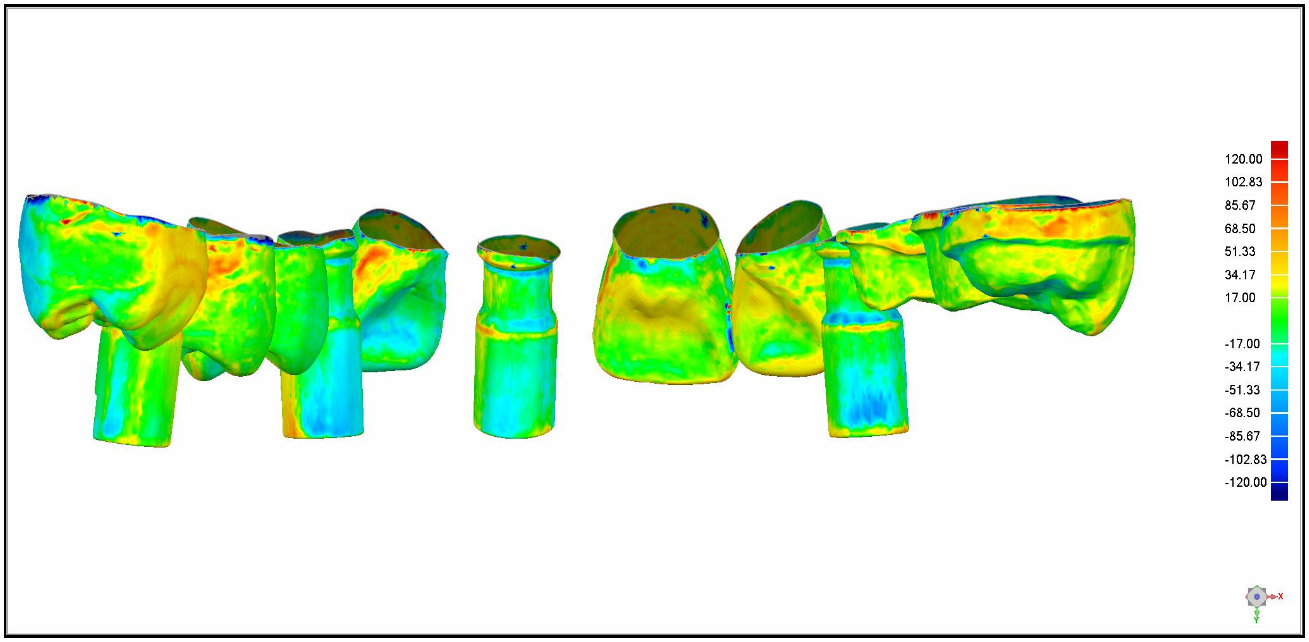

Supplement: S3 Table — Trios (scanning strategy C). (ZIP) [file pone.0202916.s003.zip › S3/3S5C.pdf]
