# Supplementary material for: Accuracy of four digital scanners according to scanning strategy in complete-arch impressions
Source: PLoS One. 2018 Sep 13;13(9):e0202916. doi: 10.1371/journal.pone.0202916 (PMC6136706; doi:10.1371/journal.pone.0202916)

3D Comparación Resultados

Modelo referencia	MRC
Modelo test	3S2C
Nº de puntos de datos	112486
# Aislados	115

Tipo tolerancia	3D desviación
Unidades	u
Máx. crítico	120.00
Máx. nominal	20.00
Mín. nominal	-20.00
Mín. crítico	-120.00

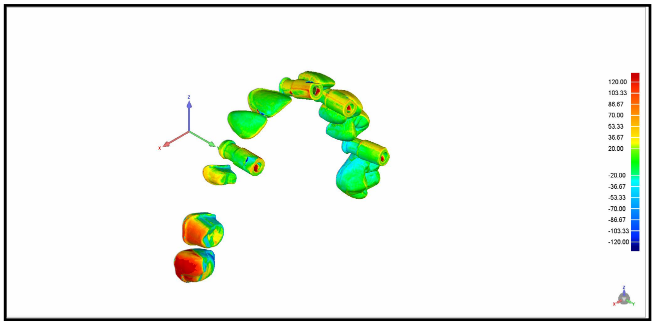

Desviación	
Desviación superior máx.	3014.88
Desviación inferior máx.	-3154.62
Desviación media	63.55 /-56.93
Desviación estándar	201.93

Distribución desviación

>=Min	<Max	# Puntos	%
-120.00	-103.33	629	0.56
-103.33	-86.67	1341	1.19
-86.67	-70.00	1487	1.32
-70.00	-53.33	2033	1.81
-53.33	-36.67	3236	2.88
-36.67	-20.00	6574	5.84
-20.00	20.00	55667	49.49
20.00	36.67	18145	16.13
36.67	53.33	7709	6.85
53.33	70.00	3321	2.95
70.00	86.67	1988	1.77
86.67	103.33	1719	1.53
103.33	120.00	1058	0.94

Fuera del crítico superior		5346	4.75
Fuera del crítico inferior		2233	1.99

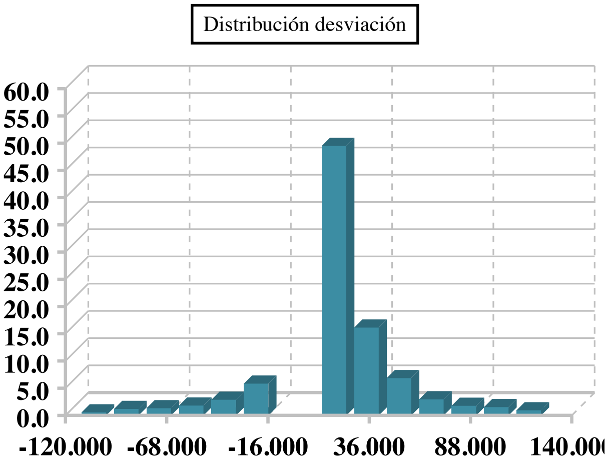

Desviaciones estándar

Distribución (+/-)	# Puntos	%
-6 * Desv. estánd.	535	0.48
-5 * Desv. estánd.	88	0.08
-4 * Desv. estánd.	108	0.10
-3 * Desv. estánd.	126	0.11
-2 * Desv. estánd.	449	0.40
-1 * Desv. estánd.	72740	64.67
1 * Desv. estánd.	35448	31.51
2 * Desv. estánd.	867	0.77
3 * Desv. estánd.	441	0.39
4 * Desv. estánd.	353	0.31
5 * Desv. estánd.	367	0.33
6 * Desv. estánd.	964	0.86

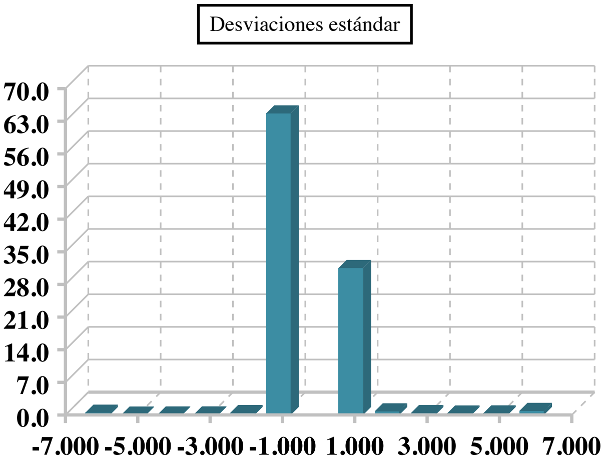

Predefinido: Isométrico

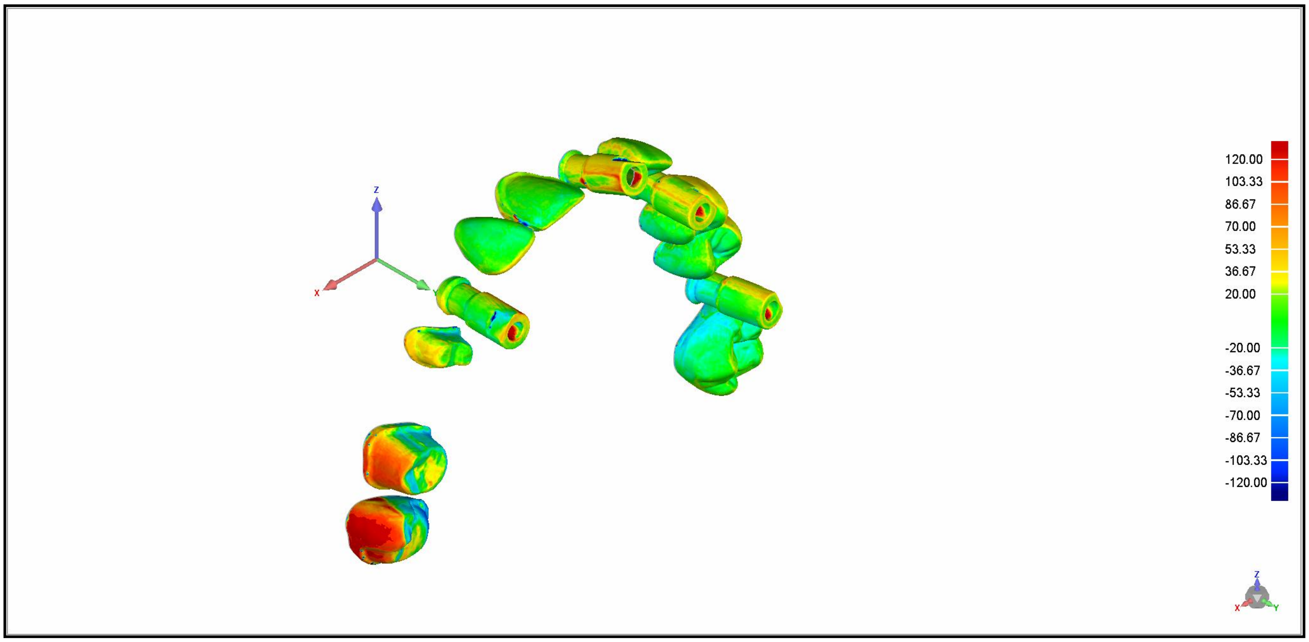

Predefinido: Frente

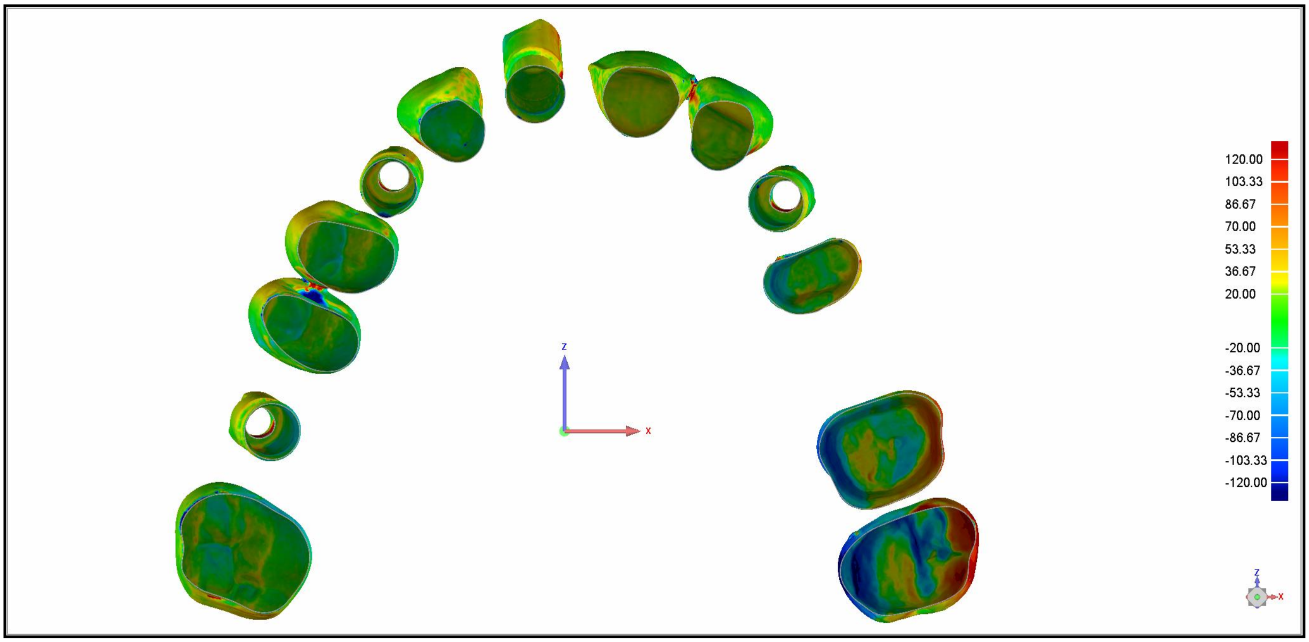

Predefinido: Atrás

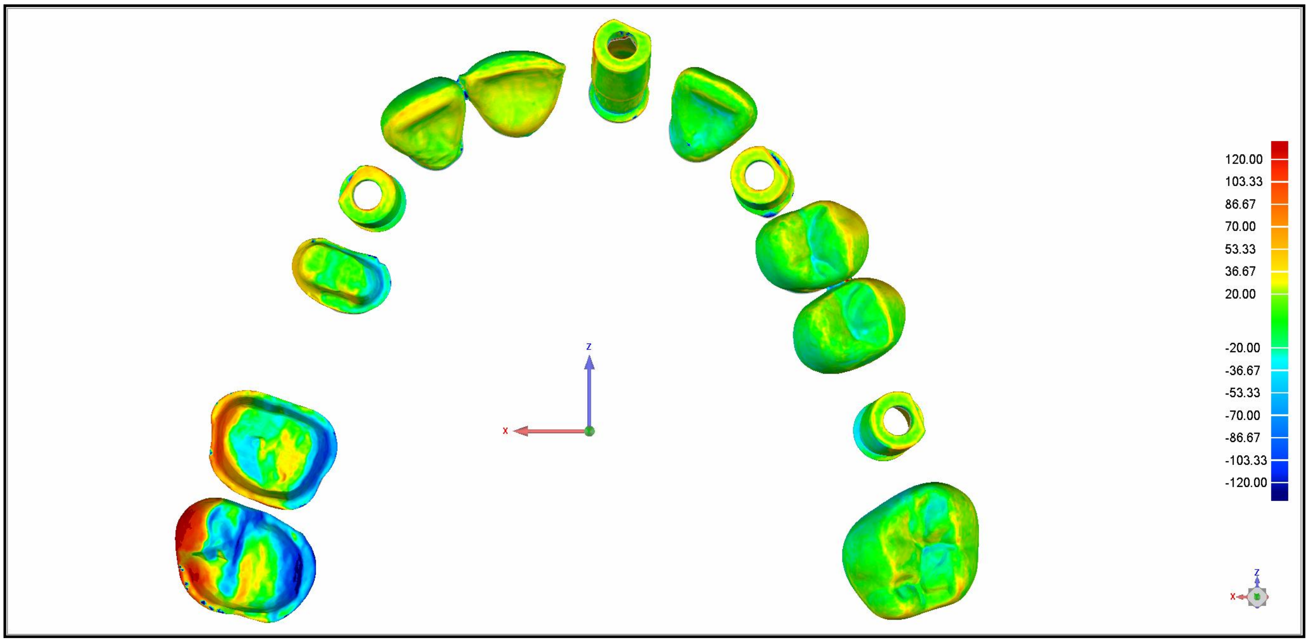

Geomagic Control™

Predefinido: Izquierda

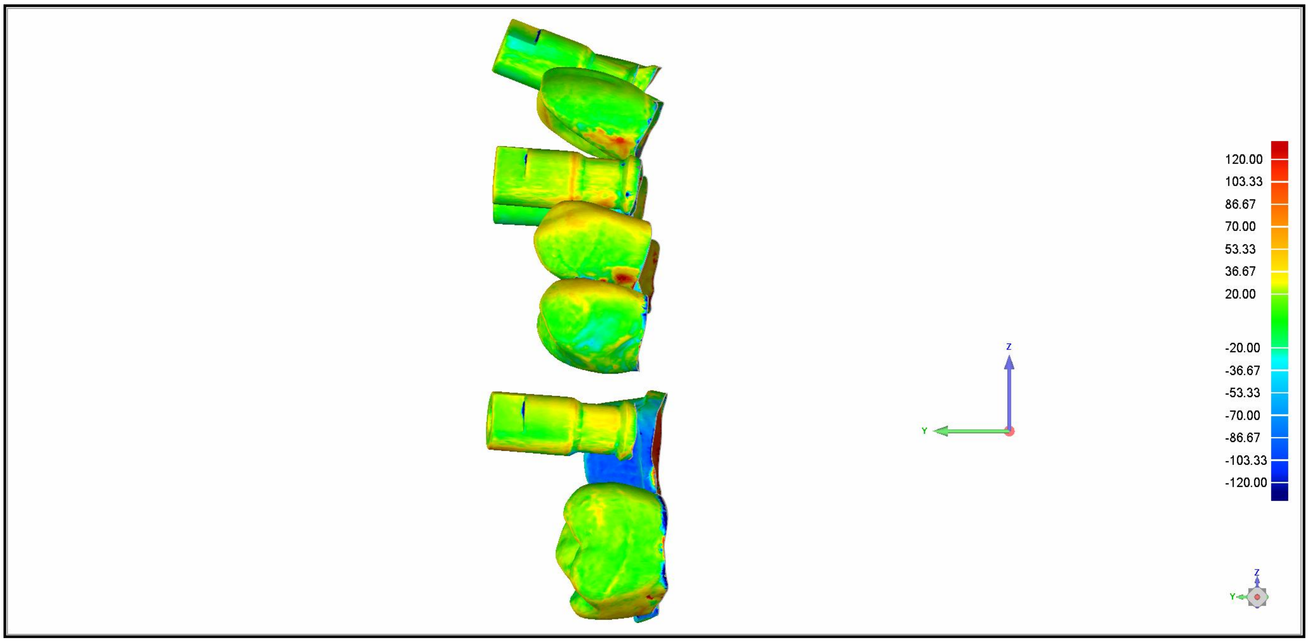

Predefinido: Derecha

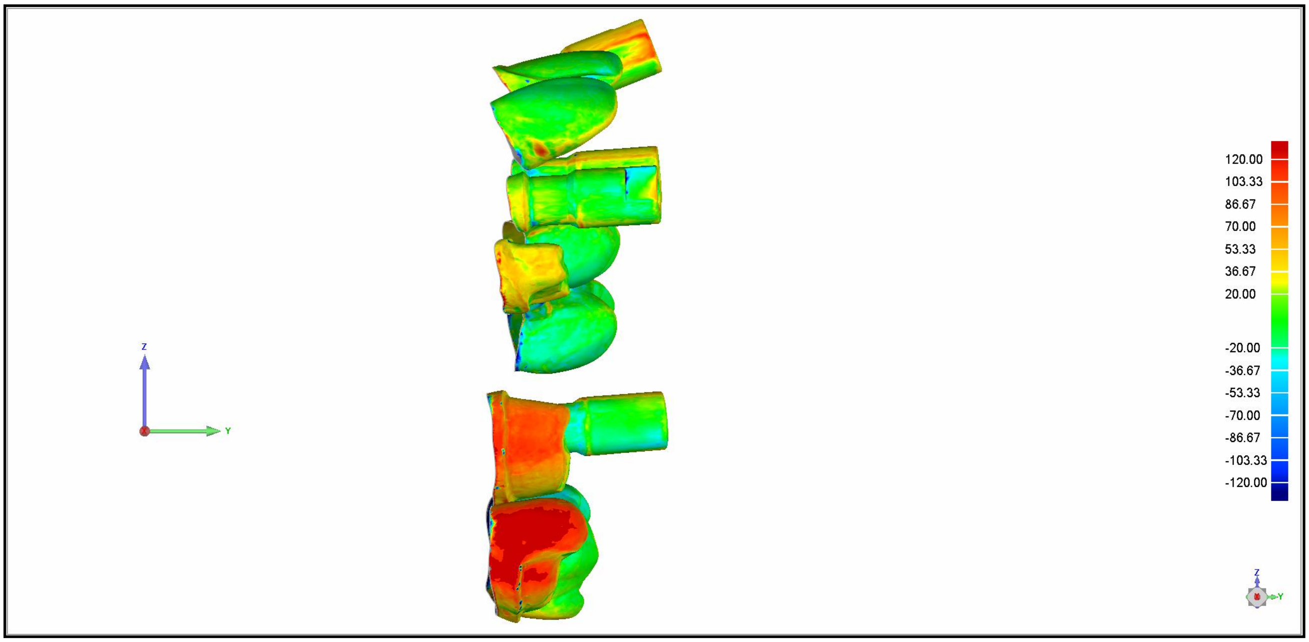

Predefinido: Superior

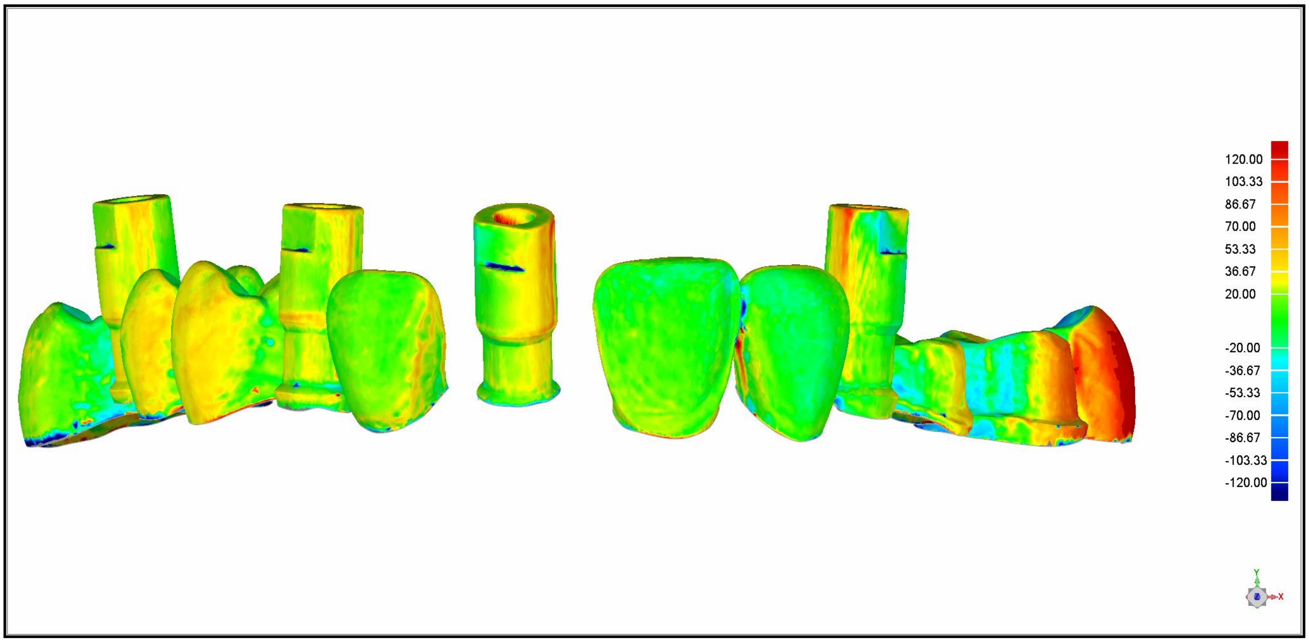

Predefinido: Inferior

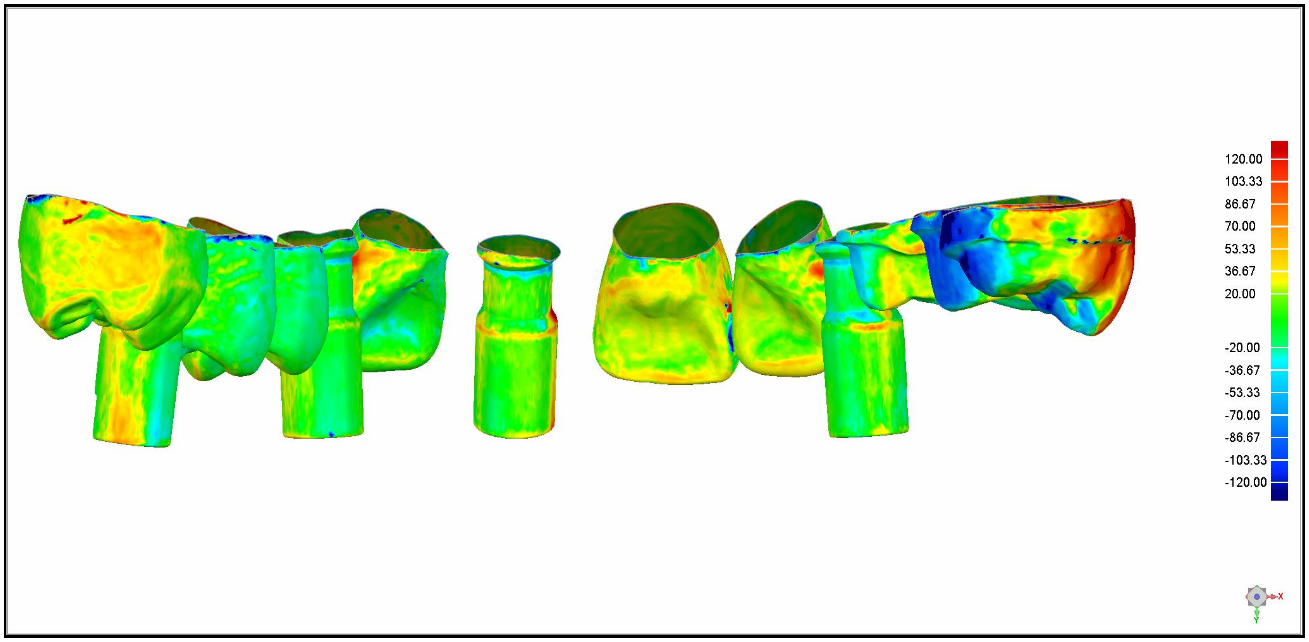

Supplement: S3 Table — Trios (scanning strategy C). (ZIP) [file pone.0202916.s003.zip › S3/3S2C.pdf]
