# Supplementary material for: Accuracy of four digital scanners according to scanning strategy in complete-arch impressions
Source: PLoS One. 2018 Sep 13;13(9):e0202916. doi: 10.1371/journal.pone.0202916 (PMC6136706; doi:10.1371/journal.pone.0202916)

Geomagic Control

Fecha: 3/6/2017, 12:18 pm

3D Comparación Resultados

Modelo referencia	MRC
Modelo test	3S3C
Nº de puntos de datos	98150
# Aislados	70

Tipo tolerancia	3D desviación
Unidades	u
Máx. crítico	120.00
Máx. nominal	20.00
Mín. nominal	-20.00
Mín. crítico	-120.00

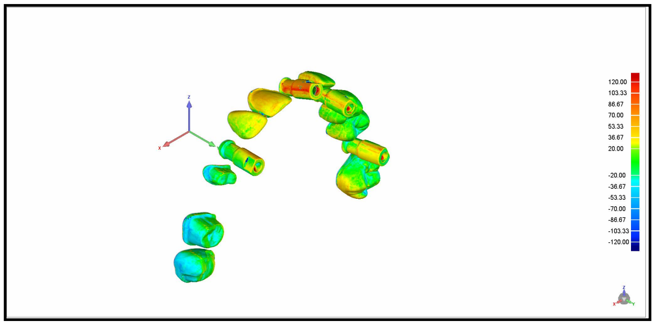

Desviación	
Desviación superior máx.	3067.57
Desviación inferior máx.	-3100.80
Desviación media	61.35 /-49.67
Desviación estándar	197.05

Distribución desviación

>=Min	<Max	# Puntos	%
-120.00	-103.33	261	0.27
-103.33	-86.67	332	0.34
-86.67	-70.00	441	0.45
-70.00	-53.33	771	0.79
-53.33	-36.67	2690	2.74
-36.67	-20.00	8152	8.31
-20.00	20.00	50816	51.77
20.00	36.67	15443	15.73
36.67	53.33	7998	8.15
53.33	70.00	3057	3.11
70.00	86.67	1351	1.38
86.67	103.33	762	0.78
103.33	120.00	479	0.49

Fuera del crítico superior	3776	3.85
Fuera del crítico inferior	1821	1.86

Distribución desviación

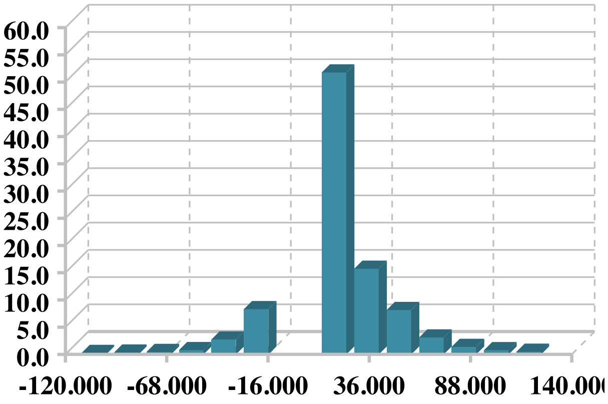

Desviaciones estándar

Distribución (+/-)	# Puntos	%
-6 * Desv. estánd.	471	0.48
-5 * Desv. estánd.	74	0.08
-4 * Desv. estánd.	128	0.13
-3 * Desv. estánd.	138	0.14
-2 * Desv. estánd.	492	0.50
-1 * Desv. estánd.	64367	65.58
1 * Desv. estánd.	29889	30.45
2 * Desv. estánd.	727	0.74
3 * Desv. estánd.	324	0.33
4 * Desv. estánd.	307	0.31
5 * Desv. estánd.	309	0.31
6 * Desv. estánd.	924	0.94

Desviaciones estándar

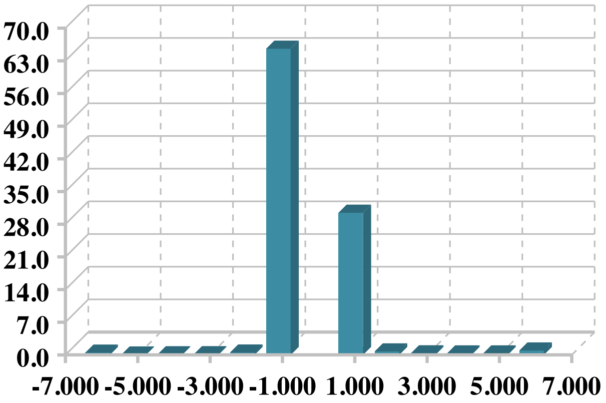

Predefinido: Isométrico

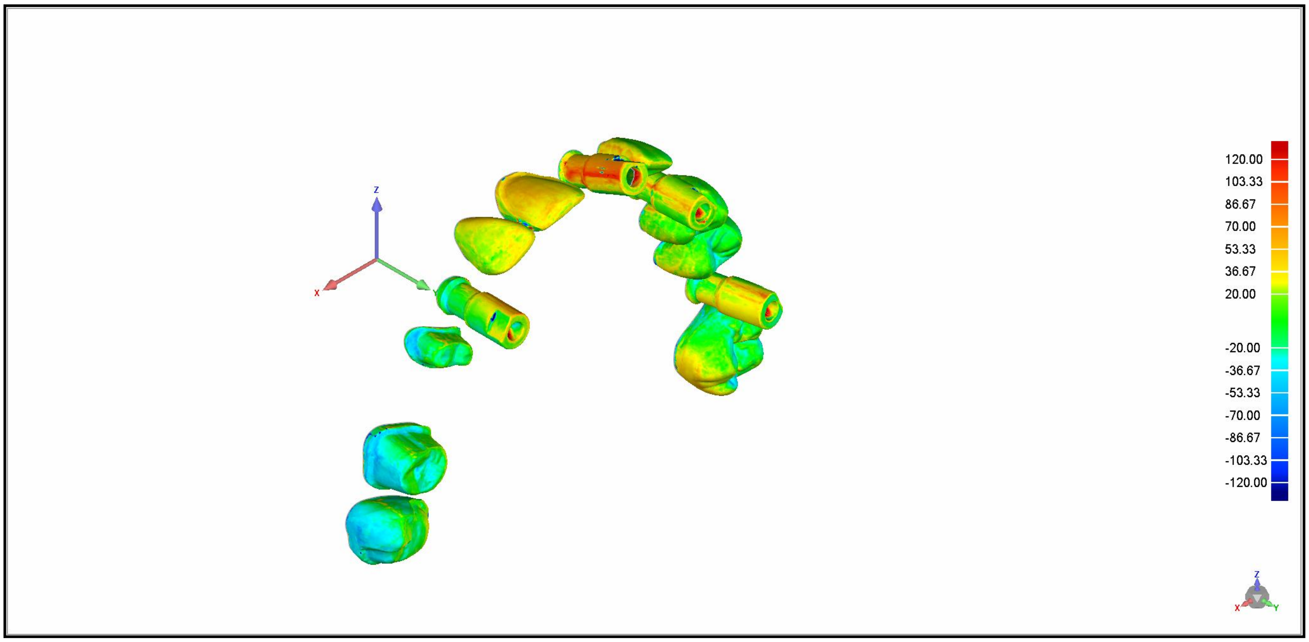

Predefinido: Frente

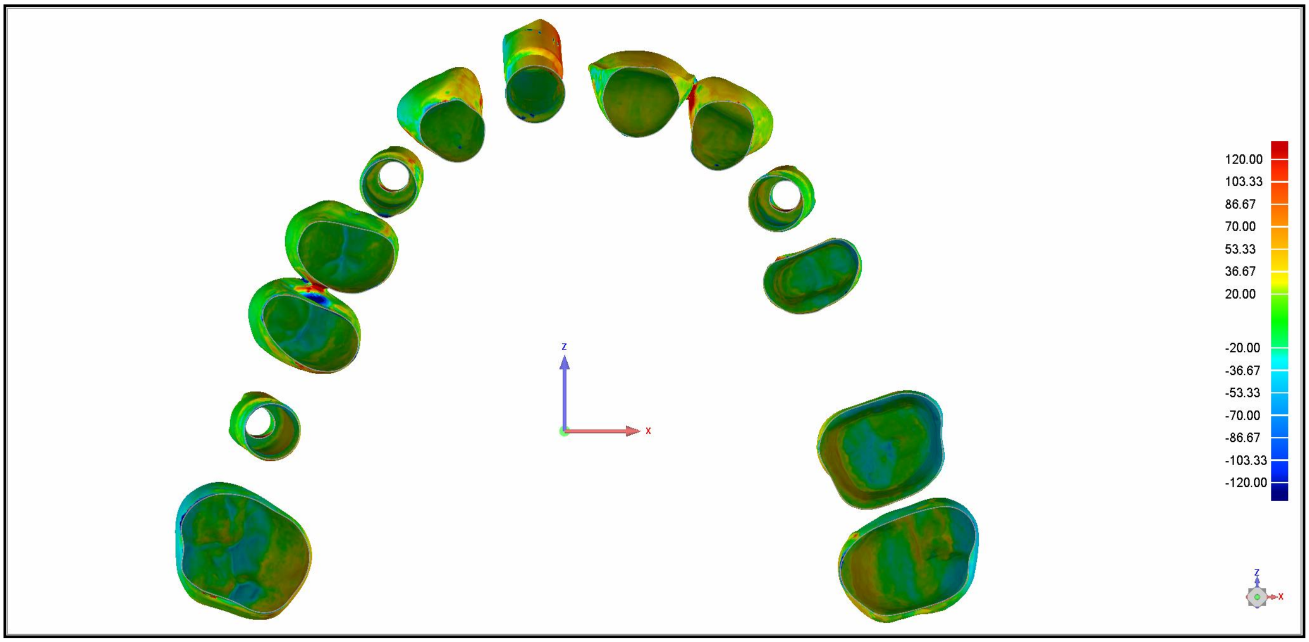

Predefinido: Atrás

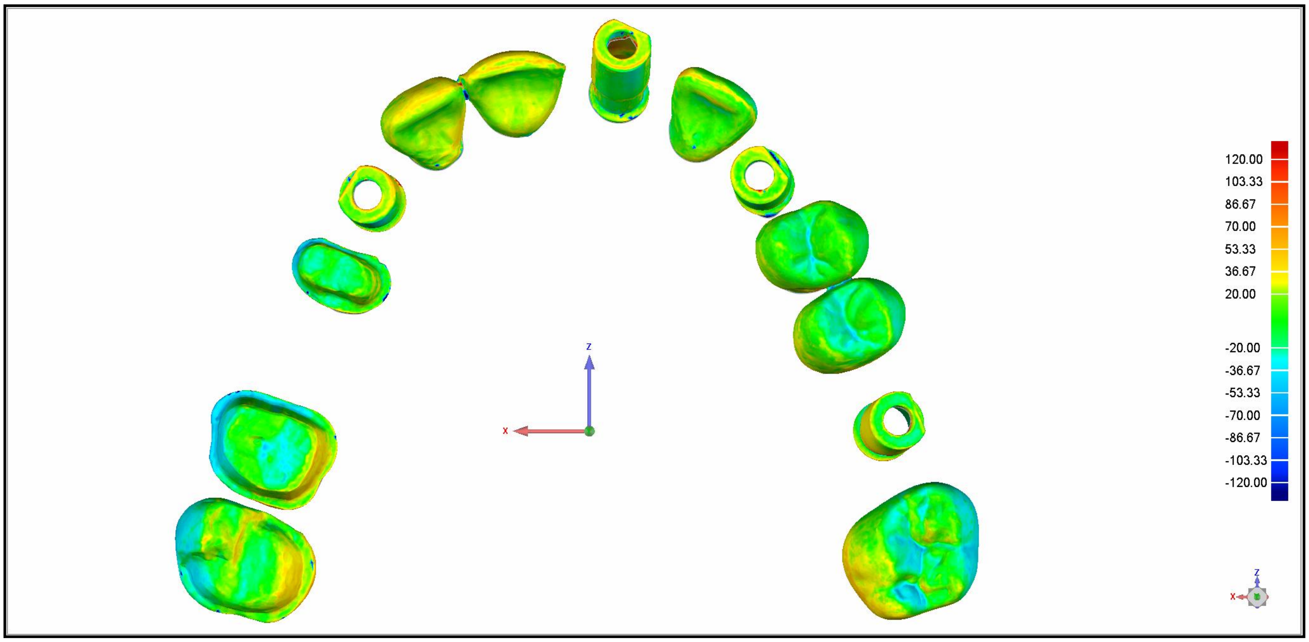

Predefinido: Izquierda

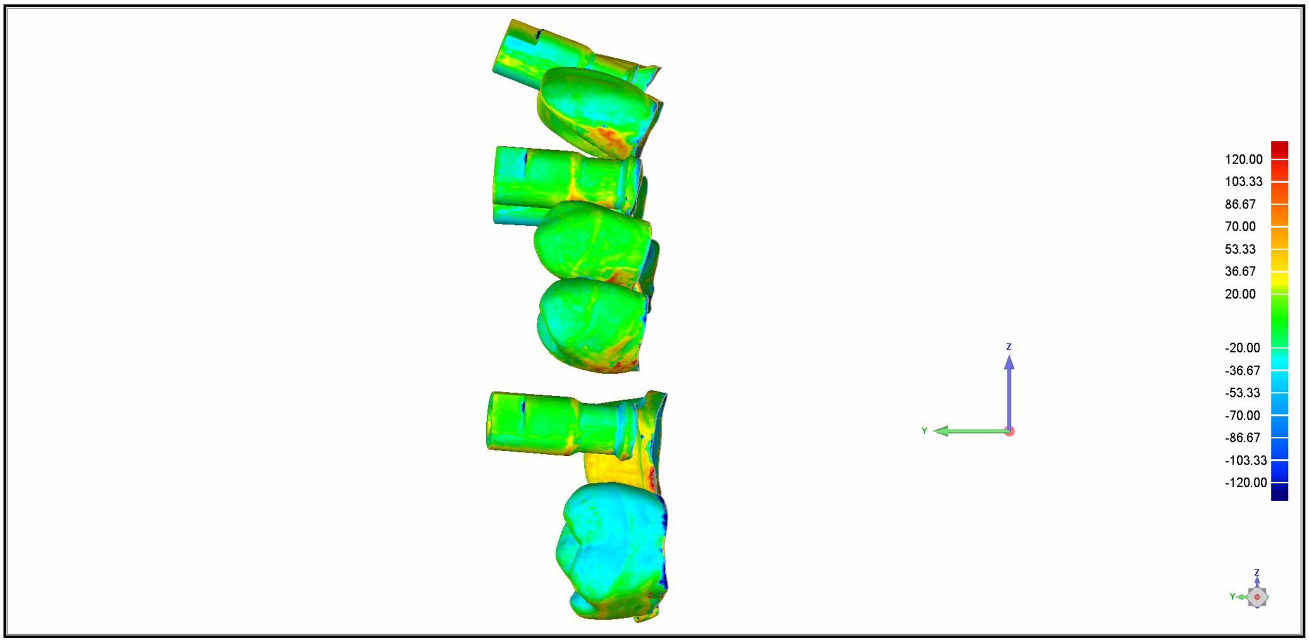

Predefinido: Derecha

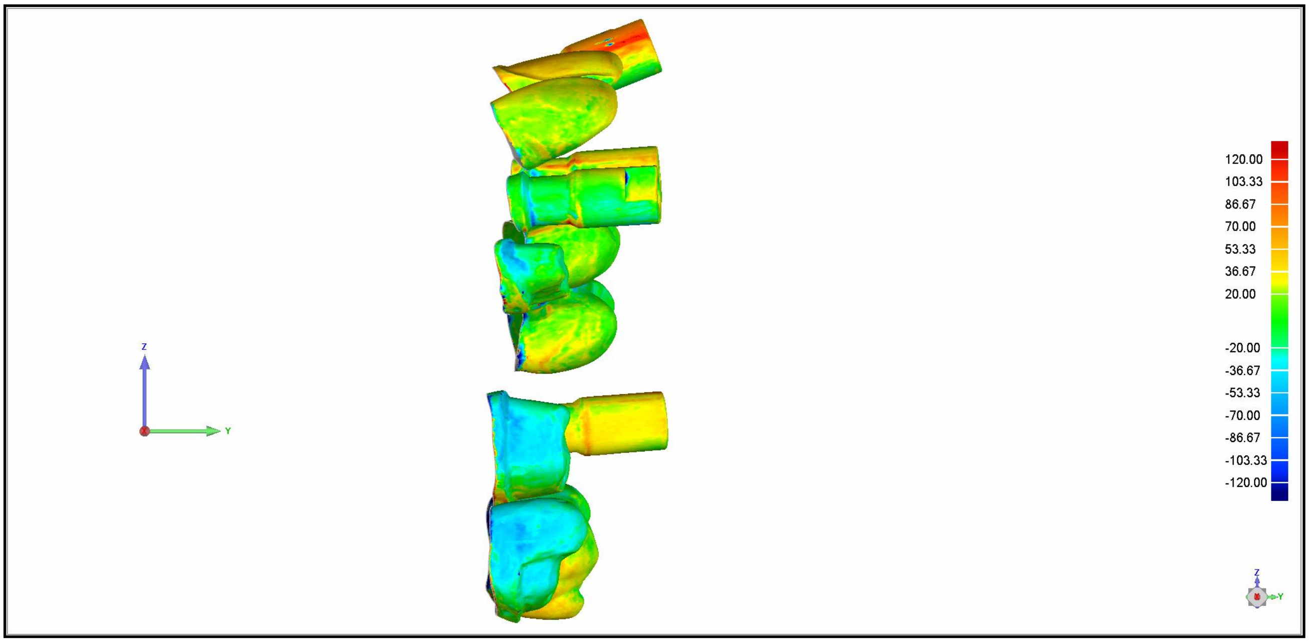

Predefinido: Superior

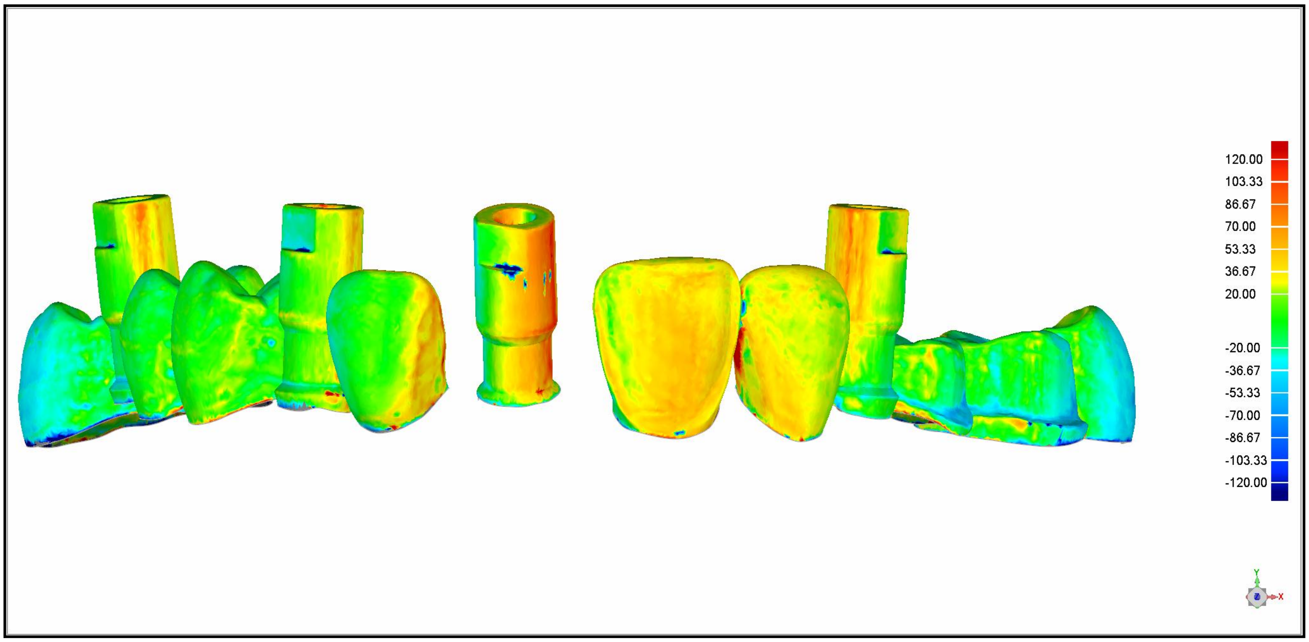

Predefinido: Inferior

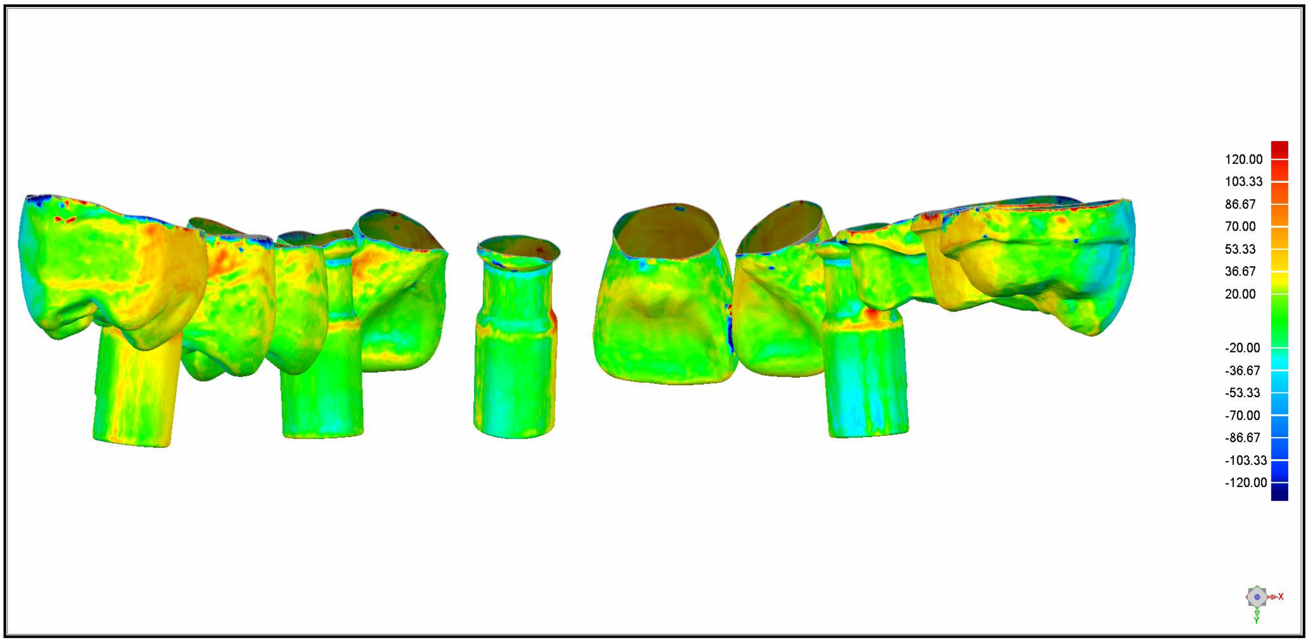

Supplement: S3 Table — Trios (scanning strategy C). (ZIP) [file pone.0202916.s003.zip › S3/3S3C.pdf]
