# Supplementary material for: Accuracy of four digital scanners according to scanning strategy in complete-arch impressions
Source: PLoS One. 2018 Sep 13;13(9):e0202916. doi: 10.1371/journal.pone.0202916 (PMC6136706; doi:10.1371/journal.pone.0202916)

3D Comparación Resultados

Modelo referencia	MRC
Modelo test	3S10C
Nº de puntos de datos	100823
# Aislados	116

Tipo tolerancia	3D desviación
Unidades	u
Máx. crítico	120.00
Máx. nominal	16.00
Mín. nominal	-16.00
Mín. crítico	-120.00

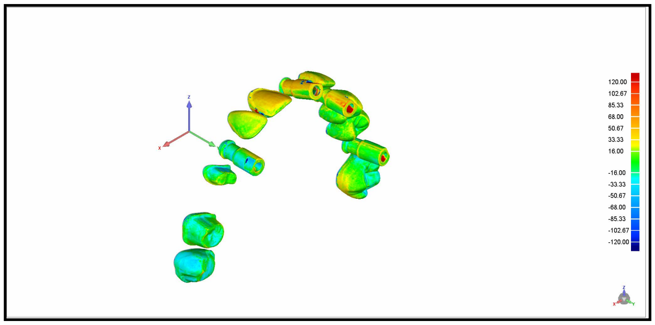

Desviación	
Desviación superior máx.	3039.11
Desviación inferior máx.	-3110.99
Desviación media	60.76 /-45.97
Desviación estándar	191.82

Distribución desviación

>=Min	<Max	# Puntos	%
-120.00	-102.67	243	0.24
-102.67	-85.33	384	0.38
-85.33	-68.00	581	0.58
-68.00	-50.67	1308	1.30
-50.67	-33.33	3579	3.55
-33.33	-16.00	11199	11.11
-16.00	16.00	49626	49.22
16.00	33.33	15962	15.83
33.33	50.67	7263	7.20
50.67	68.00	2993	2.97
68.00	85.33	1131	1.12
85.33	102.67	552	0.55
102.67	120.00	391	0.39

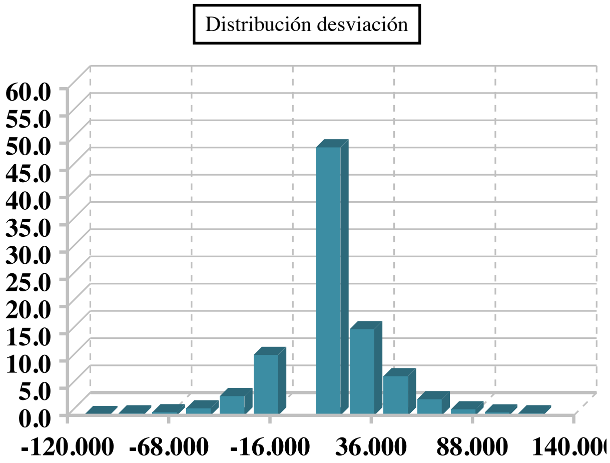

Fuera del crítico superior	3750	3.72
Fuera del crítico inferior	1861	1.85

Desviaciones estándar

Distribución (+/-)	# Puntos	%
-6 * Desv. estánd.	543	0.54
-5 * Desv. estánd.	109	0.11
-4 * Desv. estánd.	121	0.12
-3 * Desv. estánd.	151	0.15
-2 * Desv. estánd.	445	0.44
-1 * Desv. estánd.	67983	67.43
1 * Desv. estánd.	28729	28.49
2 * Desv. estánd.	678	0.67
3 * Desv. estánd.	396	0.39
4 * Desv. estánd.	401	0.40
5 * Desv. estánd.	383	0.38
6 * Desv. estánd.	884	0.88

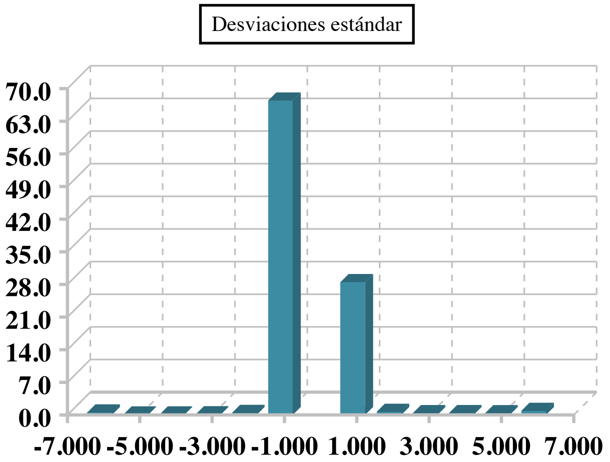

Predefinido: Isométrico

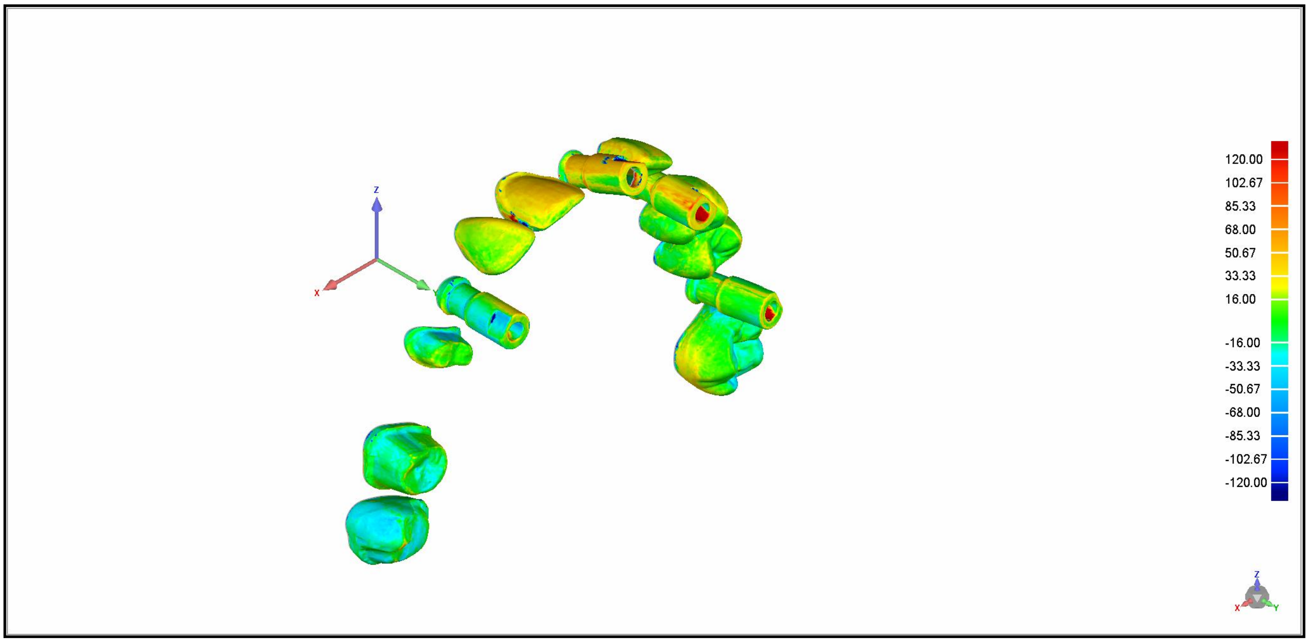

Predefinido: Frente

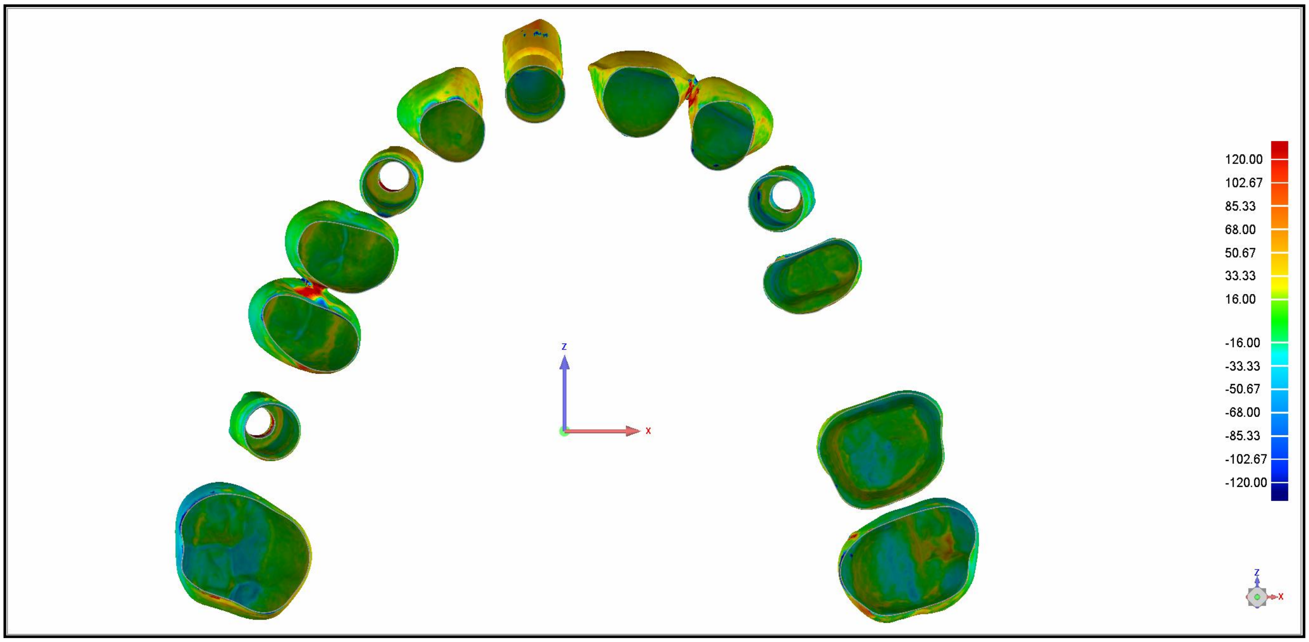

Predefinido: Atrás

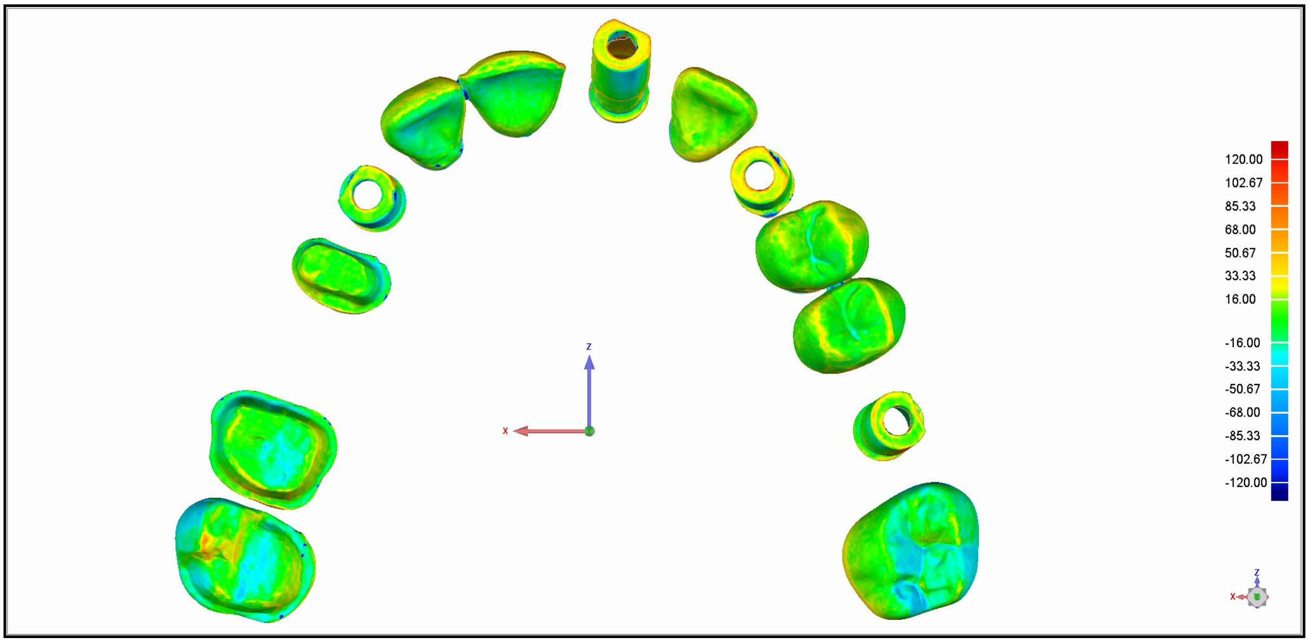

Predefinido: Izquierda

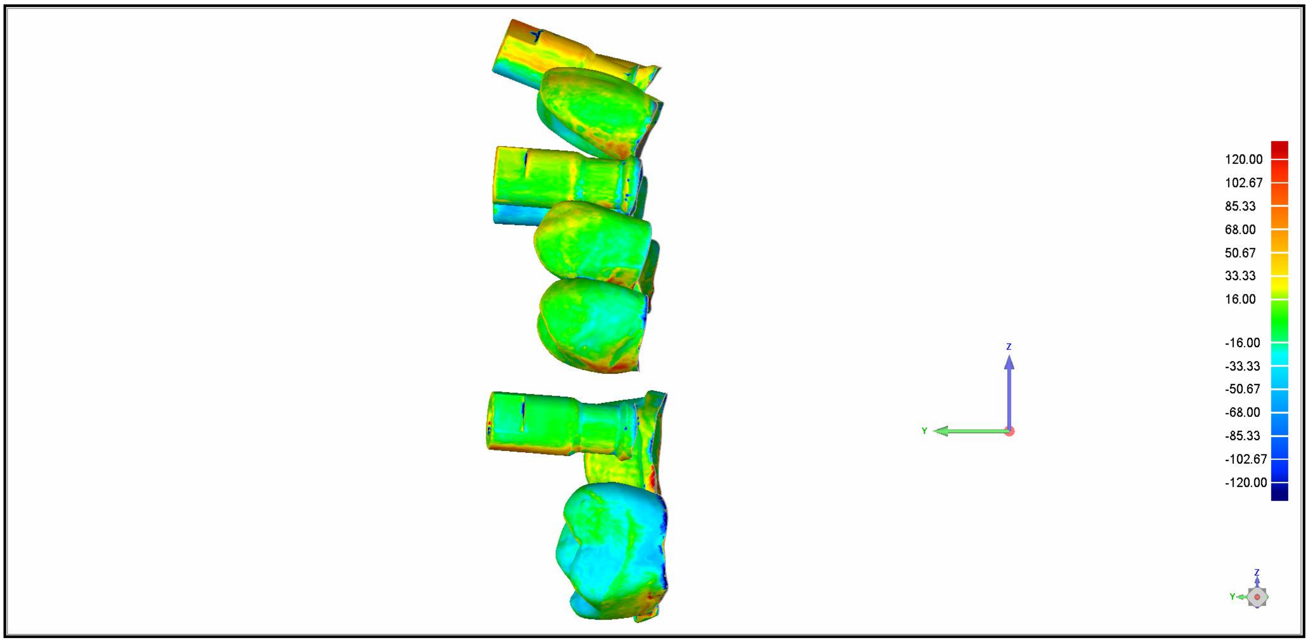

Predefinido: Derecha

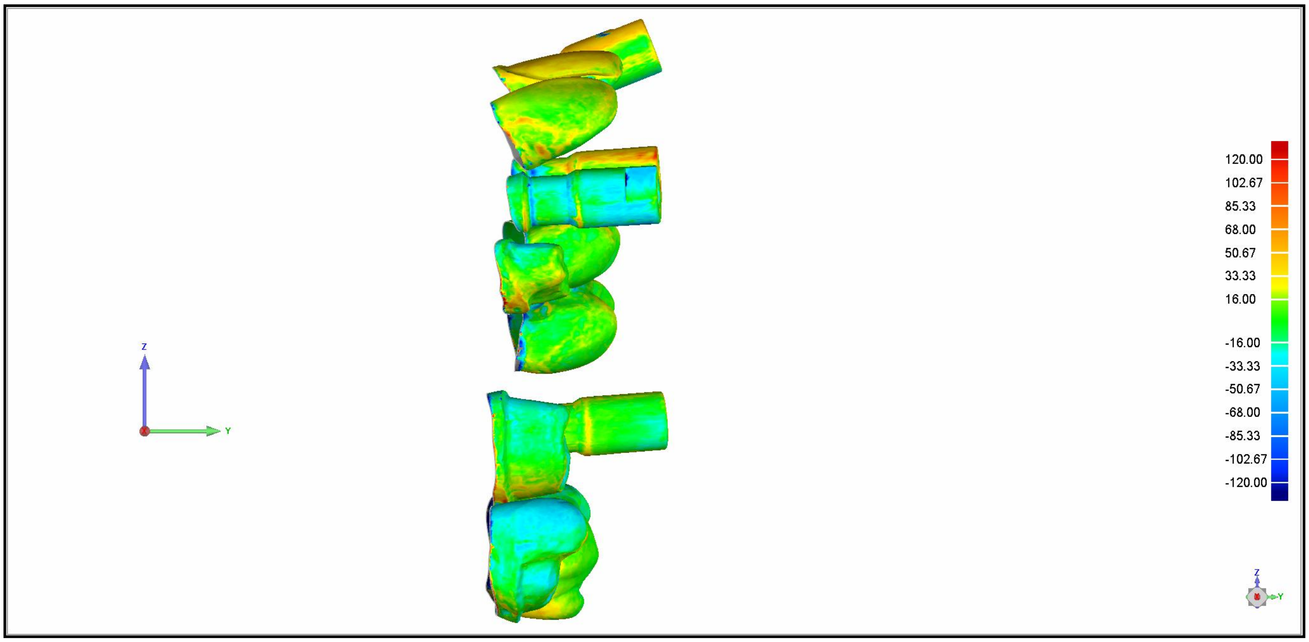

Predefinido: Superior

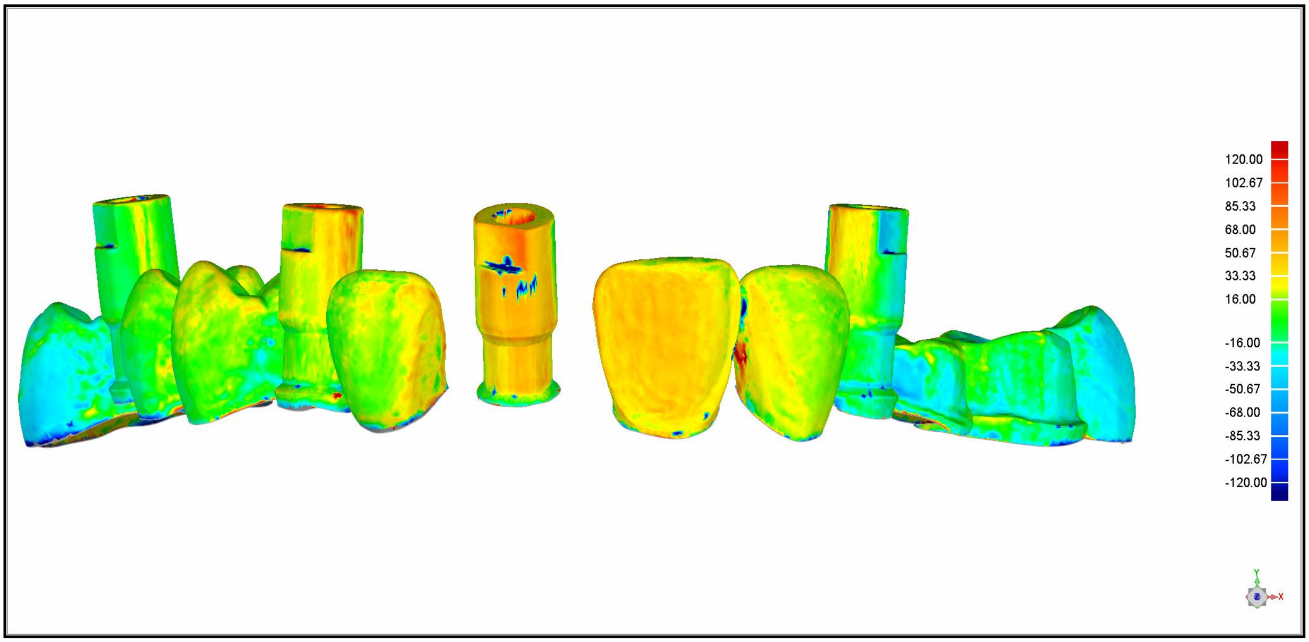

Predefinido: Inferior

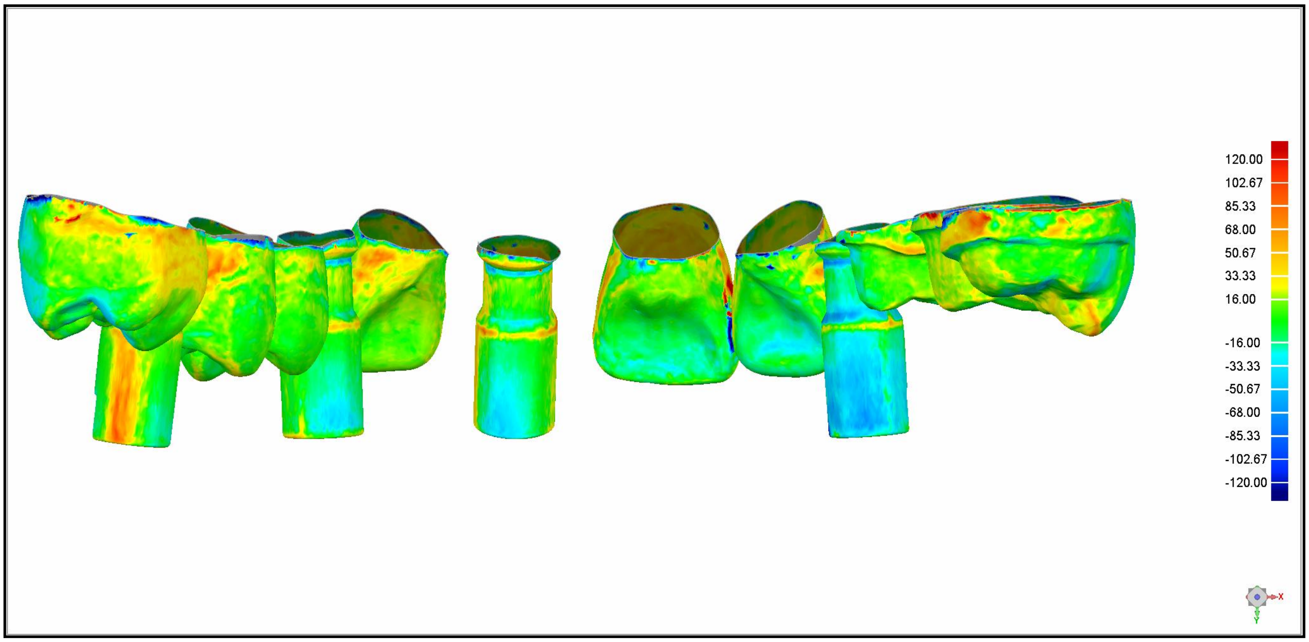

Supplement: S3 Table — Trios (scanning strategy C). (ZIP) [file pone.0202916.s003.zip › S3/3S10C.pdf]
