# Supplementary material for: Accuracy of four digital scanners according to scanning strategy in complete-arch impressions
Source: PLoS One. 2018 Sep 13;13(9):e0202916. doi: 10.1371/journal.pone.0202916 (PMC6136706; doi:10.1371/journal.pone.0202916)

Fecha: 3/6/2017, 12:13 pm

3D Comparación Resultados

Modelo referencia	MRC
Modelo test	3S1C
Nº de puntos de datos	99410
# Aislados	77

Tipo tolerancia	3D desviación
Unidades	u
Máx. crítico	120.00
Máx. nominal	13.00
Mín. nominal	-13.00
Mín. crítico	-120.00

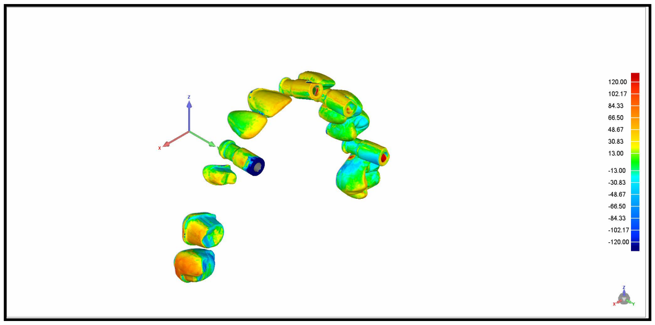

Desviación	
Desviación superior máx.	3056.90
Desviación inferior máx.	-3000.49
Desviación media	53.62 /-66.36
Desviación estándar	187.31

Distribución desviación

>=Min	<Max	# Puntos	%
-120.00	-102.17	277	0.28
-102.17	-84.33	775	0.78
-84.33	-66.50	1409	1.42
-66.50	-48.67	2615	2.63
-48.67	-30.83	5127	5.16
-30.83	-13.00	10230	10.29
-13.00	13.00	27544	27.71
13.00	30.83	24830	24.98
30.83	48.67	12749	12.82
48.67	66.50	5292	5.32
66.50	84.33	2226	2.24
84.33	102.17	826	0.83
102.17	120.00	458	0.46

Fuera del crítico superior	2955	2.97
Fuera del crítico inferior	2097	2.11

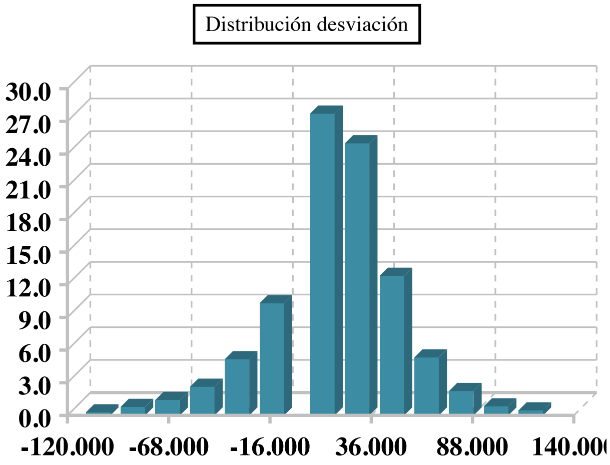

Desviaciones estándar

Distribución (+/-)	# Puntos	%
-6 * Desv. estánd.	603	0.61
-5 * Desv. estánd.	201	0.20
-4 * Desv. estánd.	191	0.19
-3 * Desv. estánd.	168	0.17
-2 * Desv. estánd.	435	0.44
-1 * Desv. estánd.	49309	49.60
1 * Desv. estánd.	46374	46.65
2 * Desv. estánd.	574	0.58
3 * Desv. estánd.	273	0.27
4 * Desv. estánd.	283	0.28
5 * Desv. estánd.	259	0.26
6 * Desv. estánd.	740	0.74

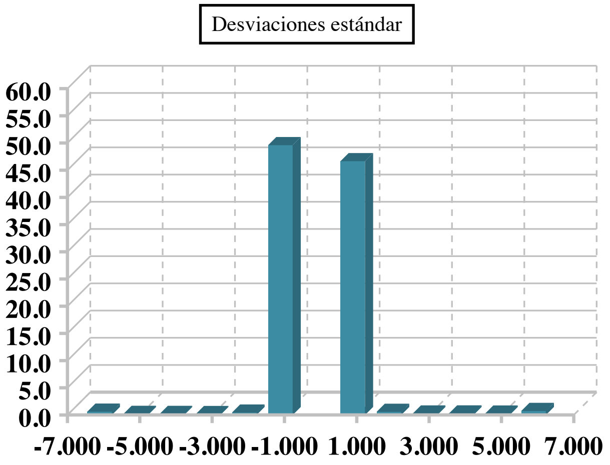

Predefinido: Isométrico

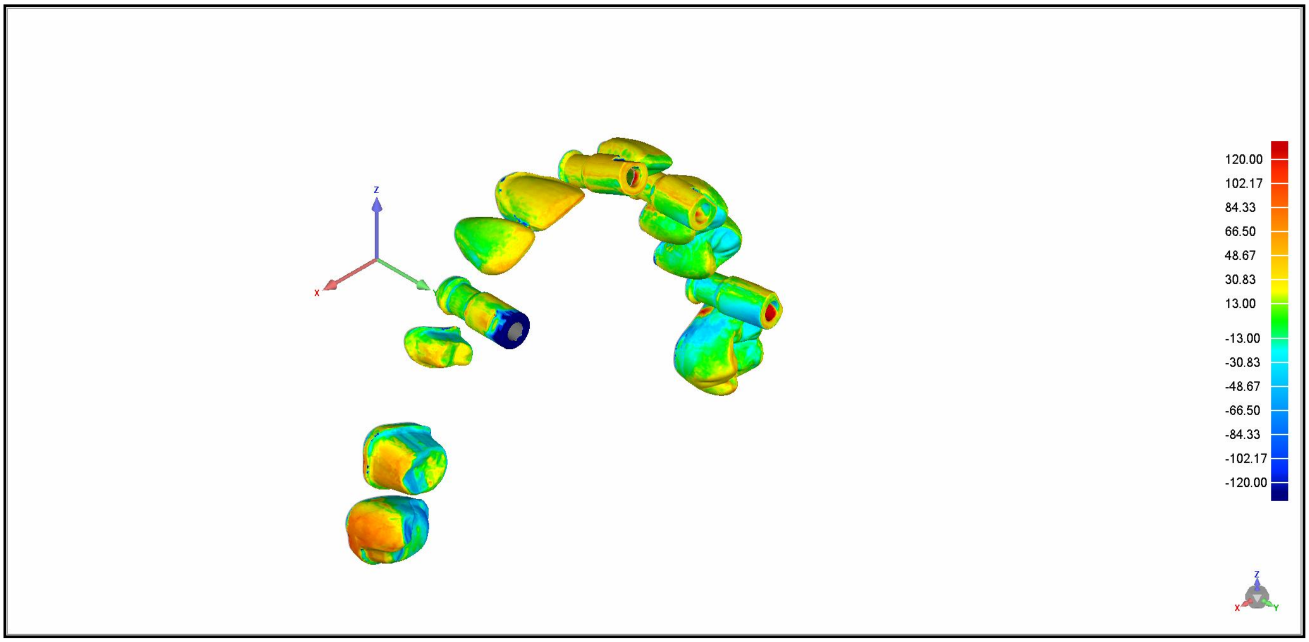

Predefinido: Frente

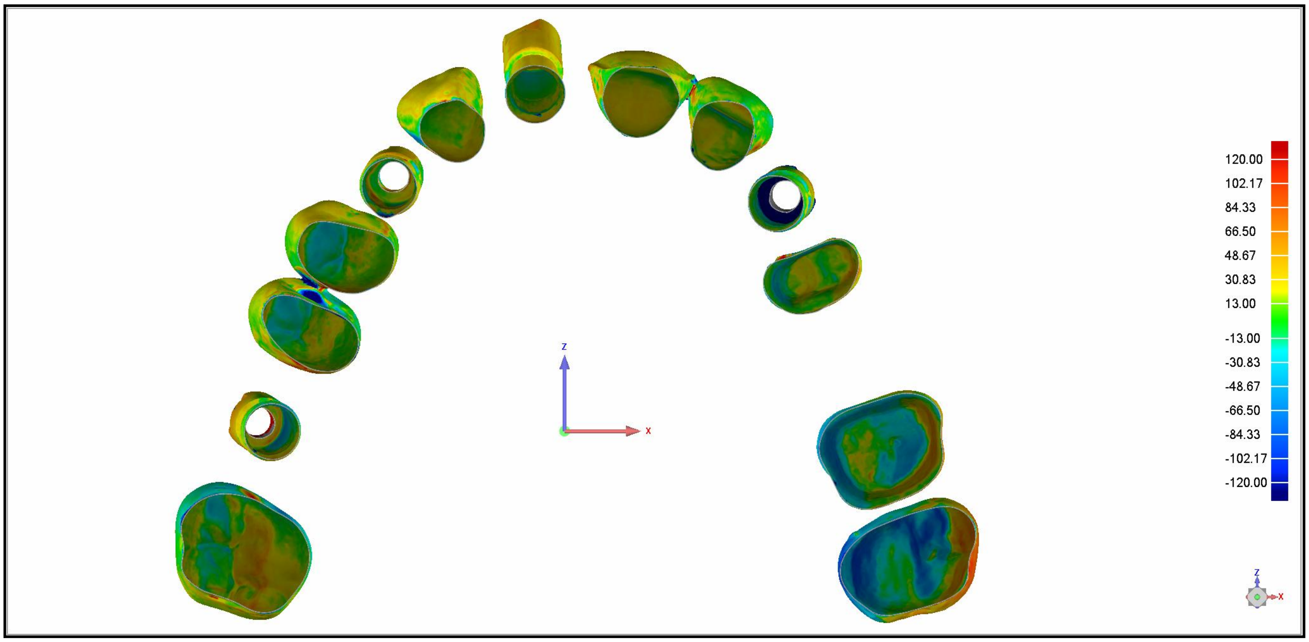

Predefinido: Atrás

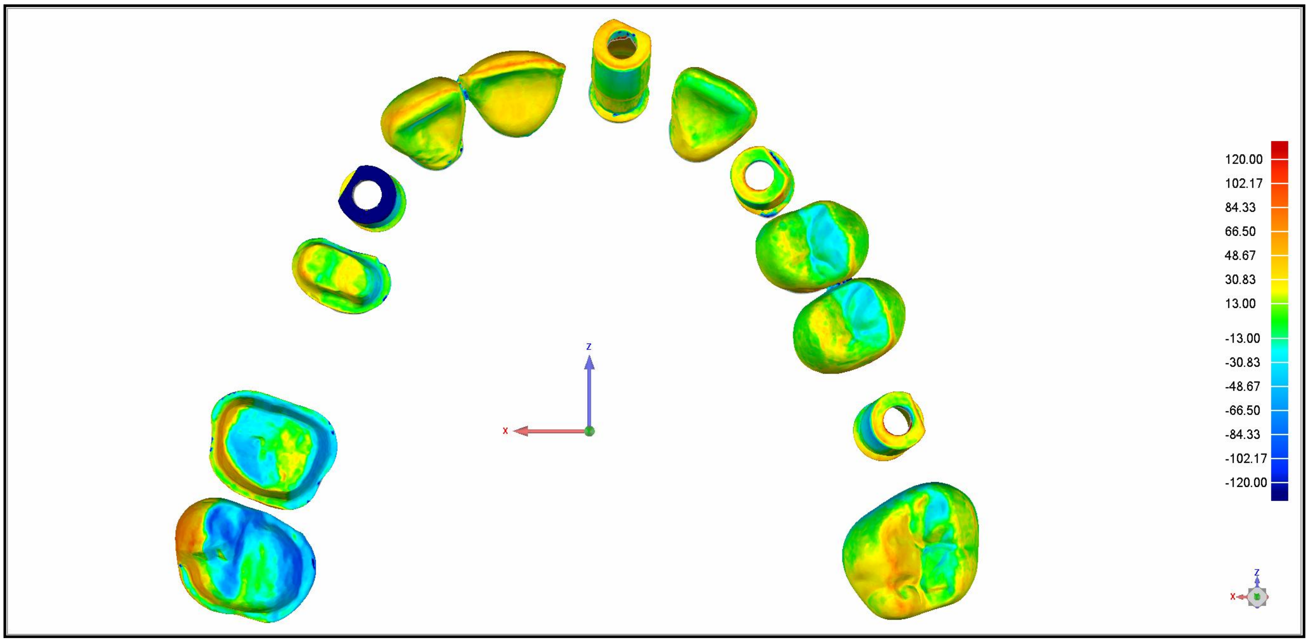

Predefinido: Izquierda

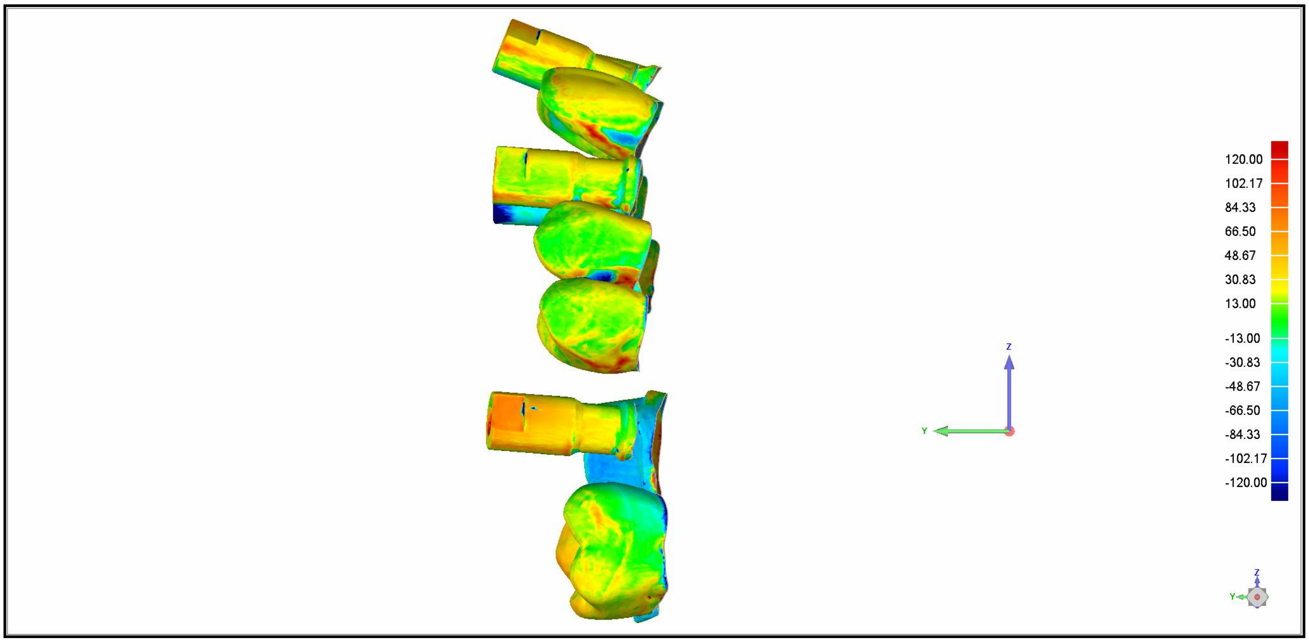

Predefinido: Derecha

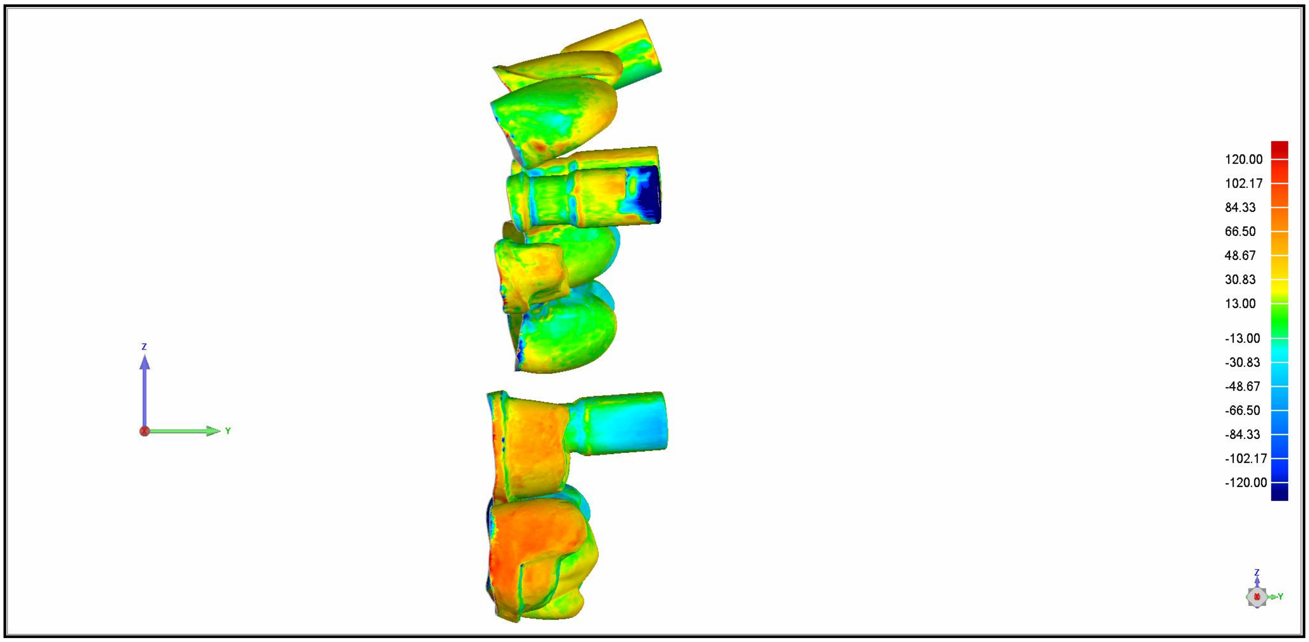

Predefinido: Superior

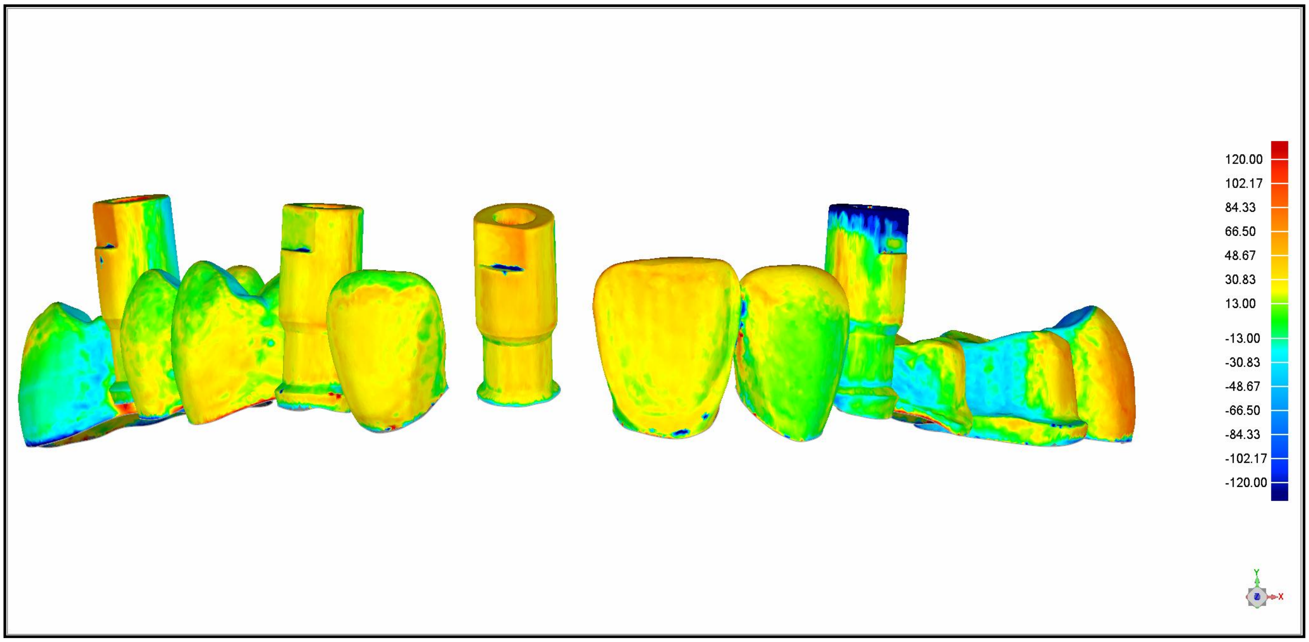

Predefinido: Inferior

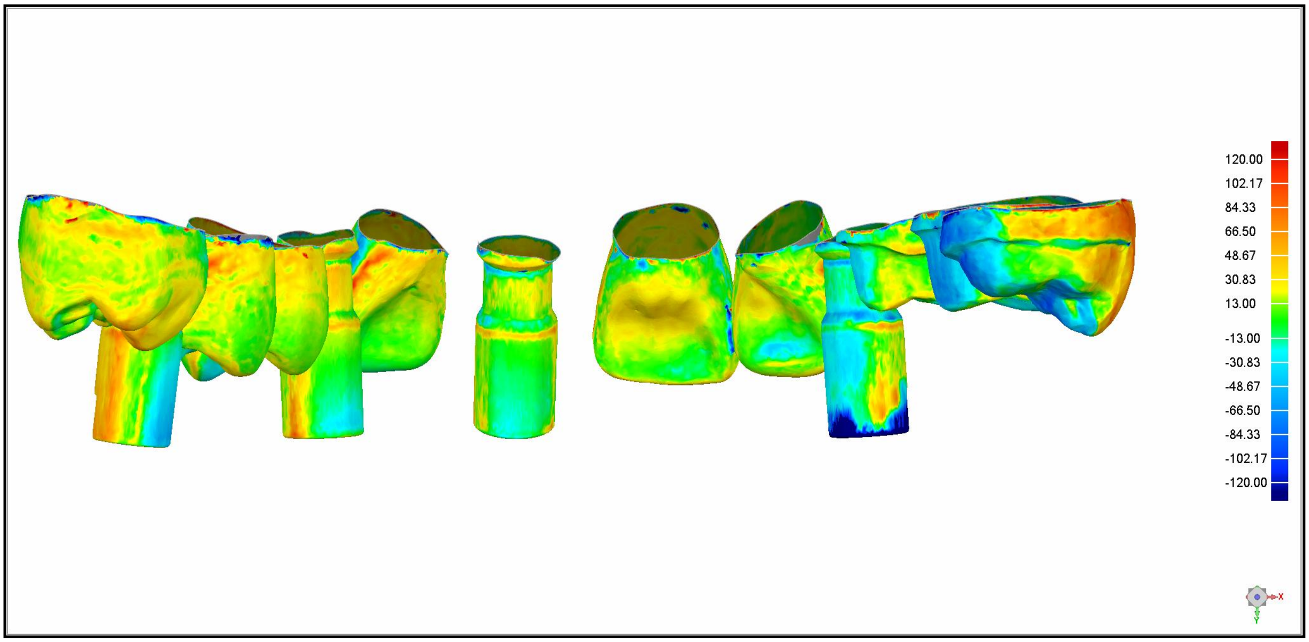

Supplement: S3 Table — Trios (scanning strategy C). (ZIP) [file pone.0202916.s003.zip › S3/3S1C.pdf]
