# Supplementary material for: Accuracy of four digital scanners according to scanning strategy in complete-arch impressions
Source: PLoS One. 2018 Sep 13;13(9):e0202916. doi: 10.1371/journal.pone.0202916 (PMC6136706; doi:10.1371/journal.pone.0202916)

Fecha: 3/6/2017, 12:37 pm

3D Comparación Resultados

Modelo referencia	MRC
Modelo test	3S6C
Nº de puntos de datos	105511
# Aislados	61

Tipo tolerancia	3D desviación
Unidades	u
Máx. crítico	120.00
Máx. nominal	17.00
Mín. nominal	-17.00
Mín. crítico	-120.00

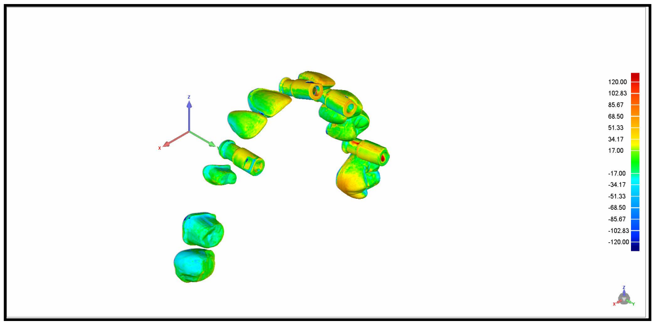

Desviación	
Desviación superior máx.	3095.71
Desviación inferior máx.	-2999.60
Desviación media	58.71 /-47.10
Desviación estándar	190.12

Distribución desviación

>=Min	<Max	# Puntos	%
-120.00	-102.83	275	0.26
-102.83	-85.67	354	0.34
-85.67	-68.50	581	0.55
-68.50	-51.33	1137	1.08
-51.33	-34.17	4052	3.84
-34.17	-17.00	10708	10.15
-17.00	17.00	51099	48.43
17.00	34.17	18039	17.10
34.17	51.33	8049	7.63
51.33	68.50	3436	3.26
68.50	85.67	1119	1.06
85.67	102.83	527	0.50
102.83	120.00	392	0.37

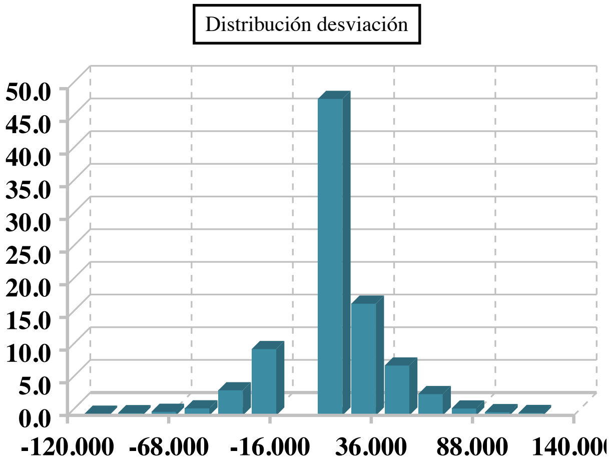

Fuera del crítico superior	3797	3.60
Fuera del crítico inferior	1946	1.84

Desviaciones estándar

Distribución (+/-)	# Puntos	%
-6 * Desv. estánd.	491	0.47
-5 * Desv. estánd.	95	0.09
-4 * Desv. estánd.	120	0.11
-3 * Desv. estánd.	173	0.16
-2 * Desv. estánd.	532	0.50
-1 * Desv. estánd.	69782	66.14
1 * Desv. estánd.	31489	29.84
2 * Desv. estánd.	731	0.69
3 * Desv. estánd.	422	0.40
4 * Desv. estánd.	429	0.41
5 * Desv. estánd.	342	0.32
6 * Desv. estánd.	905	0.86

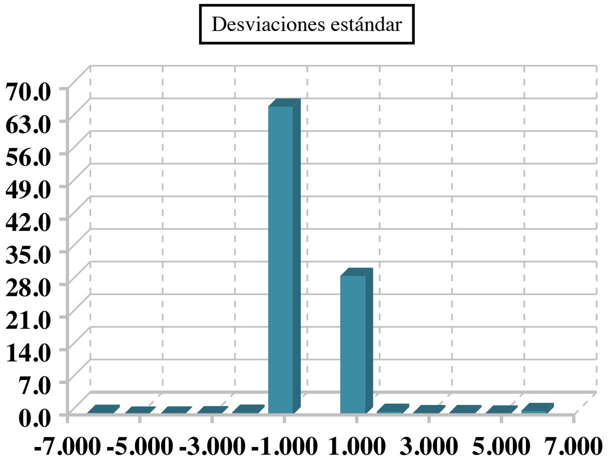

Predefinido: Isométrico

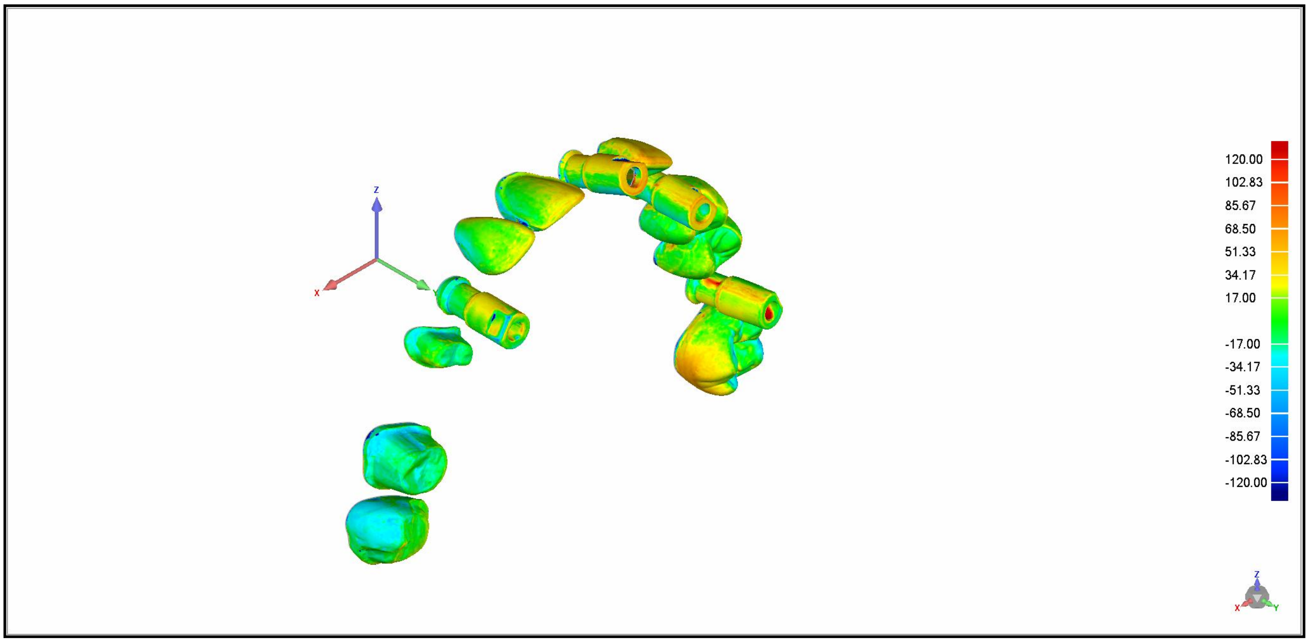

Predefinido: Frente

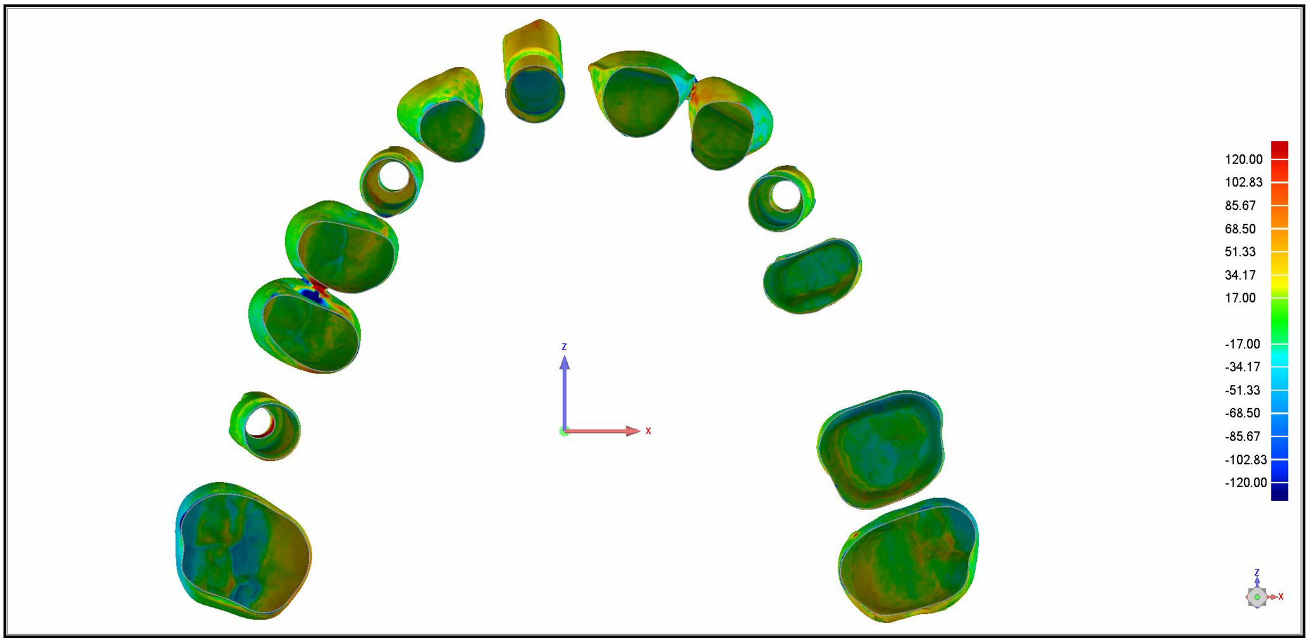

Predefinido: Atrás

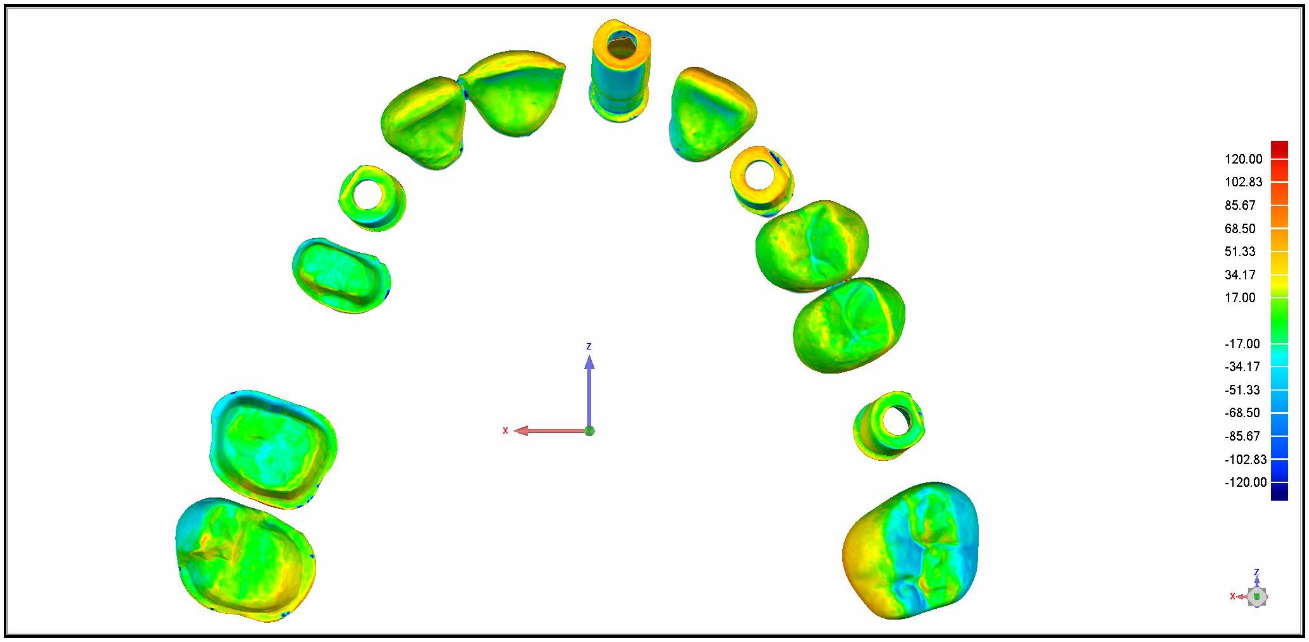

Predefinido: Izquierda

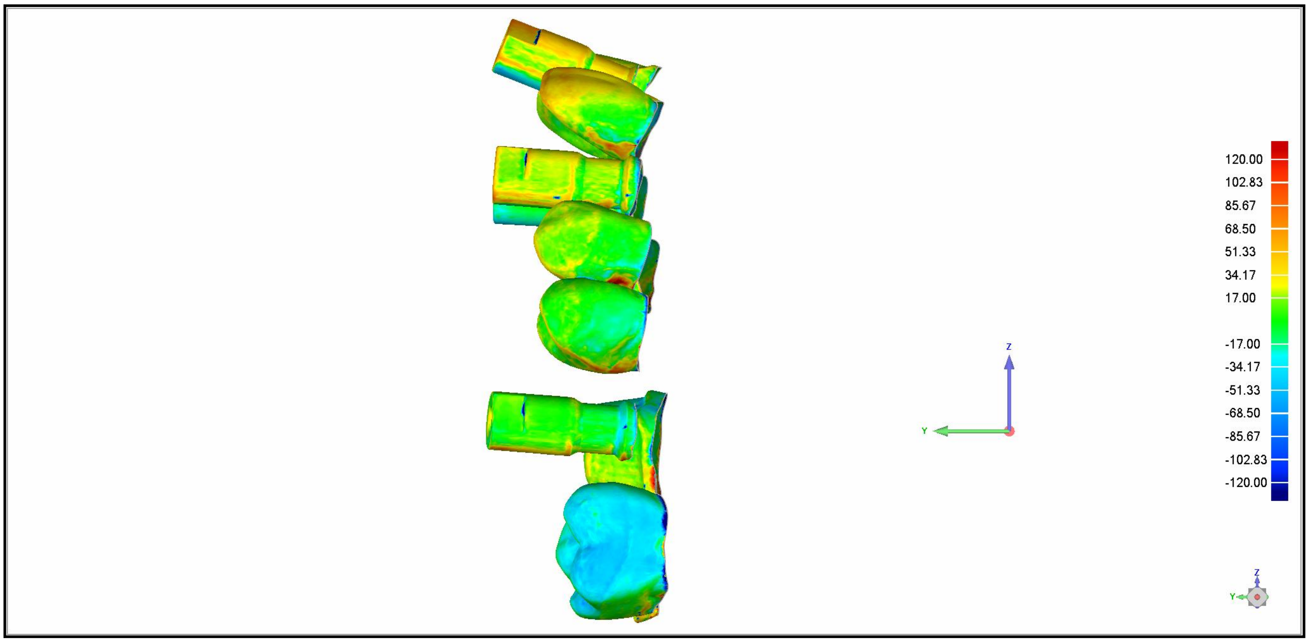

Predefinido: Derecha

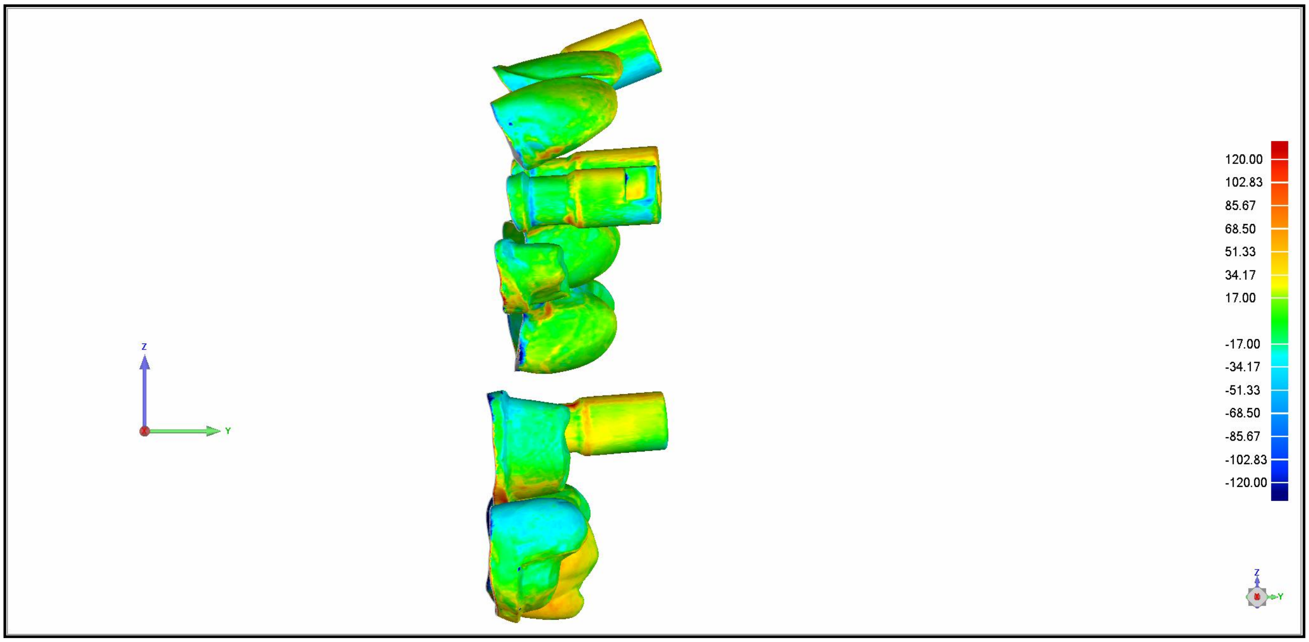

Predefinido: Superior

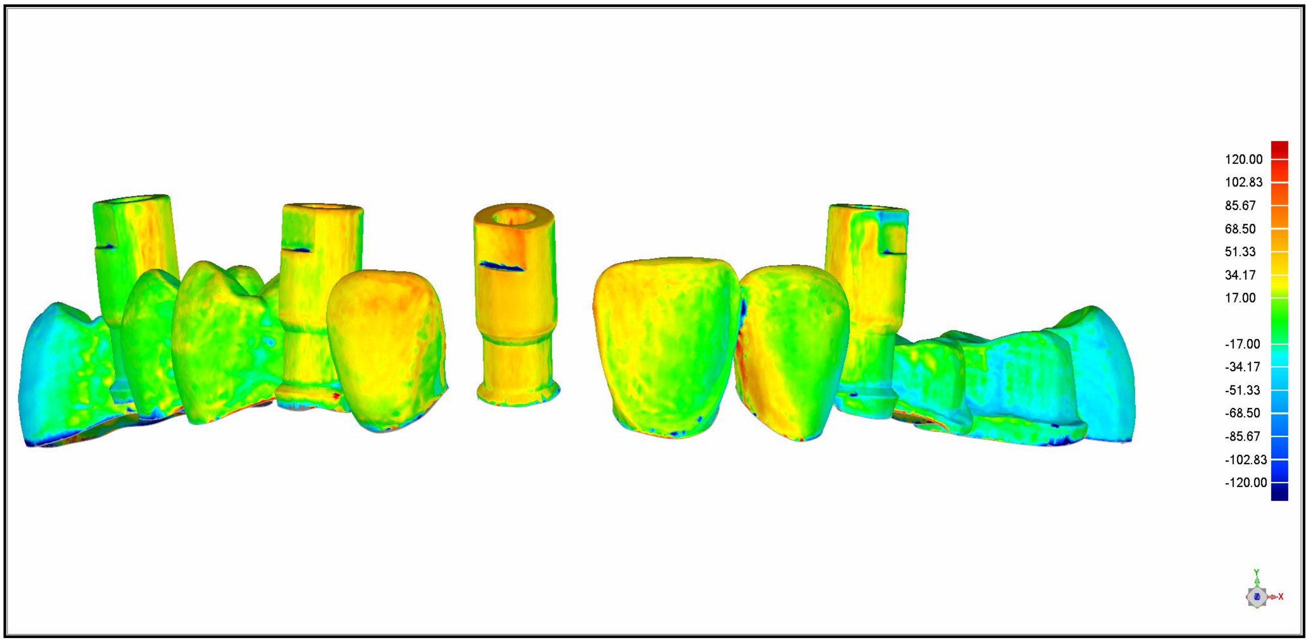

Predefinido: Inferior

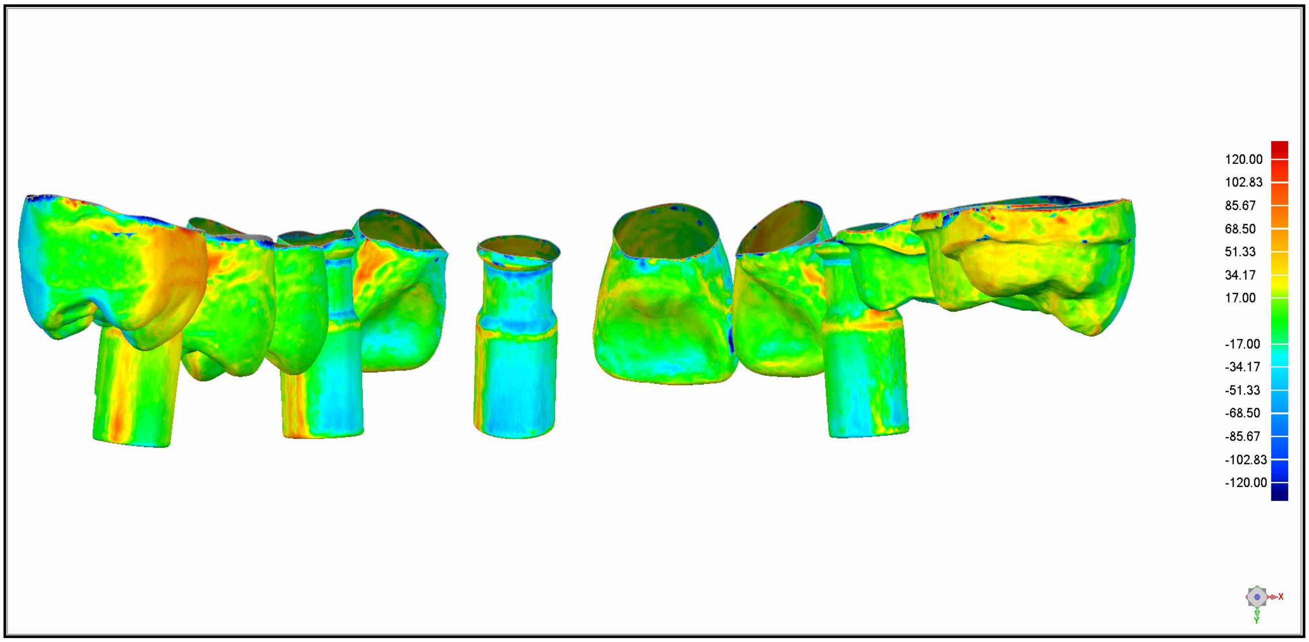

Supplement: S3 Table — Trios (scanning strategy C). (ZIP) [file pone.0202916.s003.zip › S3/3S6C.pdf]
