# Supplementary material for: Accuracy of four digital scanners according to scanning strategy in complete-arch impressions
Source: PLoS One. 2018 Sep 13;13(9):e0202916. doi: 10.1371/journal.pone.0202916 (PMC6136706; doi:10.1371/journal.pone.0202916)

Fecha: 3/6/2017, 12:39 pm

3D Comparación Resultados

Modelo referencia	MRC
Modelo test	3S7C
Nº de puntos de datos	102263
# Aislados	97

Tipo tolerancia	3D desviación
Unidades	u
Máx. crítico	120.00
Máx. nominal	15.00
Mín. nominal	-15.00
Mín. crítico	-120.00

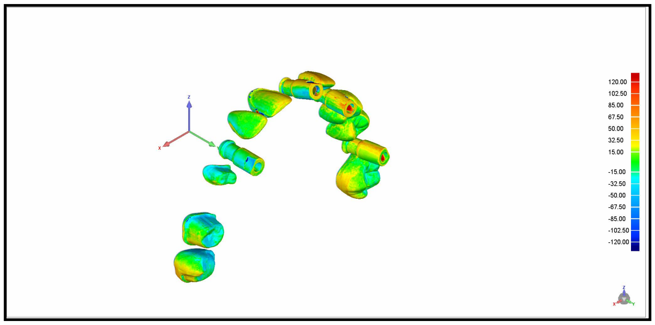

Desviación	
Desviación superior máx.	3073.71
Desviación inferior máx.	-3123.92
Desviación media	61.68 /-50.38
Desviación estándar	200.67

Distribución desviación

>=Min	<Max	# Puntos	%
-120.00	-102.50	274	0.27
-102.50	-85.00	412	0.40
-85.00	-67.50	560	0.55
-67.50	-50.00	1405	1.37
-50.00	-32.50	4903	4.79
-32.50	-15.00	12984	12.70
-15.00	15.00	41703	40.78
15.00	32.50	17737	17.34
32.50	50.00	9652	9.44
50.00	67.50	4189	4.10
67.50	85.00	1654	1.62
85.00	102.50	694	0.68
102.50	120.00	408	0.40

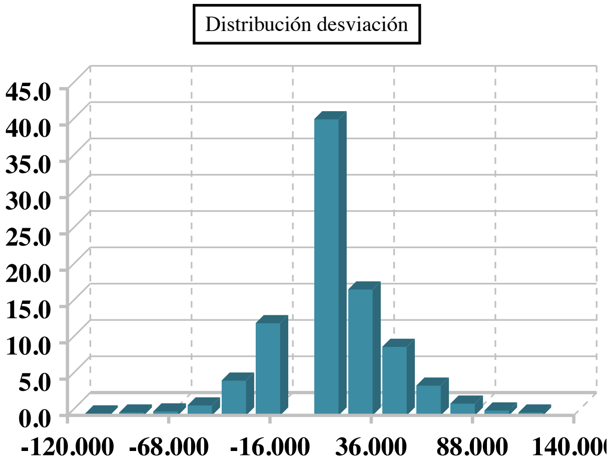

Fuera del crítico superior		3759	3.68
Fuera del crítico inferior		1929	1.89

Desviaciones estándar

Distribución (+/-)	# Puntos	%
-6 * Desv. estánd.	548	0.54
-5 * Desv. estánd.	99	0.10
-4 * Desv. estánd.	119	0.12
-3 * Desv. estánd.	161	0.16
-2 * Desv. estánd.	392	0.38
-1 * Desv. estánd.	63518	62.11
1 * Desv. estánd.	34885	34.11
2 * Desv. estánd.	715	0.70
3 * Desv. estánd.	358	0.35
4 * Desv. estánd.	334	0.33
5 * Desv. estánd.	309	0.30
6 * Desv. estánd.	825	0.81

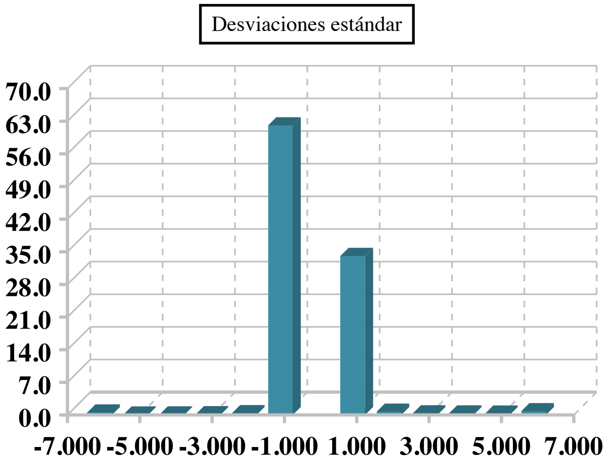

Predefinido: Isométrico

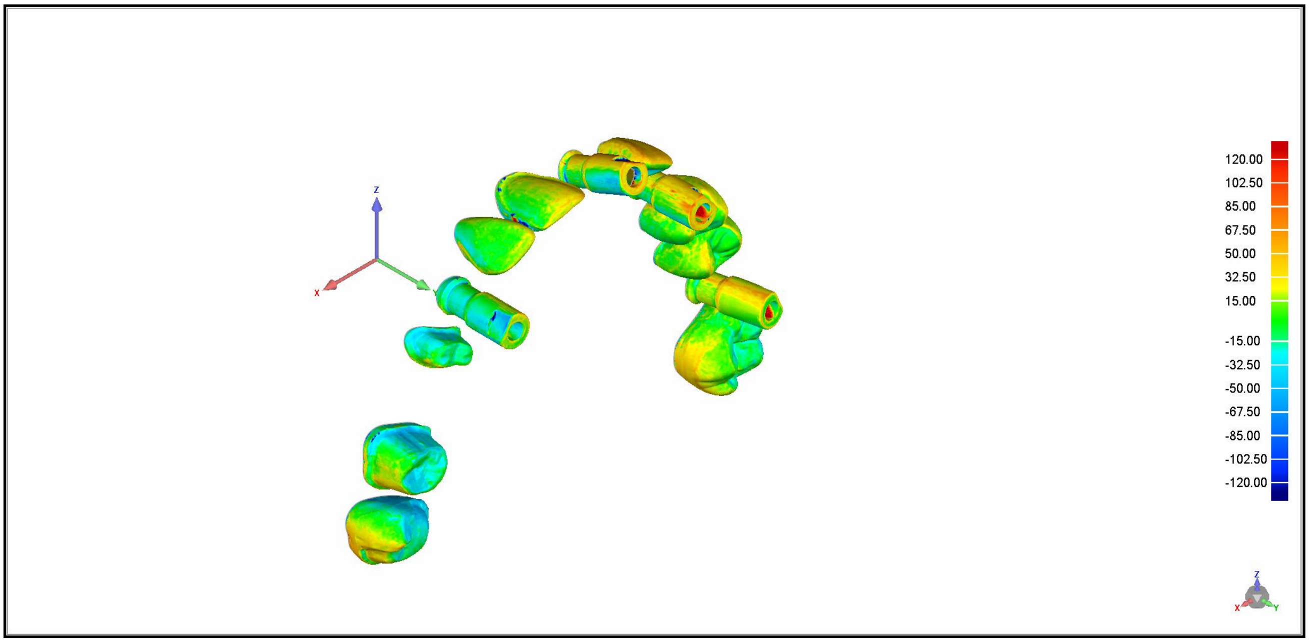

Geomagic Control™

Predefinido: Frente

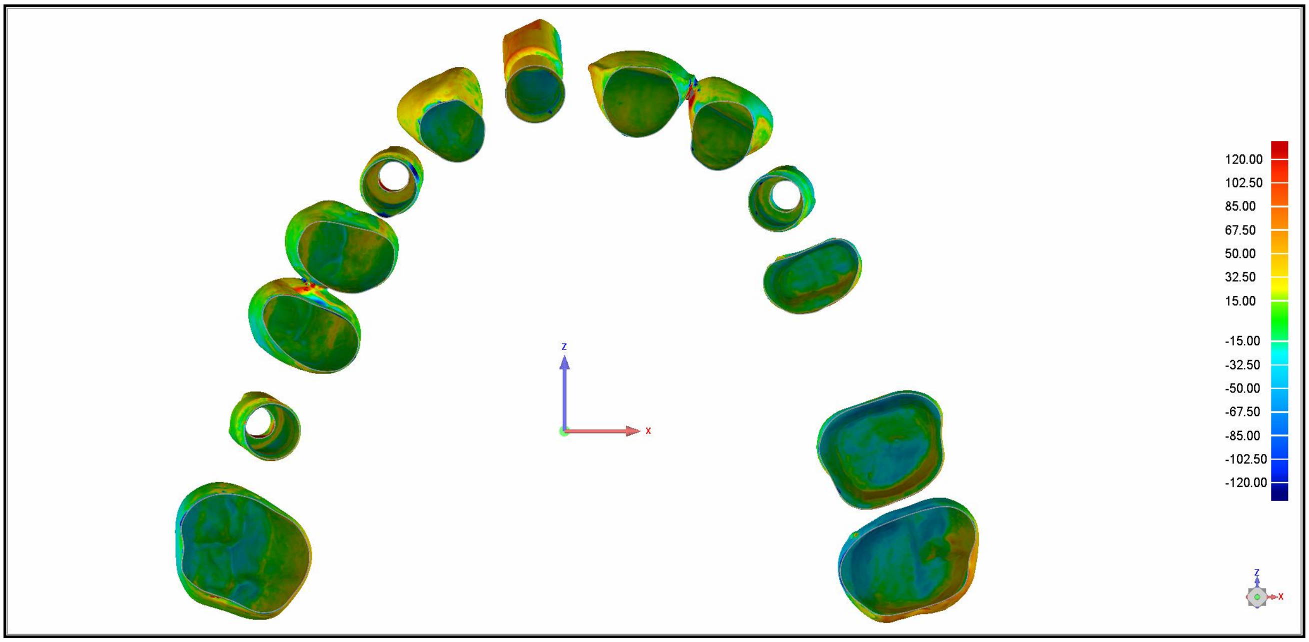

Predefinido: Atrás

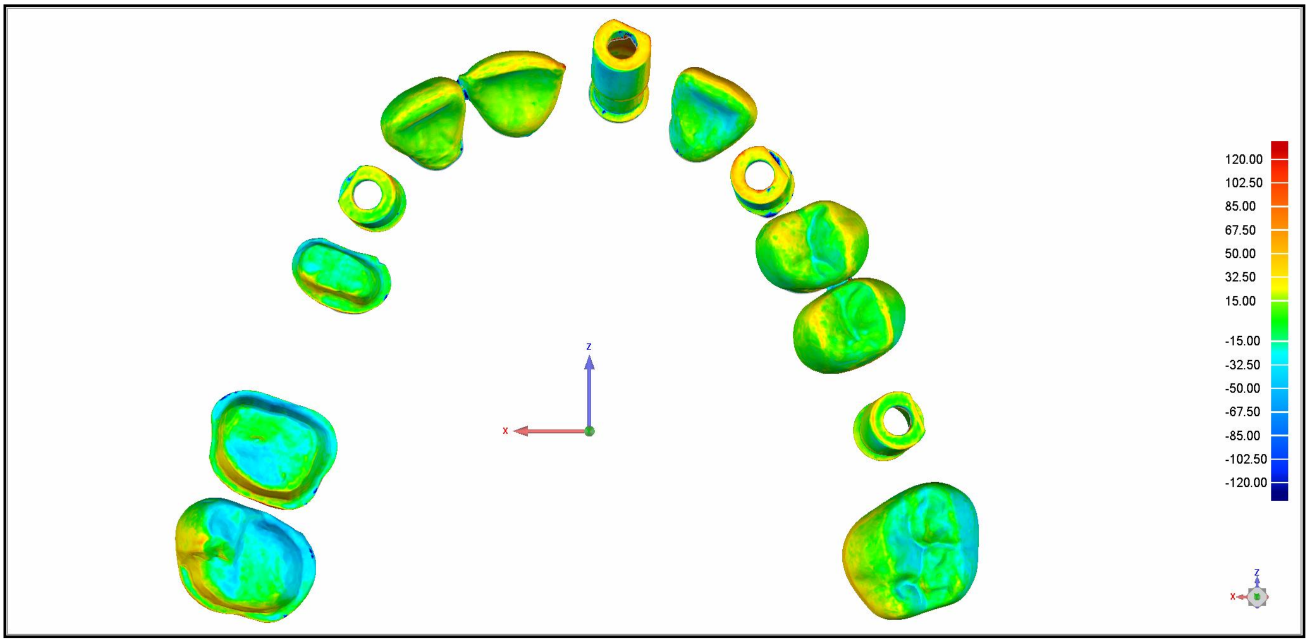

Predefinido: Izquierda

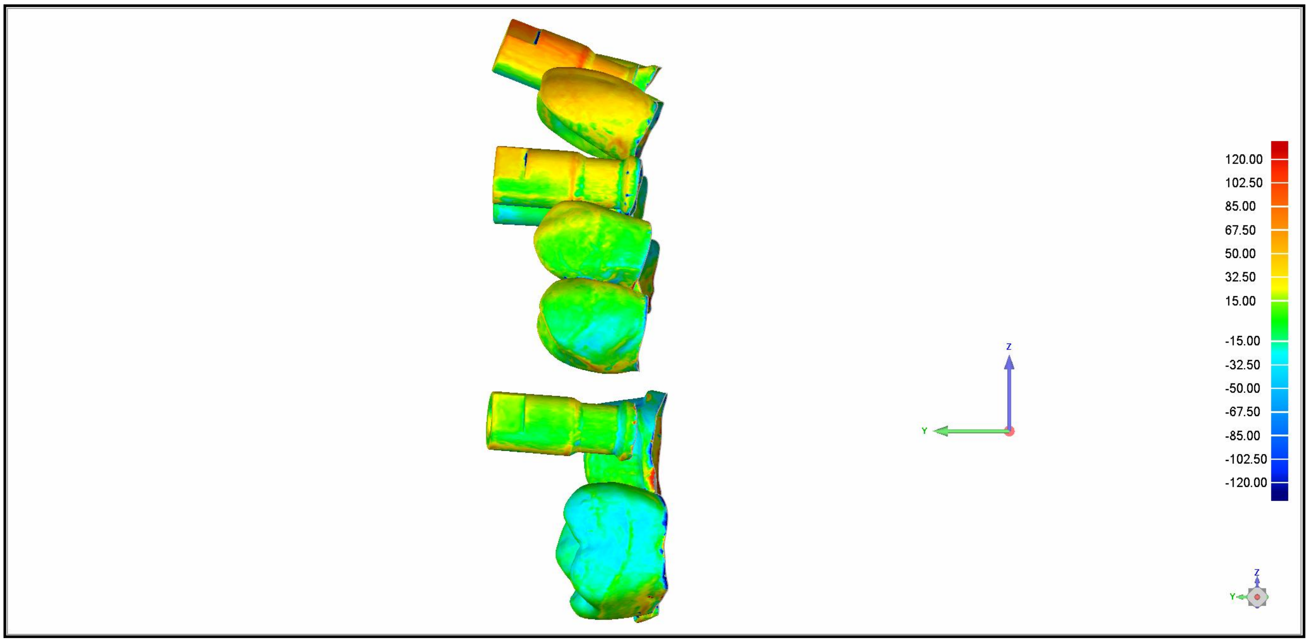

Predefinido: Derecha

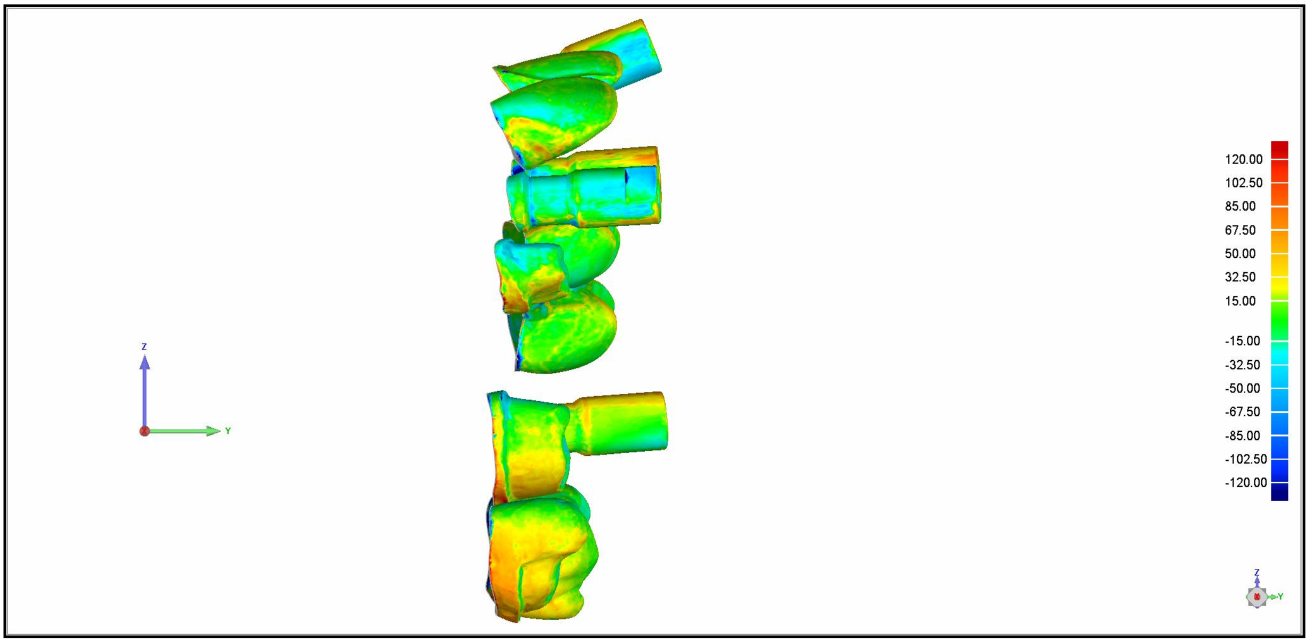

Predefinido: Superior

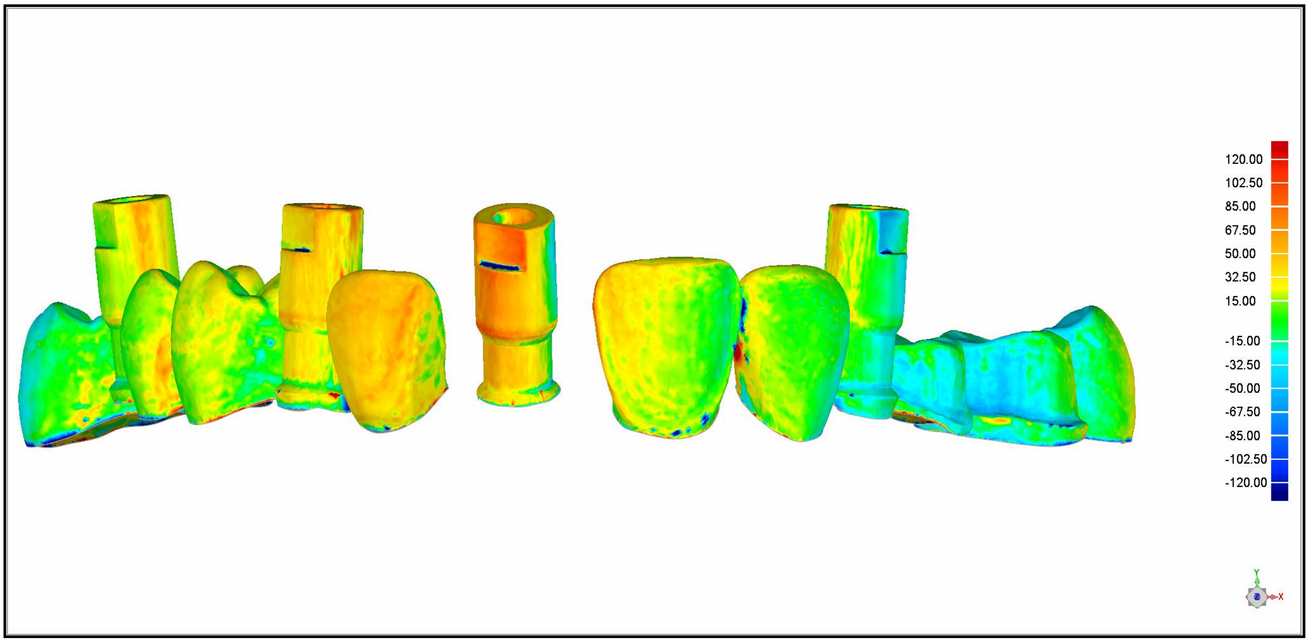

Predefinido: Inferior

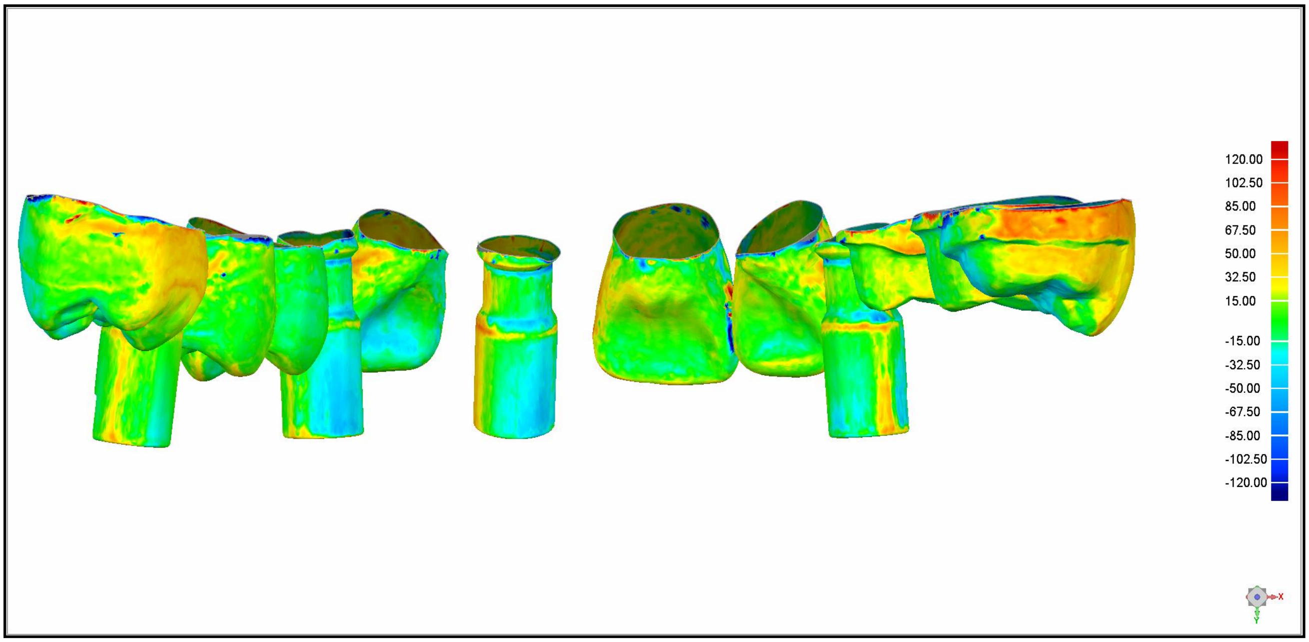

Supplement: S3 Table — Trios (scanning strategy C). (ZIP) [file pone.0202916.s003.zip › S3/3S7C.pdf]
