# Supplementary material for: Accuracy of four digital scanners according to scanning strategy in complete-arch impressions
Source: PLoS One. 2018 Sep 13;13(9):e0202916. doi: 10.1371/journal.pone.0202916 (PMC6136706; doi:10.1371/journal.pone.0202916)

Control

3D Comparación Resultados

Modelo referencia	MRC
Modelo test	3S9D
Nº de puntos de datos	108124
# Aislados	61

Tipo tolerancia	3D desviación
Unidades	u
Máx. crítico	120.00
Máx. nominal	15.00
Mín. nominal	-15.00
Mín. crítico	-120.00

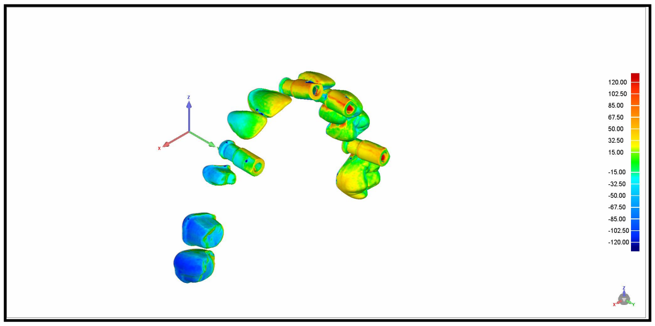

Desviación	
Desviación superior máx.	3154.10
Desviación inferior máx.	-3135.09
Desviación media	70.08 /-52.88
Desviación estándar	205.45

Distribución desviación

>=Min	<Max	# Puntos	%
-120.00	-102.50	740	0.68
-102.50	-85.00	1510	1.40
-85.00	-67.50	2377	2.20
-67.50	-50.00	3614	3.34
-50.00	-32.50	5565	5.15
-32.50	-15.00	13769	12.73
-15.00	15.00	38170	35.30
15.00	32.50	17611	16.29
32.50	50.00	9579	8.86
50.00	67.50	4214	3.90
67.50	85.00	2405	2.22
85.00	102.50	1375	1.27
102.50	120.00	803	0.74

Distribución desviación

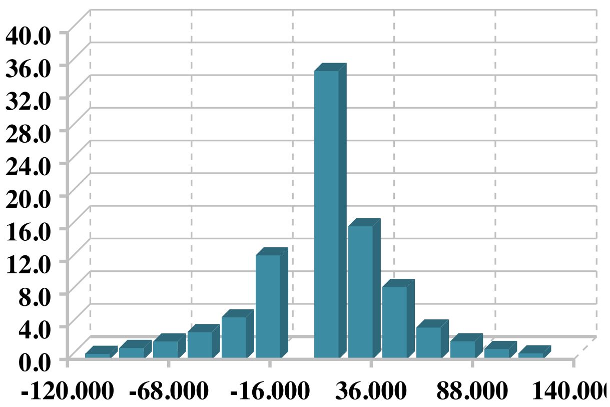

Fuera del crítico superior	4324	4.00
Fuera del crítico inferior	2068	1.91

Desviaciones estándar

Distribución (+/-)	# Puntos	%
-6 * Desv. estánd.	476	0.44
-5 * Desv. estánd.	66	0.06
-4 * Desv. estánd.	117	0.11
-3 * Desv. estánd.	173	0.16
-2 * Desv. estánd.	494	0.46
-1 * Desv. estánd.	67530	62.46
1 * Desv. estánd.	36493	33.75
2 * Desv. estánd.	733	0.68
3 * Desv. estánd.	358	0.33
4 * Desv. estánd.	348	0.32
5 * Desv. estánd.	340	0.31
6 * Desv. estánd.	996	0.92

Desviaciones estándar

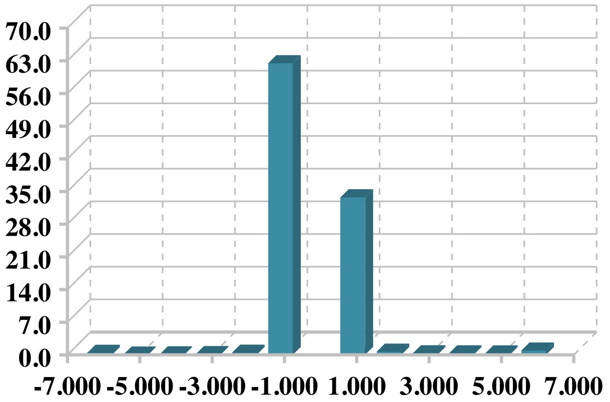

Predefinido: Isométrico

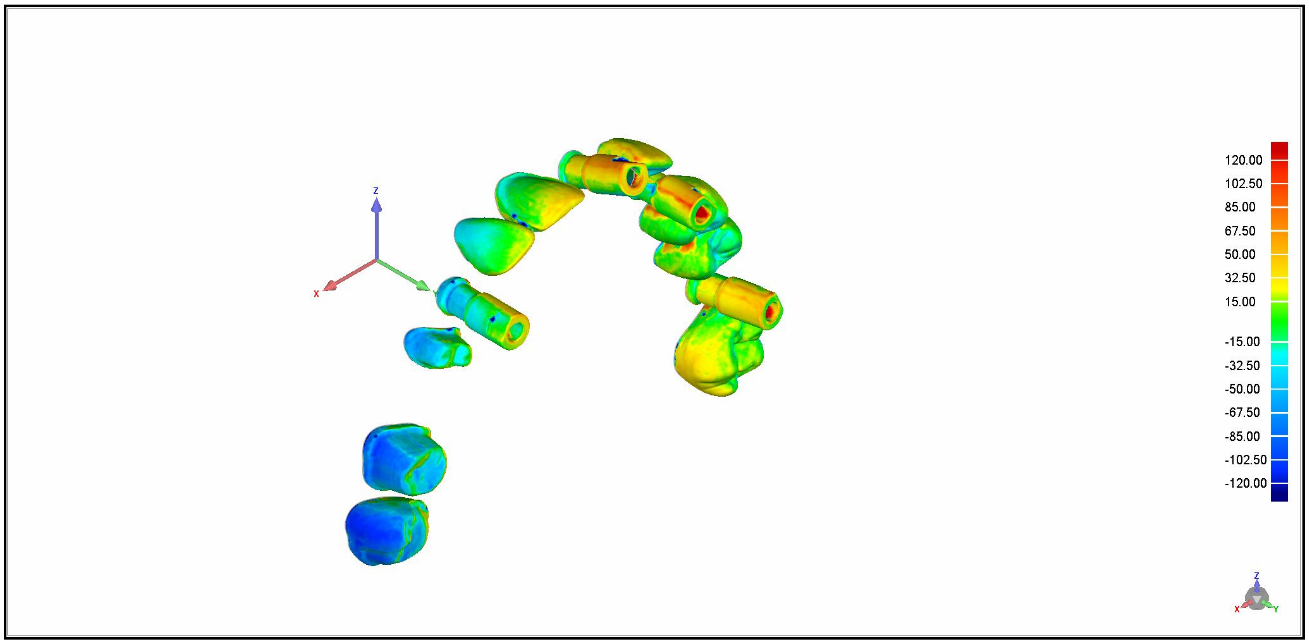

Geomagic Control™

Predefinido: Frente

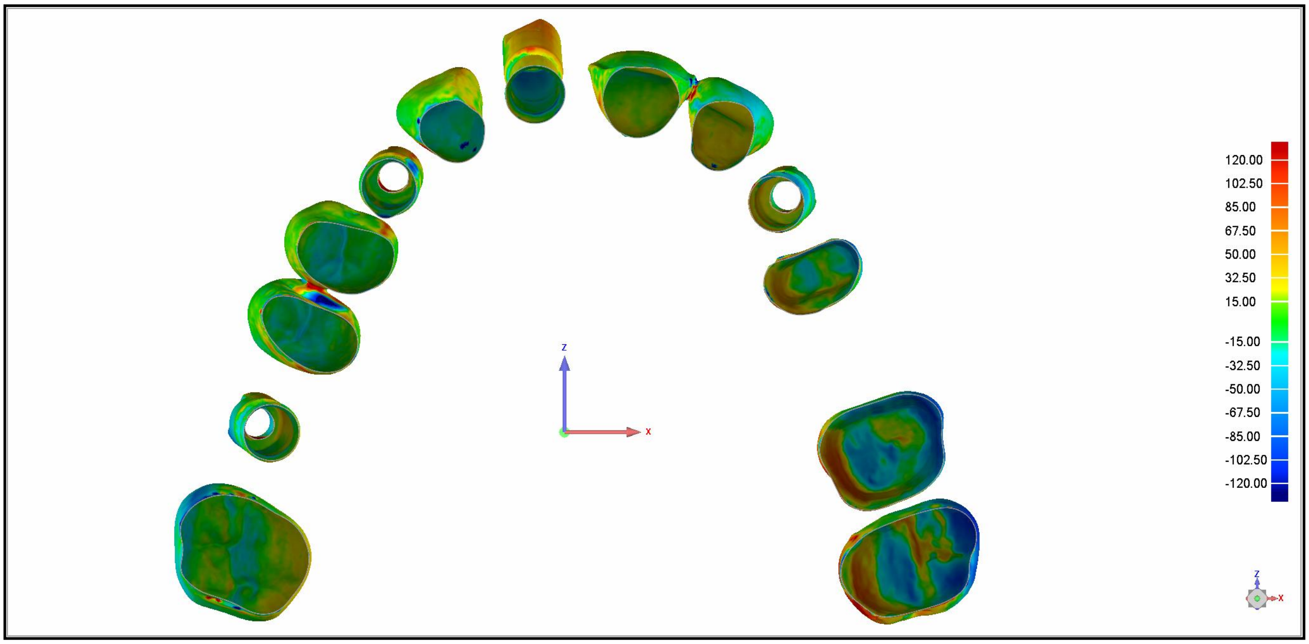

Predefinido: Atrás

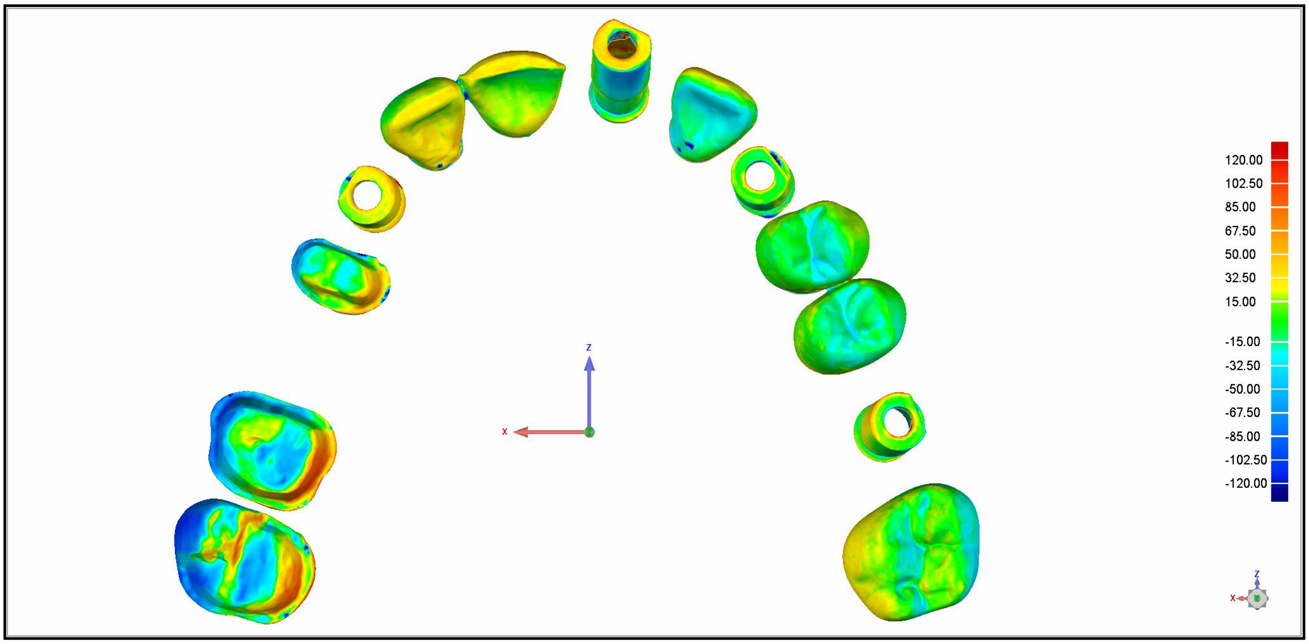

Predefinido: Izquierda

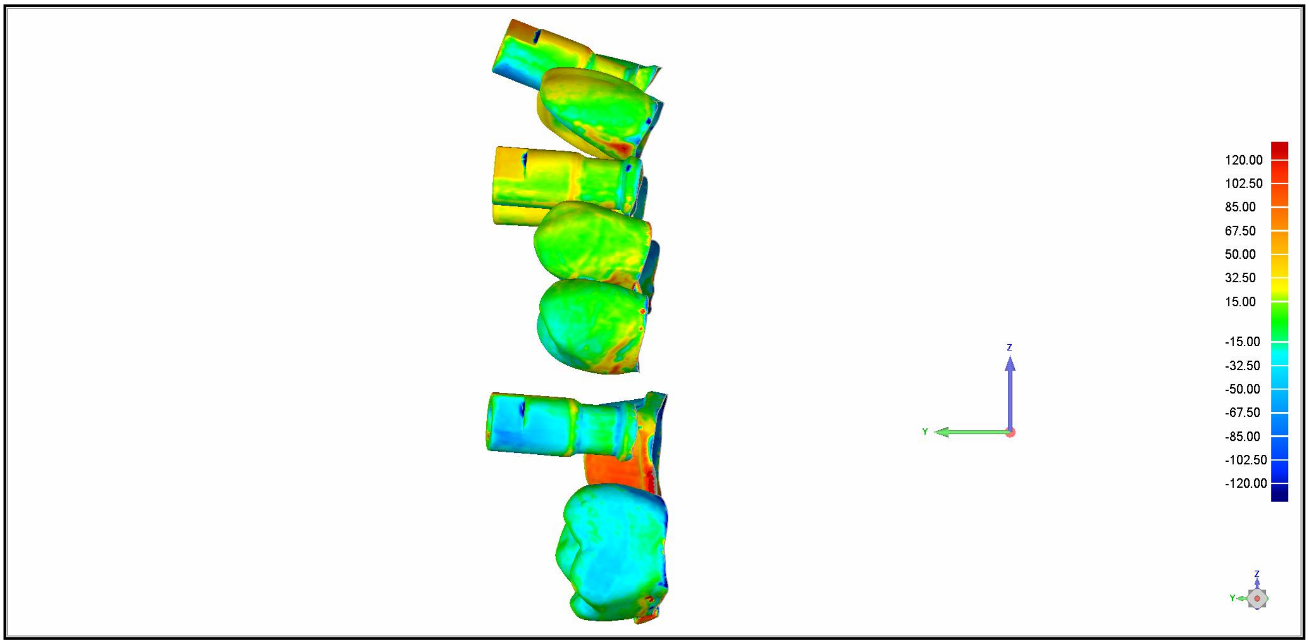

Predefinido: Derecha

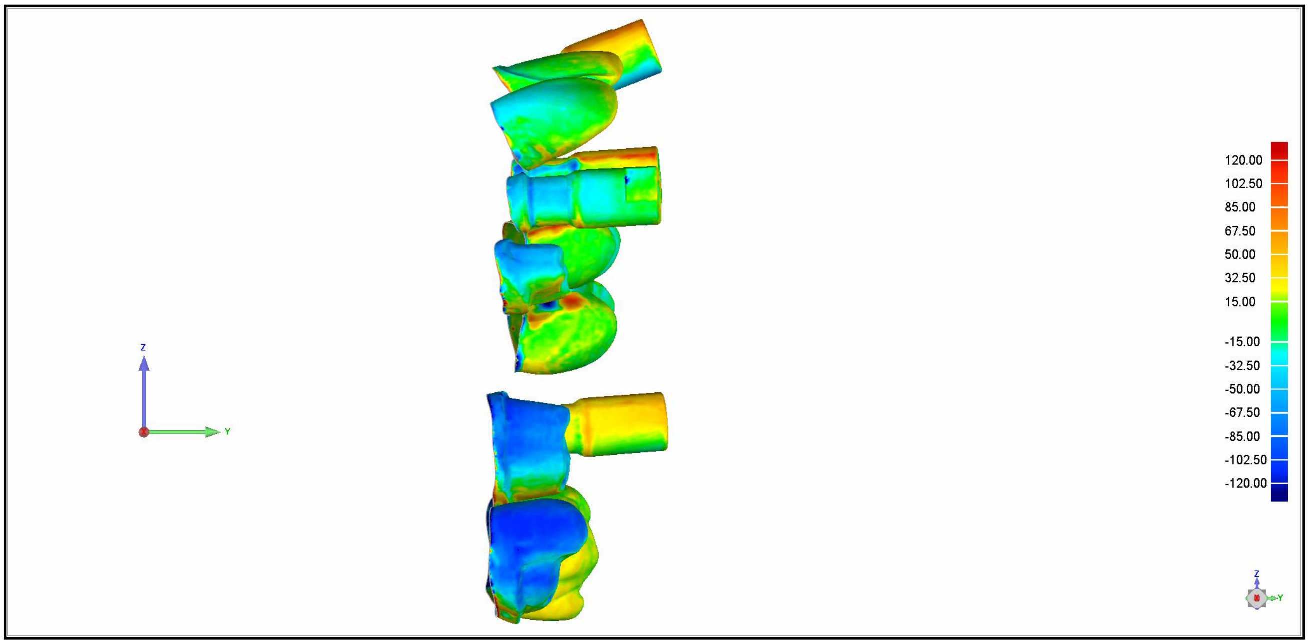

Predefinido: Superior

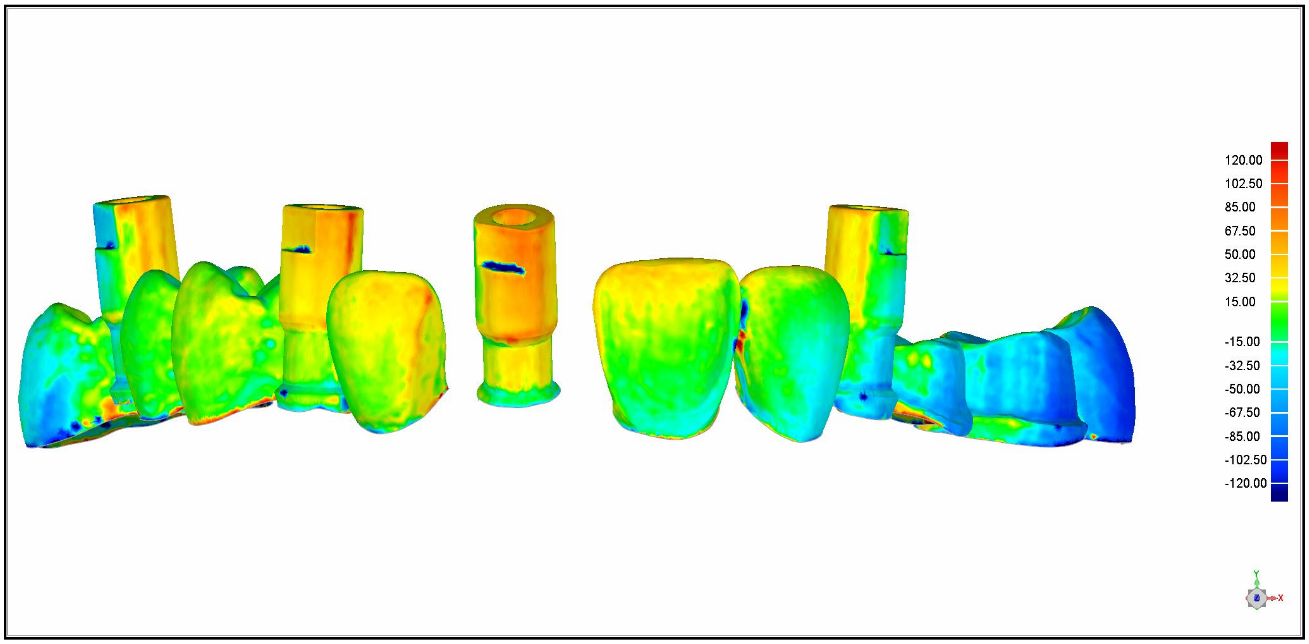

Predefinido: Inferior

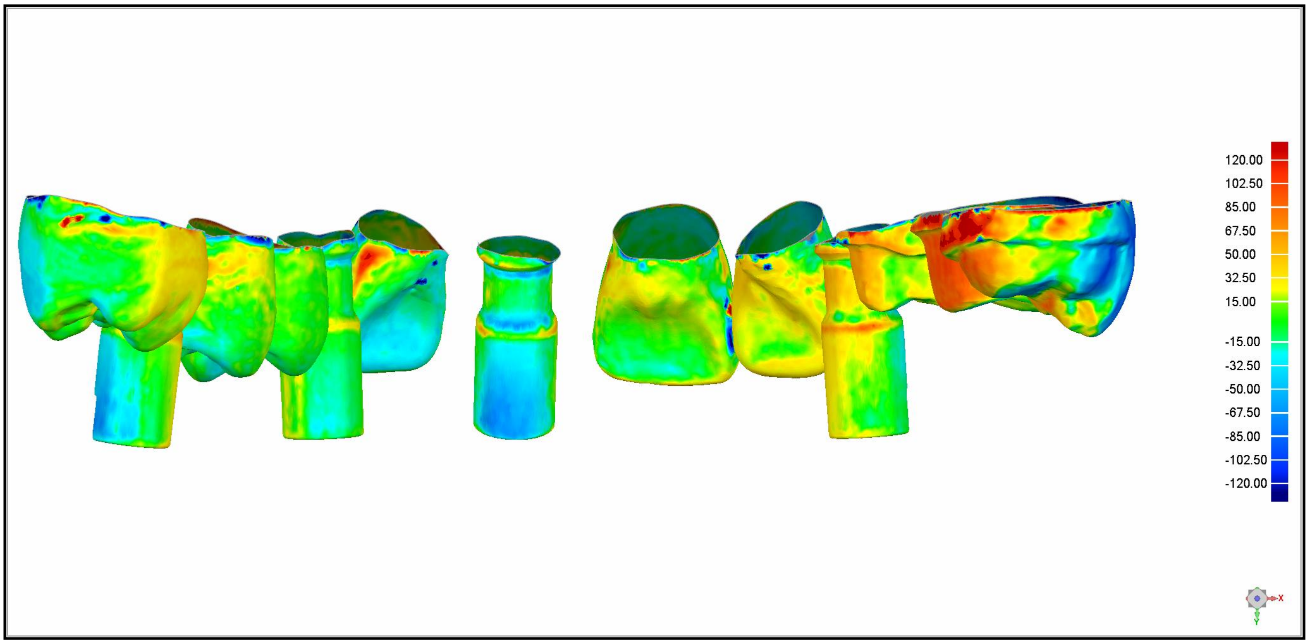

Supplement: S4 Table — Trios (scanning strategy D). (ZIP) [file pone.0202916.s004.zip › S4/3S9D.pdf]
