# Supplementary material for: Accuracy of four digital scanners according to scanning strategy in complete-arch impressions
Source: PLoS One. 2018 Sep 13;13(9):e0202916. doi: 10.1371/journal.pone.0202916 (PMC6136706; doi:10.1371/journal.pone.0202916)

3D Comparación Resultados

Modelo referencia	MRC
Modelo test	3S5D
Nº de puntos de datos	103980
# Aislados	162

Tipo tolerancia	3D desviación
Unidades	u
Máx. crítico	120.00
Máx. nominal	15.00
Mín. nominal	-15.00
Mín. crítico	-120.00

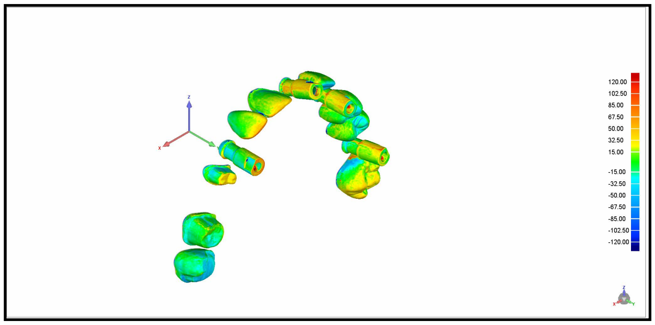

Desviación	
Desviación superior máx.	3029.34
Desviación inferior máx.	-3150.70
Desviación media	62.60 /-45.96
Desviación estándar	196.49

Distribución desviación

>=Min	<Max	# Puntos	%
-120.00	-102.50	248	0.24
-102.50	-85.00	373	0.36
-85.00	-67.50	589	0.57
-67.50	-50.00	1439	1.38
-50.00	-32.50	5553	5.34
-32.50	-15.00	14111	13.57
-15.00	15.00	43791	42.11
15.00	32.50	18327	17.63
32.50	50.00	8914	8.57
50.00	67.50	2922	2.81
67.50	85.00	1118	1.08
85.00	102.50	518	0.50
102.50	120.00	295	0.28

Fuera del crítico superior	3552	3.42
Fuera del crítico inferior	2230	2.14

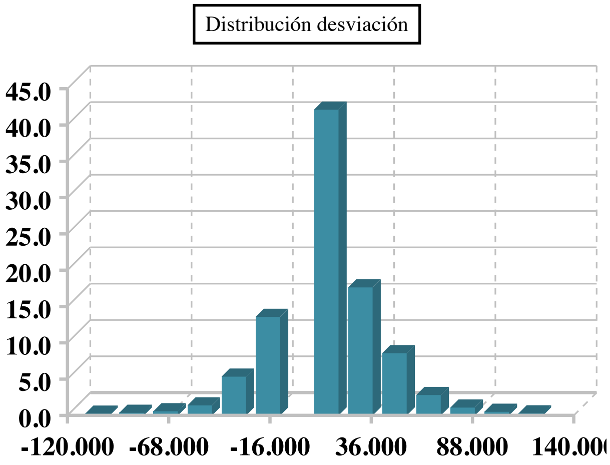

Desviaciones estándar

Distribución (+/-)	# Puntos	%
-6 * Desv. estánd.	453	0.44
-5 * Desv. estánd.	70	0.07
-4 * Desv. estánd.	134	0.13
-3 * Desv. estánd.	179	0.17
-2 * Desv. estánd.	673	0.65
-1 * Desv. estánd.	67031	64.47
1 * Desv. estánd.	32805	31.55
2 * Desv. estánd.	618	0.59
3 * Desv. estánd.	348	0.33
4 * Desv. estánd.	359	0.35
5 * Desv. estánd.	349	0.34
6 * Desv. estánd.	961	0.92

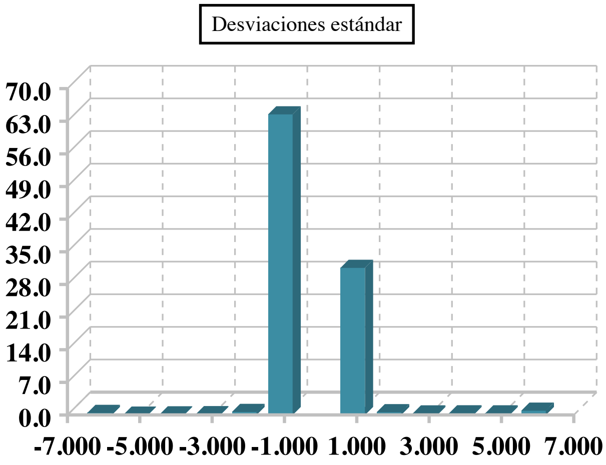

Predefinido: Isométrico

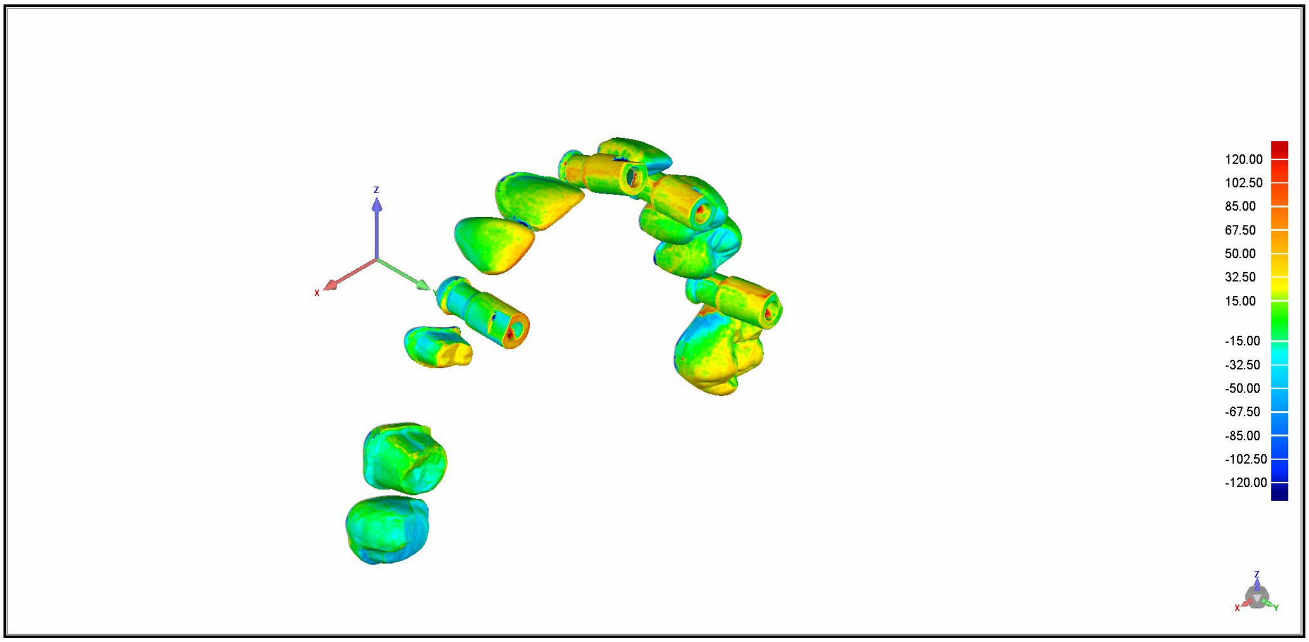

Predefinido: Frente

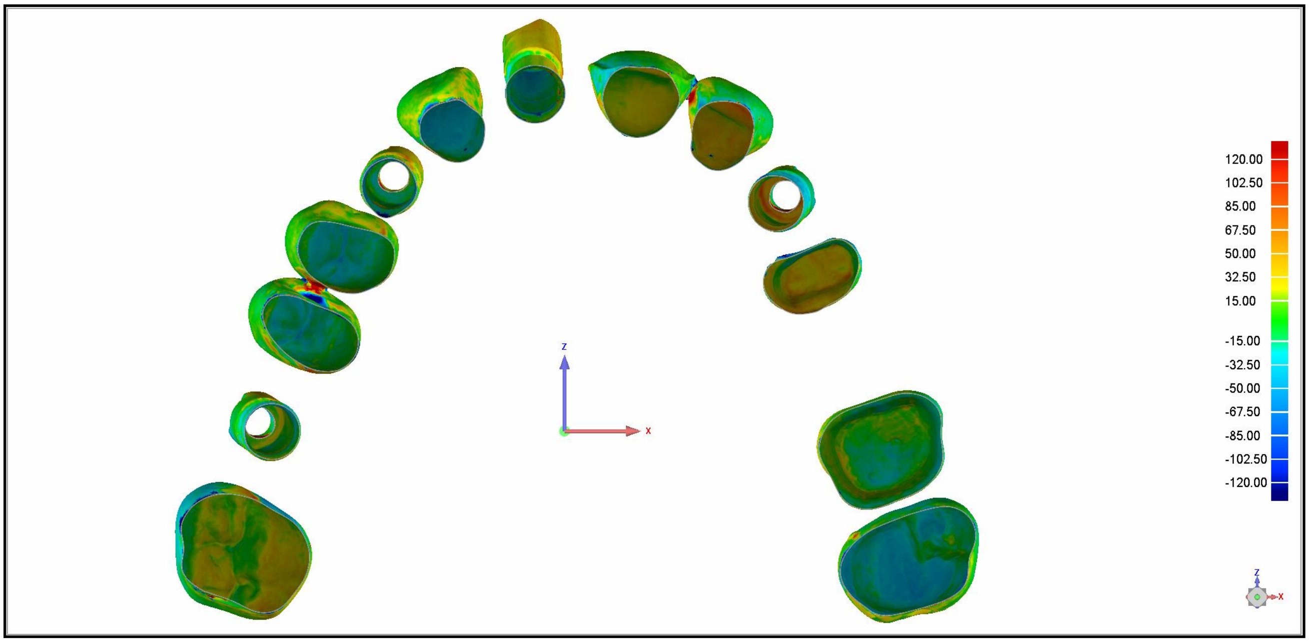

Predefinido: Atrás

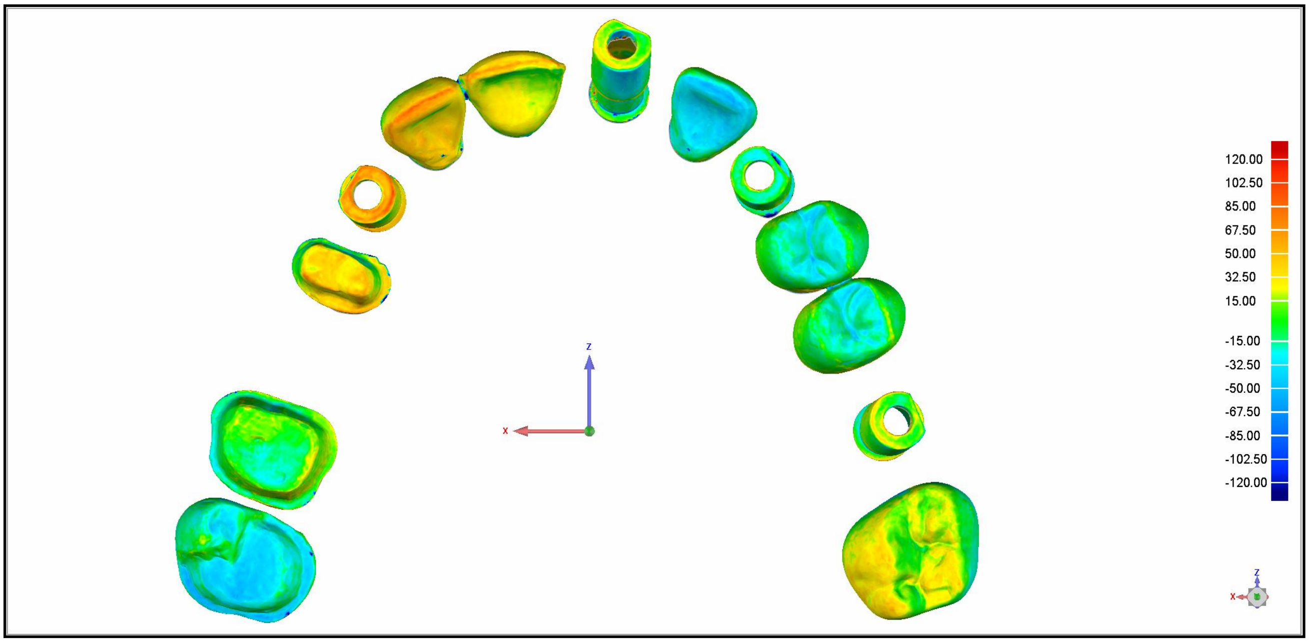

Predefinido: Izquierda

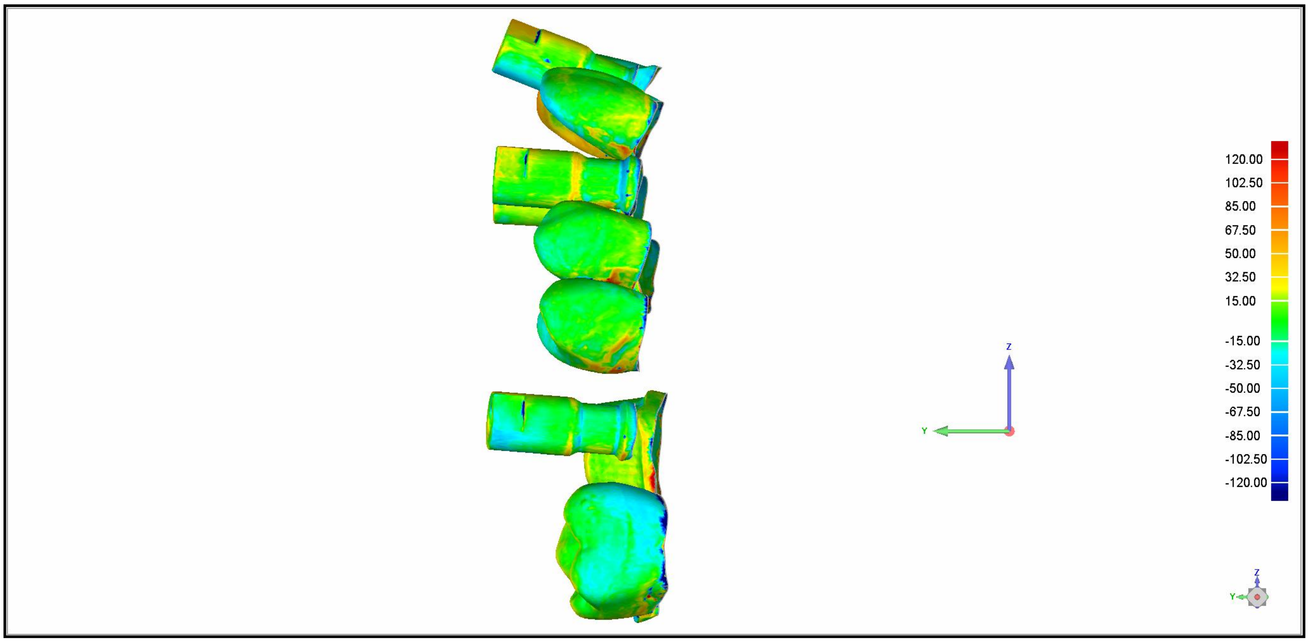

Predefinido: Derecha

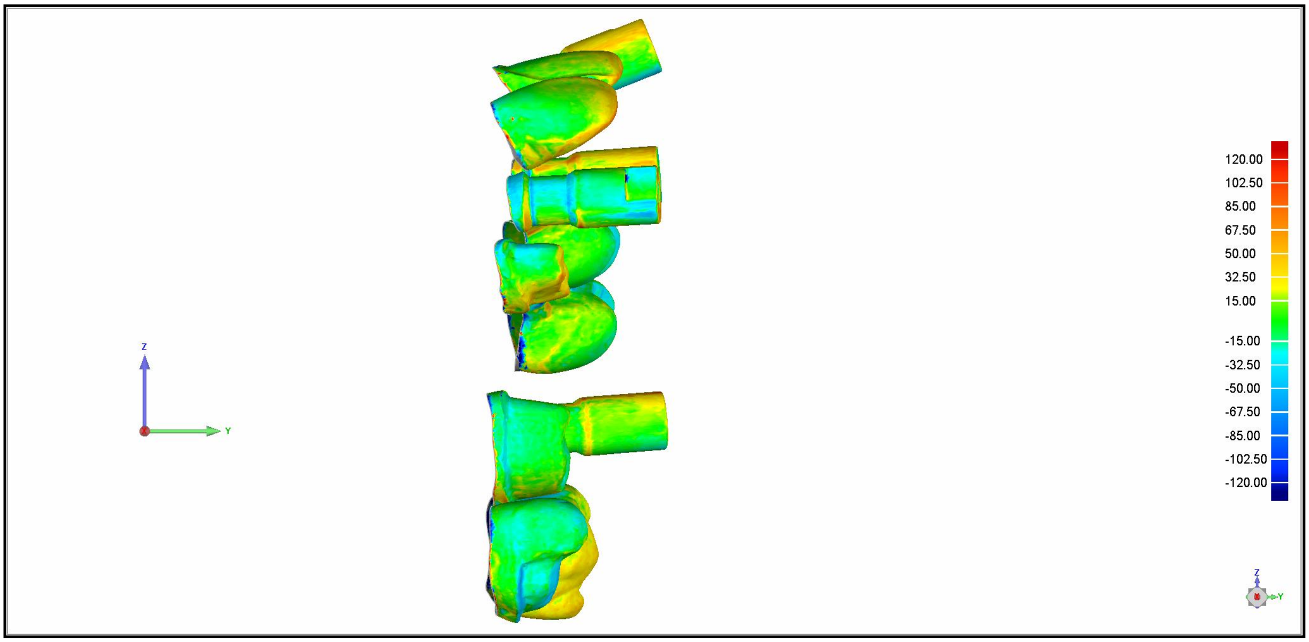

Predefinido: Superior

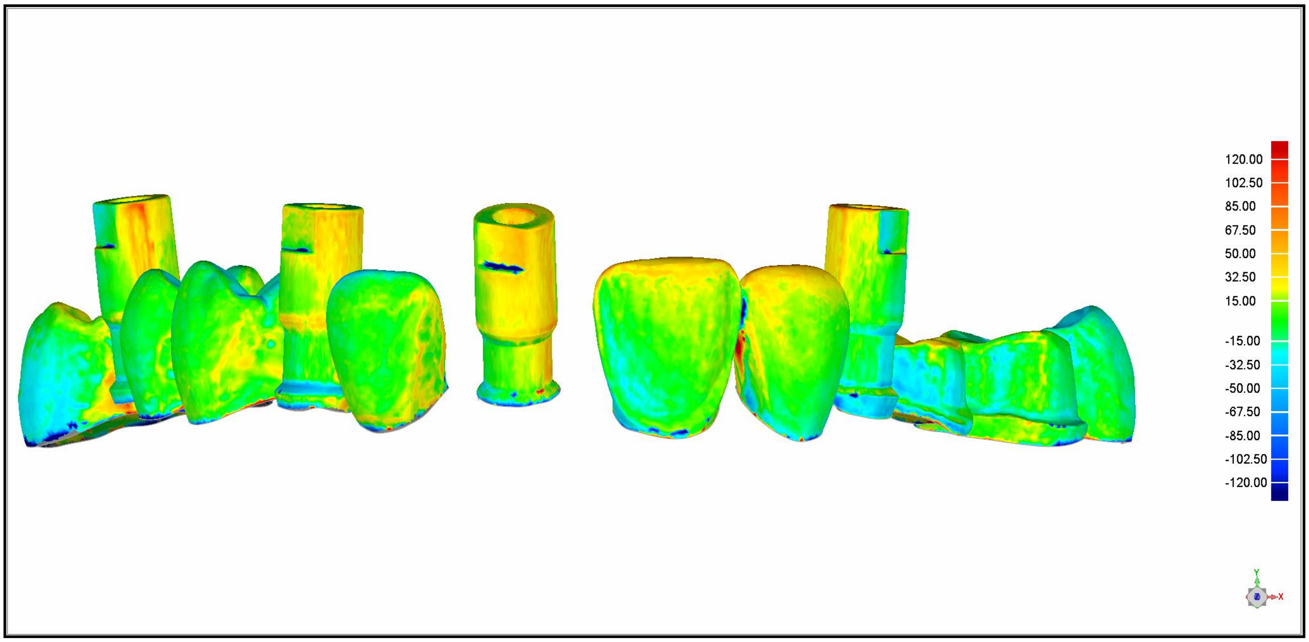

Predefinido: Inferior

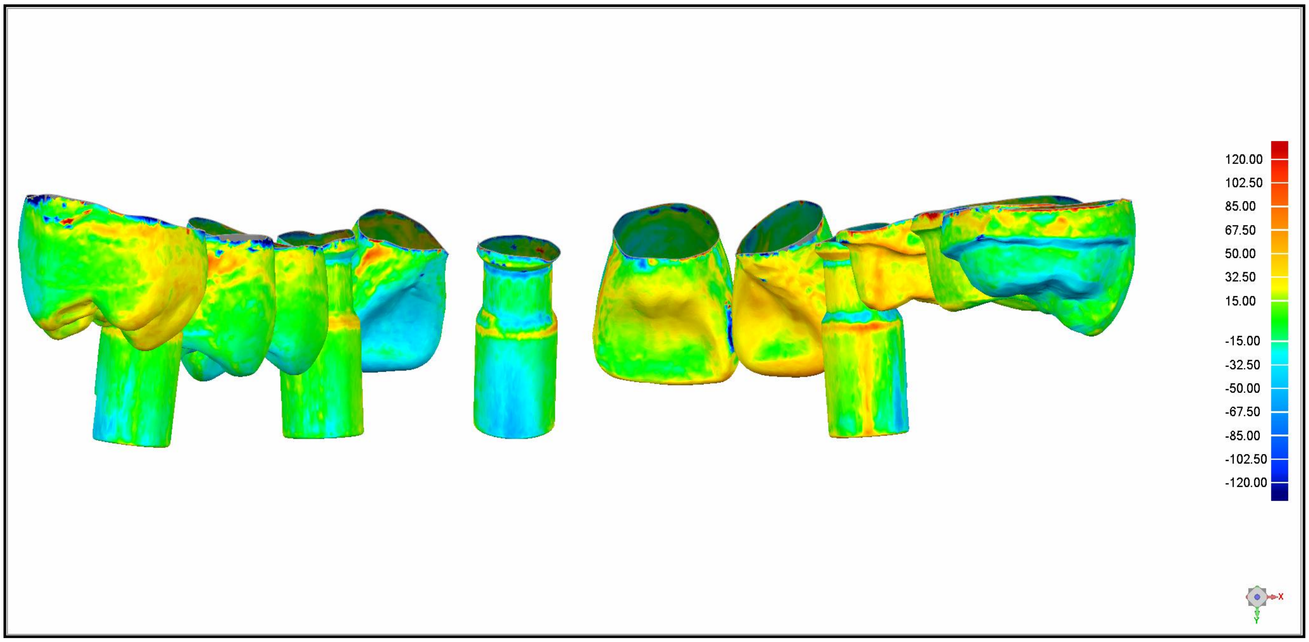

Supplement: S4 Table — Trios (scanning strategy D). (ZIP) [file pone.0202916.s004.zip › S4/3S5D.pdf]
