# Supplementary material for: Accuracy of four digital scanners according to scanning strategy in complete-arch impressions
Source: PLoS One. 2018 Sep 13;13(9):e0202916. doi: 10.1371/journal.pone.0202916 (PMC6136706; doi:10.1371/journal.pone.0202916)

3D Comparación Resultados

Modelo referencia	MRC
Modelo test	3S4D
Nº de puntos de datos	107084
# Aislados	102

Tipo tolerancia	3D desviación
Unidades	u
Máx. crítico	120.00
Máx. nominal	18.00
Mín. nominal	-18.00
Mín. crítico	-120.00

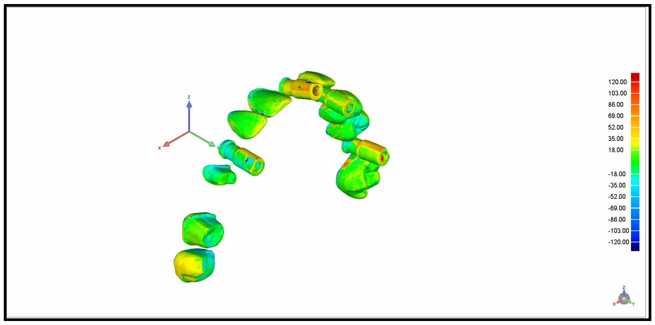

Desviación	
Desviación superior máx.	3088.22
Desviación inferior máx.	-3121.14
Desviación media	60.37 /-47.99
Desviación estándar	210.26

Distribución desviación

>=Min	<Max	# Puntos	%
-120.00	-103.00	289	0.27
-103.00	-86.00	359	0.34
-86.00	-69.00	530	0.49
-69.00	-52.00	1037	0.97
-52.00	-35.00	3090	2.89
-35.00	-18.00	9065	8.47
-18.00	18.00	60166	56.19
18.00	35.00	16977	15.85
35.00	52.00	5613	5.24
52.00	69.00	2289	2.14
69.00	86.00	983	0.92
86.00	103.00	475	0.44
103.00	120.00	328	0.31

Fuera del crítico superior	3999	3.73
Fuera del crítico inferior	1884	1.76

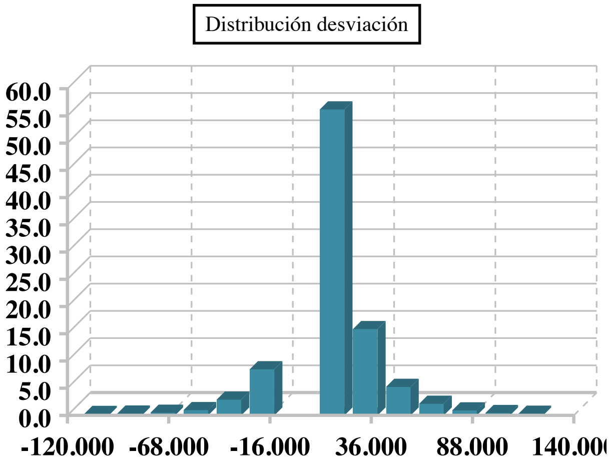

Desviaciones estándar

Distribución (+/-)	# Puntos	%
-6 * Desv. estánd.	491	0.46
-5 * Desv. estánd.	92	0.09
-4 * Desv. estánd.	86	0.08
-3 * Desv. estánd.	147	0.14
-2 * Desv. estánd.	403	0.38
-1 * Desv. estánd.	76635	71.57
1 * Desv. estánd.	26324	24.58
2 * Desv. estánd.	686	0.64
3 * Desv. estánd.	449	0.42
4 * Desv. estánd.	425	0.40
5 * Desv. estánd.	389	0.36
6 * Desv. estánd.	957	0.89

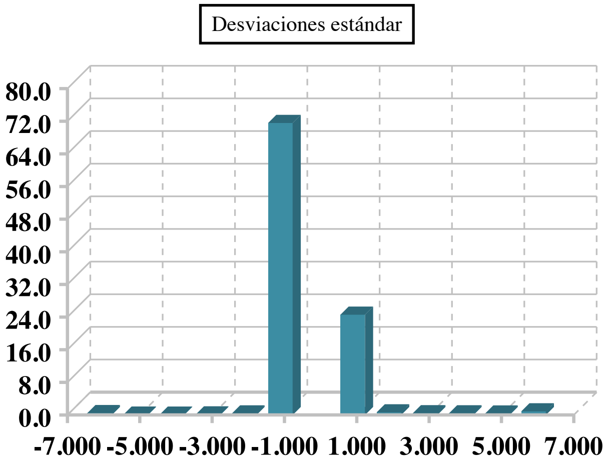

Predefinido: Isométrico

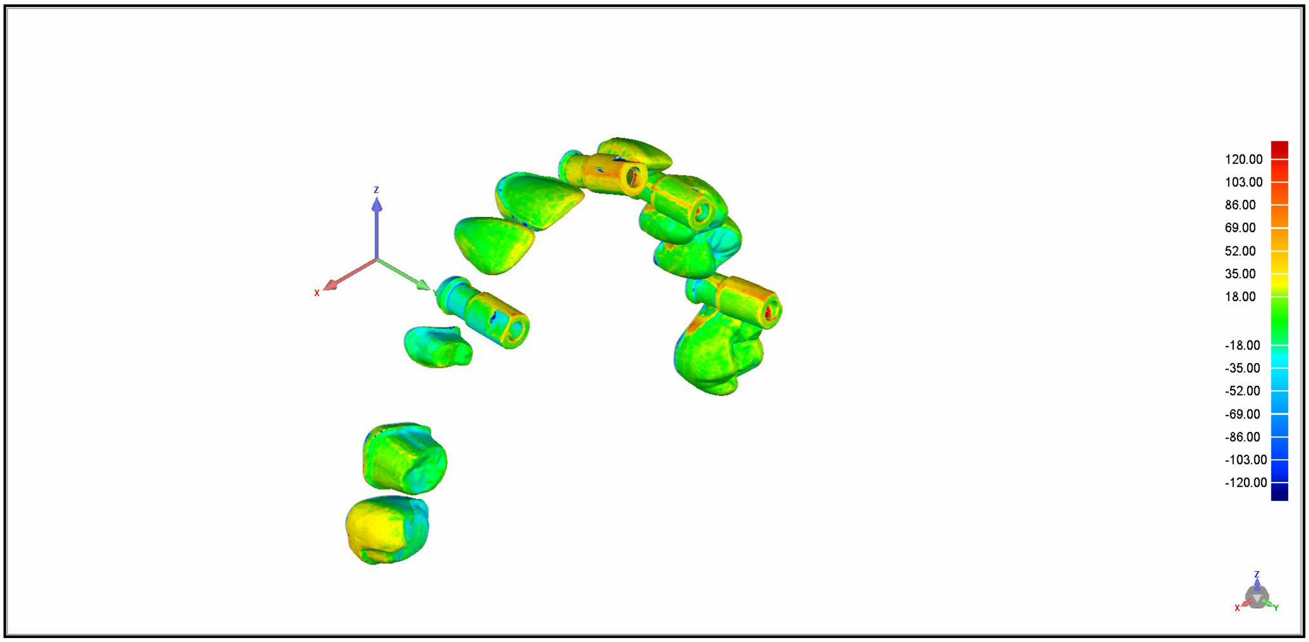

Predefinido: Frente

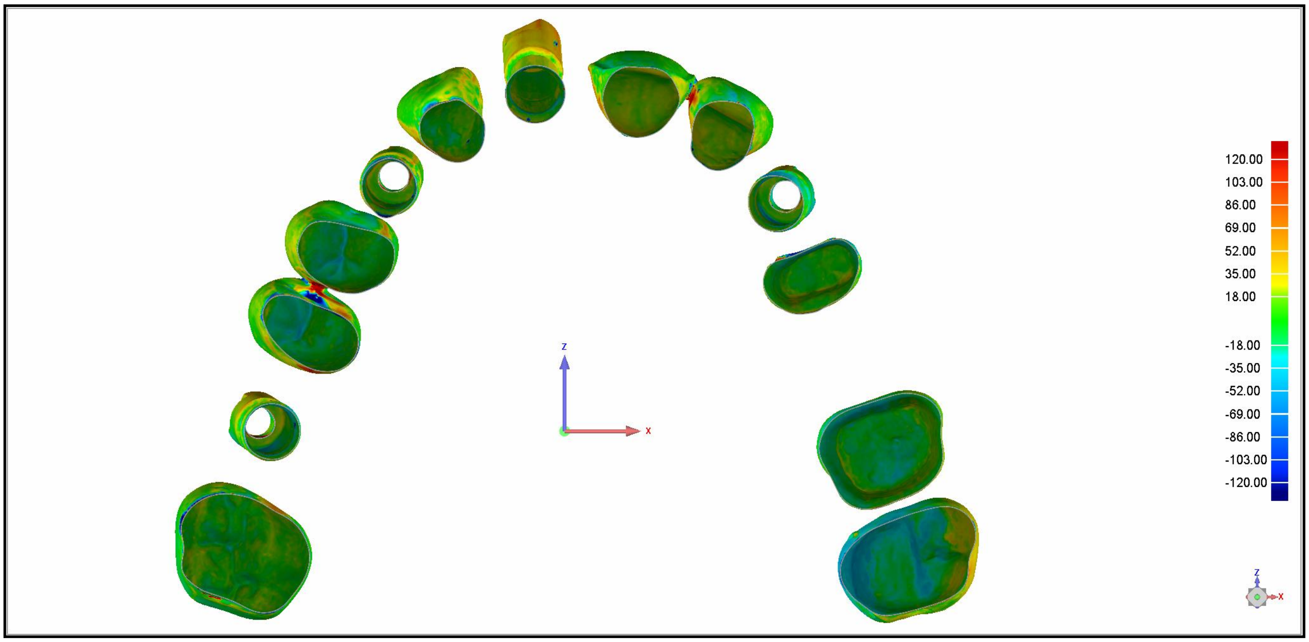

Predefinido: Atrás

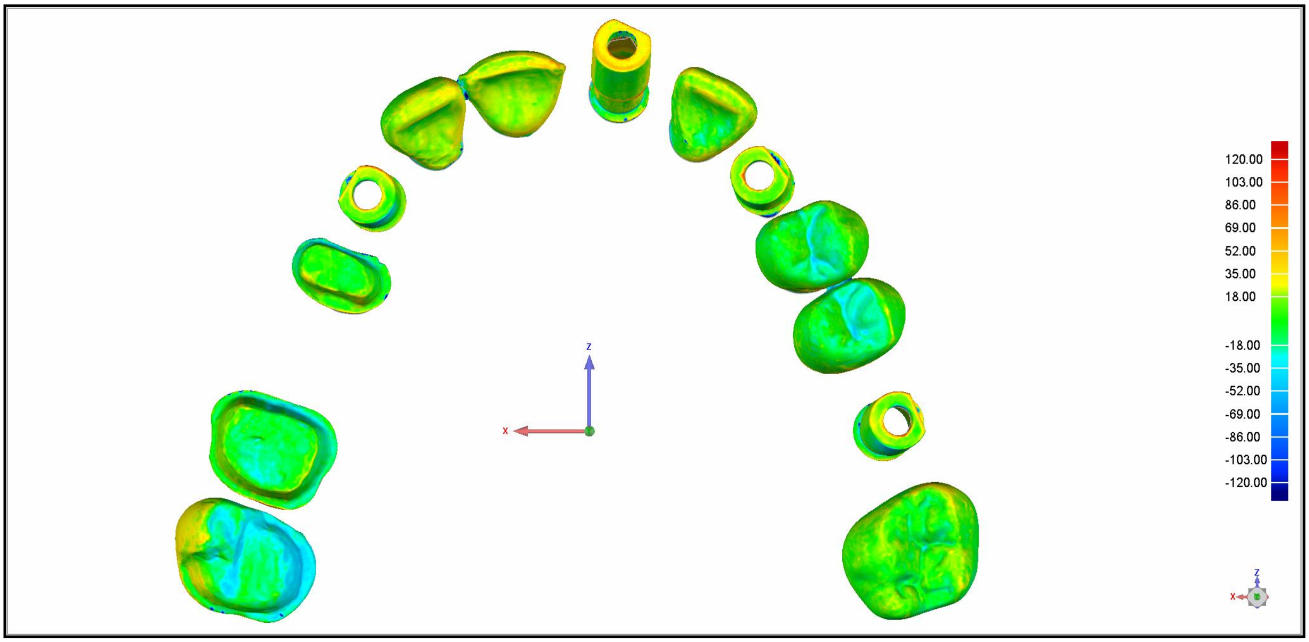

Predefinido: Izquierda

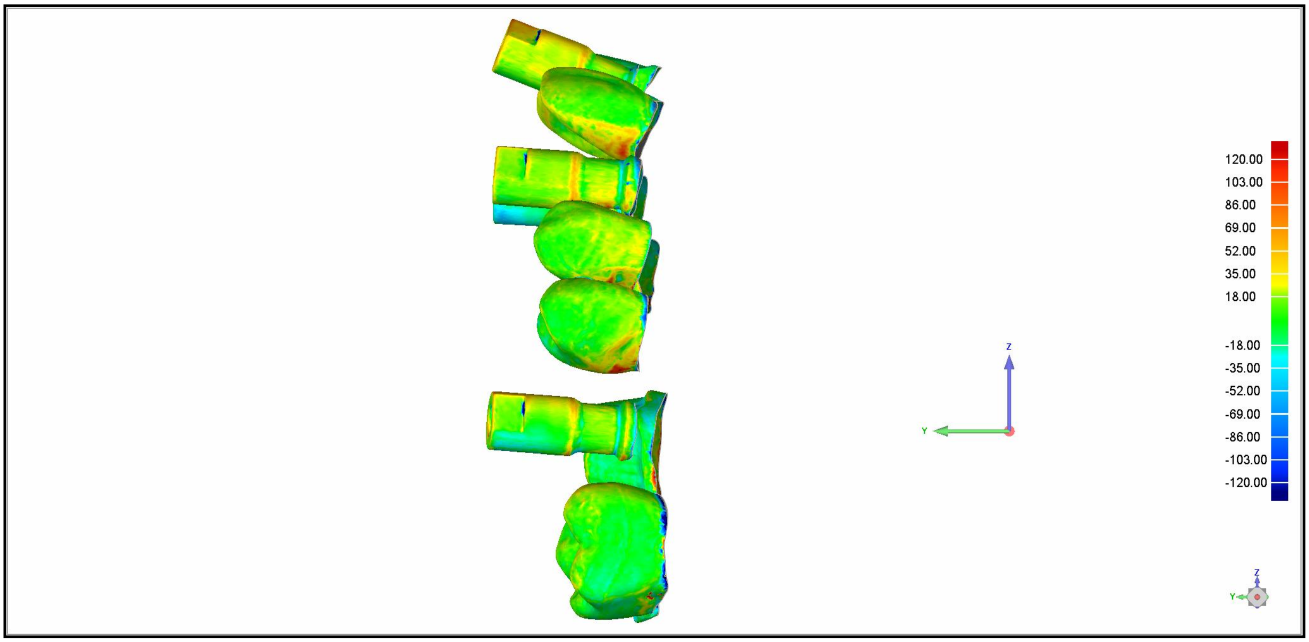

Predefinido: Derecha

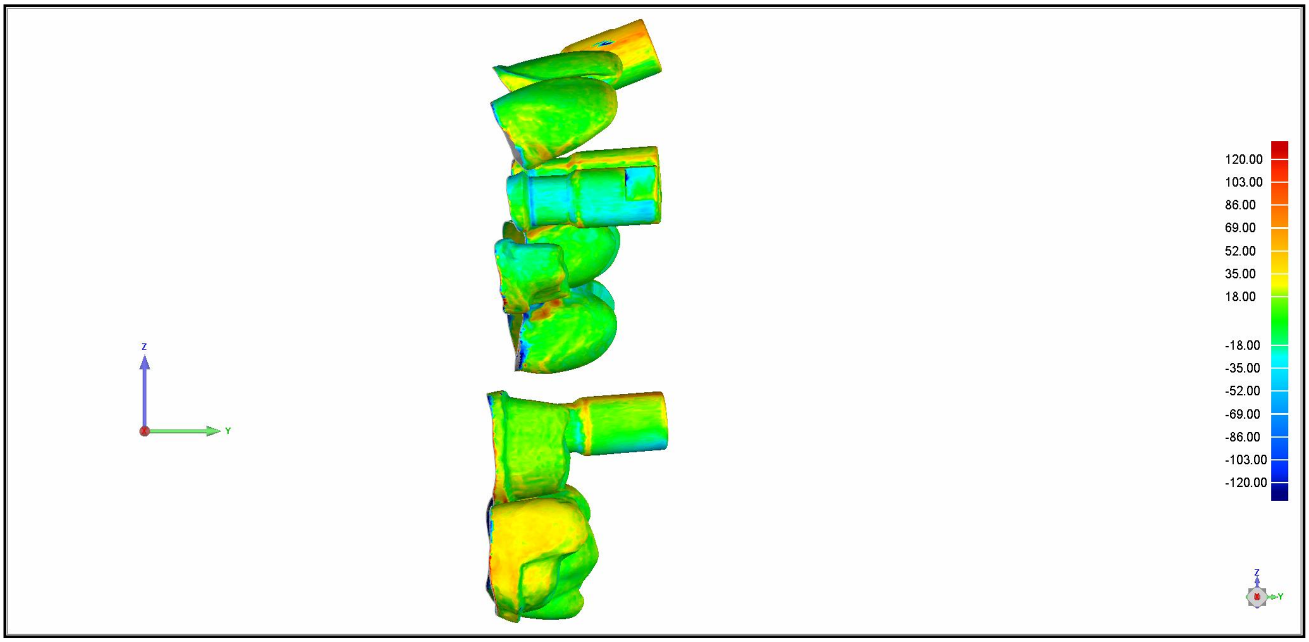

Predefinido: Superior

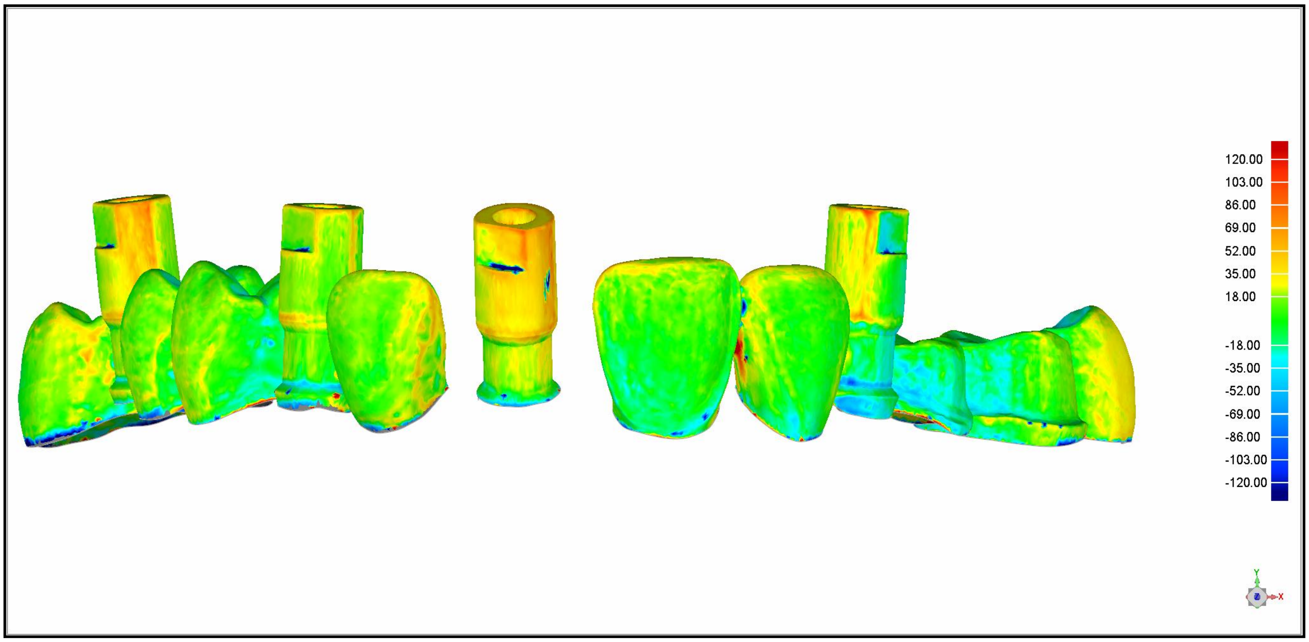

Predefinido: Inferior

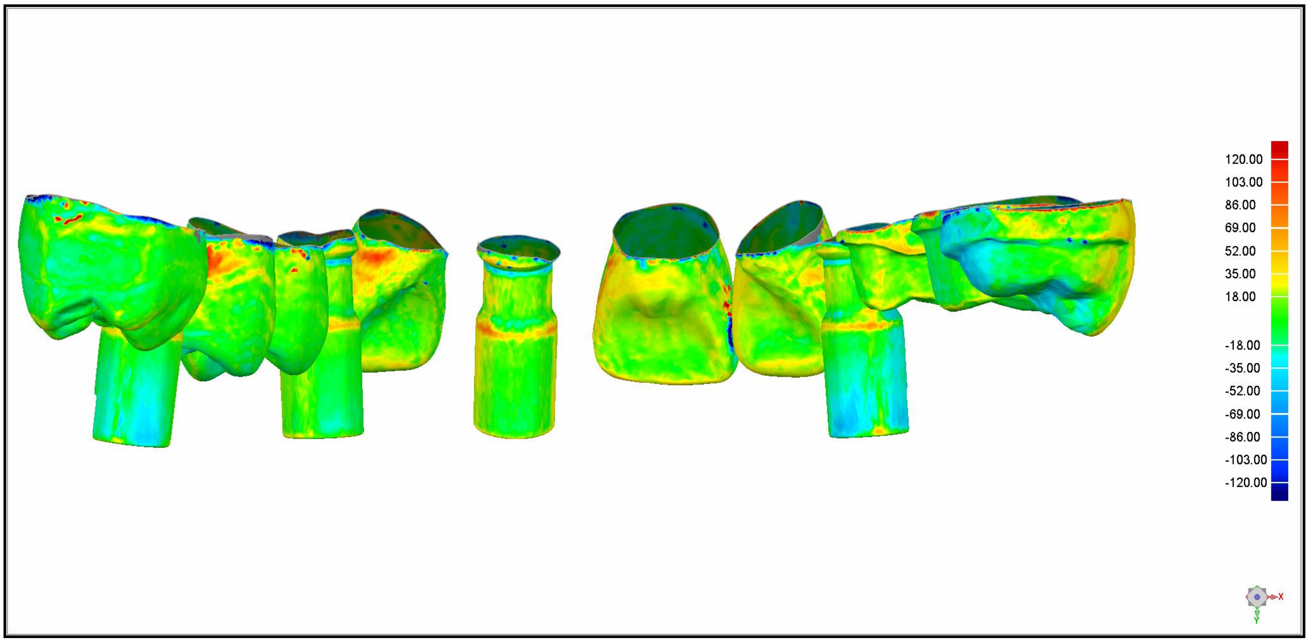

Supplement: S4 Table — Trios (scanning strategy D). (ZIP) [file pone.0202916.s004.zip › S4/3S4D.pdf]
