# Supplementary material for: Accuracy of four digital scanners according to scanning strategy in complete-arch impressions
Source: PLoS One. 2018 Sep 13;13(9):e0202916. doi: 10.1371/journal.pone.0202916 (PMC6136706; doi:10.1371/journal.pone.0202916)

Geomagic Control™

Fecha: 3/6/2017, 12:42 pm

3D Comparación Resultados

Modelo referencia	MRC
Modelo test	3S8D
Nº de puntos de datos	102361
# Aislados	87

Tipo tolerancia	3D desviación
Unidades	u
Máx. crítico	120.00
Máx. nominal	10.00
Mín. nominal	-10.00
Mín. crítico	-120.00

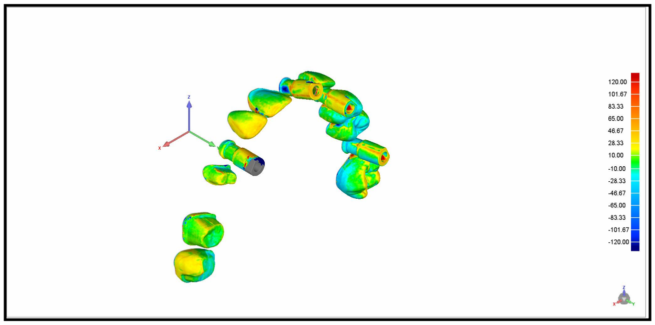

Desviación	
Desviación superior máx.	3041.64
Desviación inferior máx.	-3041.90
Desviación media	55.20 /-49.74
Desviación estándar	194.94

Distribución desviación

>=Min	<Max	# Puntos	%
-120.00	-101.67	356	0.35
-101.67	-83.33	483	0.47
-83.33	-65.00	773	0.76
-65.00	-46.67	1719	1.68
-46.67	-28.33	5479	5.35
-28.33	-10.00	17098	16.70
-10.00	10.00	32523	31.77
10.00	28.33	24071	23.52
28.33	46.67	9566	9.35
46.67	65.00	2770	2.71
65.00	83.33	1145	1.12
83.33	101.67	546	0.53
101.67	120.00	369	0.36

Fuera del crítico superior	3305	3.23
Fuera del crítico inferior	2158	2.11

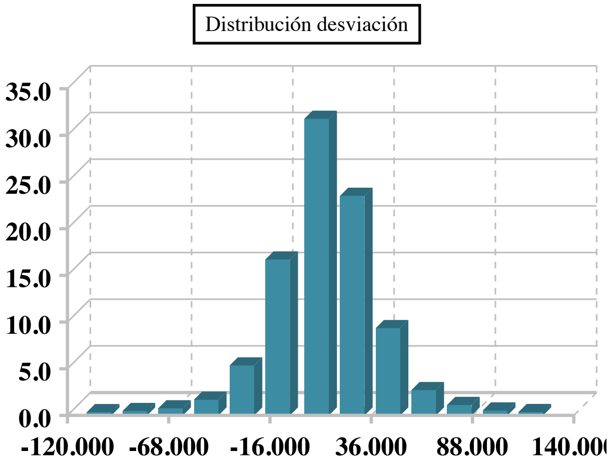

Desviaciones estándar

Distribución (+/-)	# Puntos	%
-6 * Desv. estánd.	558	0.55
-5 * Desv. estánd.	106	0.10
-4 * Desv. estánd.	135	0.13
-3 * Desv. estánd.	204	0.20
-2 * Desv. estánd.	523	0.51
-1 * Desv. estánd.	60469	59.07
1 * Desv. estánd.	38081	37.20
2 * Desv. estánd.	666	0.65
3 * Desv. estánd.	274	0.27
4 * Desv. estánd.	263	0.26
5 * Desv. estánd.	255	0.25
6 * Desv. estánd.	827	0.81

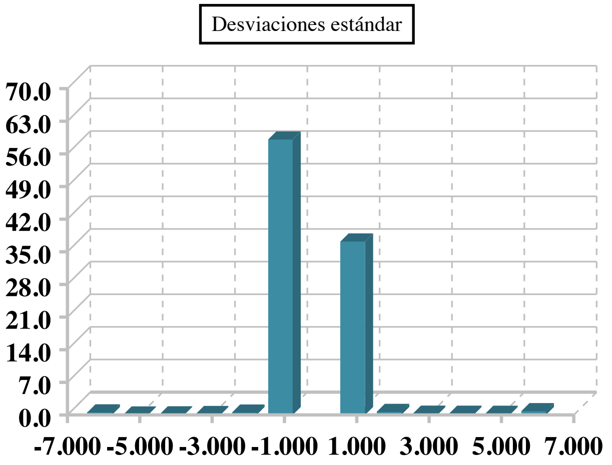

Predefinido: Isométrico

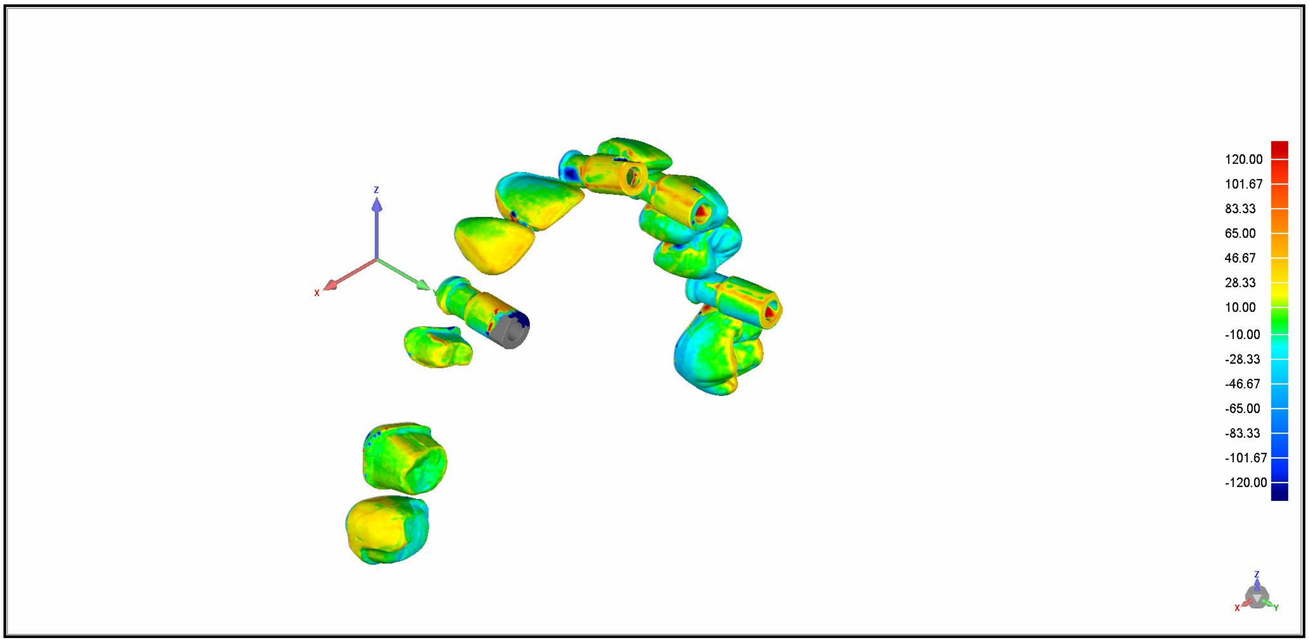

Predefinido: Frente

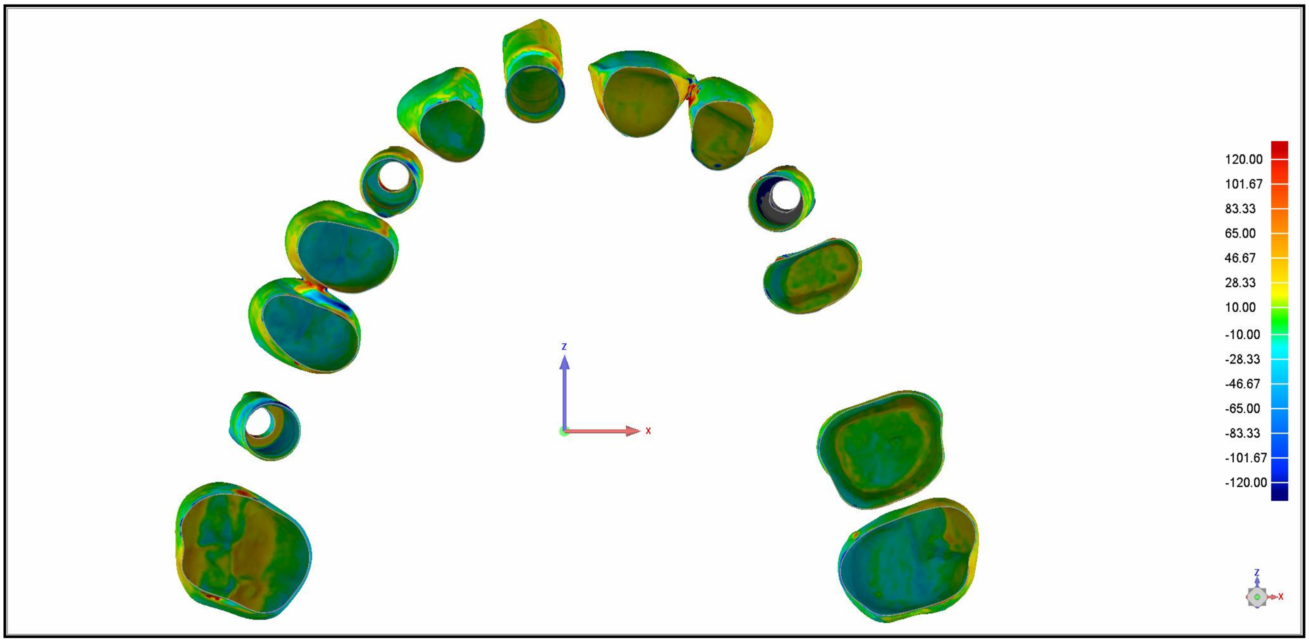

Predefinido: Atrás

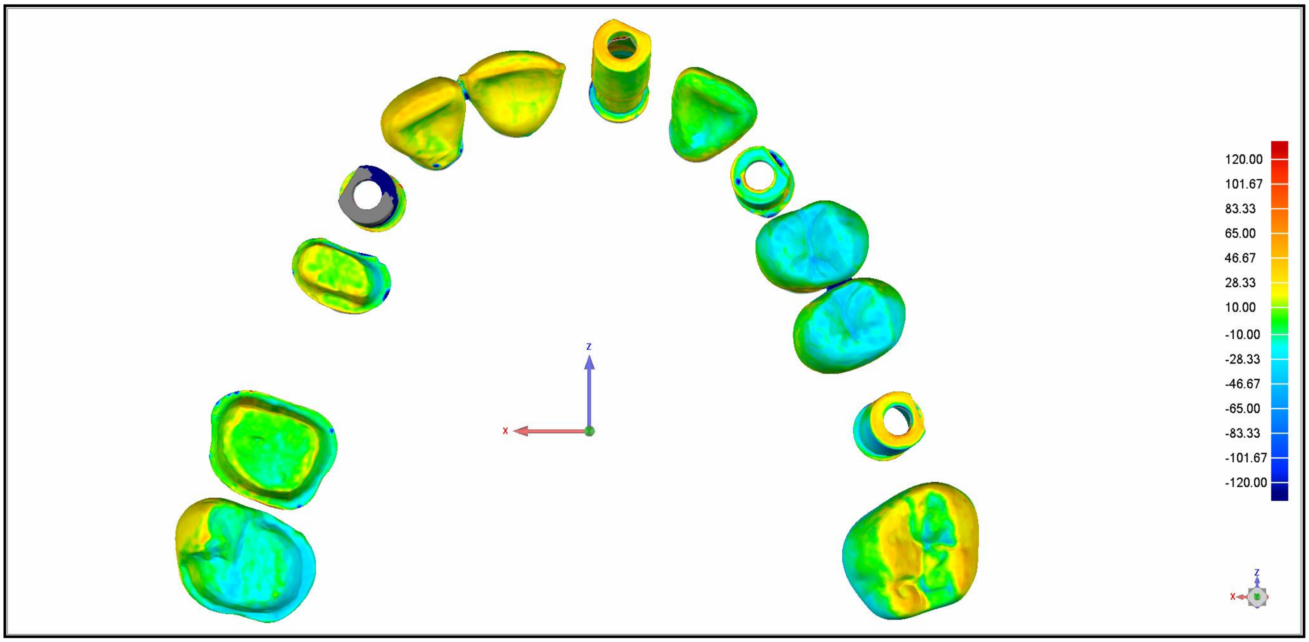

Predefinido: Izquierda

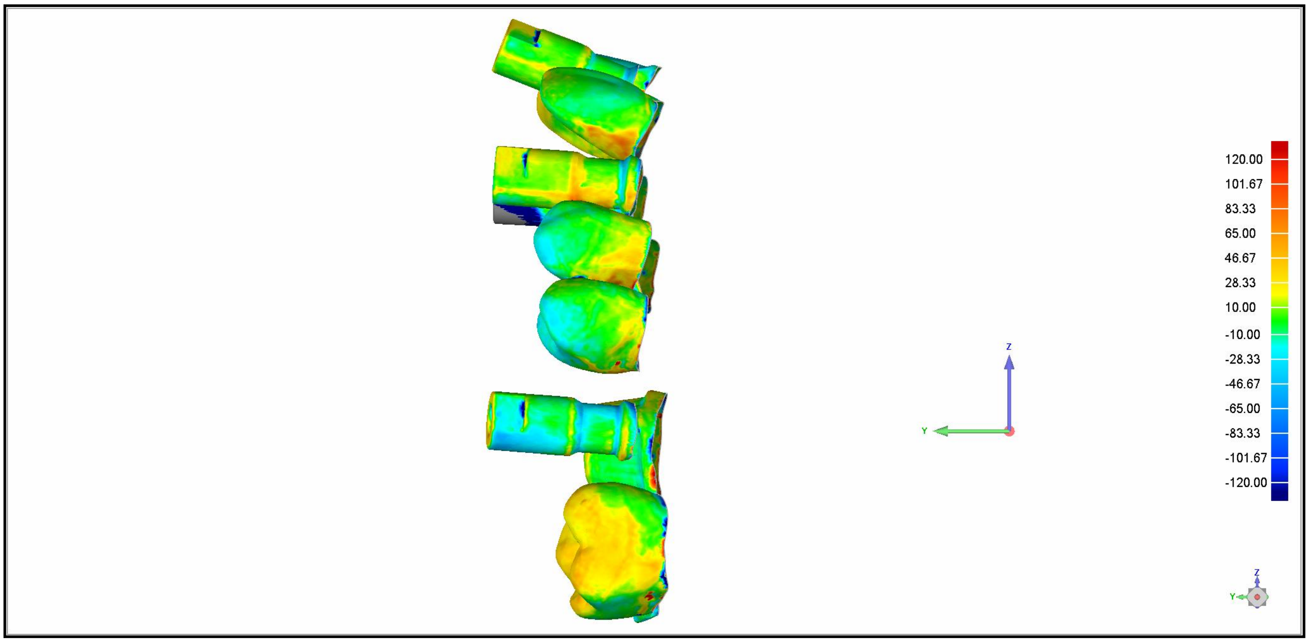

Predefinido: Derecha

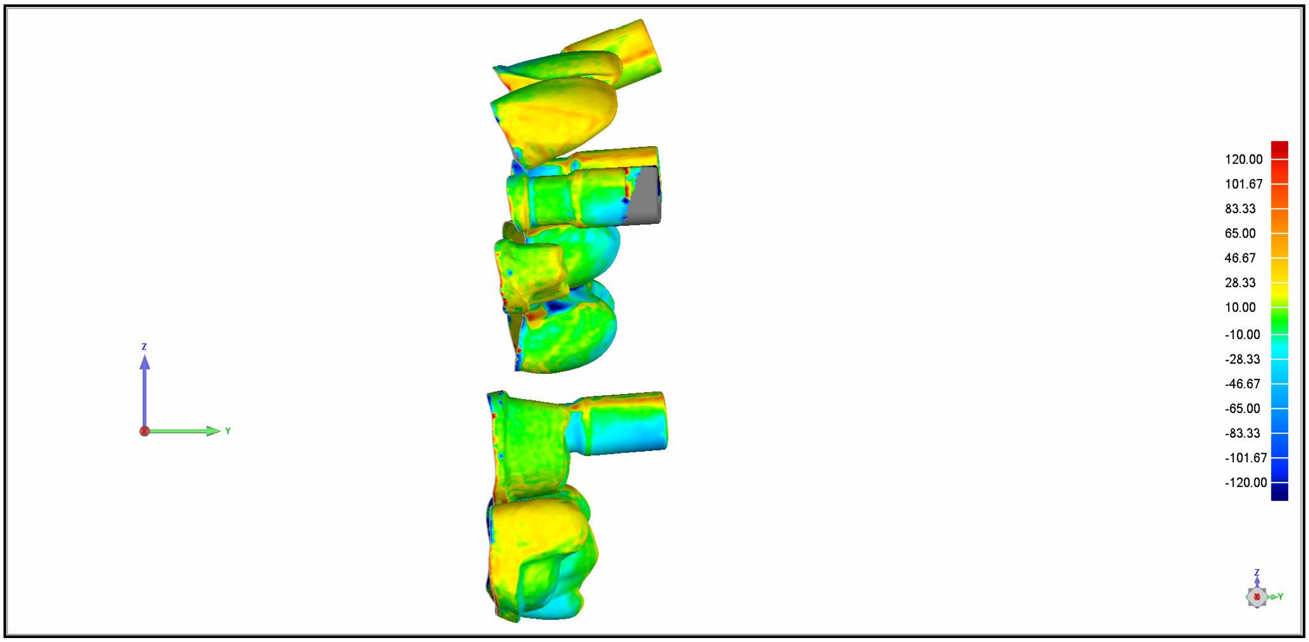

Predefinido: Superior

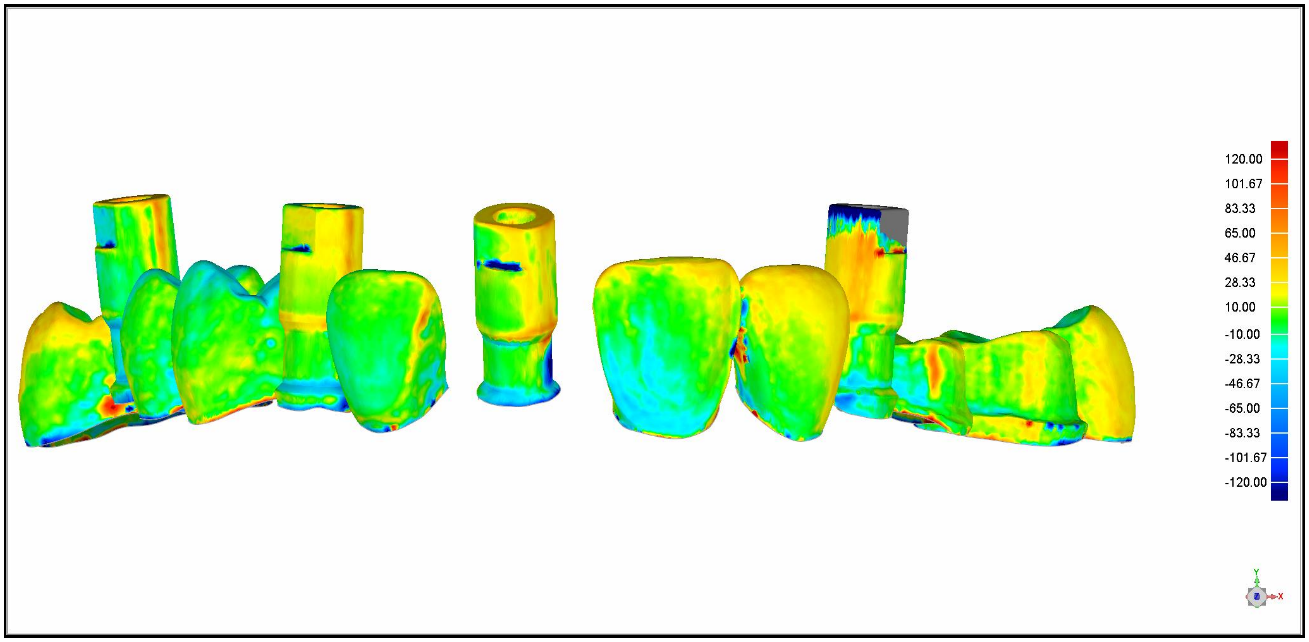

Predefinido: Inferior

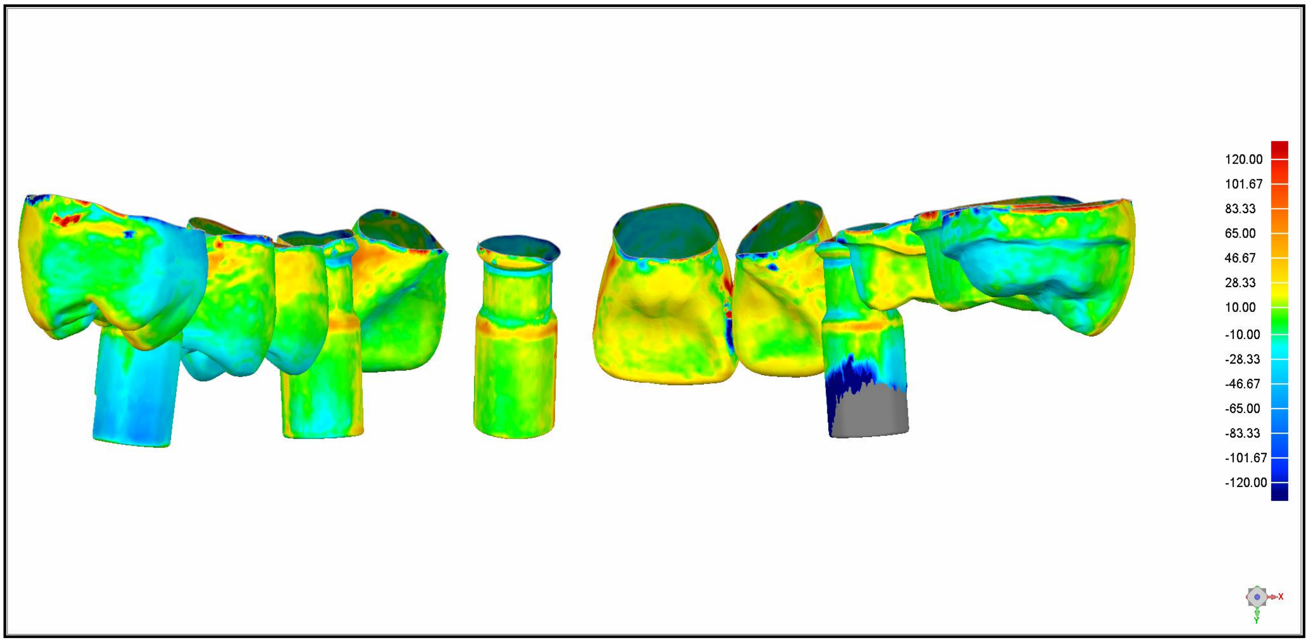

Supplement: S4 Table — Trios (scanning strategy D). (ZIP) [file pone.0202916.s004.zip › S4/3S8D.pdf]
