# Supplementary material for: Accuracy of four digital scanners according to scanning strategy in complete-arch impressions
Source: PLoS One. 2018 Sep 13;13(9):e0202916. doi: 10.1371/journal.pone.0202916 (PMC6136706; doi:10.1371/journal.pone.0202916)

3D Comparación Resultados

Modelo referencia	MRC
Modelo test	3S3D
Nº de puntos de datos	108785
# Aislados	100

Tipo tolerancia	3D desviación
Unidades	u
Máx. crítico	120.00
Máx. nominal	17.00
Mín. nominal	-17.00
Mín. crítico	-120.00

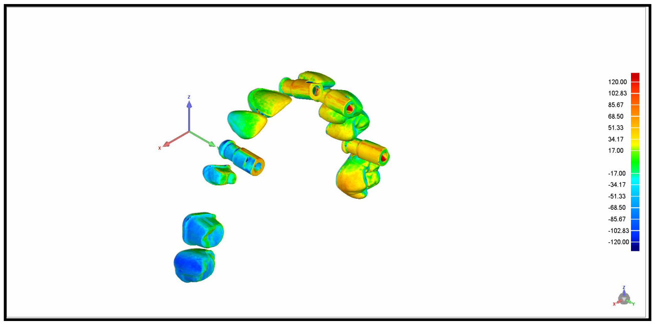

Desviación	
Desviación superior máx.	3081.99
Desviación inferior máx.	-3143.28
Desviación media	75.21 /-59.64
Desviación estándar	218.29

Distribución desviación

>=Min	<Max	# Puntos	%
-120.00	-102.83	421	0.39
-102.83	-85.67	1151	1.06
-85.67	-68.50	2626	2.41
-68.50	-51.33	5548	5.10
-51.33	-34.17	7097	6.52
-34.17	-17.00	12162	11.18
-17.00	17.00	35145	32.31
17.00	34.17	17410	16.00
34.17	51.33	11211	10.31
51.33	68.50	5732	5.27
68.50	85.67	2336	2.15
85.67	102.83	973	0.89
102.83	120.00	462	0.42

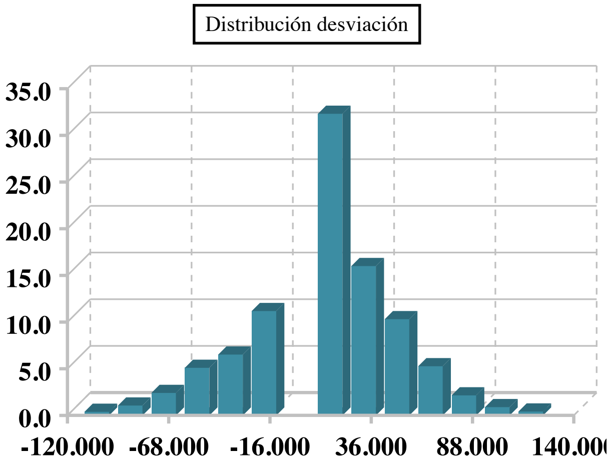

Fuera del crítico superior		4399	4.04
Fuera del crítico inferior		2112	1.94

Desviaciones estándar

Distribución (+/-)	# Puntos	%
-6 * Desv. estánd.	498	0.46
-5 * Desv. estánd.	112	0.10
-4 * Desv. estánd.	127	0.12
-3 * Desv. estánd.	189	0.17
-2 * Desv. estánd.	471	0.43
-1 * Desv. estánd.	65024	59.77
1 * Desv. estánd.	39421	36.24
2 * Desv. estánd.	710	0.65
3 * Desv. estánd.	430	0.40
4 * Desv. estánd.	397	0.36
5 * Desv. estánd.	403	0.37
6 * Desv. estánd.	1003	0.92

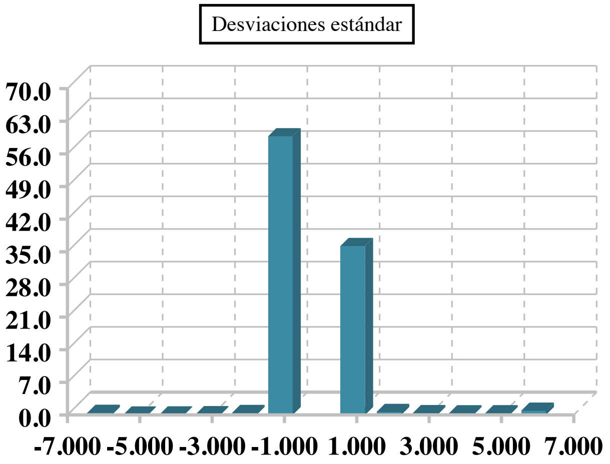

Predefinido: Isométrico

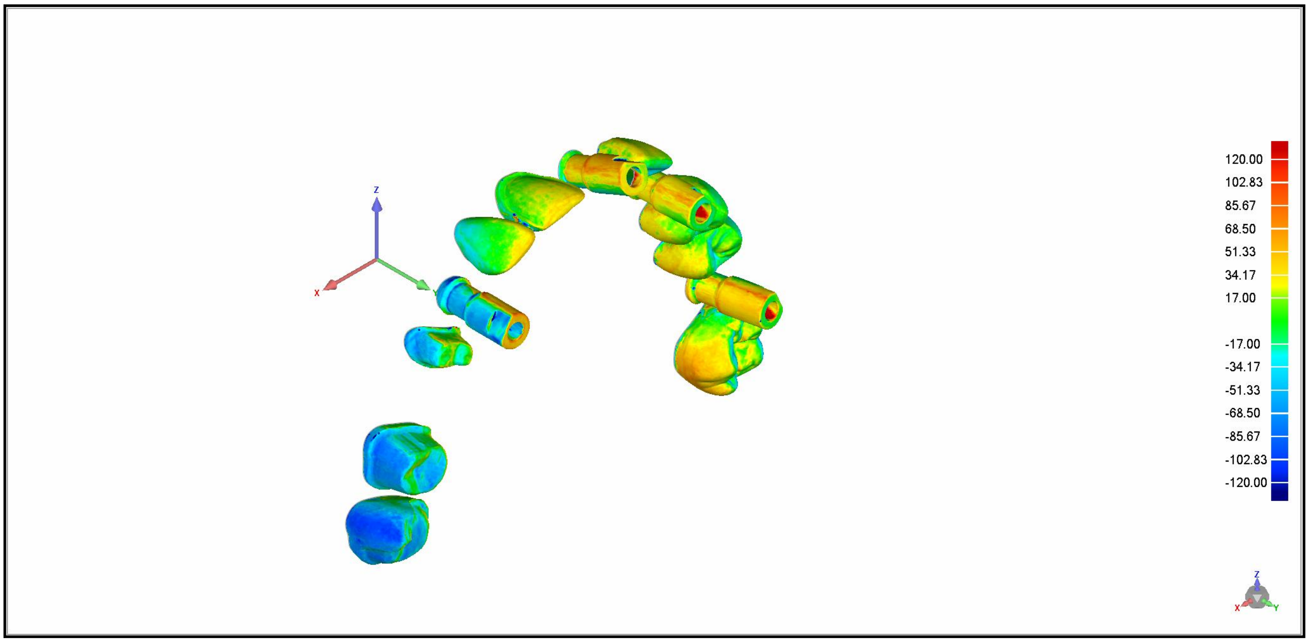

Predefinido: Frente

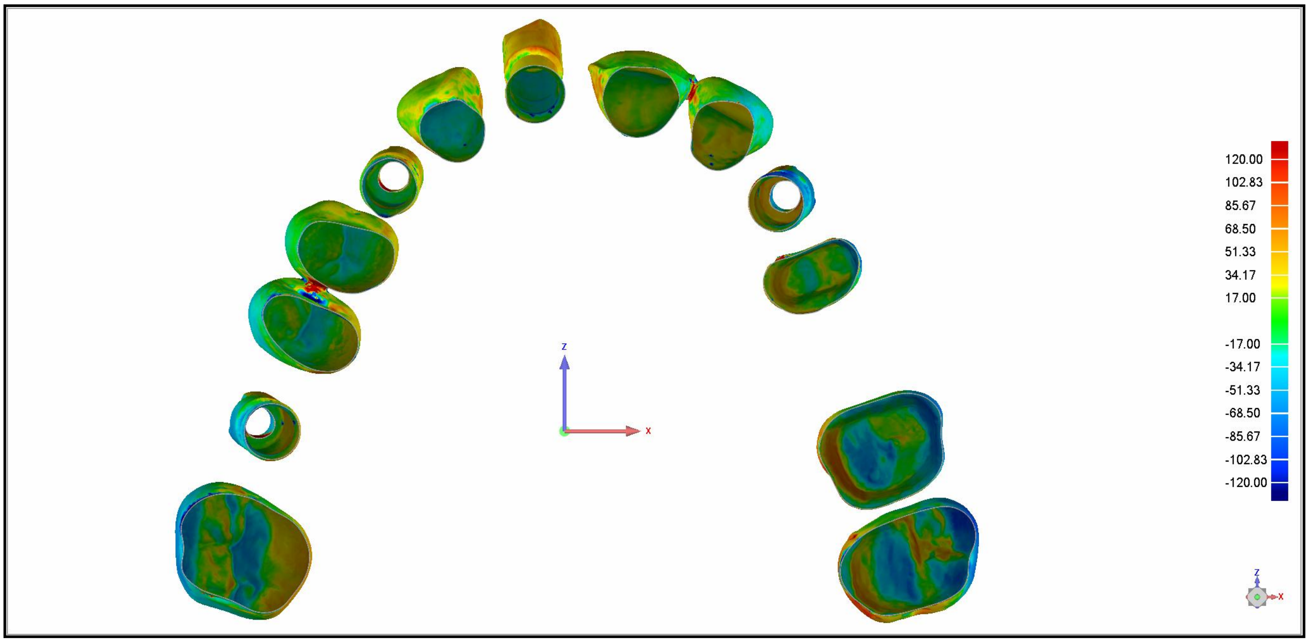

Predefinido: Atrás

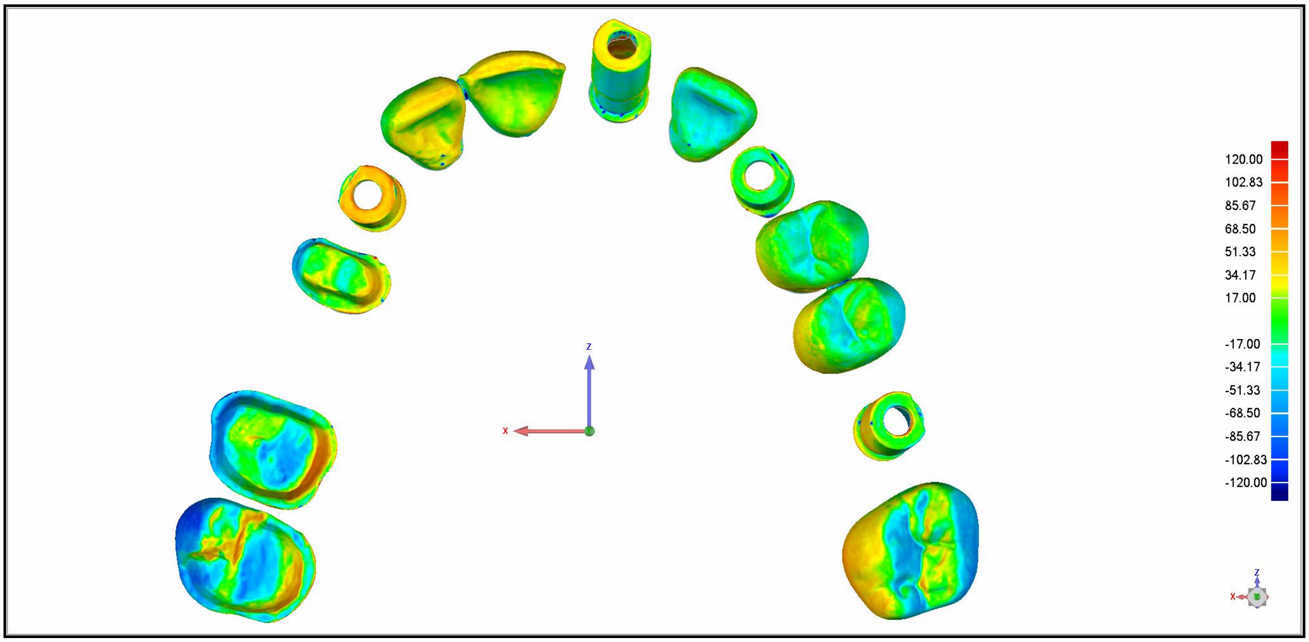

Predefinido: Izquierda

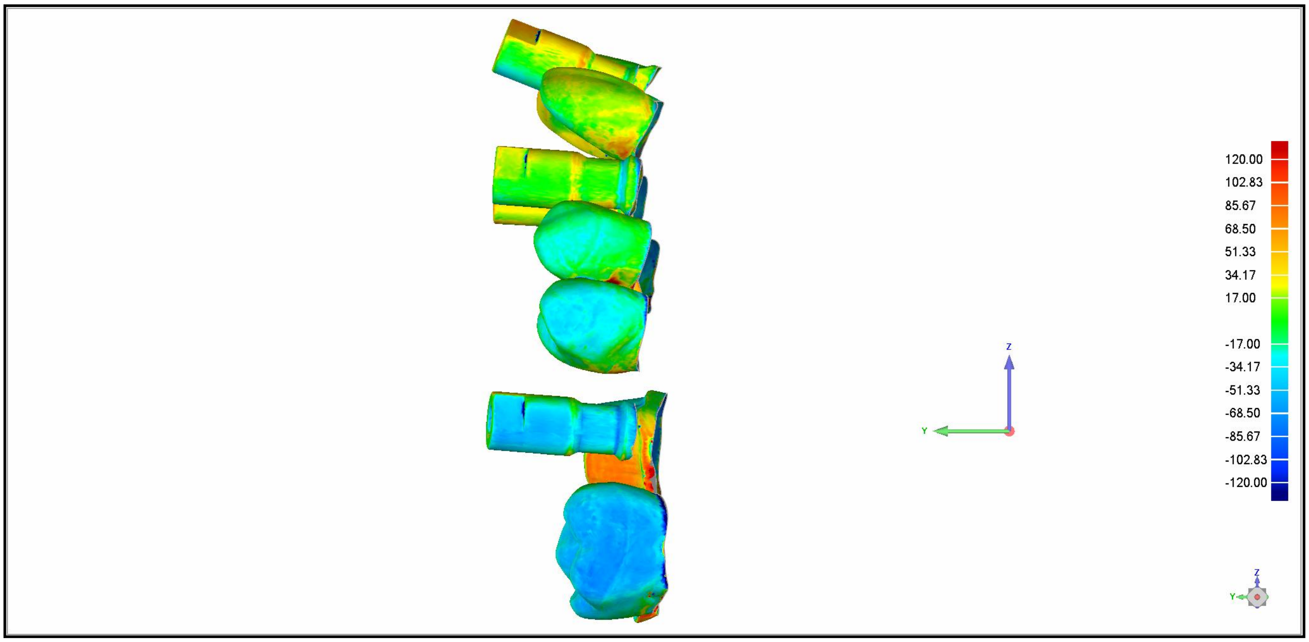

Predefinido: Derecha

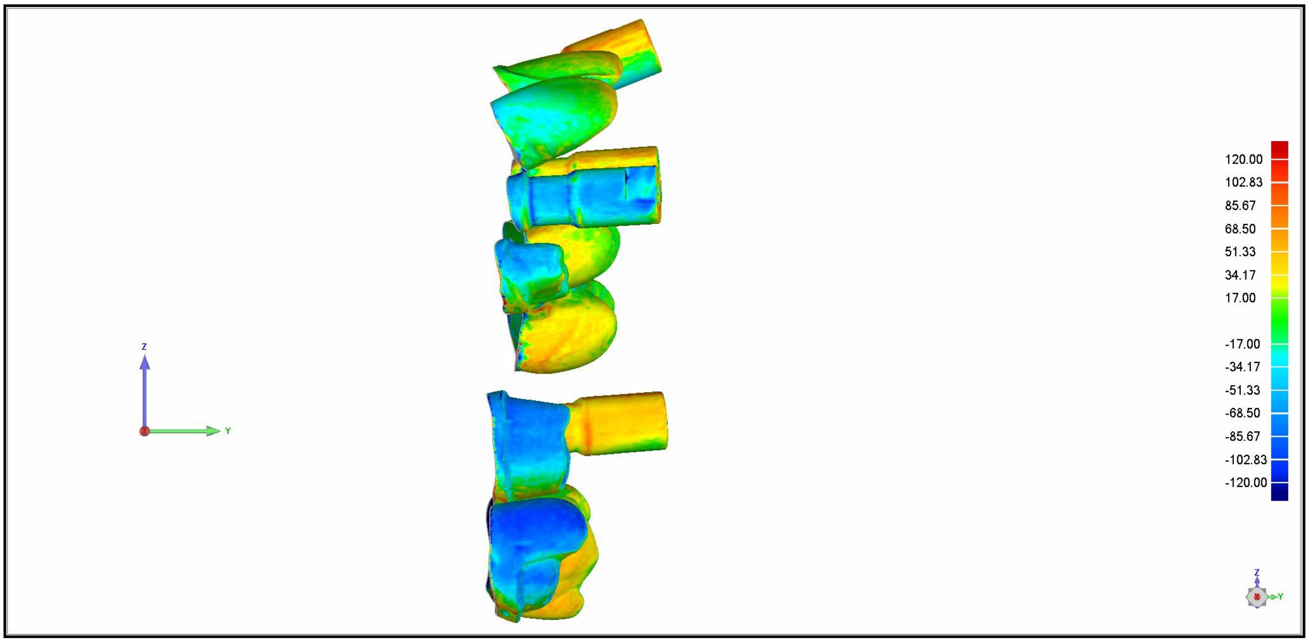

Predefinido: Superior

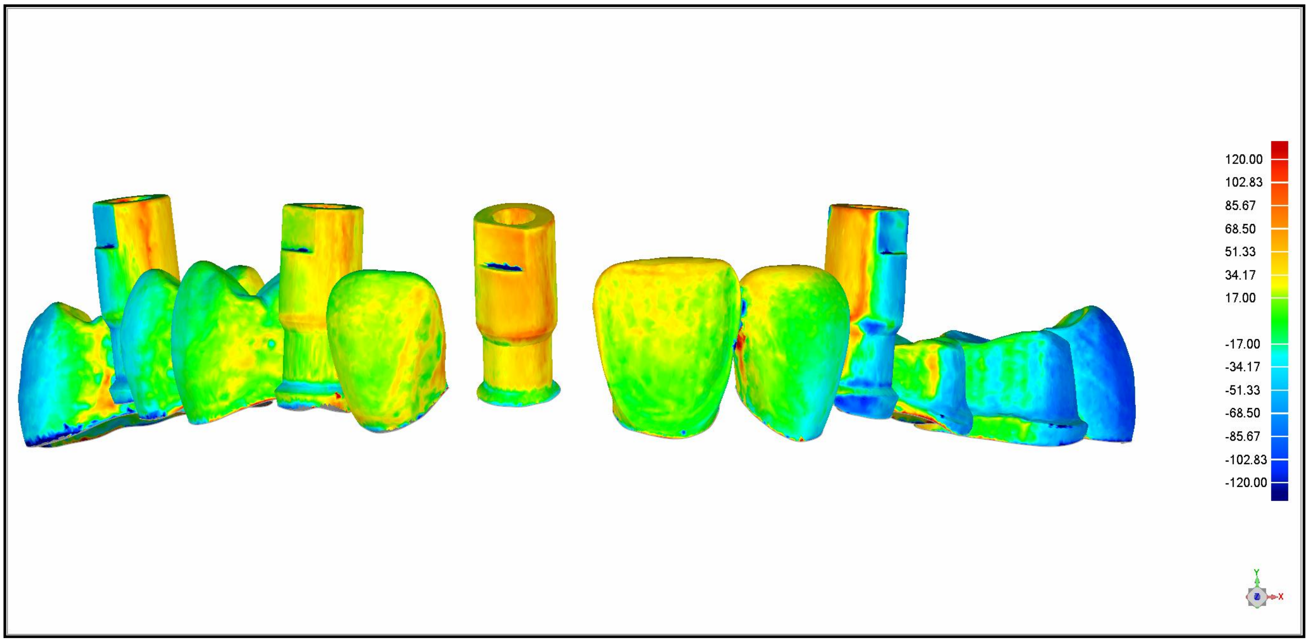

Predefinido: Inferior

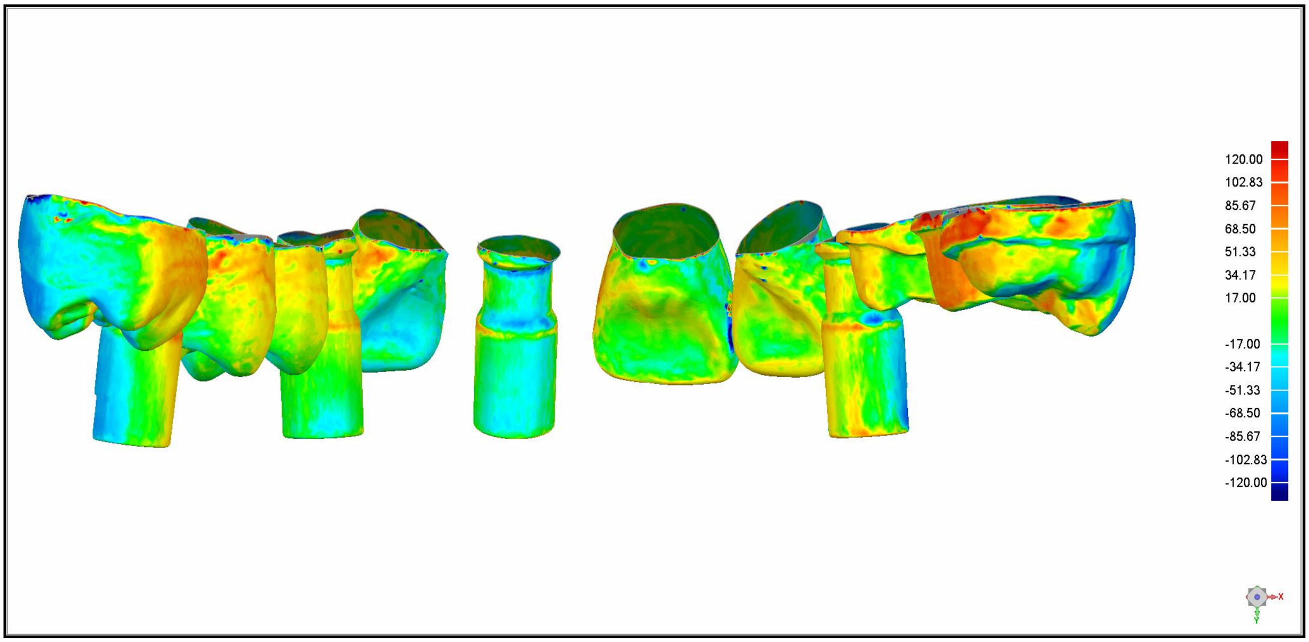

Supplement: S4 Table — Trios (scanning strategy D). (ZIP) [file pone.0202916.s004.zip › S4/3S3D.pdf]
