# Supplementary material for: Accuracy of four digital scanners according to scanning strategy in complete-arch impressions
Source: PLoS One. 2018 Sep 13;13(9):e0202916. doi: 10.1371/journal.pone.0202916 (PMC6136706; doi:10.1371/journal.pone.0202916)

Geomagic Control™

Fecha: 3/6/2017, 12:47 pm

3D Comparación Resultados

Modelo referencia	MRC
Modelo test	3S10D
Nº de puntos de datos	110189
# Aislados	87

Tipo tolerancia	3D desviación
Unidades	u
Máx. crítico	120.00
Máx. nominal	14.00
Mín. nominal	-14.00
Mín. crítico	-120.00

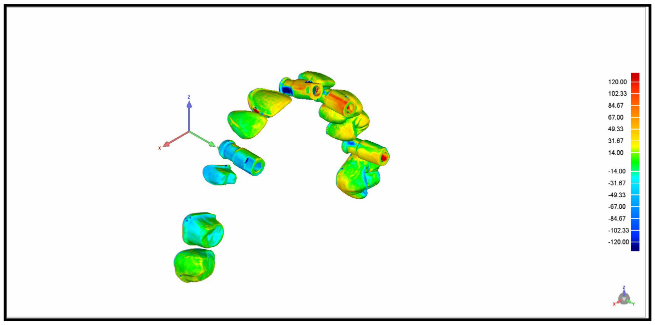

Desviación	
Desviación superior máx.	3155.44
Desviación inferior máx.	-3120.38
Desviación media	63.94 /-54.26
Desviación estándar	218.62

Distribución desviación

>=Min	<Max	# Puntos	%
-120.00	-102.33	384	0.35
-102.33	-84.67	506	0.46
-84.67	-67.00	1008	0.91
-67.00	-49.33	2897	2.63
-49.33	-31.67	6406	5.81
-31.67	-14.00	12886	11.69
-14.00	14.00	44477	40.36
14.00	31.67	22471	20.39
31.67	49.33	8042	7.30
49.33	67.00	2463	2.24
67.00	84.67	1208	1.10
84.67	102.33	640	0.58
102.33	120.00	402	0.36

Fuera del crítico superior	4088	3.71
Fuera del crítico inferior	2311	2.10

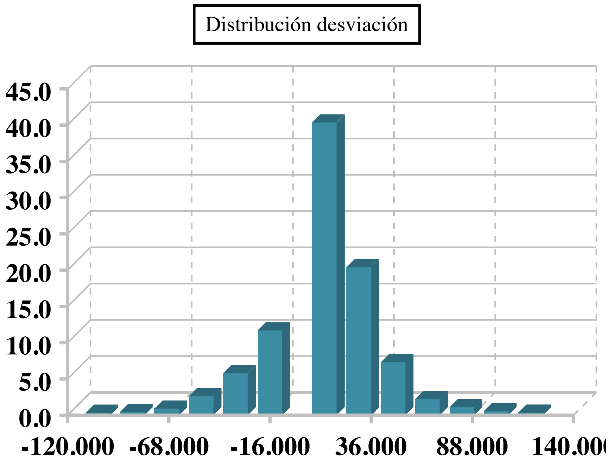

Desviaciones estándar

Distribución (+/-)	# Puntos	%
-6 * Desv. estánd.	546	0.50
-5 * Desv. estánd.	115	0.10
-4 * Desv. estánd.	130	0.12
-3 * Desv. estánd.	166	0.15
-2 * Desv. estánd.	476	0.43
-1 * Desv. estánd.	70241	63.75
1 * Desv. estánd.	35682	32.38
2 * Desv. estánd.	687	0.62
3 * Desv. estánd.	406	0.37
4 * Desv. estánd.	377	0.34
5 * Desv. estánd.	400	0.36
6 * Desv. estánd.	963	0.87

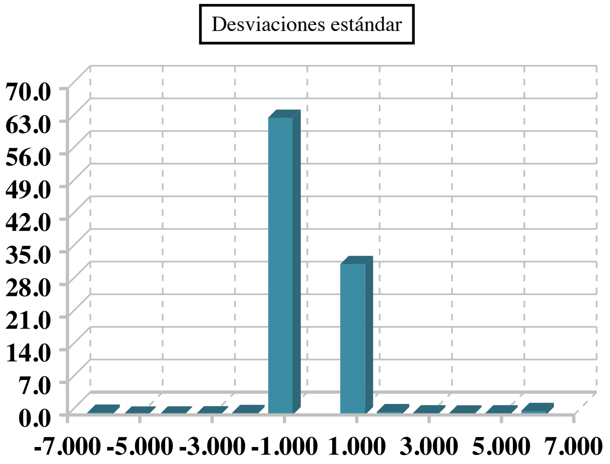

Predefinido: Isométrico

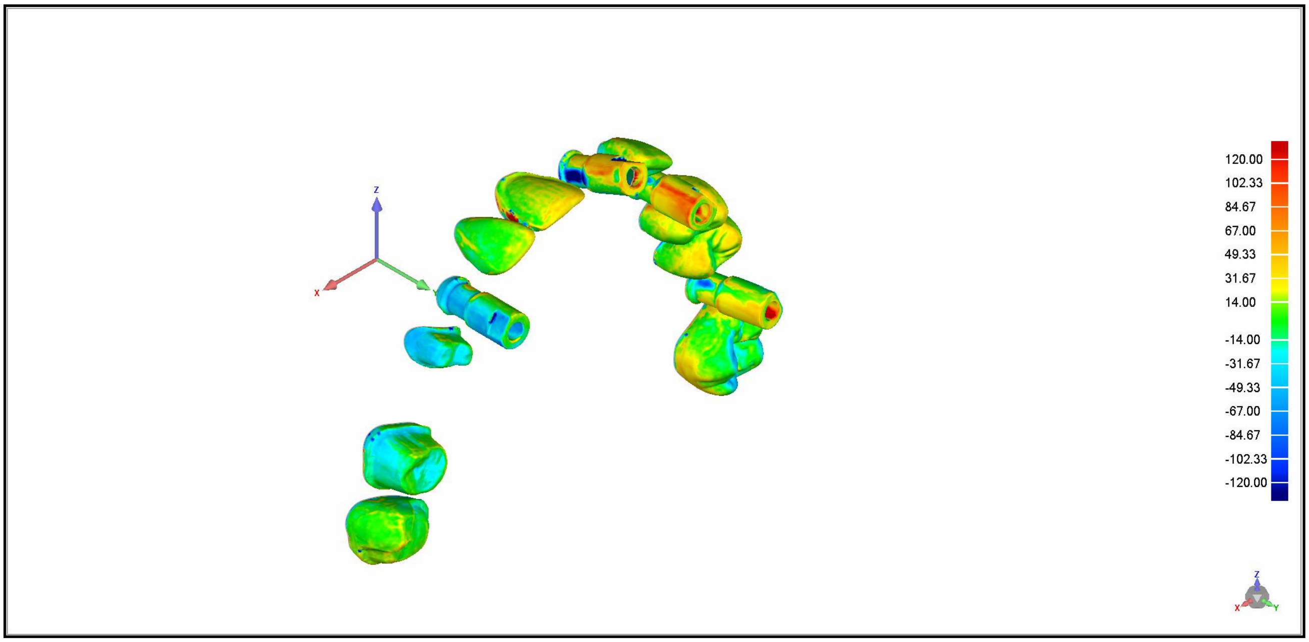

Predefinido: Frente

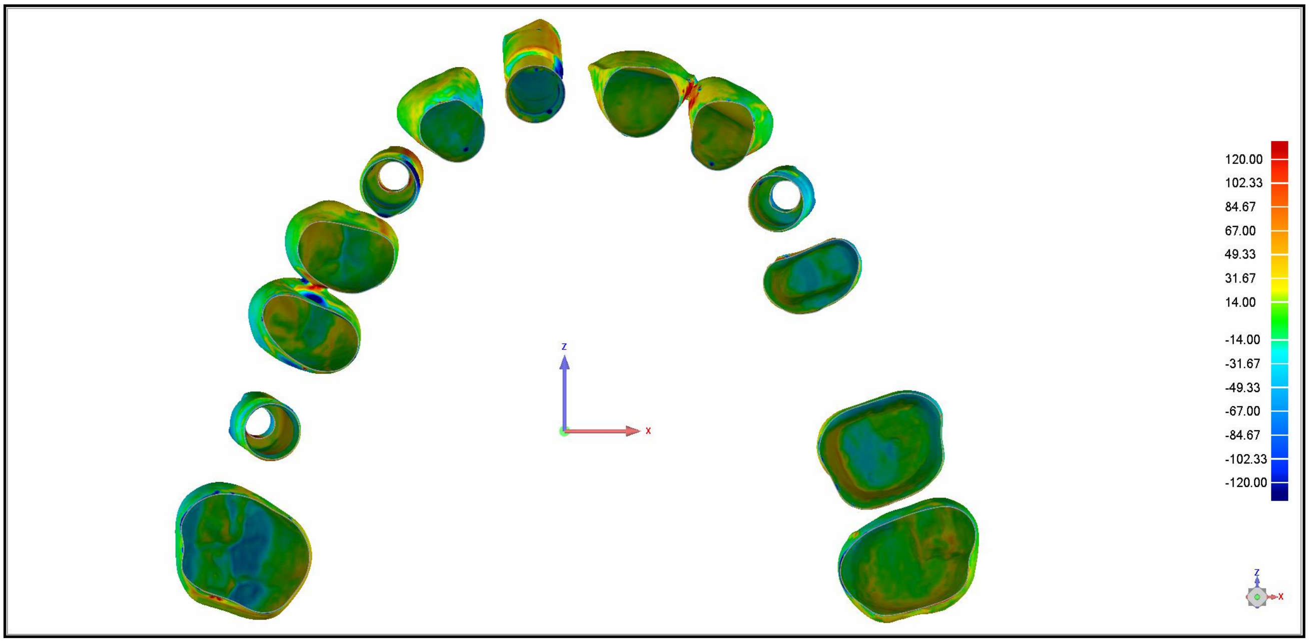

Predefinido: Atrás

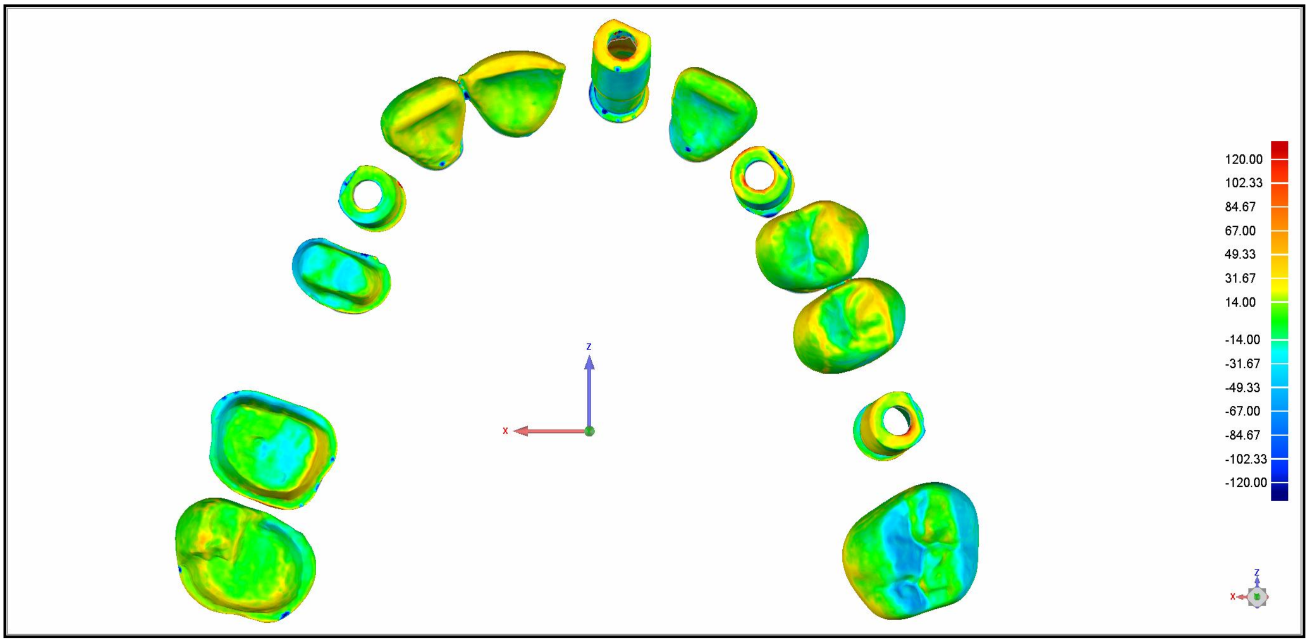

Fecha: 3/6/2017, 12:47 pm

Predefinido: Izquierda

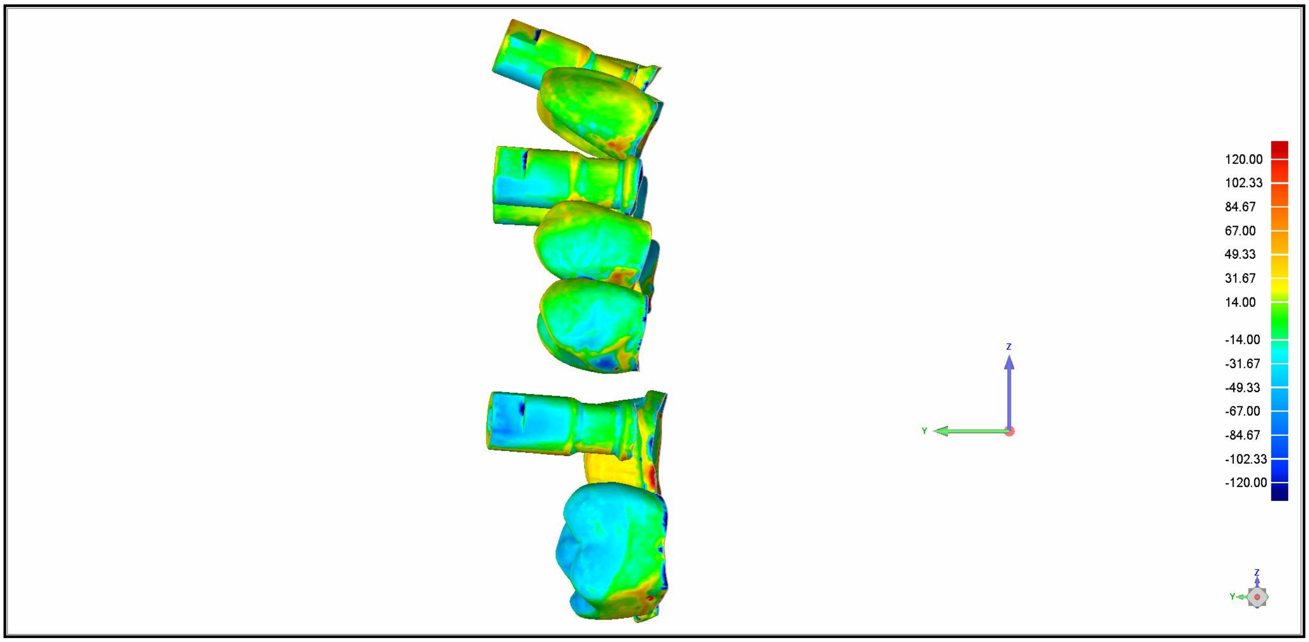

Predefinido: Derecha

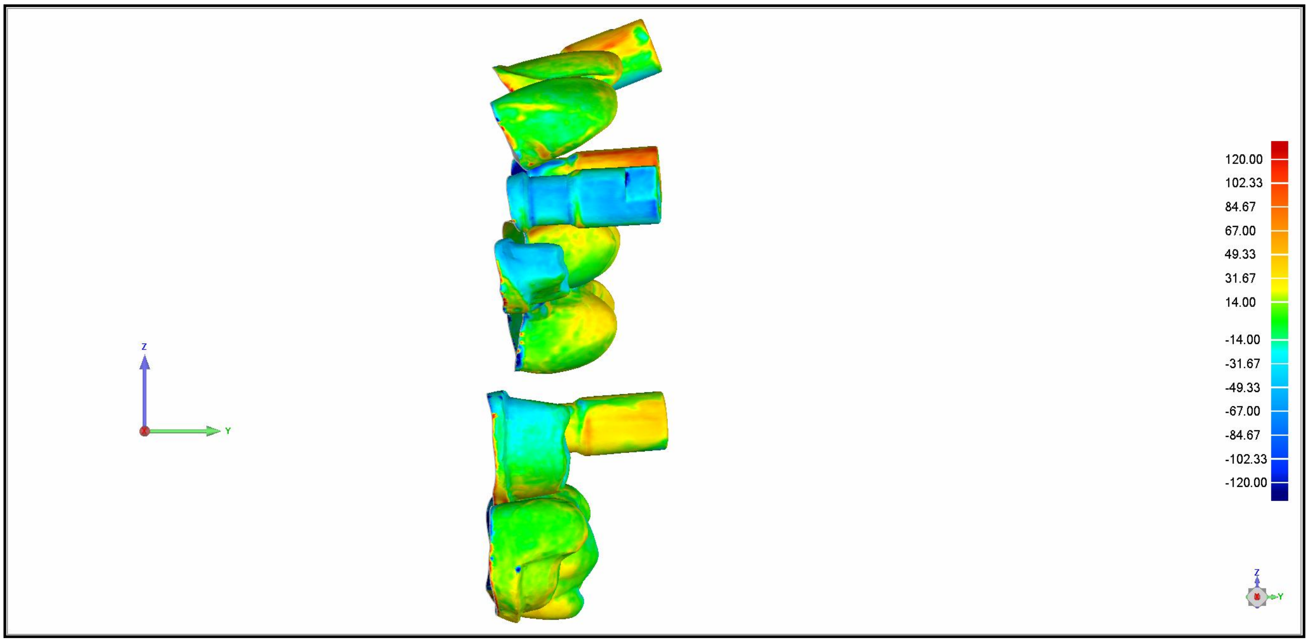

Predefinido: Superior

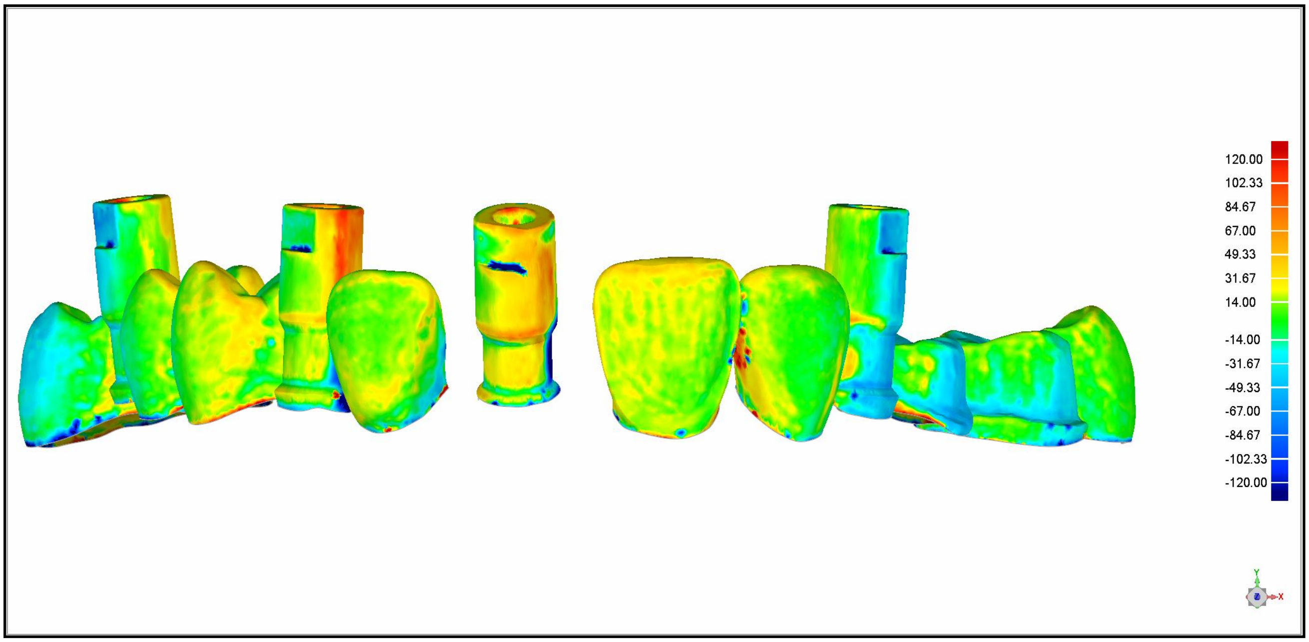

Predefinido: Inferior

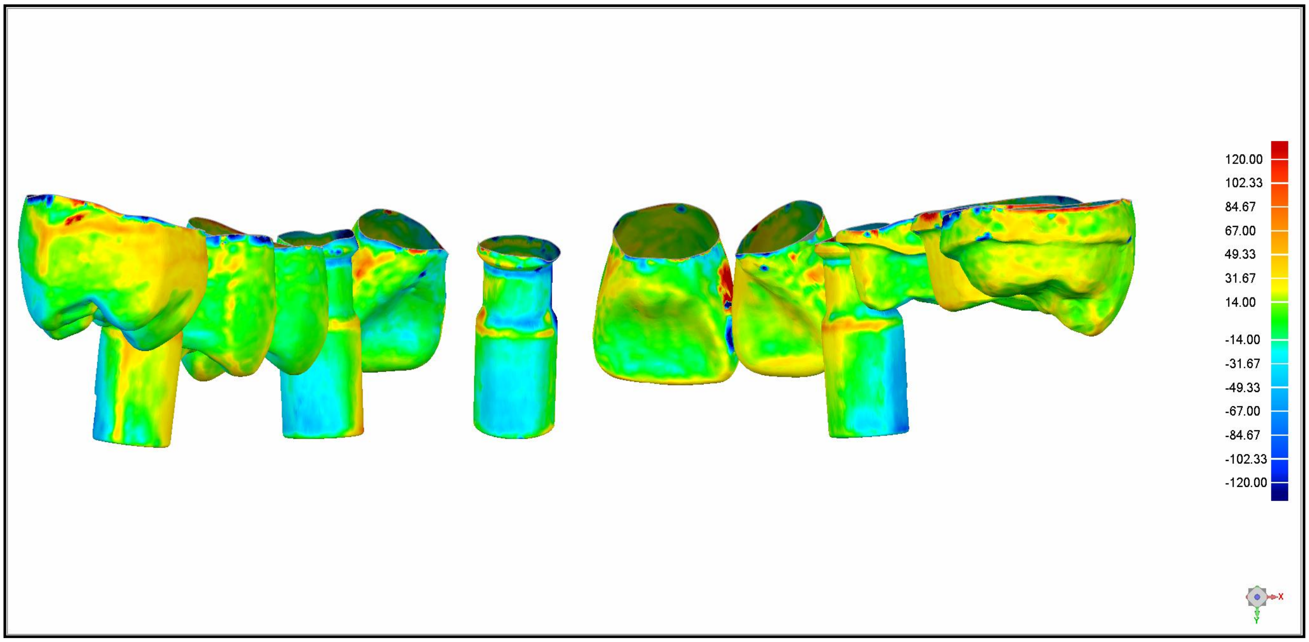

Supplement: S4 Table — Trios (scanning strategy D). (ZIP) [file pone.0202916.s004.zip › S4/3S10D.pdf]
