# Supplementary material for: Accuracy of four digital scanners according to scanning strategy in complete-arch impressions
Source: PLoS One. 2018 Sep 13;13(9):e0202916. doi: 10.1371/journal.pone.0202916 (PMC6136706; doi:10.1371/journal.pone.0202916)

3D Comparación Resultados

Modelo referencia	MRC
Modelo test	3S2D
Nº de puntos de datos	103353
# Aislados	111

Tipo tolerancia	3D desviación
Unidades	u
Máx. crítico	120.00
Máx. nominal	20.00
Mín. nominal	-20.00
Mín. crítico	-120.00

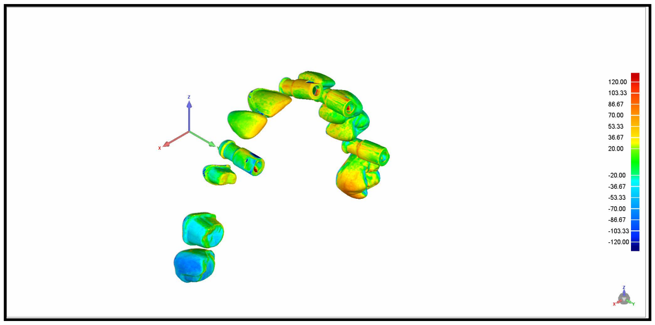

Desviación	
Desviación superior máx.	3121.81
Desviación inferior máx.	-3107.41
Desviación media	73.70 /-53.62
Desviación estándar	210.89

Distribución desviación

>=Min	<Max	# Puntos	%
-120.00	-103.33	342	0.33
-103.33	-86.67	453	0.44
-86.67	-70.00	1897	1.84
-70.00	-53.33	3095	2.99
-53.33	-36.67	4836	4.68
-36.67	-20.00	10079	9.75
-20.00	20.00	45186	43.72
20.00	36.67	16110	15.59
36.67	53.33	8718	8.44
53.33	70.00	3605	3.49
70.00	86.67	1696	1.64
86.67	103.33	497	0.48
103.33	120.00	335	0.32

Fuera del crítico superior	4354	4.21
Fuera del crítico inferior	2150	2.08

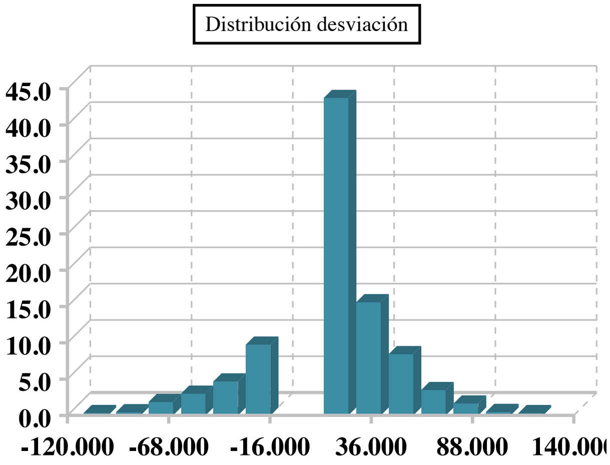

Desviaciones estándar

Distribución (+/-)	# Puntos	%
-6 * Desv. estánd.	428	0.41
-5 * Desv. estánd.	86	0.08
-4 * Desv. estánd.	121	0.12
-3 * Desv. estánd.	168	0.16
-2 * Desv. estánd.	535	0.52
-1 * Desv. estánd.	67474	65.28
1 * Desv. estánd.	31439	30.42
2 * Desv. estánd.	724	0.70
3 * Desv. estánd.	400	0.39
4 * Desv. estánd.	473	0.46
5 * Desv. estánd.	485	0.47
6 * Desv. estánd.	1020	0.99

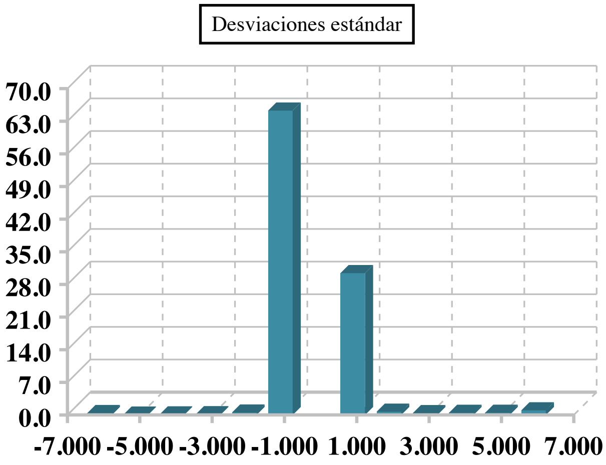

Predefinido: Isométrico

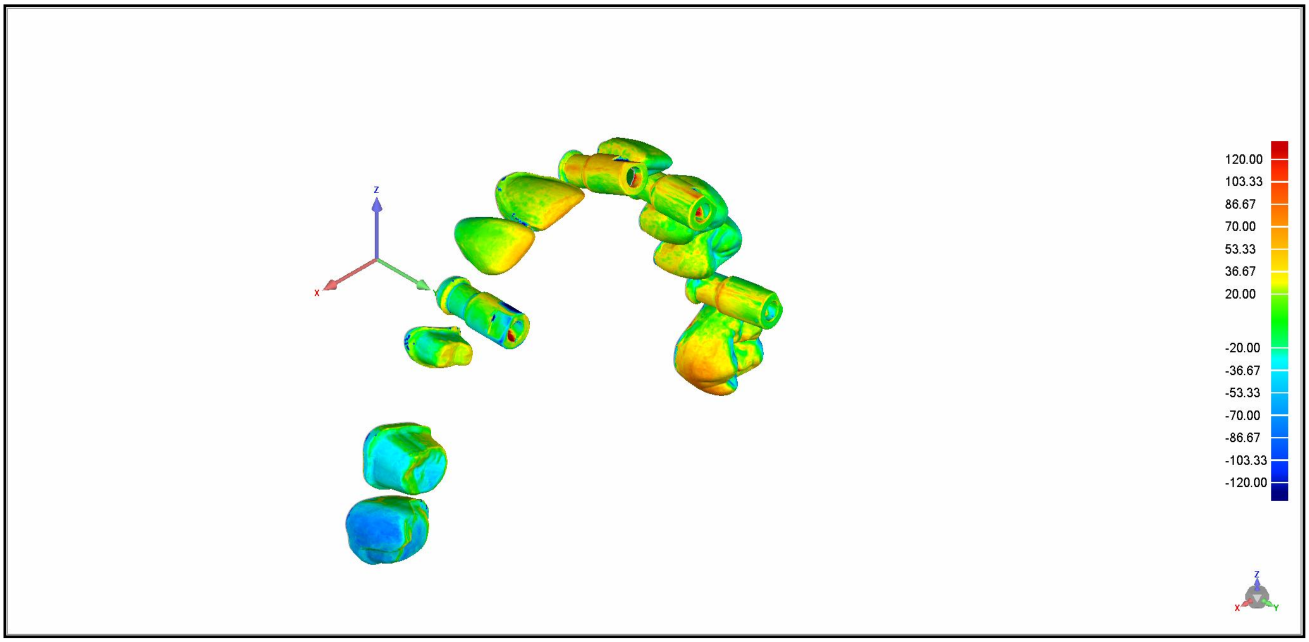

Predefinido: Frente

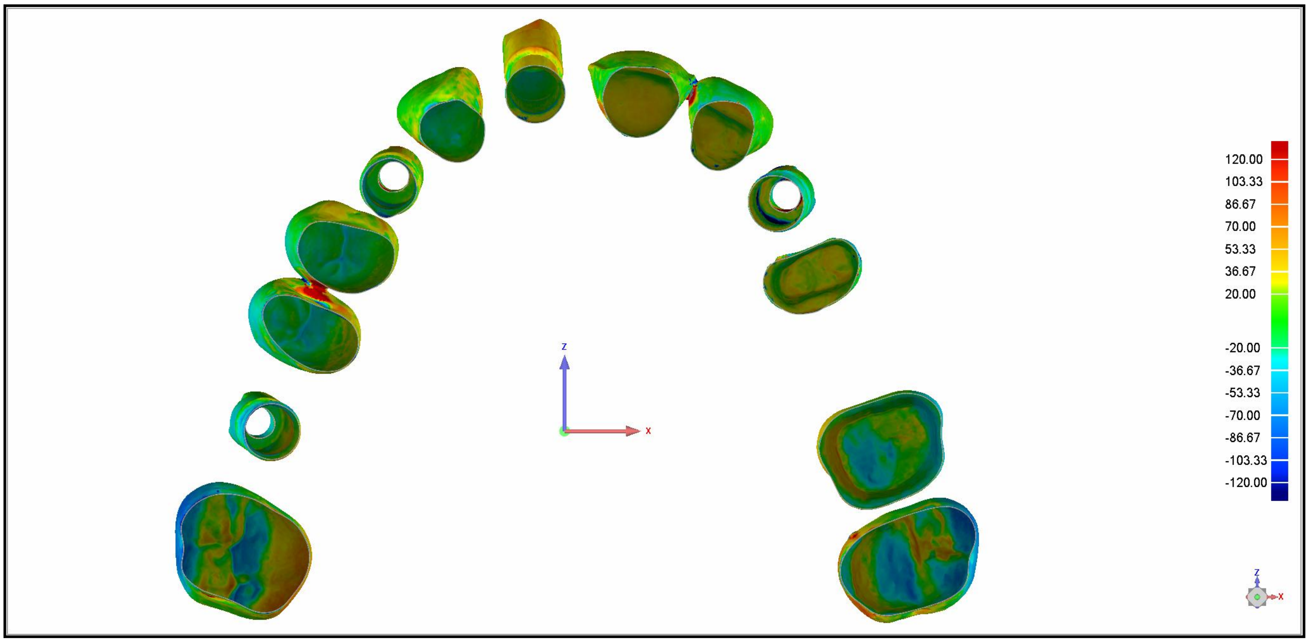

Predefinido: Atrás

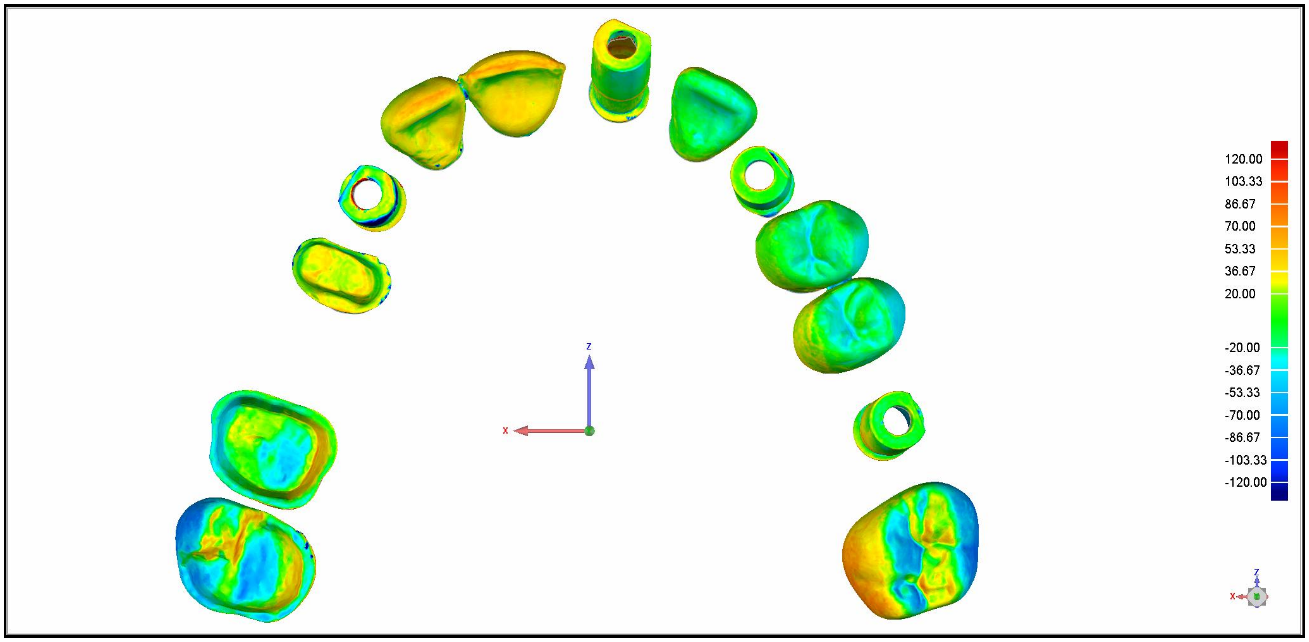

Predefinido: Izquierda

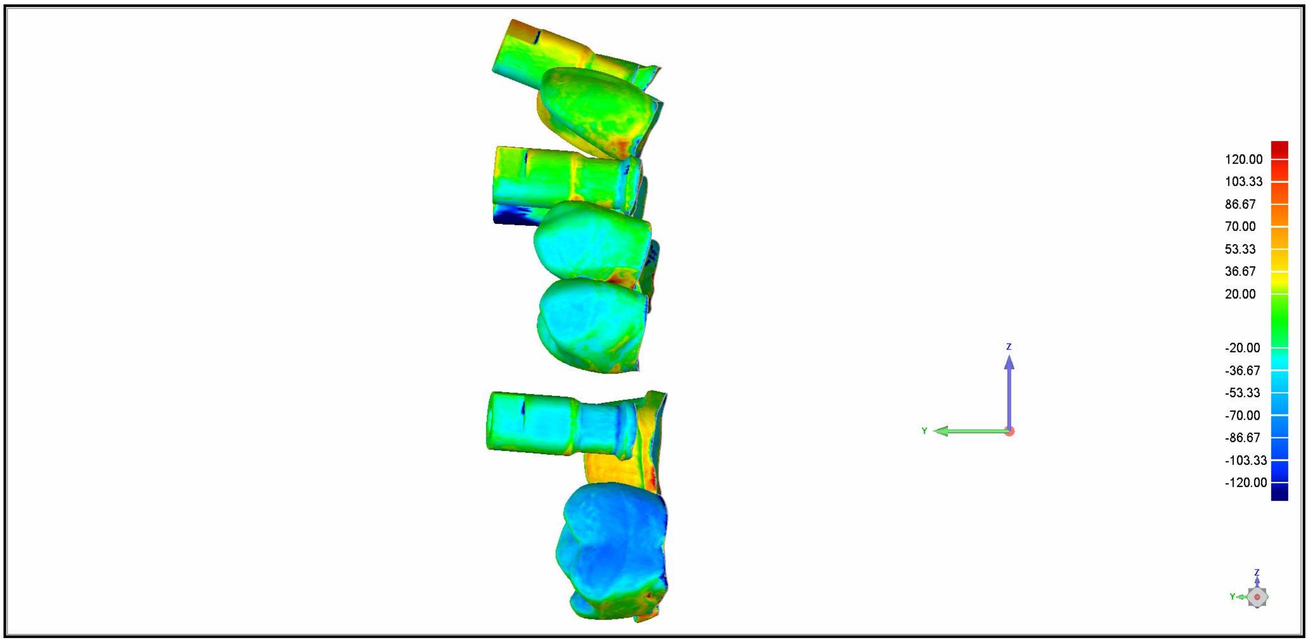

Predefinido: Derecha

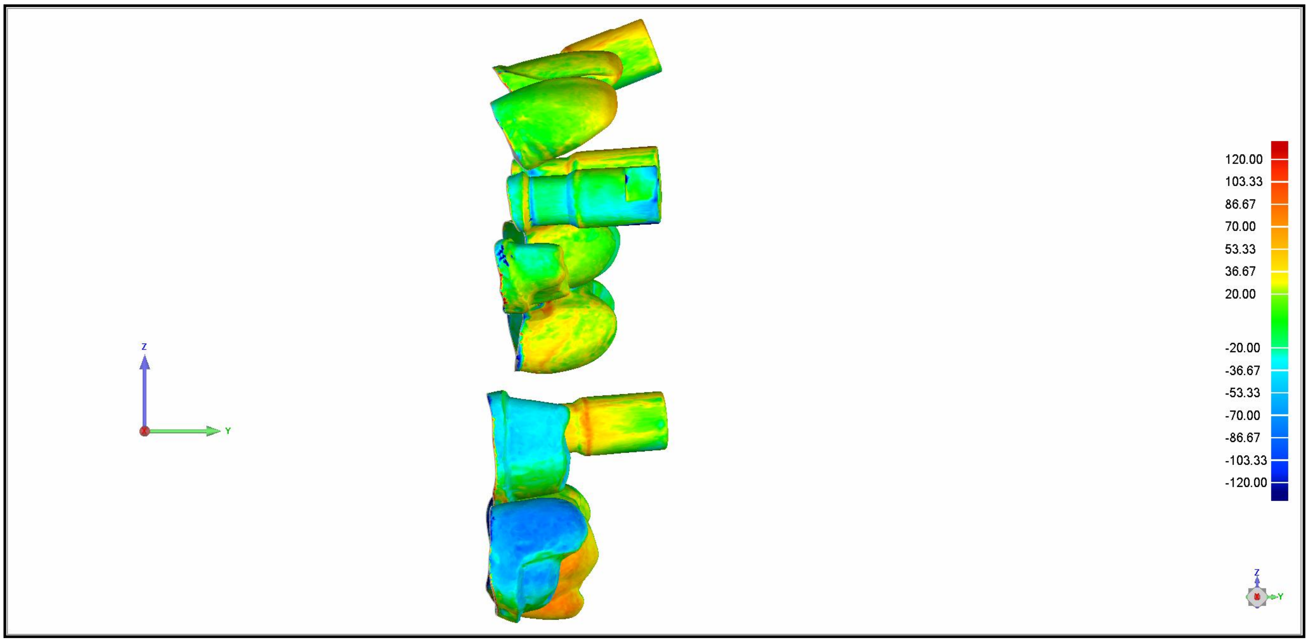

Predefinido: Superior

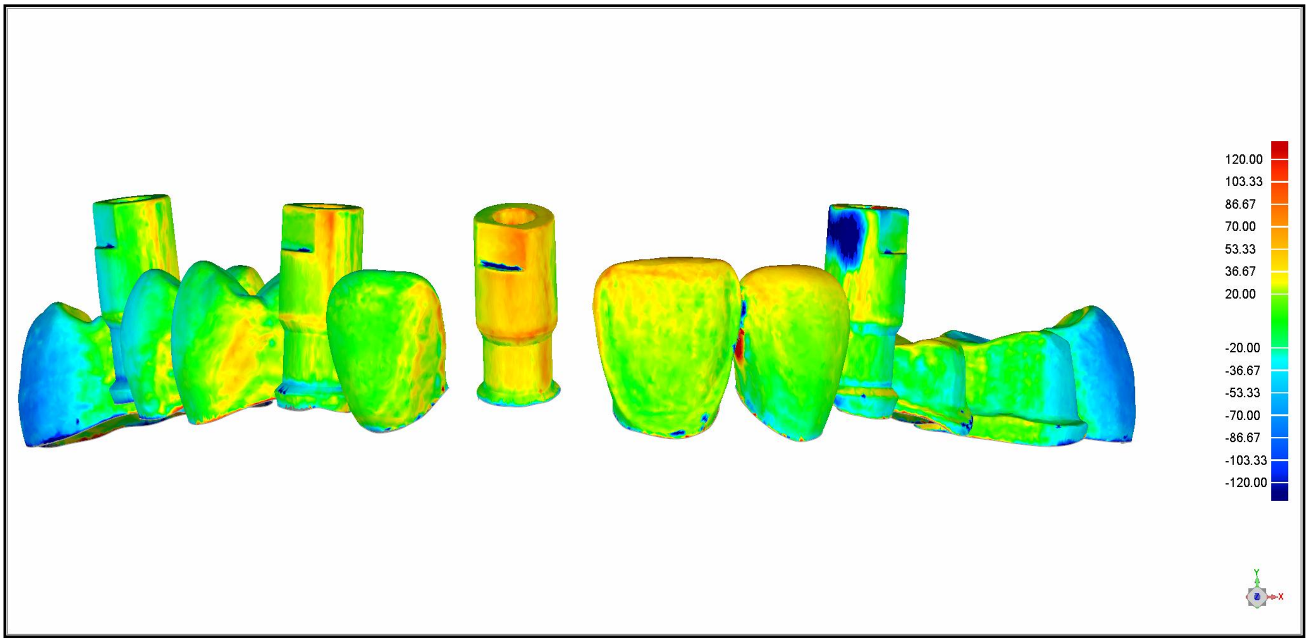

Predefinido: Inferior

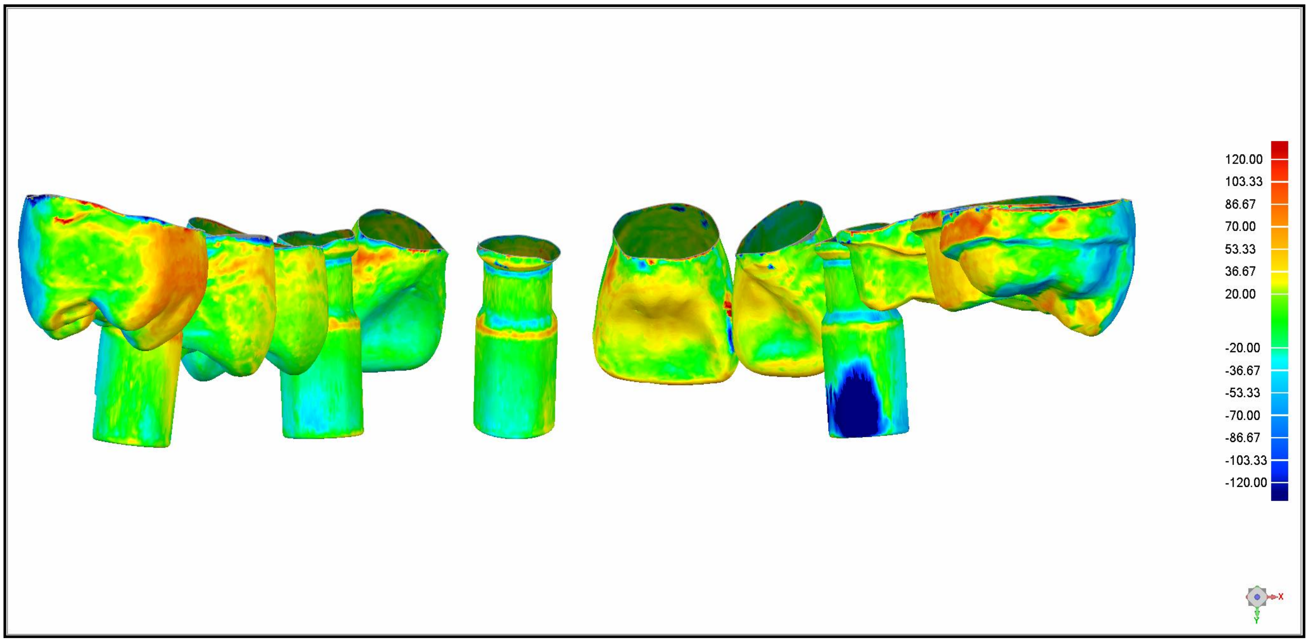

Supplement: S4 Table — Trios (scanning strategy D). (ZIP) [file pone.0202916.s004.zip › S4/3S2D.pdf]
