# Supplementary material for: Accuracy of four digital scanners according to scanning strategy in complete-arch impressions
Source: PLoS One. 2018 Sep 13;13(9):e0202916. doi: 10.1371/journal.pone.0202916 (PMC6136706; doi:10.1371/journal.pone.0202916)

Geomagic Control™

Fecha: 3/6/2017, 12:14 pm

3D Comparación Resultados

Modelo referencia	MRC
Modelo test	3S1D
Nº de puntos de datos	113072
# Aislados	94

Tipo tolerancia	3D desviación
Unidades	u
Máx. crítico	120.00
Máx. nominal	14.00
Mín. nominal	-14.00
Mín. crítico	-120.00

Desviación	
Desviación superior máx.	3031.69
Desviación inferior máx.	-3155.86
Desviación media	62.46 /-53.34
Desviación estándar	202.47

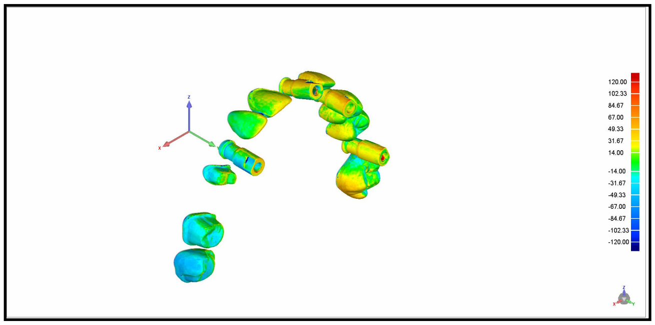

Distribución desviación

>=Min	<Max	# Puntos	%
-120.00	-102.33	295	0.26
-102.33	-84.67	430	0.38
-84.67	-67.00	811	0.72
-67.00	-49.33	2718	2.40
-49.33	-31.67	7071	6.25
-31.67	-14.00	15142	13.39
-14.00	14.00	38613	34.15
14.00	31.67	23072	20.40
31.67	49.33	12024	10.63
49.33	67.00	4280	3.79
67.00	84.67	1351	1.19
84.67	102.33	621	0.55
102.33	120.00	372	0.33

Fuera del crítico superior	3998	3.54
Fuera del crítico inferior	2274	2.01

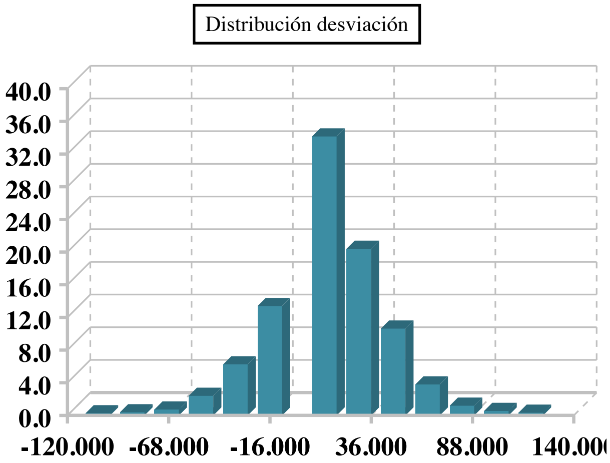

Desviaciones estándar

Distribución (+/-)	# Puntos	%
-6 * Desv. estánd.	592	0.52
-5 * Desv. estánd.	141	0.12
-4 * Desv. estánd.	171	0.15
-3 * Desv. estánd.	206	0.18
-2 * Desv. estánd.	451	0.40
-1 * Desv. estánd.	66263	58.60
1 * Desv. estánd.	42457	37.55
2 * Desv. estánd.	733	0.65
3 * Desv. estánd.	366	0.32
4 * Desv. estánd.	385	0.34
5 * Desv. estánd.	364	0.32
6 * Desv. estánd.	943	0.83

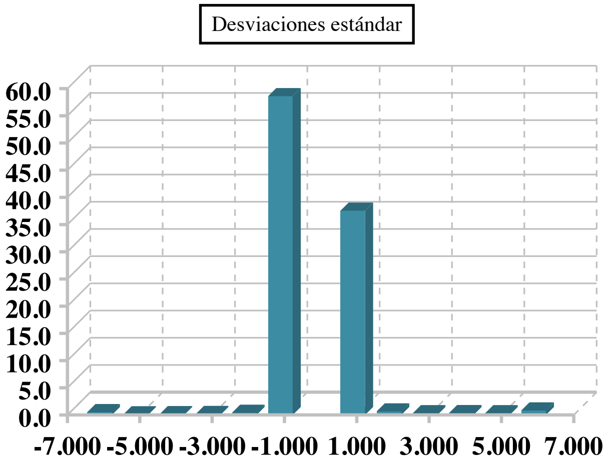

Predefinido: Isométrico

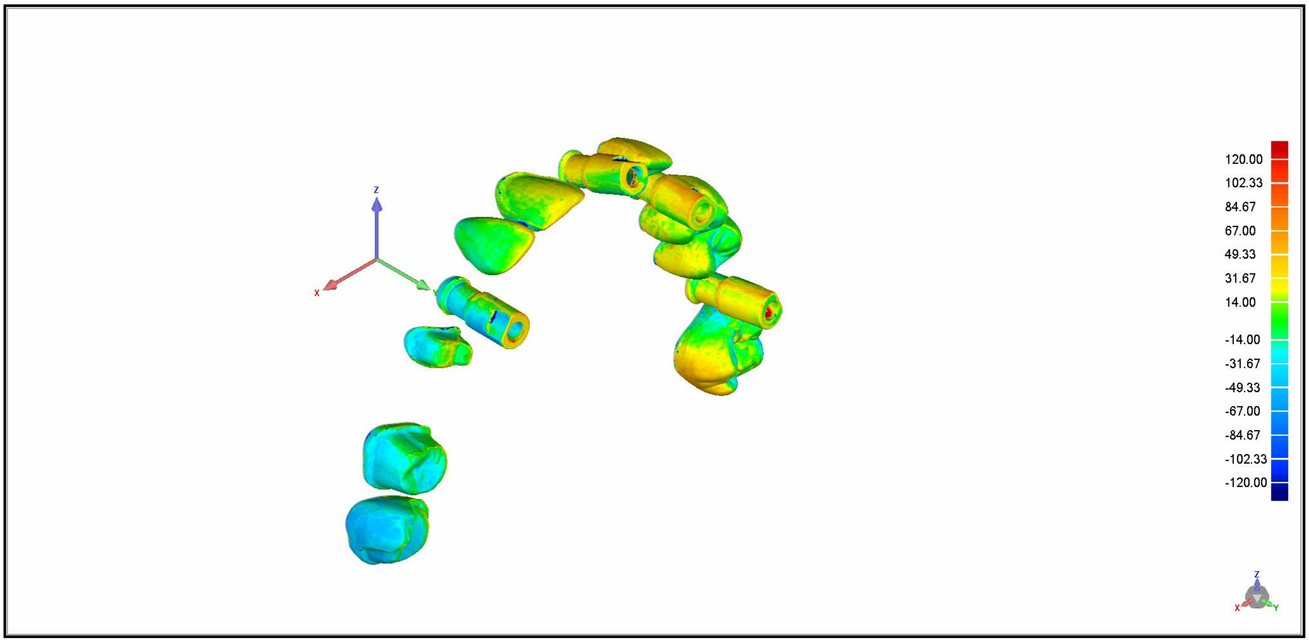

Predefinido: Frente

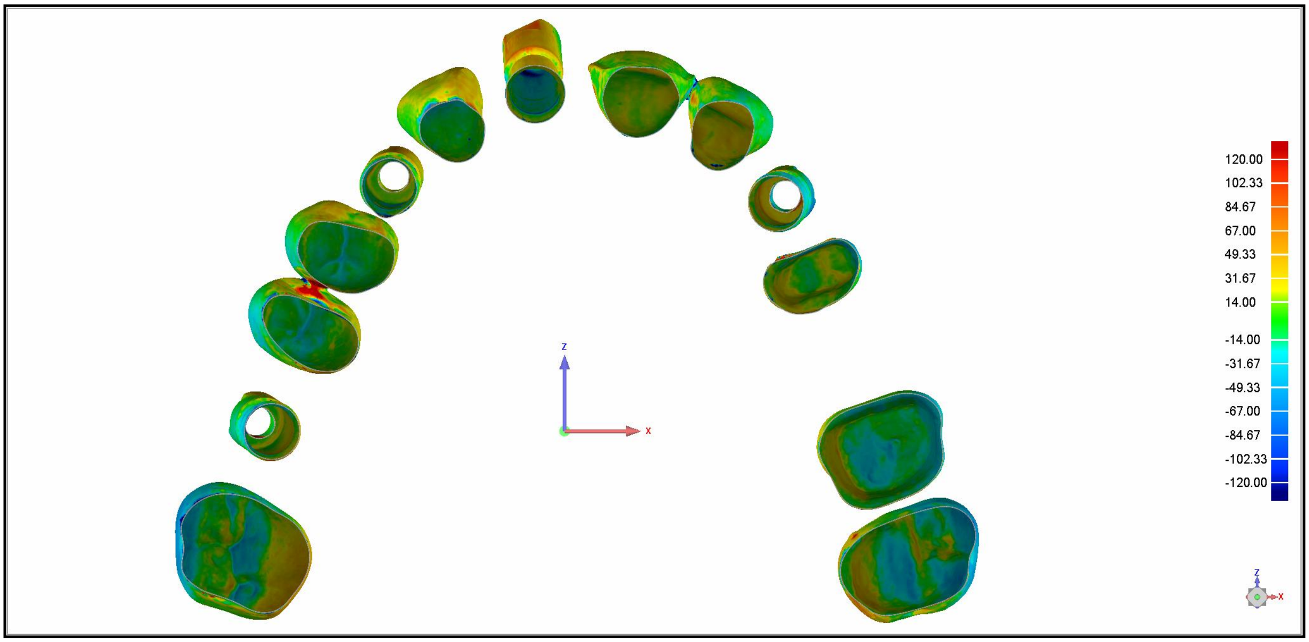

Predefinido: Atrás

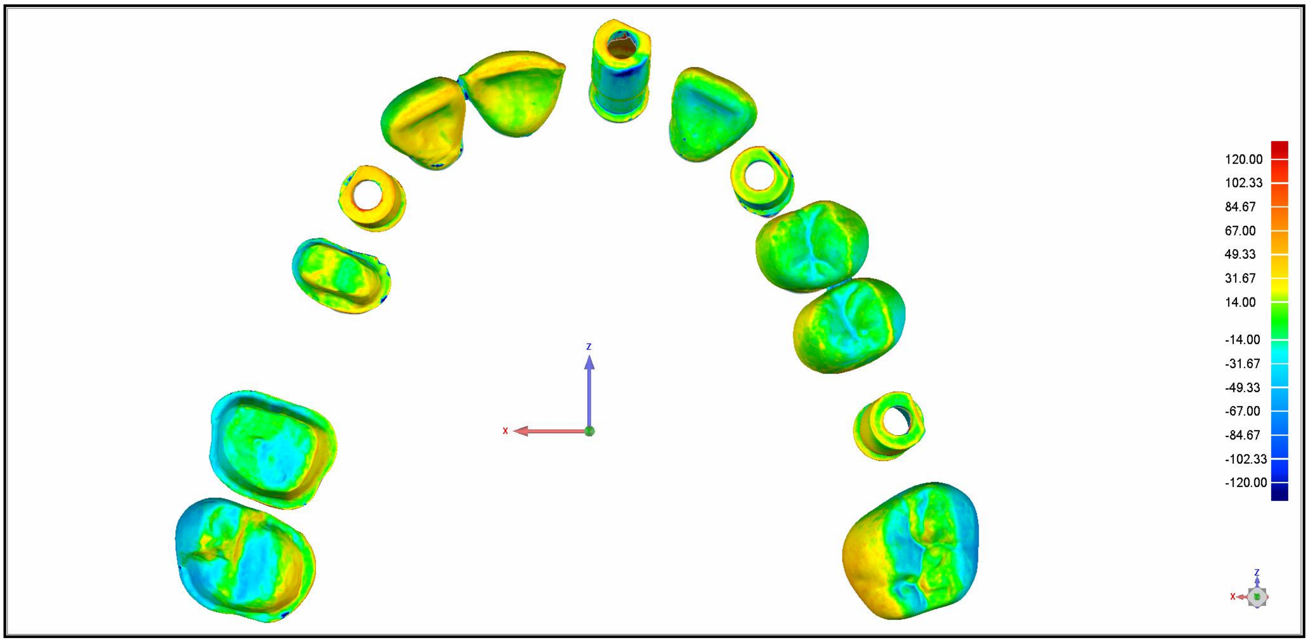

Predefinido: Izquierda

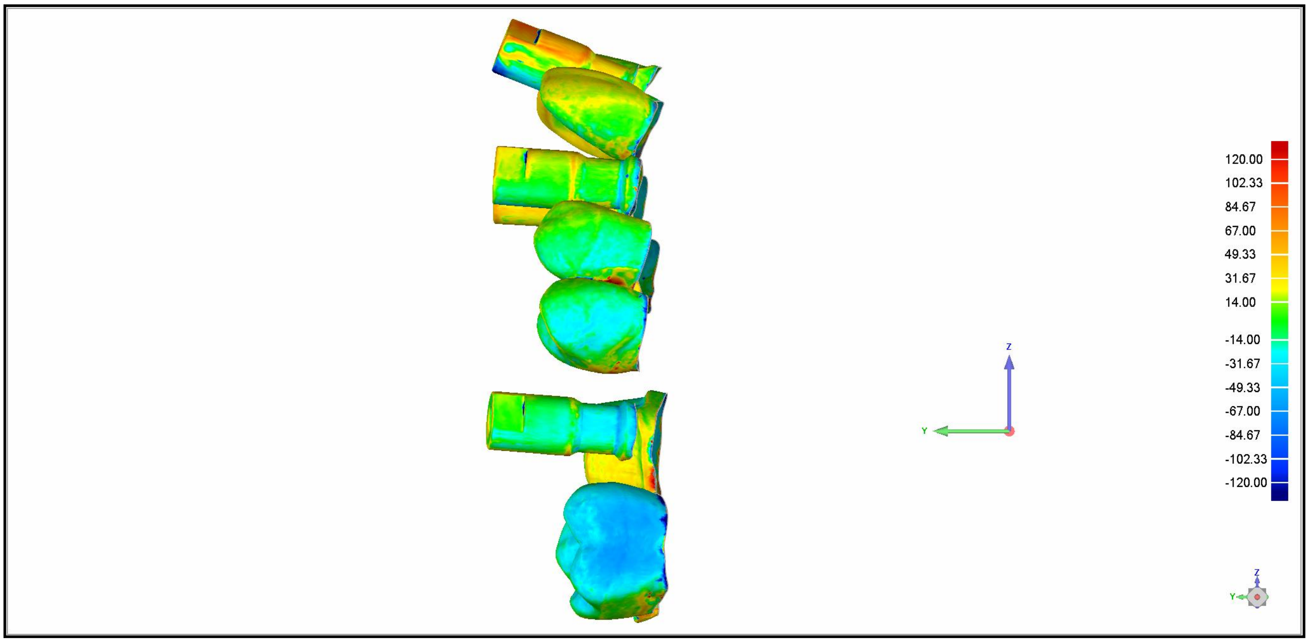

Predefinido: Derecha

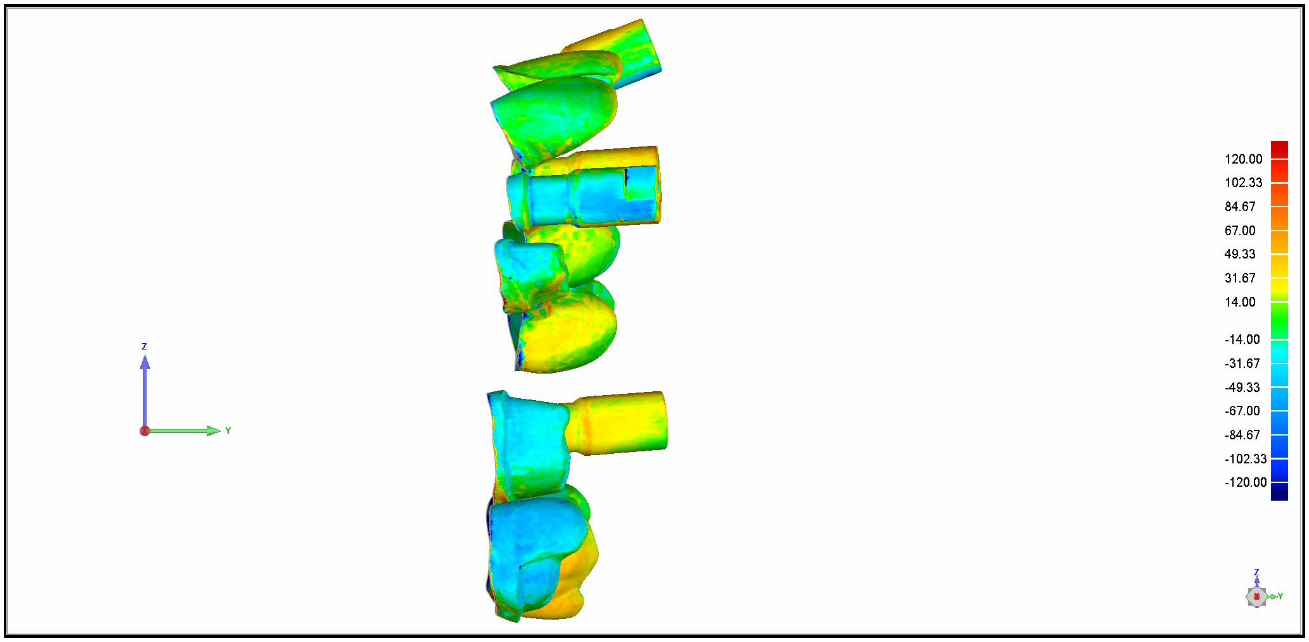

Predefinido: Superior

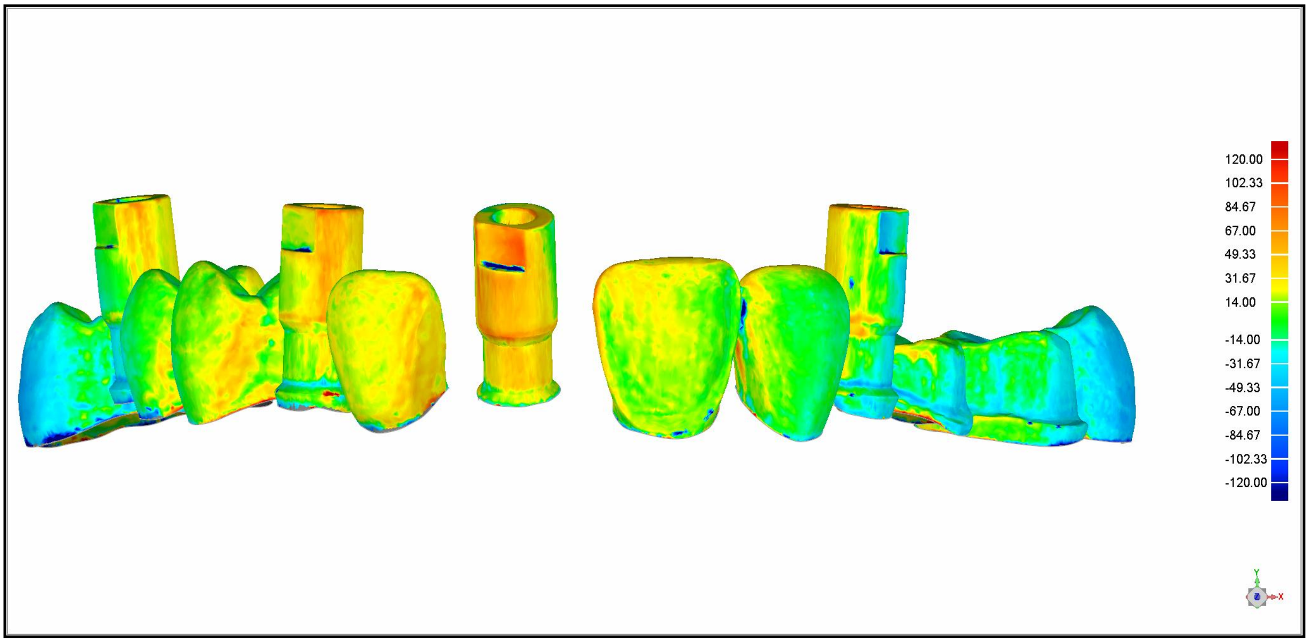

Predefinido: Inferior

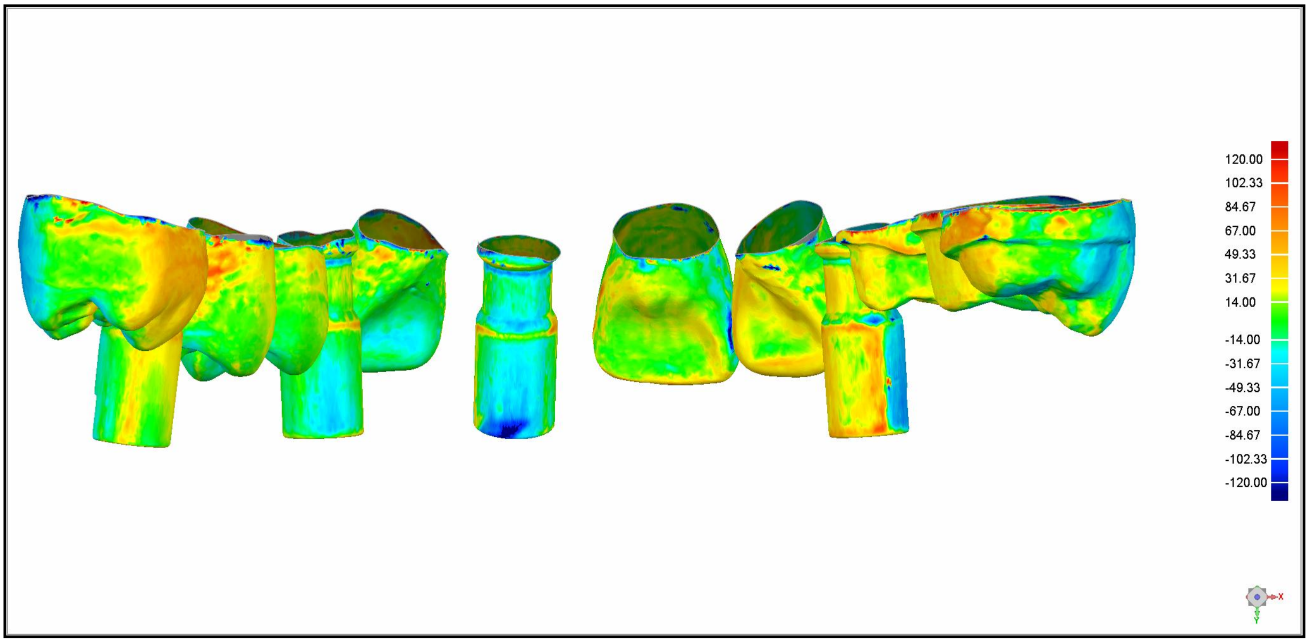

Supplement: S4 Table — Trios (scanning strategy D). (ZIP) [file pone.0202916.s004.zip › S4/3S1D.pdf]
