# Supplementary material for: Accuracy of four digital scanners according to scanning strategy in complete-arch impressions
Source: PLoS One. 2018 Sep 13;13(9):e0202916. doi: 10.1371/journal.pone.0202916 (PMC6136706; doi:10.1371/journal.pone.0202916)

3D Comparación Resultados

Modelo referencia	MRC
Modelo test	3S7D
Nº de puntos de datos	106289
# Aislados	120

Tipo tolerancia	3D desviación
Unidades	u
Máx. crítico	120.00
Máx. nominal	15.00
Mín. nominal	-15.00
Mín. crítico	-120.00

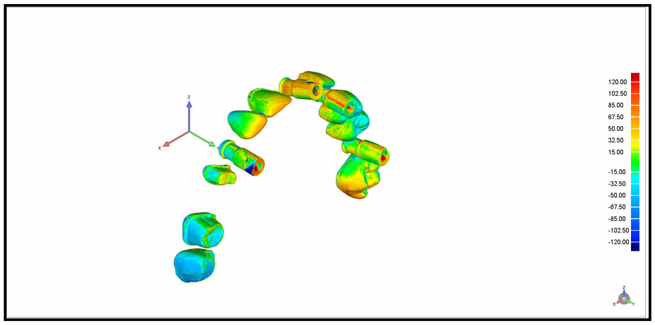

Desviación	
Desviación superior máx.	2890.23
Desviación inferior máx.	-3120.18
Desviación media	65.07 /-49.91
Desviación estándar	187.54

Distribución desviación

>=Min	<Max	# Puntos	%
-120.00	-102.50	337	0.32
-102.50	-85.00	483	0.45
-85.00	-67.50	892	0.84
-67.50	-50.00	3364	3.16
-50.00	-32.50	8256	7.77
-32.50	-15.00	14655	13.79
-15.00	15.00	33960	31.95
15.00	32.50	17904	16.84
32.50	50.00	11833	11.13
50.00	67.50	5374	5.06
67.50	85.00	2075	1.95
85.00	102.50	848	0.80
102.50	120.00	499	0.47

Fuera del crítico superior	3738	3.52
Fuera del crítico inferior	2071	1.95

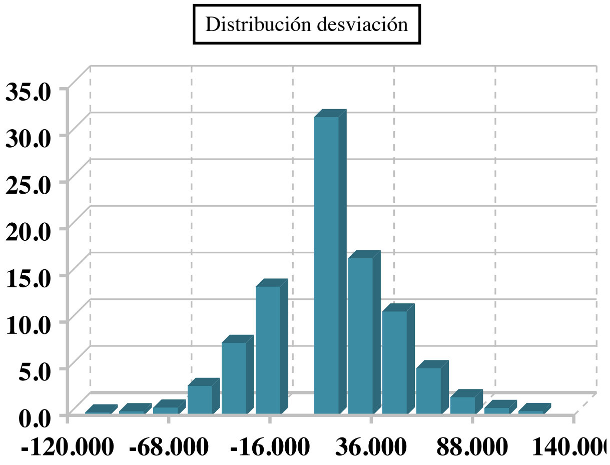

Desviaciones estándar

Distribución (+/-)	# Puntos	%
-6 * Desv. estánd.	447	0.42
-5 * Desv. estánd.	98	0.09
-4 * Desv. estánd.	156	0.15
-3 * Desv. estánd.	176	0.17
-2 * Desv. estánd.	618	0.58
-1 * Desv. estánd.	62609	58.90
1 * Desv. estánd.	39464	37.13
2 * Desv. estánd.	753	0.71
3 * Desv. estánd.	369	0.35
4 * Desv. estánd.	332	0.31
5 * Desv. estánd.	335	0.32
6 * Desv. estánd.	932	0.88

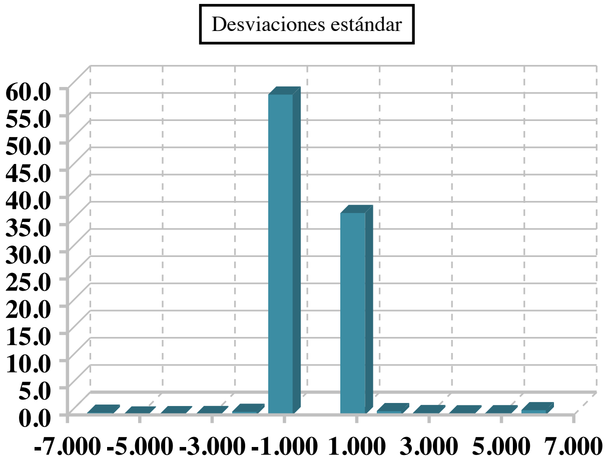

Predefinido: Isométrico

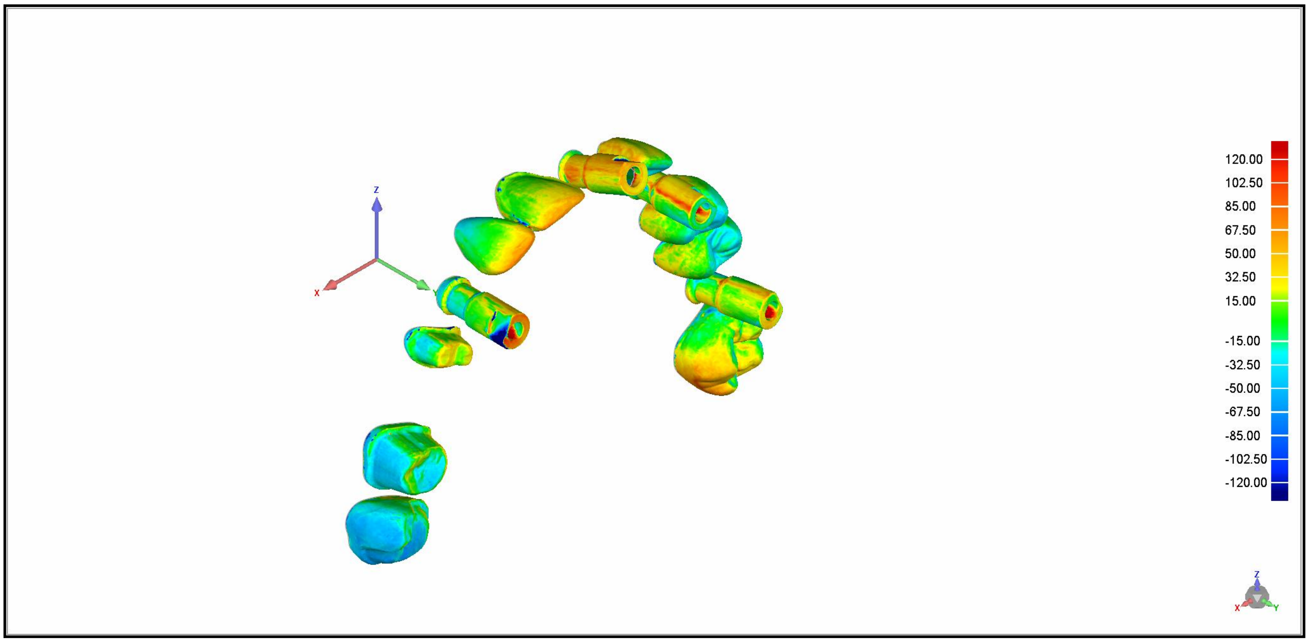

Predefinido: Frente

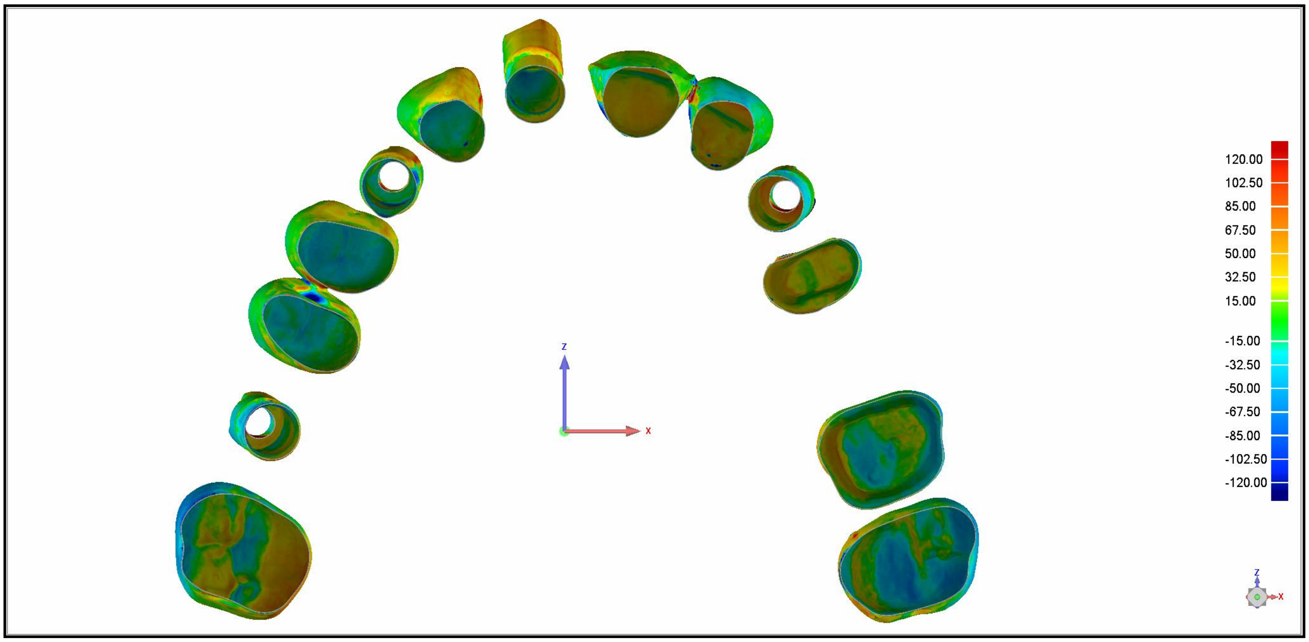

Predefinido: Atrás

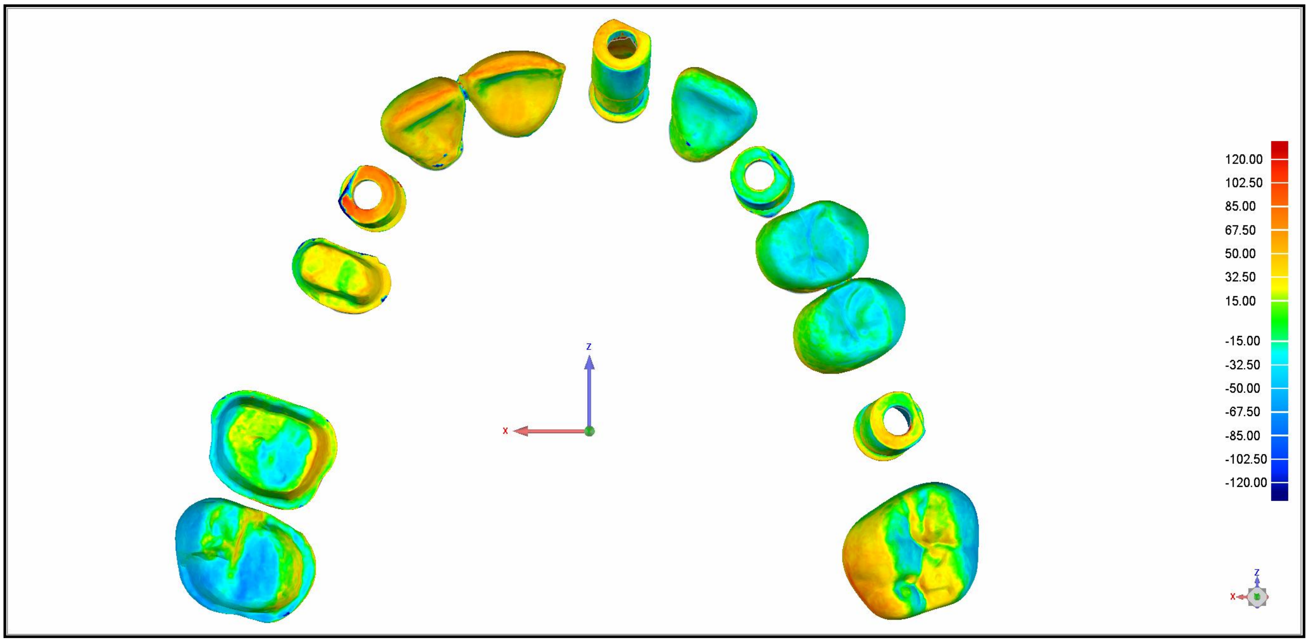

Predefinido: Izquierda

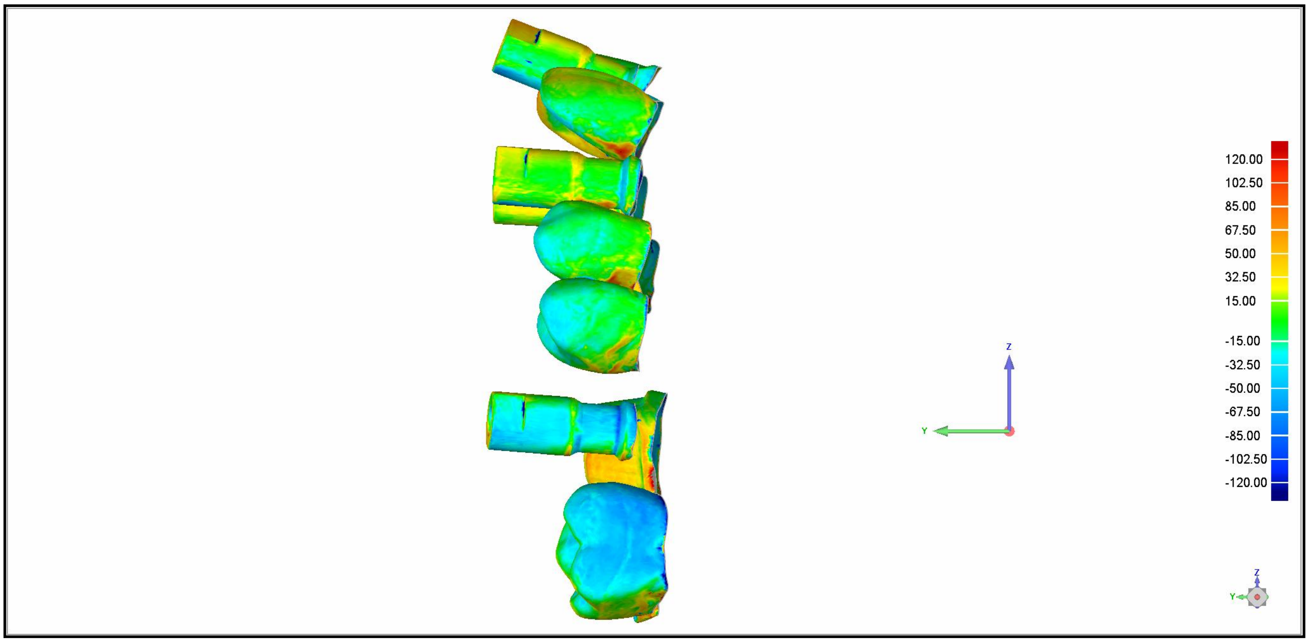

Predefinido: Derecha

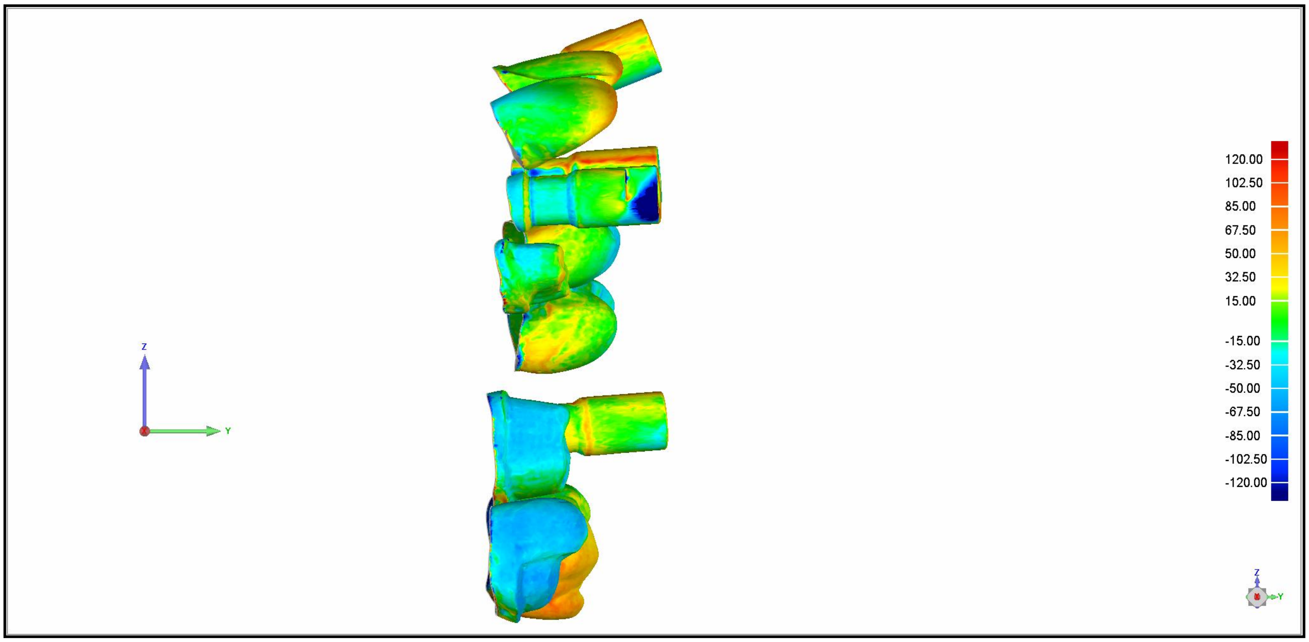

Predefinido: Superior

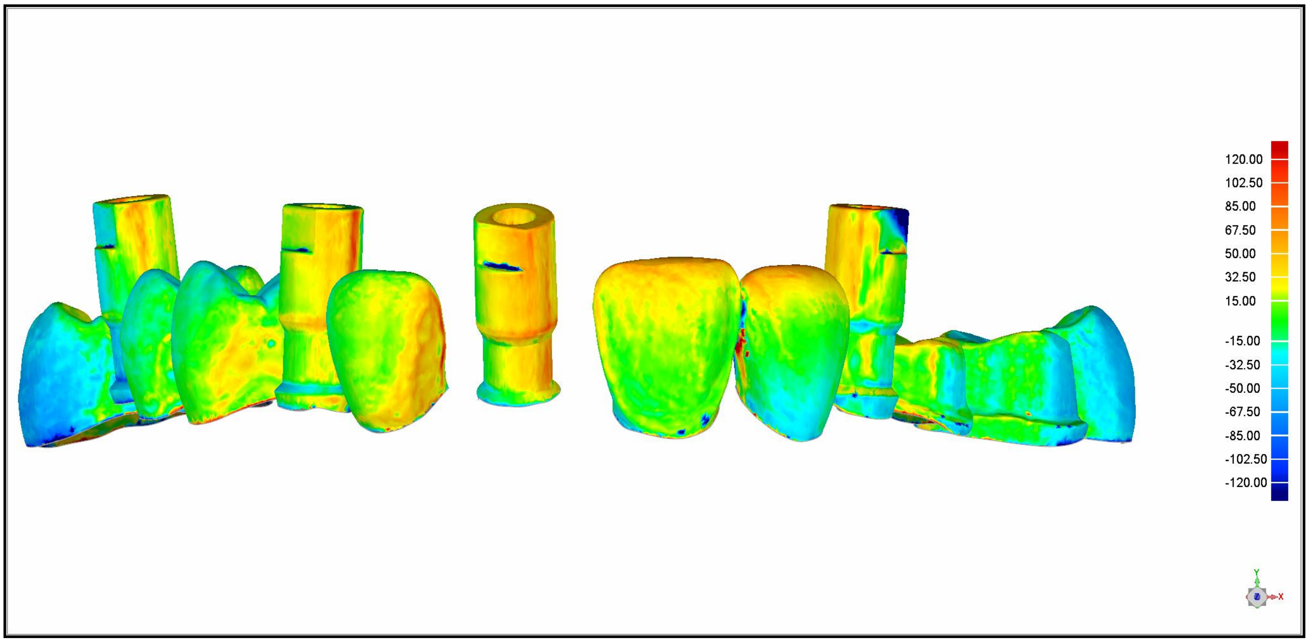

Predefinido: Inferior

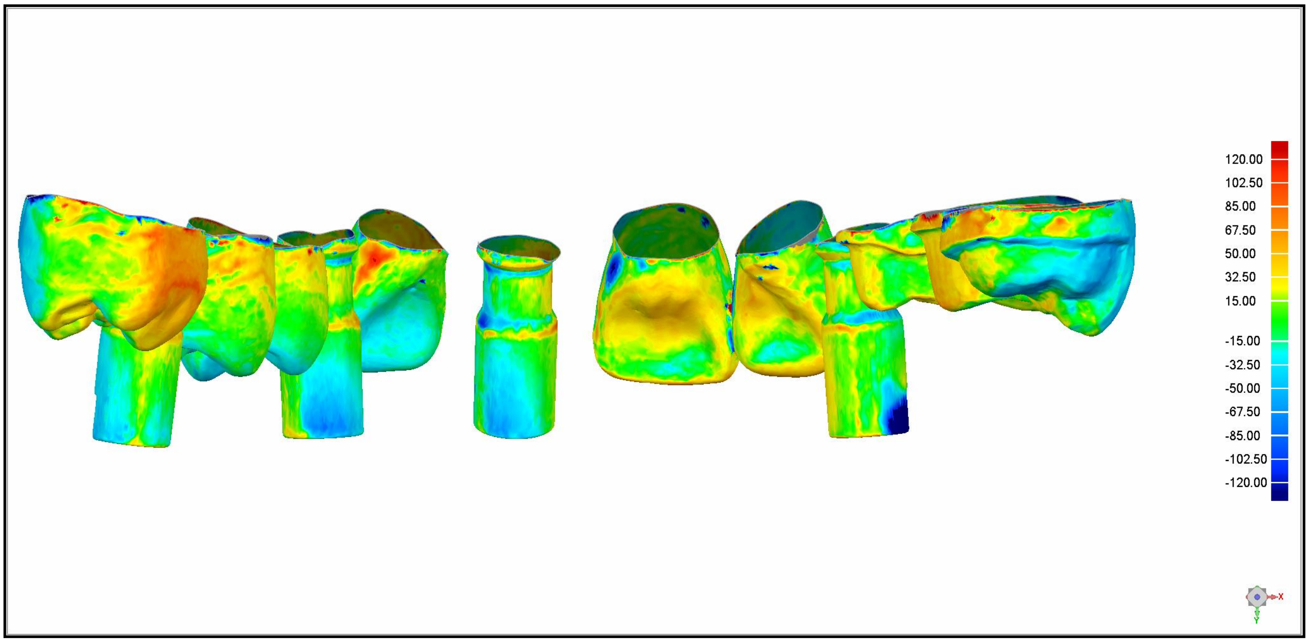

Supplement: S4 Table — Trios (scanning strategy D). (ZIP) [file pone.0202916.s004.zip › S4/3S7D.pdf]
