# Supplementary material for: Accuracy of four digital scanners according to scanning strategy in complete-arch impressions
Source: PLoS One. 2018 Sep 13;13(9):e0202916. doi: 10.1371/journal.pone.0202916 (PMC6136706; doi:10.1371/journal.pone.0202916)

Geomagic Control™

3D Comparación Resultados

Modelo referencia	MRC
Modelo test	3S6D
Nº de puntos de datos	107232
# Aislados	77

Tipo tolerancia	3D desviación
Unidades	u
Máx. crítico	120.00
Máx. nominal	18.00
Mín. nominal	-18.00
Mín. crítico	-120.00

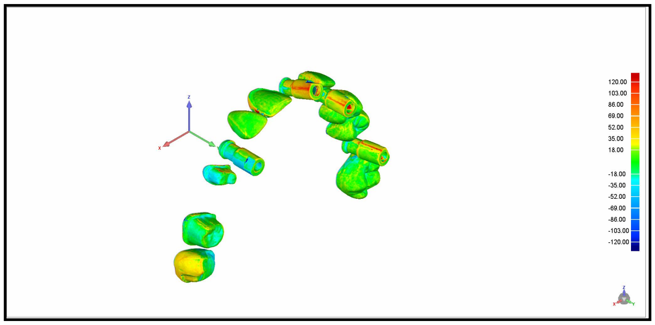

Desviación	
Desviación superior máx.	3082.98
Desviación inferior máx.	-3038.50
Desviación media	60.87 /-49.44
Desviación estándar	212.96

Distribución desviación

>=Min	<Max	# Puntos	%
-120.00	-103.00	238	0.22
-103.00	-86.00	333	0.31
-86.00	-69.00	507	0.47
-69.00	-52.00	1134	1.06
-52.00	-35.00	3245	3.03
-35.00	-18.00	8316	7.76
-18.00	18.00	60981	56.87
18.00	35.00	15652	14.60
35.00	52.00	6208	5.79
52.00	69.00	2233	2.08
69.00	86.00	1135	1.06
86.00	103.00	812	0.76
103.00	120.00	515	0.48

Fuera del crítico superior	4070	3.80
Fuera del crítico inferior	1853	1.73

Distribución desviación

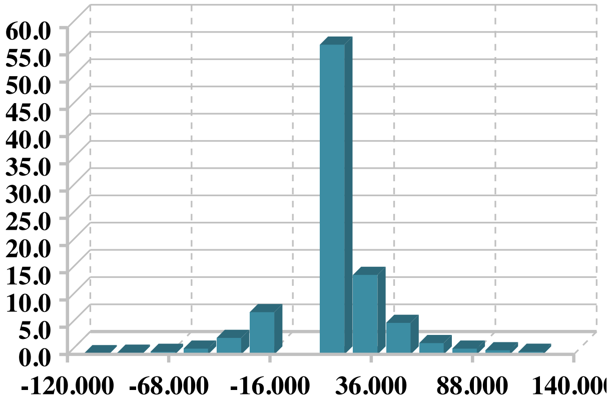

Desviaciones estándar

Distribución (+/-)	# Puntos	%
-6 * Desv. estánd.	553	0.52
-5 * Desv. estánd.	78	0.07
-4 * Desv. estánd.	102	0.10
-3 * Desv. estánd.	171	0.16
-2 * Desv. estánd.	343	0.32
-1 * Desv. estánd.	75402	70.32
1 * Desv. estánd.	27846	25.97
2 * Desv. estánd.	660	0.62
3 * Desv. estánd.	330	0.31
4 * Desv. estánd.	369	0.34
5 * Desv. estánd.	371	0.35
6 * Desv. estánd.	1007	0.94

Desviaciones estándar

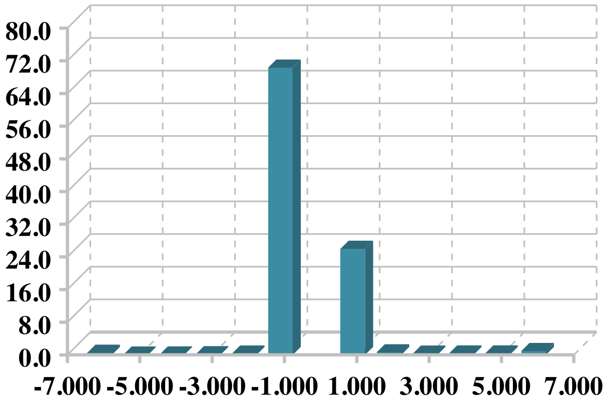

Predefinido: Isométrico

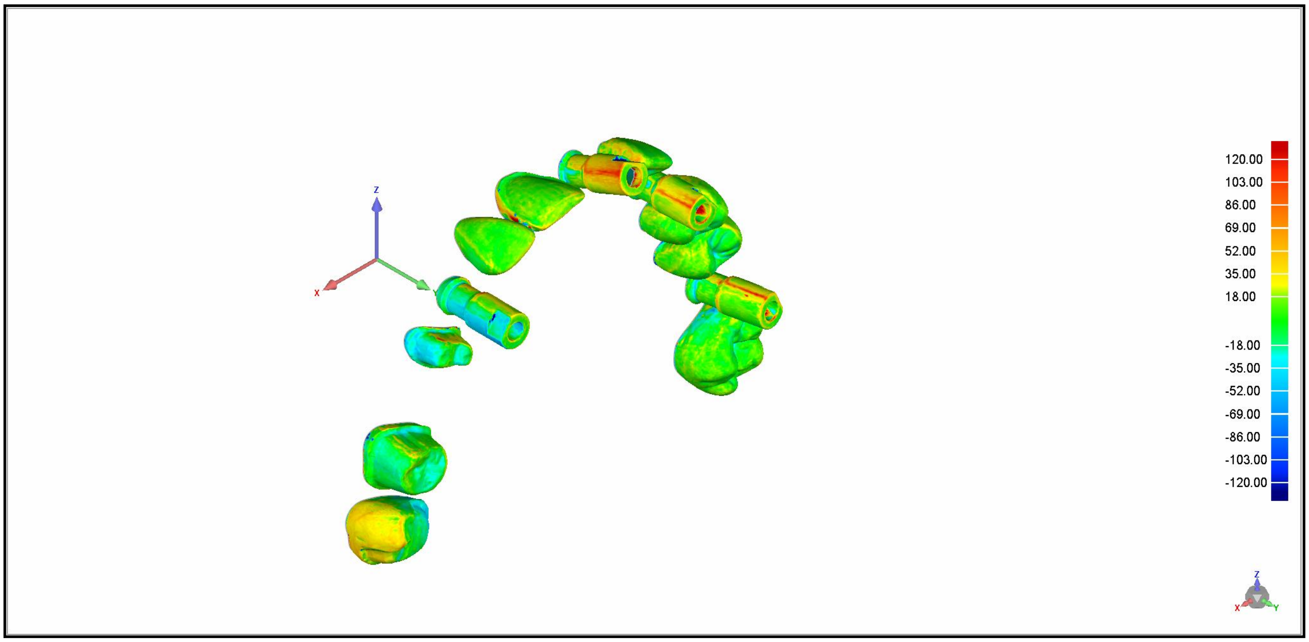

Predefinido: Frente

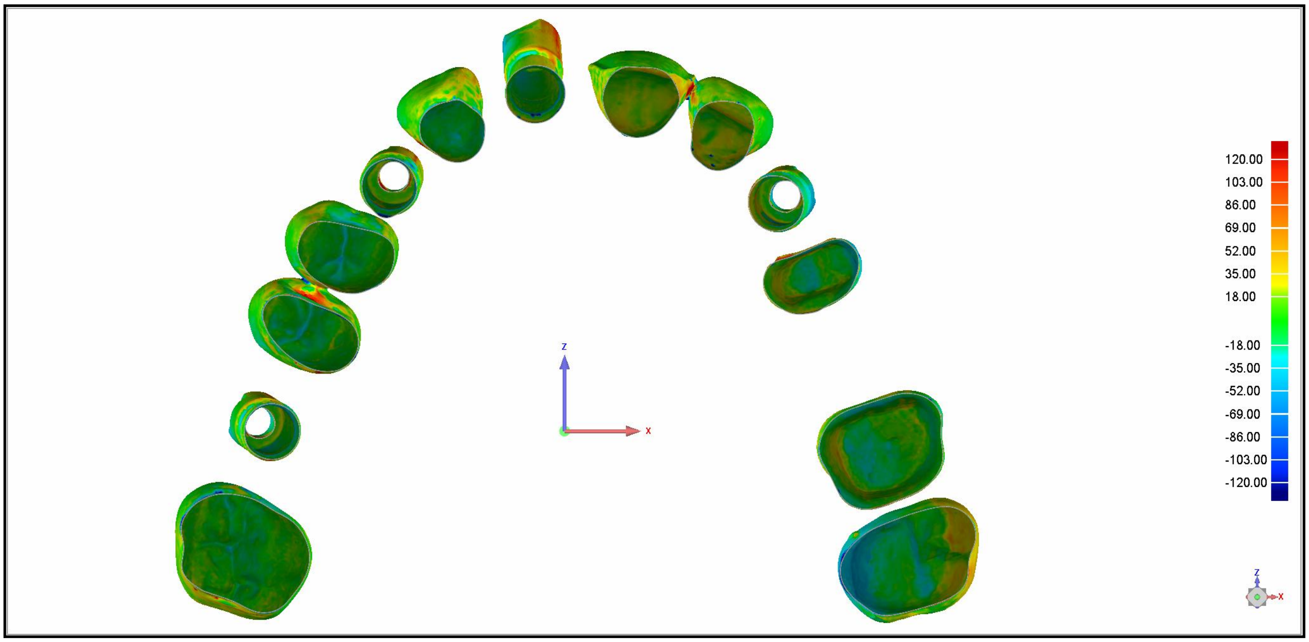

Predefinido: Atrás

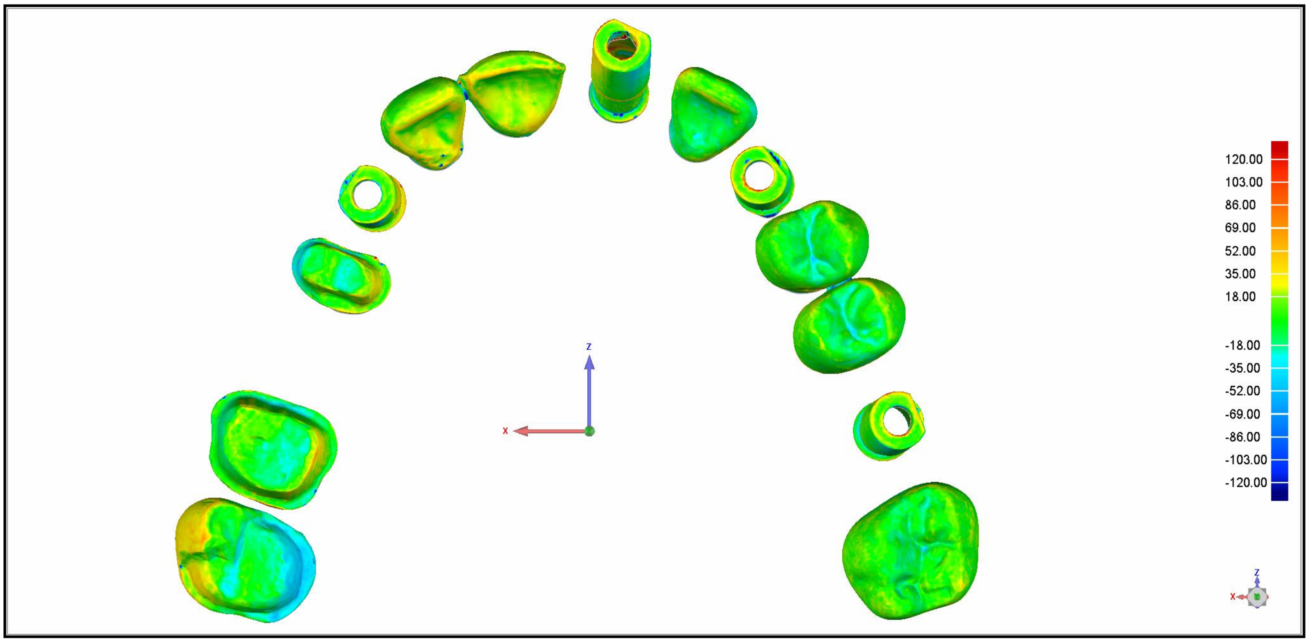

Predefinido: Izquierda

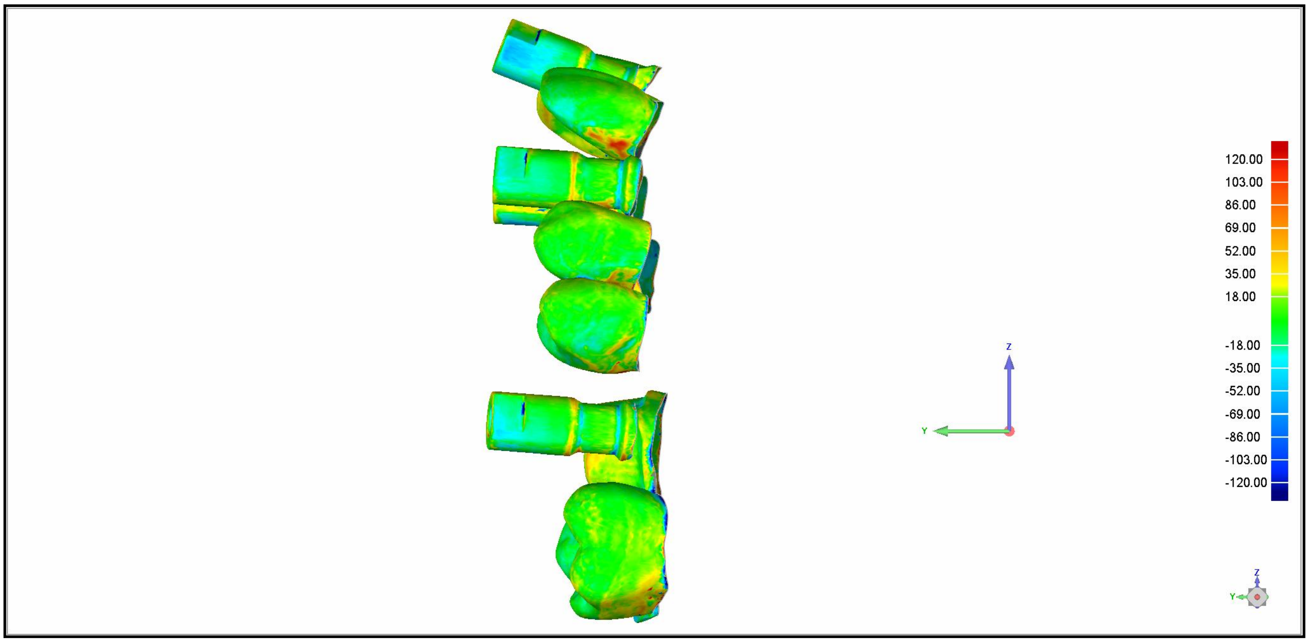

Predefinido: Derecha

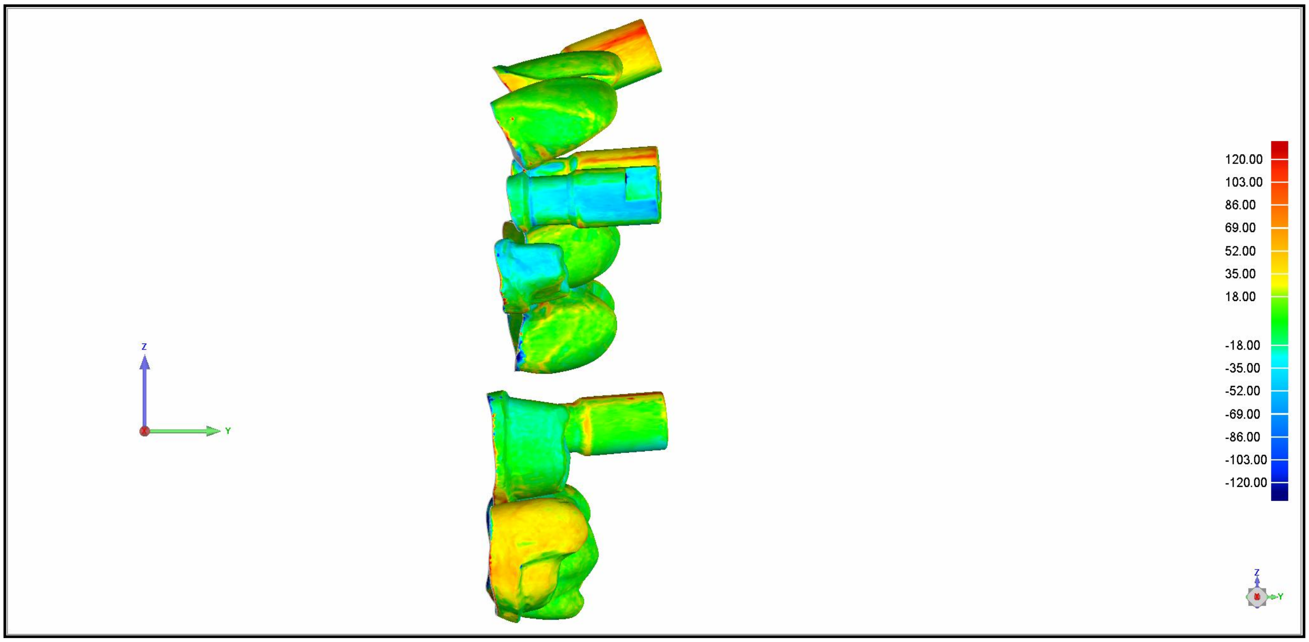

Predefinido: Superior

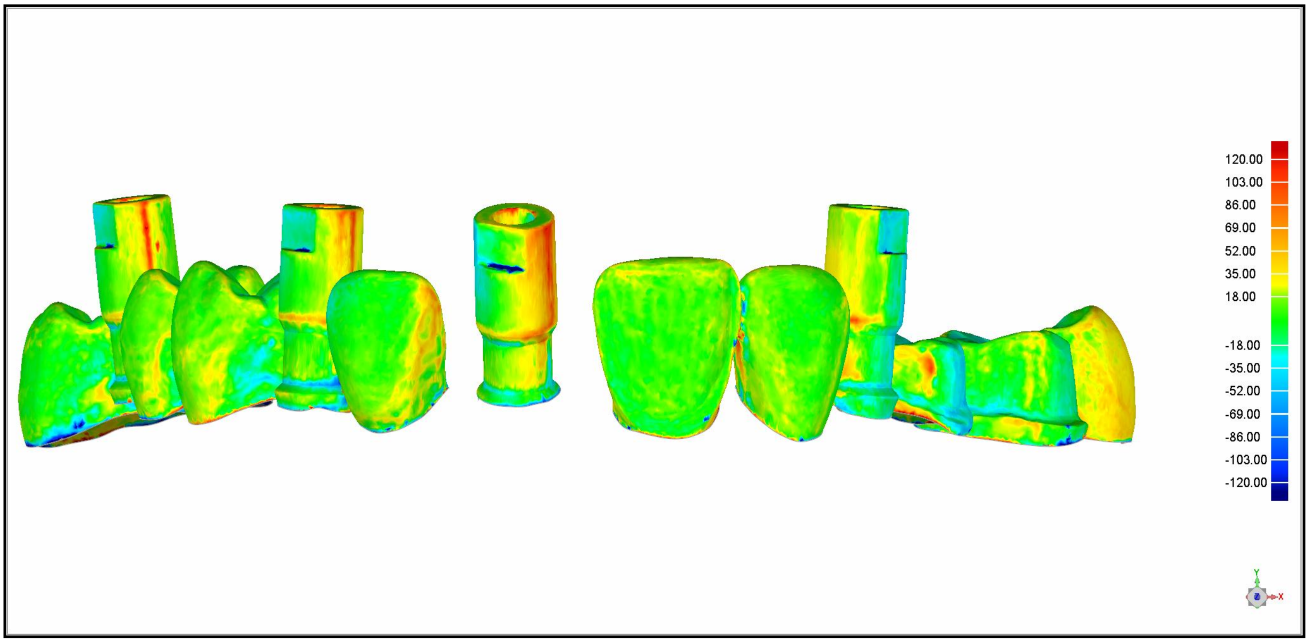

Predefinido: Inferior

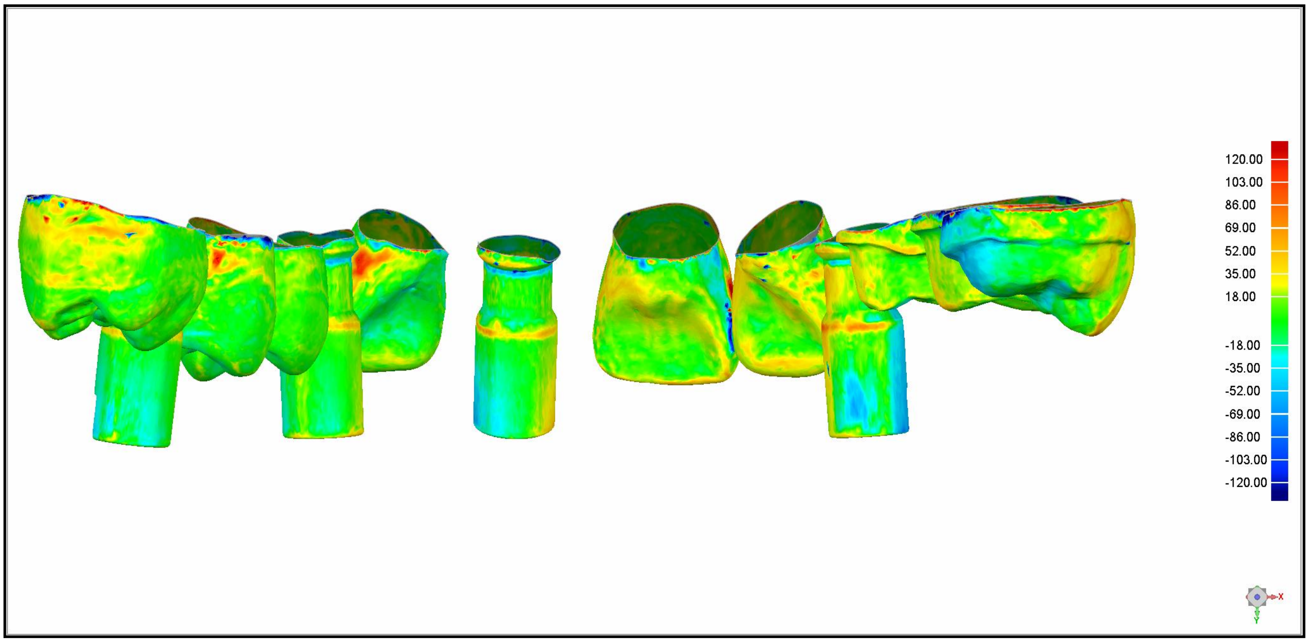

Supplement: S4 Table — Trios (scanning strategy D). (ZIP) [file pone.0202916.s004.zip › S4/3S6D.pdf]
