# Supplementary material for: Accuracy of four digital scanners according to scanning strategy in complete-arch impressions
Source: PLoS One. 2018 Sep 13;13(9):e0202916. doi: 10.1371/journal.pone.0202916 (PMC6136706; doi:10.1371/journal.pone.0202916)
Supplement: S5 Table — iTero (scanning strategy A). (ZIP) [file pone.0202916.s005.zip › S5/IT8A.pdf]

Geomagic Control

Fecha: 3/6/2017, 1:28 pm

3D Comparación Resultados

Modelo referencia	MRC
Modelo test	IT8A
Nº de puntos de datos	83207
# Aislados	781

Tipo tolerancia	3D desviación
Unidades	u
Máx. crítico	120.00
Máx. nominal	10.00
Mín. nominal	-10.00
Mín. crítico	-120.00

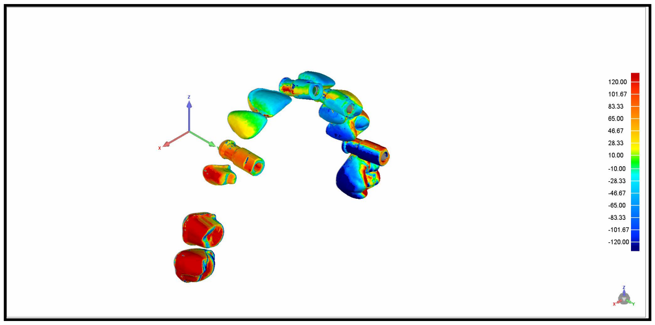

Desviación	
Desviación superior máx.	3155.17
Desviación inferior máx.	-2705.88
Desviación media	113.48 /-111.08
Desviación estándar	248.13

Distribución desviación

>=Min	<Max	# Puntos	%
-120.00	-101.67	1401	1.68
-101.67	-83.33	2016	2.42
-83.33	-65.00	2830	3.40
-65.00	-46.67	4337	5.21
-46.67	-28.33	6663	8.01
-28.33	-10.00	8159	9.81
-10.00	10.00	9037	10.86
10.00	28.33	8063	9.69
28.33	46.67	7319	8.80
46.67	65.00	4605	5.53
65.00	83.33	4059	4.88
83.33	101.67	3009	3.62
101.67	120.00	1835	2.21

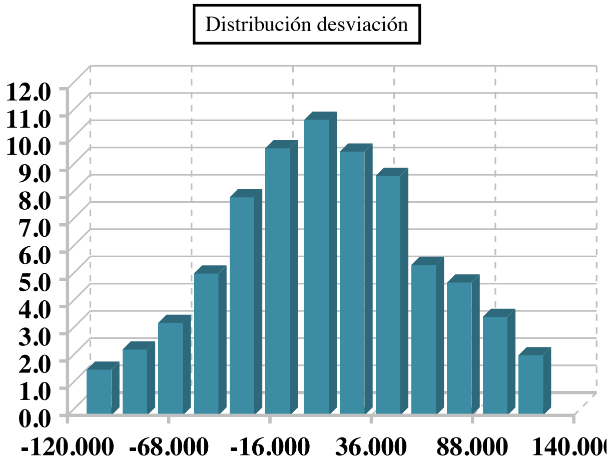

Fuera del crítico superior	11919	14.32
Fuera del crítico inferior	7955	9.56

Desviaciones estándar

Distribución (+/-)	# Puntos	%
-6 * Desv. estánd.	554	0.67
-5 * Desv. estánd.	269	0.32
-4 * Desv. estánd.	246	0.30
-3 * Desv. estánd.	326	0.39
-2 * Desv. estánd.	2107	2.53
-1 * Desv. estánd.	39163	47.07
1 * Desv. estánd.	36918	44.37
2 * Desv. estánd.	2473	2.97
3 * Desv. estánd.	287	0.34
4 * Desv. estánd.	218	0.26
5 * Desv. estánd.	198	0.24
6 * Desv. estánd.	448	0.54

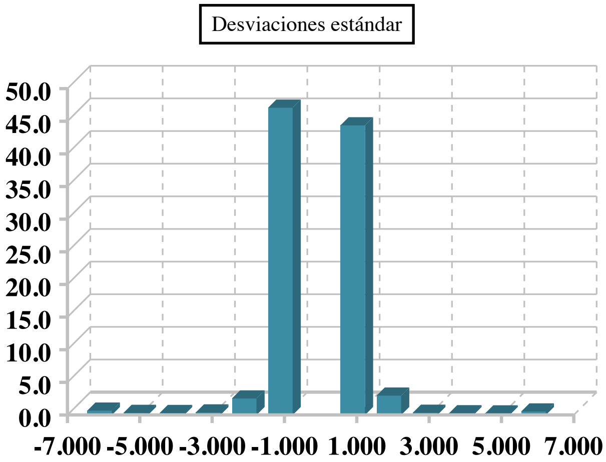

Predefinido: Isométrico

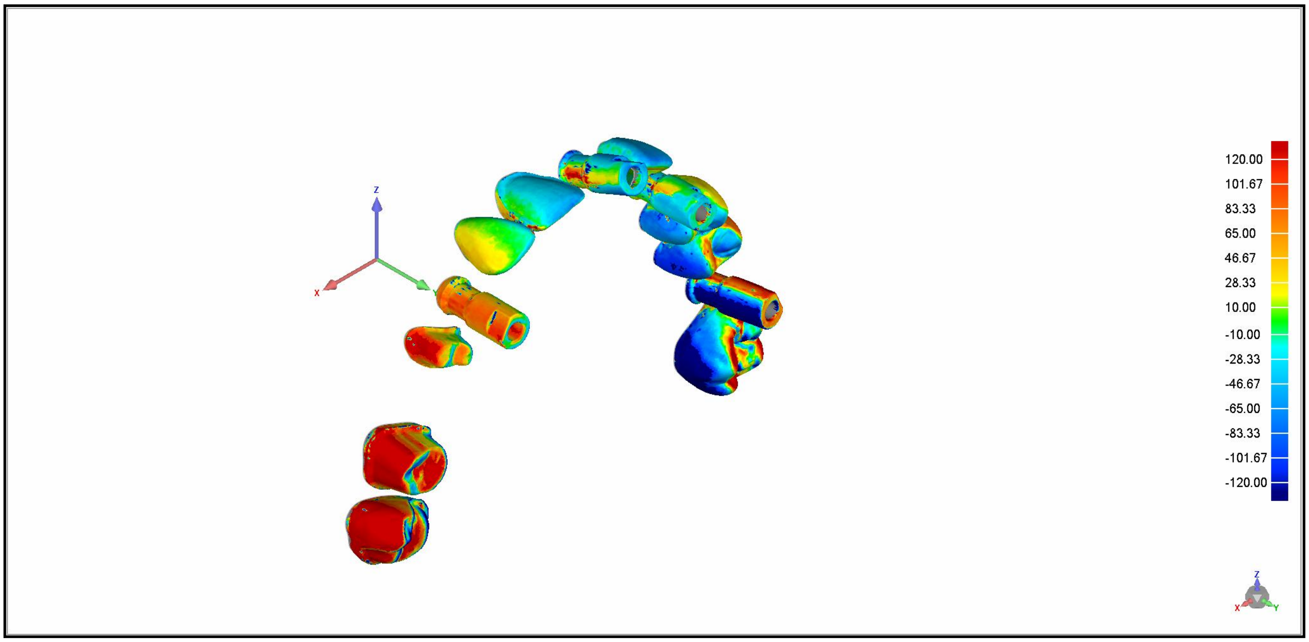

Predefinido: Frente

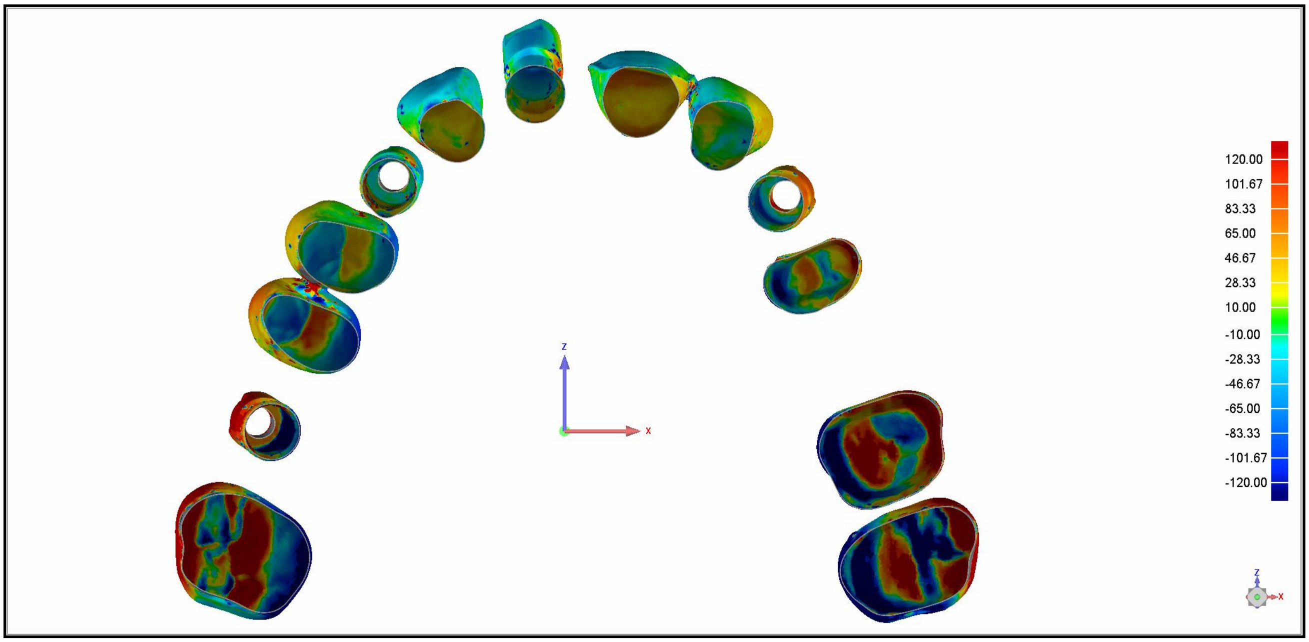

Predefinido: Atrás

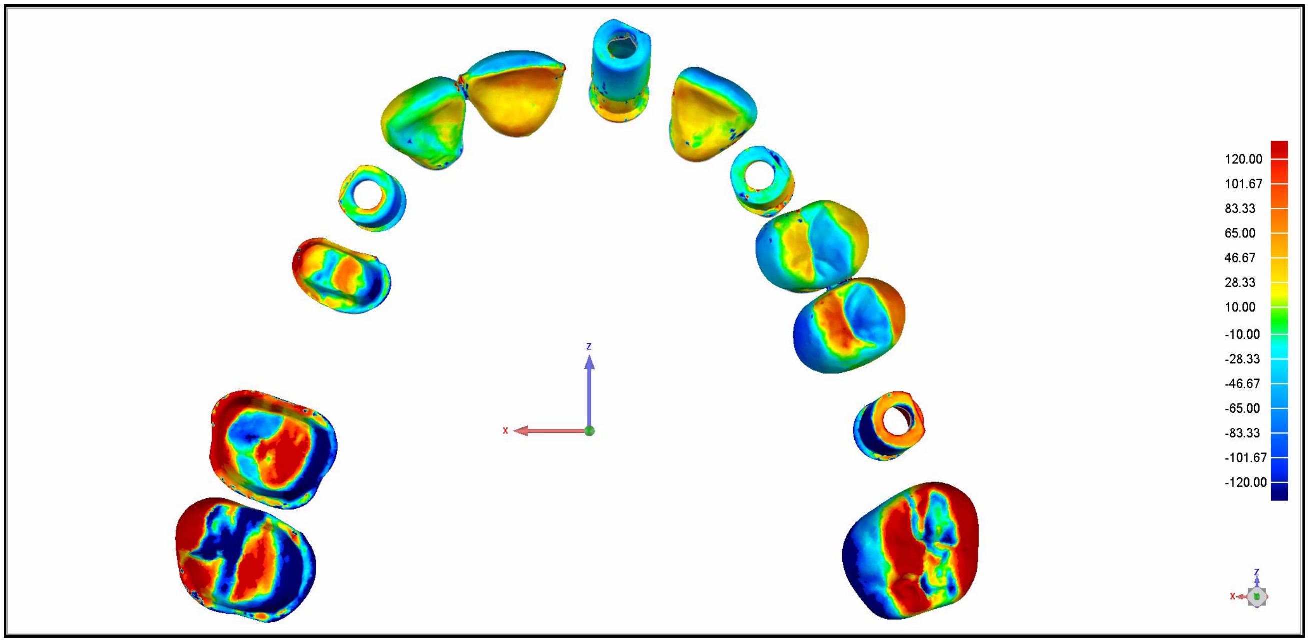

Predefinido: Izquierda

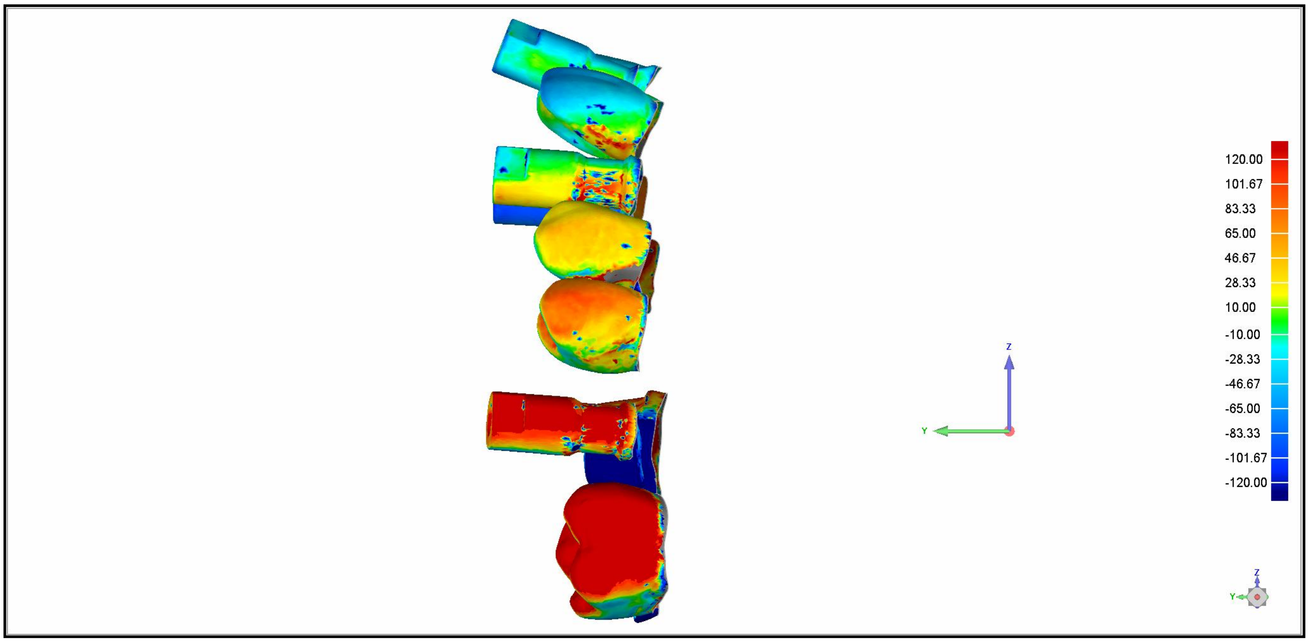

Predefinido: Derecha

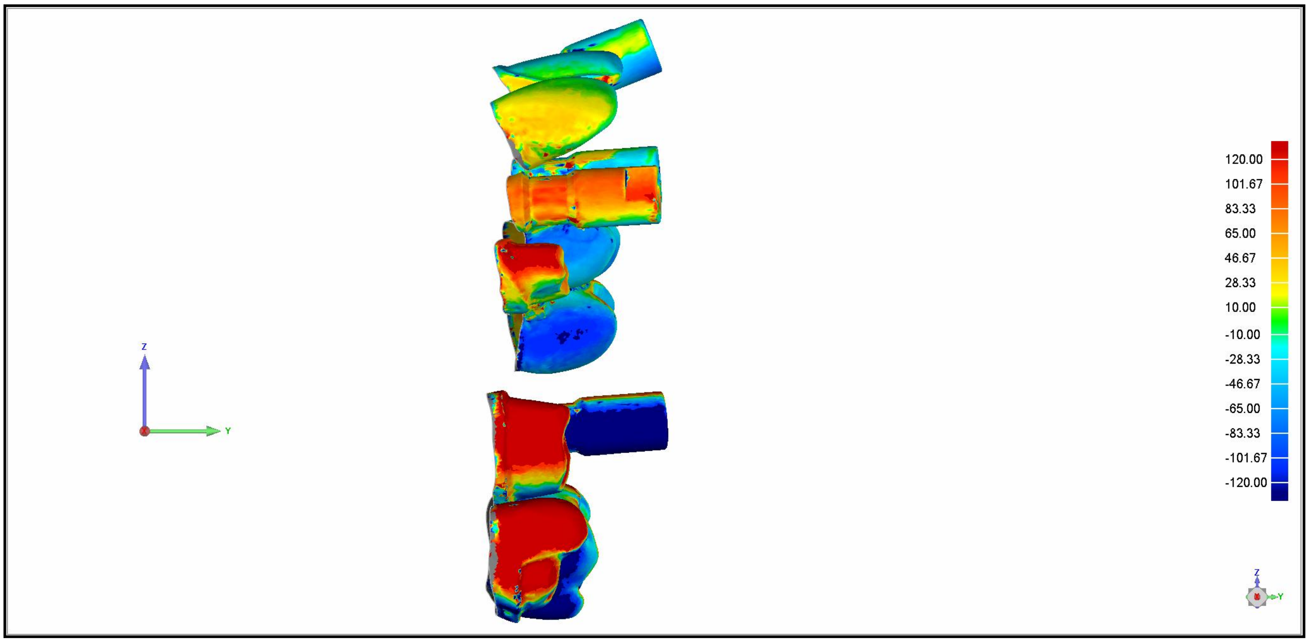

Predefinido: Superior

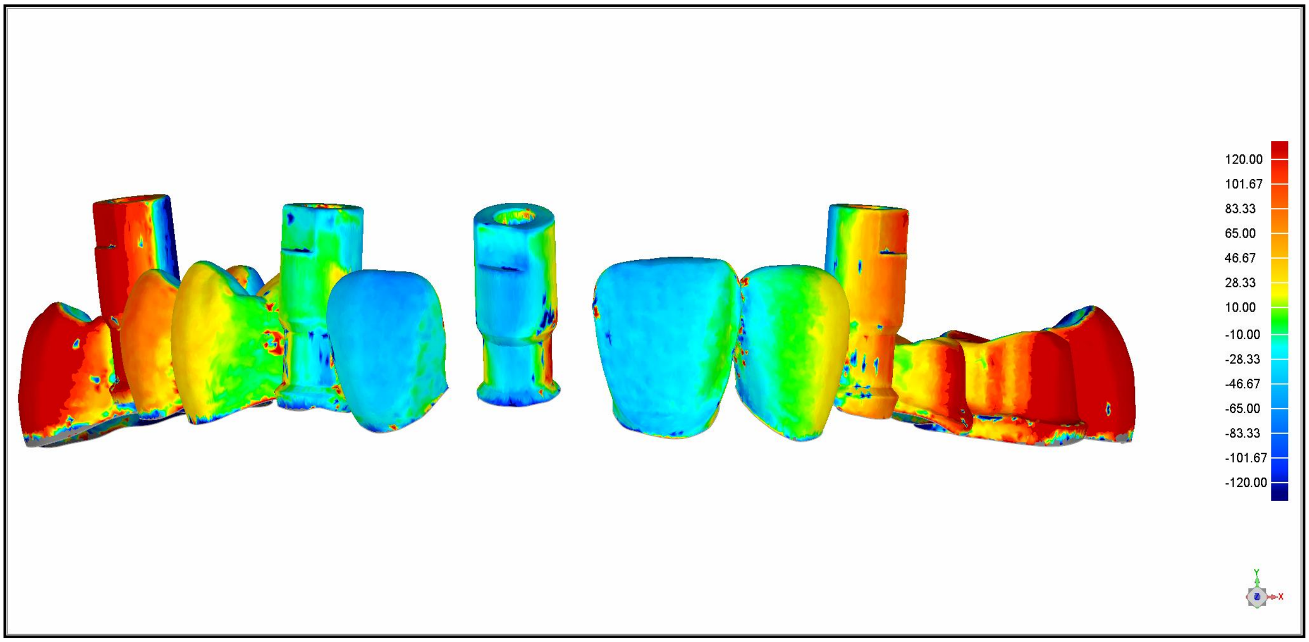

Predefinido: Inferior

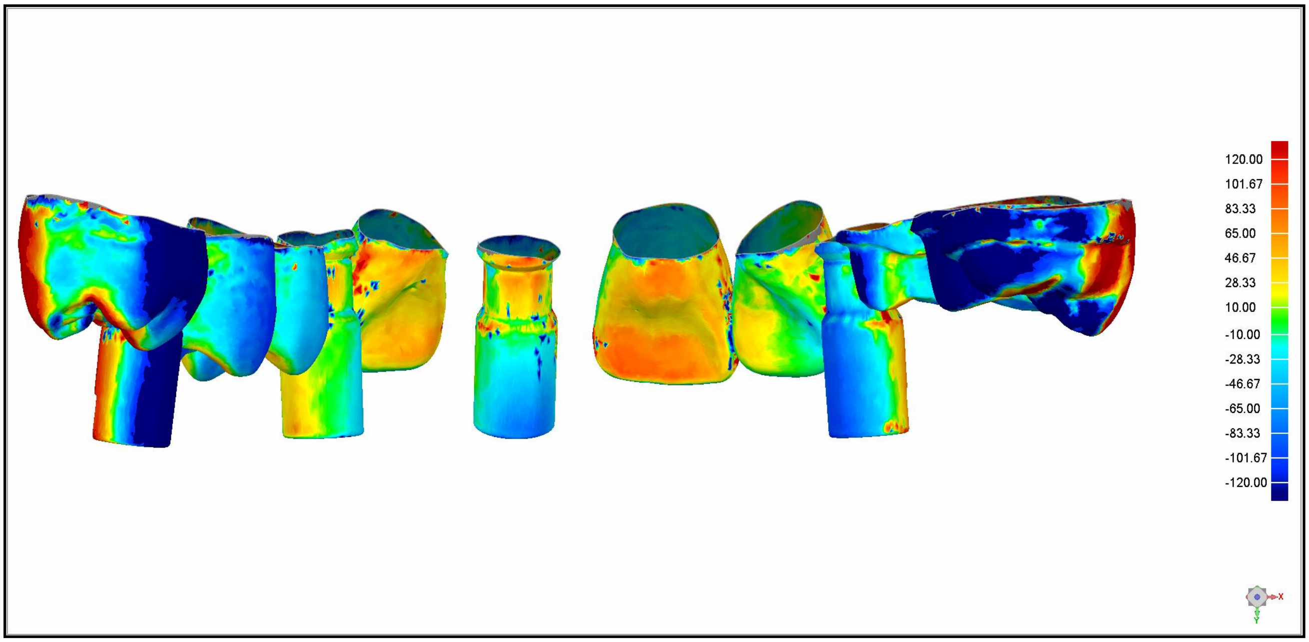

Ajuste de ubicación: Desviaciones superior e inferior

Unidades: u

Nombre	Desv	Estado	Superior Tol	Inferior Tol	Ref X	Ref Y	Ref Z	Radio	Desv X	Desv Y	Desv Z	Medido X	Medido Y	Medido Z	Dir. proy. X	Dir. proy. Y	Dir. proy. Z
Desv. inferior	-2705.88				32064.02	27122.94	-7196.92	n/a	-2224.90	316.54	1507.13	29839.12	27439.47	-5689.78	0.82	-0.12	-0.56
Desv. superior	3155.17				-24545.45	34398.07	-1717.15	n/a	-1233.72	-506.61	-2859.44	-25779.17	33891.46	-4576.60	-0.39	-0.16	-0.91
