# Supplementary material for: Accuracy of four digital scanners according to scanning strategy in complete-arch impressions
Source: PLoS One. 2018 Sep 13;13(9):e0202916. doi: 10.1371/journal.pone.0202916 (PMC6136706; doi:10.1371/journal.pone.0202916)
Supplement: S5 Table — iTero (scanning strategy A). (ZIP) [file pone.0202916.s005.zip › S5/IT4A.pdf]

Fecha: 3/6/2017, 1:18 pm

3D Comparación Resultados

Modelo referencia	MRC
Modelo test	IT4A
Nº de puntos de datos	82673
# Aislados	643

Tipo tolerancia	3D desviación
Unidades	u
Máx. crítico	120.00
Máx. nominal	1.00
Mín. nominal	-1.00
Mín. crítico	-120.00

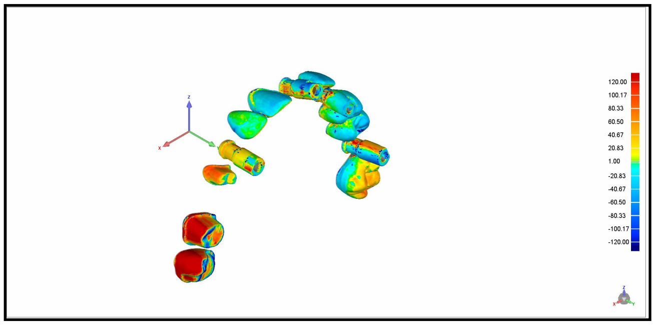

Desviación	
Desviación superior máx.	3148.78
Desviación inferior máx.	-3137.74
Desviación media	99.42 /-95.76
Desviación estándar	297.42

Distribución desviación

>=Min	<Max	# Puntos	%
-120.00	-100.17	937	1.13
-100.17	-80.33	1189	1.44
-80.33	-60.50	1964	2.38
-60.50	-40.67	3608	4.36
-40.67	-20.83	10193	12.33
-20.83	-1.00	17199	20.80
-1.00	1.00	1674	2.02
1.00	20.83	13456	16.28
20.83	40.67	9656	11.68
40.67	60.50	5498	6.65
60.50	80.33	2885	3.49
80.33	100.17	1849	2.24
100.17	120.00	1168	1.41

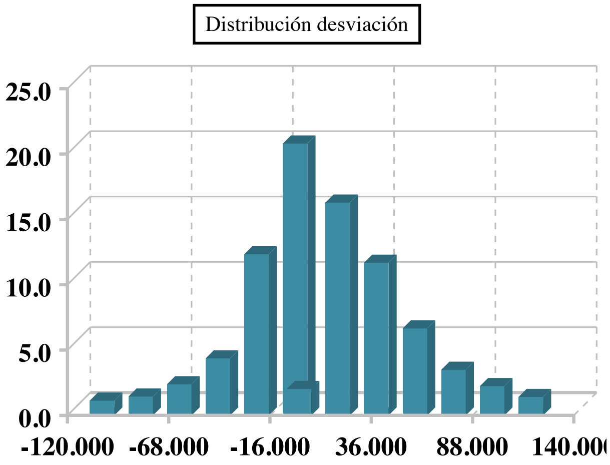

Fuera del crítico superior	5781	6.99
Fuera del crítico inferior	5616	6.79

Desviaciones estándar

Distribución (+/-)	# Puntos	%
-6 * Desv. estánd.	613	0.74
-5 * Desv. estánd.	267	0.32
-4 * Desv. estánd.	258	0.31
-3 * Desv. estánd.	248	0.30
-2 * Desv. estánd.	609	0.74
-1 * Desv. estánd.	40650	49.17
1 * Desv. estánd.	38092	46.08
2 * Desv. estánd.	558	0.67
3 * Desv. estánd.	321	0.39
4 * Desv. estánd.	281	0.34
5 * Desv. estánd.	244	0.30
6 * Desv. estánd.	532	0.64

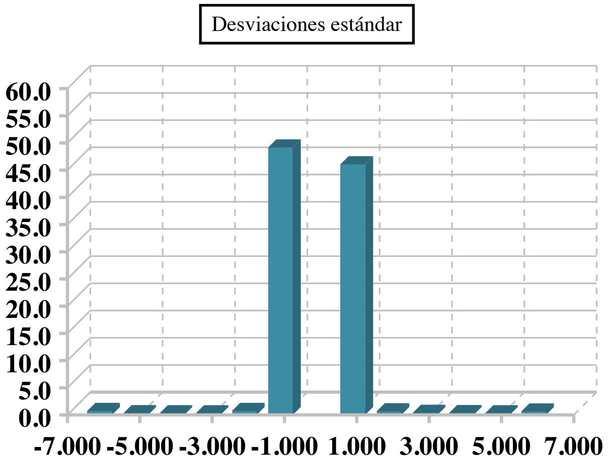

Geomagic Control™

Predefinido: Isométrico

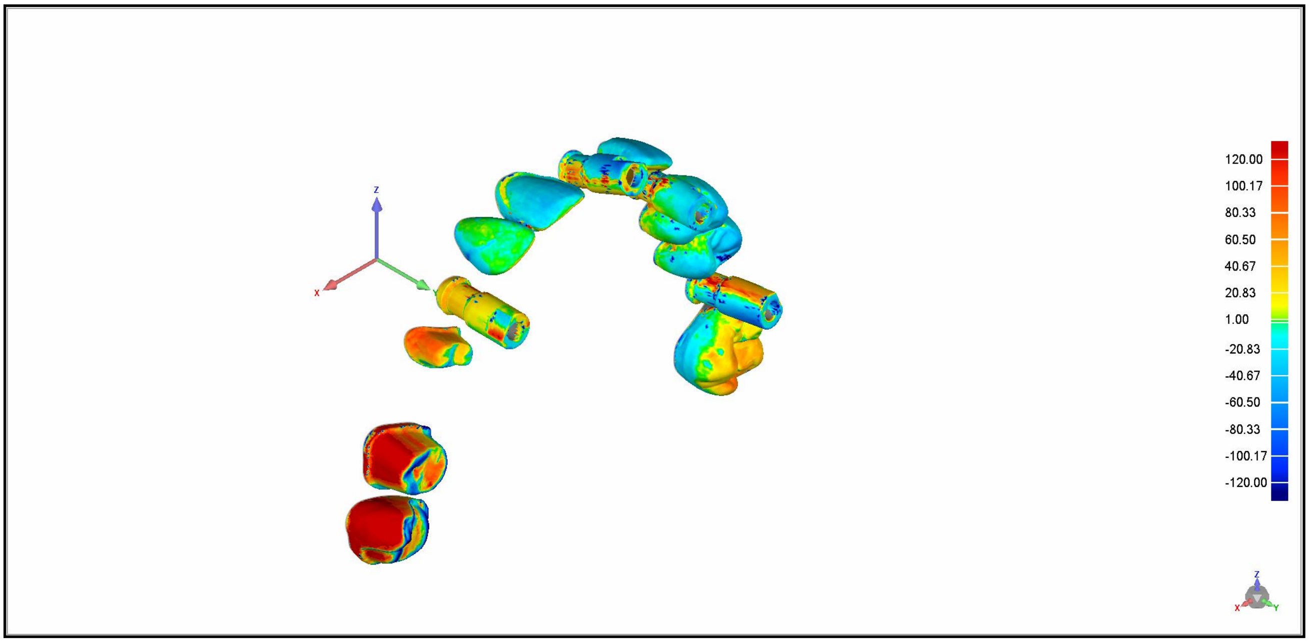

Predefinido: Frente

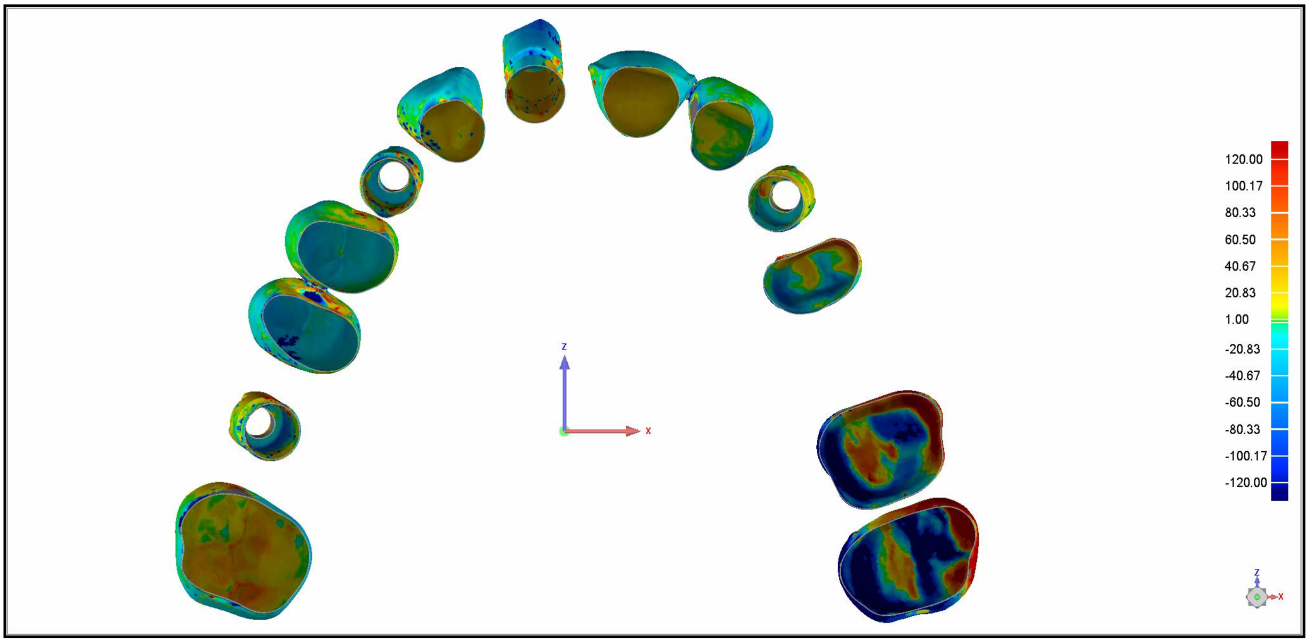

Predefinido: Atrás

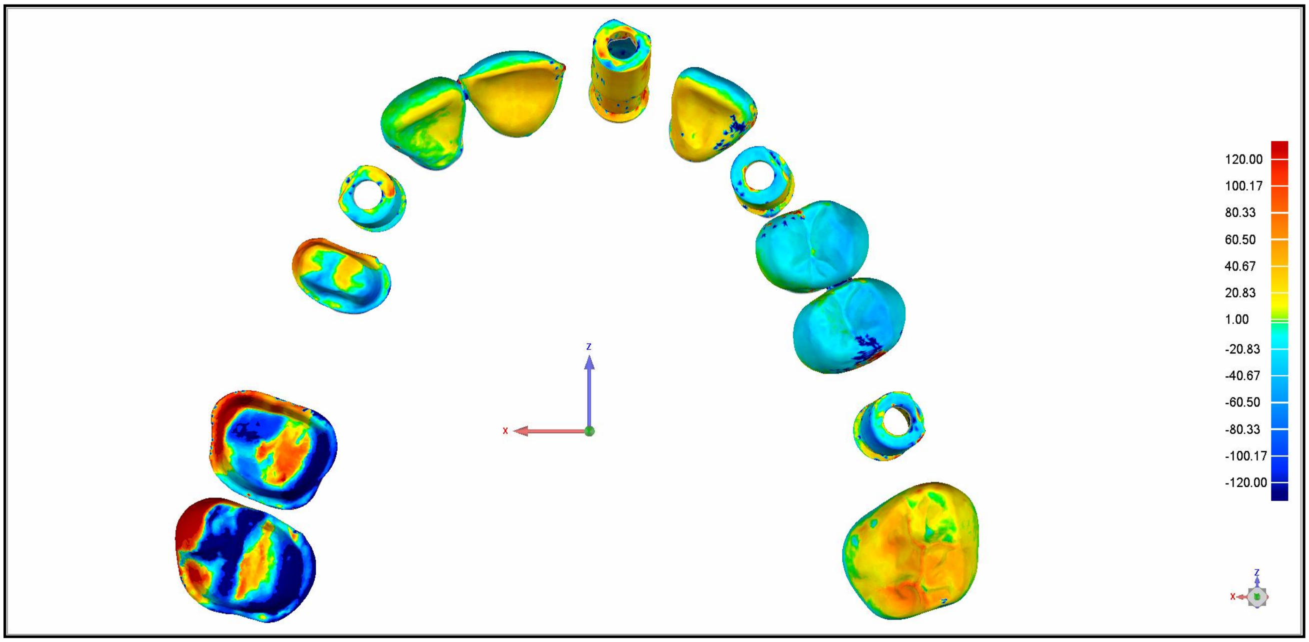

Predefinido: Izquierda

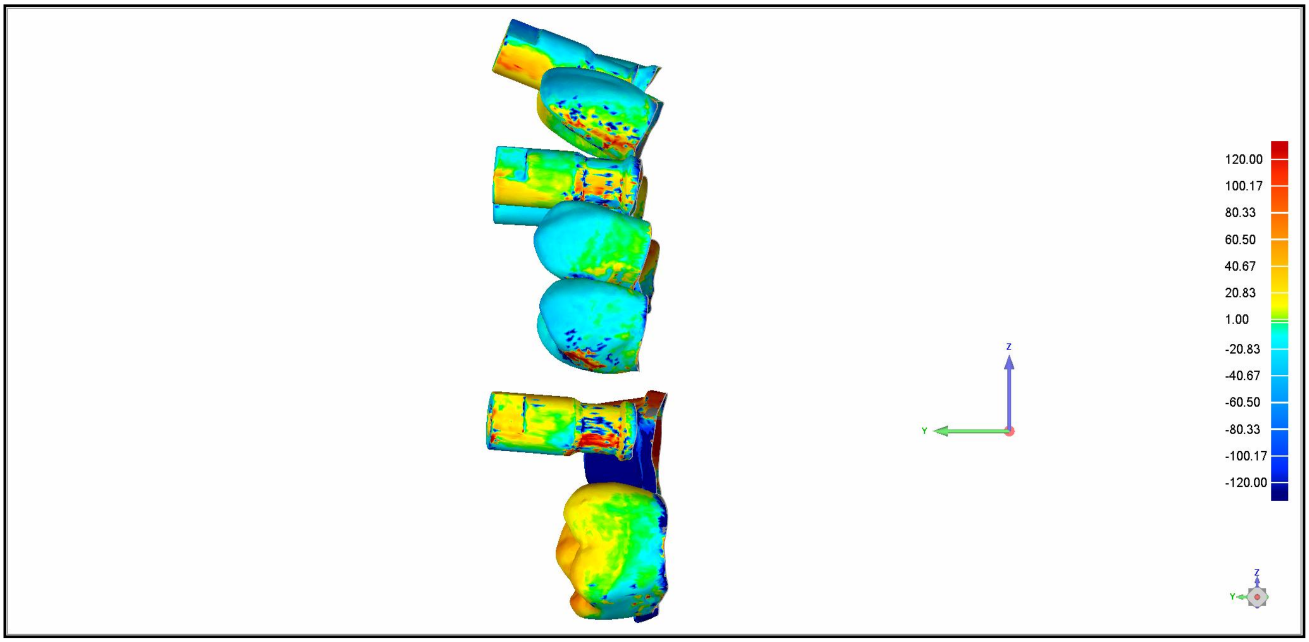

Predefinido: Derecha

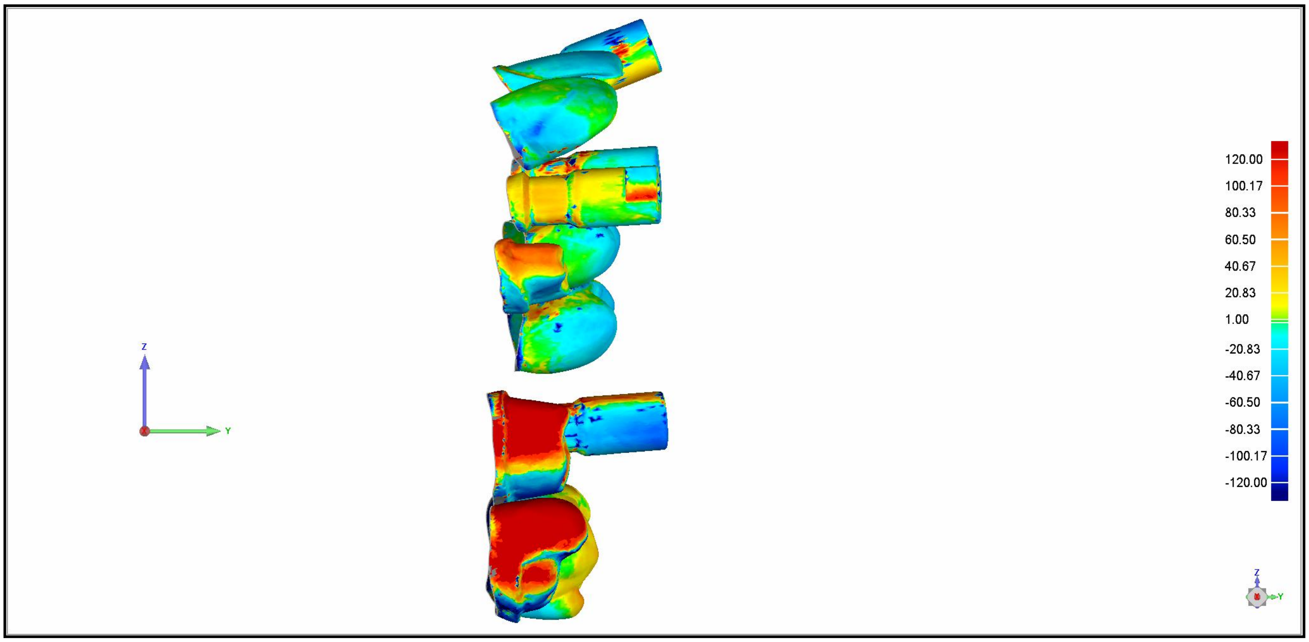

Predefinido: Superior

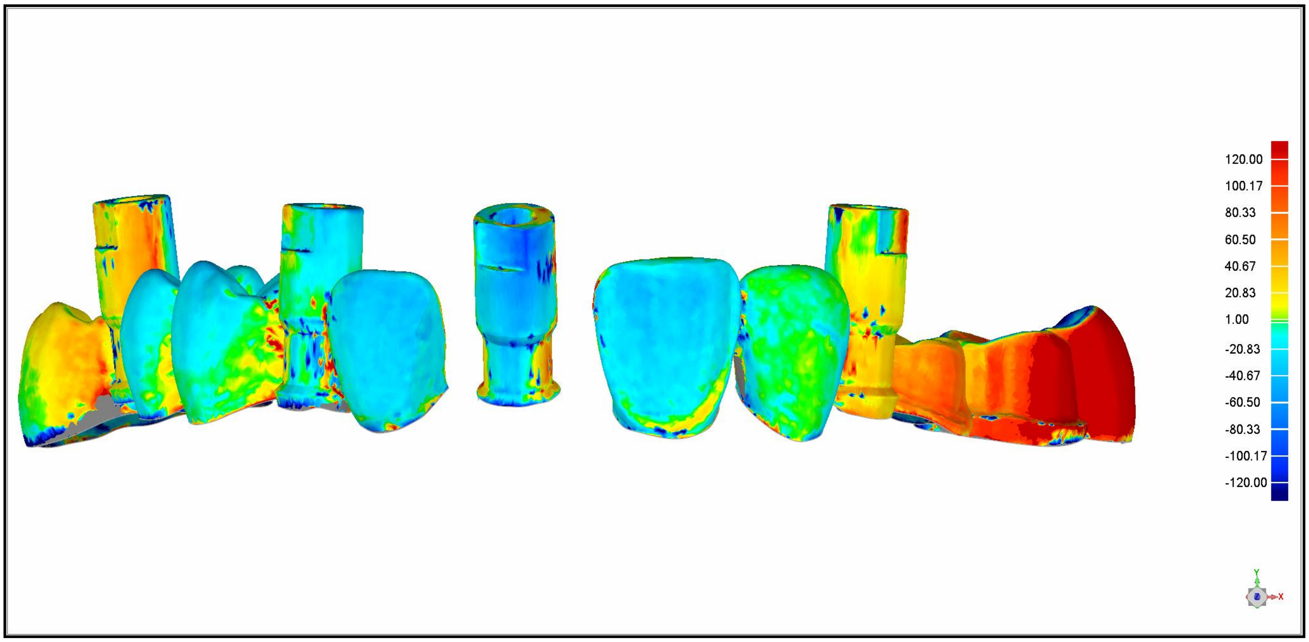

Predefinido: Inferior

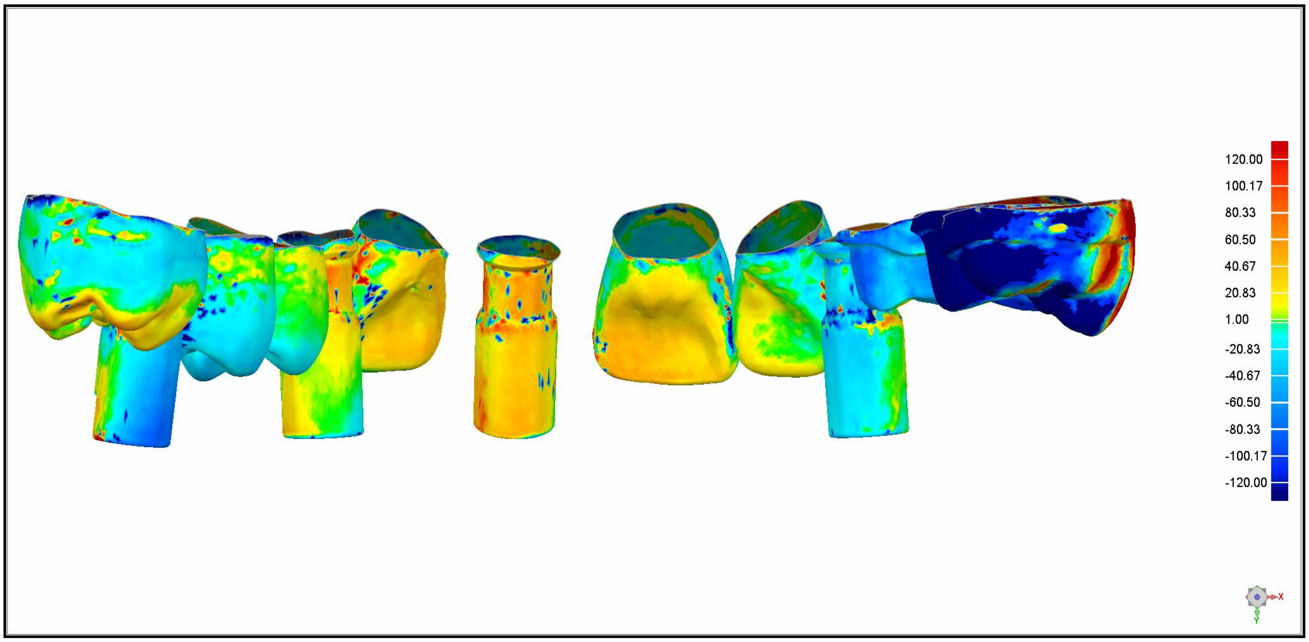

Ajuste de ubicación: Desviaciones superior e inferior

Unidades: u

Nombre	Desv	Estado	Superior Tol	Inferior Tol	Ref X	Ref Y	Ref Z	Radio	Desv X	Desv Y	Desv Z	Medido X	Medido Y	Medido Z	Dir. proy. X	Dir. proy. Y	Dir. proy. Z
Desv. inferior	-3137.74				-29525.66	27048.61	-11629.24	n/a	1708.97	1989.10	-1722.88	-27816.68	29037.71	-13352.13	-0.54	-0.63	0.55
Desv. superior	3148.78				-23263.20	34253.20	2615.95	n/a	494.92	-477.01	3072.84	-22768.27	33776.19	5688.79	0.16	-0.15	0.98
