# Supplementary material for: Accuracy of four digital scanners according to scanning strategy in complete-arch impressions
Source: PLoS One. 2018 Sep 13;13(9):e0202916. doi: 10.1371/journal.pone.0202916 (PMC6136706; doi:10.1371/journal.pone.0202916)
Supplement: S5 Table — iTero (scanning strategy A). (ZIP) [file pone.0202916.s005.zip › S5/IT5A.pdf]

3D Comparación Resultados

Modelo referencia	MRC
Modelo test	IT5A
Nº de puntos de datos	79252
# Aislados	536

Tipo tolerancia	3D desviación
Unidades	u
Máx. crítico	120.00
Máx. nominal	11.00
Mín. nominal	-11.00
Mín. crítico	-120.00

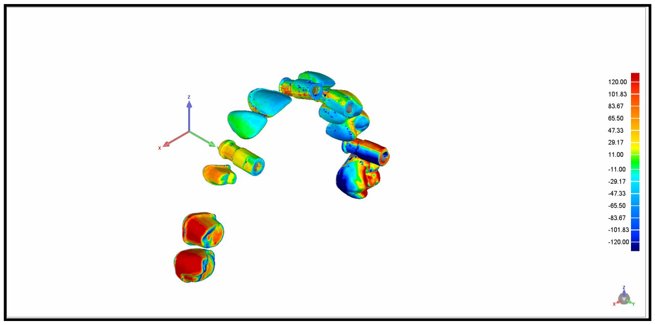

Desviación	
Desviación superior máx.	3141.18
Desviación inferior máx.	-3149.01
Desviación media	115.38 /-104.59
Desviación estándar	304.34

Distribución desviación

>=Min	<Max	# Puntos	%
-120.00	-101.83	1021	1.29
-101.83	-83.67	1317	1.66
-83.67	-65.50	2555	3.22
-65.50	-47.33	4485	5.66
-47.33	-29.17	7438	9.39
-29.17	-11.00	8462	10.68
-11.00	11.00	11830	14.93
11.00	29.17	9018	11.38
29.17	47.33	6944	8.76
47.33	65.50	4569	5.77
65.50	83.67	2915	3.68
83.67	101.83	2226	2.81
101.83	120.00	1956	2.47

Fuera del crítico superior	8502	10.73
Fuera del crítico inferior	6014	7.59

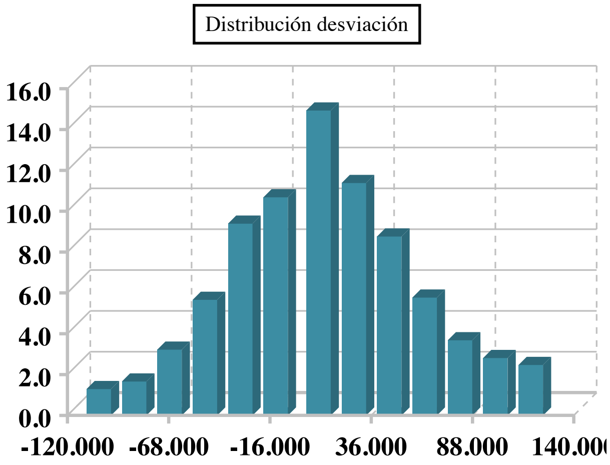

Desviaciones estándar

Distribución (+/-)	# Puntos	%
-6 * Desv. estánd.	473	0.60
-5 * Desv. estánd.	299	0.38
-4 * Desv. estánd.	249	0.31
-3 * Desv. estánd.	283	0.36
-2 * Desv. estánd.	505	0.64
-1 * Desv. estánd.	41728	52.65
1 * Desv. estánd.	33860	42.72
2 * Desv. estánd.	492	0.62
3 * Desv. estánd.	276	0.35
4 * Desv. estánd.	208	0.26
5 * Desv. estánd.	266	0.34
6 * Desv. estánd.	613	0.77

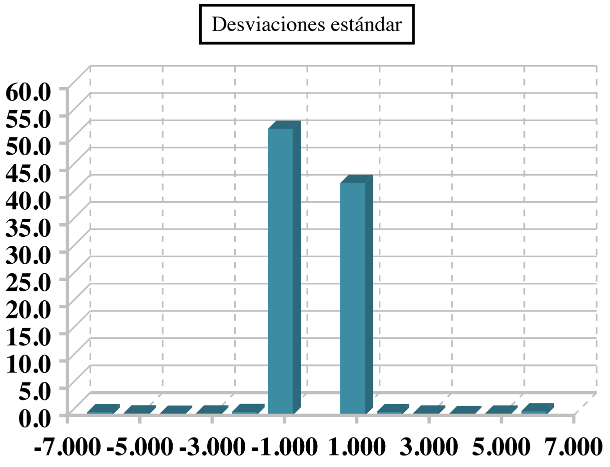

Predefinido: Isométrico

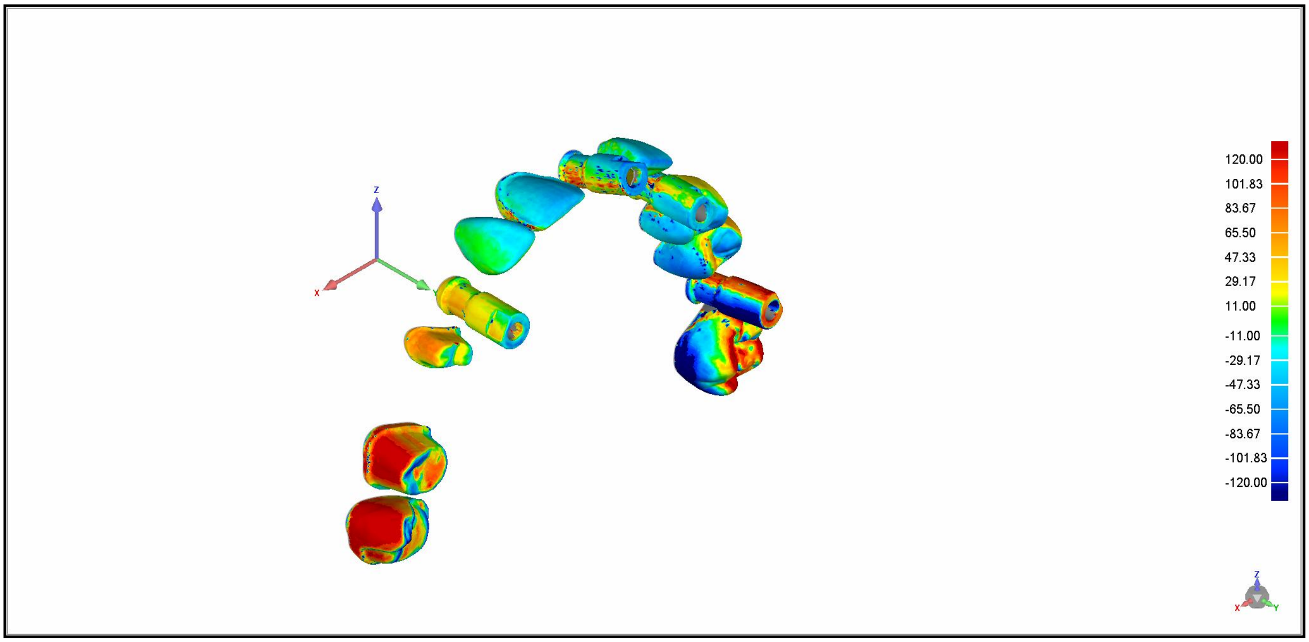

Predefinido: Frente

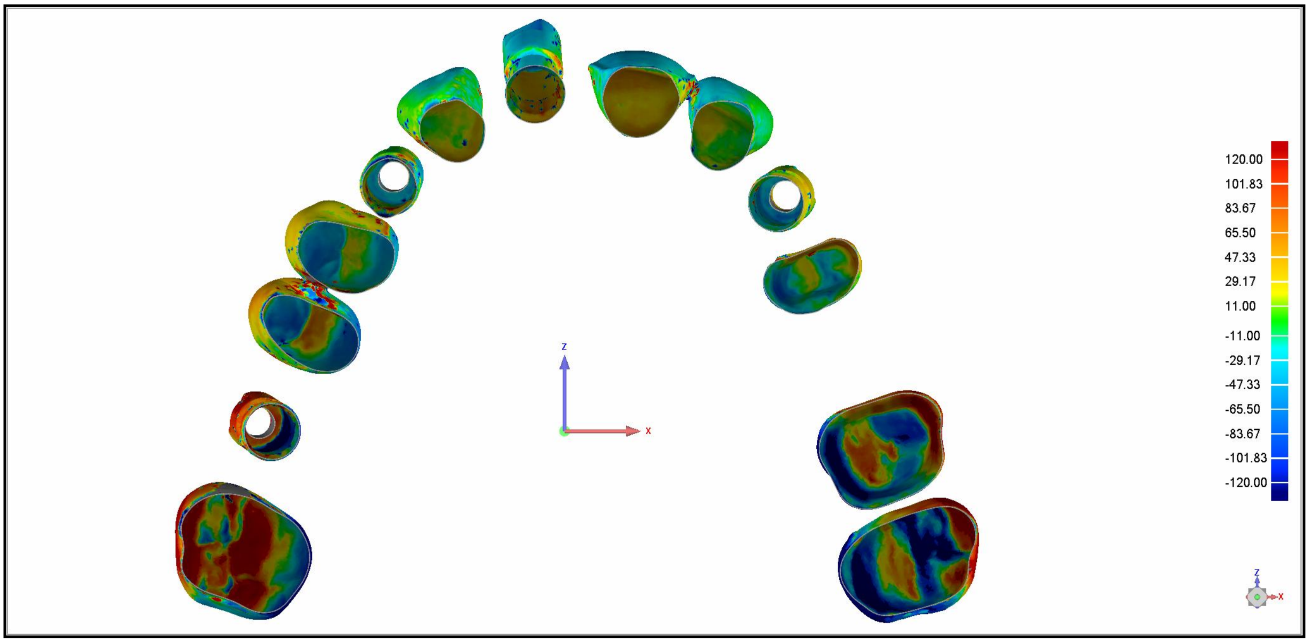

Predefinido: Atrás

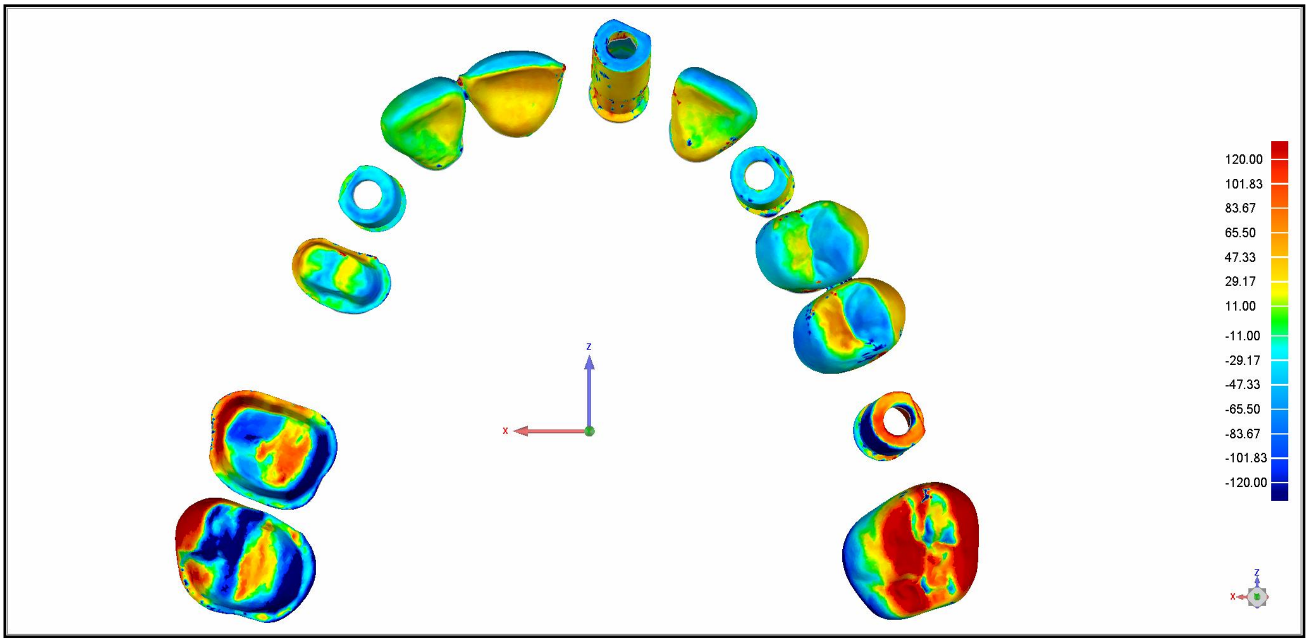

Predefinido: Izquierda

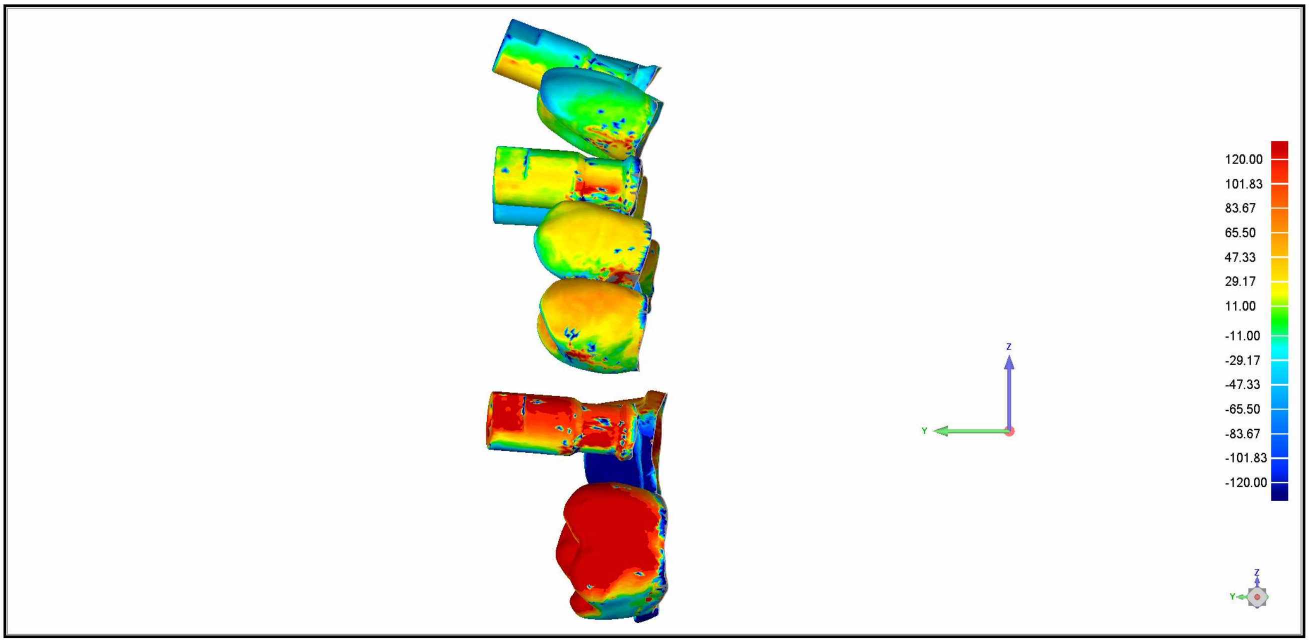

Predefinido: Derecha

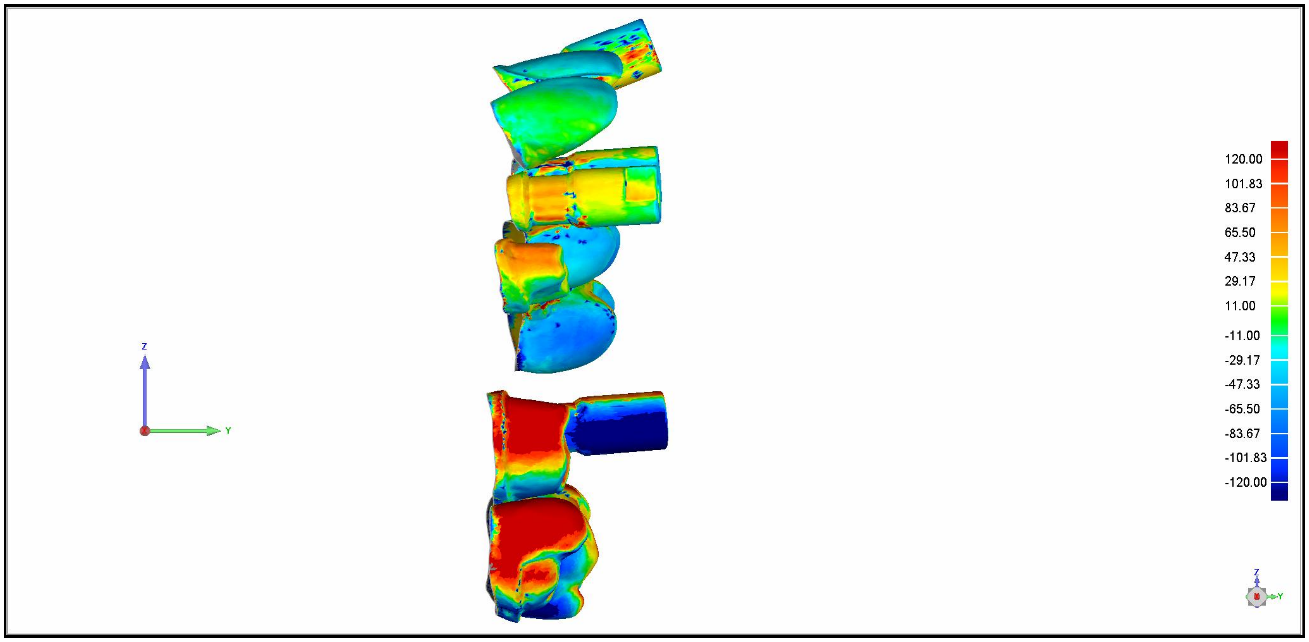

Predefinido: Superior

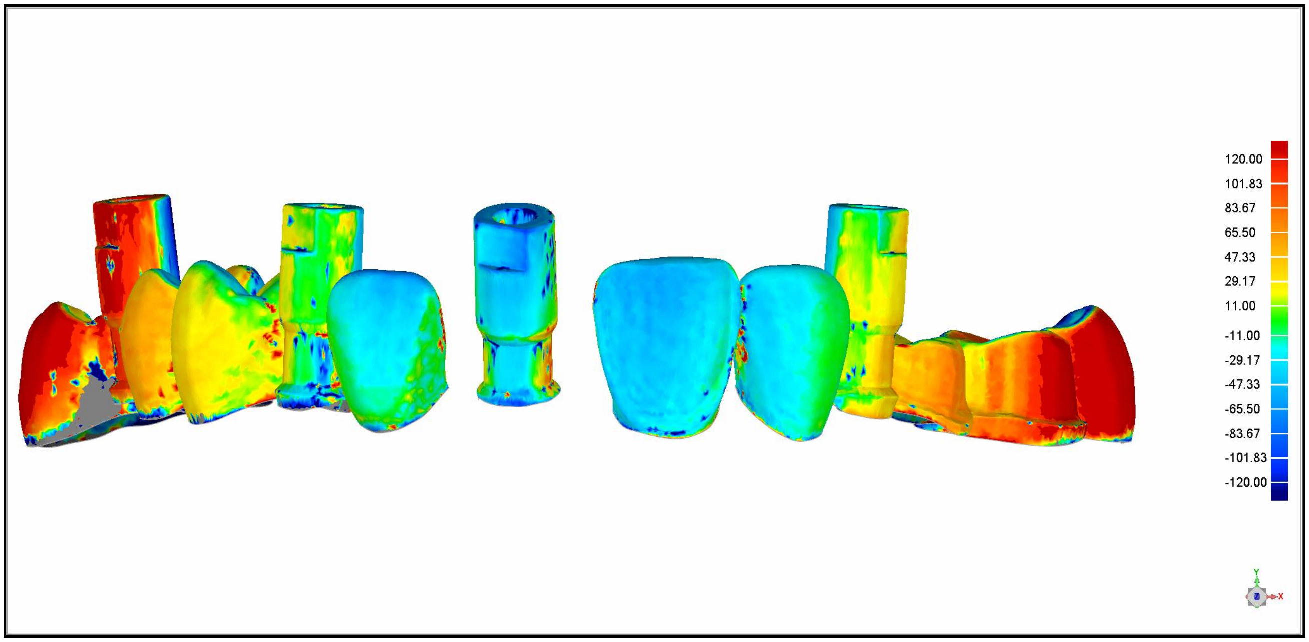

Predefinido: Inferior

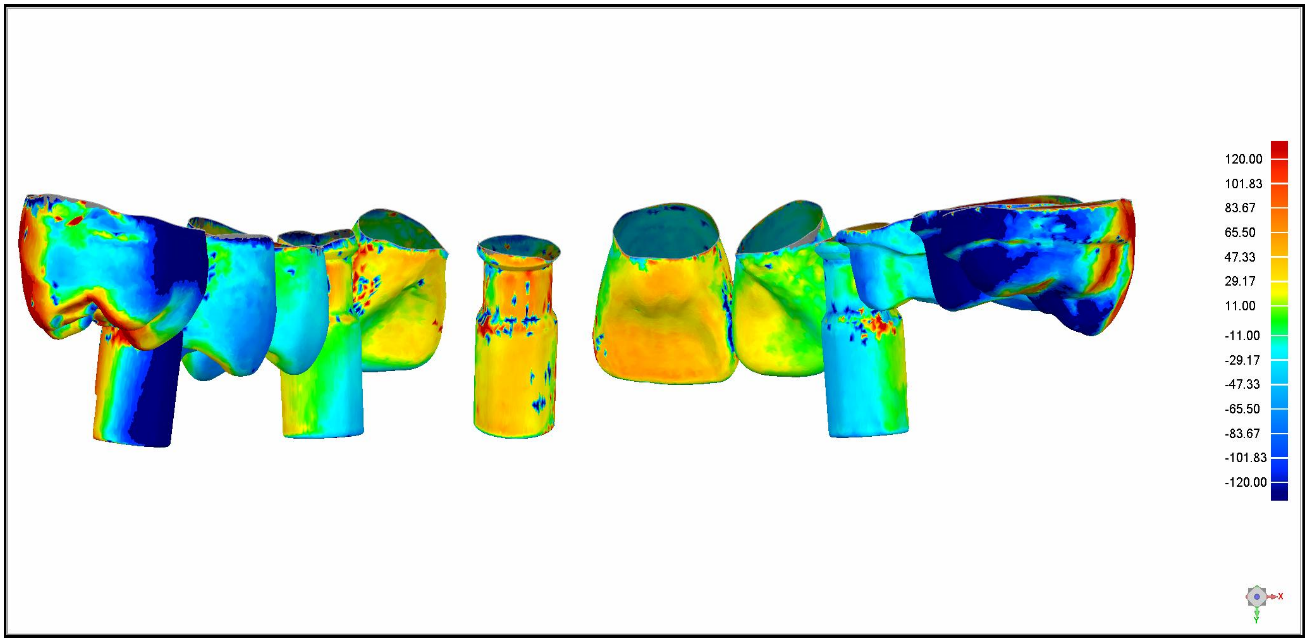

Ajuste de ubicación: Desviaciones superior e inferior

Unidades: u

Nombre	Desv	Estado	Superior Tol	Inferior Tol	Ref X	Ref Y	Ref Z	Radio	Desv X	Desv Y	Desv Z	Medido X	Medido Y	Medido Z	Dir. proy. X	Dir. proy. Y	Dir. proy. Z
Desv. inferior	-3149.01				-23318.94	38082.95	-378.92	n/a	-2172.14	2153.72	748.04	-25491.08	40236.67	369.11	0.69	-0.68	-0.24
Desv. superior	3141.18				-20319.31	30890.31	12269.01	n/a	-78.92	-2494.23	1907.77	-20398.23	28396.08	14176.78	-0.03	-0.79	0.61
