# Supplementary material for: Accuracy of four digital scanners according to scanning strategy in complete-arch impressions
Source: PLoS One. 2018 Sep 13;13(9):e0202916. doi: 10.1371/journal.pone.0202916 (PMC6136706; doi:10.1371/journal.pone.0202916)
Supplement: S5 Table — iTero (scanning strategy A). (ZIP) [file pone.0202916.s005.zip › S5/IT9A.pdf]

Fecha: 3/6/2017, 1:30 pm

3D Comparación Resultados

Modelo referencia	MRC
Modelo test	IT9A
Nº de puntos de datos	83480
# Aislados	647

Tipo tolerancia	3D desviación
Unidades	u
Máx. crítico	120.00
Máx. nominal	8.00
Mín. nominal	-8.00
Mín. crítico	-120.00

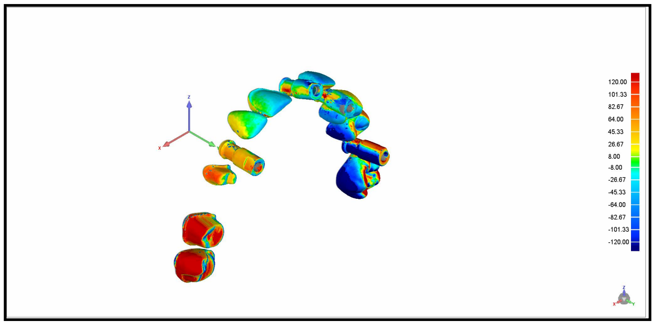

Desviación	
Desviación superior máx.	3132.51
Desviación inferior máx.	-3145.68
Desviación media	112.28 /-106.56
Desviación estándar	249.99

Distribución desviación

>=Min	<Max	# Puntos	%
-120.00	-101.33	1677	2.01
-101.33	-82.67	2073	2.48
-82.67	-64.00	3316	3.97
-64.00	-45.33	4793	5.74
-45.33	-26.67	6599	7.90
-26.67	-8.00	8901	10.66
-8.00	8.00	7525	9.01
8.00	26.67	8400	10.06
26.67	45.33	7358	8.81
45.33	64.00	5973	7.16
64.00	82.67	3773	4.52
82.67	101.33	2728	3.27
101.33	120.00	1649	1.98

Fuera del crítico superior	10430	12.49
Fuera del crítico inferior	8285	9.92

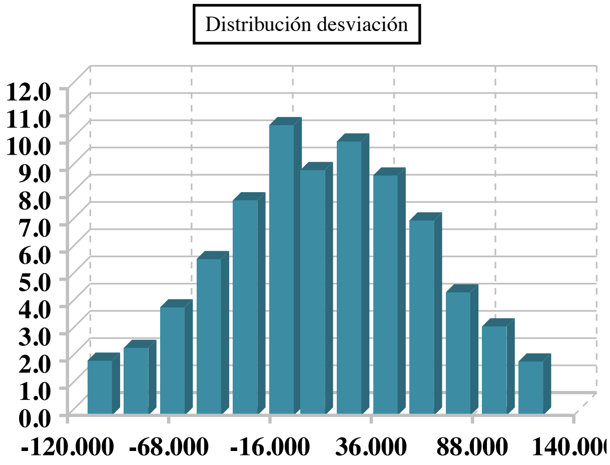

Desviaciones estándar

Distribución (+/-)	# Puntos	%
-6 * Desv. estánd.	500	0.60
-5 * Desv. estánd.	267	0.32
-4 * Desv. estánd.	232	0.28
-3 * Desv. estánd.	246	0.29
-2 * Desv. estánd.	1682	2.01
-1 * Desv. estánd.	40525	48.54
1 * Desv. estánd.	36452	43.67
2 * Desv. estánd.	2307	2.76
3 * Desv. estánd.	248	0.30
4 * Desv. estánd.	196	0.23
5 * Desv. estánd.	223	0.27
6 * Desv. estánd.	602	0.72

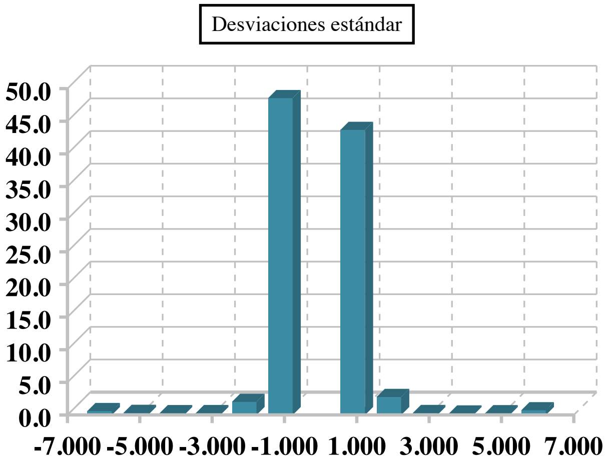

Predefinido: Isométrico

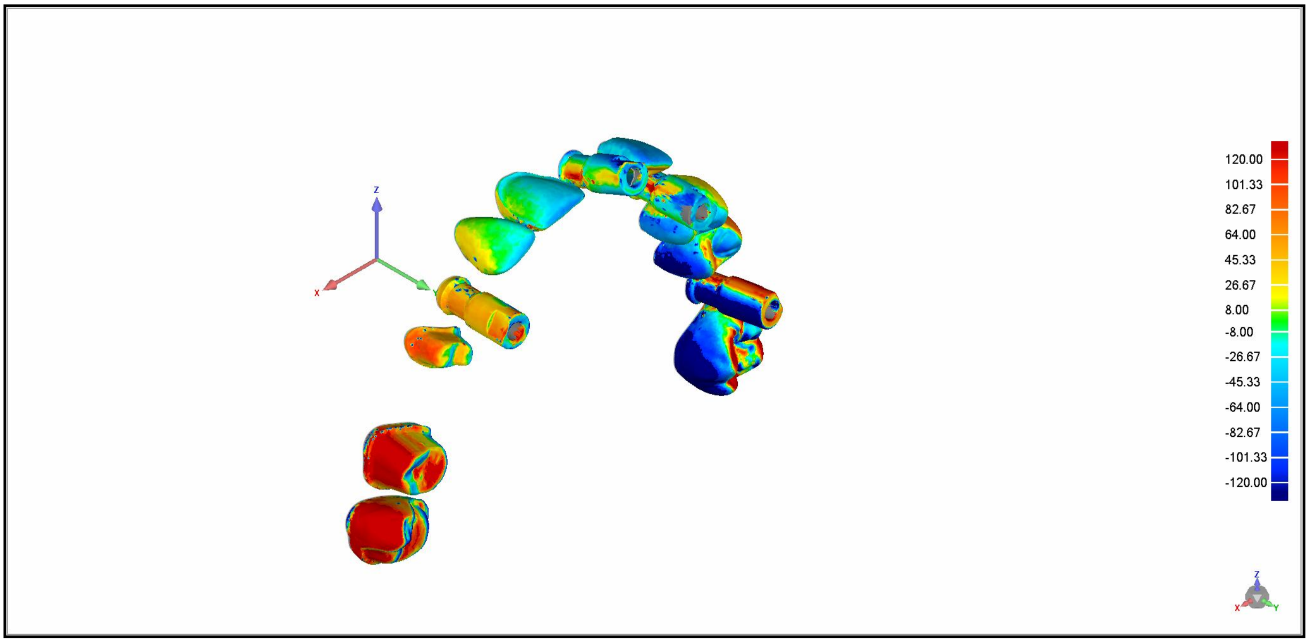

Predefinido: Frente

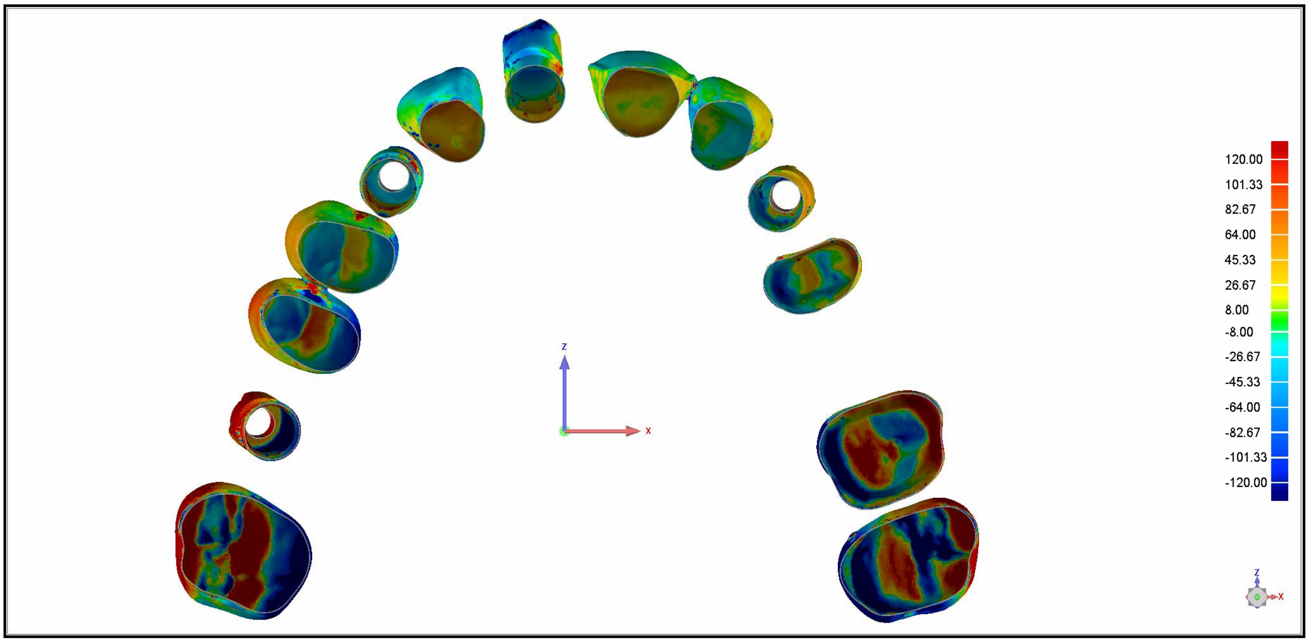

Predefinido: Atrás

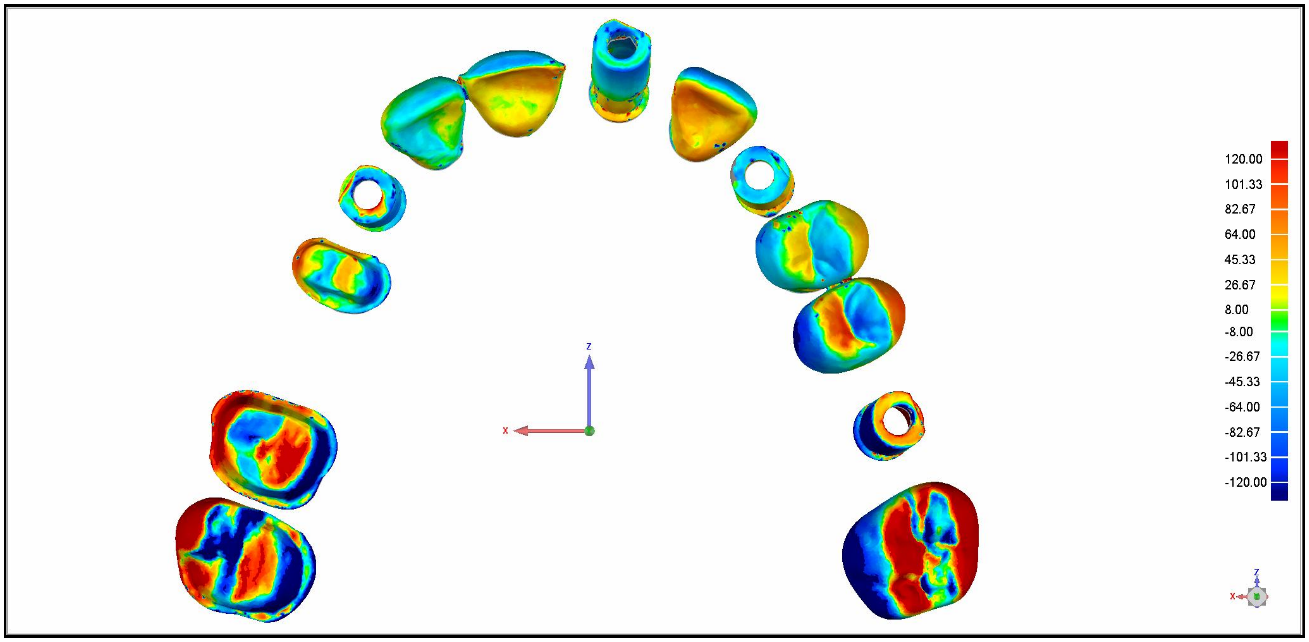

Predefinido: Izquierda

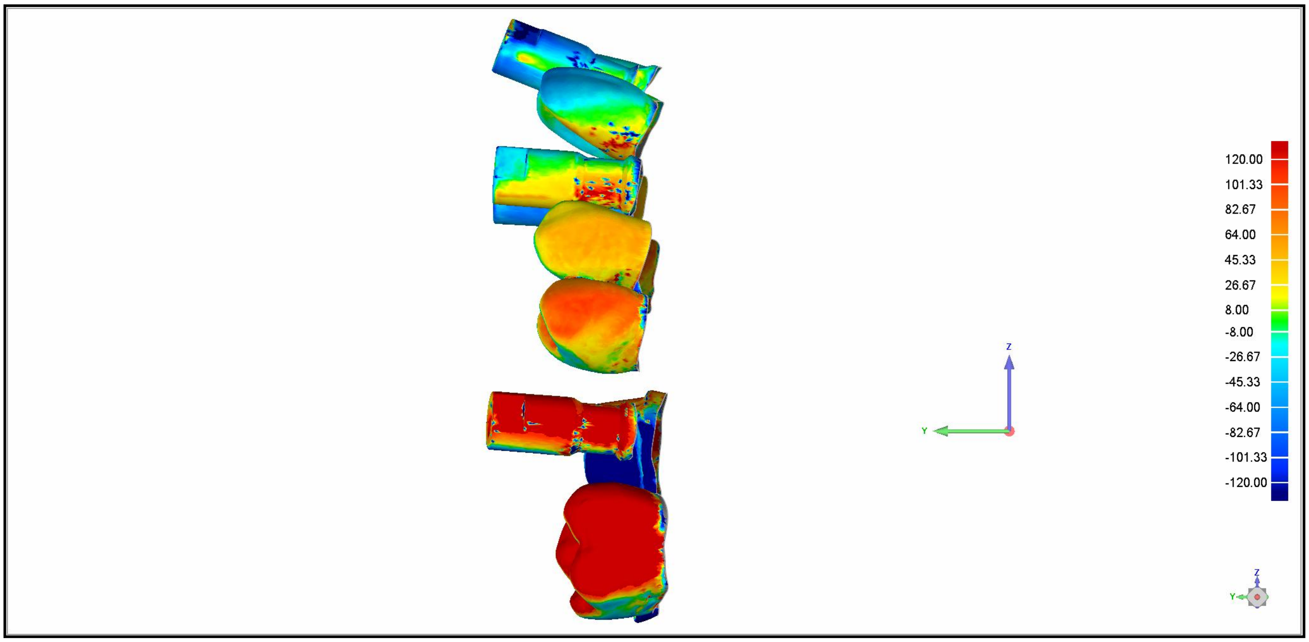

Predefinido: Derecha

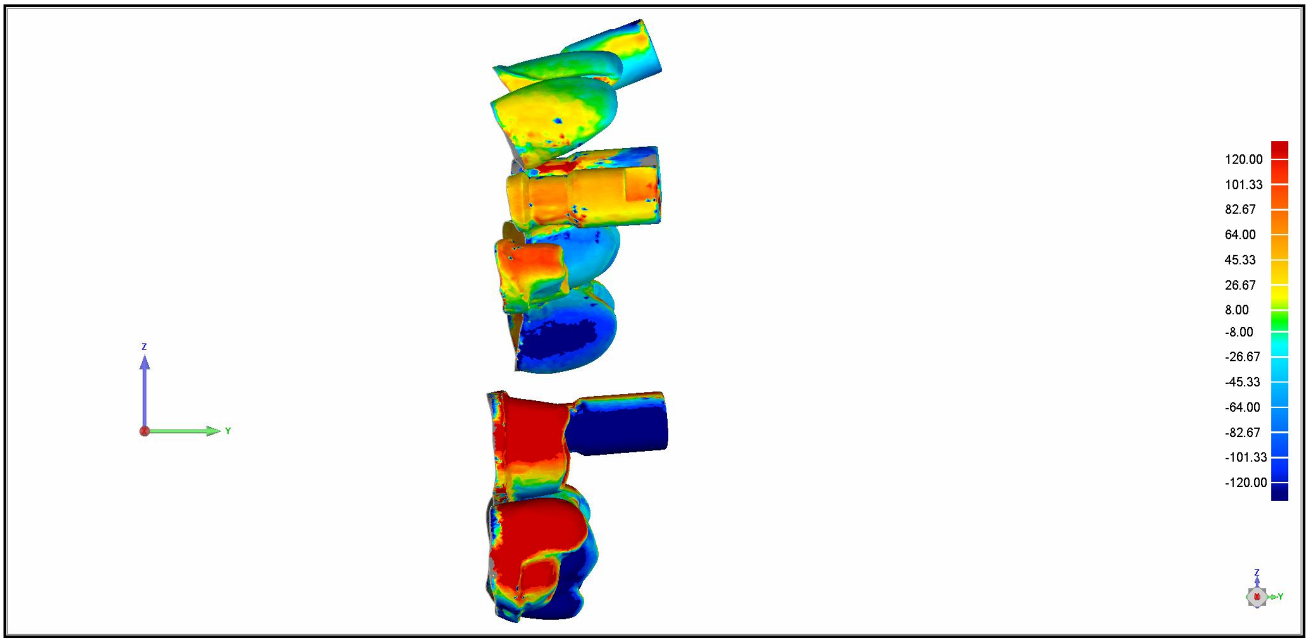

Predefinido: Superior

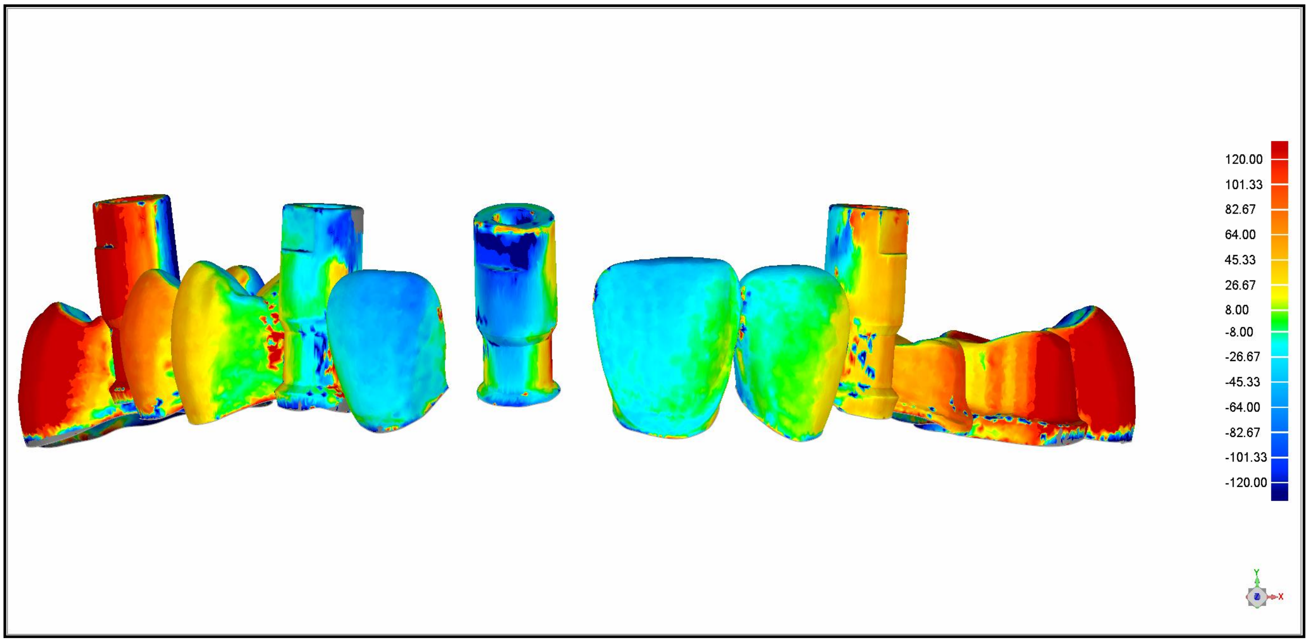

Predefinido: Inferior

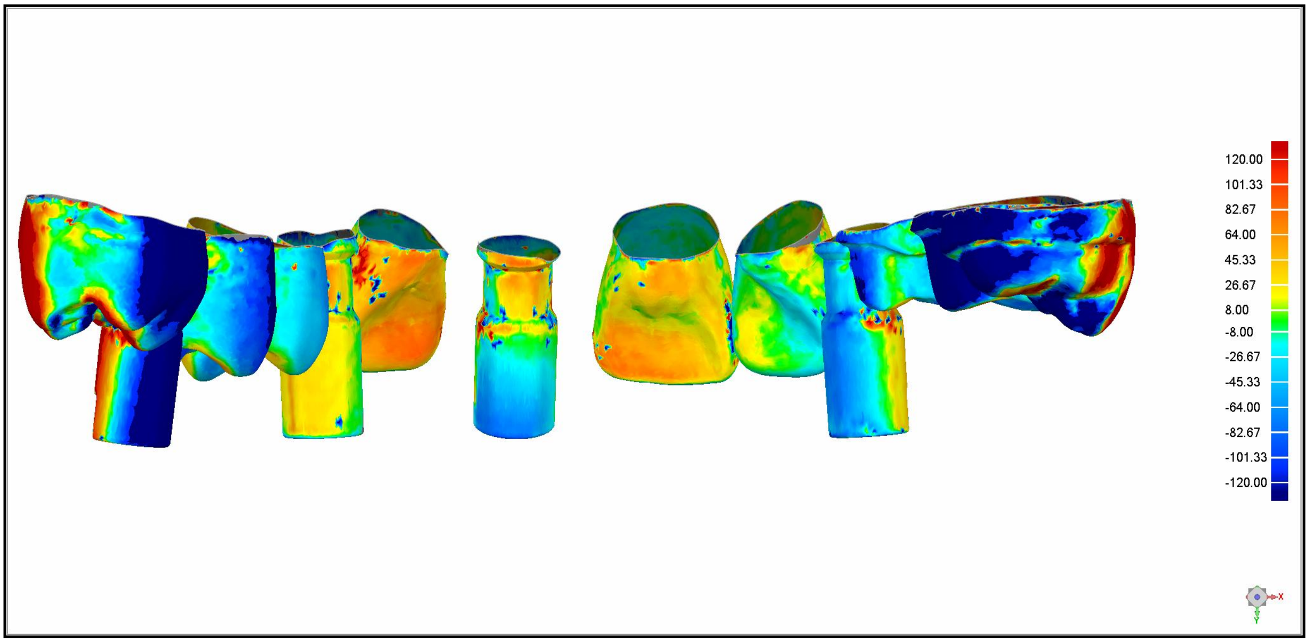

Ajuste de ubicación: Desviaciones superior e inferior

Unidades: u

Nombre	Desv	Estado	Superior Tol	Inferior Tol	Ref X	Ref Y	Ref Z	Radio	Desv X	Desv Y	Desv Z	Medido X	Medido Y	Medido Z	Dir. proy. X	Dir. proy. Y	Dir. proy. Z
Desv. inferior	-3145.68				-29208.33	26961.25	-11988.49	n/a	2723.98	435.44	-1511.83	-26484.35	27396.69	-13500.32	-0.87	-0.14	0.48
Desv. superior	3132.51				-23671.02	28734.46	9848.86	n/a	2918.16	938.68	644.83	-20752.86	29673.14	10493.69	0.93	0.30	0.21
