# Supplementary material for: Accuracy of four digital scanners according to scanning strategy in complete-arch impressions
Source: PLoS One. 2018 Sep 13;13(9):e0202916. doi: 10.1371/journal.pone.0202916 (PMC6136706; doi:10.1371/journal.pone.0202916)
Supplement: S5 Table — iTero (scanning strategy A). (ZIP) [file pone.0202916.s005.zip › S5/IT2A.pdf]

Geomagic Control™

Fecha: 3/6/2017, 1:14 pm

3D Comparación Resultados

Modelo referencia	MRC
Modelo test	IT2A
Nº de puntos de datos	79982
# Aislados	640

Tipo tolerancia	3D desviación
Unidades	u
Máx. crítico	120.00
Máx. nominal	14.00
Mín. nominal	-14.00
Mín. crítico	-120.00

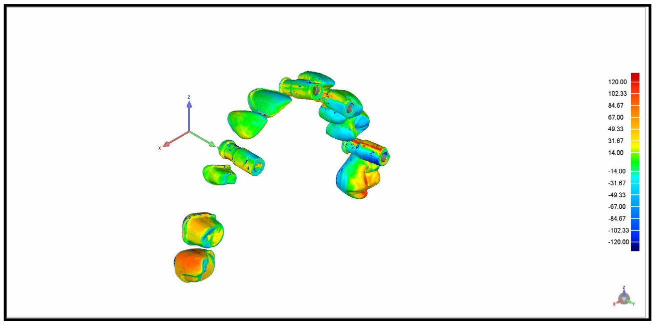

Desviación	
Desviación superior máx.	3067.66
Desviación inferior máx.	-2982.74
Desviación media	73.43 /-59.57
Desviación estándar	215.15

Distribución desviación

>=Min	<Max	# Puntos	%
-120.00	-102.33	549	0.69
-102.33	-84.67	777	0.97
-84.67	-67.00	1235	1.54
-67.00	-49.33	2364	2.96
-49.33	-31.67	5032	6.29
-31.67	-14.00	10222	12.78
-14.00	14.00	25986	32.49
14.00	31.67	12052	15.07
31.67	49.33	7800	9.75
49.33	67.00	4399	5.50
67.00	84.67	2365	2.96
84.67	102.33	1384	1.73
102.33	120.00	625	0.78

Fuera del crítico superior	2994	3.74
Fuera del crítico inferior	2198	2.75

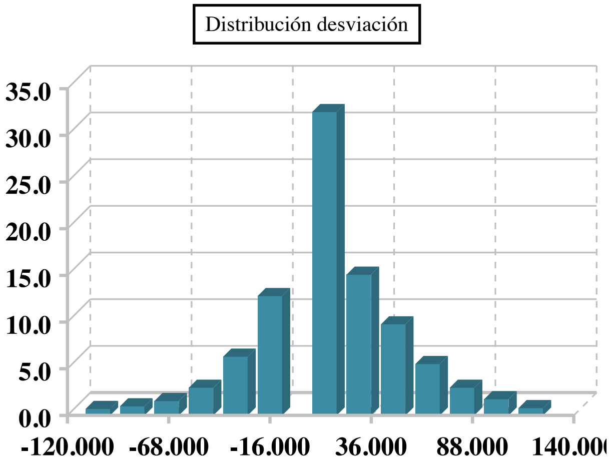

Desviaciones estándar

Distribución (+/-)	# Puntos	%
-6 * Desv. estánd.	382	0.48
-5 * Desv. estánd.	157	0.20
-4 * Desv. estánd.	196	0.25
-3 * Desv. estánd.	198	0.25
-2 * Desv. estánd.	599	0.75
-1 * Desv. estánd.	47627	59.55
1 * Desv. estánd.	28945	36.19
2 * Desv. estánd.	475	0.59
3 * Desv. estánd.	234	0.29
4 * Desv. estánd.	161	0.20
5 * Desv. estánd.	175	0.22
6 * Desv. estánd.	833	1.04

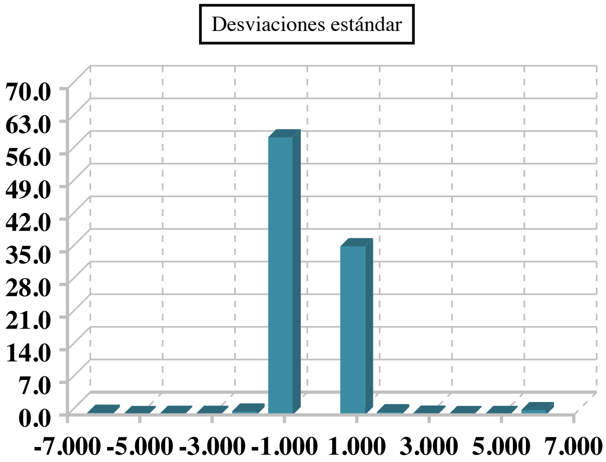

Predefinido: Isométrico

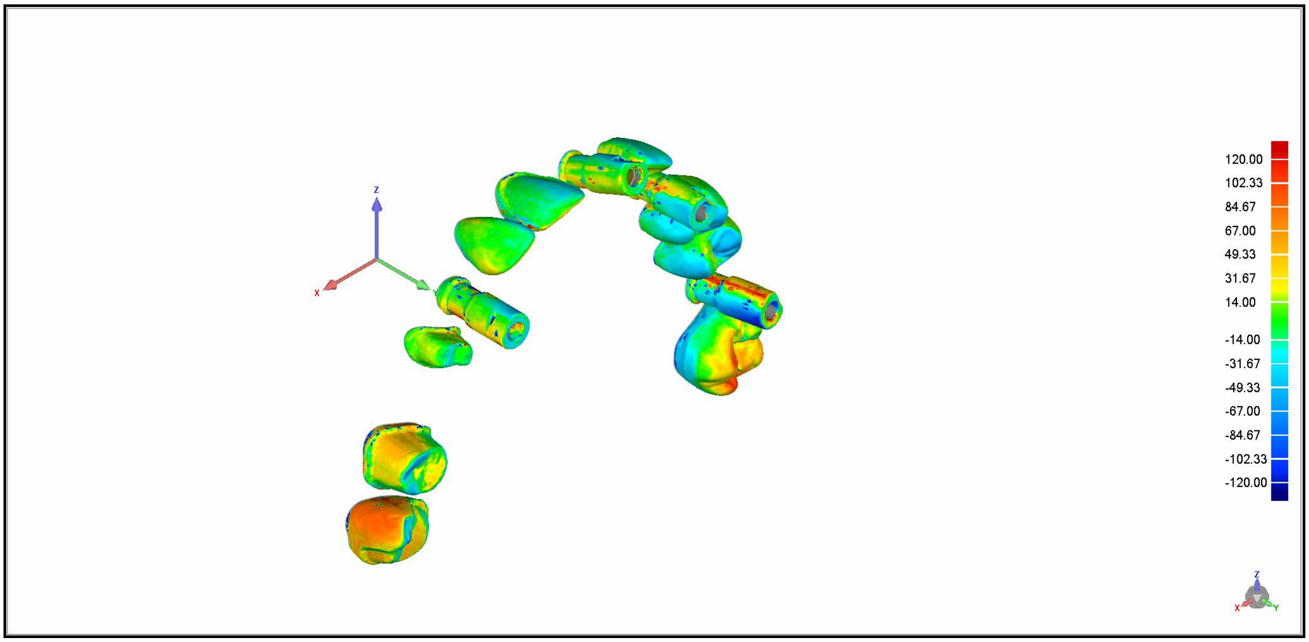

Predefinido: Frente

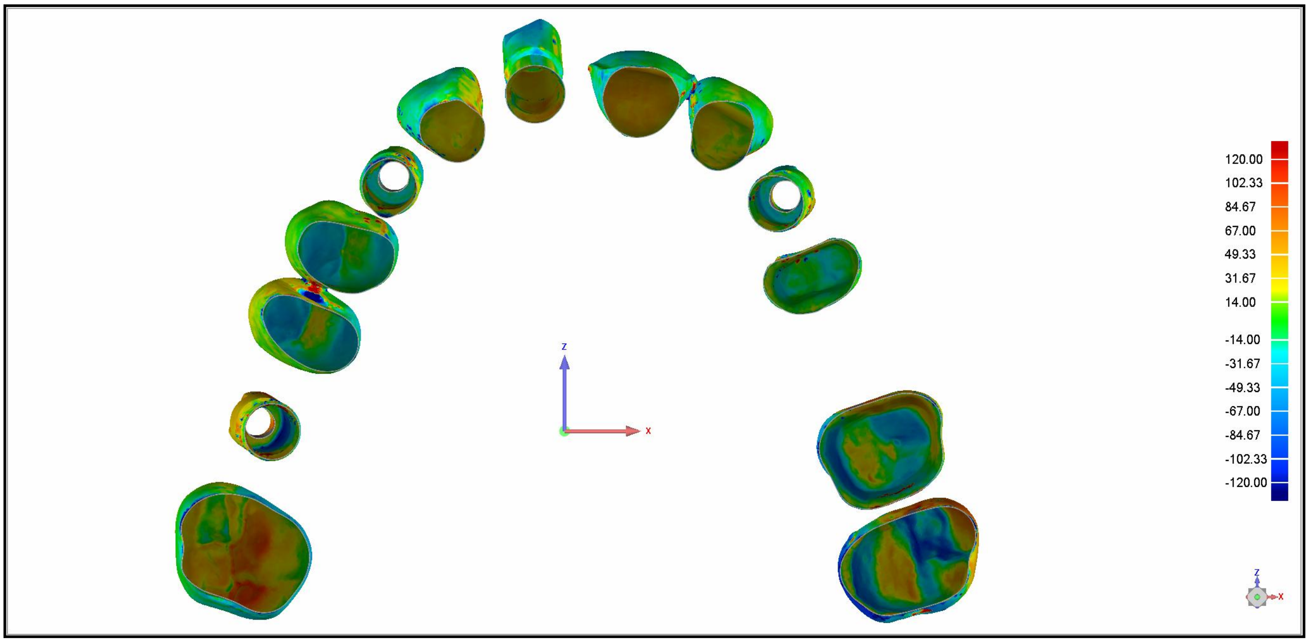

Predefinido: Atrás

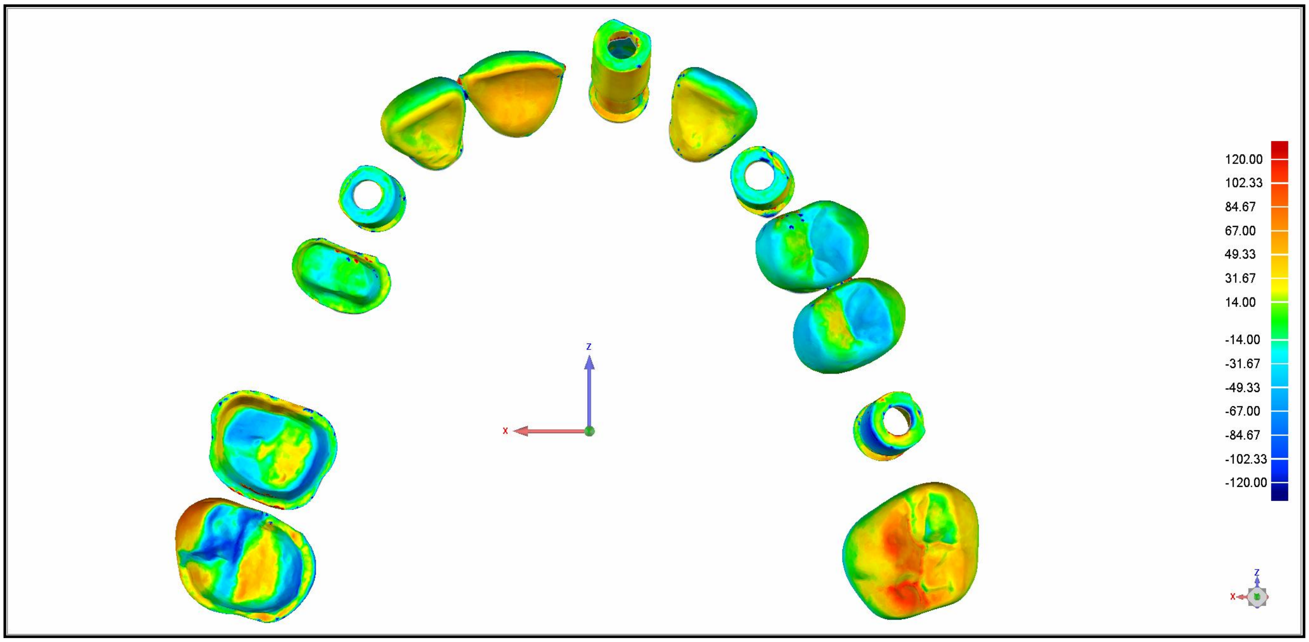

Predefinido: Izquierda

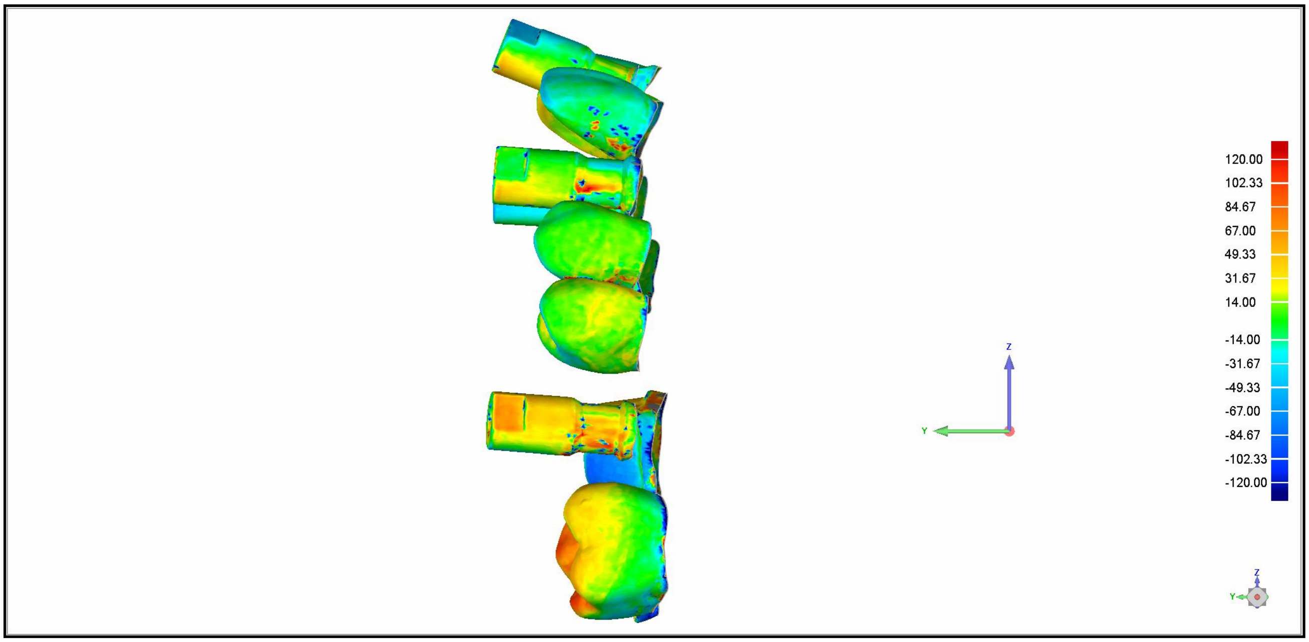

Predefinido: Derecha

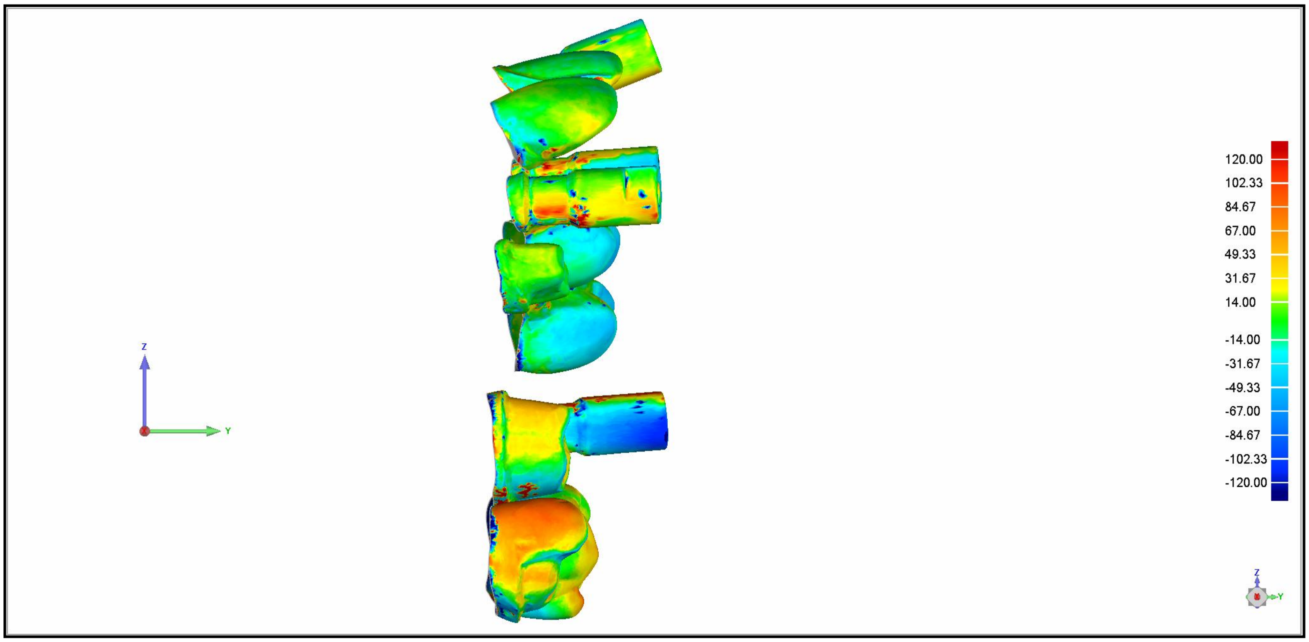

Predefinido: Superior

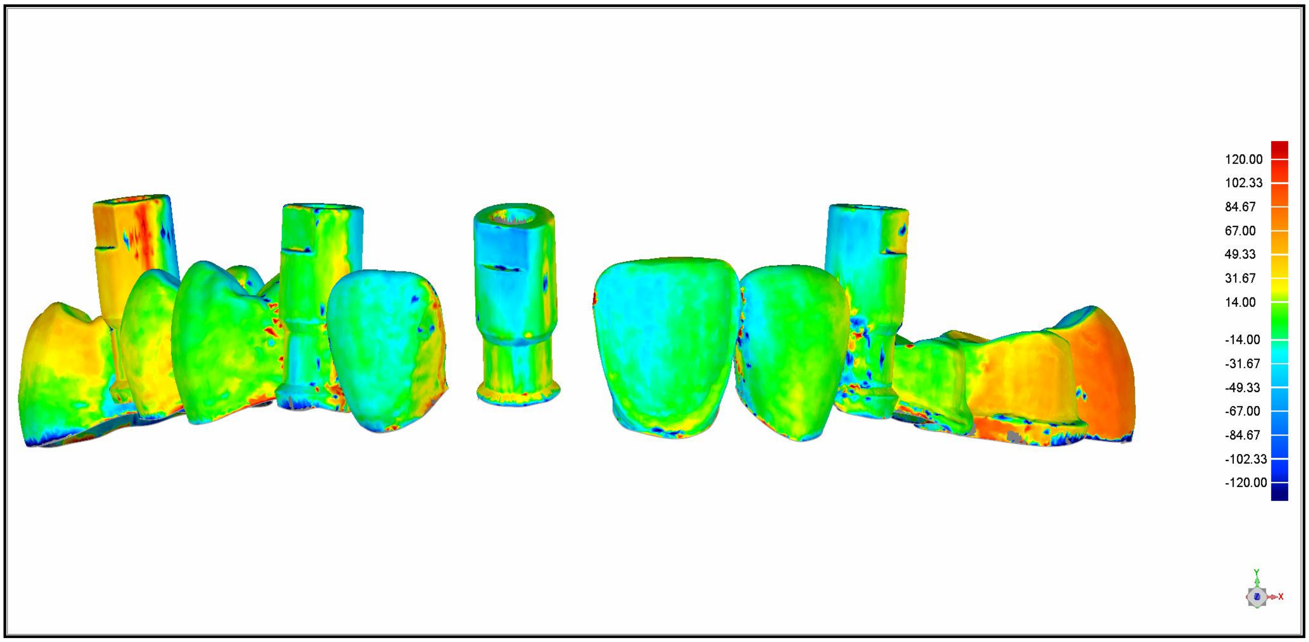

Predefinido: Inferior

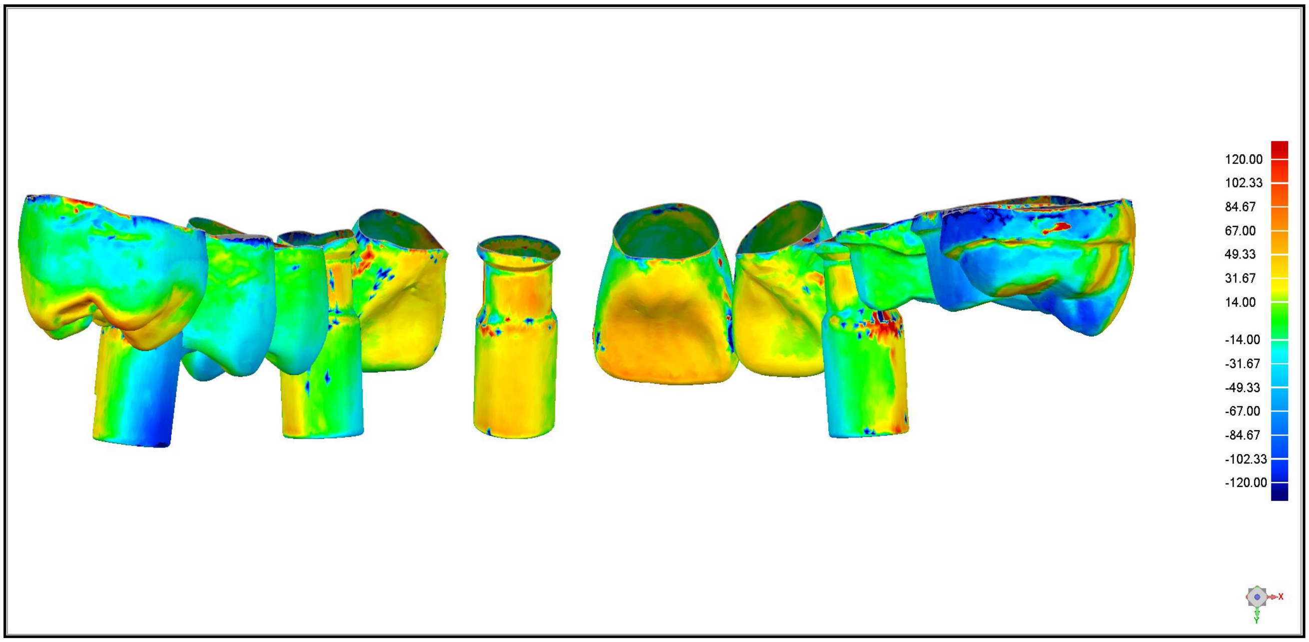

Ajuste de ubicación: Desviaciones superior e inferior

Unidades: u

Nombre	Desv	Estado	Superior Tol	Inferior Tol	Ref X	Ref Y	Ref Z	Radio	Desv X	Desv Y	Desv Z	Medido X	Medido Y	Medido Z	Dir. proy. X	Dir. proy. Y	Dir. proy. Z
Desv. inferior	-2982.74				-13661.80	38472.42	18786.03	n/a	-1529.30	2024.56	1568.16	-15191.10	40496.98	20354.20	0.51	-0.68	-0.53
Desv. superior	3067.66				19789.24	32532.00	14155.80	n/a	-902.17	1749.01	2353.21	18887.07	34281.00	16509.01	-0.29	0.57	0.77
