# Supplementary material for: Accuracy of four digital scanners according to scanning strategy in complete-arch impressions
Source: PLoS One. 2018 Sep 13;13(9):e0202916. doi: 10.1371/journal.pone.0202916 (PMC6136706; doi:10.1371/journal.pone.0202916)
Supplement: S5 Table — iTero (scanning strategy A). (ZIP) [file pone.0202916.s005.zip › S5/IT3A.pdf]

3D Comparación Resultados

Modelo referencia	MRC
Modelo test	IT3A
Nº de puntos de datos	82695
# Aislados	547

Tipo tolerancia	3D desviación
Unidades	u
Máx. crítico	120.00
Máx. nominal	18.00
Mín. nominal	-18.00
Mín. crítico	-120.00

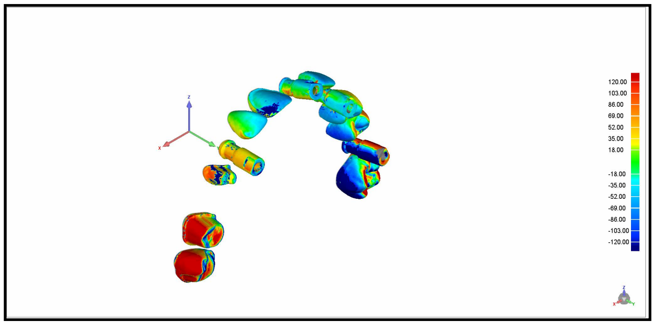

Desviación	
Desviación superior máx.	3141.74
Desviación inferior máx.	-3139.97
Desviación media	137.77 /-176.43
Desviación estándar	391.69

Distribución desviación

>=Min	<Max	# Puntos	%
-120.00	-103.00	1230	1.49
-103.00	-86.00	2019	2.44
-86.00	-69.00	2759	3.34
-69.00	-52.00	4251	5.14
-52.00	-35.00	5868	7.10
-35.00	-18.00	6926	8.38
-18.00	18.00	15498	18.74
18.00	35.00	6978	8.44
35.00	52.00	5767	6.97
52.00	69.00	3586	4.34
69.00	86.00	2683	3.24
86.00	103.00	2067	2.50
103.00	120.00	1341	1.62

Fuera del crítico superior	11337	13.71
Fuera del crítico inferior	10385	12.56

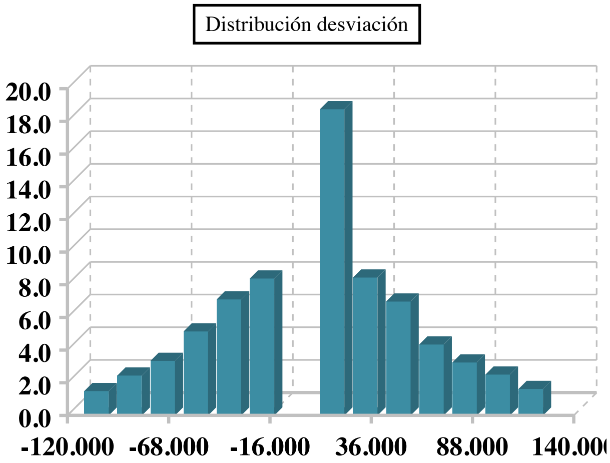

Desviaciones estándar

Distribución (+/-)	# Puntos	%
-6 * Desv. estánd.	715	0.86
-5 * Desv. estánd.	363	0.44
-4 * Desv. estánd.	603	0.73
-3 * Desv. estánd.	708	0.86
-2 * Desv. estánd.	765	0.93
-1 * Desv. estánd.	30244	36.57
1 * Desv. estánd.	47110	56.97
2 * Desv. estánd.	651	0.79
3 * Desv. estánd.	535	0.65
4 * Desv. estánd.	411	0.50
5 * Desv. estánd.	288	0.35
6 * Desv. estánd.	302	0.37

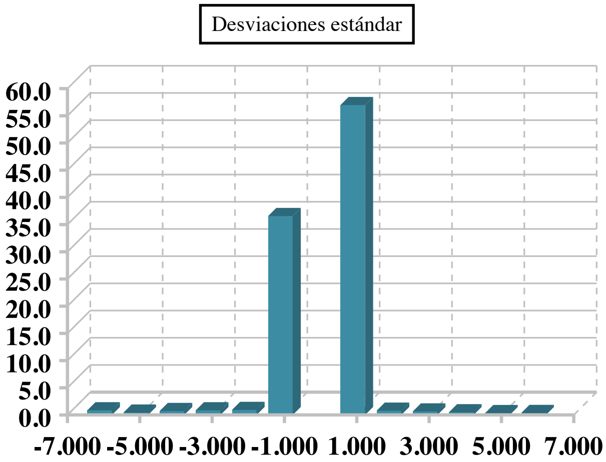

Predefinido: Isométrico

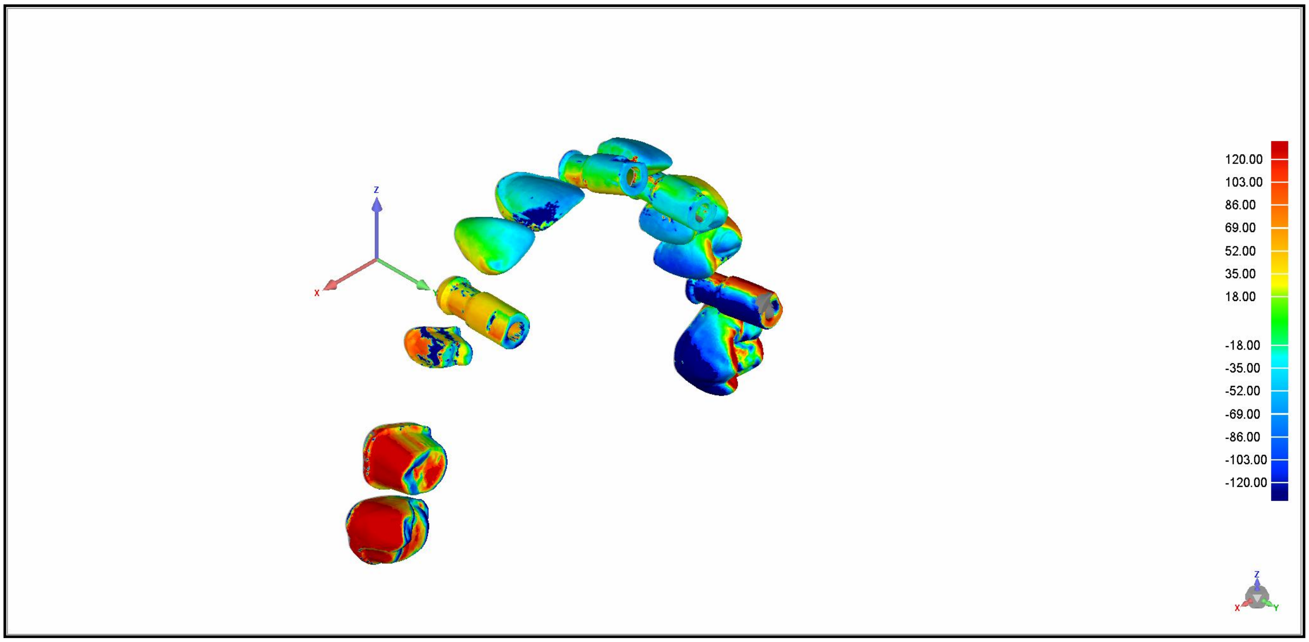

Predefinido: Frente

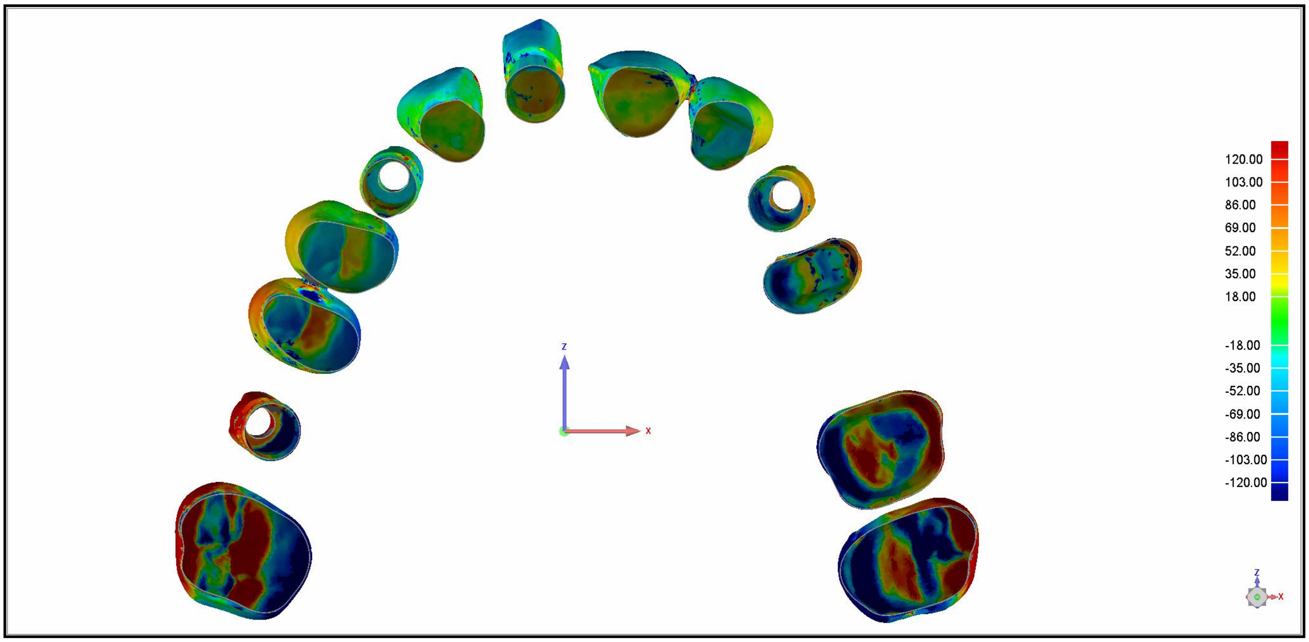

Predefinido: Atrás

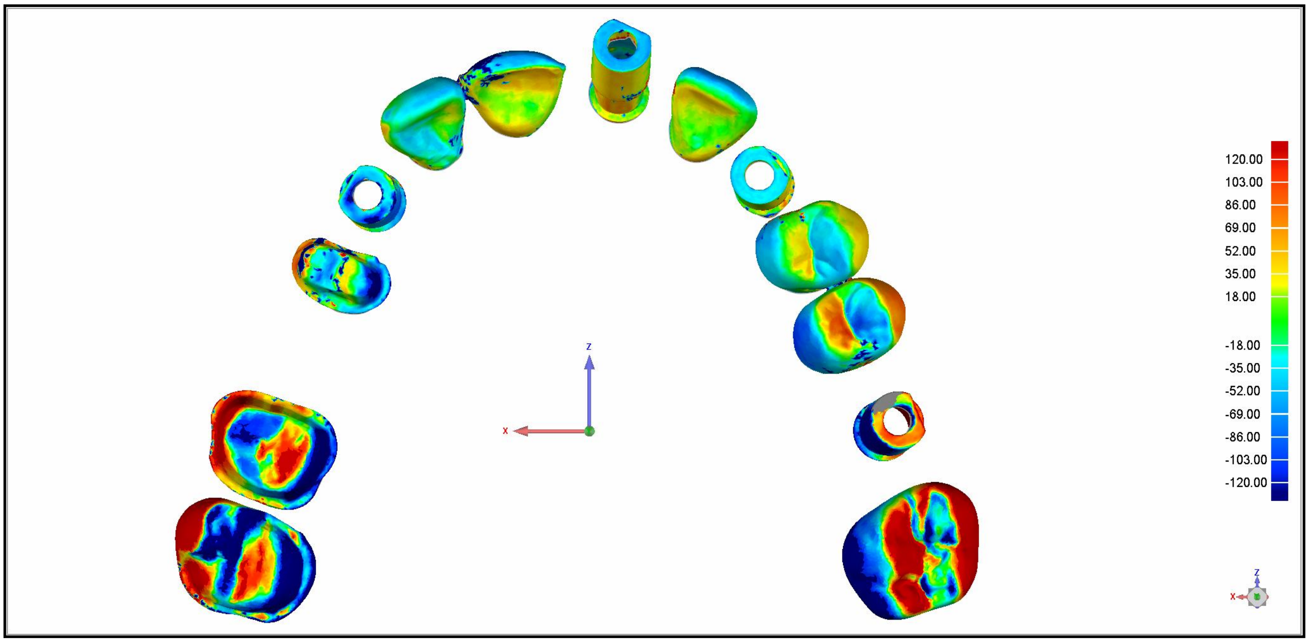

Predefinido: Izquierda

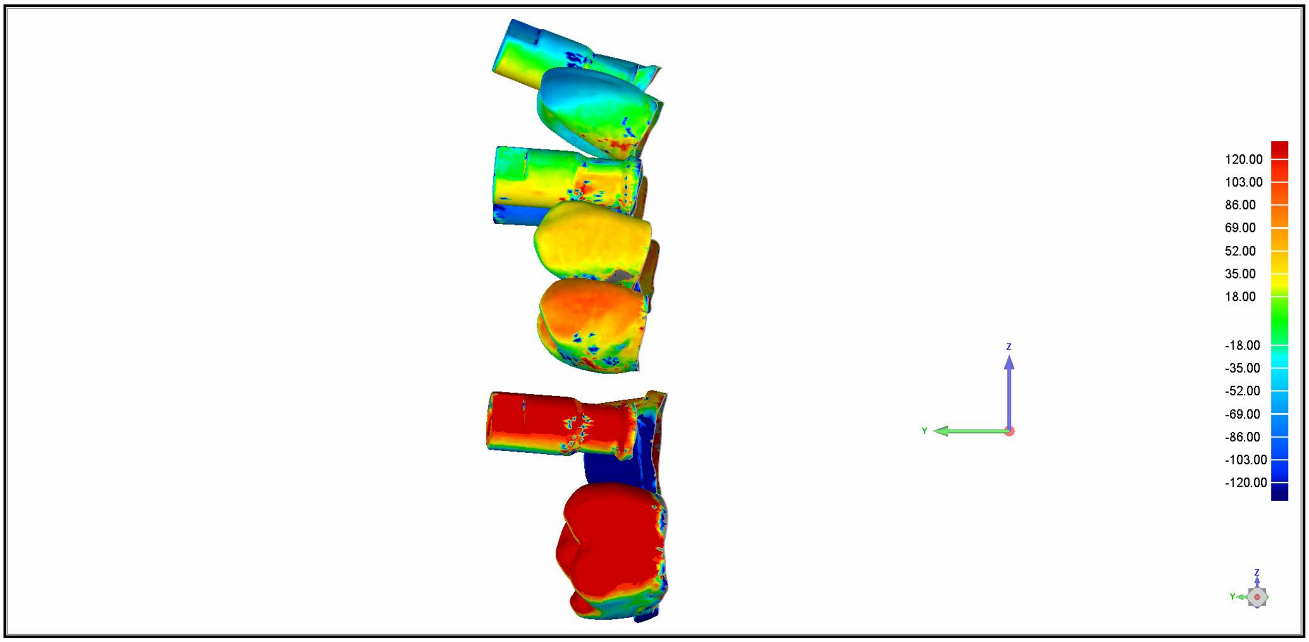

Predefinido: Derecha

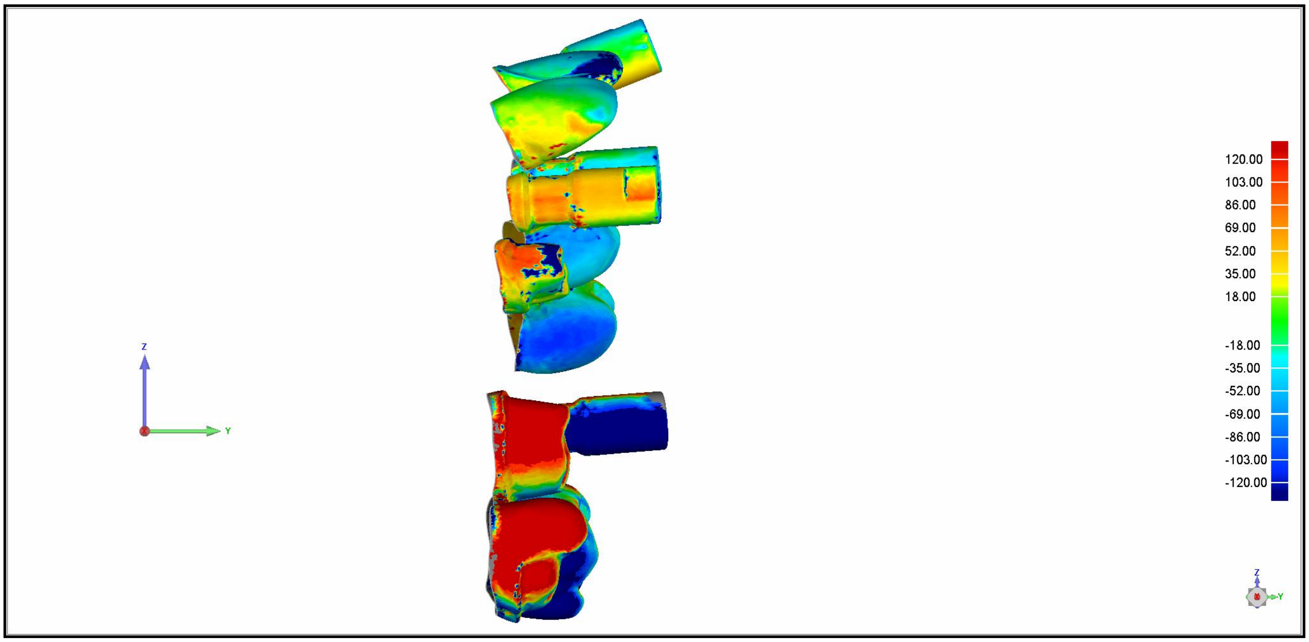

Predefinido: Superior

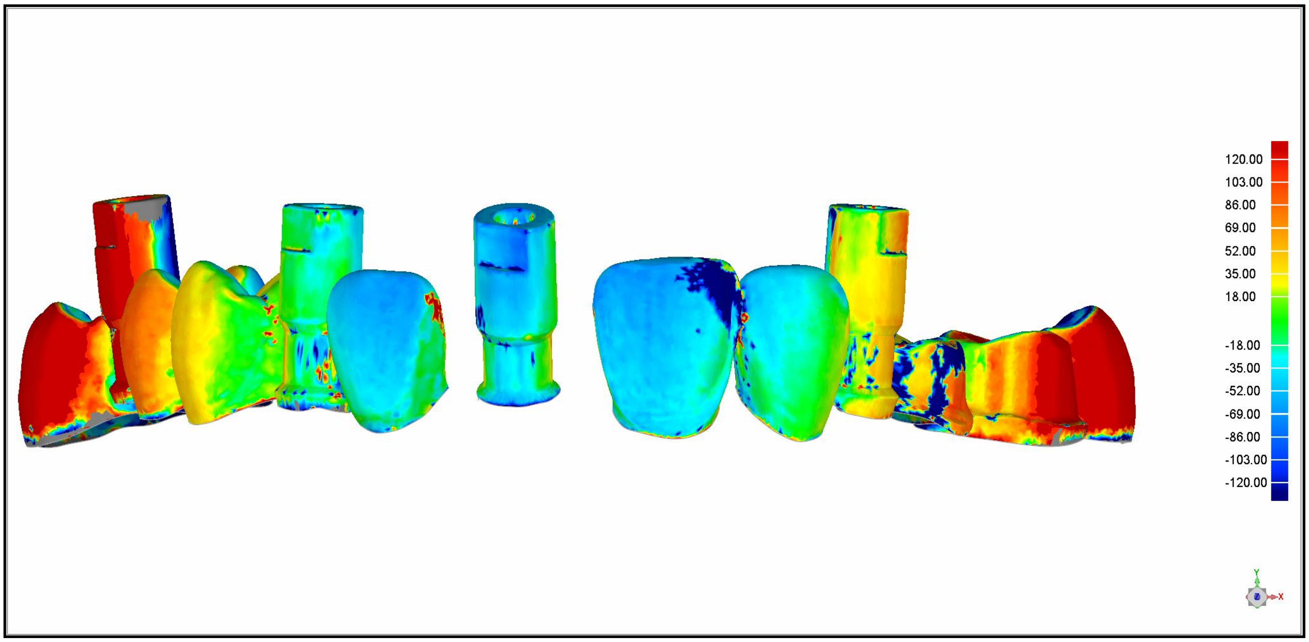

Predefinido: Inferior

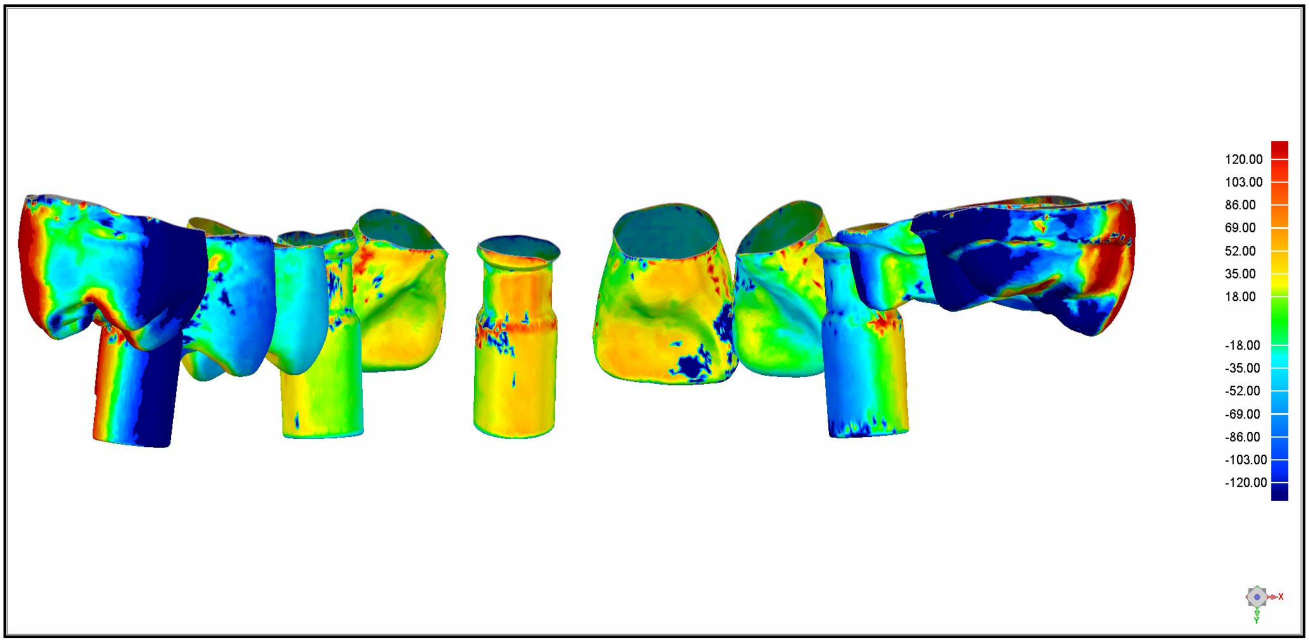

Ajuste de ubicación: Desviaciones superior e inferior

Unidades: u

Nombre	Desv	Estado	Superior Tol	Inferior Tol	Ref X	Ref Y	Ref Z	Radio	Desv X	Desv Y	Desv Z	Medido X	Medido Y	Medido Z	Dir. proy. X	Dir. proy. Y	Dir. proy. Z
Desv. inferior	-3139.97				10100.45	35491.65	27340.07	n/a	-2395.34	1260.34	1591.64	7705.11	36751.99	28931.70	0.76	-0.40	-0.51
Desv. superior	3141.74				-23083.85	34232.53	2576.56	n/a	789.06	-1344.48	2727.69	-22294.78	32888.05	5304.25	0.25	-0.43	0.87
