# Supplementary material for: Accuracy of four digital scanners according to scanning strategy in complete-arch impressions
Source: PLoS One. 2018 Sep 13;13(9):e0202916. doi: 10.1371/journal.pone.0202916 (PMC6136706; doi:10.1371/journal.pone.0202916)
Supplement: S5 Table — iTero (scanning strategy A). (ZIP) [file pone.0202916.s005.zip › S5/IT1A.pdf]

Geomagic Control

Fecha: 3/6/2017, 1:12 pm

3D Comparación Resultados

Modelo referencia	MRC
Modelo test	IT1A
Nº de puntos de datos	79979
# Aislados	648

Tipo tolerancia	3D desviación
Unidades	u
Máx. crítico	120.00
Máx. nominal	14.00
Mín. nominal	-14.00
Mín. crítico	-120.00

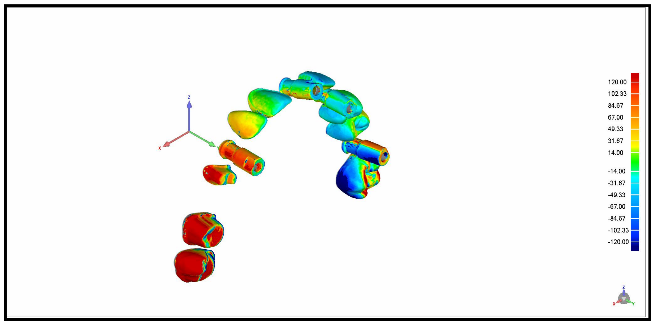

Desviación	
Desviación superior máx.	3110.02
Desviación inferior máx.	-3107.53
Desviación media	98.18 /-94.23
Desviación estándar	210.03

Distribución desviación

>=Min	<Max	# Puntos	%
-120.00	-102.33	1400	1.75
-102.33	-84.67	1610	2.01
-84.67	-67.00	1942	2.43
-67.00	-49.33	2943	3.68
-49.33	-31.67	5527	6.91
-31.67	-14.00	7804	9.76
-14.00	14.00	14920	18.65
14.00	31.67	9628	12.04
31.67	49.33	5641	7.05
49.33	67.00	4232	5.29
67.00	84.67	3282	4.10
84.67	102.33	2884	3.61
102.33	120.00	1951	2.44

Fuera del crítico superior	9899	12.38
Fuera del crítico inferior	6316	7.90

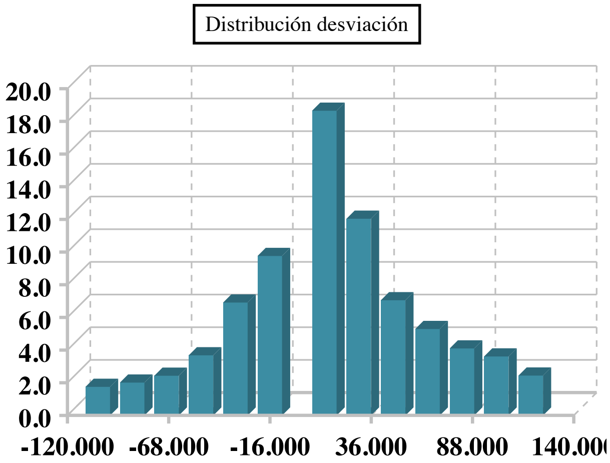

Desviaciones estándar

Distribución (+/-)	# Puntos	%
-6 * Desv. estánd.	455	0.57
-5 * Desv. estánd.	165	0.21
-4 * Desv. estánd.	171	0.21
-3 * Desv. estánd.	451	0.56
-2 * Desv. estánd.	2240	2.80
-1 * Desv. estánd.	39346	49.20
1 * Desv. estánd.	33142	41.44
2 * Desv. estánd.	2851	3.56
3 * Desv. estánd.	382	0.48
4 * Desv. estánd.	172	0.22
5 * Desv. estánd.	132	0.17
6 * Desv. estánd.	472	0.59

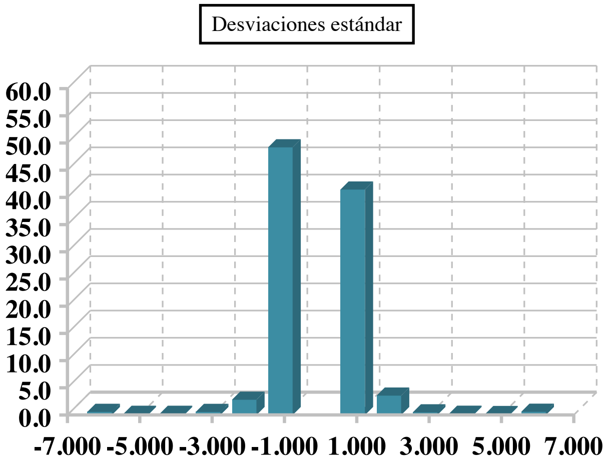

Predefinido: Isométrico

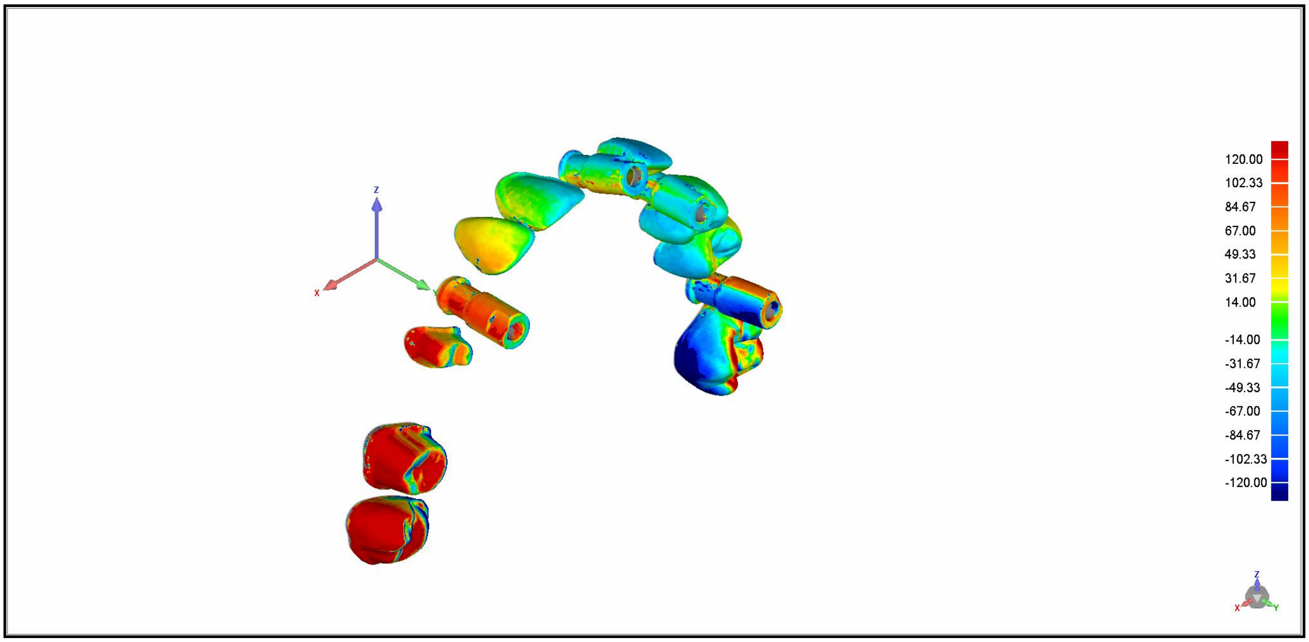

Predefinido: Frente

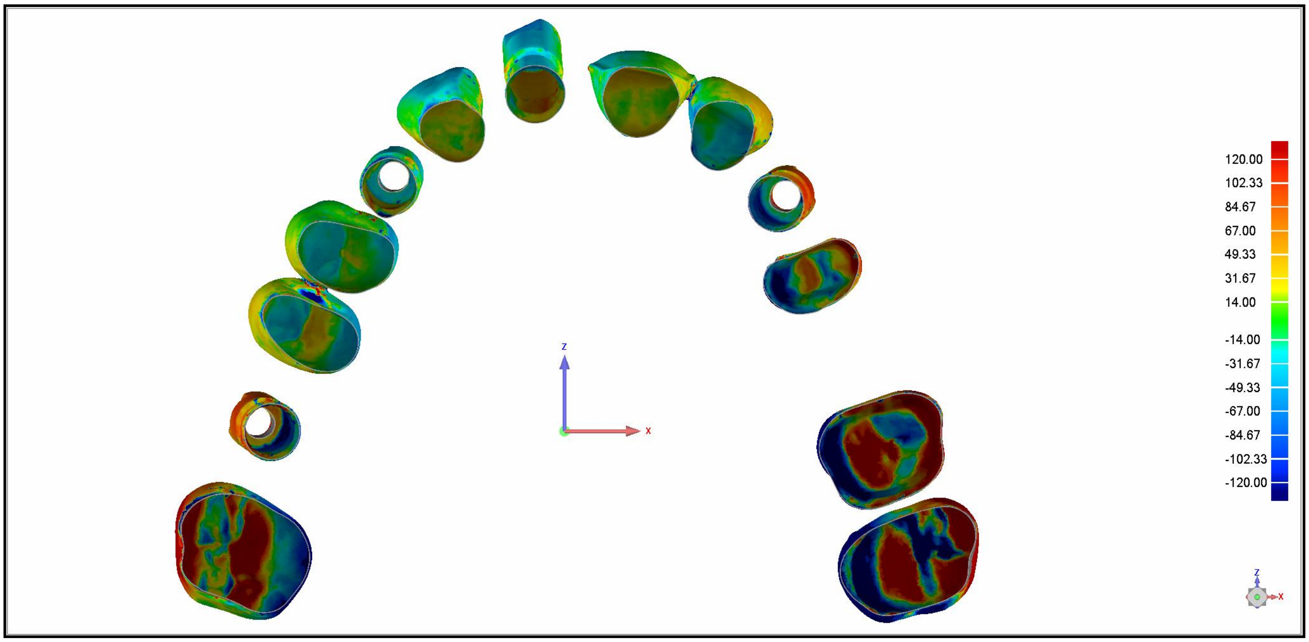

Predefinido: Atrás

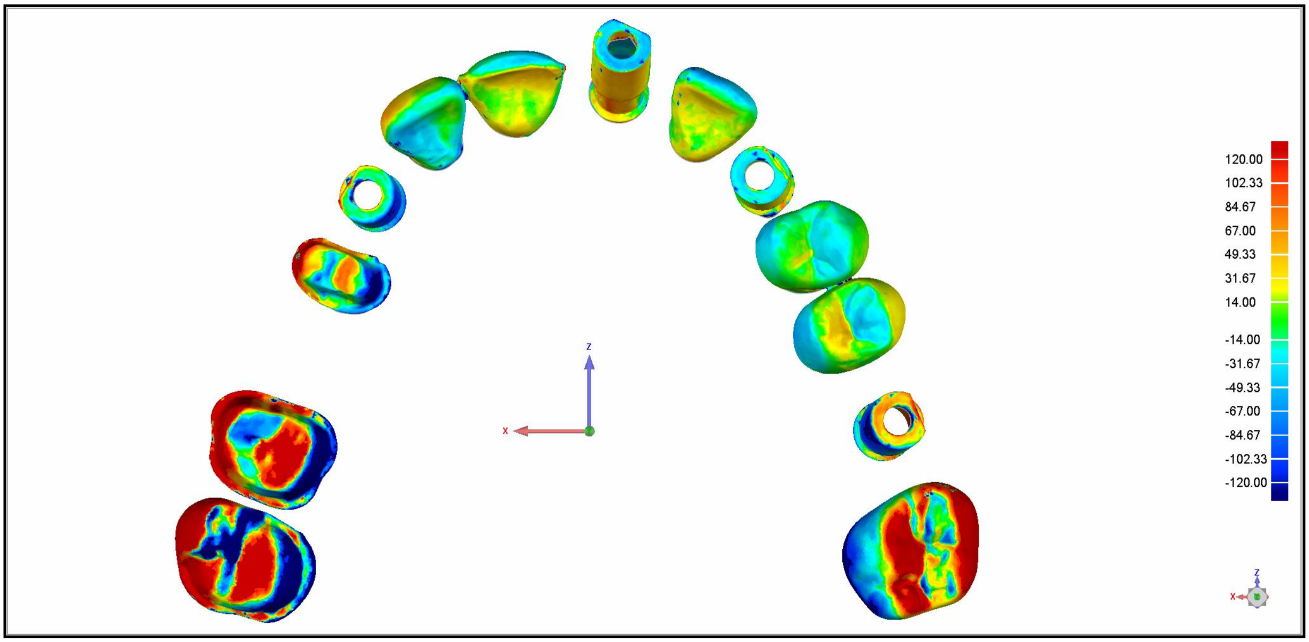

Predefinido: Izquierda

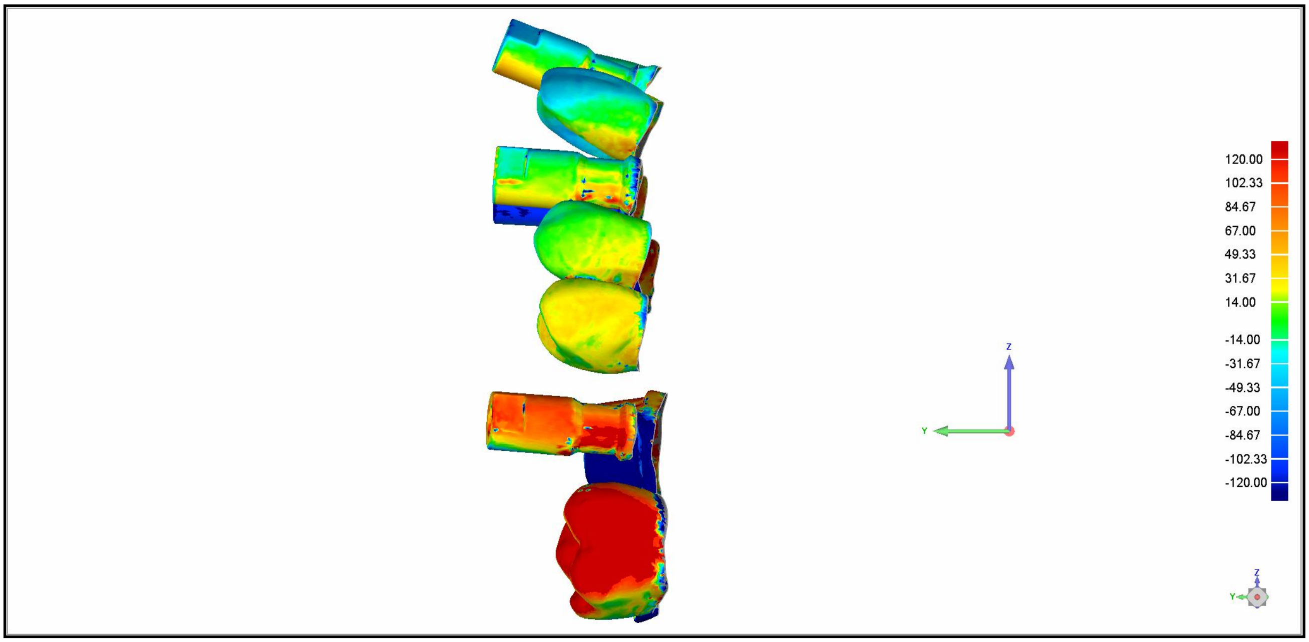

Predefinido: Derecha

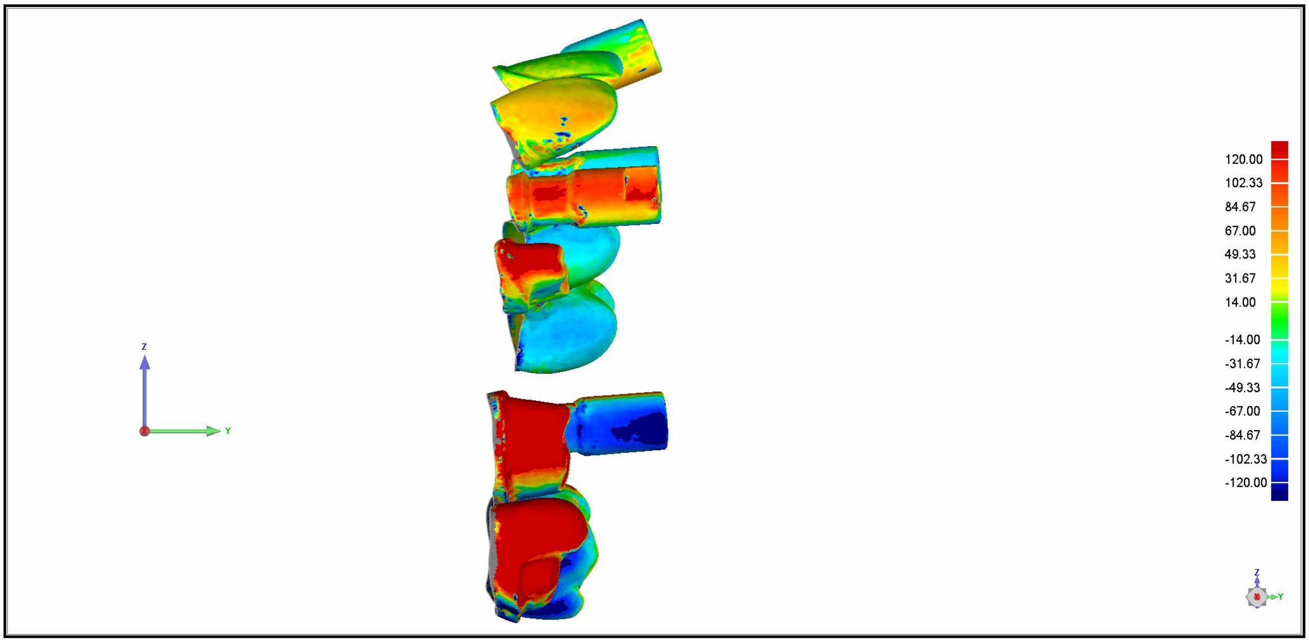

Predefinido: Superior

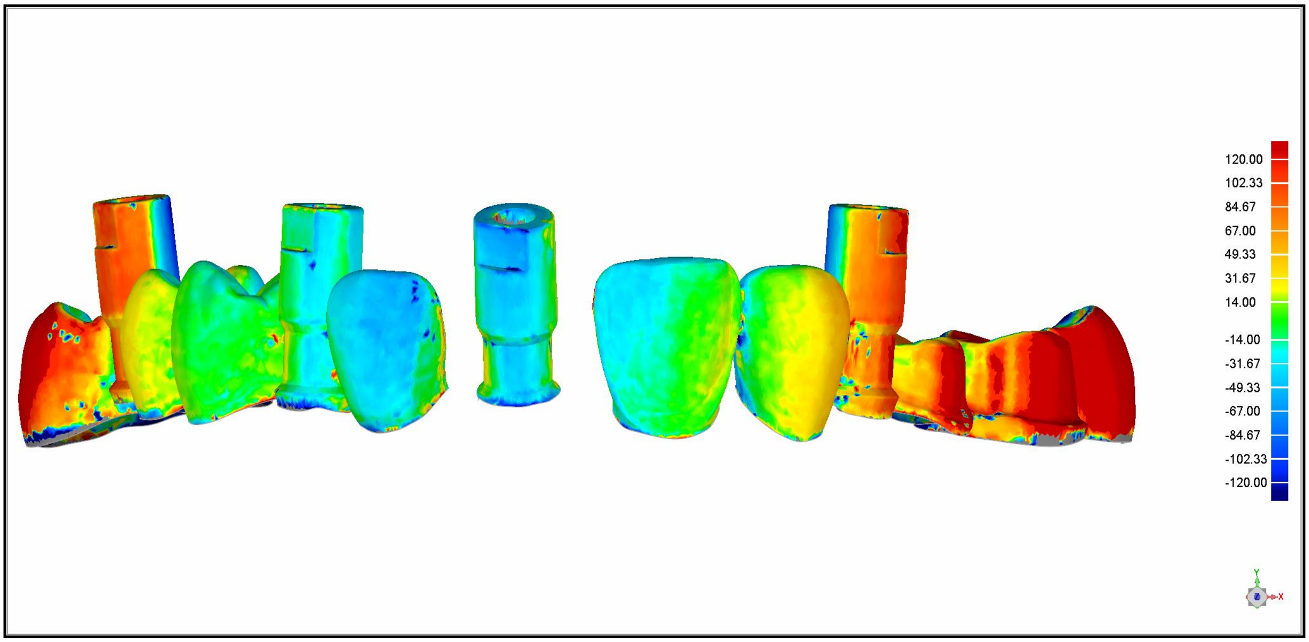

Predefinido: Inferior

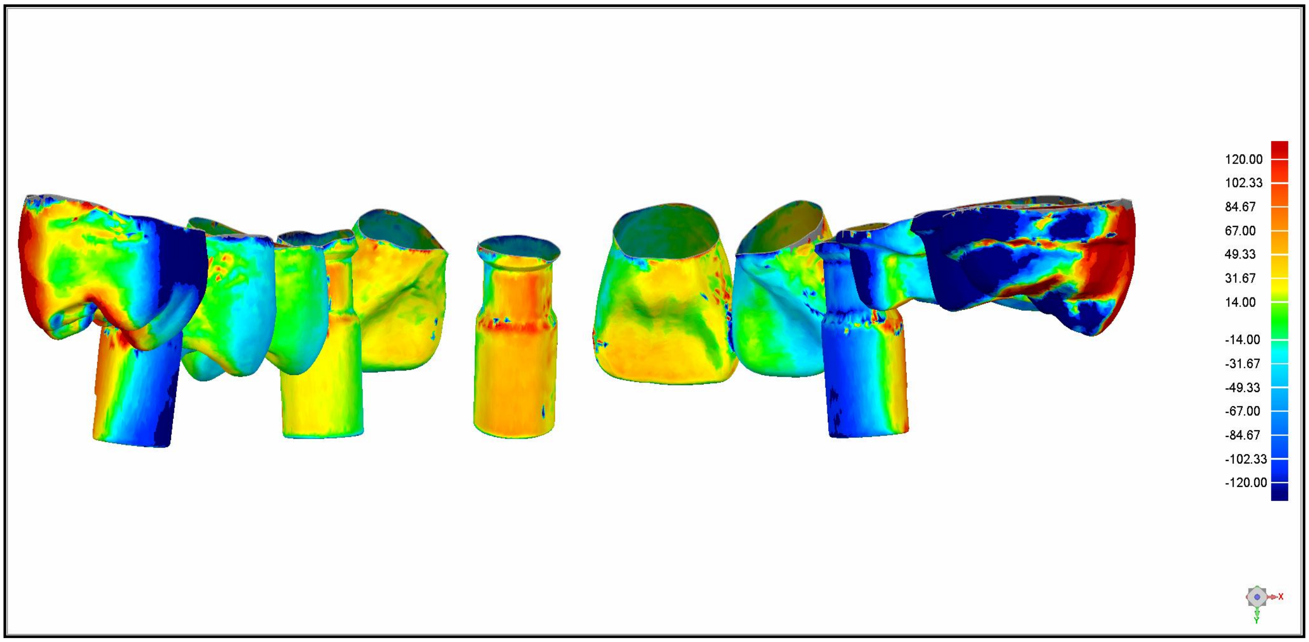

Ajuste de ubicación: Desviaciones superior e inferior

Unidades: u

Nombre	Desv	Estado	Superior Tol	Inferior Tol	Ref X	Ref Y	Ref Z	Radio	Desv X	Desv Y	Desv Z	Medido X	Medido Y	Medido Z	Dir. proy. X	Dir. proy. Y	Dir. proy. Z
Desv. inferior	-3107.53				-21736.25	35495.65	-886.27	n/a	-2934.40	-218.29	-999.18	-24670.65	35277.36	-1885.44	0.94	0.07	0.32
Desv. superior	3110.02				-24522.47	30239.06	-1849.11	n/a	-1711.33	-656.47	-2512.50	-26233.79	29582.59	-4361.61	-0.55	-0.21	-0.81
