# Supplementary material for: Accuracy of four digital scanners according to scanning strategy in complete-arch impressions
Source: PLoS One. 2018 Sep 13;13(9):e0202916. doi: 10.1371/journal.pone.0202916 (PMC6136706; doi:10.1371/journal.pone.0202916)
Supplement: S5 Table — iTero (scanning strategy A). (ZIP) [file pone.0202916.s005.zip › S5/IT6A.pdf]

3D Comparación Resultados

Modelo referencia	MRC
Modelo test	IT6A
Nº de puntos de datos	79700
# Aislados	776

Tipo tolerancia	3D desviación
Unidades	u
Máx. crítico	120.00
Máx. nominal	12.00
Mín. nominal	-12.00
Mín. crítico	-120.00

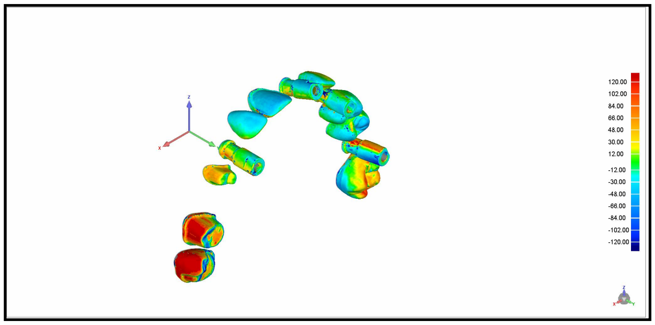

Desviación	
Desviación superior máx.	3136.93
Desviación inferior máx.	-3095.59
Desviación media	91.90 /-74.81
Desviación estándar	236.41

Distribución desviación

>=Min	<Max	# Puntos	%
-120.00	-102.00	723	0.91
-102.00	-84.00	973	1.22
-84.00	-66.00	1405	1.76
-66.00	-48.00	2609	3.27
-48.00	-30.00	6901	8.66
-30.00	-12.00	11608	14.56
-12.00	12.00	18140	22.76
12.00	30.00	10868	13.64
30.00	48.00	7582	9.51
48.00	66.00	4317	5.42
66.00	84.00	2358	2.96
84.00	102.00	1643	2.06
102.00	120.00	1090	1.37

Fuera del crítico superior	5067	6.36
Fuera del crítico inferior	4416	5.54

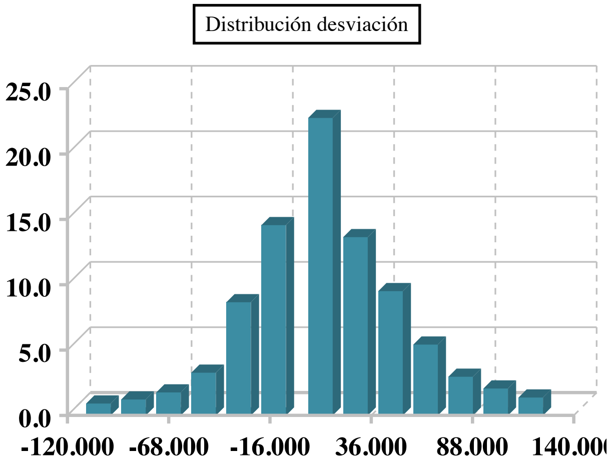

Desviaciones estándar

Distribución (+/-)	# Puntos	%
-6 * Desv. estánd.	418	0.52
-5 * Desv. estánd.	220	0.28
-4 * Desv. estánd.	238	0.30
-3 * Desv. estánd.	309	0.39
-2 * Desv. estánd.	720	0.90
-1 * Desv. estánd.	44950	56.40
1 * Desv. estánd.	30714	38.54
2 * Desv. estánd.	472	0.59
3 * Desv. estánd.	296	0.37
4 * Desv. estánd.	255	0.32
5 * Desv. estánd.	303	0.38
6 * Desv. estánd.	805	1.01

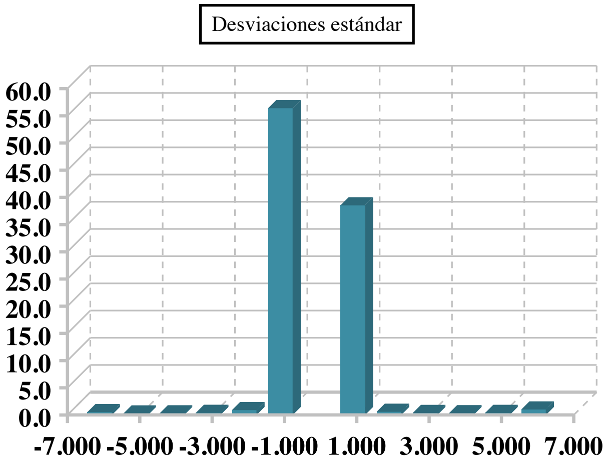

Predefinido: Isométrico

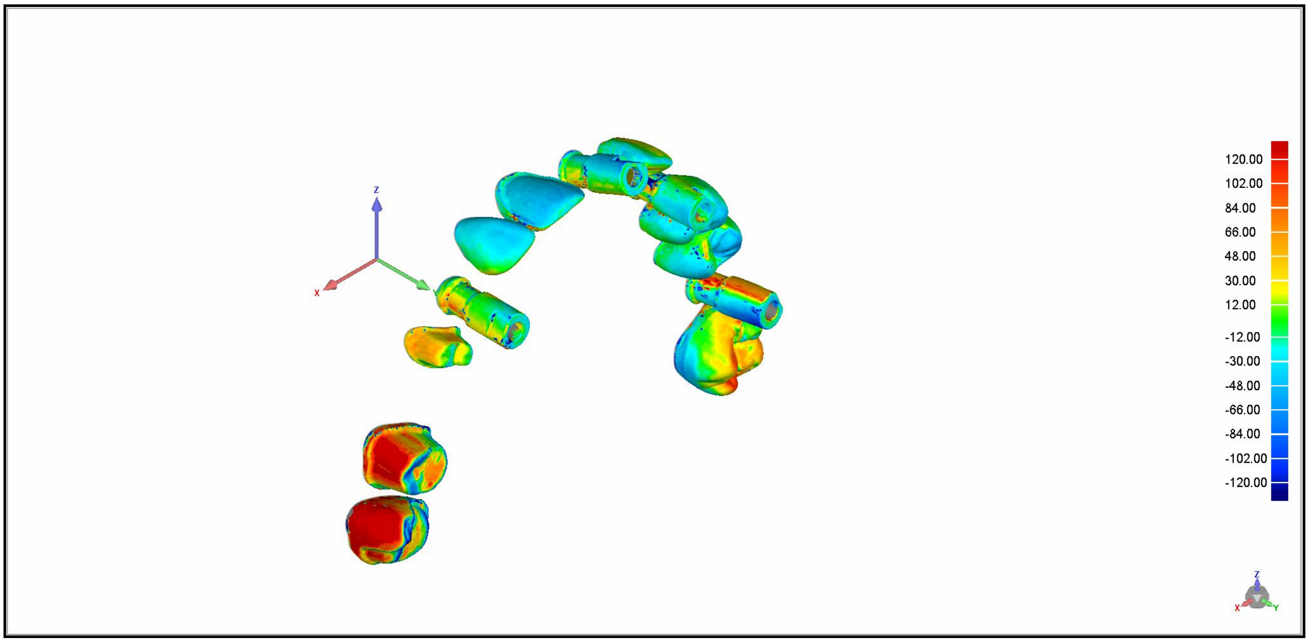

Predefinido: Frente

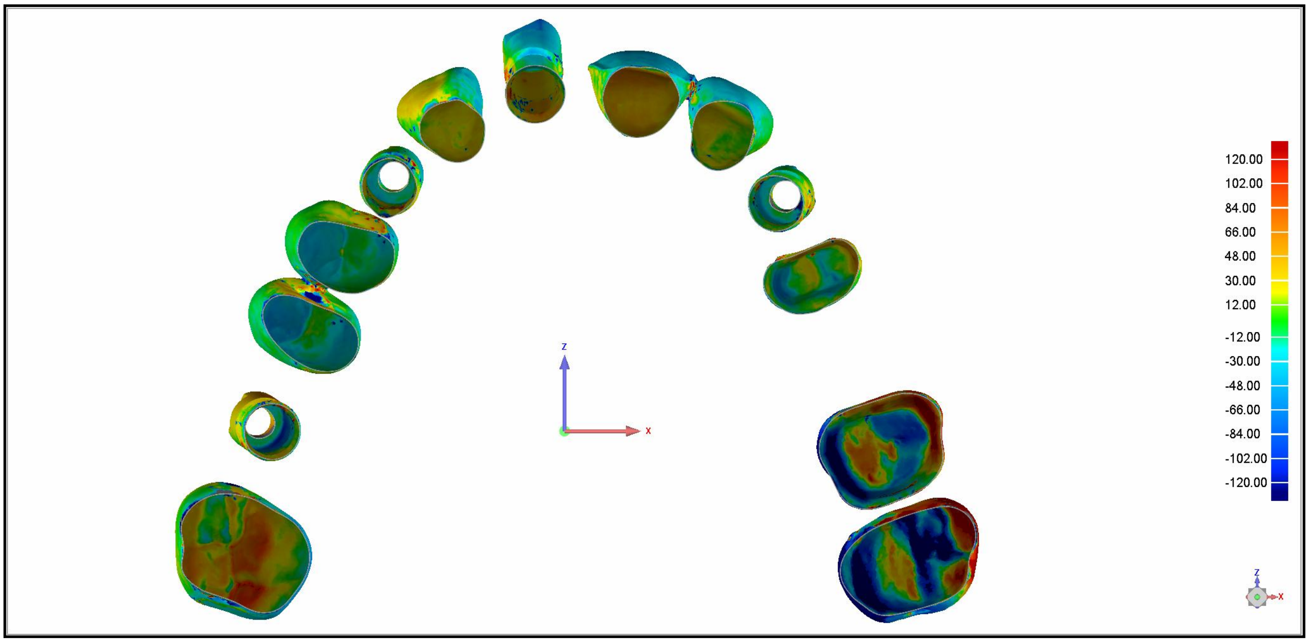

Predefinido: Atrás

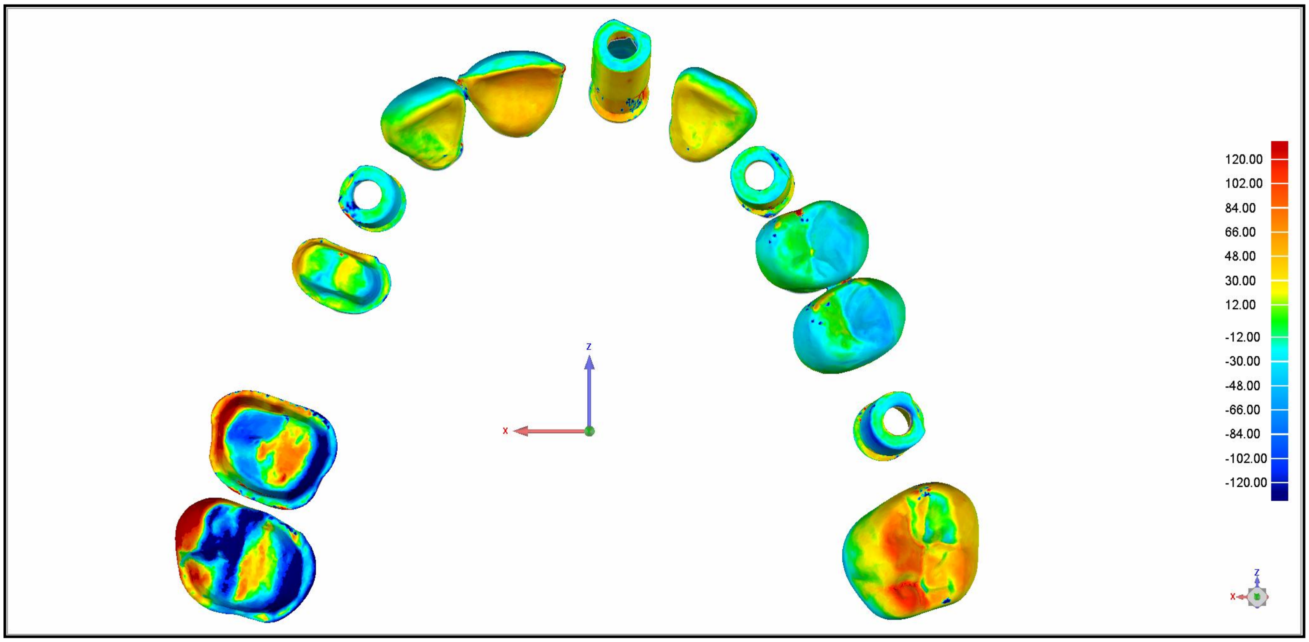

Fecha: 3/6/2017, 1:24 pm

Predefinido: Izquierda

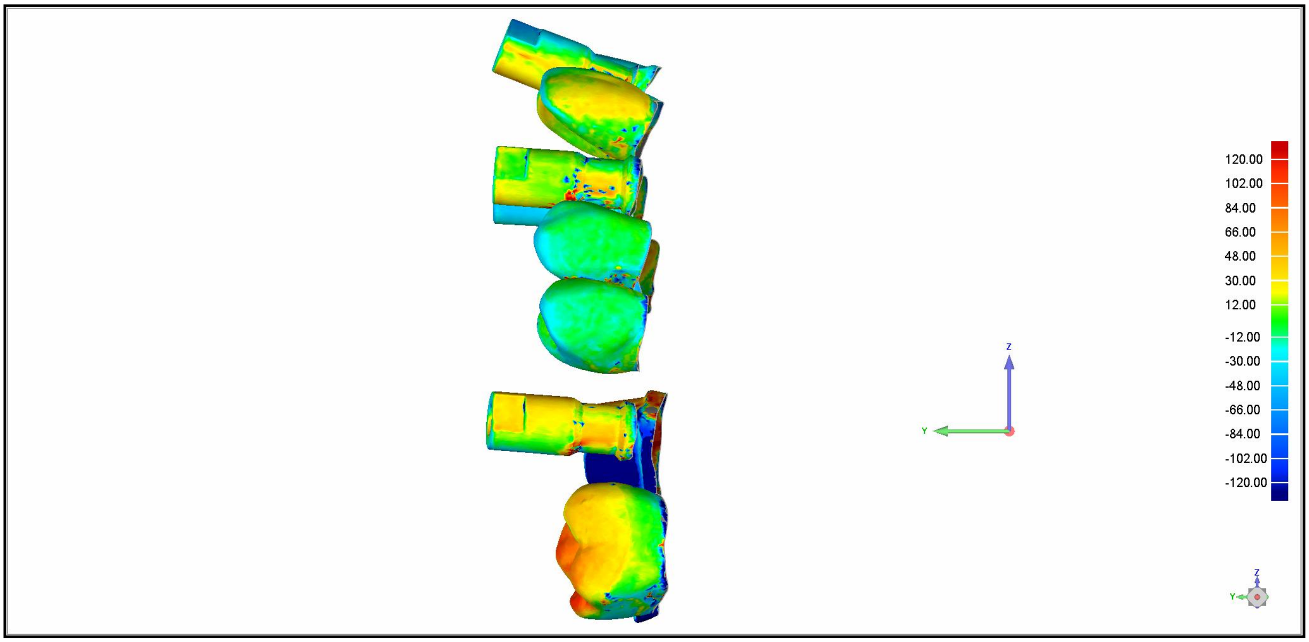

Predefinido: Derecha

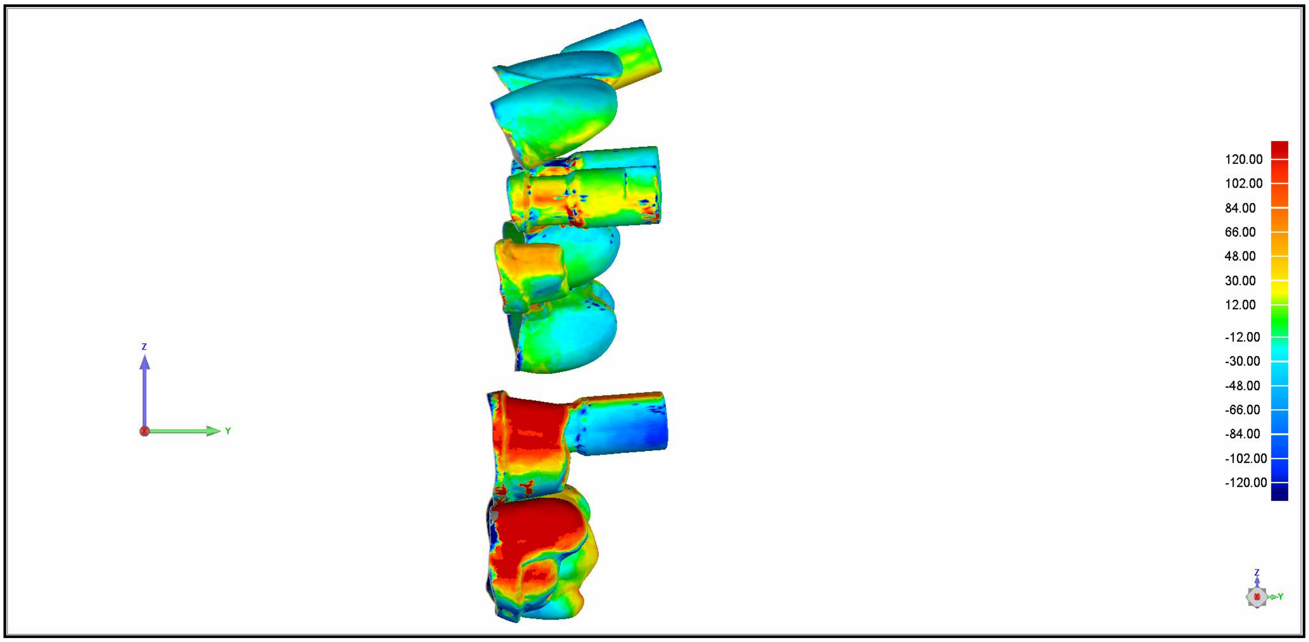

Predefinido: Superior

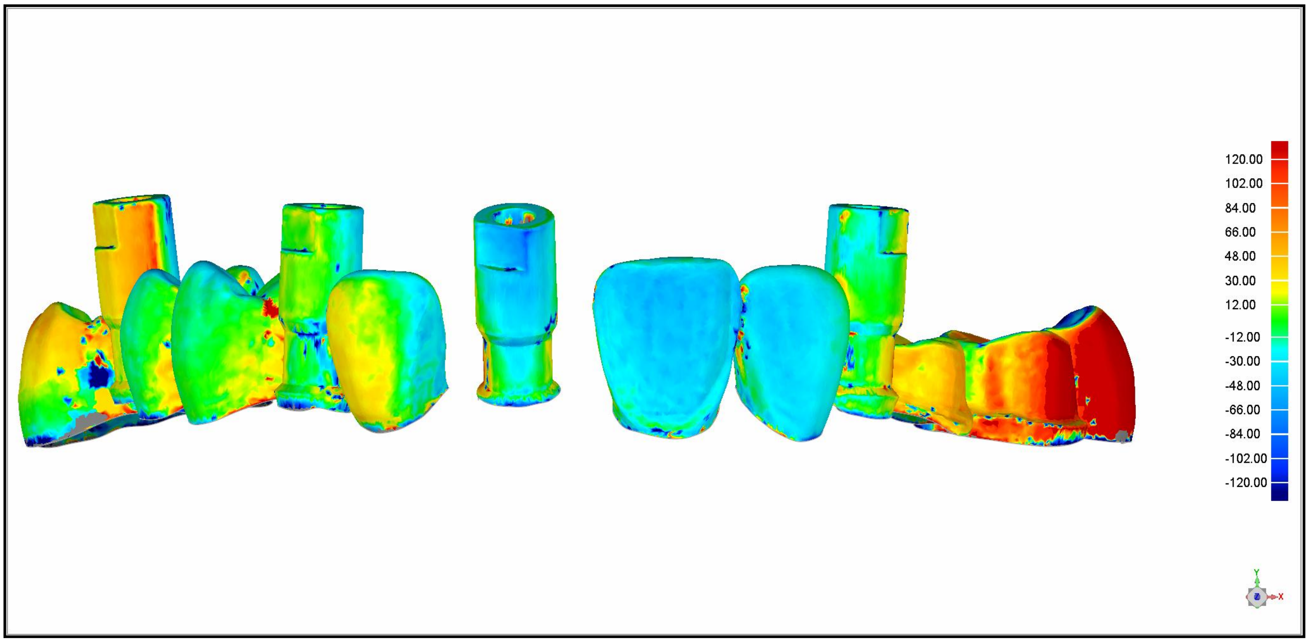

Predefinido: Inferior

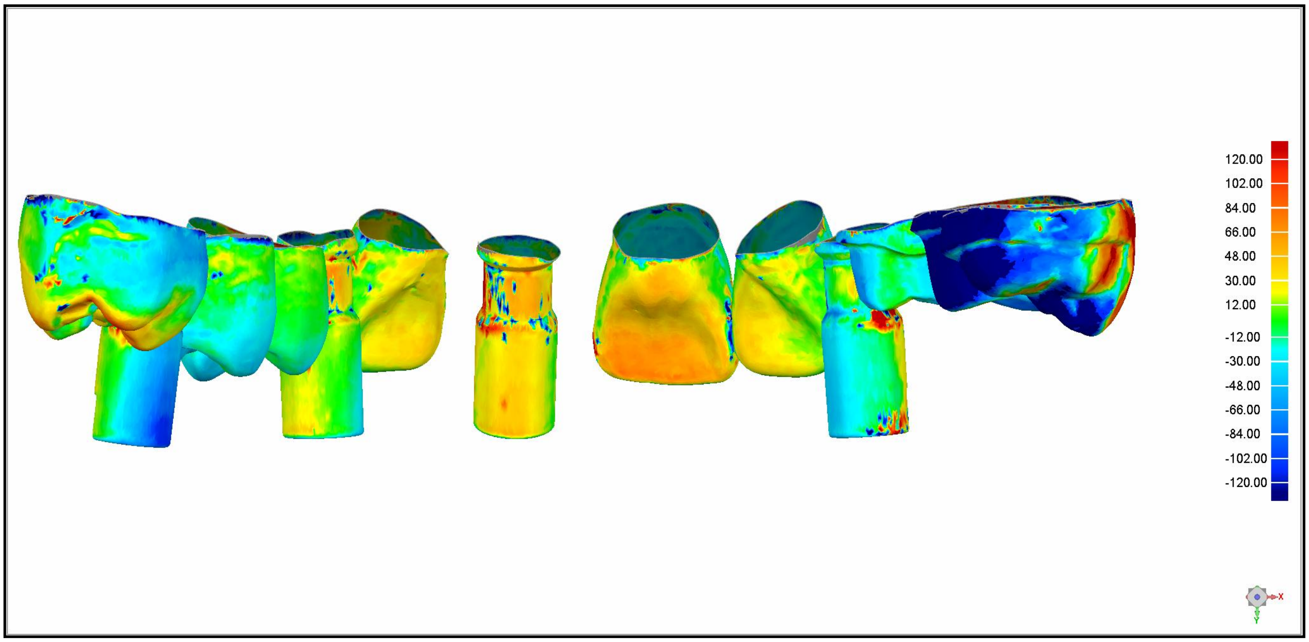

Fecha: 3/6/2017, 1:24 pm

Ajuste de ubicación: Desviaciones superior e inferior

Unidades: u

Nombre	Desv	Estado	Superior Tol	Inferior Tol	Ref X	Ref Y	Ref Z	Radio	Desv X	Desv Y	Desv Z	Medido X	Medido Y	Medido Z	Dir. proy. X	Dir. proy. Y	Dir. proy. Z
Desv. inferior	-3095.59				-29292.33	26884.28	-11910.36	n/a	2652.22	418.67	-1540.50	-26640.11	27302.95	-13450.86	-0.86	-0.14	0.50
Desv. superior	3136.93				-24424.76	34420.95	-1773.28	n/a	-1114.06	-852.49	-2805.79	-25538.83	33568.46	-4579.07	-0.36	-0.27	-0.89
