# Supplementary material for: Accuracy of four digital scanners according to scanning strategy in complete-arch impressions
Source: PLoS One. 2018 Sep 13;13(9):e0202916. doi: 10.1371/journal.pone.0202916 (PMC6136706; doi:10.1371/journal.pone.0202916)
Supplement: S5 Table — iTero (scanning strategy A). (ZIP) [file pone.0202916.s005.zip › S5/IT7A.pdf]

Fecha: 3/6/2017, 1:26 pm

3D Comparación Resultados

Modelo referencia	MRC
Modelo test	IT7A
Nº de puntos de datos	80244
# Aislados	753

Tipo tolerancia	3D desviación
Unidades	u
Máx. crítico	120.00
Máx. nominal	1.00
Mín. nominal	-1.00
Mín. crítico	-120.00

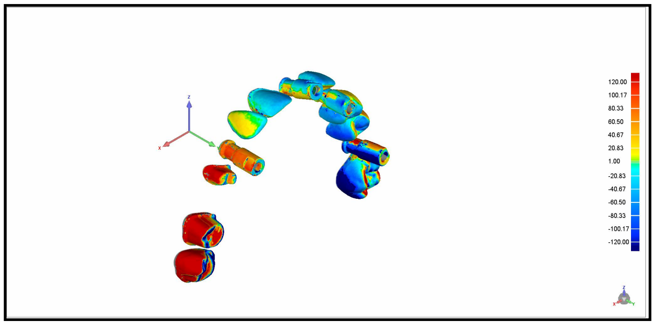

Desviación	
Desviación superior máx.	3139.95
Desviación inferior máx.	-3126.84
Desviación media	120.67 /-122.14
Desviación estándar	253.13

Distribución desviación

>=Min	<Max	# Puntos	%
-120.00	-100.17	1837	2.29
-100.17	-80.33	2584	3.22
-80.33	-60.50	3317	4.13
-60.50	-40.67	5259	6.55
-40.67	-20.83	7405	9.23
-20.83	-1.00	8941	11.14
-1.00	1.00	844	1.05
1.00	20.83	8206	10.23
20.83	40.67	7863	9.80
40.67	60.50	5131	6.39
60.50	80.33	3642	4.54
80.33	100.17	2412	3.01
100.17	120.00	1904	2.37

Fuera del crítico superior	11275	14.05
Fuera del crítico inferior	9624	11.99

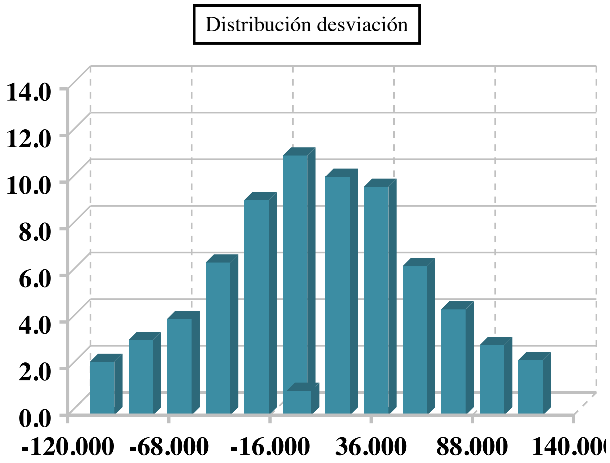

Desviaciones estándar

Distribución (+/-)	# Puntos	%
-6 * Desv. estánd.	452	0.56
-5 * Desv. estánd.	271	0.34
-4 * Desv. estánd.	220	0.27
-3 * Desv. estánd.	743	0.93
-2 * Desv. estánd.	2648	3.30
-1 * Desv. estánd.	35685	44.47
1 * Desv. estánd.	36081	44.96
2 * Desv. estánd.	2516	3.14
3 * Desv. estánd.	755	0.94
4 * Desv. estánd.	241	0.30
5 * Desv. estánd.	233	0.29
6 * Desv. estánd.	399	0.50

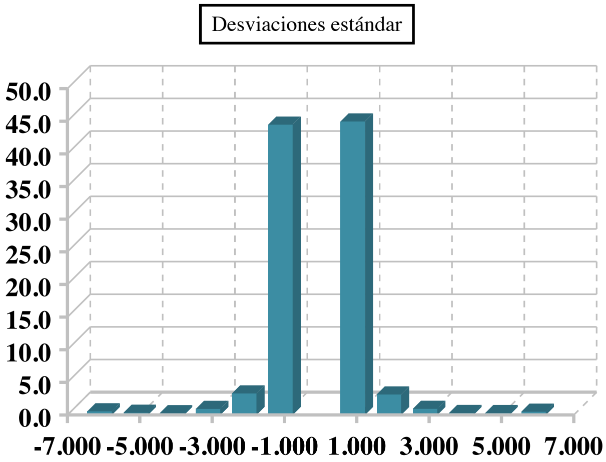

Predefinido: Isométrico

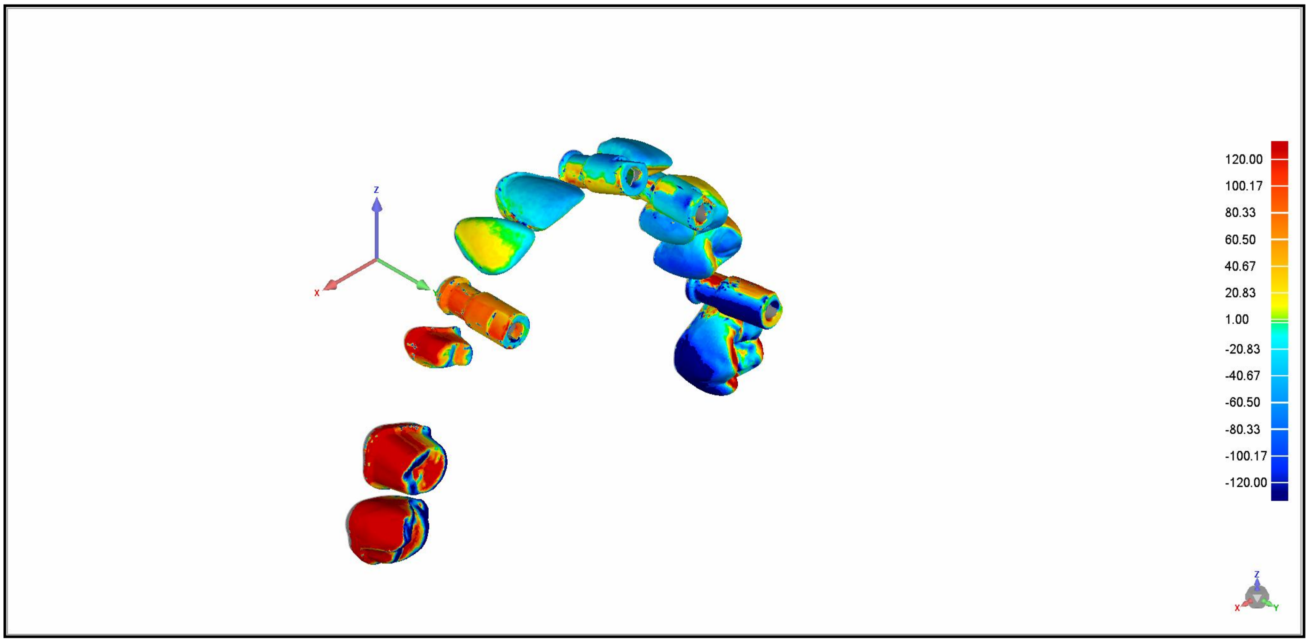

Predefinido: Frente

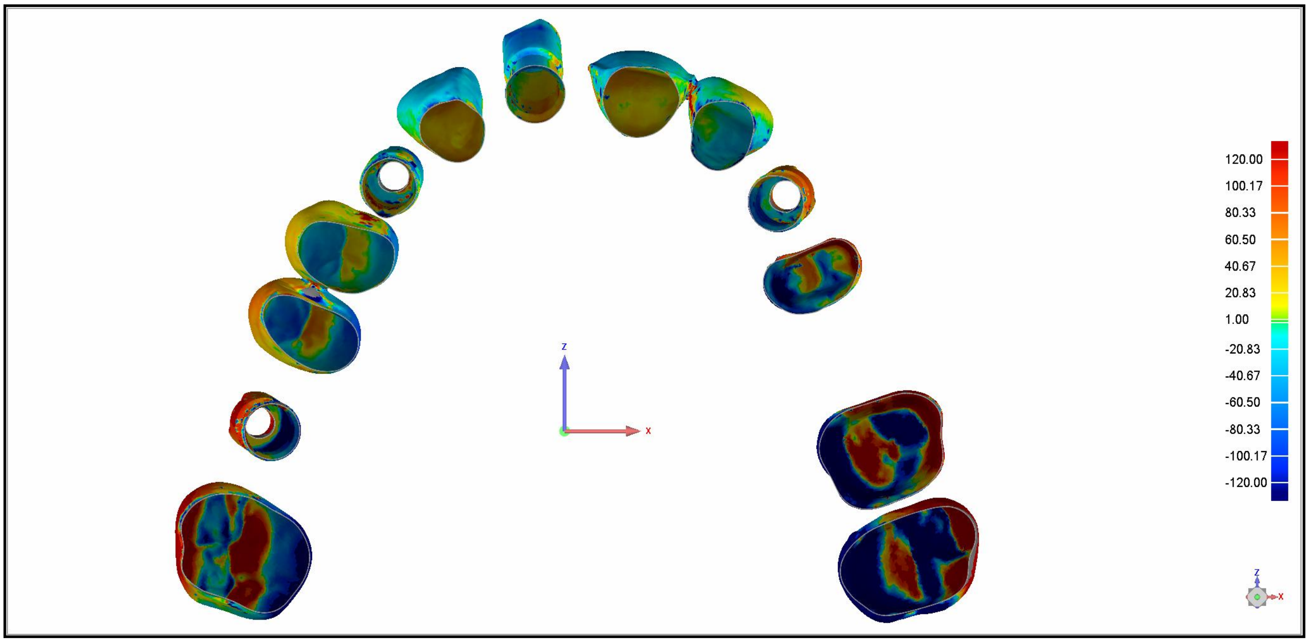

Predefinido: Atrás

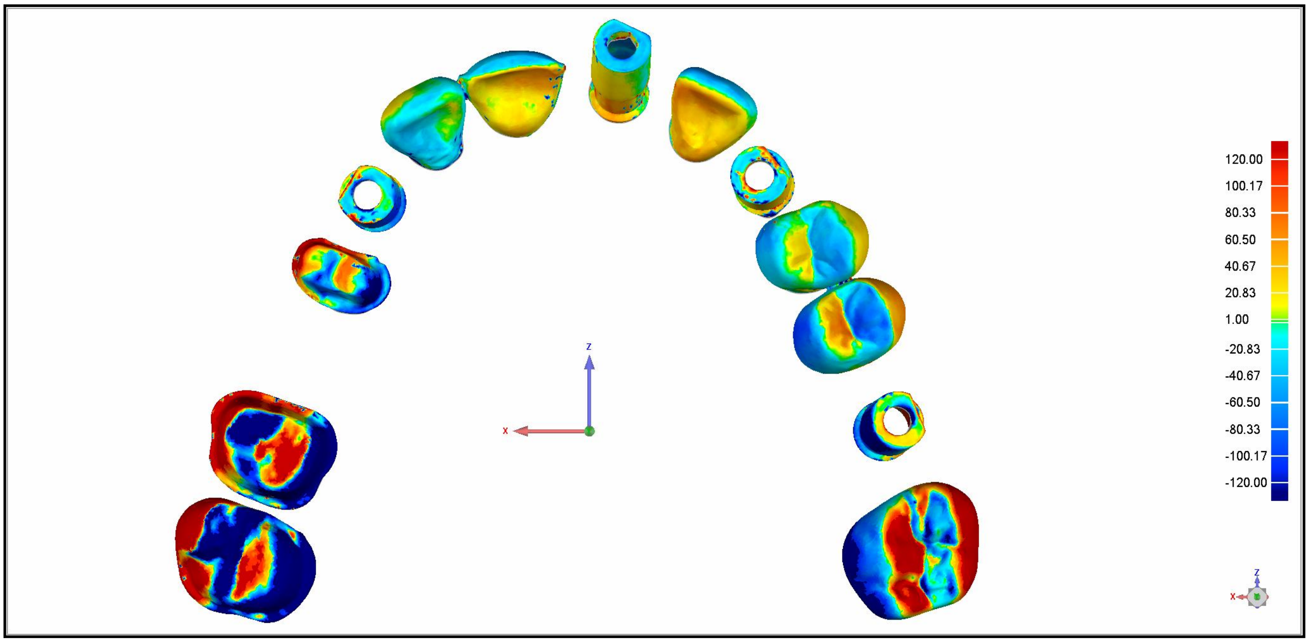

Predefinido: Izquierda

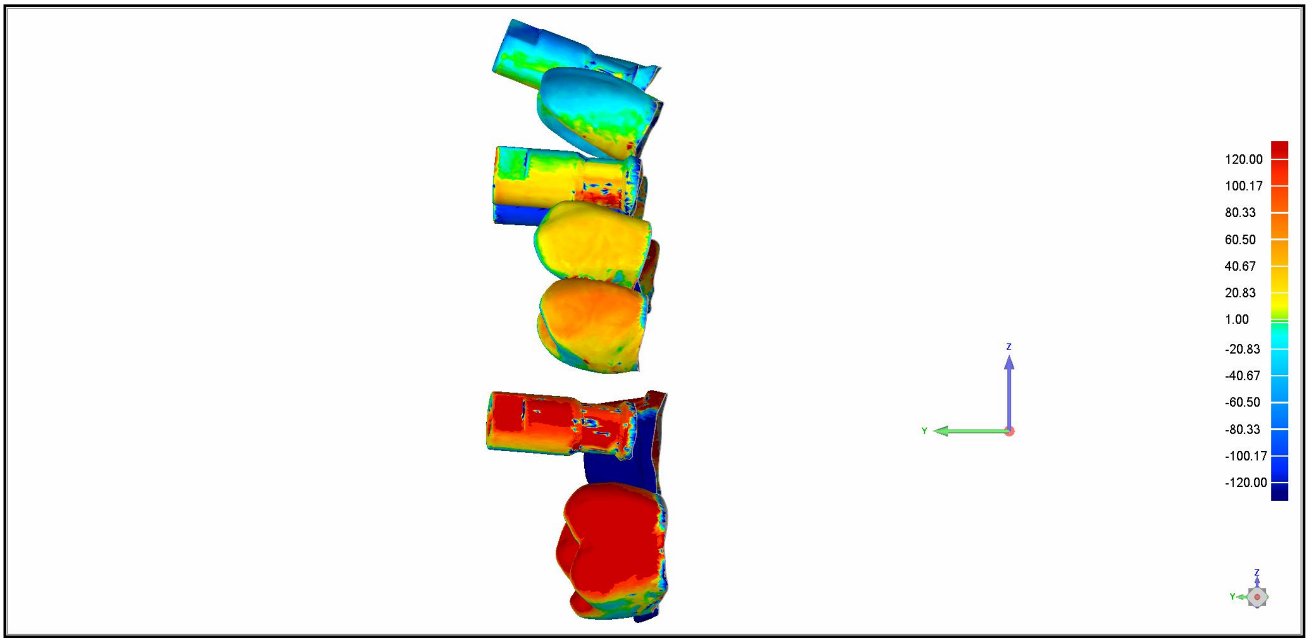

Predefinido: Derecha

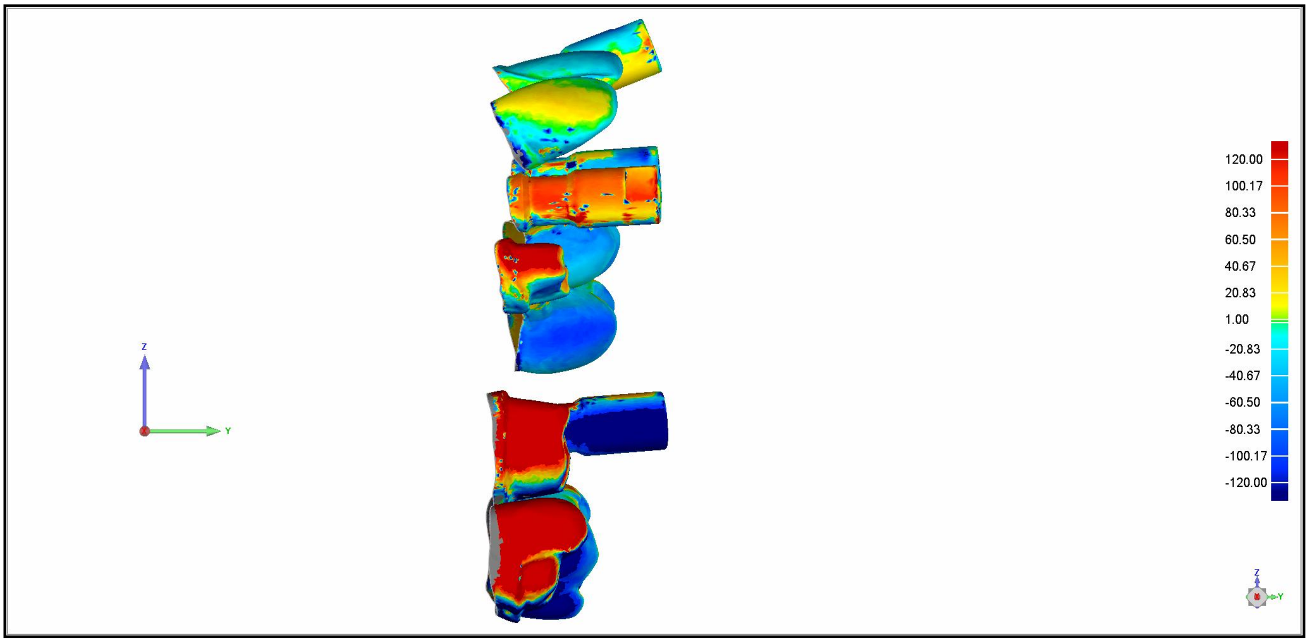

Predefinido: Superior

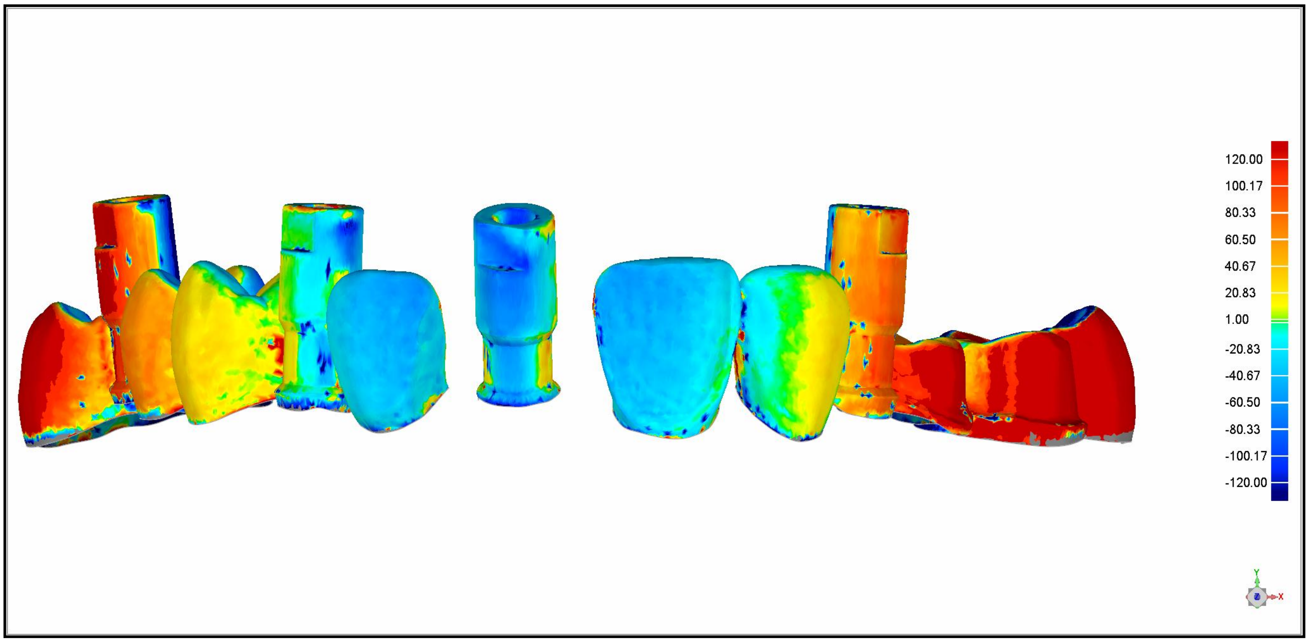

Predefinido: Inferior

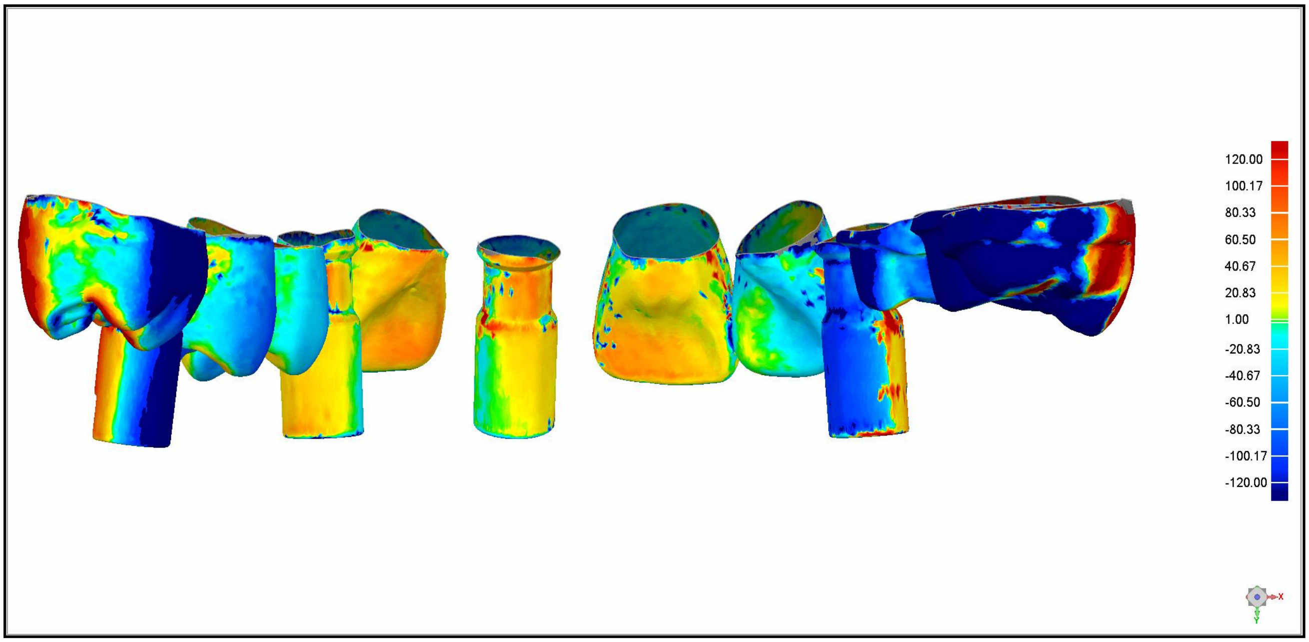

Ajuste de ubicación: Desviaciones superior e inferior

Unidades: u

Nombre	Desv	Estado	Superior Tol	Inferior Tol	Ref X	Ref Y	Ref Z	Radio	Desv X	Desv Y	Desv Z	Medido X	Medido Y	Medido Z	Dir. proy. X	Dir. proy. Y	Dir. proy. Z
Desv. inferior	-3126.84				16989.02	37628.06	17251.36	n/a	221.85	2857.79	-1249.38	17210.86	40485.85	16001.98	-0.07	-0.91	0.40
Desv. superior	3139.95				-22112.39	33245.19	5415.75	n/a	-1161.23	420.59	-2886.86	-23273.63	33665.78	2528.89	-0.37	0.13	-0.92
