# Supplementary material for: Accuracy of four digital scanners according to scanning strategy in complete-arch impressions
Source: PLoS One. 2018 Sep 13;13(9):e0202916. doi: 10.1371/journal.pone.0202916 (PMC6136706; doi:10.1371/journal.pone.0202916)
Supplement: S5 Table — iTero (scanning strategy A). (ZIP) [file pone.0202916.s005.zip › S5/IT10A.pdf]

3D Comparación Resultados

Modelo referencia	MRC
Modelo test	IT10A
Nº de puntos de datos	79265
# Aislados	737

Tipo tolerancia	3D desviación
Unidades	u
Máx. crítico	120.00
Máx. nominal	7.00
Mín. nominal	-7.00
Mín. crítico	-120.00

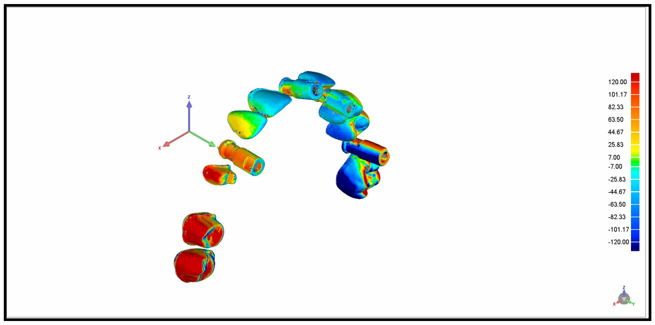

Desviación	
Desviación superior máx.	3063.25
Desviación inferior máx.	-3151.99
Desviación media	130.45 /-115.06
Desviación estándar	293.17

Distribución desviación

>=Min	<Max	# Puntos	%
-120.00	-101.17	1612	2.03
-101.17	-82.33	2251	2.84
-82.33	-63.50	2995	3.78
-63.50	-44.67	4771	6.02
-44.67	-25.83	7946	10.02
-25.83	-7.00	8481	10.70
-7.00	7.00	6097	7.69
7.00	25.83	7583	9.57
25.83	44.67	6474	8.17
44.67	63.50	4763	6.01
63.50	82.33	3564	4.50
82.33	101.17	2311	2.92
101.17	120.00	1563	1.97

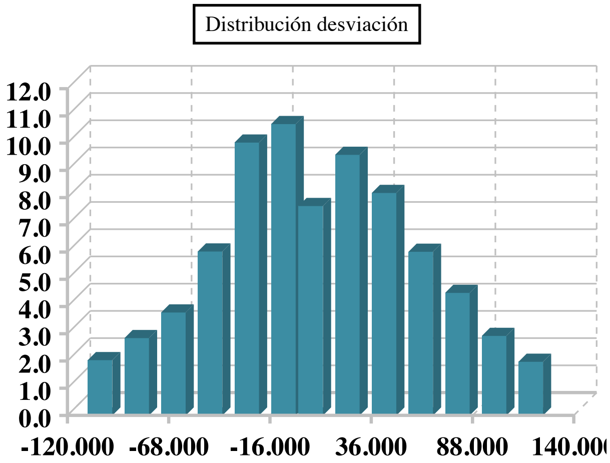

Fuera del crítico superior		10439	13.17
Fuera del crítico inferior		8415	10.62

Desviaciones estándar

Distribución (+/-)	# Puntos	%
-6 * Desv. estánd.	436	0.55
-5 * Desv. estánd.	253	0.32
-4 * Desv. estánd.	260	0.33
-3 * Desv. estánd.	333	0.42
-2 * Desv. estánd.	1531	1.93
-1 * Desv. estánd.	40021	50.49
1 * Desv. estánd.	33848	42.70
2 * Desv. estánd.	1213	1.53
3 * Desv. estánd.	287	0.36
4 * Desv. estánd.	236	0.30
5 * Desv. estánd.	303	0.38
6 * Desv. estánd.	544	0.69

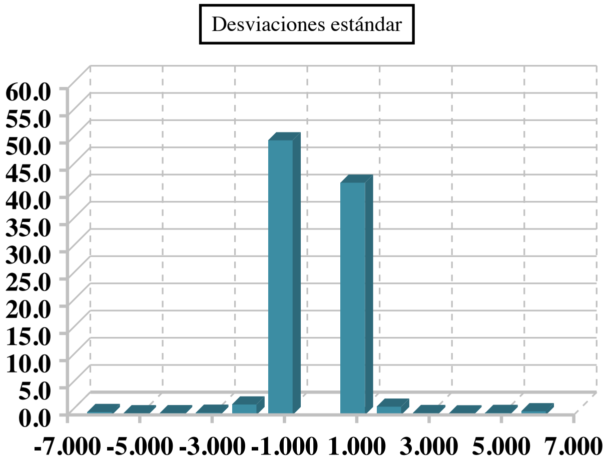

Predefinido: Isométrico

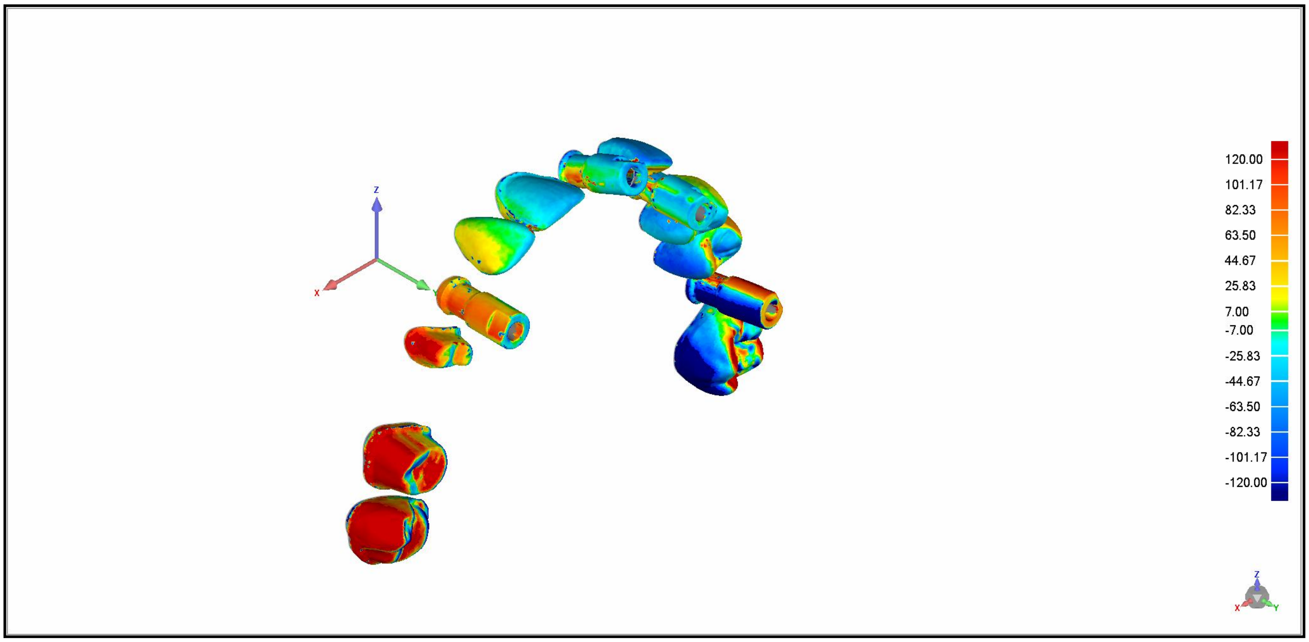

Predefinido: Frente

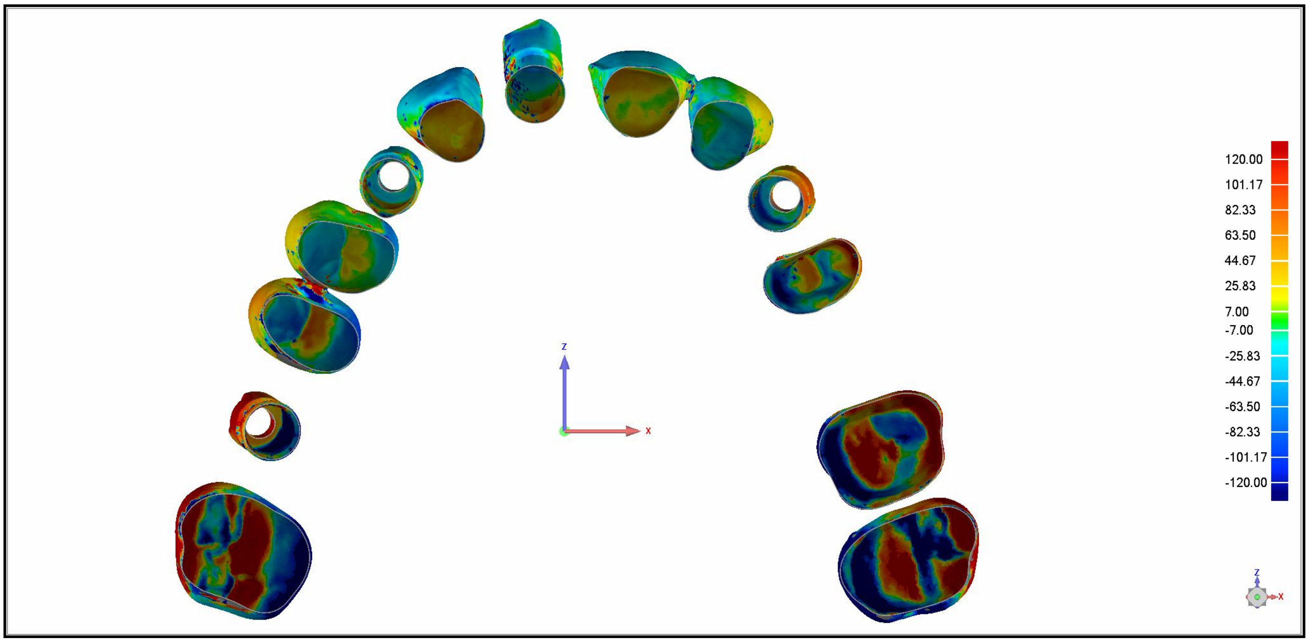

Predefinido: Atrás

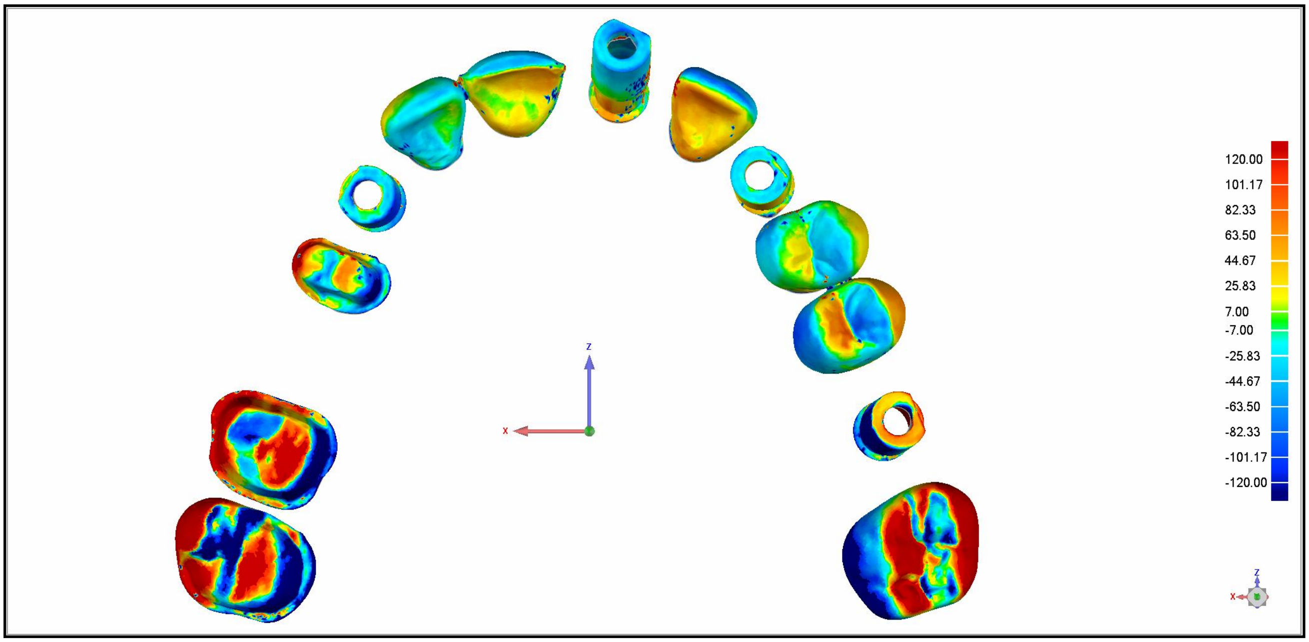

Predefinido: Izquierda

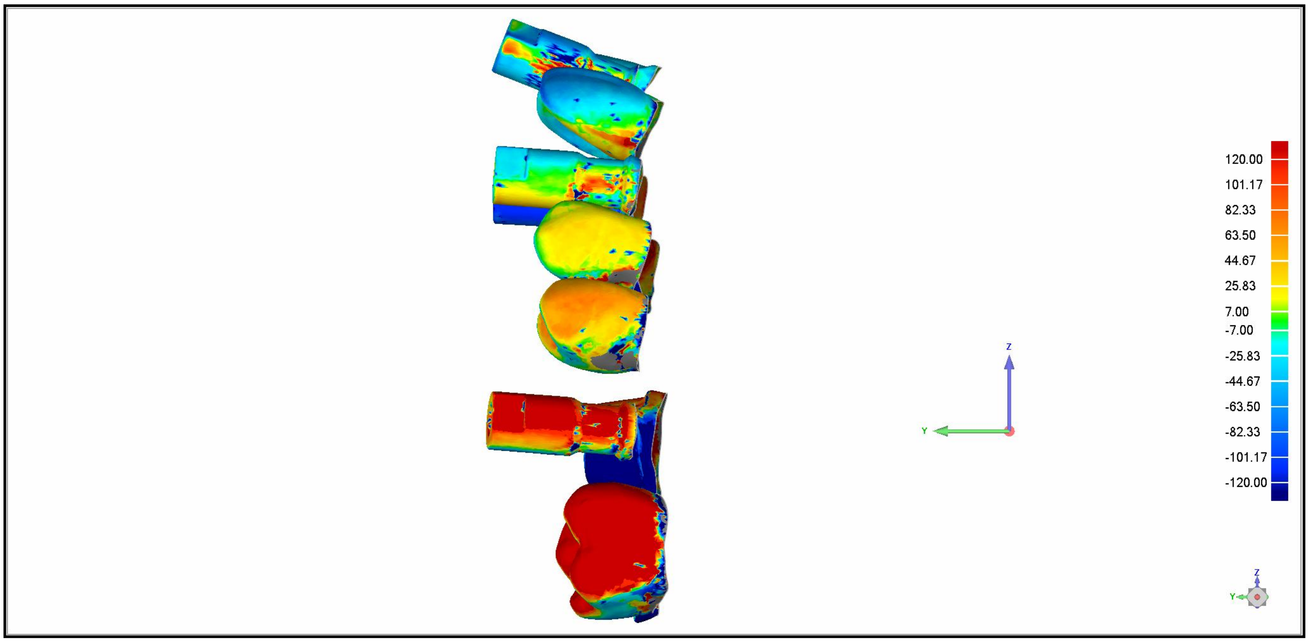

Predefinido: Derecha

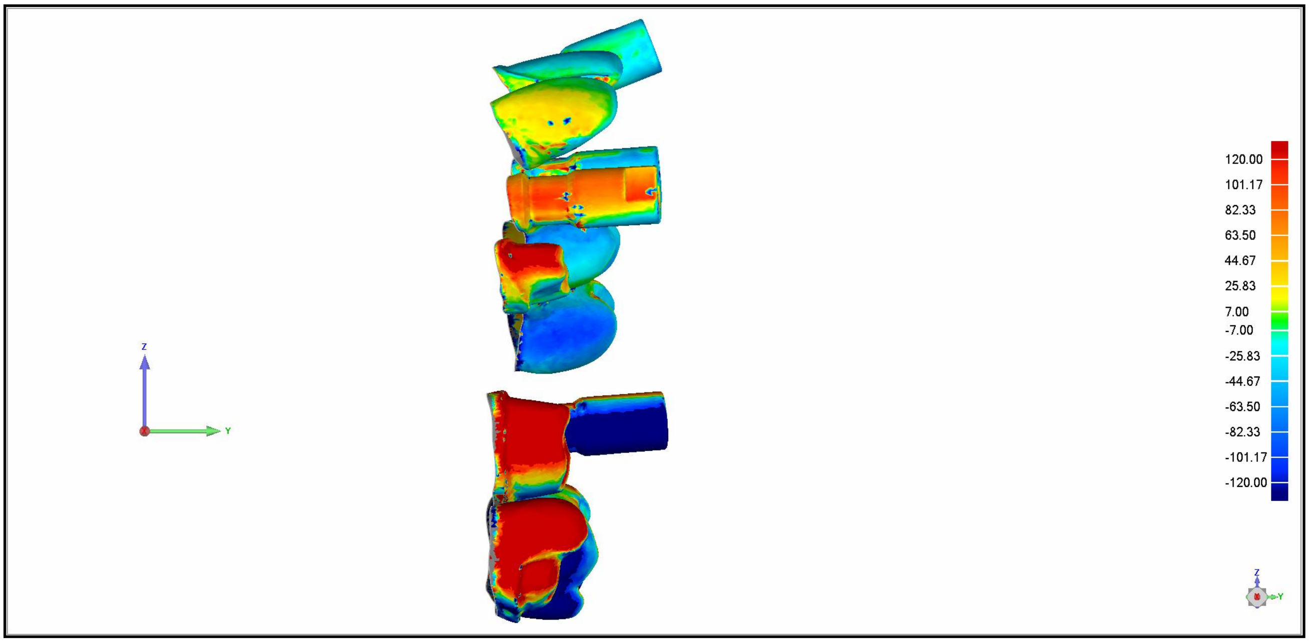

Predefinido: Superior

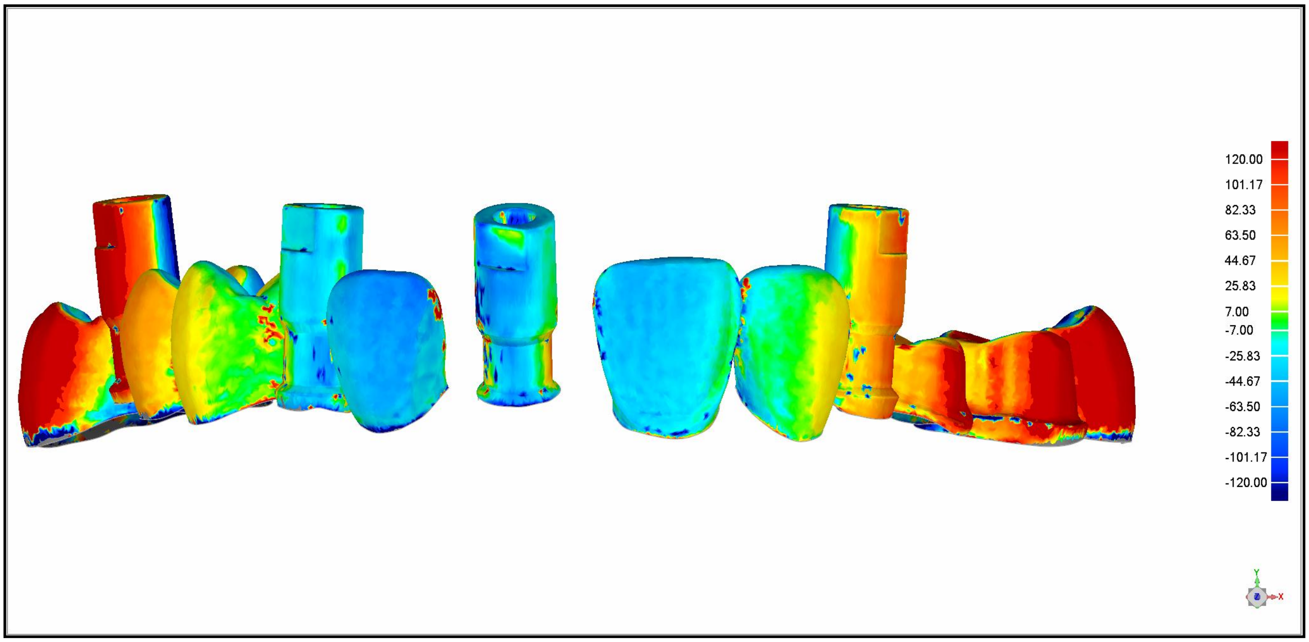

Predefinido: Inferior

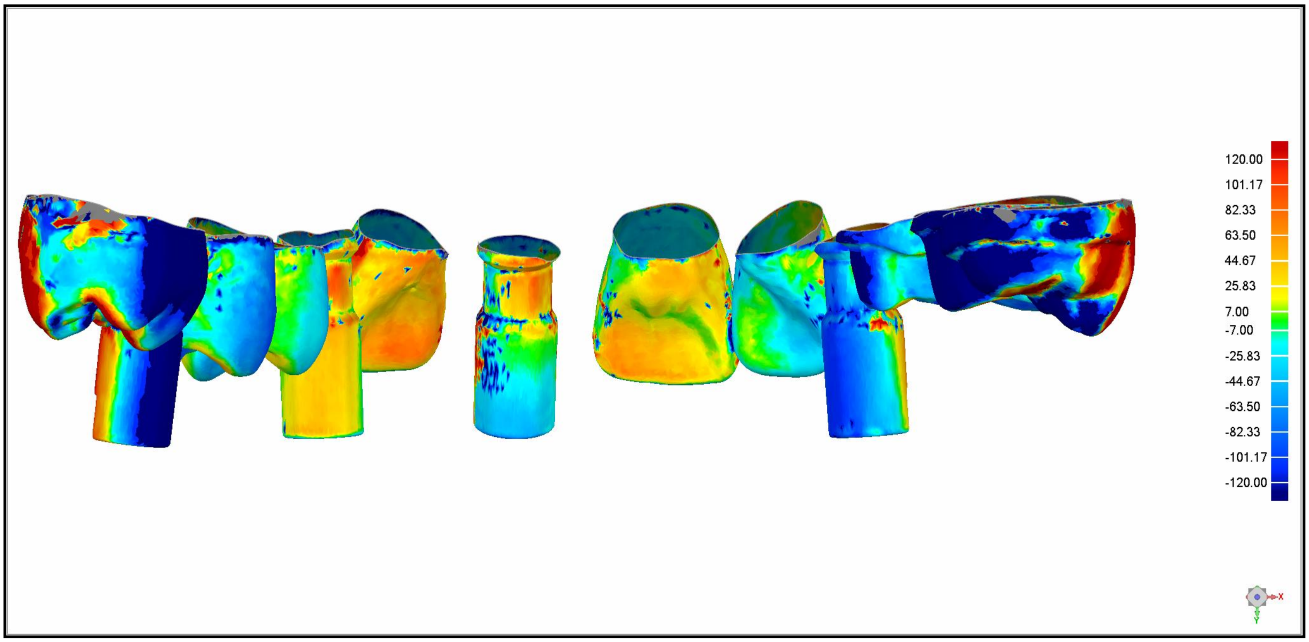

Ajuste de ubicación: Desviaciones superior e inferior

Unidades: u

Nombre	Desv	Estado	Superior Tol	Inferior Tol	Ref X	Ref Y	Ref Z	Radio	Desv X	Desv Y	Desv Z	Medido X	Medido Y	Medido Z	Dir. proy. X	Dir. proy. Y	Dir. proy. Z
Desv. inferior	-3151.99				-21214.71	35233.48	7138.15	n/a	-1647.99	-2392.17	-1223.40	-22862.70	32841.31	5914.75	0.52	0.76	0.39
Desv. superior	3063.25				-21354.16	30522.69	11024.30	n/a	583.27	-1637.00	2522.60	-20770.90	28885.69	13546.90	0.19	-0.53	0.82
