# Supplementary material for: Accuracy of four digital scanners according to scanning strategy in complete-arch impressions
Source: PLoS One. 2018 Sep 13;13(9):e0202916. doi: 10.1371/journal.pone.0202916 (PMC6136706; doi:10.1371/journal.pone.0202916)
Supplement: S6 Table — iTero (scanning strategy B). (ZIP) [file pone.0202916.s006.zip › S6/IT4B.pdf]

3D Comparación Resultados

Modelo referencia	MRC
Modelo test	IT4B
Nº de puntos de datos	78722
# Aislados	589

Tipo tolerancia	3D desviación
Unidades	u
Máx. crítico	120.00
Máx. nominal	6.00
Mín. nominal	-6.00
Mín. crítico	-120.00

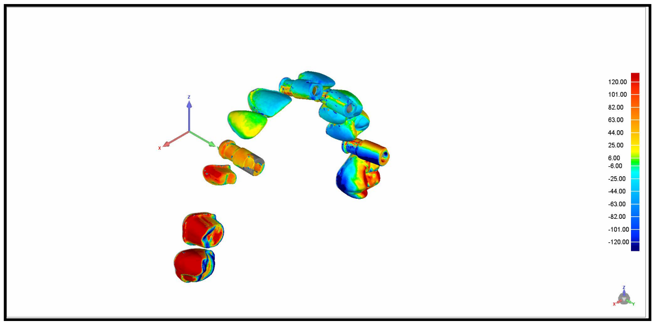

Desviación	
Desviación superior máx.	3143.93
Desviación inferior máx.	-3148.61
Desviación media	105.90 /-103.50
Desviación estándar	260.45

Distribución desviación

>=Min	<Max	# Puntos	%
-120.00	-101.00	1337	1.70
-101.00	-82.00	1623	2.06
-82.00	-63.00	2763	3.51
-63.00	-44.00	4685	5.95
-44.00	-25.00	7709	9.79
-25.00	-6.00	9003	11.44
-6.00	6.00	6641	8.44
6.00	25.00	9812	12.46
25.00	44.00	7318	9.30
44.00	63.00	4952	6.29
63.00	82.00	3191	4.05
82.00	101.00	2543	3.23
101.00	120.00	1876	2.38

Fuera del crítico superior		8310	10.56
Fuera del crítico inferior		6959	8.84

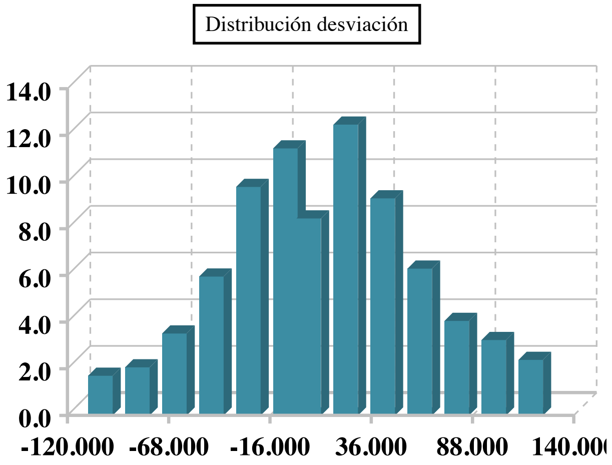

Desviaciones estándar

Distribución (+/-)	# Puntos	%
-6 * Desv. estánd.	474	0.60
-5 * Desv. estánd.	207	0.26
-4 * Desv. estánd.	167	0.21
-3 * Desv. estánd.	280	0.36
-2 * Desv. estánd.	1976	2.51
-1 * Desv. estánd.	37939	48.19
1 * Desv. estánd.	34822	44.23
2 * Desv. estánd.	1572	2.00
3 * Desv. estánd.	371	0.47
4 * Desv. estánd.	273	0.35
5 * Desv. estánd.	164	0.21
6 * Desv. estánd.	477	0.61

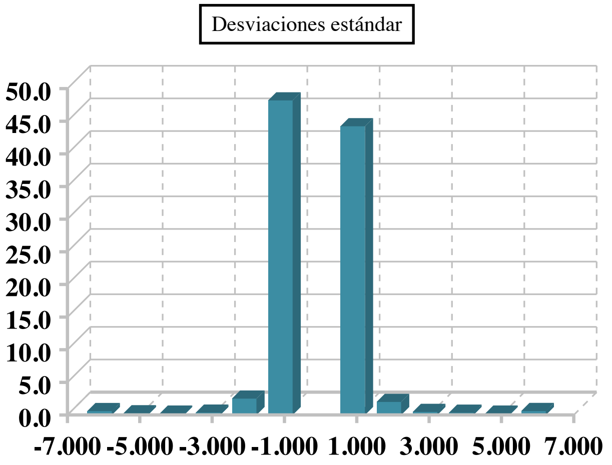

Predefinido: Isométrico

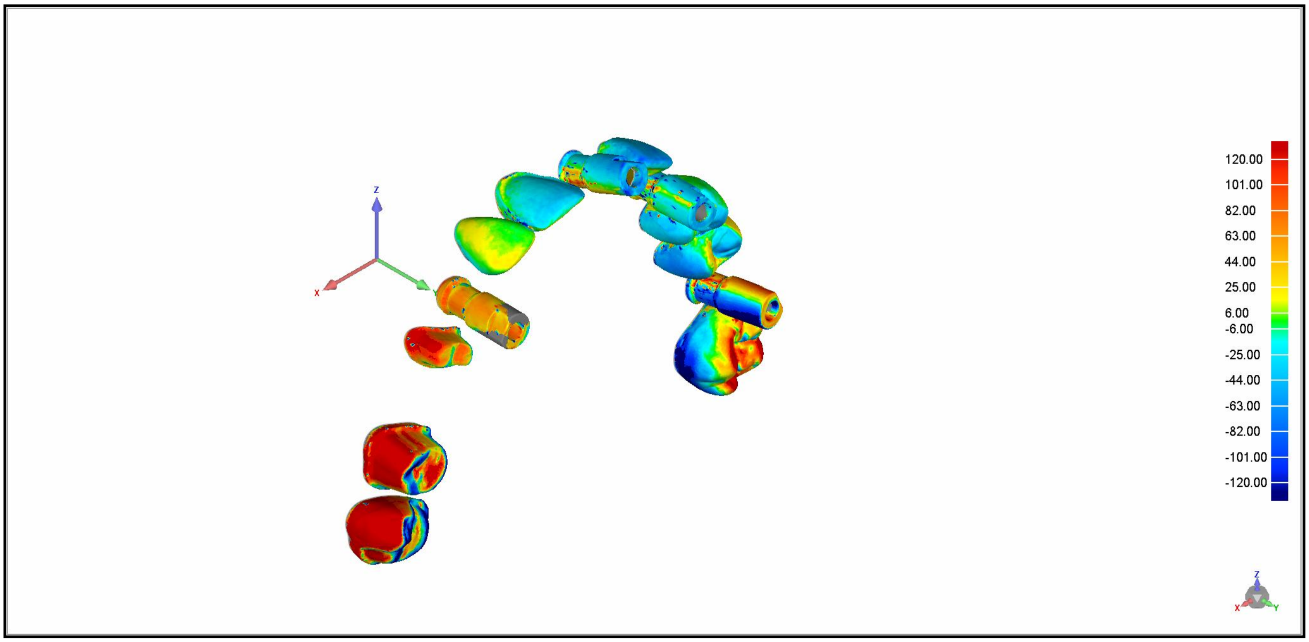

Predefinido: Frente

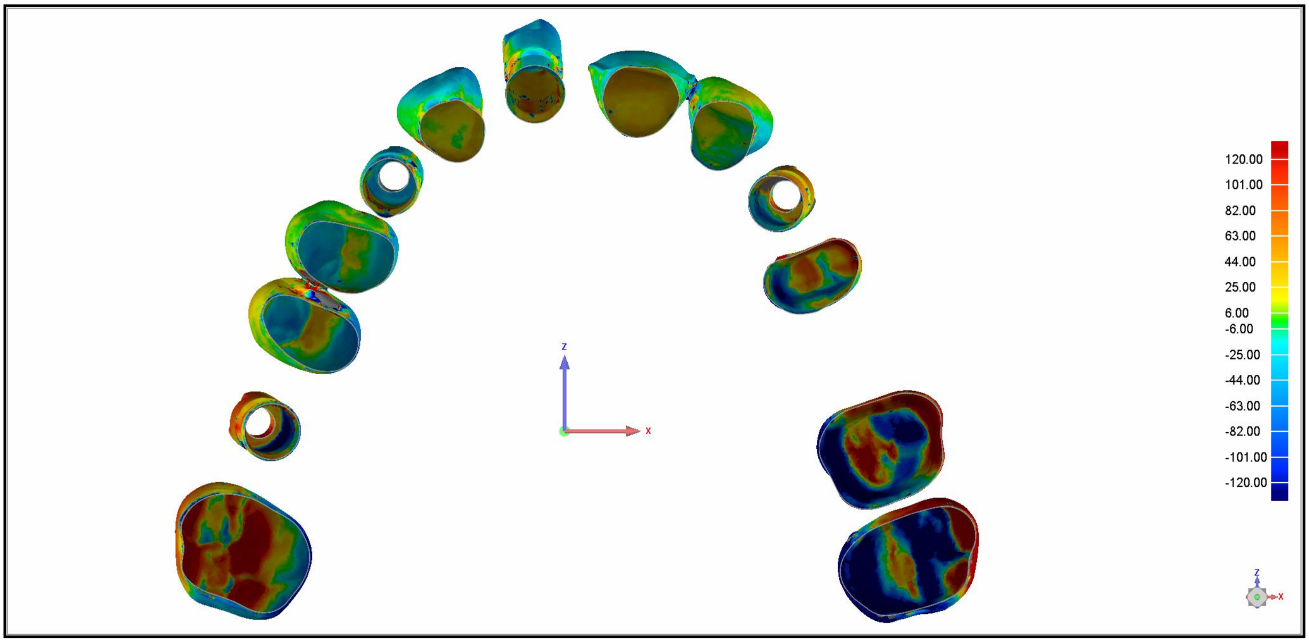

Predefinido: Atrás

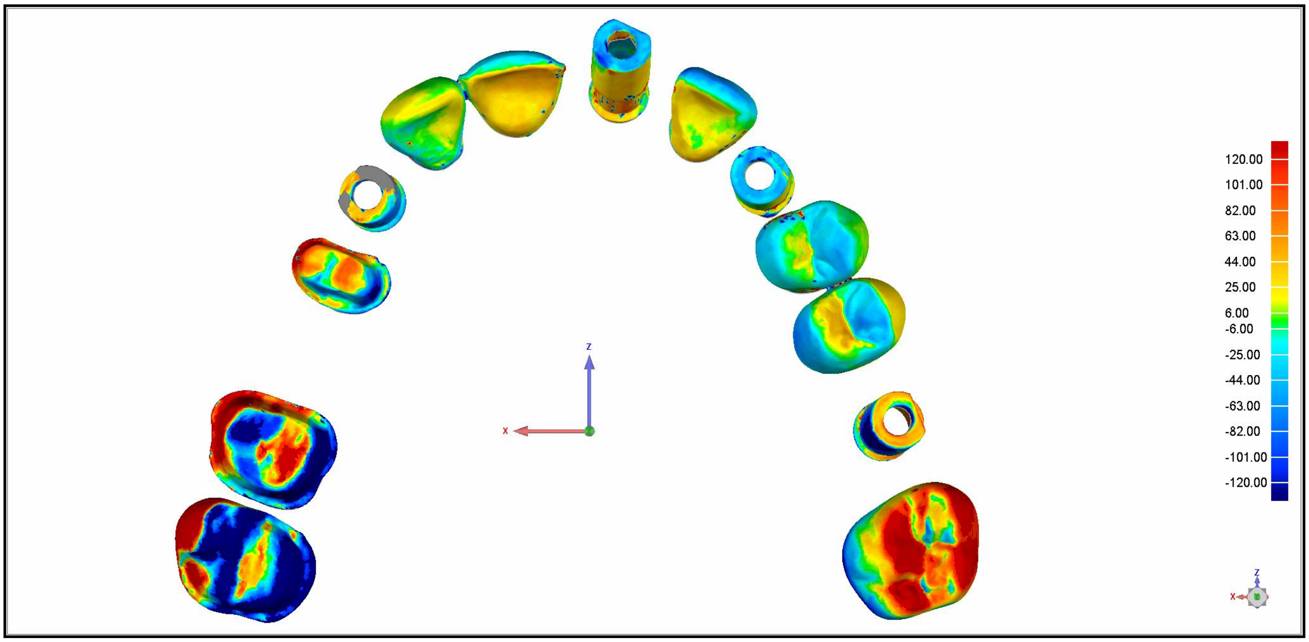

Predefinido: Izquierda

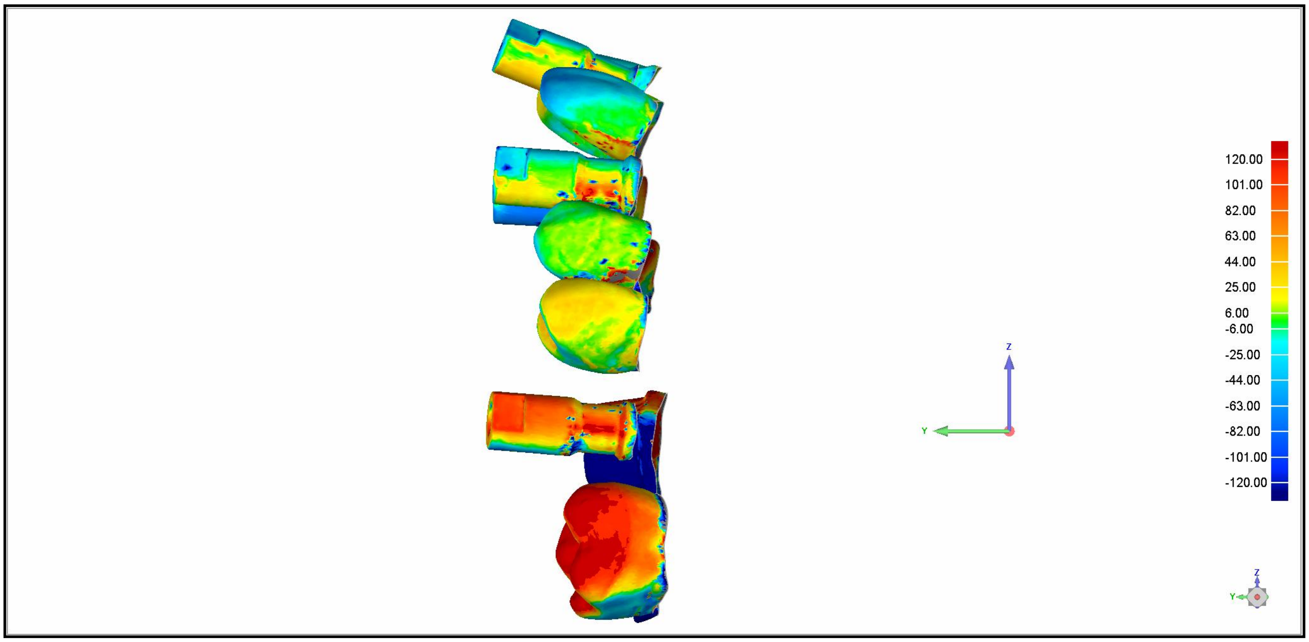

Predefinido: Derecha

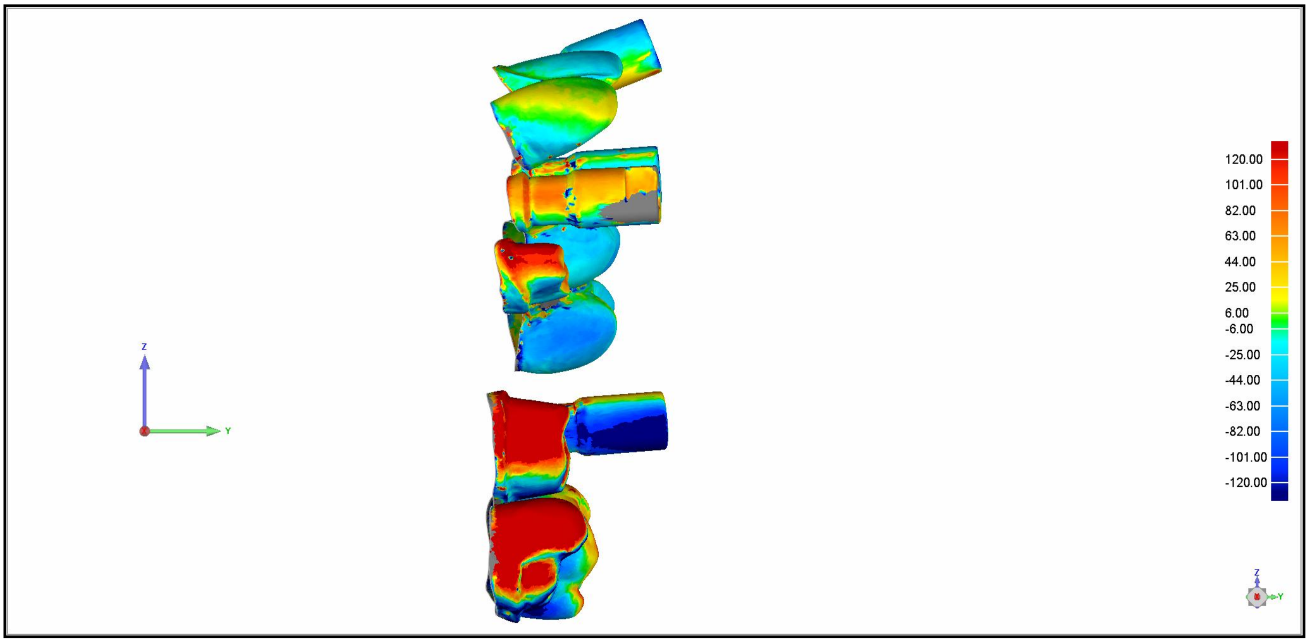

Predefinido: Superior

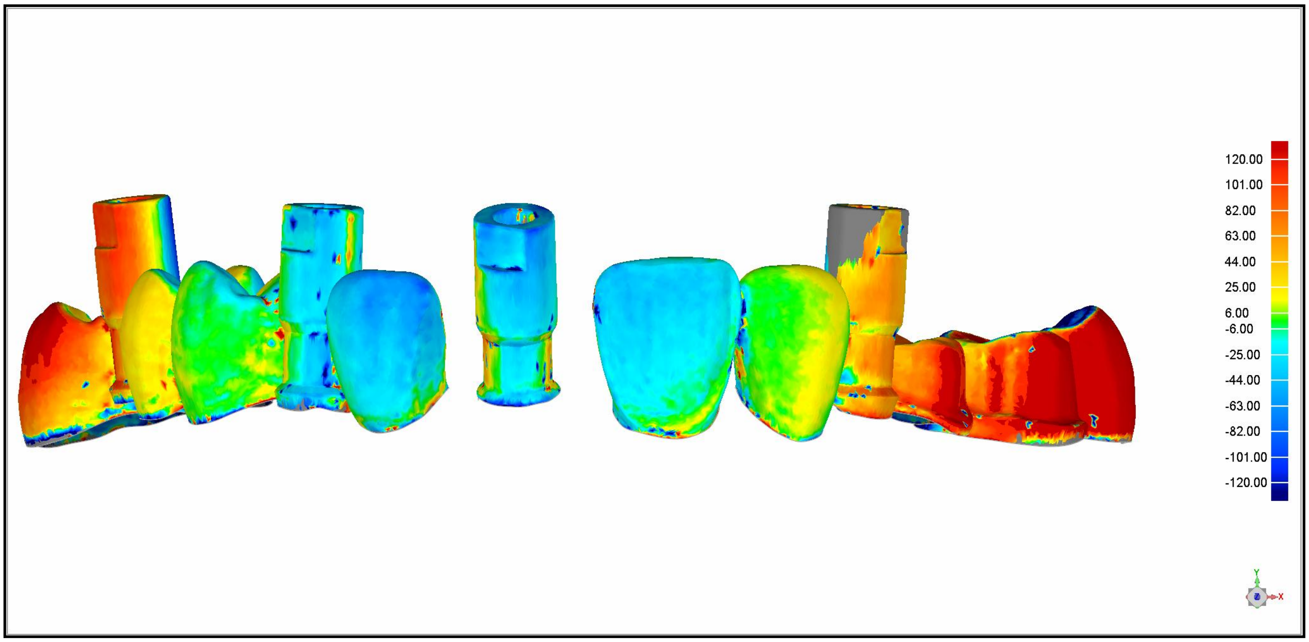

Predefinido: Inferior

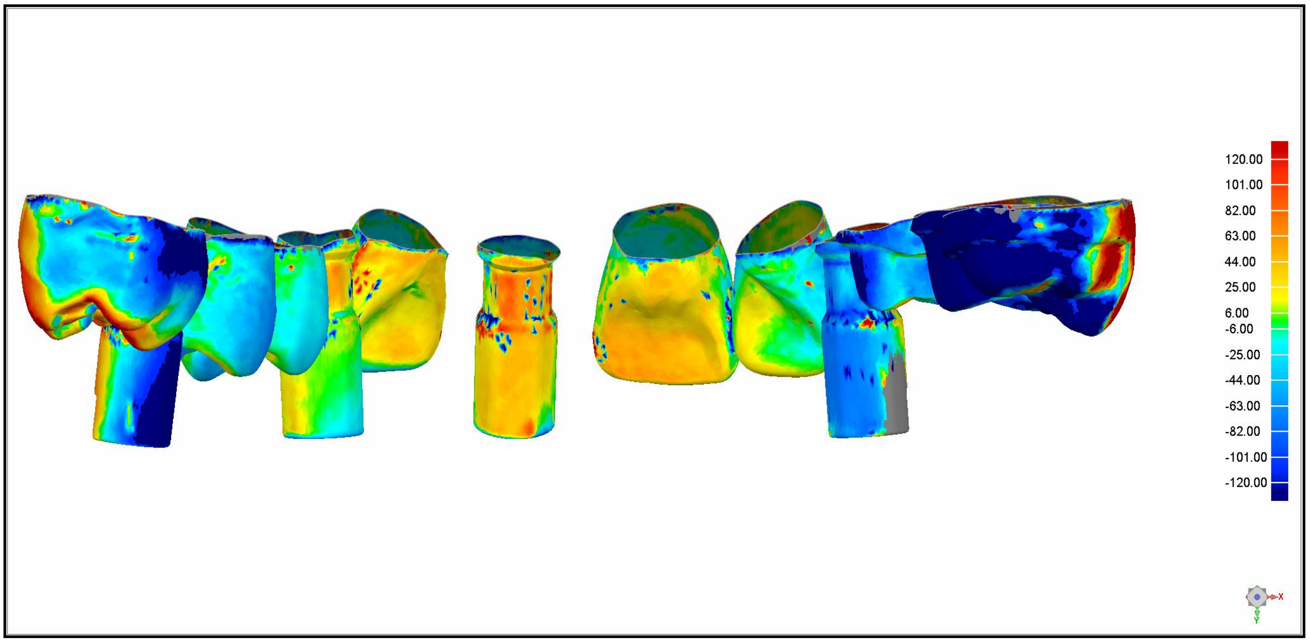

Ajuste de ubicación: Desviaciones superior e inferior

Unidades: u

Nombre	Desv	Estado	Superior Tol	Inferior Tol	Ref X	Ref Y	Ref Z	Radio	Desv X	Desv Y	Desv Z	Medido X	Medido Y	Medido Z	Dir. proy. X	Dir. proy. Y	Dir. proy. Z
Desv. inferior	-3148.61				-29208.33	26961.25	-11988.49	n/a	2727.45	406.28	-1519.77	-26480.89	27367.52	-13508.26	-0.87	-0.13	0.48
Desv. superior	3143.93				-17902.57	29727.70	11012.38	n/a	3059.91	64.67	719.07	-14842.66	29792.38	11731.45	0.97	0.02	0.23
