# Supplementary material for: Accuracy of four digital scanners according to scanning strategy in complete-arch impressions
Source: PLoS One. 2018 Sep 13;13(9):e0202916. doi: 10.1371/journal.pone.0202916 (PMC6136706; doi:10.1371/journal.pone.0202916)
Supplement: S6 Table — iTero (scanning strategy B). (ZIP) [file pone.0202916.s006.zip › S6/IT8B.pdf]

3D Comparación Resultados

Modelo referencia	MRC
Modelo test	IT8B
Nº de puntos de datos	80675
# Aislados	556

Tipo tolerancia	3D desviación
Unidades	u
Máx. crítico	120.00
Máx. nominal	3.00
Mín. nominal	-3.00
Mín. crítico	-120.00

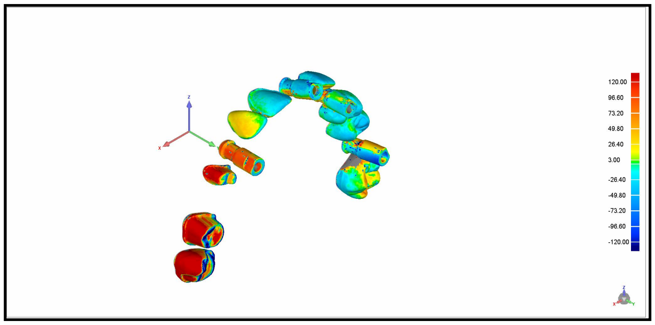

Desviación	
Desviación superior máx.	3140.14
Desviación inferior máx.	-3152.67
Desviación media	107.11 /-112.76
Desviación estándar	304.68

Distribución desviación

>=Min	<Max	# Puntos	%
-120.00	-96.60	1625	2.01
-96.60	-73.20	1939	2.40
-73.20	-49.80	2531	3.14
-49.80	-26.40	8559	10.61
-26.40	-3.00	16582	20.55
-3.00	3.00	4434	5.50
3.00	26.40	13071	16.20
26.40	49.80	8941	11.08
49.80	73.20	4364	5.41
73.20	96.60	2859	3.54
96.60	120.00	1886	2.34

Fuera del crítico superior	6875	8.52
Fuera del crítico inferior	7009	8.69

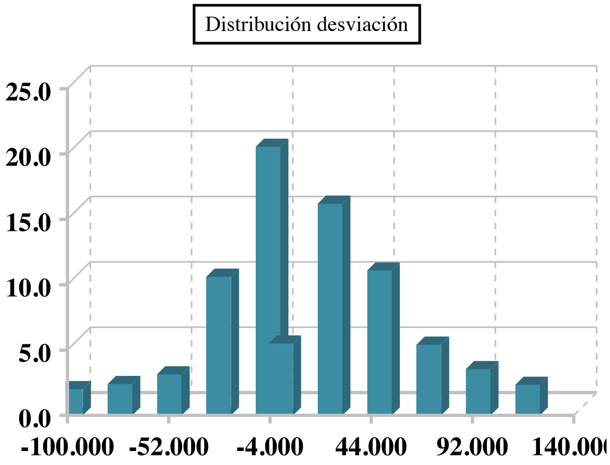

Desviaciones estándar

Distribución (+/-)	# Puntos	%
-6 * Desv. estánd.	649	0.80
-5 * Desv. estánd.	249	0.31
-4 * Desv. estánd.	253	0.31
-3 * Desv. estánd.	235	0.29
-2 * Desv. estánd.	1555	1.93
-1 * Desv. estánd.	34988	43.37
1 * Desv. estánd.	40067	49.66
2 * Desv. estánd.	1339	1.66
3 * Desv. estánd.	299	0.37
4 * Desv. estánd.	341	0.42
5 * Desv. estánd.	280	0.35
6 * Desv. estánd.	420	0.52

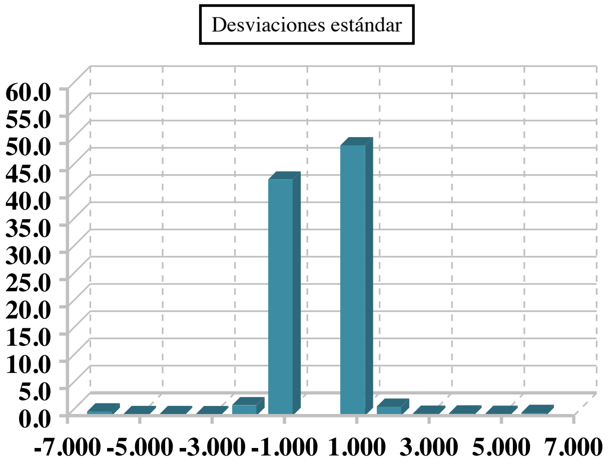

Predefinido: Isométrico

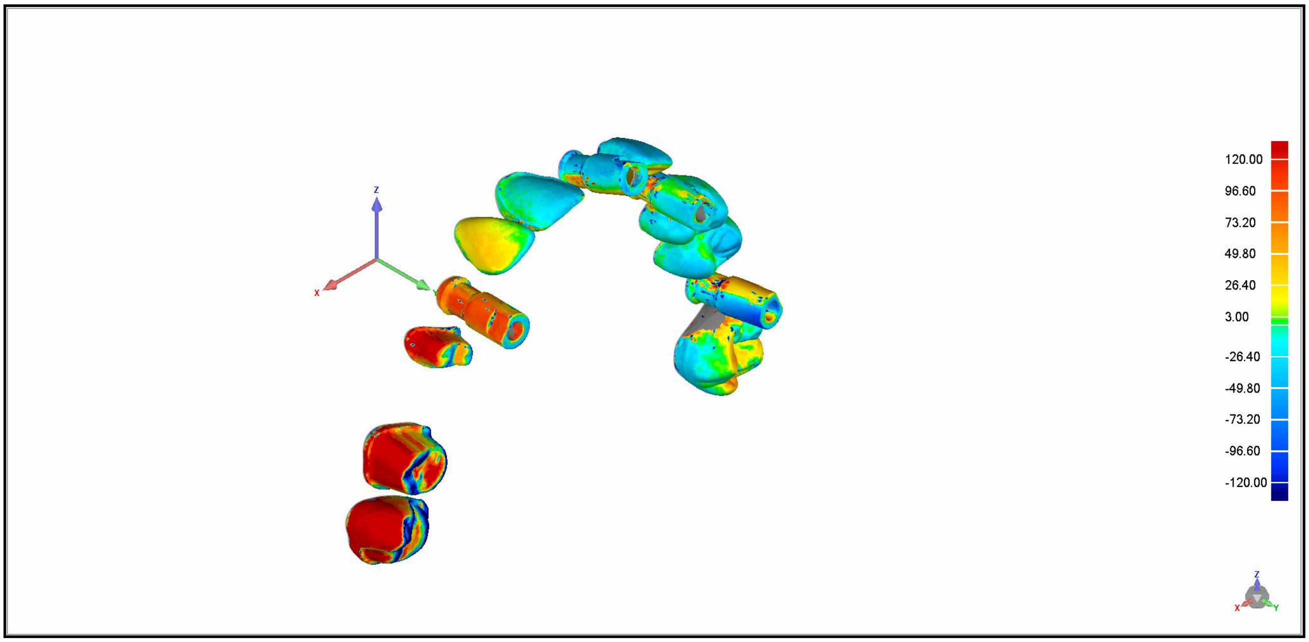

Predefinido: Frente

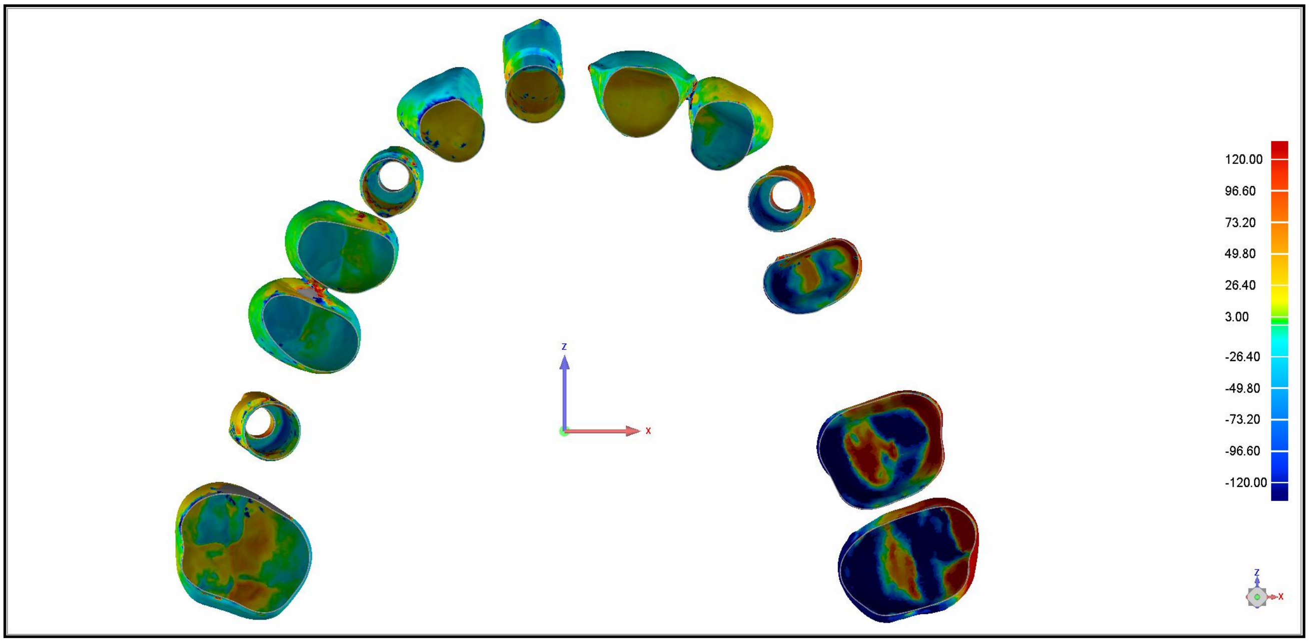

Predefinido: Atrás

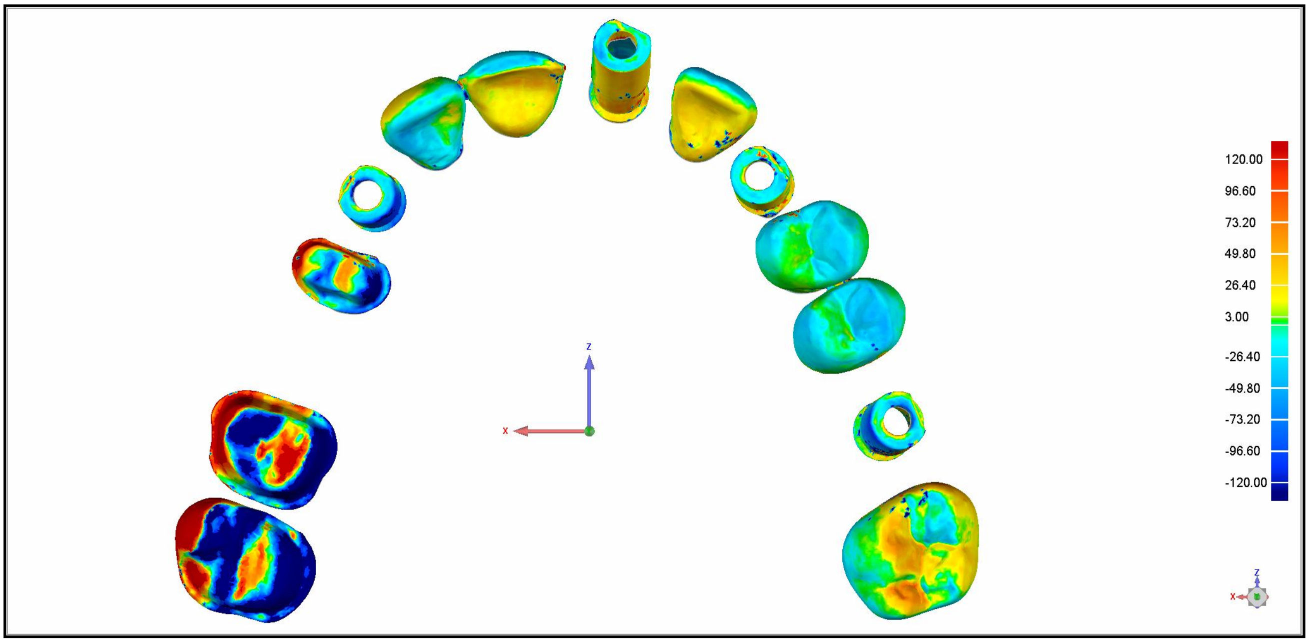

Predefinido: Izquierda

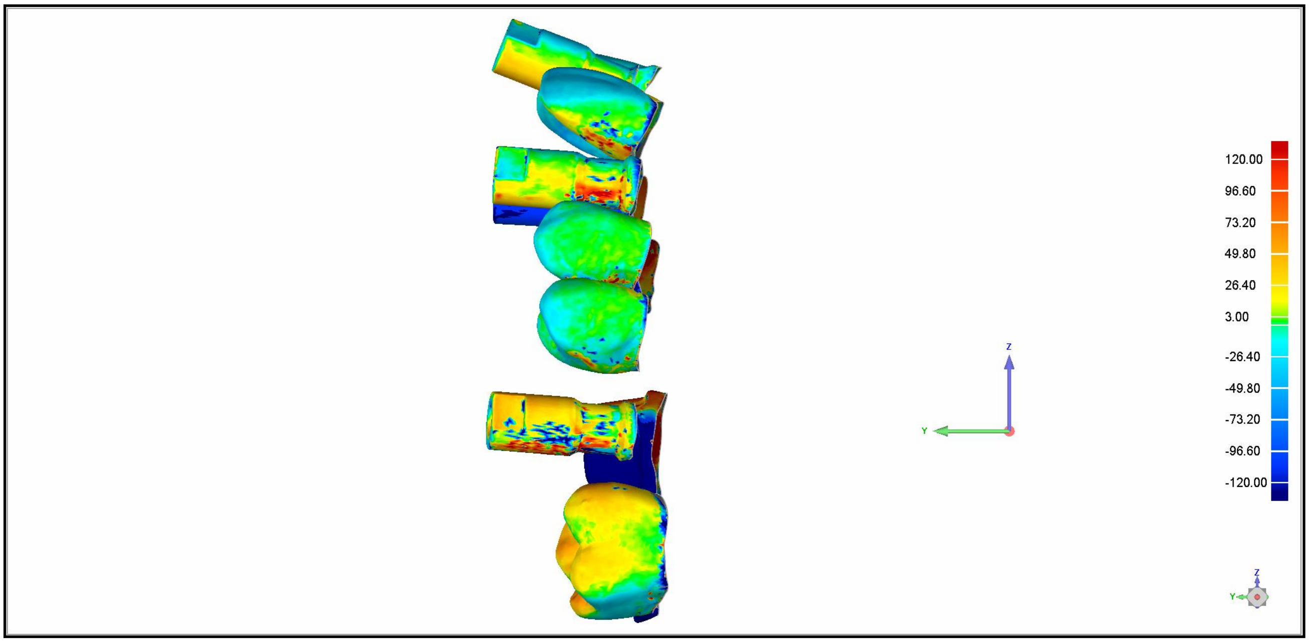

Predefinido: Derecha

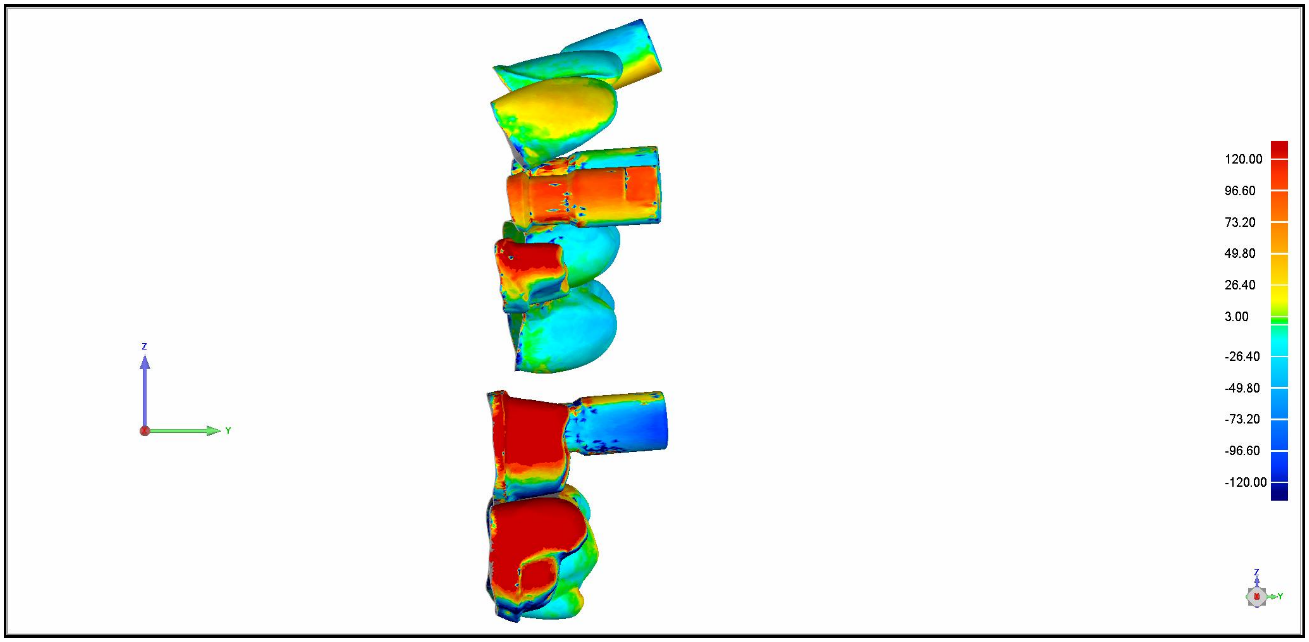

Predefinido: Superior

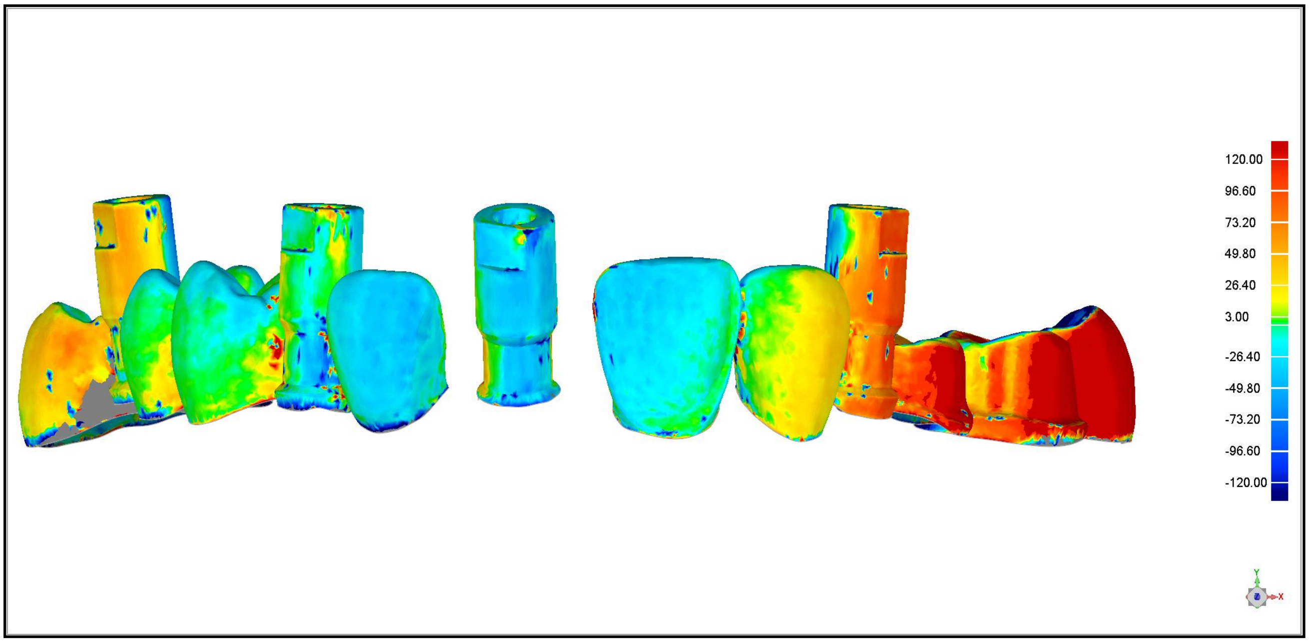

Predefinido: Inferior

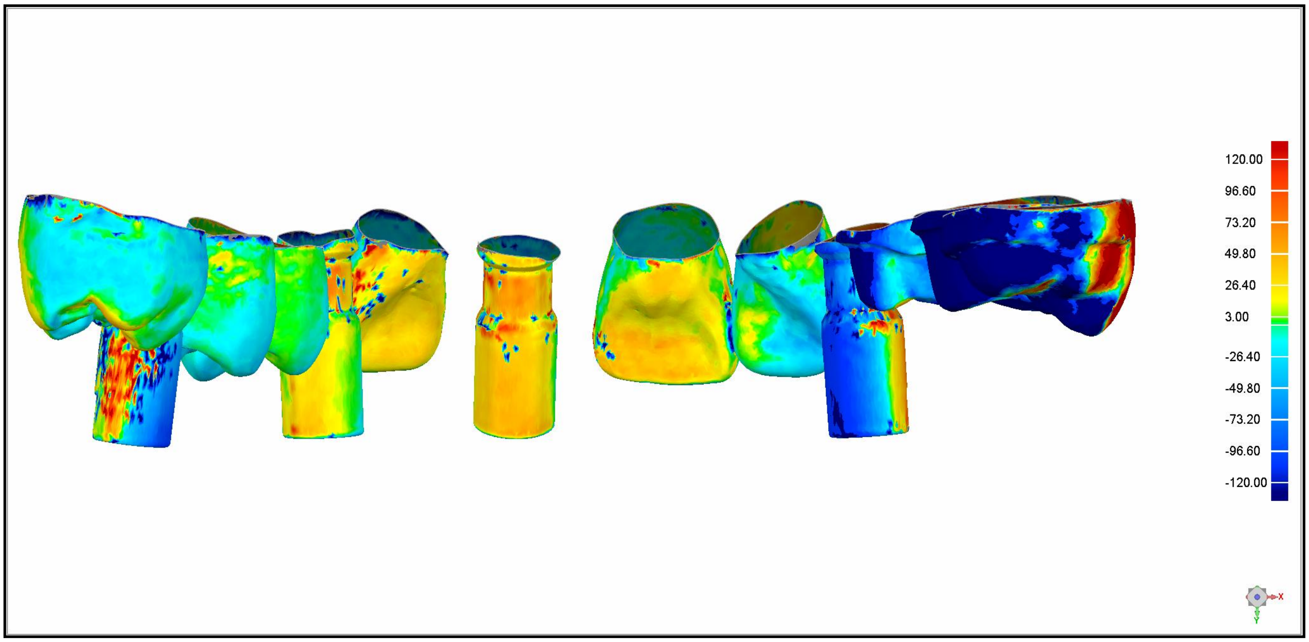

Ajuste de ubicación: Desviaciones superior e inferior

Unidades: u

Nombre	Desv	Estado	Superior Tol	Inferior Tol	Ref X	Ref Y	Ref Z	Radio	Desv X	Desv Y	Desv Z	Medido X	Medido Y	Medido Z	Dir. proy. X	Dir. proy. Y	Dir. proy. Z
Desv. inferior	-3152.67				-24238.74	38264.48	-551.15	n/a	-57.14	-2647.74	-1710.42	-24295.88	35616.74	-2261.58	0.02	0.84	0.54
Desv. superior	3140.14				-29896.55	26869.28	-6780.69	n/a	2083.72	1398.11	1887.82	-27812.83	28267.39	-4892.87	0.66	0.45	0.60
