# Supplementary material for: Accuracy of four digital scanners according to scanning strategy in complete-arch impressions
Source: PLoS One. 2018 Sep 13;13(9):e0202916. doi: 10.1371/journal.pone.0202916 (PMC6136706; doi:10.1371/journal.pone.0202916)
Supplement: S6 Table — iTero (scanning strategy B). (ZIP) [file pone.0202916.s006.zip › S6/IT9B.pdf]

Geomagic Control™

3D Comparación Resultados

Modelo referencia	MRC
Modelo test	IT9B
Nº de puntos de datos	80853
# Aislados	606

Tipo tolerancia	3D desviación
Unidades	u
Máx. crítico	120.00
Máx. nominal	5.00
Mín. nominal	-5.00
Mín. crítico	-120.00

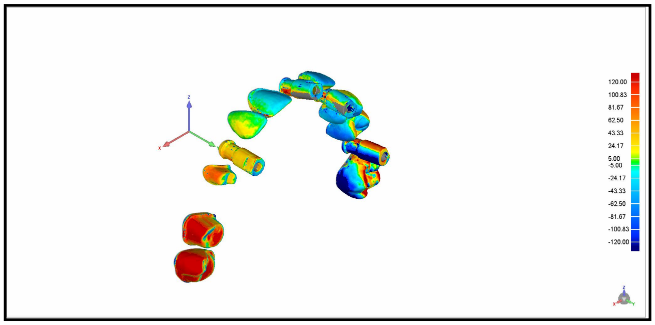

Desviación	
Desviación superior máx.	3154.25
Desviación inferior máx.	-3094.08
Desviación media	103.31 /-109.59
Desviación estándar	275.24

Distribución desviación

>=Min	<Max	# Puntos	%
-120.00	-100.83	1189	1.47
-100.83	-81.67	1622	2.01
-81.67	-62.50	3006	3.72
-62.50	-43.33	5271	6.52
-43.33	-24.17	7589	9.39
-24.17	-5.00	9885	12.23
-5.00	5.00	5350	6.62
5.00	24.17	9882	12.22
24.17	43.33	7678	9.50
43.33	62.50	5983	7.40
62.50	81.67	3505	4.34
81.67	100.83	2730	3.38
100.83	120.00	2065	2.55

Fuera del crítico superior	8959	11.08
Fuera del crítico inferior	6139	7.59

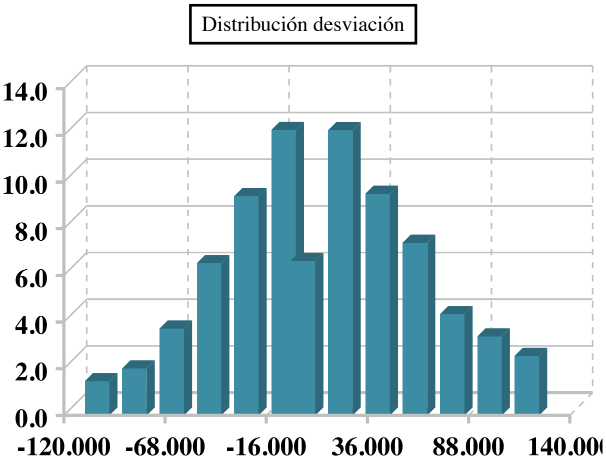

Desviaciones estándar

Distribución (+/-)	# Puntos	%
-6 * Desv. estánd.	705	0.87
-5 * Desv. estánd.	294	0.36
-4 * Desv. estánd.	267	0.33
-3 * Desv. estánd.	263	0.33
-2 * Desv. estánd.	445	0.55
-1 * Desv. estánd.	38093	47.11
1 * Desv. estánd.	38865	48.07
2 * Desv. estánd.	645	0.80
3 * Desv. estánd.	314	0.39
4 * Desv. estánd.	271	0.34
5 * Desv. estánd.	246	0.30
6 * Desv. estánd.	445	0.55

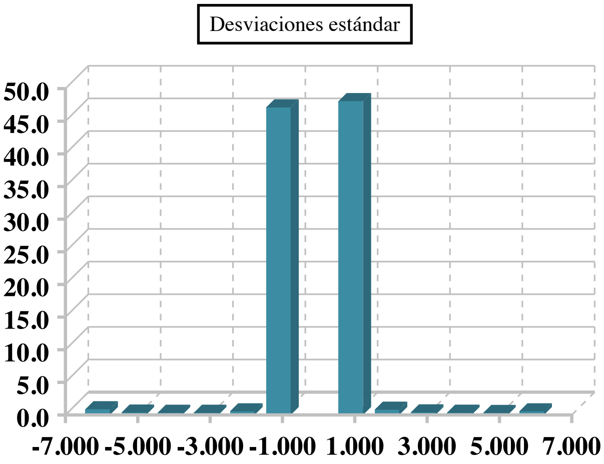

Predefinido: Isométrico

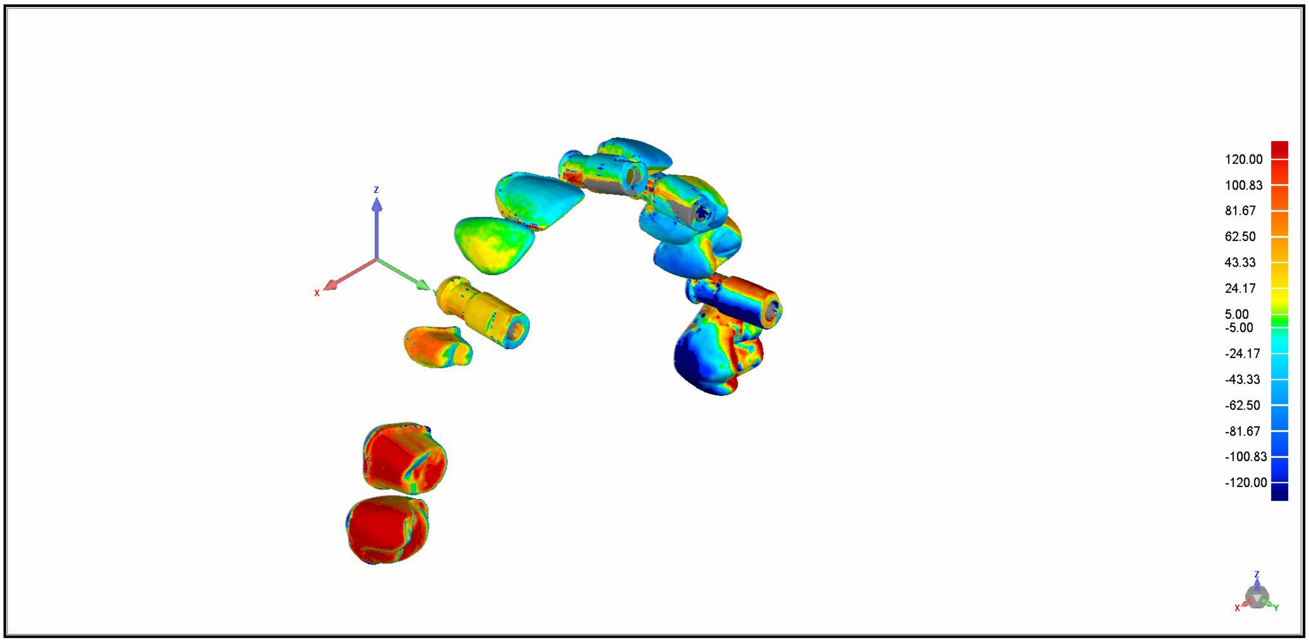

Predefinido: Frente

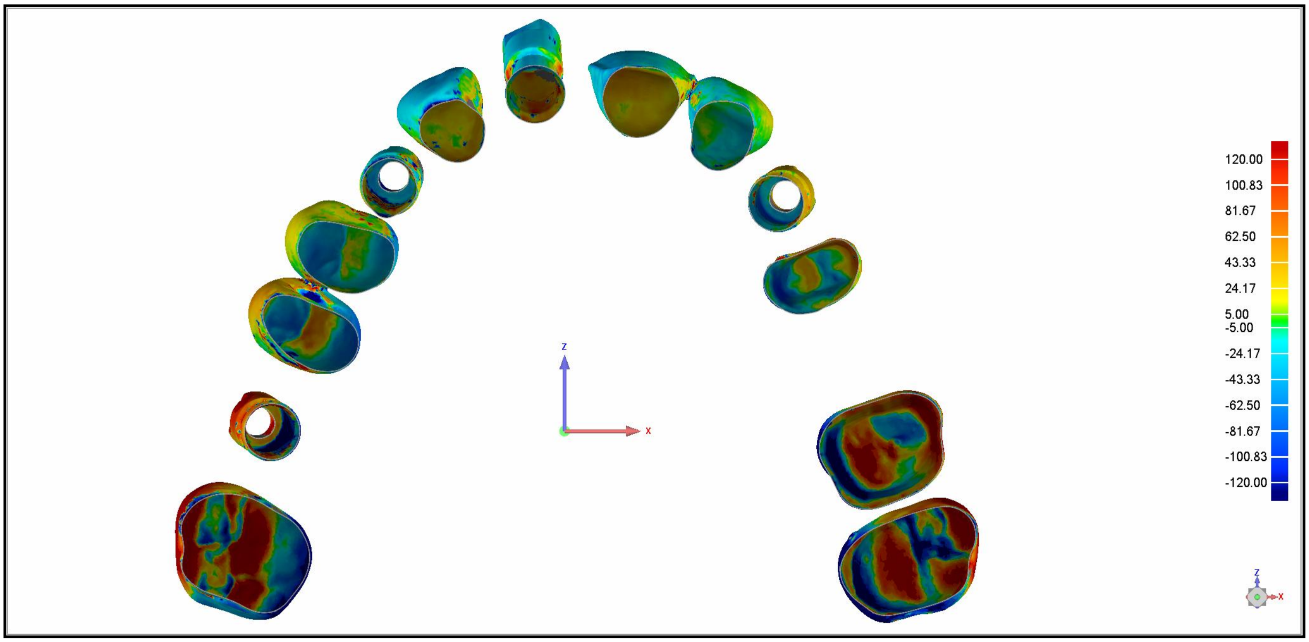

Predefinido: Atrás

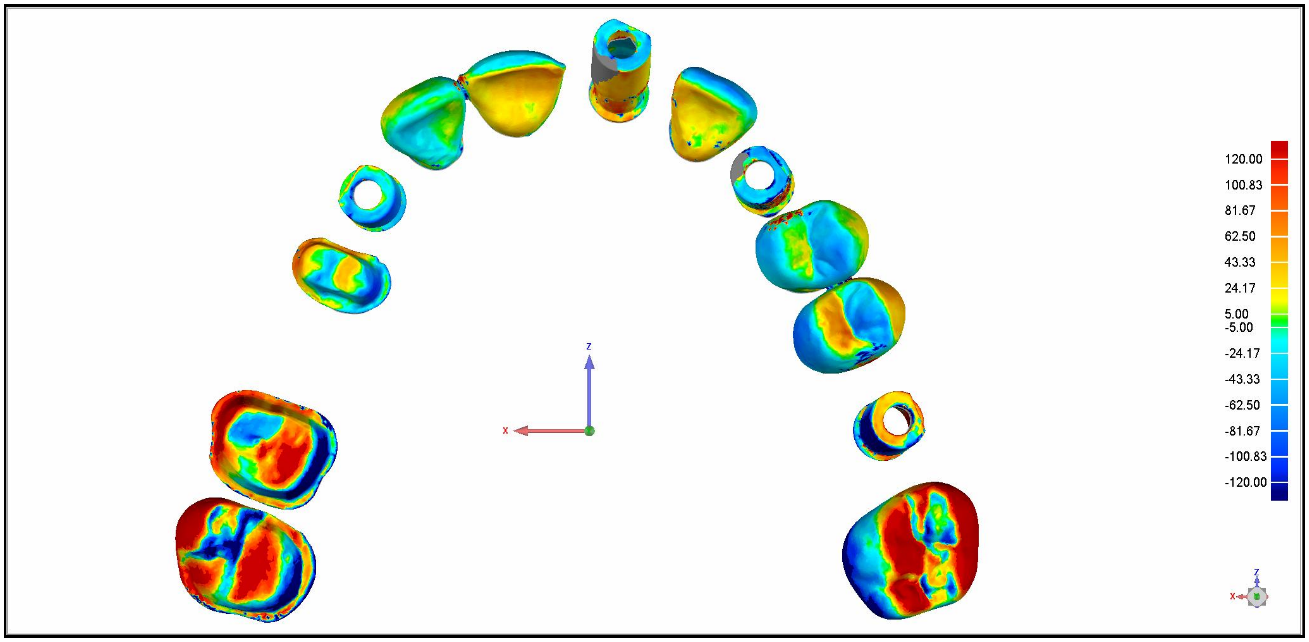

Predefinido: Izquierda

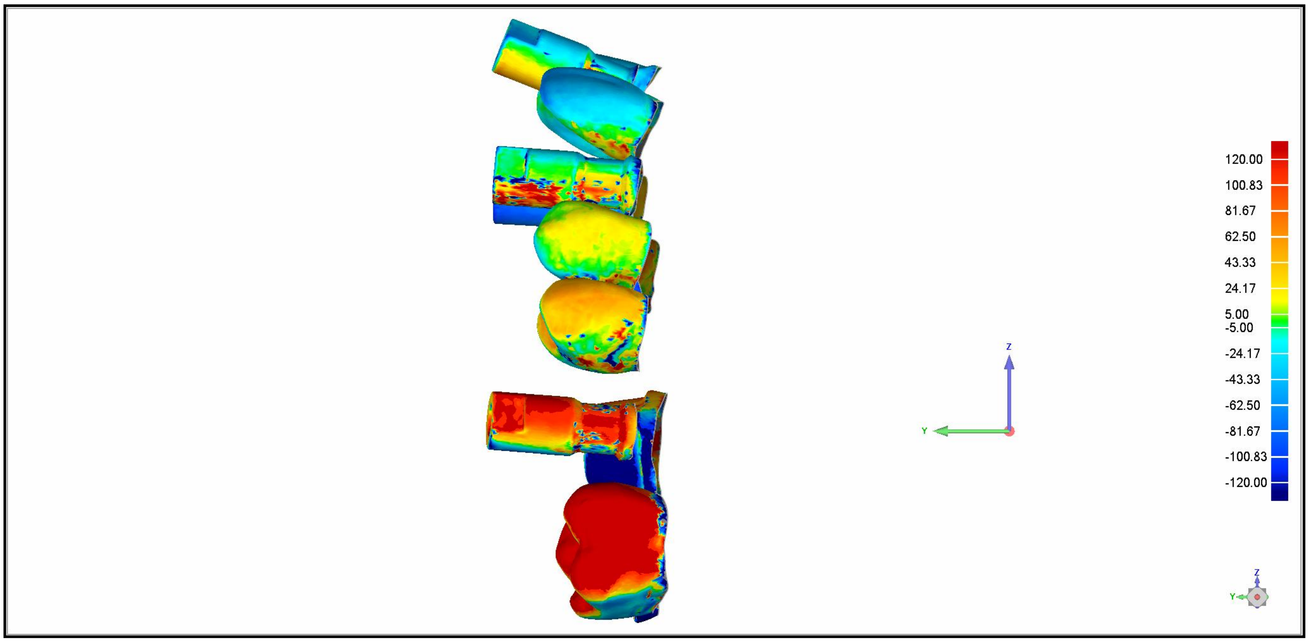

Predefinido: Derecha

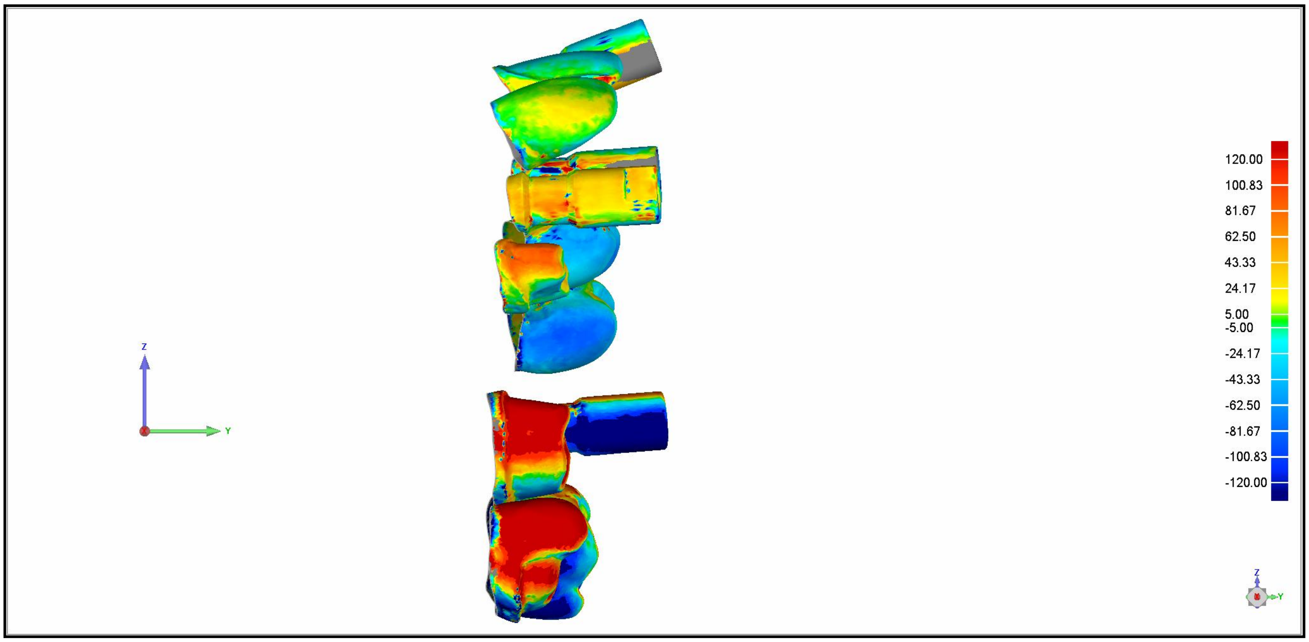

Predefinido: Superior

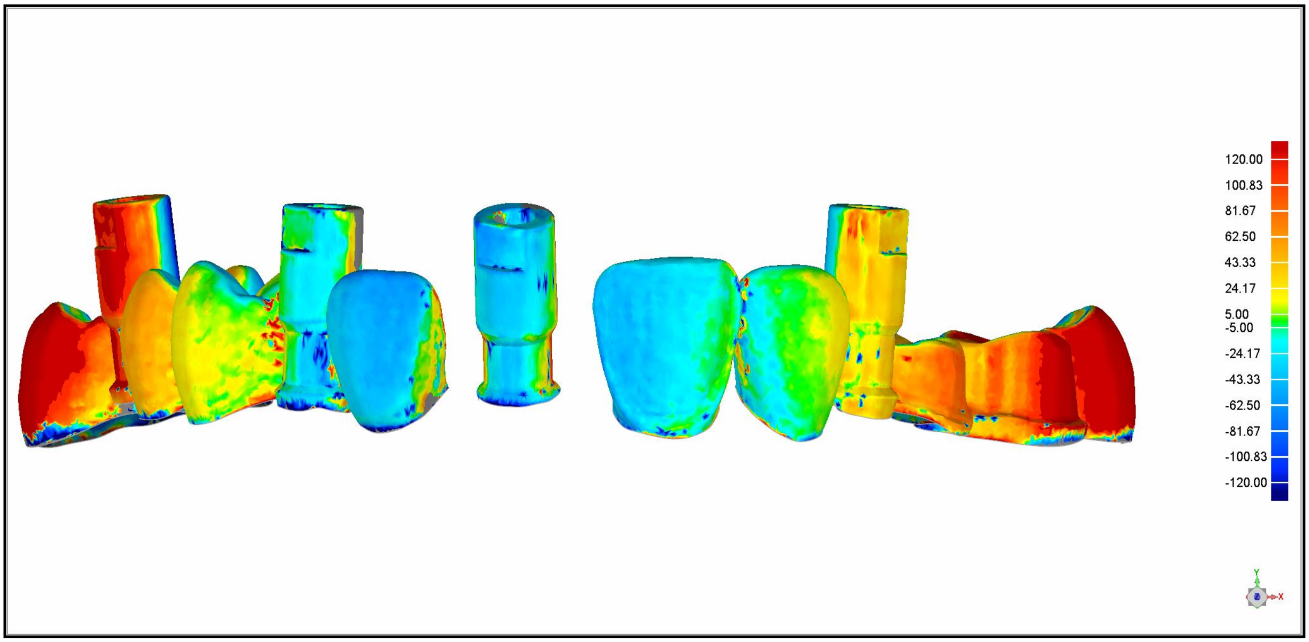

Predefinido: Inferior

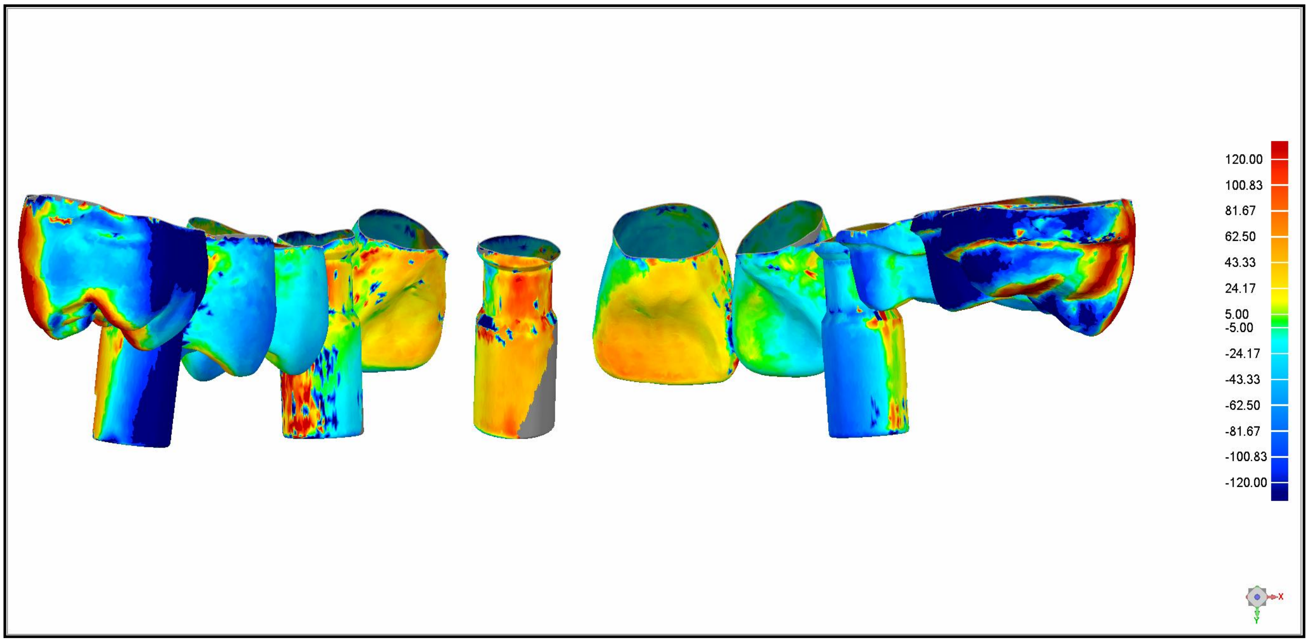

Ajuste de ubicación: Desviaciones superior e inferior

Unidades: u

Nombre	Desv	Estado	Superior Tol	Inferior Tol	Ref X	Ref Y	Ref Z	Radio	Desv X	Desv Y	Desv Z	Medido X	Medido Y	Medido Z	Dir. proy. X	Dir. proy. Y	Dir. proy. Z
Desv. inferior	-3094.08				-29208.33	26961.25	-11988.49	n/a	2663.31	412.66	-1519.81	-26545.02	27373.91	-13508.30	-0.86	-0.13	0.49
Desv. superior	3154.25				-4588.77	29542.46	26172.40	n/a	-3050.61	-678.39	-427.64	-7639.38	28864.07	25744.75	-0.97	-0.22	-0.14
