# Supplementary material for: Accuracy of four digital scanners according to scanning strategy in complete-arch impressions
Source: PLoS One. 2018 Sep 13;13(9):e0202916. doi: 10.1371/journal.pone.0202916 (PMC6136706; doi:10.1371/journal.pone.0202916)
Supplement: S6 Table — iTero (scanning strategy B). (ZIP) [file pone.0202916.s006.zip › S6/IT5B.pdf]

Fecha: 3/6/2017, 1:22 pm

3D Comparación Resultados

Modelo referencia	MRC
Modelo test	IT5B
Nº de puntos de datos	79215
# Aislados	603

Tipo tolerancia	3D desviación
Unidades	u
Máx. crítico	120.00
Máx. nominal	17.00
Mín. nominal	-17.00
Mín. crítico	-120.00

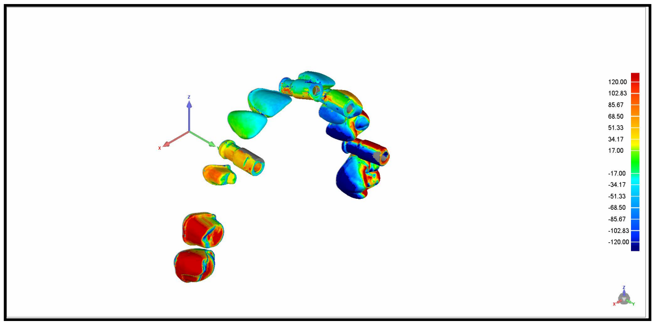

Desviación	
Desviación superior máx.	3093.77
Desviación inferior máx.	-3121.70
Desviación media	124.74 /-108.07
Desviación estándar	258.56

Distribución desviación

>=Min	<Max	# Puntos	%
-120.00	-102.83	1202	1.52
-102.83	-85.67	1742	2.20
-85.67	-68.50	2502	3.16
-68.50	-51.33	3367	4.25
-51.33	-34.17	5494	6.94
-34.17	-17.00	7183	9.07
-17.00	17.00	13901	17.55
17.00	34.17	6470	8.17
34.17	51.33	5438	6.86
51.33	68.50	4743	5.99
68.50	85.67	3608	4.55
85.67	102.83	2025	2.56
102.83	120.00	1471	1.86

Fuera del crítico superior	12144	15.33
Fuera del crítico inferior	7925	10.00

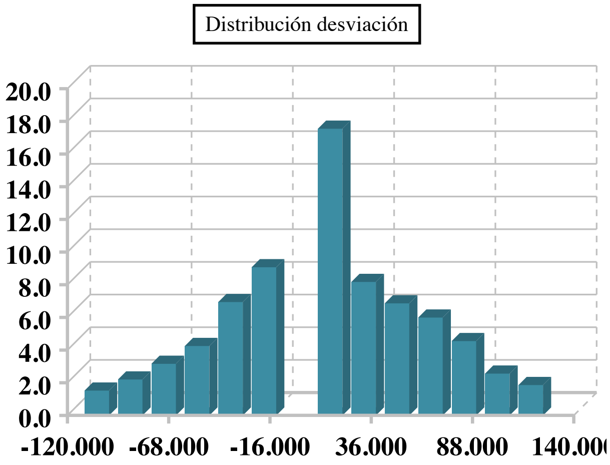

Desviaciones estándar

Distribución (+/-)	# Puntos	%
-6 * Desv. estánd.	427	0.54
-5 * Desv. estánd.	236	0.30
-4 * Desv. estánd.	258	0.33
-3 * Desv. estánd.	266	0.34
-2 * Desv. estánd.	2011	2.54
-1 * Desv. estánd.	40165	50.70
1 * Desv. estánd.	32619	41.18
2 * Desv. estánd.	1902	2.40
3 * Desv. estánd.	235	0.30
4 * Desv. estánd.	223	0.28
5 * Desv. estánd.	267	0.34
6 * Desv. estánd.	606	0.77

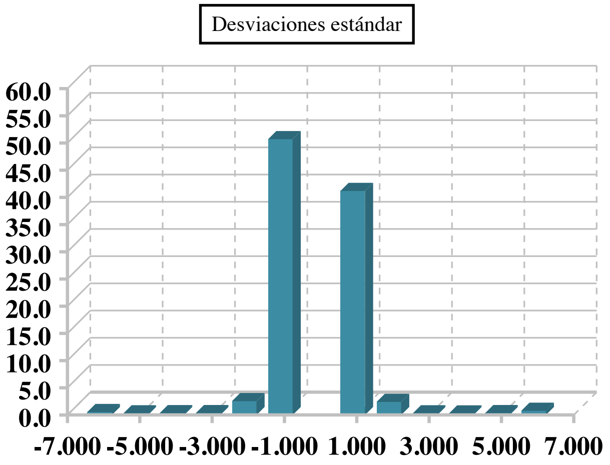

Predefinido: Isométrico

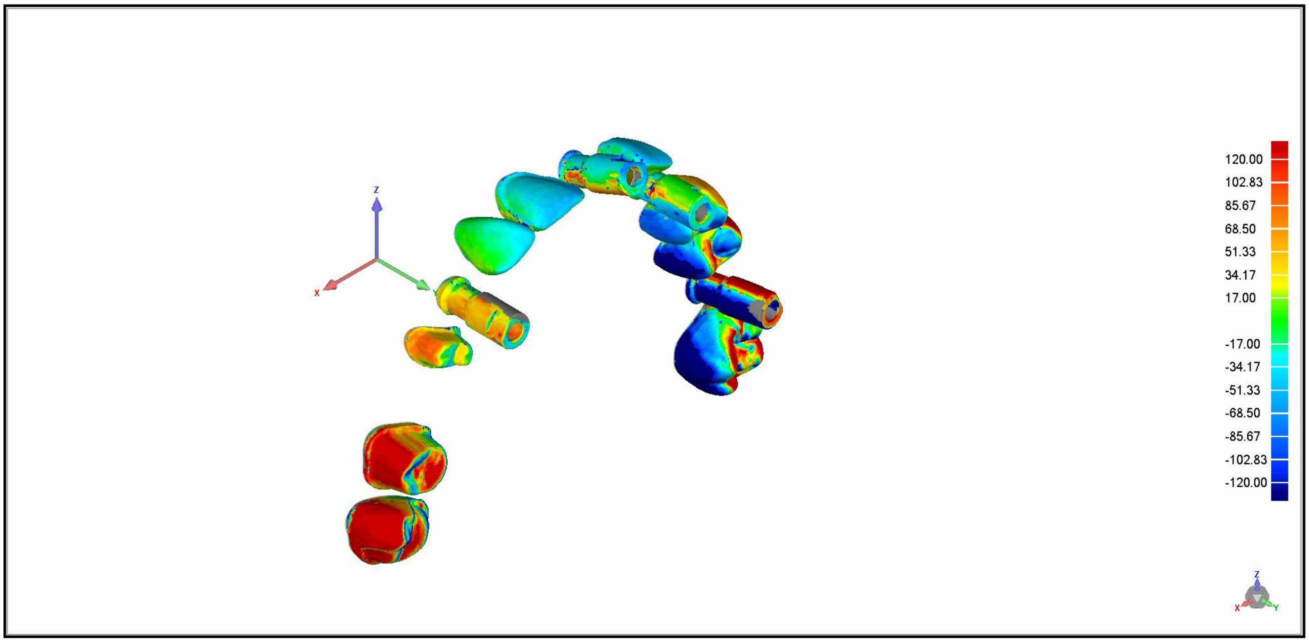

Predefinido: Frente

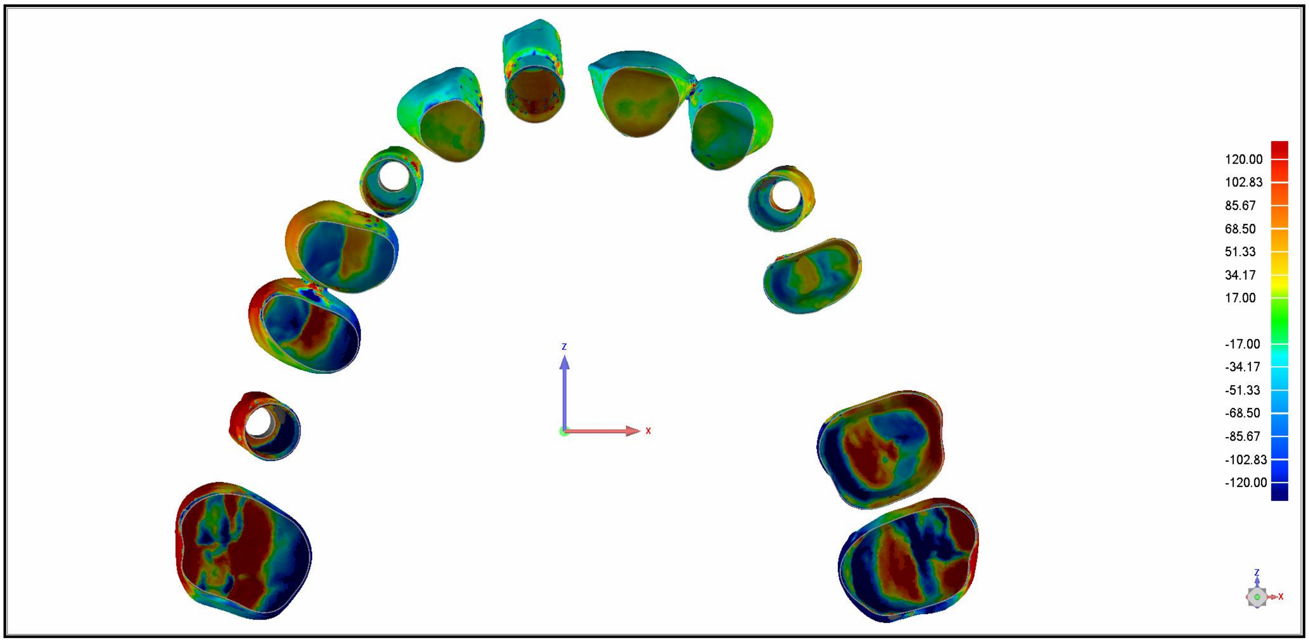

Predefinido: Atrás

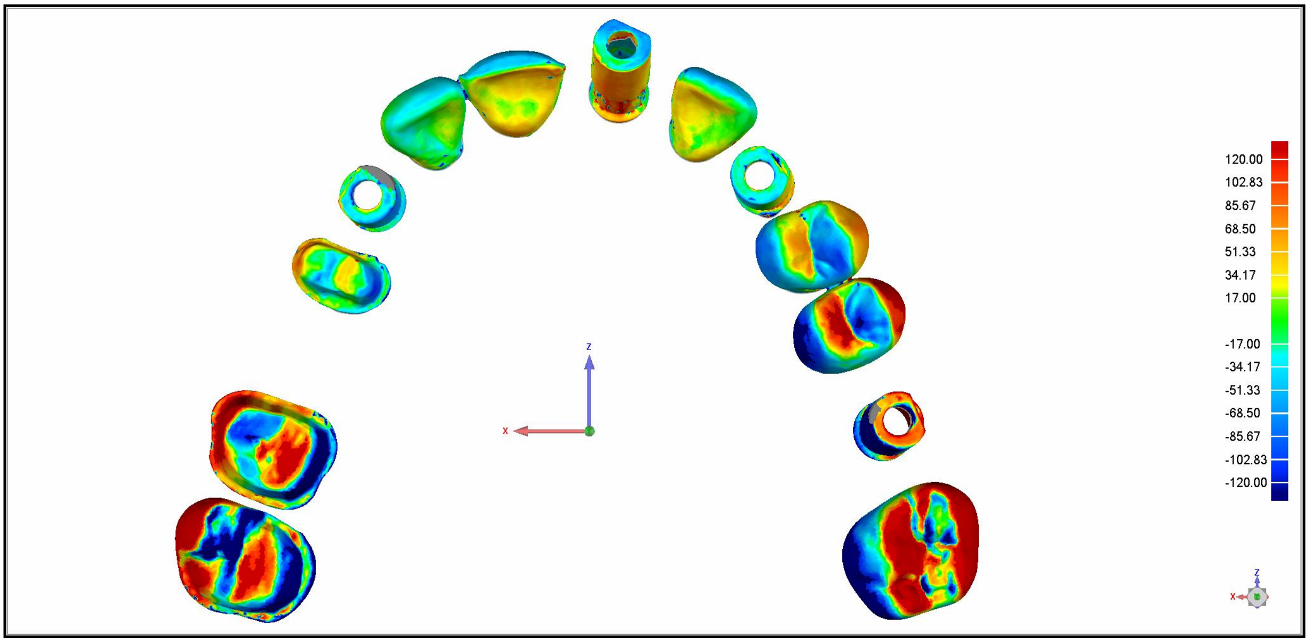

Geomagic Control™

Predefinido: Izquierda

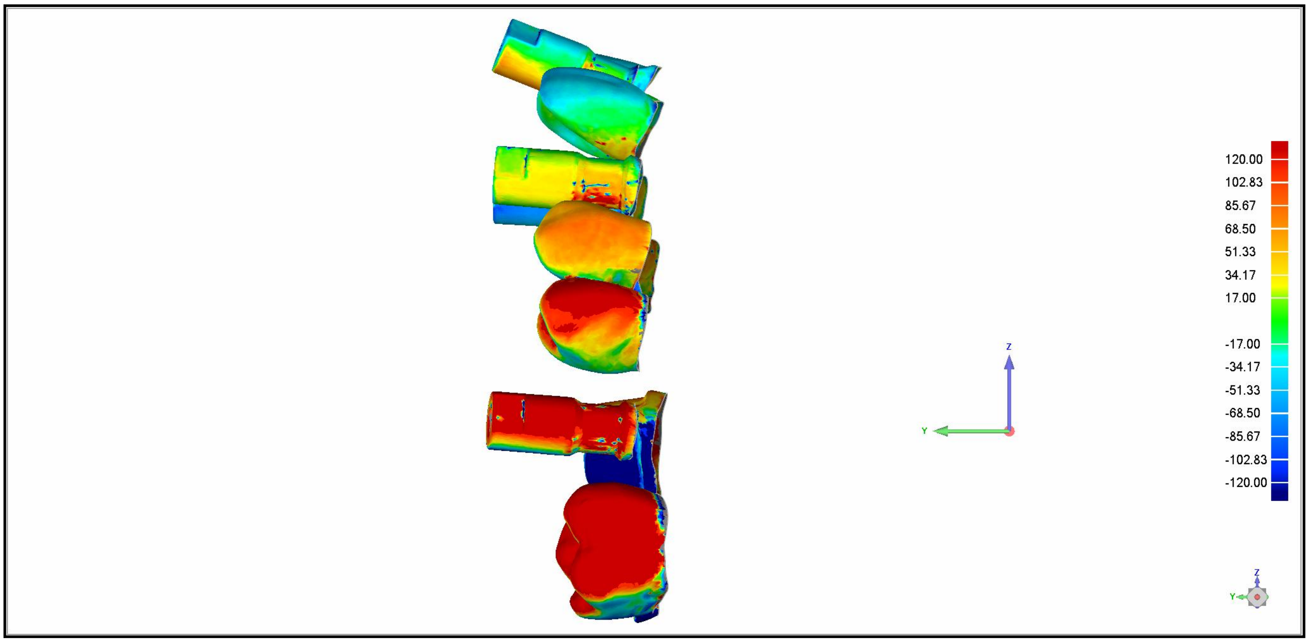

Predefinido: Derecha

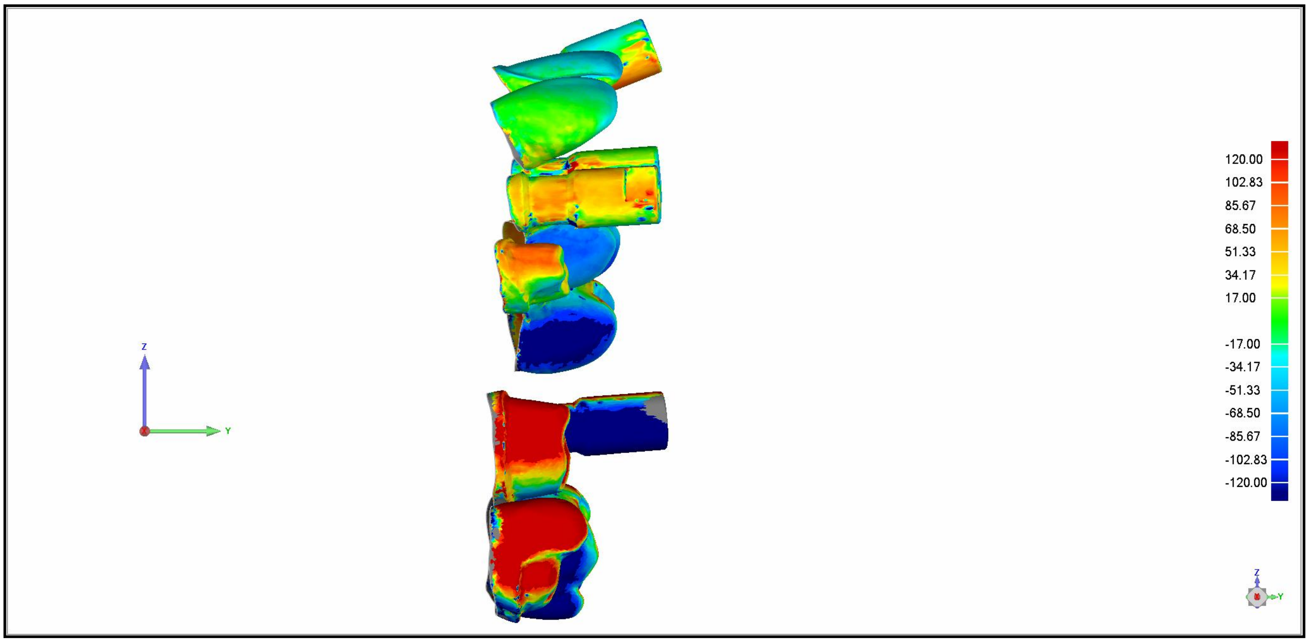

Predefinido: Superior

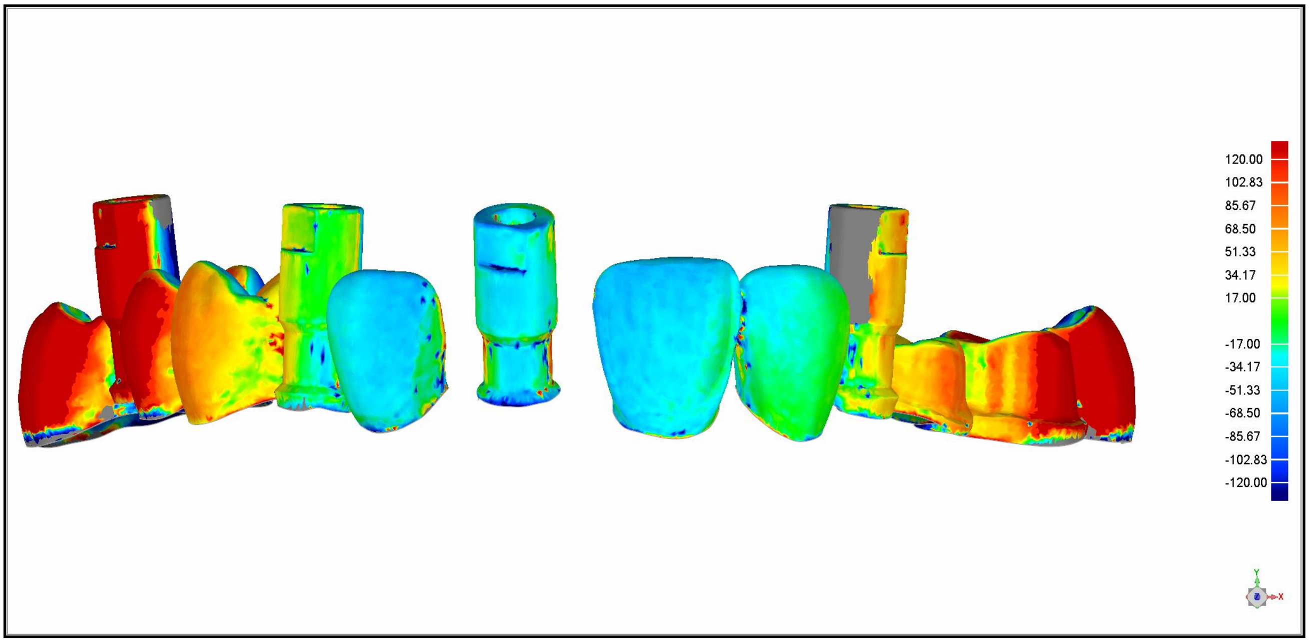

Predefinido: Inferior

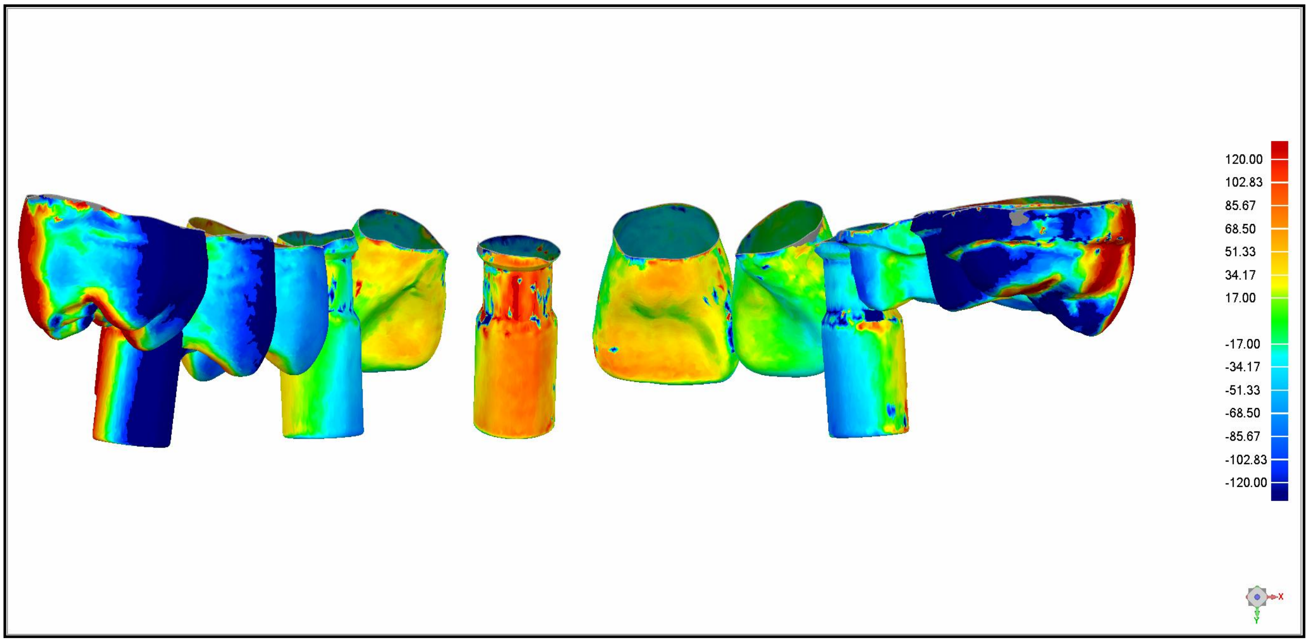

Ajuste de ubicación: Desviaciones superior e inferior

Unidades: u

Nombre	Desv	Estado	Superior Tol	Inferior Tol	Ref X	Ref Y	Ref Z	Radio	Desv X	Desv Y	Desv Z	Medido X	Medido Y	Medido Z	Dir. proy. X	Dir. proy. Y	Dir. proy. Z
Desv. inferior	-3121.70				-29208.33	26961.25	-11988.49	n/a	2700.05	495.08	-1486.48	-26508.28	27456.33	-13474.96	-0.86	-0.16	0.48
Desv. superior	3093.77				18191.95	32839.05	13375.05	n/a	-1304.84	1183.41	2543.29	16887.10	34022.45	15918.33	-0.42	0.38	0.82
