# Supplementary material for: Accuracy of four digital scanners according to scanning strategy in complete-arch impressions
Source: PLoS One. 2018 Sep 13;13(9):e0202916. doi: 10.1371/journal.pone.0202916 (PMC6136706; doi:10.1371/journal.pone.0202916)
Supplement: S6 Table — iTero (scanning strategy B). (ZIP) [file pone.0202916.s006.zip › S6/IT2B.pdf]

Geomagic Control™

Fecha: 3/6/2017, 1:14 pm

3D Comparación Resultados

Modelo referencia	MRC
Modelo test	IT2B
Nº de puntos de datos	81528
# Aislados	496

Tipo tolerancia	3D desviación
Unidades	u
Máx. crítico	120.00
Máx. nominal	14.00
Mín. nominal	-14.00
Mín. crítico	-120.00

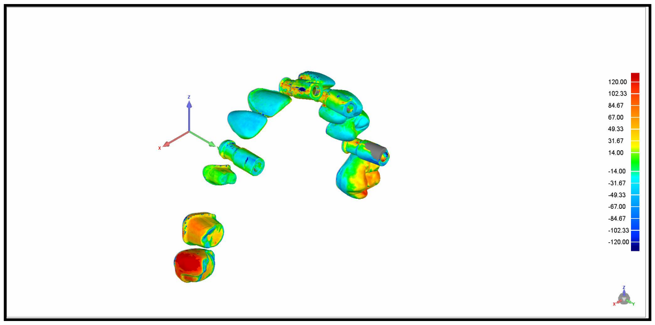

Desviación	
Desviación superior máx.	3139.42
Desviación inferior máx.	-3147.73
Desviación media	87.26 /-62.70
Desviación estándar	236.07

Distribución desviación

>=Min	<Max	# Puntos	%
-120.00	-102.33	652	0.80
-102.33	-84.67	944	1.16
-84.67	-67.00	1285	1.58
-67.00	-49.33	2187	2.68
-49.33	-31.67	5400	6.62
-31.67	-14.00	12322	15.11
-14.00	14.00	25550	31.34
14.00	31.67	11048	13.55
31.67	49.33	6998	8.58
49.33	67.00	4126	5.06
67.00	84.67	2129	2.61
84.67	102.33	989	1.21
102.33	120.00	801	0.98

Fuera del crítico superior	3997	4.90
Fuera del crítico inferior	3100	3.80

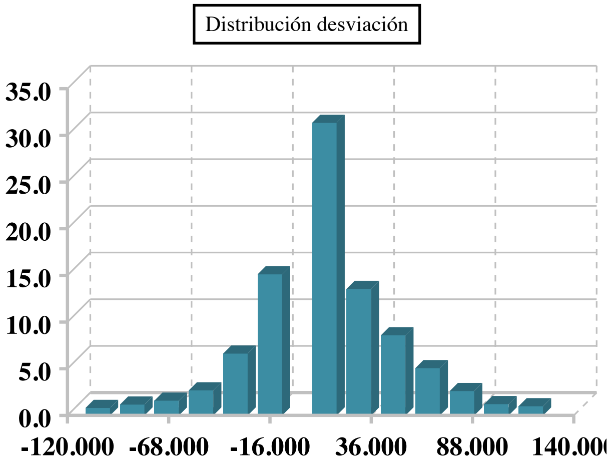

Desviaciones estándar

Distribución (+/-)	# Puntos	%
-6 * Desv. estánd.	393	0.48
-5 * Desv. estánd.	157	0.19
-4 * Desv. estánd.	180	0.22
-3 * Desv. estánd.	245	0.30
-2 * Desv. estánd.	627	0.77
-1 * Desv. estánd.	50224	61.60
1 * Desv. estánd.	27394	33.60
2 * Desv. estánd.	587	0.72
3 * Desv. estánd.	384	0.47
4 * Desv. estánd.	295	0.36
5 * Desv. estánd.	265	0.33
6 * Desv. estánd.	777	0.95

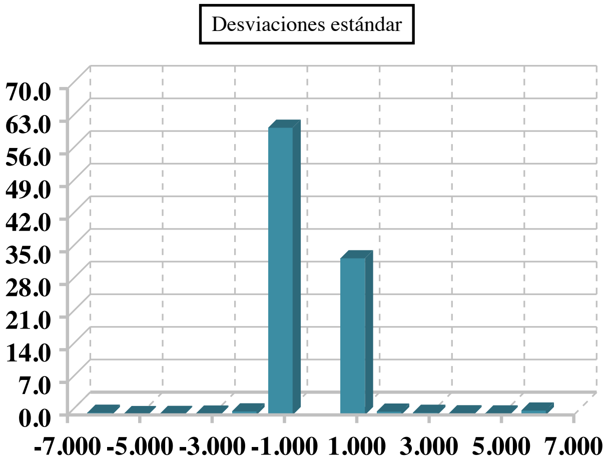

Predefinido: Isométrico

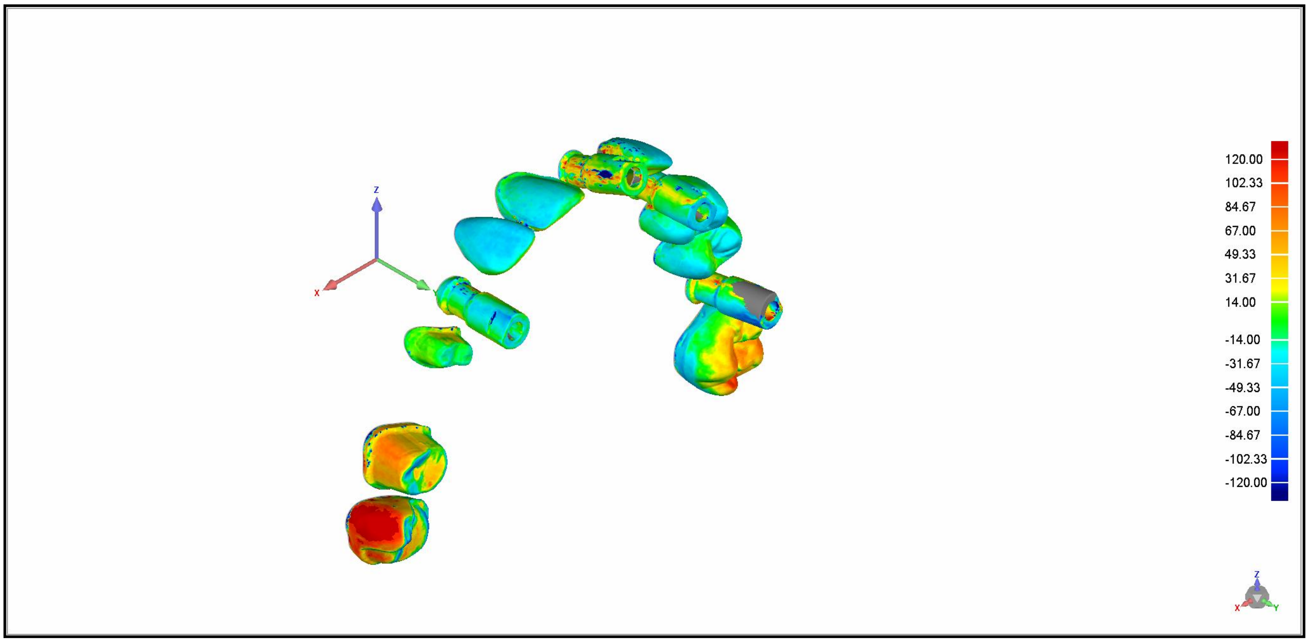

Predefinido: Frente

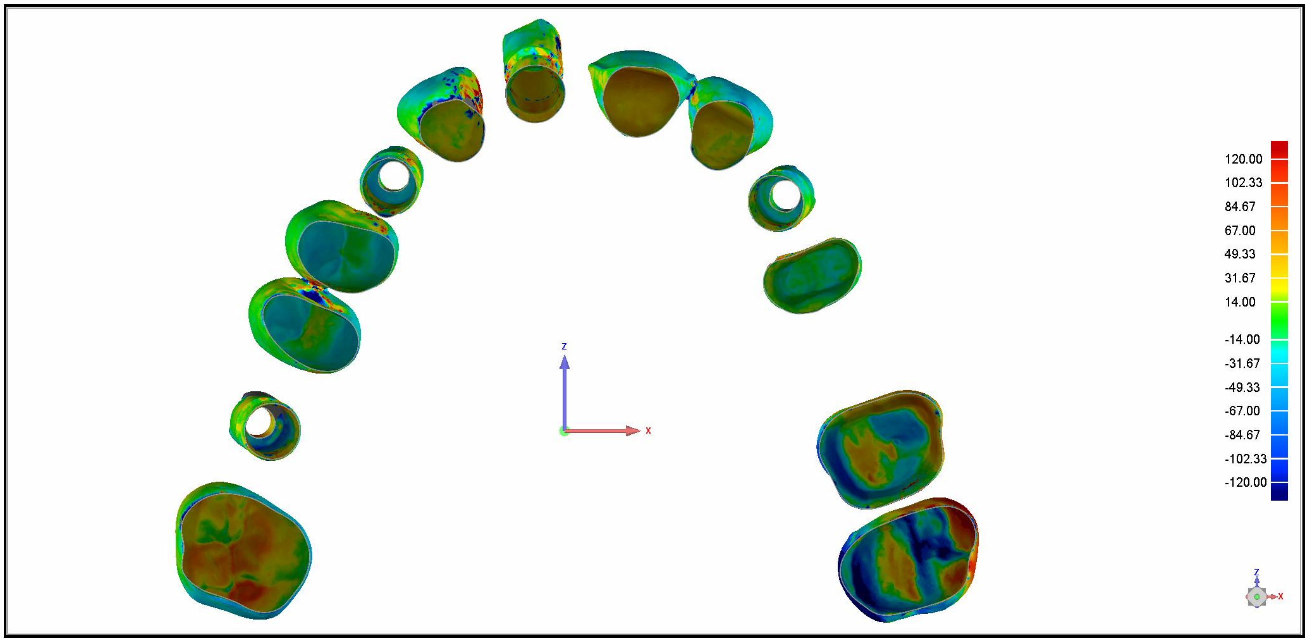

Predefinido: Atrás

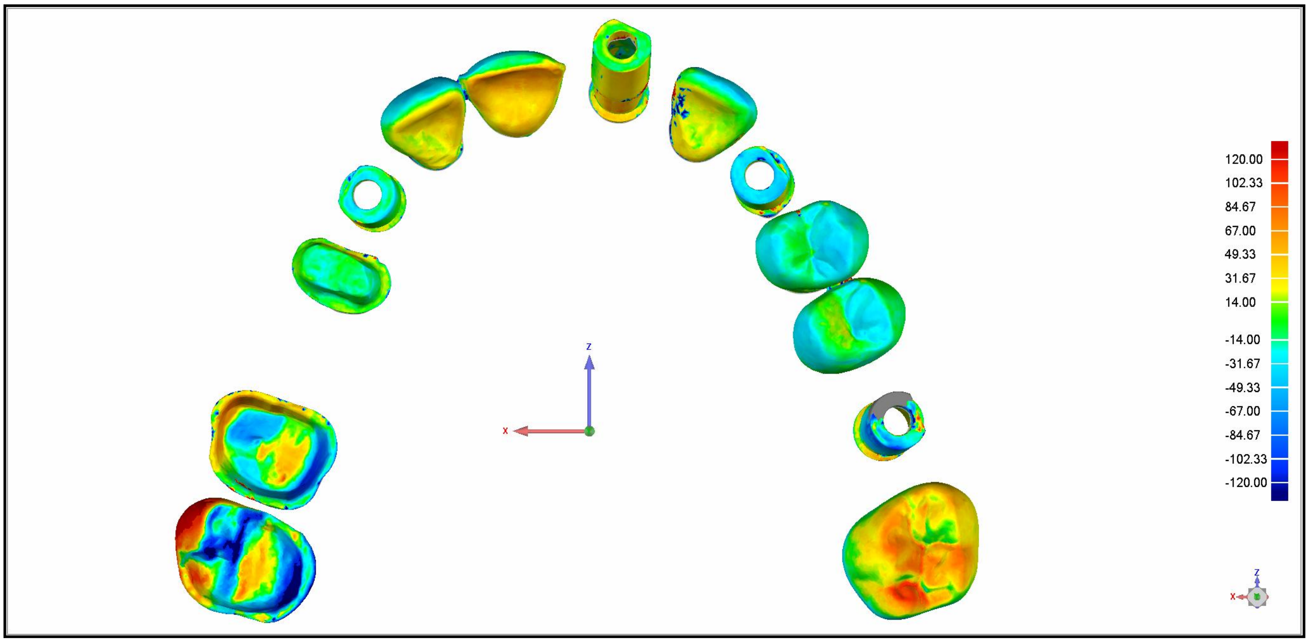

Predefinido: Izquierda

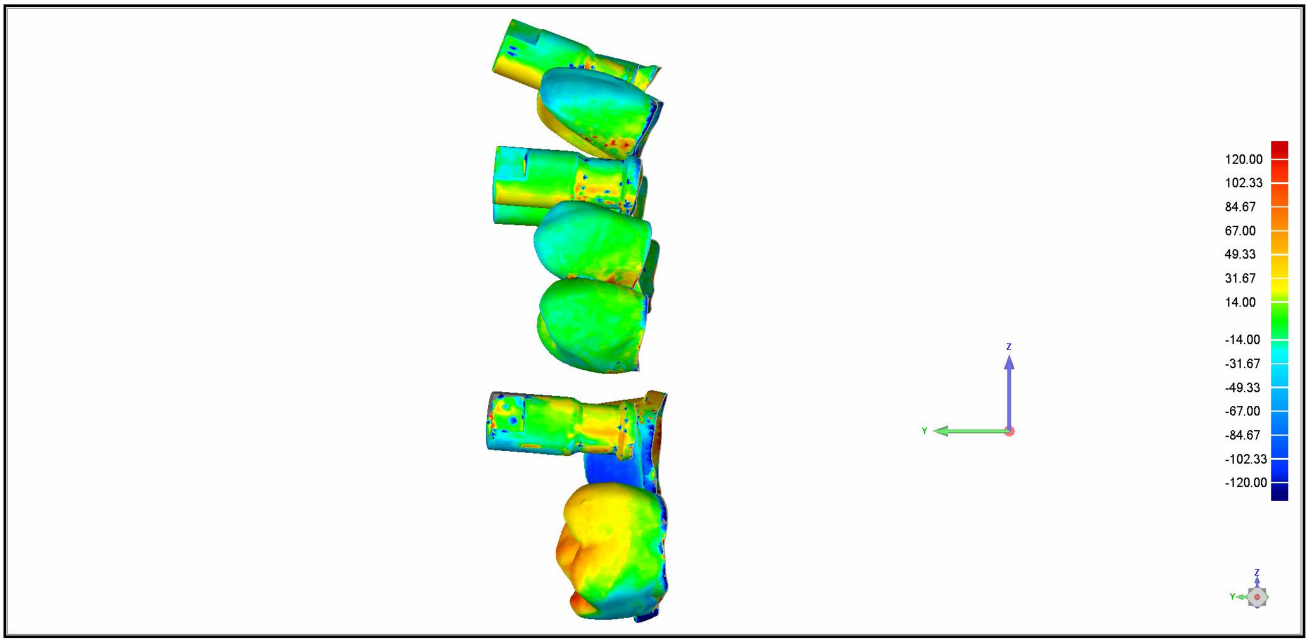

Predefinido: Derecha

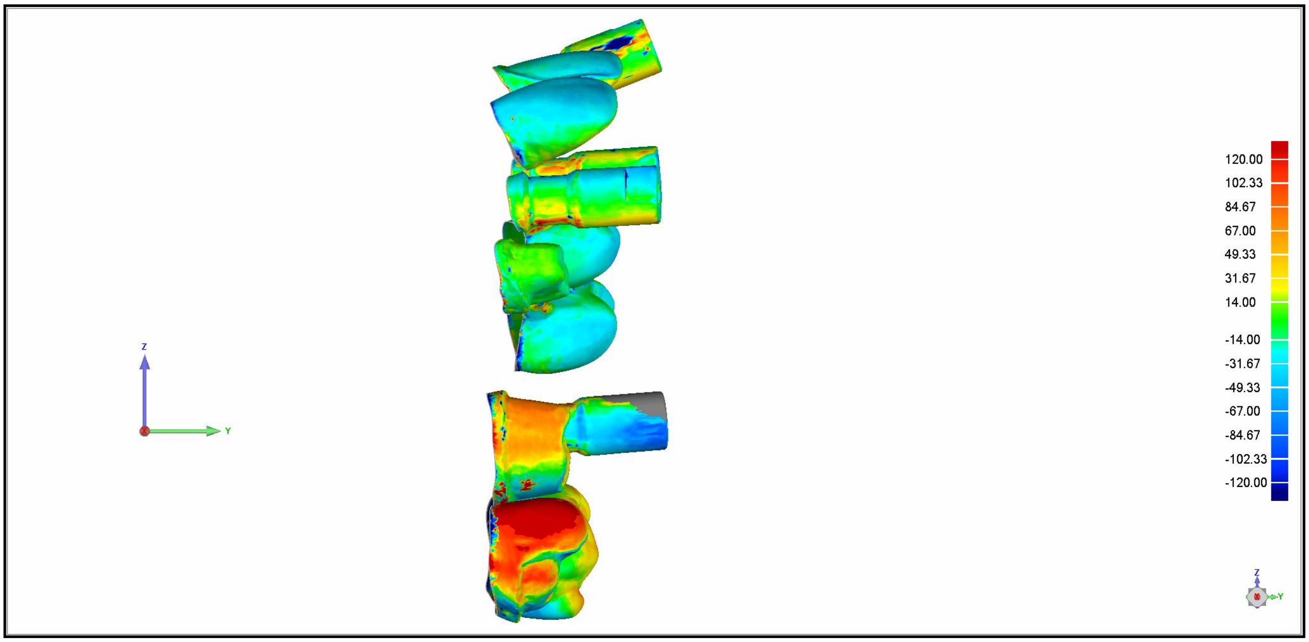

Predefinido: Superior

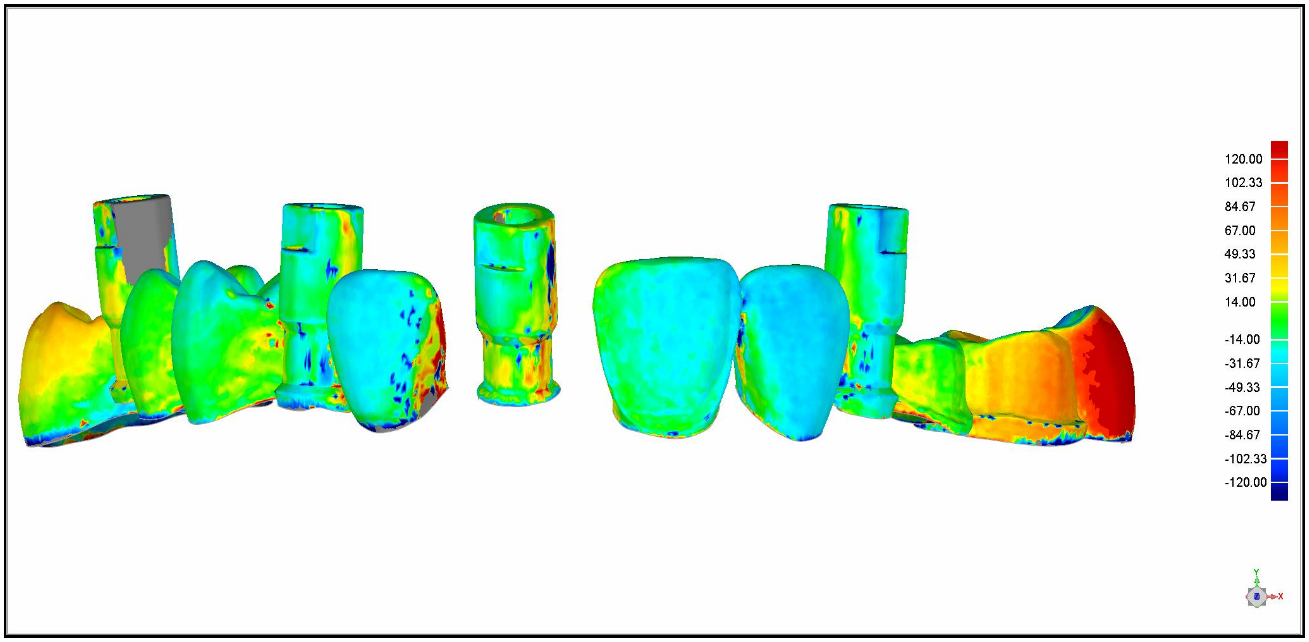

Predefinido: Inferior

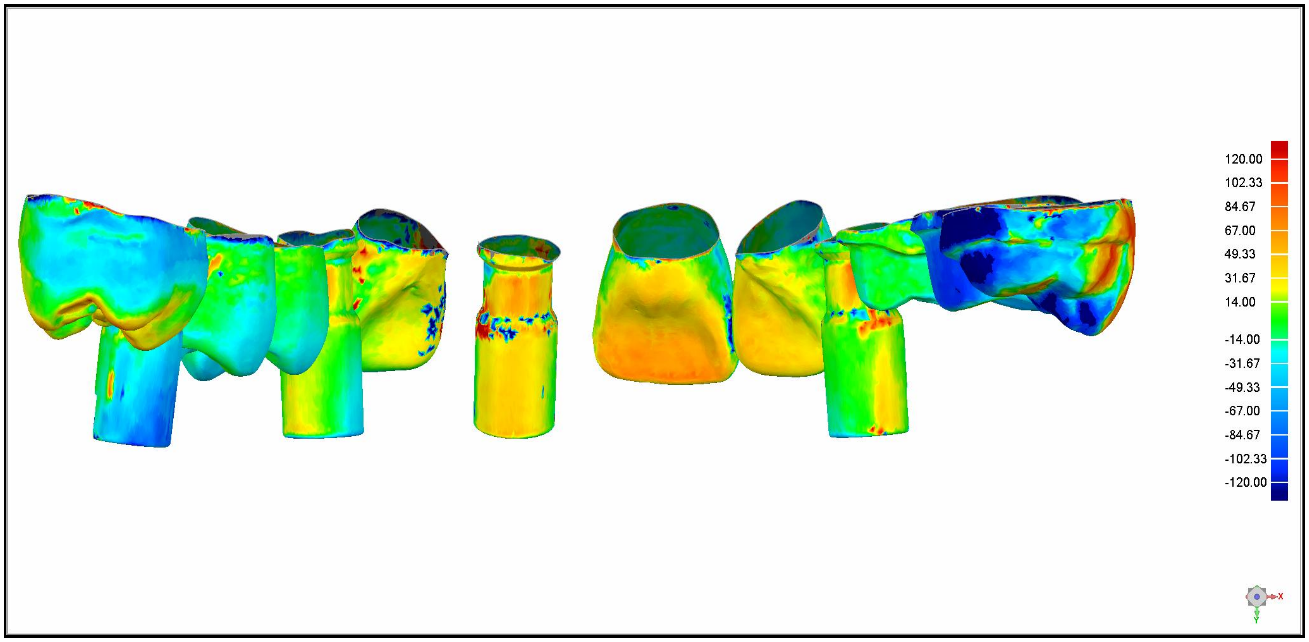

Ajuste de ubicación: Desviaciones superior e inferior

Unidades: u

Nombre	Desv	Estado	Superior Tol	Inferior Tol	Ref X	Ref Y	Ref Z	Radio	Desv X	Desv Y	Desv Z	Medido X	Medido Y	Medido Z	Dir. proy. X	Dir. proy. Y	Dir. proy. Z
Desv. inferior	-3147.73				-11322.55	28141.01	24480.51	n/a	2777.50	132.70	1475.15	-8545.05	28273.72	25955.67	-0.88	-0.04	-0.47
Desv. superior	3139.42				-4122.37	29983.27	25046.23	n/a	-2420.44	1788.36	893.98	-6542.81	31771.63	25940.21	-0.77	0.57	0.28
