# Supplementary material for: Accuracy of four digital scanners according to scanning strategy in complete-arch impressions
Source: PLoS One. 2018 Sep 13;13(9):e0202916. doi: 10.1371/journal.pone.0202916 (PMC6136706; doi:10.1371/journal.pone.0202916)
Supplement: S6 Table — iTero (scanning strategy B). (ZIP) [file pone.0202916.s006.zip › S6/IT3B.pdf]

3D Comparación Resultados

Modelo referencia	MRC
Modelo test	IT3B
Nº de puntos de datos	80499
# Aislados	647

Tipo tolerancia	3D desviación
Unidades	u
Máx. crítico	120.00
Máx. nominal	3.00
Mín. nominal	-3.00
Mín. crítico	-120.00

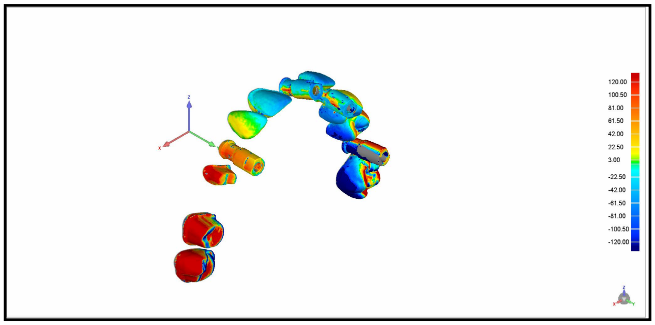

Desviación	
Desviación superior máx.	3138.42
Desviación inferior máx.	-3082.57
Desviación media	127.68 /-131.88
Desviación estándar	286.43

Distribución desviación

>=Min	<Max	# Puntos	%
-120.00	-100.50	1541	1.91
-100.50	-81.00	2065	2.57
-81.00	-61.50	3506	4.36
-61.50	-42.00	5333	6.62
-42.00	-22.50	7032	8.74
-22.50	-3.00	8493	10.55
-3.00	3.00	2672	3.32
3.00	22.50	8203	10.19
22.50	42.00	6746	8.38
42.00	61.50	5186	6.44
61.50	81.00	4004	4.97
81.00	100.50	2979	3.70
100.50	120.00	1895	2.35

Fuera del crítico superior	11775	14.63
Fuera del crítico inferior	9069	11.27

Distribución desviación

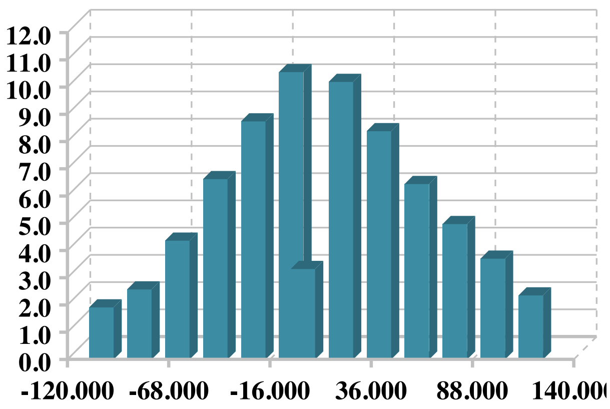

Desviaciones estándar

Distribución (+/-)	# Puntos	%
-6 * Desv. estánd.	561	0.70
-5 * Desv. estánd.	336	0.42
-4 * Desv. estánd.	311	0.39
-3 * Desv. estánd.	305	0.38
-2 * Desv. estánd.	2236	2.78
-1 * Desv. estánd.	36331	45.13
1 * Desv. estánd.	36326	45.13
2 * Desv. estánd.	2899	3.60
3 * Desv. estánd.	271	0.34
4 * Desv. estánd.	272	0.34
5 * Desv. estánd.	284	0.35
6 * Desv. estánd.	367	0.46

Desviaciones estándar

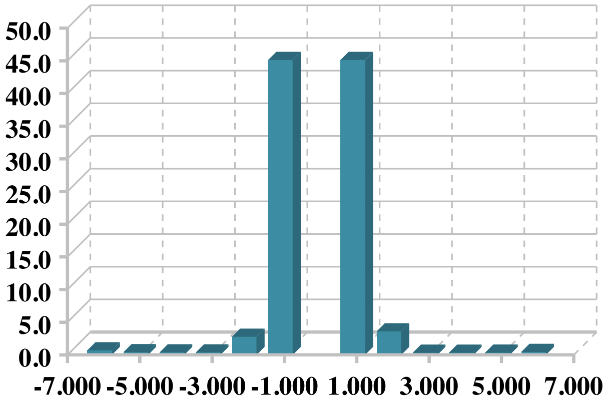

Predefinido: Isométrico

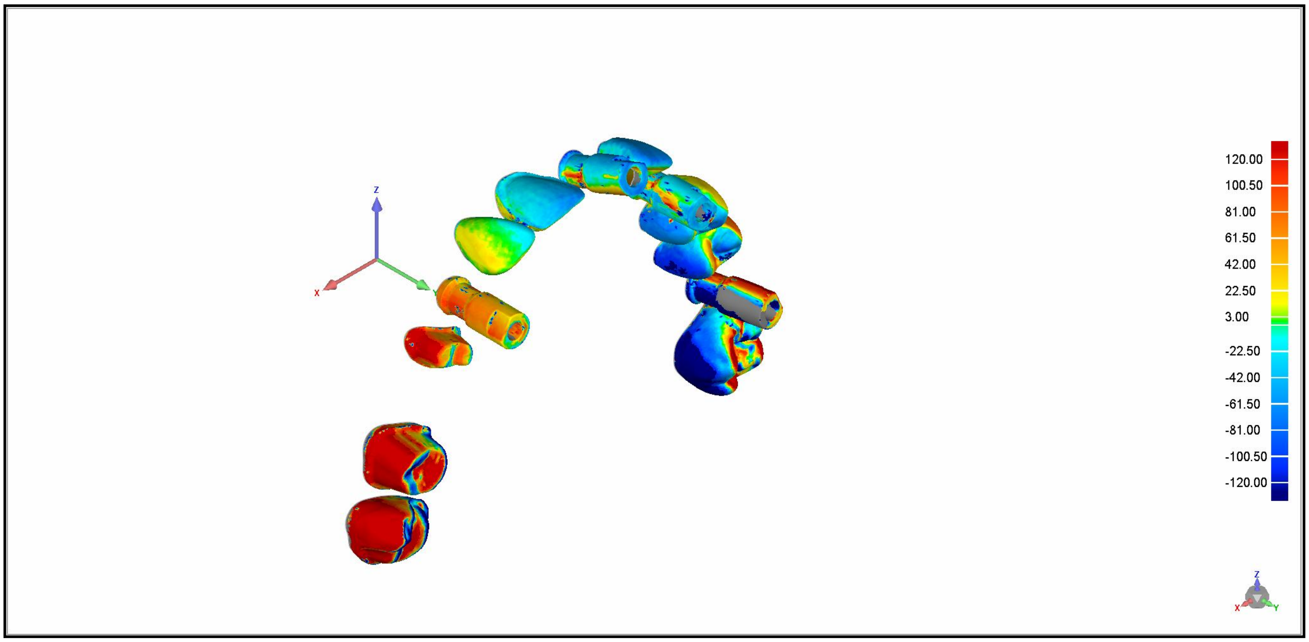

Predefinido: Frente

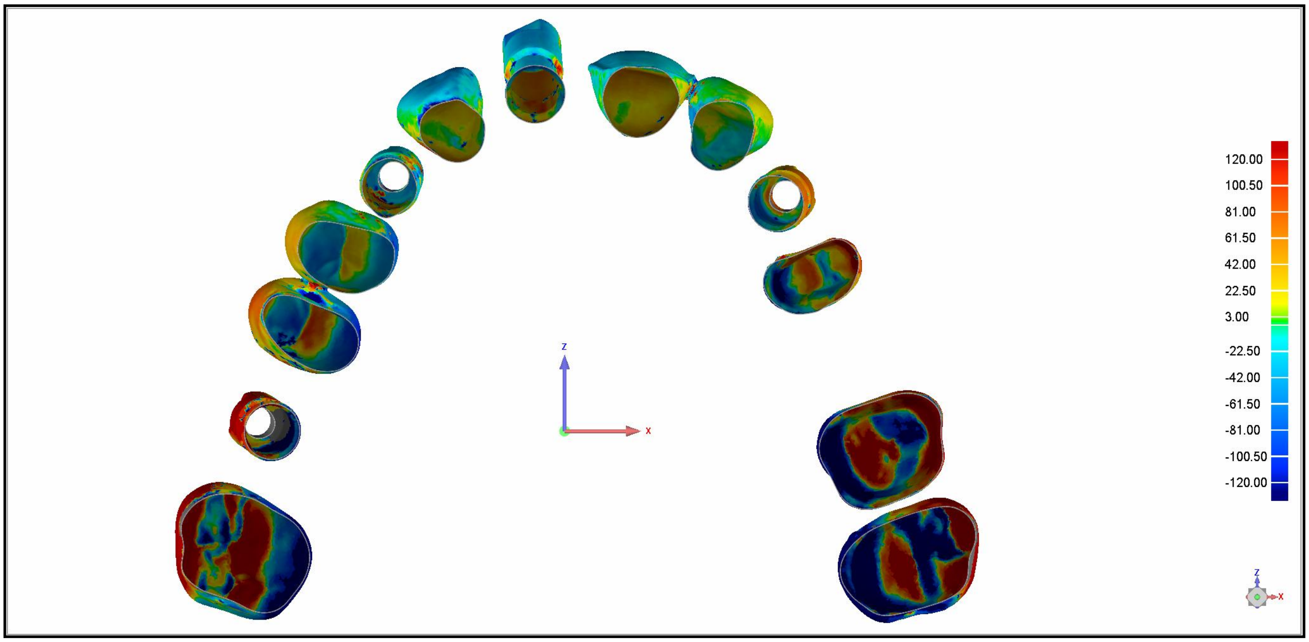

Predefinido: Atrás

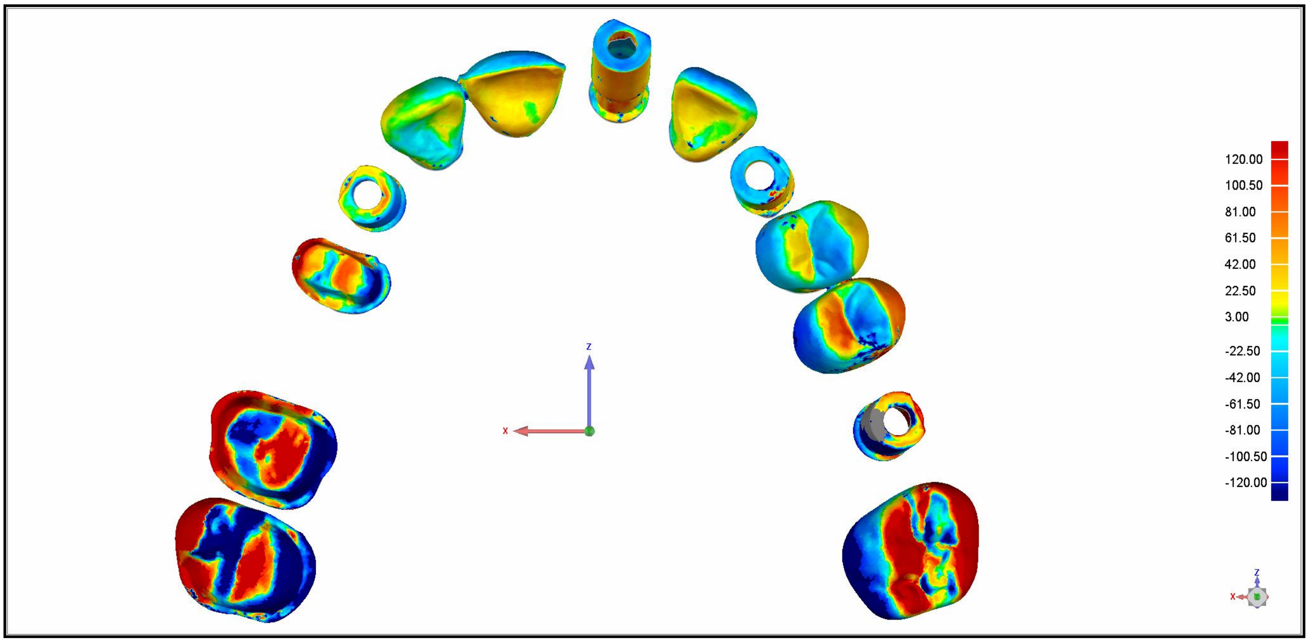

Predefinido: Izquierda

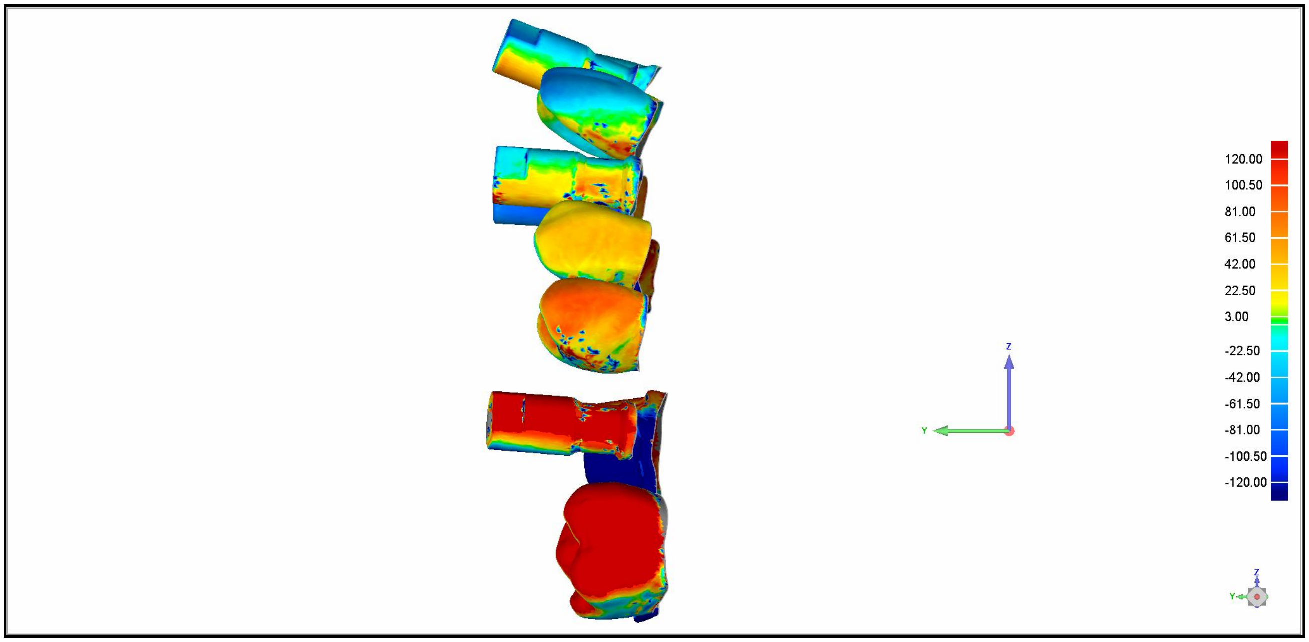

Predefinido: Derecha

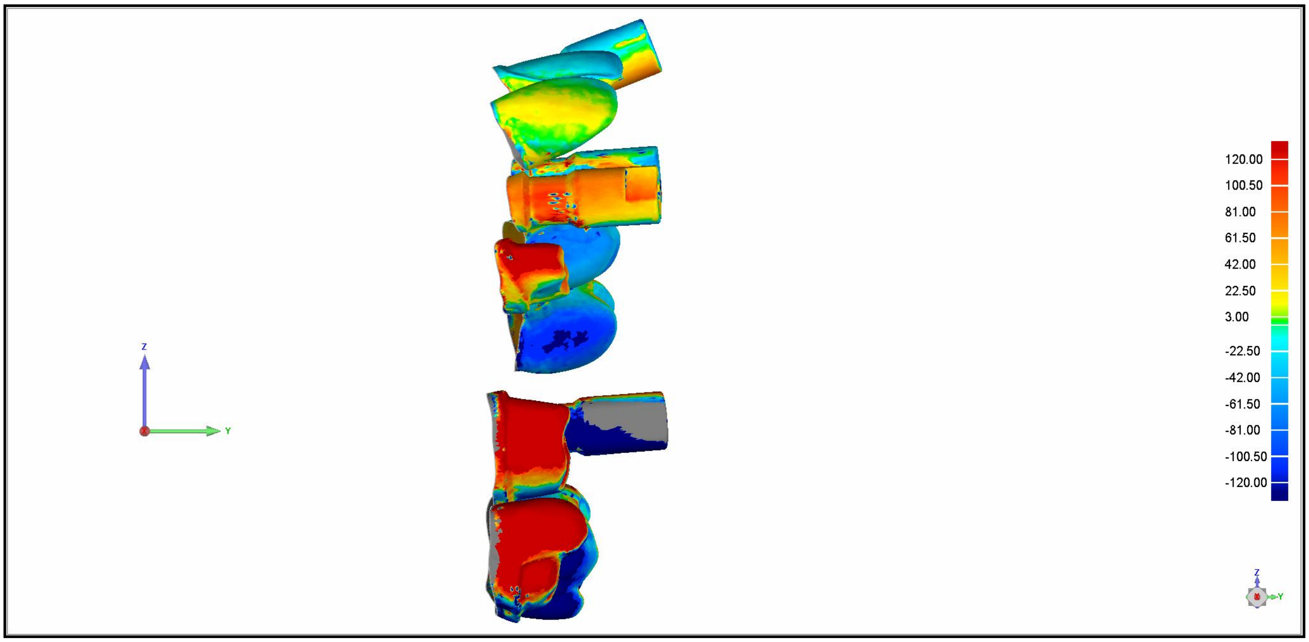

Predefinido: Superior

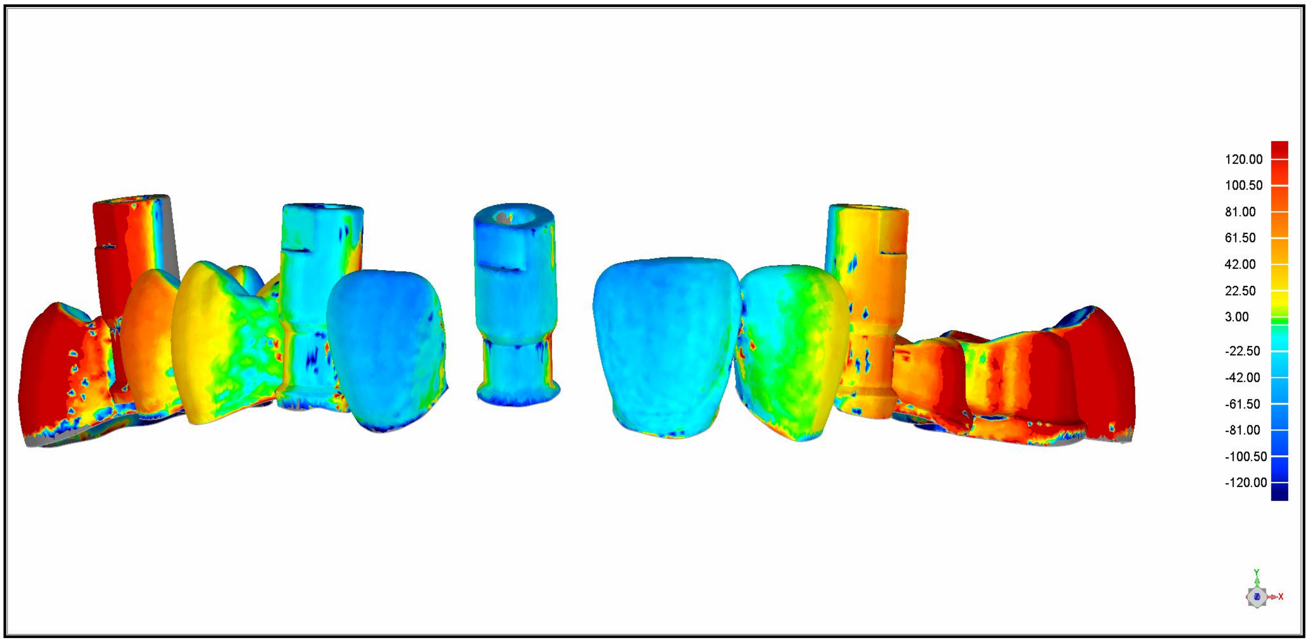

Predefinido: Inferior

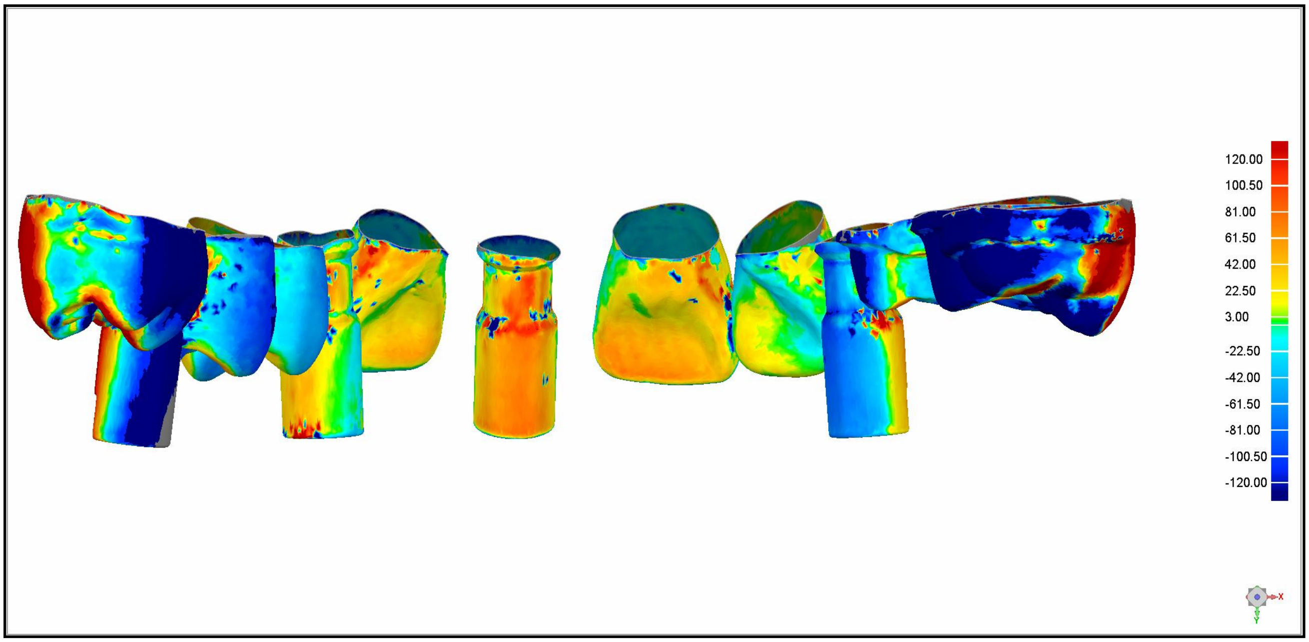

Ajuste de ubicación: Desviaciones superior e inferior

Unidades: u

Nombre	Desv	Estado	Superior Tol	Inferior Tol	Ref X	Ref Y	Ref Z	Radio	Desv X	Desv Y	Desv Z	Medido X	Medido Y	Medido Z	Dir. proy. X	Dir. proy. Y	Dir. proy. Z
Desv. inferior	-3082.57				-22979.30	37924.84	-95.54	n/a	-377.39	3040.43	-339.94	-23356.69	40965.27	-435.49	0.12	-0.99	0.11
Desv. superior	3138.42				19367.98	32192.36	14088.38	n/a	-510.31	1530.23	2692.15	18857.67	33722.59	16780.53	-0.16	0.49	0.86
