# Supplementary material for: Accuracy of four digital scanners according to scanning strategy in complete-arch impressions
Source: PLoS One. 2018 Sep 13;13(9):e0202916. doi: 10.1371/journal.pone.0202916 (PMC6136706; doi:10.1371/journal.pone.0202916)
Supplement: S6 Table — iTero (scanning strategy B). (ZIP) [file pone.0202916.s006.zip › S6/IT1B.pdf]

3D Comparación Resultados

Modelo referencia	MRC
Modelo test	IT1B
Nº de puntos de datos	82342
# Aislados	605

Tipo tolerancia	3D desviación
Unidades	u
Máx. crítico	120.00
Máx. nominal	3.00
Mín. nominal	-3.00
Mín. crítico	-120.00

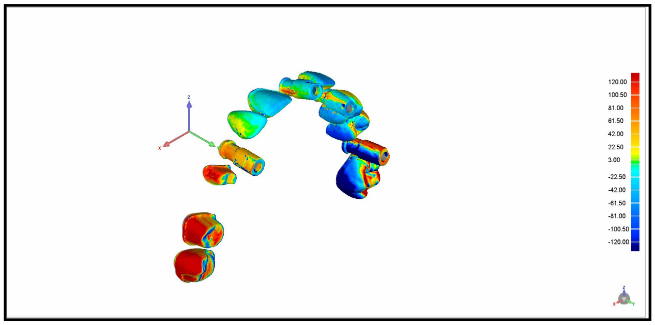

Desviación	
Desviación superior máx.	3140.90
Desviación inferior máx.	-3140.18
Desviación media	117.20 /-128.78
Desviación estándar	311.30

Distribución desviación

>=Min	<Max	# Puntos	%
-120.00	-100.50	1568	1.90
-100.50	-81.00	2508	3.05
-81.00	-61.50	3175	3.86
-61.50	-42.00	5219	6.34
-42.00	-22.50	7900	9.59
-22.50	-3.00	10346	12.56
-3.00	3.00	2902	3.52
3.00	22.50	8980	10.91
22.50	42.00	7835	9.52
42.00	61.50	5256	6.38
61.50	81.00	3715	4.51
81.00	100.50	2734	3.32
100.50	120.00	2227	2.70

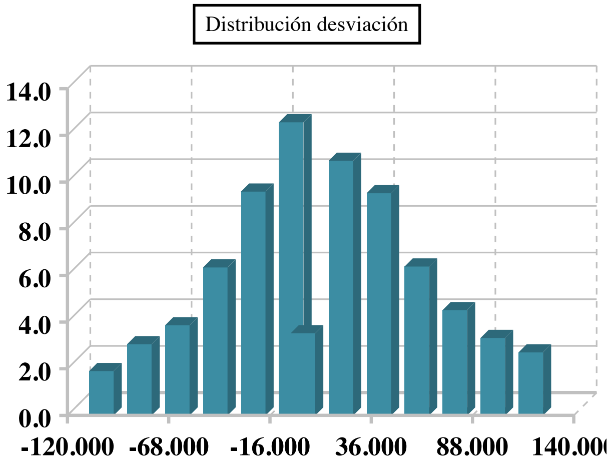

Fuera del crítico superior	9774	11.87
Fuera del crítico inferior	8203	9.96

Desviaciones estándar

Distribución (+/-)	# Puntos	%
-6 * Desv. estánd.	735	0.89
-5 * Desv. estánd.	369	0.45
-4 * Desv. estánd.	307	0.37
-3 * Desv. estánd.	305	0.37
-2 * Desv. estánd.	540	0.66
-1 * Desv. estánd.	36412	44.22
1 * Desv. estánd.	41778	50.74
2 * Desv. estánd.	674	0.82
3 * Desv. estánd.	308	0.37
4 * Desv. estánd.	179	0.22
5 * Desv. estánd.	267	0.32
6 * Desv. estánd.	468	0.57

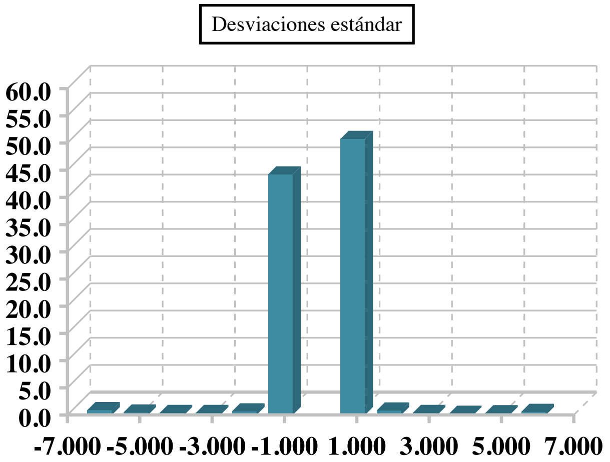

Predefinido: Isométrico

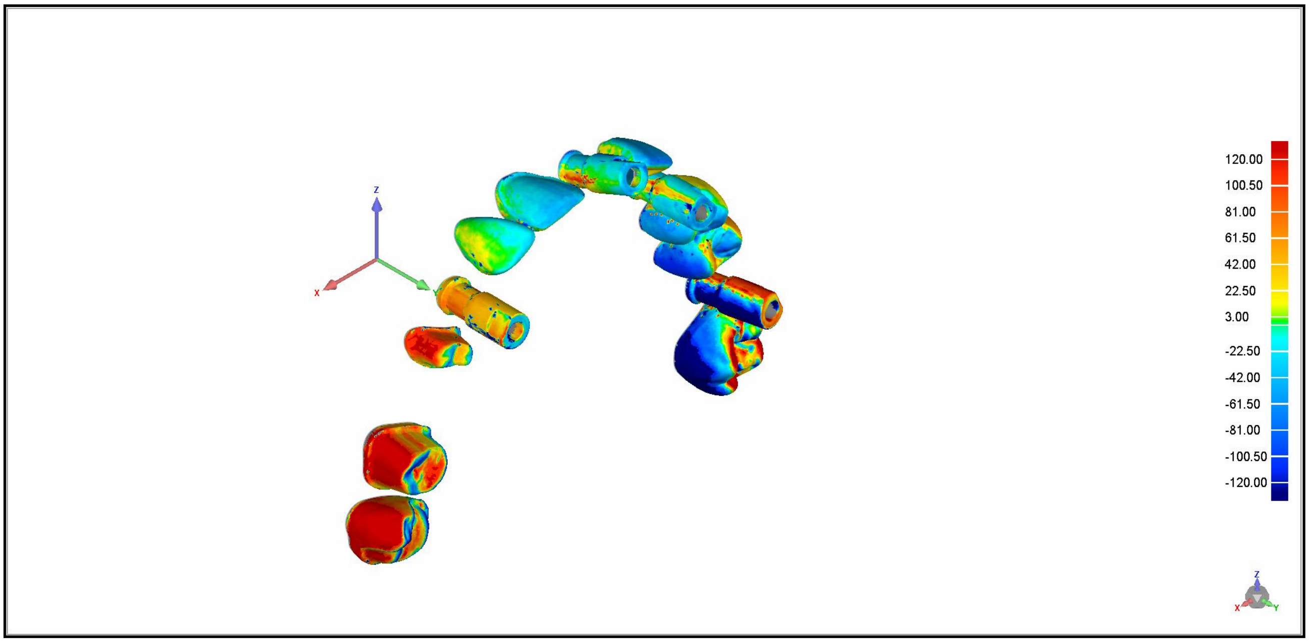

Predefinido: Frente

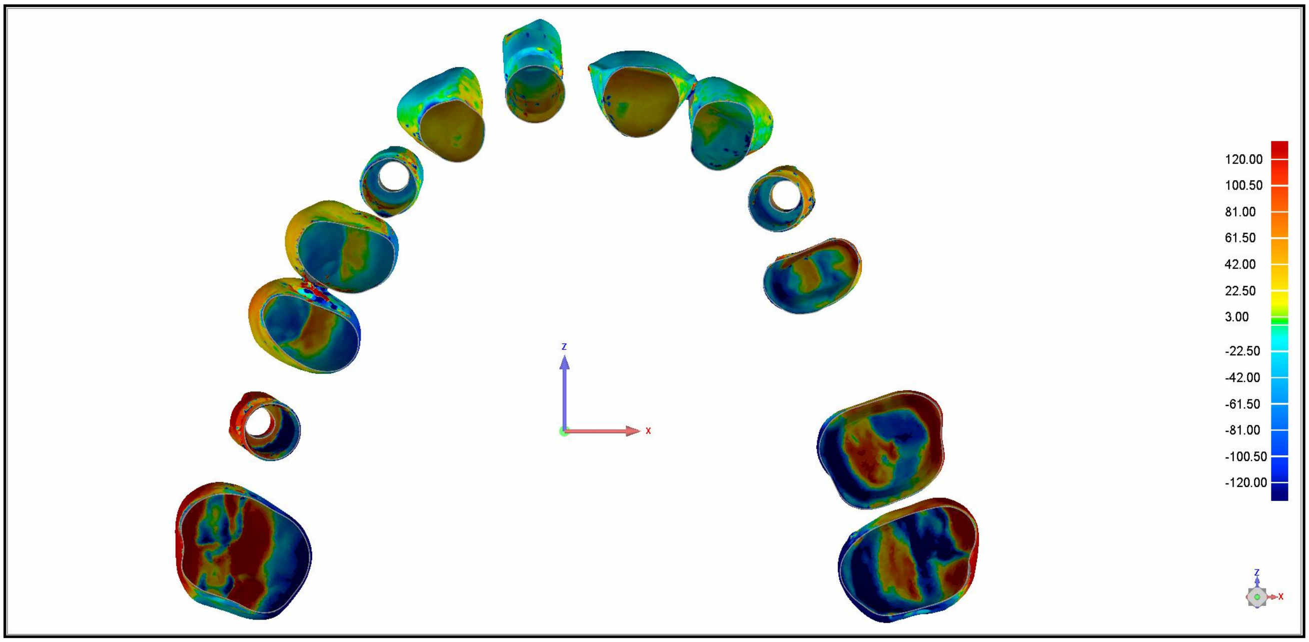

Predefinido: Atrás

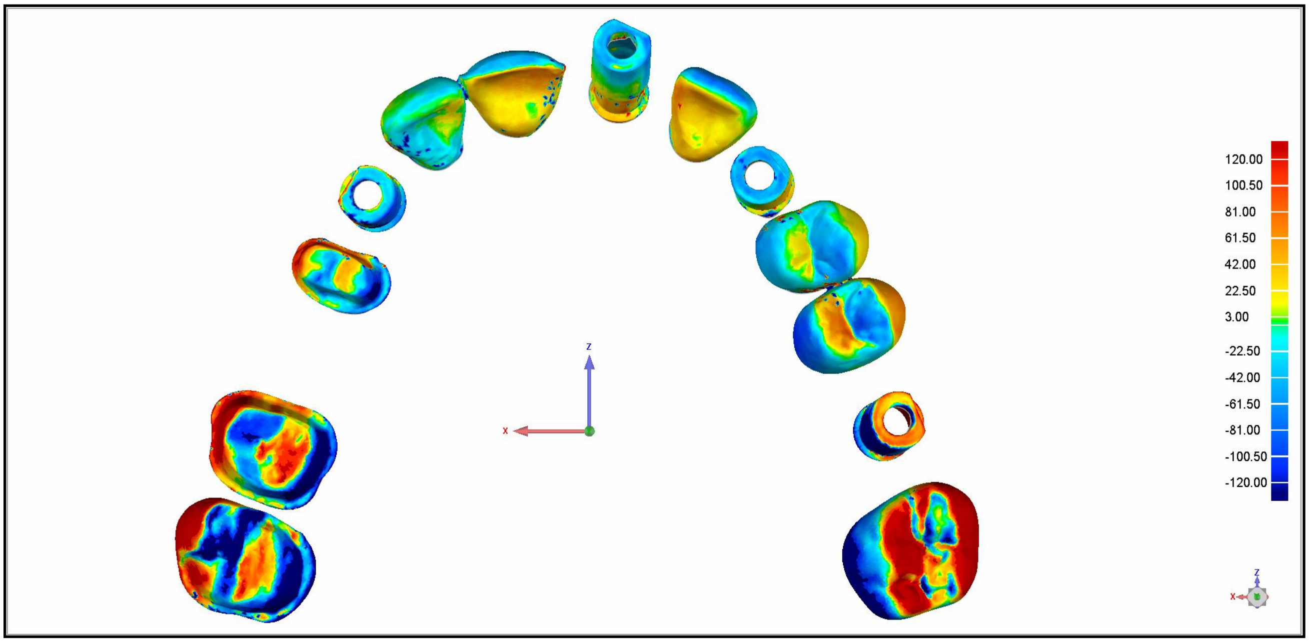

Predefinido: Izquierda

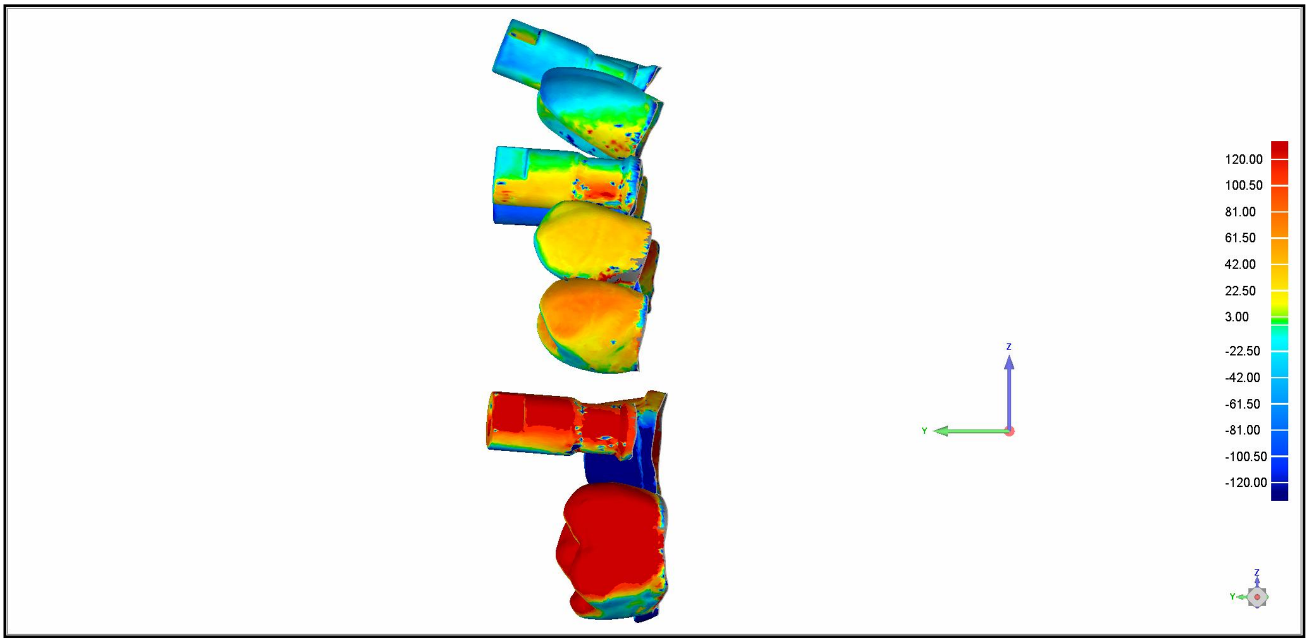

Predefinido: Derecha

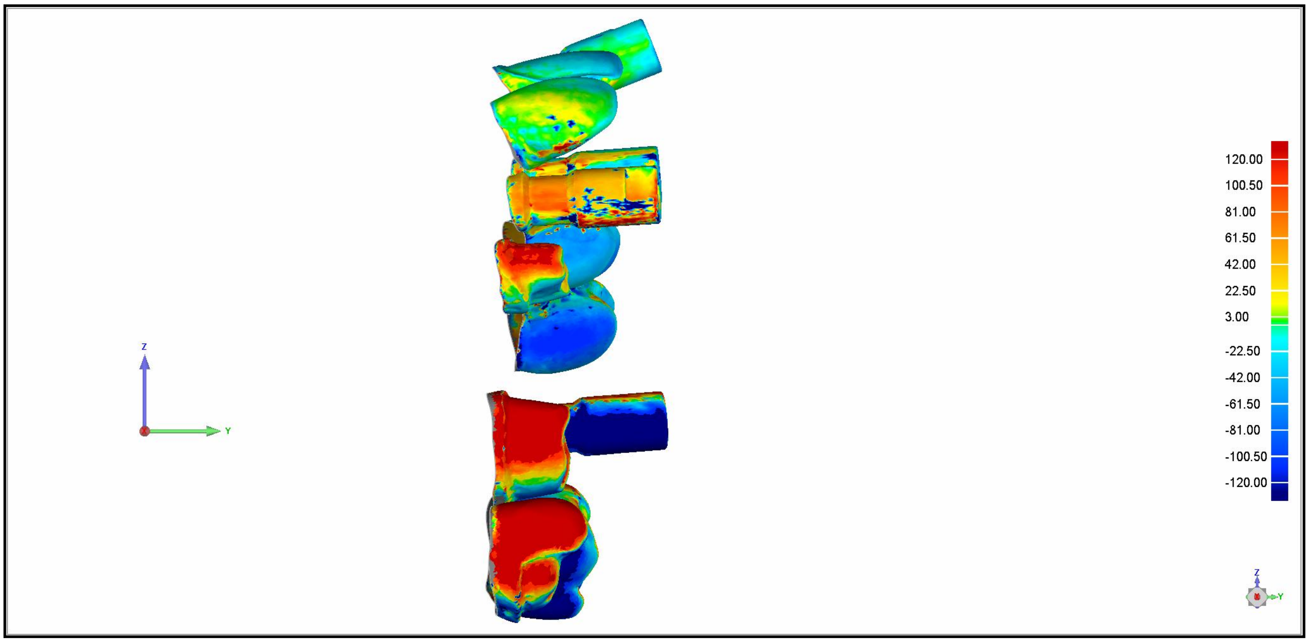

Predefinido: Superior

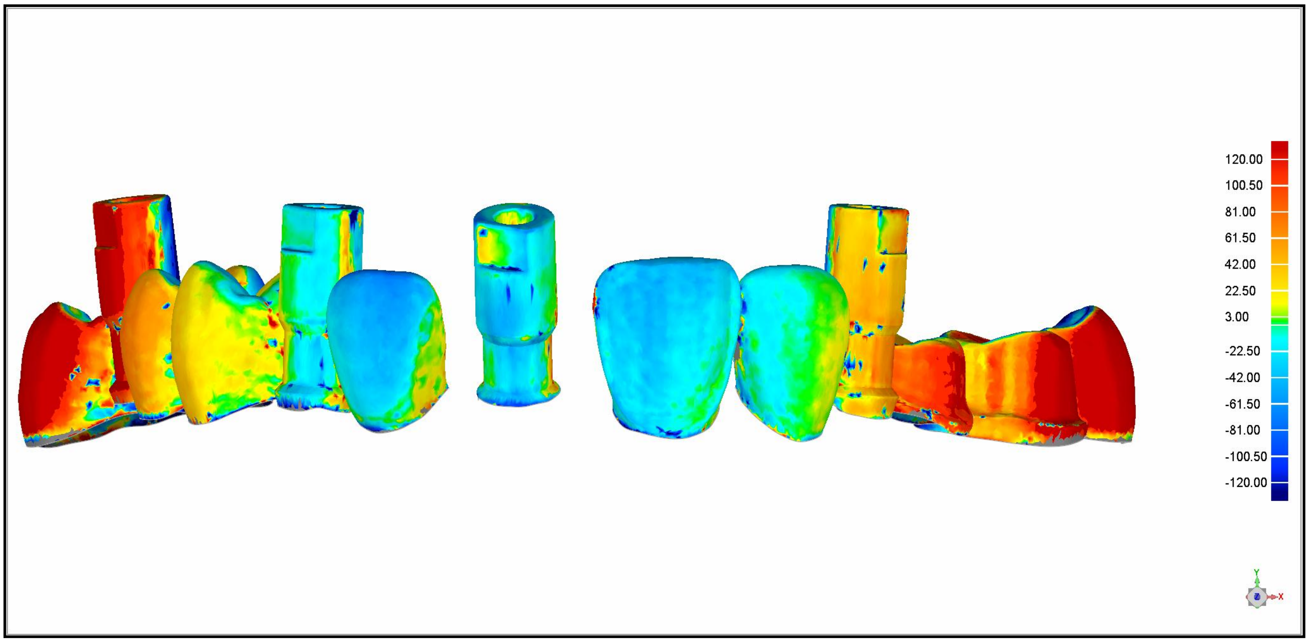

Predefinido: Inferior

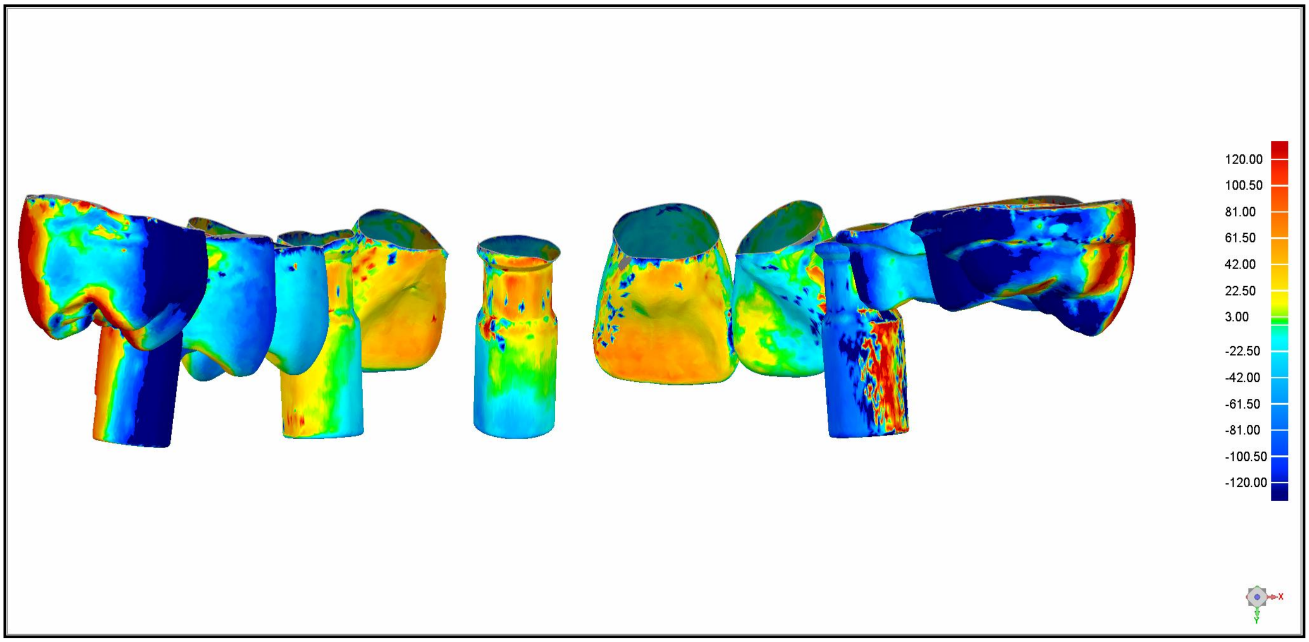

Ajuste de ubicación: Desviaciones superior e inferior

Unidades: u

Nombre	Desv	Estado	Superior Tol	Inferior Tol	Ref X	Ref Y	Ref Z	Radio	Desv X	Desv Y	Desv Z	Medido X	Medido Y	Medido Z	Dir. proy. X	Dir. proy. Y	Dir. proy. Z
Desv. inferior	-3140.18				17725.09	37493.08	17274.82	n/a	853.22	-2818.12	-1091.32	18578.30	34674.96	16183.50	-0.27	0.90	0.35
Desv. superior	3140.90				-7053.08	33836.14	25576.13	n/a	2744.13	879.16	1249.83	-4308.95	34715.30	26825.96	0.87	0.28	0.40
