# Supplementary material for: Accuracy of four digital scanners according to scanning strategy in complete-arch impressions
Source: PLoS One. 2018 Sep 13;13(9):e0202916. doi: 10.1371/journal.pone.0202916 (PMC6136706; doi:10.1371/journal.pone.0202916)
Supplement: S6 Table — iTero (scanning strategy B). (ZIP) [file pone.0202916.s006.zip › S6/IT6B.pdf]

Fecha: 3/6/2017, 1:25 pm

3D Comparación Resultados

Modelo referencia	MRC
Modelo test	IT6B
Nº de puntos de datos	80637
# Aislados	607

Tipo tolerancia	3D desviación
Unidades	u
Máx. crítico	120.00
Máx. nominal	1.00
Mín. nominal	-1.00
Mín. crítico	-120.00

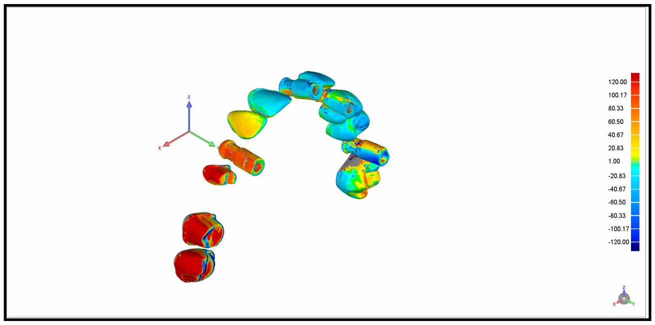

Desviación	
Desviación superior máx.	3141.80
Desviación inferior máx.	-3151.63
Desviación media	107.12 /-116.86
Desviación estándar	309.77

Distribución desviación

>=Min	<Max	# Puntos	%
-120.00	-100.17	1352	1.68
-100.17	-80.33	1602	1.99
-80.33	-60.50	1807	2.24
-60.50	-40.67	3687	4.57
-40.67	-20.83	9508	11.79
-20.83	-1.00	14053	17.43
-1.00	1.00	1523	1.89
1.00	20.83	11920	14.78
20.83	40.67	9386	11.64
40.67	60.50	5125	6.36
60.50	80.33	3125	3.88
80.33	100.17	2476	3.07
100.17	120.00	1402	1.74

Fuera del crítico superior	7323	9.08
Fuera del crítico inferior	6348	7.87

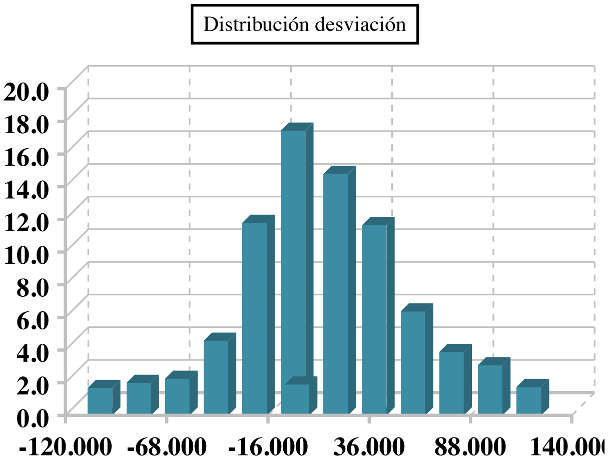

Desviaciones estándar

Distribución (+/-)	# Puntos	%
-6 * Desv. estánd.	665	0.82
-5 * Desv. estánd.	294	0.36
-4 * Desv. estánd.	278	0.34
-3 * Desv. estánd.	308	0.38
-2 * Desv. estánd.	1317	1.63
-1 * Desv. estánd.	35073	43.49
1 * Desv. estánd.	39982	49.58
2 * Desv. estánd.	1353	1.68
3 * Desv. estánd.	328	0.41
4 * Desv. estánd.	350	0.43
5 * Desv. estánd.	285	0.35
6 * Desv. estánd.	404	0.50

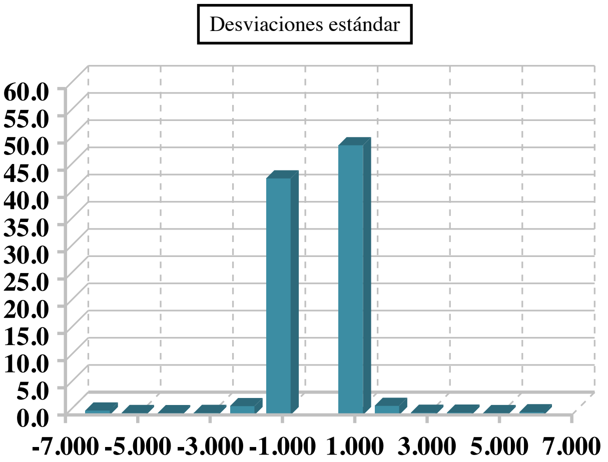

Predefinido: Isométrico

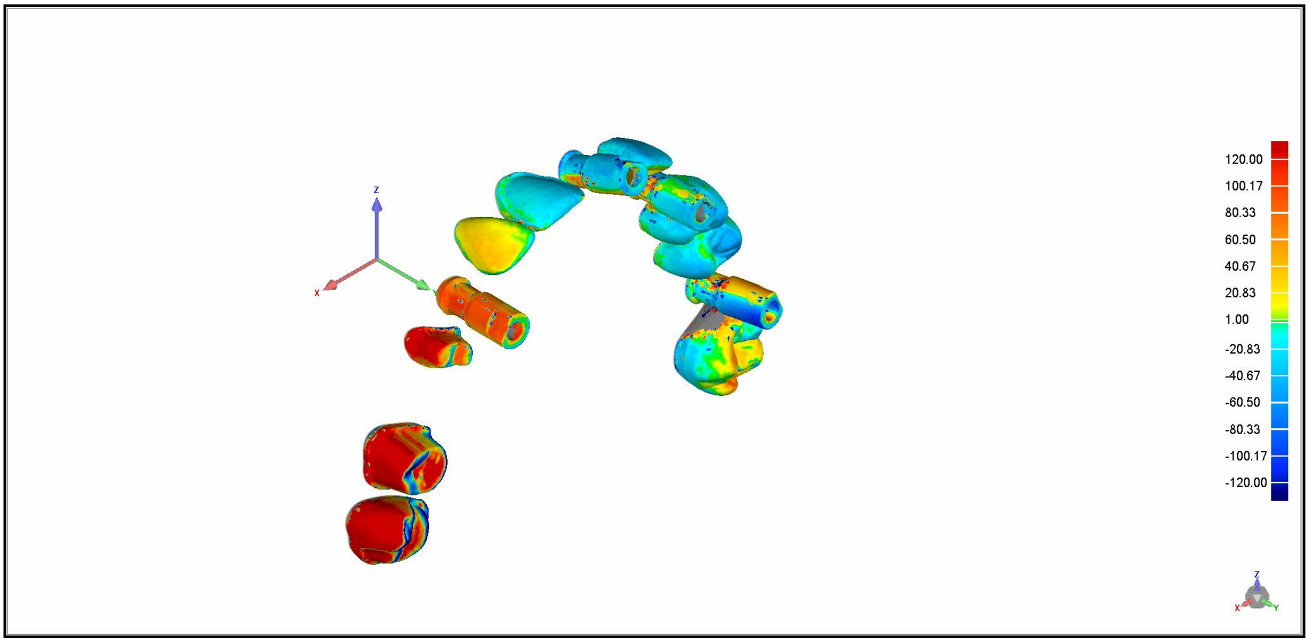

Predefinido: Frente

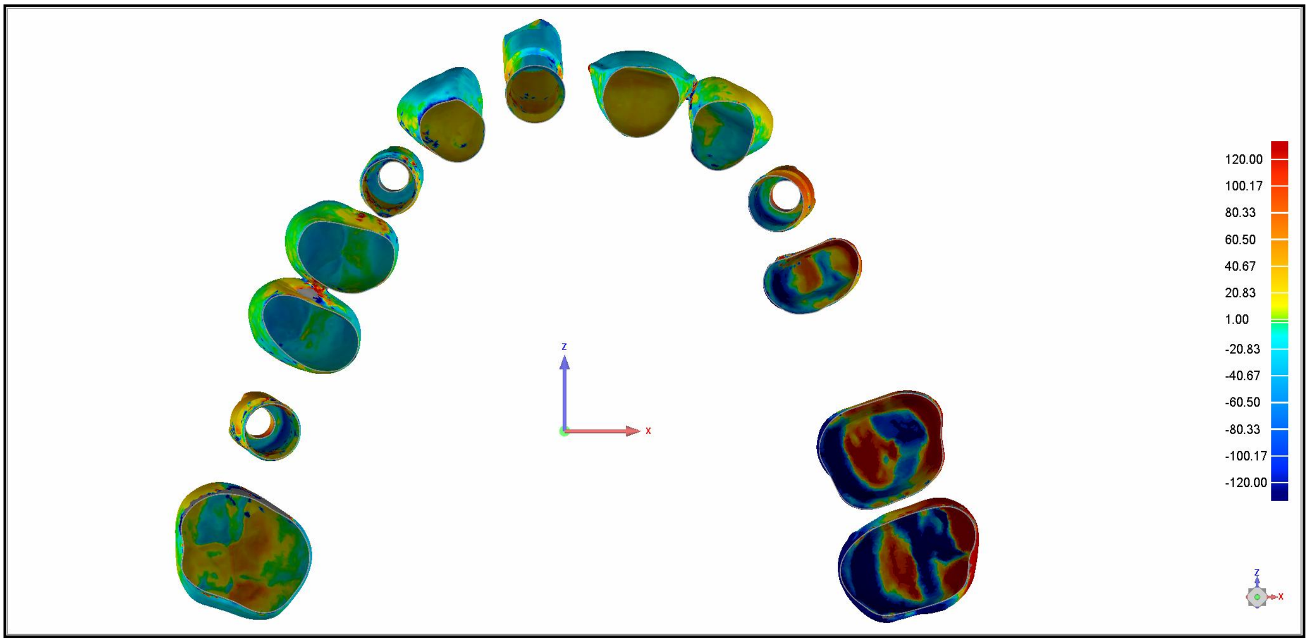

Predefinido: Atrás

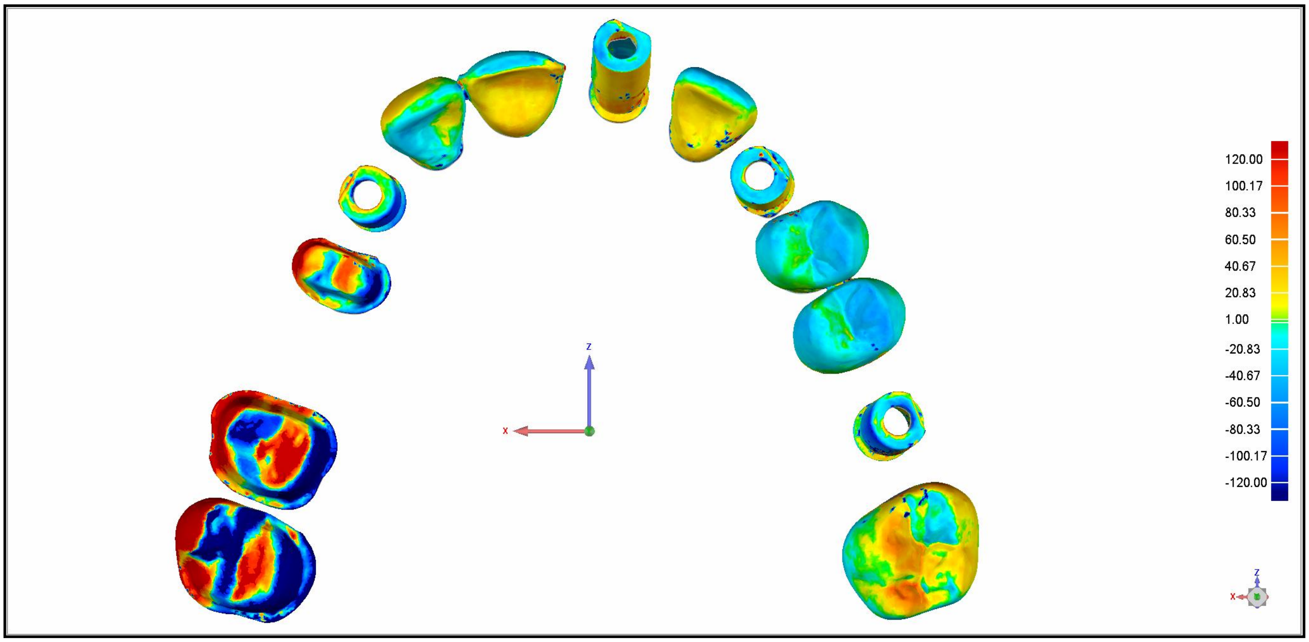

Predefinido: Izquierda

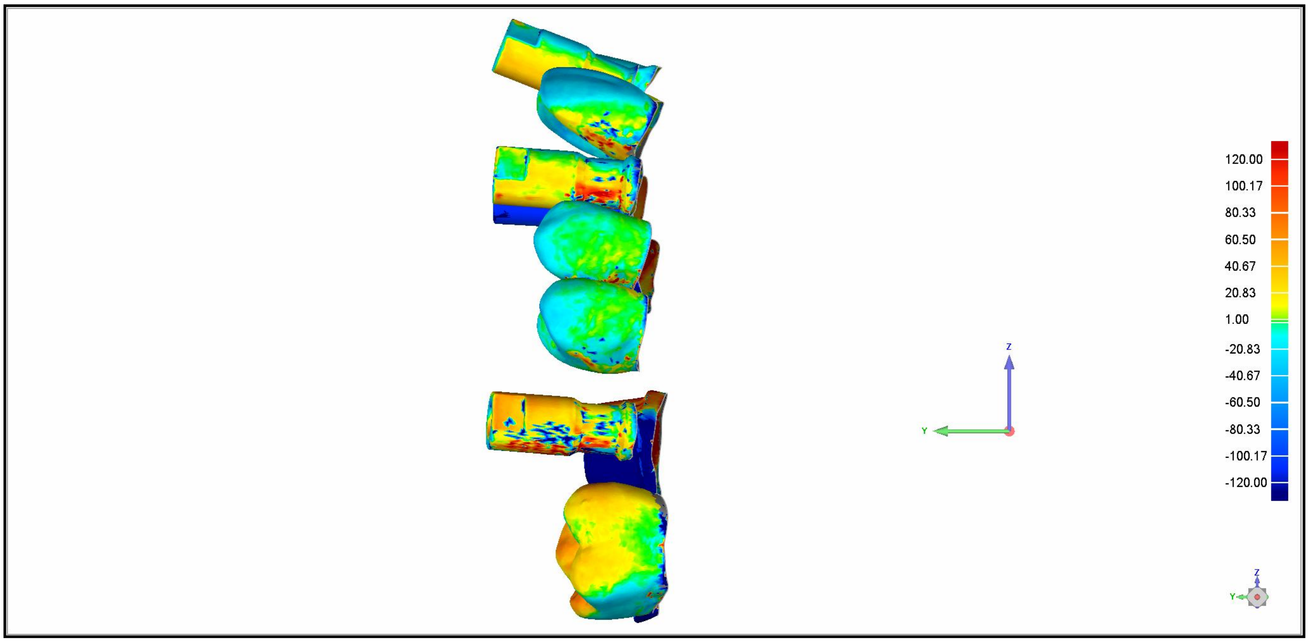

Predefinido: Derecha

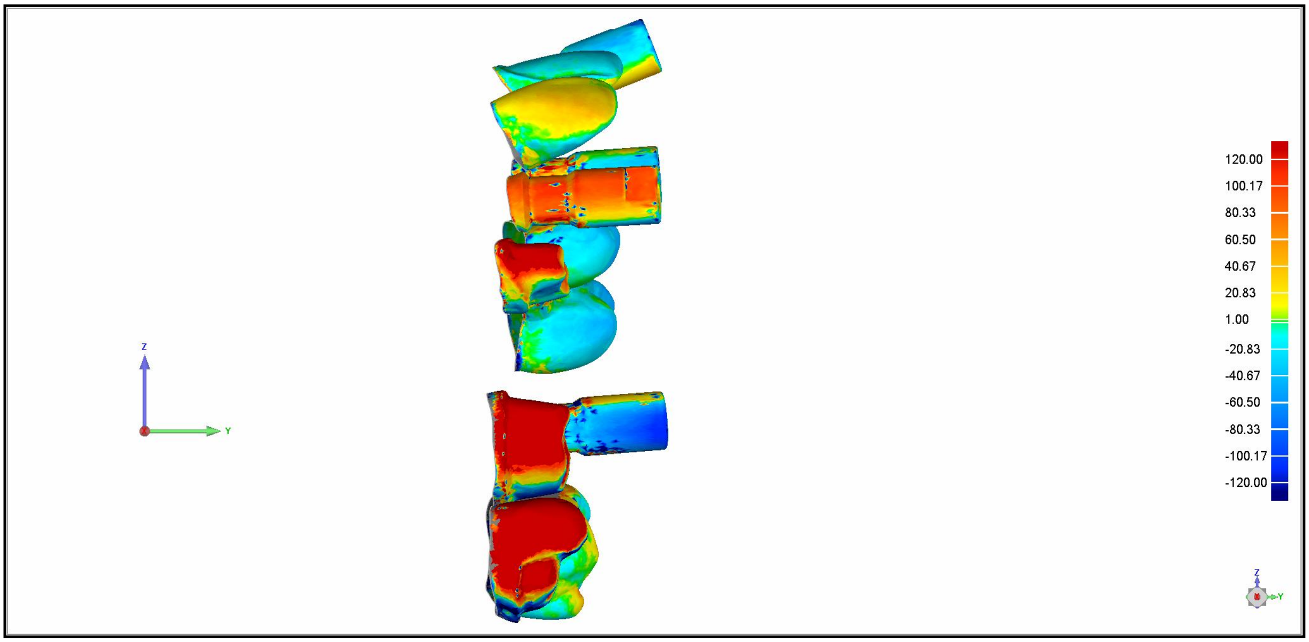

Predefinido: Superior

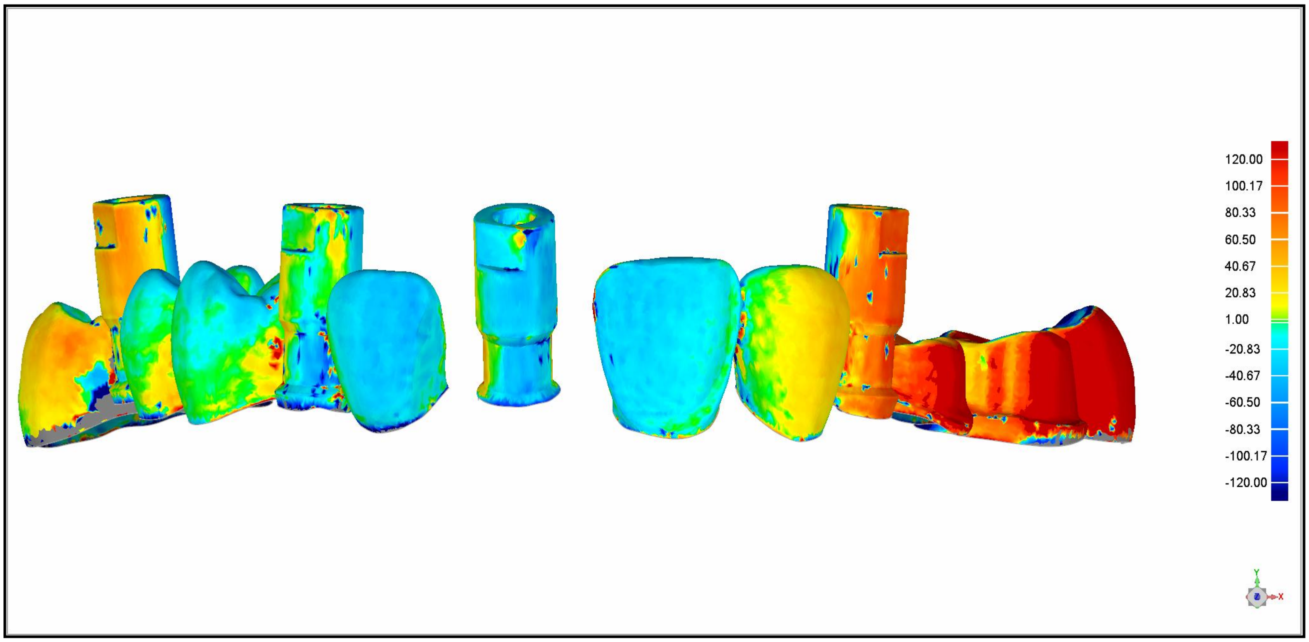

Predefinido: Inferior

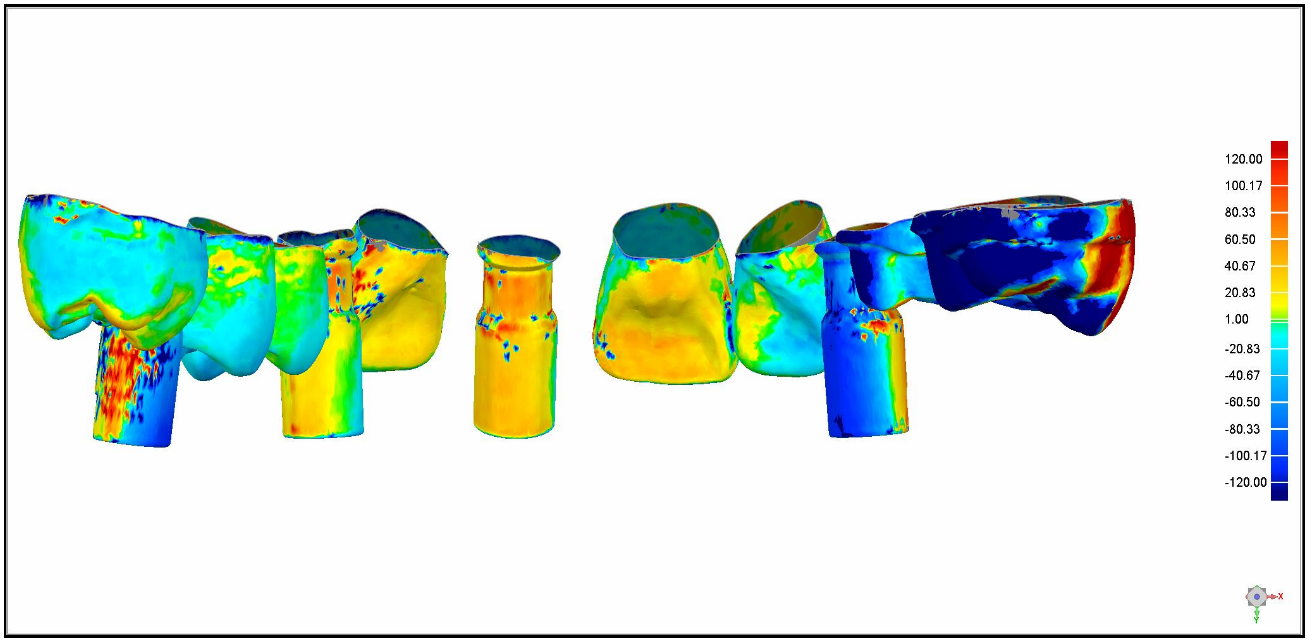

Ajuste de ubicación: Desviaciones superior e inferior

Unidades: u

Nombre	Desv	Estado	Superior Tol	Inferior Tol	Ref X	Ref Y	Ref Z	Radio	Desv X	Desv Y	Desv Z	Medido X	Medido Y	Medido Z	Dir. proy. X	Dir. proy. Y	Dir. proy. Z
Desv. inferior	-3151.63				-24238.74	38264.47	-551.15	n/a	-58.94	-2647.19	-1709.30	-24297.68	35617.29	-2260.46	0.02	0.84	0.54
Desv. superior	3141.80				-29896.55	26869.28	-6780.69	n/a	2121.53	1353.79	1880.76	-27775.02	28223.07	-4899.93	0.68	0.43	0.60
