# Supplementary material for: Accuracy of four digital scanners according to scanning strategy in complete-arch impressions
Source: PLoS One. 2018 Sep 13;13(9):e0202916. doi: 10.1371/journal.pone.0202916 (PMC6136706; doi:10.1371/journal.pone.0202916)
Supplement: S6 Table — iTero (scanning strategy B). (ZIP) [file pone.0202916.s006.zip › S6/IT7B.pdf]

Geomagic Control

Fecha: 3/6/2017, 1:27 pm

3D Comparación Resultados

Modelo referencia	MRC
Modelo test	IT7B
Nº de puntos de datos	75768
# Aislados	586

Tipo tolerancia	3D desviación
Unidades	u
Máx. crítico	120.00
Máx. nominal	15.00
Mín. nominal	-15.00
Mín. crítico	-120.00

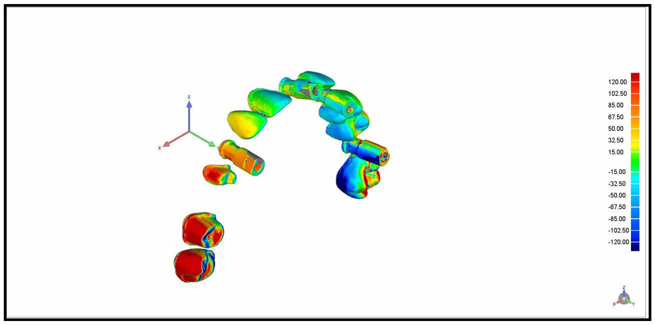

Desviación	
Desviación superior máx.	3035.99
Desviación inferior máx.	-3118.16
Desviación media	101.21 /-91.39
Desviación estándar	231.29

Distribución desviación

>=Min	<Max	# Puntos	%
-120.00	-102.50	1086	1.43
-102.50	-85.00	1449	1.91
-85.00	-67.50	1924	2.54
-67.50	-50.00	2842	3.75
-50.00	-32.50	4918	6.49
-32.50	-15.00	8011	10.57
-15.00	15.00	16194	21.37
15.00	32.50	9114	12.03
32.50	50.00	6002	7.92
50.00	67.50	3687	4.87
67.50	85.00	2654	3.50
85.00	102.50	2000	2.64
102.50	120.00	1759	2.32

Fuera del crítico superior	8301	10.96
Fuera del crítico inferior	5827	7.69

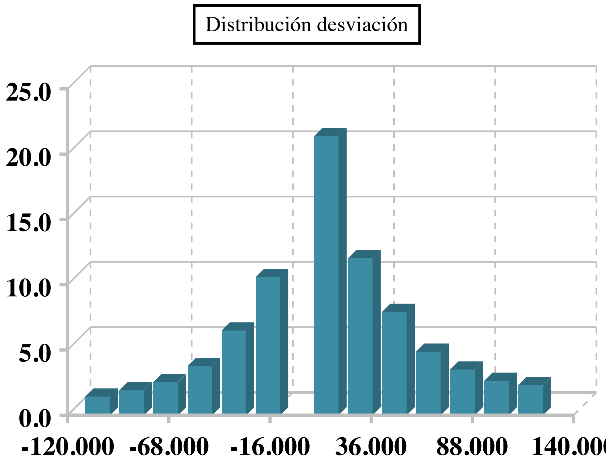

Desviaciones estándar

Distribución (+/-)	# Puntos	%
-6 * Desv. estánd.	396	0.52
-5 * Desv. estánd.	138	0.18
-4 * Desv. estánd.	164	0.22
-3 * Desv. estánd.	268	0.35
-2 * Desv. estánd.	2109	2.78
-1 * Desv. estánd.	39503	52.14
1 * Desv. estánd.	30225	39.89
2 * Desv. estánd.	1639	2.16
3 * Desv. estánd.	246	0.32
4 * Desv. estánd.	228	0.30
5 * Desv. estánd.	256	0.34
6 * Desv. estánd.	596	0.79

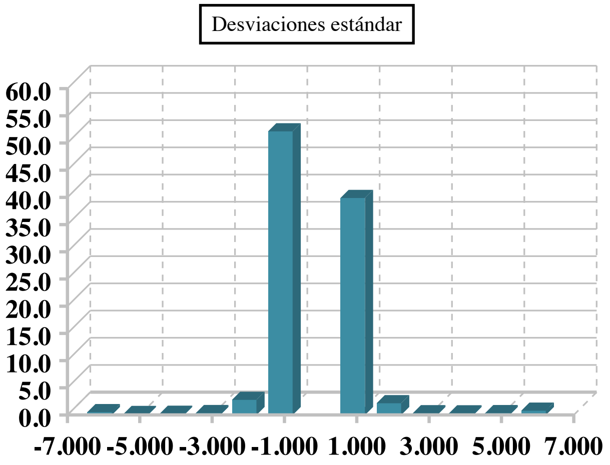

Predefinido: Isométrico

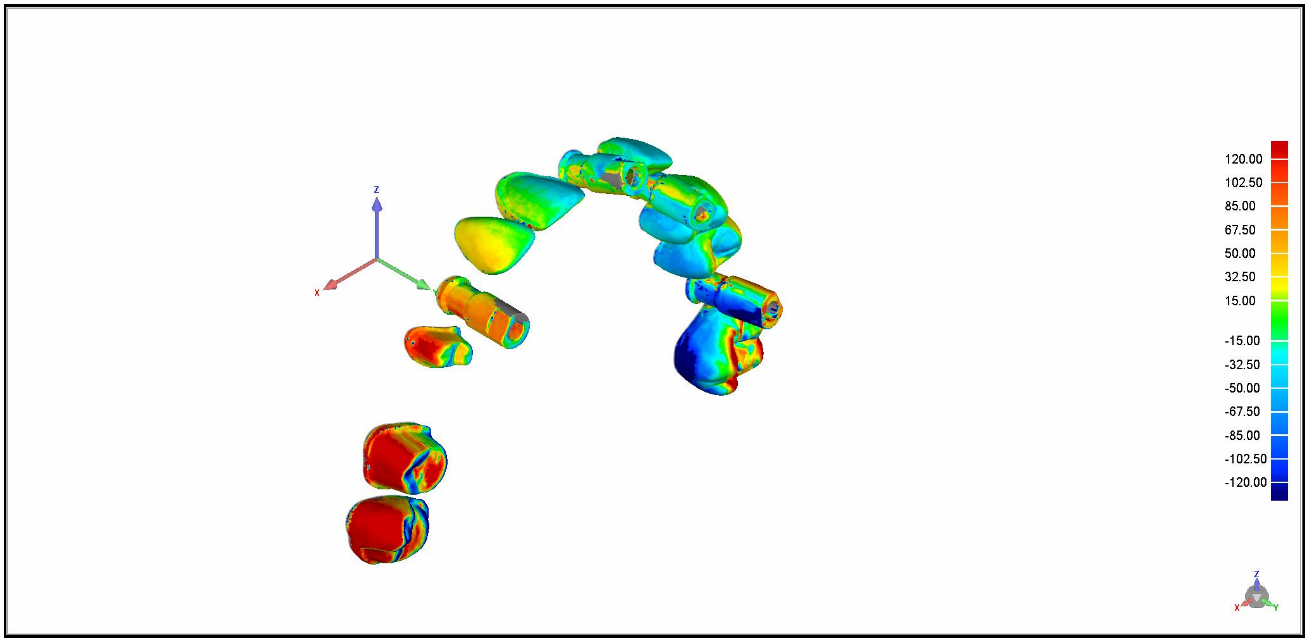

Predefinido: Frente

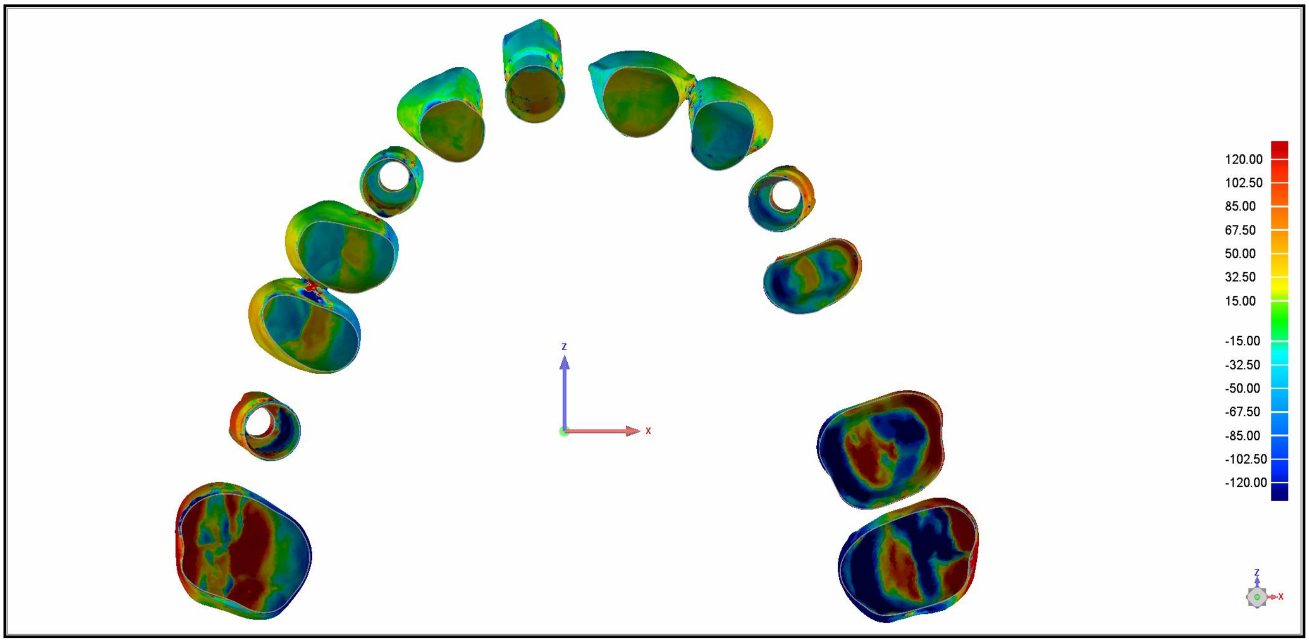

Predefinido: Atrás

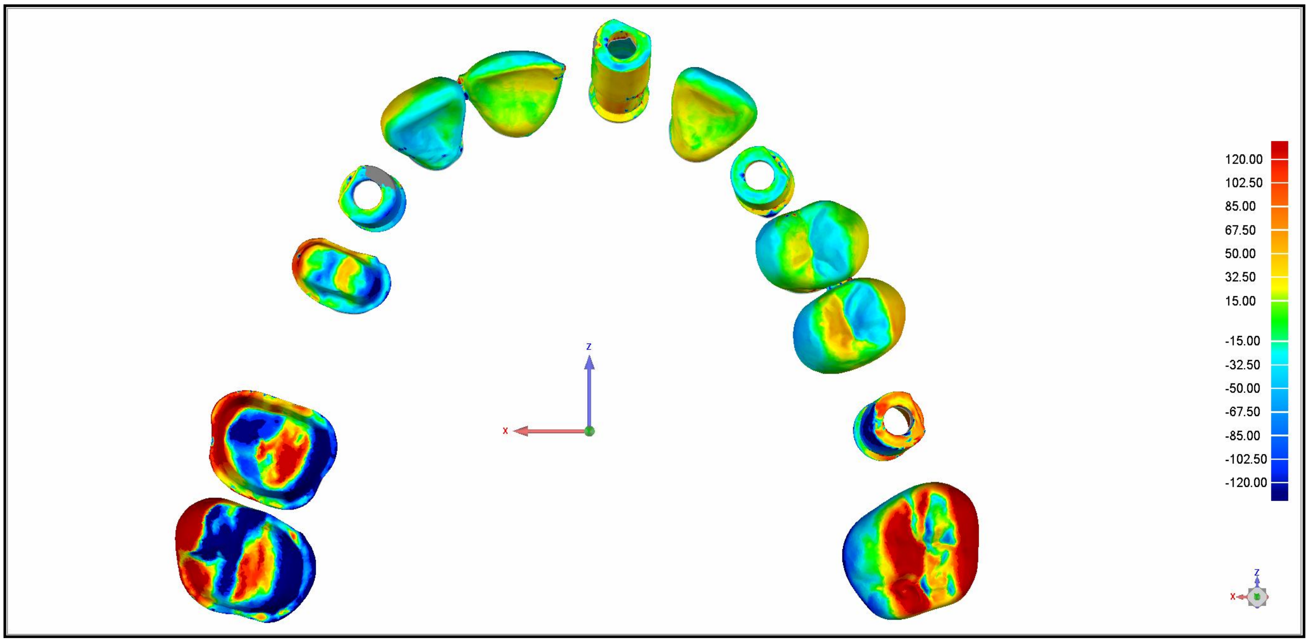

Predefinido: Izquierda

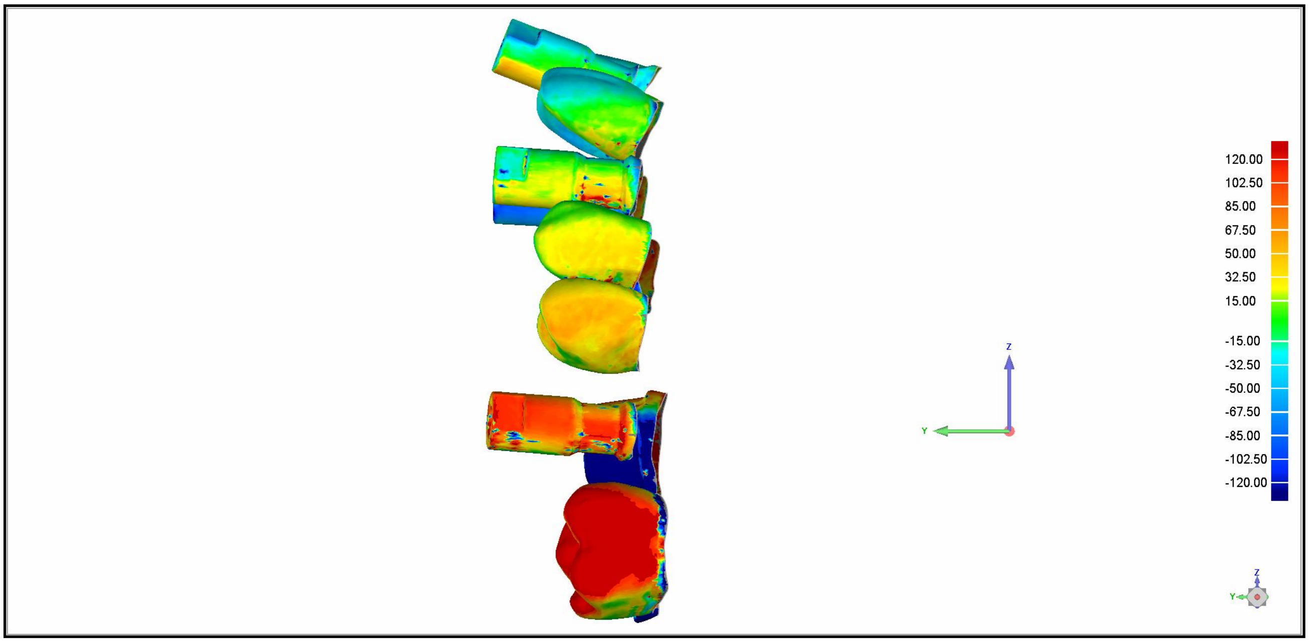

Predefinido: Derecha

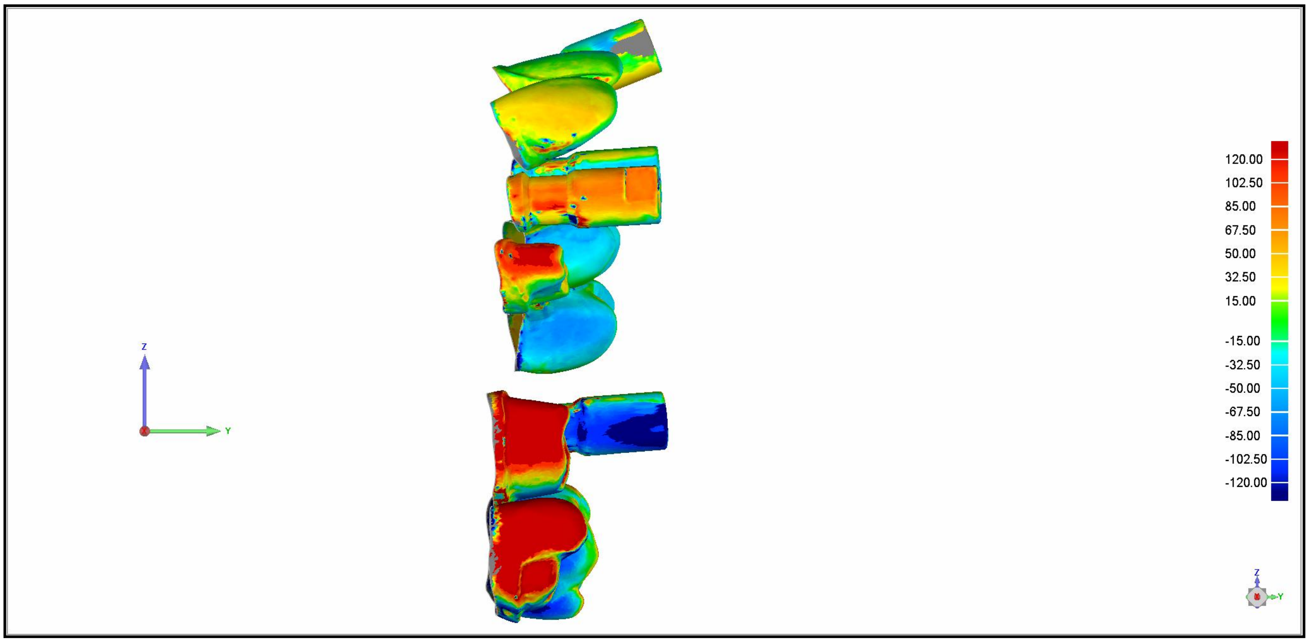

Predefinido: Superior

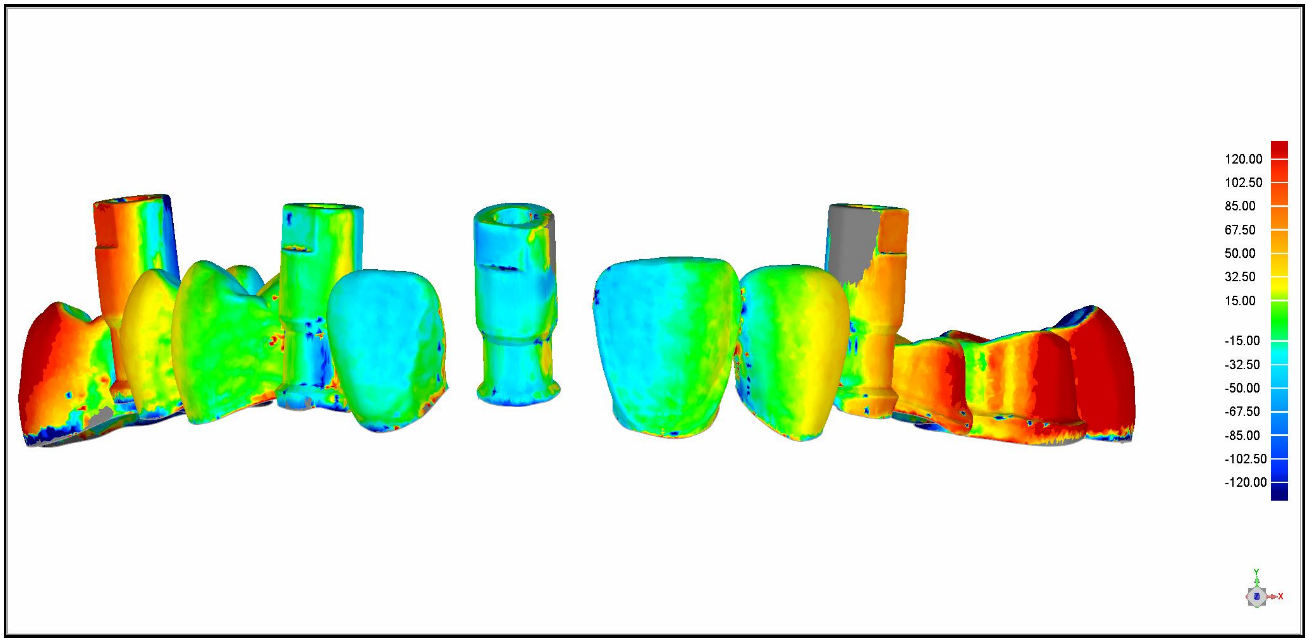

Predefinido: Inferior

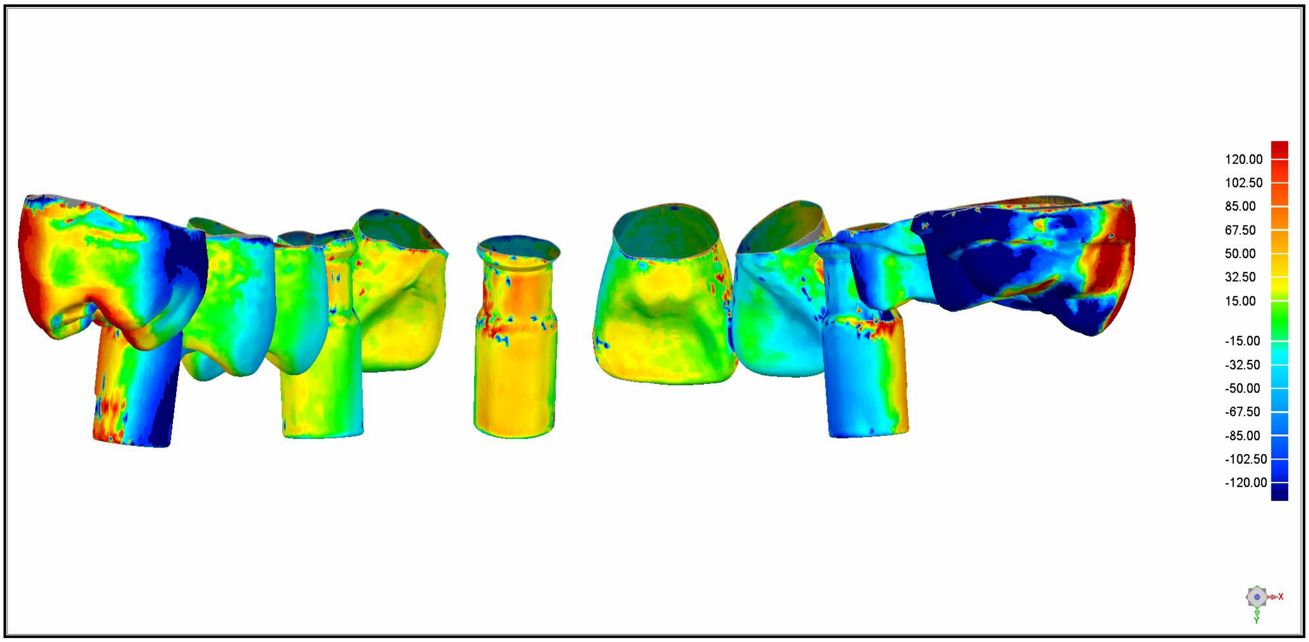

Ajuste de ubicación: Desviaciones superior e inferior

Unidades: u

Nombre	Desv	Estado	Superior Tol	Inferior Tol	Ref X	Ref Y	Ref Z	Radio	Desv X	Desv Y	Desv Z	Medido X	Medido Y	Medido Z	Dir. proy. X	Dir. proy. Y	Dir. proy. Z
Desv. inferior	-3118.16				-29208.33	26961.25	-11988.49	n/a	2755.36	631.05	-1316.31	-26452.97	27592.29	-13304.79	-0.88	-0.20	0.42
Desv. superior	3035.99				-24542.29	30268.52	-1843.31	n/a	-1649.92	-443.25	-2509.69	-26192.20	29825.26	-4353.00	-0.54	-0.15	-0.83
