# Supplementary material for: Accuracy of four digital scanners according to scanning strategy in complete-arch impressions
Source: PLoS One. 2018 Sep 13;13(9):e0202916. doi: 10.1371/journal.pone.0202916 (PMC6136706; doi:10.1371/journal.pone.0202916)
Supplement: S6 Table — iTero (scanning strategy B). (ZIP) [file pone.0202916.s006.zip › S6/IT10B.pdf]

3D Comparación Resultados

Modelo referencia	MRC
Modelo test	IT10B
Nº de puntos de datos	81665
# Aislados	553

Tipo tolerancia	3D desviación
Unidades	u
Máx. crítico	120.00
Máx. nominal	7.00
Mín. nominal	-7.00
Mín. crítico	-120.00

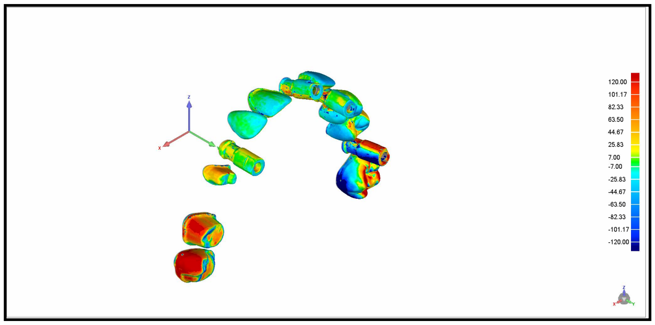

Desviación	
Desviación superior máx.	3058.84
Desviación inferior máx.	-3131.42
Desviación media	96.01 /-82.67
Desviación estándar	248.38

Distribución desviación

>=Min	<Max	# Puntos	%
-120.00	-101.17	1042	1.28
-101.17	-82.33	1442	1.77
-82.33	-63.50	1878	2.30
-63.50	-44.67	4102	5.02
-44.67	-25.83	7702	9.43
-25.83	-7.00	13199	16.16
-7.00	7.00	10783	13.20
7.00	25.83	9992	12.24
25.83	44.67	7354	9.01
44.67	63.50	4527	5.54
63.50	82.33	3060	3.75
82.33	101.17	2069	2.53
101.17	120.00	2063	2.53

Fuera del crítico superior	7186	8.80
Fuera del crítico inferior	5266	6.45

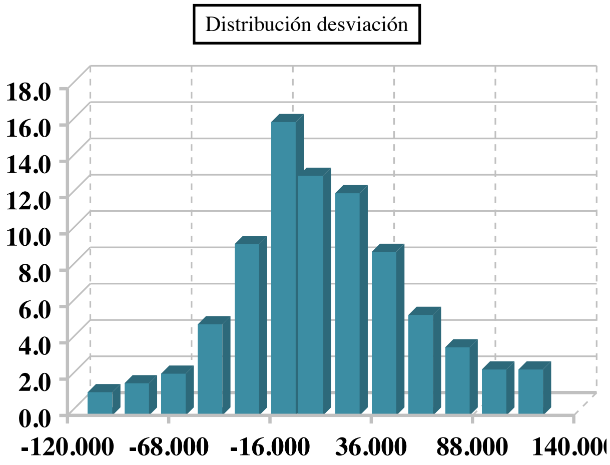

Desviaciones estándar

Distribución (+/-)	# Puntos	%
-6 * Desv. estánd.	594	0.73
-5 * Desv. estánd.	210	0.26
-4 * Desv. estánd.	222	0.27
-3 * Desv. estánd.	186	0.23
-2 * Desv. estánd.	381	0.47
-1 * Desv. estánd.	44240	54.17
1 * Desv. estánd.	33991	41.62
2 * Desv. estánd.	592	0.72
3 * Desv. estánd.	264	0.32
4 * Desv. estánd.	179	0.22
5 * Desv. estánd.	204	0.25
6 * Desv. estánd.	602	0.74

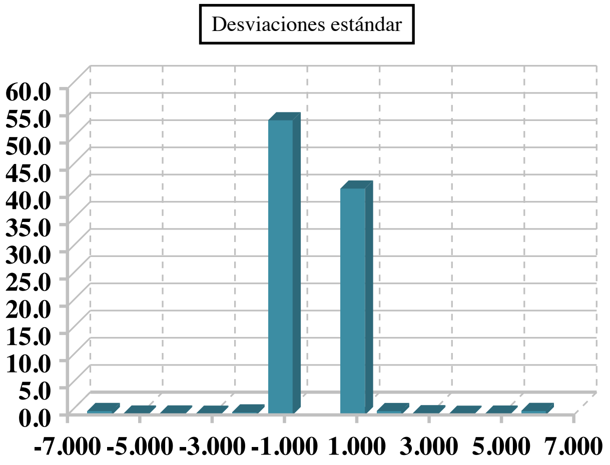

Predefinido: Isométrico

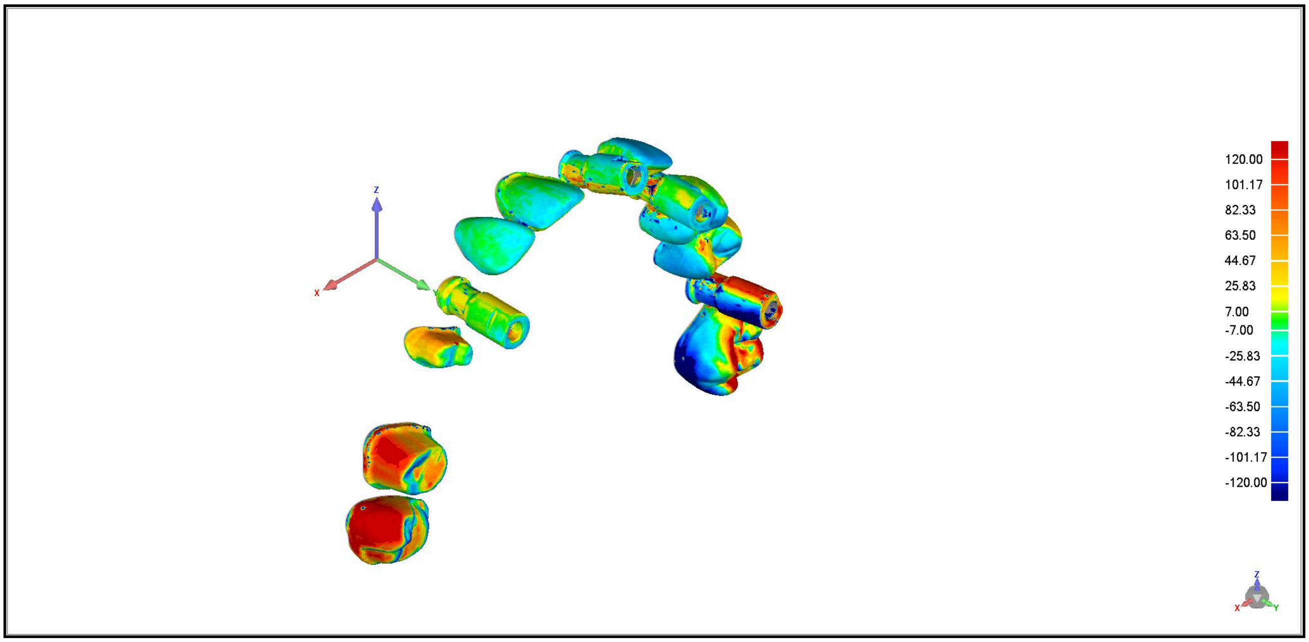

Predefinido: Frente

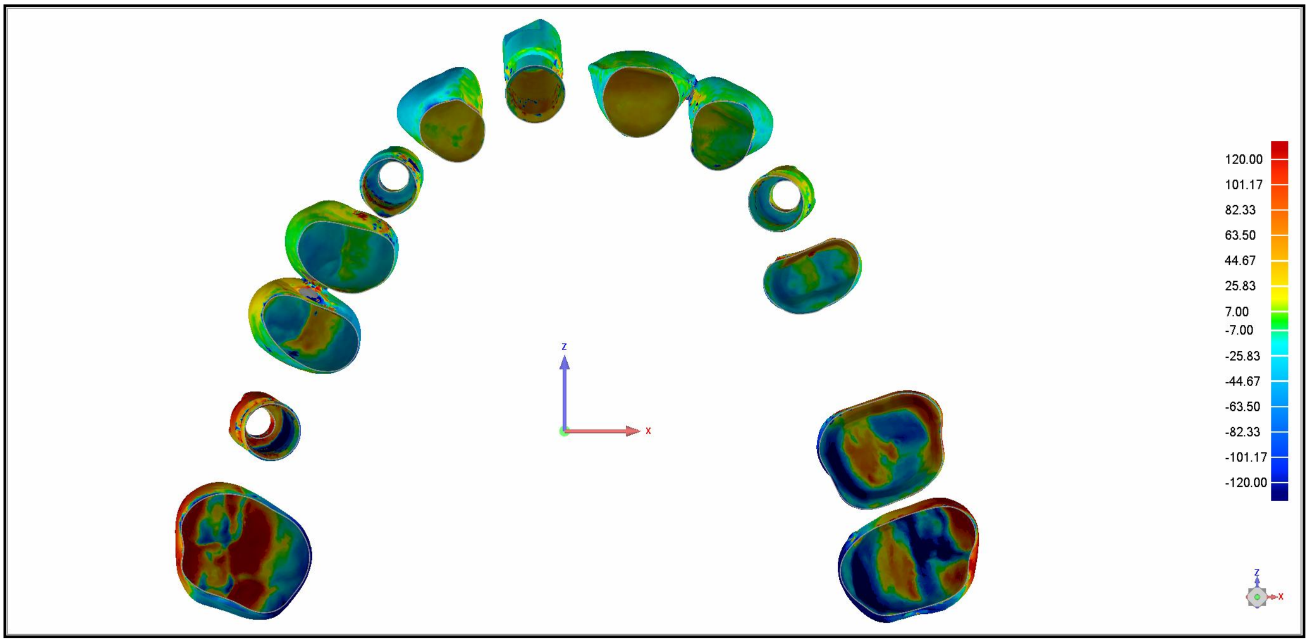

Predefinido: Atrás

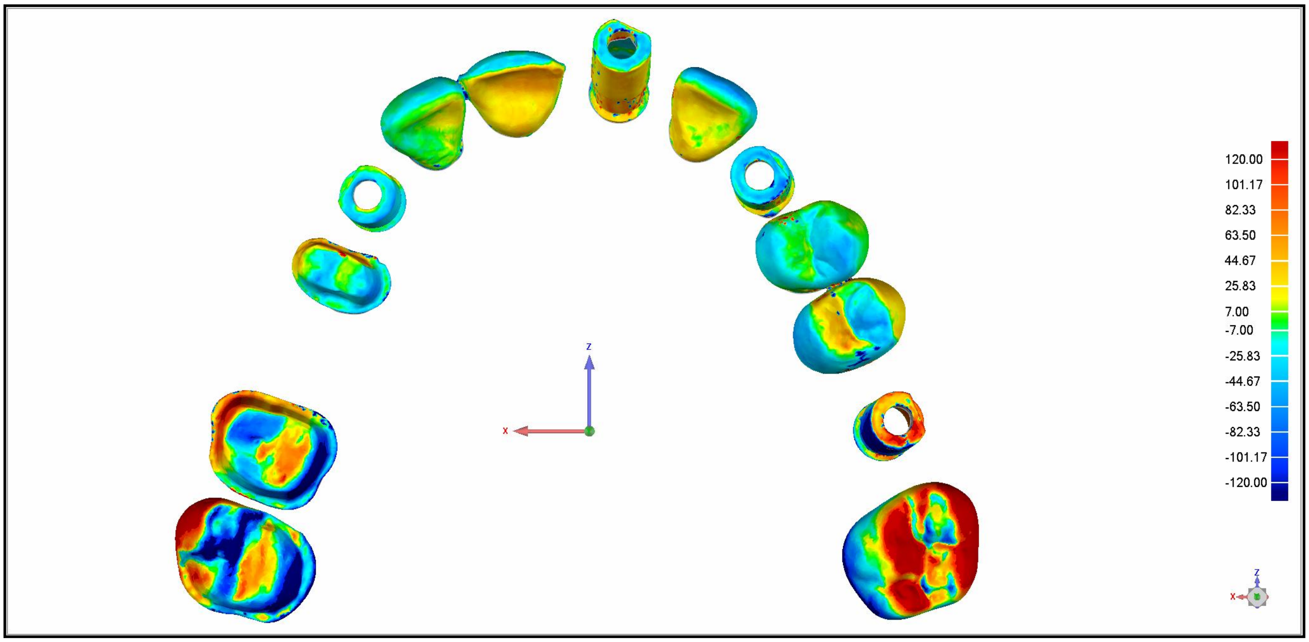

Predefinido: Izquierda

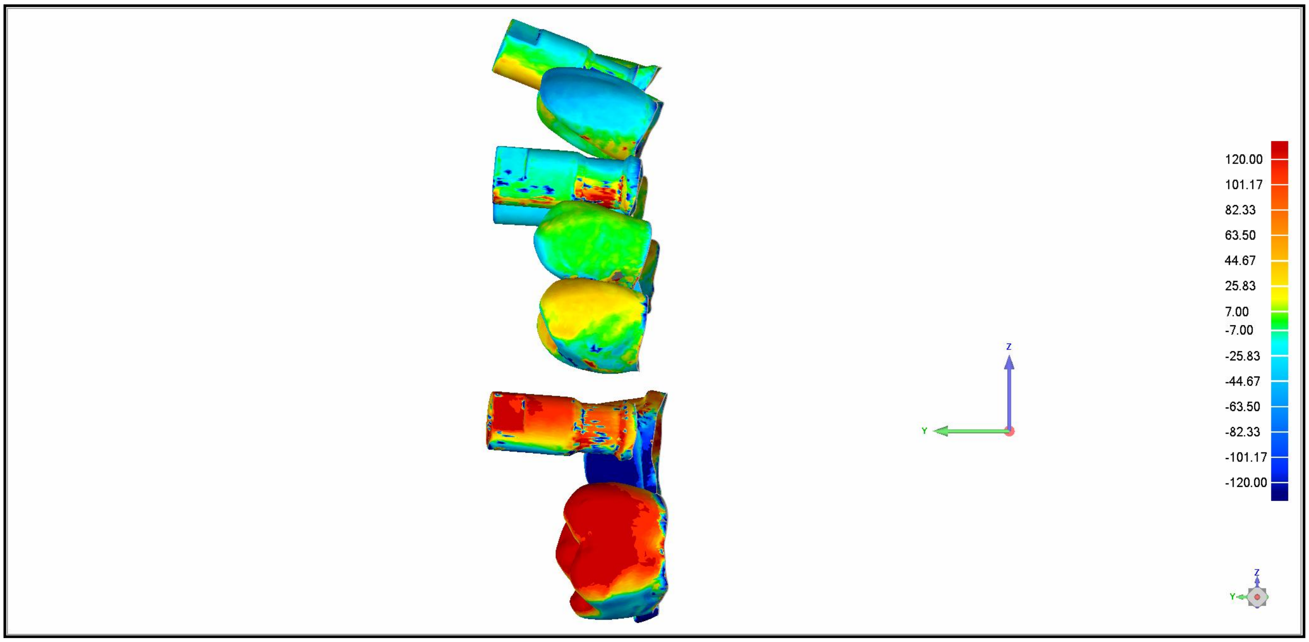

Predefinido: Derecha

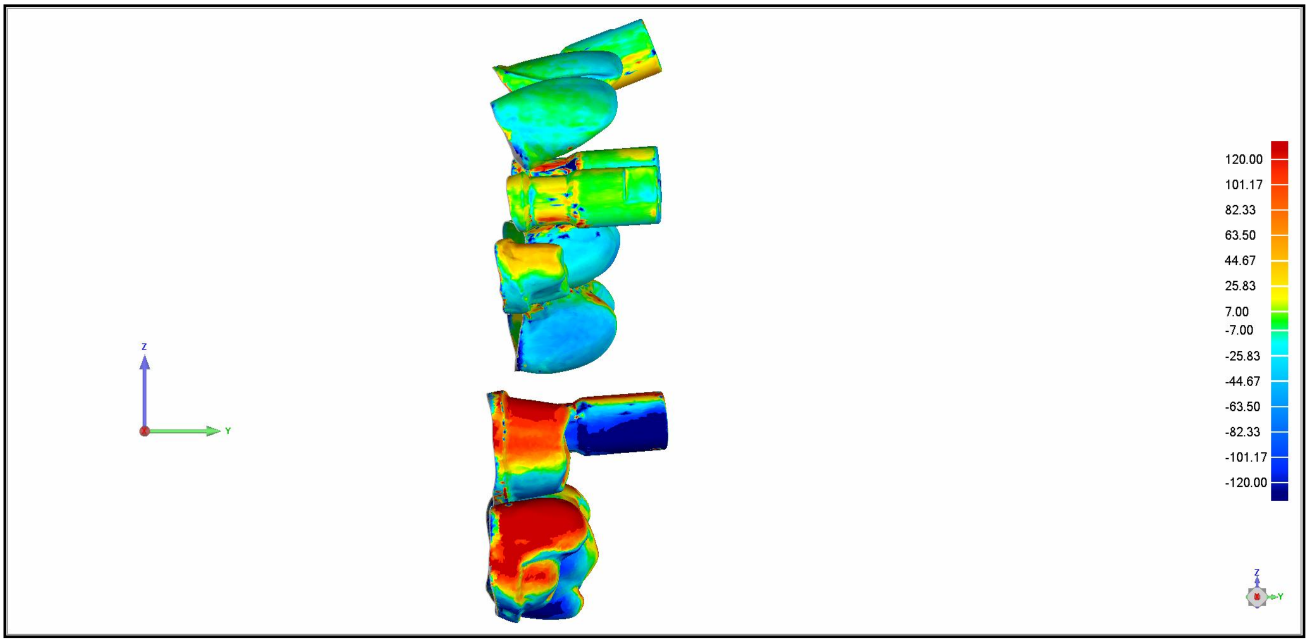

Predefinido: Superior

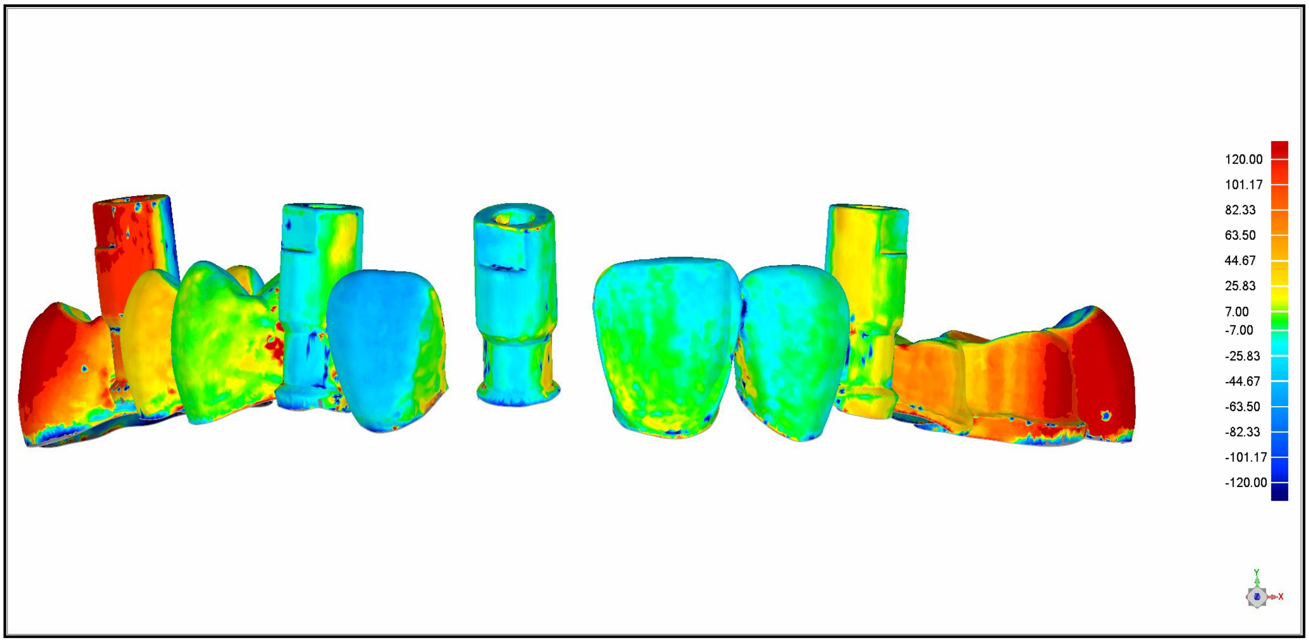

Predefinido: Inferior

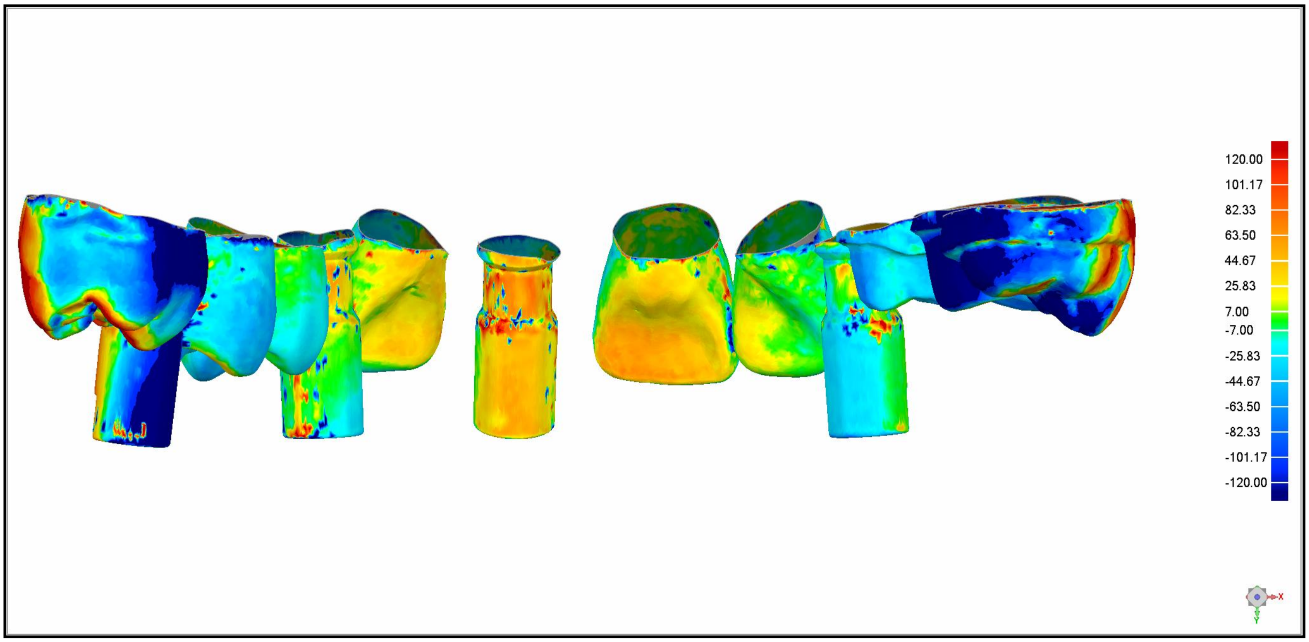

Ajuste de ubicación: Desviaciones superior e inferior

Unidades: u

Nombre	Desv	Estado	Superior Tol	Inferior Tol	Ref X	Ref Y	Ref Z	Radio	Desv X	Desv Y	Desv Z	Medido X	Medido Y	Medido Z	Dir. proy. X	Dir. proy. Y	Dir. proy. Z
Desv. inferior	-3131.42				-22762.84	38034.02	441.63	n/a	629.83	2874.61	1070.40	-22133.01	40908.62	1512.03	-0.20	-0.92	-0.34
Desv. superior	3058.84				19439.97	32593.27	13956.80	n/a	-961.40	1725.34	2335.68	18478.57	34318.61	16292.48	-0.31	0.56	0.76
