# Supplementary material for: Accuracy of four digital scanners according to scanning strategy in complete-arch impressions
Source: PLoS One. 2018 Sep 13;13(9):e0202916. doi: 10.1371/journal.pone.0202916 (PMC6136706; doi:10.1371/journal.pone.0202916)
Supplement: S7 Table — iTero (scanning strategy C). (ZIP) [file pone.0202916.s007.zip › S7/IT4C.pdf]

Fecha: 3/6/2017, 1:21 pm

3D Comparación Resultados

Modelo referencia	MRC
Modelo test	IT4C
Nº de puntos de datos	82486
# Aislados	544

Tipo tolerancia	3D desviación
Unidades	u
Máx. crítico	120.00
Máx. nominal	5.00
Mín. nominal	-5.00
Mín. crítico	-120.00

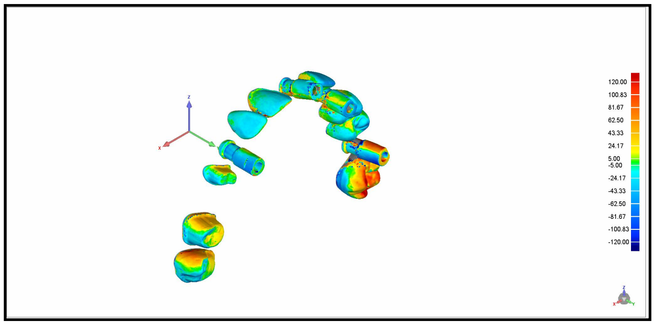

Desviación	
Desviación superior máx.	3135.34
Desviación inferior máx.	-3149.27
Desviación media	82.88 /-61.89
Desviación estándar	233.64

Distribución desviación

>=Min	<Max	# Puntos	%
-120.00	-100.83	454	0.55
-100.83	-81.67	885	1.07
-81.67	-62.50	2279	2.76
-62.50	-43.33	5755	6.98
-43.33	-24.17	11995	14.54
-24.17	-5.00	16162	19.59
-5.00	5.00	8149	9.88
5.00	24.17	13277	16.10
24.17	43.33	7876	9.55
43.33	62.50	4285	5.19
62.50	81.67	2552	3.09
81.67	100.83	1869	2.27
100.83	120.00	1047	1.27

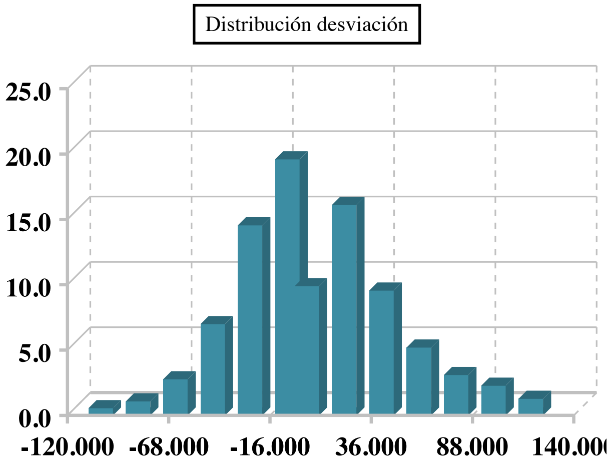

Fuera del crítico superior	3435	4.16
Fuera del crítico inferior	2466	2.99

Desviaciones estándar

Distribución (+/-)	# Puntos	%
-6 * Desv. estánd.	456	0.55
-5 * Desv. estánd.	179	0.22
-4 * Desv. estánd.	133	0.16
-3 * Desv. estánd.	209	0.25
-2 * Desv. estánd.	590	0.72
-1 * Desv. estánd.	46950	56.92
1 * Desv. estánd.	32167	39.00
2 * Desv. estánd.	507	0.61
3 * Desv. estánd.	231	0.28
4 * Desv. estánd.	267	0.32
5 * Desv. estánd.	246	0.30
6 * Desv. estánd.	551	0.67

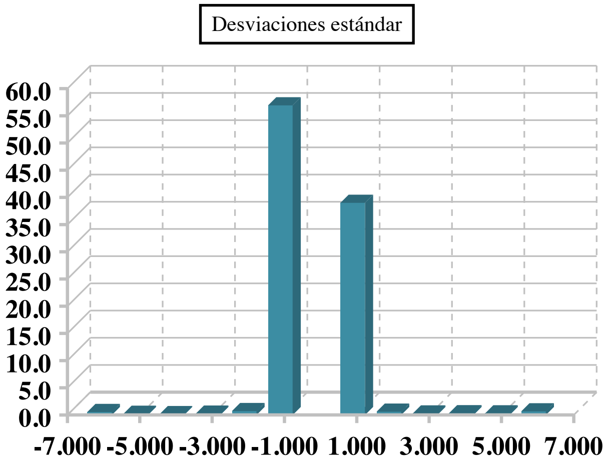

Predefinido: Isométrico

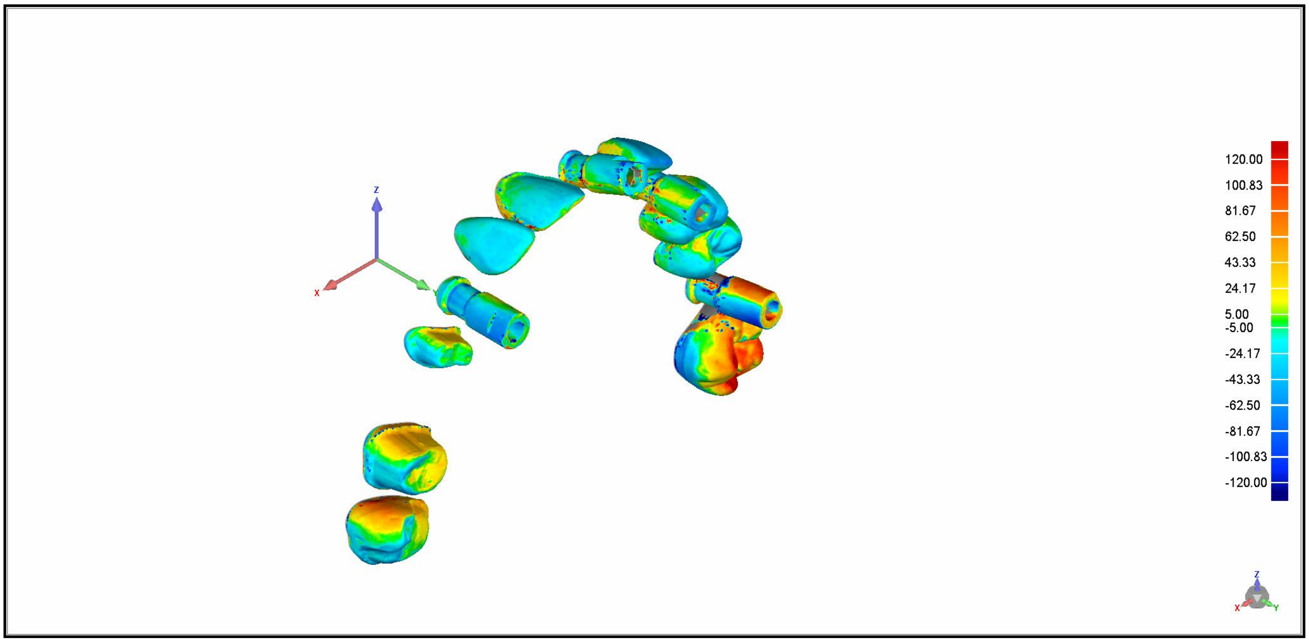

Predefinido: Frente

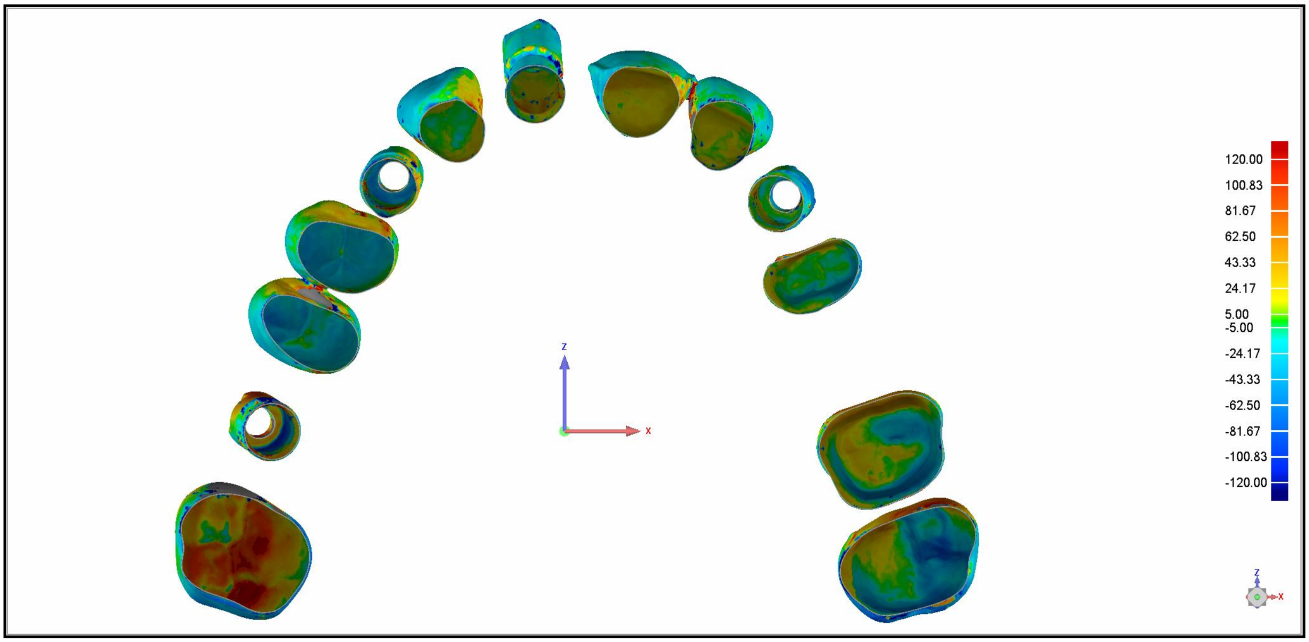

Predefinido: Atrás

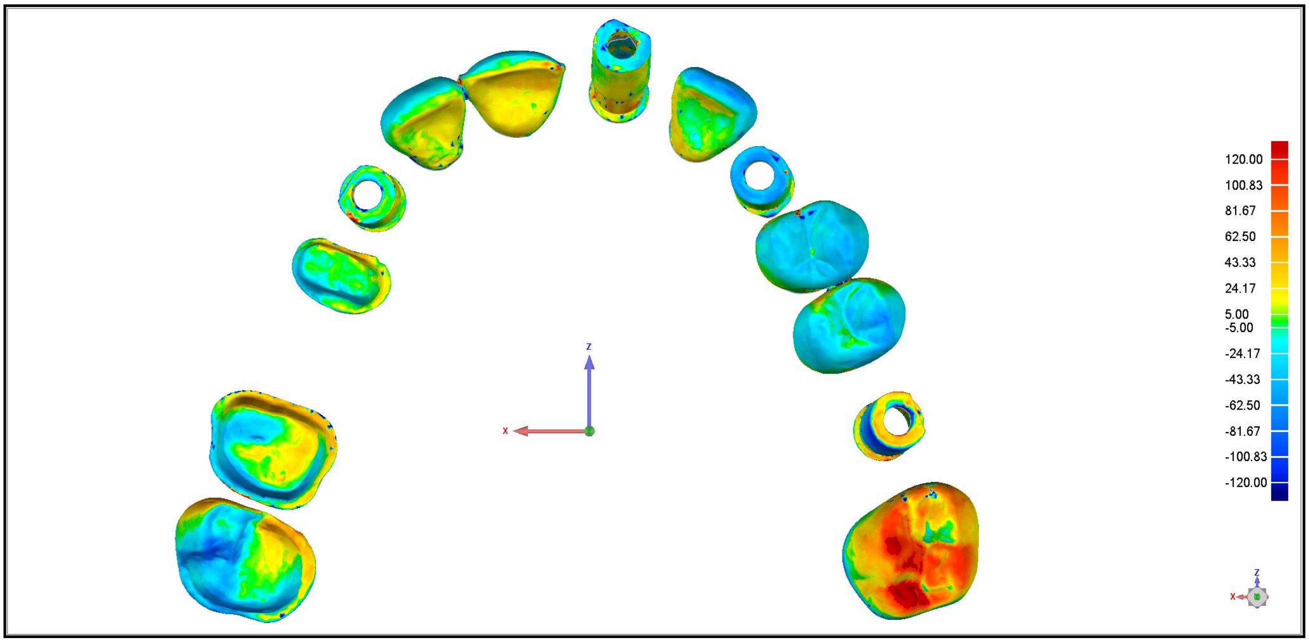

Predefinido: Izquierda

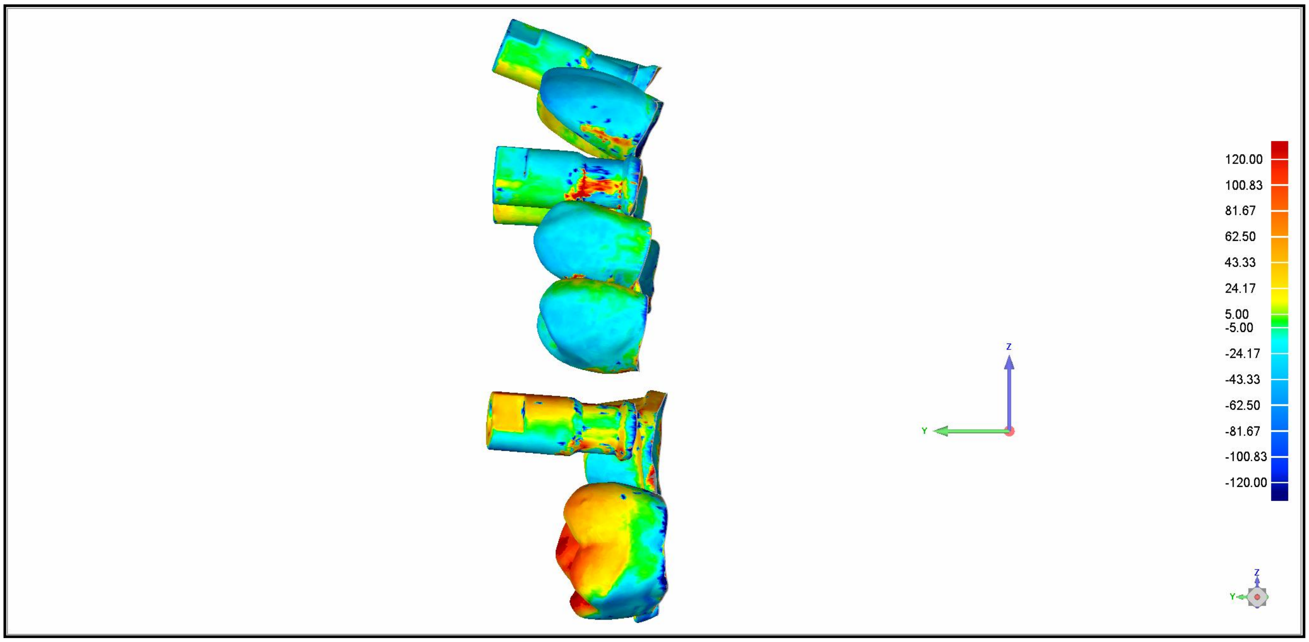

Predefinido: Derecha

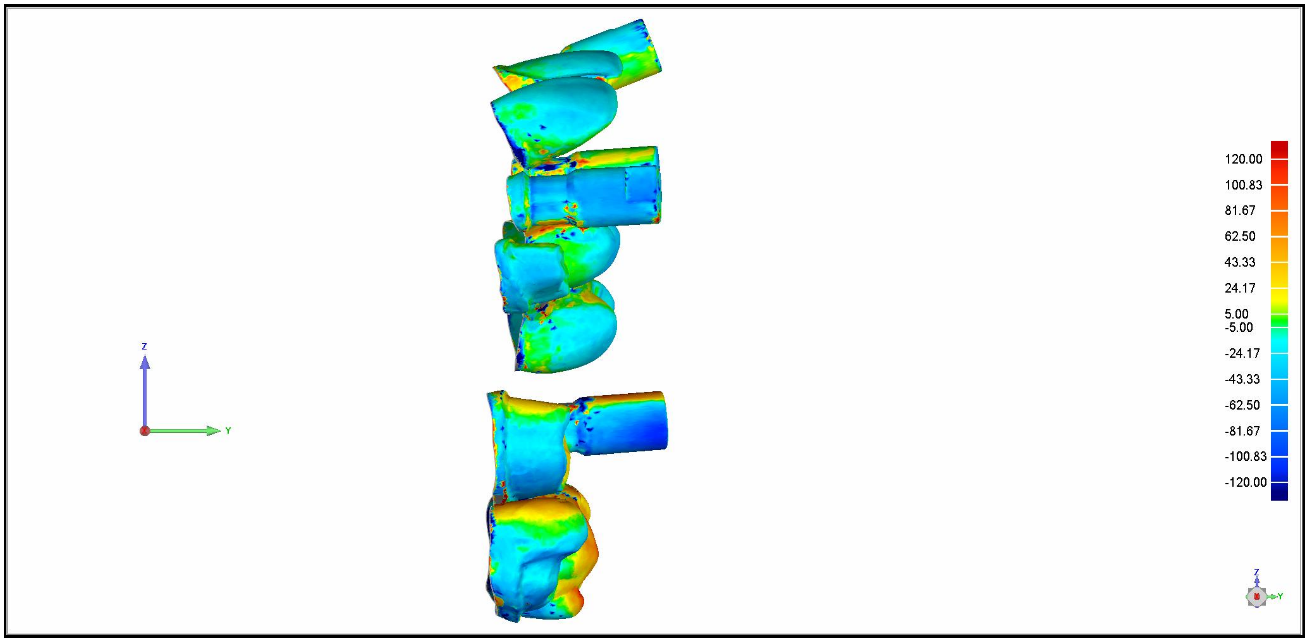

Predefinido: Superior

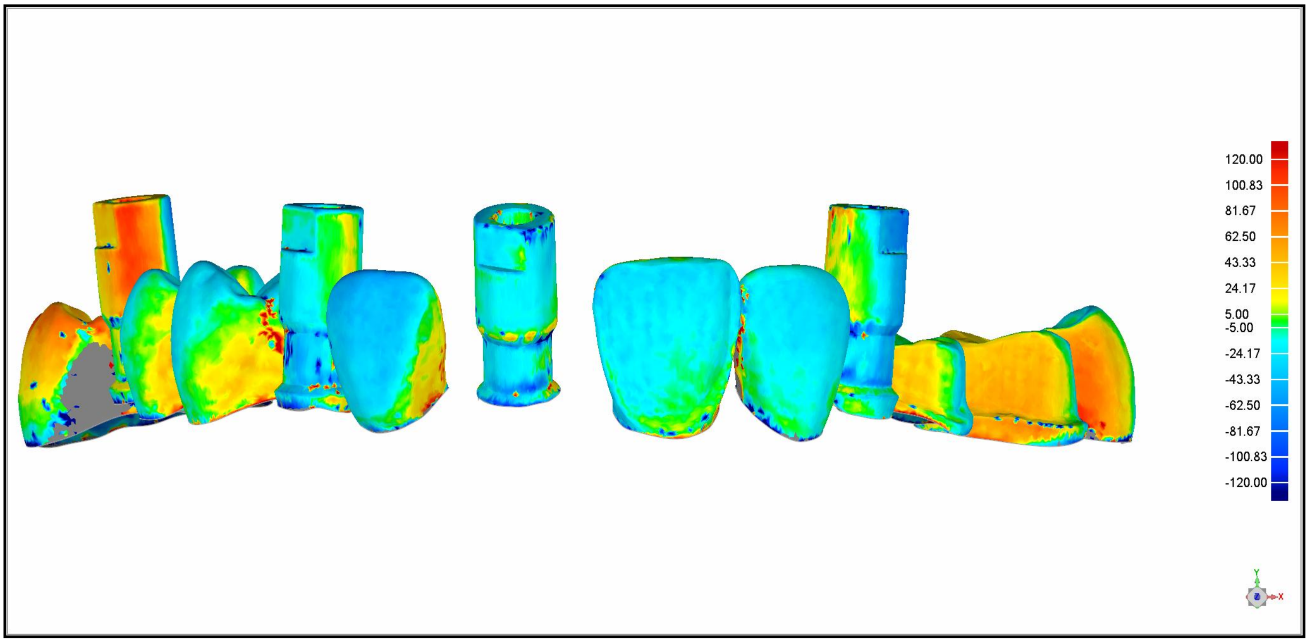

Predefinido: Inferior

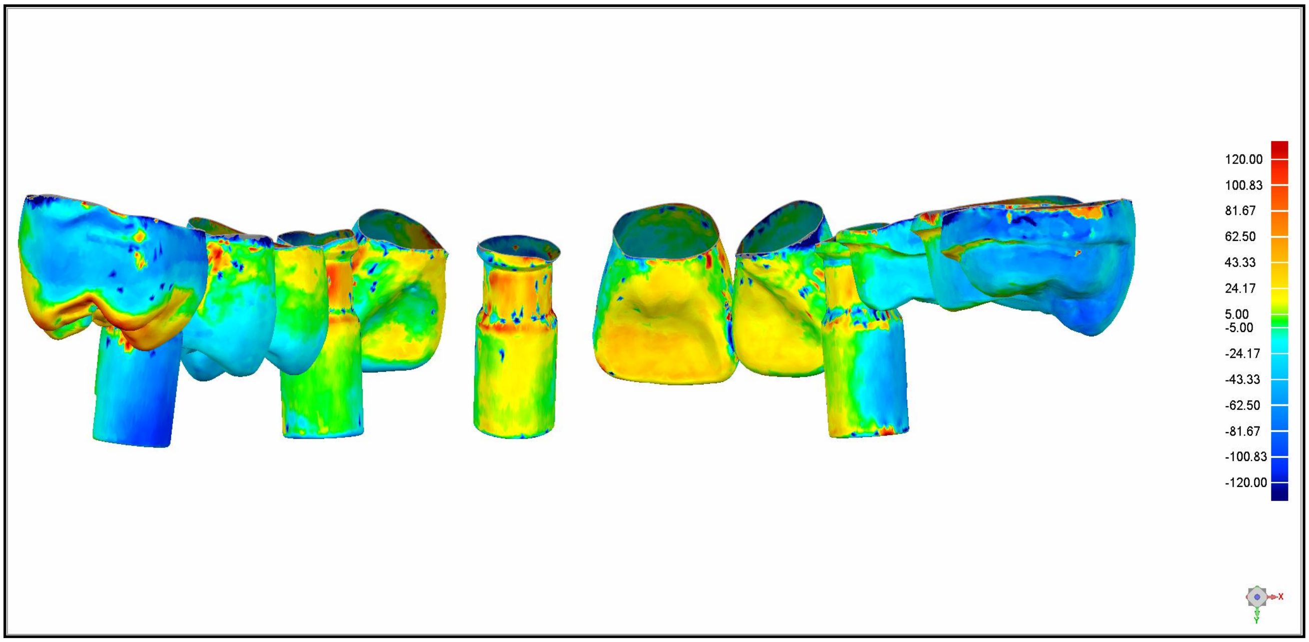

Ajuste de ubicación: Desviaciones superior e inferior

Unidades: u

Nombre	Desv	Estado	Superior Tol	Inferior Tol	Ref X	Ref Y	Ref Z	Radio	Desv X	Desv Y	Desv Z	Medido X	Medido Y	Medido Z	Dir. proy. X	Dir. proy. Y	Dir. proy. Z
Desv. inferior	-3149.27				16989.02	37628.06	17251.36	n/a	1389.65	2696.88	-844.72	18378.67	40324.95	16406.64	-0.44	-0.86	0.27
Desv. superior	3135.34				28638.90	28169.90	-4177.71	n/a	2282.48	-898.96	-1952.56	30921.38	27270.93	-6130.27	0.73	-0.29	-0.62
