# Supplementary material for: Accuracy of four digital scanners according to scanning strategy in complete-arch impressions
Source: PLoS One. 2018 Sep 13;13(9):e0202916. doi: 10.1371/journal.pone.0202916 (PMC6136706; doi:10.1371/journal.pone.0202916)
Supplement: S7 Table — iTero (scanning strategy C). (ZIP) [file pone.0202916.s007.zip › S7/IT8C.pdf]

Geomagic Control™

3D Comparación Resultados

Modelo referencia	MRC
Modelo test	IT8C
Nº de puntos de datos	80034
# Aislados	595

Tipo tolerancia	3D desviación
Unidades	u
Máx. crítico	120.00
Máx. nominal	6.00
Mín. nominal	-6.00
Mín. crítico	-120.00

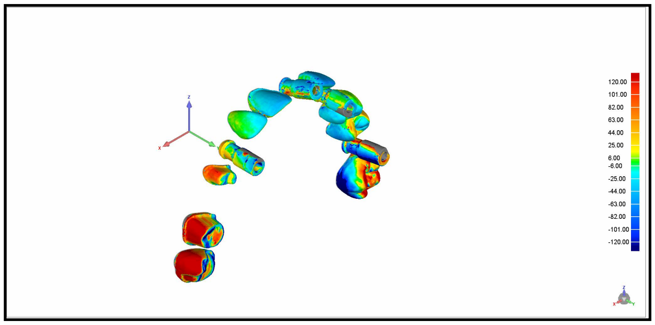

Desviación	
Desviación superior máx.	3089.98
Desviación inferior máx.	-3128.10
Desviación media	114.75 /-98.11
Desviación estándar	262.22

Distribución desviación

>=Min	<Max	# Puntos	%
-120.00	-101.00	1610	2.01
-101.00	-82.00	2197	2.75
-82.00	-63.00	2698	3.37
-63.00	-44.00	4856	6.07
-44.00	-25.00	8001	10.00
-25.00	-6.00	9912	12.38
-6.00	6.00	7708	9.63
6.00	25.00	9185	11.48
25.00	44.00	6631	8.29
44.00	63.00	4062	5.08
63.00	82.00	2657	3.32
82.00	101.00	2095	2.62
101.00	120.00	1763	2.20

Fuera del crítico superior	9016	11.27
Fuera del crítico inferior	7643	9.55

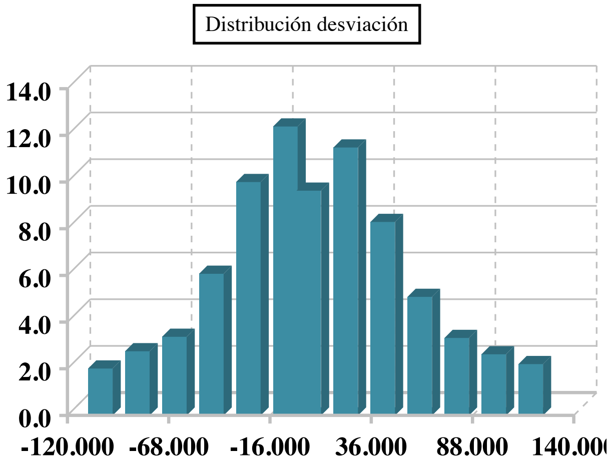

Desviaciones estándar

Distribución (+/-)	# Puntos	%
-6 * Desv. estánd.	395	0.49
-5 * Desv. estánd.	243	0.30
-4 * Desv. estánd.	262	0.33
-3 * Desv. estánd.	312	0.39
-2 * Desv. estánd.	1485	1.86
-1 * Desv. estánd.	42372	52.94
1 * Desv. estánd.	32511	40.62
2 * Desv. estánd.	1075	1.34
3 * Desv. estánd.	252	0.31
4 * Desv. estánd.	261	0.33
5 * Desv. estánd.	206	0.26
6 * Desv. estánd.	660	0.82

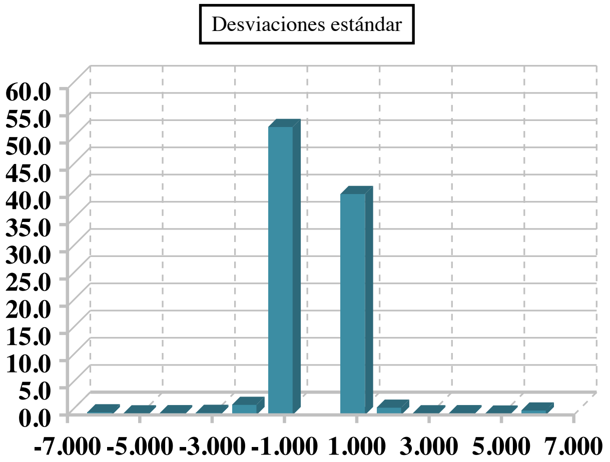

Predefinido: Isométrico

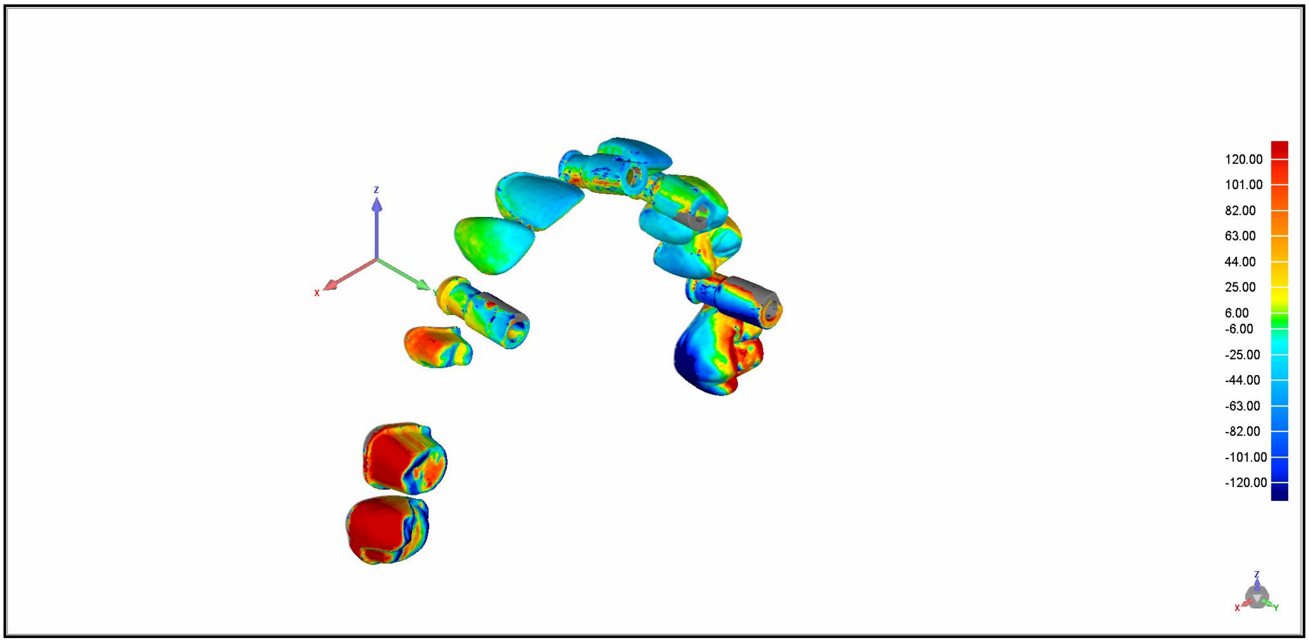

Predefinido: Frente

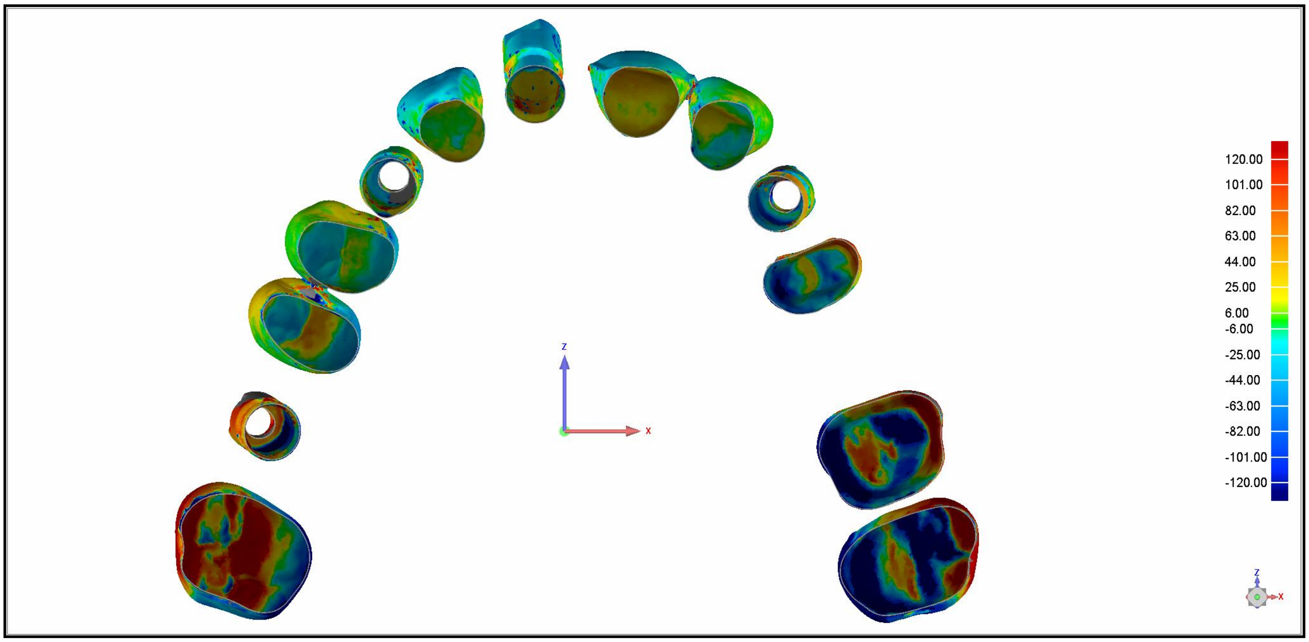

Predefinido: Atrás

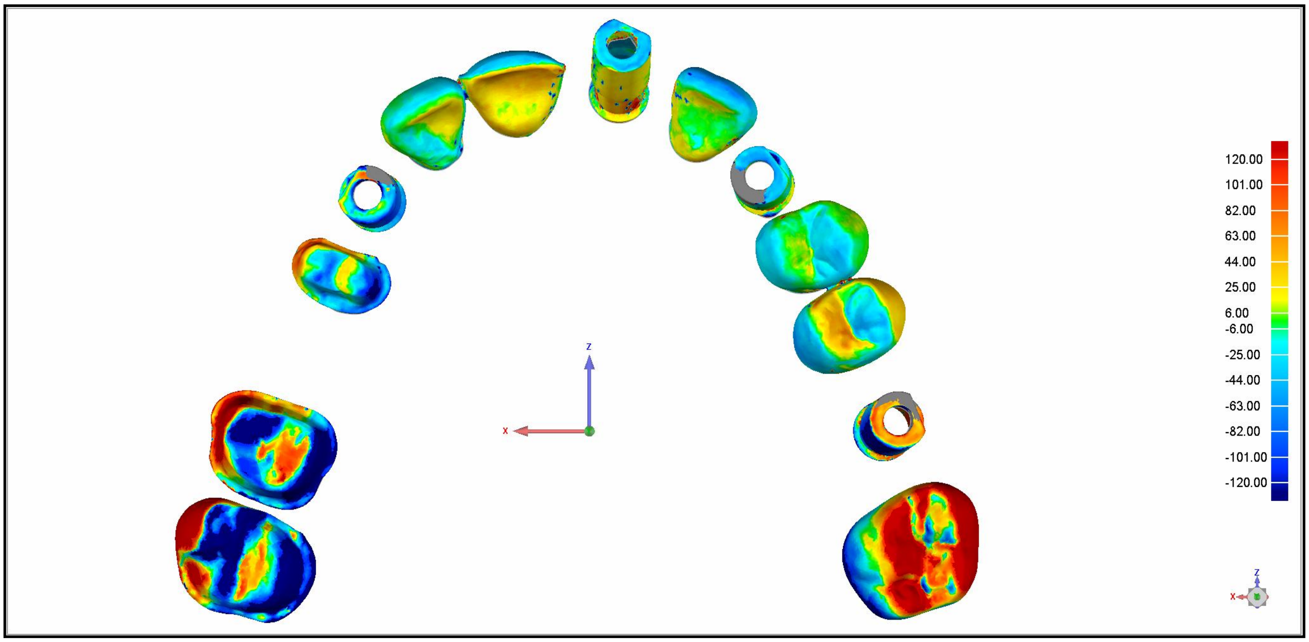

Predefinido: Izquierda

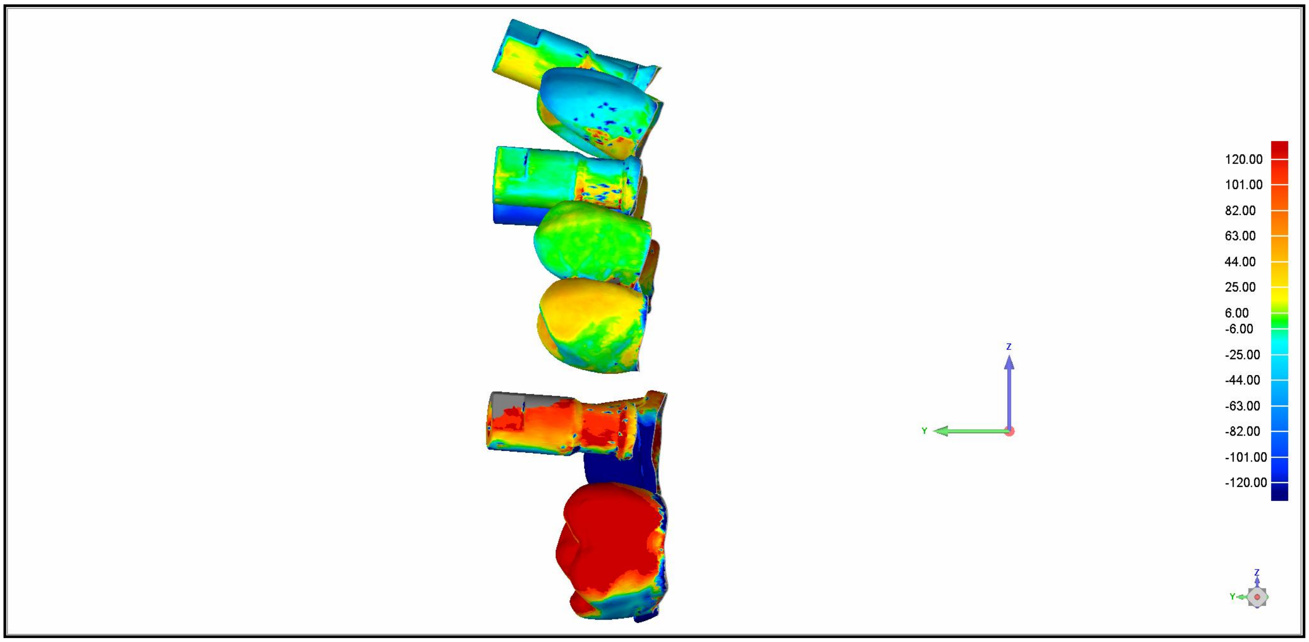

Predefinido: Derecha

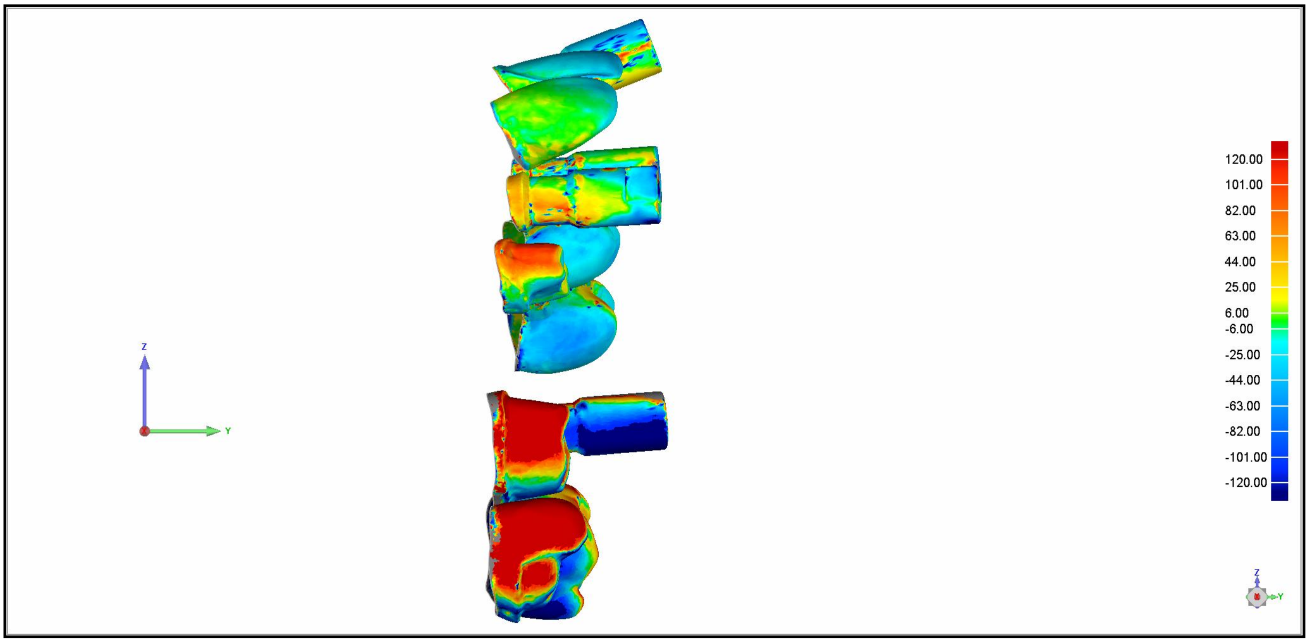

Predefinido: Superior

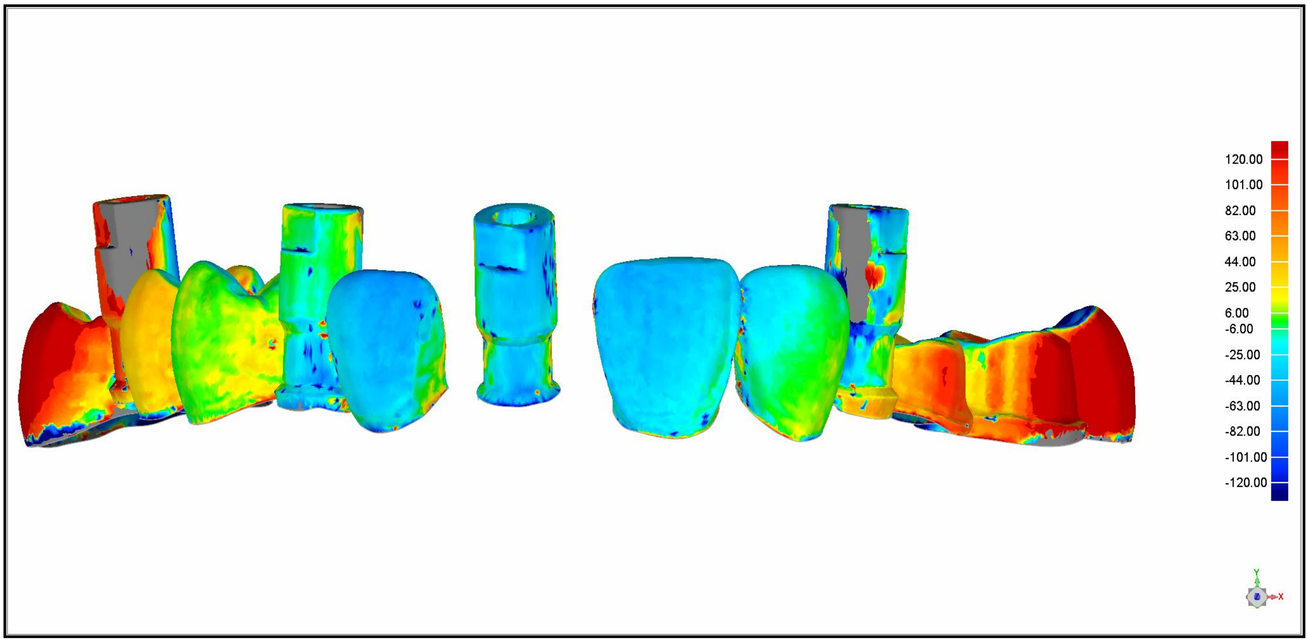

Predefinido: Inferior

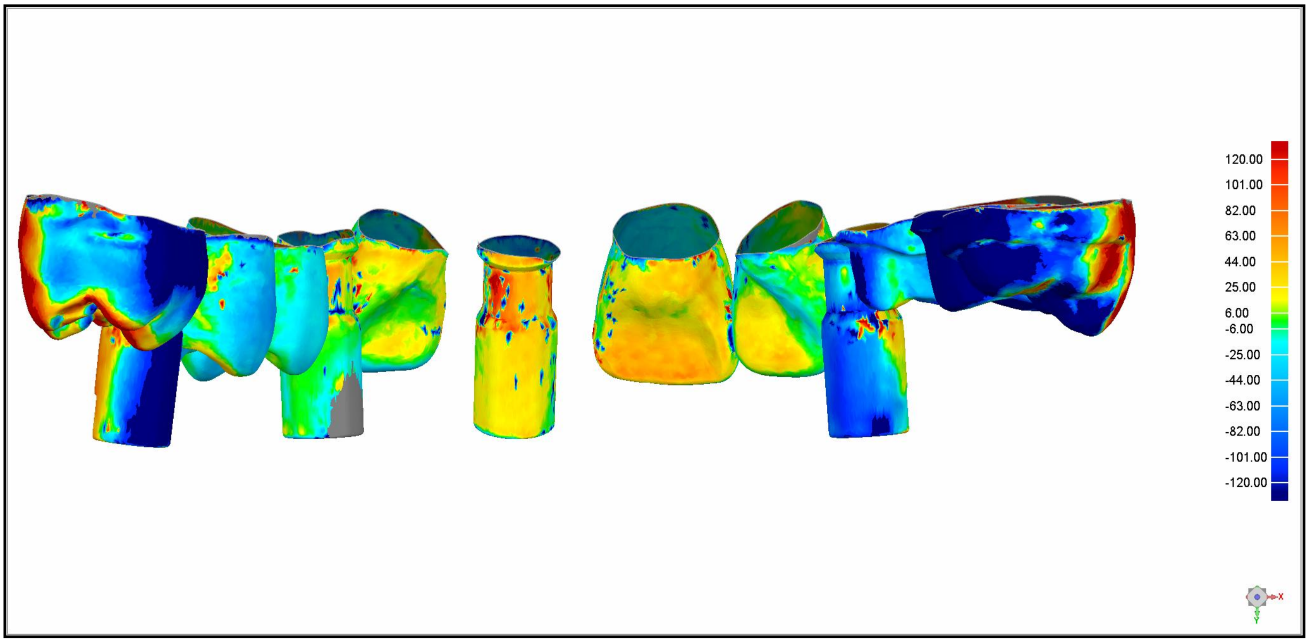

Ajuste de ubicación: Desviaciones superior e inferior

Unidades: u

Nombre	Desv	Estado	Superior Tol	Inferior Tol	Ref X	Ref Y	Ref Z	Radio	Desv X	Desv Y	Desv Z	Medido X	Medido Y	Medido Z	Dir. proy. X	Dir. proy. Y	Dir. proy. Z
Desv. inferior	-3128.10				-23261.17	38062.66	-344.09	n/a	884.14	2988.05	273.61	-22377.03	41050.71	-70.48	-0.28	-0.96	-0.09
Desv. superior	3089.98				-23866.15	29962.44	-2111.67	n/a	-984.32	-712.40	-2841.05	-24850.47	29250.04	-4952.71	-0.32	-0.23	-0.92
