# Supplementary material for: Accuracy of four digital scanners according to scanning strategy in complete-arch impressions
Source: PLoS One. 2018 Sep 13;13(9):e0202916. doi: 10.1371/journal.pone.0202916 (PMC6136706; doi:10.1371/journal.pone.0202916)
Supplement: S7 Table — iTero (scanning strategy C). (ZIP) [file pone.0202916.s007.zip › S7/IT9C.pdf]

Geomagic Control

3D Comparación Resultados

Modelo referencia	MRC
Modelo test	IT9C
Nº de puntos de datos	82180
# Aislados	682

Tipo tolerancia	3D desviación
Unidades	u
Máx. crítico	120.00
Máx. nominal	8.00
Mín. nominal	-8.00
Mín. crítico	-120.00

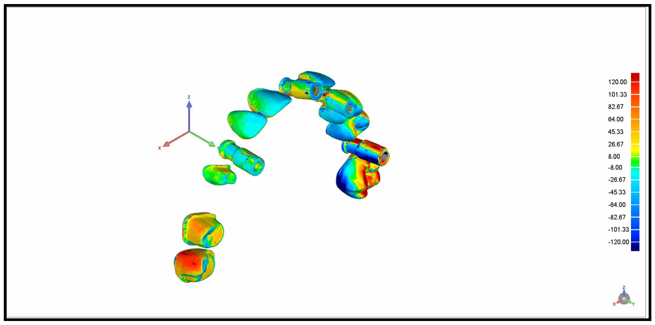

Desviación	
Desviación superior máx.	3109.55
Desviación inferior máx.	-3092.22
Desviación media	91.16 /-78.96
Desviación estándar	238.29

Distribución desviación

>=Min	<Max	# Puntos	%
-120.00	-101.33	887	1.08
-101.33	-82.67	1327	1.61
-82.67	-64.00	2729	3.32
-64.00	-45.33	5284	6.43
-45.33	-26.67	8932	10.87
-26.67	-8.00	11200	13.63
-8.00	8.00	10760	13.09
8.00	26.67	11088	13.49
26.67	45.33	7905	9.62
45.33	64.00	4772	5.81
64.00	82.67	2825	3.44
82.67	101.33	2260	2.75
101.33	120.00	2196	2.67

Fuera del crítico superior	5980	7.28
Fuera del crítico inferior	4035	4.91

Distribución desviación

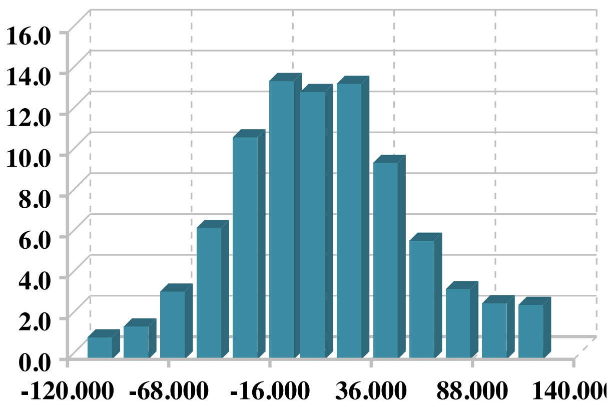

Desviaciones estándar

Distribución (+/-)	# Puntos	%
-6 * Desv. estánd.	508	0.62
-5 * Desv. estánd.	190	0.23
-4 * Desv. estánd.	185	0.23
-3 * Desv. estánd.	289	0.35
-2 * Desv. estánd.	744	0.91
-1 * Desv. estánd.	43616	53.07
1 * Desv. estánd.	34822	42.37
2 * Desv. estánd.	502	0.61
3 * Desv. estánd.	221	0.27
4 * Desv. estánd.	224	0.27
5 * Desv. estánd.	227	0.28
6 * Desv. estánd.	652	0.79

Desviaciones estándar

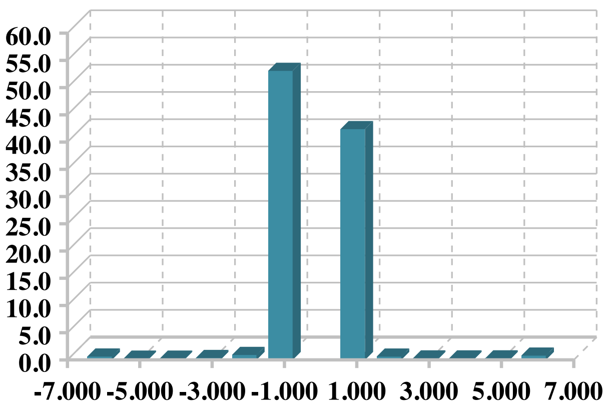

Predefinido: Isométrico

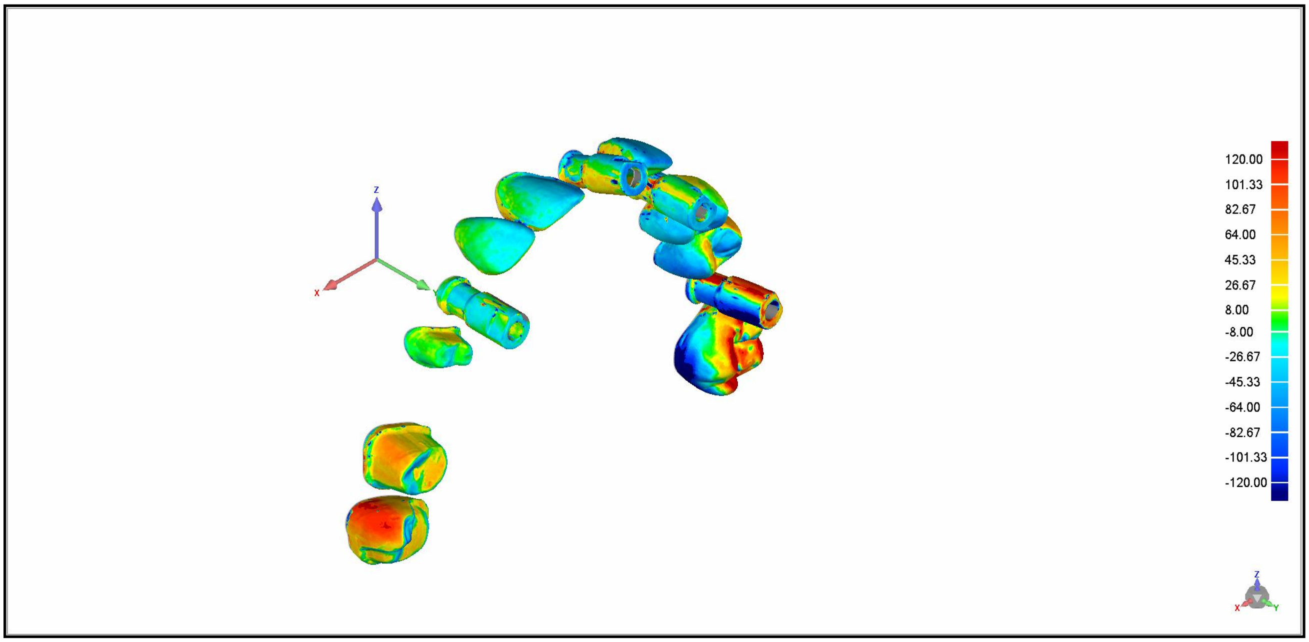

Predefinido: Frente

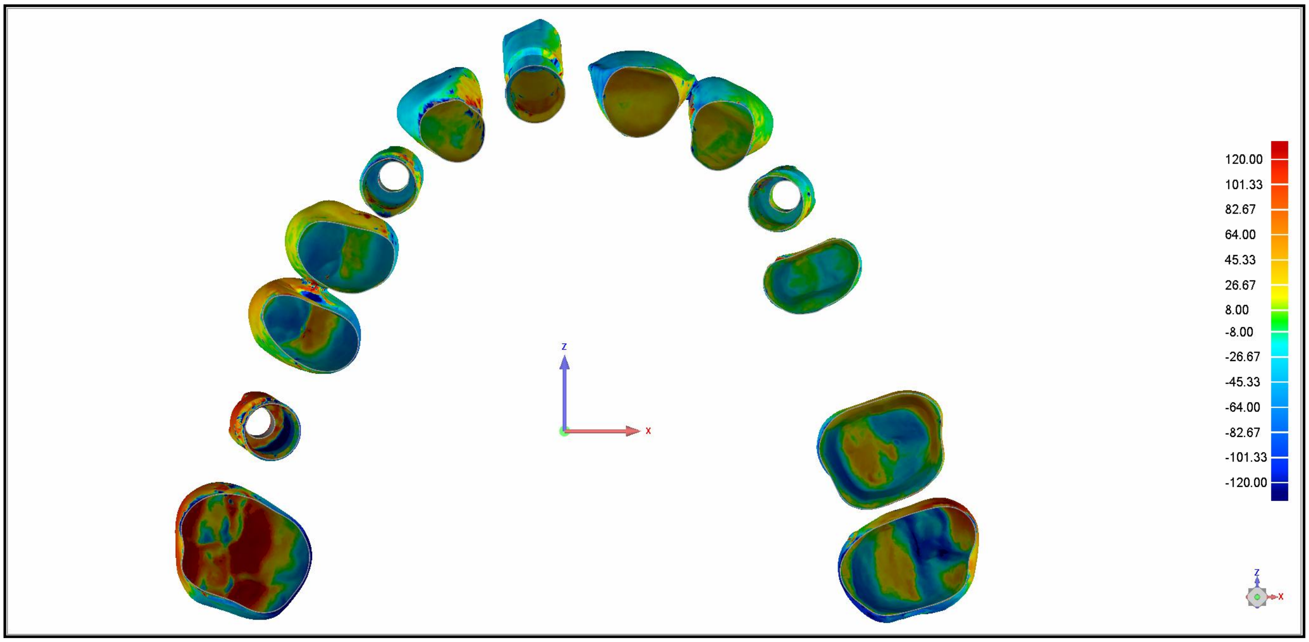

Predefinido: Atrás

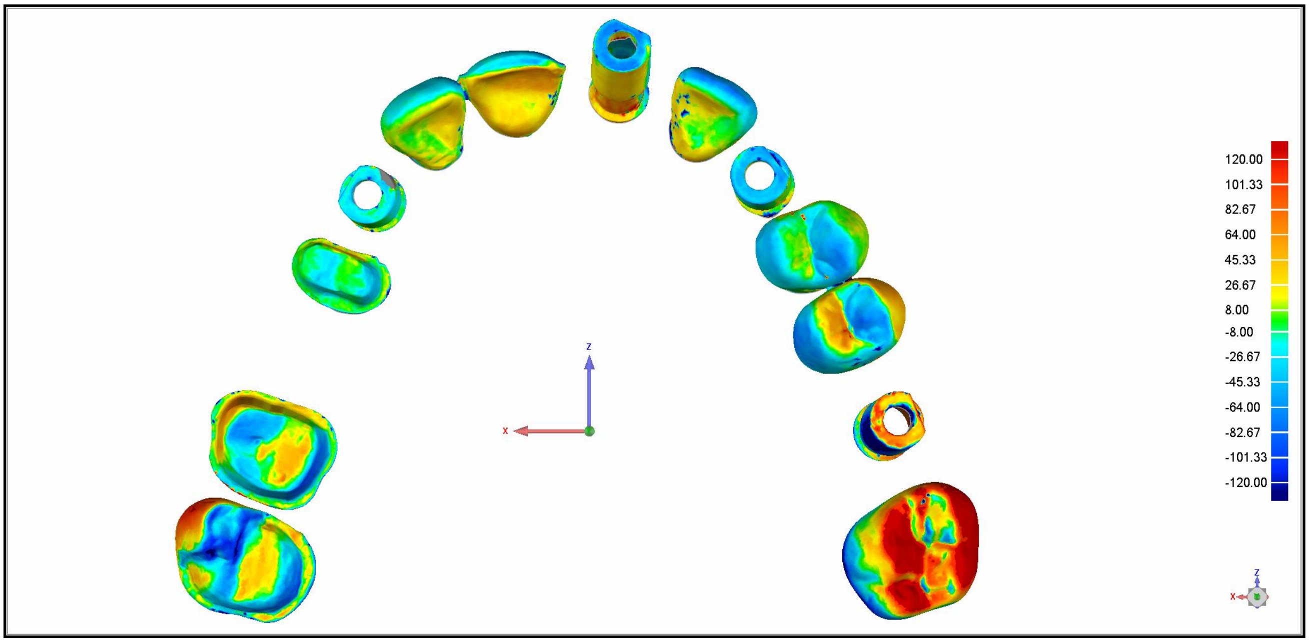

Predefinido: Izquierda

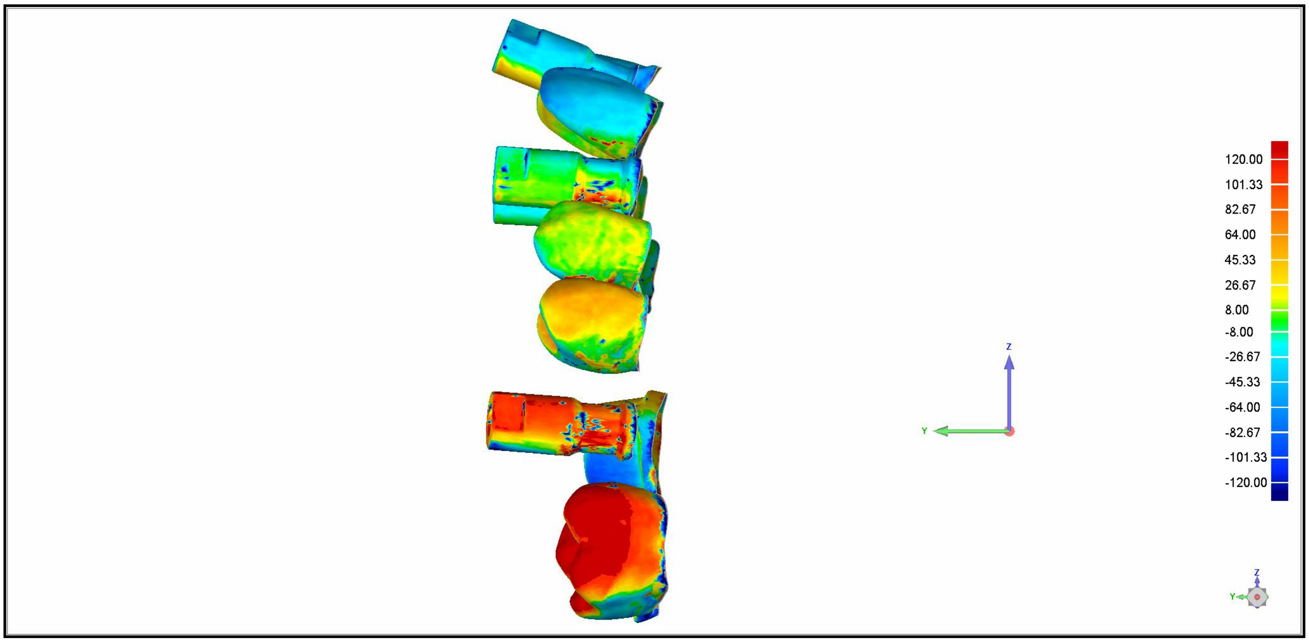

Predefinido: Derecha

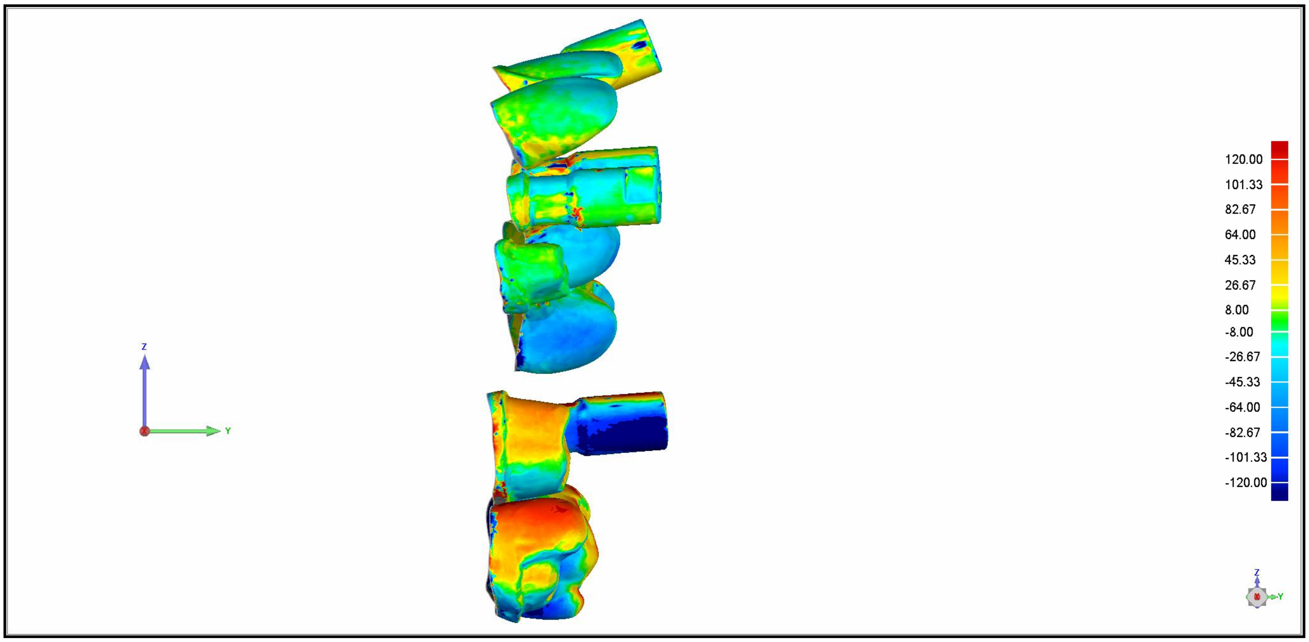

Predefinido: Superior

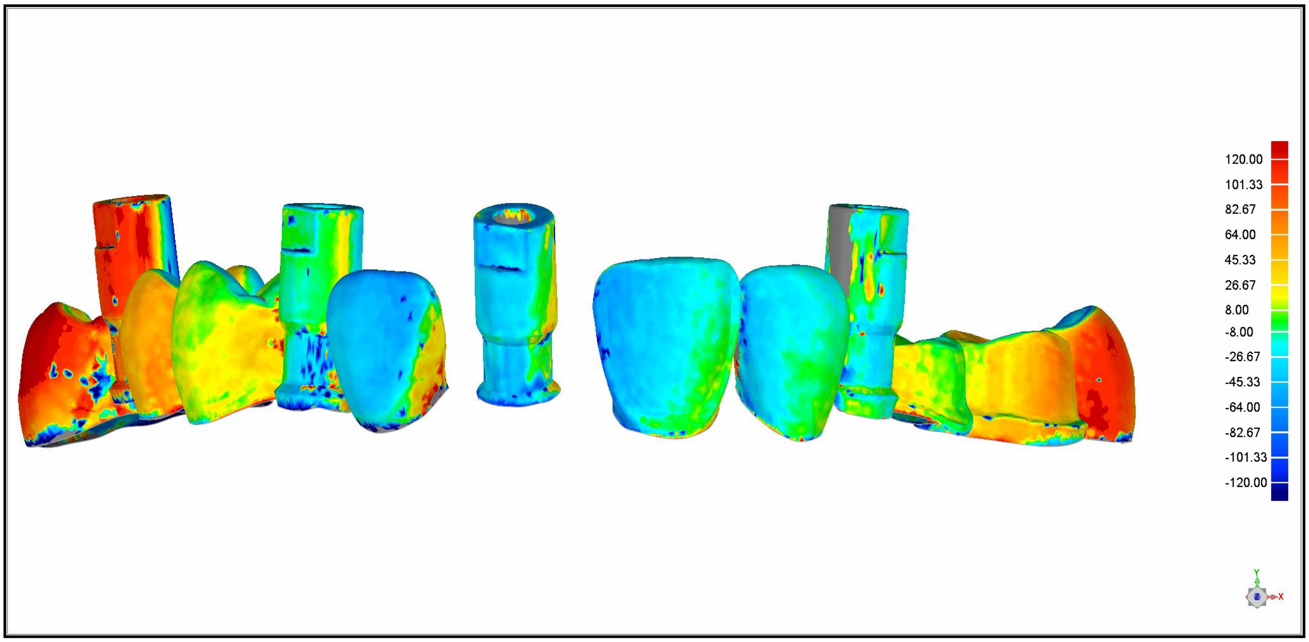

Predefinido: Inferior

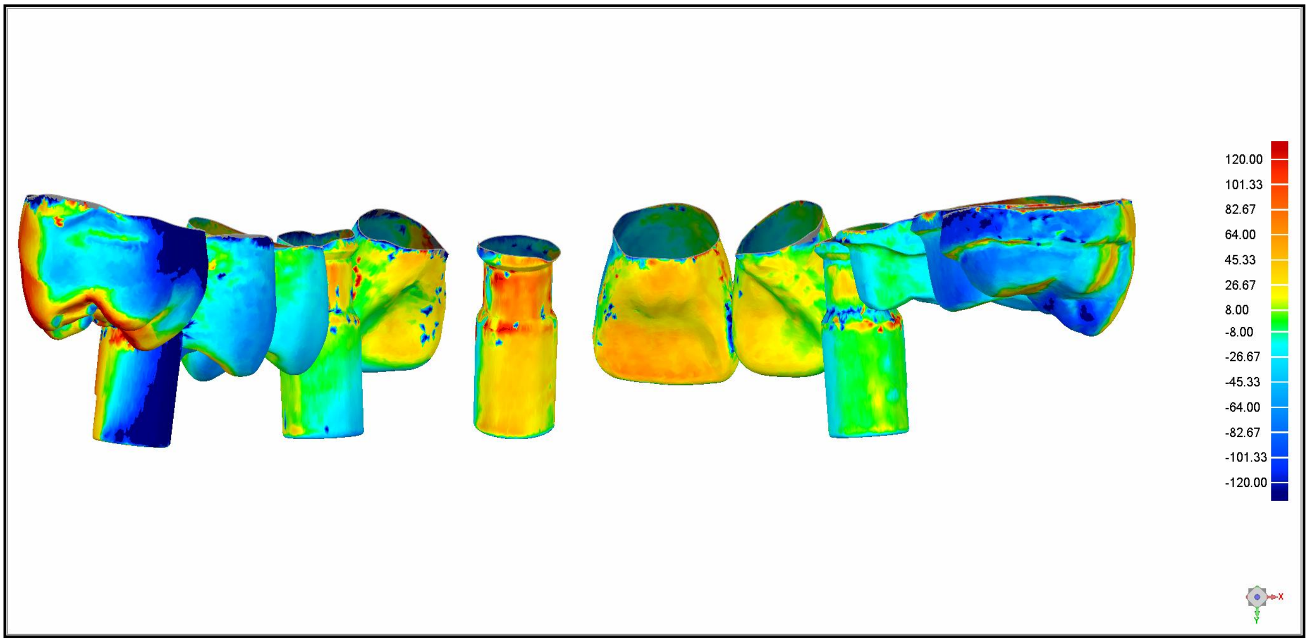

Ajuste de ubicación: Desviaciones superior e inferior

Unidades: u

Nombre	Desv	Estado	Superior Tol	Inferior Tol	Ref X	Ref Y	Ref Z	Radio	Desv X	Desv Y	Desv Z	Medido X	Medido Y	Medido Z	Dir. proy. X	Dir. proy. Y	Dir. proy. Z
Desv. inferior	-3092.22				-29208.33	26961.25	-11988.49	n/a	2671.48	495.41	-1476.34	-26536.85	27456.66	-13464.83	-0.86	-0.16	0.48
Desv. superior	3109.55				-22957.79	34285.30	2555.18	n/a	137.65	-701.99	3026.14	-22820.14	33583.31	5581.32	0.04	-0.23	0.97
