# Supplementary material for: Accuracy of four digital scanners according to scanning strategy in complete-arch impressions
Source: PLoS One. 2018 Sep 13;13(9):e0202916. doi: 10.1371/journal.pone.0202916 (PMC6136706; doi:10.1371/journal.pone.0202916)
Supplement: S7 Table — iTero (scanning strategy C). (ZIP) [file pone.0202916.s007.zip › S7/IT5C.pdf]

Fecha: 3/6/2017, 1:23 pm

3D Comparación Resultados

Modelo referencia	MRC
Modelo test	IT5C
Nº de puntos de datos	82550
# Aislados	597

Tipo tolerancia	3D desviación
Unidades	u
Máx. crítico	120.00
Máx. nominal	0.00
Mín. nominal	0.00
Mín. crítico	-120.00

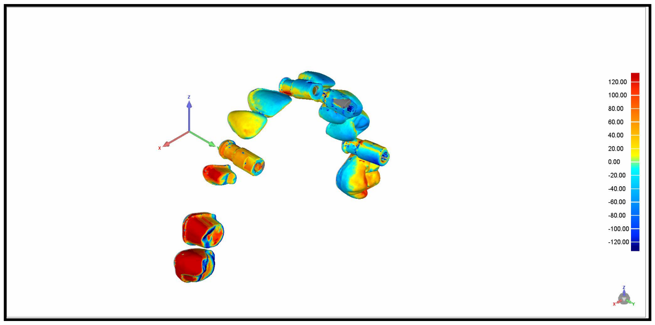

Desviación	
Desviación superior máx.	3153.90
Desviación inferior máx.	-3119.98
Desviación media	87.52 /-94.39
Desviación estándar	238.05

Distribución desviación

>=Min	<Max	# Puntos	%
-120.00	-100.00	1440	1.74
-100.00	-80.00	2027	2.46
-80.00	-60.00	3126	3.79
-60.00	-40.00	6359	7.70
-40.00	-20.00	8917	10.80
-20.00	0.00	11953	14.48
0.00	20.00	14224	17.23
20.00	40.00	9386	11.37
40.00	60.00	6004	7.27
60.00	80.00	3415	4.14
80.00	100.00	2053	2.49
100.00	120.00	1495	1.81

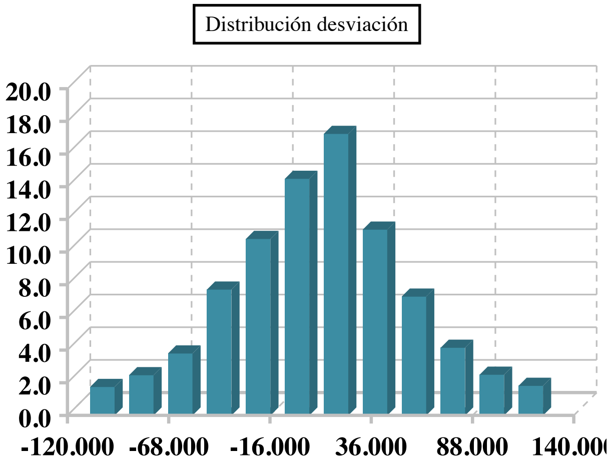

Fuera del crítico superior	6152	7.45
Fuera del crítico inferior	5999	7.27

Desviaciones estándar

Distribución (+/-)	# Puntos	%
-6 * Desv. esténd.	623	0.75
-5 * Desv. esténd.	184	0.22
-4 * Desv. esténd.	197	0.24
-3 * Desv. esténd.	225	0.27
-2 * Desv. esténd.	1649	2.00
-1 * Desv. esténd.	36794	44.57
1 * Desv. esténd.	40072	48.54
2 * Desv. esténd.	1589	1.92
3 * Desv. esténd.	234	0.28
4 * Desv. esténd.	236	0.29
5 * Desv. esténd.	238	0.29
6 * Desv. esténd.	509	0.62

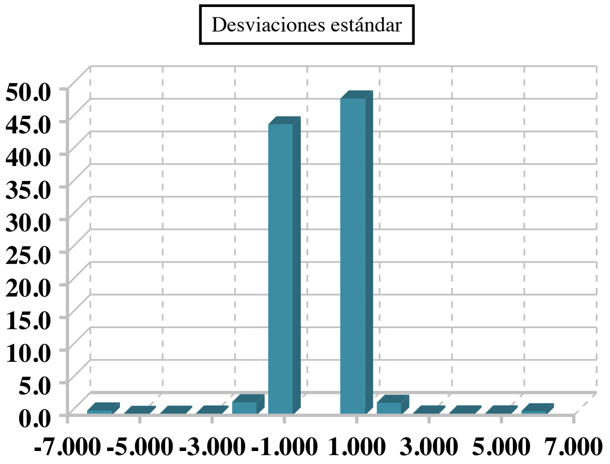

Predefinido: Isométrico

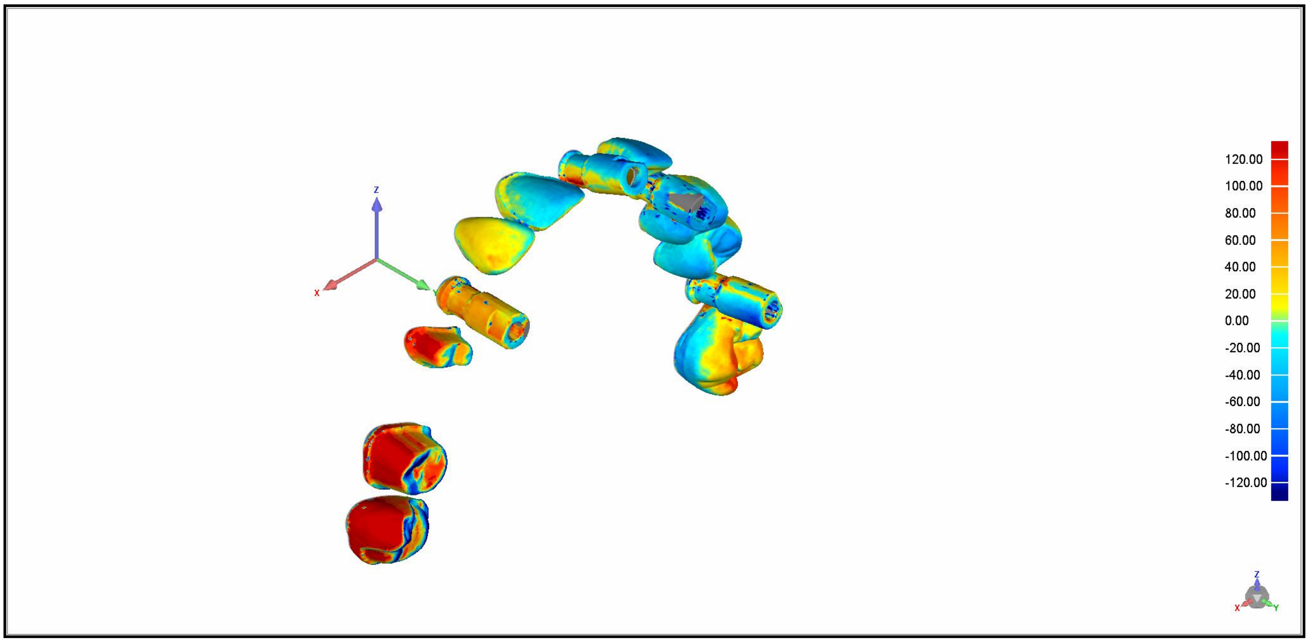

Predefinido: Frente

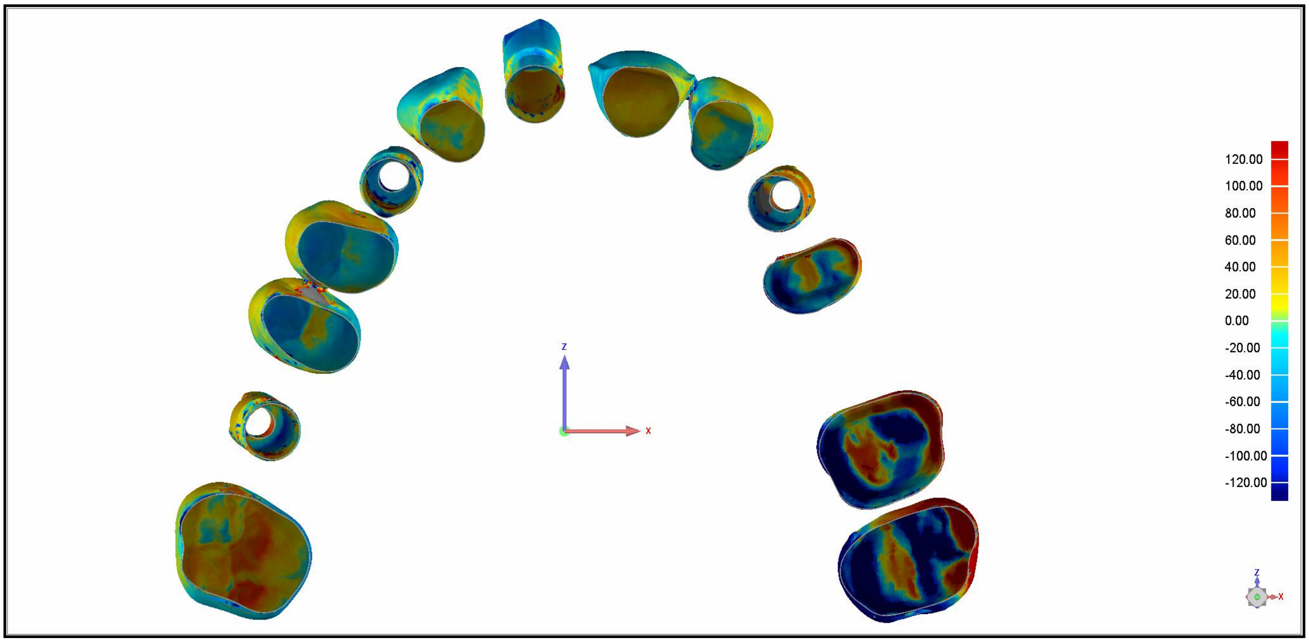

Predefinido: Atrás

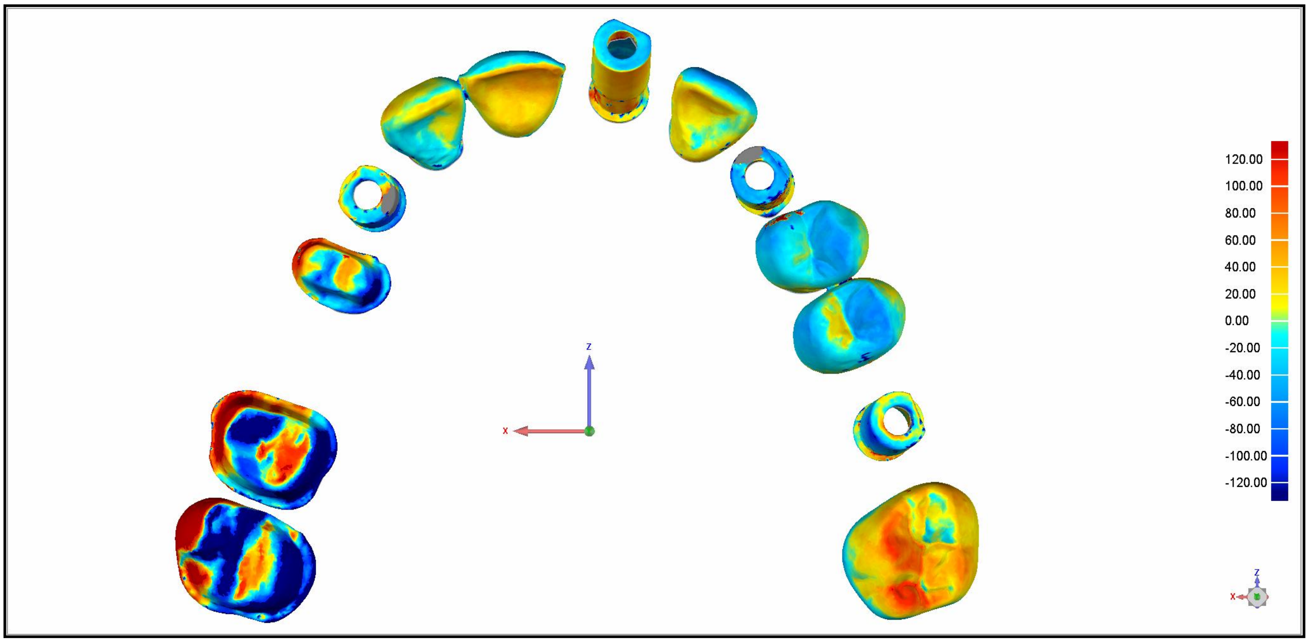

Predefinido: Izquierda

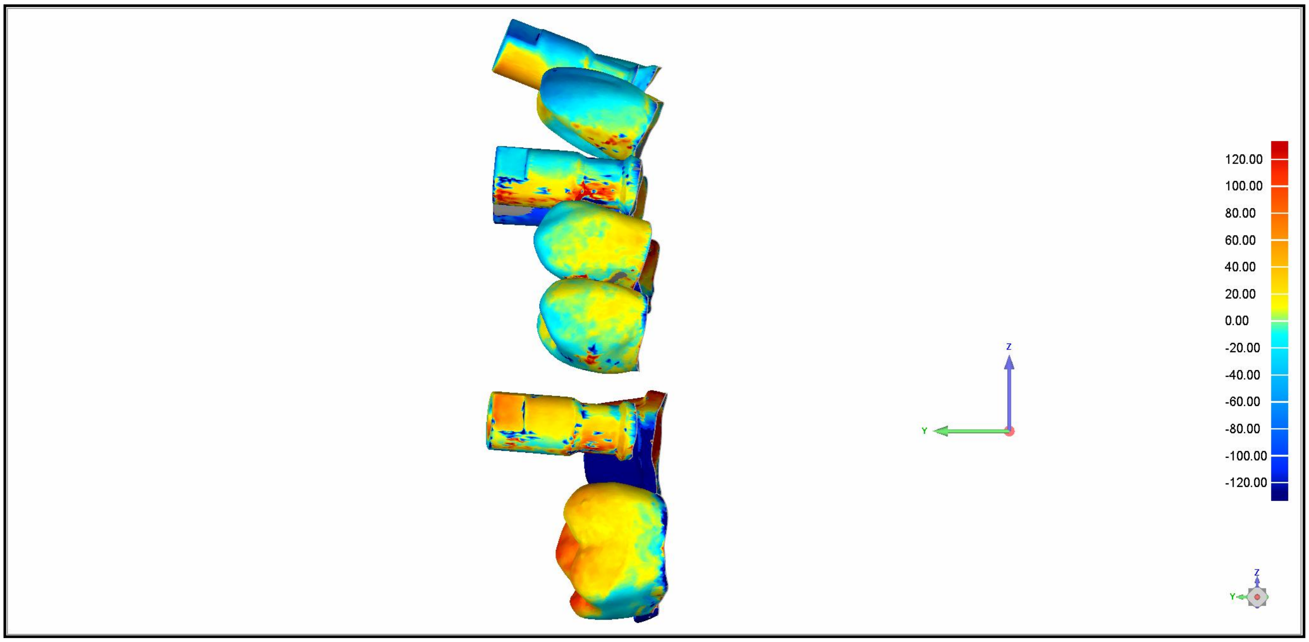

Predefinido: Derecha

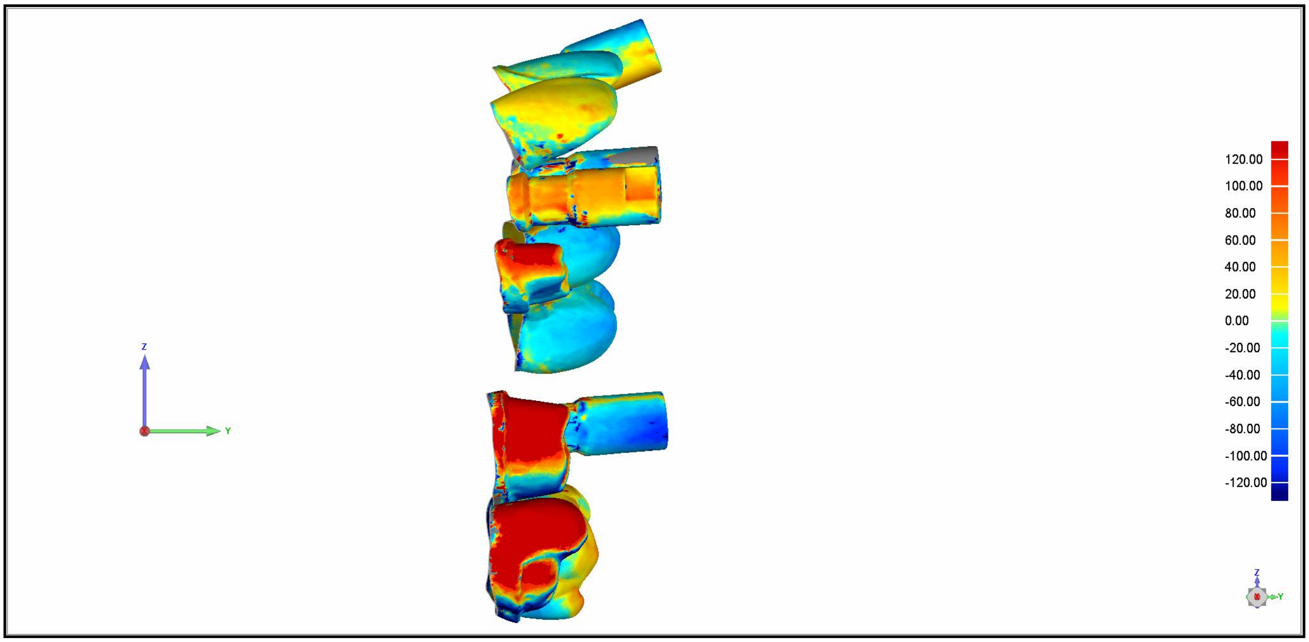

Predefinido: Superior

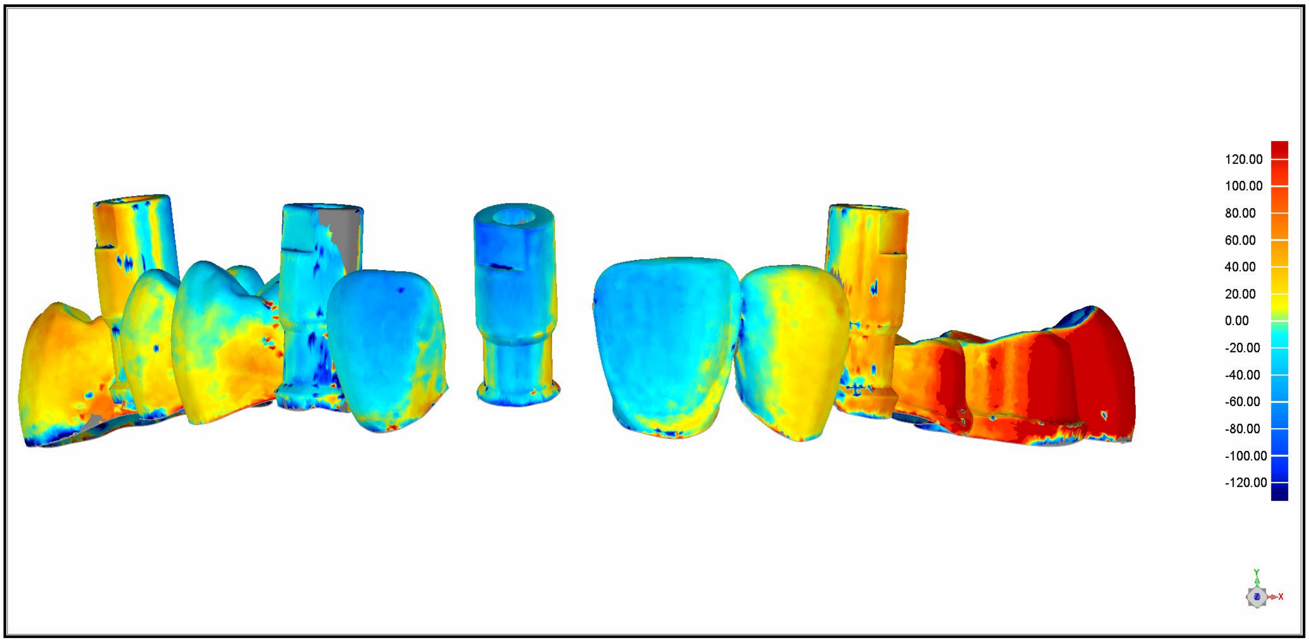

Predefinido: Inferior

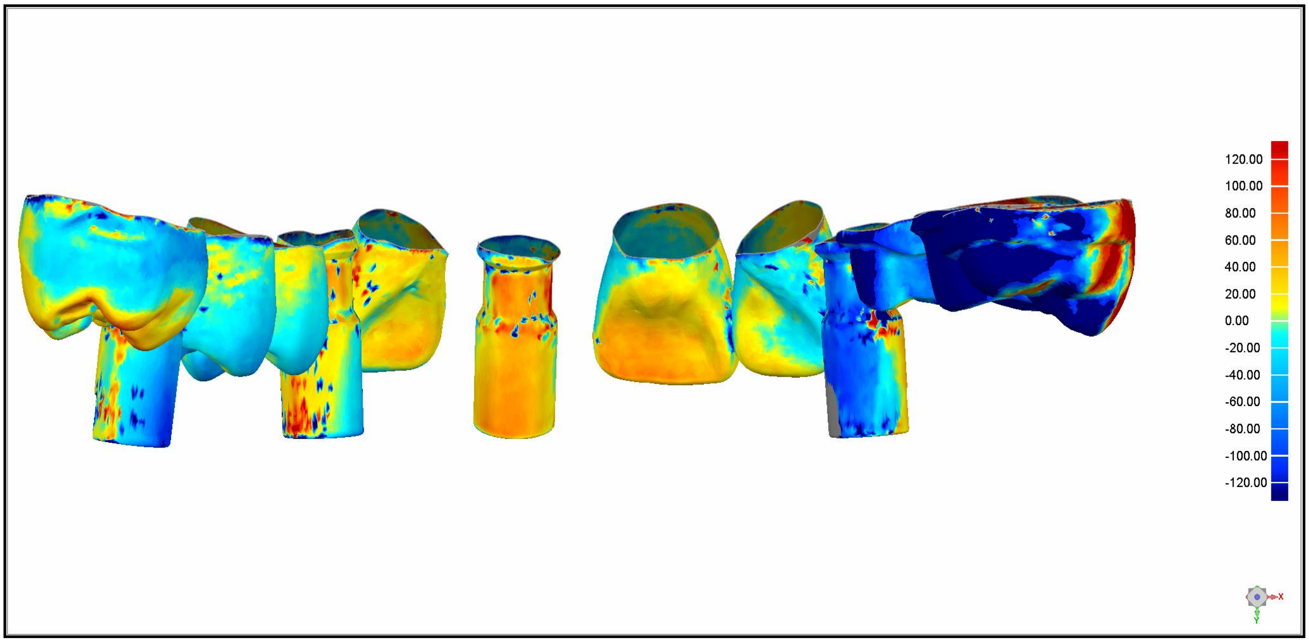

Ajuste de ubicación: Desviaciones superior e inferior

Unidades: u

Nombre	Desv	Estado	Superior Tol	Inferior Tol	Ref X	Ref Y	Ref Z	Radio	Desv X	Desv Y	Desv Z	Medido X	Medido Y	Medido Z	Dir. proy. X	Dir. proy. Y	Dir. proy. Z
Desv. inferior	-3119.98				17725.09	37493.08	17274.82	n/a	795.00	-2813.23	-1089.95	18520.09	34679.85	16184.87	-0.25	0.90	0.35
Desv. superior	3153.90				-22859.28	34269.97	2523.80	n/a	926.31	-1513.63	2607.29	-21932.98	32756.34	5131.08	0.29	-0.48	0.83
