# Supplementary material for: Accuracy of four digital scanners according to scanning strategy in complete-arch impressions
Source: PLoS One. 2018 Sep 13;13(9):e0202916. doi: 10.1371/journal.pone.0202916 (PMC6136706; doi:10.1371/journal.pone.0202916)
Supplement: S7 Table — iTero (scanning strategy C). (ZIP) [file pone.0202916.s007.zip › S7/IT2C.pdf]

Geomagic Control™

Fecha: 3/6/2017, 1:15 pm

3D Comparación Resultados

Modelo referencia	MRC
Modelo test	IT2C
Nº de puntos de datos	80041
# Aislados	605

Tipo tolerancia	3D desviación
Unidades	u
Máx. crítico	120.00
Máx. nominal	3.00
Mín. nominal	-3.00
Mín. crítico	-120.00

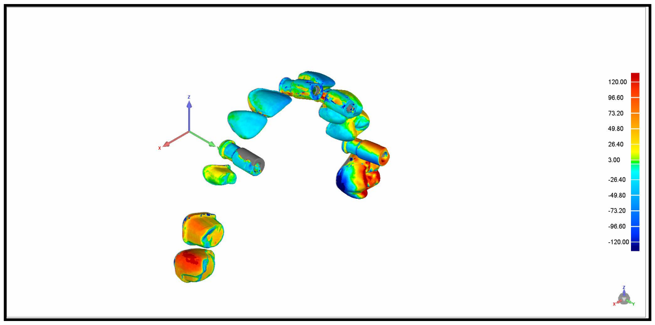

Desviación	
Desviación superior máx.	3136.54
Desviación inferior máx.	-3153.30
Desviación media	94.94 /-81.22
Desviación estándar	261.25

Distribución desviación

>=Min	<Max	# Puntos	%
-120.00	-96.60	1508	1.88
-96.60	-73.20	2402	3.00
-73.20	-49.80	4259	5.32
-49.80	-26.40	10361	12.94
-26.40	-3.00	16971	21.20
-3.00	3.00	4493	5.61
3.00	26.40	14337	17.91
26.40	49.80	7185	8.98
49.80	73.20	4357	5.44
73.20	96.60	2978	3.72
96.60	120.00	1855	2.32

Fuera del crítico superior	5579	6.97
Fuera del crítico inferior	3756	4.69

Distribución desviación

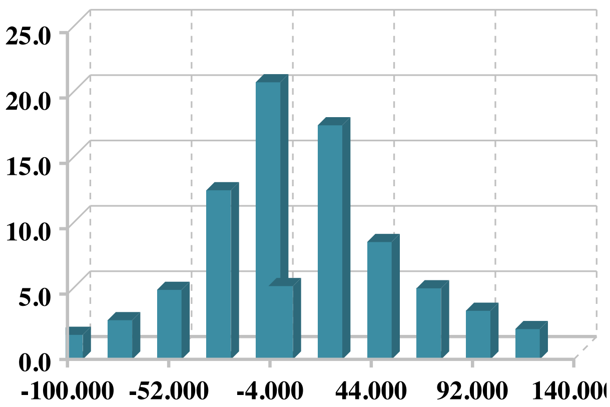

Desviaciones estándar

Distribución (+/-)	# Puntos	%
-6 * Desv. estánd.	488	0.61
-5 * Desv. estánd.	262	0.33
-4 * Desv. estánd.	264	0.33
-3 * Desv. estánd.	277	0.35
-2 * Desv. estánd.	627	0.78
-1 * Desv. estánd.	42280	52.82
1 * Desv. estánd.	34173	42.69
2 * Desv. estánd.	434	0.54
3 * Desv. estánd.	207	0.26
4 * Desv. estánd.	180	0.22
5 * Desv. estánd.	249	0.31
6 * Desv. estánd.	600	0.75

Desviaciones estándar

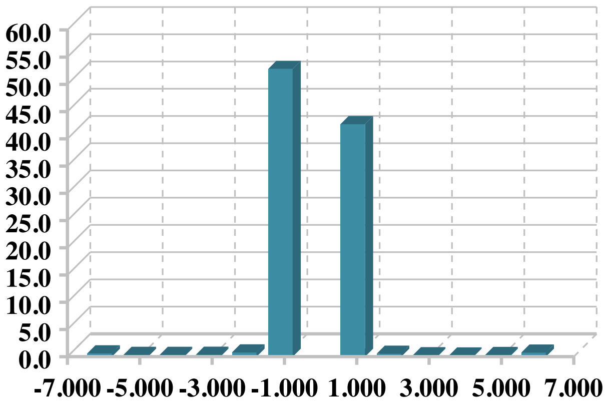

Predefinido: Isométrico

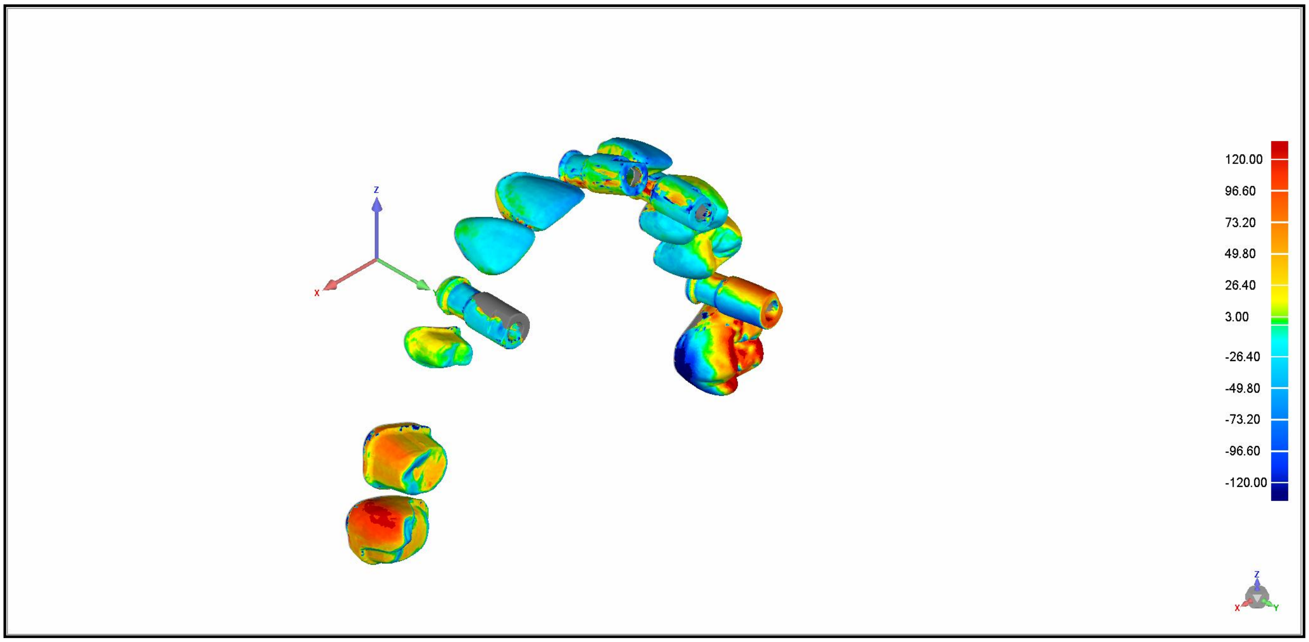

Predefinido: Frente

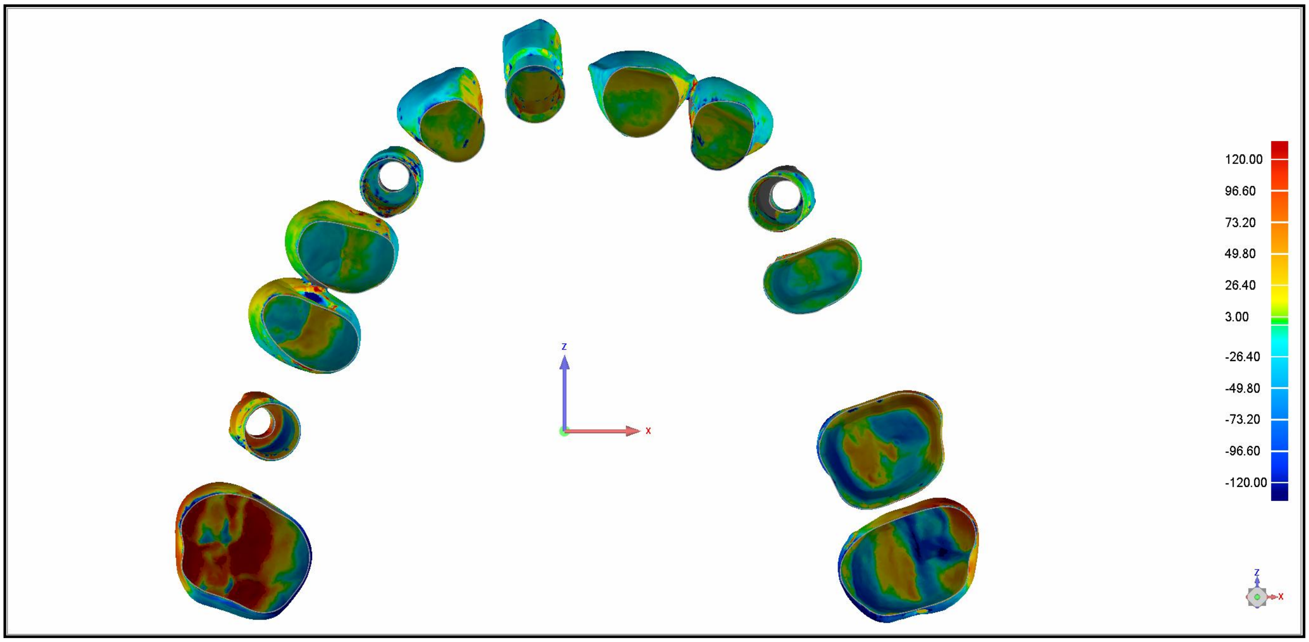

Predefinido: Atrás

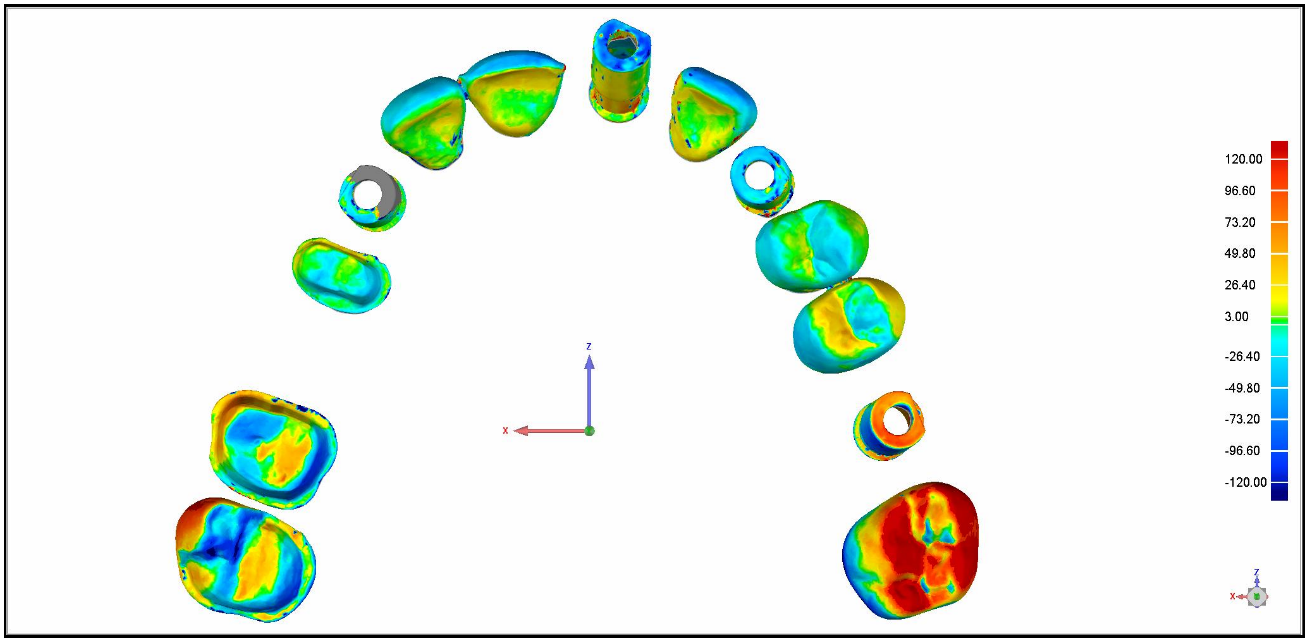

Predefinido: Izquierda

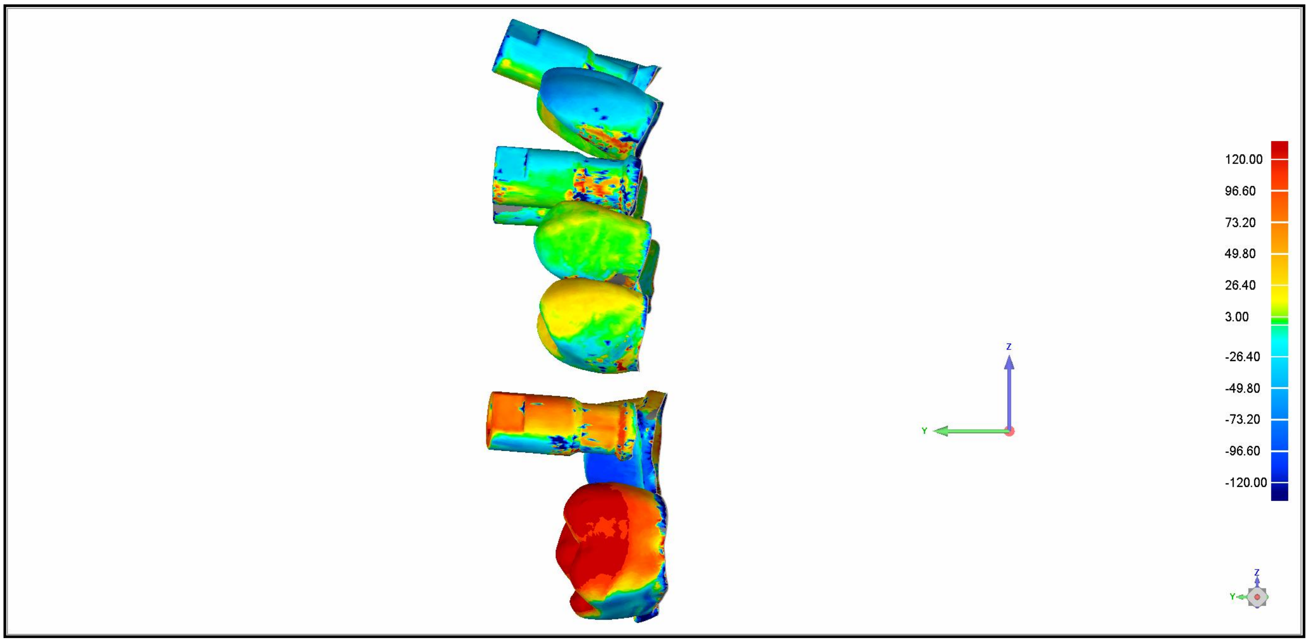

Predefinido: Derecha

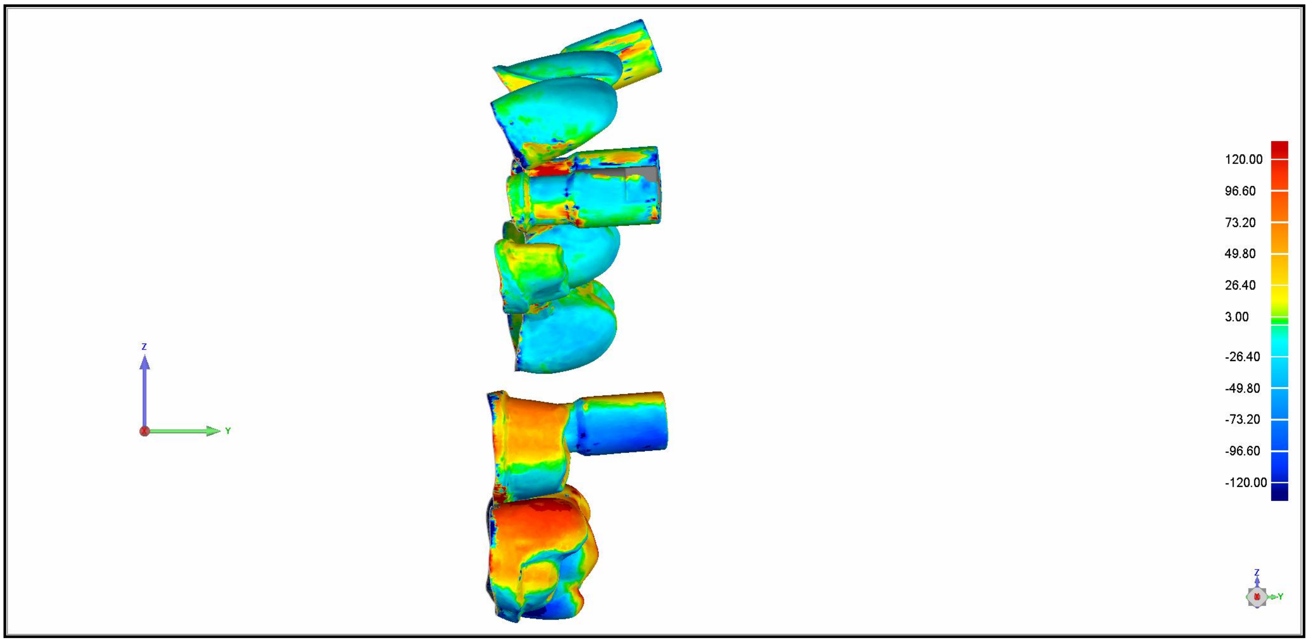

Predefinido: Superior

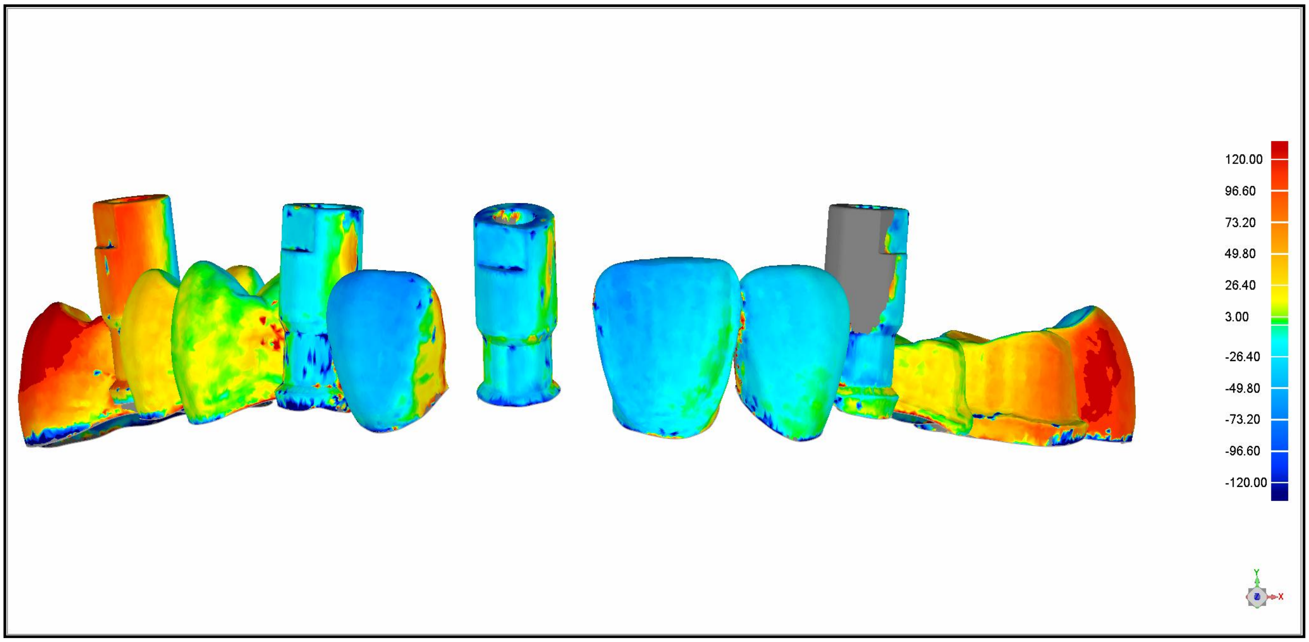

Predefinido: Inferior

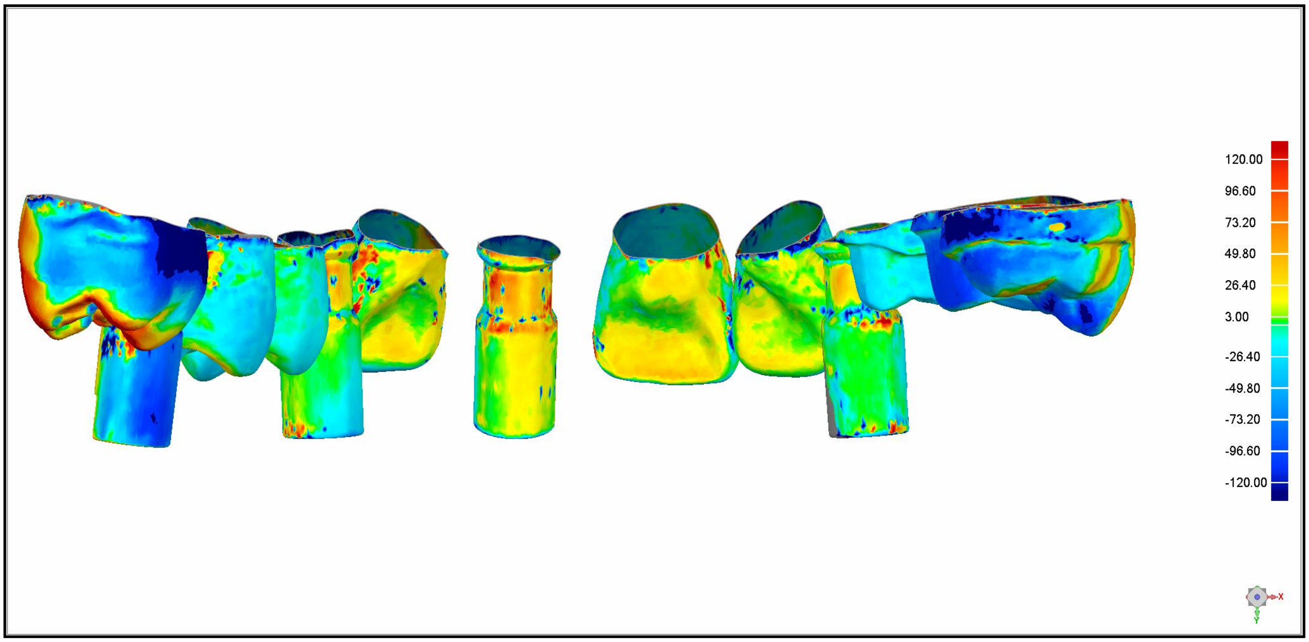

Geomagic Control™

Fecha: 3/6/2017, 1:15 pm

Ajuste de ubicación: Desviaciones superior e inferior

Unidades: u

Nombre	Desv	Estado	Superior Tol	Inferior Tol	Ref X	Ref Y	Ref Z	Radio	Desv X	Desv Y	Desv Z	Medido X	Medido Y	Medido Z	Dir. proy. X	Dir. proy. Y	Dir. proy. Z
Desv. inferior	-3153.30				16989.02	37628.06	17251.36	n/a	2341.11	2112.14	37.01	19330.13	39740.20	17288.37	-0.74	-0.67	-0.01
Desv. superior	3136.54				-25522.63	33821.41	-4757.40	n/a	1063.27	1555.51	2507.54	-24459.36	35376.92	-2249.87	0.34	0.50	0.80
