# Supplementary material for: Accuracy of four digital scanners according to scanning strategy in complete-arch impressions
Source: PLoS One. 2018 Sep 13;13(9):e0202916. doi: 10.1371/journal.pone.0202916 (PMC6136706; doi:10.1371/journal.pone.0202916)
Supplement: S7 Table — iTero (scanning strategy C). (ZIP) [file pone.0202916.s007.zip › S7/IT3C.pdf]

Geomagic Control™

3D Comparación Resultados

Modelo referencia	MRC
Modelo test	IT3C
Nº de puntos de datos	82284
# Aislados	626

Tipo tolerancia	3D desviación
Unidades	u
Máx. crítico	120.00
Máx. nominal	7.00
Mín. nominal	-7.00
Mín. crítico	-120.00

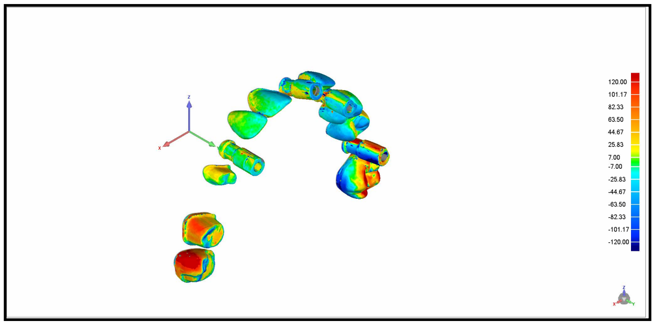

Desviación	
Desviación superior máx.	3082.23
Desviación inferior máx.	-3100.14
Desviación media	91.92 /-78.99
Desviación estándar	243.10

Distribución desviación

>=Min	<Max	# Puntos	%
-120.00	-101.17	1413	1.72
-101.17	-82.33	1658	2.01
-82.33	-63.50	2414	2.93
-63.50	-44.67	5614	6.82
-44.67	-25.83	8942	10.87
-25.83	-7.00	11342	13.78
-7.00	7.00	9911	12.04
7.00	25.83	11031	13.41
25.83	44.67	7932	9.64
44.67	63.50	4616	5.61
63.50	82.33	3402	4.13
82.33	101.17	2653	3.22
101.17	120.00	1901	2.31

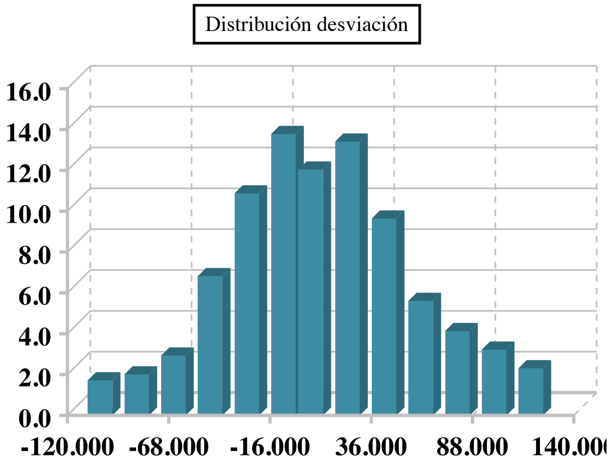

Fuera del crítico superior		5360	6.51
Fuera del crítico inferior		4095	4.98

Desviaciones estándar

Distribución (+/-)	# Puntos	%
-6 * Desv. estánd.	475	0.58
-5 * Desv. estánd.	203	0.25
-4 * Desv. estánd.	185	0.22
-3 * Desv. estánd.	320	0.39
-2 * Desv. estánd.	689	0.84
-1 * Desv. estánd.	43999	53.47
1 * Desv. estánd.	34576	42.02
2 * Desv. estánd.	508	0.62
3 * Desv. estánd.	204	0.25
4 * Desv. estánd.	215	0.26
5 * Desv. estánd.	248	0.30
6 * Desv. estánd.	662	0.80

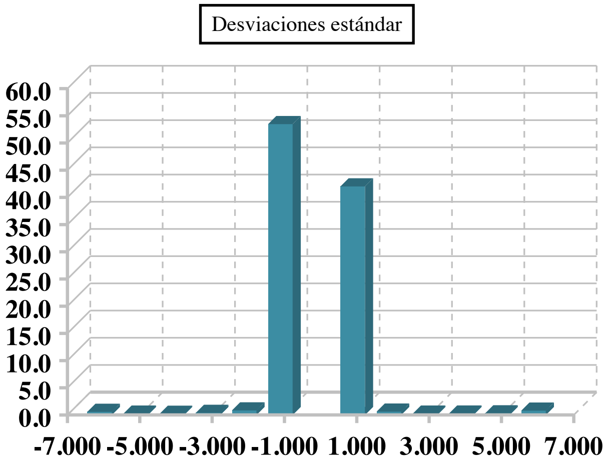

Predefinido: Isométrico

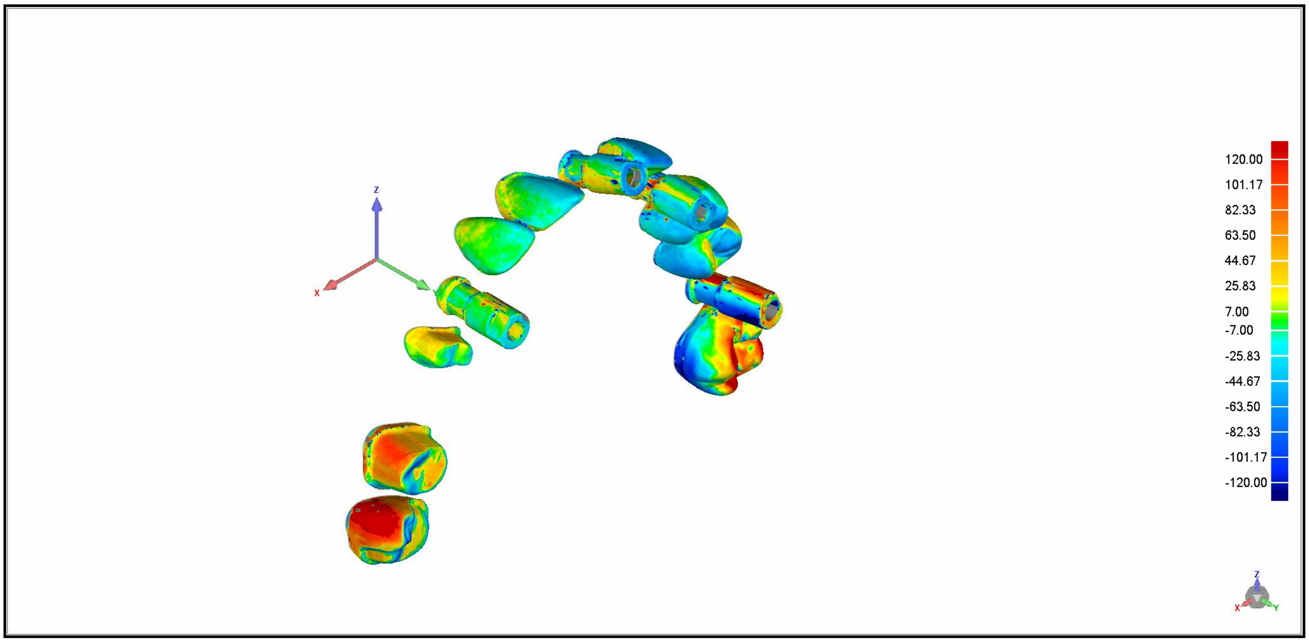

Predefinido: Frente

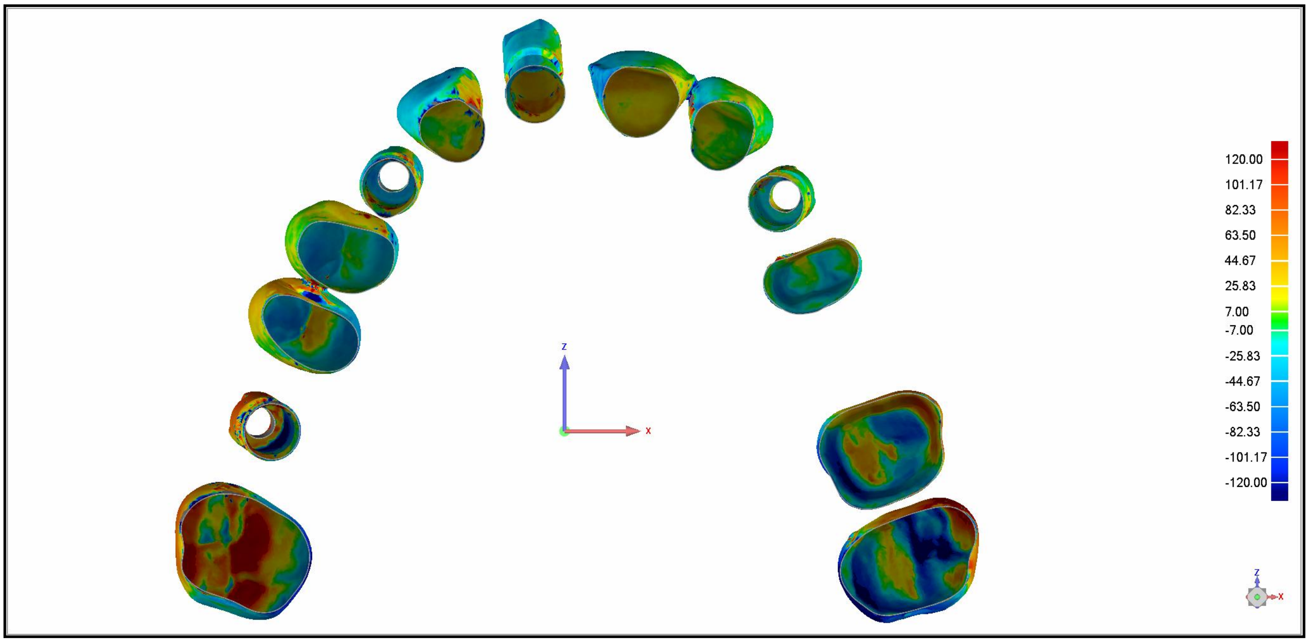

Predefinido: Atrás

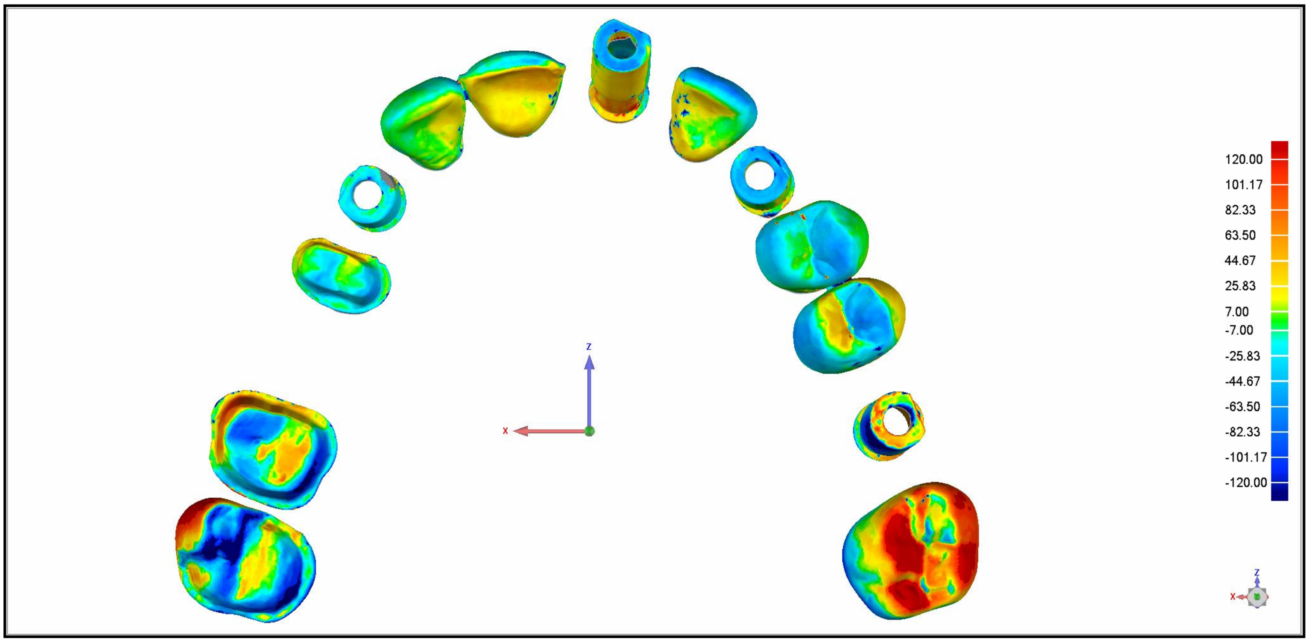

Predefinido: Izquierda

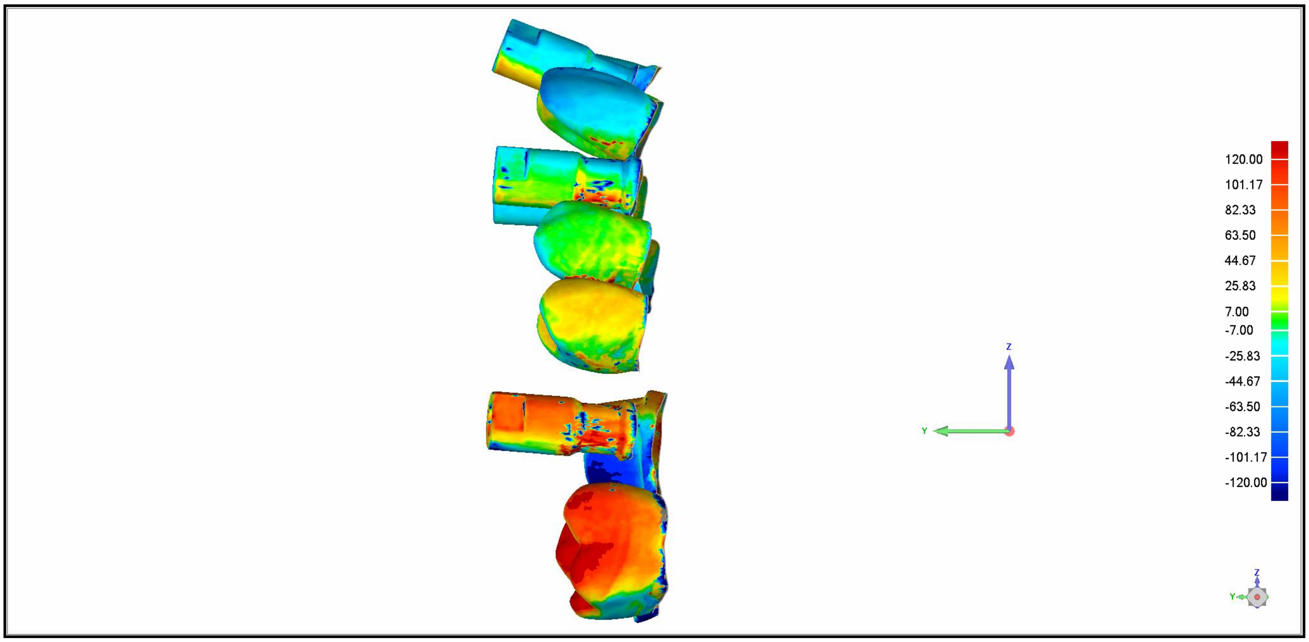

Predefinido: Derecha

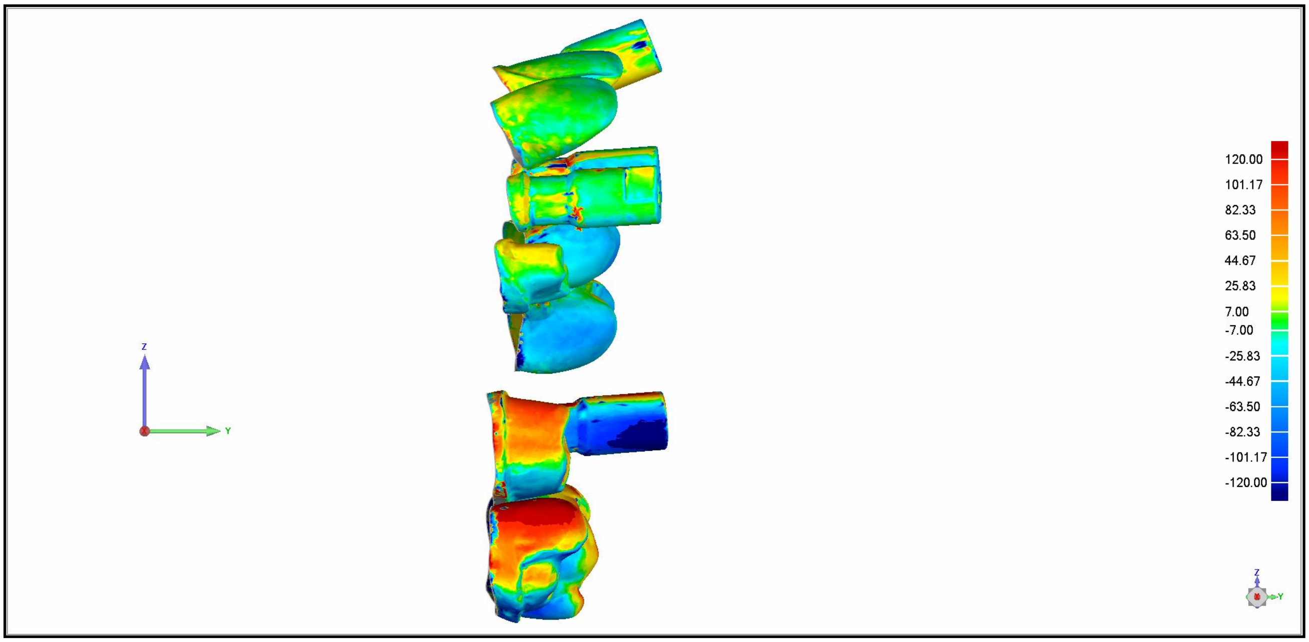

Predefinido: Superior

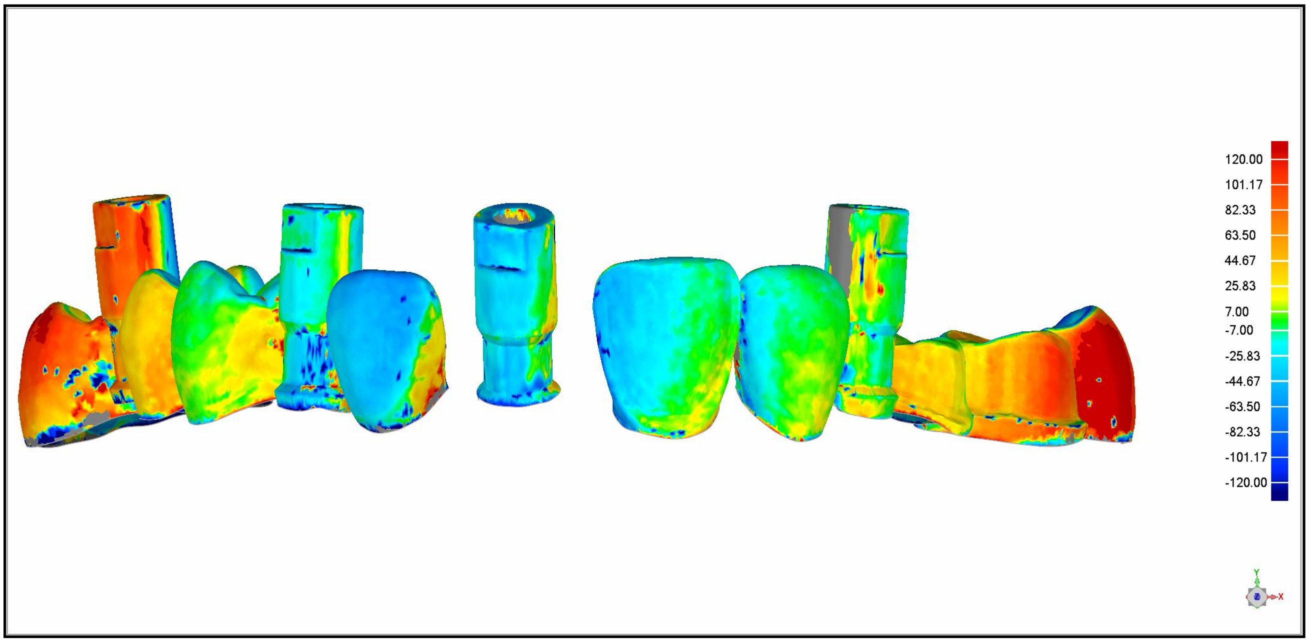

Predefinido: Inferior

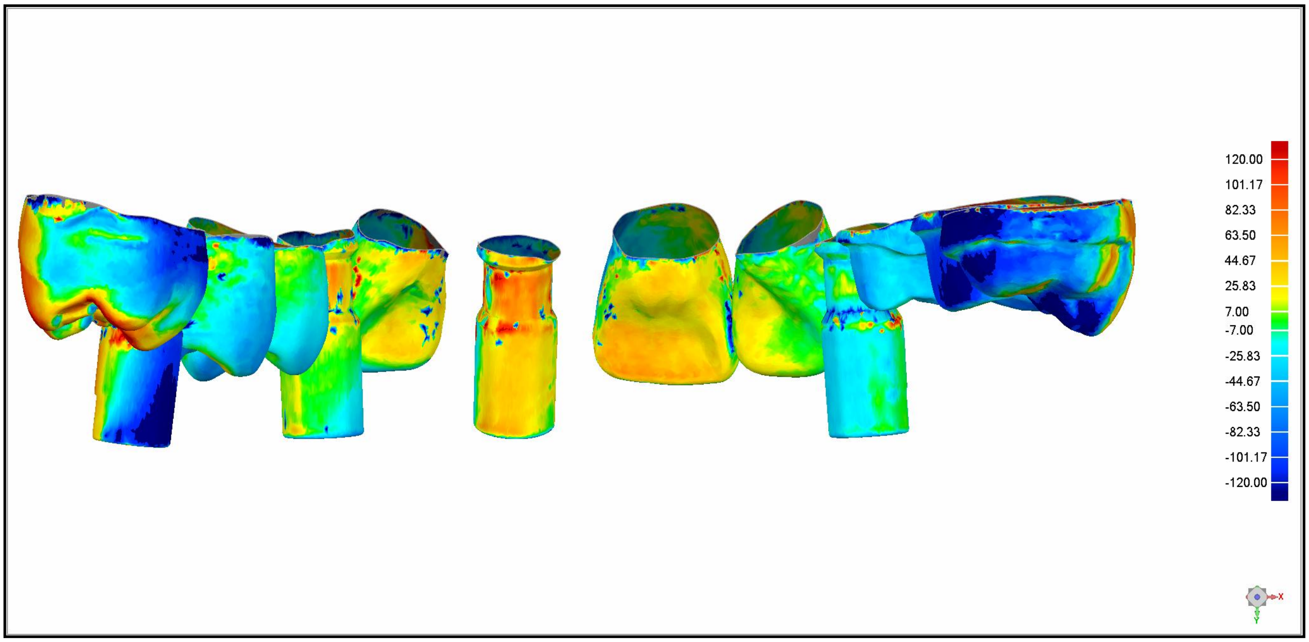

Ajuste de ubicación: Desviaciones superior e inferior

Unidades: u

Nombre	Desv	Estado	Superior Tol	Inferior Tol	Ref X	Ref Y	Ref Z	Radio	Desv X	Desv Y	Desv Z	Medido X	Medido Y	Medido Z	Dir. proy. X	Dir. proy. Y	Dir. proy. Z
Desv. inferior	-3100.14				-29208.33	26961.25	-11988.49	n/a	2710.61	546.31	-1401.78	-26497.72	27507.56	-13390.27	-0.87	-0.18	0.45
Desv. superior	3082.23				-24887.28	29763.40	-1296.73	n/a	-552.76	3010.51	362.46	-25440.05	32773.92	-934.28	-0.18	0.98	0.12
