# Supplementary material for: Accuracy of four digital scanners according to scanning strategy in complete-arch impressions
Source: PLoS One. 2018 Sep 13;13(9):e0202916. doi: 10.1371/journal.pone.0202916 (PMC6136706; doi:10.1371/journal.pone.0202916)
Supplement: S7 Table — iTero (scanning strategy C). (ZIP) [file pone.0202916.s007.zip › S7/IT1C.pdf]

Geomagic Control™

3D Comparación Resultados

Modelo referencia	MRC
Modelo test	IT1C
Nº de puntos de datos	80166
# Aislados	620

Tipo tolerancia	3D desviación
Unidades	u
Máx. crítico	120.00
Máx. nominal	8.00
Mín. nominal	-8.00
Mín. crítico	-120.00

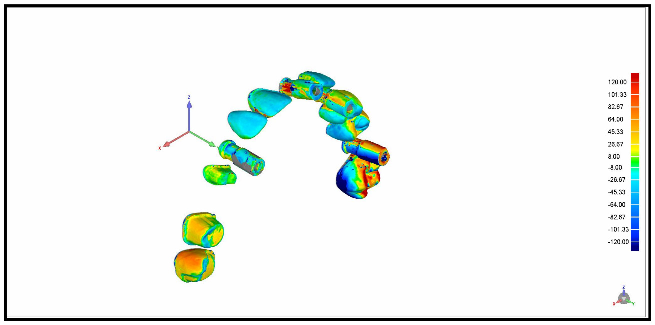

Desviación	
Desviación superior máx.	3153.09
Desviación inferior máx.	-3132.13
Desviación media	100.91 /-86.01
Desviación estándar	283.84

Distribución desviación

>=Min	<Max	# Puntos	%
-120.00	-101.33	607	0.76
-101.33	-82.67	1099	1.37
-82.67	-64.00	1884	2.35
-64.00	-45.33	3938	4.91
-45.33	-26.67	8818	11.00
-26.67	-8.00	13361	16.67
-8.00	8.00	11531	14.38
8.00	26.67	12087	15.08
26.67	45.33	7953	9.92
45.33	64.00	3671	4.58
64.00	82.67	2247	2.80
82.67	101.33	1485	1.85
101.33	120.00	1376	1.72

Fuera del crítico superior	5976	7.45
Fuera del crítico inferior	4133	5.16

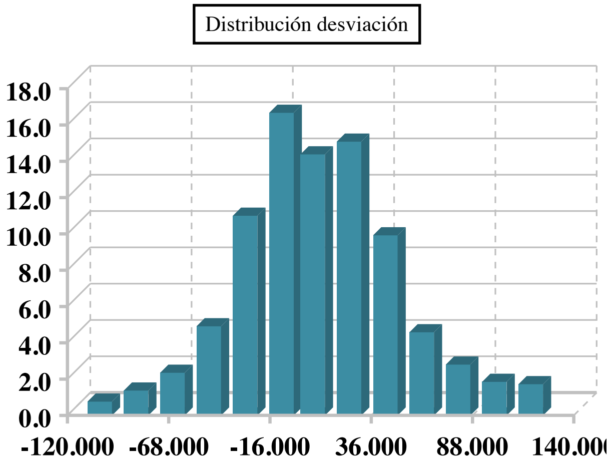

Desviaciones estándar

Distribución (+/-)	# Puntos	%
-6 * Desv. estánd.	493	0.61
-5 * Desv. estánd.	229	0.29
-4 * Desv. estánd.	278	0.35
-3 * Desv. estánd.	330	0.41
-2 * Desv. estánd.	652	0.81
-1 * Desv. estánd.	43912	54.78
1 * Desv. estánd.	32399	40.41
2 * Desv. estánd.	392	0.49
3 * Desv. estánd.	275	0.34
4 * Desv. estánd.	258	0.32
5 * Desv. estánd.	389	0.49
6 * Desv. estánd.	559	0.70

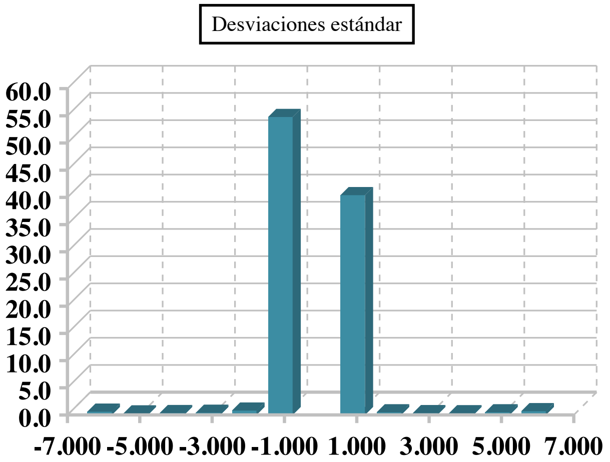

Predefinido: Isométrico

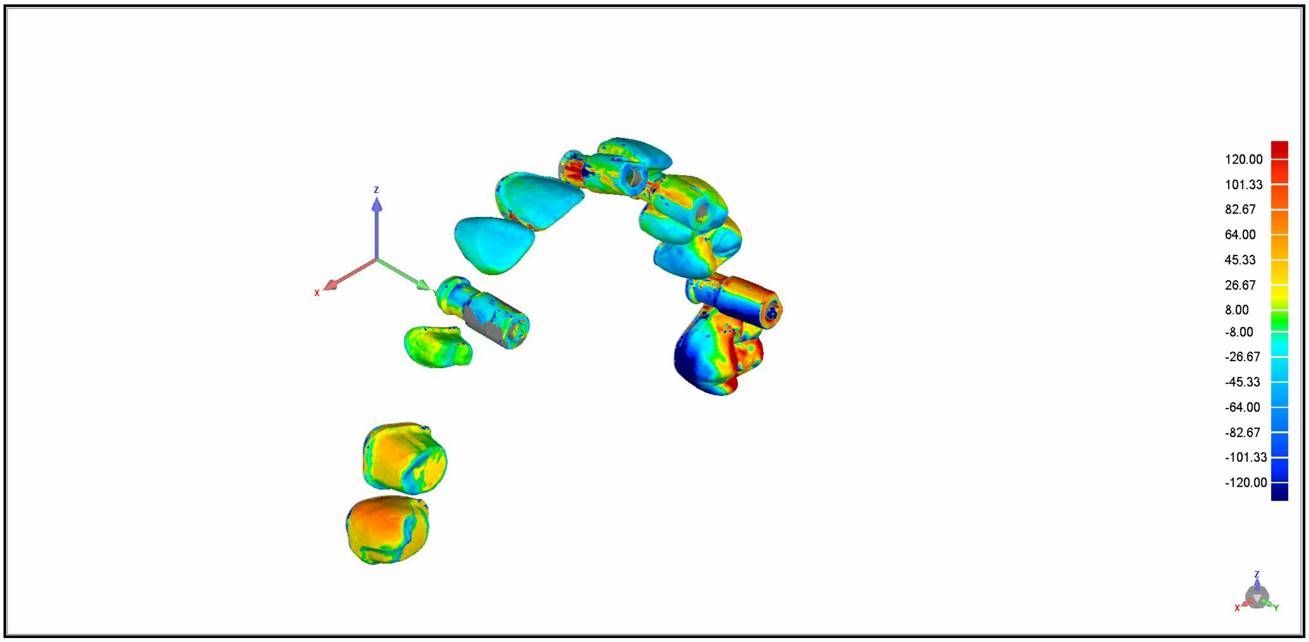

Predefinido: Frente

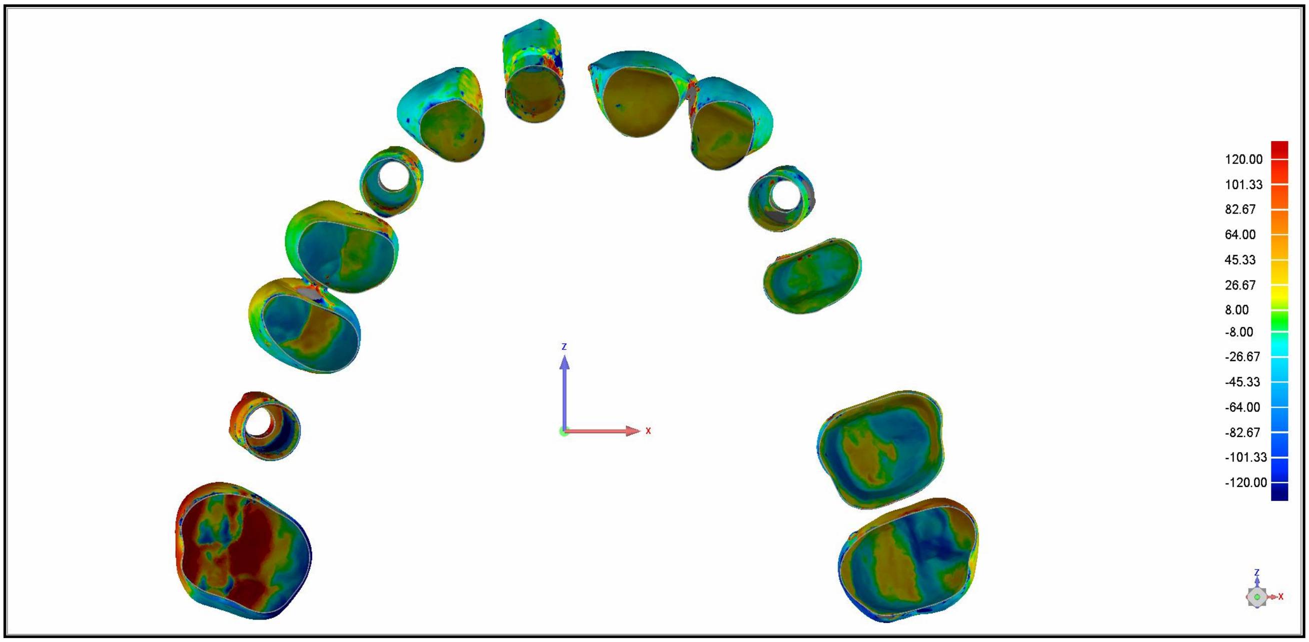

Predefinido: Atrás

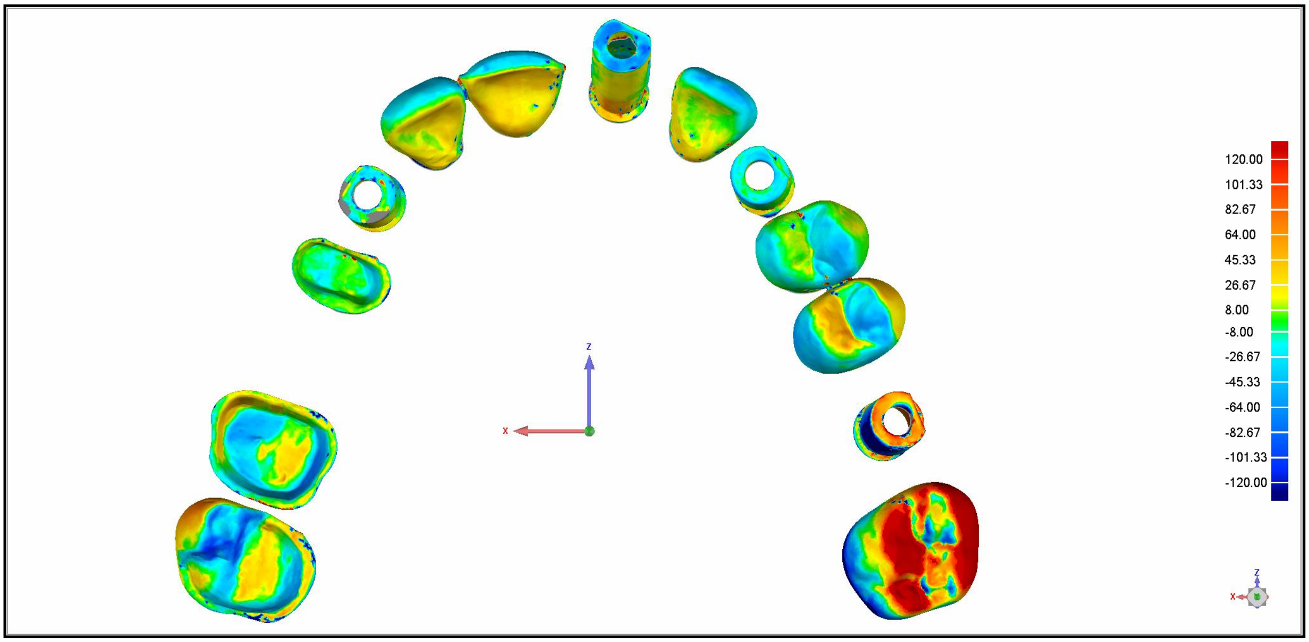

Predefinido: Izquierda

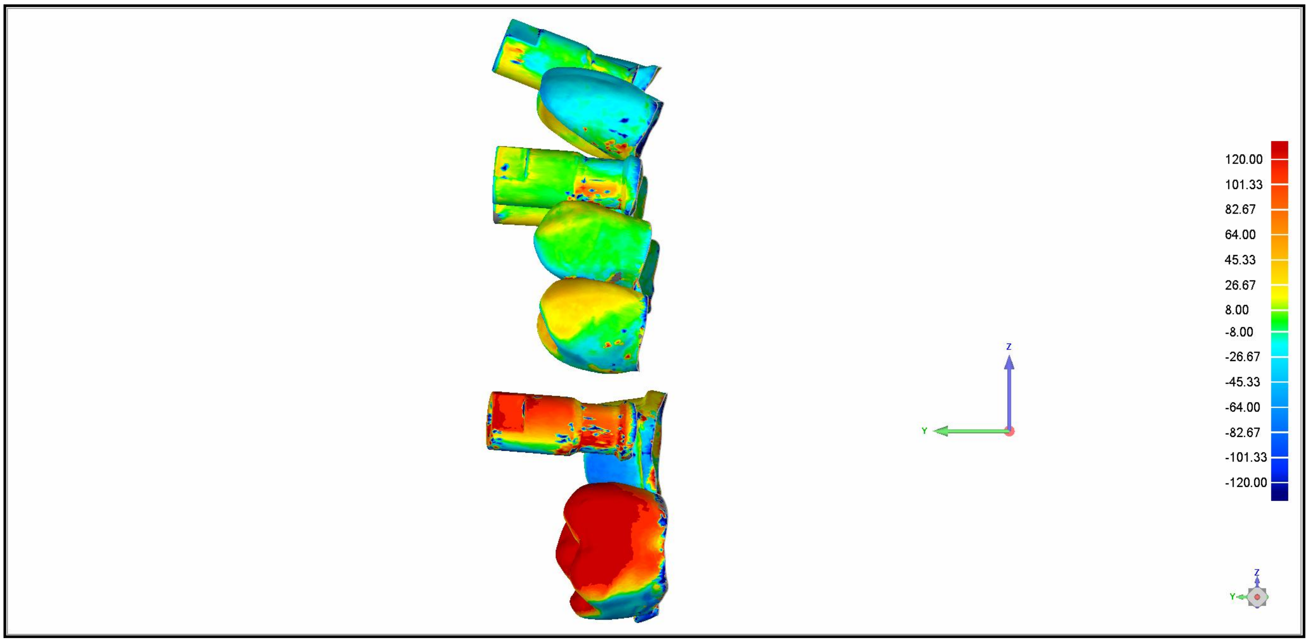

Predefinido: Derecha

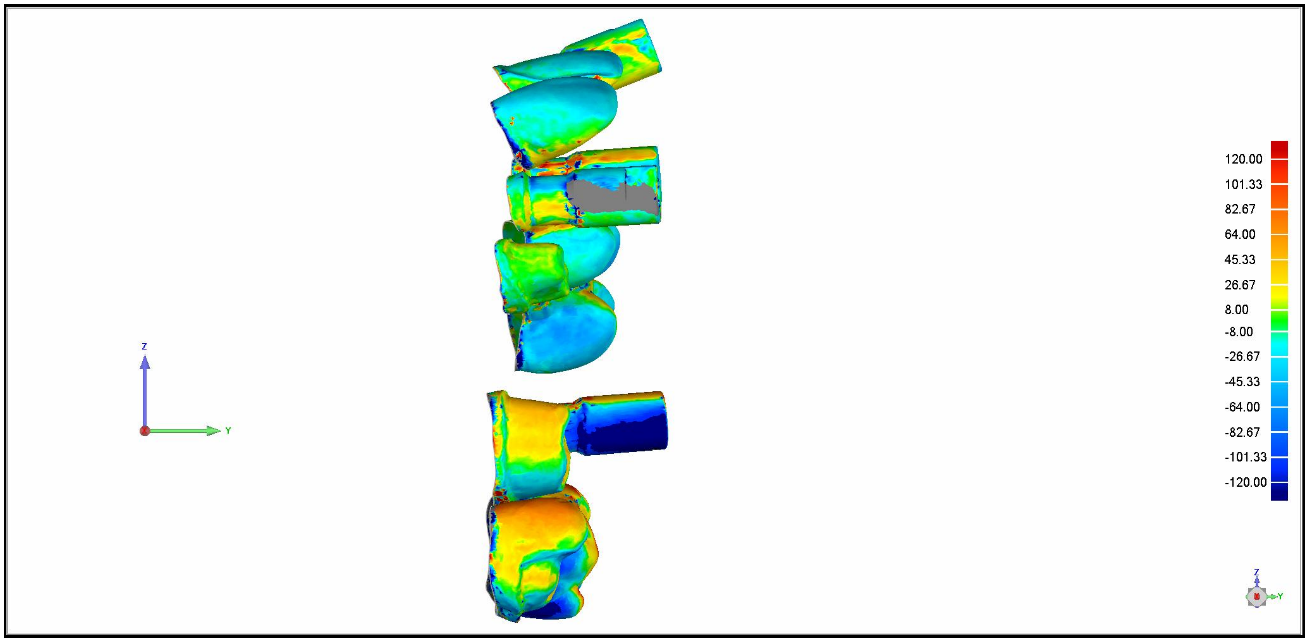

Predefinido: Superior

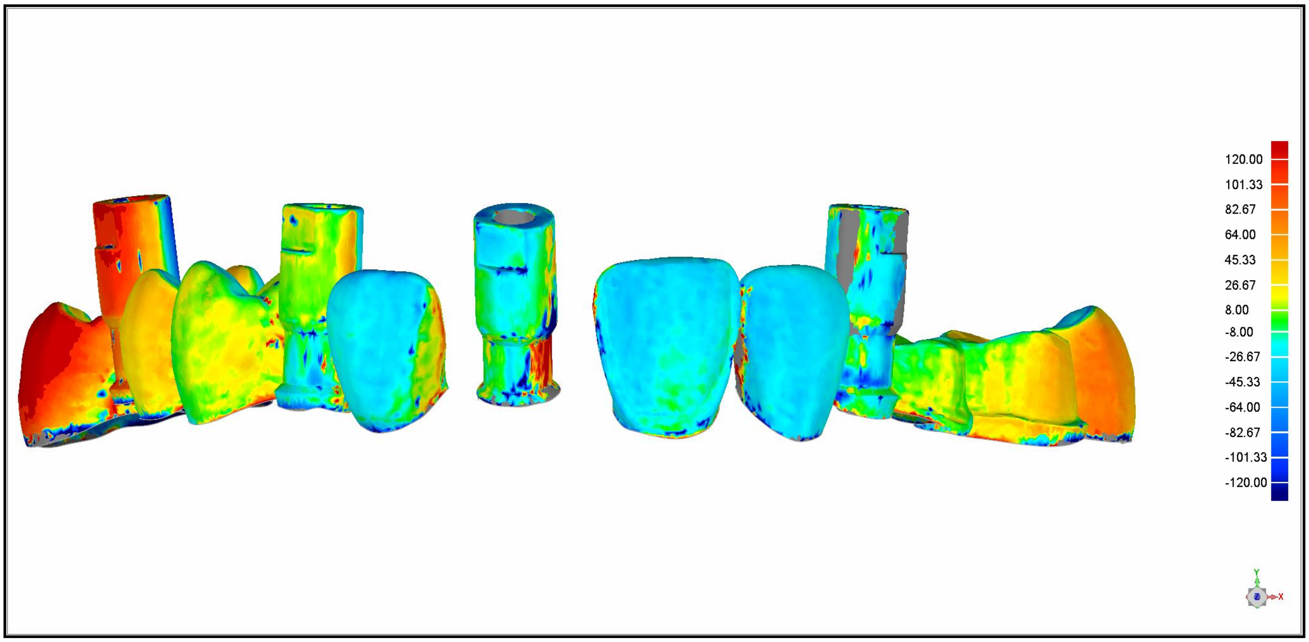

Predefinido: Inferior

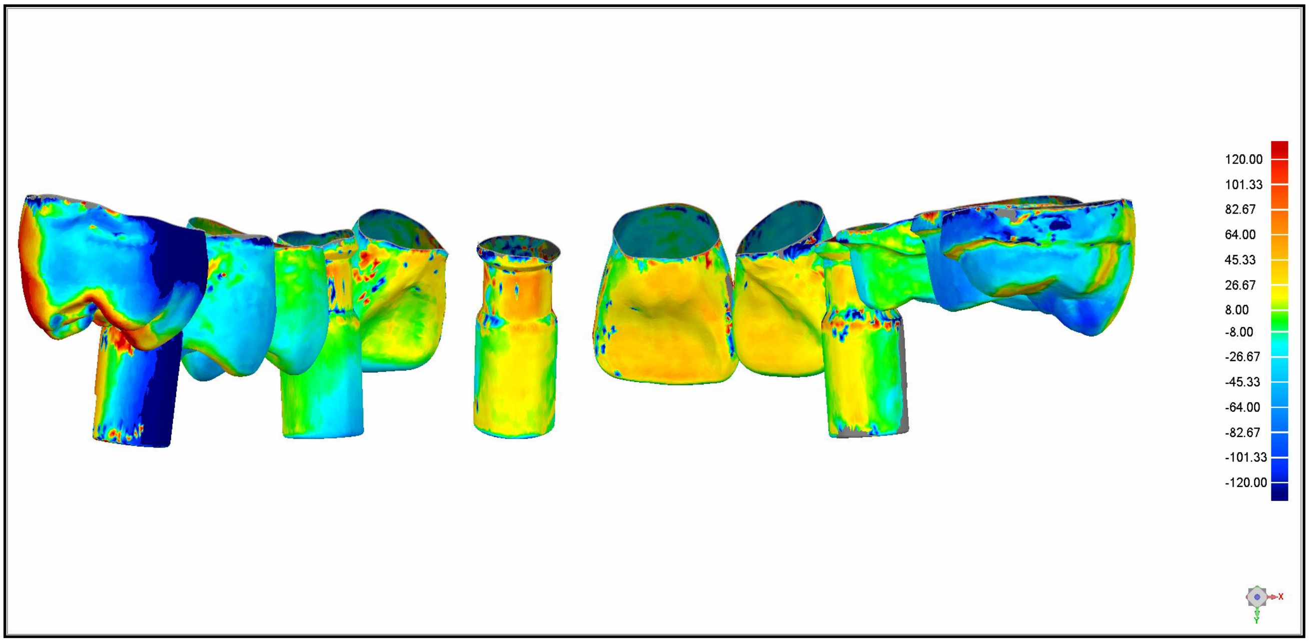

Ajuste de ubicación: Desviaciones superior e inferior

Unidades: u

Nombre	Desv	Estado	Superior Tol	Inferior Tol	Ref X	Ref Y	Ref Z	Radio	Desv X	Desv Y	Desv Z	Medido X	Medido Y	Medido Z	Dir. proy. X	Dir. proy. Y	Dir. proy. Z
Desv. inferior	-3132.13				-23318.94	38082.95	-378.93	n/a	-1818.57	2364.79	-954.38	-25137.51	40447.73	-1333.30	0.58	-0.76	0.30
Desv. superior	3153.09				-23170.64	30472.07	-2360.70	n/a	-115.43	217.83	-3143.43	-23286.07	30689.90	-5504.14	-0.04	0.07	-1.00
