# Supplementary material for: Accuracy of four digital scanners according to scanning strategy in complete-arch impressions
Source: PLoS One. 2018 Sep 13;13(9):e0202916. doi: 10.1371/journal.pone.0202916 (PMC6136706; doi:10.1371/journal.pone.0202916)
Supplement: S7 Table — iTero (scanning strategy C). (ZIP) [file pone.0202916.s007.zip › S7/IT6C.pdf]

Fecha: 3/6/2017, 1:25 pm

3D Comparación Resultados

Modelo referencia	MRC
Modelo test	IT6C
Nº de puntos de datos	81363
# Aislados	622

Tipo tolerancia	3D desviación
Unidades	u
Máx. crítico	120.00
Máx. nominal	9.00
Mín. nominal	-9.00
Mín. crítico	-120.00

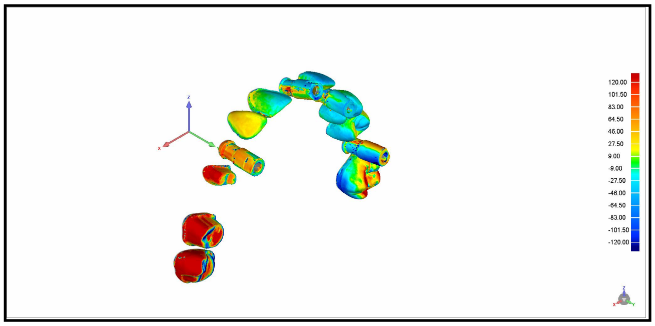

Desviación	
Desviación superior máx.	3020.04
Desviación inferior máx.	-2936.00
Desviación media	106.41 /-91.26
Desviación estándar	238.28

Distribución desviación

>=Min	<Max	# Puntos	%
-120.00	-101.50	1586	1.95
-101.50	-83.00	1878	2.31
-83.00	-64.50	2086	2.56
-64.50	-46.00	3666	4.51
-46.00	-27.50	7673	9.43
-27.50	-9.00	10342	12.71
-9.00	9.00	11056	13.59
9.00	27.50	9423	11.58
27.50	46.00	7106	8.73
46.00	64.50	4666	5.73
64.50	83.00	3273	4.02
83.00	101.50	2149	2.64
101.50	120.00	1478	1.82

Fuera del crítico superior	8264	10.16
Fuera del crítico inferior	6717	8.26

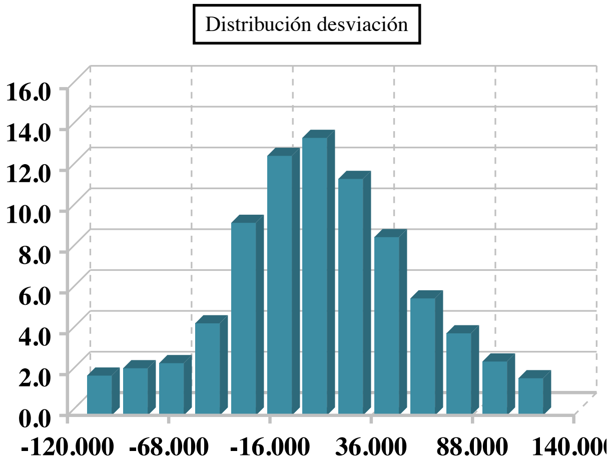

Desviaciones estándar

Distribución (+/-)	# Puntos	%
-6 * Desv. estánd.	439	0.54
-5 * Desv. estánd.	168	0.21
-4 * Desv. estánd.	256	0.31
-3 * Desv. estánd.	325	0.40
-2 * Desv. estánd.	2047	2.52
-1 * Desv. estánd.	42120	51.77
1 * Desv. estánd.	32254	39.64
2 * Desv. estánd.	2478	3.05
3 * Desv. estánd.	226	0.28
4 * Desv. estánd.	202	0.25
5 * Desv. estánd.	203	0.25
6 * Desv. estánd.	645	0.79

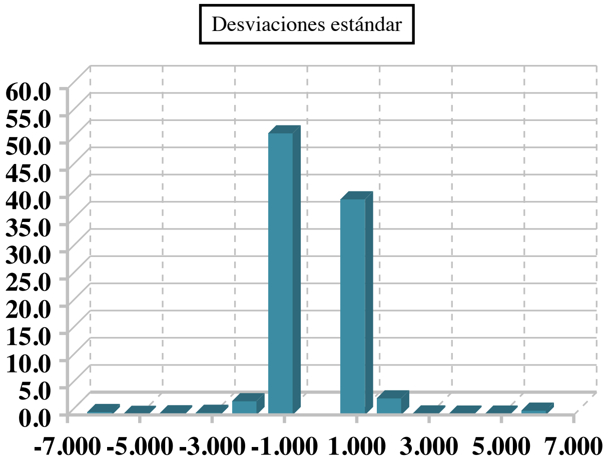

Predefinido: Isométrico

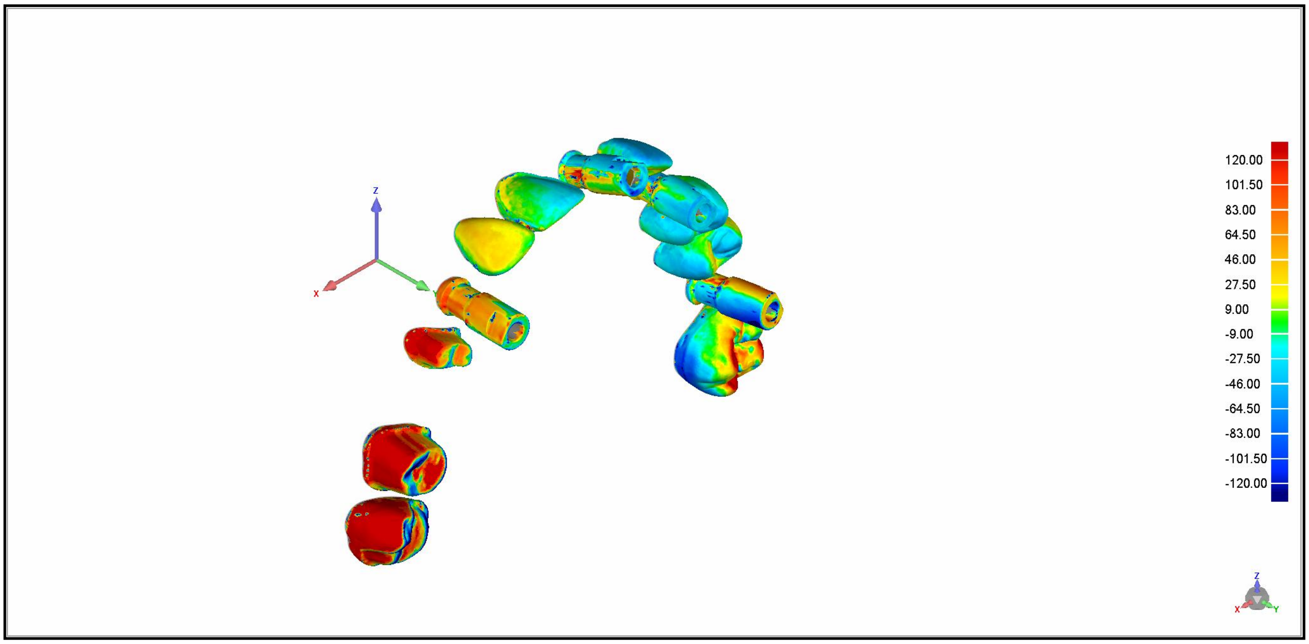

Predefinido: Frente

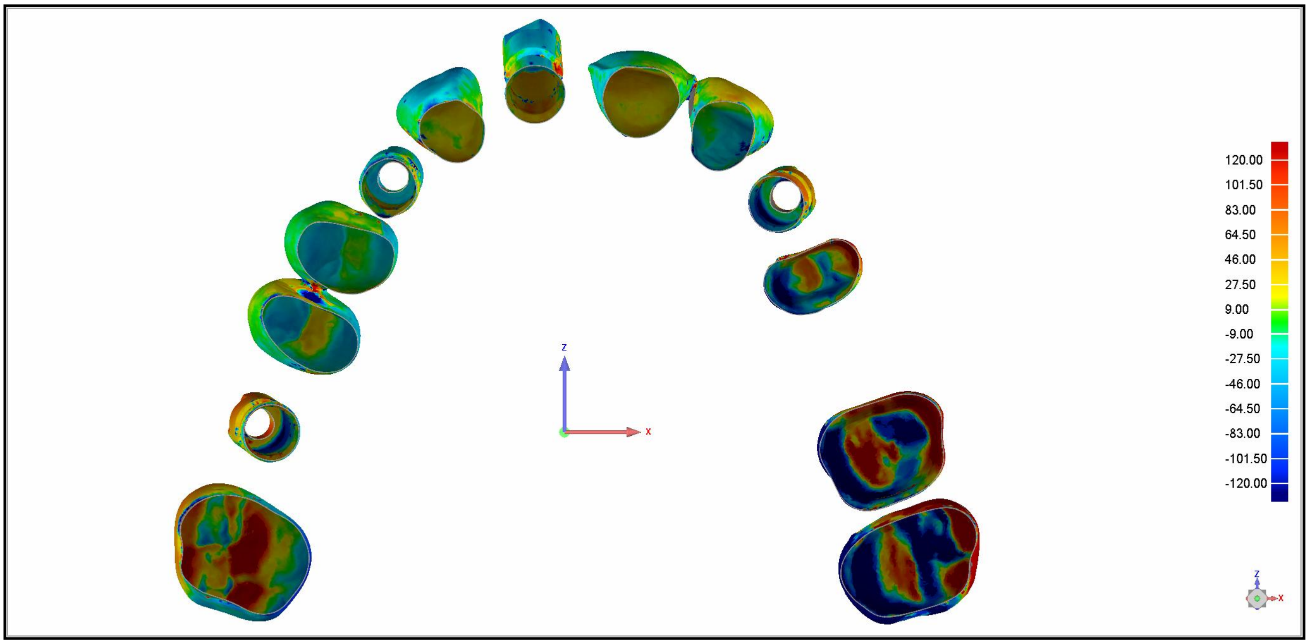

Predefinido: Atrás

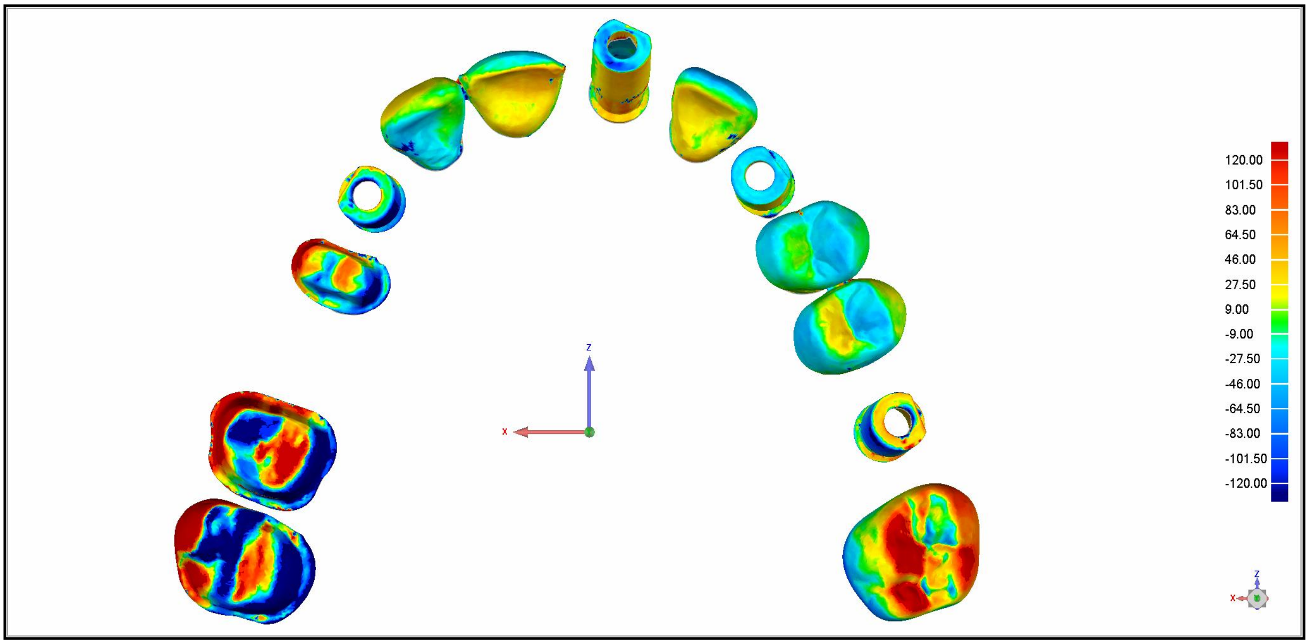

Control™

Predefinido: Izquierda

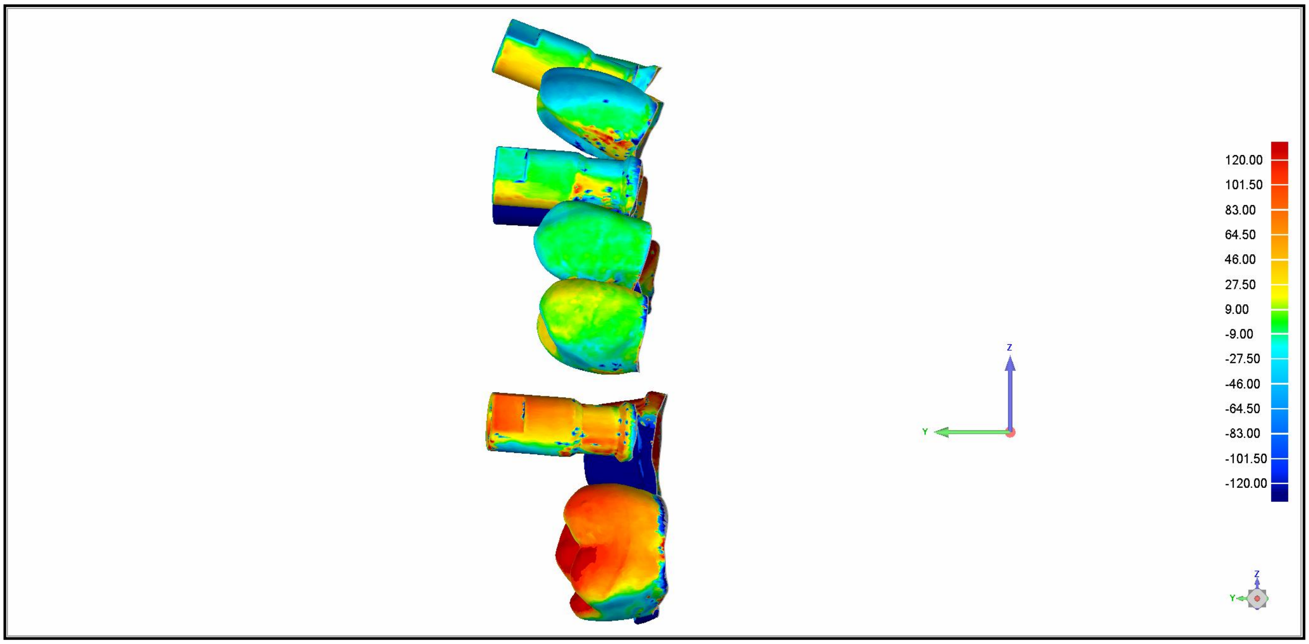

Predefinido: Derecha

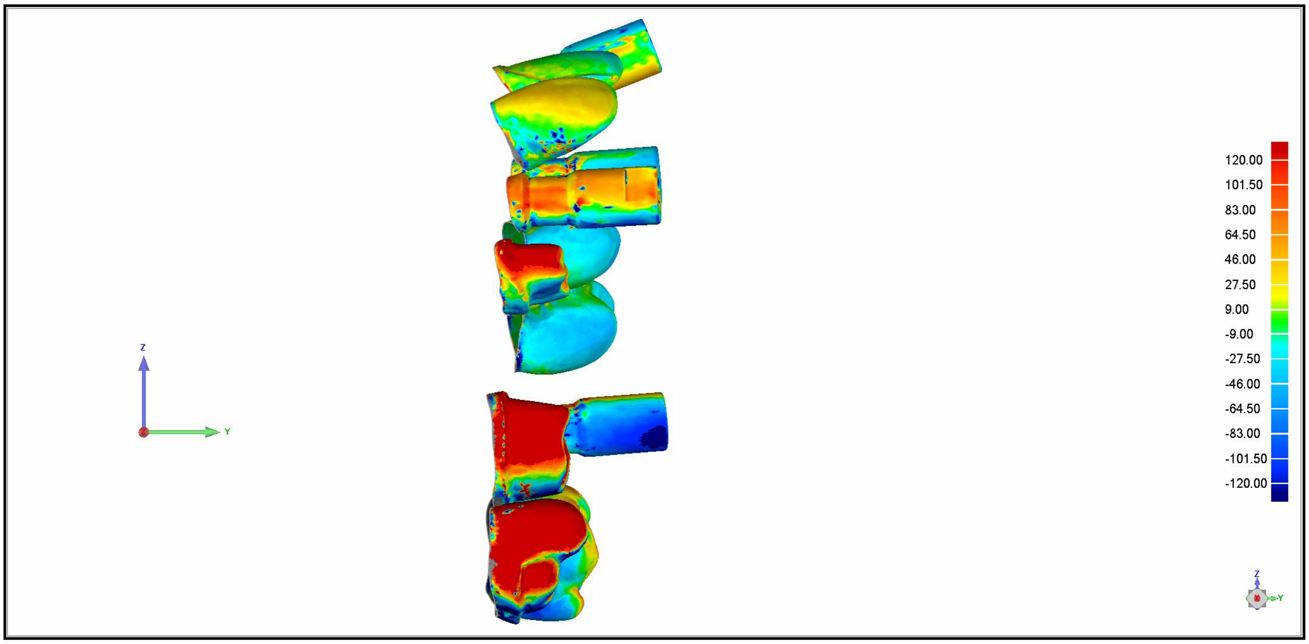

Predefinido: Superior

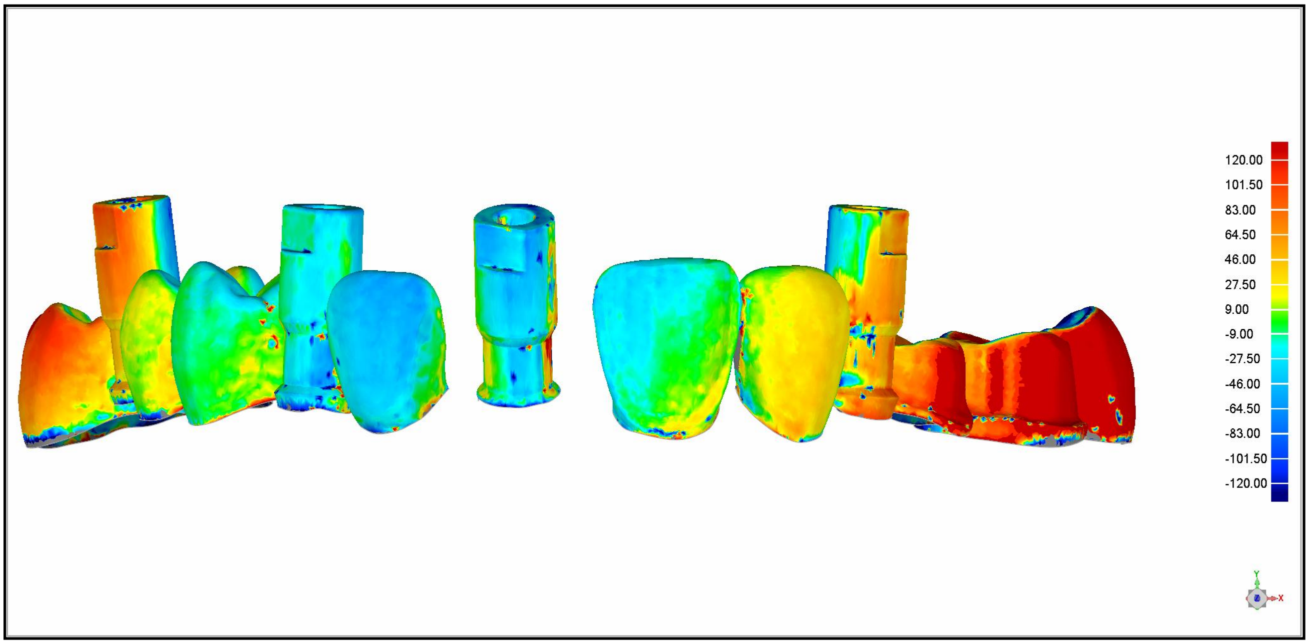

Predefinido: Inferior

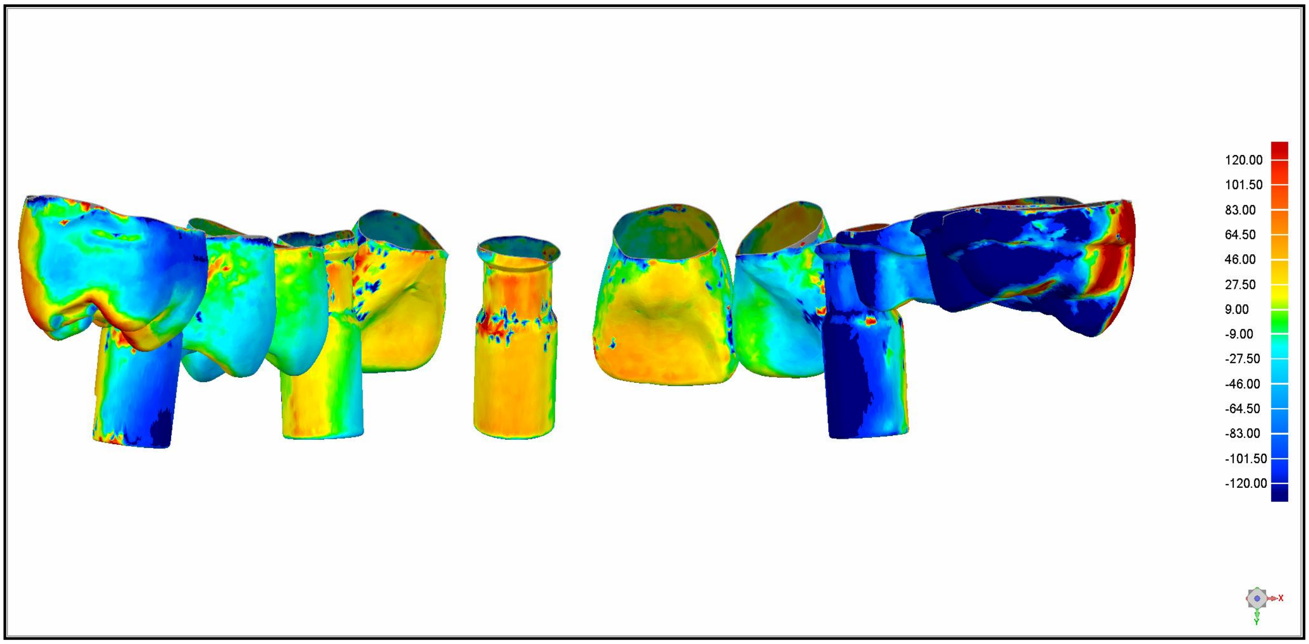

Ajuste de ubicación: Desviaciones superior e inferior

Unidades: u

Nombre	Desv	Estado	Superior Tol	Inferior Tol	Ref X	Ref Y	Ref Z	Radio	Desv X	Desv Y	Desv Z	Medido X	Medido Y	Medido Z	Dir. proy. X	Dir. proy. Y	Dir. proy. Z
Desv. inferior	-2936.00				-16597.07	29132.89	5701.00	n/a	-2818.76	112.48	-813.65	-19415.83	29245.37	4887.34	0.96	-0.04	0.28
Desv. superior	3020.04				27731.36	28303.98	-4830.46	n/a	2870.86	-514.13	-783.90	30602.22	27789.84	-5614.36	0.95	-0.17	-0.26
