# Supplementary material for: Accuracy of four digital scanners according to scanning strategy in complete-arch impressions
Source: PLoS One. 2018 Sep 13;13(9):e0202916. doi: 10.1371/journal.pone.0202916 (PMC6136706; doi:10.1371/journal.pone.0202916)
Supplement: S7 Table — iTero (scanning strategy C). (ZIP) [file pone.0202916.s007.zip › S7/IT10C.pdf]

3D Comparación Resultados

Modelo referencia	MRC
Modelo test	IT10C
Nº de puntos de datos	82518
# Aislados	492

Tipo tolerancia	3D desviación
Unidades	u
Máx. crítico	120.00
Máx. nominal	5.00
Mín. nominal	-5.00
Mín. crítico	-120.00

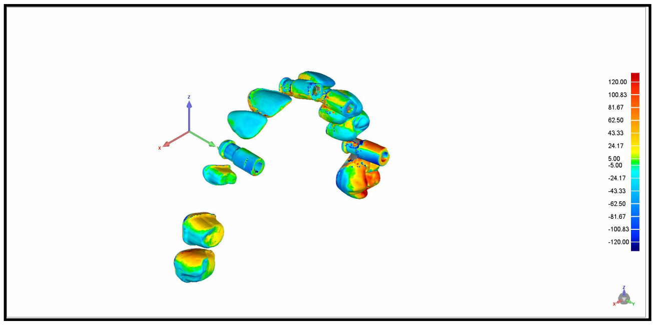

Desviación	
Desviación superior máx.	3135.48
Desviación inferior máx.	-3129.83
Desviación media	83.61 /-63.78
Desviación estándar	240.24

Distribución desviación

>=Min	<Max	# Puntos	%
-120.00	-100.83	469	0.57
-100.83	-81.67	805	0.98
-81.67	-62.50	2022	2.45
-62.50	-43.33	5782	7.01
-43.33	-24.17	13166	15.96
-24.17	-5.00	15188	18.41
-5.00	5.00	7785	9.43
5.00	24.17	12924	15.66
24.17	43.33	9098	11.03
43.33	62.50	4197	5.09
62.50	81.67	2489	3.02
81.67	100.83	1670	2.02
100.83	120.00	963	1.17

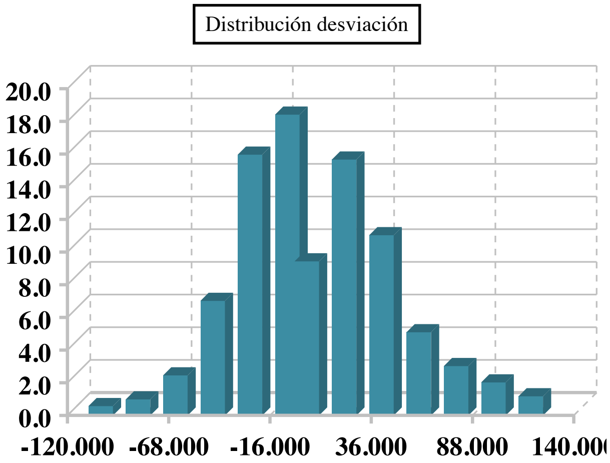

Fuera del crítico superior	3381	4.10
Fuera del crítico inferior	2579	3.13

Desviaciones estándar

Distribución (+/-)	# Puntos	%
-6 * Desv. estánd.	450	0.55
-5 * Desv. estánd.	186	0.23
-4 * Desv. estánd.	152	0.18
-3 * Desv. estánd.	216	0.26
-2 * Desv. estánd.	535	0.65
-1 * Desv. estánd.	46327	56.14
1 * Desv. estánd.	32864	39.83
2 * Desv. estánd.	475	0.58
3 * Desv. estánd.	256	0.31
4 * Desv. estánd.	256	0.31
5 * Desv. estánd.	240	0.29
6 * Desv. estánd.	561	0.68

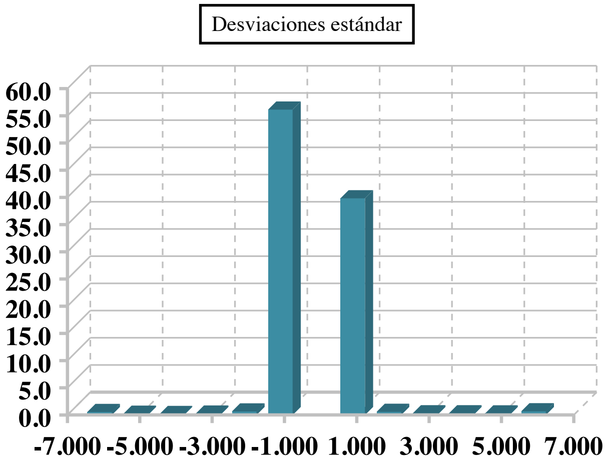

Predefinido: Isométrico

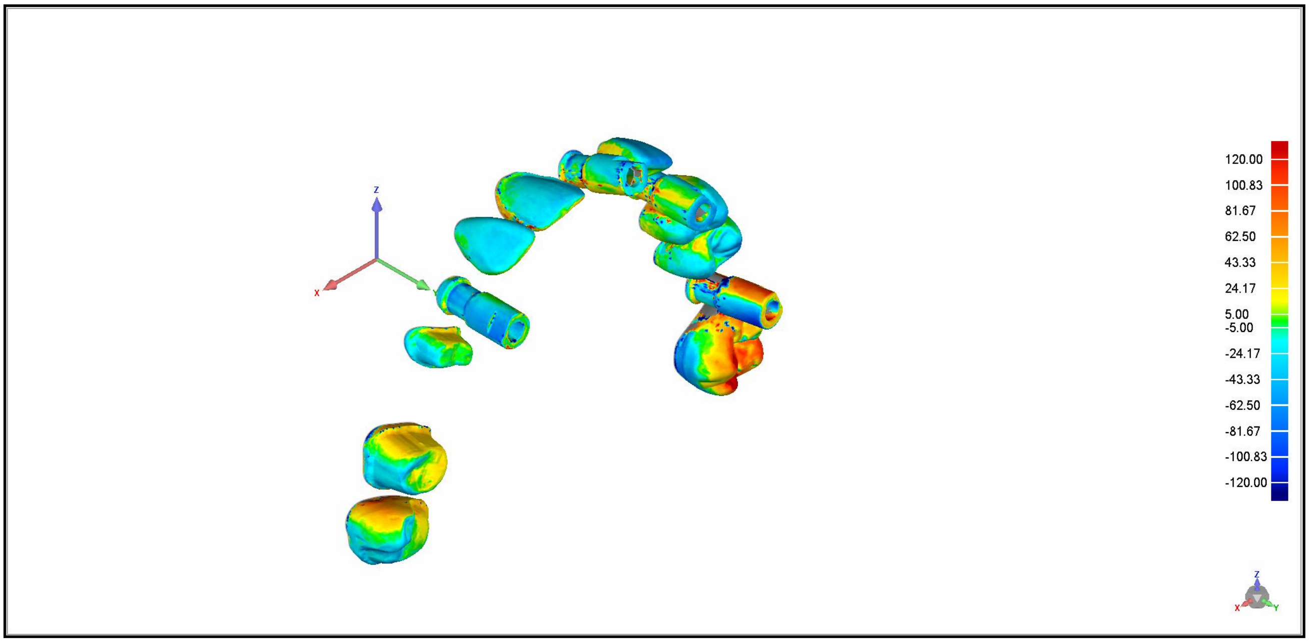

Predefinido: Frente

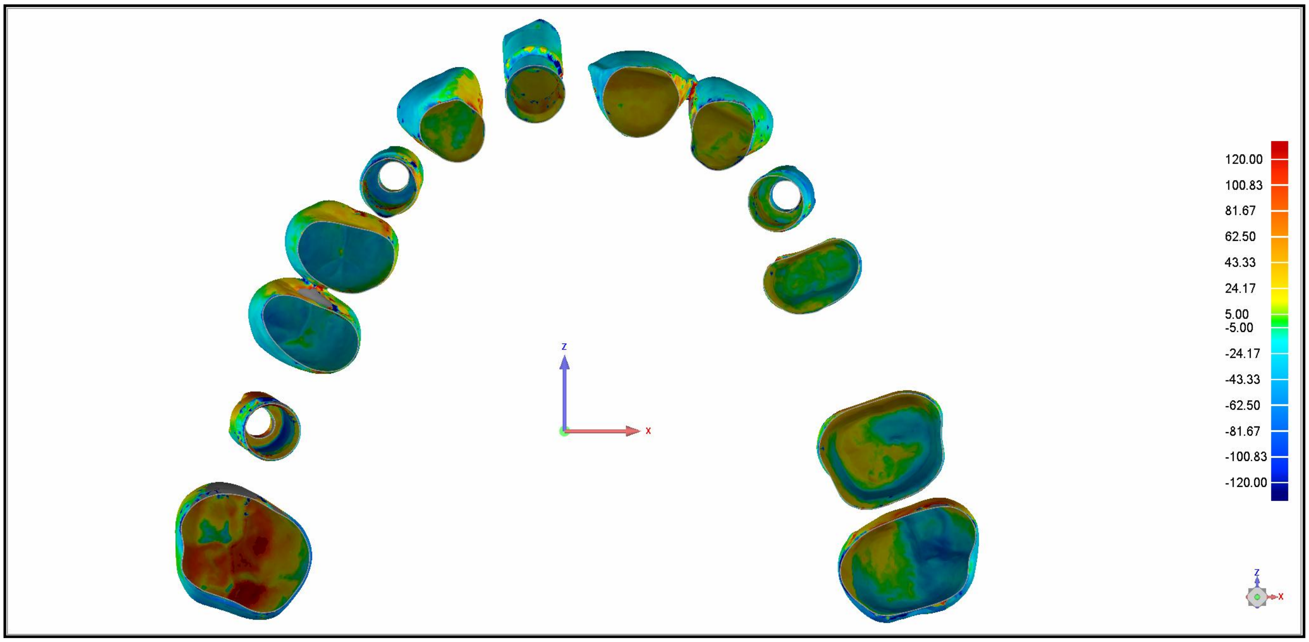

Predefinido: Atrás

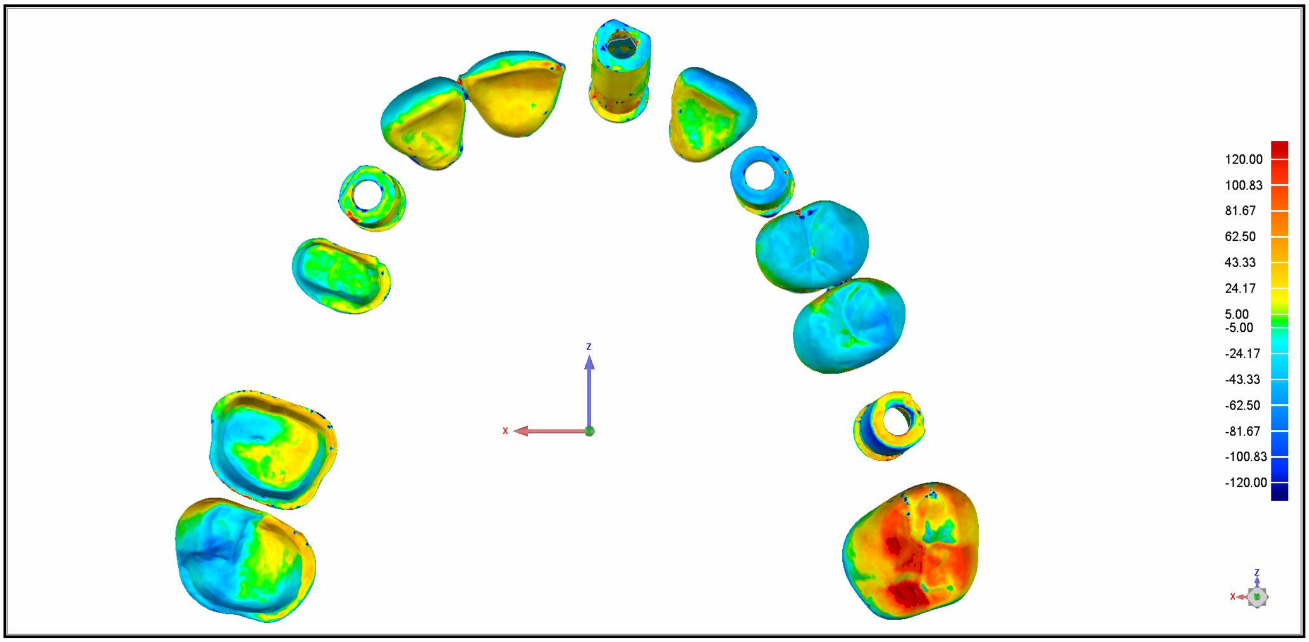

Predefinido: Izquierda

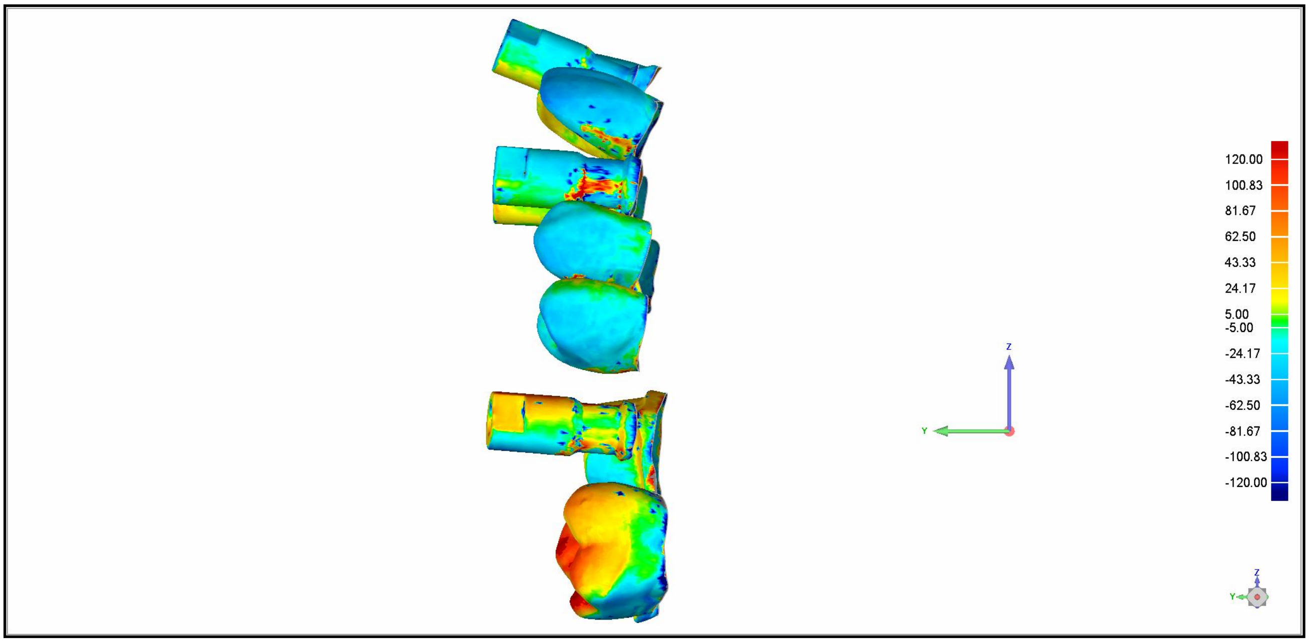

Predefinido: Derecha

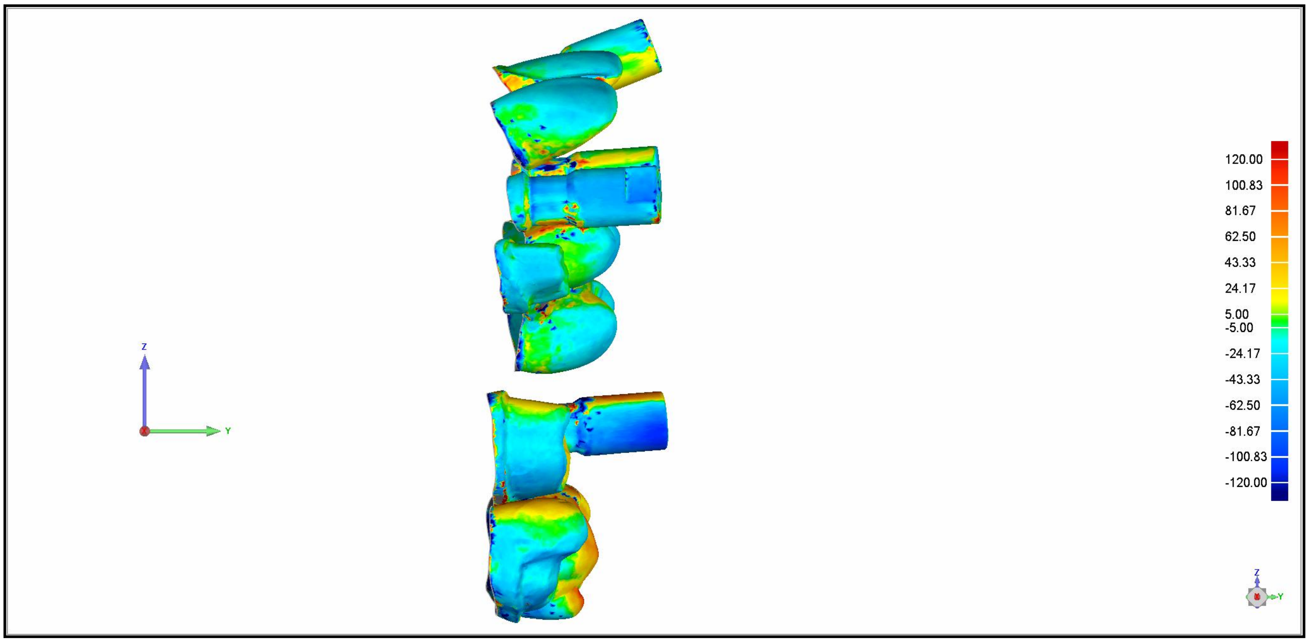

Predefinido: Superior

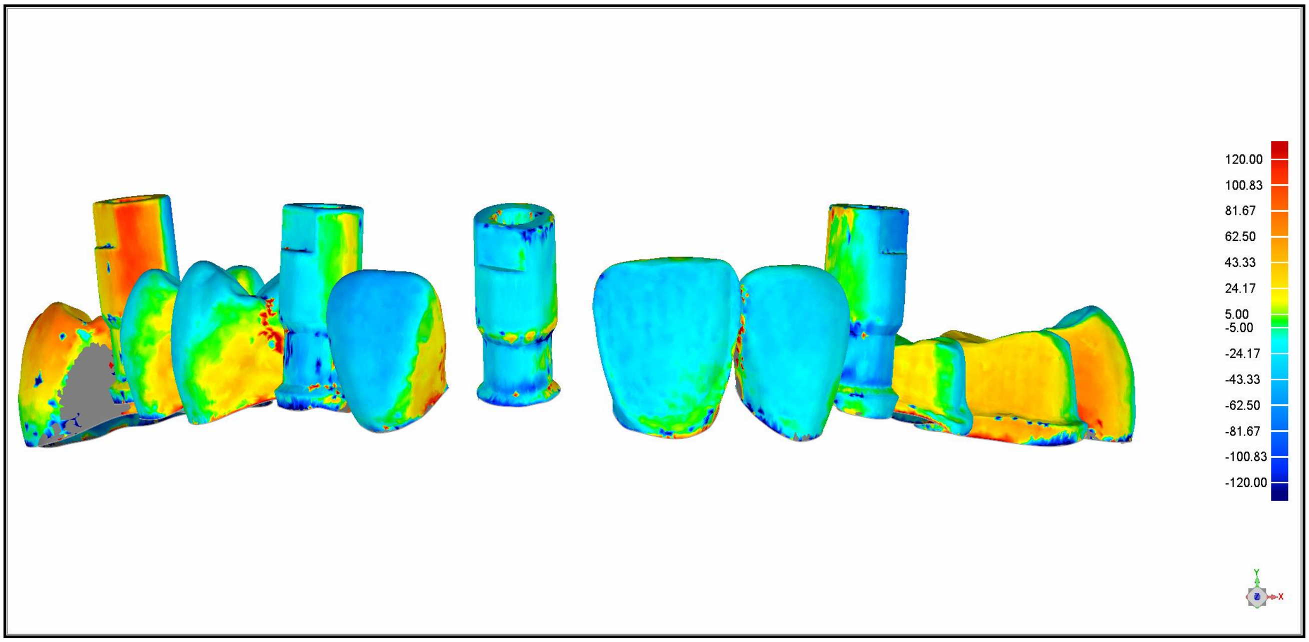

Predefinido: Inferior

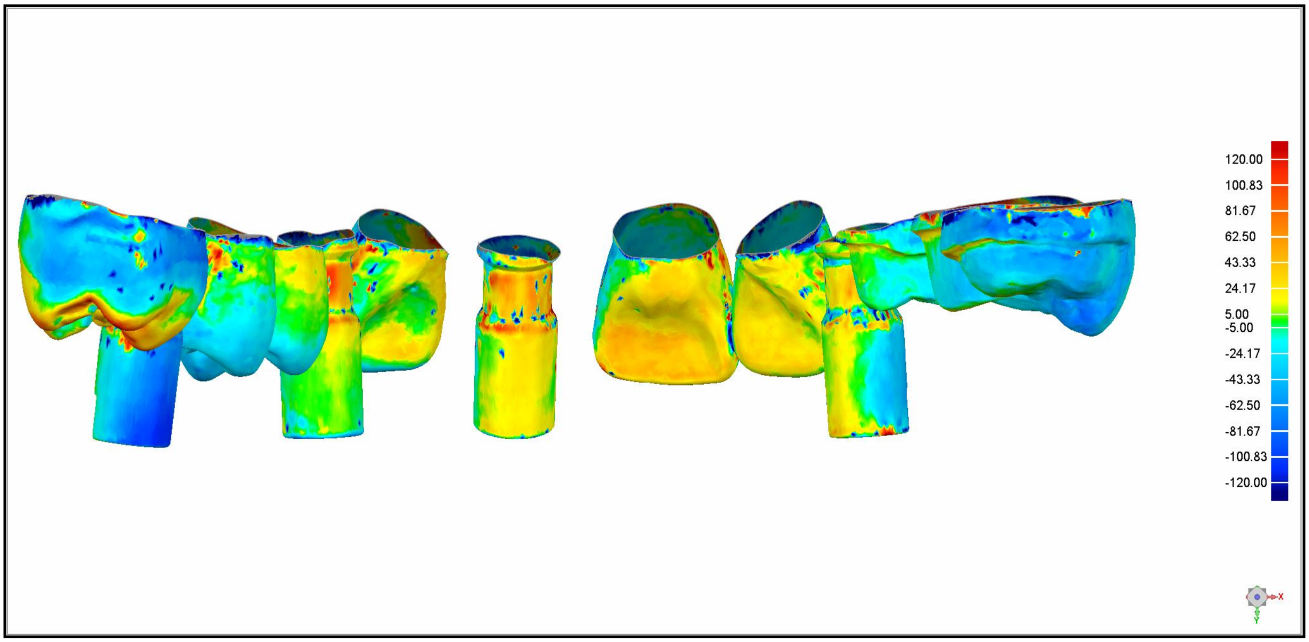

Ajuste de ubicación: Desviaciones superior e inferior

Unidades: u

Nombre	Desv	Estado	Superior Tol	Inferior Tol	Ref X	Ref Y	Ref Z	Radio	Desv X	Desv Y	Desv Z	Medido X	Medido Y	Medido Z	Dir. proy. X	Dir. proy. Y	Dir. proy. Z
Desv. inferior	-3129.83				-29896.55	26869.28	-6780.69	n/a	1460.12	1881.27	2030.95	-28436.43	28750.55	-4749.74	-0.47	-0.60	-0.65
Desv. superior	3135.48				28638.90	28169.90	-4177.71	n/a	2275.60	-891.77	-1964.09	30914.50	27278.13	-6141.80	0.73	-0.28	-0.63
