# Supplementary material for: Accuracy of four digital scanners according to scanning strategy in complete-arch impressions
Source: PLoS One. 2018 Sep 13;13(9):e0202916. doi: 10.1371/journal.pone.0202916 (PMC6136706; doi:10.1371/journal.pone.0202916)
Supplement: S7 Table — iTero (scanning strategy C). (ZIP) [file pone.0202916.s007.zip › S7/IT7C.pdf]

3D Comparación Resultados

Modelo referencia	MRC
Modelo test	IT7C
Nº de puntos de datos	79409
# Aislados	640

Tipo tolerancia	3D desviación
Unidades	u
Máx. crítico	120.00
Máx. nominal	2.00
Mín. nominal	-2.00
Mín. crítico	-120.00

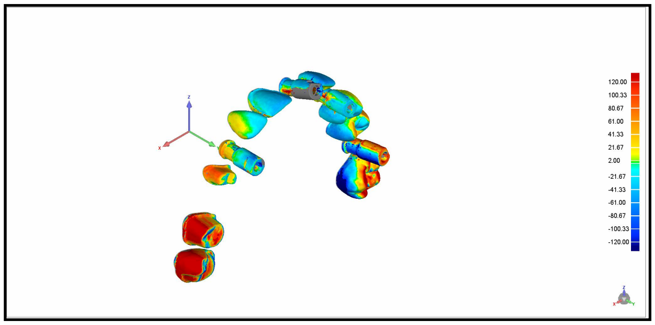

Desviación	
Desviación superior máx.	3078.35
Desviación inferior máx.	-3126.61
Desviación media	100.10 /-94.38
Desviación estándar	241.49

Distribución desviación

>=Min	<Max	# Puntos	%
-120.00	-100.33	1404	1.77
-100.33	-80.67	1837	2.31
-80.67	-61.00	3010	3.79
-61.00	-41.33	6034	7.60
-41.33	-21.67	9334	11.75
-21.67	-2.00	11182	14.08
-2.00	2.00	2206	2.78
2.00	21.67	10758	13.55
21.67	41.33	6671	8.40
41.33	61.00	4620	5.82
61.00	80.67	3379	4.26
80.67	100.33	2698	3.40
100.33	120.00	1935	2.44

Fuera del crítico superior	8285	10.43
Fuera del crítico inferior	6056	7.63

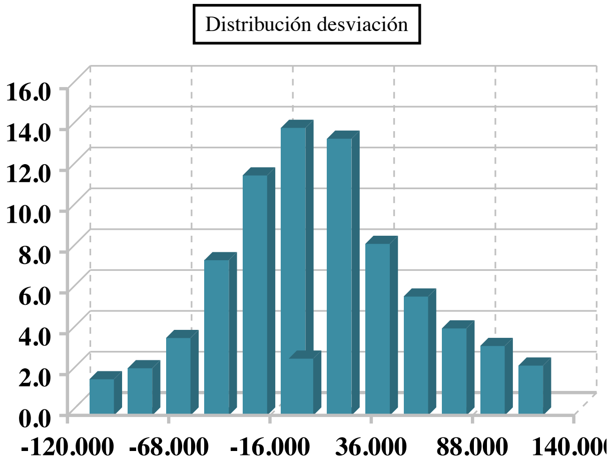

Desviaciones estándar

Distribución (+/-)	# Puntos	%
-6 * Desv. esténd.	586	0.74
-5 * Desv. esténd.	223	0.28
-4 * Desv. esténd.	237	0.30
-3 * Desv. esténd.	292	0.37
-2 * Desv. esténd.	907	1.14
-1 * Desv. esténd.	38885	48.97
1 * Desv. esténd.	35840	45.13
2 * Desv. esténd.	1261	1.59
3 * Desv. esténd.	280	0.35
4 * Desv. esténd.	214	0.27
5 * Desv. esténd.	259	0.33
6 * Desv. esténd.	425	0.54

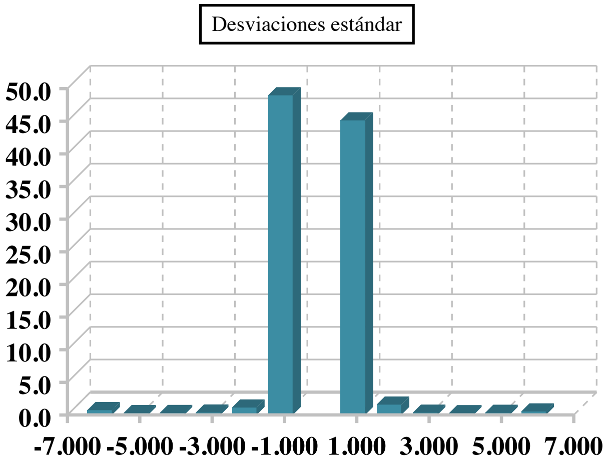

Predefinido: Isométrico

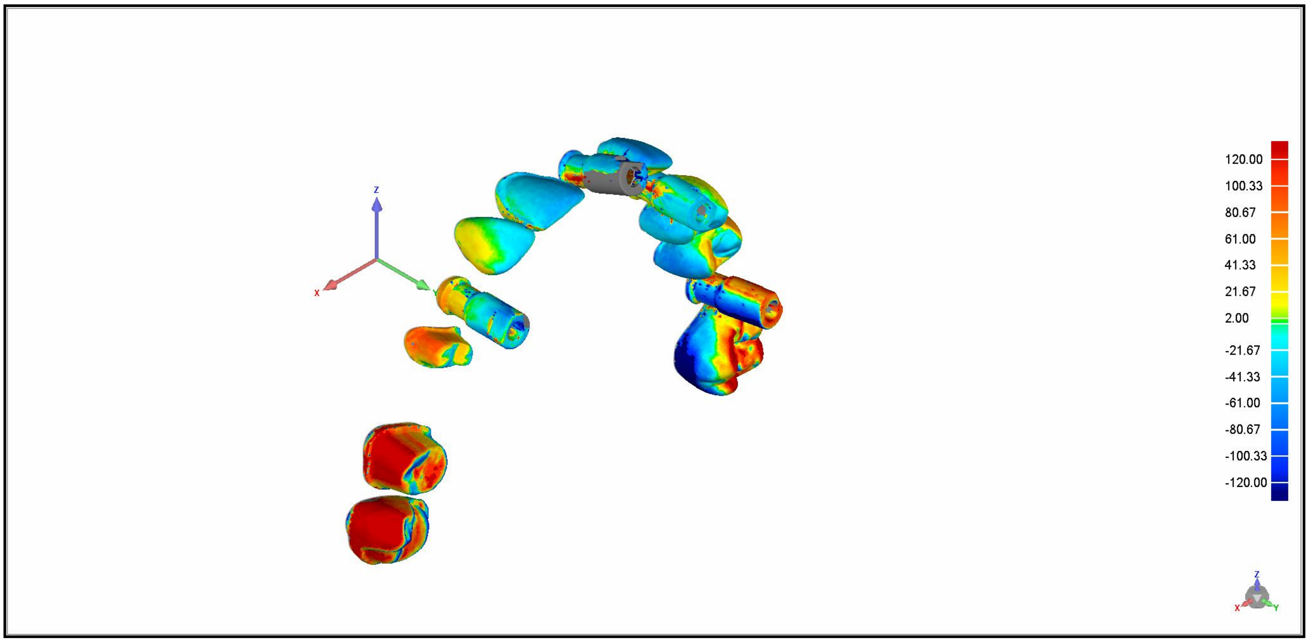

Predefinido: Frente

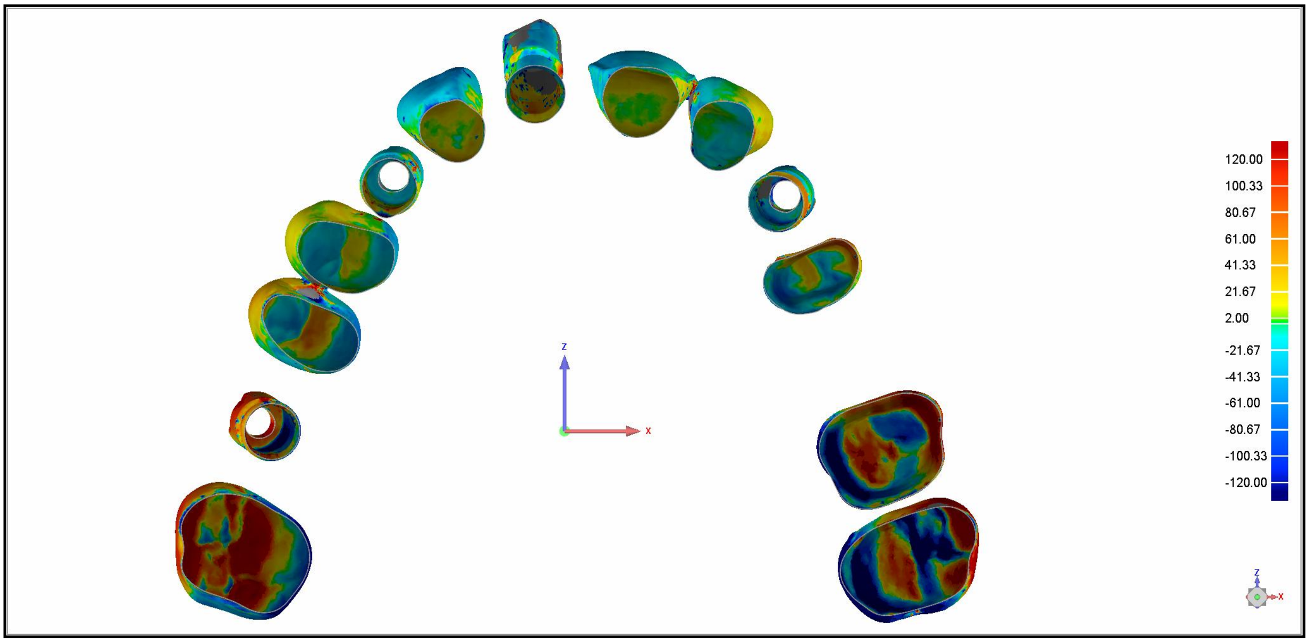

Predefinido: Atrás

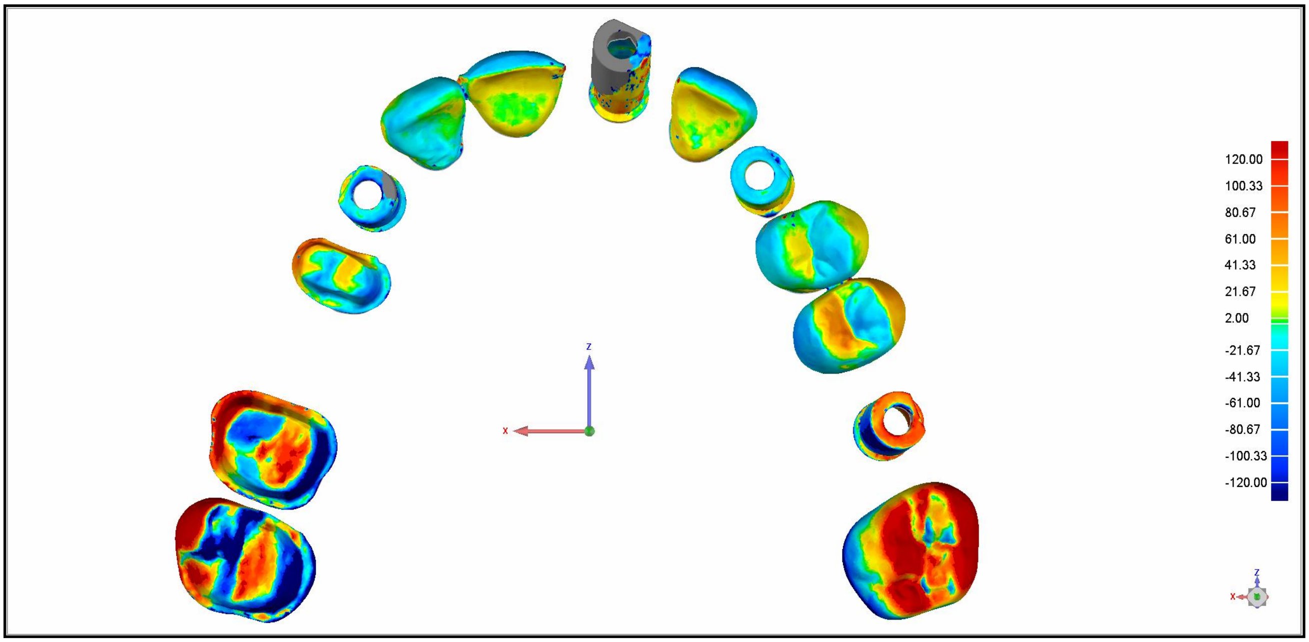

Predefinido: Izquierda

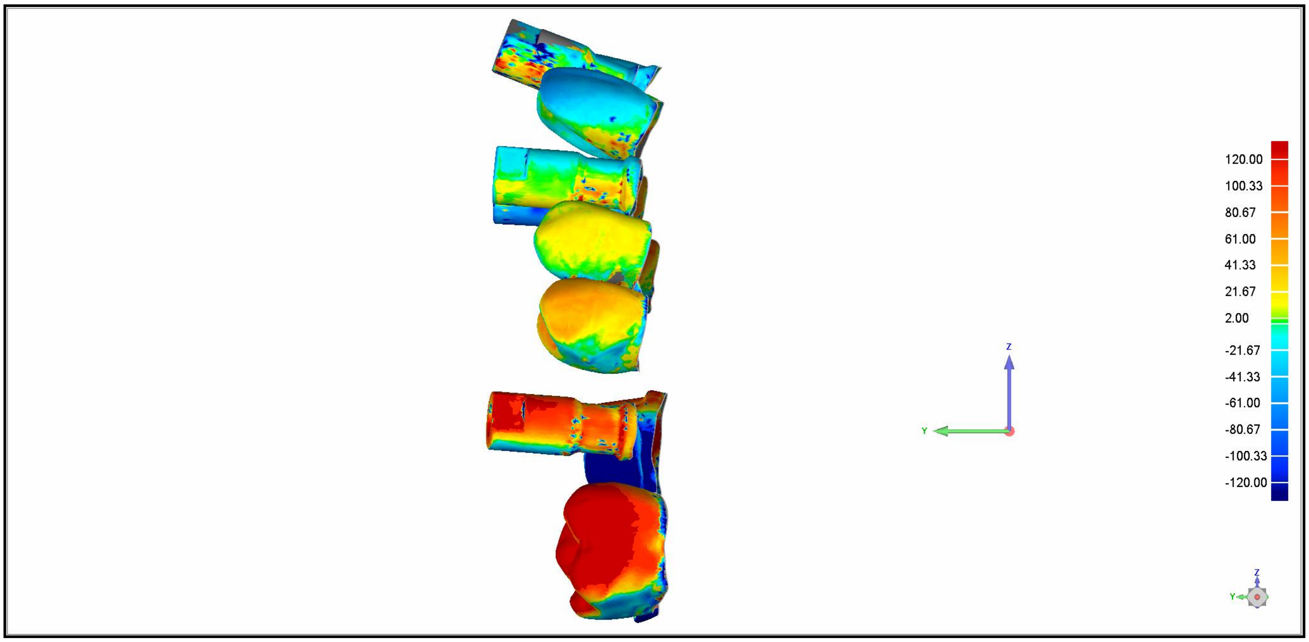

Predefinido: Derecha

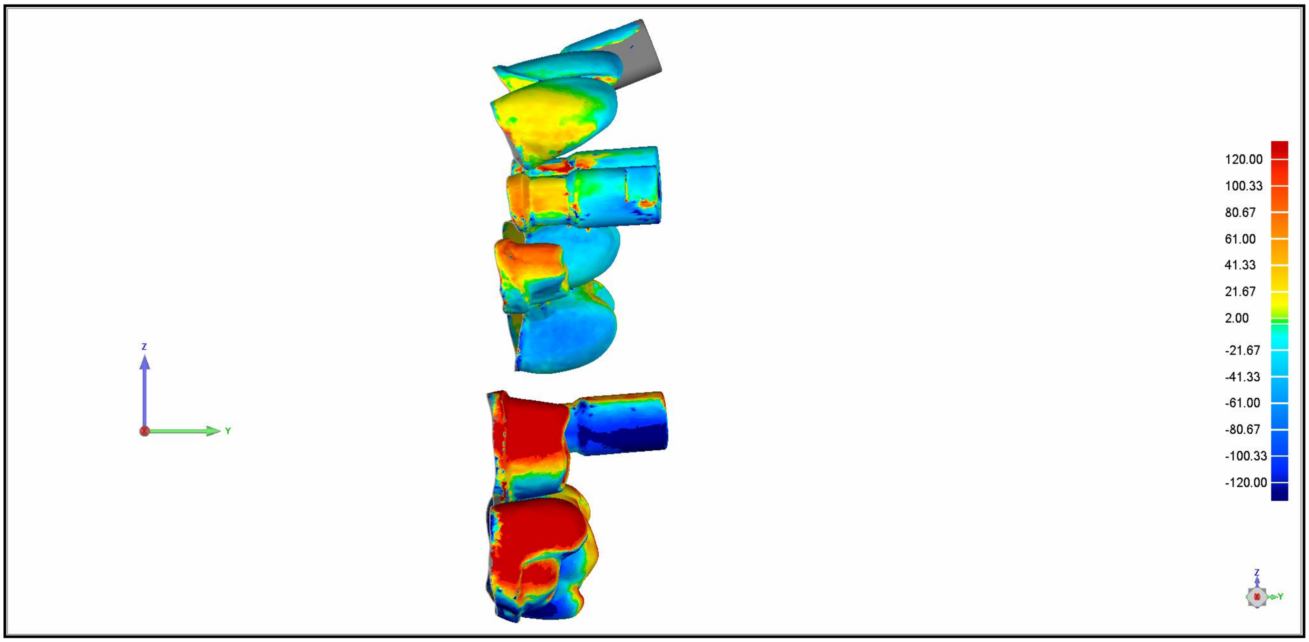

Predefinido: Superior

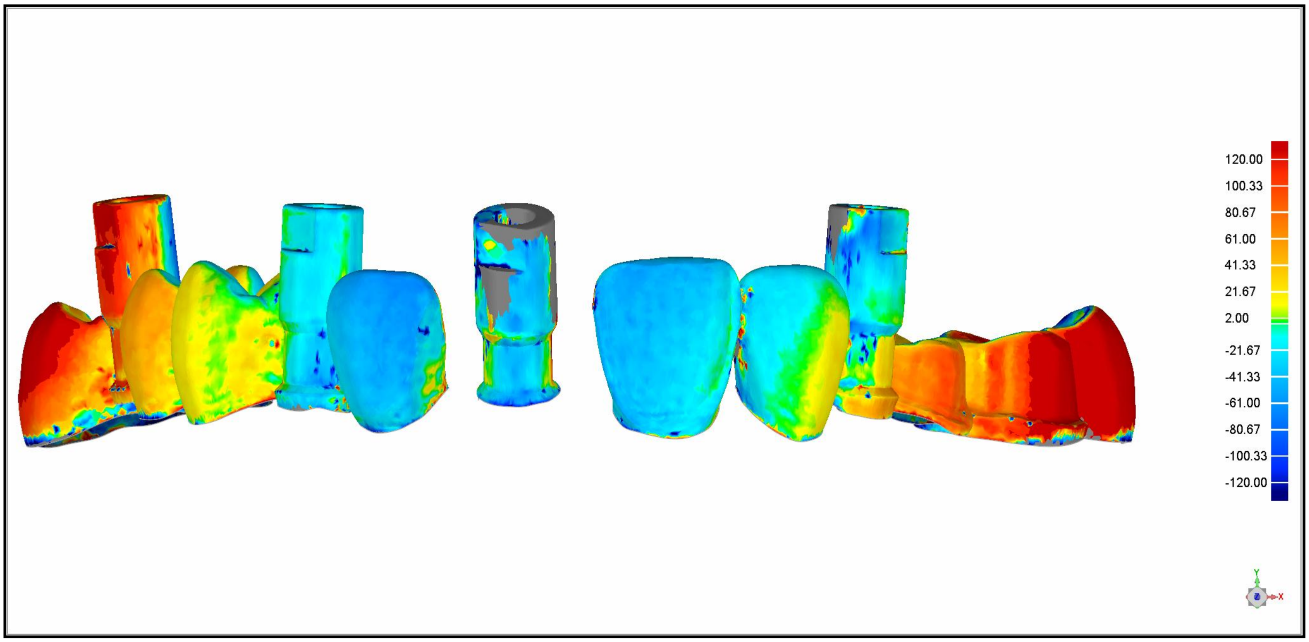

Predefinido: Inferior

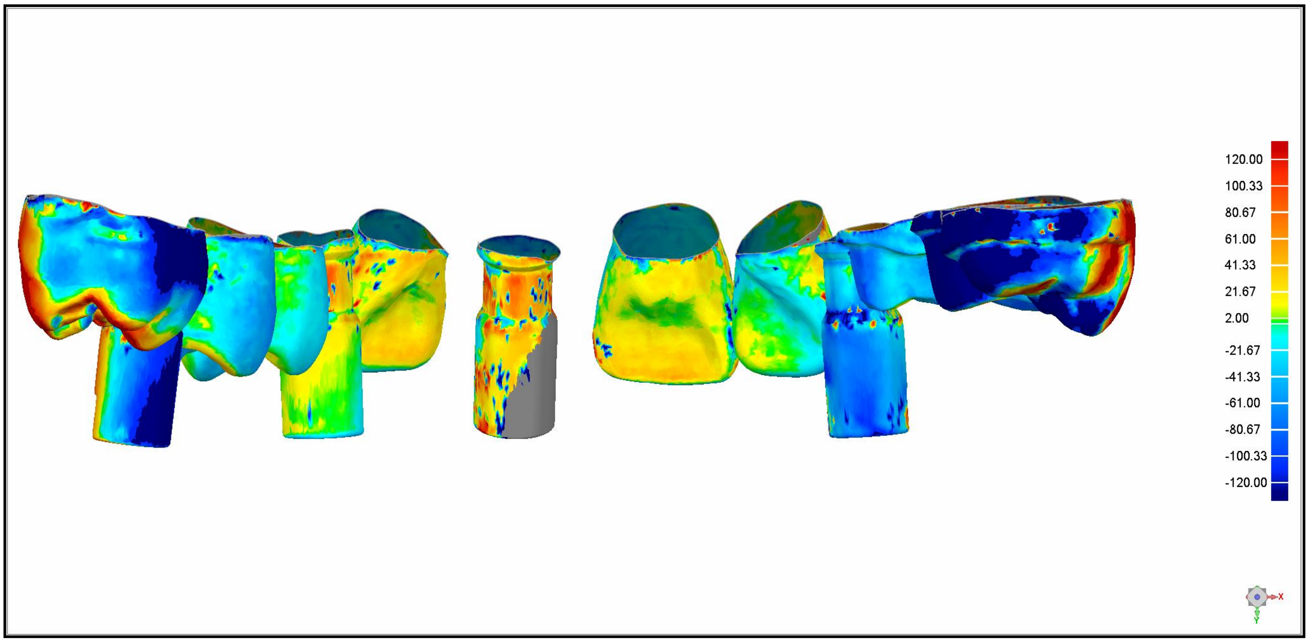

Geomagic Control™

Ajuste de ubicación: Desviaciones superior e inferior

Unidades: u

Nombre	Desv	Estado	Superior Tol	Inferior Tol	Ref X	Ref Y	Ref Z	Radio	Desv X	Desv Y	Desv Z	Medido X	Medido Y	Medido Z	Dir. proy. X	Dir. proy. Y	Dir. proy. Z
Desv. inferior	-3126.61				-20620.17	28746.73	-7981.87	n/a	-2357.48	23.87	2053.62	-22977.65	28770.60	-5928.25	0.75	-0.01	-0.66
Desv. superior	3078.35				-24812.05	31736.01	-4676.80	n/a	1369.87	258.27	2744.63	-23442.17	31994.28	-1932.18	0.45	0.08	0.89
