# Supplementary material for: Accuracy of four digital scanners according to scanning strategy in complete-arch impressions
Source: PLoS One. 2018 Sep 13;13(9):e0202916. doi: 10.1371/journal.pone.0202916 (PMC6136706; doi:10.1371/journal.pone.0202916)
Supplement: S8 Table — iTero (scanning strategy D). (ZIP) [file pone.0202916.s008.zip › S8/IT9D.pdf]

3D Comparación Resultados

Modelo referencia	MRC
Modelo test	IT9D
Nº de puntos de datos	77842
# Aislados	565

Tipo tolerancia	3D desviación
Unidades	u
Máx. crítico	120.00
Máx. nominal	6.00
Mín. nominal	-6.00
Mín. crítico	-120.00

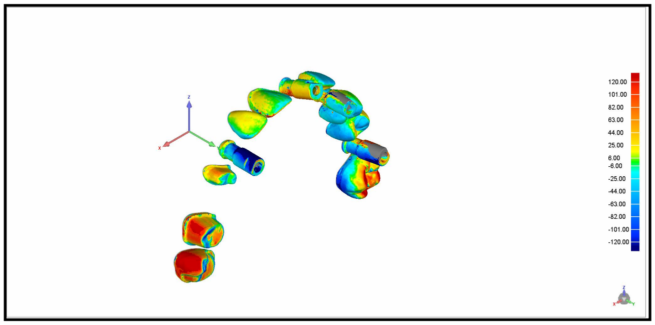

Desviación	
Desviación superior máx.	3145.24
Desviación inferior máx.	-3084.76
Desviación media	72.46 /-67.03
Desviación estándar	165.49

Distribución desviación

>=Min	<Max	# Puntos	%
-120.00	-101.00	1361	1.75
-101.00	-82.00	1676	2.15
-82.00	-63.00	2125	2.73
-63.00	-44.00	4370	5.61
-44.00	-25.00	8347	10.72
-25.00	-6.00	10560	13.57
-6.00	6.00	7696	9.89
6.00	25.00	10619	13.64
25.00	44.00	7306	9.39
44.00	63.00	5348	6.87
63.00	82.00	4393	5.64
82.00	101.00	3298	4.24
101.00	120.00	1728	2.22

Fuera del crítico superior	4585	5.89
Fuera del crítico inferior	4430	5.69

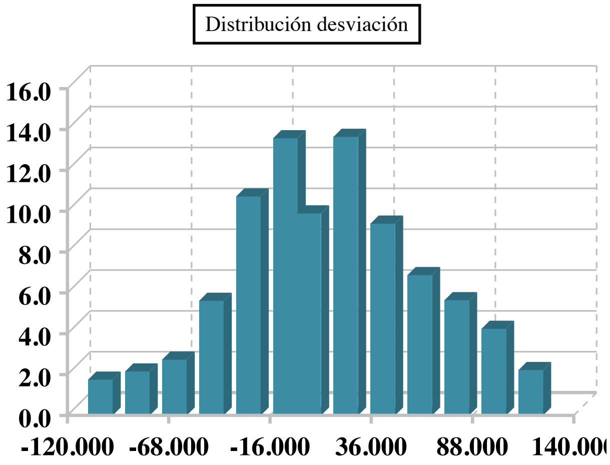

Desviaciones estándar

Distribución (+/-)	# Puntos	%
-6 * Desv. estánd.	380	0.49
-5 * Desv. estánd.	84	0.11
-4 * Desv. estánd.	143	0.18
-3 * Desv. estánd.	187	0.24
-2 * Desv. estánd.	1825	2.34
-1 * Desv. estánd.	38501	49.46
1 * Desv. estánd.	34473	44.29
2 * Desv. estánd.	1287	1.65
3 * Desv. estánd.	193	0.25
4 * Desv. estánd.	175	0.22
5 * Desv. estánd.	137	0.18
6 * Desv. estánd.	457	0.59

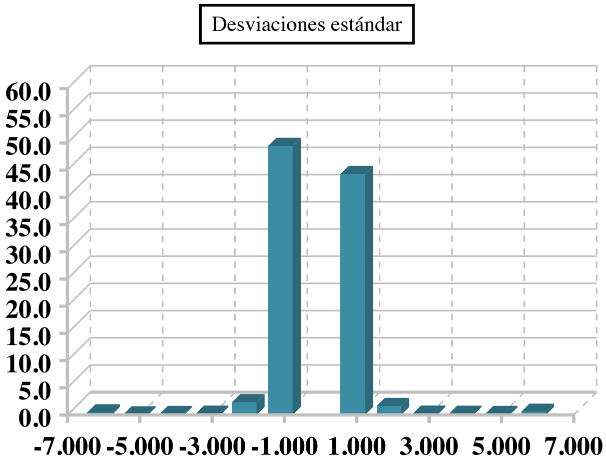

Predefinido: Isométrico

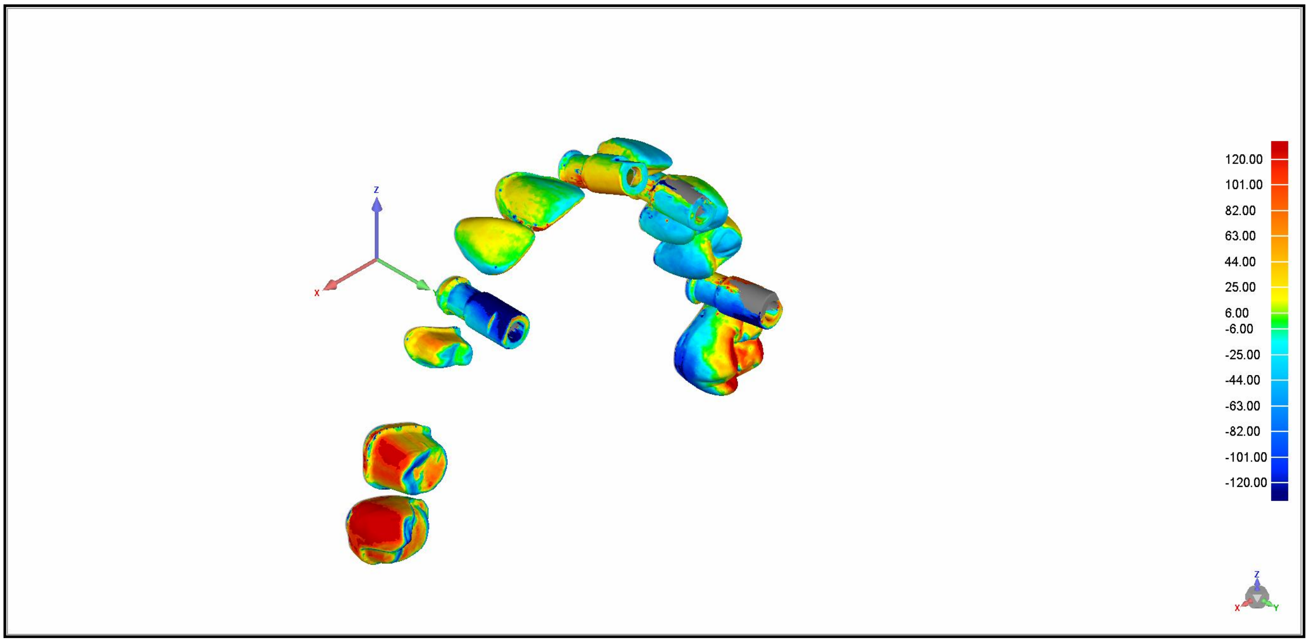

Predefinido: Frente

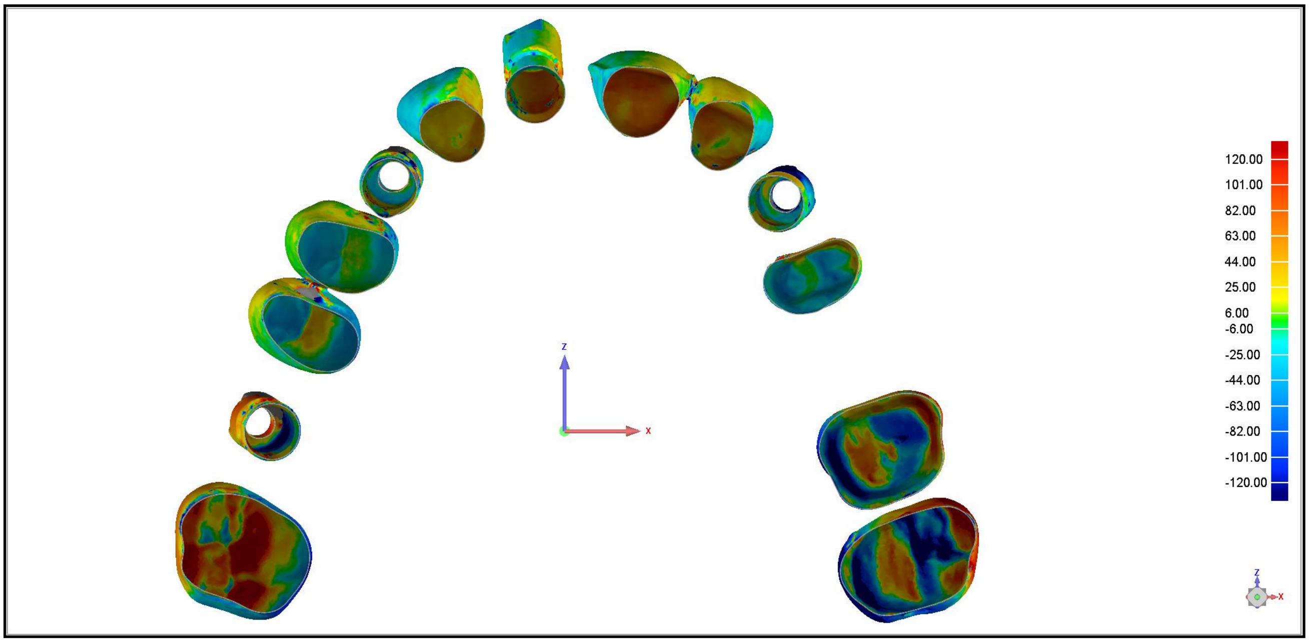

Predefinido: Atrás

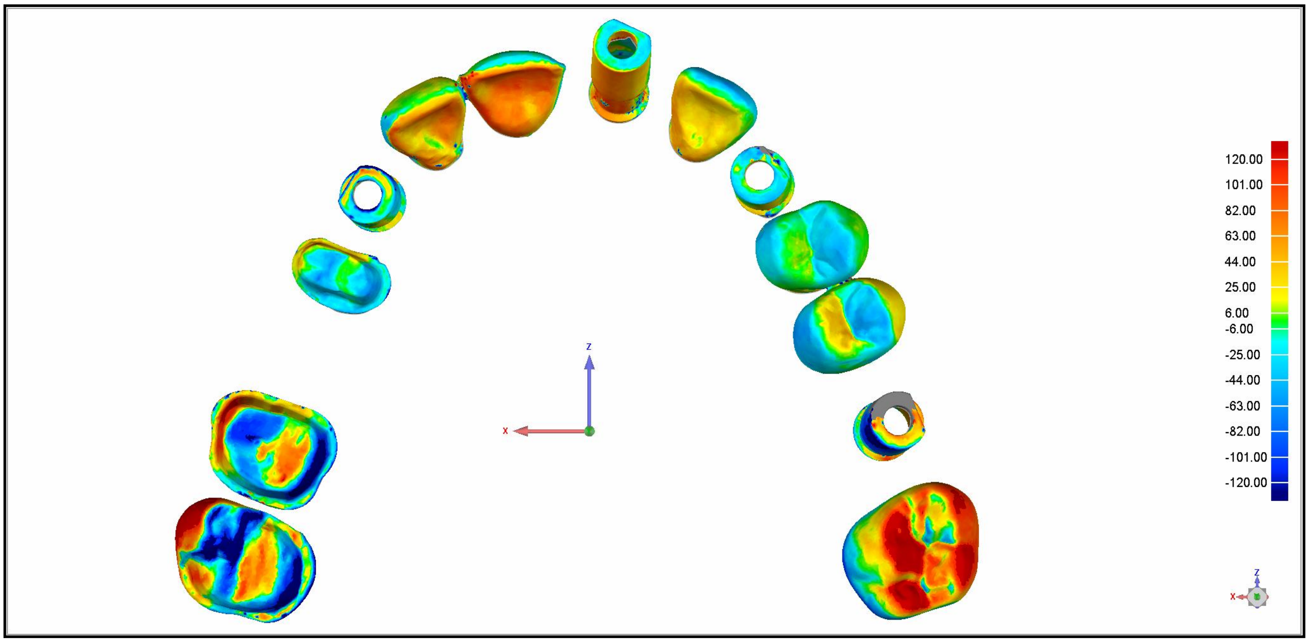

Predefinido: Izquierda

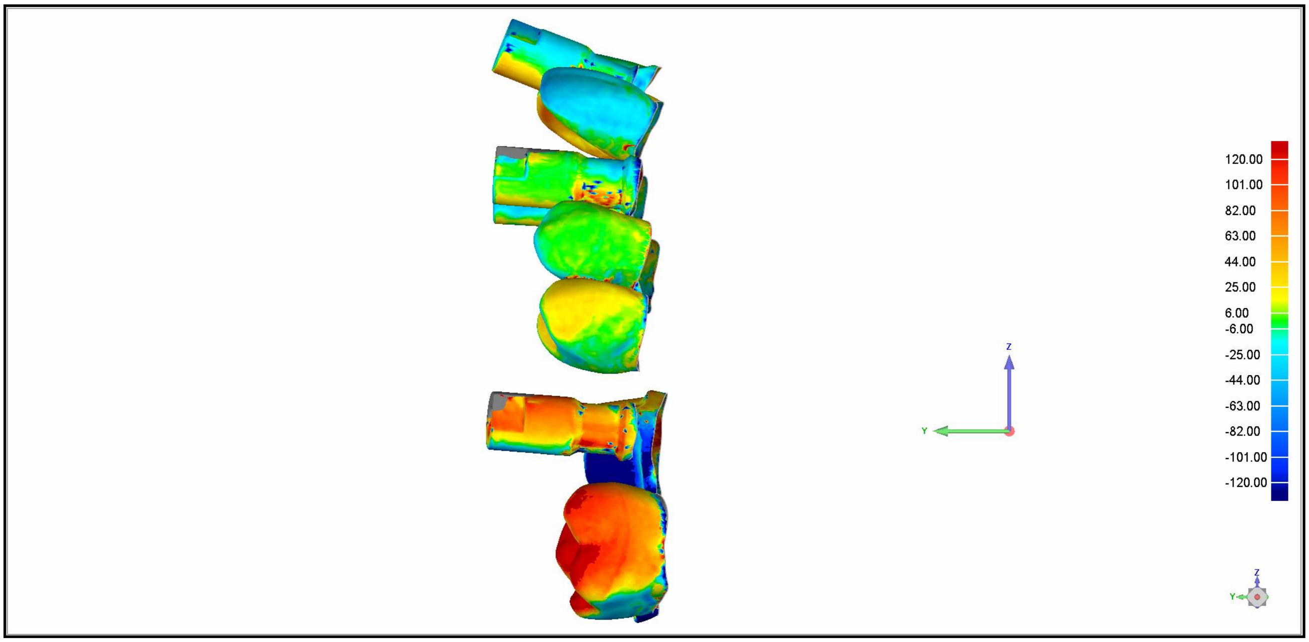

Predefinido: Derecha

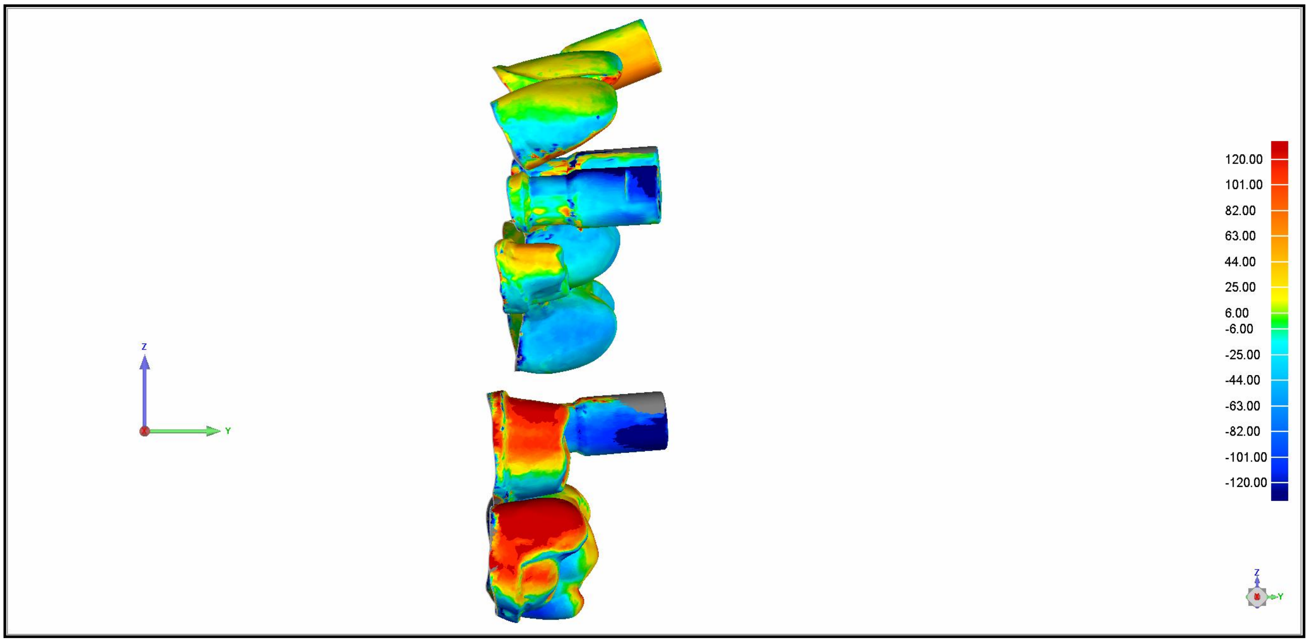

Predefinido: Superior

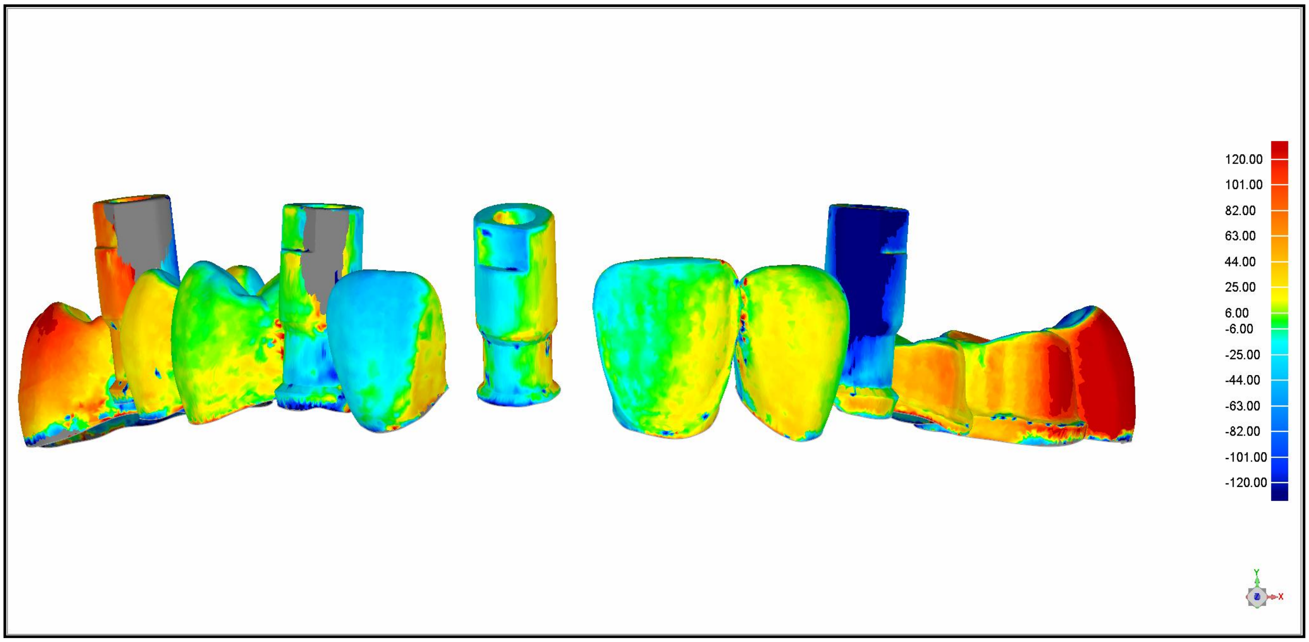

Predefinido: Inferior

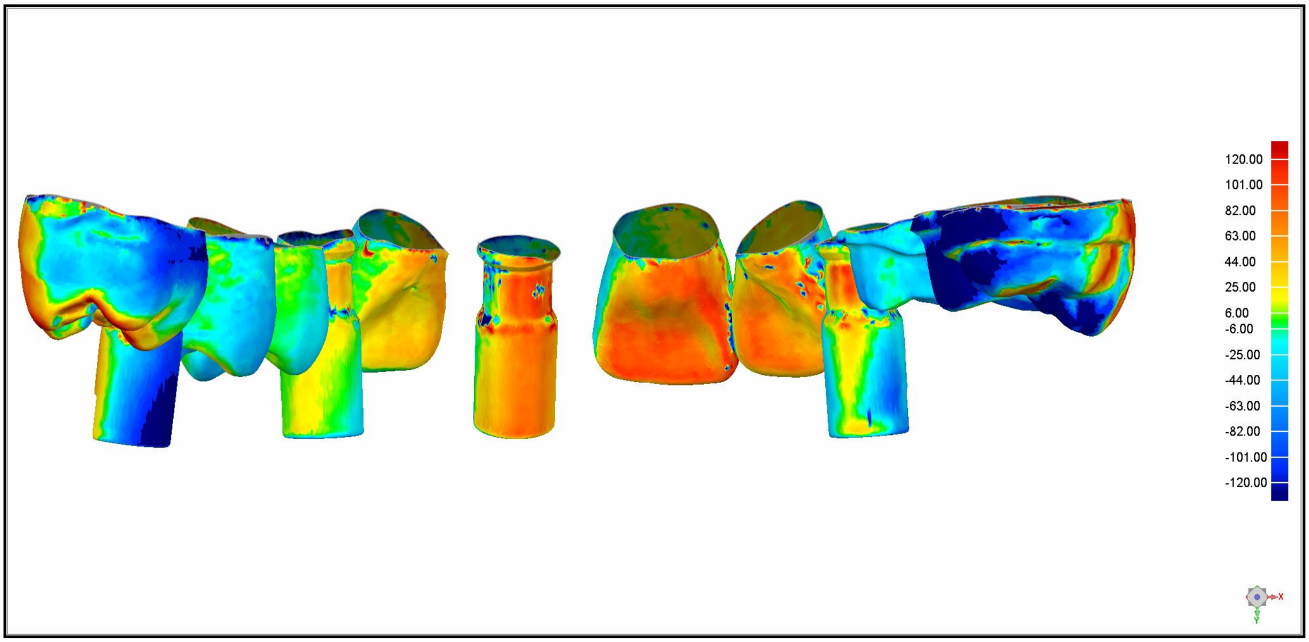

Ajuste de ubicación: Desviaciones superior e inferior

Unidades: u

Nombre	Desv	Estado	Superior Tol	Inferior Tol	Ref X	Ref Y	Ref Z	Radio	Desv X	Desv Y	Desv Z	Medido X	Medido Y	Medido Z	Dir. proy. X	Dir. proy. Y	Dir. proy. Z
Desv. inferior	-3084.76				-29208.33	26961.25	-11988.49	n/a	2659.56	359.17	-1521.02	-26548.77	27320.42	-13509.51	-0.86	-0.12	0.49
Desv. superior	3145.24				-29896.55	26869.28	-6780.69	n/a	2366.00	929.90	1851.99	-27530.55	27799.18	-4928.70	0.75	0.30	0.59
