# Supplementary material for: Accuracy of four digital scanners according to scanning strategy in complete-arch impressions
Source: PLoS One. 2018 Sep 13;13(9):e0202916. doi: 10.1371/journal.pone.0202916 (PMC6136706; doi:10.1371/journal.pone.0202916)
Supplement: S8 Table — iTero (scanning strategy D). (ZIP) [file pone.0202916.s008.zip › S8/IT5D.pdf]

3D Comparación Resultados

Modelo referencia	MRC
Modelo test	IT5D
Nº de puntos de datos	82072
# Aislados	646

Tipo tolerancia	3D desviación
Unidades	u
Máx. crítico	120.00
Máx. nominal	6.00
Mín. nominal	-6.00
Mín. crítico	-120.00

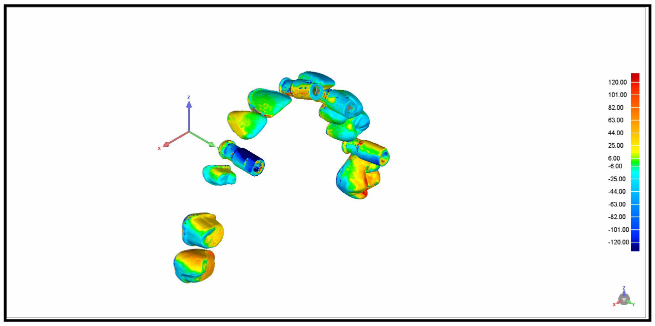

Desviación	
Desviación superior máx.	3154.97
Desviación inferior máx.	-3152.97
Desviación media	81.84 /-76.90
Desviación estándar	246.49

Distribución desviación

>=Min	<Max	# Puntos	%
-120.00	-101.00	880	1.07
-101.00	-82.00	1485	1.81
-82.00	-63.00	2267	2.76
-63.00	-44.00	4757	5.80
-44.00	-25.00	10750	13.10
-25.00	-6.00	16346	19.92
-6.00	6.00	8987	10.95
6.00	25.00	12636	15.40
25.00	44.00	8909	10.86
44.00	63.00	4393	5.35
63.00	82.00	1879	2.29
82.00	101.00	931	1.13
101.00	120.00	548	0.67

Fuera del crítico superior	2878	3.51
Fuera del crítico inferior	4426	5.39

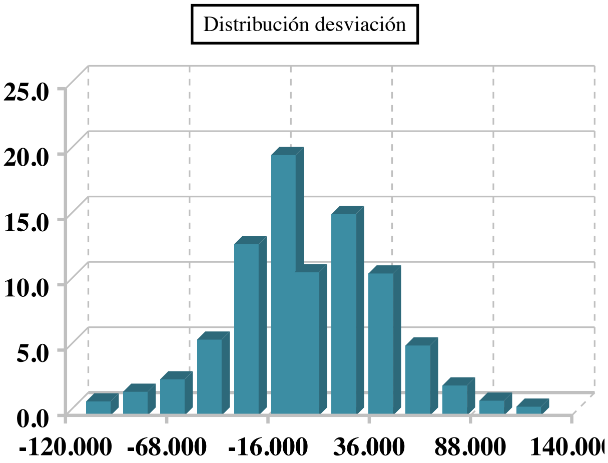

Desviaciones estándar

Distribución (+/-)	# Puntos	%
-6 * Desv. estánd.	509	0.62
-5 * Desv. estánd.	249	0.30
-4 * Desv. estánd.	272	0.33
-3 * Desv. estánd.	318	0.39
-2 * Desv. estánd.	729	0.89
-1 * Desv. estánd.	38662	47.11
1 * Desv. estánd.	39506	48.14
2 * Desv. estánd.	473	0.58
3 * Desv. estánd.	268	0.33
4 * Desv. estánd.	238	0.29
5 * Desv. estánd.	250	0.30
6 * Desv. estánd.	598	0.73

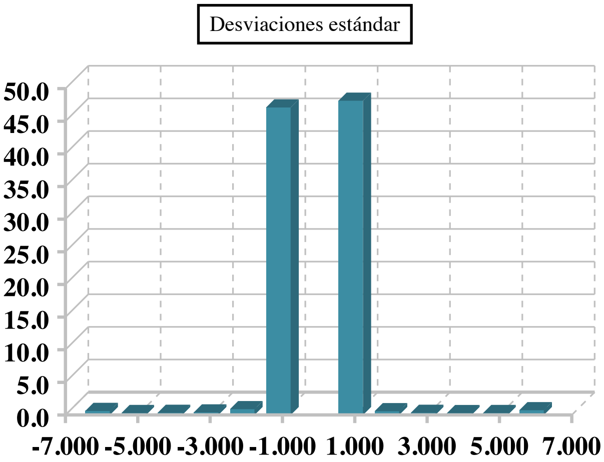

Predefinido: Isométrico

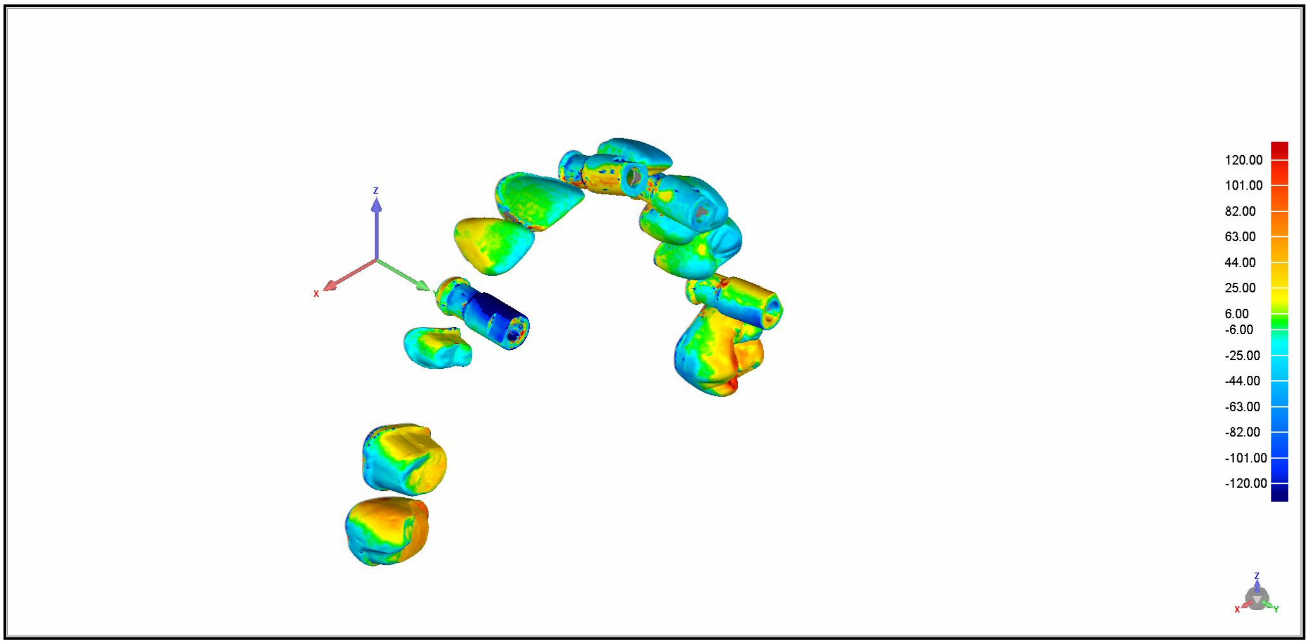

Predefinido: Frente

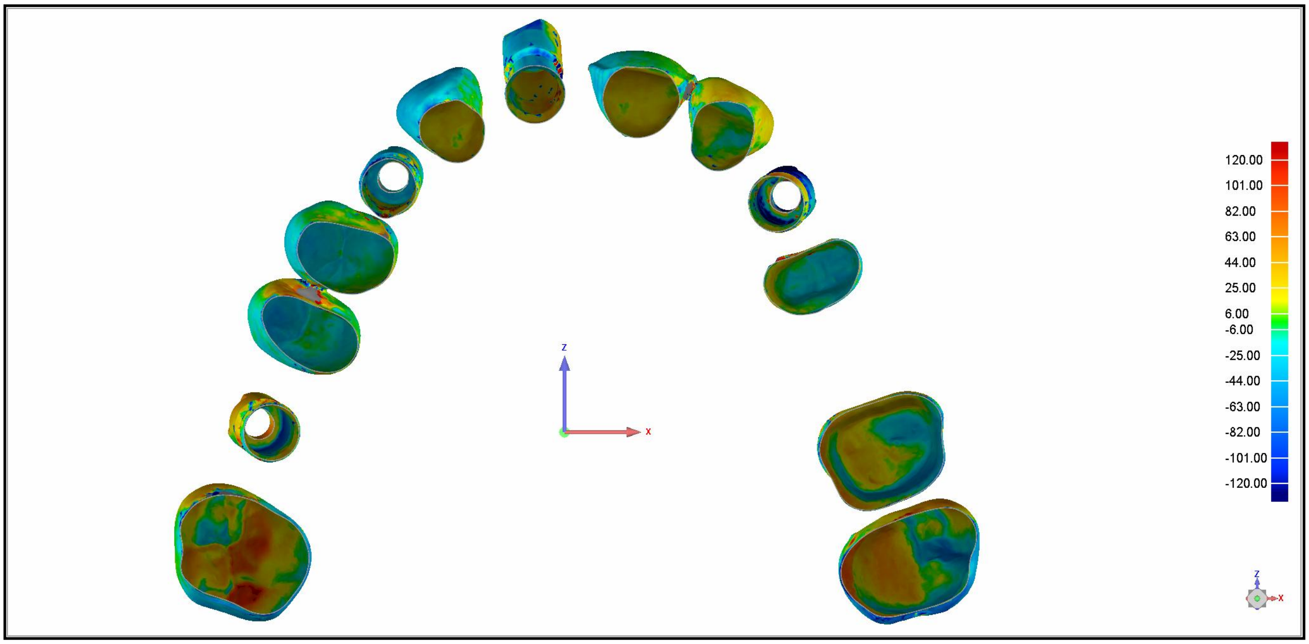

Predefinido: Atrás

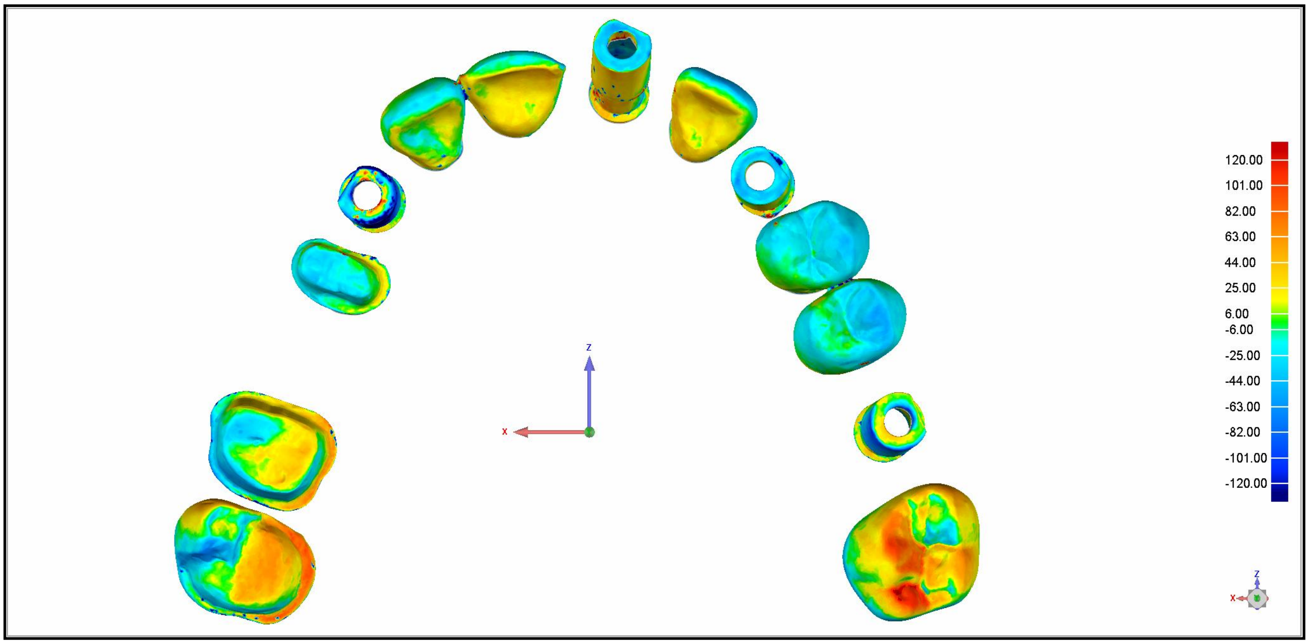

Predefinido: Izquierda

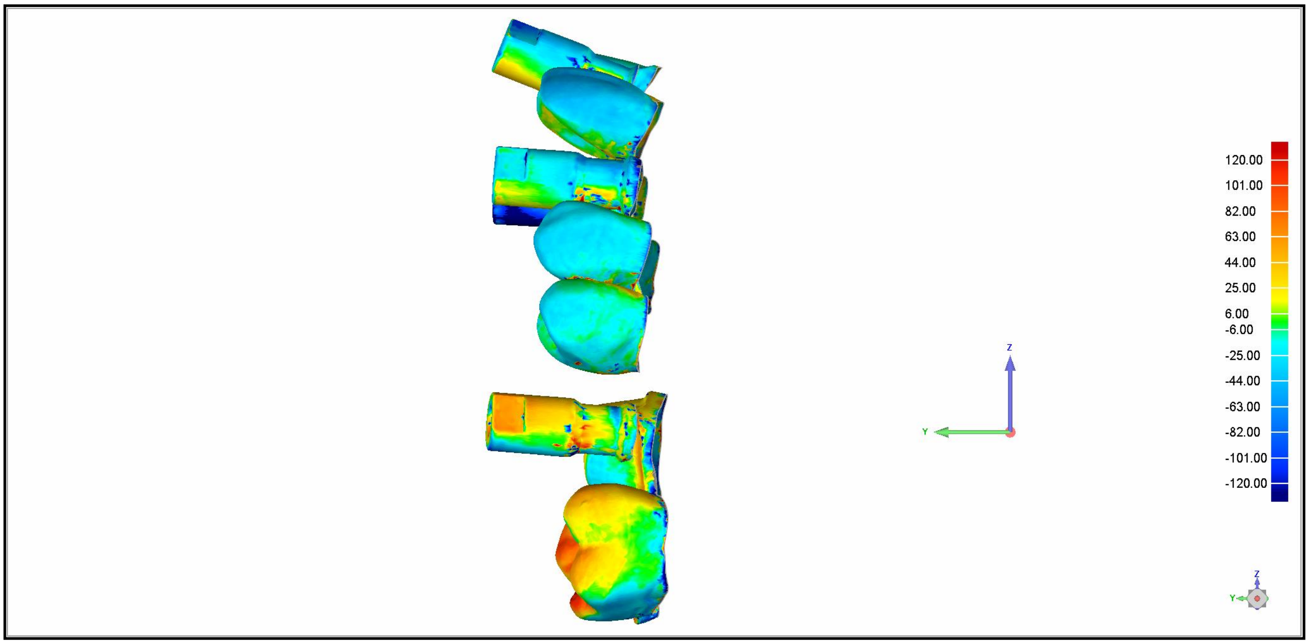

Predefinido: Derecha

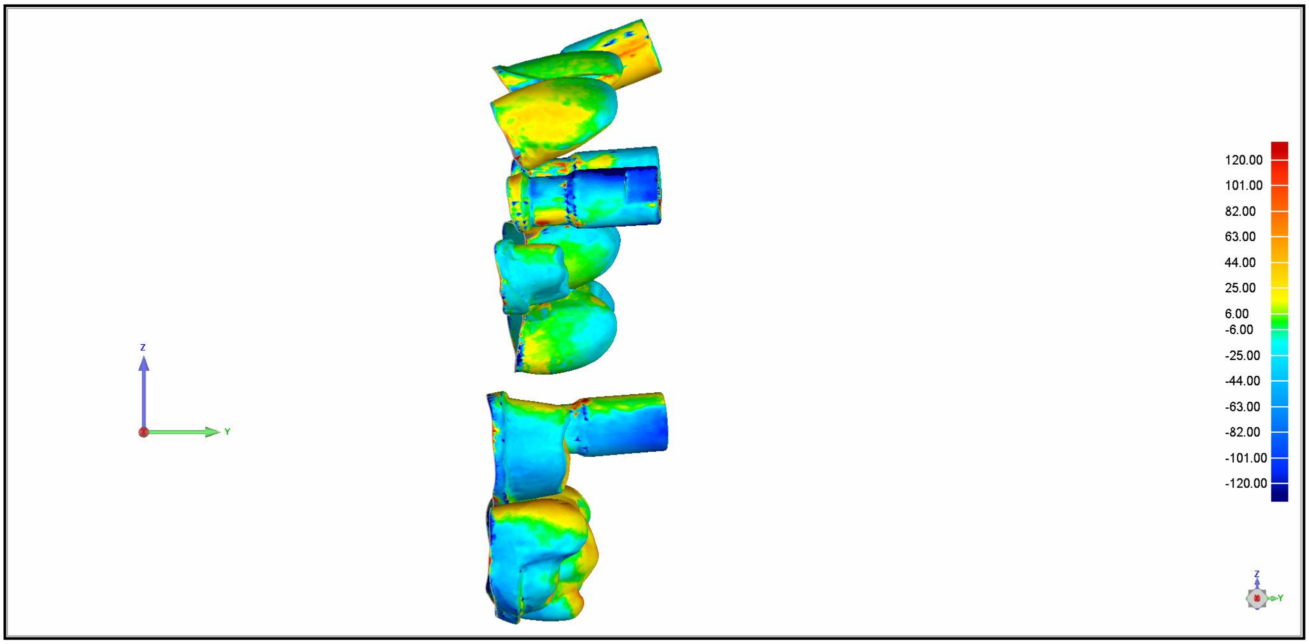

Predefinido: Superior

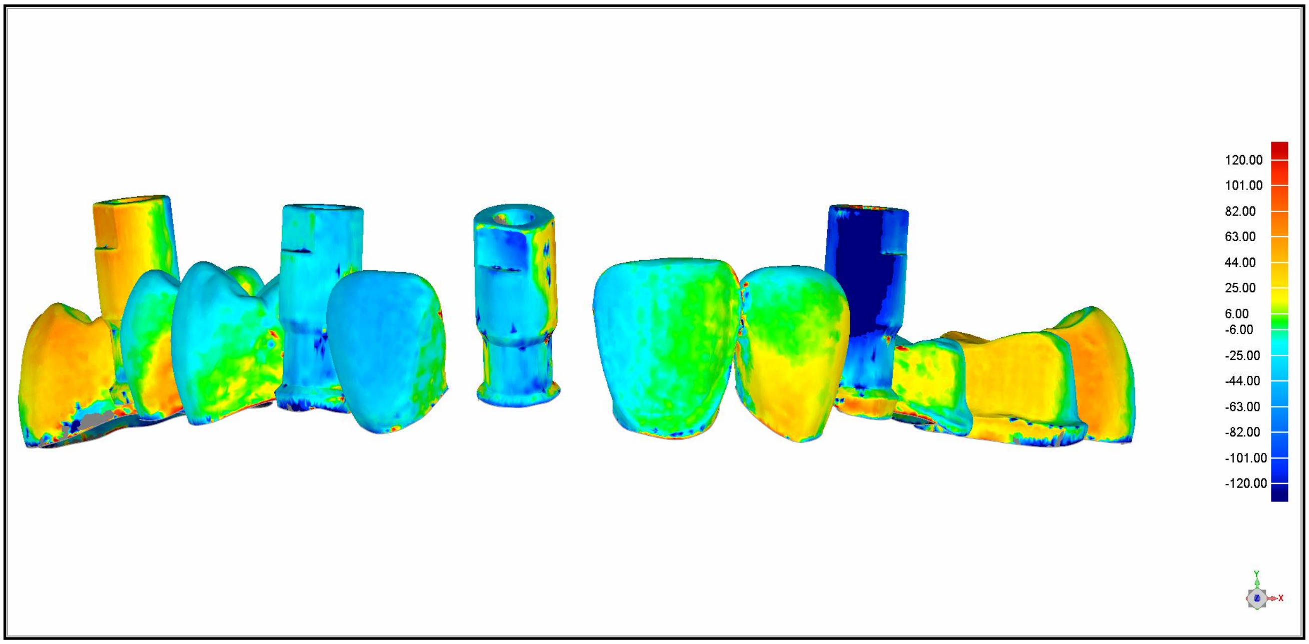

Predefinido: Inferior

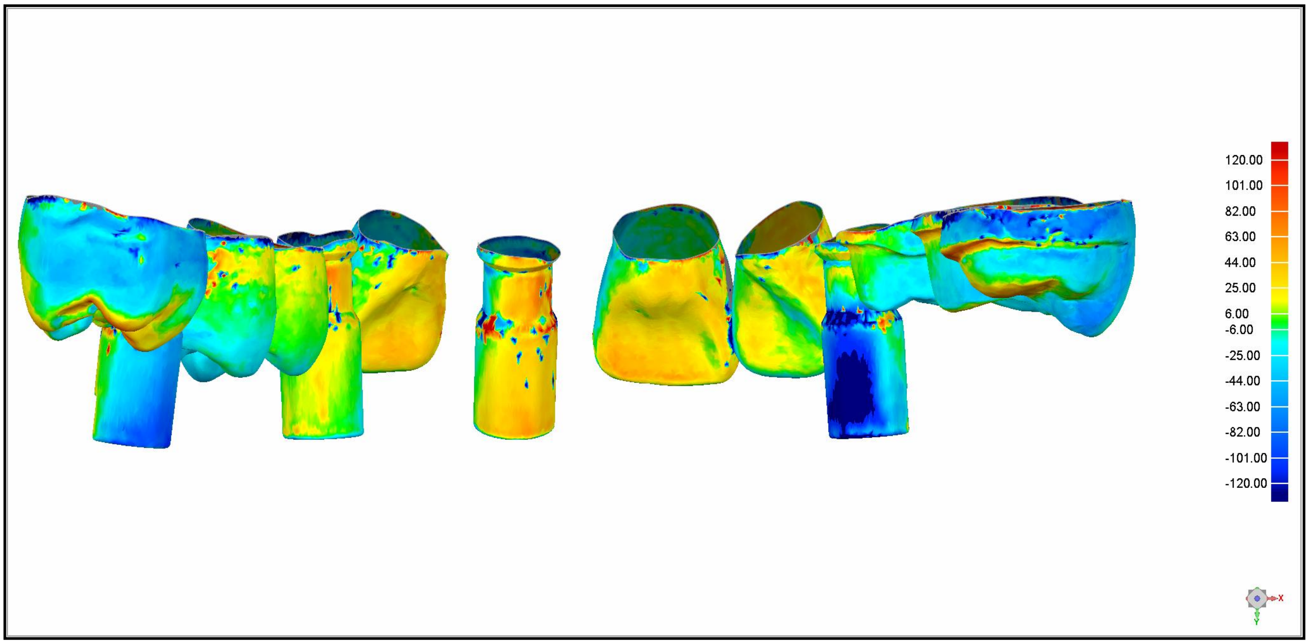

Ajuste de ubicación: Desviaciones superior e inferior

Unidades: u

Nombre	Desv	Estado	Superior Tol	Inferior Tol	Ref X	Ref Y	Ref Z	Radio	Desv X	Desv Y	Desv Z	Medido X	Medido Y	Medido Z	Dir. proy. X	Dir. proy. Y	Dir. proy. Z
Desv. inferior	-3152.97				16989.02	37628.06	17251.36	n/a	-474.93	2870.73	1214.32	16514.09	40498.80	18465.68	0.15	-0.91	-0.39
Desv. superior	3154.97				-21620.21	33965.87	5419.07	n/a	-980.73	809.07	-2887.46	-22600.94	34774.94	2531.61	-0.31	0.26	-0.92
