# Supplementary material for: Accuracy of four digital scanners according to scanning strategy in complete-arch impressions
Source: PLoS One. 2018 Sep 13;13(9):e0202916. doi: 10.1371/journal.pone.0202916 (PMC6136706; doi:10.1371/journal.pone.0202916)
Supplement: S8 Table — iTero (scanning strategy D). (ZIP) [file pone.0202916.s008.zip › S8/IT4D.pdf]

3D Comparación Resultados

Modelo referencia	MRC
Modelo test	IT4D
Nº de puntos de datos	77792
# Aislados	561

Tipo tolerancia	3D desviación
Unidades	u
Máx. crítico	120.00
Máx. nominal	5.00
Mín. nominal	-5.00
Mín. crítico	-120.00

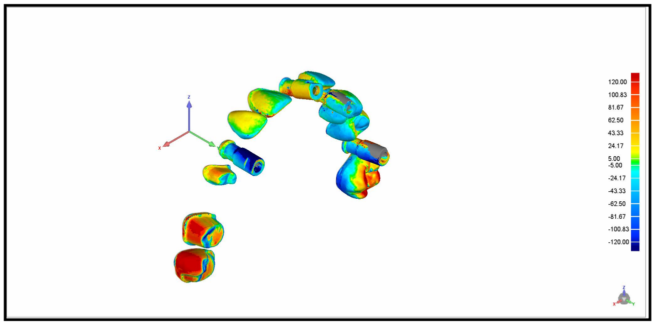

Desviación	
Desviación superior máx.	2553.33
Desviación inferior máx.	-2786.84
Desviación media	71.08 /-65.48
Desviación estándar	157.17

Distribución desviación

>=Min	<Max	# Puntos	%
-120.00	-100.83	1297	1.67
-100.83	-81.67	1778	2.29
-81.67	-62.50	2321	2.98
-62.50	-43.33	5081	6.53
-43.33	-24.17	8592	11.04
-24.17	-5.00	10672	13.72
-5.00	5.00	6339	8.15
5.00	24.17	10994	14.13
24.17	43.33	7220	9.28
43.33	62.50	5518	7.09
62.50	81.67	4493	5.78
81.67	100.83	2943	3.78
100.83	120.00	1571	2.02

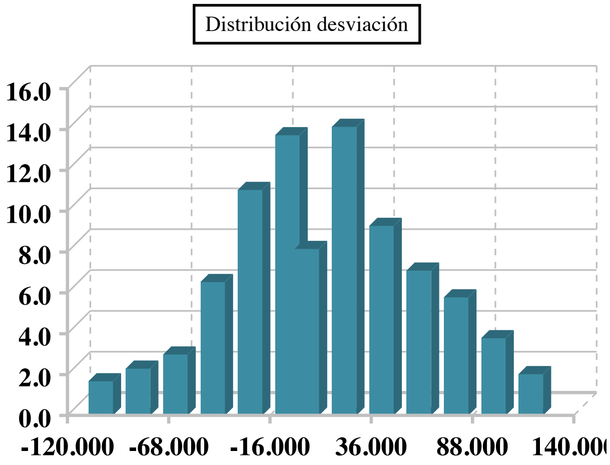

Fuera del crítico superior	4499	5.78
Fuera del crítico inferior	4474	5.75

Desviaciones estándar

Distribución (+/-)	# Puntos	%
-6 * Desv. estánd.	363	0.47
-5 * Desv. estánd.	75	0.10
-4 * Desv. estánd.	133	0.17
-3 * Desv. estánd.	193	0.25
-2 * Desv. estánd.	2257	2.90
-1 * Desv. estánd.	37844	48.65
1 * Desv. estánd.	34498	44.35
2 * Desv. estánd.	1477	1.90
3 * Desv. estánd.	182	0.23
4 * Desv. estánd.	162	0.21
5 * Desv. estánd.	146	0.19
6 * Desv. estánd.	462	0.59

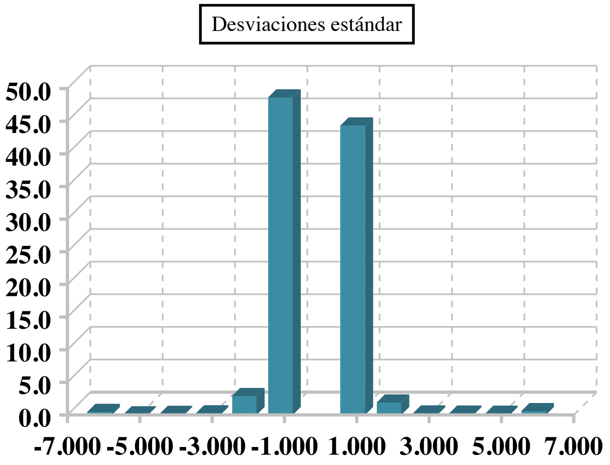

Predefinido: Isométrico

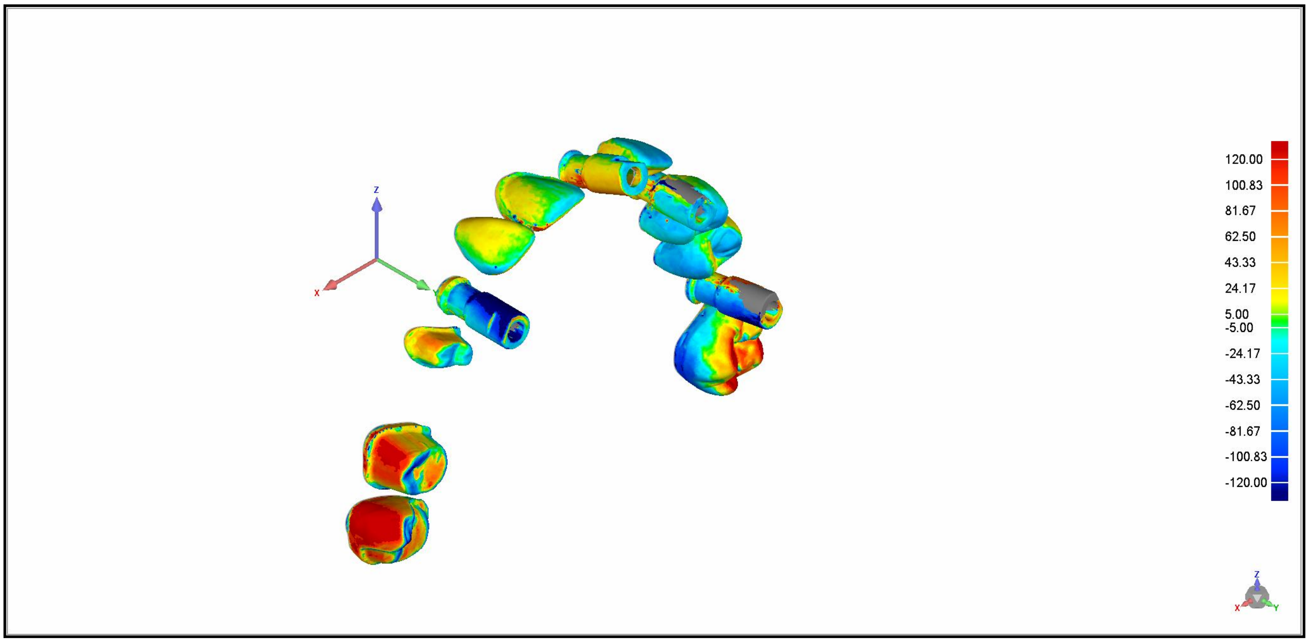

Predefinido: Frente

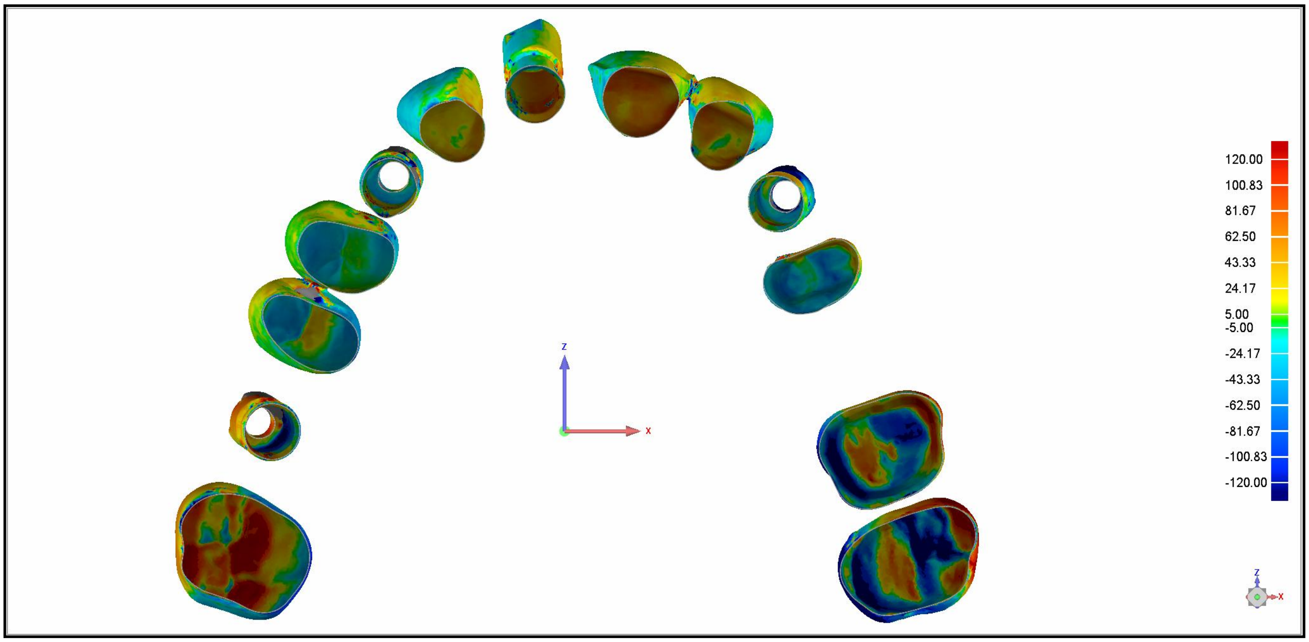

Predefinido: Atrás

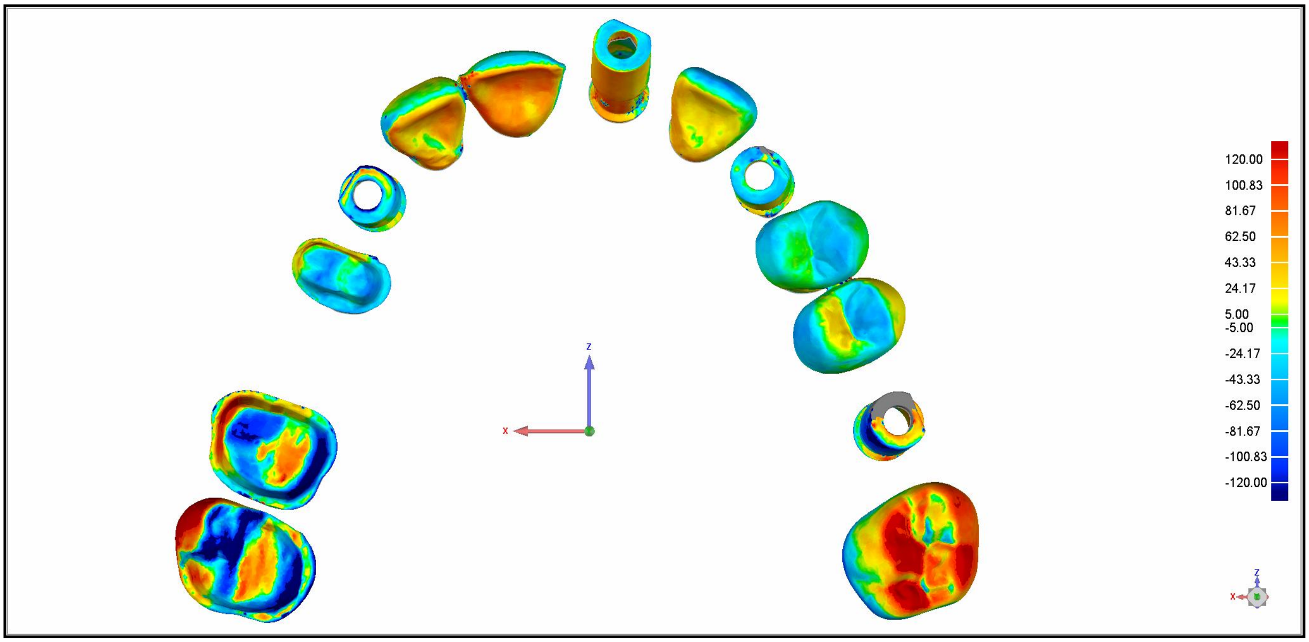

Predefinido: Izquierda

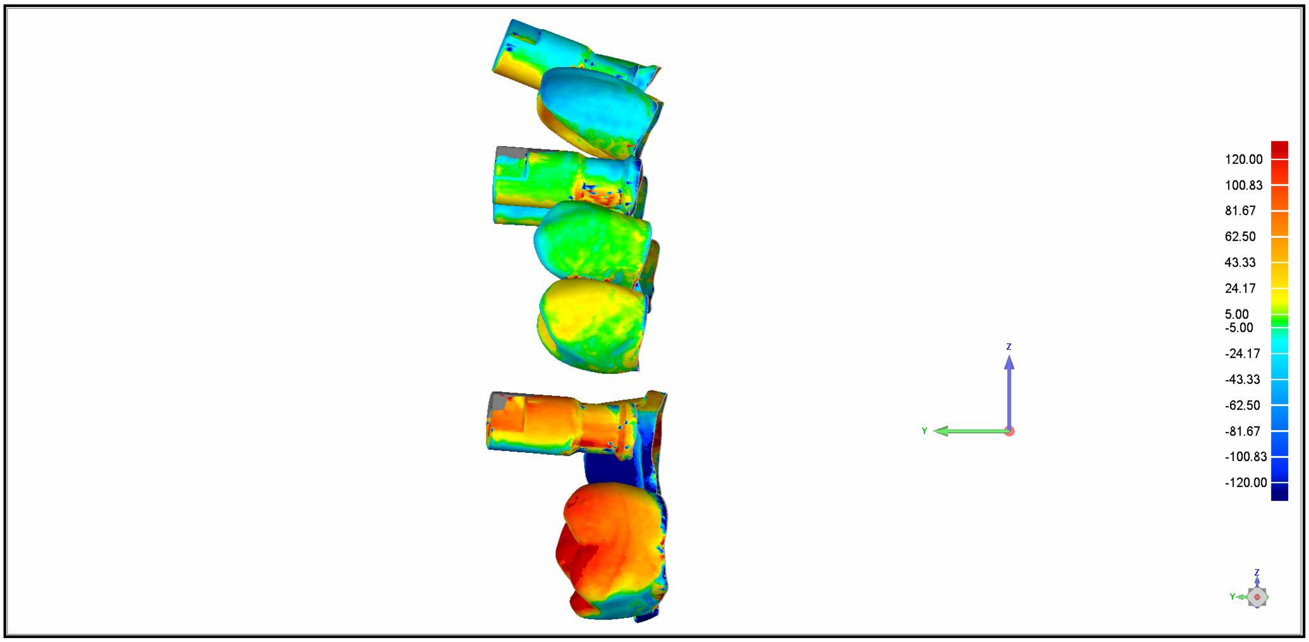

Predefinido: Derecha

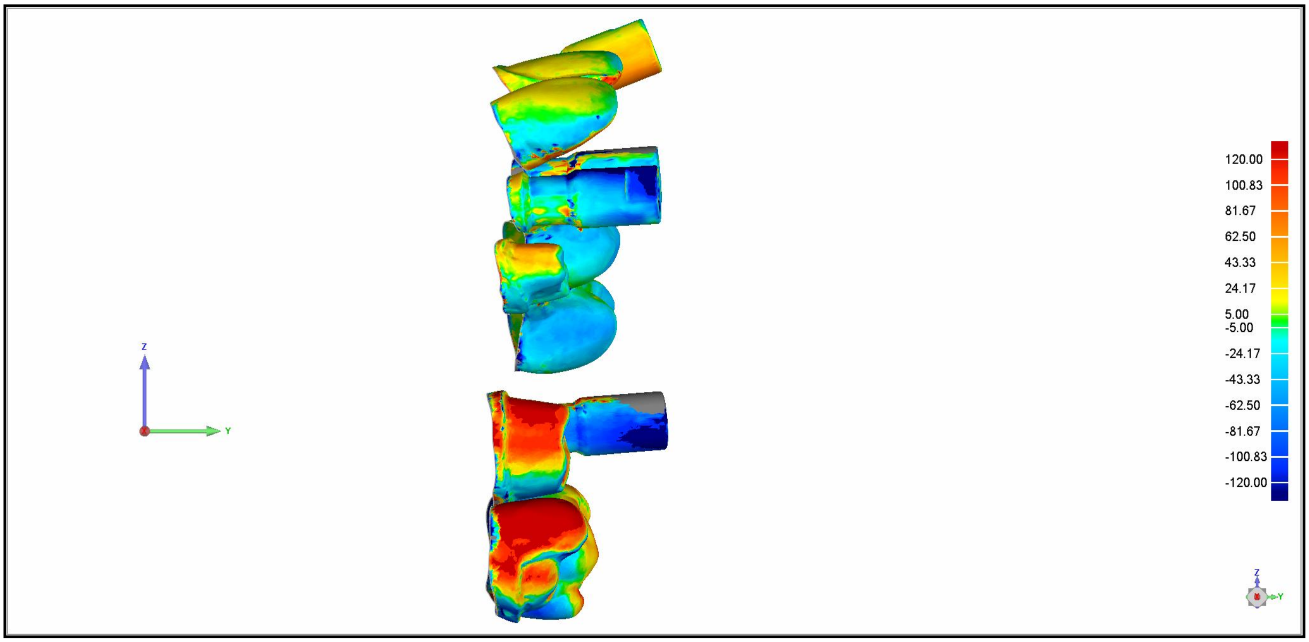

Predefinido: Superior

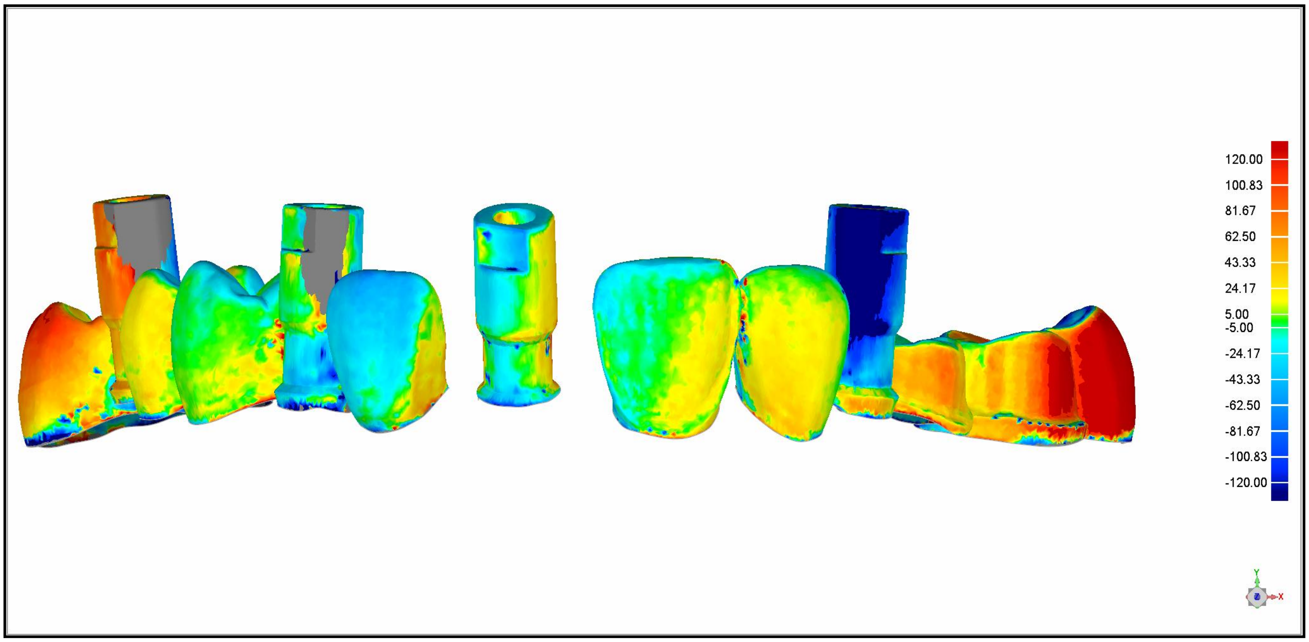

Predefinido: Inferior

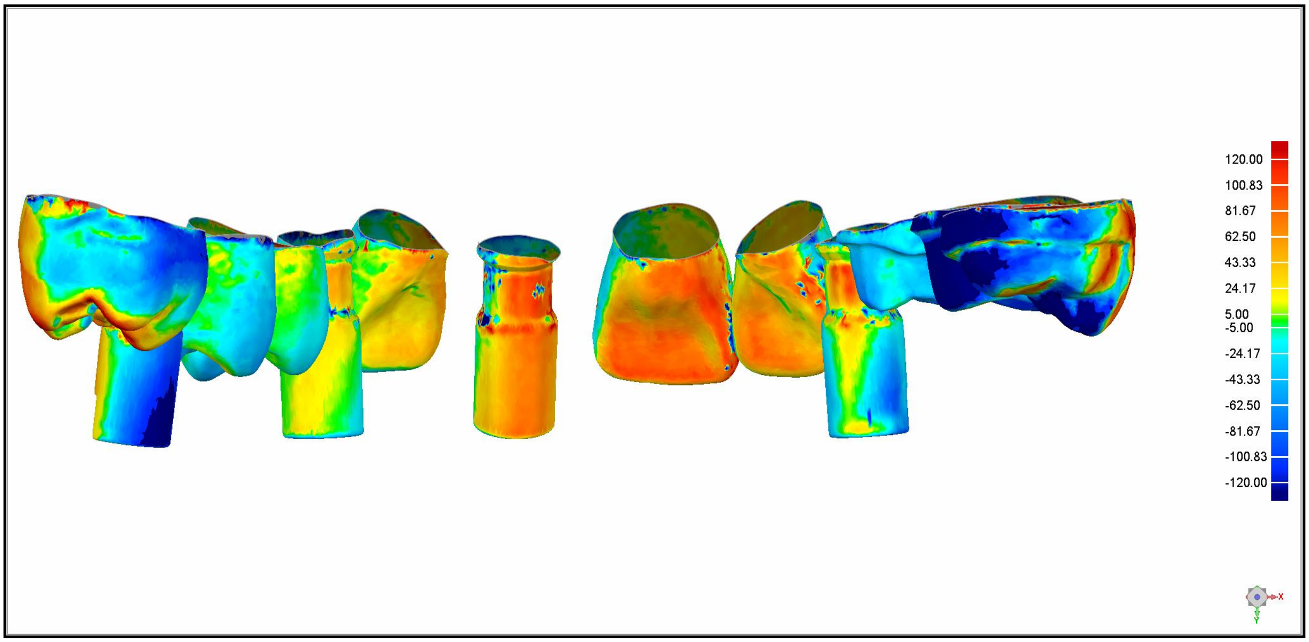

Ajuste de ubicación: Desviaciones superior e inferior

Unidades: u

Nombre	Desv	Estado	Superior Tol	Inferior Tol	Ref X	Ref Y	Ref Z	Radio	Desv X	Desv Y	Desv Z	Medido X	Medido Y	Medido Z	Dir. proy. X	Dir. proy. Y	Dir. proy. Z
Desv. inferior	-2786.84				-16597.07	29132.89	5701.00	n/a	-2654.71	131.42	-837.71	-19251.78	29264.31	4863.29	0.95	-0.05	0.30
Desv. superior	2553.33				2122.85	32866.10	27623.33	n/a	-2499.31	-215.58	-475.88	-376.46	32650.52	27147.45	-0.98	-0.08	-0.19
