# Supplementary material for: Accuracy of four digital scanners according to scanning strategy in complete-arch impressions
Source: PLoS One. 2018 Sep 13;13(9):e0202916. doi: 10.1371/journal.pone.0202916 (PMC6136706; doi:10.1371/journal.pone.0202916)
Supplement: S8 Table — iTero (scanning strategy D). (ZIP) [file pone.0202916.s008.zip › S8/IT8D.pdf]

3D Comparación Resultados

Modelo referencia	MRC
Modelo test	IT8D
Nº de puntos de datos	79678
# Aislados	725

Tipo tolerancia	3D desviación
Unidades	u
Máx. crítico	120.00
Máx. nominal	2.00
Mín. nominal	-2.00
Mín. crítico	-120.00

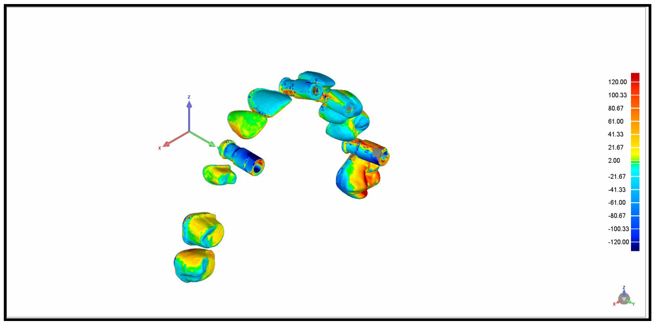

Desviación	
Desviación superior máx.	2912.14
Desviación inferior máx.	-3123.49
Desviación media	67.42 /-66.57
Desviación estándar	192.50

Distribución desviación

>=Min	<Max	# Puntos	%
-120.00	-100.33	892	1.12
-100.33	-80.67	1299	1.63
-80.67	-61.00	2060	2.59
-61.00	-41.33	5018	6.30
-41.33	-21.67	12531	15.73
-21.67	-2.00	14785	18.56
-2.00	2.00	3044	3.82
2.00	21.67	14370	18.04
21.67	41.33	9487	11.91
41.33	61.00	4486	5.63
61.00	80.67	2481	3.11
80.67	100.33	1495	1.88
100.33	120.00	908	1.14

Fuera del crítico superior	3421	4.29
Fuera del crítico inferior	3401	4.27

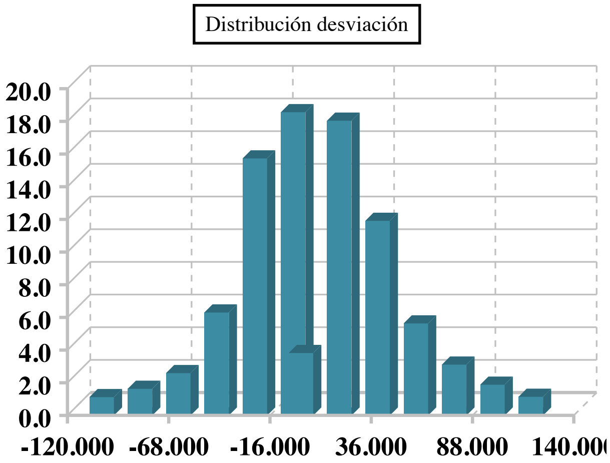

Desviaciones estándar

Distribución (+/-)	# Puntos	%
-6 * Desv. estánd.	595	0.75
-5 * Desv. estánd.	153	0.19
-4 * Desv. estánd.	178	0.22
-3 * Desv. estánd.	241	0.30
-2 * Desv. estánd.	808	1.01
-1 * Desv. estánd.	37683	47.29
1 * Desv. estánd.	38213	47.96
2 * Desv. estánd.	717	0.90
3 * Desv. estánd.	287	0.36
4 * Desv. estánd.	182	0.23
5 * Desv. estánd.	122	0.15
6 * Desv. estánd.	499	0.63

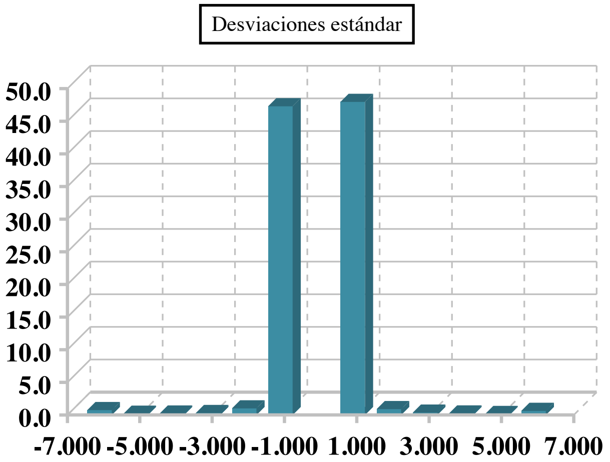

Predefinido: Isométrico

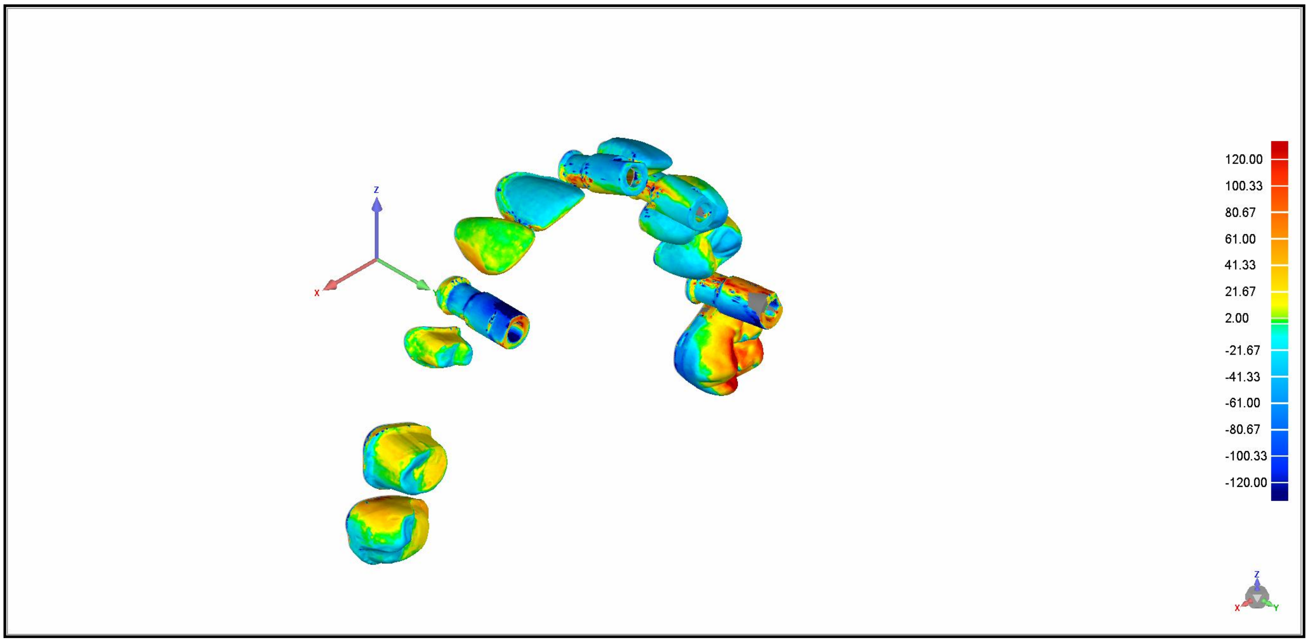

Predefinido: Frente

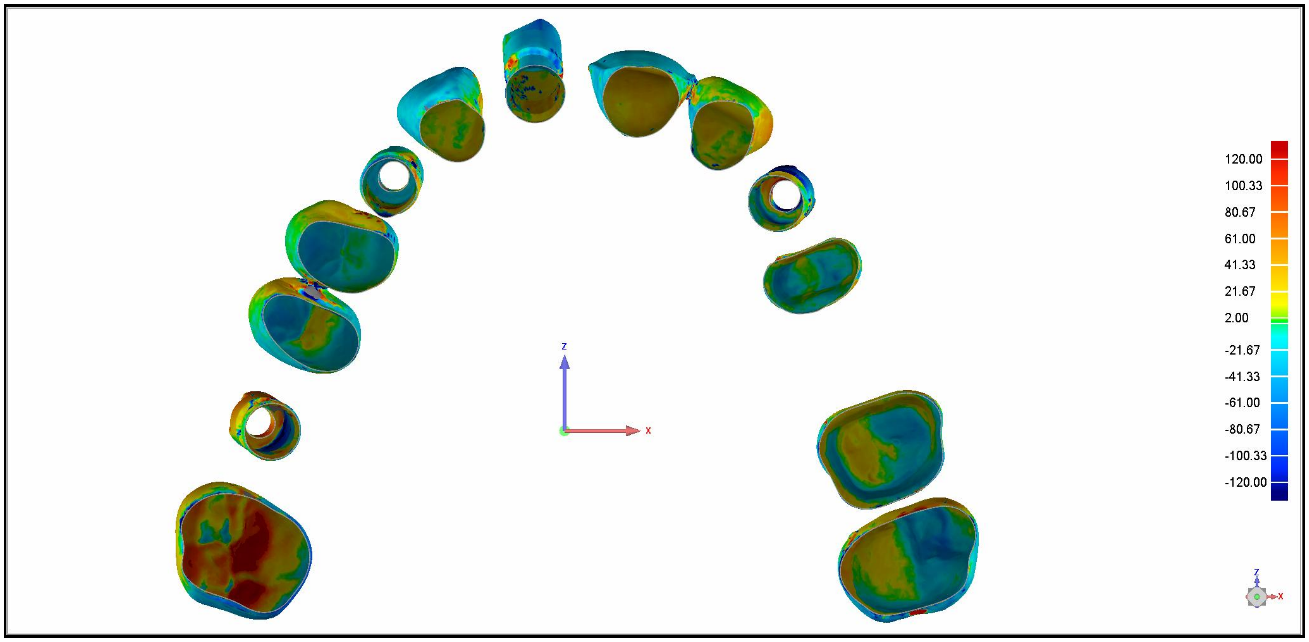

Predefinido: Atrás

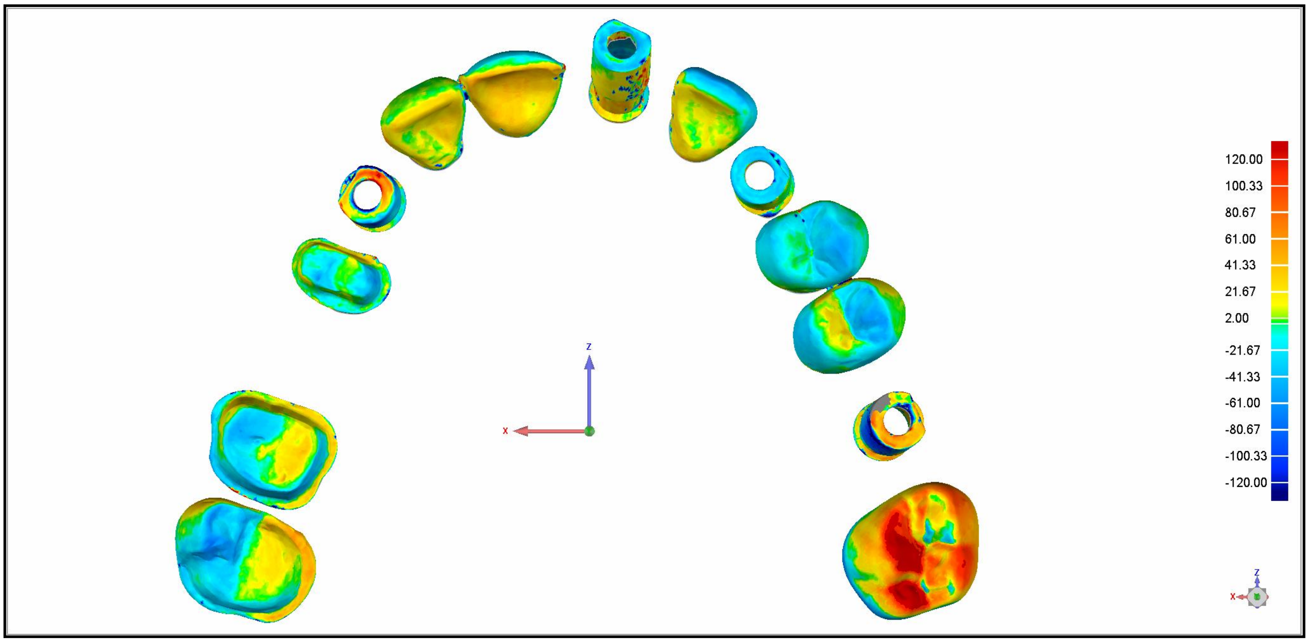

Predefinido: Izquierda

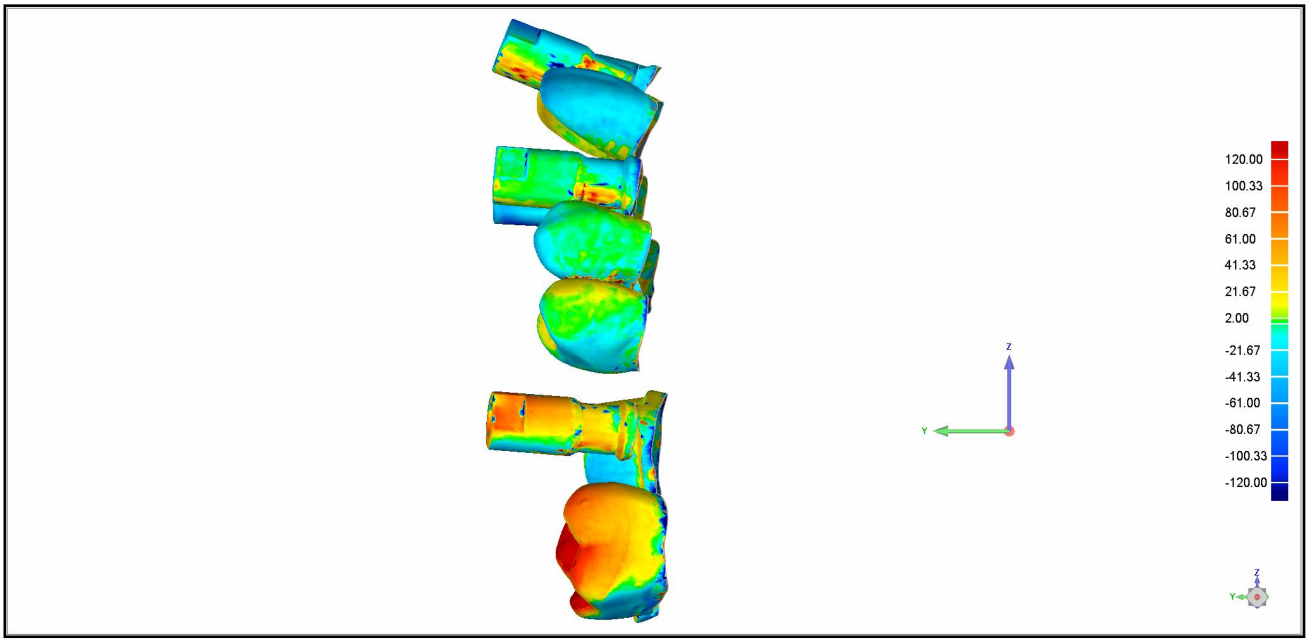

Predefinido: Derecha

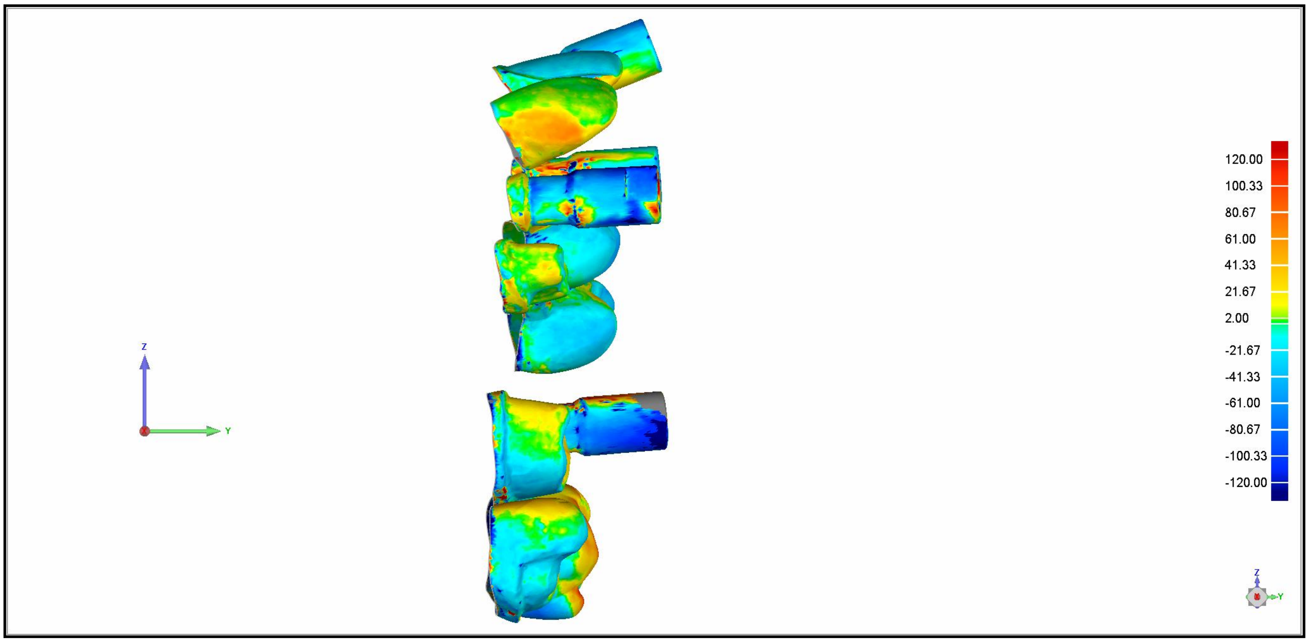

Predefinido: Superior

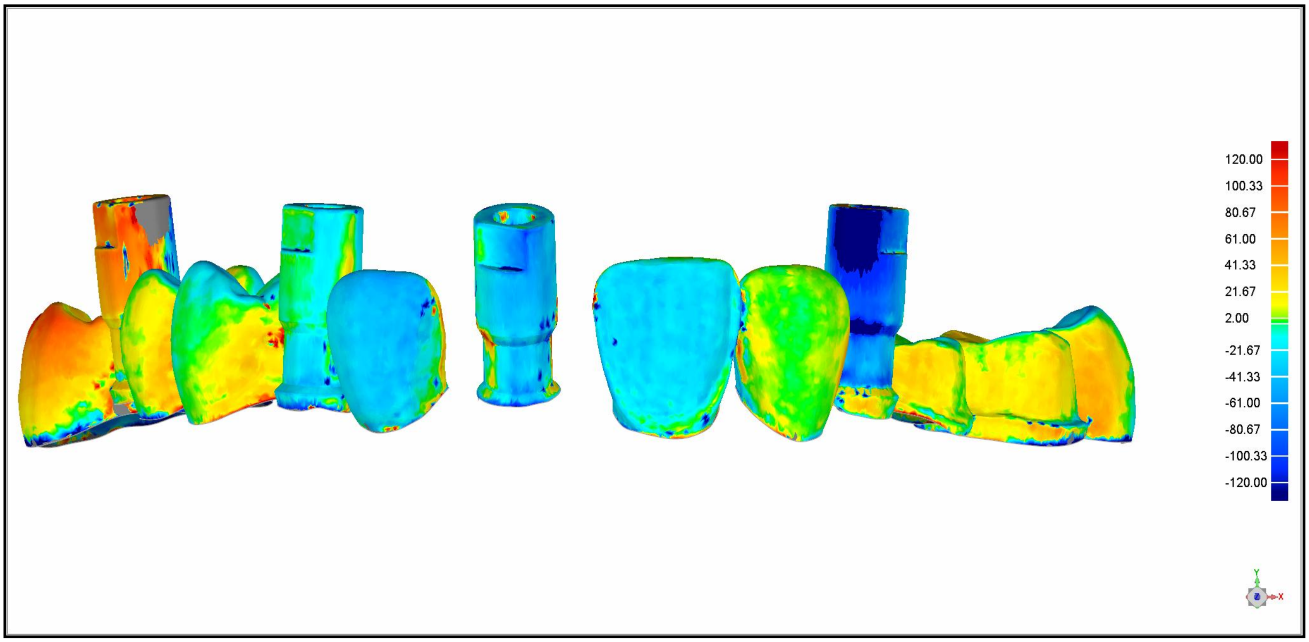

Predefinido: Inferior

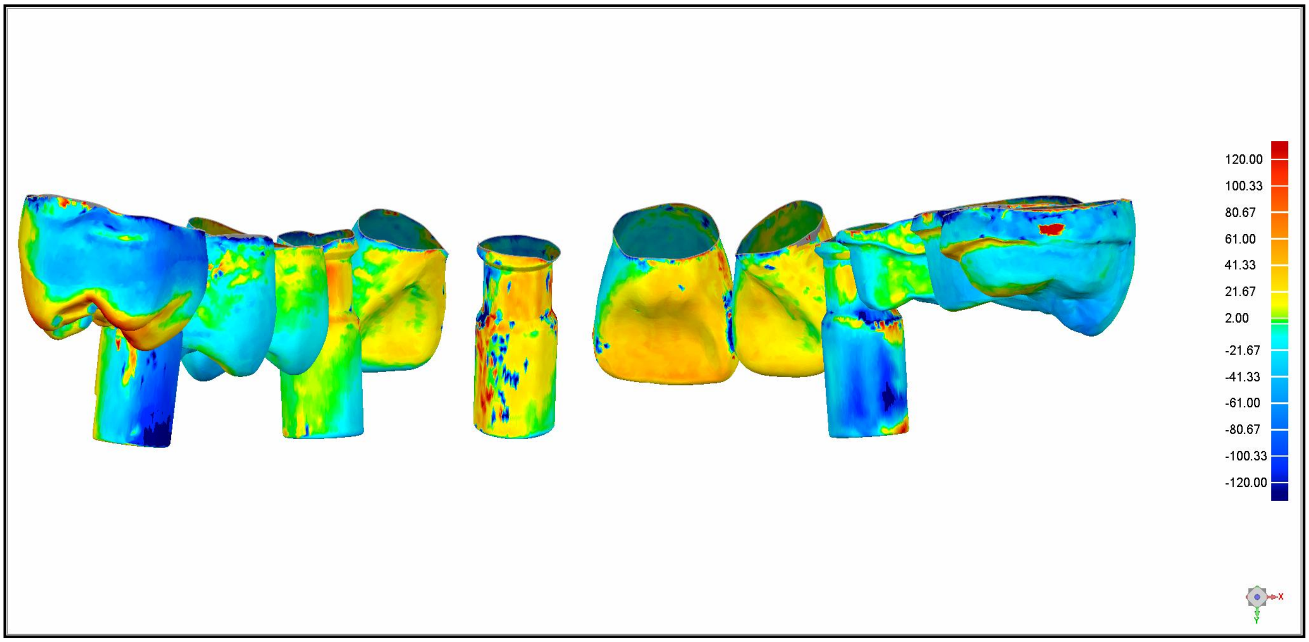

Ajuste de ubicación: Desviaciones superior e inferior

Unidades: u

Nombre	Desv	Estado	Superior Tol	Inferior Tol	Ref X	Ref Y	Ref Z	Radio	Desv X	Desv Y	Desv Z	Medido X	Medido Y	Medido Z	Dir. proy. X	Dir. proy. Y	Dir. proy. Z
Desv. inferior	-3123.49				-16597.07	29132.89	5701.00	n/a	-3016.30	92.38	-805.99	-19613.37	29225.28	4895.01	0.97	-0.03	0.26
Desv. superior	2912.14				-23704.23	30356.53	-2263.69	n/a	-809.77	-712.09	-2705.13	-24514.00	29644.44	-4968.82	-0.28	-0.24	-0.93
