# Supplementary material for: Accuracy of four digital scanners according to scanning strategy in complete-arch impressions
Source: PLoS One. 2018 Sep 13;13(9):e0202916. doi: 10.1371/journal.pone.0202916 (PMC6136706; doi:10.1371/journal.pone.0202916)
Supplement: S8 Table — iTero (scanning strategy D). (ZIP) [file pone.0202916.s008.zip › S8/IT3D.pdf]

Fecha: 3/6/2017, 1:18 pm

# 3D Comparación  Resultados

| | |
|---|---|
| Modelo referencia | MRC |
| Modelo test | IT3D |
| Nº de puntos de datos | 78743 |
| # Aislados | 599 |

| | |
|---|---|
| Tipo tolerancia | 3D desviación |
| Unidades | u |
| Máx. crítico | 120.00 |
| Máx. nominal | 4.00 |
| Mín. nominal | -4.00 |
| Mín. crítico | -120.00 |

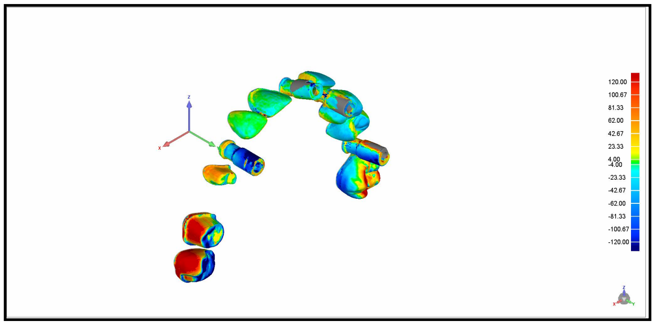

| Desviación | |
|---|---|
| Desviación superior máx. | 3036.73 |
| Desviación inferior máx. | -3112.52 |
| Desviación media | 80.09 /-83.01 |
| Desviación estándar | 202.61 |

## Distribución desviación

| >=Min | <Max | # Puntos | % |
|---|---|---|---|
| -120.00 | -100.67 | 1628 | 2.07 |
| -100.67 | -81.33 | 1969 | 2.50 |
| -81.33 | -62.00 | 2166 | 2.75 |
| -62.00 | -42.67 | 4050 | 5.14 |
| -42.67 | -23.33 | 7641 | 9.70 |
| -23.33 | -4.00 | 12495 | 15.87 |
| -4.00 | 4.00 | 6299 | 8.00 |
| 4.00 | 23.33 | 11949 | 15.17 |
| 23.33 | 42.67 | 7611 | 9.67 |
| 42.67 | 62.00 | 4283 | 5.44 |
| 62.00 | 81.33 | 2709 | 3.44 |
| 81.33 | 100.67 | 1727 | 2.19 |
| 100.67 | 120.00 | 1213 | 1.54 |

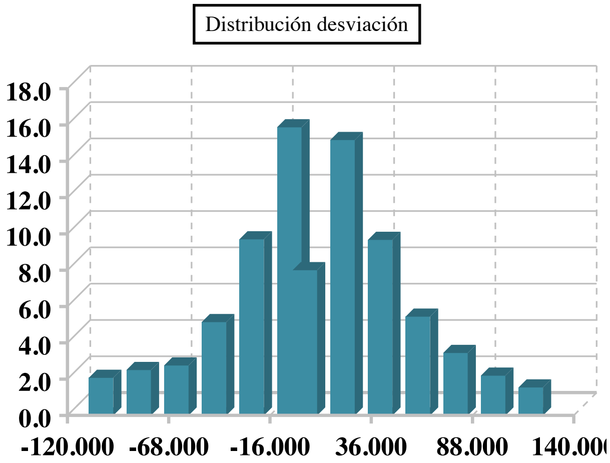


| | | | |
|---|---|---|---|
| Fuera del crítico superior | | 5405 | 6.86 |
| Fuera del crítico inferior | | 7598 | 9.65 |

## Desviaciones estándar

| Distribución (+/-) | # Puntos | % |
|---|---|---|
| -6 * Desv. esténd. | 454 | 0.58 |
| -5 * Desv. esténd. | 165 | 0.21 |
| -4 * Desv. esténd. | 188 | 0.24 |
| -3 * Desv. esténd. | 184 | 0.23 |
| -2 * Desv. esténd. | 1959 | 2.49 |
| -1 * Desv. esténd. | 34105 | 43.31 |
| 1 * Desv. esténd. | 39379 | 50.01 |
| 2 * Desv. esténd. | 1163 | 1.48 |
| 3 * Desv. esténd. | 259 | 0.33 |
| 4 * Desv. esténd. | 209 | 0.27 |
| 5 * Desv. esténd. | 169 | 0.21 |
| 6 * Desv. esténd. | 509 | 0.65 |

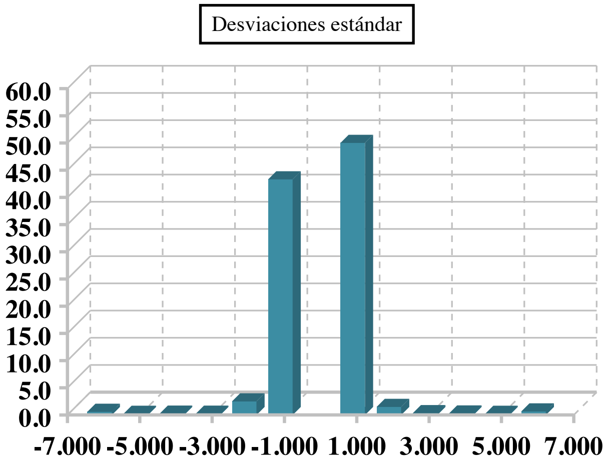

Predefinido: Isométrico

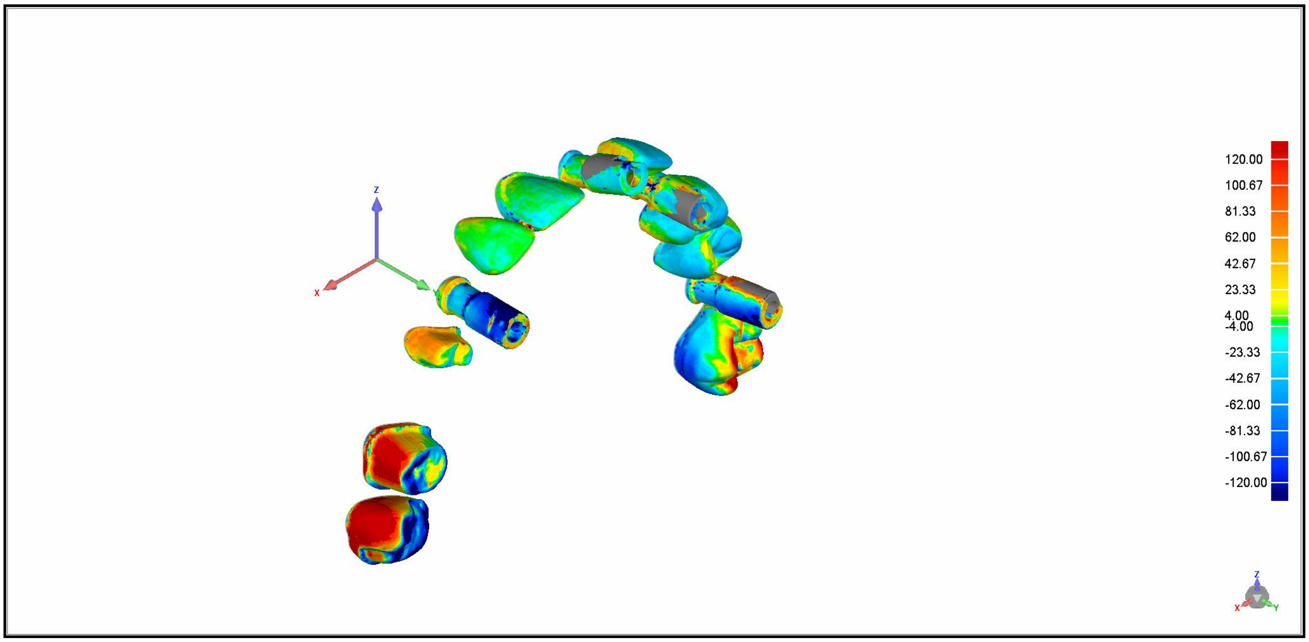

Predefinido: Frente

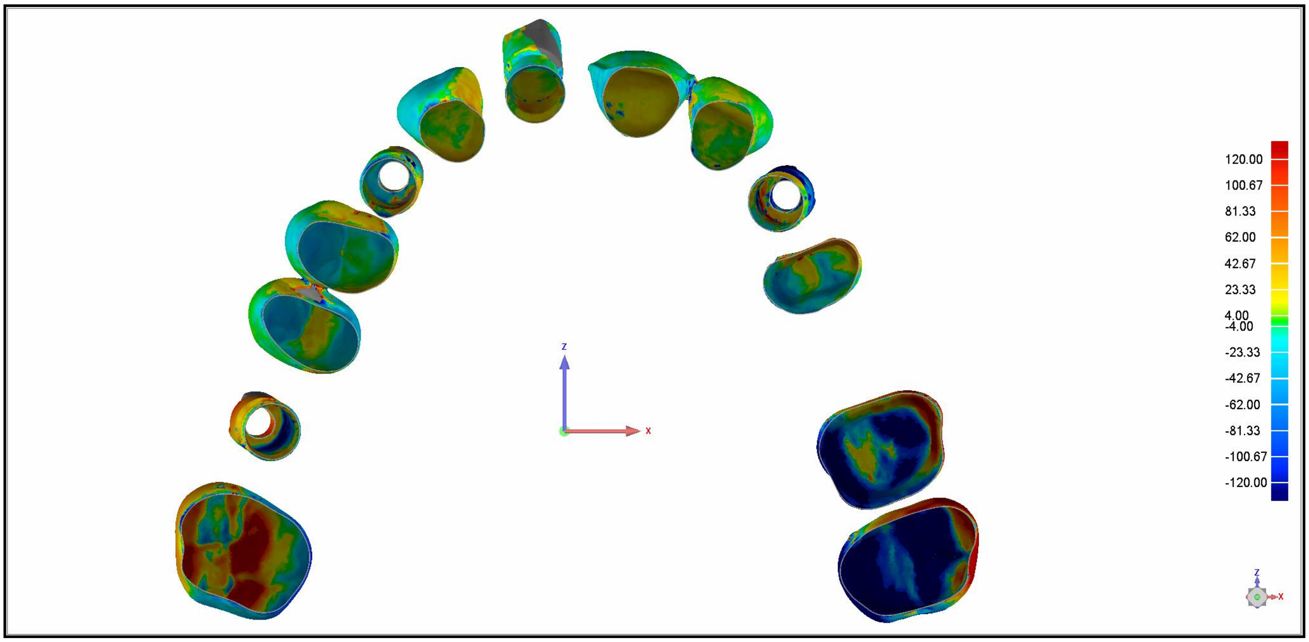

Predefinido: Atrás

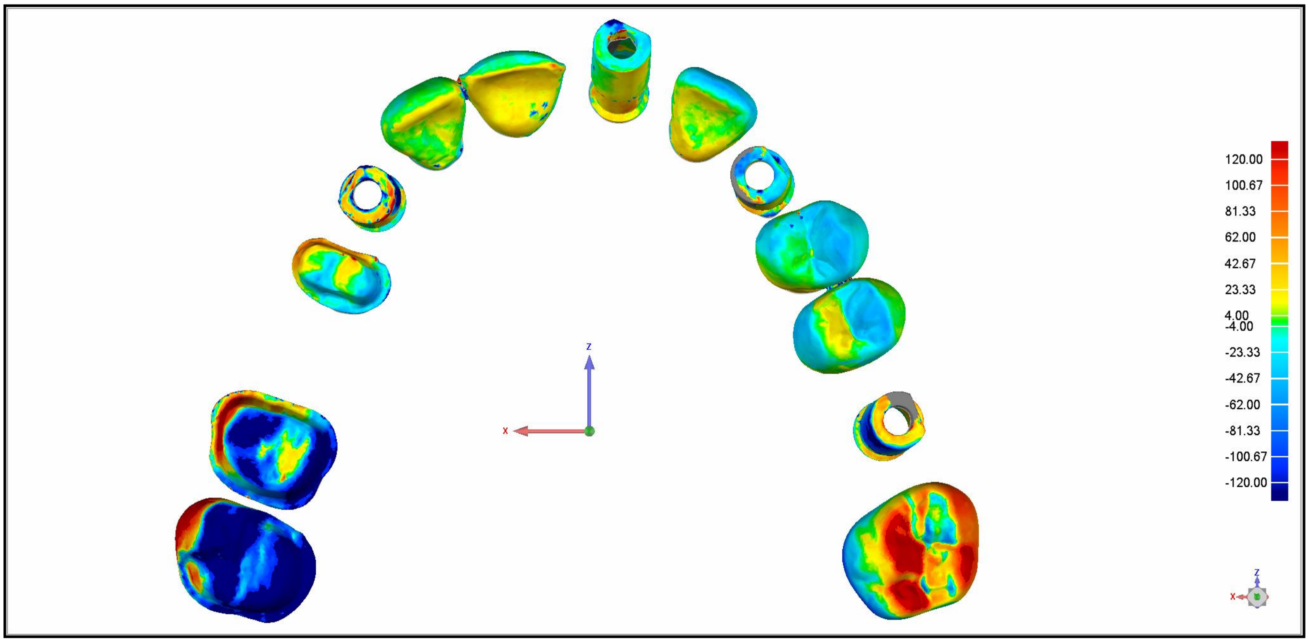

Predefinido: Izquierda

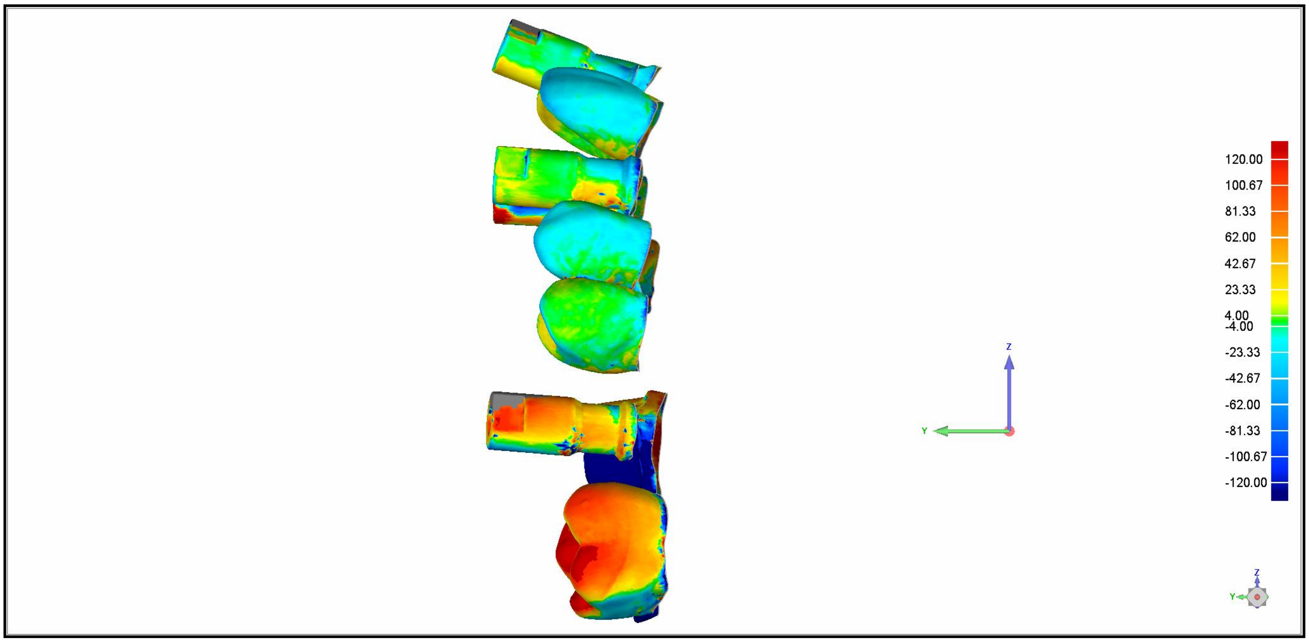

Predefinido: Derecha

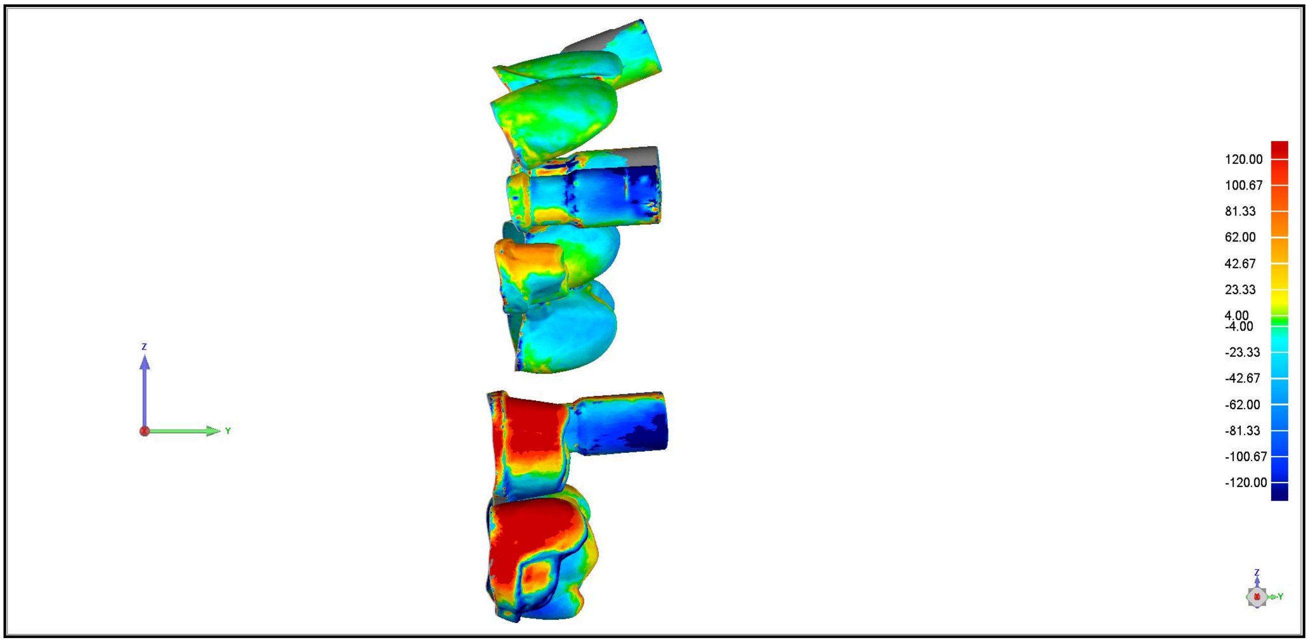

Predefinido: Superior

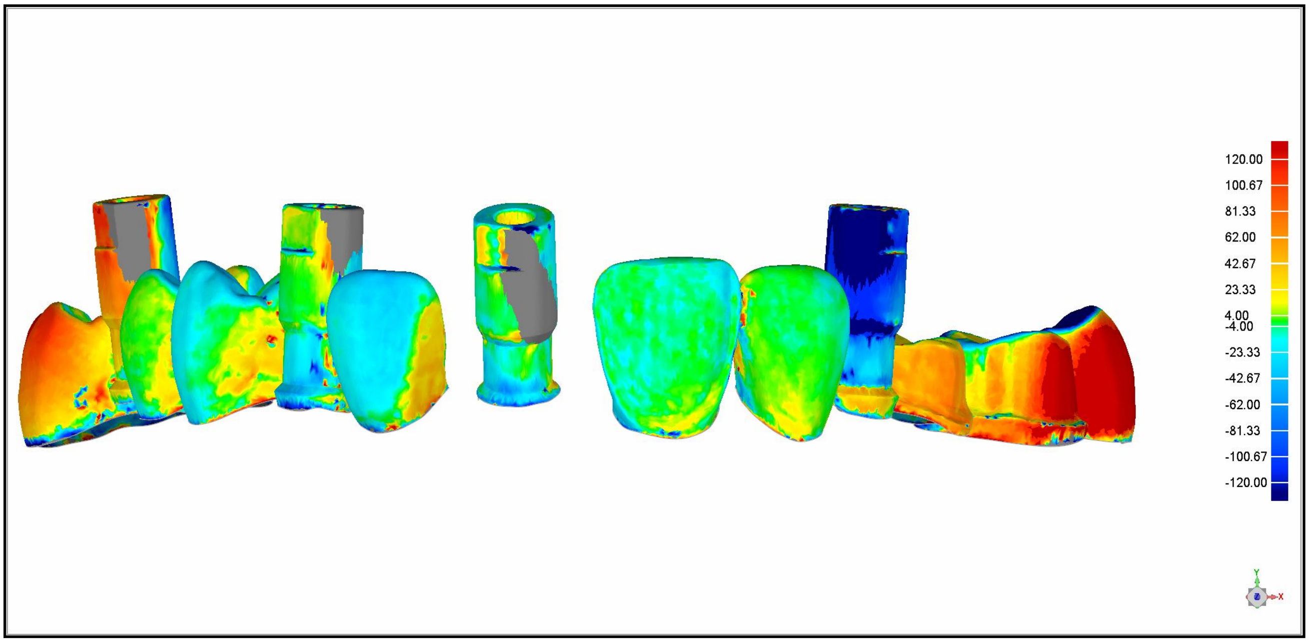

Predefinido: Inferior

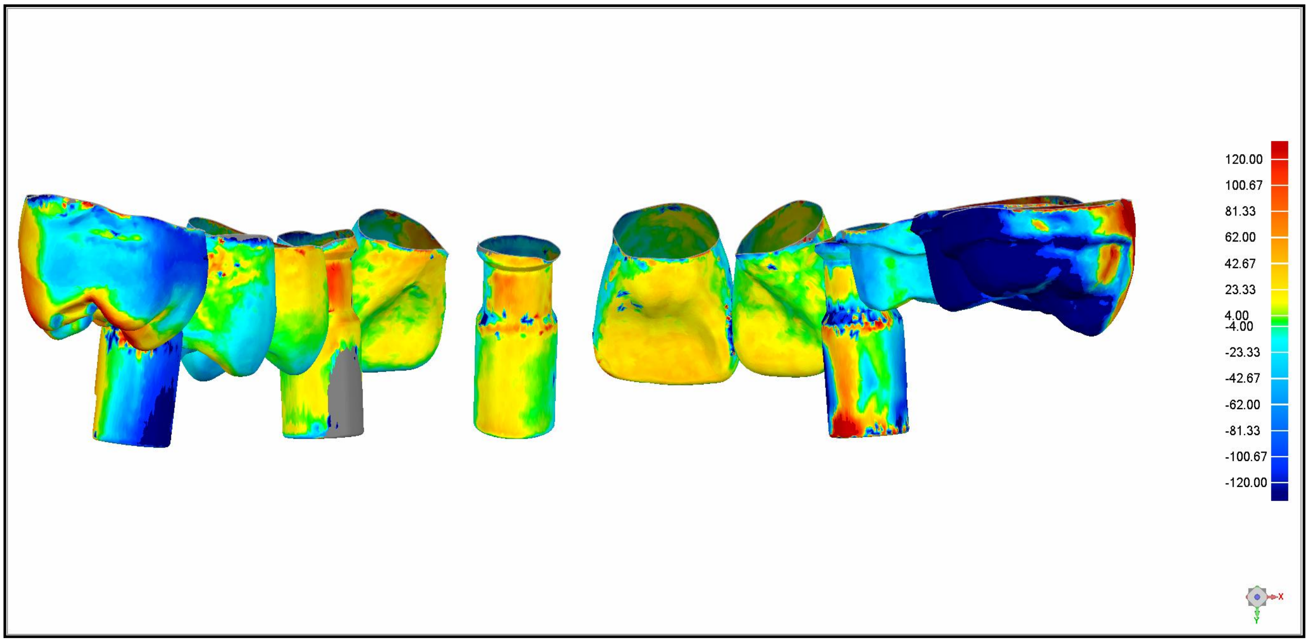

# Ajuste de ubicación: Desviaciones superior e inferior

## Unidades: u

| Nombre | Desv | Estado | Superior Tol | Inferior Tol | Ref X | Ref Y | Ref Z | Radio | Desv X | Desv Y | Desv Z | Medido X | Medido Y | Medido Z | Dir. proy. X | Dir. proy. Y | Dir. proy. Z |
|---|---|---|---|---|---|---|---|---|---|---|---|---|---|---|---|---|---|
| Desv. inferior | -3112.52 | | | | 16989.02 | 37628.06 | 17251.36 | n/a | 2068.88 | 2307.75 | -286.07 | 19057.90 | 39935.81 | 16965.29 | -0.66 | -0.74 | 0.09 |
| Desv. superior | 3036.73 | | | | -2362.14 | 39181.60 | 31346.02 | n/a | -277.15 | -2770.93 | -1211.15 | -2639.29 | 36410.67 | 30134.87 | -0.09 | -0.91 | -0.40 |
