# Supplementary material for: Accuracy of four digital scanners according to scanning strategy in complete-arch impressions
Source: PLoS One. 2018 Sep 13;13(9):e0202916. doi: 10.1371/journal.pone.0202916 (PMC6136706; doi:10.1371/journal.pone.0202916)
Supplement: S8 Table — iTero (scanning strategy D). (ZIP) [file pone.0202916.s008.zip › S8/IT2D.pdf]

3D Comparación Resultados

Modelo referencia	MRC
Modelo test	IT2D
Nº de puntos de datos	79753
# Aislados	651

Tipo tolerancia	3D desviación
Unidades	u
Máx. crítico	120.00
Máx. nominal	3.00
Mín. nominal	-3.00
Mín. crítico	-120.00

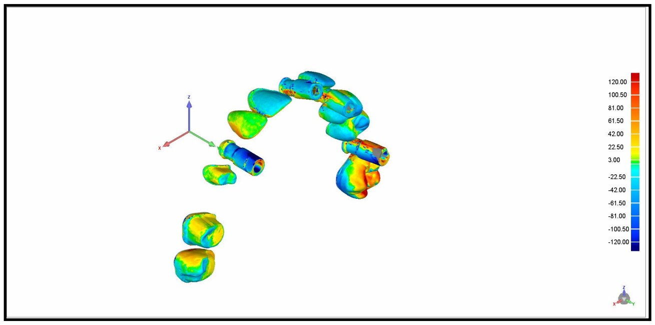

Desviación	
Desviación superior máx.	3044.83
Desviación inferior máx.	-3136.99
Desviación media	68.51 /-66.07
Desviación estándar	195.70

Distribución desviación

>=Min	<Max	# Puntos	%
-120.00	-100.50	891	1.12
-100.50	-81.00	1292	1.62
-81.00	-61.50	2146	2.69
-61.50	-42.00	5363	6.72
-42.00	-22.50	12422	15.58
-22.50	-3.00	14711	18.45
-3.00	3.00	4710	5.91
3.00	22.50	13913	17.45
22.50	42.00	8677	10.88
42.00	61.50	4108	5.15
61.50	81.00	2402	3.01
81.00	100.50	1415	1.77
100.50	120.00	936	1.17

Fuera del crítico superior	3426	4.30
Fuera del crítico inferior	3341	4.19

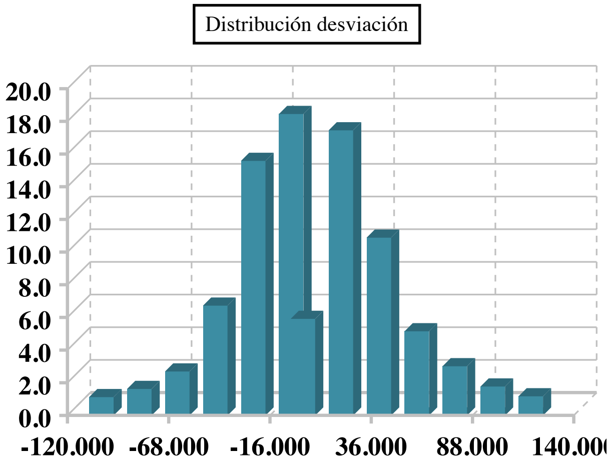

Desviaciones estándar

Distribución (+/-)	# Puntos	%
-6 * Desv. estánd.	588	0.74
-5 * Desv. estánd.	141	0.18
-4 * Desv. estánd.	159	0.20
-3 * Desv. estánd.	245	0.31
-2 * Desv. estánd.	753	0.94
-1 * Desv. estánd.	38185	47.88
1 * Desv. estánd.	37863	47.48
2 * Desv. estánd.	742	0.93
3 * Desv. estánd.	277	0.35
4 * Desv. estánd.	183	0.23
5 * Desv. estánd.	118	0.15
6 * Desv. estánd.	499	0.63

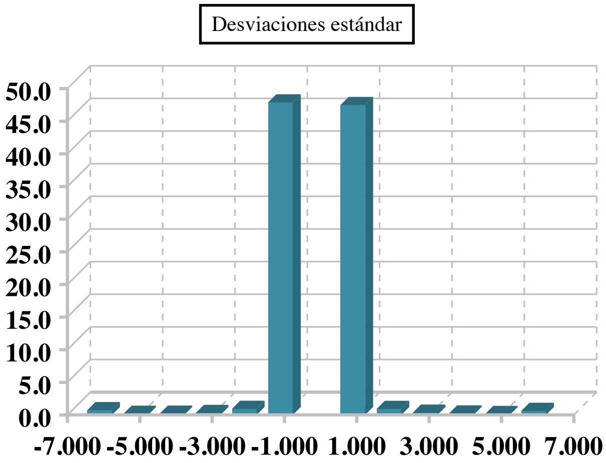

Predefinido: Isométrico

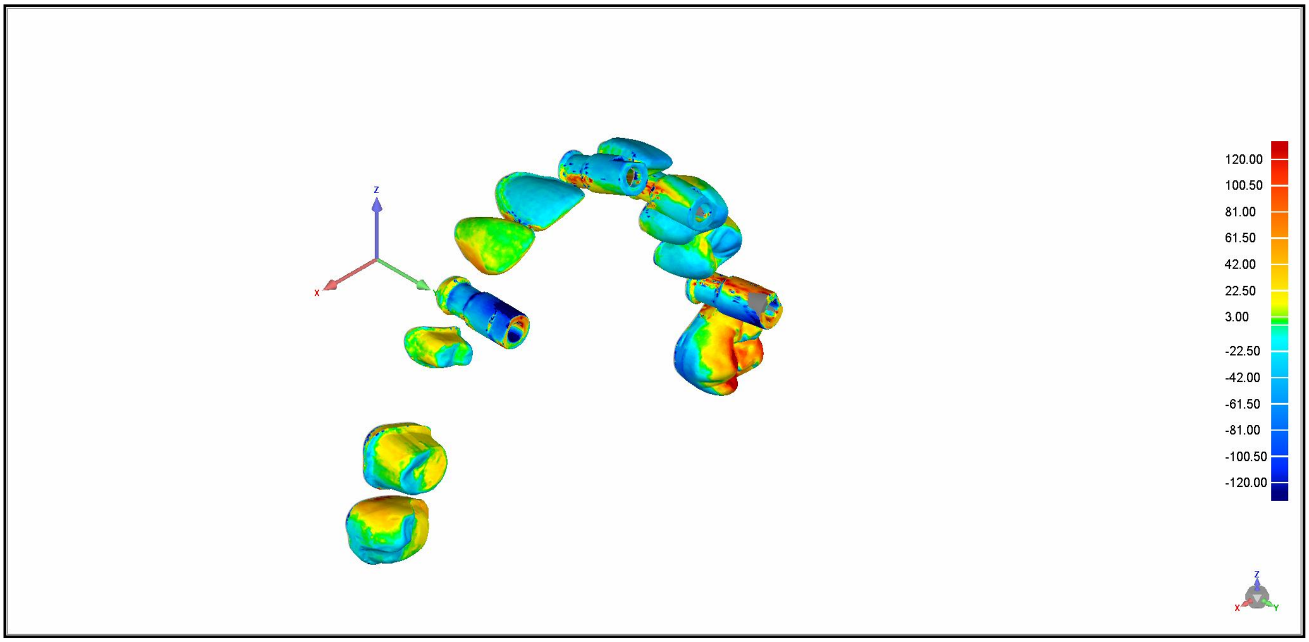

Predefinido: Frente

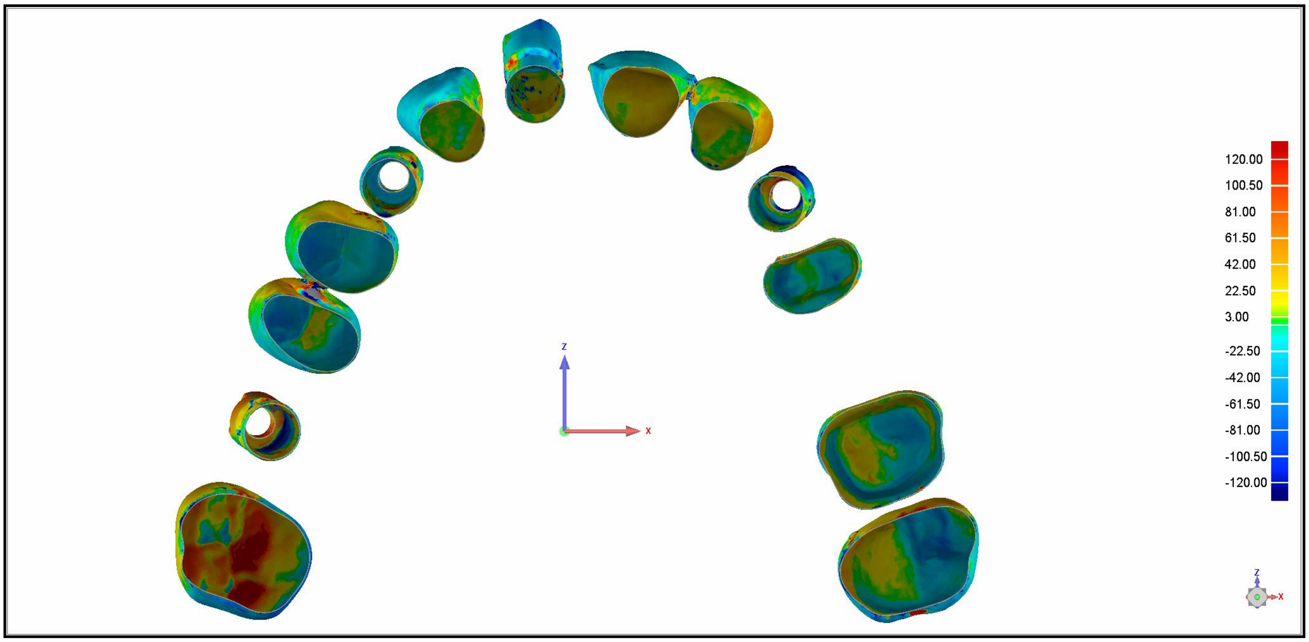

Predefinido: Atrás

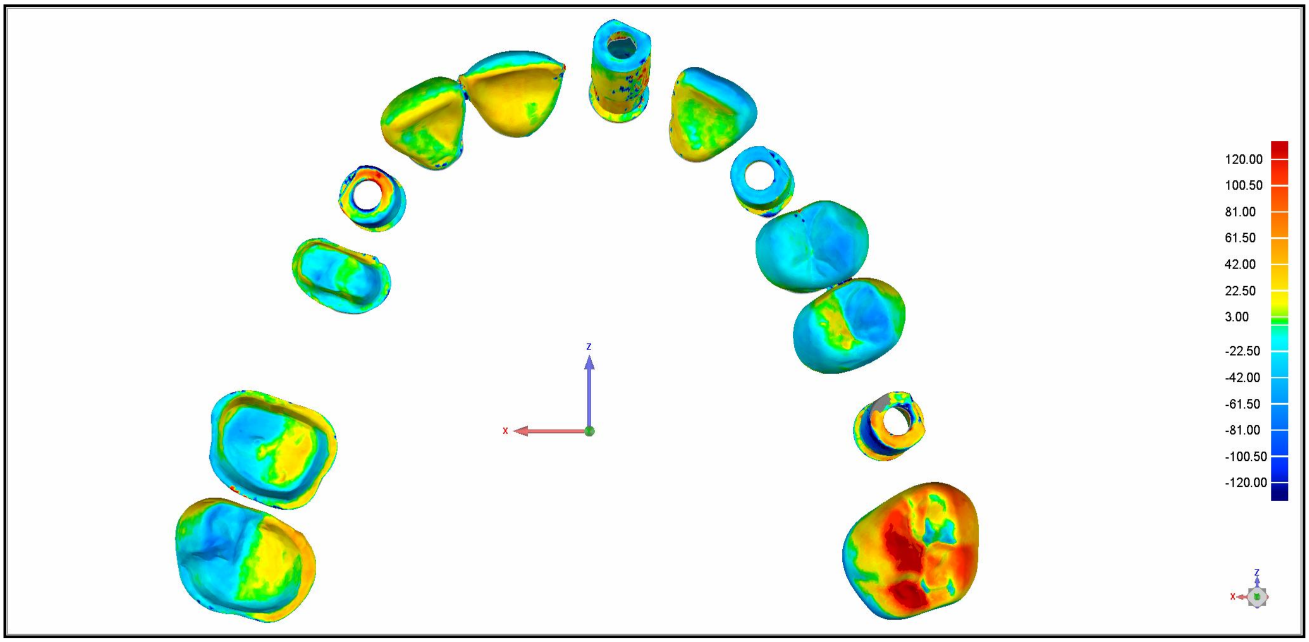

Predefinido: Izquierda

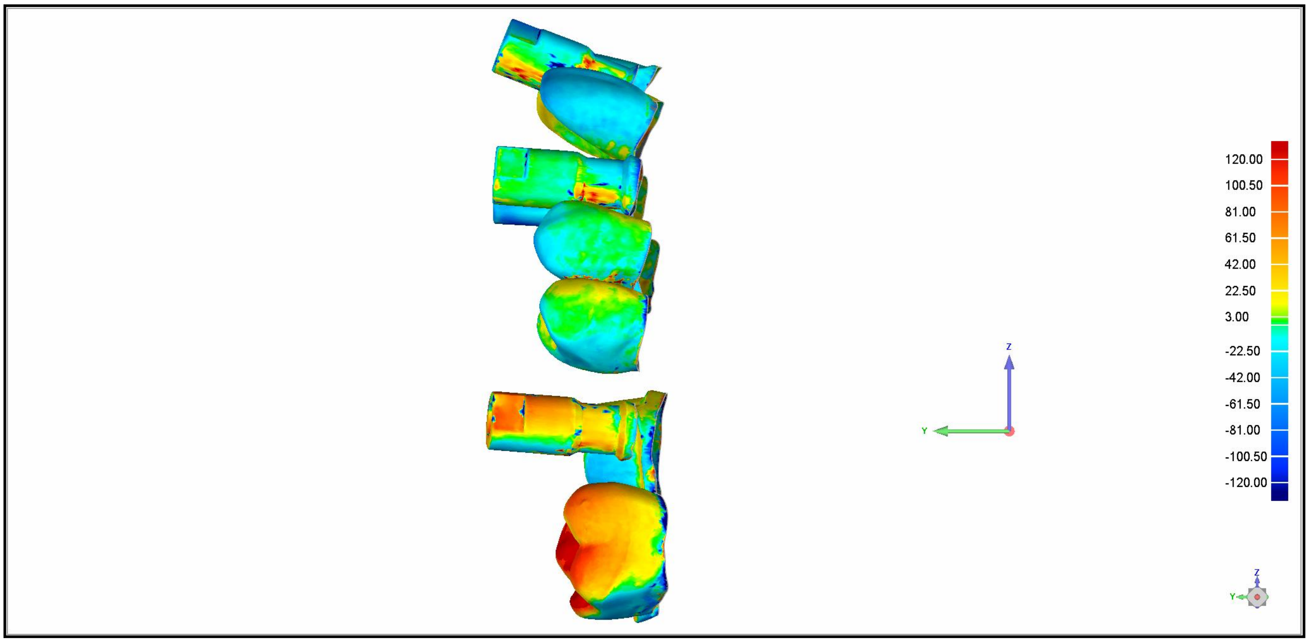

Predefinido: Derecha

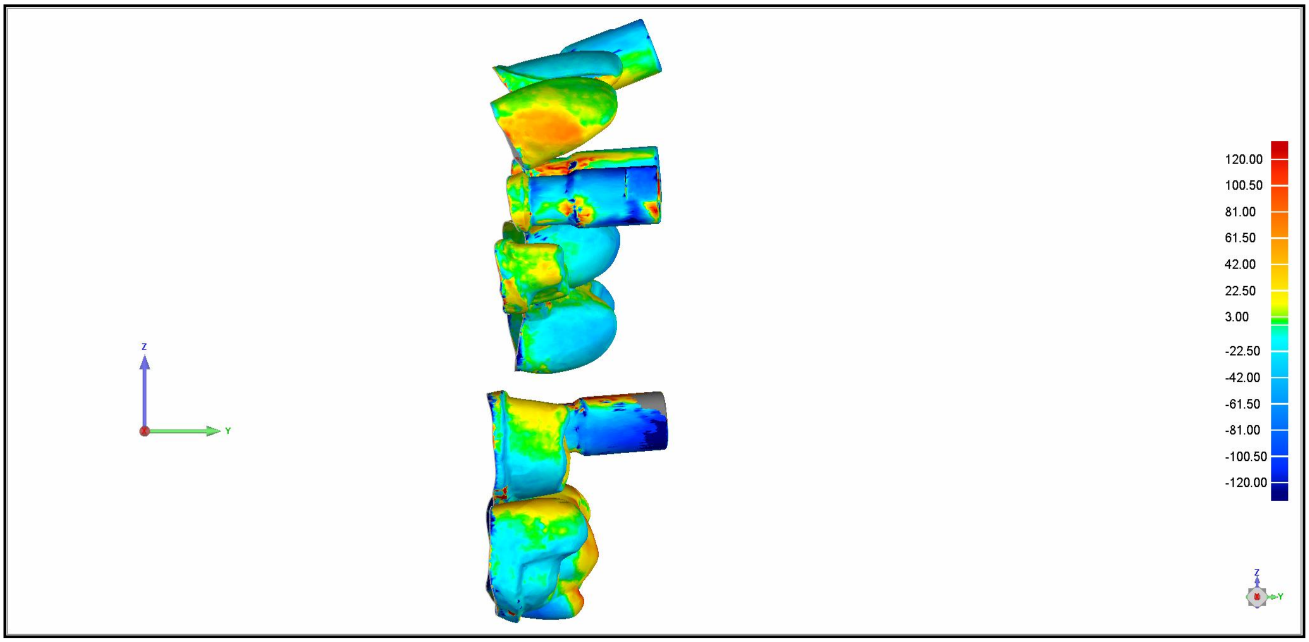

Fecha: 3/6/2017, 1:15 pm

Predefinido: Superior

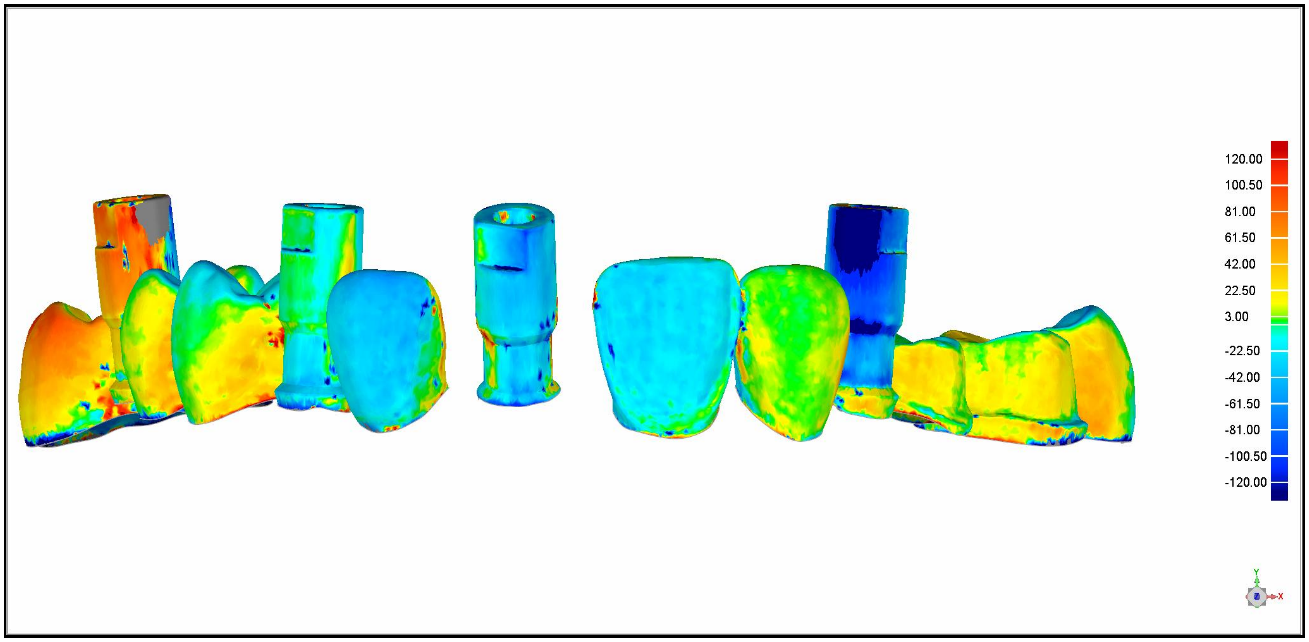

Predefinido: Inferior

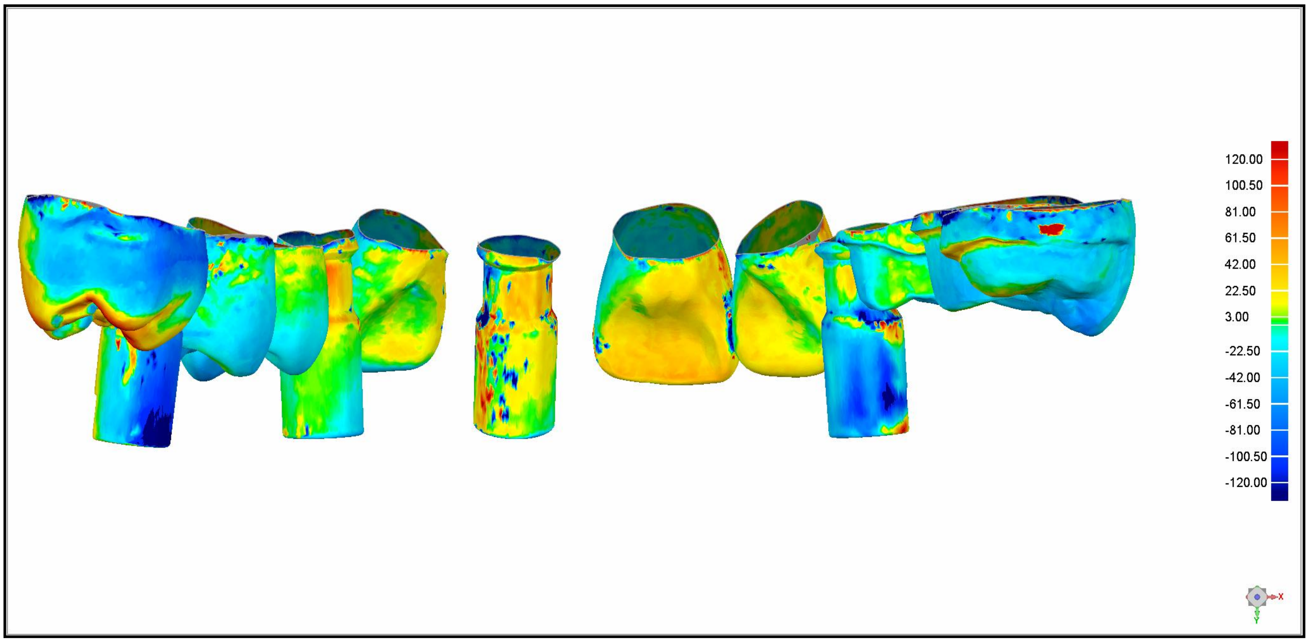

Ajuste de ubicación: Desviaciones superior e inferior

Unidades: u

Nombre	Desv	Estado	Superior Tol	Inferior Tol	Ref X	Ref Y	Ref Z	Radio	Desv X	Desv Y	Desv Z	Medido X	Medido Y	Medido Z	Dir. proy. X	Dir. proy. Y	Dir. proy. Z
Desv. inferior	-3136.99				-29208.33	26961.25	-11988.49	n/a	2714.87	406.43	-1518.21	-26493.46	27367.68	-13506.70	-0.87	-0.13	0.48
Desv. superior	3044.83				-23627.76	30241.63	-2252.01	n/a	-761.15	-795.46	-2838.82	-24388.91	29446.18	-5090.83	-0.25	-0.26	-0.93
