# Supplementary material for: Accuracy of four digital scanners according to scanning strategy in complete-arch impressions
Source: PLoS One. 2018 Sep 13;13(9):e0202916. doi: 10.1371/journal.pone.0202916 (PMC6136706; doi:10.1371/journal.pone.0202916)
Supplement: S8 Table — iTero (scanning strategy D). (ZIP) [file pone.0202916.s008.zip › S8/IT1D.pdf]

3D Comparación Resultados

Modelo referencia	MRC
Modelo test	IT1D
Nº de puntos de datos	76528
# Aislados	603

Tipo tolerancia	3D desviación
Unidades	u
Máx. crítico	120.00
Máx. nominal	1.00
Mín. nominal	-1.00
Mín. crítico	-120.00

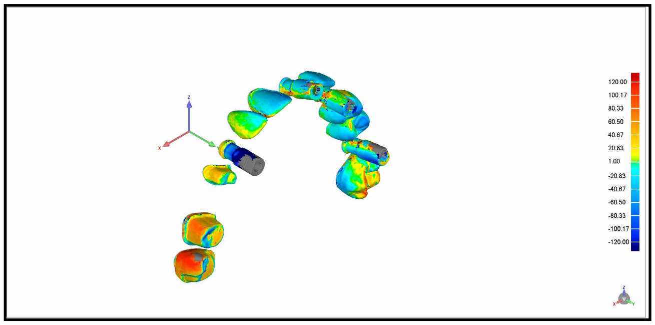

Desviación	
Desviación superior máx.	3154.62
Desviación inferior máx.	-3068.41
Desviación media	68.70 /-60.42
Desviación estándar	182.67

Distribución desviación

>=Min	<Max	# Puntos	%
-120.00	-100.17	1273	1.66
-100.17	-80.33	1720	2.25
-80.33	-60.50	2715	3.55
-60.50	-40.67	5739	7.50
-40.67	-20.83	9748	12.74
-20.83	-1.00	15618	20.41
-1.00	1.00	1792	2.34
1.00	20.83	15118	19.75
20.83	40.67	8324	10.88
40.67	60.50	3765	4.92
60.50	80.33	2007	2.62
80.33	100.17	1365	1.78
100.17	120.00	796	1.04

Fuera del crítico superior	2719	3.55
Fuera del crítico inferior	3829	5.00

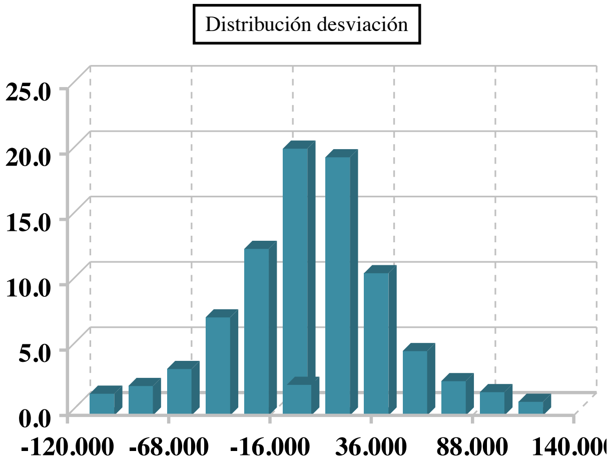

Desviaciones estándar

Distribución (+/-)	# Puntos	%
-6 * Desv. estánd.	406	0.53
-5 * Desv. estánd.	92	0.12
-4 * Desv. estánd.	103	0.13
-3 * Desv. estánd.	161	0.21
-2 * Desv. estánd.	1040	1.36
-1 * Desv. estánd.	38466	50.26
1 * Desv. estánd.	34300	44.82
2 * Desv. estánd.	804	1.05
3 * Desv. estánd.	182	0.24
4 * Desv. estánd.	154	0.20
5 * Desv. estánd.	180	0.24
6 * Desv. estánd.	640	0.84

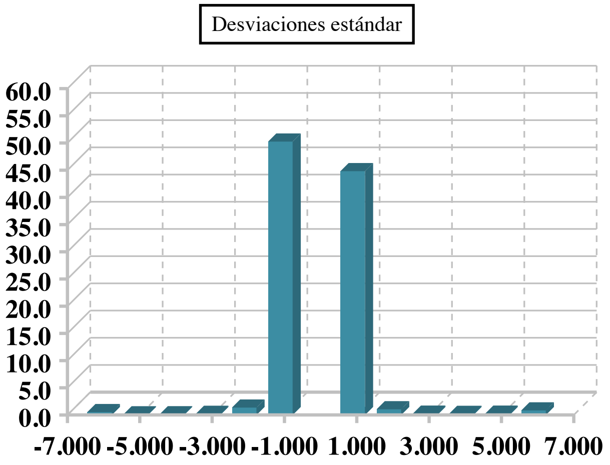

Predefinido: Isométrico

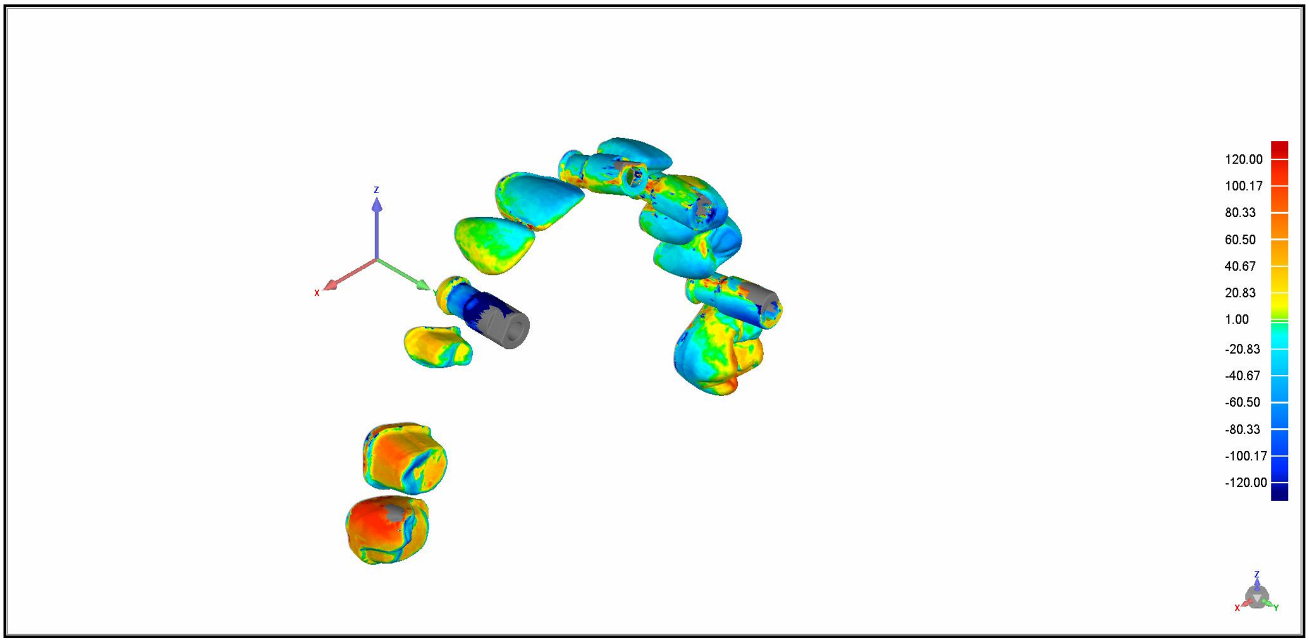

Predefinido: Frente

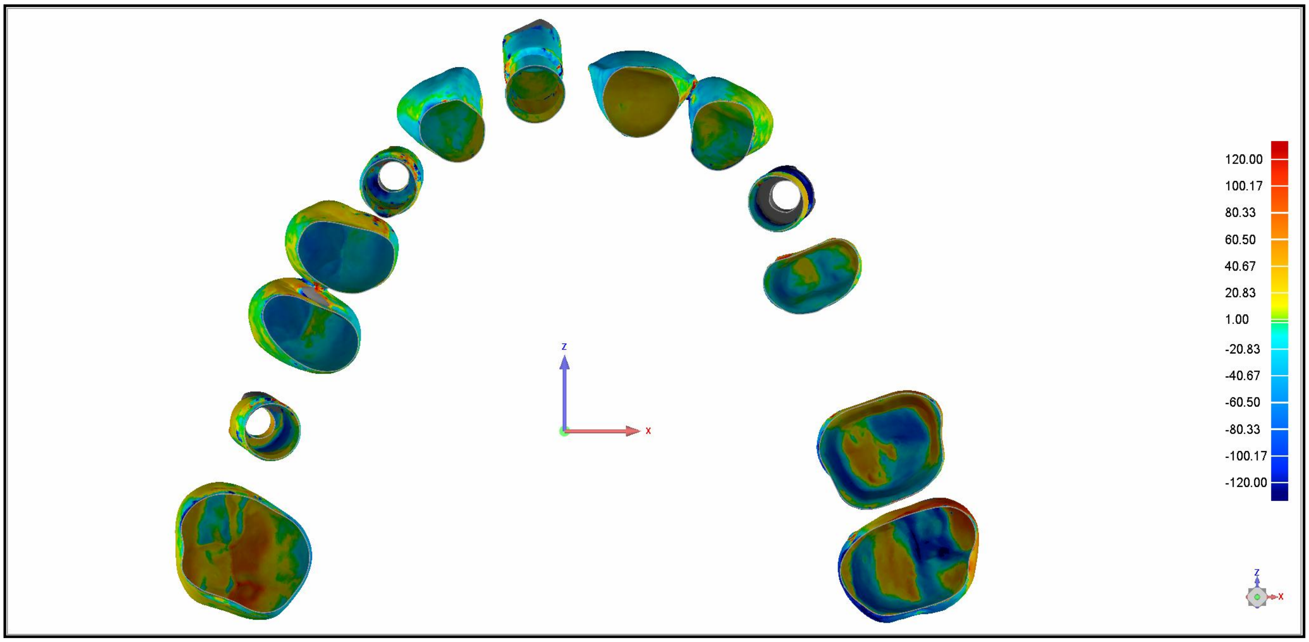

Predefinido: Atrás

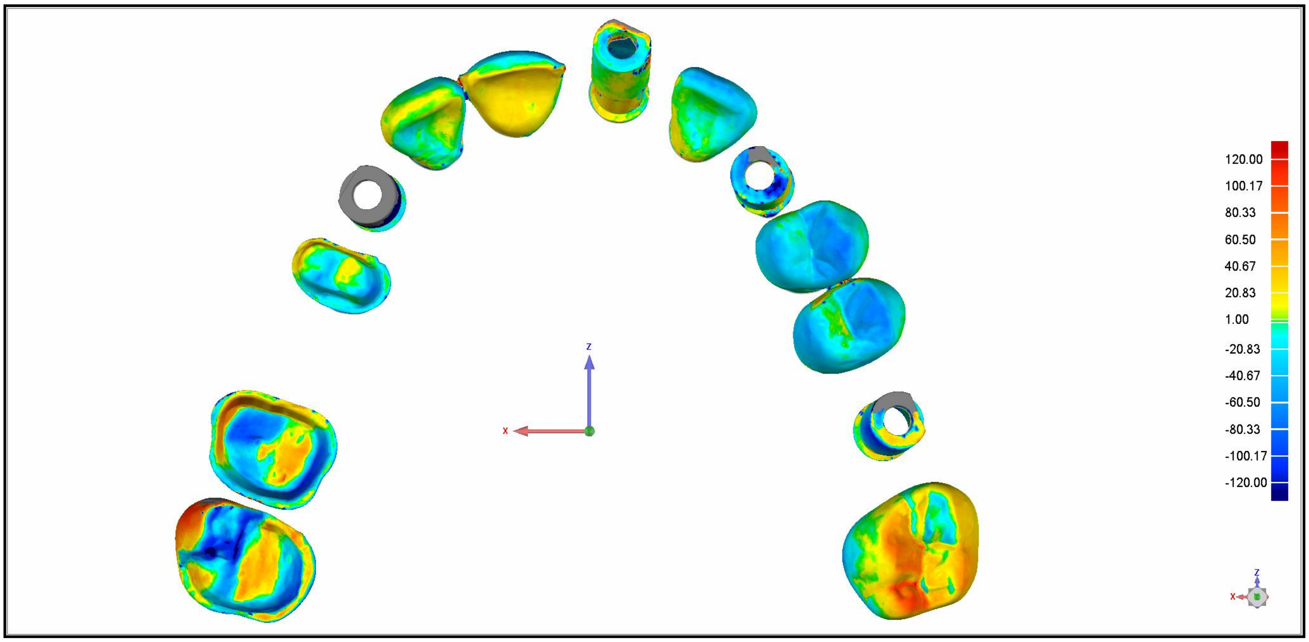

Predefinido: Izquierda

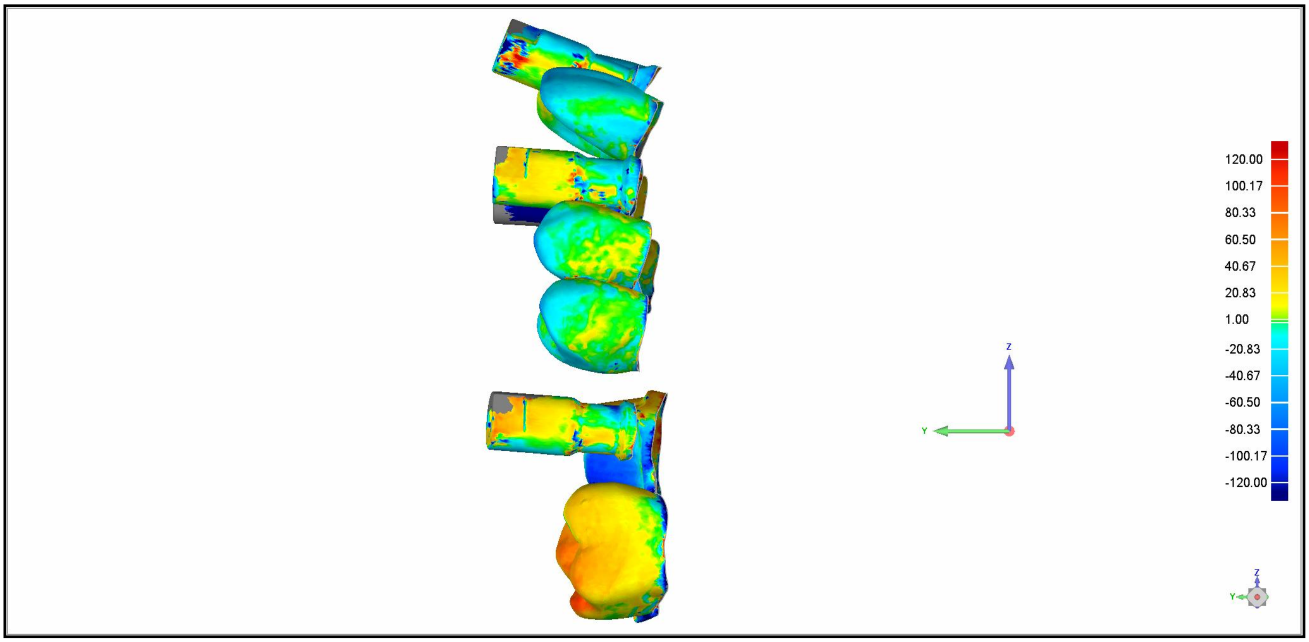

Predefinido: Derecha

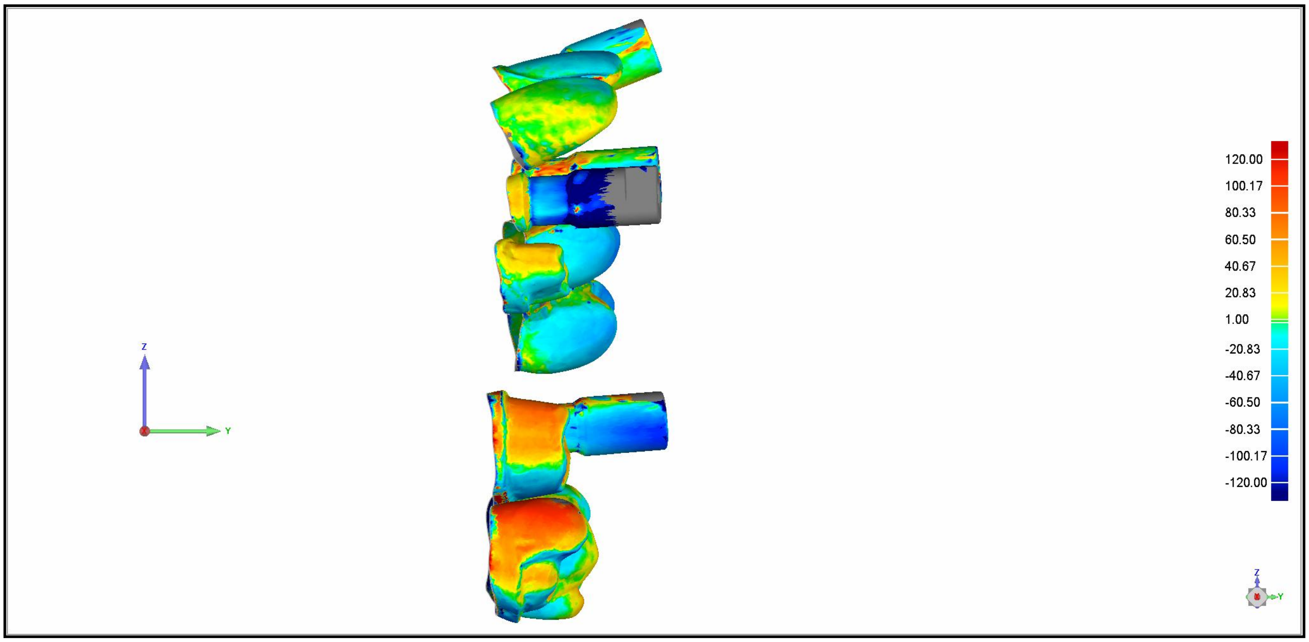

Predefinido: Superior

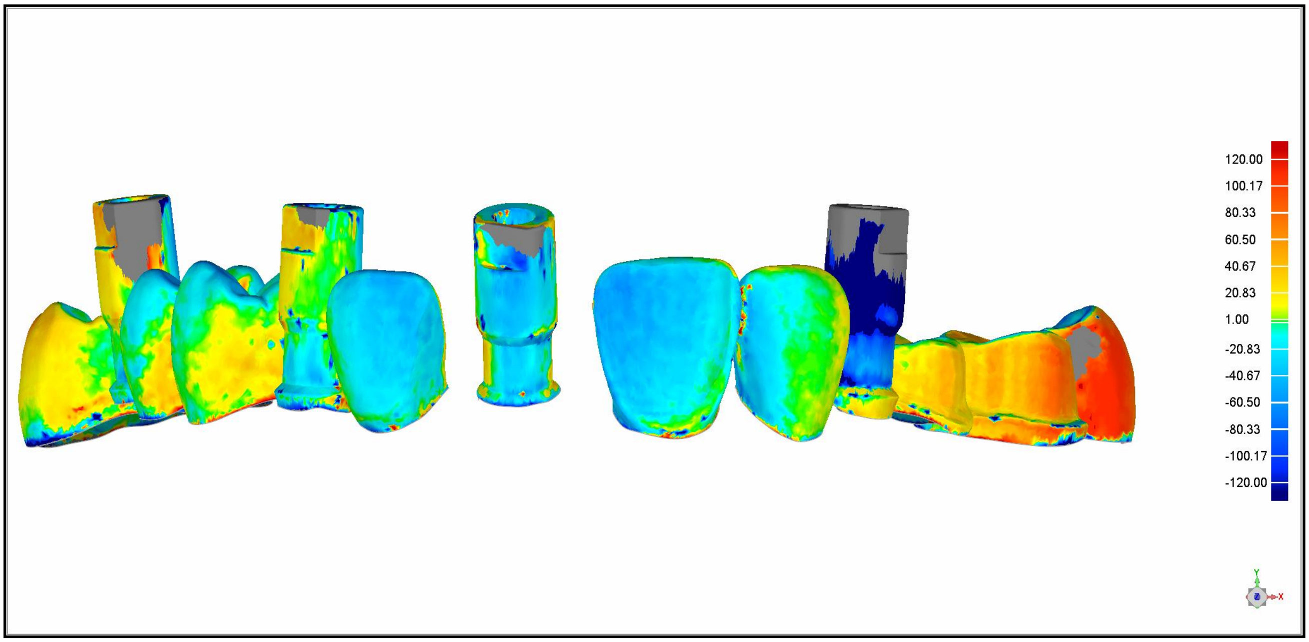

Predefinido: Inferior

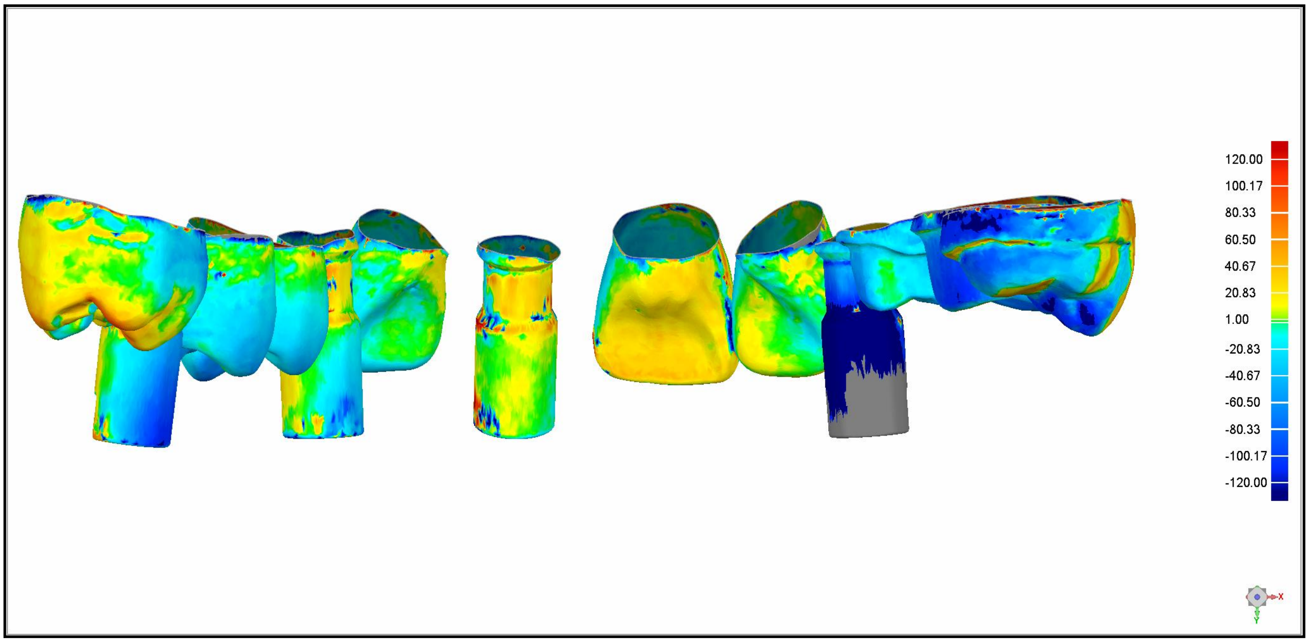

Ajuste de ubicación: Desviaciones superior e inferior

Unidades: u

Nombre	Desv	Estado	Superior Tol	Inferior Tol	Ref X	Ref Y	Ref Z	Radio	Desv X	Desv Y	Desv Z	Medido X	Medido Y	Medido Z	Dir. proy. X	Dir. proy. Y	Dir. proy. Z
Desv. inferior	-3068.41				-29155.64	26913.15	-12045.72	n/a	2712.00	516.70	-1339.12	-26443.65	27429.85	-13384.84	-0.88	-0.17	0.44
Desv. superior	3154.62				-20553.64	28741.29	-8096.88	n/a	-2395.45	103.14	2050.07	-22949.09	28844.43	-6046.81	-0.76	0.03	0.65
