# Supplementary material for: Accuracy of four digital scanners according to scanning strategy in complete-arch impressions
Source: PLoS One. 2018 Sep 13;13(9):e0202916. doi: 10.1371/journal.pone.0202916 (PMC6136706; doi:10.1371/journal.pone.0202916)
Supplement: S8 Table — iTero (scanning strategy D). (ZIP) [file pone.0202916.s008.zip › S8/IT7D.pdf]

Geomagic Control™

Fecha: 3/6/2017, 1:28 pm

3D Comparación Resultados

Modelo referencia	MRC
Modelo test	IT7D
Nº de puntos de datos	81756
# Aislados	522

Tipo tolerancia	3D desviación
Unidades	u
Máx. crítico	120.00
Máx. nominal	1.00
Mín. nominal	-1.00
Mín. crítico	-120.00

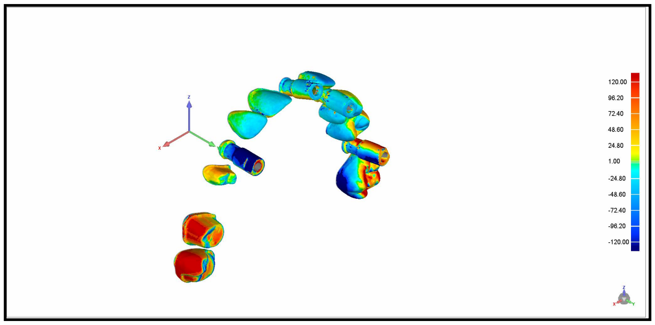

Desviación	
Desviación superior máx.	3021.46
Desviación inferior máx.	-3134.08
Desviación media	96.59 /-83.06
Desviación estándar	211.62

Distribución desviación

>=Min	<Max	# Puntos	%
-120.00	-96.20	2145	2.62
-96.20	-72.40	3024	3.70
-72.40	-48.60	5125	6.27
-48.60	-24.80	11652	14.25
-24.80	-1.00	13489	16.50
-1.00	1.00	1133	1.39
1.00	24.80	12356	15.11
24.80	48.60	7597	9.29
48.60	72.40	4850	5.93
72.40	96.20	2687	3.29
96.20	120.00	2156	2.64

Fuera del crítico superior		7485	9.16
Fuera del crítico inferior		8057	9.85

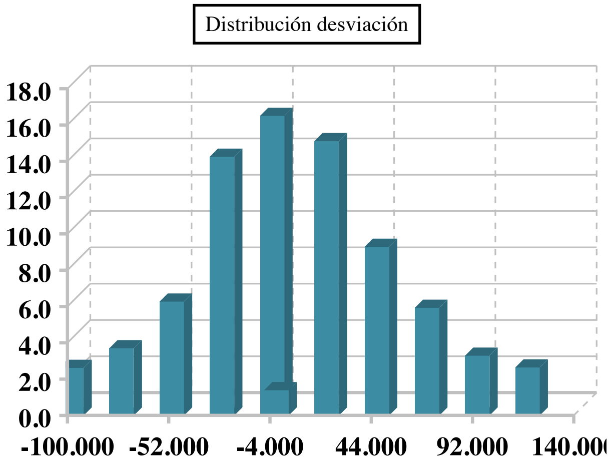

Desviaciones estándar

Distribución (+/-)	# Puntos	%
-6 * Desv. estánd.	385	0.47
-5 * Desv. estánd.	133	0.16
-4 * Desv. estánd.	151	0.18
-3 * Desv. estánd.	215	0.26
-2 * Desv. estánd.	1889	2.31
-1 * Desv. estánd.	41155	50.34
1 * Desv. estánd.	35091	42.92
2 * Desv. estánd.	1507	1.84
3 * Desv. estánd.	232	0.28
4 * Desv. estánd.	201	0.25
5 * Desv. estánd.	189	0.23
6 * Desv. estánd.	608	0.74

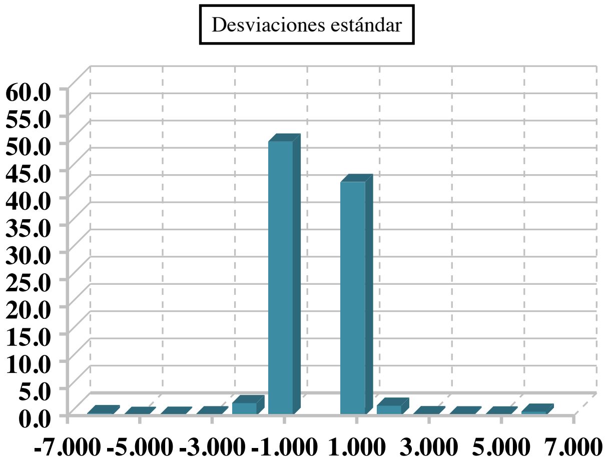

Predefinido: Isométrico

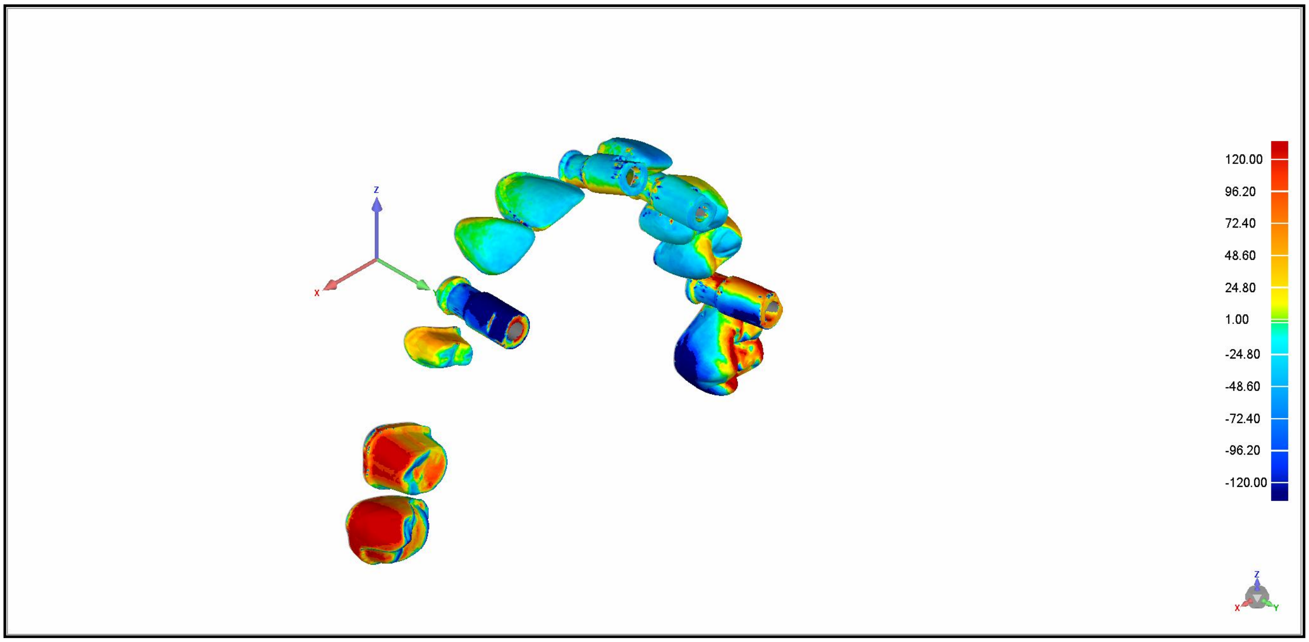

Predefinido: Frente

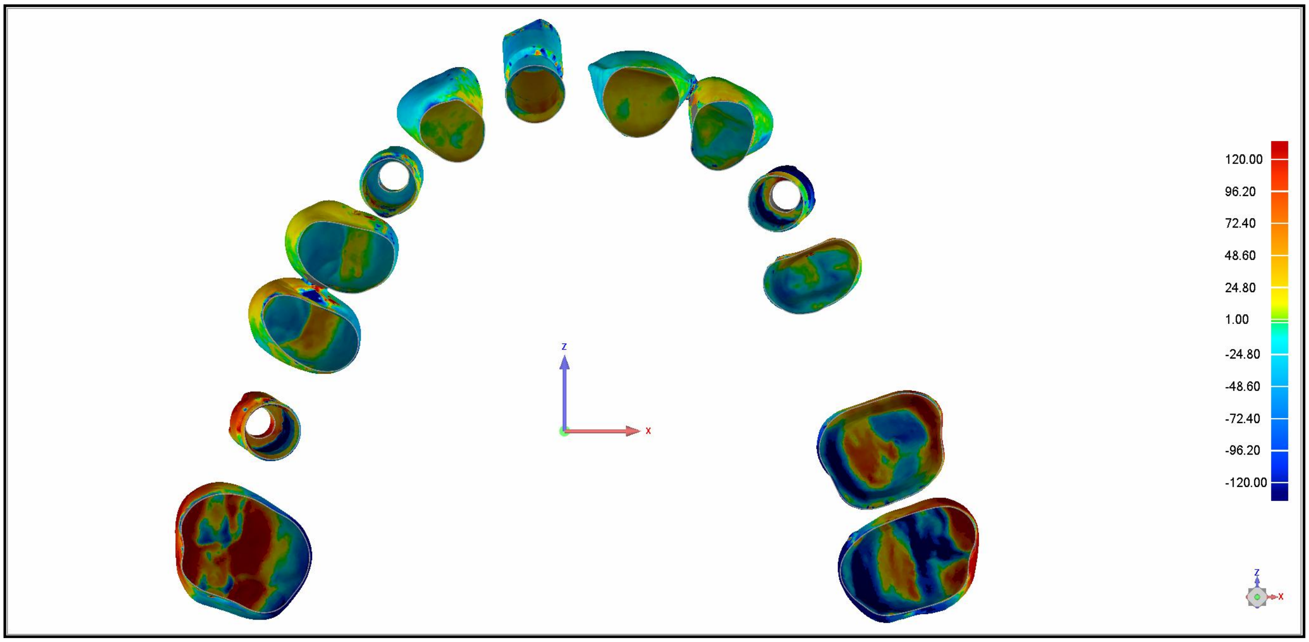

Predefinido: Atrás

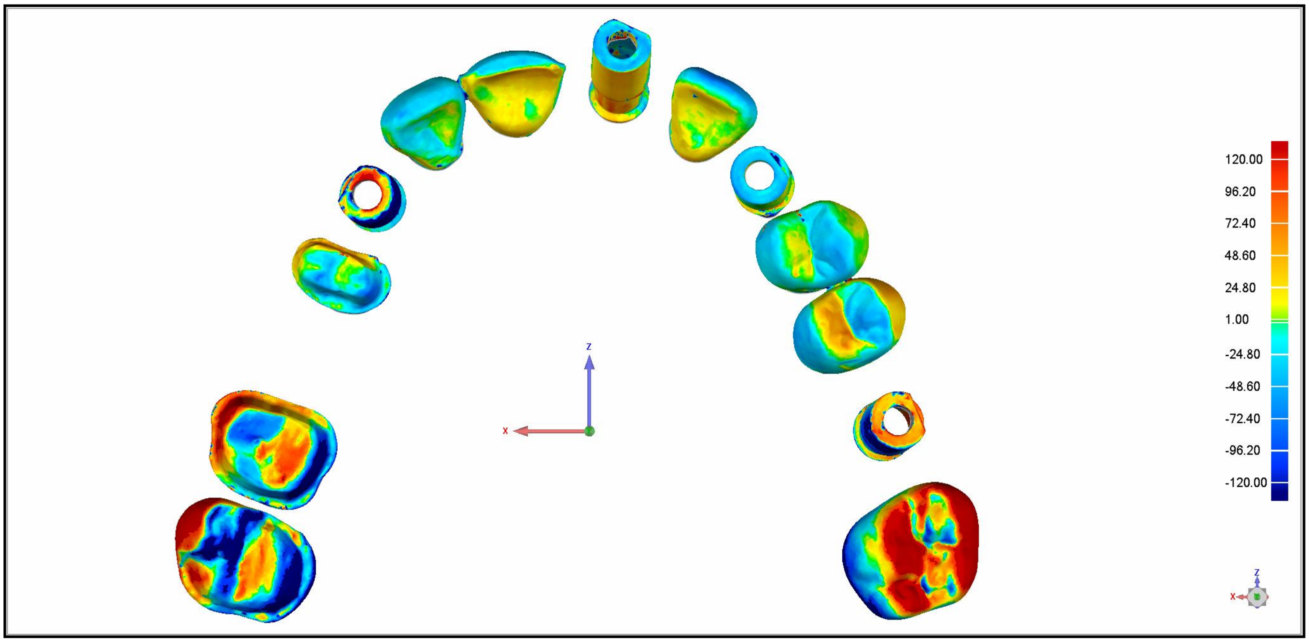

Predefinido: Izquierda

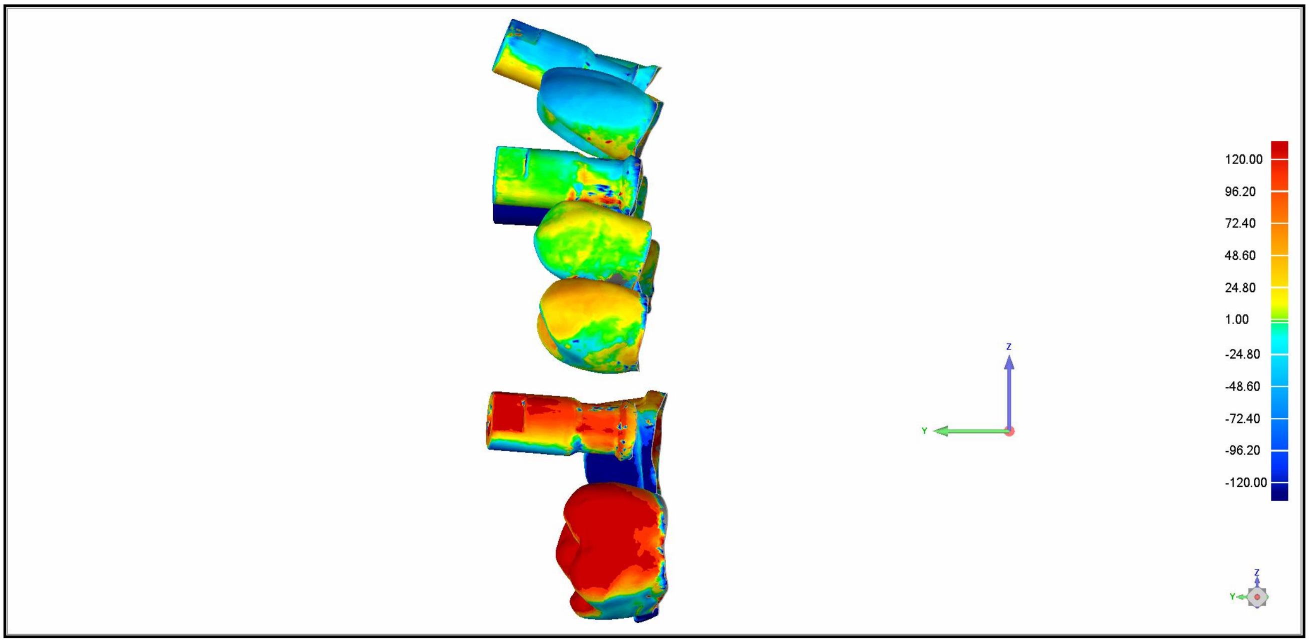

Predefinido: Derecha

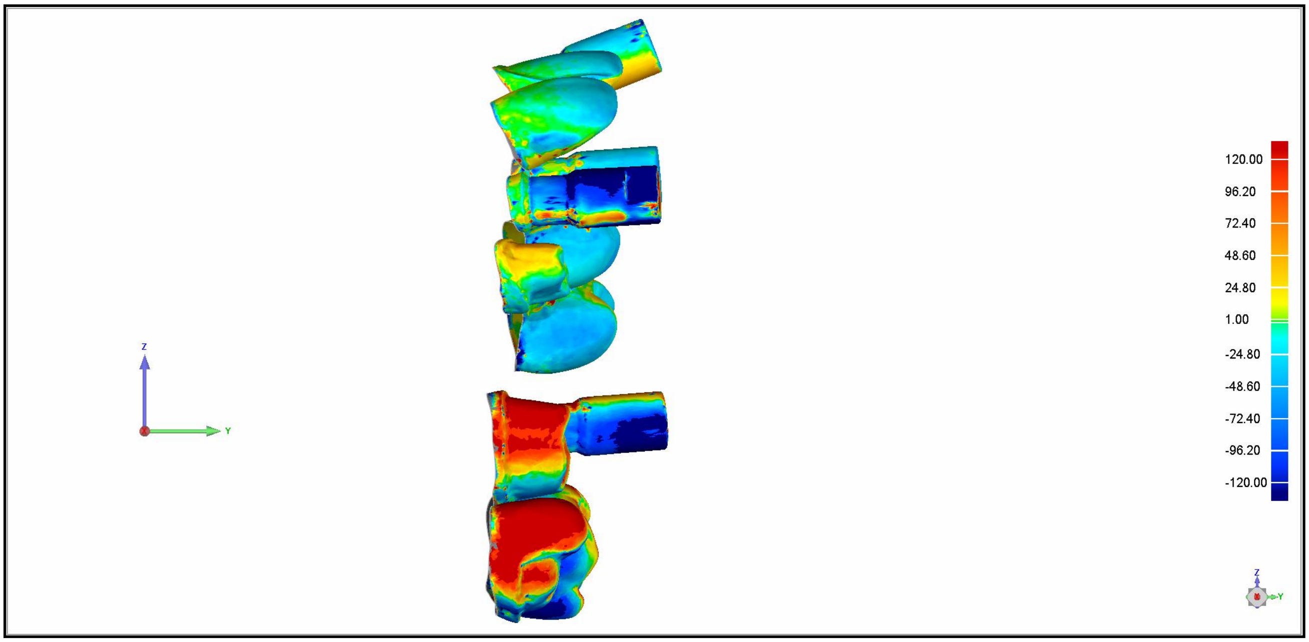

Geomagic Control™

Predefinido: Superior

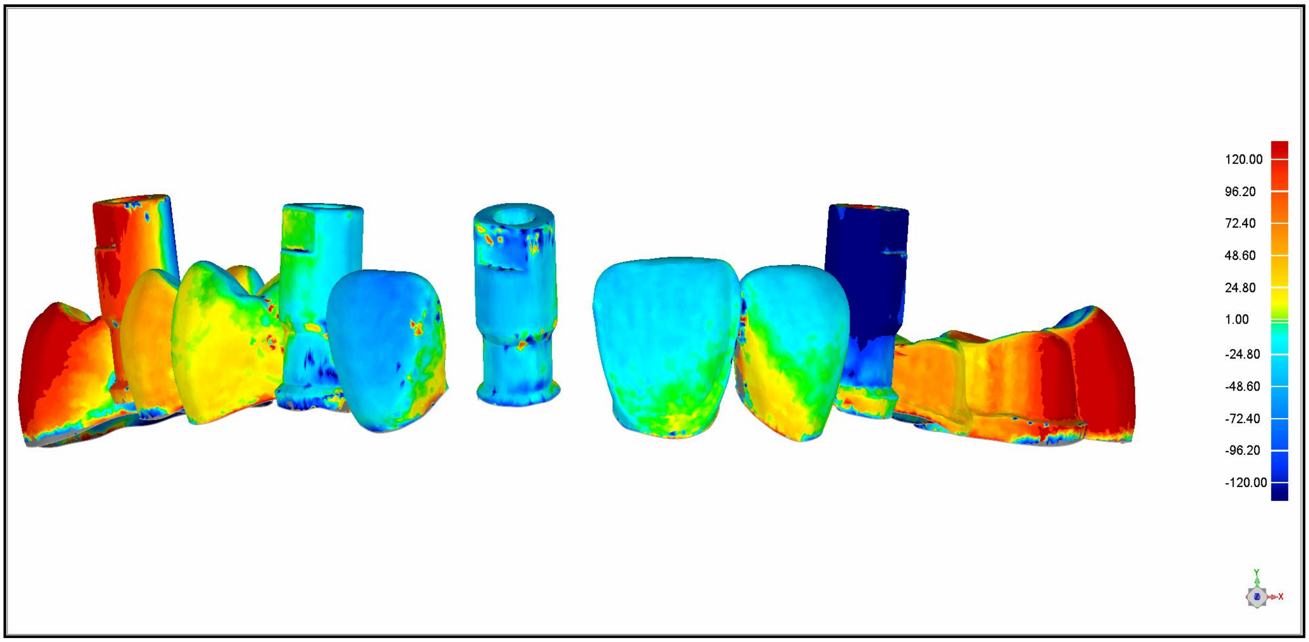

Predefinido: Inferior

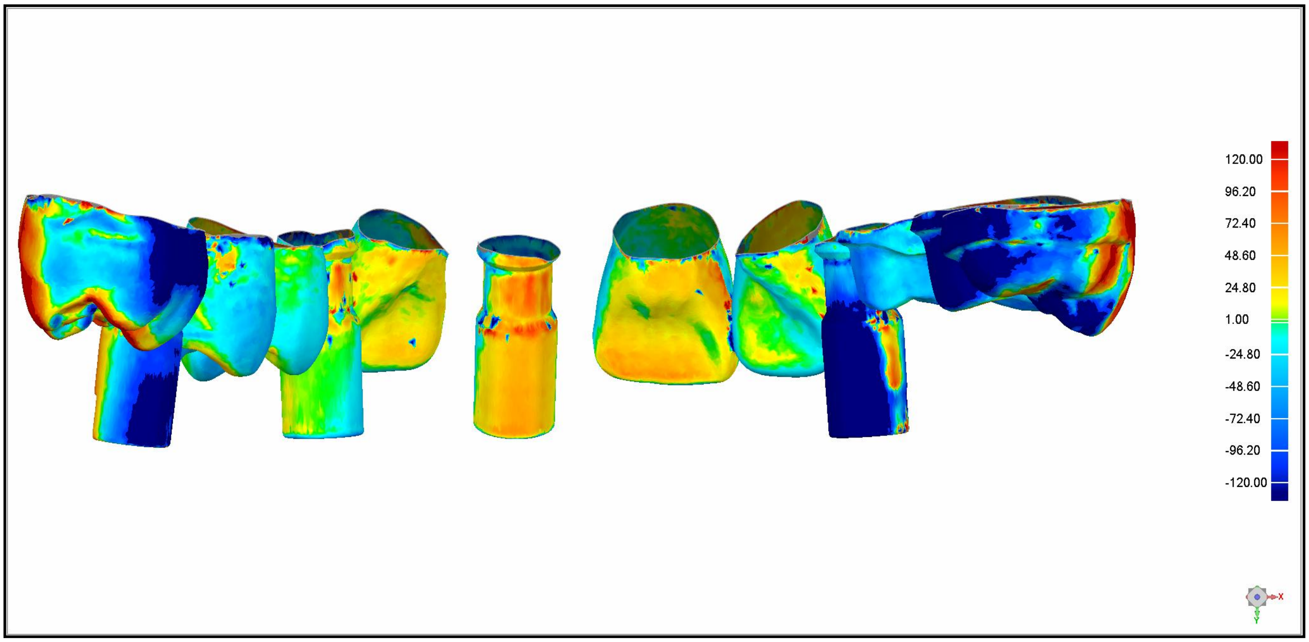

Ajuste de ubicación: Desviaciones superior e inferior

Unidades: u

Nombre	Desv	Estado	Superior Tol	Inferior Tol	Ref X	Ref Y	Ref Z	Radio	Desv X	Desv Y	Desv Z	Medido X	Medido Y	Medido Z	Dir. proy. X	Dir. proy. Y	Dir. proy. Z
Desv. inferior	-3134.08				16989.02	37628.06	17251.36	n/a	1994.39	2385.34	-393.73	18983.40	40013.40	16857.63	-0.64	-0.76	0.13
Desv. superior	3021.46				19351.36	32568.74	13953.26	n/a	-885.73	1808.65	2252.45	18465.64	34377.38	16205.71	-0.29	0.60	0.75
