# Supplementary material for: Accuracy of four digital scanners according to scanning strategy in complete-arch impressions
Source: PLoS One. 2018 Sep 13;13(9):e0202916. doi: 10.1371/journal.pone.0202916 (PMC6136706; doi:10.1371/journal.pone.0202916)
Supplement: S8 Table — iTero (scanning strategy D). (ZIP) [file pone.0202916.s008.zip › S8/IT10D.pdf]

# 3D Comparación  Resultados

| | |
|---|---|
| Modelo referencia | MRC |
| Modelo test | IT10D |
| Nº de puntos de datos | 81782 |
| # Aislados | 607 |

| | |
|---|---|
| Tipo tolerancia | 3D desviación |
| Unidades | u |
| Máx. crítico | 120.00 |
| Máx. nominal | 1.00 |
| Mín. nominal | -1.00 |
| Mín. crítico | -120.00 |

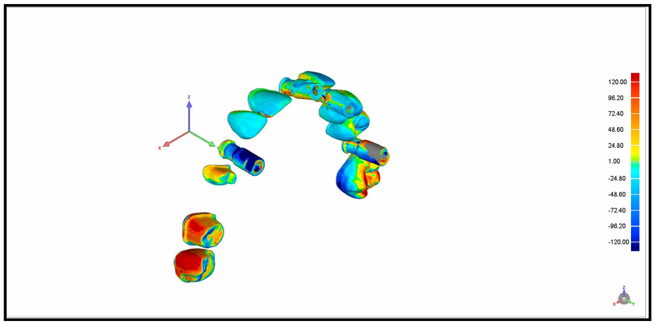

| | |
|---|---|
| Desviación | |
| Desviación superior máx. | 2681.40 |
| Desviación inferior máx. | -3145.40 |
| Desviación media | 87.87 /-70.55 |
| Desviación estándar | 201.28 |

## Distribución desviación

| >=Min | <Max | # Puntos | % |
|---|---|---|---|
| -120.00 | -96.20 | 2459 | 3.01 |
| -96.20 | -72.40 | 2875 | 3.52 |
| -72.40 | -48.60 | 4626 | 5.66 |
| -48.60 | -24.80 | 11610 | 14.20 |
| -24.80 | -1.00 | 17446 | 21.33 |
| -1.00 | 1.00 | 1424 | 1.74 |
| 1.00 | 24.80 | 12631 | 15.44 |
| 24.80 | 48.60 | 8123 | 9.93 |
| 48.60 | 72.40 | 5000 | 6.11 |
| 72.40 | 96.20 | 3099 | 3.79 |
| 96.20 | 120.00 | 1968 | 2.41 |

| | | |
|---|---|---|
| Fuera del crítico superior | 5006 | 6.12 |
| Fuera del crítico inferior | 5515 | 6.74 |

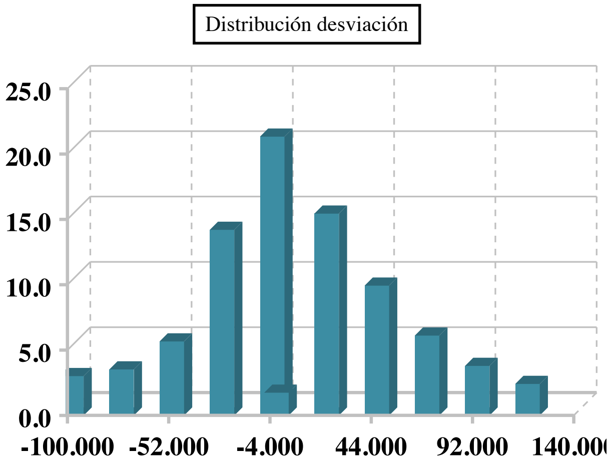


## Desviaciones estándar

| Distribución (+/-) | # Puntos | % |
|---|---|---|
| -6 * Desv. estánd. | 451 | 0.55 |
| -5 * Desv. estánd. | 128 | 0.16 |
| -4 * Desv. estánd. | 145 | 0.18 |
| -3 * Desv. estánd. | 234 | 0.29 |
| -2 * Desv. estánd. | 946 | 1.16 |
| -1 * Desv. estánd. | 43515 | 53.21 |
| 1 * Desv. estánd. | 34321 | 41.97 |
| 2 * Desv. estánd. | 673 | 0.82 |
| 3 * Desv. estánd. | 277 | 0.34 |
| 4 * Desv. estánd. | 241 | 0.29 |
| 5 * Desv. estánd. | 204 | 0.25 |
| 6 * Desv. estánd. | 647 | 0.79 |

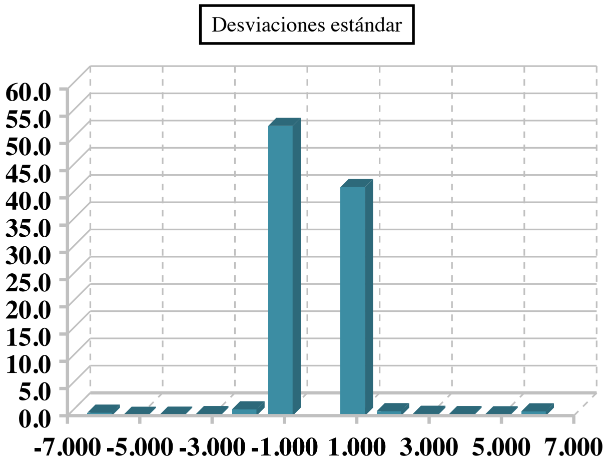

Predefinido: Isométrico

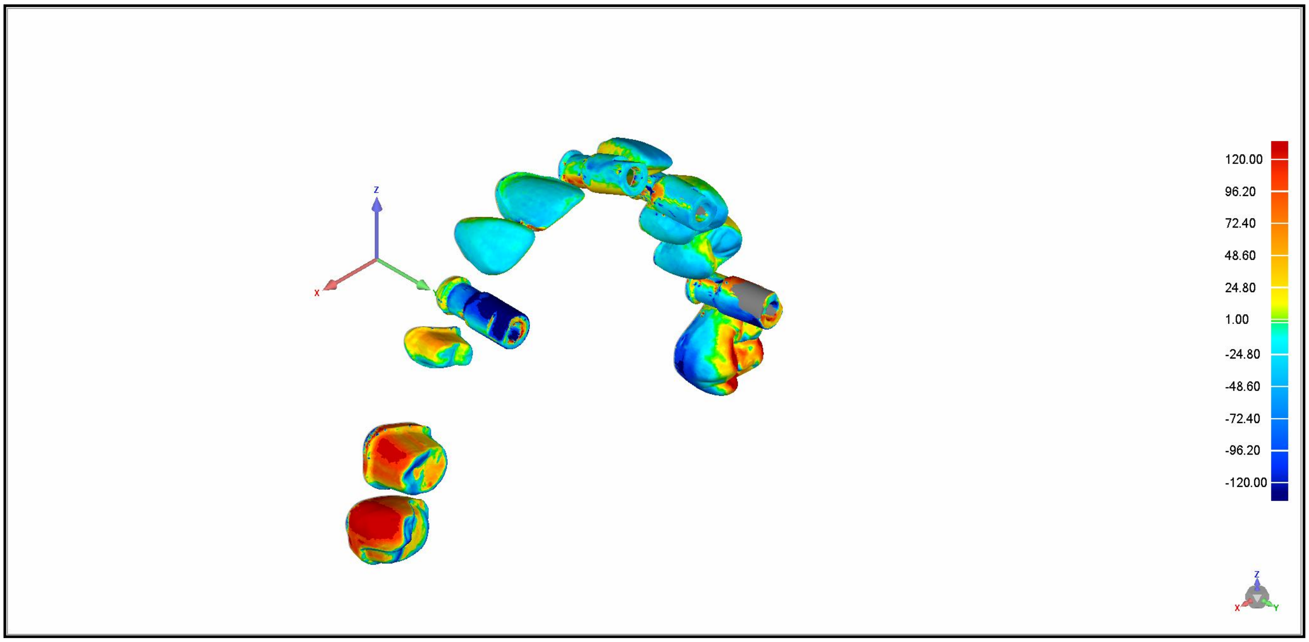

Predefinido: Frente

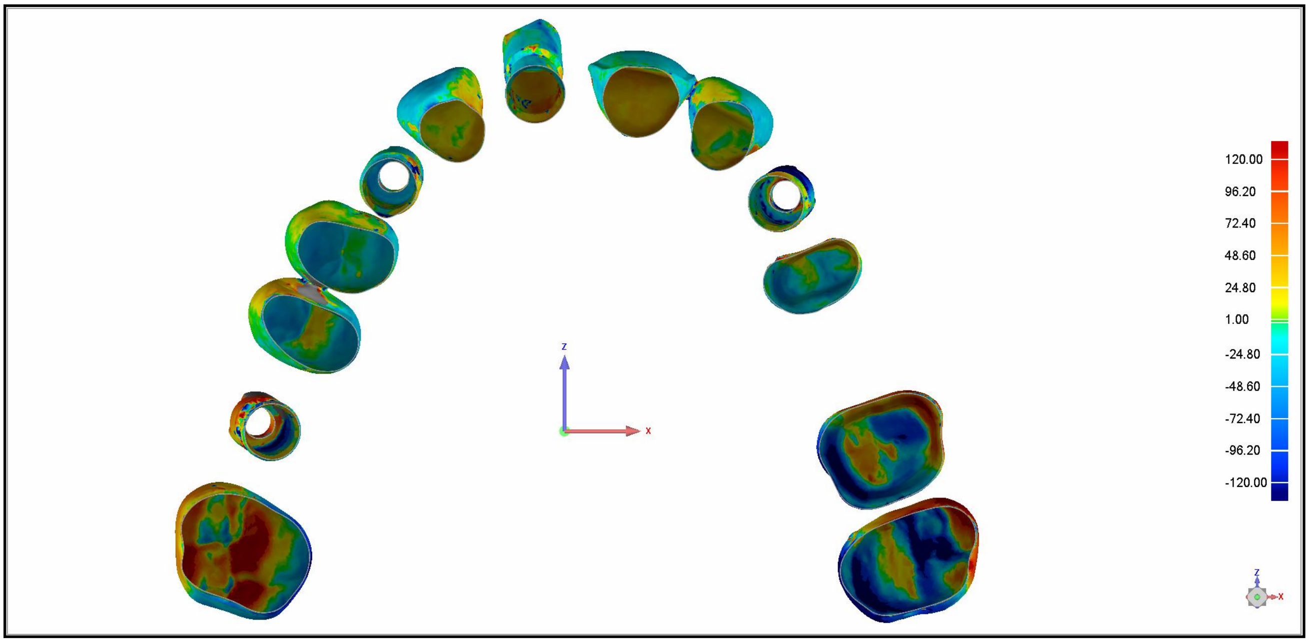

Predefinido: Atrás

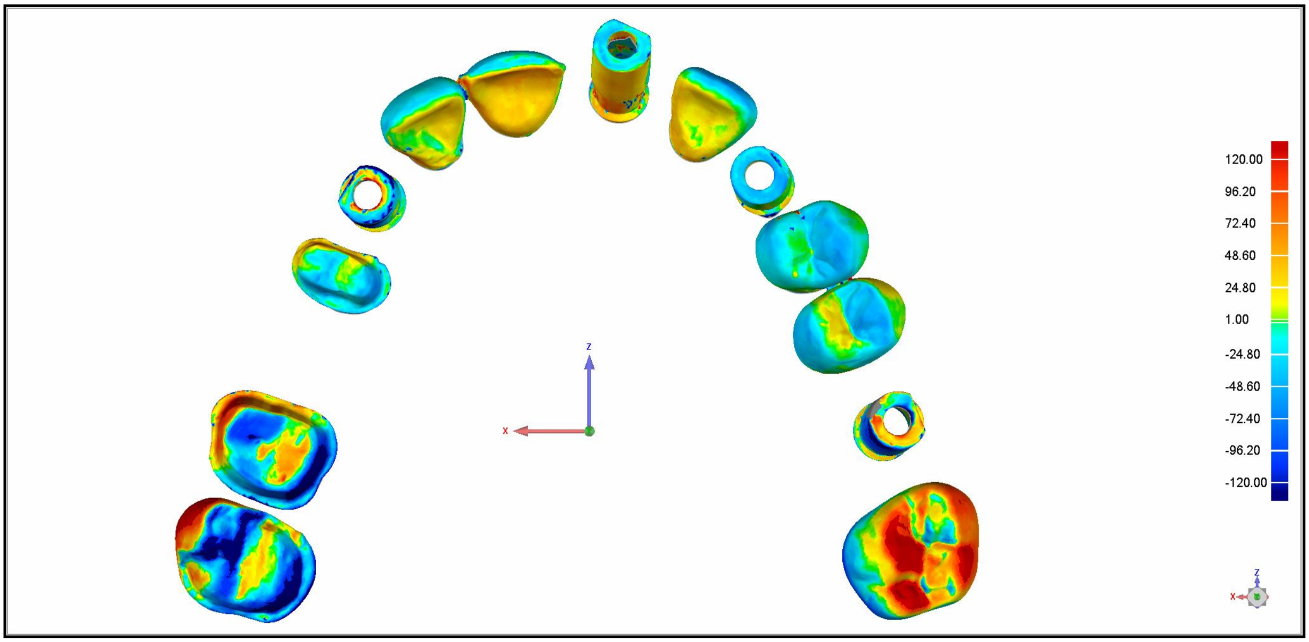

Predefinido: Izquierda

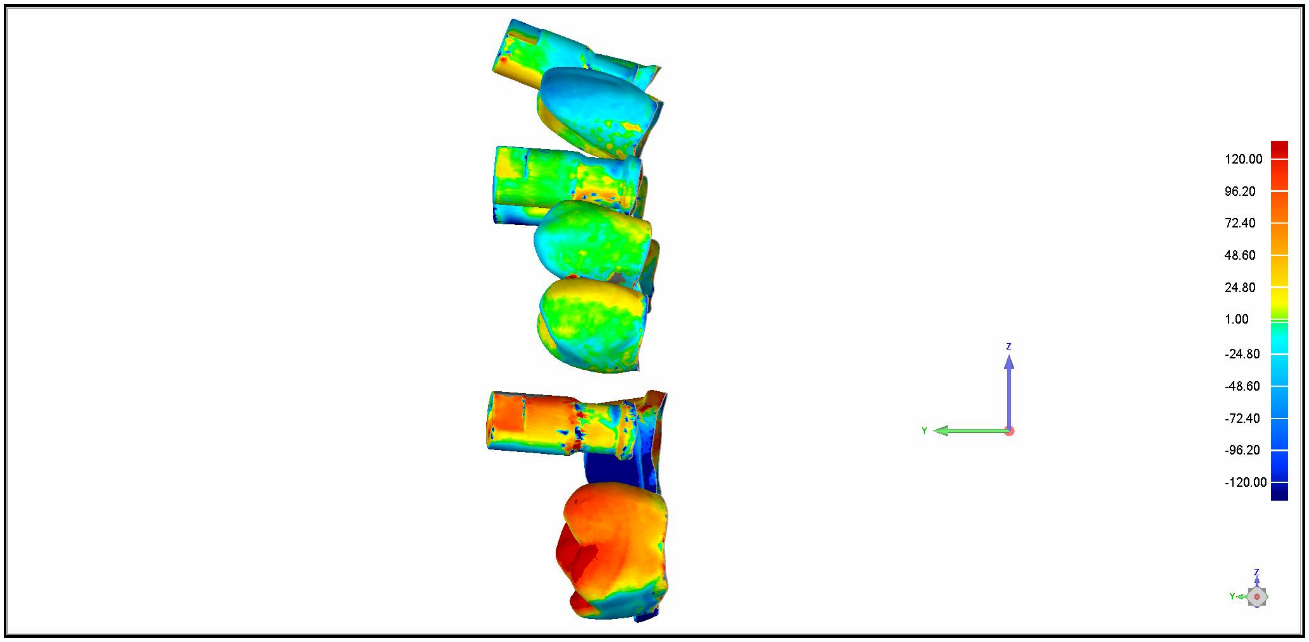

Predefinido: Derecha

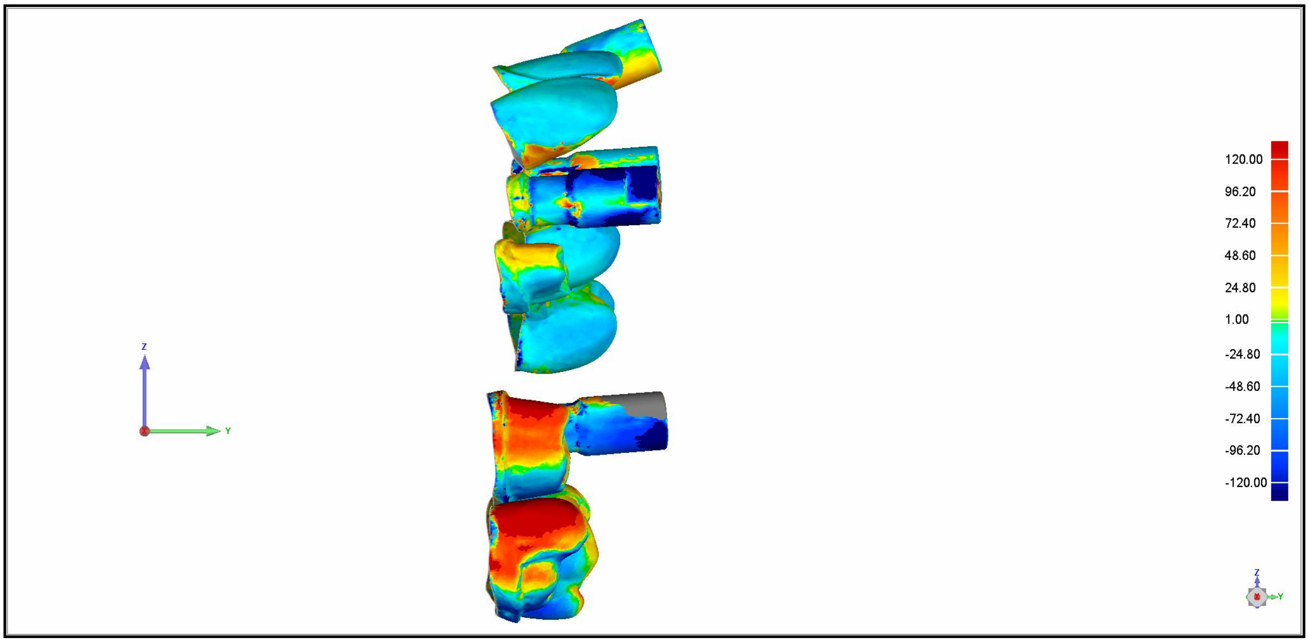

Predefinido: Superior

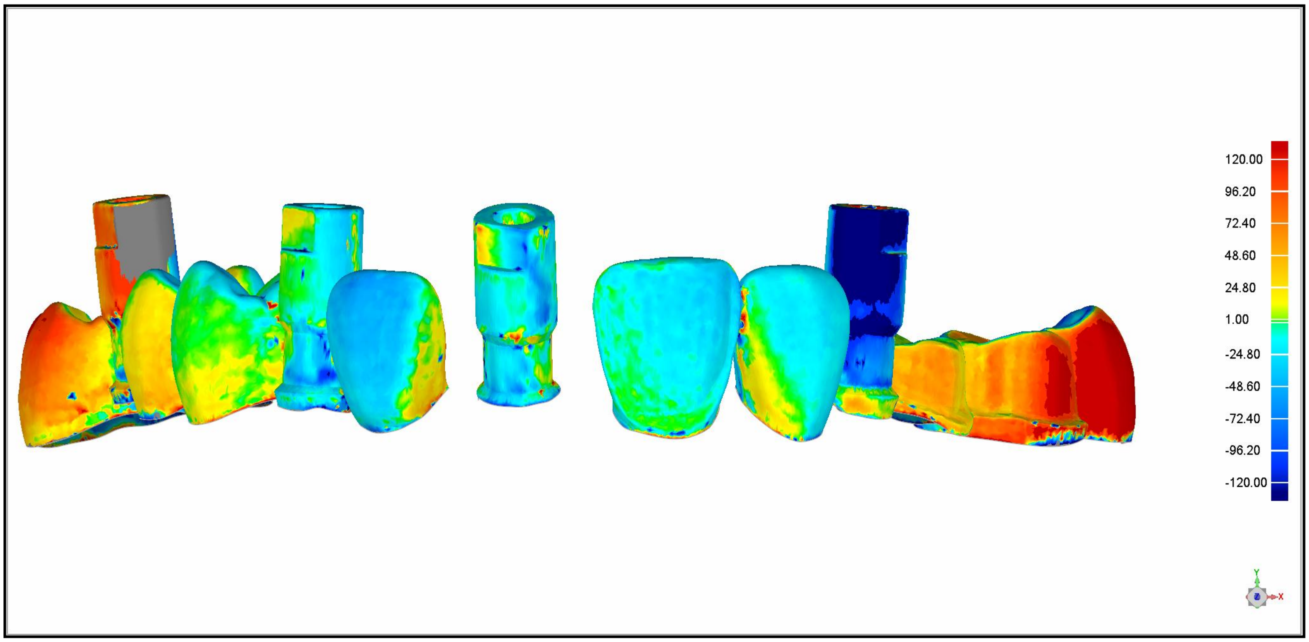

Predefinido: Inferior

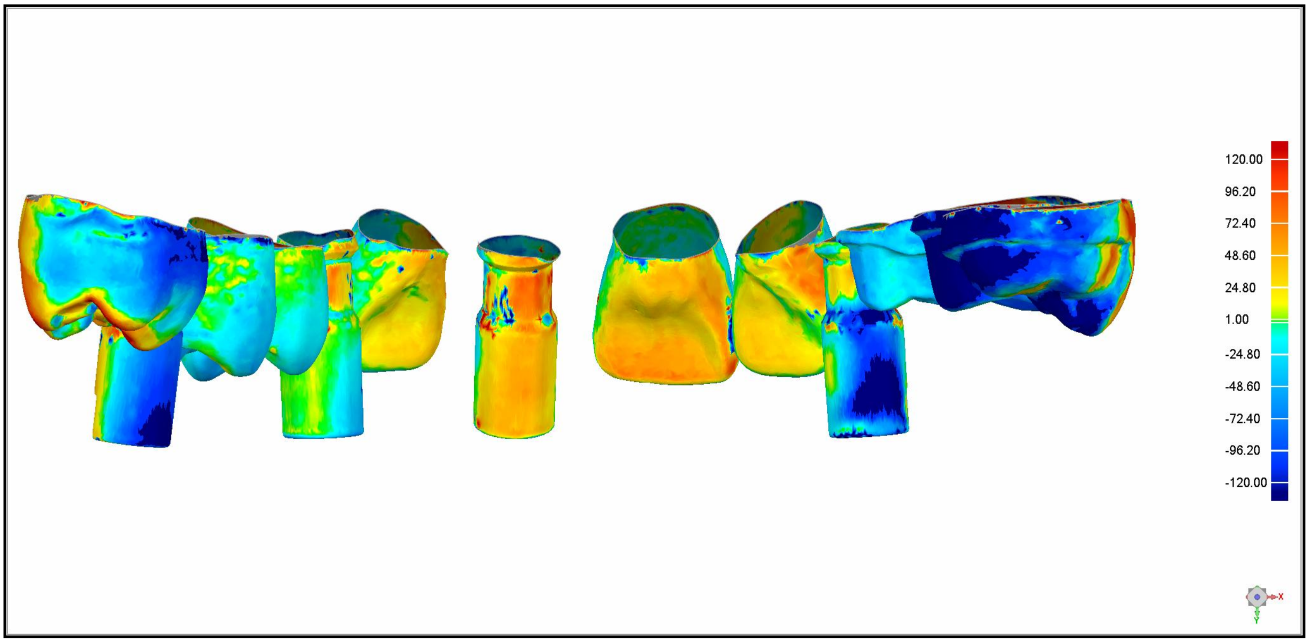

# Ajuste de ubicación: Desviaciones superior e inferior

## Unidades: u

| Nombre | Desv | Estado | Superior Tol | Inferior Tol | Ref X | Ref Y | Ref Z | Radio | Desv X | Desv Y | Desv Z | Medido X | Medido Y | Medido Z | Dir. proy. X | Dir. proy. Y | Dir. proy. Z |
|---|---|---|---|---|---|---|---|---|---|---|---|---|---|---|---|---|---|
| Desv. inferior | -3145.40 | | | | 16989.02 | 37628.06 | 17251.36 | n/a | -286.00 | 2913.27 | 1150.91 | 16703.02 | 40541.33 | 18402.27 | 0.09 | -0.93 | -0.37 |
| Desv. superior | 2681.40 | | | | 2184.43 | 32622.61 | 27334.95 | n/a | -2638.05 | -289.75 | -382.97 | -453.62 | 32332.86 | 26951.98 | -0.98 | -0.11 | -0.14 |
