# Supplementary material for: Accuracy of four digital scanners according to scanning strategy in complete-arch impressions
Source: PLoS One. 2018 Sep 13;13(9):e0202916. doi: 10.1371/journal.pone.0202916 (PMC6136706; doi:10.1371/journal.pone.0202916)
Supplement: S8 Table — iTero (scanning strategy D). (ZIP) [file pone.0202916.s008.zip › S8/IT6D.pdf]

Geomagic Control™

3D Comparación Resultados

Modelo referencia	MRC
Modelo test	IT6D
Nº de puntos de datos	81635
# Aislados	730

Tipo tolerancia	3D desviación
Unidades	u
Máx. crítico	120.00
Máx. nominal	1.00
Mín. nominal	-1.00
Mín. crítico	-120.00

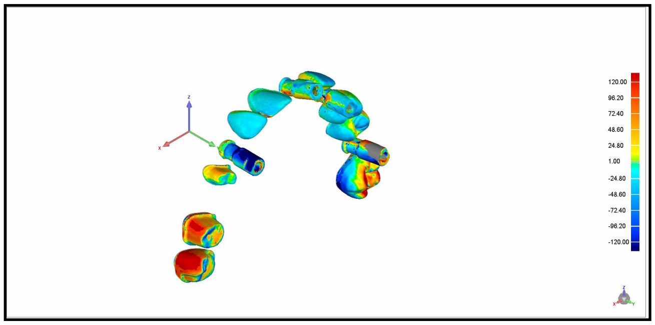

Desviación	
Desviación superior máx.	3143.08
Desviación inferior máx.	-3139.71
Desviación media	93.18 /-72.82
Desviación estándar	216.13

Distribución desviación

>=Min	<Max	# Puntos	%
-120.00	-96.20	2405	2.95
-96.20	-72.40	2939	3.60
-72.40	-48.60	4693	5.75
-48.60	-24.80	11568	14.17
-24.80	-1.00	17643	21.61
-1.00	1.00	1404	1.72
1.00	24.80	12319	15.09
24.80	48.60	7996	9.79
48.60	72.40	4661	5.71
72.40	96.20	3126	3.83
96.20	120.00	1971	2.41

Fuera del crítico superior	5141	6.30
Fuera del crítico inferior	5769	7.07

Distribución desviación

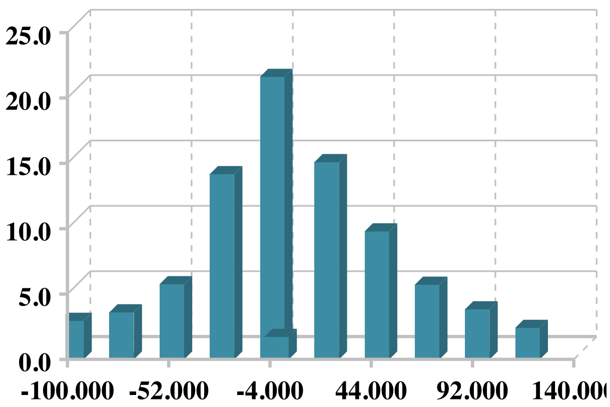

Desviaciones estándar

Distribución (+/-)	# Puntos	%
-6 * Desv. estánd.	459	0.56
-5 * Desv. estánd.	135	0.17
-4 * Desv. estánd.	163	0.20
-3 * Desv. estánd.	239	0.29
-2 * Desv. estánd.	914	1.12
-1 * Desv. estánd.	43975	53.87
1 * Desv. estánd.	33731	41.32
2 * Desv. estánd.	621	0.76
3 * Desv. estánd.	293	0.36
4 * Desv. estánd.	245	0.30
5 * Desv. estánd.	232	0.28
6 * Desv. estánd.	628	0.77

Desviaciones estándar

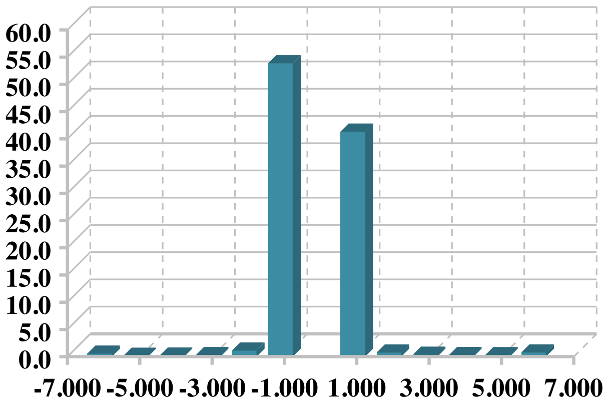

Predefinido: Isométrico

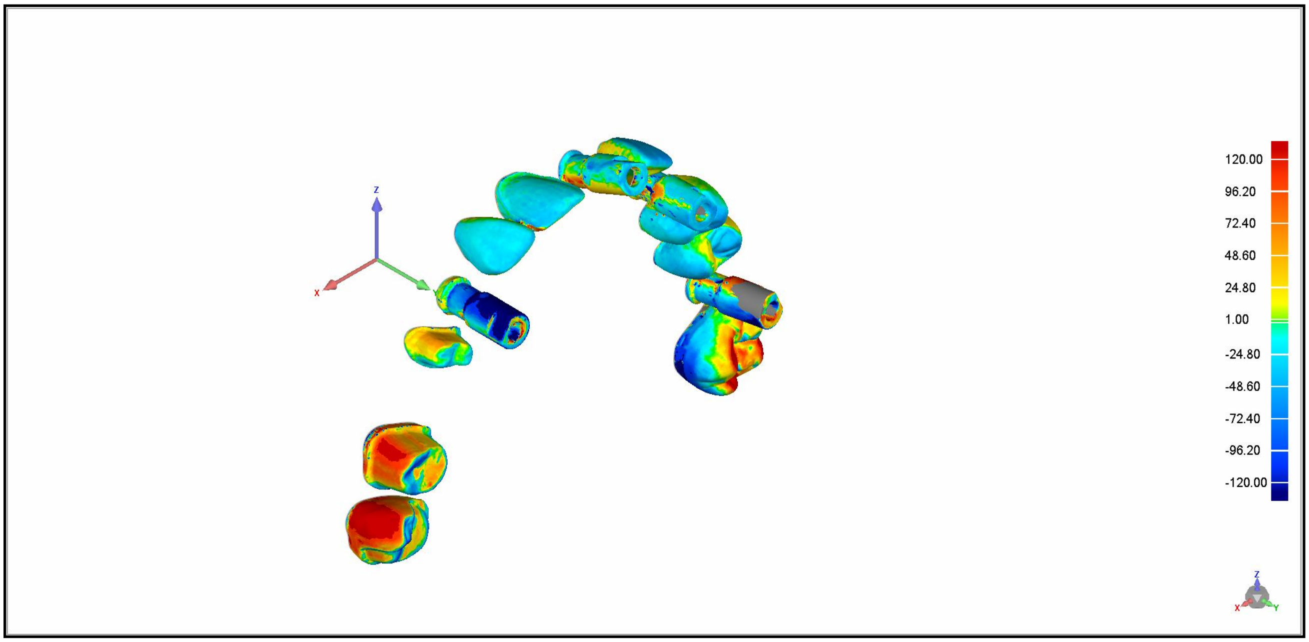

Predefinido: Frente

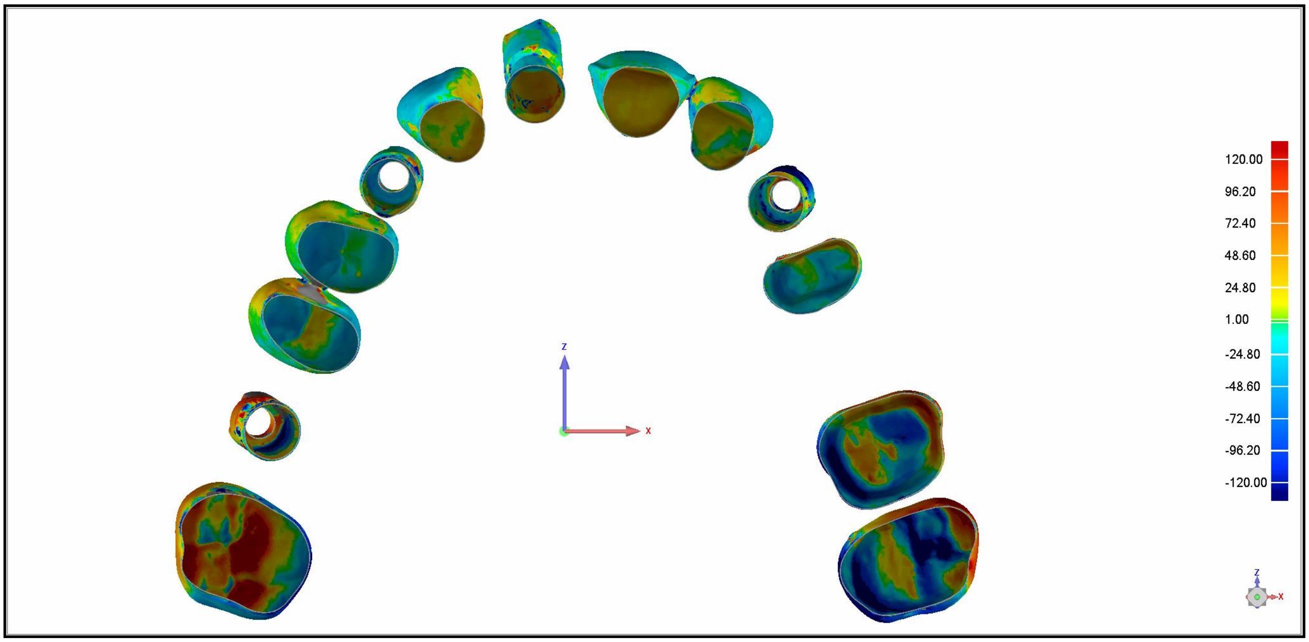

Predefinido: Atrás

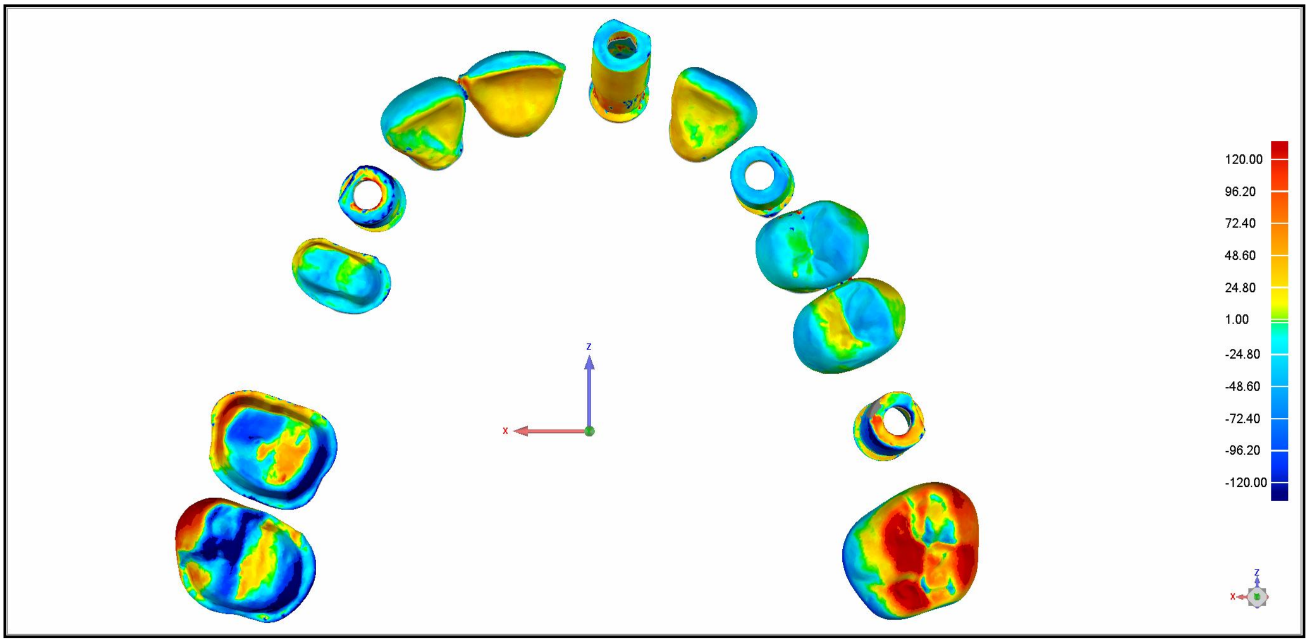

Predefinido: Izquierda

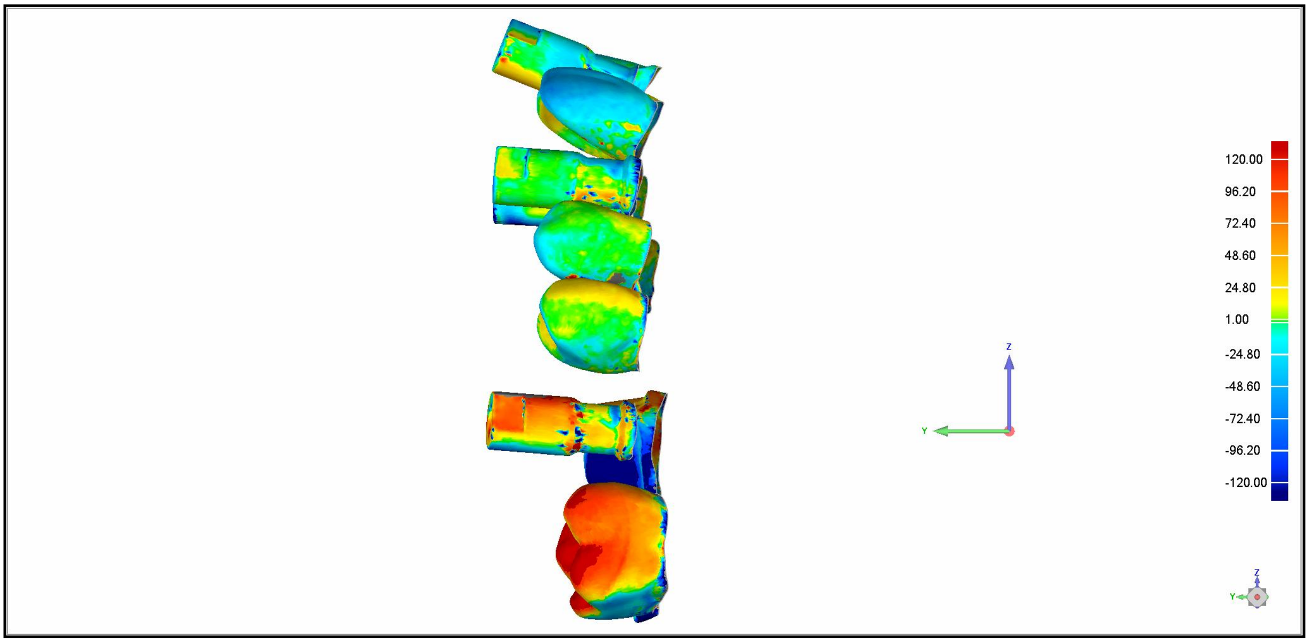

Predefinido: Derecha

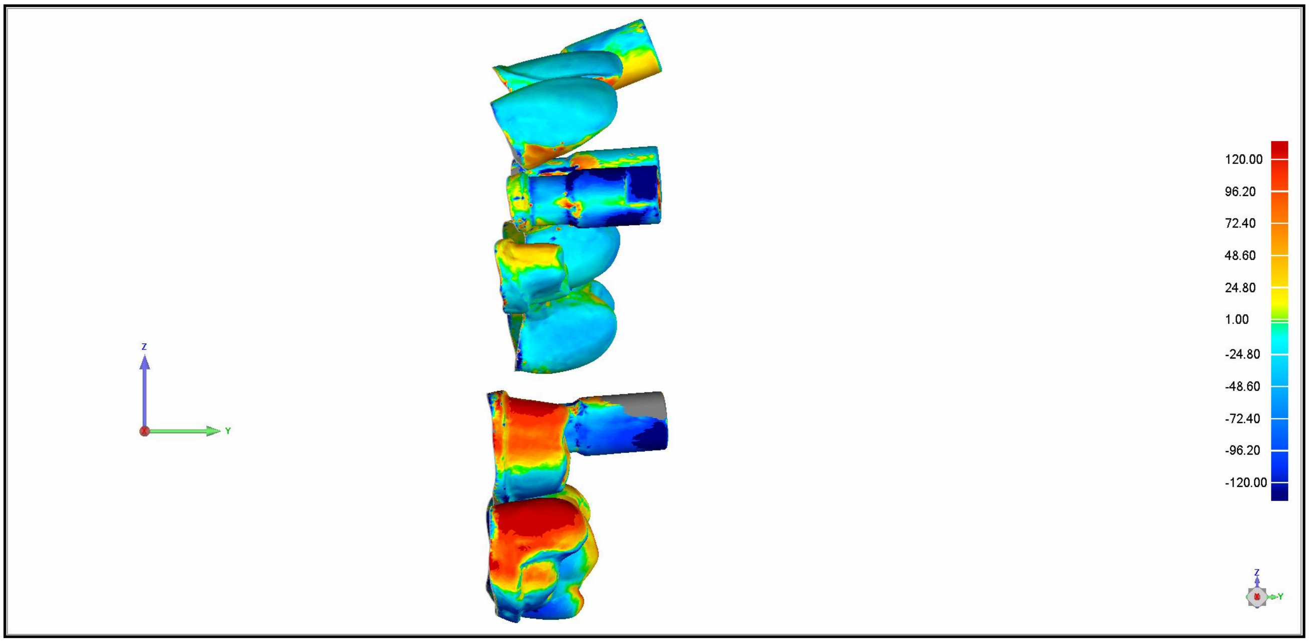

Predefinido: Superior

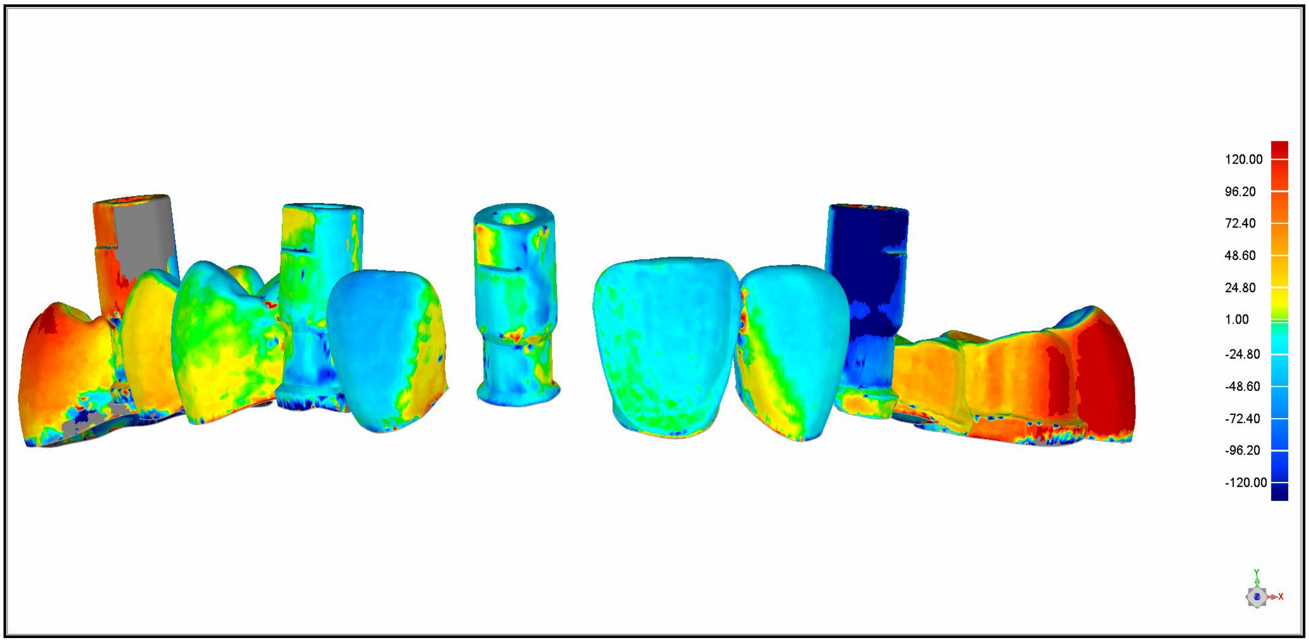

Predefinido: Inferior

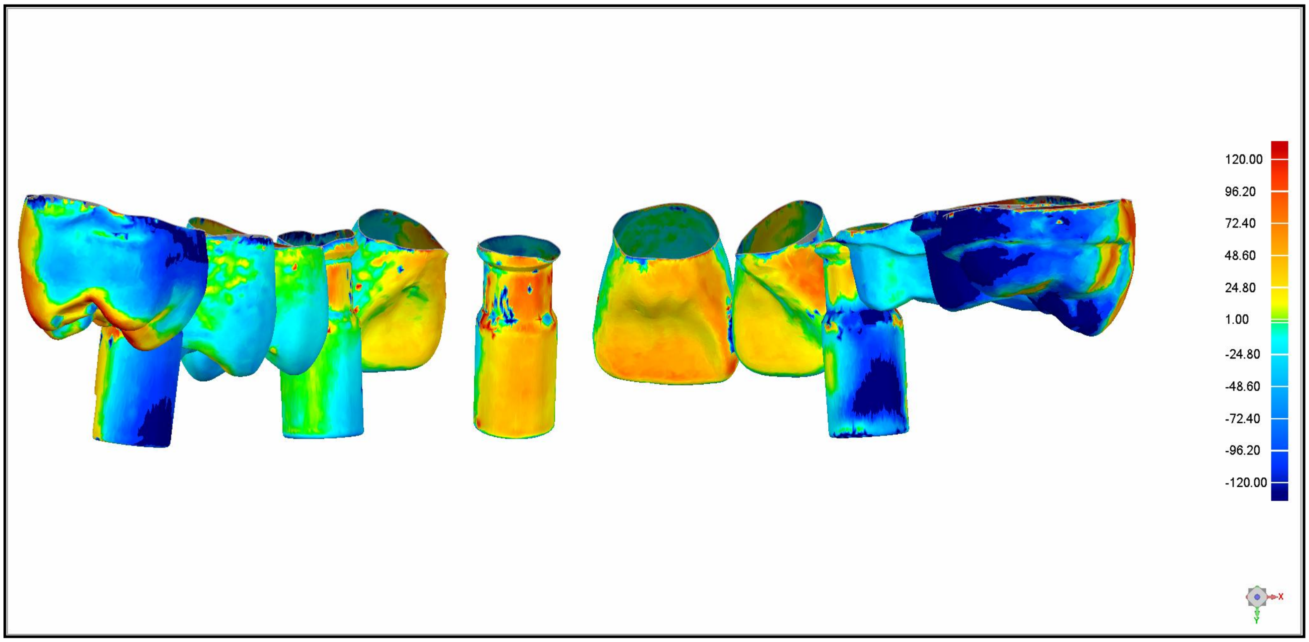

Ajuste de ubicación: Desviaciones superior e inferior

Unidades: u

Nombre	Desv	Estado	Superior Tol	Inferior Tol	Ref X	Ref Y	Ref Z	Radio	Desv X	Desv Y	Desv Z	Medido X	Medido Y	Medido Z	Dir. proy. X	Dir. proy. Y	Dir. proy. Z
Desv. inferior	-3139.71				16989.02	37628.06	17251.36	n/a	-284.10	2907.79	1149.72	16704.92	40535.85	18401.08	0.09	-0.93	-0.37
Desv. superior	3143.08				-29824.91	26937.61	-11348.70	n/a	2607.69	325.62	-1724.21	-27217.22	27263.23	-13072.91	0.83	0.10	-0.55
