# Supplementary material for: Accuracy of four digital scanners according to scanning strategy in complete-arch impressions
Source: PLoS One. 2018 Sep 13;13(9):e0202916. doi: 10.1371/journal.pone.0202916 (PMC6136706; doi:10.1371/journal.pone.0202916)
Supplement: S9 Table — Omnicam (scanning strategy A). (ZIP) [file pone.0202916.s009.zip › S9/OM2A.pdf]

# 3D Comparación  Resultados

| | |
|---|---|
| Modelo referencia | MRC |
| Modelo test | OM2A |
| Nº de puntos de datos | 197028 |
| # Aislados | 730 |

| | |
|---|---|
| Tipo tolerancia | 3D desviación |
| Unidades | u |
| Máx. crítico | 120.00 |
| Máx. nominal | 16.00 |
| Mín. nominal | -16.00 |
| Mín. crítico | -120.00 |

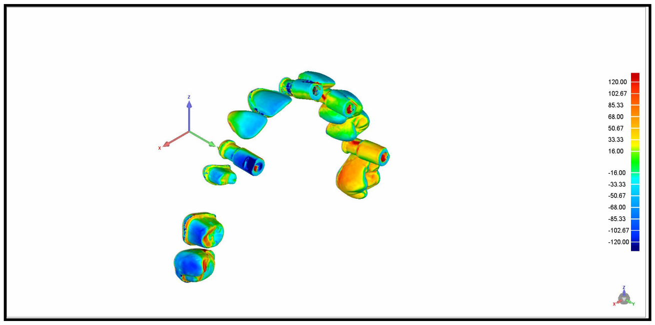

| | |
|---|---|
| Desviación | |
| Desviación superior máx. | 3075.82 |
| Desviación inferior máx. | -3150.40 |
| Desviación media | 101.82 /-81.87 |
| Desviación estándar | 268.01 |

## Distribución desviación

| >=Min | <Max | # Puntos | % |
|---|---|---|---|
| -120.00 | -102.67 | 1499 | 0.76 |
| -102.67 | -85.33 | 2522 | 1.28 |
| -85.33 | -68.00 | 4013 | 2.04 |
| -68.00 | -50.67 | 8954 | 4.54 |
| -50.67 | -33.33 | 17995 | 9.13 |
| -33.33 | -16.00 | 23253 | 11.80 |
| -16.00 | 16.00 | 54699 | 27.76 |
| 16.00 | 33.33 | 23790 | 12.07 |
| 33.33 | 50.67 | 17582 | 8.92 |
| 50.67 | 68.00 | 9778 | 4.96 |
| 68.00 | 85.33 | 5494 | 2.79 |
| 85.33 | 102.67 | 3384 | 1.72 |
| 102.67 | 120.00 | 2177 | 1.10 |

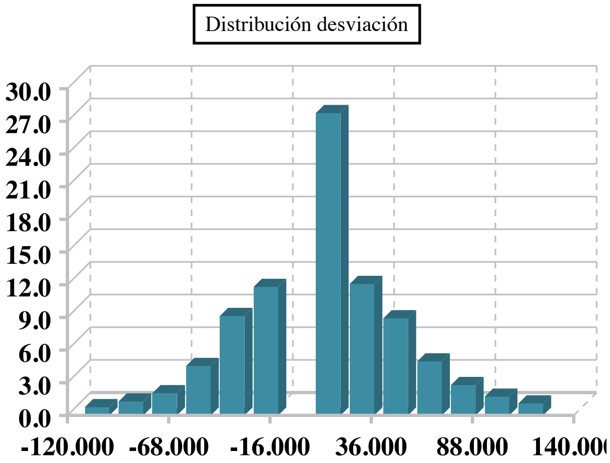


| | | | |
|---|---|---|---|
| Fuera del crítico superior | | 15280 | 7.76 |
| Fuera del crítico inferior | | 6608 | 3.35 |

## Desviaciones estándar

| Distribución (+/-) | # Puntos | % |
|---|---|---|
| -6 * Desv. estánd. | 1375 | 0.70 |
| -5 * Desv. estánd. | 528 | 0.27 |
| -4 * Desv. estánd. | 512 | 0.26 |
| -3 * Desv. estánd. | 586 | 0.30 |
| -2 * Desv. estánd. | 830 | 0.42 |
| -1 * Desv. estánd. | 117000 | 59.38 |
| 1 * Desv. estánd. | 68372 | 34.70 |
| 2 * Desv. estánd. | 3360 | 1.71 |
| 3 * Desv. estánd. | 1415 | 0.72 |
| 4 * Desv. estánd. | 1107 | 0.56 |
| 5 * Desv. estánd. | 799 | 0.41 |
| 6 * Desv. estánd. | 1144 | 0.58 |

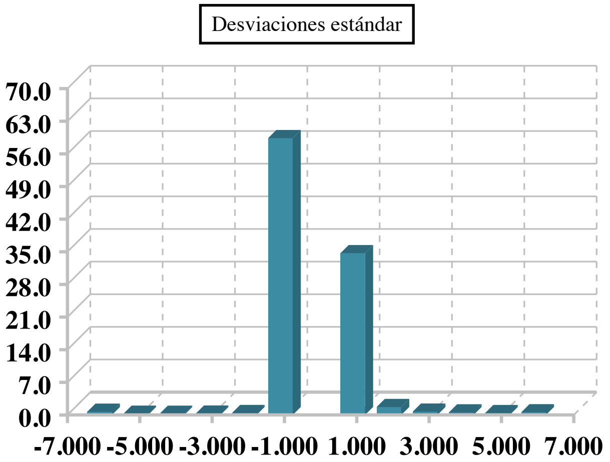

Predefinido: Isométrico

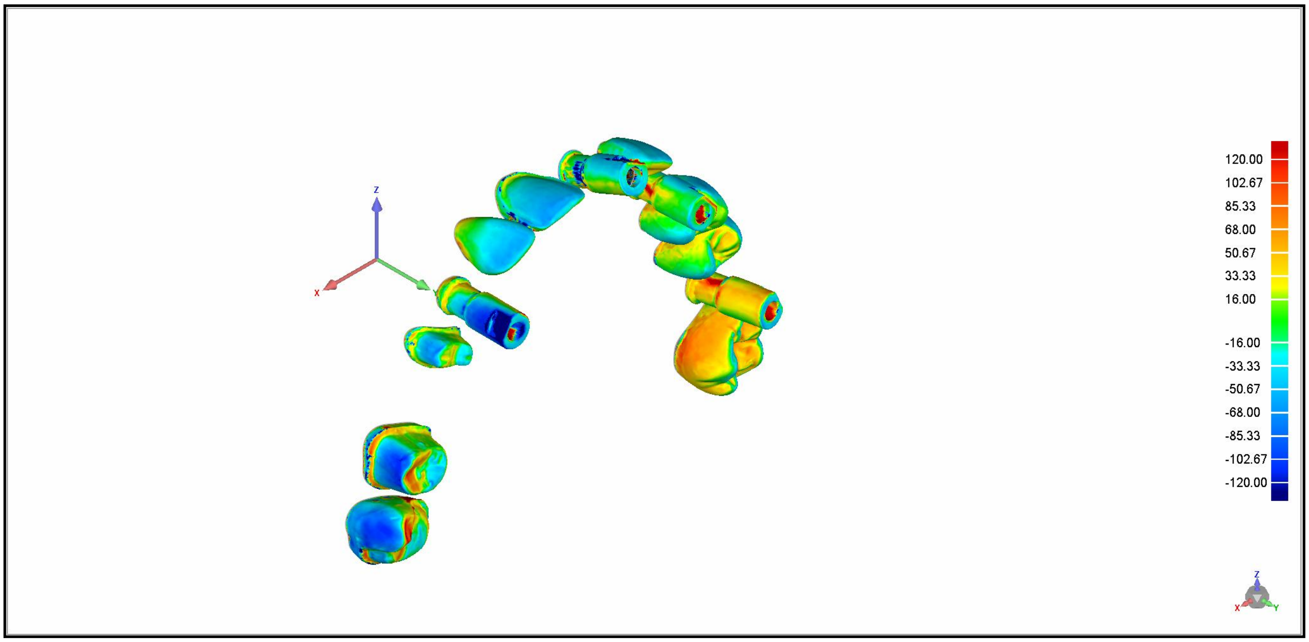

Predefinido: Frente

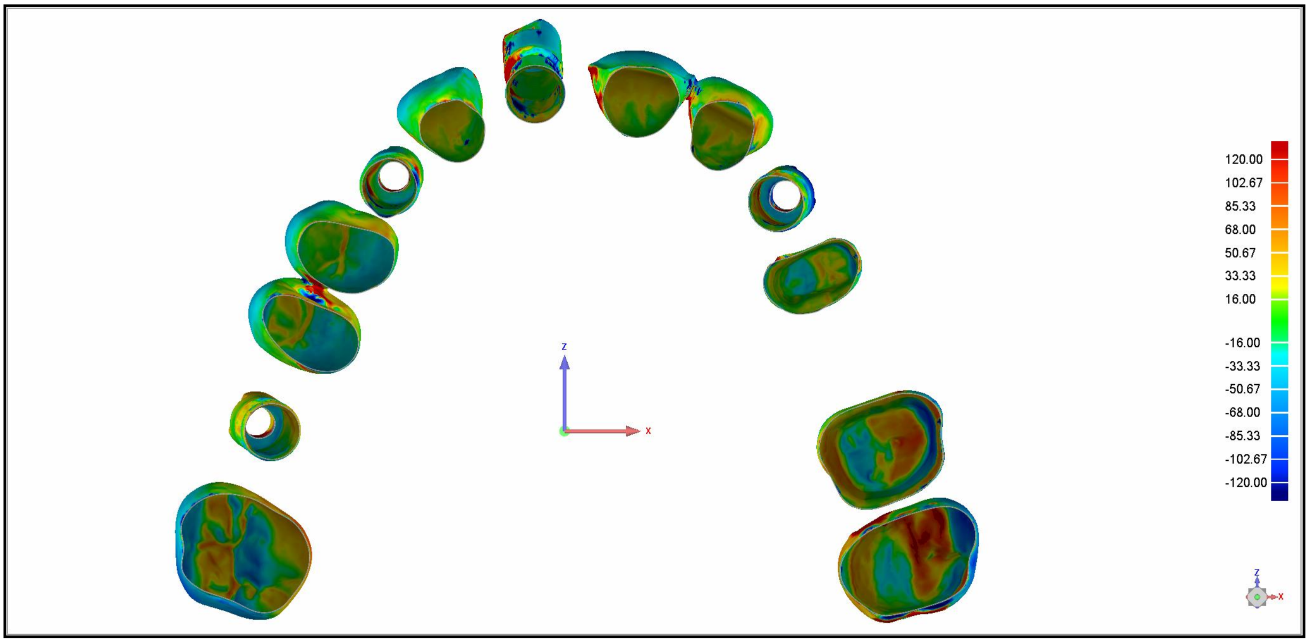

Predefinido: Atrás

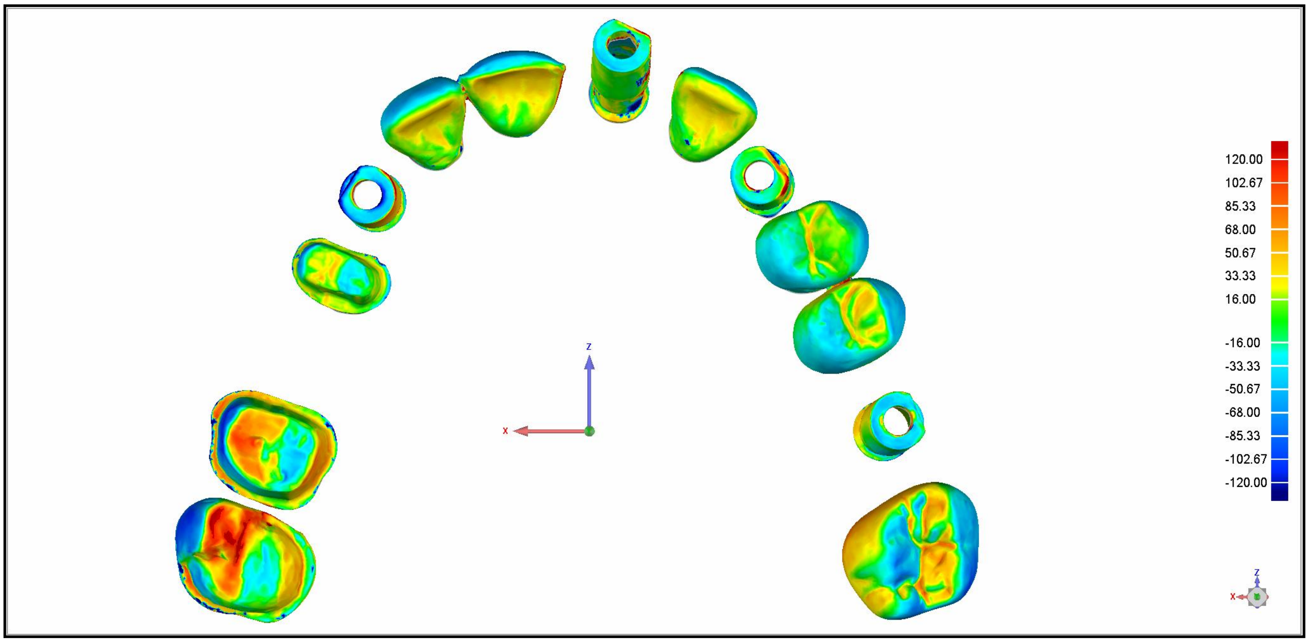

Predefinido: Izquierda

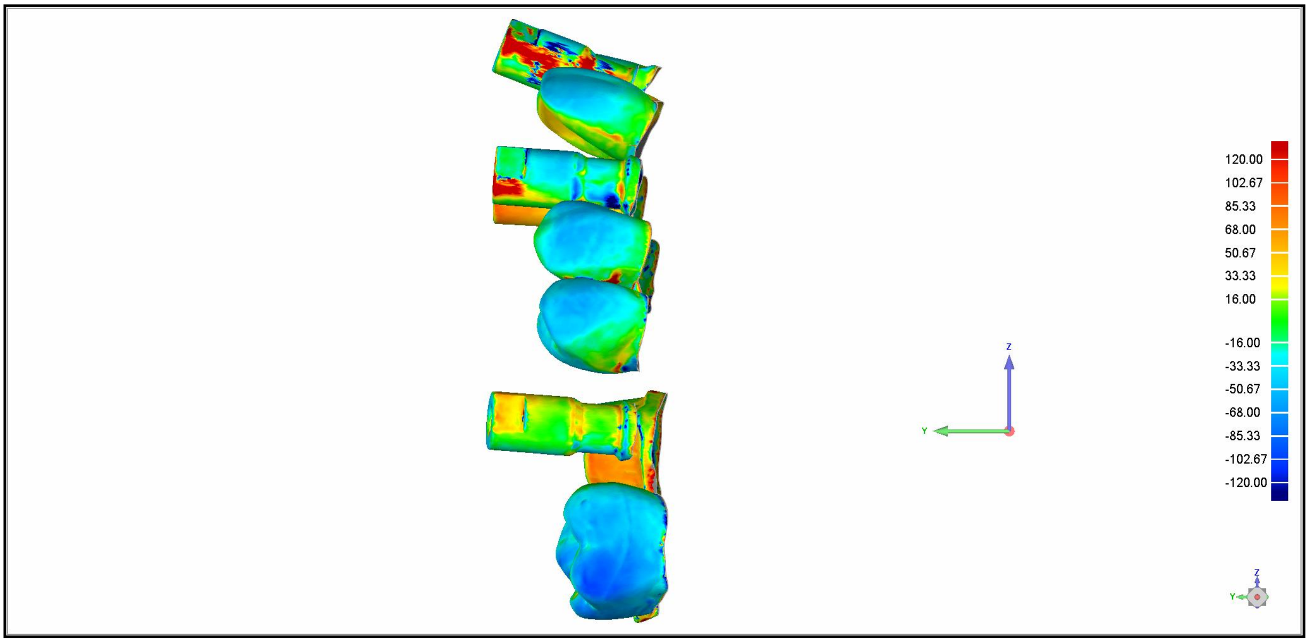

Predefinido: Derecha

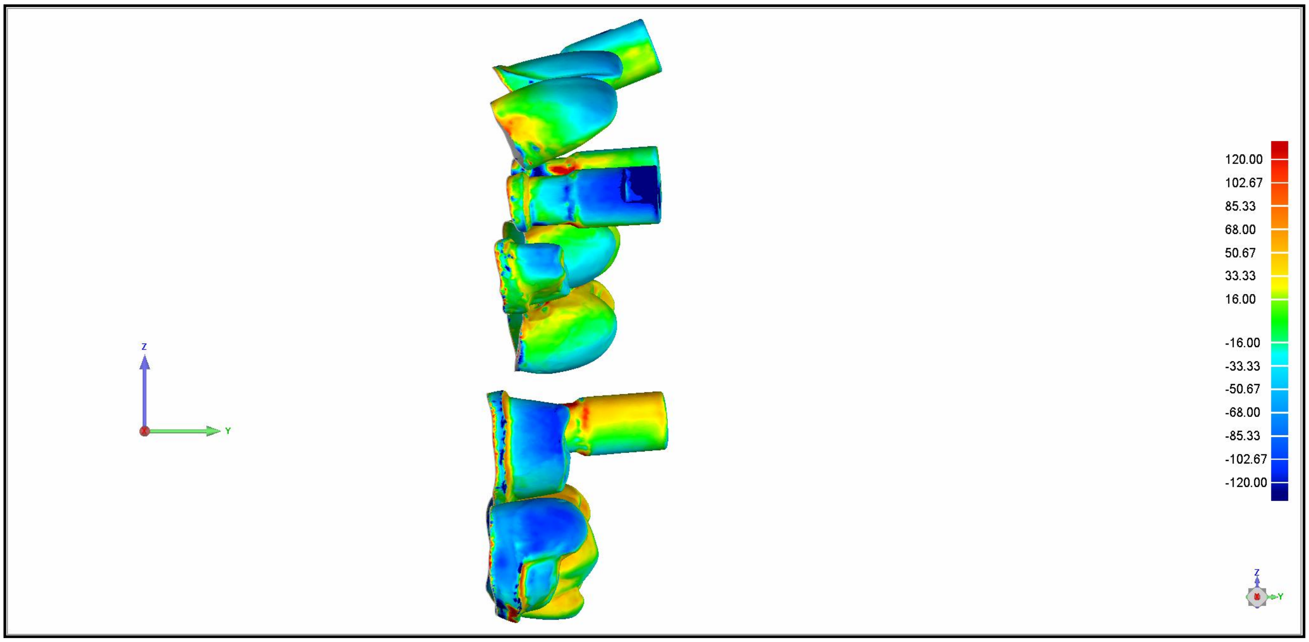

Predefinido: Superior

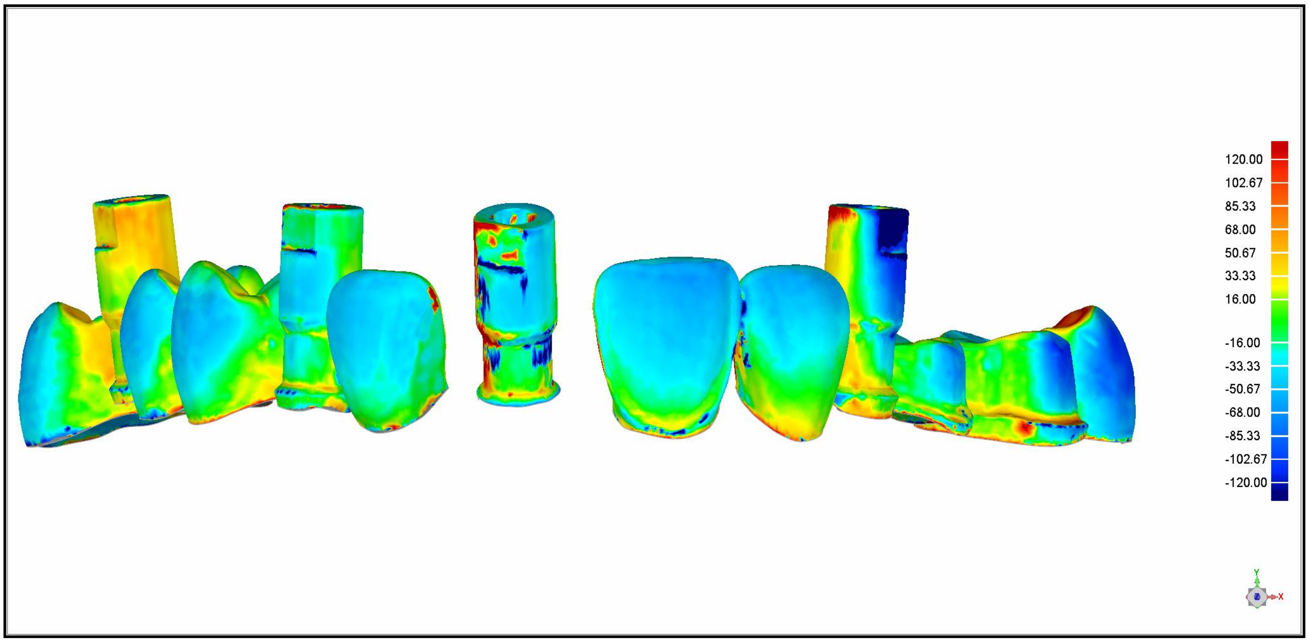

Predefinido: Inferior

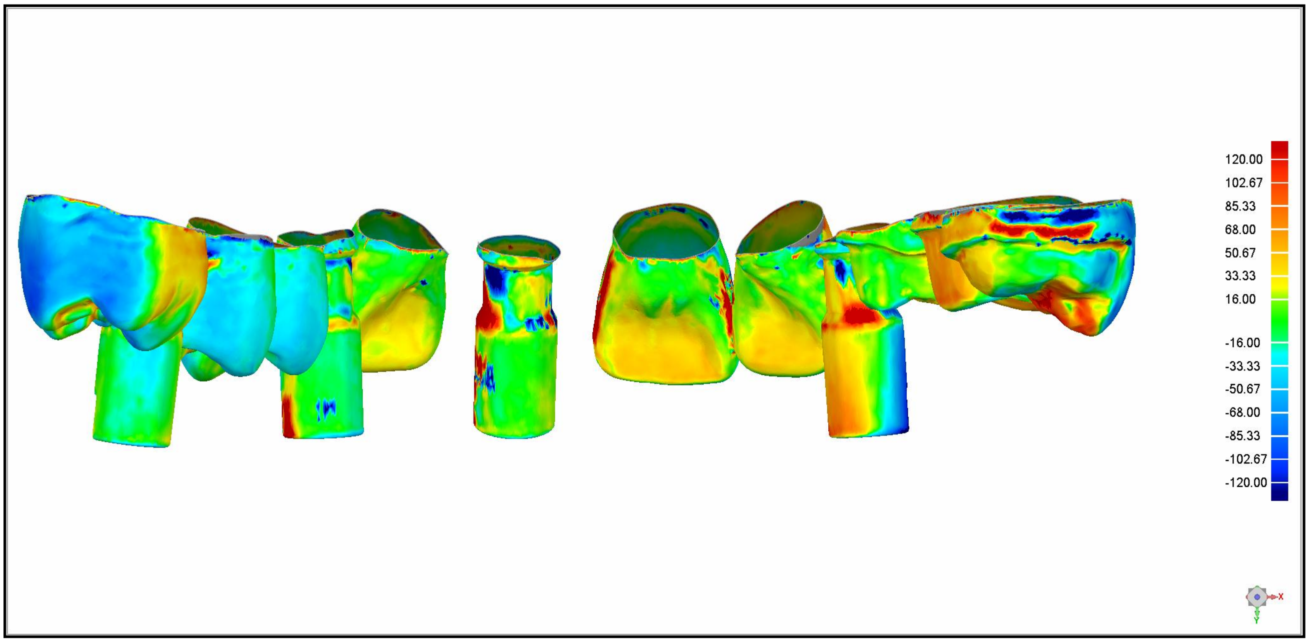

# Ajuste de ubicación: Desviaciones superior e inferior

## Unidades: u

| Nombre | Desv | Estado | Superior Tol | Inferior Tol | Ref X | Ref Y | Ref Z | Radio | Desv X | Desv Y | Desv Z | Medido X | Medido Y | Medido Z | Dir. proy. X | Dir. proy. Y | Dir. proy. Z |
|---|---|---|---|---|---|---|---|---|---|---|---|---|---|---|---|---|---|
| Desv. inferior | -3150.40 | | | | -1396.96 | 38555.79 | 30191.84 | n/a | -2695.08 | 505.45 | 1551.15 | -4092.05 | 39061.24 | 31742.99 | 0.86 | -0.16 | -0.49 |
| Desv. superior | 3075.82 | | | | 22997.99 | 29782.64 | -7852.21 | n/a | 2510.64 | 447.61 | 1719.59 | 25508.63 | 30230.25 | -6132.62 | 0.82 | 0.15 | 0.56 |
