# Supplementary material for: Accuracy of four digital scanners according to scanning strategy in complete-arch impressions
Source: PLoS One. 2018 Sep 13;13(9):e0202916. doi: 10.1371/journal.pone.0202916 (PMC6136706; doi:10.1371/journal.pone.0202916)
Supplement: S9 Table — Omnicam (scanning strategy A). (ZIP) [file pone.0202916.s009.zip › S9/OM3A.pdf]

3D Comparación Resultados

Modelo referencia	MRC
Modelo test	OM3A
Nº de puntos de datos	201109
# Aislados	435

Tipo tolerancia	3D desviación
Unidades	u
Máx. crítico	120.00
Máx. nominal	2.00
Mín. nominal	-2.00
Mín. crítico	-120.00

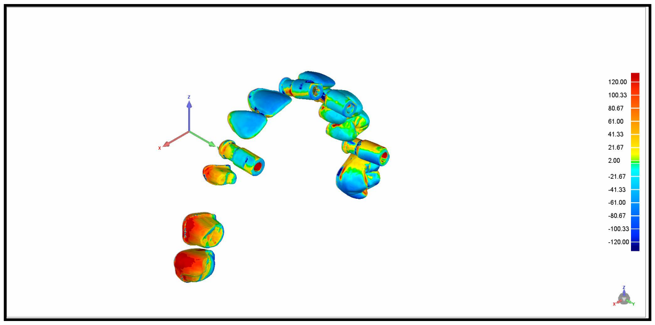

Desviación	
Desviación superior máx.	3041.95
Desviación inferior máx.	-3125.26
Desviación media	106.36 /-102.87
Desviación estándar	282.41

Distribución desviación

>=Min	<Max	# Puntos	%
-120.00	-100.33	2295	1.14
-100.33	-80.67	4091	2.03
-80.67	-61.00	9326	4.64
-61.00	-41.33	14598	7.26
-41.33	-21.67	22409	11.14
-21.67	-2.00	31946	15.88
-2.00	2.00	7038	3.50
2.00	21.67	28204	14.02
21.67	41.33	19752	9.82
41.33	61.00	14035	6.98
61.00	80.67	9056	4.50
80.67	100.33	5462	2.72
100.33	120.00	4446	2.21

Fuera del crítico superior	17209	8.56
Fuera del crítico inferior	11242	5.59

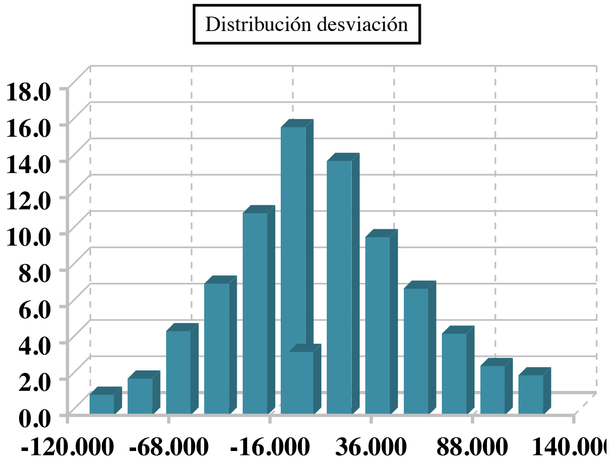

Desviaciones estándar

Distribución (+/-)	# Puntos	%
-6 * Desv. estánd.	1660	0.83
-5 * Desv. estánd.	736	0.37
-4 * Desv. estánd.	712	0.35
-3 * Desv. estánd.	1028	0.51
-2 * Desv. estánd.	2002	1.00
-1 * Desv. estánd.	98408	48.93
1 * Desv. estánd.	89322	44.41
2 * Desv. estánd.	2655	1.32
3 * Desv. estánd.	1876	0.93
4 * Desv. estánd.	1068	0.53
5 * Desv. estánd.	866	0.43
6 * Desv. estánd.	776	0.39

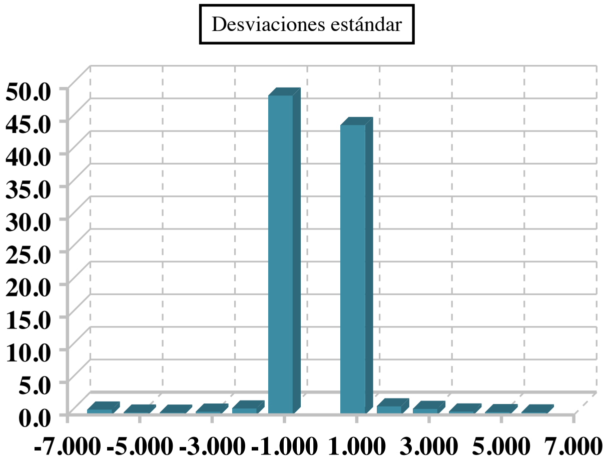

Predefinido: Isométrico

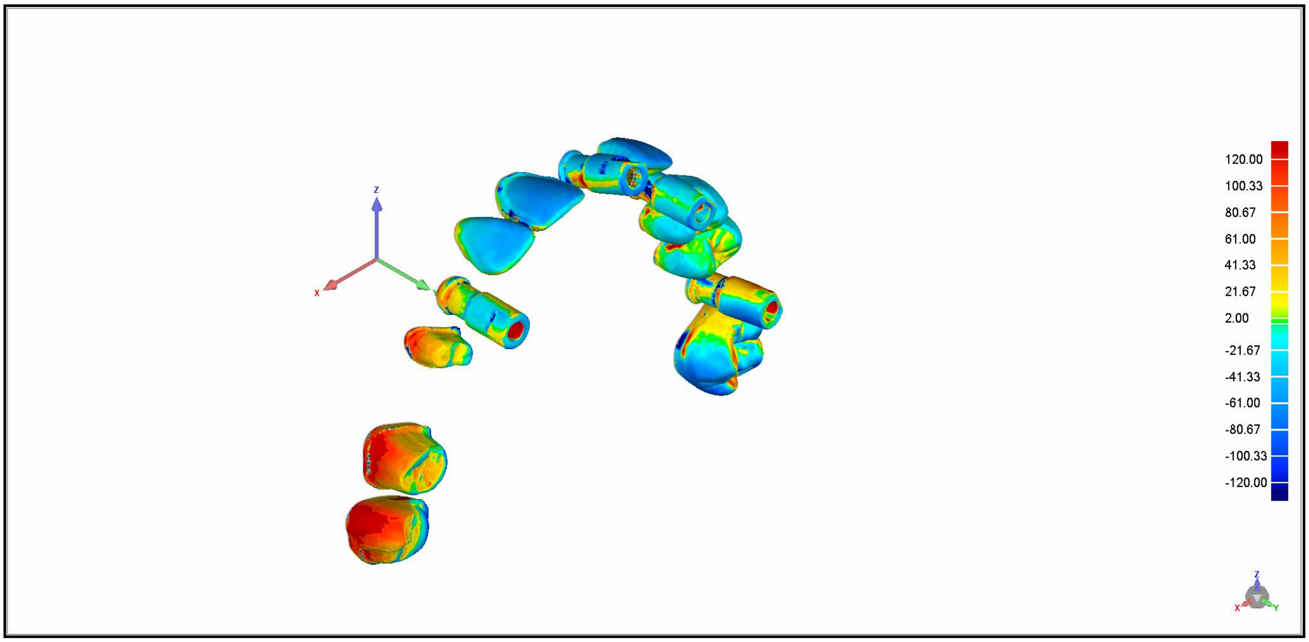

Predefinido: Frente

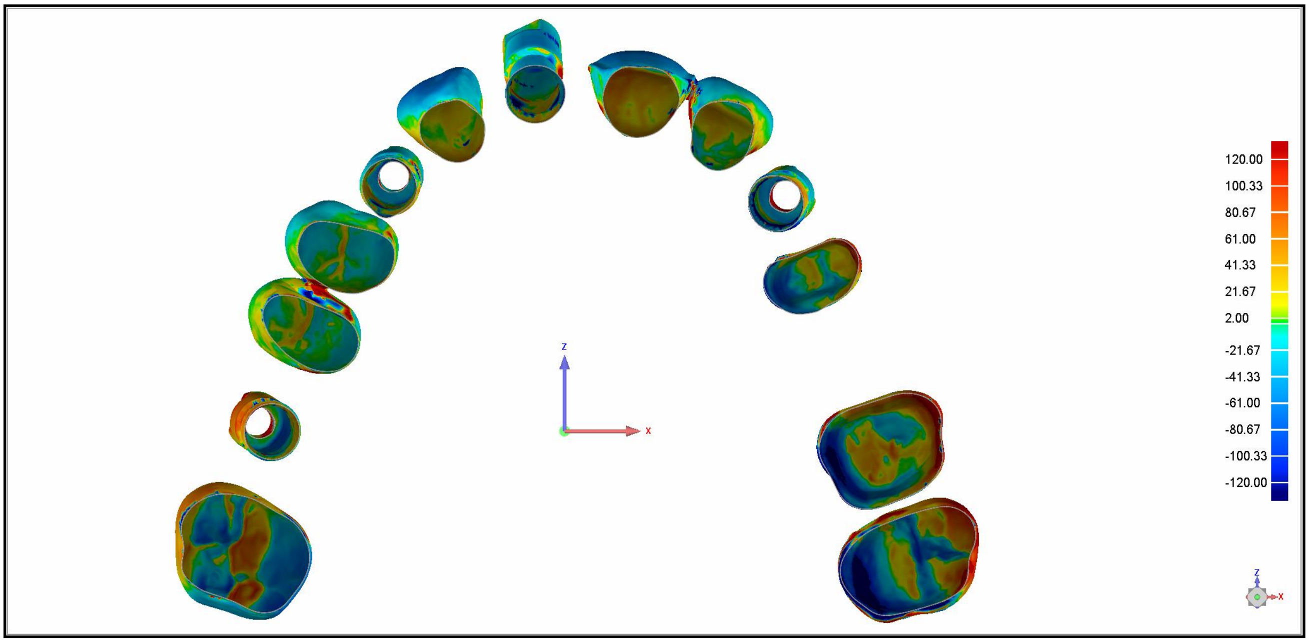

Predefinido: Atrás

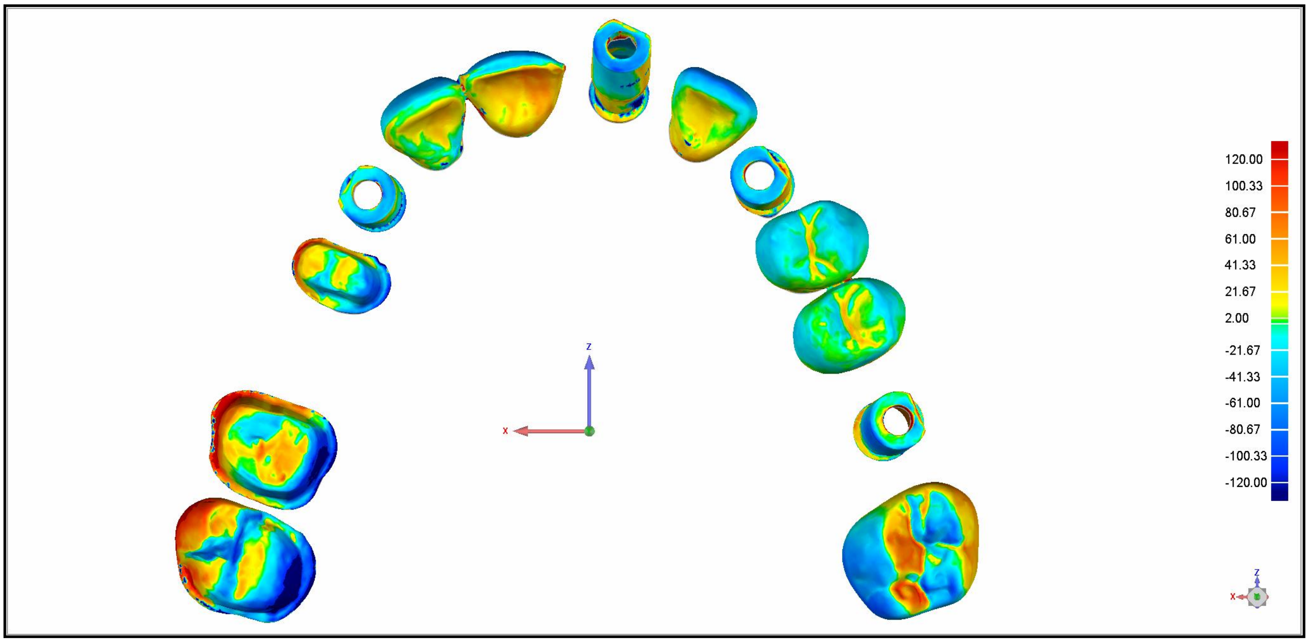

Predefinido: Izquierda

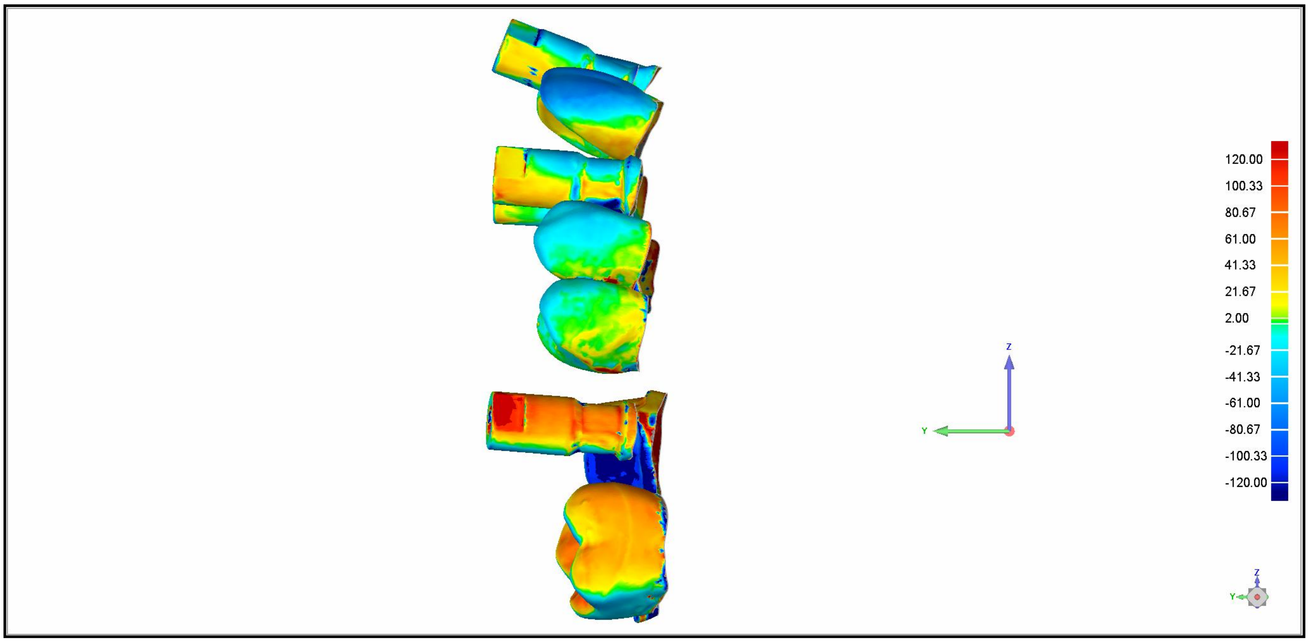

Predefinido: Derecha

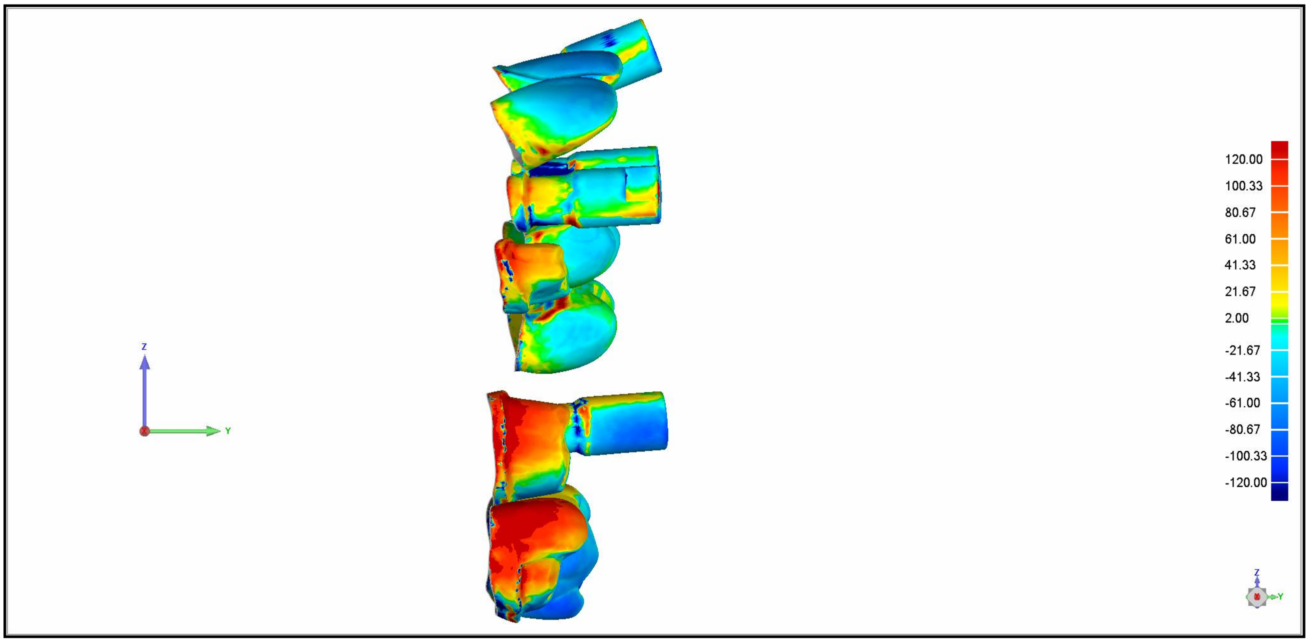

Predefinido: Superior

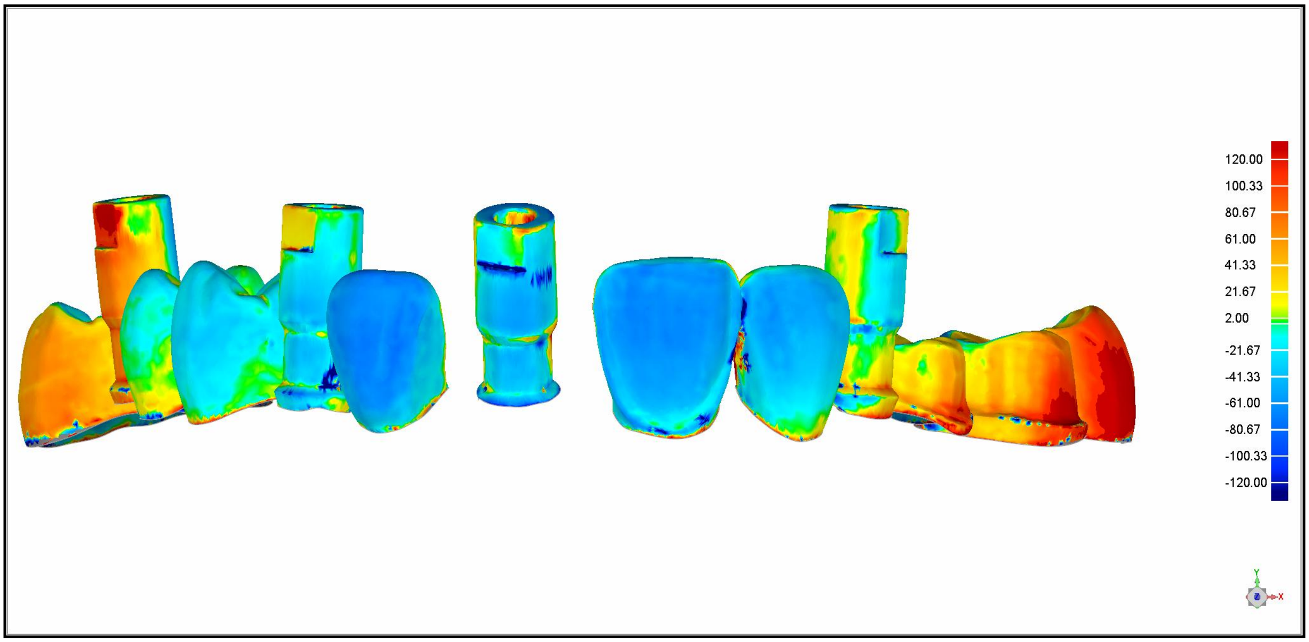

Predefinido: Inferior

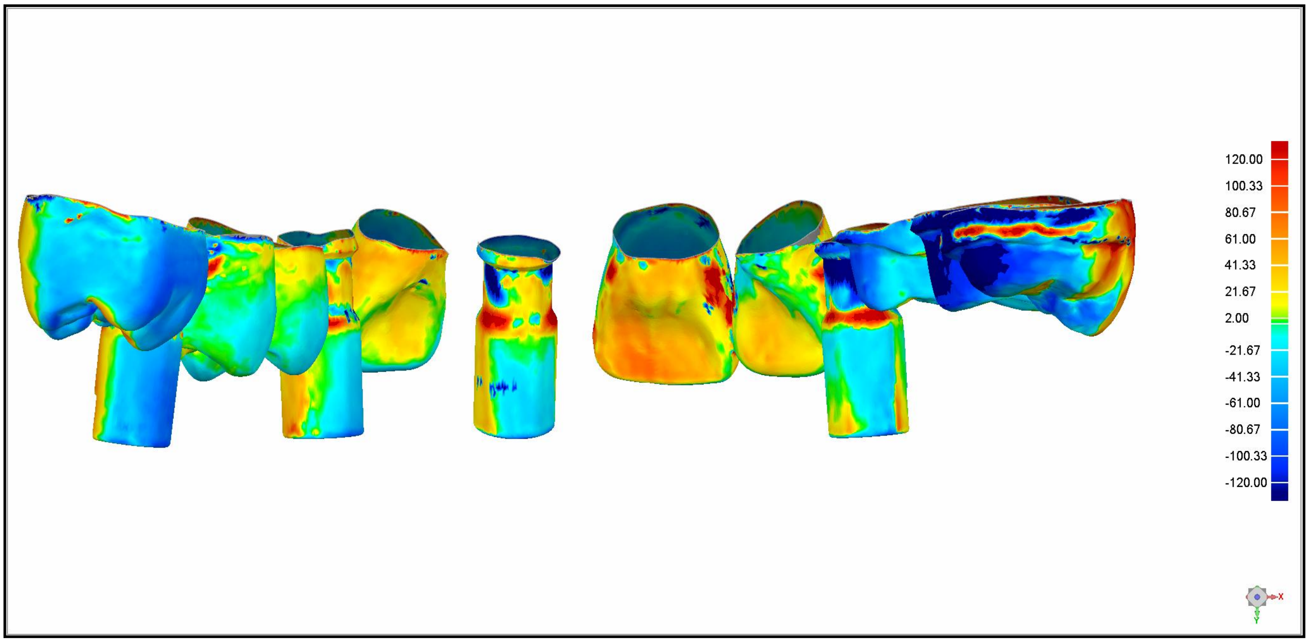

Ajuste de ubicación: Desviaciones superior e inferior

Unidades: u

Nombre	Desv	Estado	Superior Tol	Inferior Tol	Ref X	Ref Y	Ref Z	Radio	Desv X	Desv Y	Desv Z	Medido X	Medido Y	Medido Z	Dir. proy. X	Dir. proy. Y	Dir. proy. Z
Desv. inferior	-3125.26				-16597.07	29132.89	5701.00	n/a	-3001.90	41.77	-868.38	-19598.97	29174.66	4832.61	0.96	-0.01	0.28
Desv. superior	3041.95				29547.11	27336.30	1208.53	n/a	-2345.96	-385.29	1897.76	27201.15	26951.00	3106.29	-0.77	-0.13	0.62
