# Supplementary material for: Accuracy of four digital scanners according to scanning strategy in complete-arch impressions
Source: PLoS One. 2018 Sep 13;13(9):e0202916. doi: 10.1371/journal.pone.0202916 (PMC6136706; doi:10.1371/journal.pone.0202916)
Supplement: S9 Table — Omnicam (scanning strategy A). (ZIP) [file pone.0202916.s009.zip › S9/OM8A.pdf]

3D Comparación Resultados

Modelo referencia	MRC
Modelo test	OM8A
Nº de puntos de datos	192749
# Aislados	676

Tipo tolerancia	3D desviación
Unidades	u
Máx. crítico	120.00
Máx. nominal	15.00
Mín. nominal	-15.00
Mín. crítico	-120.00

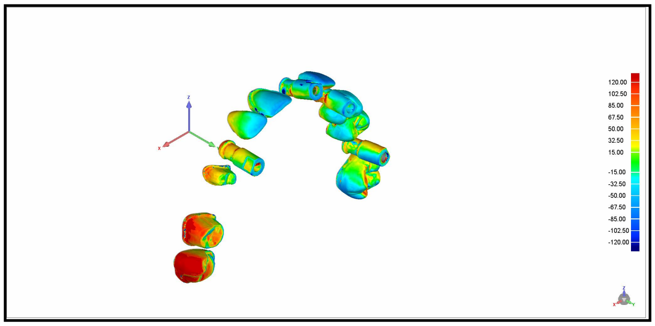

Desviación	
Desviación superior máx.	3131.71
Desviación inferior máx.	-3115.08
Desviación media	93.69 /-72.76
Desviación estándar	212.76

Distribución desviación

>=Min	<Max	# Puntos	%
-120.00	-102.50	1650	0.86
-102.50	-85.00	2423	1.26
-85.00	-67.50	5279	2.74
-67.50	-50.00	10428	5.41
-50.00	-32.50	16604	8.61
-32.50	-15.00	23398	12.14
-15.00	15.00	44629	23.15
15.00	32.50	20076	10.42
32.50	50.00	13865	7.19
50.00	67.50	10935	5.67
67.50	85.00	7418	3.85
85.00	102.50	5438	2.82
102.50	120.00	4398	2.28

Fuera del crítico superior	17721	9.19
Fuera del crítico inferior	8487	4.40

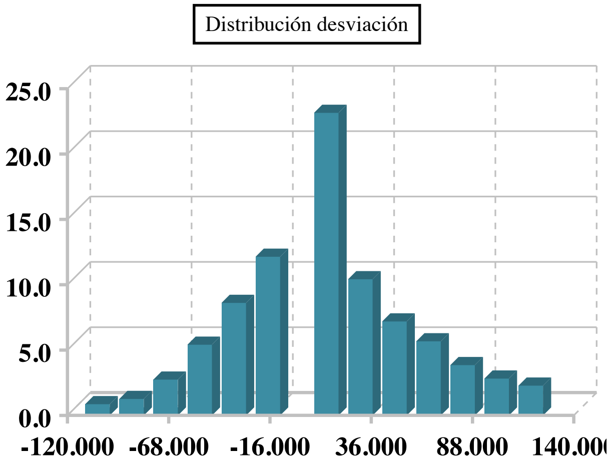

Desviaciones estándar

Distribución (+/-)	# Puntos	%
-6 * Desv. estánd.	1082	0.56
-5 * Desv. estánd.	335	0.17
-4 * Desv. estánd.	468	0.24
-3 * Desv. estánd.	573	0.30
-2 * Desv. estánd.	1907	0.99
-1 * Desv. estánd.	109190	56.65
1 * Desv. estánd.	72448	37.59
2 * Desv. estánd.	2912	1.51
3 * Desv. estánd.	1167	0.61
4 * Desv. estánd.	973	0.50
5 * Desv. estánd.	649	0.34
6 * Desv. estánd.	1045	0.54

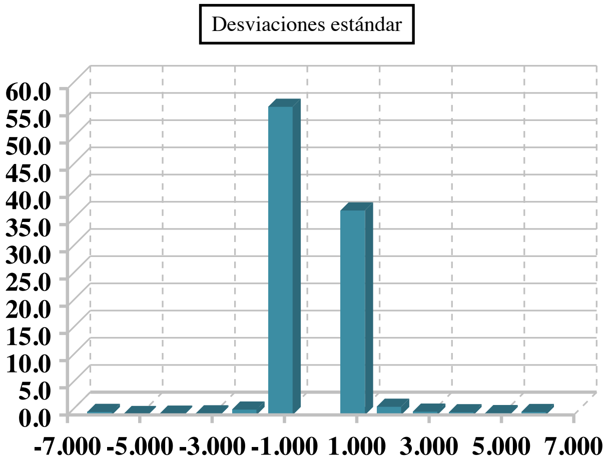

Predefinido: Isométrico

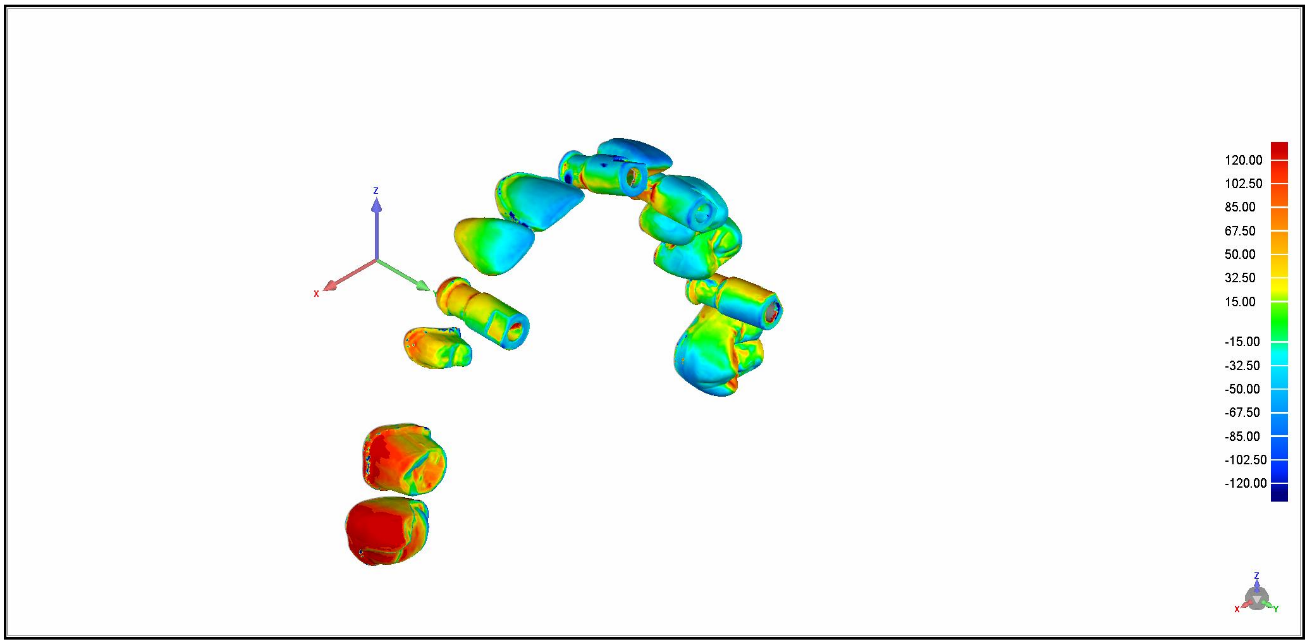

Predefinido: Frente

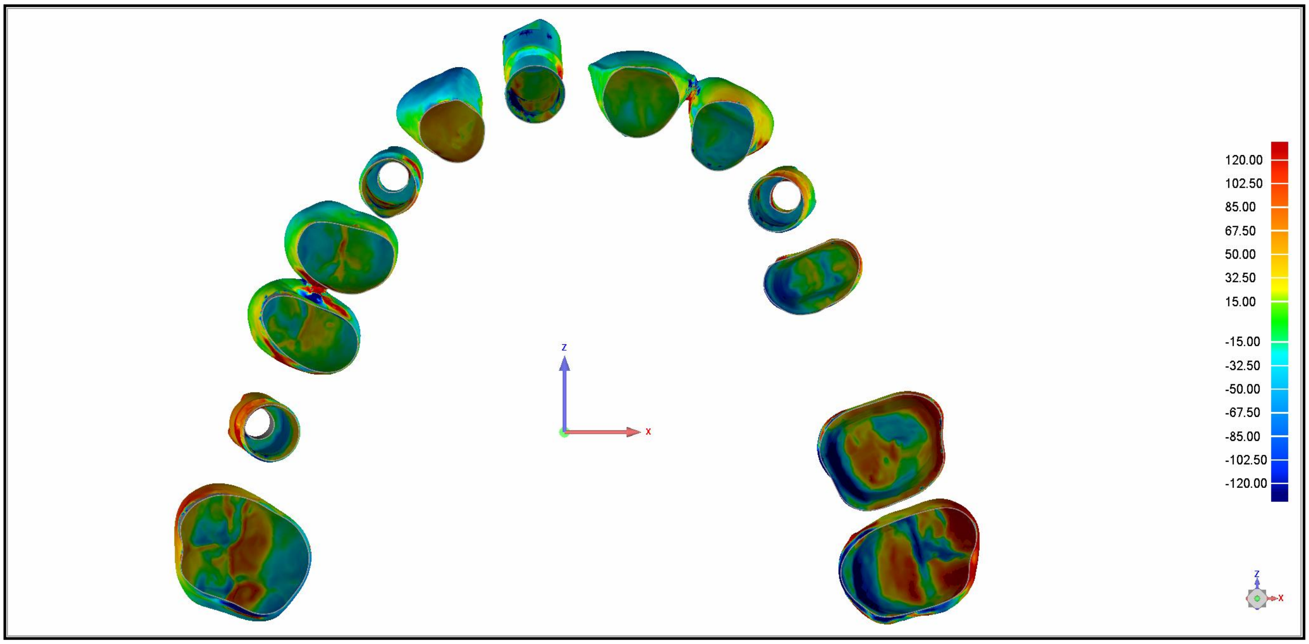

Predefinido: Atrás

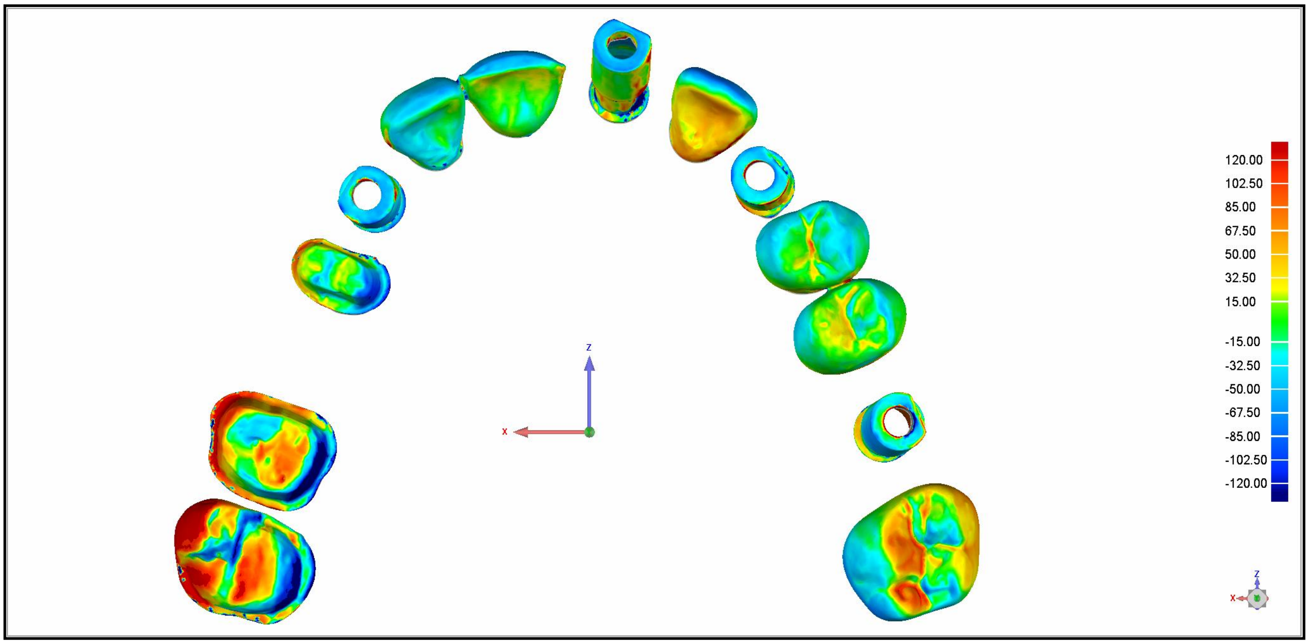

Predefinido: Izquierda

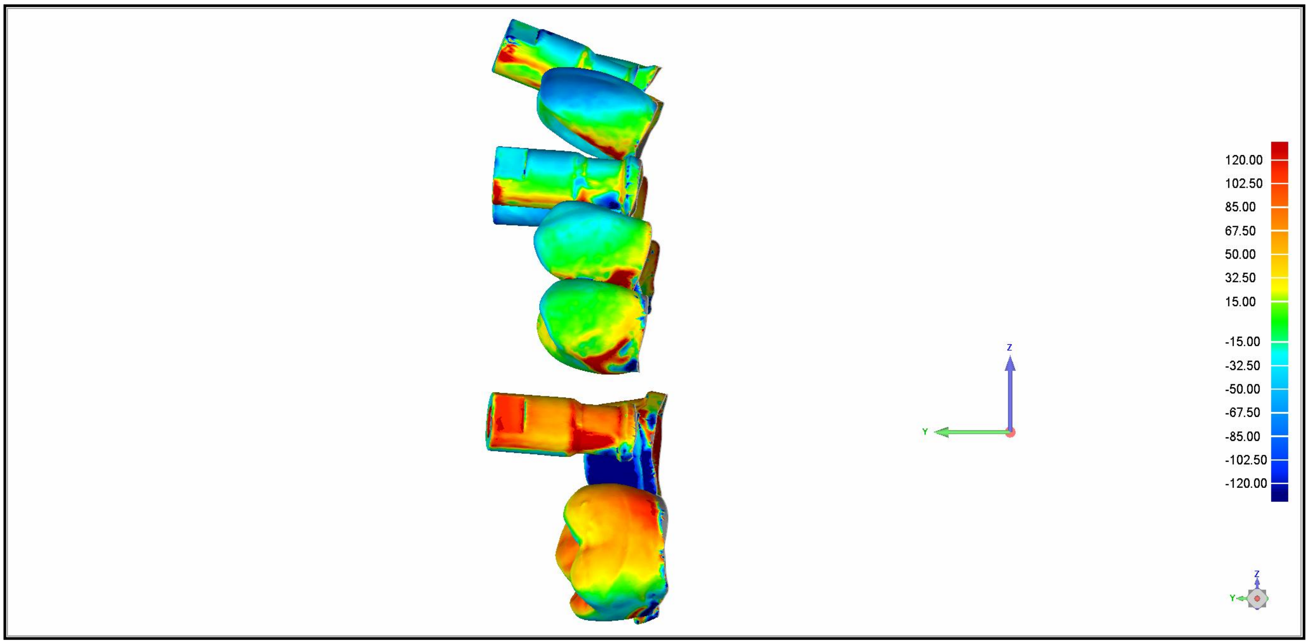

Predefinido: Derecha

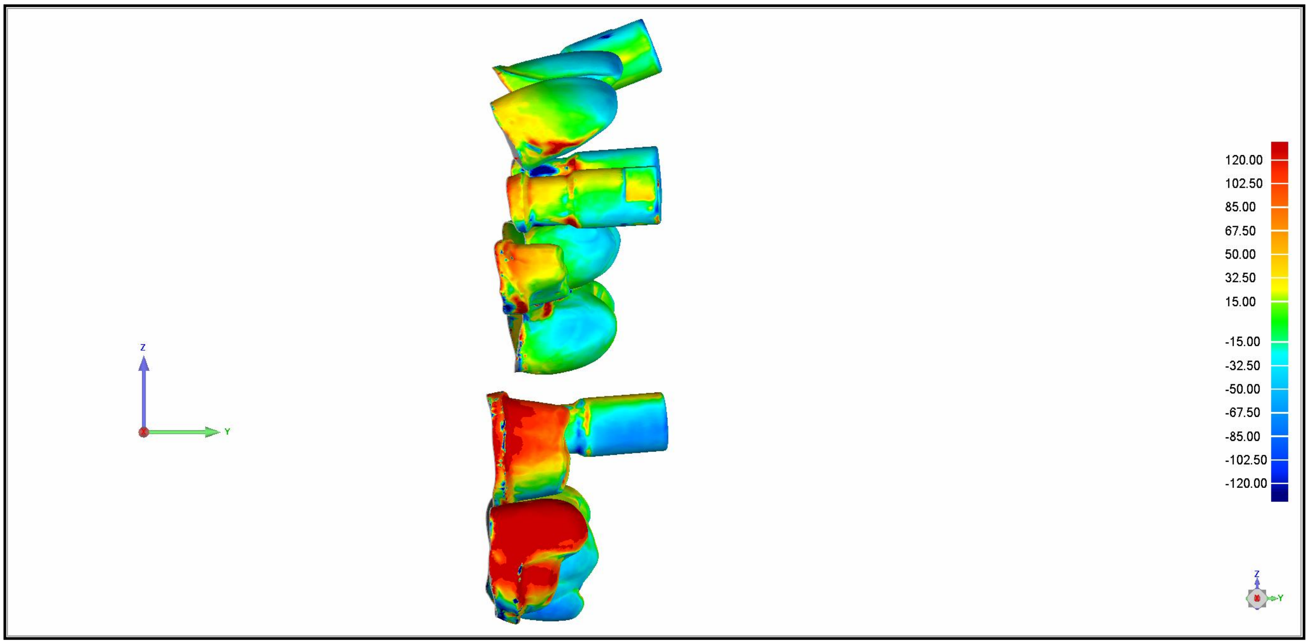

Predefinido: Superior

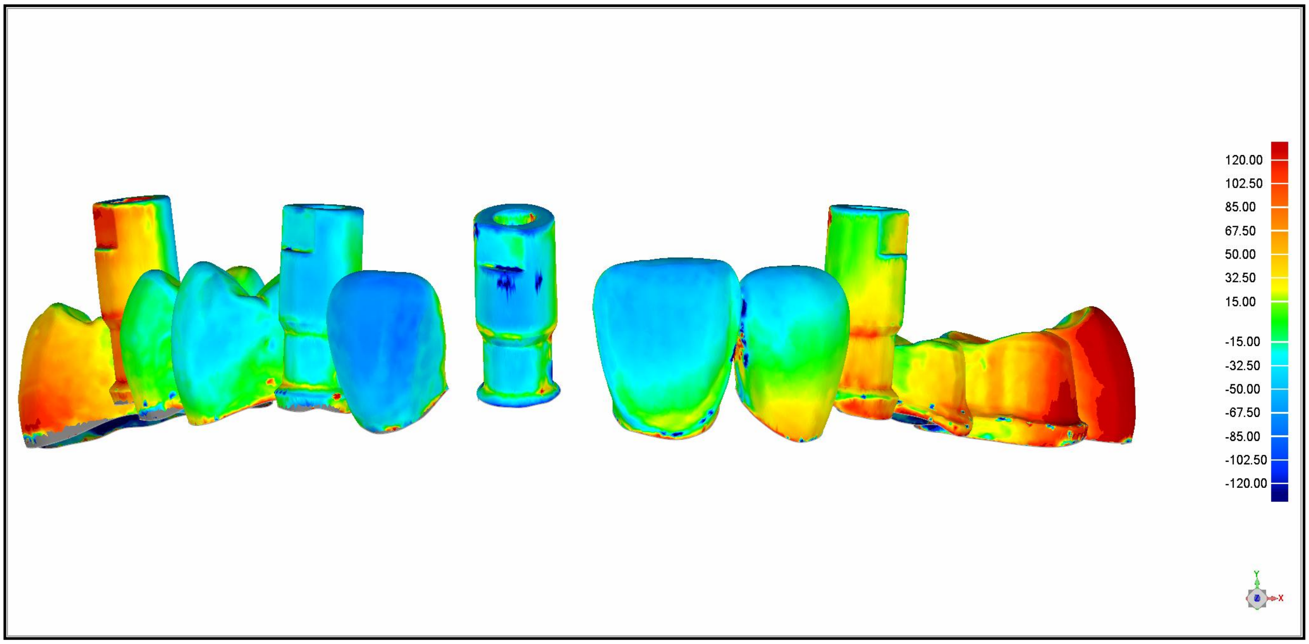

Predefinido: Inferior

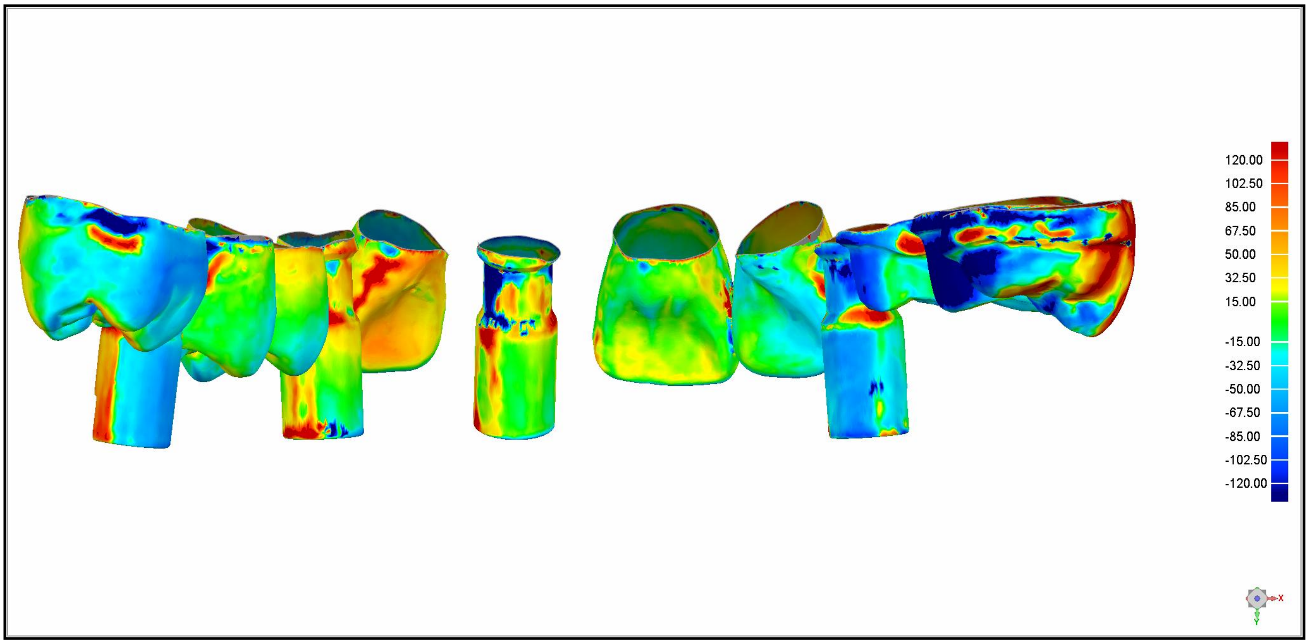

Ajuste de ubicación: Desviaciones superior e inferior

Unidades: u

Nombre	Desv	Estado	Superior Tol	Inferior Tol	Ref X	Ref Y	Ref Z	Radio	Desv X	Desv Y	Desv Z	Medido X	Medido Y	Medido Z	Dir. proy. X	Dir. proy. Y	Dir. proy. Z
Desv. inferior	-3115.08				-29208.33	26961.25	-11988.49	n/a	2799.47	354.30	-1319.52	-26408.86	27315.54	-13308.00	-0.90	-0.11	0.42
Desv. superior	3131.71				-12553.67	29785.74	21343.12	n/a	1085.87	-1394.20	2585.48	-11467.80	28391.53	23928.60	0.35	-0.45	0.83
