# Supplementary material for: Accuracy of four digital scanners according to scanning strategy in complete-arch impressions
Source: PLoS One. 2018 Sep 13;13(9):e0202916. doi: 10.1371/journal.pone.0202916 (PMC6136706; doi:10.1371/journal.pone.0202916)
Supplement: S9 Table — Omnicam (scanning strategy A). (ZIP) [file pone.0202916.s009.zip › S9/OM4A.pdf]

3D Comparación Resultados

Modelo referencia	MRC
Modelo test	OM4A
Nº de puntos de datos	200319
# Aislados	721

Tipo tolerancia	3D desviación
Unidades	u
Máx. crítico	120.00
Máx. nominal	6.00
Mín. nominal	-6.00
Mín. crítico	-120.00

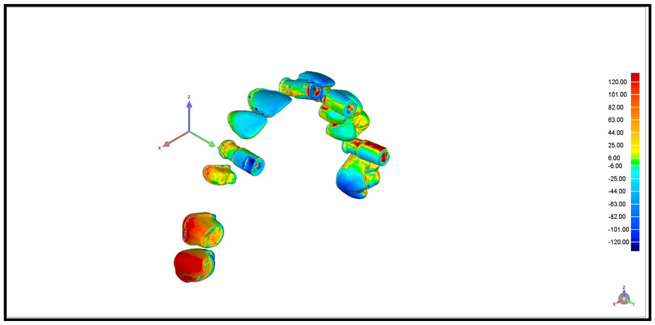

Desviación	
Desviación superior máx.	3132.29
Desviación inferior máx.	-3071.90
Desviación media	105.72 /-116.45
Desviación estándar	298.41

Distribución desviación

>=Min	<Max	# Puntos	%
-120.00	-101.00	2468	1.23
-101.00	-82.00	4051	2.02
-82.00	-63.00	5837	2.91
-63.00	-44.00	9512	4.75
-44.00	-25.00	19099	9.53
-25.00	-6.00	26588	13.27
-6.00	6.00	19822	9.90
6.00	25.00	29255	14.60
25.00	44.00	22042	11.00
44.00	63.00	13434	6.71
63.00	82.00	7987	3.99
82.00	101.00	4469	2.23
101.00	120.00	3377	1.69

Fuera del crítico superior	19871	9.92
Fuera del crítico inferior	12507	6.24

Distribución desviación

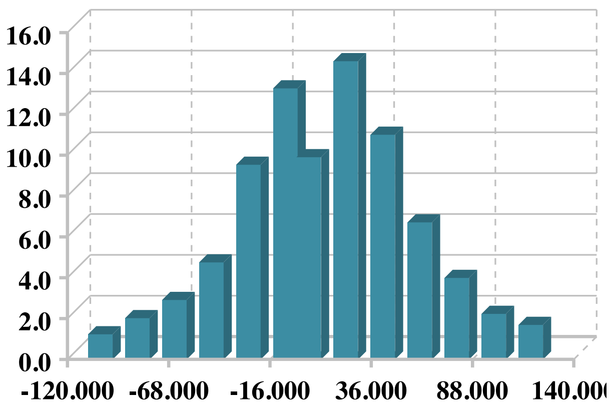

Desviaciones estándar

Distribución (+/-)	# Puntos	%
-6 * Desv. estánd.	1661	0.83
-5 * Desv. estánd.	1128	0.56
-4 * Desv. estánd.	661	0.33
-3 * Desv. estánd.	921	0.46
-2 * Desv. estánd.	1793	0.90
-1 * Desv. estánd.	94079	46.96
1 * Desv. estánd.	93387	46.62
2 * Desv. estánd.	2467	1.23
3 * Desv. estánd.	1493	0.75
4 * Desv. estánd.	1100	0.55
5 * Desv. estánd.	675	0.34
6 * Desv. estánd.	954	0.48

Desviaciones estándar

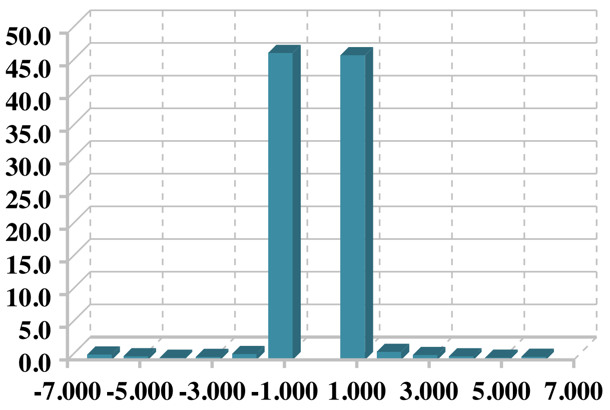

Predefinido: Isométrico

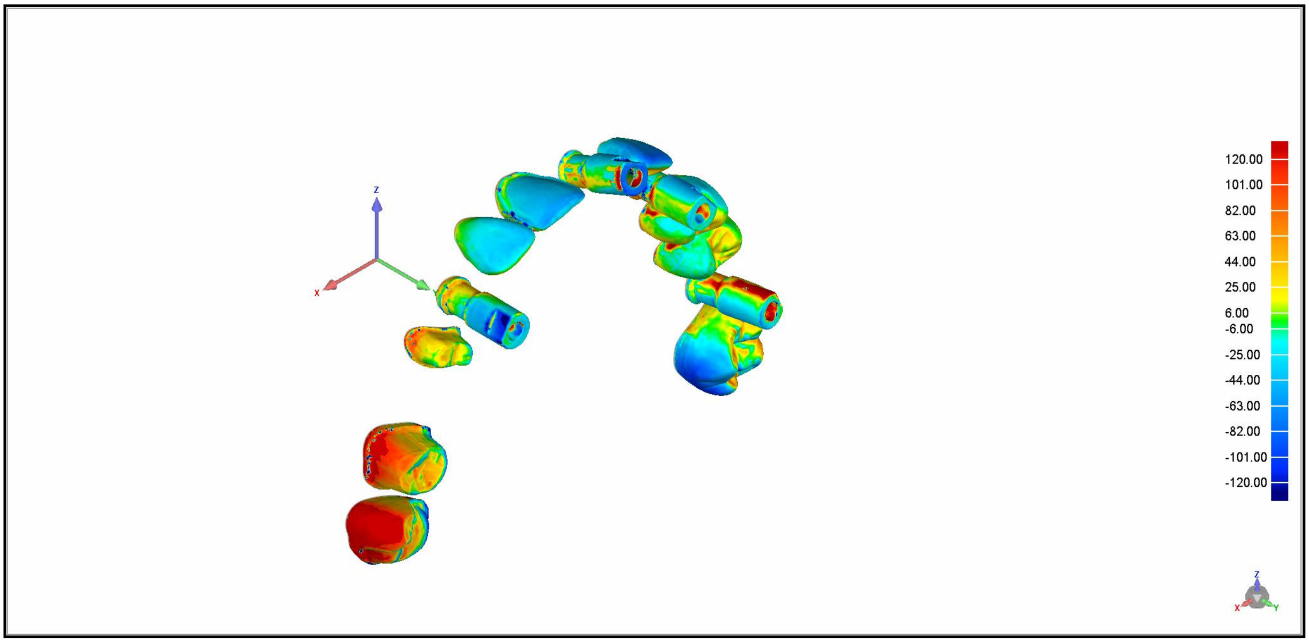

Predefinido: Frente

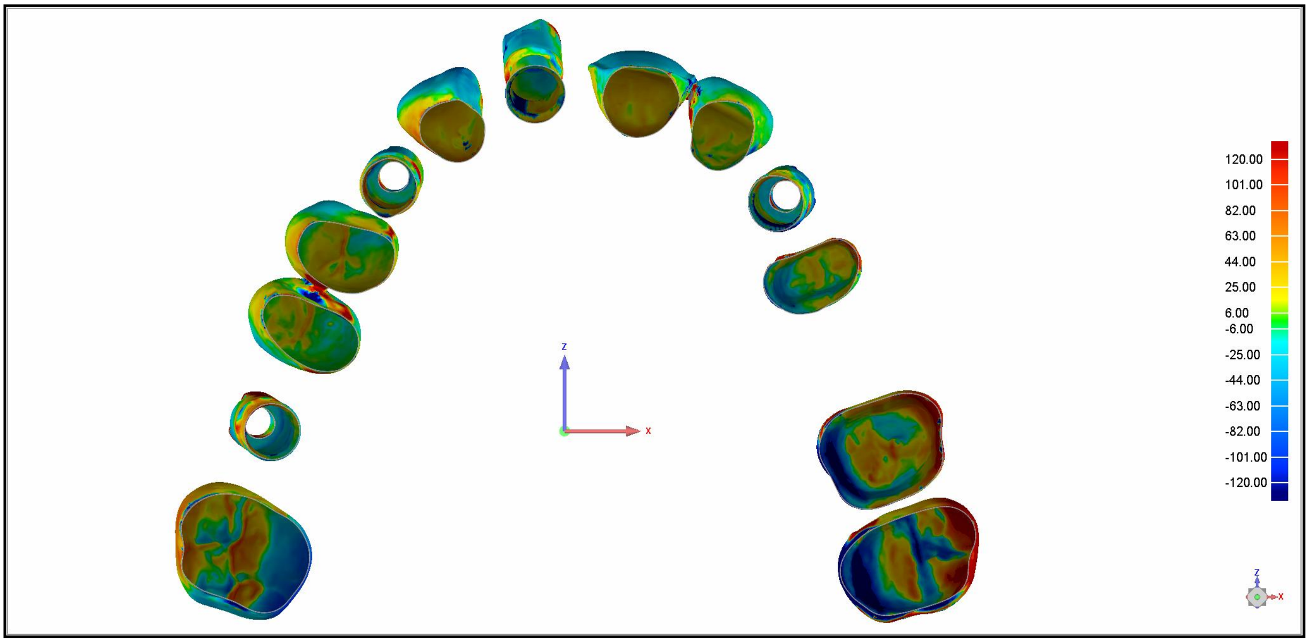

Predefinido: Atrás

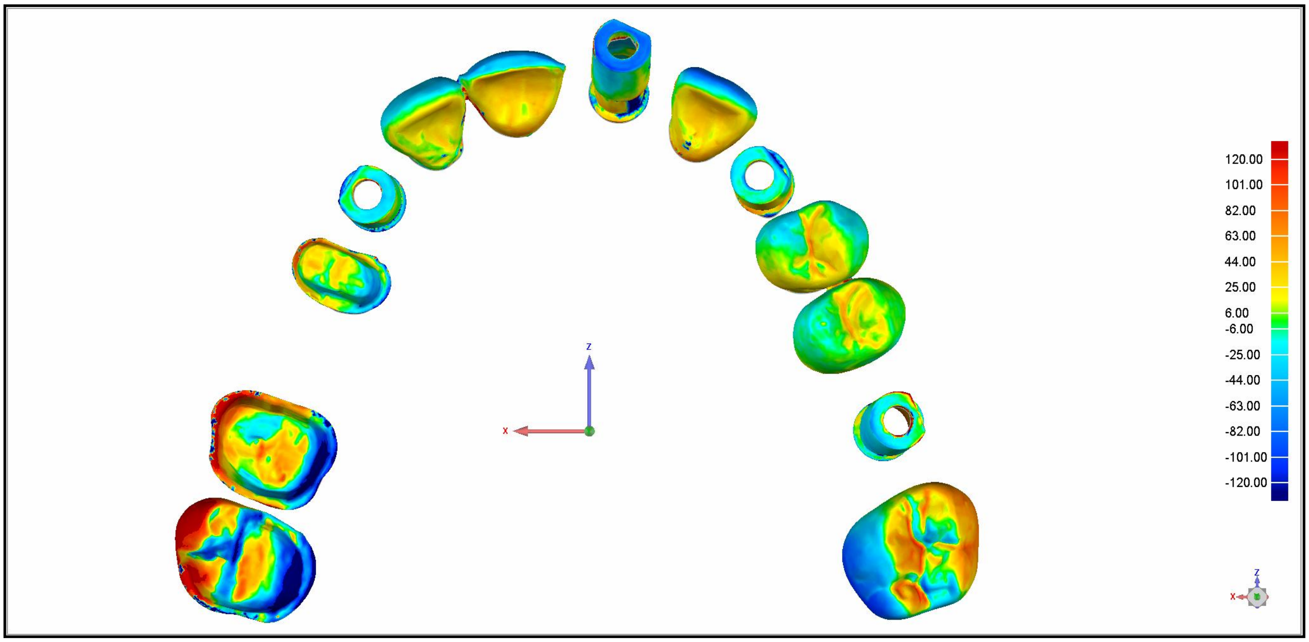

Predefinido: Izquierda

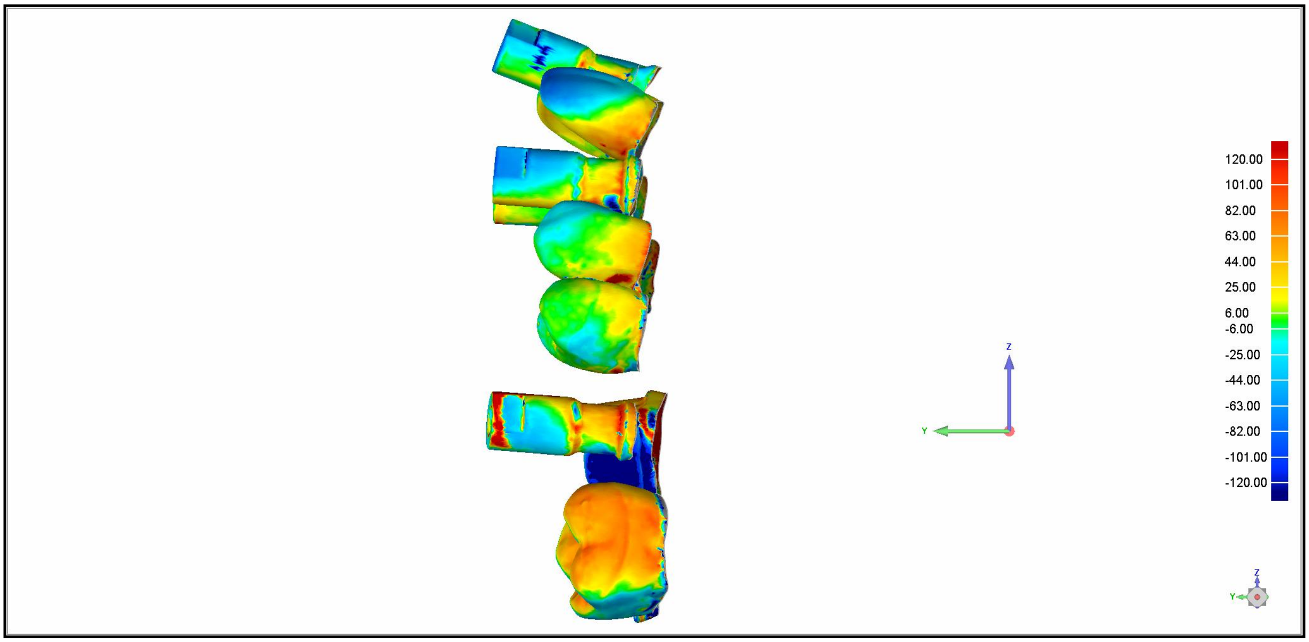

Predefinido: Derecha

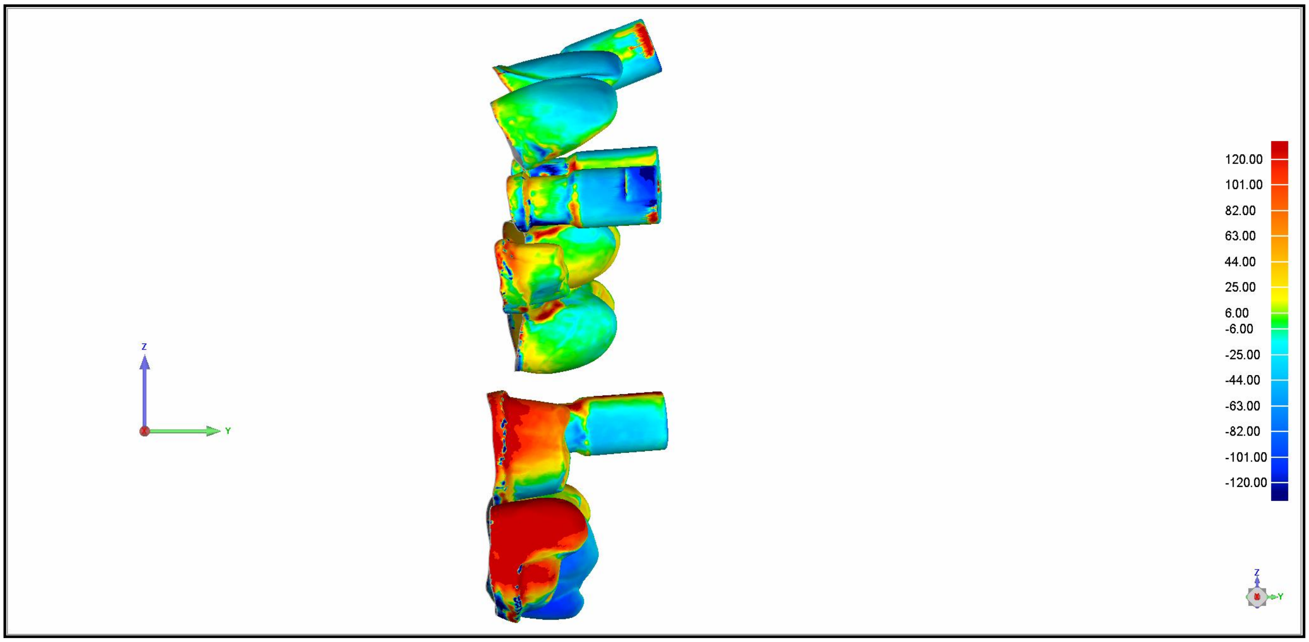

Predefinido: Superior

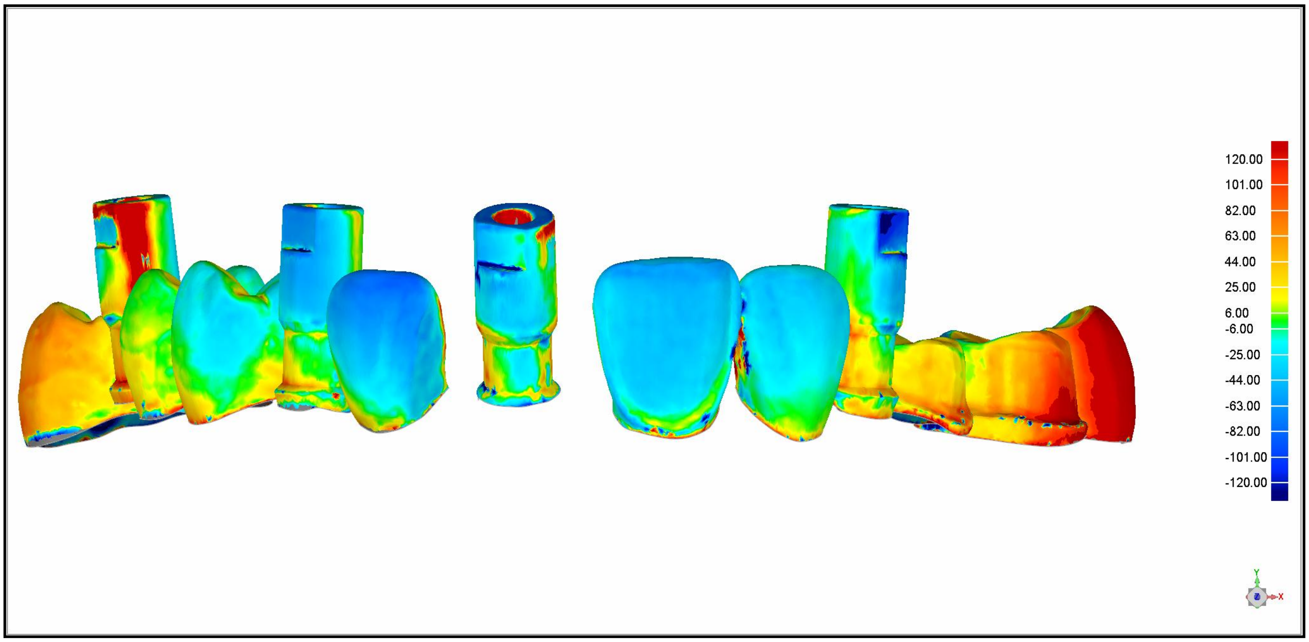

Predefinido: Inferior

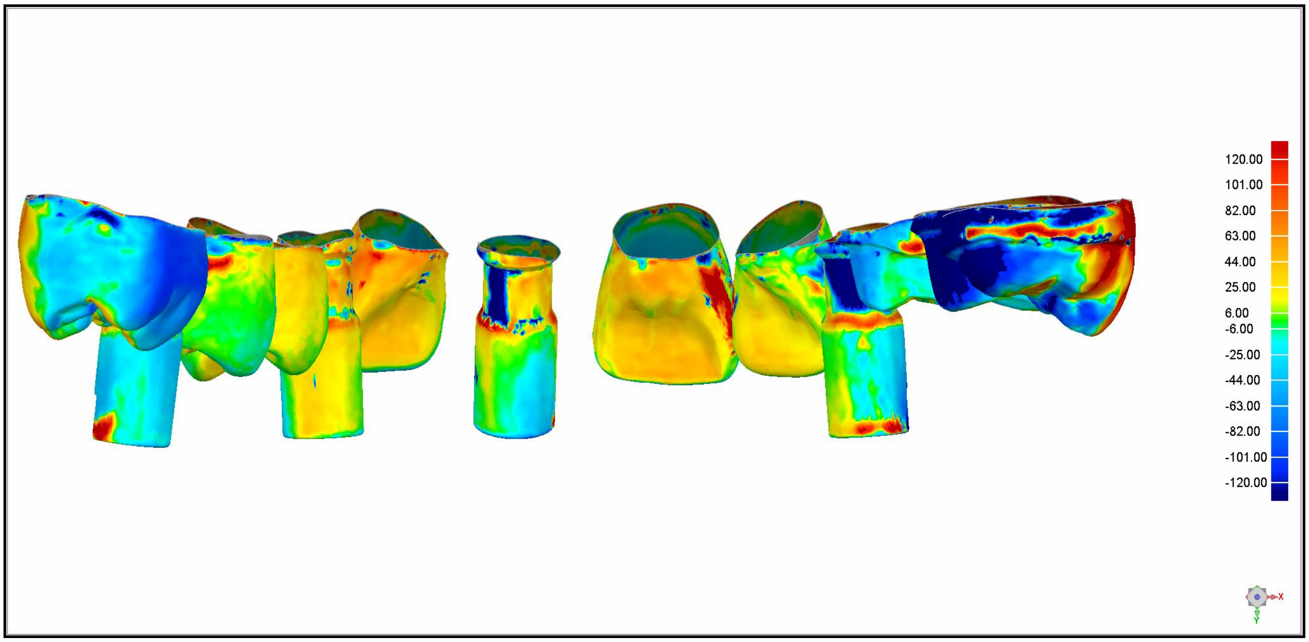

Ajuste de ubicación: Desviaciones superior e inferior

Unidades: u

Nombre	Desv	Estado	Superior Tol	Inferior Tol	Ref X	Ref Y	Ref Z	Radio	Desv X	Desv Y	Desv Z	Medido X	Medido Y	Medido Z	Dir. proy. X	Dir. proy. Y	Dir. proy. Z
Desv. inferior	-3071.90				18729.30	30224.44	17943.32	n/a	-2786.01	40.79	-1293.46	15943.28	30265.23	16649.86	0.91	-0.01	0.42
Desv. superior	3132.29				-20342.69	30903.90	12278.62	n/a	-683.84	-2459.76	1814.71	-21026.54	28444.14	14093.33	-0.22	-0.79	0.58
