# Supplementary material for: Accuracy of four digital scanners according to scanning strategy in complete-arch impressions
Source: PLoS One. 2018 Sep 13;13(9):e0202916. doi: 10.1371/journal.pone.0202916 (PMC6136706; doi:10.1371/journal.pone.0202916)
Supplement: S9 Table — Omnicam (scanning strategy A). (ZIP) [file pone.0202916.s009.zip › S9/OM5A.pdf]

Fecha: 3/6/2017, 3:01 pm

3D Comparación Resultados

Modelo referencia	MRC
Modelo test	OM5A
Nº de puntos de datos	198531
# Aislados	1164

Tipo tolerancia	3D desviación
Unidades	u
Máx. crítico	120.00
Máx. nominal	15.00
Mín. nominal	-15.00
Mín. crítico	-120.00

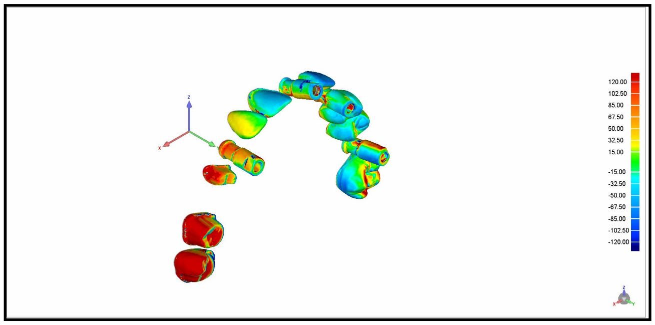

Desviación	
Desviación superior máx.	3143.44
Desviación inferior máx.	-3138.92
Desviación media	128.10 /-127.36
Desviación estándar	299.87

Distribución desviación

>=Min	<Max	# Puntos	%
-120.00	-102.50	1707	0.86
-102.50	-85.00	2341	1.18
-85.00	-67.50	5507	2.77
-67.50	-50.00	9110	4.59
-50.00	-32.50	14110	7.11
-32.50	-15.00	18921	9.53
-15.00	15.00	39092	19.69
15.00	32.50	17385	8.76
32.50	50.00	13878	6.99
50.00	67.50	11656	5.87
67.50	85.00	8240	4.15
85.00	102.50	6637	3.34
102.50	120.00	4830	2.43

Fuera del crítico superior	29417	14.82
Fuera del crítico inferior	15700	7.91

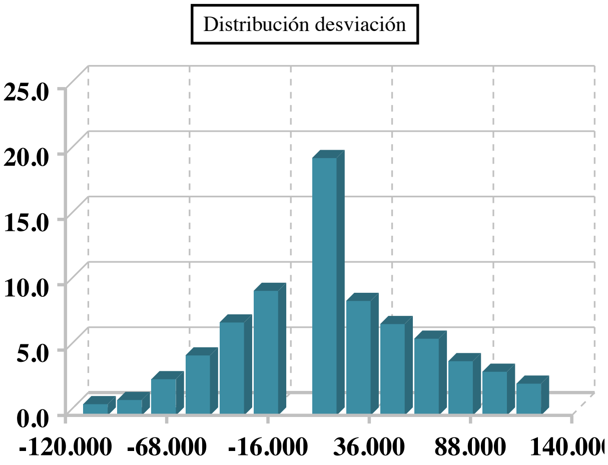

Desviaciones estándar

Distribución (+/-)	# Puntos	%
-6 * Desv. estánd.	1674	0.84
-5 * Desv. estánd.	777	0.39
-4 * Desv. estánd.	886	0.45
-3 * Desv. estánd.	1006	0.51
-2 * Desv. estánd.	3460	1.74
-1 * Desv. estánd.	99398	50.07
1 * Desv. estánd.	81901	41.25
2 * Desv. estánd.	5240	2.64
3 * Desv. estánd.	1777	0.90
4 * Desv. estánd.	1248	0.63
5 * Desv. estánd.	574	0.29
6 * Desv. estánd.	590	0.30

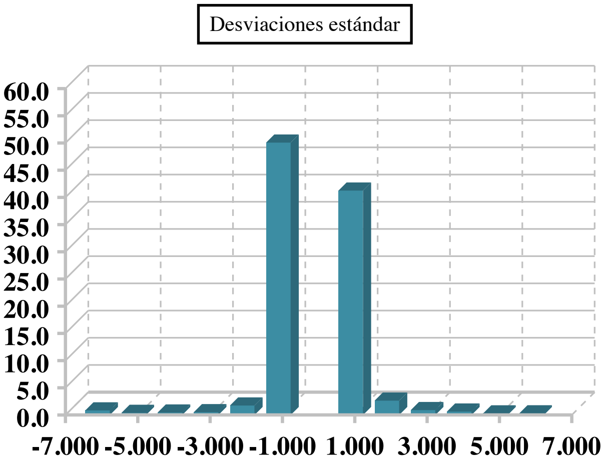

Predefinido: Isométrico

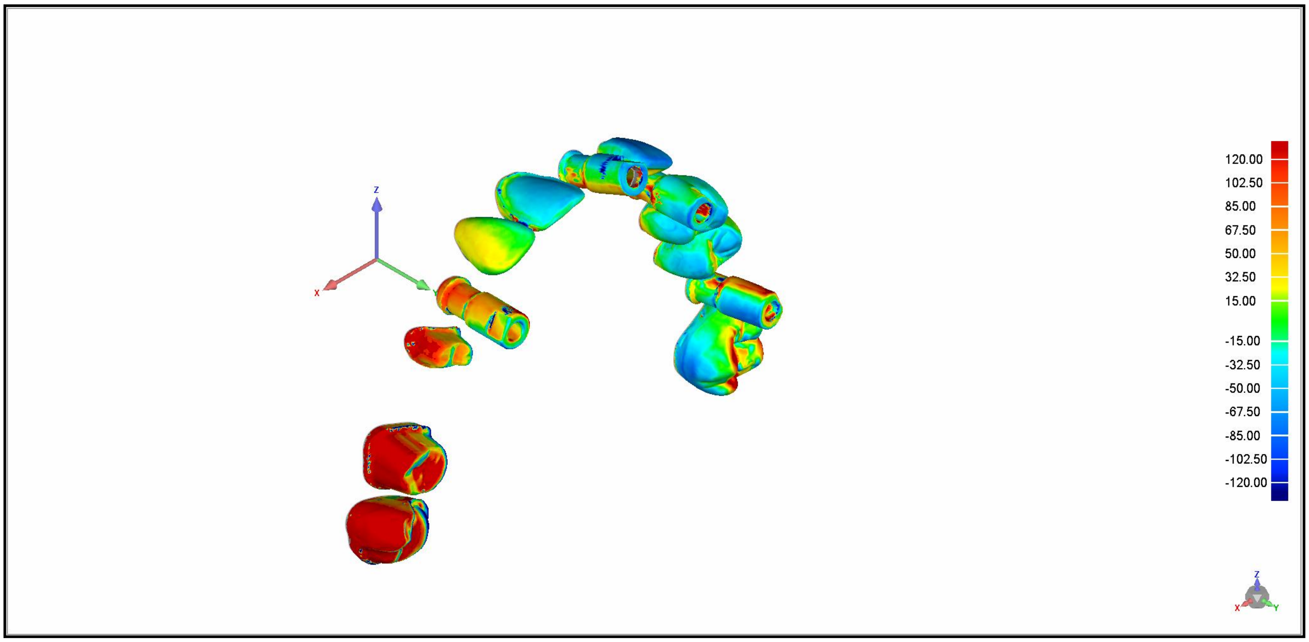

Predefinido: Frente

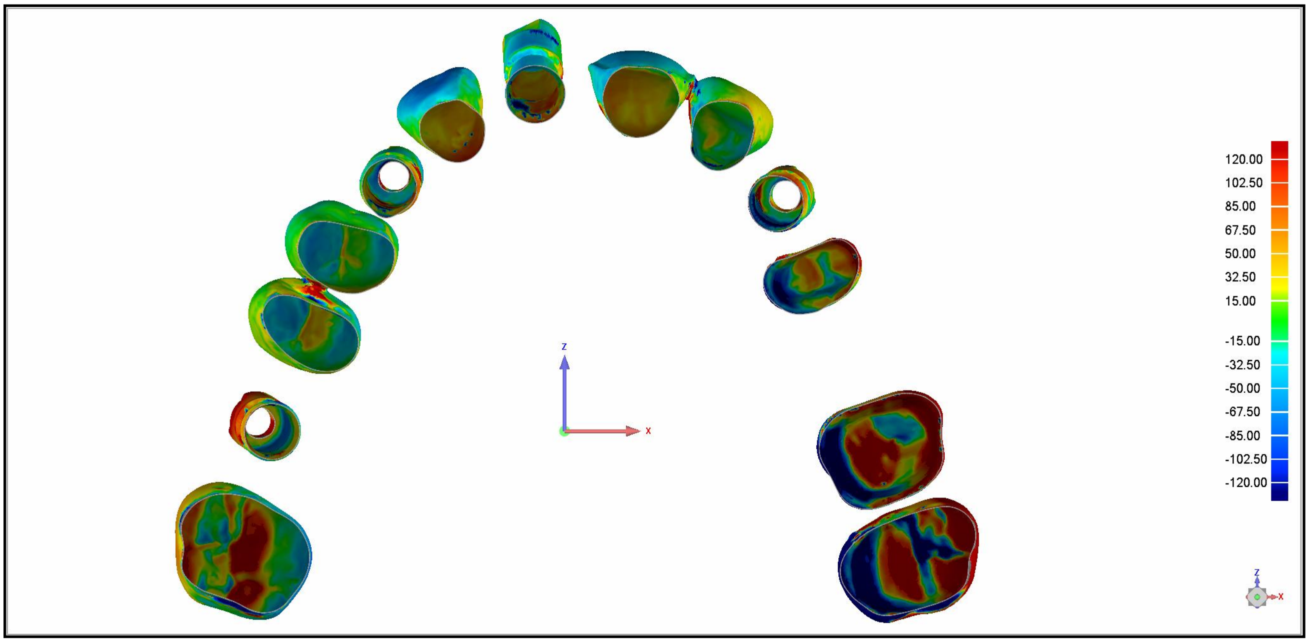

Predefinido: Atrás

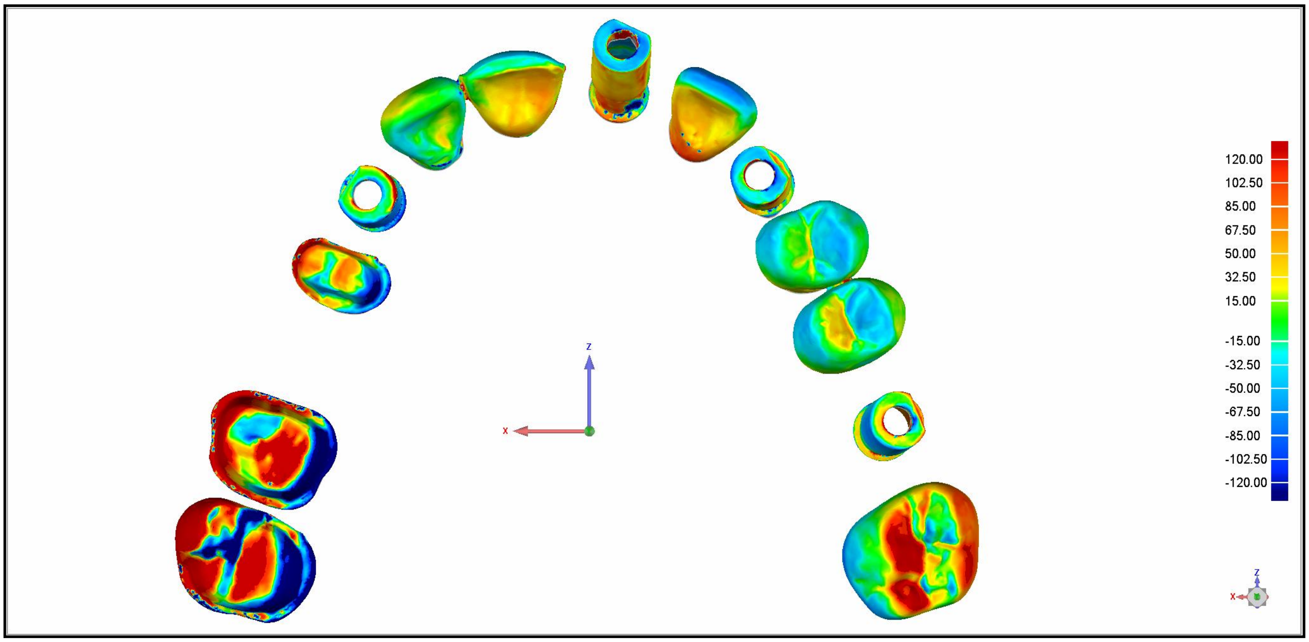

Predefinido: Izquierda

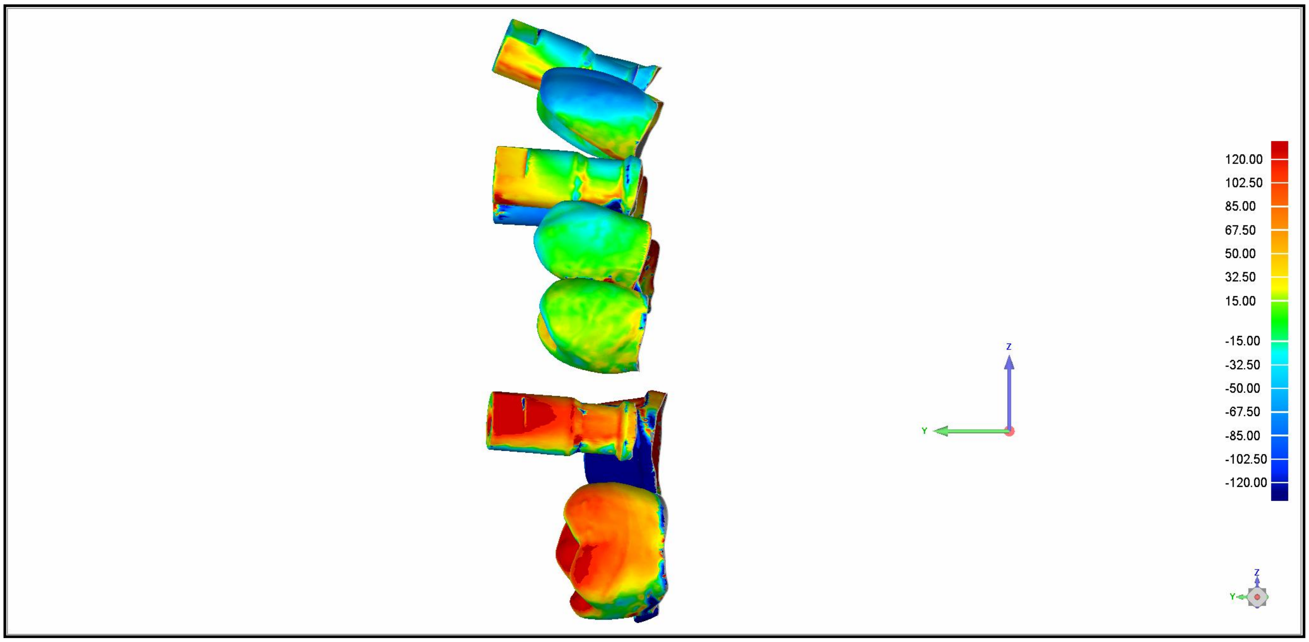

Predefinido: Derecha

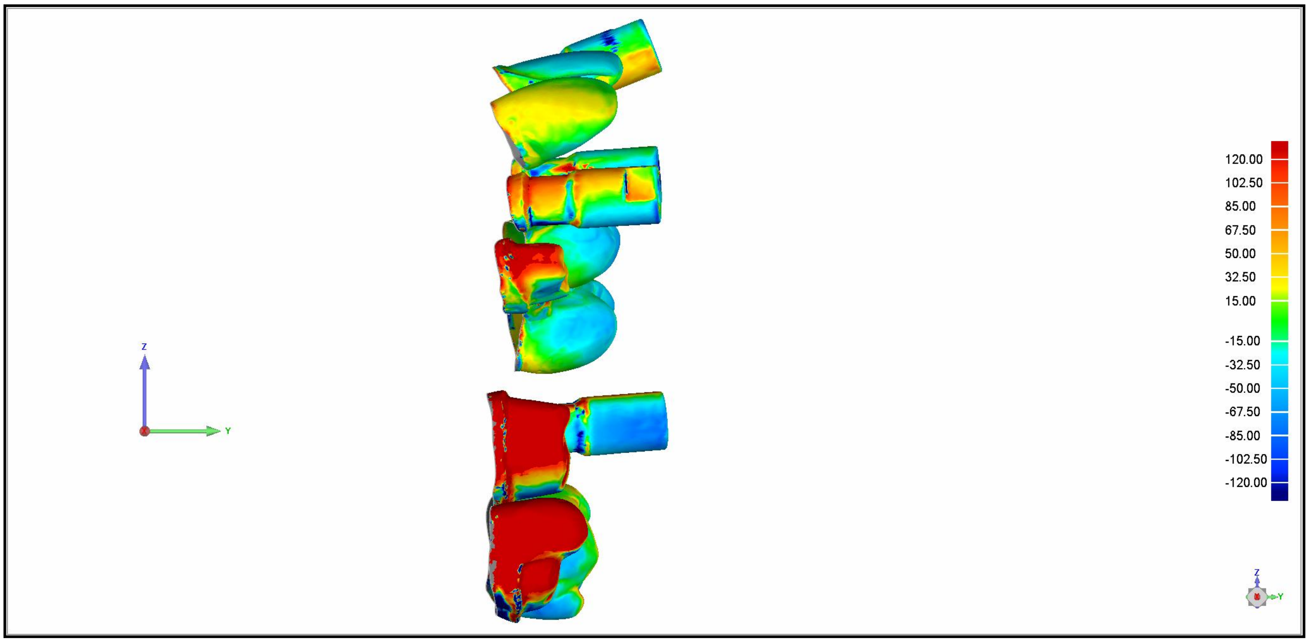

Predefinido: Superior

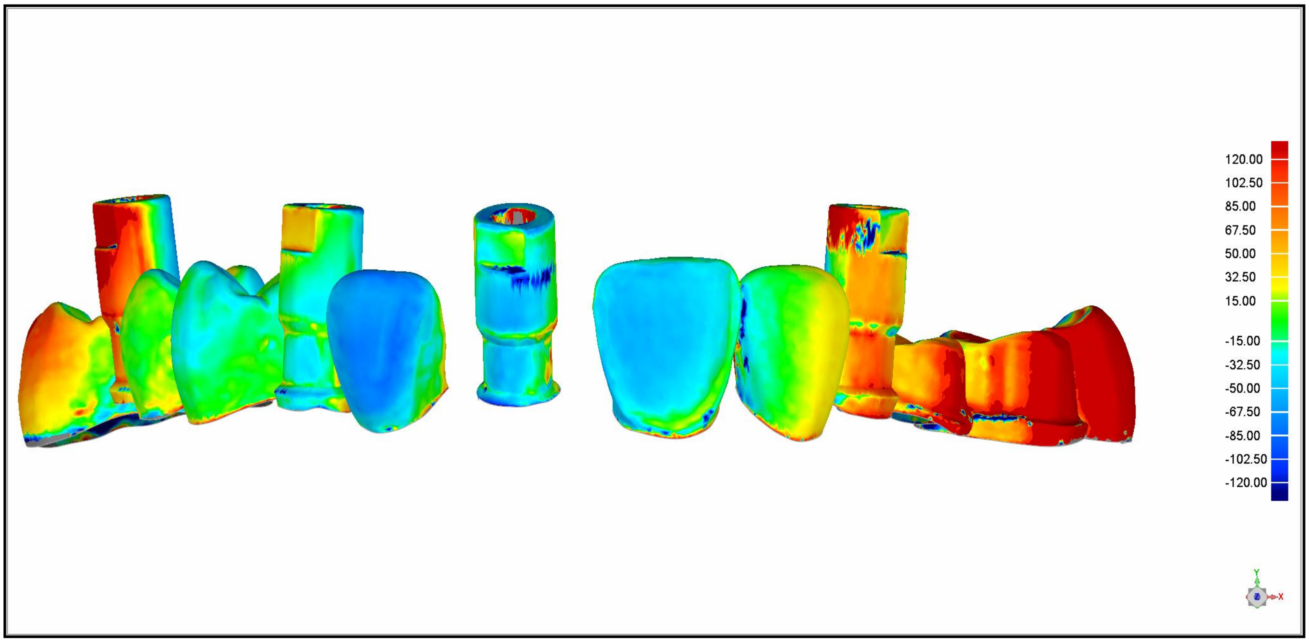

Predefinido: Inferior

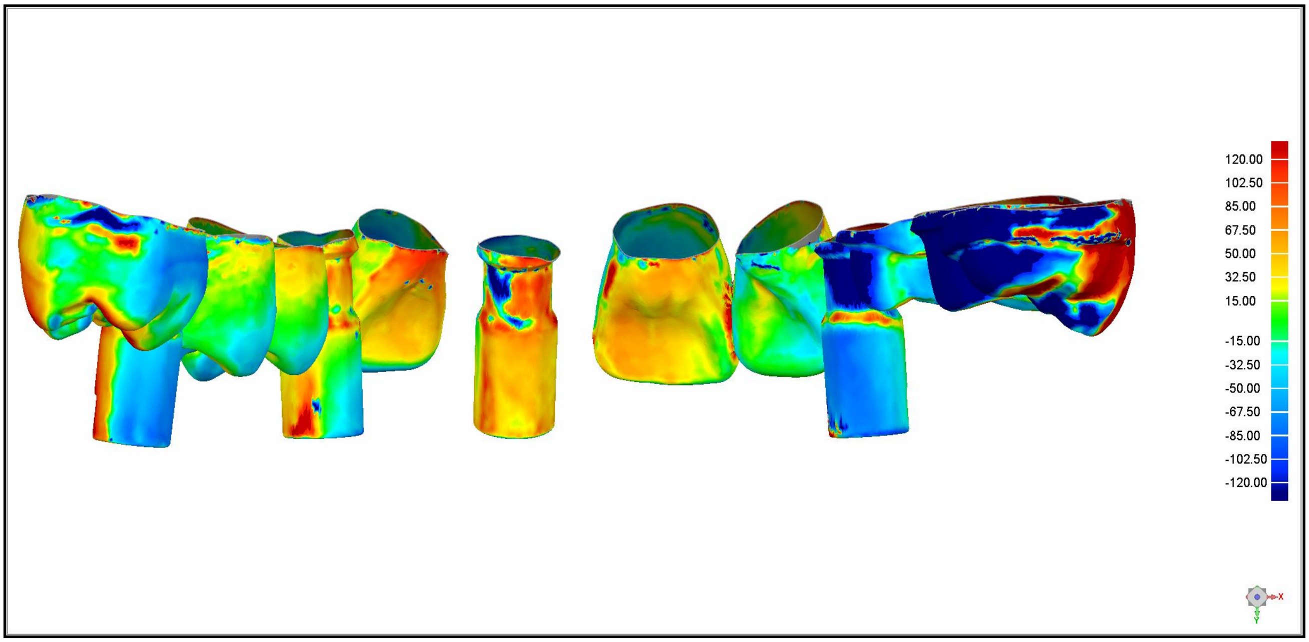

Ajuste de ubicación: Desviaciones superior e inferior

Unidades: u

Nombre	Desv	Estado	Superior Tol	Inferior Tol	Ref X	Ref Y	Ref Z	Radio	Desv X	Desv Y	Desv Z	Medido X	Medido Y	Medido Z	Dir. proy. X	Dir. proy. Y	Dir. proy. Z
Desv. inferior	-3138.92				-22607.19	28955.77	6808.03	n/a	-975.28	-407.73	2955.57	-23582.47	28548.04	9763.61	0.31	0.13	-0.94
Desv. superior	3143.44				-30.00	30038.48	26868.26	n/a	-1609.37	379.87	-2673.37	-1639.36	30418.35	24194.89	-0.51	0.12	-0.85
