# Supplementary material for: Accuracy of four digital scanners according to scanning strategy in complete-arch impressions
Source: PLoS One. 2018 Sep 13;13(9):e0202916. doi: 10.1371/journal.pone.0202916 (PMC6136706; doi:10.1371/journal.pone.0202916)
Supplement: S9 Table — Omnicam (scanning strategy A). (ZIP) [file pone.0202916.s009.zip › S9/OM9A.pdf]

Geomagic Control

Fecha: 3/6/2017, 3:11 pm

3D Comparación Resultados

Modelo referencia	MRC
Modelo test	OM9A
Nº de puntos de datos	194381
# Aislados	695

Tipo tolerancia	3D desviación
Unidades	u
Máx. crítico	120.00
Máx. nominal	2.00
Mín. nominal	-2.00
Mín. crítico	-120.00

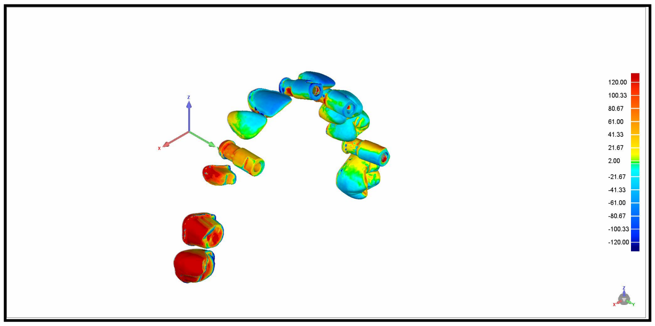

Desviación	
Desviación superior máx.	3149.70
Desviación inferior máx.	-3129.55
Desviación media	101.18 /-122.10
Desviación estándar	283.96

Distribución desviación

>=Min	<Max	# Puntos	%
-120.00	-100.33	2286	1.18
-100.33	-80.67	2831	1.46
-80.67	-61.00	5604	2.88
-61.00	-41.33	11415	5.87
-41.33	-21.67	19432	10.00
-21.67	-2.00	26029	13.39
-2.00	2.00	5789	2.98
2.00	21.67	27660	14.23
21.67	41.33	23091	11.88
41.33	61.00	16012	8.24
61.00	80.67	8932	4.60
80.67	100.33	5618	2.89
100.33	120.00	3463	1.78

Fuera del crítico superior	20732	10.67
Fuera del crítico inferior	15487	7.97

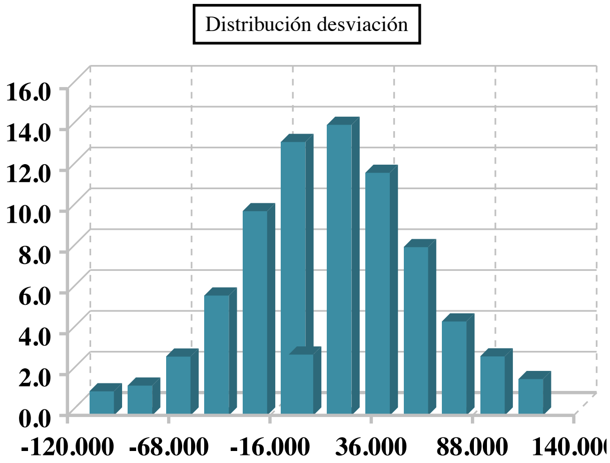

Desviaciones estándar

Distribución (+/-)	# Puntos	%
-6 * Desv. (estánd.	1904	0.98
-5 * Desv. estánd.	913	0.47
-4 * Desv. estánd.	626	0.32
-3 * Desv. estánd.	657	0.34
-2 * Desv. estánd.	2250	1.16
-1 * Desv. estánd.	83017	42.71
1 * Desv. estánd.	98641	50.75
2 * Desv. estánd.	3180	1.64
3 * Desv. estánd.	1074	0.55
4 * Desv. estánd.	824	0.42
5 * Desv. estánd.	523	0.27
6 * Desv. estánd.	772	0.40

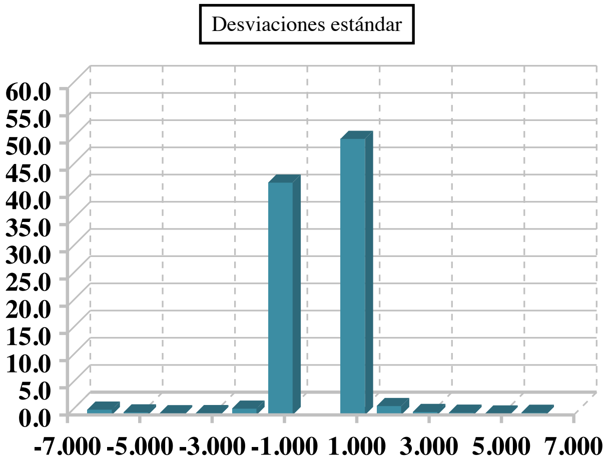

Predefinido: Isométrico

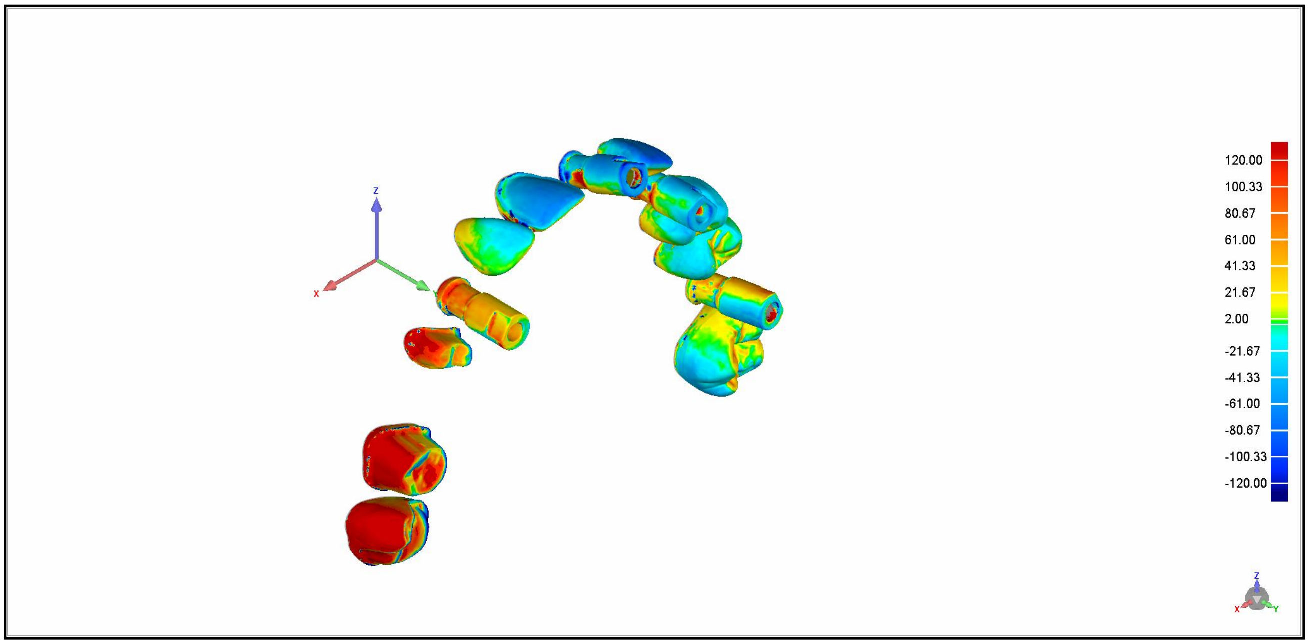

Predefinido: Frente

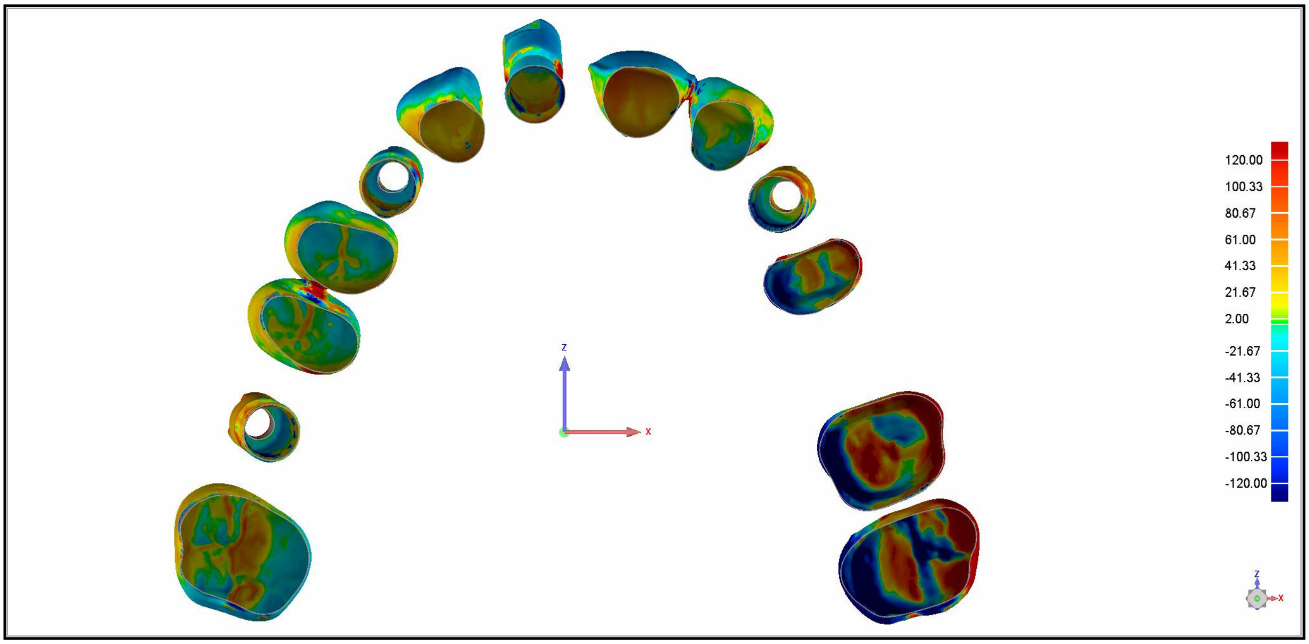

Predefinido: Atrás

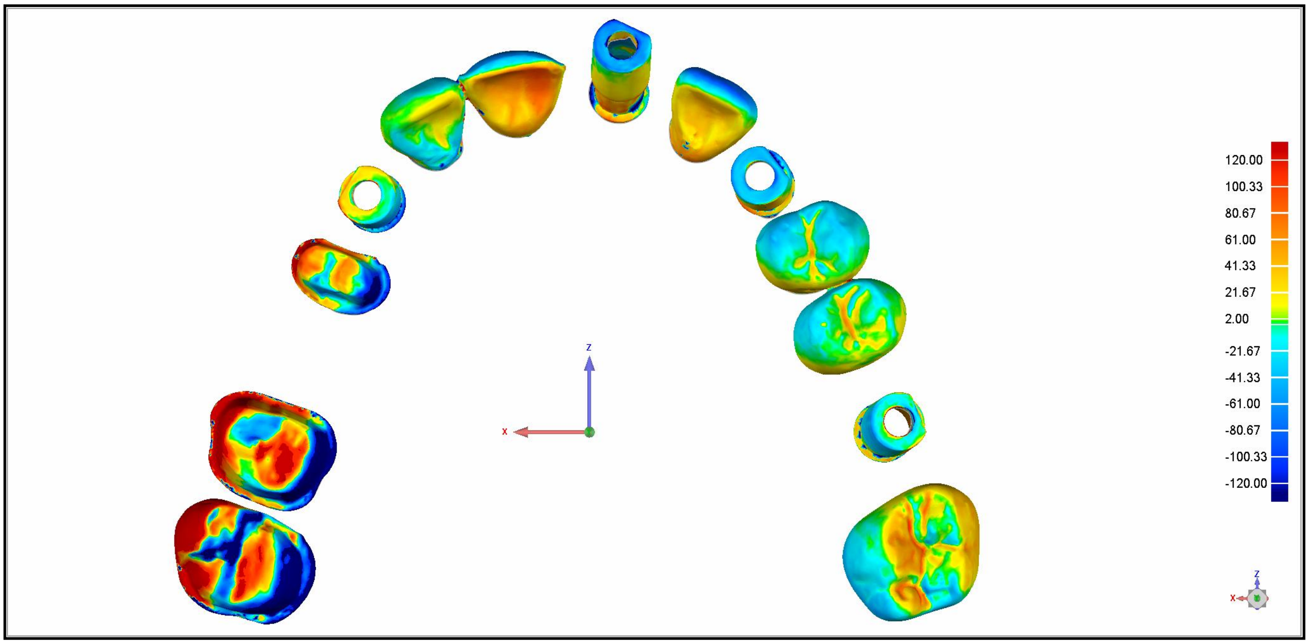

Predefinido: Izquierda

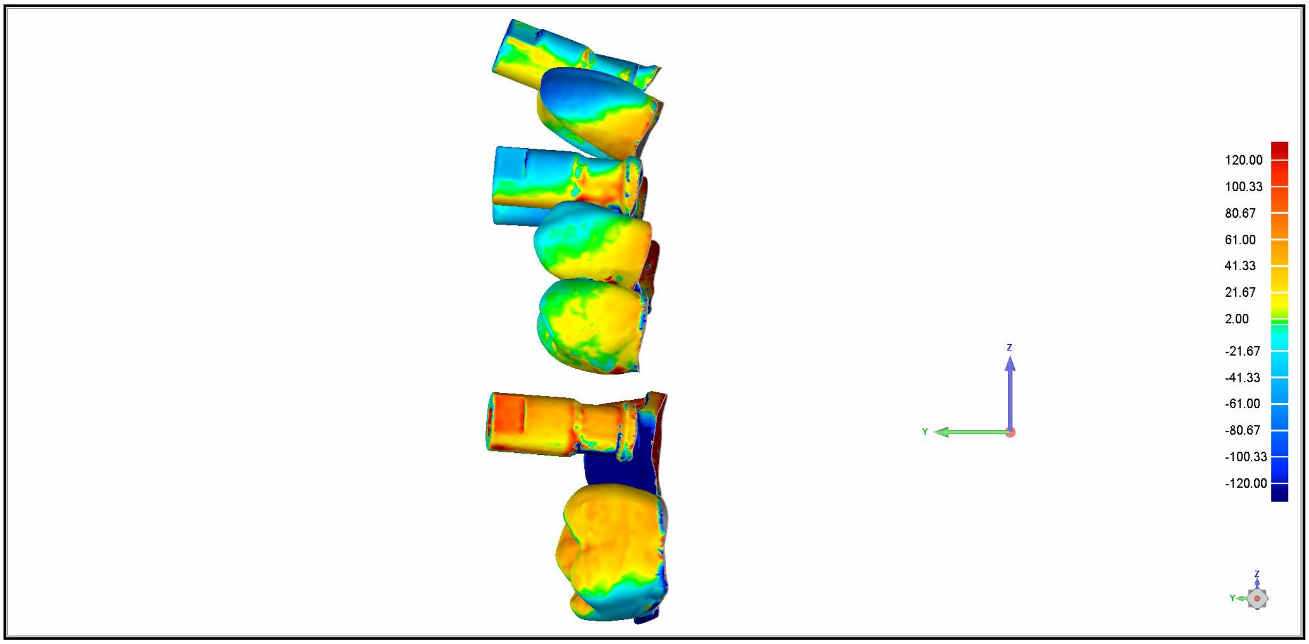

Predefinido: Derecha

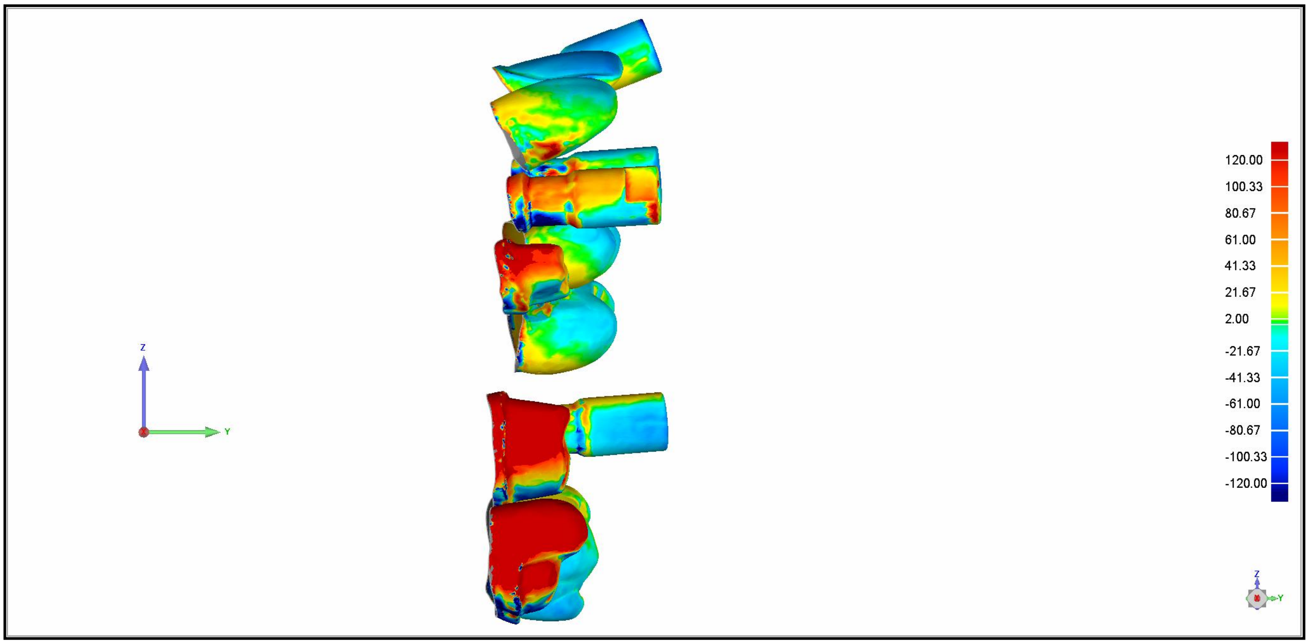

Predefinido: Superior

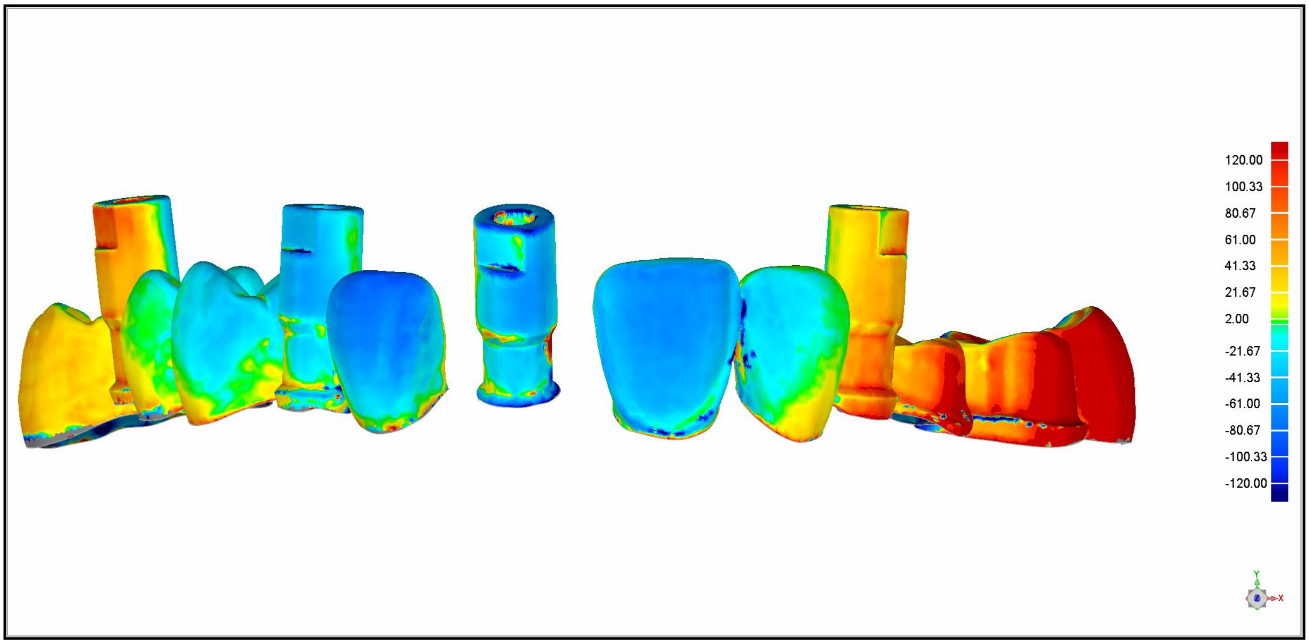

Predefinido: Inferior

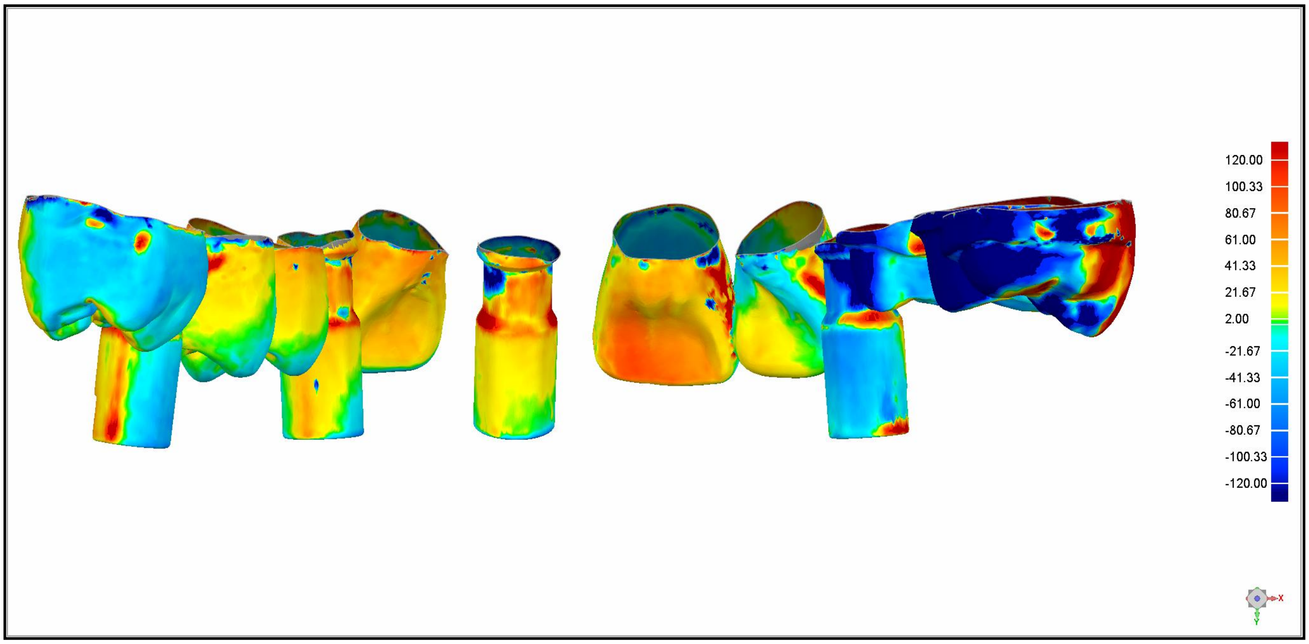

Ajuste de ubicación: Desviaciones superior e inferior

Unidades: u

Nombre	Desv	Estado	Superior Tol	Inferior Tol	Ref X	Ref Y	Ref Z	Radio	Desv X	Desv Y	Desv Z	Medido X	Medido Y	Medido Z	Dir. proy. X	Dir. proy. Y	Dir. proy. Z
Desv. inferior	-3129.55				-29208.33	26961.25	-11988.49	n/a	2786.43	289.44	-1395.03	-26421.90	27250.68	-13383.51	-0.89	-0.09	0.45
Desv. superior	3149.70				18471.59	28896.56	16745.52	n/a	-953.27	369.82	-2979.11	17518.32	29266.39	13766.40	-0.30	0.12	-0.95
