# Supplementary material for: Accuracy of four digital scanners according to scanning strategy in complete-arch impressions
Source: PLoS One. 2018 Sep 13;13(9):e0202916. doi: 10.1371/journal.pone.0202916 (PMC6136706; doi:10.1371/journal.pone.0202916)
Supplement: S9 Table — Omnicam (scanning strategy A). (ZIP) [file pone.0202916.s009.zip › S9/OM6A.pdf]

Fecha: 3/6/2017, 3:03 pm

3D Comparación Resultados

Modelo referencia	MRC
Modelo test	OM6A
Nº de puntos de datos	197800
# Aislados	849

Tipo tolerancia	3D desviación
Unidades	u
Máx. crítico	120.00
Máx. nominal	4.00
Mín. nominal	-4.00
Mín. crítico	-120.00

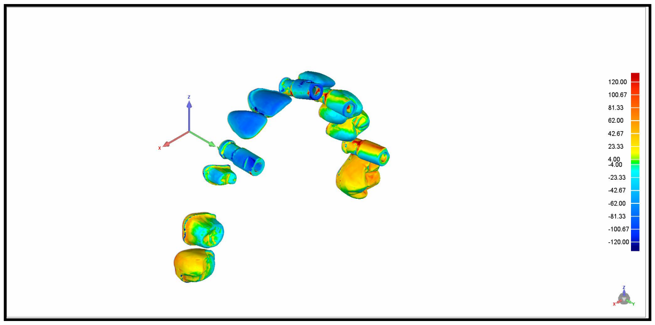

Desviación	
Desviación superior máx.	3154.90
Desviación inferior máx.	-3134.85
Desviación media	95.25 /-91.27
Desviación estándar	288.64

Distribución desviación

>=Min	<Max	# Puntos	%
-120.00	-100.67	1216	0.61
-100.67	-81.33	3141	1.59
-81.33	-62.00	8748	4.42
-62.00	-42.67	14440	7.30
-42.67	-23.33	24623	12.45
-23.33	-4.00	39070	19.75
-4.00	4.00	14817	7.49
4.00	23.33	29939	15.14
23.33	42.67	20786	10.51
42.67	62.00	11935	6.03
62.00	81.33	5364	2.71
81.33	100.67	2644	1.34
100.67	120.00	1702	0.86

Fuera del crítico superior	12161	6.15
Fuera del crítico inferior	7214	3.65

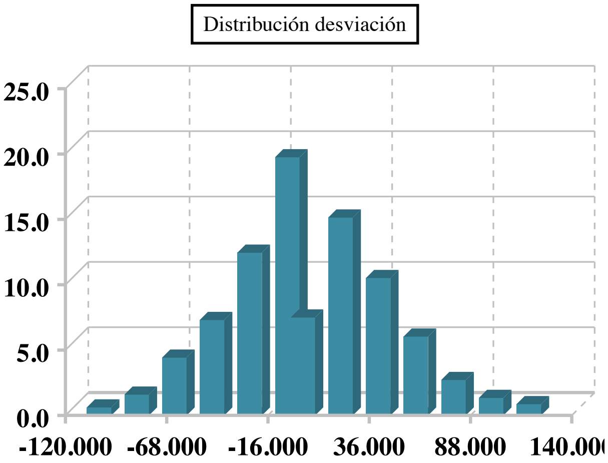

Desviaciones estándar

Distribución (+/-)	# Puntos	%
-6 * Desv. estánd.	1910	0.97
-5 * Desv. estánd.	465	0.24
-4 * Desv. estánd.	636	0.32
-3 * Desv. estánd.	935	0.47
-2 * Desv. estánd.	1110	0.56
-1 * Desv. estánd.	92010	46.52
1 * Desv. estánd.	94738	47.90
2 * Desv. estánd.	2105	1.06
3 * Desv. estánd.	1528	0.77
4 * Desv. estánd.	1058	0.53
5 * Desv. estánd.	500	0.25
6 * Desv. estánd.	805	0.41

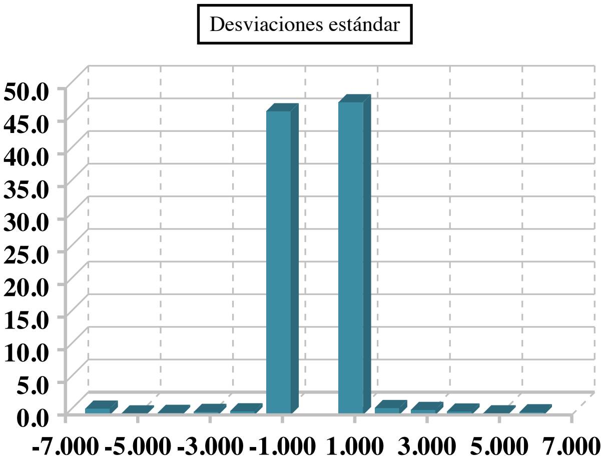

Predefinido: Isométrico

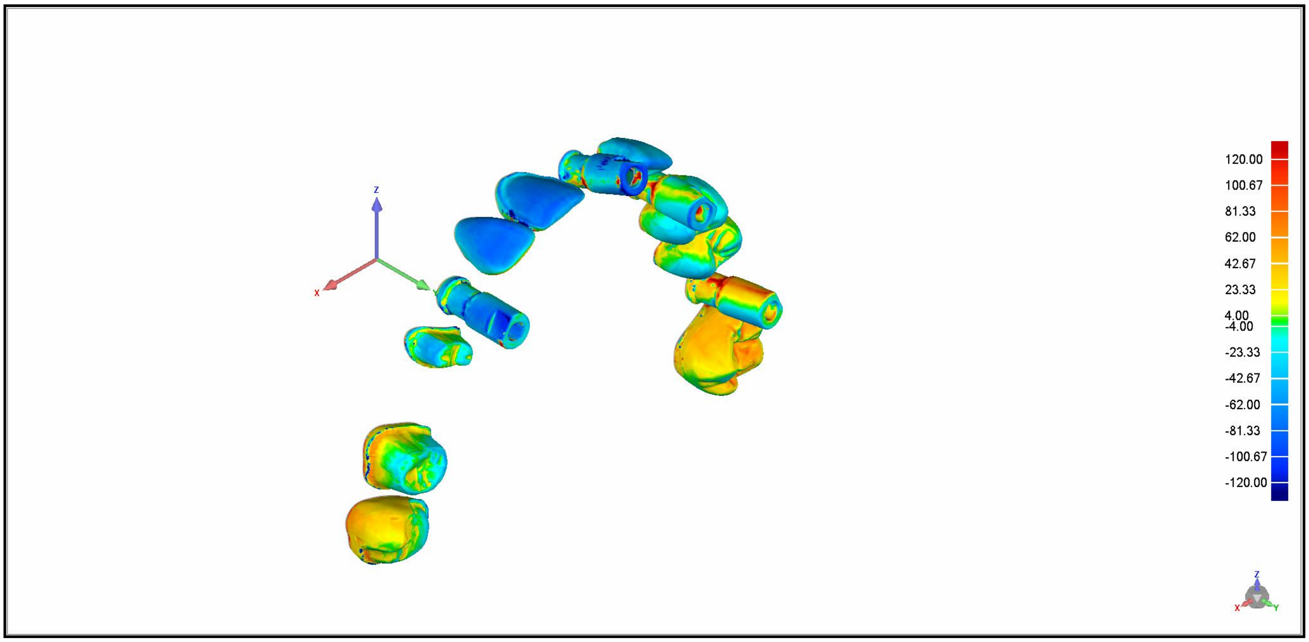

Predefinido: Frente

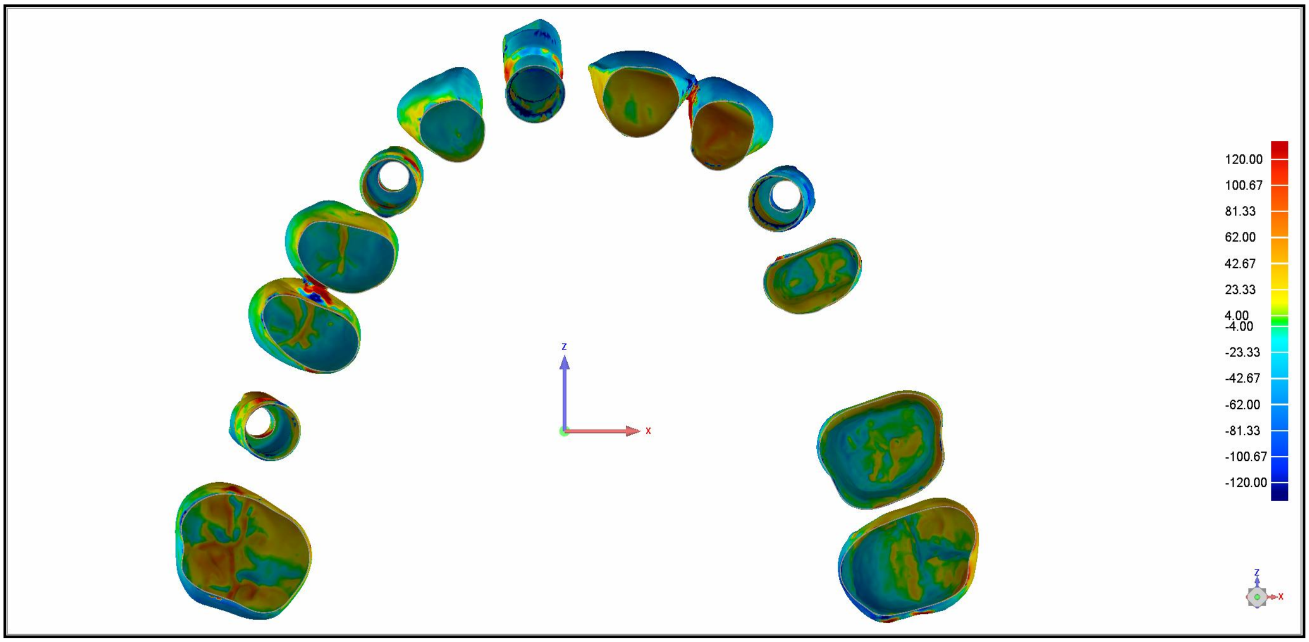

Predefinido: Atrás

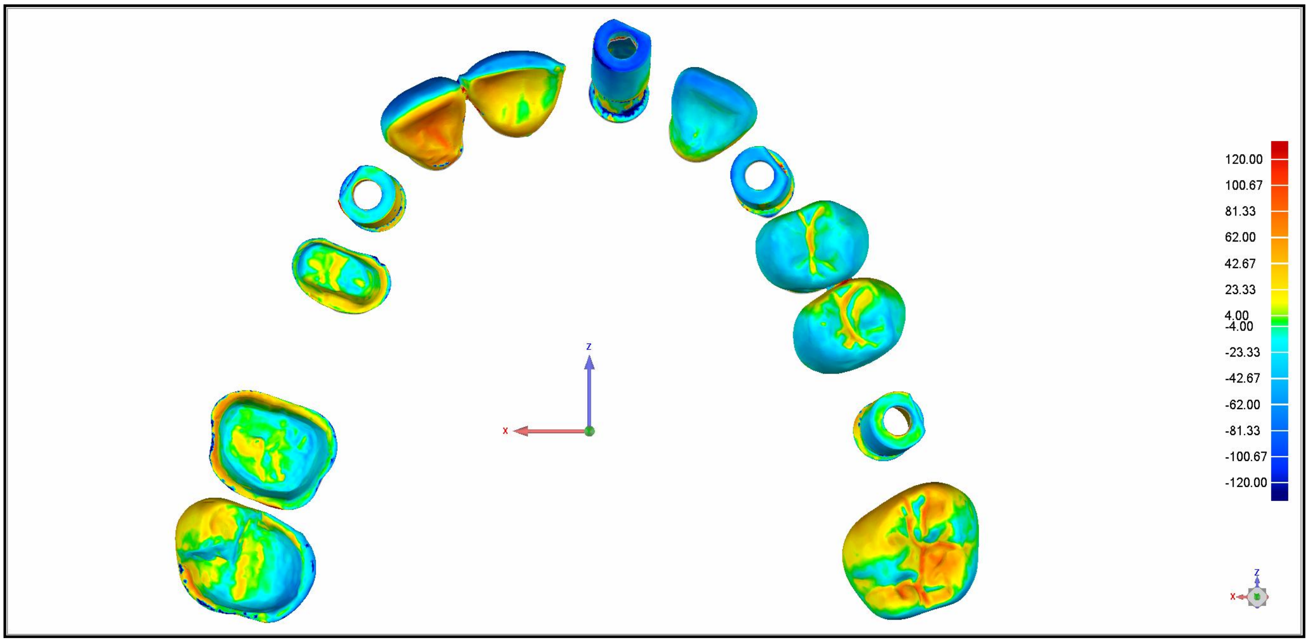

Fecha: 3/6/2017, 3:03 pm

Predefinido: Izquierda

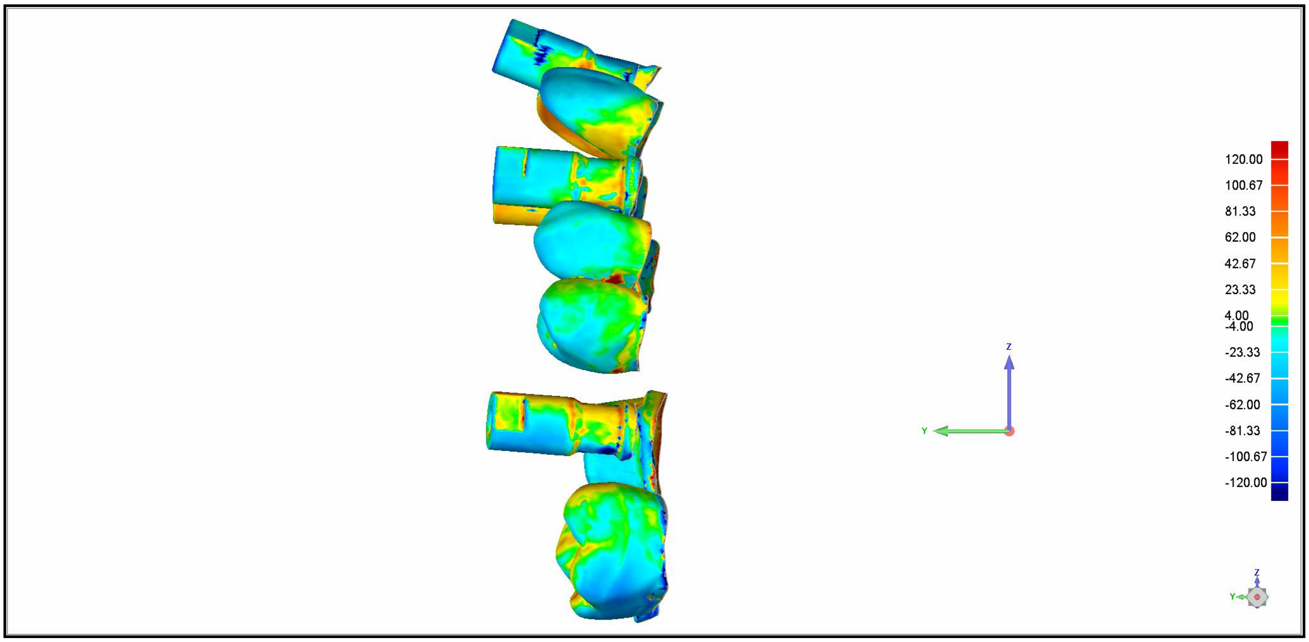

Predefinido: Derecha

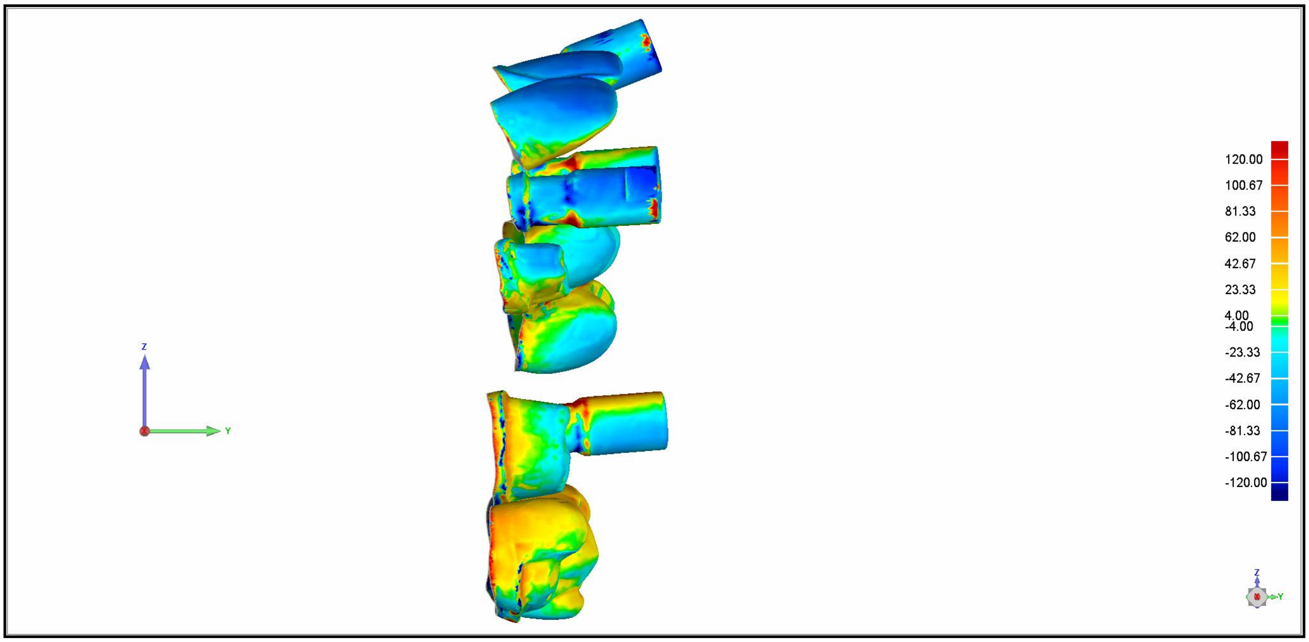

Predefinido: Superior

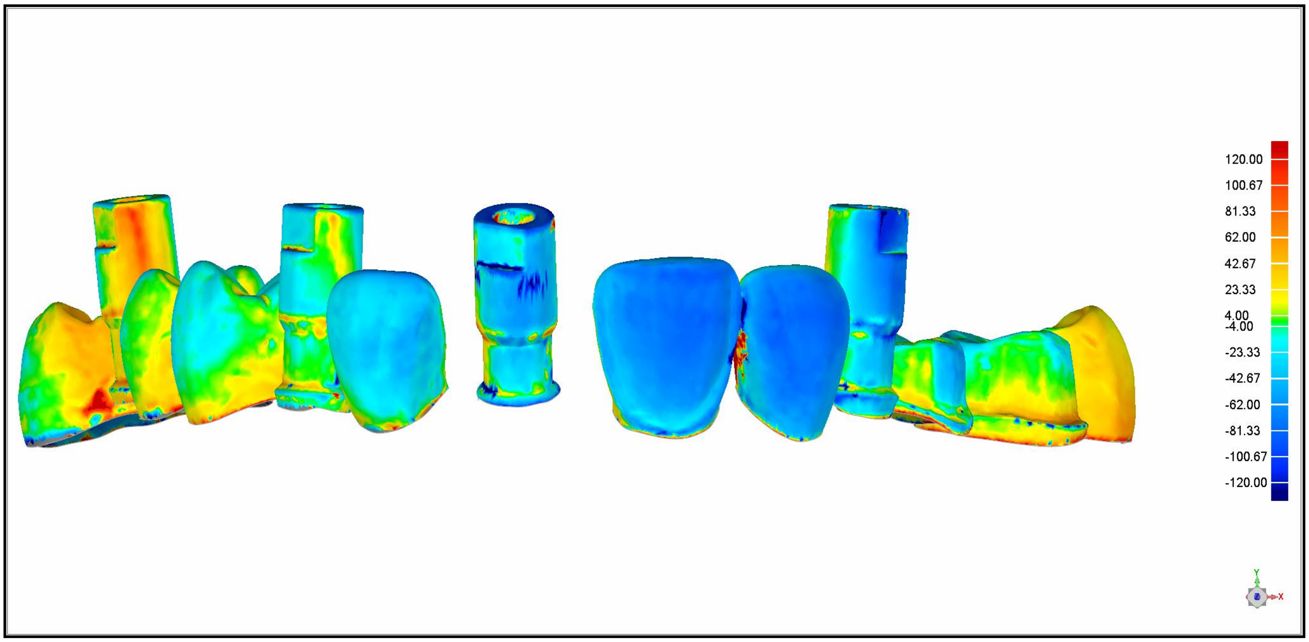

Predefinido: Inferior

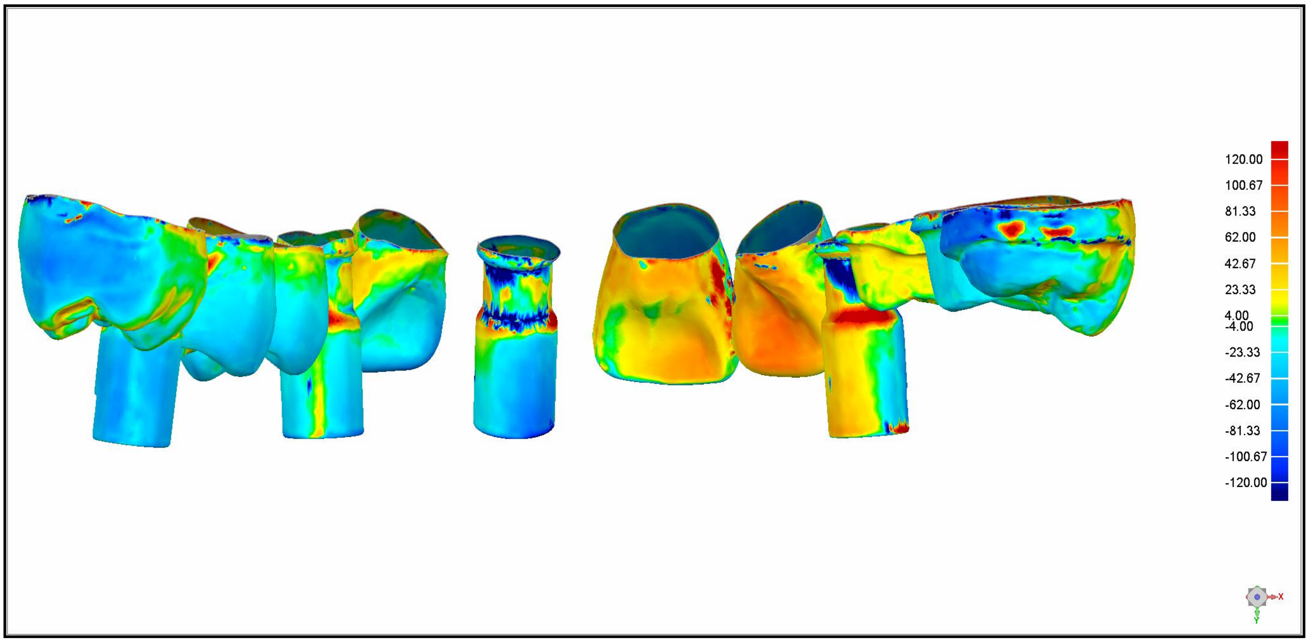

Ajuste de ubicación: Desviaciones superior e inferior

Unidades: u

Nombre	Desv	Estado	Superior Tol	Inferior Tol	Ref X	Ref Y	Ref Z	Radio	Desv X	Desv Y	Desv Z	Medido X	Medido Y	Medido Z	Dir. proy. X	Dir. proy. Y	Dir. proy. Z
Desv. inferior	-3134.85				-22607.19	28955.77	6808.03	n/a	-938.62	-344.49	2971.12	-23545.82	28611.28	9779.16	0.30	0.11	-0.95
Desv. superior	3154.90				-12601.26	29768.36	21377.11	n/a	1139.67	-1376.68	2599.87	-11461.59	28391.69	23976.97	0.36	-0.44	0.82
