# Supplementary material for: Accuracy of four digital scanners according to scanning strategy in complete-arch impressions
Source: PLoS One. 2018 Sep 13;13(9):e0202916. doi: 10.1371/journal.pone.0202916 (PMC6136706; doi:10.1371/journal.pone.0202916)
Supplement: S9 Table — Omnicam (scanning strategy A). (ZIP) [file pone.0202916.s009.zip › S9/OM7A.pdf]

3D Comparación Resultados

Modelo referencia	MRC
Modelo test	OM7A
Nº de puntos de datos	193050
# Aislados	576

Tipo tolerancia	3D desviación
Unidades	u
Máx. crítico	120.00
Máx. nominal	1.00
Mín. nominal	1.00
Mín. crítico	-120.00

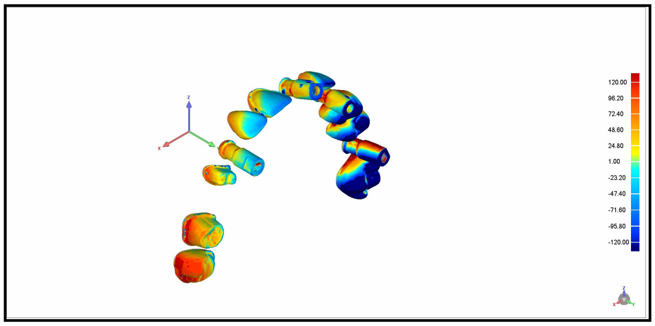

Desviación	
Desviación superior máx.	2728.10
Desviación inferior máx.	-2945.49
Desviación media	111.48 /-100.39
Desviación estándar	209.95

Distribución desviación

>=Min	<Max	# Puntos	%
-120.00	-95.80	8269	4.28
-95.80	-71.60	9305	4.82
-71.60	-47.40	13645	7.07
-47.40	-23.20	21185	10.97
-23.20	1.00	24296	12.59
1.00	24.80	22344	11.57
24.80	48.60	16507	8.55
48.60	72.40	12293	6.37
72.40	96.20	8671	4.49
96.20	120.00	7679	3.98

Fuera del crítico superior		22959	11.89
Fuera del crítico inferior		25897	13.41

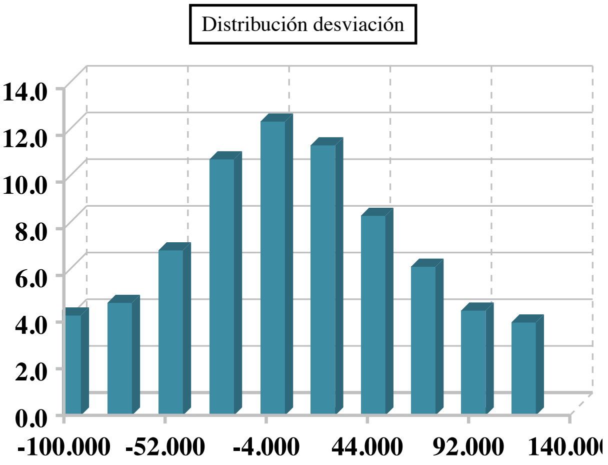

Desviaciones estándar

Distribución (+/-)	# Puntos	%
-6 * Desv. estánd.	922	0.48
-5 * Desv. estánd.	288	0.15
-4 * Desv. estánd.	499	0.26
-3 * Desv. estánd.	521	0.27
-2 * Desv. estánd.	6962	3.61
-1 * Desv. estánd.	92388	47.86
1 * Desv. estánd.	81753	42.35
2 * Desv. estánd.	5755	2.98
3 * Desv. estánd.	1335	0.69
4 * Desv. estánd.	970	0.50
5 * Desv. estánd.	723	0.37
6 * Desv. estánd.	934	0.48

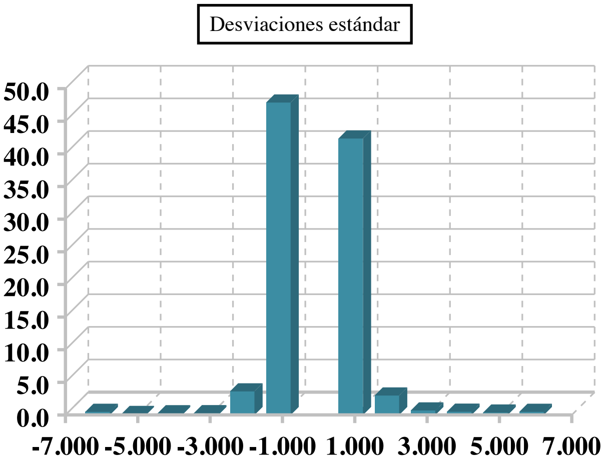

Predefinido: Isométrico

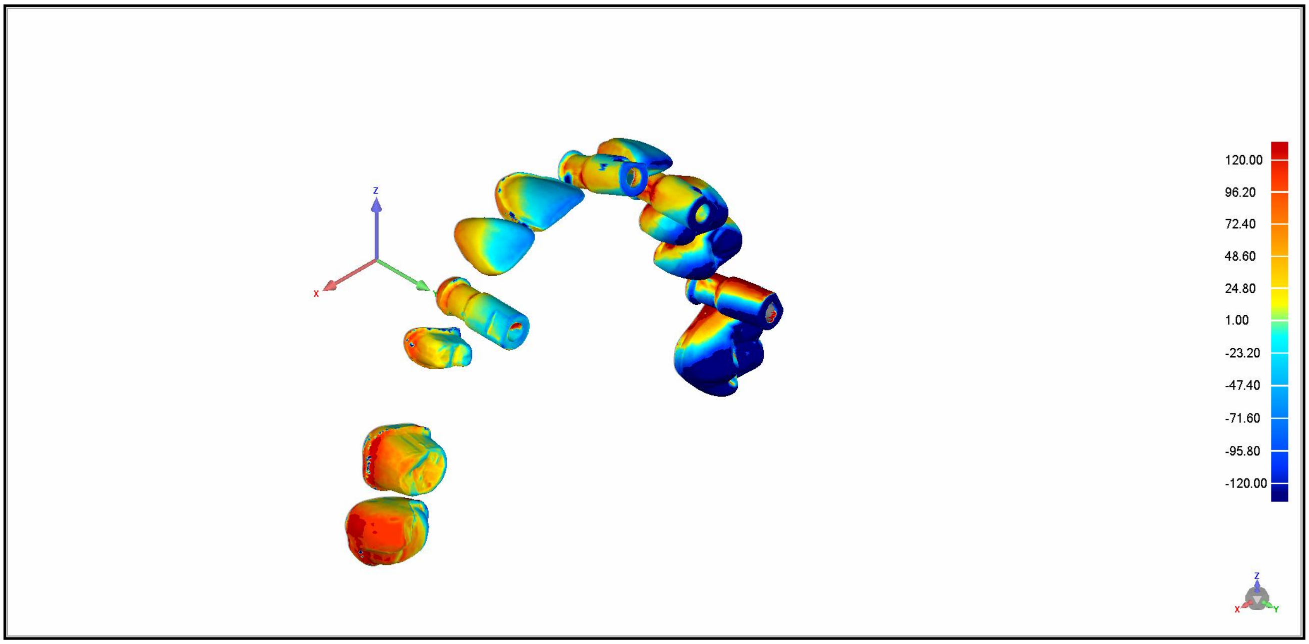

Predefinido: Frente

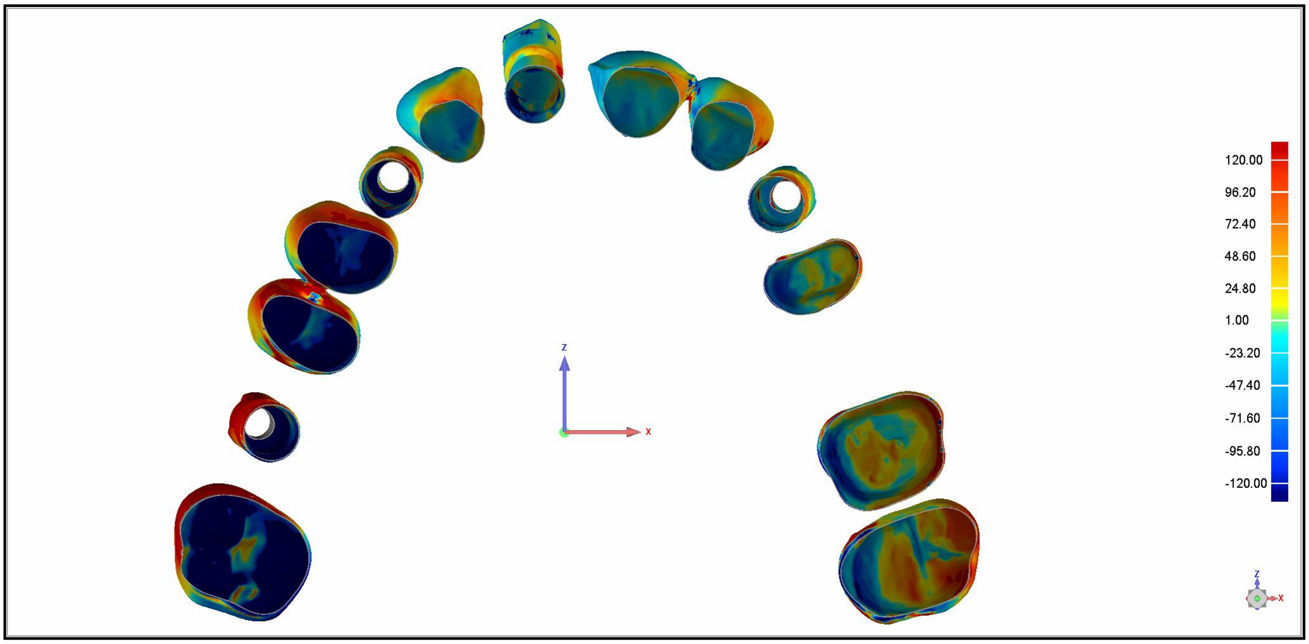

Predefinido: Atrás

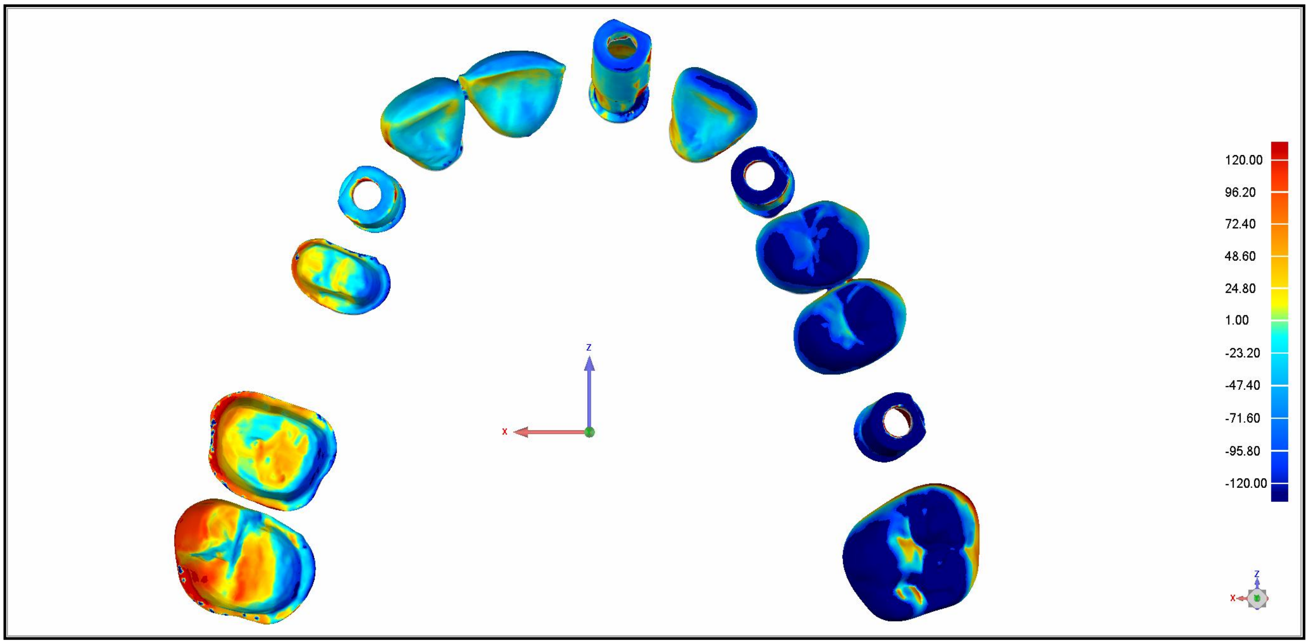

Predefinido: Izquierda

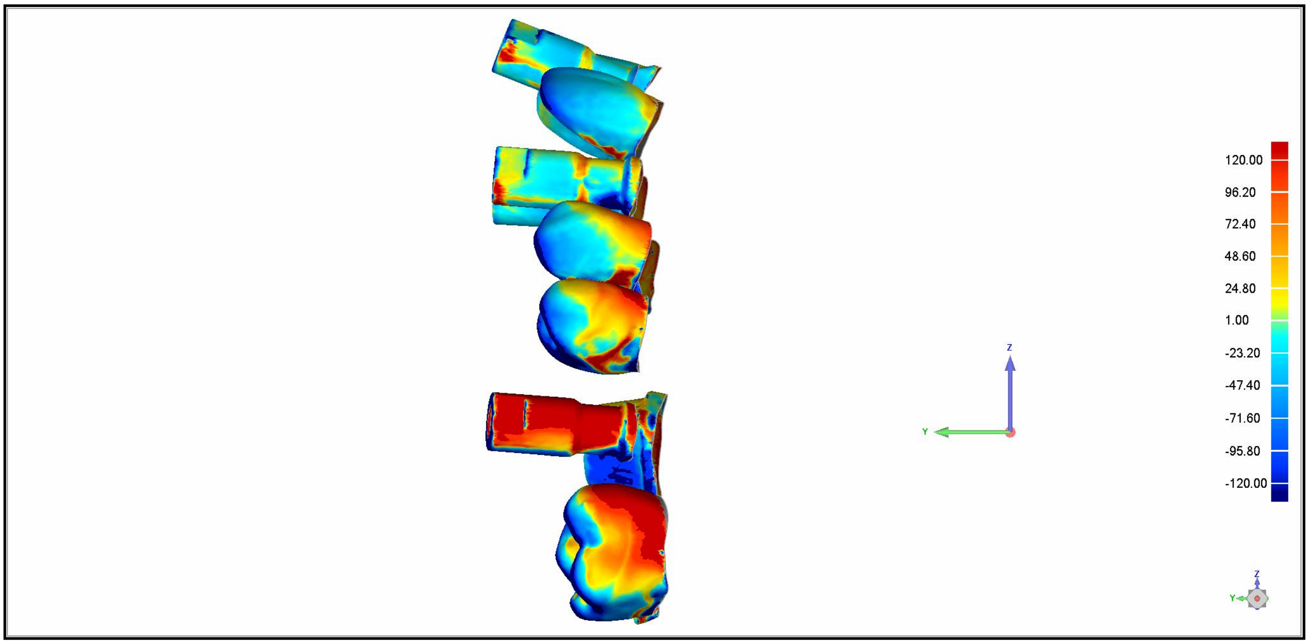

Predefinido: Derecha

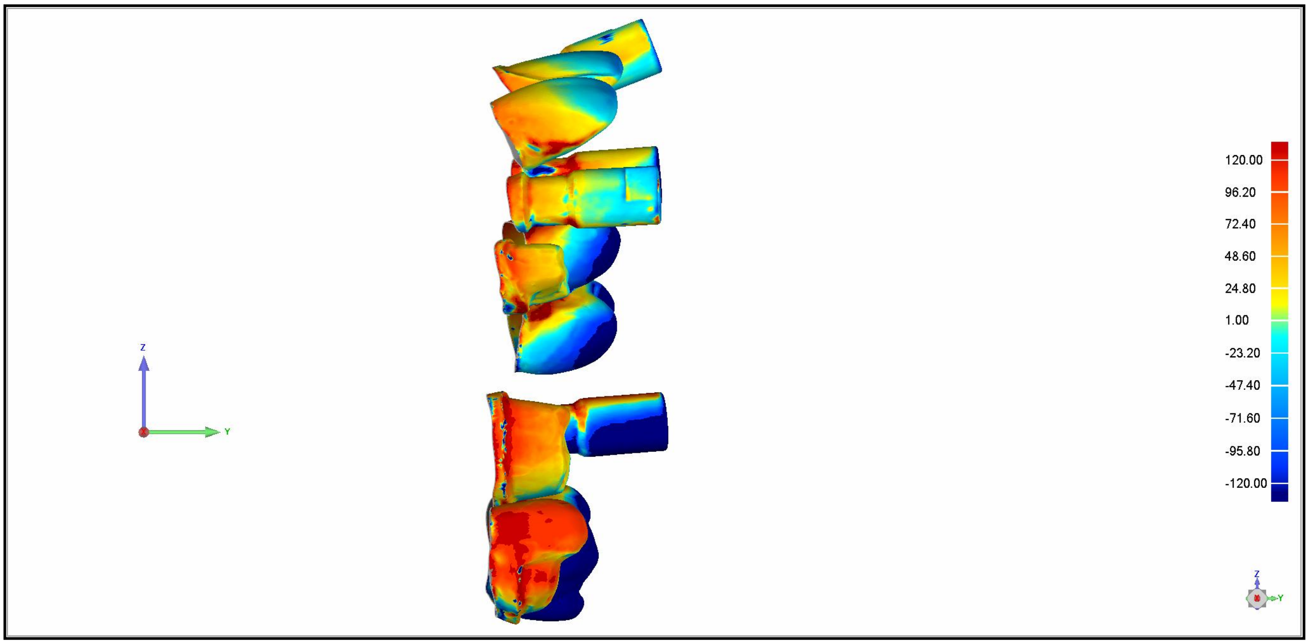

Predefinido: Superior

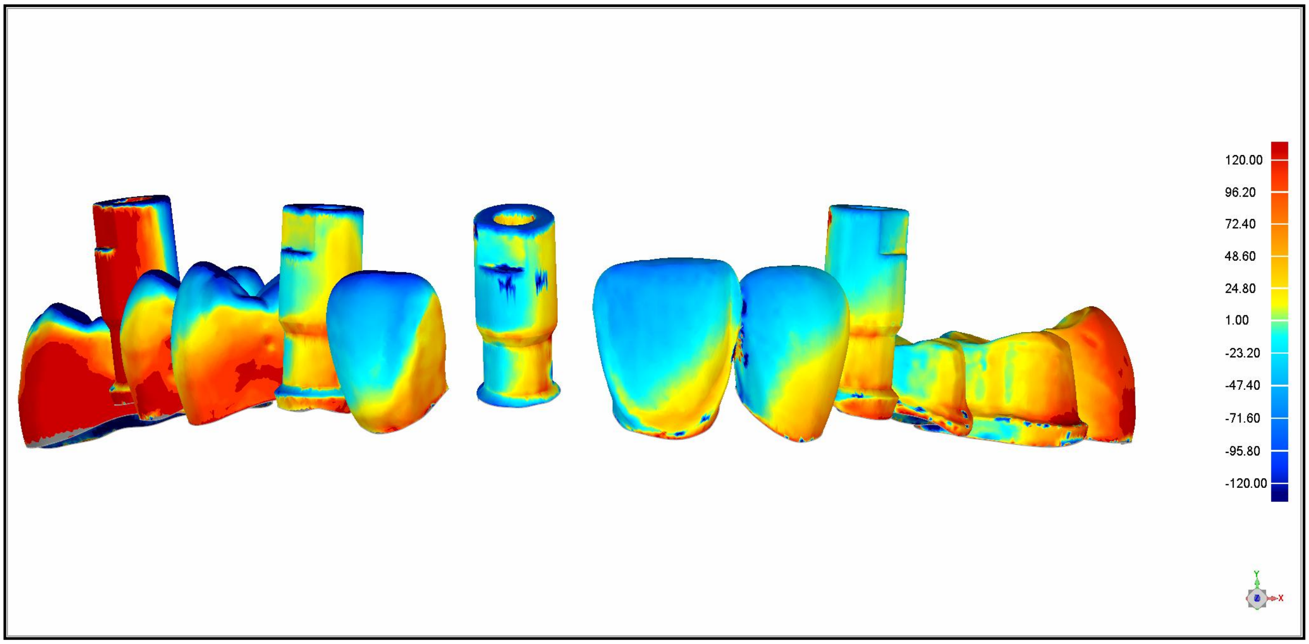

Predefinido: Inferior

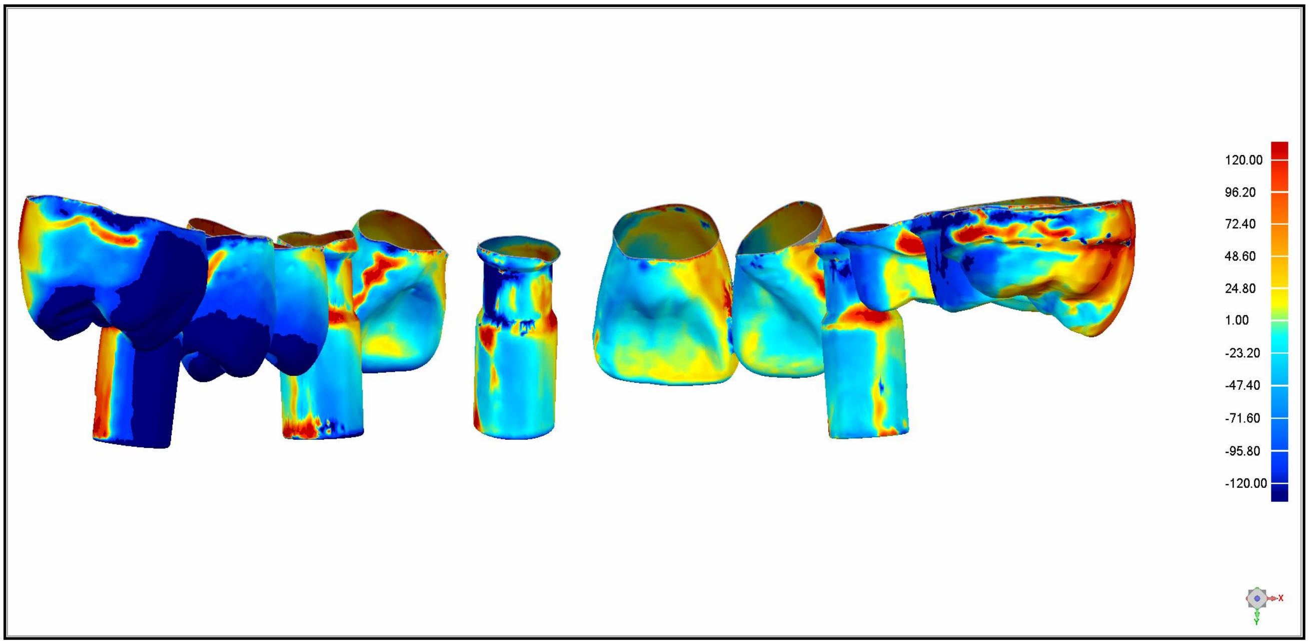

Ajuste de ubicación: Desviaciones superior e inferior

Unidades: u

Nombre	Desv	Estado	Superior Tol	Inferior Tol	Ref X	Ref Y	Ref Z	Radio	Desv X	Desv Y	Desv Z	Medido X	Medido Y	Medido Z	Dir. proy. X	Dir. proy. Y	Dir. proy. Z
Desv. inferior	-2945.49				31351.83	27101.88	-6377.73	n/a	-2908.72	346.90	308.02	28443.10	27448.77	-6069.71	0.99	-0.12	-0.10
Desv. superior	2728.10				-3314.37	31084.83	25125.78	n/a	-68.45	2446.82	1204.55	-3382.82	33531.65	26330.33	-0.03	0.90	0.44
