# Supplementary material for: Accuracy of four digital scanners according to scanning strategy in complete-arch impressions
Source: PLoS One. 2018 Sep 13;13(9):e0202916. doi: 10.1371/journal.pone.0202916 (PMC6136706; doi:10.1371/journal.pone.0202916)
Supplement: S9 Table — Omnicam (scanning strategy A). (ZIP) [file pone.0202916.s009.zip › S9/OM10A.pdf]

3D Comparación Resultados

Modelo referencia	MRC
Modelo test	OM10A
Nº de puntos de datos	192252
# Aislados	676

Tipo tolerancia	3D desviación
Unidades	u
Máx. crítico	120.00
Máx. nominal	2.00
Mín. nominal	-2.00
Mín. crítico	-120.00

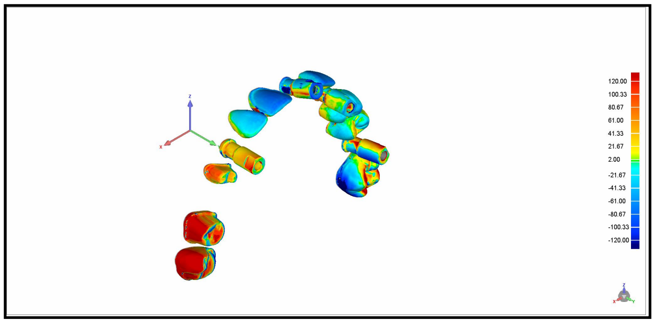

Desviación	
Desviación superior máx.	3147.10
Desviación inferior máx.	-3132.06
Desviación media	95.26 /-99.39
Desviación estándar	235.28

Distribución desviación

>=Min	<Max	# Puntos	%
-120.00	-100.33	3393	1.76
-100.33	-80.67	4912	2.55
-80.67	-61.00	9638	5.01
-61.00	-41.33	12012	6.25
-41.33	-21.67	18377	9.56
-21.67	-2.00	25128	13.07
-2.00	2.00	5484	2.85
2.00	21.67	27242	14.17
21.67	41.33	18203	9.47
41.33	61.00	12052	6.27
61.00	80.67	7987	4.15
80.67	100.33	6344	3.30
100.33	120.00	5068	2.64

Fuera del crítico superior	21432	11.15
Fuera del crítico inferior	14980	7.79

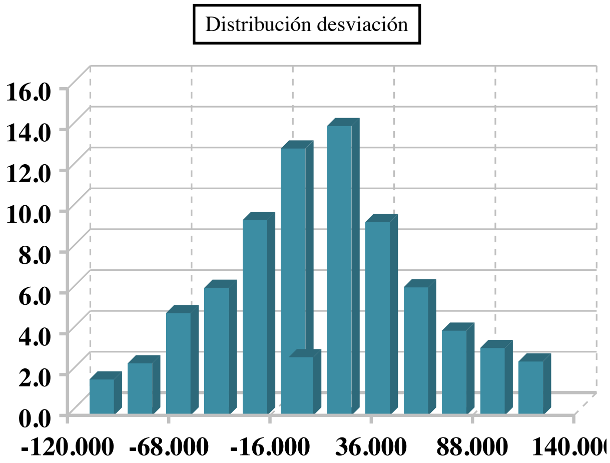

Desviaciones estándar

Distribución (+/-)	# Puntos	%
-6 * Desv. estánd.	1375	0.72
-5 * Desv. estánd.	425	0.22
-4 * Desv. estánd.	526	0.27
-3 * Desv. estánd.	779	0.41
-2 * Desv. estánd.	3826	1.99
-1 * Desv. estánd.	88270	45.91
1 * Desv. estánd.	90954	47.31
2 * Desv. estánd.	3345	1.74
3 * Desv. estánd.	850	0.44
4 * Desv. estánd.	474	0.25
5 * Desv. estánd.	399	0.21
6 * Desv. estánd.	1029	0.54

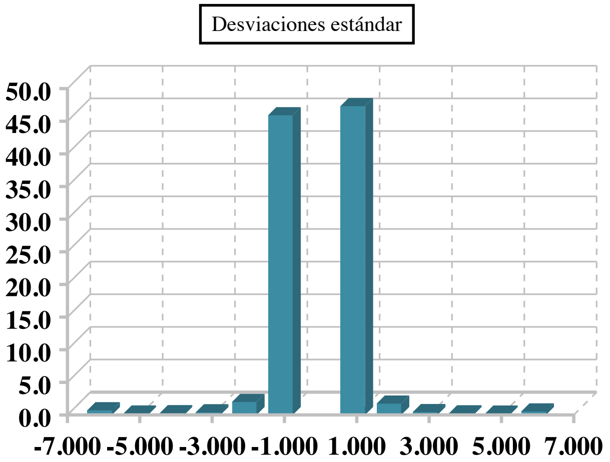

Predefinido: Isométrico

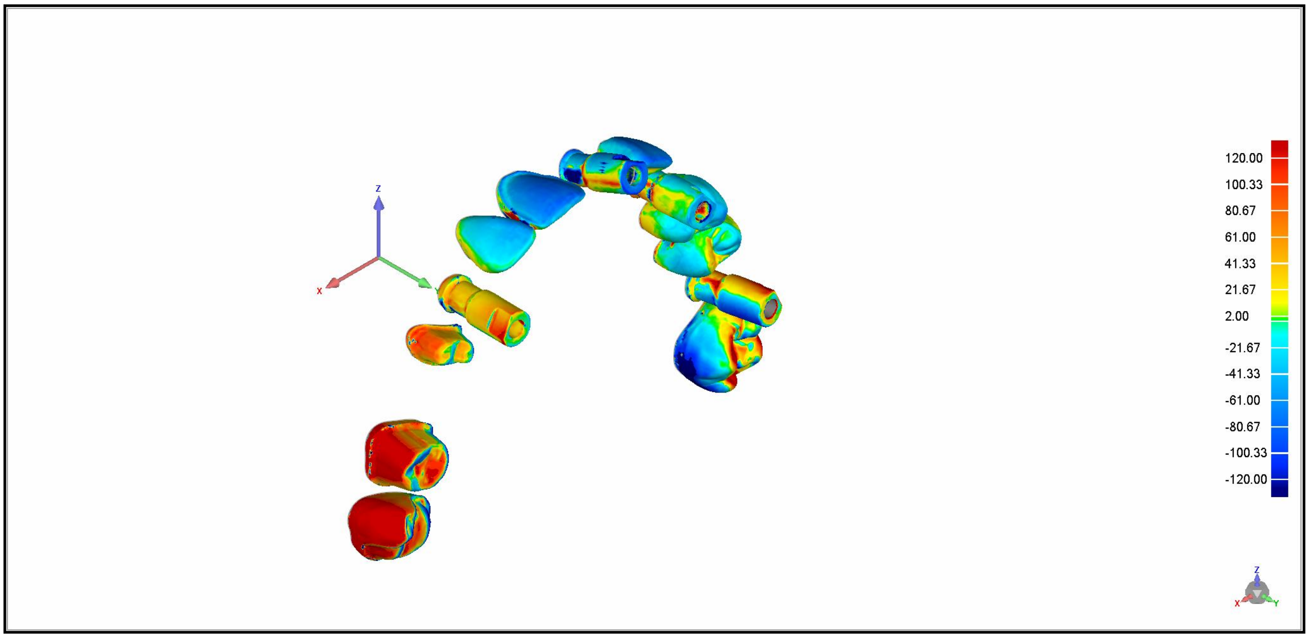

Predefinido: Frente

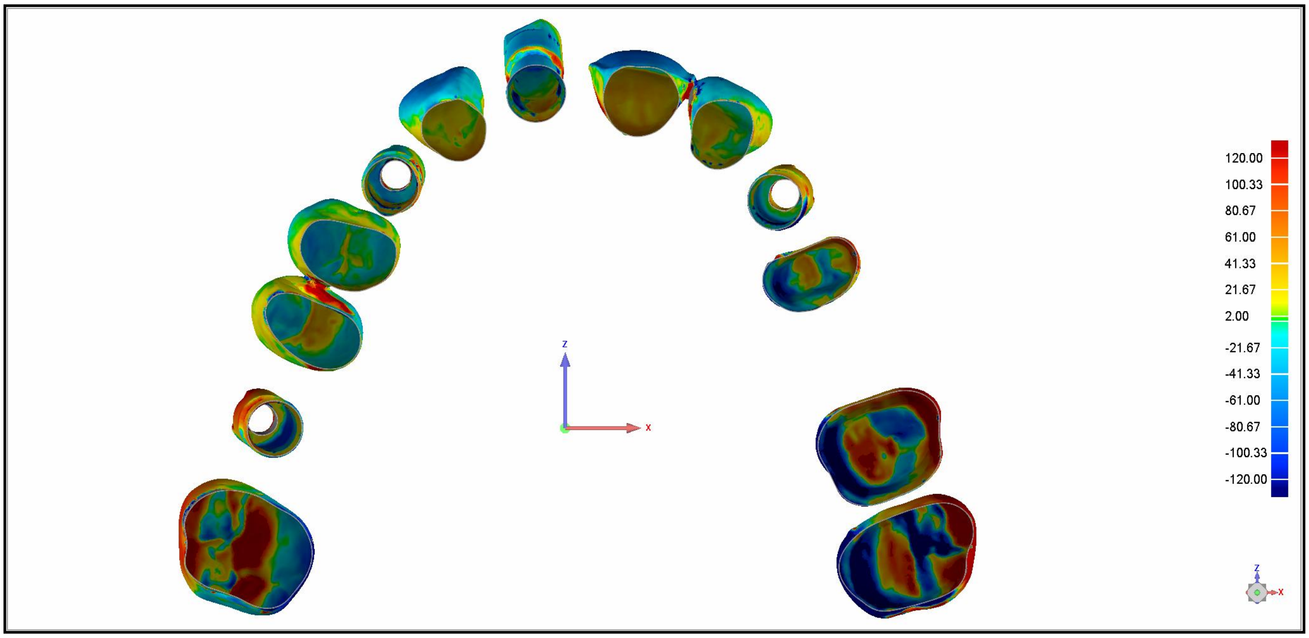

Predefinido: Atrás

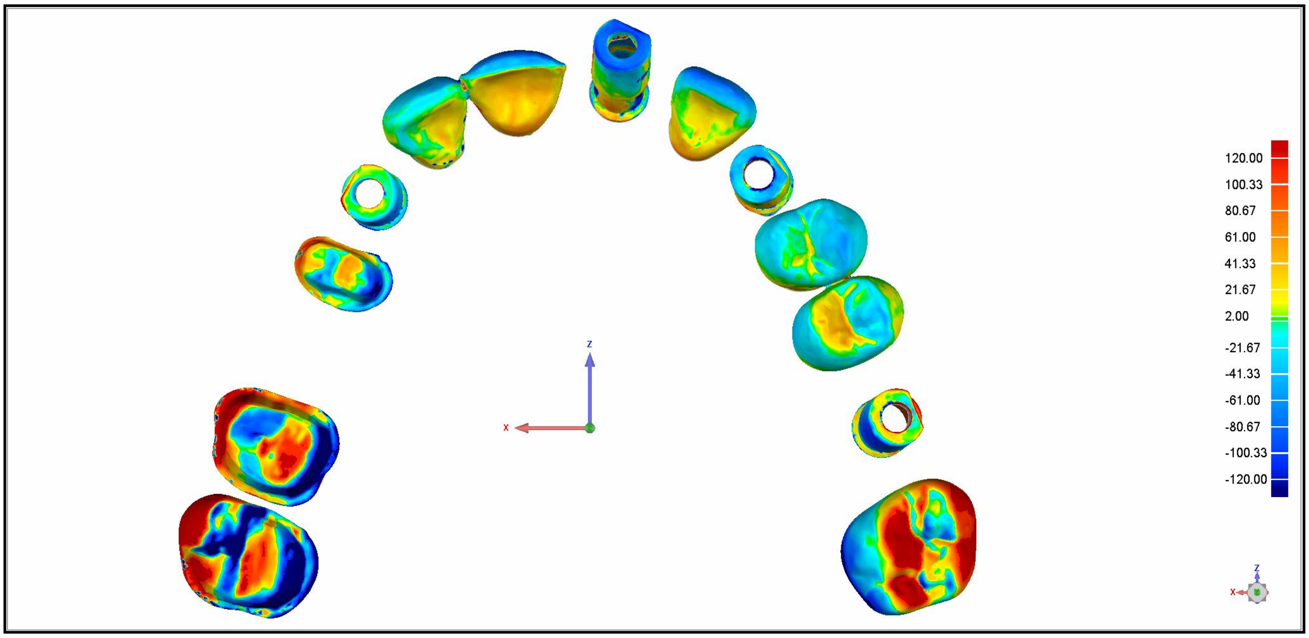

Predefinido: Izquierda

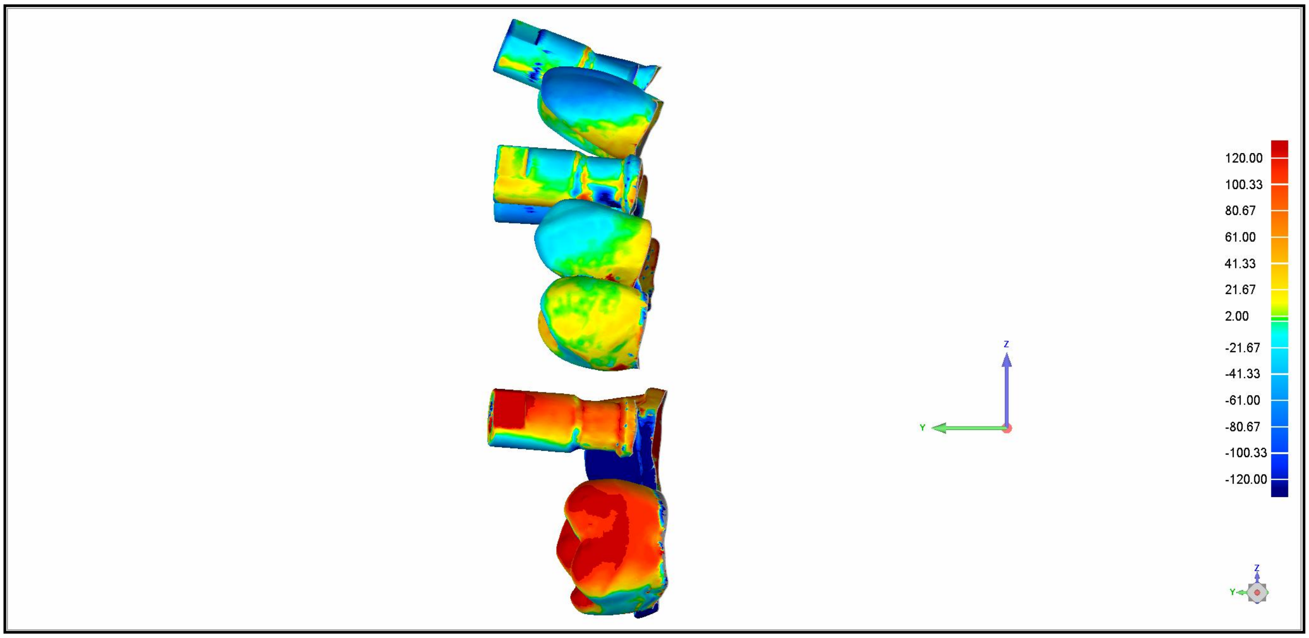

Predefinido: Derecha

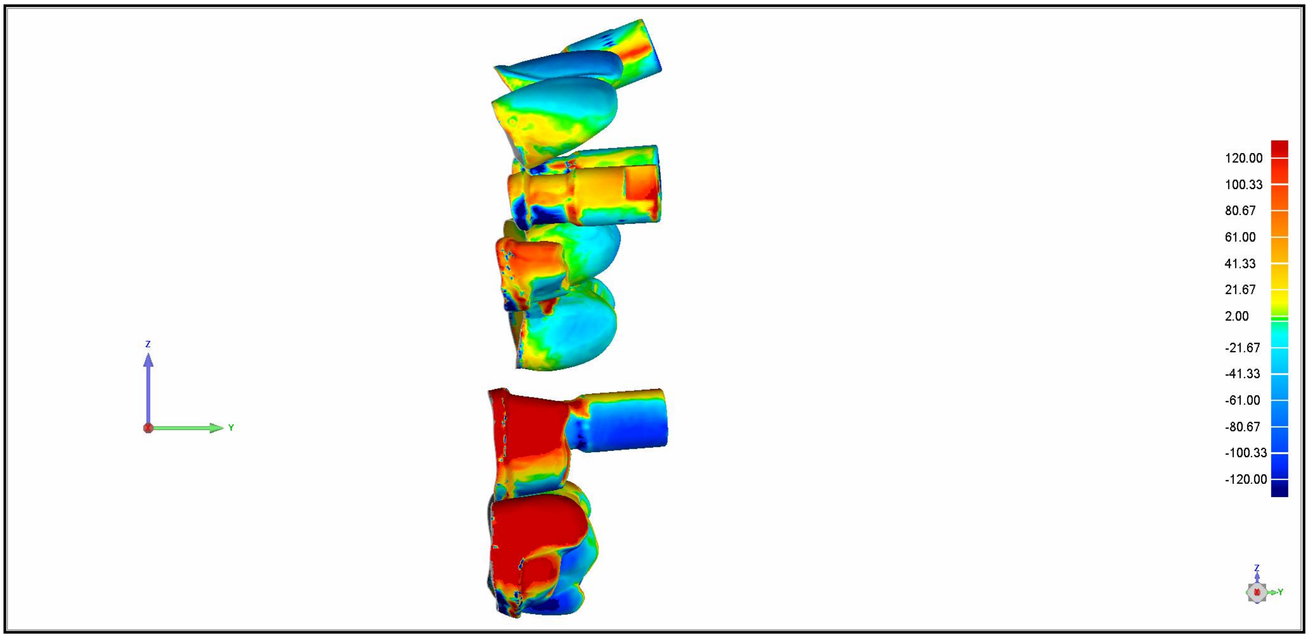

Predefinido: Superior

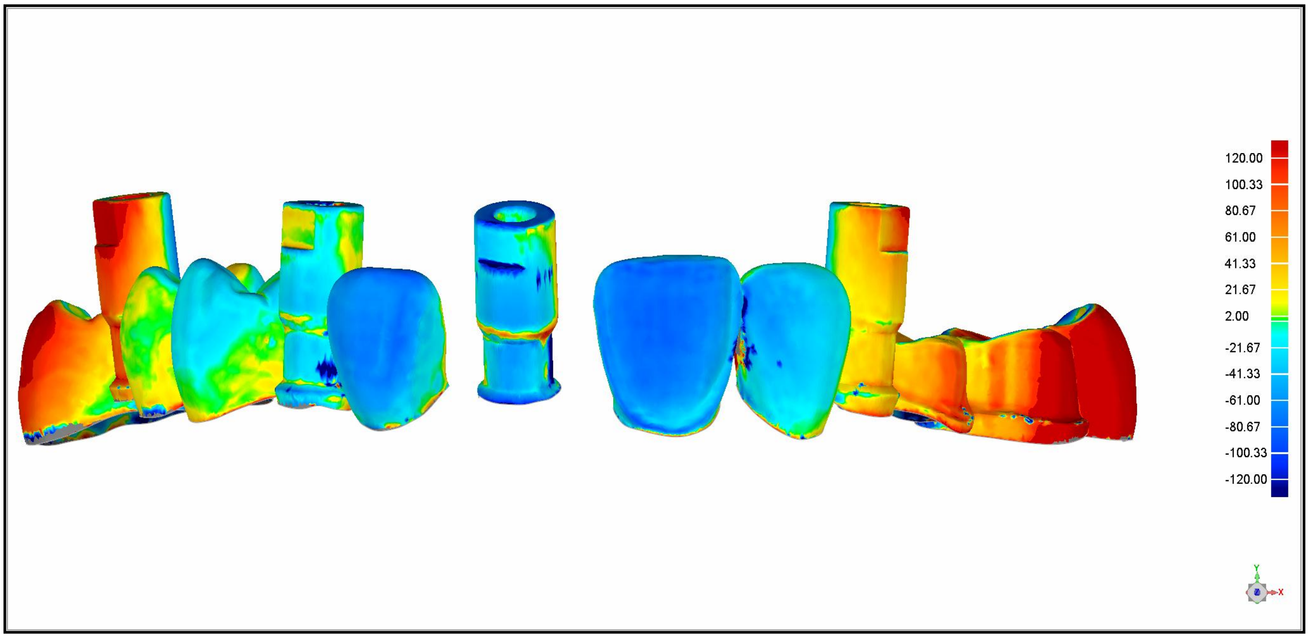

Predefinido: Inferior

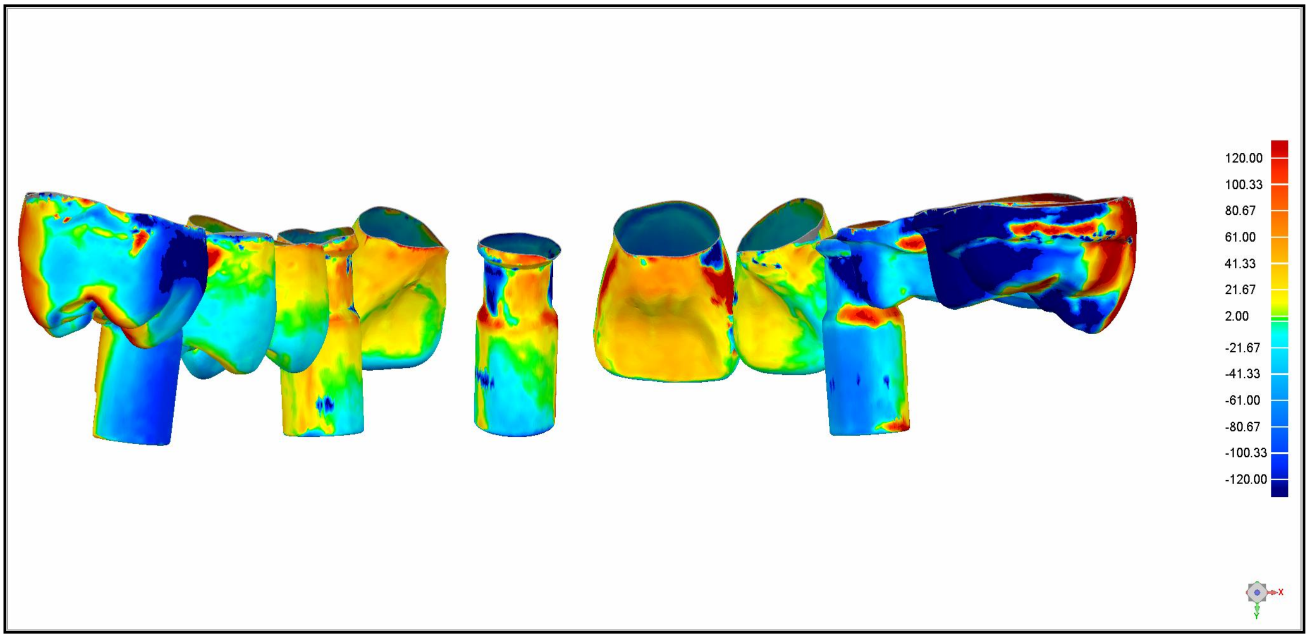

Ajuste de ubicación: Desviaciones superior e inferior

Unidades: u

Nombre	Desv	Estado	Superior Tol	Inferior Tol	Ref X	Ref Y	Ref Z	Radio	Desv X	Desv Y	Desv Z	Medido X	Medido Y	Medido Z	Dir. proy. X	Dir. proy. Y	Dir. proy. Z
Desv. inferior	-3132.06				-22607.19	28955.77	6808.03	n/a	-939.28	-287.32	2974.05	-23546.47	28668.45	9782.08	0.30	0.09	-0.95
Desv. superior	3147.10				32263.48	27229.24	-7694.37	n/a	-2466.06	59.30	1954.30	29797.42	27288.54	-5740.07	-0.78	0.02	0.62
