# Supplementary material for: Accuracy of four digital scanners according to scanning strategy in complete-arch impressions
Source: PLoS One. 2018 Sep 13;13(9):e0202916. doi: 10.1371/journal.pone.0202916 (PMC6136706; doi:10.1371/journal.pone.0202916)
Supplement: S9 Table — Omnicam (scanning strategy A). (ZIP) [file pone.0202916.s009.zip › S9/OM1A.pdf]

3D Comparación Resultados

Modelo referencia	MRC
Modelo test	OM1A
Nº de puntos de datos	194798
# Aislados	508

Tipo tolerancia	3D desviación
Unidades	u
Máx. crítico	120.00
Máx. nominal	4.00
Mín. nominal	-4.00
Mín. crítico	-120.00

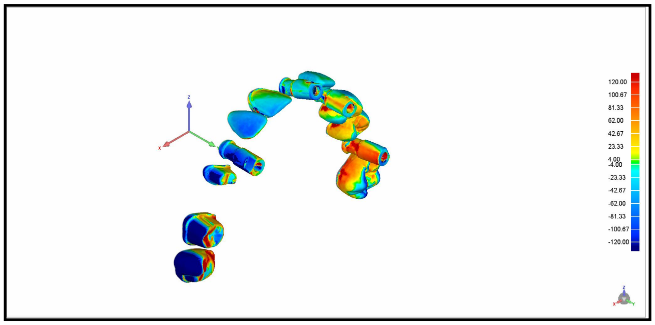

Desviación	
Desviación superior máx.	3111.40
Desviación inferior máx.	-3057.69
Desviación media	108.77 /-89.68
Desviación estándar	221.99

Distribución desviación

>=Min	<Max	# Puntos	%
-120.00	-100.67	4138	2.12
-100.67	-81.33	6210	3.19
-81.33	-62.00	8332	4.28
-62.00	-42.67	16368	8.40
-42.67	-23.33	22134	11.36
-23.33	-4.00	24339	12.49
-4.00	4.00	8672	4.45
4.00	23.33	18372	9.43
23.33	42.67	15457	7.93
42.67	62.00	12341	6.34
62.00	81.33	8036	4.13
81.33	100.67	7089	3.64
100.67	120.00	4587	2.35

Fuera del crítico superior	22851	11.73
Fuera del crítico inferior	15872	8.15

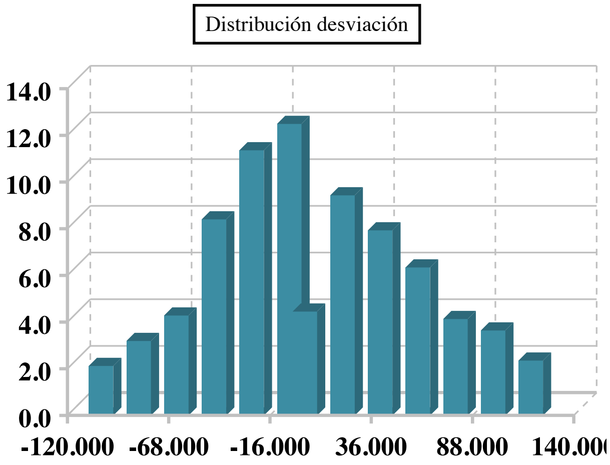

Desviaciones estándar

Distribución (+/-)	# Puntos	%
-6 * Desv. estánd.	1118	0.57
-5 * Desv. estánd.	399	0.20
-4 * Desv. estánd.	503	0.26
-3 * Desv. estánd.	773	0.40
-2 * Desv. estánd.	4466	2.29
-1 * Desv. estánd.	99763	51.21
1 * Desv. estánd.	79551	40.84
2 * Desv. estánd.	4678	2.40
3 * Desv. estánd.	1350	0.69
4 * Desv. estánd.	959	0.49
5 * Desv. estánd.	420	0.22
6 * Desv. estánd.	818	0.42

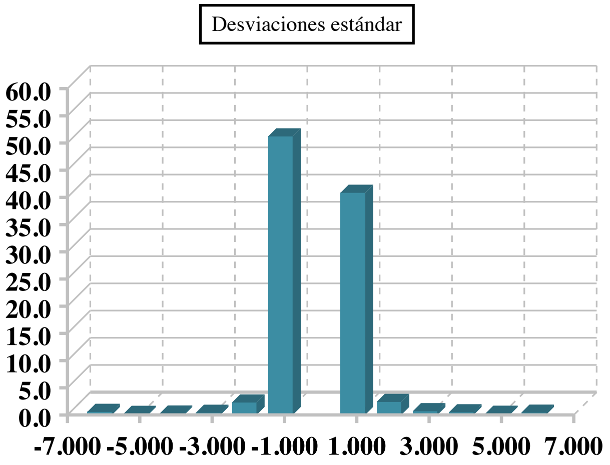

Predefinido: Isométrico

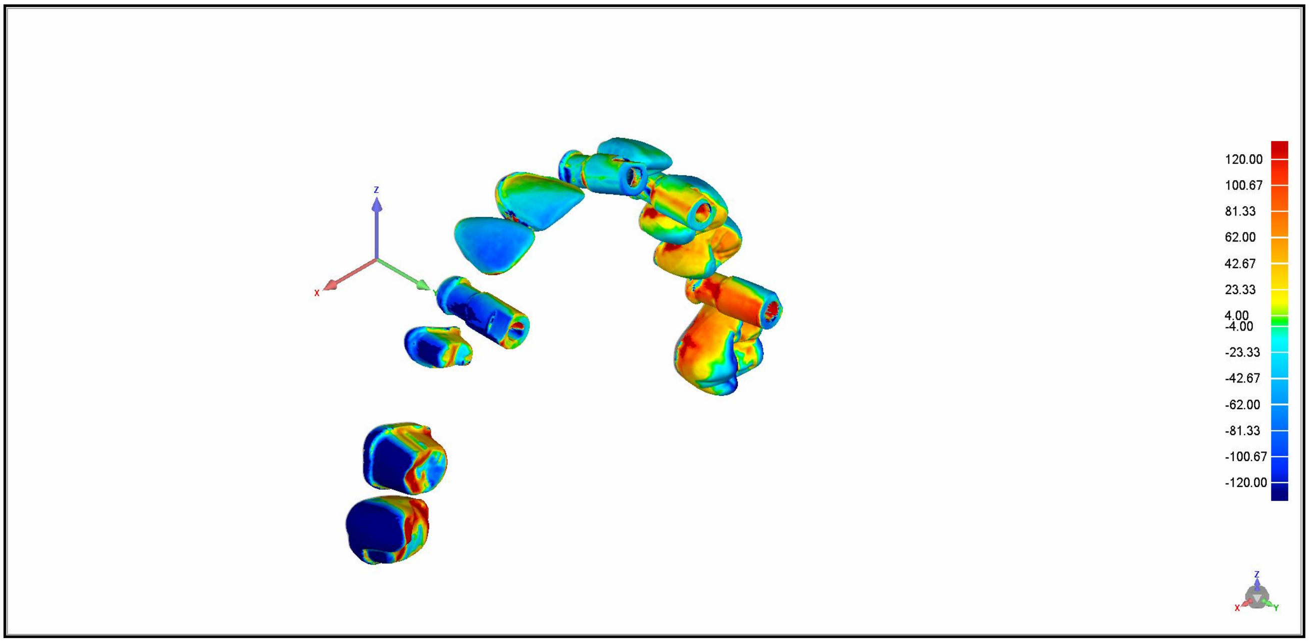

Predefinido: Frente

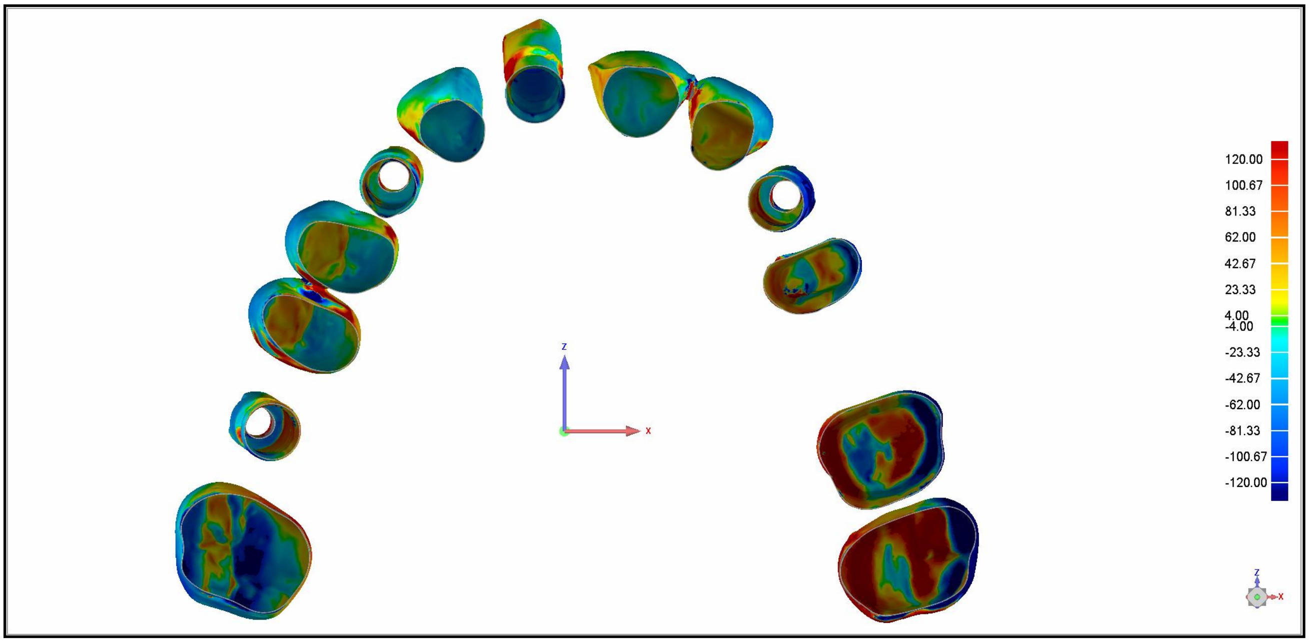

Predefinido: Atrás

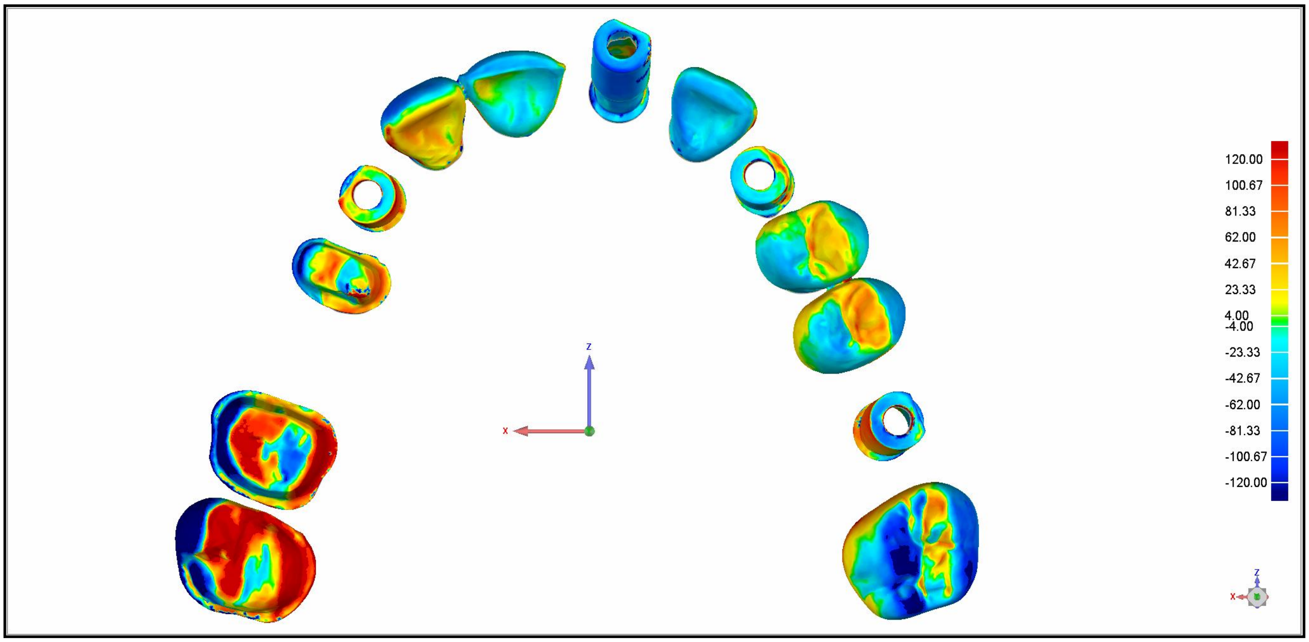

Predefinido: Izquierda

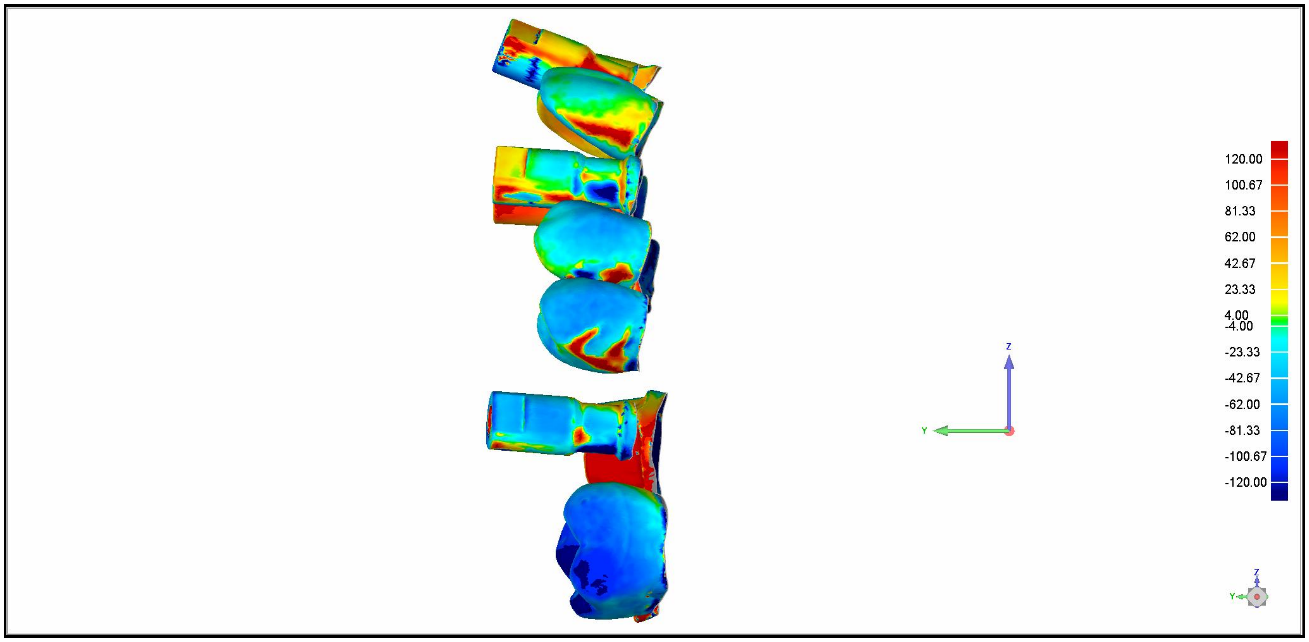

Predefinido: Derecha

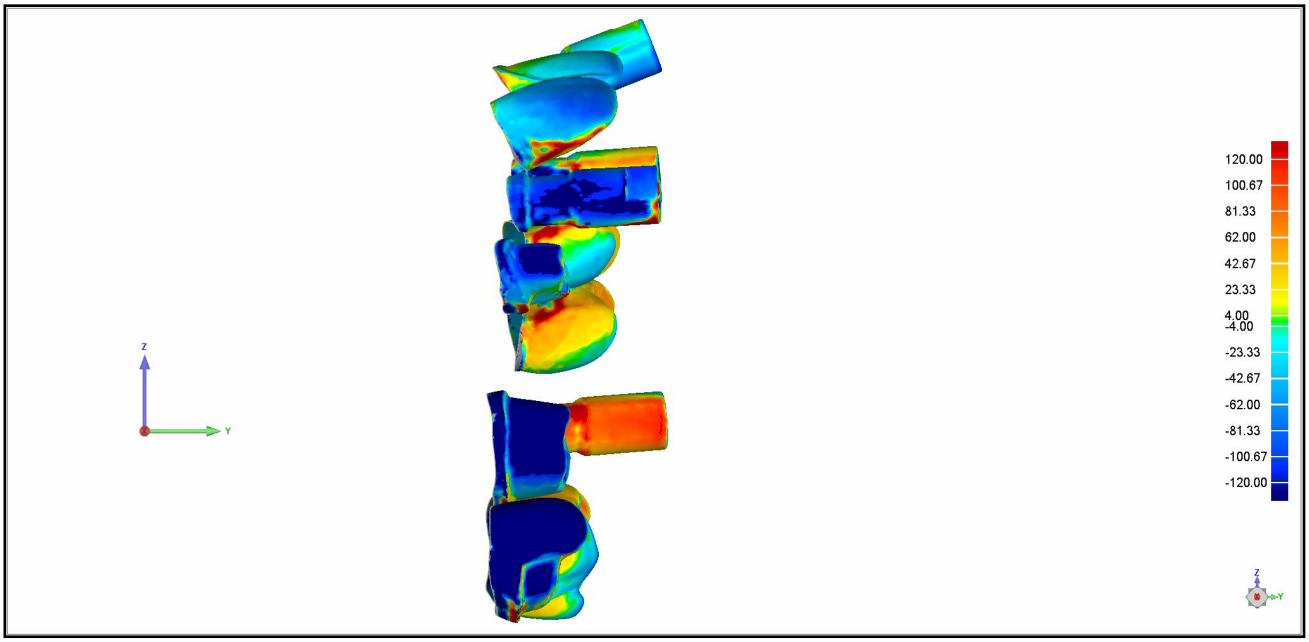

Predefinido: Superior

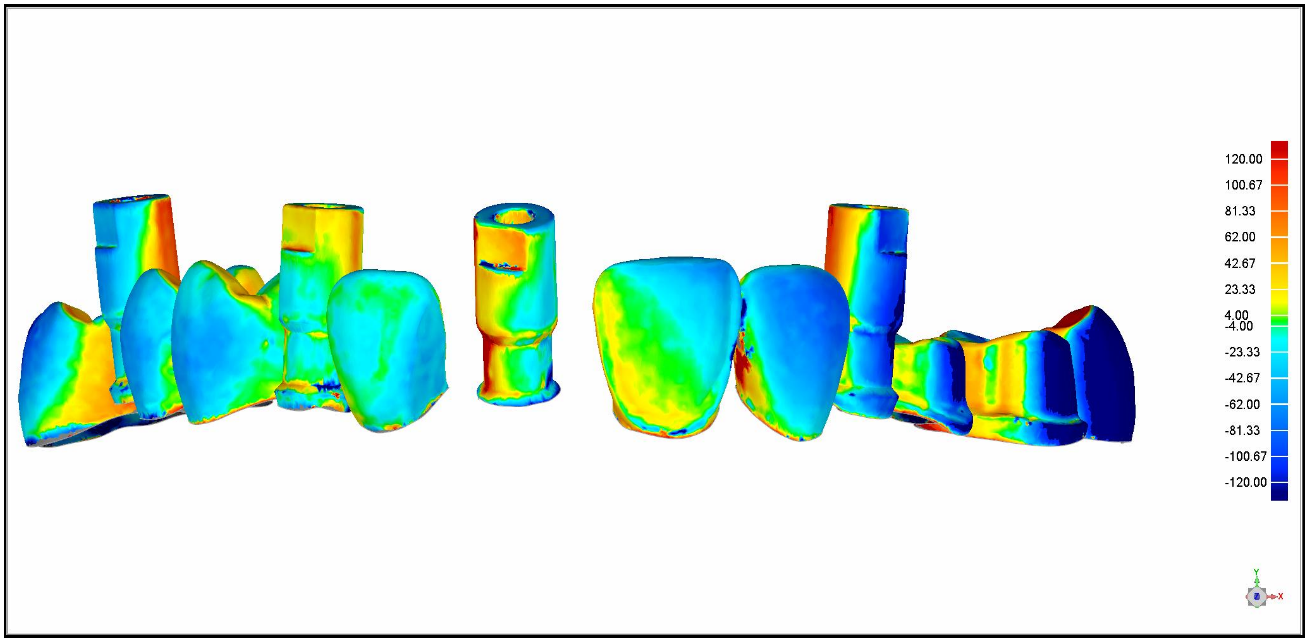

Predefinido: Inferior

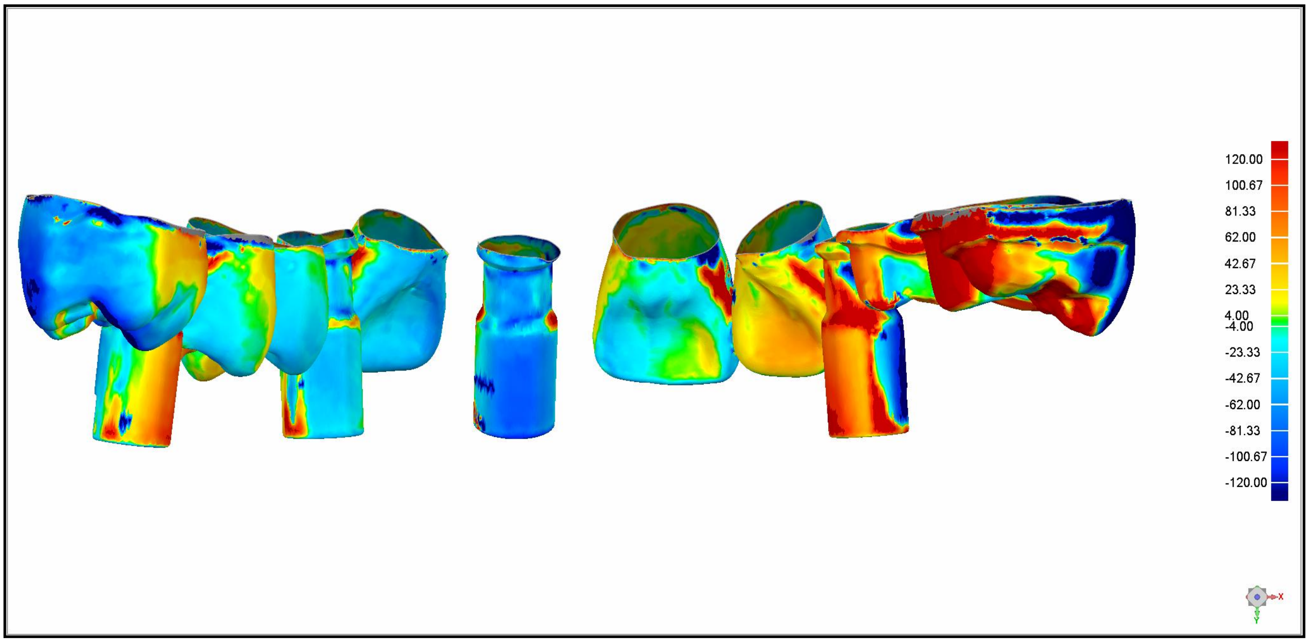

Ajuste de ubicación: Desviaciones superior e inferior

Unidades: u

Nombre	Desv	Estado	Superior Tol	Inferior Tol	Ref X	Ref Y	Ref Z	Radio	Desv X	Desv Y	Desv Z	Medido X	Medido Y	Medido Z	Dir. proy. X	Dir. proy. Y	Dir. proy. Z
Desv. inferior	-3057.69				-22607.19	28955.77	6808.03	n/a	-998.86	-513.32	2843.99	-23606.05	28442.45	9652.02	0.33	0.17	-0.93
Desv. superior	3111.40				-12540.11	29731.12	21354.82	n/a	1069.91	-1360.11	2585.78	-11470.20	28371.02	23940.60	0.34	-0.44	0.83
