# Supplementary material for: Accuracy of four digital scanners according to scanning strategy in complete-arch impressions
Source: PLoS One. 2018 Sep 13;13(9):e0202916. doi: 10.1371/journal.pone.0202916 (PMC6136706; doi:10.1371/journal.pone.0202916)
Supplement: S10 Table — Omnicam (scanning strategy B). (ZIP) [file pone.0202916.s010.zip › S10/OM2B.pdf]

Geomagic Control™

Fecha: 3/6/2017, 2:51 pm

# 3D Comparación  Resultados

| | |
|---|---|
| Modelo referencia | MRC |
| Modelo test | OM2B |
| Nº de puntos de datos | 195295 |
| # Aislados | 537 |

| | |
|---|---|
| Tipo tolerancia | 3D desviación |
| Unidades | u |
| Máx. crítico | 120.00 |
| Máx. nominal | 1.00 |
| Mín. nominal | -1.00 |
| Mín. crítico | -120.00 |

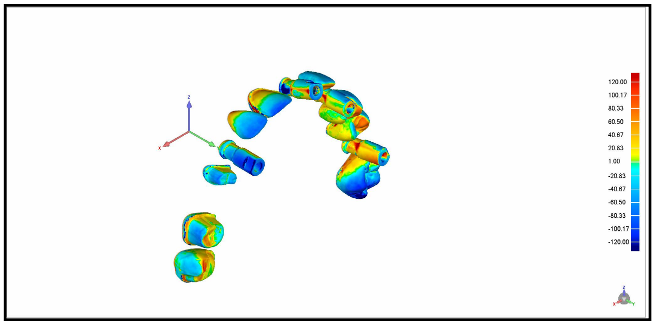

| | |
|---|---|
| Desviación | |
| Desviación superior máx. | 2918.70 |
| Desviación inferior máx. | -3154.80 |
| Desviación media | 94.76 /-99.11 |
| Desviación estándar | 283.63 |

## Distribución desviación

| >=Min | <Max | # Puntos | % |
|---|---|---|---|
| -120.00 | -100.17 | 3076 | 1.58 |
| -100.17 | -80.33 | 4992 | 2.56 |
| -80.33 | -60.50 | 9661 | 4.95 |
| -60.50 | -40.67 | 16017 | 8.20 |
| -40.67 | -20.83 | 23619 | 12.09 |
| -20.83 | -1.00 | 29176 | 14.94 |
| -1.00 | 1.00 | 3454 | 1.77 |
| 1.00 | 20.83 | 32635 | 16.71 |
| 20.83 | 40.67 | 20978 | 10.74 |
| 40.67 | 60.50 | 14638 | 7.50 |
| 60.50 | 80.33 | 9379 | 4.80 |
| 80.33 | 100.17 | 4520 | 2.31 |
| 100.17 | 120.00 | 2213 | 1.13 |

| | | |
|---|---|---|
| Fuera del crítico superior | 12497 | 6.40 |
| Fuera del crítico inferior | 8440 | 4.32 |

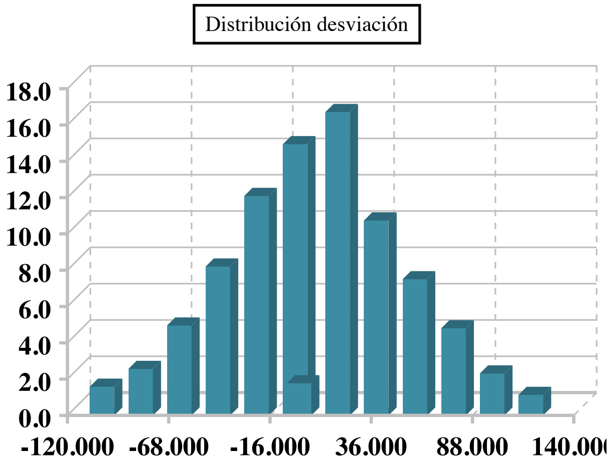


## Desviaciones estándar

| Distribución (+/-) | # Puntos | % |
|---|---|---|
| -6 * Desv. estánd. | 1761 | 0.90 |
| -5 * Desv. estánd. | 551 | 0.28 |
| -4 * Desv. estánd. | 562 | 0.29 |
| -3 * Desv. estánd. | 730 | 0.37 |
| -2 * Desv. estánd. | 1228 | 0.63 |
| -1 * Desv. estánd. | 89908 | 46.04 |
| 1 * Desv. estánd. | 94444 | 48.36 |
| 2 * Desv. estánd. | 2221 | 1.14 |
| 3 * Desv. estánd. | 1608 | 0.82 |
| 4 * Desv. estánd. | 840 | 0.43 |
| 5 * Desv. estánd. | 565 | 0.29 |
| 6 * Desv. estánd. | 877 | 0.45 |

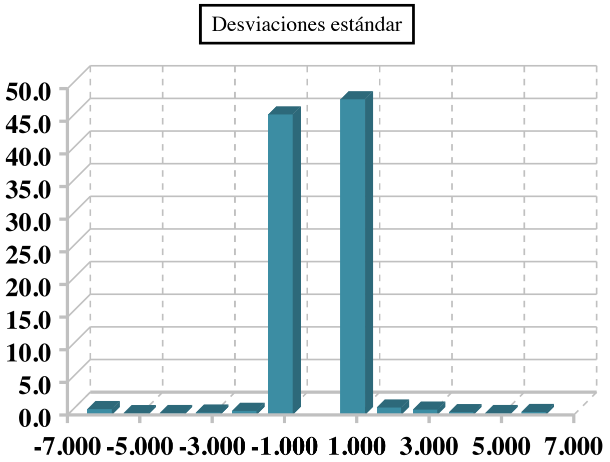

Predefinido: Isométrico

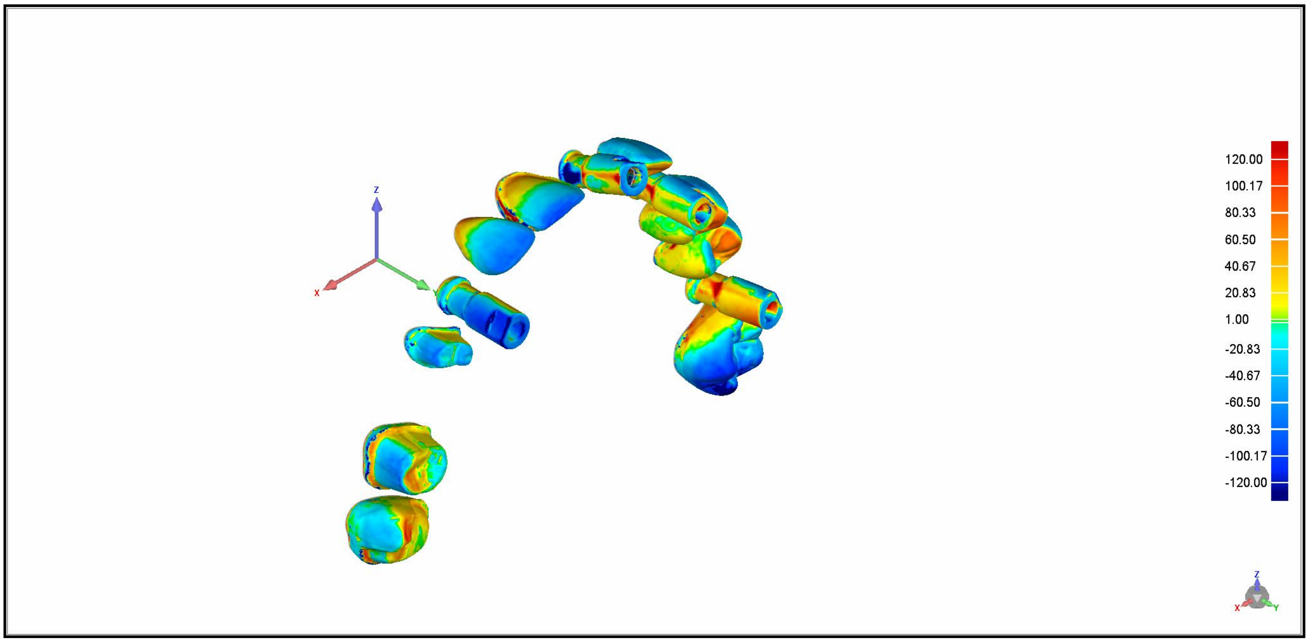

Predefinido: Frente

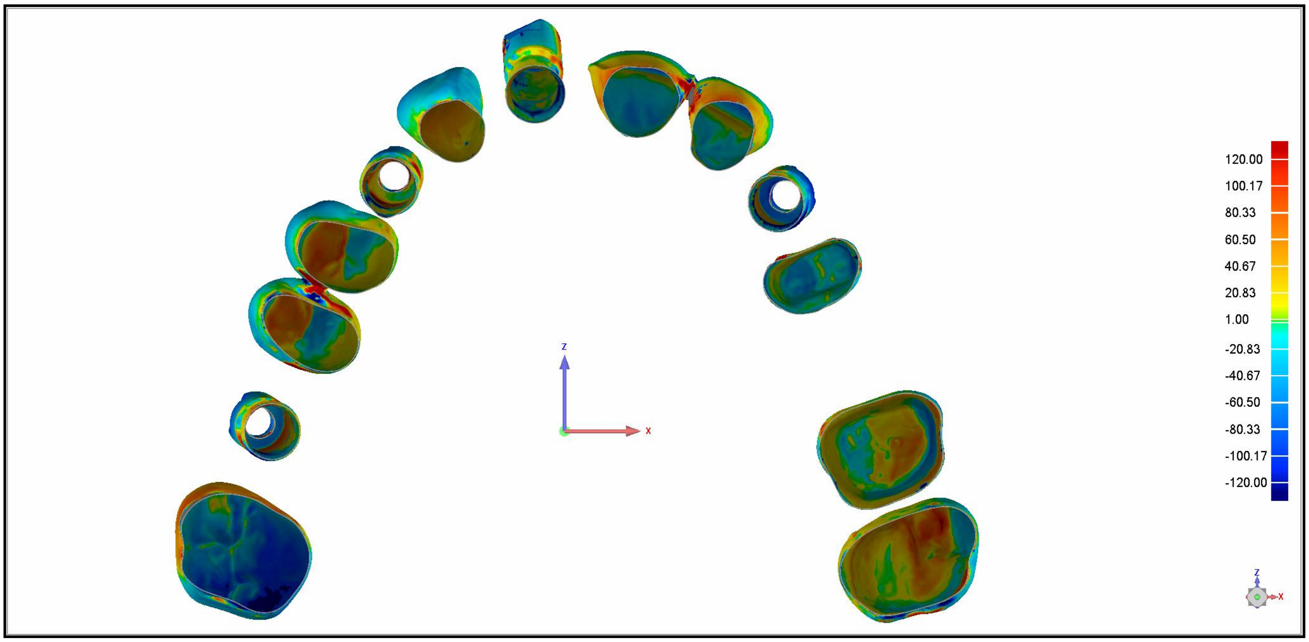

Predefinido: Atrás

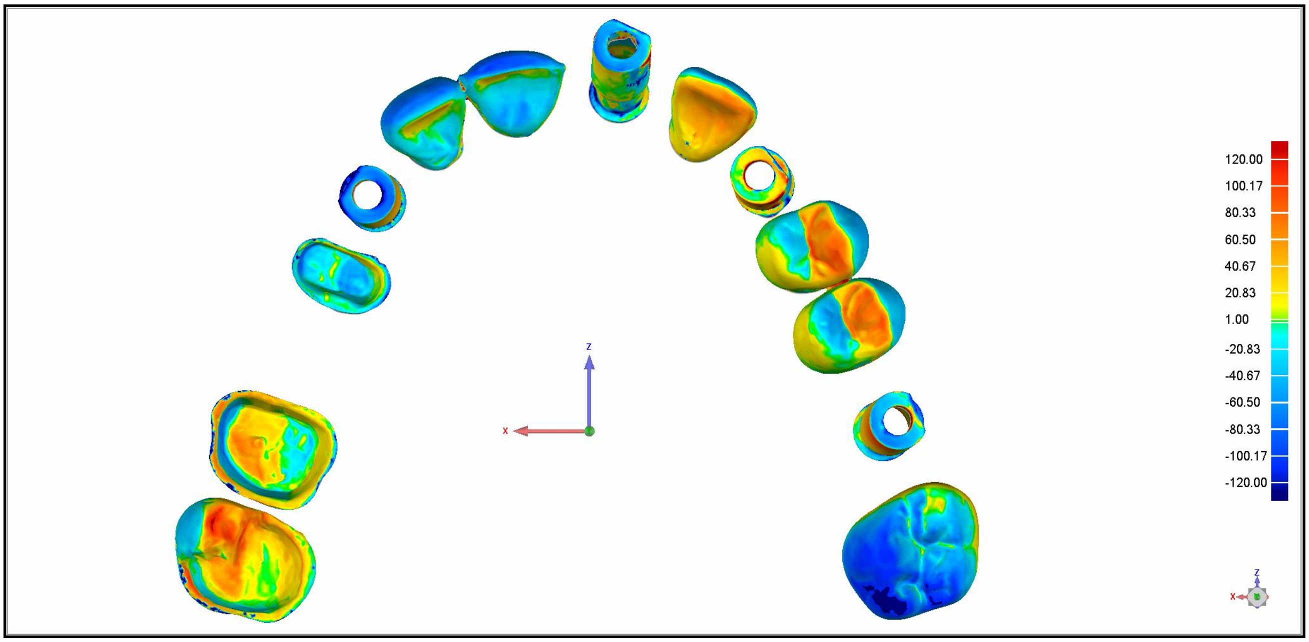

Predefinido: Izquierda

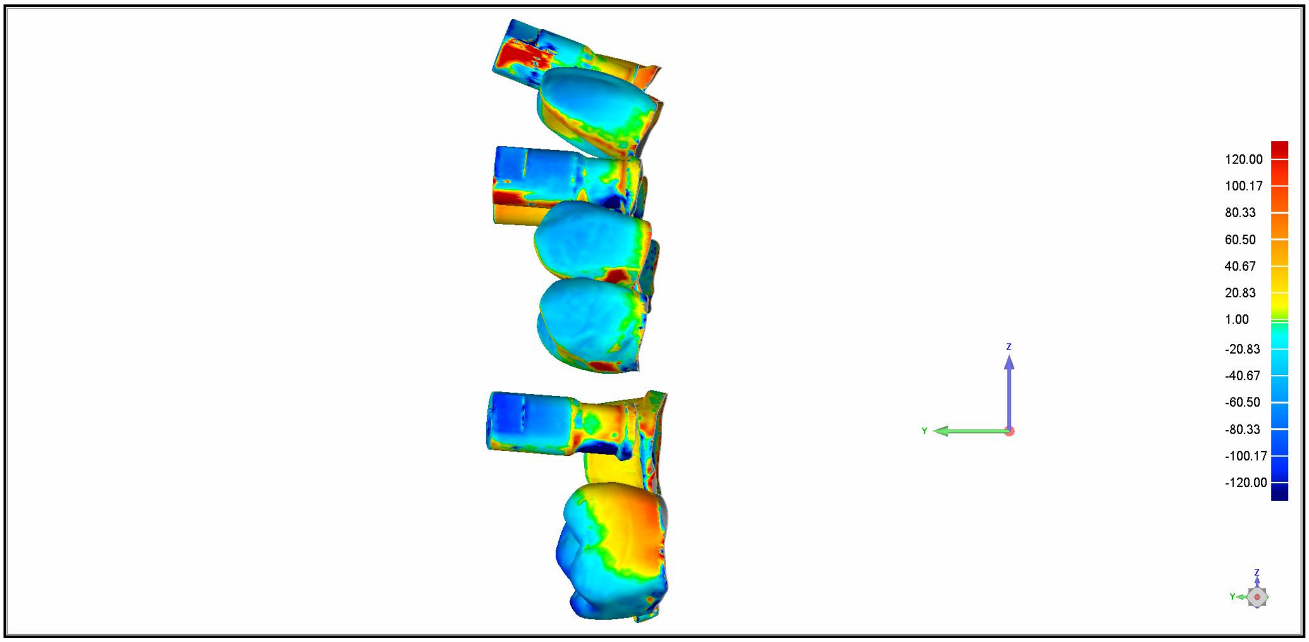

Predefinido: Derecha

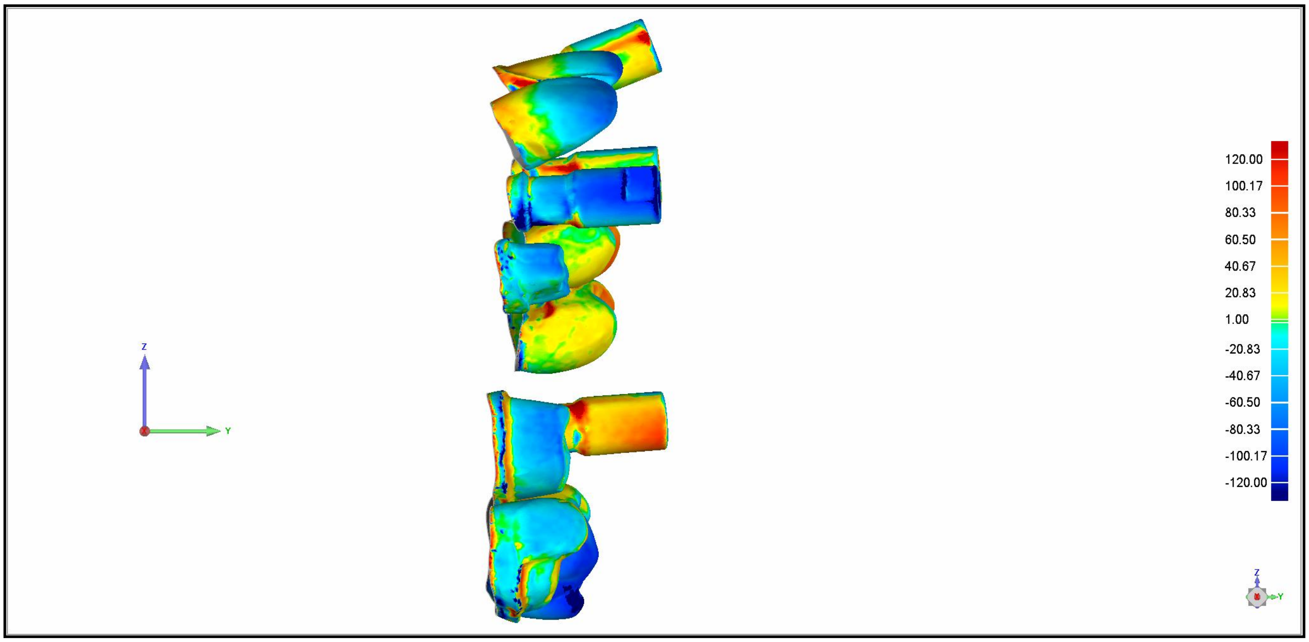

Predefinido: Superior

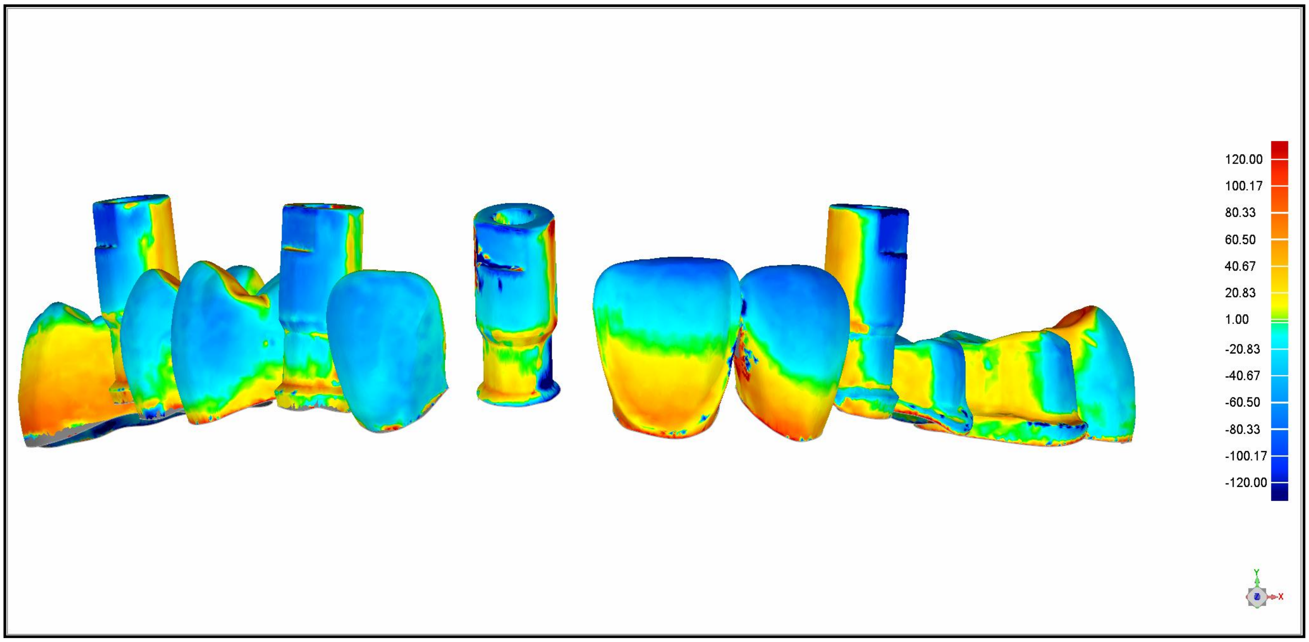

Predefinido: Inferior

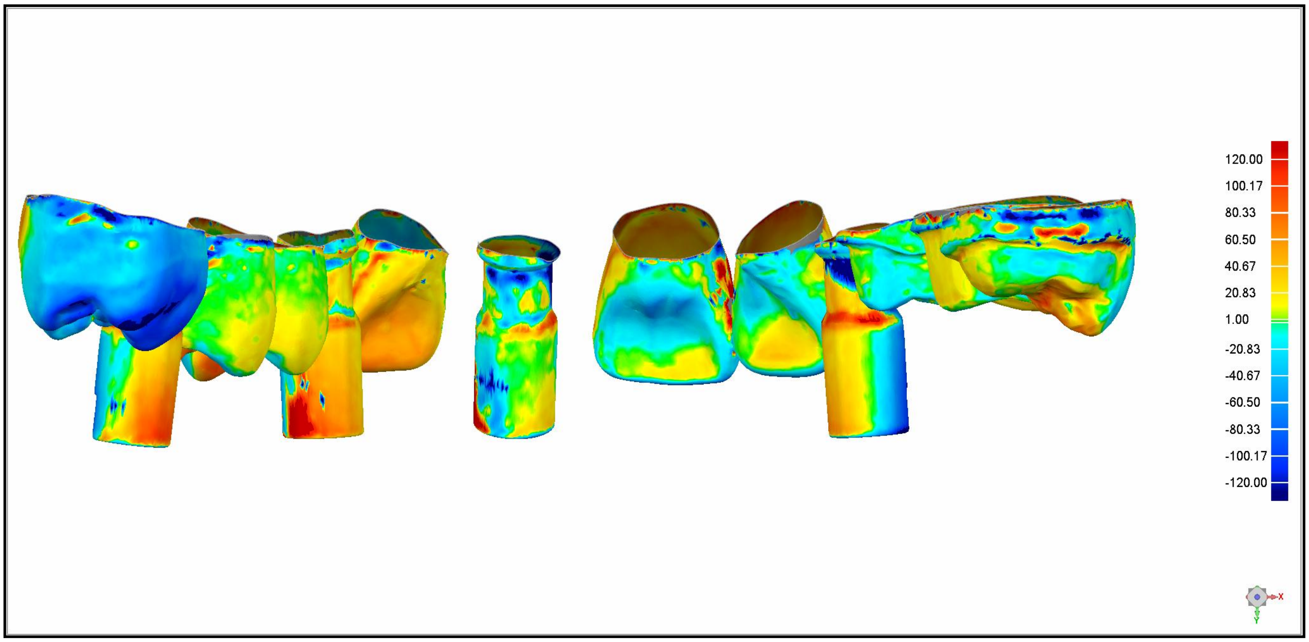

# Ajuste de ubicación: Desviaciones superior e inferior

## Unidades: u

| Nombre | Desv | Estado | Superior Tol | Inferior Tol | Ref X | Ref Y | Ref Z | Radio | Desv X | Desv Y | Desv Z | Medido X | Medido Y | Medido Z | Dir. proy. X | Dir. proy. Y | Dir. proy. Z |
|---|---|---|---|---|---|---|---|---|---|---|---|---|---|---|---|---|---|
| Desv. inferior | -3154.80 | | | | -22607.19 | 28955.77 | 6808.03 | n/a | -945.98 | -266.90 | 2997.77 | -23553.17 | 28688.87 | 9805.80 | 0.30 | 0.08 | -0.95 |
| Desv. superior | 2918.70 | | | | 25410.11 | 27462.45 | -5764.99 | n/a | 2896.25 | -11.69 | -361.14 | 28306.36 | 27450.76 | -6126.14 | 0.99 | -0.00 | -0.12 |
