# Supplementary material for: Accuracy of four digital scanners according to scanning strategy in complete-arch impressions
Source: PLoS One. 2018 Sep 13;13(9):e0202916. doi: 10.1371/journal.pone.0202916 (PMC6136706; doi:10.1371/journal.pone.0202916)
Supplement: S10 Table — Omnicam (scanning strategy B). (ZIP) [file pone.0202916.s010.zip › S10/OM3B.pdf]

3D Comparación Resultados

Modelo referencia	MRC
Modelo test	OM3B
Nº de puntos de datos	201843
# Aislados	817

Tipo tolerancia	3D desviación
Unidades	u
Máx. crítico	120.00
Máx. nominal	1.00
Mín. nominal	-1.00
Mín. crítico	-120.00

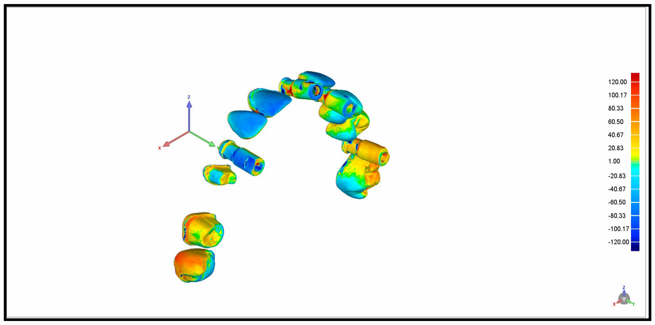

Desviación	
Desviación superior máx.	3034.78
Desviación inferior máx.	-3143.34
Desviación media	100.42 /-101.57
Desviación estándar	274.37

Distribución desviación

>=Min	<Max	# Puntos	%
-120.00	-100.17	1927	0.95
-100.17	-80.33	3065	1.52
-80.33	-60.50	8178	4.05
-60.50	-40.67	15634	7.75
-40.67	-20.83	22582	11.19
-20.83	-1.00	35772	17.72
-1.00	1.00	3874	1.92
1.00	20.83	31589	15.65
20.83	40.67	25131	12.45
40.67	60.50	14723	7.29
60.50	80.33	7221	3.58
80.33	100.17	4184	2.07
100.17	120.00	2581	1.28

Fuera del crítico superior	15037	7.45
Fuera del crítico inferior	10345	5.13

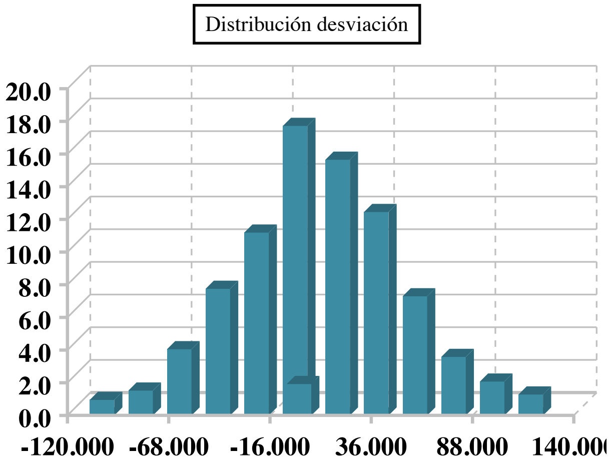

Desviaciones estándar

Distribución (+/-)	# Puntos	%
-6 * Desv. estánd.	1580	0.78
-5 * Desv. estánd.	1271	0.63
-4 * Desv. estánd.	949	0.47
-3 * Desv. estánd.	1189	0.59
-2 * Desv. estánd.	1699	0.84
-1 * Desv. estánd.	94332	46.74
1 * Desv. estánd.	93592	46.37
2 * Desv. estánd.	2698	1.34
3 * Desv. estánd.	1623	0.80
4 * Desv. estánd.	976	0.48
5 * Desv. estánd.	952	0.47
6 * Desv. estánd.	982	0.49

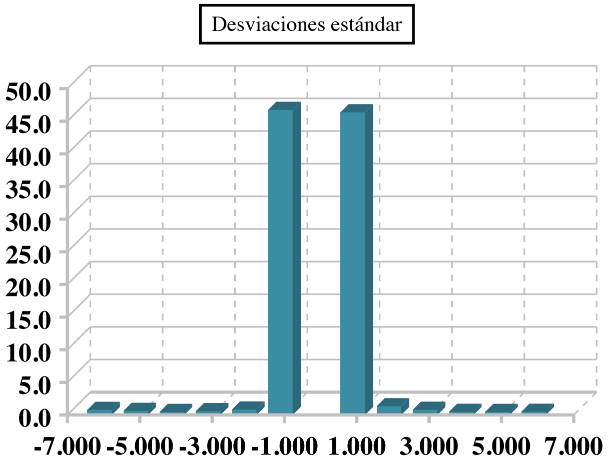

Geomagic Control™

Predefinido: Isométrico

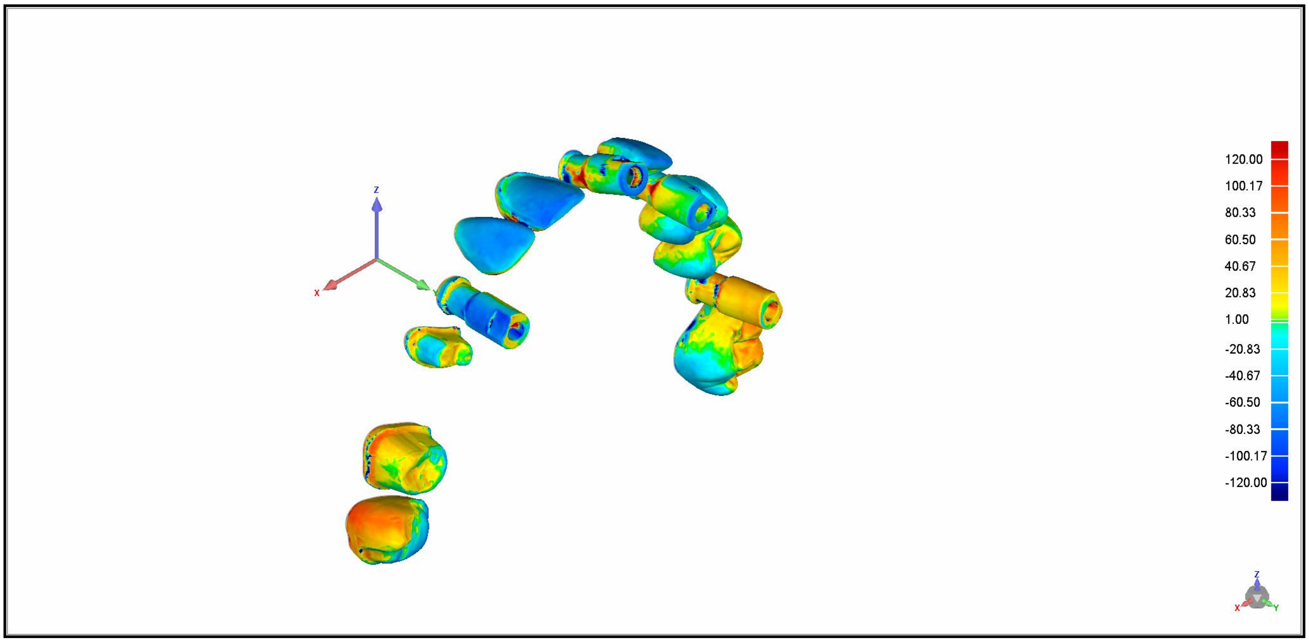

Predefinido: Frente

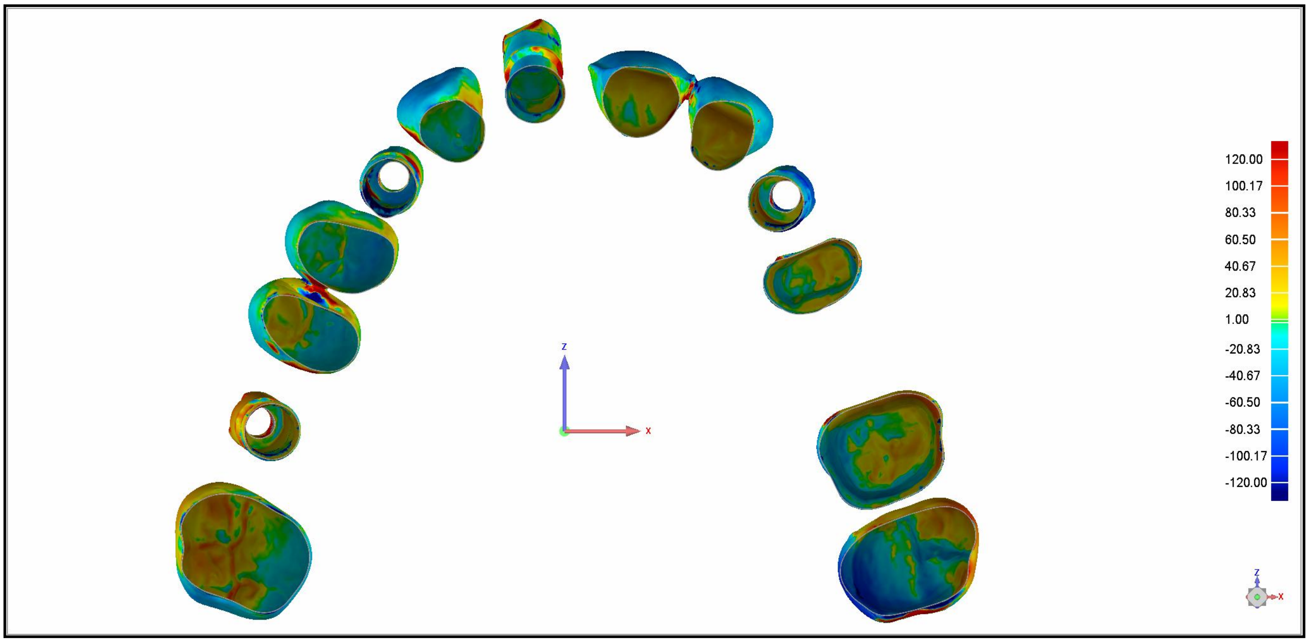

Fecha: 3/6/2017, 2:56 pm

Predefinido: Atrás

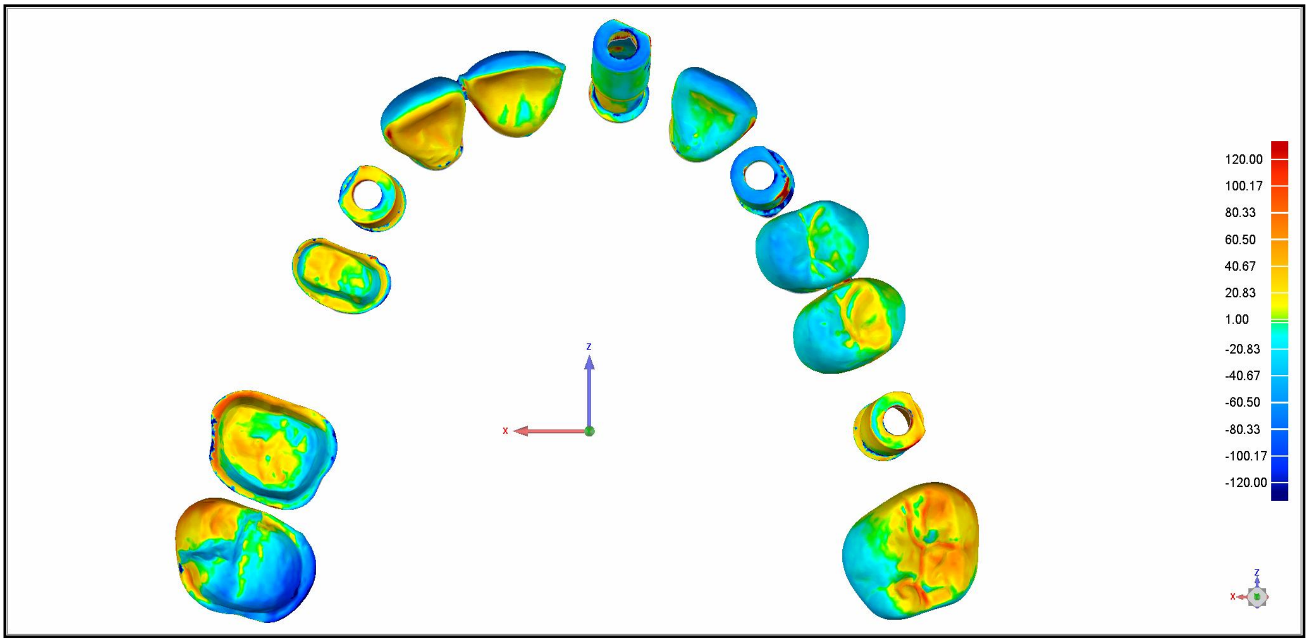

Predefinido: Izquierda

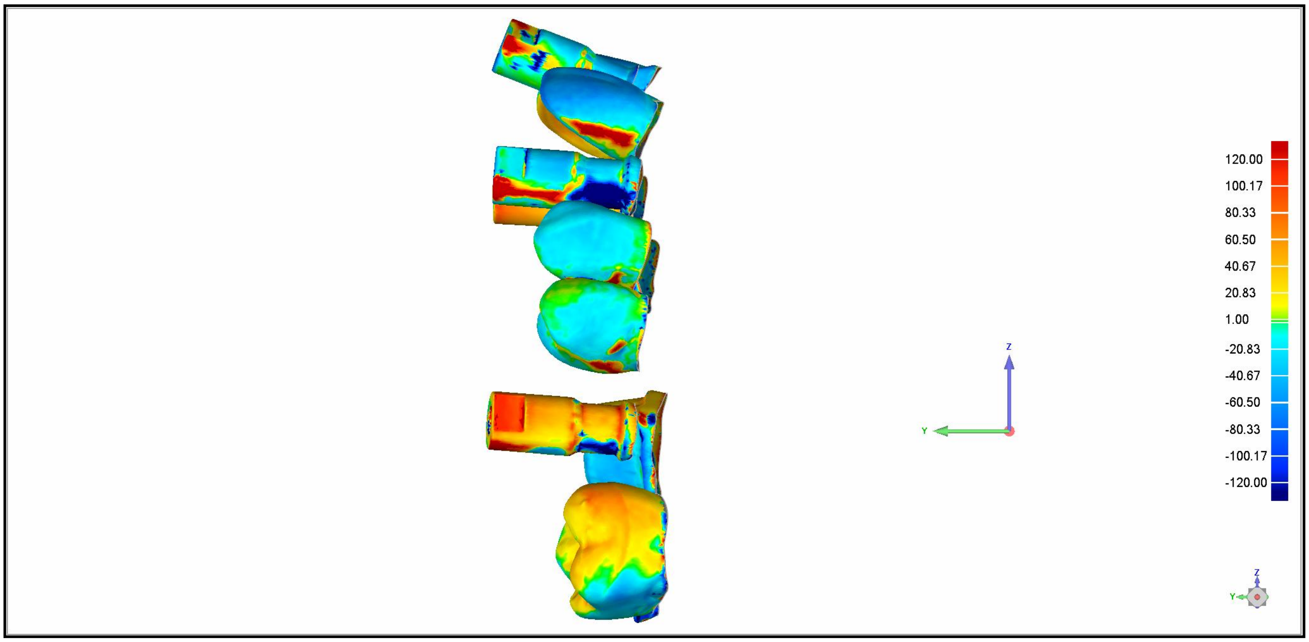

Predefinido: Derecha

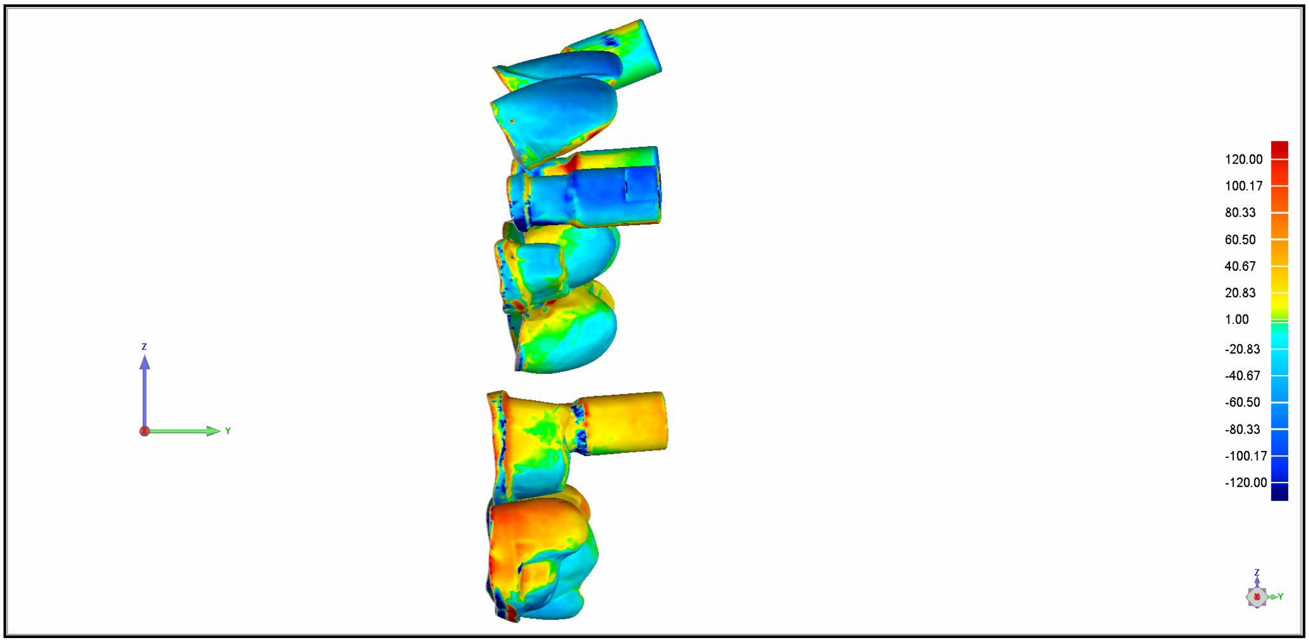

Predefinido: Superior

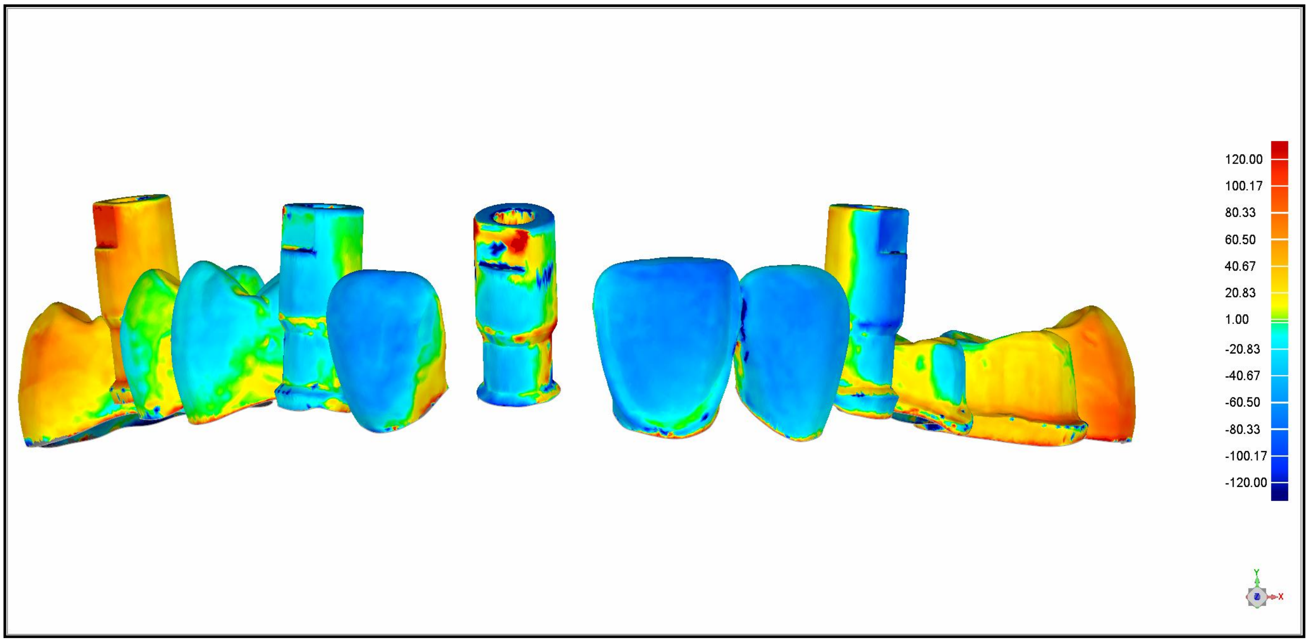

Predefinido: Inferior

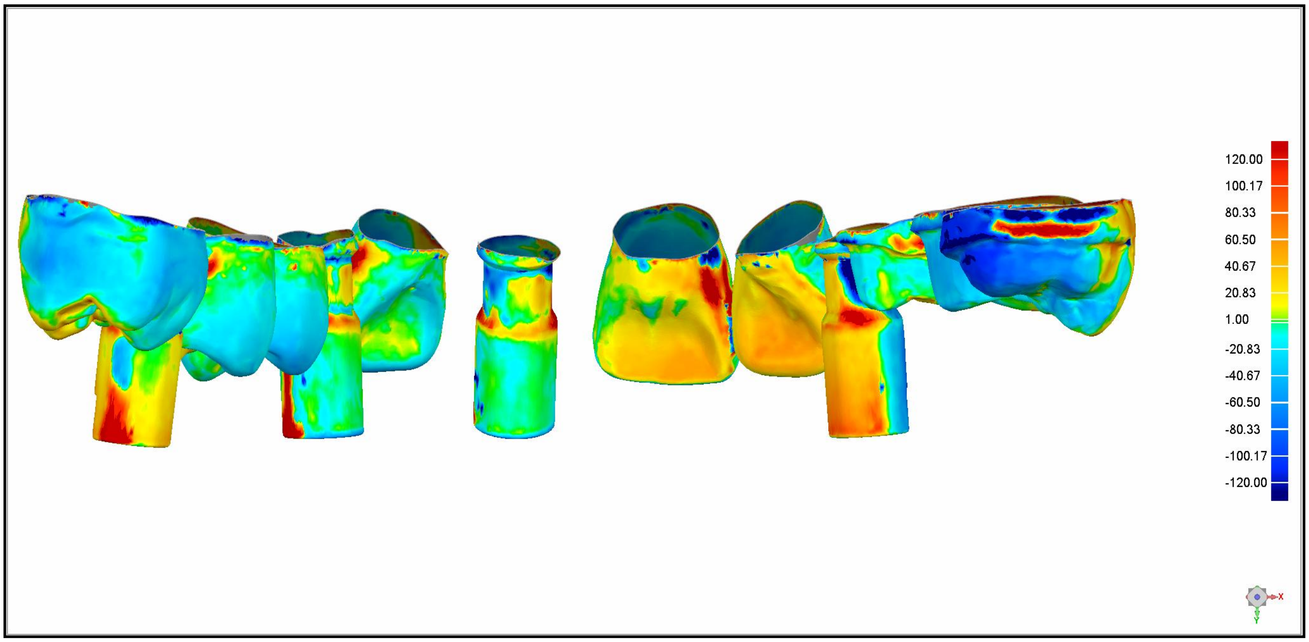

Ajuste de ubicación: Desviaciones superior e inferior

Unidades: u

Nombre	Desv	Estado	Superior Tol	Inferior Tol	Ref X	Ref Y	Ref Z	Radio	Desv X	Desv Y	Desv Z	Medido X	Medido Y	Medido Z	Dir. proy. X	Dir. proy. Y	Dir. proy. Z
Desv. inferior	-3143.34				-22607.19	28955.77	6808.03	n/a	-842.15	-495.07	2987.69	-23449.34	28460.70	9795.72	0.27	0.16	-0.95
Desv. superior	3034.78				29547.11	27336.30	1208.53	n/a	-2329.76	-419.75	1898.93	27217.35	26916.55	3107.46	-0.77	-0.14	0.63
