# Supplementary material for: Accuracy of four digital scanners according to scanning strategy in complete-arch impressions
Source: PLoS One. 2018 Sep 13;13(9):e0202916. doi: 10.1371/journal.pone.0202916 (PMC6136706; doi:10.1371/journal.pone.0202916)
Supplement: S10 Table — Omnicam (scanning strategy B). (ZIP) [file pone.0202916.s010.zip › S10/OM4B.pdf]

Geomagic Control™

Fecha: 3/6/2017, 2:59 pm

3D Comparación Resultados

Modelo referencia	MRC
Modelo test	OM4B
Nº de puntos de datos	200403
# Aislados	801

Tipo tolerancia	3D desviación
Unidades	u
Máx. crítico	120.00
Máx. nominal	1.00
Mín. nominal	-1.00
Mín. crítico	-120.00

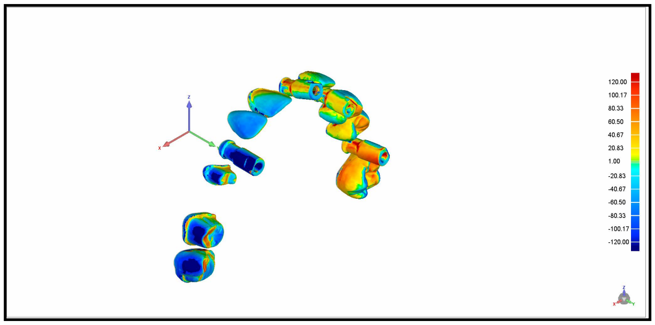

Desviación	
Desviación superior máx.	3154.69
Desviación inferior máx.	-3155.11
Desviación media	108.68 /-115.36
Desviación estándar	307.49

Distribución desviación

>=Min	<Max	# Puntos	%
-120.00	-100.17	3654	1.82
-100.17	-80.33	5647	2.82
-80.33	-60.50	9510	4.75
-60.50	-40.67	14273	7.12
-40.67	-20.83	21192	10.57
-20.83	-1.00	28394	14.17
-1.00	1.00	3055	1.52
1.00	20.83	27444	13.69
20.83	40.67	20993	10.48
40.67	60.50	15159	7.56
60.50	80.33	10576	5.28
80.33	100.17	6374	3.18
100.17	120.00	4272	2.13

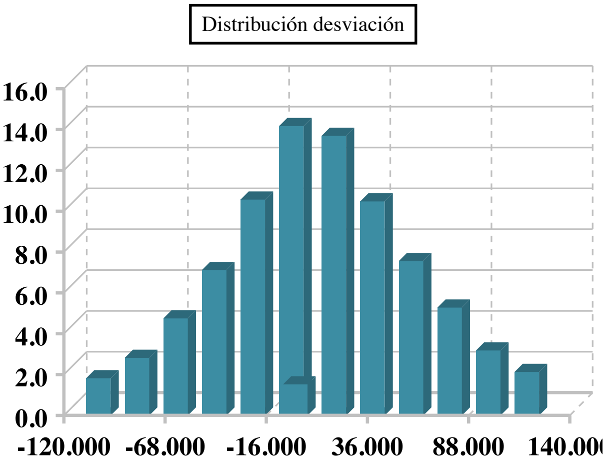

Fuera del crítico superior	16940	8.45
Fuera del crítico inferior	12920	6.45

Desviaciones estándar

Distribución (+/-)	# Puntos	%
-6 * Desv. eständ.	1613	0.80
-5 * Desv. eständ.	722	0.36
-4 * Desv. eständ.	925	0.46
-3 * Desv. eständ.	1030	0.51
-2 * Desv. eständ.	1148	0.57
-1 * Desv. eständ.	91857	45.84
1 * Desv. eständ.	96169	47.99
2 * Desv. eständ.	2971	1.48
3 * Desv. eständ.	1619	0.81
4 * Desv. eständ.	1003	0.50
5 * Desv. eständ.	634	0.32
6 * Desv. eständ.	712	0.36

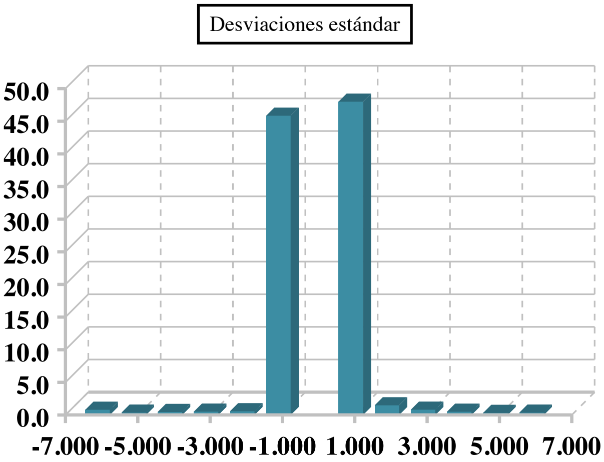

Predefinido: Isométrico

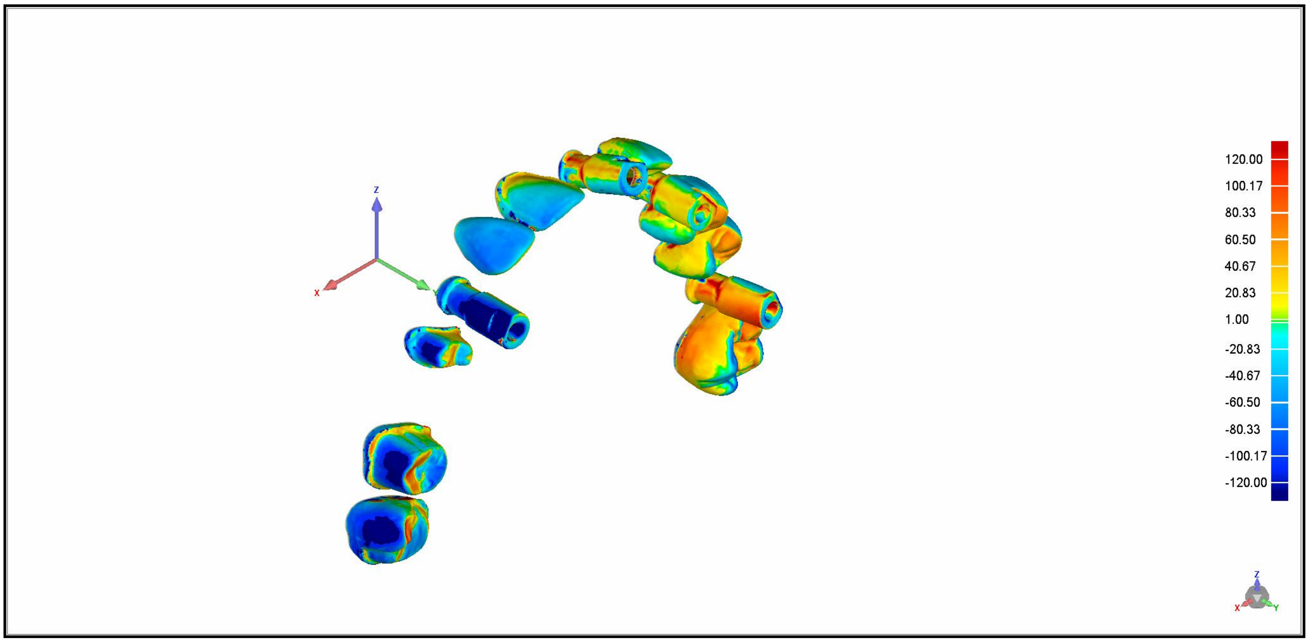

Predefinido: Frente

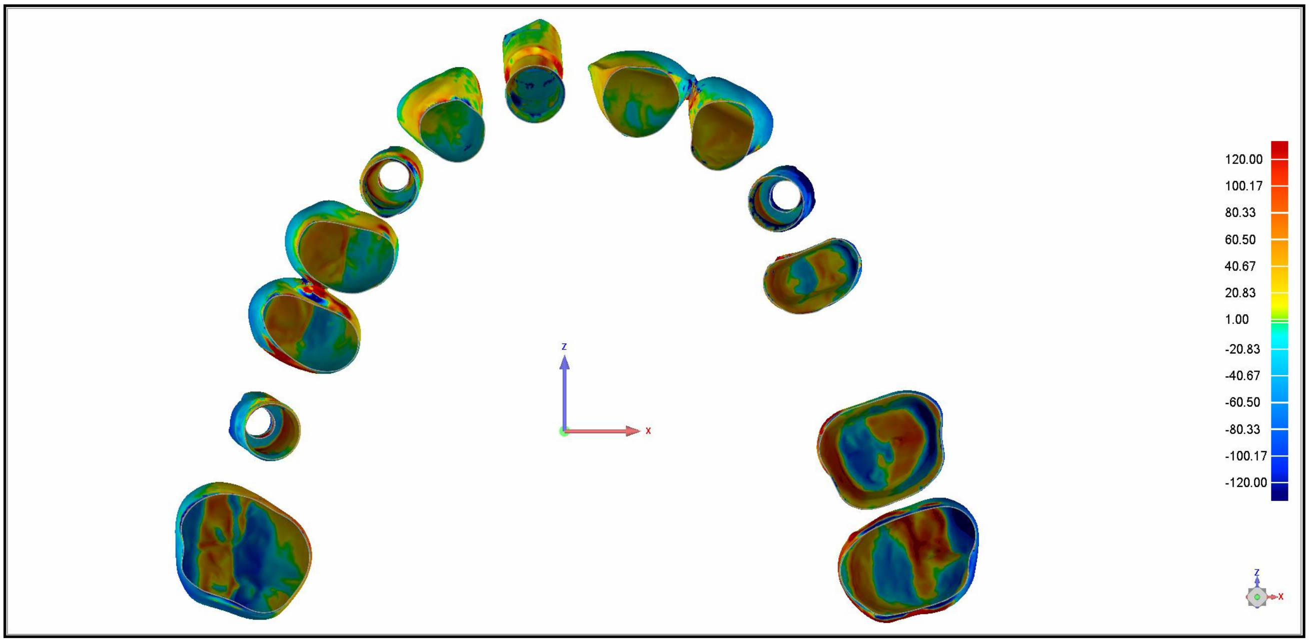

Predefinido: Atrás

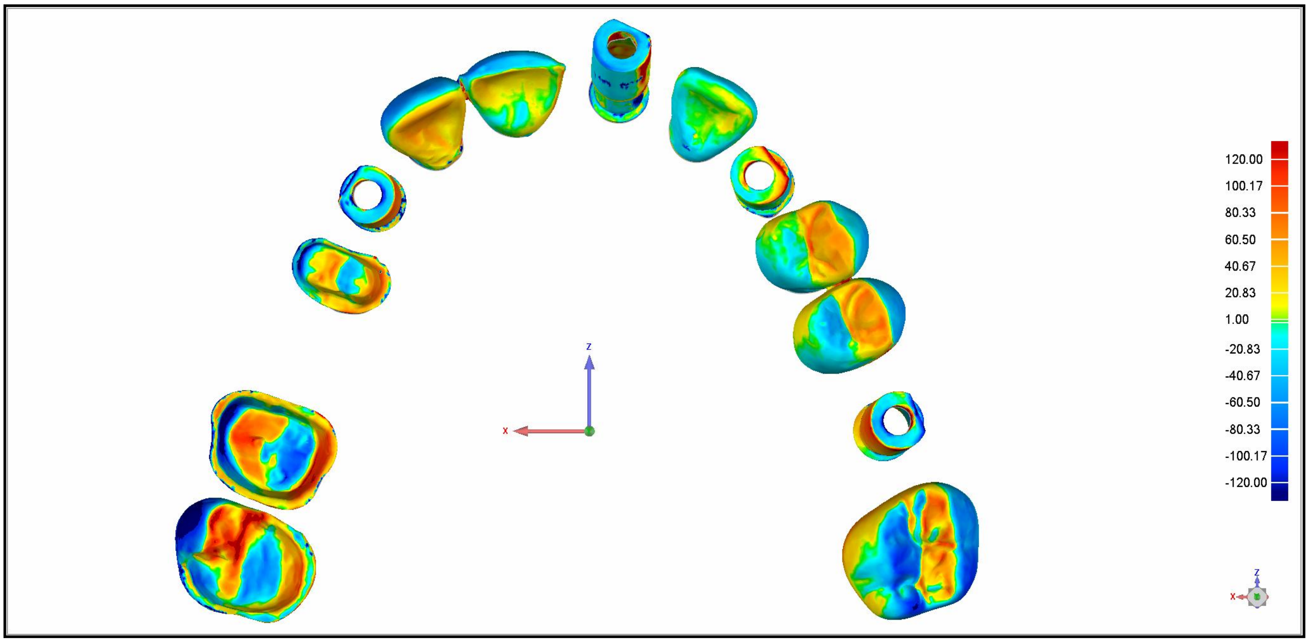

Predefinido: Izquierda

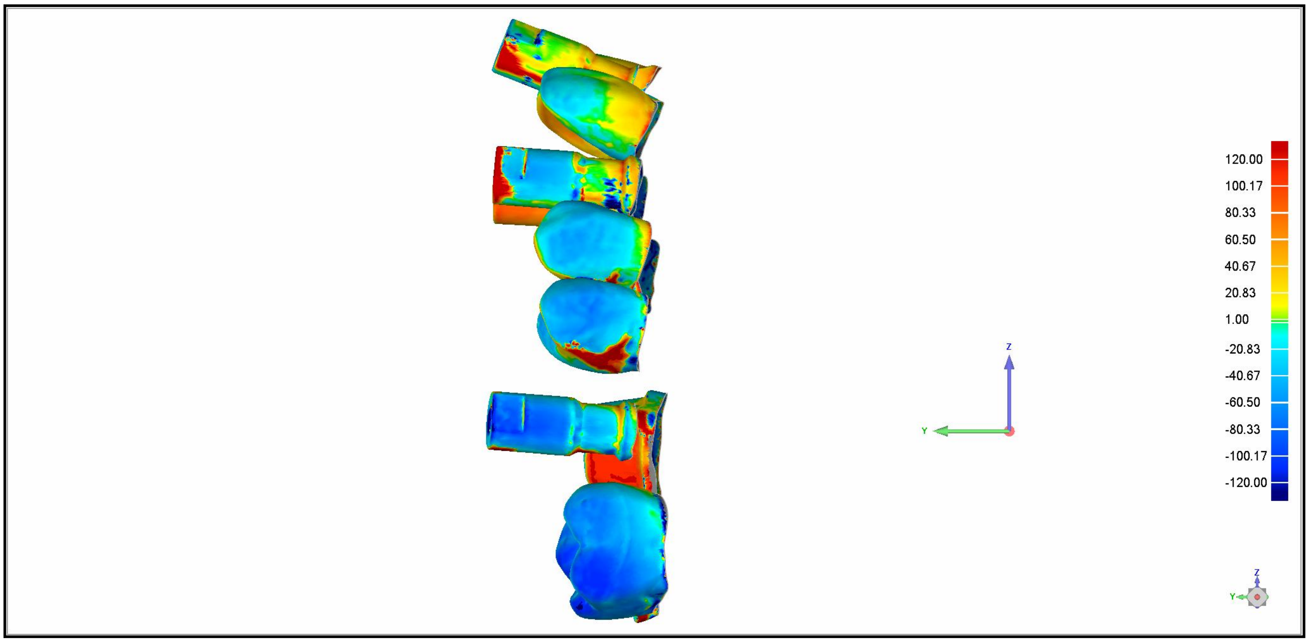

Predefinido: Derecha

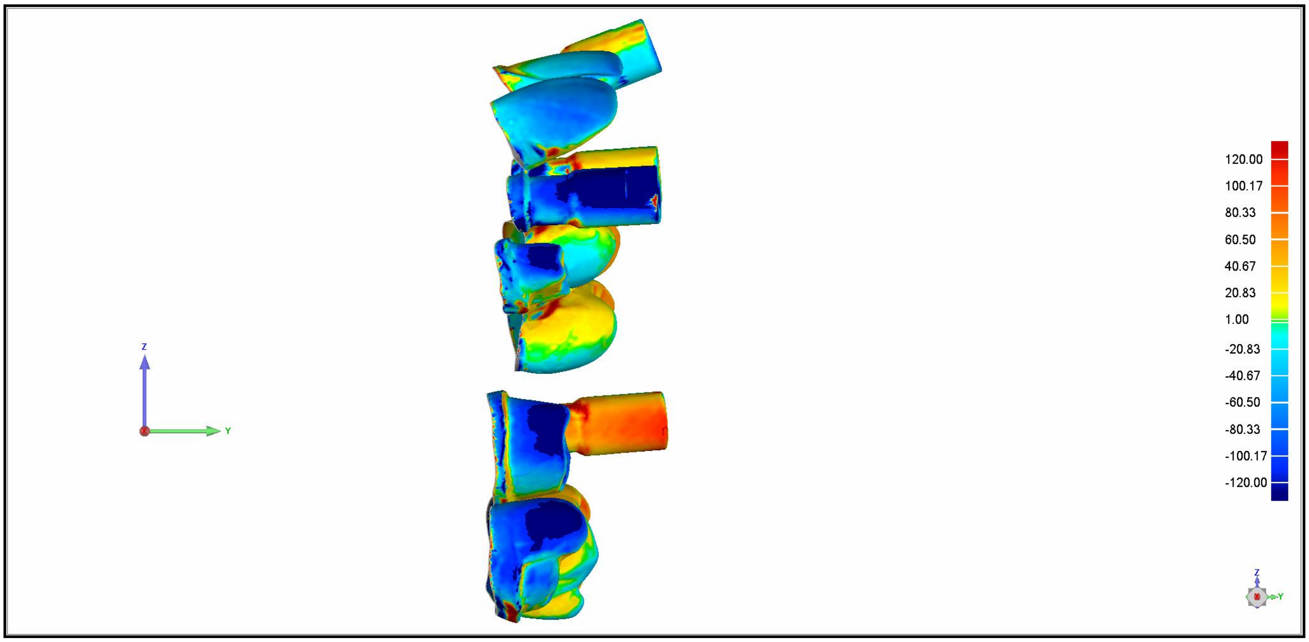

Predefinido: Superior

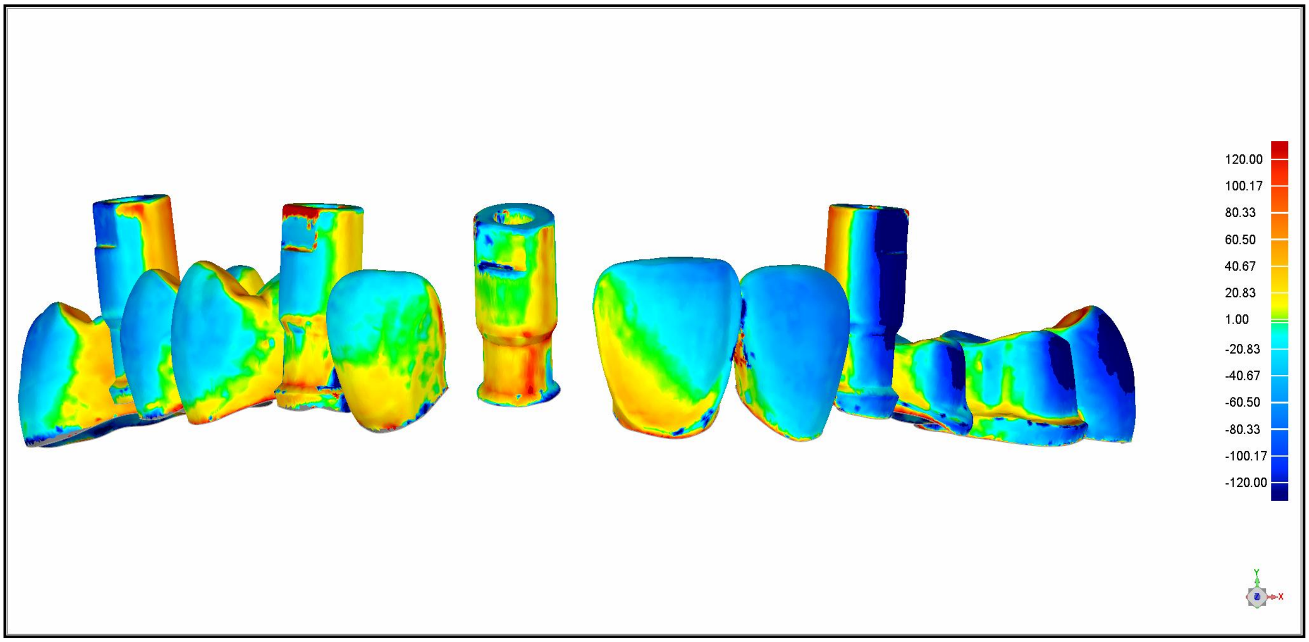

Predefinido: Inferior

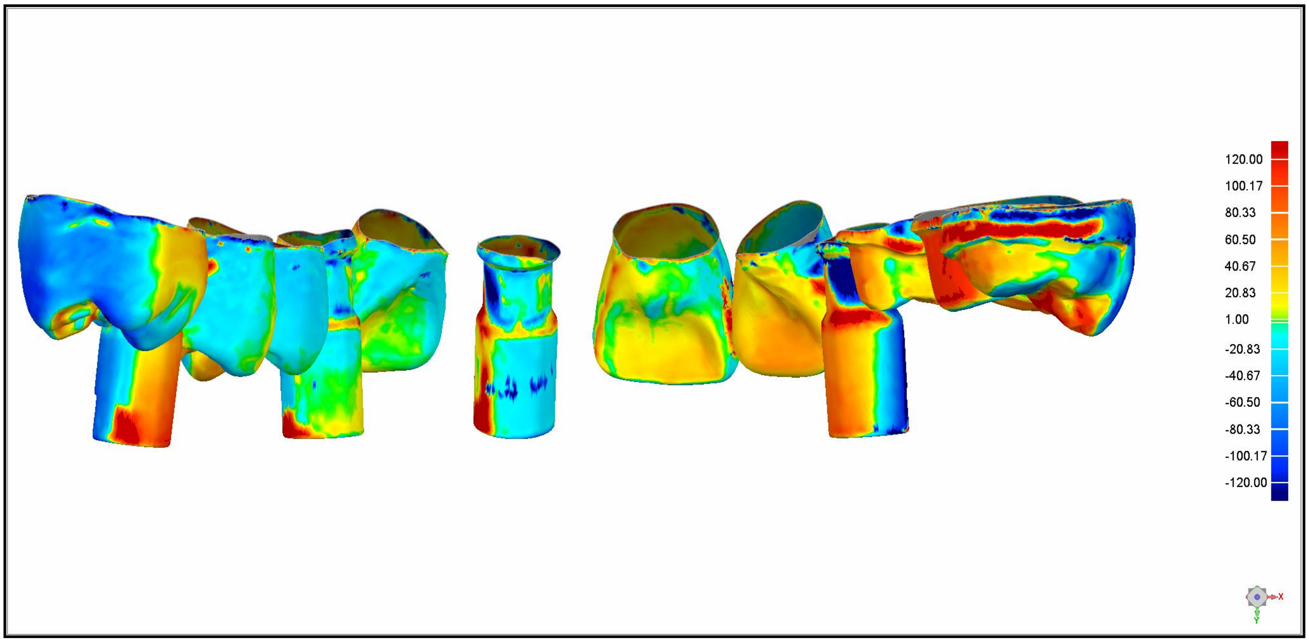

Ajuste de ubicación: Desviaciones superior e inferior

Unidades: u

Nombre	Desv	Estado	Superior Tol	Inferior Tol	Ref X	Ref Y	Ref Z	Radio	Desv X	Desv Y	Desv Z	Medido X	Medido Y	Medido Z	Dir. proy. X	Dir. proy. Y	Dir. proy. Z
Desv. inferior	-3155.11				-13203.90	38507.15	18811.31	n/a	-1381.09	1238.62	2552.09	-14584.99	39745.77	21363.39	0.44	-0.39	-0.81
Desv. superior	3154.69				-2214.15	38916.92	28794.18	n/a	-2702.63	539.90	1535.04	-4916.78	39456.83	30329.23	-0.86	0.17	0.49
