# Supplementary material for: Accuracy of four digital scanners according to scanning strategy in complete-arch impressions
Source: PLoS One. 2018 Sep 13;13(9):e0202916. doi: 10.1371/journal.pone.0202916 (PMC6136706; doi:10.1371/journal.pone.0202916)
Supplement: S10 Table — Omnicam (scanning strategy B). (ZIP) [file pone.0202916.s010.zip › S10/OM8B.pdf]

# 3D Comparación  Resultados

| | |
|---|---|
| Modelo referencia | MRC |
| Modelo test | OM8B |
| Nº de puntos de datos | 196001 |
| # Aislados | 871 |

| | |
|---|---|
| Tipo tolerancia | 3D desviación |
| Unidades | u |
| Máx. crítico | 120.00 |
| Máx. nominal | 12.00 |
| Mín. nominal | -12.00 |
| Mín. crítico | -123.00 |

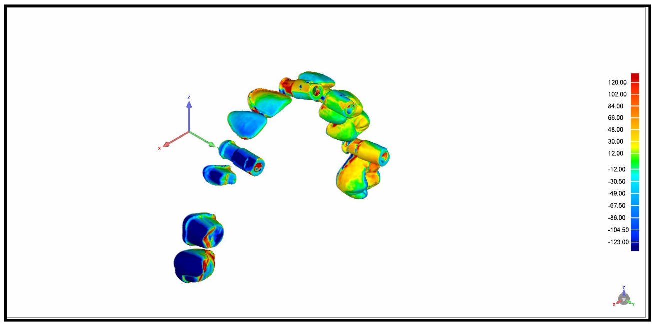

| | |
|---|---|
| Desviación | |
| Desviación superior máx. | 2999.42 |
| Desviación inferior máx. | -3134.65 |
| Desviación media | 100.62 /-91.54 |
| Desviación estándar | 237.10 |

## Distribución desviación

| >=Min | <Max | # Puntos | % |
|---|---|---|---|
| -123.00 | -104.50 | 2517 | 1.28 |
| -104.50 | -86.00 | 2993 | 1.53 |
| -86.00 | -67.50 | 5122 | 2.61 |
| -67.50 | -49.00 | 9042 | 4.61 |
| -49.00 | -30.50 | 13252 | 6.76 |
| -30.50 | -12.00 | 22461 | 11.46 |
| -12.00 | 12.00 | 39232 | 20.02 |
| 12.00 | 30.00 | 23026 | 11.75 |
| 30.00 | 48.00 | 16787 | 8.56 |
| 48.00 | 66.00 | 9536 | 4.87 |
| 66.00 | 84.00 | 6967 | 3.55 |
| 84.00 | 102.00 | 5566 | 2.84 |
| 102.00 | 120.00 | 4471 | 2.28 |

| | | |
|---|---|---|
| Fuera del crítico superior | 20974 | 10.70 |
| Fuera del crítico inferior | 14055 | 7.17 |

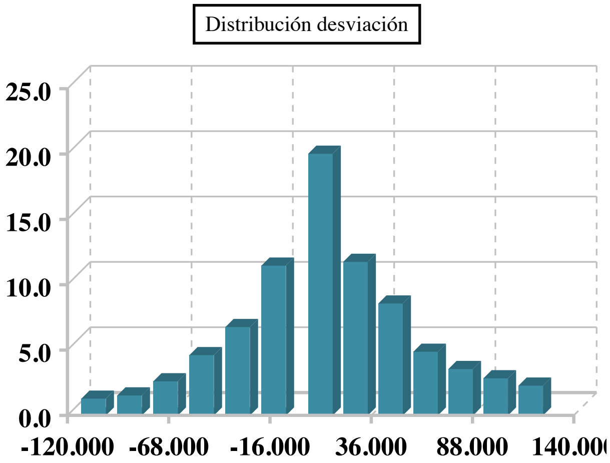


## Desviaciones estándar

| Distribución (+/-) | # Puntos | % |
|---|---|---|
| -6 * Desv. estánd. | 1227 | 0.63 |
| -5 * Desv. estánd. | 354 | 0.18 |
| -4 * Desv. estánd. | 595 | 0.30 |
| -3 * Desv. estánd. | 913 | 0.47 |
| -2 * Desv. estánd. | 3351 | 1.71 |
| -1 * Desv. estánd. | 103514 | 52.81 |
| 1 * Desv. estánd. | 79074 | 40.34 |
| 2 * Desv. estánd. | 2791 | 1.42 |
| 3 * Desv. estánd. | 1277 | 0.65 |
| 4 * Desv. estánd. | 1105 | 0.56 |
| 5 * Desv. estánd. | 806 | 0.41 |
| 6 * Desv. estánd. | 994 | 0.51 |

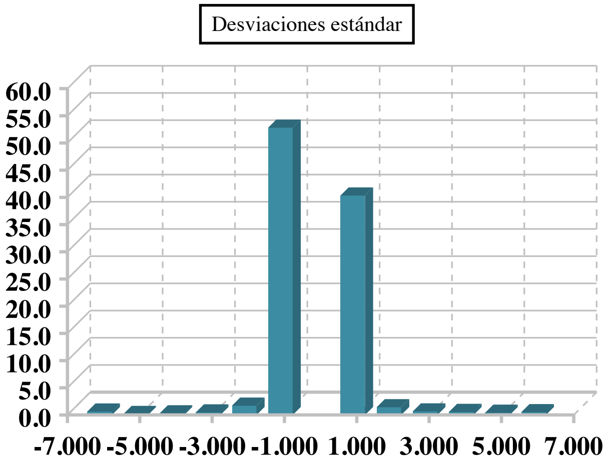

Predefinido: Isométrico

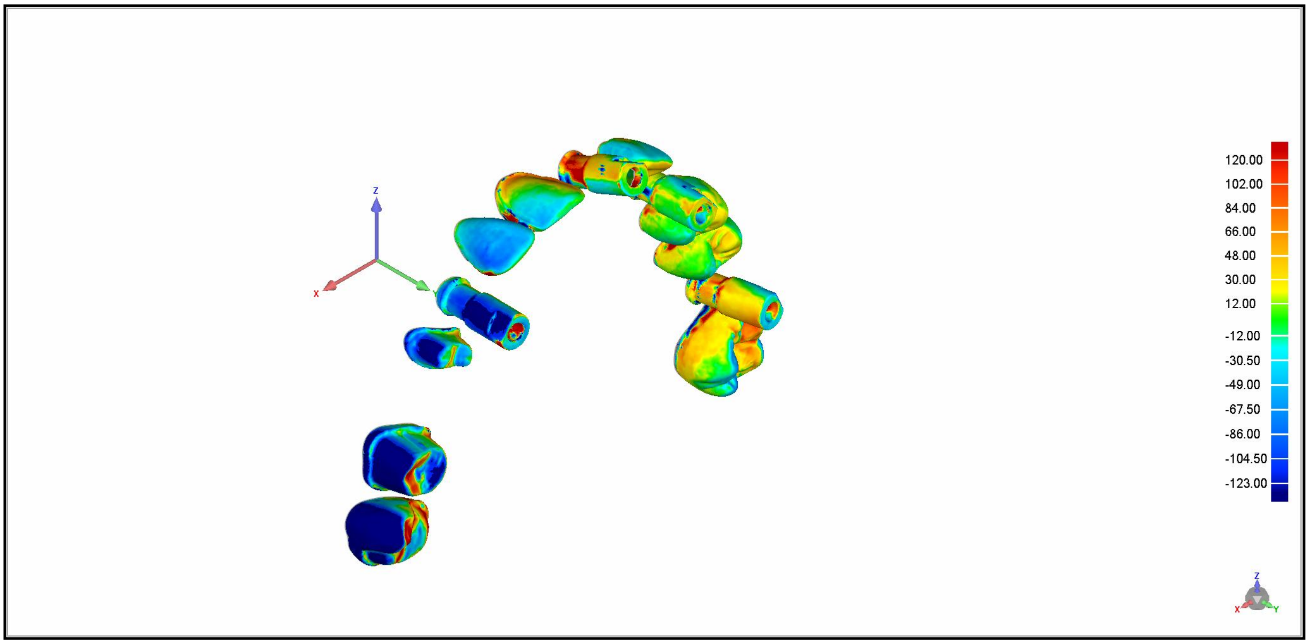

Predefinido: Frente

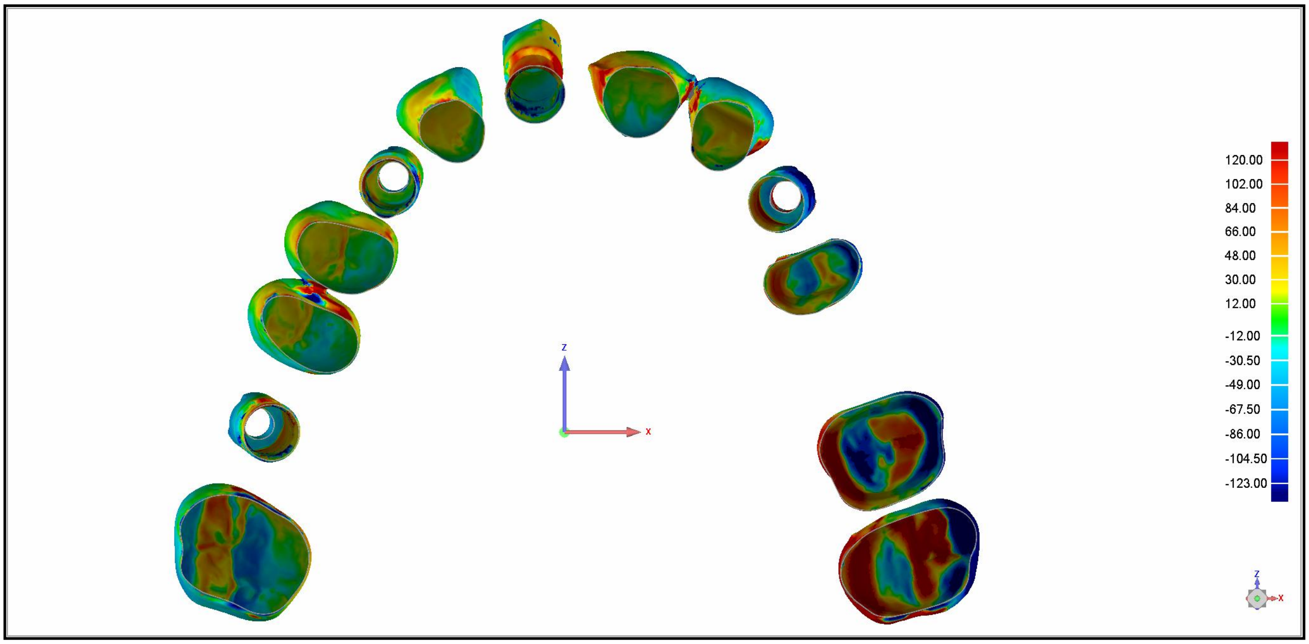

Fecha: 3/6/2017, 3:09 pm

Predefinido: Atrás

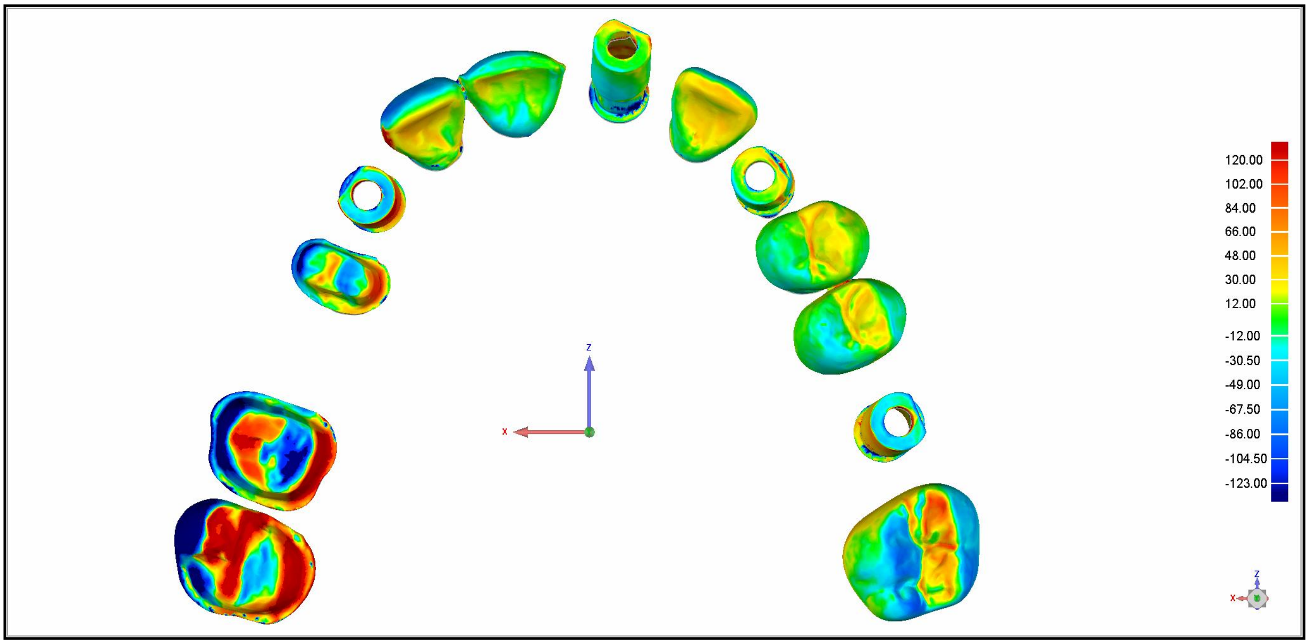

Predefinido: Izquierda

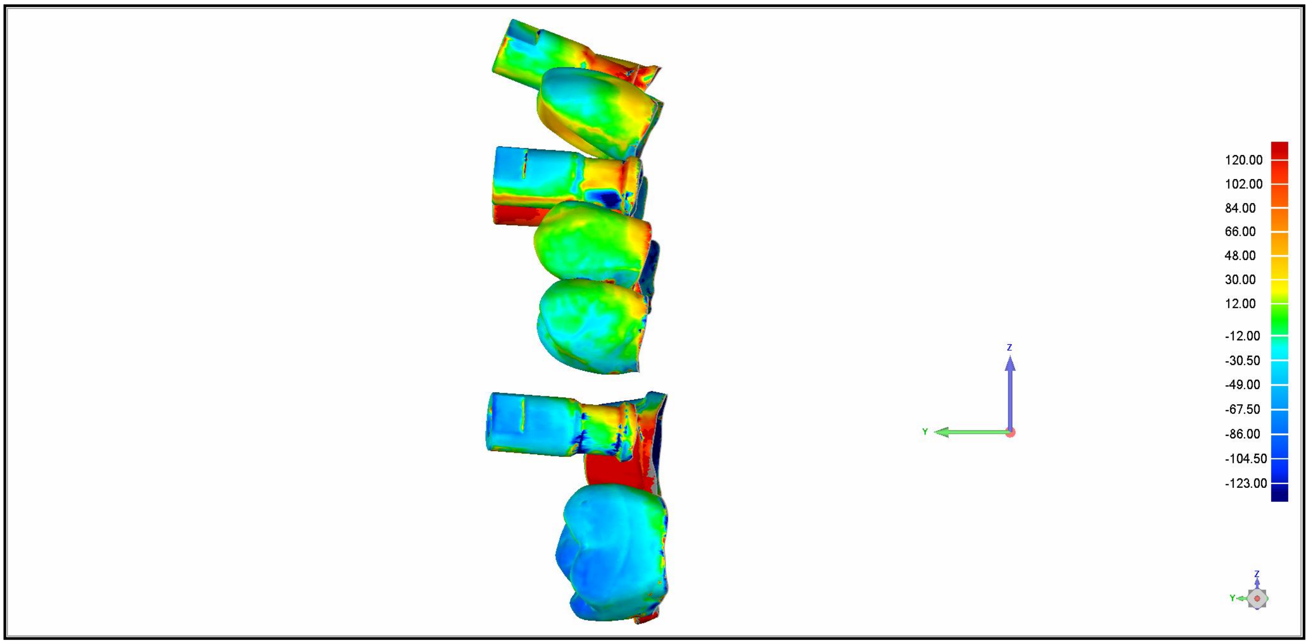

Predefinido: Derecha

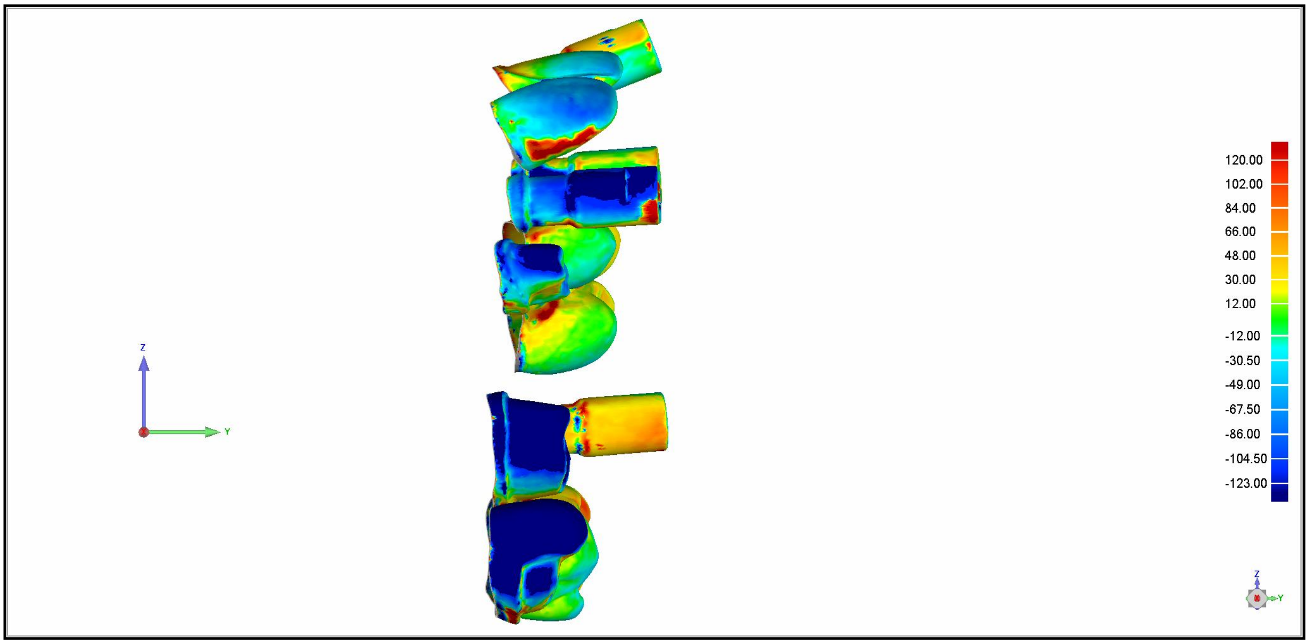

Predefinido: Superior

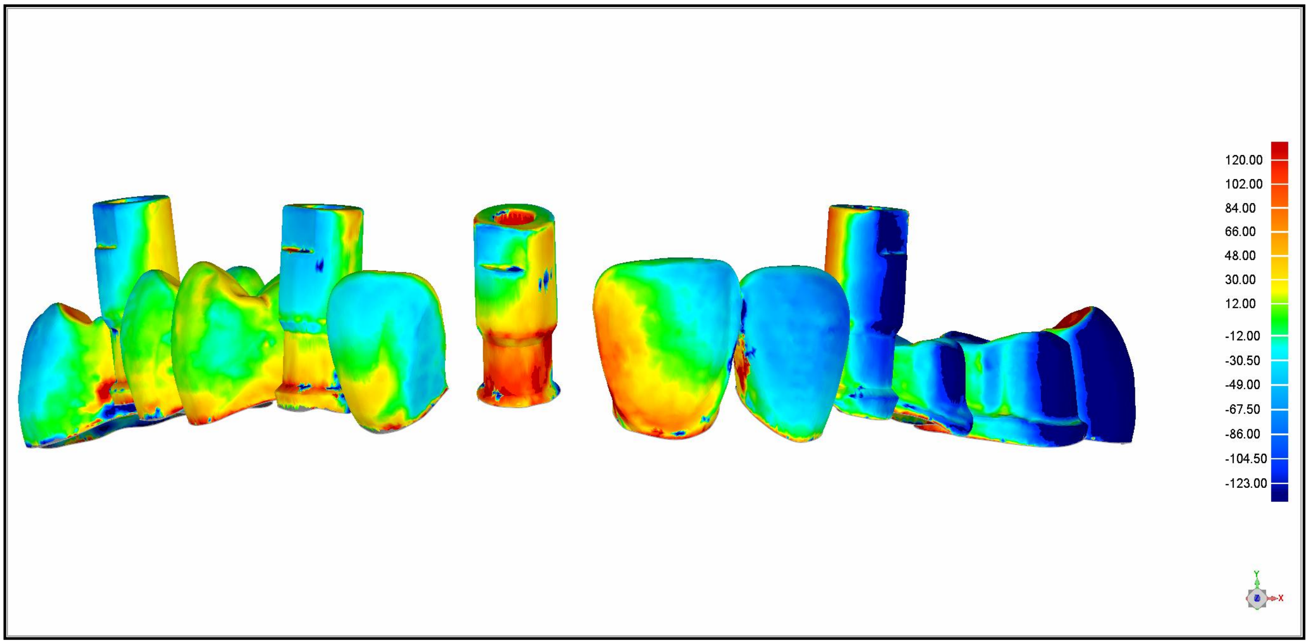

Predefinido: Inferior

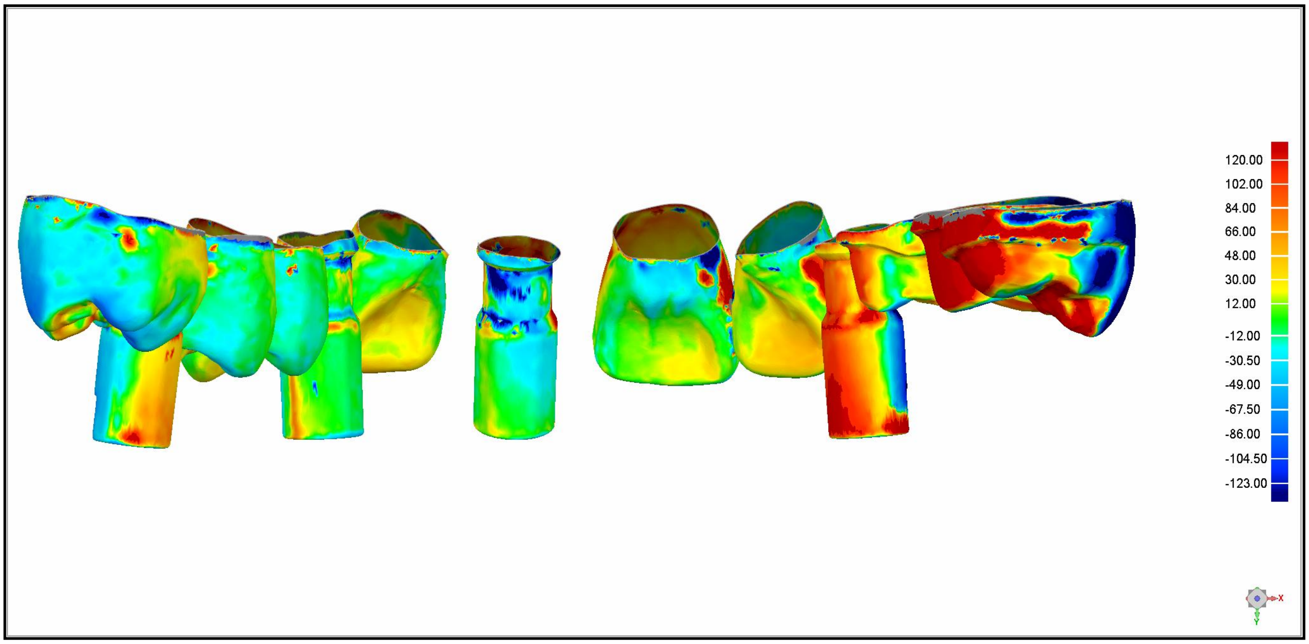

# Ajuste de ubicación: Desviaciones superior e inferior

## Unidades: u

| Nombre | Desv | Estado | Superior Tol | Inferior Tol | Ref X | Ref Y | Ref Z | Radio | Desv X | Desv Y | Desv Z | Medido X | Medido Y | Medido Z | Dir. proy. X | Dir. proy. Y | Dir. proy. Z |
|---|---|---|---|---|---|---|---|---|---|---|---|---|---|---|---|---|---|
| Desv. inferior | -3134.65 | | | | -16498.53 | 29426.69 | 5746.14 | n/a | -3011.88 | -160.63 | -853.70 | -19510.40 | 29266.06 | 4892.44 | 0.96 | 0.05 | 0.27 |
| Desv. superior | 2999.42 | | | | -6338.50 | 30079.10 | 24316.83 | n/a | 2997.46 | 101.51 | 38.48 | -3341.05 | 30180.61 | 24355.31 | 1.00 | 0.03 | 0.01 |
