# Supplementary material for: Accuracy of four digital scanners according to scanning strategy in complete-arch impressions
Source: PLoS One. 2018 Sep 13;13(9):e0202916. doi: 10.1371/journal.pone.0202916 (PMC6136706; doi:10.1371/journal.pone.0202916)
Supplement: S10 Table — Omnicam (scanning strategy B). (ZIP) [file pone.0202916.s010.zip › S10/OM9B.pdf]

Geomagic Control™

Fecha: 3/6/2017, 3:12 pm

3D Comparación Resultados

Modelo referencia	MRC
Modelo test	OM9B
Nº de puntos de datos	196988
# Aislados	611

Tipo tolerancia	3D desviación
Unidades	u
Máx. crítico	120.00
Máx. nominal	5.00
Mín. nominal	-5.00
Mín. crítico	-120.00

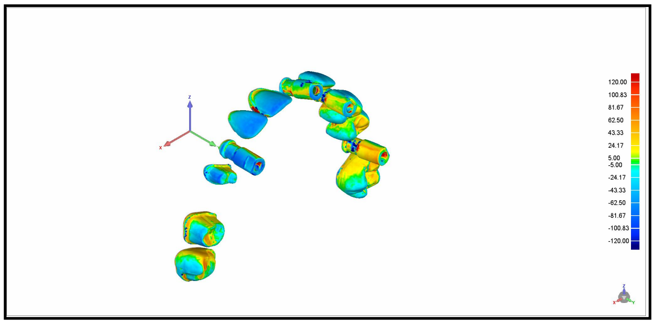

Desviación	
Desviación superior máx.	3146.26
Desviación inferior máx.	-3151.53
Desviación media	83.64 /-66.01
Desviación estándar	221.41

Distribución desviación

>=Min	<Max	# Puntos	%
-120.00	-100.83	804	0.41
-100.83	-81.67	1561	0.79
-81.67	-62.50	4992	2.53
-62.50	-43.33	14063	7.14
-43.33	-24.17	27486	13.95
-24.17	-5.00	37450	19.01
-5.00	5.00	20191	10.25
5.00	24.17	34831	17.68
24.17	43.33	19446	9.87
43.33	62.50	9243	4.69
62.50	81.67	4064	2.06
81.67	100.83	2621	1.33
100.83	120.00	2029	1.03

Fuera del crítico superior	11949	6.07
Fuera del crítico inferior	6258	3.18

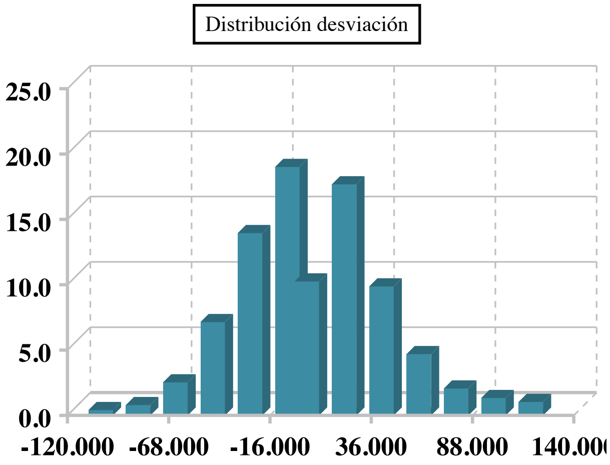

Desviaciones estándar

Distribución (+/-)	# Puntos	%
-6 * Desv. estánd.	1356	0.69
-5 * Desv. estánd.	364	0.18
-4 * Desv. estánd.	524	0.27
-3 * Desv. estánd.	704	0.36
-2 * Desv. estánd.	1469	0.75
-1 * Desv. estánd.	109479	55.58
1 * Desv. estánd.	76467	38.82
2 * Desv. estánd.	2553	1.30
3 * Desv. estánd.	1290	0.65
4 * Desv. estánd.	963	0.49
5 * Desv. estánd.	603	0.31
6 * Desv. estánd.	1216	0.62

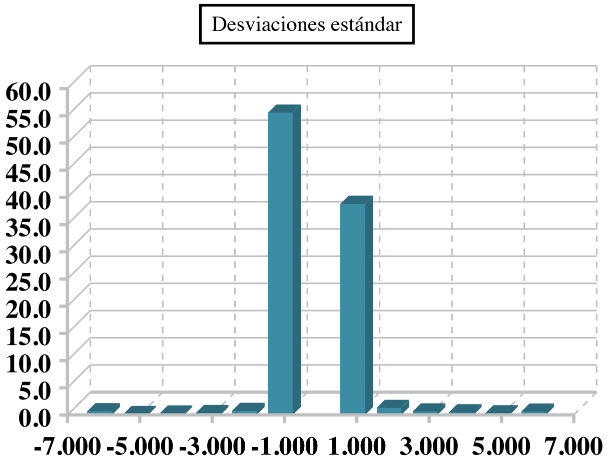

Geomagic Control™

Predefinido: Isométrico

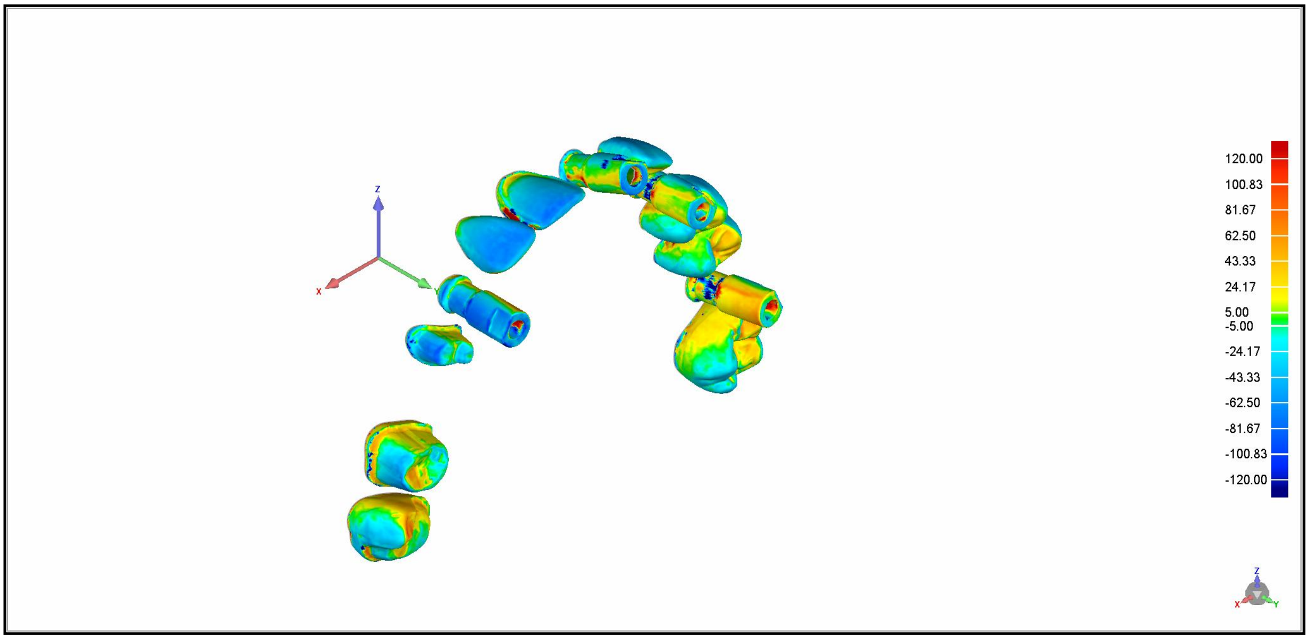

Predefinido: Frente

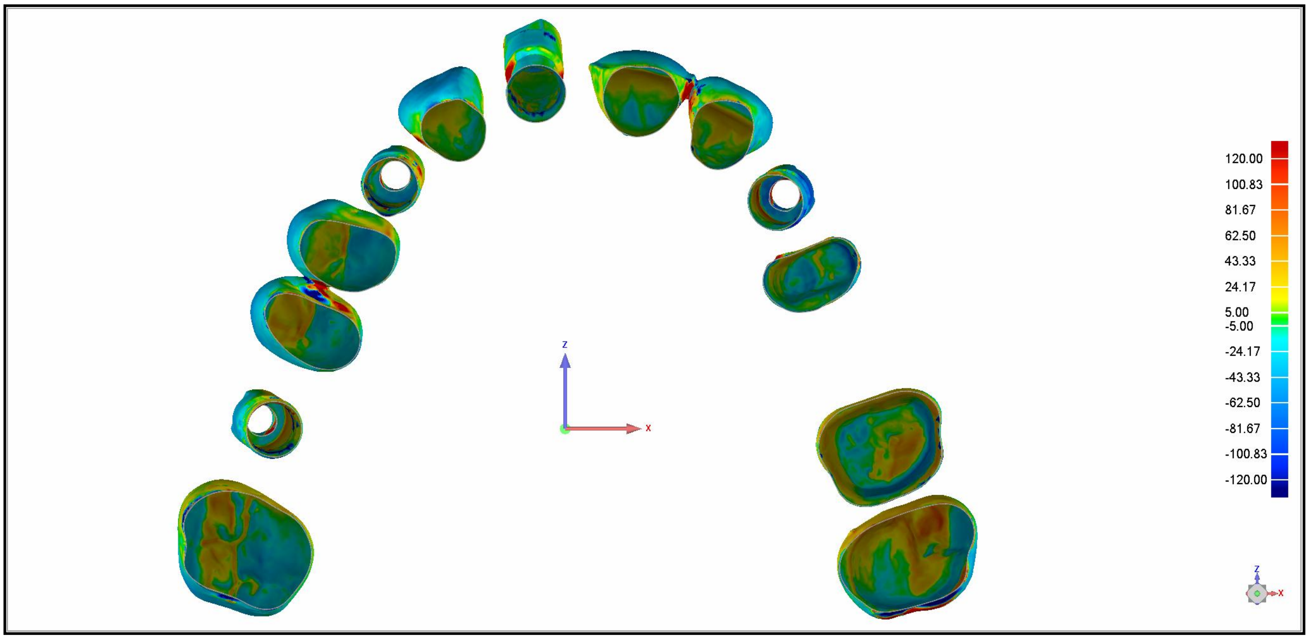

Predefinido: Atrás

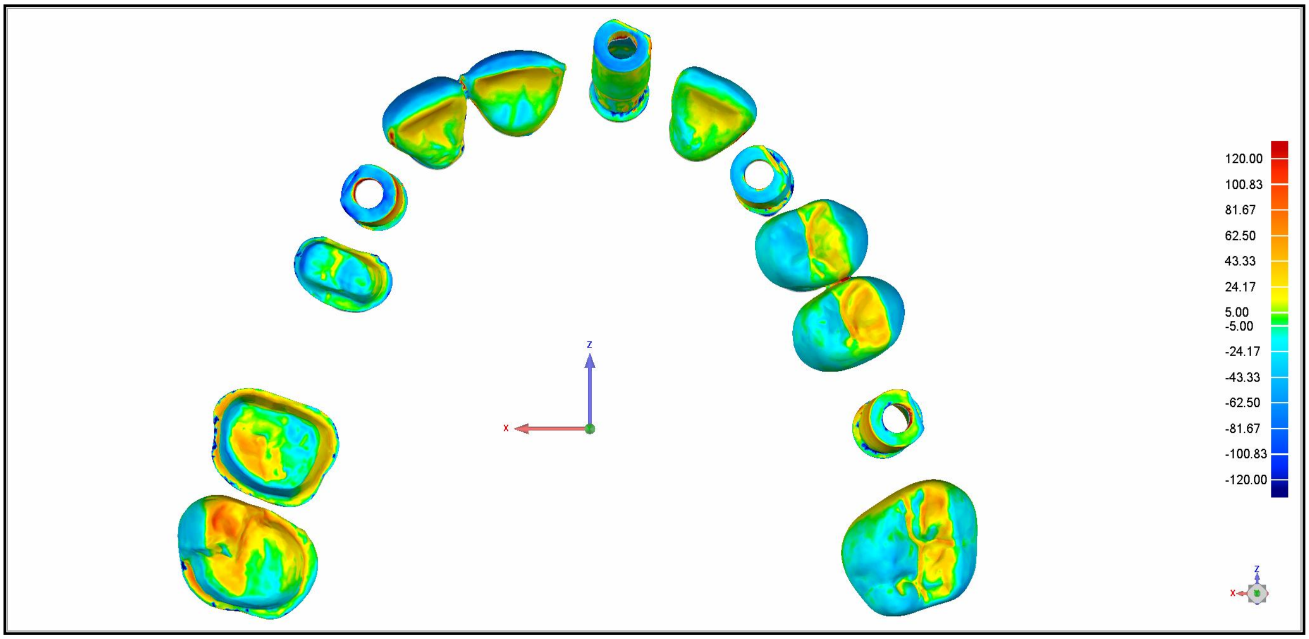

Predefinido: Izquierda

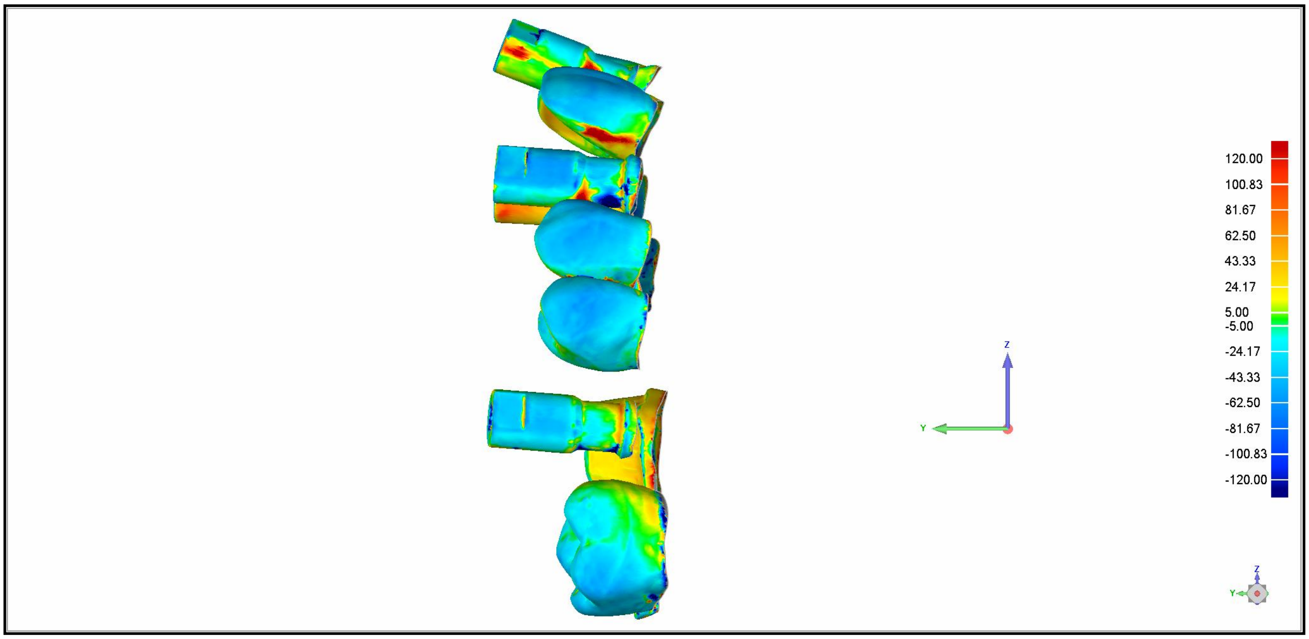

Predefinido: Derecha

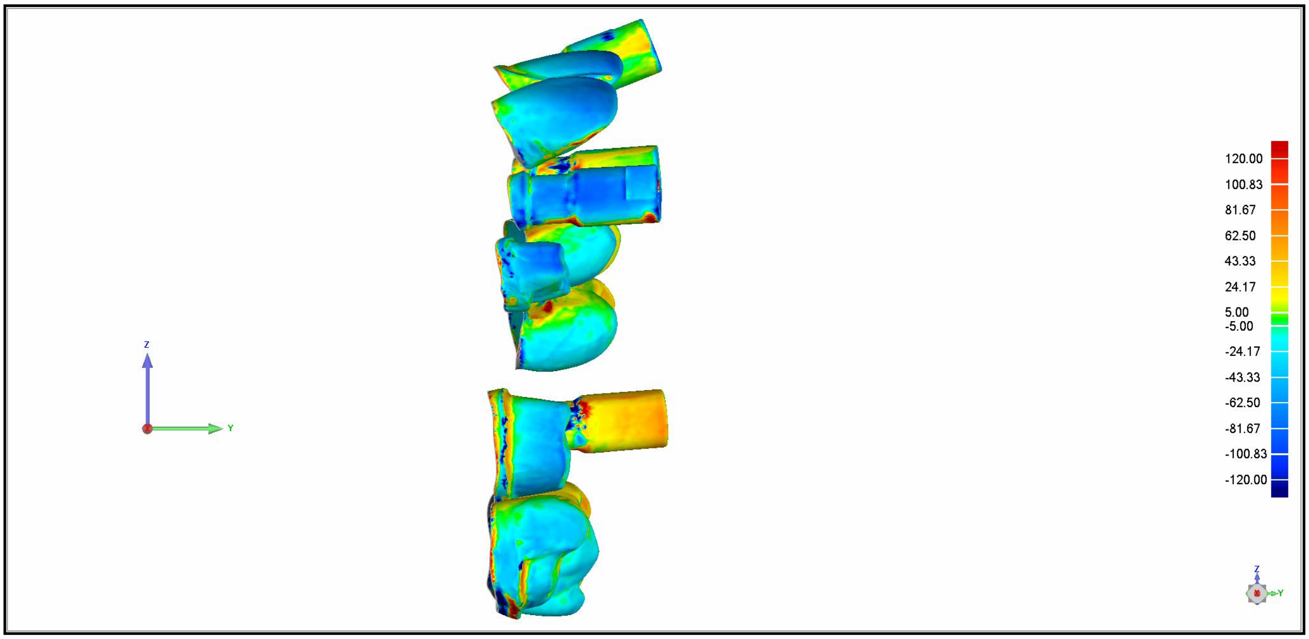

Predefinido: Superior

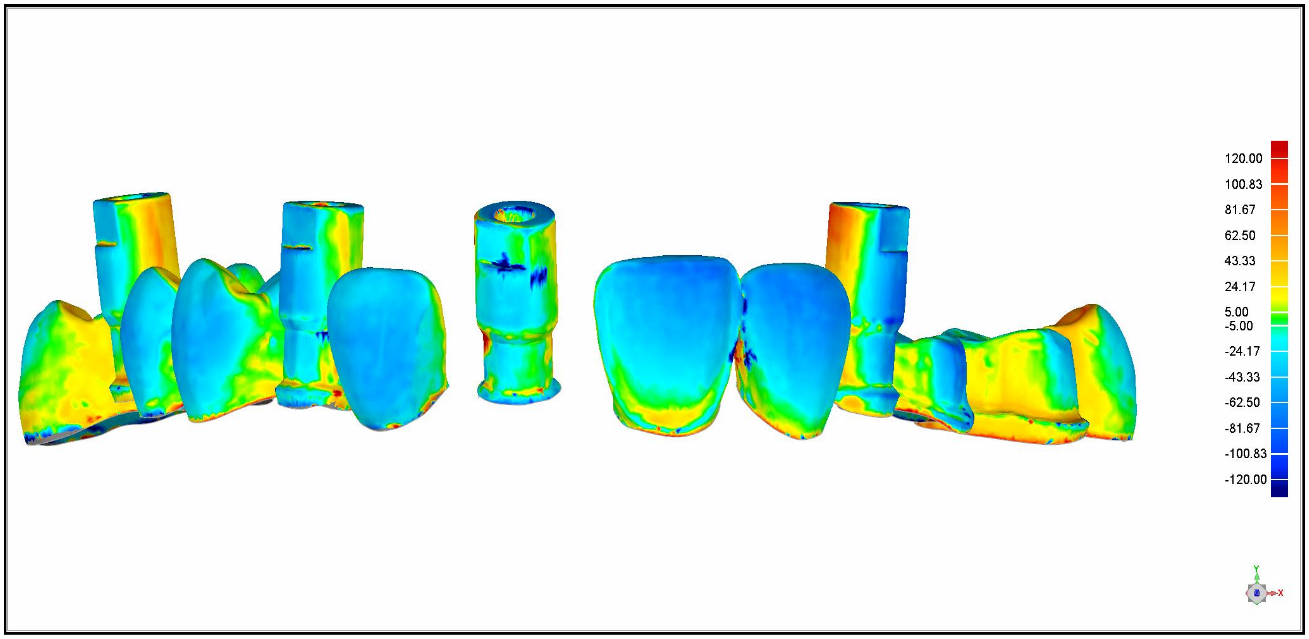

Predefinido: Inferior

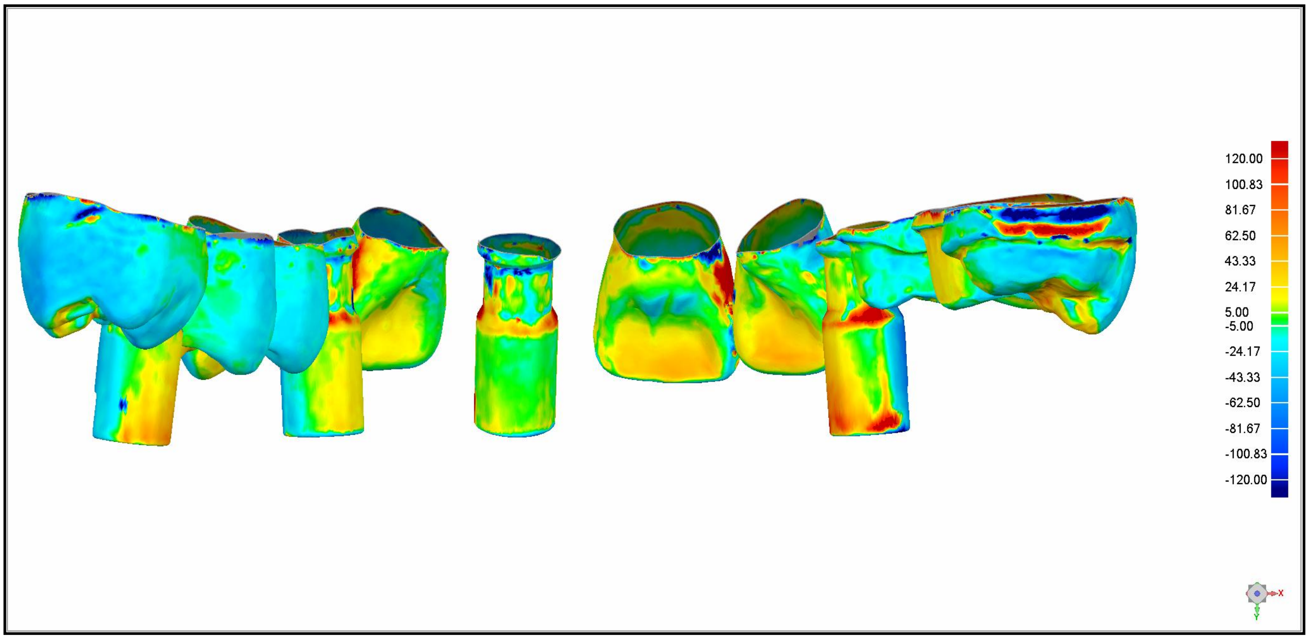

Ajuste de ubicación: Desviaciones superior e inferior

Unidades: u

Nombre	Desv	Estado	Superior Tol	Inferior Tol	Ref X	Ref Y	Ref Z	Radio	Desv X	Desv Y	Desv Z	Medido X	Medido Y	Medido Z	Dir. proy. X	Dir. proy. Y	Dir. proy. Z
Desv. inferior	-3151.53				-22607.19	28955.77	6808.03	n/a	-872.68	-449.57	2994.74	-23479.87	28506.20	9802.77	0.28	0.14	-0.95
Desv. superior	3146.26				-29824.91	26937.61	-11348.70	n/a	2659.51	231.11	-1665.11	-27165.40	27168.72	-13013.81	0.85	0.07	-0.53
