# Supplementary material for: Accuracy of four digital scanners according to scanning strategy in complete-arch impressions
Source: PLoS One. 2018 Sep 13;13(9):e0202916. doi: 10.1371/journal.pone.0202916 (PMC6136706; doi:10.1371/journal.pone.0202916)
Supplement: S10 Table — Omnicam (scanning strategy B). (ZIP) [file pone.0202916.s010.zip › S10/OM5B.pdf]

3D Comparación Resultados

Modelo referencia	MRC
Modelo test	OM5B
Nº de puntos de datos	194957
# Aislados	939

Tipo tolerancia	3D desviación
Unidades	u
Máx. crítico	120.00
Máx. nominal	10.00
Mín. nominal	-10.00
Mín. crítico	-120.00

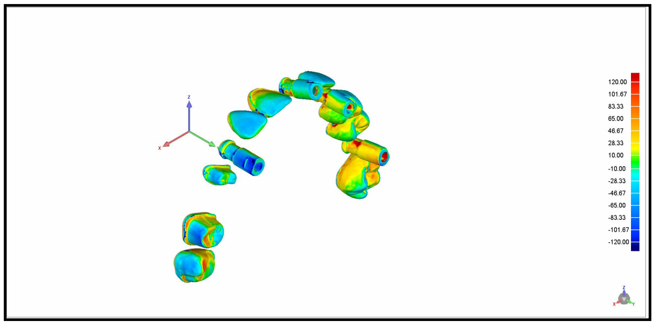

Desviación	
Desviación superior máx.	3062.45
Desviación inferior máx.	-3106.46
Desviación media	82.15 /-68.44
Desviación estándar	215.85

Distribución desviación

>=Min	<Max	# Puntos	%
-120.00	-101.67	1190	0.61
-101.67	-83.33	1722	0.88
-83.33	-65.00	3747	1.92
-65.00	-46.67	9204	4.72
-46.67	-28.33	20089	10.30
-28.33	-10.00	29957	15.37
-10.00	10.00	41705	21.39
10.00	28.33	32437	16.64
28.33	46.67	19161	9.83
46.67	65.00	8958	4.59
65.00	83.33	4525	2.32
83.33	101.67	2650	1.36
101.67	120.00	1604	0.82

Fuera del crítico superior	11549	5.92
Fuera del crítico inferior	6459	3.31

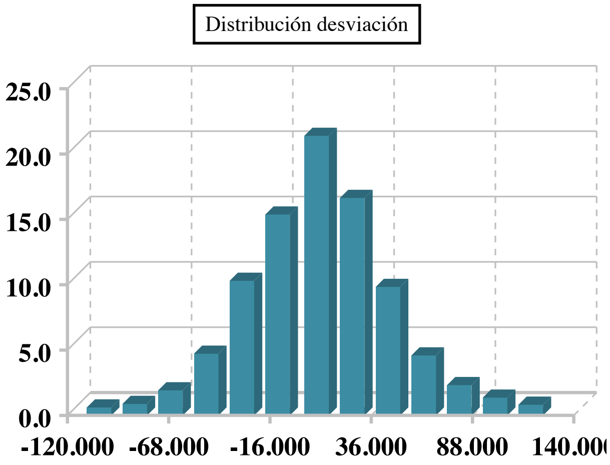

Desviaciones estándar

Distribución (+/-)	# Puntos	%
-6 * Desv. estánd.	1030	0.53
-5 * Desv. estánd.	643	0.33
-4 * Desv. estánd.	971	0.50
-3 * Desv. estánd.	802	0.41
-2 * Desv. estánd.	1095	0.56
-1 * Desv. estánd.	110471	56.66
1 * Desv. estánd.	72901	37.39
2 * Desv. estánd.	2427	1.24
3 * Desv. estánd.	1343	0.69
4 * Desv. estánd.	1207	0.62
5 * Desv. estánd.	808	0.41
6 * Desv. estánd.	1259	0.65

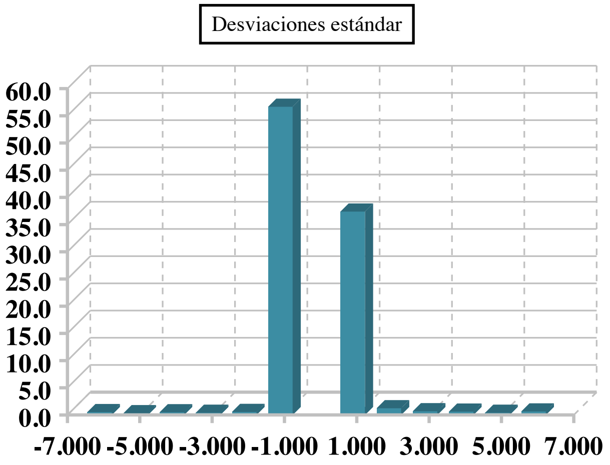

Predefinido: Isométrico

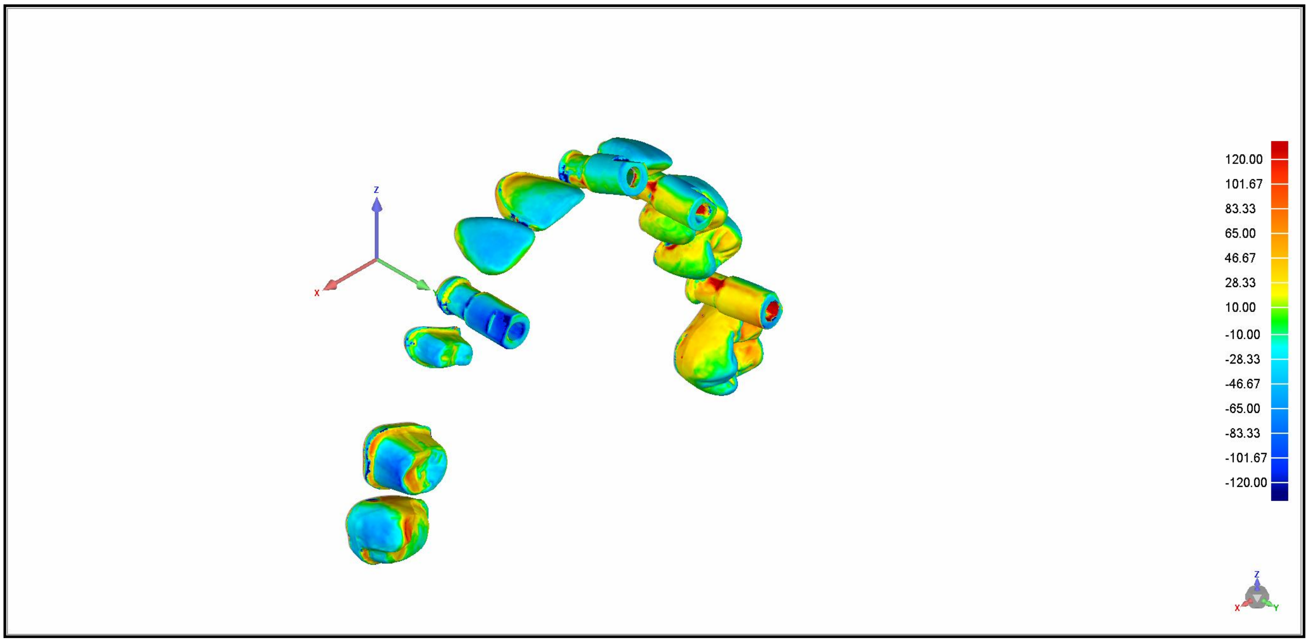

Predefinido: Frente

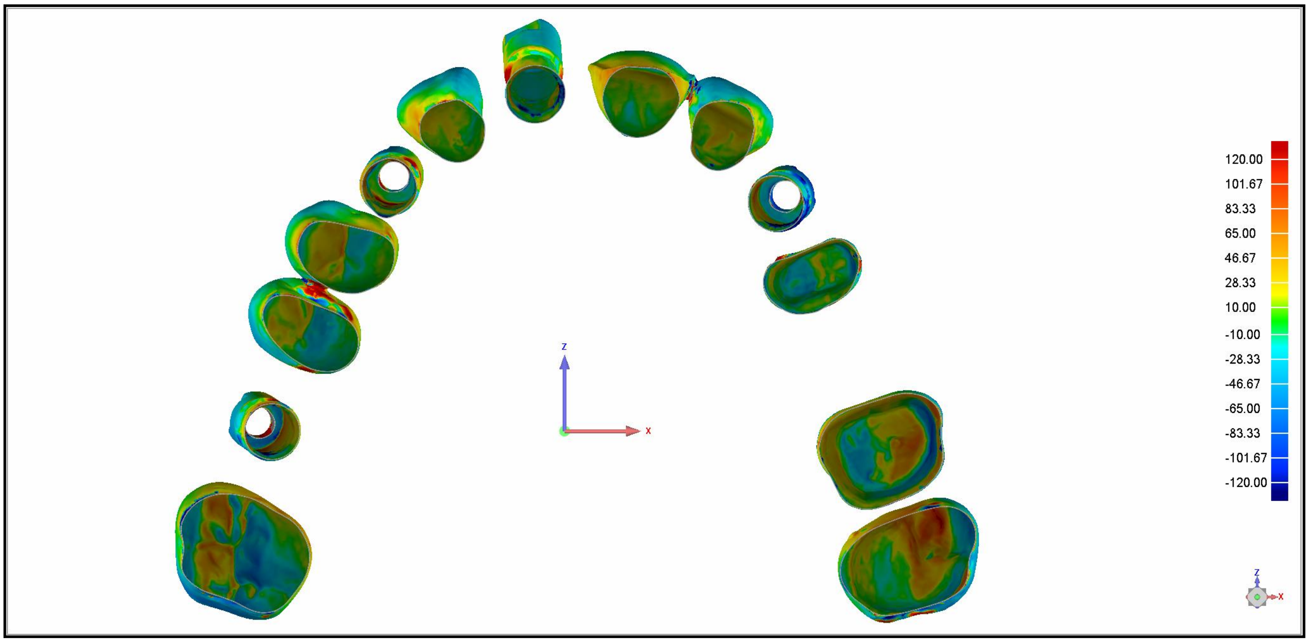

Predefinido: Atrás

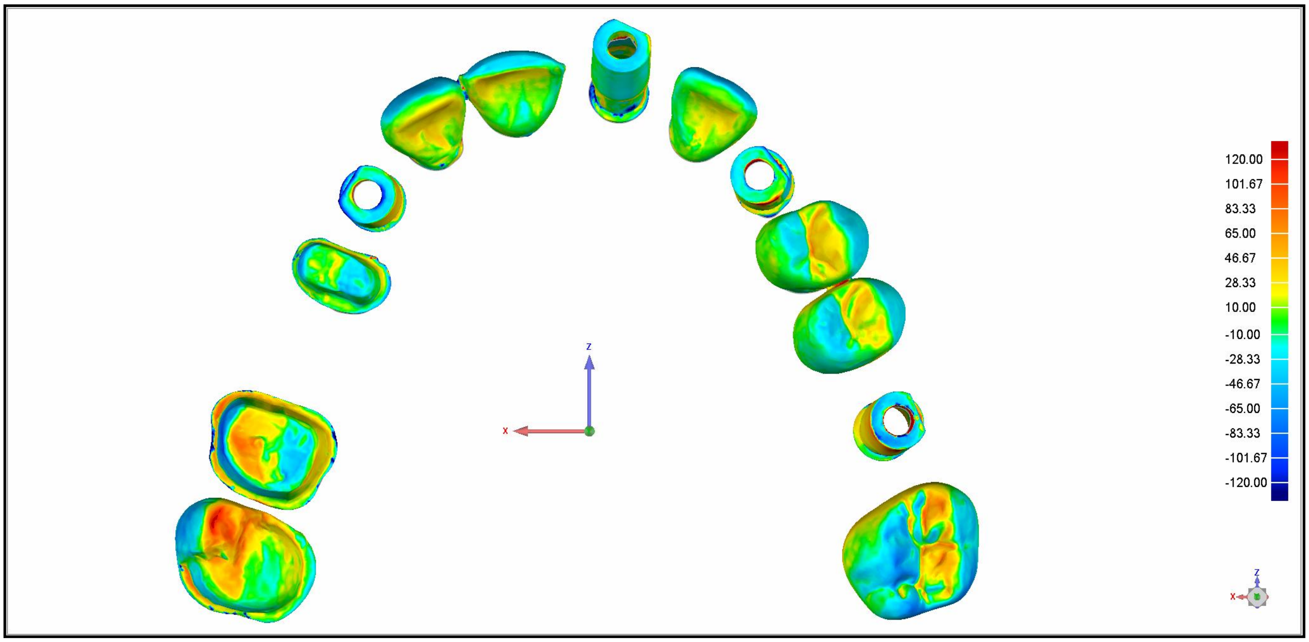

Predefinido: Izquierda

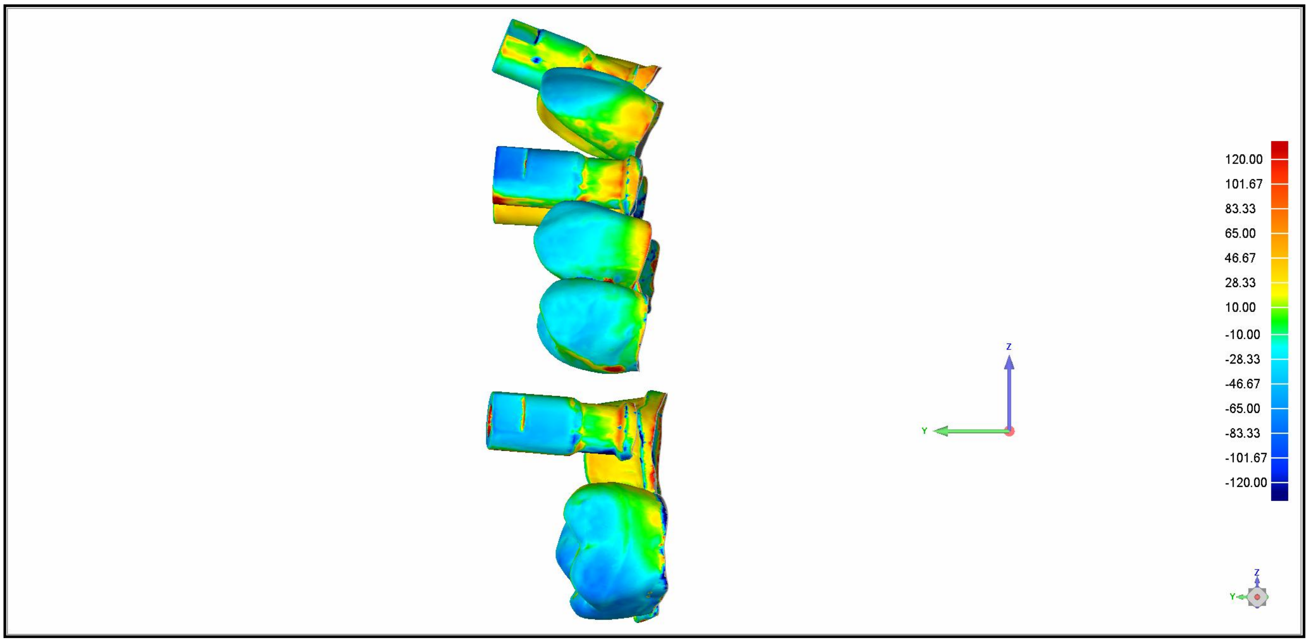

Predefinido: Derecha

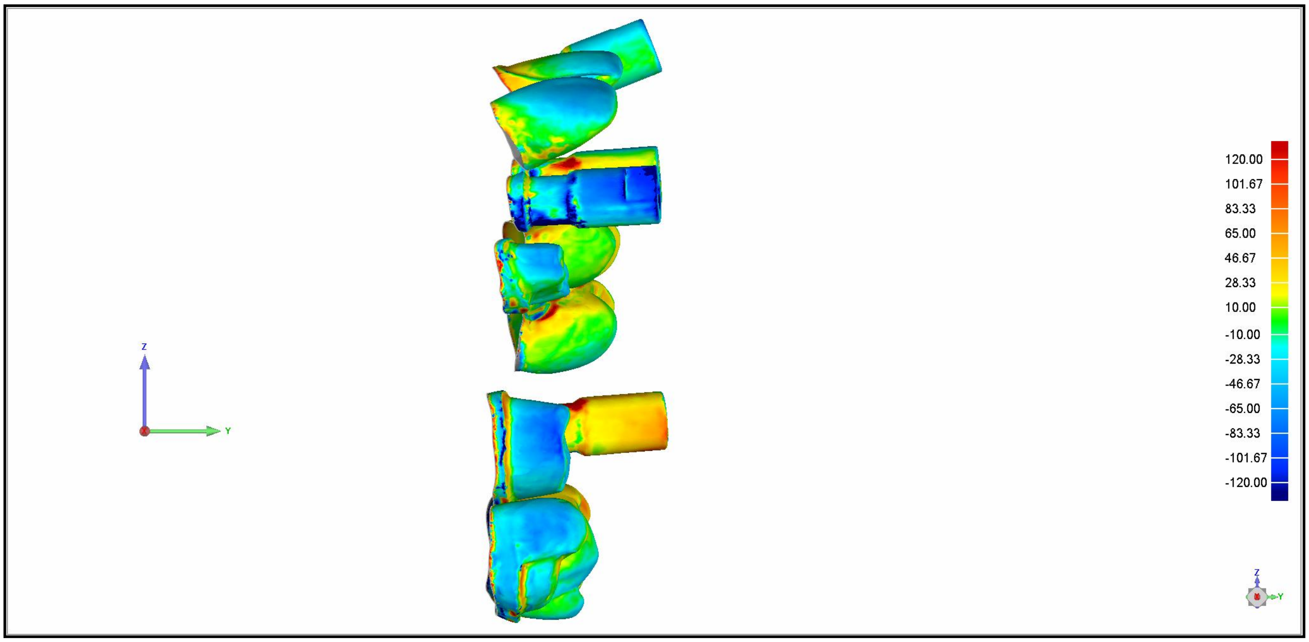

Predefinido: Superior

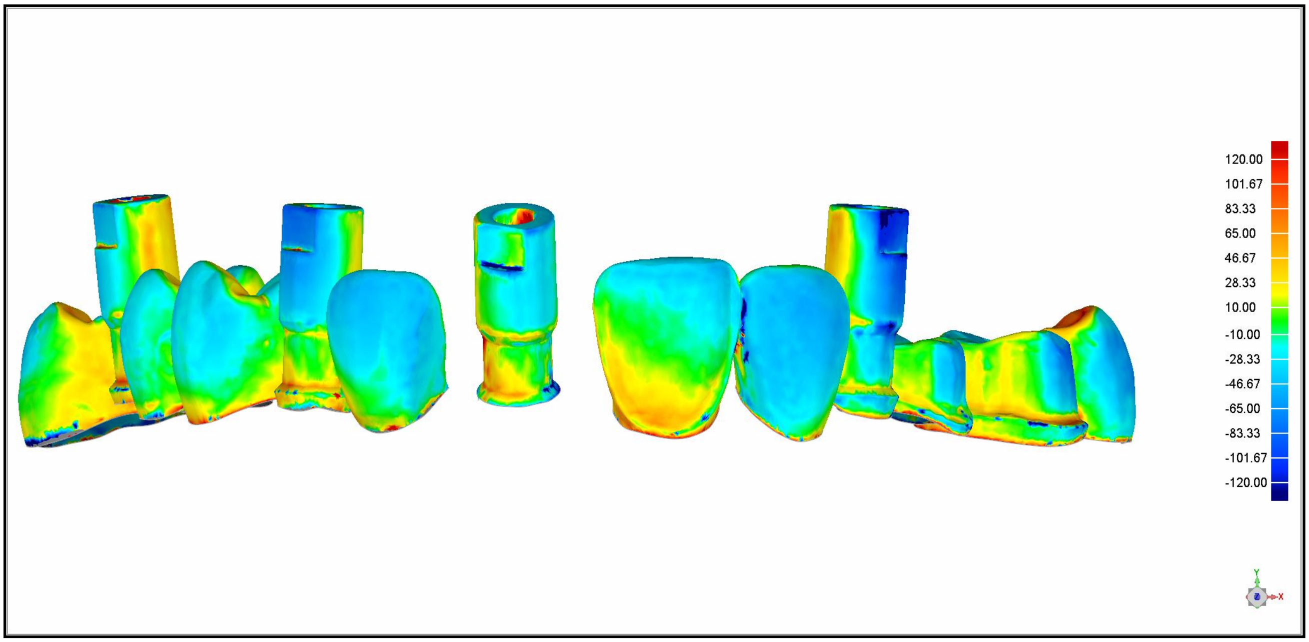

Predefinido: Inferior

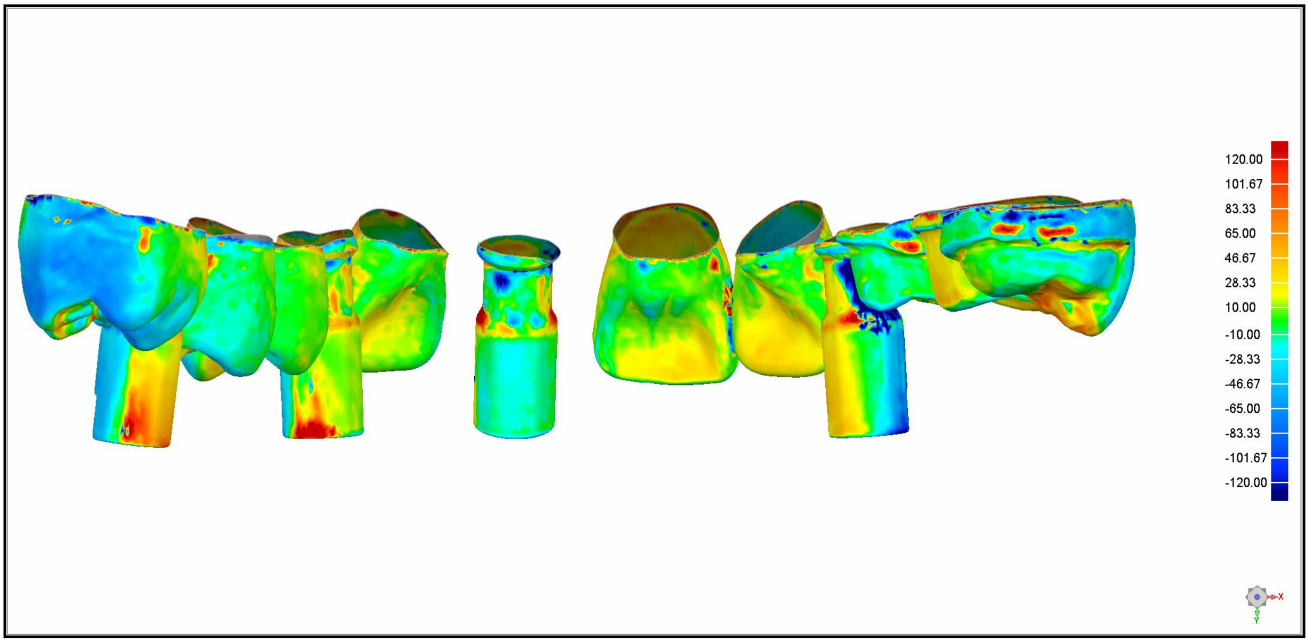

Ajuste de ubicación: Desviaciones superior e inferior

Unidades: u

Nombre	Desv	Estado	Superior Tol	Inferior Tol	Ref X	Ref Y	Ref Z	Radio	Desv X	Desv Y	Desv Z	Medido X	Medido Y	Medido Z	Dir. proy. X	Dir. proy. Y	Dir. proy. Z
Desv. inferior	-3106.46				-22607.19	28955.77	6808.03	n/a	-955.90	-451.40	2921.06	-23563.09	28504.37	9729.09	0.31	0.15	-0.94
Desv. superior	3062.45				-20553.64	28741.29	-8096.88	n/a	-2284.37	35.67	2039.36	-22838.02	28776.95	-6057.52	-0.75	0.01	0.67
