# Supplementary material for: Accuracy of four digital scanners according to scanning strategy in complete-arch impressions
Source: PLoS One. 2018 Sep 13;13(9):e0202916. doi: 10.1371/journal.pone.0202916 (PMC6136706; doi:10.1371/journal.pone.0202916)
Supplement: S10 Table — Omnicam (scanning strategy B). (ZIP) [file pone.0202916.s010.zip › S10/OM6B.pdf]

3D Comparación Resultados

Modelo referencia	MRC
Modelo test	OM6B
Nº de puntos de datos	196176
# Aislados	647

Tipo tolerancia	3D desviación
Unidades	u
Máx. crítico	120.00
Máx. nominal	9.00
Mín. nominal	-9.00
Mín. crítico	-120.00

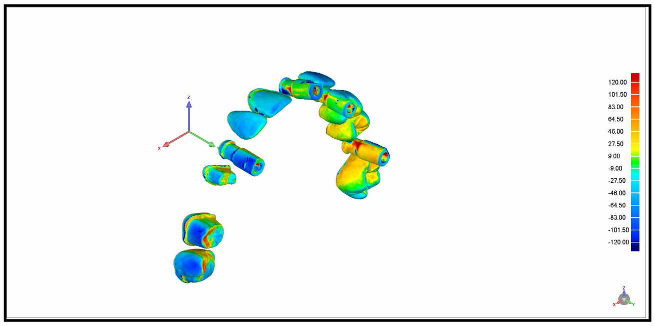

Desviación	
Desviación superior máx.	3116.20
Desviación inferior máx.	-3144.68
Desviación media	100.08 /-71.72
Desviación estándar	235.39

Distribución desviación

>=Min	<Max	# Puntos	%
-120.00	-101.50	1989	1.01
-101.50	-83.00	3610	1.84
-83.00	-64.50	7500	3.82
-64.50	-46.00	16005	8.16
-46.00	-27.50	22718	11.58
-27.50	-9.00	28252	14.40
-9.00	9.00	30745	15.67
9.00	27.50	26734	13.63
27.50	46.00	17866	9.11
46.00	64.50	9048	4.61
64.50	83.00	5353	2.73
83.00	101.50	3236	1.65
101.50	120.00	2217	1.13

Fuera del crítico superior	13684	6.98
Fuera del crítico inferior	7219	3.68

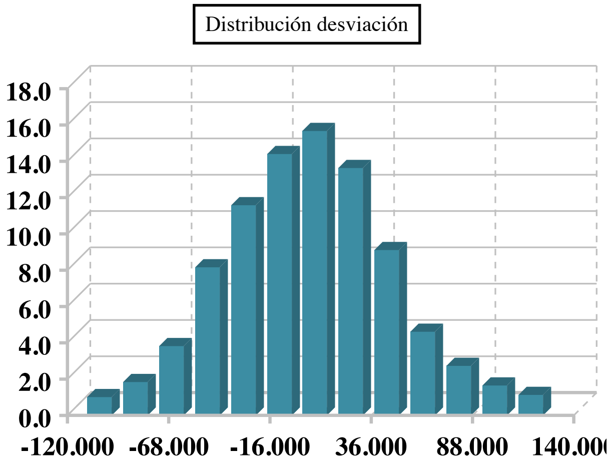

Desviaciones estándar

Distribución (+/-)	# Puntos	%
-6 * Desv. estánd.	1261	0.64
-5 * Desv. estánd.	478	0.24
-4 * Desv. estánd.	434	0.22
-3 * Desv. estánd.	504	0.26
-2 * Desv. estánd.	1179	0.60
-1 * Desv. estánd.	115567	58.91
1 * Desv. estánd.	69156	35.25
2 * Desv. estánd.	2688	1.37
3 * Desv. estánd.	1602	0.82
4 * Desv. estánd.	1222	0.62
5 * Desv. estánd.	855	0.44
6 * Desv. estánd.	1230	0.63

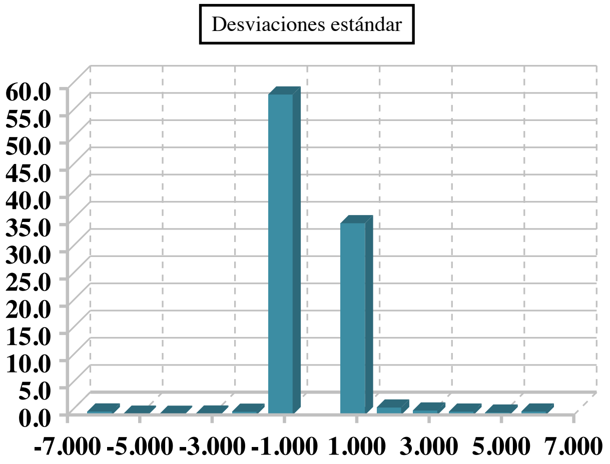

Predefinido: Isométrico

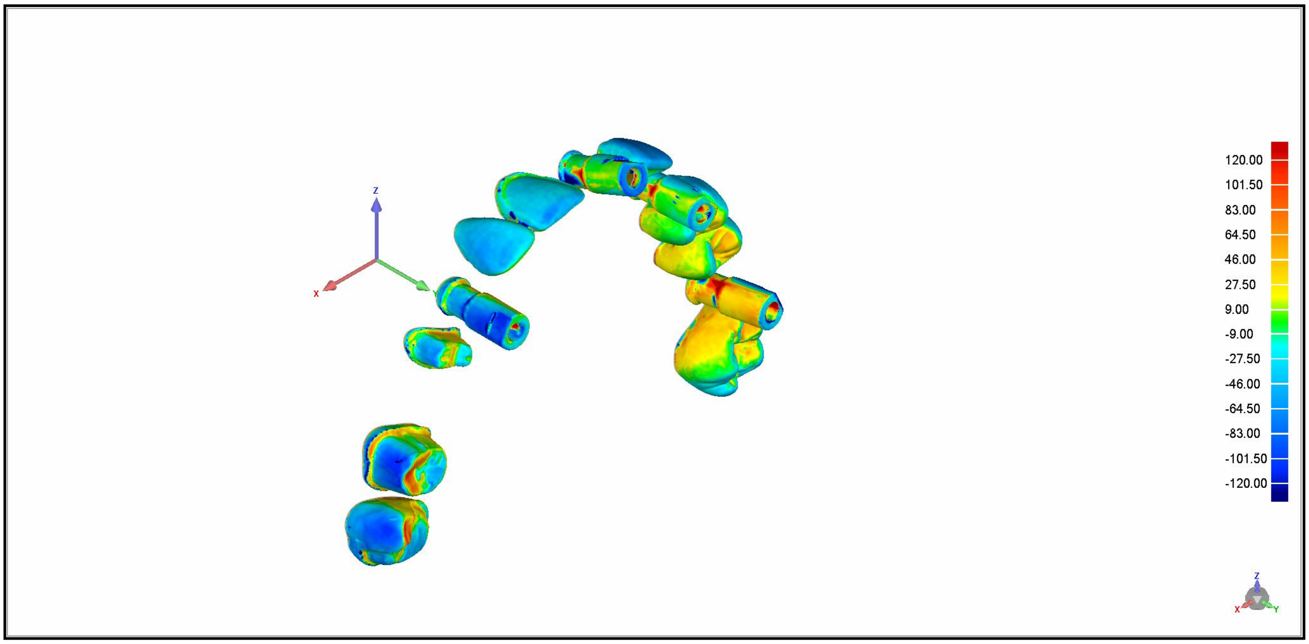

Predefinido: Frente

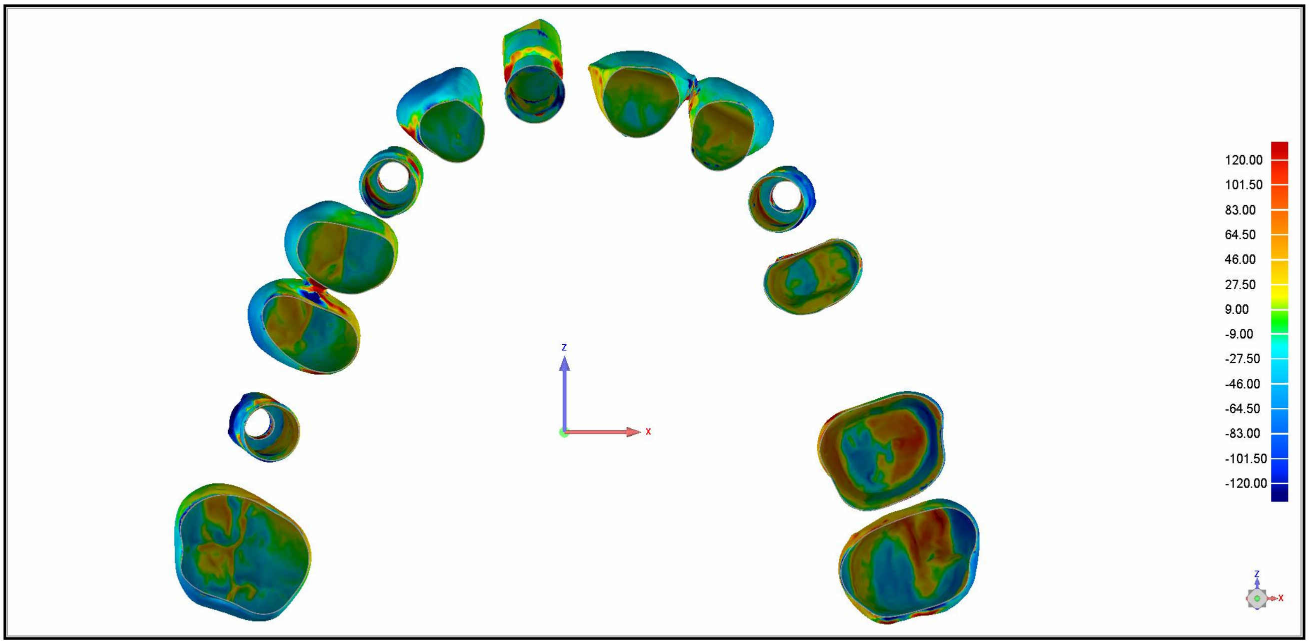

Predefinido: Atrás

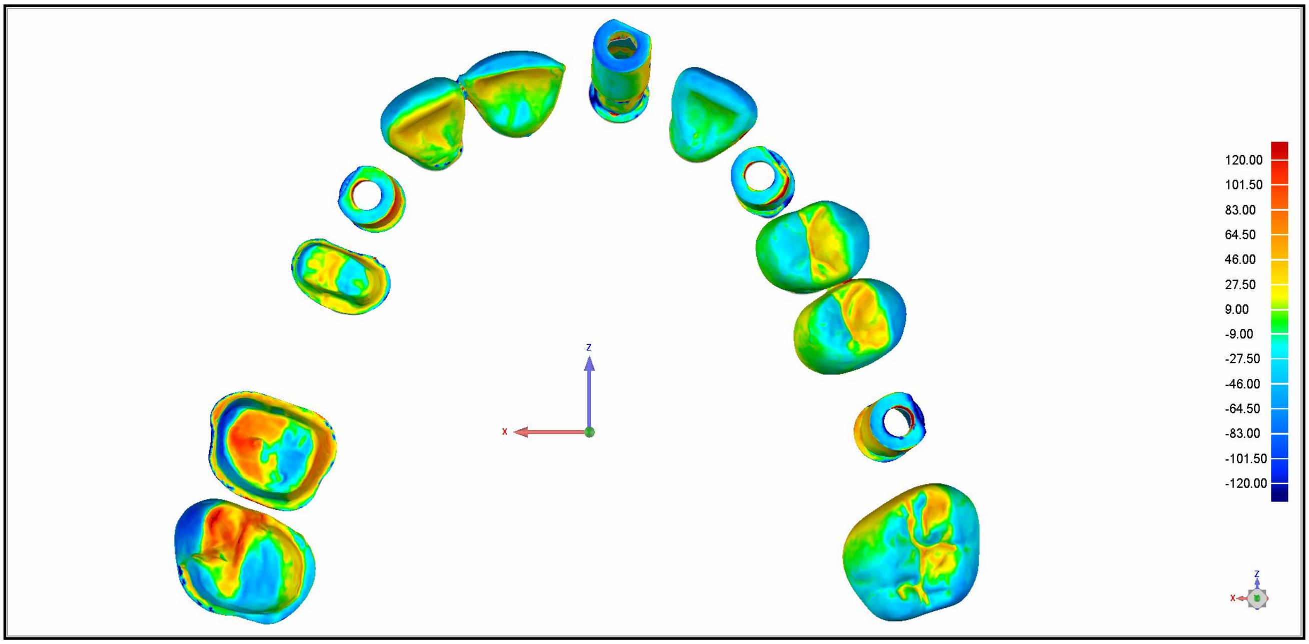

Predefinido: Izquierda

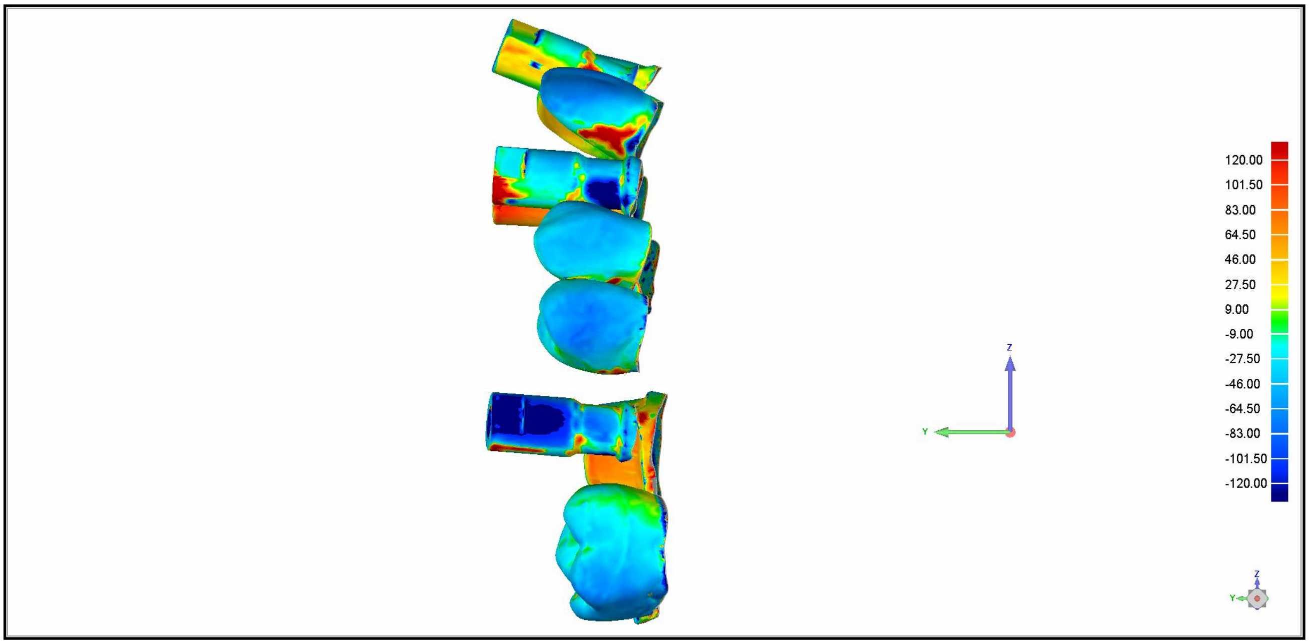

Predefinido: Derecha

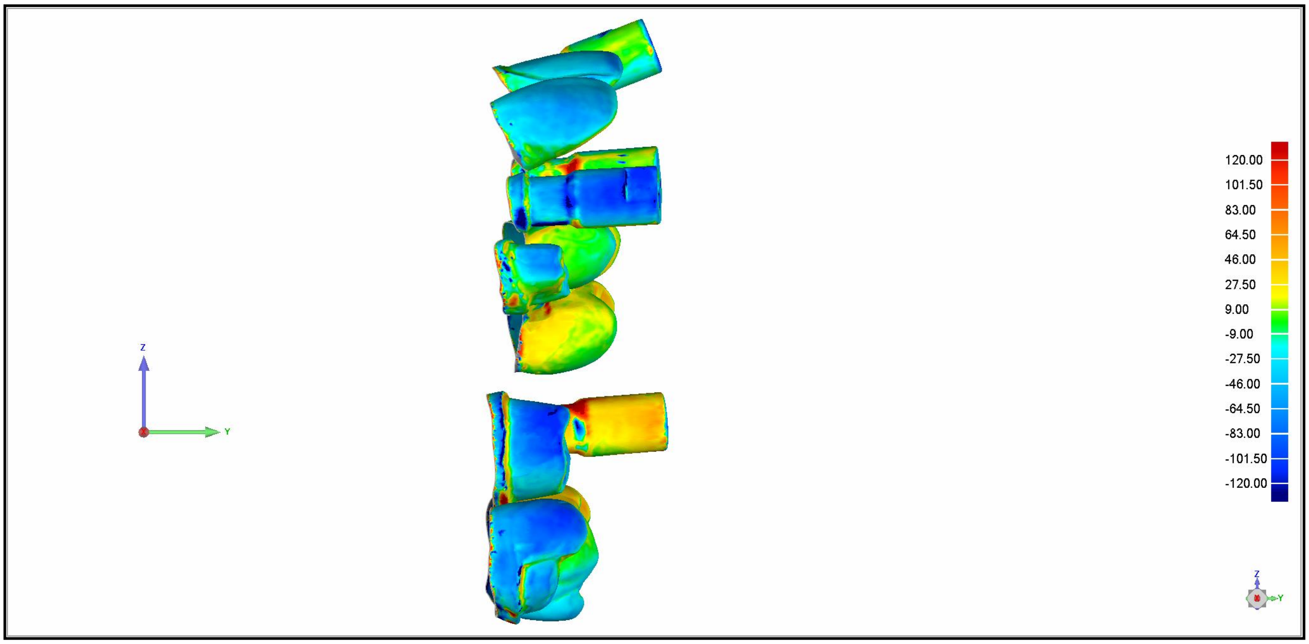

Predefinido: Superior

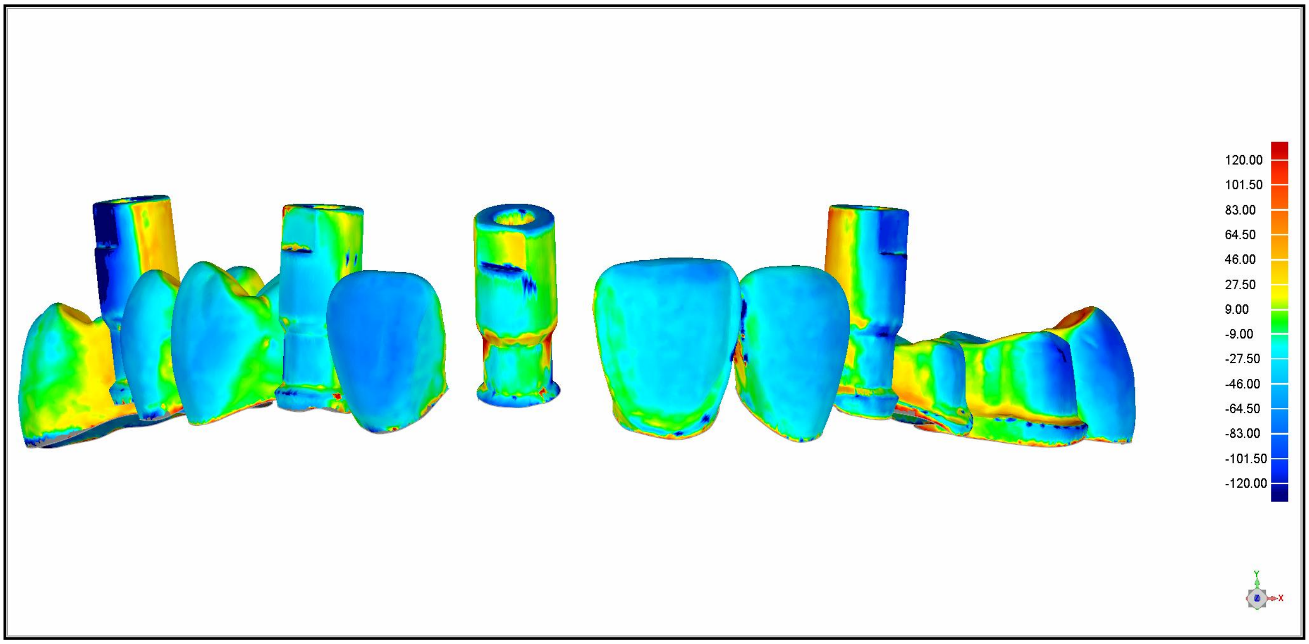

Predefinido: Inferior

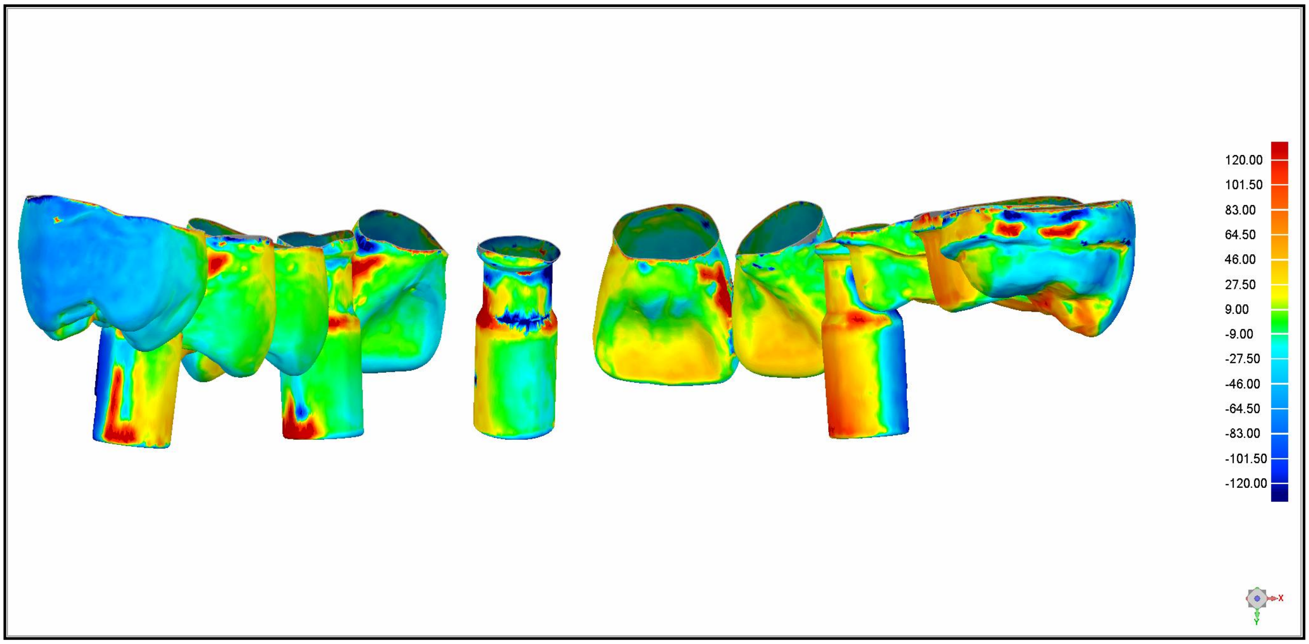

Ajuste de ubicación: Desviaciones superior e inferior

Unidades: u

Nombre	Desv	Estado	Superior Tol	Inferior Tol	Ref X	Ref Y	Ref Z	Radio	Desv X	Desv Y	Desv Z	Medido X	Medido Y	Medido Z	Dir. proy. X	Dir. proy. Y	Dir. proy. Z
Desv. inferior	-3144.68				-16498.53	29426.69	5746.14	n/a	-3023.99	-194.09	-840.72	-19522.52	29232.60	4905.43	0.96	0.06	0.27
Desv. superior	3116.20				-12601.26	29768.36	21377.11	n/a	1196.21	-1380.58	2524.64	-11405.05	28387.78	23901.75	0.38	-0.44	0.81
