# Supplementary material for: Accuracy of four digital scanners according to scanning strategy in complete-arch impressions
Source: PLoS One. 2018 Sep 13;13(9):e0202916. doi: 10.1371/journal.pone.0202916 (PMC6136706; doi:10.1371/journal.pone.0202916)
Supplement: S10 Table — Omnicam (scanning strategy B). (ZIP) [file pone.0202916.s010.zip › S10/OM7B.pdf]

3D Comparación Resultados

Modelo referencia	MRC
Modelo test	OM7B
Nº de puntos de datos	190465
# Aislados	668

Tipo tolerancia	3D desviación
Unidades	u
Máx. crítico	120.00
Máx. nominal	21.00
Mín. nominal	-21.00
Mín. crítico	-120.00

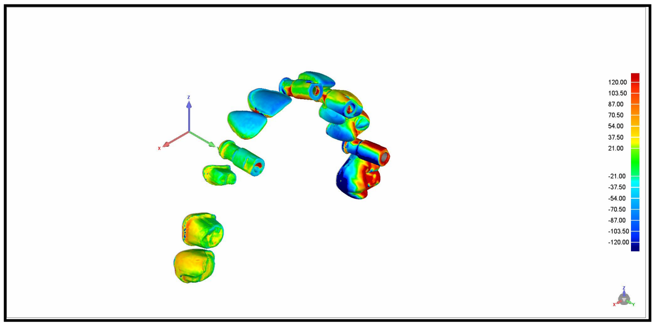

Desviación	
Desviación superior máx.	3092.85
Desviación inferior máx.	-3121.55
Desviación media	95.56 /-75.69
Desviación estándar	191.23

Distribución desviación

>=Min	<Max	# Puntos	%
-120.00	-103.50	1847	0.97
-103.50	-87.00	3072	1.61
-87.00	-70.50	6554	3.44
-70.50	-54.00	10566	5.55
-54.00	-37.50	12232	6.42
-37.50	-21.00	14602	7.67
-21.00	21.00	52428	27.53
21.00	37.50	18767	9.85
37.50	54.00	13868	7.28
54.00	70.50	9401	4.94
70.50	87.00	6204	3.26
87.00	103.50	4706	2.47
103.50	120.00	3421	1.80

Fuera del crítico superior		23506	12.34
Fuera del crítico inferior		9291	4.88

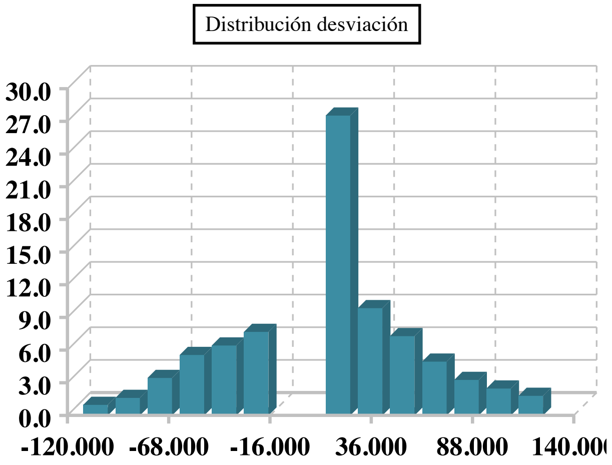

Desviaciones estándar

Distribución (+/-)	# Puntos	%
-6 * Desv. estánd.	804	0.42
-5 * Desv. estánd.	326	0.17
-4 * Desv. estánd.	551	0.29
-3 * Desv. estánd.	815	0.43
-2 * Desv. estánd.	3280	1.72
-1 * Desv. estánd.	105362	55.32
1 * Desv. estánd.	68248	35.83
2 * Desv. estánd.	8061	4.23
3 * Desv. estánd.	921	0.48
4 * Desv. estánd.	681	0.36
5 * Desv. estánd.	359	0.19
6 * Desv. estánd.	1057	0.55

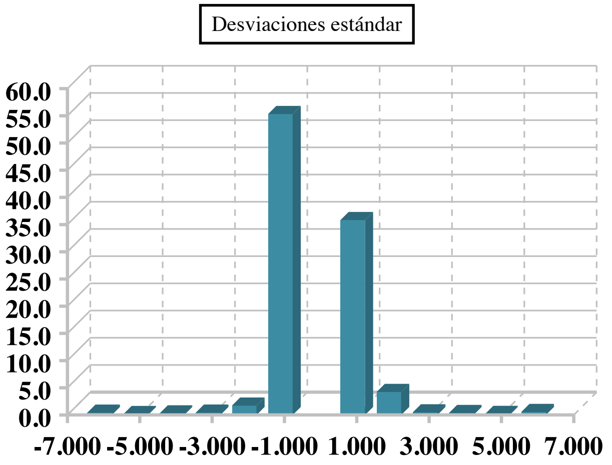

Predefinido: Isométrico

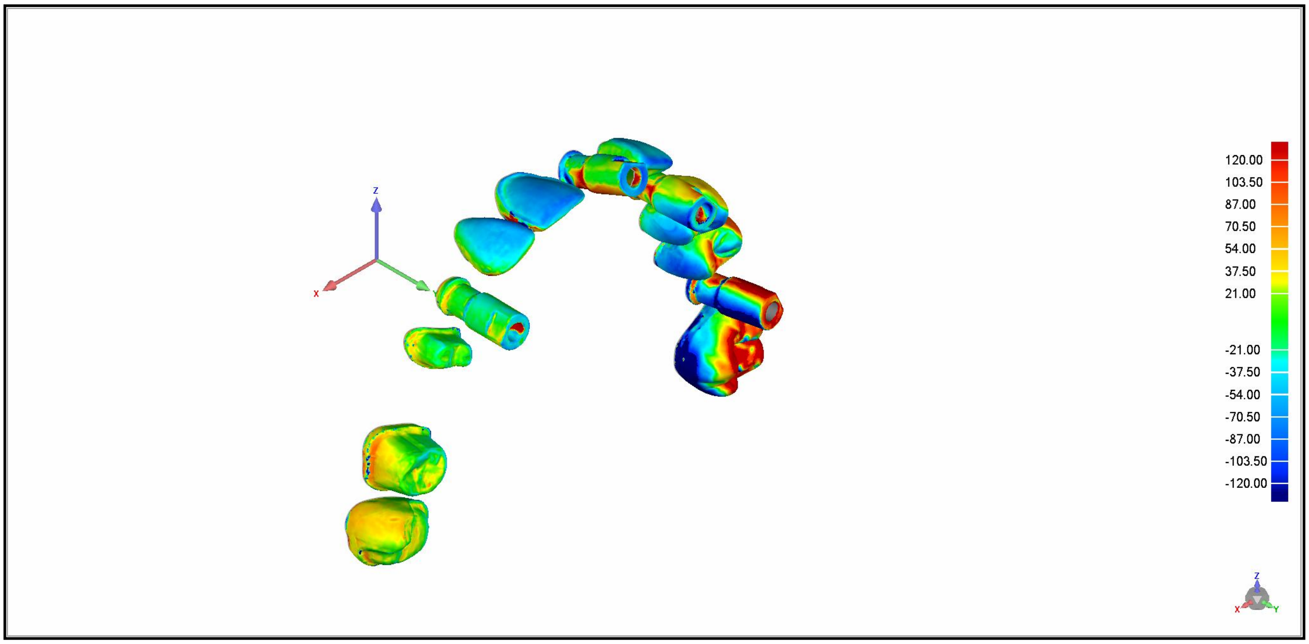

Predefinido: Frente

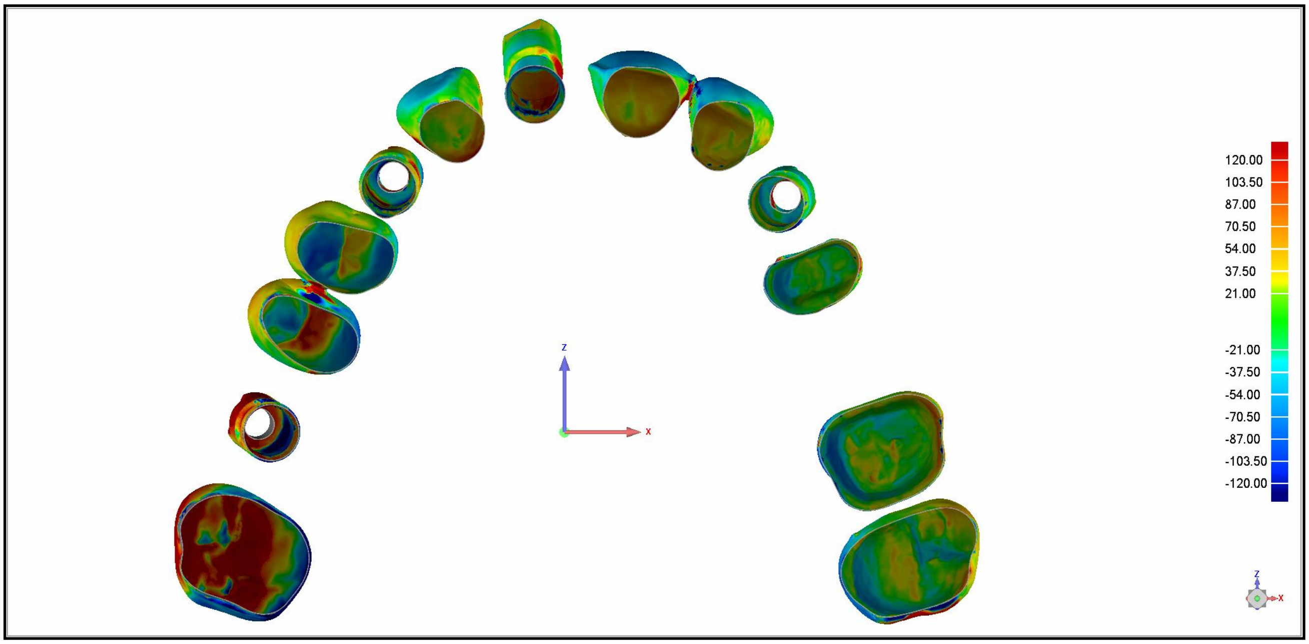

Predefinido: Atrás

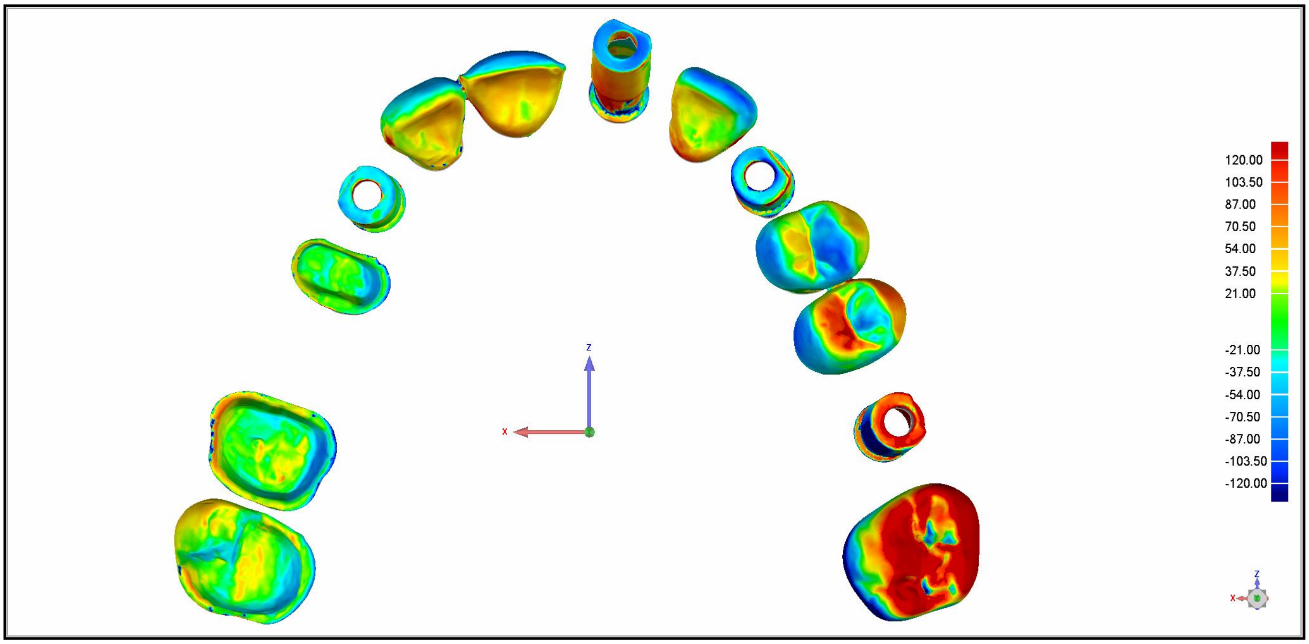

Predefinido: Izquierda

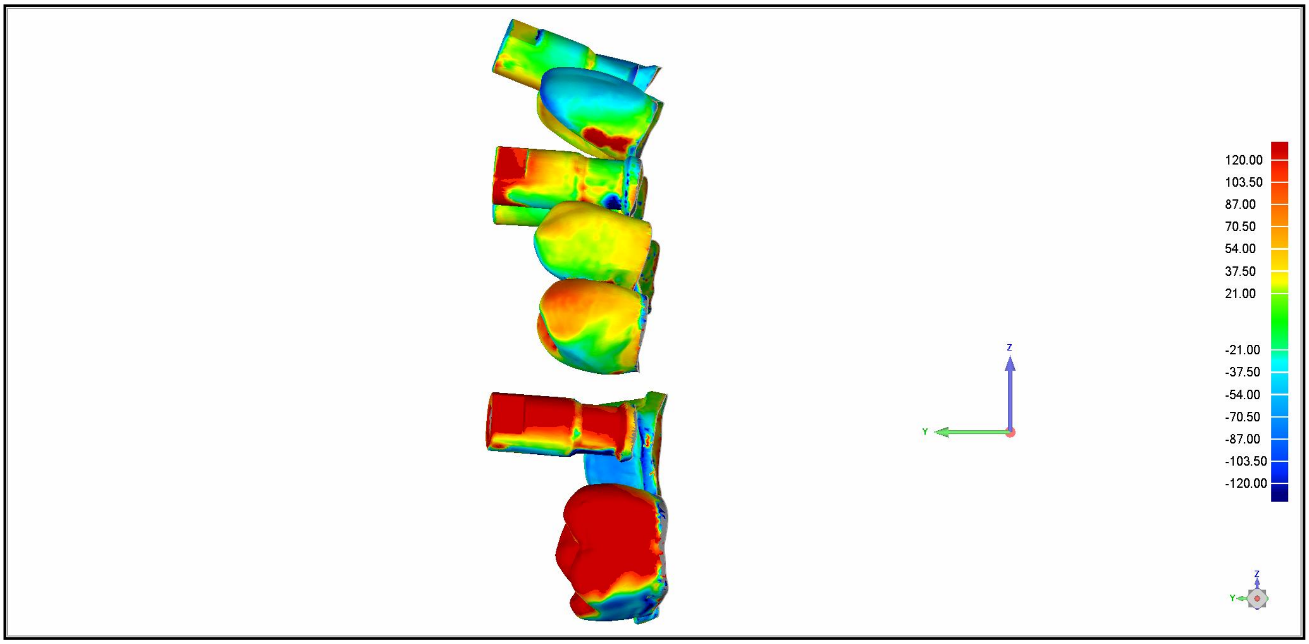

Predefinido: Derecha

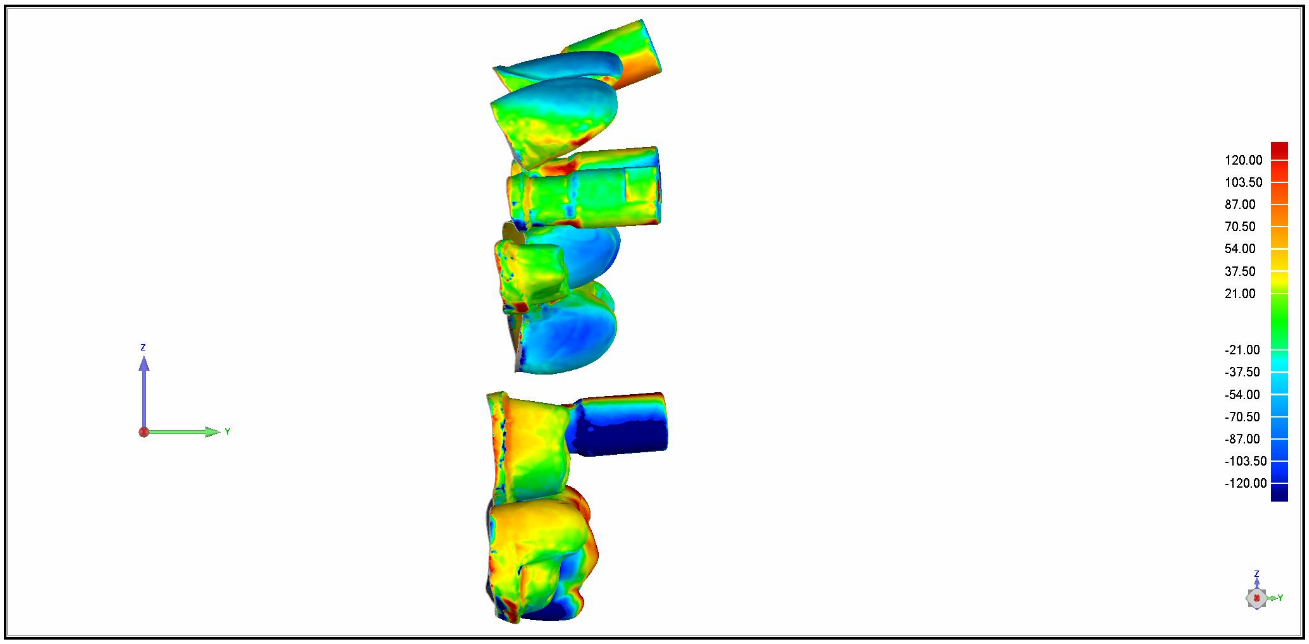

Predefinido: Superior

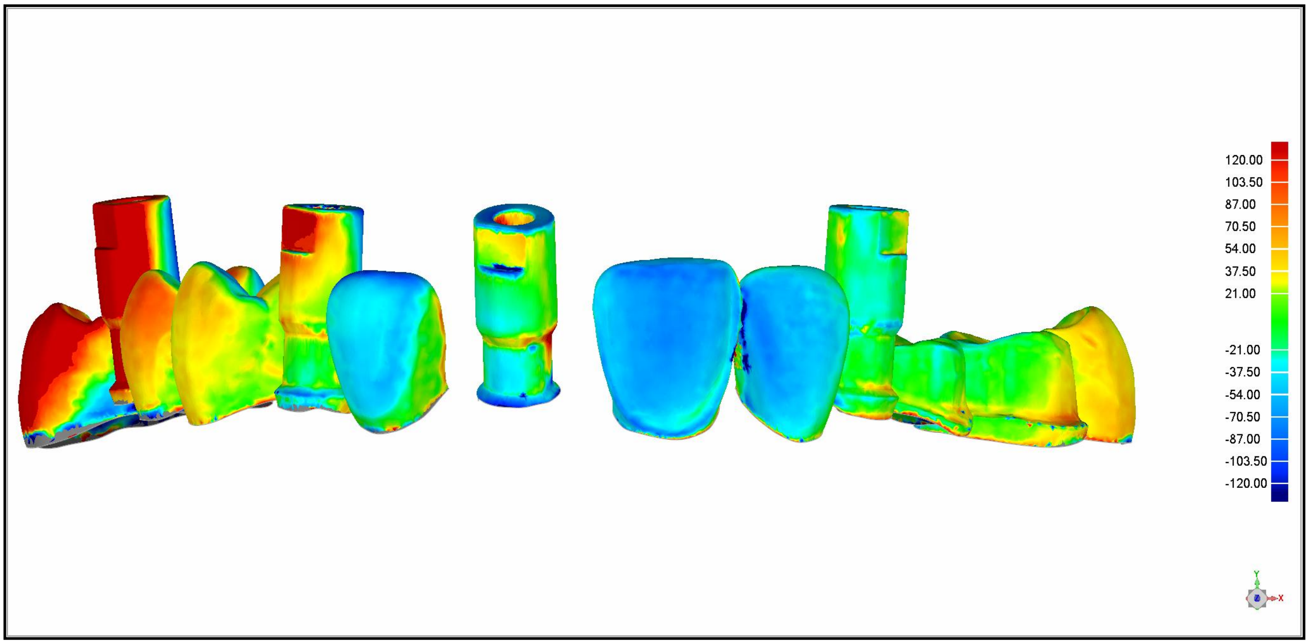

Predefinido: Inferior

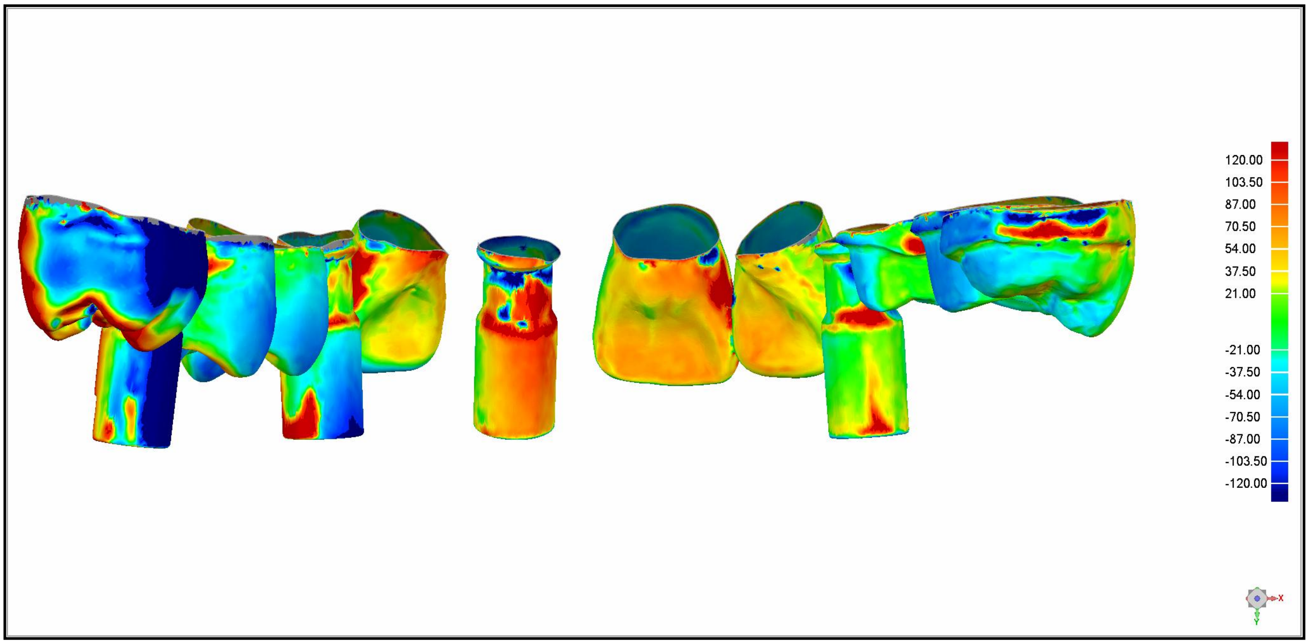

Ajuste de ubicación: Desviaciones superior e inferior

Unidades: u

Nombre	Desv	Estado	Superior Tol	Inferior Tol	Ref X	Ref Y	Ref Z	Radio	Desv X	Desv Y	Desv Z	Medido X	Medido Y	Medido Z	Dir. proy. X	Dir. proy. Y	Dir. proy. Z
Desv. inferior	-3121.55				-22607.19	28955.77	6808.03	n/a	-1036.64	-177.57	2939.03	-23643.83	28778.20	9747.06	0.33	0.06	-0.94
Desv. superior	3092.85				-20553.64	28741.29	-8096.88	n/a	-2342.70	178.20	2011.39	-22896.35	28919.48	-6085.49	-0.76	0.06	0.65
