# Supplementary material for: Accuracy of four digital scanners according to scanning strategy in complete-arch impressions
Source: PLoS One. 2018 Sep 13;13(9):e0202916. doi: 10.1371/journal.pone.0202916 (PMC6136706; doi:10.1371/journal.pone.0202916)
Supplement: S10 Table — Omnicam (scanning strategy B). (ZIP) [file pone.0202916.s010.zip › S10/OM10B.pdf]

3D Comparación Resultados

Modelo referencia	MRC
Modelo test	OM10B
Nº de puntos de datos	197606
# Aislados	631

Tipo tolerancia	3D desviación
Unidades	u
Máx. crítico	120.00
Máx. nominal	5.00
Mín. nominal	-5.00
Mín. crítico	-120.00

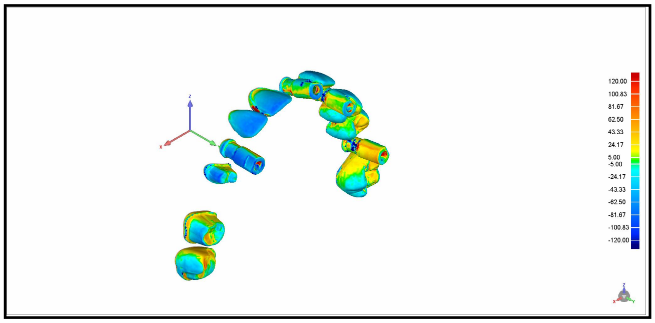

Desviación	
Desviación superior máx.	3146.97
Desviación inferior máx.	-3144.80
Desviación media	84.18 /-65.25
Desviación estándar	218.71

Distribución desviación

>=Min	<Max	# Puntos	%
-120.00	-100.83	836	0.42
-100.83	-81.67	1785	0.90
-81.67	-62.50	5461	2.76
-62.50	-43.33	13248	6.70
-43.33	-24.17	28085	14.21
-24.17	-5.00	38016	19.24
-5.00	5.00	19954	10.10
5.00	24.17	34689	17.55
24.17	43.33	19168	9.70
43.33	62.50	9067	4.59
62.50	81.67	4022	2.04
81.67	100.83	2629	1.33
100.83	120.00	2028	1.03

Fuera del crítico superior	12322	6.24
Fuera del crítico inferior	6296	3.19

Distribución desviación

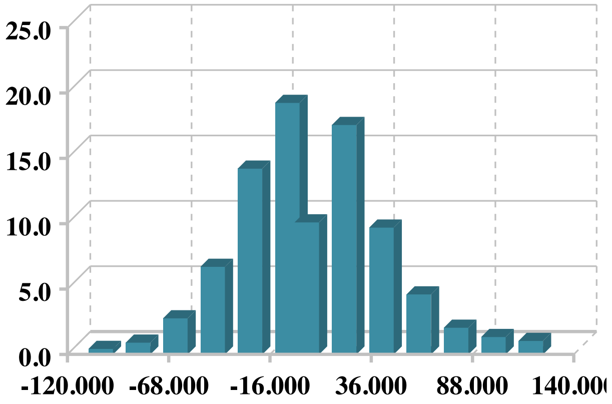

Desviaciones estándar

Distribución (+/-)	# Puntos	%
-6 * Desv. estánd.	1330	0.67
-5 * Desv. estánd.	368	0.19
-4 * Desv. estánd.	517	0.26
-3 * Desv. estánd.	726	0.37
-2 * Desv. estánd.	1476	0.75
-1 * Desv. estánd.	110657	56.00
1 * Desv. estánd.	75751	38.33
2 * Desv. estánd.	2637	1.33
3 * Desv. estánd.	1322	0.67
4 * Desv. estánd.	999	0.51
5 * Desv. estánd.	602	0.30
6 * Desv. estánd.	1221	0.62

Desviaciones estándar

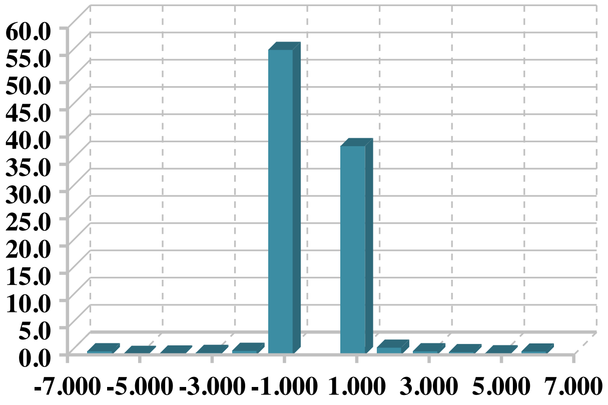

Predefinido: Isométrico

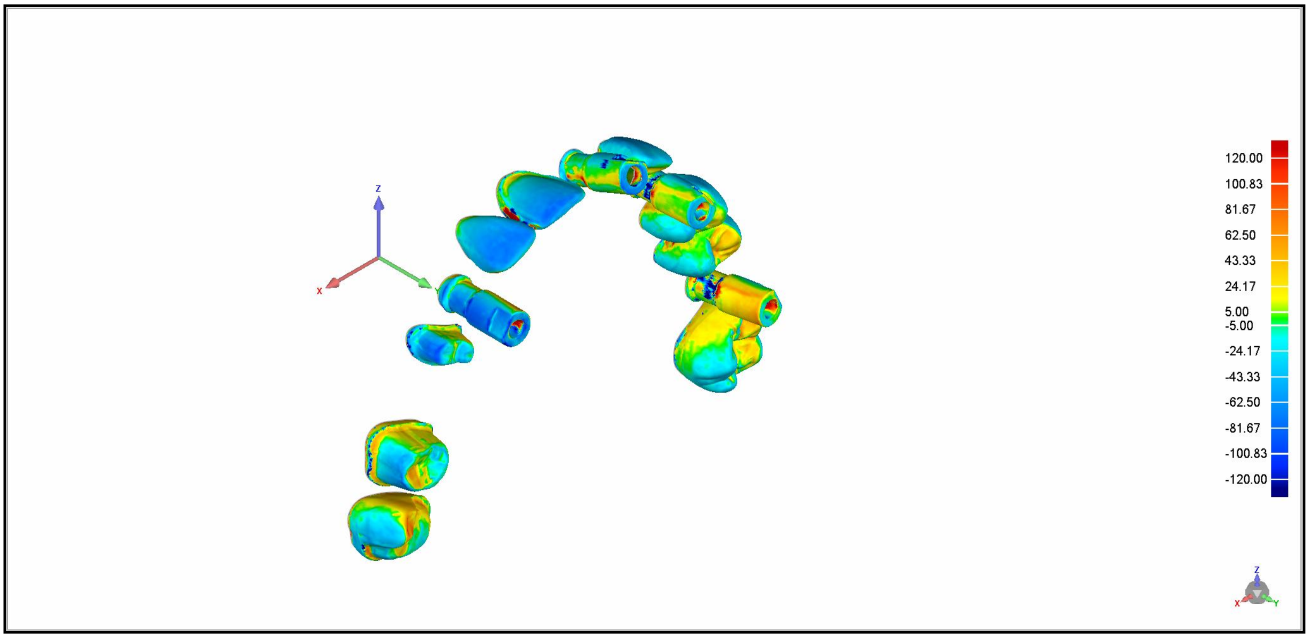

Predefinido: Frente

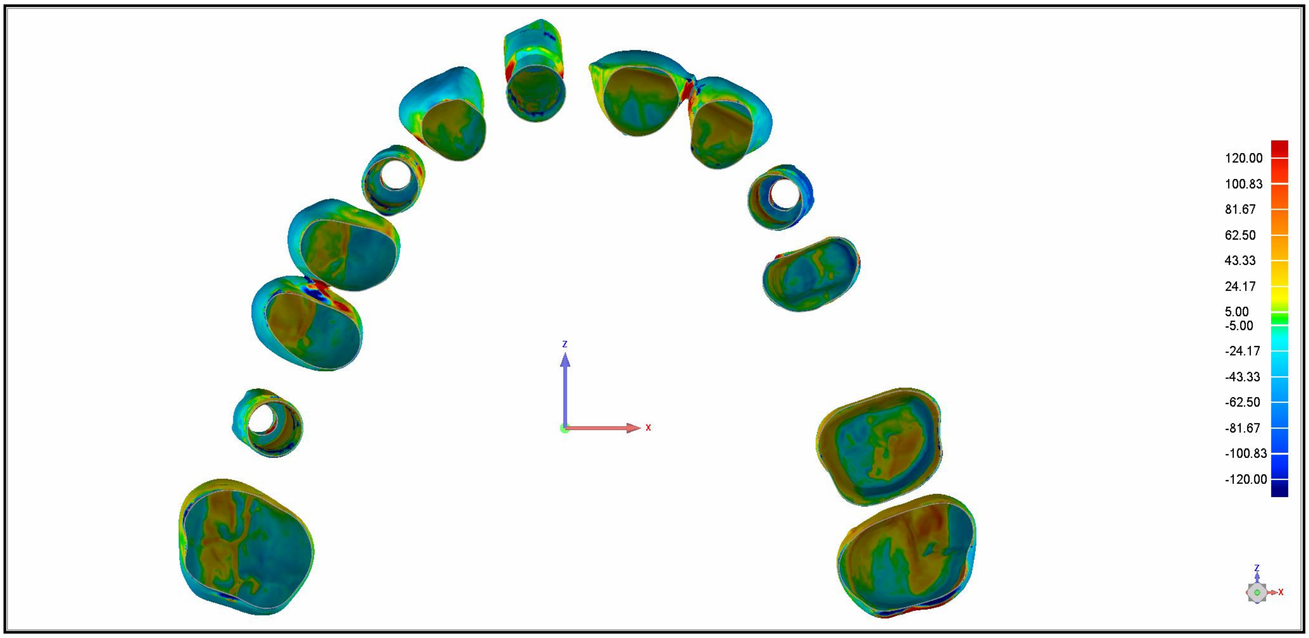

Predefinido: Atrás

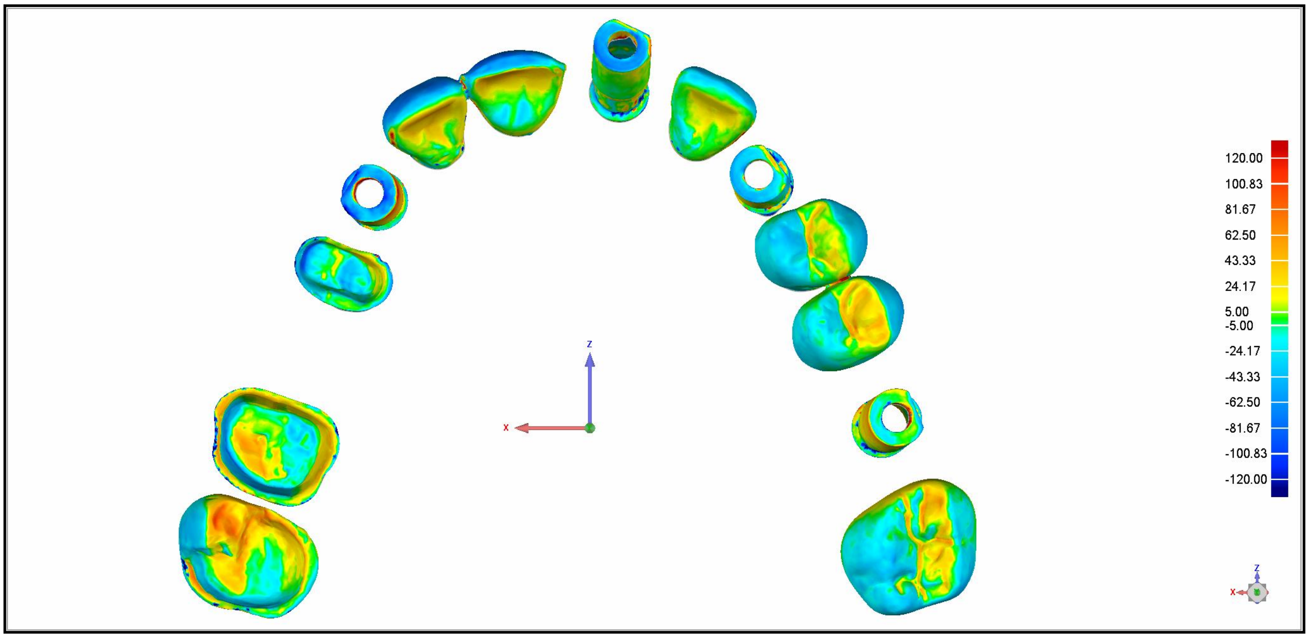

Predefinido: Izquierda

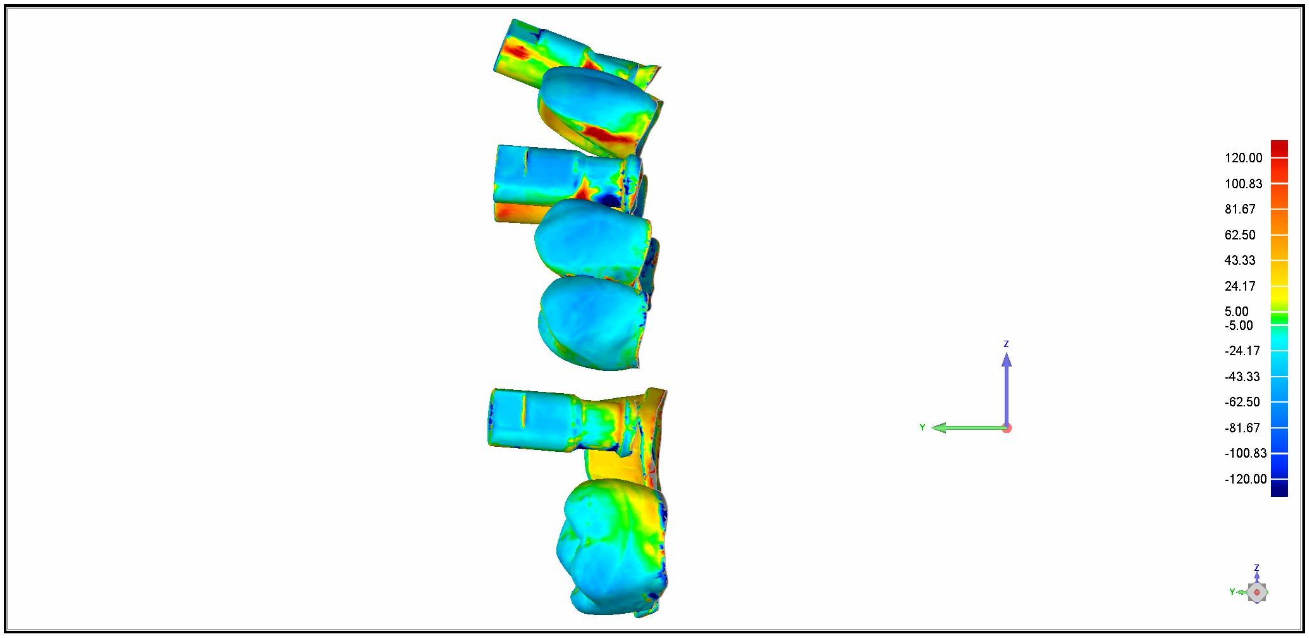

Predefinido: Derecha

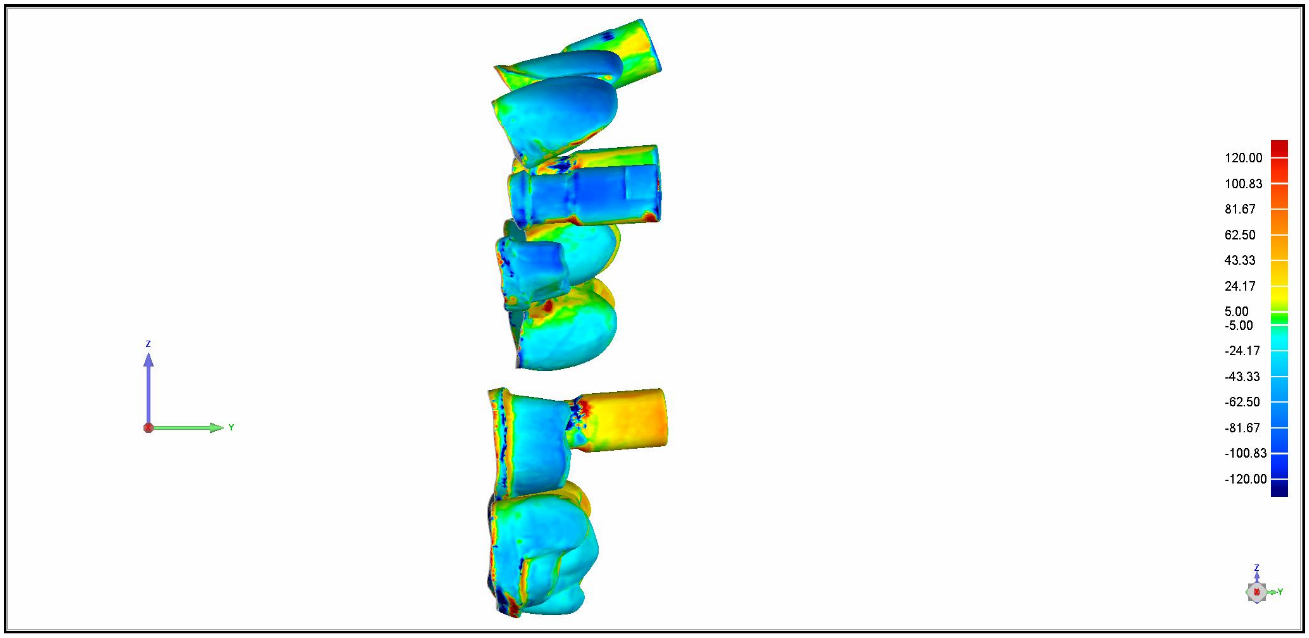

Predefinido: Superior

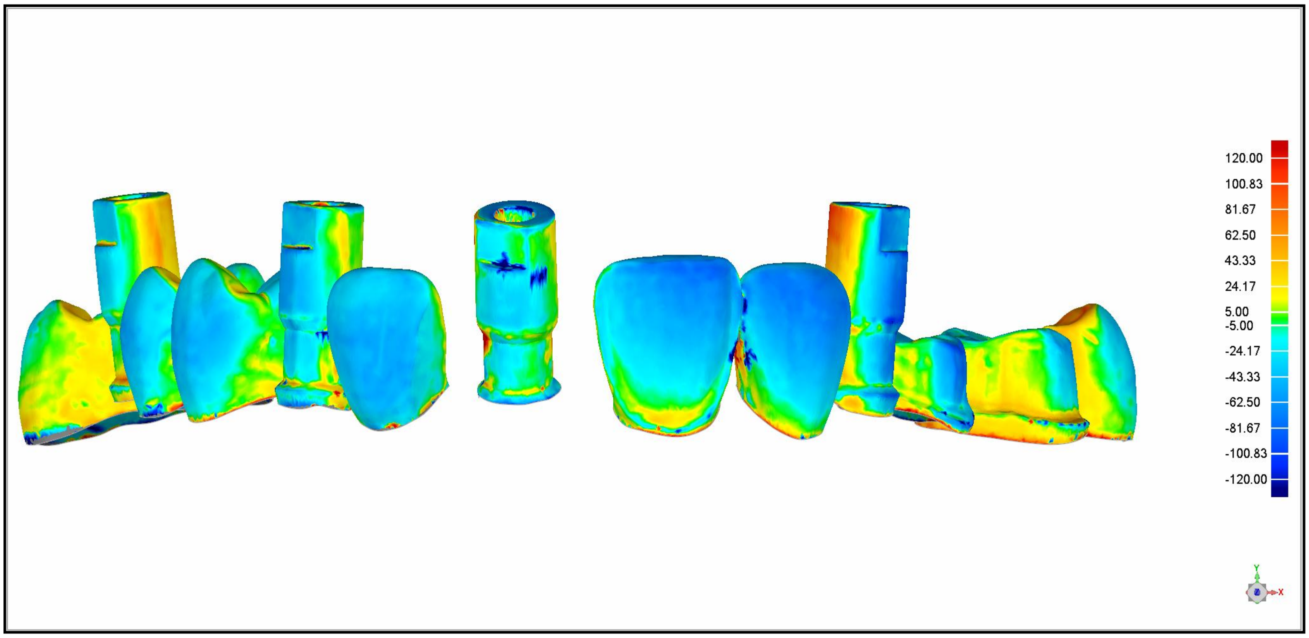

Predefinido: Inferior

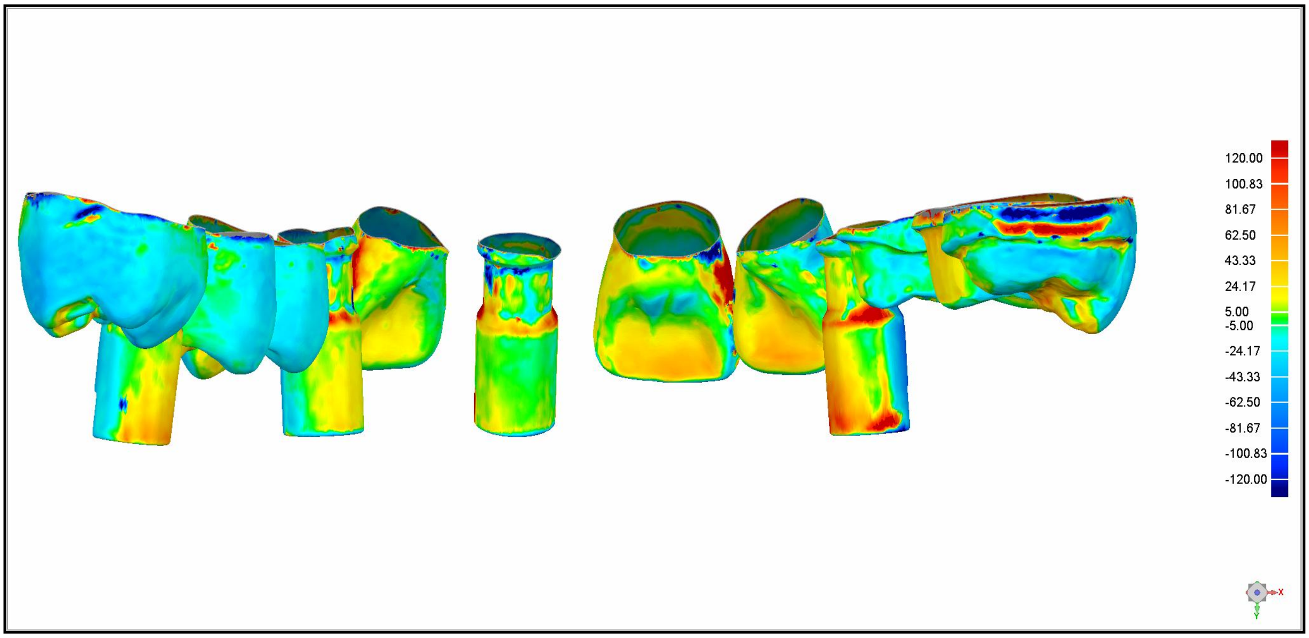

Ajuste de ubicación: Desviaciones superior e inferior

Unidades: u

Nombre	Desv	Estado	Superior Tol	Inferior Tol	Ref X	Ref Y	Ref Z	Radio	Desv X	Desv Y	Desv Z	Medido X	Medido Y	Medido Z	Dir. proy. X	Dir. proy. Y	Dir. proy. Z
Desv. inferior	-3144.80				-22607.19	28955.77	6808.03	n/a	-827.69	-388.61	3008.94	-23434.88	28567.16	9816.97	0.26	0.12	-0.96
Desv. superior	3146.97				-20553.64	28741.29	-8096.88	n/a	-2426.47	64.25	2002.88	-22980.11	28805.54	-6094.00	-0.77	0.02	0.64
