# Supplementary material for: Accuracy of four digital scanners according to scanning strategy in complete-arch impressions
Source: PLoS One. 2018 Sep 13;13(9):e0202916. doi: 10.1371/journal.pone.0202916 (PMC6136706; doi:10.1371/journal.pone.0202916)
Supplement: S10 Table — Omnicam (scanning strategy B). (ZIP) [file pone.0202916.s010.zip › S10/OM1B.pdf]

3D Comparación Resultados

Modelo referencia	MRC
Modelo test	OM1B
Nº de puntos de datos	196723
# Aislados	600

Tipo tolerancia	3D desviación
Unidades	u
Máx. crítico	120.00
Máx. nominal	13.00
Mín. nominal	-13.00
Mín. crítico	-120.00

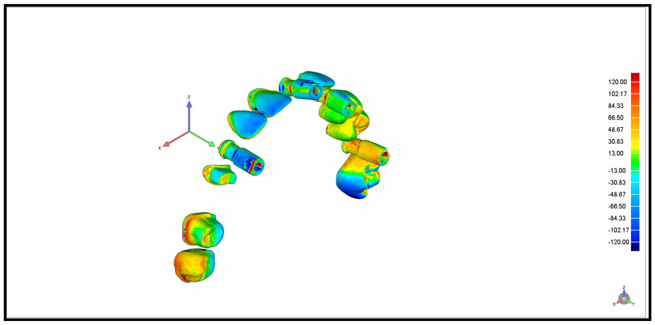

Desviación	
Desviación superior máx.	3097.97
Desviación inferior máx.	-3145.62
Desviación media	90.74 / -88.02
Desviación estándar	251.71

Distribución desviación

>=Min	<Max	# Puntos	%
-120.00	-102.17	1913	0.97
-102.17	-84.33	2974	1.51
-84.33	-66.50	5857	2.98
-66.50	-48.67	8816	4.48
-48.67	-30.83	13440	6.83
-30.83	-13.00	20765	10.56
-13.00	13.00	49223	25.02
13.00	30.83	25578	13.00
30.83	48.67	19157	9.74
48.67	66.50	11788	5.99
66.50	84.33	7885	4.01
84.33	102.17	4640	2.36
102.17	120.00	2777	1.41

Fuera del crítico superior	14574	7.41
Fuera del crítico inferior	7336	3.73

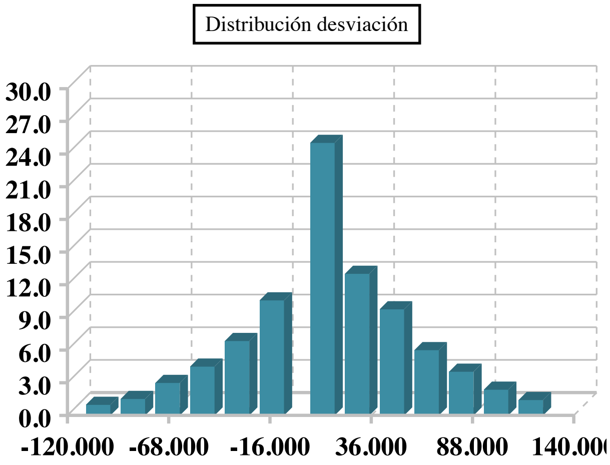

Desviaciones estándar

Distribución (+/-)	# Puntos	%
-6 * Desv. estánd.	1434	0.73
-5 * Desv. estánd.	546	0.28
-4 * Desv. estánd.	733	0.37
-3 * Desv. estánd.	617	0.31
-2 * Desv. estánd.	1188	0.60
-1 * Desv. estánd.	107289	54.54
1 * Desv. estánd.	78175	39.74
2 * Desv. estánd.	2428	1.23
3 * Desv. estánd.	1435	0.73
4 * Desv. estánd.	1059	0.54
5 * Desv. estánd.	764	0.39
6 * Desv. estánd.	1055	0.54

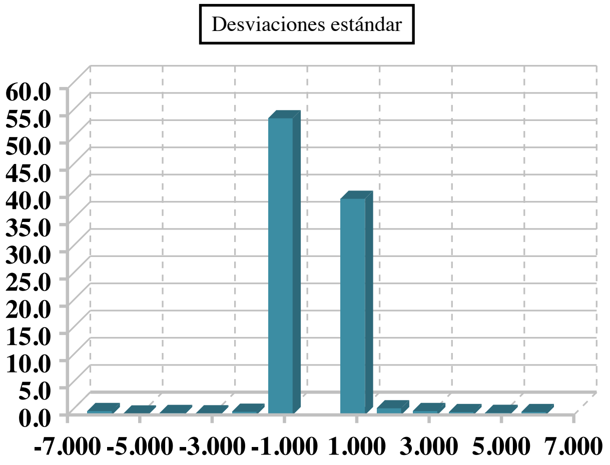

Predefinido: Isométrico

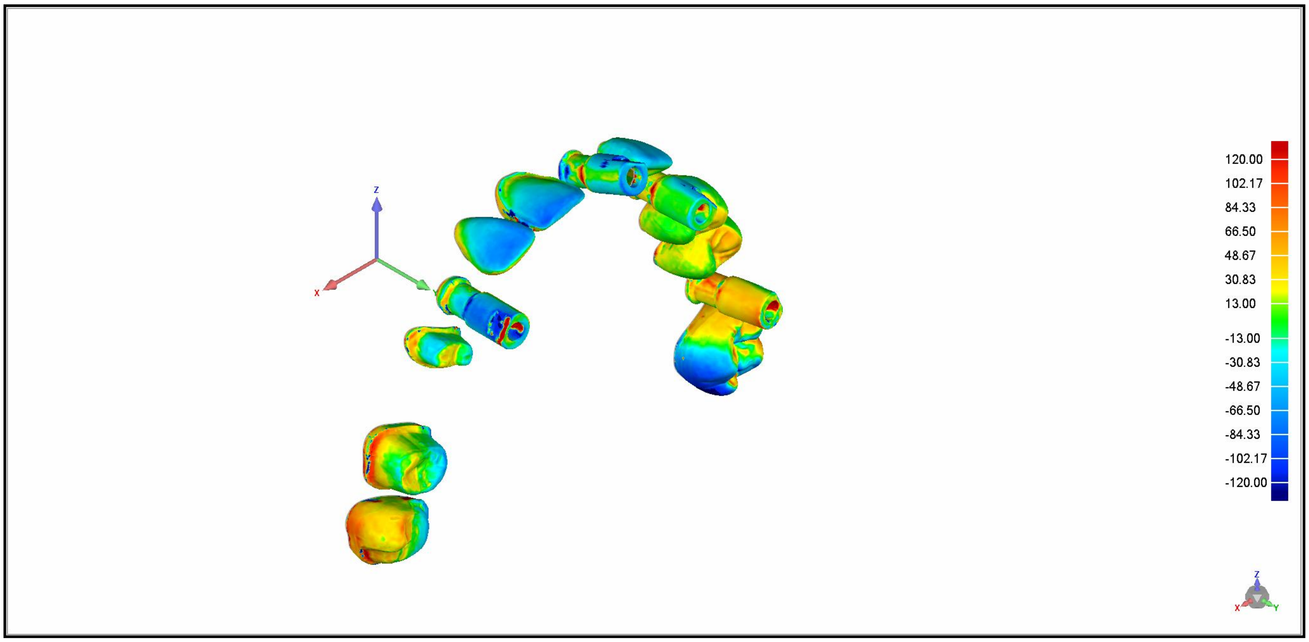

Predefinido: Frente

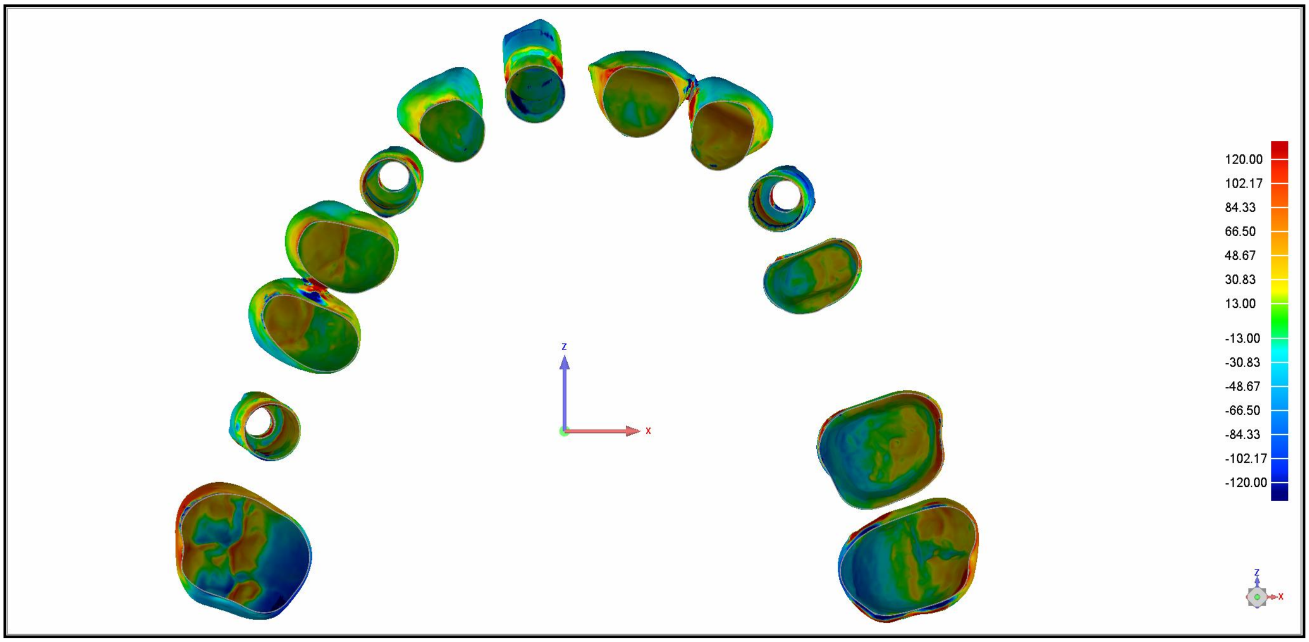

Predefinido: Atrás

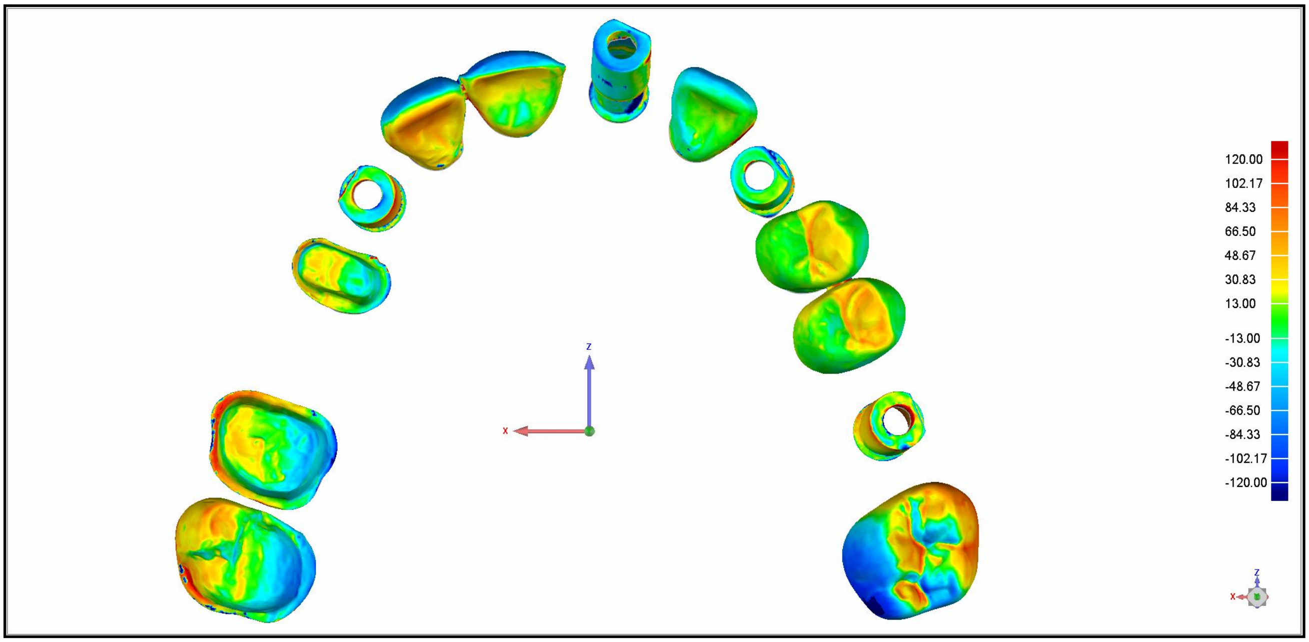

Predefinido: Izquierda

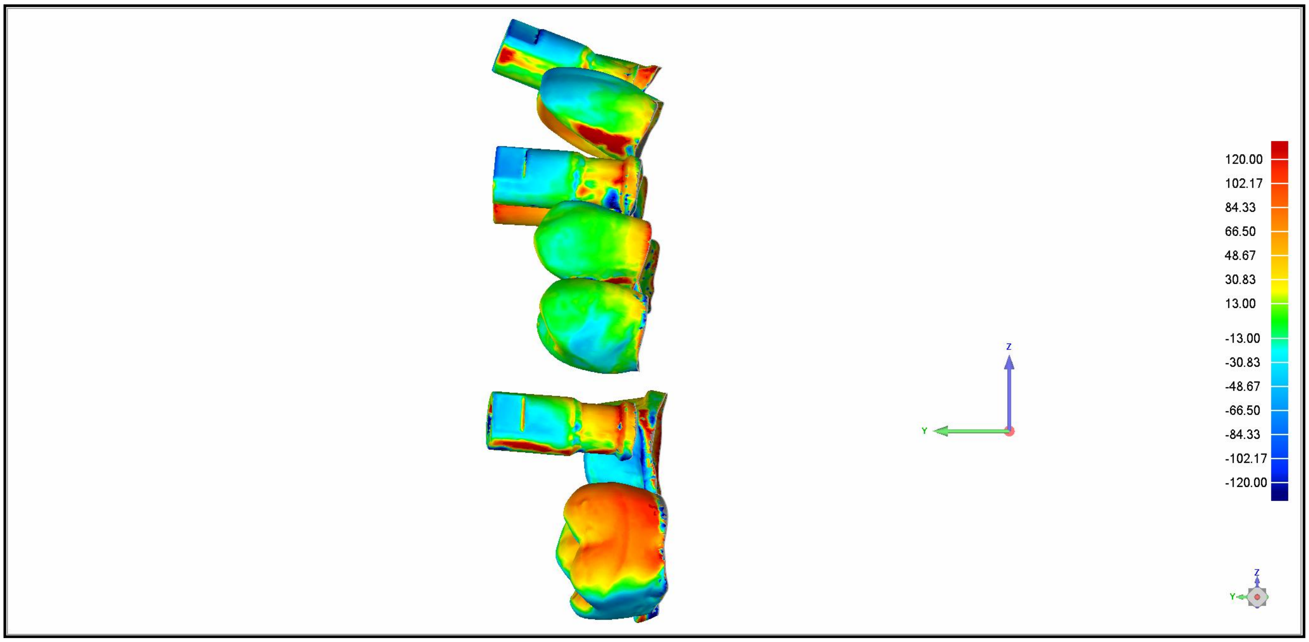

Predefinido: Derecha

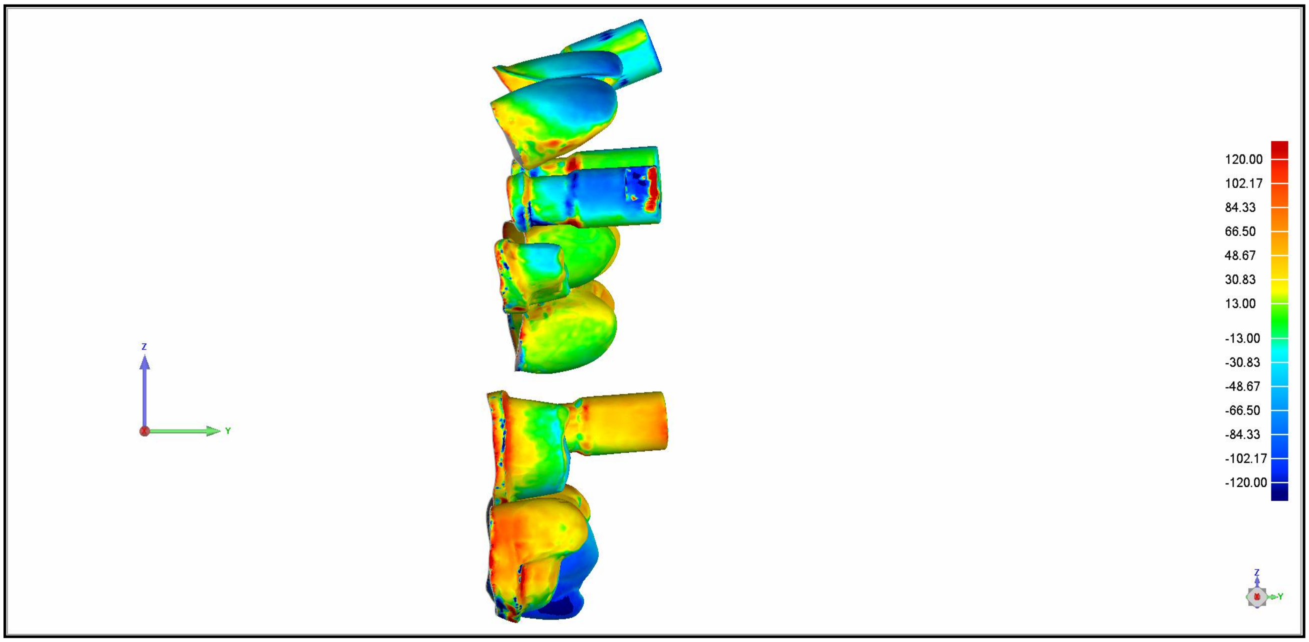

Predefinido: Superior

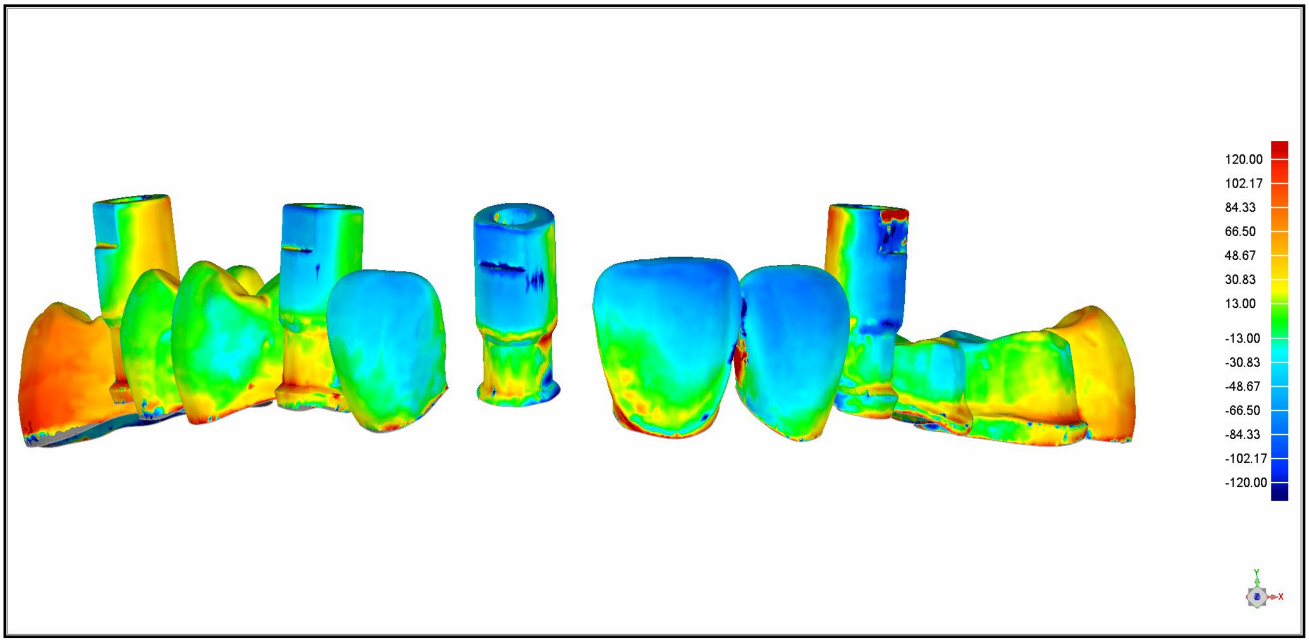

Predefinido: Inferior

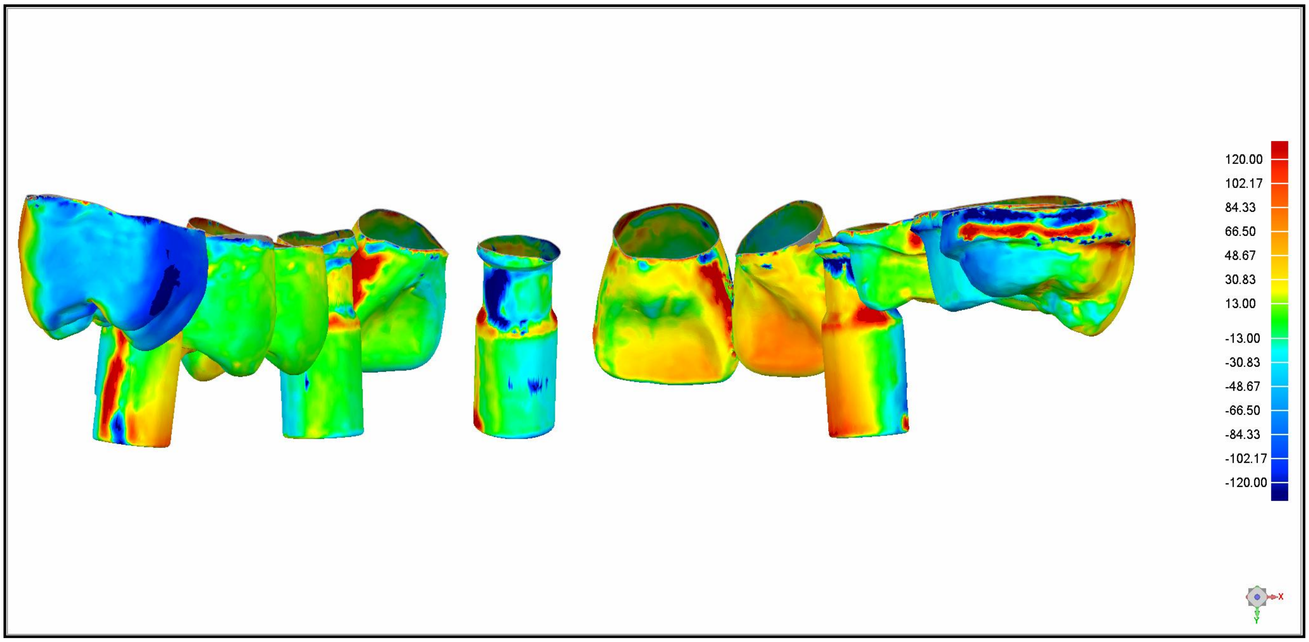

Ajuste de ubicación: Desviaciones superior e inferior

Unidades: u

Nombre	Desv	Estado	Superior Tol	Inferior Tol	Ref X	Ref Y	Ref Z	Radio	Desv X	Desv Y	Desv Z	Medido X	Medido Y	Medido Z	Dir. proy. X	Dir. proy. Y	Dir. proy. Z
Desv. inferior	-3145.62				-22607.19	28955.77	6808.03	n/a	-939.37	-289.00	2988.15	-23546.56	28666.77	9796.18	0.30	0.09	-0.95
Desv. superior	3097.97				-12601.26	29768.36	21377.11	n/a	1095.68	-1347.88	2565.17	-11505.58	28420.48	23942.28	0.35	-0.44	0.83
