# Supplementary material for: Accuracy of four digital scanners according to scanning strategy in complete-arch impressions
Source: PLoS One. 2018 Sep 13;13(9):e0202916. doi: 10.1371/journal.pone.0202916 (PMC6136706; doi:10.1371/journal.pone.0202916)
Supplement: S11 Table — Omnicam (scanning strategy C). (ZIP) [file pone.0202916.s011.zip › S11/OM2C.pdf]

3D Comparación Resultados

Modelo referencia	MRC
Modelo test	OM2C
Nº de puntos de datos	199383
# Aislados	794

Tipo tolerancia	3D desviación
Unidades	u
Máx. crítico	120.00
Máx. nominal	18.00
Mín. nominal	-18.00
Mín. crítico	-120.00

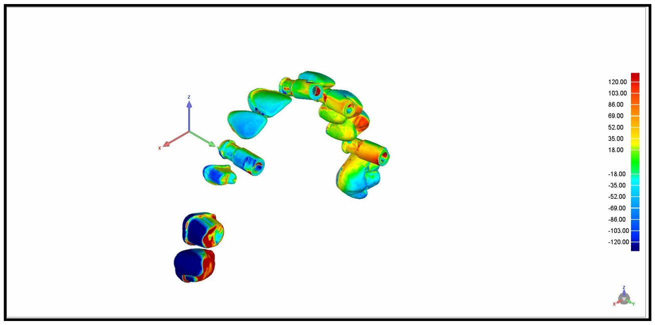

Desviación	
Desviación superior máx.	3134.81
Desviación inferior máx.	-3147.44
Desviación media	121.90 /-103.40
Desviación estándar	273.72

Distribución desviación

>=Min	<Max	# Puntos	%
-120.00	-103.00	1485	0.74
-103.00	-86.00	1998	1.00
-86.00	-69.00	2638	1.32
-69.00	-52.00	5622	2.82
-52.00	-35.00	11975	6.01
-35.00	-18.00	24210	12.14
-18.00	18.00	56843	28.51
18.00	35.00	19844	9.95
35.00	52.00	11749	5.89
52.00	69.00	8769	4.40
69.00	86.00	6513	3.27
86.00	103.00	4244	2.13
103.00	120.00	3049	1.53

Fuera del crítico superior	26112	13.10
Fuera del crítico inferior	14332	7.19

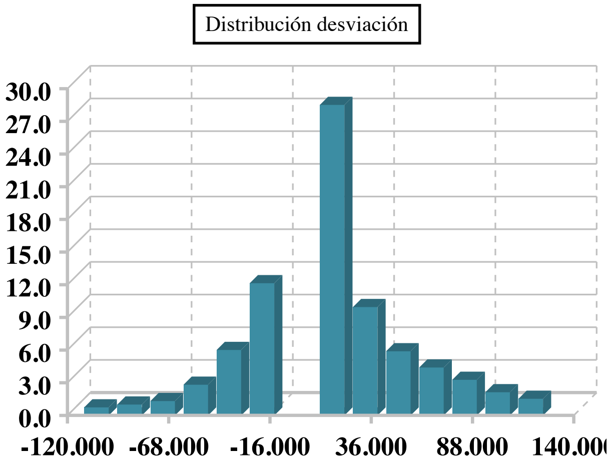

Desviaciones estándar

Distribución (+/-)	# Puntos	%
-6 * Desv. estánd.	1364	0.68
-5 * Desv. estánd.	837	0.42
-4 * Desv. estánd.	834	0.42
-3 * Desv. estánd.	1213	0.61
-2 * Desv. estánd.	3260	1.64
-1 * Desv. estánd.	112134	56.24
1 * Desv. estánd.	70387	35.30
2 * Desv. estánd.	4712	2.36
3 * Desv. estánd.	1432	0.72
4 * Desv. estánd.	1080	0.54
5 * Desv. estánd.	1169	0.59
6 * Desv. estánd.	961	0.48

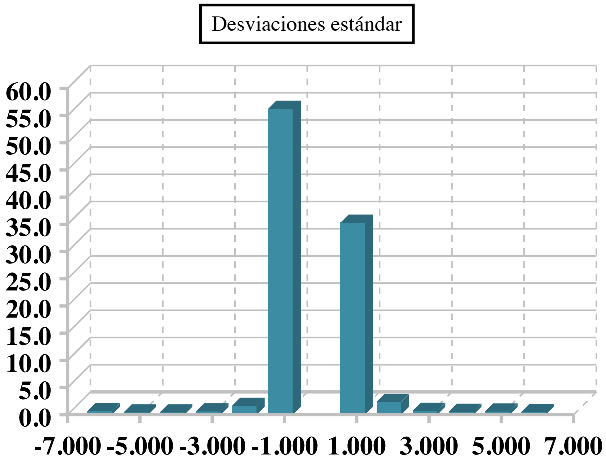

Predefinido: Isométrico

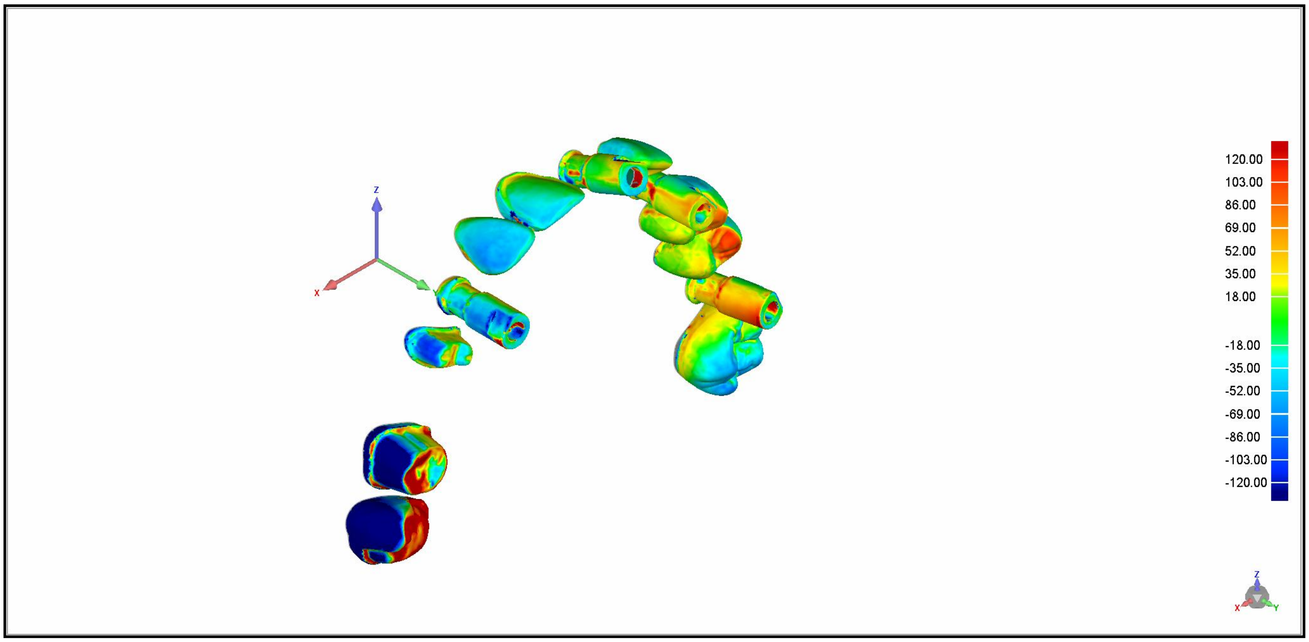

Predefinido: Frente

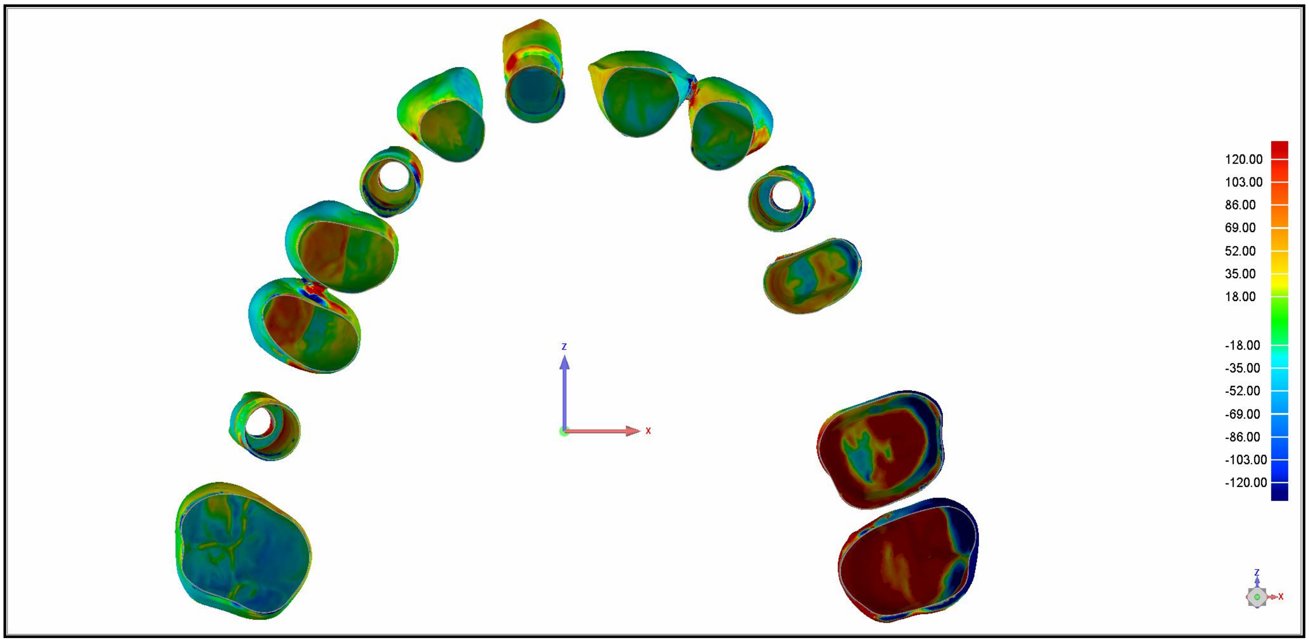

Predefinido: Atrás

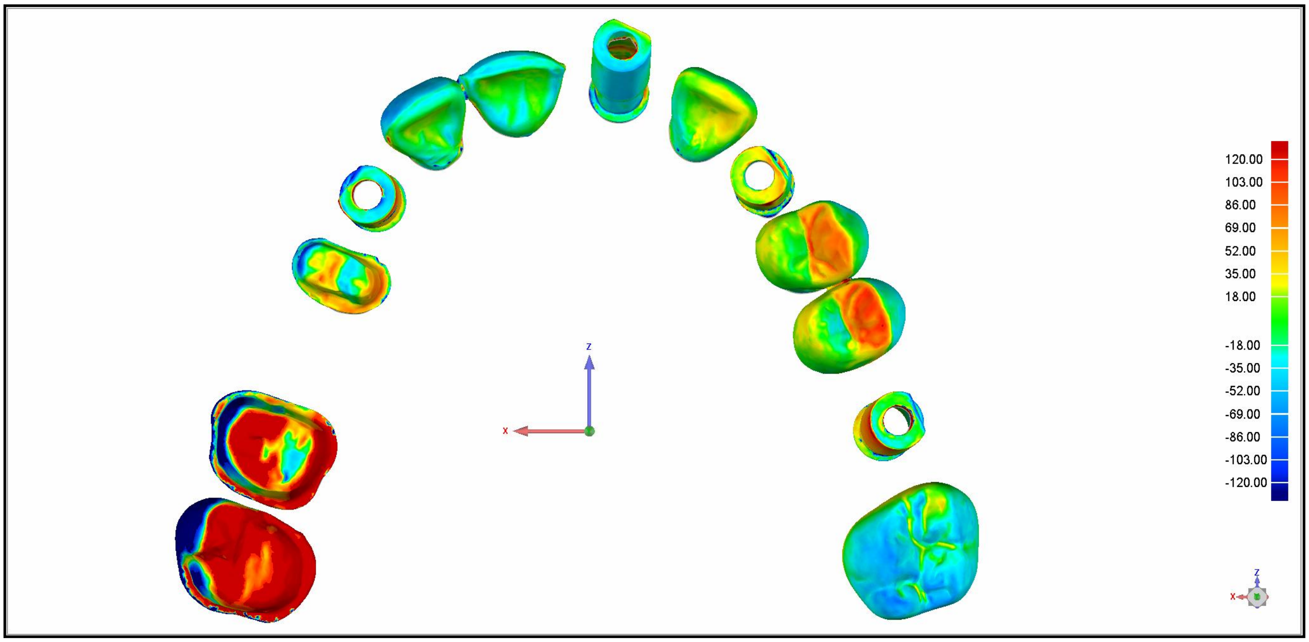

Predefinido: Izquierda

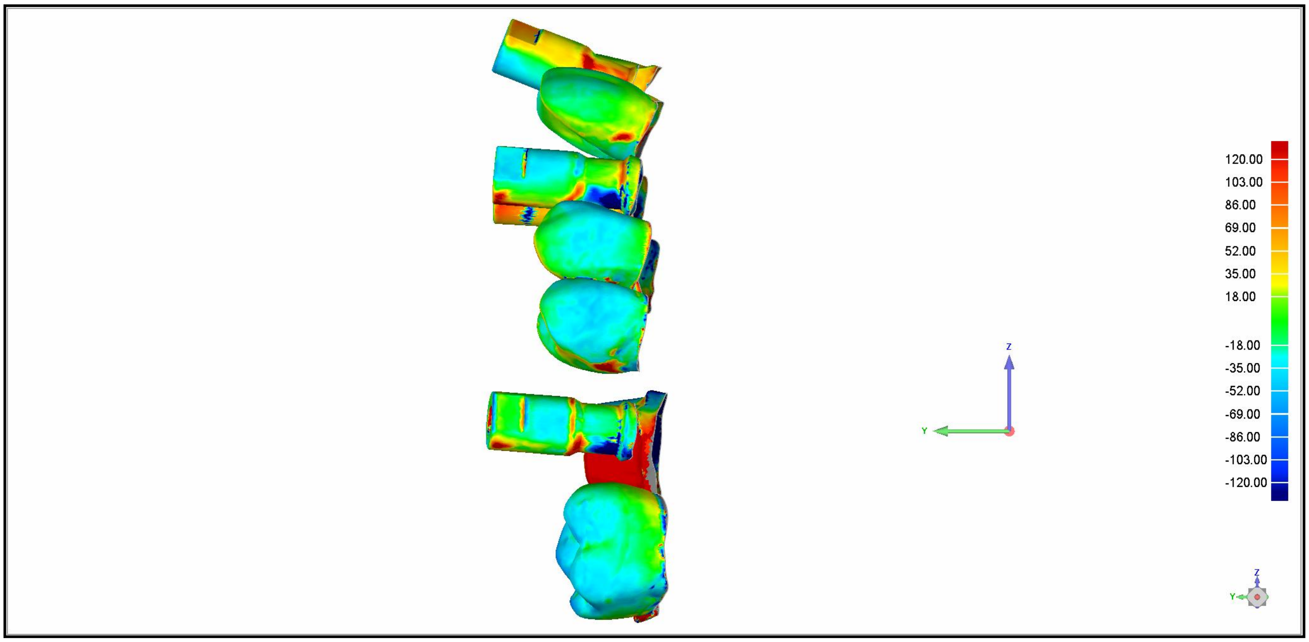

Predefinido: Derecha

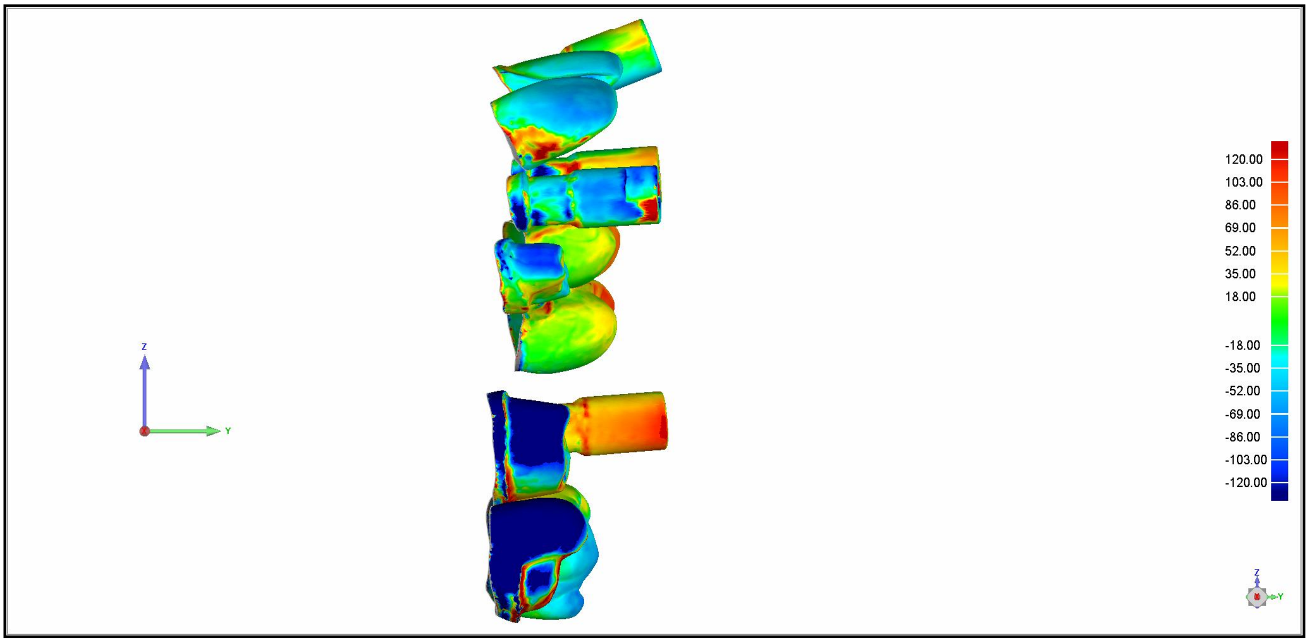

Predefinido: Superior

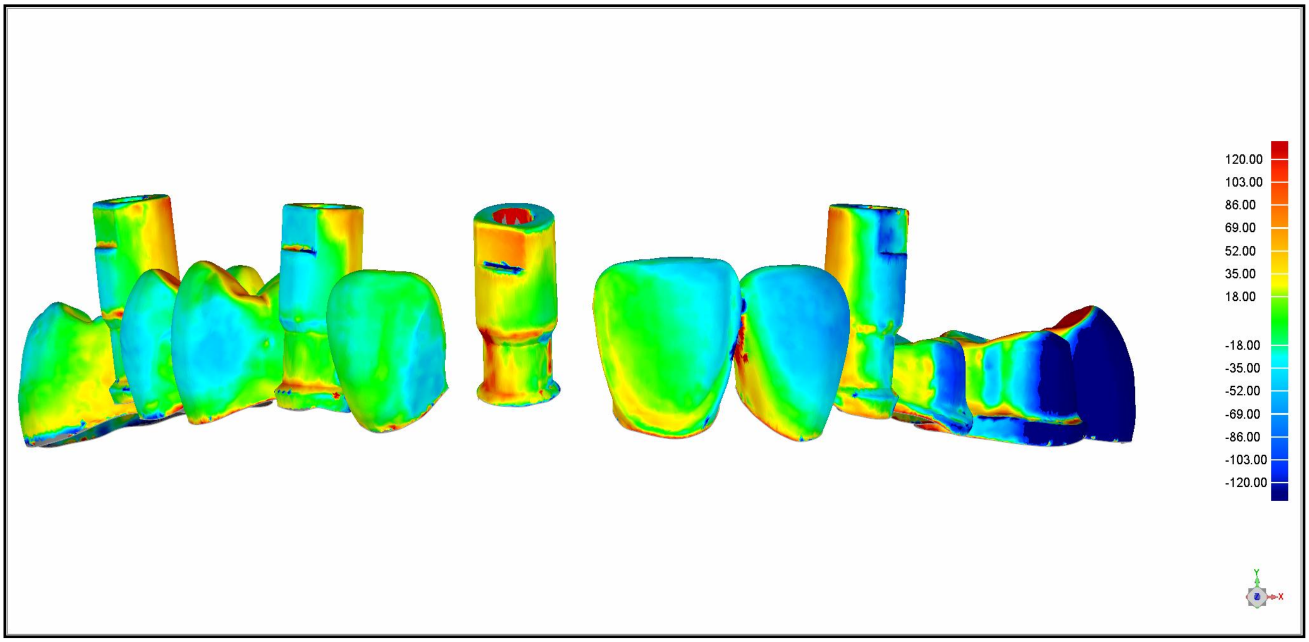

Predefinido: Inferior

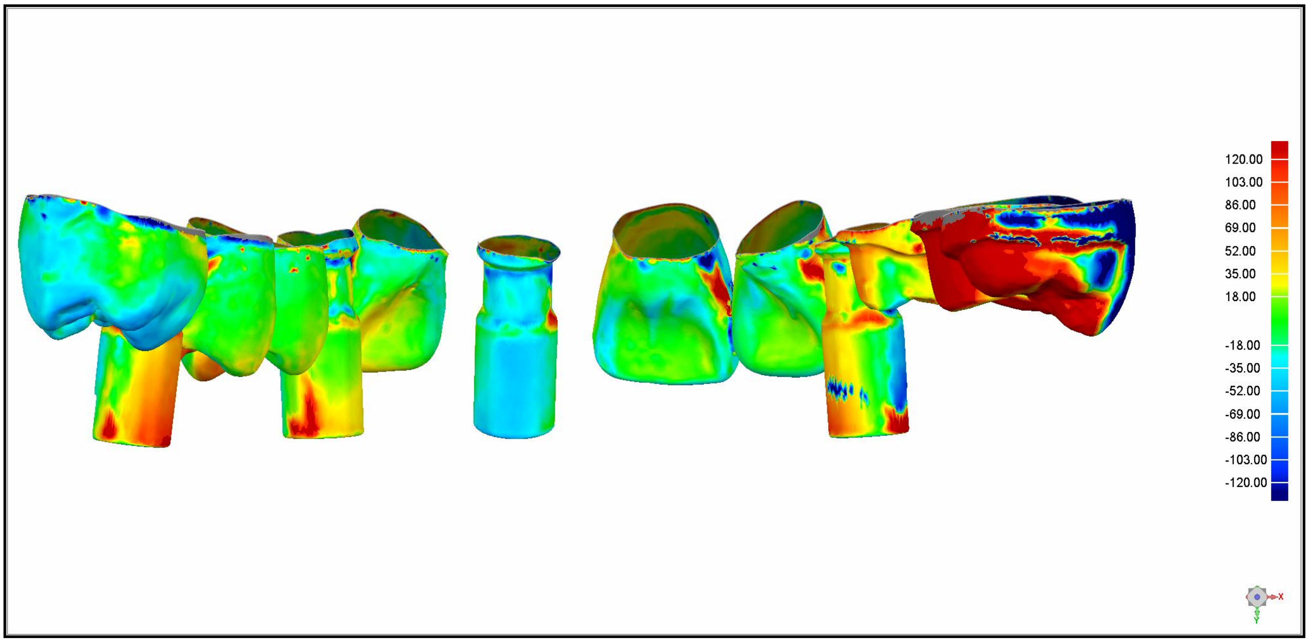

Ajuste de ubicación: Desviaciones superior e inferior

Unidades: u

Nombre	Desv	Estado	Superior Tol	Inferior Tol	Ref X	Ref Y	Ref Z	Radio	Desv X	Desv Y	Desv Z	Medido X	Medido Y	Medido Z	Dir. proy. X	Dir. proy. Y	Dir. proy. Z
Desv. inferior	-3147.44				-22607.19	28955.77	6808.03	n/a	-848.98	-494.75	2990.12	-23456.17	28461.01	9798.16	0.27	0.16	-0.95
Desv. superior	3134.81				-12553.67	29785.74	21343.12	n/a	1050.06	-1412.55	2594.05	-11503.61	28373.19	23937.17	0.33	-0.45	0.83
