# Supplementary material for: Accuracy of four digital scanners according to scanning strategy in complete-arch impressions
Source: PLoS One. 2018 Sep 13;13(9):e0202916. doi: 10.1371/journal.pone.0202916 (PMC6136706; doi:10.1371/journal.pone.0202916)
Supplement: S11 Table — Omnicam (scanning strategy C). (ZIP) [file pone.0202916.s011.zip › S11/OM3C.pdf]

3D Comparación Resultados

Modelo referencia	MRC
Modelo test	OM3C
Nº de puntos de datos	199430
# Aislados	634

Tipo tolerancia	3D desviación
Unidades	u
Máx. crítico	120.00
Máx. nominal	1.00
Mín. nominal	-1.00
Mín. crítico	-120.00

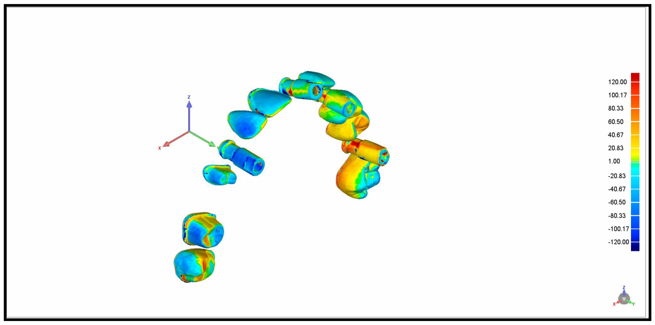

Desviación	
Desviación superior máx.	3140.05
Desviación inferior máx.	-3155.28
Desviación media	93.59 /-91.96
Desviación estándar	277.08

Distribución desviación

>=Min	<Max	# Puntos	%
-120.00	-100.17	985	0.49
-100.17	-80.33	2239	1.12
-80.33	-60.50	5010	2.51
-60.50	-40.67	12300	6.17
-40.67	-20.83	26384	13.23
-20.83	-1.00	41653	20.89
-1.00	1.00	4040	2.03
1.00	20.83	37624	18.87
20.83	40.67	25280	12.68
40.67	60.50	11309	5.67
60.50	80.33	6124	3.07
80.33	100.17	3301	1.66
100.17	120.00	2045	1.03

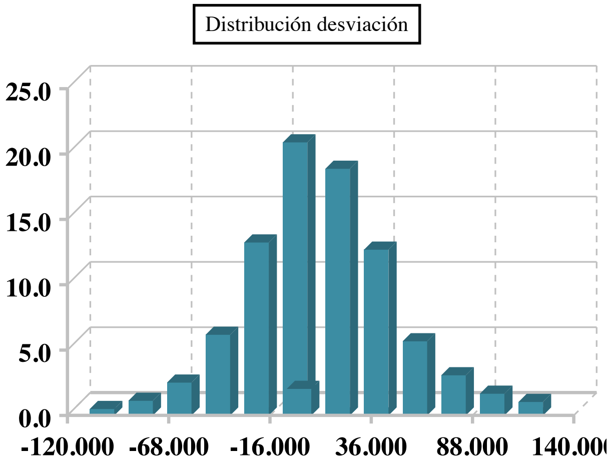

Fuera del crítico superior		12884	6.46
Fuera del crítico inferior		8252	4.14

Desviaciones estándar

Distribución (+/-)	# Puntos	%
-6 * Desv. estánd.	1427	0.72
-5 * Desv. estánd.	741	0.37
-4 * Desv. estánd.	1078	0.54
-3 * Desv. estánd.	1359	0.68
-2 * Desv. estánd.	1379	0.69
-1 * Desv. estánd.	95982	48.13
1 * Desv. estánd.	91163	45.71
2 * Desv. estánd.	1705	0.85
3 * Desv. estánd.	1423	0.71
4 * Desv. estánd.	1461	0.73
5 * Desv. estánd.	1012	0.51
6 * Desv. estánd.	700	0.35

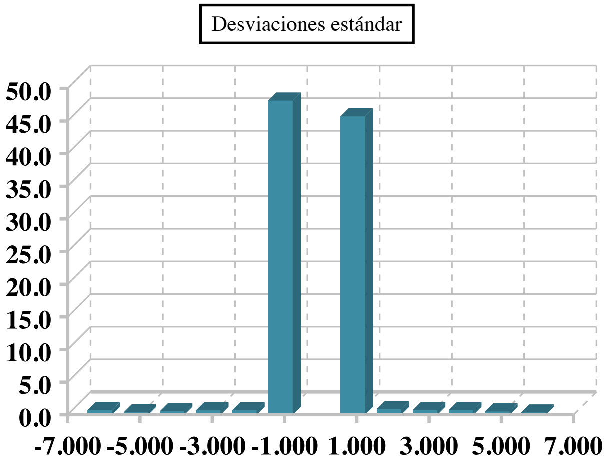

Predefinido: Isométrico

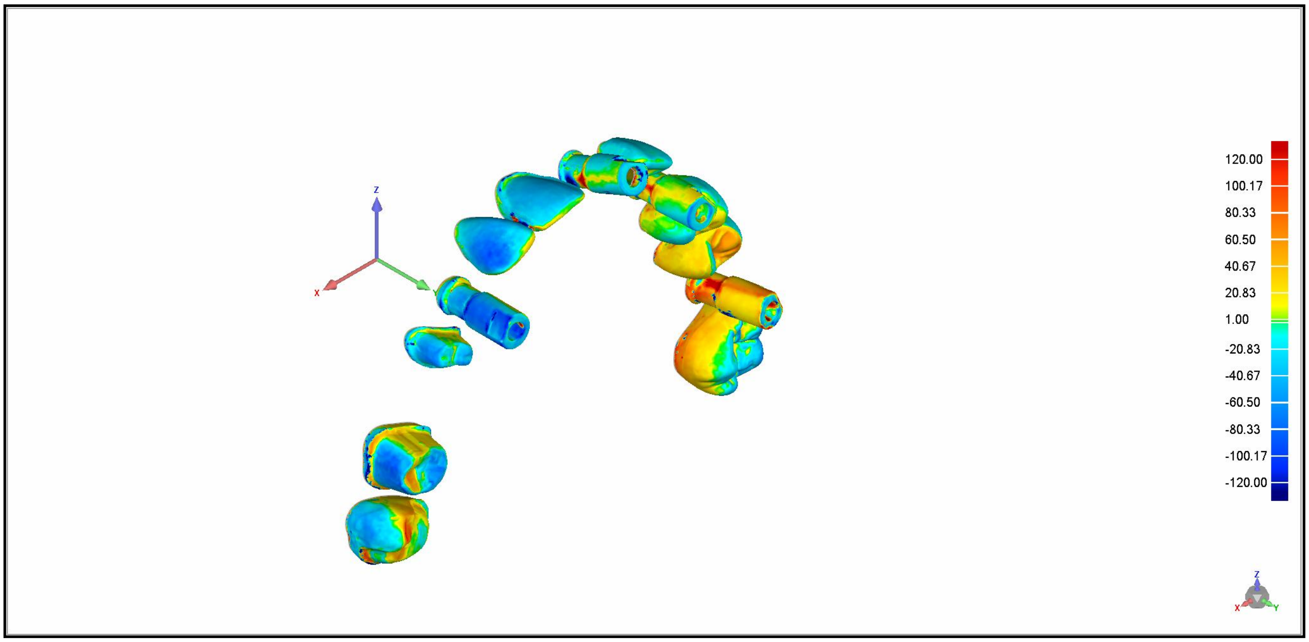

Predefinido: Frente

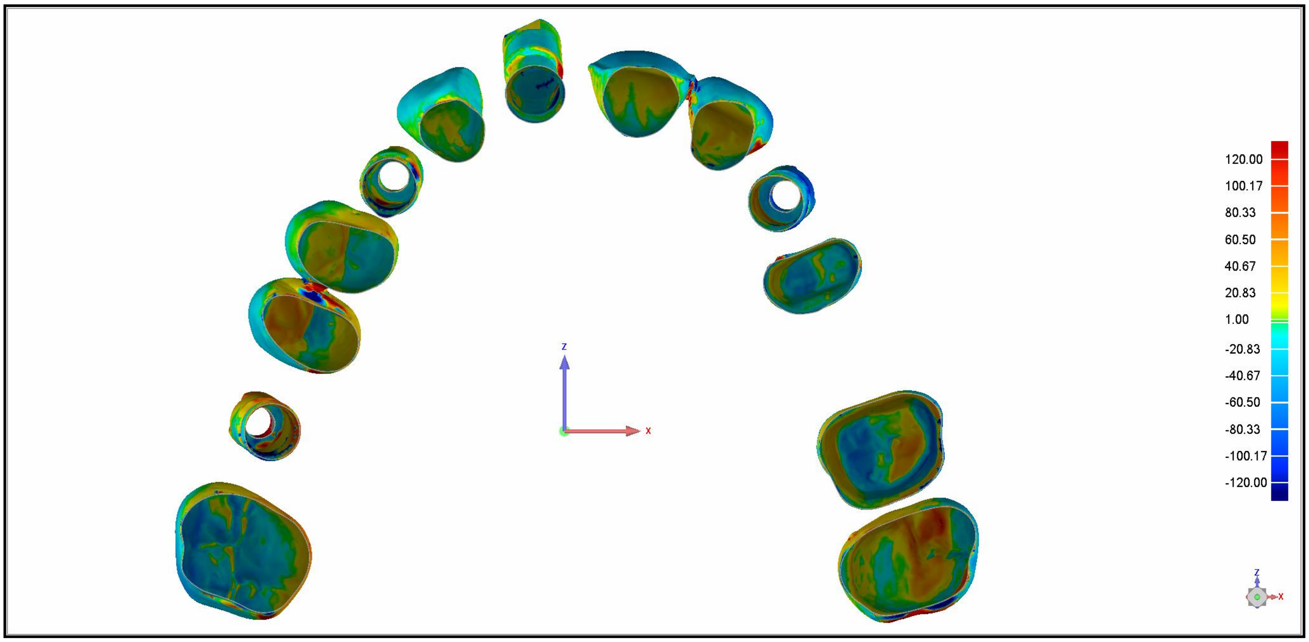

Predefinido: Atrás

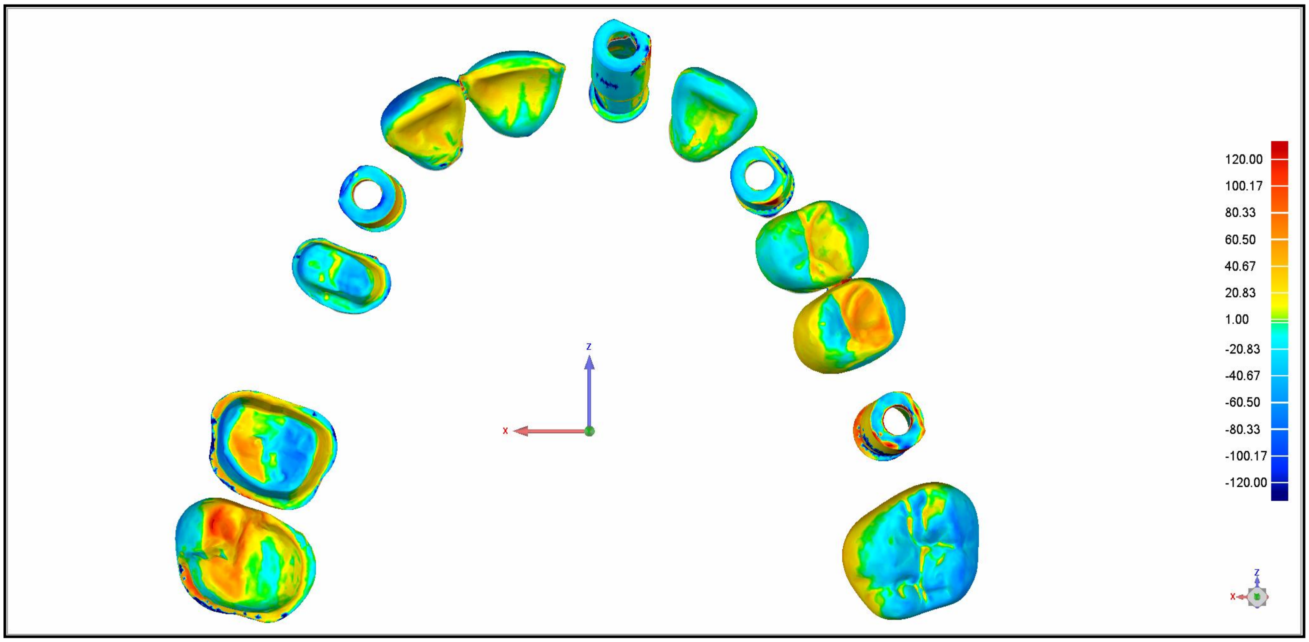

Predefinido: Izquierda

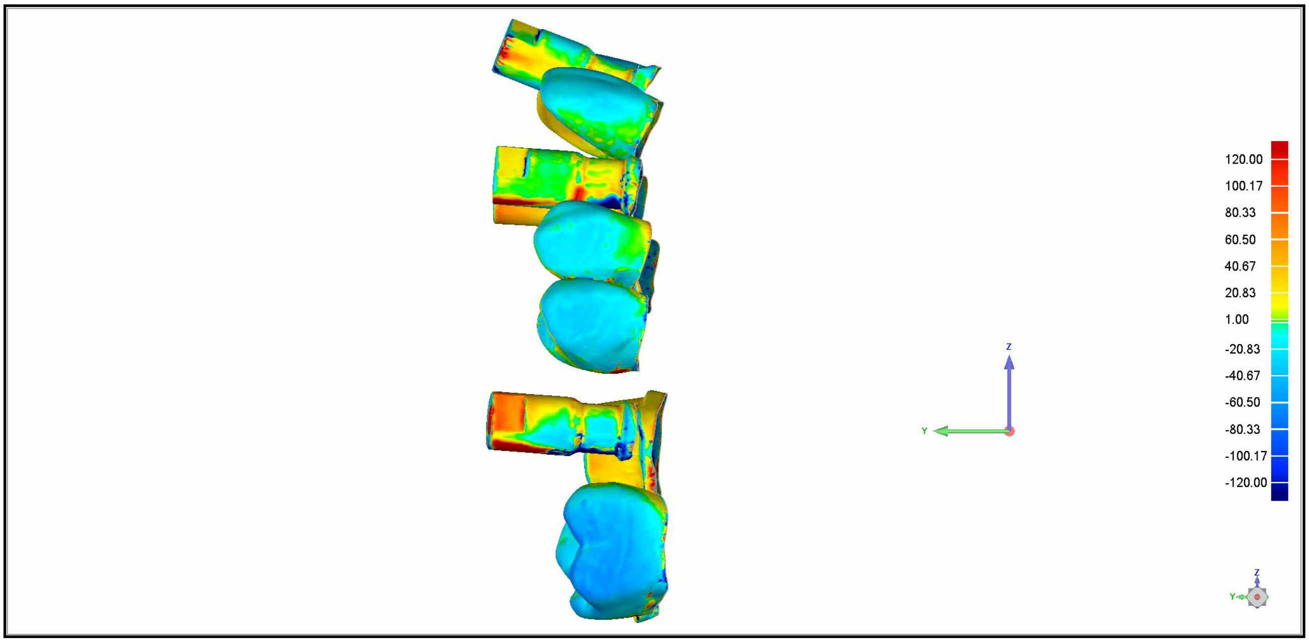

Predefinido: Derecha

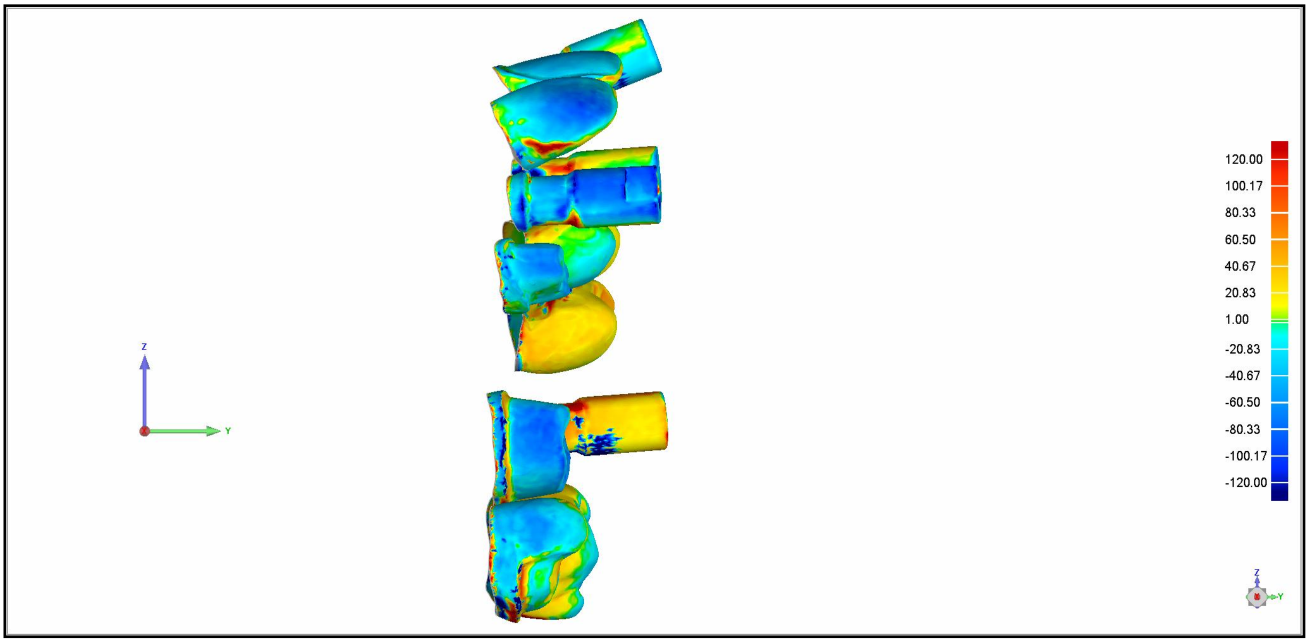

Predefinido: Superior

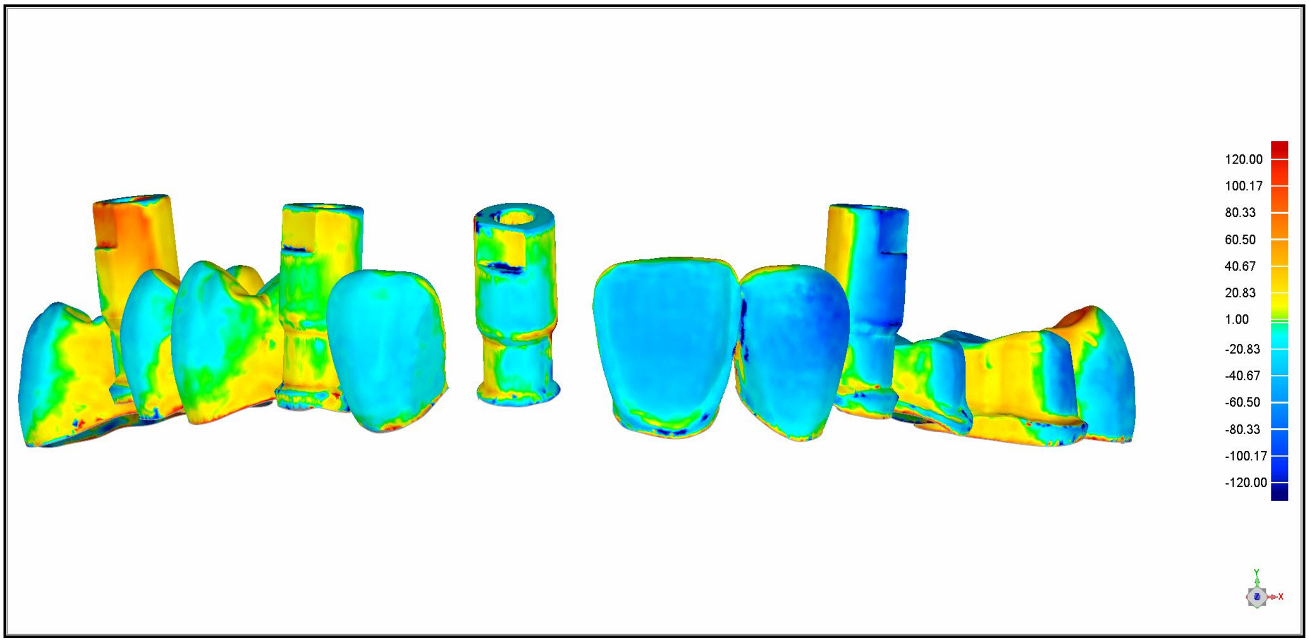

Predefinido: Inferior

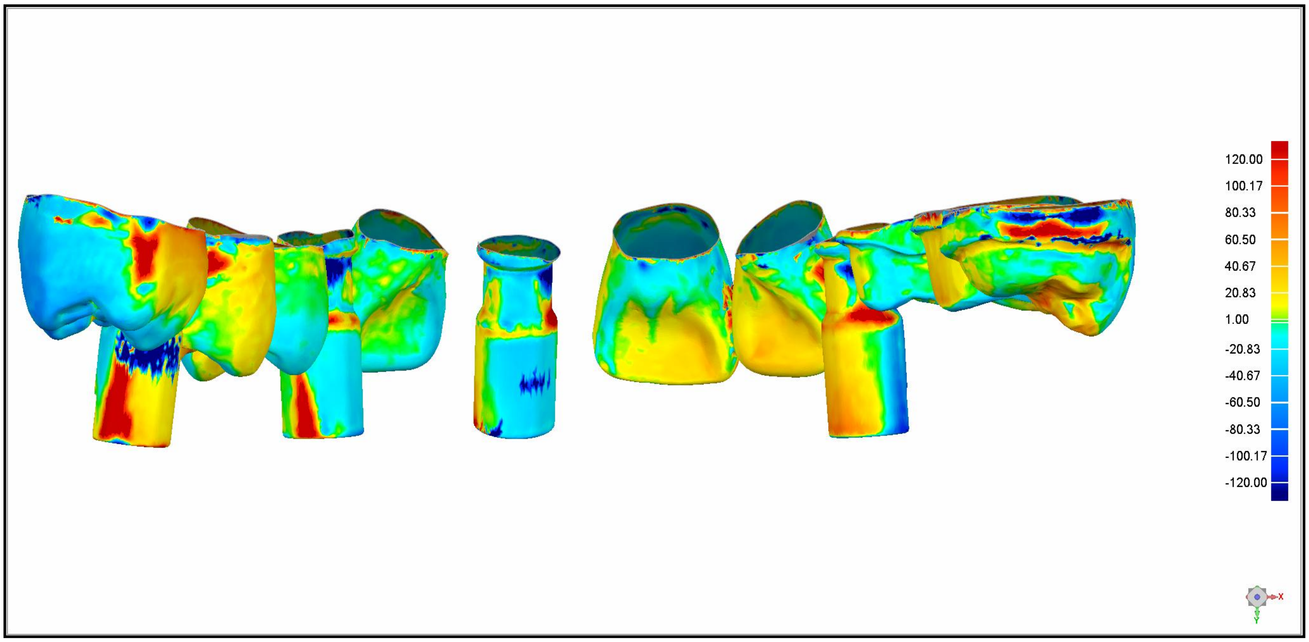

Ajuste de ubicación: Desviaciones superior e inferior

Unidades: u

Nombre	Desv	Estado	Superior Tol	Inferior Tol	Ref X	Ref Y	Ref Z	Radio	Desv X	Desv Y	Desv Z	Medido X	Medido Y	Medido Z	Dir. proy. X	Dir. proy. Y	Dir. proy. Z
Desv. inferior	-3155.28				-23066.00	37963.82	-193.73	n/a	-12.95	-2953.63	-1109.80	-23078.95	35010.19	-1303.53	0.00	0.94	0.35
Desv. superior	3140.05				29547.11	27336.30	1208.53	n/a	-2528.84	-415.70	1814.41	27018.27	26920.60	3022.94	-0.81	-0.13	0.58
