# Supplementary material for: Accuracy of four digital scanners according to scanning strategy in complete-arch impressions
Source: PLoS One. 2018 Sep 13;13(9):e0202916. doi: 10.1371/journal.pone.0202916 (PMC6136706; doi:10.1371/journal.pone.0202916)
Supplement: S11 Table — Omnicam (scanning strategy C). (ZIP) [file pone.0202916.s011.zip › S11/OM4C.pdf]

Geomagic Control™

Fecha: 3/6/2017, 2:59 pm

3D Comparación Resultados

Modelo referencia	MRC
Modelo test	OM4C
Nº de puntos de datos	196005
# Aislados	777

Tipo tolerancia	3D desviación
Unidades	u
Máx. crítico	120.00
Máx. nominal	14.00
Mín. nominal	-14.00
Mín. crítico	-120.00

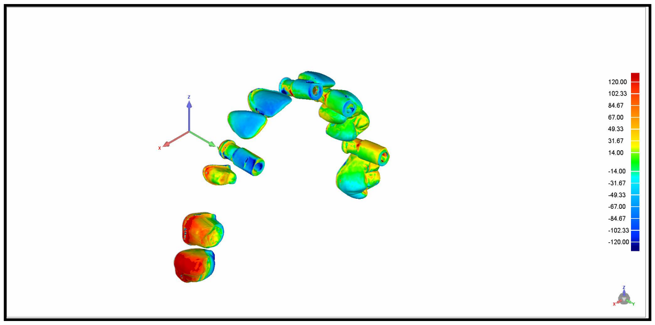

Desviación	
Desviación superior máx.	3145.69
Desviación inferior máx.	-3148.88
Desviación media	99.67 /-74.76
Desviación estándar	235.64

Distribución desviación

>=Min	<Max	# Puntos	%
-120.00	-102.33	2235	1.14
-102.33	-84.67	2696	1.38
-84.67	-67.00	4529	2.31
-67.00	-49.33	9524	4.86
-49.33	-31.67	15934	8.13
-31.67	-14.00	25806	13.17
-14.00	14.00	49970	25.49
14.00	31.67	23699	12.09
31.67	49.33	14438	7.37
49.33	67.00	9554	4.87
67.00	84.67	5540	2.83
84.67	102.33	3878	1.98
102.33	120.00	2895	1.48

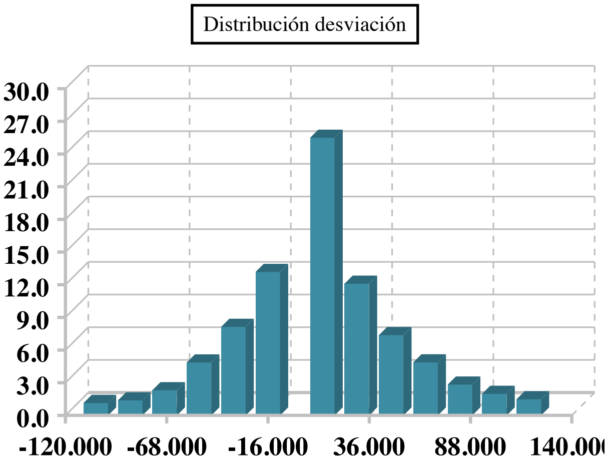

Fuera del crítico superior		16292	8.31
Fuera del crítico inferior		9015	4.60

Desviaciones estándar

Distribución (+/-)	# Puntos	%
-6 * Desv. estánd.	1295	0.66
-5 * Desv. estánd.	421	0.21
-4 * Desv. estánd.	475	0.24
-3 * Desv. estánd.	550	0.28
-2 * Desv. estánd.	1702	0.87
-1 * Desv. estánd.	115827	59.09
1 * Desv. estánd.	68592	35.00
2 * Desv. estánd.	2398	1.22
3 * Desv. estánd.	1475	0.75
4 * Desv. estánd.	971	0.50
5 * Desv. estánd.	889	0.45
6 * Desv. estánd.	1410	0.72

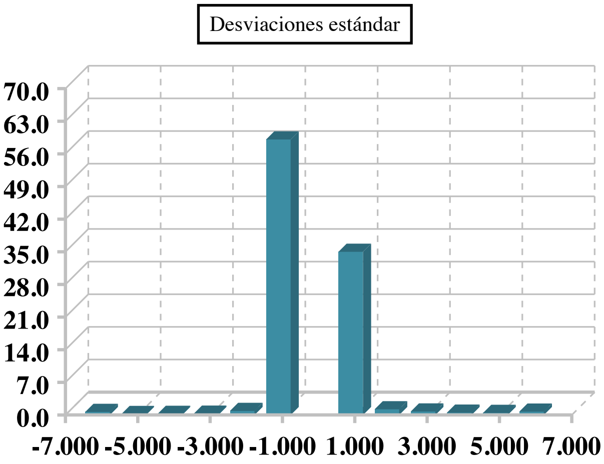

Predefinido: Isométrico

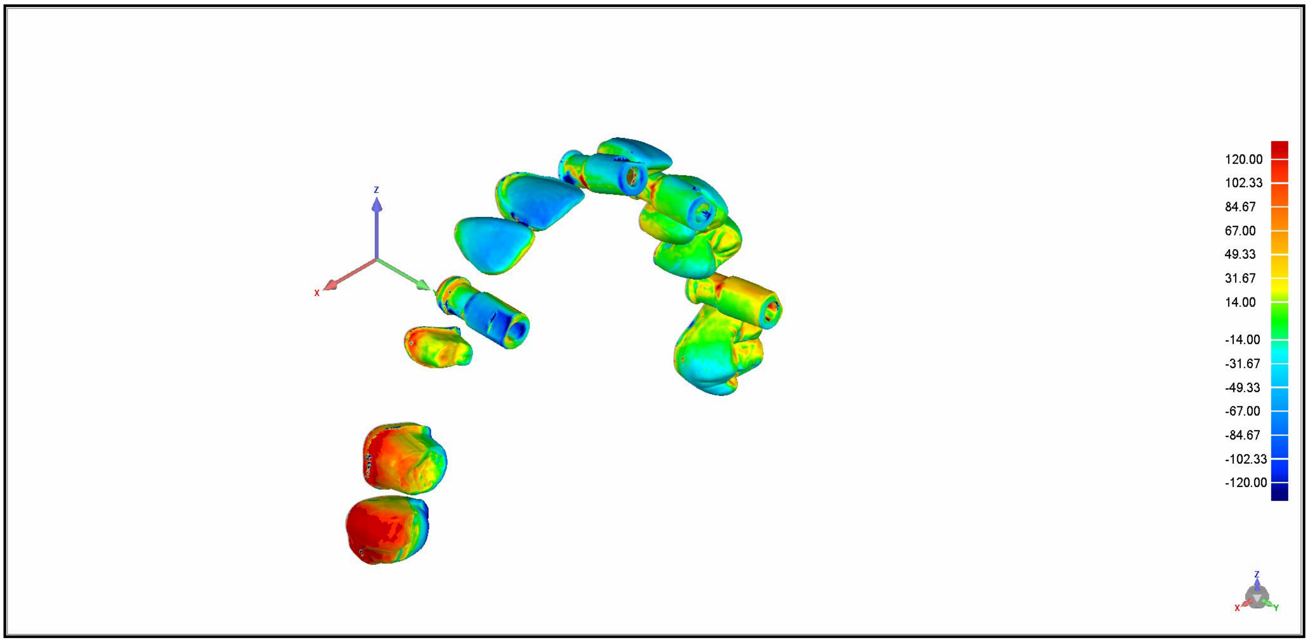

Predefinido: Frente

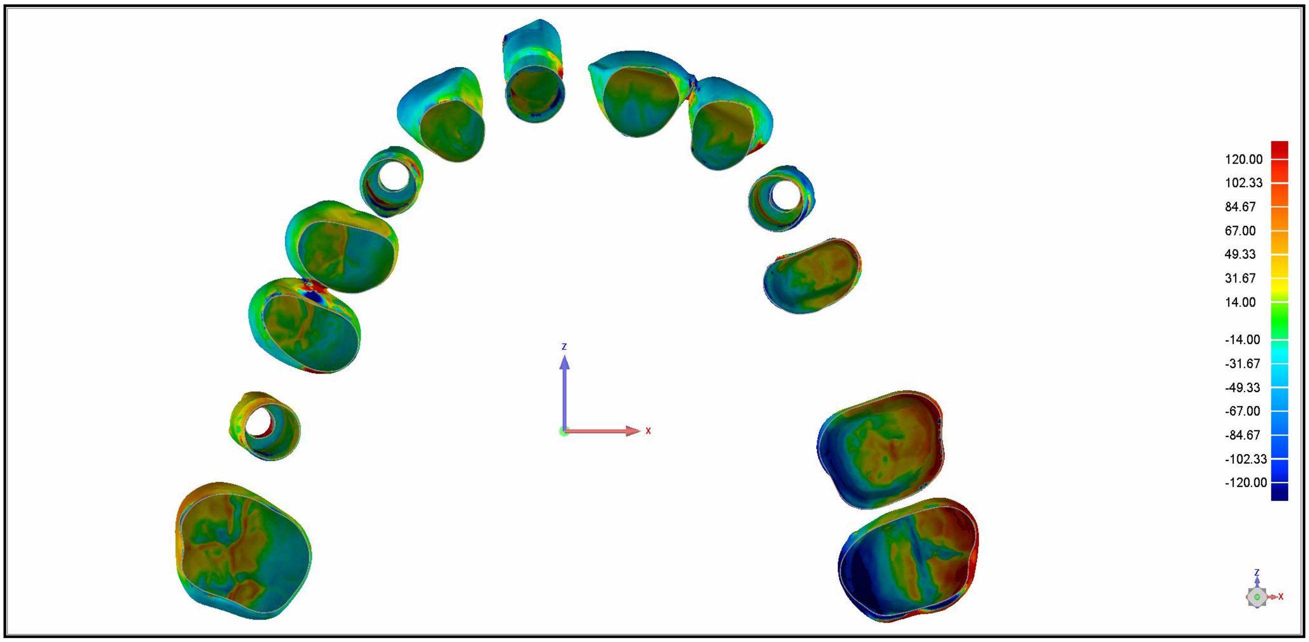

Predefinido: Atrás

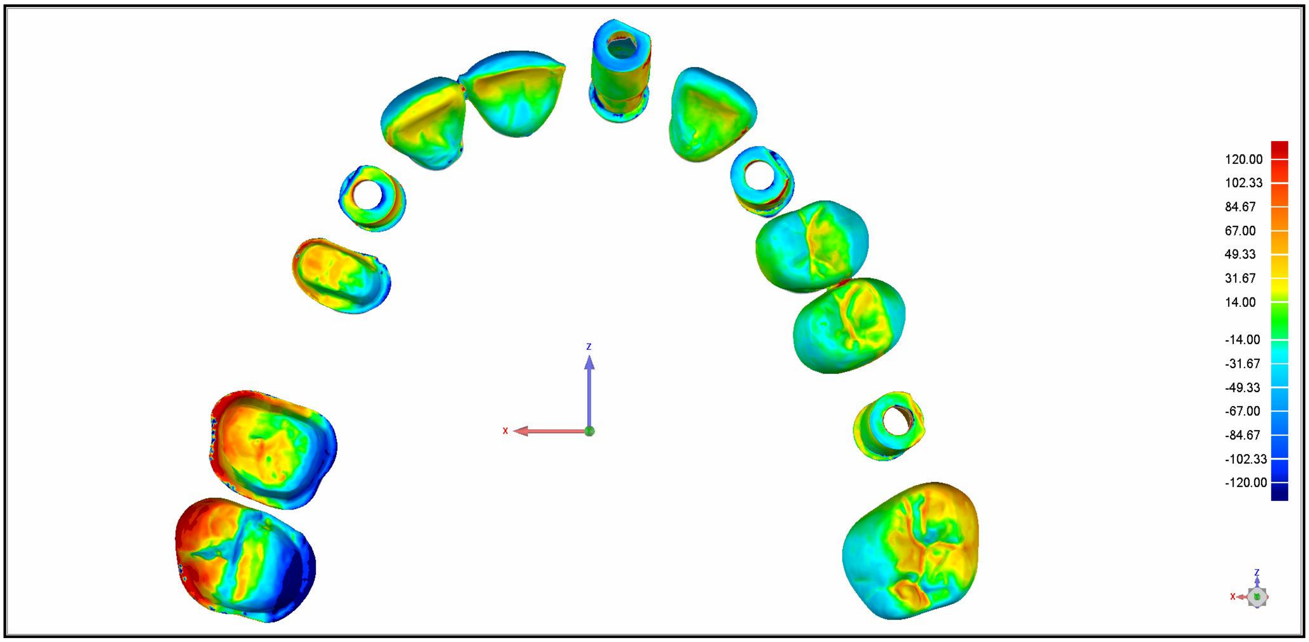

Predefinido: Izquierda

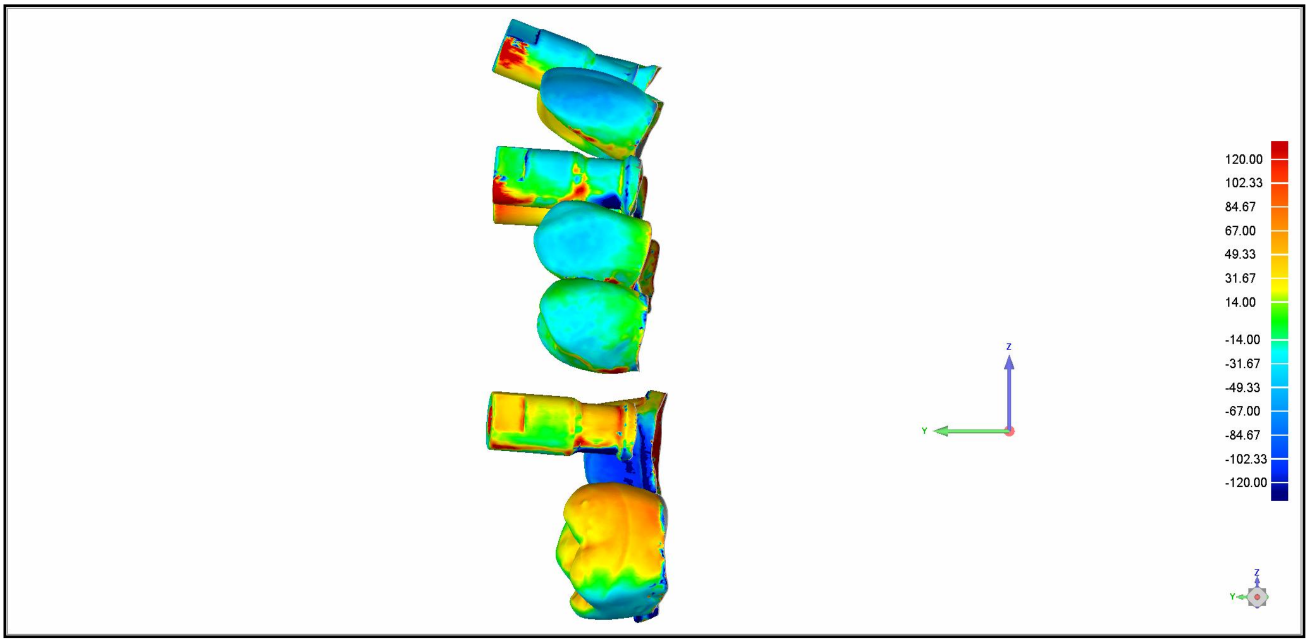

Predefinido: Derecha

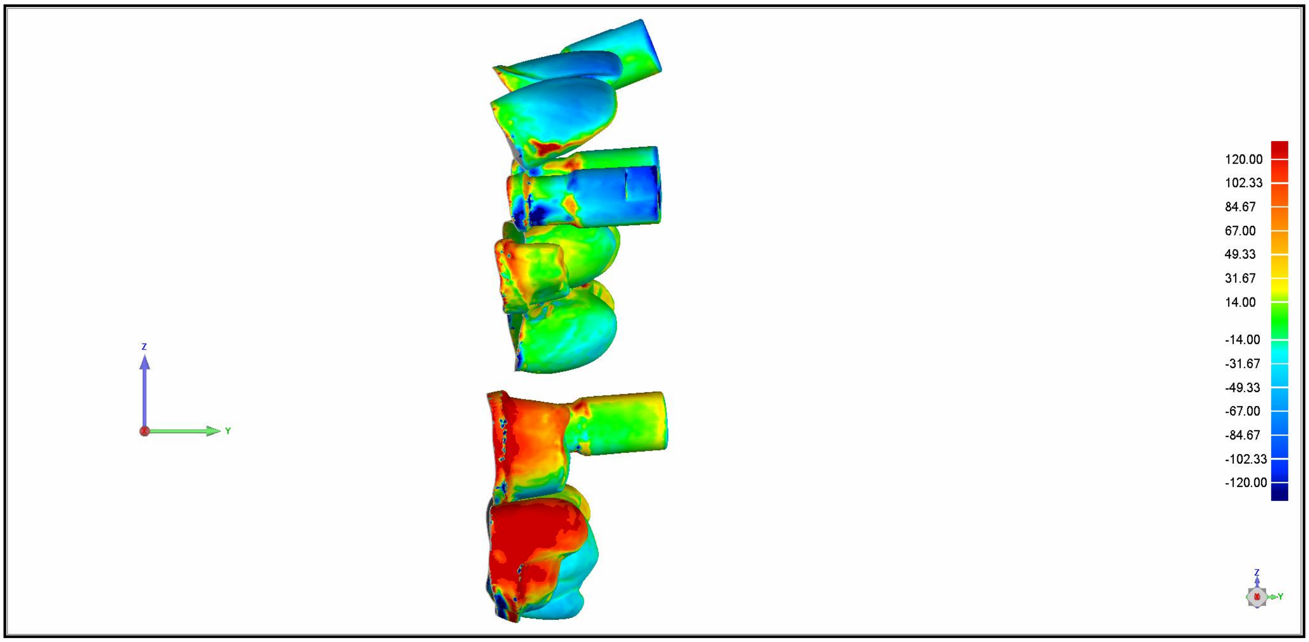

Predefinido: Superior

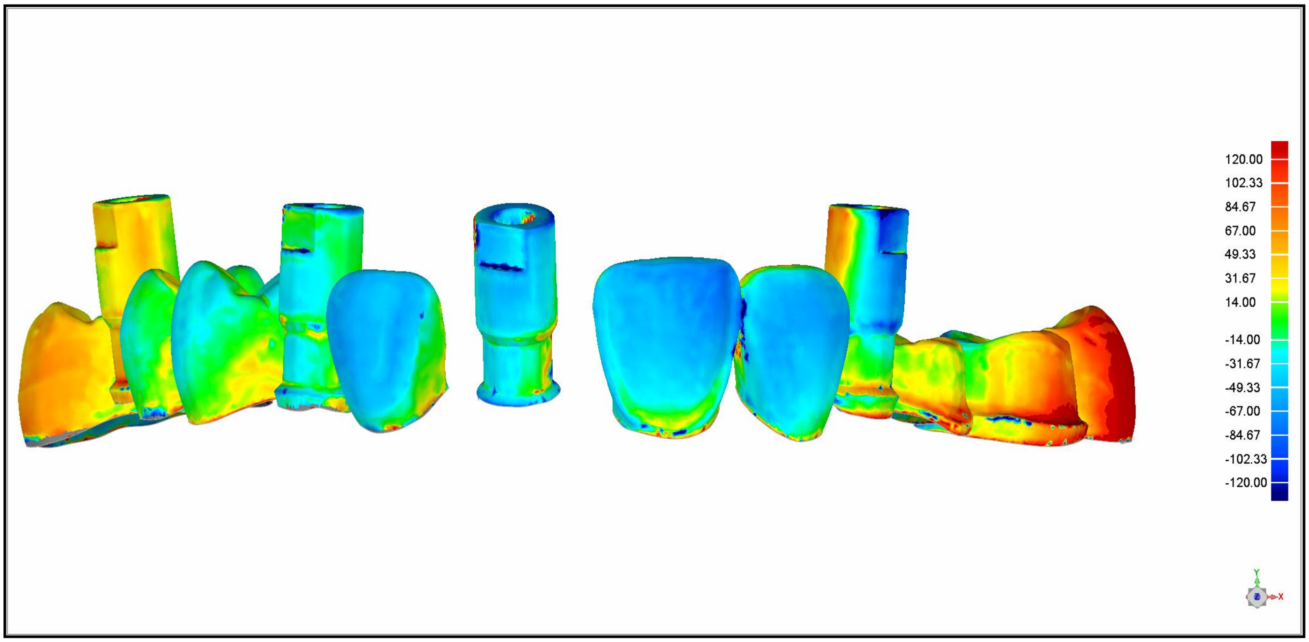

Predefinido: Inferior

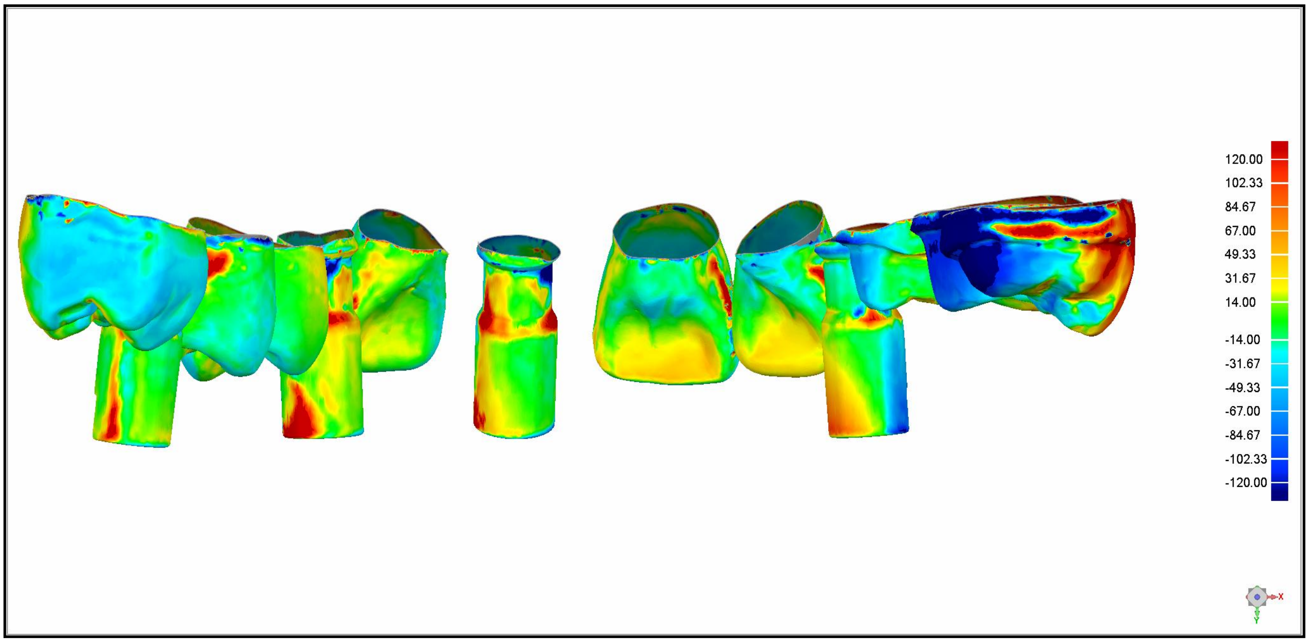

Ajuste de ubicación: Desviaciones superior e inferior

Unidades: u

Nombre	Desv	Estado	Superior Tol	Inferior Tol	Ref X	Ref Y	Ref Z	Radio	Desv X	Desv Y	Desv Z	Medido X	Medido Y	Medido Z	Dir. proy. X	Dir. proy. Y	Dir. proy. Z
Desv. inferior	-3148.88				-22607.19	28955.77	6808.03	n/a	-996.03	-397.55	2960.62	-23603.23	28558.22	9768.66	0.32	0.13	-0.94
Desv. superior	3145.69				-13203.90	38507.15	18811.31	n/a	-2748.17	1418.42	575.36	-15952.06	39925.57	19386.67	-0.87	0.45	0.18
