# Supplementary material for: Accuracy of four digital scanners according to scanning strategy in complete-arch impressions
Source: PLoS One. 2018 Sep 13;13(9):e0202916. doi: 10.1371/journal.pone.0202916 (PMC6136706; doi:10.1371/journal.pone.0202916)
Supplement: S11 Table — Omnicam (scanning strategy C). (ZIP) [file pone.0202916.s011.zip › S11/OM8C.pdf]

3D Comparación Resultados

Modelo referencia	MRC
Modelo test	OM8C
Nº de puntos de datos	196630
# Aislados	790

Tipo tolerancia	3D desviación
Unidades	u
Máx. crítico	120.00
Máx. nominal	13.00
Mín. nominal	-13.00
Mín. crítico	-120.00

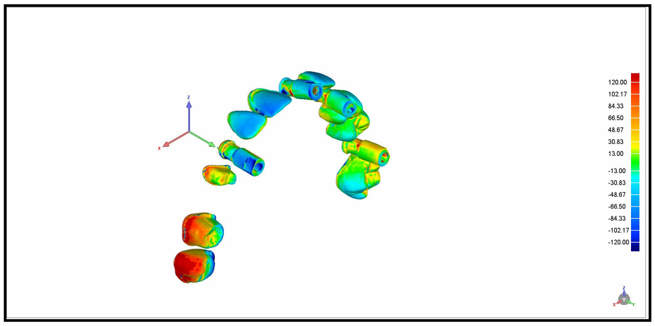

Desviación	
Desviación superior máx.	3146.31
Desviación inferior máx.	-3154.86
Desviación media	99.17 /-74.00
Desviación estándar	232.79

Distribución desviación

>=Min	<Max	# Puntos	%
-120.00	-102.17	2400	1.22
-102.17	-84.33	2947	1.50
-84.33	-66.50	4953	2.52
-66.50	-48.67	10055	5.11
-48.67	-30.83	15544	7.91
-30.83	-13.00	27983	14.23
-13.00	13.00	46730	23.77
13.00	30.83	24614	12.52
30.83	48.67	14562	7.41
48.67	66.50	8965	4.56
66.50	84.33	5447	2.77
84.33	102.17	3786	1.93
102.17	120.00	2875	1.46

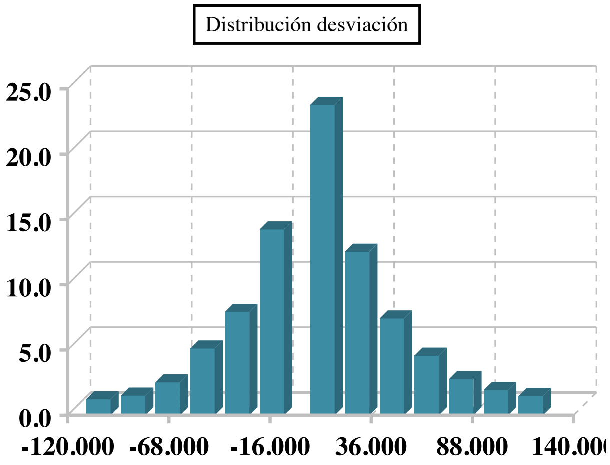

Fuera del crítico superior		16241	8.26
Fuera del crítico inferior		9528	4.85

Desviaciones estándar

Distribución (+/-)	# Puntos	%
-6 * Desv. estánd.	1244	0.63
-5 * Desv. estánd.	422	0.21
-4 * Desv. estánd.	466	0.24
-3 * Desv. estánd.	560	0.28
-2 * Desv. estánd.	1727	0.88
-1 * Desv. estánd.	116048	59.02
1 * Desv. estánd.	68954	35.07
2 * Desv. estánd.	2465	1.25
3 * Desv. estánd.	1486	0.76
4 * Desv. estánd.	936	0.48
5 * Desv. estánd.	869	0.44
6 * Desv. estánd.	1453	0.74

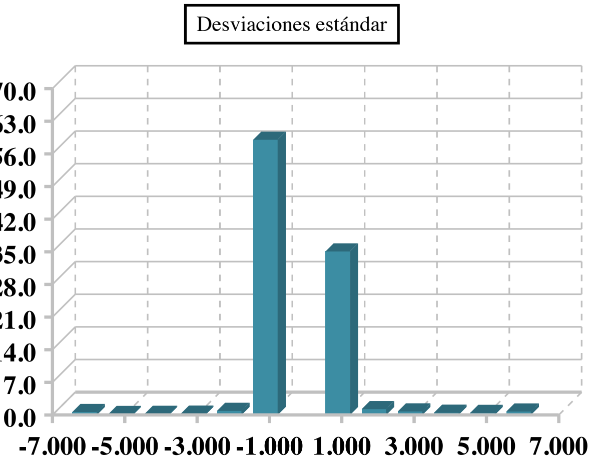

Predefinido: Isométrico

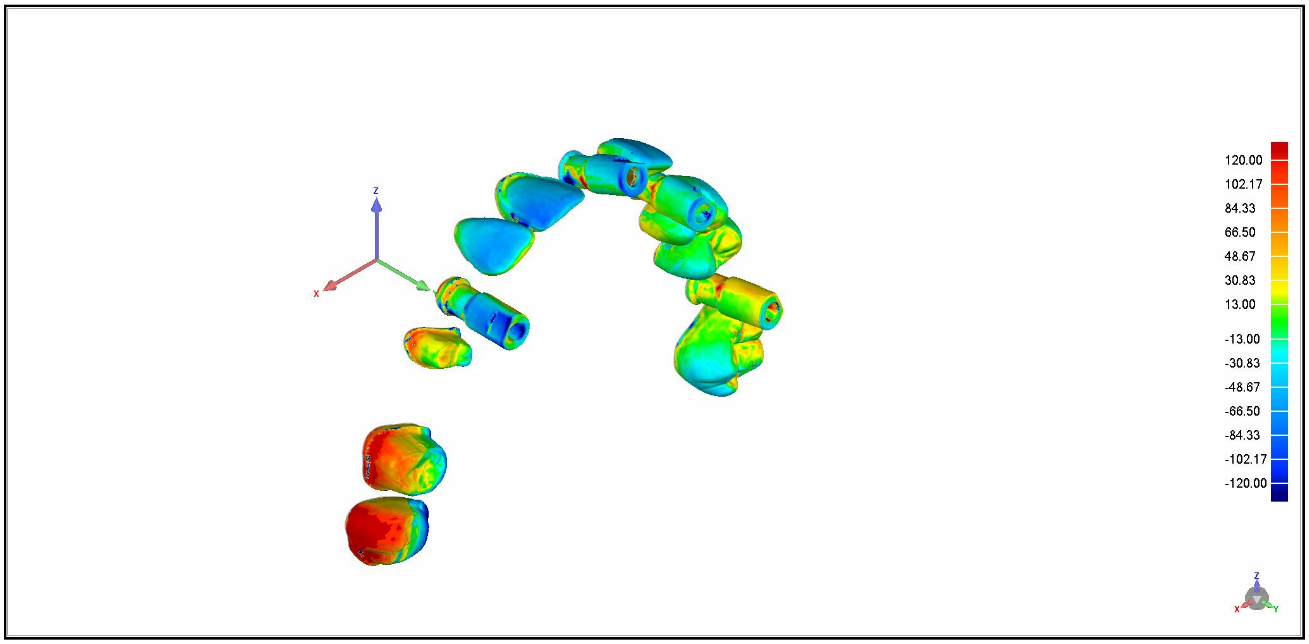

Predefinido: Frente

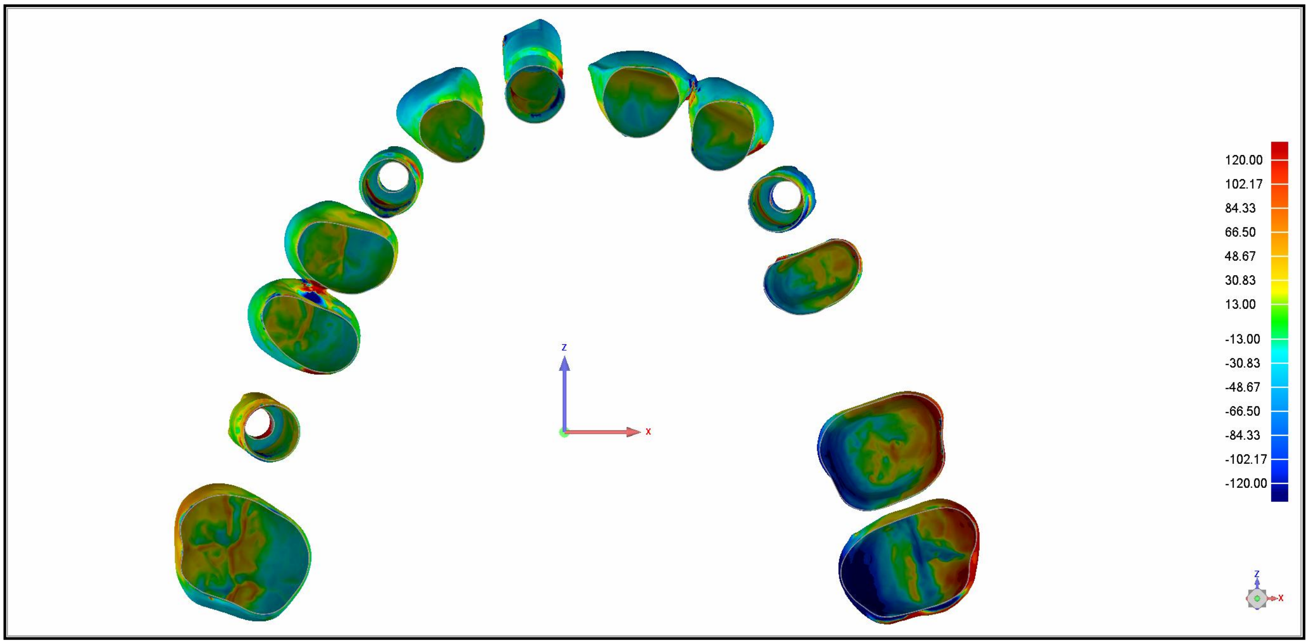

Predefinido: Atrás

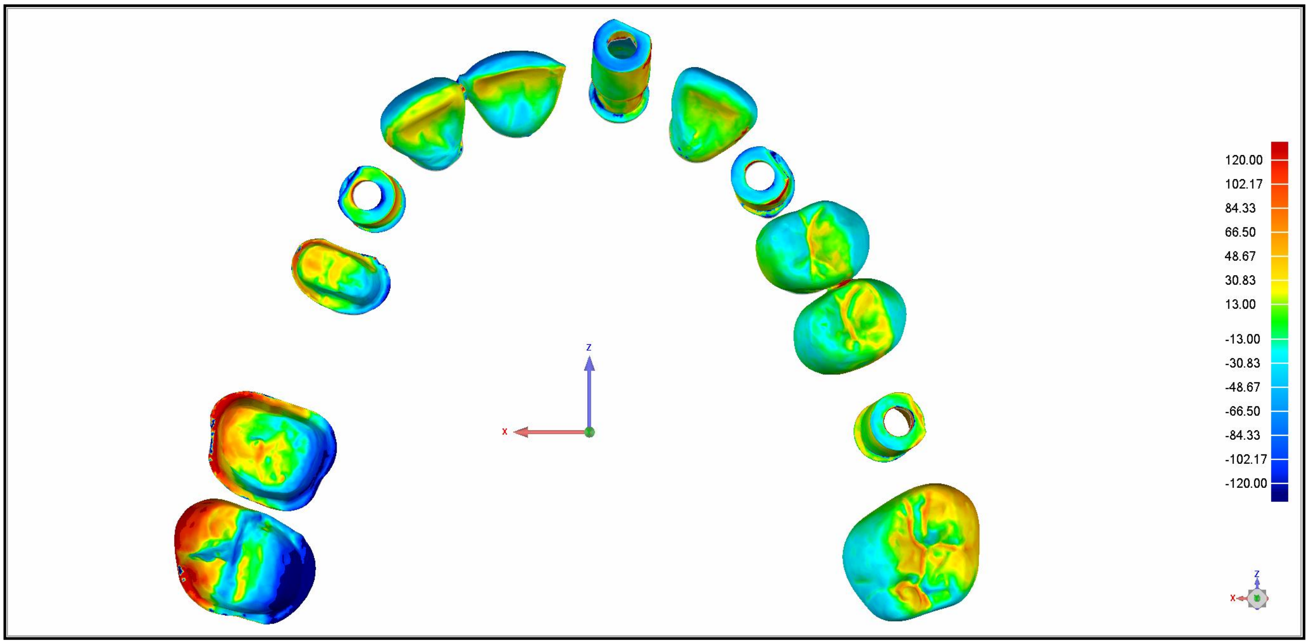

Predefinido: Izquierda

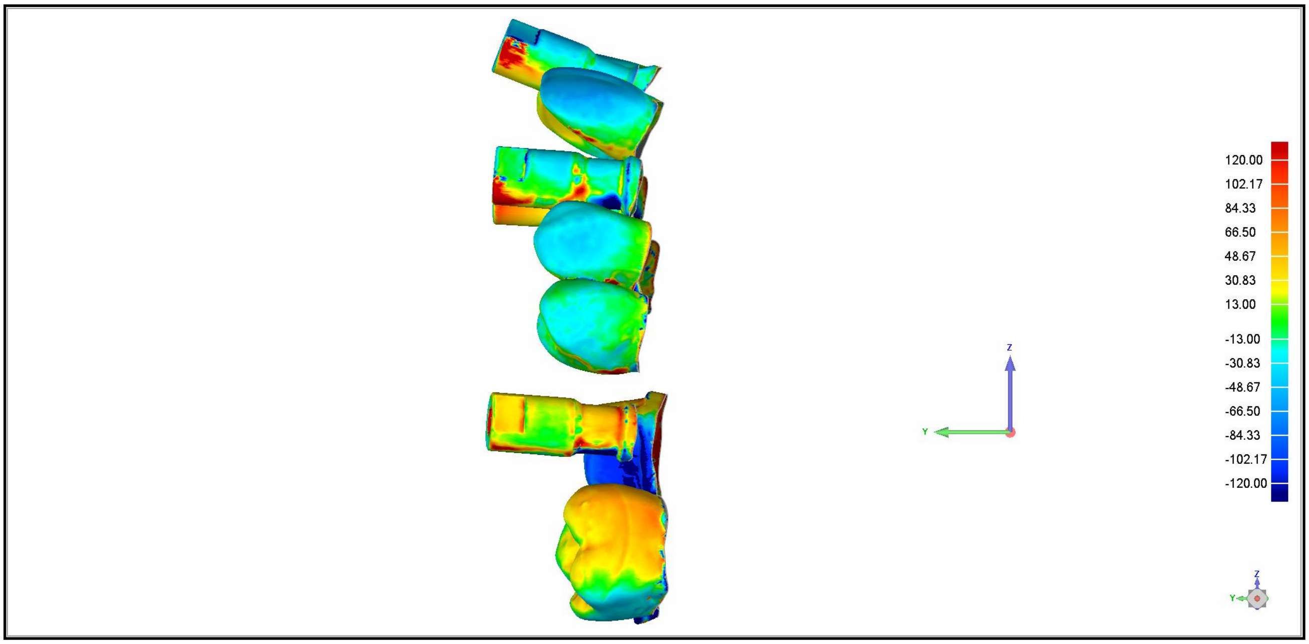

Predefinido: Derecha

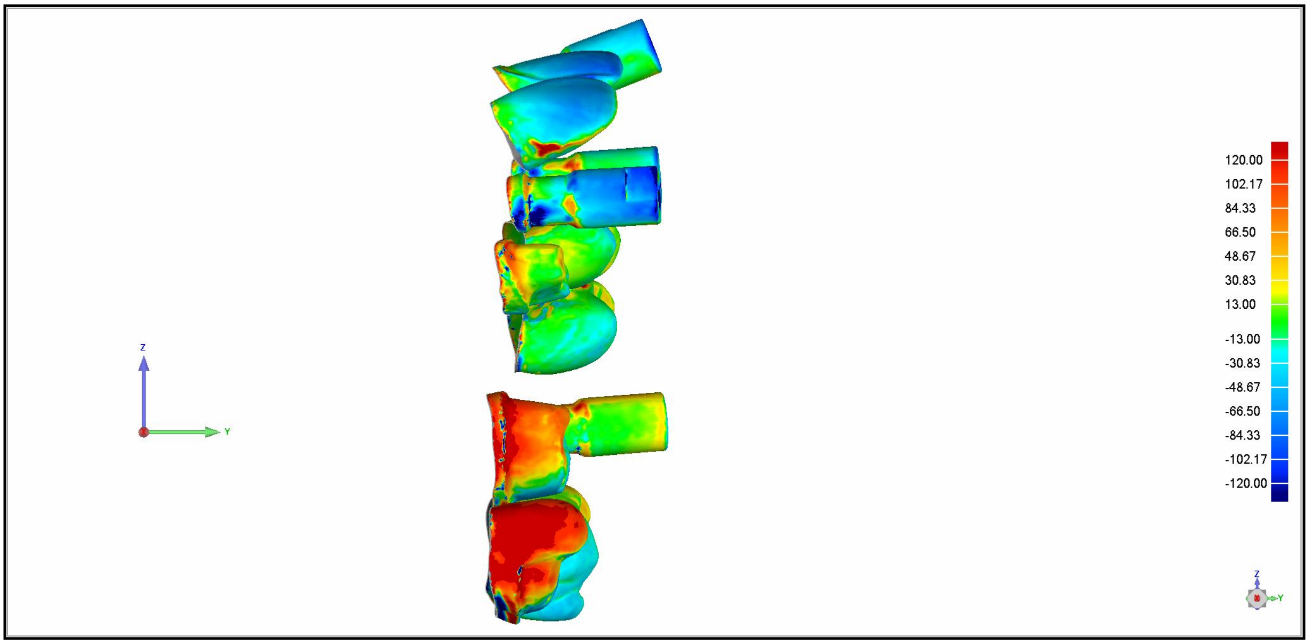

Geomagic Control™

Predefinido: Superior

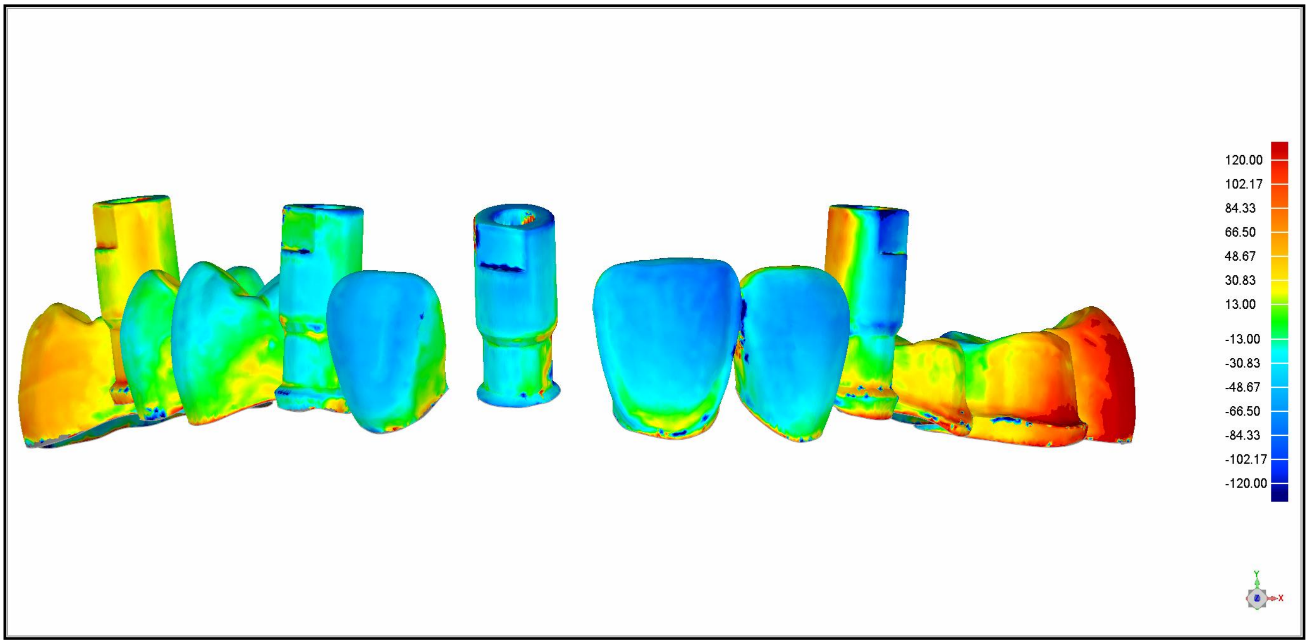

Predefinido: Inferior

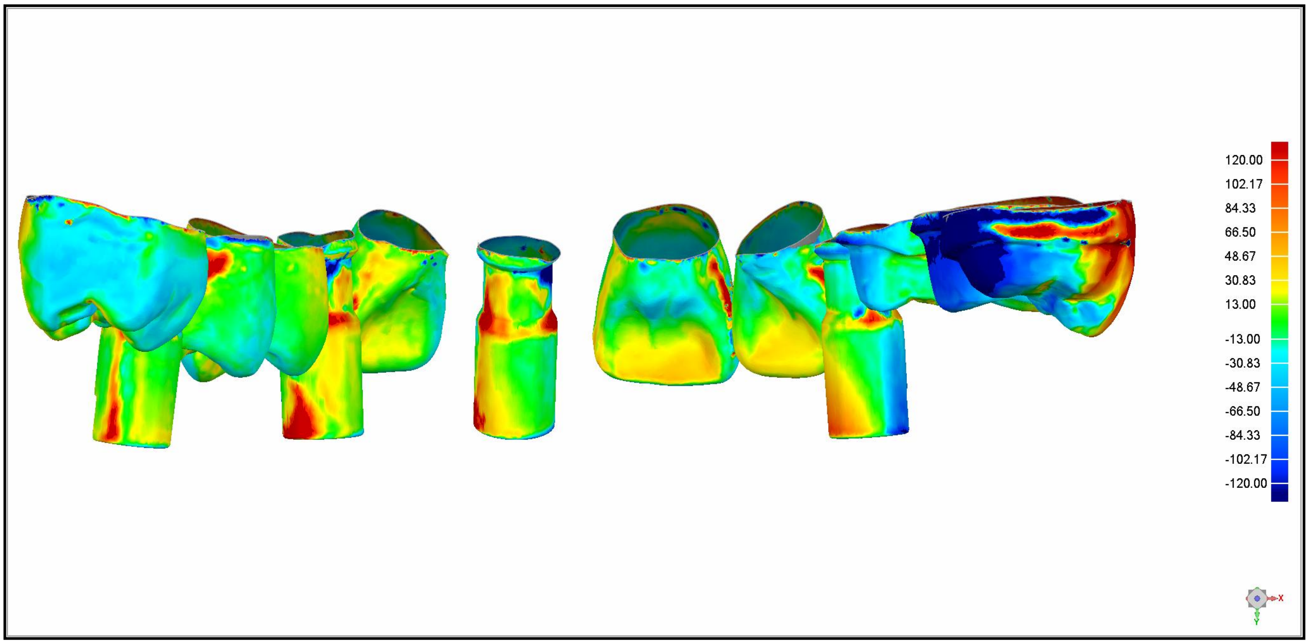

Ajuste de ubicación: Desviaciones superior e inferior

Unidades: u

Nombre	Desv	Estado	Superior Tol	Inferior Tol	Ref X	Ref Y	Ref Z	Radio	Desv X	Desv Y	Desv Z	Medido X	Medido Y	Medido Z	Dir. proy. X	Dir. proy. Y	Dir. proy. Z
Desv. inferior	-3154.86				-22607.19	28955.77	6808.03	n/a	-913.57	-493.04	2979.17	-23520.77	28462.72	9787.20	0.29	0.16	-0.94
Desv. superior	3146.31				-13203.89	38507.14	18811.31	n/a	-2749.96	1418.63	569.65	-15953.86	39925.78	19380.95	-0.87	0.45	0.18
