# Supplementary material for: Accuracy of four digital scanners according to scanning strategy in complete-arch impressions
Source: PLoS One. 2018 Sep 13;13(9):e0202916. doi: 10.1371/journal.pone.0202916 (PMC6136706; doi:10.1371/journal.pone.0202916)
Supplement: S11 Table — Omnicam (scanning strategy C). (ZIP) [file pone.0202916.s011.zip › S11/OM9C.pdf]

Geomagic Control™



# 3D Comparación Resultados

| | |
|---|---|
| Modelo referencia | MRC |
| Modelo test | OM9C |
| Nº de puntos de datos | 202997 |
| # Aislados | 1002 |

| | |
|---|---|
| Tipo tolerancia | 3D desviación |
| Unidades | u |
| Máx. crítico | 120.00 |
| Máx. nominal | 1.00 |
| Mín. nominal | -1.00 |
| Mín. crítico | -120.00 |

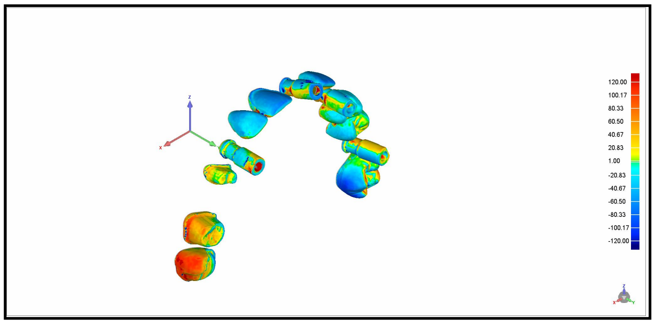


| | |
|---|---|
| Desviación | |
| Desviación superior máx. | 2992.71 |
| Desviación inferior máx. | -3086.35 |
| Desviación media | 103.04 /-109.73 |
| Desviación estándar | 294.70 |

## Distribución desviación

| >=Min | <Max | # Puntos | % |
|---|---|---|---|
| -120.00 | -100.17 | 1848 | 0.91 |
| -100.17 | -80.33 | 4303 | 2.12 |
| -80.33 | -60.50 | 6930 | 3.41 |
| -60.50 | -40.67 | 14120 | 6.96 |
| -40.67 | -20.83 | 23900 | 11.77 |
| -20.83 | -1.00 | 34970 | 17.23 |
| -1.00 | 1.00 | 3794 | 1.87 |
| 1.00 | 20.83 | 35411 | 17.44 |
| 20.83 | 40.67 | 23950 | 11.80 |
| 40.67 | 60.50 | 12959 | 6.38 |
| 60.50 | 80.33 | 8275 | 4.08 |
| 80.33 | 100.17 | 5383 | 2.65 |
| 100.17 | 120.00 | 3478 | 1.71 |

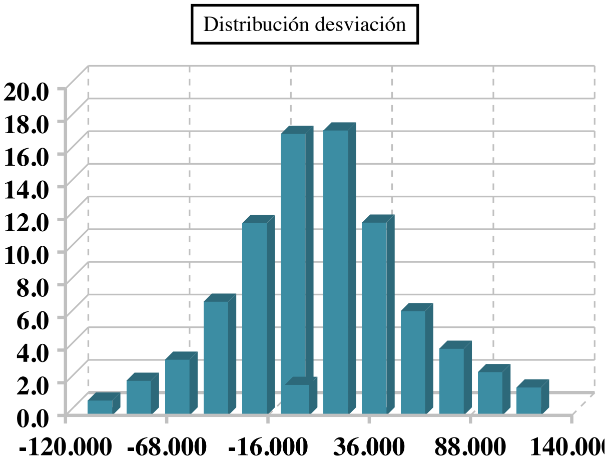


| | | | |
|---|---|---|---|
| Fuera del crítico superior | | 13218 | 6.51 |
| Fuera del crítico inferior | | 10458 | 5.15 |

## Desviaciones estándar

| Distribución (+/-) | # Puntos | % |
|---|---|---|
| -6 * Desv. estánd. | 1333 | 0.66 |
| -5 * Desv. estánd. | 1212 | 0.60 |
| -4 * Desv. estánd. | 1302 | 0.64 |
| -3 * Desv. estánd. | 1647 | 0.81 |
| -2 * Desv. estánd. | 1878 | 0.93 |
| -1 * Desv. estánd. | 90727 | 44.69 |
| 1 * Desv. estánd. | 97858 | 48.21 |
| 2 * Desv. estánd. | 1960 | 0.97 |
| 3 * Desv. estánd. | 1670 | 0.82 |
| 4 * Desv. estánd. | 1406 | 0.69 |
| 5 * Desv. estánd. | 1227 | 0.60 |
| 6 * Desv. estánd. | 777 | 0.38 |

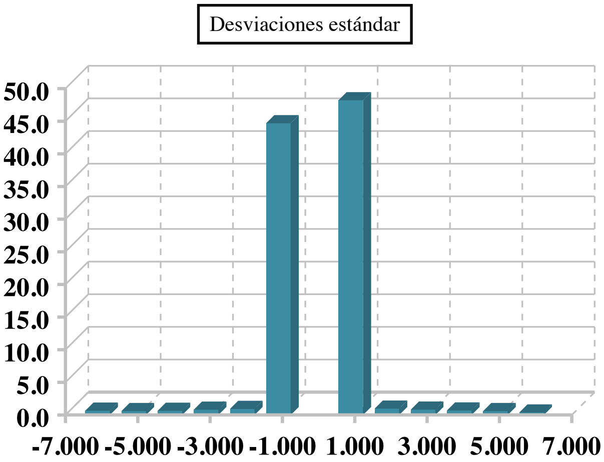

Predefinido: Isométrico

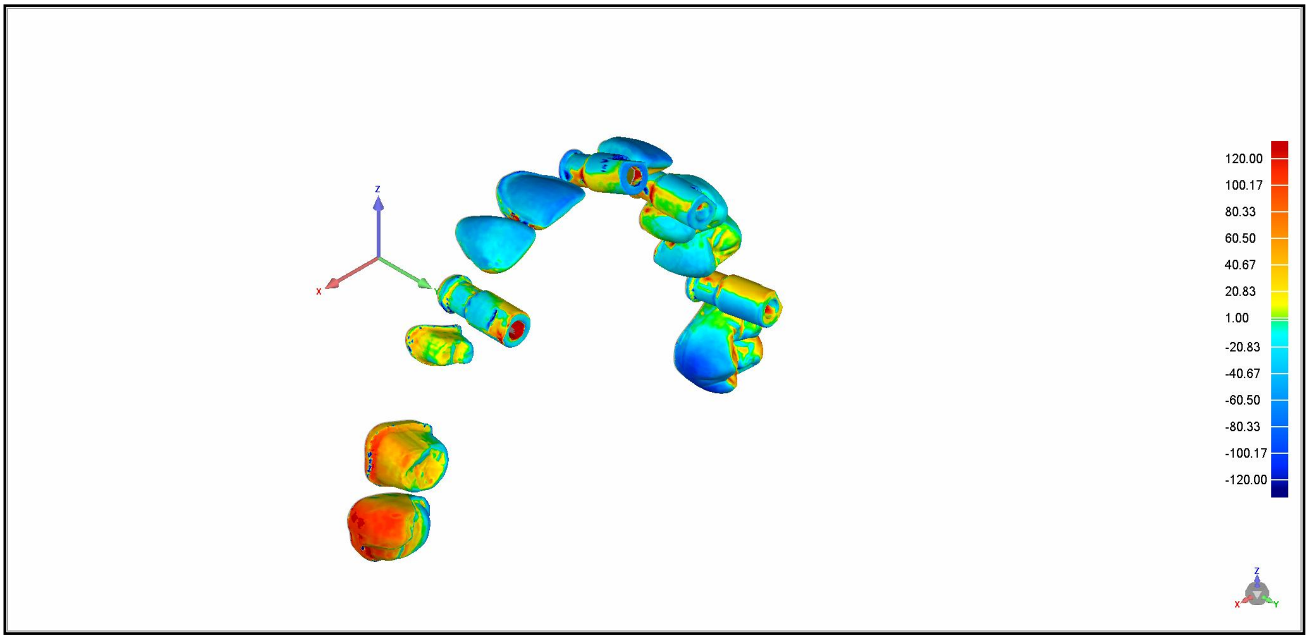

Predefinido: Frente

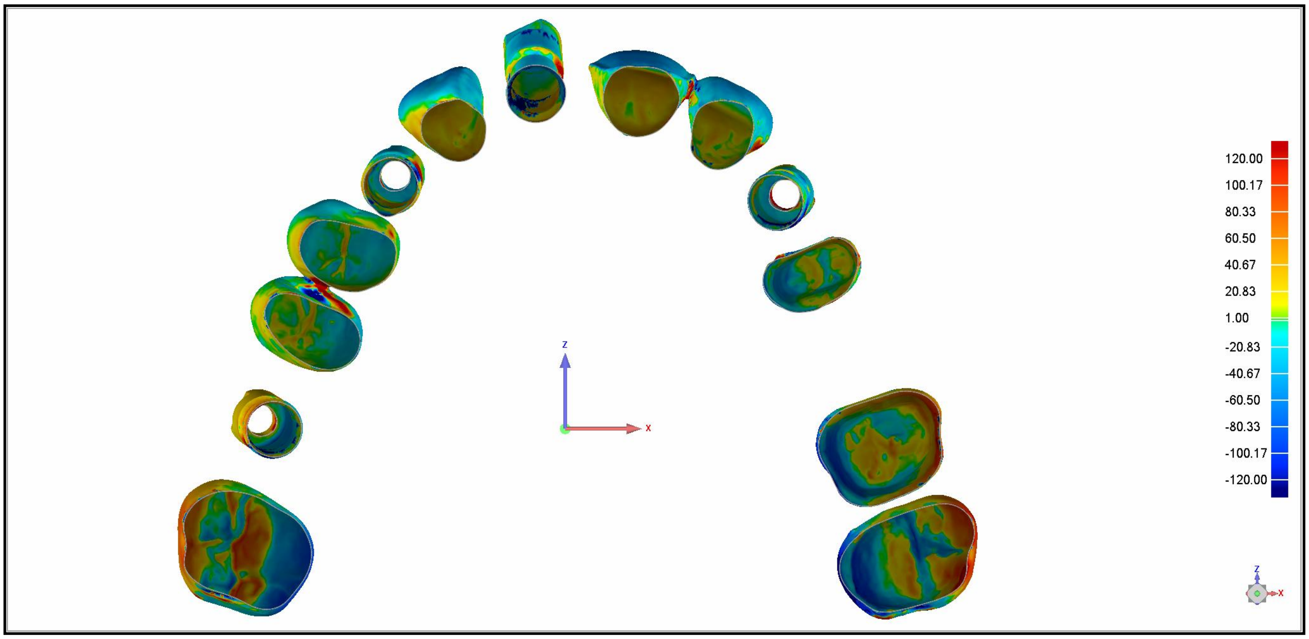

Predefinido: Atrás

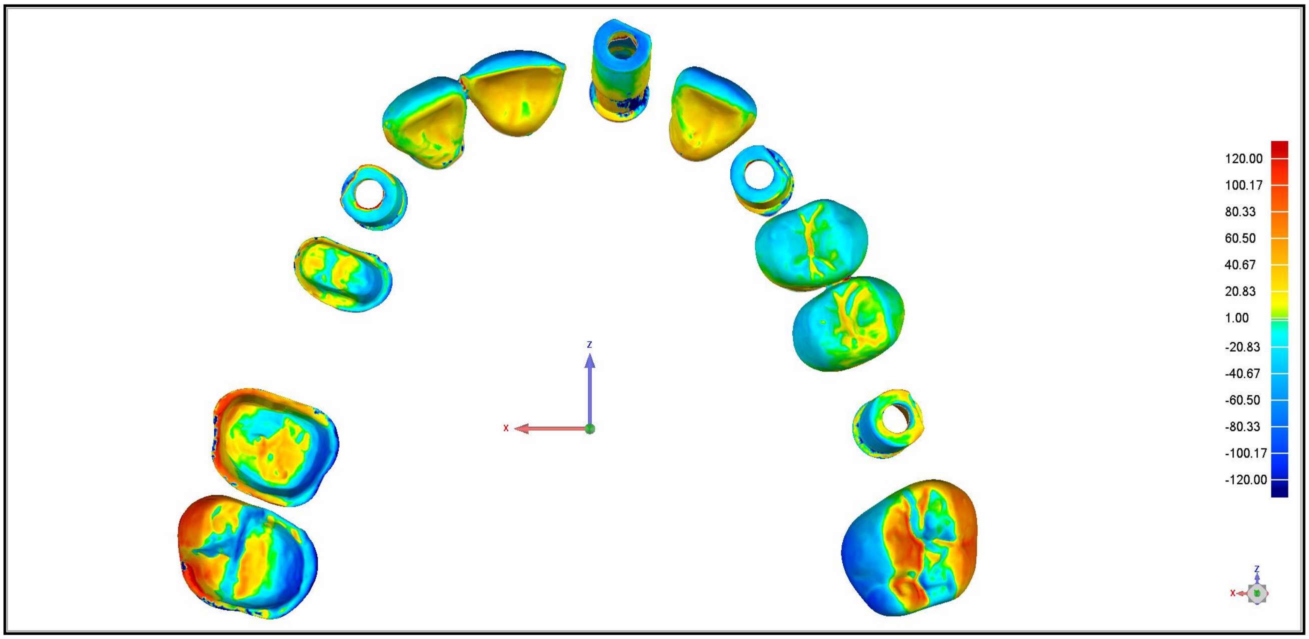

Predefinido: Izquierda

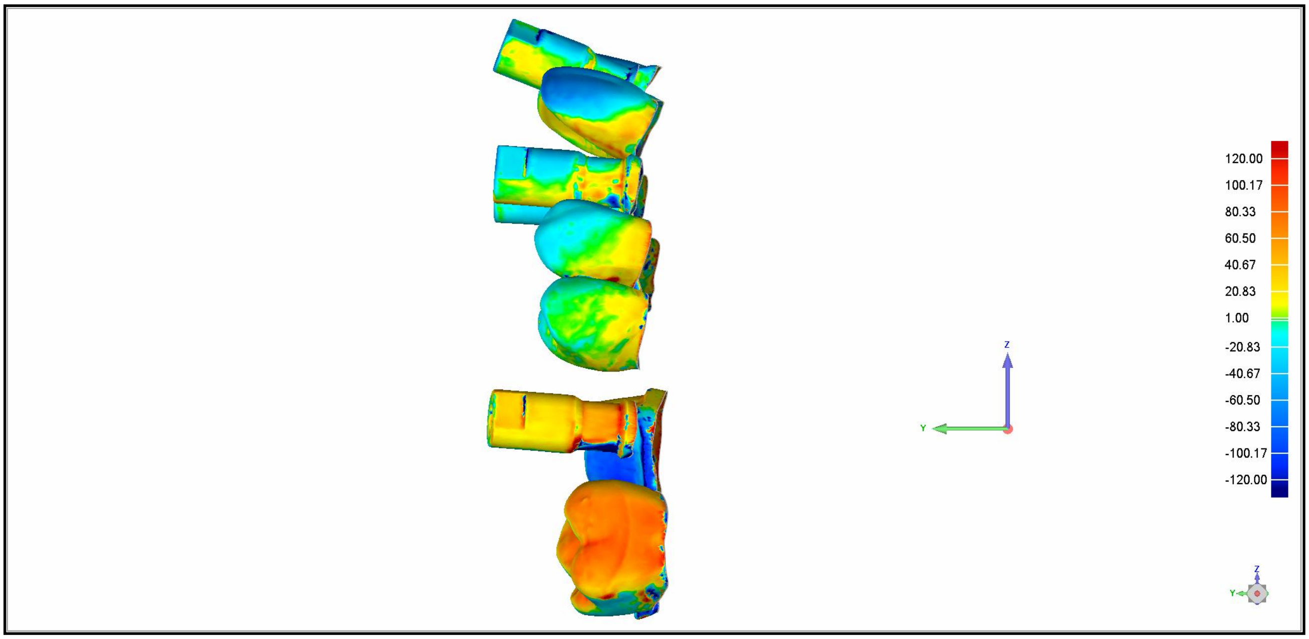

Predefinido: Derecha

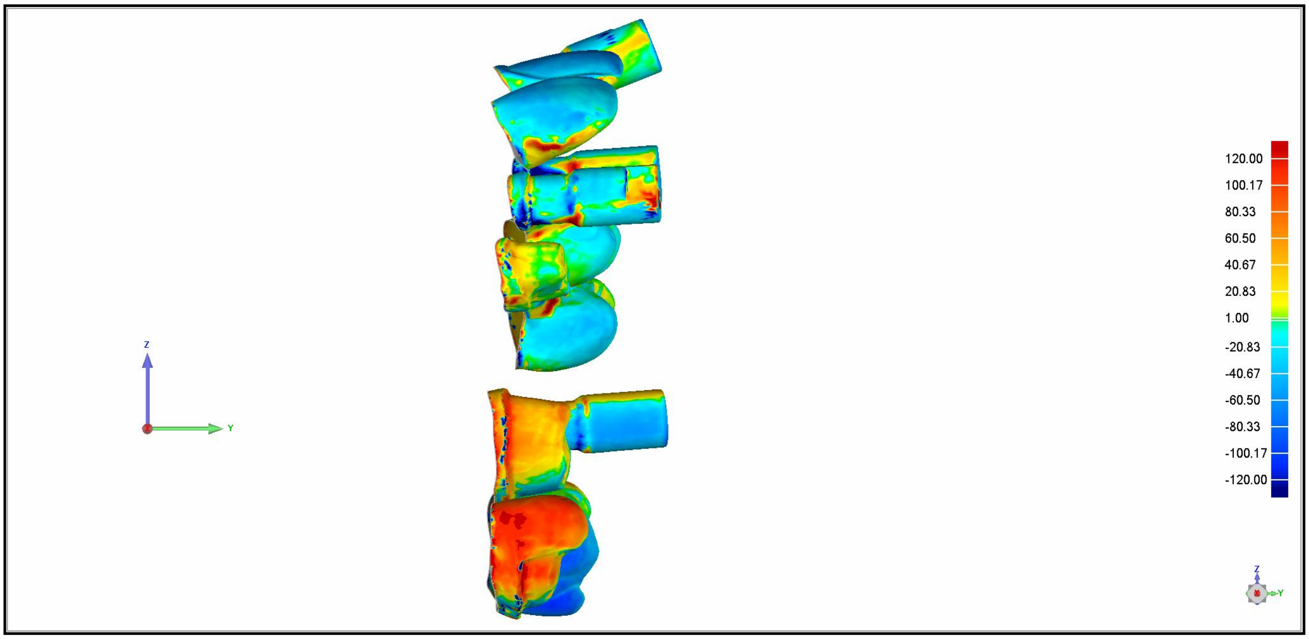

Predefinido: Superior

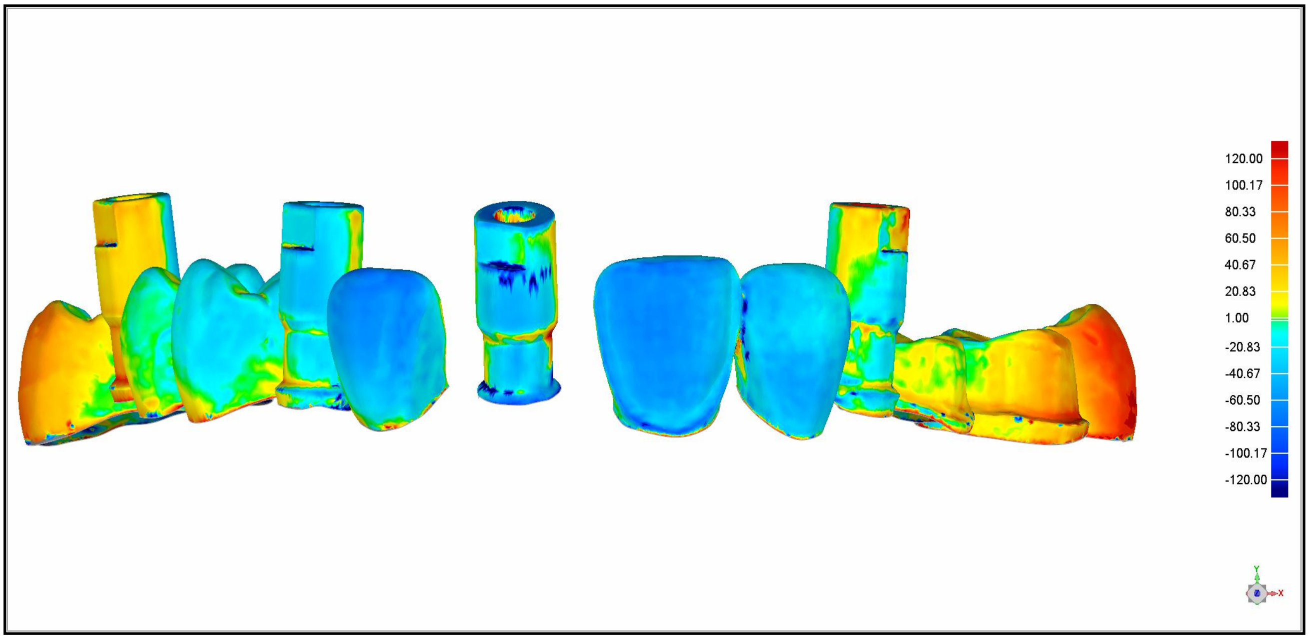

Predefinido: Inferior

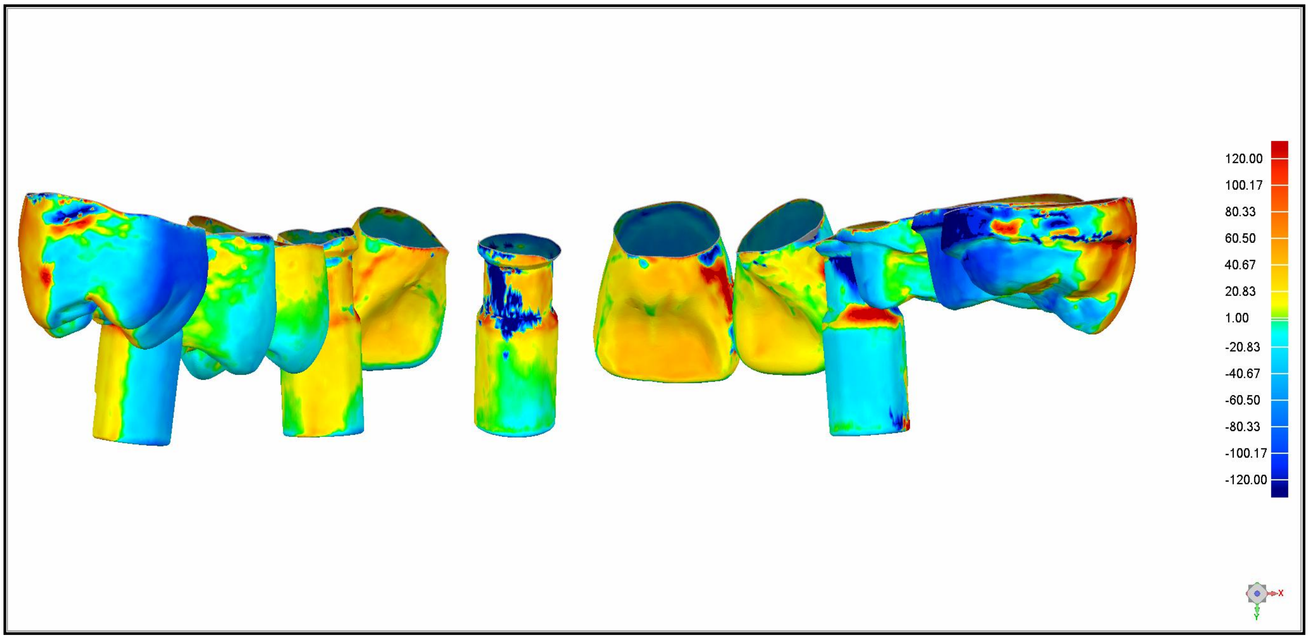

# Ajuste de ubicación: Desviaciones superior e inferior

## Unidades: u

| Nombre | Desv | Estado | Superior Tol | Inferior Tol | Ref X | Ref Y | Ref Z | Radio | Desv X | Desv Y | Desv Z | Medido X | Medido Y | Medido Z | Dir. proy. X | Dir. proy. Y | Dir. proy. Z |
|---|---|---|---|---|---|---|---|---|---|---|---|---|---|---|---|---|---|
| Desv. inferior | -3086.35 | | | | -447.85 | 33320.03 | 27712.93 | n/a | -2593.08 | 1132.94 | -1232.05 | -3040.93 | 34452.97 | 26480.89 | 0.84 | -0.37 | 0.40 |
| Desv. superior | 2992.71 | | | | -3007.03 | 31117.97 | 24973.88 | n/a | 152.59 | 2786.82 | 1080.13 | -2854.44 | 33904.79 | 26054.00 | 0.05 | 0.93 | 0.36 |
