# Supplementary material for: Accuracy of four digital scanners according to scanning strategy in complete-arch impressions
Source: PLoS One. 2018 Sep 13;13(9):e0202916. doi: 10.1371/journal.pone.0202916 (PMC6136706; doi:10.1371/journal.pone.0202916)
Supplement: S11 Table — Omnicam (scanning strategy C). (ZIP) [file pone.0202916.s011.zip › S11/OM5C.pdf]

Geomagic Control

Fecha: 3/6/2017, 3:02 pm

3D Comparación Resultados

Modelo referencia	MRC
Modelo test	OM5C
Nº de puntos de datos	196562
# Aislados	717

Tipo tolerancia	3D desviación
Unidades	u
Máx. crítico	120.00
Máx. nominal	13.00
Mín. nominal	-13.00
Mín. crítico	-120.00

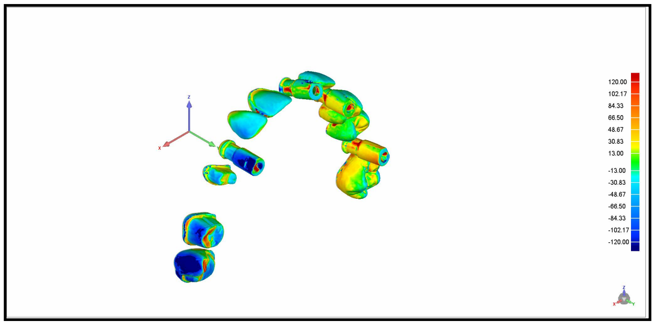

Desviación	
Desviación superior máx.	3124.30
Desviación inferior máx.	-3149.24
Desviación media	86.78 /-78.50
Desviación estándar	243.41

Distribución desviación

>=Min	<Max	# Puntos	%
-120.00	-102.17	1992	1.01
-102.17	-84.33	2220	1.13
-84.33	-66.50	3267	1.66
-66.50	-48.67	5334	2.71
-48.67	-30.83	11527	5.86
-30.83	-13.00	27168	13.82
-13.00	13.00	54134	27.54
13.00	30.83	27485	13.98
30.83	48.67	17922	9.12
48.67	66.50	9804	4.99
66.50	84.33	6401	3.26
84.33	102.17	4718	2.40
102.17	120.00	3035	1.54

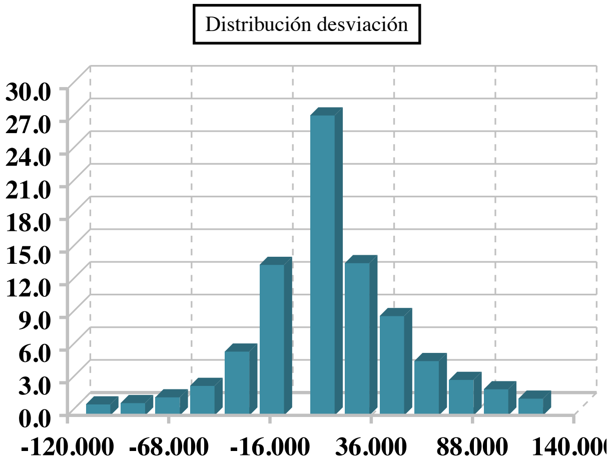

Fuera del crítico superior		12735	6.48
Fuera del crítico inferior		8820	4.49

Desviaciones estándar

Distribución (+/-)	# Puntos	%
-6 * Desv. estánd.	1238	0.63
-5 * Desv. estánd.	408	0.21
-4 * Desv. estánd.	582	0.30
-3 * Desv. estánd.	968	0.49
-2 * Desv. estánd.	1346	0.68
-1 * Desv. estánd.	110135	56.03
1 * Desv. estánd.	75990	38.66
2 * Desv. estánd.	1710	0.87
3 * Desv. estánd.	1130	0.57
4 * Desv. estánd.	997	0.51
5 * Desv. estánd.	833	0.42
6 * Desv. estánd.	1225	0.62

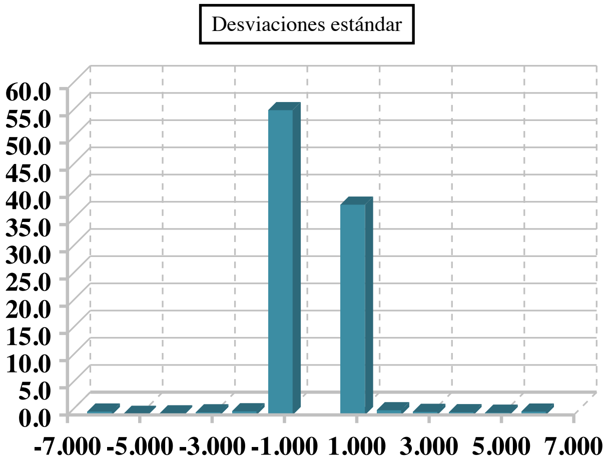

Predefinido: Isométrico

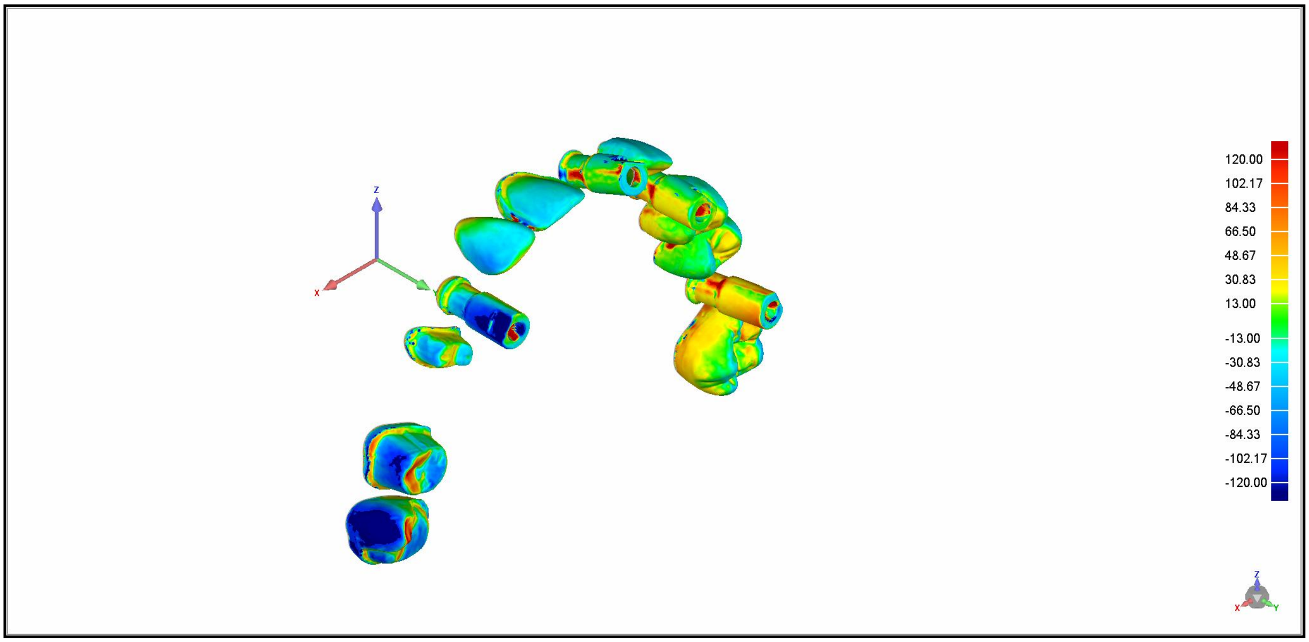

Predefinido: Frente

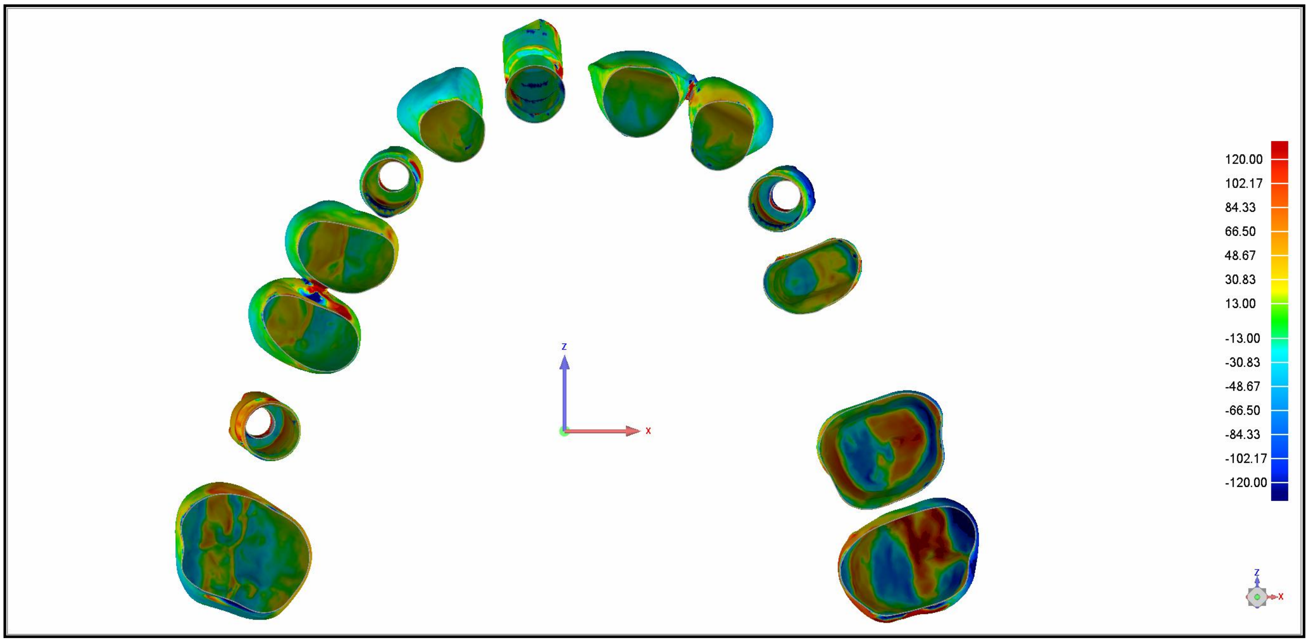

Predefinido: Atrás

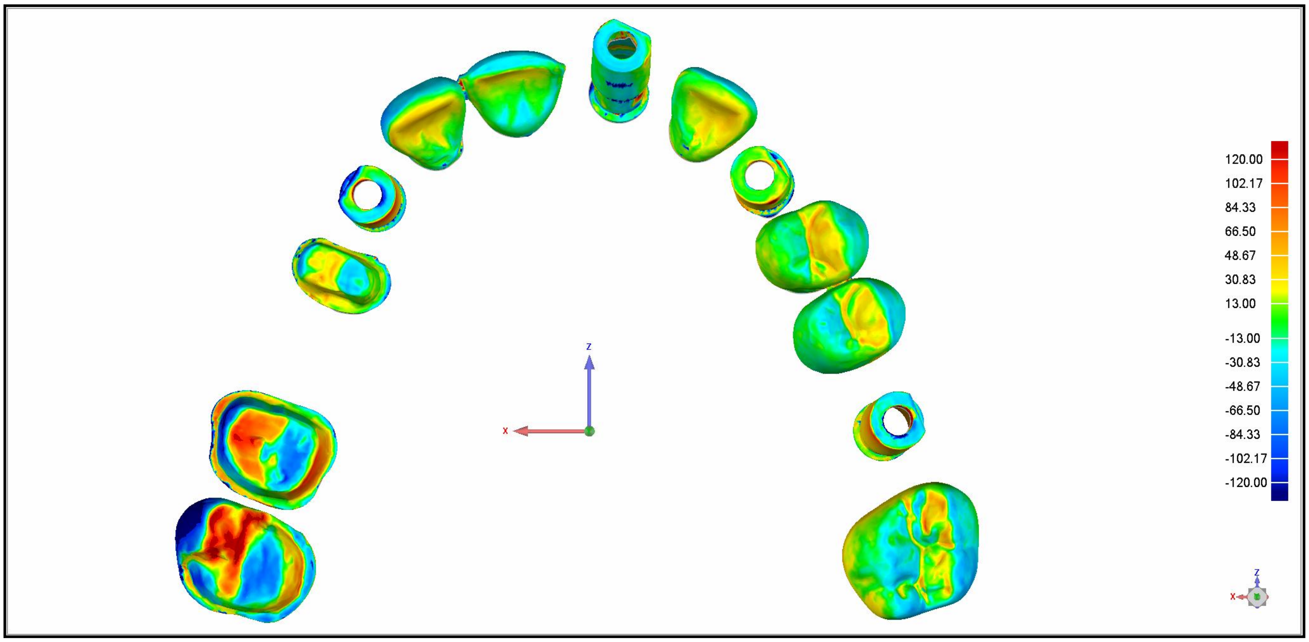

Predefinido: Izquierda

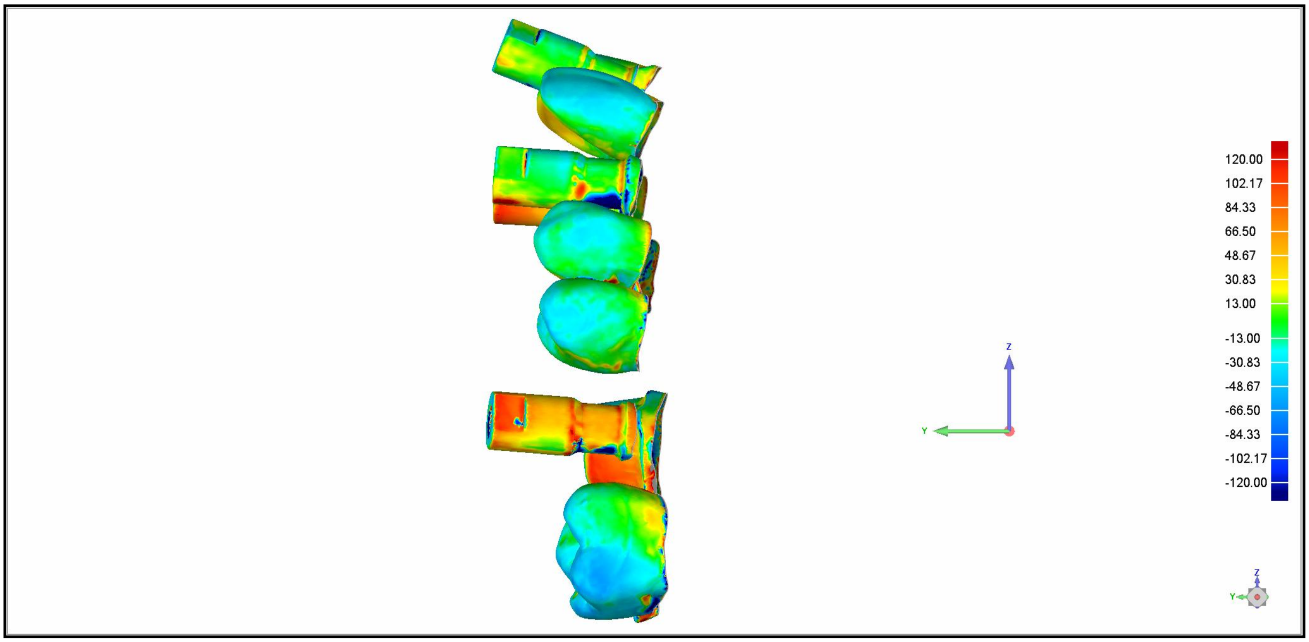

Predefinido: Derecha

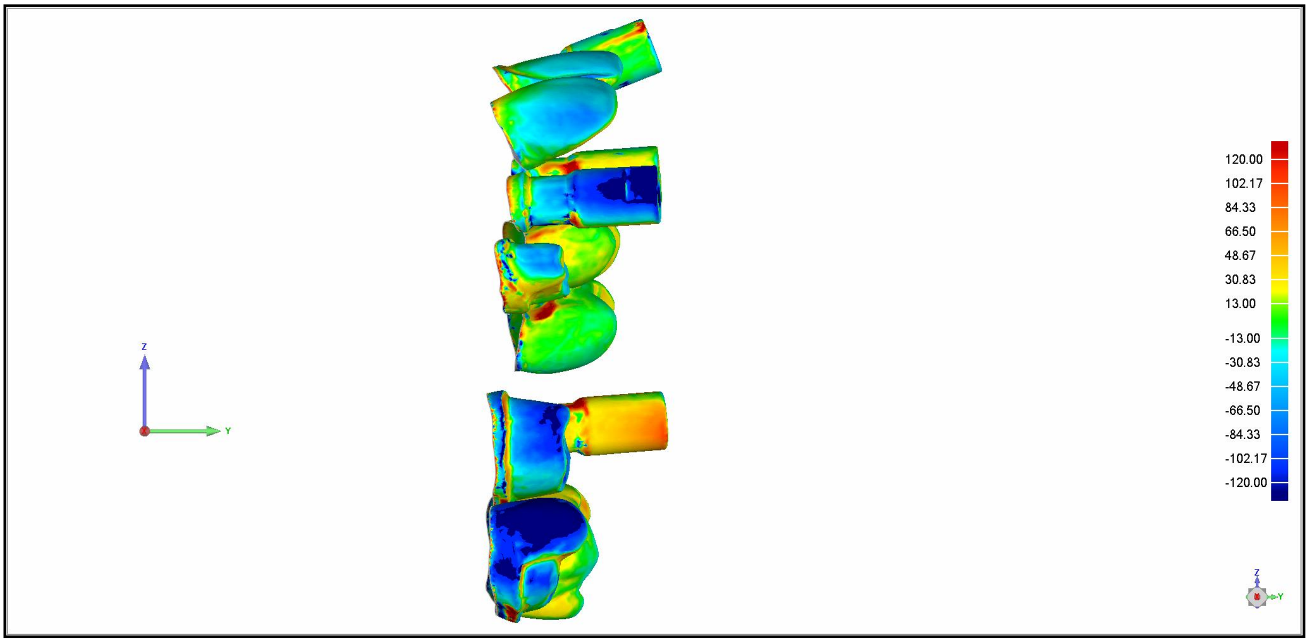

Predefinido: Superior

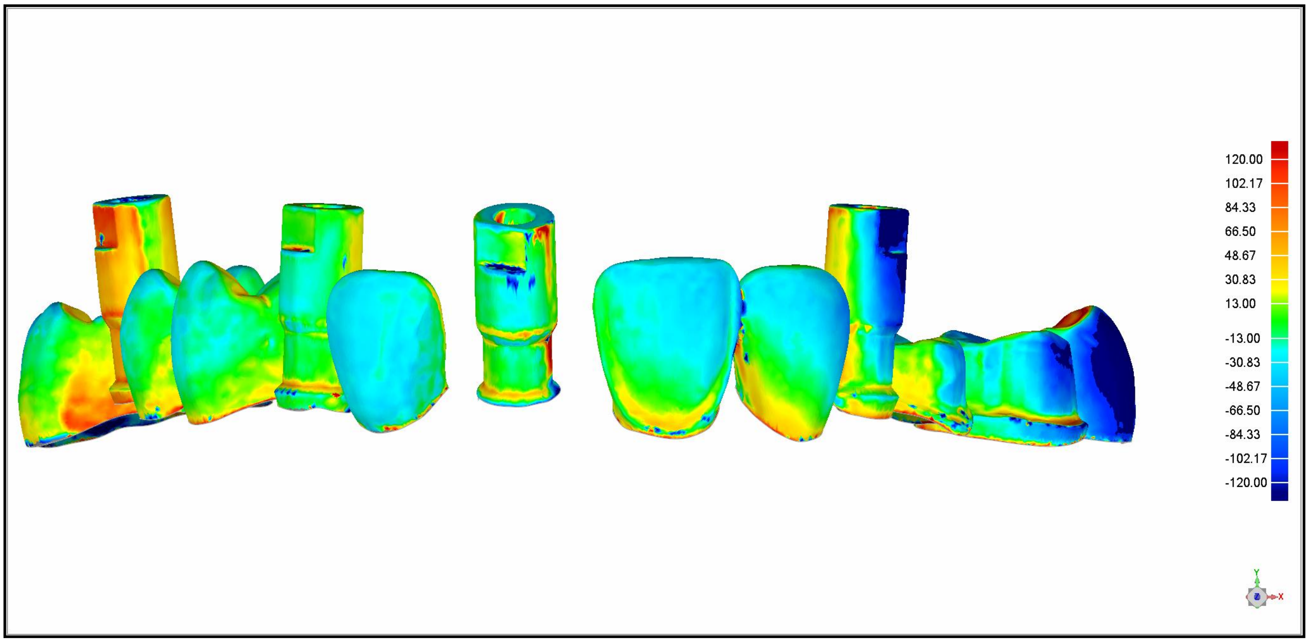

Predefinido: Inferior

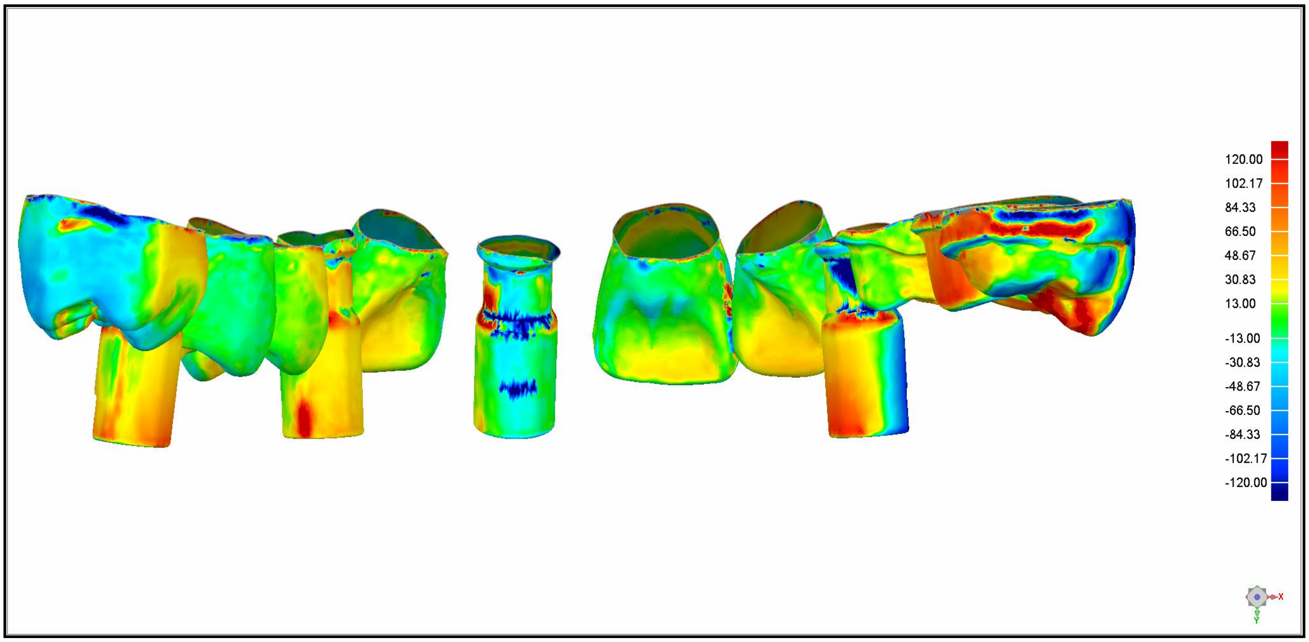

Ajuste de ubicación: Desviaciones superior e inferior

Unidades: u

Nombre	Desv	Estado	Superior Tol	Inferior Tol	Ref X	Ref Y	Ref Z	Radio	Desv X	Desv Y	Desv Z	Medido X	Medido Y	Medido Z	Dir. proy. X	Dir. proy. Y	Dir. proy. Z
Desv. inferior	-3149.24				-22607.19	28955.77	6808.03	n/a	-881.36	-509.80	2980.11	-23488.56	28445.97	9788.14	0.28	0.16	-0.95
Desv. superior	3124.30				-2206.13	31227.25	24916.67	n/a	-86.83	2799.68	1384.01	-2292.96	34026.93	26300.68	-0.03	0.90	0.44
