# Supplementary material for: Accuracy of four digital scanners according to scanning strategy in complete-arch impressions
Source: PLoS One. 2018 Sep 13;13(9):e0202916. doi: 10.1371/journal.pone.0202916 (PMC6136706; doi:10.1371/journal.pone.0202916)
Supplement: S11 Table — Omnicam (scanning strategy C). (ZIP) [file pone.0202916.s011.zip › S11/OM6C.pdf]

Geomagic Control

Fecha: 3/6/2017, 3:05 pm

3D Comparación Resultados

Modelo referencia	MRC
Modelo test	OM6C
Nº de puntos de datos	199838
# Aislados	636

Tipo tolerancia	3D desviación
Unidades	u
Máx. crítico	120.00
Máx. nominal	7.00
Mín. nominal	-7.00
Mín. crítico	-120.00

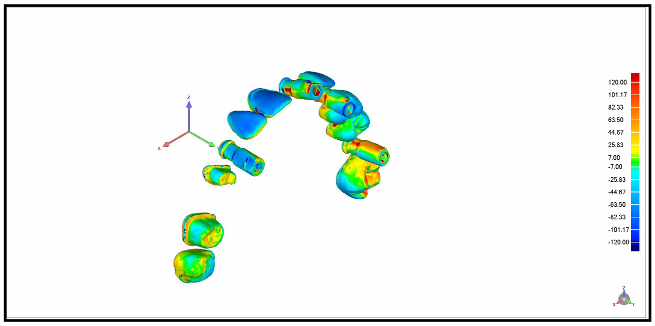

Desviación	
Desviación superior máx.	3111.58
Desviación inferior máx.	-3151.89
Desviación media	95.33 /-84.47
Desviación estándar	262.22

Distribución desviación

>=Min	<Max	# Puntos	%
-120.00	-101.17	751	0.38
-101.17	-82.33	1603	0.80
-82.33	-63.50	7479	3.74
-63.50	-44.67	14308	7.16
-44.67	-25.83	20999	10.51
-25.83	-7.00	32477	16.25
-7.00	7.00	27583	13.80
7.00	25.83	31072	15.55
25.83	44.67	20118	10.07
44.67	63.50	11165	5.59
63.50	82.33	6208	3.11
82.33	101.17	3065	1.53
101.17	120.00	2160	1.08

Fuera del crítico superior	14194	7.10
Fuera del crítico inferior	6656	3.33

Distribución desviación

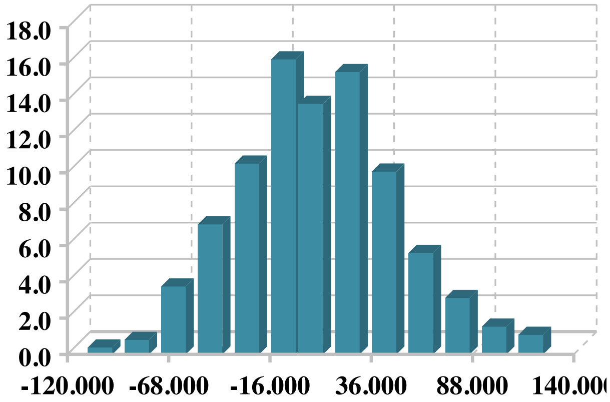

Desviaciones estándar

Distribución (+/-)	# Puntos	%
-6 * Desv. estánd.	1657	0.83
-5 * Desv. estánd.	840	0.42
-4 * Desv. estánd.	687	0.34
-3 * Desv. estánd.	584	0.29
-2 * Desv. estánd.	997	0.50
-1 * Desv. estánd.	107667	53.88
1 * Desv. estánd.	80123	40.09
2 * Desv. estánd.	2957	1.48
3 * Desv. estánd.	1566	0.78
4 * Desv. estánd.	920	0.46
5 * Desv. estánd.	792	0.40
6 * Desv. estánd.	1048	0.52

Desviaciones estándar

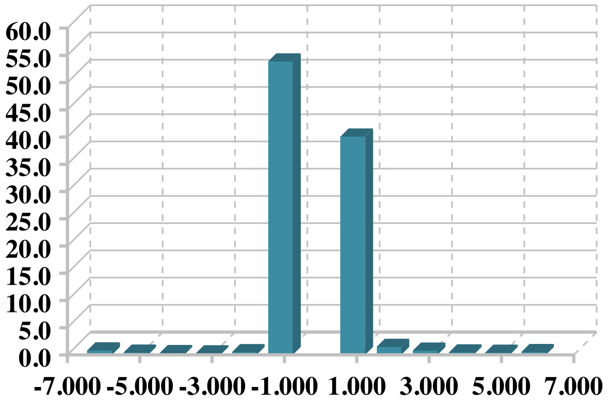

Predefinido: Isométrico

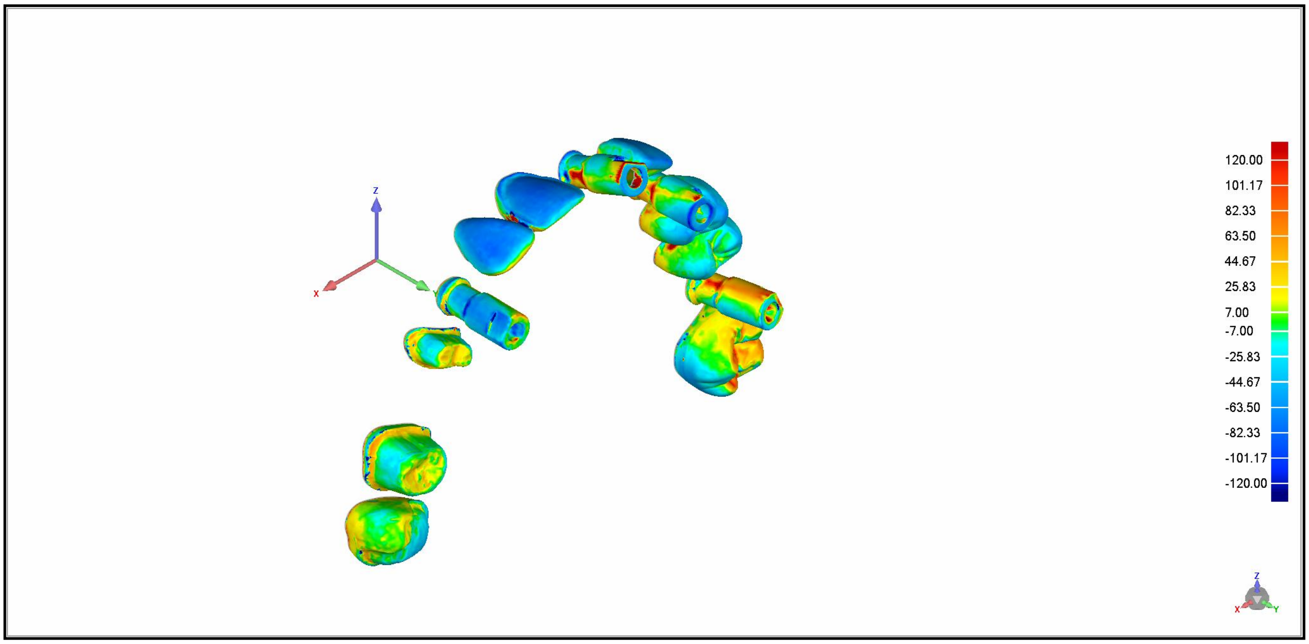

Predefinido: Frente

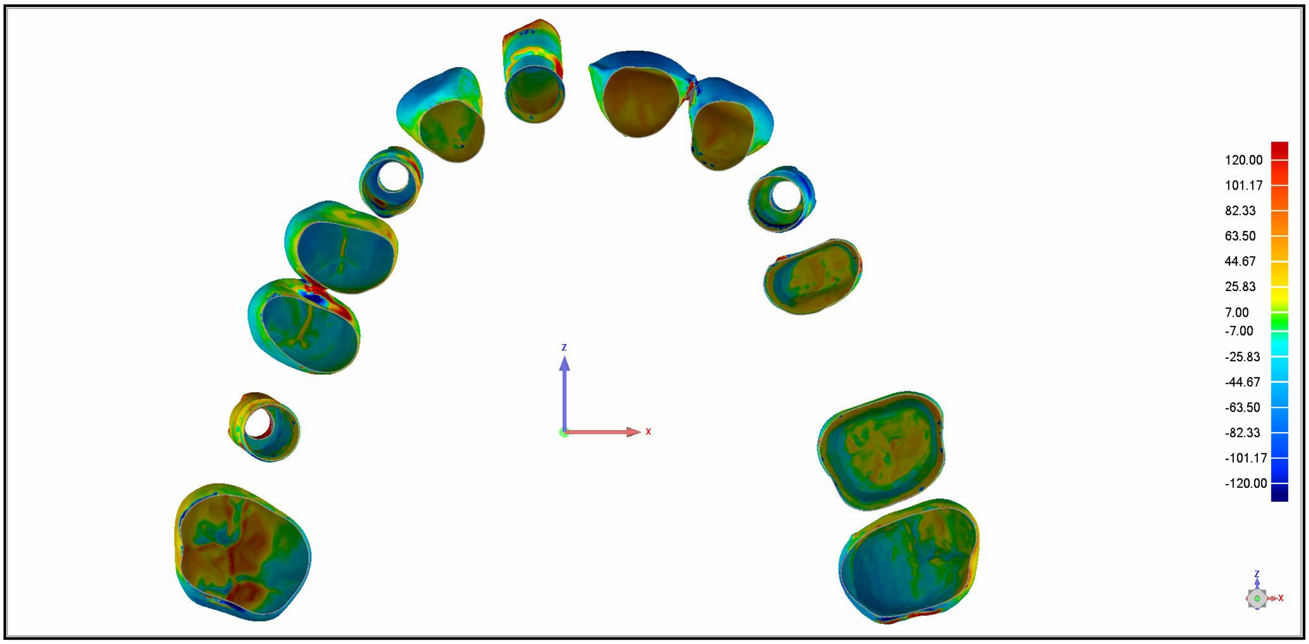

Predefinido: Atrás

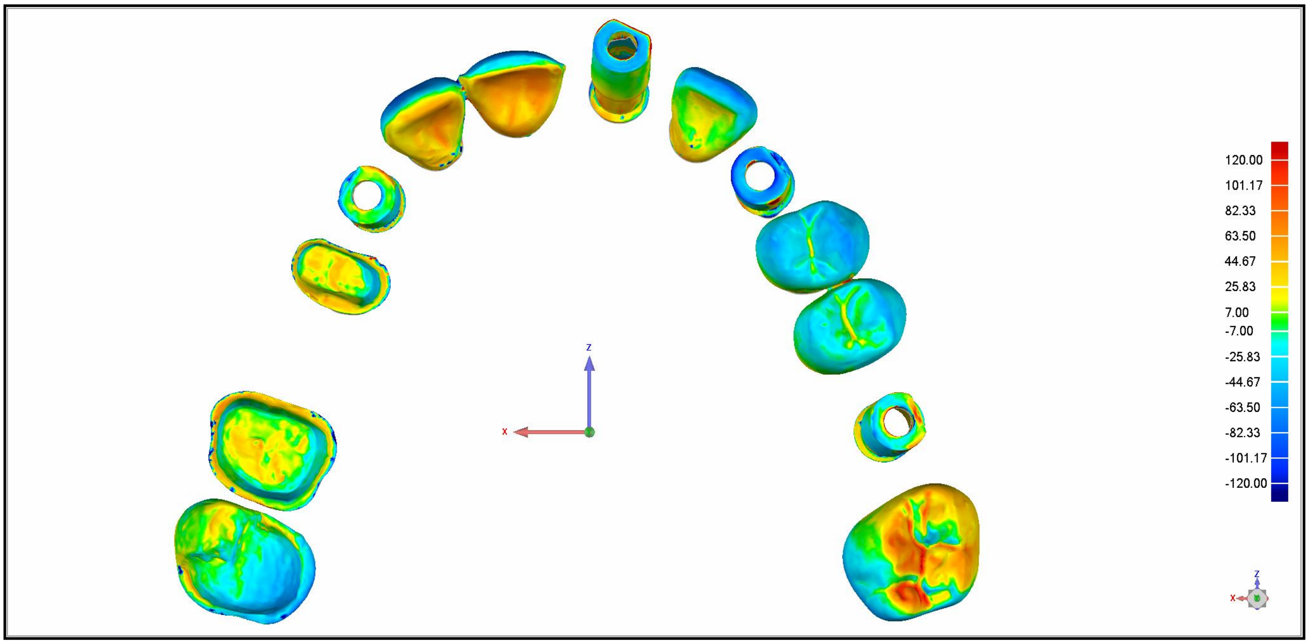

Predefinido: Izquierda

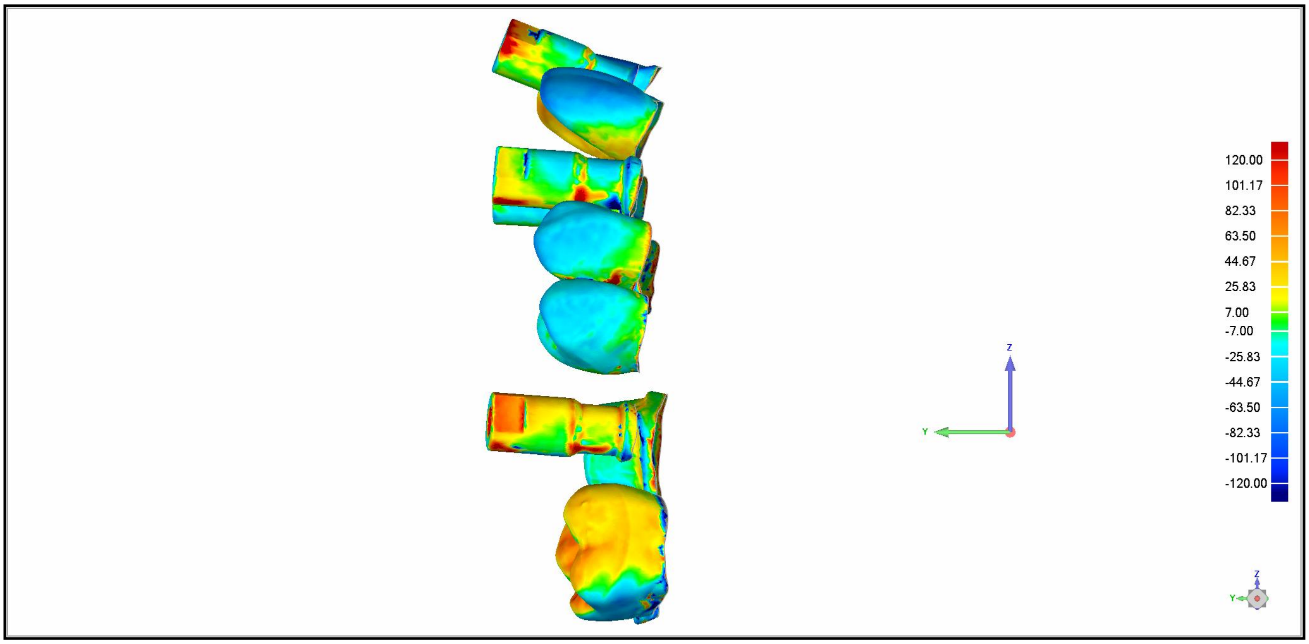

Predefinido: Derecha

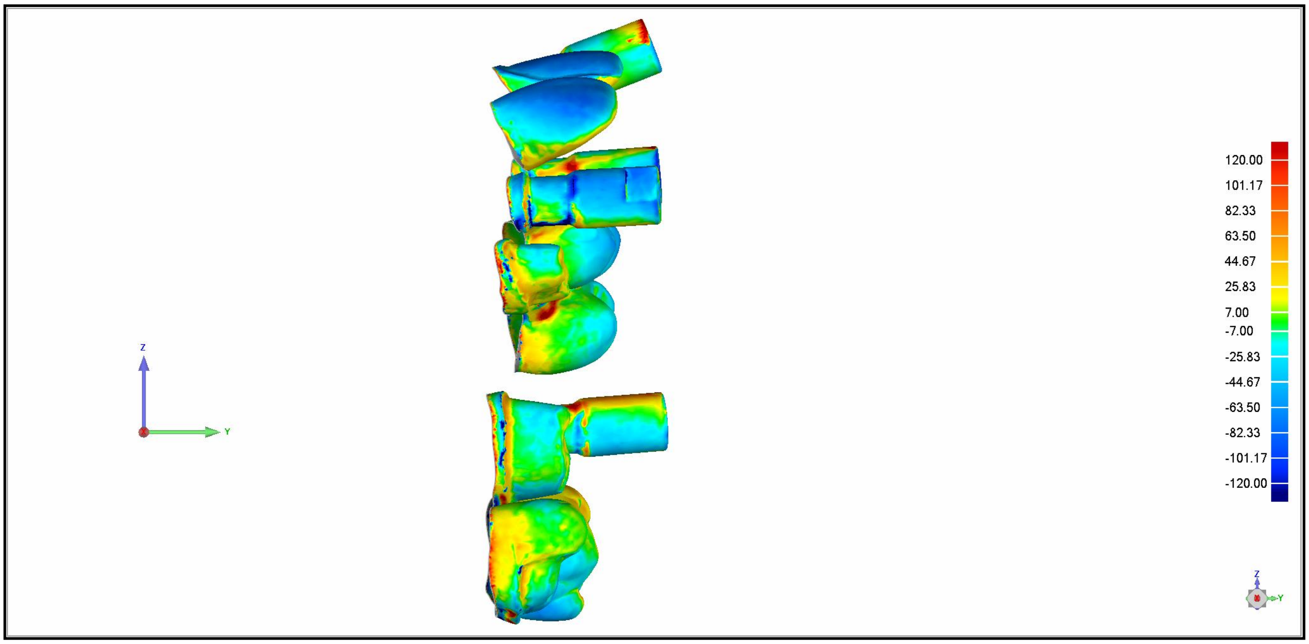

Predefinido: Superior

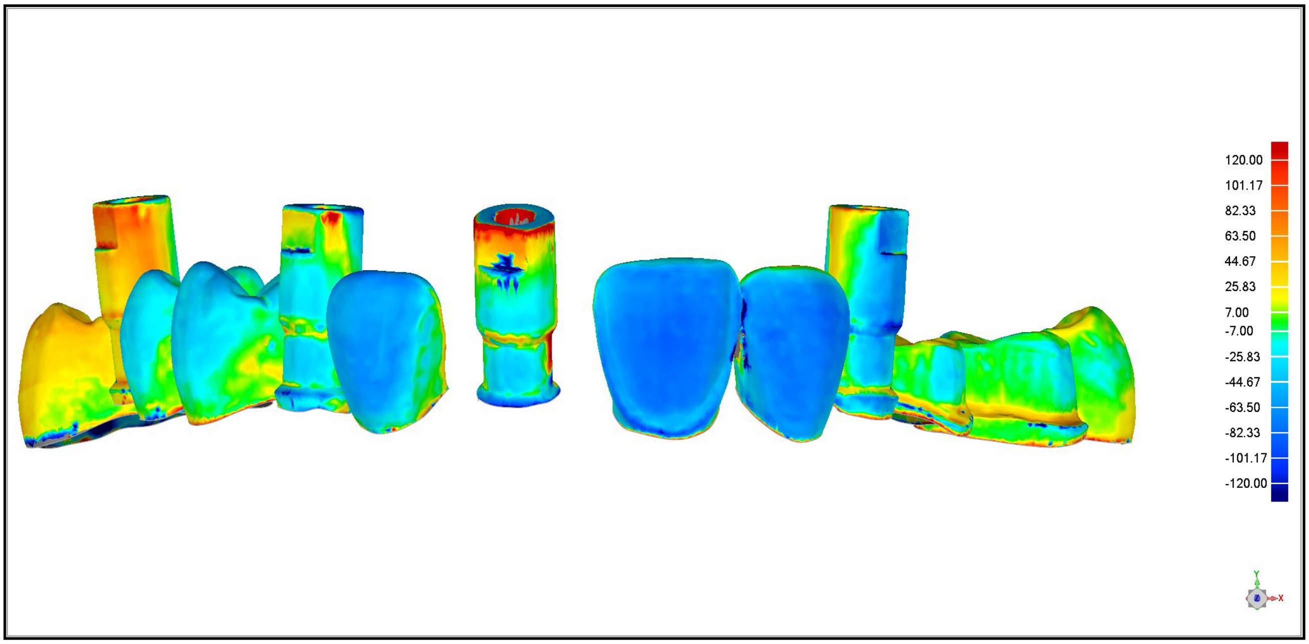

Predefinido: Inferior

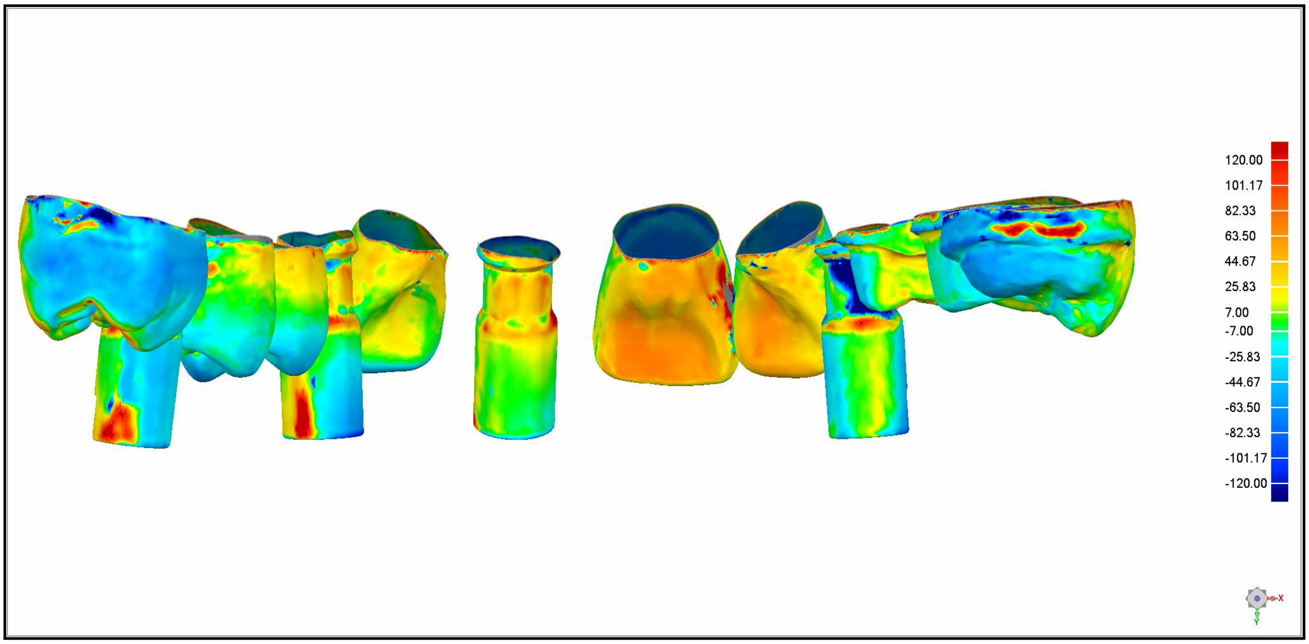

Ajuste de ubicación: Desviaciones superior e inferior

Unidades: u

Nombre	Desv	Estado	Superior Tol	Inferior Tol	Ref X	Ref Y	Ref Z	Radio	Desv X	Desv Y	Desv Z	Medido X	Medido Y	Medido Z	Dir. proy. X	Dir. proy. Y	Dir. proy. Z
Desv. inferior	-3151.89				-16498.53	29426.69	5746.14	n/a	-3016.36	-208.89	-890.13	-19514.88	29217.80	4856.01	0.96	0.07	0.28
Desv. superior	3111.58				-20553.64	28741.29	-8096.88	n/a	-2343.69	38.99	2046.34	-22897.33	28780.28	-6050.54	-0.75	0.01	0.66
