# Supplementary material for: Accuracy of four digital scanners according to scanning strategy in complete-arch impressions
Source: PLoS One. 2018 Sep 13;13(9):e0202916. doi: 10.1371/journal.pone.0202916 (PMC6136706; doi:10.1371/journal.pone.0202916)
Supplement: S11 Table — Omnicam (scanning strategy C). (ZIP) [file pone.0202916.s011.zip › S11/OM7C.pdf]

Geomagic Control™

3D Comparación Resultados

Modelo referencia	MRC
Modelo test	OM7C
Nº de puntos de datos	200955
# Aislados	830

Tipo tolerancia	3D desviación
Unidades	u
Máx. crítico	120.00
Máx. nominal	6.00
Mín. nominal	-6.00
Mín. crítico	-120.00

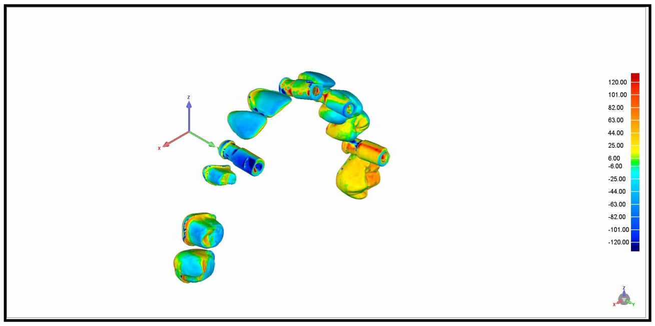

Desviación	
Desviación superior máx.	3014.69
Desviación inferior máx.	-2982.42
Desviación media	90.41 /-77.52
Desviación estándar	238.92

Distribución desviación

>=Min	<Max	# Puntos	%
-120.00	-101.00	1393	0.69
-101.00	-82.00	1885	0.94
-82.00	-63.00	2984	1.48
-63.00	-44.00	10451	5.20
-44.00	-25.00	26967	13.42
-25.00	-6.00	36629	18.23
-6.00	6.00	23113	11.50
6.00	25.00	33020	16.43
25.00	44.00	21039	10.47
44.00	63.00	11257	5.60
63.00	82.00	6228	3.10
82.00	101.00	3272	1.63
101.00	120.00	1958	0.97

Fuera del crítico superior	12642	6.29
Fuera del crítico inferior	8117	4.04

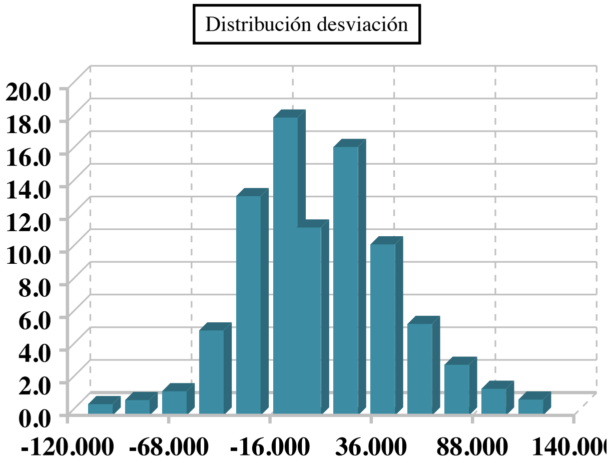

Desviaciones estándar

Distribución (+/-)	# Puntos	%
-6 * Desv. estánd.	1352	0.67
-5 * Desv. estánd.	592	0.29
-4 * Desv. estánd.	1040	0.52
-3 * Desv. estánd.	1237	0.62
-2 * Desv. estánd.	1423	0.71
-1 * Desv. estánd.	107496	53.49
1 * Desv. estánd.	80853	40.23
2 * Desv. estánd.	2424	1.21
3 * Desv. estánd.	1175	0.58
4 * Desv. estánd.	1174	0.58
5 * Desv. estánd.	1112	0.55
6 * Desv. estánd.	1077	0.54

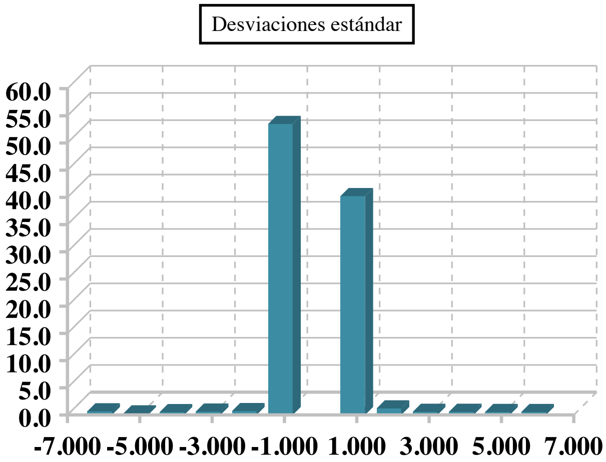

Predefinido: Isométrico

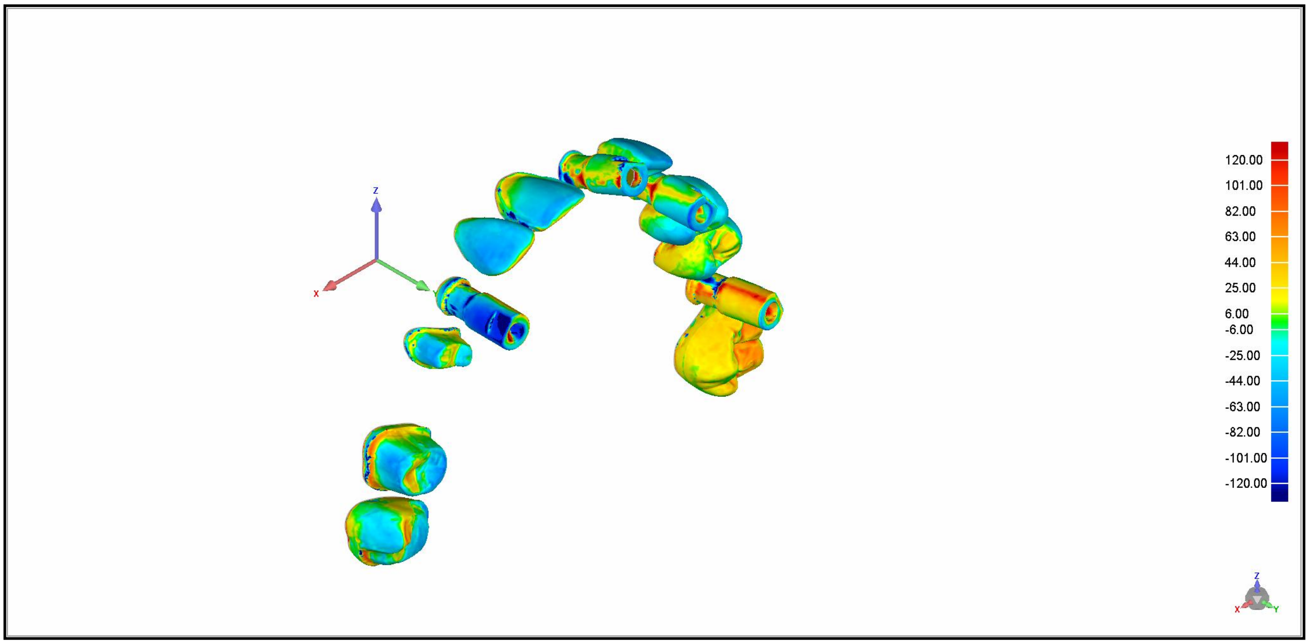

Predefinido: Frente

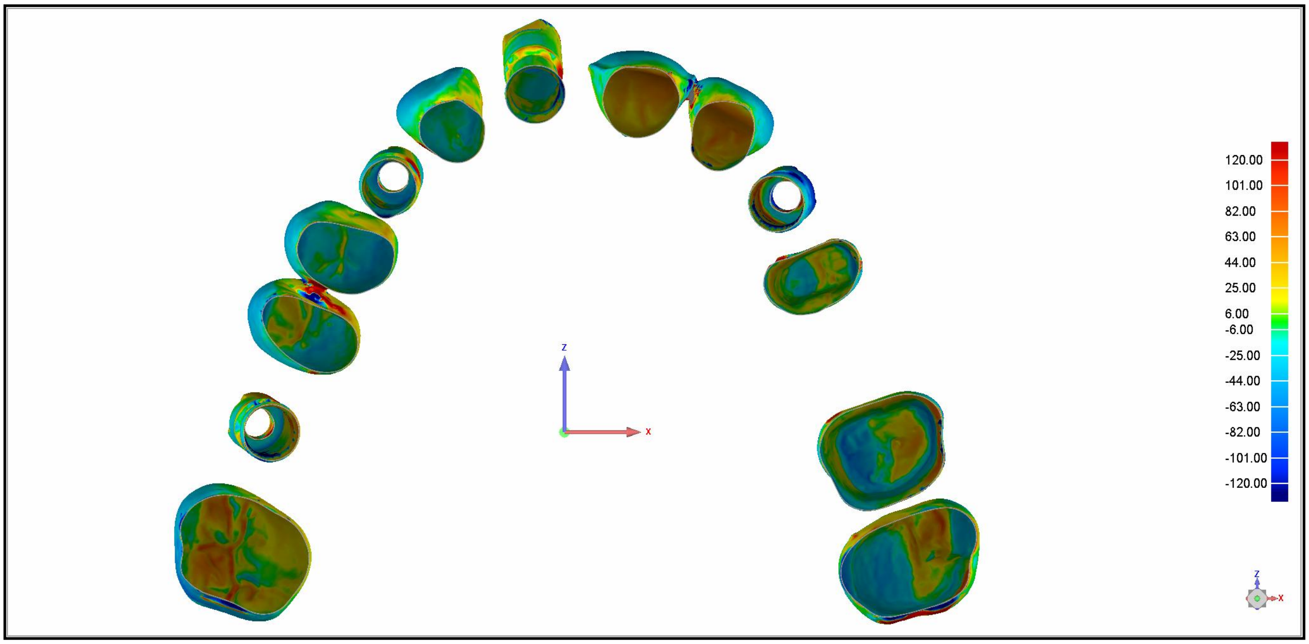

Geomagic Control™

Predefinido: Atrás

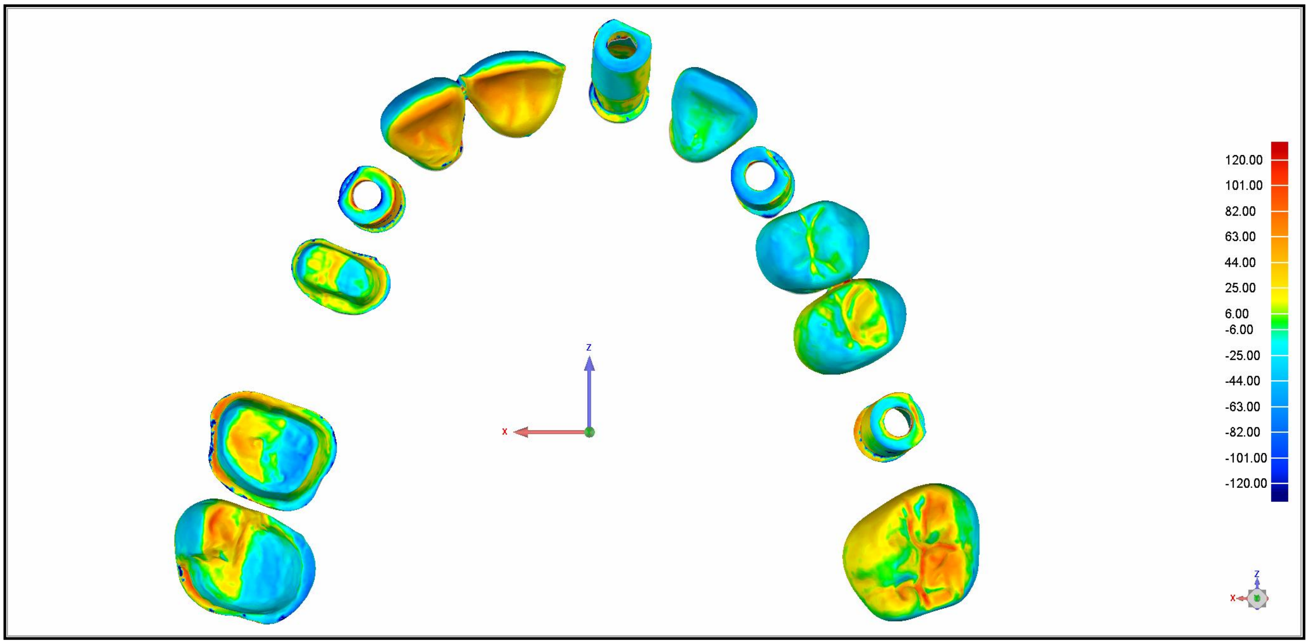

Predefinido: Izquierda

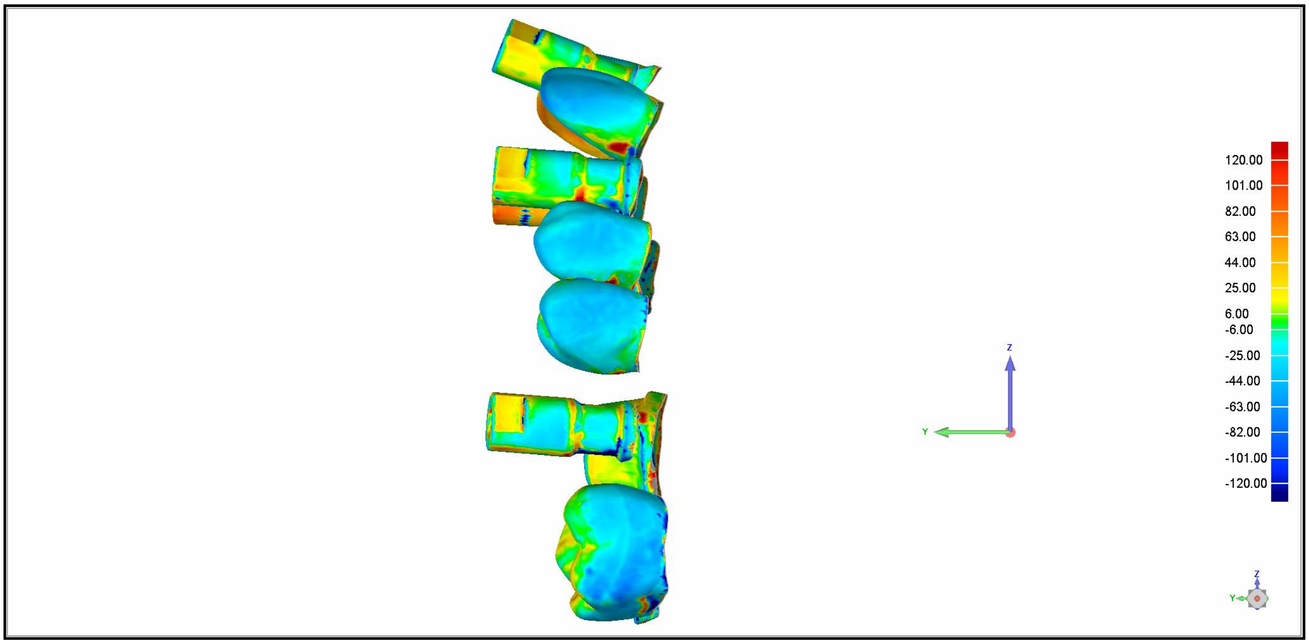

Predefinido: Derecha

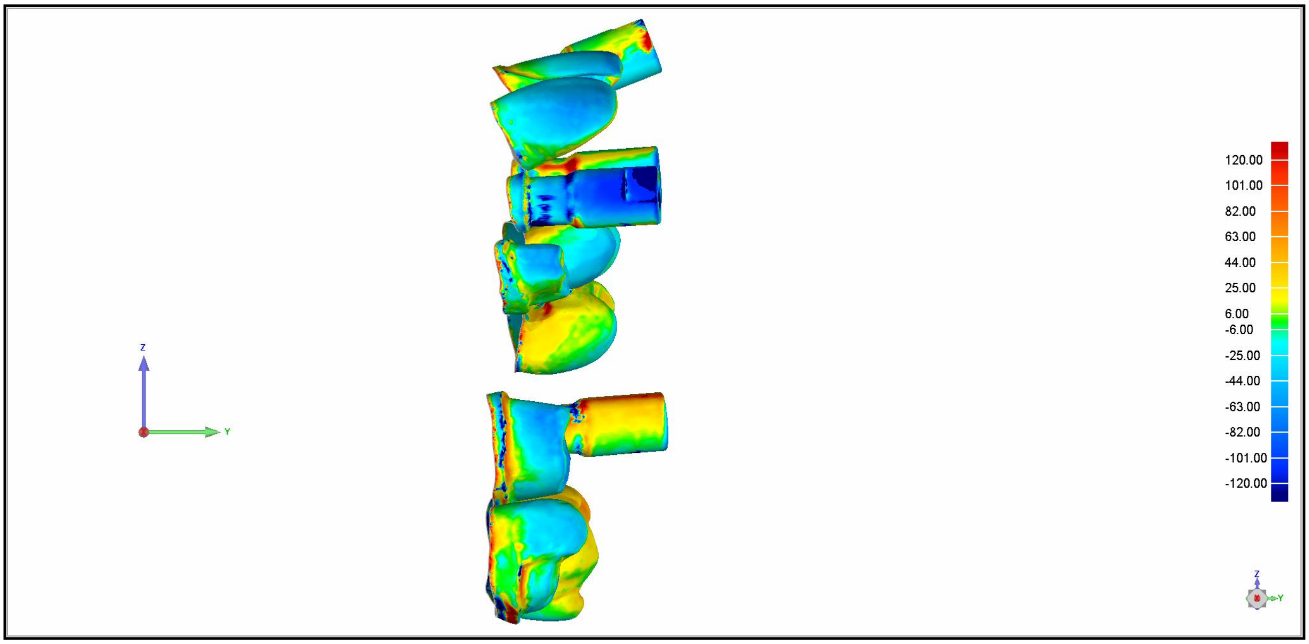

Predefinido: Superior

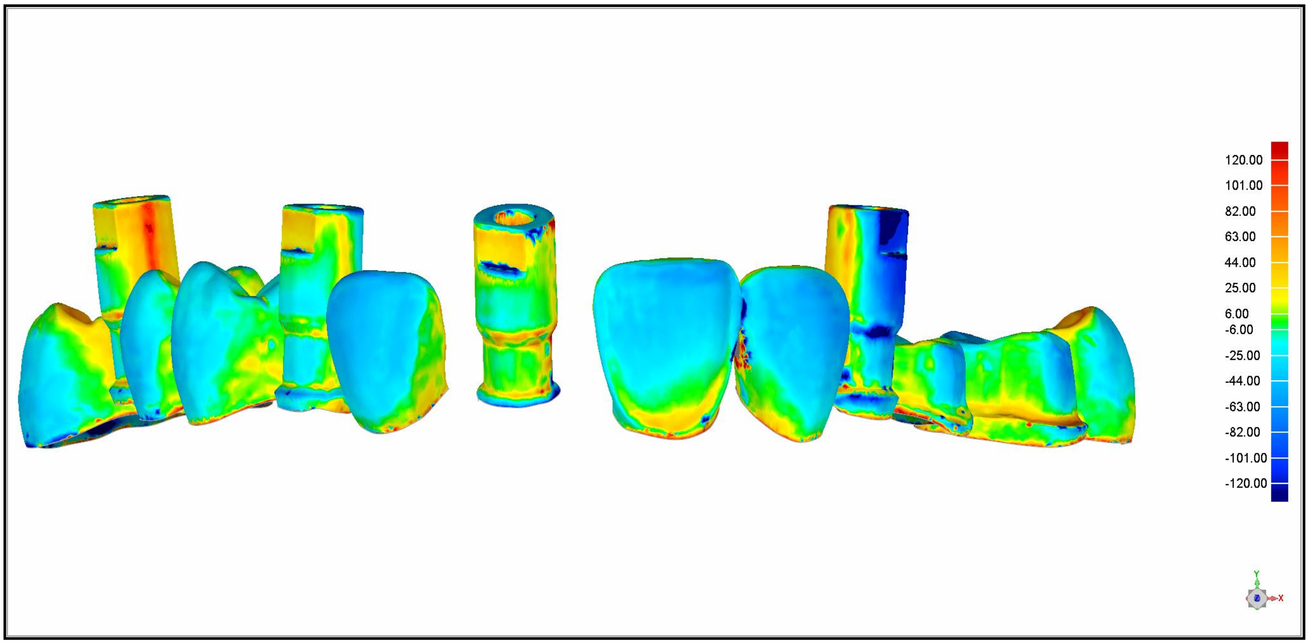

Predefinido: Inferior

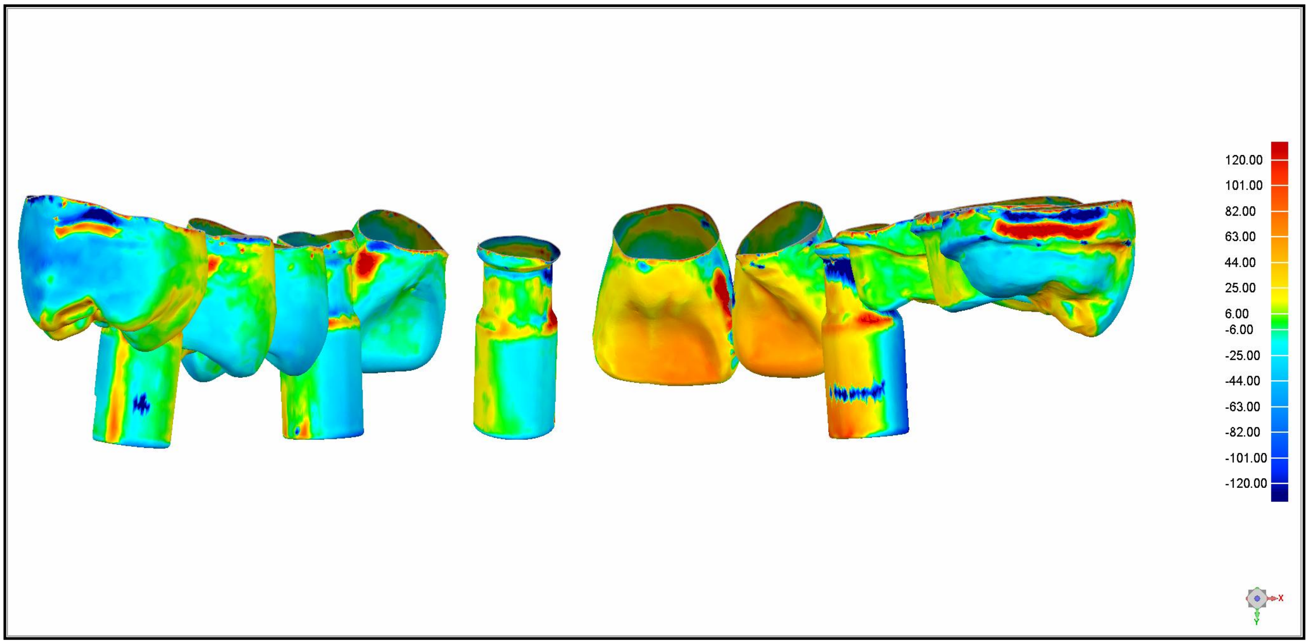

Ajuste de ubicación: Desviaciones superior e inferior

Unidades: u

Nombre	Desv	Estado	Superior Tol	Inferior Tol	Ref X	Ref Y	Ref Z	Radio	Desv X	Desv Y	Desv Z	Medido X	Medido Y	Medido Z	Dir. proy. X	Dir. proy. Y	Dir. proy. Z
Desv. inferior	-2982.42				20524.03	27891.24	-2916.66	n/a	-197.29	13.34	2975.85	20326.74	27904.58	59.19	0.07	-0.00	-1.00
Desv. superior	3014.69				29547.11	27336.30	1208.53	n/a	-2360.03	-420.99	1827.95	27187.08	26915.30	3036.48	-0.78	-0.14	0.61
