# Supplementary material for: Accuracy of four digital scanners according to scanning strategy in complete-arch impressions
Source: PLoS One. 2018 Sep 13;13(9):e0202916. doi: 10.1371/journal.pone.0202916 (PMC6136706; doi:10.1371/journal.pone.0202916)
Supplement: S11 Table — Omnicam (scanning strategy C). (ZIP) [file pone.0202916.s011.zip › S11/OM10C.pdf]

3D Comparación Resultados

Modelo referencia	MRC
Modelo test	OM10C
Nº de puntos de datos	200539
# Aislados	810

Tipo tolerancia	3D desviación
Unidades	u
Máx. crítico	120.00
Máx. nominal	5.00
Mín. nominal	-5.00
Mín. crítico	-120.00

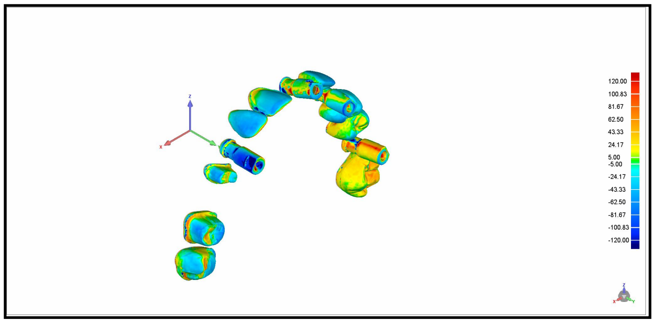

Desviación	
Desviación superior máx.	2978.00
Desviación inferior máx.	-3102.62
Desviación media	91.95 /-78.59
Desviación estándar	243.24

Distribución desviación

>=Min	<Max	# Puntos	%
-120.00	-100.83	1440	0.72
-100.83	-81.67	1923	0.96
-81.67	-62.50	3354	1.67
-62.50	-43.33	11459	5.71
-43.33	-24.17	28203	14.06
-24.17	-5.00	37376	18.64
-5.00	5.00	19491	9.72
5.00	24.17	33549	16.73
24.17	43.33	19926	9.94
43.33	62.50	11343	5.66
62.50	81.67	6353	3.17
81.67	100.83	3118	1.55
100.83	120.00	2014	1.00

Fuera del crítico superior	12712	6.34
Fuera del crítico inferior	8278	4.13

Distribución desviación

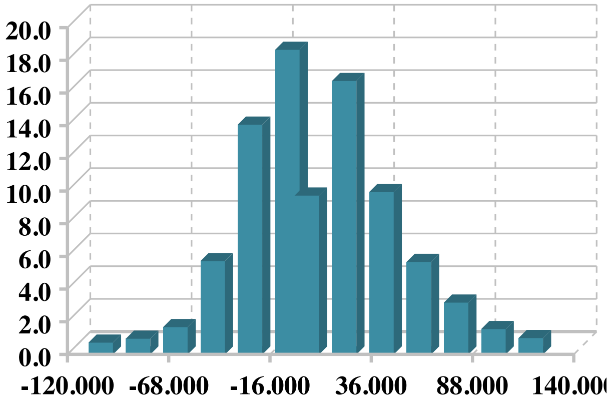

Desviaciones estándar

Distribución (+/-)	# Puntos	%
-6 * Desv. estánd.	1398	0.70
-5 * Desv. estánd.	580	0.29
-4 * Desv. estánd.	1032	0.51
-3 * Desv. estánd.	1233	0.61
-2 * Desv. estánd.	1443	0.72
-1 * Desv. estánd.	106638	53.18
1 * Desv. estánd.	81273	40.53
2 * Desv. estánd.	2433	1.21
3 * Desv. estánd.	1157	0.58
4 * Desv. estánd.	1218	0.61
5 * Desv. estánd.	1064	0.53
6 * Desv. estánd.	1070	0.53

Desviaciones estándar

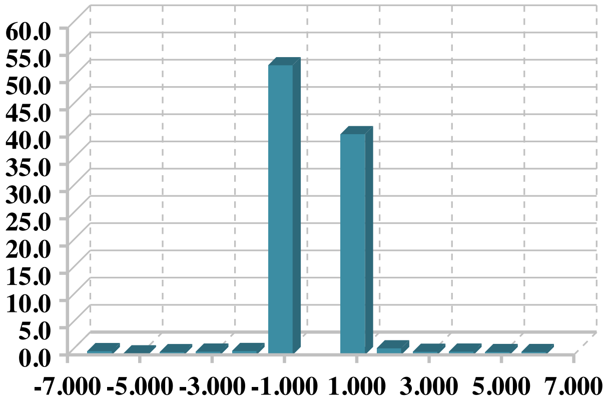

Predefinido: Isométrico

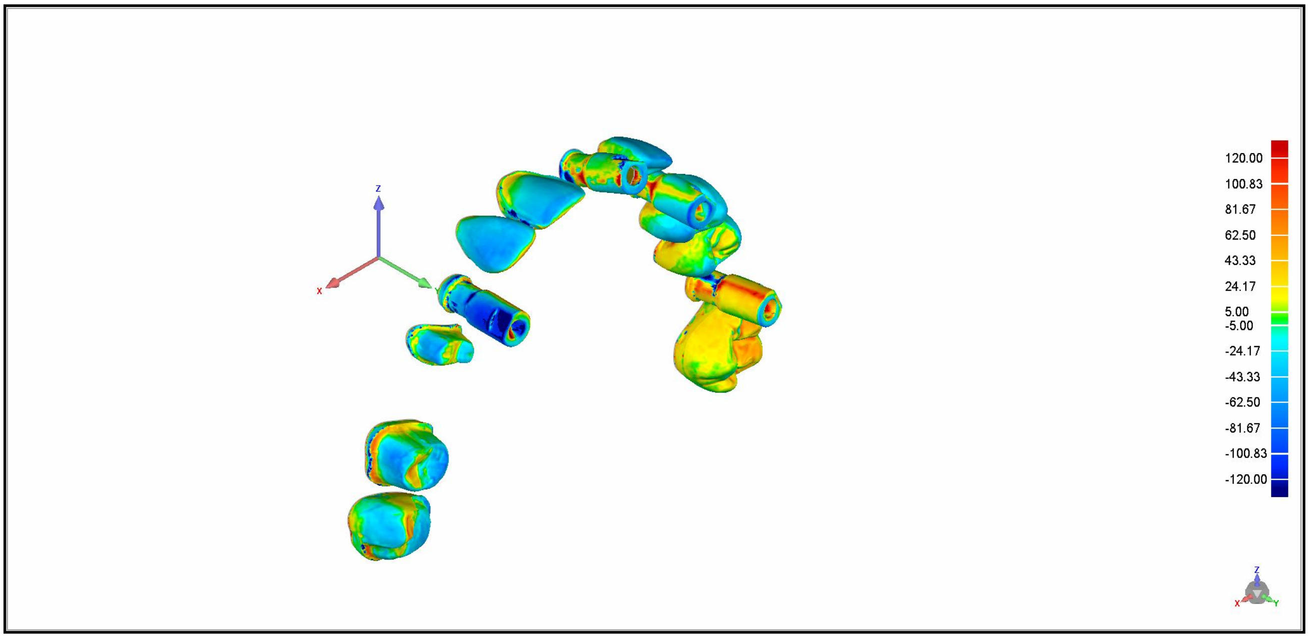

Predefinido: Frente

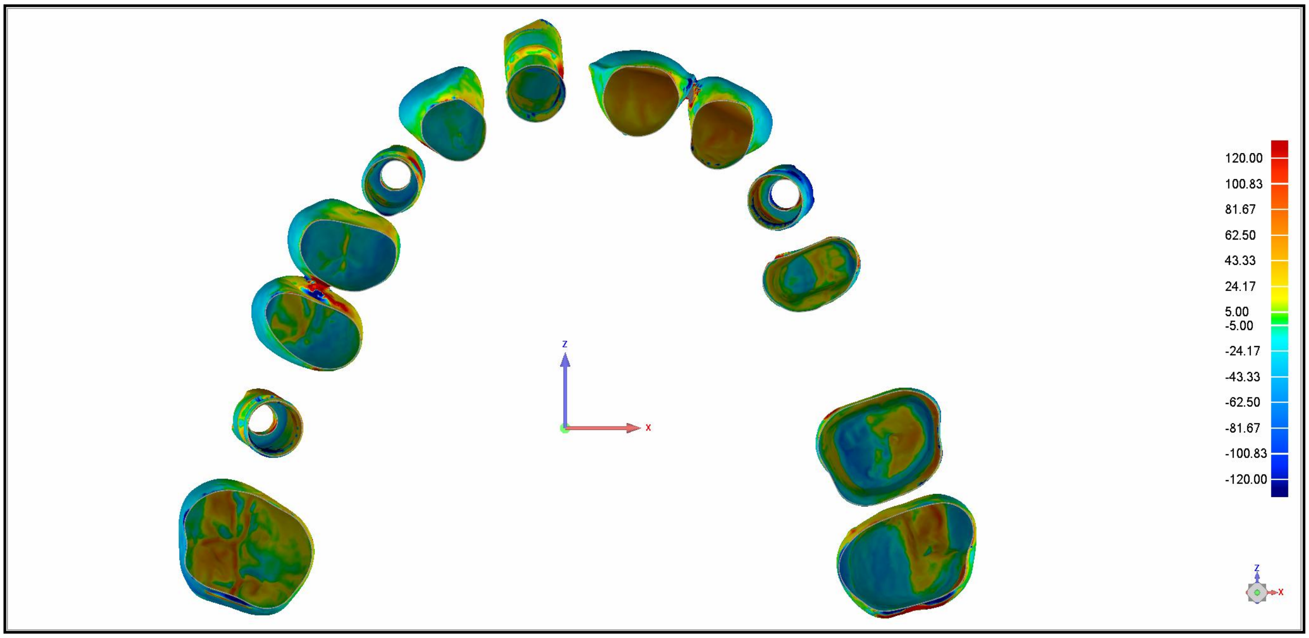

Predefinido: Atrás

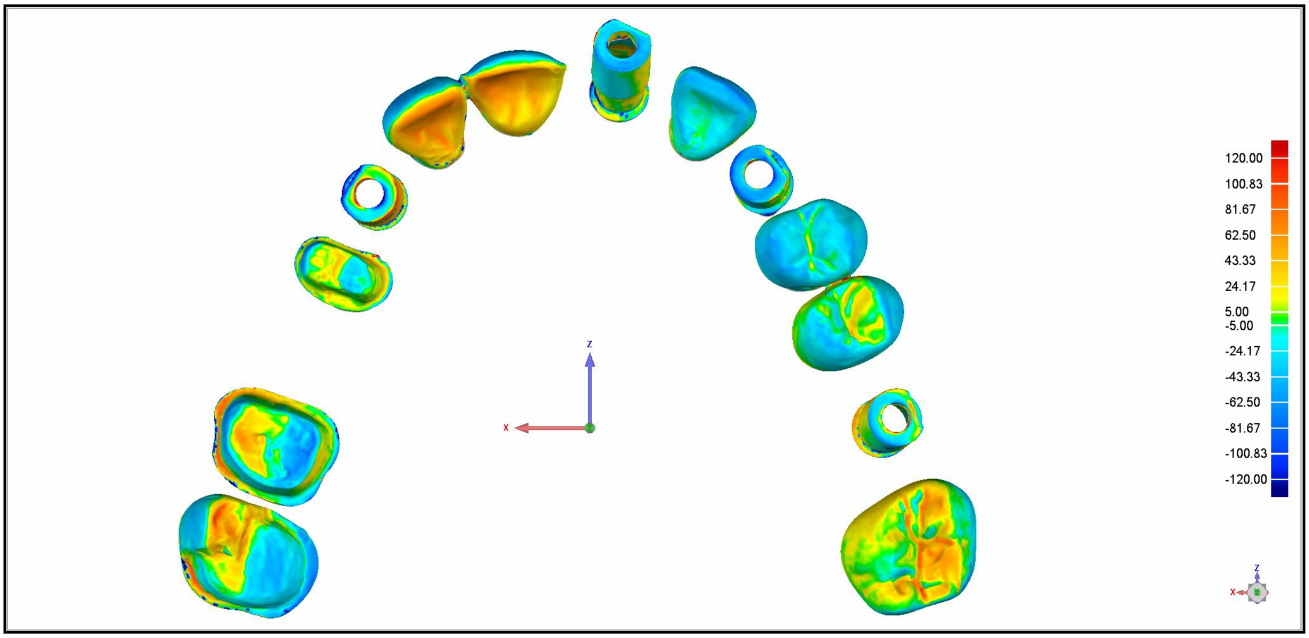

Predefinido: Izquierda

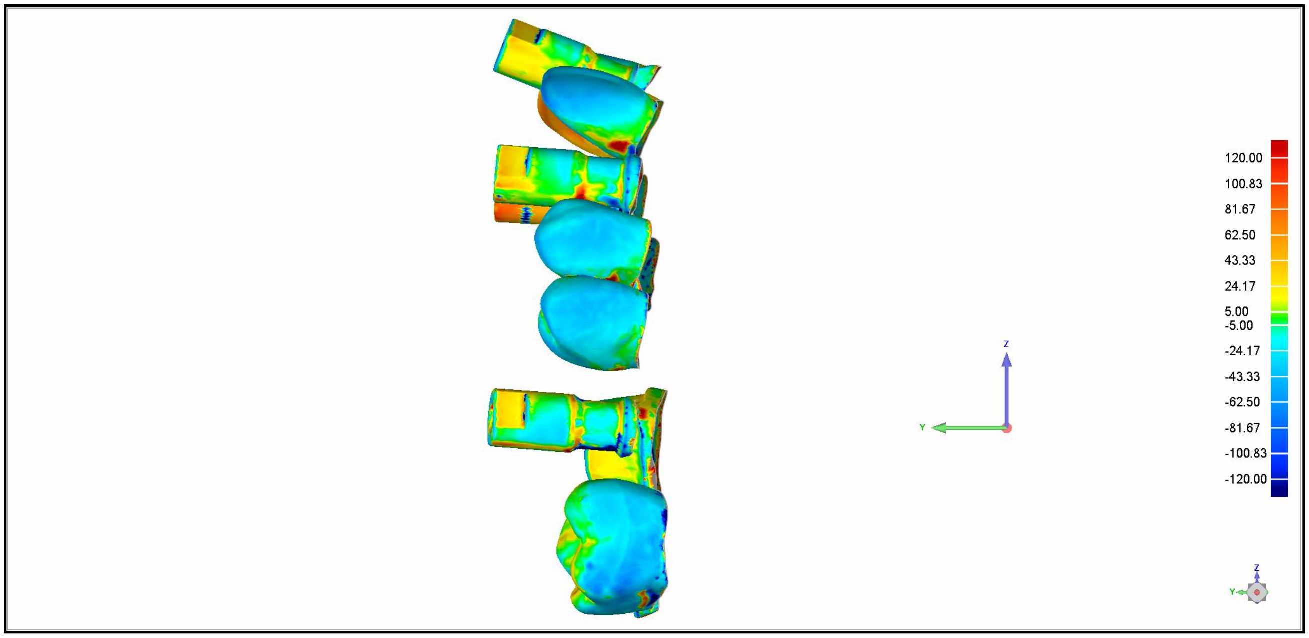

Predefinido: Derecha

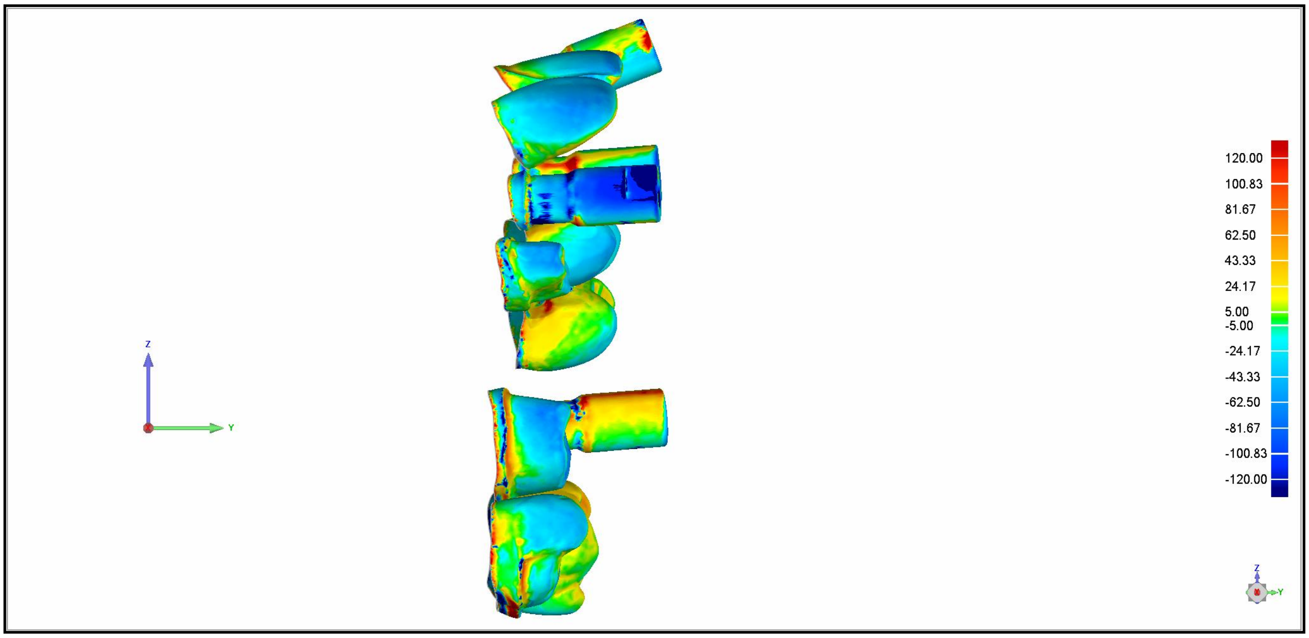

Predefinido: Superior

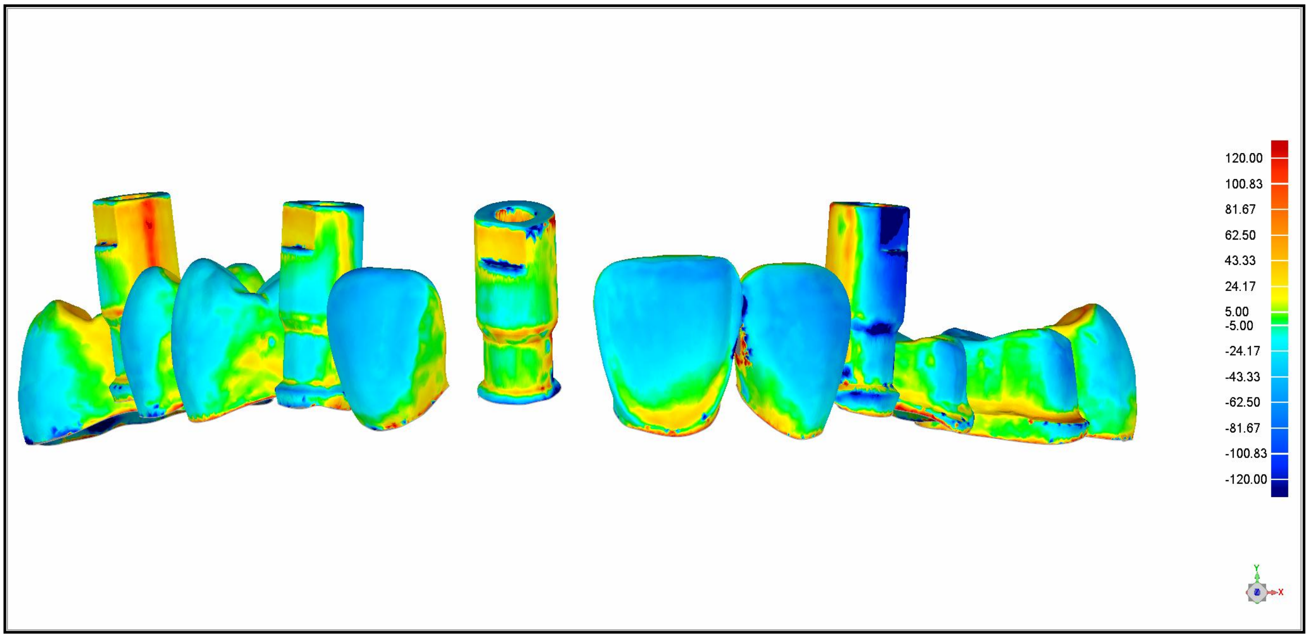

Predefinido: Inferior

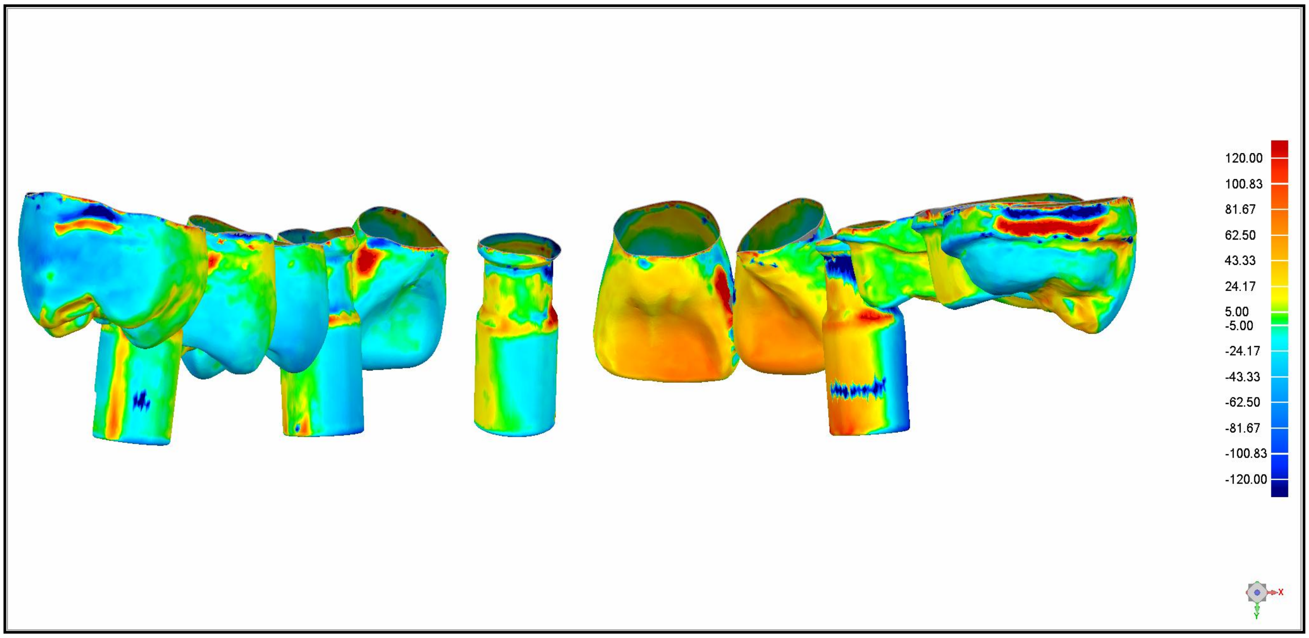

Ajuste de ubicación: Desviaciones superior e inferior

Unidades: u

Nombre	Desv	Estado	Superior Tol	Inferior Tol	Ref X	Ref Y	Ref Z	Radio	Desv X	Desv Y	Desv Z	Medido X	Medido Y	Medido Z	Dir. proy. X	Dir. proy. Y	Dir. proy. Z
Desv. inferior	-3102.62				32061.00	27268.70	-9389.56	n/a	-695.66	-40.38	-3023.36	31365.34	27228.33	-12412.92	0.22	0.01	0.97
Desv. superior	2978.00				29547.11	27336.30	1208.53	n/a	-2288.98	-449.42	1851.24	27258.13	26886.87	3059.77	-0.77	-0.15	0.62
