# Supplementary material for: Accuracy of four digital scanners according to scanning strategy in complete-arch impressions
Source: PLoS One. 2018 Sep 13;13(9):e0202916. doi: 10.1371/journal.pone.0202916 (PMC6136706; doi:10.1371/journal.pone.0202916)
Supplement: S11 Table — Omnicam (scanning strategy C). (ZIP) [file pone.0202916.s011.zip › S11/OM1C.pdf]

# 3D Comparación  Resultados

| | |
|---|---|
| Modelo referencia | MRC |
| Modelo test | OM1C |
| Nº de puntos de datos | 202983 |
| # Aislados | 860 |

| | |
|---|---|
| Tipo tolerancia | 3D desviación |
| Unidades | u |
| Máx. crítico | 120.00 |
| Máx. nominal | 0.00 |
| Mín. nominal | 0.00 |
| Mín. crítico | -120.00 |

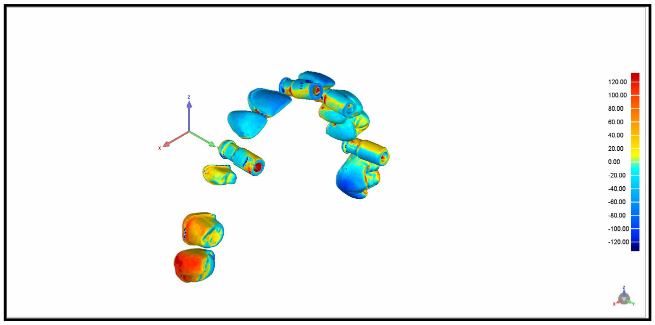

| | |
|---|---|
| Desviación | |
| Desviación superior máx. | 3109.52 |
| Desviación inferior máx. | -3154.15 |
| Desviación media | 105.23 /-105.92 |
| Desviación estándar | 293.56 |

## Distribución desviación

| >=Min | <Max | # Puntos | % |
|---|---|---|---|
| -120.00 | -100.00 | 2024 | 1.00 |
| -100.00 | -80.00 | 4565 | 2.25 |
| -80.00 | -60.00 | 7609 | 3.75 |
| -60.00 | -40.00 | 15086 | 7.43 |
| -40.00 | -20.00 | 26310 | 12.96 |
| -20.00 | 0.00 | 34998 | 17.24 |
| 0.00 | 20.00 | 34945 | 17.22 |
| 20.00 | 40.00 | 24609 | 12.12 |
| 40.00 | 60.00 | 12419 | 6.12 |
| 60.00 | 80.00 | 8390 | 4.13 |
| 80.00 | 100.00 | 4735 | 2.33 |
| 100.00 | 120.00 | 3451 | 1.70 |

| | | |
|---|---|---|
| Fuera del crítico superior | 13343 | 6.57 |
| Fuera del crítico inferior | 10499 | 5.17 |

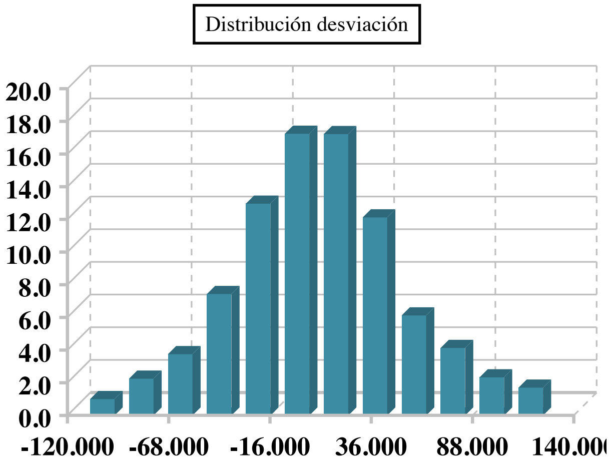


## Desviaciones estándar

| Distribución (+/-) | # Puntos | % |
|---|---|---|
| -6 * Desv. estánd. | 1258 | 0.62 |
| -5 * Desv. estánd. | 1176 | 0.58 |
| -4 * Desv. estánd. | 1261 | 0.62 |
| -3 * Desv. estánd. | 1632 | 0.80 |
| -2 * Desv. estánd. | 1845 | 0.91 |
| -1 * Desv. estánd. | 94058 | 46.34 |
| 1 * Desv. estánd. | 94734 | 46.67 |
| 2 * Desv. estánd. | 1941 | 0.96 |
| 3 * Desv. estánd. | 1661 | 0.82 |
| 4 * Desv. estánd. | 1381 | 0.68 |
| 5 * Desv. estánd. | 1202 | 0.59 |
| 6 * Desv. estánd. | 834 | 0.41 |

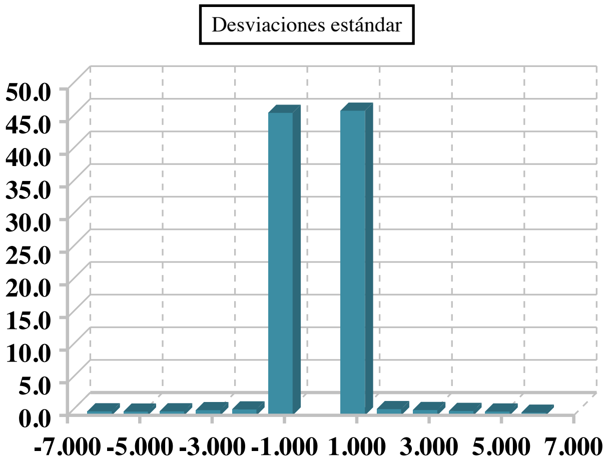

Predefinido: Isométrico

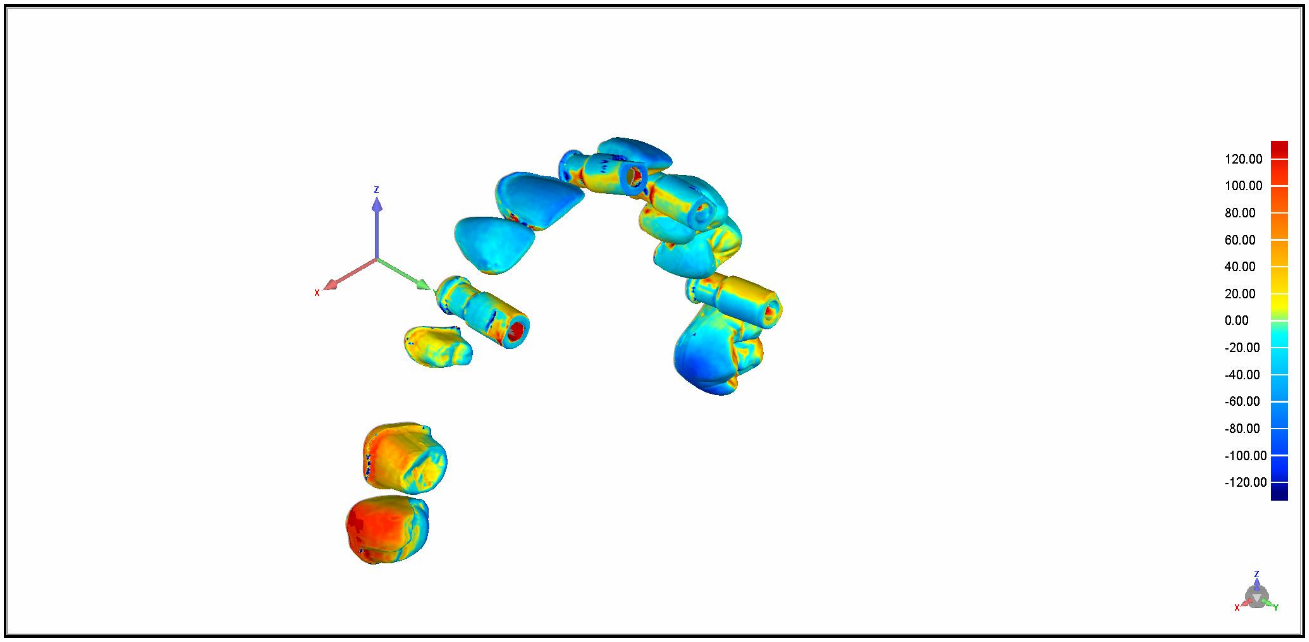

Predefinido: Frente

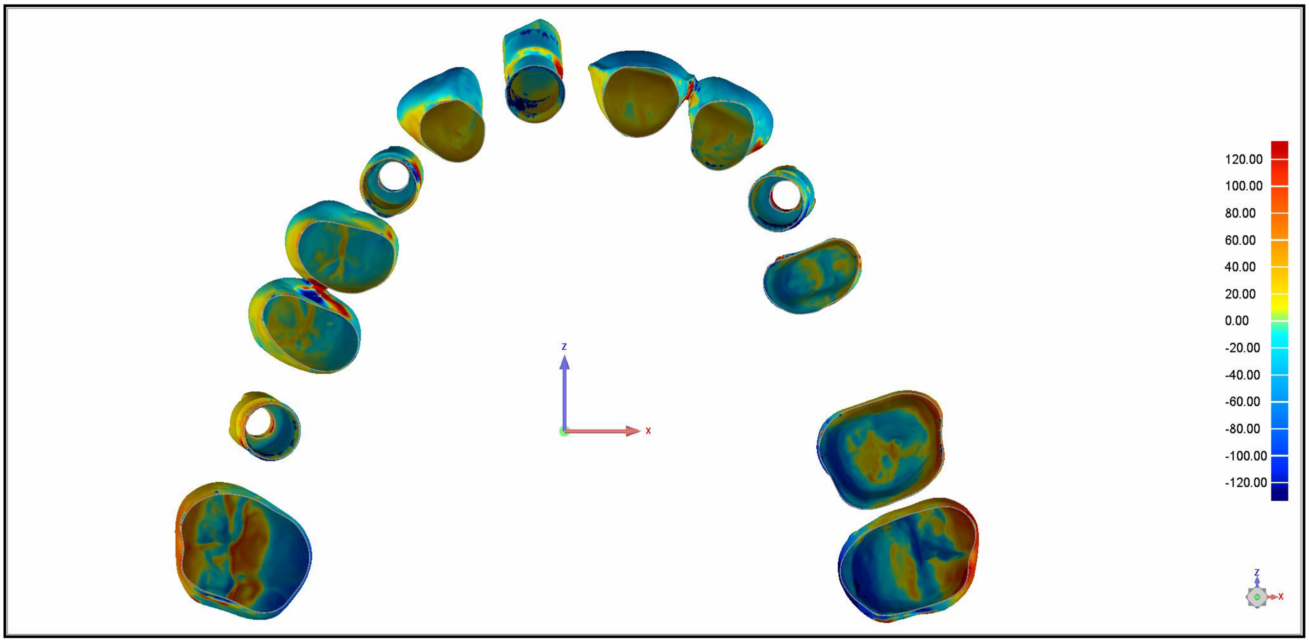

Predefinido: Atrás

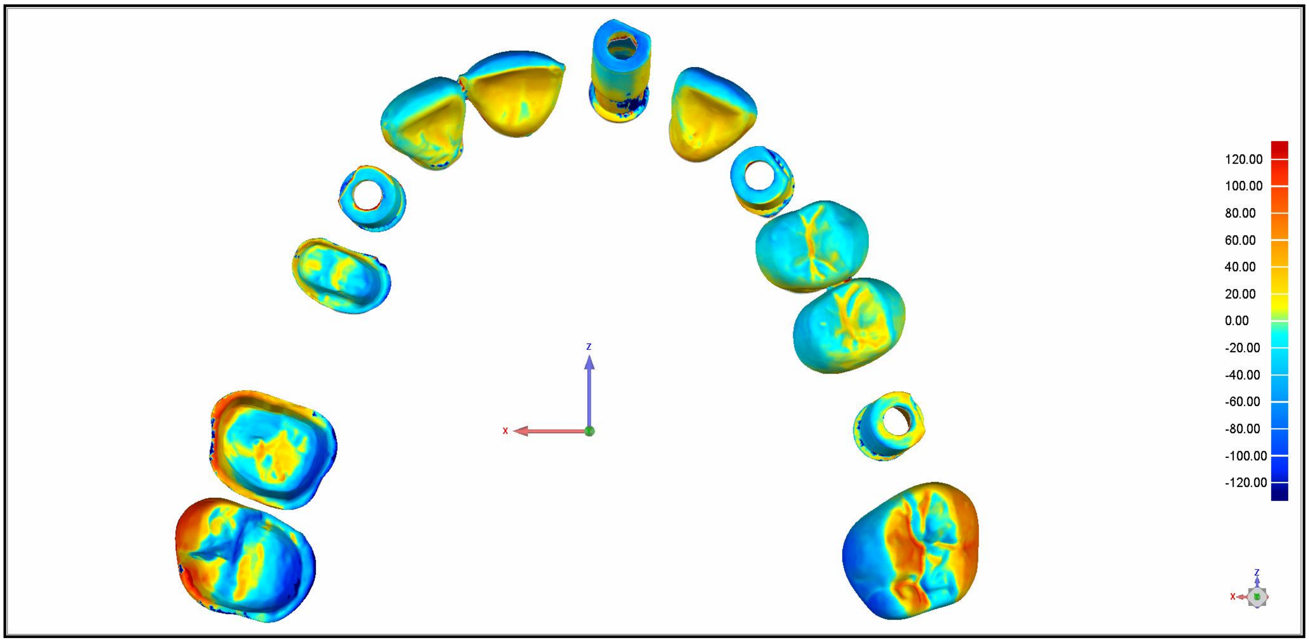

Predefinido: Izquierda

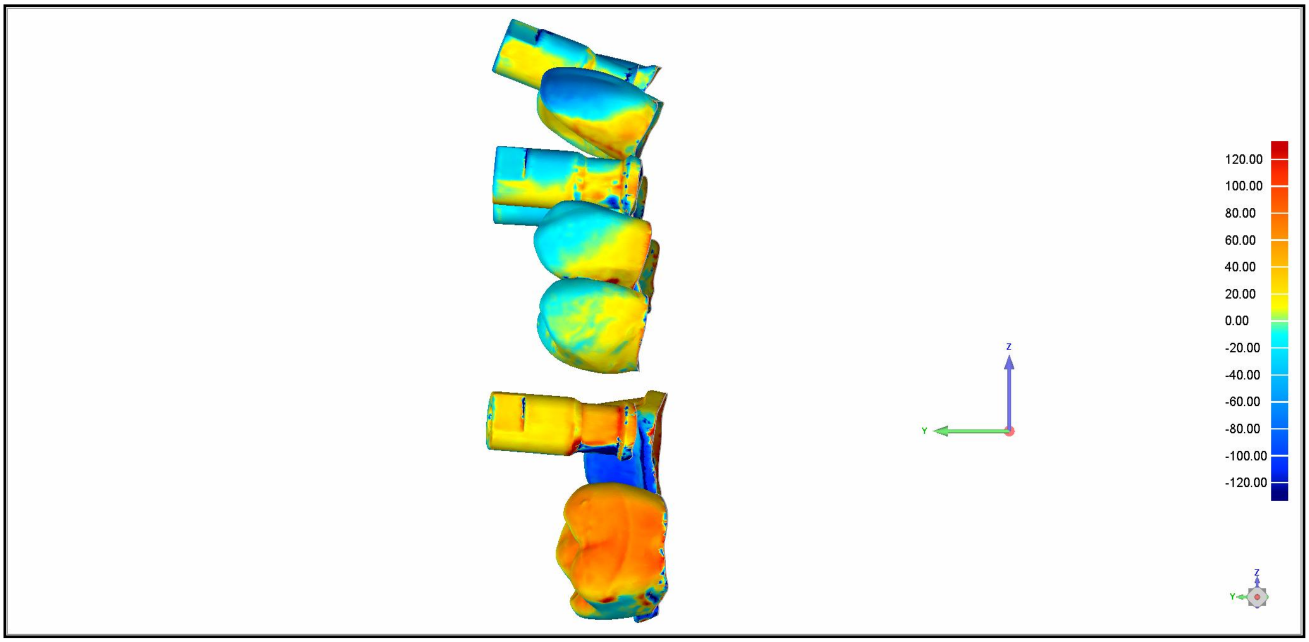

Predefinido: Derecha

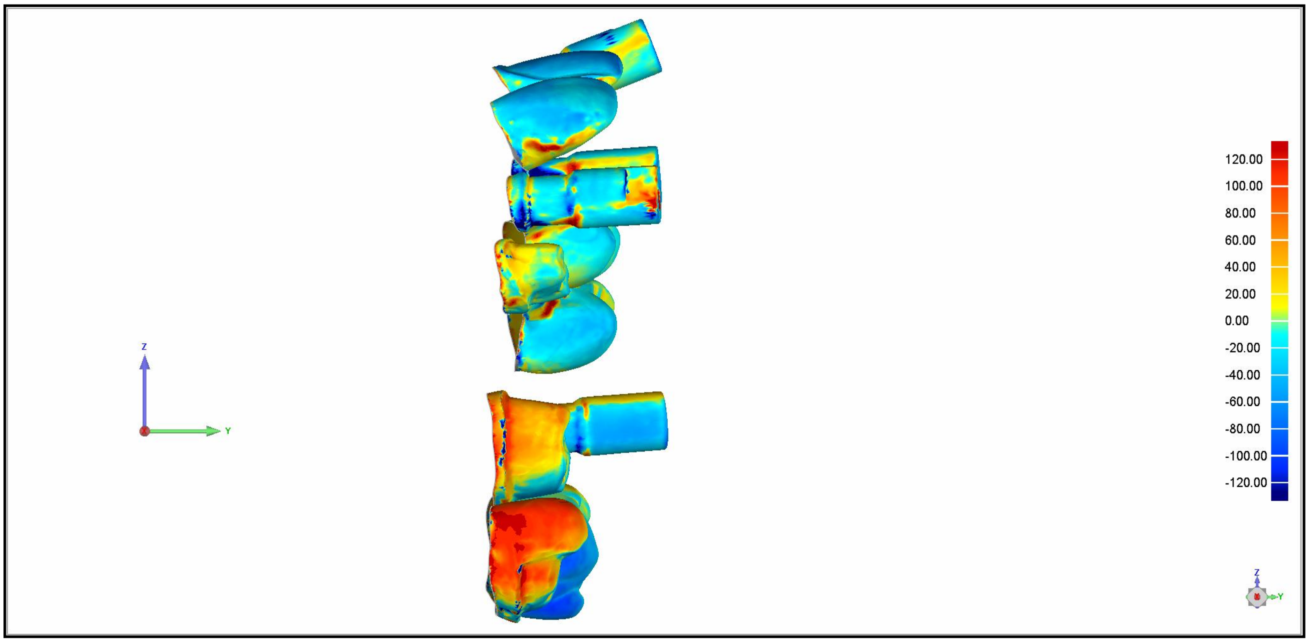

Predefinido: Superior

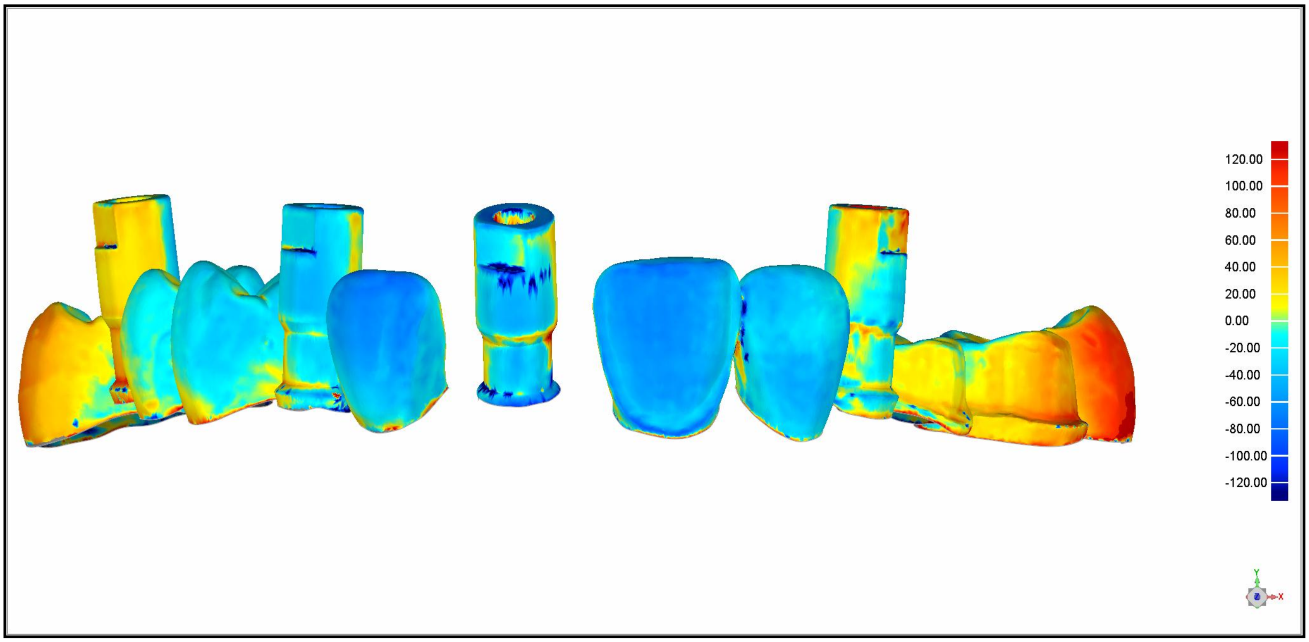

Predefinido: Inferior

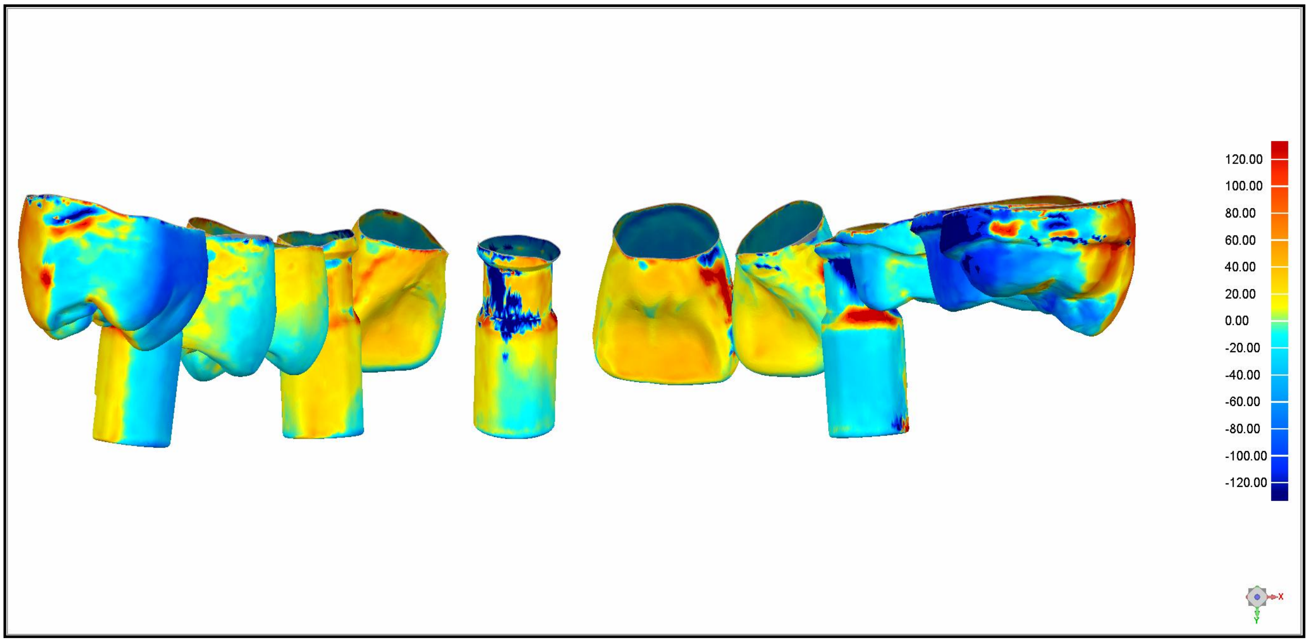

# Ajuste de ubicación: Desviaciones superior e inferior

## Unidades: u

| Nombre | Desv | Estado | Superior Tol | Inferior Tol | Ref X | Ref Y | Ref Z | Radio | Desv X | Desv Y | Desv Z | Medido X | Medido Y | Medido Z | Dir. proy. X | Dir. proy. Y | Dir. proy. Z |
|---|---|---|---|---|---|---|---|---|---|---|---|---|---|---|---|---|---|
| Desv. inferior | -3154.15 | | | | -22607.19 | 28955.77 | 6808.03 | n/a | -1000.27 | -505.96 | 2948.25 | -23607.46 | 28449.81 | 9756.28 | 0.32 | 0.16 | -0.93 |
| Desv. superior | 3109.52 | | | | -22452.73 | 28971.74 | 6644.19 | n/a | -1194.34 | -481.08 | 2830.41 | -23647.06 | 28490.65 | 9474.60 | -0.38 | -0.15 | 0.91 |
