# Supplementary material for: Accuracy of four digital scanners according to scanning strategy in complete-arch impressions
Source: PLoS One. 2018 Sep 13;13(9):e0202916. doi: 10.1371/journal.pone.0202916 (PMC6136706; doi:10.1371/journal.pone.0202916)
Supplement: S12 Table — Omnicam (scanning strategy D). (ZIP) [file pone.0202916.s012.zip › S12/OM3D.pdf]

3D Comparación Resultados

Modelo referencia	MRC
Modelo test	OM3D
Nº de puntos de datos	195270
# Aislados	745

Tipo tolerancia	3D desviación
Unidades	u
Máx. crítico	120.00
Máx. nominal	15.00
Mín. nominal	-15.00
Mín. crítico	-120.00

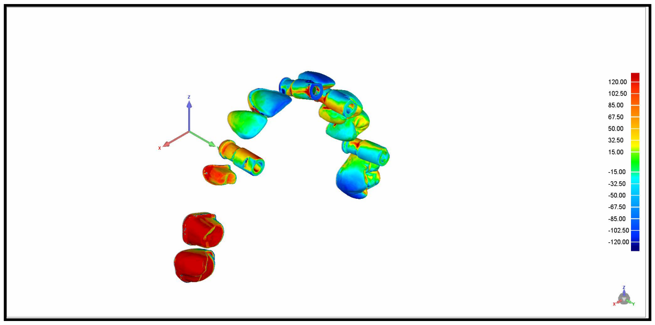

Desviación	
Desviación superior máx.	3150.07
Desviación inferior máx.	-3154.29
Desviación media	128.42 /-112.71
Desviación estándar	284.17

Distribución desviación

>=Min	<Max	# Puntos	%
-120.00	-102.50	2645	1.35
-102.50	-85.00	6111	3.13
-85.00	-67.50	6789	3.48
-67.50	-50.00	9182	4.70
-50.00	-32.50	12137	6.22
-32.50	-15.00	20202	10.35
-15.00	15.00	38829	19.88
15.00	32.50	16935	8.67
32.50	50.00	12601	6.45
50.00	67.50	10618	5.44
67.50	85.00	8193	4.20
85.00	102.50	5655	2.90
102.50	120.00	4081	2.09

Fuera del crítico superior	27587	14.13
Fuera del crítico inferior	13705	7.02

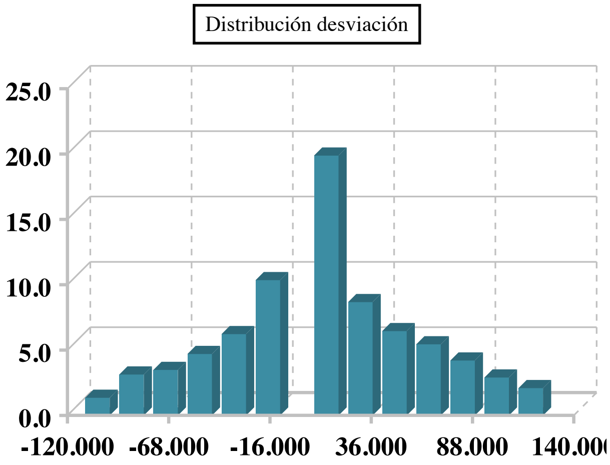

Desviaciones estándar

Distribución (+/-)	# Puntos	%
-6 * Desv. estánd.	1531	0.78
-5 * Desv. estánd.	635	0.33
-4 * Desv. estánd.	582	0.30
-3 * Desv. estánd.	836	0.43
-2 * Desv. estánd.	3487	1.79
-1 * Desv. estánd.	102919	52.71
1 * Desv. estánd.	75500	38.66
2 * Desv. estánd.	5925	3.03
3 * Desv. estánd.	1377	0.71
4 * Desv. estánd.	819	0.42
5 * Desv. estánd.	747	0.38
6 * Desv. estánd.	912	0.47

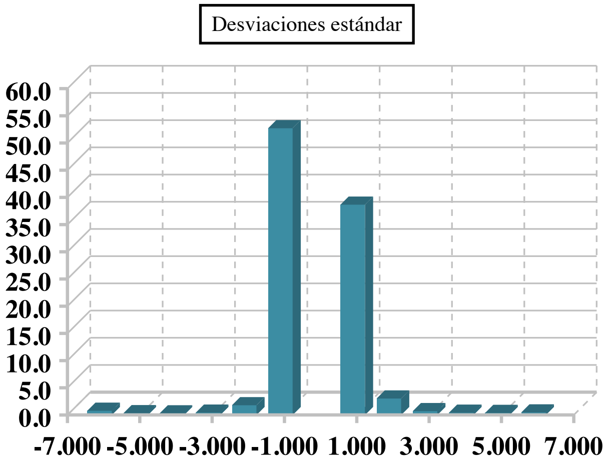

Predefinido: Isométrico

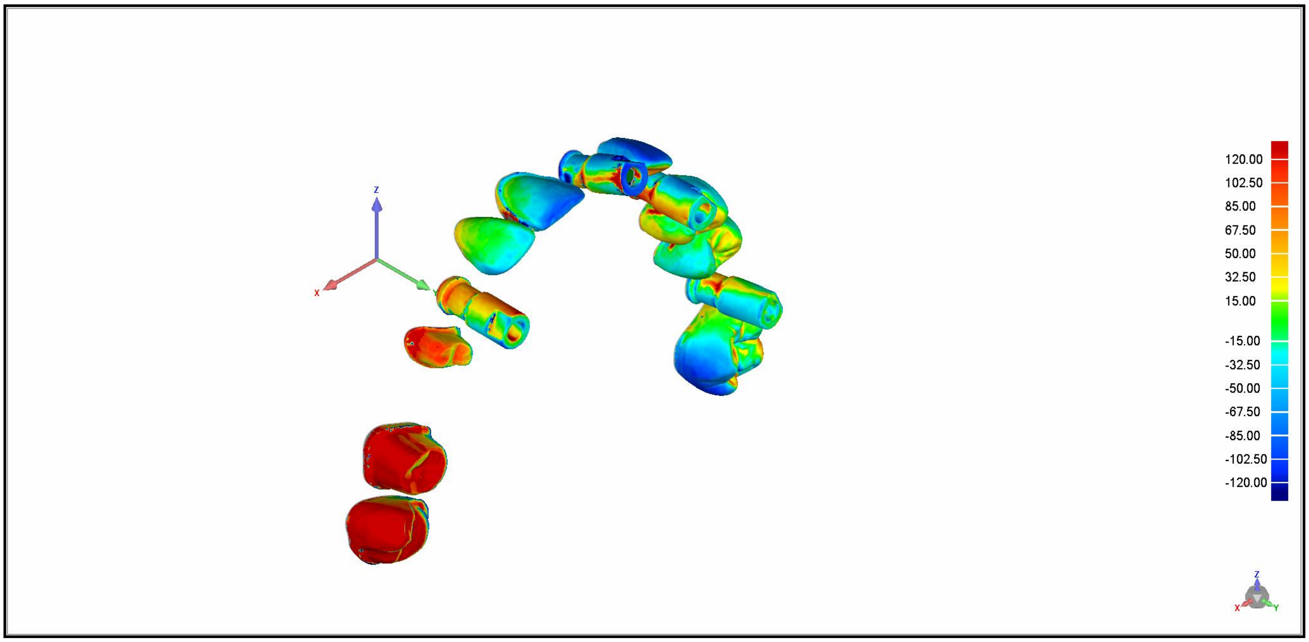

Predefinido: Frente

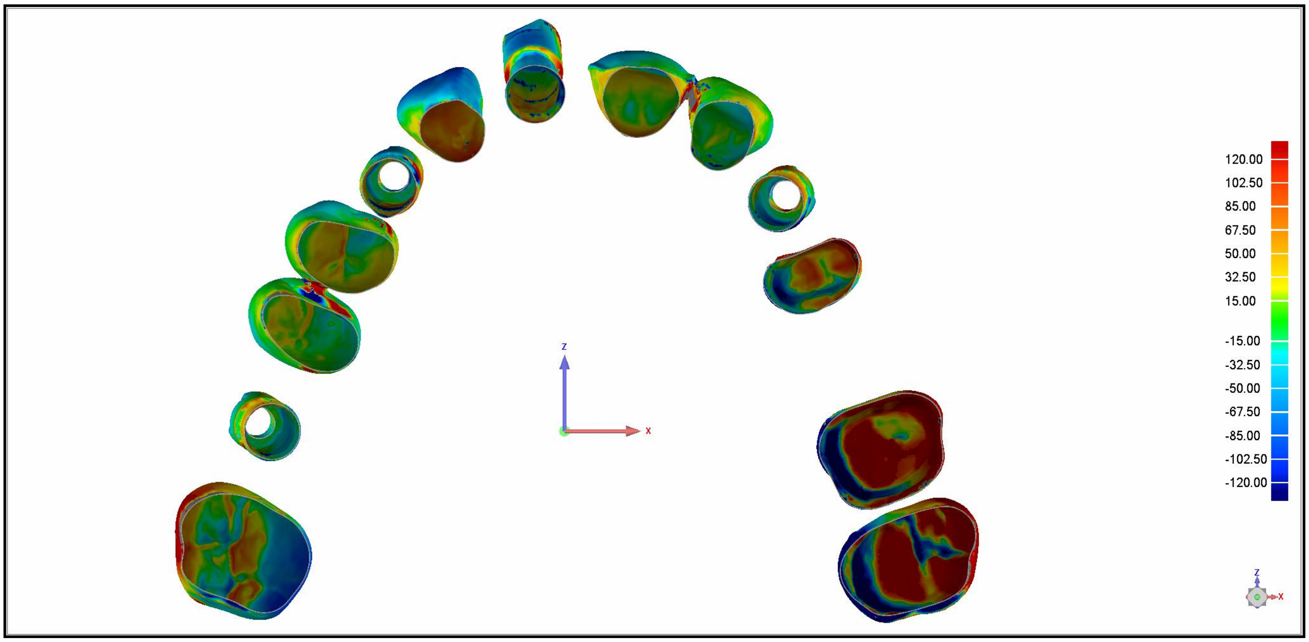

Predefinido: Atrás

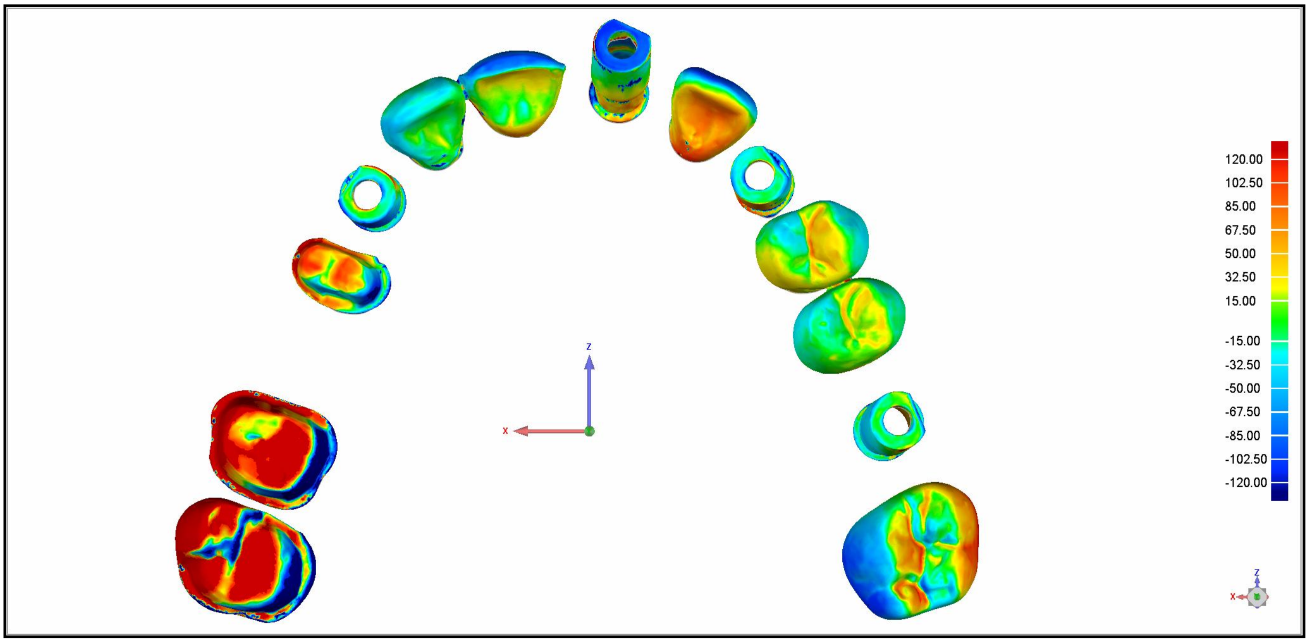

Predefinido: Izquierda

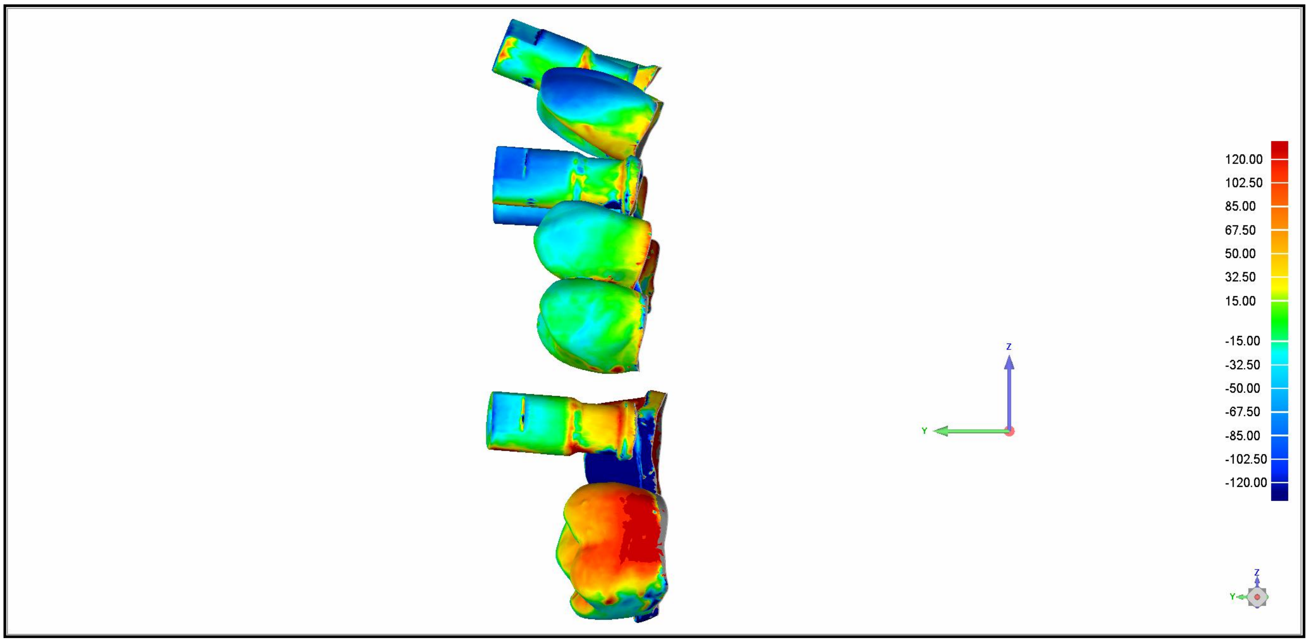

Predefinido: Derecha

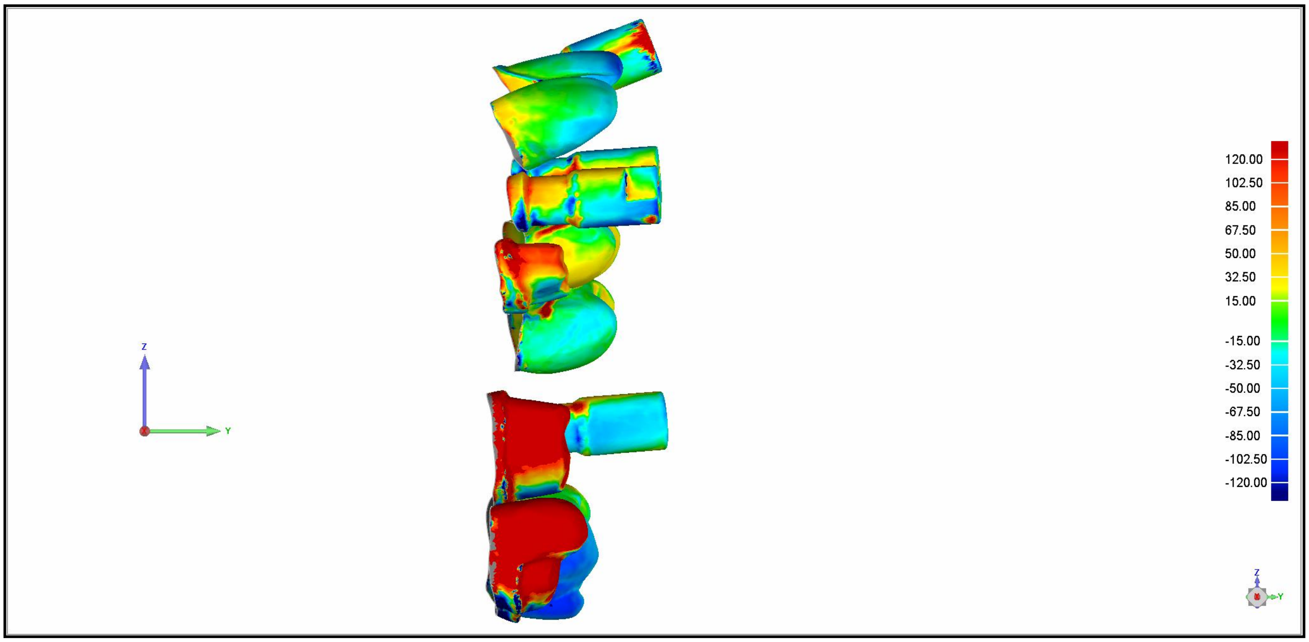

Predefinido: Superior

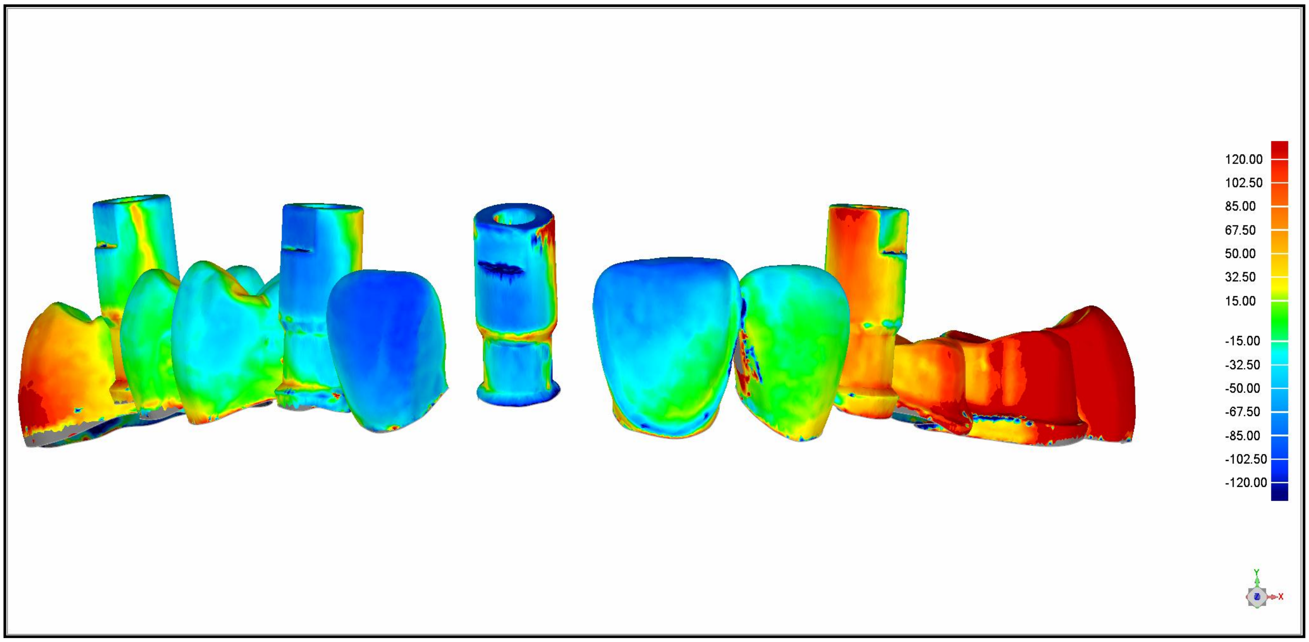

Predefinido: Inferior

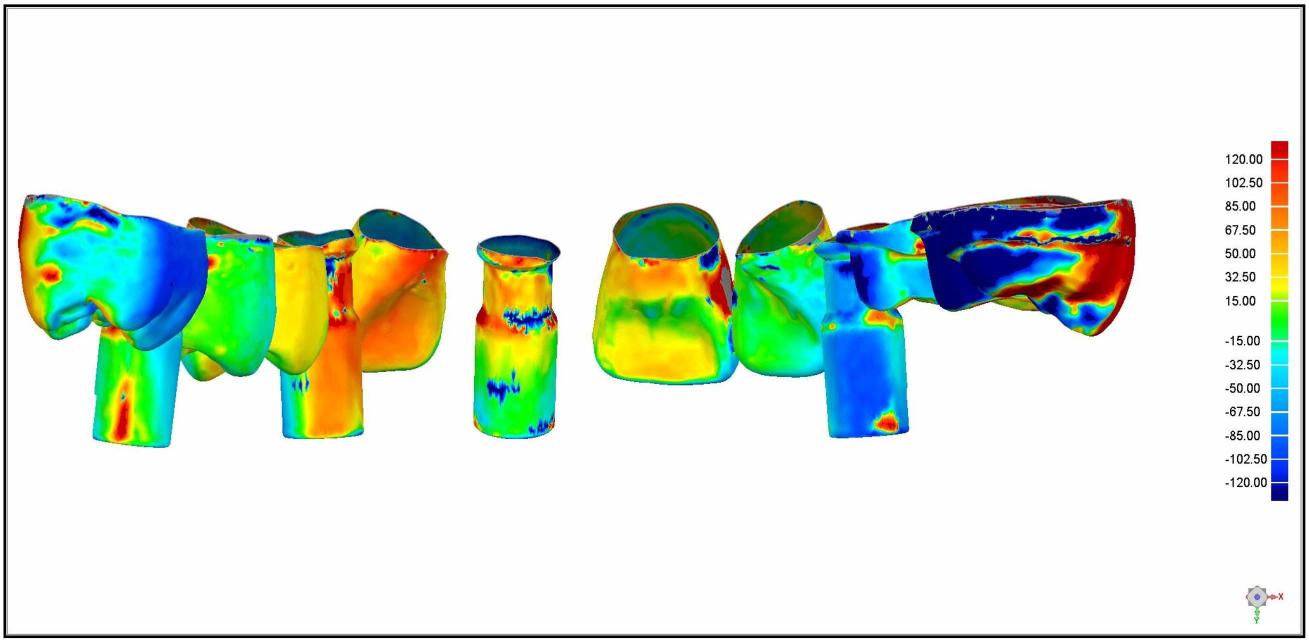

Ajuste de ubicación: Desviaciones superior e inferior

Unidades: u

Nombre	Desv	Estado	Superior Tol	Inferior Tol	Ref X	Ref Y	Ref Z	Radio	Desv X	Desv Y	Desv Z	Medido X	Medido Y	Medido Z	Dir. proy. X	Dir. proy. Y	Dir. proy. Z
Desv. inferior	-3154.29				32064.02	27122.94	-7196.92	n/a	-2788.22	421.10	1413.53	29275.80	27544.04	-5783.38	0.88	-0.13	-0.45
Desv. superior	3150.07				-29824.91	26937.61	-11348.70	n/a	2610.29	186.07	-1753.47	-27214.62	27123.67	-13102.17	0.83	0.06	-0.56
