# Supplementary material for: Accuracy of four digital scanners according to scanning strategy in complete-arch impressions
Source: PLoS One. 2018 Sep 13;13(9):e0202916. doi: 10.1371/journal.pone.0202916 (PMC6136706; doi:10.1371/journal.pone.0202916)
Supplement: S12 Table — Omnicam (scanning strategy D). (ZIP) [file pone.0202916.s012.zip › S12/OM2D.pdf]

3D Comparación Resultados

Modelo referencia	MRC
Modelo test	OM2D
Nº de puntos de datos	201034
# Aislados	790

Tipo tolerancia	3D desviación
Unidades	u
Máx. crítico	120.00
Máx. nominal	8.00
Mín. nominal	-8.00
Mín. crítico	-120.00

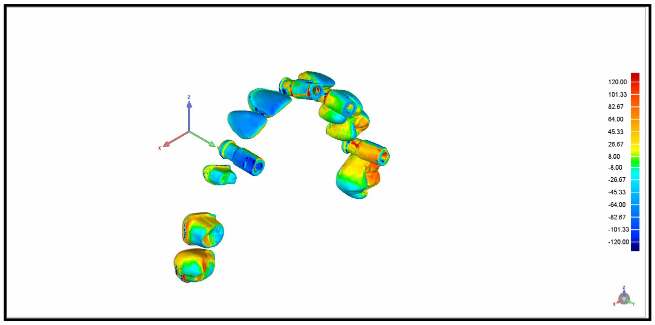

Desviación	
Desviación superior máx.	3113.18
Desviación inferior máx.	-3147.63
Desviación media	101.33 /-89.23
Desviación estándar	278.59

Distribución desviación

>=Min	<Max	# Puntos	%
-120.00	-101.33	1693	0.84
-101.33	-82.67	2915	1.45
-82.67	-64.00	5688	2.83
-64.00	-45.33	11856	5.90
-45.33	-26.67	23009	11.45
-26.67	-8.00	29253	14.55
-8.00	8.00	29435	14.64
8.00	26.67	28628	14.24
26.67	45.33	20032	9.96
45.33	64.00	13619	6.77
64.00	82.67	7312	3.64
82.67	101.33	3952	1.97
101.33	120.00	1800	0.90

Fuera del crítico superior	12348	6.14
Fuera del crítico inferior	9494	4.72

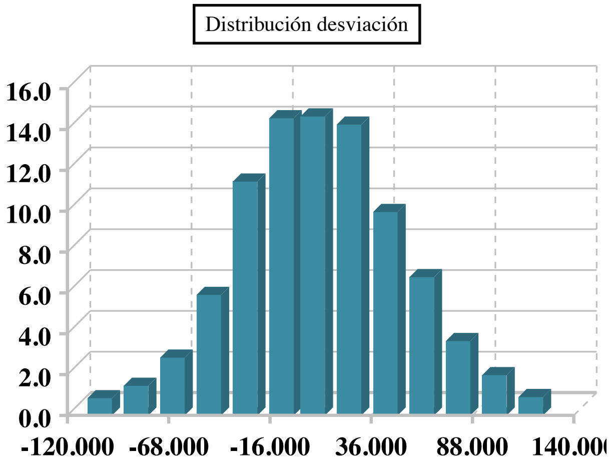

Desviaciones estándar

Distribución (+/-)	# Puntos	%
-6 * Desv. estánd.	1315	0.65
-5 * Desv. estánd.	517	0.26
-4 * Desv. estánd.	712	0.35
-3 * Desv. estánd.	895	0.45
-2 * Desv. estánd.	1878	0.93
-1 * Desv. estánd.	108193	53.82
1 * Desv. estánd.	80887	40.24
2 * Desv. estánd.	1801	0.90
3 * Desv. estánd.	1262	0.63
4 * Desv. estánd.	1336	0.66
5 * Desv. estánd.	1128	0.56
6 * Desv. estánd.	1110	0.55

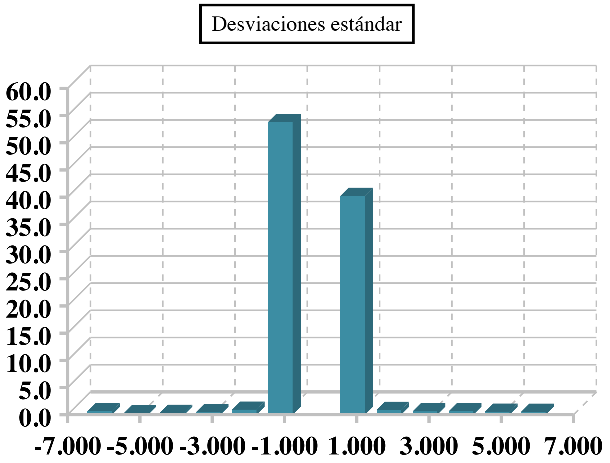

Predefinido: Isométrico

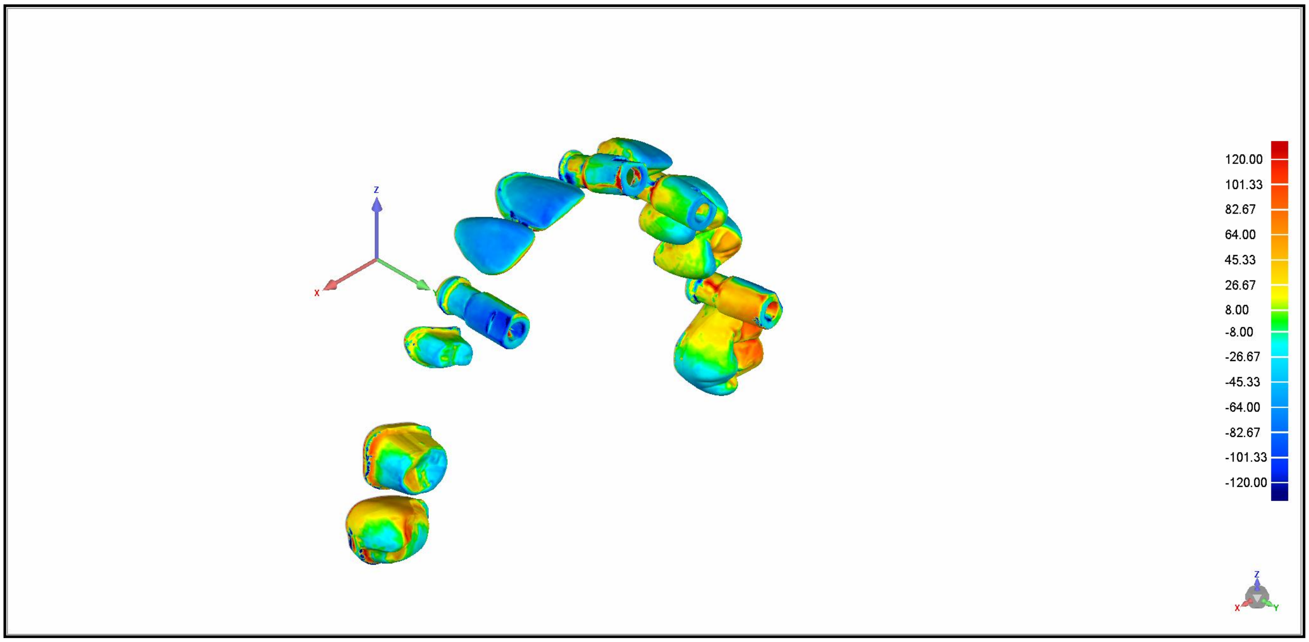

Predefinido: Frente

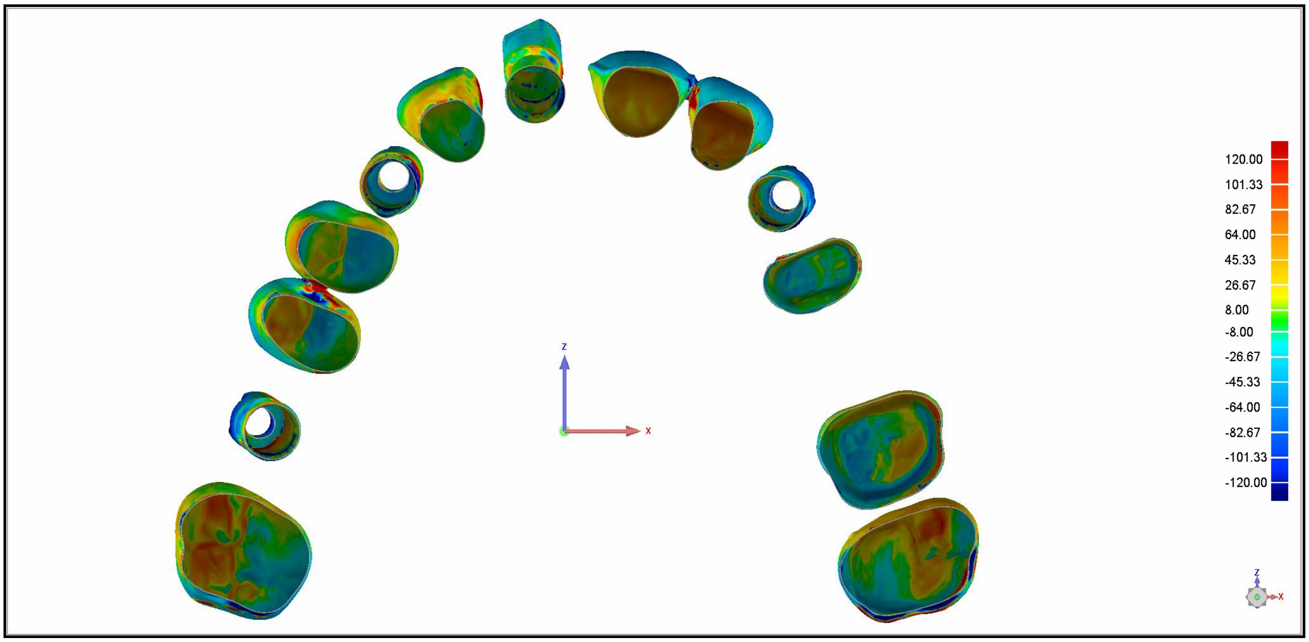

Predefinido: Atrás

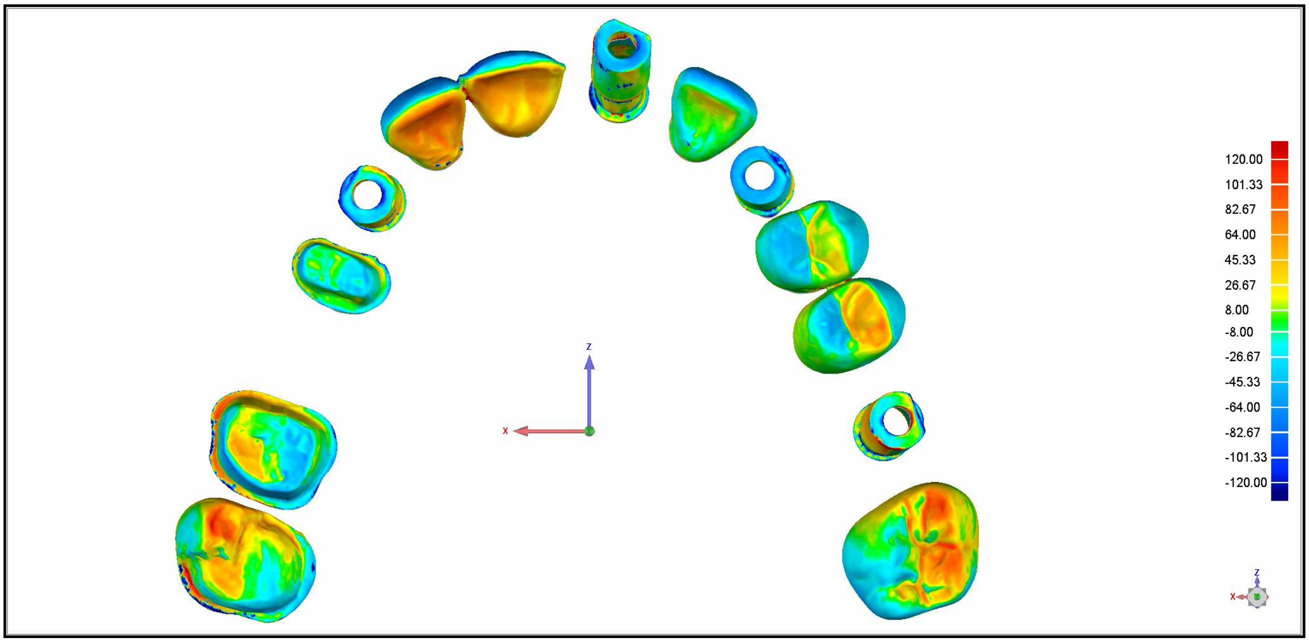

Predefinido: Izquierda

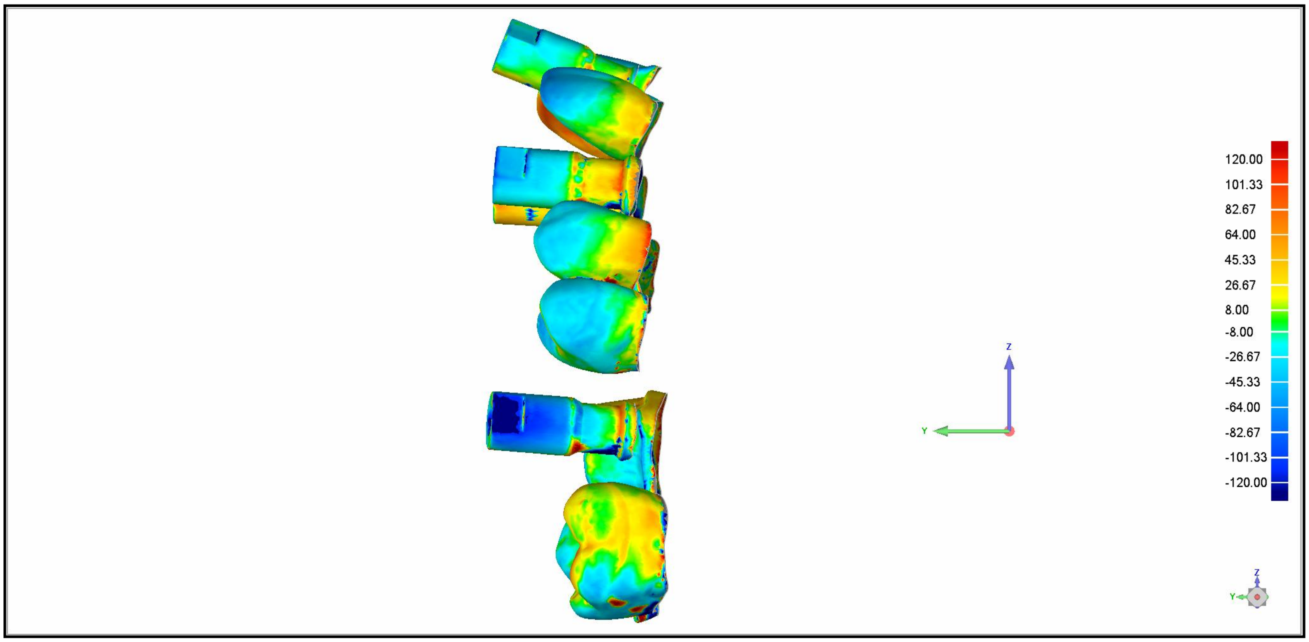

Predefinido: Derecha

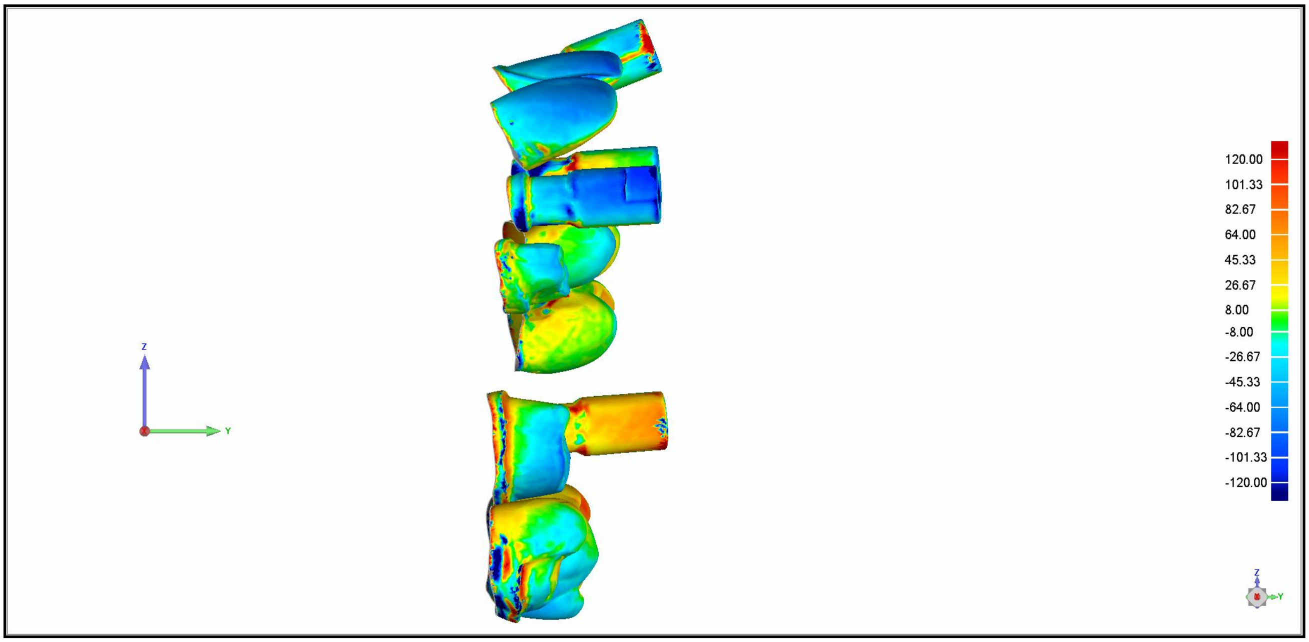

Predefinido: Superior

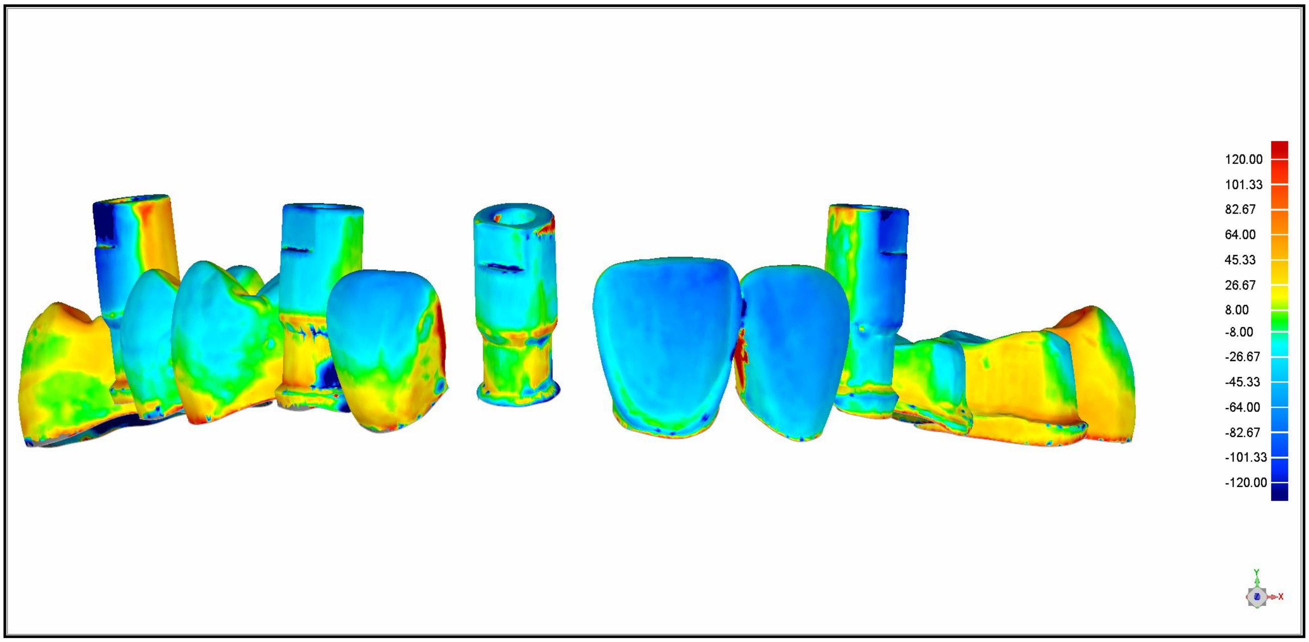

Predefinido: Inferior

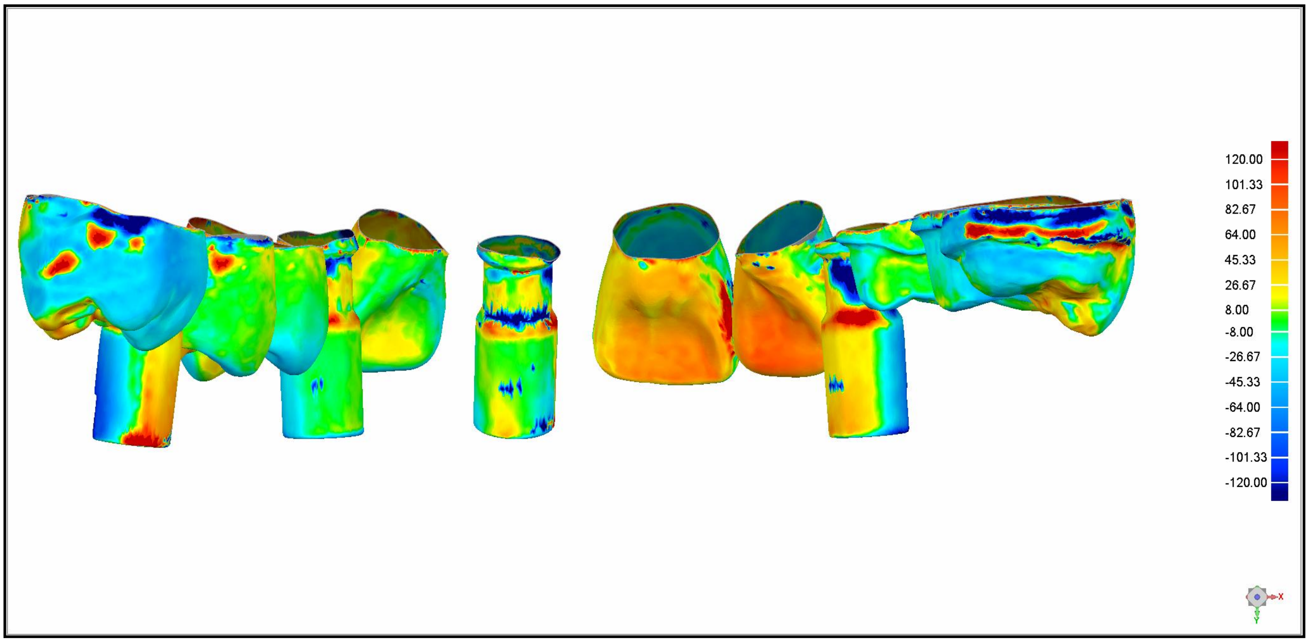

Ajuste de ubicación: Desviaciones superior e inferior

Unidades: u

Nombre	Desv	Estado	Superior Tol	Inferior Tol	Ref X	Ref Y	Ref Z	Radio	Desv X	Desv Y	Desv Z	Medido X	Medido Y	Medido Z	Dir. proy. X	Dir. proy. Y	Dir. proy. Z
Desv. inferior	-3147.63				-29727.32	27158.09	-11408.24	n/a	2635.16	-36.21	-1721.10	-27092.16	27121.88	-13129.34	-0.84	0.01	0.55
Desv. superior	3113.18				-20351.46	30909.00	12282.23	n/a	-732.89	-2459.15	1762.77	-21084.36	28449.85	14045.00	-0.24	-0.79	0.57
