# Supplementary material for: Accuracy of four digital scanners according to scanning strategy in complete-arch impressions
Source: PLoS One. 2018 Sep 13;13(9):e0202916. doi: 10.1371/journal.pone.0202916 (PMC6136706; doi:10.1371/journal.pone.0202916)
Supplement: S12 Table — Omnicam (scanning strategy D). (ZIP) [file pone.0202916.s012.zip › S12/OM9D.pdf]

Geomagic Control

3D Comparación Resultados

Modelo referencia	MRC
Modelo test	OM9D
Nº de puntos de datos	199064
# Aislados	859

Tipo tolerancia	3D desviación
Unidades	u
Máx. crítico	120.00
Máx. nominal	5.00
Mín. nominal	-5.00
Mín. crítico	-120.00

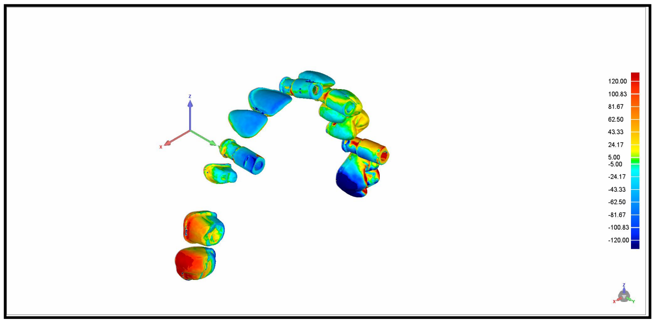

Desviación	
Desviación superior máx.	3150.17
Desviación inferior máx.	-3155.38
Desviación media	110.84 /-116.68
Desviación estándar	318.45

Distribución desviación

>=Min	<Max	# Puntos	%
-120.00	-100.83	2757	1.38
-100.83	-81.67	4835	2.43
-81.67	-62.50	6931	3.48
-62.50	-43.33	10044	5.05
-43.33	-24.17	17245	8.66
-24.17	-5.00	28972	14.55
-5.00	5.00	17945	9.01
5.00	24.17	29130	14.63
24.17	43.33	18443	9.26
43.33	62.50	10684	5.37
62.50	81.67	8248	4.14
81.67	100.83	5577	2.80
100.83	120.00	4489	2.26

Fuera del crítico superior	21073	10.59
Fuera del crítico inferior	12691	6.38

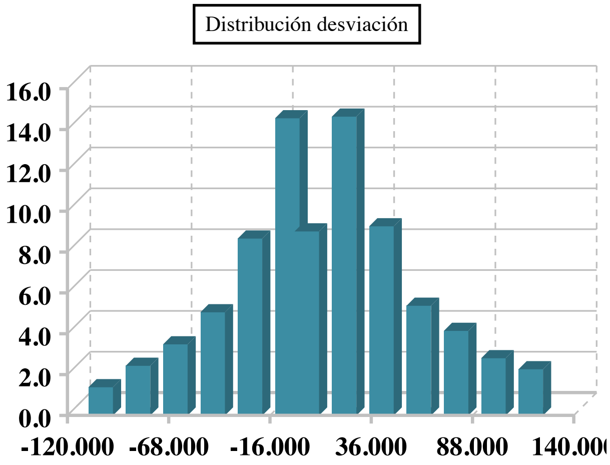

Desviaciones estándar

Distribución (+/-)	# Puntos	%
-6 * Desv. estánd.	1907	0.96
-5 * Desv. estánd.	695	0.35
-4 * Desv. estánd.	596	0.30
-3 * Desv. estánd.	632	0.32
-2 * Desv. estánd.	1526	0.77
-1 * Desv. estánd.	96337	48.39
1 * Desv. estánd.	91499	45.96
2 * Desv. estánd.	1850	0.93
3 * Desv. estánd.	1251	0.63
4 * Desv. estánd.	1085	0.55
5 * Desv. estánd.	784	0.39
6 * Desv. estánd.	902	0.45

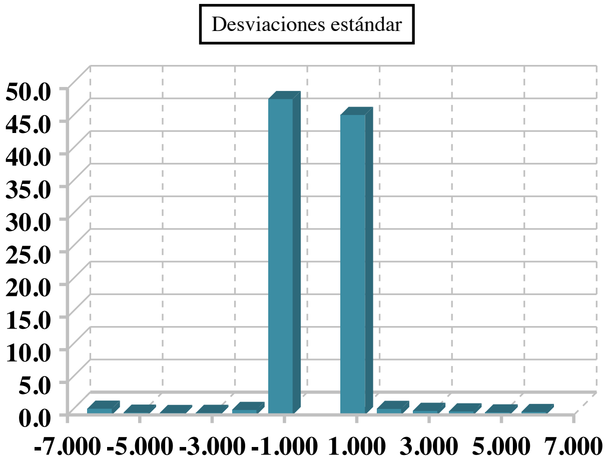

Predefinido: Isométrico

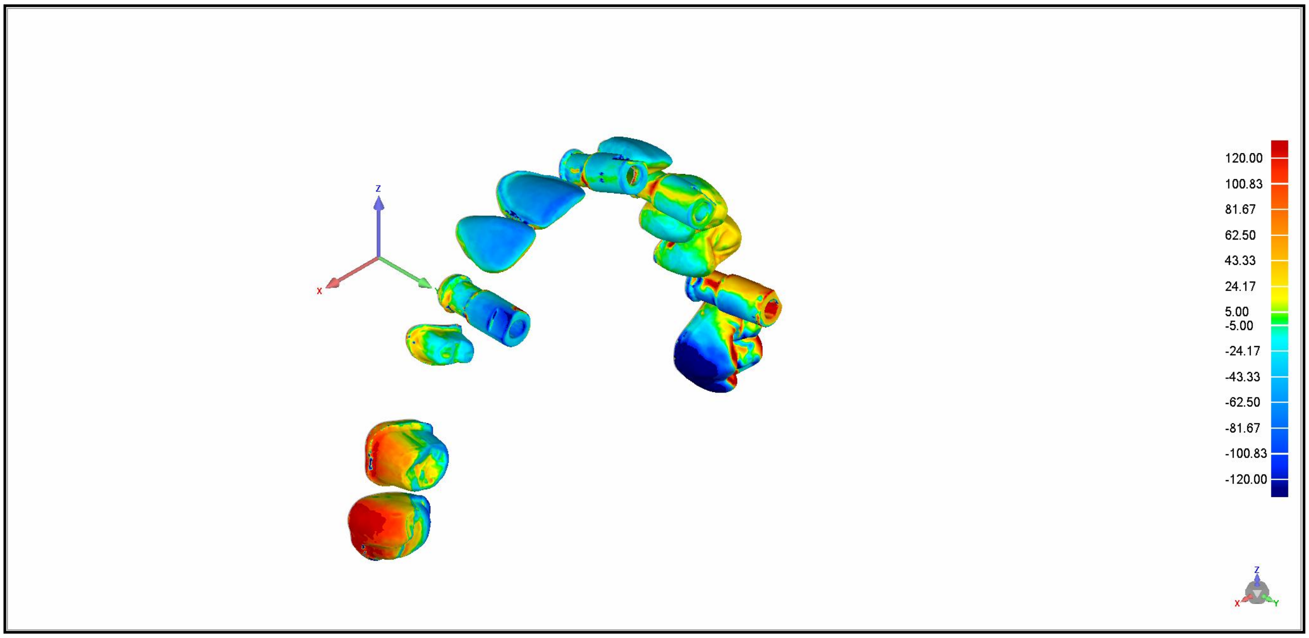

Predefinido: Frente

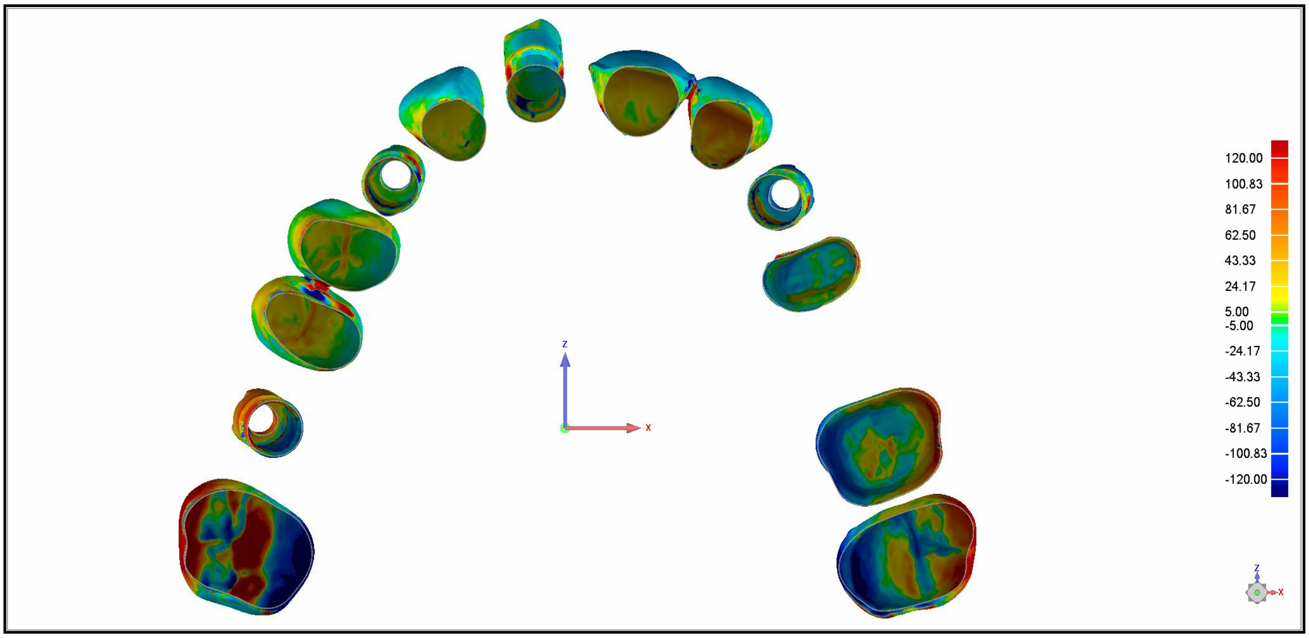

Predefinido: Atrás

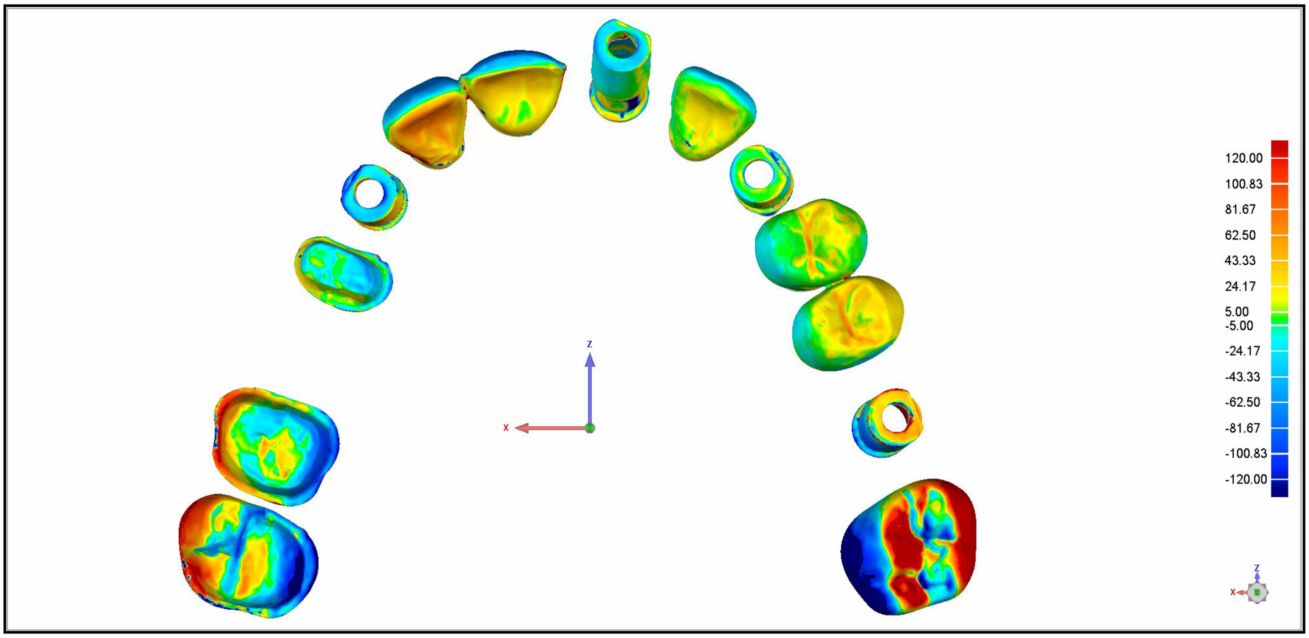

Predefinido: Izquierda

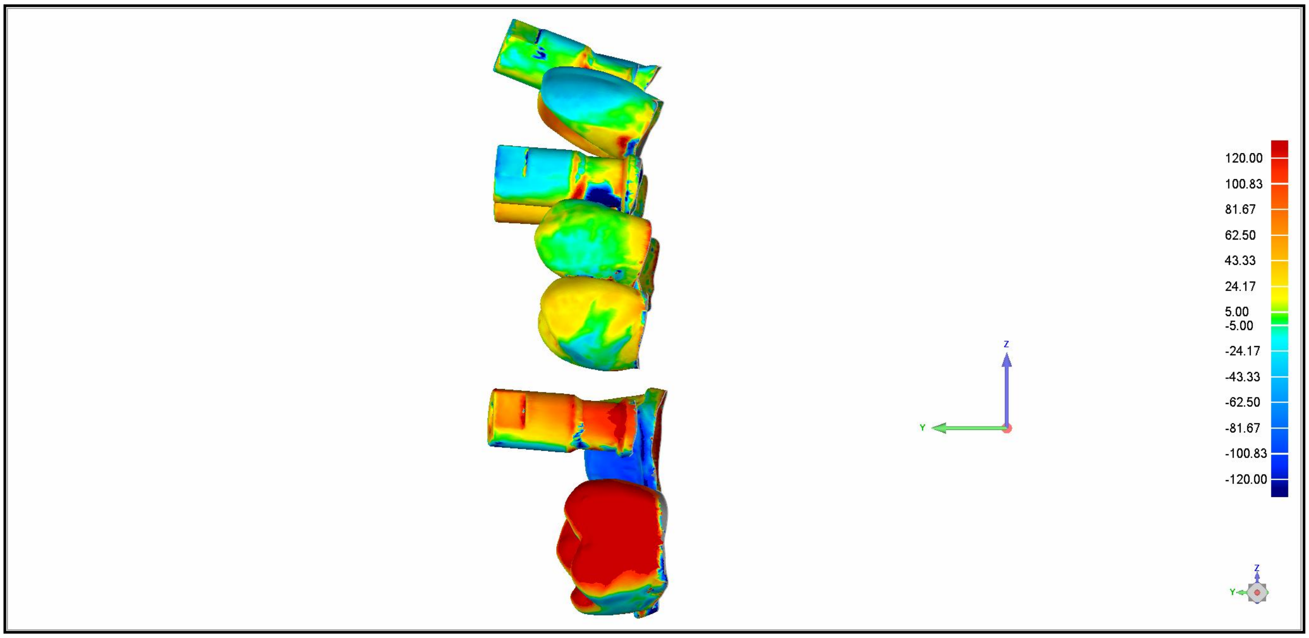

Predefinido: Derecha

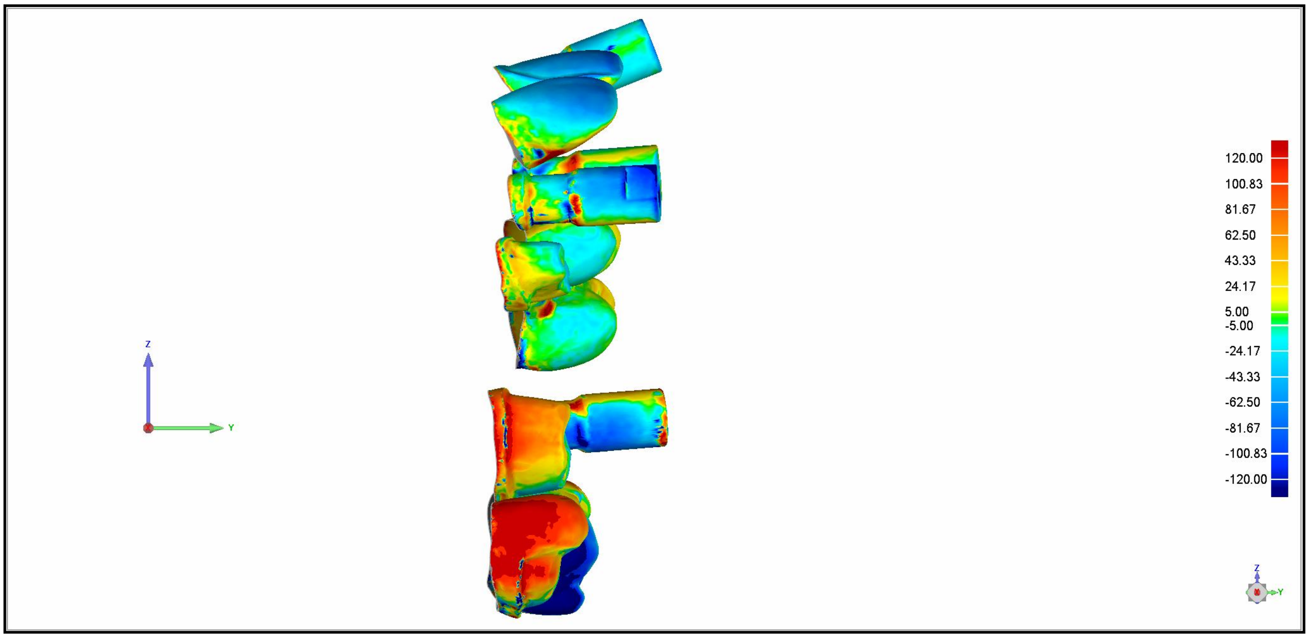

Predefinido: Superior

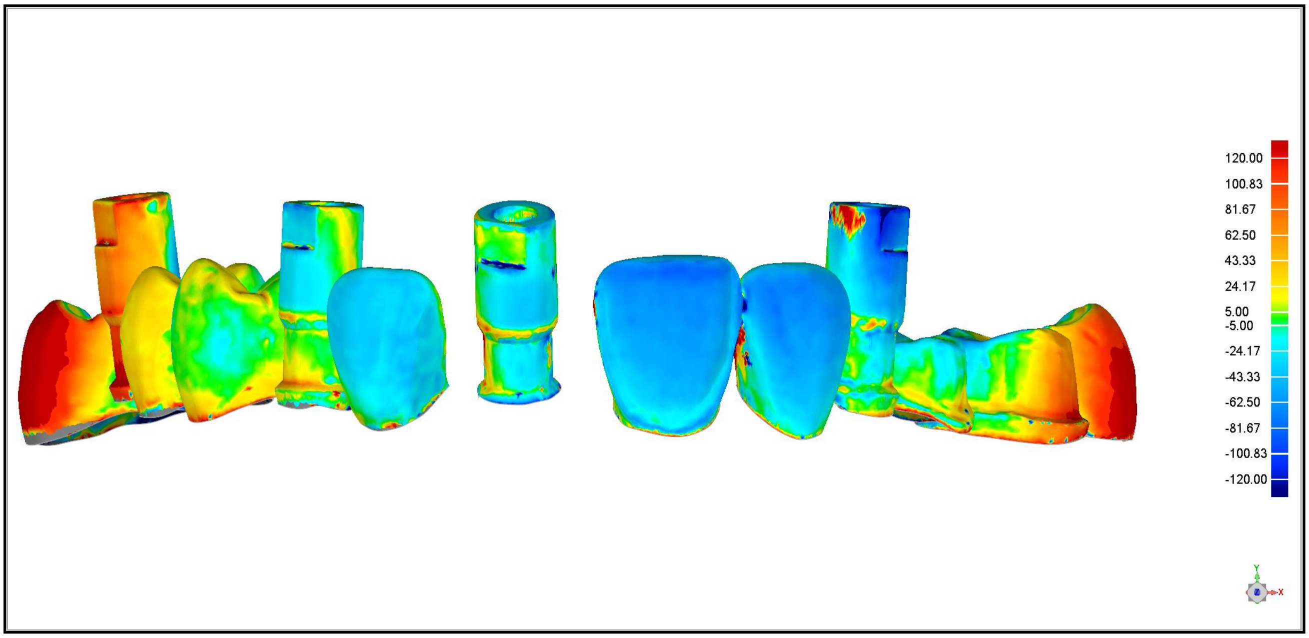

Predefinido: Inferior

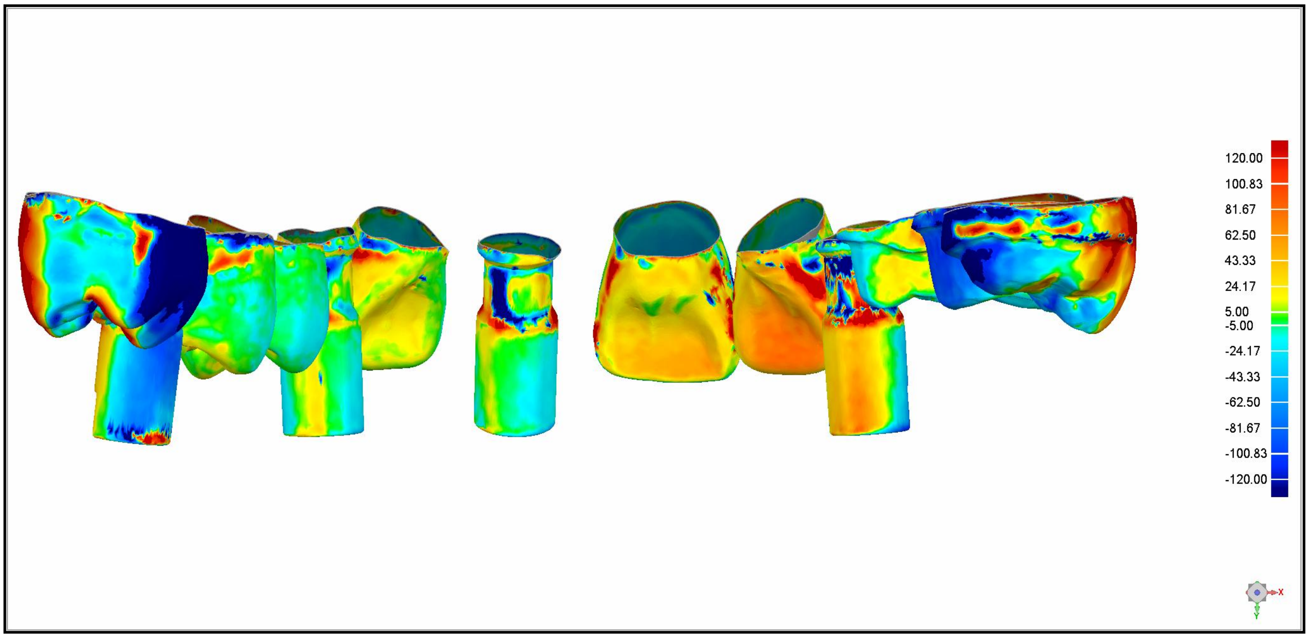

Ajuste de ubicación: Desviaciones superior e inferior

Unidades: u

Nombre	Desv	Estado	Superior Tol	Inferior Tol	Ref X	Ref Y	Ref Z	Radio	Desv X	Desv Y	Desv Z	Medido X	Medido Y	Medido Z	Dir. proy. X	Dir. proy. Y	Dir. proy. Z
Desv. inferior	-3155.38				-23318.94	38082.95	-378.92	n/a	-1366.45	1988.91	2033.09	-24685.39	40071.86	1654.17	0.43	-0.63	-0.64
Desv. superior	3150.17				-12553.67	29785.74	21343.12	n/a	1078.33	-1404.21	2605.56	-11475.33	28381.53	23948.68	0.34	-0.45	0.83
