# Supplementary material for: Accuracy of four digital scanners according to scanning strategy in complete-arch impressions
Source: PLoS One. 2018 Sep 13;13(9):e0202916. doi: 10.1371/journal.pone.0202916 (PMC6136706; doi:10.1371/journal.pone.0202916)
Supplement: S12 Table — Omnicam (scanning strategy D). (ZIP) [file pone.0202916.s012.zip › S12/OM5D.pdf]

3D Comparación Resultados

Modelo referencia	MRC
Modelo test	OM5D
Nº de puntos de datos	200882
# Aislados	863

Tipo tolerancia	3D desviación
Unidades	u
Máx. crítico	120.00
Máx. nominal	4.00
Mín. nominal	-4.00
Mín. crítico	-120.00

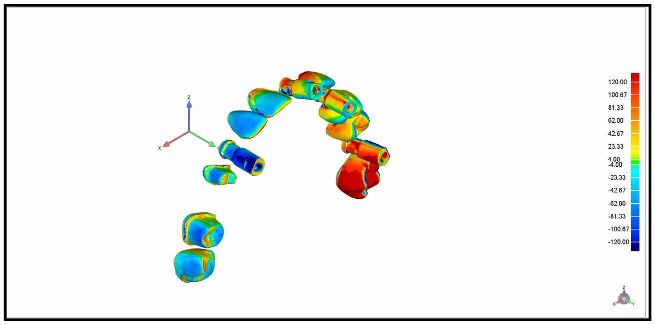

Desviación	
Desviación superior máx.	3148.61
Desviación inferior máx.	-3145.94
Desviación media	116.01 /-121.55
Desviación estándar	291.14

Distribución desviación

>=Min	<Max	# Puntos	%
-120.00	-100.67	2825	1.41
-100.67	-81.33	3335	1.66
-81.33	-62.00	7105	3.54
-62.00	-42.67	12263	6.10
-42.67	-23.33	20814	10.36
-23.33	-4.00	24240	12.07
-4.00	4.00	9774	4.87
4.00	23.33	23775	11.84
23.33	42.67	19676	9.79
42.67	62.00	14058	7.00
62.00	81.33	9988	4.97
81.33	100.67	6727	3.35
100.67	120.00	4931	2.45

Fuera del crítico superior	22518	11.21
Fuera del crítico inferior	18853	9.39

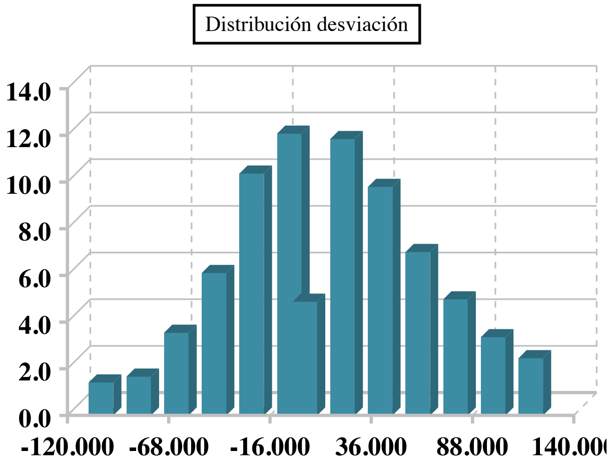

Desviaciones estándar

Distribución (+/-)	# Puntos	%
-6 * Desv. estánd.	1365	0.68
-5 * Desv. estánd.	816	0.41
-4 * Desv. estánd.	883	0.44
-3 * Desv. estánd.	1282	0.64
-2 * Desv. estánd.	2280	1.13
-1 * Desv. estánd.	93301	46.45
1 * Desv. estánd.	94310	46.95
2 * Desv. estánd.	2056	1.02
3 * Desv. estánd.	1641	0.82
4 * Desv. estánd.	1238	0.62
5 * Desv. estánd.	792	0.39
6 * Desv. estánd.	918	0.46

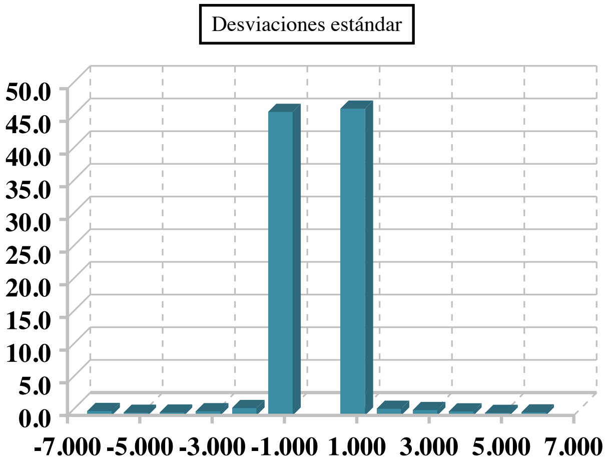

Predefinido: Isométrico

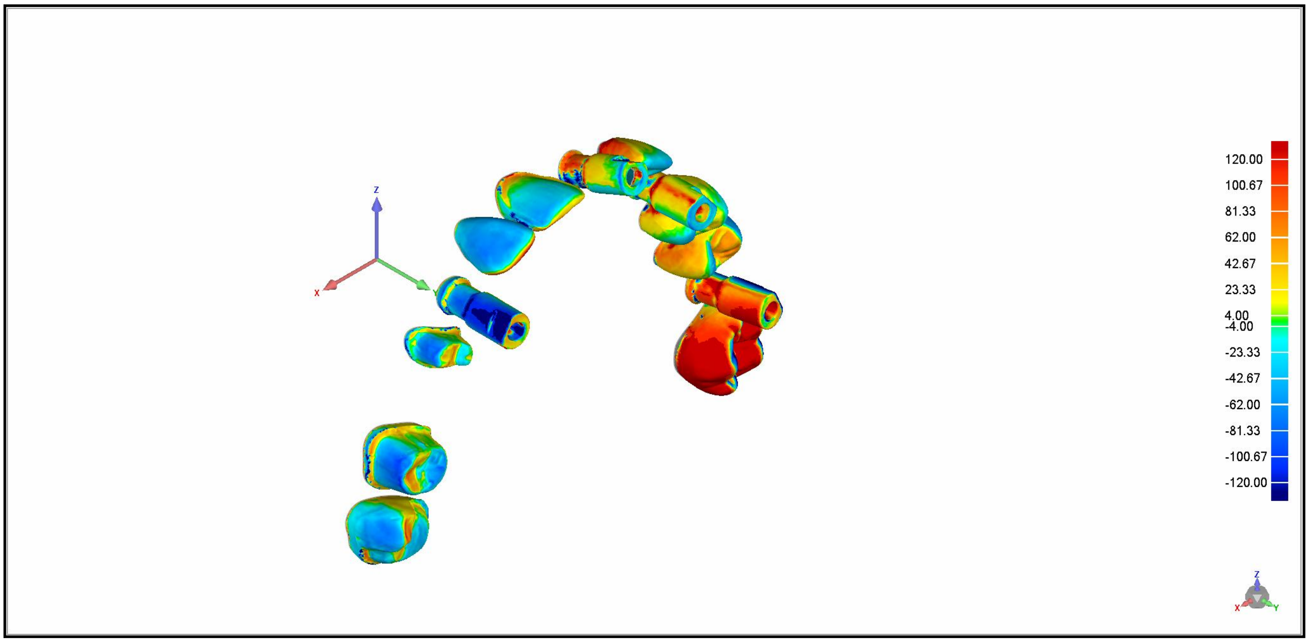

Predefinido: Frente

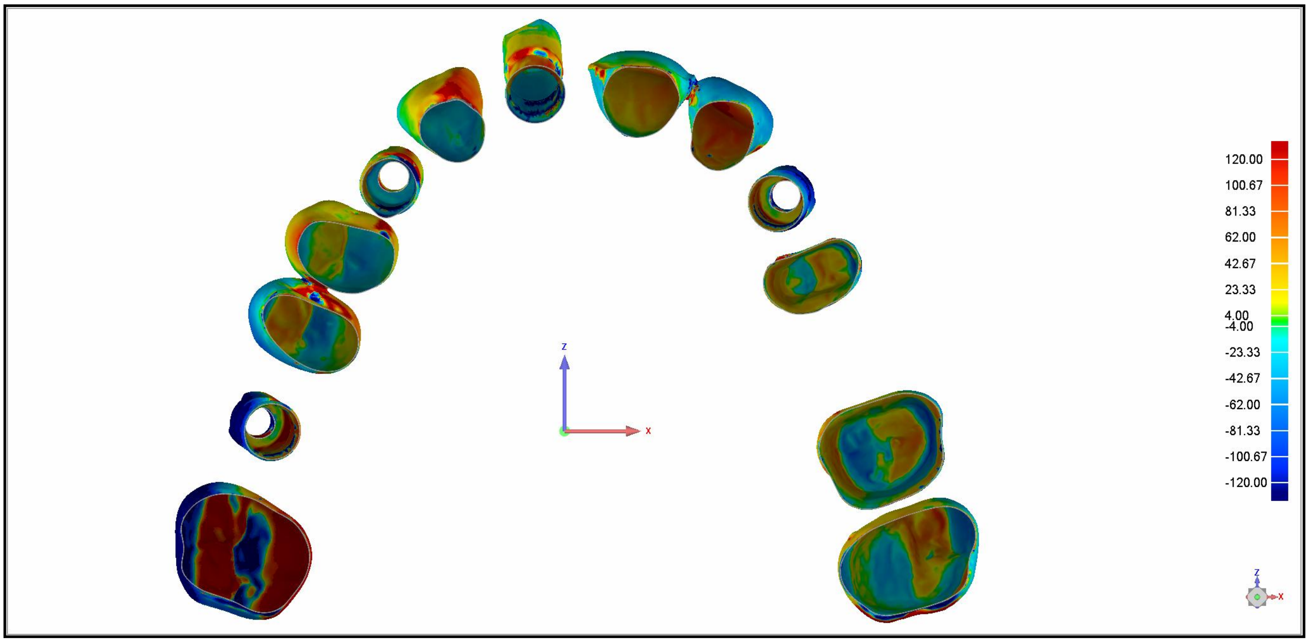

Predefinido: Atrás

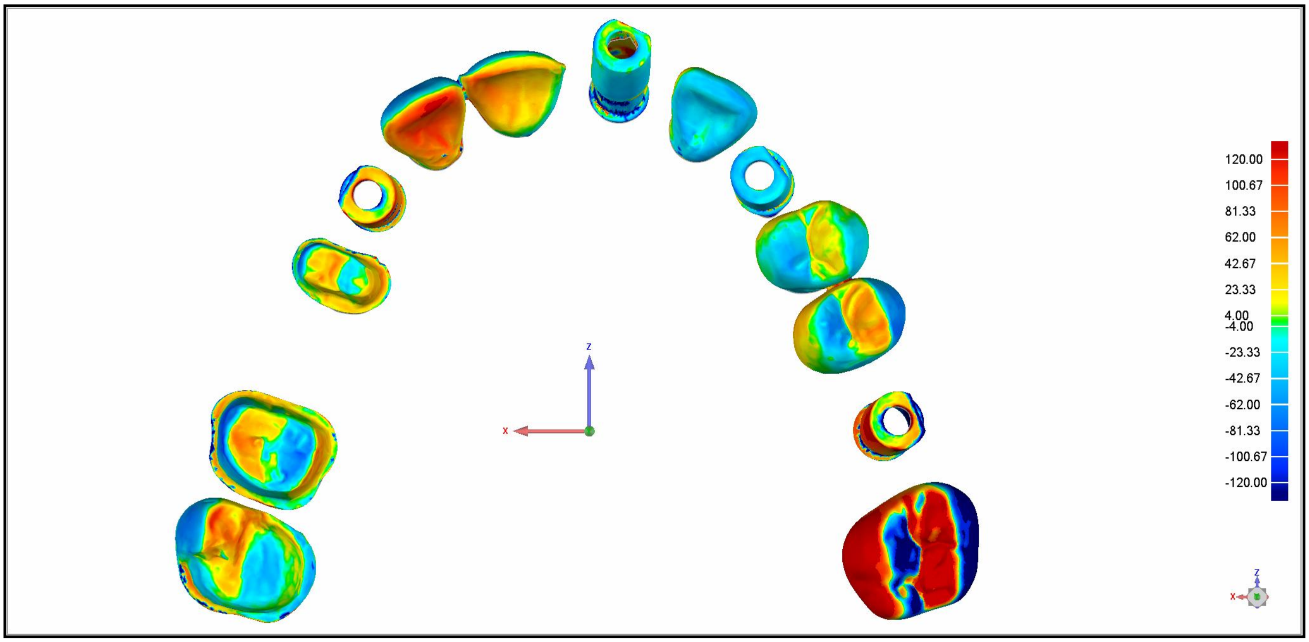

Predefinido: Izquierda

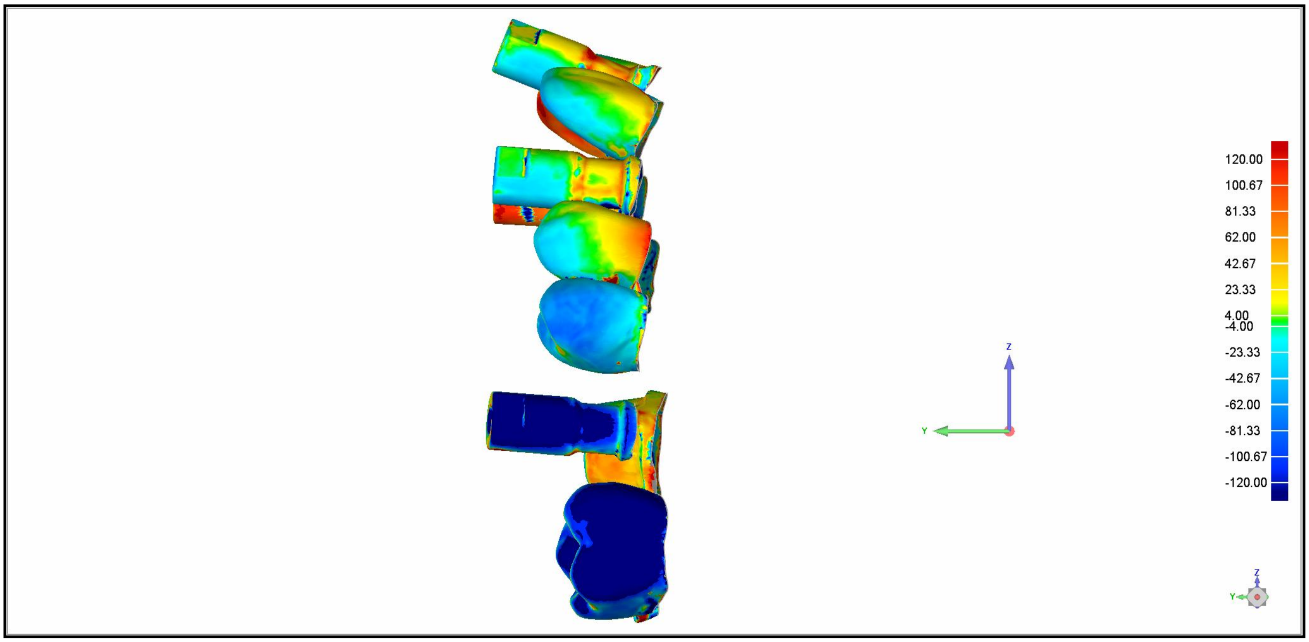

Predefinido: Derecha

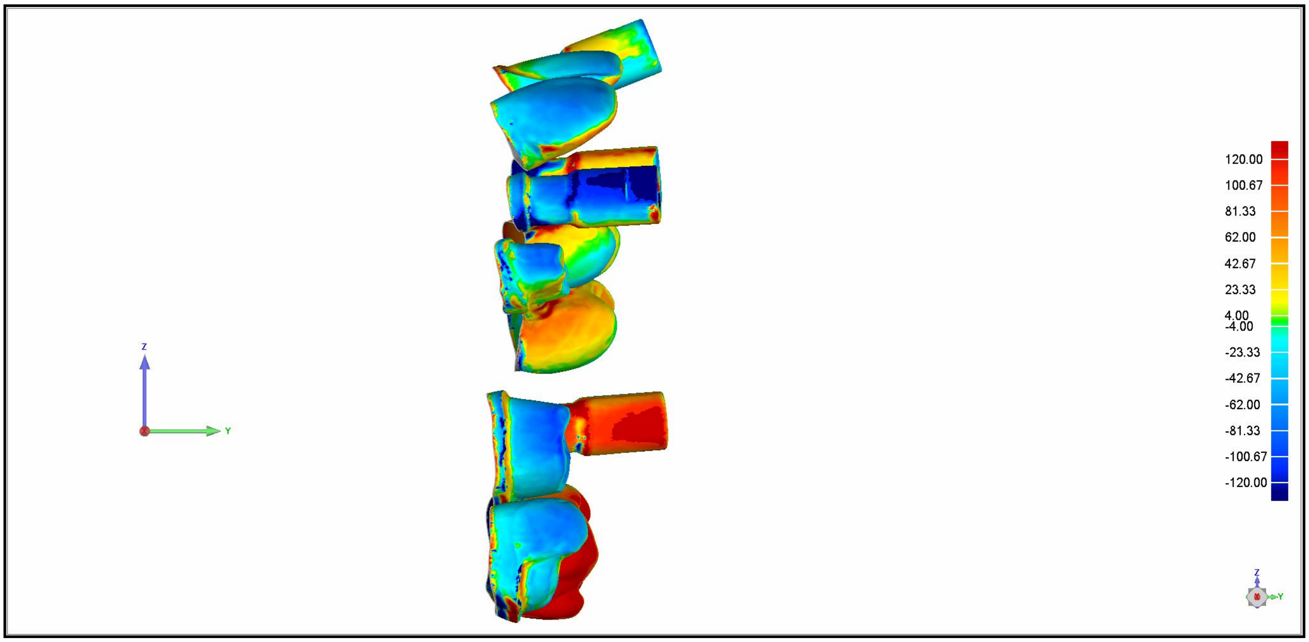

Predefinido: Superior

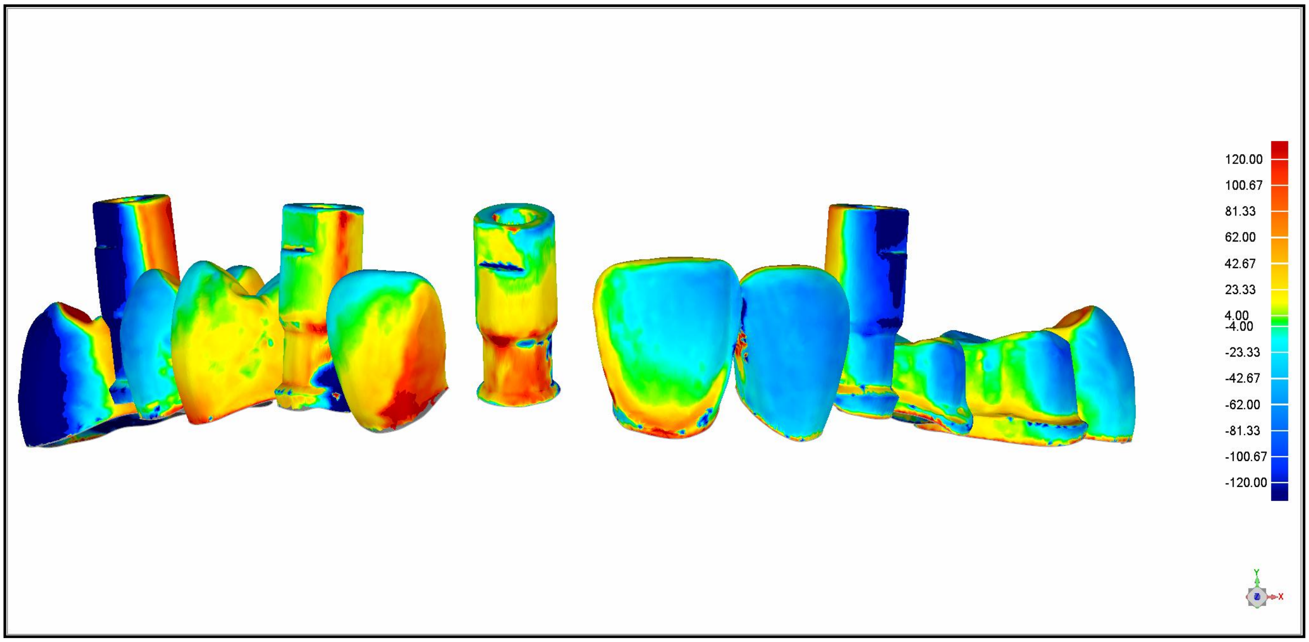

Predefinido: Inferior

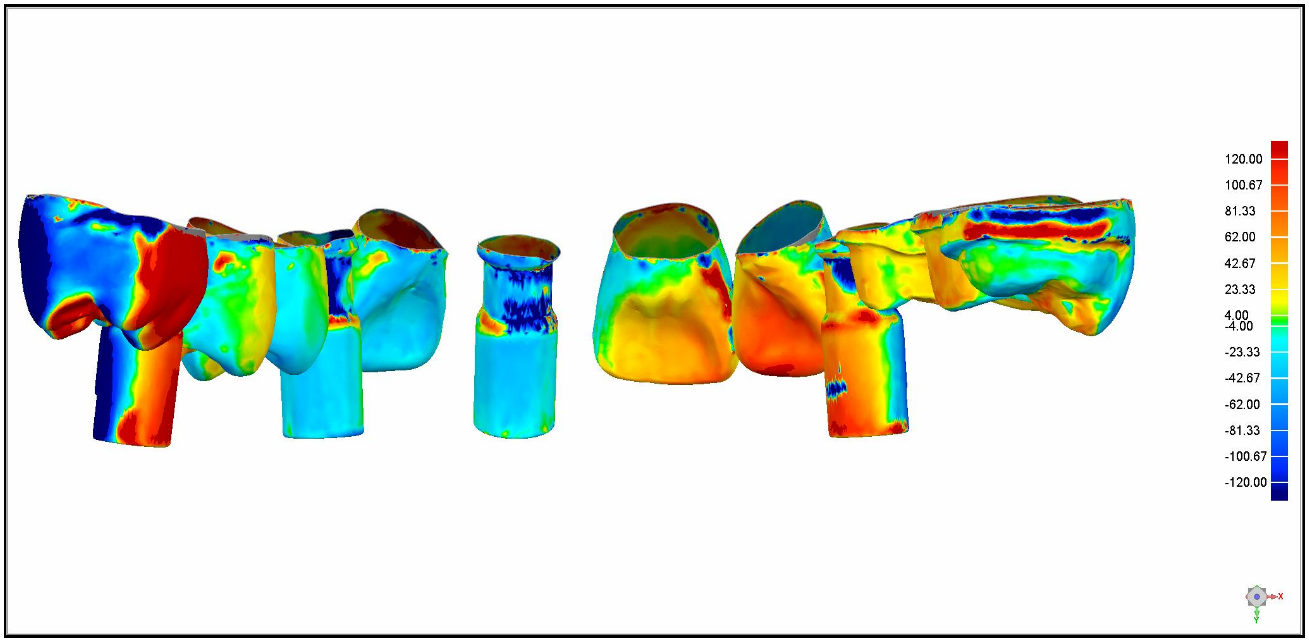

Ajuste de ubicación: Desviaciones superior e inferior

Unidades: u

Nombre	Desv	Estado	Superior Tol	Inferior Tol	Ref X	Ref Y	Ref Z	Radio	Desv X	Desv Y	Desv Z	Medido X	Medido Y	Medido Z	Dir. proy. X	Dir. proy. Y	Dir. proy. Z
Desv. inferior	-3145.94				-16498.53	29426.69	5746.14	n/a	-3025.17	-126.80	-853.96	-19523.69	29299.89	4892.18	0.96	0.04	0.27
Desv. superior	3148.61				-20336.80	30900.48	12276.20	n/a	-702.64	-2483.80	1802.99	-21039.44	28416.68	14079.19	-0.22	-0.79	0.57
