# Supplementary material for: Accuracy of four digital scanners according to scanning strategy in complete-arch impressions
Source: PLoS One. 2018 Sep 13;13(9):e0202916. doi: 10.1371/journal.pone.0202916 (PMC6136706; doi:10.1371/journal.pone.0202916)
Supplement: S12 Table — Omnicam (scanning strategy D). (ZIP) [file pone.0202916.s012.zip › S12/OM4D.pdf]

3D Comparación Resultados

Modelo referencia	MRC
Modelo test	OM4D
Nº de puntos de datos	197995
# Aislados	931

Tipo tolerancia	3D desviación
Unidades	u
Máx. crítico	120.00
Máx. nominal	12.00
Mín. nominal	-12.00
Mín. crítico	-120.00

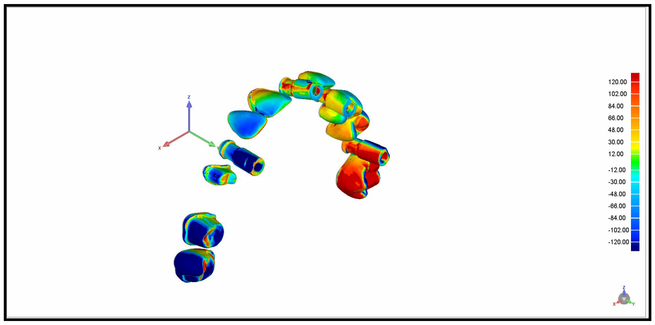

Desviación	
Desviación superior máx.	3142.13
Desviación inferior máx.	-2922.04
Desviación media	126.40 /-112.24
Desviación estándar	253.29

Distribución desviación

>=Min	<Max	# Puntos	%
-120.00	-102.00	3860	1.95
-102.00	-84.00	5169	2.61
-84.00	-66.00	7589	3.83
-66.00	-48.00	10053	5.08
-48.00	-30.00	12906	6.52
-30.00	-12.00	17374	8.77
-12.00	12.00	24883	12.57
12.00	30.00	16458	8.31
30.00	48.00	13596	6.87
48.00	66.00	11403	5.76
66.00	84.00	7903	3.99
84.00	102.00	6392	3.23
102.00	120.00	5861	2.96

Fuera del crítico superior	29971	15.14
Fuera del crítico inferior	24577	12.41

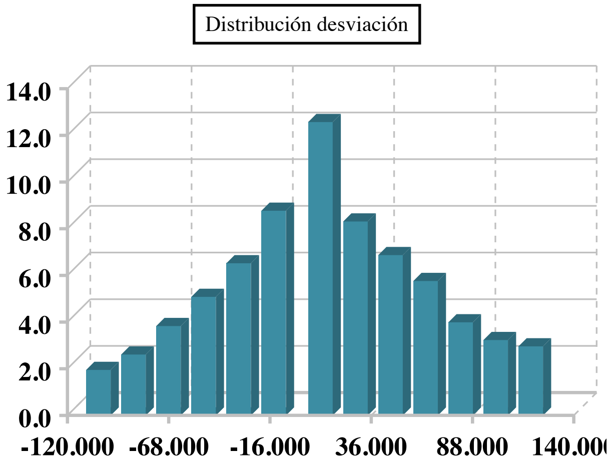

Desviaciones estándar

Distribución (+/-)	# Puntos	%
-6 * Desv. estánd.	1060	0.54
-5 * Desv. estánd.	399	0.20
-4 * Desv. estánd.	490	0.25
-3 * Desv. estánd.	976	0.49
-2 * Desv. estánd.	4728	2.39
-1 * Desv. estánd.	99458	50.23
1 * Desv. estánd.	83088	41.96
2 * Desv. estánd.	3353	1.69
3 * Desv. estánd.	1258	0.64
4 * Desv. estánd.	1115	0.56
5 * Desv. estánd.	970	0.49
6 * Desv. estánd.	1100	0.56

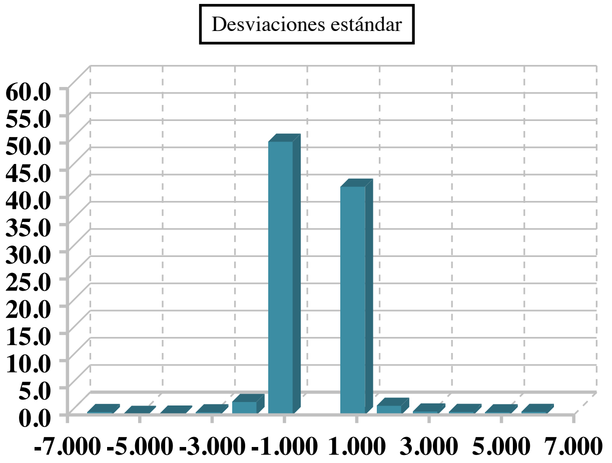

Predefinido: Isométrico

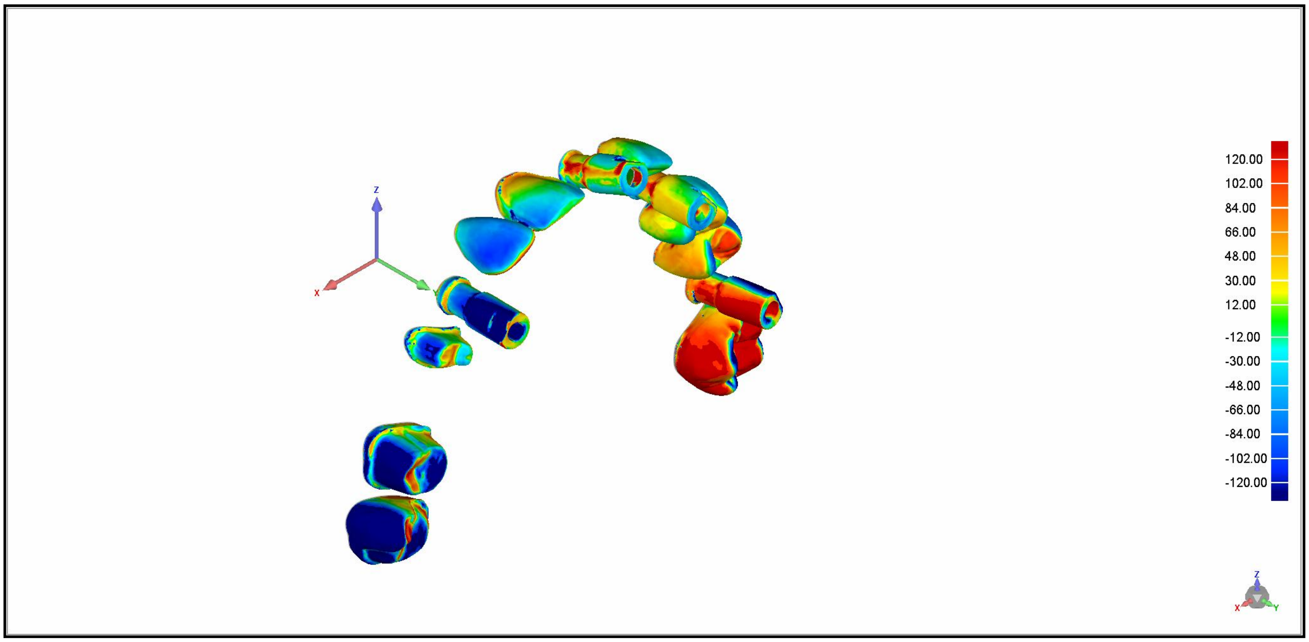

Predefinido: Frente

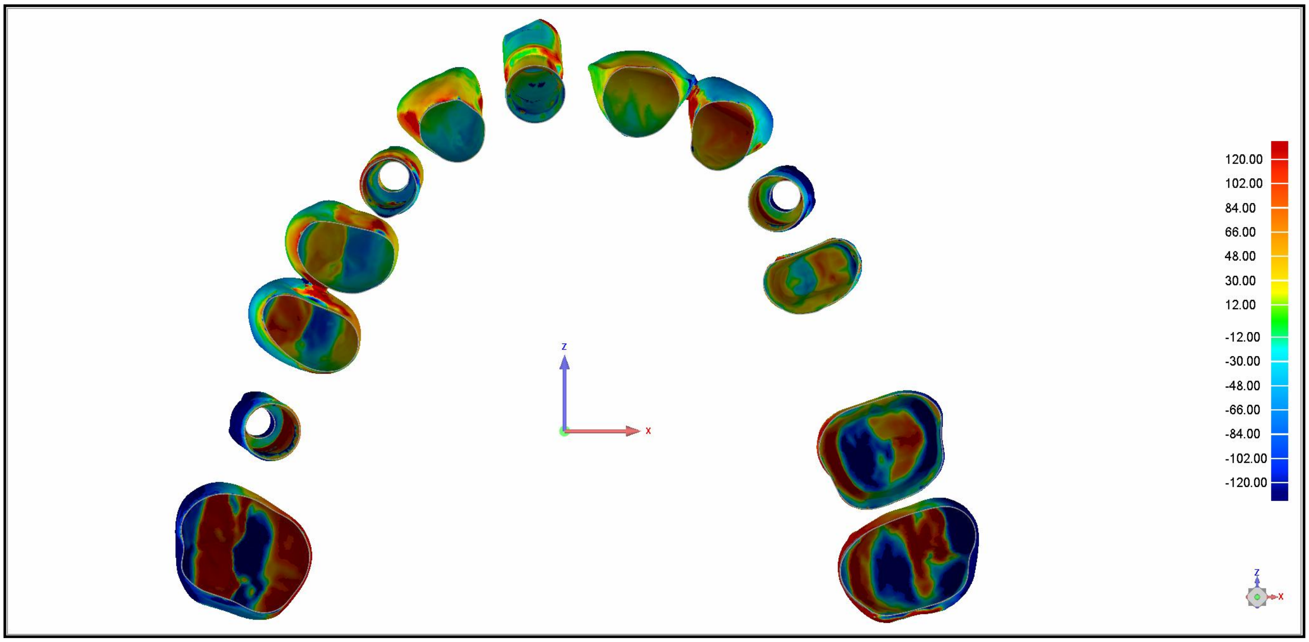

Predefinido: Atrás

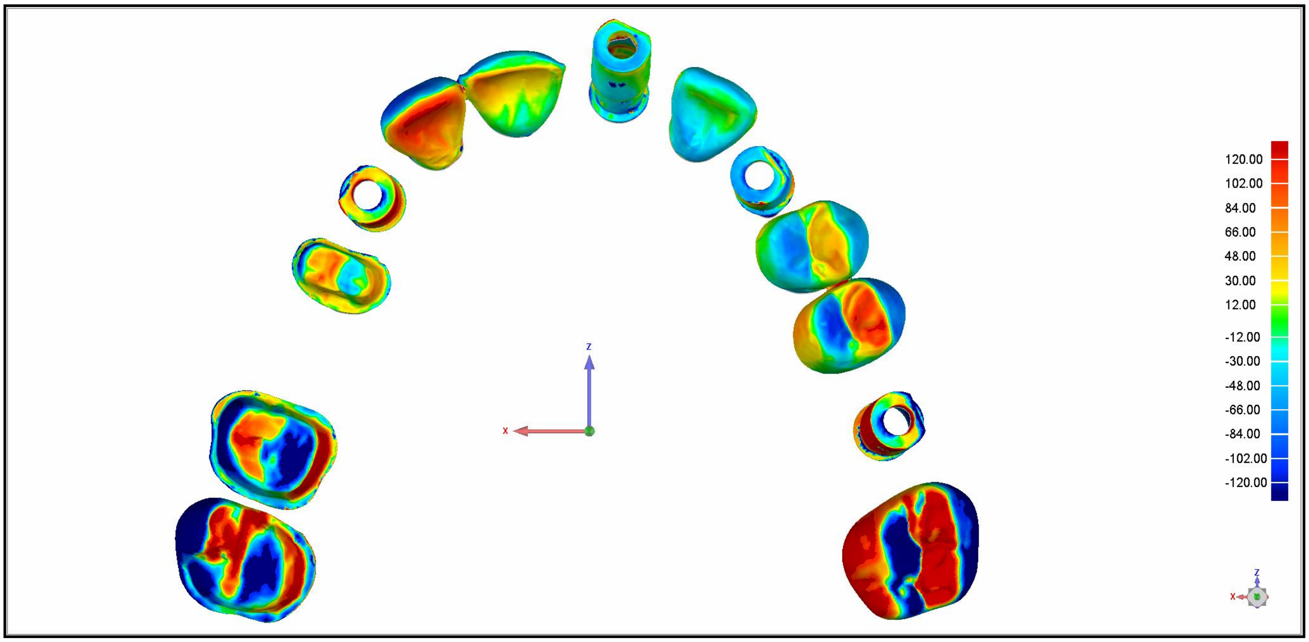

Predefinido: Izquierda

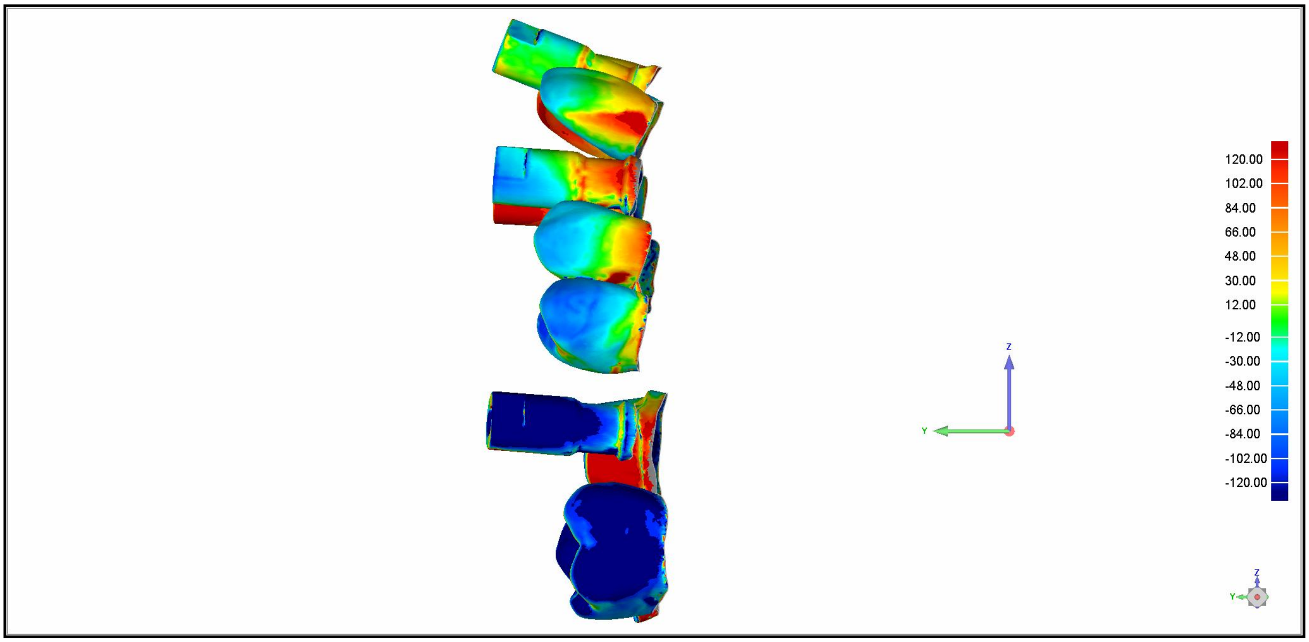

Predefinido: Derecha

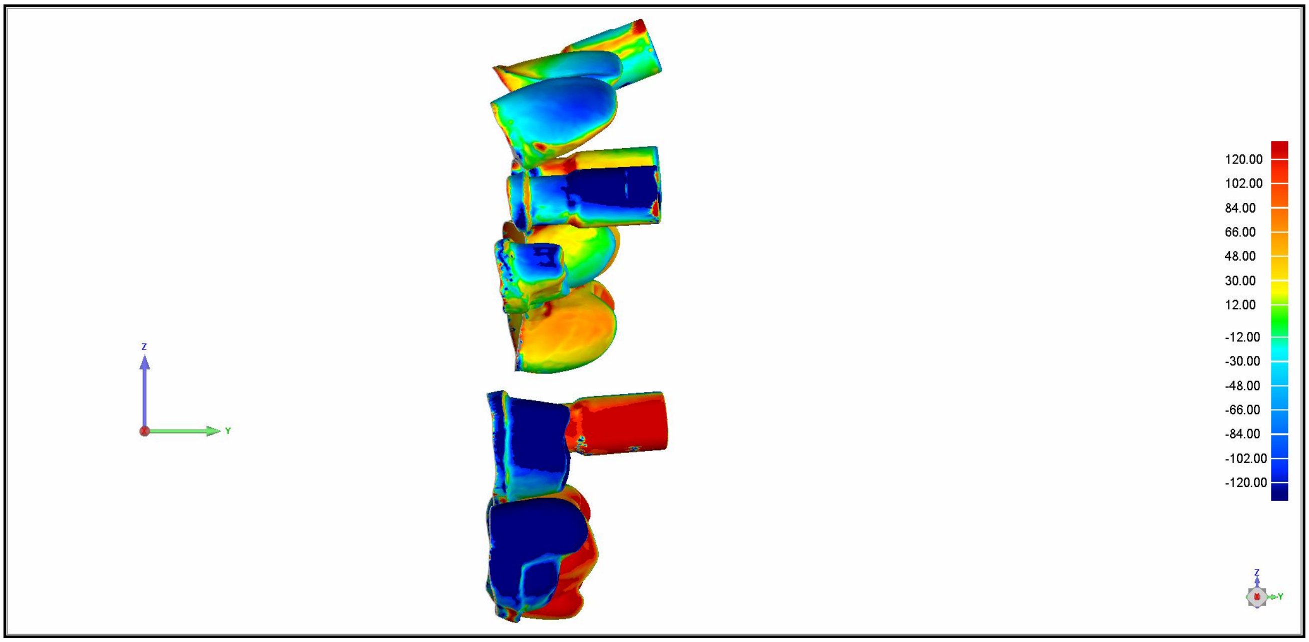

Predefinido: Superior

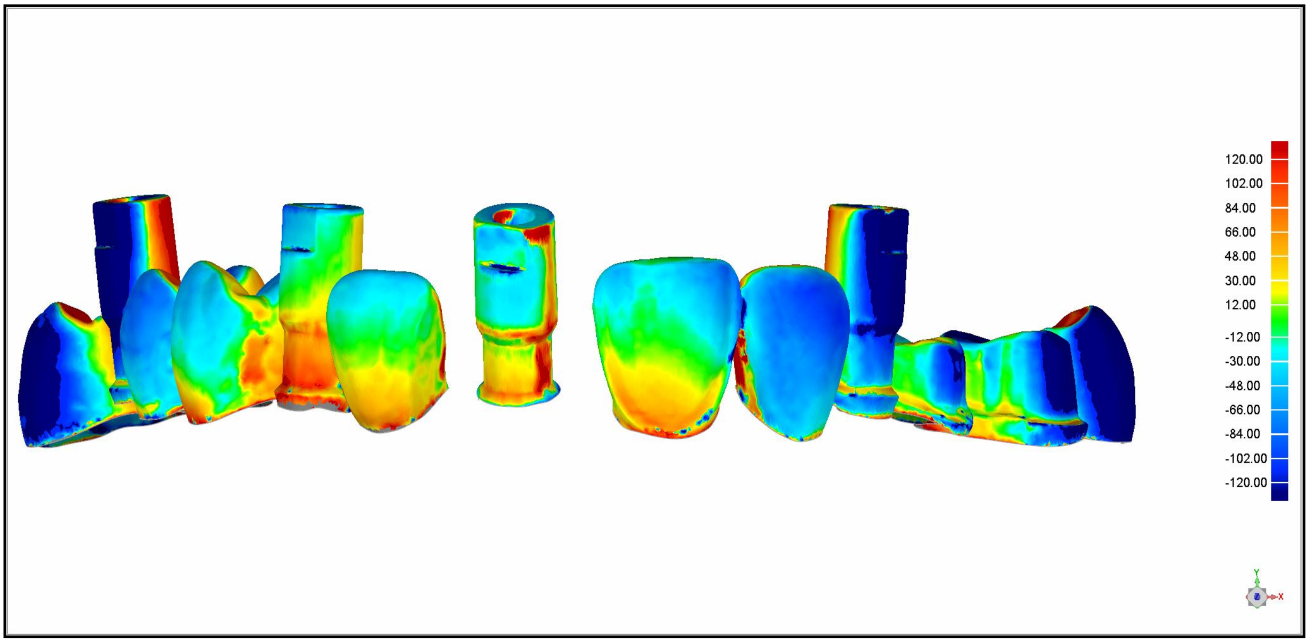

Predefinido: Inferior

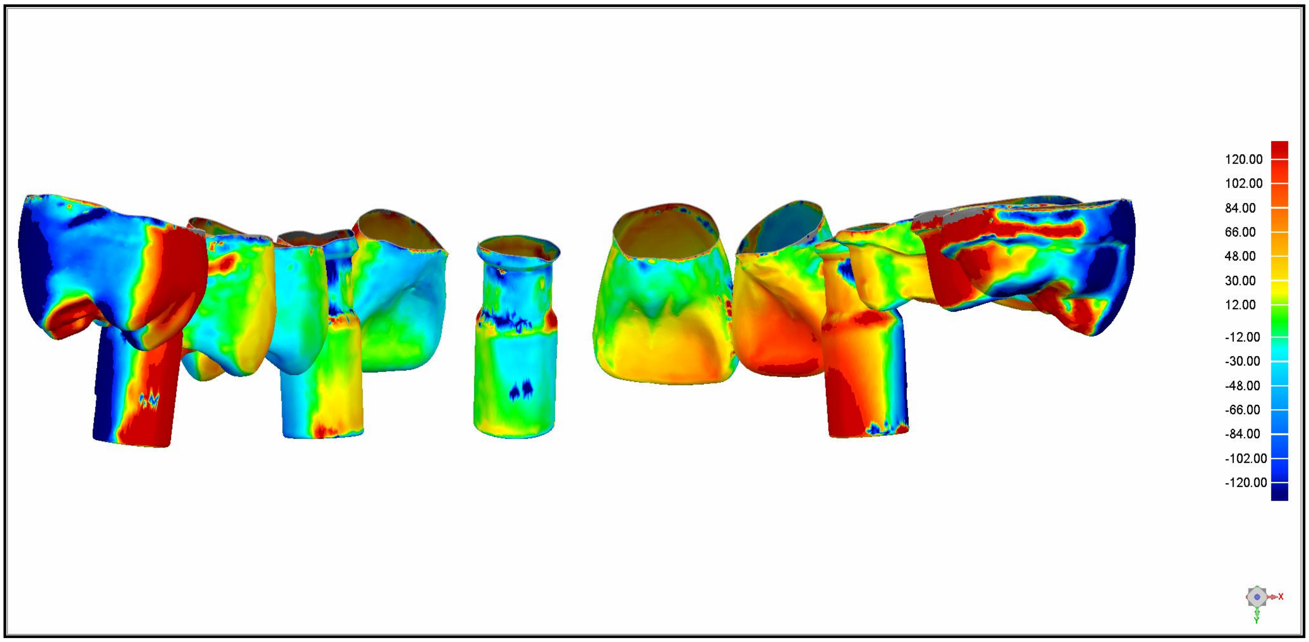

Ajuste de ubicación: Desviaciones superior e inferior

Unidades: u

Nombre	Desv	Estado	Superior Tol	Inferior Tol	Ref X	Ref Y	Ref Z	Radio	Desv X	Desv Y	Desv Z	Medido X	Medido Y	Medido Z	Dir. proy. X	Dir. proy. Y	Dir. proy. Z
Desv. inferior	-2922.04				-3155.88	37011.66	31001.10	n/a	1159.00	115.44	-2679.88	-1996.88	37127.10	28321.22	-0.40	-0.04	0.92
Desv. superior	3142.13				29547.11	27336.30	1208.53	n/a	-2564.49	-410.52	1768.56	26982.61	26925.77	2977.09	-0.82	-0.13	0.56
