# Supplementary material for: Accuracy of four digital scanners according to scanning strategy in complete-arch impressions
Source: PLoS One. 2018 Sep 13;13(9):e0202916. doi: 10.1371/journal.pone.0202916 (PMC6136706; doi:10.1371/journal.pone.0202916)
Supplement: S12 Table — Omnicam (scanning strategy D). (ZIP) [file pone.0202916.s012.zip › S12/OM8D.pdf]

3D Comparación Resultados

Modelo referencia	MRC
Modelo test	OM8D
Nº de puntos de datos	200587
# Aislados	575

Tipo tolerancia	3D desviación
Unidades	u
Máx. crítico	120.00
Máx. nominal	7.00
Mín. nominal	-7.00
Mín. crítico	-120.00

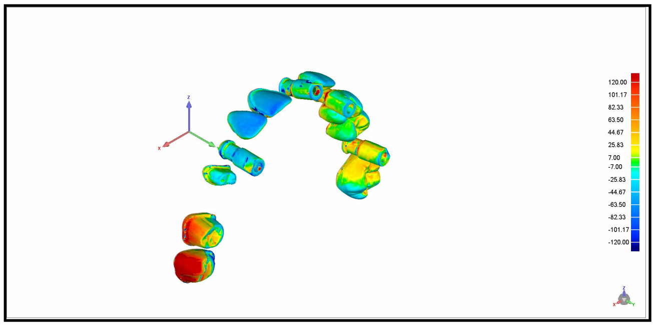

Desviación	
Desviación superior máx.	3091.72
Desviación inferior máx.	-3153.91
Desviación media	93.64 /-92.82
Desviación estándar	261.86

Distribución desviación

>=Min	<Max	# Puntos	%
-120.00	-101.17	1909	0.95
-101.17	-82.33	2620	1.31
-82.33	-63.50	4609	2.30
-63.50	-44.67	9309	4.64
-44.67	-25.83	19302	9.62
-25.83	-7.00	32065	15.99
-7.00	7.00	28189	14.05
7.00	25.83	33649	16.78
25.83	44.67	19622	9.78
44.67	63.50	9147	4.56
63.50	82.33	5001	2.49
82.33	101.17	4017	2.00
101.17	120.00	3288	1.64

Fuera del crítico superior	18383	9.16
Fuera del crítico inferior	9477	4.72

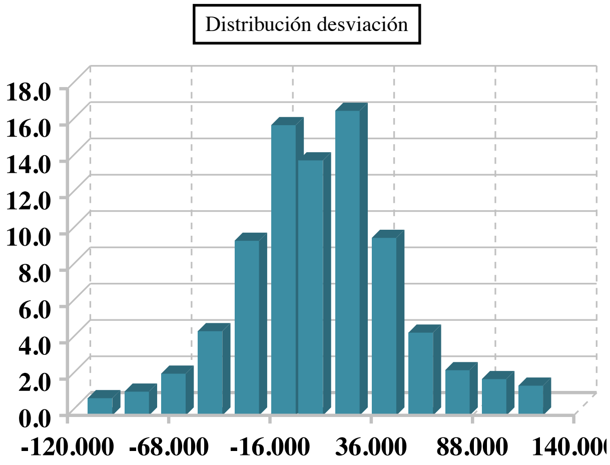

Desviaciones estándar

Distribución (+/-)	# Puntos	%
-6 * Desv. estánd.	1515	0.76
-5 * Desv. estánd.	702	0.35
-4 * Desv. estánd.	858	0.43
-3 * Desv. estánd.	939	0.47
-2 * Desv. estánd.	1586	0.79
-1 * Desv. estánd.	101935	50.82
1 * Desv. estánd.	86210	42.98
2 * Desv. estánd.	2629	1.31
3 * Desv. estánd.	1516	0.76
4 * Desv. estánd.	1166	0.58
5 * Desv. estánd.	701	0.35
6 * Desv. estánd.	830	0.41

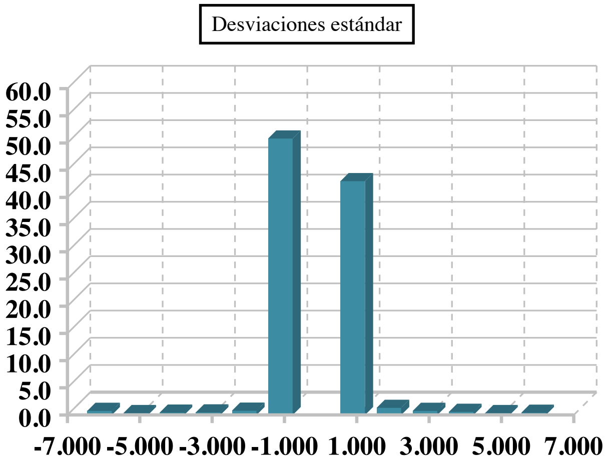

Predefinido: Isométrico

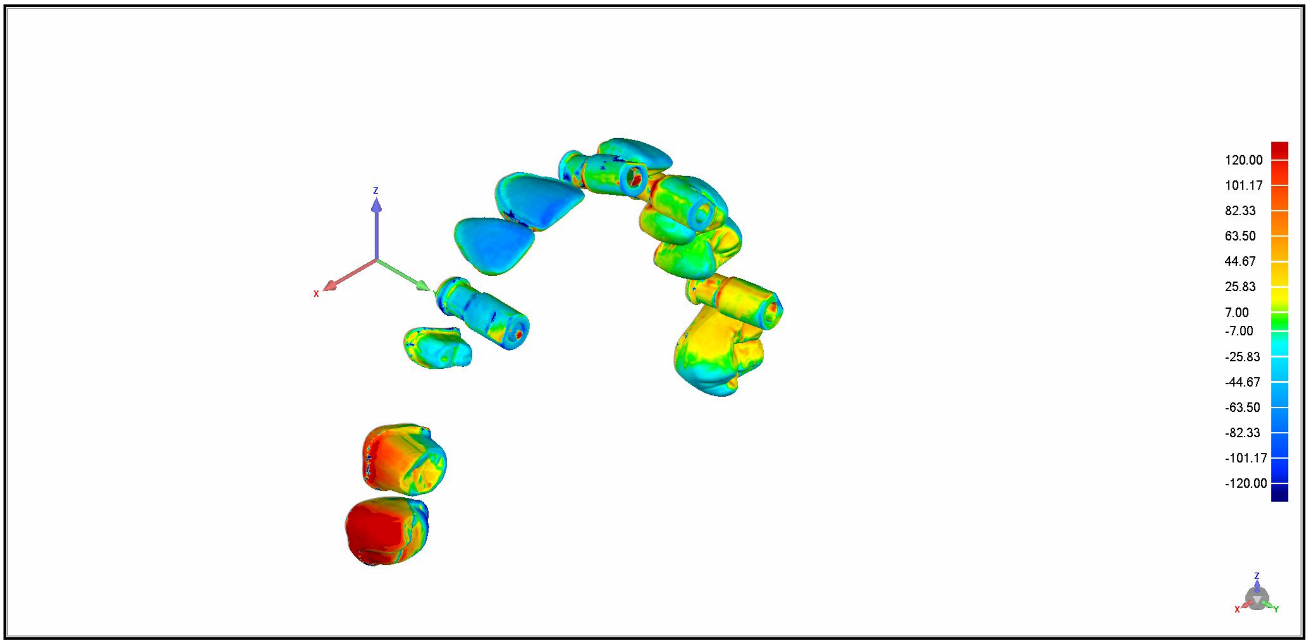

Predefinido: Frente

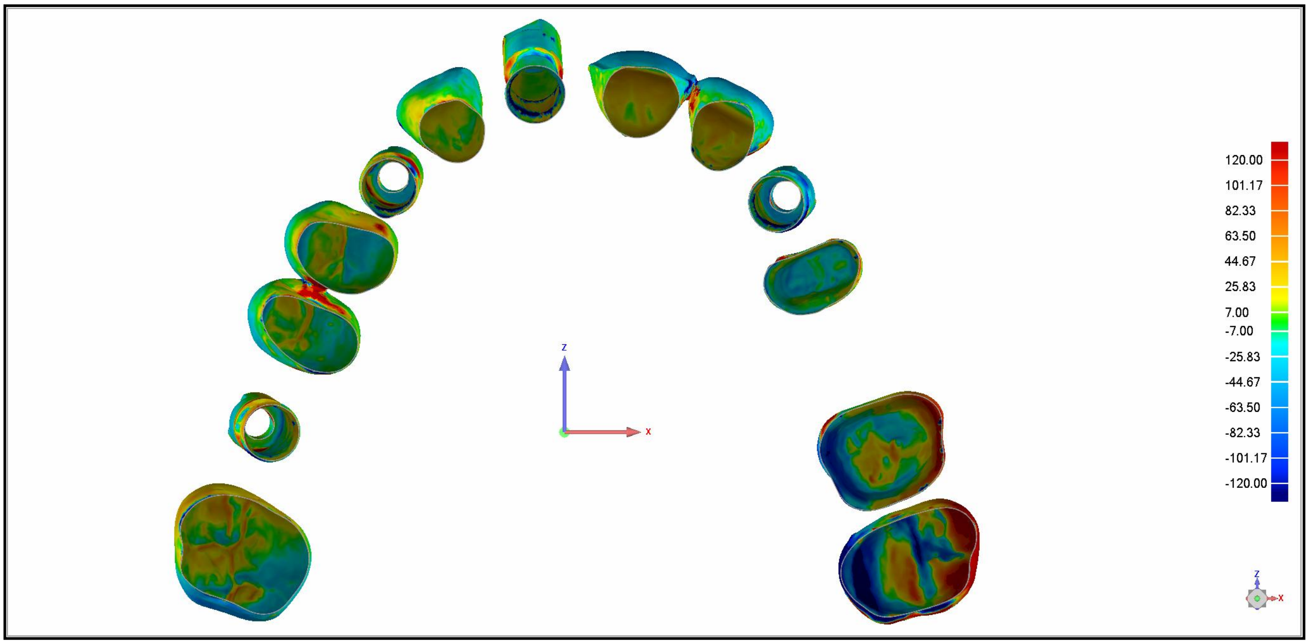

Predefinido: Atrás

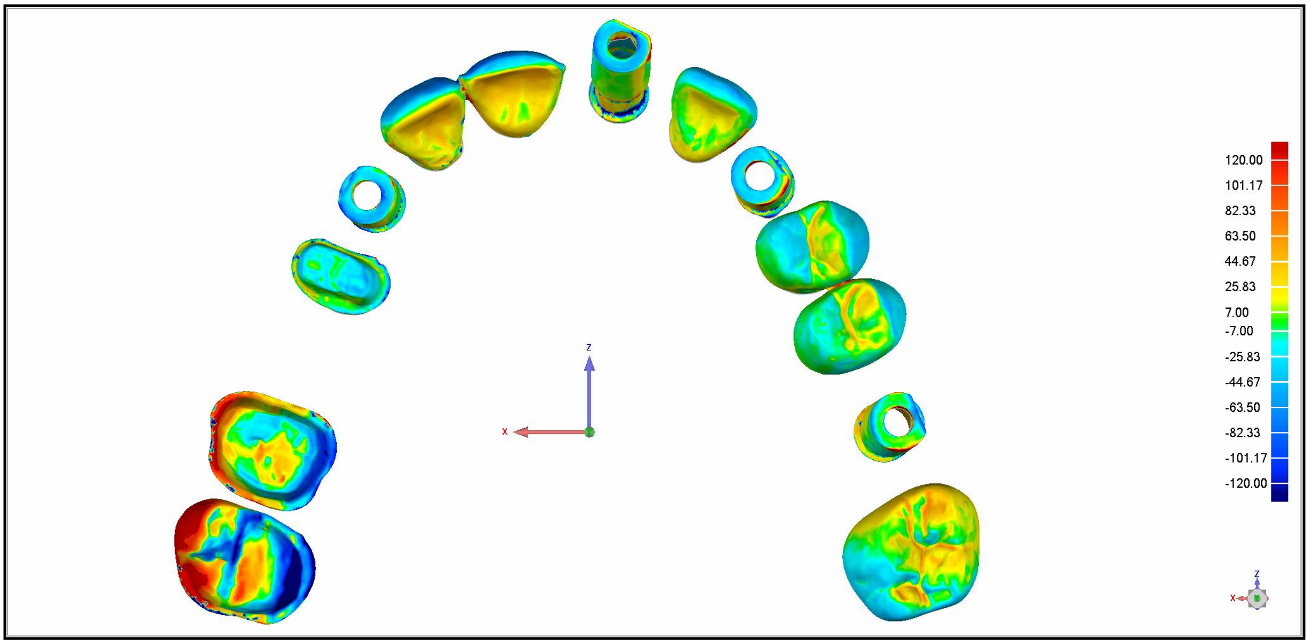

Fecha: 3/6/2017, 3:10 pm

Predefinido: Izquierda

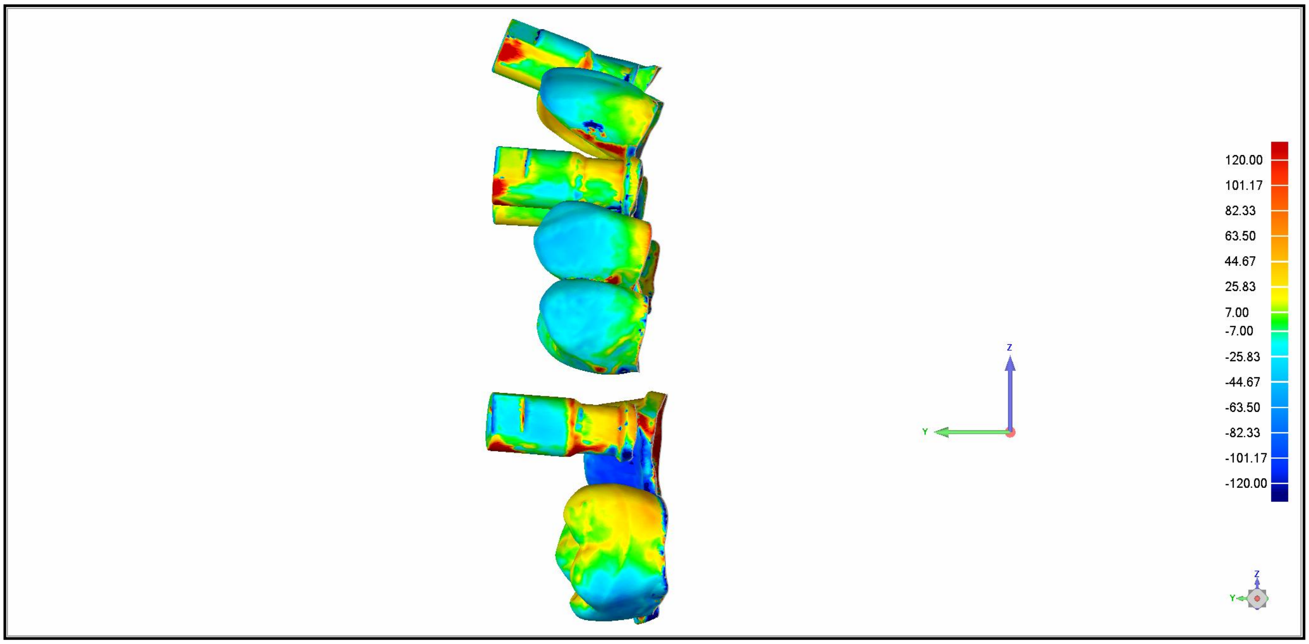

Predefinido: Derecha

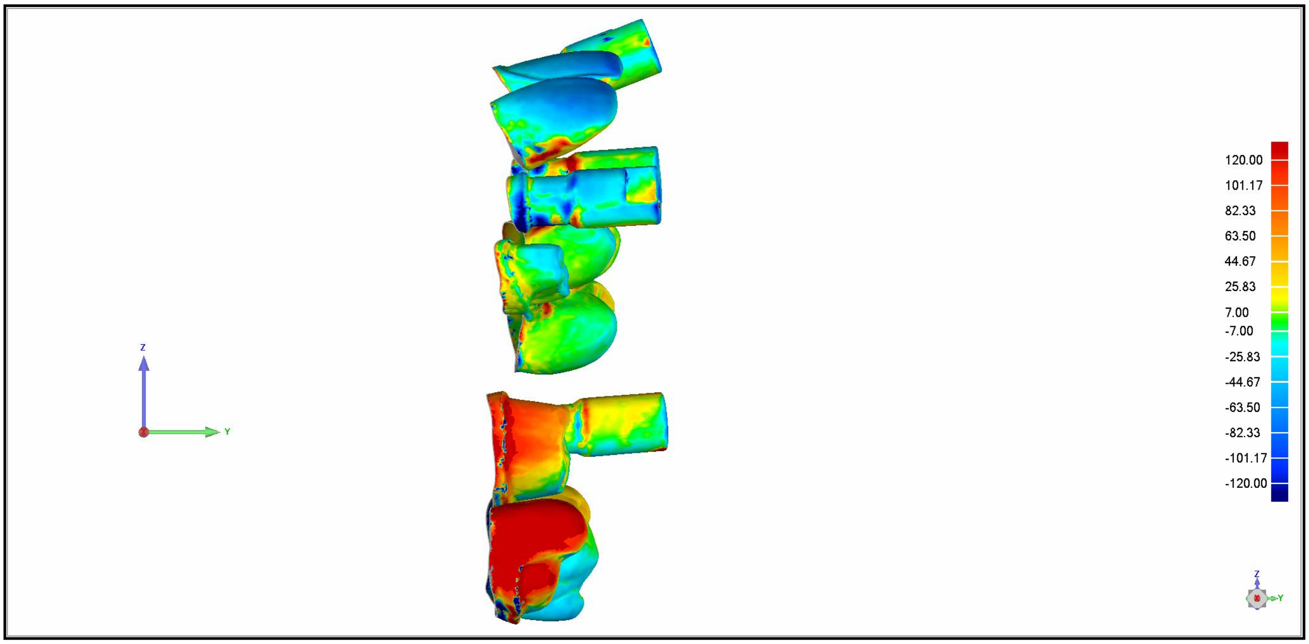

Predefinido: Superior

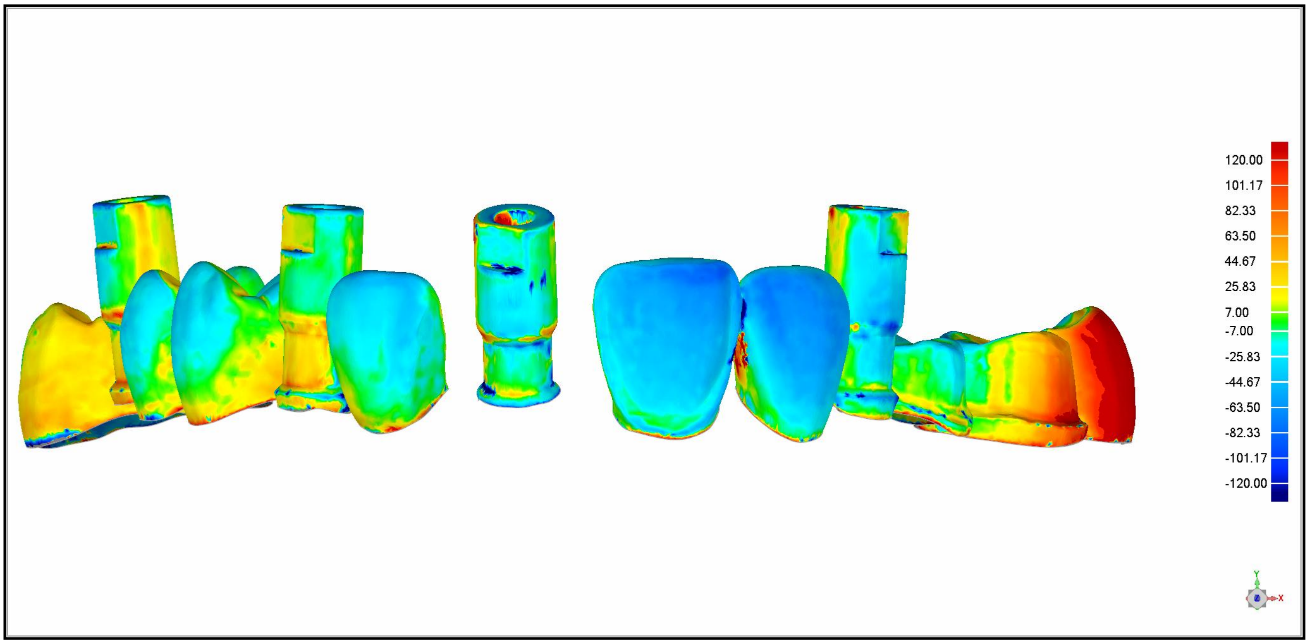

Predefinido: Inferior

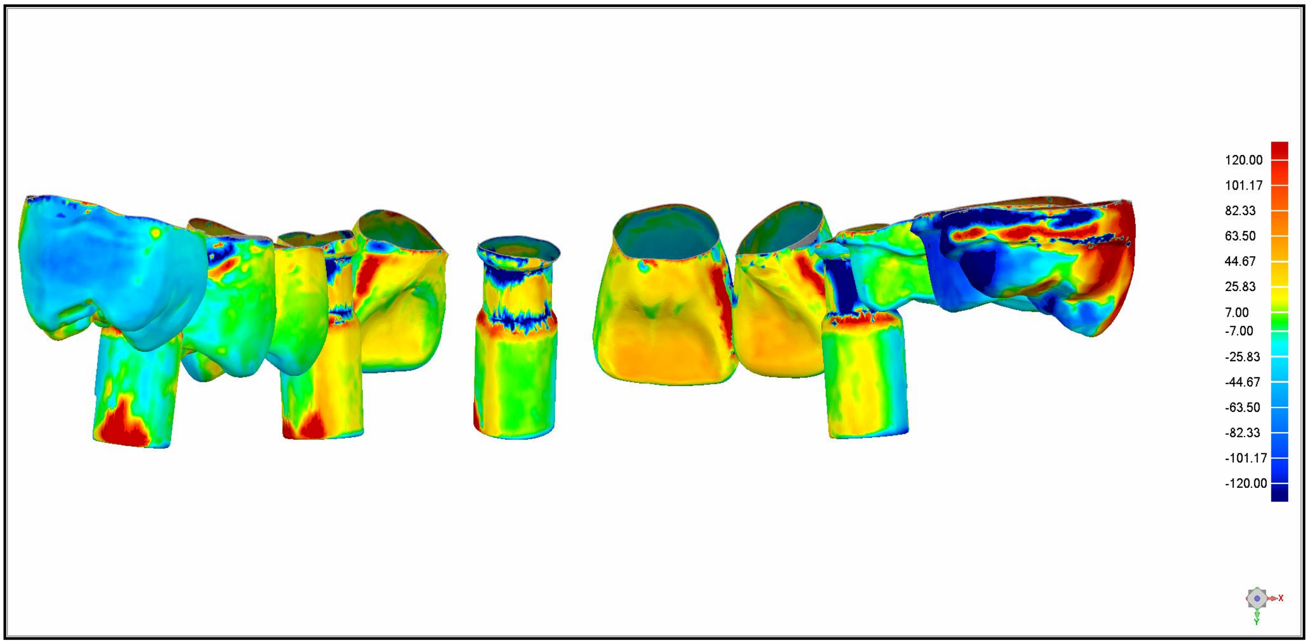

Ajuste de ubicación: Desviaciones superior e inferior

Unidades: u

Nombre	Desv	Estado	Superior Tol	Inferior Tol	Ref X	Ref Y	Ref Z	Radio	Desv X	Desv Y	Desv Z	Medido X	Medido Y	Medido Z	Dir. proy. X	Dir. proy. Y	Dir. proy. Z
Desv. inferior	-3153.91				-22607.19	28955.77	6808.03	n/a	-843.48	-497.43	2998.04	-23450.68	28458.34	9806.07	0.27	0.16	-0.95
Desv. superior	3091.72				-1817.06	31198.05	24975.22	n/a	-340.72	2754.61	1361.90	-2157.79	33952.65	26337.12	-0.11	0.89	0.44
