# Supplementary material for: Accuracy of four digital scanners according to scanning strategy in complete-arch impressions
Source: PLoS One. 2018 Sep 13;13(9):e0202916. doi: 10.1371/journal.pone.0202916 (PMC6136706; doi:10.1371/journal.pone.0202916)
Supplement: S12 Table — Omnicam (scanning strategy D). (ZIP) [file pone.0202916.s012.zip › S12/OM7D.pdf]

Fecha: 3/6/2017, 3:08 pm

3D Comparación Resultados

Modelo referencia	MRC
Modelo test	OM7D
Nº de puntos de datos	200039
# Aislados	585

Tipo tolerancia	3D desviación
Unidades	u
Máx. crítico	120.00
Máx. nominal	3.00
Mín. nominal	-3.00
Mín. crítico	-120.00

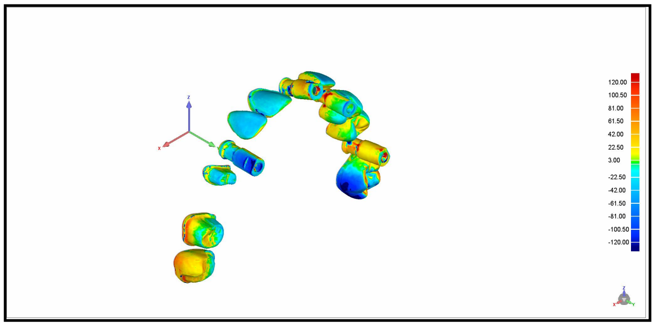

Desviación	
Desviación superior máx.	2666.87
Desviación inferior máx.	-3152.31
Desviación media	82.66 /-100.49
Desviación estándar	277.80

Distribución desviación

>=Min	<Max	# Puntos	%
-120.00	-100.50	2345	1.17
-100.50	-81.00	2725	1.36
-81.00	-61.50	3934	1.97
-61.50	-42.00	6493	3.25
-42.00	-22.50	18907	9.45
-22.50	-3.00	36285	18.14
-3.00	3.00	13730	6.86
3.00	22.50	41484	20.74
22.50	42.00	27448	13.72
42.00	61.50	11495	5.75
61.50	81.00	6738	3.37
81.00	100.50	4632	2.32
100.50	120.00	2576	1.29

Fuera del crítico superior	12036	6.02
Fuera del crítico inferior	9211	4.60

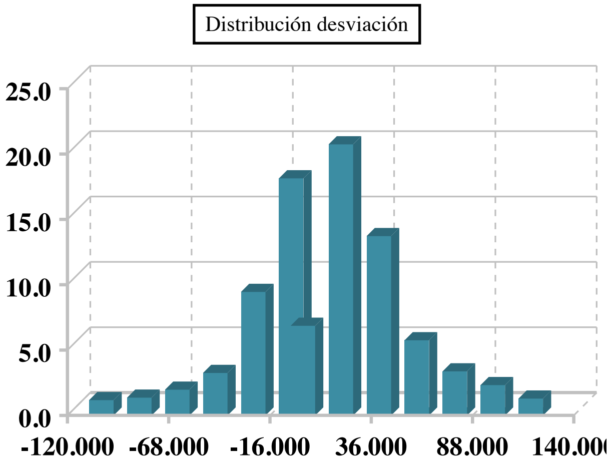

Desviaciones estándar

Distribución (+/-)	# Puntos	%
-6 * Desv. estánd.	1763	0.88
-5 * Desv. estánd.	851	0.43
-4 * Desv. estánd.	587	0.29
-3 * Desv. estánd.	919	0.46
-2 * Desv. estánd.	1032	0.52
-1 * Desv. estánd.	88658	44.32
1 * Desv. estánd.	100639	50.31
2 * Desv. estánd.	1718	0.86
3 * Desv. estánd.	1238	0.62
4 * Desv. estánd.	772	0.39
5 * Desv. estánd.	777	0.39
6 * Desv. estánd.	1085	0.54

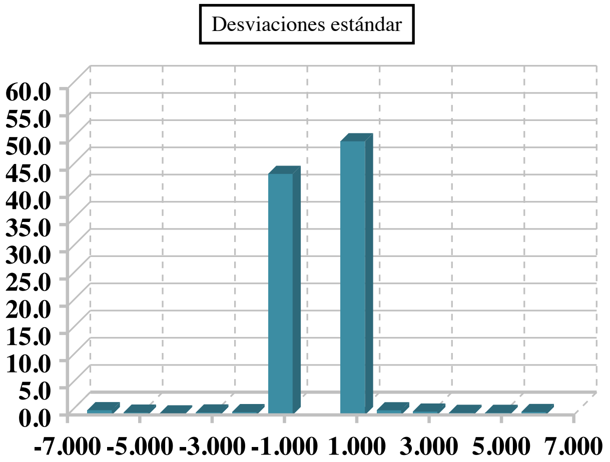

Predefinido: Isométrico

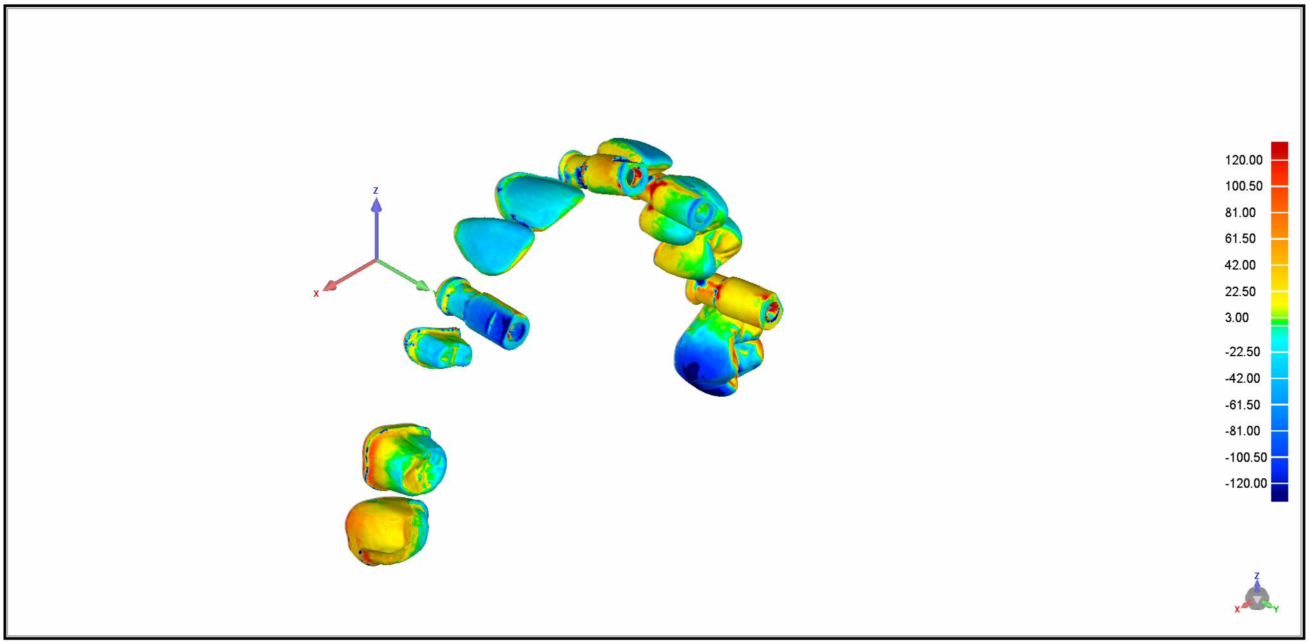

Predefinido: Frente

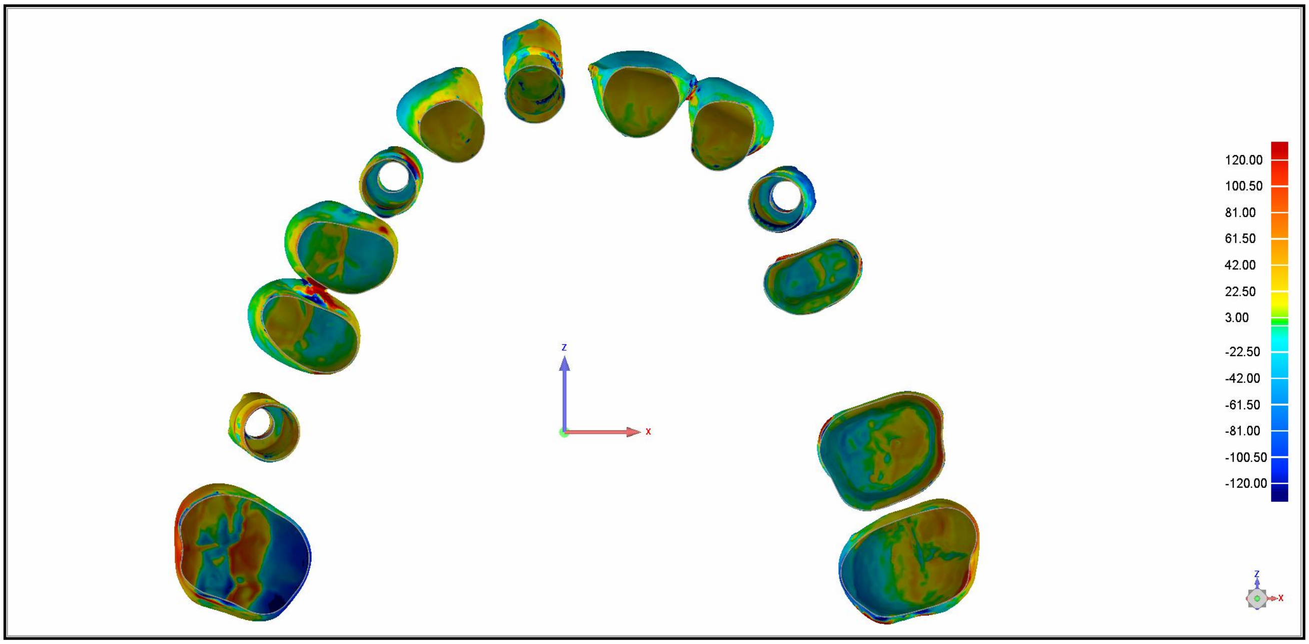

Predefinido: Atrás

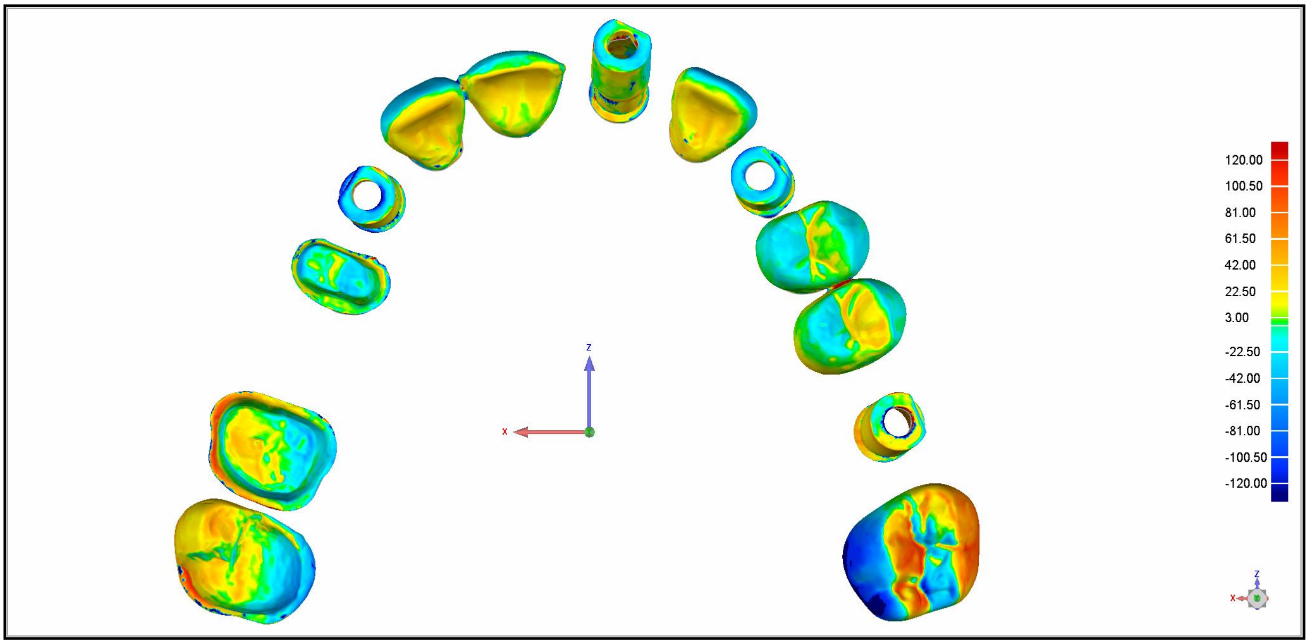

Predefinido: Izquierda

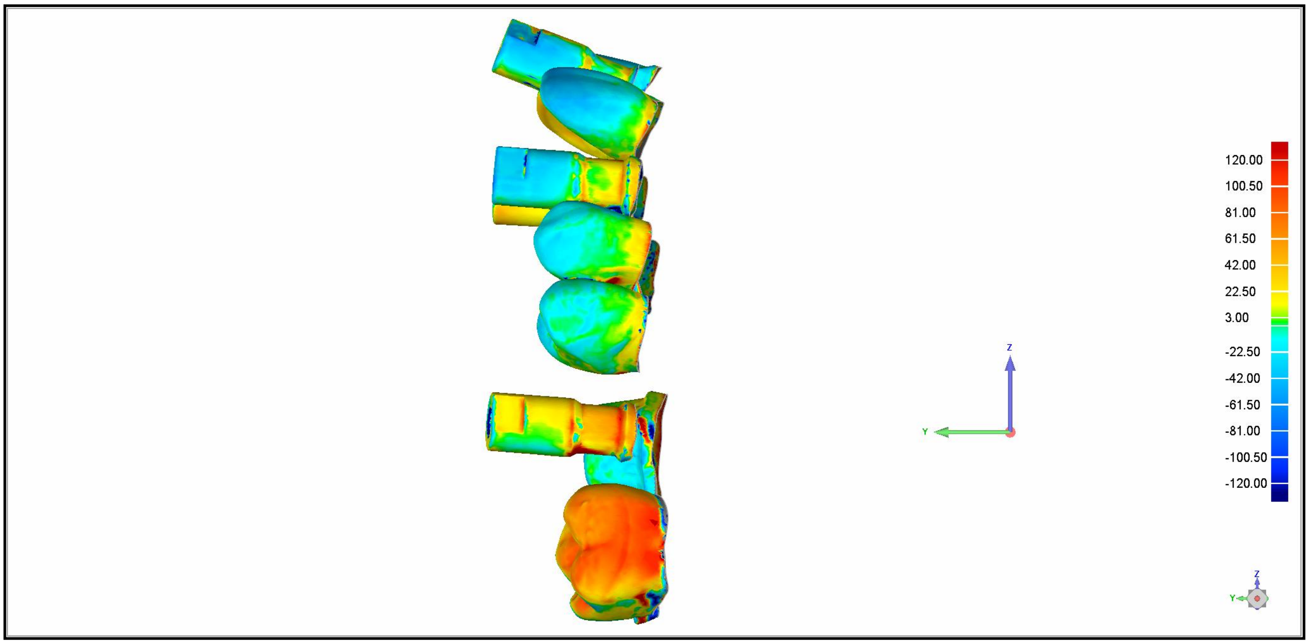

Predefinido: Derecha

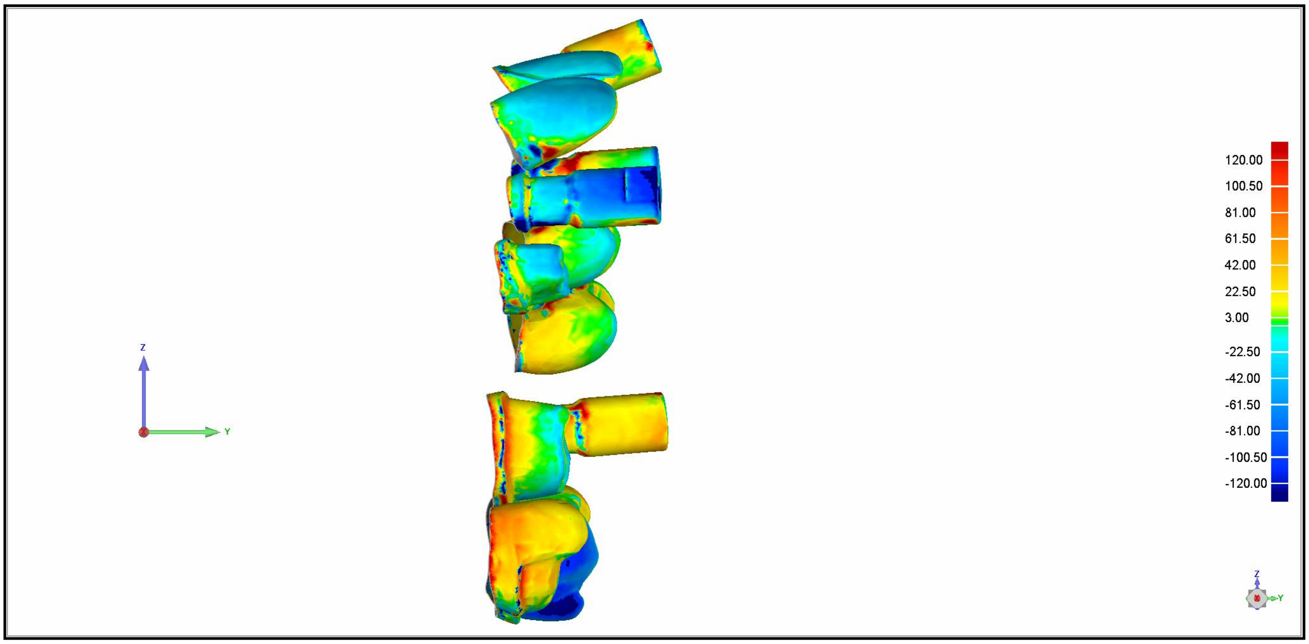

Predefinido: Superior

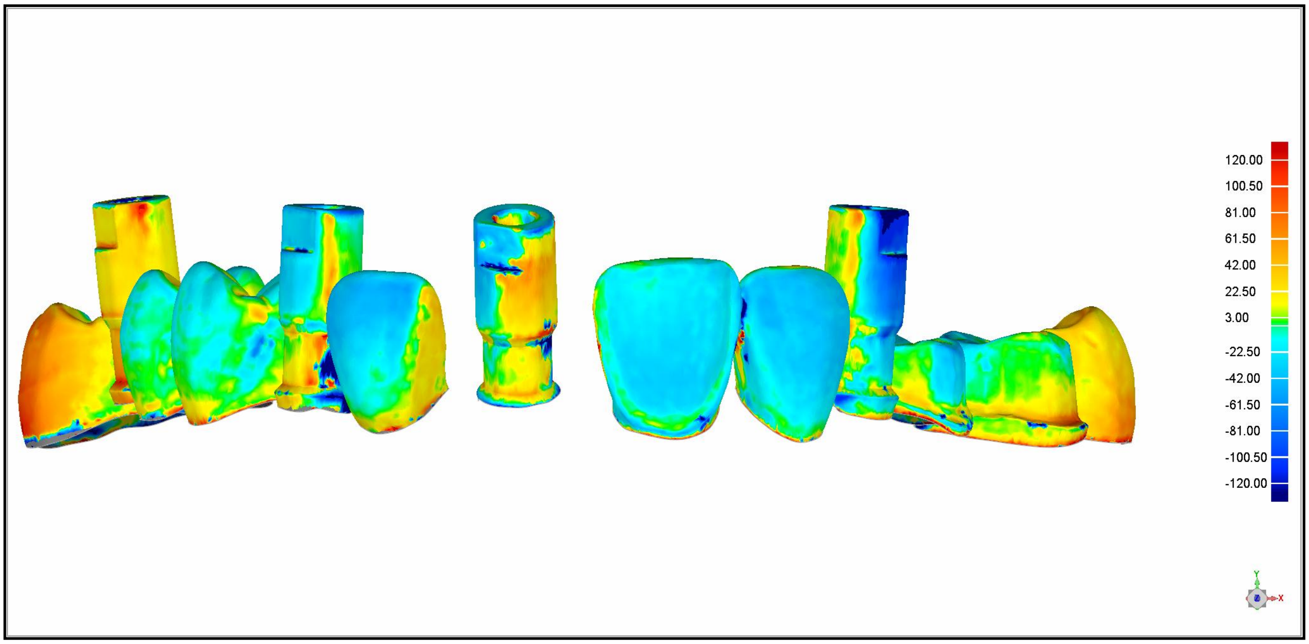

Predefinido: Inferior

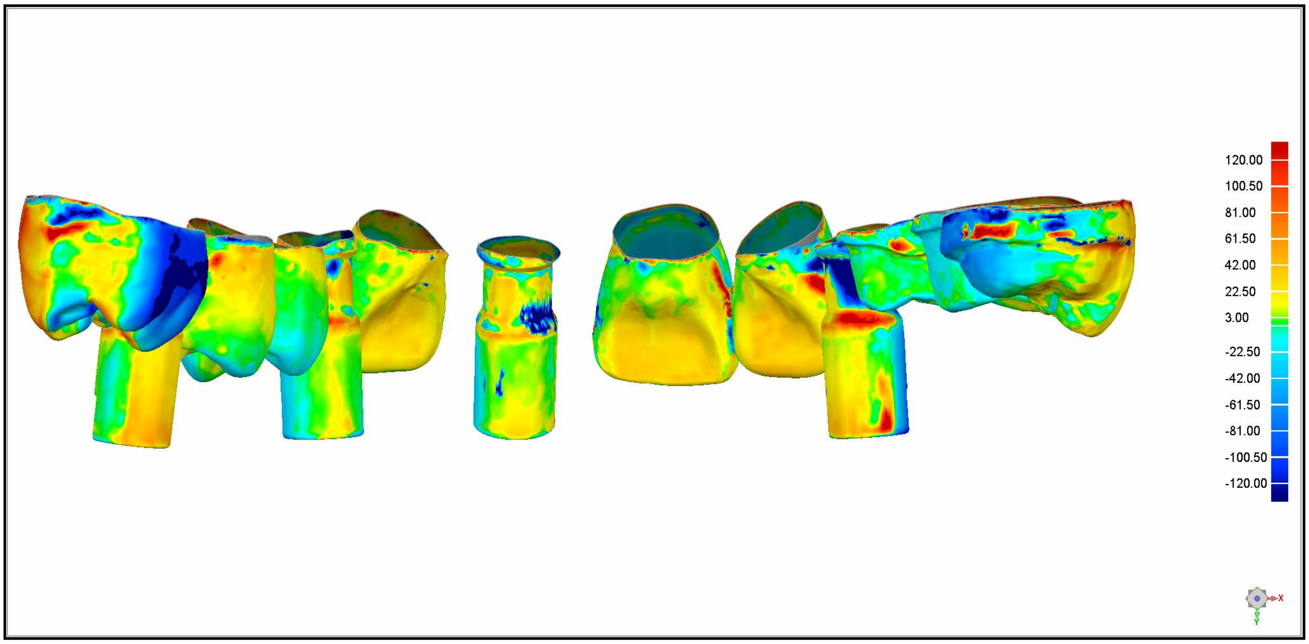

Ajuste de ubicación: Desviaciones superior e inferior

Unidades: u

Nombre	Desv	Estado	Superior Tol	Inferior Tol	Ref X	Ref Y	Ref Z	Radio	Desv X	Desv Y	Desv Z	Medido X	Medido Y	Medido Z	Dir. proy. X	Dir. proy. Y	Dir. proy. Z
Desv. inferior	-3152.31				-16597.07	29132.89	5701.00	n/a	-3030.75	71.53	-864.02	-19627.82	29204.43	4836.97	0.96	-0.02	0.27
Desv. superior	2666.87				14759.35	29872.92	19291.21	n/a	-2316.53	72.46	1319.34	12442.83	29945.38	20610.54	-0.87	0.03	0.49
