# Supplementary material for: Accuracy of four digital scanners according to scanning strategy in complete-arch impressions
Source: PLoS One. 2018 Sep 13;13(9):e0202916. doi: 10.1371/journal.pone.0202916 (PMC6136706; doi:10.1371/journal.pone.0202916)
Supplement: S12 Table — Omnicam (scanning strategy D). (ZIP) [file pone.0202916.s012.zip › S12/OM6D.pdf]

Geomagic Control™

Fecha: 3/6/2017, 3:05 pm

3D Comparación Resultados

Modelo referencia	MRC
Modelo test	OM6D
Nº de puntos de datos	202456
# Aislados	433

Tipo tolerancia	3D desviación
Unidades	u
Máx. crítico	120.00
Máx. nominal	14.00
Mín. nominal	-14.00
Mín. crítico	-120.00

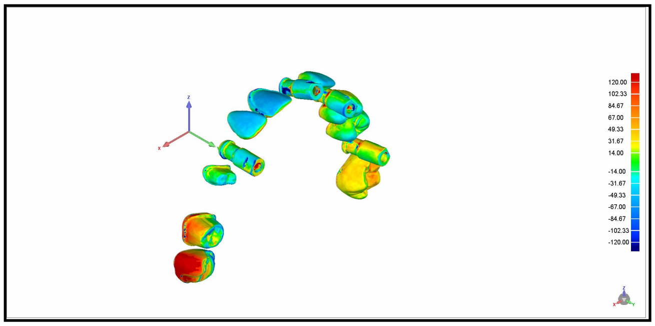

Desviación	
Desviación superior máx.	3123.67
Desviación inferior máx.	-3145.33
Desviación media	98.67 /-88.22
Desviación estándar	272.25

Distribución desviación

>=Min	<Max	# Puntos	%
-120.00	-102.33	1346	0.66
-102.33	-84.67	2516	1.24
-84.67	-67.00	3070	1.52
-67.00	-49.33	7296	3.60
-49.33	-31.67	14298	7.06
-31.67	-14.00	28859	14.25
-14.00	14.00	49486	24.44
14.00	31.67	29291	14.47
31.67	49.33	17221	8.51
49.33	67.00	10628	5.25
67.00	84.67	6162	3.04
84.67	102.33	3720	1.84
102.33	120.00	2786	1.38

Fuera del crítico superior	16511	8.16
Fuera del crítico inferior	9266	4.58

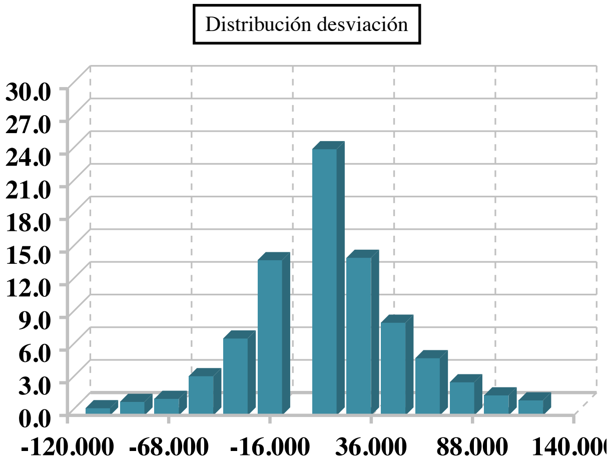

Desviaciones estándar

Distribución (+/-)	# Puntos	%
-6 * Desv. estánd.	1523	0.75
-5 * Desv. estánd.	543	0.27
-4 * Desv. estánd.	420	0.21
-3 * Desv. estánd.	644	0.32
-2 * Desv. estánd.	1402	0.69
-1 * Desv. estánd.	111615	55.13
1 * Desv. estánd.	79494	39.26
2 * Desv. estánd.	2153	1.06
3 * Desv. estánd.	1459	0.72
4 * Desv. estánd.	1132	0.56
5 * Desv. estánd.	932	0.46
6 * Desv. estánd.	1139	0.56

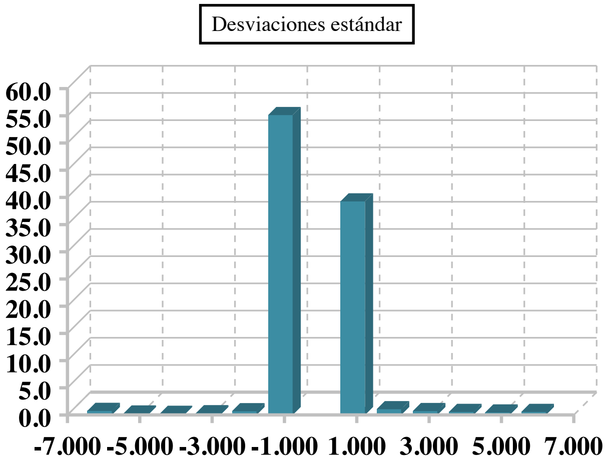

Predefinido: Isométrico

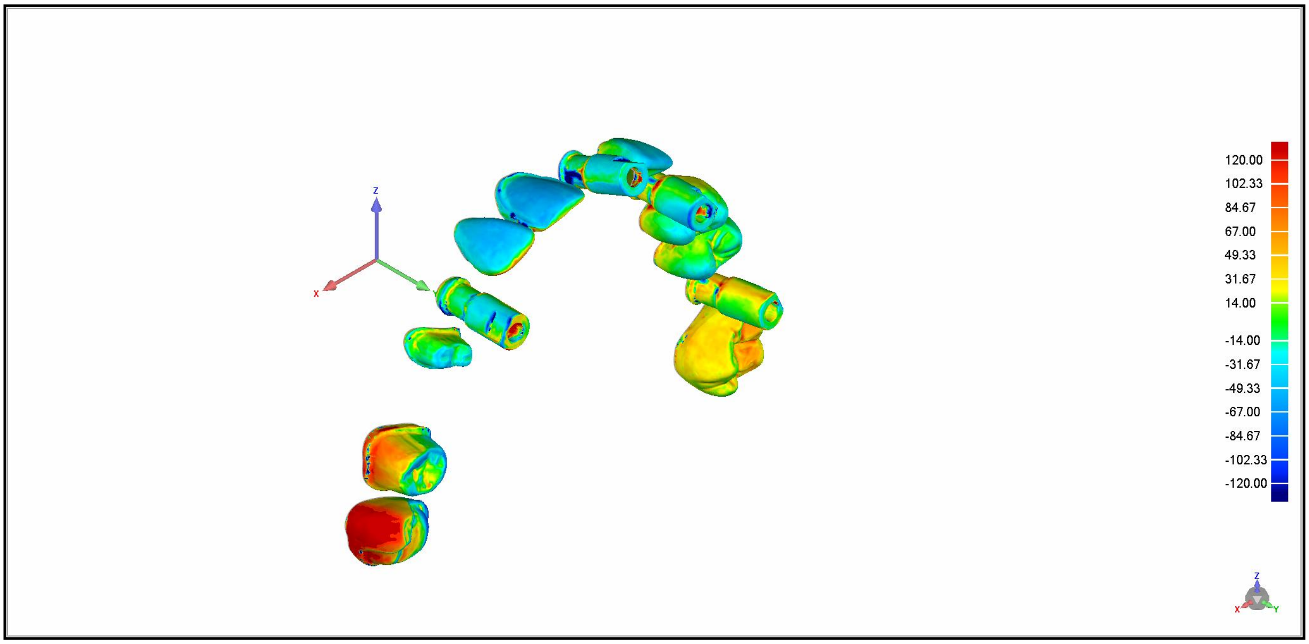

Predefinido: Frente

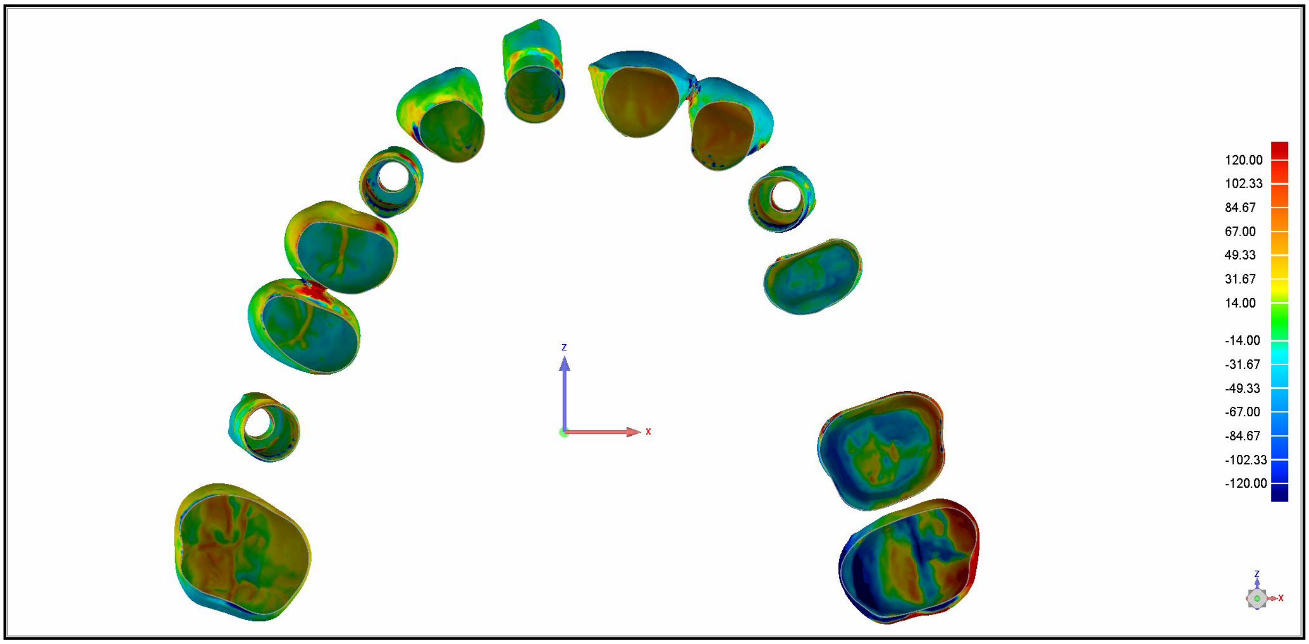

Predefinido: Atrás

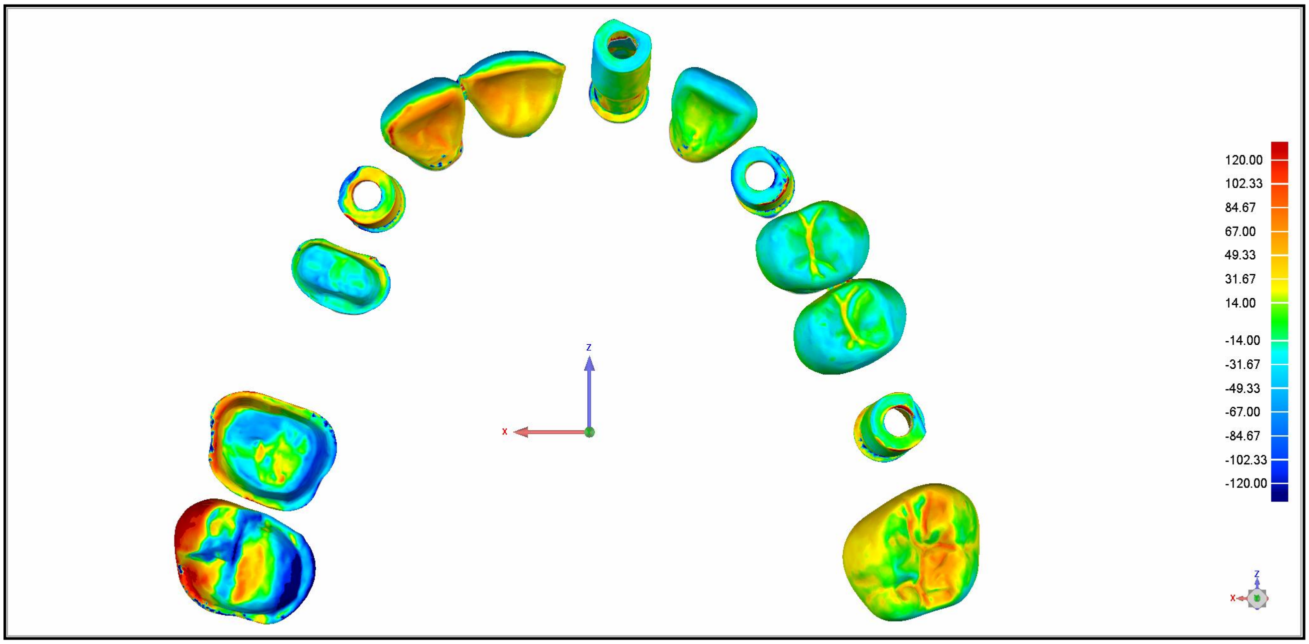

Predefinido: Izquierda

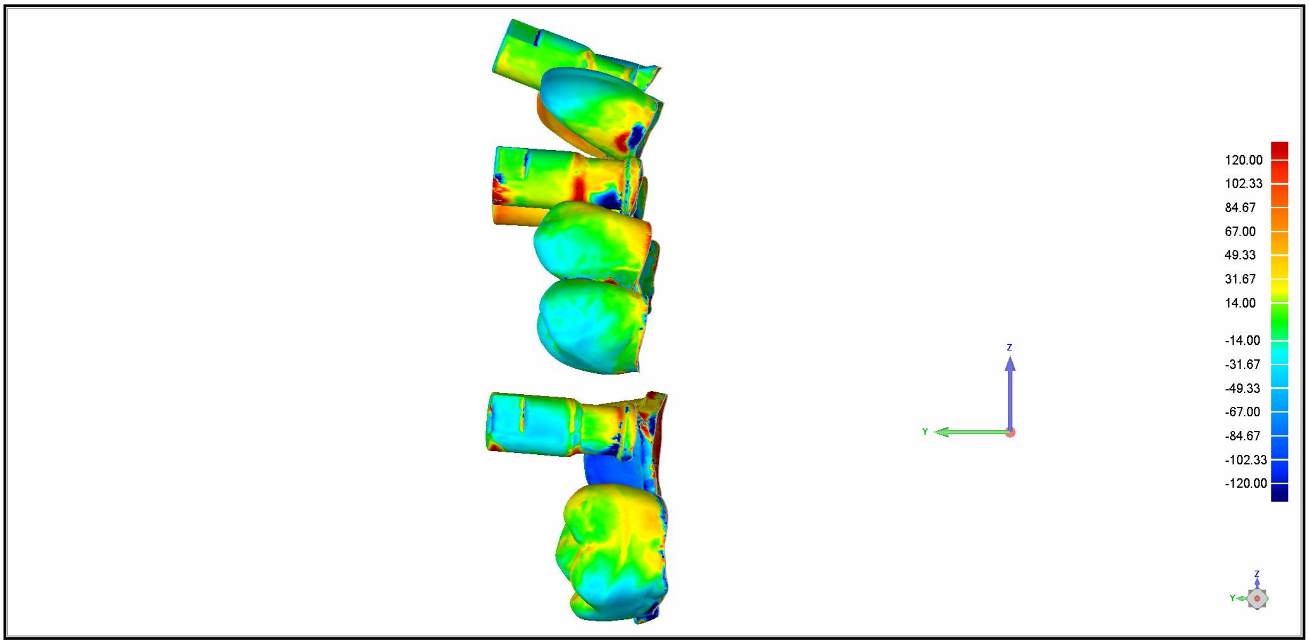

Predefinido: Derecha

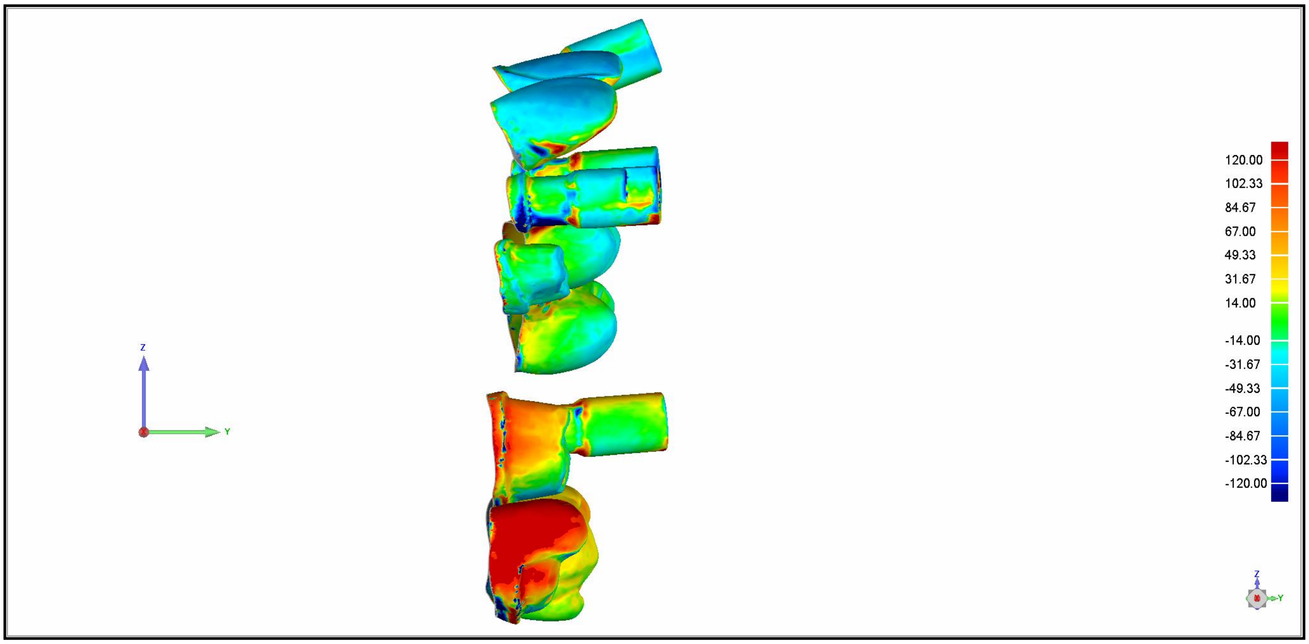

Predefinido: Superior

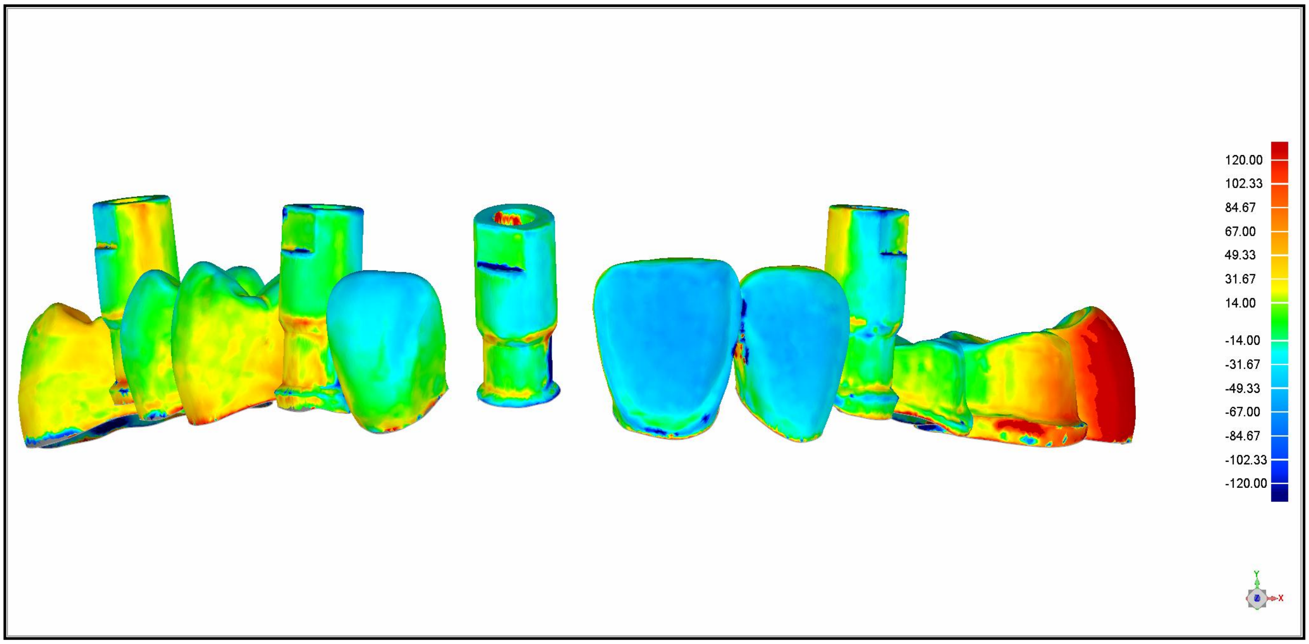

Predefinido: Inferior

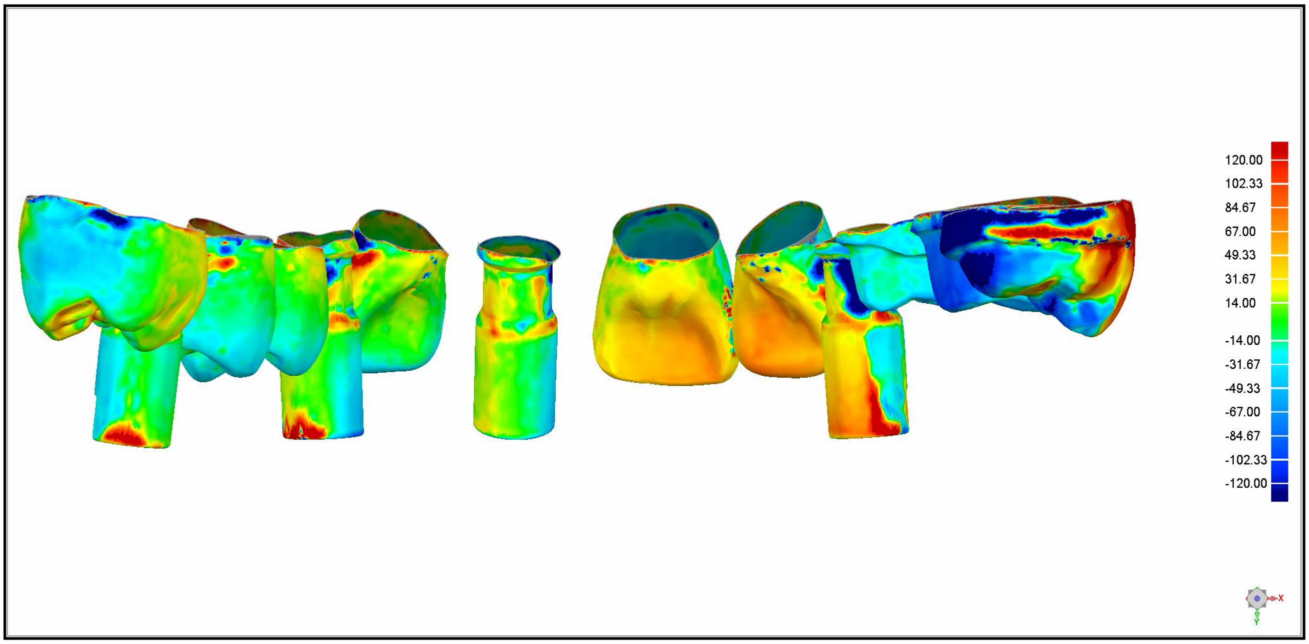

Ajuste de ubicación: Desviaciones superior e inferior

Unidades: u

Nombre	Desv	Estado	Superior Tol	Inferior Tol	Ref X	Ref Y	Ref Z	Radio	Desv X	Desv Y	Desv Z	Medido X	Medido Y	Medido Z	Dir. proy. X	Dir. proy. Y	Dir. proy. Z
Desv. inferior	-3145.33				-13203.90	38507.15	18811.31	n/a	-2448.64	1857.02	669.86	-15652.54	40364.16	19481.17	0.78	-0.59	-0.21
Desv. superior	3123.67				-20553.64	28741.29	-8096.88	n/a	-2342.71	12.66	2066.12	-22896.35	28753.95	-6030.76	-0.75	0.00	0.66
