# Supplementary material for: Accuracy of four digital scanners according to scanning strategy in complete-arch impressions
Source: PLoS One. 2018 Sep 13;13(9):e0202916. doi: 10.1371/journal.pone.0202916 (PMC6136706; doi:10.1371/journal.pone.0202916)
Supplement: S12 Table — Omnicam (scanning strategy D). (ZIP) [file pone.0202916.s012.zip › S12/OM1D.pdf]

Fecha: 3/6/2017, 2:50 pm

3D Comparación Resultados

Modelo referencia	MRC
Modelo test	OM1D
Nº de puntos de datos	200749
# Aislados	705

Tipo tolerancia	3D desviación
Unidades	u
Máx. crítico	120.00
Máx. nominal	4.00
Mín. nominal	-4.00
Mín. crítico	-120.00

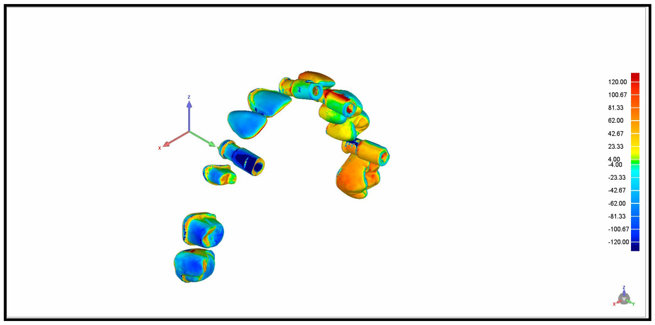

Desviación	
Desviación superior máx.	3143.30
Desviación inferior máx.	-3153.77
Desviación media	110.43 /-128.60
Desviación estándar	327.42

Distribución desviación

>=Min	<Max	# Puntos	%
-120.00	-100.67	1839	0.92
-100.67	-81.33	3670	1.83
-81.33	-62.00	8881	4.42
-62.00	-42.67	12347	6.15
-42.67	-23.33	17818	8.88
-23.33	-4.00	26500	13.20
-4.00	4.00	12730	6.34
4.00	23.33	26661	13.28
23.33	42.67	22694	11.30
42.67	62.00	17537	8.74
62.00	81.33	12235	6.09
81.33	100.67	6374	3.18
100.67	120.00	3839	1.91

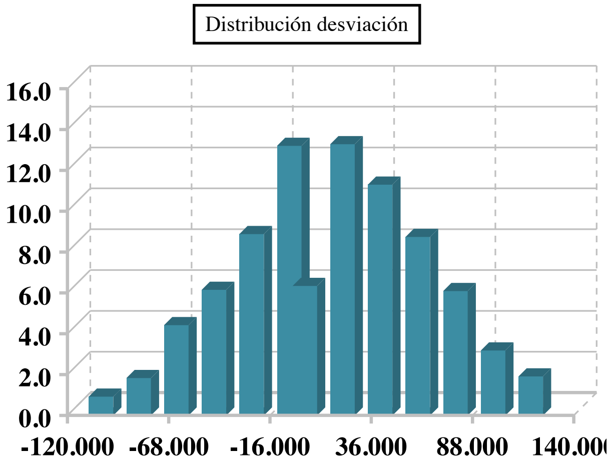

Fuera del crítico superior	16671	8.30
Fuera del crítico inferior	10953	5.46

Desviaciones estándar

Distribución (+/-)	# Puntos	%
-6 * Desv. estánd.	1694	0.84
-5 * Desv. estánd.	962	0.48
-4 * Desv. estánd.	835	0.42
-3 * Desv. estánd.	1090	0.54
-2 * Desv. estánd.	2374	1.18
-1 * Desv. estánd.	89096	44.38
1 * Desv. estánd.	97890	48.76
2 * Desv. estánd.	2131	1.06
3 * Desv. estánd.	1545	0.77
4 * Desv. estánd.	1530	0.76
5 * Desv. estánd.	875	0.44
6 * Desv. estánd.	727	0.36

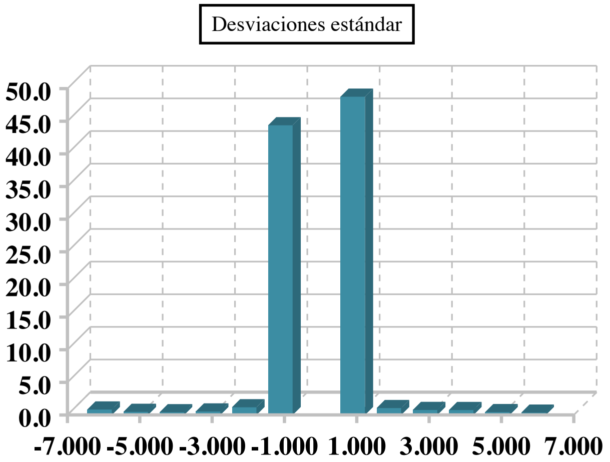

Predefinido: Isométrico

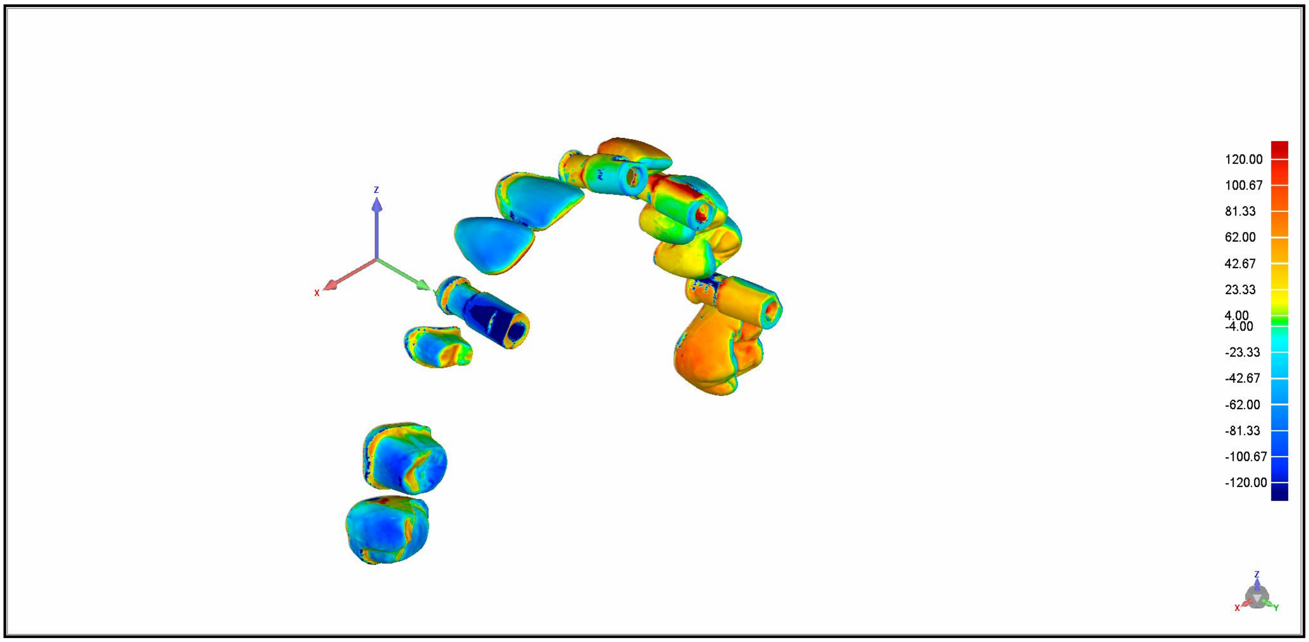

Predefinido: Frente

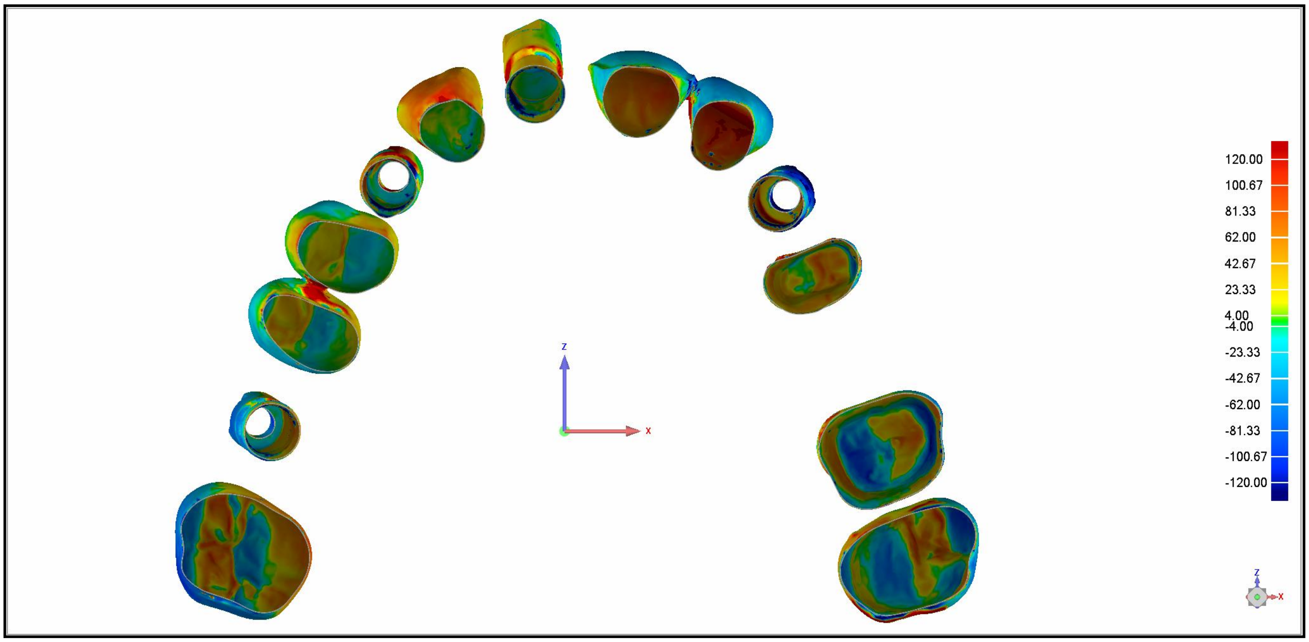

Predefinido: Atrás

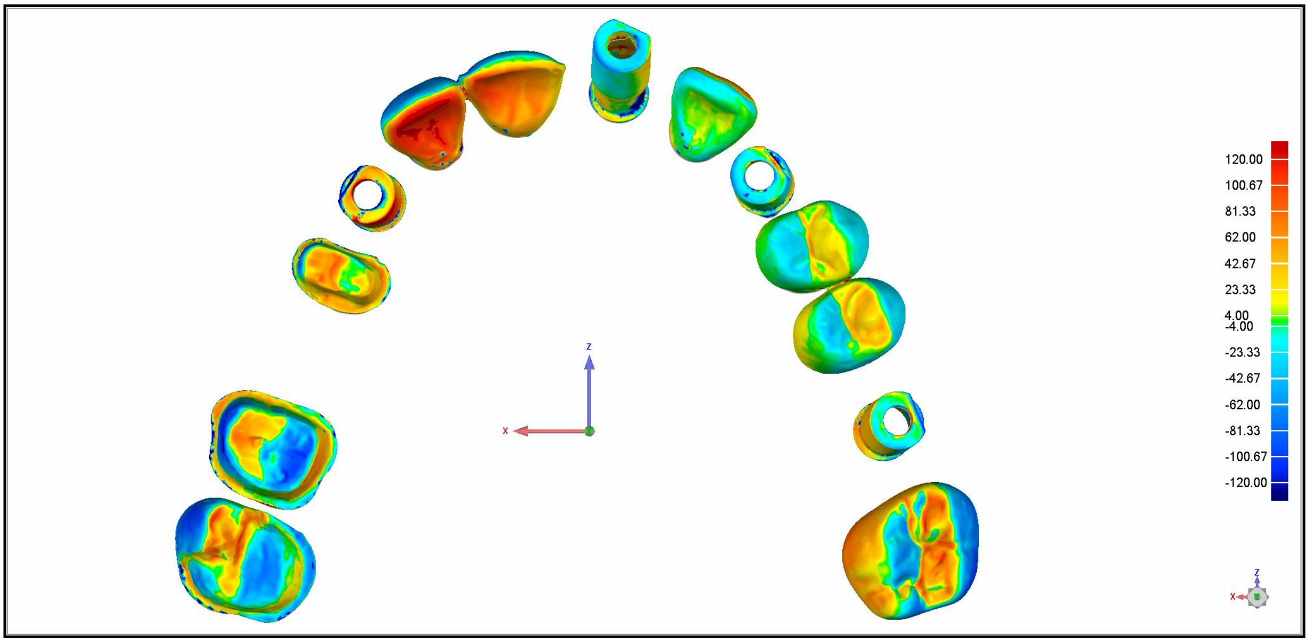

Predefinido: Izquierda

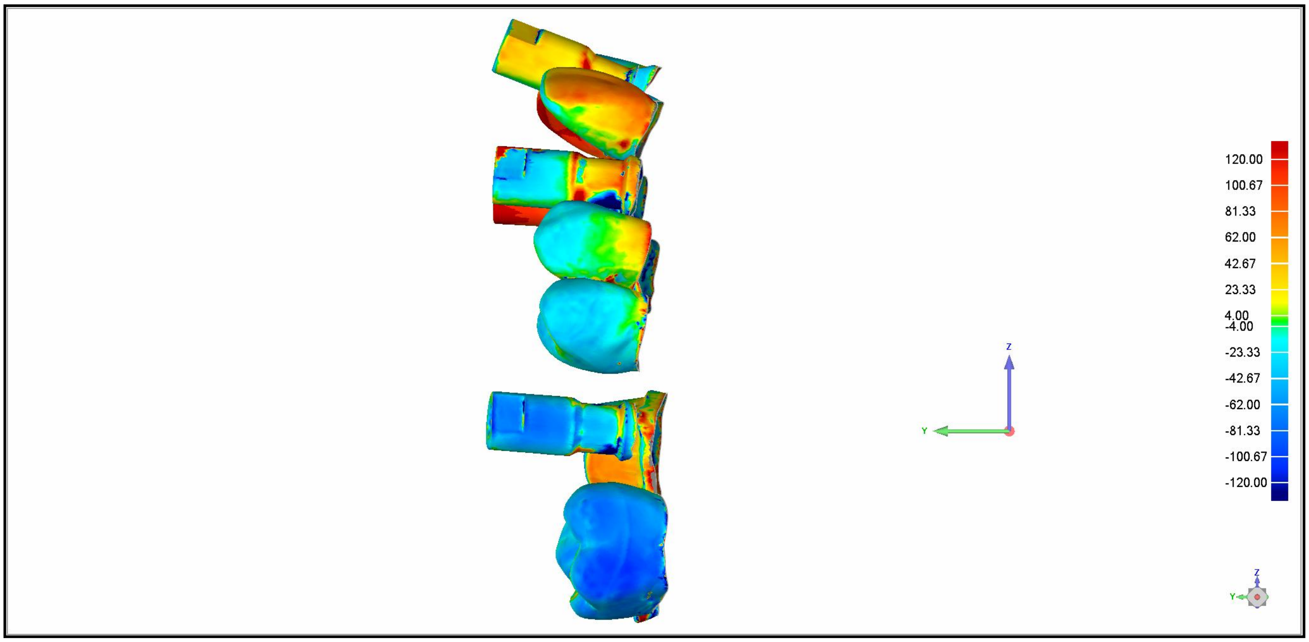

Predefinido: Derecha

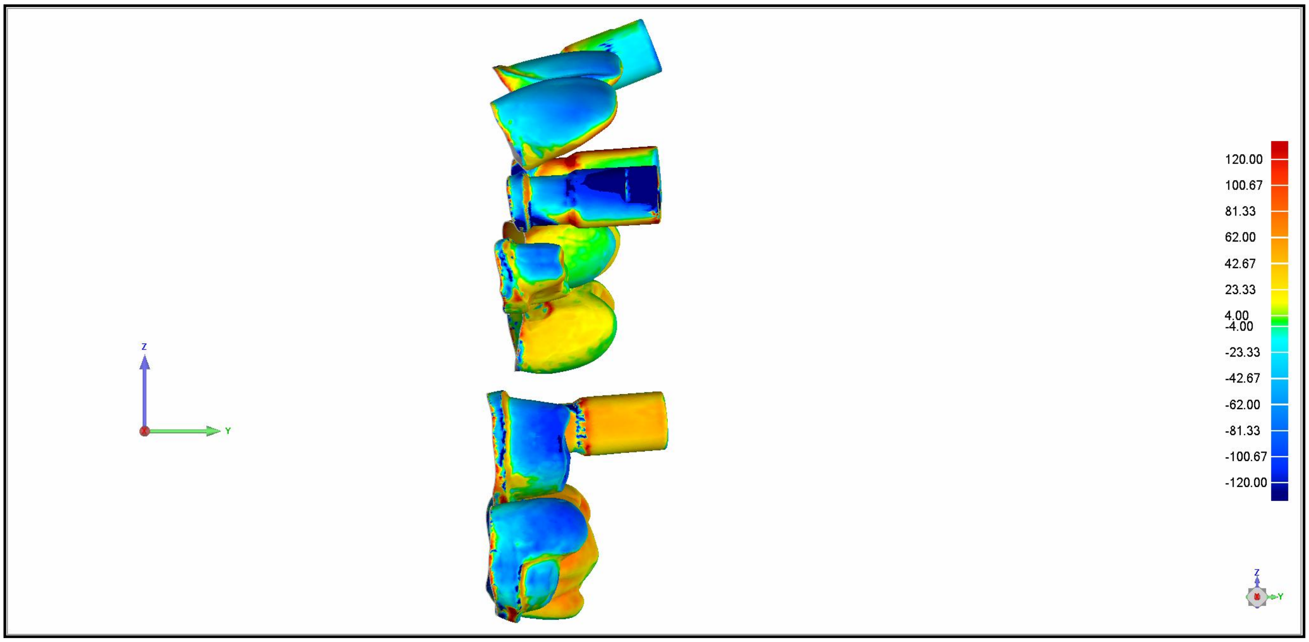

Predefinido: Superior

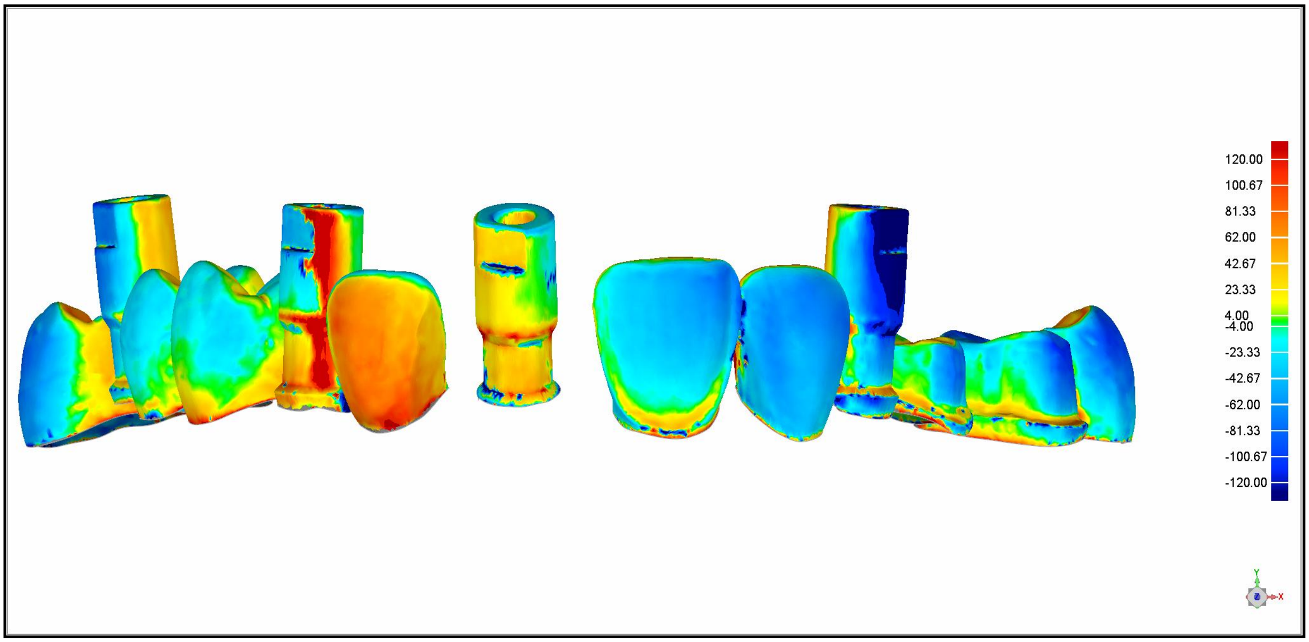

Predefinido: Inferior

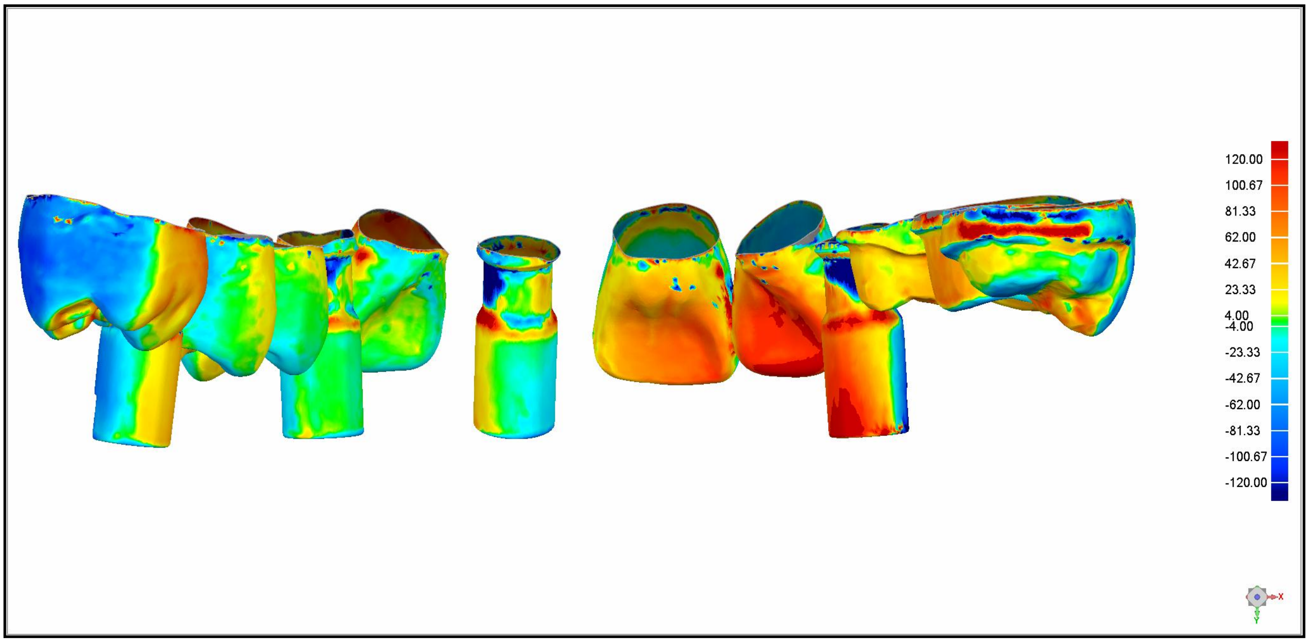

Ajuste de ubicación: Desviaciones superior e inferior

Unidades: u

Nombre	Desv	Estado	Superior Tol	Inferior Tol	Ref X	Ref Y	Ref Z	Radio	Desv X	Desv Y	Desv Z	Medido X	Medido Y	Medido Z	Dir. proy. X	Dir. proy. Y	Dir. proy. Z
Desv. inferior	-3153.77				16989.02	37628.06	17251.36	n/a	1694.43	2484.33	-950.40	18683.45	40112.39	16300.96	-0.54	-0.79	0.30
Desv. superior	3143.30				-20553.64	28741.29	-8096.88	n/a	-2363.03	15.72	2072.72	-22916.67	28757.01	-6024.16	-0.75	0.01	0.66
