# Supplementary material for: Accuracy of four digital scanners according to scanning strategy in complete-arch impressions
Source: PLoS One. 2018 Sep 13;13(9):e0202916. doi: 10.1371/journal.pone.0202916 (PMC6136706; doi:10.1371/journal.pone.0202916)
Supplement: S12 Table — Omnicam (scanning strategy D). (ZIP) [file pone.0202916.s012.zip › S12/OM10D.pdf]

3D Comparación Resultados

Modelo referencia	MRC
Modelo test	OM10D
Nº de puntos de datos	202901
# Aislados	918

Tipo tolerancia	3D desviación
Unidades	u
Máx. crítico	120.00
Máx. nominal	7.00
Mín. nominal	-7.00
Mín. crítico	-120.00

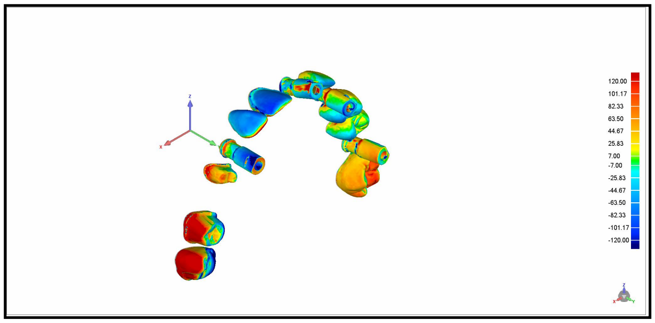

Desviación	
Desviación superior máx.	3117.92
Desviación inferior máx.	-3130.03
Desviación media	116.83 /-117.91
Desviación estándar	272.42

Distribución desviación

>=Min	<Max	# Puntos	%
-120.00	-101.17	3154	1.55
-101.17	-82.33	5416	2.67
-82.33	-63.50	8311	4.10
-63.50	-44.67	11854	5.84
-44.67	-25.83	19558	9.64
-25.83	-7.00	20150	9.93
-7.00	7.00	15123	7.45
7.00	25.83	19839	9.78
25.83	44.67	17708	8.73
44.67	63.50	14631	7.21
63.50	82.33	9601	4.73
82.33	101.17	7570	3.73
101.17	120.00	5928	2.92

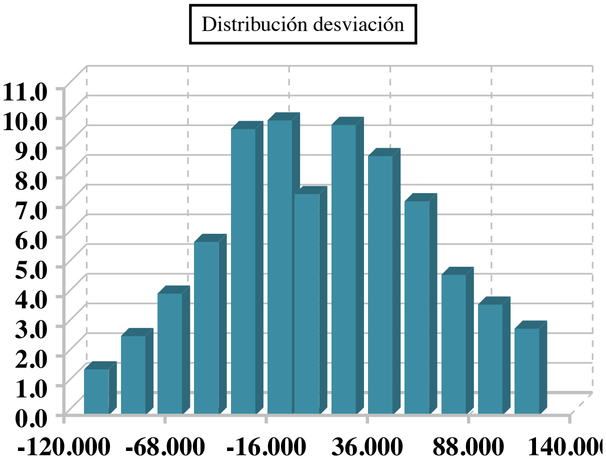

Fuera del crítico superior		25539	12.59
Fuera del crítico inferior		18519	9.13

Desviaciones estándar

Distribución (+/-)	# Puntos	%
-6 * Desv. estánd.	1426	0.70
-5 * Desv. estánd.	547	0.27
-4 * Desv. estánd.	716	0.35
-3 * Desv. estánd.	1427	0.70
-2 * Desv. estánd.	3310	1.63
-1 * Desv. estánd.	95476	47.06
1 * Desv. estánd.	93086	45.88
2 * Desv. estánd.	2676	1.32
3 * Desv. estánd.	1383	0.68
4 * Desv. estánd.	1005	0.50
5 * Desv. estánd.	788	0.39
6 * Desv. estánd.	1061	0.52

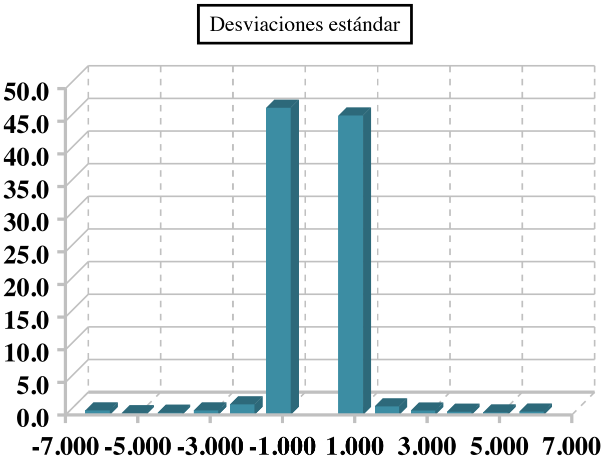

Fecha: 3/6/2017, 3:15 pm

Predefinido: Isométrico

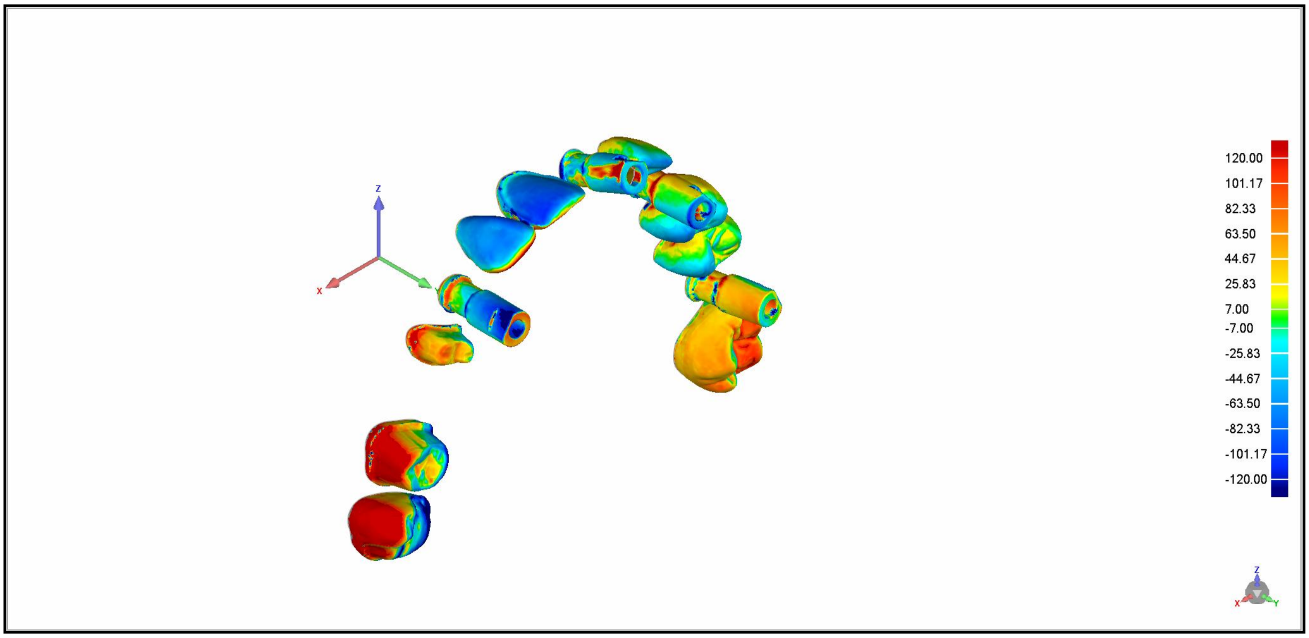

Predefinido: Frente

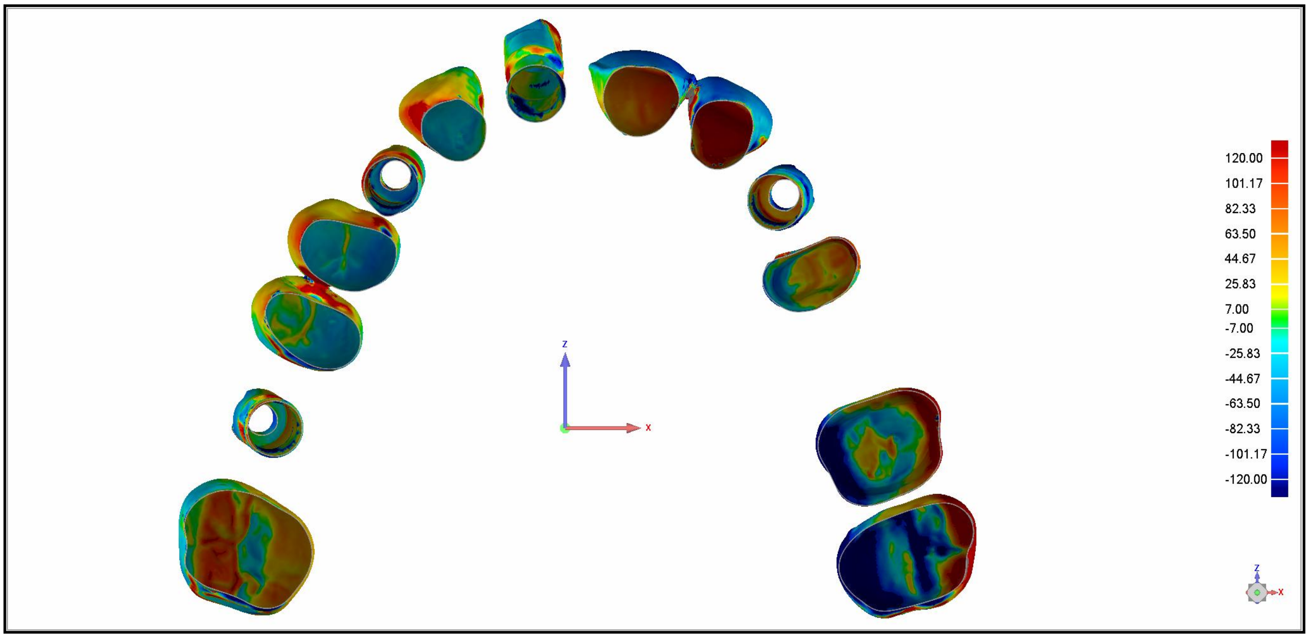

Predefinido: Atrás

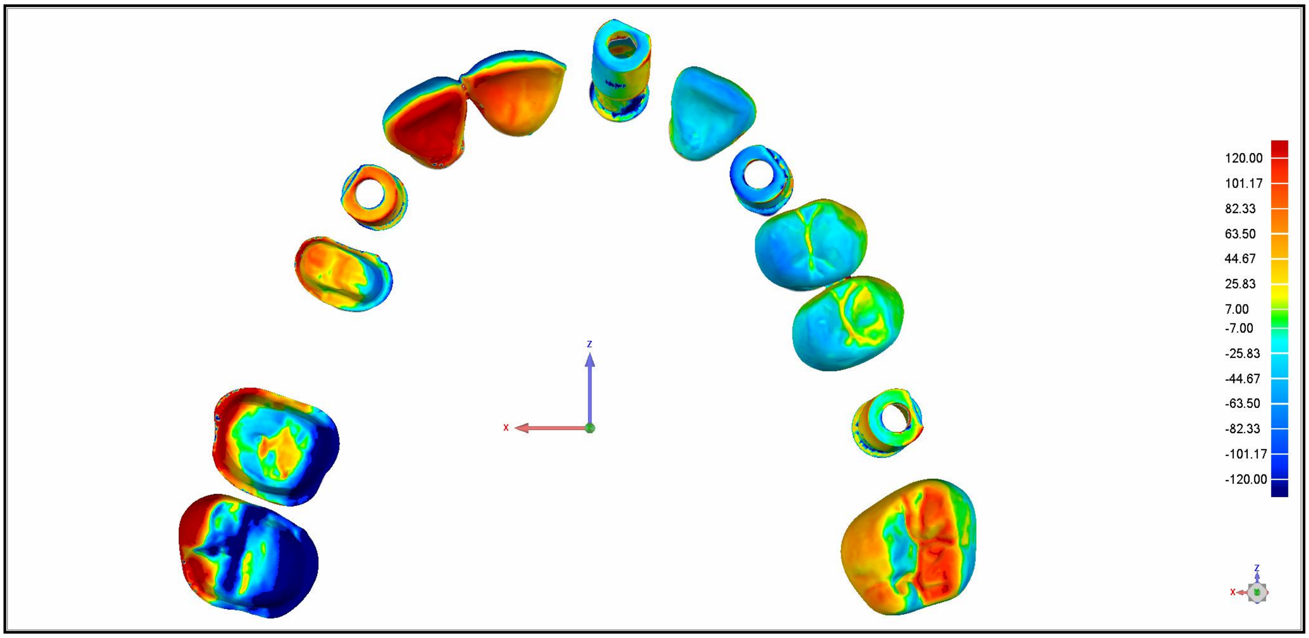

Predefinido: Izquierda

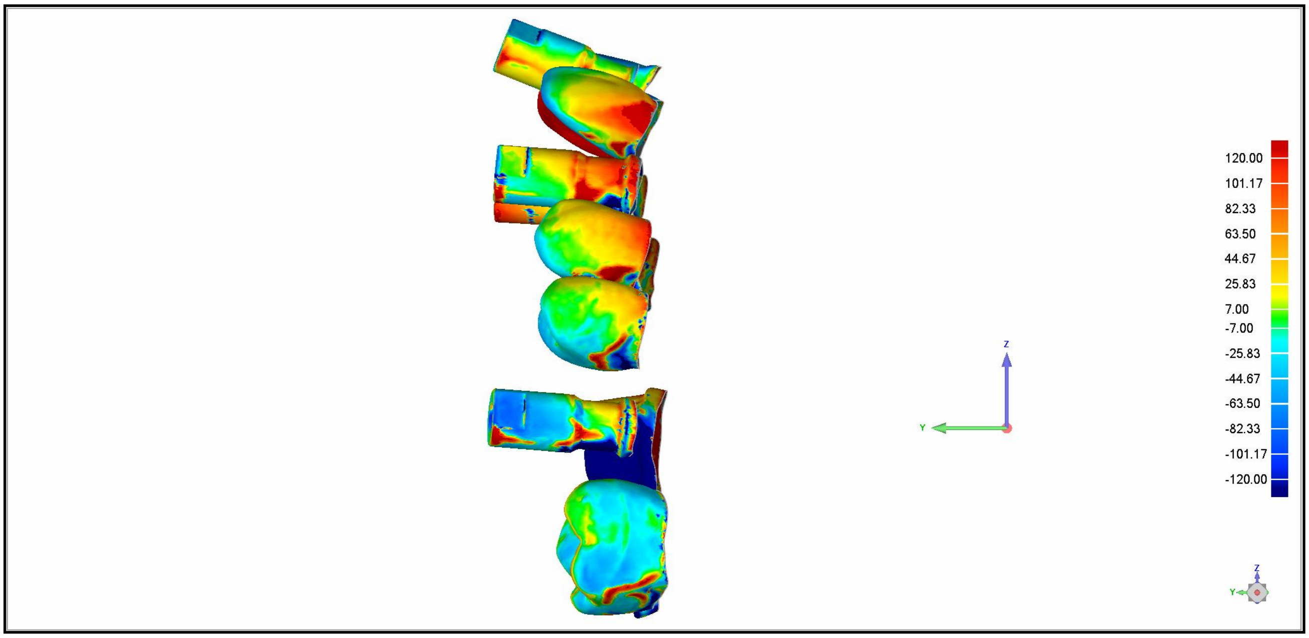

Predefinido: Derecha

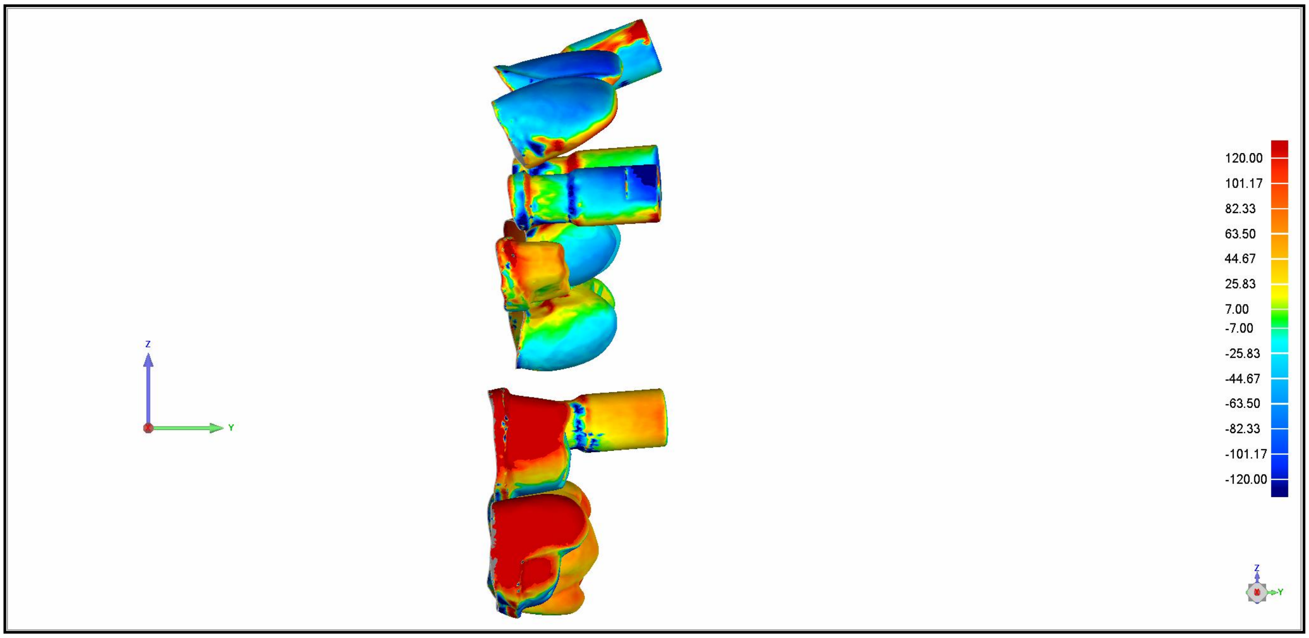

Predefinido: Superior

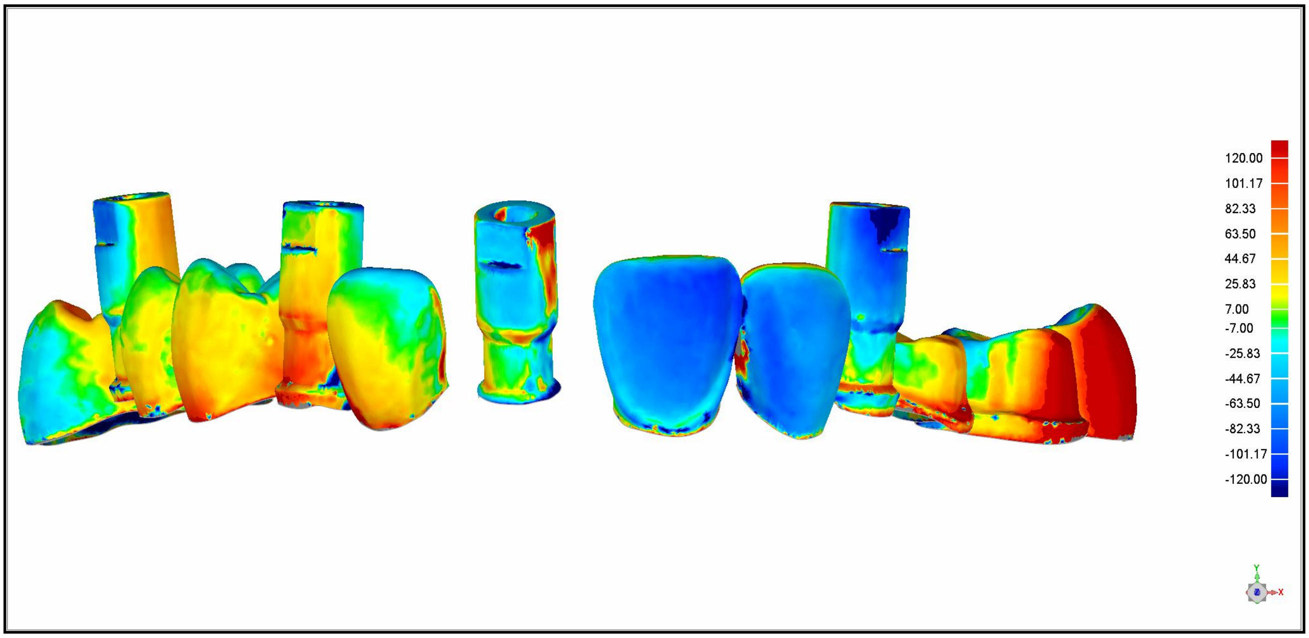

Predefinido: Inferior

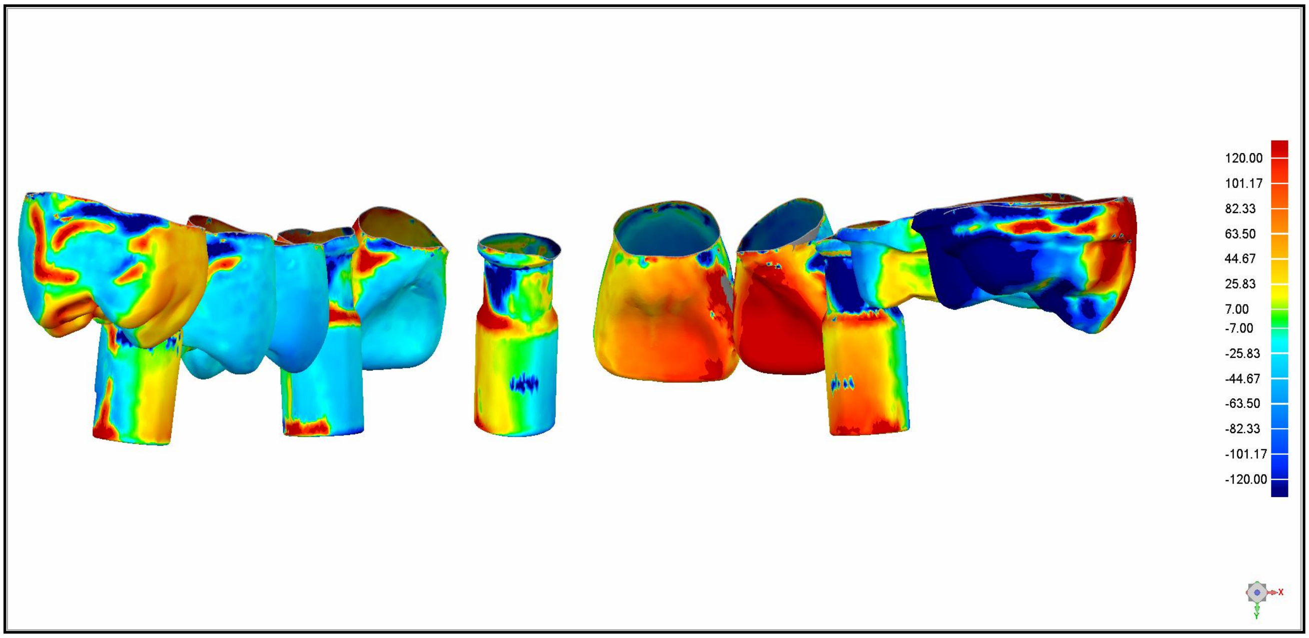

Ajuste de ubicación: Desviaciones superior e inferior

Unidades: u

Nombre	Desv	Estado	Superior Tol	Inferior Tol	Ref X	Ref Y	Ref Z	Radio	Desv X	Desv Y	Desv Z	Medido X	Medido Y	Medido Z	Dir. proy. X	Dir. proy. Y	Dir. proy. Z
Desv. inferior	-3130.03				-21889.71	33882.36	1456.70	n/a	-1135.55	544.59	-2865.49	-23025.27	34426.95	-1408.79	0.36	-0.17	0.92
Desv. superior	3117.92				-12685.55	29759.31	21419.26	n/a	1065.68	-1368.84	2590.75	-11619.87	28390.47	24010.02	0.34	-0.44	0.83
