# Supplementary material for: Accuracy of four digital scanners according to scanning strategy in complete-arch impressions
Source: PLoS One. 2018 Sep 13;13(9):e0202916. doi: 10.1371/journal.pone.0202916 (PMC6136706; doi:10.1371/journal.pone.0202916)
Supplement: S13 Table — True definition (scanning strategy A). (ZIP) [file pone.0202916.s013.zip › S13/TD3A.pdf]

3D Comparación Resultados

Modelo referencia	MRC
Modelo test	TD3A
Nº de puntos de datos	129233
# Aislados	418

Tipo tolerancia	3D desviación
Unidades	u
Máx. crítico	120.00
Máx. nominal	7.00
Mín. nominal	-7.00
Mín. crítico	-120.00

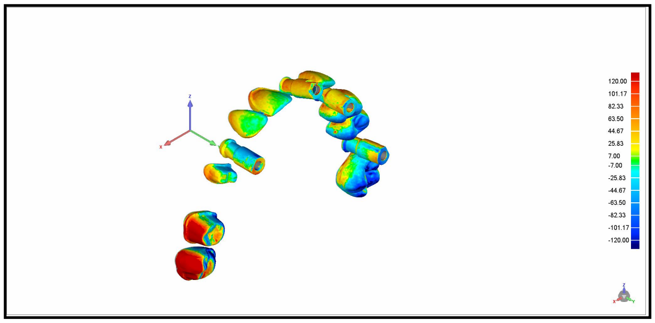

Desviación	
Desviación superior máx.	2924.25
Desviación inferior máx.	-2699.40
Desviación media	63.14 /-53.46
Desviación estándar	86.91

Distribución desviación

>=Min	<Max	# Puntos	%
-120.00	-101.17	2513	1.94
-101.17	-82.33	4646	3.60
-82.33	-63.50	7473	5.78
-63.50	-44.67	10179	7.88
-44.67	-25.83	12425	9.61
-25.83	-7.00	14198	10.99
-7.00	7.00	11324	8.76
7.00	25.83	15067	11.66
25.83	44.67	12909	9.99
44.67	63.50	10594	8.20
63.50	82.33	7005	5.42
82.33	101.17	4492	3.48
101.17	120.00	3116	2.41

Fuera del crítico superior	8486	6.57
Fuera del crítico inferior	4806	3.72

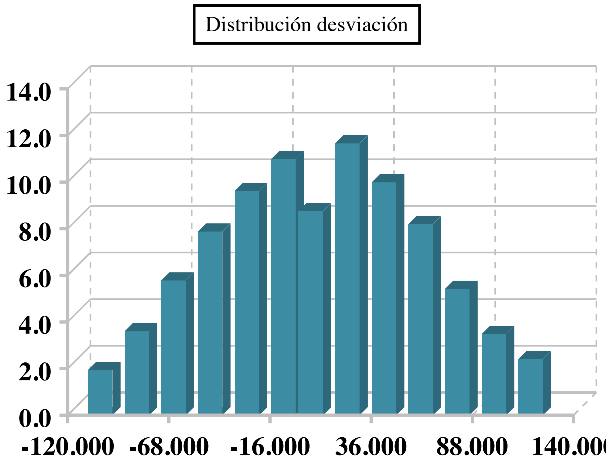

Desviaciones estándar

Distribución (+/-)	# Puntos	%
-6 * Desv. estánd.	38	0.03
-5 * Desv. estánd.	79	0.06
-4 * Desv. estánd.	146	0.11
-3 * Desv. estánd.	1767	1.37
-2 * Desv. estánd.	10798	8.36
-1 * Desv. estánd.	54961	42.53
1 * Desv. estánd.	48381	37.44
2 * Desv. estánd.	9510	7.36
3 * Desv. estánd.	2272	1.76
4 * Desv. estánd.	984	0.76
5 * Desv. estánd.	93	0.07
6 * Desv. estánd.	204	0.16

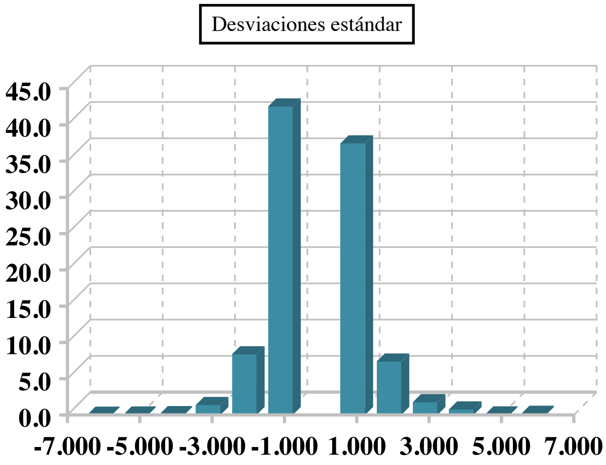

Predefinido: Isométrico

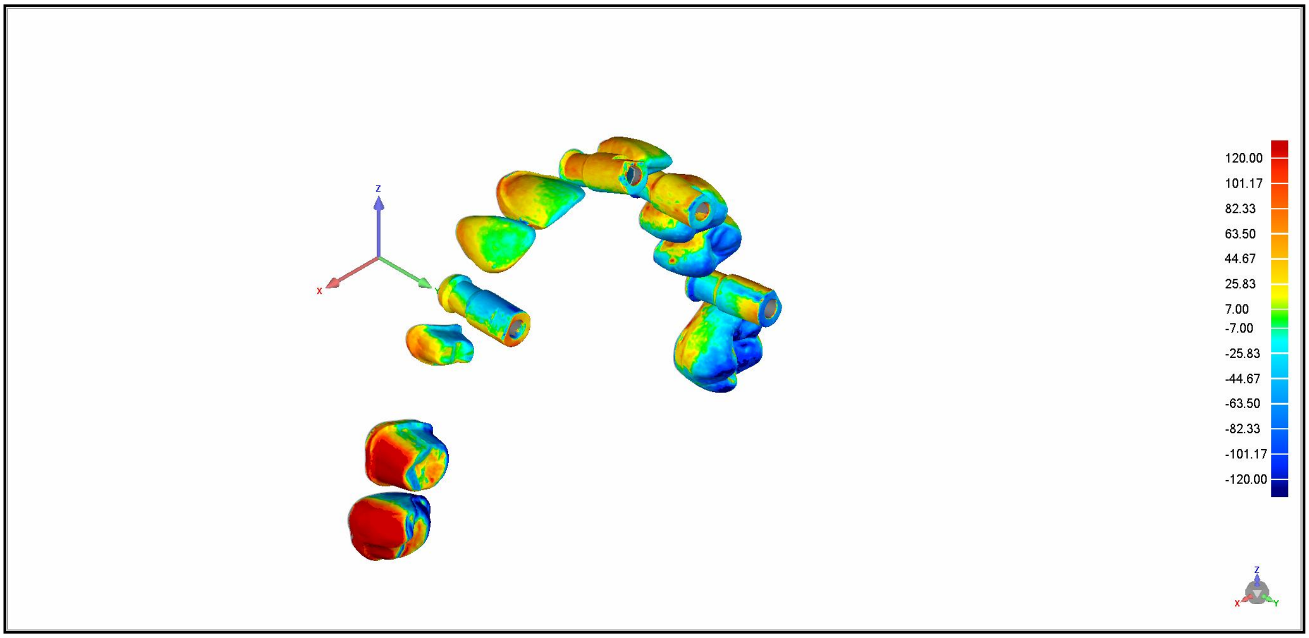

Predefinido: Frente

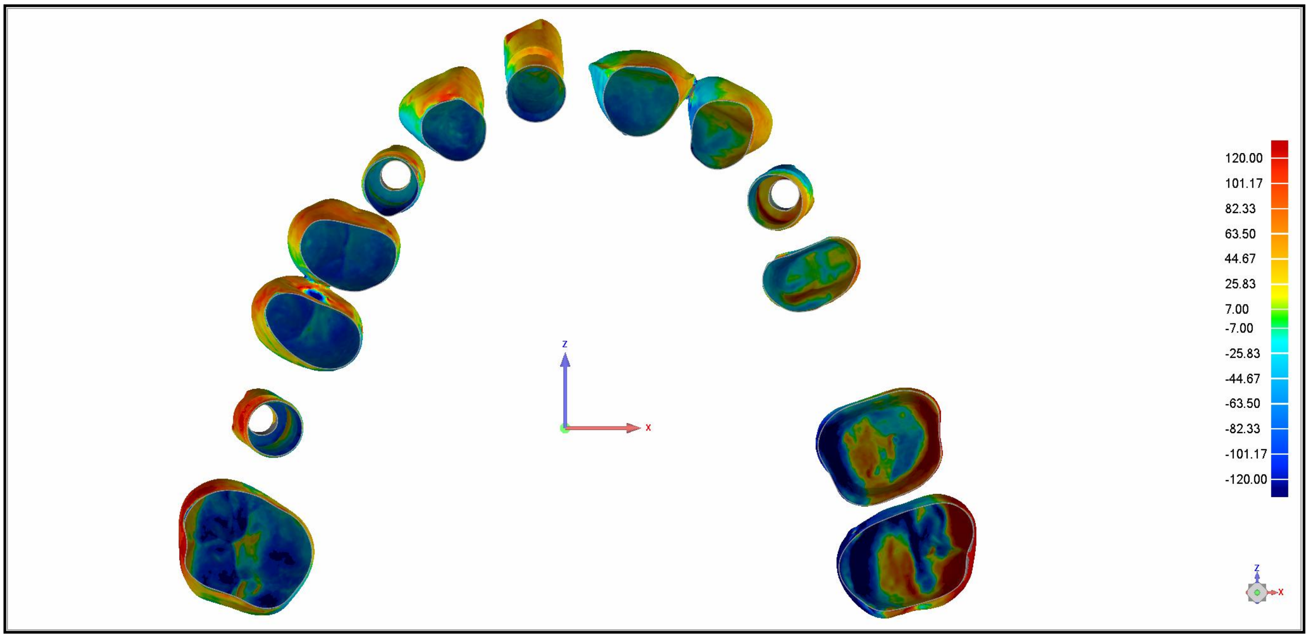

Predefinido: Atrás

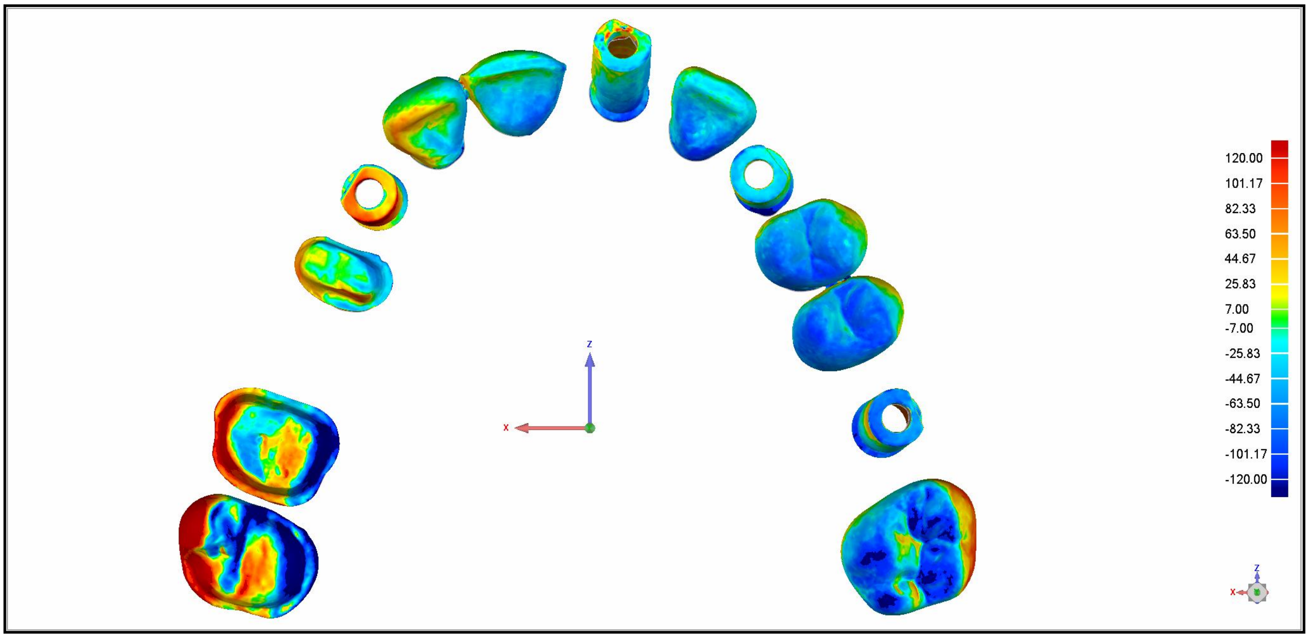

Predefinido: Izquierda

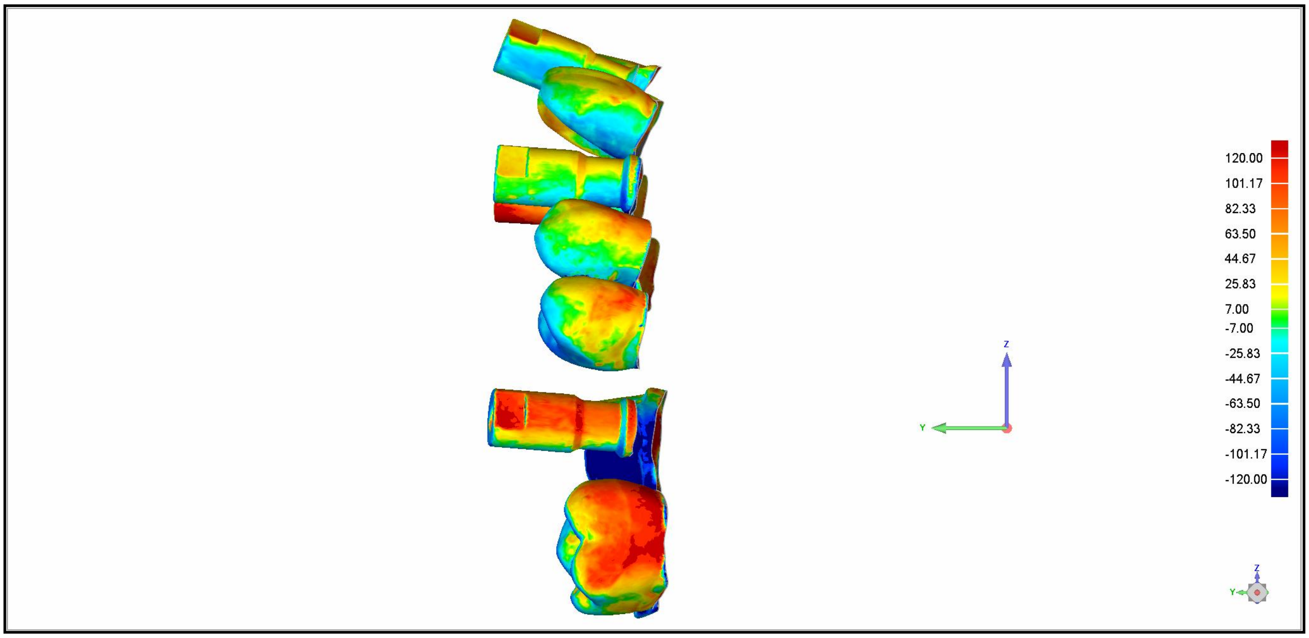

Predefinido: Derecha

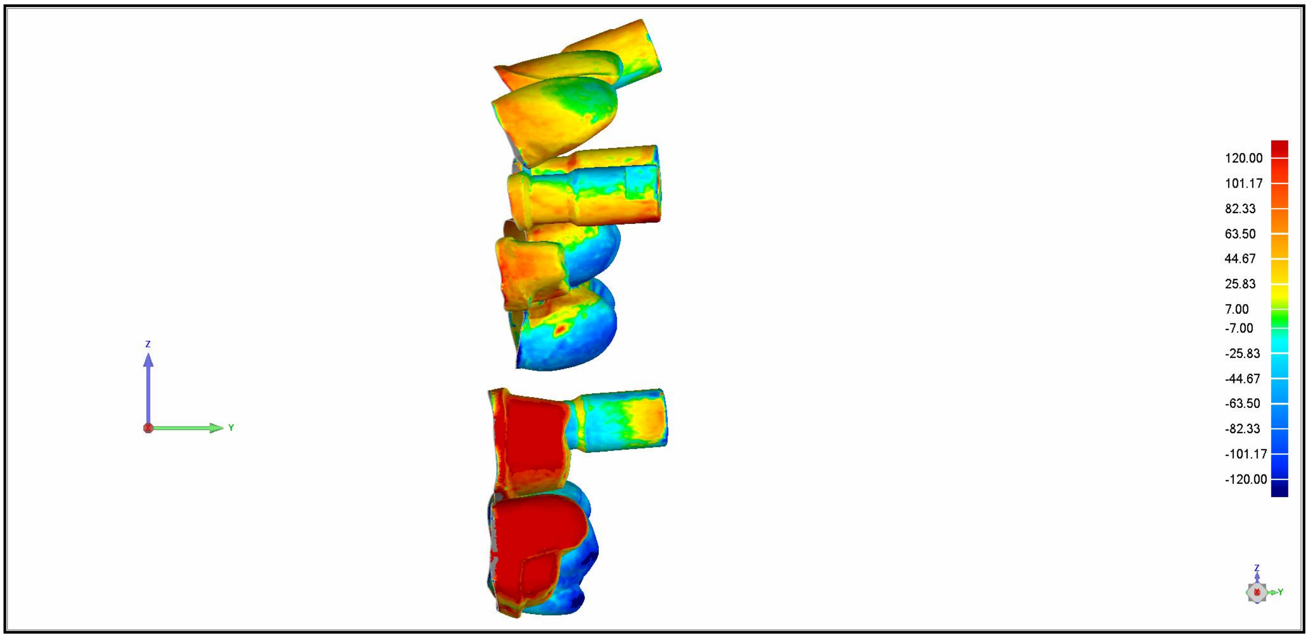

Predefinido: Superior

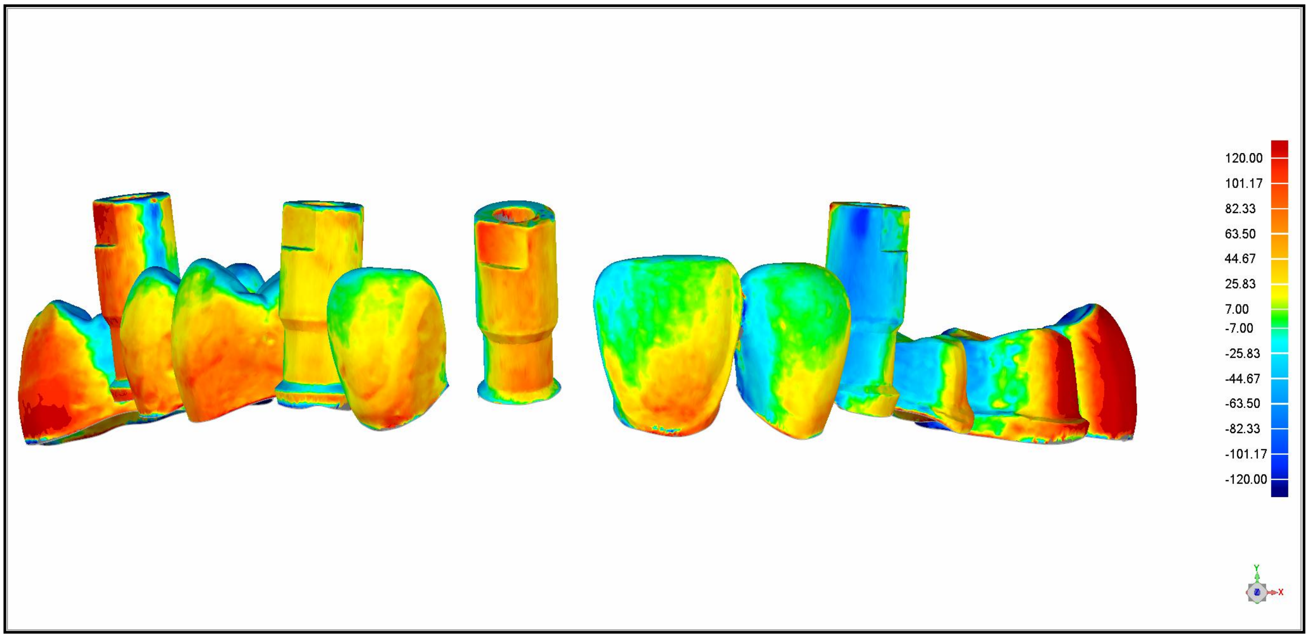

Predefinido: Inferior

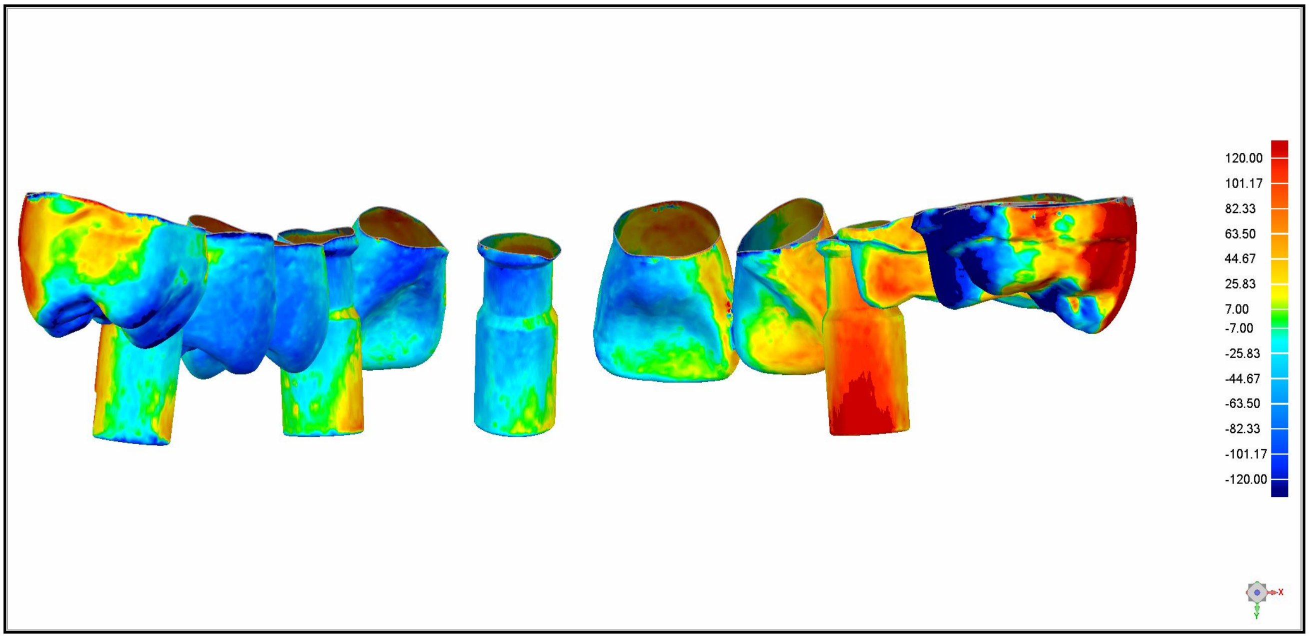

Ajuste de ubicación: Desviaciones superior e inferior

Unidades: u

Nombre	Desv	Estado	Superior Tol	Inferior Tol	Ref X	Ref Y	Ref Z	Radio	Desv X	Desv Y	Desv Z	Medido X	Medido Y	Medido Z	Dir. proy. X	Dir. proy. Y	Dir. proy. Z
Desv. inferior	-2699.40				32064.02	27122.94	-7196.92	n/a	-2364.64	164.79	1291.53	29699.38	27287.72	-5905.39	0.88	-0.06	-0.48
Desv. superior	2924.25				25410.11	27462.45	-5764.99	n/a	2840.74	-20.67	-693.56	28250.85	27441.78	-6458.55	0.97	-0.01	-0.24
