# Supplementary material for: Accuracy of four digital scanners according to scanning strategy in complete-arch impressions
Source: PLoS One. 2018 Sep 13;13(9):e0202916. doi: 10.1371/journal.pone.0202916 (PMC6136706; doi:10.1371/journal.pone.0202916)
Supplement: S13 Table — True definition (scanning strategy A). (ZIP) [file pone.0202916.s013.zip › S13/TD2A.pdf]

3D Comparación Resultados

Modelo referencia	MRC
Modelo test	TD2A
Nº de puntos de datos	129107
# Aislados	318

Tipo tolerancia	3D desviación
Unidades	u
Máx. crítico	120.00
Máx. nominal	32.00
Mín. nominal	-32.00
Mín. crítico	-120.00

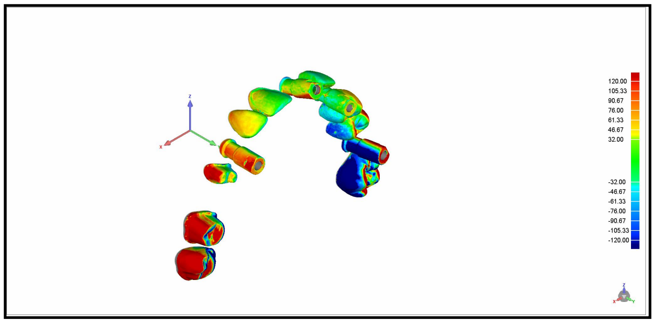

Desviación	
Desviación superior máx.	2880.78
Desviación inferior máx.	-3111.94
Desviación media	115.96 /-95.90
Desviación estándar	172.84

Distribución desviación

>=Min	<Max	# Puntos	%
-120.00	-105.33	1419	1.10
-105.33	-90.67	1869	1.45
-90.67	-76.00	2266	1.76
-76.00	-61.33	2746	2.13
-61.33	-46.67	4011	3.11
-46.67	-32.00	5479	4.24
-32.00	32.00	42576	32.98
32.00	46.67	9176	7.11
46.67	61.33	7763	6.01
61.33	76.00	6162	4.77
76.00	90.67	4222	3.27
90.67	105.33	3151	2.44
105.33	120.00	2690	2.08

Fuera del crítico superior		22530	17.45
Fuera del crítico inferior		13047	10.11

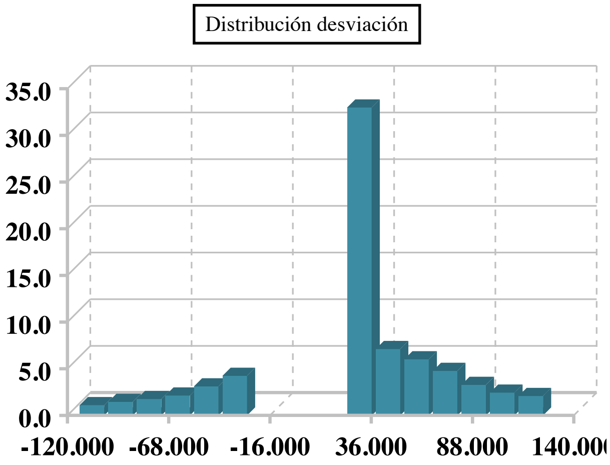

Desviaciones estándar

Distribución (+/-)	# Puntos	%
-6 * Desv. estánd.	96	0.07
-5 * Desv. estánd.	71	0.05
-4 * Desv. estánd.	493	0.38
-3 * Desv. estánd.	2999	2.32
-2 * Desv. estánd.	7909	6.13
-1 * Desv. estánd.	61923	47.96
1 * Desv. estánd.	41475	32.12
2 * Desv. estánd.	8112	6.28
3 * Desv. estánd.	5637	4.37
4 * Desv. estánd.	142	0.11
5 * Desv. estánd.	66	0.05
6 * Desv. estánd.	184	0.14

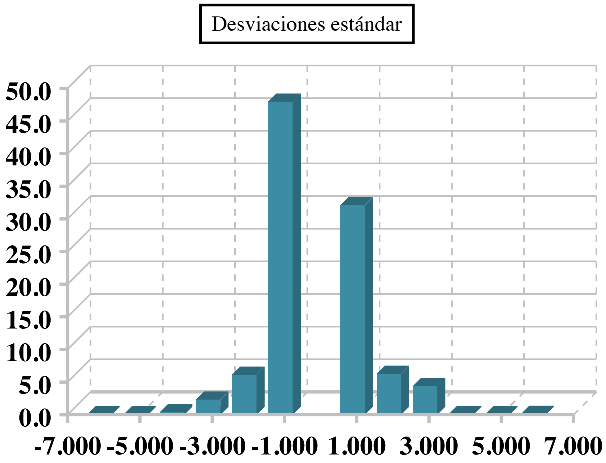

Predefinido: Isométrico

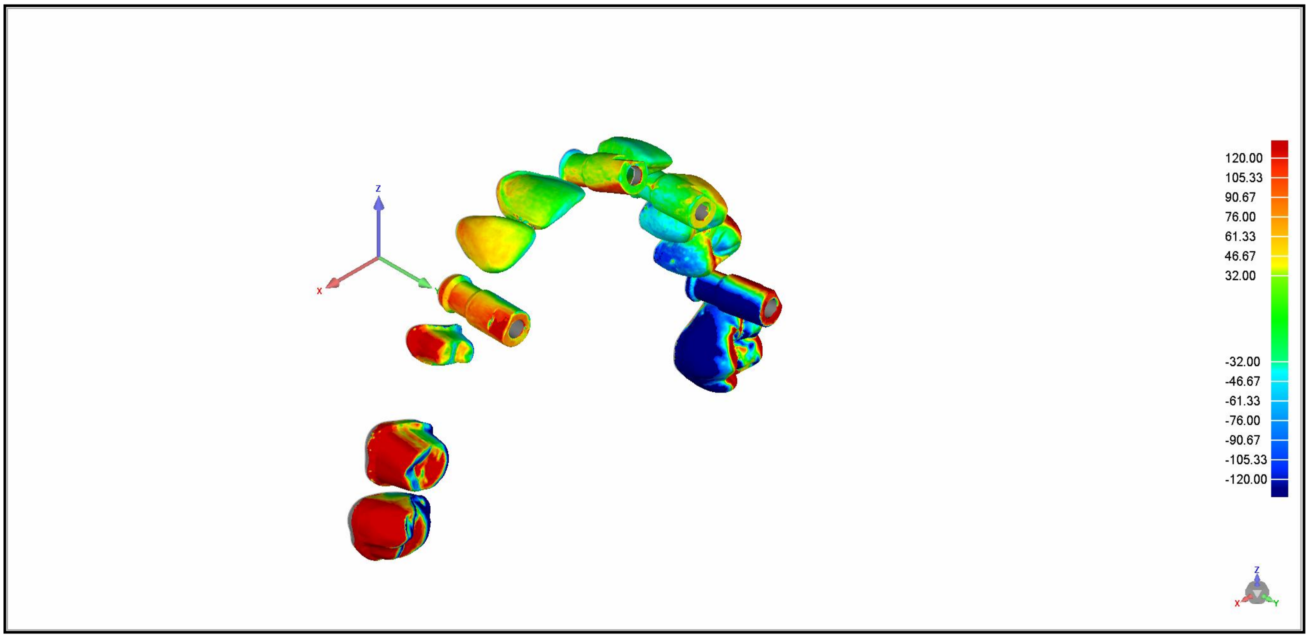

Predefinido: Frente

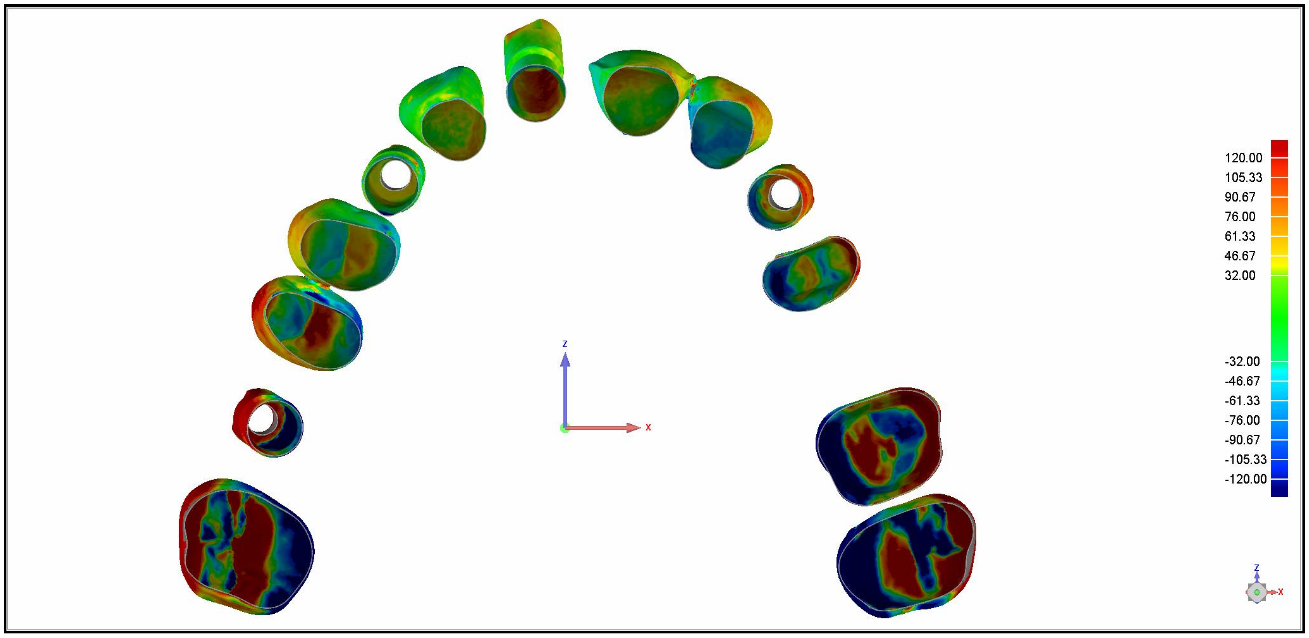

Predefinido: Atrás

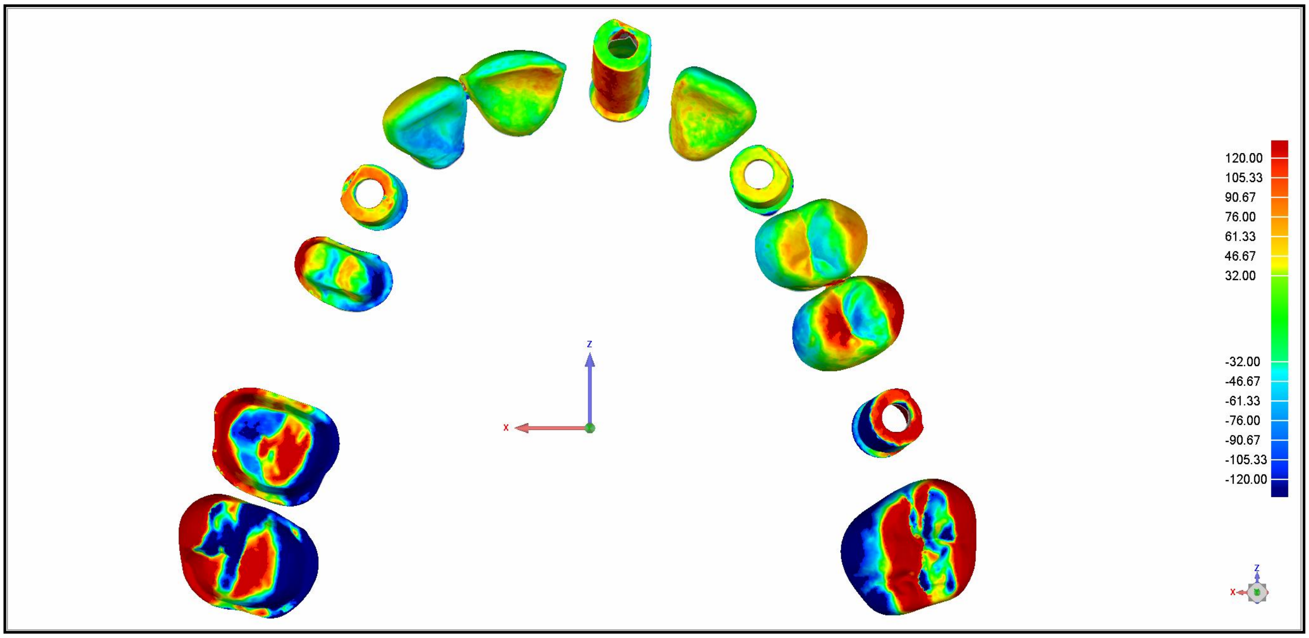

Predefinido: Izquierda

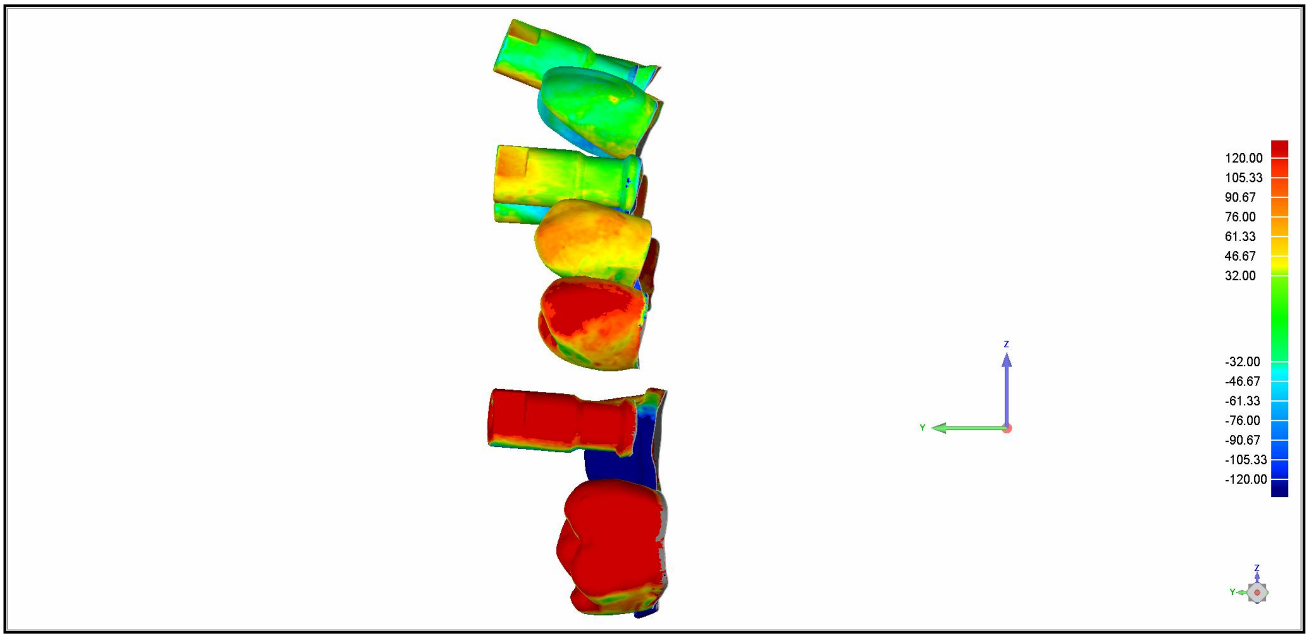

Predefinido: Derecha

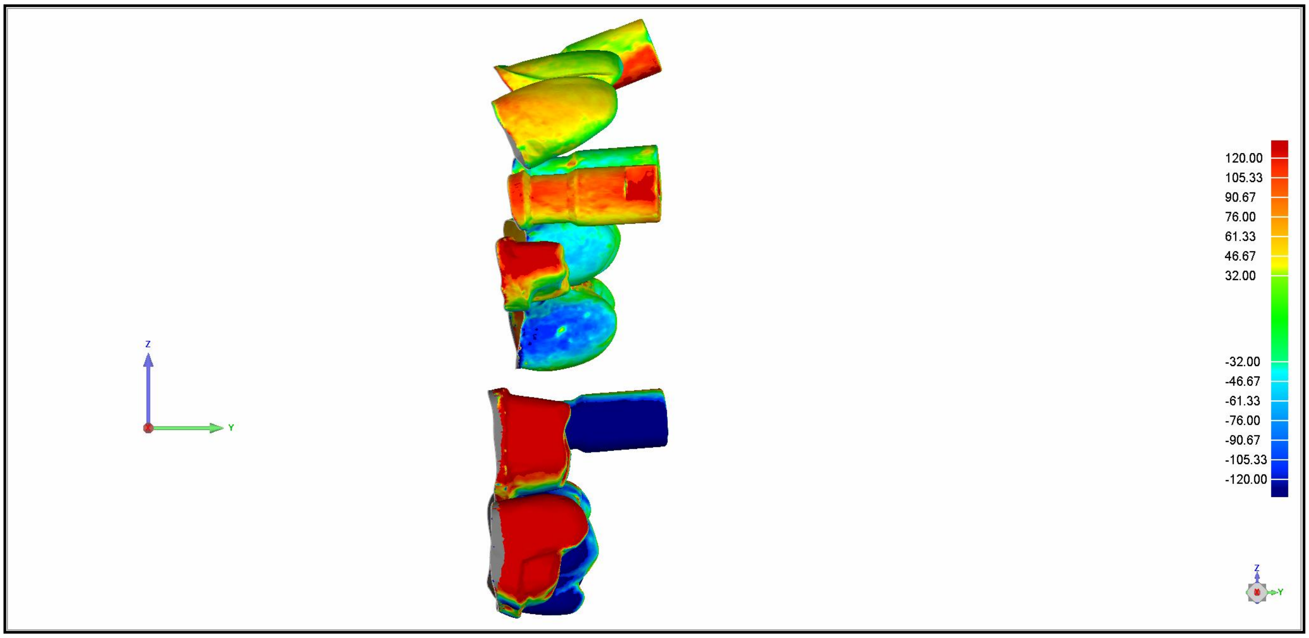

Predefinido: Superior

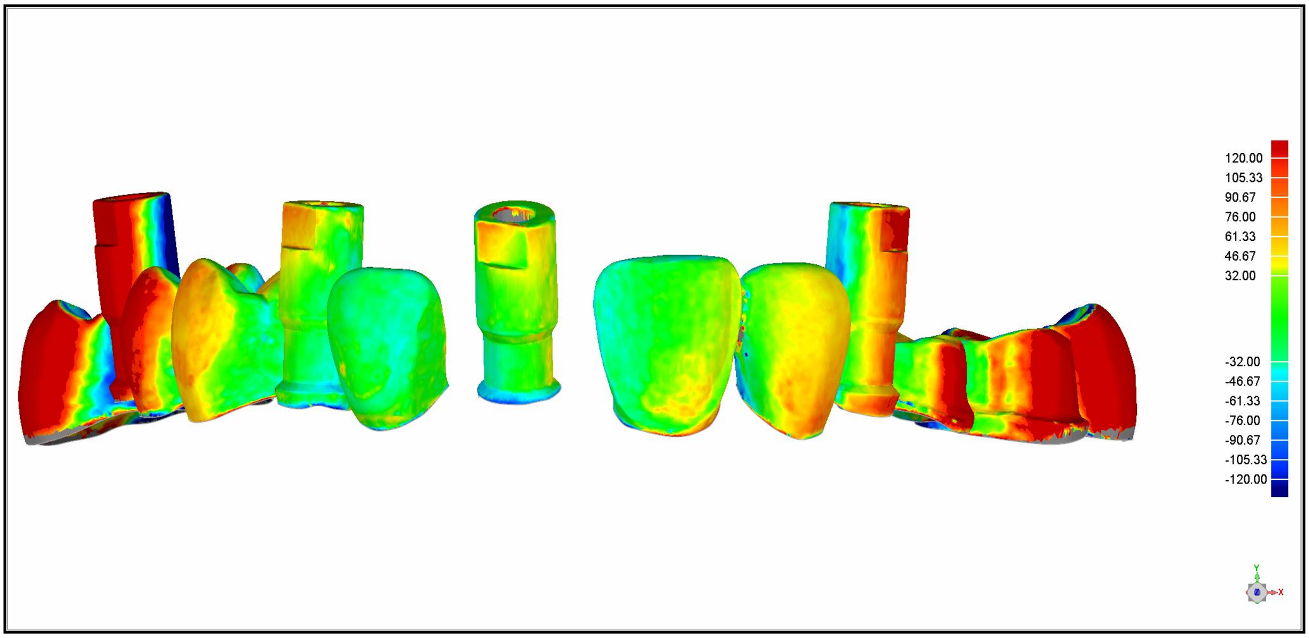

Predefinido: Inferior

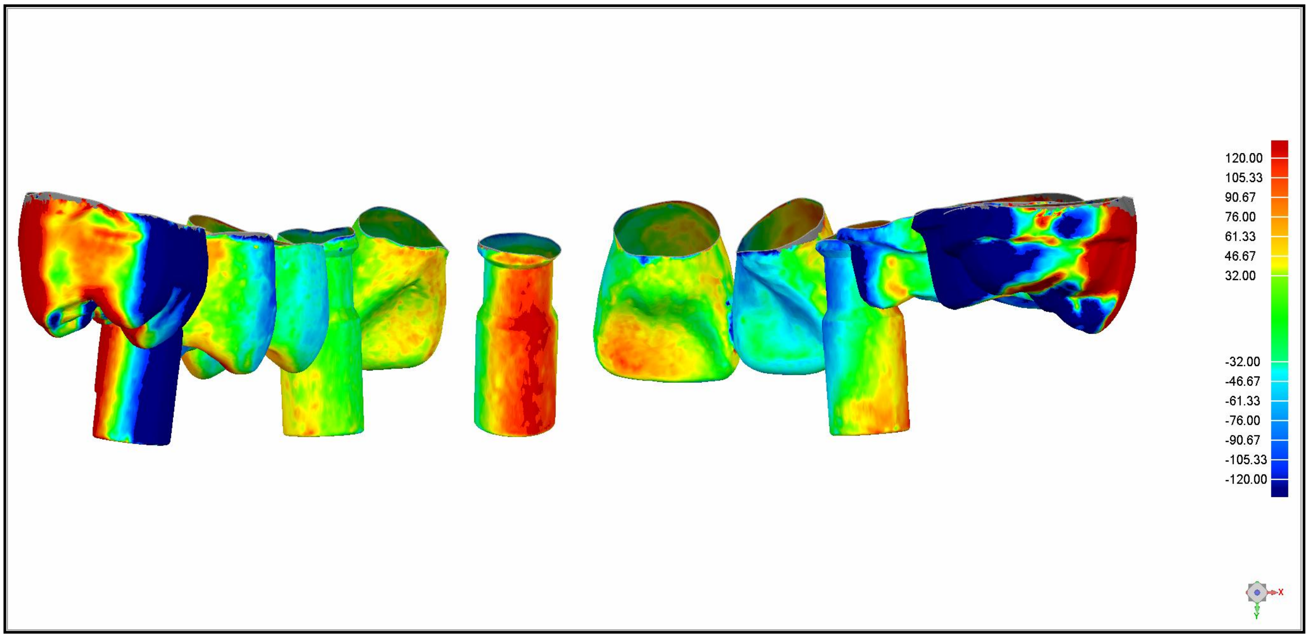

Ajuste de ubicación: Desviaciones superior e inferior

Unidades: u

Nombre	Desv	Estado	Superior Tol	Inferior Tol	Ref X	Ref Y	Ref Z	Radio	Desv X	Desv Y	Desv Z	Medido X	Medido Y	Medido Z	Dir. proy. X	Dir. proy. Y	Dir. proy. Z
Desv. inferior	-3111.94				32061.00	27268.70	-9389.56	n/a	-505.91	486.14	-3031.81	31555.09	27754.85	-12421.38	0.16	-0.16	0.97
Desv. superior	2880.78				32263.48	27229.24	-7694.37	n/a	-2119.88	283.63	1929.91	30143.59	27512.87	-5764.46	-0.74	0.10	0.67
