# Supplementary material for: Accuracy of four digital scanners according to scanning strategy in complete-arch impressions
Source: PLoS One. 2018 Sep 13;13(9):e0202916. doi: 10.1371/journal.pone.0202916 (PMC6136706; doi:10.1371/journal.pone.0202916)
Supplement: S13 Table — True definition (scanning strategy A). (ZIP) [file pone.0202916.s013.zip › S13/TD9A.pdf]

3D Comparación Resultados

Modelo referencia	MRC
Modelo test	TD9A
Nº de puntos de datos	129287
# Aislados	388

Tipo tolerancia	3D desviación
Unidades	u
Máx. crítico	120.00
Máx. nominal	13.00
Mín. nominal	-13.00
Mín. crítico	-120.00

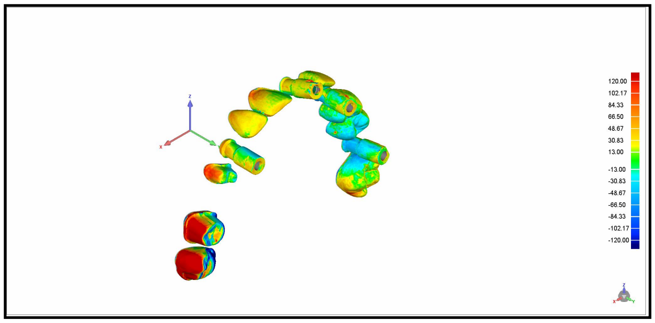

Desviación	
Desviación superior máx.	1866.02
Desviación inferior máx.	-3019.21
Desviación media	50.81 /-45.61
Desviación estándar	93.62

Distribución desviación

>=Min	<Max	# Puntos	%
-120.00	-102.17	651	0.50
-102.17	-84.33	746	0.58
-84.33	-66.50	1360	1.05
-66.50	-48.67	3510	2.71
-48.67	-30.83	7901	6.11
-30.83	-13.00	15645	12.10
-13.00	13.00	32071	24.81
13.00	30.83	24767	19.16
30.83	48.67	16776	12.98
48.67	66.50	6850	5.30
66.50	84.33	3120	2.41
84.33	102.17	1982	1.53
102.17	120.00	1341	1.04

Fuera del crítico superior	7277	5.63
Fuera del crítico inferior	5290	4.09

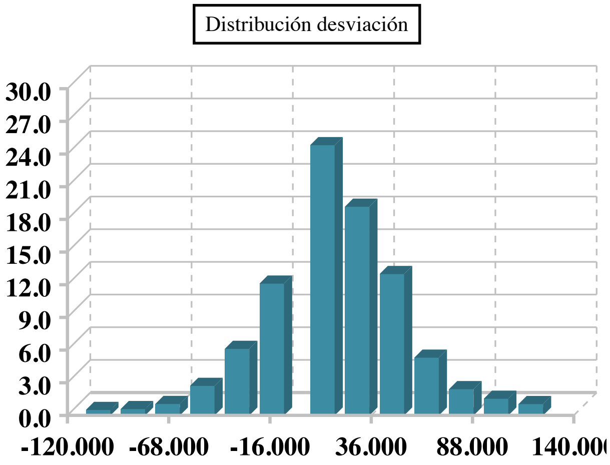

Desviaciones estándar

Distribución (+/-)	# Puntos	%
-6 * Desv. estánd.	147	0.11
-5 * Desv. estánd.	64	0.05
-4 * Desv. estánd.	458	0.35
-3 * Desv. estánd.	2547	1.97
-2 * Desv. estánd.	3706	2.87
-1 * Desv. estánd.	61285	47.40
1 * Desv. estánd.	52912	40.93
2 * Desv. estánd.	4677	3.62
3 * Desv. estánd.	2549	1.97
4 * Desv. estánd.	432	0.33
5 * Desv. estánd.	90	0.07
6 * Desv. estánd.	420	0.32

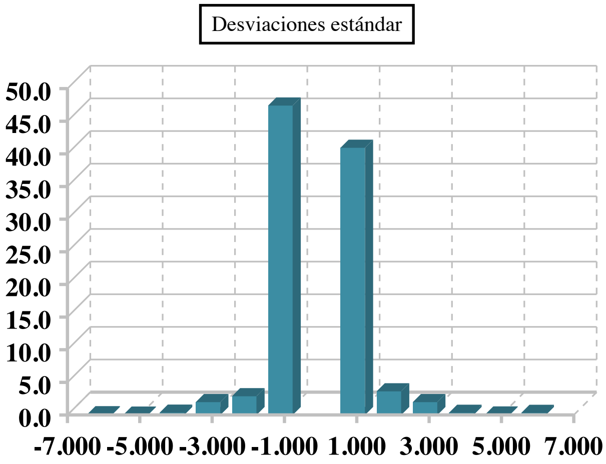

Predefinido: Isométrico

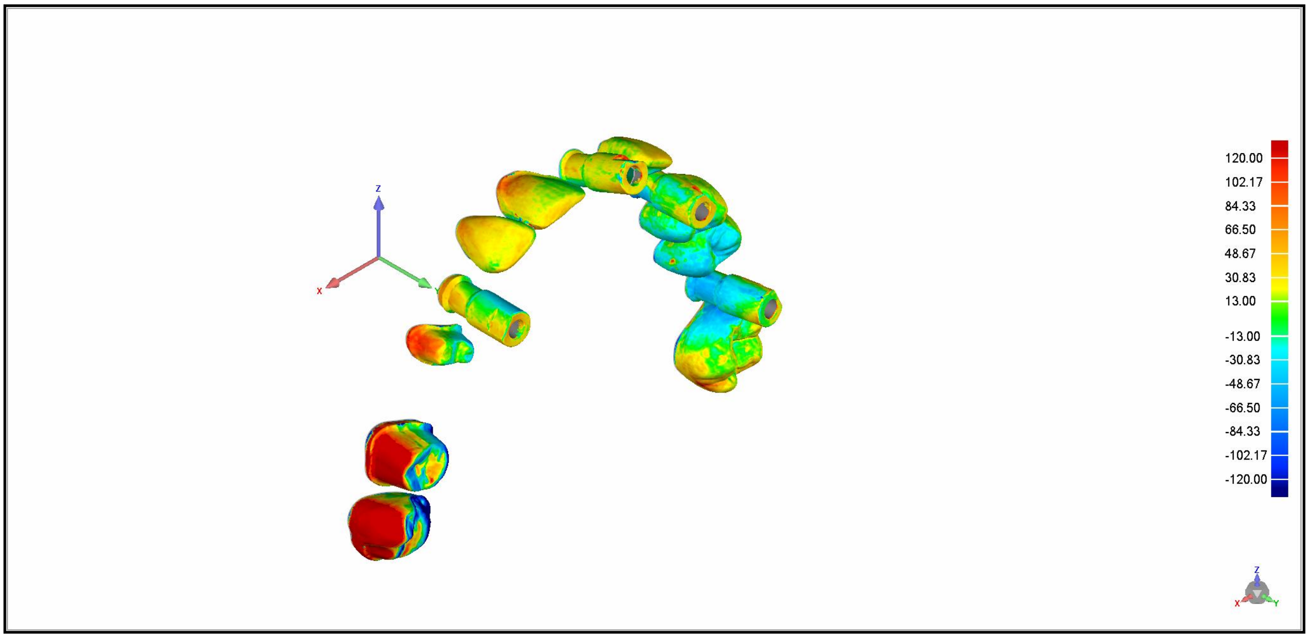

Predefinido: Frente

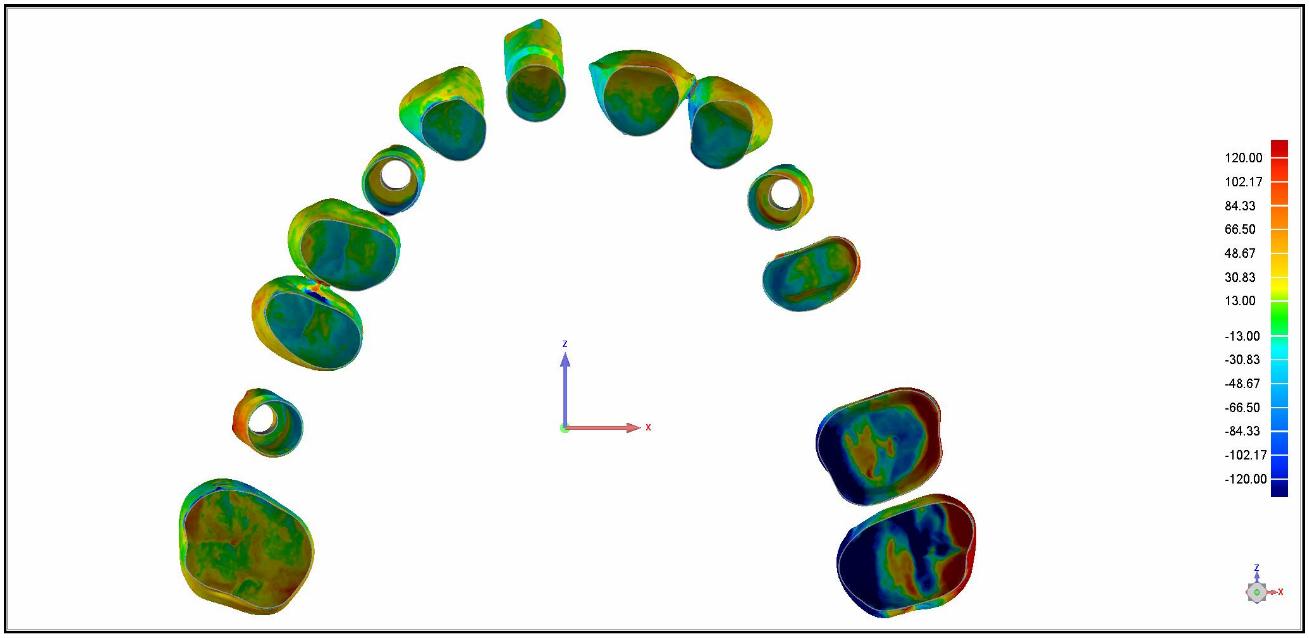

Predefinido: Atrás

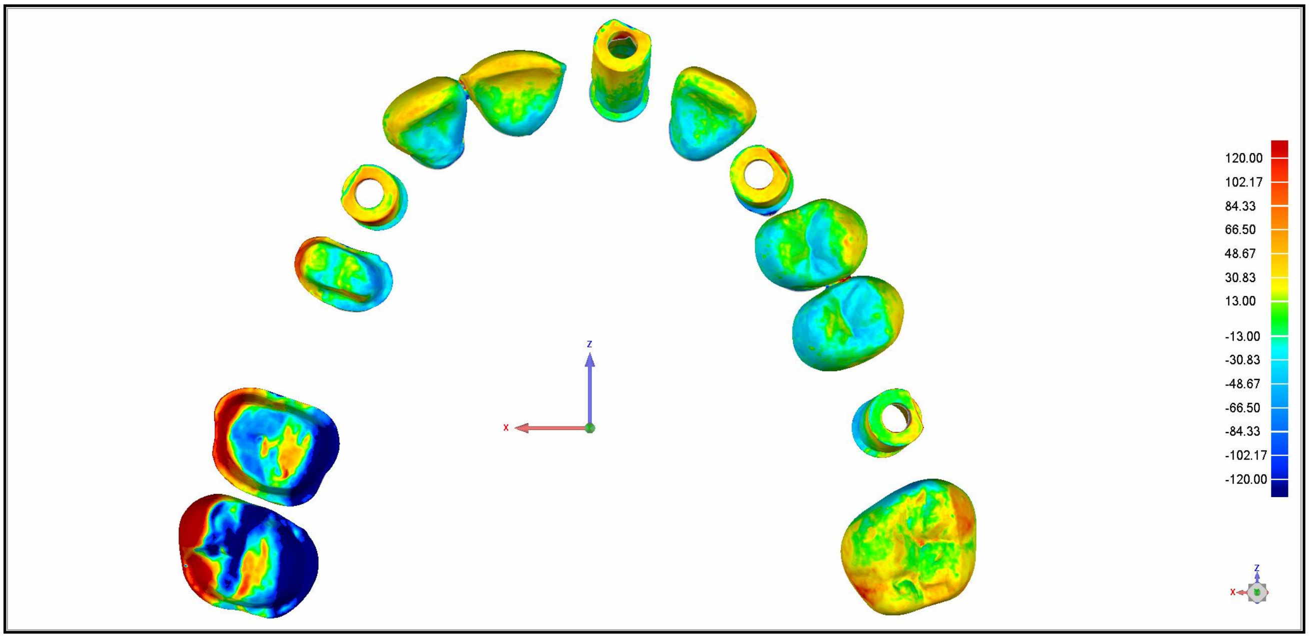

Predefinido: Izquierda

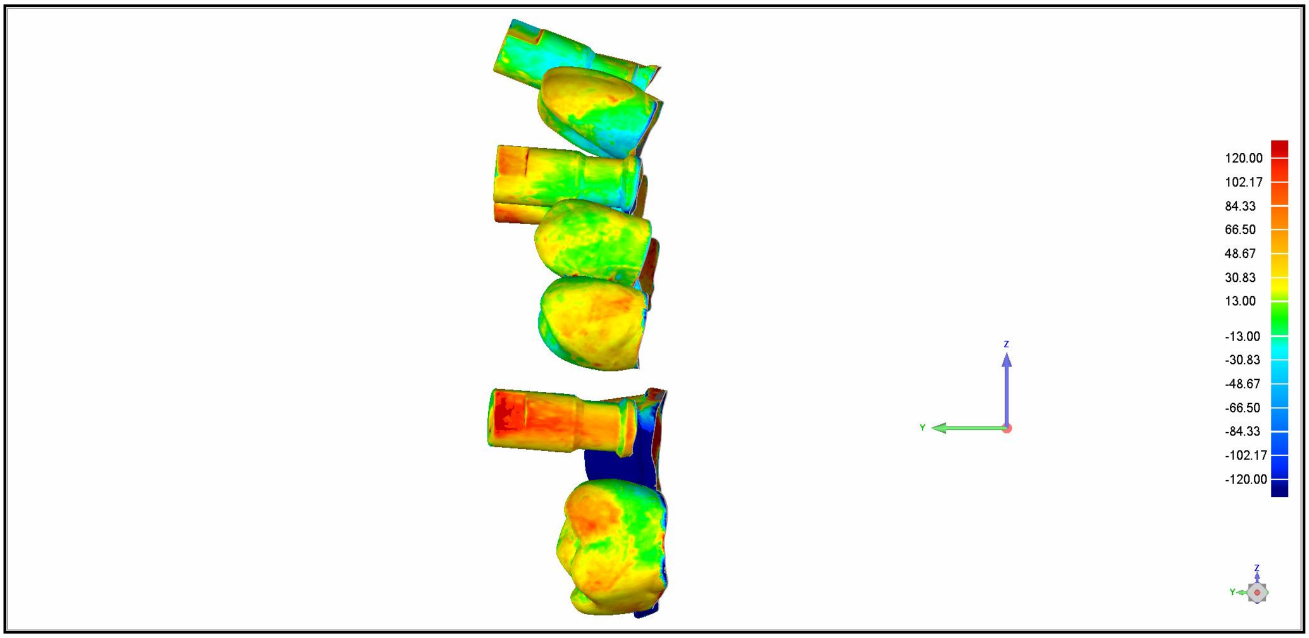

Predefinido: Derecha

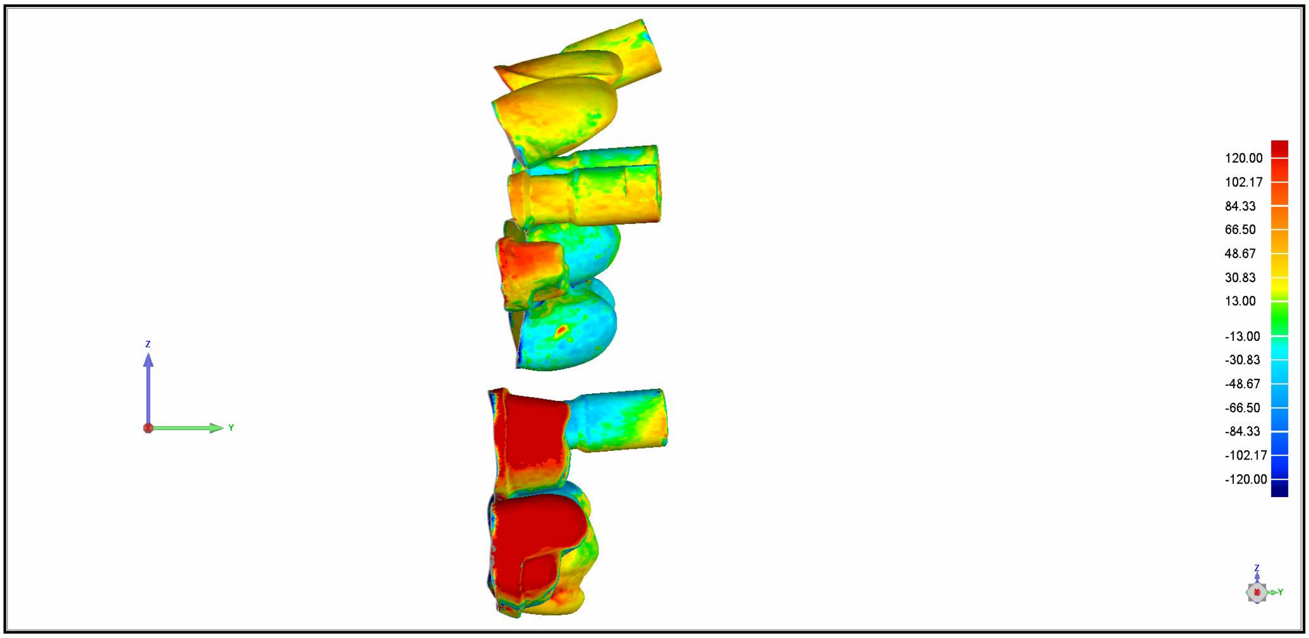

Predefinido: Superior

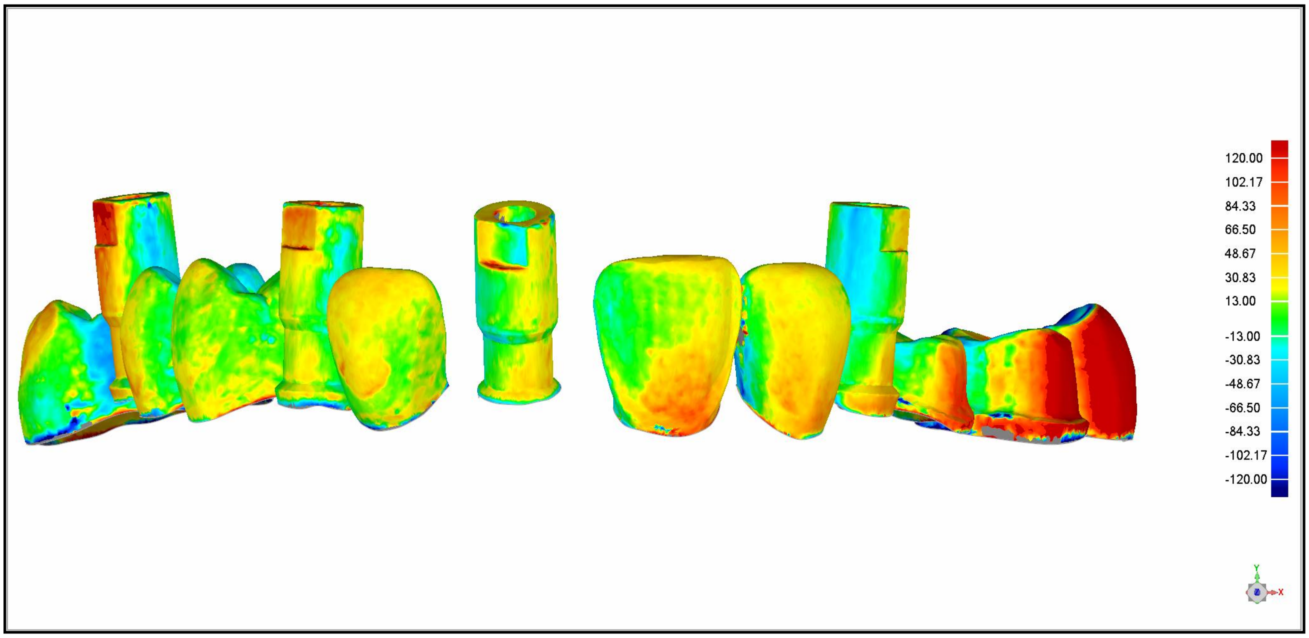

Predefinido: Inferior

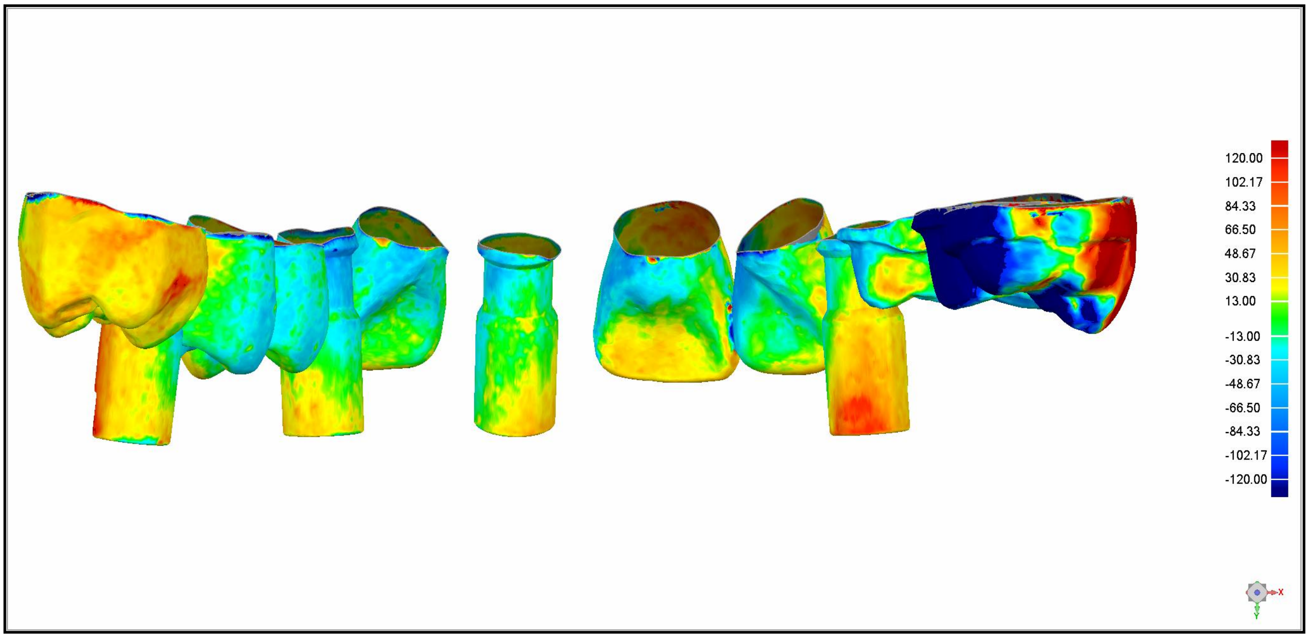

Ajuste de ubicación: Desviaciones superior e inferior

Unidades: u

Nombre	Desv	Estado	Superior Tol	Inferior Tol	Ref X	Ref Y	Ref Z	Radio	Desv X	Desv Y	Desv Z	Medido X	Medido Y	Medido Z	Dir. proy. X	Dir. proy. Y	Dir. proy. Z
Desv. inferior	-3019.21				16989.02	37628.06	17251.36	n/a	1337.67	2531.95	-956.82	18326.68	40160.02	16294.54	-0.44	-0.84	0.32
Desv. superior	1866.02				31723.91	27105.47	-10369.29	n/a	18.63	21.59	-1865.81	31742.53	27127.07	-12235.10	0.01	0.01	-1.00
