# Supplementary material for: Accuracy of four digital scanners according to scanning strategy in complete-arch impressions
Source: PLoS One. 2018 Sep 13;13(9):e0202916. doi: 10.1371/journal.pone.0202916 (PMC6136706; doi:10.1371/journal.pone.0202916)
Supplement: S13 Table — True definition (scanning strategy A). (ZIP) [file pone.0202916.s013.zip › S13/TD5A.pdf]

Geomagic Control™

Fecha: 3/6/2017, 3:42 pm

3D Comparación Resultados

Modelo referencia	MRC
Modelo test	TD5A
Nº de puntos de datos	129491
# Aislados	447

Tipo tolerancia	3D desviación
Unidades	u
Máx. crítico	120.00
Máx. nominal	31.00
Mín. nominal	-31.00
Mín. crítico	-120.00

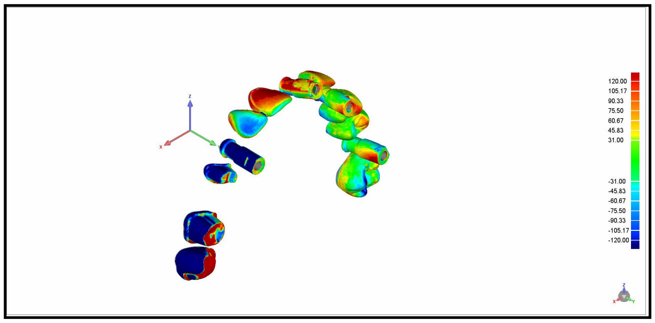

Desviación	
Desviación superior máx.	2976.55
Desviación inferior máx.	-3096.48
Desviación media	126.77 /-106.47
Desviación estándar	209.94

Distribución desviación

>=Min	<Max	# Puntos	%
-120.00	-105.17	1347	1.04
-105.17	-90.33	2218	1.71
-90.33	-75.50	2853	2.20
-75.50	-60.67	3541	2.73
-60.67	-45.83	4205	3.25
-45.83	-31.00	5894	4.55
-31.00	31.00	43826	33.84
31.00	45.83	8345	6.44
45.83	60.67	7138	5.51
60.67	75.50	5448	4.21
75.50	90.33	4268	3.30
90.33	105.17	3503	2.71
105.17	120.00	2697	2.08

Fuera del crítico superior	22259	17.19
Fuera del crítico inferior	11949	9.23

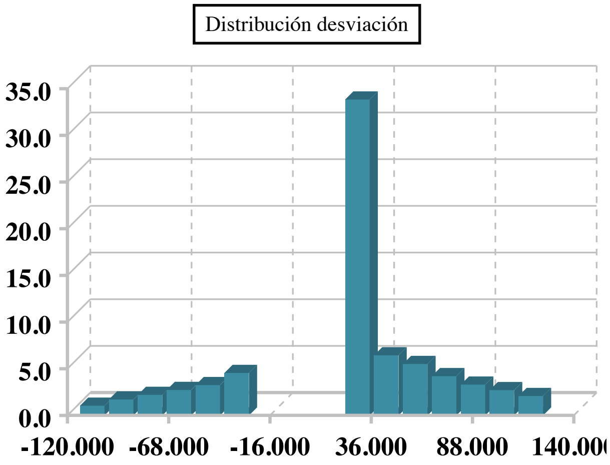

Desviaciones estándar

Distribución (+/-)	# Puntos	%
-6 * Desv. esténd.	140	0.11
-5 * Desv. esténd.	64	0.05
-4 * Desv. esténd.	1141	0.88
-3 * Desv. esténd.	2382	1.84
-2 * Desv. esténd.	5322	4.11
-1 * Desv. esténd.	67159	51.86
1 * Desv. esténd.	41984	32.42
2 * Desv. esténd.	5753	4.44
3 * Desv. esténd.	3713	2.87
4 * Desv. esténd.	1517	1.17
5 * Desv. esténd.	143	0.11
6 * Desv. esténd.	173	0.13

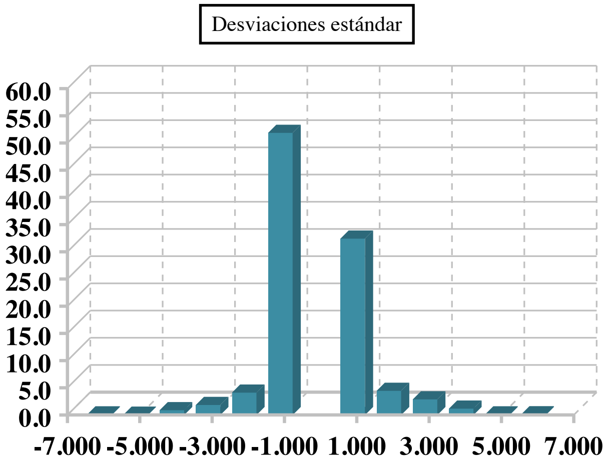

Geomagic Control™

Predefinido: Isométrico

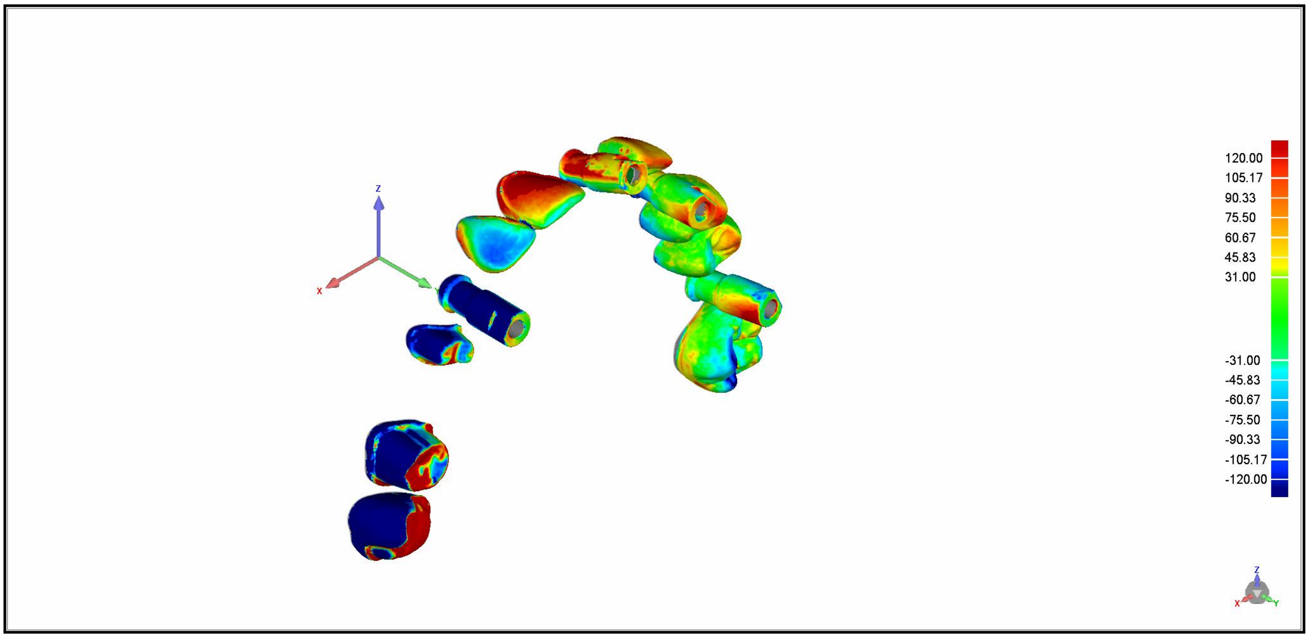

Predefinido: Frente

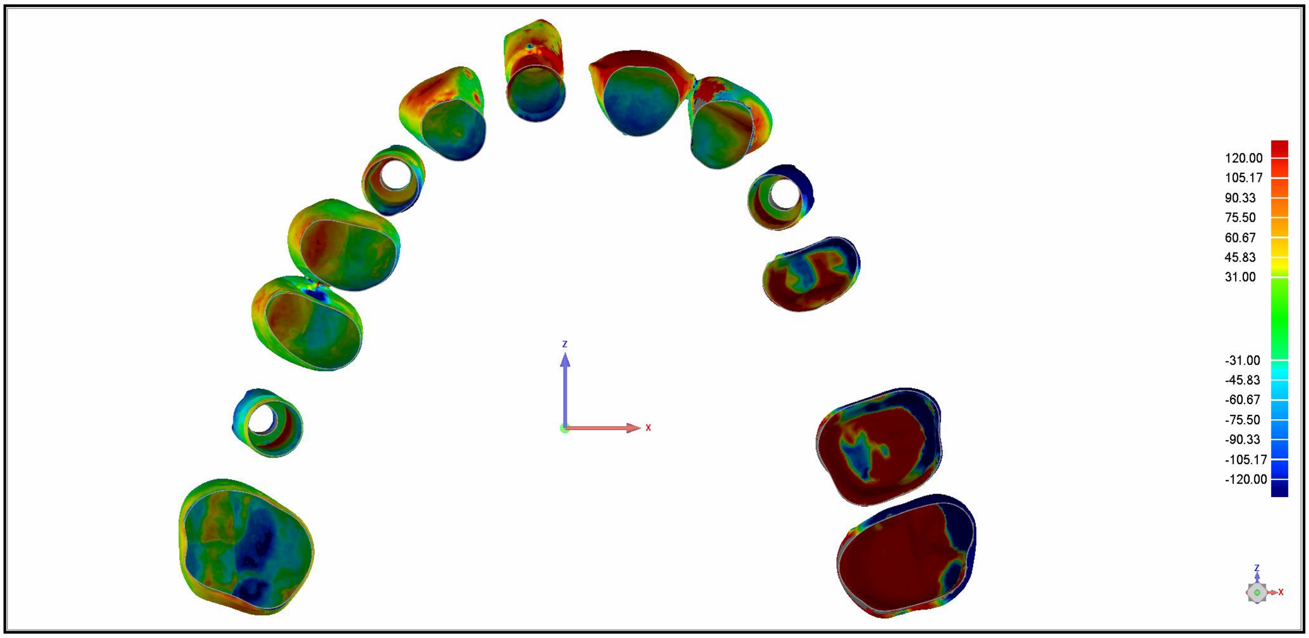

Predefinido: Atrás

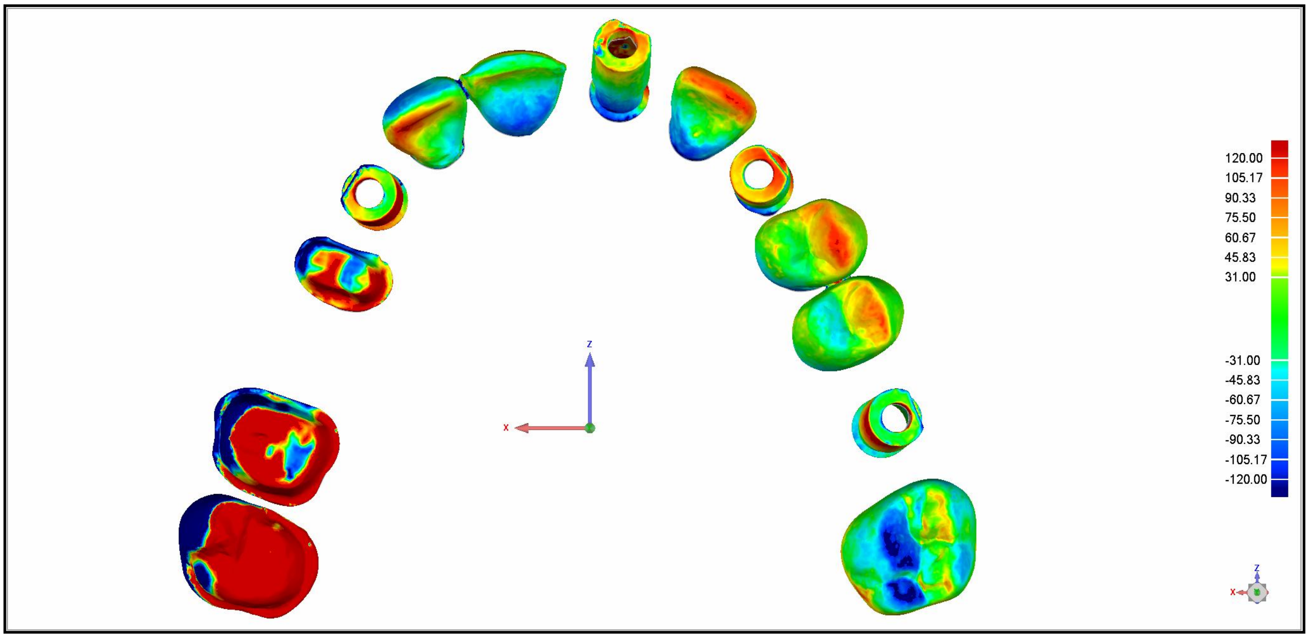

Geomagic Control™

Predefinido: Izquierda

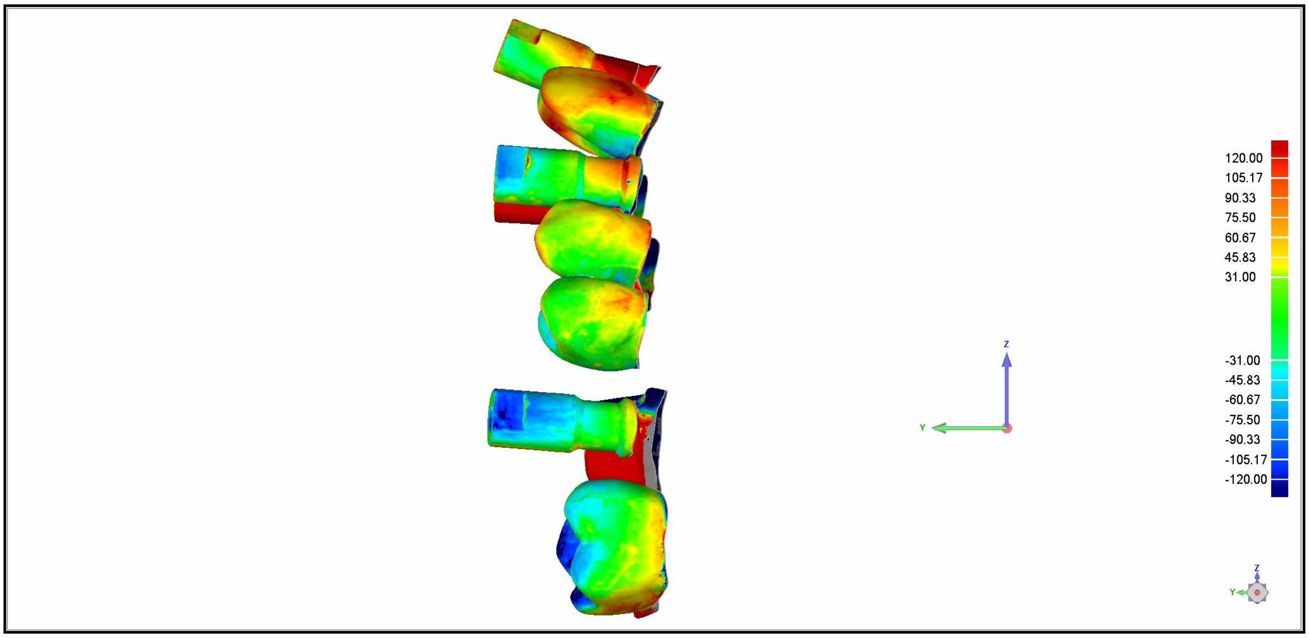

Predefinido: Derecha

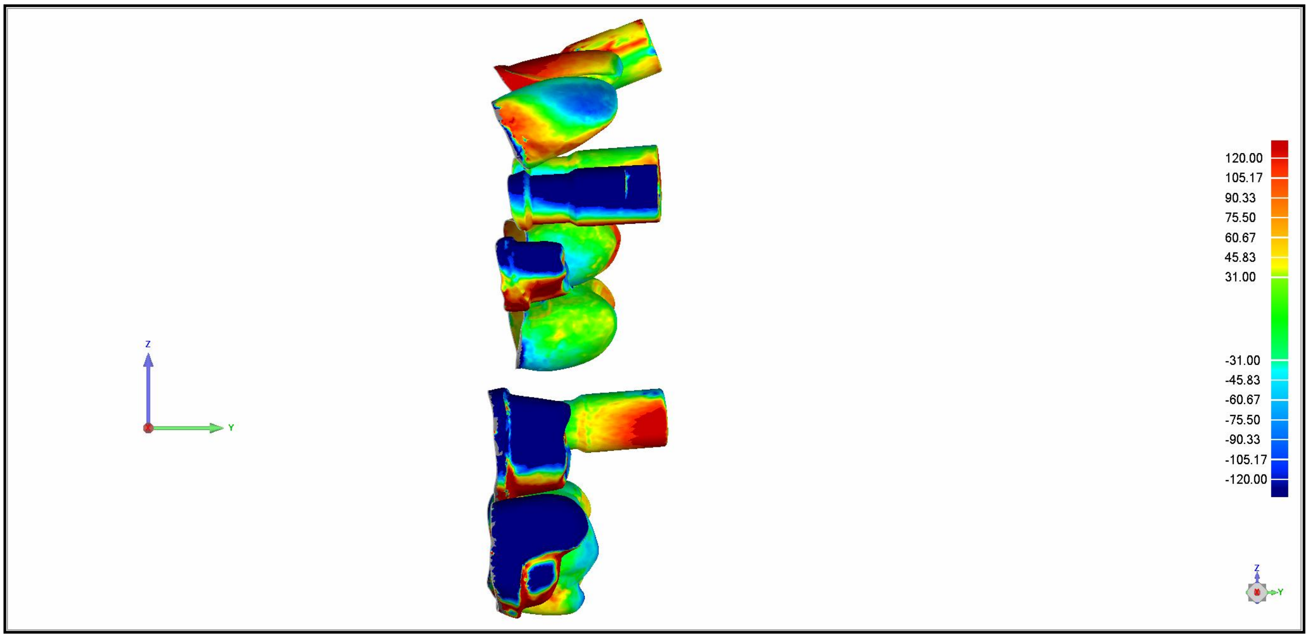

Predefinido: Superior

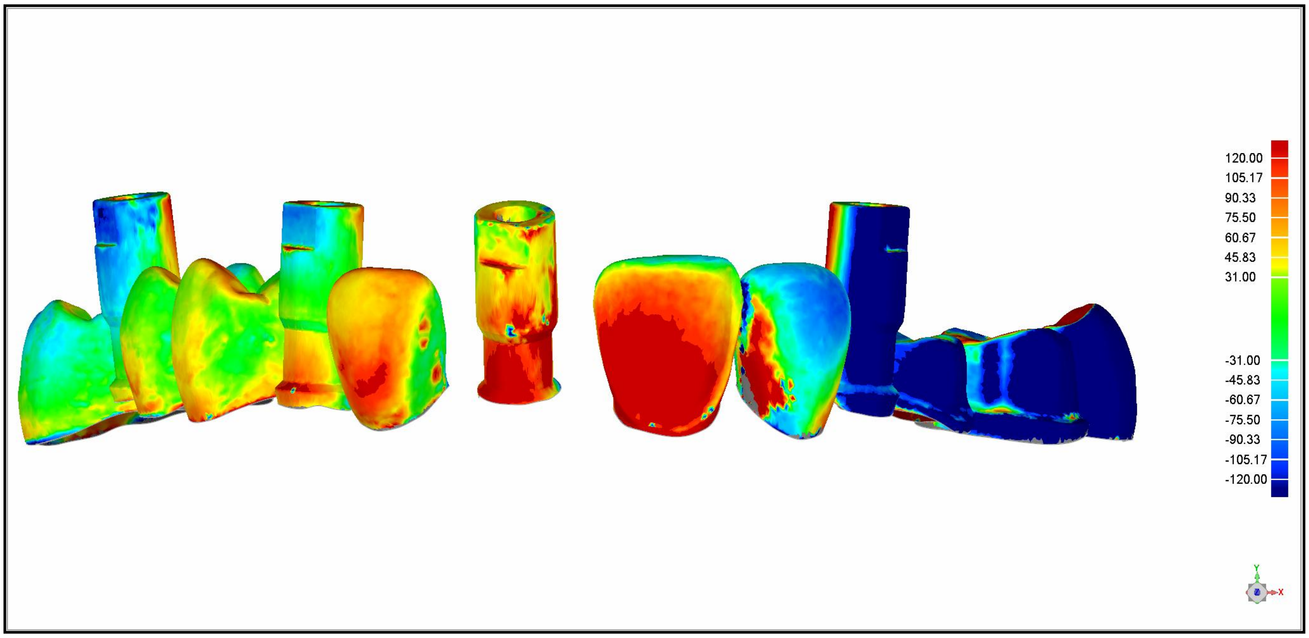

Predefinido: Inferior

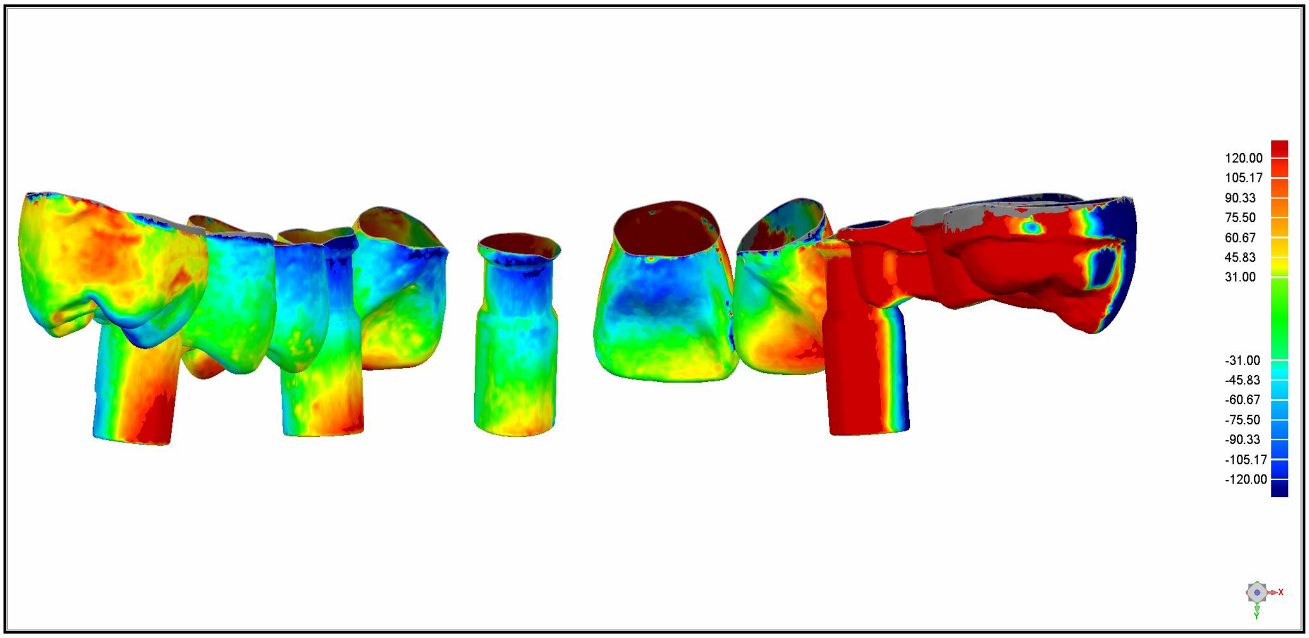

Ajuste de ubicación: Desviaciones superior e inferior

Unidades: u

Nombre	Desv	Estado	Superior Tol	Inferior Tol	Ref X	Ref Y	Ref Z	Radio	Desv X	Desv Y	Desv Z	Medido X	Medido Y	Medido Z	Dir. proy. X	Dir. proy. Y	Dir. proy. Z
Desv. inferior	-3096.48				32061.00	27268.70	-9389.57	n/a	-867.70	282.17	-2959.00	31193.30	27550.87	-12348.57	0.28	-0.09	0.96
Desv. superior	2976.55				25410.11	27462.45	-5764.99	n/a	2916.56	130.17	-580.12	28326.67	27592.62	-6345.12	0.98	0.04	-0.19
