# Supplementary material for: Accuracy of four digital scanners according to scanning strategy in complete-arch impressions
Source: PLoS One. 2018 Sep 13;13(9):e0202916. doi: 10.1371/journal.pone.0202916 (PMC6136706; doi:10.1371/journal.pone.0202916)
Supplement: S13 Table — True definition (scanning strategy A). (ZIP) [file pone.0202916.s013.zip › S13/TD4A.pdf]

Geomagic Control™

Fecha: 3/6/2017, 3:39 pm

# 3D Comparación  Resultados

| | |
|---|---|
| Modelo referencia | MRC |
| Modelo test | TD4A |
| Nº de puntos de datos | 128482 |
| # Aislados | 197 |

| | |
|---|---|
| Tipo tolerancia | 3D desviación |
| Unidades | u |
| Máx. crítico | 120.00 |
| Máx. nominal | 10.00 |
| Mín. nominal | -10.00 |
| Mín. crítico | -120.00 |

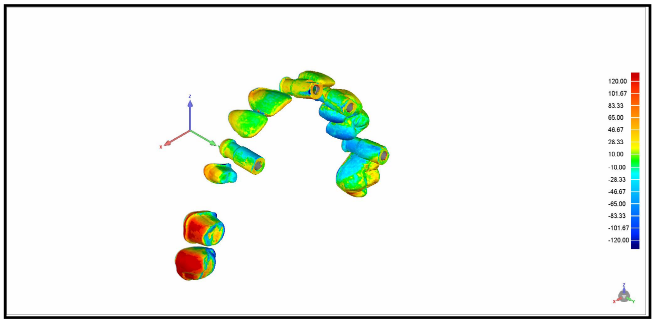

| | |
|---|---|
| Desviación | |
| Desviación superior máx. | 2413.14 |
| Desviación inferior máx. | -2930.92 |
| Desviación media | 43.93 /-36.33 |
| Desviación estándar | 89.59 |

## Distribución desviación

| >=Min | <Max | # Puntos | % |
|---|---|---|---|
| -120.00 | -101.67 | 958 | 0.75 |
| -101.67 | -83.33 | 1306 | 1.02 |
| -83.33 | -65.00 | 3213 | 2.50 |
| -65.00 | -46.67 | 5443 | 4.24 |
| -46.67 | -28.33 | 8987 | 6.99 |
| -28.33 | -10.00 | 15995 | 12.45 |
| -10.00 | 10.00 | 30848 | 24.01 |
| 10.00 | 28.33 | 27243 | 21.20 |
| 28.33 | 46.67 | 14169 | 11.03 |
| 46.67 | 65.00 | 5887 | 4.58 |
| 65.00 | 83.33 | 2578 | 2.01 |
| 83.33 | 101.67 | 1884 | 1.47 |
| 101.67 | 120.00 | 1910 | 1.49 |

| | | |
|---|---|---|
| Fuera del crítico superior | 5177 | 4.03 |
| Fuera del crítico inferior | 2884 | 2.24 |

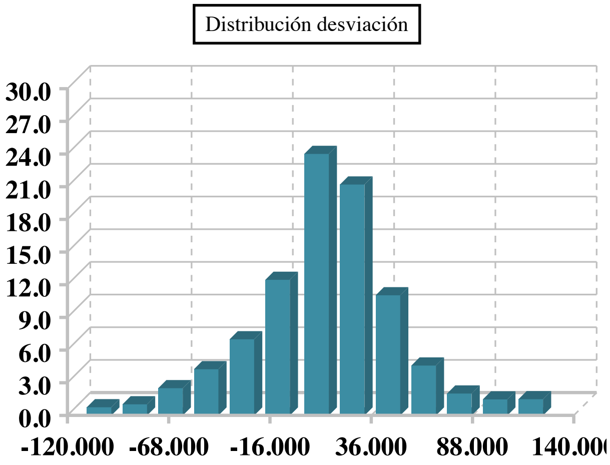


## Desviaciones estándar

| Distribución (+/-) | # Puntos | % |
|---|---|---|
| -6 * Desv. estánd. | 70 | 0.05 |
| -5 * Desv. estánd. | 40 | 0.03 |
| -4 * Desv. estánd. | 74 | 0.06 |
| -3 * Desv. estánd. | 313 | 0.24 |
| -2 * Desv. estánd. | 5190 | 4.04 |
| -1 * Desv. estánd. | 65153 | 50.71 |
| 1 * Desv. estánd. | 50396 | 39.22 |
| 2 * Desv. estánd. | 5810 | 4.52 |
| 3 * Desv. estánd. | 643 | 0.50 |
| 4 * Desv. estánd. | 164 | 0.13 |
| 5 * Desv. estánd. | 104 | 0.08 |
| 6 * Desv. estánd. | 525 | 0.41 |

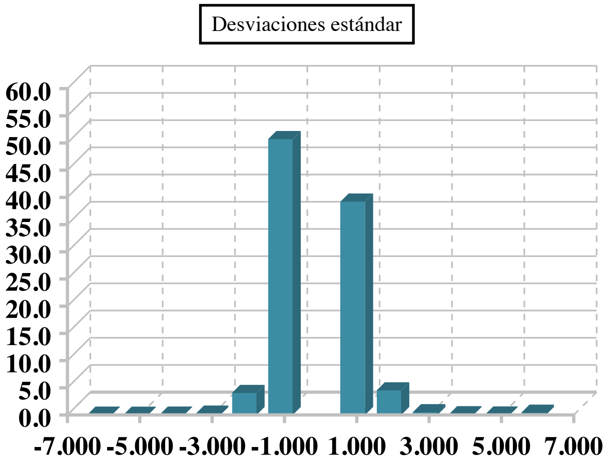

Predefinido: Isométrico

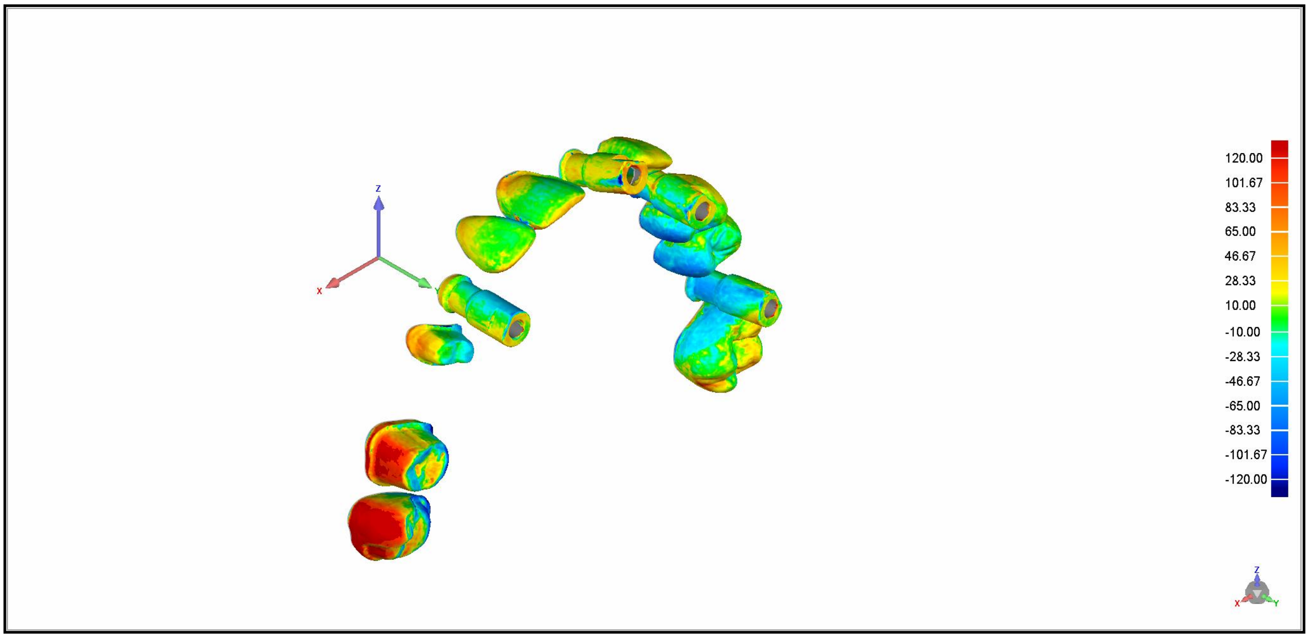

Predefinido: Frente

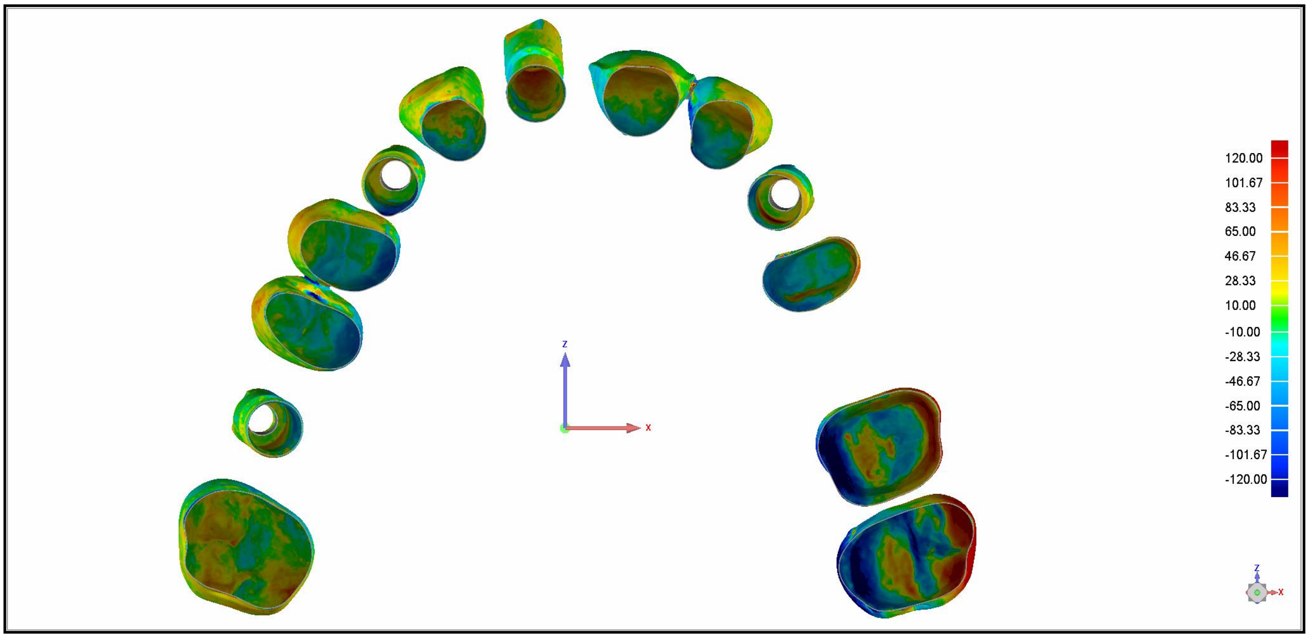

Predefinido: Atrás

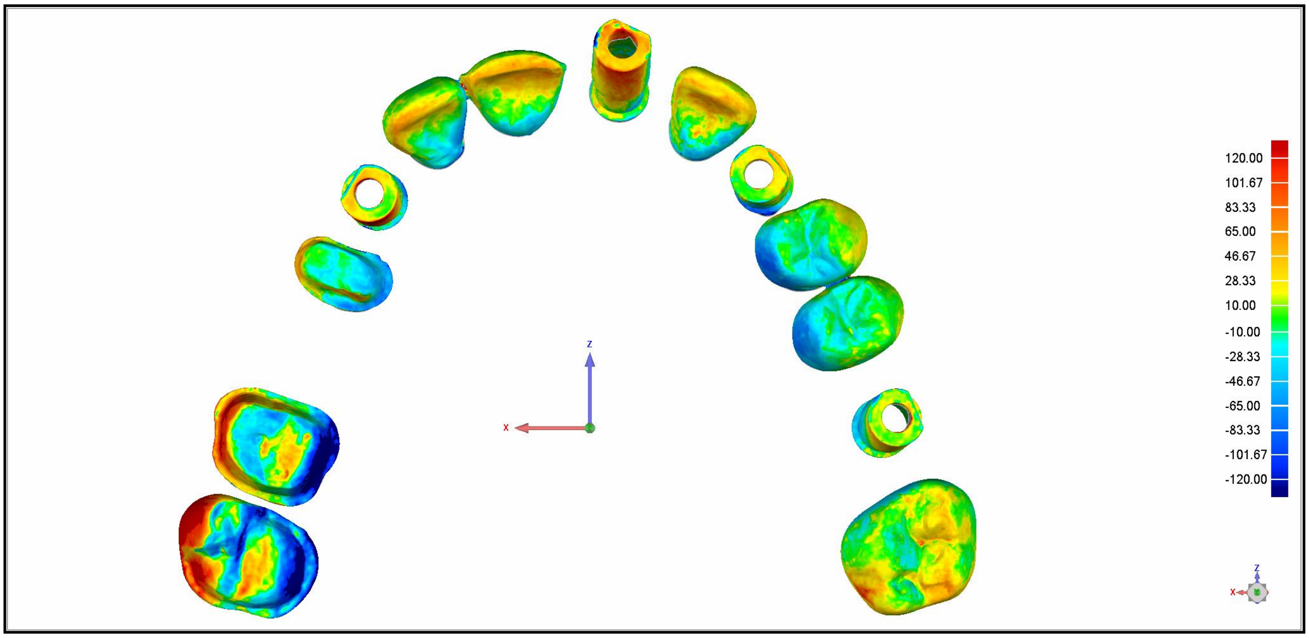

Predefinido: Izquierda

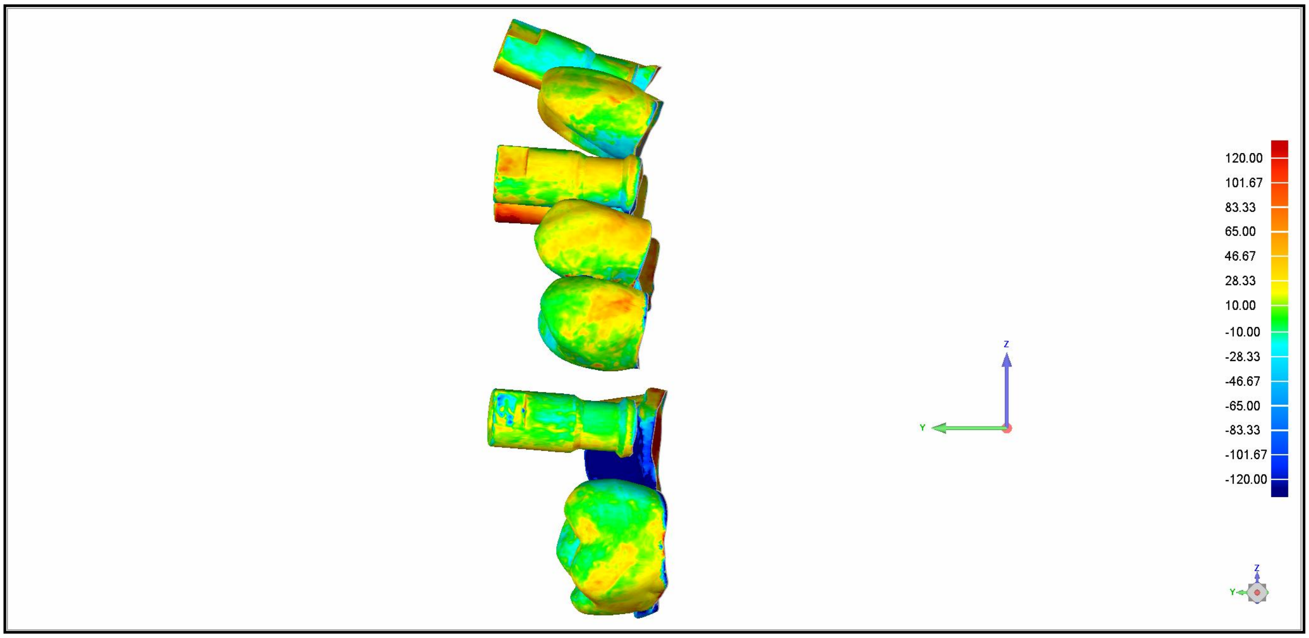

Predefinido: Derecha

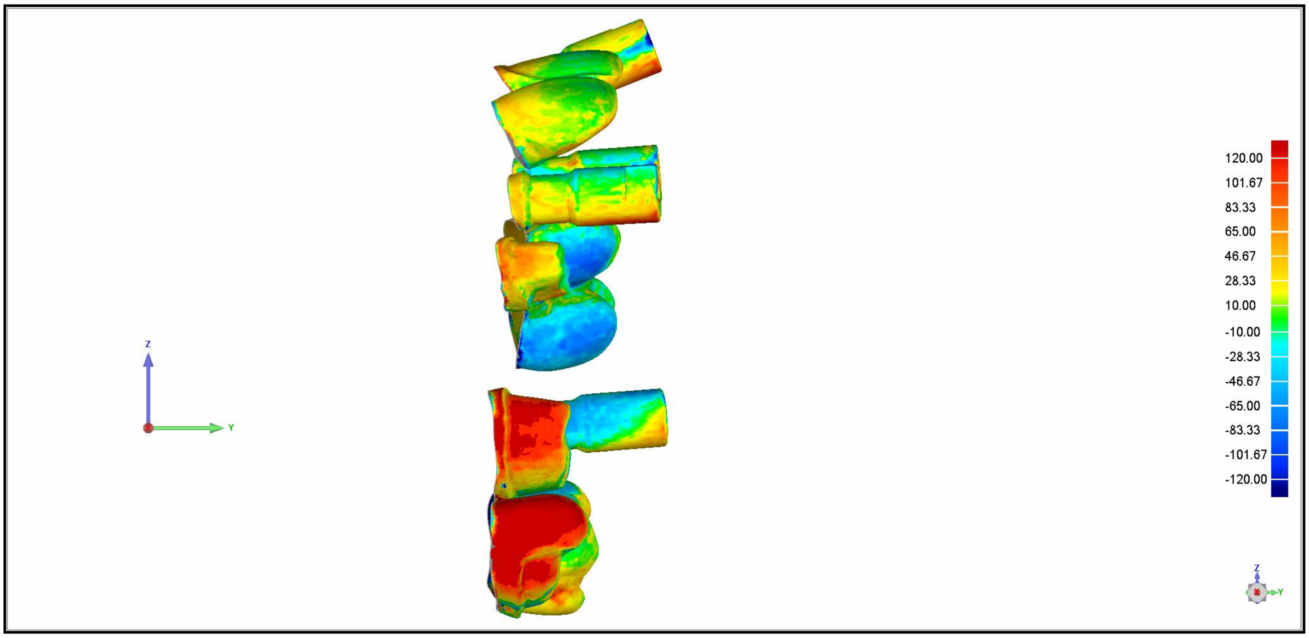

Predefinido: Superior

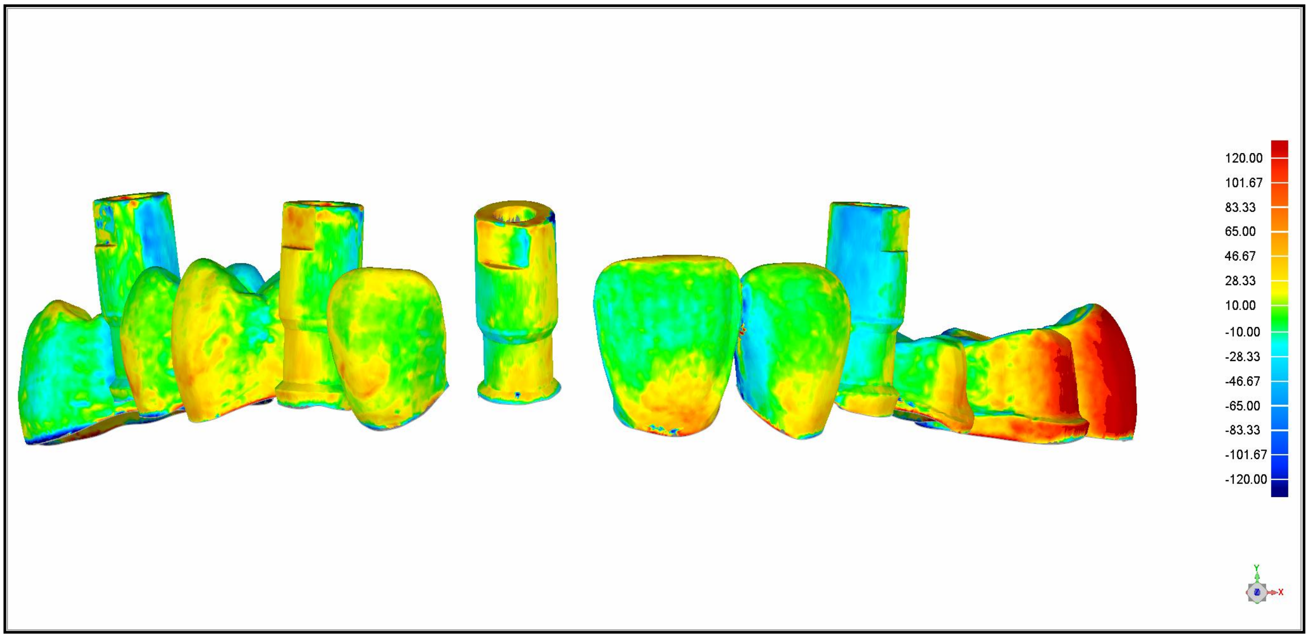

Predefinido: Inferior

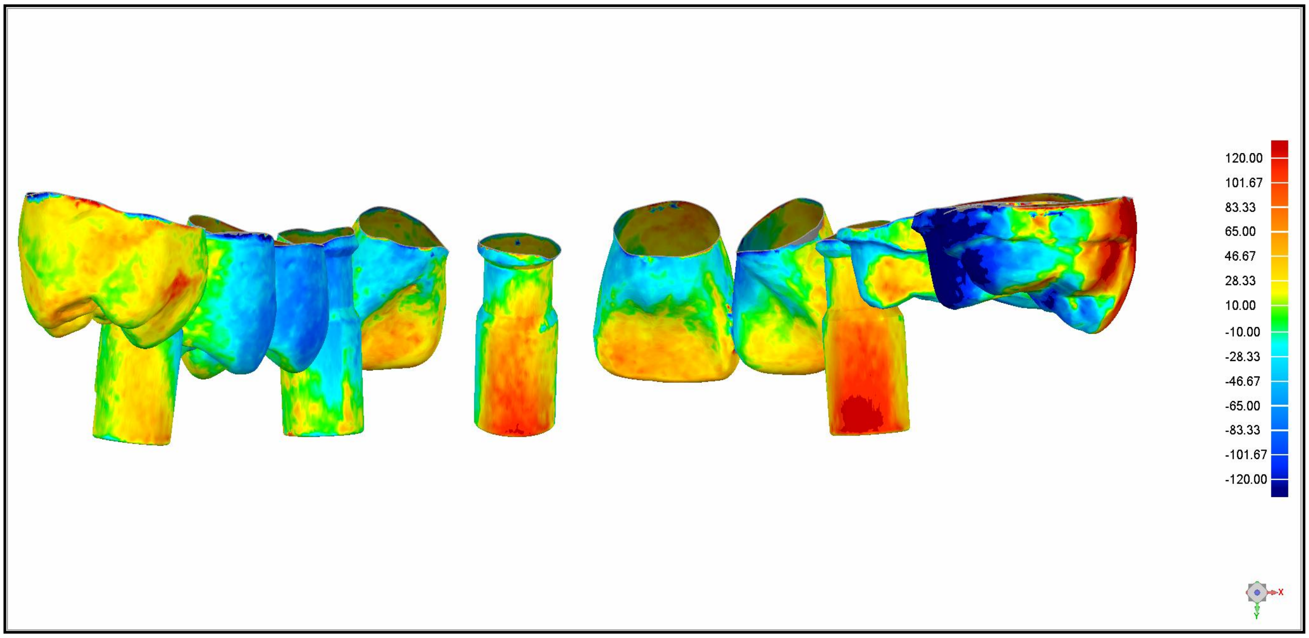

# Ajuste de ubicación: Desviaciones superior e inferior

## Unidades: u

| Nombre | Desv | Estado | Superior Tol | Inferior Tol | Ref X | Ref Y | Ref Z | Radio | Desv X | Desv Y | Desv Z | Medido X | Medido Y | Medido Z | Dir. proy. X | Dir. proy. Y | Dir. proy. Z |
|---|---|---|---|---|---|---|---|---|---|---|---|---|---|---|---|---|---|
| Desv. inferior | -2930.92 | | | | -13203.90 | 38507.15 | 18811.31 | n/a | -2203.44 | 1903.44 | 334.78 | -15407.33 | 40410.59 | 19146.09 | 0.75 | -0.65 | -0.11 |
| Desv. superior | 2413.14 | | | | 25410.11 | 27462.45 | -5764.99 | n/a | 2288.76 | 31.04 | -764.09 | 27698.87 | 27493.50 | -6529.08 | 0.95 | 0.01 | -0.32 |
