# Supplementary material for: Accuracy of four digital scanners according to scanning strategy in complete-arch impressions
Source: PLoS One. 2018 Sep 13;13(9):e0202916. doi: 10.1371/journal.pone.0202916 (PMC6136706; doi:10.1371/journal.pone.0202916)
Supplement: S13 Table — True definition (scanning strategy A). (ZIP) [file pone.0202916.s013.zip › S13/TD8A.pdf]

Geomagic Control™

Fecha: 3/6/2017, 3:54 pm

3D Comparación Resultados

Modelo referencia	MRC
Modelo test	TD8A
Nº de puntos de datos	129317
# Aislados	399

Tipo tolerancia	3D desviación
Unidades	u
Máx. crítico	120.00
Máx. nominal	5.00
Mín. nominal	-5.00
Mín. crítico	-120.00

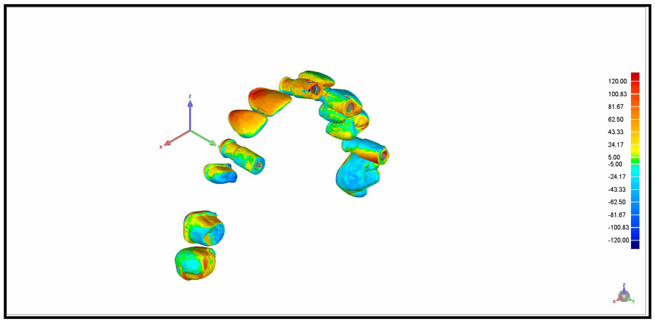

Desviación	
Desviación superior máx.	2503.71
Desviación inferior máx.	-2268.21
Desviación media	39.82 /-32.82
Desviación estándar	73.49

Distribución desviación

>=Min	<Max	# Puntos	%
-120.00	-100.83	1053	0.81
-100.83	-81.67	1679	1.30
-81.67	-62.50	2512	1.94
-62.50	-43.33	6738	5.21
-43.33	-24.17	17260	13.35
-24.17	-5.00	23895	18.48
-5.00	5.00	13019	10.07
5.00	24.17	22747	17.59
24.17	43.33	17324	13.40
43.33	62.50	11900	9.20
62.50	81.67	5298	4.10
81.67	100.83	2155	1.67
100.83	120.00	941	0.73

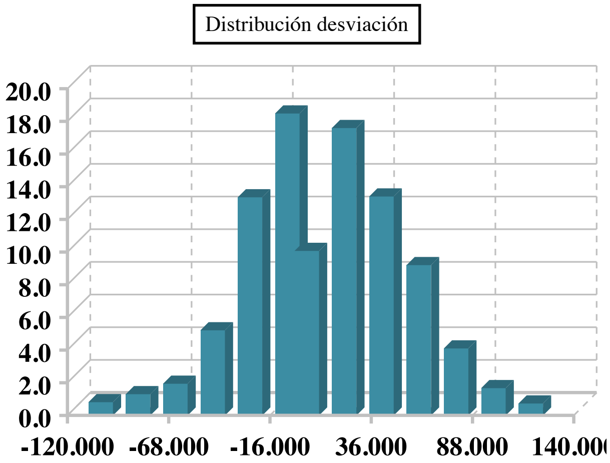

Fuera del crítico superior		1582	1.22
Fuera del crítico inferior		1214	0.94

Desviaciones estándar

Distribución (+/-)	# Puntos	%
-6 * Desv. estánd.	130	0.10
-5 * Desv. estánd.	77	0.06
-4 * Desv. estánd.	160	0.12
-3 * Desv. estánd.	401	0.31
-2 * Desv. estánd.	4859	3.76
-1 * Desv. estánd.	62306	48.18
1 * Desv. estánd.	56264	43.51
2 * Desv. estánd.	4102	3.17
3 * Desv. estánd.	397	0.31
4 * Desv. estánd.	213	0.16
5 * Desv. estánd.	102	0.08
6 * Desv. estánd.	306	0.24

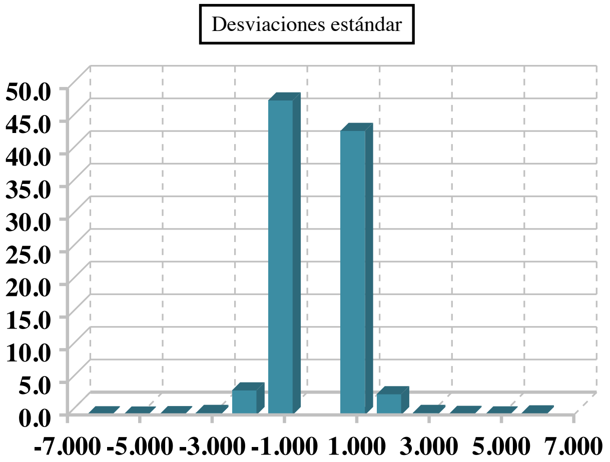

Predefinido: Isométrico

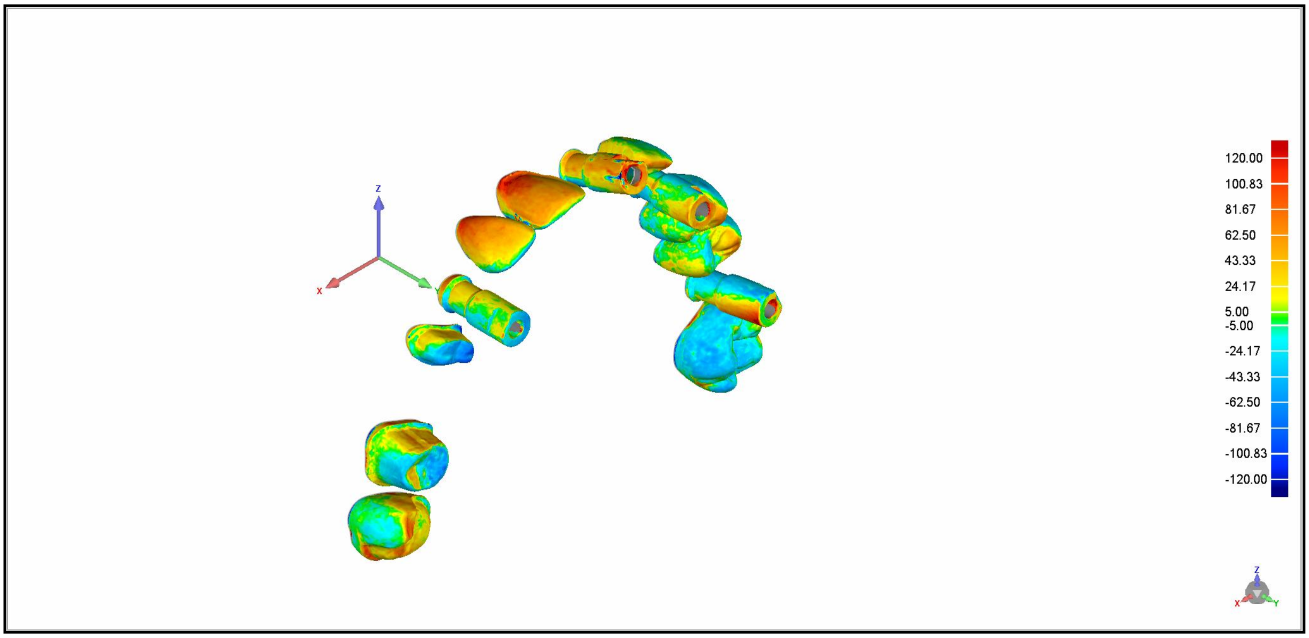

Predefinido: Frente

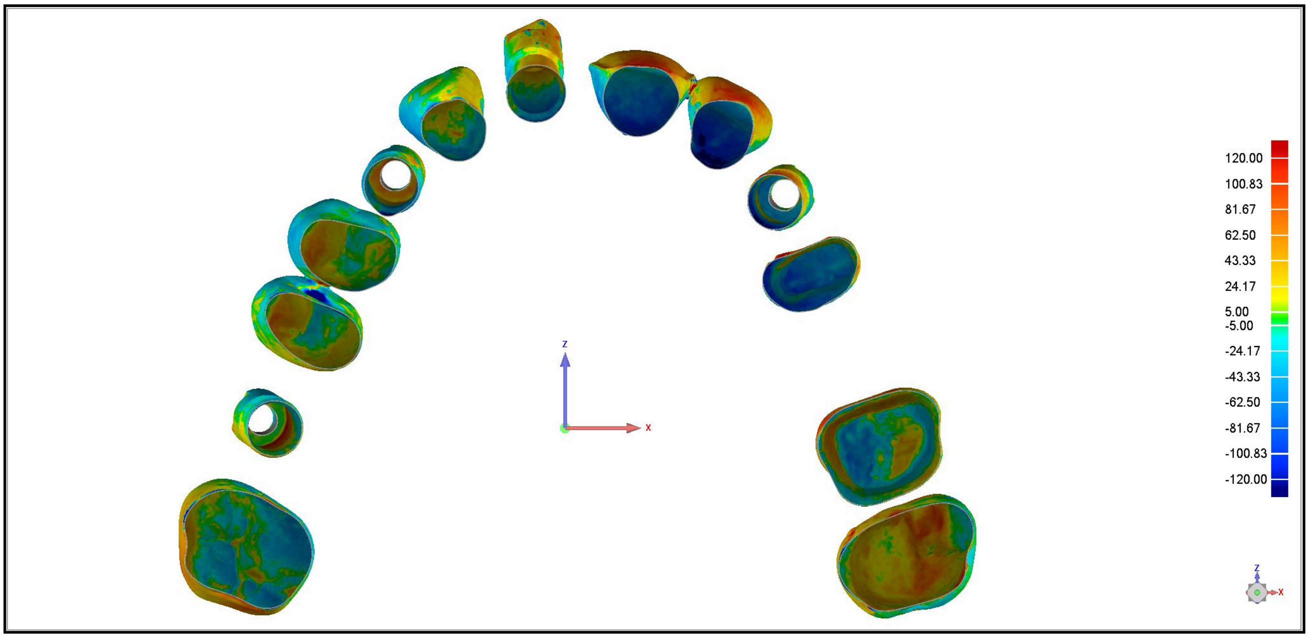

Predefinido: Atrás

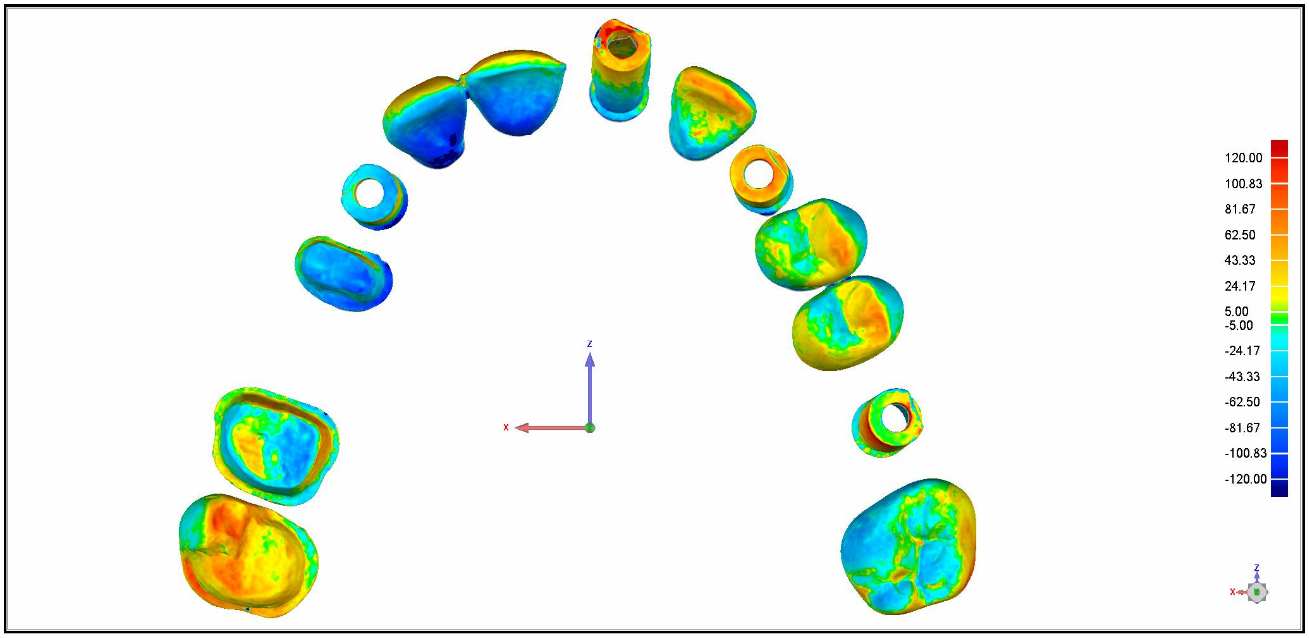

Predefinido: Izquierda

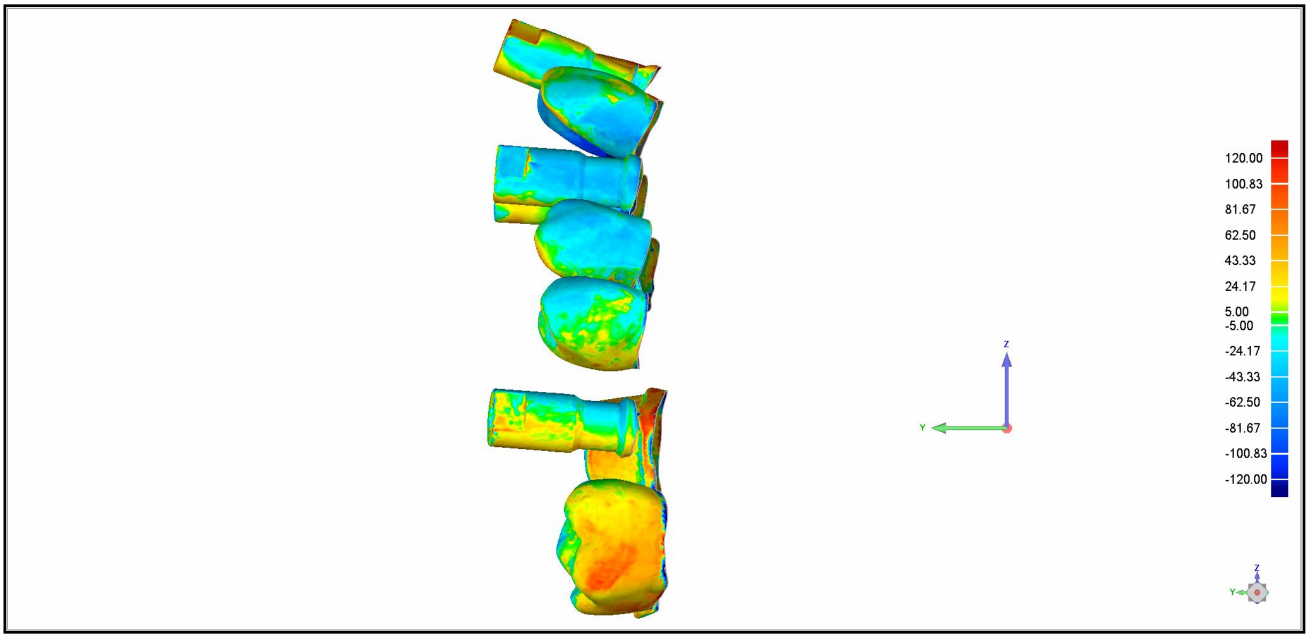

Predefinido: Derecha

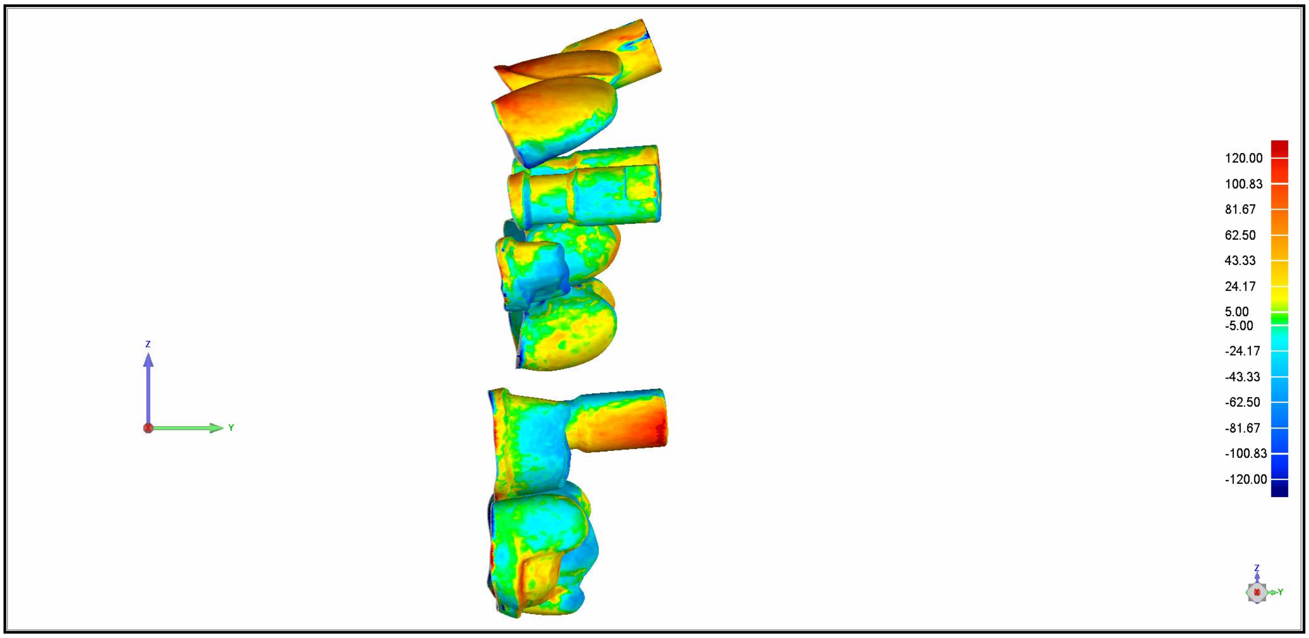

Predefinido: Superior

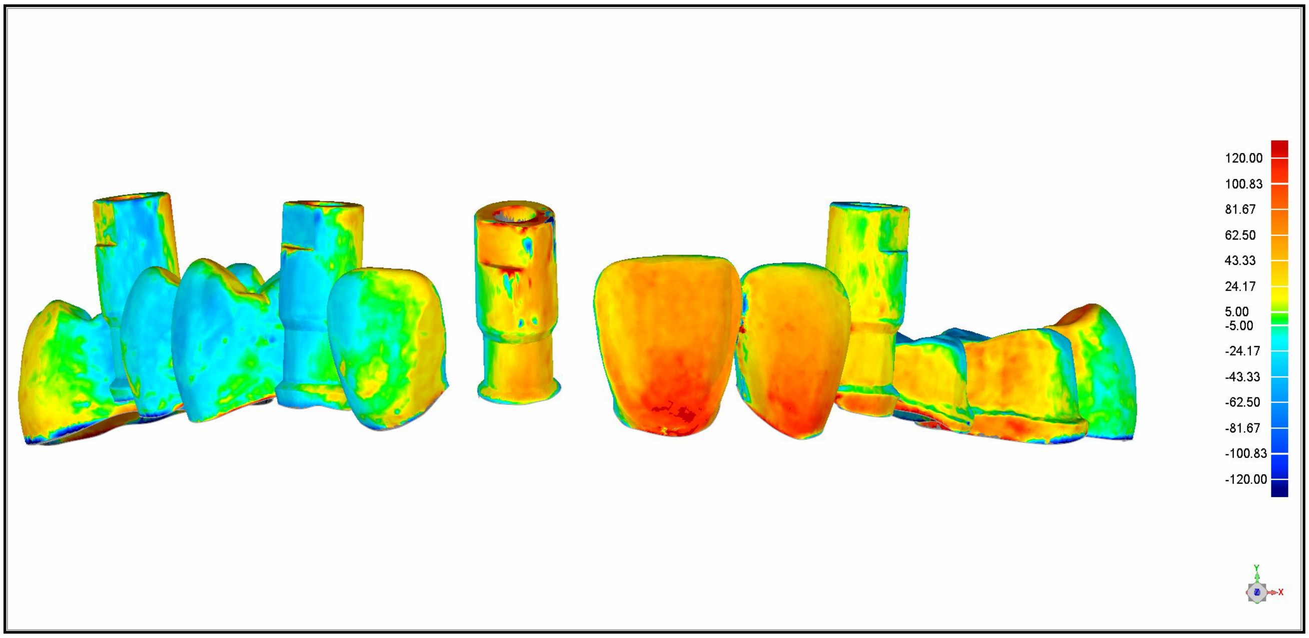

Predefinido: Inferior

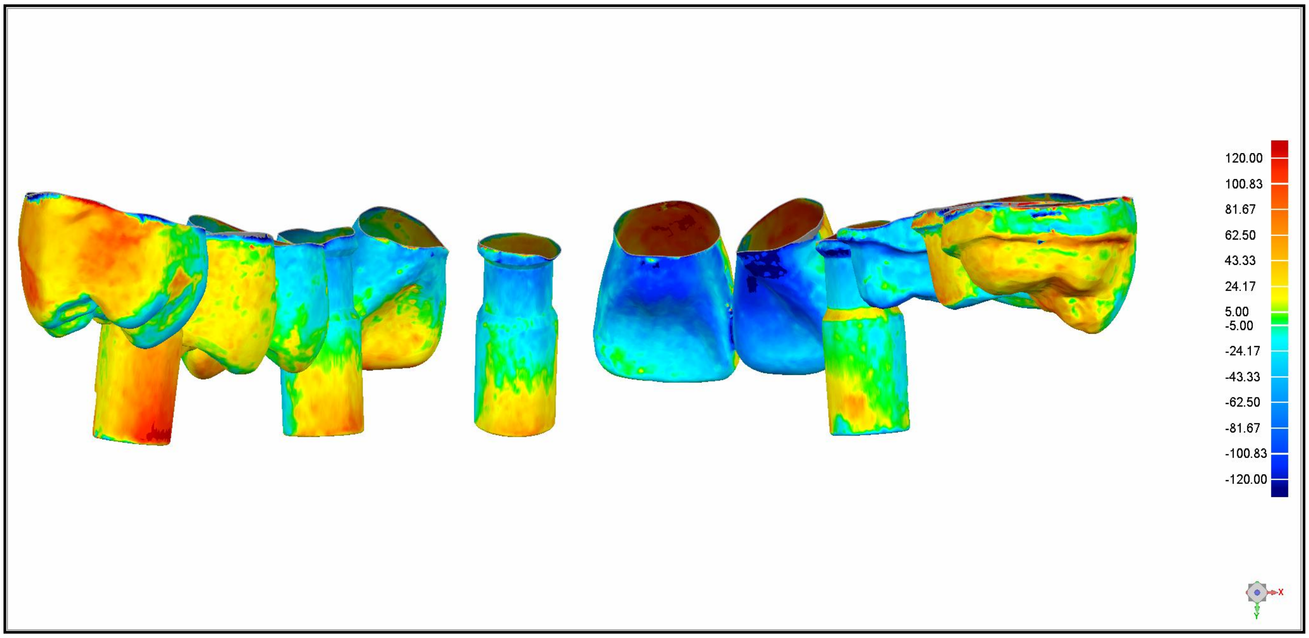

Ajuste de ubicación: Desviaciones superior e inferior

Unidades: u

Nombre	Desv	Estado	Superior Tol	Inferior Tol	Ref X	Ref Y	Ref Z	Radio	Desv X	Desv Y	Desv Z	Medido X	Medido Y	Medido Z	Dir. proy. X	Dir. proy. Y	Dir. proy. Z
Desv. inferior	-2268.21				-16597.07	29132.89	5701.00	n/a	-2063.82	168.49	-925.74	-18660.90	29301.39	4775.26	0.91	-0.07	0.41
Desv. superior	2503.71				25491.12	27467.20	-5778.68	n/a	2464.59	144.62	-416.45	27955.71	27611.82	-6195.13	0.98	0.06	-0.17
