# Supplementary material for: Accuracy of four digital scanners according to scanning strategy in complete-arch impressions
Source: PLoS One. 2018 Sep 13;13(9):e0202916. doi: 10.1371/journal.pone.0202916 (PMC6136706; doi:10.1371/journal.pone.0202916)
Supplement: S13 Table — True definition (scanning strategy A). (ZIP) [file pone.0202916.s013.zip › S13/TD7A.pdf]

3D Comparación Resultados

Modelo referencia	MRC
Modelo test	TD7A
Nº de puntos de datos	130011
# Aislados	324

Tipo tolerancia	3D desviación
Unidades	u
Máx. crítico	120.00
Máx. nominal	28.00
Mín. nominal	-28.00
Mín. crítico	-120.00

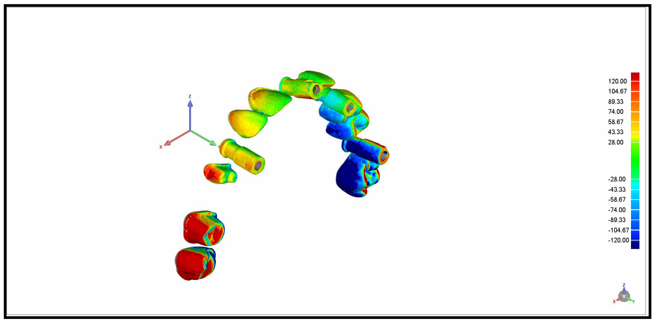

Desviación	
Desviación superior máx.	2137.69
Desviación inferior máx.	-3087.47
Desviación media	93.53 /-85.24
Desviación estándar	145.95

Distribución desviación

>=Min	<Max	# Puntos	%
-120.00	-104.67	1919	1.48
-104.67	-89.33	2668	2.05
-89.33	-74.00	3467	2.67
-74.00	-58.67	3730	2.87
-58.67	-43.33	4532	3.49
-43.33	-28.00	5772	4.44
-28.00	28.00	37162	28.58
28.00	43.33	12450	9.58
43.33	58.67	10524	8.09
58.67	74.00	7601	5.85
74.00	89.33	4549	3.50
89.33	104.67	2923	2.25
104.67	120.00	2584	1.99

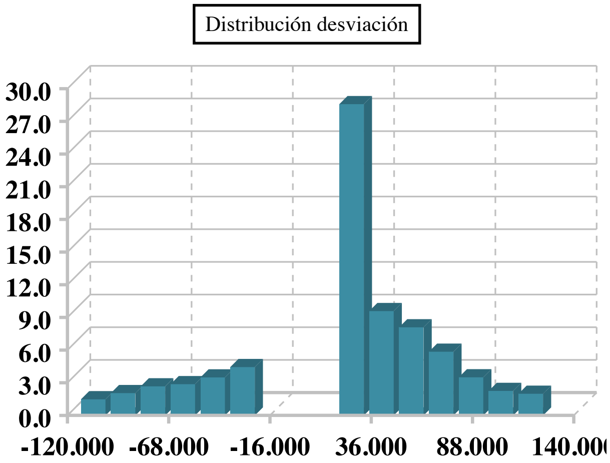

Fuera del crítico superior	19057	14.66
Fuera del crítico inferior	11073	8.52

Desviaciones estándar

Distribución (+/-)	# Puntos	%
-6 * Desv. estánd.	117	0.09
-5 * Desv. estánd.	52	0.04
-4 * Desv. estánd.	309	0.24
-3 * Desv. estánd.	1897	1.46
-2 * Desv. estánd.	9009	6.93
-1 * Desv. estánd.	59665	45.89
1 * Desv. estánd.	45746	35.19
2 * Desv. estánd.	9925	7.63
3 * Desv. estánd.	2635	2.03
4 * Desv. estánd.	157	0.12
5 * Desv. estánd.	86	0.07
6 * Desv. estánd.	413	0.32

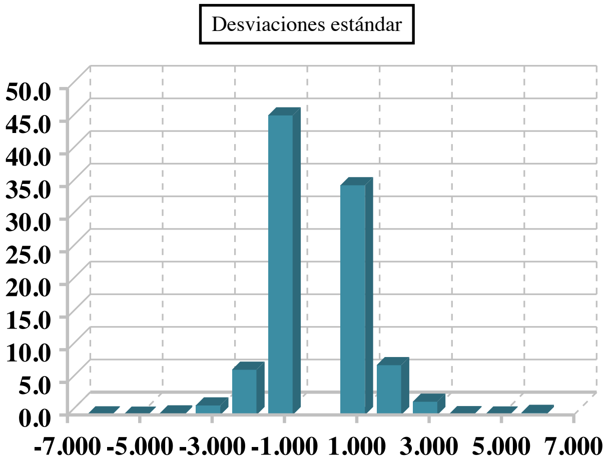

Predefinido: Isométrico

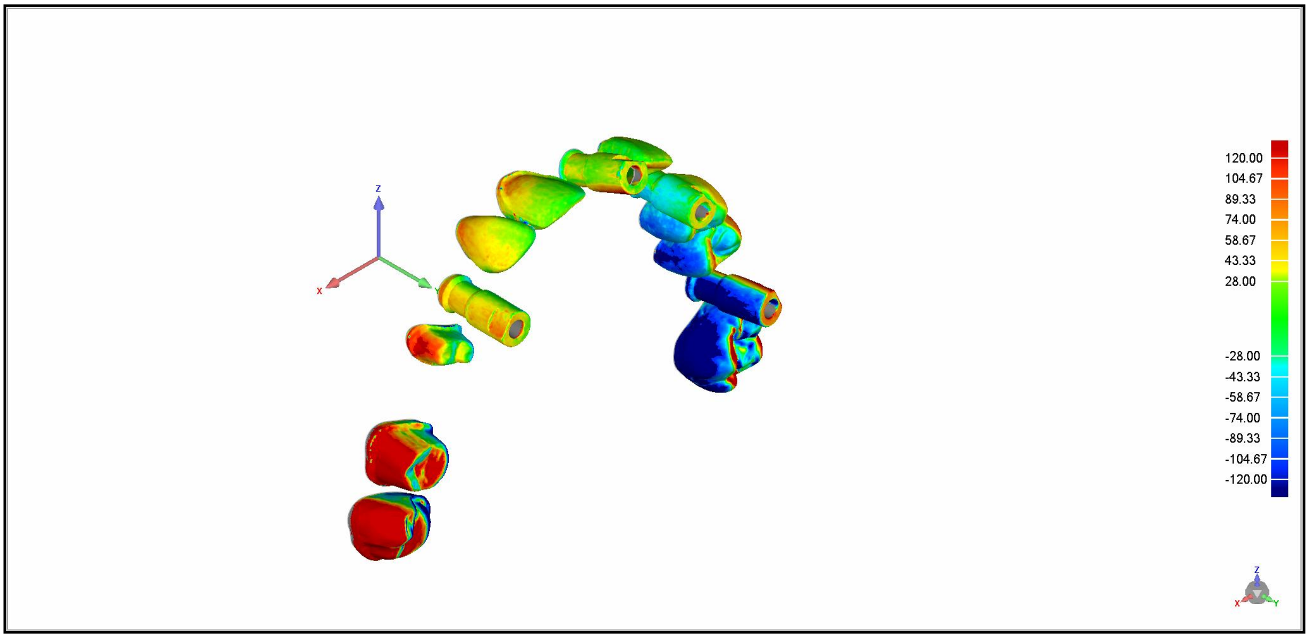

Predefinido: Frente

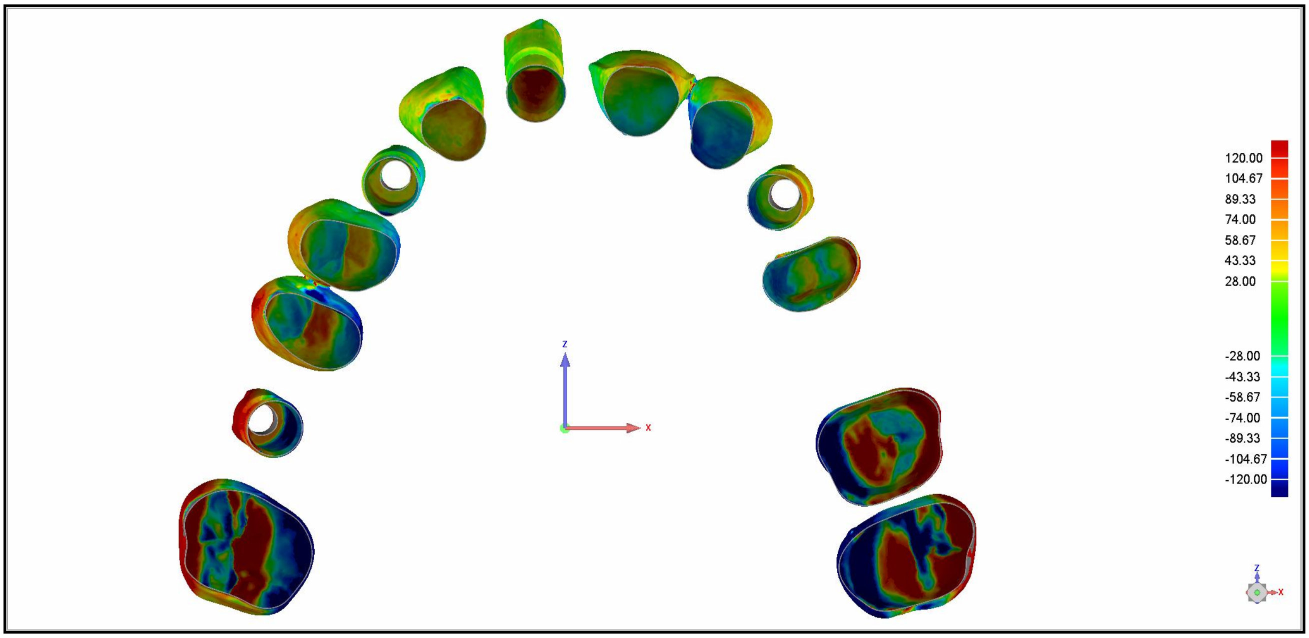

Predefinido: Atrás

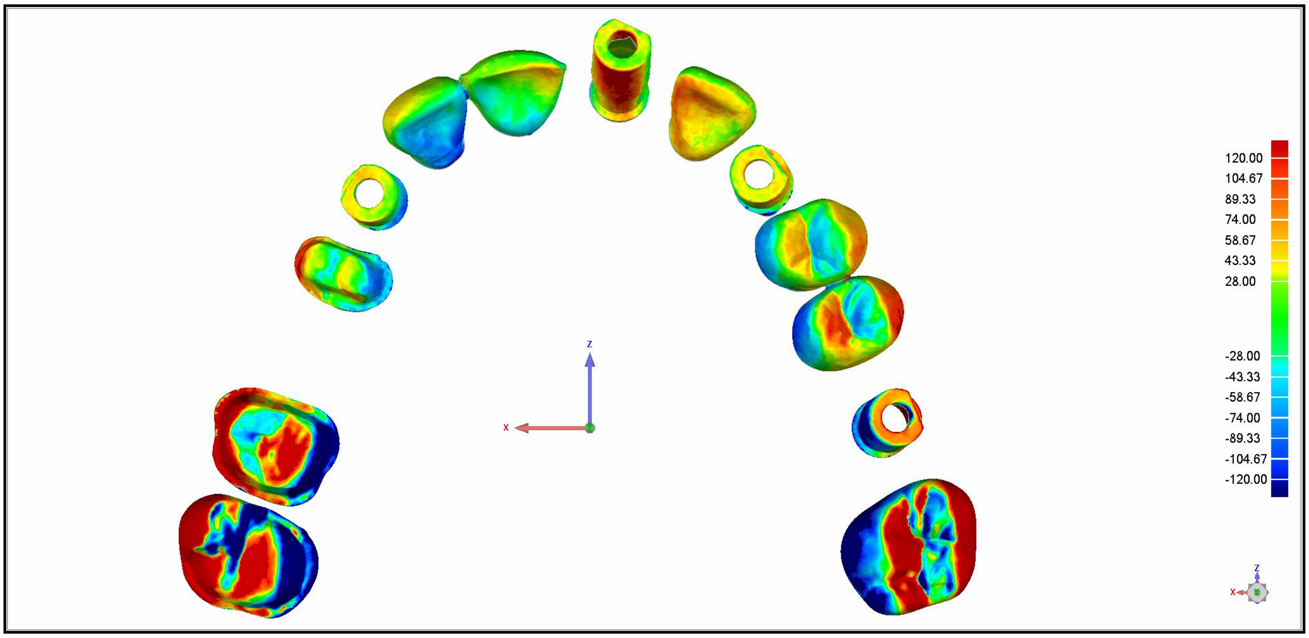

Predefinido: Izquierda

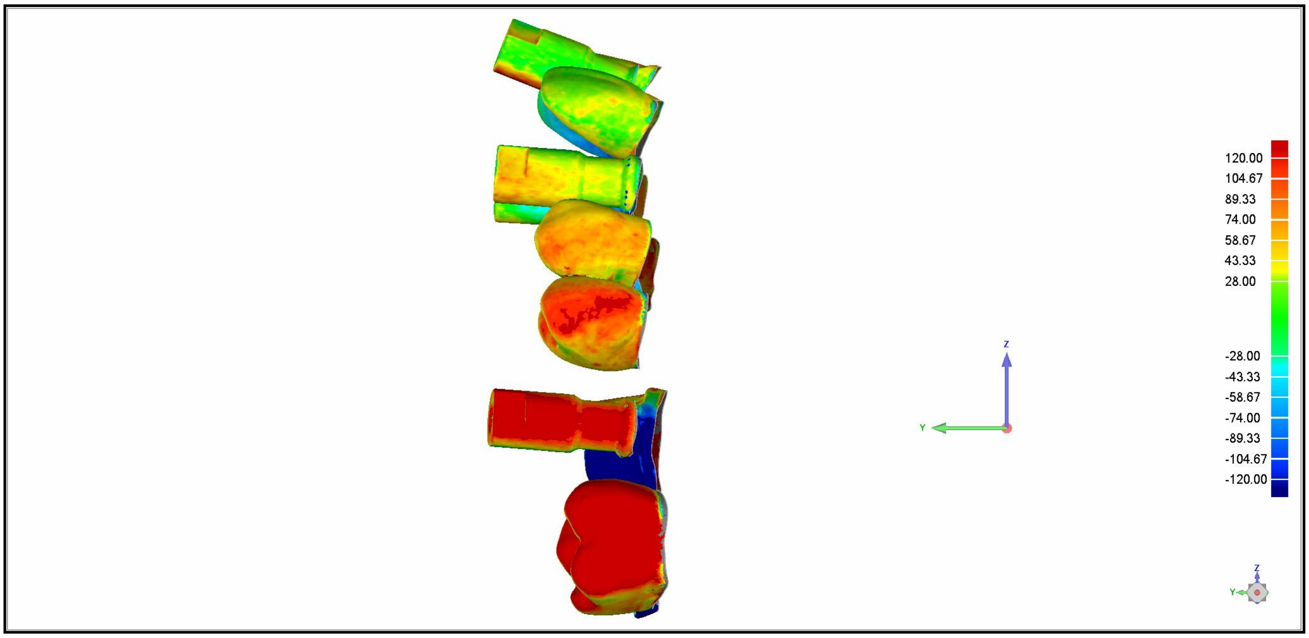

Predefinido: Derecha

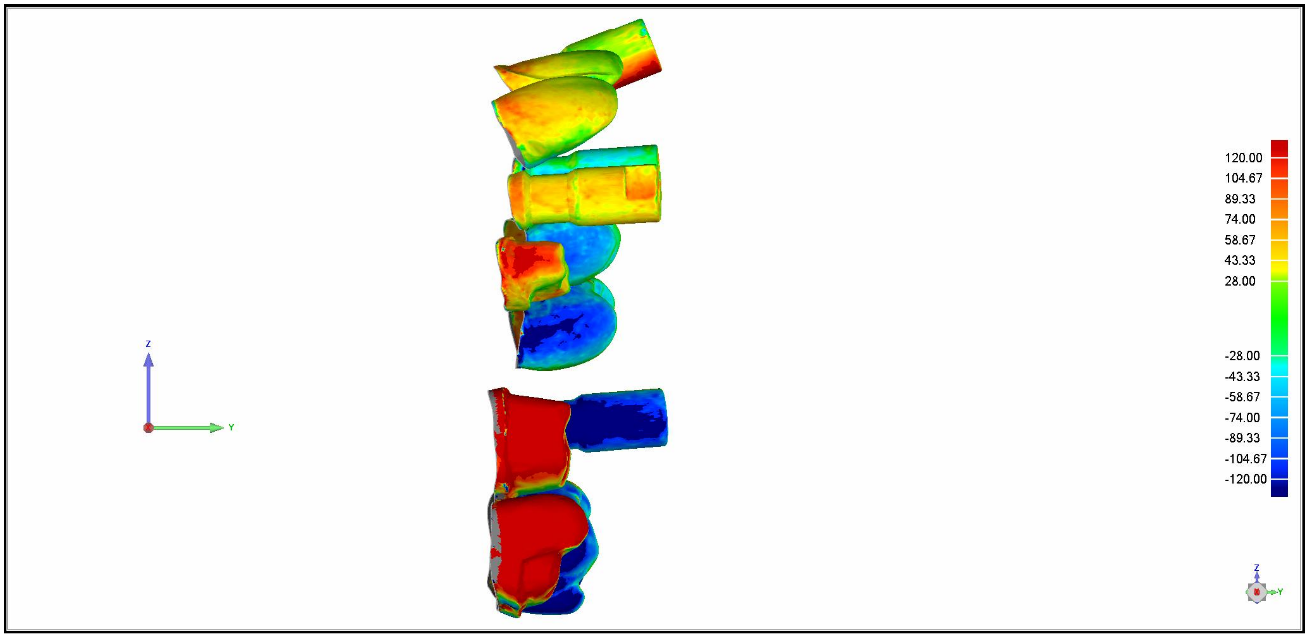

Predefinido: Superior

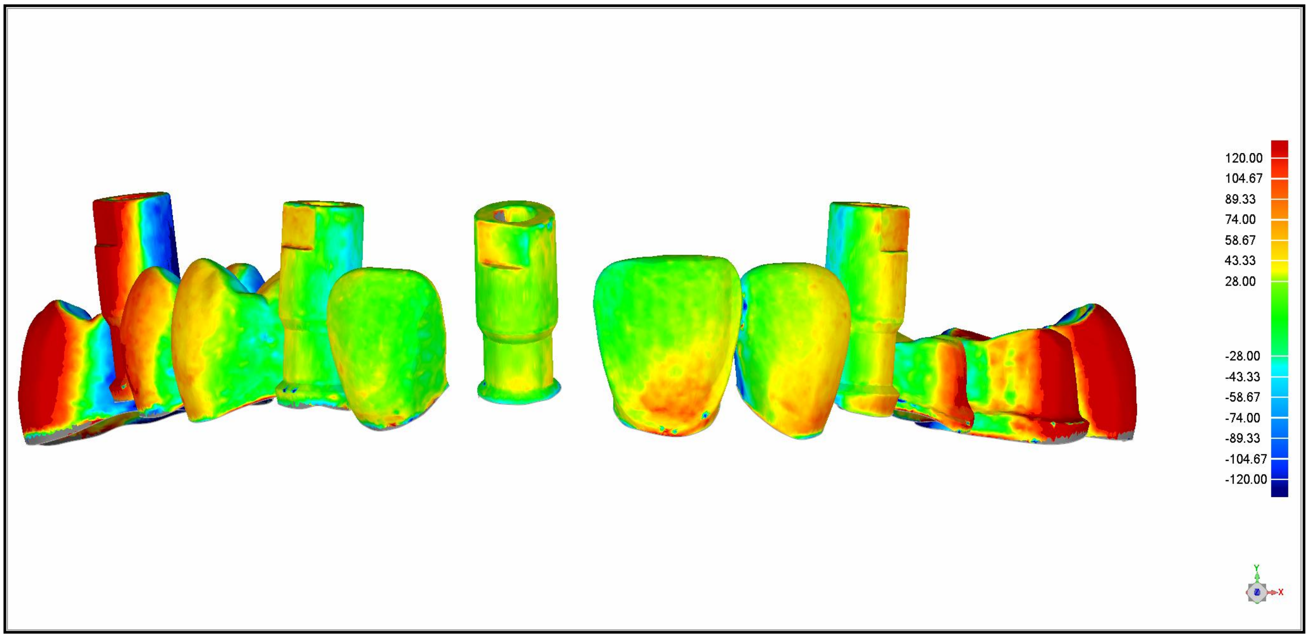

Predefinido: Inferior

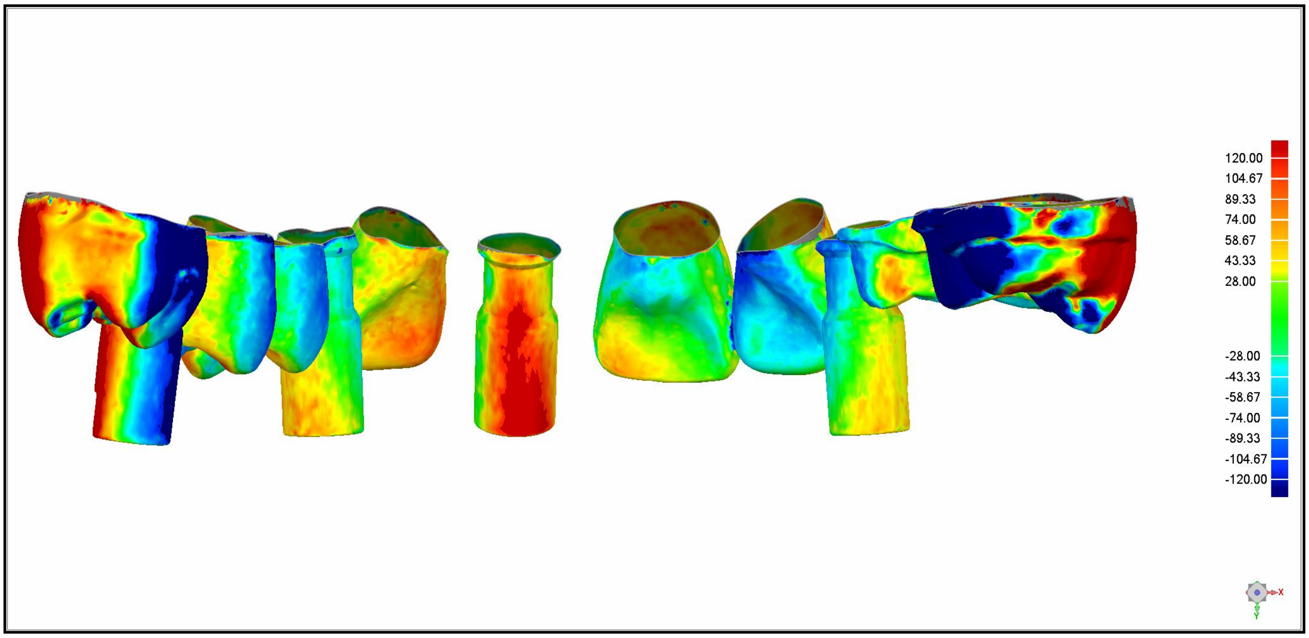

Ajuste de ubicación: Desviaciones superior e inferior

Unidades: u

Nombre	Desv	Estado	Superior Tol	Inferior Tol	Ref X	Ref Y	Ref Z	Radio	Desv X	Desv Y	Desv Z	Medido X	Medido Y	Medido Z	Dir. proy. X	Dir. proy. Y	Dir. proy. Z
Desv. inferior	-3087.47				32061.00	27268.70	-9389.57	n/a	-556.97	310.39	-3020.91	31504.03	27579.10	-12410.47	0.18	-0.10	0.98
Desv. superior	2137.69				16660.54	38373.51	19471.23	n/a	651.89	-2029.59	-159.84	17312.42	36343.93	19311.39	0.30	-0.95	-0.07
