# Supplementary material for: Accuracy of four digital scanners according to scanning strategy in complete-arch impressions
Source: PLoS One. 2018 Sep 13;13(9):e0202916. doi: 10.1371/journal.pone.0202916 (PMC6136706; doi:10.1371/journal.pone.0202916)
Supplement: S13 Table — True definition (scanning strategy A). (ZIP) [file pone.0202916.s013.zip › S13/TD10A.pdf]

3D Comparación Resultados

Modelo referencia	MRC
Modelo test	TD10A
Nº de puntos de datos	129082
# Aislados	371

Tipo tolerancia	3D desviación
Unidades	u
Máx. crítico	120.00
Máx. nominal	11.00
Mín. nominal	-11.00
Mín. crítico	-120.00

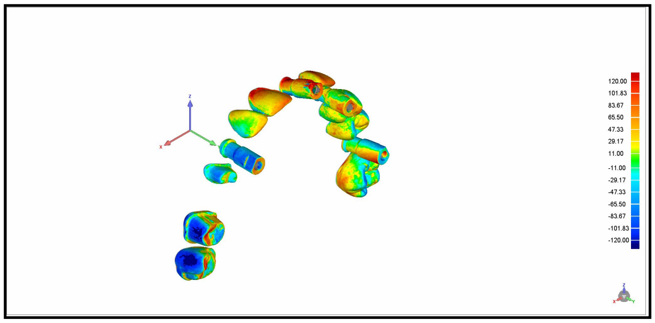

Desviación	
Desviación superior máx.	2763.99
Desviación inferior máx.	-2509.23
Desviación media	49.80 /-38.07
Desviación estándar	77.36

Distribución desviación

>=Min	<Max	# Puntos	%
-120.00	-101.83	1665	1.29
-101.83	-83.67	2531	1.96
-83.67	-65.50	4483	3.47
-65.50	-47.33	6184	4.79
-47.33	-29.17	9783	7.58
-29.17	-11.00	16252	12.59
-11.00	11.00	27660	21.43
11.00	29.17	18748	14.52
29.17	47.33	12407	9.61
47.33	65.50	8561	6.63
65.50	83.67	6677	5.17
83.67	101.83	4322	3.35
101.83	120.00	2947	2.28

Fuera del crítico superior	5302	4.11
Fuera del crítico inferior	1560	1.21

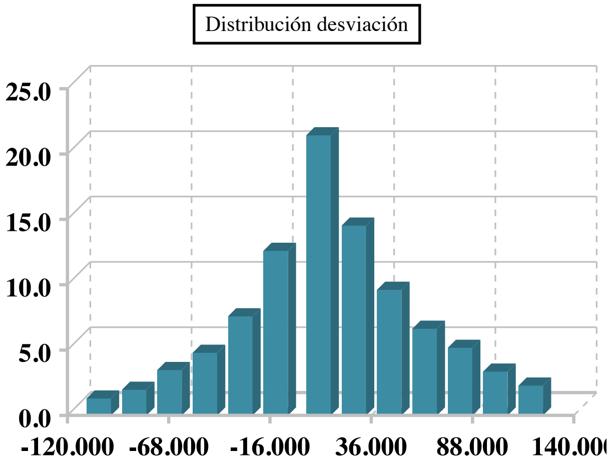

Desviaciones estándar

Distribución (+/-)	# Puntos	%
-6 * Desv. estánd.	80	0.06
-5 * Desv. estánd.	39	0.03
-4 * Desv. estánd.	125	0.10
-3 * Desv. estánd.	348	0.27
-2 * Desv. estánd.	9549	7.40
-1 * Desv. estánd.	60558	46.91
1 * Desv. estánd.	47223	36.58
2 * Desv. estánd.	9888	7.66
3 * Desv. estánd.	727	0.56
4 * Desv. estánd.	169	0.13
5 * Desv. estánd.	110	0.09
6 * Desv. estánd.	266	0.21

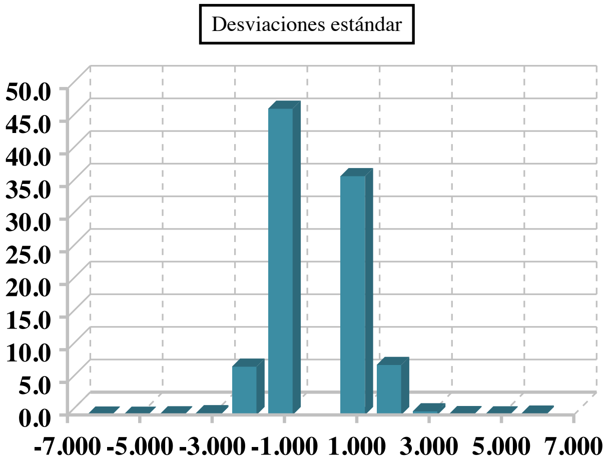

Predefinido: Isométrico

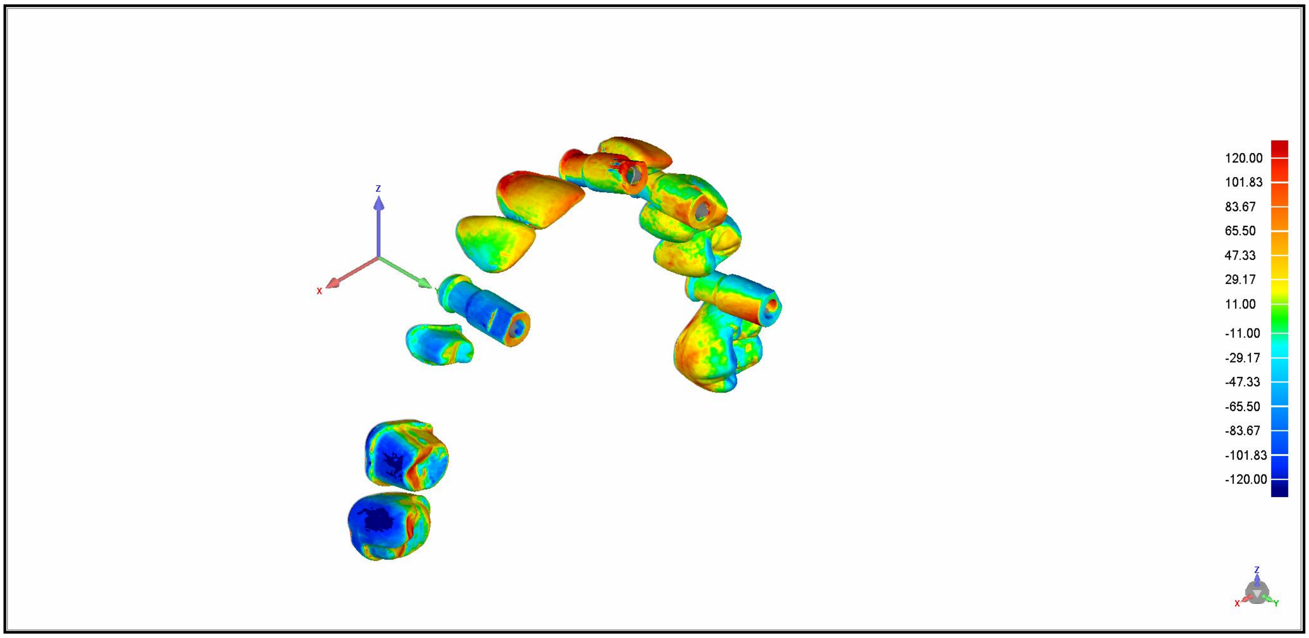

Predefinido: Frente

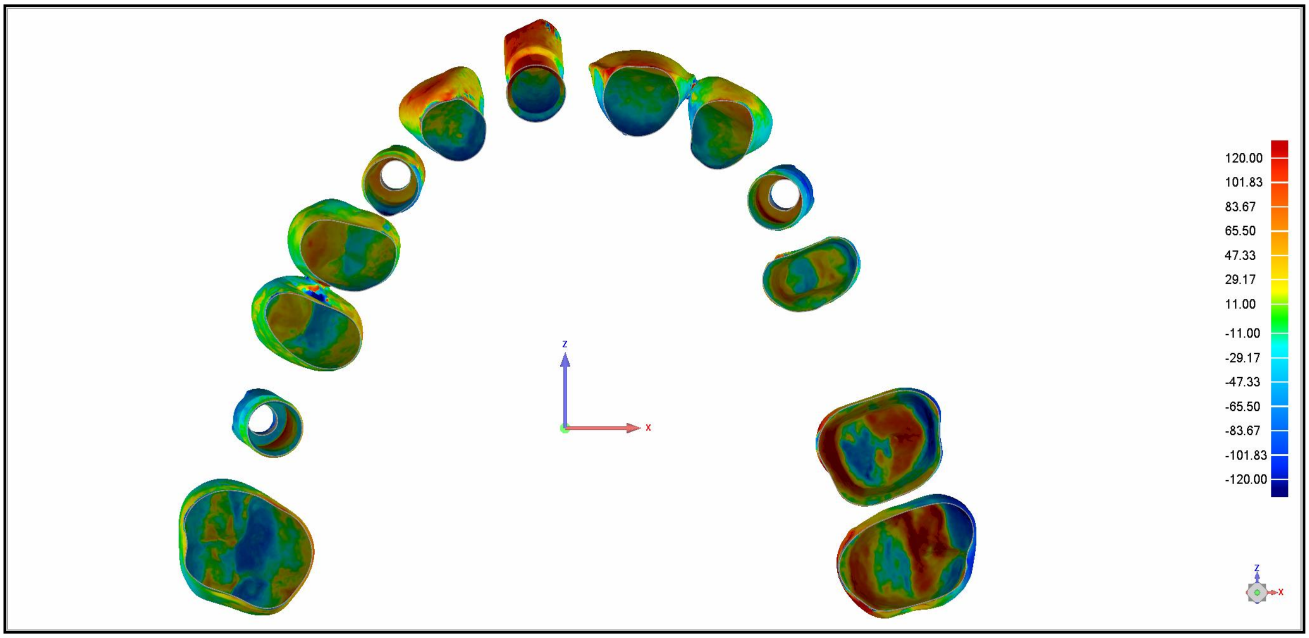

Predefinido: Atrás

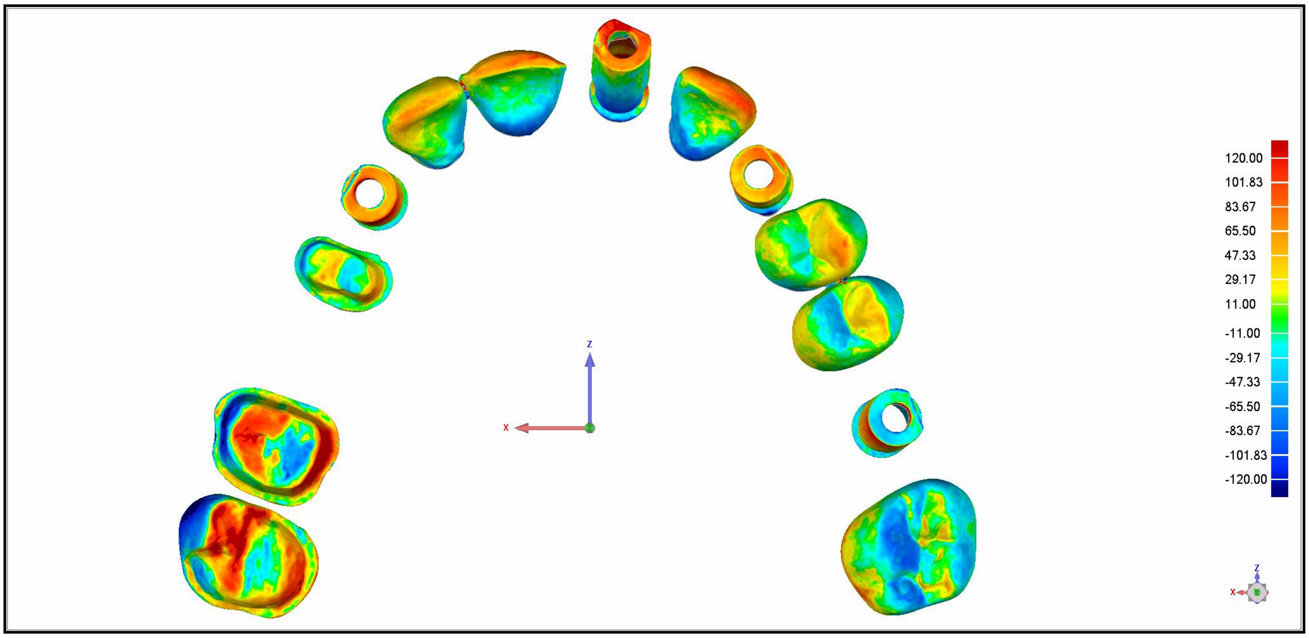

Predefinido: Izquierda

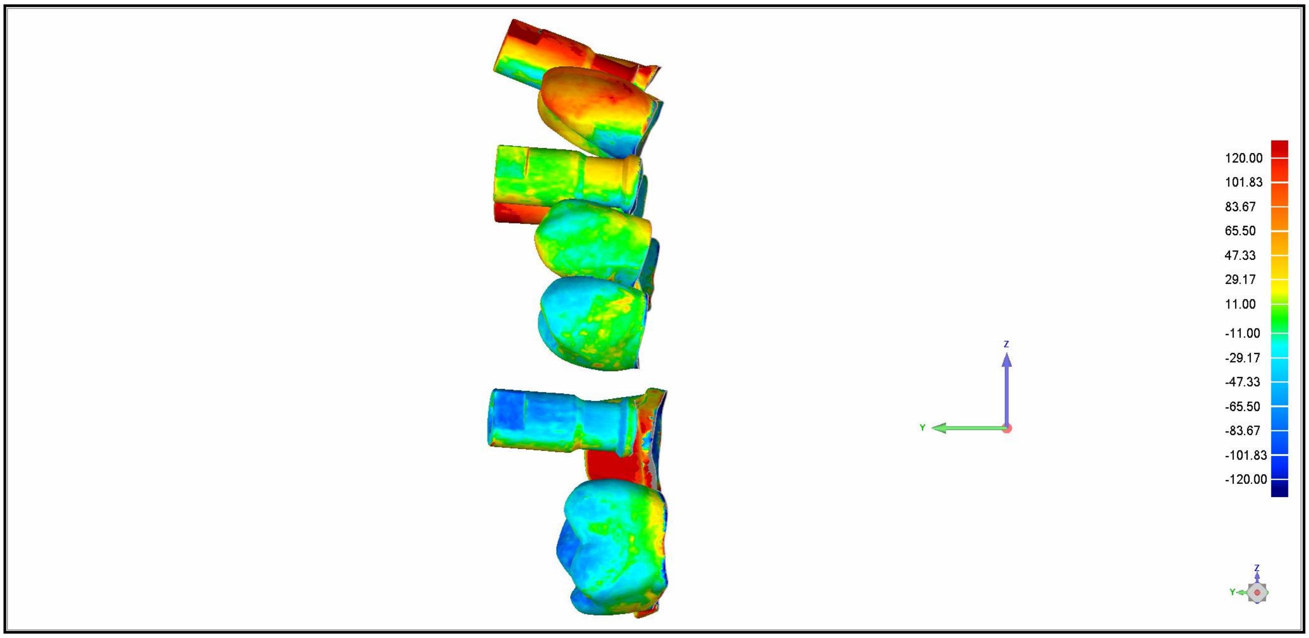

Predefinido: Derecha

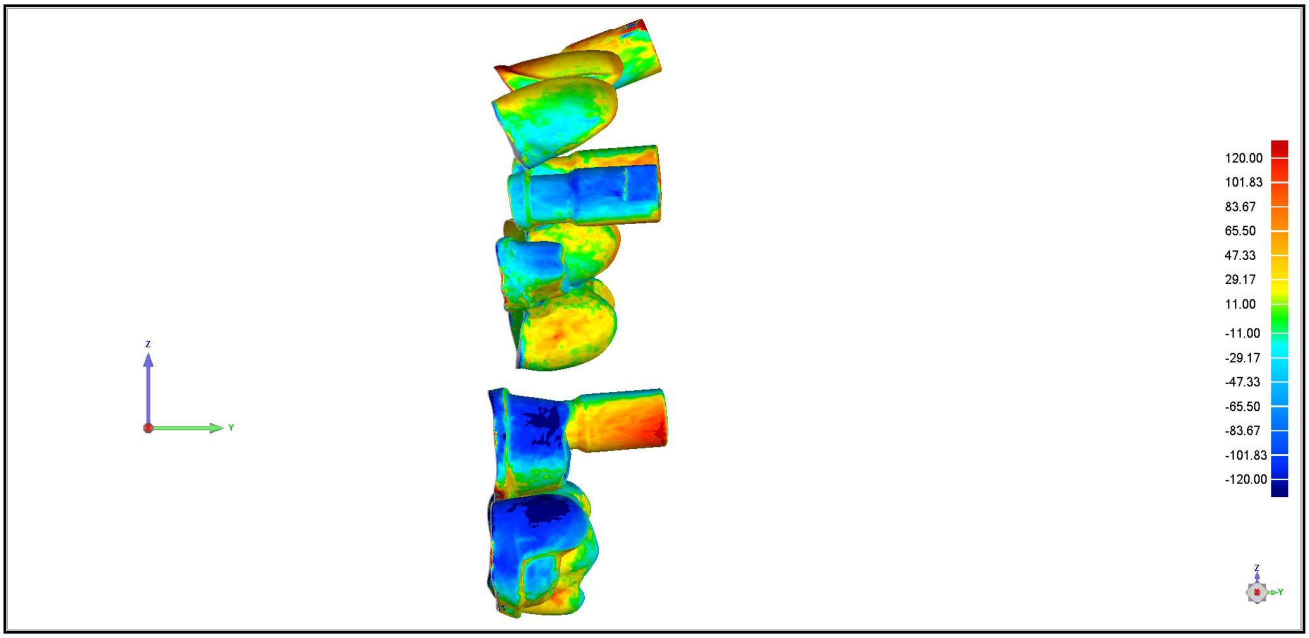

Predefinido: Superior

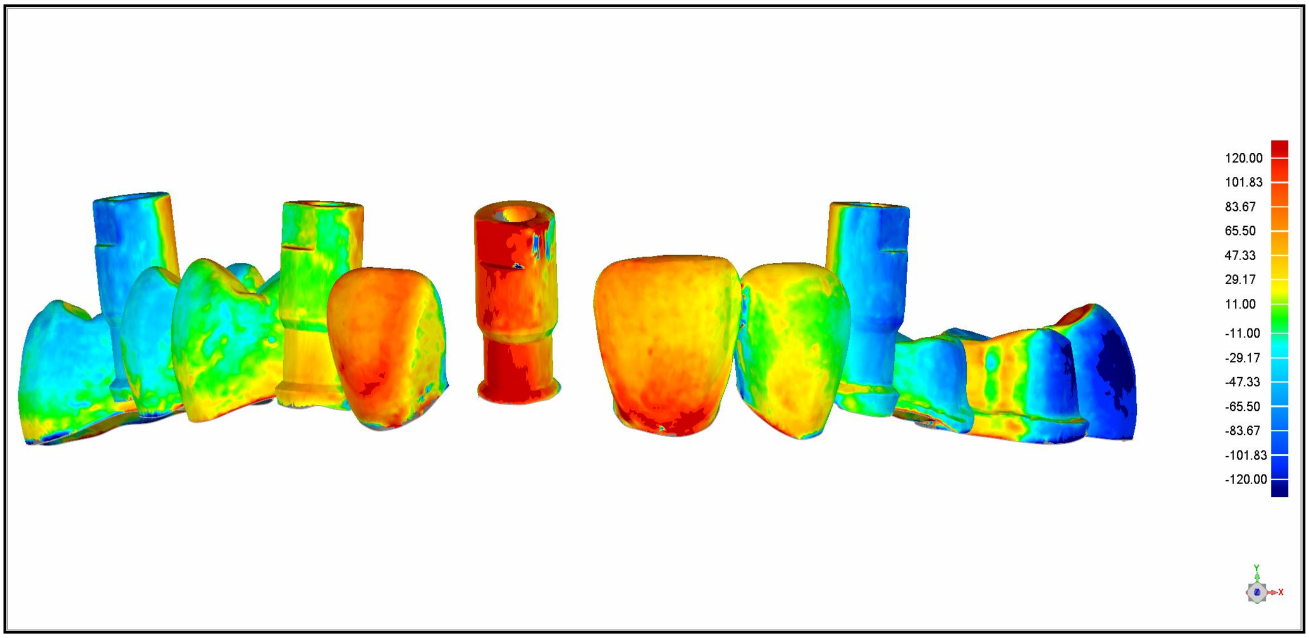

Predefinido: Inferior

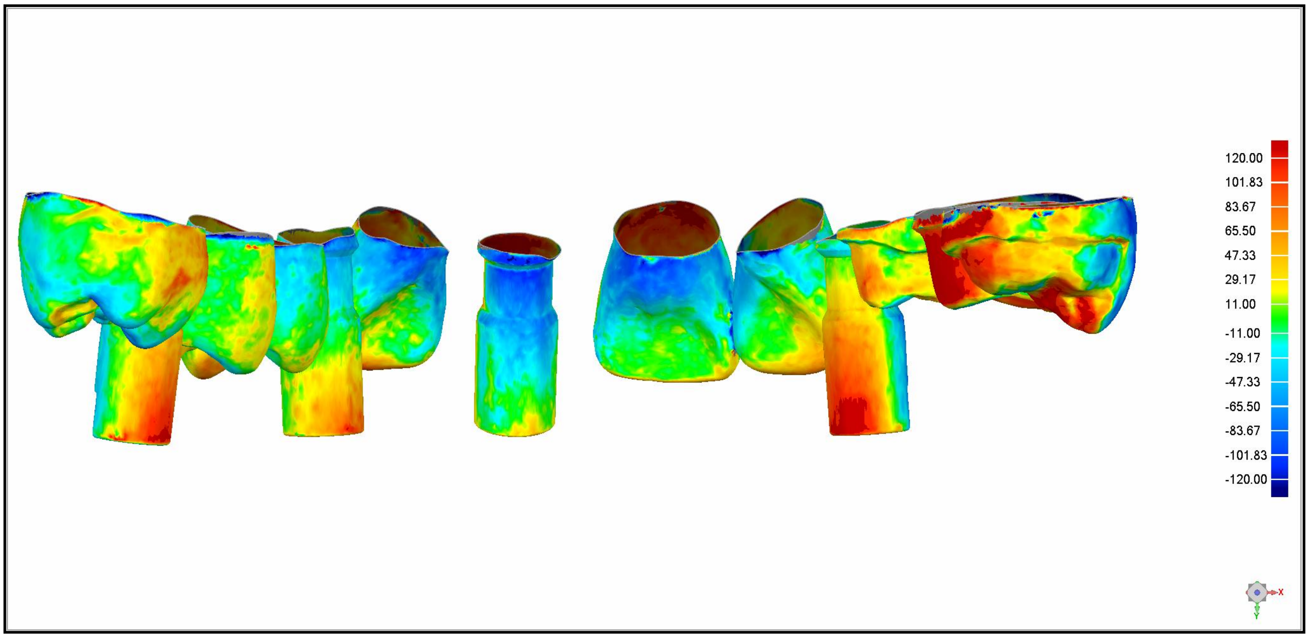

Ajuste de ubicación: Desviaciones superior e inferior

Unidades: u

Nombre	Desv	Estado	Superior Tol	Inferior Tol	Ref X	Ref Y	Ref Z	Radio	Desv X	Desv Y	Desv Z	Medido X	Medido Y	Medido Z	Dir. proy. X	Dir. proy. Y	Dir. proy. Z
Desv. inferior	-2509.23				-2424.02	36916.12	31336.11	n/a	1003.97	186.67	-2292.04	-1420.05	37102.79	29044.07	-0.40	-0.07	0.91
Desv. superior	2763.99				16989.02	37628.06	17251.36	n/a	2612.64	-58.25	900.20	19601.65	37569.81	18151.56	0.95	-0.02	0.33
