# Supplementary material for: Accuracy of four digital scanners according to scanning strategy in complete-arch impressions
Source: PLoS One. 2018 Sep 13;13(9):e0202916. doi: 10.1371/journal.pone.0202916 (PMC6136706; doi:10.1371/journal.pone.0202916)
Supplement: S13 Table — True definition (scanning strategy A). (ZIP) [file pone.0202916.s013.zip › S13/TD6A.pdf]

Geomagic Control™

Fecha: 3/6/2017, 3:44 pm

3D Comparación Resultados

Modelo referencia	MRC
Modelo test	TD6A
Nº de puntos de datos	130600
# Aislados	396

Tipo tolerancia	3D desviación
Unidades	u
Máx. crítico	120.00
Máx. nominal	9.00
Mín. nominal	-9.00
Mín. crítico	-120.00

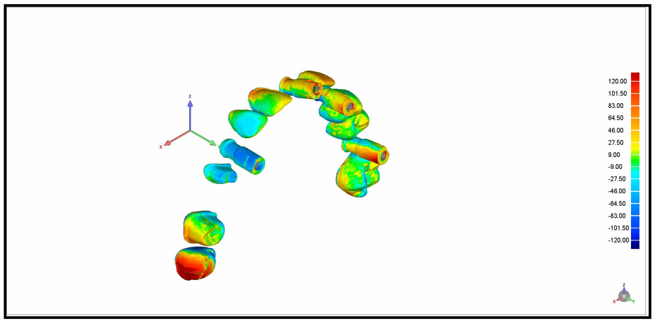

Desviación	
Desviación superior máx.	1717.20
Desviación inferior máx.	-1517.69
Desviación media	40.59 /-30.44
Desviación estándar	64.89

Distribución desviación

>=Min	<Max	# Puntos	%
-120.00	-101.50	475	0.36
-101.50	-83.00	1164	0.89
-83.00	-64.50	3246	2.49
-64.50	-46.00	5783	4.43
-46.00	-27.50	11431	8.75
-27.50	-9.00	21069	16.13
-9.00	9.00	27244	20.86
9.00	27.50	23810	18.23
27.50	46.00	15396	11.79
46.00	64.50	8176	6.26
64.50	83.00	3844	2.94
83.00	101.50	2454	1.88
101.50	120.00	1913	1.46

Fuera del crítico superior	3505	2.68
Fuera del crítico inferior	1090	0.83

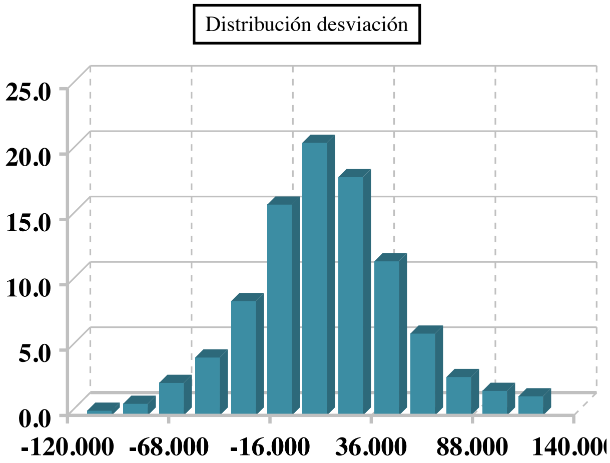

Desviaciones estándar

Distribución (+/-)	# Puntos	%
-6 * Desv. estánd.	98	0.08
-5 * Desv. estánd.	99	0.08
-4 * Desv. estánd.	246	0.19
-3 * Desv. estánd.	634	0.49
-2 * Desv. estánd.	7095	5.43
-1 * Desv. estánd.	63505	48.63
1 * Desv. estánd.	49499	37.90
2 * Desv. estánd.	7238	5.54
3 * Desv. estánd.	1472	1.13
4 * Desv. estánd.	208	0.16
5 * Desv. estánd.	102	0.08
6 * Desv. estánd.	404	0.31

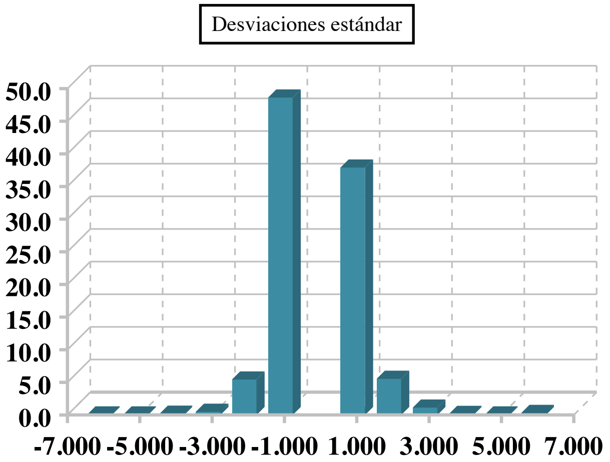

Predefinido: Isométrico

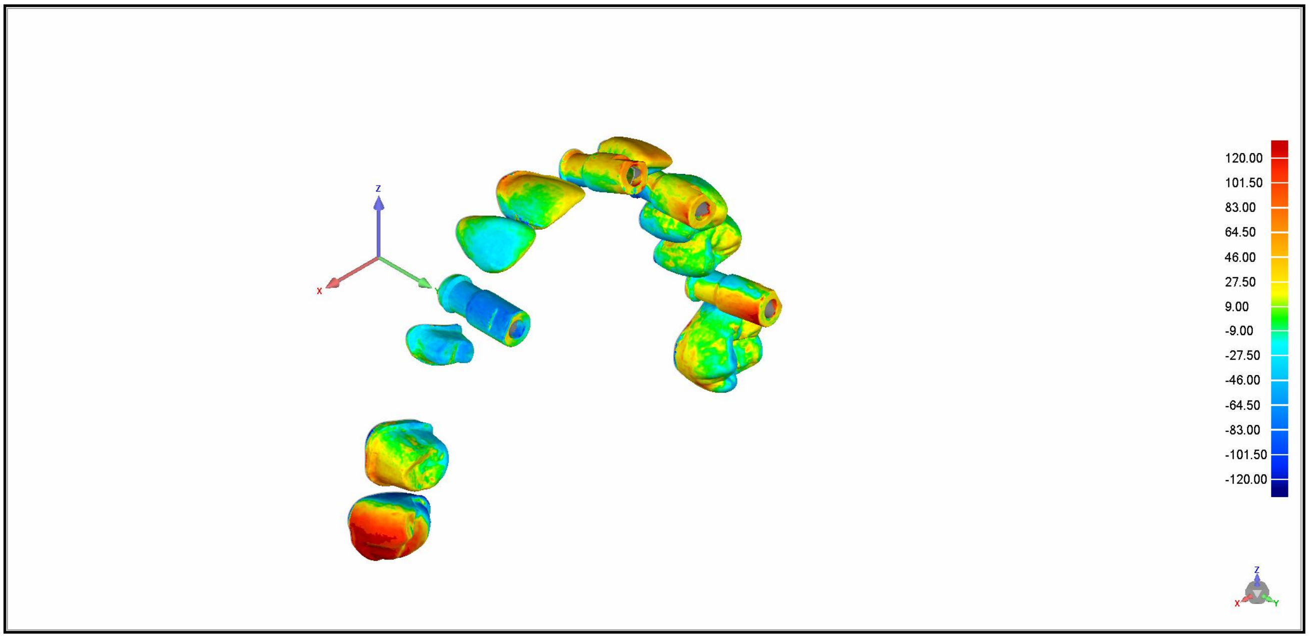

Predefinido: Frente

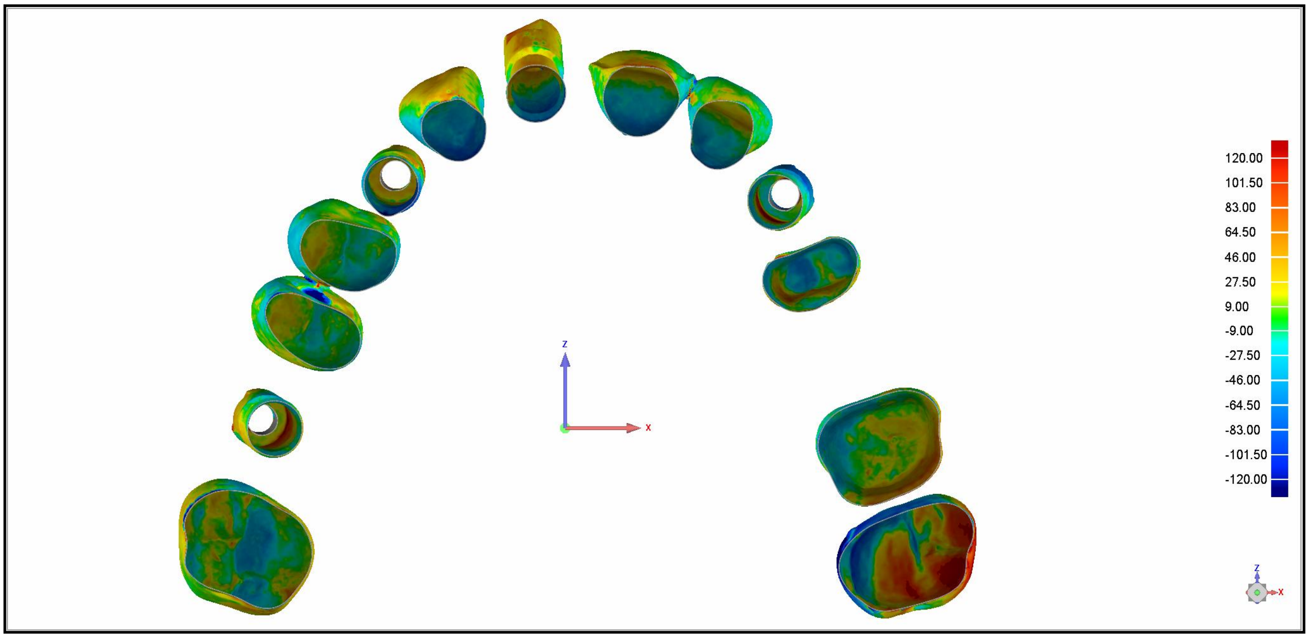

Predefinido: Atrás

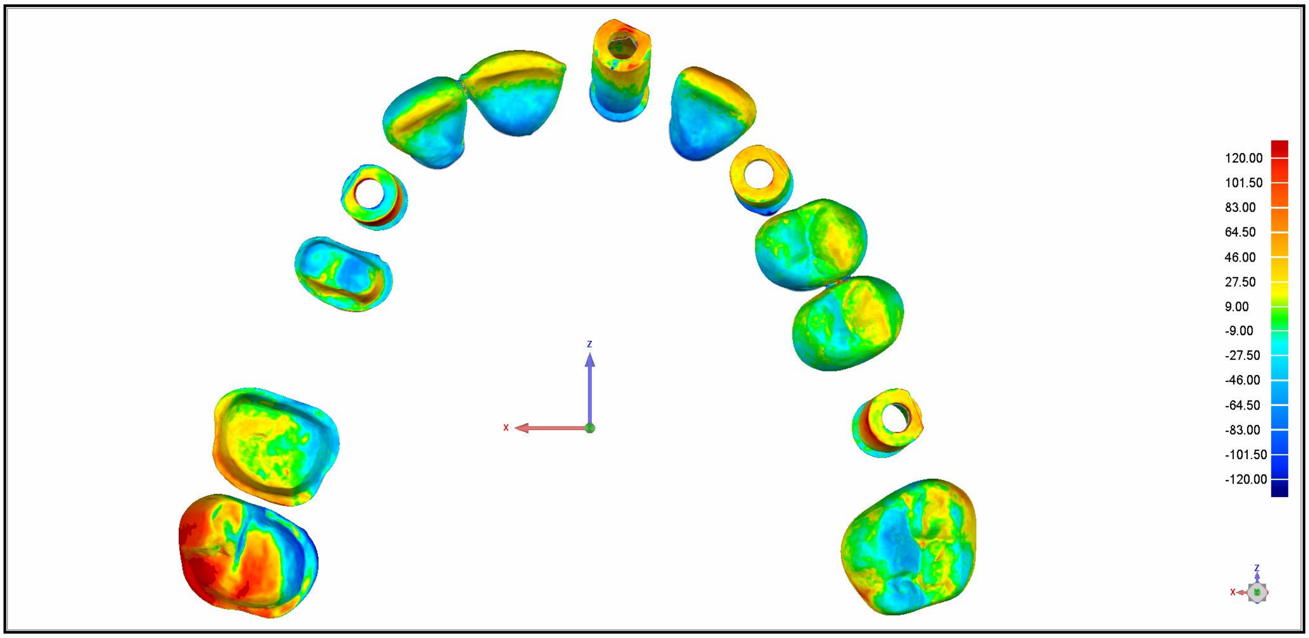

Predefinido: Izquierda

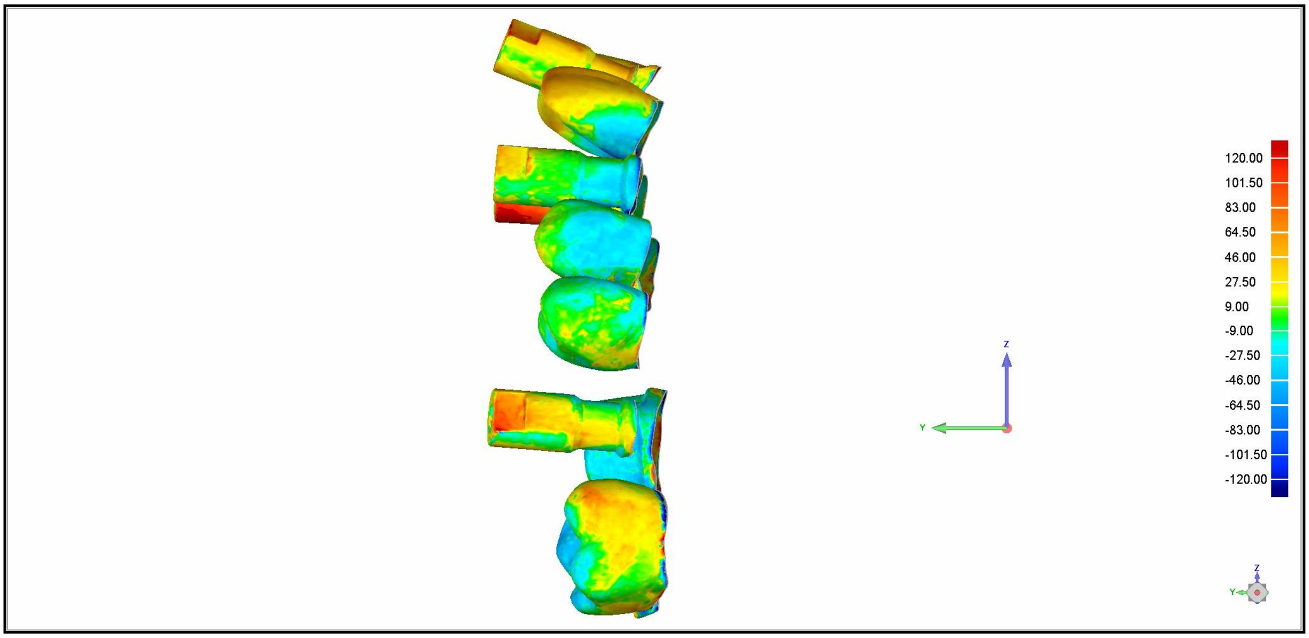

Predefinido: Derecha

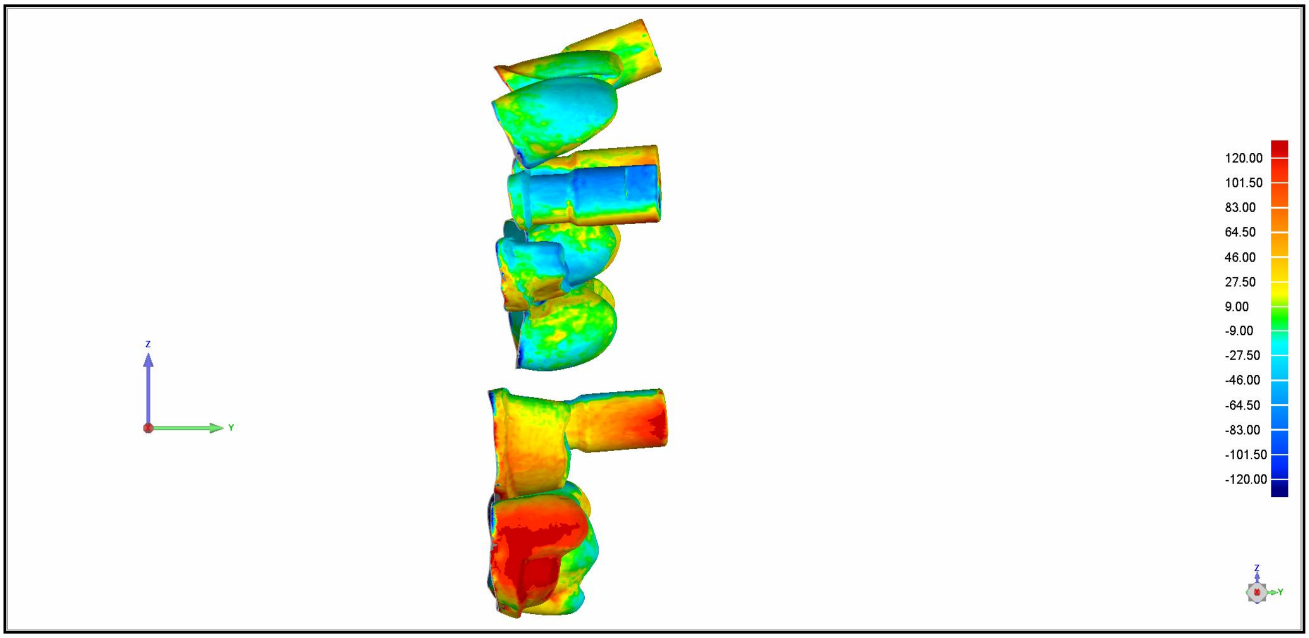

Predefinido: Superior

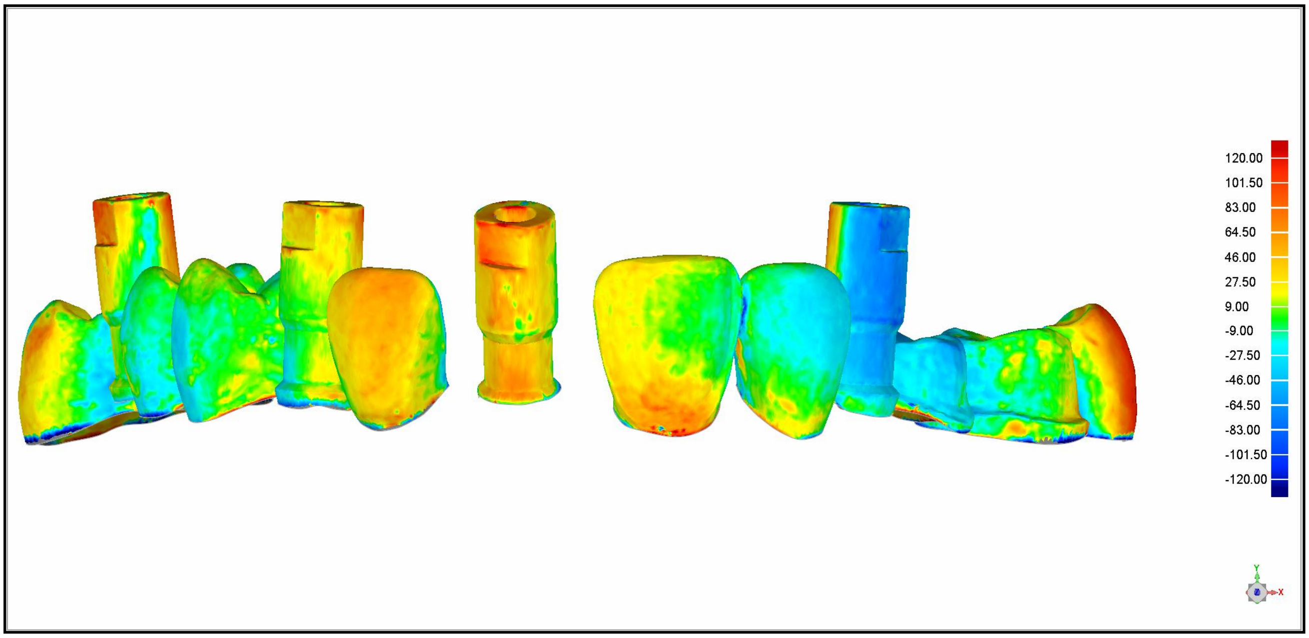

Predefinido: Inferior

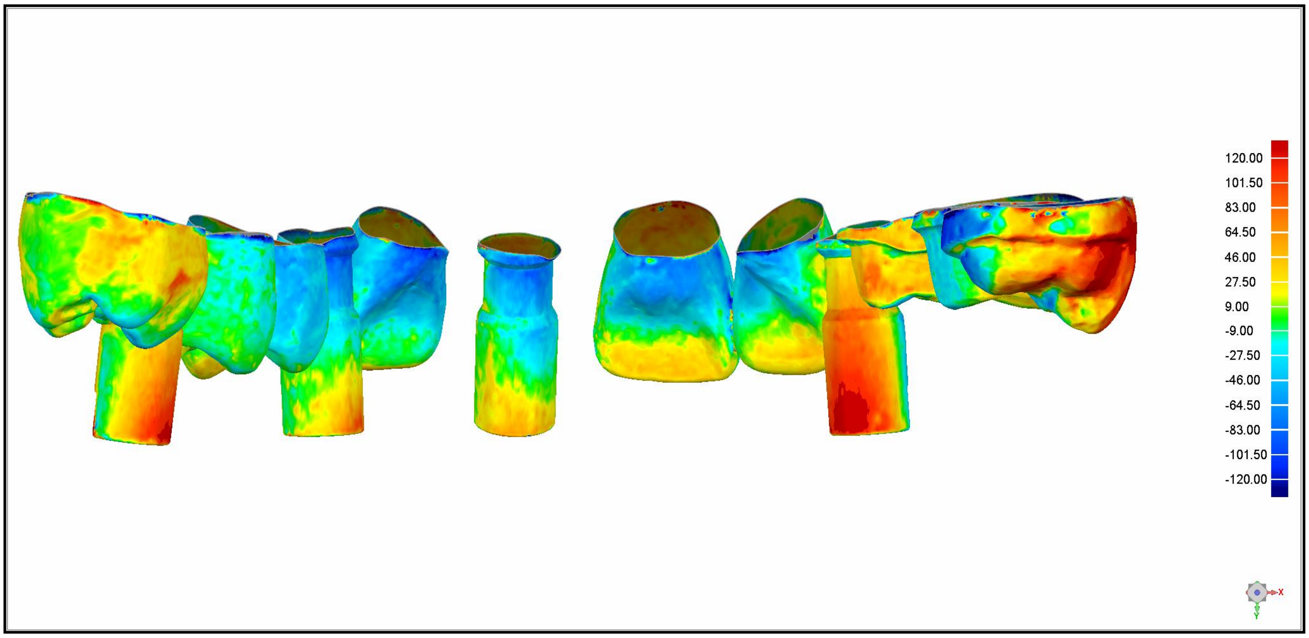

Ajuste de ubicación: Desviaciones superior e inferior

Unidades: u

Nombre	Desv	Estado	Superior Tol	Inferior Tol	Ref X	Ref Y	Ref Z	Radio	Desv X	Desv Y	Desv Z	Medido X	Medido Y	Medido Z	Dir. proy. X	Dir. proy. Y	Dir. proy. Z
Desv. inferior	-1517.69				32064.02	27122.94	-7196.92	n/a	-1249.59	63.30	859.02	30814.43	27186.23	-6337.89	0.82	-0.04	-0.57
Desv. superior	1717.20				16520.24	38298.02	19351.69	n/a	842.59	-1494.06	-81.34	17362.83	36803.96	19270.35	0.49	-0.87	-0.05
