# Supplementary material for: Accuracy of four digital scanners according to scanning strategy in complete-arch impressions
Source: PLoS One. 2018 Sep 13;13(9):e0202916. doi: 10.1371/journal.pone.0202916 (PMC6136706; doi:10.1371/journal.pone.0202916)
Supplement: S13 Table — True definition (scanning strategy A). (ZIP) [file pone.0202916.s013.zip › S13/TD1A.pdf]

3D Comparación Resultados

Modelo referencia	MRC
Modelo test	TD1A
Nº de puntos de datos	130778
# Aislados	284

Tipo tolerancia	3D desviación
Unidades	u
Máx. crítico	120.00
Máx. nominal	12.00
Mín. nominal	-12.00
Mín. crítico	-120.00

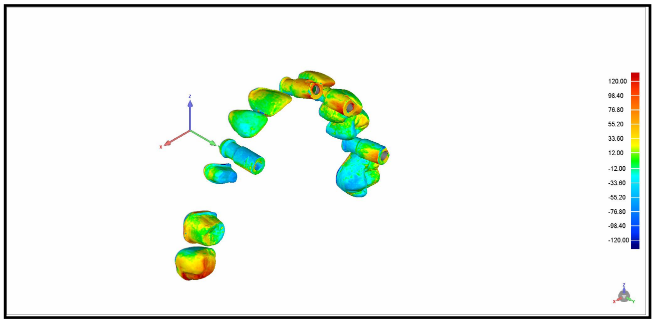

Desviación	
Desviación superior máx.	1985.89
Desviación inferior máx.	-2119.41
Desviación media	40.77 /-26.64
Desviación estándar	83.77

Distribución desviación

>=Min	<Max	# Puntos	%
-120.00	-98.40	298	0.23
-98.40	-76.80	676	0.52
-76.80	-55.20	2563	1.96
-55.20	-33.60	8464	6.47
-33.60	-12.00	22292	17.05
-12.00	12.00	40246	30.77
12.00	33.60	26571	20.32
33.60	55.20	14136	10.81
55.20	76.80	6811	5.21
76.80	98.40	3711	2.84
98.40	120.00	1964	1.50

Fuera del crítico superior	2138	1.63
Fuera del crítico inferior	908	0.69

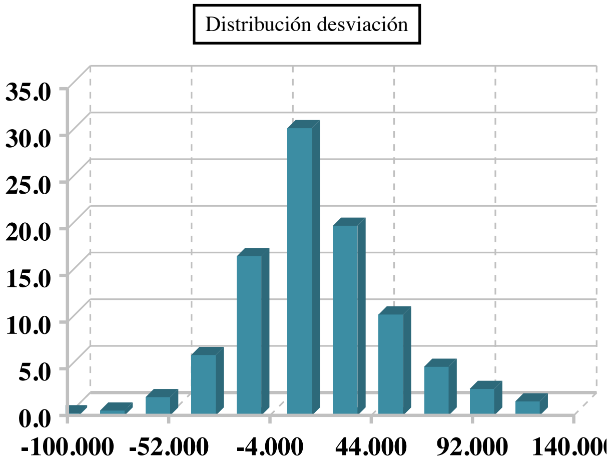

Desviaciones estándar

Distribución (+/-)	# Puntos	%
-6 * Desv. estánd.	164	0.13
-5 * Desv. estánd.	41	0.03
-4 * Desv. estánd.	98	0.07
-3 * Desv. estánd.	291	0.22
-2 * Desv. estánd.	1663	1.27
-1 * Desv. estánd.	73340	56.08
1 * Desv. estánd.	50777	38.83
2 * Desv. estánd.	3327	2.54
3 * Desv. estánd.	270	0.21
4 * Desv. estánd.	168	0.13
5 * Desv. estánd.	94	0.07
6 * Desv. estánd.	545	0.42

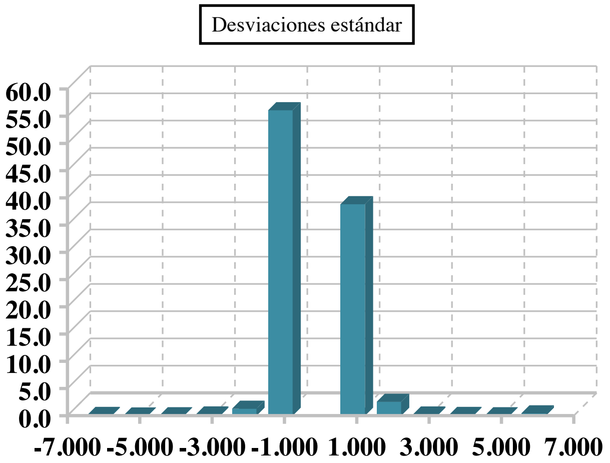

Predefinido: Isométrico

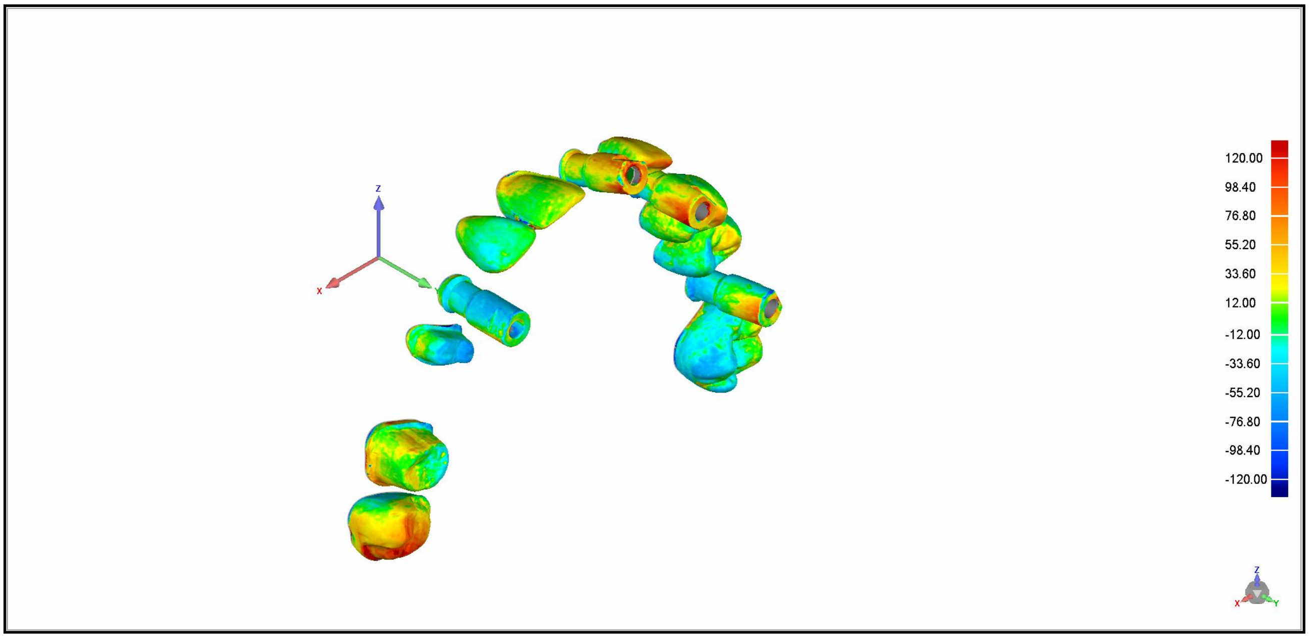

Predefinido: Frente

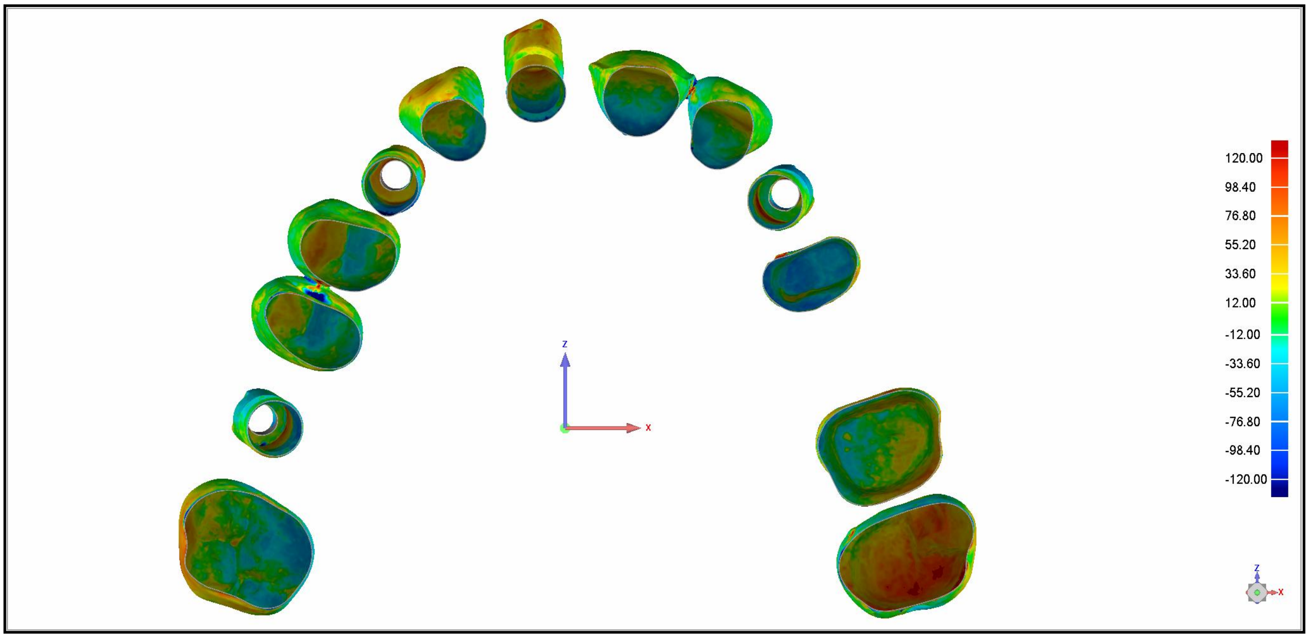

Predefinido: Atrás

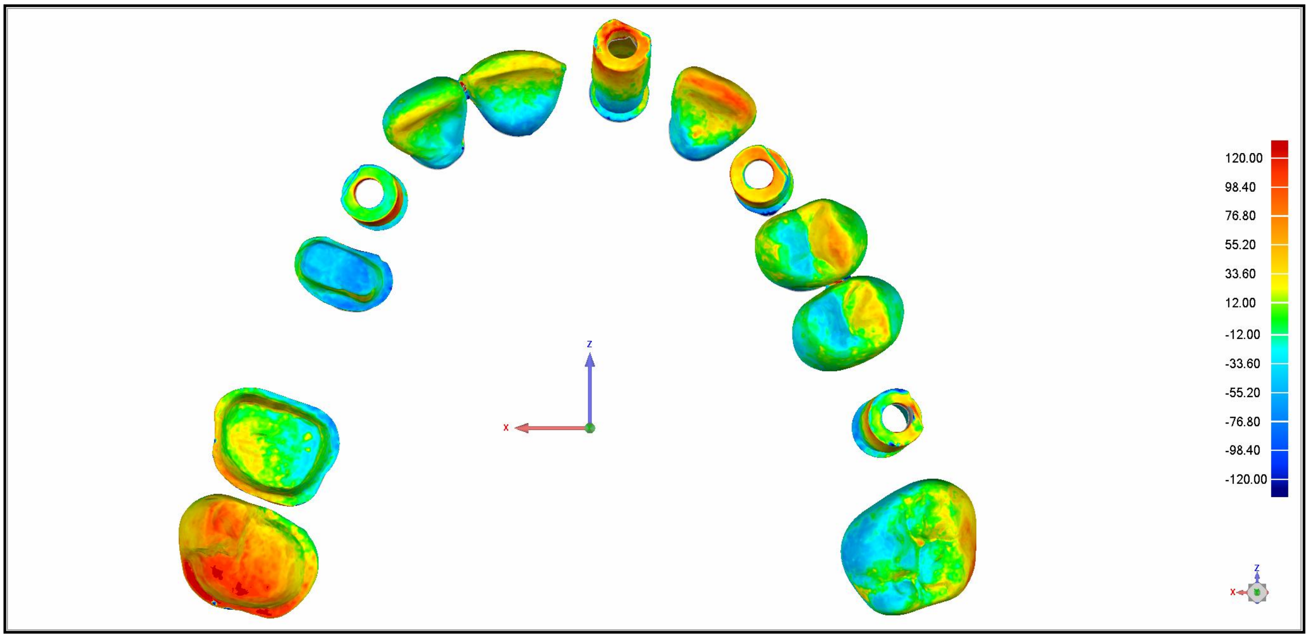

Predefinido: Izquierda

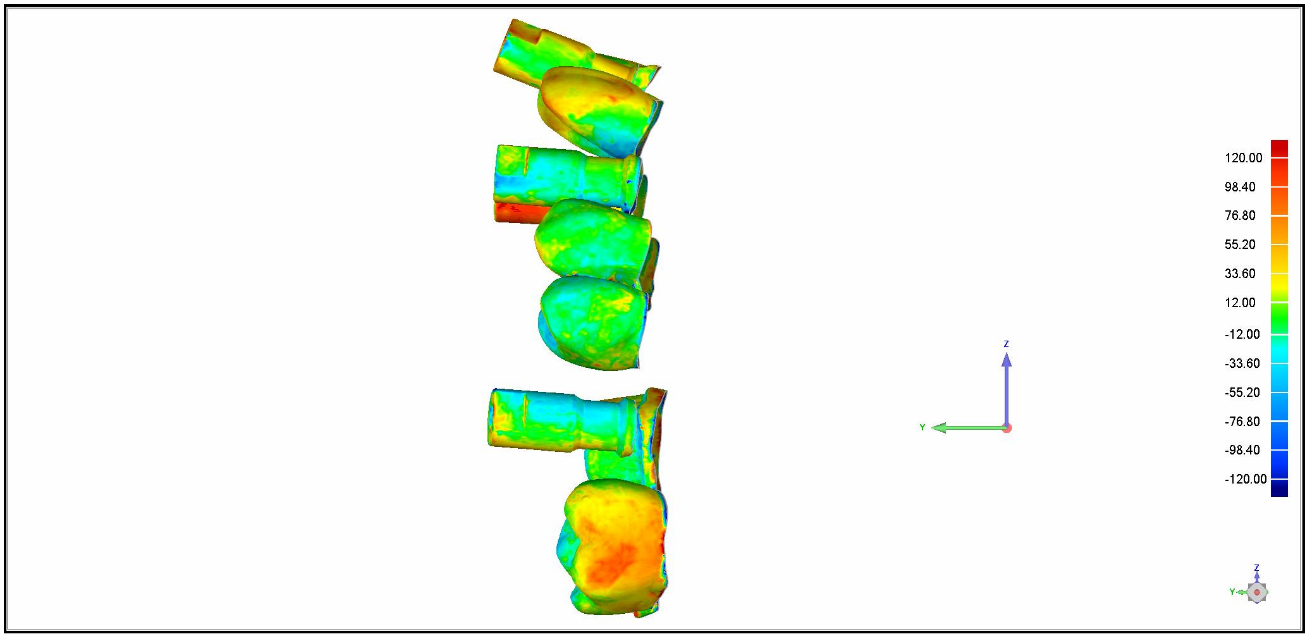

Predefinido: Derecha

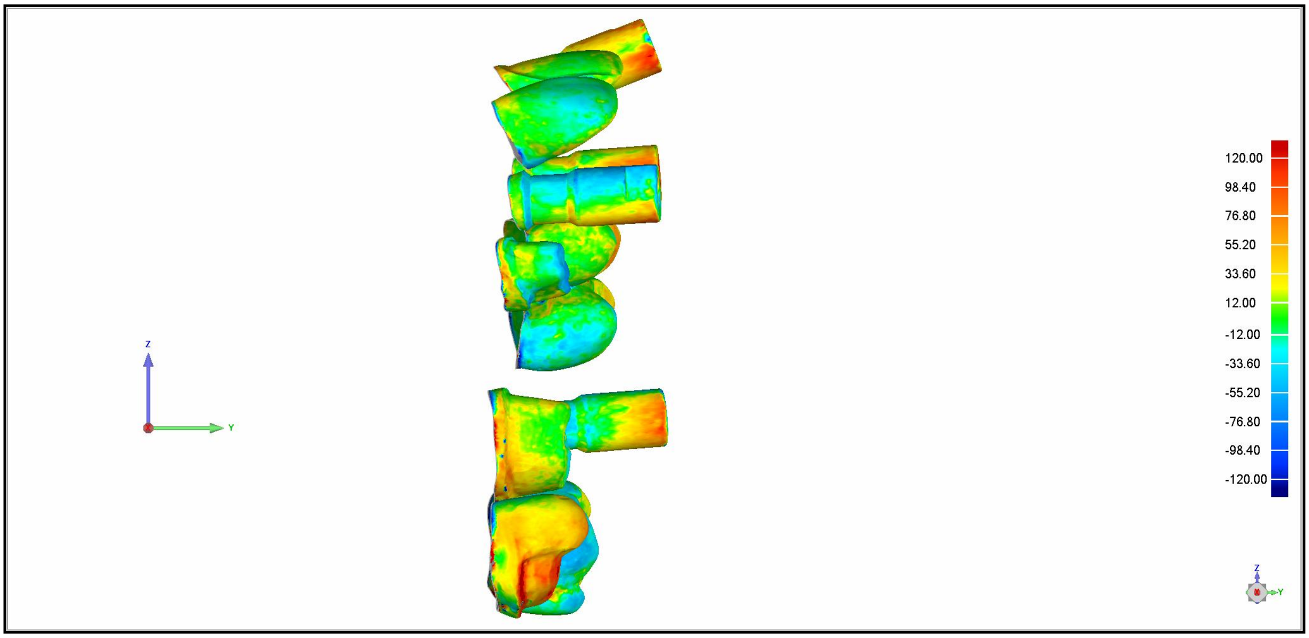

Predefinido: Superior

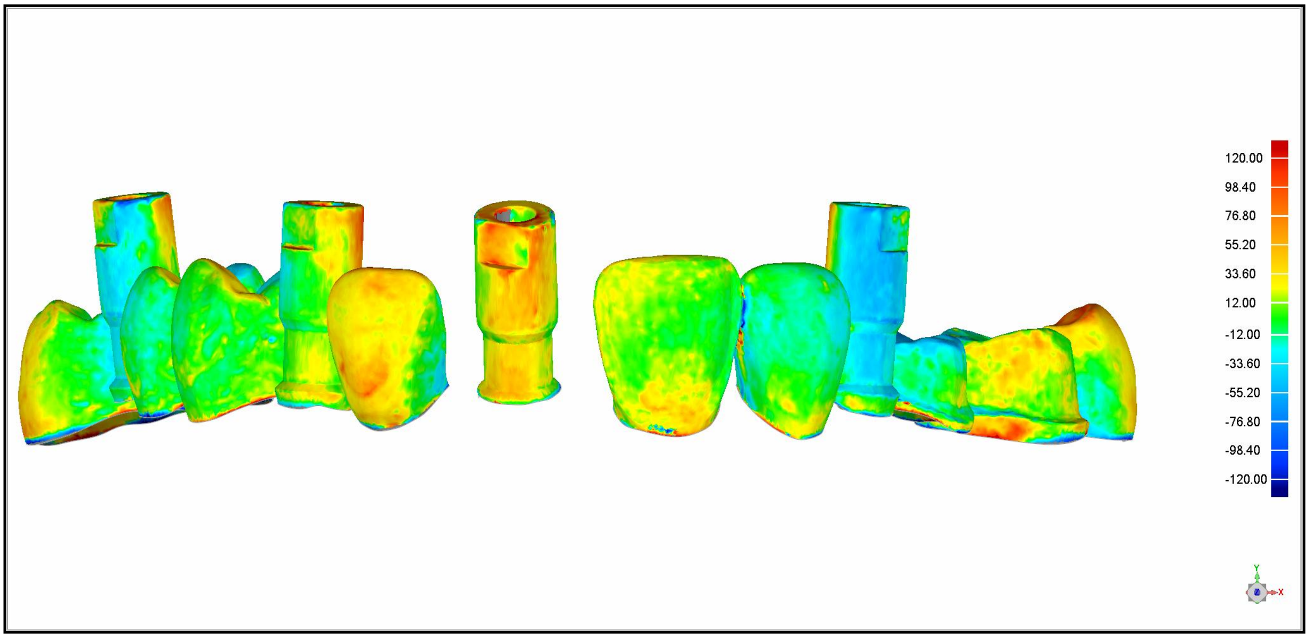

Predefinido: Inferior

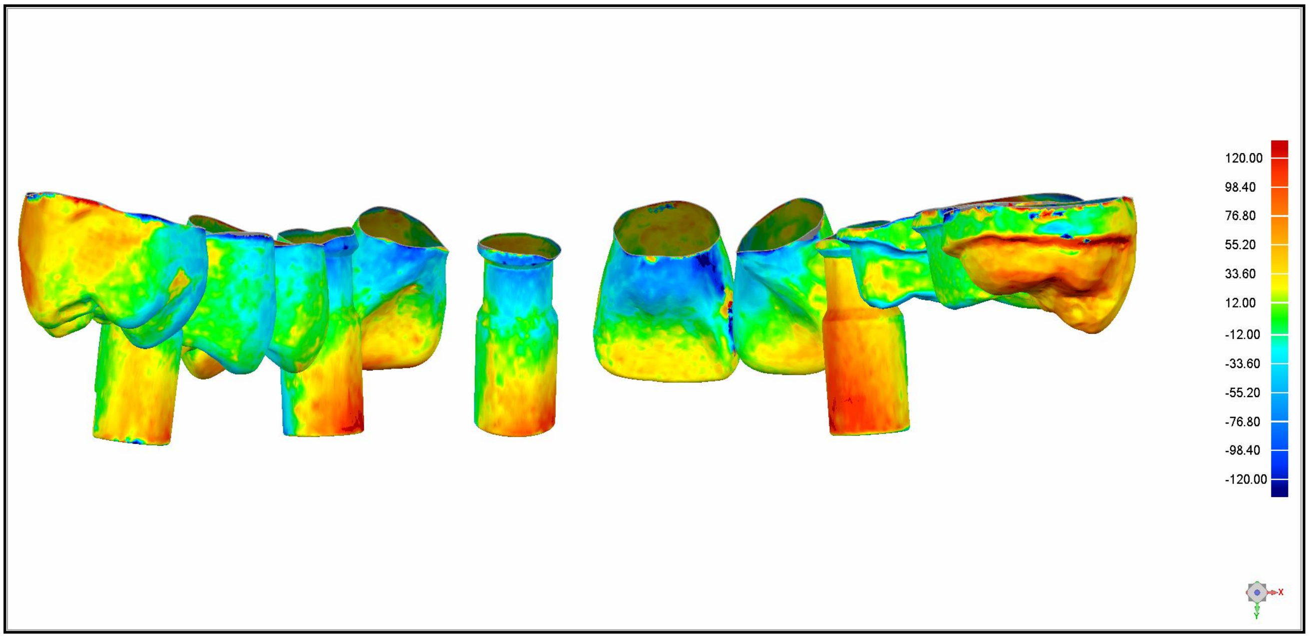

Ajuste de ubicación: Desviaciones superior e inferior

Unidades: u

Nombre	Desv	Estado	Superior Tol	Inferior Tol	Ref X	Ref Y	Ref Z	Radio	Desv X	Desv Y	Desv Z	Medido X	Medido Y	Medido Z	Dir. proy. X	Dir. proy. Y	Dir. proy. Z
Desv. inferior	-2119.41				29715.55	29554.58	-13882.37	n/a	-519.63	-2053.68	65.52	29195.92	27500.90	-13816.84	0.25	0.97	-0.03
Desv. superior	1985.89				17773.89	38478.39	19616.81	n/a	-74.62	-1936.70	-432.87	17699.27	36541.69	19183.94	-0.04	-0.98	-0.22
