# Supplementary material for: Accuracy of four digital scanners according to scanning strategy in complete-arch impressions
Source: PLoS One. 2018 Sep 13;13(9):e0202916. doi: 10.1371/journal.pone.0202916 (PMC6136706; doi:10.1371/journal.pone.0202916)
Supplement: S14 Table — True definition (scanning strategy B). (ZIP) [file pone.0202916.s014.zip › S14/TD3B.pdf]

3D Comparación Resultados

Modelo referencia	MRC
Modelo test	TD3B
Nº de puntos de datos	126974
# Aislados	323

Tipo tolerancia	3D desviación
Unidades	u
Máx. crítico	120.00
Máx. nominal	36.00
Mín. nominal	-36.00
Mín. crítico	-120.00

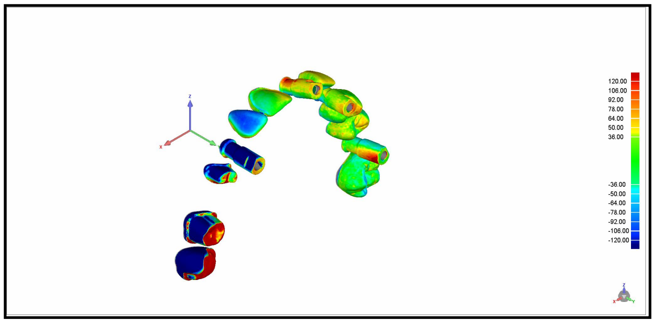

Desviación	
Desviación superior máx.	2993.89
Desviación inferior máx.	-3083.23
Desviación media	124.88 /-80.30
Desviación estándar	203.24

Distribución desviación

>=Min	<Max	# Puntos	%
-120.00	-106.00	796	0.63
-106.00	-92.00	1730	1.36
-92.00	-78.00	2054	1.62
-78.00	-64.00	2235	1.76
-64.00	-50.00	3485	2.74
-50.00	-36.00	5686	4.48
-36.00	36.00	62779	49.44
36.00	50.00	7983	6.29
50.00	64.00	4972	3.92
64.00	78.00	2937	2.31
78.00	92.00	1874	1.48
92.00	106.00	1508	1.19
106.00	120.00	1111	0.87

Fuera del crítico superior	18181	14.32
Fuera del crítico inferior	9643	7.59

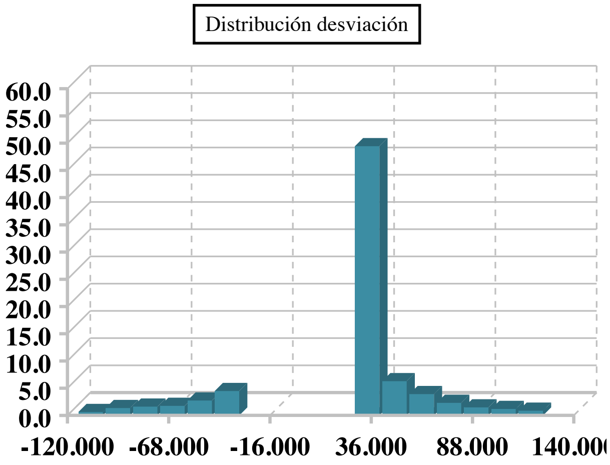

Desviaciones estándar

Distribución (+/-)	# Puntos	%
-6 * Desv. estánd.	95	0.07
-5 * Desv. estánd.	43	0.03
-4 * Desv. estánd.	593	0.47
-3 * Desv. estánd.	1957	1.54
-2 * Desv. estánd.	4516	3.56
-1 * Desv. estánd.	81250	63.99
1 * Desv. estánd.	27010	21.27
2 * Desv. estánd.	4316	3.40
3 * Desv. estánd.	4421	3.48
4 * Desv. estánd.	2398	1.89
5 * Desv. estánd.	155	0.12
6 * Desv. estánd.	220	0.17

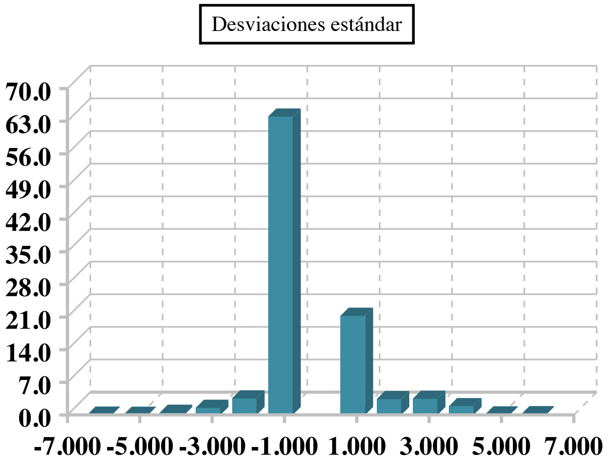

Predefinido: Isométrico

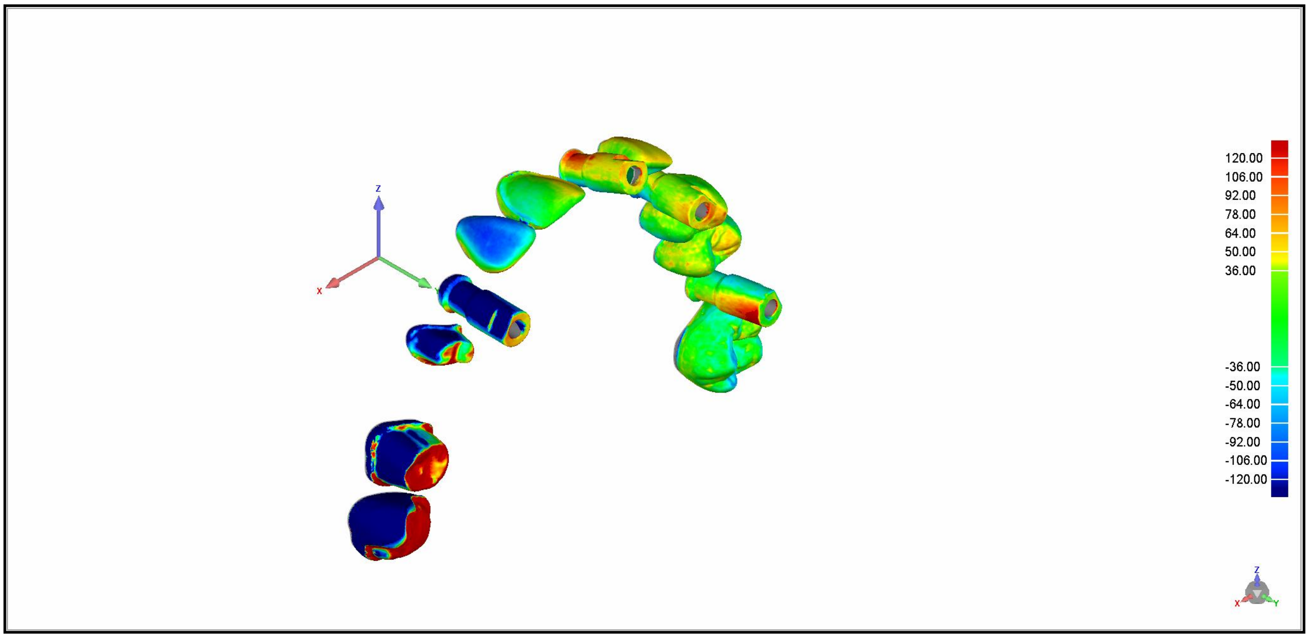

Predefinido: Frente

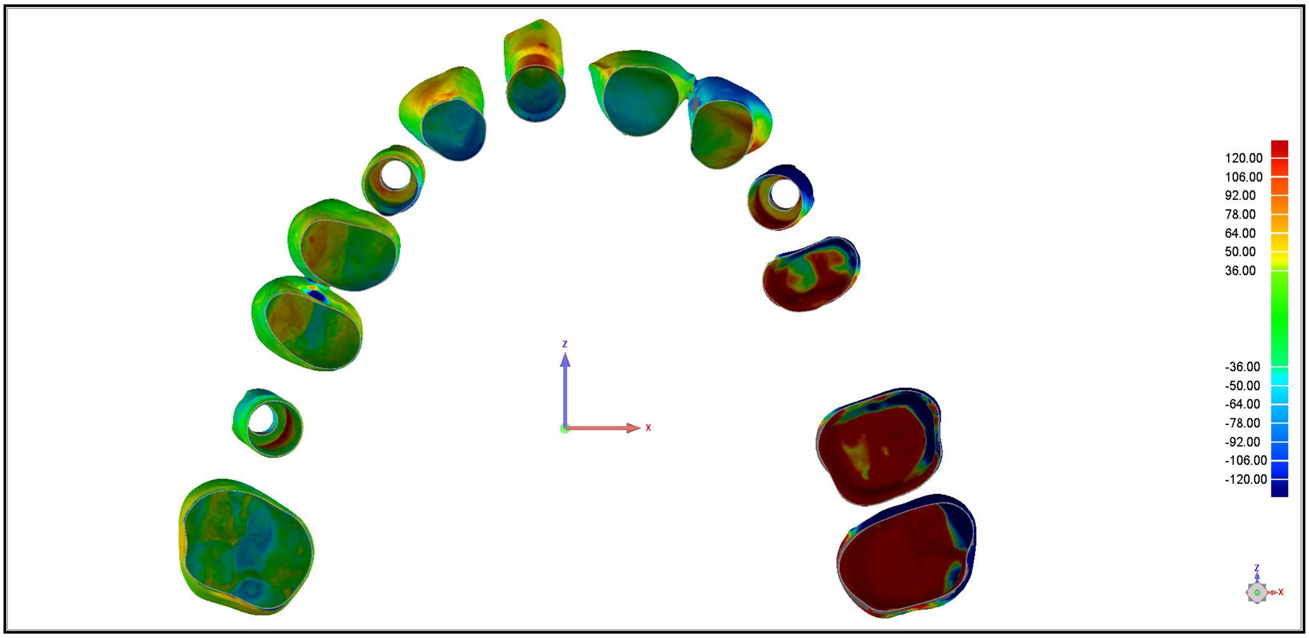

Predefinido: Atrás

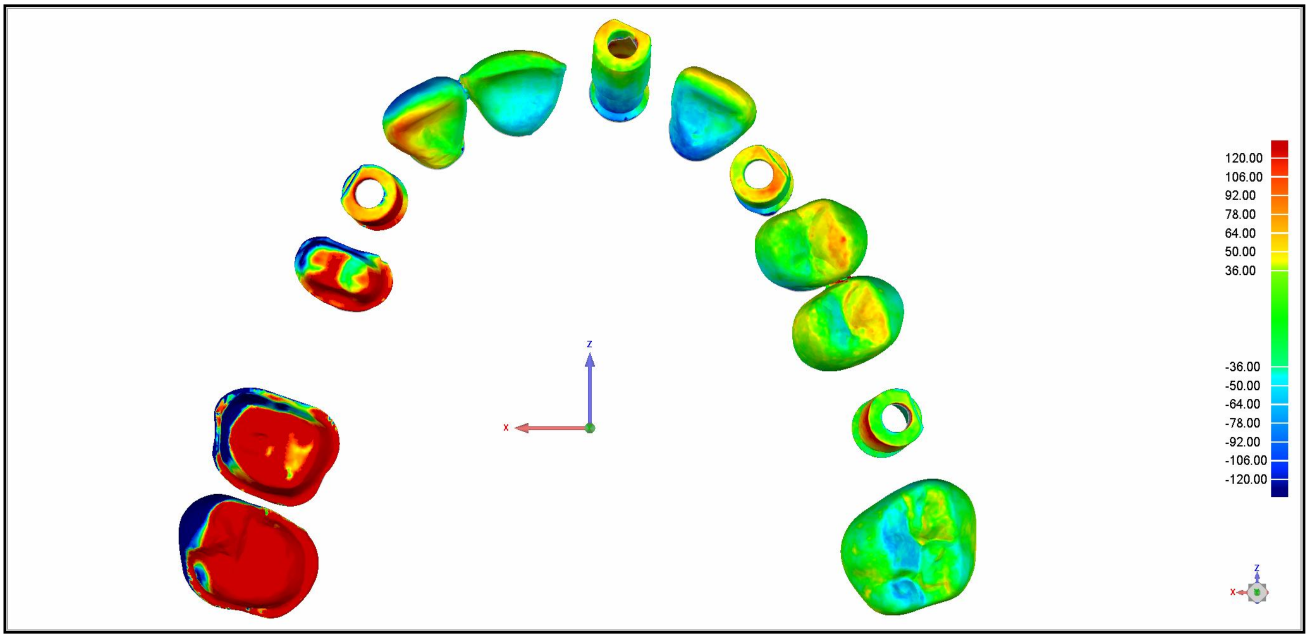

Predefinido: Izquierda

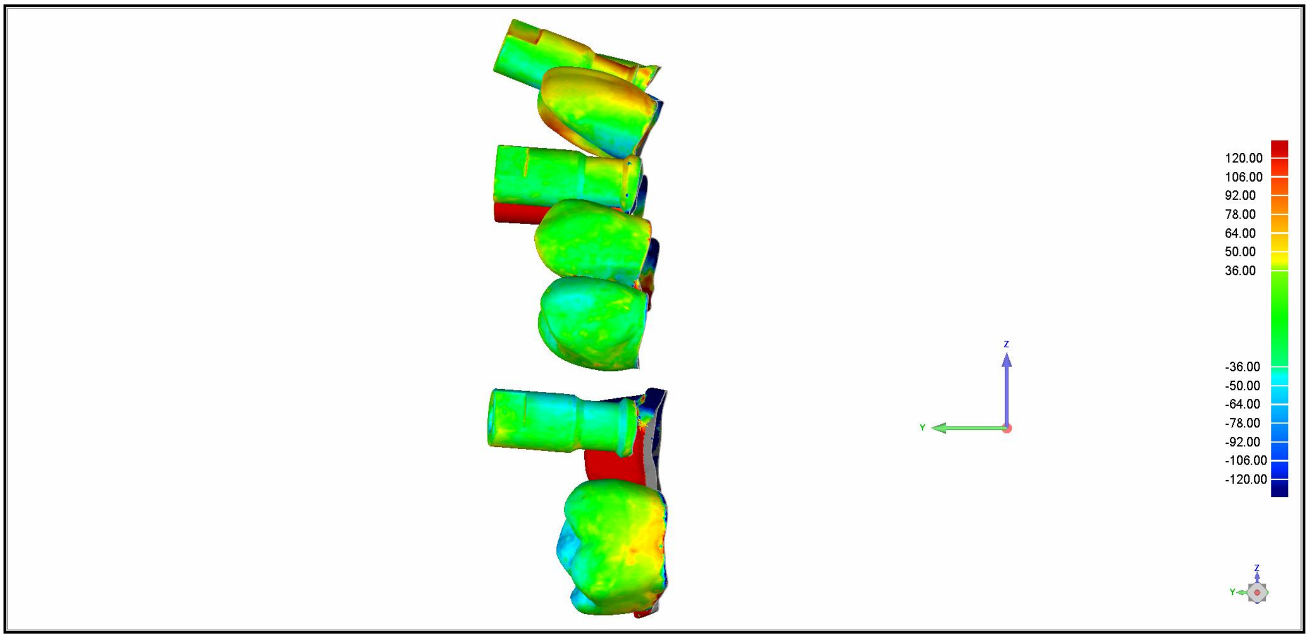

Predefinido: Derecha

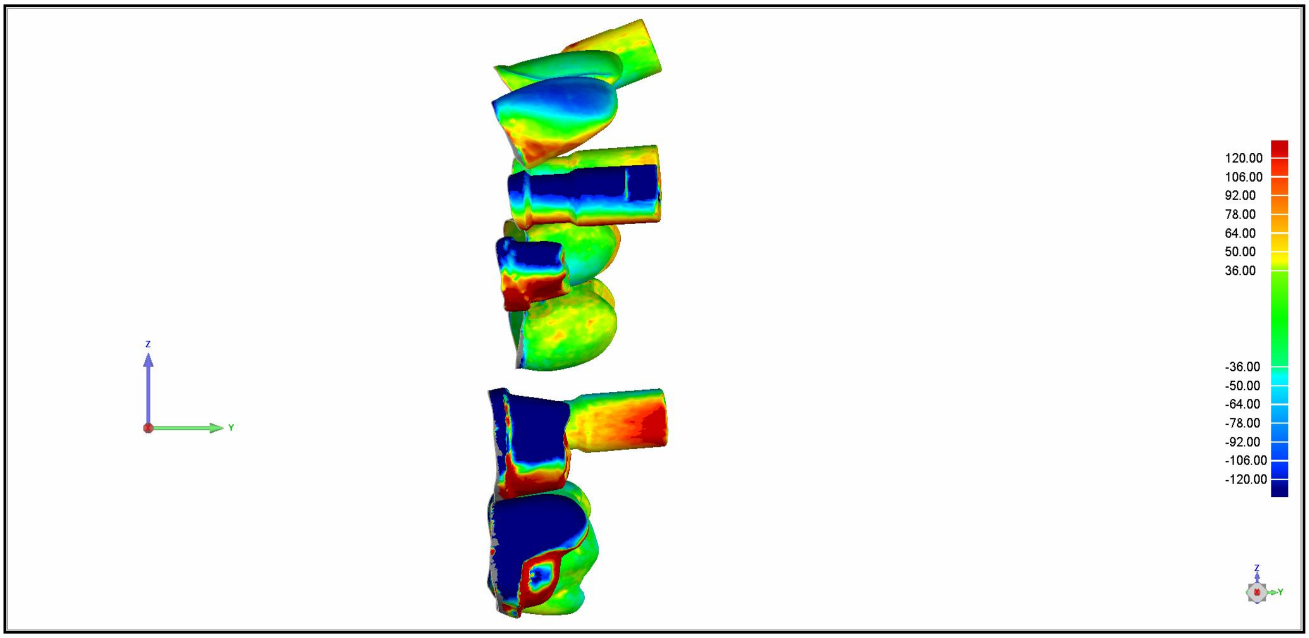

Predefinido: Superior

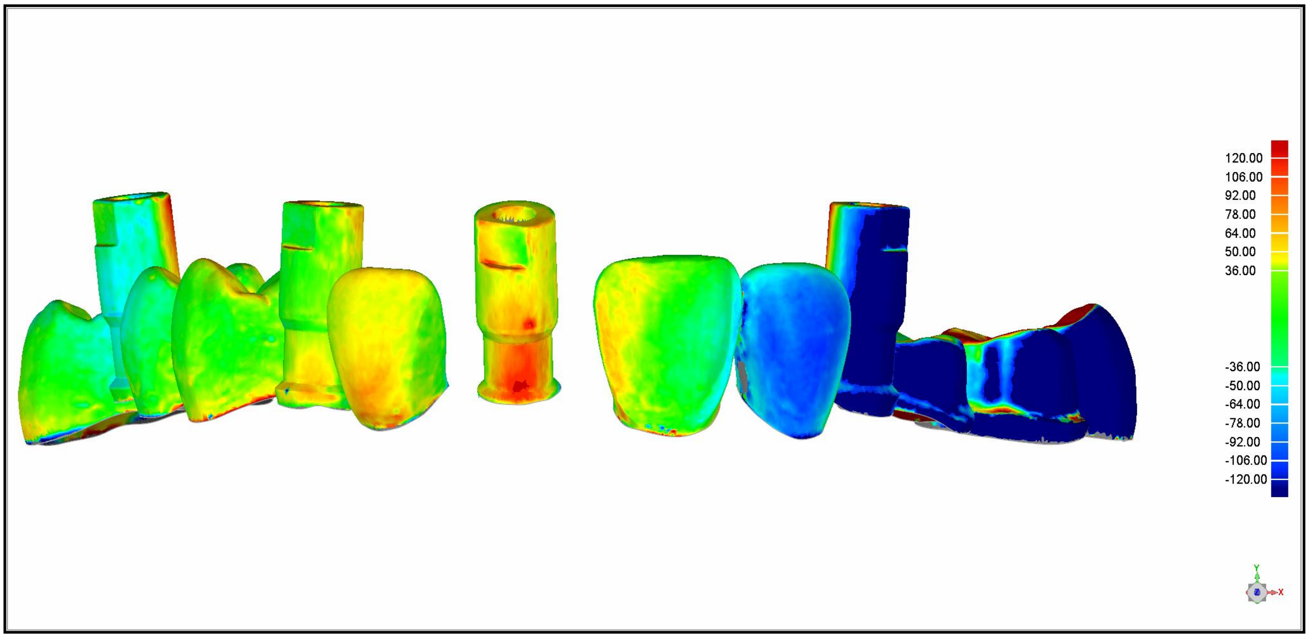

Predefinido: Inferior

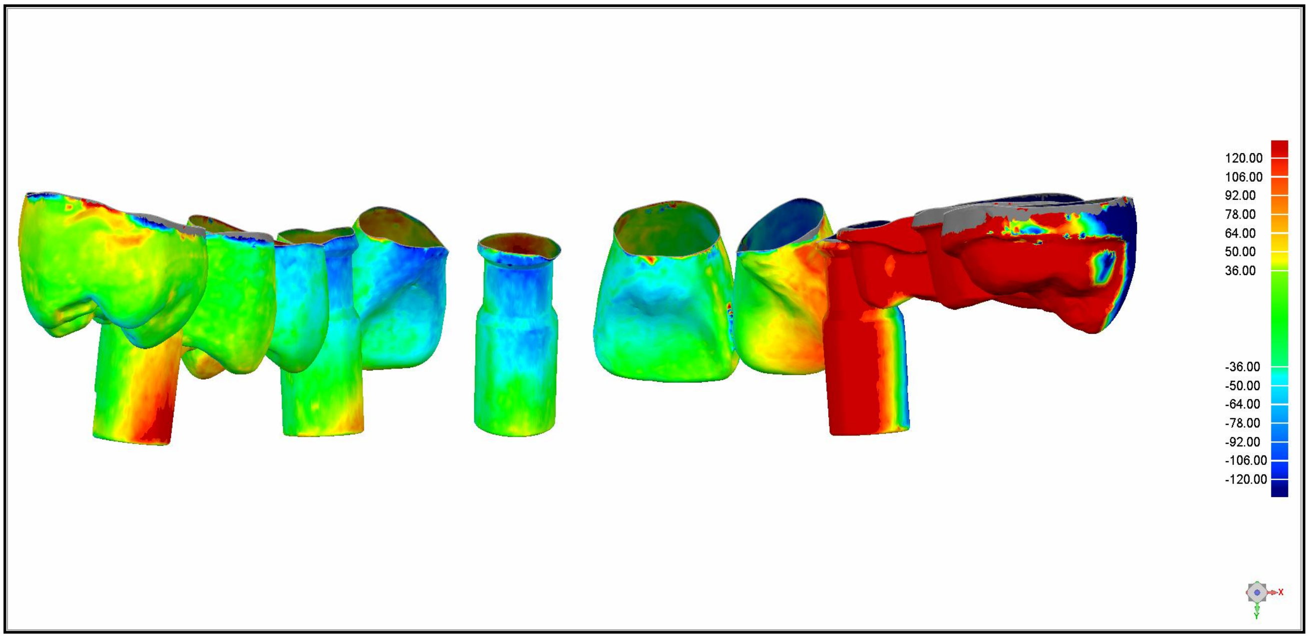

Ajuste de ubicación: Desviaciones superior e inferior

Unidades: u

Nombre	Desv	Estado	Superior Tol	Inferior Tol	Ref X	Ref Y	Ref Z	Radio	Desv X	Desv Y	Desv Z	Medido X	Medido Y	Medido Z	Dir. proy. X	Dir. proy. Y	Dir. proy. Z
Desv. inferior	-3083.23				32061.00	27268.70	-9389.56	n/a	-802.94	484.80	-2937.10	31258.06	27753.50	-12326.67	0.26	-0.16	0.95
Desv. superior	2993.89				25410.11	27462.45	-5764.99	n/a	2932.11	260.37	-546.17	28342.22	27722.82	-6311.16	0.98	0.09	-0.18
