# Supplementary material for: Accuracy of four digital scanners according to scanning strategy in complete-arch impressions
Source: PLoS One. 2018 Sep 13;13(9):e0202916. doi: 10.1371/journal.pone.0202916 (PMC6136706; doi:10.1371/journal.pone.0202916)
Supplement: S14 Table — True definition (scanning strategy B). (ZIP) [file pone.0202916.s014.zip › S14/TD2B.pdf]

3D Comparación Resultados

Modelo referencia	MRC
Modelo test	TD2B
Nº de puntos de datos	130062
# Aislados	347

Tipo tolerancia	3D desviación
Unidades	u
Máx. crítico	120.00
Máx. nominal	17.00
Mín. nominal	-17.00
Mín. crítico	-120.00

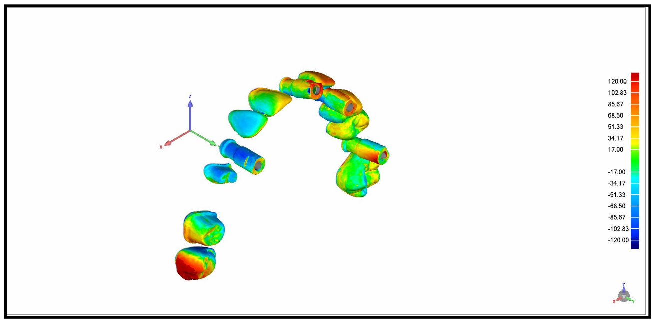

Desviación	
Desviación superior máx.	2396.34
Desviación inferior máx.	-1159.73
Desviación media	53.11 /-34.34
Desviación estándar	77.20

Distribución desviación

>=Min	<Max	# Puntos	%
-120.00	-102.83	414	0.32
-102.83	-85.67	1824	1.40
-85.67	-68.50	3143	2.42
-68.50	-51.33	6046	4.65
-51.33	-34.17	9710	7.47
-34.17	-17.00	12748	9.80
-17.00	17.00	38532	29.63
17.00	34.17	16722	12.86
34.17	51.33	11906	9.15
51.33	68.50	7797	5.99
68.50	85.67	7344	5.65
85.67	102.83	4461	3.43
102.83	120.00	2559	1.97

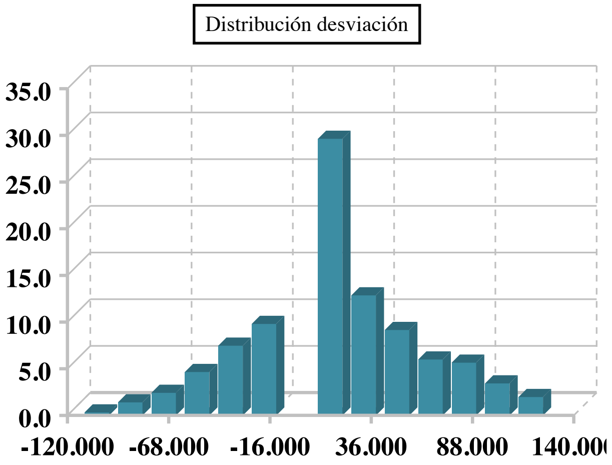

Fuera del crítico superior		6226	4.79
Fuera del crítico inferior		630	0.48

Desviaciones estándar

Distribución (+/-)	# Puntos	%
-6 * Desv. estánd.	55	0.04
-5 * Desv. estánd.	29	0.02
-4 * Desv. estánd.	121	0.09
-3 * Desv. estánd.	281	0.22
-2 * Desv. estánd.	7995	6.15
-1 * Desv. estánd.	64767	49.80
1 * Desv. estánd.	46350	35.64
2 * Desv. estánd.	8760	6.74
3 * Desv. estánd.	1107	0.85
4 * Desv. estánd.	147	0.11
5 * Desv. estánd.	83	0.06
6 * Desv. estánd.	367	0.28

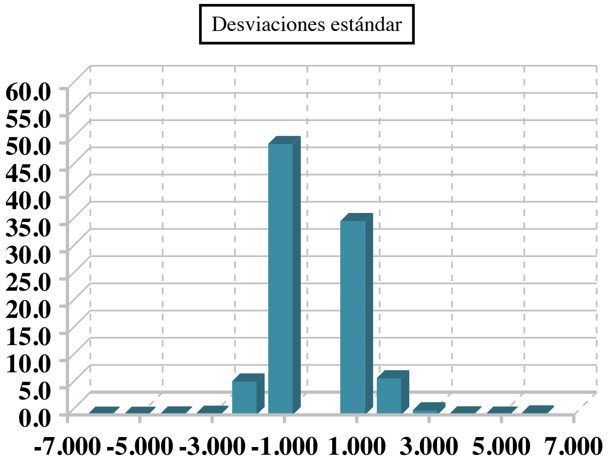

Predefinido: Isométrico

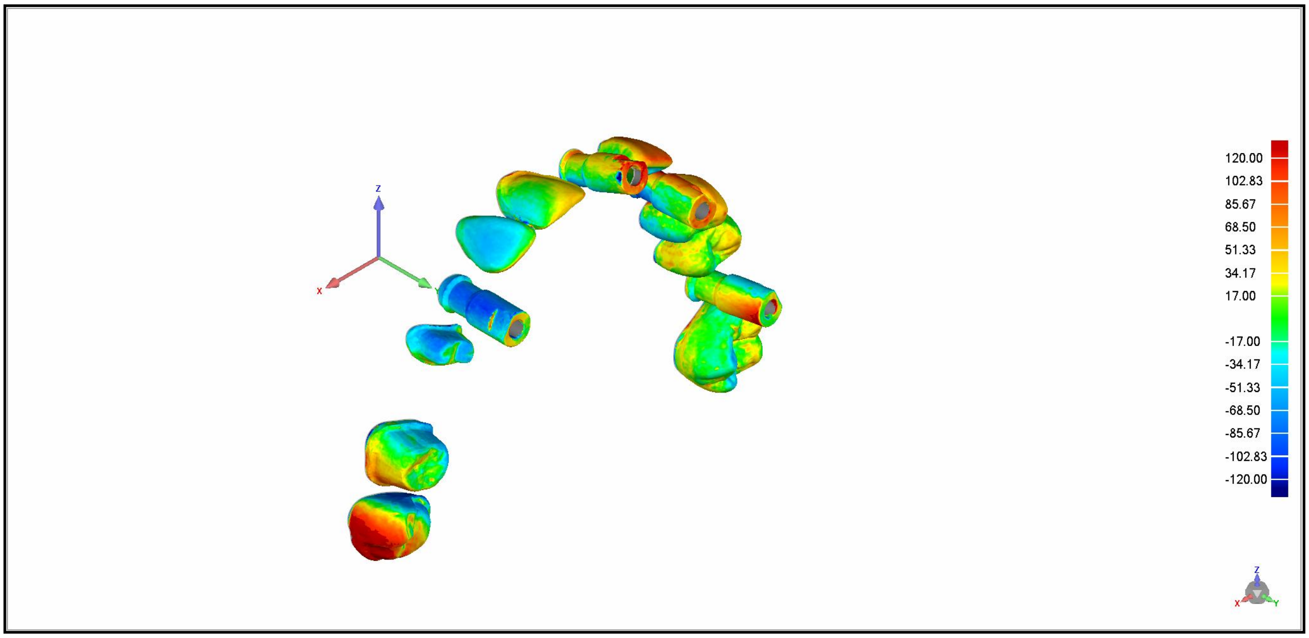

Predefinido: Frente

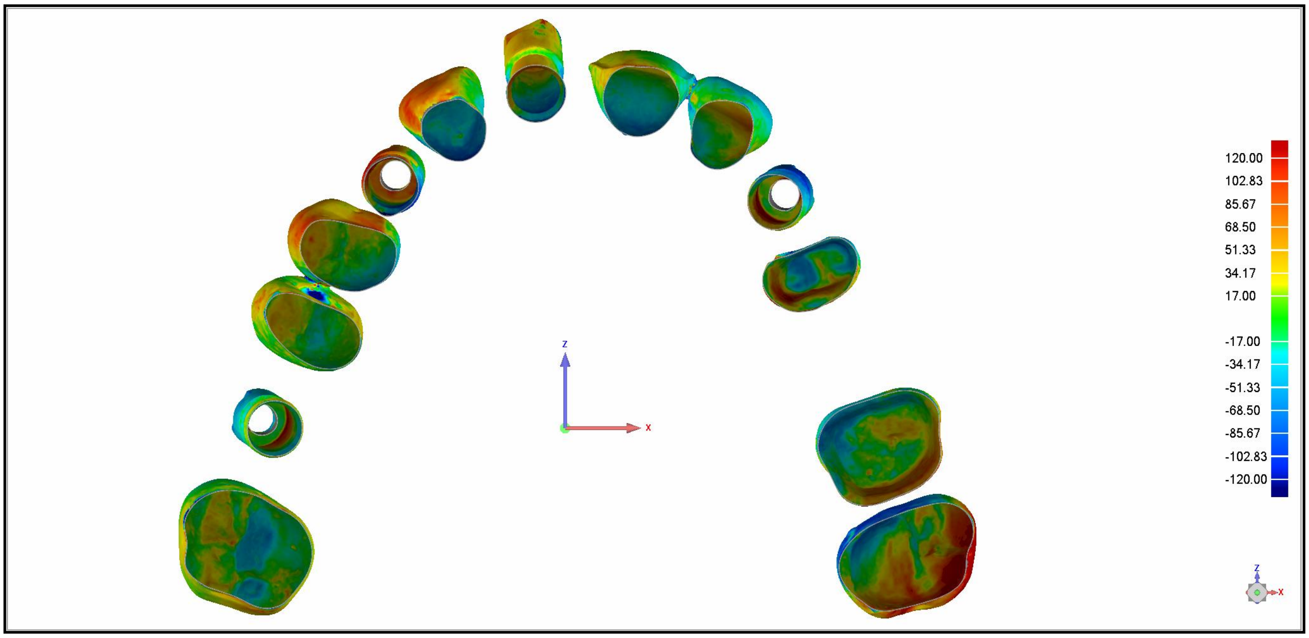

Predefinido: Atrás

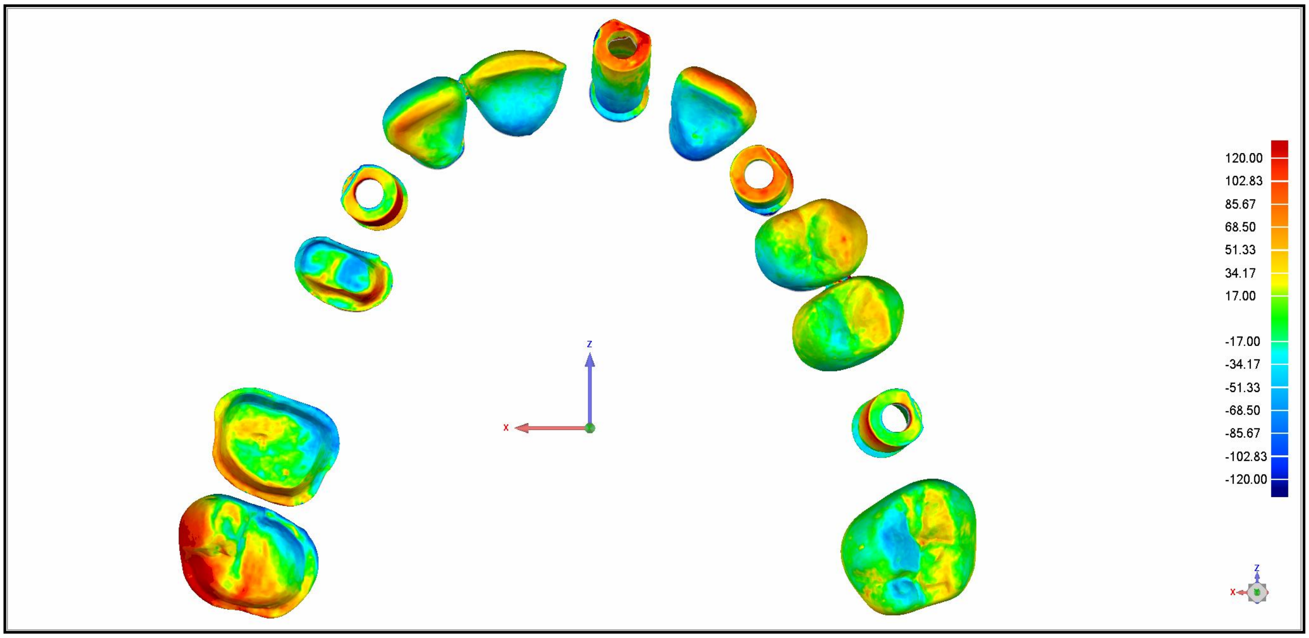

Predefinido: Izquierda

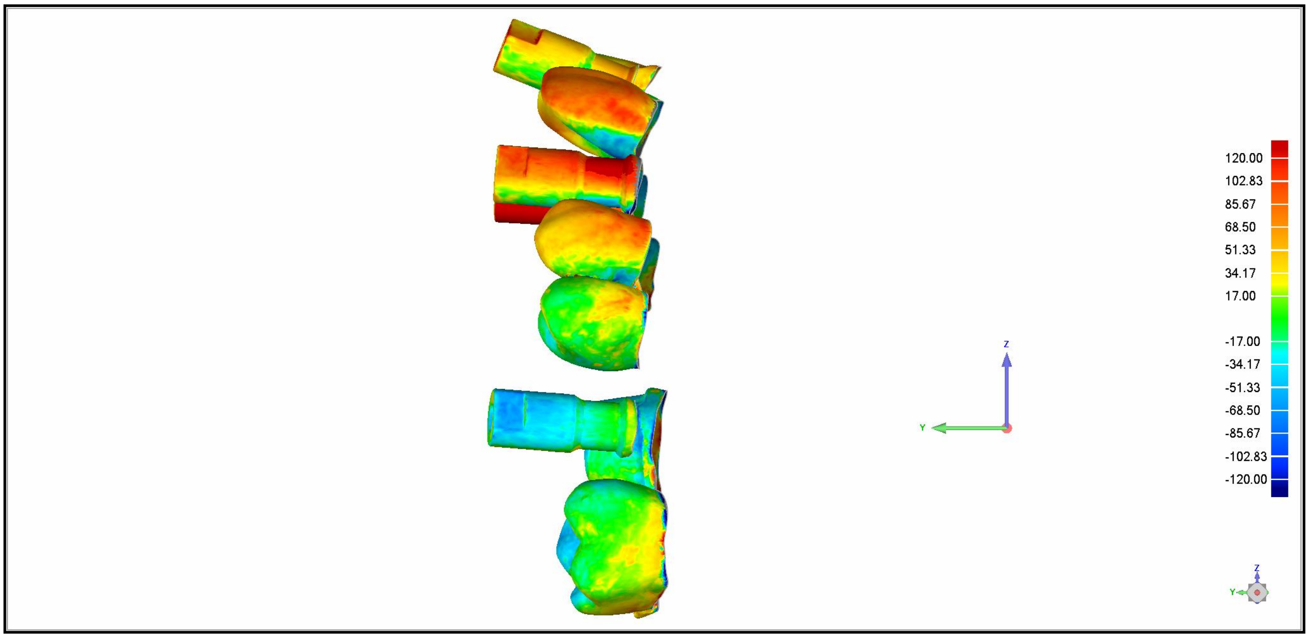

Predefinido: Derecha

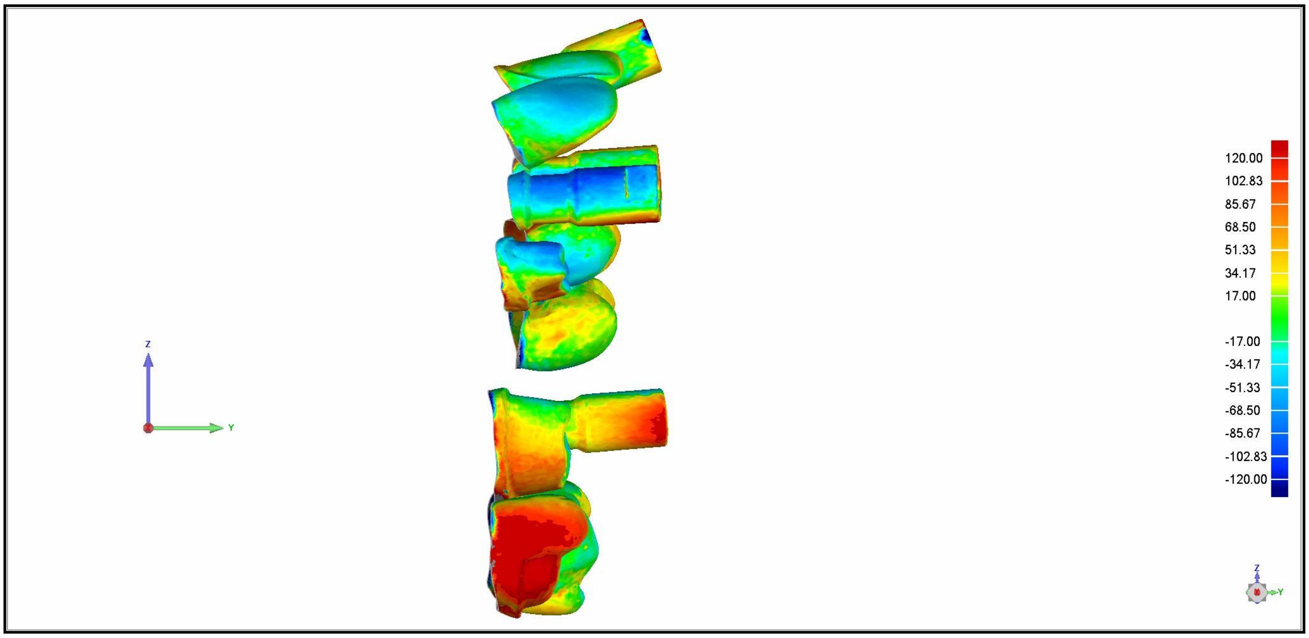

Predefinido: Superior

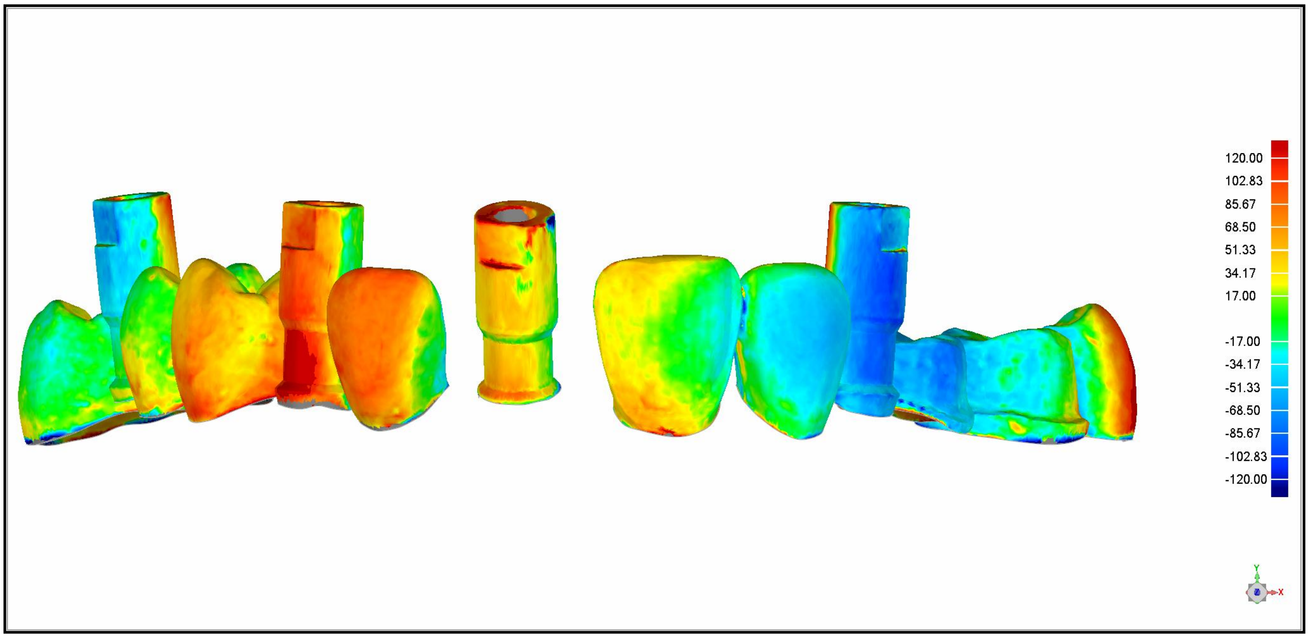

Predefinido: Inferior

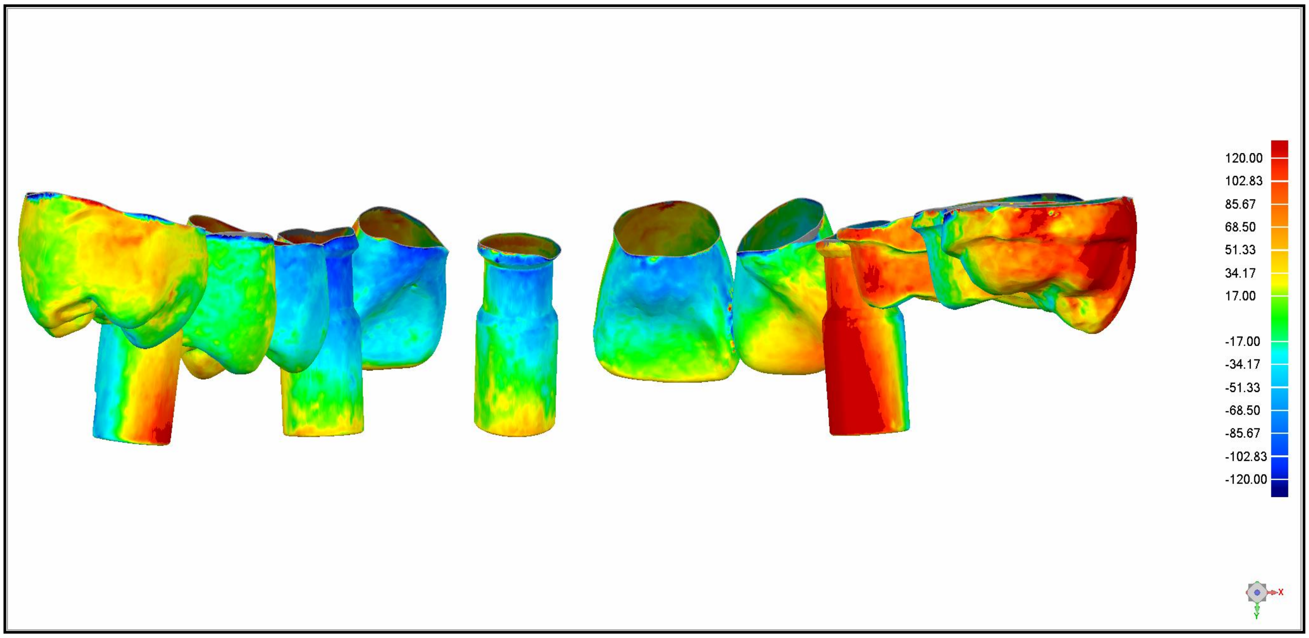

Ajuste de ubicación: Desviaciones superior e inferior

Unidades: u

Nombre	Desv	Estado	Superior Tol	Inferior Tol	Ref X	Ref Y	Ref Z	Radio	Desv X	Desv Y	Desv Z	Medido X	Medido Y	Medido Z	Dir. proy. X	Dir. proy. Y	Dir. proy. Z
Desv. inferior	-1159.73				23969.71	27759.43	-5958.84	n/a	-1130.96	240.59	89.55	22838.75	28000.02	-5869.29	0.98	-0.21	-0.08
Desv. superior	2396.34				-13871.23	29023.09	21431.13	n/a	2017.42	-215.37	-1275.17	-11853.81	28807.72	20155.96	0.84	-0.09	-0.53
