# Supplementary material for: Accuracy of four digital scanners according to scanning strategy in complete-arch impressions
Source: PLoS One. 2018 Sep 13;13(9):e0202916. doi: 10.1371/journal.pone.0202916 (PMC6136706; doi:10.1371/journal.pone.0202916)
Supplement: S14 Table — True definition (scanning strategy B). (ZIP) [file pone.0202916.s014.zip › S14/TD5B.pdf]

3D Comparación Resultados

Modelo referencia	MRC
Modelo test	TD5B
Nº de puntos de datos	128078
# Aislados	401

Tipo tolerancia	3D desviación
Unidades	u
Máx. crítico	120.00
Máx. nominal	36.00
Mín. nominal	-36.00
Mín. crítico	-120.00

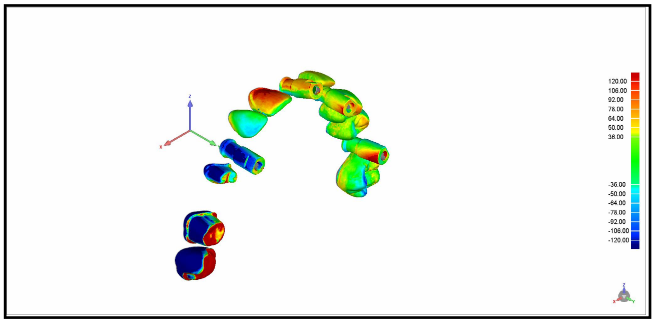

Desviación	
Desviación superior máx.	2180.49
Desviación inferior máx.	-2921.27
Desviación media	105.85 /-69.31
Desviación estándar	168.12

Distribución desviación

>=Min	<Max	# Puntos	%
-120.00	-106.00	1051	0.82
-106.00	-92.00	1095	0.85
-92.00	-78.00	1315	1.03
-78.00	-64.00	2241	1.75
-64.00	-50.00	4025	3.14
-50.00	-36.00	5380	4.20
-36.00	36.00	61692	48.17
36.00	50.00	8522	6.65
50.00	64.00	6150	4.80
64.00	78.00	4237	3.31
78.00	92.00	2773	2.17
92.00	106.00	2251	1.76
106.00	120.00	1923	1.50

Fuera del crítico superior	17561	13.71
Fuera del crítico inferior	7862	6.14

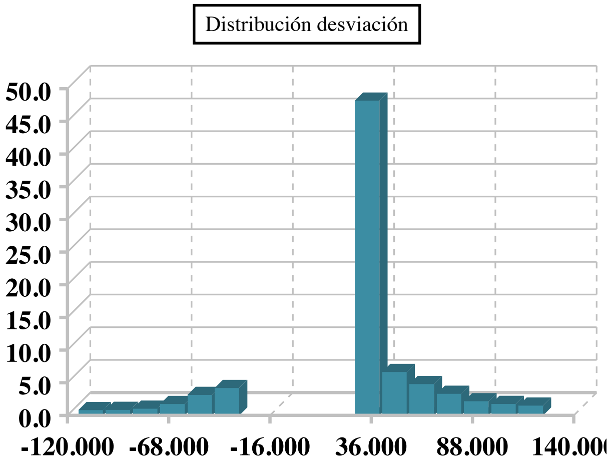

Desviaciones estándar

Distribución (+/-)	# Puntos	%
-6 * Desv. estánd.	85	0.07
-5 * Desv. estánd.	78	0.06
-4 * Desv. estánd.	471	0.37
-3 * Desv. estánd.	1955	1.53
-2 * Desv. estánd.	4557	3.56
-1 * Desv. estánd.	77643	60.62
1 * Desv. estánd.	32091	25.06
2 * Desv. estánd.	4492	3.51
3 * Desv. estánd.	3925	3.06
4 * Desv. estánd.	2383	1.86
5 * Desv. estánd.	160	0.12
6 * Desv. estánd.	238	0.19

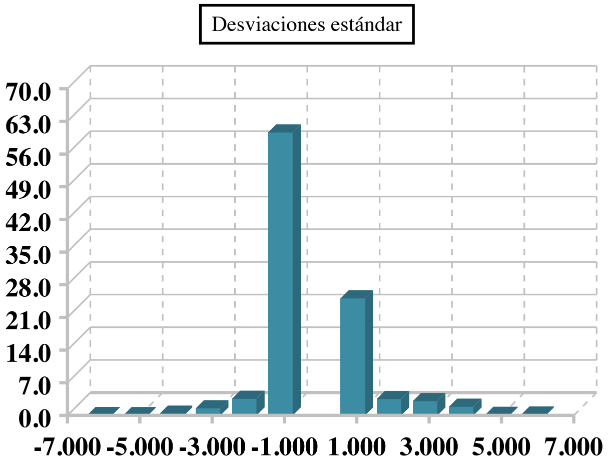

Predefinido: Isométrico

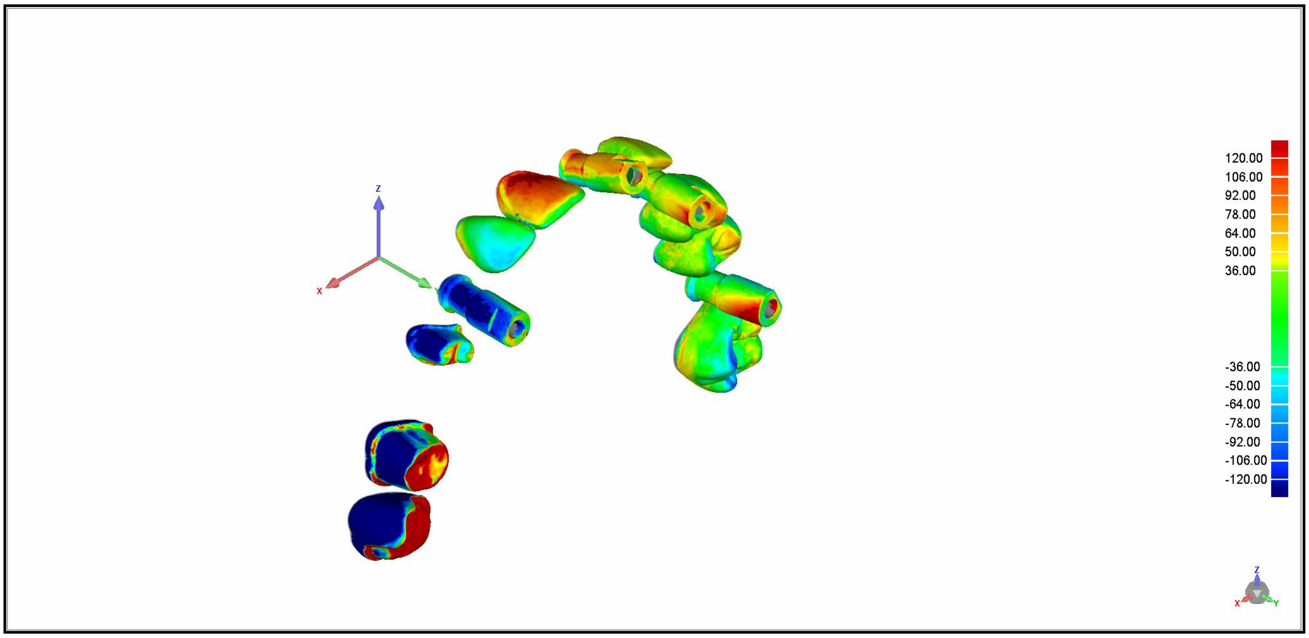

Predefinido: Frente

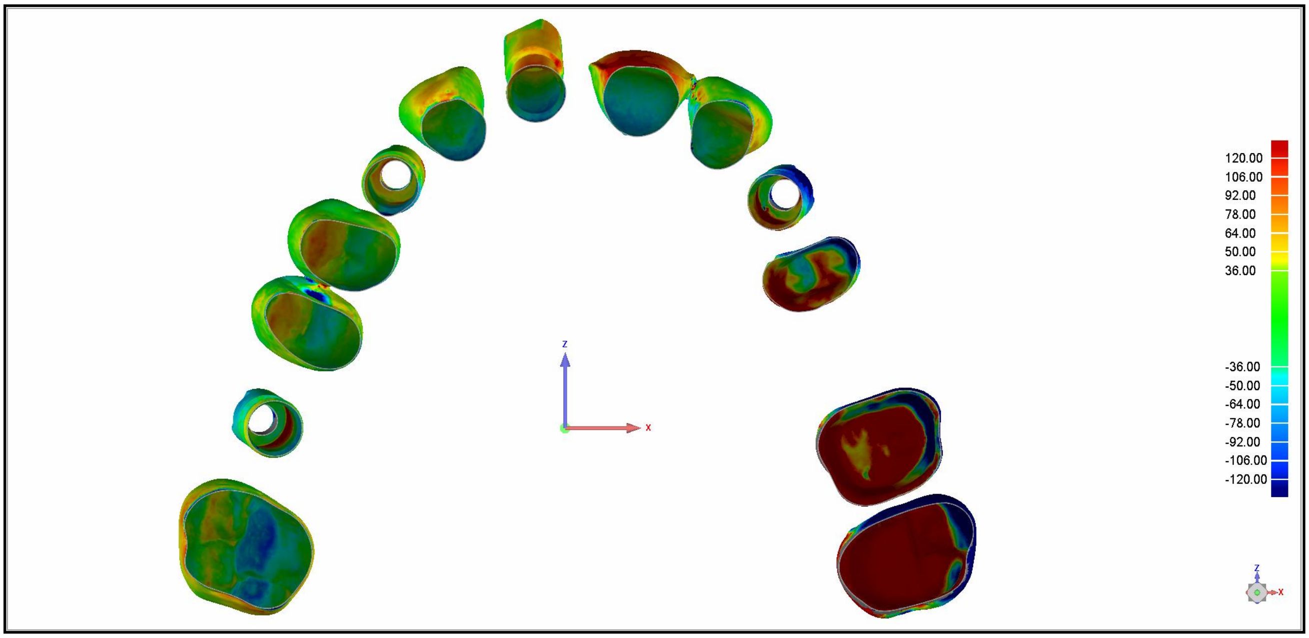

Predefinido: Atrás

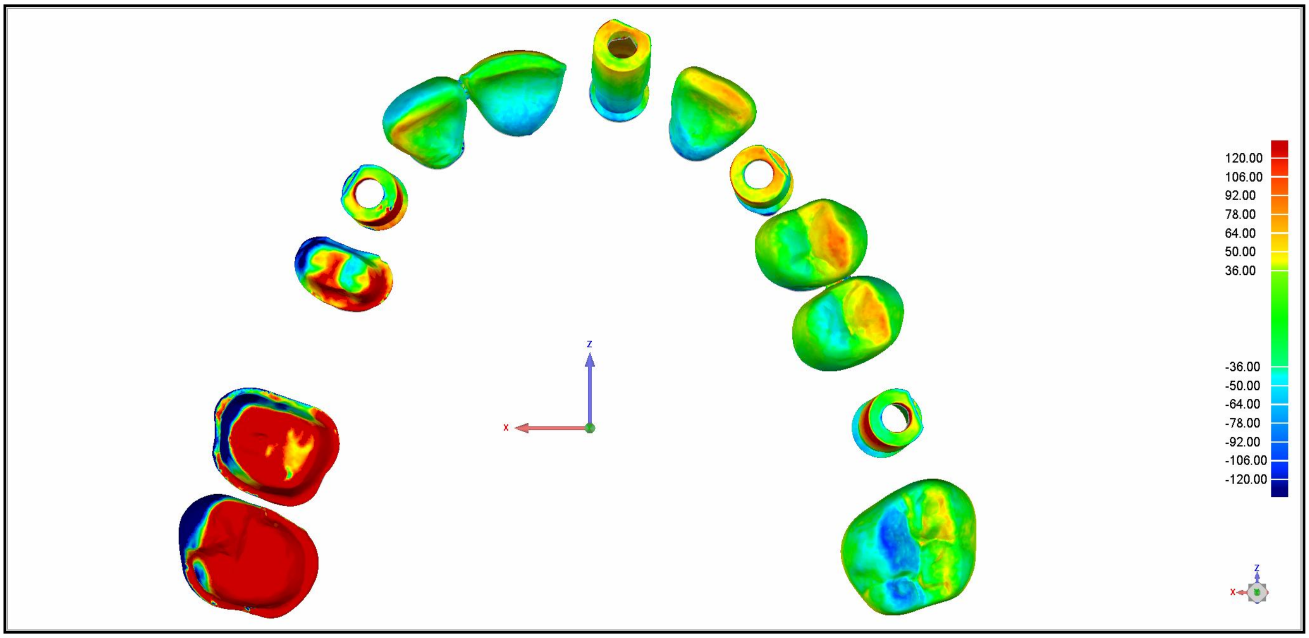

Predefinido: Izquierda

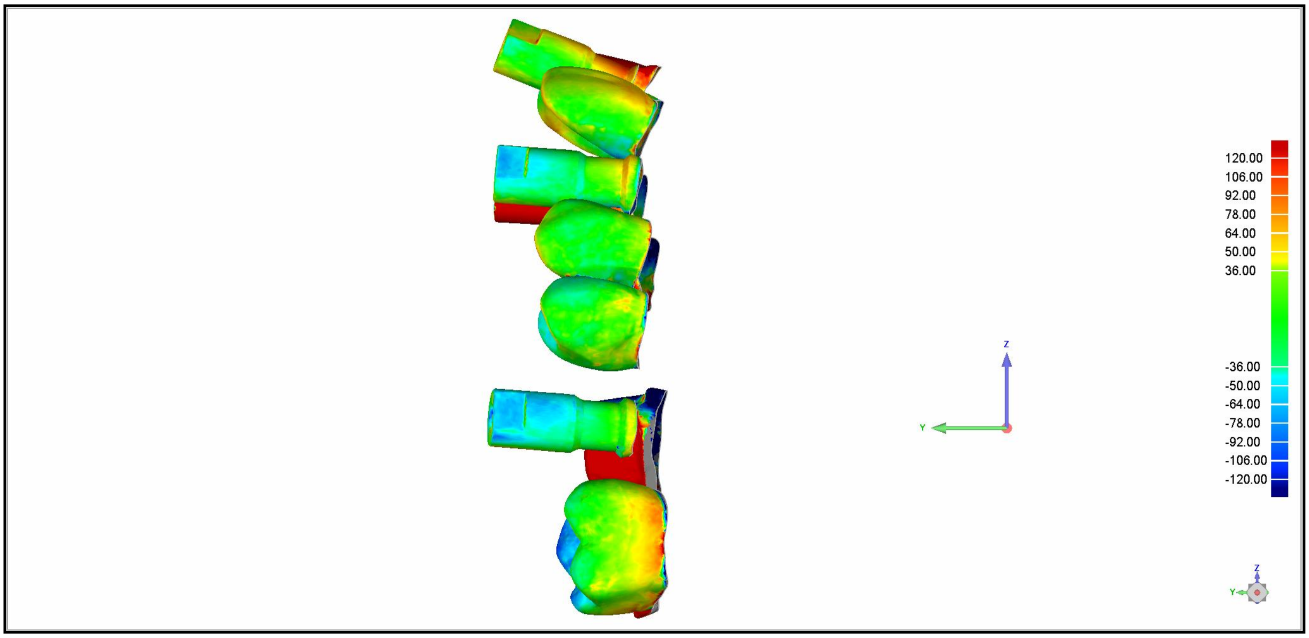

Predefinido: Derecha

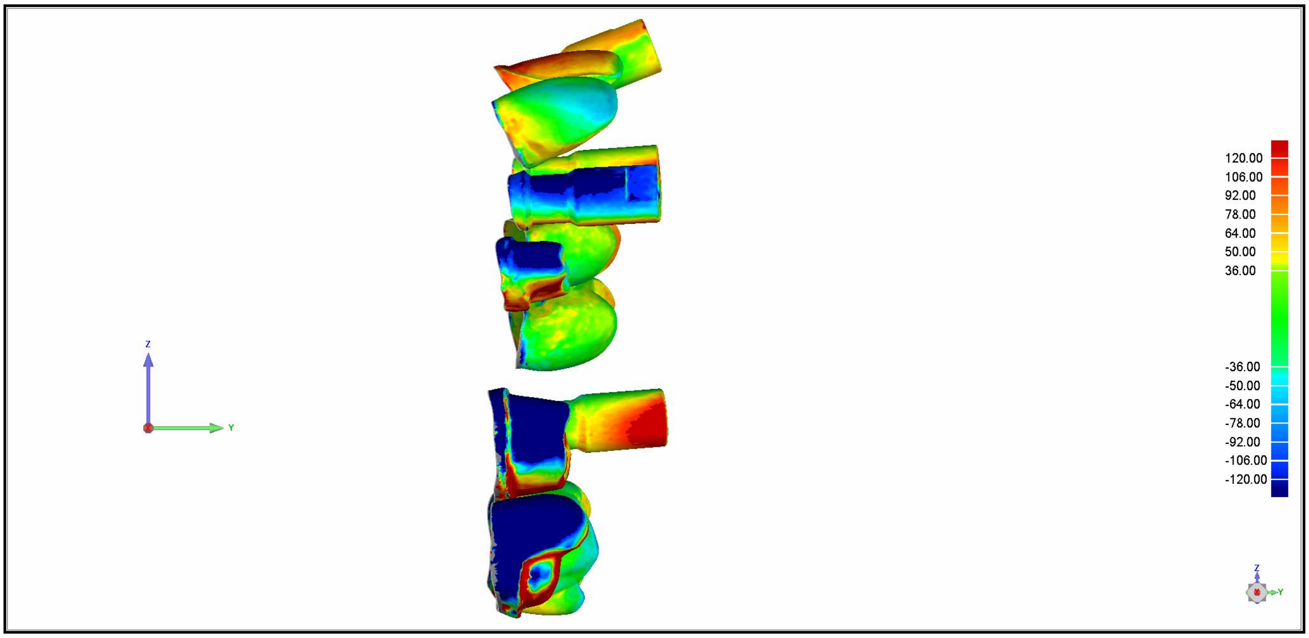

Predefinido: Superior

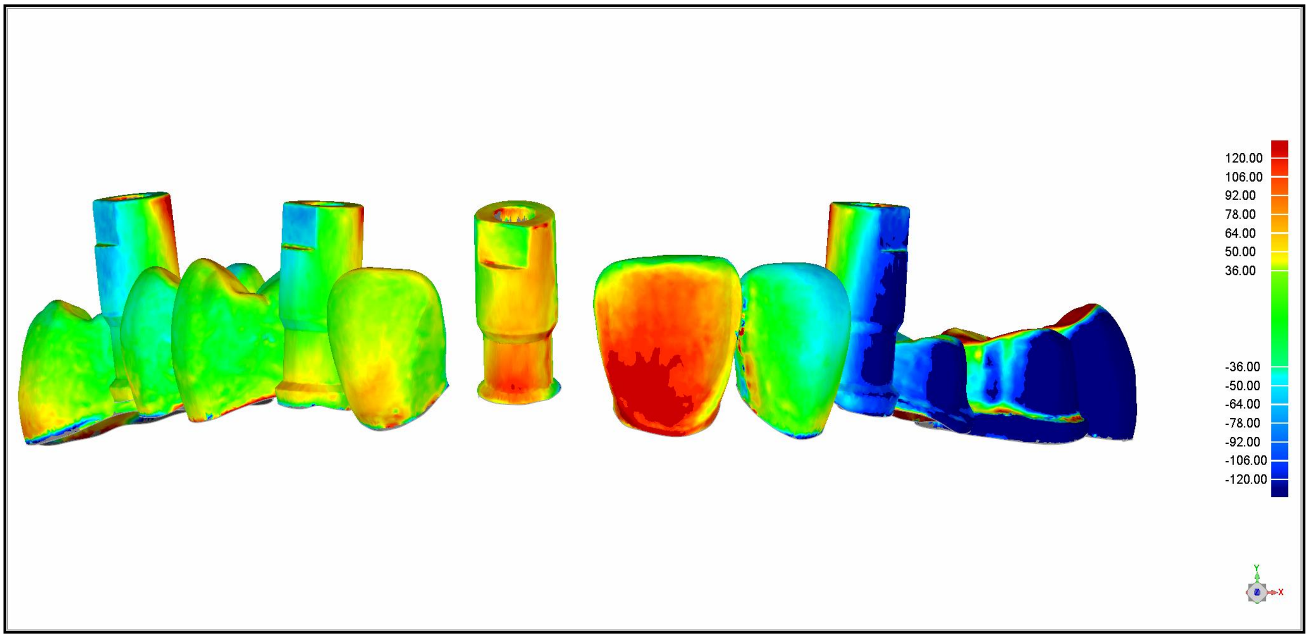

Predefinido: Inferior

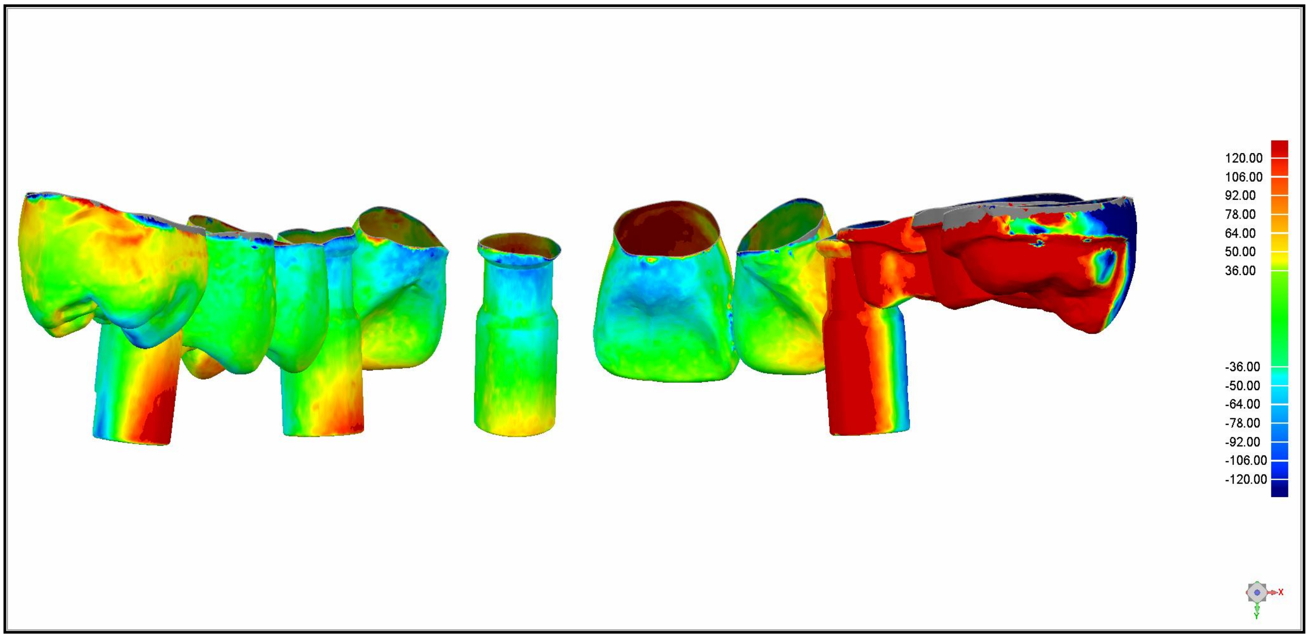

Ajuste de ubicación: Desviaciones superior e inferior

Unidades: u

Nombre	Desv	Estado	Superior Tol	Inferior Tol	Ref X	Ref Y	Ref Z	Radio	Desv X	Desv Y	Desv Z	Medido X	Medido Y	Medido Z	Dir. proy. X	Dir. proy. Y	Dir. proy. Z
Desv. inferior	-2921.27				32061.00	27268.70	-9389.57	n/a	-708.06	331.45	-2814.71	31352.93	27600.16	-12204.28	0.24	-0.11	0.96
Desv. superior	2180.49				17773.89	38478.38	19616.81	n/a	-369.94	-2115.68	-376.23	17403.94	36362.70	19240.57	-0.17	-0.97	-0.17
