# Supplementary material for: Accuracy of four digital scanners according to scanning strategy in complete-arch impressions
Source: PLoS One. 2018 Sep 13;13(9):e0202916. doi: 10.1371/journal.pone.0202916 (PMC6136706; doi:10.1371/journal.pone.0202916)
Supplement: S14 Table — True definition (scanning strategy B). (ZIP) [file pone.0202916.s014.zip › S14/TD9B.pdf]

3D Comparación Resultados

Modelo referencia	MRC
Modelo test	TD9B
Nº de puntos de datos	127425
# Aislados	353

Tipo tolerancia	3D desviación
Unidades	u
Máx. crítico	120.00
Máx. nominal	16.00
Mín. nominal	-16.00
Mín. crítico	-120.00

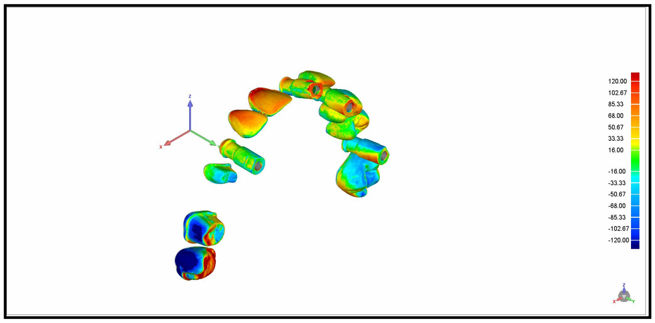

Desviación	
Desviación superior máx.	2174.04
Desviación inferior máx.	-2089.97
Desviación media	57.19 /-42.64
Desviación estándar	89.84

Distribución desviación

>=Min	<Max	# Puntos	%
-120.00	-102.67	1476	1.16
-102.67	-85.33	2063	1.62
-85.33	-68.00	3287	2.58
-68.00	-50.67	5533	4.34
-50.67	-33.33	7128	5.59
-33.33	-16.00	12425	9.75
-16.00	16.00	35924	28.19
16.00	33.33	17030	13.36
33.33	50.67	9868	7.74
50.67	68.00	8146	6.39
68.00	85.33	6218	4.88
85.33	102.67	4333	3.40
102.67	120.00	2763	2.17

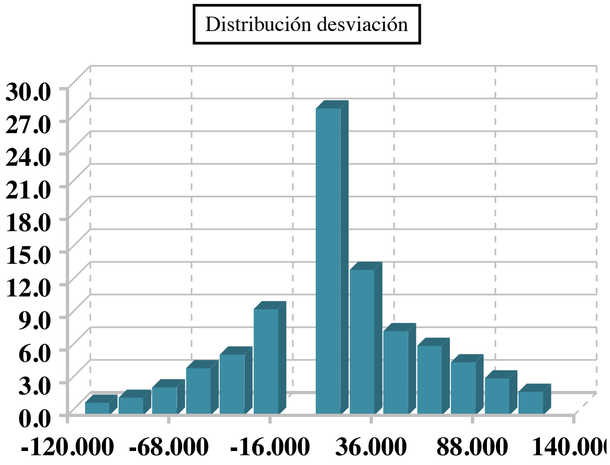

Fuera del crítico superior	8346	6.55
Fuera del crítico inferior	2885	2.26

Desviaciones estándar

Distribución (+/-)	# Puntos	%
-6 * Desv. estánd.	118	0.09
-5 * Desv. estánd.	61	0.05
-4 * Desv. estánd.	116	0.09
-3 * Desv. estánd.	552	0.43
-2 * Desv. estánd.	7690	6.03
-1 * Desv. estánd.	63000	49.44
1 * Desv. estánd.	45517	35.72
2 * Desv. estánd.	7896	6.20
3 * Desv. estánd.	1920	1.51
4 * Desv. estánd.	115	0.09
5 * Desv. estánd.	87	0.07
6 * Desv. estánd.	353	0.28

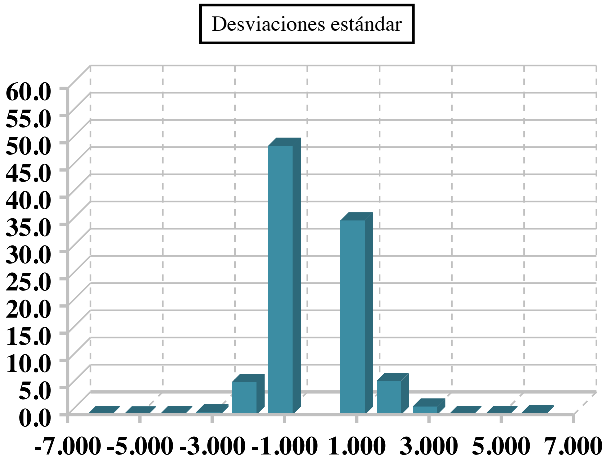

Predefinido: Isométrico

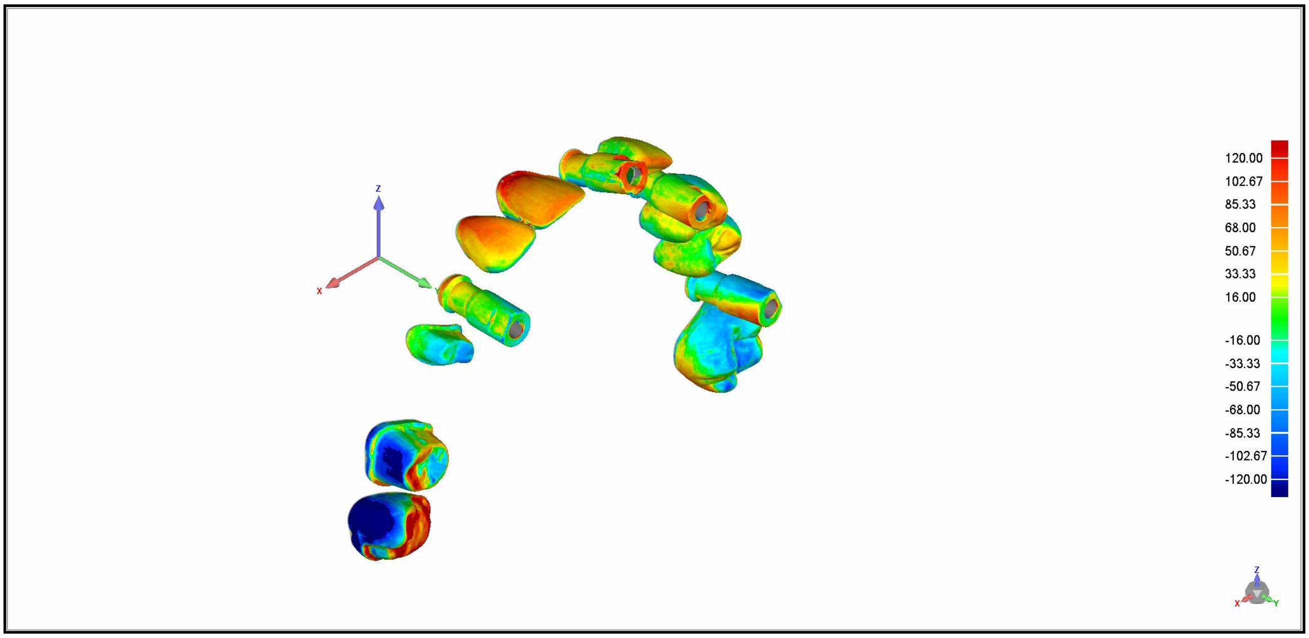

Predefinido: Frente

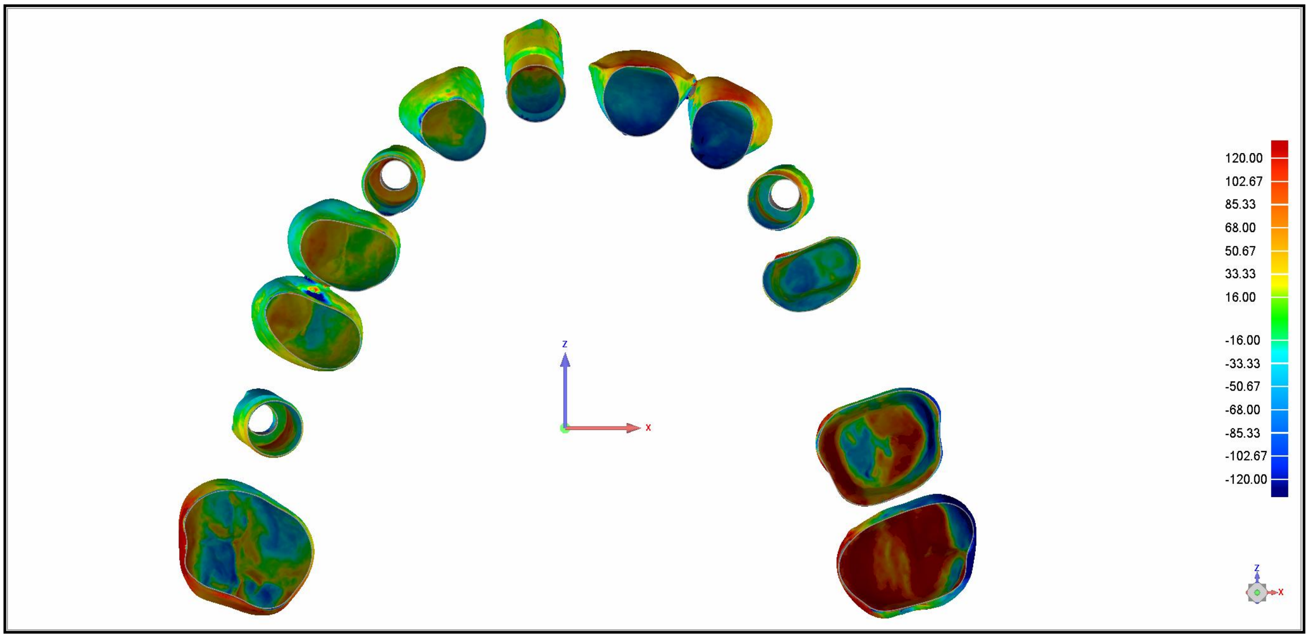

Predefinido: Atrás

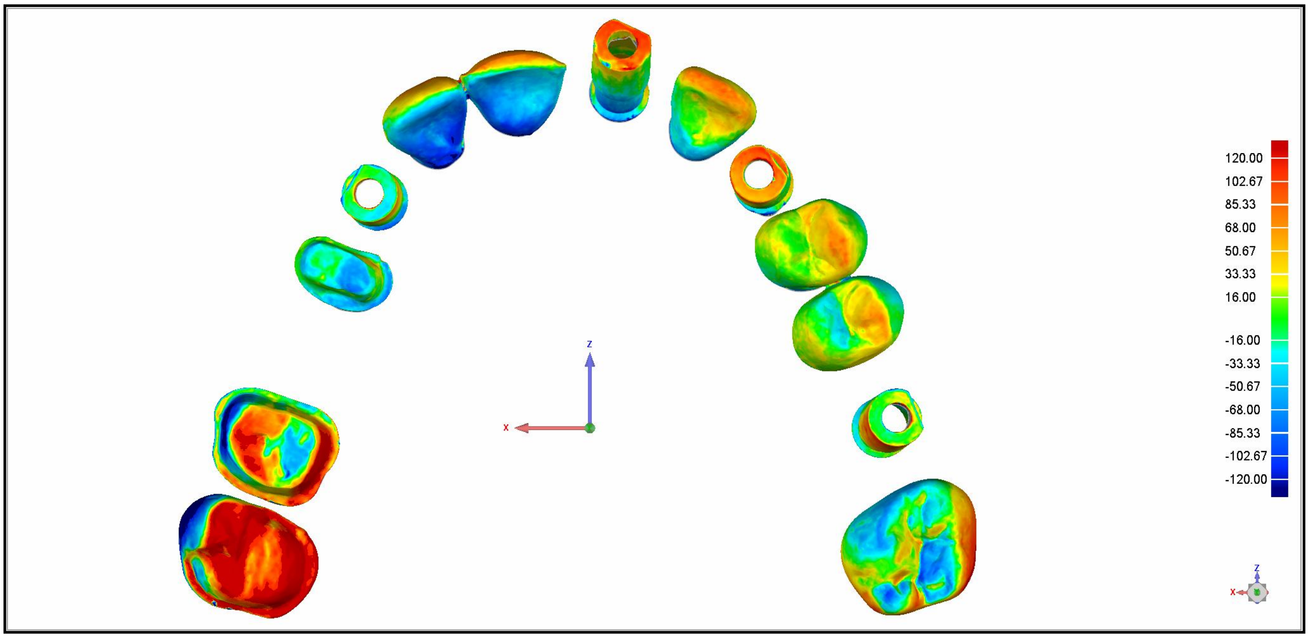

Predefinido: Izquierda

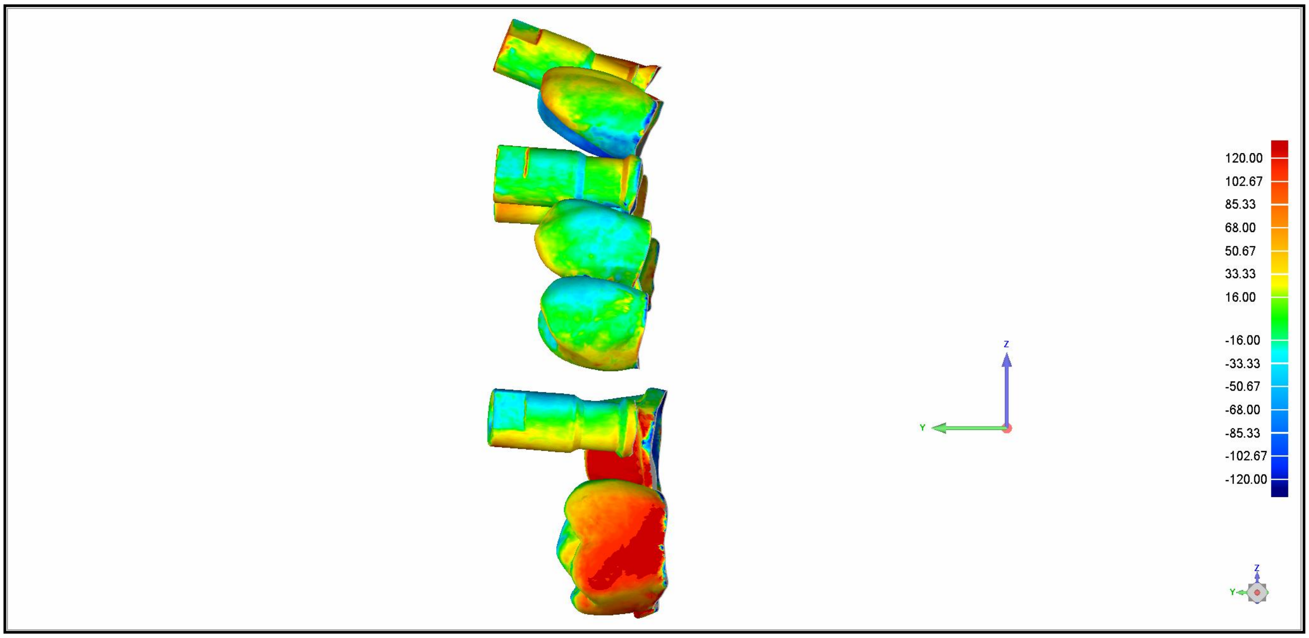

Predefinido: Derecha

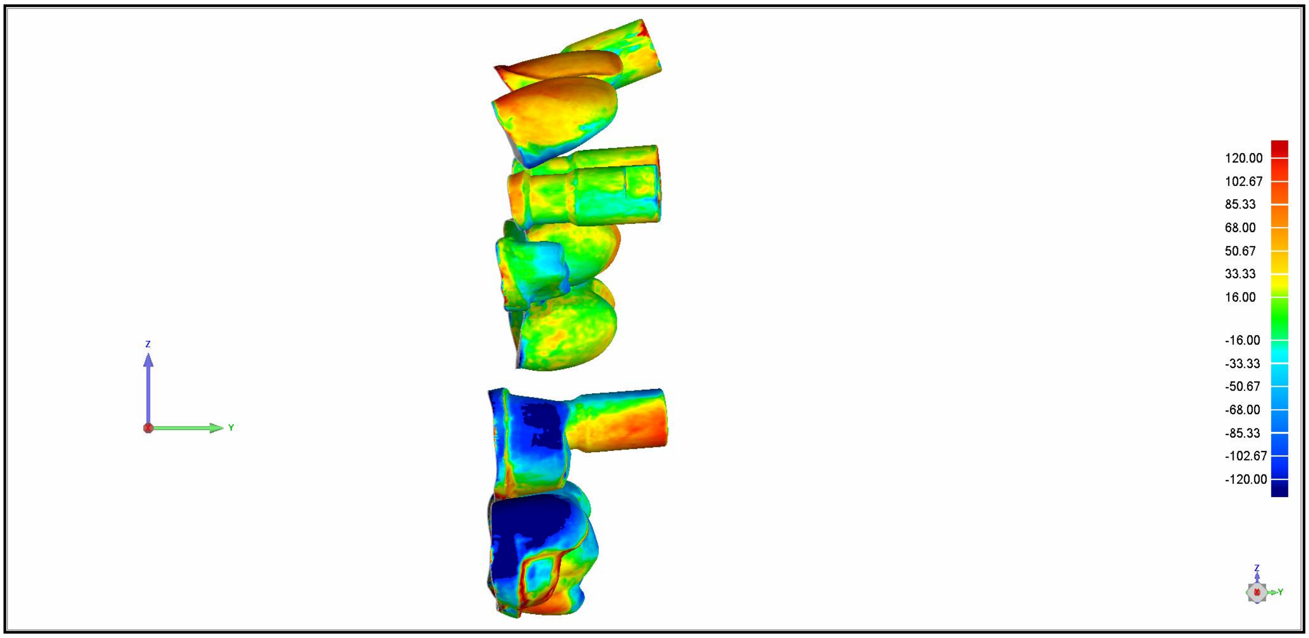

Predefinido: Superior

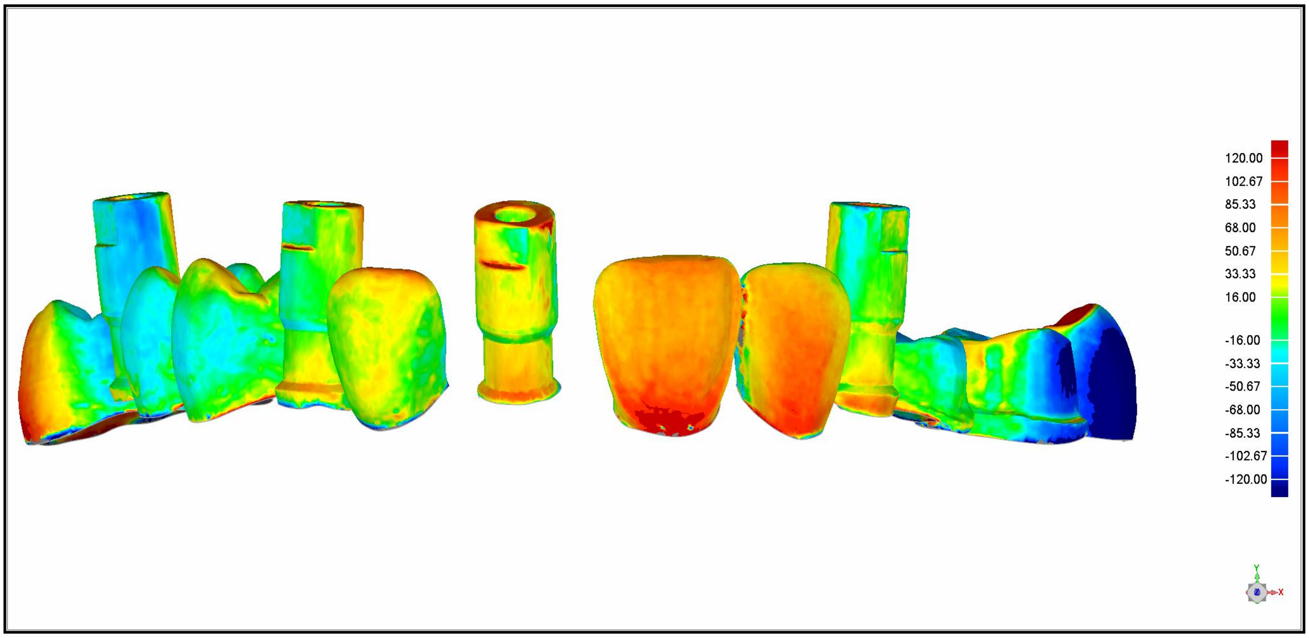

Predefinido: Inferior

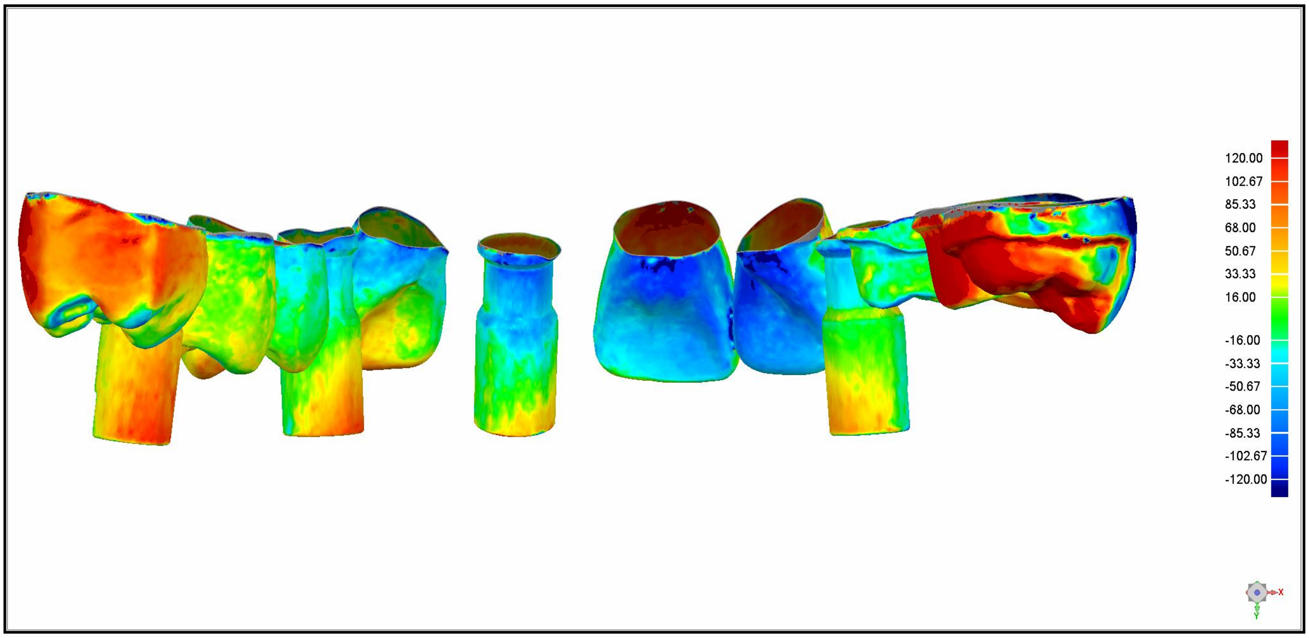

Ajuste de ubicación: Desviaciones superior e inferior

Unidades: u

Nombre	Desv	Estado	Superior Tol	Inferior Tol	Ref X	Ref Y	Ref Z	Radio	Desv X	Desv Y	Desv Z	Medido X	Medido Y	Medido Z	Dir. proy. X	Dir. proy. Y	Dir. proy. Z
Desv. inferior	-2089.97				29658.20	29476.92	-13998.63	n/a	209.81	-2008.74	537.52	29868.00	27468.18	-13461.11	-0.10	0.96	-0.26
Desv. superior	2174.04				17773.89	38478.38	19616.81	n/a	-27.94	-2152.20	-306.08	17745.95	36326.18	19310.73	-0.01	-0.99	-0.14
