# Supplementary material for: Accuracy of four digital scanners according to scanning strategy in complete-arch impressions
Source: PLoS One. 2018 Sep 13;13(9):e0202916. doi: 10.1371/journal.pone.0202916 (PMC6136706; doi:10.1371/journal.pone.0202916)
Supplement: S14 Table — True definition (scanning strategy B). (ZIP) [file pone.0202916.s014.zip › S14/TD8B.pdf]

Geomagic Control™

Fecha: 3/6/2017, 3:55 pm

3D Comparación Resultados

Modelo referencia	MRC
Modelo test	TD8B
Nº de puntos de datos	126265
# Aislados	324

Tipo tolerancia	3D desviación
Unidades	u
Máx. crítico	120.00
Máx. nominal	17.00
Mín. nominal	-17.00
Mín. crítico	-120.00

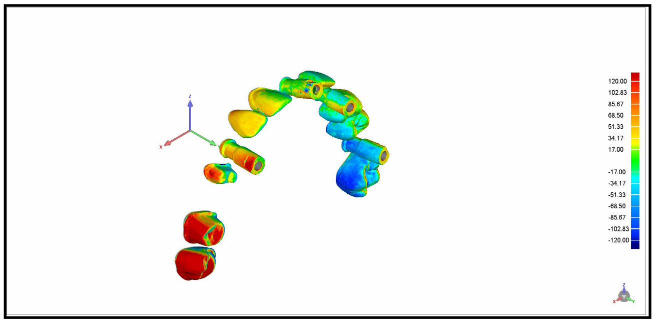

Desviación	
Desviación superior máx.	1913.26
Desviación inferior máx.	-3146.49
Desviación media	67.40 /-48.75
Desviación estándar	104.07

Distribución desviación

>=Min	<Max	# Puntos	%
-120.00	-102.83	1265	1.00
-102.83	-85.67	2078	1.65
-85.67	-68.50	3744	2.97
-68.50	-51.33	5624	4.45
-51.33	-34.17	7893	6.25
-34.17	-17.00	12998	10.29
-17.00	17.00	31781	25.17
17.00	34.17	13805	10.93
34.17	51.33	13146	10.41
51.33	68.50	7768	6.15
68.50	85.67	5892	4.67
85.67	102.83	3578	2.83
102.83	120.00	2575	2.04

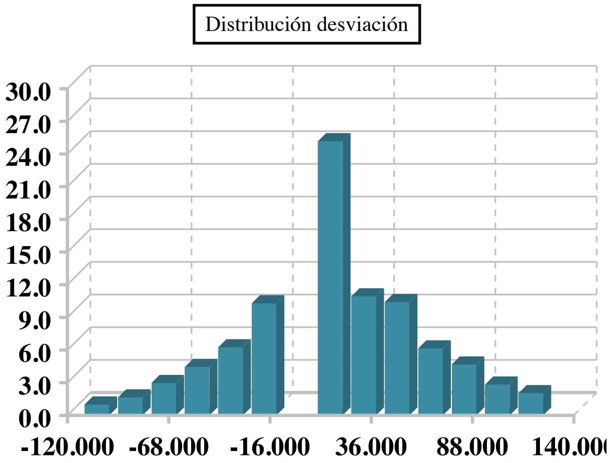

Fuera del crítico superior		10226	8.10
Fuera del crítico inferior		3892	3.08

Desviaciones estándar

Distribución (+/-)	# Puntos	%
-6 * Desv. estánd.	133	0.11
-5 * Desv. estánd.	47	0.04
-4 * Desv. estánd.	202	0.16
-3 * Desv. estánd.	1486	1.18
-2 * Desv. estánd.	5258	4.16
-1 * Desv. estánd.	62768	49.71
1 * Desv. estánd.	46452	36.79
2 * Desv. estánd.	6554	5.19
3 * Desv. estánd.	2761	2.19
4 * Desv. estánd.	185	0.15
5 * Desv. estánd.	97	0.08
6 * Desv. estánd.	322	0.26

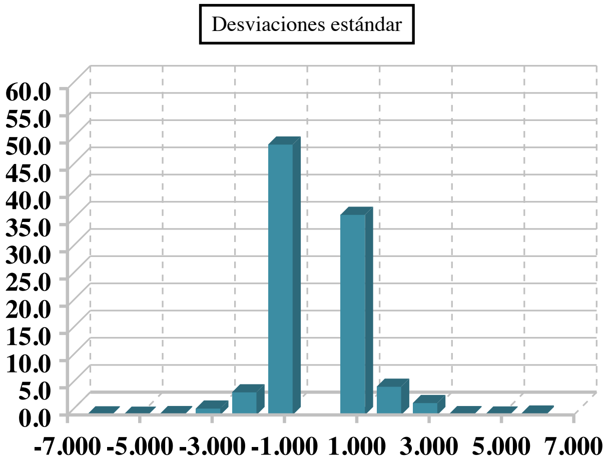

Predefinido: Isométrico

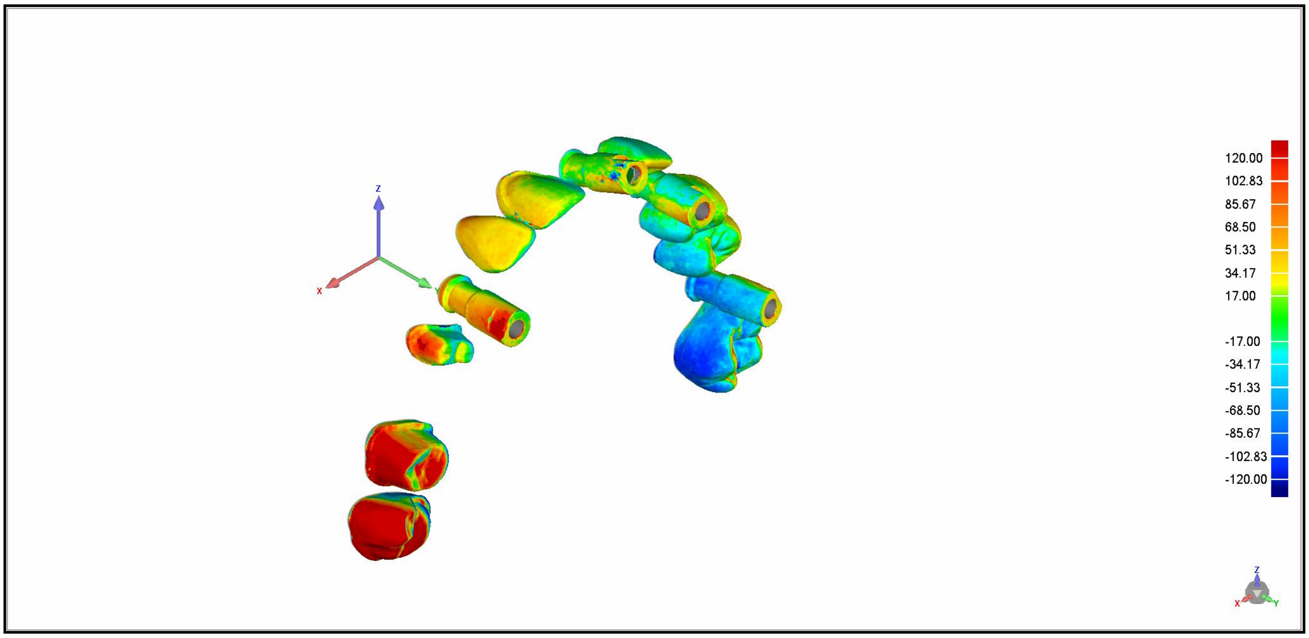

Predefinido: Frente

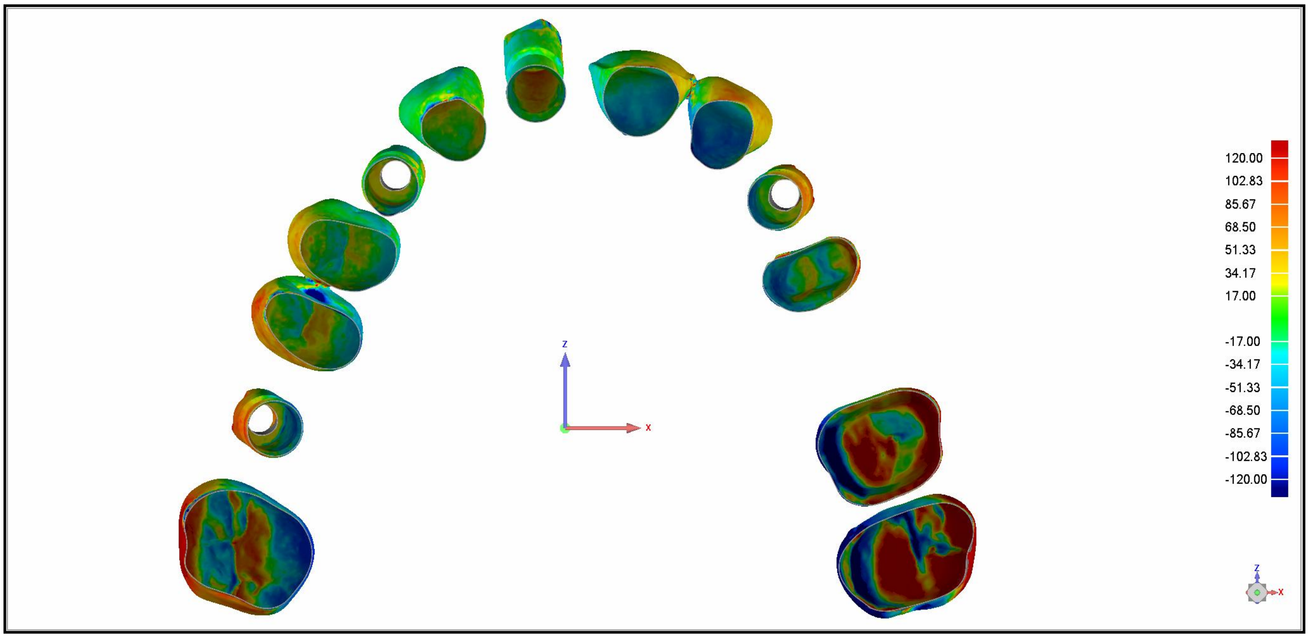

Predefinido: Atrás

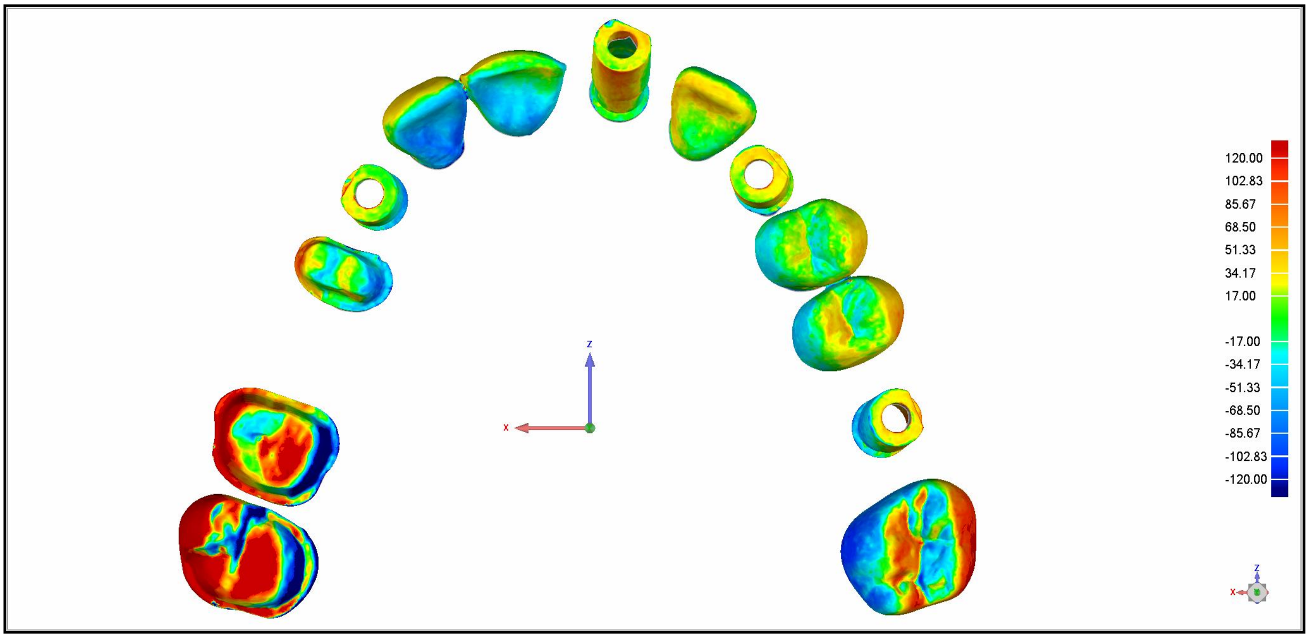

Predefinido: Izquierda

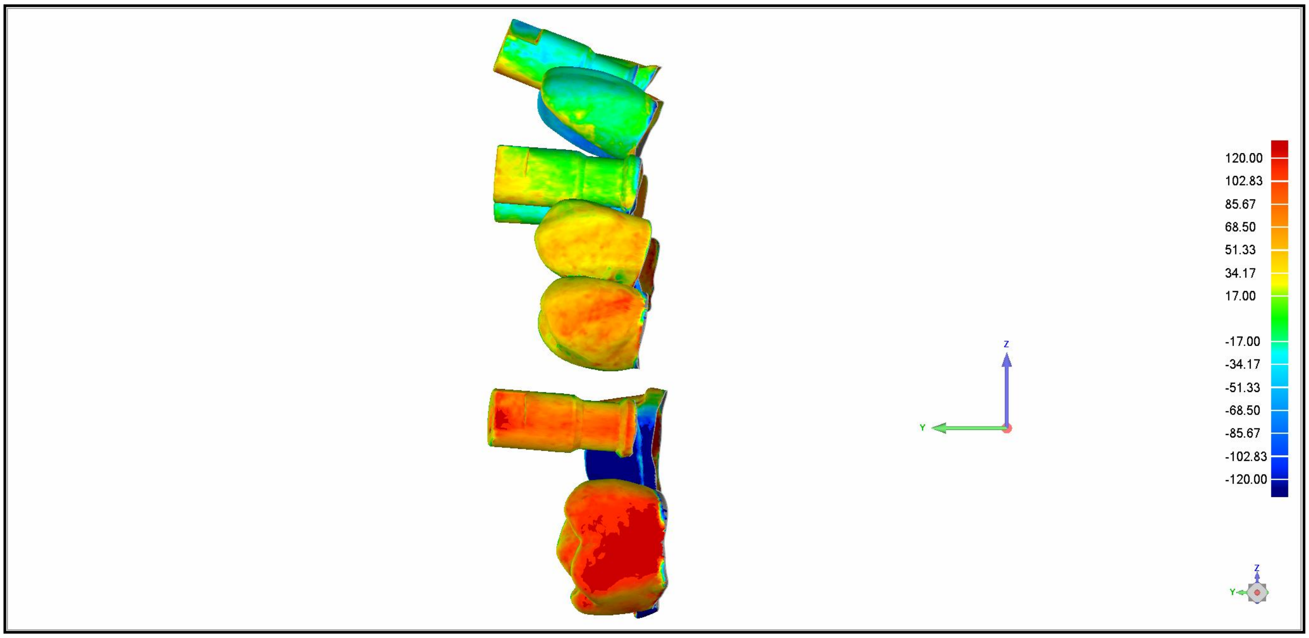

Predefinido: Derecha

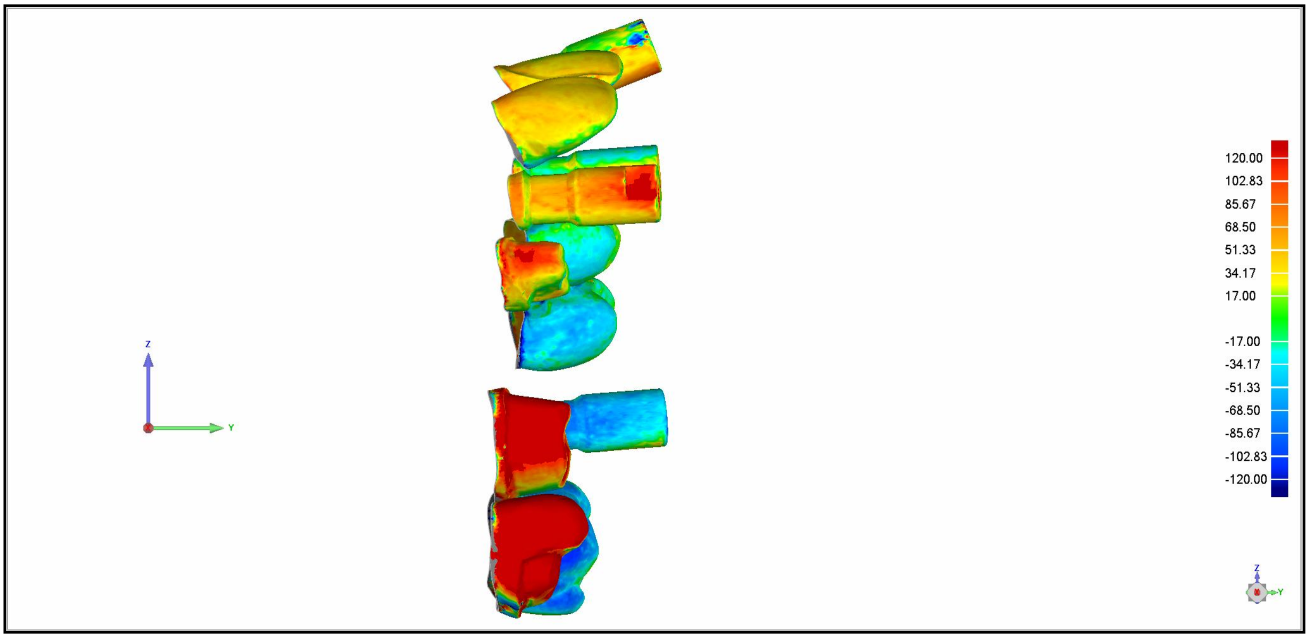

Predefinido: Superior

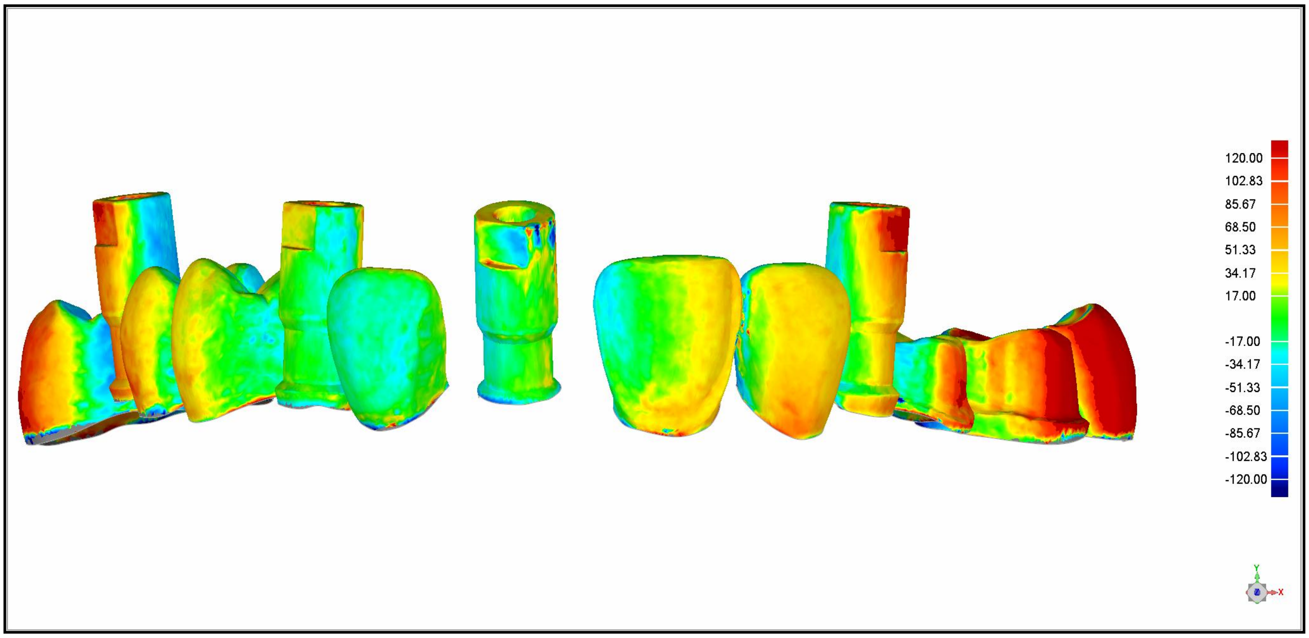

Predefinido: Inferior

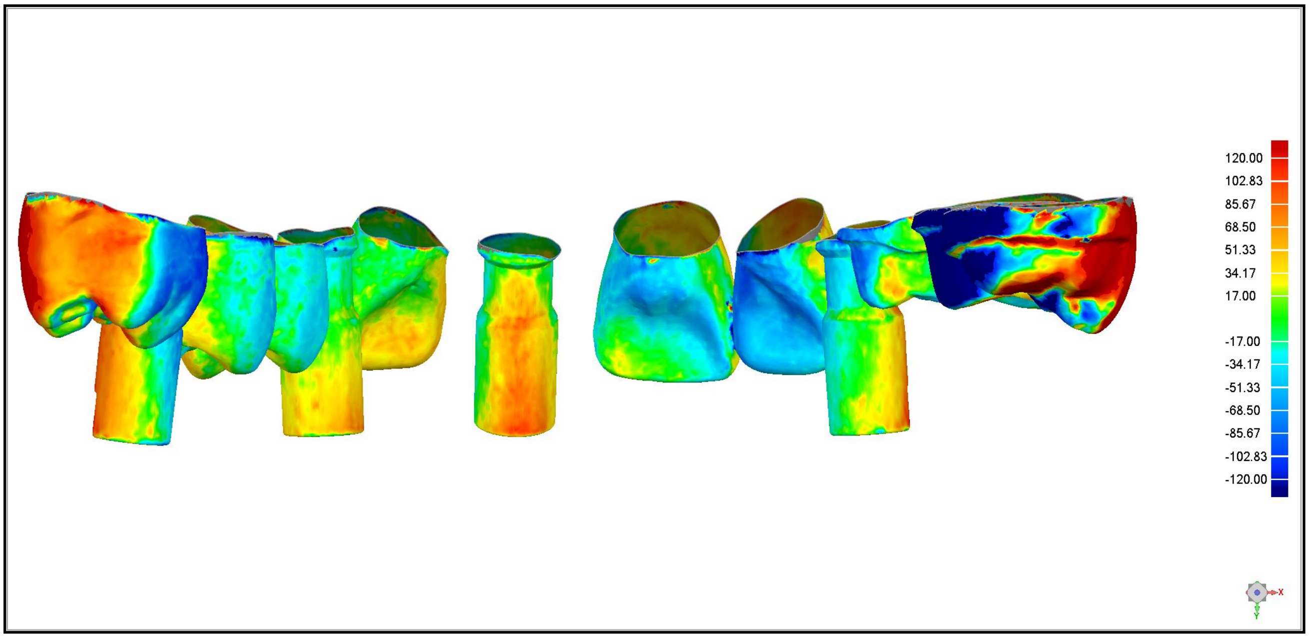

Ajuste de ubicación: Desviaciones superior e inferior

Unidades: u

Nombre	Desv	Estado	Superior Tol	Inferior Tol	Ref X	Ref Y	Ref Z	Radio	Desv X	Desv Y	Desv Z	Medido X	Medido Y	Medido Z	Dir. proy. X	Dir. proy. Y	Dir. proy. Z
Desv. inferior	-3146.49				-22607.19	28955.77	6808.03	n/a	-1016.93	-309.22	2961.52	-23624.13	28646.55	9769.56	0.32	0.10	-0.94
Desv. superior	1913.26				-13006.02	38540.95	21105.34	n/a	-638.89	-1778.33	-299.92	-13644.91	36762.62	20805.42	-0.33	-0.93	-0.16
