# Supplementary material for: Accuracy of four digital scanners according to scanning strategy in complete-arch impressions
Source: PLoS One. 2018 Sep 13;13(9):e0202916. doi: 10.1371/journal.pone.0202916 (PMC6136706; doi:10.1371/journal.pone.0202916)
Supplement: S14 Table — True definition (scanning strategy B). (ZIP) [file pone.0202916.s014.zip › S14/TD4B.pdf]

Geomagic Control™

Fecha: 3/6/2017, 3:40 pm

3D Comparación Resultados

Modelo referencia	MRC
Modelo test	TD4B
Nº de puntos de datos	130850
# Aislados	338

Tipo tolerancia	3D desviación
Unidades	u
Máx. crítico	120.00
Máx. nominal	15.00
Mín. nominal	-15.00
Mín. crítico	-120.00

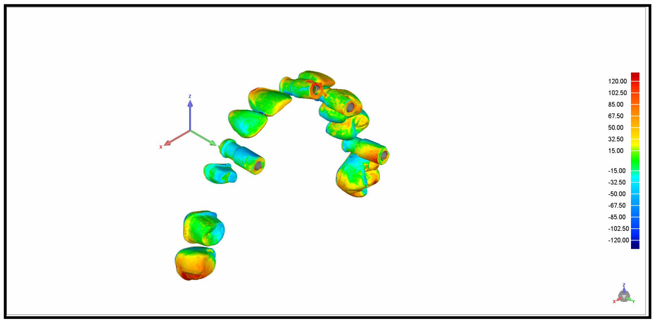

Desviación	
Desviación superior máx.	2074.58
Desviación inferior máx.	-1214.69
Desviación media	40.78 /-24.63
Desviación estándar	81.07

Distribución desviación

>=Min	<Max	# Puntos	%
-120.00	-102.50	136	0.10
-102.50	-85.00	194	0.15
-85.00	-67.50	546	0.42
-67.50	-50.00	2368	1.81
-50.00	-32.50	7360	5.62
-32.50	-15.00	15629	11.94
-15.00	15.00	49131	37.55
15.00	32.50	21880	16.72
32.50	50.00	13862	10.59
50.00	67.50	8915	6.81
67.50	85.00	4587	3.51
85.00	102.50	2019	1.54
102.50	120.00	1072	0.82

Fuera del crítico superior	2392	1.83
Fuera del crítico inferior	759	0.58

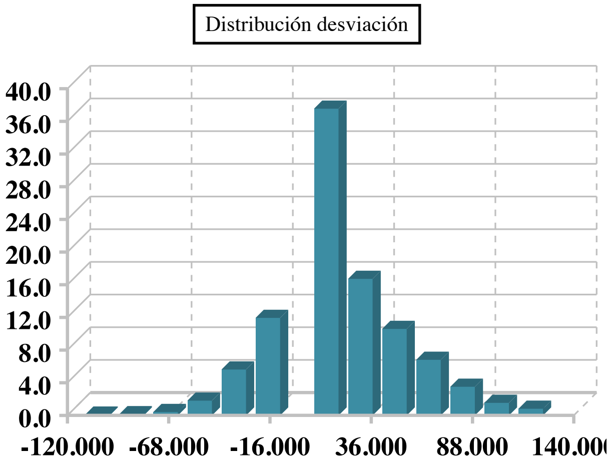

Desviaciones estándar

Distribución (+/-)	# Puntos	%
-6 * Desv. estánd.	171	0.13
-5 * Desv. estánd.	42	0.03
-4 * Desv. estánd.	95	0.07
-3 * Desv. estánd.	282	0.22
-2 * Desv. estánd.	1171	0.89
-1 * Desv. estánd.	75222	57.49
1 * Desv. estánd.	49896	38.13
2 * Desv. estánd.	2789	2.13
3 * Desv. estánd.	321	0.25
4 * Desv. estánd.	189	0.14
5 * Desv. estánd.	100	0.08
6 * Desv. estánd.	572	0.44

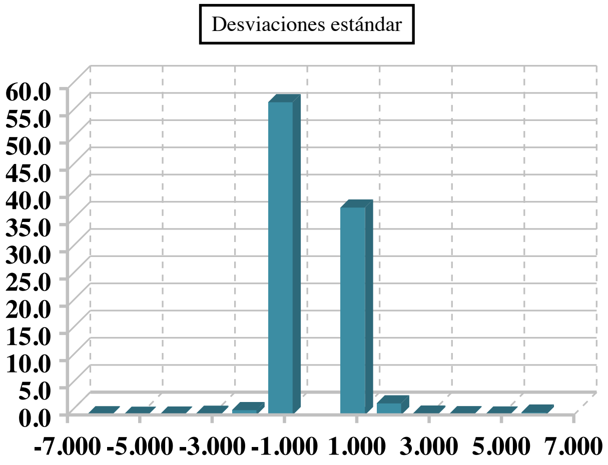

Predefinido: Isométrico

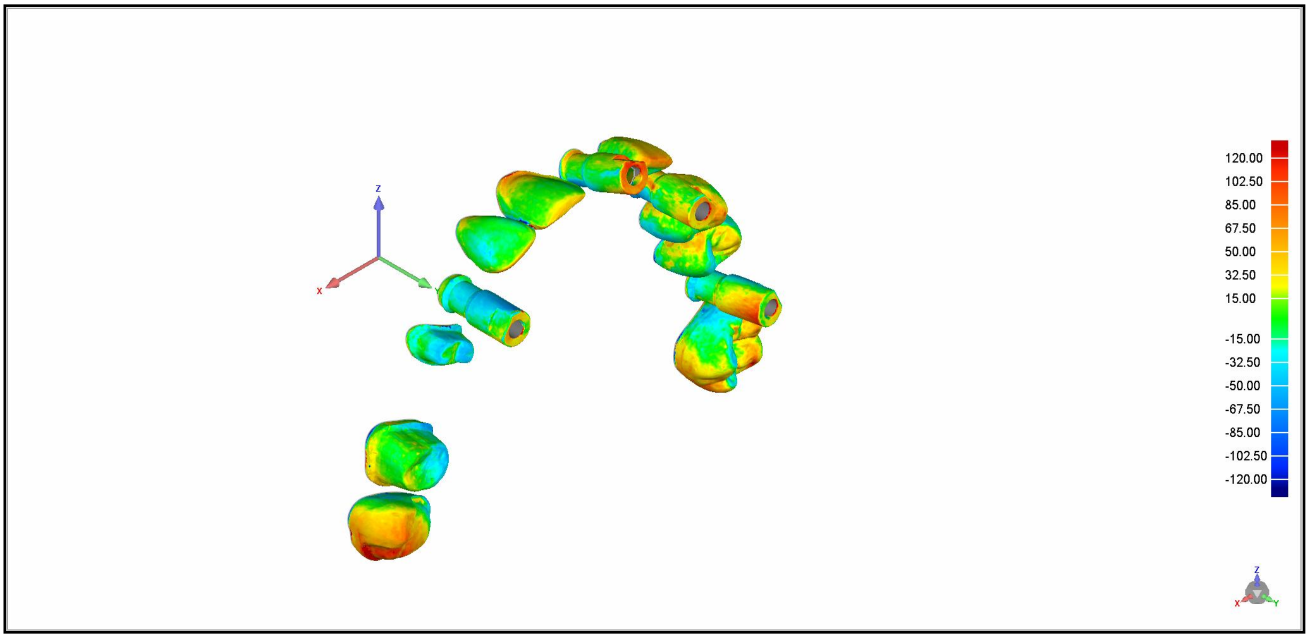

Predefinido: Frente

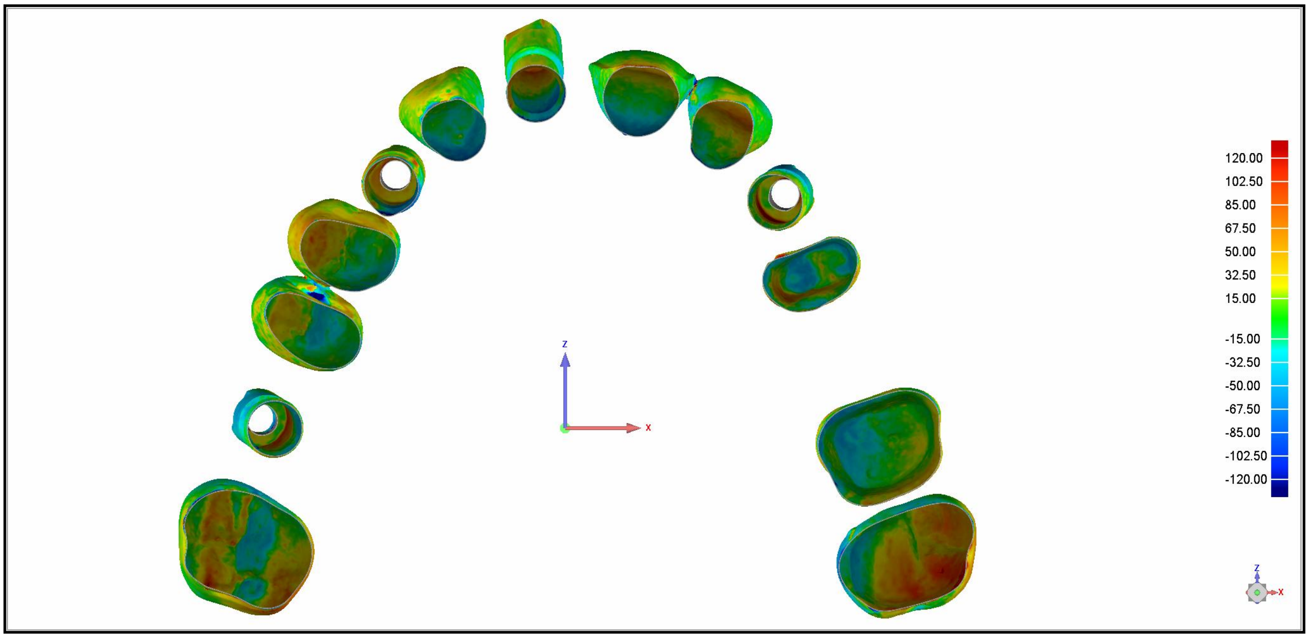

Predefinido: Atrás

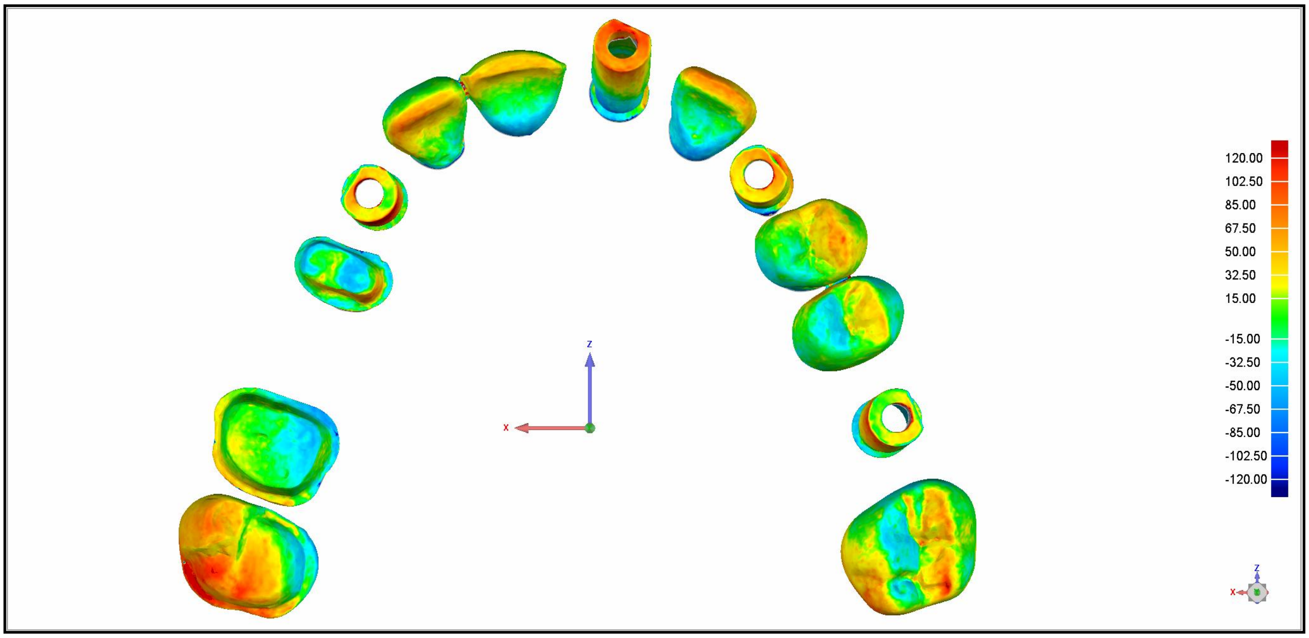

Predefinido: Izquierda

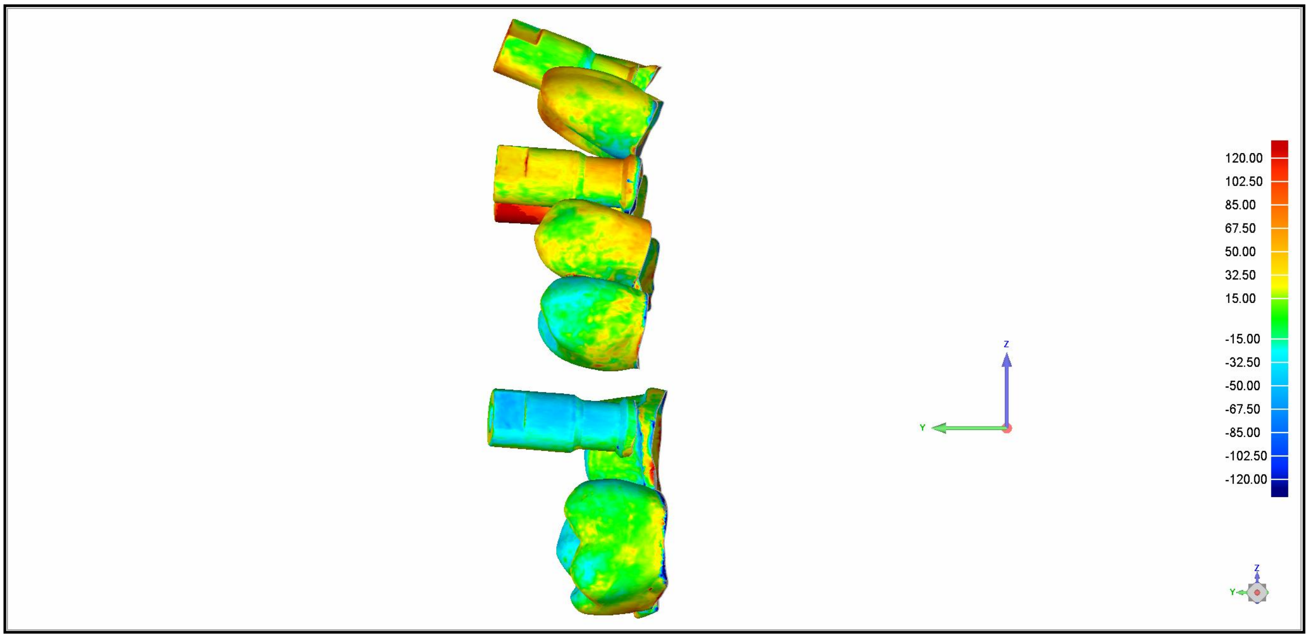

Predefinido: Derecha

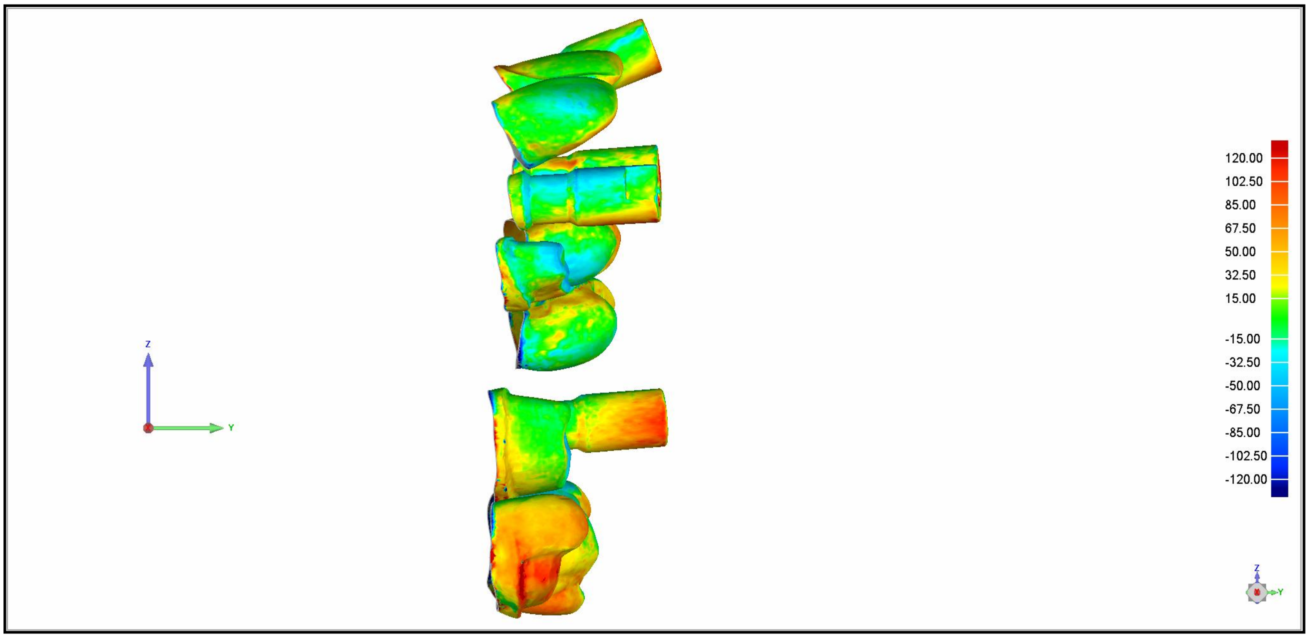

Predefinido: Superior

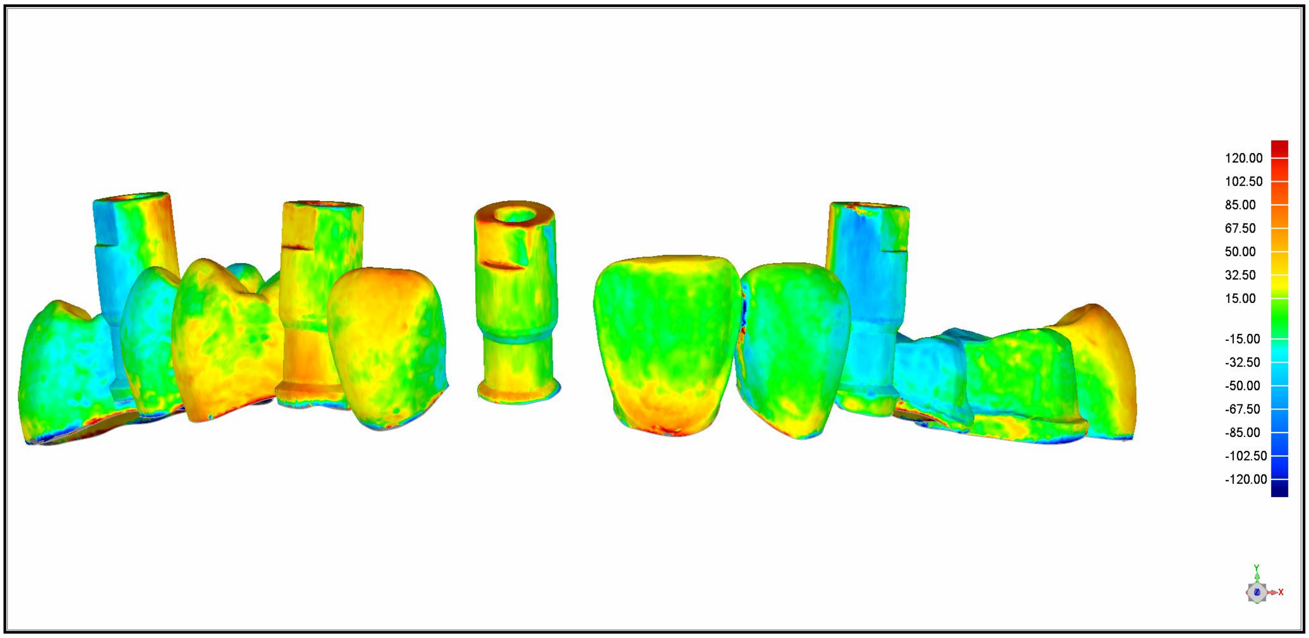

Predefinido: Inferior

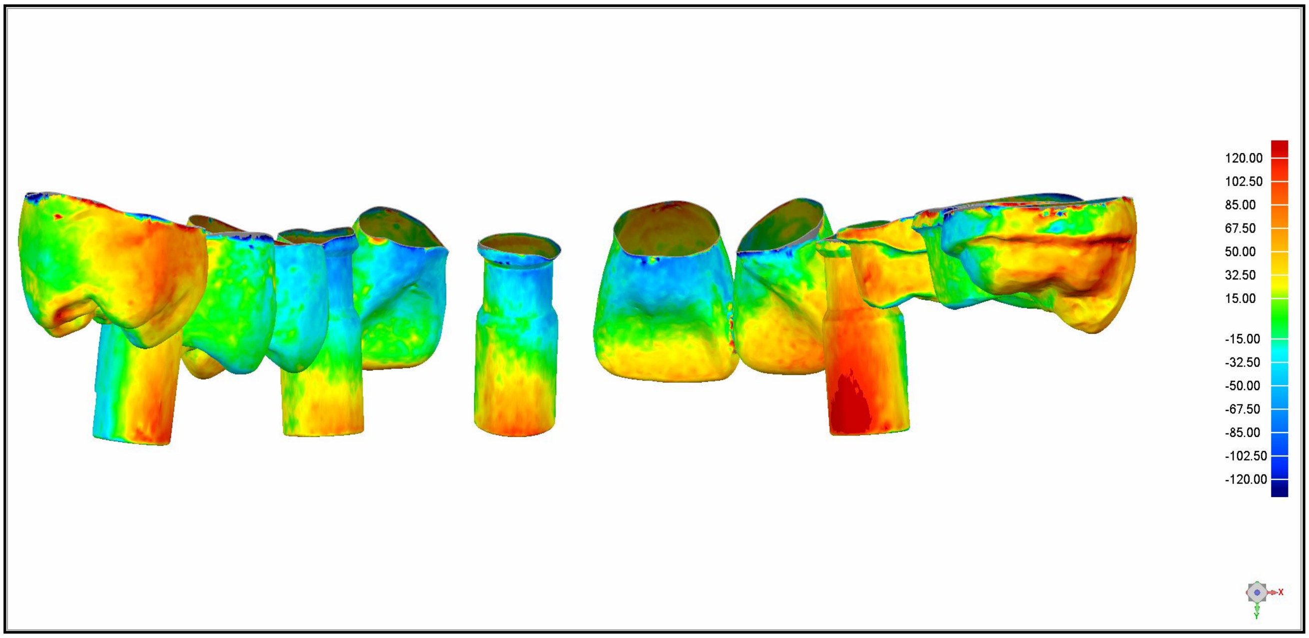

Ajuste de ubicación: Desviaciones superior e inferior

Unidades: u

Nombre	Desv	Estado	Superior Tol	Inferior Tol	Ref X	Ref Y	Ref Z	Radio	Desv X	Desv Y	Desv Z	Medido X	Medido Y	Medido Z	Dir. proy. X	Dir. proy. Y	Dir. proy. Z
Desv. inferior	-1214.69				-27983.79	27113.85	-12612.88	n/a	1057.65	185.56	-567.82	-26926.14	27299.41	-13180.69	-0.87	-0.15	0.47
Desv. superior	2074.58				17773.89	38478.39	19616.81	n/a	-235.28	-2014.32	-437.12	17538.61	36464.07	19179.69	-0.11	-0.97	-0.21
