# Supplementary material for: Accuracy of four digital scanners according to scanning strategy in complete-arch impressions
Source: PLoS One. 2018 Sep 13;13(9):e0202916. doi: 10.1371/journal.pone.0202916 (PMC6136706; doi:10.1371/journal.pone.0202916)
Supplement: S14 Table — True definition (scanning strategy B). (ZIP) [file pone.0202916.s014.zip › S14/TD7B.pdf]

Geomagic Control

Fecha: 3/6/2017, 3:52 pm

3D Comparación Resultados

Modelo referencia	MRC
Modelo test	TD7B
Nº de puntos de datos	132755
# Aislados	239

Tipo tolerancia	3D desviación
Unidades	u
Máx. crítico	120.00
Máx. nominal	22.00
Mín. nominal	-22.00
Mín. crítico	-120.00

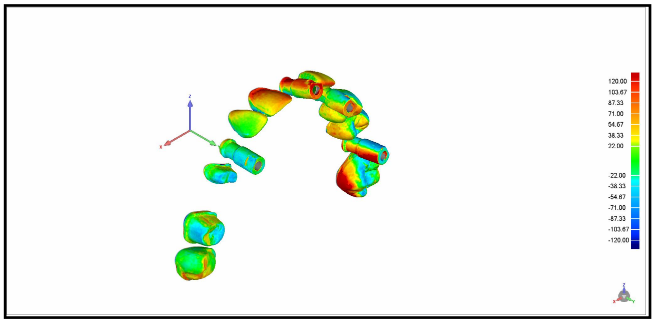

Desviación	
Desviación superior máx.	2537.14
Desviación inferior máx.	-2400.03
Desviación media	54.52 /-31.77
Desviación estándar	90.87

Distribución desviación

>=Min	<Max	# Puntos	%
-120.00	-103.67	239	0.18
-103.67	-87.33	605	0.46
-87.33	-71.00	1486	1.12
-71.00	-54.67	3824	2.88
-54.67	-38.33	7353	5.54
-38.33	-22.00	12148	9.15
-22.00	22.00	48909	36.84
22.00	38.33	17018	12.82
38.33	54.67	12811	9.65
54.67	71.00	8621	6.49
71.00	87.33	5643	4.25
87.33	103.67	4427	3.33
103.67	120.00	2821	2.12

Fuera del crítico superior	6213	4.68
Fuera del crítico inferior	637	0.48

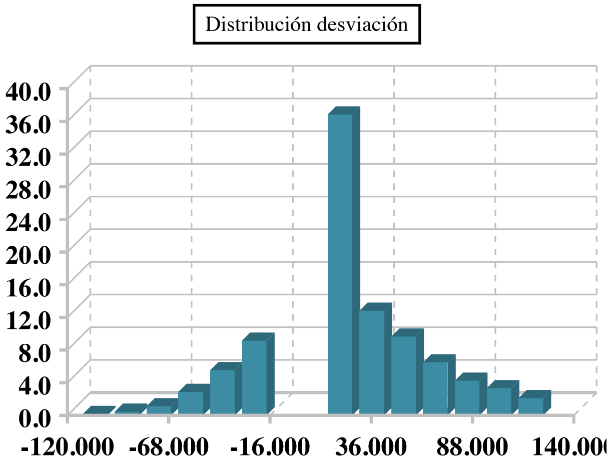

Desviaciones estándar

Distribución (+/-)	# Puntos	%
-6 * Desv. estánd.	98	0.07
-5 * Desv. estánd.	30	0.02
-4 * Desv. estánd.	44	0.03
-3 * Desv. estánd.	226	0.17
-2 * Desv. estánd.	2974	2.24
-1 * Desv. estánd.	72187	54.38
1 * Desv. estánd.	49967	37.64
2 * Desv. estánd.	5279	3.98
3 * Desv. estánd.	1377	1.04
4 * Desv. estánd.	112	0.08
5 * Desv. estánd.	73	0.05
6 * Desv. estánd.	388	0.29

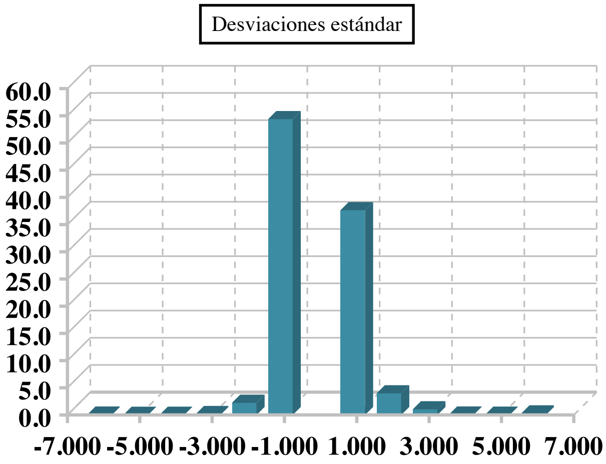

Predefinido: Isométrico

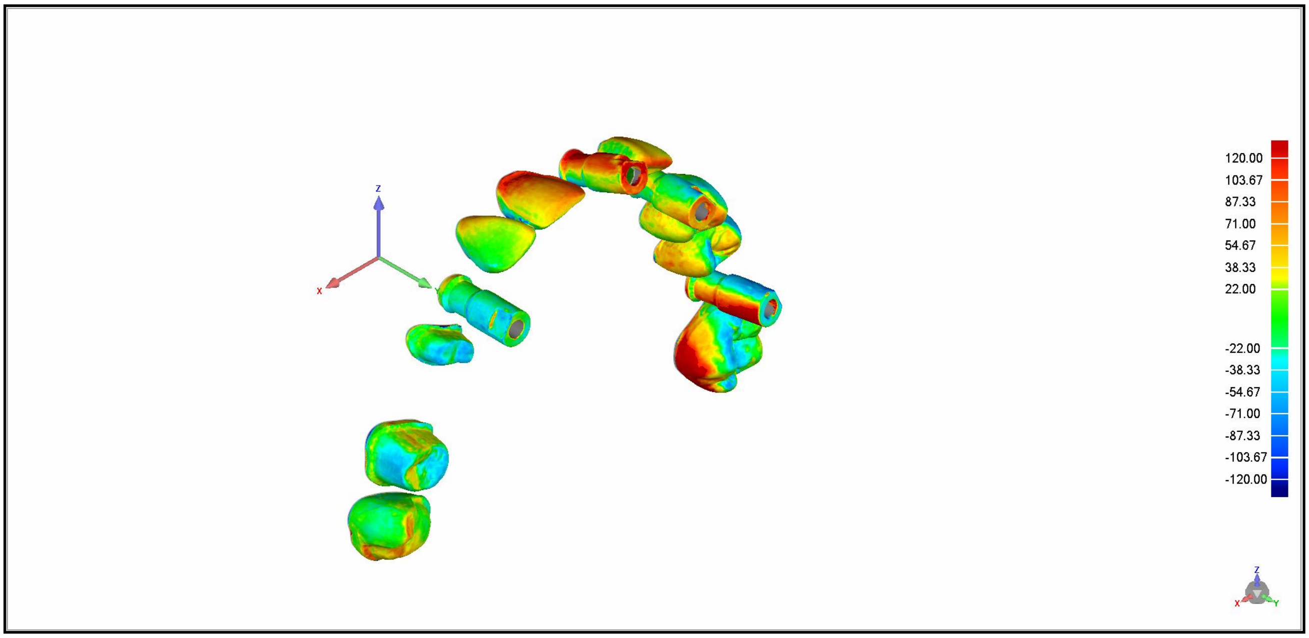

Predefinido: Frente

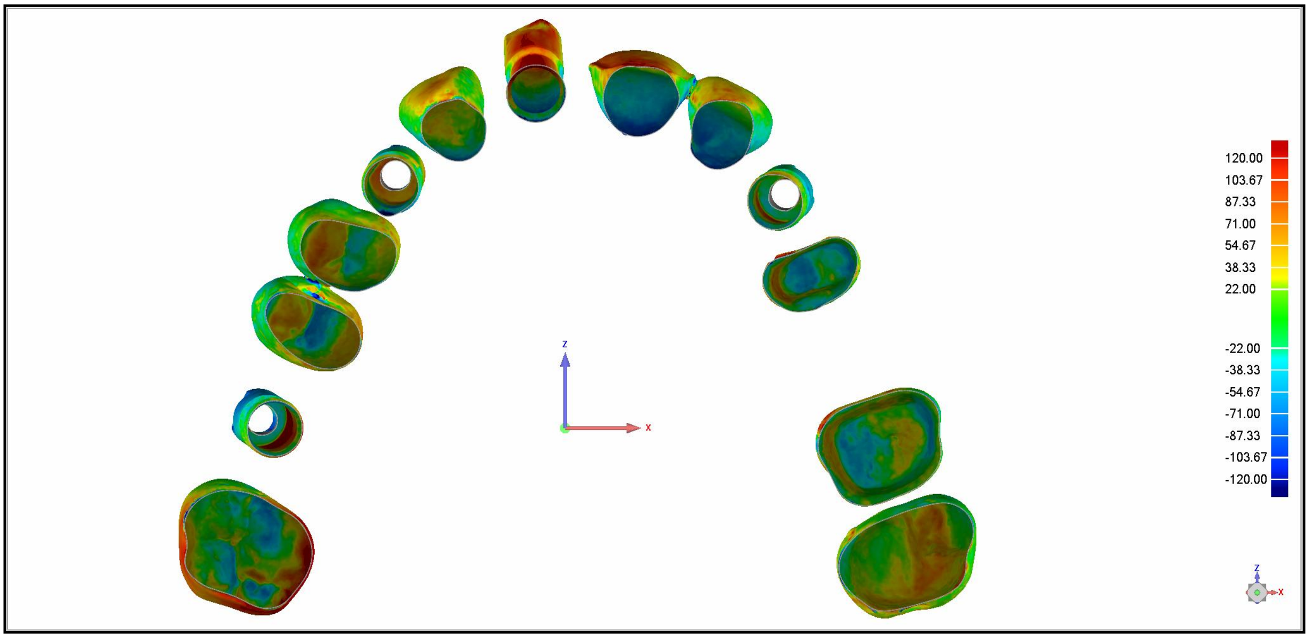

Predefinido: Atrás

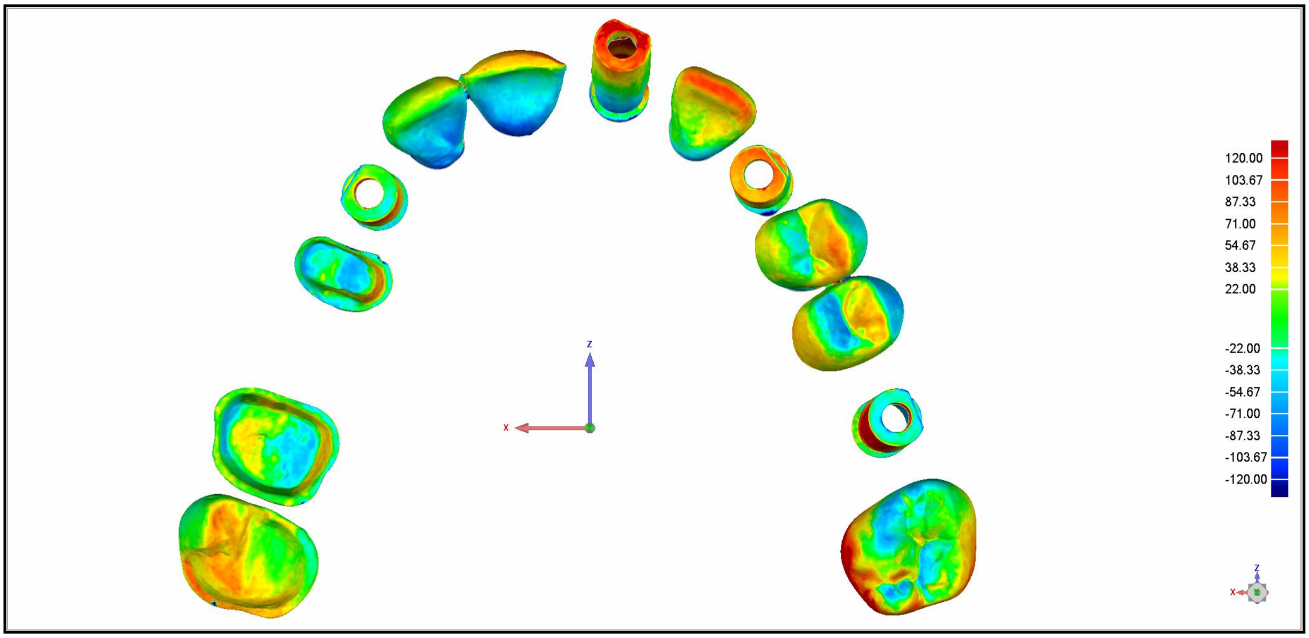

Predefinido: Izquierda

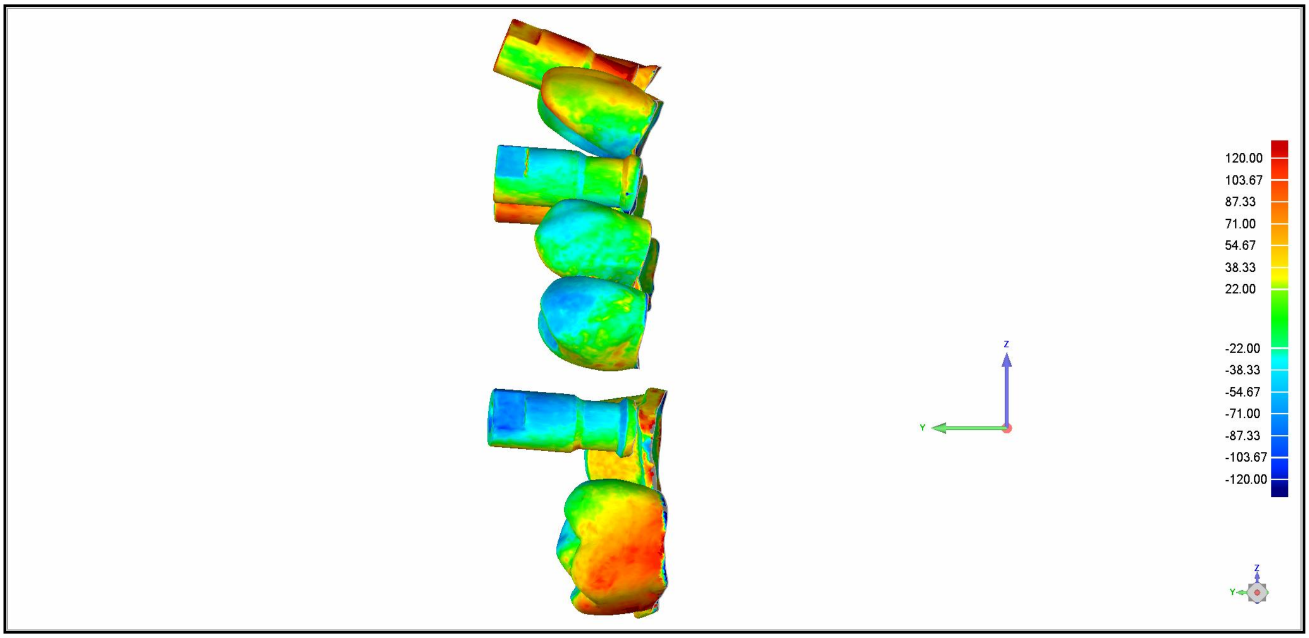

Predefinido: Derecha

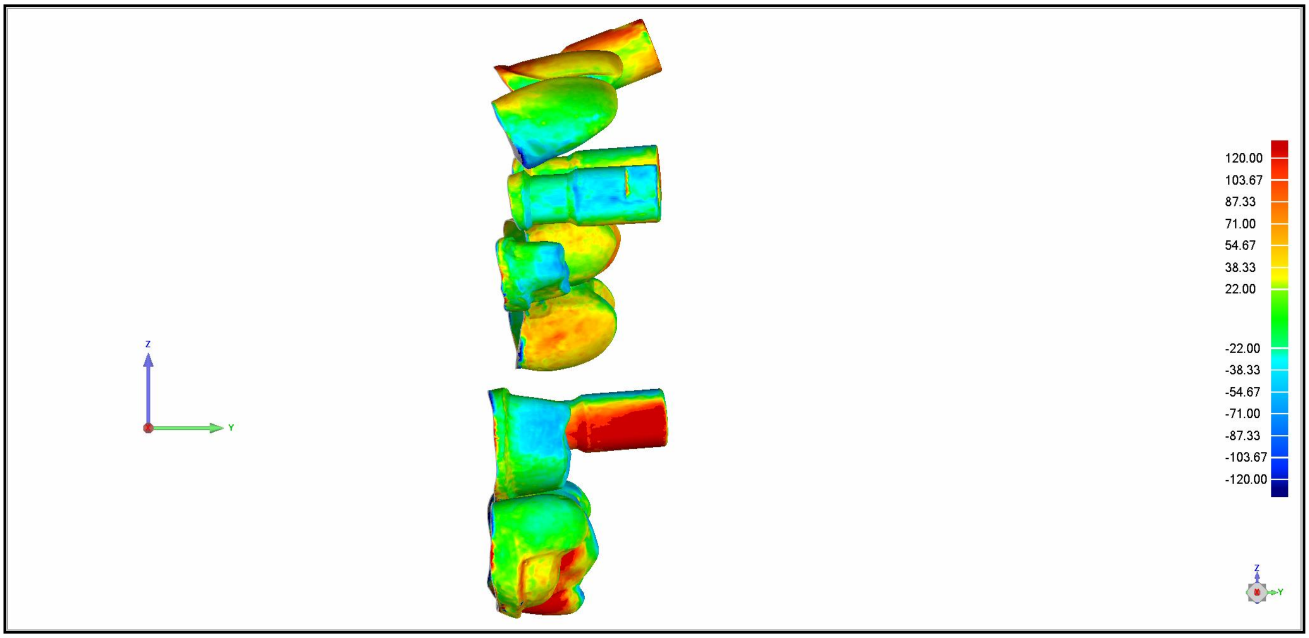

Predefinido: Superior

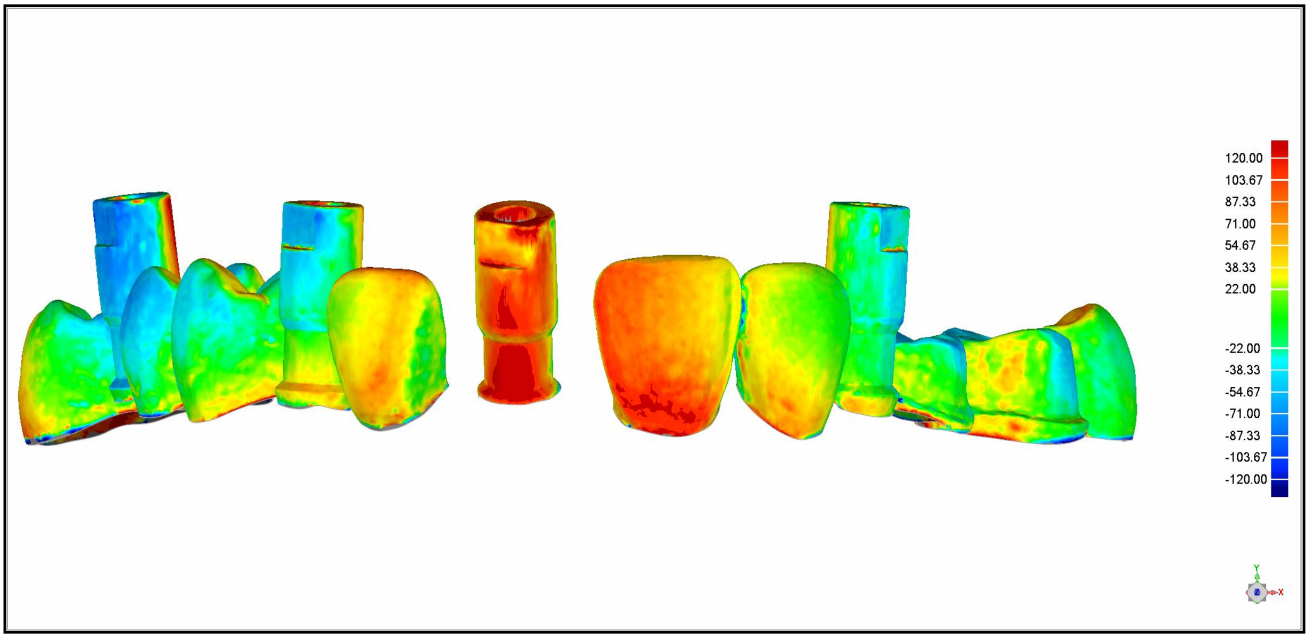

Predefinido: Inferior

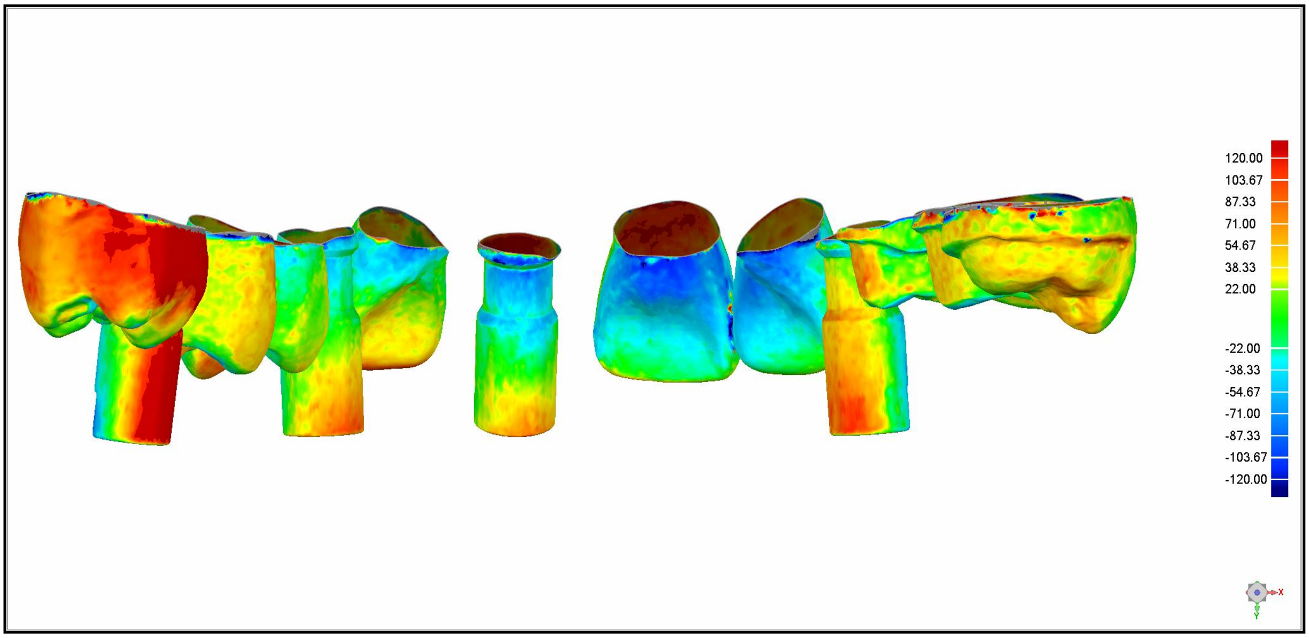

Ajuste de ubicación: Desviaciones superior e inferior

Unidades: u

Nombre	Desv	Estado	Superior Tol	Inferior Tol	Ref X	Ref Y	Ref Z	Radio	Desv X	Desv Y	Desv Z	Medido X	Medido Y	Medido Z	Dir. proy. X	Dir. proy. Y	Dir. proy. Z
Desv. inferior	-2400.03				-29208.33	26961.25	-11988.49	n/a	2061.20	283.13	-1196.41	-27147.13	27244.38	-13184.90	-0.86	-0.12	0.50
Desv. superior	2537.14				-20553.64	28741.29	-8096.88	n/a	-1778.45	80.96	1807.66	-22332.09	28822.24	-6289.23	-0.70	0.03	0.71
