# Supplementary material for: Accuracy of four digital scanners according to scanning strategy in complete-arch impressions
Source: PLoS One. 2018 Sep 13;13(9):e0202916. doi: 10.1371/journal.pone.0202916 (PMC6136706; doi:10.1371/journal.pone.0202916)
Supplement: S14 Table — True definition (scanning strategy B). (ZIP) [file pone.0202916.s014.zip › S14/TD10B.pdf]

3D Comparación Resultados

Modelo referencia	MRC
Modelo test	TD10B
Nº de puntos de datos	126400
# Aislados	357

Tipo tolerancia	3D desviación
Unidades	u
Máx. crítico	120.00
Máx. nominal	11.00
Mín. nominal	-11.00
Mín. crítico	-120.00

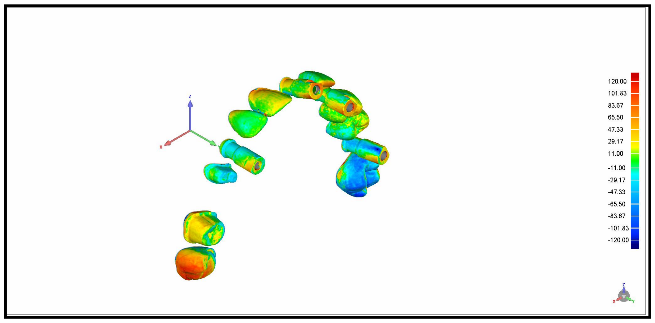

Desviación	
Desviación superior máx.	2311.62
Desviación inferior máx.	-2576.28
Desviación media	39.95 /-28.72
Desviación estándar	73.91

Distribución desviación

>=Min	<Max	# Puntos	%
-120.00	-101.83	234	0.19
-101.83	-83.67	792	0.63
-83.67	-65.50	2398	1.90
-65.50	-47.33	4834	3.82
-47.33	-29.17	8513	6.73
-29.17	-11.00	16266	12.87
-11.00	11.00	36735	29.06
11.00	29.17	22710	17.97
29.17	47.33	12641	10.00
47.33	65.50	7667	6.07
65.50	83.67	5241	4.15
83.67	101.83	3372	2.67
101.83	120.00	1847	1.46

Fuera del crítico superior	2312	1.83
Fuera del crítico inferior	838	0.66

Distribución desviación

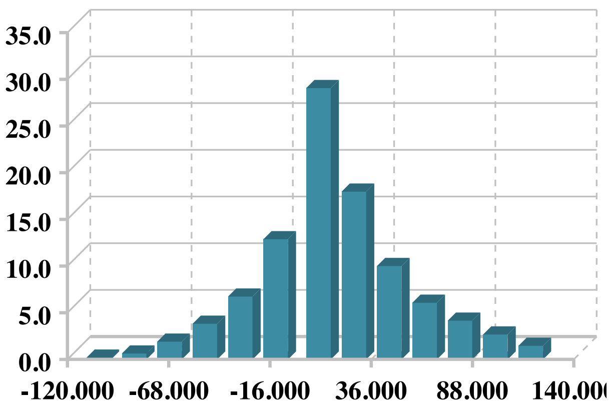

Desviaciones estándar

Distribución (+/-)	# Puntos	%
-6 * Desv. estánd.	115	0.09
-5 * Desv. estánd.	82	0.06
-4 * Desv. estánd.	208	0.16
-3 * Desv. estánd.	307	0.24
-2 * Desv. estánd.	4252	3.36
-1 * Desv. estánd.	66951	52.97
1 * Desv. estánd.	47435	37.53
2 * Desv. estánd.	6042	4.78
3 * Desv. estánd.	367	0.29
4 * Desv. estánd.	178	0.14
5 * Desv. estánd.	81	0.06
6 * Desv. estánd.	382	0.30

Desviaciones estándar

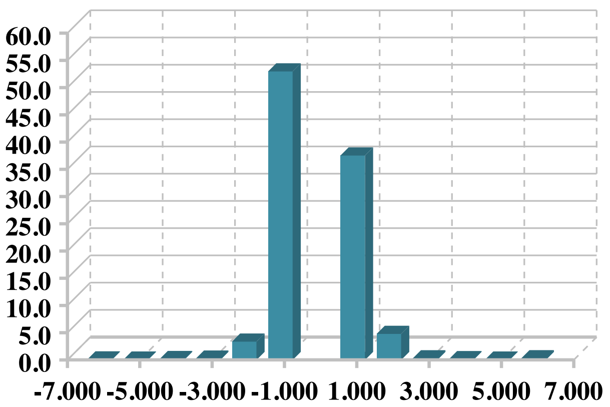

Predefinido: Isométrico

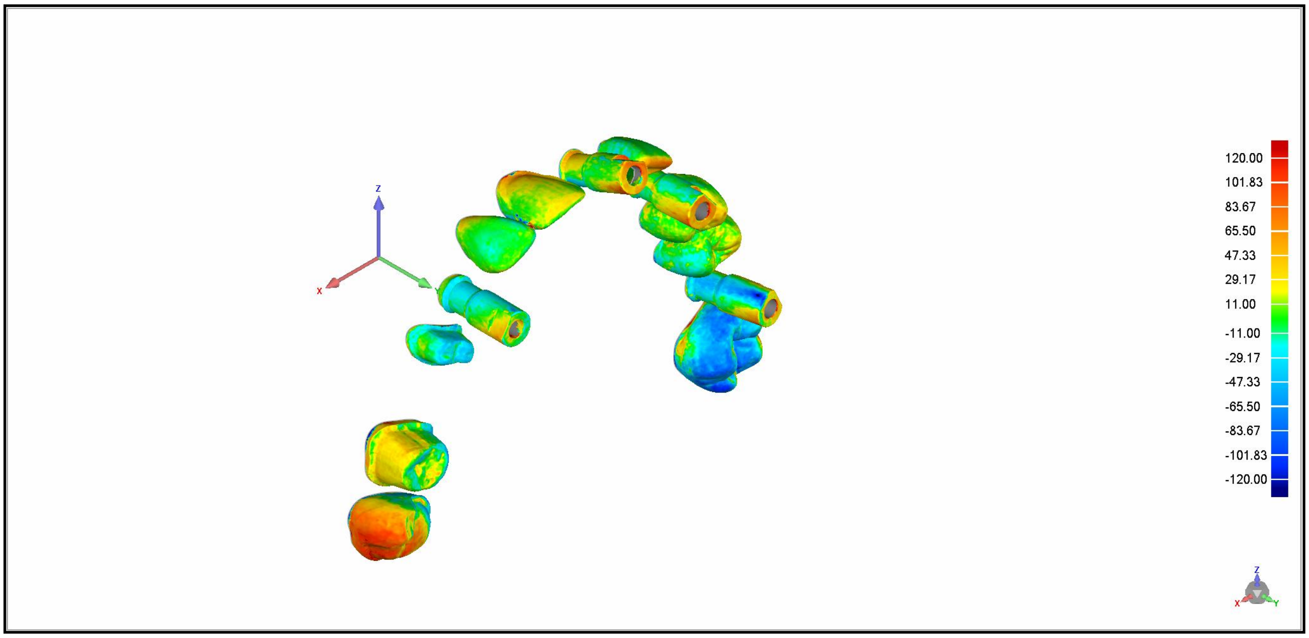

Predefinido: Frente

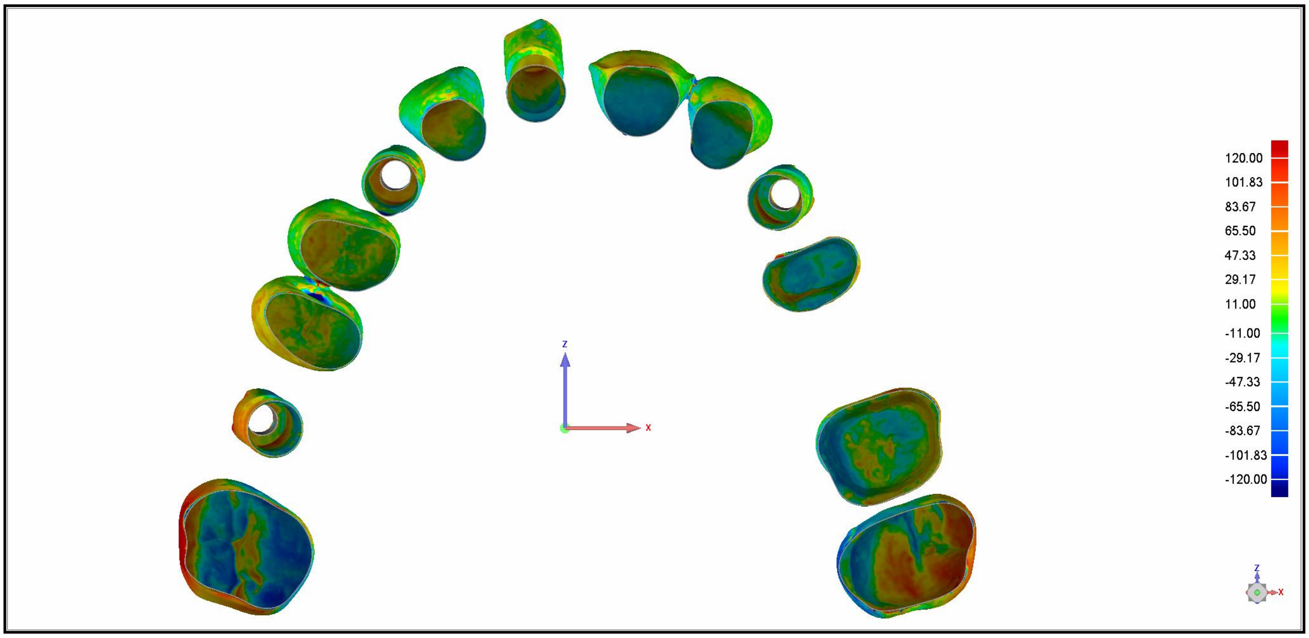

Predefinido: Atrás

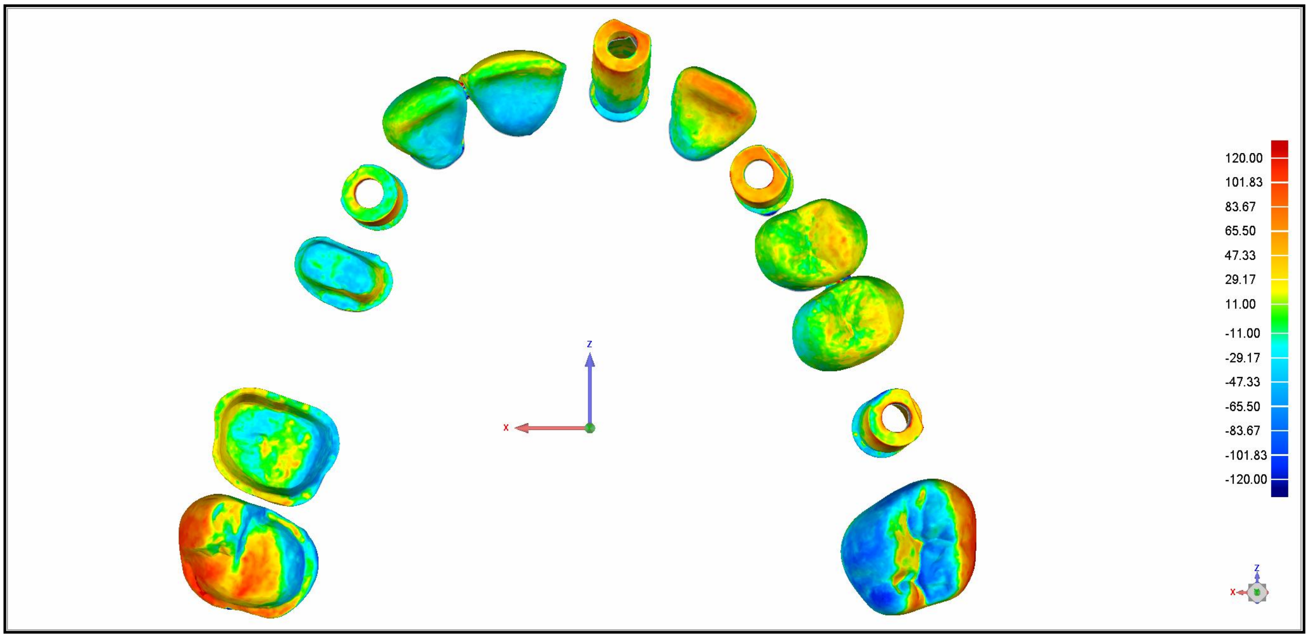

Predefinido: Izquierda

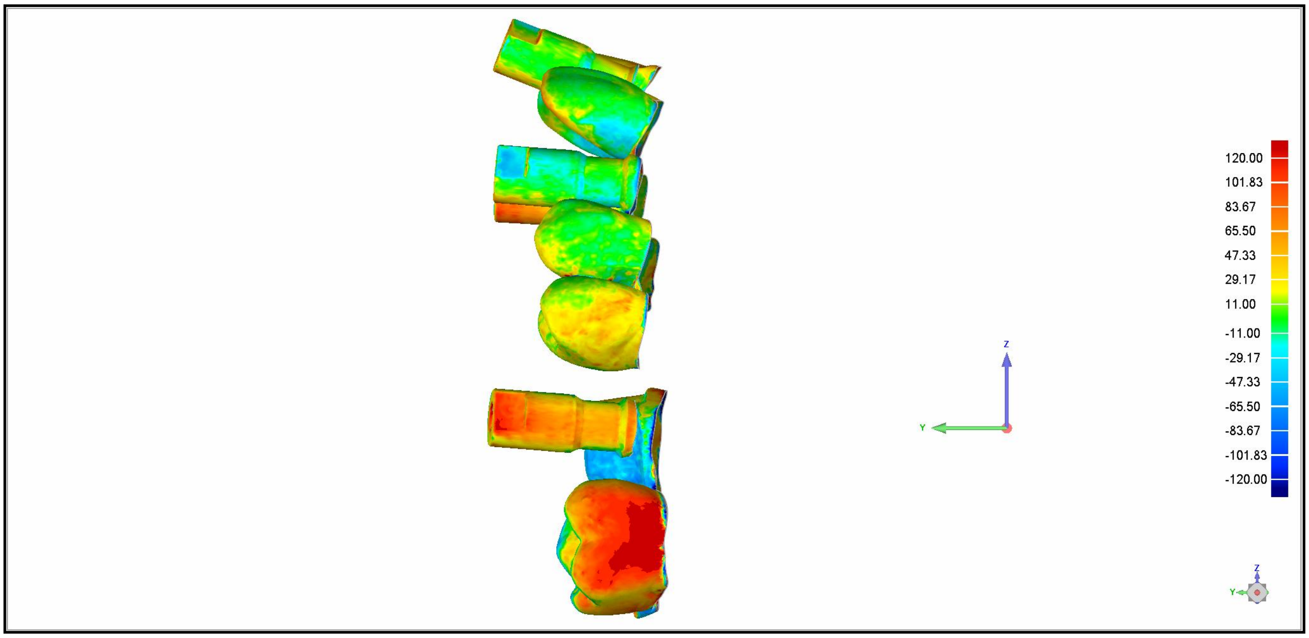

Predefinido: Derecha

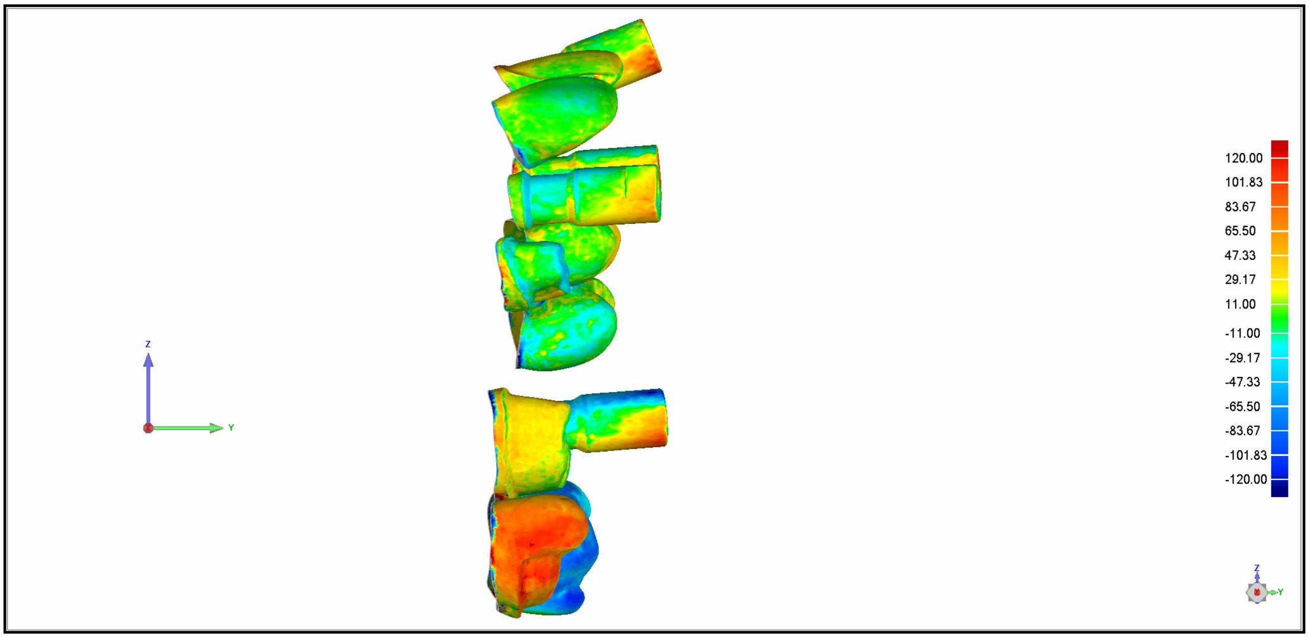

Predefinido: Superior

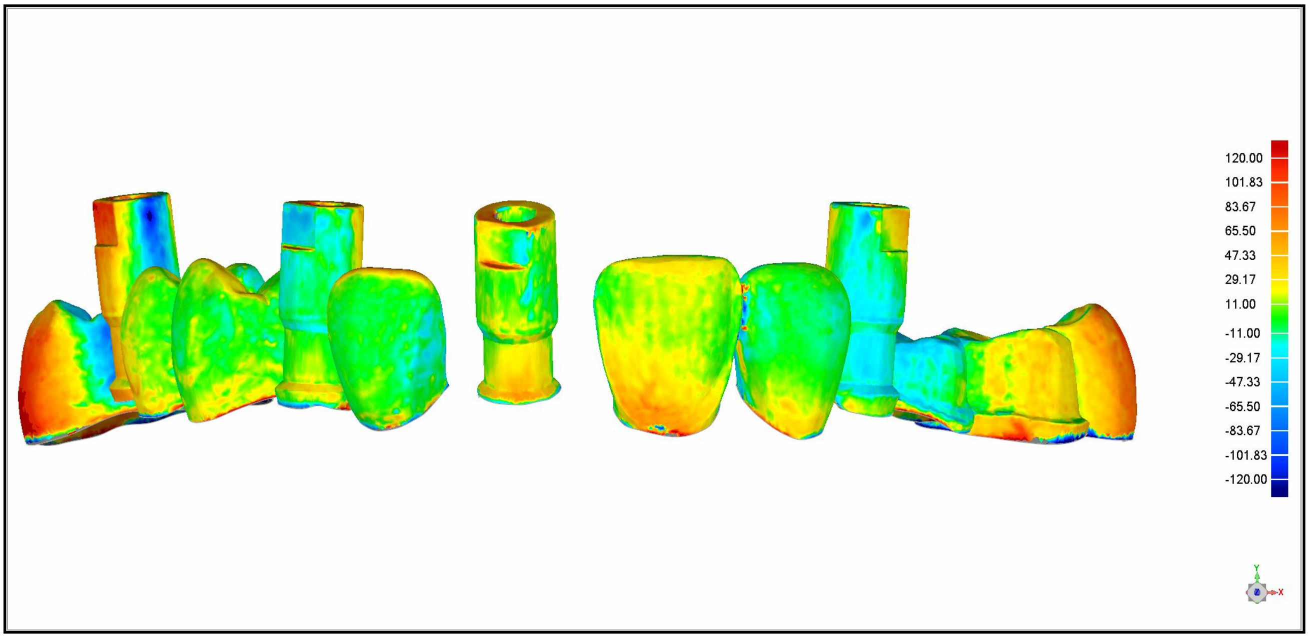

Predefinido: Inferior

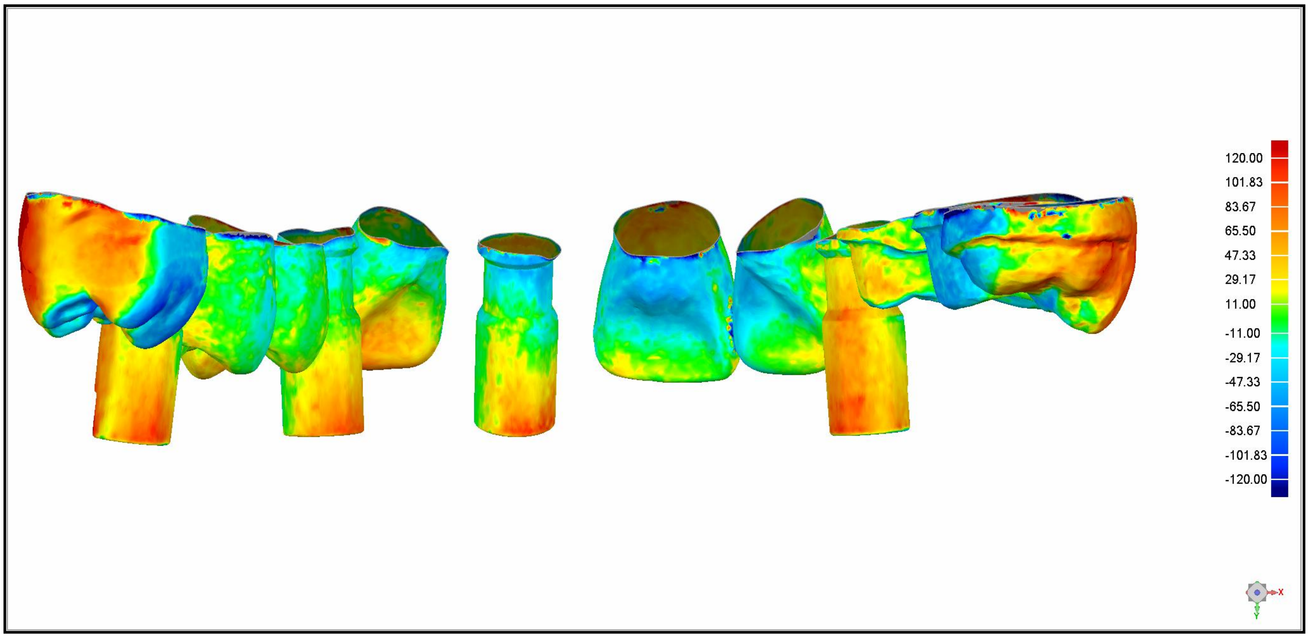

Ajuste de ubicación: Desviaciones superior e inferior

Unidades: u

Nombre	Desv	Estado	Superior Tol	Inferior Tol	Ref X	Ref Y	Ref Z	Radio	Desv X	Desv Y	Desv Z	Medido X	Medido Y	Medido Z	Dir. proy. X	Dir. proy. Y	Dir. proy. Z
Desv. inferior	-2576.28				-22762.84	38034.02	441.63	n/a	-470.37	2514.83	-302.66	-23233.22	40548.85	138.97	0.18	-0.98	0.12
Desv. superior	2311.62				31358.31	27225.24	-12185.59	n/a	-1754.44	217.17	-1489.42	29603.87	27442.42	-13675.01	-0.76	0.09	-0.64
