# Supplementary material for: Accuracy of four digital scanners according to scanning strategy in complete-arch impressions
Source: PLoS One. 2018 Sep 13;13(9):e0202916. doi: 10.1371/journal.pone.0202916 (PMC6136706; doi:10.1371/journal.pone.0202916)
Supplement: S14 Table — True definition (scanning strategy B). (ZIP) [file pone.0202916.s014.zip › S14/TD6B.pdf]

Geomagic Control™

3D Comparación Resultados

Modelo referencia	MRC
Modelo test	TD6B
Nº de puntos de datos	127318
# Aislados	330

Tipo tolerancia	3D desviación
Unidades	u
Máx. crítico	120.00
Máx. nominal	24.00
Mín. nominal	-24.00
Mín. crítico	-120.00

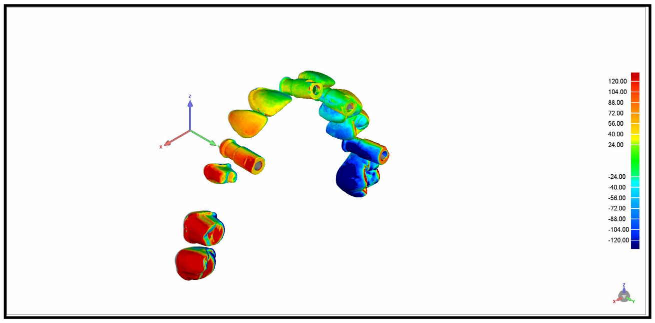

Desviación	
Desviación superior máx.	2680.46
Desviación inferior máx.	-3142.68
Desviación media	92.88 /-74.91
Desviación estándar	141.58

Distribución desviación

>=Min	<Max	# Puntos	%
-120.00	-104.00	1850	1.45
-104.00	-88.00	2535	1.99
-88.00	-72.00	3706	2.91
-72.00	-56.00	4088	3.21
-56.00	-40.00	4784	3.76
-40.00	-24.00	7187	5.64
-24.00	24.00	37088	29.13
24.00	40.00	10722	8.42
40.00	56.00	9288	7.30
56.00	72.00	6991	5.49
72.00	88.00	5449	4.28
88.00	104.00	3401	2.67
104.00	120.00	2519	1.98

Fuera del crítico superior	17735	13.93
Fuera del crítico inferior	9975	7.83

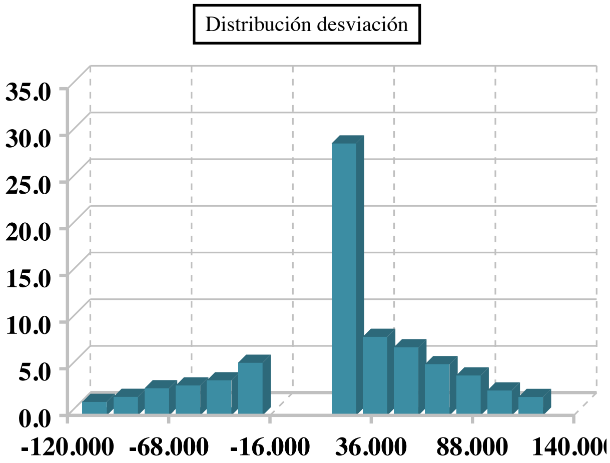

Desviaciones estándar

Distribución (+/-)	# Puntos	%
-6 * Desv. estánd.	144	0.11
-5 * Desv. estánd.	73	0.06
-4 * Desv. estánd.	311	0.24
-3 * Desv. estánd.	2048	1.61
-2 * Desv. estánd.	7732	6.07
-1 * Desv. estánd.	61479	48.29
1 * Desv. estánd.	43129	33.88
2 * Desv. estánd.	8551	6.72
3 * Desv. estánd.	2633	2.07
4 * Desv. estánd.	867	0.68
5 * Desv. estánd.	70	0.05
6 * Desv. estánd.	281	0.22

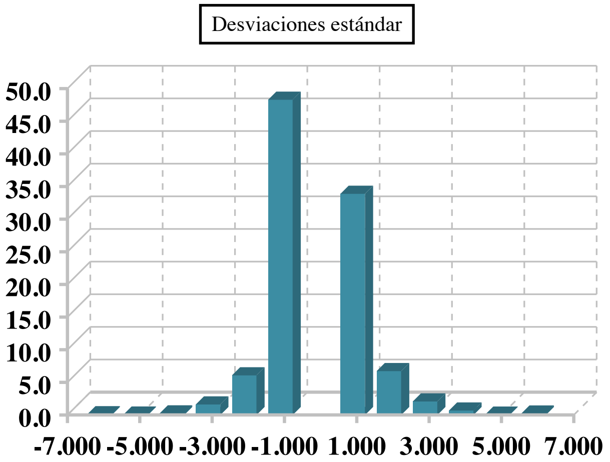

Predefinido: Isométrico

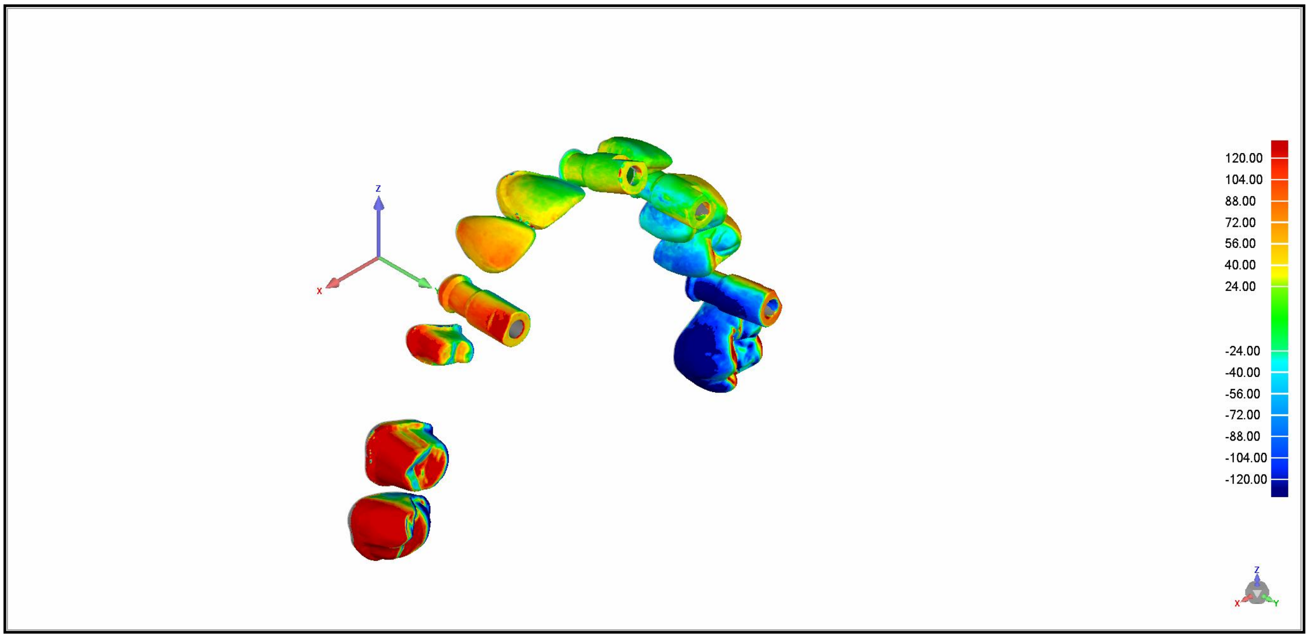

Predefinido: Frente

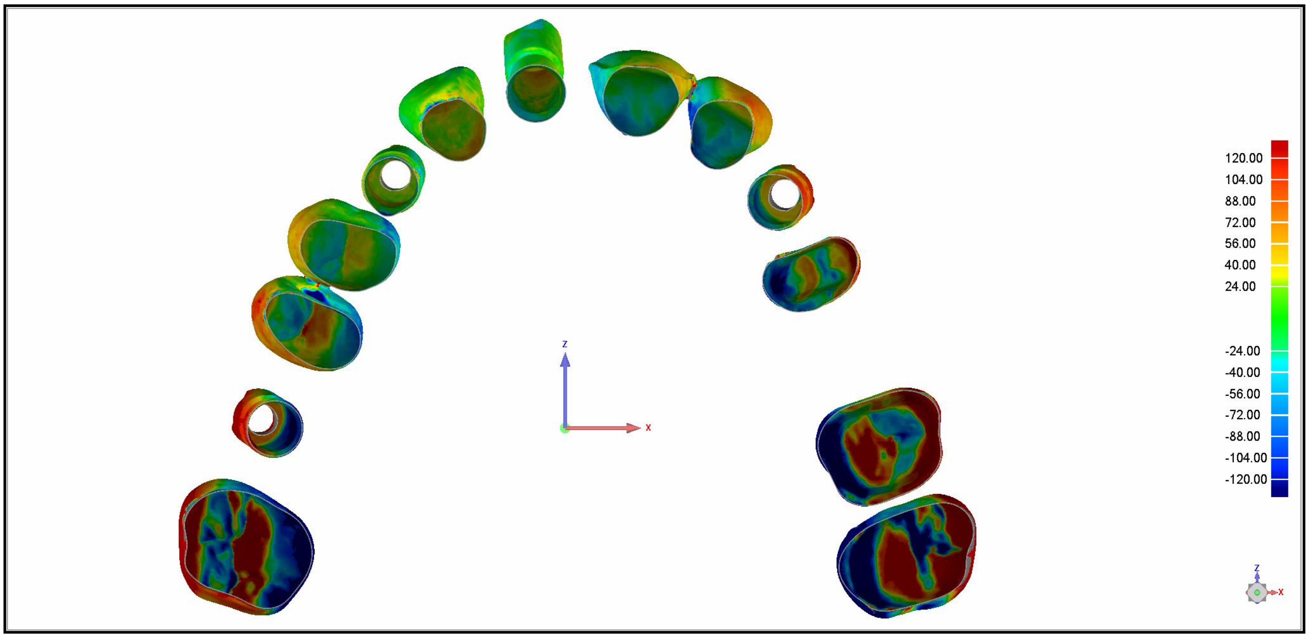

Predefinido: Atrás

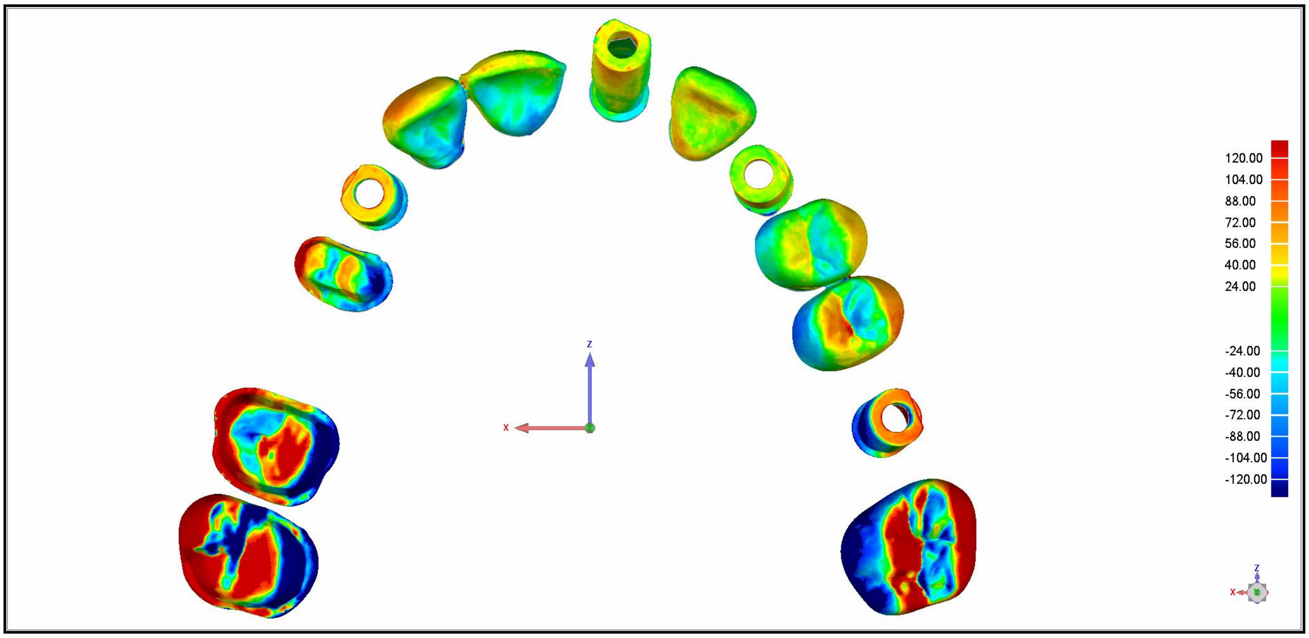

Predefinido: Izquierda

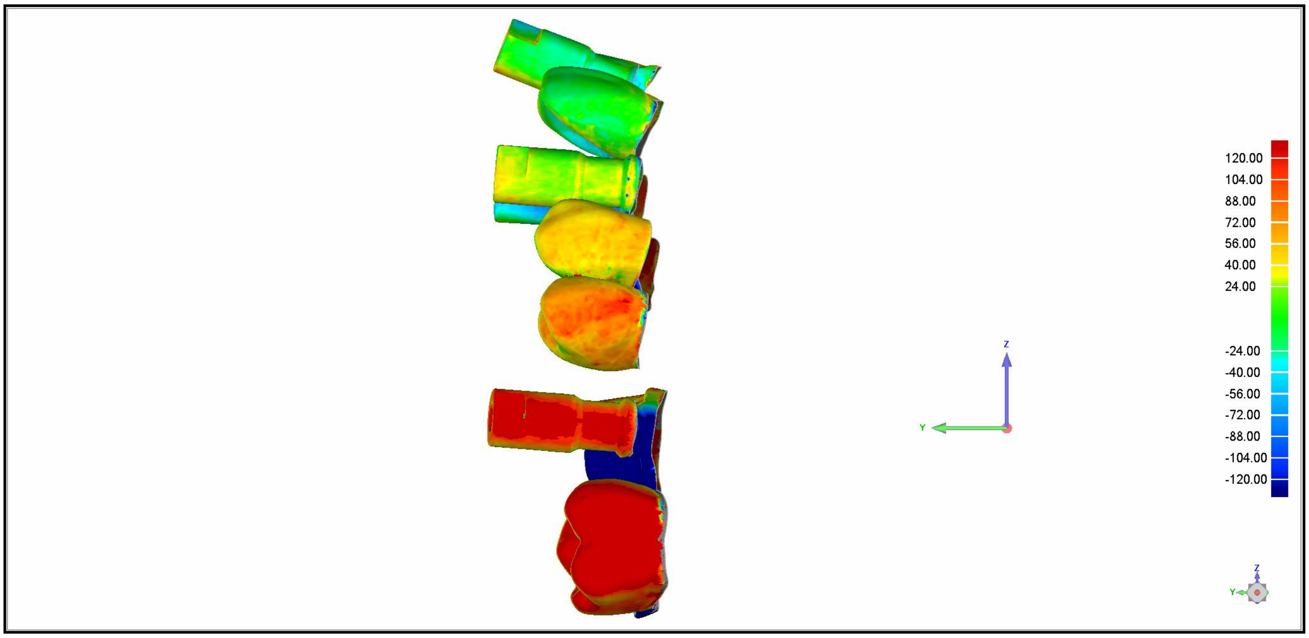

Predefinido: Derecha

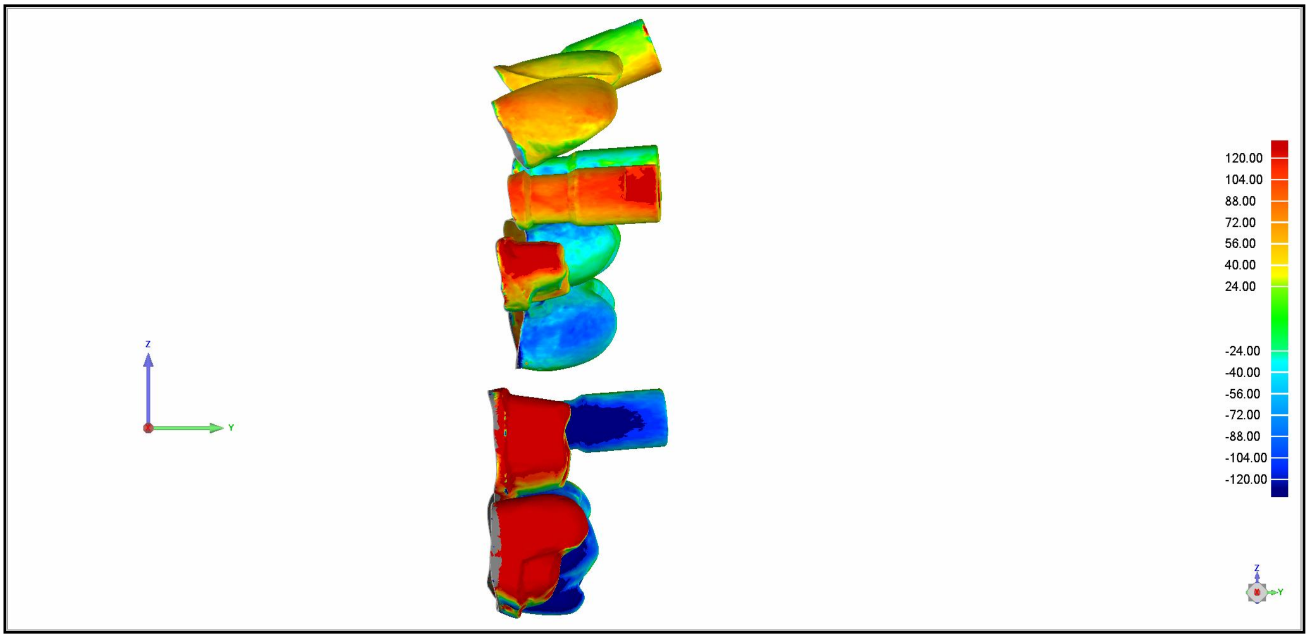

Predefinido: Superior

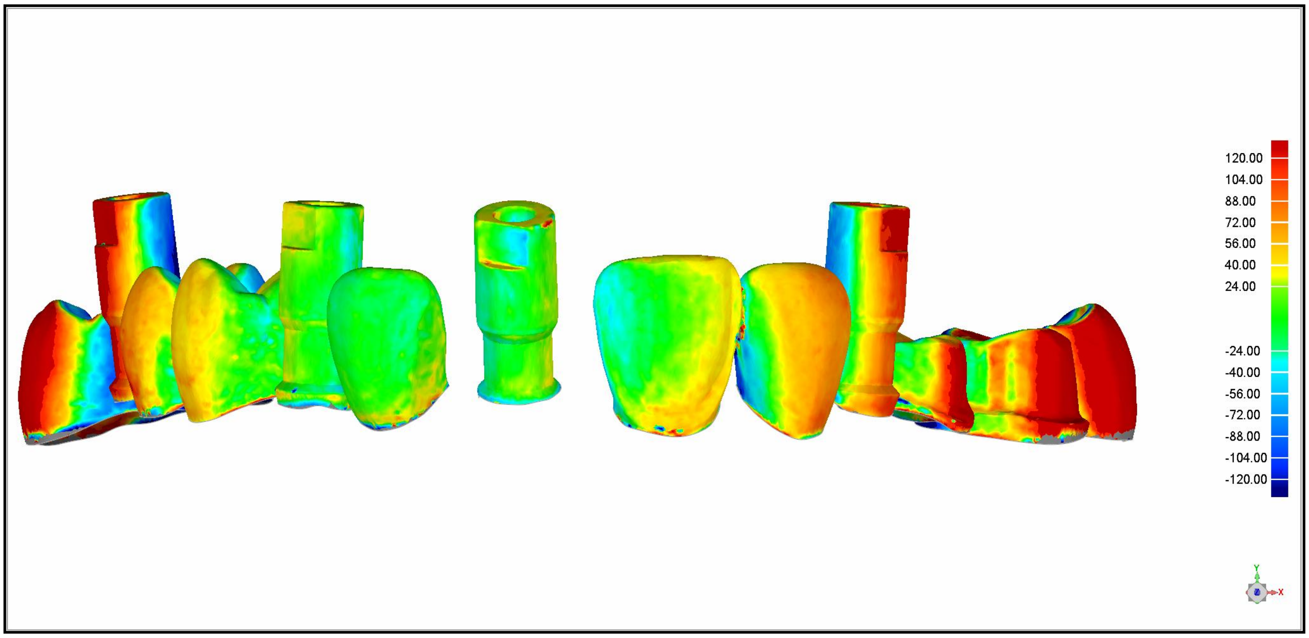

Predefinido: Inferior

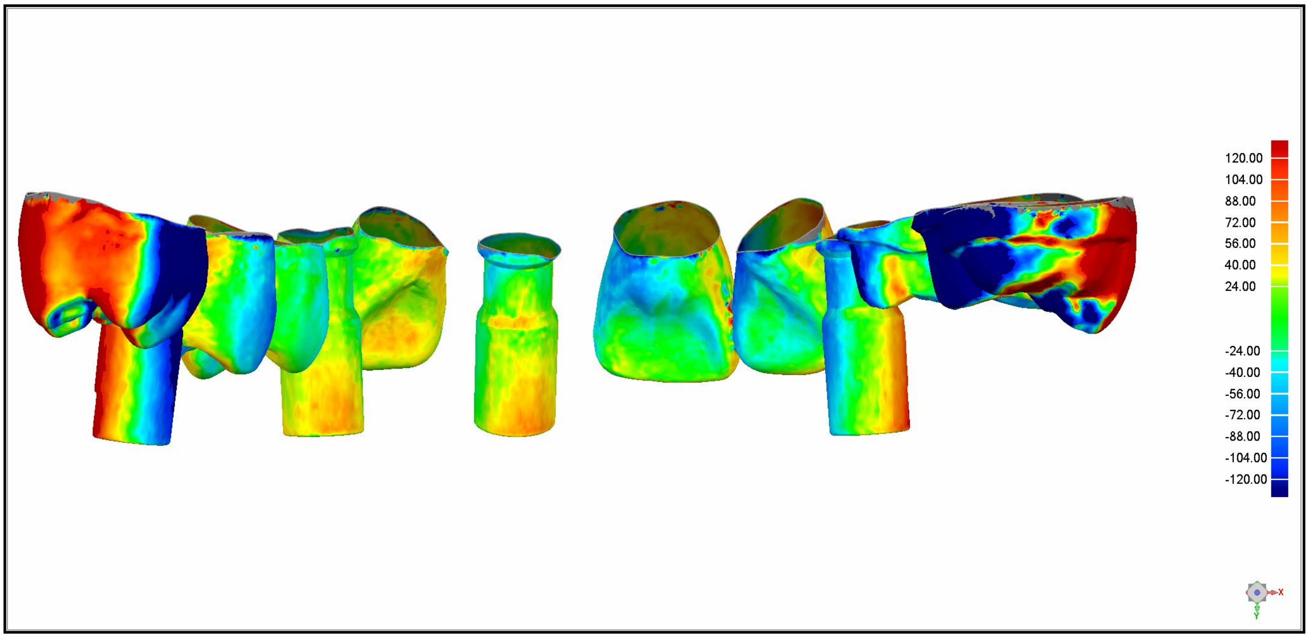

Ajuste de ubicación: Desviaciones superior e inferior

Unidades: u

Nombre	Desv	Estado	Superior Tol	Inferior Tol	Ref X	Ref Y	Ref Z	Radio	Desv X	Desv Y	Desv Z	Medido X	Medido Y	Medido Z	Dir. proy. X	Dir. proy. Y	Dir. proy. Z
Desv. inferior	-3142.68				-22607.19	28955.77	6808.03	n/a	-933.88	-332.73	2982.21	-23541.07	28623.04	9790.24	0.30	0.11	-0.95
Desv. superior	2680.46				-27689.92	27388.00	-5061.48	n/a	-2217.15	-39.11	-1505.84	-29907.07	27348.89	-6567.33	-0.83	-0.01	-0.56
