# Supplementary material for: Accuracy of four digital scanners according to scanning strategy in complete-arch impressions
Source: PLoS One. 2018 Sep 13;13(9):e0202916. doi: 10.1371/journal.pone.0202916 (PMC6136706; doi:10.1371/journal.pone.0202916)
Supplement: S14 Table — True definition (scanning strategy B). (ZIP) [file pone.0202916.s014.zip › S14/TD1B.pdf]

Geomagic Control™

Fecha: 3/6/2017, 3:32 pm

3D Comparación Resultados

Modelo referencia	MRC
Modelo test	TD1B
Nº de puntos de datos	131362
# Aislados	395

Tipo tolerancia	3D desviación
Unidades	u
Máx. crítico	120.00
Máx. nominal	14.00
Mín. nominal	-14.00
Mín. crítico	-120.00

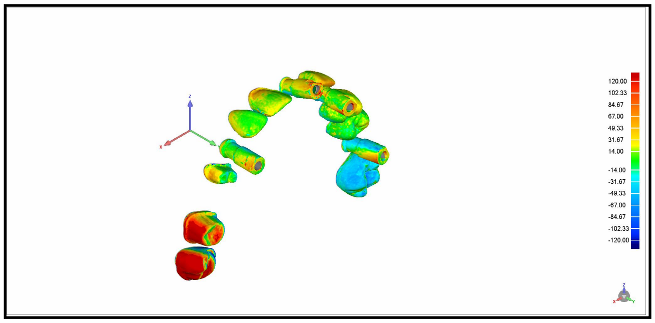

Desviación	
Desviación superior máx.	2217.98
Desviación inferior máx.	-2513.38
Desviación media	44.30 /-37.01
Desviación estándar	87.94

Distribución desviación

>=Min	<Max	# Puntos	%
-120.00	-102.33	943	0.72
-102.33	-84.67	847	0.64
-84.67	-67.00	1277	0.97
-67.00	-49.33	3379	2.57
-49.33	-31.67	7936	6.04
-31.67	-14.00	13126	9.99
-14.00	14.00	44997	34.25
14.00	31.67	23867	18.17
31.67	49.33	11088	8.44
49.33	67.00	6667	5.08
67.00	84.67	4183	3.18
84.67	102.33	2059	1.57
102.33	120.00	1502	1.14

Fuera del crítico superior	6936	5.28
Fuera del crítico inferior	2555	1.95

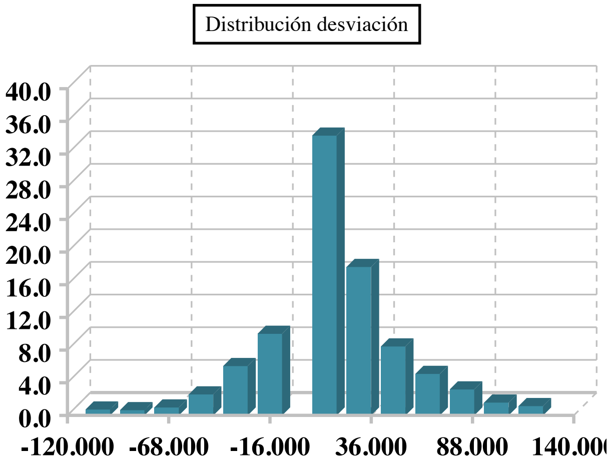

Desviaciones estándar

Distribución (+/-)	# Puntos	%
-6 * Desv. estánd.	171	0.13
-5 * Desv. estánd.	70	0.05
-4 * Desv. estánd.	146	0.11
-3 * Desv. estánd.	945	0.72
-2 * Desv. estánd.	3712	2.83
-1 * Desv. estánd.	70682	53.81
1 * Desv. estánd.	47198	35.93
2 * Desv. estánd.	5485	4.18
3 * Desv. estánd.	2461	1.87
4 * Desv. estánd.	120	0.09
5 * Desv. estánd.	89	0.07
6 * Desv. estánd.	283	0.22

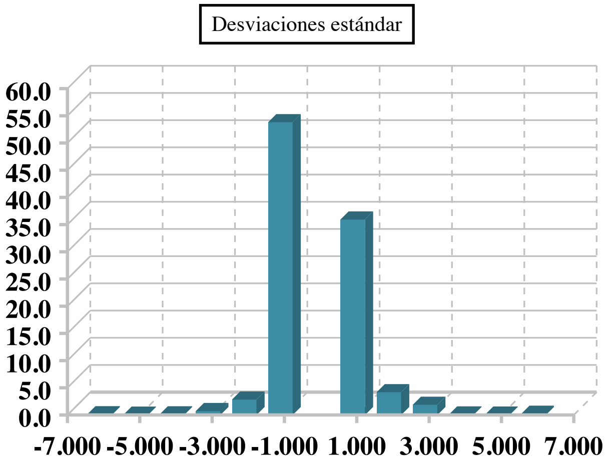

Predefinido: Isométrico

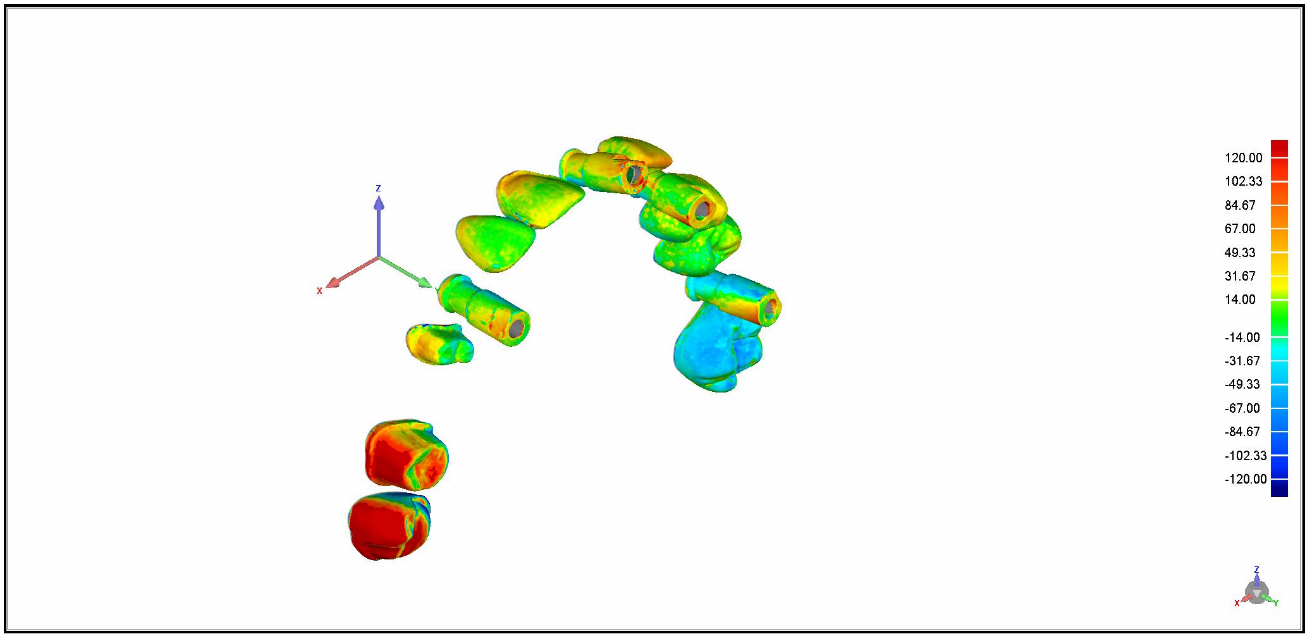

Predefinido: Frente

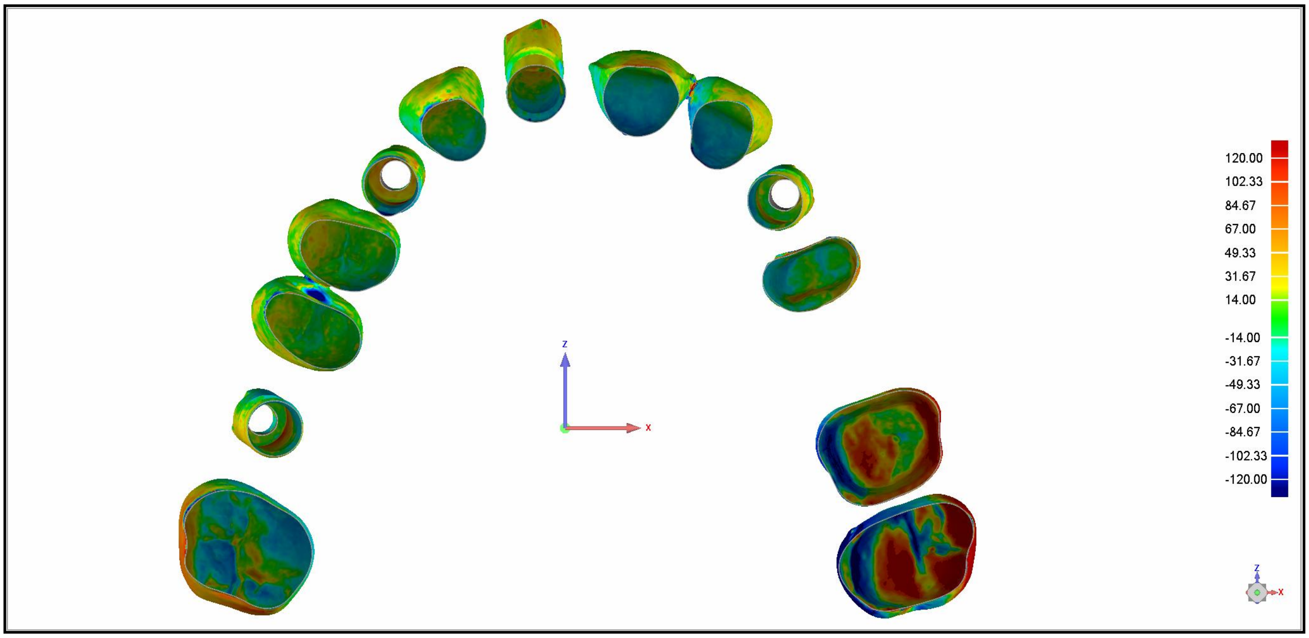

Predefinido: Atrás

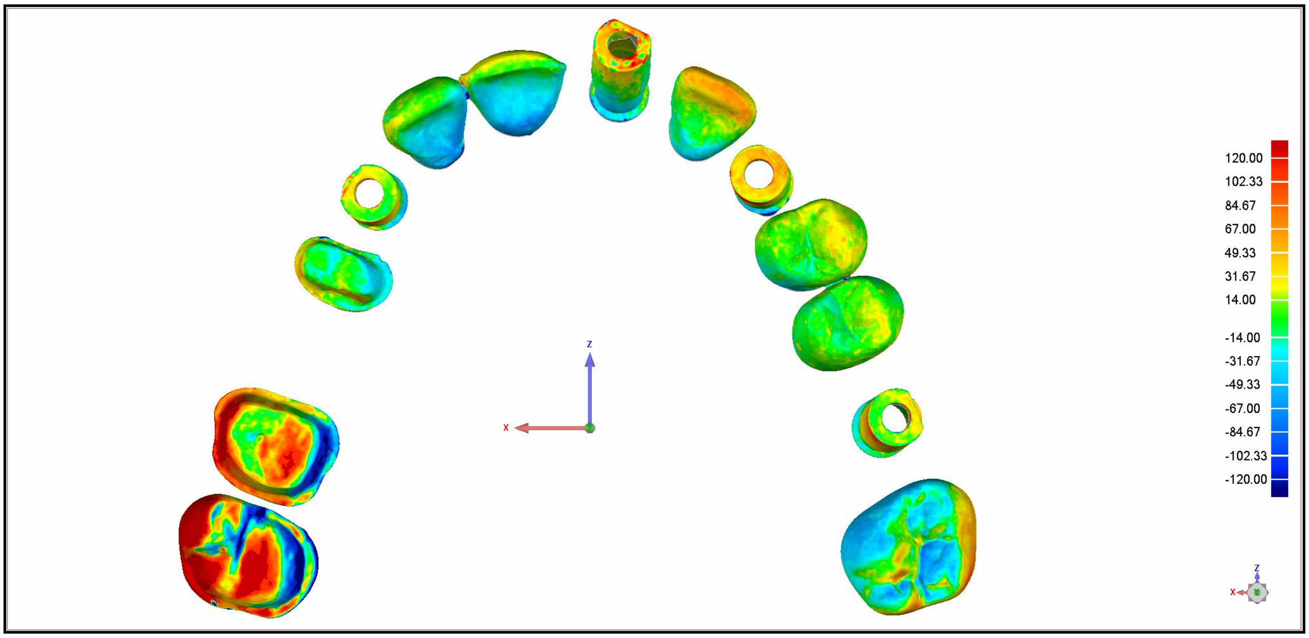

Predefinido: Izquierda

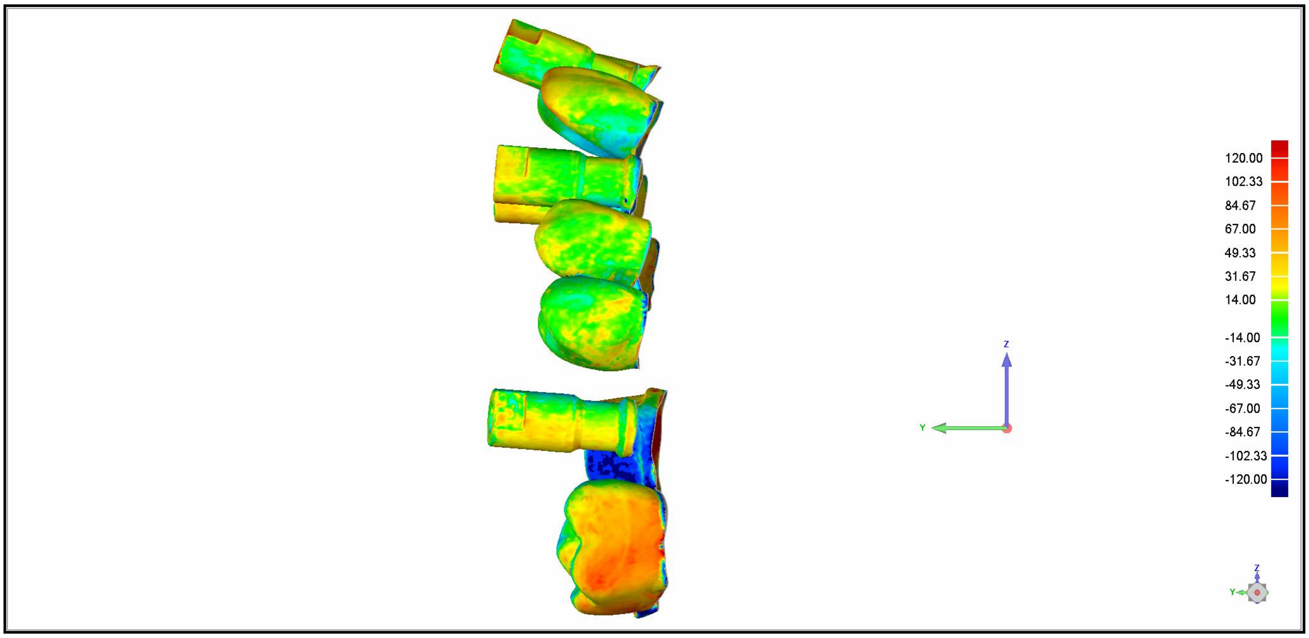

Predefinido: Derecha

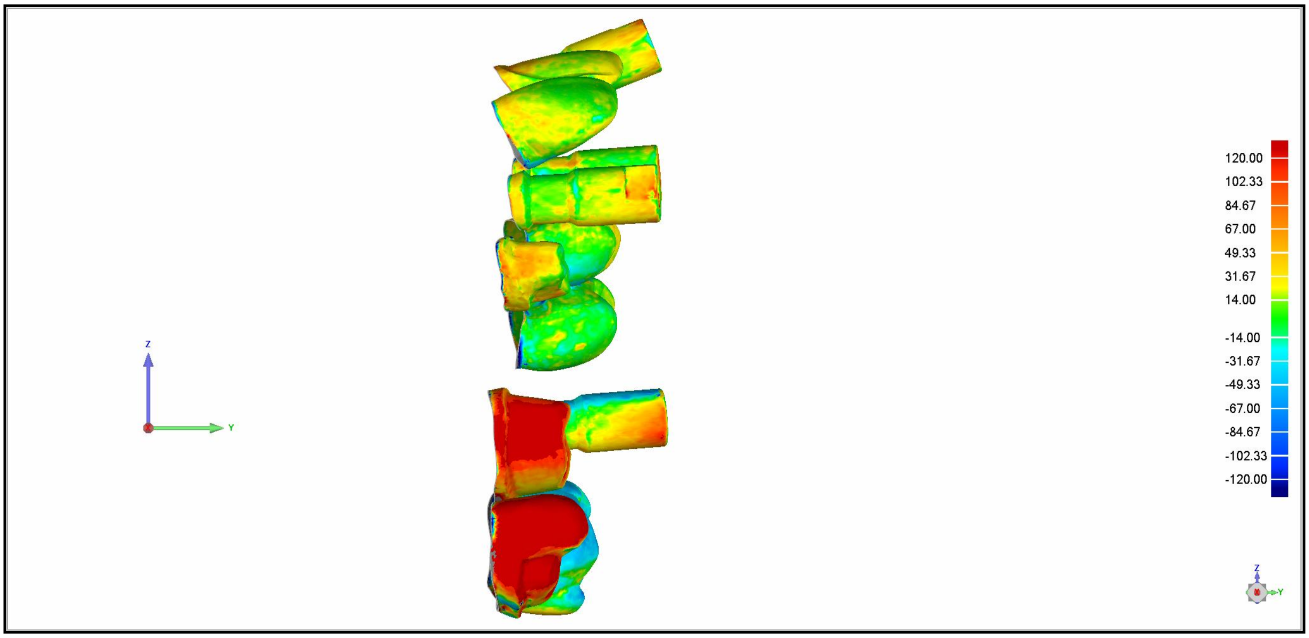

Geomagic Control™

Fecha: 3/6/2017, 3:32 pm

Predefinido: Superior

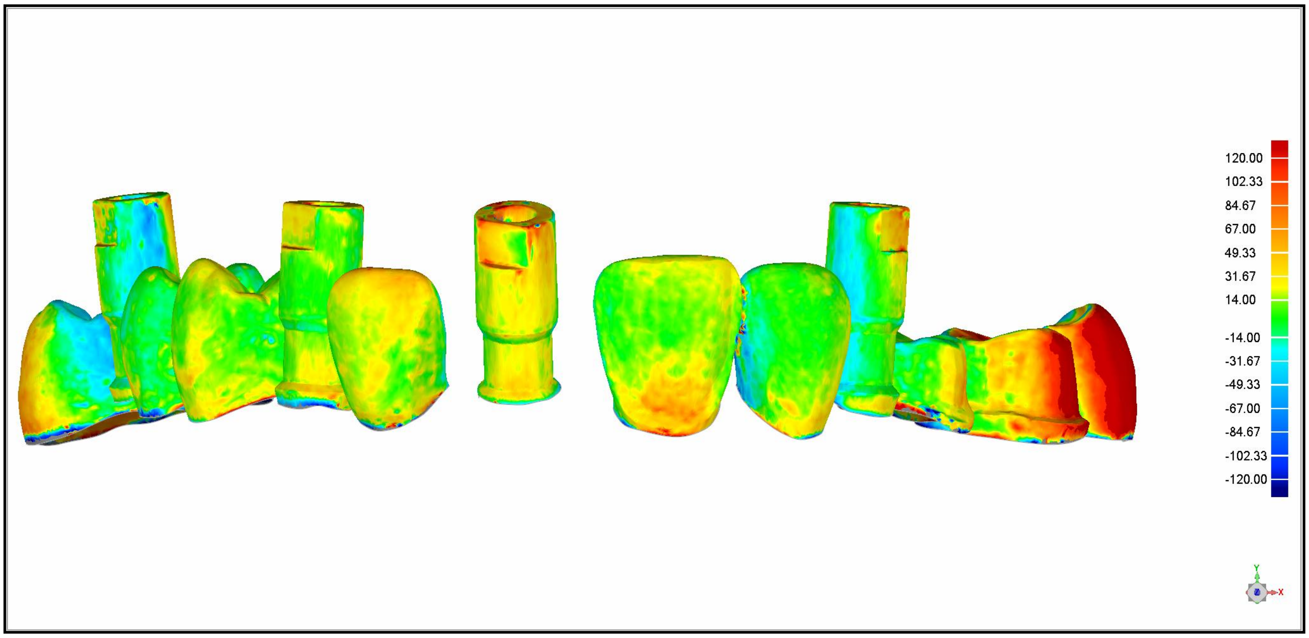

Predefinido: Inferior

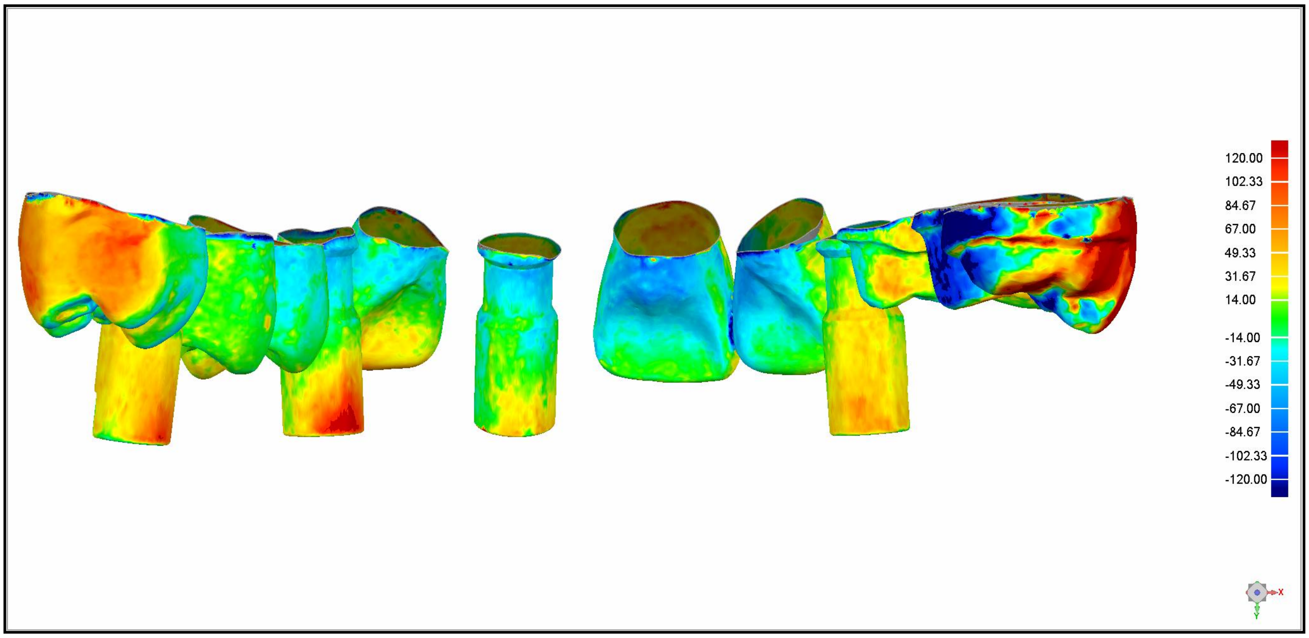

Ajuste de ubicación: Desviaciones superior e inferior

Unidades: u

Nombre	Desv	Estado	Superior Tol	Inferior Tol	Ref X	Ref Y	Ref Z	Radio	Desv X	Desv Y	Desv Z	Medido X	Medido Y	Medido Z	Dir. proy. X	Dir. proy. Y	Dir. proy. Z
Desv. inferior	-2513.38				-29292.33	26884.28	-11910.36	n/a	2156.66	329.66	-1247.89	-27135.68	27213.94	-13158.25	-0.86	-0.13	0.50
Desv. superior	2217.98				17581.30	38164.54	17270.81	n/a	677.54	2020.84	613.65	18258.84	40185.38	17884.46	0.31	0.91	0.28
