# Supplementary material for: Accuracy of four digital scanners according to scanning strategy in complete-arch impressions
Source: PLoS One. 2018 Sep 13;13(9):e0202916. doi: 10.1371/journal.pone.0202916 (PMC6136706; doi:10.1371/journal.pone.0202916)
Supplement: S15 Table — True definition (scanning strategy C). (ZIP) [file pone.0202916.s015.zip › S15/TD3C.pdf]

Geomagic Control™

Fecha: 3/6/2017, 3:38 pm

3D Comparación Resultados

Modelo referencia	MRC
Modelo test	TD3C
Nº de puntos de datos	130529
# Aislados	330

Tipo tolerancia	3D desviación
Unidades	u
Máx. crítico	120.00
Máx. nominal	15.00
Mín. nominal	-15.00
Mín. crítico	-120.00

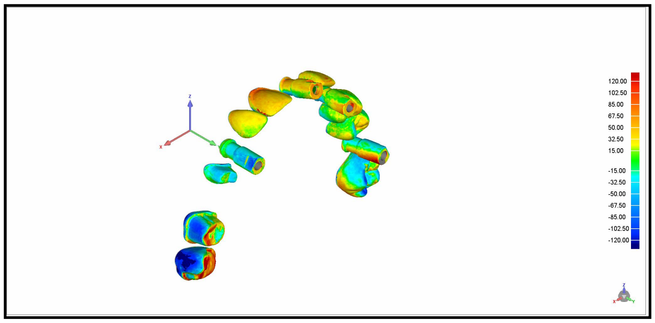

Desviación	
Desviación superior máx.	2308.04
Desviación inferior máx.	-2127.31
Desviación media	48.63 /-36.21
Desviación estándar	84.49

Distribución desviación

>=Min	<Max	# Puntos	%
-120.00	-102.50	1174	0.90
-102.50	-85.00	1594	1.22
-85.00	-67.50	2225	1.70
-67.50	-50.00	4641	3.56
-50.00	-32.50	9591	7.35
-32.50	-15.00	14235	10.91
-15.00	15.00	37025	28.37
15.00	32.50	21308	16.32
32.50	50.00	14306	10.96
50.00	67.50	6694	5.13
67.50	85.00	4374	3.35
85.00	102.50	3242	2.48
102.50	120.00	2541	1.95

Fuera del crítico superior	6125	4.69
Fuera del crítico inferior	1454	1.11

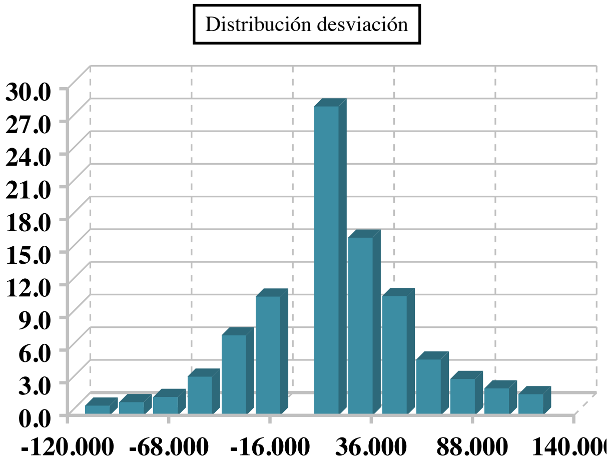

Desviaciones estándar

Distribución (+/-)	# Puntos	%
-6 * Desv. estánd.	109	0.08
-5 * Desv. estánd.	49	0.04
-4 * Desv. estánd.	99	0.08
-3 * Desv. estánd.	285	0.22
-2 * Desv. estánd.	5638	4.32
-1 * Desv. estánd.	65932	50.51
1 * Desv. estánd.	49334	37.80
2 * Desv. estánd.	7709	5.91
3 * Desv. estánd.	629	0.48
4 * Desv. estánd.	155	0.12
5 * Desv. estánd.	113	0.09
6 * Desv. estánd.	477	0.37

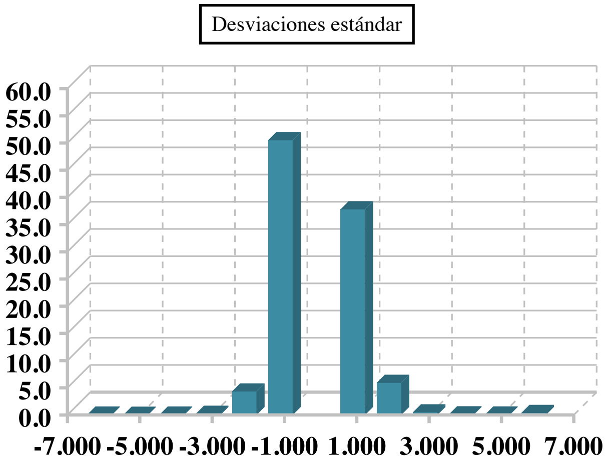

Predefinido: Isométrico

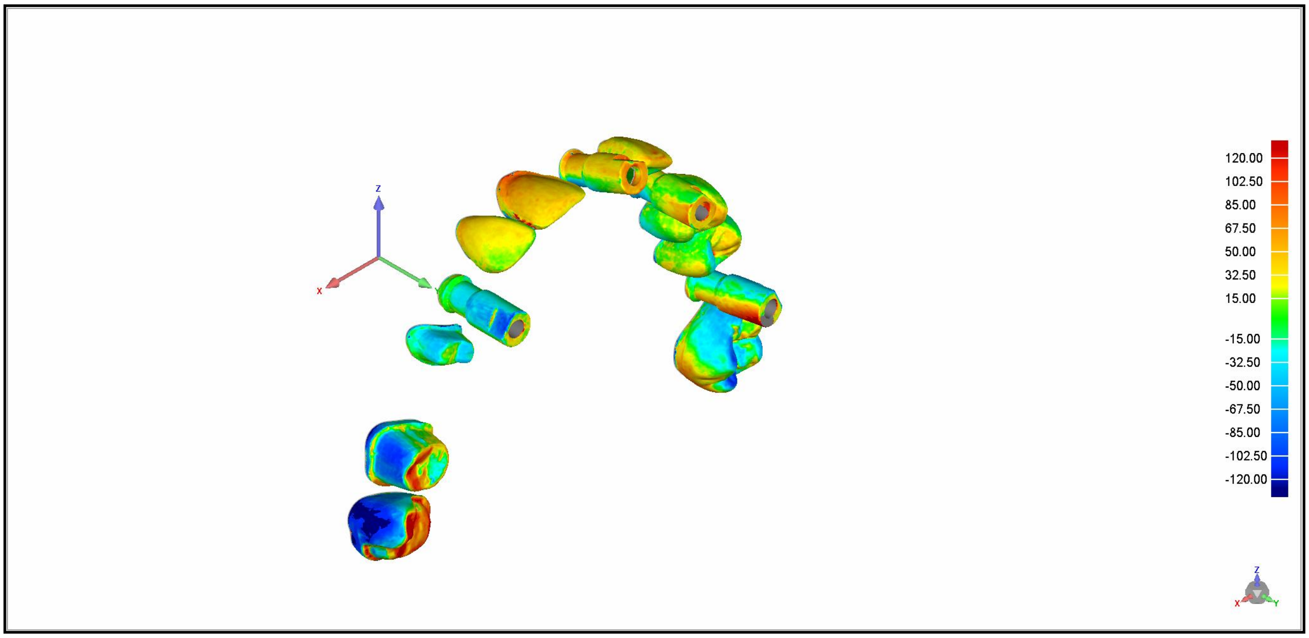

Predefinido: Frente

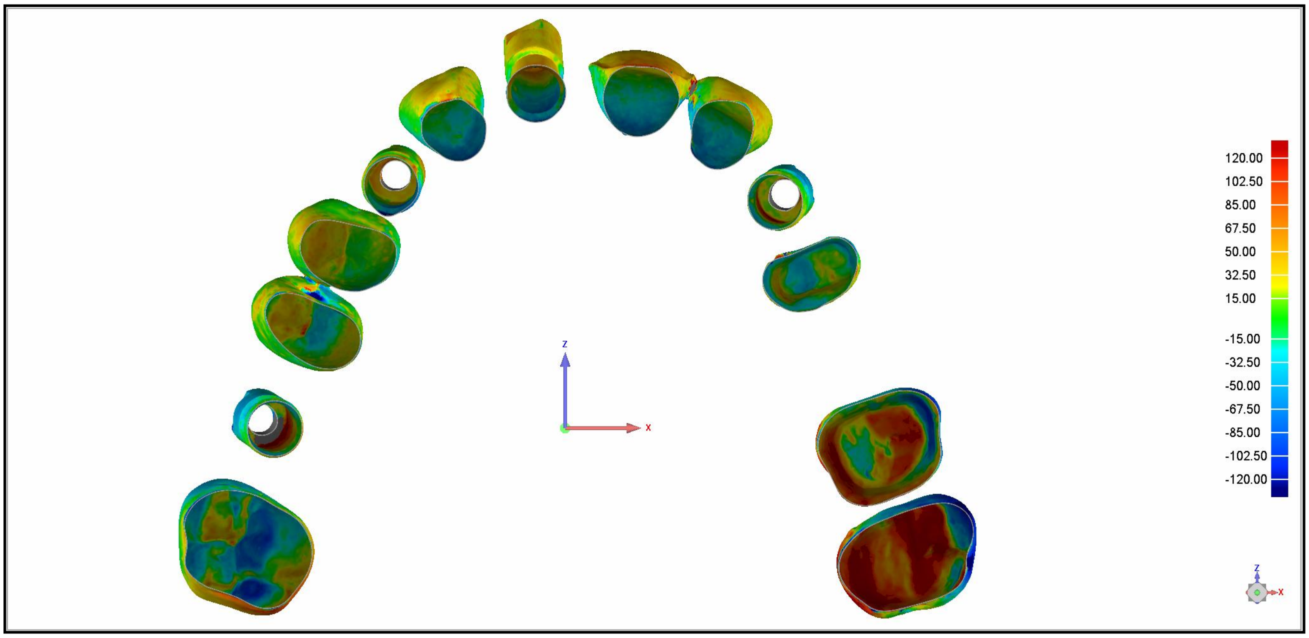

Predefinido: Atrás

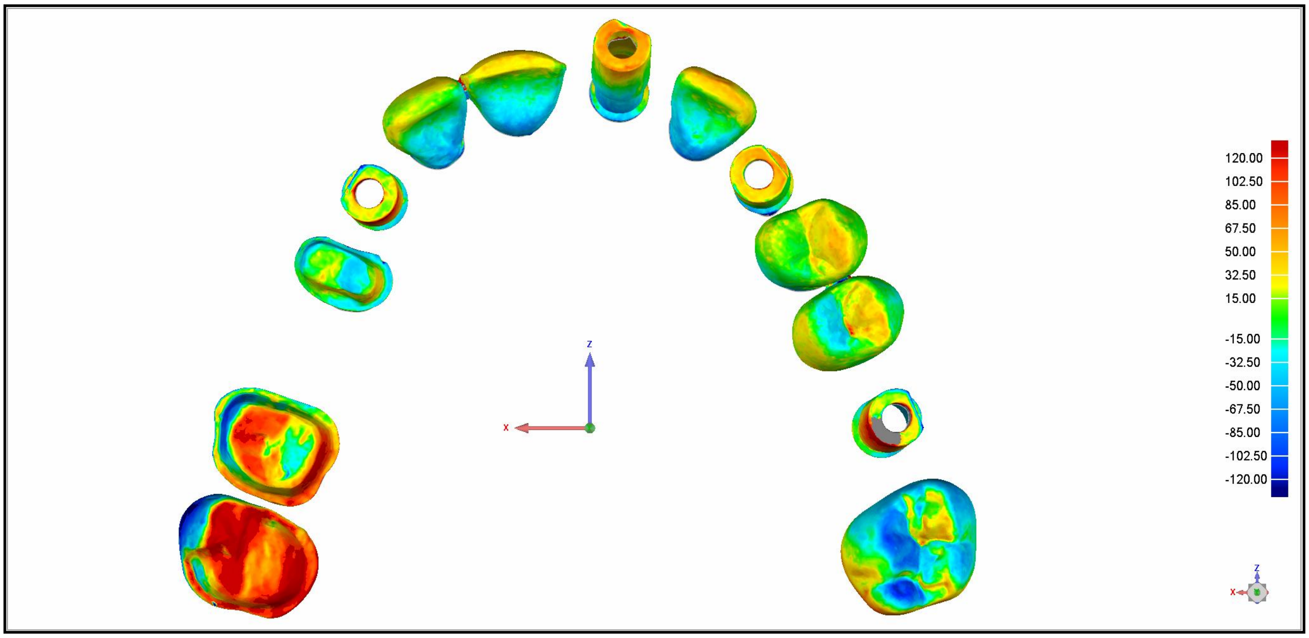

Predefinido: Izquierda

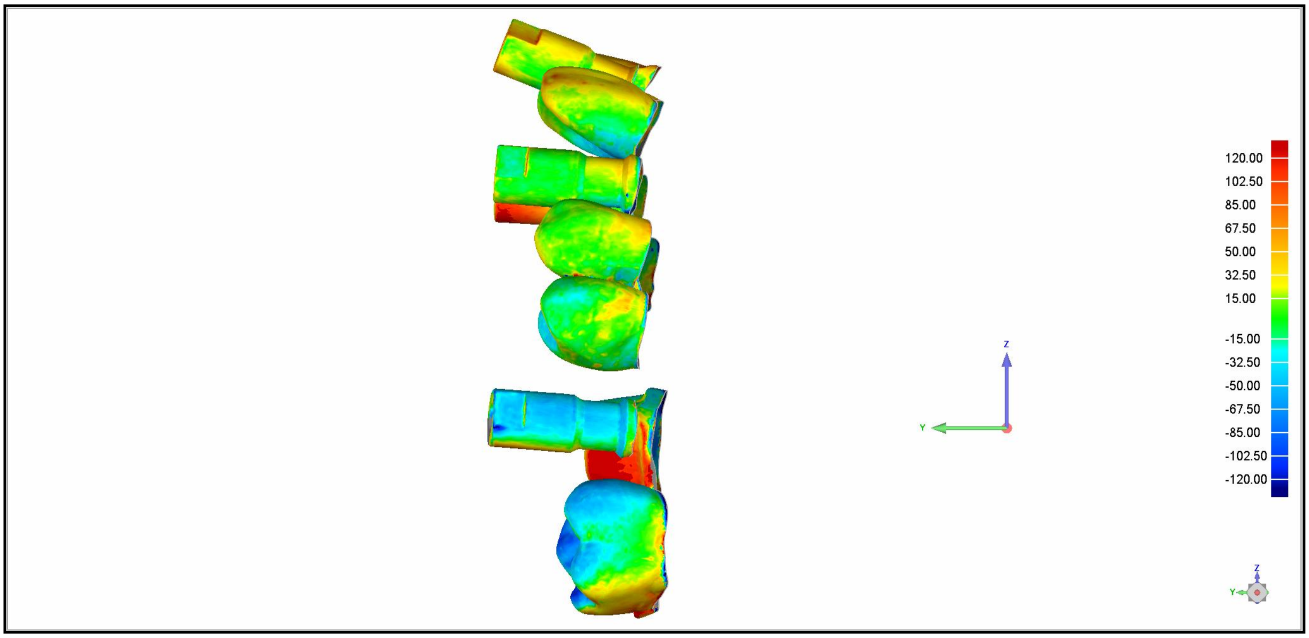

Predefinido: Derecha

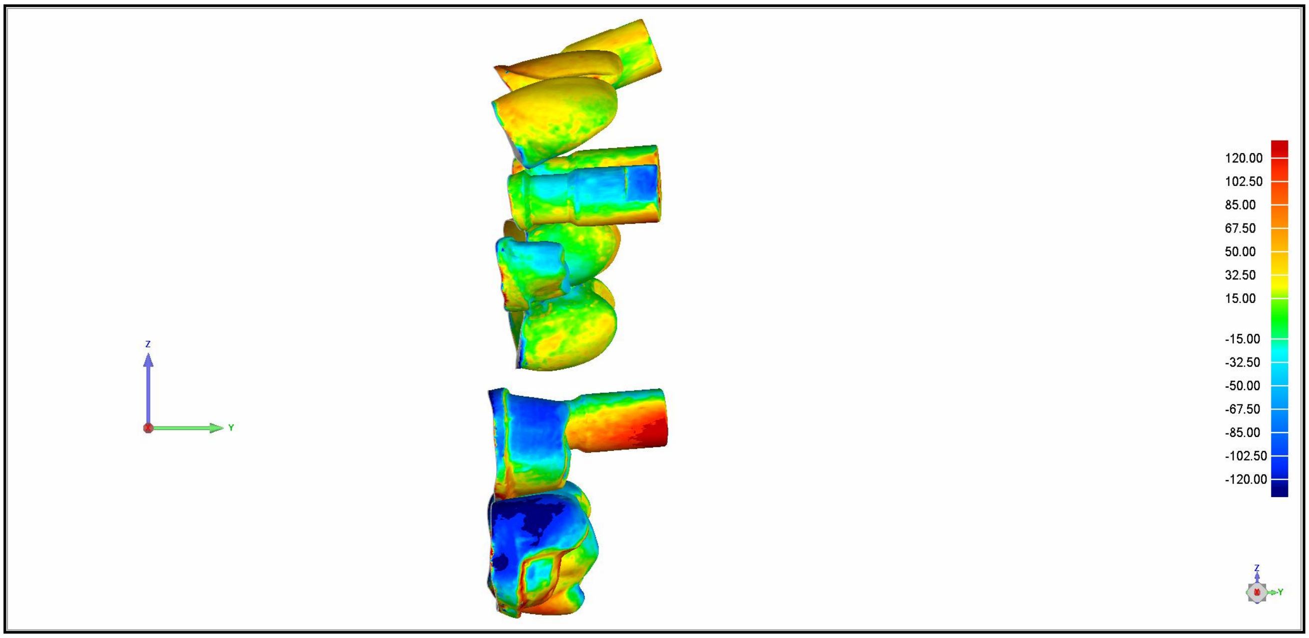

Predefinido: Superior

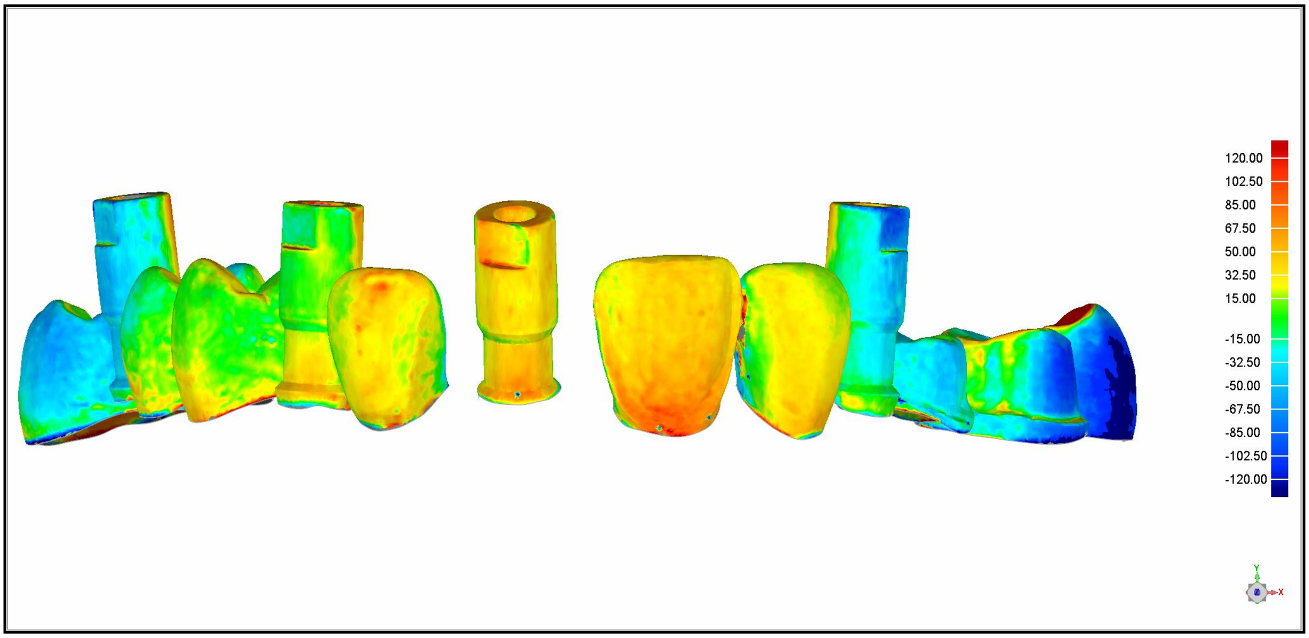

Predefinido: Inferior

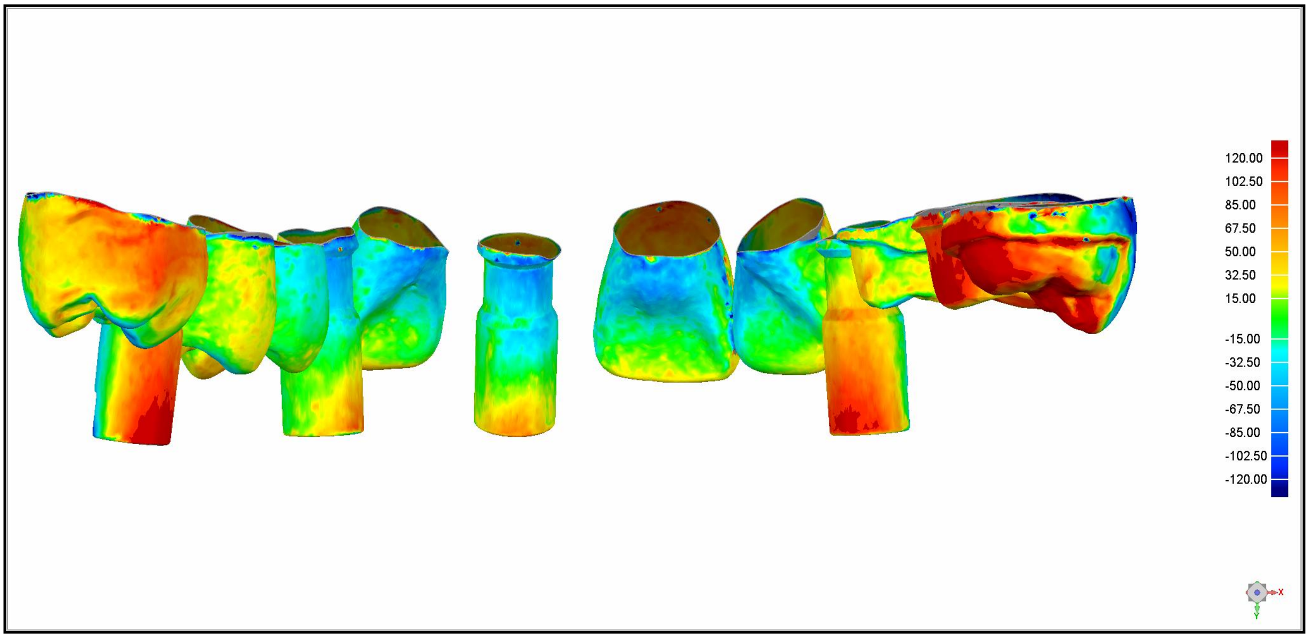

Ajuste de ubicación: Desviaciones superior e inferior

Unidades: u

Nombre	Desv	Estado	Superior Tol	Inferior Tol	Ref X	Ref Y	Ref Z	Radio	Desv X	Desv Y	Desv Z	Medido X	Medido Y	Medido Z	Dir. proy. X	Dir. proy. Y	Dir. proy. Z
Desv. inferior	-2127.31				29715.55	29554.58	-13882.37	n/a	-322.48	-2098.34	135.63	29393.07	27456.23	-13746.74	0.15	0.99	-0.06
Desv. superior	2308.04				25410.11	27462.45	-5764.99	n/a	2248.57	116.24	-507.40	27658.68	27578.69	-6272.39	0.97	0.05	-0.22
