# Supplementary material for: Accuracy of four digital scanners according to scanning strategy in complete-arch impressions
Source: PLoS One. 2018 Sep 13;13(9):e0202916. doi: 10.1371/journal.pone.0202916 (PMC6136706; doi:10.1371/journal.pone.0202916)
Supplement: S15 Table — True definition (scanning strategy C). (ZIP) [file pone.0202916.s015.zip › S15/TD2C.pdf]

Geomagic Control

3D Comparación Resultados

Modelo referencia	MRC
Modelo test	TD2C
Nº de puntos de datos	130547
# Aislados	273

Tipo tolerancia	3D desviación
Unidades	u
Máx. crítico	120.00
Máx. nominal	10.00
Mín. nominal	-10.00
Mín. crítico	-120.00

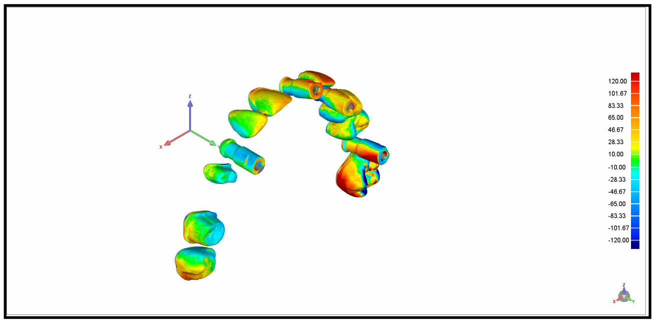

Desviación	
Desviación superior máx.	2230.83
Desviación inferior máx.	-2607.87
Desviación media	50.49 /-43.74
Desviación estándar	91.97

Distribución desviación

>=Min	<Max	# Puntos	%
-120.00	-101.67	1759	1.35
-101.67	-83.33	2109	1.62
-83.33	-65.00	3090	2.37
-65.00	-46.67	5994	4.59
-46.67	-28.33	10884	8.34
-28.33	-10.00	17632	13.51
-10.00	10.00	21866	16.75
10.00	28.33	20399	15.63
28.33	46.67	16467	12.61
46.67	65.00	10642	8.15
65.00	83.33	5631	4.31
83.33	101.67	3788	2.90
101.67	120.00	1852	1.42

Fuera del crítico superior	4429	3.39
Fuera del crítico inferior	4005	3.07

Distribución desviación

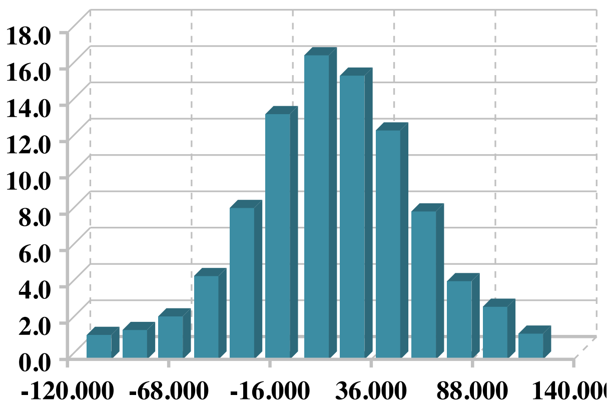

Desviaciones estándar

Distribución (+/-)	# Puntos	%
-6 * Desv. estánd.	97	0.07
-5 * Desv. estánd.	36	0.03
-4 * Desv. estánd.	90	0.07
-3 * Desv. estánd.	1022	0.78
-2 * Desv. estánd.	6853	5.25
-1 * Desv. estánd.	59514	45.59
1 * Desv. estánd.	56731	43.46
2 * Desv. estánd.	4999	3.83
3 * Desv. estánd.	387	0.30
4 * Desv. estánd.	147	0.11
5 * Desv. estánd.	118	0.09
6 * Desv. estánd.	553	0.42

Desviaciones estándar

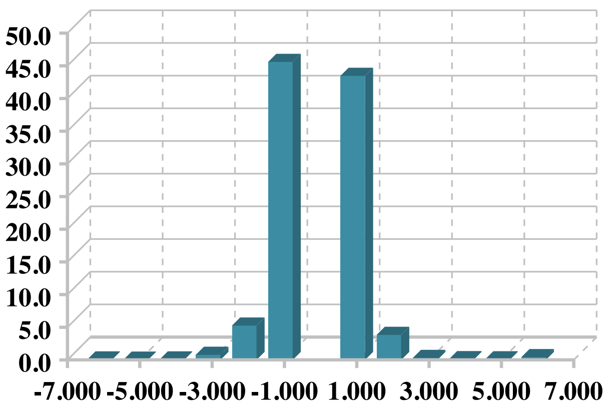

Predefinido: Isométrico

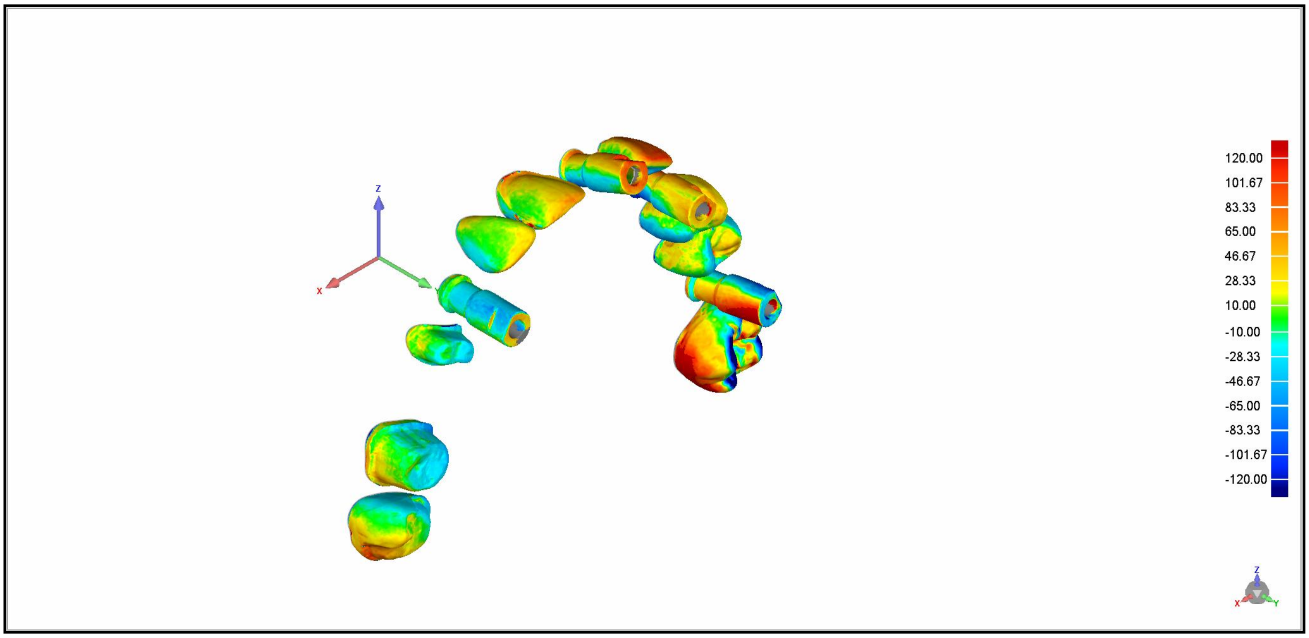

Predefinido: Frente

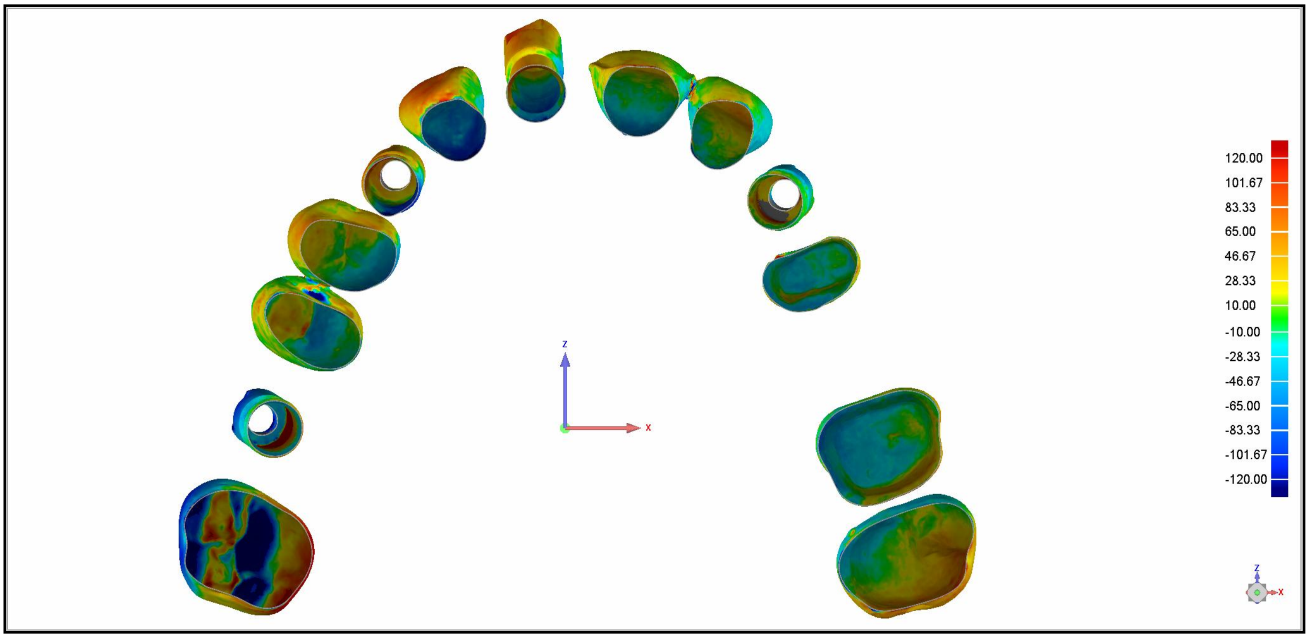

Predefinido: Atrás

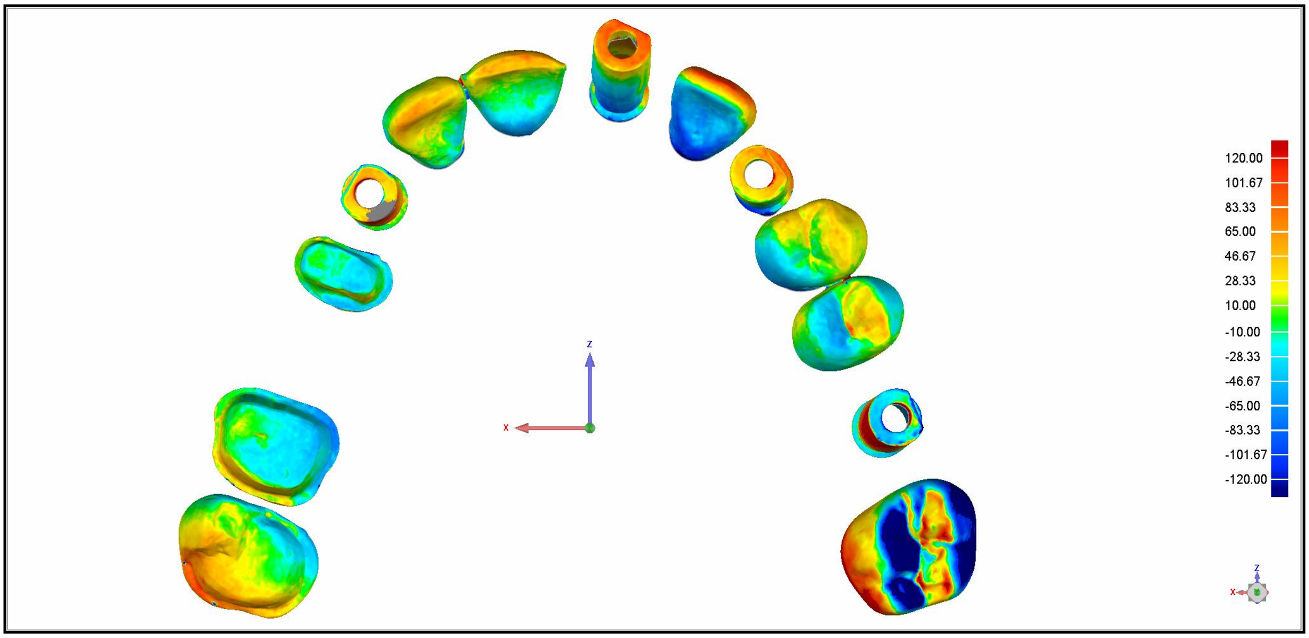

Predefinido: Izquierda

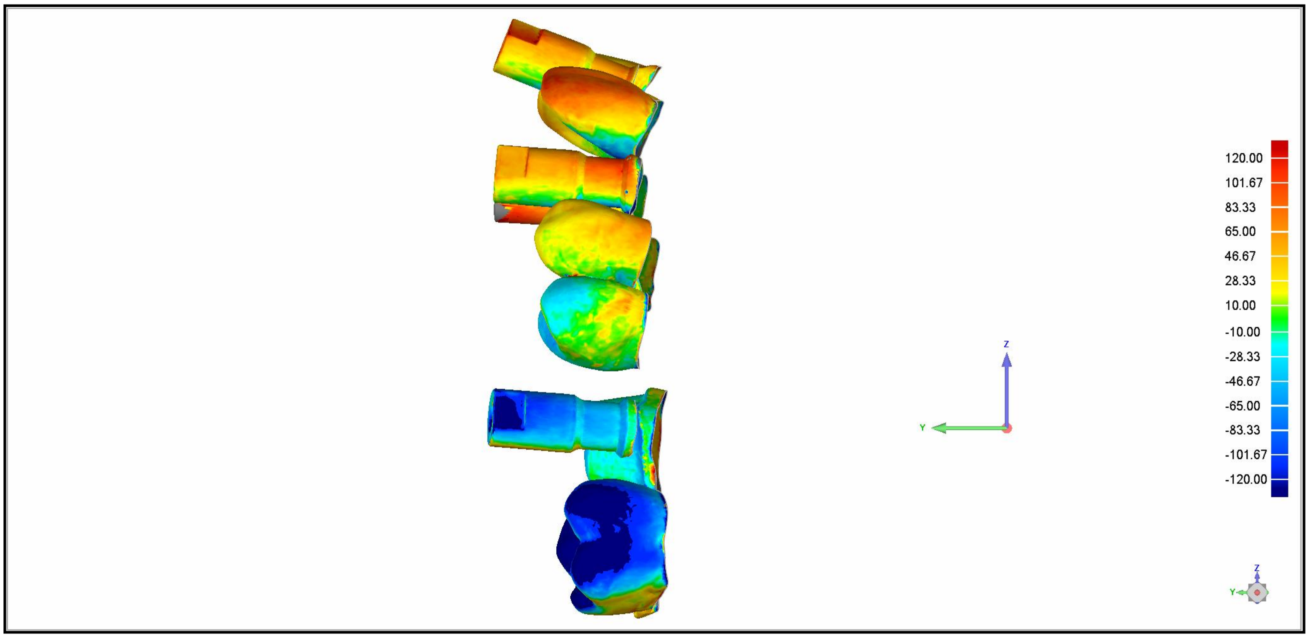

Predefinido: Derecha

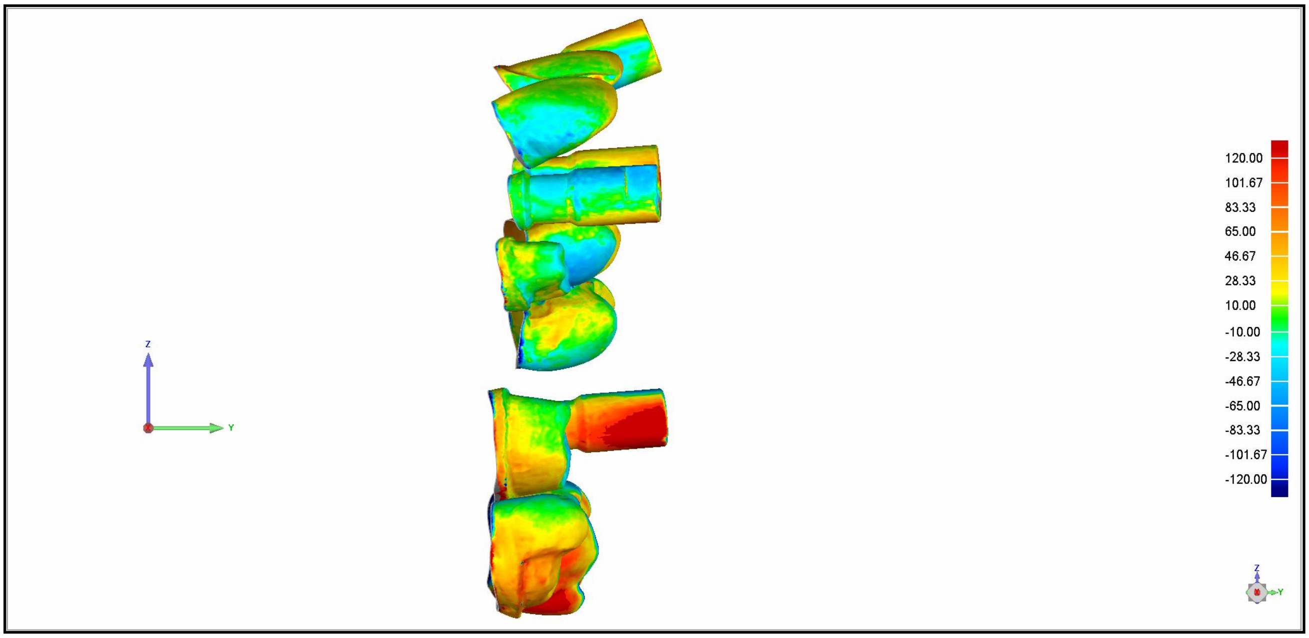

Predefinido: Superior

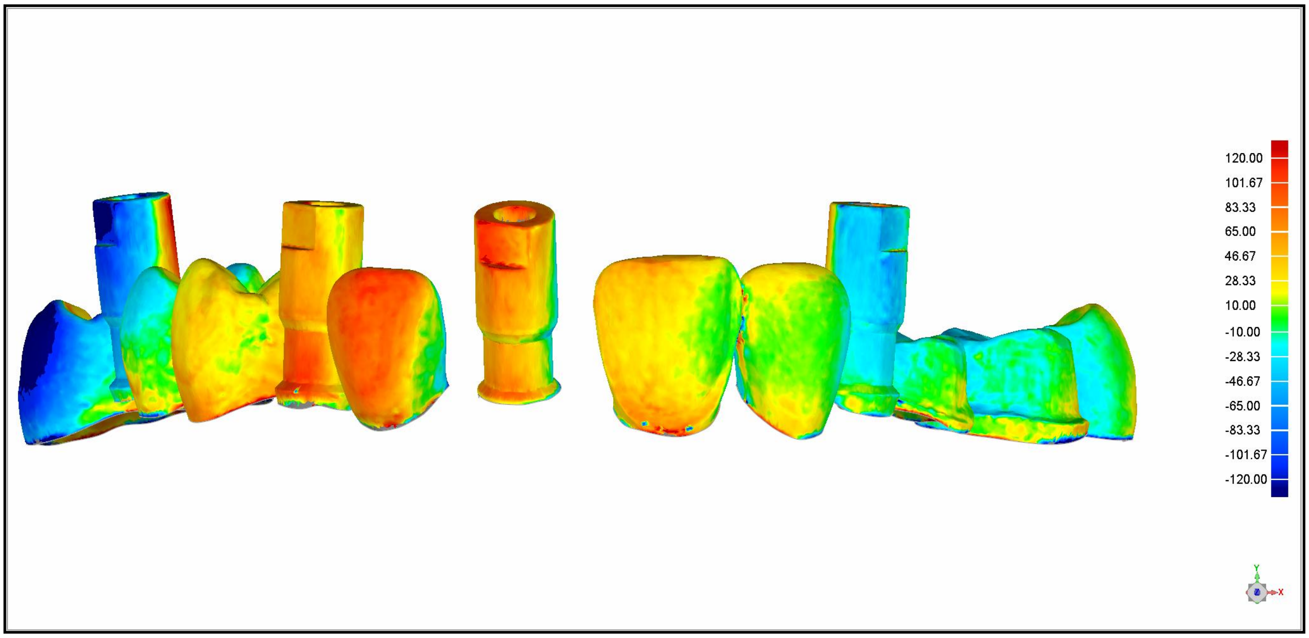

Predefinido: Inferior

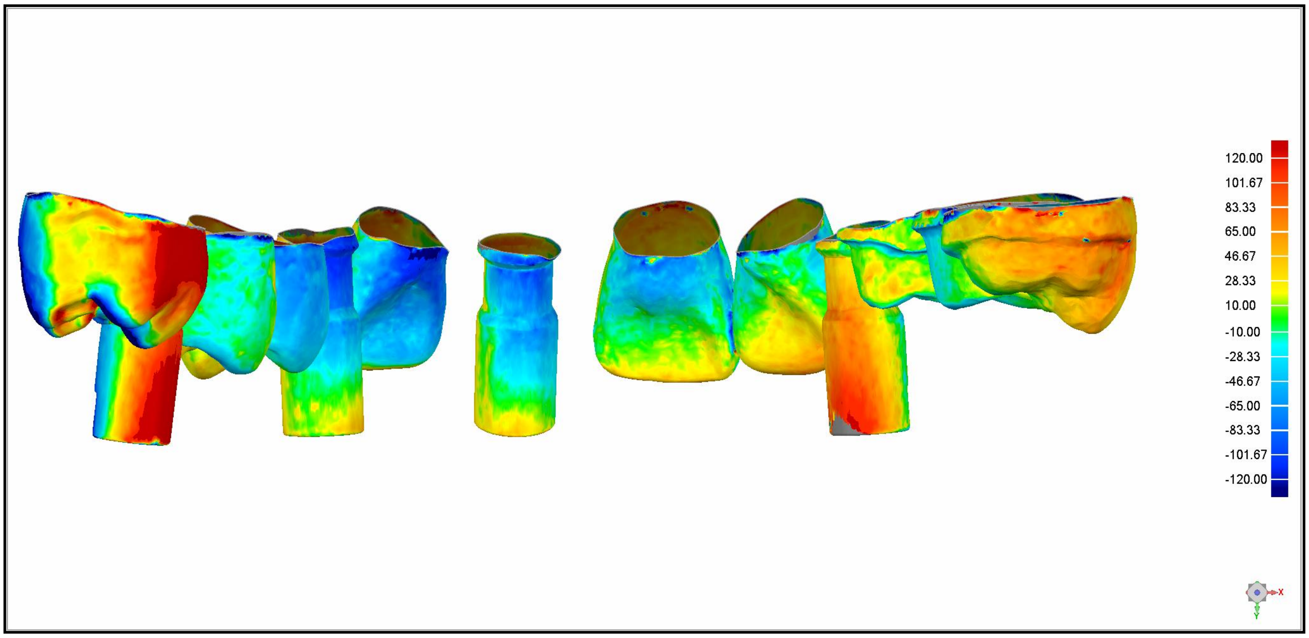

Ajuste de ubicación: Desviaciones superior e inferior

Unidades: u

Nombre	Desv	Estado	Superior Tol	Inferior Tol	Ref X	Ref Y	Ref Z	Radio	Desv X	Desv Y	Desv Z	Medido X	Medido Y	Medido Z	Dir. proy. X	Dir. proy. Y	Dir. proy. Z
Desv. inferior	-2607.87				-14204.33	37896.38	18990.13	n/a	-276.26	2200.16	1372.57	-14480.59	40096.54	20362.70	0.11	-0.84	-0.53
Desv. superior	2230.83				-25226.45	38719.99	790.08	n/a	950.64	-1966.95	451.67	-24275.80	36753.04	1241.74	0.43	-0.88	0.20
