# Supplementary material for: Accuracy of four digital scanners according to scanning strategy in complete-arch impressions
Source: PLoS One. 2018 Sep 13;13(9):e0202916. doi: 10.1371/journal.pone.0202916 (PMC6136706; doi:10.1371/journal.pone.0202916)
Supplement: S15 Table — True definition (scanning strategy C). (ZIP) [file pone.0202916.s015.zip › S15/TD5C.pdf]

Geomagic Control™

Fecha: 3/6/2017, 3:43 pm

3D Comparación Resultados

Modelo referencia	MRC
Modelo test	TD5C
Nº de puntos de datos	131420
# Aislados	354

Tipo tolerancia	3D desviación
Unidades	u
Máx. crítico	120.00
Máx. nominal	18.00
Mín. nominal	-18.00
Mín. crítico	-120.00

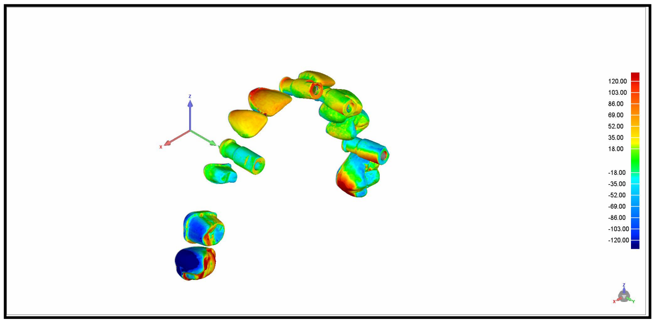

Desviación	
Desviación superior máx.	3154.56
Desviación inferior máx.	-3096.94
Desviación media	51.61 /-39.99
Desviación estándar	96.91

Distribución desviación

>=Min	<Max	# Puntos	%
-120.00	-103.00	822	0.63
-103.00	-86.00	1100	0.84
-86.00	-69.00	1322	1.01
-69.00	-52.00	2996	2.28
-52.00	-35.00	6496	4.94
-35.00	-18.00	11767	8.95
-18.00	18.00	46185	35.14
18.00	35.00	17259	13.13
35.00	52.00	12795	9.74
52.00	69.00	8393	6.39
69.00	86.00	5714	4.35
86.00	103.00	3924	2.99
103.00	120.00	2905	2.21

Fuera del crítico superior	6898	5.25
Fuera del crítico inferior	2844	2.16

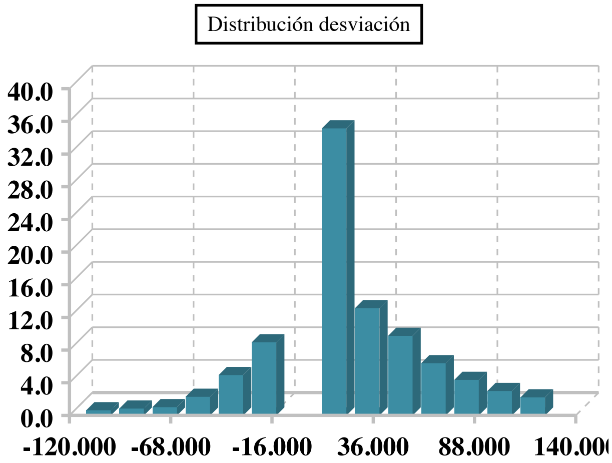

Desviaciones estándar

Distribución (+/-)	# Puntos	%
-6 * Desv. estánd.	209	0.16
-5 * Desv. estánd.	45	0.03
-4 * Desv. estánd.	100	0.08
-3 * Desv. estánd.	777	0.59
-2 * Desv. estánd.	4135	3.15
-1 * Desv. estánd.	68753	52.32
1 * Desv. estánd.	49836	37.92
2 * Desv. estánd.	6313	4.80
3 * Desv. estánd.	669	0.51
4 * Desv. estánd.	143	0.11
5 * Desv. estánd.	108	0.08
6 * Desv. estánd.	332	0.25

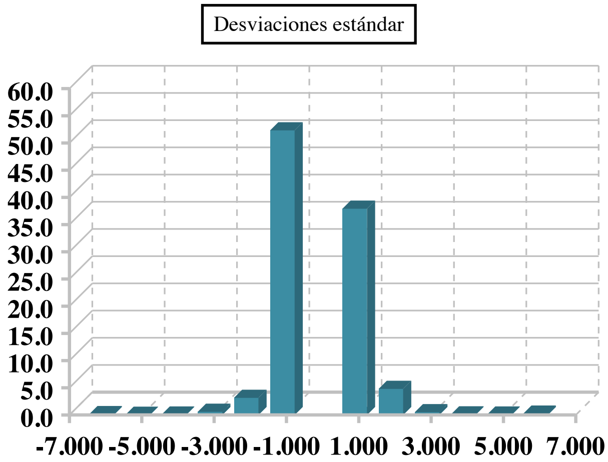

Predefinido: Isométrico

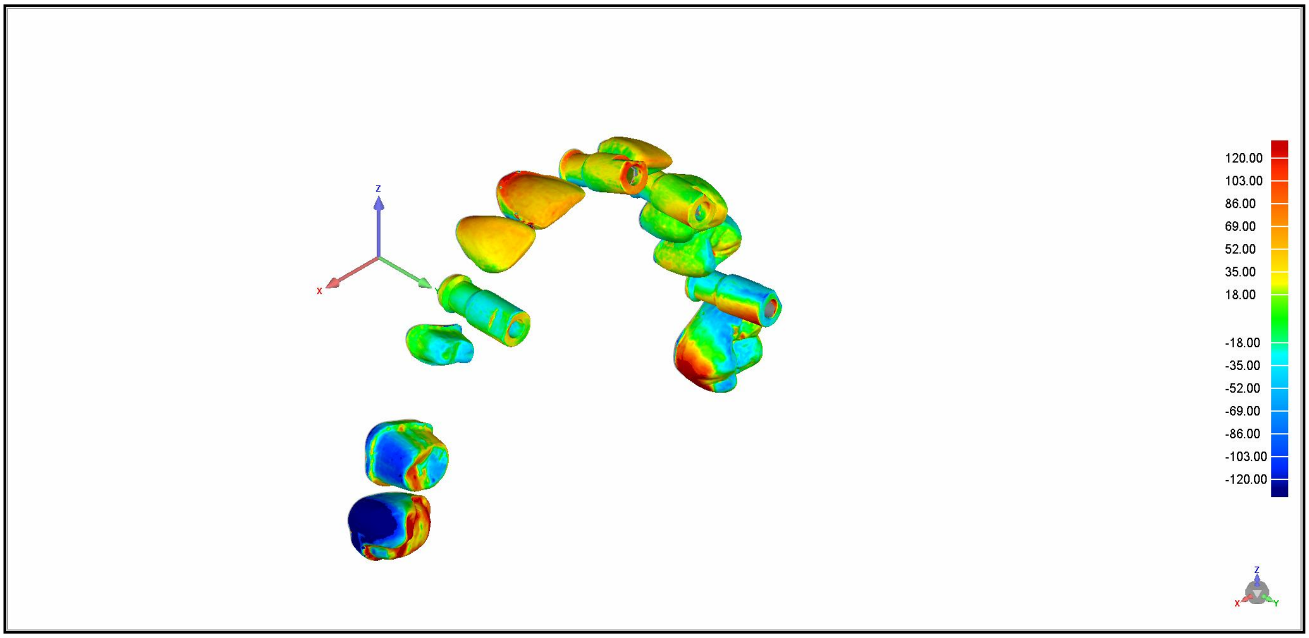

Predefinido: Frente

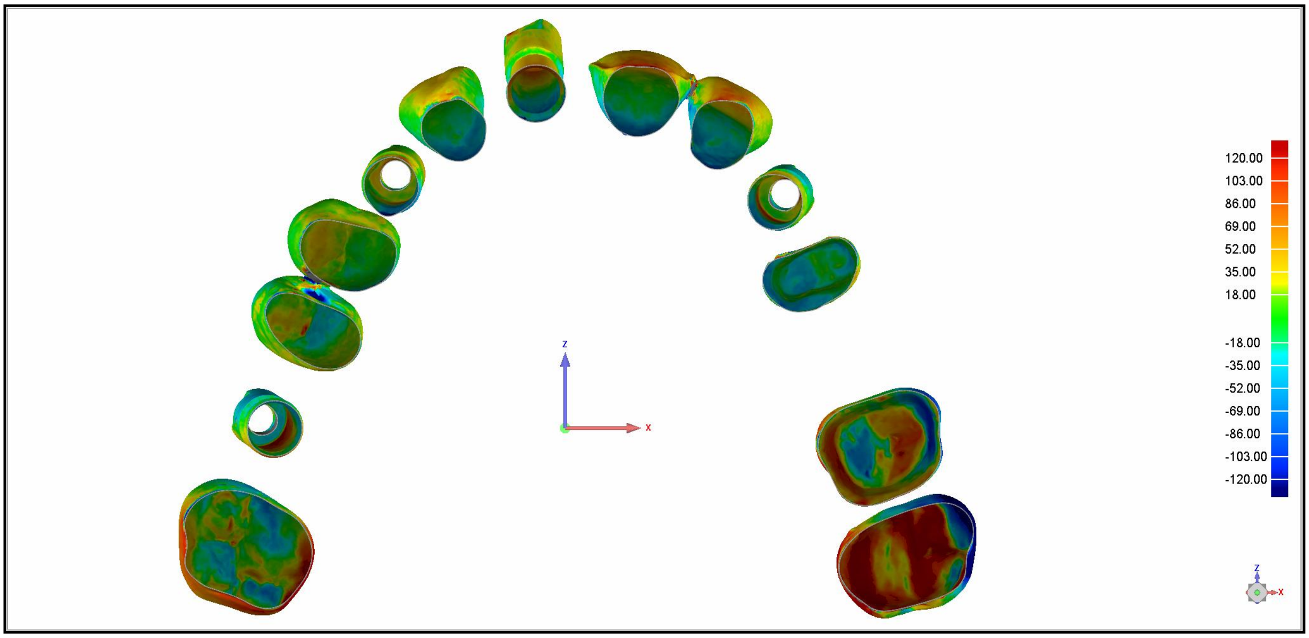

Predefinido: Atrás

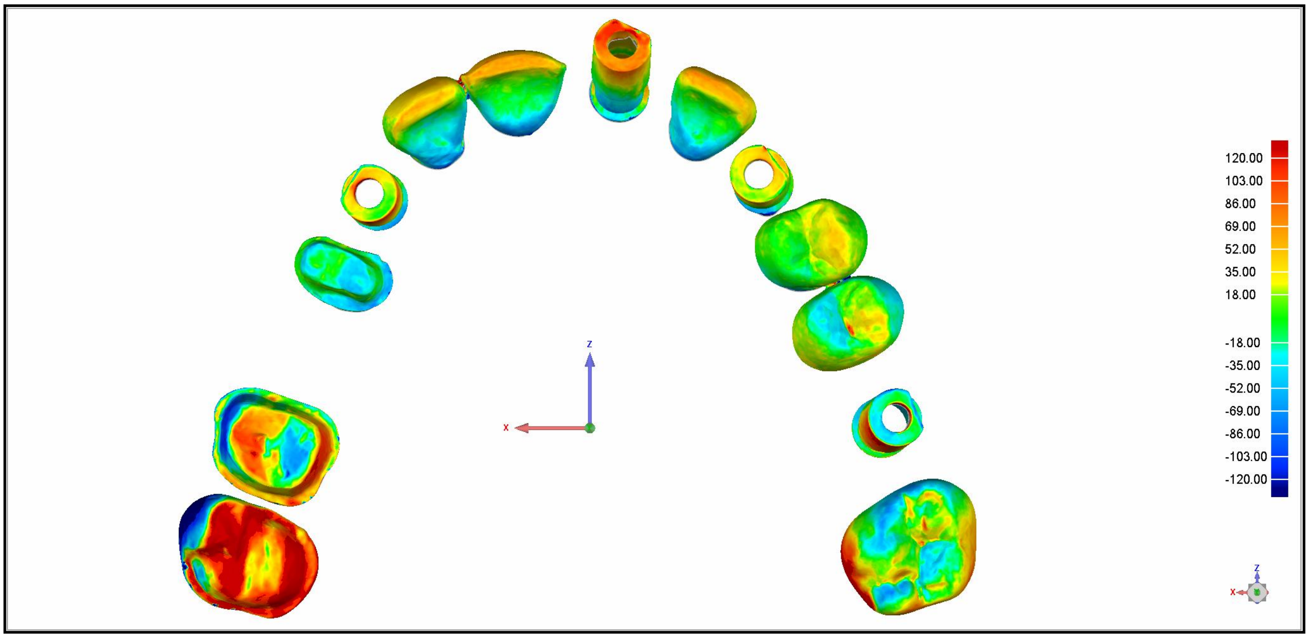

Predefinido: Izquierda

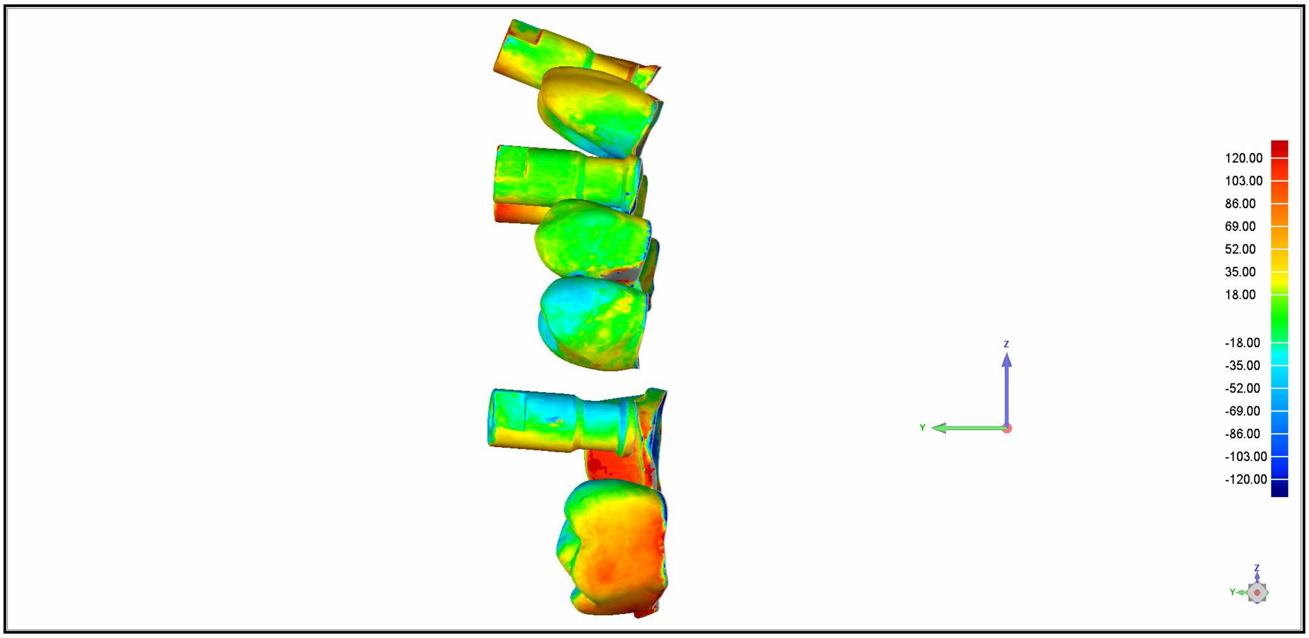

Predefinido: Derecha

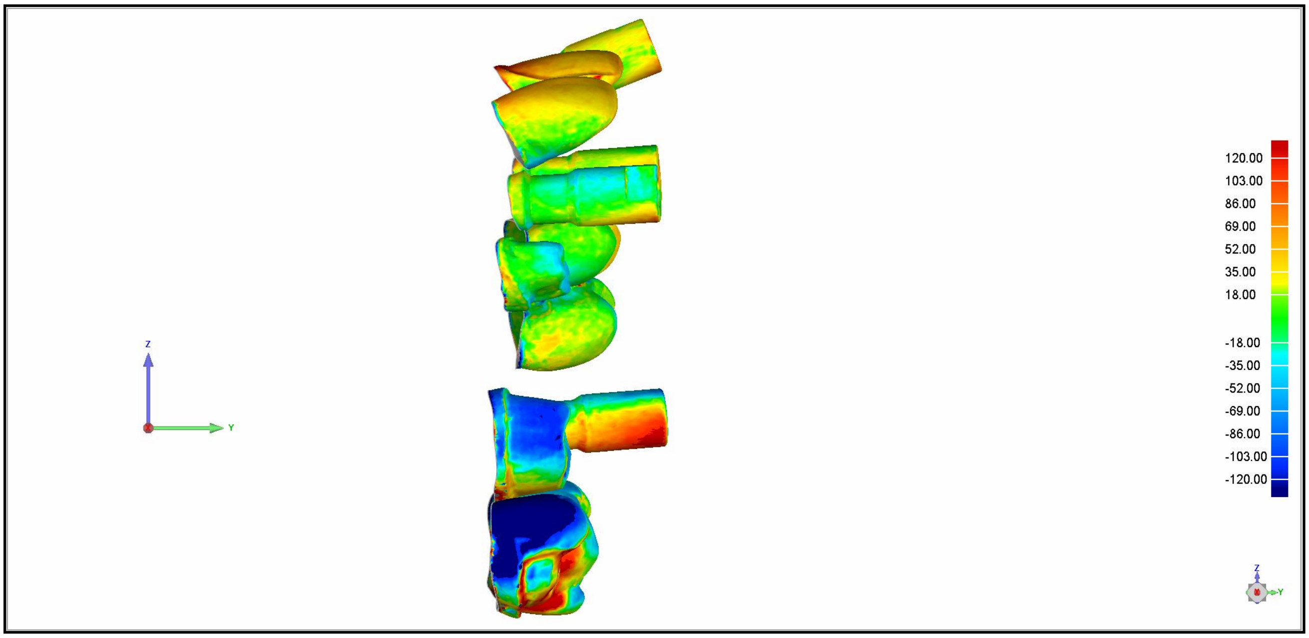

Predefinido: Superior

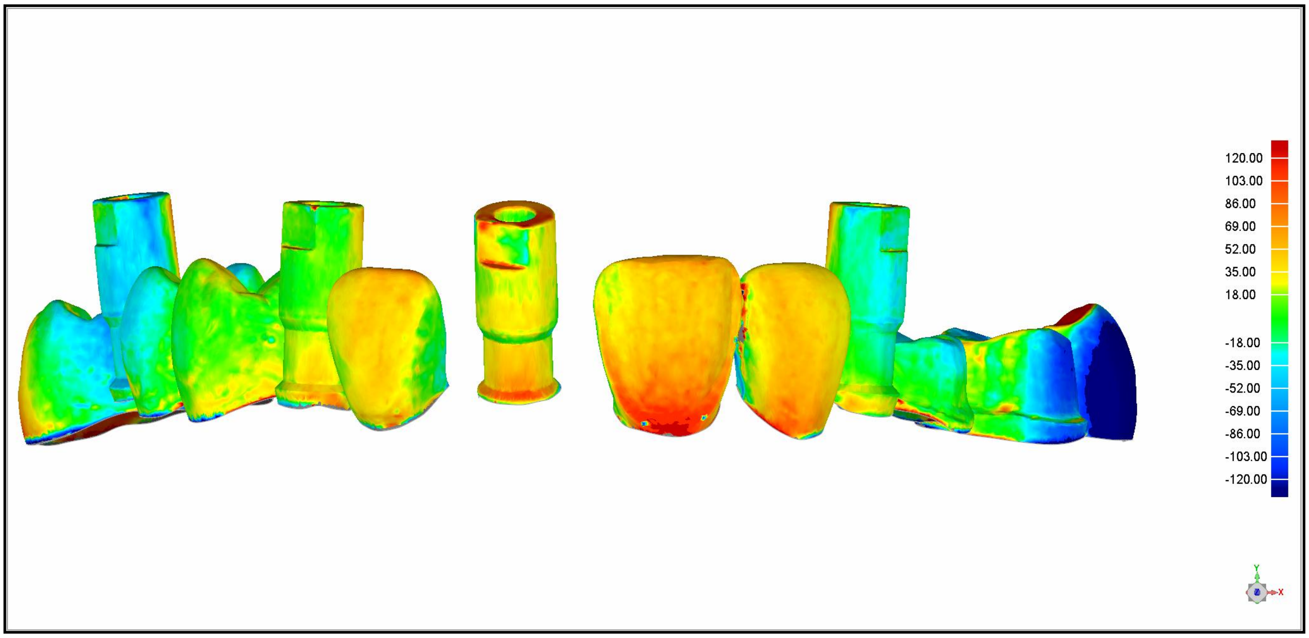

Predefinido: Inferior

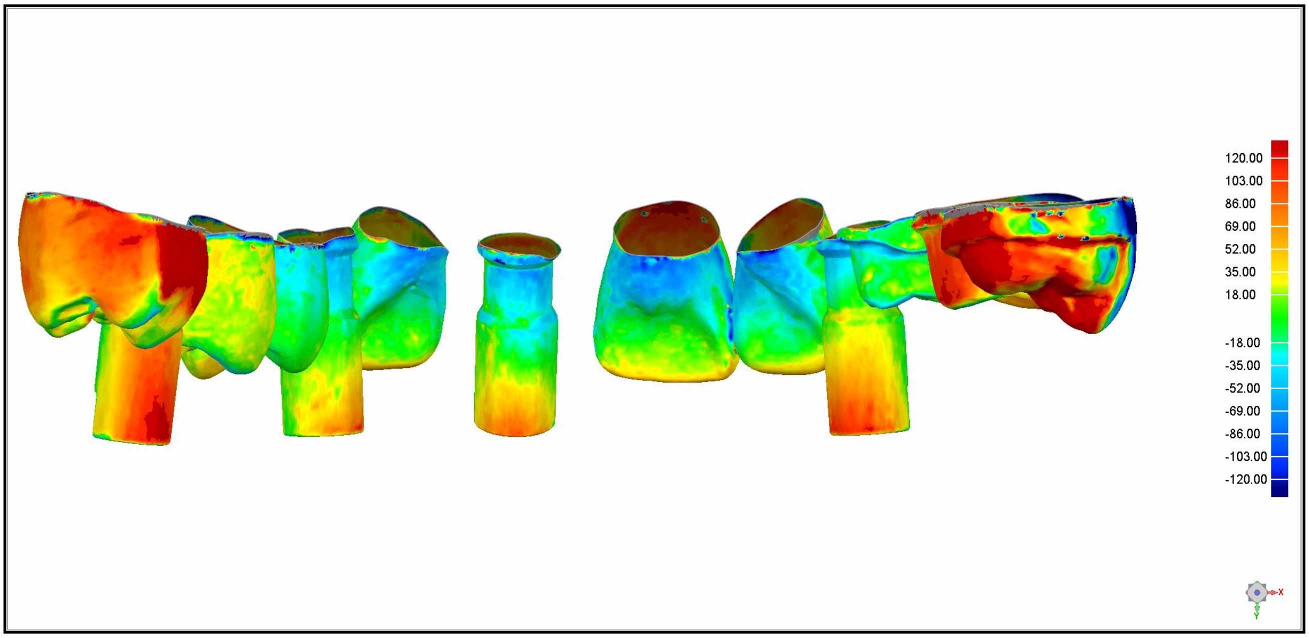

Ajuste de ubicación: Desviaciones superior e inferior

Unidades: u

Nombre	Desv	Estado	Superior Tol	Inferior Tol	Ref X	Ref Y	Ref Z	Radio	Desv X	Desv Y	Desv Z	Medido X	Medido Y	Medido Z	Dir. proy. X	Dir. proy. Y	Dir. proy. Z
Desv. inferior	-3096.94				-13501.21	29913.37	14588.79	n/a	-1337.46	-77.39	-2792.17	-14838.67	29835.98	11796.62	0.43	0.02	0.90
Desv. superior	3154.56				-17902.57	29727.70	11012.38	n/a	3104.95	200.88	519.77	-14797.62	29928.58	11532.15	0.98	0.06	0.16
