# Supplementary material for: Accuracy of four digital scanners according to scanning strategy in complete-arch impressions
Source: PLoS One. 2018 Sep 13;13(9):e0202916. doi: 10.1371/journal.pone.0202916 (PMC6136706; doi:10.1371/journal.pone.0202916)
Supplement: S15 Table — True definition (scanning strategy C). (ZIP) [file pone.0202916.s015.zip › S15/TD9C.pdf]

3D Comparación Resultados

Modelo referencia	MRC
Modelo test	TD9C
Nº de puntos de datos	130601
# Aislados	386

Tipo tolerancia	3D desviación
Unidades	u
Máx. crítico	120.00
Máx. nominal	17.00
Mín. nominal	-17.00
Mín. crítico	-120.00

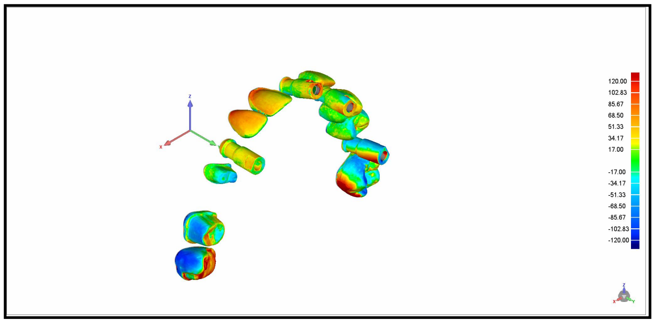

Desviación	
Desviación superior máx.	3136.55
Desviación inferior máx.	-2471.00
Desviación media	50.23 /-38.24
Desviación estándar	78.54

Distribución desviación

>=Min	<Max	# Puntos	%
-120.00	-102.83	894	0.68
-102.83	-85.67	2190	1.68
-85.67	-68.50	3767	2.88
-68.50	-51.33	5554	4.25
-51.33	-34.17	7660	5.87
-34.17	-17.00	10910	8.35
-17.00	17.00	38478	29.46
17.00	34.17	19684	15.07
34.17	51.33	13218	10.12
51.33	68.50	8870	6.79
68.50	85.67	5680	4.35
85.67	102.83	3803	2.91
102.83	120.00	2373	1.82

Fuera del crítico superior	6757	5.17
Fuera del crítico inferior	763	0.58

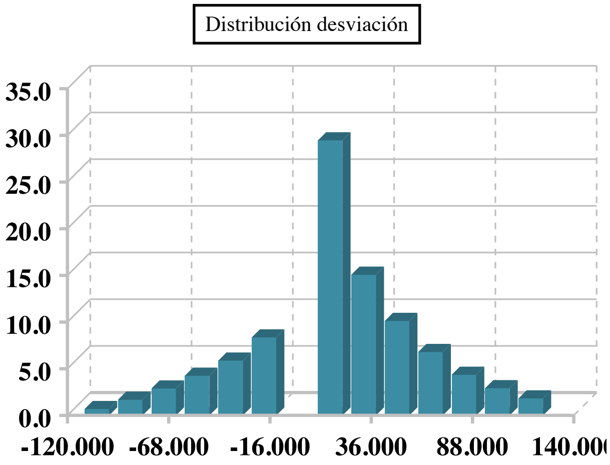

Desviaciones estándar

Distribución (+/-)	# Puntos	%
-6 * Desv. estánd.	106	0.08
-5 * Desv. estánd.	51	0.04
-4 * Desv. estánd.	148	0.11
-3 * Desv. estánd.	307	0.24
-2 * Desv. estánd.	9263	7.09
-1 * Desv. estánd.	60845	46.59
1 * Desv. estánd.	49331	37.77
2 * Desv. estánd.	8484	6.50
3 * Desv. estánd.	1493	1.14
4 * Desv. estánd.	213	0.16
5 * Desv. estánd.	95	0.07
6 * Desv. estánd.	265	0.20

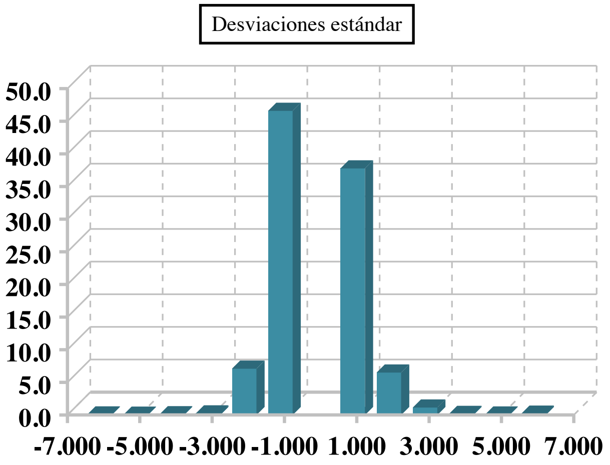

Predefinido: Isométrico

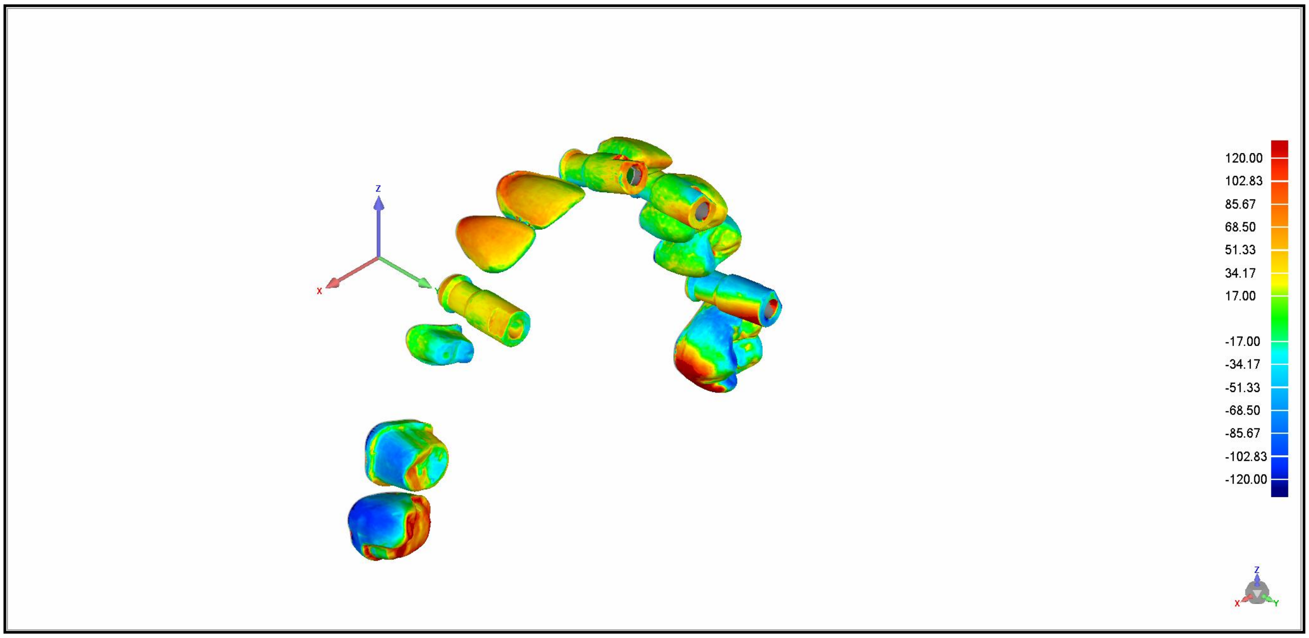

Predefinido: Frente

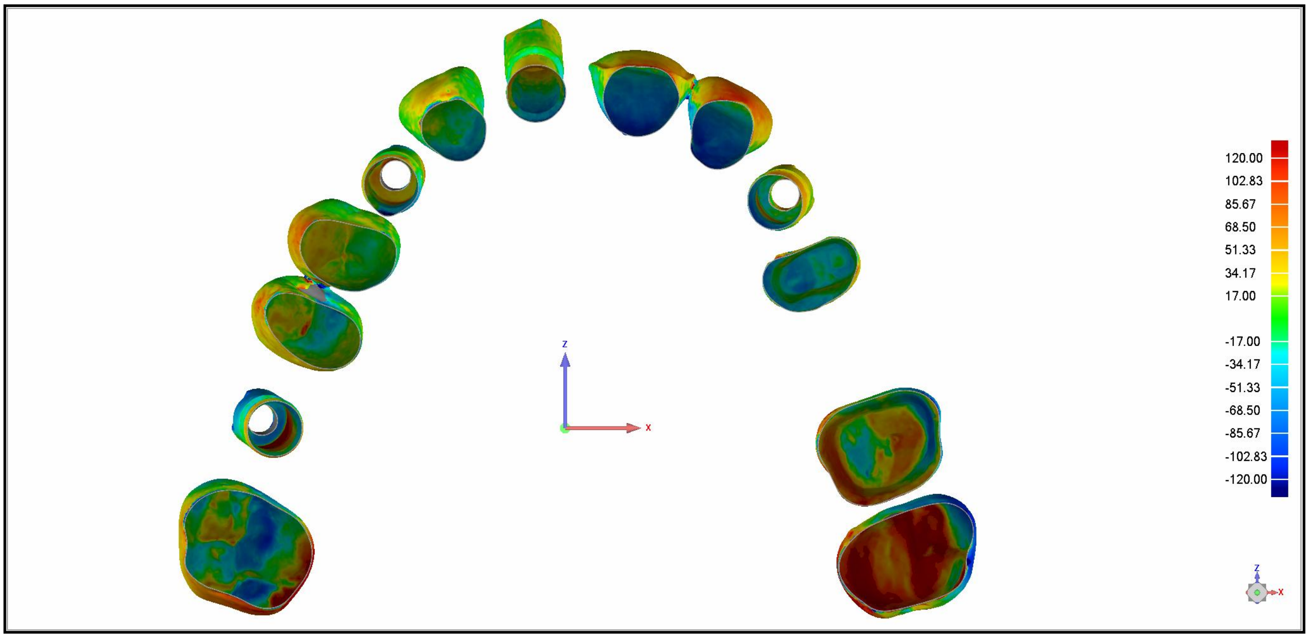

Predefinido: Atrás

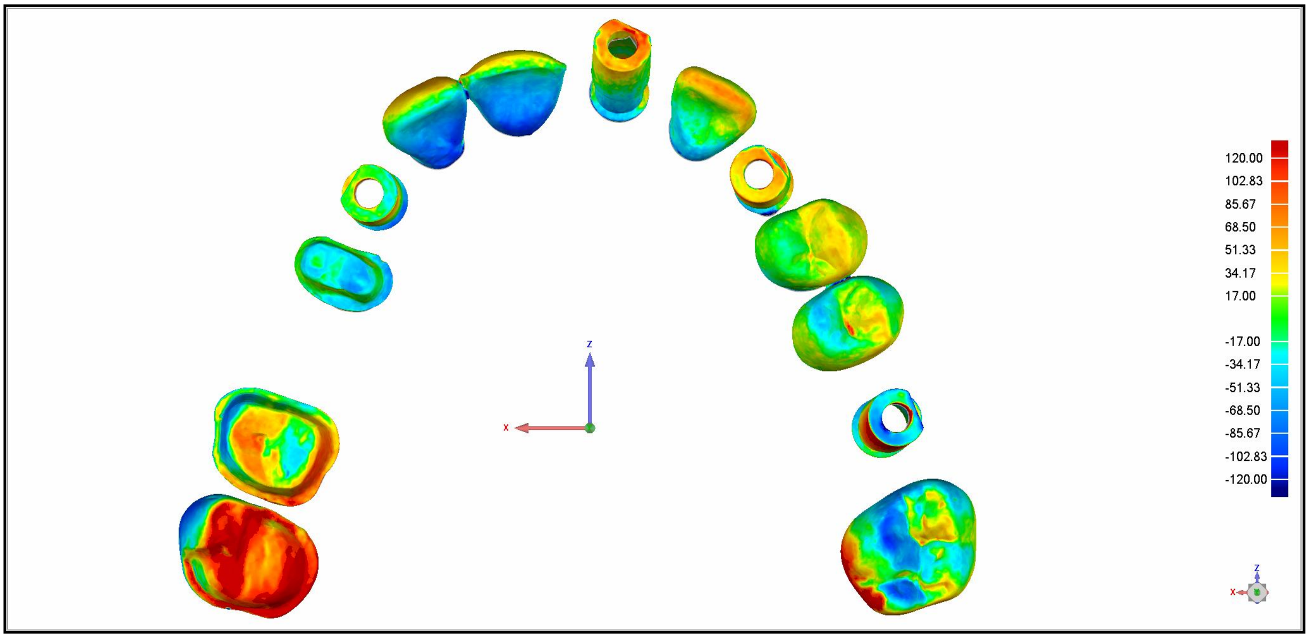

Predefinido: Izquierda

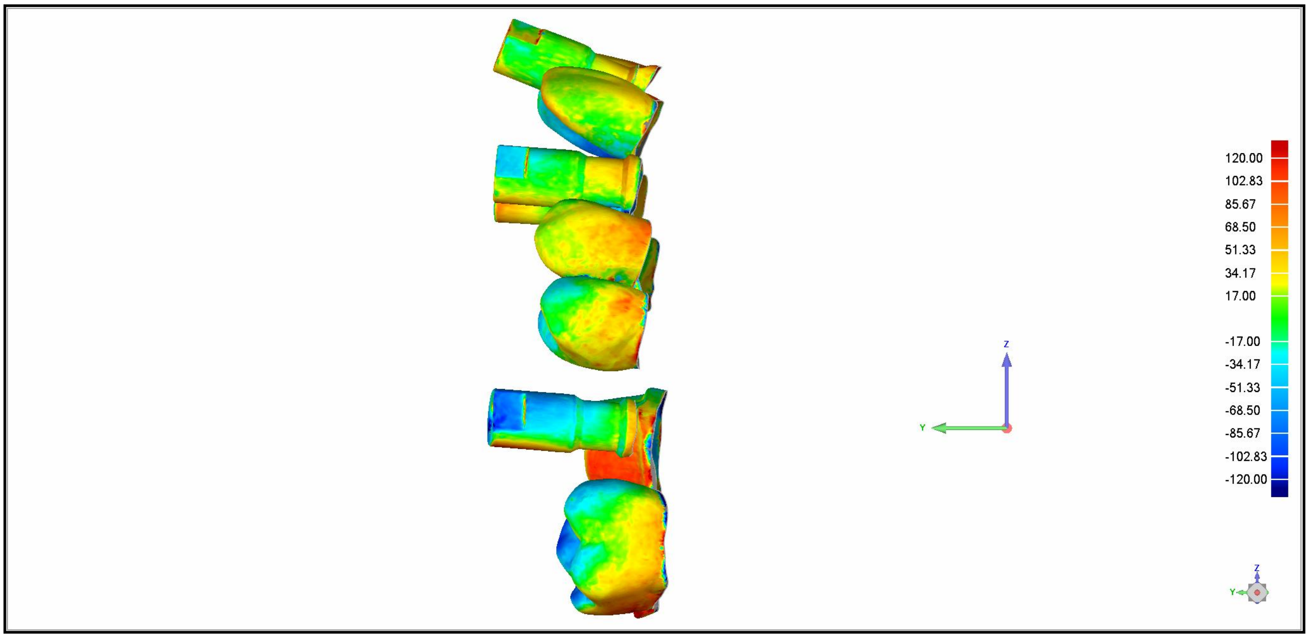

Predefinido: Derecha

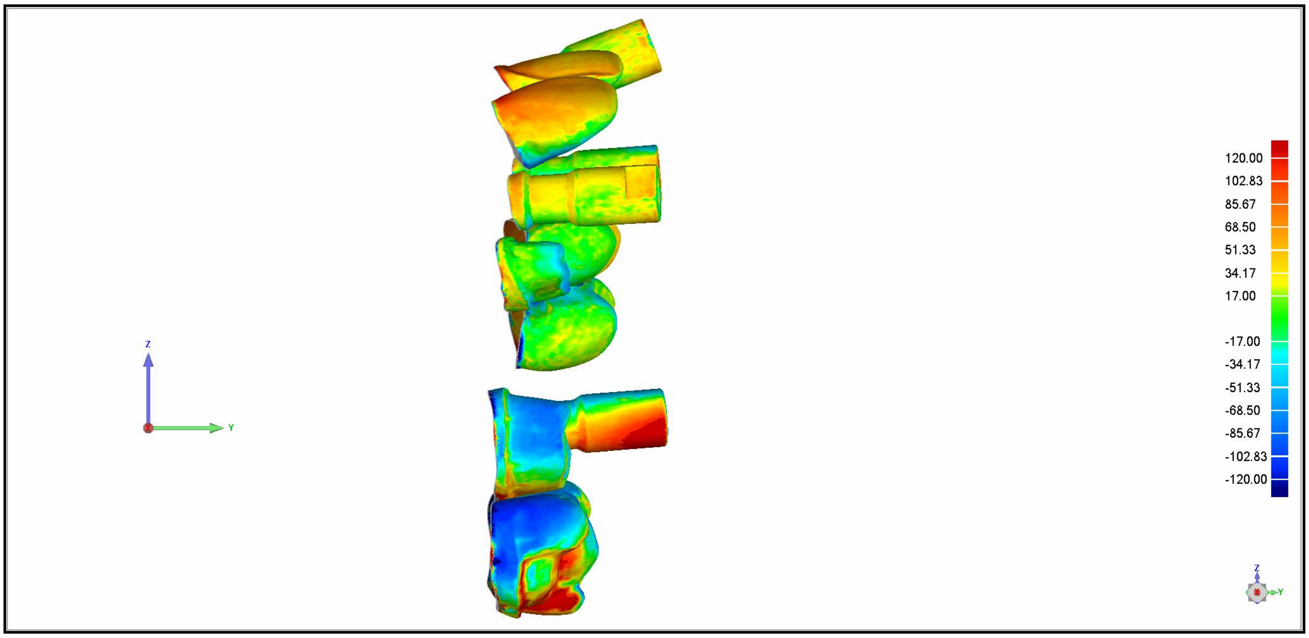

Predefinido: Superior

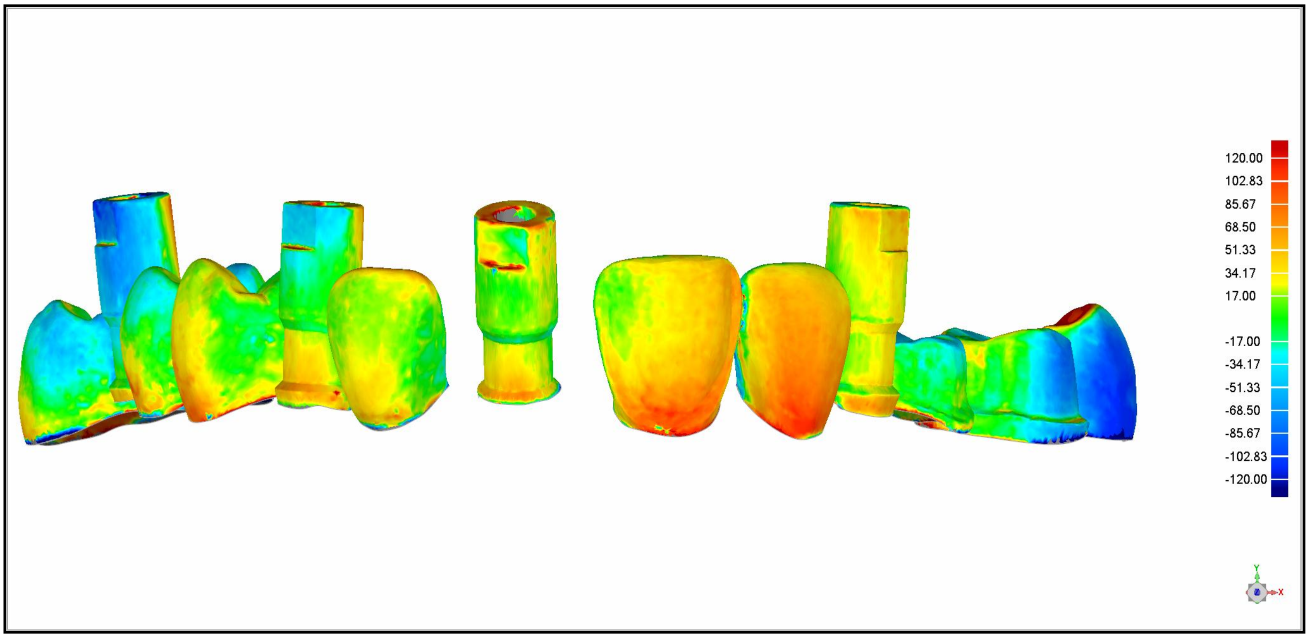

Predefinido: Inferior

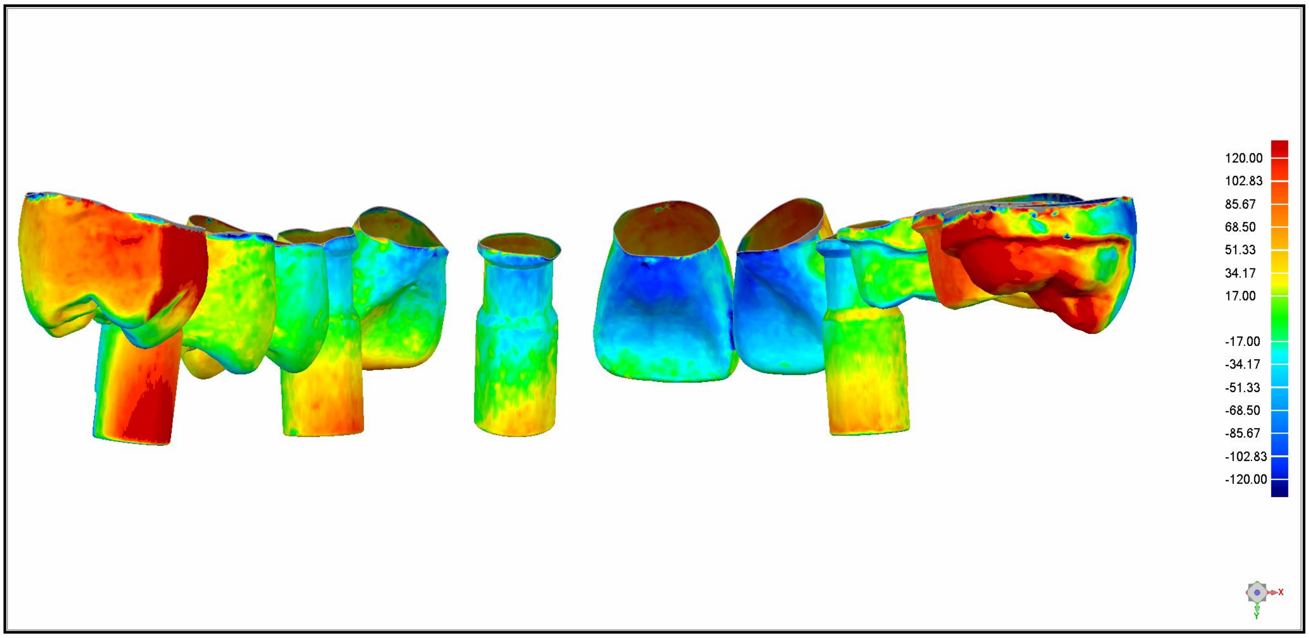

Ajuste de ubicación: Desviaciones superior e inferior

Unidades: u

Nombre	Desv	Estado	Superior Tol	Inferior Tol	Ref X	Ref Y	Ref Z	Radio	Desv X	Desv Y	Desv Z	Medido X	Medido Y	Medido Z	Dir. proy. X	Dir. proy. Y	Dir. proy. Z
Desv. inferior	-2471.00				-29292.33	26884.28	-11910.36	n/a	2102.48	361.61	-1246.86	-27189.85	27245.89	-13157.21	-0.85	-0.15	0.50
Desv. superior	3136.55				-12601.26	29768.36	21377.11	n/a	1018.42	-1299.41	2666.89	-11582.84	28468.95	24044.00	0.32	-0.41	0.85
