# Supplementary material for: Accuracy of four digital scanners according to scanning strategy in complete-arch impressions
Source: PLoS One. 2018 Sep 13;13(9):e0202916. doi: 10.1371/journal.pone.0202916 (PMC6136706; doi:10.1371/journal.pone.0202916)
Supplement: S15 Table — True definition (scanning strategy C). (ZIP) [file pone.0202916.s015.zip › S15/TD8C.pdf]

3D Comparación Resultados

Modelo referencia	MRC
Modelo test	TD8C
Nº de puntos de datos	129696
# Aislados	541

Tipo tolerancia	3D desviación
Unidades	u
Máx. crítico	120.00
Máx. nominal	14.00
Mín. nominal	-14.00
Mín. crítico	-120.00

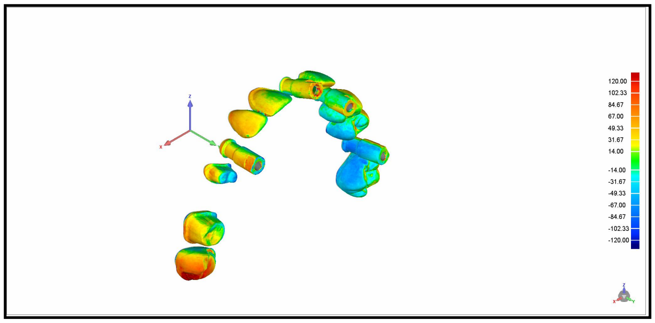

Desviación	
Desviación superior máx.	3121.59
Desviación inferior máx.	-2082.09
Desviación media	43.78 /-29.73
Desviación estándar	59.47

Distribución desviación

>=Min	<Max	# Puntos	%
-120.00	-102.33	169	0.13
-102.33	-84.67	433	0.33
-84.67	-67.00	2241	1.73
-67.00	-49.33	6090	4.70
-49.33	-31.67	9323	7.19
-31.67	-14.00	16528	12.74
-14.00	14.00	32321	24.92
14.00	31.67	17738	13.68
31.67	49.33	16473	12.70
49.33	67.00	12783	9.86
67.00	84.67	7675	5.92
84.67	102.33	3717	2.87
102.33	120.00	1742	1.34

Fuera del crítico superior	1877	1.45
Fuera del crítico inferior	586	0.45

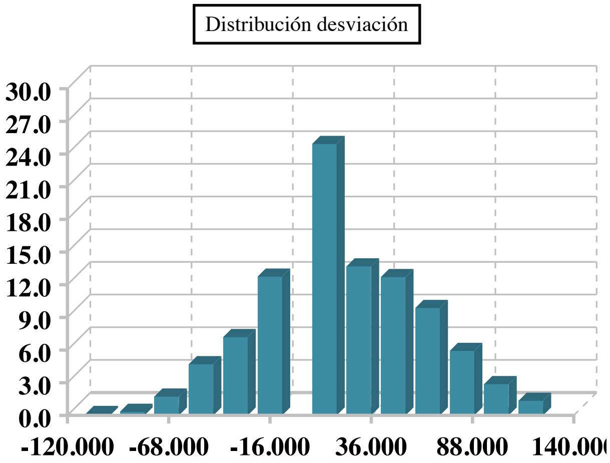

Desviaciones estándar

Distribución (+/-)	# Puntos	%
-6 * Desv. estánd.	32	0.02
-5 * Desv. estánd.	82	0.06
-4 * Desv. estánd.	177	0.14
-3 * Desv. estánd.	431	0.33
-2 * Desv. estánd.	10562	8.14
-1 * Desv. estánd.	56541	43.60
1 * Desv. estánd.	50161	38.68
2 * Desv. estánd.	10471	8.07
3 * Desv. estánd.	831	0.64
4 * Desv. estánd.	206	0.16
5 * Desv. estánd.	87	0.07
6 * Desv. estánd.	115	0.09

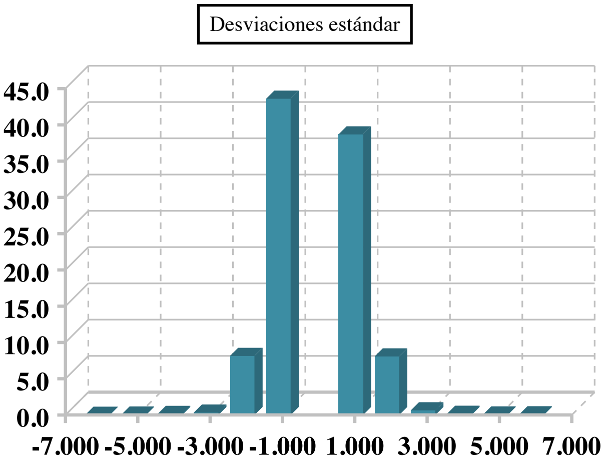

Predefinido: Isométrico

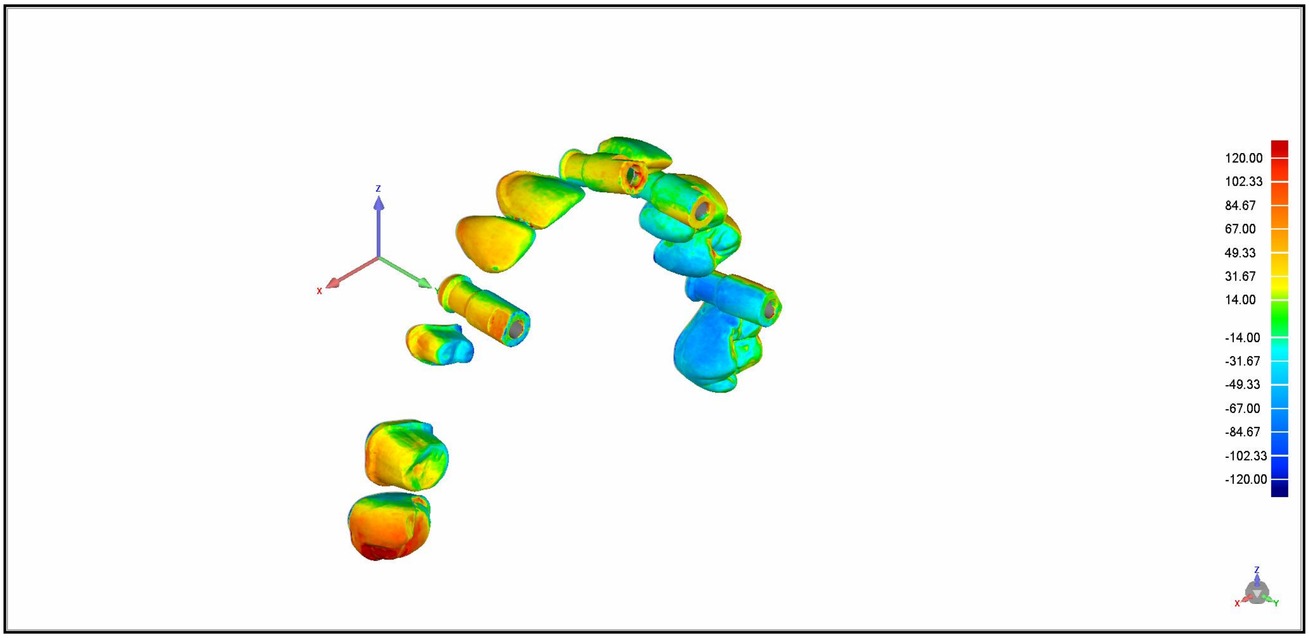

Predefinido: Frente

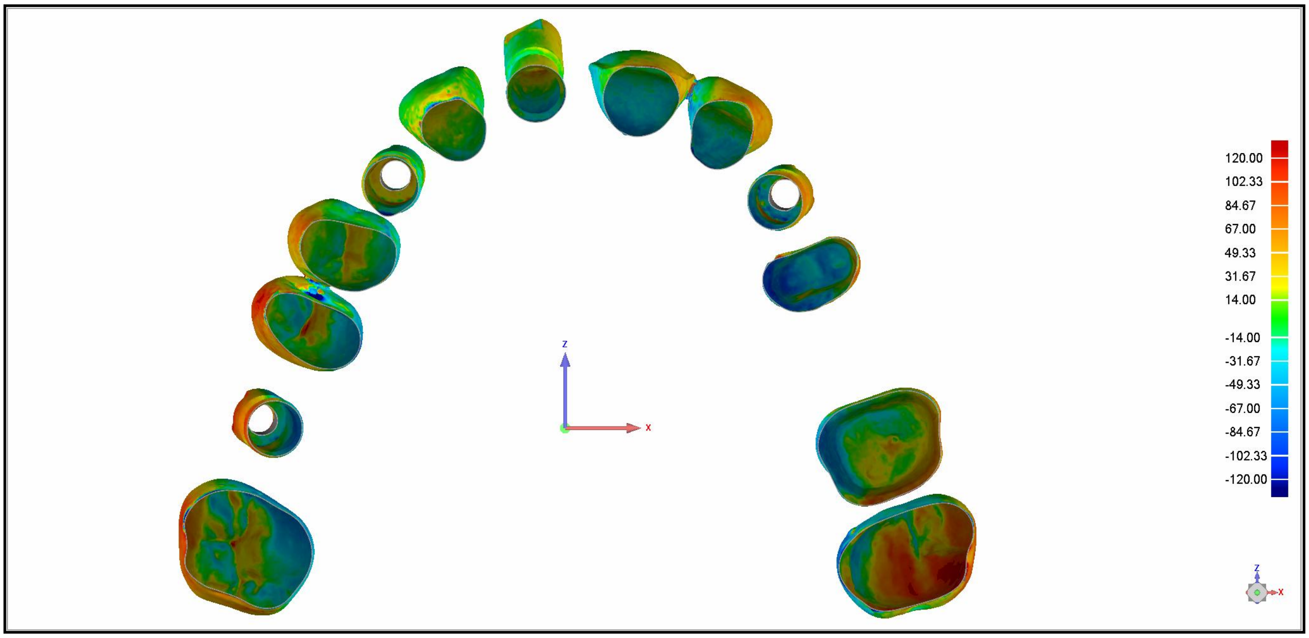

Predefinido: Atrás

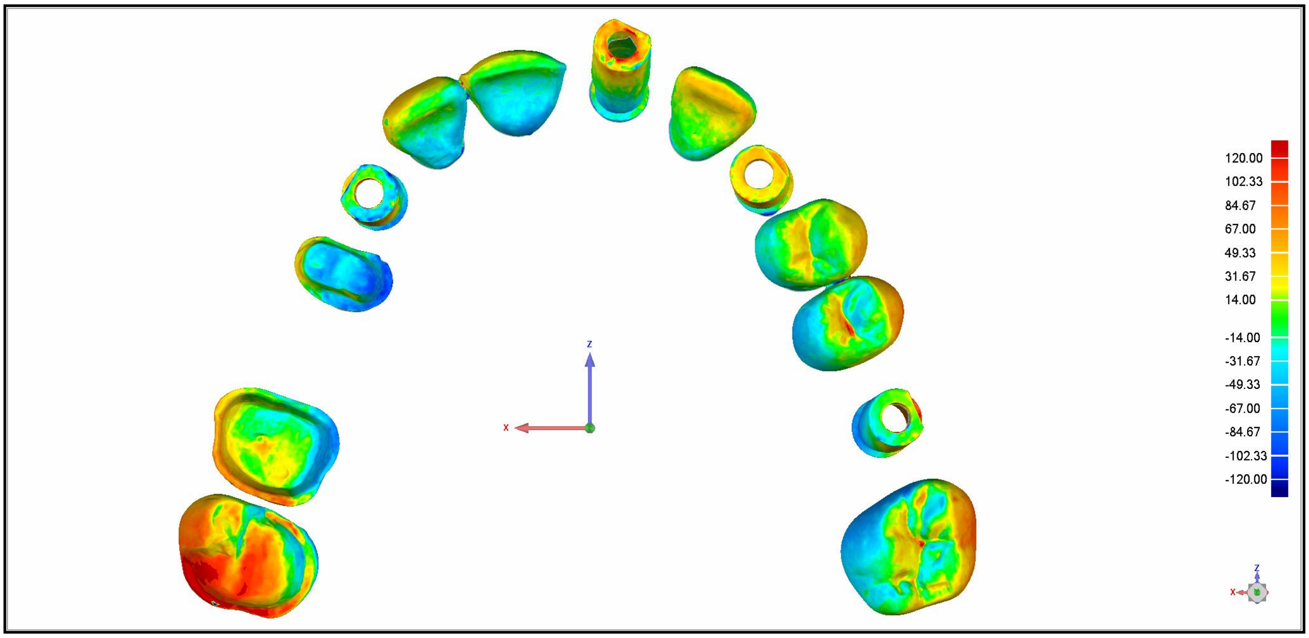

Predefinido: Izquierda

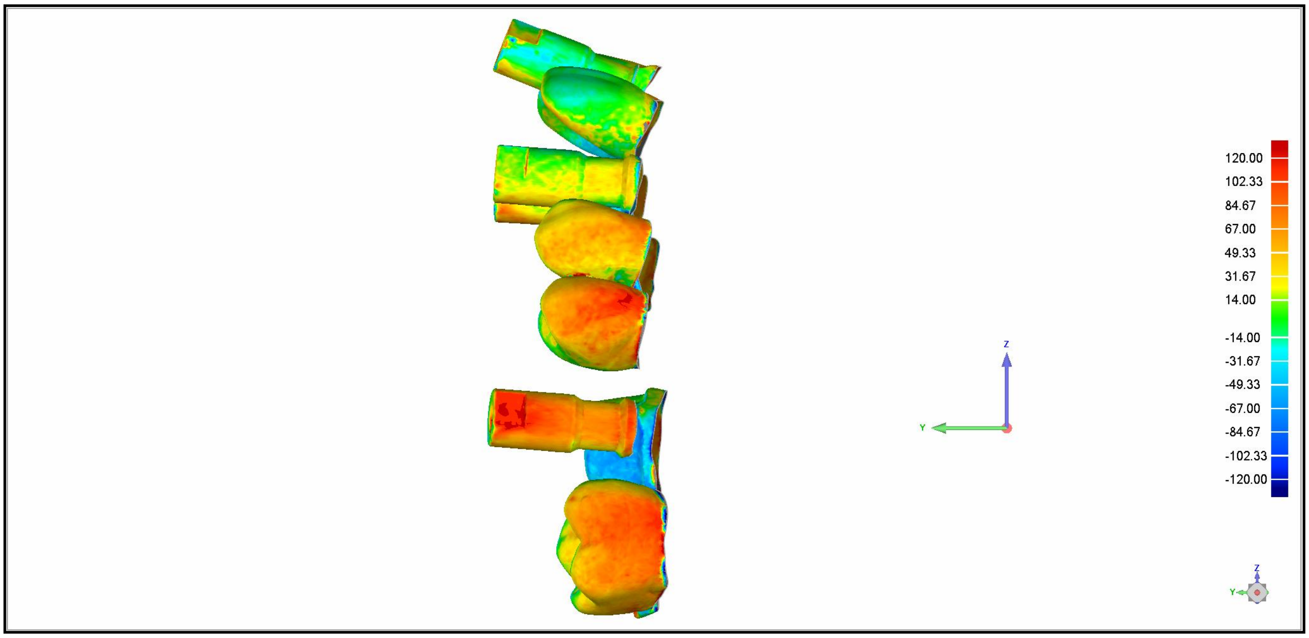

Predefinido: Derecha

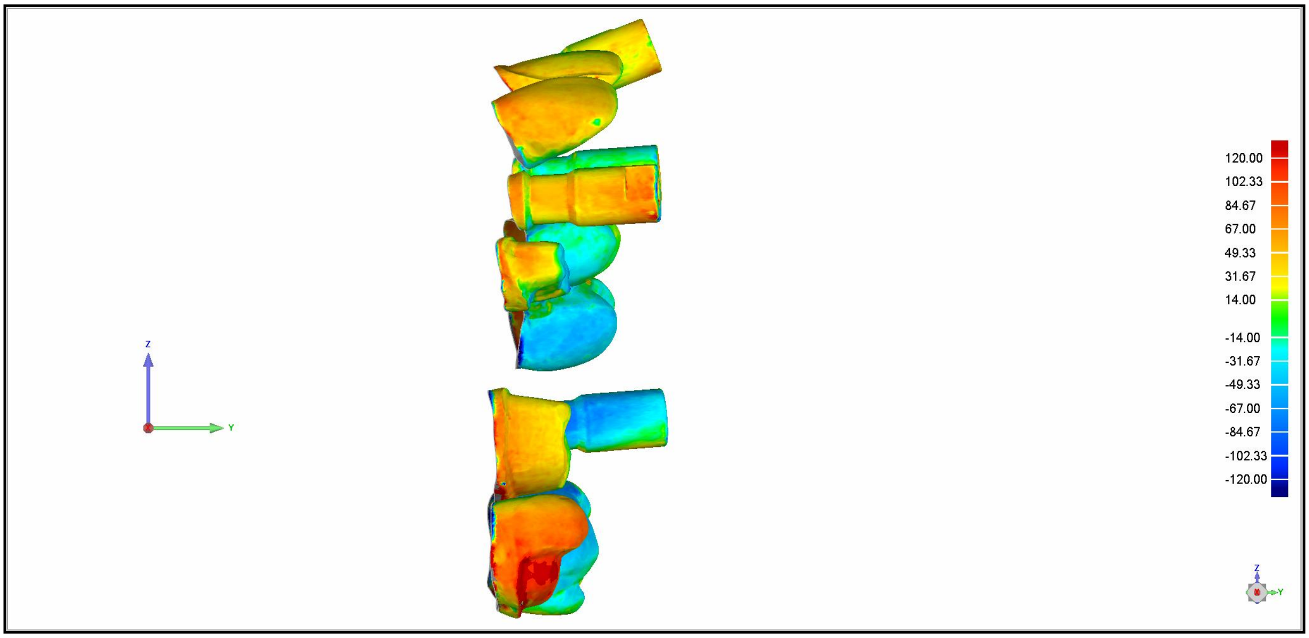

Predefinido: Superior

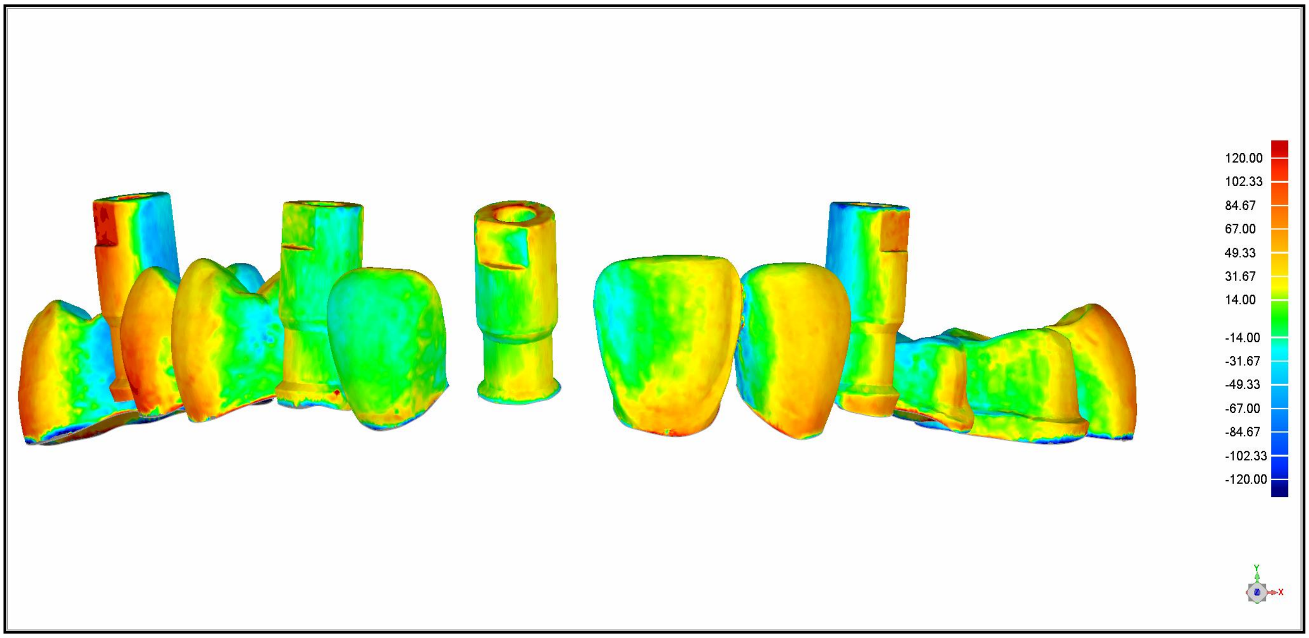

Predefinido: Inferior

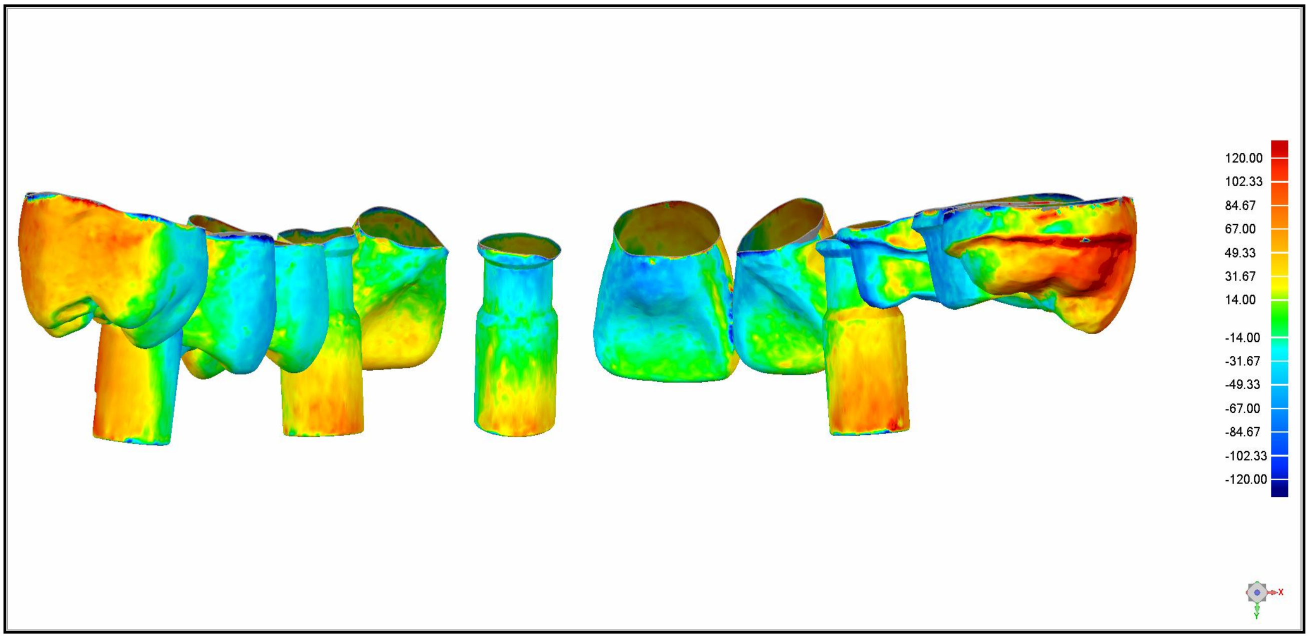

Ajuste de ubicación: Desviaciones superior e inferior

Unidades: u

Nombre	Desv	Estado	Superior Tol	Inferior Tol	Ref X	Ref Y	Ref Z	Radio	Desv X	Desv Y	Desv Z	Medido X	Medido Y	Medido Z	Dir. proy. X	Dir. proy. Y	Dir. proy. Z
Desv. inferior	-2082.09				29707.64	29539.35	-13912.95	n/a	-629.75	-1984.28	-34.39	29077.89	27555.08	-13947.35	0.30	0.95	0.02
Desv. superior	3121.59				-12601.26	29768.36	21377.11	n/a	1030.77	-1297.51	2645.44	-11570.49	28470.85	24022.55	0.33	-0.42	0.85
