# Supplementary material for: Accuracy of four digital scanners according to scanning strategy in complete-arch impressions
Source: PLoS One. 2018 Sep 13;13(9):e0202916. doi: 10.1371/journal.pone.0202916 (PMC6136706; doi:10.1371/journal.pone.0202916)
Supplement: S15 Table — True definition (scanning strategy C). (ZIP) [file pone.0202916.s015.zip › S15/TD4C.pdf]

3D Comparación Resultados

Modelo referencia	MRC
Modelo test	TD4C
Nº de puntos de datos	129133
# Aislados	464

Tipo tolerancia	3D desviación
Unidades	u
Máx. crítico	120.00
Máx. nominal	15.00
Mín. nominal	-15.00
Mín. crítico	-120.00

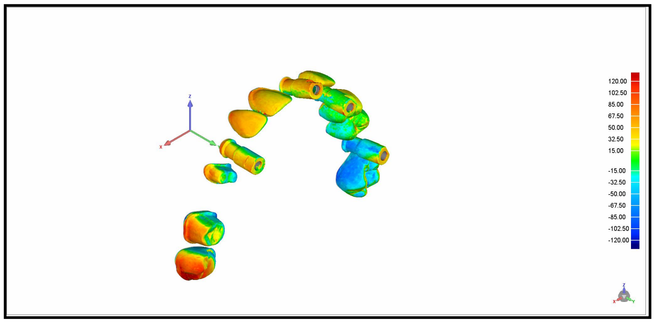

Desviación	
Desviación superior máx.	1808.04
Desviación inferior máx.	-2540.77
Desviación media	43.53 /-35.60
Desviación estándar	68.88

Distribución desviación

>=Min	<Max	# Puntos	%
-120.00	-102.50	496	0.38
-102.50	-85.00	1169	0.91
-85.00	-67.50	3484	2.70
-67.50	-50.00	5101	3.95
-50.00	-32.50	7915	6.13
-32.50	-15.00	12049	9.33
-15.00	15.00	33751	26.14
15.00	32.50	20434	15.82
32.50	50.00	16067	12.44
50.00	67.50	12517	9.69
67.50	85.00	6847	5.30
85.00	102.50	4591	3.56
102.50	120.00	2199	1.70

Fuera del crítico superior	1775	1.37
Fuera del crítico inferior	738	0.57

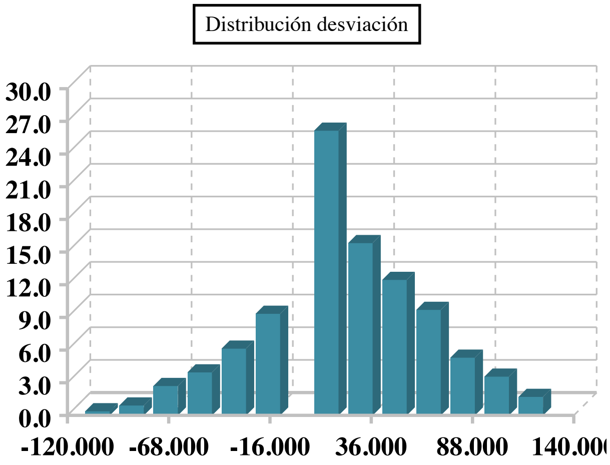

Desviaciones estándar

Distribución (+/-)	# Puntos	%
-6 * Desv. estánd.	111	0.09
-5 * Desv. estánd.	76	0.06
-4 * Desv. estánd.	188	0.15
-3 * Desv. estánd.	340	0.26
-2 * Desv. estánd.	9024	6.99
-1 * Desv. estánd.	55203	42.75
1 * Desv. estánd.	55292	42.82
2 * Desv. estánd.	8056	6.24
3 * Desv. estánd.	401	0.31
4 * Desv. estánd.	146	0.11
5 * Desv. estánd.	92	0.07
6 * Desv. estánd.	204	0.16

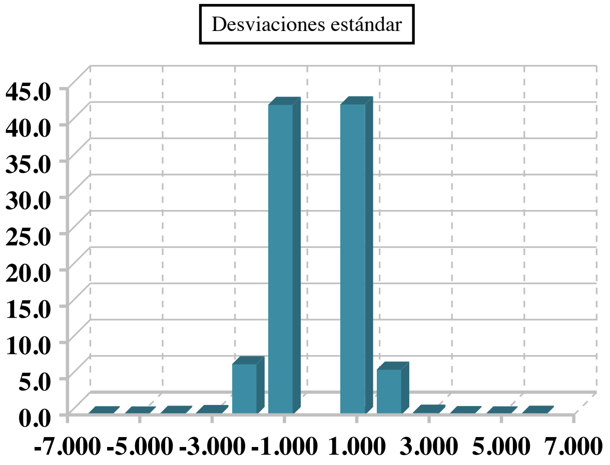

Predefinido: Isométrico

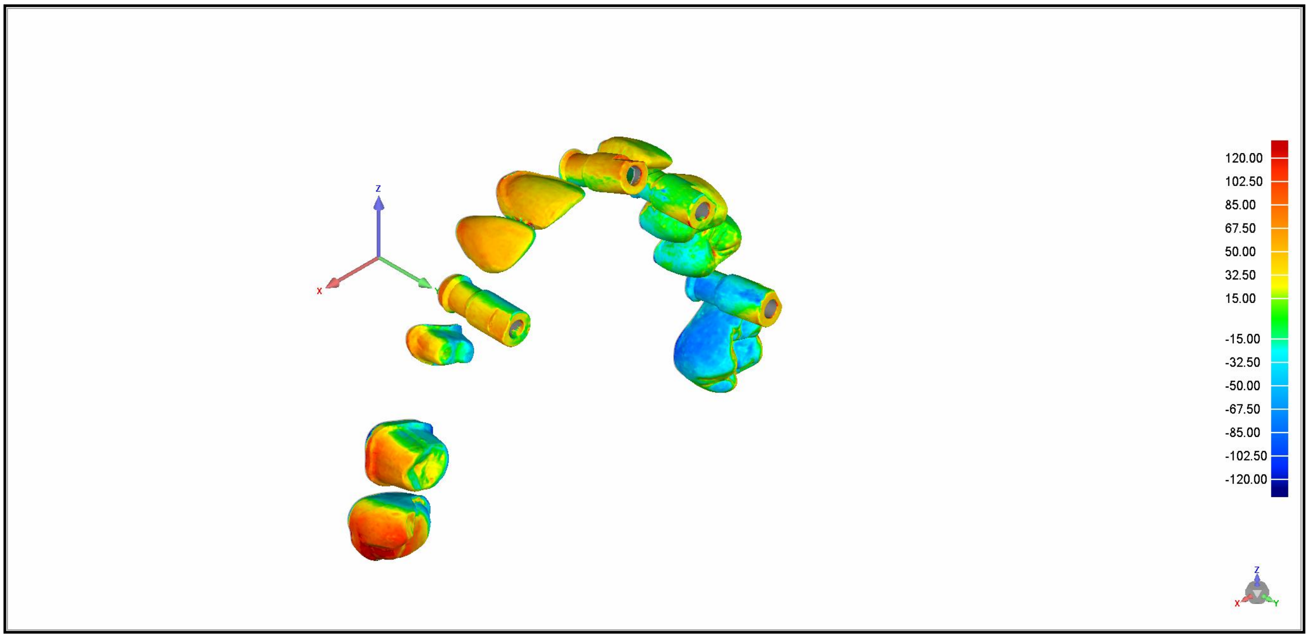

Predefinido: Frente

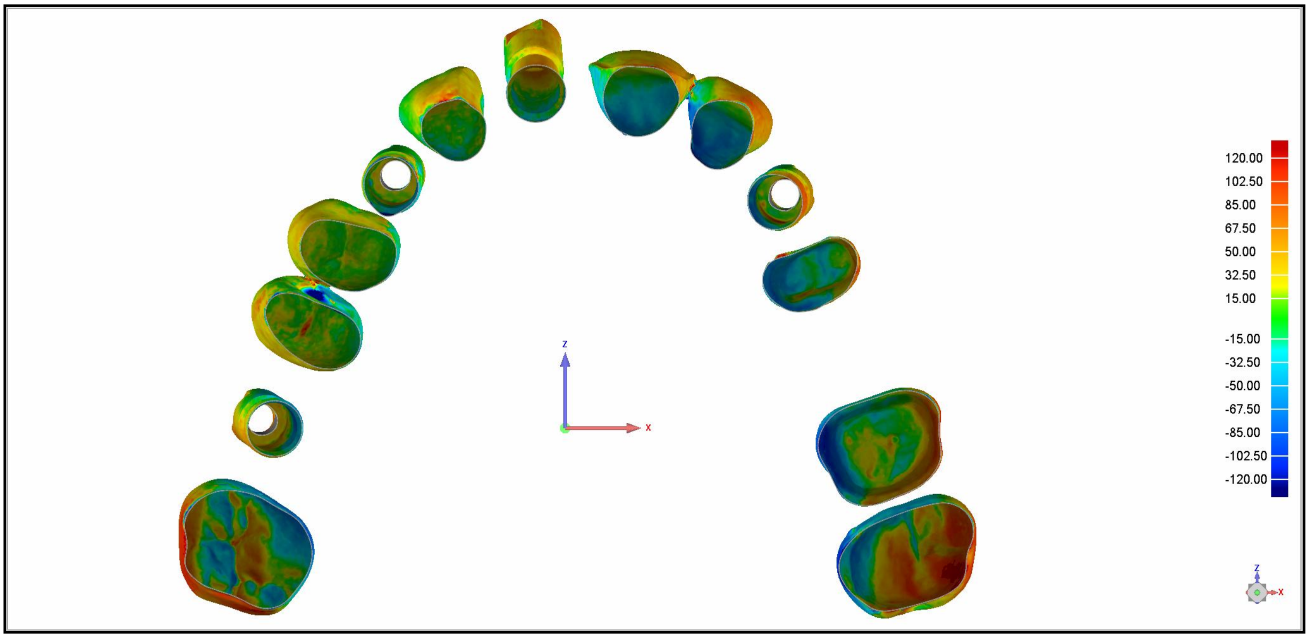

Predefinido: Atrás

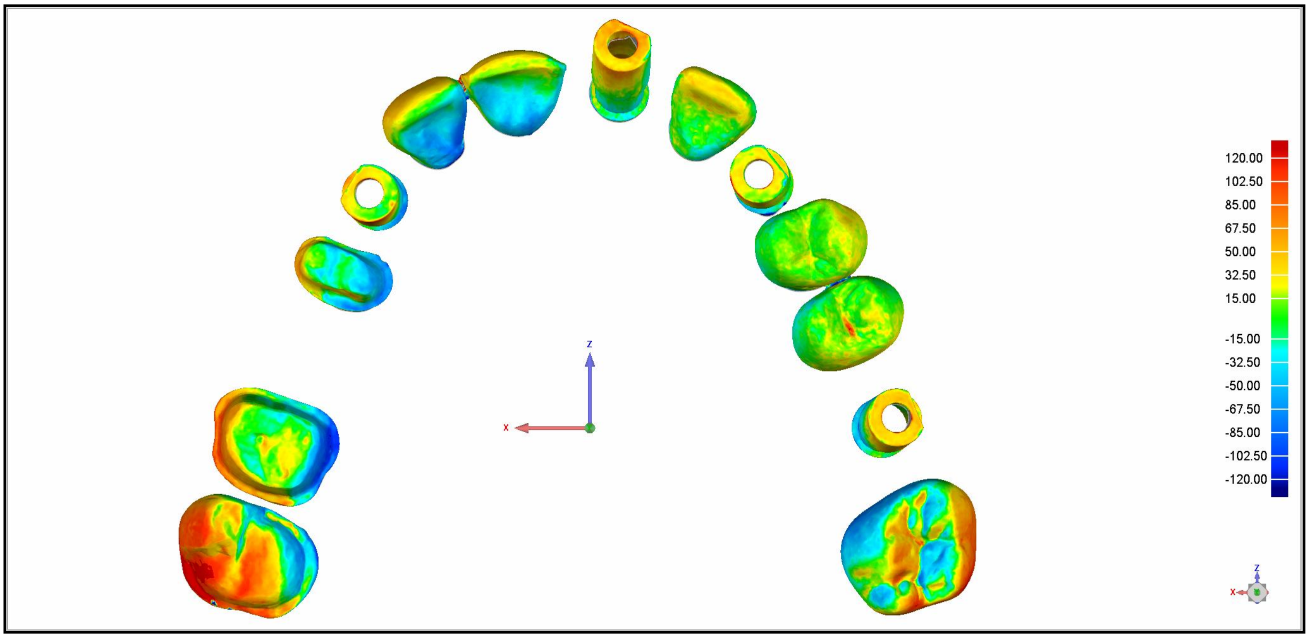

Predefinido: Izquierda

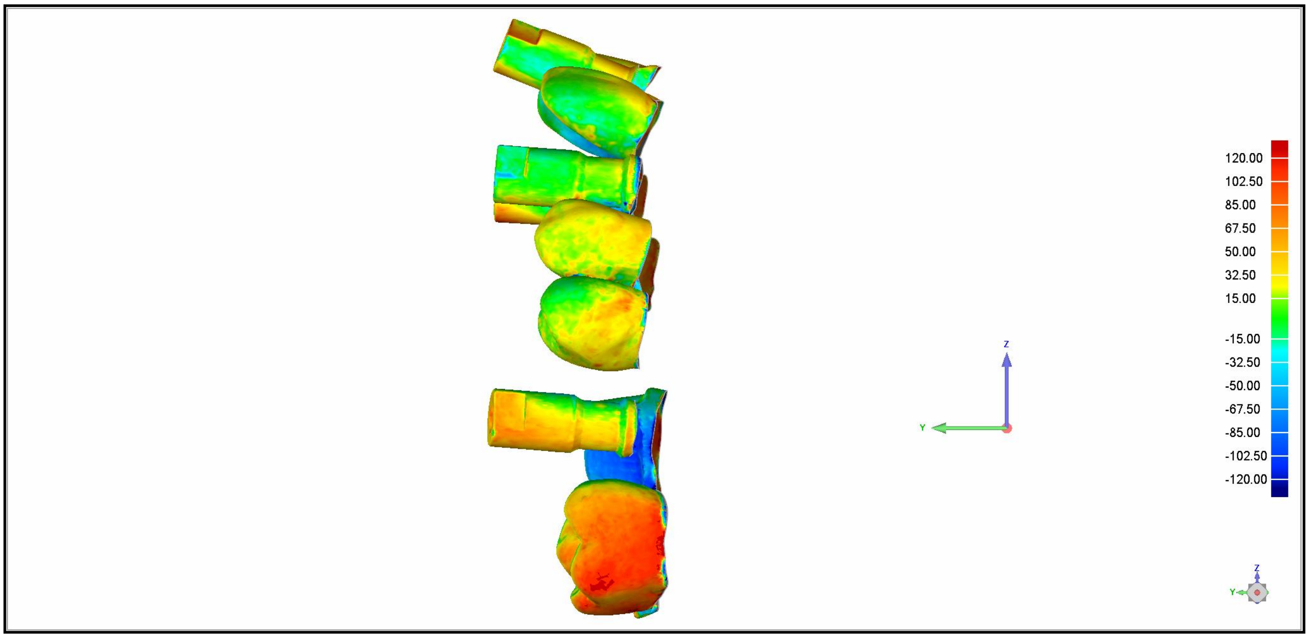

Predefinido: Derecha

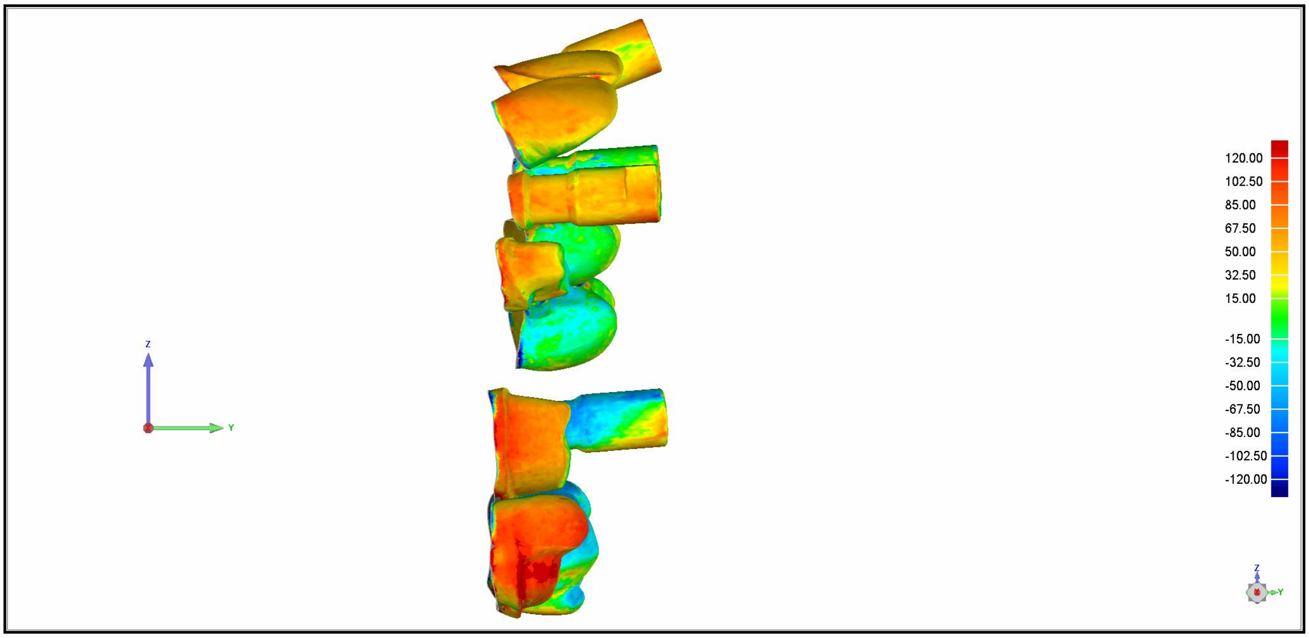

Predefinido: Superior

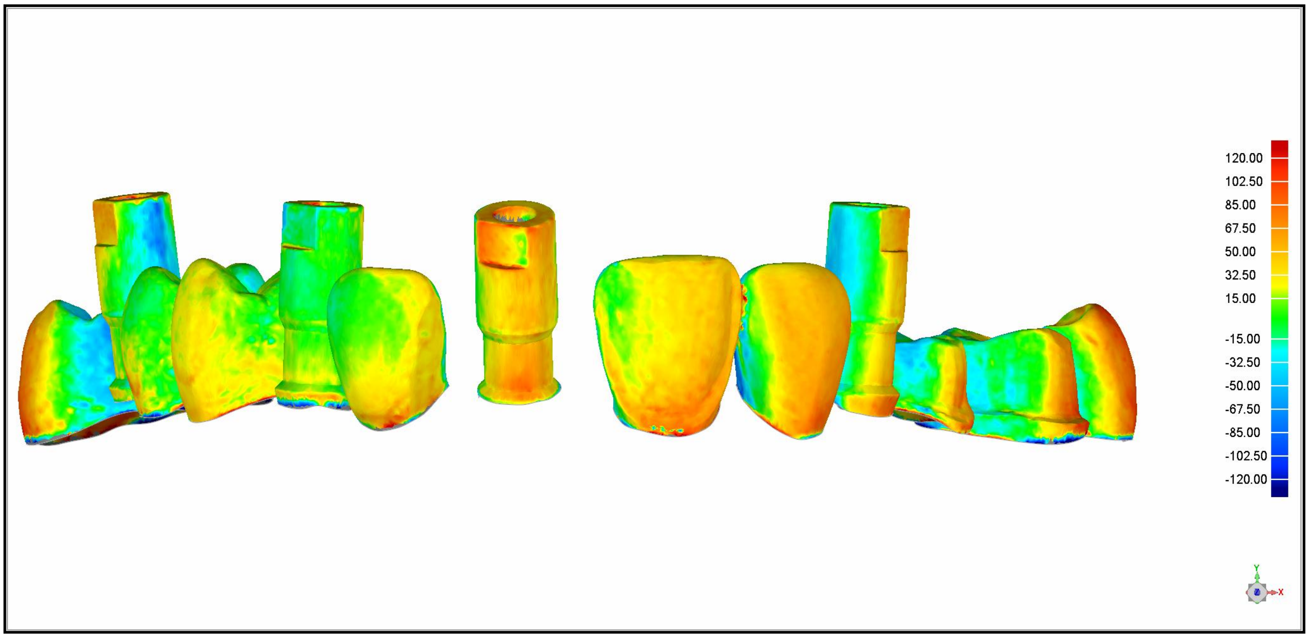

Predefinido: Inferior

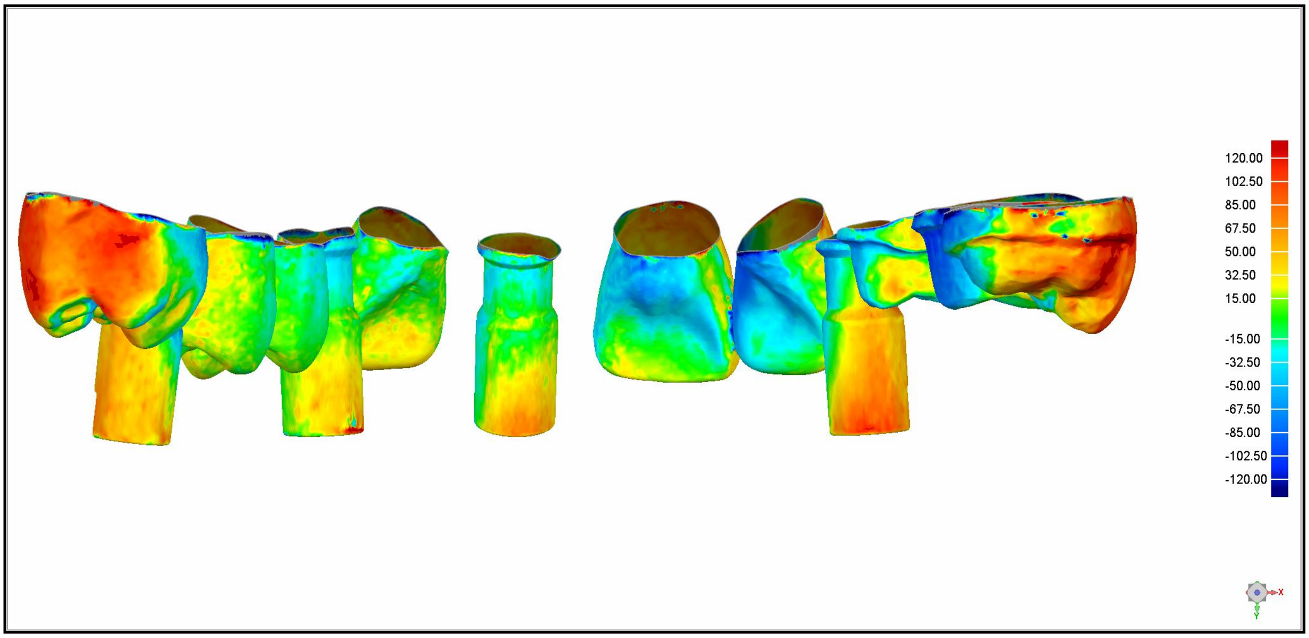

Geomagic Control

Ajuste de ubicación: Desviaciones superior e inferior

Unidades: u

Nombre	Desv	Estado	Superior Tol	Inferior Tol	Ref X	Ref Y	Ref Z	Radio	Desv X	Desv Y	Desv Z	Medido X	Medido Y	Medido Z	Dir. proy. X	Dir. proy. Y	Dir. proy. Z
Desv. inferior	-2540.77				-29208.33	26961.25	-11988.49	n/a	2185.47	360.95	-1244.58	-27022.87	27322.20	-13233.06	-0.86	-0.14	0.49
Desv. superior	1808.04				-23282.49	38564.87	1627.32	n/a	-713.45	-1658.72	-93.04	-23995.94	36906.15	1534.28	-0.39	-0.92	-0.05
