# Supplementary material for: Accuracy of four digital scanners according to scanning strategy in complete-arch impressions
Source: PLoS One. 2018 Sep 13;13(9):e0202916. doi: 10.1371/journal.pone.0202916 (PMC6136706; doi:10.1371/journal.pone.0202916)
Supplement: S15 Table — True definition (scanning strategy C). (ZIP) [file pone.0202916.s015.zip › S15/TD7C.pdf]

Geomagic Control™

Fecha: 3/6/2017, 3:53 pm

3D Comparación Resultados

Modelo referencia	MRC
Modelo test	TD7C
Nº de puntos de datos	196957
# Aislados	517

Tipo tolerancia	3D desviación
Unidades	u
Máx. crítico	120.00
Máx. nominal	15.00
Mín. nominal	-15.00
Mín. crítico	-120.00

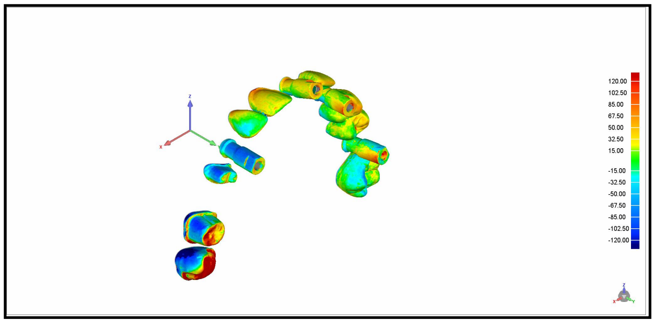

Desviación	
Desviación superior máx.	2646.03
Desviación inferior máx.	-2692.01
Desviación media	45.23 /-30.55
Desviación estándar	84.07

Distribución desviación

>=Min	<Max	# Puntos	%
-120.00	-102.50	906	0.46
-102.50	-85.00	1685	0.86
-85.00	-67.50	3641	1.85
-67.50	-50.00	5134	2.61
-50.00	-32.50	8122	4.12
-32.50	-15.00	22866	11.61
-15.00	15.00	71380	36.24
15.00	32.50	33728	17.12
32.50	50.00	17991	9.13
50.00	67.50	8431	4.28
67.50	85.00	4851	2.46
85.00	102.50	3761	1.91
102.50	120.00	3205	1.63

Fuera del crítico superior	9604	4.88
Fuera del crítico inferior	1652	0.84

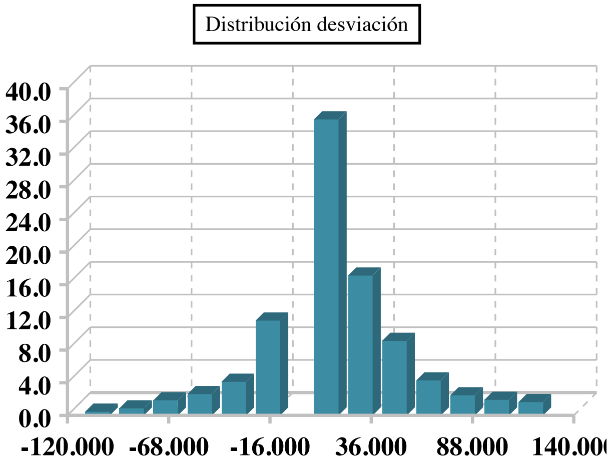

Desviaciones estándar

Distribución (+/-)	# Puntos	%
-6 * Desv. estánd.	234	0.12
-5 * Desv. estánd.	30	0.02
-4 * Desv. estánd.	106	0.05
-3 * Desv. estánd.	380	0.19
-2 * Desv. estánd.	6874	3.49
-1 * Desv. estánd.	109009	55.35
1 * Desv. estánd.	66949	33.99
2 * Desv. estánd.	10565	5.36
3 * Desv. estánd.	1764	0.90
4 * Desv. estánd.	198	0.10
5 * Desv. estánd.	131	0.07
6 * Desv. estánd.	717	0.36

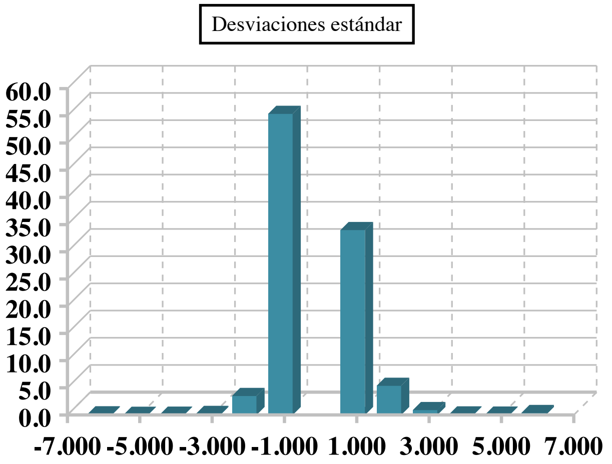

Geomagic Control™

Predefinido: Isométrico

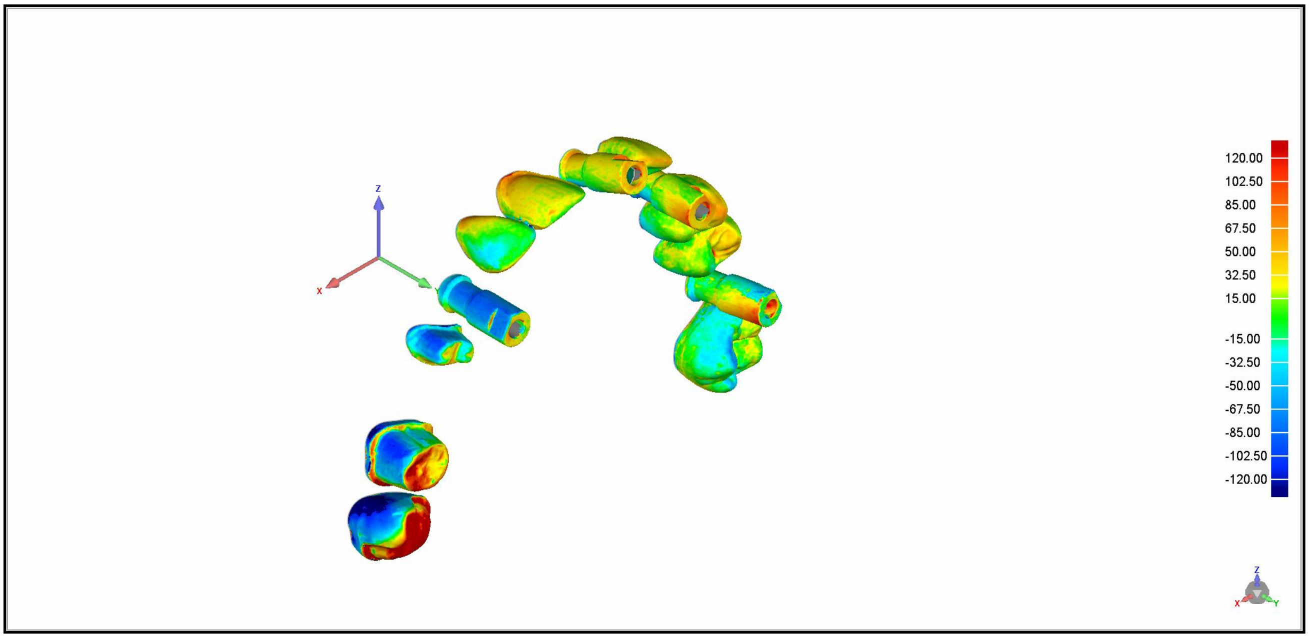

Predefinido: Frente

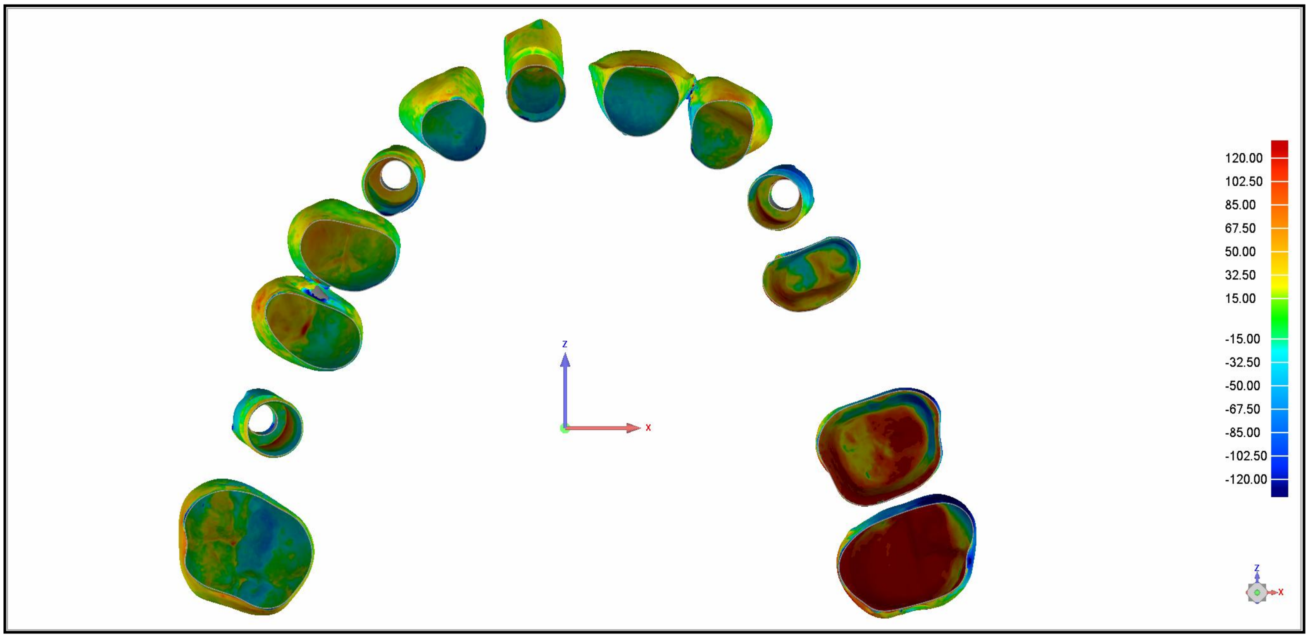

Predefinido: Atrás

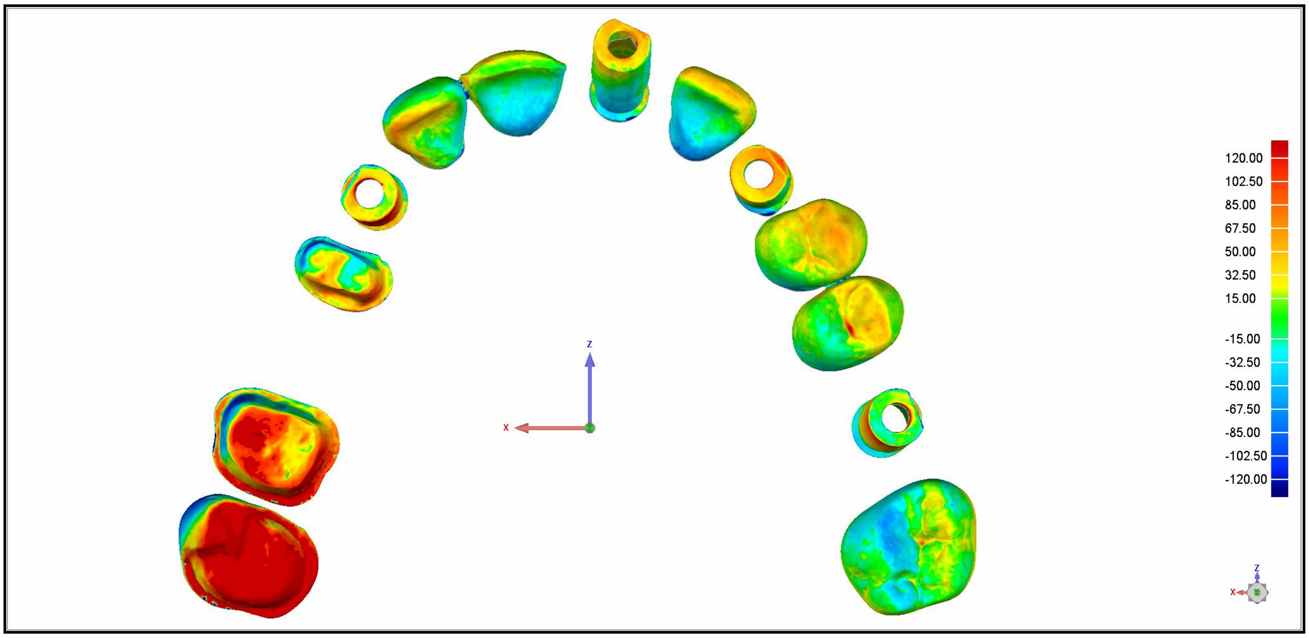

Predefinido: Izquierda

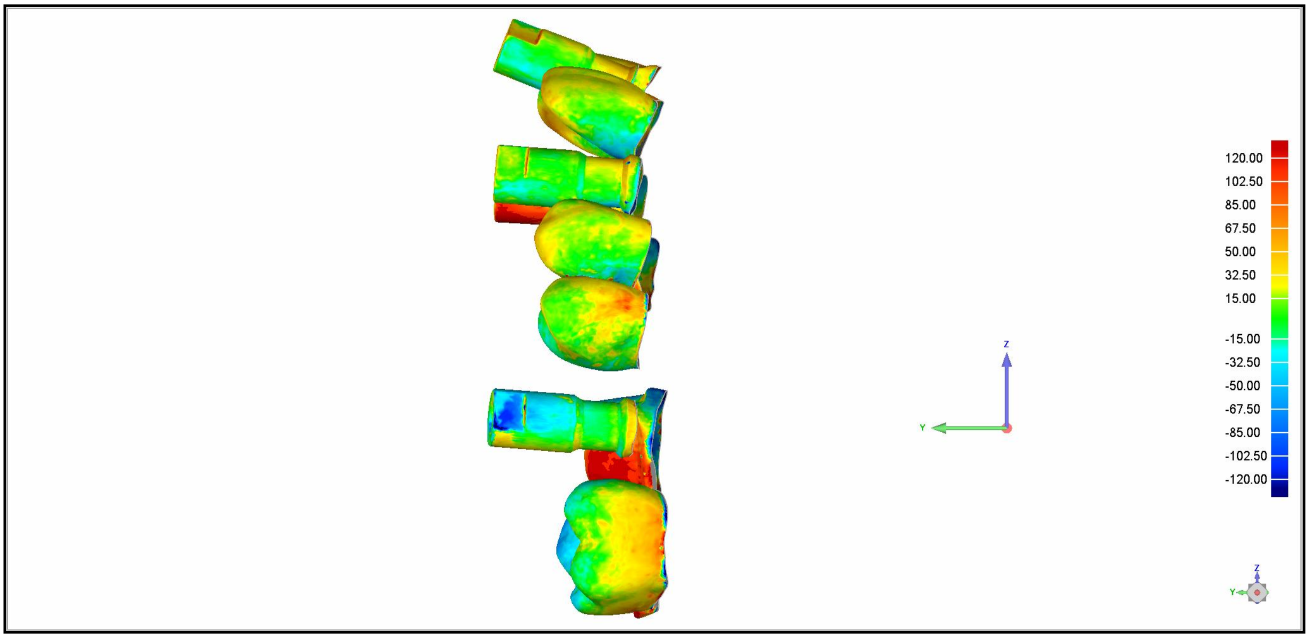

Predefinido: Derecha

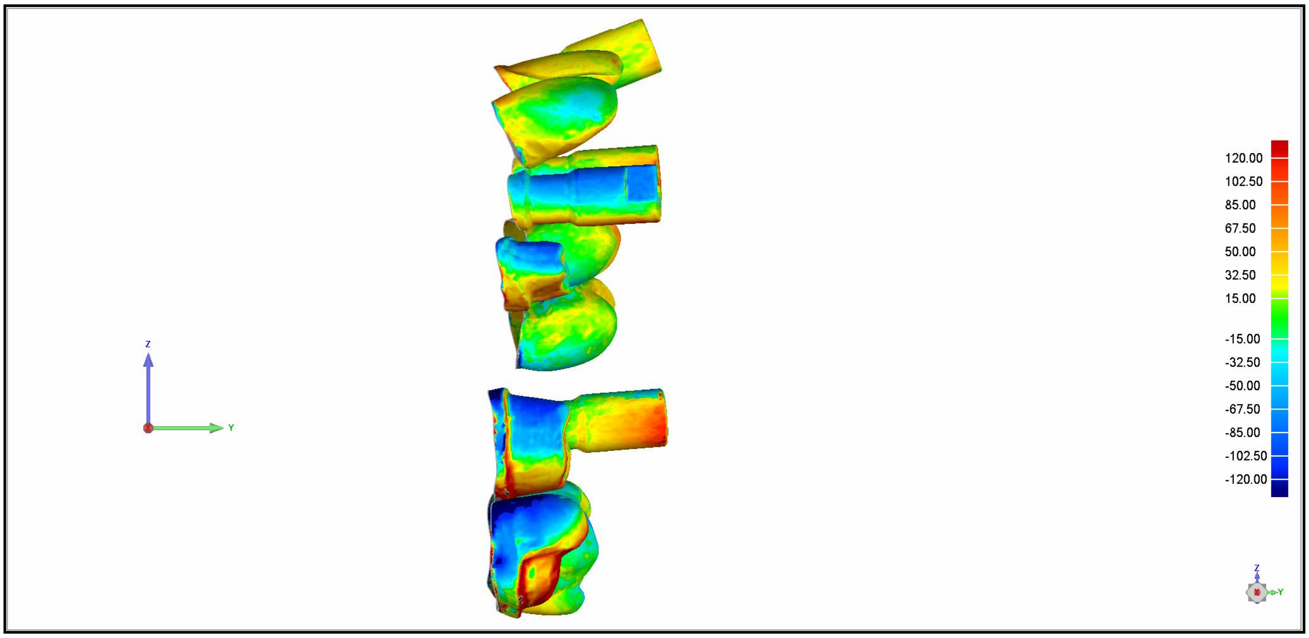

Predefinido: Superior

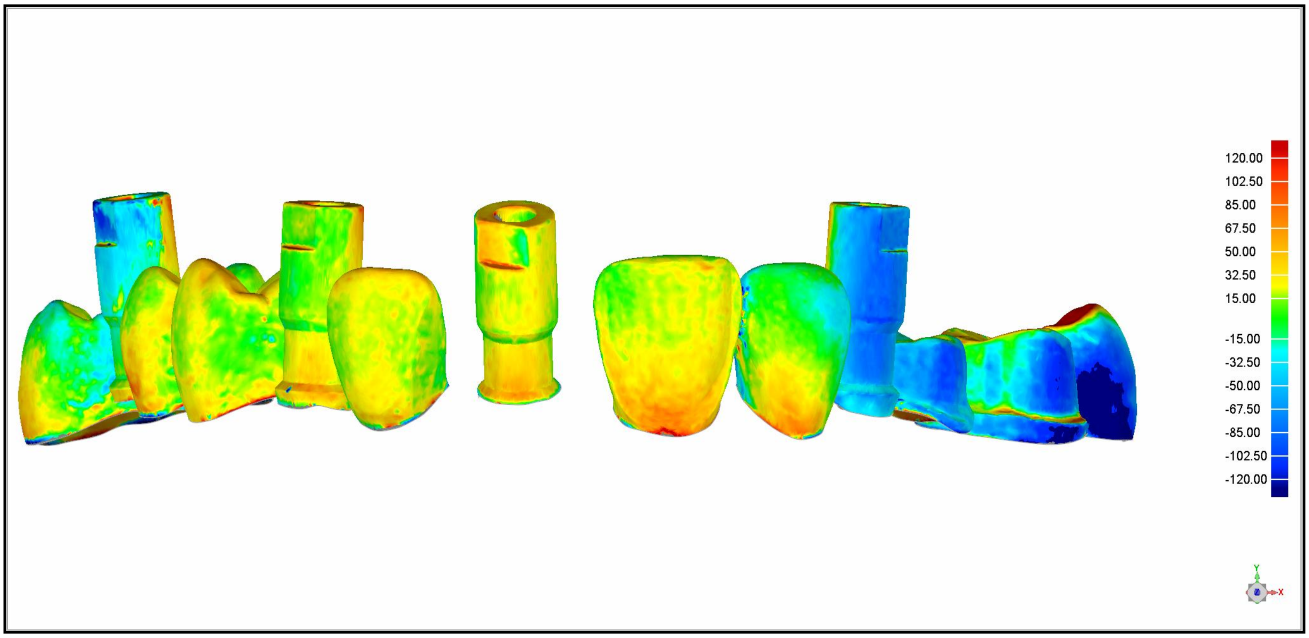

Predefinido: Inferior

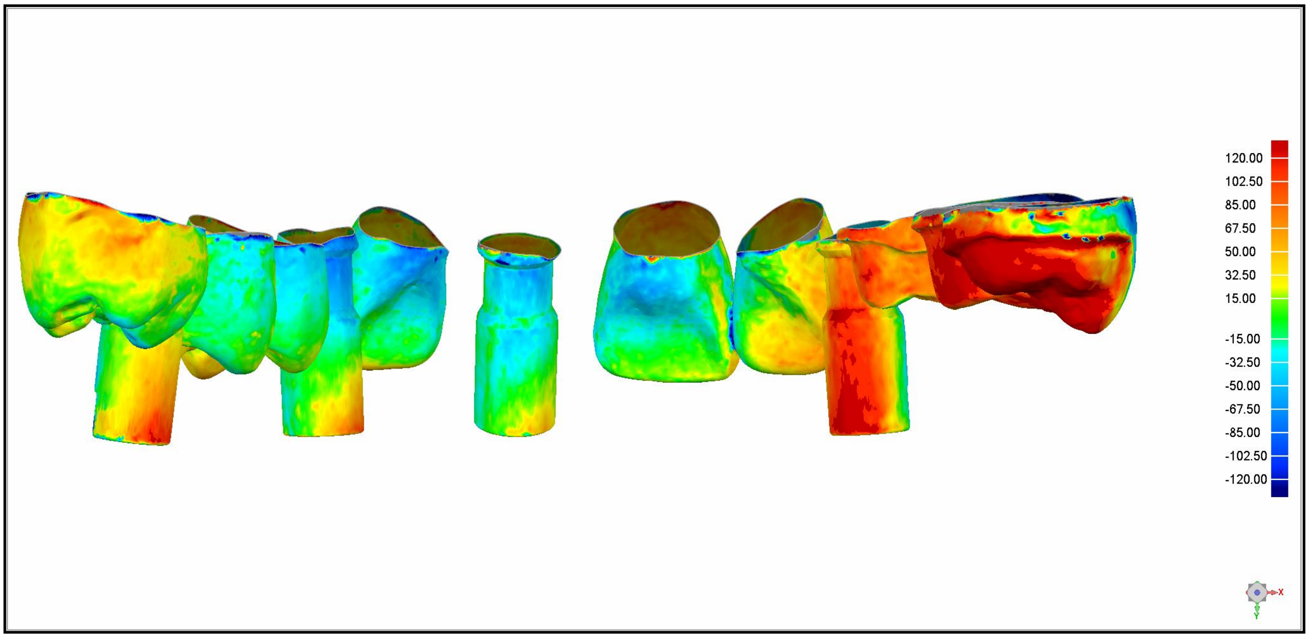

Ajuste de ubicación: Desviaciones superior e inferior

Unidades: u

Nombre	Desv	Estado	Superior Tol	Inferior Tol	Ref X	Ref Y	Ref Z	Radio	Desv X	Desv Y	Desv Z	Medido X	Medido Y	Medido Z	Dir. proy. X	Dir. proy. Y	Dir. proy. Z
Desv. inferior	-2692.01				-24685.43	38551.34	-340.81	n/a	-1252.63	1890.38	1450.63	-25938.05	40441.72	1109.82	0.47	-0.70	-0.54
Desv. superior	2646.03				25410.10	27462.45	-5764.99	n/a	2605.48	147.27	-437.35	28015.58	27609.72	-6202.34	0.98	0.06	-0.17
