# Supplementary material for: Accuracy of four digital scanners according to scanning strategy in complete-arch impressions
Source: PLoS One. 2018 Sep 13;13(9):e0202916. doi: 10.1371/journal.pone.0202916 (PMC6136706; doi:10.1371/journal.pone.0202916)
Supplement: S15 Table — True definition (scanning strategy C). (ZIP) [file pone.0202916.s015.zip › S15/TD6C.pdf]

Fecha: 3/6/2017, 3:45 pm

3D Comparación Resultados

Modelo referencia	MRC
Modelo test	TD6C
Nº de puntos de datos	130384
# Aislados	280

Tipo tolerancia	3D desviación
Unidades	u
Máx. crítico	120.00
Máx. nominal	35.00
Mín. nominal	-35.00
Mín. crítico	-120.00

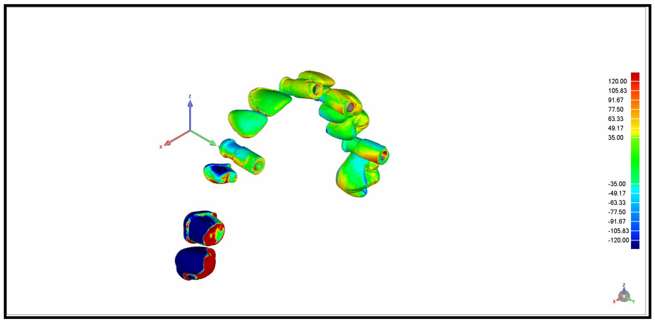

Desviación	
Desviación superior máx.	2340.64
Desviación inferior máx.	-2690.39
Desviación media	106.12 /-79.70
Desviación estándar	193.31

Distribución desviación

>=Min	<Max	# Puntos	%
-120.00	-105.83	407	0.31
-105.83	-91.67	619	0.47
-91.67	-77.50	1027	0.79
-77.50	-63.33	1957	1.50
-63.33	-49.17	3172	2.43
-49.17	-35.00	5392	4.14
-35.00	35.00	67864	52.05
35.00	49.17	11433	8.77
49.17	63.33	6216	4.77
63.33	77.50	3083	2.36
77.50	91.67	1934	1.48
91.67	105.83	1349	1.03
105.83	120.00	1232	0.94

Fuera del crítico superior	17108	13.12
Fuera del crítico inferior	7591	5.82

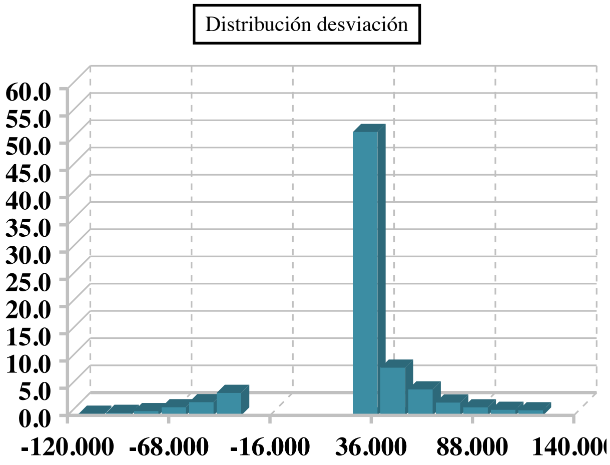

Desviaciones estándar

Distribución (+/-)	# Puntos	%
-6 * Desv. estánd.	144	0.11
-5 * Desv. estánd.	100	0.08
-4 * Desv. estánd.	1057	0.81
-3 * Desv. estánd.	1952	1.50
-2 * Desv. estánd.	3263	2.50
-1 * Desv. estánd.	82211	63.05
1 * Desv. estánd.	30505	23.40
2 * Desv. estánd.	4802	3.68
3 * Desv. estánd.	3935	3.02
4 * Desv. estánd.	1936	1.48
5 * Desv. estánd.	160	0.12
6 * Desv. estánd.	319	0.24

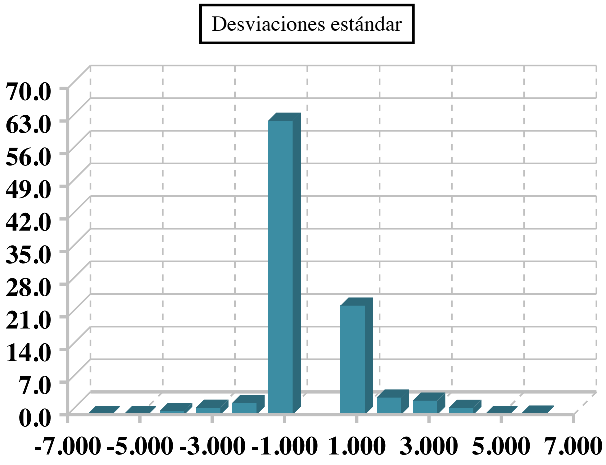

Predefinido: Isométrico

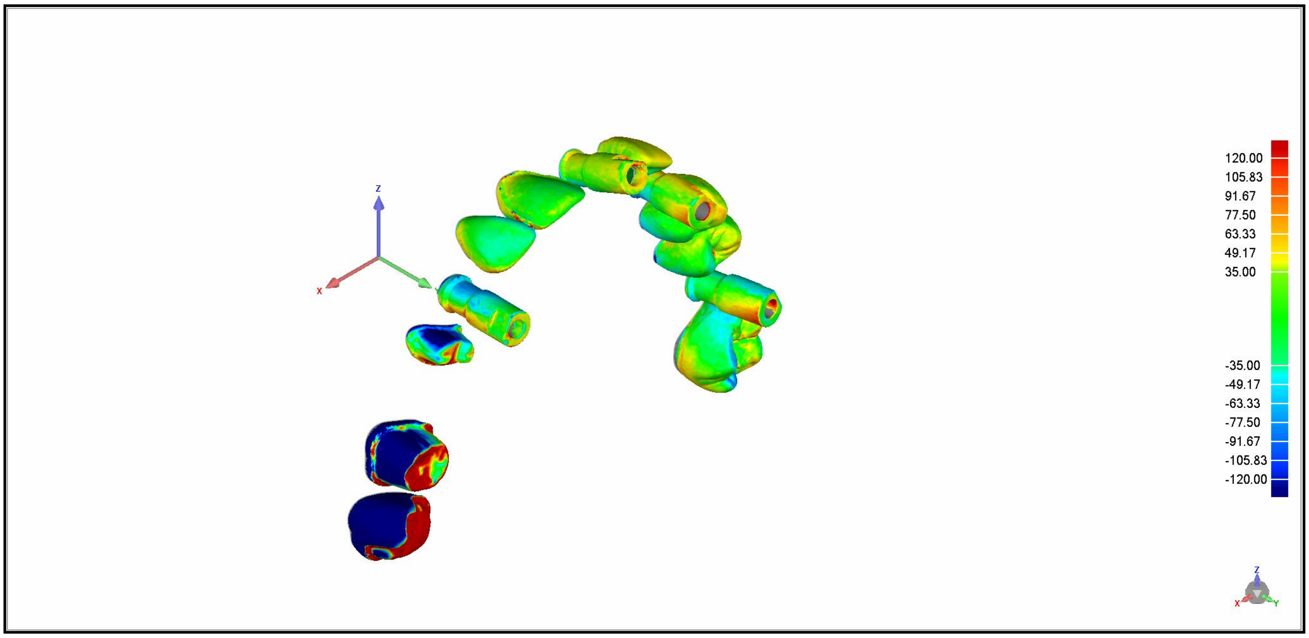

Predefinido: Frente

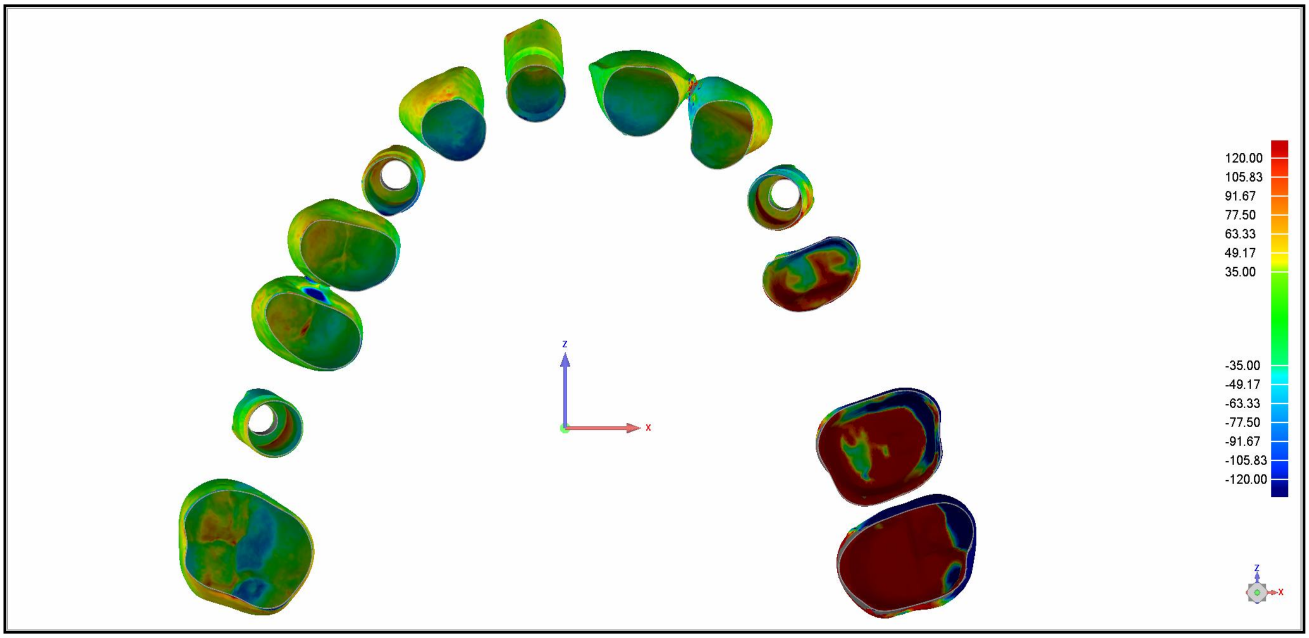

Predefinido: Atrás

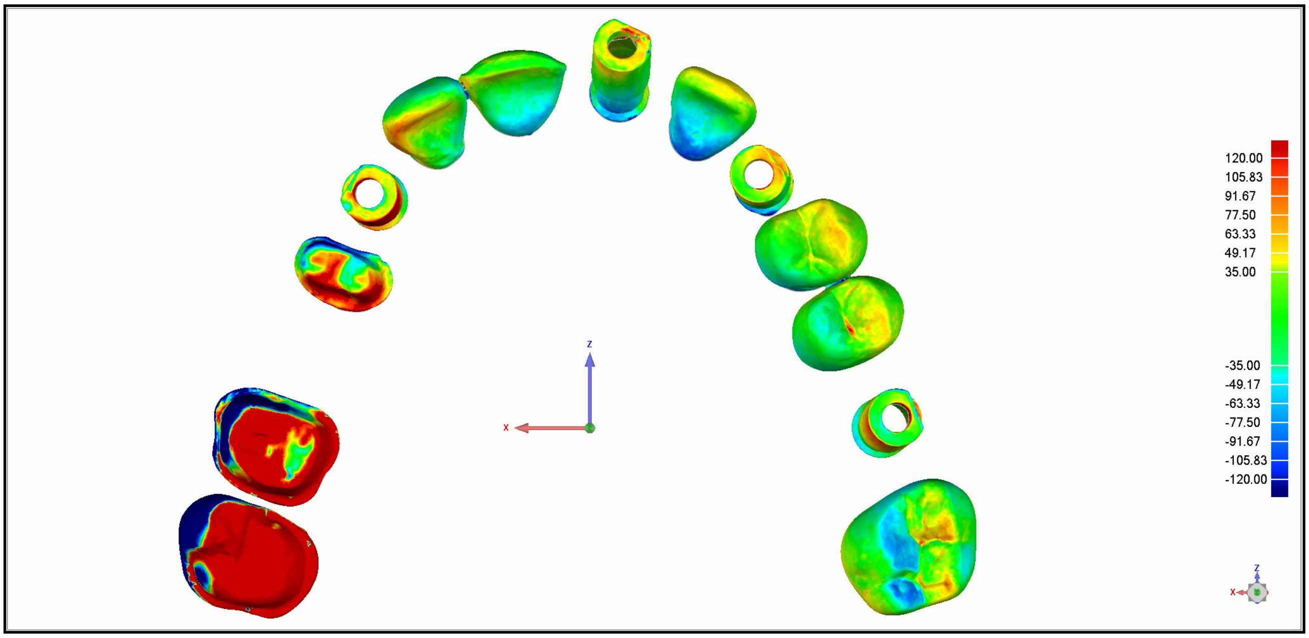

Predefinido: Izquierda

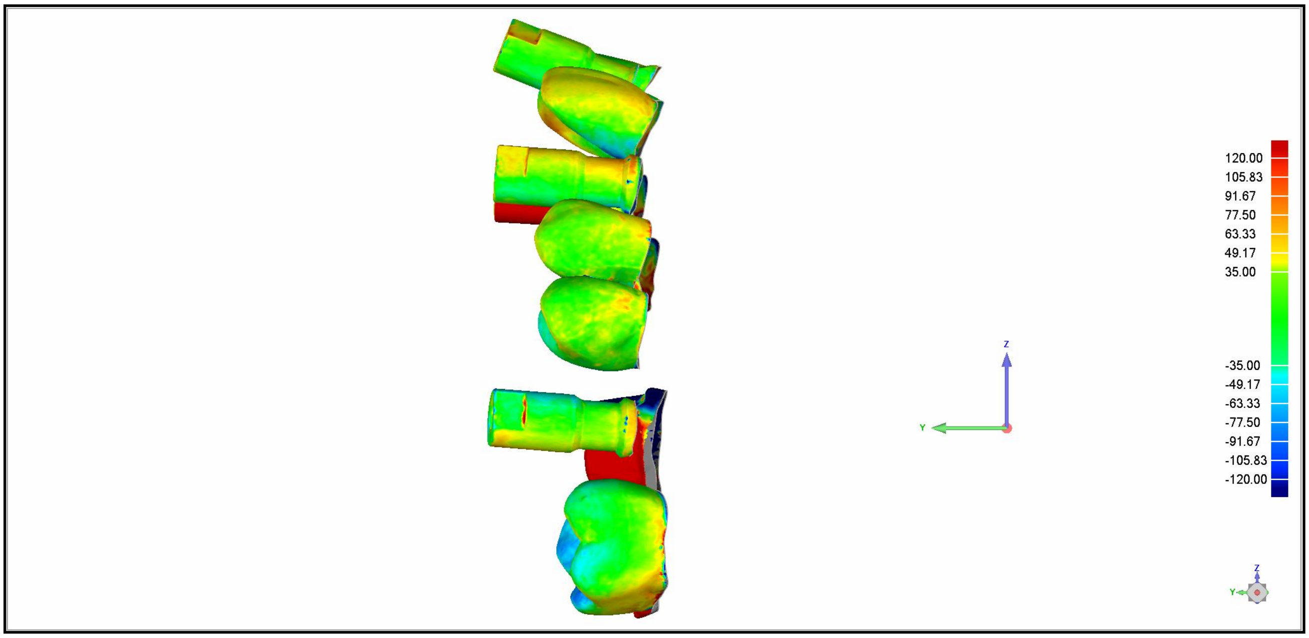

Predefinido: Derecha

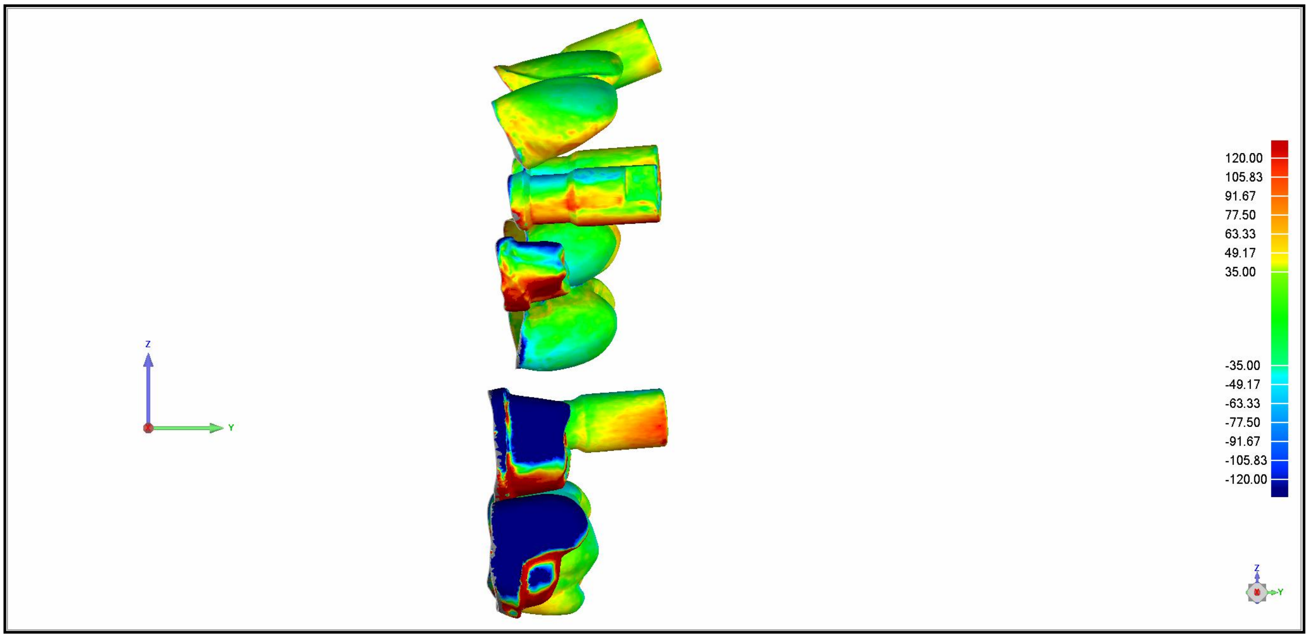

Predefinido: Superior

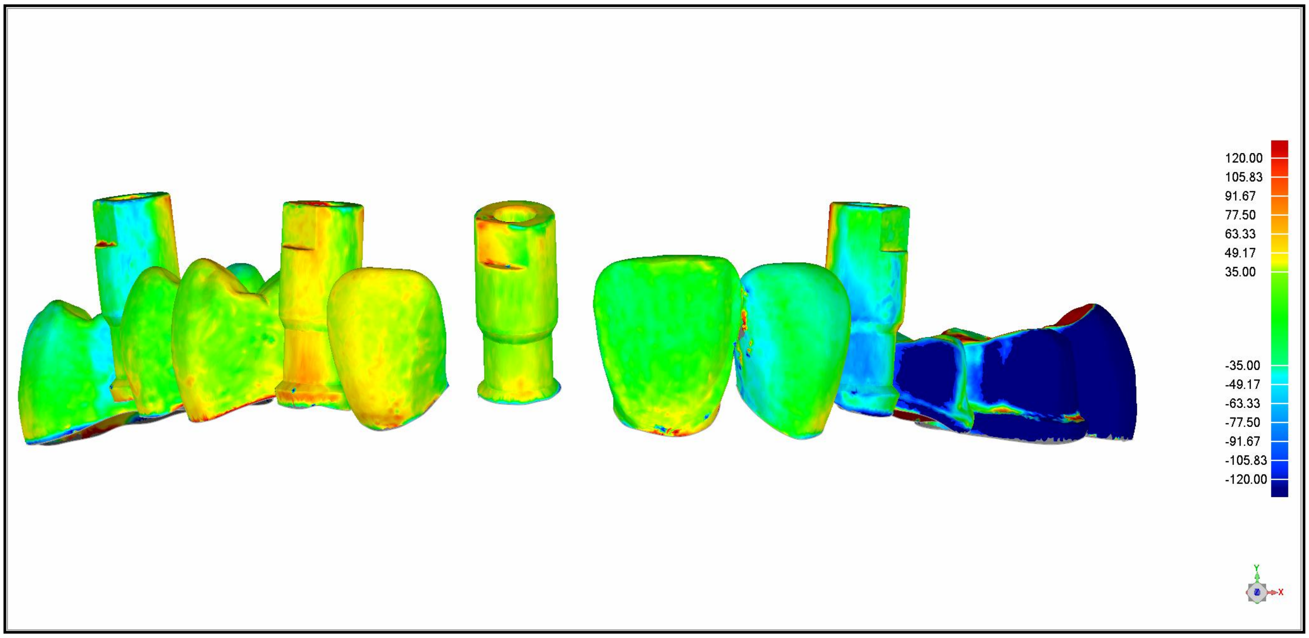

Predefinido: Inferior

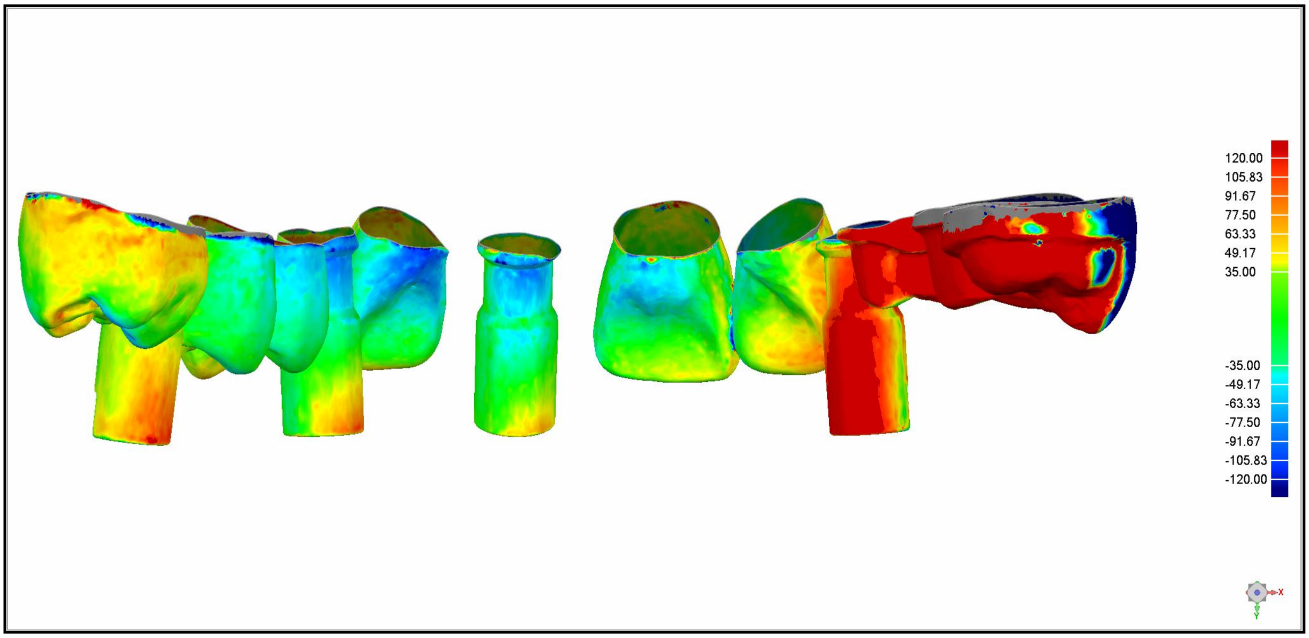

Ajuste de ubicación: Desviaciones superior e inferior

Unidades: u

Nombre	Desv	Estado	Superior Tol	Inferior Tol	Ref X	Ref Y	Ref Z	Radio	Desv X	Desv Y	Desv Z	Medido X	Medido Y	Medido Z	Dir. proy. X	Dir. proy. Y	Dir. proy. Z
Desv. inferior	-2690.39				24355.26	27602.30	-14349.49	n/a	-2171.20	1535.73	406.96	22184.06	29138.03	-13942.53	0.81	-0.57	-0.15
Desv. superior	2340.64				31351.83	27101.88	-6377.73	n/a	-2274.70	519.07	186.79	29077.12	27620.95	-6190.94	-0.97	0.22	0.08
