# Supplementary material for: Accuracy of four digital scanners according to scanning strategy in complete-arch impressions
Source: PLoS One. 2018 Sep 13;13(9):e0202916. doi: 10.1371/journal.pone.0202916 (PMC6136706; doi:10.1371/journal.pone.0202916)
Supplement: S15 Table — True definition (scanning strategy C). (ZIP) [file pone.0202916.s015.zip › S15/TD10C.pdf]

Geomagic Control™

Fecha: 3/6/2017, 4:02 pm

3D Comparación Resultados

Modelo referencia	MRC
Modelo test	TD10C
Nº de puntos de datos	129902
# Aislados	447

Tipo tolerancia	3D desviación
Unidades	u
Máx. crítico	120.00
Máx. nominal	17.00
Mín. nominal	-17.00
Mín. crítico	-120.00

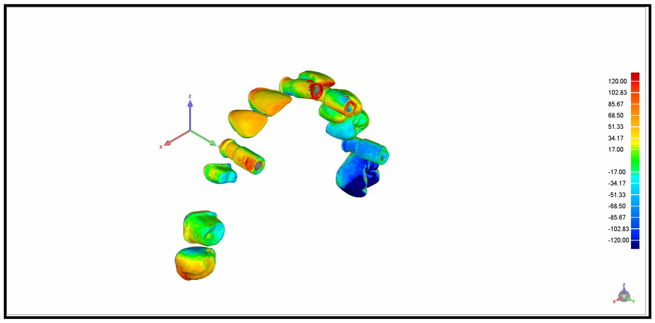

Desviación	
Desviación superior máx.	2999.04
Desviación inferior máx.	-1892.75
Desviación media	49.71 /-43.27
Desviación estándar	76.32

Distribución desviación

>=Min	<Max	# Puntos	%
-120.00	-102.83	1041	0.80
-102.83	-85.67	1671	1.29
-85.67	-68.50	2850	2.19
-68.50	-51.33	3921	3.02
-51.33	-34.17	6243	4.81
-34.17	-17.00	9899	7.62
-17.00	17.00	36868	28.38
17.00	34.17	20940	16.12
34.17	51.33	16124	12.41
51.33	68.50	10106	7.78
68.50	85.67	5125	3.95
85.67	102.83	3568	2.75
102.83	120.00	2399	1.85

Fuera del crítico superior	5918	4.56
Fuera del crítico inferior	3229	2.49

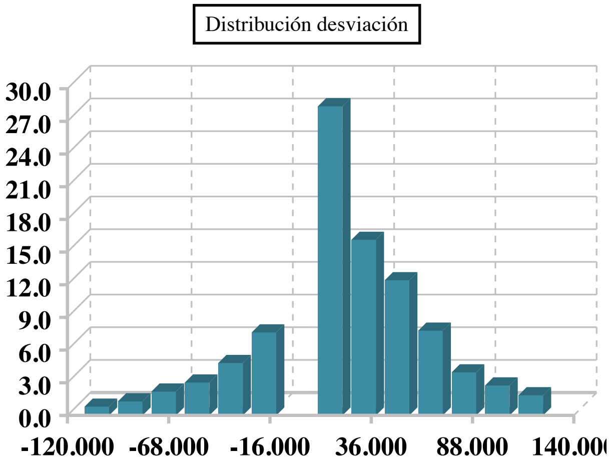

Desviaciones estándar

Distribución (+/-)	# Puntos	%
-6 * Desv. esténd.	81	0.06
-5 * Desv. esténd.	73	0.06
-4 * Desv. esténd.	303	0.23
-3 * Desv. esténd.	2118	1.63
-2 * Desv. esténd.	8300	6.39
-1 * Desv. esténd.	55818	42.97
1 * Desv. esténd.	53156	40.92
2 * Desv. esténd.	6546	5.04
3 * Desv. esténd.	2812	2.16
4 * Desv. esténd.	349	0.27
5 * Desv. esténd.	86	0.07
6 * Desv. esténd.	260	0.20

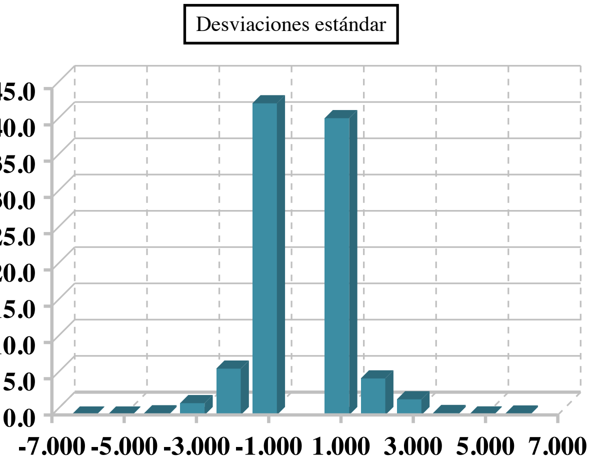

Predefinido: Isométrico

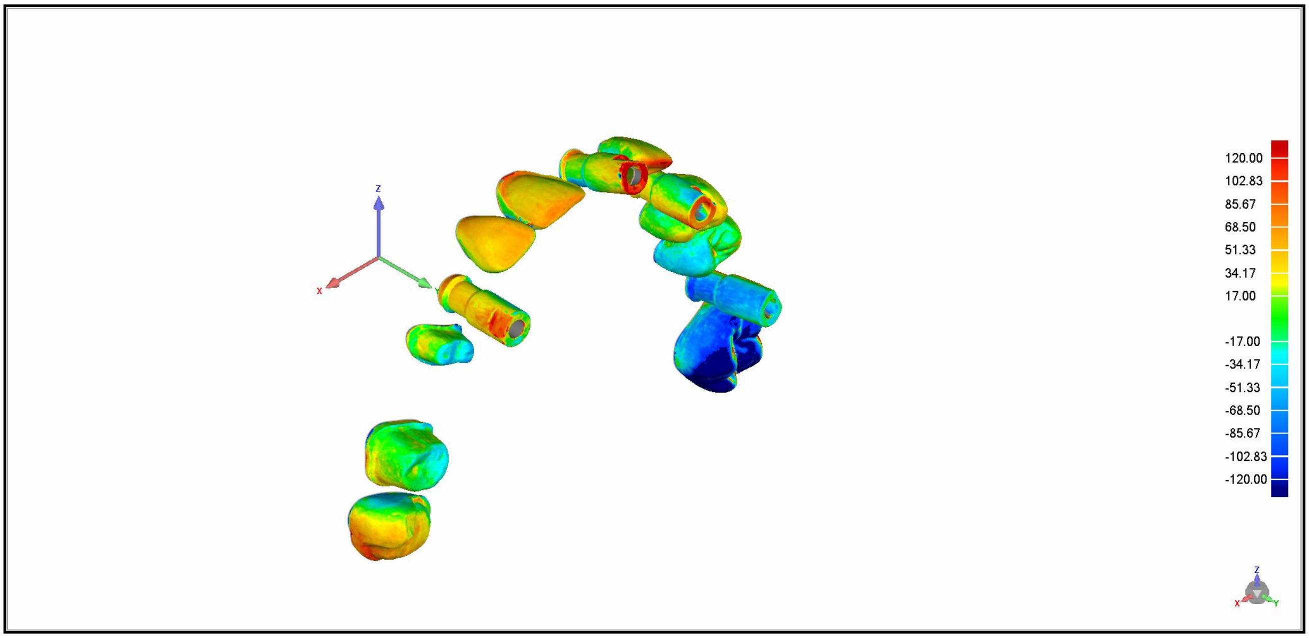

Predefinido: Frente

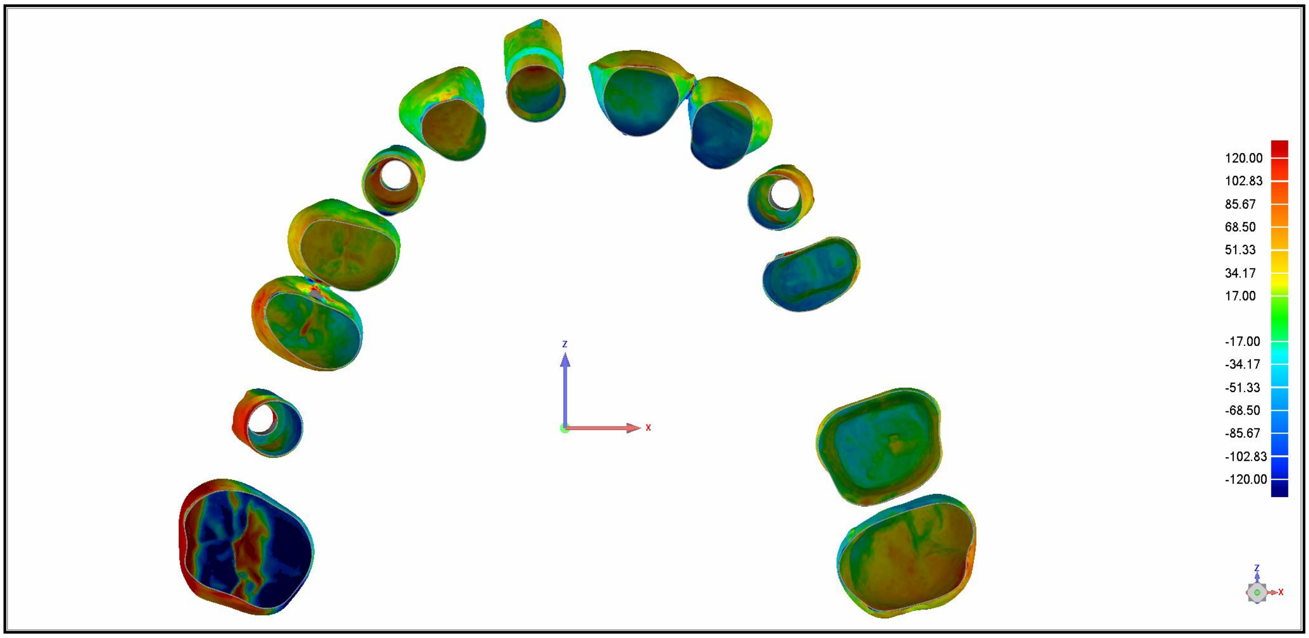

Predefinido: Atrás

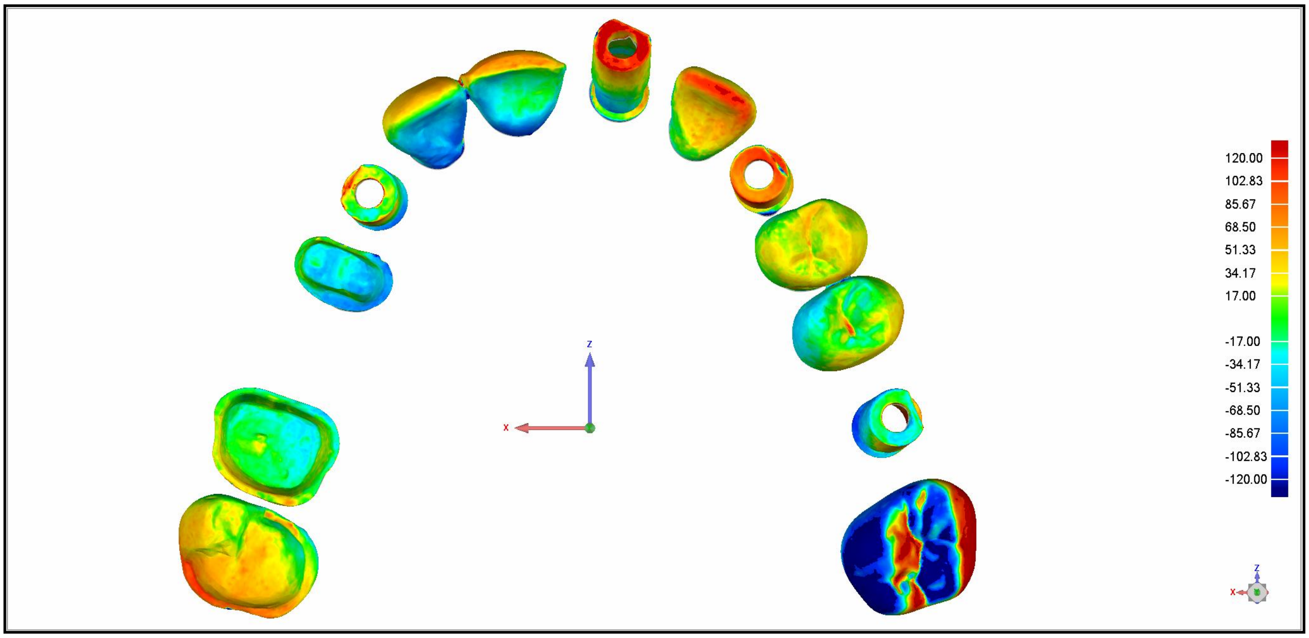

Predefinido: Izquierda

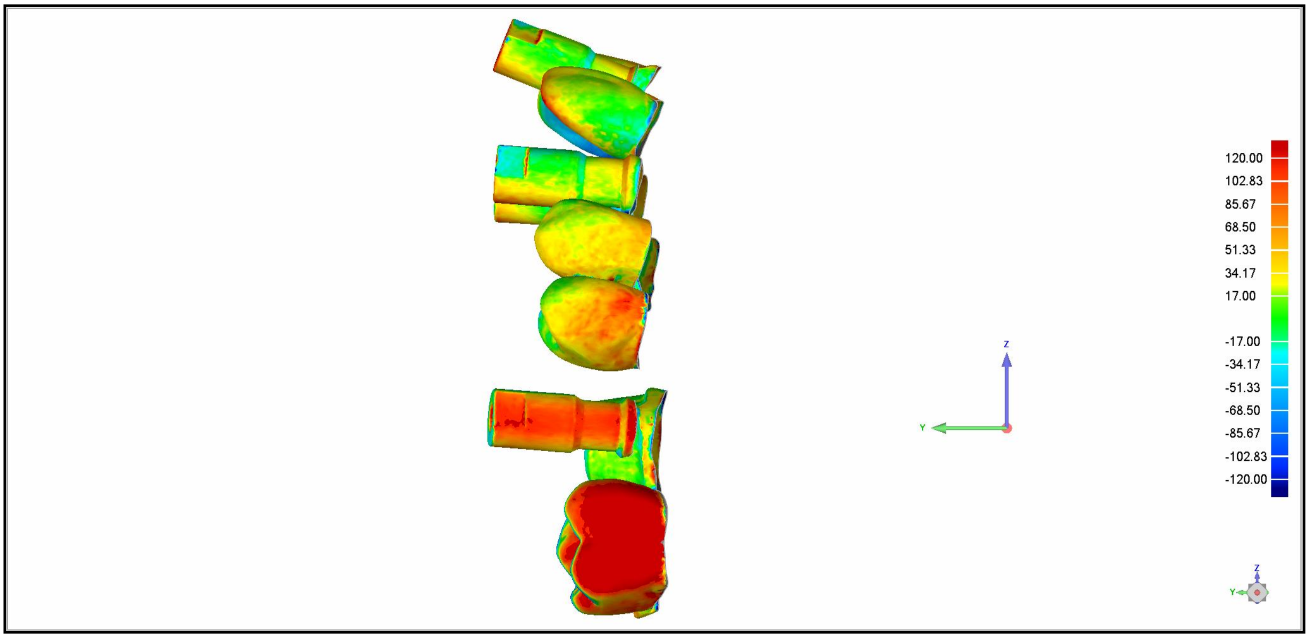

Predefinido: Derecha

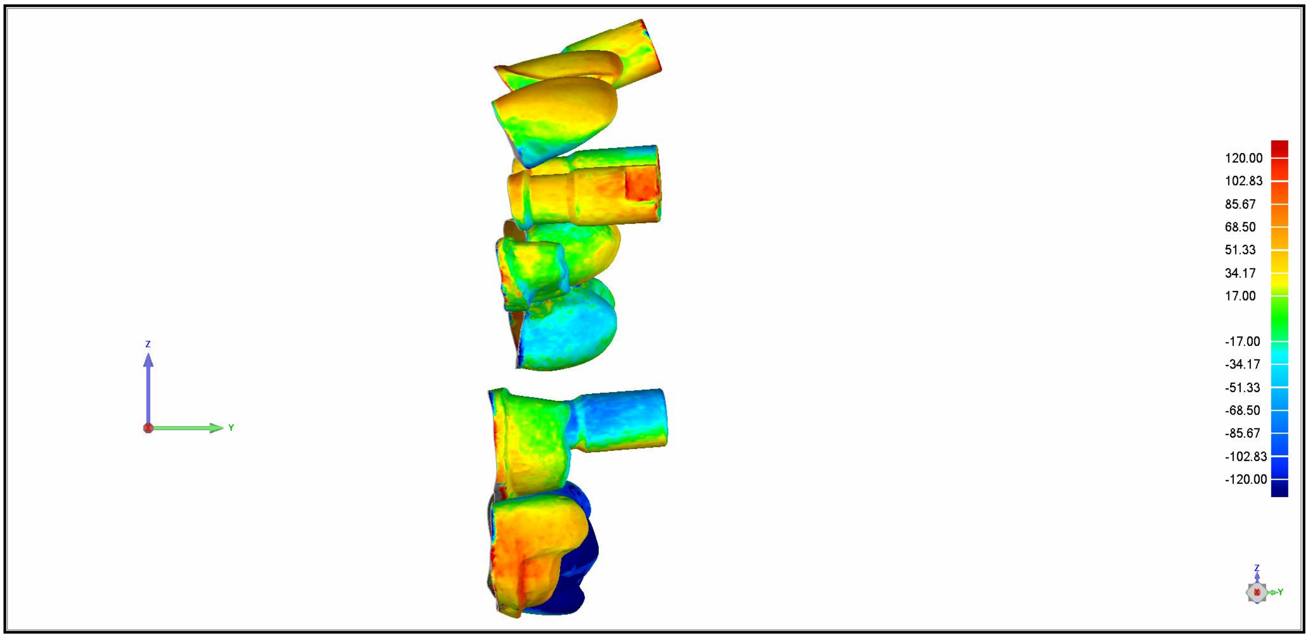

Predefinido: Superior

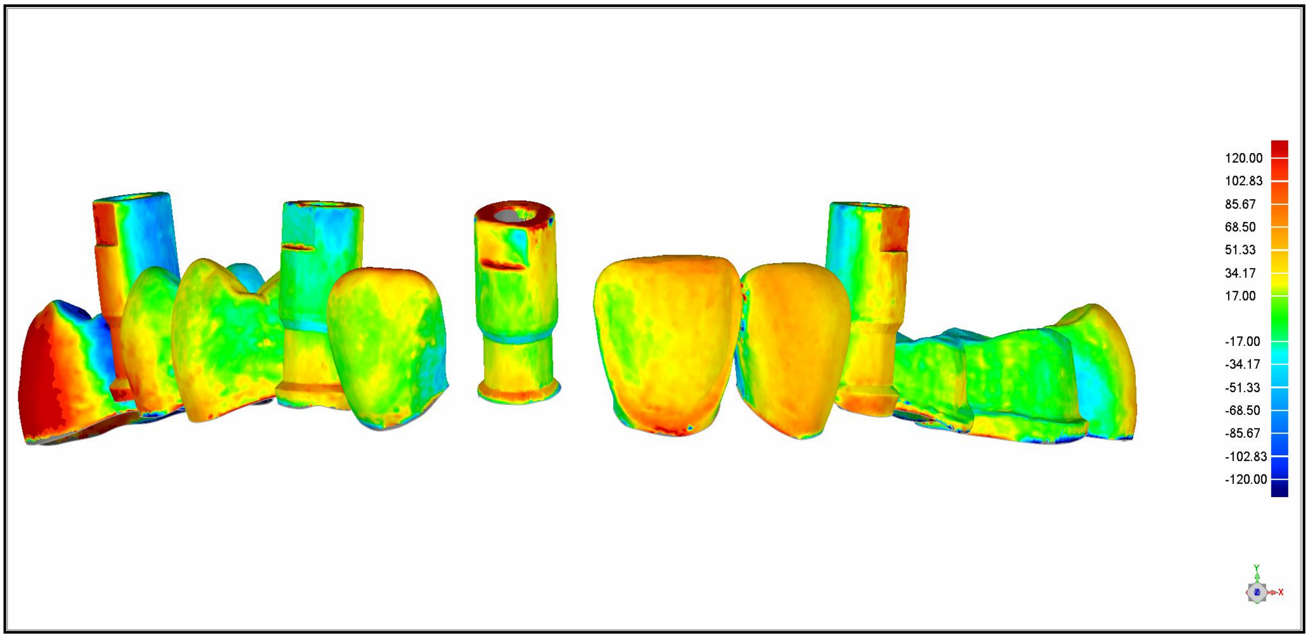

Predefinido: Inferior

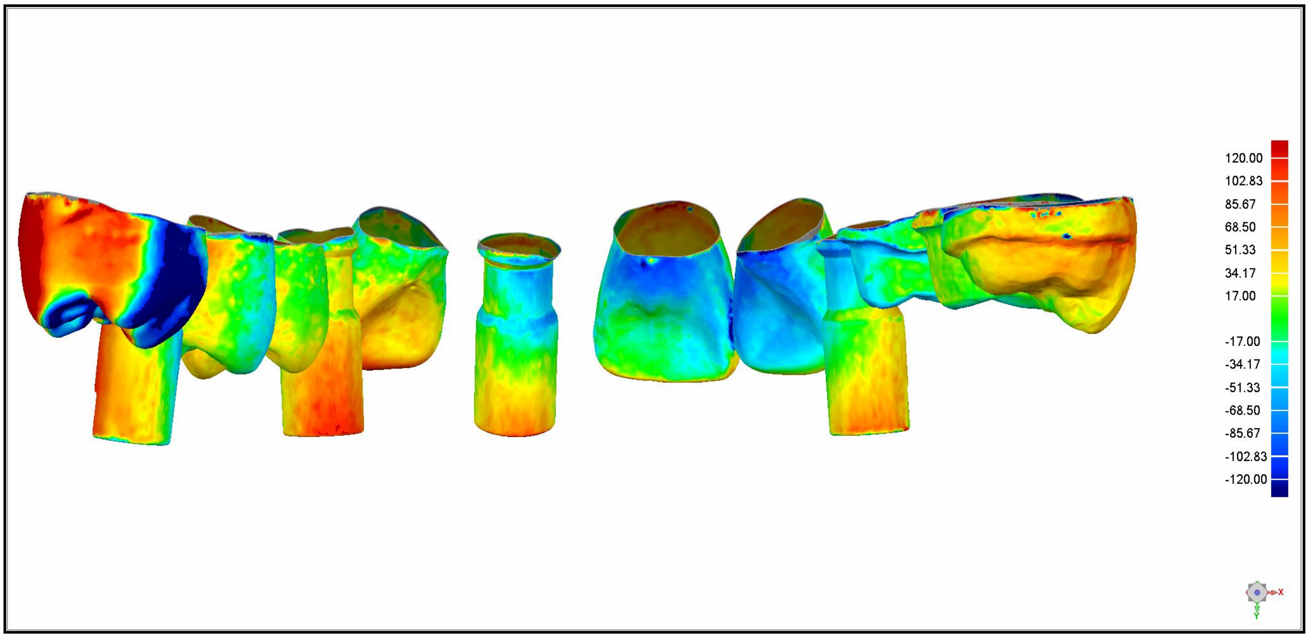

Ajuste de ubicación: Desviaciones superior e inferior

Unidades: u

Nombre	Desv	Estado	Superior Tol	Inferior Tol	Ref X	Ref Y	Ref Z	Radio	Desv X	Desv Y	Desv Z	Medido X	Medido Y	Medido Z	Dir. proy. X	Dir. proy. Y	Dir. proy. Z
Desv. inferior	-1892.75				28702.77	29351.58	-14300.82	n/a	392.08	-1846.80	134.55	29094.85	27504.78	-14166.27	-0.21	0.98	-0.07
Desv. superior	2999.04				25410.11	27462.45	-5764.99	n/a	2781.23	286.38	1084.90	28191.33	27748.83	-4680.09	0.93	0.10	0.36
