# Supplementary material for: Accuracy of four digital scanners according to scanning strategy in complete-arch impressions
Source: PLoS One. 2018 Sep 13;13(9):e0202916. doi: 10.1371/journal.pone.0202916 (PMC6136706; doi:10.1371/journal.pone.0202916)
Supplement: S15 Table — True definition (scanning strategy C). (ZIP) [file pone.0202916.s015.zip › S15/TD1C.pdf]

Geomagic Control™

Fecha: 3/6/2017, 3:33 pm

3D Comparación Resultados

Modelo referencia	MRC
Modelo test	TD1C
Nº de puntos de datos	129663
# Aislados	422

Tipo tolerancia	3D desviación
Unidades	u
Máx. crítico	120.00
Máx. nominal	6.00
Mín. nominal	-6.00
Mín. crítico	-120.00

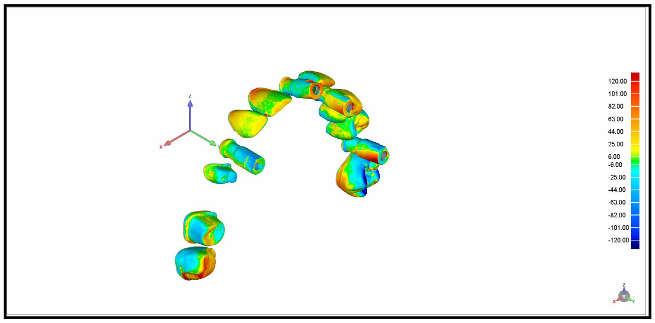

Desviación	
Desviación superior máx.	2399.42
Desviación inferior máx.	-2481.72
Desviación media	35.34 /-32.99
Desviación estándar	74.02

Distribución desviación

>=Min	<Max	# Puntos	%
-120.00	-101.00	528	0.41
-101.00	-82.00	1147	0.88
-82.00	-63.00	2691	2.08
-63.00	-44.00	6075	4.69
-44.00	-25.00	12373	9.54
-25.00	-6.00	21834	16.84
-6.00	6.00	18944	14.61
6.00	25.00	29006	22.37
25.00	44.00	14965	11.54
44.00	63.00	9400	7.25
63.00	82.00	5490	4.23
82.00	101.00	2699	2.08
101.00	120.00	1228	0.95

Fuera del crítico superior	1938	1.49
Fuera del crítico inferior	1345	1.04

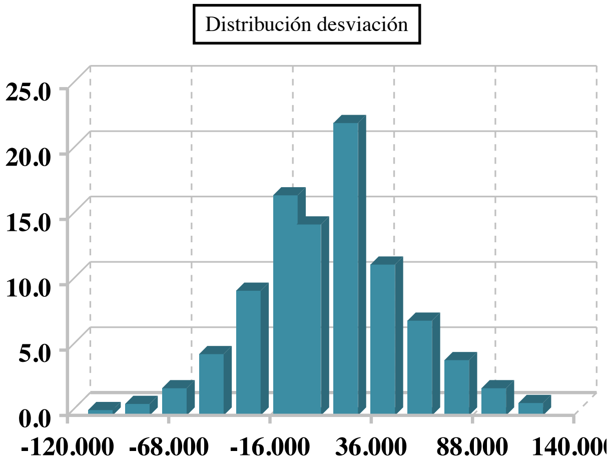

Desviaciones estándar

Distribución (+/-)	# Puntos	%
-6 * Desv. estánd.	121	0.09
-5 * Desv. estánd.	30	0.02
-4 * Desv. estánd.	76	0.06
-3 * Desv. estánd.	405	0.31
-2 * Desv. estánd.	4210	3.25
-1 * Desv. estánd.	60698	46.81
1 * Desv. estánd.	57921	44.67
2 * Desv. estánd.	5193	4.00
3 * Desv. estánd.	561	0.43
4 * Desv. estánd.	185	0.14
5 * Desv. estánd.	112	0.09
6 * Desv. estánd.	151	0.12

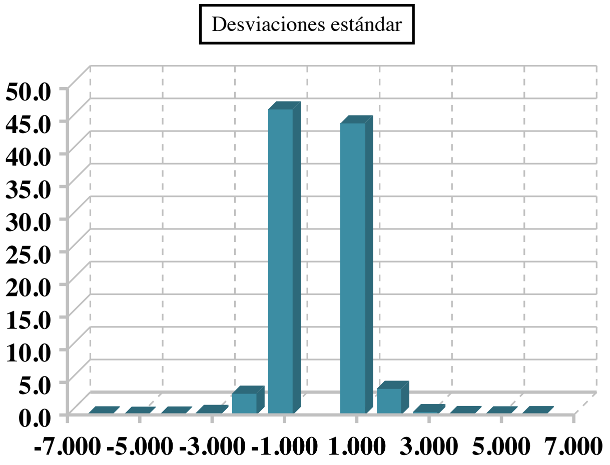

Predefinido: Isométrico

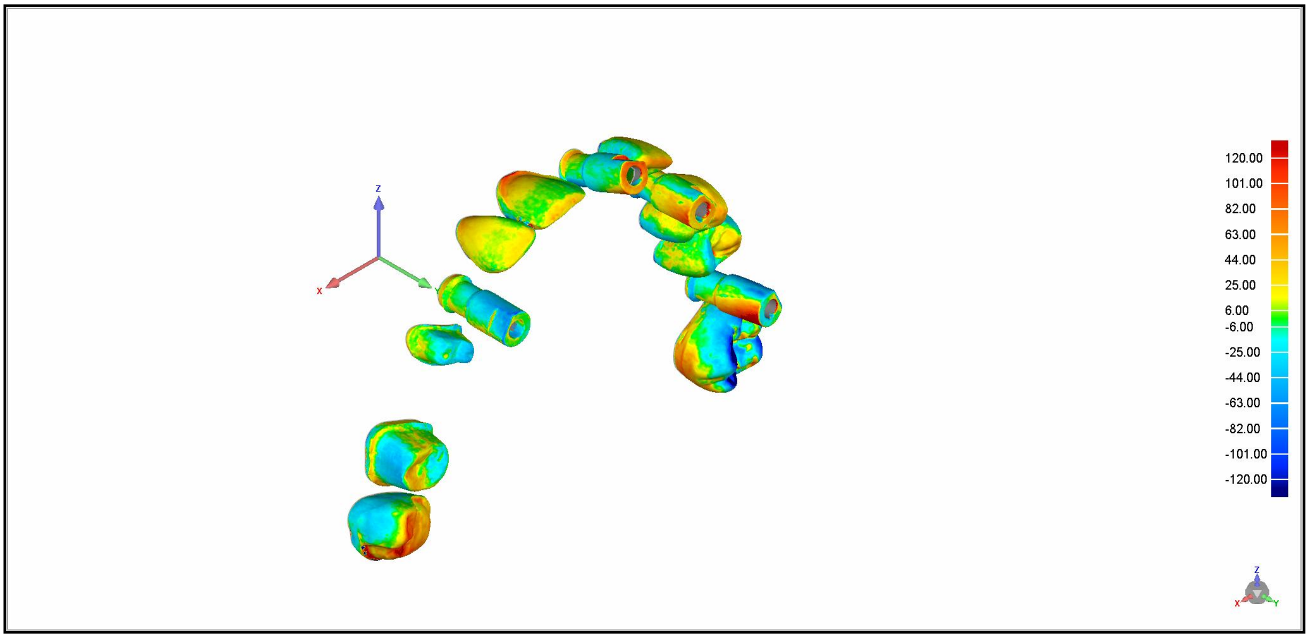

Predefinido: Frente

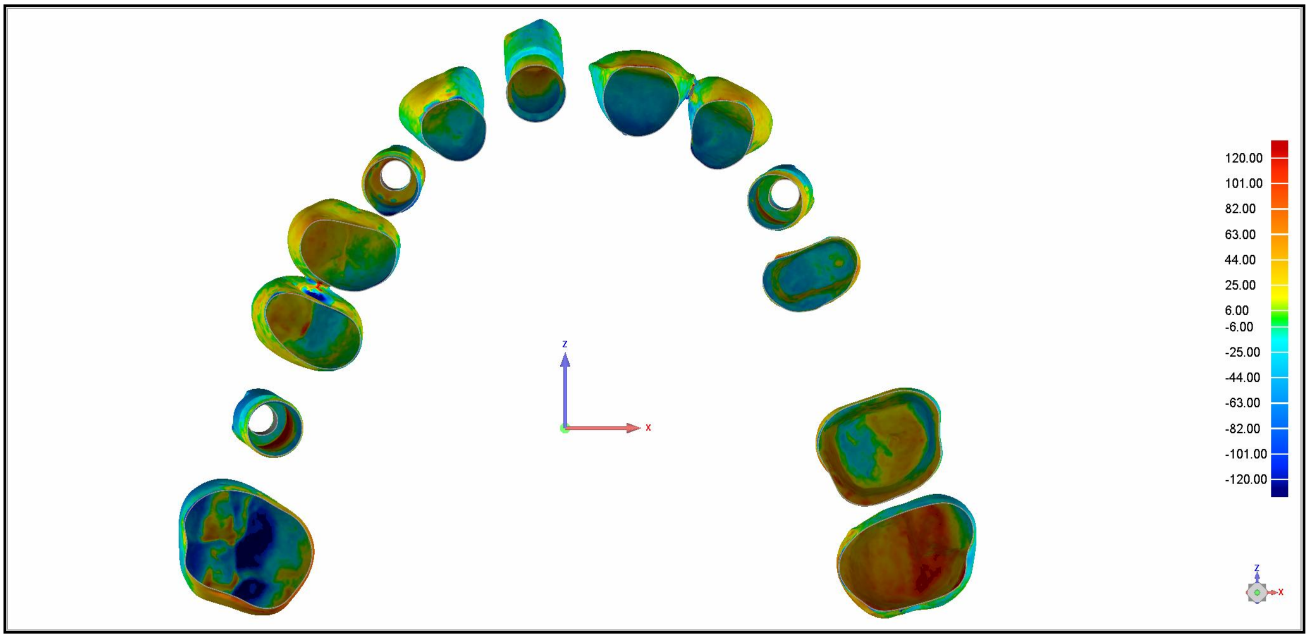

Predefinido: Atrás

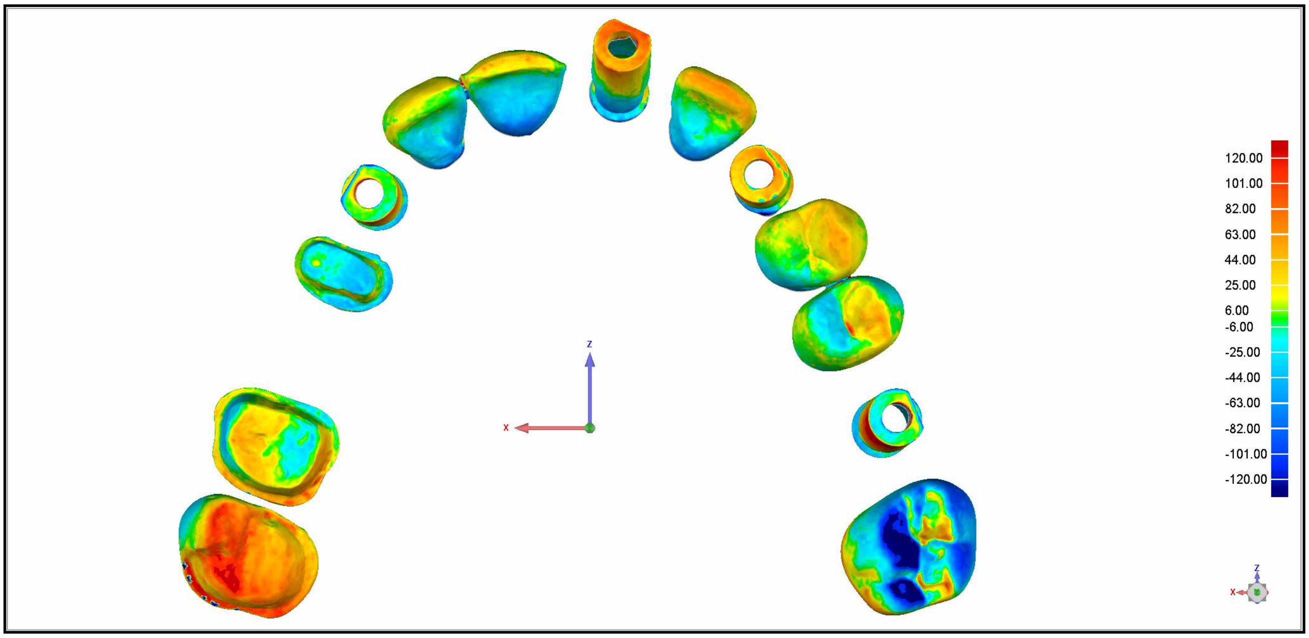

Predefinido: Izquierda

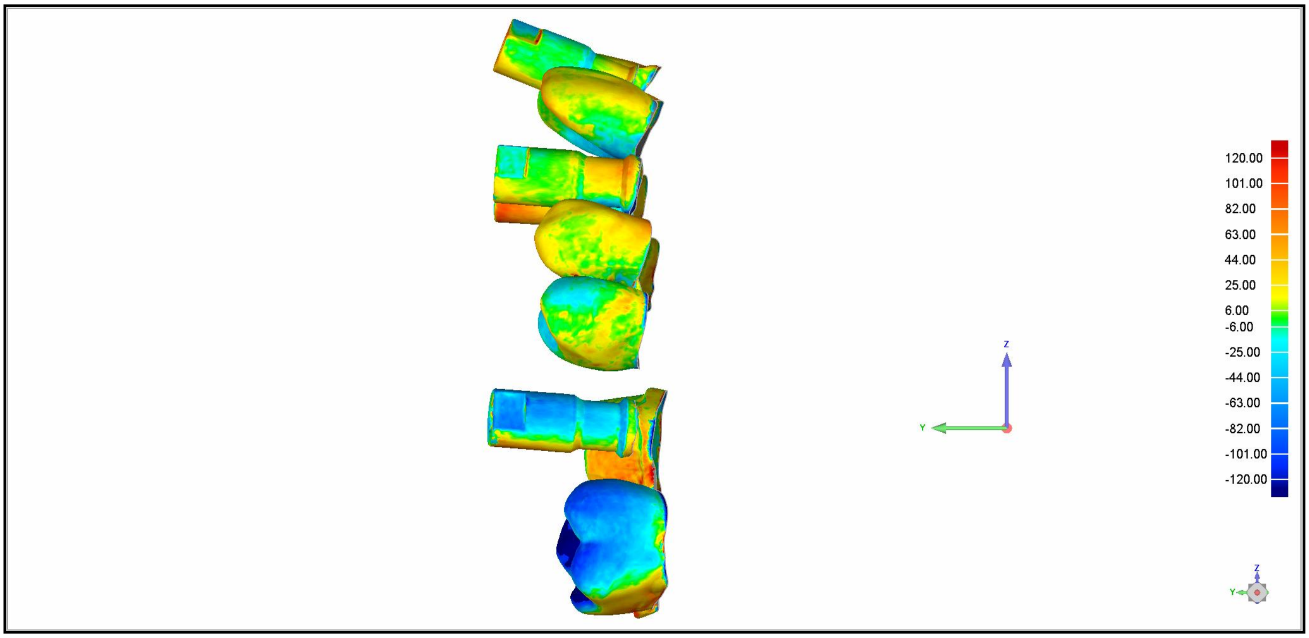

Predefinido: Derecha

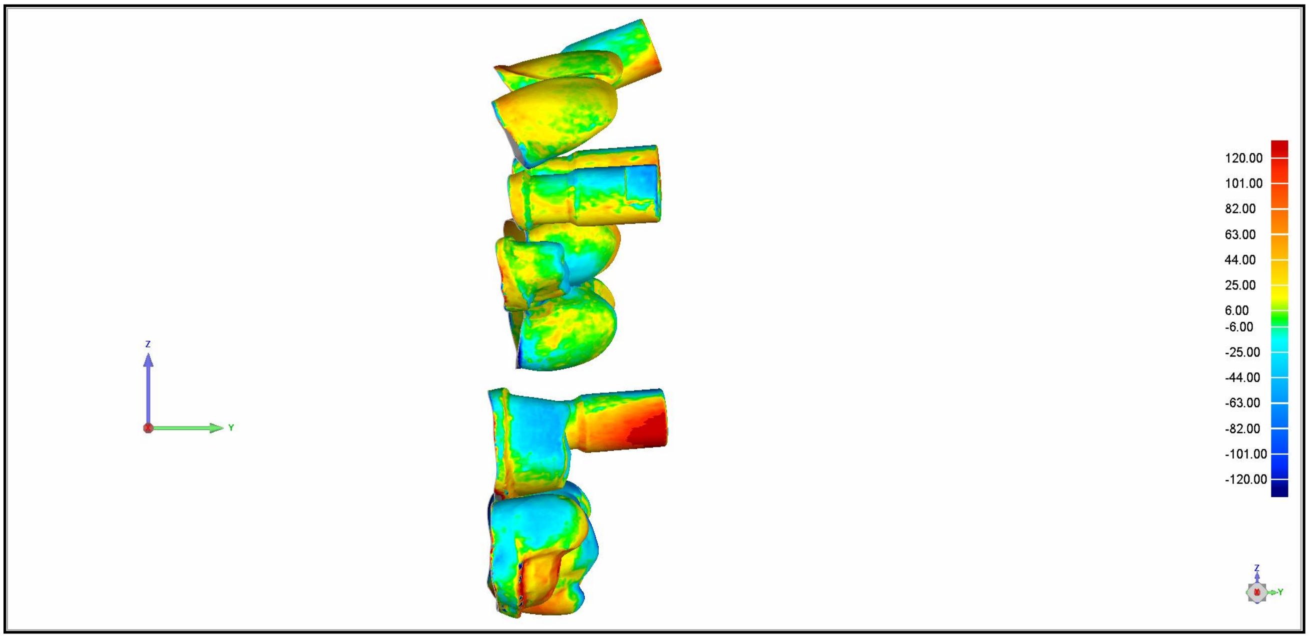

Predefinido: Superior

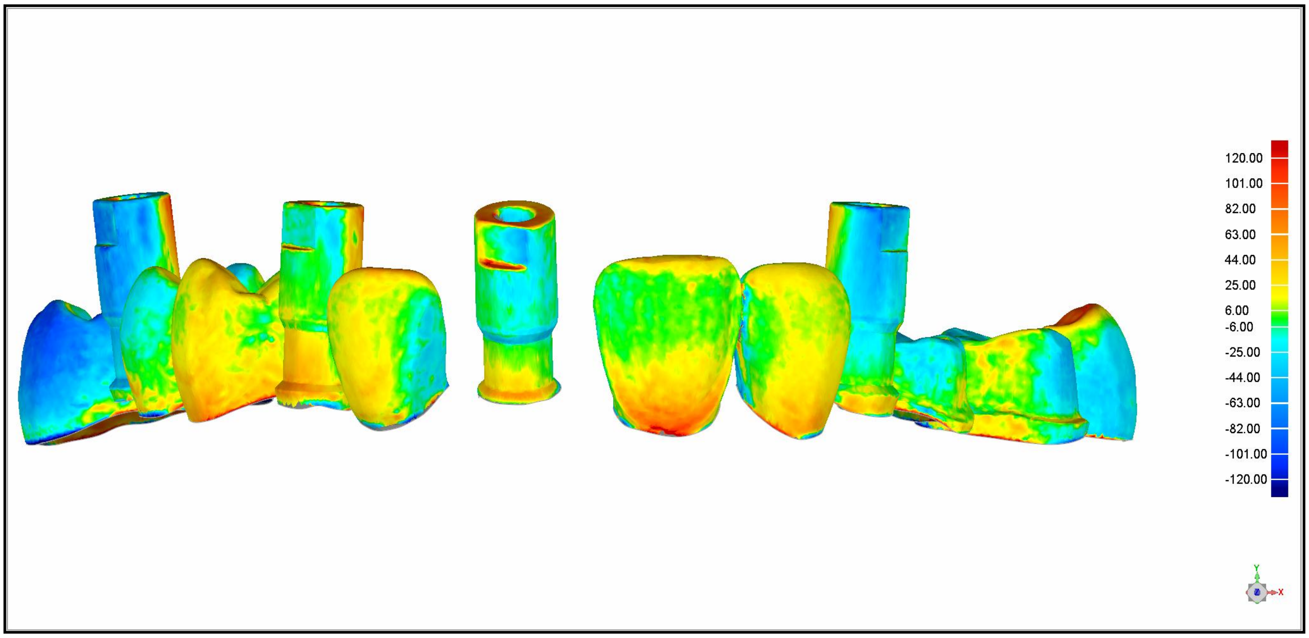

Predefinido: Inferior

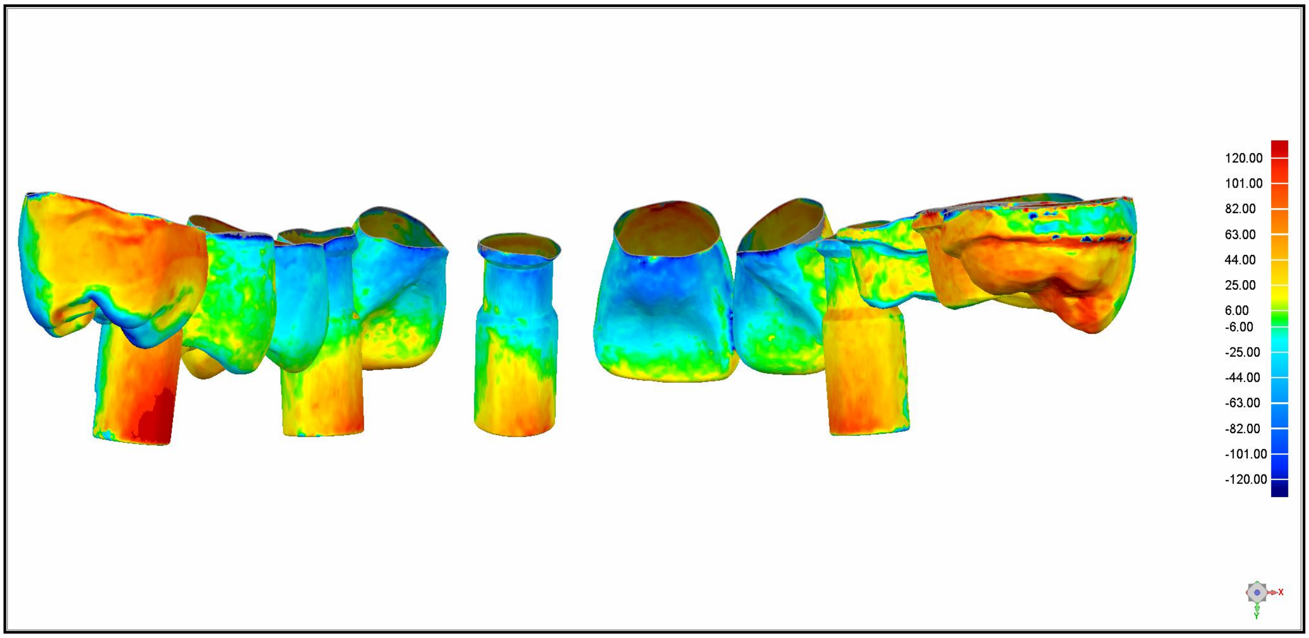

Ajuste de ubicación: Desviaciones superior e inferior

Unidades: u

Nombre	Desv	Estado	Superior Tol	Inferior Tol	Ref X	Ref Y	Ref Z	Radio	Desv X	Desv Y	Desv Z	Medido X	Medido Y	Medido Z	Dir. proy. X	Dir. proy. Y	Dir. proy. Z
Desv. inferior	-2481.72				32072.67	29477.54	-10894.63	n/a	-11.13	-2361.62	762.58	32061.54	27115.91	-10132.04	0.00	0.95	-0.31
Desv. superior	2399.42				-14593.33	38035.67	19570.06	n/a	617.79	2283.47	-401.64	-13975.54	40319.14	19168.42	0.26	0.95	-0.17
