# Supplementary material for: Accuracy of four digital scanners according to scanning strategy in complete-arch impressions
Source: PLoS One. 2018 Sep 13;13(9):e0202916. doi: 10.1371/journal.pone.0202916 (PMC6136706; doi:10.1371/journal.pone.0202916)
Supplement: S16 Table — True definition (scanning strategy D). (ZIP) [file pone.0202916.s016.zip › S16/TD2D.pdf]

3D Comparación Resultados

Modelo referencia	MRC
Modelo test	TD2D
Nº de puntos de datos	130780
# Aislados	477

Tipo tolerancia	3D desviación
Unidades	u
Máx. crítico	120.00
Máx. nominal	17.00
Mín. nominal	-17.00
Mín. crítico	-120.00

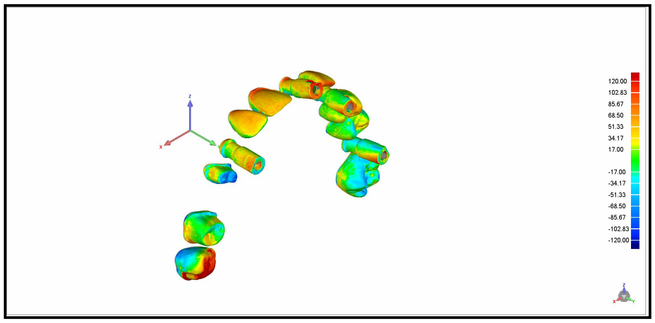

Desviación	
Desviación superior máx.	2039.06
Desviación inferior máx.	-2714.69
Desviación media	40.23 /-29.50
Desviación estándar	70.57

Distribución desviación

>=Min	<Max	# Puntos	%
-120.00	-102.83	277	0.21
-102.83	-85.67	583	0.45
-85.67	-68.50	1199	0.92
-68.50	-51.33	2588	1.98
-51.33	-34.17	5054	3.86
-34.17	-17.00	11445	8.75
-17.00	17.00	47099	36.01
17.00	34.17	22042	16.85
34.17	51.33	16555	12.66
51.33	68.50	10390	7.94
68.50	85.67	4610	3.53
85.67	102.83	2938	2.25
102.83	120.00	1871	1.43

Fuera del crítico superior	3384	2.59
Fuera del crítico inferior	745	0.57

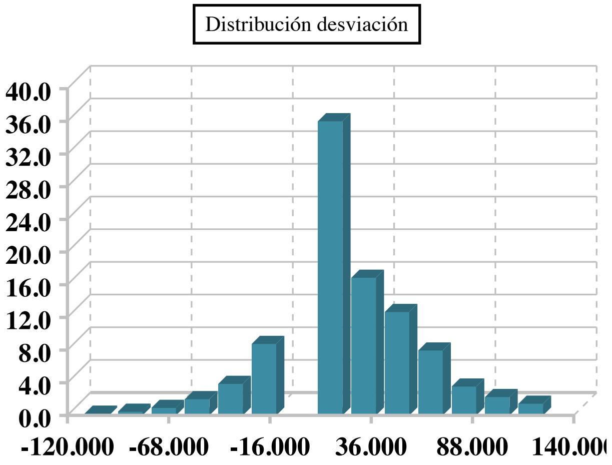

Desviaciones estándar

Distribución (+/-)	# Puntos	%
-6 * Desv. estánd.	111	0.08
-5 * Desv. estánd.	68	0.05
-4 * Desv. estánd.	149	0.11
-3 * Desv. estánd.	403	0.31
-2 * Desv. estánd.	4395	3.36
-1 * Desv. estánd.	65100	49.78
1 * Desv. estánd.	52901	40.45
2 * Desv. estánd.	6360	4.86
3 * Desv. estánd.	891	0.68
4 * Desv. estánd.	156	0.12
5 * Desv. estánd.	99	0.08
6 * Desv. estánd.	147	0.11

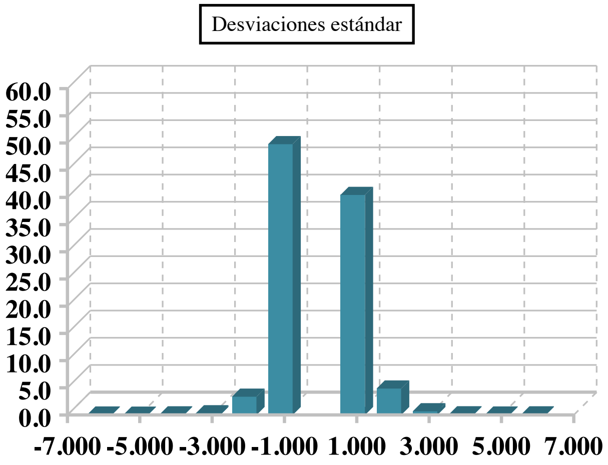

Predefinido: Isométrico

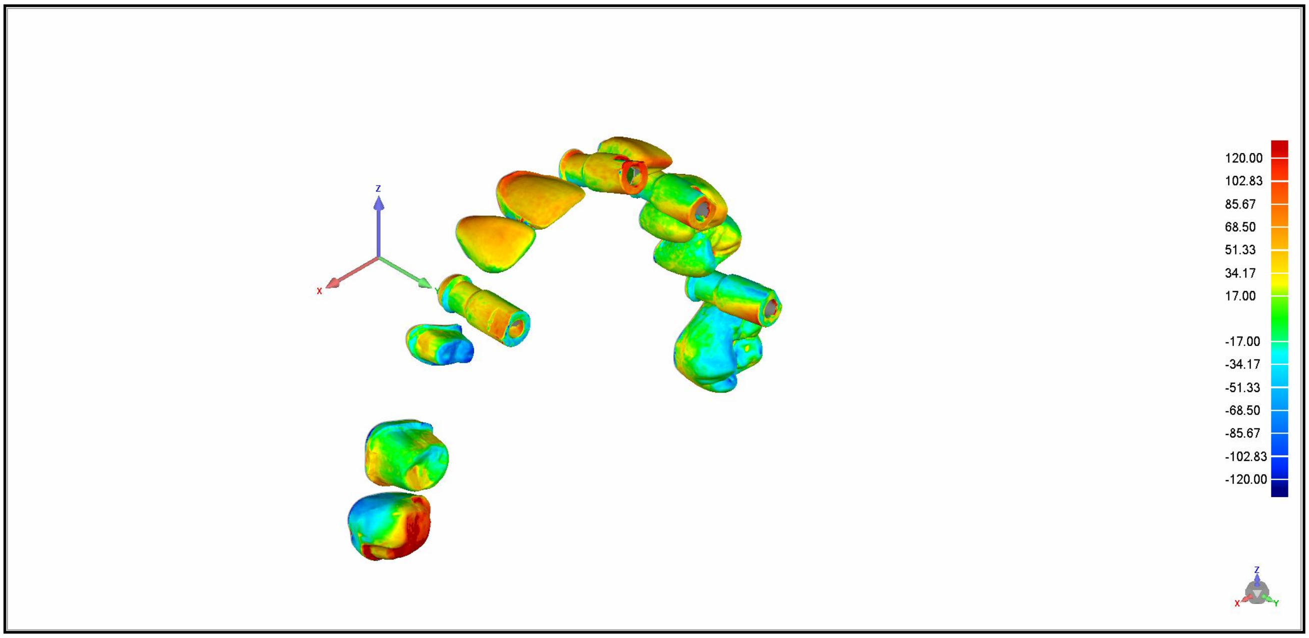

Predefinido: Frente

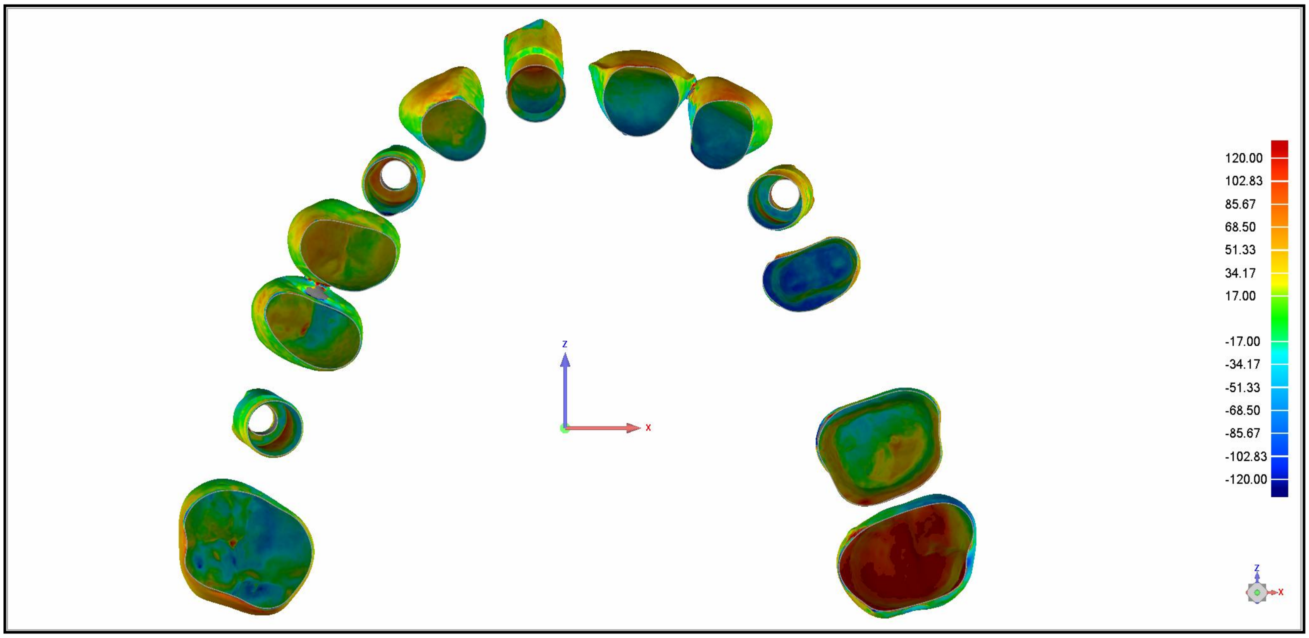

Predefinido: Atrás

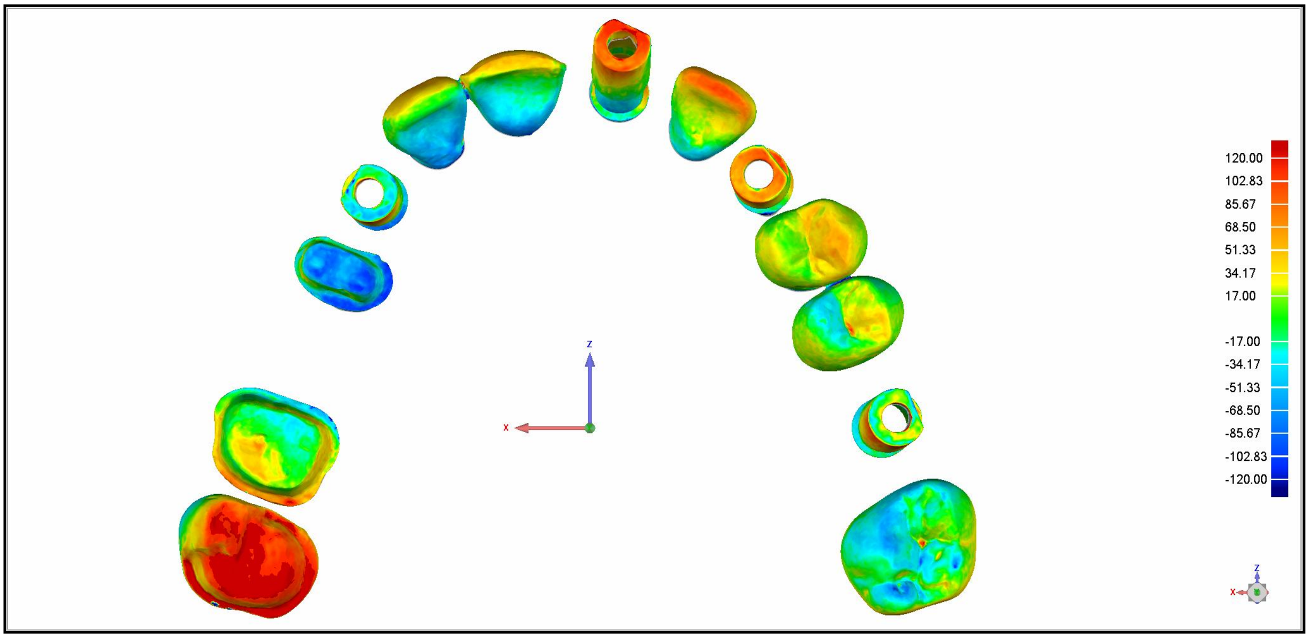

Predefinido: Izquierda

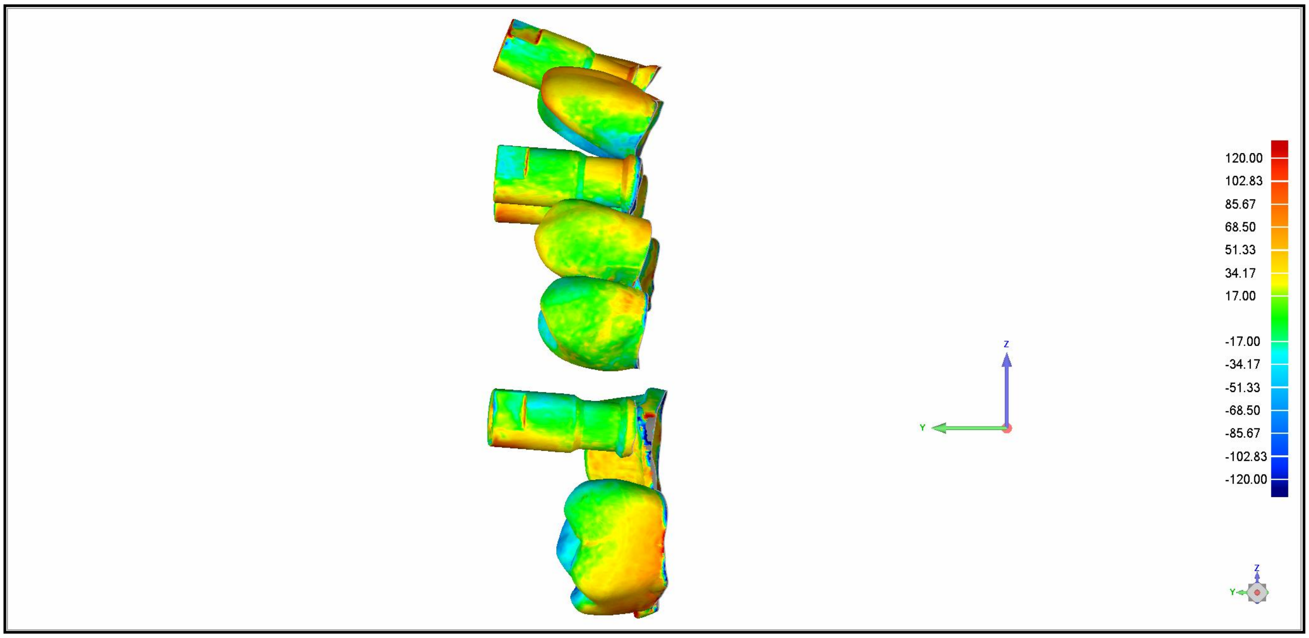

Predefinido: Derecha

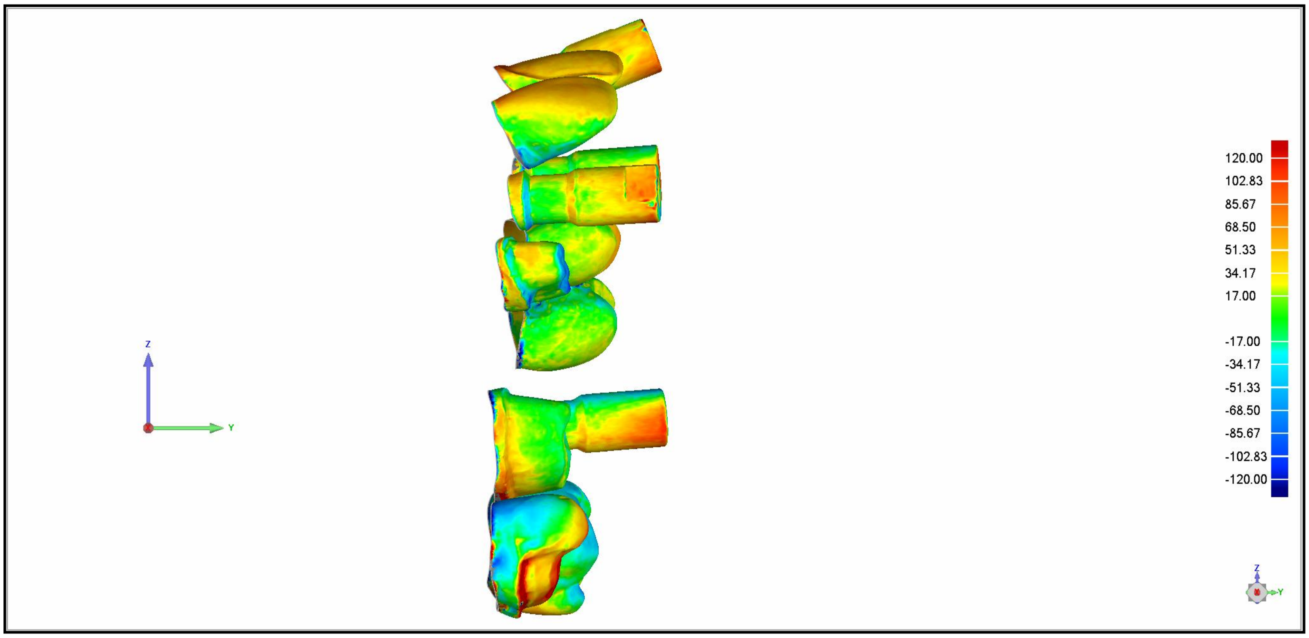

Predefinido: Superior

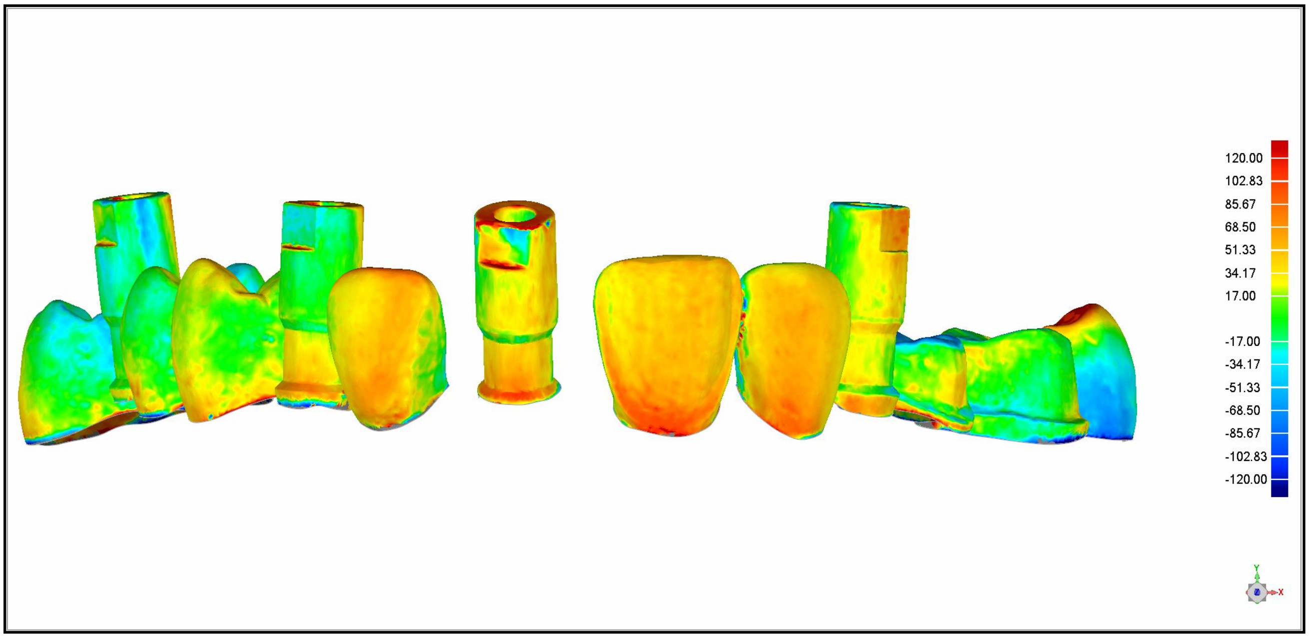

Predefinido: Inferior

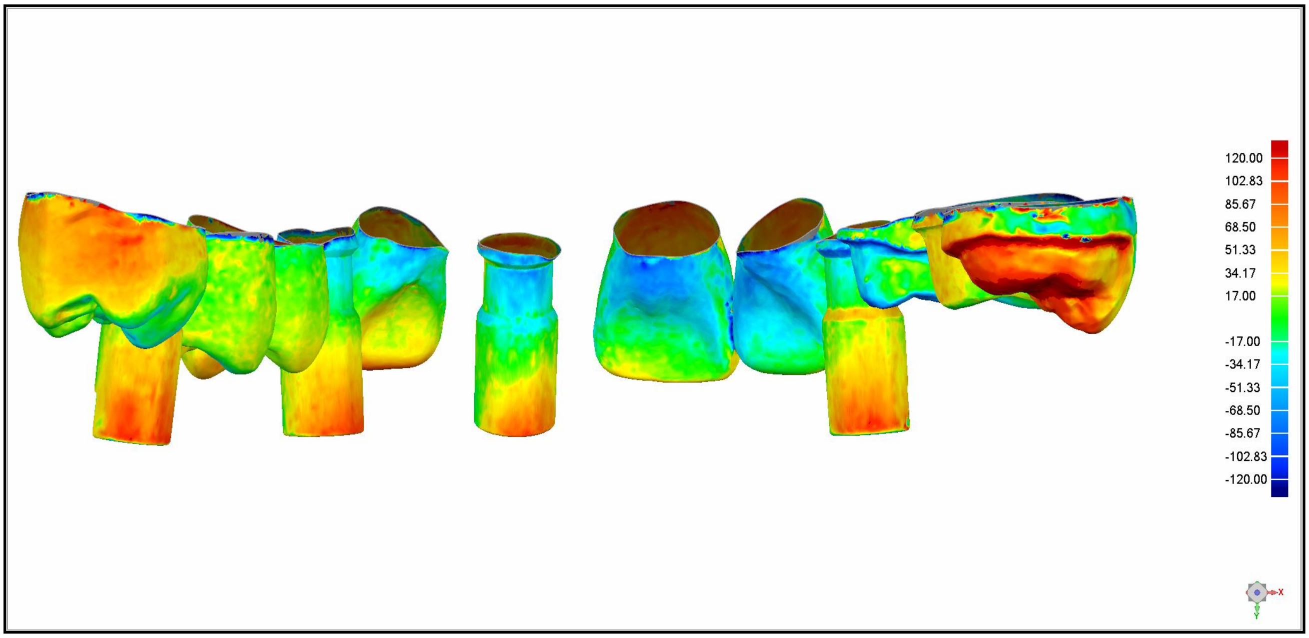

Ajuste de ubicación: Desviaciones superior e inferior

Unidades: u

Nombre	Desv	Estado	Superior Tol	Inferior Tol	Ref X	Ref Y	Ref Z	Radio	Desv X	Desv Y	Desv Z	Medido X	Medido Y	Medido Z	Dir. proy. X	Dir. proy. Y	Dir. proy. Z
Desv. inferior	-2714.69				-15830.08	29141.25	19869.75	n/a	760.39	699.00	-2510.53	-15069.69	29840.25	17359.21	-0.28	-0.26	0.92
Desv. superior	2039.06				23737.64	28001.17	-7987.62	n/a	-171.83	-64.42	2030.78	23565.81	27936.75	-5956.84	-0.08	-0.03	1.00
