# Supplementary material for: Accuracy of four digital scanners according to scanning strategy in complete-arch impressions
Source: PLoS One. 2018 Sep 13;13(9):e0202916. doi: 10.1371/journal.pone.0202916 (PMC6136706; doi:10.1371/journal.pone.0202916)
Supplement: S16 Table — True definition (scanning strategy D). (ZIP) [file pone.0202916.s016.zip › S16/TD3D.pdf]

3D Comparación Resultados

Modelo referencia	MRC
Modelo test	TD3D
Nº de puntos de datos	129447
# Aislados	424

Tipo tolerancia	3D desviación
Unidades	u
Máx. crítico	120.00
Máx. nominal	19.00
Mín. nominal	-19.00
Mín. crítico	-120.00

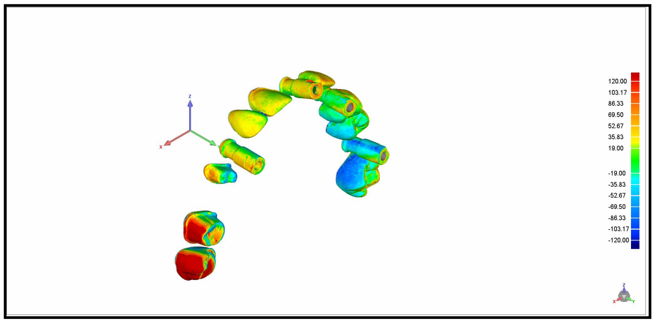

Desviación	
Desviación superior máx.	2517.39
Desviación inferior máx.	-3135.62
Desviación media	51.56 /-41.11
Desviación estándar	85.28

Distribución desviación

>=Min	<Max	# Puntos	%
-120.00	-103.17	919	0.71
-103.17	-86.33	1463	1.13
-86.33	-69.50	2841	2.19
-69.50	-52.67	3304	2.55
-52.67	-35.83	5763	4.45
-35.83	-19.00	10046	7.76
-19.00	19.00	40737	31.47
19.00	35.83	21382	16.52
35.83	52.67	14524	11.22
52.67	69.50	8313	6.42
69.50	86.33	4010	3.10
86.33	103.17	3127	2.42
103.17	120.00	2708	2.09

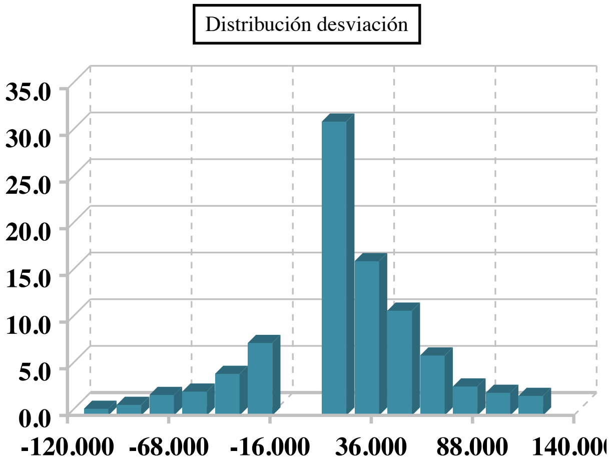

Fuera del crítico superior	8265	6.38
Fuera del crítico inferior	2045	1.58

Desviaciones estándar

Distribución (+/-)	# Puntos	%
-6 * Desv. estánd.	203	0.16
-5 * Desv. estánd.	86	0.07
-4 * Desv. estánd.	136	0.11
-3 * Desv. estánd.	641	0.50
-2 * Desv. estánd.	6816	5.27
-1 * Desv. estánd.	59676	46.10
1 * Desv. estánd.	51170	39.53
2 * Desv. estánd.	8164	6.31
3 * Desv. estánd.	2195	1.70
4 * Desv. estánd.	130	0.10
5 * Desv. estánd.	68	0.05
6 * Desv. estánd.	162	0.13

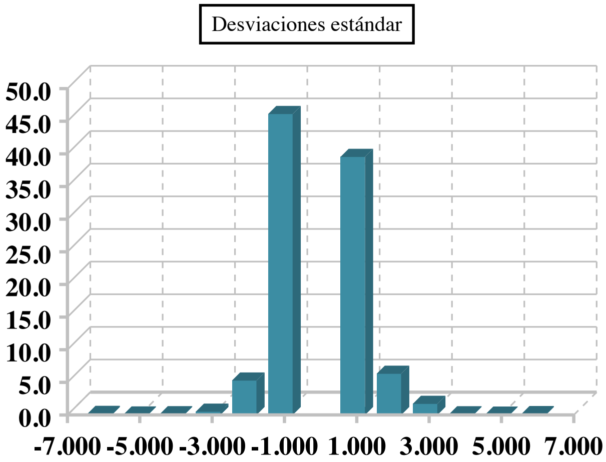

Predefinido: Isométrico

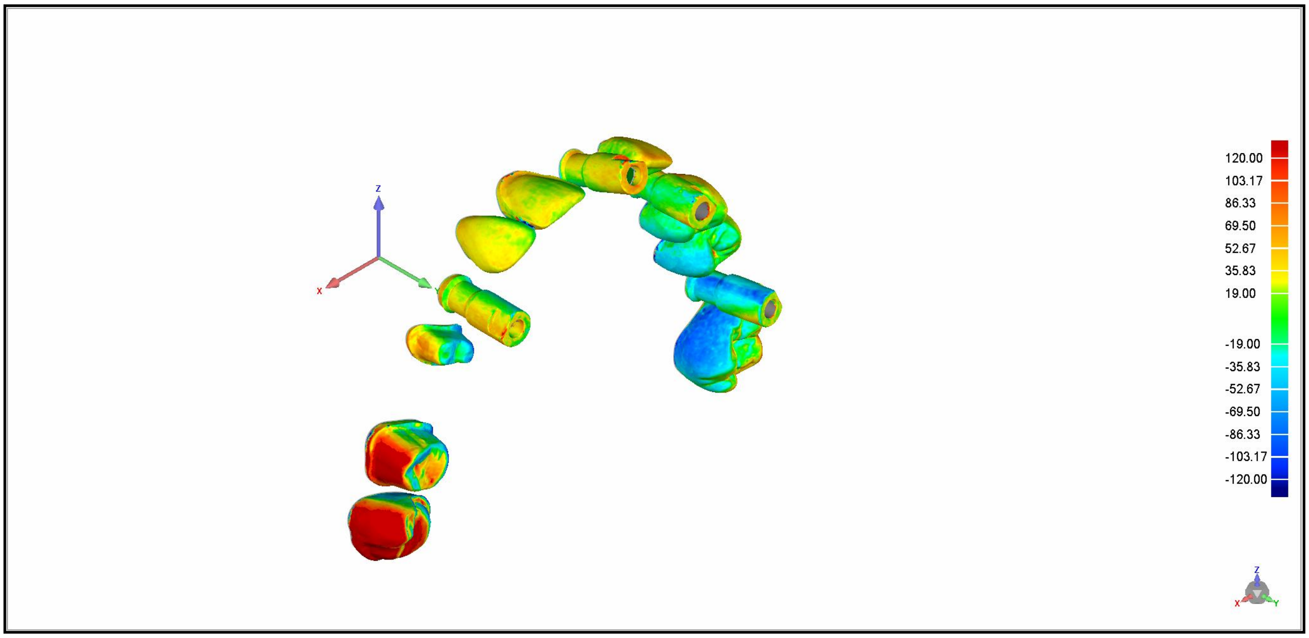

Predefinido: Frente

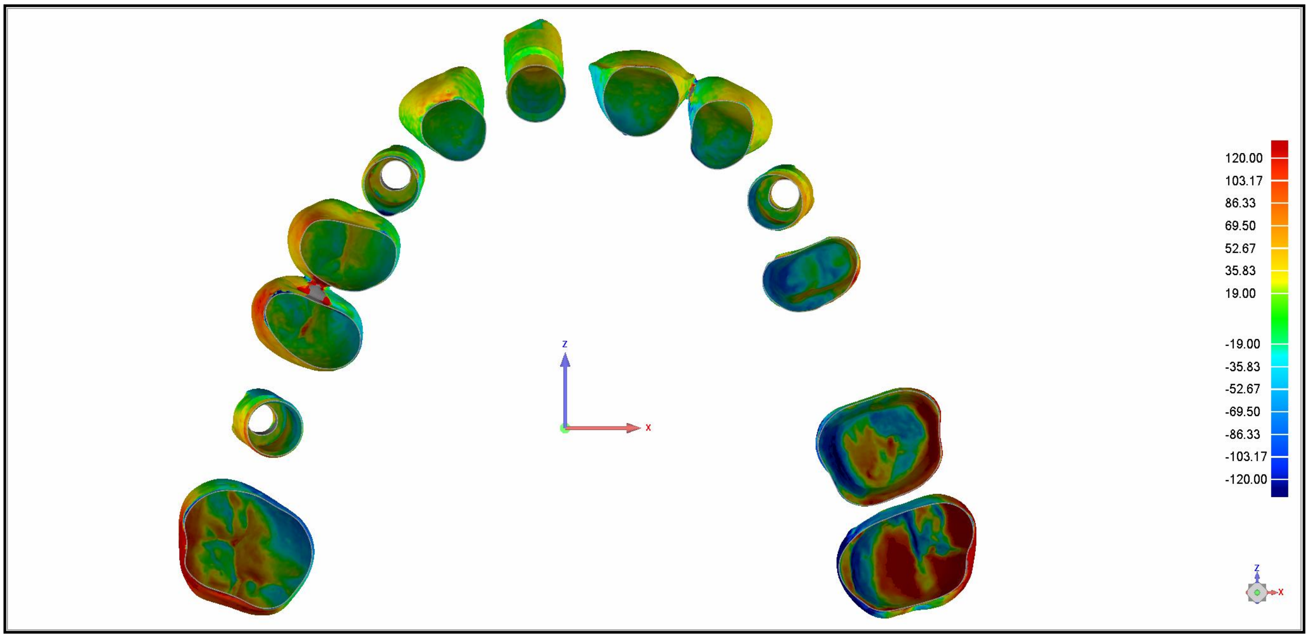

Predefinido: Atrás

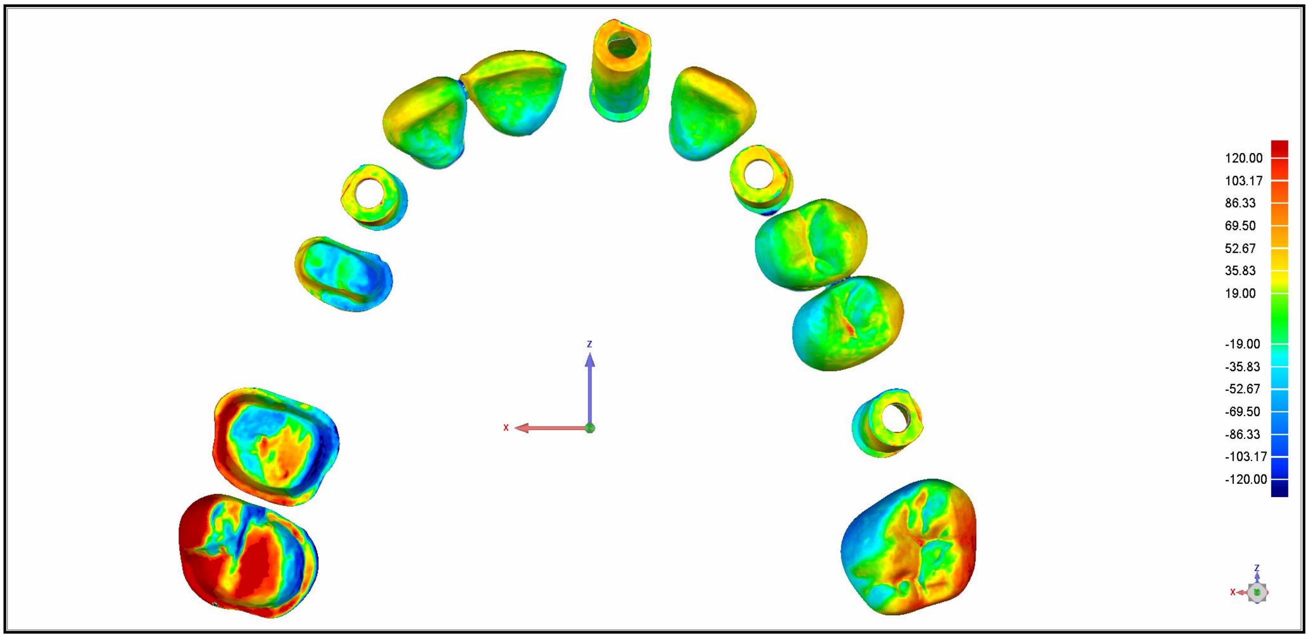

Predefinido: Izquierda

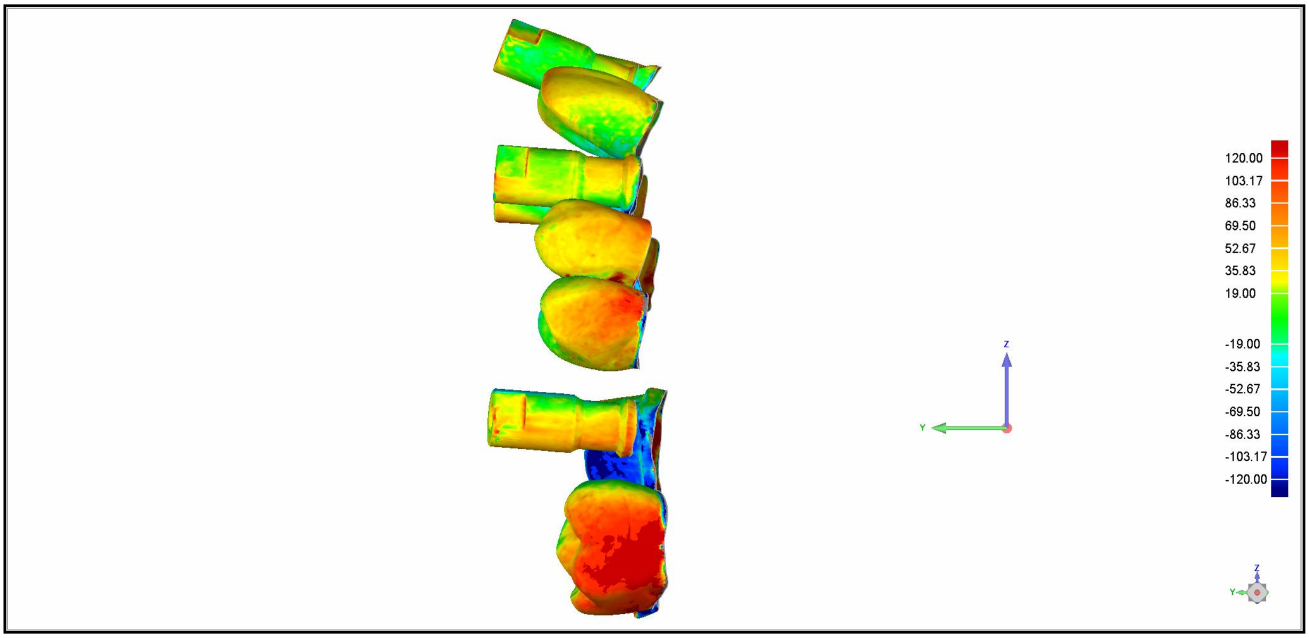

Predefinido: Derecha

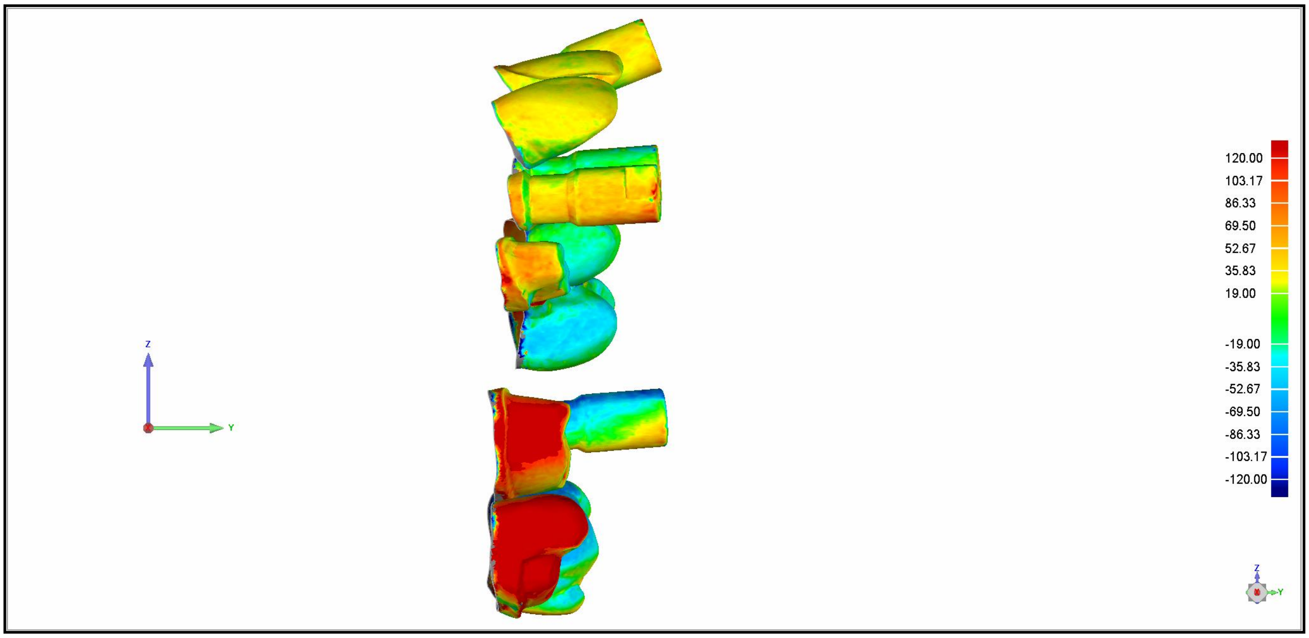

Predefinido: Superior

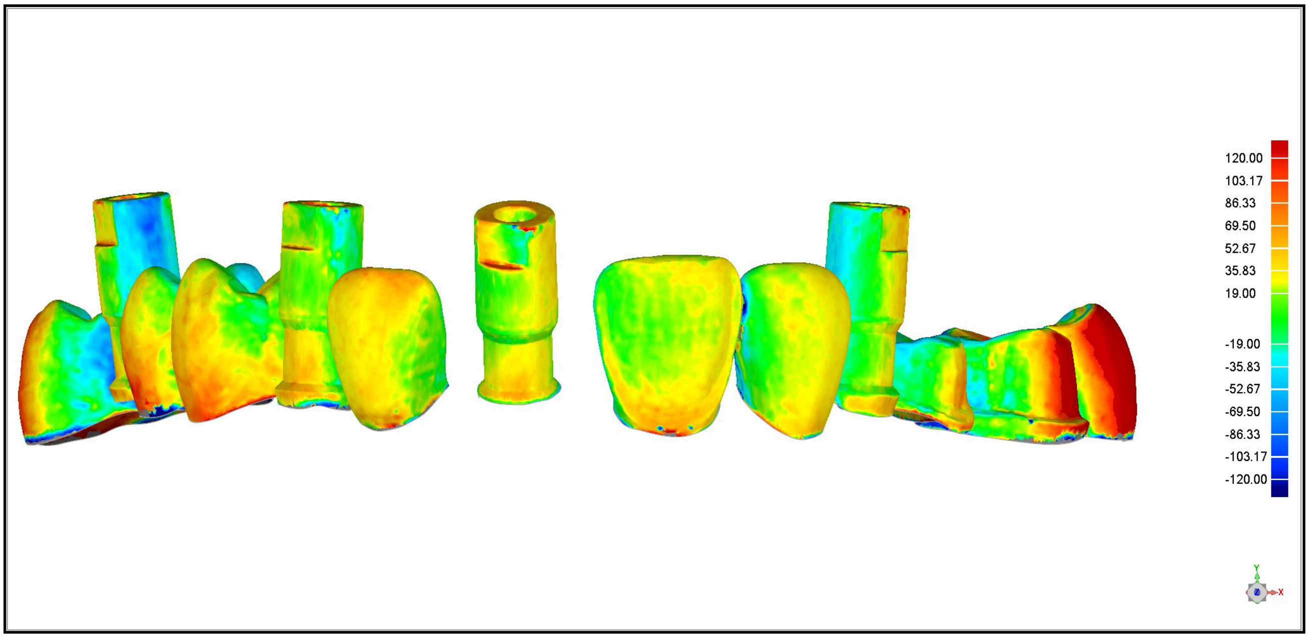

Predefinido: Inferior

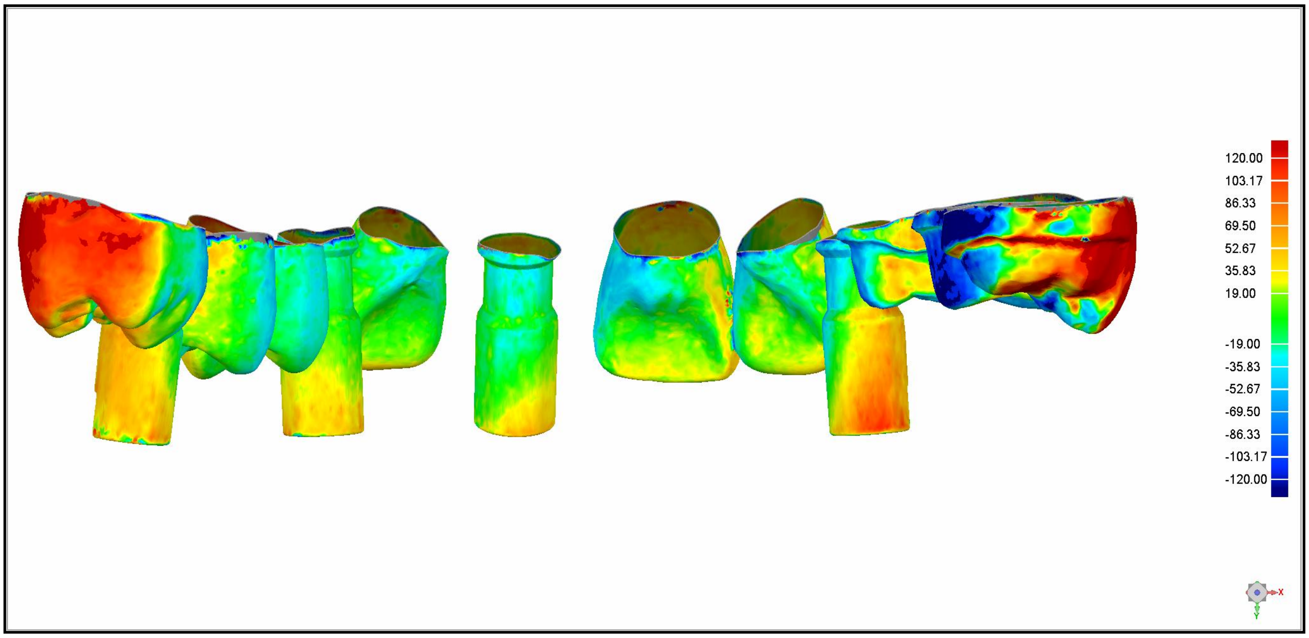

Ajuste de ubicación: Desviaciones superior e inferior

Unidades: u

Nombre	Desv	Estado	Superior Tol	Inferior Tol	Ref X	Ref Y	Ref Z	Radio	Desv X	Desv Y	Desv Z	Medido X	Medido Y	Medido Z	Dir. proy. X	Dir. proy. Y	Dir. proy. Z
Desv. inferior	-3135.62				-22607.19	28955.77	6808.03	n/a	-911.56	-329.56	2982.04	-23518.76	28626.21	9790.07	0.29	0.11	-0.95
Desv. superior	2517.39				-29292.33	26884.28	-11910.36	n/a	2066.36	406.57	-1379.17	-27225.97	27290.84	-13289.53	0.82	0.16	-0.55
