# Supplementary material for: Accuracy of four digital scanners according to scanning strategy in complete-arch impressions
Source: PLoS One. 2018 Sep 13;13(9):e0202916. doi: 10.1371/journal.pone.0202916 (PMC6136706; doi:10.1371/journal.pone.0202916)
Supplement: S16 Table — True definition (scanning strategy D). (ZIP) [file pone.0202916.s016.zip › S16/TD8D.pdf]

# 3D Comparación Resultados

| | |
|---|---|
| Modelo referencia | MRC |
| Modelo test | TD8D |
| Nº de puntos de datos | 129135 |
| # Aislados | 324 |

| | |
|---|---|
| Tipo tolerancia | 3D desviación |
| Unidades | u |
| Máx. crítico | 120.00 |
| Máx. nominal | 9.00 |
| Mín. nominal | -9.00 |
| Mín. crítico | -120.00 |

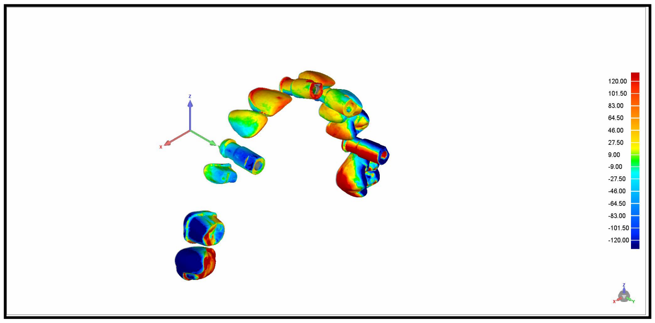

| | |
|---|---|
| Desviación | |
| Desviación superior máx. | 3056.40 |
| Desviación inferior máx. | -2940.45 |
| Desviación media | 82.69 /-80.90 |
| Desviación estándar | 132.36 |

## Distribución desviación

| >=Min | <Max | # Puntos | % |
|---|---|---|---|
| -120.00 | -101.50 | 2719 | 2.11 |
| -101.50 | -83.00 | 3246 | 2.51 |
| -83.00 | -64.50 | 4052 | 3.14 |
| -64.50 | -46.00 | 5786 | 4.48 |
| -46.00 | -27.50 | 9068 | 7.02 |
| -27.50 | -9.00 | 12805 | 9.92 |
| -9.00 | 9.00 | 14138 | 10.95 |
| 9.00 | 27.50 | 14338 | 11.10 |
| 27.50 | 46.00 | 11187 | 8.66 |
| 46.00 | 64.50 | 8028 | 6.22 |
| 64.50 | 83.00 | 6196 | 4.80 |
| 83.00 | 101.50 | 4762 | 3.69 |
| 101.50 | 120.00 | 3266 | 2.53 |

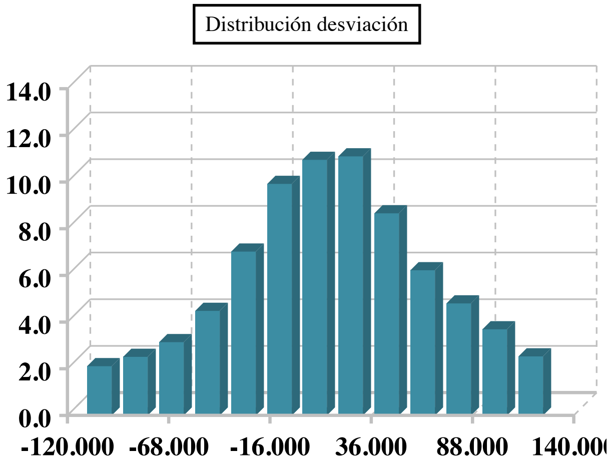


| | | |
|---|---|---|
| Fuera del crítico superior | 15981 | 12.38 |
| Fuera del crítico inferior | 13563 | 10.50 |

## Desviaciones estándar

| Distribución (+/-) | # Puntos | % |
|---|---|---|
| -6 * Desv. estánd. | 122 | 0.09 |
| -5 * Desv. estánd. | 60 | 0.05 |
| -4 * Desv. estánd. | 69 | 0.05 |
| -3 * Desv. estánd. | 2454 | 1.90 |
| -2 * Desv. estánd. | 10351 | 8.02 |
| -1 * Desv. estánd. | 52337 | 40.53 |
| 1 * Desv. estánd. | 50338 | 38.98 |
| 2 * Desv. estánd. | 10371 | 8.03 |
| 3 * Desv. estánd. | 2690 | 2.08 |
| 4 * Desv. estánd. | 114 | 0.09 |
| 5 * Desv. estánd. | 76 | 0.06 |
| 6 * Desv. estánd. | 153 | 0.12 |

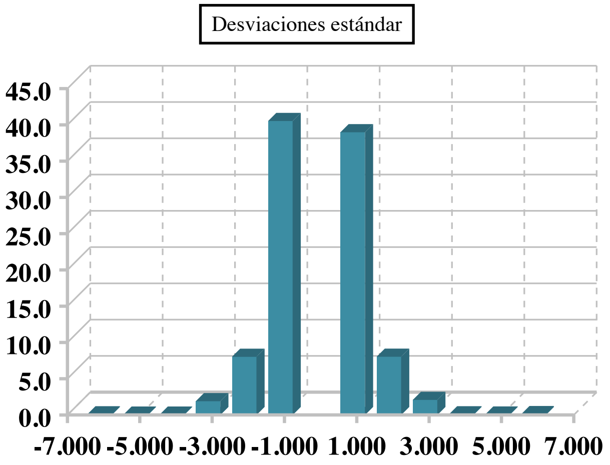

Predefinido: Isométrico

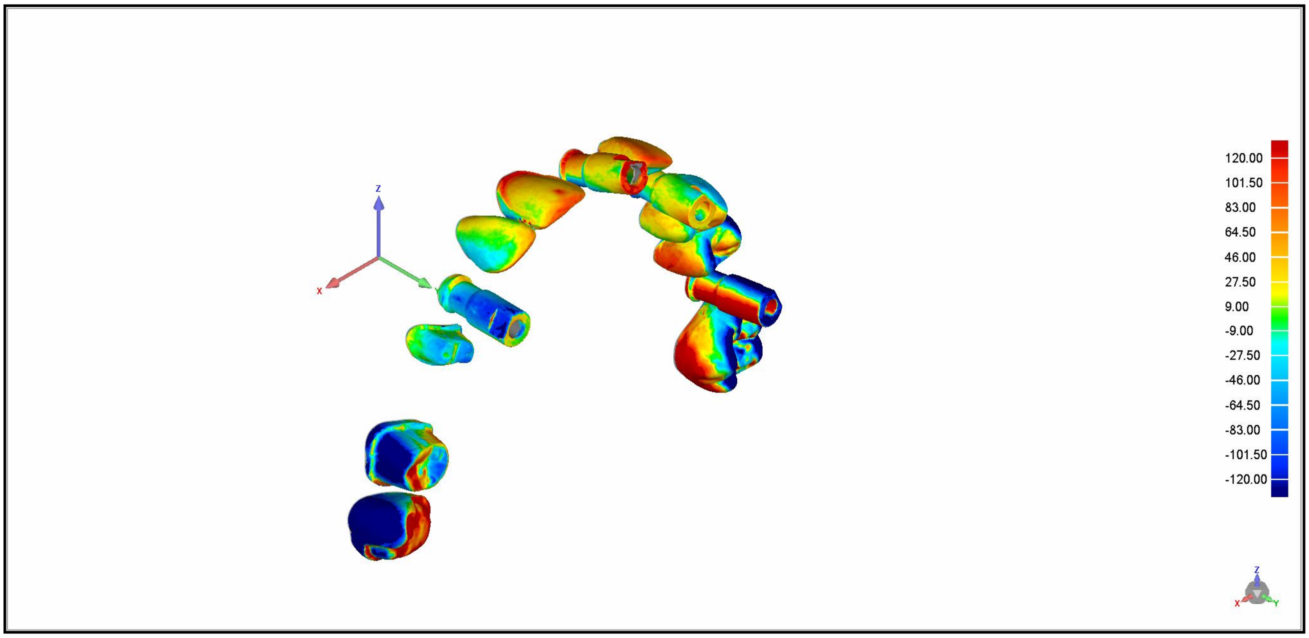

Predefinido: Frente

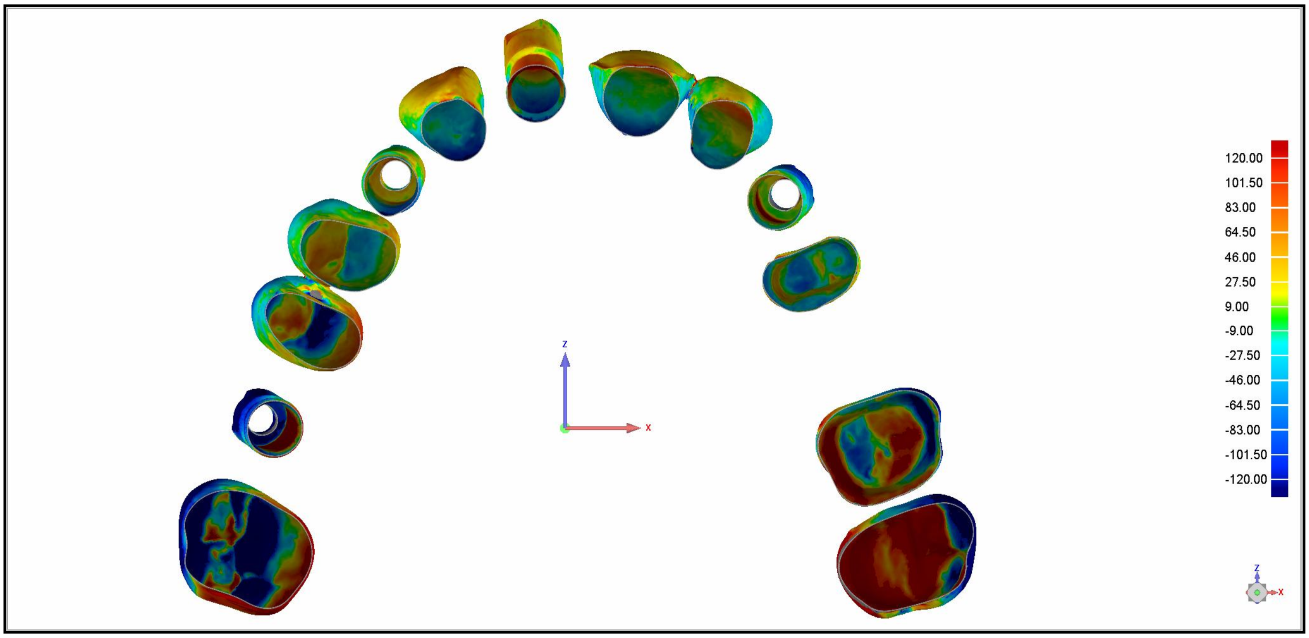

Predefinido: Atrás

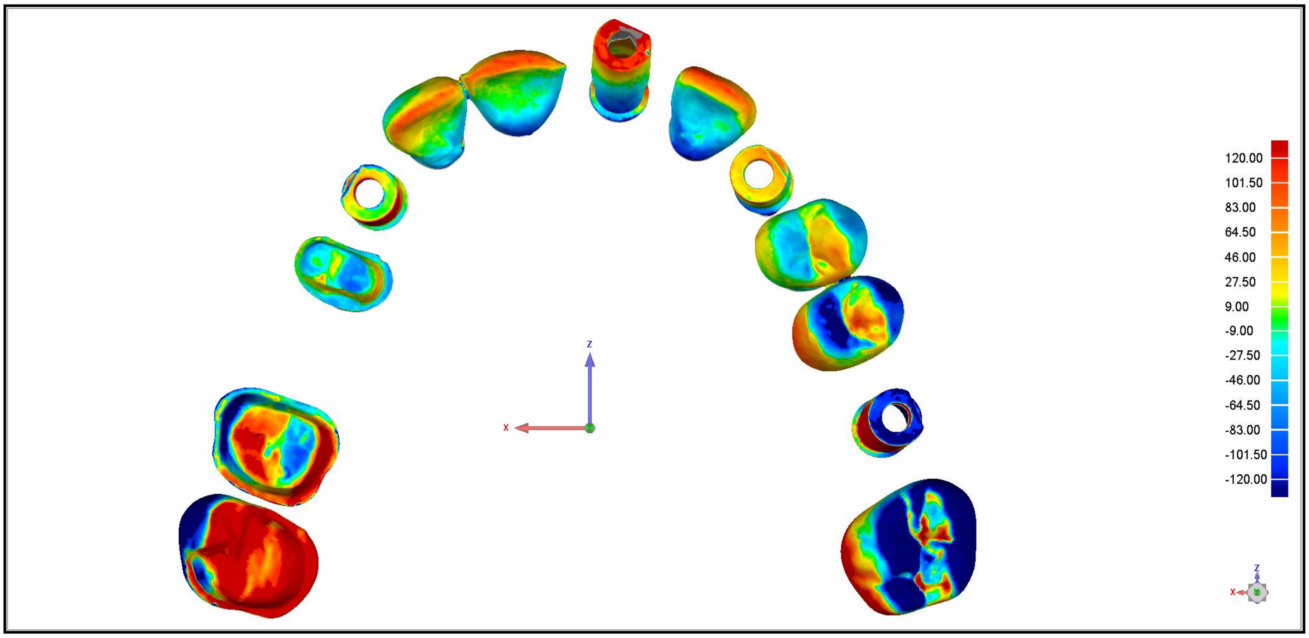

Predefinido: Izquierda

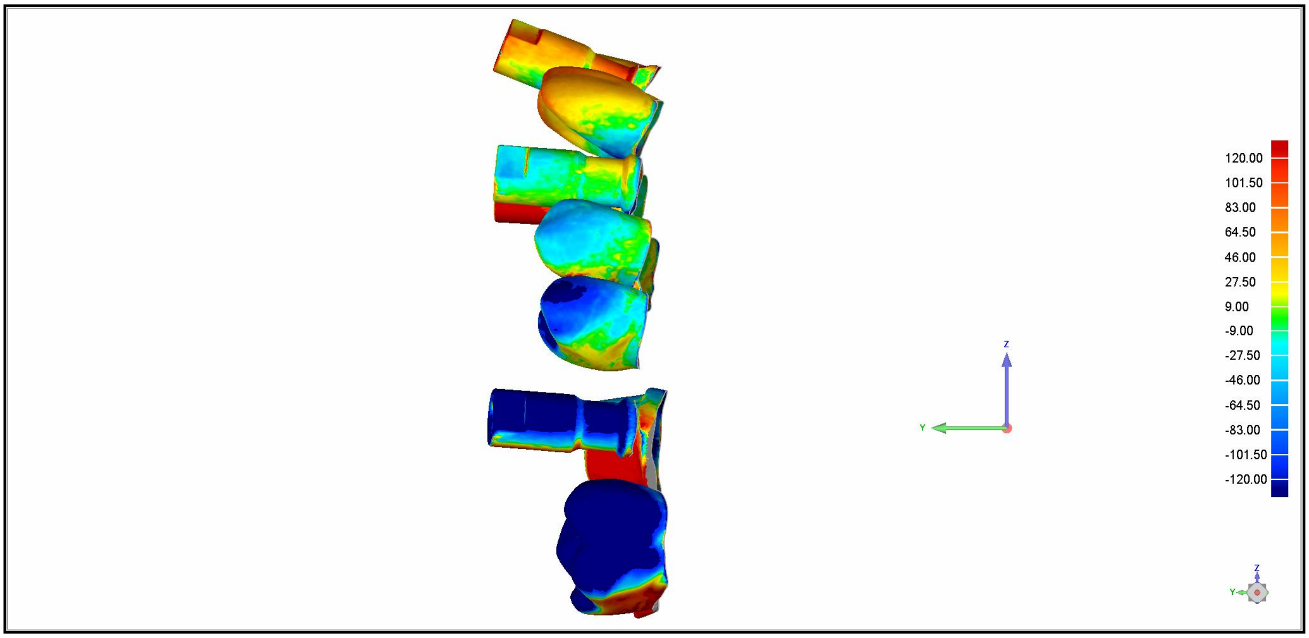

Fecha: 3/6/2017, 3:58 pm

Predefinido: Derecha

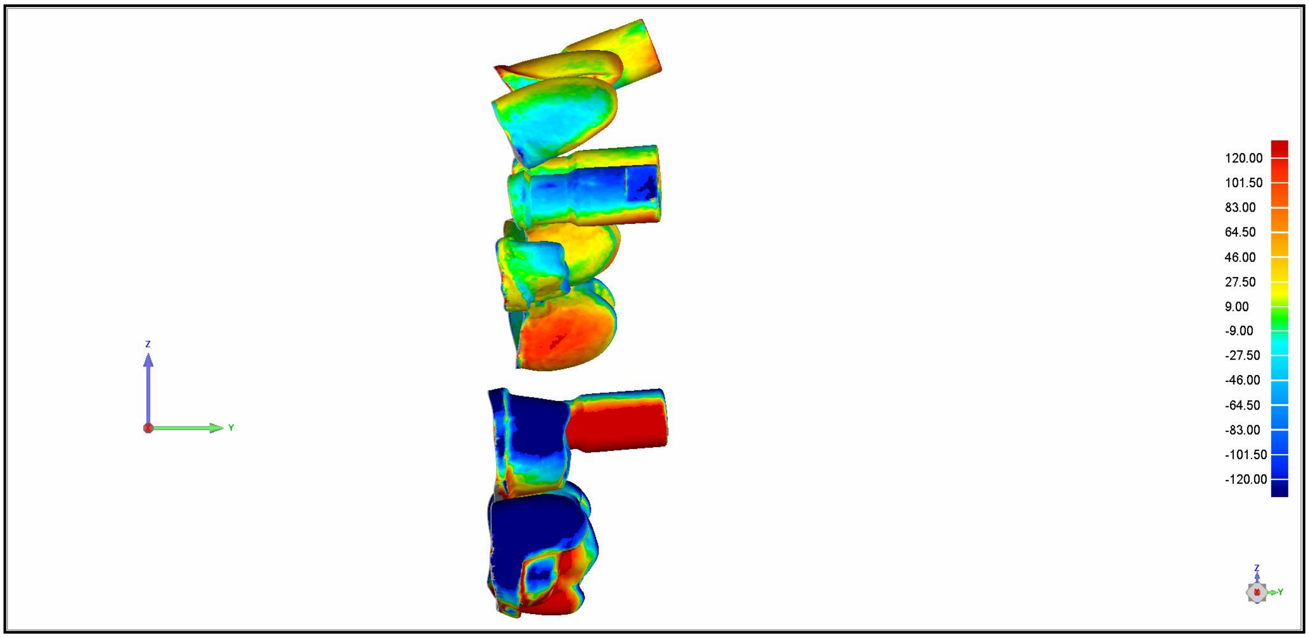

Predefinido: Superior

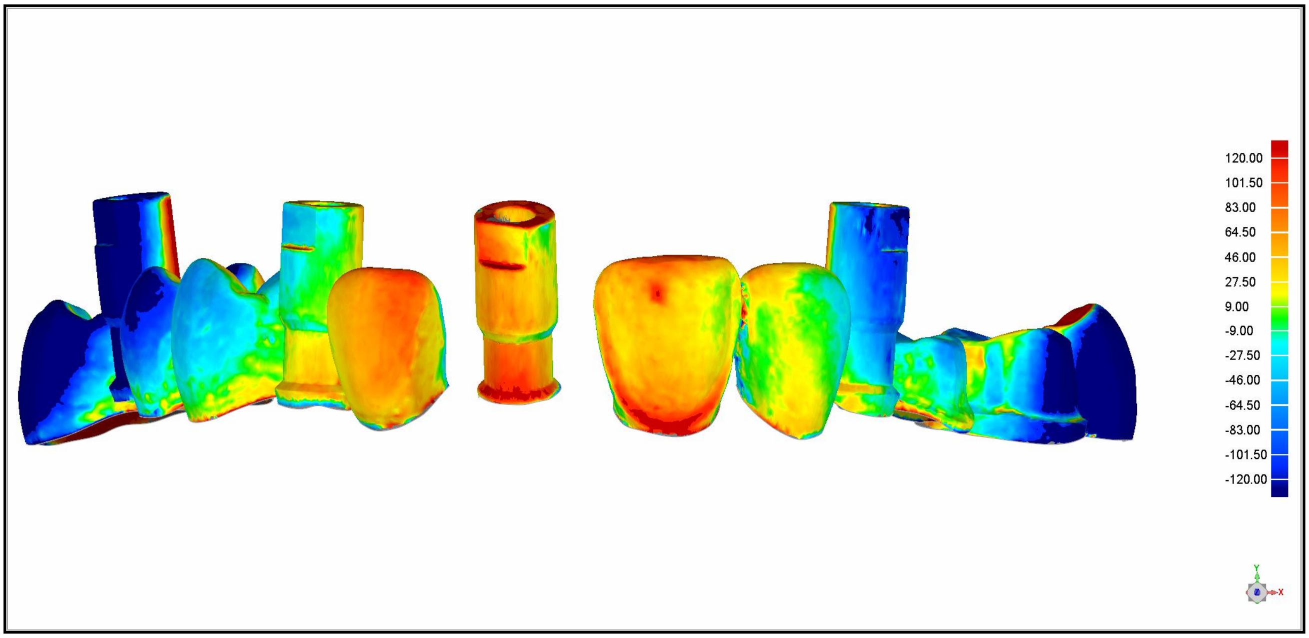

Predefinido: Inferior

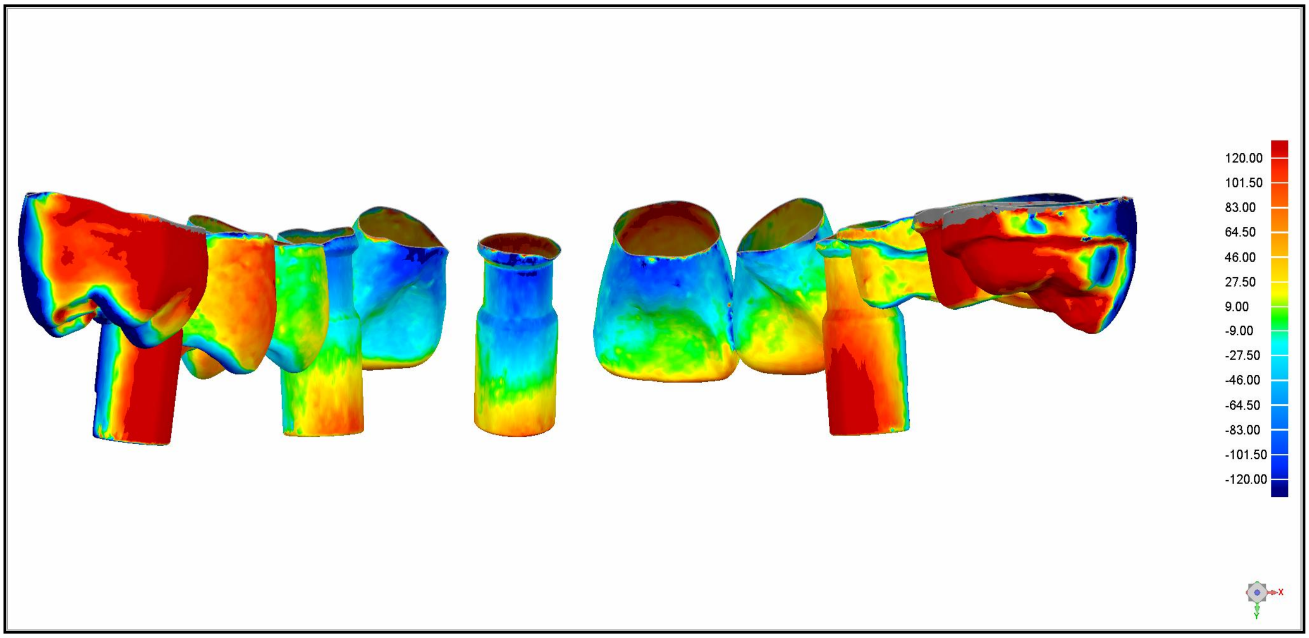

# Ajuste de ubicación: Desviaciones superior e inferior

## Unidades: u

| Nombre | Desv | Estado | Superior Tol | Inferior Tol | Ref X | Ref Y | Ref Z | Radio | Desv X | Desv Y | Desv Z | Medido X | Medido Y | Medido Z | Dir. proy. X | Dir. proy. Y | Dir. proy. Z |
|---|---|---|---|---|---|---|---|---|---|---|---|---|---|---|---|---|---|
| Desv. inferior | -2940.45 | | | | 32061.00 | 27268.70 | -9389.56 | n/a | -651.20 | 71.83 | -2866.54 | 31409.80 | 27340.54 | -12256.10 | 0.22 | -0.02 | 0.97 |
| Desv. superior | 3056.40 | | | | -6666.84 | 29713.26 | 24874.31 | n/a | -241.96 | 201.40 | -3040.14 | -6908.80 | 29914.66 | 21834.17 | -0.08 | 0.07 | -0.99 |
