# Supplementary material for: Accuracy of four digital scanners according to scanning strategy in complete-arch impressions
Source: PLoS One. 2018 Sep 13;13(9):e0202916. doi: 10.1371/journal.pone.0202916 (PMC6136706; doi:10.1371/journal.pone.0202916)
Supplement: S16 Table — True definition (scanning strategy D). (ZIP) [file pone.0202916.s016.zip › S16/TD4D.pdf]

3D Comparación Resultados

Modelo referencia	MRC
Modelo test	TD4D
Nº de puntos de datos	128713
# Aislados	541

Tipo tolerancia	3D desviación
Unidades	u
Máx. crítico	120.00
Máx. nominal	9.00
Mín. nominal	-9.00
Mín. crítico	-120.00

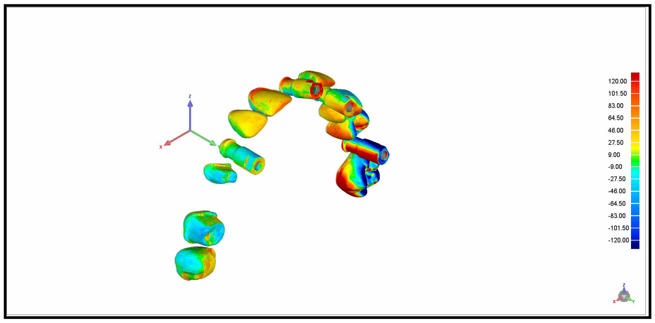

Desviación	
Desviación superior máx.	2729.57
Desviación inferior máx.	-2172.87
Desviación media	53.22 /-51.21
Desviación estándar	88.72

Distribución desviación

>=Min	<Max	# Puntos	%
-120.00	-101.50	1785	1.39
-101.50	-83.00	2282	1.77
-83.00	-64.50	2694	2.09
-64.50	-46.00	4021	3.12
-46.00	-27.50	9440	7.33
-27.50	-9.00	17838	13.86
-9.00	9.00	18315	14.23
9.00	27.50	20138	15.65
27.50	46.00	18512	14.38
46.00	64.50	8940	6.95
64.50	83.00	5003	3.89
83.00	101.50	4004	3.11
101.50	120.00	2287	1.78

Fuera del crítico superior	7074	5.50
Fuera del crítico inferior	6380	4.96

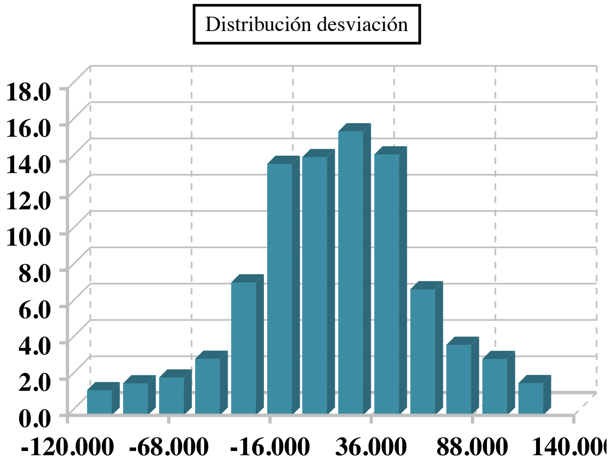

Desviaciones estándar

Distribución (+/-)	# Puntos	%
-6 * Desv. estánd.	62	0.05
-5 * Desv. estánd.	33	0.03
-4 * Desv. estánd.	451	0.35
-3 * Desv. estánd.	2905	2.26
-2 * Desv. estánd.	7573	5.88
-1 * Desv. estánd.	52643	40.90
1 * Desv. estánd.	55187	42.88
2 * Desv. estánd.	6484	5.04
3 * Desv. estánd.	2814	2.19
4 * Desv. estánd.	391	0.30
5 * Desv. estánd.	41	0.03
6 * Desv. estánd.	129	0.10

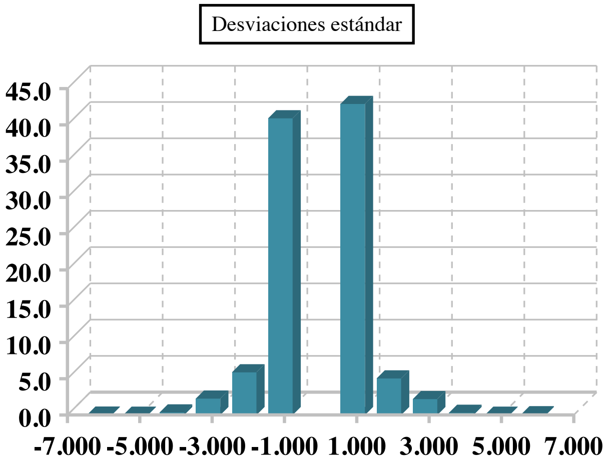

Predefinido: Isométrico

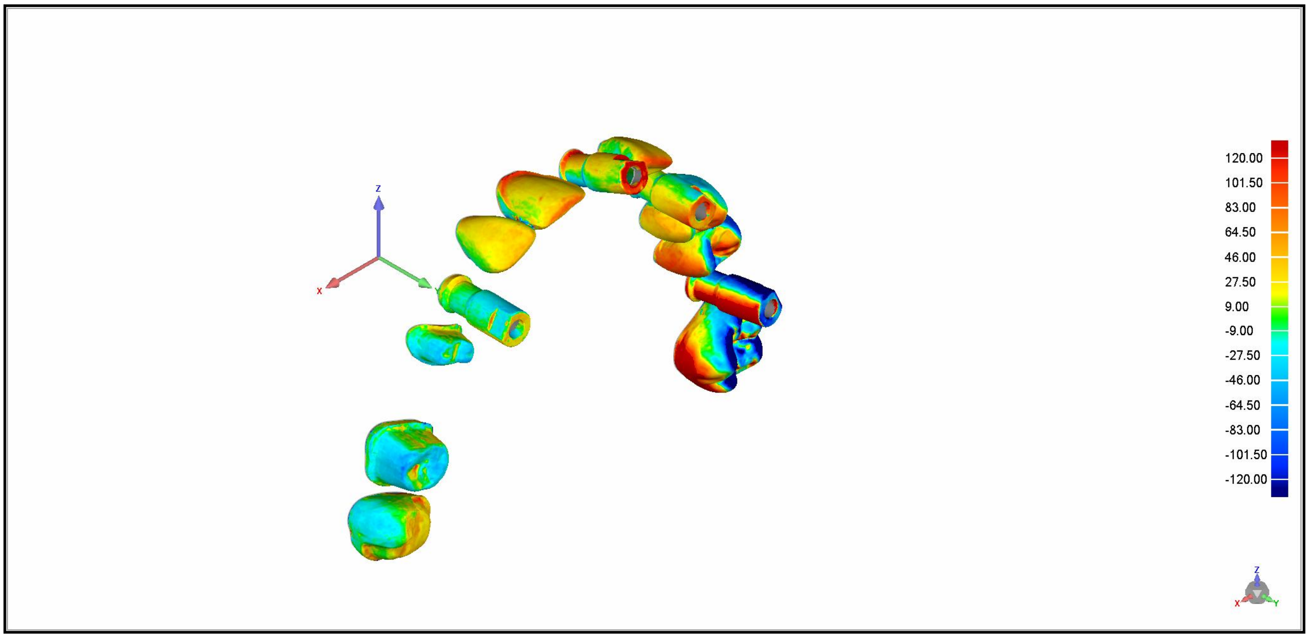

Predefinido: Frente

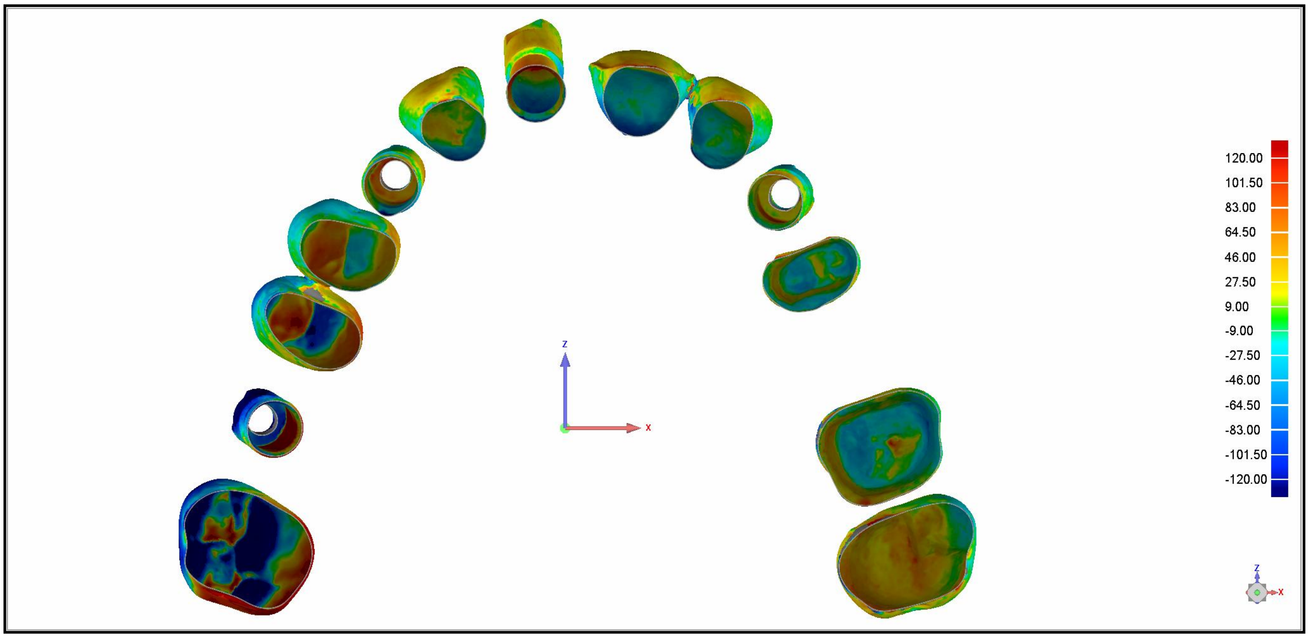

Predefinido: Atrás

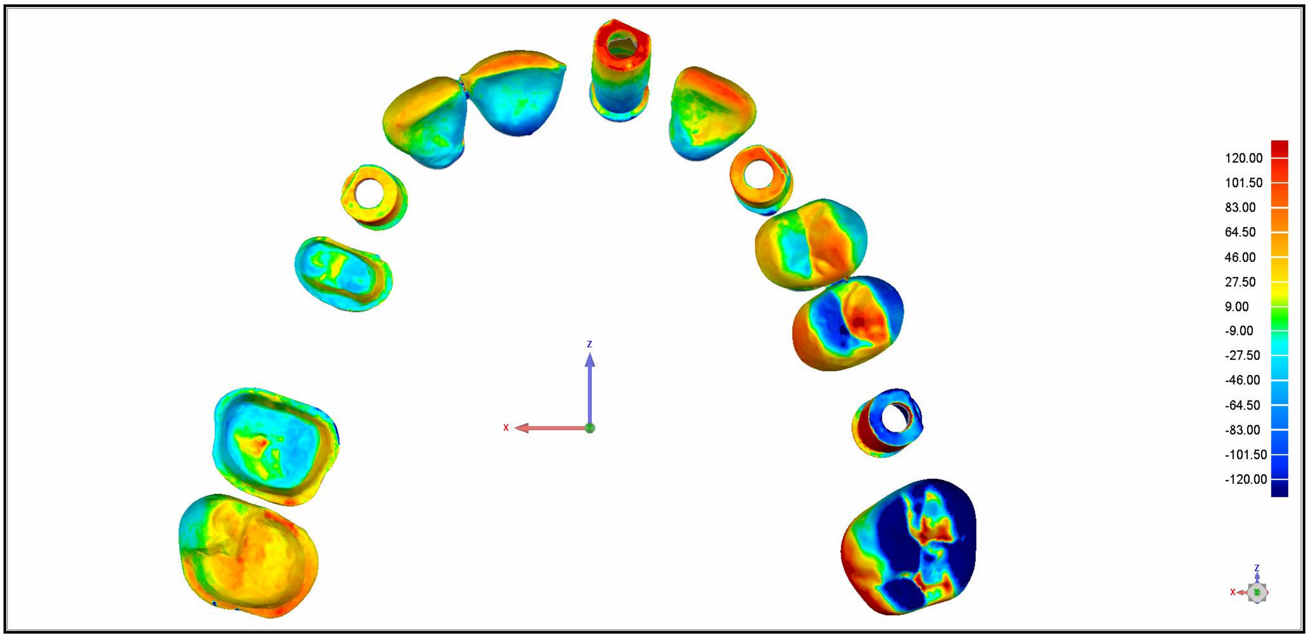

Predefinido: Izquierda

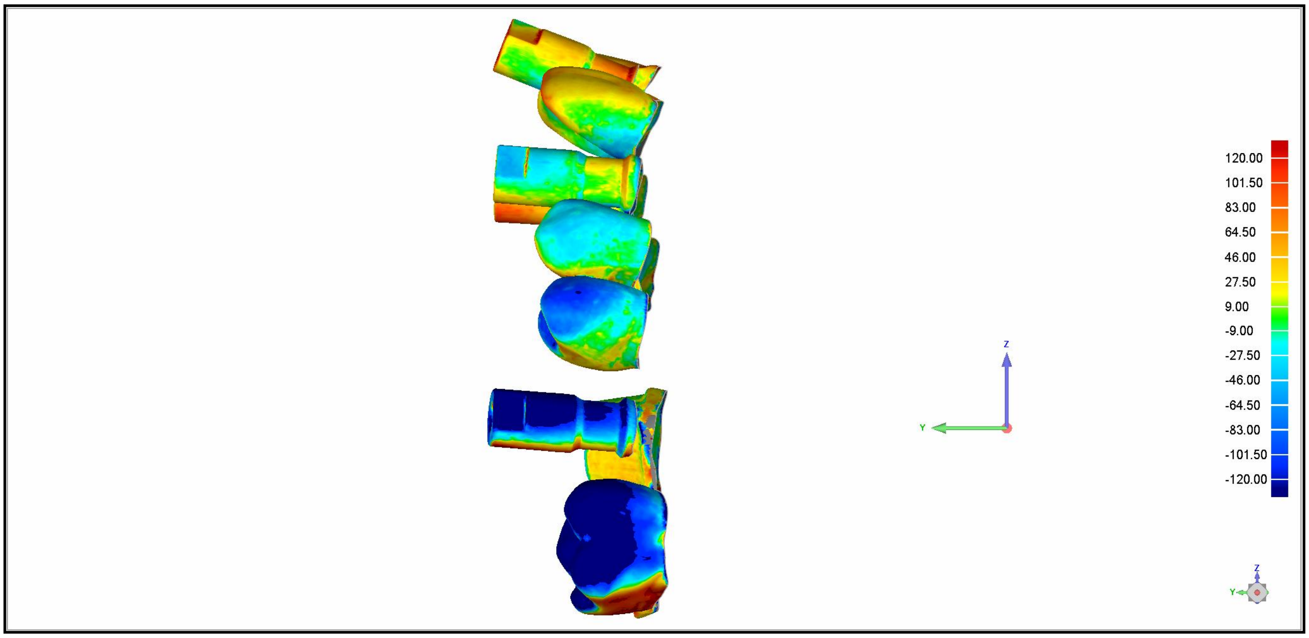

Predefinido: Derecha

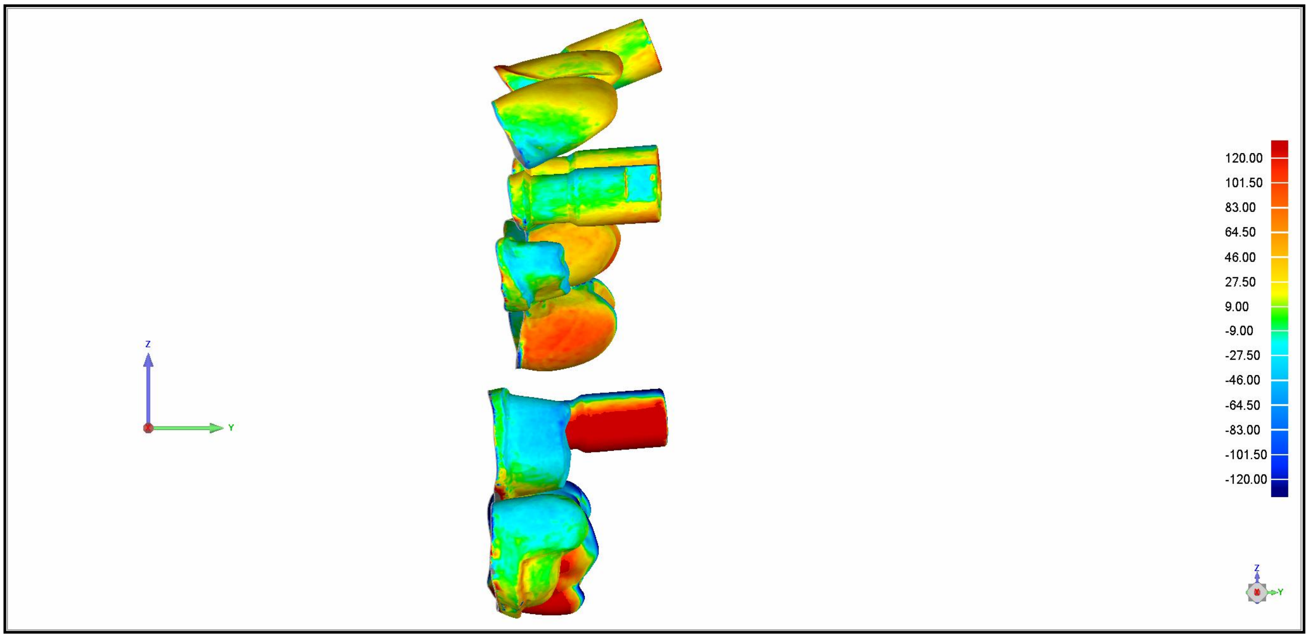

Predefinido: Superior

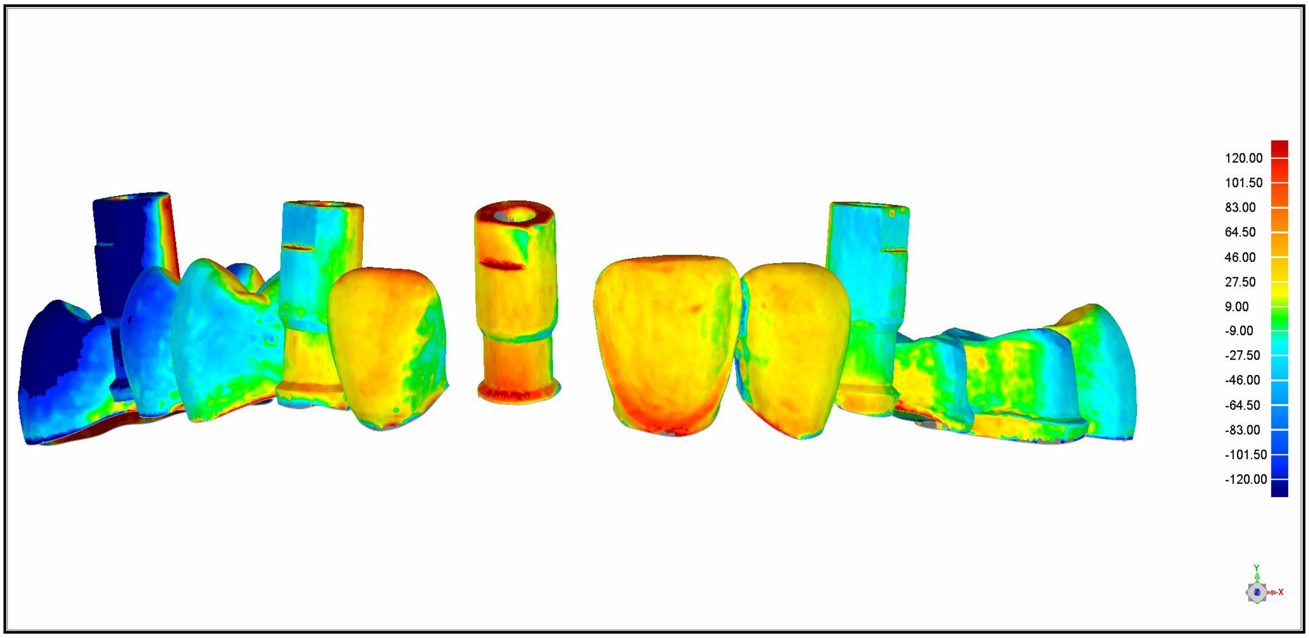

Predefinido: Inferior

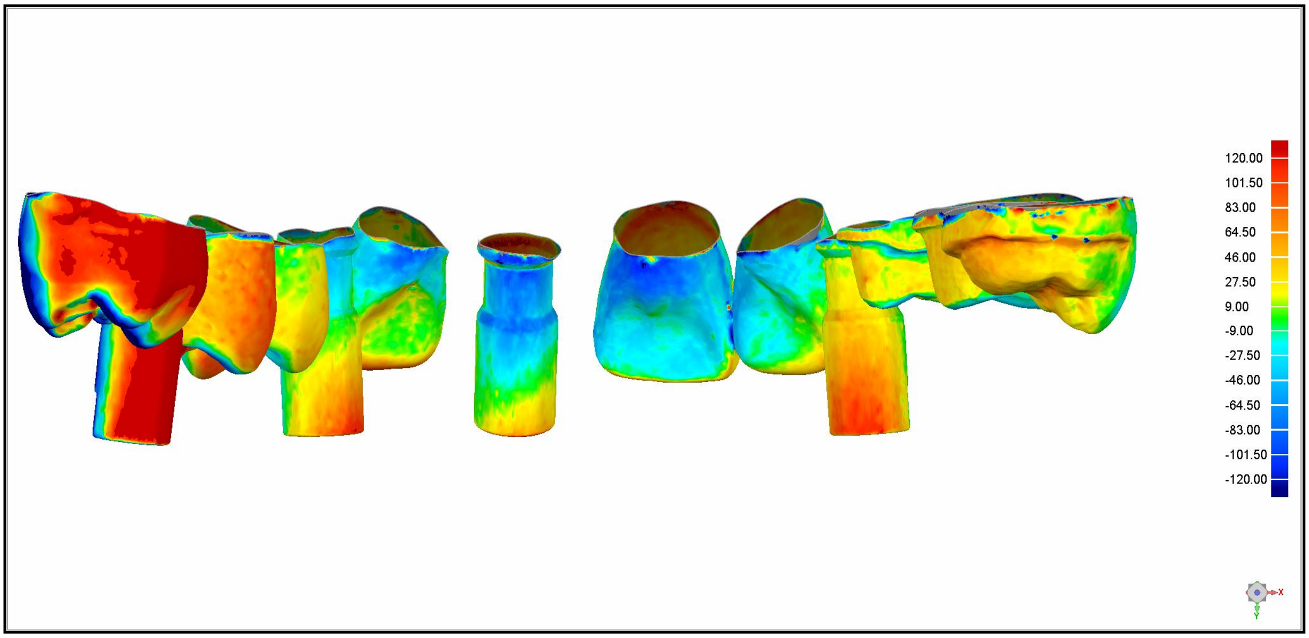

Ajuste de ubicación: Desviaciones superior e inferior

Unidades: u

Nombre	Desv	Estado	Superior Tol	Inferior Tol	Ref X	Ref Y	Ref Z	Radio	Desv X	Desv Y	Desv Z	Medido X	Medido Y	Medido Z	Dir. proy. X	Dir. proy. Y	Dir. proy. Z
Desv. inferior	-2172.87				29707.64	29539.35	-13912.95	n/a	-682.35	-2062.95	1.99	29025.29	27476.41	-13910.96	0.31	0.95	-0.00
Desv. superior	2729.57				20008.28	28145.06	-832.97	n/a	1215.01	993.17	2233.36	21223.29	29138.23	1400.39	0.45	0.36	0.82
