# Supplementary material for: Accuracy of four digital scanners according to scanning strategy in complete-arch impressions
Source: PLoS One. 2018 Sep 13;13(9):e0202916. doi: 10.1371/journal.pone.0202916 (PMC6136706; doi:10.1371/journal.pone.0202916)
Supplement: S16 Table — True definition (scanning strategy D). (ZIP) [file pone.0202916.s016.zip › S16/TD5D.pdf]

3D Comparación Resultados

Modelo referencia	MRC
Modelo test	TD5D
Nº de puntos de datos	128310
# Aislados	562

Tipo tolerancia	3D desviación
Unidades	u
Máx. crítico	120.00
Máx. nominal	15.00
Mín. nominal	-15.00
Mín. crítico	-120.00

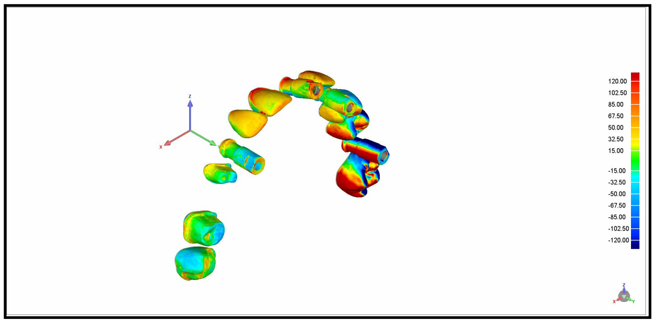

Desviación	
Desviación superior máx.	2269.42
Desviación inferior máx.	-3040.20
Desviación media	68.91 /-70.26
Desviación estándar	108.56

Distribución desviación

>=Min	<Max	# Puntos	%
-120.00	-102.50	1069	0.83
-102.50	-85.00	1433	1.12
-85.00	-67.50	2239	1.74
-67.50	-50.00	4407	3.43
-50.00	-32.50	7810	6.09
-32.50	-15.00	10071	7.85
-15.00	15.00	26808	20.89
15.00	32.50	16641	12.97
32.50	50.00	14366	11.20
50.00	67.50	9367	7.30
67.50	85.00	5488	4.28
85.00	102.50	3880	3.02
102.50	120.00	2774	2.16

Fuera del crítico superior	12176	9.49
Fuera del crítico inferior	9781	7.62

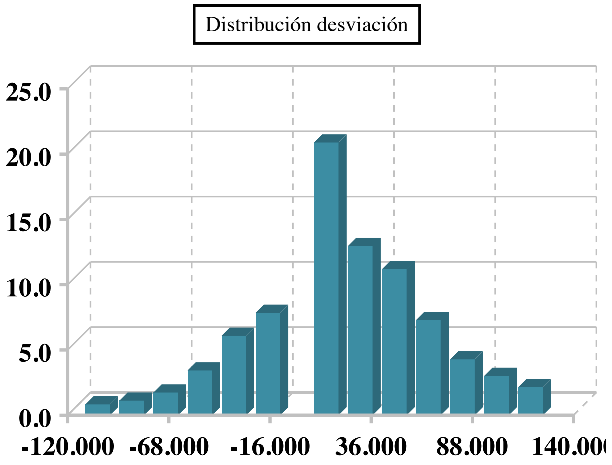

Desviaciones estándar

Distribución (+/-)	# Puntos	%
-6 * Desv. estánd.	44	0.03
-5 * Desv. estánd.	27	0.02
-4 * Desv. estánd.	644	0.50
-3 * Desv. estánd.	5149	4.01
-2 * Desv. estánd.	5630	4.39
-1 * Desv. estánd.	52141	40.64
1 * Desv. estánd.	52965	41.28
2 * Desv. estánd.	7057	5.50
3 * Desv. estánd.	3363	2.62
4 * Desv. estánd.	1192	0.93
5 * Desv. estánd.	20	0.02
6 * Desv. estánd.	78	0.06

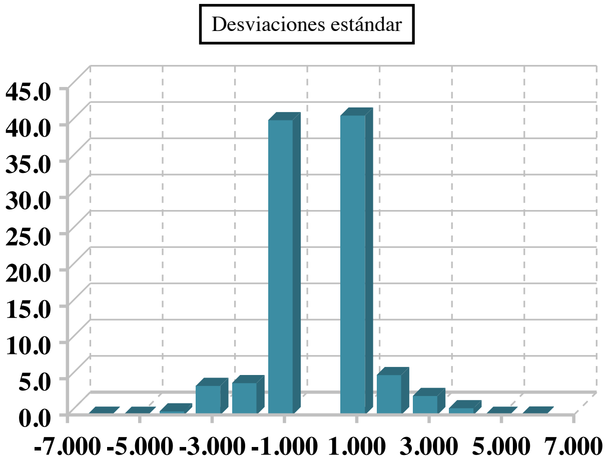

Predefinido: Isométrico

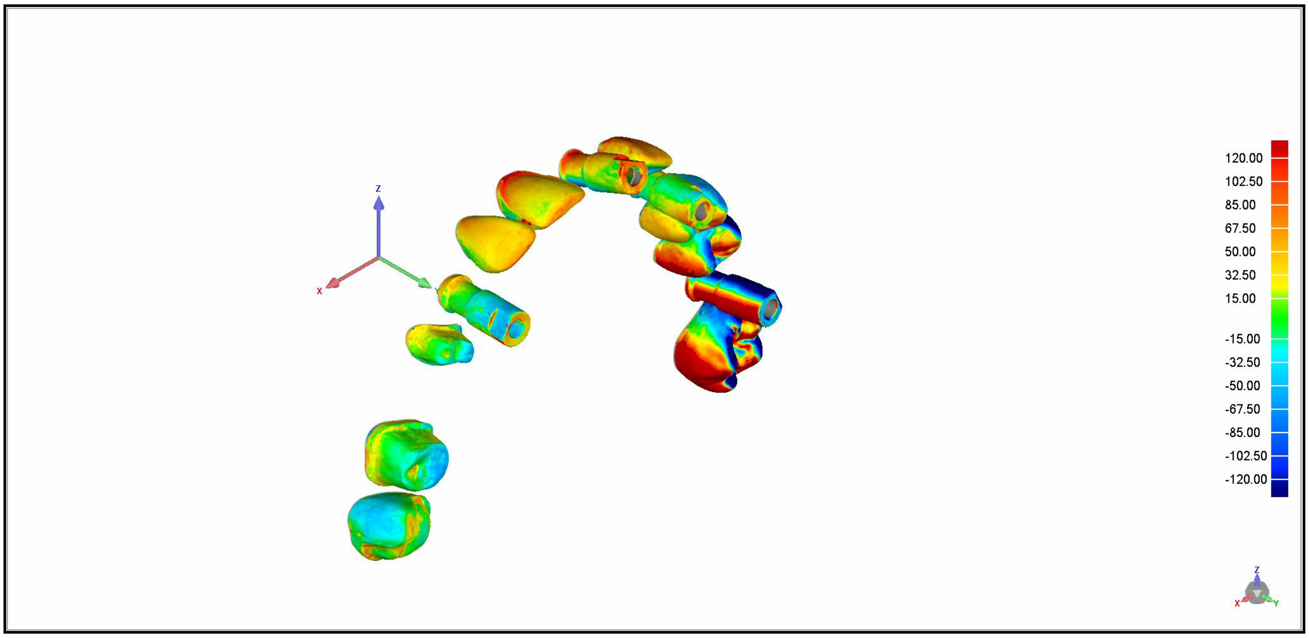

Predefinido: Frente

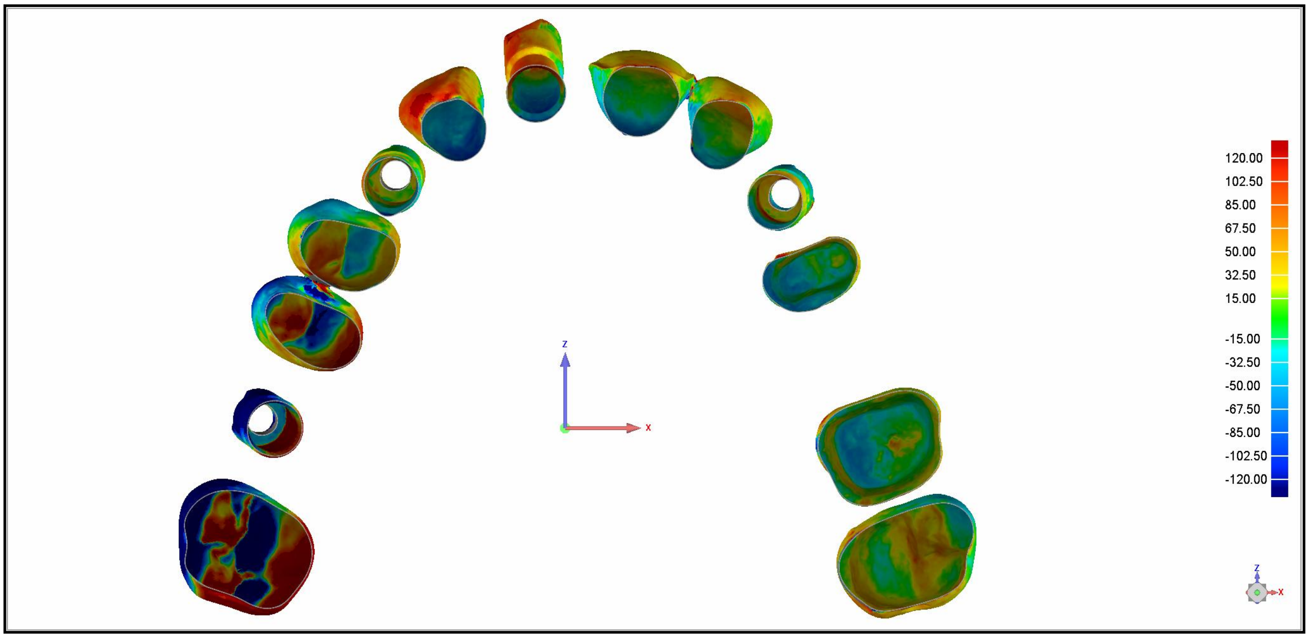

Predefinido: Atrás

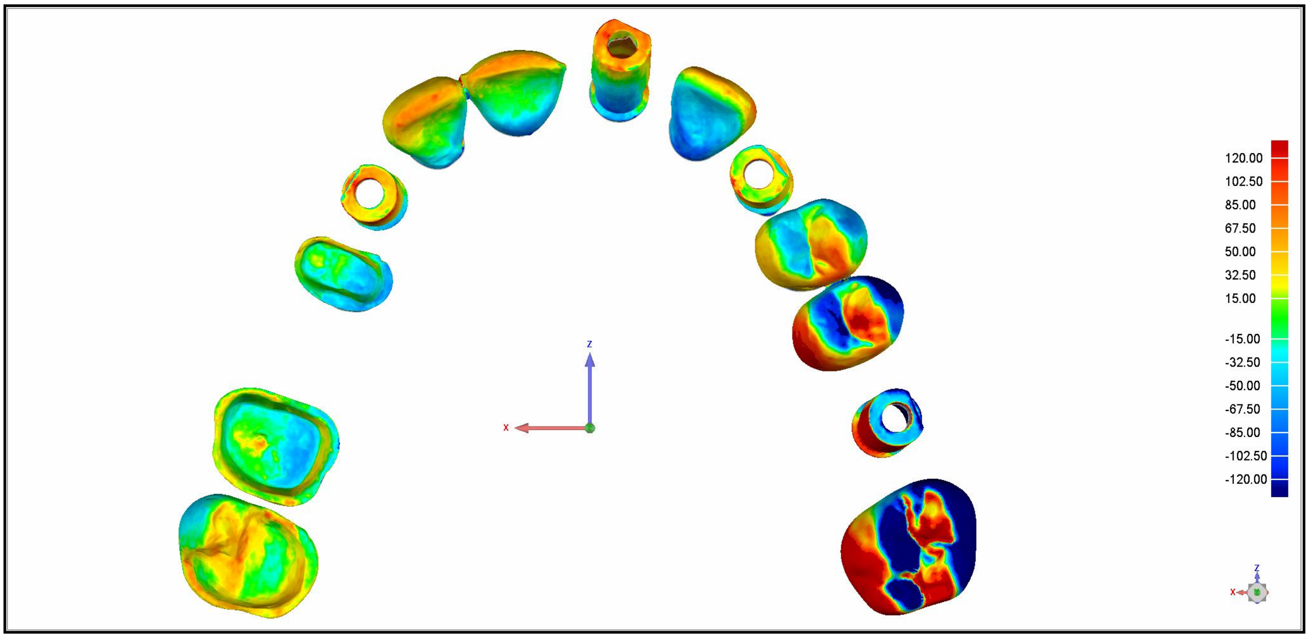

Predefinido: Izquierda

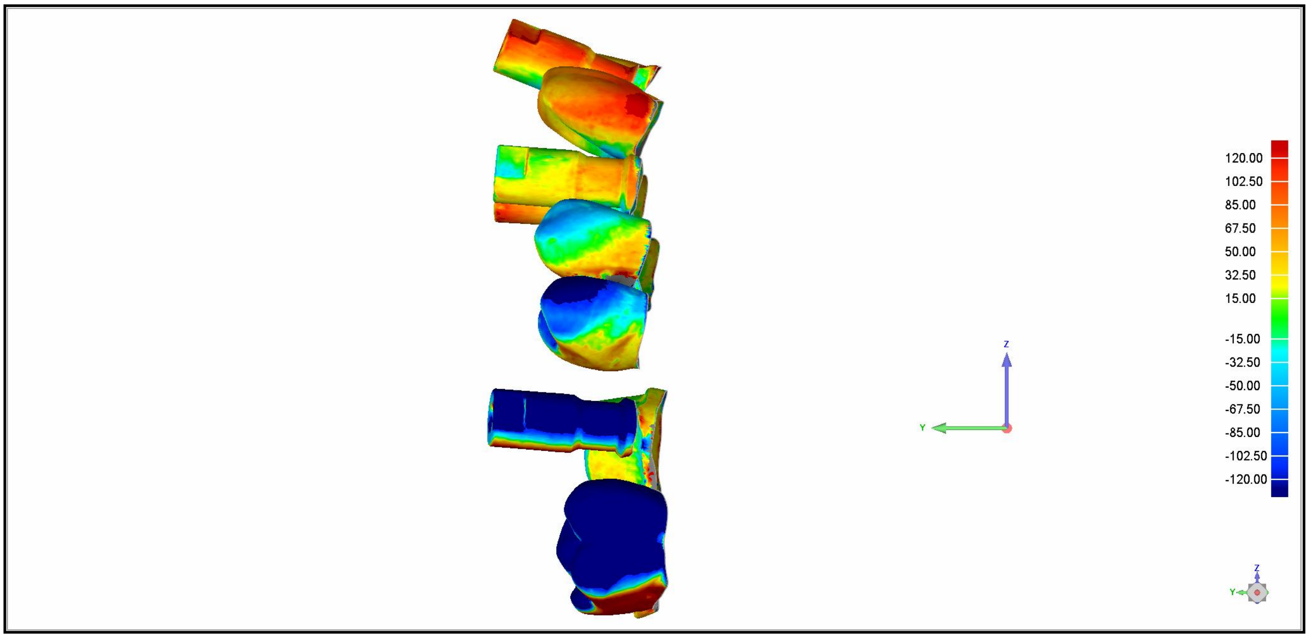

Predefinido: Derecha

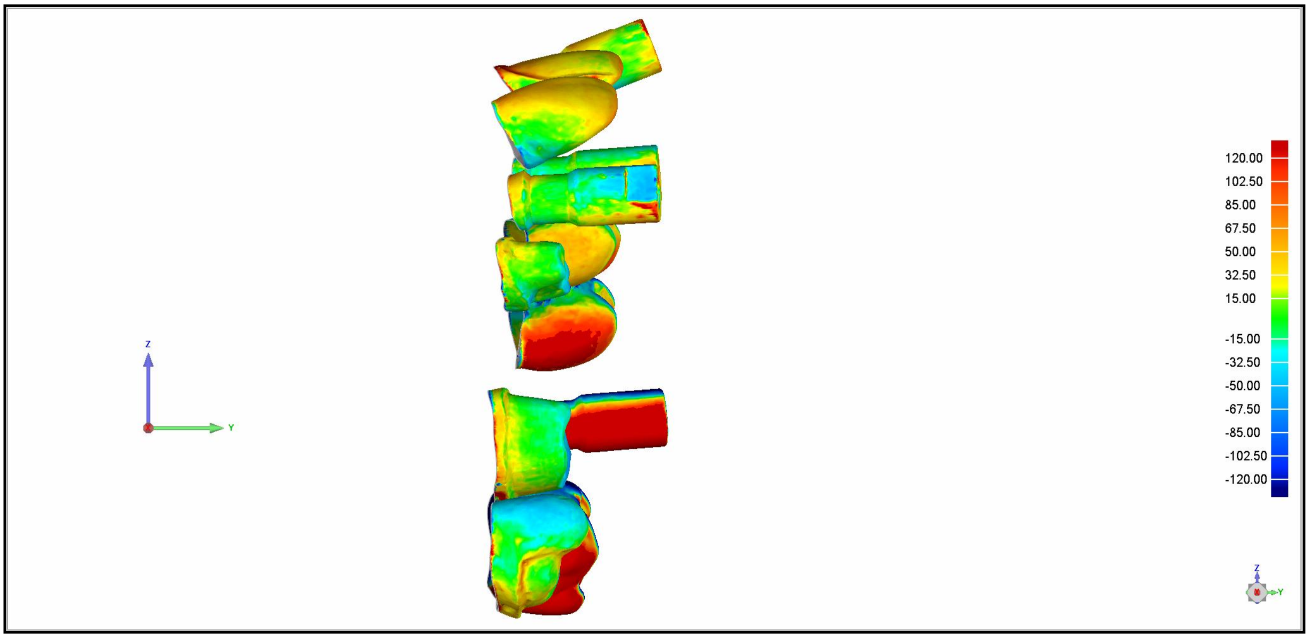

Predefinido: Superior

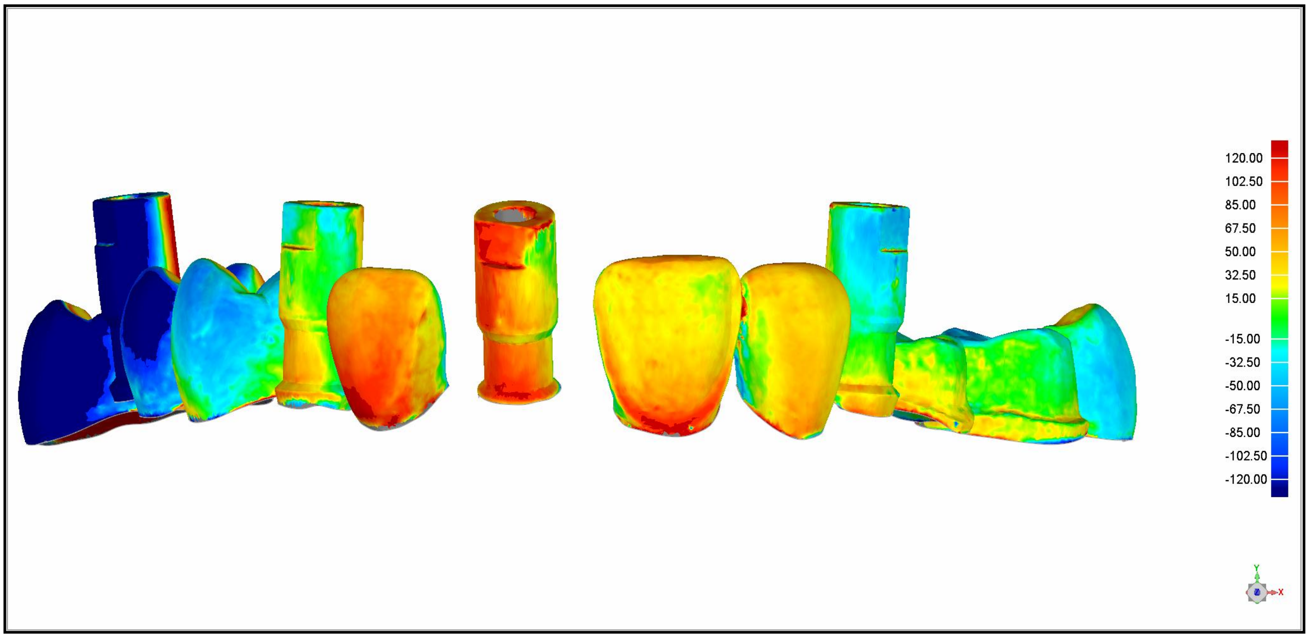

Predefinido: Inferior

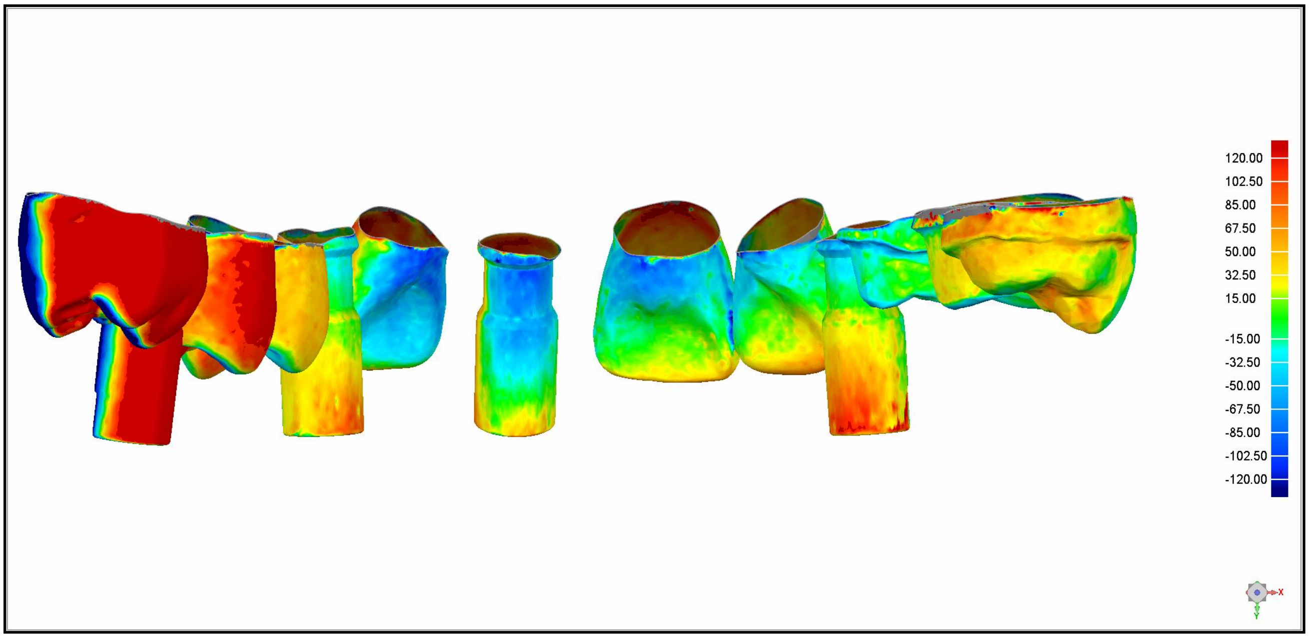

Geomagic Control™

Ajuste de ubicación: Desviaciones superior e inferior

Unidades: u

Nombre	Desv	Estado	Superior Tol	Inferior Tol	Ref X	Ref Y	Ref Z	Radio	Desv X	Desv Y	Desv Z	Medido X	Medido Y	Medido Z	Dir. proy. X	Dir. proy. Y	Dir. proy. Z
Desv. inferior	-3040.20				20451.44	27940.78	-2791.89	n/a	23.86	70.34	3039.29	20475.30	28011.12	247.39	-0.01	-0.02	-1.00
Desv. superior	2269.42				23193.66	28248.20	-8244.19	n/a	741.86	-429.83	2101.23	23935.51	27818.37	-6142.96	0.33	-0.19	0.93
