# Supplementary material for: Accuracy of four digital scanners according to scanning strategy in complete-arch impressions
Source: PLoS One. 2018 Sep 13;13(9):e0202916. doi: 10.1371/journal.pone.0202916 (PMC6136706; doi:10.1371/journal.pone.0202916)
Supplement: S16 Table — True definition (scanning strategy D). (ZIP) [file pone.0202916.s016.zip › S16/TD9D.pdf]

Geomagic Control™

Fecha: 3/6/2017, 4:01 pm

3D Comparación Resultados

Modelo referencia	MRC
Modelo test	TD9D
Nº de puntos de datos	134261
# Aislados	363

Tipo tolerancia	3D desviación
Unidades	u
Máx. crítico	120.00
Máx. nominal	23.00
Mín. nominal	-23.00
Mín. crítico	-120.00

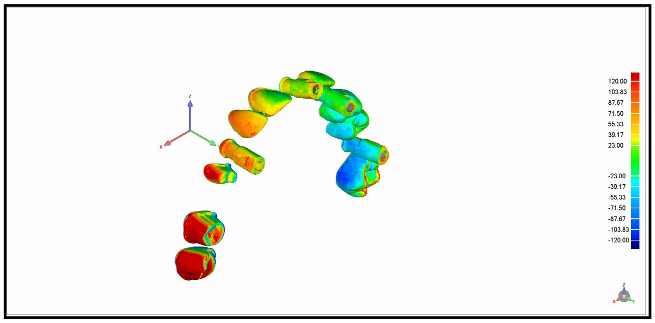

Desviación	
Desviación superior máx.	2179.83
Desviación inferior máx.	-2038.72
Desviación media	66.52 /-44.13
Desviación estándar	97.59

Distribución desviación

>=Min	<Max	# Puntos	%
-120.00	-103.83	861	0.64
-103.83	-87.67	1401	1.04
-87.67	-71.50	2420	1.80
-71.50	-55.33	3536	2.63
-55.33	-39.17	5892	4.39
-39.17	-23.00	9389	6.99
-23.00	23.00	51570	38.41
23.00	39.17	13118	9.77
39.17	55.33	9867	7.35
55.33	71.50	6916	5.15
71.50	87.67	5578	4.15
87.67	103.83	3922	2.92
103.83	120.00	3067	2.28

Fuera del crítico superior	12666	9.43
Fuera del crítico inferior	4058	3.02

Distribución desviación

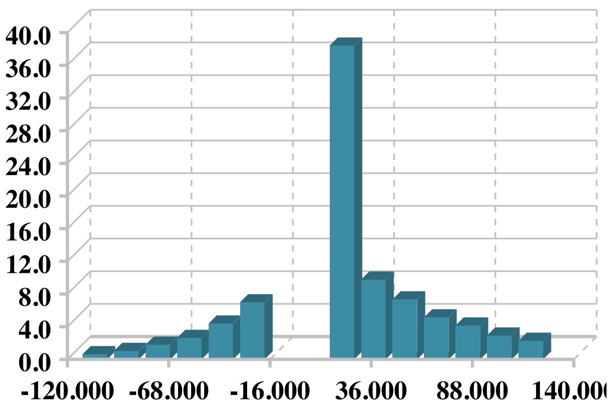

Desviaciones estándar

Distribución (+/-)	# Puntos	%
-6 * Desv. estánd.	99	0.07
-5 * Desv. estánd.	80	0.06
-4 * Desv. estánd.	158	0.12
-3 * Desv. estánd.	2264	1.69
-2 * Desv. estánd.	5666	4.22
-1 * Desv. estánd.	71314	53.12
1 * Desv. estánd.	42186	31.42
2 * Desv. estánd.	8098	6.03
3 * Desv. estánd.	3734	2.78
4 * Desv. estánd.	178	0.13
5 * Desv. estánd.	118	0.09
6 * Desv. estánd.	366	0.27

Desviaciones estándar

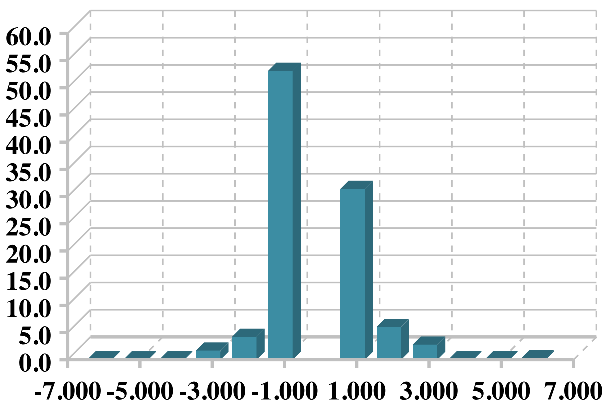

Predefinido: Isométrico

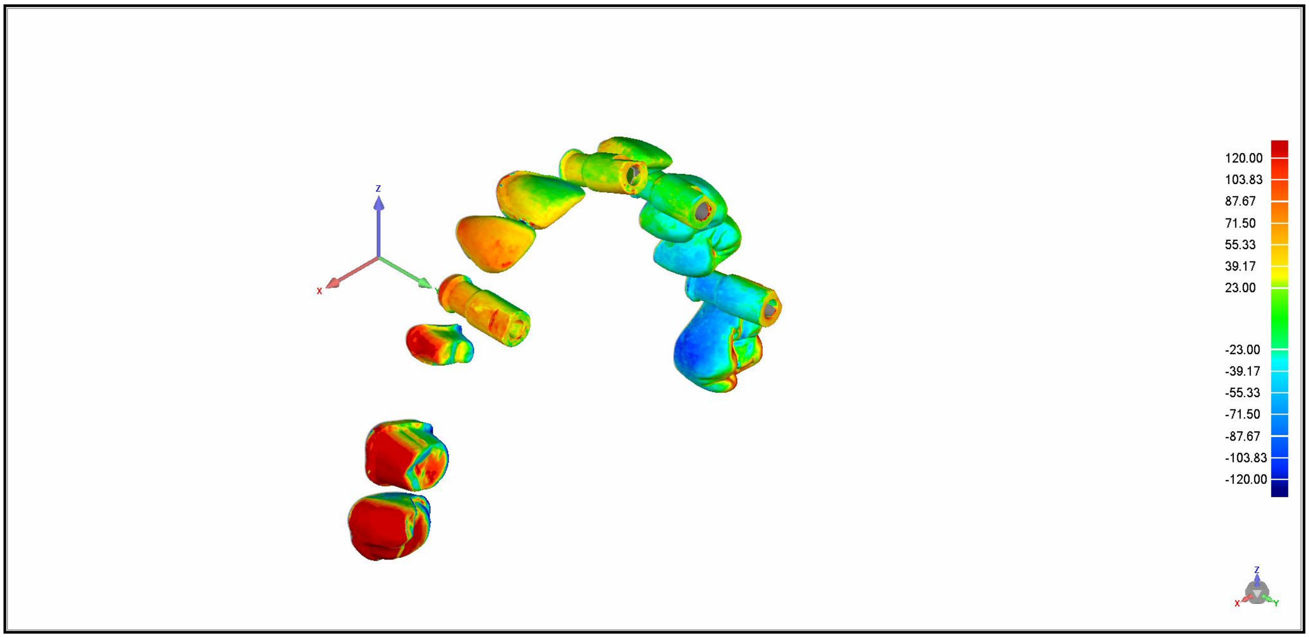

Predefinido: Frente

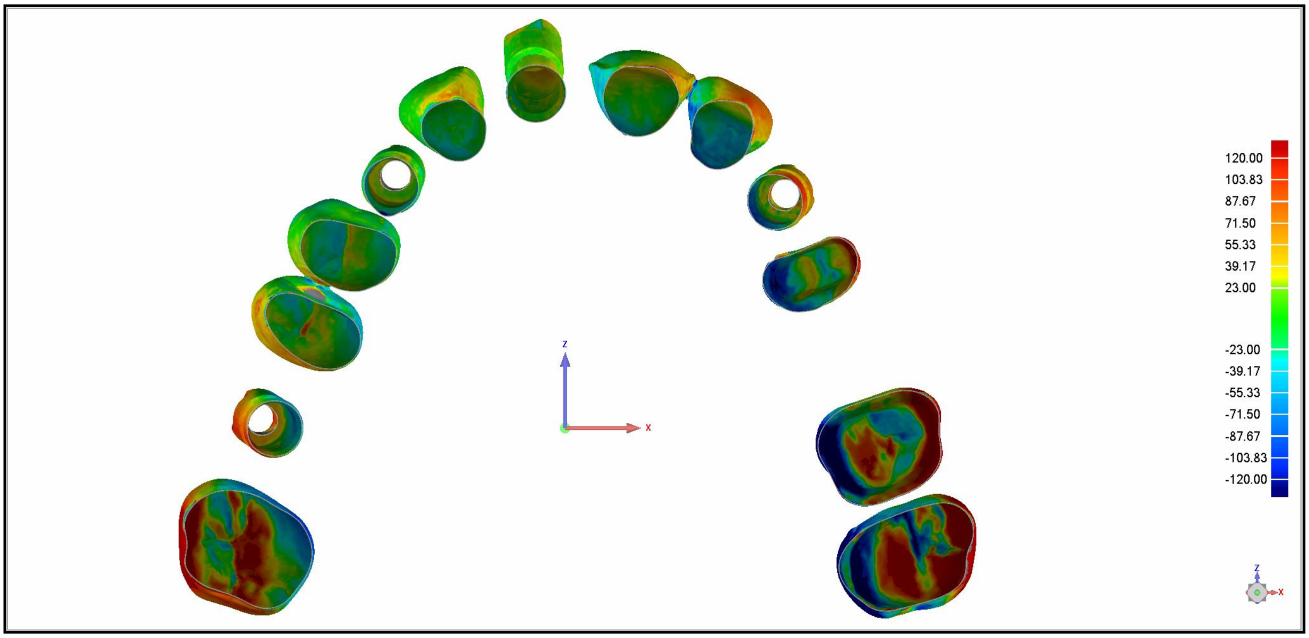

Predefinido: Atrás

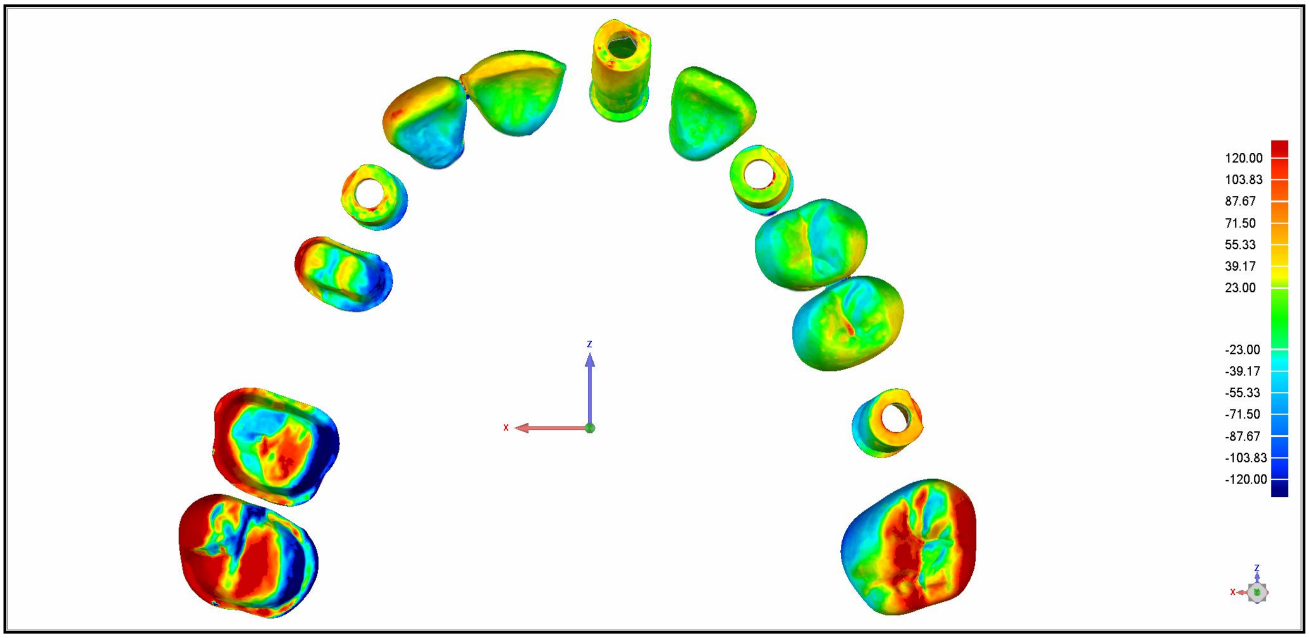

Predefinido: Izquierda

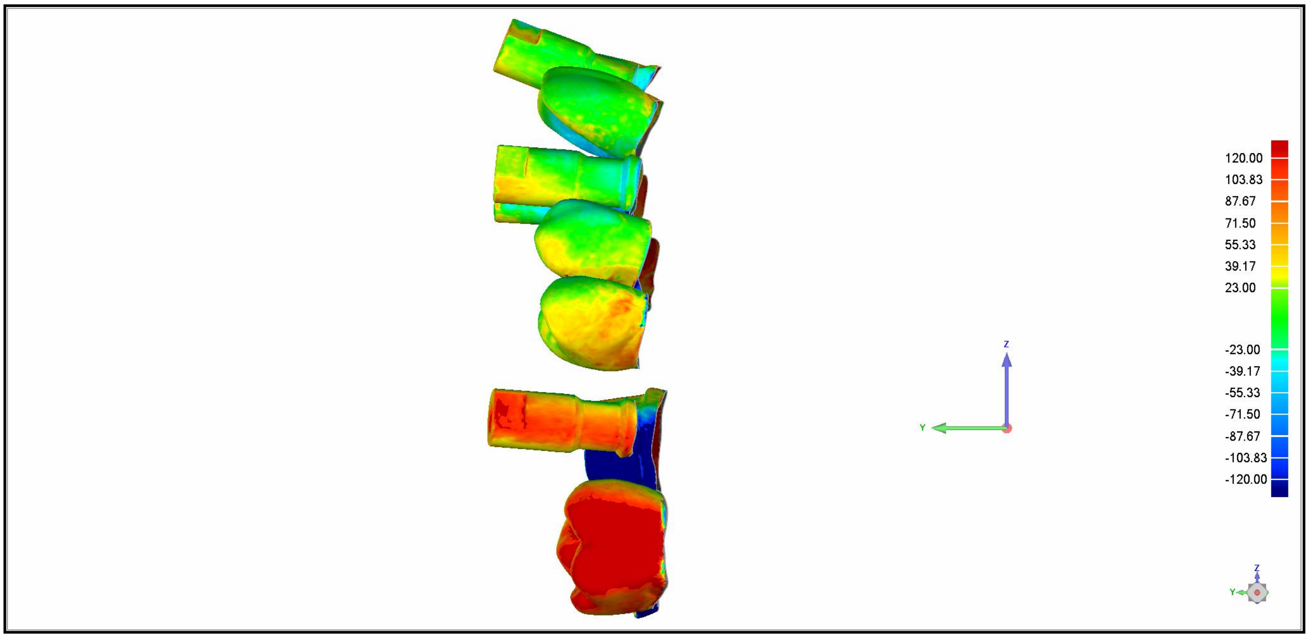

Predefinido: Derecha

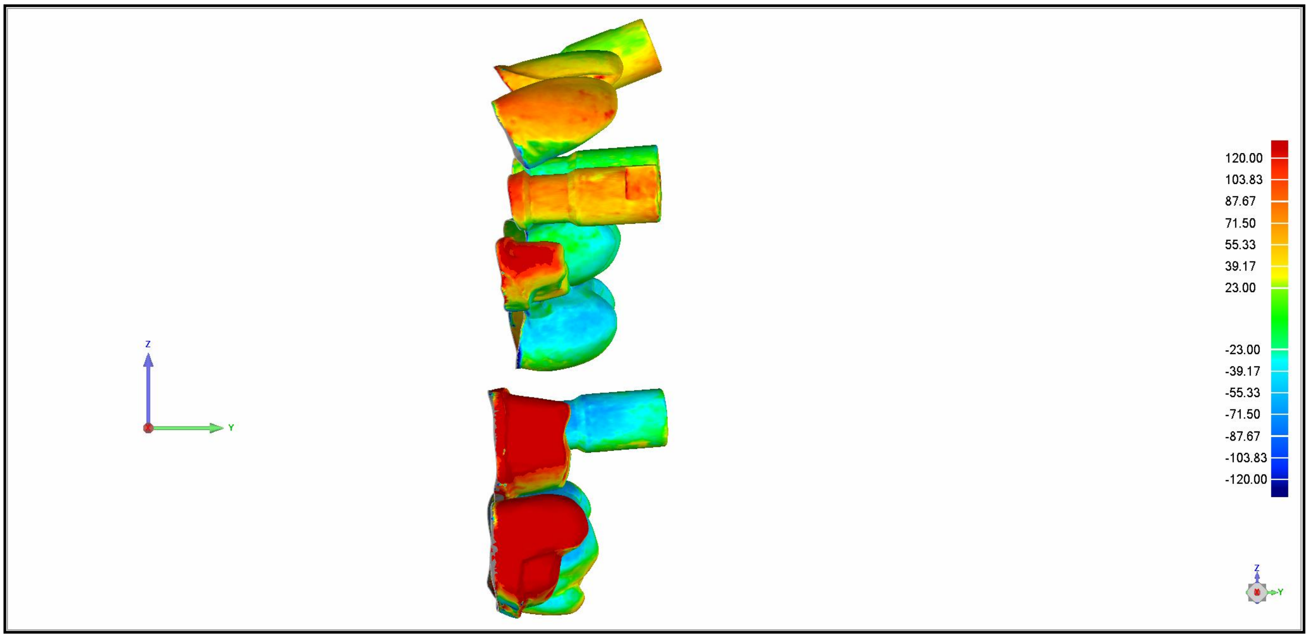

Predefinido: Superior

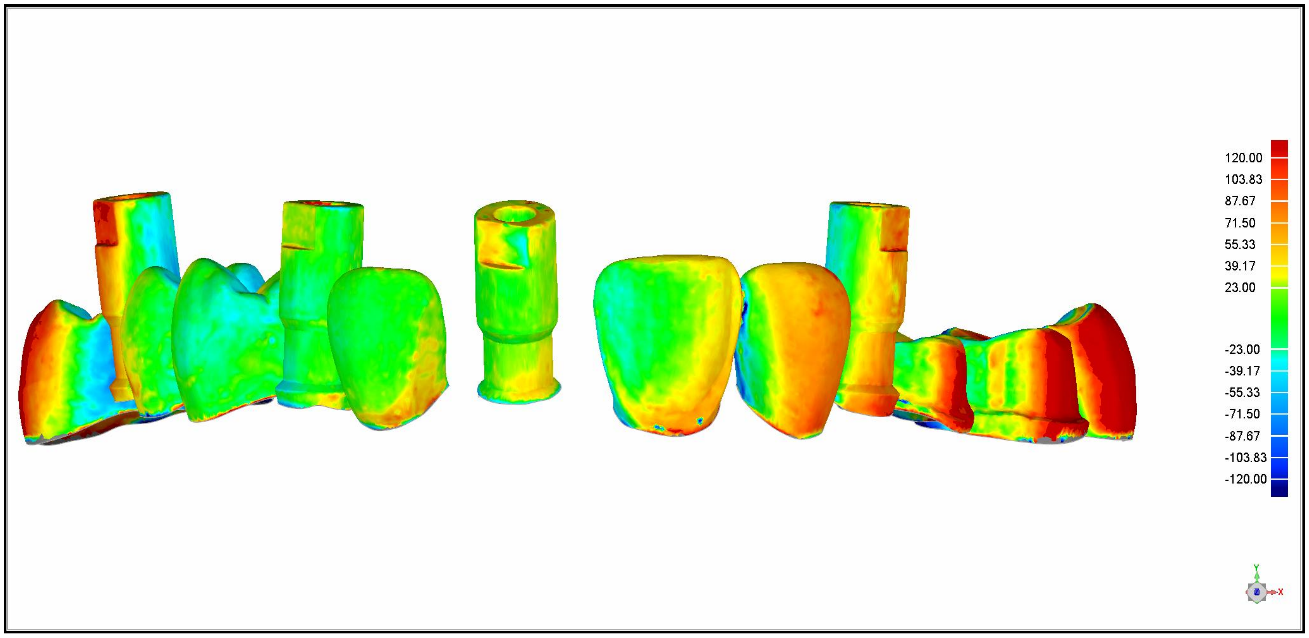

Predefinido: Inferior

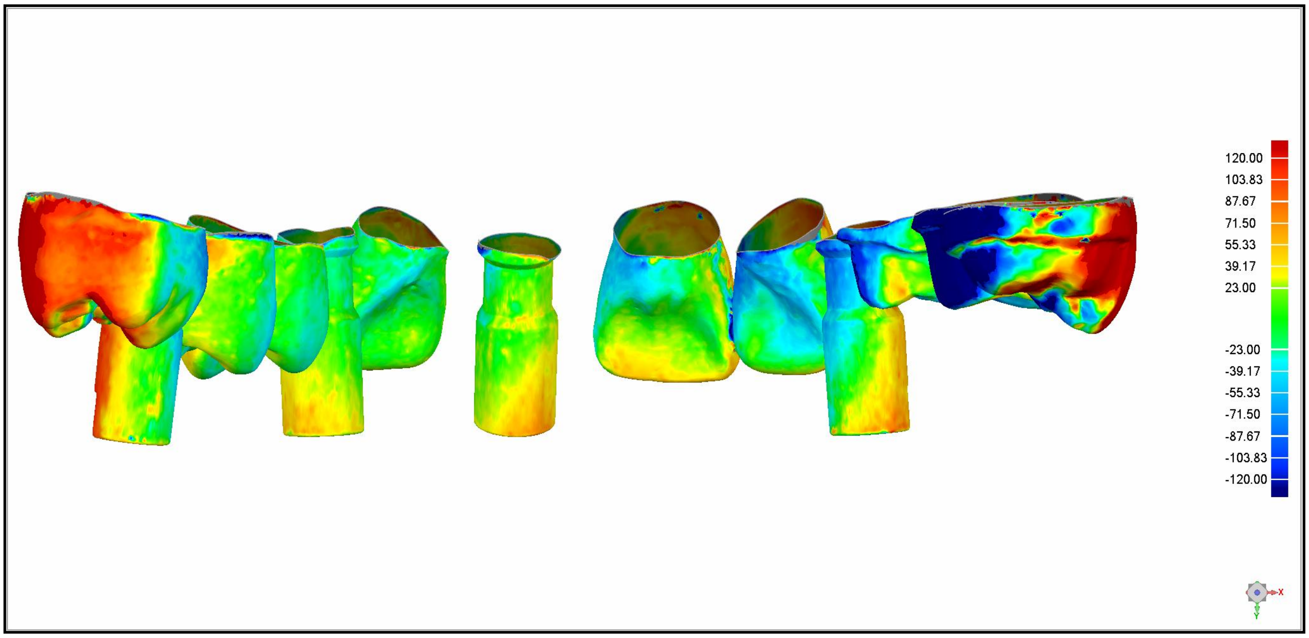

Ajuste de ubicación: Desviaciones superior e inferior

Unidades: u

Nombre	Desv	Estado	Superior Tol	Inferior Tol	Ref X	Ref Y	Ref Z	Radio	Desv X	Desv Y	Desv Z	Medido X	Medido Y	Medido Z	Dir. proy. X	Dir. proy. Y	Dir. proy. Z
Desv. inferior	-2038.72				29715.55	29554.58	-13882.37	n/a	-273.99	-2013.78	161.25	29441.56	27540.80	-13721.12	0.13	0.99	-0.08
Desv. superior	2179.83				-27689.92	27388.00	-5061.48	n/a	-1877.60	-231.71	-1082.87	-29567.53	27156.29	-6144.35	-0.86	-0.11	-0.50
