# Supplementary material for: Accuracy of four digital scanners according to scanning strategy in complete-arch impressions
Source: PLoS One. 2018 Sep 13;13(9):e0202916. doi: 10.1371/journal.pone.0202916 (PMC6136706; doi:10.1371/journal.pone.0202916)
Supplement: S16 Table — True definition (scanning strategy D). (ZIP) [file pone.0202916.s016.zip › S16/TD10D.pdf]

3D Comparación Resultados

Modelo referencia	MRC
Modelo test	TD10D
Nº de puntos de datos	126022
# Aislados	440

Tipo tolerancia	3D desviación
Unidades	u
Máx. crítico	120.00
Máx. nominal	12.00
Mín. nominal	-12.00
Mín. crítico	-120.00

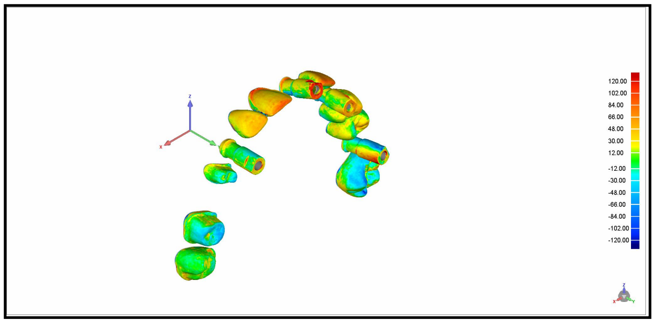

Desviación	
Desviación superior máx.	2224.78
Desviación inferior máx.	-2716.46
Desviación media	33.51 /-25.34
Desviación estándar	57.30

Distribución desviación

>=Min	<Max	# Puntos	%
-120.00	-102.00	139	0.11
-102.00	-84.00	274	0.22
-84.00	-66.00	791	0.63
-66.00	-48.00	2867	2.27
-48.00	-30.00	7947	6.31
-30.00	-12.00	14652	11.63
-12.00	12.00	36843	29.24
12.00	30.00	27186	21.57
30.00	48.00	17541	13.92
48.00	66.00	8405	6.67
66.00	84.00	4230	3.36
84.00	102.00	2151	1.71
102.00	120.00	942	0.75

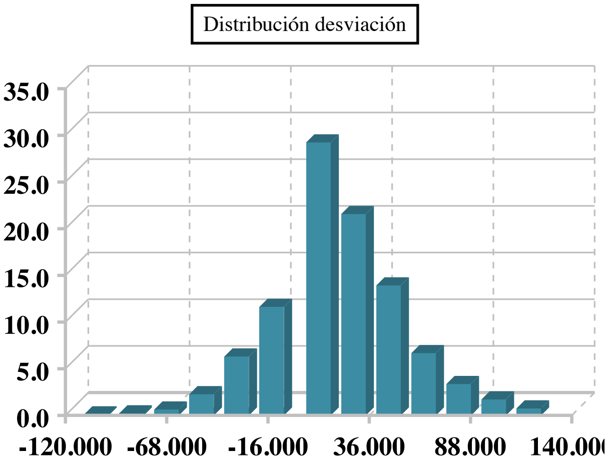

Fuera del crítico superior	1399	1.11
Fuera del crítico inferior	655	0.52

Desviaciones estándar

Distribución (+/-)	# Puntos	%
-6 * Desv. estánd.	90	0.07
-5 * Desv. estánd.	98	0.08
-4 * Desv. estánd.	215	0.17
-3 * Desv. estánd.	391	0.31
-2 * Desv. estánd.	5040	4.00
-1 * Desv. estánd.	59670	47.35
1 * Desv. estánd.	52894	41.97
2 * Desv. estánd.	6456	5.12
3 * Desv. estánd.	702	0.56
4 * Desv. estánd.	219	0.17
5 * Desv. estánd.	131	0.10
6 * Desv. estánd.	116	0.09

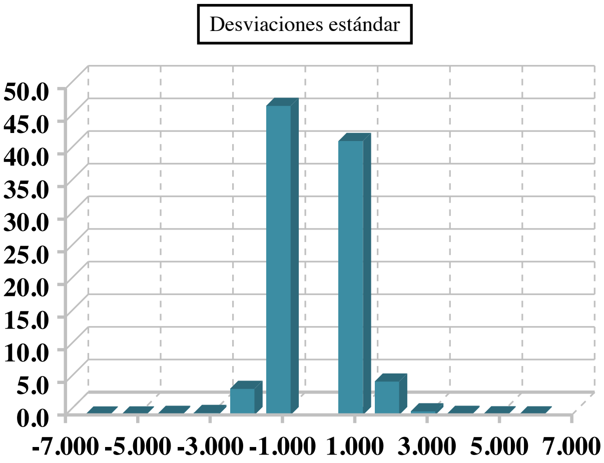

Predefinido: Isométrico

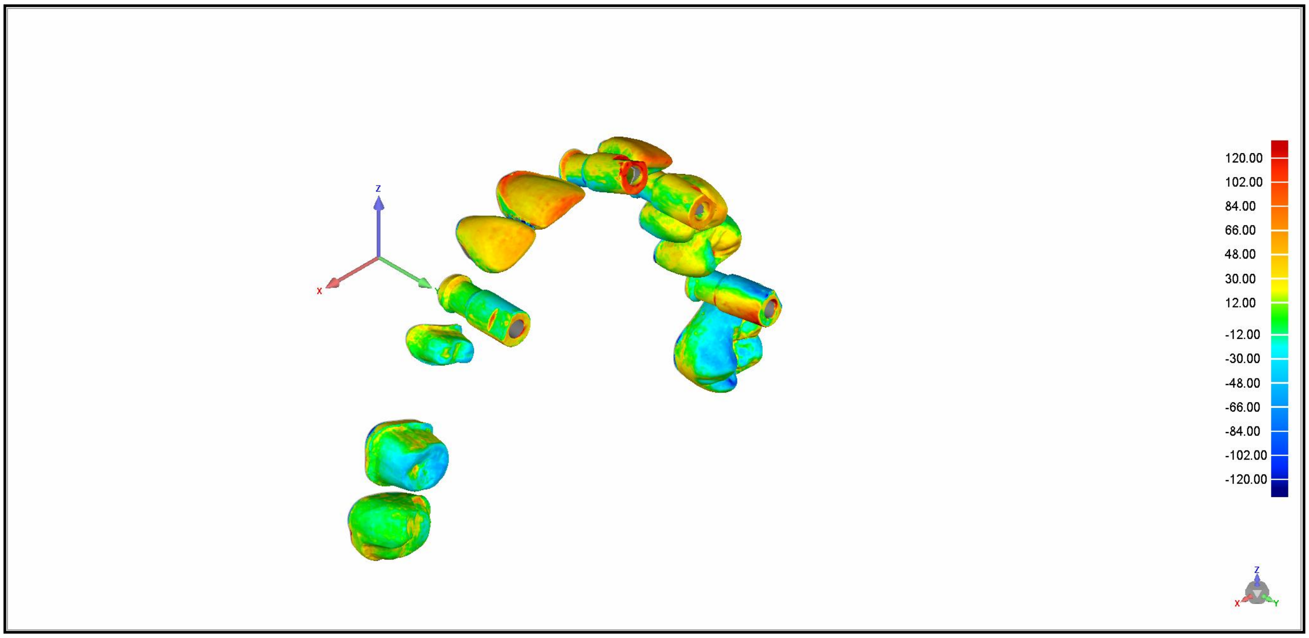

Predefinido: Frente

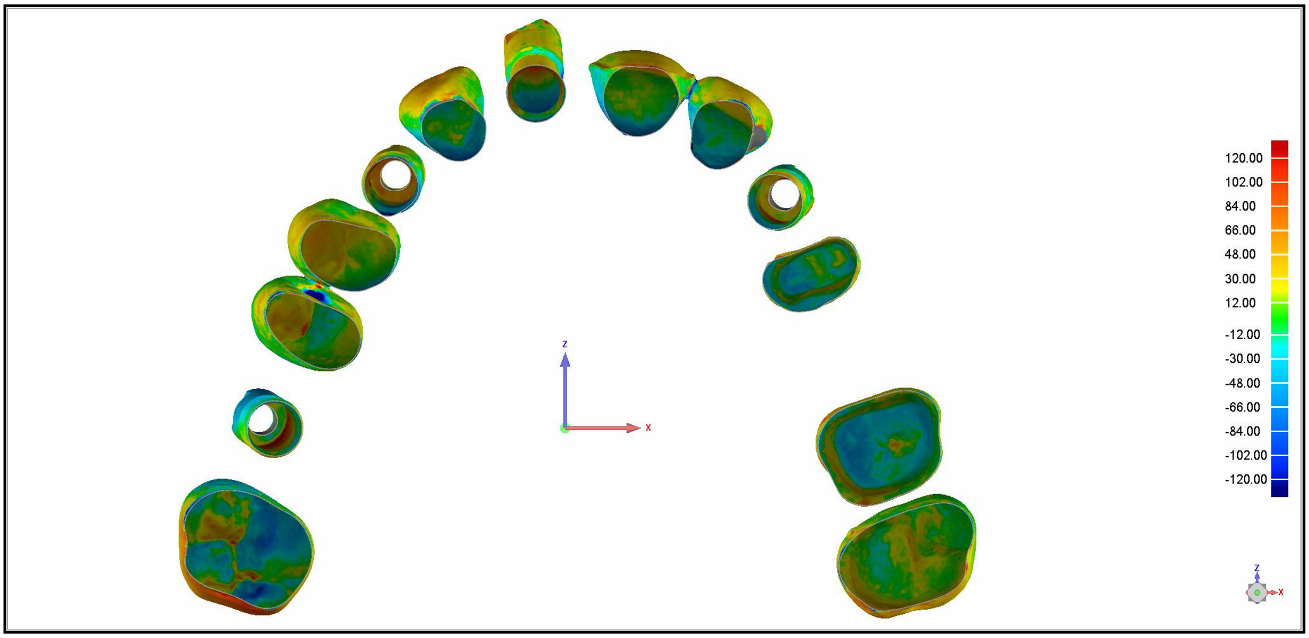

Predefinido: Atrás

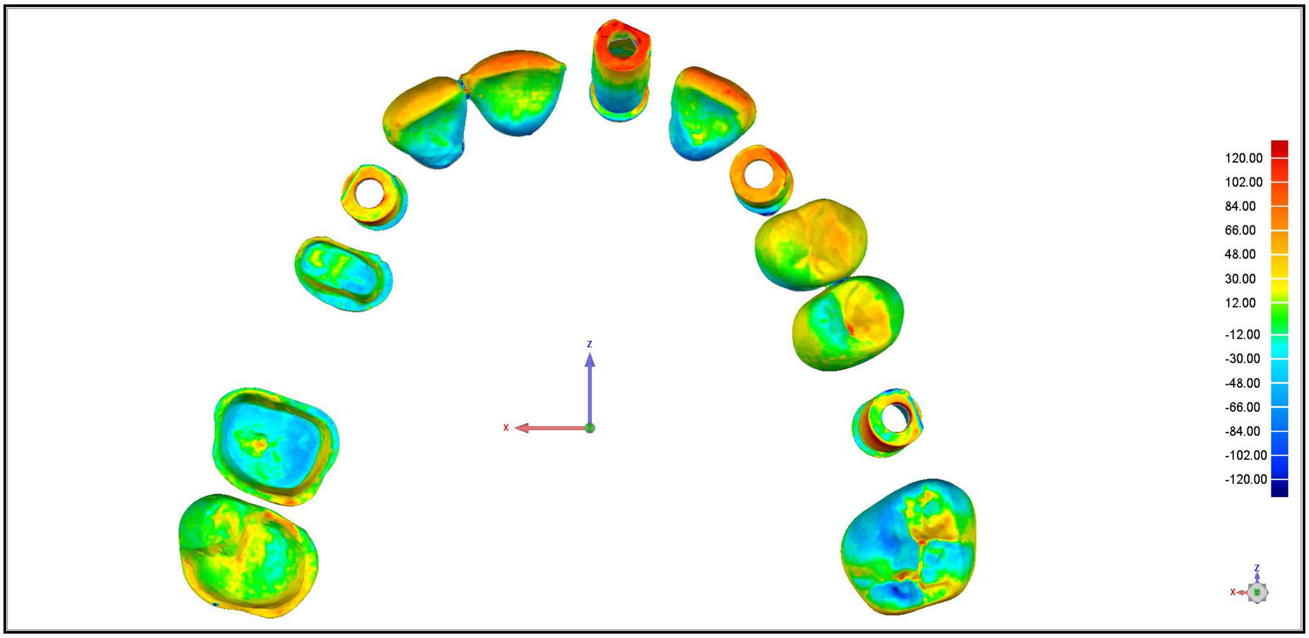

Predefinido: Izquierda

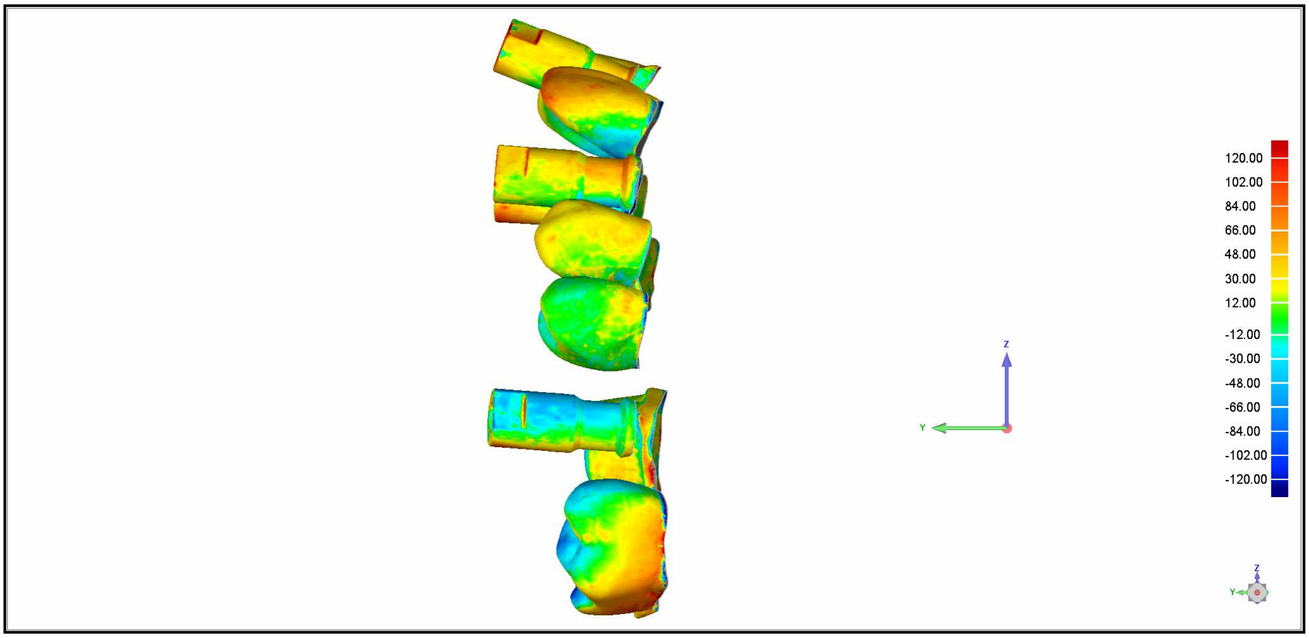

Predefinido: Derecha

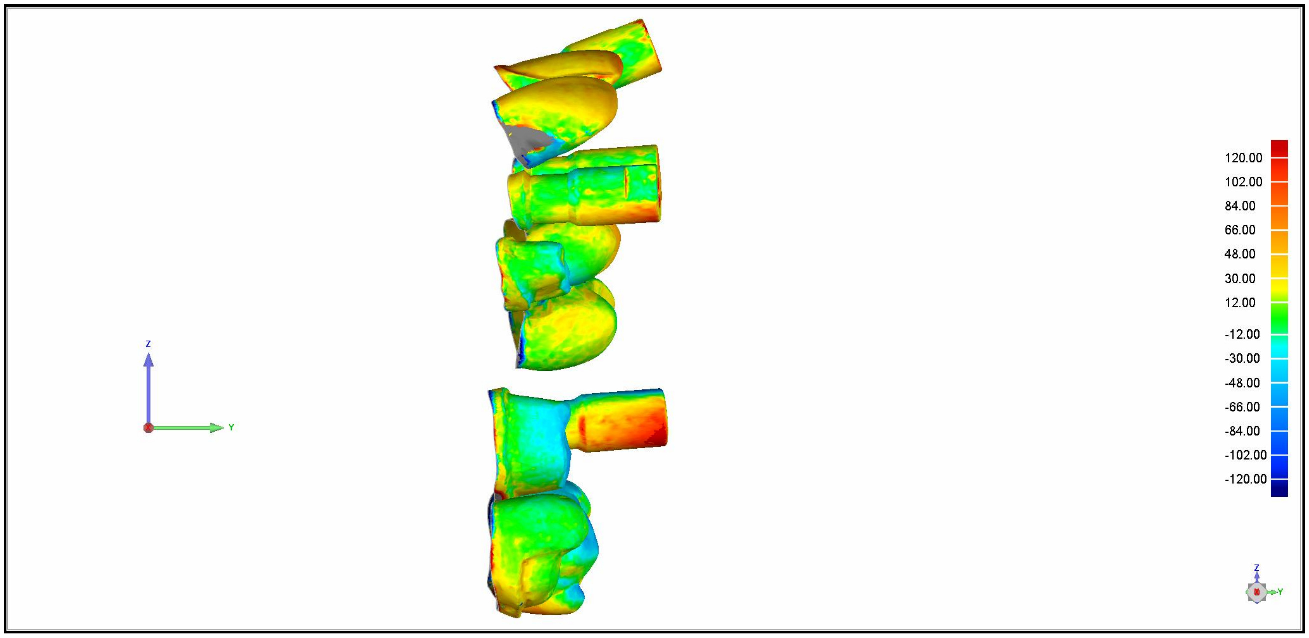

Predefinido: Superior

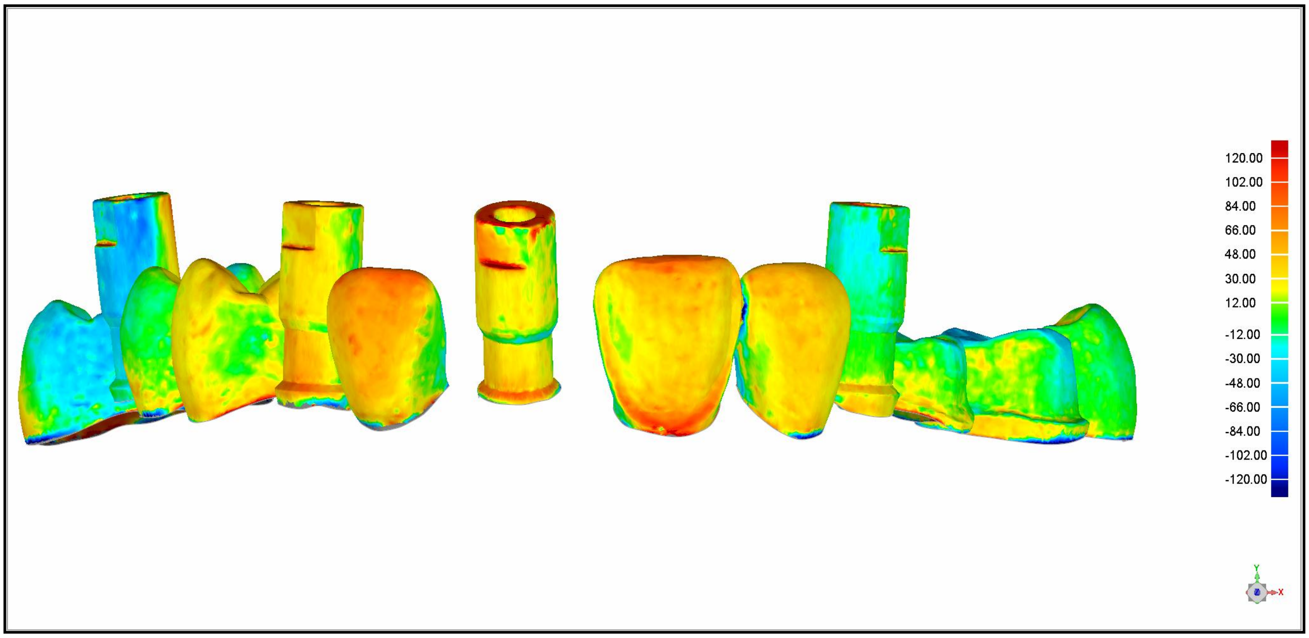

Predefinido: Inferior

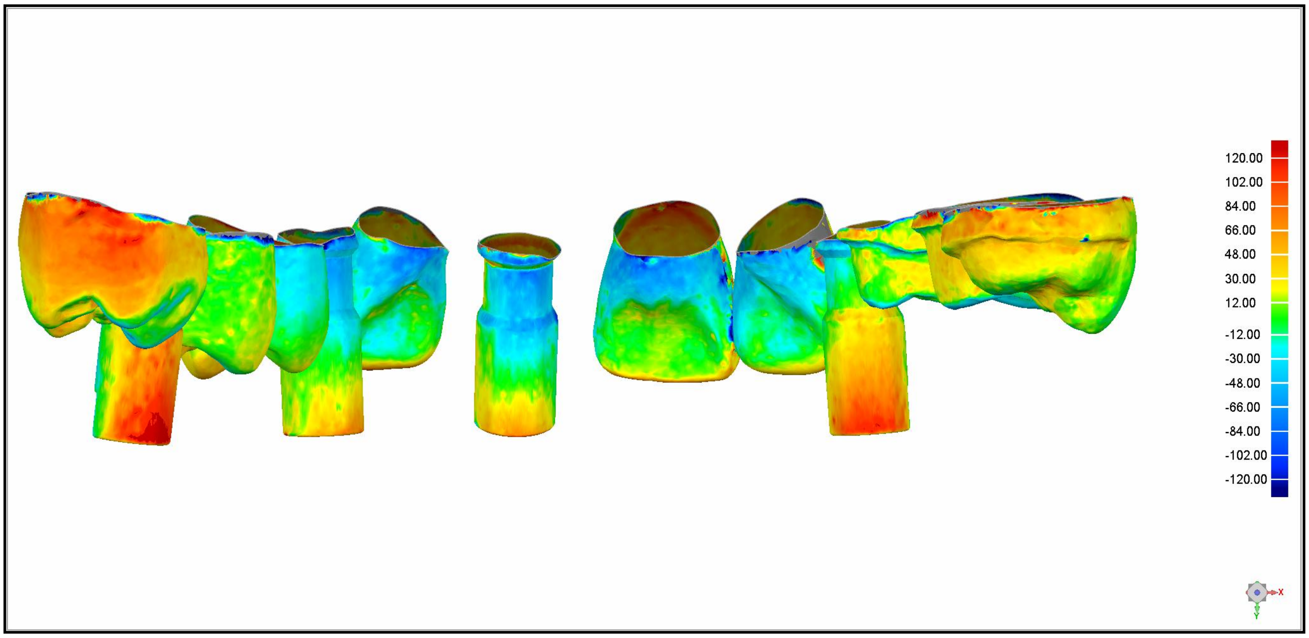

Ajuste de ubicación: Desviaciones superior e inferior

Unidades: u

Nombre	Desv	Estado	Superior Tol	Inferior Tol	Ref X	Ref Y	Ref Z	Radio	Desv X	Desv Y	Desv Z	Medido X	Medido Y	Medido Z	Dir. proy. X	Dir. proy. Y	Dir. proy. Z
Desv. inferior	-2716.46				-29292.33	26884.28	-11910.36	n/a	2315.94	446.53	-1347.67	-26976.39	27330.80	-13258.02	-0.85	-0.16	0.50
Desv. superior	2224.78				15390.07	29830.43	19962.85	n/a	-1066.44	265.38	1934.41	14323.63	30095.81	21897.25	-0.48	0.12	0.87
