# Supplementary material for: Accuracy of four digital scanners according to scanning strategy in complete-arch impressions
Source: PLoS One. 2018 Sep 13;13(9):e0202916. doi: 10.1371/journal.pone.0202916 (PMC6136706; doi:10.1371/journal.pone.0202916)
Supplement: S16 Table — True definition (scanning strategy D). (ZIP) [file pone.0202916.s016.zip › S16/TD6D.pdf]

Fecha: 3/6/2017, 3:46 pm

3D Comparación Resultados

Modelo referencia	MRC
Modelo test	TD6D
Nº de puntos de datos	125805
# Aislados	360

Tipo tolerancia	3D desviación
Unidades	u
Máx. crítico	120.00
Máx. nominal	16.00
Mín. nominal	-16.00
Mín. crítico	-120.00

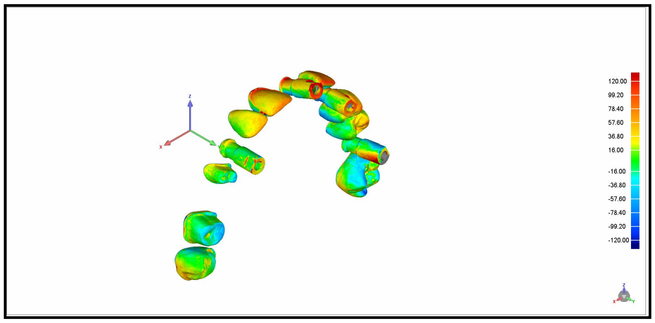

Desviación	
Desviación superior máx.	1885.22
Desviación inferior máx.	-1993.48
Desviación media	39.79 /-28.22
Desviación estándar	57.83

Distribución desviación

>=Min	<Max	# Puntos	%
-120.00	-99.20	314	0.25
-99.20	-78.40	886	0.70
-78.40	-57.60	2892	2.30
-57.60	-36.80	6816	5.42
-36.80	-16.00	13143	10.45
-16.00	16.00	40431	32.14
16.00	36.80	26488	21.05
36.80	57.60	17786	14.14
57.60	78.40	8868	7.05
78.40	99.20	3556	2.83
99.20	120.00	1674	1.33

Fuera del crítico superior	2373	1.89
Fuera del crítico inferior	578	0.46

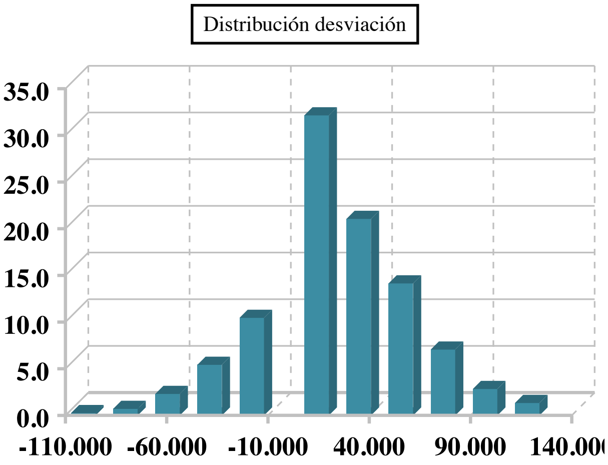

Desviaciones estándar

Distribución (+/-)	# Puntos	%
-6 * Desv. estánd.	77	0.06
-5 * Desv. estánd.	89	0.07
-4 * Desv. estánd.	217	0.17
-3 * Desv. estánd.	517	0.41
-2 * Desv. estánd.	8765	6.97
-1 * Desv. estánd.	56528	44.93
1 * Desv. estánd.	50758	40.35
2 * Desv. estánd.	7093	5.64
3 * Desv. estánd.	1025	0.81
4 * Desv. estánd.	247	0.20
5 * Desv. estánd.	123	0.10
6 * Desv. estánd.	366	0.29

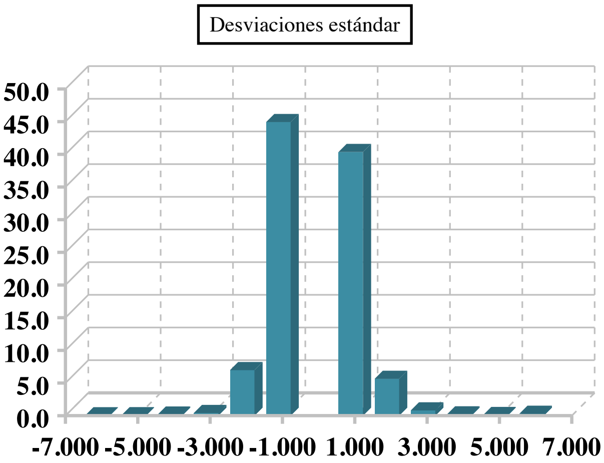

Predefinido: Isométrico

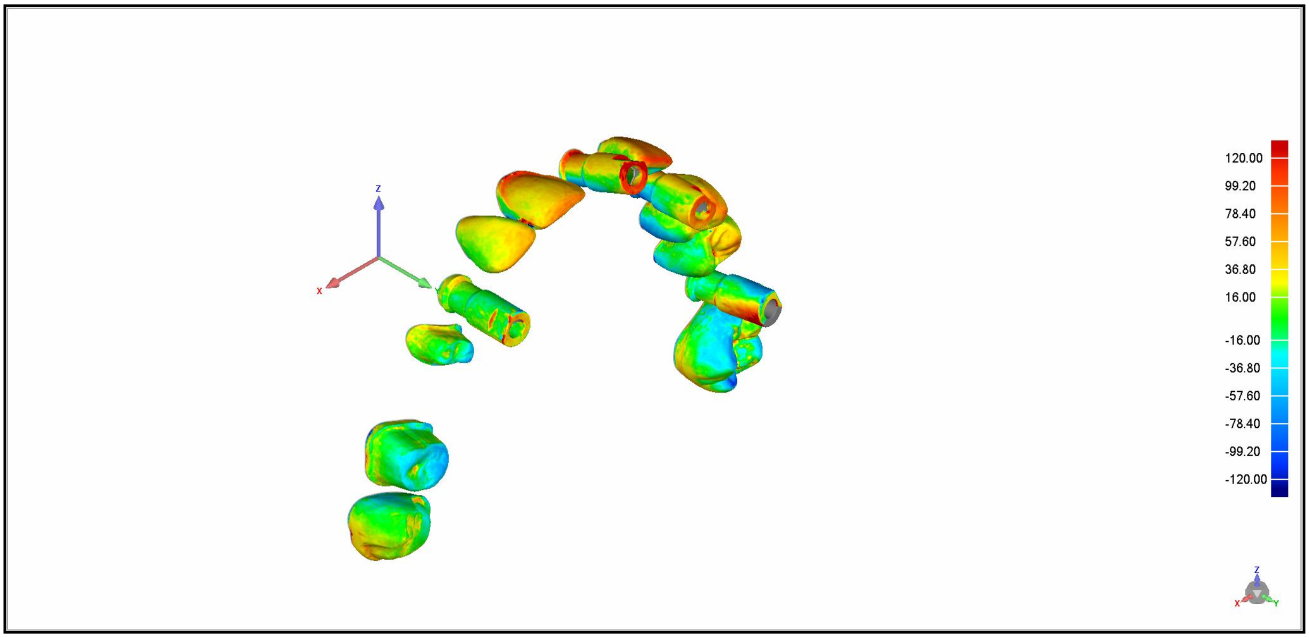

Predefinido: Frente

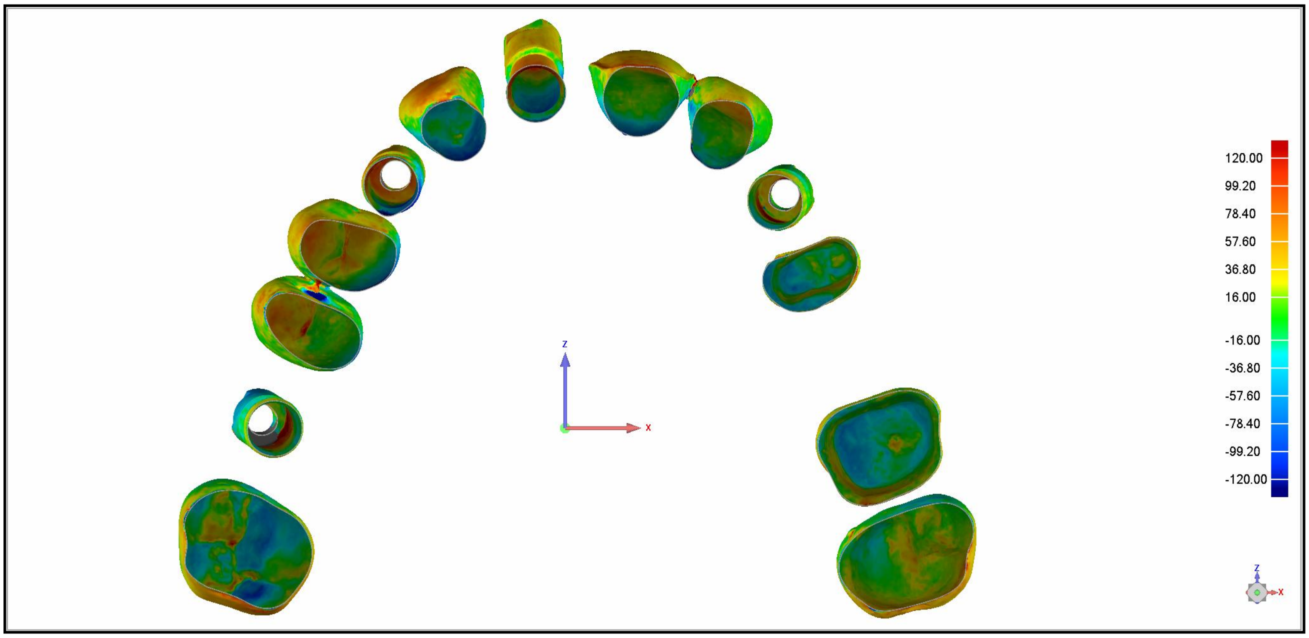

Predefinido: Atrás

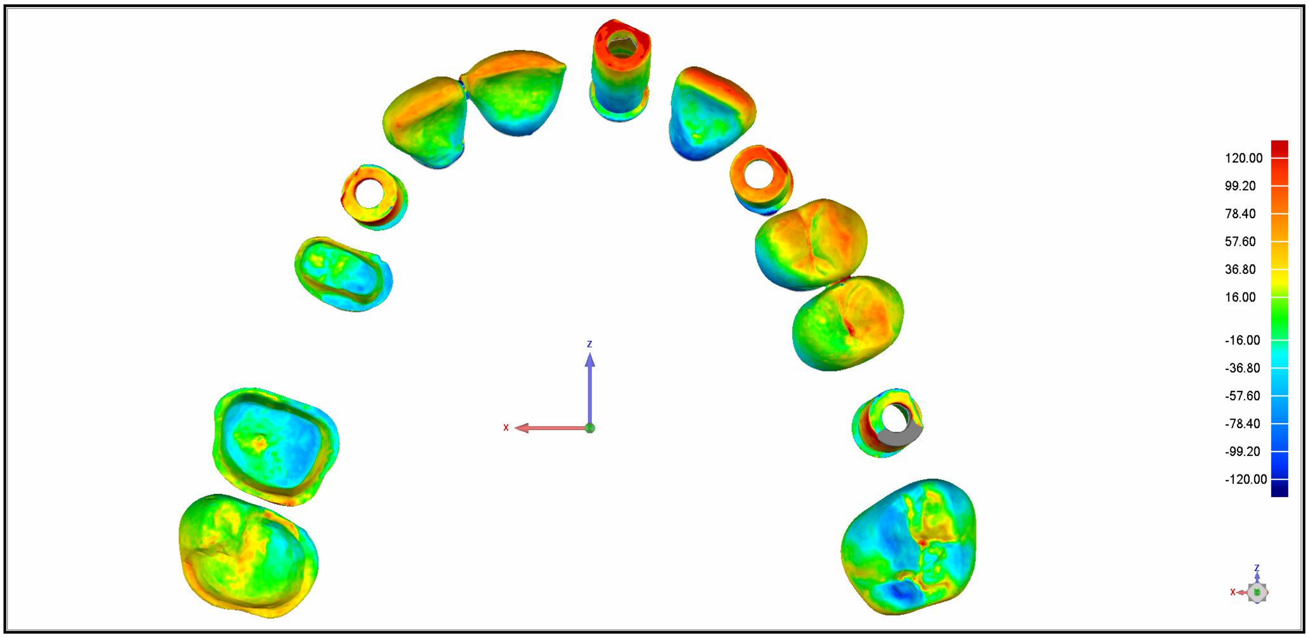

Predefinido: Izquierda

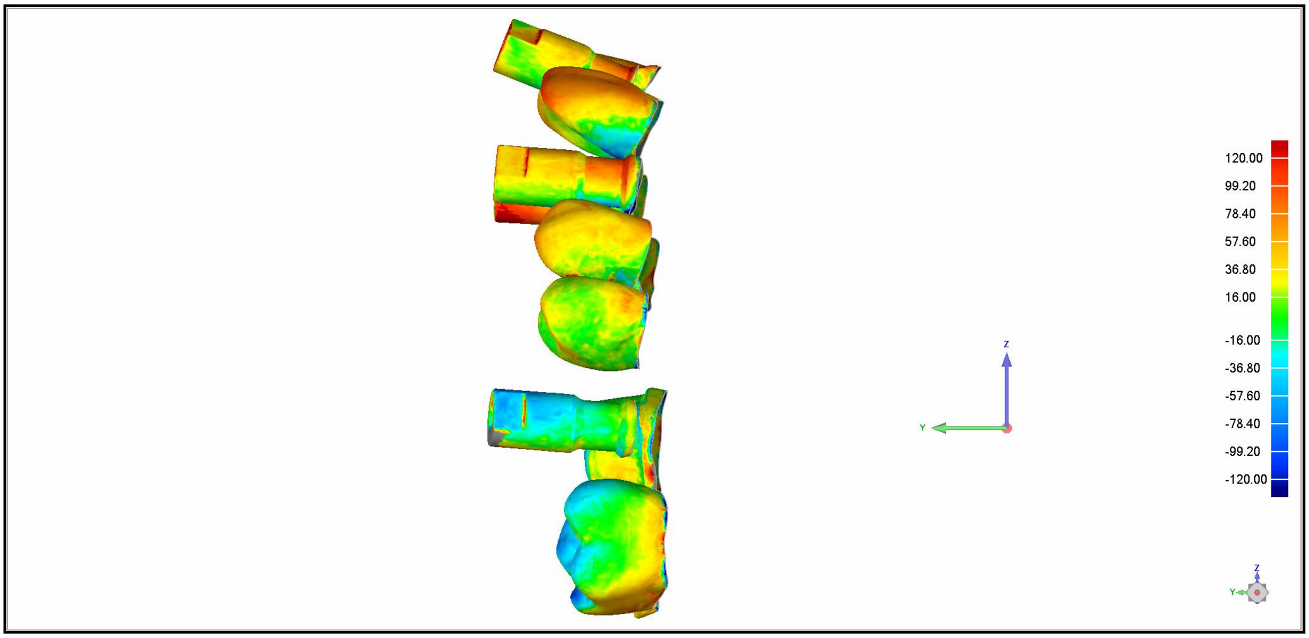

Predefinido: Derecha

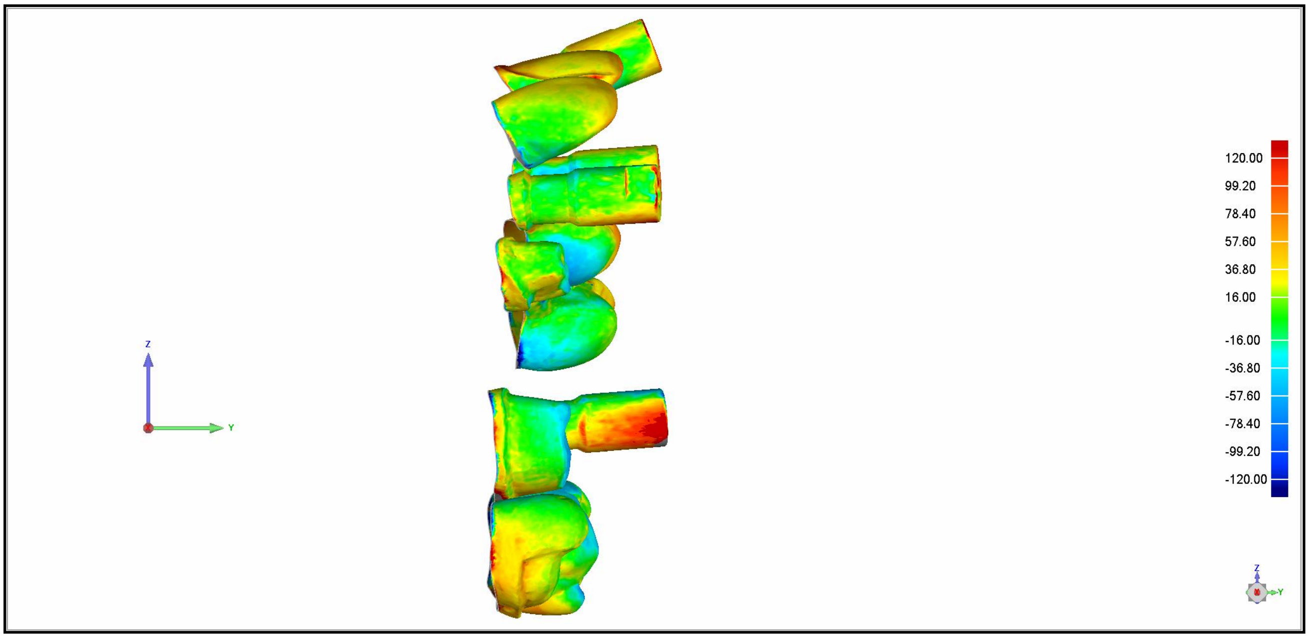

Predefinido: Superior

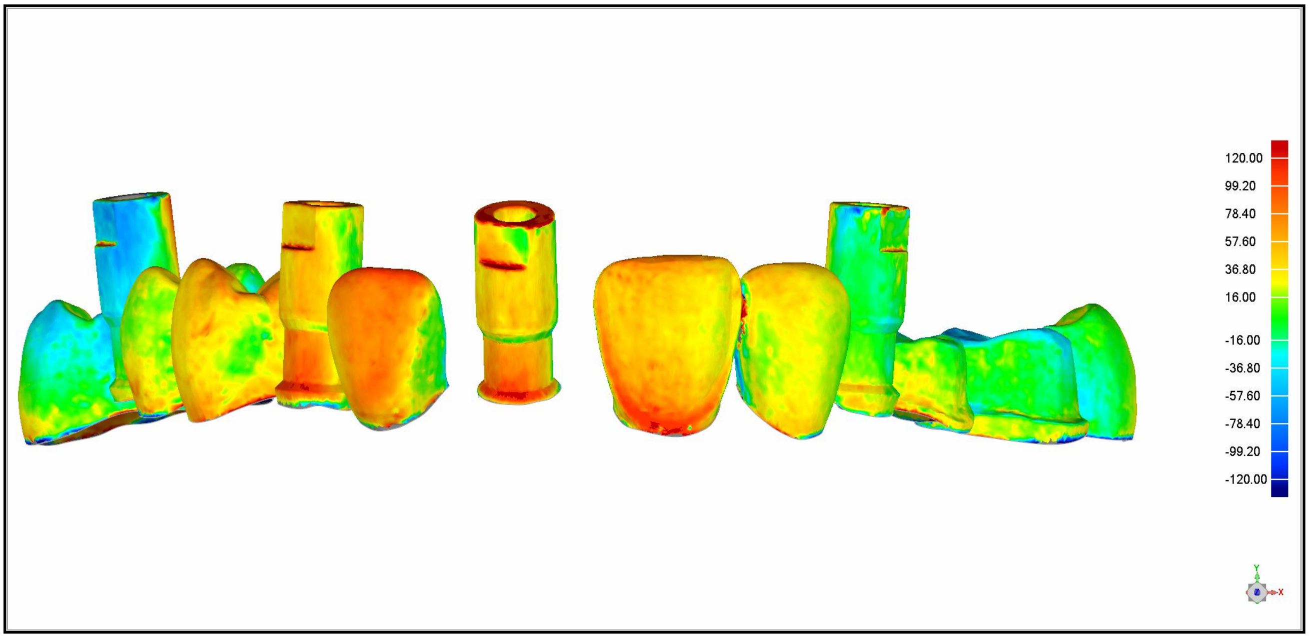

Predefinido: Inferior

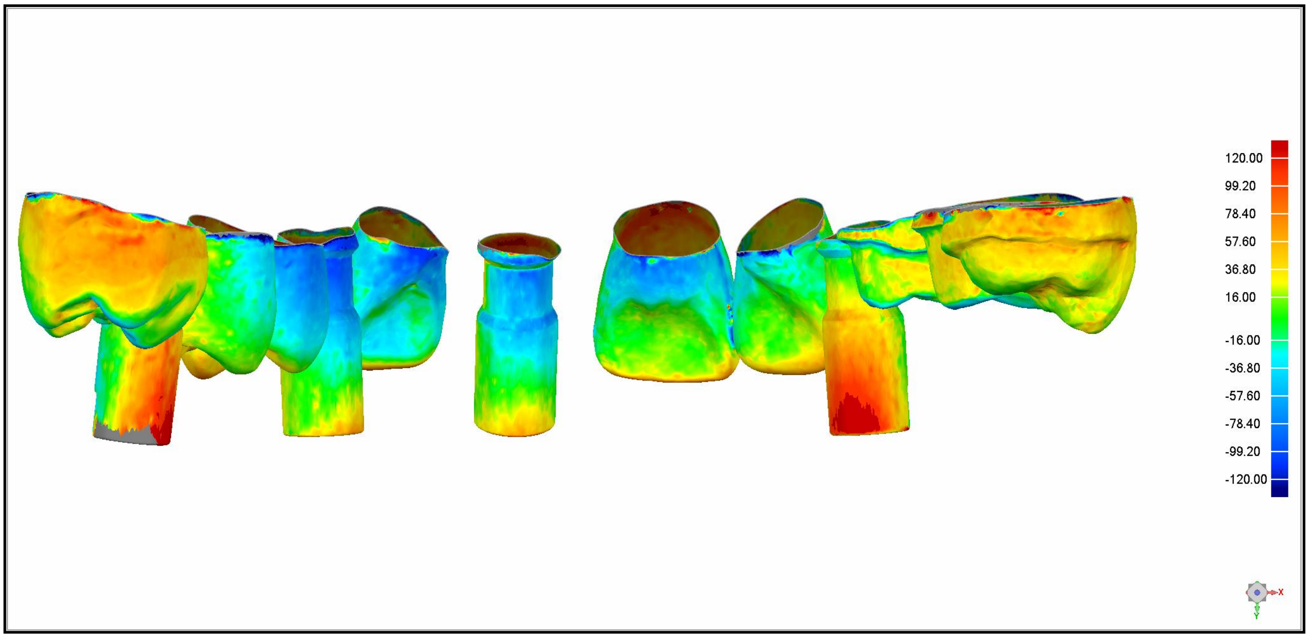

Ajuste de ubicación: Desviaciones superior e inferior

Unidades: u

Nombre	Desv	Estado	Superior Tol	Inferior Tol	Ref X	Ref Y	Ref Z	Radio	Desv X	Desv Y	Desv Z	Medido X	Medido Y	Medido Z	Dir. proy. X	Dir. proy. Y	Dir. proy. Z
Desv. inferior	-1993.48				-16478.80	29167.93	5886.93	n/a	147.27	346.00	1957.69	-16331.54	29513.93	7844.62	-0.07	-0.17	-0.98
Desv. superior	1885.22				-1530.56	38605.60	29213.33	n/a	-13.83	-1884.63	-45.20	-1544.39	36720.97	29168.13	-0.01	-1.00	-0.02
