# Supplementary material for: Accuracy of four digital scanners according to scanning strategy in complete-arch impressions
Source: PLoS One. 2018 Sep 13;13(9):e0202916. doi: 10.1371/journal.pone.0202916 (PMC6136706; doi:10.1371/journal.pone.0202916)
Supplement: S16 Table — True definition (scanning strategy D). (ZIP) [file pone.0202916.s016.zip › S16/TD7D.pdf]

3D Comparación Resultados

Modelo referencia	MRC
Modelo test	TD7D
Nº de puntos de datos	130902
# Aislados	502

Tipo tolerancia	3D desviación
Unidades	u
Máx. crítico	120.00
Máx. nominal	11.00
Mín. nominal	-11.00
Mín. crítico	-120.00

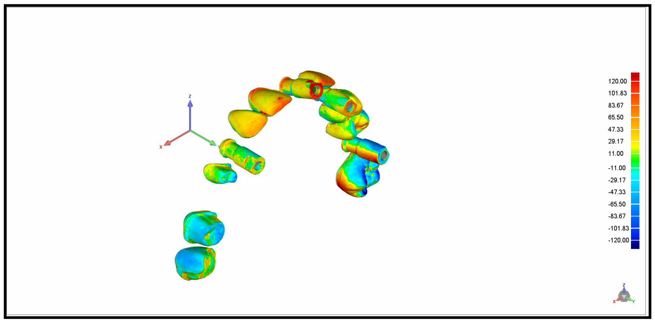

Desviación	
Desviación superior máx.	1386.23
Desviación inferior máx.	-2158.66
Desviación media	39.15 /-33.24
Desviación estándar	56.64

Distribución desviación

>=Min	<Max	# Puntos	%
-120.00	-101.83	626	0.48
-101.83	-83.67	1167	0.89
-83.67	-65.50	2765	2.11
-65.50	-47.33	6068	4.64
-47.33	-29.17	11023	8.42
-29.17	-11.00	15790	12.06
-11.00	11.00	25966	19.84
11.00	29.17	27389	20.92
29.17	47.33	15930	12.17
47.33	65.50	10068	7.69
65.50	83.67	5974	4.56
83.67	101.83	3203	2.45
101.83	120.00	1816	1.39

Fuera del crítico superior	2423	1.85
Fuera del crítico inferior	694	0.53

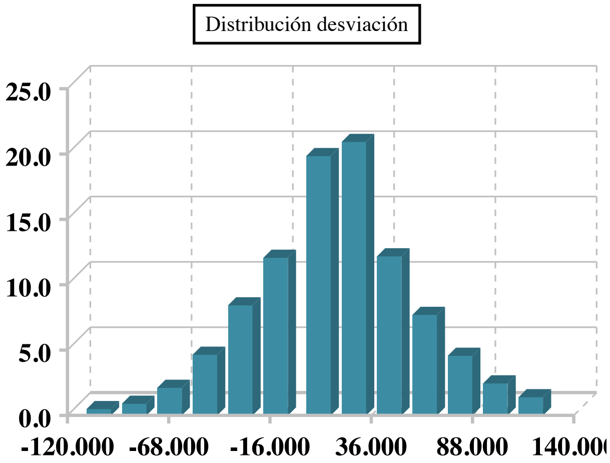

Desviaciones estándar

Distribución (+/-)	# Puntos	%
-6 * Desv. estánd.	83	0.06
-5 * Desv. estánd.	111	0.08
-4 * Desv. estánd.	207	0.16
-3 * Desv. estánd.	914	0.70
-2 * Desv. estánd.	10915	8.34
-1 * Desv. estánd.	52258	39.92
1 * Desv. estánd.	54037	41.28
2 * Desv. estánd.	10315	7.88
3 * Desv. estánd.	1448	1.11
4 * Desv. estánd.	298	0.23
5 * Desv. estánd.	138	0.11
6 * Desv. estánd.	178	0.14

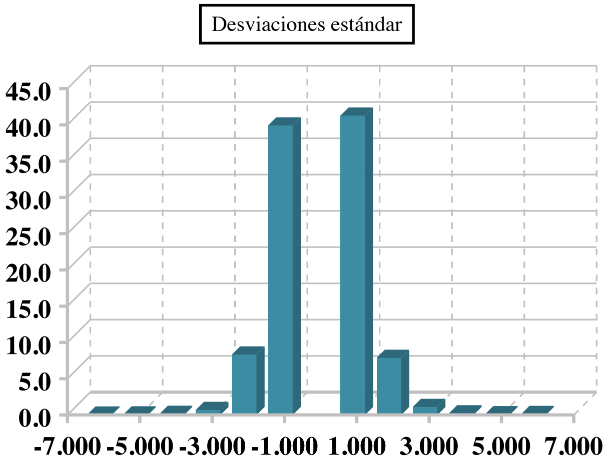

Predefinido: Isométrico

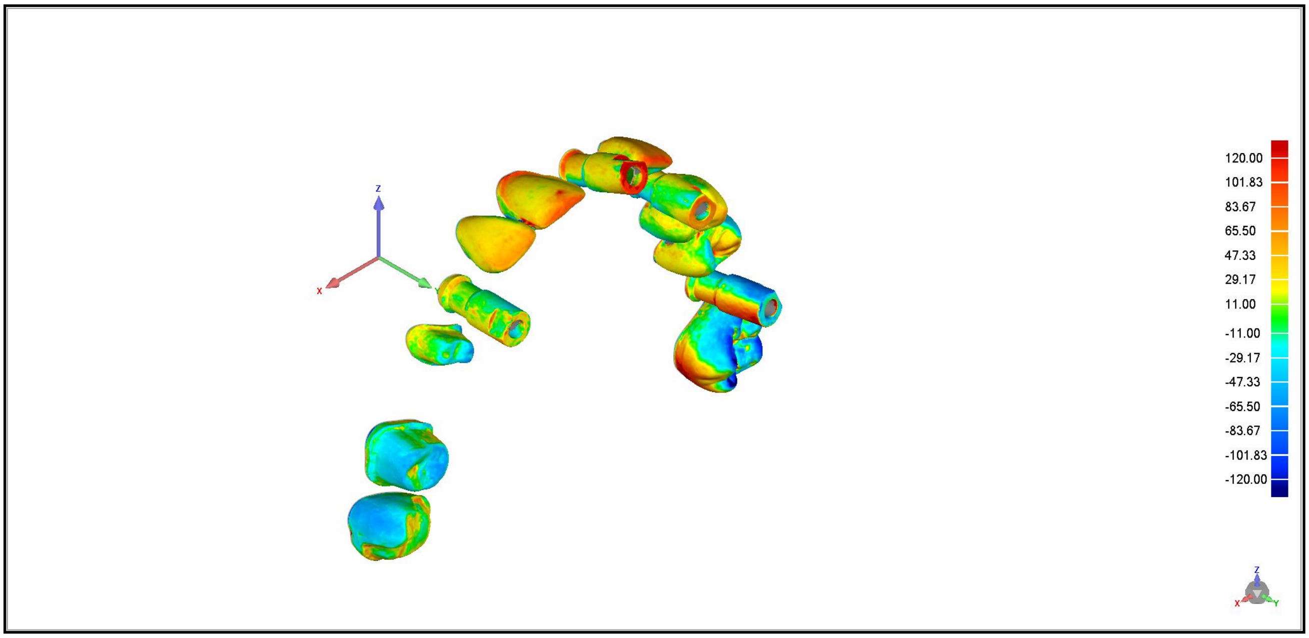

Predefinido: Frente

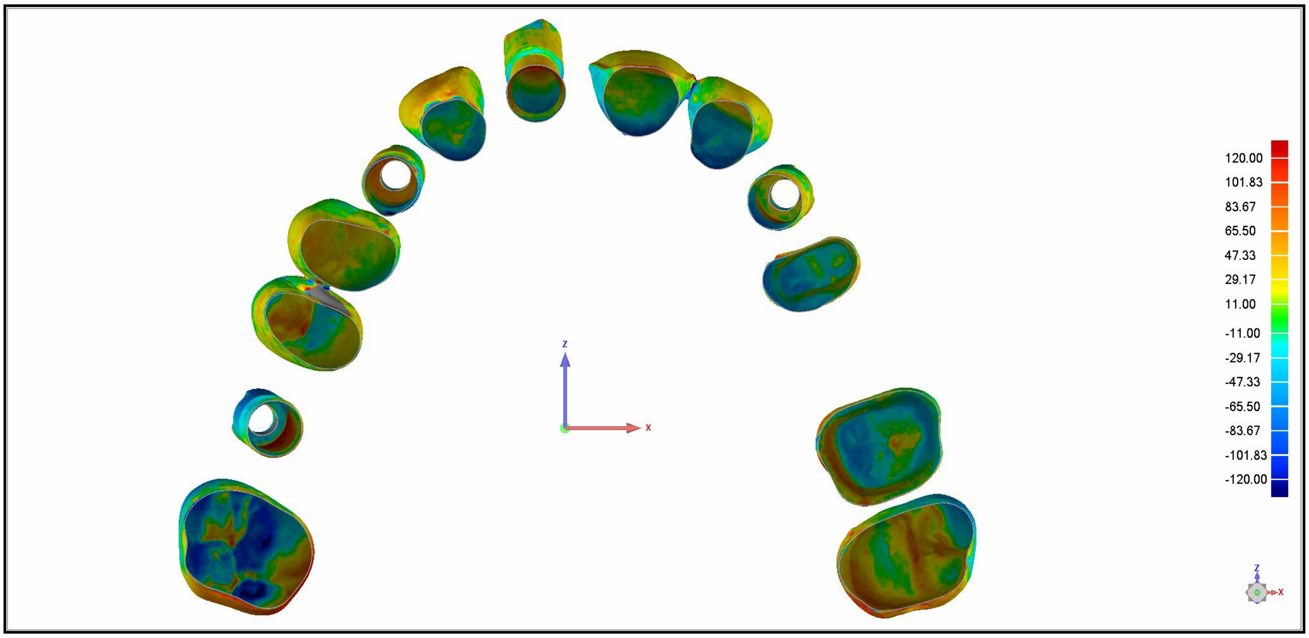

Predefinido: Atrás

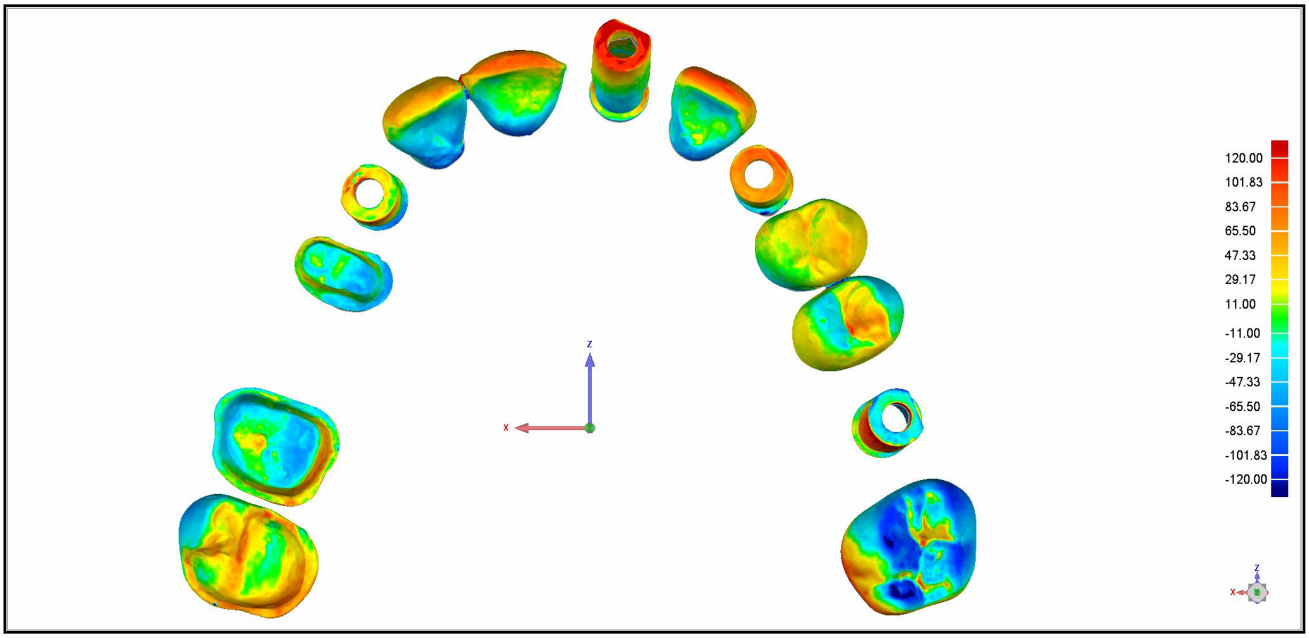

Predefinido: Izquierda

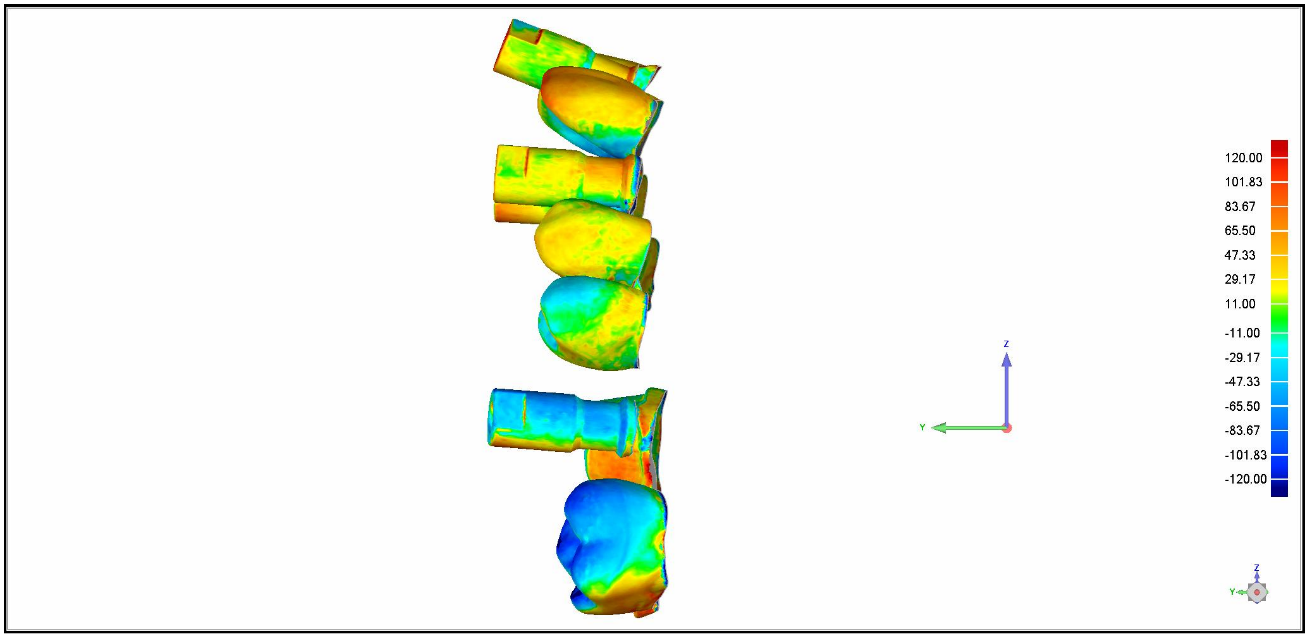

Predefinido: Derecha

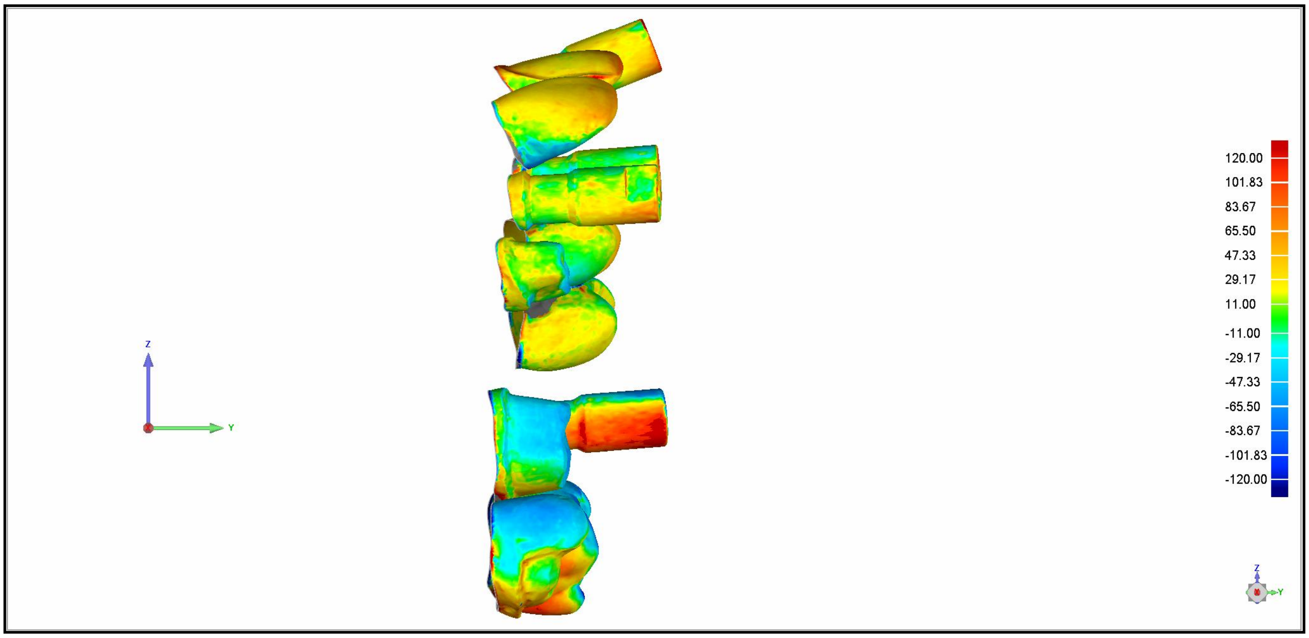

Predefinido: Superior

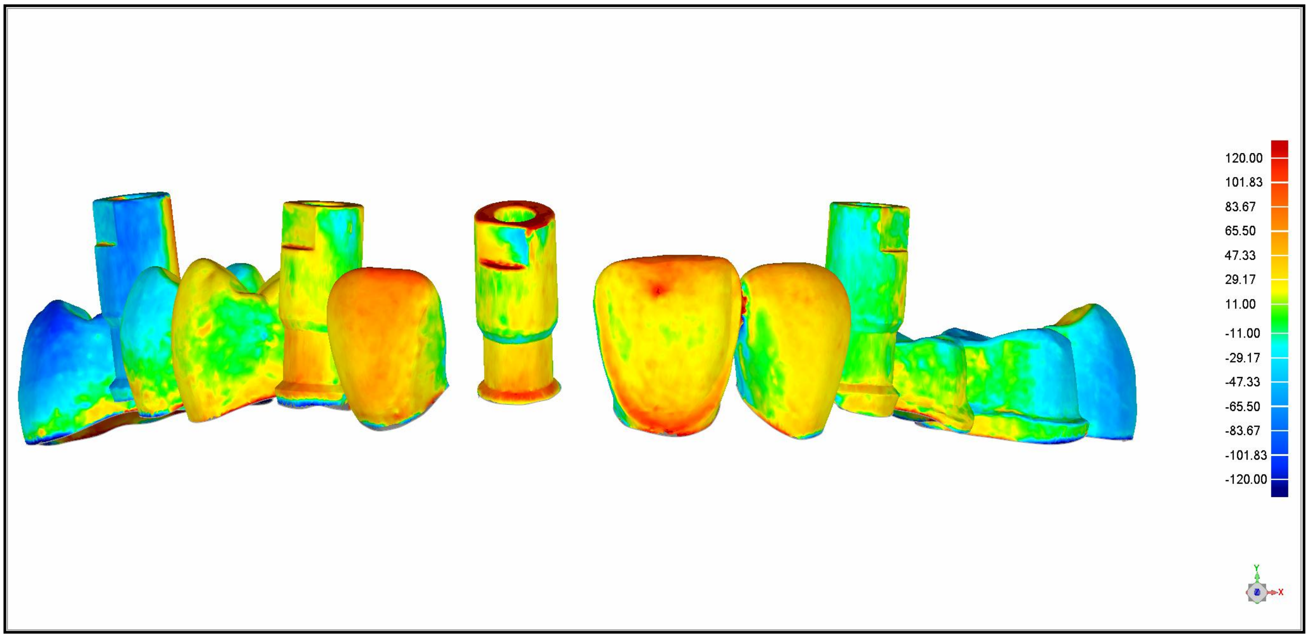

Predefinido: Inferior

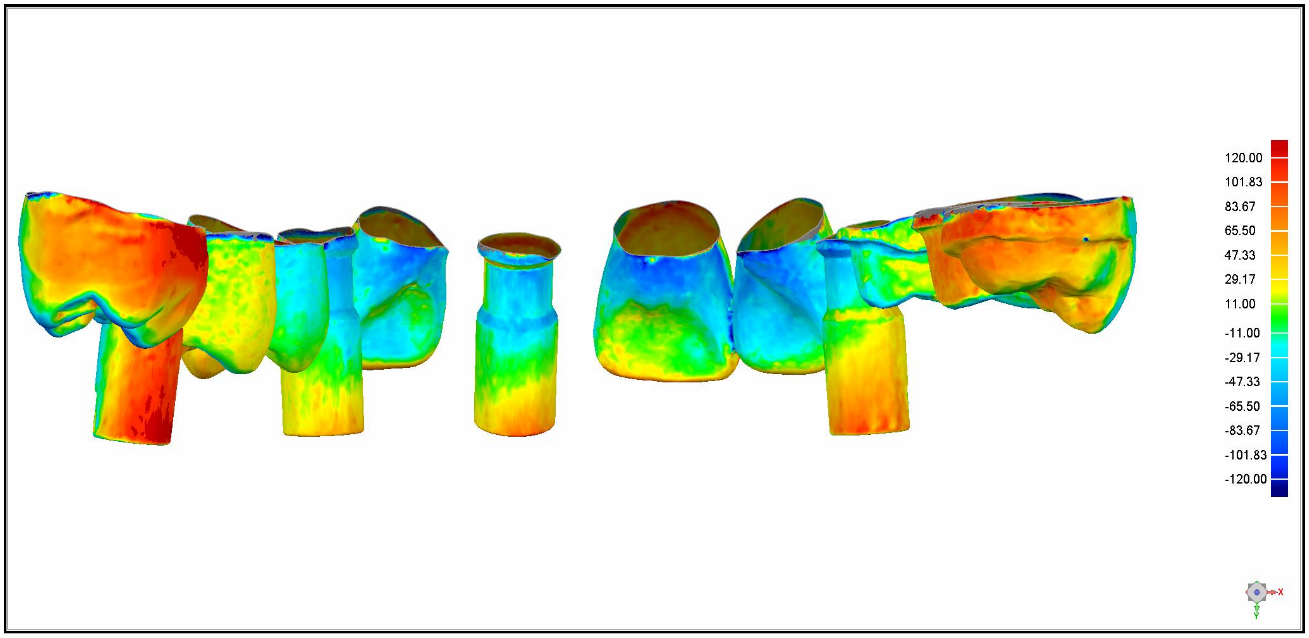

Ajuste de ubicación: Desviaciones superior e inferior

Unidades: u

Nombre	Desv	Estado	Superior Tol	Inferior Tol	Ref X	Ref Y	Ref Z	Radio	Desv X	Desv Y	Desv Z	Medido X	Medido Y	Medido Z	Dir. proy. X	Dir. proy. Y	Dir. proy. Z
Desv. inferior	-2158.66				29707.64	29539.35	-13912.95	n/a	-449.79	-2111.23	13.65	29257.85	27428.12	-13899.30	0.21	0.98	-0.01
Desv. superior	1386.23				-2091.03	38812.77	28805.89	n/a	-642.52	-1118.13	-508.50	-2733.55	37694.63	28297.39	-0.46	-0.81	-0.37
