# Supplementary material for: Accuracy of four digital scanners according to scanning strategy in complete-arch impressions
Source: PLoS One. 2018 Sep 13;13(9):e0202916. doi: 10.1371/journal.pone.0202916 (PMC6136706; doi:10.1371/journal.pone.0202916)
Supplement: S16 Table — True definition (scanning strategy D). (ZIP) [file pone.0202916.s016.zip › S16/TD1D.pdf]

3D Comparación Resultados

Modelo referencia	MRC
Modelo test	TD1D
Nº de puntos de datos	199438
# Aislados	691

Tipo tolerancia	3D desviación
Unidades	u
Máx. crítico	120.00
Máx. nominal	17.00
Mín. nominal	-17.00
Mín. crítico	-120.00

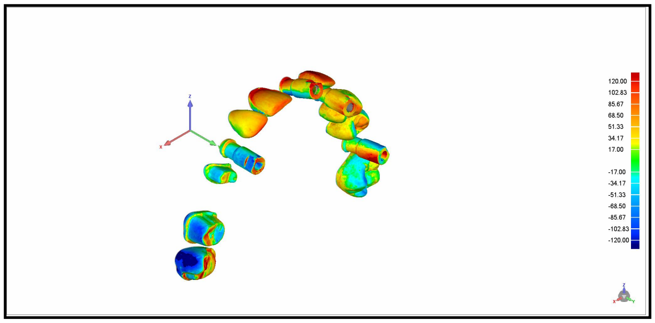

Desviación	
Desviación superior máx.	2472.29
Desviación inferior máx.	-2522.96
Desviación media	51.67 /-35.77
Desviación estándar	73.49

Distribución desviación

>=Min	<Max	# Puntos	%
-120.00	-102.83	1514	0.76
-102.83	-85.67	3096	1.55
-85.67	-68.50	4736	2.37
-68.50	-51.33	7004	3.51
-51.33	-34.17	11476	5.75
-34.17	-17.00	19688	9.87
-17.00	17.00	59824	30.00
17.00	34.17	25151	12.61
34.17	51.33	20514	10.29
51.33	68.50	13491	6.76
68.50	85.67	9285	4.66
85.67	102.83	6872	3.45
102.83	120.00	5188	2.60

Fuera del crítico superior	9816	4.92
Fuera del crítico inferior	1783	0.89

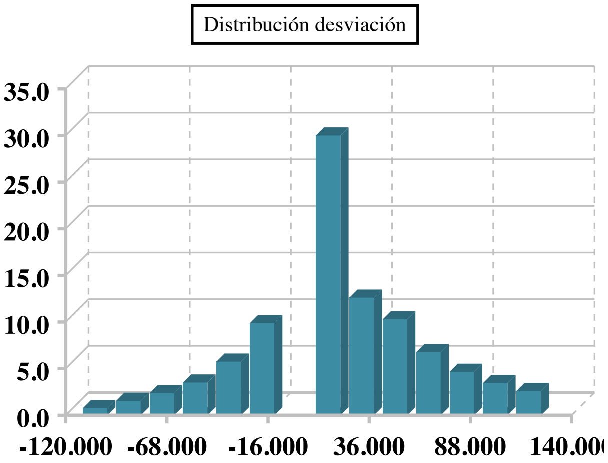

Desviaciones estándar

Distribución (+/-)	# Puntos	%
-6 * Desv. estánd.	143	0.07
-5 * Desv. estánd.	55	0.03
-4 * Desv. estánd.	149	0.07
-3 * Desv. estánd.	764	0.38
-2 * Desv. estánd.	14718	7.38
-1 * Desv. estánd.	93362	46.81
1 * Desv. estánd.	70597	35.40
2 * Desv. estánd.	16650	8.35
3 * Desv. estánd.	2181	1.09
4 * Desv. estánd.	246	0.12
5 * Desv. estánd.	160	0.08
6 * Desv. estánd.	413	0.21

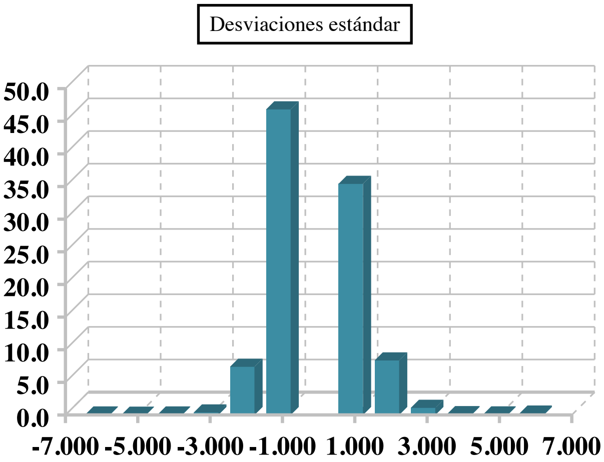

Predefinido: Isométrico

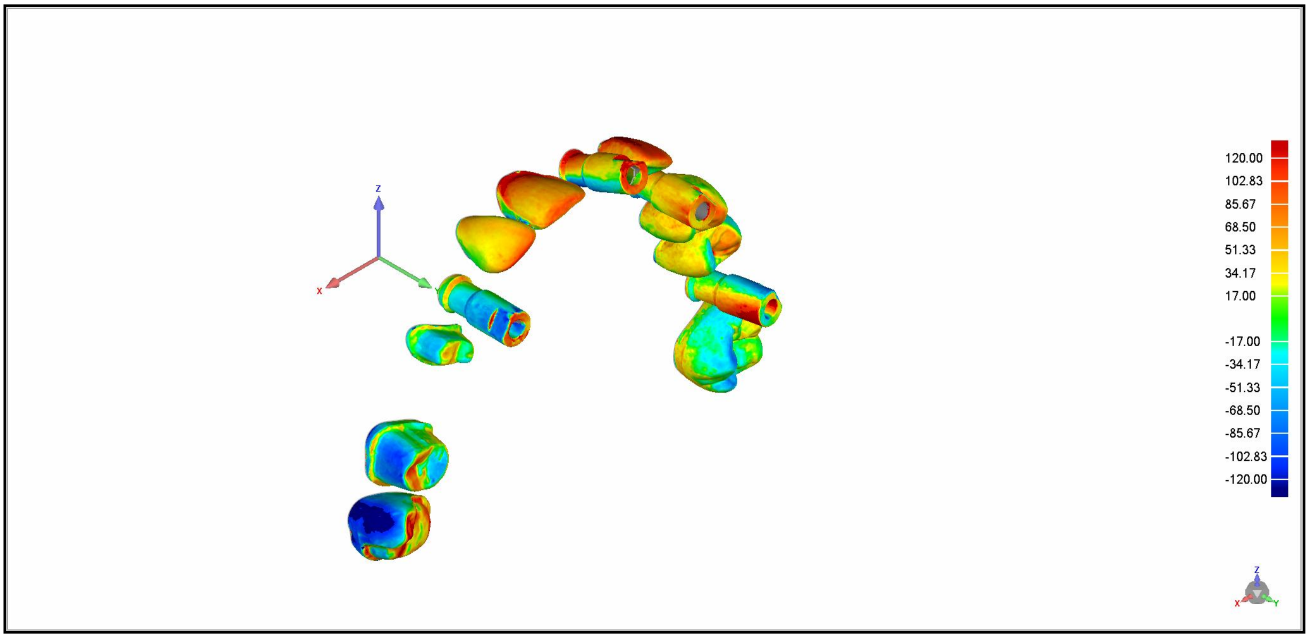

Predefinido: Frente

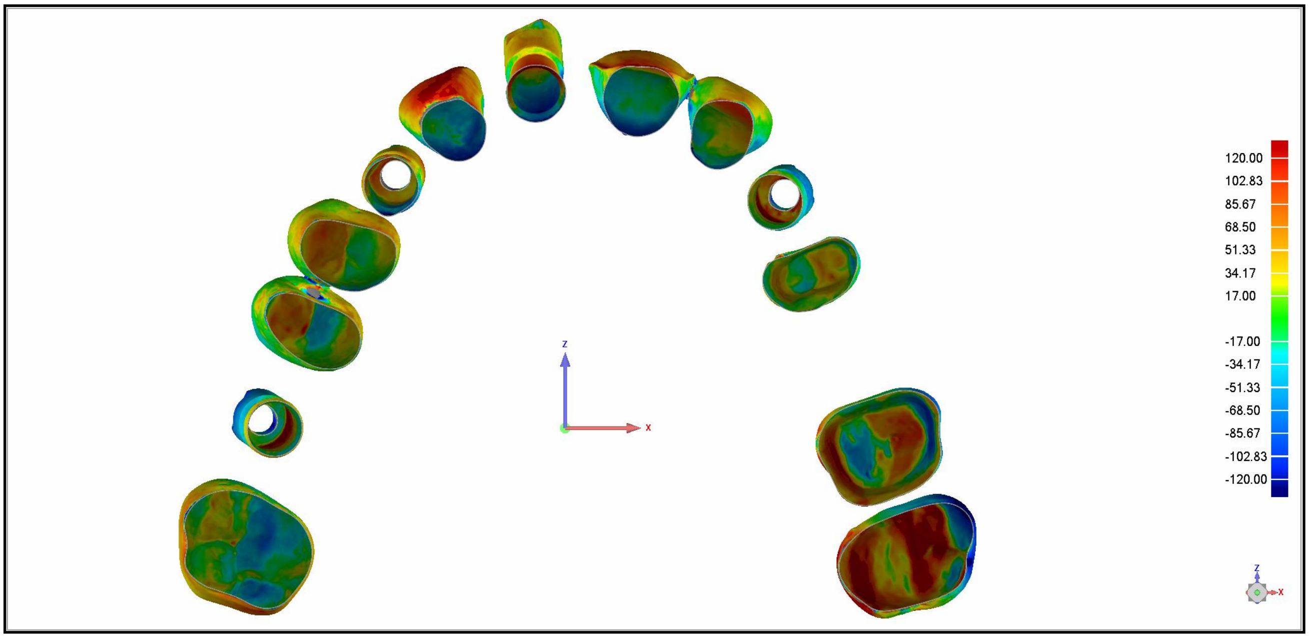

Predefinido: Atrás

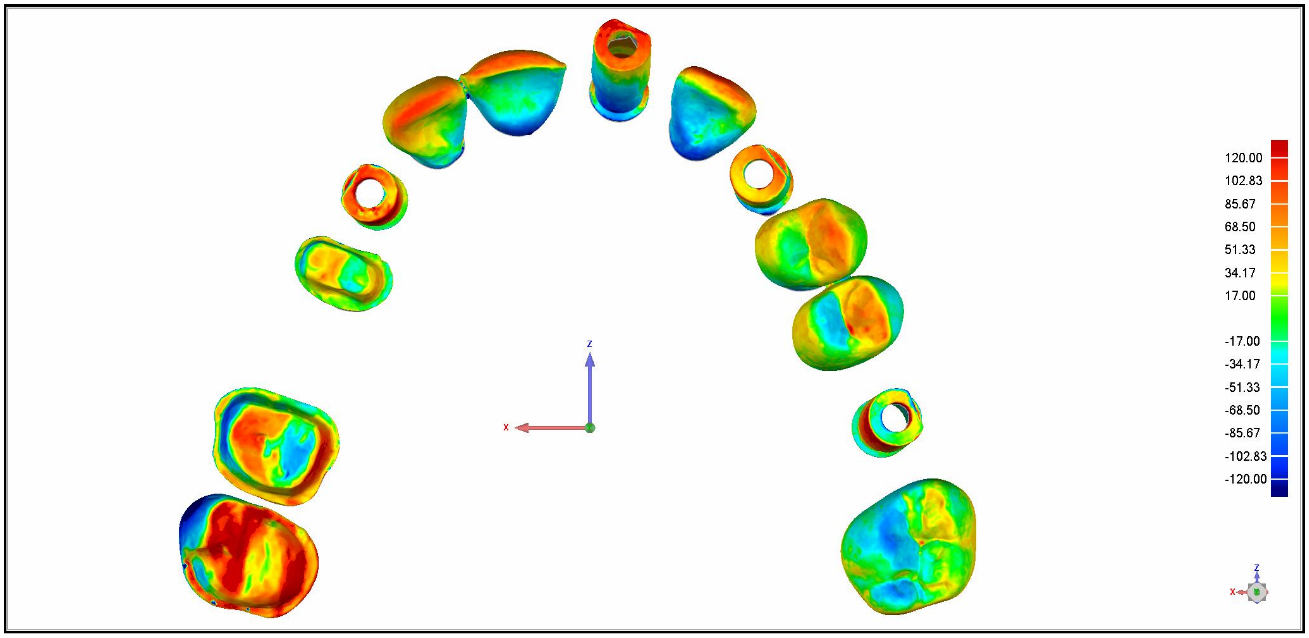

Predefinido: Izquierda

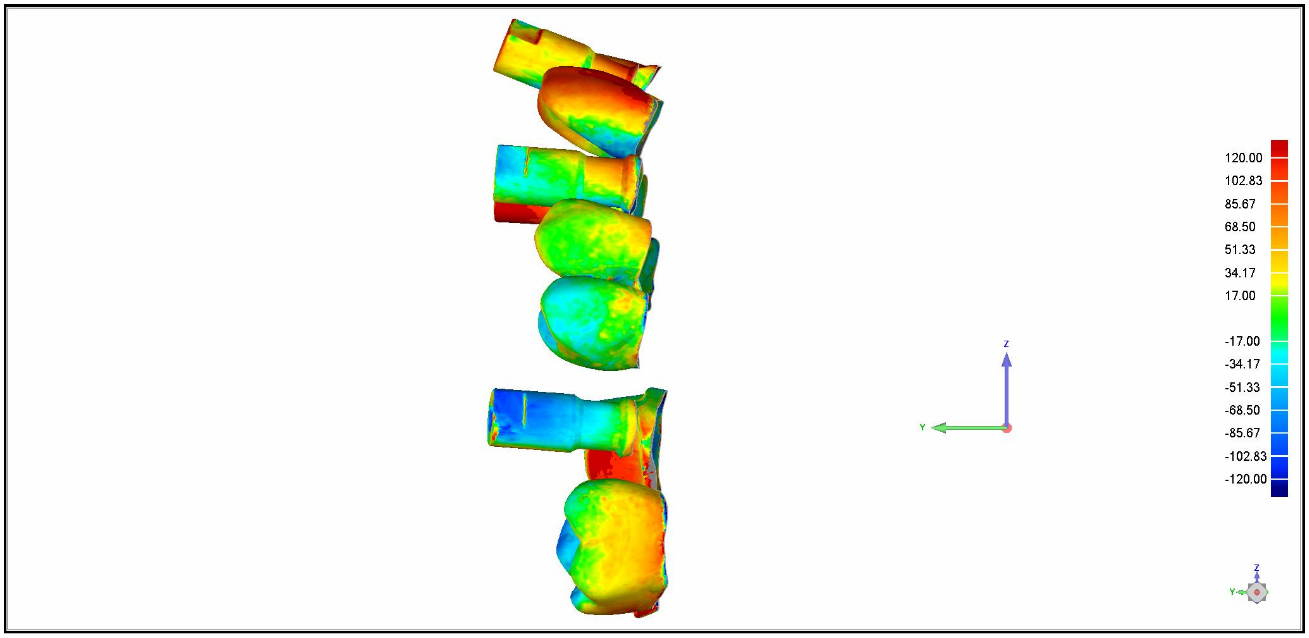

Predefinido: Derecha

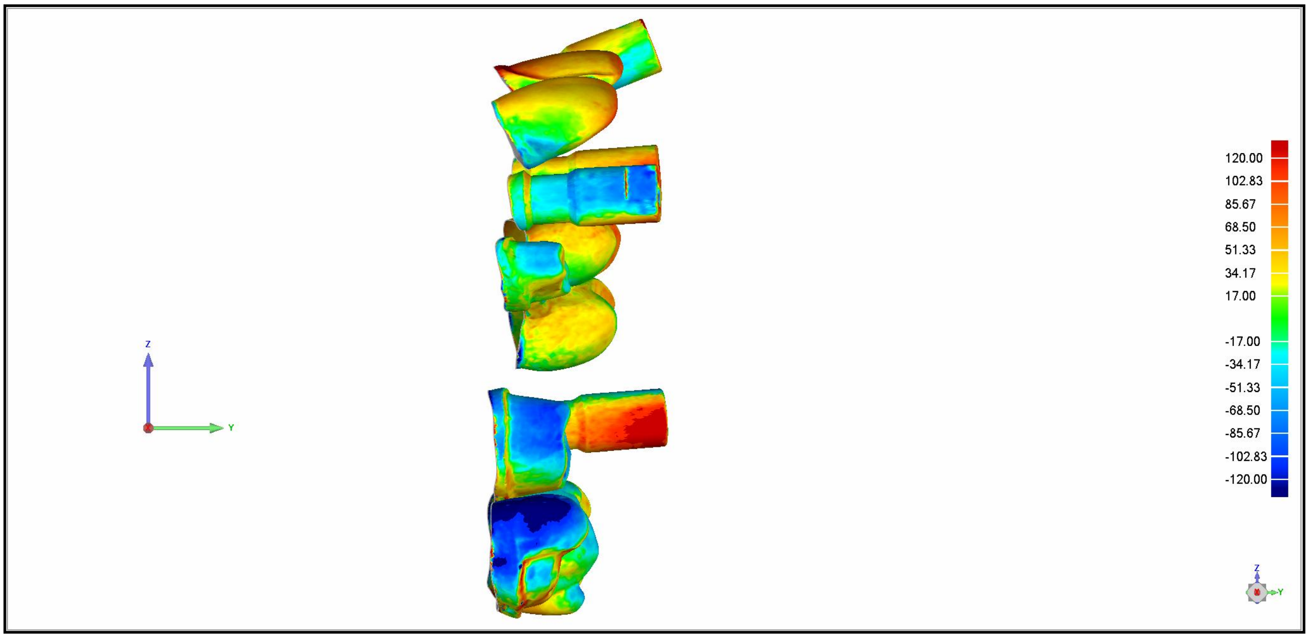

Predefinido: Superior

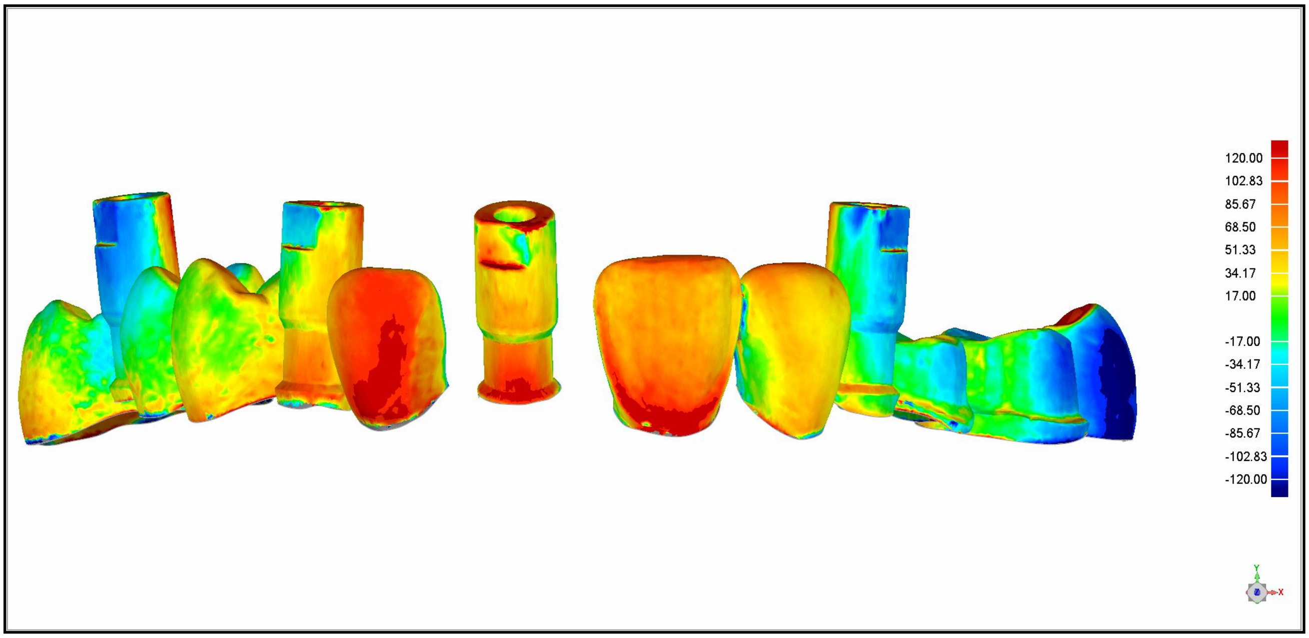

Predefinido: Inferior

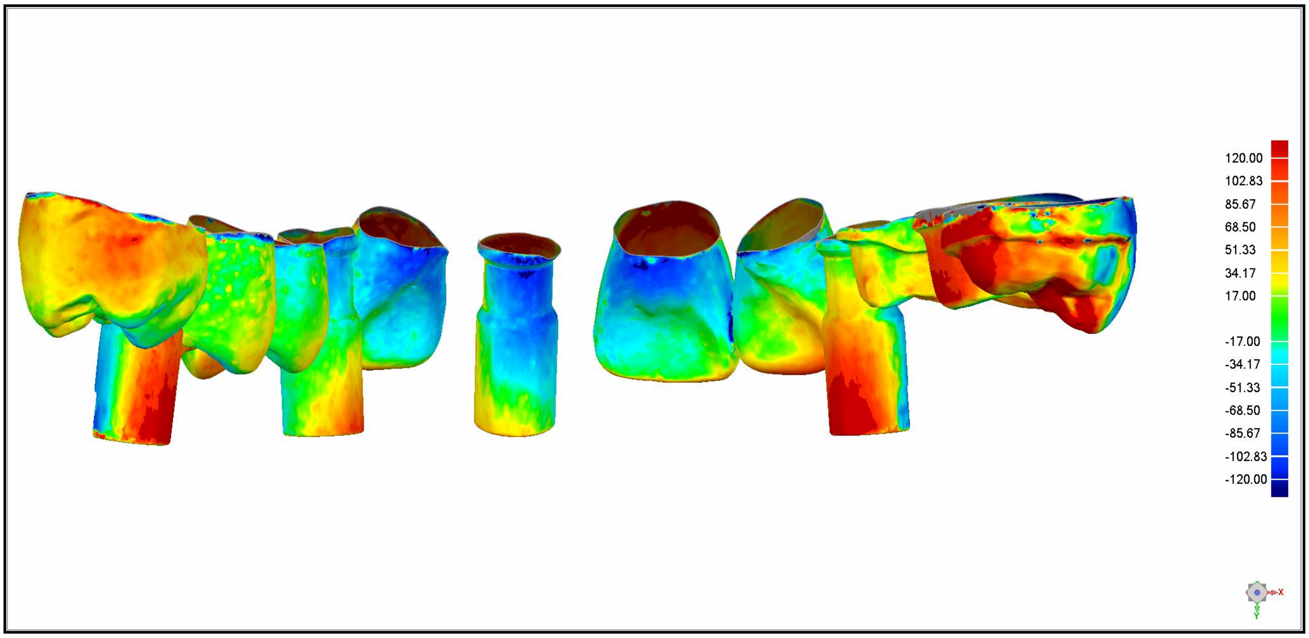

Ajuste de ubicación: Desviaciones superior e inferior

Unidades: u

Nombre	Desv	Estado	Superior Tol	Inferior Tol	Ref X	Ref Y	Ref Z	Radio	Desv X	Desv Y	Desv Z	Medido X	Medido Y	Medido Z	Dir. proy. X	Dir. proy. Y	Dir. proy. Z
Desv. inferior	-2522.96				-9707.88	27813.18	25813.63	n/a	-1875.95	603.31	-1575.49	-11583.83	28416.49	24238.14	0.74	-0.24	0.62
Desv. superior	2472.29				-13871.23	29023.09	21431.13	n/a	2080.22	-213.66	-1318.81	-11791.01	28809.43	20112.31	0.84	-0.09	-0.53
