# Supplementary material for: Accuracy of four digital scanners according to scanning strategy in complete-arch impressions
Source: PLoS One. 2018 Sep 13;13(9):e0202916. doi: 10.1371/journal.pone.0202916 (PMC6136706; doi:10.1371/journal.pone.0202916)
Supplement: S17 Table — N, Mean, Median, SD, Minimum and Maximum. (PDF) [file pone.0202916.s017.pdf]

Raw data manuscript: **"Accuracy of four digital scanners according to scanning strategy in complete arch impressions"**

Unprocess data of trueness and precisión (µm)

							TRUENESS	PRECISION
**Scanner**	**Scanning strategy**	**Sample**	**Maximum external discrepancy**	**Maximum internal discrepancy**	**Average internal discrepancy**	**Average external discrepancy**	**Average of the mean internal and external discrepancy (no signs)**	**Standard deviation**
**TRIOS**	a	1	2753,80	-3006,85	-52,65	62,78	57,72	174,43
**TRIOS**	a	2	2605,45	-2895,40	-43,99	56,69	50,34	185,78
**TRIOS**	a	3	2964,60	-2527,09	-49,42	54,58	52,00	181,86
**TRIOS**	a	4	3121,77	-2710,45	-45,71	60,16	52,94	184,59
**TRIOS**	a	5	2741,78	-2649,12	-64,30	75,94	70,12	198,42
**TRIOS**	a	6	2091,17	-2144,61	-49,22	69,66	59,44	174,54
**TRIOS**	a	7	3068,01	-2140,78	-42,41	57,64	50,03	167,15
**TRIOS**	a	8	2660,05	-2984,26	-59,63	73,43	66,53	198,55
**TRIOS**	a	9	3133,91	-3104,33	-47,28	61,42	54,35	196,25
**TRIOS**	a	10	3129,77	-2992,71	-48,39	66,59	57,49	183,59
**TRIOS**	b	1	3067,01	-3130,49	-46,72	63,31	55,02	191,06
**TRIOS**	b	2	3154,45	-2991,27	-51,50	58,78	55,14	203,51
**TRIOS**	b	3	3140,06	-3124,96	-60,61	70,57	65,59	196,52
**TRIOS**	b	4	3122,01	-3091,48	-46,75	61,04	53,90	181,53
**TRIOS**	b	5	2980,90	-3075,60	-48,11	61,16	54,64	194,01
**TRIOS**	b	6	3104,44	-3147,02	-50,28	60,70	55,49	190,74
**TRIOS**	b	7	3154,33	-3111,81	-47,21	59,83	53,52	193,62
**TRIOS**	b	8	3043,07	-3086,29	-44,54	58,61	51,58	188,58

**TRIOS**	b	9	2968,28	-3143,26	-50,54	60,22	55,38	200,28
**TRIOS**	b	10	3151,20	-3132,83	-49,87	62,39	56,13	205,47
**TRIOS**	c	1	3056,90	-3000,49	-66,36	53,62	59,99	187,31
**TRIOS**	c	2	3014,88	3154,62	-56,93	63,55	60,24	201,93
**TRIOS**	c	3	3067,57	-3100,80	-49,67	61,35	55,51	197,05
**TRIOS**	c	4	3153,79	-3123,78	-50,21	55,38	52,80	175,21
**TRIOS**	c	5	3133,67	-3126,26	-53,08	61,05	57,07	202,45
**TRIOS**	c	6	3095,71	-2999,60	-47,10	58,71	52,91	190,12
**TRIOS**	c	7	3073,71	-3123,92	-50,38	61,68	56,03	200,67
**TRIOS**	c	8	3110,88	-3106,42	-51,23	61,74	56,49	196,19
**TRIOS**	c	9	3153,61	3155,89	-42,95	59,81	51,38	190,14
**TRIOS**	c	10	3039,11	-3110,99	-45,97	60,76	53,37	191,82
**TRIOS**	d	1	3031,69	-3155,86	-53,43	62,46	57,95	202,47
**TRIOS**	d	2	3121,81	-3107,41	53,62	73,70	10,04	210,89
**TRIOS**	d	3	3081,99	-3143,28	-59,64	75,21	67,43	218,29
**TRIOS**	d	4	3088,22	-3121,14	-47,99	60,37	54,18	210,26
**TRIOS**	d	5	3029,34	-3150,70	-45,96	62,60	54,28	196,49
**TRIOS**	d	6	3083,98	-3038,50	-49,44	60,87	55,16	212,96
**TRIOS**	d	7	2890,23	-3120,18	-49,91	65,07	57,49	187,54
**TRIOS**	d	8	3041,64	-3041,90	-49,74	55,20	52,47	194,94
**TRIOS**	d	9	3154,10	-3135,09	-52,88	70,08	61,48	205,45
**TRIOS**	d	10	3155,44	-3120,38	-54,26	63,94	59,10	218,62
**iTERO**	a	1	3110,02	-3107,53	-94,23	98,18	96,21	210,03
**iTERO**	a	2	3067,66	-2982,74	-59,57	73,43	66,50	215,15
**iTERO**	a	3	3141,74	-3139,97	-176,43	137,77	157,10	391,69

**iTERO**	a	4	3148,78	-3137,74	-95,76	99,42	97,59	297,42
**iTERO**	a	5	3141,18	-3149,01	-104,59	115,38	109,99	304,34
**iTERO**	a	6	3136,93	-3095,59	-74,81	91,90	83,36	236,41
**iTERO**	a	7	3139,95	-3126,84	-122,67	120,67	121,67	252,13
**iTERO**	a	8	3155,17	-2705,88	-111,08	113,48	112,28	248,13
**iTERO**	a	9	3132,51	-3145,68	-106,56	112,28	109,42	249,99
**iTERO**	a	10	3063,25	-3151,99	-115,06	130,45	122,76	293,17
**iTERO**	b	1	3140,90	-3140,18	-128,78	117,20	122,99	311,30
**iTERO**	b	2	3139,42	-3147,73	-62,70	87,26	74,98	236,07
**iTERO**	b	3	3138,42	-3082,57	-131,88	127,68	129,78	286,43
**iTERO**	b	4	3143,93	-3148,61	-103,50	105,90	104,70	260,45
**iTERO**	b	5	3093,77	-3121,70	-108,07	124,74	116,41	258,56
**iTERO**	b	6	3141,80	-3151,63	-116,86	107,12	111,99	309,77
**iTERO**	b	7	3035,99	-3118,16	-91,39	101,21	96,30	231,29
**iTERO**	b	8	3140,14	-3152,67	-112,76	107,11	109,94	304,68
**iTERO**	b	9	3154,25	-3094,08	-109,59	103,31	106,45	275,24
**iTERO**	b	10	3058,84	-3131,42	-82,67	96,01	89,34	248,38
**iTERO**	c	1	3153,09	-3132,13	-86,01	100,91	93,46	283,84
**iTERO**	c	2	3136,54	-3153,30	-81,22	94,94	88,08	261,25
**iTERO**	c	3	3082,23	-3100,14	-78,99	91,92	85,46	243,10
**iTERO**	c	4	3135,34	-3149,27	-61,89	82,88	72,39	233,64
**iTERO**	c	5	3153,90	-3119,98	-94,39	87,52	90,96	238,05
**iTERO**	c	6	3020,04	-2936,00	-91,26	106,41	98,84	238,28
**iTERO**	c	7	3078,35	-3126,61	-94,38	100,10	97,24	241,49
**iTERO**	c	8	3089,98	-3128,10	-98,11	114,75	106,43	262,22

**iTERO**	c	9	3109,55	-3092,22	-78,96	91,16	85,06	238,29
**iTERO**	c	10	3135,48	-3129,83	-63,78	83,61	73,70	240,24
**iTERO**	d	1	3154,62	-3068,41	-60,42	68,70	64,56	182,67
**iTERO**	d	2	3044,83	-3136,98	-66,07	68,51	67,29	195,70
**iTERO**	d	3	3036,73	-3112,52	-83,01	80,09	81,55	202,61
**iTERO**	d	4	2553,33	-2786,84	-65,48	71,08	68,28	157,17
**iTERO**	d	5	3154,97	-3152,97	-76,90	81,84	79,37	246,49
**iTERO**	d	6	3143,08	-3139,71	-72,82	93,18	83,00	216,13
**iTERO**	d	7	3021,46	-3134,08	-83,06	96,59	89,83	211,62
**iTERO**	d	8	2912,14	-3123,49	-66,57	67,42	67,00	192,50
**iTERO**	d	9	3145,24	-3084,76	-67,03	72,46	69,75	165,49
**iTERO**	d	10	2681,40	-3145,40	-70,55	87,87	79,21	201,28
**OMNICAM**	a	1	3111,40	-3057,69	-89,68	108,77	99,23	221,99
**OMNICAM**	a	2	3075,82	-3150,40	-81,87	101,82	91,85	268,01
**OMNICAM**	a	3	3041,26	-3125,26	-102,87	106,36	104,62	282,41
**OMNICAM**	a	4	3132,29	-3071,90	-116,45	105,72	111,09	298,41
**OMNICAM**	a	5	3143,44	-3138,92	-127,36	128,10	127,73	299,87
**OMNICAM**	a	6	3154,90	-3134,85	-91,27	95,25	93,26	288,64
**OMNICAM**	a	7	2728,10	-2945,49	-100,39	111,48	105,94	209,95
**OMNICAM**	a	8	3131,71	-3115,08	-72,76	93,69	83,23	212,76
**OMNICAM**	a	9	3149,70	-3129,55	-122,10	101,18	111,64	283,96
**OMNICAM**	a	10	3147,10	-3132,06	-99,39	95,26	97,33	235,28
**OMNICAM**	b	1	3097,97	-3145,62	-88,02	90,74	89,38	251,71
**OMNICAM**	b	2	2918,70	-3154,80	-99,11	94,76	96,94	283,63
**OMNICAM**	b	3	3034,78	-3143,34	-101,57	100,42	101,00	274,37

**OMNICAM**	b	4	3154,69	-3155,11	-115,36	108,68	112,02	307,49
**OMNICAM**	b	5	3062,45	-3106,46	-68,44	82,15	75,30	215,85
**OMNICAM**	b	6	3116,20	-3144,68	-71,72	100,08	85,90	235,39
**OMNICAM**	b	7	3092,85	-3121,55	-75,69	95,56	85,63	191,23
**OMNICAM**	b	8	2999,42	-3134,65	-91,54	100,62	96,08	237,10
**OMNICAM**	b	9	3146,26	-3153,53	-66,01	83,64	74,83	221,41
**OMNICAM**	b	10	3146,97	-3144,80	-65,25	84,18	74,72	218,71
**OMNICAM**	c	1	3109,52	-3154,15	-105,92	105,23	105,58	293,56
**OMNICAM**	c	2	3134,81	-3147,44	-103,40	121,90	112,65	273,72
**OMNICAM**	c	3	3140,05	-3155,28	-91,59	93,59	92,59	277,08
**OMNICAM**	c	4	3145,69	-3148,88	-74,76	99,67	87,22	235,64
**OMNICAM**	c	5	3124,30	-3149,24	-78,50	86,78	82,64	243,41
**OMNICAM**	c	6	3111,58	-3151,89	-84,47	95,33	89,90	262,22
**OMNICAM**	c	7	3014,69	-2982,42	-77,52	90,41	83,97	238,92
**OMNICAM**	c	8	3146,31	-3154,86	-74,00	99,17	86,59	232,79
**OMNICAM**	c	9	2992,71	-3086,35	-109,73	103,04	106,39	294,70
**OMNICAM**	c	10	2978,00	-3102,62	-78,59	91,95	85,27	243,24
**OMNICAM**	d	1	3143,30	-3153,77	-128,60	110,43	119,52	327,42
**OMNICAM**	d	2	3113,18	-3147,63	-89,23	101,33	95,28	278,59
**OMNICAM**	d	3	3150,07	-3154,29	-112,71	128,42	120,57	284,17
**OMNICAM**	d	4	3142,13	-2922,04	-112,24	126,40	119,32	253,29
**OMNICAM**	d	5	3148,61	-3145,94	-121,55	116,01	118,78	291,14
**OMNICAM**	d	6	3123,67	-3145,33	-88,22	98,67	93,45	272,25
**OMNICAM**	d	7	2666,87	-3152,31	-100,49	82,66	91,58	277,80
**OMNICAM**	d	8	3091,72	-3153,91	-92,82	93,64	93,23	261,86

**OMNICAM**	d	9	3150,17	-3155,38	-116,68	110,84	113,76	318,45
**OMNICAM**	d	10	3117,92	-3130,03	-117,91	116,83	117,37	272,42
**TRUE DEFINITION**	a	1	1985,89	-2119,41	40,77	-26,64	-33,71	83,77
**TRUE DEFINITION**	a	2	2880,78	-3111,94	115,96	-95,90	-105,93	172,84
**TRUE DEFINITION**	a	3	2924,25	-2699,40	63,14	-53,46	-58,30	86,91
**TRUE DEFINITION**	a	4	2413,14	-2930,92	43,93	-36,33	-40,13	89,59
**TRUE DEFINITION**	a	5	2976,55	-3096,48	126,77	-106,47	-116,62	209,94
**TRUE DEFINITION**	a	6	1717,20	-1517,69	40,59	-30,44	-35,52	64,89
**TRUE DEFINITION**	a	7	2137,69	-3087,47	93,53	-85,24	-89,39	145,95
**TRUE DEFINITION**	a	8	2503,71	-2268,21	39,82	-32,82	-36,32	73,49
**TRUE DEFINITION**	a	9	1866,02	-3019,21	50,81	-45,61	-48,21	93,62
**TRUE DEFINITION**	a	10	2763,99	-2509,23	49,80	-38,07	-43,94	77,36
**TRUE DEFINITION**	b	1	2217,98	-2513,38	44,30	-37,01	-40,66	87,94
**TRUE DEFINITION**	b	2	2396,34	-1159,73	53,11	-34,34	-43,73	77,20
**TRUE DEFINITION**	b	3	2993,89	-3083,23	124,88	-80,30	-102,59	203,24
**TRUE DEFINITION**	b	4	2074,58	-1214,69	40,78	-24,63	-32,71	81,07
**TRUE DEFINITION**	b	5	2180,49	-2921,27	105,85	-69,31	-87,58	168,12
**TRUE DEFINITION**	b	6	2680,46	-3142,68	92,88	-74,91	-83,90	141,58

**TRUE DEFINITION**	b	7	2537,14	-2400,03	54,52	-31,77	-43,15	90,87
**TRUE DEFINITION**	b	8	1913,26	-3146,49	67,40	-48,75	-58,08	104,07
**TRUE DEFINITION**	b	9	2174,04	-2089,97	57,19	-42,64	-49,92	89,84
**TRUE DEFINITION**	b	10	2311,62	-2576,28	39,95	-28,72	-34,34	73,91
**TRUE DEFINITION**	c	1	2399,42	-2481,72	35,34	-32,99	-34,17	74,02
**TRUE DEFINITION**	c	2	2230,83	-2607,87	50,49	-43,74	-47,12	91,97
**TRUE DEFINITION**	c	3	2308,04	-2127,31	48,63	-36,21	-42,42	84,49
**TRUE DEFINITION**	c	4	1808,04	-2540,77	43,53	-35,60	-39,57	68,88
**TRUE DEFINITION**	c	5	3154,56	-3096,94	51,61	-39,99	-45,80	96,91
**TRUE DEFINITION**	c	6	2340,64	-2690,39	106,12	-79,70	-92,91	193,31
**TRUE DEFINITION**	c	7	2646,03	-2692,01	45,23	-30,55	-37,89	84,07
**TRUE DEFINITION**	c	8	3121,59	-2082,09	43,78	-29,73	-36,76	59,47
**TRUE DEFINITION**	c	9	3136,55	-2471,00	50,23	-38,24	-44,24	78,54
**TRUE DEFINITION**	c	10	2999,04	-1892,75	49,71	-43,27	-46,49	76,32
**TRUE DEFINITION**	d	1	2472,29	-2522,96	51,67	-35,77	-43,72	73,49
**TRUE DEFINITION**	d	2	2039,06	-2714,69	40,23	-29,50	-34,87	70,57
**TRUE DEFINITION**	d	3	2517,39	-3135,62	51,56	-41,11	-46,34	85,28
**TRUE DEFINITION**	d	4	2729,57	-2172,87	53,22	-51,21	-52,22	88,72

**TRUE DEFINITION**	d	5	2269,42	-3040,20	68,91	-70,26	-69,59	108,56
**TRUE DEFINITION**	d	6	1885,22	-1993,48	39,79	-28,22	-34,01	57,83
**TRUE DEFINITION**	d	7	1386,23	-2158,66	39,15	-33,24	-36,20	56,64
**TRUE DEFINITION**	d	8	3056,40	-2940,45	82,69	-80,90	-81,80	132,36
**TRUE DEFINITION**	d	9	2179,83	-2038,72	66,52	-44,13	-55,33	97,59
**TRUE DEFINITION**	d	10	2224,78	-2716,46	33,51	-25,34	-29,43	57,30
